# Whole-Blood MicroRNA Sequence Profiling and Identification of Specific miR-21 for Adolescents With Postural Tachycardia Syndrome

**DOI:** 10.3389/fnins.2022.920477

**Published:** 2022-06-30

**Authors:** Jing Lin, Jie Shen, Juan Liu, Wenjie Cheng, Lintian Li, Fuyong Jiao

**Affiliations:** ^1^Key Laboratory for Disease Prevention and Control and Health Promotion of Shaanxi Province, School of Public Health, Xi'an Jiaotong University, Xi'an, China; ^2^Department of Cardiology, National Clinical Research Center for Child Health, Children's Hospital, Zhejiang University School of Medicine, Hangzhou, China; ^3^Department of Pediatrics, Shenmu County Hospital, Yulin, China; ^4^School of Public Health, Xi'an Jiaotong University, Xi'an, China; ^5^Department of Pediatrics, The Third Affiliated Hospital of Medical College, Xi'an Jiaotong University, Xi'an, China

**Keywords:** postural tachycardia syndrome, whole blood, high-throughput sequencing, miR-21, adolescents

## Abstract

**Objective:**

The aim of the study was to establish whether whole-blood microRNA (miRNA) profiles differ between postural tachycardia syndrome (POTS) sufferers and control subjects and to identify the miRNA that regulates plasma H_2_S.

**Study Design:**

High-throughput sequencing was used to obtain whole-blood miRNA expression profiles for 20 POTS sufferers and 20 normal children.The thresholds for defining differentially expressed miRNAs (DEmiRNAs) were an adjusted DESeq *P* of <0.05 and a log2 fold variation of ≥3. The DEmiRNA target genes were identified using RNAhybrid and miRanda, and only those identified by both were considered. The combined effects of the DEmiRNAs were determined using KEGG pathway analysis. Another 40 POTS and 20 normal patients were used as validation subjects. Plasma H_2_S was determined with a sulfide electrode, and flow-mediated vasodilation (FMD) was performed with a color Doppler ultrasound system. miRNAs were analyzed using qRT-PCR.

**Results:**

Totally, 13 DEmiRNAs were identified through high-throughput sequencing. In the 60-member validation group, the 13 miRNAs were verified again, and it turned out that miR-21 was significantly elevated and could diagnose POTS with a 100% specificity and 92.5% sensitivity. Overall, 198 and 481 genes, respectively, were shown to be targeted by the 13 DEmiRNAs when *P* values of 0.01 and 0.05 were used. The target gene of hsa-miR-21-5p was SP1 when the *P*-value is <0.01. DEmiRNAs were significantly enriched in 36 pathways (*P* < 0.05), in which PI3K/Akt signaling was closely related to vascular function. In the validation subjects, the plasma H_2_S and FMD were higher in the POTS sufferers (*P* < 0.05).

**Conclusion:**

Elevated whole-blood miR-21 levels serve as an indicator for POTS and may explain the increased plasma H_2_S observed in POTS sufferers.

## Introduction

As an orthostatic intolerance subtype, postural tachycardia syndrome (POTS) is the most frequently seen anomaly in the autonomic specialty clinics, which affects around 0.5–3 million U.S. residents, according to estimates (Robertson, 1999; Raj and Robertson, 2018). Furthermore, it is a common neurocardiovascular disease, representing approximately 32.2% of all corresponding syncope cases (Zhang et al., 2009; Johnson et al., 2010; Shen et al., 2017). In a previous study, we demonstrated about 6.8% prevalence of POTS among adolescents and children in China, some of whom present severe clinical symptoms that impact their daily lives (Lin et al., 2014).

Clinically, POTS presents with a range of symptoms of orthostatic intolerance, such as headache, dizziness, cardiopalmus, thoracic discomfort, blurring of vision, anhelation, and occasionally syncope. However, despite this condition being discovered three decades ago, its universal pathophysiologic mechanism has not yet been unambiguously identified (Streeten et al., 1988; Mar and Raj, 2014). Nevertheless, Zhang et al. reported significantly elevated plasma H_2_S, which is considered to be associated with dysfunction of peripheral vasoconstriction in patients with POTS (Zhang et al., 2012). This was later confirmed by Liao et al. who demonstrated that POTS patients present elevated flow-mediated vasodilation (FMD), implying that they suffer from poor vascular endothelial function (Liao et al., 2013). H_2_S is a vasodilative factor capable of free cytomembrane passage and quick responses to inherent environmental alterations. As a novel vascular signaling molecule, it is implicated in the origination and development of diverse cardiovascular conditions (Pan et al., 2017). However, the mechanism by which H_2_S levels in POTS patients are increased is not fully understood.

miRNAs (microRNAs) refer to 19- to 25-nucleotide-long short molecules of RNAs that are responsible for regulating target gene silencing after transcription. An individual miRNA is capable of targeting several hundreds of mRNAs and influential to multiple gene expressions, usually showing implications in the interactive pathways. miRNAs are vital for cell proliferation, differentiation, metabolism, apoptosis, development, and aging, and they are implicated in the pathophysiology of many diseases, such as cancers as well as neurologic and cardiovascular disorders (Lu and Rothenberg, 2018). At present, studies have shown that H_2_S concentration and activity are related to miRNA (Weber et al., 2016; Zhai et al., 2017). However, there are no reports about miRNA involvement in the pathogenesis of POTS, as well as whether the miRNAs participate in the regulation of H_2_S production in children and adolescents with POTS.

Therefore, in the current study, we explored whether the whole-blood miRNA profiles of patients with POTS are different from those of normal control subjects. Furthermore, we attempted to identify the miRNA that regulates plasma H_2_S levels in POTS patients.

## Materials and Methods

### Subjects

We enrolled 20 POTS children and 20 healthy controls from the pediatric outpatient division of Zhejiang University Medical College's Affiliated Children's Hospital between September 2018 and December 2020. Complete assessment of medical history, physical check-up, and laboratory testing were performed for all 20 children with POTS, including ECG, EEG, blood glucose and biochemical examinations, and cranial CT or MRI, so as to eliminate the cardiac, metabolic, neurologic, and psychogenic etiologies. Meanwhile, the recruited 20 healthy controls were matched as far as possible in terms of age, gender, height, weight, and residence location, who were screened by evaluation of medical history, physical check-up, and laboratory testing, such as ECG, Holter monitoring, and standing test. These 40 children were used as sequencing groups. Another 40 POTS patients and 20 healthy children from Shenmu County Hospital and Shaanxi Provincial People's Hospital were used as validation subjects. The enrolled children were all informed about the research objectives beforehand and agreed to participate. Their parents/guardians have all signed a written form of informed consent. For this research, approval was obtained from the ethics committees of Xi'an Jiaotong University, School of Medicine (No. 2018-84).

### Head-Up Test

Prior to the head-up test, any possible autonomic function-affecting drug was discontinued by the children. The test was conducted in a tranquil room with adequate temperature. A Multi-Lead Physiological Monitor (Dash 2000, General Electric, New York) was utilized for persistent surveillance of the cardiac rate and blood pressure during the test. The protocol constituted 10-min (at least) lying down and subsequent 10-min standing. In the event of a positive response within the 10-min standing, the test was terminated. Children with normal variation ranges of cardiac rate and blood pressure during the test underwent a head-up tilt test on the next day.

### Head-Up Tilt Test

Prior to the head-up tilt test, all children were instructed to fast for 4 h (at least) and discontinue any possible autonomic function-affecting drug. A Multi-Lead Physiological Monitor (Dash 2000, General Electric) was utilized for persistent surveillance of cardiac rates and blood pressures of these children, who were laid on a HUT-821 tilt table (Juchi, Beijing, China). Tilting of the table was performed at a 60° angle upon the cardiac rate stabilization. The surveillance of the cardiac rate and blood pressure continued until the emergence of a positive response during the first 10 min. A response would be considered positive if the cardiac rate was elevated by ≥40 beats·min^−1^, or if the maximum heart rate reaches the diagnostic criteria (≥130 beats/min for 6- to 12-year-olds, ≥125 beats/min for 12- to 18-year-olds); at the same time, blood pressure changes a little (systolic blood pressure drops <20 mmHg, diastolic blood pressure decreases <10 mmHg) and any two of the following symptoms present during tilting: dizziness/vertigo, thoracic tightness, headache, cardiopalmus, pallor, blurring of vision, weariness, or syncope. Any child with a positive response was diagnosed with POTS.

### Diagnostic Criteria for POTS

For POTS, its diagnostic criteria were (1) a normal cardiac rate in supine position; (2) over two clinical symptoms on standing, including dizziness/vertigo, aching head, lightheadedness, weariness, pallor, blurring of vision, thoracic tightness, cardiopalmus, trembling hands, and syncope; (3) cardiac rate elevation by ≥40 times/min and (or) the maximum heart rate reaches the diagnostic criteria (≥130 beats/min for 6- to 12-year olds, ≥125 beats/min for 12- to 18-year-olds), and at the same time, blood pressure changes a little (systolic blood pressure drops <20 mmHg, diastolic blood pressure decreases <10 mmHg); (4) relief or mitigation of symptoms by recumbence and ≥1-month persistence of symptoms; and (5) exclusion of other cardiovascular, metabolic, or neurologic lesions (Lin et al., 2017; Wang, 2018).

### Blood Sample Collection for Whole-Blood miRNA Sequencing

Prior to blood collection, both POTS and healthy children were instructed to fast. Blood samples (5 ml) were drawn at 8:00 am from subjects in supine position into PAXgene Blood RNA Tubes (BD). Immediately following blood sampling, the tubes were inverted 8–10 times mildly. In every tube, whole blood (2.5 ml) and additives (7 ml) were added. The blood samples were allowed to stand for 4 h at ambient temperature and subsequently preserved at −80°C.

### Total RNA Extraction

RNA extraction was accomplished from the Blood RNA tubes (PAXgene), followed by quality control. Central analysis was performed on all the quality control outcomes at the end of 2020. After ambient temperature thawing and agitation, the samples were all observed for identifying whether the tubes contained any blood clot. For the automatic RNA extraction from the samples, the Blood RNA kit (PAXgene) was utilized in combination with a QIA cube. Following elution with buffer, the RNA was preserved at −80°C.

### Small RNA Library Construction and High-Throughput Sequencing

Regarding the miRNA library input material, 2 μg of RNA was used per sample. A Small RNA Sample Prep Kit (TruSeq, Illumina, San Diego, USA) was utilized for the creation of miRNA sequencing libraries, and the sequences were assigned to every sample through the addition of index codes. Initially, the total RNA was ligated to a 3′ adaptor, and the resulting mixture was subjected to a 2-min incubation at 70°C for the secondary RNA structure breakage, followed by instant placement on ice. The next step was a 1-h specific ligation of the 3′ adaptor to the miRNA 3′ end by the T4 Rnl2 (T4 RNA Ligase 2) truncated at 28°C. An additional 1-h ligation of the ligated fragment to a 5′ adapter proceeded at 28°C by T4RNA ligase. Utilizing SS IV (SuperScript IV), the synthesis of the first-strand cDNA was accomplished as per the manufacturer's instructions. For the polymerase chain reaction (PCR) amplification, 5-fold Phusion HF buffer, RP1 (RNA PCR primer), RPLX (RNA PCR primer index) primer, dNTP, and Phusion DNA polymerase were utilized. Subsequently, a polyacrylamide gel (6%) was used to purify the PCR products. Thereafter, the 140- to 160-bp-long RNA fragments (corresponding to the length of small non-coding RNA plus 3′/5′ adaptors) were recovered and then dissolved using elution buffer (8 μL). Lastly, a Qubit spectrophotometer was utilized to assess the library concentration, while a Bioanalyzer system (Agilent 2100) and a High-Sensitivity DNA Kit were employed to examine the library quality. The index-coded samples were clustered on a cBot station with a TruSeq PE Cluster Kit v3-cBot-HS (Illumina) as per the manufacturer's instructions. Following cluster generation, sequencing of the prepared libraries proceeded on a HiSeq 2000 platform (Illumina), and single-end 50-bp reads were produced to assess transcriptomics. The sample quality control, experimentation, and data analysis were all accomplished by Genesky Biotechnologies Inc. in Shanghai, China.

### Known miRNA Expression Profile Generation, Normalization, and Clustering

Exploiting the miRDeep2's quantifier module, the expression profile was generated for the known mature miRNA, which offers read counts for known miRNAs. The generated expression profiles of raw reads for all the sample replicates were normalized by the trimmed mean of the M-value approach *via* the Bioconductor's edgeR. The resulting normalized profiles for all the sample replicates were subsequently analyzed by hierarchical clustering and principal component analysis (PCA) to control quality and evaluate inter-sample similarities.

### Quantification of miRNA Expression Levels and Differentially Expressed miRNA (DEmiRNA) Analysis

Aided by HTSeq (v0.6.1), the quantity of reads mapped to every miRNA was subjected to normalization. Then, the expected fragments per kilobase million (FPKM) number was computed for every miRNA according to the gene length and the read quantity mapped to miRNA. It is common practice to predict the levels of miRNA expression in FPKM, a value reflecting the effects of both gene length and sequencing depth for the read quantity. DESeq2's R package was exploited to analyze the DEmiRNAs. Utilizing the digital miRNA expression data, this package enables routine statistical determination of differential expression with a negative binomial distribution-based model. For the false discovery rate (FDR) control, the Benjamini–Hochberg method was employed to adjust the resulting *P* values. The thresholds for defining DEmiRNAs were an adjusted DESeq *P* of <0.05 and a log2 fold variation of ≥3.

### DEmiRNA Target Gene Identification

The miRanda and RNAhybrid algorithms were used for the potential target gene recognition for the dysregulated miRNAs. We considered the miRNA target genes recognized by both algorithms.

### Pathway Analysis of Related Target Genes

DEmiRNA joint effects on pathways were evaluated by KEGG analysis based on their associated target genes. The threshold parameters for pathway significance were *P-*value and FDR. Any KEGG pathway was regarded as significant when *P* < 0.01.

### Quantitative Real-Time PCR

For placental isolation of total RNA in humans, an RNeasy Micro Kit (Qiagen, Hilden, Germany) was utilized. A Nanodrop spectrophotometer (Wilmington, USA) was used for the quantification of RNA. Initially, reverse transcription of total RNA (20 mg) per sample was accomplished with reverse transcriptase (SuperScript II, Invitrogen)-involving a master mix. Real-time PCR was carried out with a PRISM 7700 Sequence Detector (ABI, Warrington, UK) as per the instructions of the manufacturer. All the Assays-on-Demand primers and probes were procured from ABI. All PCRs were run in triplicate. The reaction system contains forward primers and reverse primers (0.3 μM, respectively) and 2 × Maxima SYBR Green qPCR Master Mix (12.5 μL), cDNA(200 ng), and nuclease-free water reaches 25 μL totally. U6 small nuclear RNA (snRNA) was used as an internal control of miR-21 levels. The expression of miR-21 was normalized to snRNA U6, and relative expression was calculated using the 2^−ΔΔCT^ method. The primers of miR-21 and U6 were as follows: 5'-TAGCTTATCAGAC TGATGTTGA-3' (hsa-mir21-5p, forward); and 5'-CTCAACTGGTGTCGTGGA-3'(hsa-mir21-5p, reverse); 5'-CTCGCTTCGGCAGCACA-3' (U6, forward); 5'-AACGCTTCACGAATTTGCGT-3' (U6, reverse).

### Plasma H_2_S Concentration

Prior to the examination, the subjects were asked to fast for 8 h, avoid vasoactive agents, high-lipid foods, caffeine, and vitamin C for 24 h, and evade exercise for 4–6 h. In the case of adolescent women, the menstrual period was also avoided. A blood sample (1 ml) was collected at the cubital vein from every subject in the morning (7:00 am to 8:00 am) in a tube containing ethylenediaminetetraacetic acid and aprotinin. After mixing 0.5 mL of plasma with an equal volume of antioxidant buffer, a PXS-270 sulfide electrode (Shanghai, China) was used to determine the plasma H_2_S. After washing with distilled water and drying, the electrode was soaked in the sample. Upon stabilization of readout, we documented the electrode potential. The standard curve method was employed for the computation of the H_2_S concentration (Zhang et al., 2012).

### FMD

An HP2500 color Doppler ultrasonographer (Philips Healthcare, Andover, USA) was utilized for the FMD test, whose transducer frequency was 7.5 MHz. The American College of Cardiology guideline was followed in this study to achieve brachial arterial assessment of the endothelium-dependent FMD (Corretti et al., 2002; Donald et al., 2008). The participant was laid down with their arm in a location suitable for the brachial arterial imaging. The transducer was arranged vertically on the upper brachial skin 5–10 cm above the antecubital fossa, while a cuff of mercurial sphygmomanometer was placed on the forearm. For the baseline vascular lumen diameter determination, the baseline brachial arterial image was acquired, while for the blood flow assessment, the pulsed wave Doppler velocity signal was analyzed (detection angle 40°). Next, arterial occlusion was established by inflating the cuff for 5 min to 40–50 mm Hg higher than the systolic pressure. Upon deflation of the cuff, there emerged a high-flow stimulus, and dilation of the brachial artery was noted due to shear stress enhancement. The brachial arterial image and blood flow assessment was attained at the identical position within 2 min of the cuff deflation. The maximum vascular lumen diameter during dilation was documented. The computational formula for FMD (%) was [(maximum diameter – baseline diameter)/baseline diameter]× 100% (Liao et al., 2013).

### Statistical Analyses

SPSS (v 13.0; Chicago, USA) was used for analyzing data. The continuous data displayed are means ± SDs, while the categorical data displayed are quantities of cases. The inter-group differences were examined by χ^2^ and *t*-tests. For the fold variation computation of miRNA expression, the levels of expression were compared between POTS and control groups. The screening criteria for DEmiRNAs adopted herein were a fold variation of ≥2 and *P* < 0.05. The quantile normalization was conducted by R software (LC-Bio, Hangzhou, China), which was also used for the GO (gene ontology) and KEGG analyses. Differences were regarded as significant when *P* < 0.05. The datasets for this study can be found in the [ArrayExpress] [https://www.ebi.ac.uk/arrayexpress/]. The accession number is E-MTAB-10961.

## Results

### Baseline Characteristics of POTS Patients and Control Subjects

The 20 POTS patients consisted of 12 boys and eight girls, aged 10.8–13.9 years, with a mean of (12.7 ± 1.21) years. The 20 healthy controls also comprised 12 boys and eight girls, aged 11.2–14.1 years, with a mean of (13.1 ± 1.13) years. Insignificant inter-group differences were noted regarding age, height, blood pressure, or weight (*P* >0.05), while the cardiac rate varied significantly (*P* <0.05) (Table 1).

**Table 1:** Demographic and hemodynamic parameters between patients with POTS and comparison subjects.

	**POTS patients**	**Control group**	***t***	***P***
Age (years)	12.78 ± 1.21	13.12 ± 1.13	0.458	0.659
Height (cm)	155.8 ± 13.44	157.6 ± 10.73	0.234	0.821
Weight (kg)	48.3 ± 11.91	48.74 ± 9.91	0.063	0.951
Supine HR (beats/min)	72 ± 3.80	87.4 ± 9.83	3.26	0.011
Supine SBP (mmHg)	102 ± 4.41	107.8 ± 10.83	1.11	0.299
Supine DBP (mmHg)	68.80 ± 10.61	66.40 ± 4.67	0.463	0.656
Upright HR (beats/min)	130.40 ± 6.80	104.8 ± 12.93	3.918	0.004
Upright SBP (mmHg)	116.6 ± 4.87	119.40 ± 15.13	0.394	0.704
Upright DBP (mmHg)	77.20 ± 3.27	76.40 ± 8.96	0.88	0.856
HR increment (beats/min)	58.40 ± 8.38	17.40 ± 7.19	8.297	<0.001

*DBP, diastolic blood pressure; HR, heart rate; POTS, postural tachycardia syndrome; SBP, systolic blood pressure*.

### DEmiRNAs

**Figure 1:**
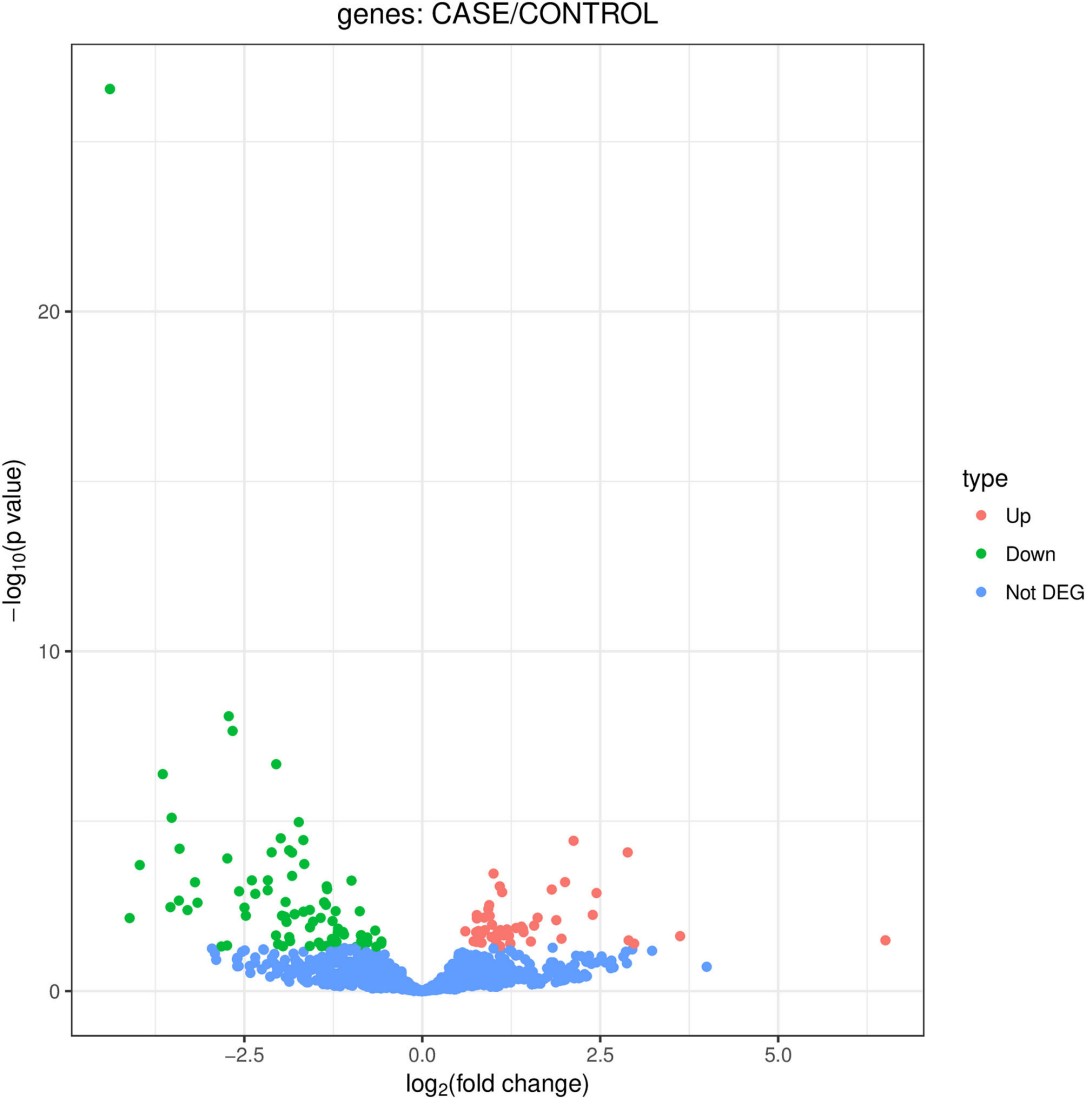
Volcano map of DEmiRNAs. DEmiRNAs between POTS patients and control subjects. Red spots show upregulated miRNAs, while the green spots show downregulated miRNAs (not DEG, not differently expressed genes).

We identified 135 DEmiRNAs between the two groups. Among them, 49 are upregulated and 86 are downregulated (Figure 1). The 32 miRNAs for which a fold change ≥2 and *P* < 0.05 are shown in Table 2. Among the miRNAs, 13 miRNAs show a log2 fold change ≥3. They are hsa-miR-4707-3p, hsa-miR-21-5p, hsa-miR-151b, hsa-miR-151a-5p, hsa-miR-548b-5p, hsa-miR-32-3p, hsa-miR-15a-5p, hsa-miR-3613-5p, hsa-miR-574-5p, hsa-miR-18b-5p, hsa-let-7g-3p, hsa-miR-1278, and hsa-miR-1-3p.

**Table 2:** List of 32 differently expressed miRNAs in POTS patients, in comparison with healthy controls (*P* < 0.05, log2 fold change ≥ 2).

**No**.	**miRNA-ID**	**Log2 fold_change**	**Regulation**	***P-*value**
**Upregulated miRNAs**				
1	hsa-miR-4707-3p	6.51	Up	0.0318
2	hsa-miR-21-5p	3.78	Up	0.0000
3	hsa-miR-409-5p	2.89	Up	0.0001
4	hsa-miR-4747-3p	2.45	Up	0.0013
5	hsa-miR-4676-5p	2.40	Up	0.0057
6	hsa-miR-16-2-3p	2.13	Up	0.0000
**Downregulated miRNAs**				
1	hsa-miR-151b	−4.39	Down	0.0000
2	hsa-miR-151a-5p	−4.39	Down	0.0000
3	hsa-miR-548b-5p	−4.11	Down	0.0071
4	hsa-miR-32-3p	−3.97	Down	0.0002
5	hsa-miR-15a-5p	−3.65	Down	0.0001
6	hsa-miR-3613-5p	−3.54	Down	0.0034
7	hsa-miR-574-5p	−3.52	Down	0.0000
8	hsa-miR-18b-5p	−3.41	Down	0.0001
9	hsa-let-7g-3p	−3.30	Down	0.0041
10	hsa-miR-1278	−3.19	Down	0.0006
11	hsa-miR-1-3p	−3.16	Down	0.0025
12	hsa-miR-548	−2.74	Down	0.0455
13	hsa-miR-450b-5p	−2.74	Down	0.0001
14	hsa-miR-26b-5p	−2.72	Down	0.0000
15	hsa-miR-16-5p	−2.66	Down	0.0000
16	hsa-miR-18a-5p	−2.57	Down	0.0012
17	hsa-miR-424-5p	−2.50	Down	0.0035
18	hsa-miR-338-3p	−2.48	Down	0.0060
19	hsa-miR-374a-5p	−2.39	Down	0.0005
20	hsa-miR-181c-5p	−2.35	Down	0.0014
21	hsa-miR-5582-3p	−2.17	Down	0.0011
22	hsa-miR-17-3p	−2.17	Down	0.0005
23	hsa-miR-98-5p	−2.12	Down	0.0001
24	hsa-miR-4775	−2.06	Down	0.0229
25	hsa-miR-450a-5p	−2.05	Down	0.0001
26	hsa-miR-4482-3p	−2.03	Down	0.0415

### Identification of miRNA-Regulated Target Genes

The target genes were identified by RNAhybrid and miRanda, and only those identified by both algorithms were considered. In total, 3,820 genes were targeted by the 135 DEmiRNAs (data not shown). The target gene counts of the 13 miRNA for which a log2 fold change ≥3 is shown in Table 3. When the *P*-value is 0.01 and 0.05, 198 and 481 genes, respectively, are shown to be targeted by the 13 miRNAs. The target gene of hsa-miR-21-5p was SP1 when the *P*-value is <0.01 (Supplementary Table 1).

**Table 3:** Target gene count of the 13 miRNAs with a log2 fold change ≥3.

**miRNA**	**Target genes count (*P* < 0.01)**	**Target genes count (*P* < 0.05)**	**Regulation**	**Log2 fold_change**
hsa-miR-4707-3p	86	215	Up	6.51
hsa-miR-21-5p	1	2	Up	3.78
hsa-miR-151b	10	19	Down	−4.39
hsa-miR-151a-5p	12	22	Down	−4.39
hsa-miR-548b-5p	22	33	Down	−4.11
hsa-miR-32-3p	1	1	Down	−3.97
hsa-miR-15a-5p	3	19	Down	−3.65
has-miR-3613-5p	0	2	Down	−3.54
hsa-miR-574-5p	49	119	Down	−3.52
hsa-miR-18b-5p	1	13	Down	−3.41
hsa-let-7g-3p	11	28	Down	−3.30
hsa-miR-1278	0	4	Down	−3.19
hsa-miR-1-3p	2	4	Down	−3.16

### GO Analysis

**Figure 2:**
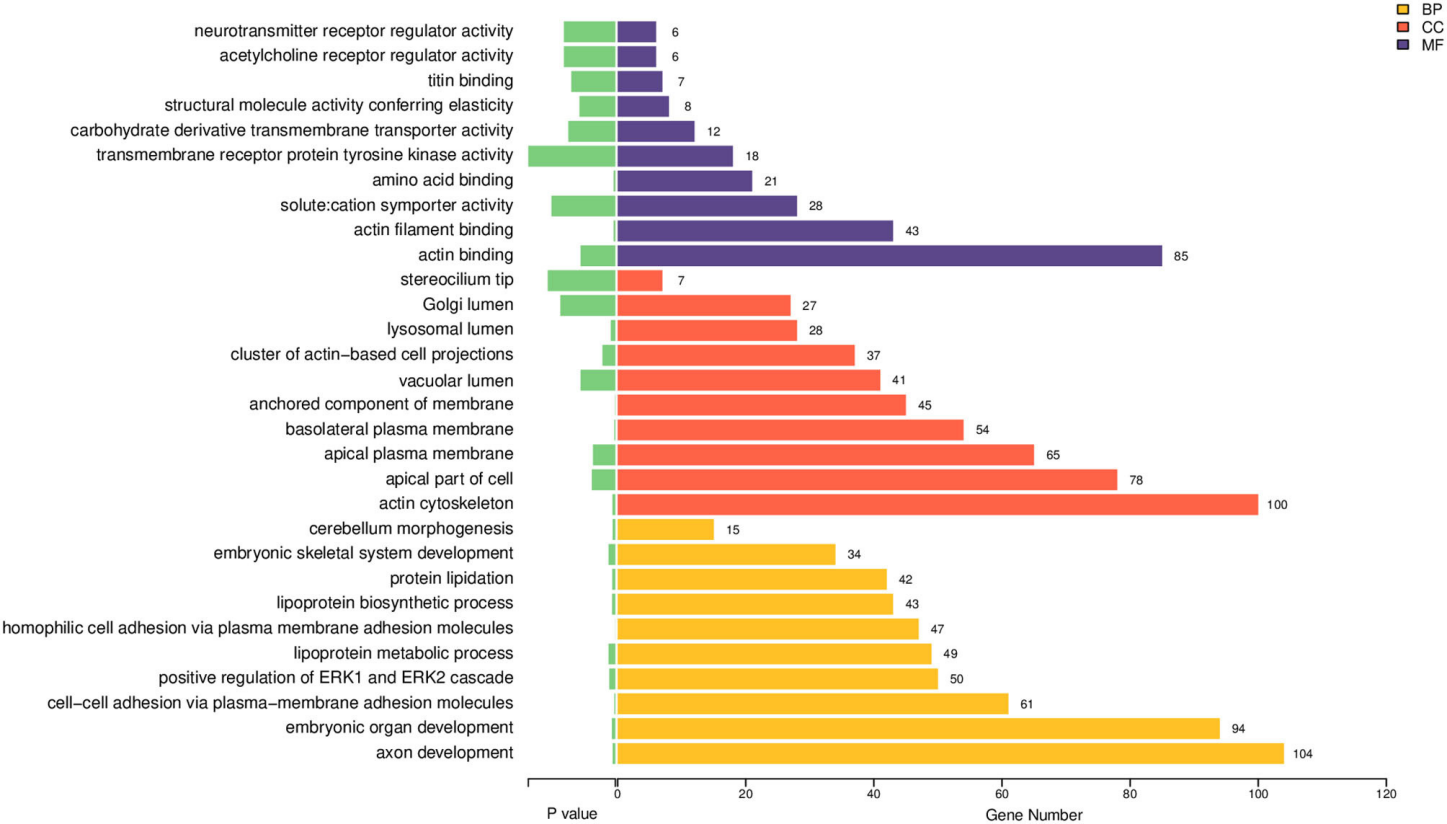
GO enrichment bar chart for the target genes of DEmiRNAs. Varying categories of GO function are indicated by different colors, while the quantity of differential genes enriched per GO category is denoted by the number shown at each bar end (BP, biological process; CC, cellular component; MF, molecular function).

GO functional enrichment analyses were performed on the DEmiRNAs. The 10 most enriched functions of GO were chosen from the following three categories: CC (cellular component), BP (biological process), and MF (molecular function), according to the *P-*value (Figure 2, Supplementary Table 2). The main enriched BP entries are axon development, embryonic organ development, intercellular adhesion *via* plasma membrane adhesion molecules, and positive ERK1 and ERK2 regulation. Meanwhile, the primary enriched MF entries include actin filament binding and actin binding. As for the chief enriched CC entries, they include the actin cytoskeleton, apical plasma membrane, apical part of the cell, and basolateral plasma membrane.

### KEGG Analysis

**Figure 3:**
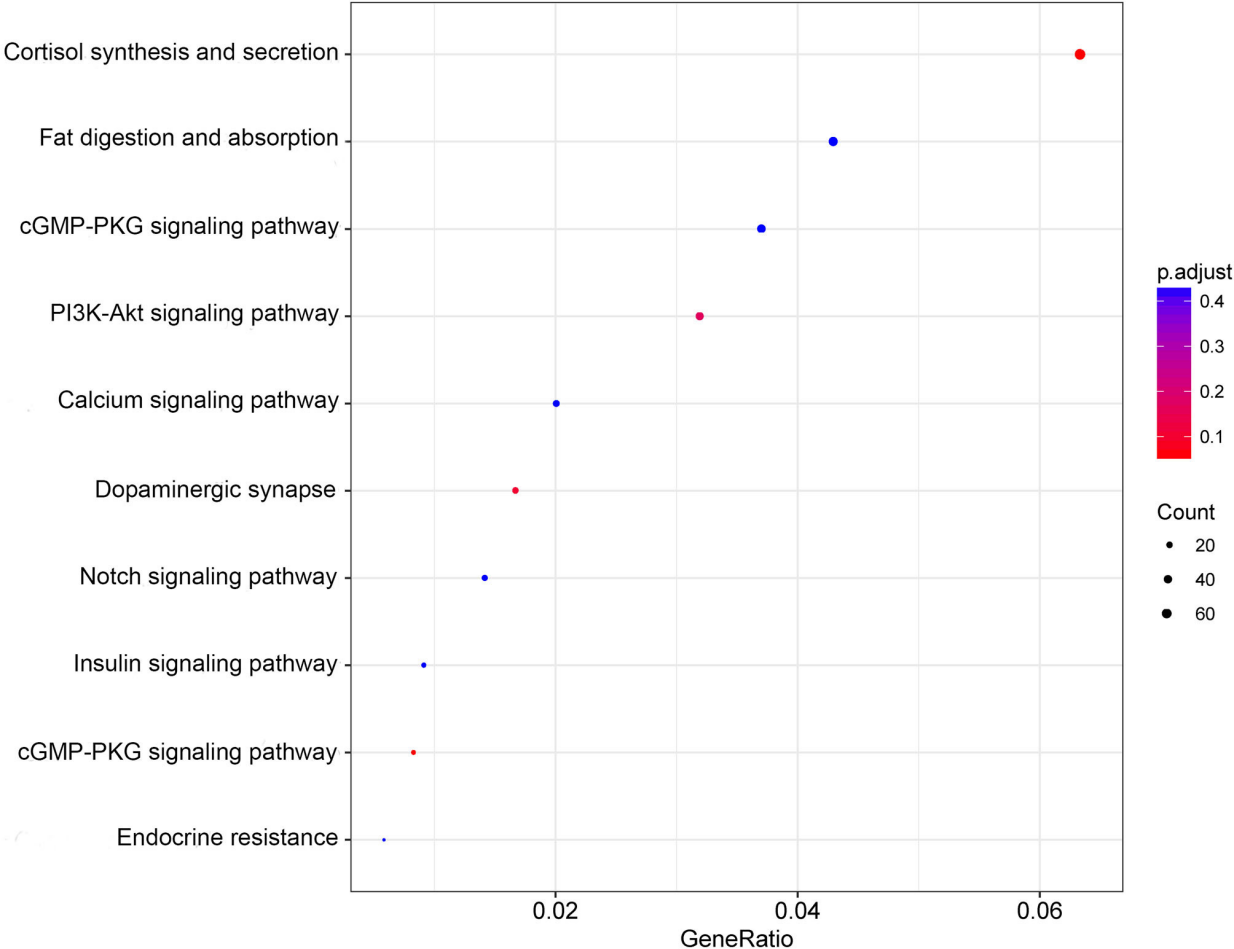
Scatter plot of KEGG pathway enrichment. The abscissa indicates the GeneRatio, which refers to the pathway-enriched genes as a percentage of the overall enriched genes. Meanwhile, the ordinate indicates -log10 (Q-value), an adjusted value of *P*. The quantity of pathway enriched genes is reflected by the size of dot, while the *P* value is indicated by the color.

The pathway enrichment evaluation for the DEmiRNAs in POTS patients was accomplished by utilizing the KEGG database, which revealed prominent enrichment of the DEmiRNAs in 36 pathways (*P* < 0.05) (Supplementary Table 3). Figure 3 shows a scatter chart that presents the top 10 pathways, of which the PI3K/Akt signaling pathway is closely related to vascular function.

### External Validation

**Figure 4:**
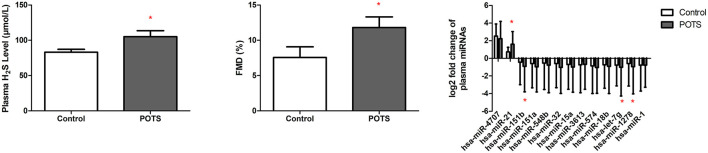
Plasma H_2_S, FMD, FMD, and the level of the 13 whole-blood DEmiRNAs in POTS patients and the control. *P<0.05 when compare with control group.

On the basis of the foregoing findings, we recruited another 60 children and adolescents as validation participants to externally confirm the findings. The blood pressure and cardiac rate of POTS patients differed significantly from those of the healthy participants (Table 4). The plasma H_2_S and FMD are higher in POTS patients. Furthermore, the fold increase in the expression level of whole-blood miR-21 is higher in POTS patients, while miR-151b, miR-let7g, and miR-1278 were reduced (*P* < 0.05) (Figure 4).

**Table 4:** Distribution of characteristics of subjects in the validation group.

	**POTS patients**	**Control group**	**χ^2^/ *t***	***P***
Gender(M/F)	17/23	7/13	0.313	0.576
Age(years)	12.63 ± 1.59	13.35 ± 1.04	1.841	0.071
Height(cm)	151.28 ± 12.91	156.95 ± 9.774	1.73	0.089
Weight(kg)	43.76 ± 7.668	47.15 ± 8.916	1.527	0.132
Supine HR(mmHg)	78.50 ± 8.412	82.40 ± 10.826	1.536	0.13
Supine SBP(mmHg)	103.18 ± 8.161	108.90 ± 9.503	2.424	0.018
Supine DBP(mmHg)	62.50 ± 7.383	67.40 ± 6.099	2.56	0.013
Upright HR(beats/min)	117.53 ± 13.435	103.95 ± 10.724	3.93	<0.001
Upright SBP(mmHg)	106.73 ± 11.327	114.55 ± 10.175	2.606	0.012
Upright DBP(mmHg)	69.05 ± 8.280	74.75 ± 7.793	2.562	0.013
HR increament(beats/min)	39.02 ± 10.56	21.55 ± 10.45	6.061	<0.001

*DBP, diastolic blood pressure; HR, heart rate; POTS, postural tachycardia syndrome; SBP, systolic blood pressure*.

Next, the validation participants were diagnosed by our standard criteria for defining POTS sufferers as described earlier. The levels of whole-blood miR-21 were determined by qRT-PCR. The results in Table 5 show that according to the defining standard criteria, 40 of the 60 children were diagnosed with POTS. Of these 40 cases, 37 were predicted correctly using whole-blood miR-21 levels. Thus, whole-blood miR-21 was used to diagnose POTS, where the specificity was 100% and the sensitivity was 92.5%.

**Table 5:** Diagnostic test of miR-21.

		**Gold Standard**		
		**POTS**	**Control**	**Totally**
Plasma miR-21	POTS	37	0	37
	Control	3	20	23
Totally		40	20	60

## Discussion

To our knowledge, this is the first study to focus on differences in the expression of miRNAs between POTS sufferers and healthy subjects. The whole-blood miRNA sequencing approach revealed that 13 miRNAs are expressed significantly differently between POTS sufferers and control subjects. In a 60-member validation group, the 13 miRNAs were verified again, and it turned out that the miR-21, miR-151b, miR-let7g, and miR-1278 were significantly elevated or reduced in POTS patients compared with those of normal subjects. It was further discovered that miR-21could diagnose POTS with a 100% specificity and 92.5% sensitivity. As was expected, we established that the plasma H_2_S level in POTS patients was higher than that in the normal subjects, and the FMD of POTS patients was also increased due to the influence of plasma H_2_S. The increment of plasma H_2_S and FMD was in line with the previous study (Zhang et al., 2012; Liao et al., 2013). According to RNAhybrid and miRanda, SP1 was the target gene of miR-21. GO and KEGG analyses revealed that the PI3K/Akt signaling pathway may be involved in the mechanism of POTS. Thus, we propose that whole-blood miR-21 levels are a potential POTS biomarker which may affect the plasma H_2_S level through the PI3K/Akt pathway.

From L-cysteine, the synthesis of endogenous H_2_S is accomplished by cystathionine β-synthase (CBS) and cystathionine γ-lyase (CSE), two enzymes relying on pyridoxal phosphate (vitamin B6) (Kabil and Banerjee, 2014). Implication of H_2_S in the regulation of multiple bioprocesses has been widely demonstrated, including neurotransmission, immune/inflammatory reactions, gastrointestinal functionality, and vascular homeostasis. The vascular implication of H_2_S in the regulation of vasomotor tone, angiogenesis, proliferation and apoptosis of smooth muscle cells, and atherosclerotic plaque lipid deposition has been noted, suggesting its possible usage as a therapeutic target for such diseases as atherosclerosis and arterial hypertension (Wang, 2012). Furthermore, H_2_S facilitates the endothelium-dependent vasorelaxation (Yang et al., 2008) and plays a regulatory role in the pathogenesis of diverse conditions, including shock, pulmonary hypertension, and hypertension (Du et al., 2006; Tang et al., 2006; Szabo, 2007). In the present research, we have demonstrated that POTS is associated with abnormal vasodilation (higher FMD) and higher plasma H_2_S, which is in accordance with the results published by Prof. Du (Liao et al., 2013; Yang et al., 2013). However, the increment of H_2_S in POTS patients has not been hitherto explained.

The expression of miR-21, a well-characterized miRNA, was upregulated prominently in cardiovascular cells, serves as an activator for the Akt pathway by regulating the expression of phosphatase and tensin homolog (PTEN), and also exhibits anti-inflammatory and anti-apoptotic effects. A cell model of hypoxia/reoxygenation has suggested that incubation of sodium hydrosulfide (H_2_S donor) increased the expression of miR-21, which could protect the cell from injury. Anti-miR-21 abolished the protective effects of sodium hydrosulfide by inactivating the Akt pathway. Hence, the activation of the Akt pathway regulated by miR-21 participates in the protective effects of H_2_S against hypoxia/reoxygenation-induced injury (Lu et al., 2018). Animal experiments have also suggested that H_2_S plays an active role in improving thyroxin-induced myocardial fibrosis in rats through the upregulation of expression of the PI3K/AKT signaling pathway and downregulation of expressions of miR-21 (Liu et al., 2018). Therefore, in our present study, through the whole-blood miRNA sequencing and the data analysis, we propose that miR-21 regulates plasma H_2_S of POTS patients through the PI3K/Akt pathway.

The PI3K/Akt pathway is also proved to be related to peripheral vascular diastole, which is further support for our speculation. A study from a cohort of Bartter's and Gitelman's syndrome (BS/GS) patients revealed that the adipokine retinol-binding protein 4 (RBP4) stimulates the PI3K/Akt pathway, which increased NO-mediated vasodilation. As a measure of the endothelium (NO)-dependent response, FMD was increased as expected (Calò et al., 2014). In the present study, the H_2_S and FMD of POTS patients were all increased. Of the top 10 pathways, the PI3K/Akt signaling pathway ranks second; hence, we highly suspect that elevated H_2_S in patients is associated with this pathway.

miRNA inhibits transcription and translation mainly by binding to target genes. The present study showed that the predicted target gene of miR-21 is SP1, which also makes sense in theory. To evaluate the role of miR-21 in response to low-dose ionizing radiation, a study was carried out on 38 volunteer patients, and bioinformatics analysis indicated that miR-21 can contribute to the response to acute low-dose ionizing radiation by targeting the SP1 (Mahmoudi et al., 2022). Yang et al. found that miR-21 regulates CSE protein expression in vascular smooth muscles by regulating SP1 genes (Yang et al., 2012). In vascular endothelial tissues, whether miR-21 regulates the transcription of CSE mRNA through SP1 genes and regulates the expression of CSE protein/H_2_S needs to be further clarified.

In addition to the aforementioned possibility, miR-21 may also affect the concentration and activity of endogenous H_2_S by regulating the endogenous NO level and activity directly. According to a prior report, excessive miR-21 expression in cardiovascular patients has been observed, like those with vascular obliteration, ischemic cardiopathy, and ventricular hypertrophy (Ji et al., 2007; Matsumoto and Hwang, 2007). Furthermore, research has shown that miR-21 is an important regulator of vascular smooth muscle apoptosis (Ji et al., 2007). Weber et al. proved that the overexpression of miR-21 suppresses phosphatase and tensin homolog (PTEN) expression and increases Akt phosphorylation, eNOS phosphorylation, and NO levels (Weber et al., 2010). Furthermore, NO and CO have been shown to exhibit dilatory actions *in vitro* (Myatt, 1992; Barber et al., 2001). However, the mechanism underlying the vasodilating effect of H_2_S has been reported to be mediated chiefly by the KATP (ATP-sensitive potassium) channels, which can be enhanced by NO. The associations between NO and H_2_S have been emphasized in the experiments in which treatment using a NO donor enhanced the endogenous production of H_2_S in rat aortic tissues (Zhao et al., 2001). Through the CSE expression regulation and with cGMP-dependent protein kinases, NO is also capable of enhancing the vascular tissue activity of CSE (Zhao et al., 2001). In addition, NO can potentiate the vascular functionality of H_2_S (Hosoki et al., 1997; Zhao et al., 2001). Collectively, the increment of miR-21 suppresses PTEN expression but increases NO levels, which could increase the activity of CSE and promote the function of H_2_S.

Despite powerful support by evidence in statistical and biological terms, there remain a few shortcomings with our study. First, although matching of the patients and the control subjects was ensured as much as possible, the sample size used for the whole-blood miRNA sequencing was small. Even though the association between miRNA and POTS was shown to be statistically significant, extra miRNA associations were probably missed since the sample size was small, and power issues were present. However, as a small sample of a pre-experimental study, 13 DEmiRNAs were identified, which was satisfactory. Second, during the validation process, it would have been advisable to ascertain the levels of plasma NO for the patients and control subjects, which would have proven our results more reliable, but corresponding evidence can be confirmed in other literature. Third, cell experiments and vascular ring tests to establish whether miR-21 mediates the expression of H_2_S through the PI3K/Akt/eNOS pathway were needed, as well as research on how the level of H_2_S changes if the expression of miR-21 was suppressed or overexpressed. In fact, relevant experiments have already been carried out, and the present study only provides timely sequencing data (all the data could be found on the ArrayExpress platform) for relevant researchers to study. Finally, the cause of miR-21 elevation in POTS patients cannot be answered by the current study. Further studies are apparently needed to confirm our findings on miR-21 in relation to POTS and to elucidate underlying mechanisms.

## Data Availability Statement

The raw sequencing data for this study can be found on the ArrayExpress database://www.ebi.ac.uk/arrayexpress/. The accession number is E-MTAB-10961. For more information, contact the corresponding author at linjing0127@xjtu.edu.cn.

## Ethics Statement

The studies involving human participants were reviewed and approved by Xi'an Jiaotong University, school of medicine. Written informed consent to participate in this study was provided by the participants' legal guardian/next of kin.

## Author Contributions

JLin contributed to conceptualization, writing the original draft, and reviewing and editing, as well as reading, and approving the final manuscript. JS and WC assisted with conceptualization and acquisition of data and read and approved the final manuscript. JLiu contributed to conceptualization and acquisition of data and read and approved the final manuscript. LL helped with the acquisition of data and read and approved the final manuscript. FJ contributed to writing, reviewing and editing, and as well as read and approved the final manuscript. All authors contributed to the article and approved the submitted version.

## Funding

The work was supported by the National Natural Science Foundation of China (grant number 81803263 [to JLin]).

## Conflict of Interest

The authors declare that the research was conducted in the absence of any commercial or financial relationships that could be construed as a potential conflict of interest.

## Publisher's Note

All claims expressed in this article are solely those of the authors and do not necessarily represent those of their affiliated organizations, or those of the publisher, the editors and the reviewers. Any product that may be evaluated in this article, or claim that may be made by its manufacturer, is not guaranteed or endorsed by the publisher.

## Supplementary Material

Table S1 Known_MicrorRNA_Target_Gene_Prediction_Summary.pdf.

Table S2 GO_Enrichment_Summary.pdf.

Table S3 KEGG_Enrichment_Summary.pdf.
