# Supplementary material for: Whole-Blood MicroRNA Sequence Profiling and Identification of Specific miR-21 for Adolescents With Postural Tachycardia Syndrome
Source: Front Neurosci. 2022 Jun 30;16:920477. doi: 10.3389/fnins.2022.920477 (PMC9281551; doi:10.3389/fnins.2022.920477)
Supplement: Table S1 — Known_MicrorRNA_Target_Gene_Prediction_Summary.pdf. [file Data_Sheet_1.ZIP › supp table 2 GO_Enrichment_Summary.pdf]

ID	Description	GeneRat	BgRatio	pvalue	p.adjus	qvalue	geneID	Coun	Typ
GO:0007156	homophilic cell adhesion via plasma membrane adhesion molecules	47/2734	157/17381	5.2E-06	0.0309	0.03039	AMIGO1/CDH12/CDH13/CDH15/CDH16/CDH23/CDH24/CDH5/CDHR1/CDHR5/CELSR2/CELSR3/CLSTN1/CLSTN3/FAT1/FAT2/KIRREL3/NCR3/PCDH10/PCDH11Y/PCDH12/PCDHB1/PCDHGA1/PCDHGA10/PCDHGA11/PCDHGA12/PCDHGA2/PCDHGA3/PCDHGA4/PCDHGA5/PCDHGA6/PCDHGA7/PCDHGA8/PCDHGA9/PCDHGB1/PCDHGB2/PCDHGB3/PCDHGB4/PCDHGB5/PCDHGB6/PCDHGB7/PCDHGC3/PCDHGC4/PCDHGC5/PKD1/PLXNB3/PTP[illegible]	47	BP
GO:0098742	cell-cell adhesion via plasma-membrane adhesion molecules	61/2734	239/17381	5.9E-05	0.1748	0.17197	ALCAM/AMIGO1/CD6/CDH12/CDH13/CDH15/CDH16/CDH23/CDH24/CDH5/CDHR1/CDHR5/CELSR2/CELSR3/CLDN15/CLDN2/CLDN23/CLDN4/CLDN6/CLSTN1/CLSTN3/DAB1/FAT1/FAT2/FGFRL1/IL1RN/ITGAL/ITGAM/KIRREL3/MIR221/NCR3/PCDH10/PCDH11Y/PCDH12/PCDHB1/PCDHGA1/PCDHGA10/PCDHGA11/PCDHGA12/PCDHGA2/PCDHGA3/PCDHGA4/PCDHGA5/PCDHGA6/PCDHGA7/PCDHGA8/PCDHGA9/PCDHGB1/PCDHGB2/PCDHGB3/PCDHGB4/PCDHGB5/PCDHGB6/PCDHGB7/PCDHGC3/PCDHGC4/PCDH[illegible]	61	BP
GO:0061564	axon development	104/2734	473/17381	0.00018	0.1862	0.18312	ABL1/ADGRB1/ALCAM/AMIGO1/ANAPC2/APBB1/ARHGAP4/ARTN/BARHL2/BOC/BRSK2/CDK5/CELSR3/CSF1R/CTNNA2/CTTN/DAB1/DISC1/DOCK7/DOK1/DOK2/DRAXIN/DRD2/EFNA1/EFNA3/EFNA4/EFNB1/EPHA8/EPHB3/ERBB2/EVL/FGFR2/FN1/GBX2/GPC1/GRIN1/HRAS/ILK/INPP5F/ISLR2/L1CAM/LAMA2/LHX1/LHX3/LHX4/LIMK1/LINGO1/LINGO4/LLGL1/LRIG2/LYPLA2/MAPK3/MARK2/METRN/MIR221/MIR222/NKX2-8/NLGN3/NOTCH1/NRG1/NTNG2/PARD3/PDLIM7/PHOX2B/PLXNB1/PLXNB3/PLXND1/PTPN11/PTPRF/RAP1GAP/RGMA/RTN4RL1/RTN4RL2/S100A6/SCARF1/SEMA3F/SEMA4A/SEMA5B/SEMA6C/SEMA7A/SHC1/SHH/SLITRK3/SPON2/SPTAN1/SPTB/SPTBN2/STXBP1/TBR1/THY1/TLR9/TNC/TNFRSF12A/TNN/TRIM46/TRPV2/TSKU/ULK1/VAX1/VAX2/WNT3A/WNT7A/ZFY[illegible]	104	BP
GO:0021587	cerebellum morphogenesis	15/2734	36/17381	0.00018	0.1862	0.18312	ABL1/CDK5/CEND1/DAB1/GNPAT/HSPA5/KNDC1/LDB1/LHX1/LHX5/PTPN11/SMO/SPTBN2/WNT1/WNT7A	15	BP
GO:0006497	protein lipidation	42/2734	155/17381	0.0002	0.1862	0.18312	ALPPL2/ATG10/CWH43/DPM2/FOLR2/GBA/GP2/GPAA1/GPIHBP1/LY6D/LY6E/LY6H/LYPD1/LYPD2/LYPD6B/LYPD8/MAP6D1/MSLN/NRN1/NRN1L/NTM/NTNG2/PIGC/PIGG/PIGO/PIGQ/PIGY/PIGZ/PORCN/PSCA/PYURF/RAET1G/RTN4RL1/RTN4RL2/THY1/ULBP2/VNN3/ZDHHC12/ZDHHC14/ZDHH[illegible]	42	BP

GO:0042158	lipoprotein biosynthetic process	43/2734	160/17381	0.00021	0.1862	0.18312	ALPPL2/APOB/ATG10/CWH43/DPM2/FOLR2/GBA/GP2/GPAA1/GPIHBP1/LY6D/LY6E/LY6H/LYPD1/LYPD2/LYPD6B/LYPD8/MAP6D1/MSLN/NRN1/NRN1L/NTM/NTNG2/PIGC/PIGG/PIGO/PIGQ/PIGY/PIGZ/PORCN/PSCA/PYURF/RAET1G/RTN4RL1/RTN4RL2/THY1/ULBP2/VNN3/ZDHHC12/ZDHHC14	43	BP
GO:0048568	embryonic organ development	94/2734	422/17381	0.00022	0.1862	0.18312	ADM/ALX3/ASCL2/ATP6V1B1/AXIN1/BMP4/C2CD3/CC2D2A/CCDC103/CCDC40/CDX2/CRB2/DVL2/E2F8/EFNA1/EGFR/EN1/ENG/EYA1/FGFR2/FZD2/GATA4/GBX2/GCM2/GRHL3/HEY1/HLX/HMX2/HOXA3/HOXB1/HOXB2/HOXB3/HOXB4/HOXB7/HOXB8/HOXC11/HOXC4/HOXD3/HOXD4/HSF1/IFT140/KCNQ4/KDR/LHFPL5/LHX1/MAPK3/MICAL2/MTHFD1/MYCN/MYO3A/MYO7A/NAGLU/NES/NKX2-5/NKX3-2/NOTCH1/NOTO/NSDHL/OSR1/PCDH12/PCGF2/PDGFA/PHLDA2/PKD1/PKDCC/PLCD1/POLE/RARA/RARRES2/RBPMS2/SCRIB/SETD2/SHH/SLC44A4/SMO/SPINT1/ST14/STRA6/SUFU/TBX15/TBX2/TCAP/TEAD4/TFEB/TH/[illegible]	94	BP
GO:0070374	positive regulation of ERK1 and ERK2 cascade	50/2734	199/17381	0.00039	0.2588	0.25458	ABL1/ADRA1A/ARAF/ARRB2/BMP4/CCL1/CCL19/CCL21/CCL5/CD74/CIB1/CSF1R/DRD2/EGFR/F2RL1/FGA/FGFR2/FGFR4/FLT4/GCG/GLIPR2/GPER1/HAVCR2/HRAS/KARS/KDR/LGALS9/MAPK3/MIR221/MIR222/NECAB2/NOD1/NOTCH1/NOX4/NQO2/OPRM1/PDGFA/PDGFRB/PLA2G5/PTK2B/PTPN11/RAF1/SEMA7A/SERPINF2/SHC1/THPO/TIRAP/TNFAIP8L3/TREM2/TRPV	50	BP
GO:0042157	lipoprotein metabolic process	49/2734	195/17381	0.00044	0.2588	0.25458	ALPPL2/APOA5/APOB/APOC3/ATG10/CWH43/DPM2/FOLR2/GBA/GP2/GPAA1/GPIHBP1/LRP1/LY6D/LY6E/LY6H/LYPD1/LYPD2/LYPD6B/LYPD8/LYPLA2/MAP6D1/MSLN/NPC1L1/NRN1/NRN1L/NTM/NTNG2/PEMT/PIGC/PIGG/PIGO/PIGQ/PIGY/PIGZ/PORCN/PSCA/PYURF/RAET1G/RTN4RL1/RTN4RL2/THY1/ULBP2/VNN3/ZDHHC12/ZDHHC14/ZDHHC15/ZDHHC18/ZDHH	49	BP
GO:0048706	embryonic skeletal system development	34/2734	122/17381	0.00044	0.2588	0.25458	ALX3/AXIN1/BMP4/COL1A1/DEAF1/EYA1/FGFR2/HOXA3/HOXB1/HOXB2/HOXB3/HOXB4/HOXB7/HOXB8/HOXB9/HOXC11/HOXC5/HOXC6/HOXD1/HOXD3/HOXD4/IFT140/LHX1/MTHFD1/MYCN/NKX3-2/OSR1/PCGF2/SCX/SETD2/SHH/TBX15/TULP3/WNT11	34	BP

GO:0048562	embryonic organ morphogenesis	66/2734	283/17381	0.0005	0.2588	0.25458	ALX3/ATP6V1B1/AXIN1/BMP4/C2CD3/CCDC103/CCDC40/CRB2/DVL2/EFNA1/ENG/EYA1/FGFR2/FZD2/GATA4/GBX2/GRHL3/HLX/HMX2/HOXA3/HOXB1/HOXB2/HOXB3/HOXB4/HOXB7/HOXB8/HOXC11/HOXC4/HOXD3/HOXD4/IFT140/KCNQ4/LHFPL5/LHX1/MAPK3/MICAL2/MTHFD1/MYCN/MYO3A/MYO7A/NAGLU/NKX2-5/NKX3-2/NOTCH1/NOTO/OSR1/PCGF2/RBPMS2/SCRIB/SETD2/SHH/SLC44A4/SMO/STRA6/SUFU/TBX15/TBX2/TCAP/TH/TMIE/TULP3/VAX2/WDPCP/WNT1[illegible]	66	BP
GO:0022037	metencephalon development	29/2734	100/17381	0.00056	0.2588	0.25458	AARS/ABL1/B4GALT2/CDK5/CDK5R2/CEND1/DAB1/EN1/GBX2/GNPAT/GRIN1/HOXB1/HSPA5/KAT2A/KNDC1/LDB1/LHX1/LHX5/NAGLU/NEUROD2/OGDH/PHOX2A/PTPN11/SCRIB/SDF4/SMO/SPTBN2/WNT1/WNT7A	29	BP
GO:1902905	positive regulation of supramolecular fiber organization	46/2734	182/17381	0.00056	0.2588	0.25458	ABL1/ANKRD53/ARFIP1/ARHGEF10/ARPC1A/ARPC1B/ARPC2/ARPC4/BAIAP2L1/BRK1/CAV3/CCL21/CDC42EP2/CFL1/CLU/CORO1A/CORO1B/CSF3/CTTN/EVL/F2RL1/FES/FHOD1/GPX1/HIP1R/KATNB1/LIMK1/LMOD1/MLST8/MTOR/MYO1C/MYOC/NOX4/NUMA1/PFN1/PTK2B/SCIN/SERPINF2/SH3PXD2B/SORBS3/TACR1/TRPV4/WAS/WDR1/WHAMM/WNT11	46	BP
GO:0021575	hindbrain morphogenesis	15/2734	40/17381	0.00068	0.2891	0.28434	ABL1/CDK5/CEND1/DAB1/GNPAT/HSPA5/KNDC1/LDB1/LHX1/LHX5/PTPN11/SMO/SPTBN2/WNT1/WNT7A	15	BP
GO:0033622	integrin activation	9/2734	18/17381	0.00073	0.2891	0.28434	COL16A1/FARP2/FBLIM1/FERMT2/FERMT3/FN1/MZB1/SELP/TLN1	9	BP
GO:0007409	axonogenesis	93/2734	433/17381	0.00084	0.3141	0.3089	ABL1/ADGRB1/ALCAM/AMIGO1/ANAPC2/APBB1/ARHGAP4/ARTN/BARHL2/BOC/BRSK2/CDK5/CELSR3/CSF1R/CTNNA2/CTTN/DAB1/DISC1/DOCK7/DOK1/DOK2/DRAXIN/DRD2/EFNA1/EFNA3/EFNA4/EFNB1/EPHA8/EPHB3/ERBB2/EVL/FGFR2/FN1/GBX2/GPC1/GRIN1/HRAS/ILK/ISLR2/L1CAM/LAMA2/LHX1/LHX3/LHX4/LIMK1/LINGO1/LINGO4/LLGL1/LYPLA2/MAPK3/MARK2/METRN/NKX2-8/NLGN3/NOTCH1/NRG1/NTNG2/PARD3/PDLIM7/PHOX2B/PLXNB1/PLXNB3/PLXND1/PTPN11/RAP1GAP/S100A6/SEMA3F/SEMA4A/SEMA5B/SEMA6C/SEMA7A/SHC1/SHH/SLITRK3/SPON2/SPTAN1/SPTB/SPTBN2/STXBP1/[illegible]	93	BP
GO:0021695	cerebellar cortex	16/2734	45/17381	0.0009	0.3159	0.31071	AARS/B4GALT2/CDK5/CEND1/HSPA5/KNDC1/LDB1/LHX1/LHX5/NAGLU/NEUROD2/OGDH/PTPN11/SMO/SPTBN2/WNT7A	16	BP

GO:0030902	hindbrain development	37/2734	142/17381	0.00103	0.3245	0.31919	AARS/ABL1/B4GALT2/CDK5/CDK5R2/CEND1/CTNNA2/DAB1/EN1/FLNA/GBX2/GNPAT/GRIN1/HOXB1/HOXB2/HOXB3/HSPA5/KAT2A/KNDC1/LDB1/LHX1/LHX5/NAGLU/NEUROD2/OGDH/PHOX2A/PHOX2B/PTPN11/SCRIB/SDF4/SHH/SMO/SPTBN2/TBR1/WLS/WNT1/WNT7A	37	BP
GO:0042692	muscle cell differentiation	87/2734	403/17381	0.00103	0.3245	0.31919	ABL1/ACTA1/ADAM12/ADGRB1/ADM/AGT/ANKRD2/ARRB2/AXIN1/BARX2/BCL9/BIN1/BIN3/BMP4/BOC/CACNA1H/CACYBP/CAV3/CDH15/CDK9/CHRNB1/CMTM5/CTNNA2/EHD1/EIF5A/ENG/EPC1/FGFR2/FLNC/FOXO4/GATA4/GPER1/GPX1/HEY1/HINFP/IFT20/IGFBP3/IGSF22/IL18/IL4R/ILK/KAT2A/KDM6B/KLHL40/LMNA/LMOD1/MIR195/MIR221/MIR222/MTOR/MYBPC1/MYBPC3/MYBPH/MYBPHL/MYOD1/NKX2-5/NOTCH1/NOX4/NPPA/NRG1/OBSCN/OBSL1/P2RX2/PDCD4/PDGFRB/PDLIM5/PITX1/PPARD/PRKAR1A/RARA/RBM4/RBPMS2/SDC1/SHH/SMO/SMYD3/SOX8/SUPT6H/TBX2/TCAP/THRA/TRIM54/TRIM72/WDR1/WFIKKN2/[illegible]	87	BP
GO:0006501	C-terminal protein lipidation	23/2734	77/17381	0.00129	0.3852	0.37888	ALPPL2/FOLR2/GP2/GPIHBP1/LY6D/LY6E/LY6H/LYPD1/LYPD2/LYPD6B/LYPD8/MSLN/NRN1/NRN1L/NTM/NTNG2/PSCA/RAET1G/RTN4RL1/RTN4RL2/THY1/ULBP2/VNN3	23	BP
GO:0051495	positive regulation of cytoskeleton organization	49/2734	205/17381	0.00143	0.4057	0.39905	ABL1/ANKRD53/ARFIP1/ARHGEF10/ARPC1A/ARPC1B/ARPC2/ARPC4/BAIAP2L1/BCAS3/BRK1/CAV3/CCL21/CDC42EP2/CDK5/CFL1/CORO1A/CORO1B/CSF3/CTTN/DYNC1H1/EVL/F2RL1/FES/FHOD1/HIP1R/HRAS/KATNB1/LIMK1/LMOD1/MLST8/MTOR/MYO1C/MYOC/NES/NOX4/NUMA1/PFN1/PTK2B/SCIN/SERPINF2/SH3PXD2B/SORBS3/TACR1/TRPV4/WAS/WDR1/W[illegible]	49	BP
GO:0070372	regulation of ERK1 and ERK2 cascade	62/2734	274/17381	0.00161	0.4092	0.40245	ABL1/ADRA1A/ARAF/ARRB2/ATF3/BMP4/C1QL4/CCL1/CCL19/CCL21/CCL5/CD74/CIB1/CSF1R/CSK/DAB2IP/DRD2/DUSP26/EGFR/ERBB2/F2RL1/FGA/FGFR2/FGFR4/FLT4/FN1/GCG/GLIPR2/GPER1/HAVCR2/HRAS/KARS/KDR/LGALS9/MAPK3/MIR221/MIR222/NDRG2/NECAB2/NEK10/NOD1/NOTCH1/NOX4/NQO2/OPRM1/PDGFA/PDGFRB/PLA2G5/PSCA/PTK2B/PTPN11/RAF1/RGS14/SEMA7A/SERPINF2/SHC1/THPO/TIRAP/TNFAIP8L3/TREM2/T[illegible]	62	BP
GO:1901343	negative regulation of vasculature development	32/2734	121/17381	0.00167	0.4092	0.40245	ADGRB1/AGT/BMP4/CCR2/COL4A2/DAB2IP/ECSCR/EFNA3/FASLG/FOXO4/GDF2/HSPG2/MIR106B/MIR10A/MIR15A/MIR16-1/MIR212/MIR221/MIR222/MIR29C/MIR361/MIR503/MIR92A2/NOTCH1/PDCD4/SARS/SEMA4A/SPINK5/STAT1/TIE1/TNMD/XDH	32	BP

GO:0071688	striated muscle myosin thick filament	7/2734	13/17381	0.00168	0.4092	0.40245	IGSF22/MYBPC1/MYBPC3/MYBPH/MYBPHL/OBSL1/TCAP	7	BP
GO:0070471	uterine smooth muscle	6/2734	10/17381	0.00178	0.4092	0.40245	ADRA2B/ADRA2C/AGT/GPER1/TACR1/TACR2	6	BP
GO:0090066	regulation of anatomical structure size	96/2734	460/17381	0.00183	0.4092	0.40245	ABL1/ADM/ADRA1A/ADRA1B/ADRA2A/ADRA2B/ADRA2C/AGT/ANAPC2/AP2M1/AQP1/ARFIP1/ARHGAP4/ARPC1A/ARPC1B/ARPC2/ARPC4/ASIC2/ATP13A2/AVPR2/BAIAP2L1/BARHL2/BRK1/CAPZA3/CAV3/CCL21/CDC42EP2/CDHR5/CDK4/CDK5/CFL1/CHGA/CHRM1/CORO1A/CORO1B/CSF3/CTTN/DISC1/DRAXIN/E2F4/EDN2/EGFR/EPHX2/EVL/F2RL1/FGA/FN1/GCH1/GPER1/GPX1/HIP1R/HRH2/HTR1D/ILK/INS/ISLR2/KNG1/L1CAM/LARS/LIMK1/LMOD1/MIR153-1/MIR92A2/MLST8/MTOR/MYO1C/NOS3/NPPA/NRG1/PEX11A/PFN1/PLOD3/PPARD/PRKCD/PTK2B/SCIN/SEMA3F/SEMA7A/SERPINF2/SLC12A4/SLC12A7/SMTNL1/SNF8/SPTAN1/SPTB/SPTBN2/TACR1/TNFRSF12A/TRPV2/TRPV4/WAS/WDR1/WHAMM/WNT3A/WNT7A/ZFYVE27	96	BP
GO:2000181	negative regulation of blood vessel morphogenesis	30/2734	112/17381	0.00185	0.4092	0.40245	ADGRB1/AGT/CCR2/COL4A2/DAB2IP/ECSCR/EFNA3/FASLG/FOXO4/GDF2/HSPG2/MIR106B/MIR10A/MIR15A/MIR16-1/MIR212/MIR221/MIR222/MIR29C/MIR361/MIR503/MIR92A2/NOTCH1/SARS/SEMA4A/SPINK5/STAT1/TIE1/TNMD/XDH	30	BP
GO:0007015	actin filament organization	76/2734	352/17381	0.00206	0.419	0.41212	ABL1/ACTA1/ALDOA/ANG/ARAP1/ARFIP1/ARHGEF10/ARPC1A/ARPC1B/ARPC2/ARPC4/BAIAP2L1/BIN3/BRK1/CAPZA3/CATIP/CAV3/CCL21/CDC42EP2/CFL1/CORO1A/CORO1B/CSF3/CTTN/DBN1/ESPN/ESPNL/EVL/F2RL1/FAT1/FHOD1/FLNA/FSCN1/HIP1R/IGSF22/INPPL1/ITGB5/LIMK1/LMOD1/MICAL2/MLST8/MTOR/MYBPC1/MYBPC3/MYBPH/MYBPHL/MYO1C/MYOC/NOX4/OBSL1/PDLIM3/PFN1/PHACTR1/PLA2G1B/PRKCD/PTK2B/RHOD/RHOF/RND1/SCIN/SERPINF2/SH3PXD2B/SHROOM1/SORBS3/SPTAN1/SPTB/SPTBN2/TACR1/TCAP/TPM2/TRPV4/WAS/WDR1/WHAMM/WNT11/Z[illegible]	76	BP
GO:0030206	chondroitin sulfate biosynthetic	10/2734	24/17381	0.00213	0.419	0.41212	BCAN/BGN/CHPF/CHPF2/CHST12/CHST13/CHST15/CHST7/DSE/XYLT2	10	BP

GO:0030204	chondroitin sulfate	14/2734	40/17381	0.00221	0.419	0.41212	B3GAT3/BCAN/BGN/CHPF/CHPF2/CHST12/CHST13/CHST15/CHST7/DSE/HEXA/IDS/SPOCK2/XYLT2	14	BP
GO:0046328	regulation of JNK cascade	41/2734	168/17381	0.00223	0.419	0.41212	AXIN1/CARD9/CCL19/CCL21/CD27/DAB2IP/DTNBP1/DVL2/EGFR/ERN2/F2RL1/FKTN/FLT4/FZD8/GADD45G/HACD3/HRAS/LTBR/MAP3K6/MAP4K2/MAPK8IP1/MAPK8IP3/MARVELD3/MEN1/MIR92A2/NCF1/NCOR1/NOD1/PDCD4/PER1/PTK2B/SERPINF2/TAOK2/TIRAP/TLR9/TNIK/TRAF2/TRPV4/	41	BP
GO:0002576	platelet degranulation	33/2734	128/17381	0.00225	0.419	0.41212	ABCC4/AHSG/ALDOA/APLP2/CD63/CHID1/CLU/CTSW/CYB5R1/ECM1/FERMT3/FGA/FLNA/FN1/ITIH4/KNG1/LEFTY2/MAGED2/ORM1/ORM2/PDGFA/PSAP/QSOX1/RARRES2/SELP/SERPINA3/SERPINA4/SERPINF2/STXBP1/	33	BP
GO:0018410	C-terminal protein amino acid	24/2734	85/17381	0.00239	0.432	0.4249	ALPPL2/FOLR2/GP2/GPIHBP1/LCMT1/LY6D/LY6E/LY6H/LYPD1/LYPD2/LYPD6B/LYPD8/MSLN/NRN1/NRN1L/NTM/NTNG2/PSCA/RAET1G/RTN4RL1/RTN4RL2/THY1/ULBP2/VNN3	24	BP
GO:0061351	neural precursor cell proliferation	34/2734	134/17381	0.00259	0.4341	0.42696	ARTN/CEND1/DAGLB/DBN1/DISC1/DOCK7/DRD2/EML1/EMX1/FGFR2/FLNA/FZD9/GBX2/HHIP/IFT20/ILK/LHX5/LIMS2/NES/NOTCH1/OTP/POU3F3/SETD1A/SHCBP1/SHH/SLC39A5/SMO/SOX5/SPINT1/VAX1/WNT1/WNT3A/	34	BP
GO:0070371	ERK1 and ERK2 cascade	64/2734	290/17381	0.00259	0.4341	0.42696	ABL1/ADRA1A/AGT/ARAF/ARRB2/ATF3/BMP4/C1QL4/CCL1/CCL19/CCL21/CCL5/CD74/CIB1/CSF1R/CSK/CTSH/DAB2IP/DRD2/DUSP26/EGFR/ERBB2/F2RL1/FGA/FGFR2/FGFR4/FLT4/FN1/GCG/GLIPR2/GPER1/HAVCR2/HRAS/KARS/KDR/LGALS9/MAPK3/MIR221/MIR222/NDRG2/NECAB2/NEK10/NOD1/NOTCH1/NOX4/NQO2/OPRM1/PDGFA/PDGFRB/PLA2G5/PSCA/PTK2B/PTPN11/RAF1/RGS14/SEMA7A/SERPINF2/SHC1/THPO/TIRAP/TNFAIP8L	64	BP
GO:0014910	regulation of smooth muscle cell migration	20/2734	67/17381	0.00262	0.4341	0.42696	AGT/APEX1/CCL5/CORO1B/DOCK7/FOXO4/IGFBP3/ILK/LRP1/MIR15A/MIR221/MIR451A/MIR503/NOX4/P2RY6/PDGFA/PDGFRB/PLAU/PPARD/TACR1	20	BP
GO:0016525	negative regulation of angiogenesis	29/2734	110/17381	0.00283	0.4445	0.43721	ADGRB1/AGT/CCR2/COL4A2/DAB2IP/ECSCR/EFNA3/FASLG/FOXO4/GDF2/HSPG2/MIR106B/MIR10A/MIR15A/MIR16-1/MIR212/MIR221/MIR222/MIR29C/MIR361/MIR503/MIR92A2/NOTCH1/SARS/SEMA4A/SPINK5/STAT1/TIE1/TNMD	29	BP
GO:0031122	cytoplasmic microtubule organization	14/2734	41/17381	0.00287	0.4445	0.43721	AXIN1/CAV3/CCDC13/CCDC88B/CIB1/DYNC1H1/NUMA1/TRDN/TRPV4/TUBG1/TUBG2/TUBGCP2/TUBGCP3/TUBGCP5	14	BP

GO:0031034	myosin filament assembly	7/2734	14/17381	0.00291	0.4445	0.43721	IGSF22/MYBPC1/MYBPC3/MYBPH/MYBPHL/OBSL1/TCAP	7	BP
GO:0032731	positive regulation of interleukin-1	13/2734	37/17381	0.00302	0.4445	0.43721	CASP1/CASP5/CCL19/EGR1/GSDMD/HSPB1/LGALS9/NLRP1/NOD1/ORM1/ORM2/PYDC1/TLR8	13	BP
GO:0070232	regulation of T cell apoptotic	12/2734	33/17381	0.00312	0.4445	0.43721	ADAM8/BMP4/CCL5/CD27/EFNA1/IDO1/LGALS3/LGALS9/PDCD1/PRELID1/TSC22D3/TSC22D4	12	BP
GO:0050650	chondroitin sulfate proteoglycan biosynthetic	11/2734	29/17381	0.00314	0.4445	0.43721	B3GAT3/BCAN/BGN/CHPF/CHPF2/CHST12/CHST13/CHST15/CHST7/DSE/XYLT2	11	BP
GO:0002065	columnar/cuboidal epithelial cell	28/2734	106/17381	0.00321	0.4445	0.43721	ABL1/B9D1/BAD/BMP4/CDX2/DLX3/EMX1/FGFR2/GATA4/IL13/IL31RA/JAG2/LHFPL5/LRTOMT/MEN1/MYCN/MYO7A/NKX3-2/NOTCH1/RARA/RFX6/SCRIB/SIDT2/SMO/SPDEF/TMEM231/WDPCP/WN	28	BP
GO:0050654	chondroitin sulfate proteoglycan	14/2734	42/17381	0.00369	0.5001	0.49183	B3GAT3/BCAN/BGN/CHPF/CHPF2/CHST12/CHST13/CHST15/CHST7/DSE/HEXA/IDS/SPOCK2/XYLT2	14	BP
GO:0030865	cortical cytoskeleton organization	12/2734	34/17381	0.00414	0.5478	0.53882	CDK5/EPB41L2/FMNL1/FMNL2/IKBKB/KIF23/LLGL1/PPP2R3C/STRIP1/TLN1/TRPV4/WDR1	12	BP
GO:0046330	positive regulation of JNK cascade	31/2734	123/17381	0.00434	0.5522	0.54314	AXIN1/CARD9/CCL19/CCL21/CD27/DAB2IP/DVL2/ERN2/F2RL1/FLT4/FZD8/GADD45G/HACD3/HRAS/LTBR/MAP3K6/MAP4K2/MAPK8IP3/MIR92A2/NCF1/NOD1/PTK2B/SERPINF2/TAOK2/TIRAP/TLR9/TNIK/TRAF2/TRPV4/U	31	BP
GO:0043902	positive regulation of multi-organism process	41/2734	174/17381	0.00436	0.5522	0.54314	BAD/CACNA1H/CCL5/CD180/CD74/CDK9/CFL1/CHMP4C/CIB1/DDB1/DHX58/F2RL1/FKBP6/GAPDH/GLRA1/HACD3/HAVCR2/INHBB/LGALS9/LRSAM1/MAPK3/NELFB/NOD1/NOTCH1/PC/PFN1/PGC/PLB1/POLR2G/POLR2L/POMZP3/PPIB/PPIE/PRSS37/RAB7A/RSF1/TARBP2/TBK1/TRIM11/VPS37	41	BP

GO:0001501	skeletal system development	99/2734	491/17381	0.00465	0.5522	0.54314	ACAN/ACD/ACP2/ADAMTS7/AEBP1/AHSG/ALX3/ARSE/AXIN1/BARX2/BCAN/BGLAP/BMP4/CHAD/COL1A1/COL9A2/CSRNP1/DEAF1/EN1/ENG/ESRRA/EXTL1/EYA1/FBXW4/FGFR2/FGFRL1/GDF2/GUSBP3/HAPLN3/HHIP/HOXA3/HOXB1/HOXB2/HOXB3/HOXB4/HOXB7/HOXB8/HOXB9/HOXC10/HOXC11/HOXC4/HOXC5/HOXC6/HOXC8/HOXD1/HOXD3/HOXD4/IFITM5/IFT140/INPPL1/JAG2/LHX1/LOXL2/MAPK3/MCPH1/MMP2/MTHFD1/MUSTN1/MYCN/MYOC/NKX3-2/NLE1/OSR1/P3H1/PCGF2/PHOSPHO1/PITX1/PKD1/PKDCC/PLXNB1/POR/PPIB/PRDX1/PTPN11/RAI1/RARA/RIPPLY1/SCIN/SCX/SERPINH1/SETD2/SH3PXD2B/SHH/SLC38A10/SOX5/SUFU/TBX15/TEAD4/TGFBI/THBS3/THR[illegible]	99	BP
GO:0032732	positive regulation of interleukin-1	14/2734	43/17381	0.0047	0.5522	0.54314	CASP1/CASP5/CCL19/EGR1/GSDMD/HAVCR2/HSPB1/LGALS9/NLRP1/NOD1/ORM1/ORM2/PYDC1/TLR8	14	BP
GO:0010759	positive regulation of macrophage	7/2734	15/17381	0.00473	0.5522	0.54314	C3AR1/CCL5/CSF1/KARS/MAPK3/RARRES2/TRPV4	7	BP
GO:0031033	myosin filament organization	7/2734	15/17381	0.00473	0.5522	0.54314	IGSF22/MYBPC1/MYBPC3/MYBPH/MYBPHL/OBSL1/TCAP	7	BP
GO:0048193	Golgi vesicle transport	71/2734	336/17381	0.0049	0.5604	0.55122	ACTR1A/ARFGAP2/BCAP31/BGLAP/BLZF1/CAPZA3/CEP19/CNIH2/COG4/COG5/COL7A1/COMMD1/COPZ1/CSK/DCTN2/DYNC1H1/DYNC1I1/DYNLL2/GAK/GBF1/GOLGA2/GOLGA3/GORASP1/GOSR2/INS/KIF13A/KIF23/KIF26A/KIF2B/KIF4A/KLC1/KLC2/LLGL1/LMAN2/LMF1/MYO18A/NKD2/NRBP1/OSBPL5/PACS1/PKDCC/PROZ/RAB26/RAB34/RBSN/RINT1/SCAMP2/SCAMP3/SEC16A/SEC16B/SGSM2/SPTAN1/SPTB/SPTBN2/STEAP2/STX18/STX4/STX5/TMED3/TMEM115/TRAPPC1/TRAPPC2L/TRAPPC3/TRAPPC4/TR[illegible]	71	BP
GO:0032872	regulation of stress-activated MAPK cascade	48/2734	212/17381	0.00499	0.5604	0.55122	AXIN1/CARD9/CAV3/CCL19/CCL21/CD27/DAB2IP/DTNBP1/DVL2/EGFR/ERN2/F2RL1/FKTN/FLT4/FOXM1/FZD8/GADD45G/HACD3/HRAS/KARS/LTBR/MAP3K6/MAP4K2/MAPK3/MAPK8IP1/MAPK8IP3/MARVELD3/MEN1/MIR181B1/MIR92A2/NCF1/NCOR1/NOD1/PDCD4/PER1/PRDX1/PTK2B/SERPINF2/TAOK2/TIRAP/TLR9/TNIK/TRAF2/TRPV4/UNC5CL/WNT7A/XDH/Z	48	BP

GO:0051222	positive regulation of protein transport	97/2734	482/17381	0.00534	0.5678	0.55848	ABL1/ACHE/ACSM6/ADAM8/ANG/ANO1/ANP32B/APBB1/ATP13A2/B3GAT3/BAD/BCAP31/BCAS3/BLK/BMP4/CAPN10/CASP1/CASP5/CCL19/CD27/CD58/CDK5/CHRM1/CIB1/CSF1R/CSF3/CYB5R1/DOC2B/DRD2/EGFR/ELMOD1/EMD/ERBB2/F2RL1/FGA/FLNA/GAPDH/GCG/GCK/GLUD1/GPER1/GPHA2/GSDMD/HAVCR2/HCAR2/HLA-E/HNMT/IL13/IL18/IL4R/INS/ITGAM/ITGAX/KARS/KAT2A/LGALS9/LRP1/LRRC46/MAPK3/MIEF2/MYBPC1/MYO18A/MYO1C/NBPF3/NLRP1/NRG1/OAZ2/ORM1/ORM2/OSCP1/PEMT/PFKM/PLA2G1B/PPARD/PRKCD/PSMB7/PTPN23/PYDC1/RBPMS/RFX6/RNF31/SEC16B/SFN/SHH/SMO/STX4/TLR8/TLR9/TNFRSF4/TRH/TRPV4/TSGA13/UBL4B/WLS/WNT3A/ZBTB17/ZPR1	97	BP
GO:0006520	cellular amino acid metabolic process	76/2734	365/17381	0.00544	0.5678	0.55848	AARS/AARS2/AGMAT/AIMP1/AIMP2/ALDH4A1/ASL/ASNS/BCKDK/BHMT2/BLMH/BPHL/CARNS1/CRYM/DALRD3/DARS/DDC/DLST/DPEP1/FAH/GAD1/GATB/GLUD1/GLYATL1/GNMT/GOT2/GPT/GSTZ1/HAAO/HDC/HNMT/HYKK/IDO1/INS/KARS/LARS/MTHFD1/NAALAD2/NAGS/NMNAT3/NOS3/NOX4/OAT/OAZ2/OGDH/OTC/PADI4/PARS2/PEMT/PHGDH/PHYKPL/PIPOX/PLOD3/PM20D1/PPA2/PSMB11/PSMB6/PSMB7/PSMB8/PSMC3/PSMD13/PSMD3/PSMD5/PSMD7/SARS/SDSL/SHMT2/SLC6A6/SLC6A8/SLC7A7/[illegible]	76	BP
GO:0070302	regulation of stress-activated protein kinase signaling cascade	48/2734	213/17381	0.00548	0.5678	0.55848	AXIN1/CARD9/CAV3/CCL19/CCL21/CD27/DAB2IP/DTNBP1/DVL2/EGFR/ERN2/F2RL1/FKTN/FLT4/FOXM1/FZD8/GADD45G/HACD3/HRAS/KARS/LTBR/MAP3K6/MAP4K2/MAPK3/MAPK8IP1/MAPK8IP3/MARVELD3/MEN1/MIR181B1/MIR92A2/NCF1/NCOR1/NOD1/PDCD4/PER1/PRDX1/PTK2B/SERPINF2/TAOK2/TIRAP/TLR9/TNIK/TRAF2/TRPV4/UNC5CL/WNT7A/XDH/Z	48	BP
GO:0021697	cerebellar cortex	8/2734	19/17381	0.00548	0.5678	0.55848	CDK5/CEND1/KNDC1/LDB1/LHX1/LHX5/PTPN11/WNT7A	8	BP
GO:0021680	cerebellar Purkinje cell layer	9/2734	23/17381	0.00581	0.5678	0.55848	AARS/B4GALT2/CEND1/HSPA5/LDB1/LHX1/LHX5/NAGLU/SPTBN2	9	BP
GO:0006554	lysine catabolic process	6/2734	12/17381	0.0059	0.5678	0.55848	CRYM/DLST/HYKK/OGDH/PHYKPL/PIPOX	6	BP
GO:0046348	amino sugar catabolic	6/2734	12/17381	0.0059	0.5678	0.55848	AMDHD2/CHID1/CHIT1/MGAT1/NAGK/NPL	6	BP

GO:0052803	imidazole-containing compound metabolic	6/2734	12/17381	0.0059	0.5678	0.55848	CARNS1/HDC/HNMT/MTHFD1/TRH/UROC1	6	BP
GO:0042743	hydrogen peroxide	14/2734	44/17381	0.00591	0.5678	0.55848	CYP1A1/CYP1A2/DUOXA1/EGFR/EPX/GPX1/GPX3/LPO/MMP3/MPO/NOXO1/PRDX1/PRDX6/ZNF205	14	BP
GO:0040017	positive regulation of locomotion	98/2734	490/17381	0.00615	0.5678	0.55848	ABL1/ADAM8/ADRA2A/AGT/AQP1/ARTN/BCAS3/BMP4/C3AR1/CAMK1D/CCL1/CCL19/CCL21/CCL5/CCR2/CD74/CDH13/CHMP4C/CIB1/COL1A1/CORO1A/CREB3/CSF1/CSF1R/CTSH/DAB2IP/DAPK2/DDB1/DDR2/DOCK1/DOCK7/EDN2/EGFR/F2RL1/FAM83H/FAM89B/FERMT3/FLNA/FLT4/FN1/FOXO4/GLIPR2/GPER1/HMCN2/HRAS/HSPA5/HSPB1/ILK/INS/KARS/KDR/LAMC2/LGALS3/LGALS9/LGR6/MAPK3/MCAM/MIR181B1/MIR221/MIR222/MIR29A/MIR451A/MTOR/MYO1C/MYOC/NOTCH1/NOX4/NSMF/P2RY6/PC/PDGFA/PDGFRB/PF4V1/PFN1/PGAM4/PLAU/PRDM14/PTK2B/RAB25/RARRES2/RHOD/RNASE9/SCARB1/SELP/SPOCK2/STX4/TACR1/TACR2/TDGF1/[illegible]	98	BP
GO:0021549	cerebellum development	24/2734	91/17381	0.00619	0.5678	0.55848	AARS/ABL1/B4GALT2/CDK5/CDK5R2/CEND1/DAB1/EN1/GBX2/GNPAT/HSPA5/KNDC1/LDB1/LHX1/LHX5/NAGLU/NEUROD2/OGDH/PTPN11/SDF4/SMO/SPTBN2/WNT1/WNT7A	24	BP
GO:0030838	positive regulation of actin filament	24/2734	91/17381	0.00619	0.5678	0.55848	ARFIP1/ARPC1A/ARPC1B/ARPC2/ARPC4/BAIAP2L1/BRK1/CCL21/CDC42EP2/CORO1A/CORO1B/CSF3/CTTN/EVL/HIP1R/LMOD1/MLST8/MTOR/MYO1C/PFN1/PTK2B/SCIN/WAS/WHAMM	24	BP
GO:0001525	angiogenesis	94/2734	469/17381	0.00676	0.5941	0.58435	ABL1/ADAM8/ADGRB1/ADM/AGT/AIMP1/ANG/ANPEP/AQP1/ARHGAP22/ATPIF1/BCAS3/BMP4/BRCA1/C3AR1/CCR2/CDH13/CIB1/COL4A2/CTSH/CXCR3/DAB2IP/E2F8/ECM1/ECSCR/EFNA1/EFNA3/EGFL7/ENG/EPHB3/EPHB4/ERBB2/FASLG/FGFR2/FLT4/FN1/FOXO4/FZD8/GATA4/GBX2/GDF2/GPX1/HEY1/HOXA3/HOXB3/HSPB1/HSPG2/HTATIP2/IL18/KDR/LEPR/LOXL2/MCAM/MIR106B/MIR10A/MIR15A/MIR16-1/MIR210/MIR212/MIR221/MIR222/MIR29C/MIR361/MIR451A/MIR503/MIR92A2/MMP2/NOS3/NOTCH1/NR4A1/PDGFA/PDGFRB/PIK3R6/PLCD1/PLXND1/PRCP/PTK2B/RNH1/SARS/SEMA4A/SETD2/SH2D2A/SHC1/SHH/SPIN[illegible]	94	BP

GO:0010821	regulation of mitochondrion organization	50/2734	226/17381	0.00677	0.5941	0.58435	ACSM6/APOPT1/ARRB2/ATP13A2/ATPIF1/BAD/BOK/CLU/CTTN/CYB5R1/DHODH/DYNLL2/ELMOD1/FZD9/GBA/GPER1/GPHA2/GPX1/HIP1R/HNMT/HRK/ITGAX/KAT2A/KDR/LIG3/LMNA/LRRC46/MFN2/MIEF2/MOAP1/MYBPC1/MYO19/NBPF3/NRG1/OSCP1/PEMT/PID1/PPP1R13B/PRELID1/PSMB7/RNF31/SFN/SLC25A5/STAT2/TSGA13/UBL4B/VAT1/YWHAQ/ZBTB17/ZN	50	BP
GO:0009612	response to mechanical stimulus	47/2734	210/17381	0.00678	0.5941	0.58435	ACTA1/AGT/AQP1/ASIC2/ASIC3/ASNS/ATP1A1/BAD/BGLAP/CASP1/CASP5/CAV3/COL1A1/DRD2/EGFR/ENDOG/ENG/FOSL1/GATA4/IL13/INHBB/KCNK4/LHFPL5/LTBR/MAP3K14/MAPK3/MEIS2/MPO/NPPA/NRXN2/PDE2A/PHF24/PKD1/PTK2B/PTPN11/RAF1/SCX/SOST/STRA6/TACR1/TCAP/TLR8/TMEM150C/TNC/TRPV4/WNT11/XPC	47	BP
GO:0032460	negative regulation of protein	7/2734	16/17381	0.00727	0.6014	0.59151	CLU/CRYAB/GBA/INS/OPRD1/PEX14/PEX5	7	BP
GO:1905523	positive regulation of macrophage	7/2734	16/17381	0.00727	0.6014	0.59151	C3AR1/CCL5/CSF1/KARS/MAPK3/RARRES2/TRPV4	7	BP
GO:0048675	axon extension	26/2734	102/17381	0.00728	0.6014	0.59151	ABL1/ALCAM/ANAPC2/ARHGAP4/BARHL2/CDK5/CTTN/DISC1/DRAXIN/FN1/ILK/ISLR2/L1CAM/LIMK1/NLGN3/NRG1/PLXNB1/PLXNB3/SEMA3F/SEMA5B/SEMA7A/TNFRSF12A/TRPV2/ULK1/WNT3A/ZFYVE27	26	BP
GO:0048705	skeletal system morphogenesis	47/2734	211/17381	0.00743	0.6014	0.59151	ALX3/AXIN1/BARX2/BMP4/CHAD/COL1A1/CSRNP1/EYA1/FGFR2/HHIP/HOXA3/HOXB1/HOXB2/HOXB3/HOXB4/HOXB7/HOXB8/HOXC11/HOXC8/HOXD3/HOXD4/IFITM5/IFT140/INPPL1/LHX1/MMP2/MTHFD1/MYCN/NKX3-2/NLE1/OSR1/PCGF2/PHOSPHO1/PKD1/POR/RARA/RIPPLY1/SCX/SERPIN	47	BP

GO:0060627	regulation of vesicle-mediated transport	93/2734	465/17381	0.00746	0.6014	0.59151	ABL1/ADPRHL1/ADRA2A/AHSG/APOA5/APOC3/ARRB2/ATP13A2/BIN1/C2/CACNA1G/CACNA1H/CALY/CAMK1D/CAV3/CCL19/CCL21/CCR2/CD300LF/CD63/CDH13/CDK5/CDK5R2/CNIH2/CORO1A/CPLX1/CSK/DLG4/DNM1/DOC2A/DOC2B/DRD2/EHD1/F2RL1/FES/FGA/GRTP1/HIP1R/IL13/IL4R/INPP5F/ITGAM/LGALS3/LGALS9/LGI3/LLGL1/LMAN2/LRP1/LRRTM1/LRSAM1/MAPK3/MYO18A/NECAB2/NOTCH1/NR1H3/PACSIN3/PKDCC/PROM2/PTPN23/RAB17/RAB20/RAB25/RAB26/RAB7A/RABGAP1/RABGAP1L/RALB/RINT1/RSPO1/SCARB1/SDC1/SGSM2/SLC11A1/SNF8/SNX12/SPACA3/STON2/STX18/STX1A/STX4/STXBP1/SYTL3/TBC1D25/TBC1D28/TBC1D9/TF[illegible]	93	BP
GO:0055001	muscle cell development	39/2734	169/17381	0.00767	0.6014	0.59151	ACTA1/ADM/AGT/AXIN1/BIN3/BMP4/CAV3/CHRNB1/ENG/FLNC/GATA4/GPX1/IGSF22/KLHL40/LMNA/LMOD1/MIR195/MTOR/MYBPC1/MYBPC3/MYBPH/MYBPHL/MYOD1/NKX2-5/NOTCH1/NPPA/OBSCN/OBSL1/P2RX2/PDGFRB/PDLIM5/PRKAR1A/SDC1/SMO/SMYD3/TCAP/TRIM54/WDR1/WFIKKN2	39	BP
GO:2000147	positive regulation of cell motility	92/2734	460/17381	0.00776	0.6014	0.59151	ABL1/ADAM8/ADRA2A/AGT/AQP1/BCAS3/BMP4/C3AR1/CAMK1D/CCL1/CCL19/CCL21/CCL5/CCR2/CD74/CDH13/CIB1/COL1A1/CORO1A/CREB3/CSF1/CSF1R/CTSH/DAB2IP/DAPK2/DDR2/DOCK1/DOCK7/EDN2/EGFR/F2RL1/FAM83H/FAM89B/FERMT3/FLNA/FLT4/FN1/FOXO4/GLIPR2/GPER1/HMCN2/HRAS/HSPA5/HSPB1/ILK/INS/KARS/KDR/LAMC2/LGALS3/LGALS9/LGR6/MAPK3/MCAM/MIR181B1/MIR221/MIR222/MIR29A/MIR451A/MTOR/MYO1C/MYOC/NOTCH1/NOX4/NSMF/P2RY6/PDGFA/PDGFRB/PF4V1/PFN1/PGAM4/PLAU/PRDM14/PTK2B/RAB25/RARRES2/RHOD/RNASE9/SCARB1/SELP/SPOCK2/STX4/TACR1/TACR2/TDGF1/THY1/TIRAP/TNFRSF18[illegible]	92	BP
GO:0050718	positive regulation of interleukin-1	10/2734	28/17381	0.00783	0.6014	0.59151	CASP1/CASP5/CCL19/GSDMD/LGALS9/NLRP1/ORM1/ORM2/PYDC1/TLR8	10	BP
GO:0071260	cellular response to mechanical	21/2734	78/17381	0.00784	0.6014	0.59151	AGT/AQP1/ATP1A1/BAD/CASP1/CASP5/COL1A1/EGFR/ENG/IL13/KCNK4/LTBR/MAP3K14/MAPK3/NPPA/PDE2A/PTPN11/SCX/TLR8/TMEM150C/WNT11	21	BP
GO:0006525	arginine metabolic	8/2734	20/17381	0.0079	0.6014	0.59151	AGMAT/ASL/FAH/NAGS/NOS3/OTC/PADI4/SLC7A7	8	BP

GO:0010661	positive regulation of muscle cell	9/2734	24/17381	0.00802	0.6014	0.59151	AGT/APOPT1/CAMK2D/CDKN2A/EIF5A/LTK/MIR16-1/MIR195/PDCD4	9	BP
GO:0000226	microtubule cytoskeleton organization	94/2734	472/17381	0.00808	0.6014	0.59151	ABL1/ANKRD53/ARHGEF10/AXIN1/BCAS3/BRCA1/C2CD3/CAV3/CC2D2A/CCDC103/CCDC13/CCDC40/CCDC88B/CCNF/CCSER2/CDC14A/CDK2AP2/CEP19/CFAP157/CFAP74/CHMP1A/CHMP4C/CIB1/CKAP5/CROCC/CRYAB/DCLK2/DCTN2/DISC1/DNAJB13/DNHD1/DOCK7/DYNC1H1/E2F4/EML1/EYA1/FES/FLNA/FOPNL/GAPDH/GAS8/GOLGA2/HAUS4/HAUS7/HYDIN/INO80/KATNB1/KIF23/KIF2B/KIF4A/LMNA/LRRC6/MAP6D1/MAP7D1/MARK2/MARK3/MCPH1/MID1IP1/NCKAP5L/NCOR1/NTMT1/NUBP1/NUMA1/OBSL1/PARD6A/PARP3/PEX14/PKD1/PPP2R3C/RASSF7/RGS14/SDCCAG8/SENP6/SETD2/SPAG16/STMN4/TMEM141/TRAF3IP1/TRDN/TRIM46/TRIM54/TRPV4/TTLL5/TUBG1/TUBG2/TUBGCP2/TUBGCP3/TUBGCP5/UBE2B/UXT/WNT3A/WRAP73/XRCC2/ZPR1	94	BP
GO:0048704	embryonic skeletal system morphogenesis	24/2734	93/17381	0.00824	0.6064	0.59642	ALX3/AXIN1/BMP4/EYA1/FGFR2/HOXA3/HOXB1/HOXB2/HOXB3/HOXB4/HOXB7/HOXB8/HOXC11/HOXD3/HOXD4/IFT140/LHX1/MTHFD1/MYCN/OSR1/PCGF2/SETD2/TBX15/TULP3	24	BP
GO:0007389	pattern specification process	85/2734	422/17381	0.00848	0.6161	0.60598	ACD/ALX3/ARC/AXIN1/BMP4/C2CD3/CC2D2A/CCDC103/CCDC40/CDX2/CELSR2/CFC1/CRB2/DNAH11/DVL2/EFNB1/EMX1/EN1/ENG/EVX1/EYA1/FGFR2/GAS8/GATA4/GBX2/GRHL3/HHIP/HOXA3/HOXB1/HOXB2/HOXB3/HOXB4/HOXB7/HOXB8/HOXB9/HOXC10/HOXC11/HOXC13/HOXC4/HOXC5/HOXC6/HOXC8/HOXD3/HOXD4/IFT140/IRX3/KAT2A/LDB1/LFNG/LHX1/LHX3/LRRC6/MESP2/MICAL2/NBL1/NEK8/NKX2-5/NKX3-2/NLE1/NOTCH1/NOTO/OSR1/PBX2/PCGF2/RFNG/RIPPLY1/SEMA3F/SHH/SMAD6/SMO/SOST/SUFU/TBC1D32/TBR1/TBX2/TCAP/TDGF1/TRAF3IP1/TULP3/VAX2/WLS/WNT1/WNT3A/WNT6/WNT7A	85	BP

GO:2001233	regulation of apoptotic signaling pathway	78/2734	383/17381	0.00864	0.6199	0.60975	AGT/ANKRD2/APOPT1/ARRB2/ATF3/ATPIF1/BAD/BCAP31/BMP4/BOK/BRCA1/CCAR2/CD74/CLU/CREB3/CTSH/CTTN/DAB2IP/DAPK1/DAPK2/DYNLL2/EYA1/FASLG/FGA/FZD9/G0S2/GPER1/GPX1/HERPUD1/HIP1R/HRK/HSPB1/INCA1/INHBB/INS/LCK/LGALS3/LGALS9/LMNA/LTBR/MADD/MAPK8IP1/MIR15A/MIR16-1/MIR221/MIR222/MOAP1/MUC1/NLE1/NOC2L/NONO/NOS3/PCGF2/PEA15/PELI3/PPP1R13B/PRELID1/PRKCD/RAF1/SEPT4/SFN/SGMS1/SLC25A5/SPOP/STX4/SYVN1/TMBIM1/TNFRSF12A/TRAF2/TRAF7/TRAP1/VDAC2/WF[illegible]	78	BP
GO:0007405	neuroblast proliferation	16/2734	55/17381	0.00881	0.6246	0.61428	ARTN/DAGLB/DISC1/DOCK7/DRD2/EML1/FGFR2/FZD9/HHIP/NOTCH1/OTP/SHH/SMO/SOX5/VAX1/WNT3A	16	BP
GO:0050714	positive regulation of protein secretion	51/2734	235/17381	0.00921	0.6259	0.61565	ABL1/ACHE/ADAM8/ANG/ANO1/APBB1/ATP13A2/BAD/BLK/CAPN10/CASP1/CASP5/CCL19/CD58/CSF1R/DOC2B/DRD2/EGFR/F2RL1/FGA/GAPDH/GCG/GCK/GLUD1/GPER1/GSDMD/HAVCR2/HCAR2/HLA-E/IL13/IL4R/INS/KARS/LGALS9/MAPK3/MYO18A/NLRP1/ORM1/ORM2/PFKM/PLA2G1B/PPARD/PTPN23/PYDC1/RFX6/STX4/TLR8/TNFRSF4/TRH/TR	51	BP
GO:0022600	digestive system process	24/2734	94/17381	0.00946	0.6259	0.61565	ABCG5/ACO1/ADRA2A/APOA5/AQP1/AQP5/CHRM1/HIP1R/HRH2/KCNQ1/MUC2/MUC6/NPC1L1/NR1H3/PBLD/PTGER3/SCARB1/SERPINA3/SGK1/SLC26A6/SOAT2/TACR1/TLR9/VSIG1	24	BP
GO:0006553	lysine metabolic process	6/2734	13/17381	0.00951	0.6259	0.61565	CRYM/DLST/HYKK/OGDH/PHYKPL/PIPOX	6	BP
GO:0031282	regulation of guanylate cyclase activity	6/2734	13/17381	0.00951	0.6259	0.61565	GUCA1B/GUCA2A/GUCA2B/NOS3/PDZD3/RUNDC3A	6	BP
GO:0048246	macrophage chemotaxis	11/2734	33/17381	0.00962	0.6259	0.61565	C3AR1/CCL5/CKLF/CSF1/EDN2/KARS/LGALS3/MAPK3/PTK2B/RARRES2/TRPV4	11	BP
GO:1901264	carbohydrate derivative transport	18/2734	65/17381	0.00979	0.6259	0.61565	G6PC3/GLTP/MFSD2A/NPC2/PSAP/SCARB1/SLC25A25/SLC25A5/SLC28A1/SLC28A2/SLC29A1/SLC29A2/SLC29A3/SLC29A4/SLC35A2/SLC35C1/SLC35D2/SLC50A1	18	BP
GO:0043039	tRNA aminoacylation	15/2734	51/17381	0.0099	0.6259	0.61565	AARS/AARS2/AIMP1/AIMP2/DALRD3/DARS/GATB/KARS/LARS/PARS2/PPA2/SARS/TARS2/YARS/YARS2	15	BP

GO:0050994	regulation of lipid catabolic	15/2734	51/17381	0.0099	0.6259	0.61565	ACACB/ADRA2A/APOA5/APOC3/CDK4/HCAR2/IDH1/INS/MTOR/PNPLA2/PRKCD/RARRES2/SCARB1/THRA/TYSND1	15	BP
GO:0048660	regulation of smooth muscle cell	31/2734	130/17381	0.0101	0.6259	0.61565	ABCC4/AGT/ANG/BMP4/CCL5/CDH13/EGFR/FGFR2/GPER1/IGFBP3/IL13/IL18/ILK/IRAK1/MFN2/MIR15A/MIR221/MIR222/MIR503/MIR96/MMP2/MTOR/NDRG2/NQO2/PDGFRB/PPARD/RBPMS2/SERPINF2/SF1/STAT1/TACR1	31	BP
GO:0021696	cerebellar cortex	10/2734	29/17381	0.0103	0.6259	0.61565	CDK5/CEND1/KNDC1/LDB1/LHX1/LHX5/PTPN11/SMO/SPTBN2/WNT7A	10	BP
GO:0032273	positive regulation of protein	28/2734	115/17381	0.01055	0.6259	0.61565	ANKRD53/ARFIP1/ARPC1A/ARPC1B/ARPC2/ARPC4/BAIAP2L1/BRK1/CAV3/CCL21/CDC42EP2/CORO1A/CORO1B/CSF3/CTTN/EVL/FES/HIP1R/LMOD1/MLST8/MTOR/MYO1C/NUMA1/PFN1/PTK2B/SCIN/WAS/WHAMM	28	BP
GO:0043254	regulation of protein complex assembly	79/2734	392/17381	0.0107	0.6259	0.61565	ABL1/ANKRD53/ARFIP1/ARPC1A/ARPC1B/ARPC2/ARPC4/BAIAP2L1/BRK1/CAPZA3/CAV3/CCL21/CDC42EP2/CLU/CORO1A/CORO1B/CRYAB/CSF3/CTTN/DAB2IP/DDB1/DDB2/EIF4EBP1/EIF4G1/EVL/FARP2/FBLIM1/FES/FOSL1/FSCN1/GBA/GTF2H4/HCFC1/HIP1R/HJURP/HRK/HSP90AA1/INS/KIF9/LCMT1/LMOD1/MIEF2/MLST8/MMP3/MTOR/MYO1C/NKX2-5/NUMA1/OPRD1/PAXIP1/PEX14/PEX5/PFN1/PPP2R5B/PRKCD/PSMC3/PTK2B/PTPN11/RAF1/RALB/SCIN/SELP/SENP6/SLF2/SMAD6/SOST/SPTAN1/SPTB/SPTBN2/STX1A/STXBP1/TAF1/TAF7/THRA/TRAF3IP1/ULK1/VDAC2/WAS/WHAMM	79	BP
GO:0002577	regulation of antigen processing and	7/2734	17/17381	0.01071	0.6259	0.61565	CCL19/CCL21/CD74/NOD1/SLC11A1/TREM2/WAS	7	BP
GO:0031532	actin cytoskeleton reorganization	23/2734	90/17381	0.01079	0.6259	0.61565	ABL1/ARAP1/BAIAP2L1/BCAS3/BRSK2/CAPN10/CDC42BPG/CDK5/CSF1R/CSF3/CTTN/DTNBP1/FARP2/FES/FLNA/HRAS/PHACTR1/PTK2B/RHOD/SHC1/TNIK/TRPV4/WHAMM	23	BP
GO:0003018	vascular process in circulatory system	37/2734	162/17381	0.01089	0.6259	0.61565	ADM/ADRA1A/ADRA1B/ADRA2A/ADRA2B/ADRA2C/AGT/ASIC2/AVPR2/CHGA/CHRM1/EDN2/EGFR/EPHX2/F2RL1/FGA/FGFBP3/GCH1/GPER1/GPX1/HRH2/HTR1D/INS/KNG1/MIR153-1/MIR92A2/NOS3/NPPA/PDE2A/PLOD3/PPARD/PTP4A3/SERPINF2/SMTNL1/TACR1/TACR2/TRPV4	37	BP

GO:0051146	striated muscle cell differentiation	57/2734	270/17381	0.01101	0.6259	0.61565	ACTA1/ADAM12/ADGRB1/AGT/ANKRD2/ARRB2/BARX2/BCL9/BIN3/BMP4/CACNA1H/CACYBP/CAV3/CHRNB1/EHD1/FLNC/GATA4/GPX1/IFT20/IGSF22/IL4R/KAT2A/KDM6B/KLHL40/LMNA/LMOD1/MIR195/MIR222/MTOR/MYBPC1/MYBPC3/MYBPH/MYBPHL/MYOD1/NKX2-5/NOTCH1/NOX4/NPPA/NRG1/OBSCN/OBSL1/P2RX2/PDGFRB/PDLIM5/PRKAR1A/RARA/SDC1/SHH/SMO/SMYD3/TCAP/THRA/TRIM72/WDR1/WFIKKN2/WNT1/WNT3A	57	BP
GO:0043032	positive regulation of macrophage	8/2734	21/17381	0.01103	0.6259	0.61565	FAM19A3/HAVCR2/IL13/IL4R/ITGAM/KARS/SPACA3/TNIP2	8	BP
GO:0051496	positive regulation of stress fiber	14/2734	47/17381	0.0111	0.6259	0.61565	ABL1/ARHGEF10/EVL/FHOD1/LIMK1/MTOR/MYOC/NOX4/PFN1/SERPINF2/SH3PXD2B/SORBS3/TACR1/WNT11	14	BP
GO:1902042	negative regulation of extrinsic apoptotic signaling	12/2734	38/17381	0.01115	0.6259	0.61565	BRCA1/DAPK1/FASLG/FGA/GPX1/MIR221/MIR222/NOS3/PEA15/RAF1/TMBIM1/TRAF2	12	BP
GO:0007586	digestion	38/2734	168/17381	0.01167	0.6259	0.61565	ABCG5/ACO1/ADRA2A/APOA5/AQP1/AQP5/CAPN8/CHIT1/CHRM1/CLPS/CLPSL1/CTSE/GUCA2A/GUCA2B/HIP1R/HRH2/KCNQ1/MGAM/MUC2/MUC6/NPC1L1/NPY4R/NR1H3/PBLD/PGC/PLA2G1B/PTGER3/PYY/SCARB1/SERPINA3/SGK1/SLC26A6/SOAT2/TACR1/TLR9/UCN2/VIPR1/VSIG1	38	BP
GO:0051272	positive regulation of cellular component movement	93/2734	473/17381	0.01181	0.6259	0.61565	ABL1/ADAM8/ADRA2A/AGT/AQP1/BCAS3/BCL6/BMP4/C3AR1/CAMK1D/CCL1/CCL19/CCL21/CCL5/CCR2/CD74/CDH13/CIB1/COL1A1/CORO1A/CREB3/CSF1/CSF1R/CTSH/DAB2IP/DAPK2/DDR2/DOCK1/DOCK7/EDN2/EGFR/F2RL1/FAM83H/FAM89B/FERMT3/FLNA/FLT4/FN1/FOXO4/GLIPR2/GPER1/HMCN2/HRAS/HSPA5/HSPB1/ILK/INS/KARS/KDR/LAMC2/LGALS3/LGALS9/LGR6/MAPK3/MCAM/MIR181B1/MIR221/MIR222/MIR29A/MIR451A/MTOR/MYO1C/MYOC/NOTCH1/NOX4/NSMF/P2RY6/PDGFA/PDGFRB/PF4V1/PFN1/PGAM4/PLAU/PRDM14/PTK2B/RAB25/RARRES2/RHOD/RNASE9/SCARB1/SELP/SPOCK2/STX4/TACR1/TACR2/TDGF1/THY1/TIRAP/TNFRSF18/TRPV4/WNT11/WNT7A/ZNF703	93	BP

GO:0010631	epithelial cell migration	55/2734	260/17381	0.01185	0.6259	0.61565	ABL1/ADGRB1/AGT/AQP1/BCAS3/BMP4/CDH13/CIB1/CORO1B/CTSH/DAB2IP/DOCK1/EFNA1/EPHB4/EVL/FAT2/FLT4/GDF2/GLIPR2/GPX1/HSPB1/KDR/KRT16/LOXL2/MARVELD3/MIR10A/MIR16-1/MIR212/MIR221/MIR222/MIR29C/MIR503/MIR92A2/MTOR/NOS3/NOTCH1/NR4A1/PAXIP1/PBLD/PFN1/PLEKHG5/PLXND1/PPARD/PRCP/PTK2B/PTP4A3/PTPN11/PTPN23/RAB25/SCARB1/SEMA4A/TACR1/TDGF1/WDPCP/	55	BP
GO:0043038	amino acid activation	15/2734	52/17381	0.01193	0.6259	0.61565	AARS/AARS2/AIMP1/AIMP2/DALRD3/DARS/GATB/KARS/LARS/PARS2/PPA2/SARS/TARS2/YARS/YARS2	15	BP
GO:0002468	dendritic cell antigen processing and	5/2734	10/17381	0.01208	0.6259	0.61565	CCL19/CCL21/CD74/NOD1/SLC11A1	5	BP
GO:0006547	histidine metabolic	5/2734	10/17381	0.01208	0.6259	0.61565	CARNS1/HDC/HNMT/MTHFD1/UROC1	5	BP
GO:0021548	pons development	5/2734	10/17381	0.01208	0.6259	0.61565	CDK5R2/GRIN1/HOXB1/PHOX2A/SCRIB	5	BP
GO:0021936	regulation of cerebellar granule cell precursor	5/2734	10/17381	0.01208	0.6259	0.61565	CEND1/LHX1/LHX5/SHH/SMO	5	BP
GO:0042756	drinking behavior	5/2734	10/17381	0.01208	0.6259	0.61565	AGT/EN1/MMP17/REN/TACR1	5	BP
GO:0072203	cell proliferation involved in metanephros	5/2734	10/17381	0.01208	0.6259	0.61565	EGR1/OSR1/PDGFRB/SHH/STAT1	5	BP
GO:0014909	smooth muscle cell migration	20/2734	76/17381	0.01208	0.6259	0.61565	AGT/APEX1/CCL5/CORO1B/DOCK7/FOXO4/IGFBP3/ILK/LRP1/MIR15A/MIR221/MIR451A/MIR503/NOX4/P2RY6/PDGFA/PDGFRB/PLAU/PPARD/TAC	20	BP
GO:0030516	regulation of axon extension	20/2734	76/17381	0.01208	0.6259	0.61565	ABL1/ANAPC2/ARHGAP4/BARHL2/CDK5/CTTN/DISC1/DRAXIN/FN1/ILK/ISLR2/L1CAM/LIMK1/NRG1/SEMA3F/SEMA7A/TNFRSF12A/TRPV2/WNT3	20	BP

GO:0021915	neural tube development	36/2734	158/17381	0.01234	0.6284	0.61802	ABL1/ADM/AMBRA1/BMP4/C2CD3/CC2D2A/CFL1/DEAF1/DVL2/EN1/FERD3L/FZD2/GBX2/GRHL3/IFT140/ITPK1/KAT2A/LIAS/MTHFD1/NOTCH1/PFN1/PKD1/PLOD3/RARA/SCRIB/SETD2/SHH/SMO/SPINT1/ST14/SUFU/TBC1D32/TRAF3IP1/TULP3/WNT1/WNT3A	36	BP
GO:0031589	cell-substrate adhesion	65/2734	316/17381	0.01247	0.6284	0.61802	ABL1/ADAMTS13/AJAP1/ARPC2/BCAS3/BCL6/CCL21/CD63/CDH13/CDK5/CDKN2A/CIB1/COL16A1/COL17A1/COL1A1/CORO1A/CSF1/CTTN/DISC1/DOCK1/EFNA1/EMILIN1/EPDR1/EPHB3/FBLN2/FERMT2/FERMT3/FGA/FLNA/FN1/HOXD3/ILK/ITGA7/ITGAL/ITGB5/KDR/L1CAM/LDB1/MEN1/MIR29C/MIR503/MIR92A2/MSLN/MYOC/NOTCH1/OLFM4/PKD1/PLAU/PPARD/PTK2B/RELL2/RHOD/SMAD6/SORBS3/SPOCK2/TAOK2/THBS3/THY1/TNF[illegible]	65	BP
GO:0035296	regulation of tube diameter	31/2734	132/17381	0.01259	0.6284	0.61802	ADM/ADRA1A/ADRA1B/ADRA2A/ADRA2B/ADRA2C/AGT/ASIC2/AVPR2/CHGA/CHRM1/EDN2/EGFR/EPHX2/F2RL1/FGA/GCH1/GPER1/GPX1/HRH2/HTR1D/INS/KNG1/MIR153-	31	BP
GO:0097746	regulation of blood vessel diameter	31/2734	132/17381	0.01259	0.6284	0.61802	ADM/ADRA1A/ADRA1B/ADRA2A/ADRA2B/ADRA2C/AGT/ASIC2/AVPR2/CHGA/CHRM1/EDN2/EGFR/EPHX2/F2RL1/FGA/GCH1/GPER1/GPX1/HRH2/HTR1D/INS/KNG1/MIR153-	31	BP
GO:0009394	2'-deoxyribonucleotide metabolic process	10/2734	30/17381	0.01331	0.6284	0.61802	AK5/DGUOK/MBD4/NEIL2/NT5M/NUDT1/NUDT18/OGG1/SMUG1/TBPL1	10	BP
GO:0038084	vascular endothelial growth factor signaling	10/2734	30/17381	0.01331	0.6284	0.61802	CD63/DAB2IP/FLT4/HSPB1/KDR/MIR16-1/MYO1C/PDGFRB/PTP4A3/XDH	10	BP
GO:0070229	negative regulation of lymphocyte	10/2734	30/17381	0.01331	0.6284	0.61802	BCL6/BMP4/CCL5/CD27/CD74/EFNA1/IDO1/NOC2L/TSC22D3/TSC22D4	10	BP
GO:0006418	tRNA aminoacylation for protein	14/2734	48/17381	0.01346	0.6284	0.61802	AARS/AARS2/AIMP1/AIMP2/DALRD3/DARS/KARS/LARS/PARS2/PPA2/SARS/TARS2/YARS/YARS2	14	BP

GO:0045010	actin nucleation	14/2734	48/17381	0.01346	0.6284	0.61802	ARFIP1/ARPC1A/ARPC1B/ARPC2/ARPC4/BRK1/CORO1A/CORO1B/EVL/HIP1R/LMOD1/SCIN/WAS/WHAMM	14	BP
GO:0060563	neuroepithelial cell	14/2734	48/17381	0.01346	0.6284	0.61802	ABL1/B9D1/DLX3/EMX1/JAG2/LHFPL5/LRTOMT/MYCN/MYO7A/NOTCH1/SCRIB/TMEM231/WDPCP/WNT11	14	BP
GO:0070231	T cell apoptotic process	14/2734	48/17381	0.01346	0.6284	0.61802	ADAM8/BMP4/CCL5/CD27/DNAJA3/EFNA1/FASLG/IDO1/LGALS3/LGALS9/PDCD1/PRELID1/TSC22D3/TSC22D4	14	BP
GO:0044089	positive regulation of cellular component biogenesis	96/2734	493/17381	0.01378	0.6284	0.61802	ABL1/ADGRB1/AGRN/AGT/AMIGO1/ANKRD53/AQP1/ARAP1/ARFIP1/ARHGEF10/ARPC1A/ARPC1B/ARPC2/ARPC4/ASIC2/ATP13A2/BAIAP2L1/BCAS3/BRK1/CAV3/CCL19/CCL21/CDC42EP2/CHGA/CLSTN1/CLSTN3/CLU/COL16A1/CORO1A/CORO1B/CROCC/CSF3/CTTN/DDB1/DDB2/DEF8/DYNC1H1/EIF4G1/EPHB3/EPS8L2/ESPN/EVL/F2RL1/FES/FHOD1/FOSL1/FSCN1/GTF2H4/HIP1R/HRAS/HRK/HSF1/KDR/LIMK1/LINGO2/LMOD1/LRRTM1/LRSAM1/MIEF2/MLST8/MMP3/MTOR/MYO1C/MYOC/NKX2-5/NLGN3/NOX4/NUMA1/PAXIP1/PFN1/PLEKHM1/PPP2R5B/PSMC3/PTK2B/RAB7A/RALB/SCIN/SDC1/SEPT9/SERPINF2/SH3PXD2B/SLF2/SLITRK3/SNF8/SORBS3/STX18/TACR1/TAF1/THY1/ULK1/WAS/WHAMM/WNT1/WNT11/WNT7A/WRAP73	96	BP
GO:0009952	anterior/posterior pattern specification	44/2734	202/17381	0.01382	0.6284	0.61802	ARC/AXIN1/BMP4/CDX2/CELSR2/CRB2/EN1/GATA4/GBX2/HOXA3/HOXB1/HOXB2/HOXB3/HOXB4/HOXB7/HOXB8/HOXB9/HOXC10/HOXC11/HOXC13/HOXC4/HOXC5/HOXC6/HOXC8/HOXD3/HOXD4/KAT2A/LDB1/LFNG/LHX1/MESP2/NLE1/NOTCH1/OSR1/PCGF2/RIPPLY1/SHH/SMO/TCAP/TDG	44	BP
GO:0043030	regulation of macrophage activation	12/2734	39/17381	0.01385	0.6284	0.61802	CD74/FAM19A3/HAVCR2/IL13/IL31RA/IL4R/ITGAM/KARS/MYO18A/NR1H3/SPACA3/TNIP2	12	BP
GO:0048659	smooth muscle cell proliferation	31/2734	133/17381	0.01401	0.6284	0.61802	ABCC4/AGT/ANG/BMP4/CCL5/CDH13/EGFR/FGFR2/GPER1/IGFBP3/IL13/IL18/ILK/IRAK1/MFN2/MIR15A/MIR221/MIR222/MIR503/MIR96/MMP2/MTOR/NDRG2/NQO2/PDGFRB/PPARD/RBPMS2/SERPINF2/SF1/STAT1/TACR1	31	BP

GO:0030335	positive regulation of cell migration	87/2734	442/17381	0.0141	0.6284	0.61802	ABL1/ADAM8/ADRA2A/AGT/AQP1/BCAS3/BMP4/C3AR1/CAMK1D/CCL1/CCL19/CCL21/CCL5/CCR2/CD74/CDH13/CIB1/COL1A1/CORO1A/CREB3/CSF1/CSF1R/CTSH/DAB2IP/DAPK2/DDR2/DOCK1/DOCK7/EDN2/EGFR/F2RL1/FAM83H/FAM89B/FERMT3/FLNA/FLT4/FN1/FOXO4/GLIPR2/GPER1/HMCN2/HRAS/HSPA5/HSPB1/ILK/INS/KARS/KDR/LAMC2/LGALS3/LGALS9/LGR6/MAPK3/MCAM/MIR181B1/MIR221/MIR222/MIR29A/MIR451A/MTOR/MYO1C/MYOC/NOTCH1/NOX4/NSMF/P2RY6/PDGFA/PDGFRB/PF4V1/PFN1/PLAU/PTK2B/RAB25/RARRES2/RHOD/SCARB1/SELP/STX4/TACR1/T[illegible]	87	BP
GO:0014812	muscle cell migration	22/2734	87/17381	0.0141	0.6284	0.61802	AGT/APEX1/BIN3/CCL5/CORO1B/DOCK7/FOXO4/IGFBP3/ILK/LRP1/MIR15A/MIR221/MIR451A/MIR503/NOX4/P2RY6/PDGFA/PDGFRB/PLAU/PPARD	22	BP
GO:0001976	neurological system process involved in regulation of systemic arterial blood pressure	6/2734	14/17381	0.01446	0.6284	0.61802	ADRA1A/AGT/ASIC2/DRD2/P2RX2/TACR1	6	BP
GO:0010663	positive regulation of striated muscle cell apoptotic	6/2734	14/17381	0.01446	0.6284	0.61802	AGT/CAMK2D/EIF5A/LTK/MIR16-1/MIR195	6	BP
GO:0010666	positive regulation of cardiac muscle cell apoptotic	6/2734	14/17381	0.01446	0.6284	0.61802	AGT/CAMK2D/EIF5A/LTK/MIR16-1/MIR195	6	BP
GO:0070234	positive regulation of T cell apoptotic	6/2734	14/17381	0.01446	0.6284	0.61802	ADAM8/CCL5/IDO1/LGALS9/PDCD1/PRELID1	6	BP

GO:0071880	adenylate cyclase-activating adrenergic receptor	6/2734	14/17381	0.01446	0.6284	0.61802	ADRA1A/ADRA1B/ADRA2A/ADRA2B/ADRA2C/CHGA	6	BP
GO:0090132	epithelium migration	55/2734	263/17381	0.01481	0.6284	0.61802	ABL1/ADGRB1/AGT/AQP1/BCAS3/BMP4/CDH13/CIB1/CORO1B/CTSH/DAB2IP/DOCK1/EFNA1/EPHB4/EVL/FAT2/FLT4/GDF2/GLIPR2/GPX1/HSPB1/KDR/KRT16/LOXL2/MARVELD3/MIR10A/MIR16-1/MIR212/MIR221/MIR222/MIR29C/MIR503/MIR92A2/MTOR/NOS3/NOTCH1/NR4A1/PAXIP1/PBLD/PFN1/PLEKHG5/PLXND1/PPARD/PRCP/PTK2B/PTP4A3/PTPN11/PTPN23/RAB25/SCARB1/SEMA4A/TACR1/TDGF1/WDPCP/	55	BP
GO:0031032	actomyosin structure organization	39/2734	176/17381	0.01495	0.6284	0.61802	ABL1/ACTA1/ALKBH4/ARAP1/ARHGEF10/CAV3/EPB41L2/EVL/FHOD1/IGSF22/ITGB5/KIF23/LIMK1/LMOD1/MTOR/MYBPC1/MYBPC3/MYBPH/MYBPHL/MYO18A/MYOC/NKX2-5/NOX4/OBSCN/OBSL1/PDGFRB/PFN1/PHACTR1/PRKAR1A/PTK2B/SERPINF2/SH3PXD2B/SORBS3/TACR1/TCAP/WAS/WDR1/WNT11/ZYX	39	BP
GO:0010758	regulation of macrophage chemotaxis	8/2734	22/17381	0.01498	0.6284	0.61802	C3AR1/CCL5/CSF1/KARS/MAPK3/PTK2B/RARRES2/TRPV4	8	BP
GO:0015874	norepinephrine transport	8/2734	22/17381	0.01498	0.6284	0.61802	ADRA2A/ADRA2B/ADRA2C/AGT/NISCH/SLC22A1/SLC6A2/STX1A	8	BP
GO:0021533	cell differentiation	7/2734	18/17381	0.01519	0.6284	0.61802	CEND1/KNDC1/LDB1/LHX1/LHX5/PHOX2B/WNT7A	7	BP
GO:0044342	type B pancreatic cell	7/2734	18/17381	0.01519	0.6284	0.61802	BAD/IGFBP3/MEN1/NR4A1/SIDT2/WDR13/WNT3A	7	BP
GO:2000251	positive regulation of actin cytoskeleton	7/2734	18/17381	0.01519	0.6284	0.61802	ABL1/BAIAP2L1/BCAS3/CDK5/CSF3/FES/HRAS	7	BP

GO:0090130	tissue migration	56/2734	269/17381	0.01532	0.6285	0.61812	ABL1/ACTA1/ADGRB1/AGT/AQP1/BCAS3/BMP4/CDH13/CIB1/CORO1B/CTSH/DAB2IP/DOCK1/EFNA1/EPHB4/EVL/FAT2/FLT4/GDF2/GLIPR2/GPX1/HSPB1/KDR/KRT16/LOXL2/MARVELD3/MIR10A/MIR16-1/MIR212/MIR221/MIR222/MIR29C/MIR503/MIR92A2/MTOR/NOS3/NOTCH1/NR4A1/PAXIP1/PBLD/PFN1/PLEKHG5/PLXND1/PPARD/PRCP/PTK2B/PTP4A3/PTPN11/PTPN23/RAB25/SCARB1/SEMA4A/TACR1/TDGF1/WDPCP/	56	BP
GO:0042733	embryonic digit morphogenesis	17/2734	63/17381	0.0154	0.6285	0.61812	B9D1/BMP4/C2CD3/FBXW4/HOXC11/IFT140/MYCN/NOTCH1/OSR1/SHH/TBC1D32/TBX2/TMEM231/TRAF3IP1/TULP3/WDPCP/WNT7A	17	BP
GO:0046578	regulation of Ras protein signal transduction	45/2734	209/17381	0.01575	0.6343	0.62389	ABL1/ADRA1A/APOC3/ARHGEF10/ARHGEF16/ARHGEF17/ARHGEF25/ARHGEF4/BCL6/CSF1/DAB2IP/DGKI/EPS8L2/F2RL1/FARP2/FOXM1/GBF1/GPR17/GPR20/GPR35/HRAS/IQSEC2/MADD/MAPKAP1/MFN2/MMD2/MYOC/NOTCH1/NRG1/OBSCN/PDGFRB/PLEKHG4/PLEKHG5/PLEKHG6/PREX2/PSD/PSD2/PSD4/RAF1/RALGPS1/RASA3/RASA4B/RASGEF1A/TNK1/TRIO	45	BP
GO:1901606	alpha-amino acid catabolic process	24/2734	98/17381	0.01585	0.6343	0.62389	ALDH4A1/BLMH/CARNS1/CRYM/DLST/FAH/GAD1/GLUD1/GOT2/GPT/GSTZ1/HAAO/HDC/HNMT/HYKK/IDO1/NOS3/OGDH/OTC/PADI4/PHYKPL/PIPOX/THNSL2/UROC1	24	BP
GO:0032874	positive regulation of stress-activated MAPK cascade	34/2734	150/17381	0.01586	0.6343	0.62389	AXIN1/CARD9/CCL19/CCL21/CD27/DAB2IP/DVL2/ERN2/F2RL1/FLT4/FZD8/GADD45G/HACD3/HRAS/KARS/LTBR/MAP3K6/MAP4K2/MAPK8IP3/MIR181B1/MIR92A2/NCF1/NOD1/PTK2B/SERPINF2/TAOK2/TIRAP/TLR9/TNIK/TRAF2/TRPV4/UNC5CL/WNT7A/XDH	34	BP
GO:2000177	regulation of neural precursor cell	20/2734	78/17381	0.01608	0.6388	0.62834	CEND1/DISC1/DRD2/EMX1/FLNA/FZD9/ILK/LHX1/LHX5/LIMS2/NES/NOTCH1/OTP/SETD1A/SHCBP1/SHH/SMO/SPINT1/VAX1/WNT3A	20	BP
GO:0050880	regulation of blood vessel size	32/2734	140/17381	0.01677	0.646	0.63539	ADM/ADRA1A/ADRA1B/ADRA2A/ADRA2B/ADRA2C/AGT/ASIC2/AVPR2/CHGA/CHRM1/EDN2/EGFR/EPHX2/F2RL1/FGA/GCH1/GPER1/GPX1/HRH2/HTR1D/INS/KNG1/MIR153-	32	BP
GO:0035418	protein localization to	10/2734	31/17381	0.01694	0.646	0.63539	ASIC2/CDK5/CLSTN3/DLG4/GRIPAP1/HSPB1/LRRTM1/NRXN2/SCRIB/WNT7A	10	BP
GO:0050716	positive regulation of interleukin-1	10/2734	31/17381	0.01694	0.646	0.63539	CASP1/CASP5/CCL19/GSDMD/LGALS9/NLRP1/ORM1/ORM2/PYDC1/TLR8	10	BP

GO:0070228	regulation of lymphocyte apoptotic	15/2734	54/17381	0.01698	0.646	0.63539	ADAM8/BCL6/BMP4/CCL5/CD27/CD74/EFNA1/IDO1/LGALS3/LGALS9/NOC2L/PDCD1/PRELID1/TSC22D3/TSC22D4	15	BP
GO:0021532	neural tube patterning	12/2734	40/17381	0.01703	0.646	0.63539	BMP4/EN1/GBX2/IFT140/SHH/SMO/SUFU/TBC1D32/TRAF3IP1/TULP3/WNT1/WNT3A	12	BP
GO:0045773	positive regulation of	12/2734	40/17381	0.01703	0.646	0.63539	ANAPC2/DISC1/FN1/ILK/ISLR2/L1CAM/LIMK1/NRG1/SEMA7A/TNFRSF12A/TRPV2/ZFYVE27	12	BP
GO:0042063	gliogenesis	53/2734	254/17381	0.01718	0.646	0.63539	ABL1/ARHGEF10/ASCL2/BIN1/BOK/CCR2/CDK5/CDK5R2/CLCF1/CLU/CSF1/CSK/DAB1/DAB2IP/DISC1/EGFR/EIF2B2/EIF2B5/EMX1/ERBB2/GCM2/GPC1/HDAC11/IDH2/ILK/LAMA2/LAMC3/LRP1/LTA/MAPK3/METRN/MIR221/MIR222/MTOR/MXRA8/MYCN/MYOC/NLGN3/NOTCH1/NRG1/PARD3/PHOX2B/POU3F1/PRDM8/PTK2B/PTPN11/RNF112/SCRIB/SHH/SMO/SOX8/S	53	BP
GO:0006024	glycosaminoglycan biosynthetic process	26/2734	109/17381	0.01733	0.646	0.63539	ABCC5/ACAN/AGRN/B3GAT3/B4GALT2/B4GALT7/BCAN/BGN/CHPF/CHPF2/CHST12/CHST13/CHST15/CHST5/CHST7/DSE/GPC1/GPC2/HEXA/HS3ST6/HSPG2/PDGFRB/SDC1/SLC35D2/ST3GAL4/XYLT2	26	BP
GO:0042177	negative regulation of protein	26/2734	109/17381	0.01733	0.646	0.63539	ADGRB1/ALAD/ATP13A2/CCAR2/CDK5RAP3/DAB2IP/EFNA1/EGFR/FHIT/FLNA/FURIN/GRIN2C/INS/KLHL40/LAMP3/LRIG2/NRG1/OS9/PANO1/SHH/SNX12/SUFU/TAF1/TRIM40/USP19/WNT1	26	BP
GO:0070304	positive regulation of stress-activated protein kinase	34/2734	151/17381	0.01746	0.646	0.63539	AXIN1/CARD9/CCL19/CCL21/CD27/DAB2IP/DVL2/ERN2/F2RL1/FLT4/FZD8/GADD45G/HACD3/HRAS/KARS/LTBR/MAP3K6/MAP4K2/MAPK8IP3/MIR181B1/MIR92A2/NCF1/NOD1/PTK2B/SERPINF2/TAOK2/TIRAP/TLR9/TNIK/TRAF2/TRPV4/UNC5CL/WNT7A/XDH	34	BP
GO:0007265	Ras protein signal transduction	68/2734	338/17381	0.01748	0.646	0.63539	ABL1/ADRA1A/ADRA2A/APOC3/ARHGAP4/ARHGEF10/ARHGEF16/ARHGEF17/ARHGEF25/ARHGEF4/BCL6/BRK1/CDC42EP2/CDH13/CDKN2A/CFL1/CSF1/DAB2IP/DGKI/DOK1/DOK2/EPS8L2/F2RL1/FARP2/FOXM1/GBF1/GPR17/GPR20/GPR35/GRAP/HACD3/HRAS/IQSEC2/KSR1/LIMK1/MADD/MAPKAP1/MAPKAPK3/MFN2/MMD2/MYOC/NISCH/NOTCH1/NRG1/OBSCN/PDGFRB/PLEKHG4/PLEKHG5/PLEKHG6/PREX2/PSD/PSD2/PSD4/RAF1/RALB/RALGDS/RALGPS1/RASA3/RASA4B/RASGEF1A/RASGRP2/RHOD/RTK[illegible]	68	BP

GO:1902041	regulation of extrinsic apoptotic signaling pathway via	16/2734	59/17381	0.01757	0.646	0.63539	ATF3/BRCA1/DAPK1/FASLG/FGA/GPX1/LGALS3/MADD/MIR221/MIR222/NOS3/PEA15/RAF1/STX4/TMBIM1/TRAF2	16	BP
GO:0051403	stress-activated MAPK cascade	54/2734	260/17381	0.01776	0.6492	0.63848	AGT/AXIN1/CARD9/CAV3/CCL19/CCL21/CD27/CRYAB/DAB2IP/DTNBP1/DVL2/EGFR/ERN2/F2RL1/FKTN/FLT4/FOXM1/FZD8/GADD45G/HACD3/HRAS/IKBKB/IRAK1/KARS/LGALS9/LTBR/MAP3K6/MAP4K2/MAPK3/MAPK8IP1/MAPK8IP3/MARVELD3/MEN1/MIR181B1/MIR92A2/NCF1/NCOR1/NOD1/PDCD4/PER1/PRDX1/PTK2B/SERPINF2/TAOK2/TIRAP/TLR9/TNIK/TNIP2/TRAF2/TRPV4/UNC5CL/WNT7A/XDH/ZMYND11	54	BP
GO:1903524	positive regulation of blood	18/2734	69/17381	0.01825	0.6527	0.642	ADM/ADRA1A/ADRA1B/ADRA2C/ATP1A1/AVPR2/CHGA/EDN2/EGFR/F2RL1/FGA/GCH1/HRH2/KCNQ1/NKX2-5/NPPA/SMTNL1/TACR1	18	BP
GO:0035150	regulation of tube size	32/2734	141/17381	0.01852	0.6527	0.642	ADM/ADRA1A/ADRA1B/ADRA2A/ADRA2B/ADRA2C/AGT/ASIC2/AVPR2/CHGA/CHRM1/EDN2/EGFR/EPHX2/F2RL1/FGA/GCH1/GPER1/GPX1/HRH2/HTR1D/INS/KNG1/MIR153-	32	BP
GO:0007160	cell-matrix adhesion	44/2734	206/17381	0.01918	0.6527	0.642	ABL1/ADAMTS13/AJAP1/BCAS3/BCL6/CCL21/CD63/CDH13/CDK5/CDKN2A/CIB1/COL16A1/COL17A1/CSF1/CTTN/DISC1/EMILIN1/EPDR1/FERMT2/FGA/FN1/HOXD3/ILK/ITGA7/ITGAL/ITGB5/KDR/L1CAM/LDB1/MIR29C/MIR92A2/MSLN/MYOC/PKD1/PLAU/PTK2B/RHOD/TAOK2/THBS3/THY1/TN	44	BP
GO:0001845	phagolysosome assembly	5/2734	11/17381	0.01931	0.6527	0.642	CORO1A/MYO7A/RAB20/RAB34/RAB7A	5	BP
GO:0016322	neuron remodeling	5/2734	11/17381	0.01931	0.6527	0.642	C1QL1/EPHA8/FARP2/RND1/SCARF1	5	BP
GO:0031284	positive regulation of guanylate	5/2734	11/17381	0.01931	0.6527	0.642	GUCA1B/GUCA2A/GUCA2B/NOS3/RUNDC3A	5	BP

GO:0032463	negative regulation of protein homooligomeri	5/2734	11/17381	0.01931	0.6527	0.642	CLU/CRYAB/GBA/PEX14/PEX5	5	BP
GO:0048484	enteric nervous system development	5/2734	11/17381	0.01931	0.6527	0.642	HLX/KIF26A/PHOX2B/SOX8/TLX2	5	BP
GO:0099624	atrial cardiac muscle cell membrane repolarization	5/2734	11/17381	0.01931	0.6527	0.642	FLNA/KCNJ5/KCNQ1/MIR328/NPPA	5	BP
GO:1902093	positive regulation of flagellated	5/2734	11/17381	0.01931	0.6527	0.642	PGAM4/PRDM14/RNASE9/TACR1/TACR2	5	BP
GO:1903975	regulation of glial cell	5/2734	11/17381	0.01931	0.6527	0.642	CCR2/CSF1/IDH2/MIR221/MIR222	5	BP
GO:2000344	positive regulation of acrosome	5/2734	11/17381	0.01931	0.6527	0.642	CACNA1H/GLRA1/PLB1/POMZP3/PRSS37	5	BP
GO:0071622	regulation of granulocyte chemotaxis	14/2734	50/17381	0.01931	0.6527	0.642	C3AR1/CAMK1D/CCL19/CCL21/CCL5/CD74/CSF1/DAPK2/KARS/MAPK3/PF4V1/RARRES2/TIRAP/TRPV4	14	BP
GO:0006023	aminoglycan biosynthetic process	26/2734	110/17381	0.01939	0.6527	0.642	ABCC5/ACAN/AGRN/B3GAT3/B4GALT2/B4GALT7/BCAN/BGN/CHPF/CHPF2/CHST12/CHST13/CHST15/CHST5/CHST7/DSE/GPC1/GPC2/HEXA/HS3ST6/HSPG2/PDGFRB/SDC1/SLC35D2/ST3GAL4/XYLT2	26	BP
GO:0051882	mitochondrial depolarization	8/2734	23/17381	0.01988	0.6653	0.65433	ABL1/ATPIF1/BOK/CASP1/FZD9/KDR/MYOC/PPP2R3C	8	BP
GO:0030168	platelet activation	35/2734	158/17381	0.02038	0.6673	0.65634	ADAMTS13/ADRA2A/ADRA2B/ADRA2C/ARRB2/C1QTNF1/CLIC1/COL1A1/CSRP1/DGKI/DGKQ/DGKZ/FERMT3/FGA/FLNA/GP9/HSPB1/ILK/ITPR3/LCK/MAPK3/NOS3/PDGFA/PF4V1/PIK3R5/PIK3R6/PRKCD/PTPN11/RAF1/SELP/STXBP1/TEC/TLN1/VWF/WNT3A	35	BP

GO:0019229	regulation of vasoconstriction	16/2734	60/17381	0.02055	0.6673	0.65634	ADM/ADRA1A/ADRA1B/ADRA2A/ADRA2B/ADRA2C/AGT/ASIC2/AVPR2/CHRM1/EDN2/EGFR/FGA/HRH2/SMTNL1/TACR1	16	BP
GO:1902110	positive regulation of mitochondrial membrane permeability involved in	16/2734	60/17381	0.02055	0.6673	0.65634	ATF2/ATPIF1/BAD/BOK/DYNLL2/FZD9/HIP1R/MIR29A/MIR29C/MOAP1/PPP1R13B/RHOT2/SFN/SLC25A5/YWHAQ/ZNF205	16	BP
GO:0120034	positive regulation of plasma membrane bounded cell projection	22/2734	90/17381	0.02071	0.6673	0.65634	AGRN/AQP1/ARAP1/ARPC2/BCAS3/BRK1/CCL19/CCL21/CDC42EP2/CROCC/DEF8/EPS8L2/ESPN/F2RL1/FSCN1/HRAS/MTOR/PFN1/PLEKHM1/SEPT9/WNT1/WRAP73	22	BP
GO:0070233	negative regulation of T cell apoptotic	7/2734	19/17381	0.02085	0.6673	0.65634	BMP4/CCL5/CD27/EFNA1/IDO1/TSC22D3/TSC22D4	7	BP
GO:0009404	toxin metabolic process	6/2734	15/17381	0.02094	0.6673	0.65634	AS3MT/CYP1A1/CYP1A2/CYP2W1/DDC/TH	6	BP
GO:0018027	peptidyl-lysine dimethylation	6/2734	15/17381	0.02094	0.6673	0.65634	EHMT1/EHMT2/PRDM12/SETD2/SETD7/SUV39H1	6	BP
GO:1903358	regulation of Golgi	6/2734	15/17381	0.02094	0.6673	0.65634	MAPK3/PDE4DIP/RBSN/STX18/STX5/USP6NL	6	BP
GO:1903798	regulation of production of miRNAs involved in gene silencing	6/2734	15/17381	0.02094	0.6673	0.65634	BMP4/EGFR/MYCN/NCOR1/NCOR2/ZC3H10	6	BP
GO:0006506	GPI anchor biosynthetic process	10/2734	32/17381	0.02126	0.6702	0.65916	CWH43/DPM2/GPAA1/PIGC/PIGG/PIGO/PIGQ/PIGY/PIGZ/PYURF	10	BP

GO:0019692	deoxyribose phosphate metabolic	10/2734	32/17381	0.02126	0.6702	0.65916	AK5/DGUOK/MBD4/NEIL2/NT5M/NUDT1/NUDT18/OGG1/SMUG1/TBPL1	10	BP
GO:0035239	tube morphogenesis	69/2734	347/17381	0.02142	0.6717	0.66064	ABL1/ADM/AGT/BCAS3/BMP4/C2CD3/CAV3/CC2D2A/CCDC103/CCDC40/CFL1/CSF1/CSF1R/CTSH/DAB2IP/DEAF1/DVL2/EGFR/ENG/EPHB3/ESRP2/EYA1/FGFR2/FZD2/GATA4/GBX2/GDF2/GRHL3/HHIP/HLX/HOXB7/ILK/IRX3/KAT2A/LHX1/LIAS/MICAL2/MTHFD1/MYCN/NKX2-3/NKX2-5/NOTCH1/NOTO/OSR1/PFN1/PKD1/PLXND1/RARA/RBPMS2/SCRIB/SDCCAG8/SETD2/SFRP5/SHH/SMO/SOX8/SPINT1/ST14/STRA6/SUFU/TBX2/TCAP/TIMELESS/TNC/TULP3/WNT1/WNT11/WNT3A/WNT6	69	BP
GO:0043406	positive regulation of MAP kinase activity	54/2734	263/17381	0.02189	0.6828	0.67158	ADAM8/ADRA2A/ADRA2B/ARAF/AXIN1/BMP4/CCL19/CD74/CD81/CSK/DAB2IP/DUSP5/DVL2/EGFR/ERBB2/ERN2/FZD8/GADD45G/GNG3/HACD3/HRAS/ILK/IRAK1/KARS/MADD/MAP2K3/MAP3K14/MAP3K15/MAP3K6/MAP4K2/MAPK3/MAPK8IP3/MAPKAPK3/MIR92A2/NEK10/NOD1/NOX4/NRG1/PDGFA/PDGFRB/PEA15/PIK3R5/PIK3R6/PLA2G1B/PTK2B/PTPN11/RAF1/SHC1/TAOK2/TDGF1/TLR9/TNIK/TRAF2/TRAF7	54	BP
GO:0032535	regulation of cellular component size	65/2734	325/17381	0.02225	0.6855	0.6742	ABL1/ANAPC2/AP2M1/AQP1/ARFIP1/ARHGAP4/ARPC1A/ARPC1B/ARPC2/ARPC4/ATP13A2/BAIAP2L1/BARHL2/BRK1/CAPZA3/CAV3/CCL21/CDC42EP2/CDHR5/CDK4/CDK5/CFL1/CORO1A/CORO1B/CSF3/CTTN/DISC1/DRAXIN/E2F4/EVL/F2RL1/FN1/HIP1R/ILK/ISLR2/L1CAM/LARS/LIMK1/LMOD1/MLST8/MTOR/MYO1C/NRG1/PEX11A/PFN1/PRKCD/PTK2B/SCIN/SEMA3F/SEMA7A/SLC12A4/SLC12A7/SNF8/SPTAN1/SPTB/SPTBN2/TNFRSF12A/TRPV2/TRPV4/WAS/WDR1/WHAMM/WNT3A/WNT7A/ZFYVE27	65	BP
GO:0060541	respiratory system development	42/2734	197/17381	0.02235	0.6855	0.6742	ADAMTS2/AIMP2/AXIN1/BMP4/CCDC40/CTSH/CYP1A2/EDN2/EGFR/EIF4EBP1/ESRP2/EYA1/FGFR2/FGFRL1/FLT4/HHIP/HYDIN/IL13/LHX3/MAPK3/MSC/MYCN/NKX2-8/NOS3/NOTCH1/NUMA1/PDGFA/PDGFRB/PHOX2B/PKD1/PKDCC/PLOD3/RARA/SHH/SPDEF/STRA6/THRA/TIMELESS/TNC/TULP3/WDPCP/WNT11	42	BP

GO:0055123	digestive system development	32/2734	143/17381	0.02243	0.6855	0.6742	BMP4/CCDC103/CCDC40/CDX2/CHD8/CYP1A1/DAB1/EGFR/EPHB3/FGFR2/GATA4/HIP1R/HLX/HRH2/NKX2-3/NKX3-2/NOTCH1/OTC/PKD1/PKDCC/RARRES2/RBPMS2/SFRP5/SHH/SMO/SPDEF/STRA6/TBX2/VPS52/WDPCP/WLS/WNT11	32	BP
GO:1990138	neuron projection extension	32/2734	143/17381	0.02243	0.6855	0.6742	ABL1/ALCAM/ANAPC2/ARHGAP4/BARHL2/CDK5/CPNE5/CPNE9/CTTN/DISC1/DRAXIN/EIF2AK4/EMX1/FN1/ILK/ISLR2/L1CAM/LIMK1/LLPH/NLGN3/NRG1/NRN1L/PLXNB1/PLXNB3/SEMA3F/SEMA5B/SEMA7A/TNFRSF12A/TRPV2/ULK1/WNT3A/ZFYVE27	32	BP
GO:0050770	regulation of axonogenesis	34/2734	154/17381	0.02305	0.6973	0.68579	ABL1/AMIGO1/ANAPC2/ARHGAP4/BARHL2/CDK5/CTTN/DAB1/DISC1/DRAXIN/EFNA1/EPHB3/FN1/GRIN1/ILK/ISLR2/L1CAM/LIMK1/LINGO1/MARK2/METRN/NRG1/PLXNB1/PLXNB3/PLXND1/SEMA3F/SEMA7A/TBR1/THY1/TNFRSF12A/TRPV2/WNT3A/WNT7A/ZFYVE27	34	BP
GO:0055002	striated muscle cell development	34/2734	154/17381	0.02305	0.6973	0.68579	ACTA1/AGT/BIN3/BMP4/CAV3/CHRNB1/FLNC/GATA4/GPX1/IGSF22/KLHL40/LMNA/LMOD1/MIR195/MTOR/MYBPC1/MYBPC3/MYBPH/MYBPHL/MYOD1/NKX2-5/NPPA/OBSCN/OBSL1/P2RX2/PDGFRB/PDLIM5/PRKAR1A/SDC1/SMO/S	34	BP
GO:0031109	microtubule polymerization or	22/2734	91/17381	0.02337	0.7007	0.68918	ABL1/ANKRD53/CAV3/CIB1/CKAP5/CRYAB/FES/GOLGA2/KATNB1/KIF2B/MAP6D1/MID1IP1/NCKAP5L/NUMA1/STMN4/TRIM54/TRPV4/TUBG1/TUBG2/TUBGCP2/TUBGCP3/TUBGCP5	22	BP
GO:0030823	regulation of cGMP metabolic	9/2734	28/17381	0.02354	0.7007	0.68918	AIPL1/FZD2/GUCA1B/GUCA2A/GUCA2B/NOS3/PDZD3/PTK2B/RUNDC3A	9	BP
GO:0070570	regulation of neuron projection	9/2734	28/17381	0.02354	0.7007	0.68918	INPP5F/LRIG2/MIR221/MIR222/PTPRF/RGMA/RTN4RL1/SCARF1/THY1	9	BP

GO:0048871	multicellular organismal homeostasis	68/2734	343/17381	0.02364	0.7007	0.68918	ACACB/ACADVL/ADAM8/AIPL1/AQP1/AVPR2/AZGP1/BGLAP/CDH23/CDHR1/CIB2/CLDN4/CNGB1/CORO1A/CROCC/CSF1/CSF1R/CSK/CTSH/CUBN/CYP11B2/DEF8/DRD2/EDN2/EGFR/EIF4G1/FLG2/GBA/GPX1/GRHL3/HOMER2/HSPB1/IL1RN/IL20RB/INPP5D/KRT16/LDB1/LEPR/MLXIPL/MUC2/MUC6/NCDN/NOX4/PBLD/PLEKHM1/PM20D1/PRCP/PRDM14/PRDX1/PRKAR1A/PRKAR1B/PTGER3/PTK2B/PTPN11/RAB7A/SCNN1A/SCX/SERPINA3/SFN/SLC11A1/SMO/TLR9/TMEM79/TNS2/TRPV4/TULP1/VSIG1/WFS1	68	BP
GO:0010001	glial cell differentiation	40/2734	187/17381	0.02406	0.708	0.69633	ABL1/ARHGEF10/BIN1/BOK/CDK5/CLCF1/CLU/CSK/DAB1/EGFR/EIF2B2/EIF2B5/EMX1/ERBB2/GPC1/HDAC11/ILK/LAMA2/LAMC3/LRP1/MAPK3/METRN/MTOR/MXRA8/MYCN/MYOC/NLGN3/NOTCH1/NRG1/PARD3/PHOX2B/POU3F1/PRDM8/PTPN11/RNF112/SHH/SMO/SOX8/SPINT1/WDR1	40	BP
GO:0003002	regionalization	66/2734	332/17381	0.02412	0.708	0.69633	ACD/ARC/AXIN1/BMP4/CDX2/CELSR2/CRB2/DVL2/EMX1/EN1/EVX1/FGFR2/GATA4/GBX2/HHIP/HOXA3/HOXB1/HOXB2/HOXB3/HOXB4/HOXB7/HOXB8/HOXB9/HOXC10/HOXC11/HOXC13/HOXC4/HOXC5/HOXC6/HOXC8/HOXD3/HOXD4/IFT140/IRX3/KAT2A/LDB1/LFNG/LHX1/LHX3/MESP2/NBL1/NKX2-5/NLE1/NOTCH1/NOTO/OSR1/PBX2/PCGF2/RIPPLY1/SEMA3F/SHH/SMAD6/SMO/SOST/SUFU/TBC1D32/TBR1/TCAP/TDGF1/TRAF3IP1/TULP3/VAX2/[illegible]	66	BP
GO:0007162	negative regulation of cell adhesion	51/2734	248/17381	0.02462	0.7081	0.69641	ABL1/AJAP1/BCAS3/BCL6/BMP4/C1QTNF1/CASP3/CCL21/CD74/CDH13/CDKN2A/COL1A1/DAB1/DACT2/ERBB2/HAVCR2/HLX/IDO1/IL1RN/IL20RB/IL4R/KNG1/LGALS3/LGALS9/MAD1L1/MEN1/MIR221/MIR222/MIR29C/MIR503/MIR92A2/MUC1/MYOC/NOTCH1/PLA2G2F/PLXNB1/PLXNB3/PLXND1/PRKAR1A/PRKCD/PTPN11/RND1/SCGB1A1/SHH/SIPA1/TGFBI/TNC/TR	51	BP
GO:0071675	regulation of mononuclear cell migration	12/2734	42/17381	0.02497	0.7081	0.69641	C3AR1/CCL1/CCL5/CCR2/CREB3/CSF1/KARS/LGALS3/MAPK3/NBL1/RARRES2/TRPV4	12	BP
GO:0002793	positive regulation of peptide secretion	52/2734	254/17381	0.02535	0.7081	0.69641	ABL1/ACHE/ADAM8/ANG/ANO1/APBB1/ATP13A2/BAD/BLK/CAPN10/CASP1/CASP5/CCL19/CD58/CSF1R/DOC2B/DRD2/EGFR/F2RL1/FGA/GAPDH/GCG/GCK/GLUD1/GPER1/GSDMD/HAVCR2/HCAR2/HLA-E/IL13/IL4R/INS/KARS/LGALS9/MAPK3/MYO18A/NLRP1/ORM1/ORM2/PFKM/PLA2G1B/PPARD/PTPN23/PYDC1/RFX6/STX4/TFR2/TLR8/TNFRSF4/T	52	BP

GO:0034762	regulation of transmembrane transport	88/2734	459/17381	0.02555	0.7081	0.69641	ABL1/ADRA2A/AGT/AHNAK/AMIGO1/ANO1/ARC/ASIC2/BSND/CACNA1F/CACNA1G/CACNA1H/CACNA1S/CACNB1/CACNB3/CACNG1/CAMK2D/CATSPER1/CAV3/CLCN2/CLCNKA/CLCNKB/CLIC1/CNIH2/COMMD1/CORO1A/CRACR2A/CRHR1/DAPK1/DLG4/DPP6/DRD2/FLNA/GNB5/GPER1/GPR35/GPR89A/GRIN1/GRIN2C/GSTM2/HCN3/HSPA2/IL13/INS/JPH3/KCNAB3/KCND1/KCND3/KCNH4/KCNH6/KCNJ18/KCNJ5/KCNJ9/KCNK12/KCNK13/KCNK17/KCNK4/KCNK7/KCNQ1/KCNQ4/MIR153-1/MIR212/MIR328/MTOR/NLGN3/NPPA/NPSR1/OAZ2/OPRM1/OSR1/PM20D1/PTK2B/RRAD/SCN11A/SLC26A6/SLC34A1/THADA/THY1/TLR9/TMEM109/TMEM37/TRDN/TRPM5/VDAC2/WDR1/WNK2/WWP2/YWHAQ	88	BP
GO:0006022	aminoglycan metabolic process	36/2734	166/17381	0.02562	0.7081	0.69641	ABCC5/ACAN/AGRN/B3GAT3/B4GALT2/B4GALT7/BCAN/BGN/CHID1/CHIT1/CHPF/CHPF2/CHST12/CHST13/CHST15/CHST5/CHST7/CLN6/DSE/GPC1/GPC2/HEXA/HS3ST6/HSPG2/IDS/ITIH4/ITIH6/NAGLU/PDGFRB/SDC1/SGSH/SLC35D2/SPACA3/SPOCK2/ST3GAL4/XYLT2	36	BP
GO:0060538	skeletal muscle organ development	36/2734	166/17381	0.02562	0.7081	0.69641	ACTA1/ANKRD2/ATF3/BCL9/BIN3/CDK5/CNTFR/EGR1/EMD/FGFRL1/GPC1/GPX1/HIVEP3/HLX/KLHL40/LEMD2/MSC/MYL6B/MYOD1/NOTCH1/NUPR1/P2RX2/PHOX2B/PITX1/SCX/SHH/SMO/SOX8/SRPK3/STRA6/TSC22D3/USP19/VAMP5/VAX1/VGLL2/WNT3A	36	BP
GO:0010669	epithelial structure	8/2734	24/17381	0.0258	0.7081	0.69641	CROCC/LDB1/MUC2/MUC6/PBLD/SERPINA3/TLR9/VSIG1	8	BP
GO:0021904	dorsal/ventral neural tube patterning	8/2734	24/17381	0.0258	0.7081	0.69641	BMP4/SHH/SMO/SUFU/TBC1D32/TRAF3IP1/TULP3/WNT3A	8	BP
GO:0030878	thyroid gland development	8/2734	24/17381	0.0258	0.7081	0.69641	HOXA3/HOXB3/HOXD3/MAPK3/NKX2-5/RAF1/SHH/THRA	8	BP
GO:0046755	viral budding	8/2734	24/17381	0.0258	0.7081	0.69641	CHMP1A/CHMP4C/CHMP7/LRSAM1/SNF8/VPS37B/VPS37C/VPS37D	8	BP
GO:0048679	regulation of axon	8/2734	24/17381	0.0258	0.7081	0.69641	INPP5F/LRIG2/MIR221/MIR222/PTPRF/RGMA/RTN4RL1/SCARF1	8	BP
GO:0051125	regulation of actin nucleation	8/2734	24/17381	0.0258	0.7081	0.69641	ARFIP1/BRK1/CORO1A/CORO1B/HIP1R/SCIN/WAS/WHAMM	8	BP

GO:0007254	JNK cascade	42/2734	199/17381	0.02612	0.7081	0.69641	AXIN1/CARD9/CCL19/CCL21/CD27/DAB2IP/DTNBP1/DVL2/EGFR/ERN2/F2RL1/FKTN/FLT4/FZD8/GADD45G/HACD3/HRAS/IRAK1/LTBR/MAP3K6/MAP4K2/MAPK8IP1/MAPK8IP3/MARVELD3/MEN1/MIR92A2/NCF1/NCOR1/NOD1/PDCD4/PER1/PTK2B/SERPINF2/TAOK2/TIRAP/TLR9/TNIK/TRAF2/T	42	BP
GO:0006790	sulfur compound metabolic process	74/2734	379/17381	0.02619	0.7081	0.69641	ABHD14B/ACACB/ACAN/ACOT7/ACOT9/ACPP/ACSF3/ACSM6/B3GAT3/B4GALT2/BCAN/BGN/BHMT2/BLMH/CHAC1/CHPF/CHPF2/CHST12/CHST13/CHST15/CHST5/CHST7/CIAPIN1/CSAD/DGAT1/DLST/DPEP1/DSE/ELOVL1/ELOVL5/FAM96B/GAL3ST4/GGT6/GGTA1P/GLO1/GNMT/GPC1/GPX1/GSR/GSTM1/GSTM2/GSTZ1/HACD1/HEXA/IDH1/IDS/ISCA2/LIAS/LOC344967/LPO/MGST1/MGST3/MICAL2/MPC1/MTHFD1/NARFL/NOX4/NUBP1/NUBP2/OGDH/PC/PDHB/PEMT/PIPOX/PMVK/PODXL2/SGSH/SLC26A1/SLC35[illegible]	74	BP
GO:0006505	GPI anchor metabolic	10/2734	33/17381	0.02632	0.7081	0.69641	CWH43/DPM2/GPAA1/PIGC/PIGG/PIGO/PIGQ/PIGY/PIGZ/PYURF	10	BP
GO:0071312	cellular response to	10/2734	33/17381	0.02632	0.7081	0.69641	CACNA1S/CASP3/CASP6/DDC/GSTM2/OPRM1/PPP1R9B/RECQL5/SLC34A1/TH	10	BP
GO:0198738	cell-cell signaling by wnt	92/2734	483/17381	0.02666	0.7081	0.69641	ABL1/ANKRD10/AP2A2/AP2M1/ARRB2/AXIN1/BCL7B/BCL9/CALCOCO1/CCAR2/CELSR2/CELSR3/CHD8/COL1A1/DAB2IP/DACT2/DDB1/DISC1/DRAXIN/DRD2/DVL2/EGR1/EMD/FBXW4/FERMT2/FGFR2/FRAT1/FZD2/FZD8/FZD9/GNAO1/GRHL3/GRK6/HOXB9/IFT20/IGFBP6/ILK/KREMEN2/LDB1/LGR6/LRP1/LZTS2/MARK2/MIR222/MYOC/NAIP/NDRG2/NKD2/NKX2-5/NLE1/NOTCH1/OPRM1/PARD6A/PFN1/PLCB2/PORCN/PSMB11/PSMB6/PSMB7/PSMB8/PSMC3/PSMD13/PSMD3/PSMD5/PSMD7/PTPN23/PTPRU/PYGO2/RNF220/RSPO1/SDC1/SFRP5/SHH/SOST/SOX7/TAX1BP3/TCF7L1/TGFB1I1/TNIK/TNN/TSKU/UBE2B/VAX2/WIF1/WLS/WNT1/WNT10A/WNT11/[illegible]	92	BP
GO:1904375	regulation of protein localization to	21/2734	87/17381	0.02674	0.7081	0.69641	AP2M1/ARHGEF16/CIB1/CNPY4/CSK/EGFR/EPB41L2/GPER1/INS/LRP1/LYPD1/NKD2/NUMA1/PID1/PKDCC/STX4/STX8/TMBIM1/TREM2/VTI1B/WNT3A	21	BP
GO:0021515	cell differentiation	14/2734	52/17381	0.02691	0.7081	0.69641	DRAXIN/EVX1/HOXC10/LHX1/LHX3/LHX4/LHX5/NOTCH1/OLIG3/PHOX2A/SHH/SUFU/WNT1/WNT3A	14	BP
GO:1903902	positive regulation of	15/2734	57/17381	0.0274	0.7081	0.69641	CCL5/CD74/CHMP4C/DDB1/FKBP6/HACD3/LGALS9/NOTCH1/PC/PPIB/PPIE/TARBP2/TRIM11/VPS37B/ZNF502	15	BP

GO:0043279	response to alkaloid	25/2734	108/17381	0.02747	0.7081	0.69641	CACNA1S/CASP3/CASP6/CDK5/DDC/DHODH/DNMT3A/DRD2/EFTUD2/GNAO1/GRIN1/GSTM2/HNMT/HOMER2/HSPA5/MTOR/OPRM1/PEA15/PPP1R1B/PPP1R9B/PTK2B/RECQL5/SLC34A1/TACR1/TH	25	BP
GO:1902686	mitochondrial outer membrane permeabilization involved in programmed cell death	16/2734	62/17381	0.02766	0.7081	0.69641	ATF2/ATPIF1/BAD/BOK/DYNLL2/FZD9/HIP1R/MIR29A/MIR29C/MOAP1/PPP1R13B/RHOT2/SFN/SLC25A5/YWHAQ/ZNF205	16	BP
GO:0006783	heme biosynthetic	7/2734	20/17381	0.02782	0.7081	0.69641	ALAD/ALAS2/ATPIF1/COX10/HMBS/NFE2L1/UROS	7	BP
GO:0009068	aspartate family amino acid catabolic	7/2734	20/17381	0.02782	0.7081	0.69641	CRYM/DLST/GOT2/HYKK/OGDH/PHYKPL/PIPOX	7	BP
GO:0009264	deoxyribonucleotide catabolic process	7/2734	20/17381	0.02782	0.7081	0.69641	MBD4/NEIL2/NT5M/NUDT1/NUDT18/OGG1/SMUG1	7	BP
GO:0044241	lipid digestion	7/2734	20/17381	0.02782	0.7081	0.69641	ABCG5/APOA5/AQP1/CLPS/NPC1L1/PLA2G1B/SOAT2	7	BP
GO:0060428	lung epithelium development	11/2734	38/17381	0.02837	0.7081	0.69641	AIMP2/BMP4/EYA1/FGFR2/IL13/NUMA1/PKD1/SHH/SPDEF/STRA6/THRA	11	BP
GO:0070317	negative regulation of G0 to G1	11/2734	38/17381	0.02837	0.7081	0.69641	BRCA1/DAB2IP/DUX4/EHMT1/EHMT2/EPC1/FOXO4/MAX/PCGF2/PPP2R5B/UXT	11	BP

GO:0006936	muscle contraction	69/2734	352/17381	0.02858	0.7081	0.69641	ACTA1/ADRA1A/ADRA1B/ADRA2A/ADRA2B/ADRA2C/AGT/ALDOA/ANKRD2/ATP1A1/CACNA1G/CACNA1H/CACNA1S/CACNG1/CAMK2D/CAV3/CHGA/CHRM1/CHRNB1/CRYAB/CTTN/DES/DRD2/EDN2/EMD/FLNA/GATA4/GLRA1/GNAO1/GPER1/GSTM2/HMCN2/HTR1D/IGSF22/ITGB5/KCND3/KCNJ5/KCNQ1/LMOD1/LTB4R/MAP2K3/MIR153-1/MIR328/MTOR/MYBPC1/MYBPC3/MYBPH/MYBPHL/MYL4/MYL5/MYL6B/NKX2-5/NPPA/OBSL1/P2RX2/PPP1R12B/PTGER3/SLC6A8/SORBS3/TACR1/TACR[illegible]	69	BP
GO:0003177	pulmonary valve	5/2734	12/17381	0.02889	0.7081	0.69641	BMP4/HEY1/NOTCH1/SMAD6/STRA6	5	BP
GO:0003184	pulmonary valve	5/2734	12/17381	0.02889	0.7081	0.69641	BMP4/HEY1/NOTCH1/SMAD6/STRA6	5	BP
GO:0034331	cell junction maintenance	5/2734	12/17381	0.02889	0.7081	0.69641	CSF1R/F2RL1/KIFC3/PARD6A/RASSF8	5	BP
GO:0045628	regulation of T-helper 2 cell differentiation	5/2734	12/17381	0.02889	0.7081	0.69641	BCL6/HLX/IL18/IL4R/RARA	5	BP
GO:0051085	chaperone mediated protein folding requiring	5/2734	12/17381	0.02889	0.7081	0.69641	CD74/GAK/TOR1A/TOR1B/TOR2A	5	BP
GO:1900029	positive regulation of	5/2734	12/17381	0.02889	0.7081	0.69641	DEF8/EPS8L2/HRAS/PFN1/PLEKHM1	5	BP
GO:1905516	positive regulation of	5/2734	12/17381	0.02889	0.7081	0.69641	CACNA1H/GLRA1/PLB1/POMZP3/PRSS37	5	BP
GO:0043649	dicarboxylic acid catabolic	6/2734	16/17381	0.02915	0.7081	0.69641	ACSF3/ALDH1L1/GAD1/GLUD1/GOT2/QPRT	6	BP

GO:0070920	regulation of production of small RNA involved in gene silencing	6/2734	16/17381	0.02915	0.7081	0.69641	BMP4/EGFR/MYCN/NCOR1/NCOR2/ZC3H10	6	BP
GO:1900746	regulation of vascular endothelial growth factor signaling	6/2734	16/17381	0.02915	0.7081	0.69641	CD63/DAB2IP/MIR16-1/MYO1C/PTP4A3/XDH	6	BP
GO:1905314	semi-lunar valve	6/2734	16/17381	0.02915	0.7081	0.69641	BMP4/EFNA1/HEY1/NOTCH1/SMAD6/STRA6	6	BP
GO:2000310	regulation of NMDA receptor activity	6/2734	16/17381	0.02915	0.7081	0.69641	CNIH2/DAPK1/DLG4/NLGN3/OPRM1/PTK2B	6	BP
GO:0006888	ER to Golgi vesicle-mediated transport	39/2734	184/17381	0.02928	0.7081	0.69641	ACTR1A/ARFGAP2/BCAP31/BGLAP/CAPZA3/CNIH2/COG4/COG5/COL7A1/COPZ1/DCTN2/DYNC1H1/DYNC1I1/DYNLL2/GBF1/GOLGA2/GORASP1/GOSR2/INS/LMF1/NRBP1/PROZ/RINT1/SEC16A/SEC16B/SPTAN1/SPTB/SPTBN2/STX18/STX5/TMED3/TMEM115/TRAPPC1/TRAPPC2L/TRAPPC3/TRA	39	BP
GO:0006839	mitochondrial transport	61/2734	307/17381	0.02944	0.7081	0.69641	ACACB/ACSM6/AIP/ATF2/ATP5G1/ATP5I/ATPIF1/BAD/BOK/CPT2/CYB5R1/CYC1/DNAJC19/DNLZ/DYNLL2/ELMOD1/FZD9/GPHA2/HIP1R/HNMT/HSP90AA1/ITGAX/KAT2A/LRRC46/MFN2/MID1IP1/MIR29A/MIR29C/MOAP1/MYBPC1/NBPF3/NRG1/OSCP1/PDE2A/PEMT/PMPCA/PPP1R13B/PSMB7/RHOT2/RNF31/SFN/SLC25A14/SLC25A20/SLC25A22/SLC25A25/SLC25A43/SLC25A45/SLC25A47/SLC25A48/SLC25A5/STARD3/TIMM17B/TIMM22/TIMM23B/TIMM9/TSGA13/UBL4B/UCP3/YWHAQ/ZBTB17/ZNF205	61	BP
GO:0030866	cortical actin cytoskeleton organization	9/2734	29/17381	0.0295	0.7081	0.69641	CDK5/EPB41L2/FMNL1/FMNL2/IKBKB/KIF23/LLGL1/STRIP1/TLN1	9	BP
GO:0048730	epidermis morphogenesis	9/2734	29/17381	0.0295	0.7081	0.69641	FGFR2/FOXQ1/KRT71/NOTCH1/PLOD3/SHH/SMO/TMEM79/WNT10A	9	BP

GO:0048565	digestive tract development	29/2734	130/17381	0.02979	0.7081	0.69641	BMP4/CCDC103/CCDC40/CDX2/CHD8/CYP1A1/DAB1/EGFR/EPHB3/FGFR2/GATA4/HLX/HRH2/NKX2-3/NKX3-2/NOTCH1/OTC/PKD1/PKDCC/RARRES2/RBPMS2/SFRP5/SHH/SMO/SPDEF/STRA6/TBX2/VPS52/WNT11	29	BP
GO:0045104	intermediate filament cytoskeleton organization	12/2734	43/17381	0.02983	0.7081	0.69641	DES/FAM83H/KRT14/KRT16/KRT3/KRT6A/KRT6C/KRT71/NES/RAF1/SHH/TOR1A	12	BP
GO:0061098	positive regulation of protein tyrosine	12/2734	43/17381	0.02983	0.7081	0.69641	ADRA2A/ADRA2B/ADRA2C/AGT/CCL5/CSF1R/DOK7/DVL2/EFNA1/NCF1/UNC119/WNT3A	12	BP
GO:0072593	reactive oxygen species metabolic process	51/2734	251/17381	0.03016	0.713	0.70128	ADGRB1/AGT/ATPIF1/BRCA1/CCS/CLU/CRYAB/CYP1A1/CYP1A2/DUOXA1/EGFR/EIF5A/EPHX2/EPX/F2RL1/FOXM1/GBF1/GCH1/GCHFR/GPX1/GPX3/GRIN1/HSP90AA1/INS/ITGAM/LPO/MIR92A2/MMP3/MPO/MTOR/MYCN/NCF1/NOS3/NOX4/NOXO1/NQO2/PDGFRB/PID1/POR/PRCP/PRDX1/PRDX6/PRKCD/PTK2B/SESN1/SH3PXD2B/SOD3/TRAP1/TUSC2/XDH/ZNF205	51	BP
GO:0051493	regulation of cytoskeleton organization	87/2734	457/17381	0.03077	0.7182	0.70639	ABL1/ANKRD53/ARAP1/ARFIP1/ARHGEF10/ARPC1A/ARPC1B/ARPC2/ARPC4/BAIAP2L1/BCAS3/BRCA1/BRK1/CAPZA3/CAV3/CCL21/CCNF/CDC42EP2/CDK2AP2/CDK5/CFL1/CHMP1A/CHMP4C/CIB1/CORO1A/CORO1B/CRIPAK/CSF1R/CSF3/CTTN/DYNC1H1/ESPN/EVL/F2RL1/FES/FHOD1/FSCN1/GRHL3/HIP1R/HRAS/ILK/KATNB1/LIMK1/LMOD1/LRP1/MAP6D1/MAPK3/MARK2/MCPH1/MID1IP1/MLST8/MTOR/MYO1C/MYOC/NES/NOX4/NUBP1/NUMA1/PARP3/PDGFA/PDGFRB/PFN1/PKD1/PLXNB1/PRKCD/PTK2B/RASSF7/RHOD/SCIN/SENP6/SERPINF2/SH3PXD2B/SORBS3/SPTAN1/SPTB/SPTBN2/STMN4/TACR1/TRAF3IP1/TRIM54/TRPV4/WAS/WDR1/WHAMM/[illegible]	87	BP
GO:2000179	positive regulation of neural precursor	13/2734	48/17381	0.03082	0.7182	0.70639	DISC1/DRD2/FLNA/FZD9/LHX1/LHX5/NES/NOTCH1/OTP/SETD1A/SHH/SMO/WNT3A	13	BP
GO:2000273	positive regulation of	13/2734	48/17381	0.03082	0.7182	0.70639	ADRA2A/ADRA2B/ADRA2C/ARC/CDK5/GSTM2/NCF1/NLGN3/RWDD1/SLURP1/SRA1/TAF1/TRDN	13	BP

GO:0032388	positive regulation of intracellular transport	67/2734	342/17381	0.03094	0.7182	0.70639	ACSM6/ANP32B/ATPIF1/B3GAT3/BAD/BCAP31/BCAS3/BMP4/BOK/CAPN10/CCL19/CD27/CDK5/CHRM1/CIB1/CSF3/CYB5R1/DNM1/DYNC1H1/DYNLL2/EGFR/EHD1/ELMOD1/EMD/ERBB2/FLNA/GPHA2/HIP1R/HNMT/IL13/IL18/IL4R/ITGAM/ITGAX/KAT2A/LGALS9/LRRC46/MIEF2/MYBPC1/MYO1C/NBPF3/NRG1/NUMA1/OAZ2/OSCP1/PEMT/PPP1R13B/PRKCD/PSMB7/PTPN23/RBPMS/RNF31/SEC16B/SFN/SHH/SMO/STX18/STXBP1/TLR9/TOR1A/TSGA13/UBL4B/WNT3A/YWHAQ/ZBTB17/ZNF205/ZPR1	67	BP
GO:0048661	positive regulation of smooth muscle	19/2734	78/17381	0.0311	0.7182	0.70639	ABCC4/AGT/BMP4/CCL5/CDH13/EGFR/FGFR2/IL13/IL18/IRAK1/MIR221/MIR222/MMP2/MTOR/NQO2/PDGFRB/RBPMS2/SERPINF2/STAT1	19	BP
GO:0060560	developmental growth involved in morphogenesis	43/2734	207/17381	0.03143	0.7182	0.70639	ABL1/ALCAM/ANAPC2/ARHGAP4/BARHL2/BIN3/BMP4/CDK5/CPNE5/CPNE9/CSF1/CTTN/DISC1/DRAXIN/EIF2AK4/EMX1/FGFR2/FN1/ILK/ISLR2/L1CAM/LHX1/LIMK1/LLPH/MIR195/NLGN3/NOTCH1/NRG1/NRN1L/PLXNB1/PLXNB3/SEMA3F/SEMA5B/SEMA7A/SHH/TBX2/TNC/TNFRSF12A/TRPV2	43	BP
GO:1903363	negative regulation of cellular protein	17/2734	68/17381	0.03173	0.7182	0.70639	ALAD/ATP13A2/CCAR2/CDK5RAP3/DAB2IP/EFNA1/FHIT/FURIN/KLHL40/LAMP3/OS9/PANO1/SHH/SUFU/TAF1/USP19/WNT1	17	BP
GO:0009262	deoxyribonucleotide metabolic process	10/2734	34/17381	0.03219	0.7182	0.70639	AK5/DGUOK/MBD4/NEIL2/NT5M/NUDT1/NUDT18/OGG1/SMUG1/TBPL1	10	BP
GO:0019076	viral release from host cell	10/2734	34/17381	0.03219	0.7182	0.70639	CHMP4C/DDB1/PC/RAB7A/TRIM11/TRIM26/TRIM31/TRIM62/VPS37B/ZNF502	10	BP
GO:0032148	activation of protein kinase B activity	10/2734	34/17381	0.03219	0.7182	0.70639	ADRA2A/ADRA2B/ADRA2C/ANG/IL18/INS/MAPKAP1/MLST8/MTOR/NRG1	10	BP
GO:0035890	exit from host	10/2734	34/17381	0.03219	0.7182	0.70639	CHMP4C/DDB1/PC/RAB7A/TRIM11/TRIM26/TRIM31/TRIM62/VPS37B/ZNF502	10	BP
GO:0035891	exit from host cell	10/2734	34/17381	0.03219	0.7182	0.70639	CHMP4C/DDB1/PC/RAB7A/TRIM11/TRIM26/TRIM31/TRIM62/VPS37B/ZNF502	10	BP
GO:0052126	movement in host	10/2734	34/17381	0.03219	0.7182	0.70639	CHMP4C/DDB1/PC/RAB7A/TRIM11/TRIM26/TRIM31/TRIM62/VPS37B/ZNF502	10	BP

GO:0052192	movement in environment of other organism involved in symbiotic interaction	10/2734	34/17381	0.03219	0.7182	0.70639	CHMP4C/DDB1/PC/RAB7A/TRIM11/TRIM26/TRIM31/TRIM62/VPS37B/ZNF502	10	BP
GO:2000249	regulation of actin cytoskeleton	10/2734	34/17381	0.03219	0.7182	0.70639	ABL1/BAIAP2L1/BCAS3/CDK5/CSF1R/CSF3/FES/HRAS/PTK2B/RHOD	10	BP
GO:1903532	positive regulation of secretion by cell	69/2734	355/17381	0.03371	0.7185	0.70672	ABL1/ACHE/ADAM8/AGT/ANG/ANO1/APBB1/ATP13A2/BAD/BLK/C1QTNF1/CACNA1G/CACNA1H/CAPN10/CASP1/CASP5/CCL19/CD58/CDK5/CDK5R2/CSF1R/DOC2B/DRD2/DTNBP1/EGFR/F2RL1/FGA/GAPDH/GCG/GCK/GLUD1/GPER1/GSDMD/HAVCR2/HCAR2/HLA-E/IL13/IL4R/INHBB/INS/ITGAM/KARS/LGALS9/MAPK3/MYO18A/NLRP1/ORM1/ORM2/PFKM/PLA2G1B/PPARD/PTPN11/PTPN23/PYDC1/RAB7A/RFX6/SDC1/SNF8/STX1A/STX4/STXBP1/TACR1/TACR2/TFR2/TLR8/TNFRSF4/[illegible]	69	BP
GO:0002690	positive regulation of leukocyte	21/2734	89/17381	0.03373	0.7185	0.70672	C3AR1/CAMK1D/CCL1/CCL19/CCL21/CCL5/CCR2/CD74/CREB3/CSF1/DAPK2/EDN2/F2RL1/KARS/LGALS9/MAPK3/PF4V1/PTK2B/RARRES2/TIRAP/TRPV4	21	BP
GO:0032092	positive regulation of	21/2734	89/17381	0.03373	0.7185	0.70672	ABL1/AKTIP/BMP4/CDK5/CSF3/GCG/HERPUD1/HIP1R/HSF1/LFNG/LRP1/MAPRE3/MEN1/PKD1/PLXND1/RALB/RFNG/TAF1/TIRAP/TRAF2/WNT3A	21	BP
GO:0048145	regulation of fibroblast proliferation	21/2734	89/17381	0.03373	0.7185	0.70672	AGT/AQP1/B4GALT7/C1QL4/CD74/CDK4/DAB2IP/DDR2/EGFR/EMD/FN1/FNTB/IL13/LTA/NUPR1/PARP10/PDGFA/PDGFRB/PLA2G1B/S100A6/WNT1	21	BP
GO:0000413	protein peptidyl-prolyl isomerization	11/2734	39/17381	0.03407	0.7185	0.70672	FKBP10/FKBP6/FKBP9/PPIAL4A/PPIAL4C/PPIAL4D/PPIAL4E/PPIAL4F/PPIAL4G/PPIB/PPIE	11	BP
GO:1903959	regulation of anion transmembrane	11/2734	39/17381	0.03407	0.7185	0.70672	AGT/ANO1/BSND/CLCN2/CLCNKA/CLCNKB/GPR89A/MTOR/OSR1/SLC34A1/VDAC2	11	BP

GO:1905517	macrophage migration	11/2734	39/17381	0.03407	0.7185	0.70672	C3AR1/CCL5/CKLF/CSF1/EDN2/KARS/LGALS3/MAPK3/PTK2B/RARRES2/TRPV4	11	BP
GO:0097756	negative regulation of blood vessel	20/2734	84/17381	0.03445	0.7185	0.70672	ADM/ADRA1A/ADRA1B/ADRA2A/ADRA2B/ADRA2C/AGT/ASIC2/AVPR2/CHGA/CHRM1/EDN2/EGFR/FGA/HRH2/HTR1D/INS/MIR92A2/SMTNL1/TACR1	20	BP
GO:0030100	regulation of endocytosis	44/2734	214/17381	0.03484	0.7185	0.70672	ABL1/AHSG/APOA5/APOC3/ARRB2/BIN1/C2/CALY/CAMK1D/CAV3/CCL19/CCL21/CD300LF/CD63/CDH13/CSK/DLG4/DRD2/F2RL1/HIP1R/LGALS3/LMAN2/LRP1/LRRTM1/LRSAM1/MYO18A/NECAB2/NR1H3/PACSIN3/PROM2/RAB17/RAB20/RSPO1/SCARB1/SLC11A1/SNX12/SPACA3/STON2/TFR2/TNK2/TOR1A/TULP1/UNC119/WNT3A	44	BP
GO:1903076	regulation of protein localization to plasma	19/2734	79/17381	0.03511	0.7185	0.70672	AP2M1/ARHGEF16/CIB1/CNPY4/CSK/EGFR/GPER1/INS/LRP1/LYPD1/NKD2/PID1/PKDCC/STX4/STX8/TMBIM1/TREM2/VTI1B/WNT3A	19	BP
GO:0035924	cellular response to vascular endothelial	12/2734	44/17381	0.03533	0.7185	0.70672	CD63/DAB2IP/FLT4/HSPB1/KDR/MIR16-1/MYO1C/NOTCH1/NR4A1/PDGFRB/PTP4A3/XDH	12	BP
GO:0045103	intermediate filament-based process	12/2734	44/17381	0.03533	0.7185	0.70672	DES/FAM83H/KRT14/KRT16/KRT3/KRT6A/KRT6C/KRT71/NES/RAF1/SHH/TOR1A	12	BP
GO:0030203	glycosaminoglycan metabolic process	34/2734	159/17381	0.03539	0.7185	0.70672	ABCC5/ACAN/AGRN/B3GAT3/B4GALT2/B4GALT7/BCAN/BGN/CHPF/CHPF2/CHST12/CHST13/CHST15/CHST5/CHST7/CLN6/DSE/GPC1/GPC2/HEXA/HS3ST6/HSPG2/IDS/ITIH4/ITIH6/NAGLU/PDGFRB/SDC1/SGSH/SLC35D2/SPACA3/SPOCK2/ST3GAL4/XYLT2	34	BP
GO:0035914	skeletal muscle cell	17/2734	69/17381	0.03614	0.7185	0.70672	ANKRD2/ATF3/BCL9/EGR1/EMD/GPC1/HIVEP3/KLHL40/LEMD2/MYOD1/NOTCH1/NUPR1/PHOX2B/SCX/SOX8/VAX1/WNT3A	17	BP
GO:0070227	lymphocyte apoptotic	17/2734	69/17381	0.03614	0.7185	0.70672	ADAM8/BCL6/BMP4/CCL5/CD27/CD74/DNAJA3/EFNA1/FASLG/IDO1/LGALS3/LGALS9/NOC2L/PDCD1/PRELID1/TSC22D3/TSC22D4	17	BP
GO:0021516	dorsal spinal cord	7/2734	21/17381	0.03622	0.7185	0.70672	DRAXIN/HOXB8/LHX1/LHX3/LHX5/WNT1/WNT3A	7	BP

GO:0032462	regulation of protein homooligomerization	7/2734	21/17381	0.03622	0.7185	0.70672	CLU/CRYAB/GBA/HRK/MIEF2/PEX14/PEX5	7	BP
GO:0039702	viral budding via host ESCRT complex	7/2734	21/17381	0.03622	0.7185	0.70672	CHMP1A/CHMP4C/CHMP7/SNF8/VPS37B/VPS37C/VPS37D	7	BP
GO:0046386	deoxyribose phosphate catabolic	7/2734	21/17381	0.03622	0.7185	0.70672	MBD4/NEIL2/NT5M/NUDT1/NUDT18/OGG1/SMUG1	7	BP
GO:0070498	interleukin-1-mediated signaling	7/2734	21/17381	0.03622	0.7185	0.70672	EGR1/IKBKB/IL1RN/IRAK1/MAPK3/RPS6KA4/TNIP2	7	BP
GO:1990542	mitochondrial transmembrane transport	7/2734	21/17381	0.03622	0.7185	0.70672	ACACB/CPT2/MID1IP1/SLC25A20/TIMM22/TIMM9/UCP3	7	BP
GO:0061448	connective tissue development	48/2734	237/17381	0.03634	0.7185	0.70672	ADAMTS7/ATF2/BARX2/BMP4/CDK4/COL1A1/CRIP1/CSF1/EGR1/ESRRA/FBXW4/GDF2/HOXA3/HOXB3/HOXC4/HOXD3/LOXL2/MAPK3/MUSTN1/MYCN/NKX3-2/NOTCH1/OSR1/PAXIP1/PDGFRB/PITX1/PKD1/PKDCC/PLA2G16/POR/PPARD/RARA/RORC/SCIN/SCX/SERPINH1/SH3PXD2B/SLC25A25/SLC39A13/	48	BP
GO:0002828	regulation of type 2 immune response	9/2734	30/17381	0.03644	0.7185	0.70672	BCL6/CCR2/CD74/ECM1/HLX/IDO1/IL18/IL4R/RARA	9	BP
GO:0003382	epithelial cell morphogenesis	9/2734	30/17381	0.03644	0.7185	0.70672	DACT2/HRH2/PLOD3/RAB25/RILPL1/RILPL2/ST14/TNMD/VSIG1	9	BP
GO:0007019	microtubule depolymerizatio	9/2734	30/17381	0.03644	0.7185	0.70672	CIB1/KATNB1/KIF2B/MAP6D1/MID1IP1/NCKAP5L/STMN4/TRIM54/TRPV4	9	BP
GO:0042133	neurotransmitter metabolic	9/2734	30/17381	0.03644	0.7185	0.70672	ACHE/DAGLB/GAD1/GCHFR/HNMT/LRTOMT/NAALAD2/SLC44A4/TH	9	BP

GO:1902186	regulation of viral release from host cell	9/2734	30/17381	0.03644	0.7185	0.70672	CHMP4C/DDB1/PC/TRIM11/TRIM26/TRIM31/TRIM62/VPS37B/ZNF502	9	BP
GO:0035794	positive regulation of mitochondrial membrane	16/2734	64/17381	0.03646	0.7185	0.70672	ATF2/ATPIF1/BAD/BOK/DYNLL2/FZD9/HIP1R/MIR29A/MIR29C/MOAP1/PPP1R13B/RHOT2/SFN/SLC25A5/YWHAQ/ZNF205	16	BP
GO:0018208	peptidyl-proline modification	14/2734	54/17381	0.0365	0.7185	0.70672	FKBP10/FKBP6/FKBP9/NTMT1/P3H1/P3H3/PPIAL4A/PPIAL4C/PPIAL4D/PPIAL4E/PPIAL4F/PPIAL4G/PPIB/PPIE	14	BP
GO:0031102	neuron projection	14/2734	54/17381	0.0365	0.7185	0.70672	ADM/APOA5/INPP5F/LRIG2/MIR221/MIR222/PTPRF/RGMA/RTN4RL1/RTN4RL2/SCARF1/THY1/TNC/ULK1	14	BP
GO:0120032	regulation of plasma membrane bounded cell projection	33/2734	154/17381	0.03675	0.7185	0.70672	AGRN/AQP1/ARAP1/ARPC2/BCAS3/BIN3/BRK1/CCL19/CCL21/CDC42EP2/CROCC/DEF8/EPS8L2/ESPN/EVL/F2RL1/FSCN1/HRAS/IFT140/IFT20/MTOR/NOTO/PFN1/PLEKHM1/PLXNB3/PRKCD/RAB17/RAP1GAP/SEPT9/WAS/WDPCP/WNT1/WRAP73	33	BP
GO:0048002	antigen processing and presentation of peptide antigen	39/2734	187/17381	0.03681	0.7185	0.70672	ACTR1A/AP1S1/AP2A2/AP2M1/BCAP31/CAPZA3/CD74/CTSD/CTSE/CTSF/CTSL/DCTN2/DYNC1H1/DYNC1I1/DYNLL2/HLA-E/IKBKB/ITGB5/KIF23/KIF26A/KIF2B/KIF4A/KLC1/KLC2/NCF1/PSMB11/PSMB6/PSMB7/PSMB8/PSMC3/PSMD13/PSMD3/PSMD5/PSMD7/RAB7A/SLC11A1/SPTBN2/TAP1/TREM2	39	BP
GO:0006892	post-Golgi vesicle-	21/2734	90/17381	0.03769	0.7185	0.70672	BLZF1/COMMD1/CSK/GAK/GBF1/GOSR2/KIF13A/LLGL1/OSBPL5/PKDCC/RAB26/RAB34/RBSN/SCAMP2/SCAMP3/STEAP2/STX4/VAMP5/VPS13A/V	21	BP
GO:0048144	fibroblast proliferation	21/2734	90/17381	0.03769	0.7185	0.70672	AGT/AQP1/B4GALT7/C1QL4/CD74/CDK4/DAB2IP/DDR2/EGFR/EMD/FN1/FNTB/IL13/LTA/NUPR1/PARP10/PDGFA/PDGFRB/PLA2G1B/S100A6/WNT1	21	BP

GO:0034765	regulation of ion transmembrane transport	84/2734	444/17381	0.03799	0.7185	0.70672	ABL1/ADRA2A/AGT/AHNAK/AMIGO1/ANO1/ARC/ASIC2/BSND/CACNA1F/CACNA1G/CACNA1H/CACNA1S/CACNB1/CACNB3/CACNG1/CAMK2D/CATSPER1/CAV3/CLCN2/CLCNKA/CLCNKB/CLIC1/CNIH2/COMMD1/CORO1A/CRACR2A/CRHR1/DAPK1/DLG4/DPP6/DRD2/FLNA/GNB5/GPER1/GPR35/GPR89A/GRIN1/GRIN2C/GSTM2/HCN3/HSPA2/IL13/JPH3/KCNAB3/KCND1/KCND3/KCNH4/KCNH6/KCNJ18/KCNJ5/KCNJ9/KCNK12/KCNK13/KCNK17/KCNK4/KCNK7/KCNQ1/KCNQ4/MIR153-1/MIR212/MIR328/MTOR/NLGN3/NPPA/NPSR1/OPRM1/OSR1/PTK2B/RRAD/SCN11A/SLC34A1/THADA/THY1/TLR9/TMEM109/TMEM37/TRDN/TRPM5/VDAC2/WDR1/WNK2/WWP2/YWHAQ	84	BP
GO:0007163	establishment or maintenance of cell polarity	38/2734	182/17381	0.0383	0.7185	0.70672	AQP1/BCAS3/BRSK2/CCL19/CCL21/CDH5/CDX2/CFL1/CKAP5/CRB2/DLG4/DOCK7/EYA1/FAM89B/FAT1/FSCN1/GBF1/ILK/LMNA/LRCH4/MAPKAP1/MARK2/MARK3/MCPH1/MYO18A/NUMA1/PARD3/PARD6A/PKD1/PTK2B/SCRIB/SDCCAG8/SFRP5/SHH/WDPCP/WDR1/WNT11/WNT7A	38	BP
GO:0046329	negative regulation of	10/2734	35/17381	0.03891	0.7185	0.70672	F2RL1/FKTN/MAPK8IP1/MARVELD3/MEN1/MIR92A2/NCOR1/PDCD4/PER1/ZMYND11	10	BP
GO:0031098	stress-activated protein kinase signaling cascade	57/2734	289/17381	0.03892	0.7185	0.70672	AGT/AXIN1/CARD9/CAV3/CCL19/CCL21/CD27/CRYAB/DAB2IP/DTNBP1/DVL2/EGFR/ERN2/F2RL1/FKTN/FLT4/FOXM1/FZD8/GADD45G/HACD3/HRAS/IKBKB/IRAK1/KARS/LGALS9/LTBR/MAP2K3/MAP3K14/MAP3K6/MAP4K2/MAPK3/MAPK8IP1/MAPK8IP3/MAPKAP1/MARVELD3/MEN1/MIR181B1/MIR92A2/NCF1/NCOR1/NOD1/PDCD4/PER1/PRDX1/PTK2B/SERPINF2/TAOK2/TIRAP/TLR9/TNIK/TNIP2/TRAF2/TRPV4/UNC5CL/WNT7A/XDH/Z	57	BP
GO:0018108	peptidyl-tyrosine phosphorylation	72/2734	375/17381	0.03894	0.7185	0.70672	ABL1/ADRA2A/ADRA2B/ADRA2C/AGT/ARL2BP/ARRB2/BAZ1B/BLK/CCL5/CD74/CD81/CLCF1/CSF1R/CSF3/CSK/DDR2/DGKQ/DOK7/DVL2/EFNA1/EGFR/EPHA8/EPHB3/EPHB4/ERBB2/FES/FGF17/FGF3/FGFR2/FGFR4/FLT4/GRAP/HSF1/HSP90AA1/IL13/IL18/IL31RA/IL5RA/INPP5F/INS/KDR/LCK/LTK/MAP2K3/MAPK3/MIR221/MLST8/MTOR/NCF1/NRG1/PARP9/PDGFA/PDGFRB/PKDCC/PPP2R5B/PRKCD/PTK2B/SH3BP5/SH3BP5L/SHC1/TDGF1/TEC/THY1/TIE1/TNFRSF18/TNK1/TNK2/TREM2/UNC119/VPS25/WNT3A	72	BP

GO:0006704	glucocorticoid biosynthetic process	6/2734	17/17381	0.0392	0.7185	0.70672	ATP1A1/CACNA1H/CYP11B2/DGKQ/HSD11B2/HSD3B1	6	BP
GO:0016254	preassembly of GPI anchor in ER membrane	6/2734	17/17381	0.0392	0.7185	0.70672	DPM2/PIGC/PIGG/PIGQ/PIGY/PIGZ	6	BP
GO:0030277	maintenance of gastrointestinal epithelium	6/2734	17/17381	0.0392	0.7185	0.70672	MUC2/MUC6/PBLD/SERPINA3/TLR9/VSIG1	6	BP
GO:0033189	response to vitamin A	6/2734	17/17381	0.0392	0.7185	0.70672	CYP1A1/DNMT3A/GATA4/PPARD/RARA/SLC34A1	6	BP
GO:0033194	response to hydroperoxide	6/2734	17/17381	0.0392	0.7185	0.70672	DAPK1/GPX1/GPX3/MGST1/PRKCD/STX4	6	BP
GO:0035067	negative regulation of histone	6/2734	17/17381	0.0392	0.7185	0.70672	BRCA1/CTBP1/HDAC8/NOC2L/SPI1/TAF7	6	BP
GO:0051590	positive regulation of neurotransmitte	6/2734	17/17381	0.0392	0.7185	0.70672	DRD2/DTNBP1/GPER1/STX1A/STXBP1/TACR2	6	BP
GO:1902547	regulation of cellular response to vascular endothelial	6/2734	17/17381	0.0392	0.7185	0.70672	CD63/DAB2IP/MIR16-1/MYO1C/PTP4A3/XDH	6	BP
GO:2000738	positive regulation of stem cell	6/2734	17/17381	0.0392	0.7185	0.70672	BMP4/GATA4/HOXB4/LTBP3/NKX2-5/SOX5	6	BP

GO:0043624	cellular protein complex disassembly	41/2734	199/17381	0.0393	0.7185	0.70672	C12orf65/CAPZA3/CFL1/CIB1/EIF5A/ERAL1/F2RL1/GAK/KATNB1/KIF2B/LMOD1/MAP6D1/MICAL2/MID1IP1/MRPL10/MRPL14/MRPL28/MRPL36/MRPL37/MRPL43/MRPL52/MRPL57/MRPL9/MRPS10/MRPS11/MRPS18A/MRPS18B/MRPS21/MRPS33/MRPS5/NCKAP5L/NES/PEX14/SCIN/SPTAN1/SPTB/SPTBN2/STMN4/TRIM54/TRPV4/WDR1	41	BP
GO:0007605	sensory perception of sound	31/2734	144/17381	0.03962	0.7185	0.70672	ASIC2/ATP6V1B1/BARHL1/CASP3/CDH23/COL1A1/CRYM/DNM1/EPS8L2/ESPN/ESPNL/EYA1/GPX1/HOMER2/KCNQ1/KCNQ4/LHFPL5/LRIG2/LRTOMT/MARVELD2/MYO1A/MYO3A/MYO7A/P2RX2/PAX3/TH/TIMM9/TMIE/	31	BP
GO:0035148	tube formation	31/2734	144/17381	0.03962	0.7185	0.70672	ABL1/ADM/BCAS3/BMP4/CC2D2A/CFL1/DAB2IP/DEAF1/DVL2/FGFR2/FZD2/GRHL3/IRX3/KAT2A/LIAS/MTHFD1/NOTCH1/OSR1/PFN1/RARA/SCRIB/SDCCAG8/SETD2/SHH/SOX8/SPINT1/ST14/SUFU/TCAP/TULP3/WNT6	31	BP
GO:0002495	antigen processing and presentation of peptide antigen	23/2734	101/17381	0.03964	0.7185	0.70672	ACTR1A/AP1S1/AP2A2/AP2M1/CAPZA3/CD74/CTSD/CTSE/CTSF/CTSL/DCTN2/DYNC1H1/DYNC1I1/DYNLL2/KIF23/KIF26A/KIF2B/KIF4A/KLC1/KLC2/RAB7A/SPTBN2/TREM2	23	BP
GO:0001570	vasculogenesis	18/2734	75/17381	0.04028	0.7185	0.70672	ADM/EGFL7/ENG/GDF2/HEY1/KDR/MYO1E/NKX2-5/NOTCH1/PAXIP1/PDGFRB/SETD2/SHH/SMO/TIE1/TMEM100/WNT7A/XD	18	BP
GO:0002702	positive regulation of production of molecular mediator of	18/2734	75/17381	0.04028	0.7185	0.70672	CD74/CLCF1/F2RL1/HLA-E/IL13/IL4R/KARS/MAPK3/MZB1/PAXIP1/PGC/SEMA7A/SPON2/STX4/TLR9/TNFRSF4/TNFSF13/TRAF2	18	BP
GO:0043029	T cell homeostasis	11/2734	40/17381	0.04053	0.7185	0.70672	CACNA1F/CASP3/CCNB2/CORO1A/DNAJA3/FOXN1/LGALS9/LMO1/PPP2R3C/TSC22D3/TSC22D4	11	BP
GO:0007229	integrin-mediated	22/2734	96/17381	0.04082	0.7185	0.70672	ABL1/ADAMTS13/CD63/COL16A1/DOCK1/FERMT2/FERMT3/FLNA/ILK/ITGA7/ITGAL/ITGAM/ITGAX/ITGB5/LIMS2/PTK2B/PTPN11/SEMA7A/TEC/T	22	BP

GO:1901342	regulation of vasculature development	60/2734	307/17381	0.04095	0.7185	0.70672	ABL1/ADGRB1/ADM/AGT/AQP1/BMP4/BRCA1/C3AR1/CCR2/CIB1/COL4A2/CTSH/DAB2IP/ECM1/ECSCR/EFNA1/EFNA3/EGR1/ENG/ERBB2/FASLG/FOXO4/GATA4/GDF2/GPER1/HEY1/HSPB1/HSPG2/HTATIP2/KDR/MIR106B/MIR10A/MIR153-1/MIR15A/MIR16-1/MIR210/MIR212/MIR221/MIR222/MIR29C/MIR361/MIR451A/MIR503/MIR92A2/NOS3/NOTCH1/PDCD4/PIK3R6/PLXND1/PTK2B/RNH1/SARS/SEMA4A/SPINK5/STAT1/TIE1/TMEM100/TNFRSF12A/TNMD/XDH	60	BP
GO:0001771	immunological synapse	5/2734	13/17381	0.041	0.7185	0.70672	CCL19/CCL21/CD6/HAVCR2/LGALS3	5	BP
GO:0001956	positive regulation of neurotransmitte	5/2734	13/17381	0.041	0.7185	0.70672	DTNBP1/GPER1/STX1A/STXBP1/TACR2	5	BP
GO:0006896	Golgi to vacuole	5/2734	13/17381	0.041	0.7185	0.70672	GAK/GOSR2/RBSN/VPS52/VTI1B	5	BP
GO:0009223	pyrimidine deoxyribonucleotide catabolic process	5/2734	13/17381	0.041	0.7185	0.70672	MBD4/NEIL2/NT5M/OGG1/SMUG1	5	BP
GO:0021692	cerebellar Purkinje cell layer	5/2734	13/17381	0.041	0.7185	0.70672	CEND1/LDB1/LHX1/LHX5/SPTBN2	5	BP
GO:0033604	negative regulation of catecholamine	5/2734	13/17381	0.041	0.7185	0.70672	ADRA2A/ADRA2B/ADRA2C/CHGA/DRD2	5	BP
GO:0035791	platelet-derived growth factor receptor-beta signaling	5/2734	13/17381	0.041	0.7185	0.70672	ABL1/HIP1R/LRP1/PDGFA/PDGFRB	5	BP
GO:0061430	bone trabecula morphogenesis	5/2734	13/17381	0.041	0.7185	0.70672	CHAD/COL1A1/MMP2/PLXNB1/THBS3	5	BP

GO:0090343	positive regulation of	5/2734	13/17381	0.041	0.7185	0.70672	CDKN2A/HMGA1/LMNA/MIR10A/YPEL3	5	BP
GO:1900016	negative regulation of cytokine production involved in inflammatory	5/2734	13/17381	0.041	0.7185	0.70672	CHID1/CUEDC2/MIR221/MIR222/PDCD4	5	BP
GO:0009063	cellular amino acid catabolic process	25/2734	112/17381	0.04105	0.7185	0.70672	ALDH4A1/BCKDK/BLMH/CARNS1/CRYM/DLST/FAH/GAD1/GLUD1/GOT2/GPT/GSTZ1/HAAO/HDC/HNMT/HYKK/IDO1/NOS3/OGDH/OTC/PADI4/PHYKPL/PIPOX/THNSL2/UROC1	25	BP
GO:0031069	hair follicle morphogenesis	8/2734	26/17381	0.04112	0.7185	0.70672	FGFR2/FOXQ1/KRT71/NOTCH1/SHH/SMO/TMEM79/WNT10A	8	BP
GO:1905521	regulation of macrophage migration	8/2734	26/17381	0.04112	0.7185	0.70672	C3AR1/CCL5/CSF1/KARS/MAPK3/PTK2B/RARRES2/TRPV4	8	BP
GO:0001667	ameboidal-type cell migration	68/2734	353/17381	0.04113	0.7185	0.70672	ABL1/ADGRB1/AGT/AQP1/ARHGAP4/BCAS3/BMP4/CDH13/CFL1/CIB1/CORO1B/CRB2/CTSH/DAB2IP/DDR2/DOCK1/EFNA1/EFNB1/EPHB4/EVL/FAT2/FLT4/GBX2/GDF2/GLIPR2/GPX1/HSPB1/ILK/KDR/KRT16/LOXL2/MARVELD3/MIR10A/MIR16-1/MIR212/MIR221/MIR222/MIR29C/MIR503/MIR92A2/MTOR/NOS3/NOTCH1/NR4A1/PAXIP1/PBLD/PFN1/PHOX2B/PLEKHG5/PLXND1/PPARD/PRCP/PTK2B/PTP4A3/PTPN11/PTPN23/RAB25/SCARB1/SEMA3F/SEMA4A/SHH/S[illegible]	68	BP
GO:0045214	sarcomere organization	12/2734	45/17381	0.04152	0.7185	0.70672	CAV3/IGSF22/MYBPC1/MYBPC3/MYBPH/MYBPHL/NKX2-5/OBSCN/OBSL1/PRKAR1A/TCAP/WDR1	12	BP
GO:0050954	sensory perception of mechanical stimulus	34/2734	161/17381	0.04154	0.7185	0.70672	ASIC2/ASIC3/ATP6V1B1/BARHL1/CASP3/CDH23/COL1A1/CRYM/DNM1/EPS8L2/ESPN/ESPNL/EYA1/GPX1/HOMER2/KCNK4/KCNQ1/KCNQ4/LHFPL5/LRIG2/LRTOMT/MARVELD2/MYO1A/MYO3A/MYO7A/P2RX2/PAX3/PHF24/TH/TIMM9/TMIE/TPRN/WDR1/WFS1	34	BP
GO:0007585	respiratory gaseous	16/2734	65/17381	0.04155	0.7185	0.70672	COX11/ECEL1/ELN/FLT4/GLRA1/GRIN1/HNMT/JAG2/NLGN3/PHOX2A/PHOX2B/SFTPA1/SFTPB/TLX3/TNNC1/UCP3	16	BP

GO:0042116	macrophage activation	16/2734	65/17381	0.04155	0.7185	0.70672	CD74/CLU/EDN2/FAM19A3/HAVCR2/IL13/IL31RA/IL4R/ITGAM/KARS/MYO18A/NR1H3/SLC11A1/SPACA3/TLR8/TNIP2	16	BP
GO:2001234	negative regulation of apoptotic signaling	43/2734	211/17381	0.04164	0.7185	0.70672	ARRB2/BMP4/BOK/BRCA1/CCAR2/CD74/CLU/CREB3/CTTN/DAPK1/EYA1/FASLG/FGA/FZD9/GPX1/HERPUD1/HSPB1/INS/LGALS3/LMNA/MAPK8IP1/MIR221/MIR222/MUC1/NLE1/NOC2L/NONO/NOS3/PCGF2/PEA15/PELI3/PRELID1/RAF1/SLC25A5/SYVN1/TMBIM1/TRAF2/TRAP1/VDAC2/WFS1/ZC	43	BP
GO:0016055	Wnt signaling pathway	90/2734	481/17381	0.04166	0.7185	0.70672	ABL1/ANKRD10/AP2A2/AP2M1/ARRB2/AXIN1/BCL7B/BCL9/CALCOCO1/CCAR2/CELSR2/CELSR3/CHD8/COL1A1/DAB2IP/DACT2/DDB1/DISC1/DRAXIN/DRD2/DVL2/EGR1/EMD/FBXW4/FERMT2/FGFR2/FRAT1/FZD2/FZD8/FZD9/GNAO1/GRHL3/GRK6/HOXB9/IFT20/IGFBP6/ILK/KREMEN2/LDB1/LGR6/LRP1/LZTS2/MARK2/MIR222/MYOC/NAIP/NDRG2/NKD2/NKX2-5/NLE1/NOTCH1/PARD6A/PFN1/PLCB2/PORCN/PSMB11/PSMB6/PSMB7/PSMB8/PSMC3/PSMD13/PSMD3/PSMD5/PSMD7/PTPRU/PYGO2/RNF220/RSPO1/SDC1/SFRP5/SHH/SOST/SOX7/TAX1BP3/TCF7L1/TGFB1I1/TNIK/TNN/TSKU/UBE2B/VAX2/WIF1/WLS/WNT1/WNT10A/WNT11/WNT3A/WNT6/W[illegible]	90	BP
GO:0048546	digestive tract morphogenesis	13/2734	50/17381	0.04201	0.7185	0.70672	BMP4/EGFR/EPHB3/FGFR2/GATA4/HLX/NKX2-3/NOTCH1/RBPMS2/SFRP5/SHH/STRA6/WNT11	13	BP
GO:0051452	intracellular pH reduction	13/2734	50/17381	0.04201	0.7185	0.70672	ATP6V0B/ATP6V0E2/ATP6V1B1/ATP6V1F/ATP6V1G1/ATP6V1G2/CA7/CLN6/FASLG/GPR89A/RAB20/RAB7A/SLC11A1	13	BP
GO:0002687	positive regulation of leukocyte	27/2734	123/17381	0.04205	0.7185	0.70672	ADAM8/C3AR1/CAMK1D/CCL1/CCL19/CCL21/CCL5/CCR2/CD74/CREB3/CSF1/DAPK2/EDN2/F2RL1/KARS/LGALS3/LGALS9/MAPK3/PF4V1/PTK2B/RARRES2/SELP/TACR1/THY1/TIRAP/TNFRSF18/TRPV4	27	BP
GO:0032233	positive regulation of actin filament	14/2734	55/17381	0.04212	0.7185	0.70672	ABL1/ARHGEF10/EVL/FHOD1/LIMK1/MTOR/MYOC/NOX4/PFN1/SERPINF2/SH3PXD2B/SORBS3/TACR1/WNT11	14	BP
GO:0048146	positive regulation of fibroblast	14/2734	55/17381	0.04212	0.7185	0.70672	AGT/AQP1/CD74/CDK4/DDR2/EGFR/FN1/FNTB/IL13/PDGFA/PDGFRB/PLA2G1B/S100A6/WNT1	14	BP

GO:0071356	cellular response to tumor necrosis factor	55/2734	279/17381	0.04221	0.7185	0.70672	ADAMTS13/ADAMTS7/APOB/BRCA1/CASP1/CCDC3/CCL1/CCL19/CCL21/CCL5/CD27/CD58/CIB1/COL1A1/DAB2IP/F2RL1/GBA/GPER1/IKBKB/IL18BP/ILK/KARS/KAT2A/LTA/LTBR/MADD/MAP3K14/PELI3/PID1/PRPF8/PSMB11/PSMB6/PSMB7/PSMB8/PSMC3/PSMD13/PSMD3/PSMD5/PSMD7/PTK2B/PYDC1/RELT/RNF31/SHARPIN/SLC2A4/SMPD4/STAT1/TDGF1/TNFRSF12A/TNFRSF13B/TNFRSF18/TNFRSF4/TNFSF13/TRAF2/TRAIP	55	BP
GO:0060070	canonical Wnt signaling pathway	59/2734	302/17381	0.04263	0.7237	0.71178	ANKRD10/AXIN1/BCL9/CCAR2/CHD8/COL1A1/DAB2IP/DISC1/DRAXIN/DVL2/EGR1/EMD/FGFR2/FRAT1/FZD2/FZD8/FZD9/HOXB9/IFT20/IGFBP6/ILK/KREMEN2/LGR6/LZTS2/MIR222/NAIP/NKD2/NKX2-5/NLE1/NOTCH1/PORCN/PSMB11/PSMB6/PSMB7/PSMB8/PSMC3/PSMD13/PSMD3/PSMD5/PSMD7/PTPRU/PYGO2/RNF220/RSPO1/SDC1/SFRP5/SHH/SOST/SOX7/TCF7L1/TNN/UBE2B/WLS/WNT1/WNT10A/WNT11/WNT3A/WN	59	BP
GO:0046777	protein autophosphorylation	47/2734	234/17381	0.0431	0.724	0.7121	ABL1/ATP13A2/BLK/CAMK2D/CDK5/CSF1R/CSK/DAPK1/DAPK2/DDR2/EGFR/EIF2AK4/ENG/EPHA8/EPHB3/EPHB4/ERBB2/FES/FGFR2/FGFR4/FLT4/GRAP/INS/IRAK1/KDR/LCK/MAPK15/MAPK3/MAPKAPK3/MARK2/MEX3B/MTOR/MYO3A/NEK10/PDGFA/PDGFRB/PPP2R5B/PTK2B/STK33/TAF1/TEC/THY1/TNIK/TNK1/TNK2/ULK1/WNK2	47	BP
GO:0030038	contractile actin filament bundle assembly	20/2734	86/17381	0.04314	0.724	0.7121	ABL1/ARAP1/ARHGEF10/EVL/FHOD1/ITGB5/LIMK1/MTOR/MYOC/NOX4/PFN1/PHACTR1/PTK2B/SERPINF2/SH3PXD2B/SORBS3/TACR1/WAS/WNT11/ZYX	20	BP
GO:0043149	stress fiber assembly	20/2734	86/17381	0.04314	0.724	0.7121	ABL1/ARAP1/ARHGEF10/EVL/FHOD1/ITGB5/LIMK1/MTOR/MYOC/NOX4/PFN1/PHACTR1/PTK2B/SERPINF2/SH3PXD2B/SORBS3/TACR1/WAS/WNT	20	BP
GO:0060491	regulation of cell projection assembly	33/2734	156/17381	0.0432	0.724	0.7121	AGRN/AQP1/ARAP1/ARPC2/BCAS3/BIN3/BRK1/CCL19/CCL21/CDC42EP2/CROCC/DEF8/EPS8L2/ESPN/EVL/F2RL1/FSCN1/HRAS/IFT140/IFT20/MTOR/NOTO/PFN1/PLEKHM1/PLXNB3/PRKCD/RAB17/RAP1GAP/SEPT9/WAS/W	33	BP

GO:0051962	positive regulation of nervous system development	89/2734	476/17381	0.04326	0.724	0.7121	ADGRB1/ADRA2B/ADRA2C/AGRN/AGT/AMIGO1/ANAPC2/APBB1/ASIC2/BCL6/BIN1/BMP4/CAMK1D/CCR2/CLCF1/CLSTN1/CLSTN3/CPNE5/CPNE9/CSF1/CYB5D2/DAB1/DAB2IP/DISC1/DRD2/DUOXA1/EIF4G1/EPHB3/FES/FLNA/FN1/FOXO6/GPER1/HOXD3/HSPA5/ILK/IRX3/ISLR2/KATNB1/L1CAM/LHX1/LHX5/LIMK1/LINGO2/LLPH/LRRTM1/LTA/LTK/MARK2/METRN/MIR221/MIR222/MMD2/MTOR/NBL1/NEUROD2/NKX2-5/NLGN3/NME1/NOTCH1/NRG1/NSMF/OBSL1/OPRM1/OTP/PARD3/PHOX2B/PLXNB1/PLXNB3/PLXND1/PPP2R5B/PTK2B/RARA/RGS14/RNF112/SCARF1/SEMA7A/SERPINI1/SHH/SLITRK3/SMO/SOX8/SPEN/SPINT1/TNFRSF12A/TRPV2/WNT3A/WNT7A/ZFYVE27	89	BP
GO:0002504	antigen processing and presentation of peptide or polysaccharide antigen via [illegible]	23/2734	102/17381	0.04382	0.7297	0.71774	ACTR1A/AP1S1/AP2A2/AP2M1/CAPZA3/CD74/CTSD/CTSE/CTSF/CTSL/DCTN2/DYNC1H1/DYNC1I1/DYNLL2/KIF23/KIF26A/KIF2B/KIF4A/KLC1/KLC2/RAB7A/SPTBN2/TREM2	23	BP
GO:0019882	antigen processing and presentation	45/2734	223/17381	0.04385	0.7297	0.71774	ACTR1A/AP1S1/AP2A2/AP2M1/BCAP31/CAPZA3/CCL19/CCL21/CD74/CTSD/CTSE/CTSF/CTSH/CTSL/DCTN2/DYNC1H1/DYNC1I1/DYNLL2/HLA-E/IKBKB/ITGB5/KIF23/KIF26A/KIF2B/KIF4A/KLC1/KLC2/NCF1/NOD1/PSMB11/PSMB6/PSMB7/PSMB8/PSMC3/PSMD13/PSMD3/PSMD5/PSMD7/RAB34/RAB7A/SLC11A1/SPTBN2/TAP1/TREM2/WAS	45	BP
GO:2000106	regulation of leukocyte apoptotic	19/2734	81/17381	0.04425	0.731	0.719	ADAM8/BCL6/BMP4/CCL19/CCL21/CCL5/CD27/CD74/CDKN2A/EFNA1/HCAR2/IDO1/LGALS3/LGALS9/NOC2L/PDCD1/PRELID1/TSC22D3/TSC22D4	19	BP
GO:0006720	isoprenoid metabolic process	28/2734	129/17381	0.04431	0.731	0.719	AGRN/ALDH8A1/APOB/APOC3/CLPS/COQ2/CYP1A1/CYP1A2/DGAT1/DPM2/EGFR/GPC1/GPC2/GPIHBP1/HSPG2/LRP1/NPC2/OPN1MW/PDSS1/PLB1/PMVK/PPARD/RARRES2/RBP1/RBP2/SDC1/STRA6/TH	28	BP

GO:0034612	response to tumor necrosis factor	58/2734	297/17381	0.04439	0.731	0.719	ADAMTS13/ADAMTS7/APOB/BRCA1/CASP1/CASP3/CCDC3/CCL1/CCL19/CCL21/CCL5/CD27/CD58/CIB1/COL1A1/DAB2IP/ENDOG/F2RL1/GBA/GCH1/GPER1/IKBKB/IL18BP/ILK/KARS/KAT2A/LTA/LTBR/MADD/MAP3K14/PELI3/PID1/PRPF8/PSMB11/PSMB6/PSMB7/PSMB8/PSMC3/PSMD13/PSMD3/PSMD5/PSMD7/PTK2B/PYDC1/RELT/RNF31/SHARPIN/SLC2A4/SMPD4/STAT1/TDGF1/TNFRSF12A/TNFRSF13B/TNFRSF18/TNFRSF4/TNFSF13/TRAF	58	BP
GO:0006921	cellular component disassembly involved in execution phase	9/2734	31/17381	0.04442	0.731	0.719	BOK/CAPN10/CASP3/CDK5RAP3/ENDOG/ERN2/GPER1/HSF1/SHARPIN	9	BP
GO:0018393	internal peptidyl-lysine acetylation	32/2734	151/17381	0.04492	0.7353	0.72322	APBB1/ATF2/BRCA1/BRPF1/CRTC2/CTBP1/EPC1/HCFC1/HDAC8/ING4/KAT2A/LDB1/MAPK3/MSL3/MUC1/MYOD1/NAA60/NAT8B/NOC2L/PAXIP1/PCGF2/PER1/PIWIL2/POLE4/POR/PYGO2/RPS6KA4/SPI1/TADA3/TAF1/TAF1	32	BP
GO:0044236	multicellular organism metabolic process	32/2734	151/17381	0.04492	0.7353	0.72322	ADAMTS14/ADAMTS2/BMP4/CIITA/COL1A1/COL4A2/COL7A1/CTSD/CTSL/ENG/FURIN/MIR29A/MIR92A2/MMP15/MMP2/MMP3/MRC2/P3H1/P3H3/PDGFRB/PHYKPL/PLA2G1B/PLOD3/PM20D1/PPARD/SCX/SERPINF2/SERPINH1/THADA/TNS2/TRPV4/UCP3	32	BP
GO:0018212	peptidyl-tyrosine modification	72/2734	378/17381	0.04528	0.7355	0.72342	ABL1/ADRA2A/ADRA2B/ADRA2C/AGT/ARL2BP/ARRB2/BAZ1B/BLK/CCL5/CD74/CD81/CLCF1/CSF1R/CSF3/CSK/DDR2/DGKQ/DOK7/DVL2/EFNA1/EGFR/EPHA8/EPHB3/EPHB4/ERBB2/FES/FGF17/FGF3/FGFR2/FGFR4/FLT4/GRAP/HSF1/HSP90AA1/IL13/IL18/IL31RA/IL5RA/INPP5F/INS/KDR/LCK/LTK/MAP2K3/MAPK3/MIR221/MLST8/MTOR/NCF1/NRG1/PARP9/PDGFA/PDGFRB/PKDCC/PPP2R5B/PRKCD/PTK2B/SH3BP5/SH3BP5L/SHC1/TDGF1/TEC/THY1/TIE1/TNFRSF18/TNK1/TNK2/TREM2/UNC119/VPS25/WNT3A	72	BP
GO:0050772	positive regulation of	18/2734	76/17381	0.0453	0.7355	0.72342	AMIGO1/ANAPC2/DISC1/FN1/ILK/ISLR2/L1CAM/LIMK1/METRN/NRG1/PLXNB1/PLXNB3/PLXND1/SEMA7A/TNFRSF12A/TRPV2/WNT3A/ZFYVE27	18	BP
GO:1901605	alpha-amino acid metabolic process	44/2734	218/17381	0.04568	0.7355	0.72342	AGMAT/ALDH4A1/ASL/ASNS/BHMT2/BLMH/CARNS1/CRYM/DLST/DPEP1/FAH/GAD1/GLUD1/GLYATL1/GNMT/GOT2/GPT/GSTZ1/HAAO/HDC/HNMT/HYKK/IDO1/MTHFD1/NAGS/NMNAT3/NOS3/NOX4/OAT/OGDH/OTC/PADI4/PEMT/PHGDH/PHYKPL/PIPOX/PLOD3/SARS/SHMT2/SLC6A8/SLC7	44	BP

GO:0007398	ectoderm development	7/2734	22/17381	0.04612	0.7355	0.72342	GRHL3/ITGAM/LHX1/SHH/VAX2/VPS52/ZBTB17	7	BP
GO:0030826	regulation of cGMP biosynthetic process	7/2734	22/17381	0.04612	0.7355	0.72342	GUCA1B/GUCA2A/GUCA2B/NOS3/PDZD3/PTK2B/RUNDC3A	7	BP
GO:0033561	regulation of water loss via	7/2734	22/17381	0.04612	0.7355	0.72342	CLDN4/FLG2/GBA/GRHL3/KRT16/SFN/TMEM79	7	BP
GO:0042744	hydrogen peroxide	7/2734	22/17381	0.04612	0.7355	0.72342	EPX/GPX1/GPX3/LPO/MPO/PRDX1/PRDX6	7	BP
GO:0045956	positive regulation of calcium ion-dependent	7/2734	22/17381	0.04612	0.7355	0.72342	CACNA1G/CACNA1H/CDK5/CDK5R2/DOC2B/STX1A/STXBP1	7	BP
GO:0031346	positive regulation of cell projection organization	65/2734	338/17381	0.04634	0.7355	0.72342	AGRN/AGT/AMIGO1/ANAPC2/APBB1/AQP1/ARAP1/ARPC2/BCAS3/BRK1/CAMK1D/CCL19/CCL21/CDC42EP2/CORO1B/CPNE5/CPNE9/CROCC/DAB2IP/DEF8/DISC1/EPS8L2/ESPN/F2RL1/FES/FN1/FOXO6/FSCN1/HRAS/HSPA5/ILK/INS/ISLR2/KAT2A/KATNB1/L1CAM/LIMK1/LLPH/LTK/MARK2/METRN/MIR221/MIR222/MTOR/NME1/NRG1/OBSL1/PFN1/PLEKHM1/PLXNB1/PLXNB3/PLXND1/PPP2R5B/PROM2/PTK2B/SCARF1/SEMA7A/SEPT9/SERPINI1/TNFRSF12A/TRPV2/WNT1/WNT3A/WRAP73/ZFYVE27	65	BP
GO:0006778	porphyrin-containing compound metabolic	10/2734	36/17381	0.04654	0.7355	0.72342	ALAD/ALAS2/ATPIF1/COX10/CYP1A1/CYP1A2/HMBS/HMOX2/NFE2L1/UROS	10	BP
GO:0010464	regulation of mesenchymal cell	10/2734	36/17381	0.04654	0.7355	0.72342	BMP4/FBXW4/FGFR2/LMNA/MYCN/PDGFA/SHH/SMO/STAT1/WNT11	10	BP
GO:0034314	Arp2/3 complex-mediated actin	10/2734	36/17381	0.04654	0.7355	0.72342	ARFIP1/ARPC1A/ARPC1B/ARPC2/ARPC4/BRK1/CORO1B/HIP1R/WAS/WHAMM	10	BP

GO:0060612	adipose tissue development	10/2734	36/17381	0.04654	0.7355	0.72342	ATF2/CDK4/CSF1/PAXIP1/PLA2G16/PPARD/RORC/SH3PXD2B/SLC25A25/SOX8	10	BP
GO:0010769	regulation of cell morphogenesis involved in differentiation	51/2734	258/17381	0.04684	0.7369	0.72475	ABL1/AMIGO1/ANAPC2/ARC/ARHGAP4/ARPC2/BARHL2/CDK5/CFL1/CIB1/CTTN/DAB1/DISC1/DOCK1/DRAXIN/EFNA1/EPHB3/FGA/FLNA/FN1/GORASP1/GRIN1/ILK/ISLR2/KNDC1/L1CAM/LIMK1/LINGO1/MARK2/METRN/MYOC/NLGN3/NRG1/NSMF/OBSL1/OLFM4/PDLIM5/PLXNB1/PLXNB3/PLXND1/SEMA3F/SEMA7A/TBR1/THY1/TLX2/TNFRSF12A/TNIK/TRPV2/WN	51	BP
GO:0014068	positive regulation of phosphatidylinositol 3-kinase signaling	16/2734	66/17381	0.04712	0.7369	0.72475	AGT/CCL5/CSF3/F2RL1/GPER1/IL18/INS/KDR/MYOC/NCF1/NRG1/PDGFA/PDGFRB/PLXNB1/PPARD/SELP	16	BP
GO:1902108	regulation of mitochondrial membrane permeability involved in apoptotic	16/2734	66/17381	0.04712	0.7369	0.72475	ATF2/ATPIF1/BAD/BOK/DYNLL2/FZD9/HIP1R/MIR29A/MIR29C/MOAP1/PPP1R13B/RHOT2/SFN/SLC25A5/YWHAQ/ZNF205	16	BP
GO:1905710	positive regulation of membrane	16/2734	66/17381	0.04712	0.7369	0.72475	ATF2/ATPIF1/BAD/BOK/DYNLL2/FZD9/HIP1R/MIR29A/MIR29C/MOAP1/PPP1R13B/RHOT2/SFN/SLC25A5/YWHAQ/ZNF205	16	BP
GO:0090382	phagosome maturation	11/2734	41/17381	0.0478	0.738	0.72581	ATP6V0B/ATP6V0E2/ATP6V1B1/ATP6V1F/ATP6V1G1/ATP6V1G2/CORO1A/MYO7A/RAB20/RAB34/RAB7A	11	BP
GO:0099601	regulation of neurotransmitter receptor	11/2734	41/17381	0.0478	0.738	0.72581	ARC/CNIH2/DAPK1/DLG4/FNTA/LYNX1/LYPD6B/NLGN3/OPRM1/PSCA/PTK2B	11	BP
GO:0048013	ephrin receptor signaling	20/2734	87/17381	0.04803	0.738	0.72581	AP2A2/AP2M1/APH1A/ARPC1A/ARPC1B/ARPC2/ARPC4/DNM1/EFNA1/EFNA3/EFNA4/EFNB1/EPHA8/EPHB3/EPHB4/EPHB6/GRIN1/HRAS/MMP2/PT	20	BP

GO:0002478	antigen processing and presentation of exogenous	36/2734	174/17381	0.04812	0.738	0.72581	ACTR1A/AP1S1/AP2A2/AP2M1/CAPZA3/CD74/CTSD/CTSE/CTSF/CTSL/DCTN2/DYNC1H1/DYNC1I1/DYNLL2/HLA-E/IKBKB/ITGB5/KIF23/KIF26A/KIF2B/KIF4A/KLC1/KLC2/NCF1/PSMB11/PSMB6/PSMB7/PSMB8/PSMC3/PSMD13/PSMD3/PSMD5/PSMD7/RAB7A/SPTB	36	BP
GO:0050731	positive regulation of peptidyl-tyrosine	36/2734	174/17381	0.04812	0.738	0.72581	ABL1/ADRA2A/ADRA2B/ADRA2C/AGT/ARL2BP/ARRB2/CCL5/CD74/CD81/CLCF1/CSF1R/CSF3/DGKQ/DOK7/DVL2/EFNA1/HSF1/IL13/IL18/IL31RA/INS/MIR221/MLST8/MTOR/NCF1/NRG1/PARP9/PTK2B/TDGF1/TEC/TNFRSF18/TNK2/TREM2/UNC119/WNT3A	36	BP
GO:0001954	positive regulation of cell-matrix	12/2734	46/17381	0.04841	0.738	0.72581	ABL1/CCL21/CDH13/CIB1/COL16A1/CSF1/DISC1/ILK/KDR/MYOC/PTK2B/THY1	12	BP
GO:2000107	negative regulation of leukocyte	12/2734	46/17381	0.04841	0.738	0.72581	BCL6/BMP4/CCL19/CCL21/CCL5/CD27/CD74/EFNA1/IDO1/NOC2L/TSC22D3/TSC22D4	12	BP
GO:0045851	pH reduction	13/2734	51/17381	0.04855	0.738	0.72581	ATP6V0B/ATP6V0E2/ATP6V1B1/ATP6V1F/ATP6V1G1/ATP6V1G2/CA7/CLN6/FASLG/GPR89A/RAB20/RAB7A/SLC11A1	13	BP
GO:1905330	regulation of morphogenesis of an epithelium	37/2734	180/17381	0.04964	0.738	0.72581	ABL1/AGT/AJAP1/AP2A2/AP2M1/ARRB2/BMP4/CAV3/CELSR2/CELSR3/DVL2/FGFR2/FZD2/GRHL3/HOXB7/LHX1/MIR221/MTOR/PARD6A/PDGFA/PFN1/PSMB11/PSMB6/PSMB7/PSMB8/PSMC3/PSMD13/PSMD3/PSMD5/PSMD7/SHH/SMO/SOX8/STAT1/TBX2/WNT1/WNT11	37	BP
GO:0019886	antigen processing and presentation of exogenous peptide antigen	22/2734	98/17381	0.04997	0.738	0.72581	ACTR1A/AP1S1/AP2A2/AP2M1/CAPZA3/CD74/CTSD/CTSE/CTSF/CTSL/DCTN2/DYNC1H1/DYNC1I1/DYNLL2/KIF23/KIF26A/KIF2B/KIF4A/KLC1/KLC2/RAB7A/SPTBN2	22	BP
GO:0032963	collagen metabolic process	27/2734	125/17381	0.05017	0.738	0.72581	ADAMTS14/ADAMTS2/BMP4/CIITA/COL1A1/COL4A2/COL7A1/CTSD/CTSL/ENG/FURIN/MIR29A/MIR92A2/MMP15/MMP2/MMP3/MRC2/P3H1/P3H3/PDGFRB/PHYKPL/PLOD3/PPARD/SCX/SERPINF2/SERPINH1/TNS2	27	BP

GO:0070838	divalent metal ion transport	85/2734	456/17381	0.05029	0.738	0.72581	ABL1/ADRA1A/ADRA2A/AGT/AHNAK/ARRB2/ATP13A2/ATP2A3/ATP2C2/BSPRY/CACNA1F/CACNA1G/CACNA1H/CACNA1S/CACNB1/CACNB3/CACNG1/CAMK2D/CATSPER1/CAV3/CCL19/CCL21/CCL5/CDH23/CDK5/CNNM2/CORO1A/CRACR2A/CRHR1/DRD2/FASLG/GCG/GCK/GNAO1/GNB5/GPER1/GPR35/GRIN1/GRIN2C/GSTM2/HSPA2/IL13/ITPR3/JPH3/LCK/LGALS3/MIR328/NIPA2/NIPAL1/NIPAL2/NOS3/NPPA/NPSR1/OPRD1/OPRM1/ORAI1/ORAI3/P2RX2/PACSIN3/PDE2A/PDGFRB/PKD1/PLA2G1B/PMPCA/PTK2B/RASA3/RRAD/SLC11A1/SLC25A25/SLC30A3/SLC39A13/SLC39A5/TFR2/THADA/THY1/TLR9/TMEM37/TRDN/TRPM5/TRPV2/TRPV4/TRPV6/WFS1 [illegible]	85	BP
GO:0007519	skeletal muscle tissue development	33/2734	158/17381	0.05045	0.738	0.72581	ACTA1/ANKRD2/ATF3/BCL9/BIN3/CDK5/EGR1/EMD/GPC1/GPX1/HIVEP3/HLX/KLHL40/LEMD2/MSC/MYL6B/MYOD1/NOTCH1/NUPR1/P2RX2/PHOX2B/PITX1/SCX/SHH/SMO/SOX8/SRPK3/TSC22D3/USP19/VAMP5/VAX1/V	33	BP
GO:0006359	regulation of transcription from RNA polymerase III	8/2734	27/17381	0.05064	0.738	0.72581	BRCA1/BRF1/CHD8/ERBB2/GTF3C1/MAF1/MTOR/ZNF76	8	BP
GO:0032647	regulation of interferon-alpha production	8/2734	27/17381	0.05064	0.738	0.72581	HAVCR2/IRF5/NMI/SETD2/STAT1/TBK1/TLR8/TLR9	8	BP
GO:0090383	phagosome acidification	8/2734	27/17381	0.05064	0.738	0.72581	ATP6V0B/ATP6V0E2/ATP6V1B1/ATP6V1F/ATP6V1G1/ATP6V1G2/RAB20/RAB7A	8	BP
GO:0016226	iron-sulfur cluster	6/2734	18/17381	0.05121	0.738	0.72581	CIAPIN1/FAM96B/ISCA2/NARFL/NUBP1/NUBP2	6	BP
GO:0031163	metallo-sulfur cluster	6/2734	18/17381	0.05121	0.738	0.72581	CIAPIN1/FAM96B/ISCA2/NARFL/NUBP1/NUBP2	6	BP
GO:0048243	norepinephrine secretion	6/2734	18/17381	0.05121	0.738	0.72581	ADRA2A/ADRA2B/ADRA2C/AGT/NISCH/STX1A	6	BP
GO:1901881	positive regulation of protein	6/2734	18/17381	0.05121	0.738	0.72581	CFL1/F2RL1/KATNB1/NES/TRPV4/WDR1	6	BP

GO:0014902	myotube differentiation	24/2734	109/17381	0.05128	0.738	0.72581	ACTA1/ADAM12/ADGRB1/ANKRD2/BARX2/BCL9/BIN3/CACNA1H/CAV3/EHD1/GPX1/IL4R/KLHL40/MTOR/MYOD1/NKX2-5/NOTCH1/P2RX2/SHH/SMO/SMYD3/THRA/TRIM72/WNT1	24	BP
GO:0051261	protein depolymerizatio	21/2734	93/17381	0.05163	0.738	0.72581	CAPZA3/CFL1/CIB1/F2RL1/GAK/KATNB1/KIF2B/LMOD1/MAP6D1/MICAL2/MID1IP1/NCKAP5L/NES/SCIN/SPTAN1/SPTB/SPTBN2/STMN4/TRIM54/T	21	BP
GO:0032956	regulation of actin cytoskeleton organization	57/2734	294/17381	0.05165	0.738	0.72581	ABL1/ARAP1/ARFIP1/ARHGEF10/ARPC1A/ARPC1B/ARPC2/ARPC4/BAIAP2L1/BCAS3/BRK1/CAPZA3/CAV3/CCL21/CDC42EP2/CDK5/CFL1/CORO1A/CORO1B/CSF1R/CSF3/CTTN/EVL/F2RL1/FES/FHOD1/FSCN1/GRHL3/HIP1R/HRAS/ILK/LIMK1/LMOD1/LRP1/MLST8/MTOR/MYO1C/MYOC/NOX4/PDGFA/PDGFRB/PFN1/PRKCD/PTK2B/RHOD/SCIN/SERPINF2/SH3PXD2B/SORBS3/SPTAN1/SPTB/SPTBN2/TACR1/WAS/WDR1/WHAMM/WNT11	57	BP
GO:0016049	cell growth	90/2734	486/17381	0.05175	0.738	0.72581	ABL1/ADRA1A/AGT/ALCAM/ANAPC2/APBB1/ARHGAP4/ATAD3A/BARHL2/BCL6/BIN3/BLZF1/BRAT1/CAMK2D/CAV3/CCAR2/CCDC85B/CDA/CDK5/CDKN2A/CIB1/CISH/CLSTN1/CLSTN3/CPNE5/CPNE9/CRYAB/CTTN/DISC1/DNPH1/DRAXIN/EGFR/EIF2AK4/EIF4G1/EMX1/ERBB2/ESR2/EXOSC2/FN1/GATA4/GDF2/HSPG2/HTRA3/IGFBP3/IGFBP6/ILK/INO80/INS/ISLR2/KIF26A/L1CAM/LEFTY2/LIMK1/LLPH/MIR195/MTOR/NLGN3/NPPA/NRG1/NRN1L/NUBP1/NUPR1/OSGIN1/PDLIM5/PLXNB1/PLXNB3/PPARD/PPP1R9B/PTK2B/RASGRP2/RPS6KA1/RRAGC/SEMA3F/SEMA5B/SEMA7A/SFN/SGK1/SIPA1/SLC44A4/TAOK2/TNFRSF12A/TNK1/TNN/TRIM40/TRPV2/TSPXL2/ULK1/WNT11/WNT3A/ZFYVE27	90	BP
GO:0009582	detection of abiotic stimulus	28/2734	131/17381	0.05254	0.738	0.72581	AIPL1/ANO1/ARRB2/ASIC2/ASIC3/CACNA1F/CAV3/CNGB1/FNTA/FNTB/GNAT1/GUCA1B/GUCY2D/KCNK4/LHFPL5/OPN1MW/OPN4/PHF24/PITPNM1/PKD1/PPEF1/PRDM12/SEMA5B/TACR1/TCAP/TIMELESS/TULP1/UNC1	28	BP
GO:0048524	positive regulation of viral process	23/2734	104/17381	0.05315	0.738	0.72581	CCL5/CD74/CDK9/CFL1/CHMP4C/DDB1/FKBP6/HACD3/LGALS9/NELFB/NOTCH1/PC/PFN1/POLR2G/POLR2L/PPIB/PPIE/RAB7A/RSF1/TARBP2/TRIM11/VPS37B/ZNF502	23	BP
GO:0048839	inner ear development	37/2734	181/17381	0.05327	0.738	0.72581	ANP32B/ATP6V1B1/BMP4/C1QB/CDH23/DVL2/EYA1/FGFR2/FZD2/GBX2/GRHL3/HMX2/IFT20/JAG2/KCNQ1/KCNQ4/LHFPL5/LHX3/LRP10/LRTOMT/MCM2/MYCN/MYO3A/MYO7A/NAGLU/NOTCH1/PDGFRB/PHOX2B/PTPN11/SCRIB/SHH/SLC44A4/TCAP/TMIE/WDPCP/WNT1/WNT3A	37	BP

GO:0000305	response to oxygen radical	9/2734	32/17381	0.05348	0.738	0.72581	ADPRHL2/GCH1/MPO/NOS3/PRDX1/SOD3/TXNRD1/TXNRD3/UCP3	9	BP
GO:0010591	regulation of lamellipodium assembly	9/2734	32/17381	0.05348	0.738	0.72581	AQP1/ARPC2/BIN3/BRK1/FSCN1/MTOR/PLXNB3/WAS/WNT1	9	BP
GO:0014072	response to isoquinoline alkaloid	9/2734	32/17381	0.05348	0.738	0.72581	DRD2/GNAO1/GRIN1/MTOR/OPRM1/PEA15/PPP1R1B/PPP1R9B/TACR1	9	BP
GO:0043278	response to morphine	9/2734	32/17381	0.05348	0.738	0.72581	DRD2/GNAO1/GRIN1/MTOR/OPRM1/PEA15/PPP1R1B/PPP1R9B/TACR1	9	BP
GO:1902624	positive regulation of neutrophil	9/2734	32/17381	0.05348	0.738	0.72581	ADAM8/C3AR1/CAMK1D/CCL19/CCL21/CD74/DAPK2/PF4V1/TIRAP	9	BP
GO:0010822	positive regulation of mitochondrion organization	35/2734	170/17381	0.0539	0.738	0.72581	ACSM6/APOPT1/ATPIF1/BAD/BOK/CYB5R1/DYNLL2/ELMOD1/GBA/GPER1/GPHA2/HIP1R/HNMT/HRK/ITGAX/KAT2A/KDR/LRRC46/MFN2/MIEF2/MOAP1/MYBPC1/NBPF3/NRG1/OSCP1/PEMT/PPP1R13B/PSMB7/RNF31/SFN/TSGA13/UBL4B/YWHAQ/ZBTB17/ZNF205	35	BP
GO:0042246	tissue regeneration	15/2734	62/17381	0.05426	0.738	0.72581	APOA5/BCL9/BIN3/FZD9/GJD4/GPX1/LGR6/MUSTN1/MYOD1/NINJ2/NOTCH1/PKM/PPARD/TEC/WNT7A	15	BP
GO:0001578	microtubule bundle	19/2734	83/17381	0.05498	0.738	0.72581	CC2D2A/CCDC103/CCDC40/CCSER2/CFAP157/CFAP74/DNAJB13/DNHD1/FES/GAS8/HYDIN/LRRC6/NCKAP5L/NUMA1/SPAG16/TMEM141/TRIM46/	19	BP
GO:0006029	proteoglycan metabolic	19/2734	83/17381	0.05498	0.738	0.72581	B3GAT3/B4GALT7/BCAN/BGN/CHPF/CHPF2/CHST12/CHST13/CHST15/CHST7/DSE/GAL3ST4/GPC1/HEXA/IDS/PPARD/SGSH/SPOCK2/XYLT2	19	BP
GO:0008625	extrinsic apoptotic signaling pathway via	19/2734	83/17381	0.05498	0.738	0.72581	ATF3/BAD/BRCA1/DAB2IP/DAPK1/FASLG/FGA/GPX1/LGALS3/MADD/MIR221/MIR222/MOAP1/NOS3/PEA15/RAF1/STX4/TMBIM1/TRAF2	19	BP
GO:0060191	regulation of lipase activity	19/2734	83/17381	0.05498	0.738	0.72581	ABL1/ADRA1A/AGT/ANG/APOA5/APOC3/CCL5/EGFR/FGFR2/FURIN/GPIHBP1/LMF1/LRP1/NR1H3/PDGFRB/PLA2G1B/PLA2G5/PLCB2/POR	19	BP
GO:0019068	virion assembly	10/2734	37/17381	0.05512	0.738	0.72581	CHMP1A/CHMP4C/CHMP7/LRSAM1/PC/SNF8/USP6NL/VPS37B/VPS37C/VPS37D	10	BP

GO:0042092	type 2 immune response	10/2734	37/17381	0.05512	0.738	0.72581	BCL6/CCR2/CD74/ECM1/HLX/IDO1/IL18/IL31RA/IL4R/RARA	10	BP
GO:0045742	positive regulation of epidermal growth factor receptor	10/2734	37/17381	0.05512	0.738	0.72581	ADRA2A/ADRA2B/ADRA2C/AGT/ARAP1/DOK1/FASLG/GPER1/HIP1R/NCF1	10	BP
GO:0042490	mechanoreceptor differentiation	14/2734	57/17381	0.05513	0.738	0.72581	BMP4/CDH23/FZD2/IFT20/JAG2/LHFPL5/LRTOMT/MYCN/MYO7A/NAGLU/NOTCH1/SCRIB/SLC44A4/WDPCP	14	BP
GO:0006244	pyrimidine nucleotide catabolic	5/2734	14/17381	0.05574	0.738	0.72581	MBD4/NEIL2/NT5M/OGG1/SMUG1	5	BP
GO:0006684	sphingomyelin metabolic	5/2734	14/17381	0.05574	0.738	0.72581	PEMT/PRKCD/SGMS1/SMPD4/SPTLC1	5	BP
GO:0021527	spinal cord association neuron	5/2734	14/17381	0.05574	0.738	0.72581	LHX1/LHX3/LHX5/WNT1/WNT3A	5	BP
GO:0021535	cell migration in hindbrain	5/2734	14/17381	0.05574	0.738	0.72581	CEND1/CTNNA2/DAB1/FLNA/PHOX2B	5	BP
GO:0032930	positive regulation of superoxide	5/2734	14/17381	0.05574	0.738	0.72581	AGT/EGFR/F2RL1/ITGAM/PRKCD	5	BP
GO:0035864	response to potassium ion	5/2734	14/17381	0.05574	0.738	0.72581	ADAMTS13/CACNA1H/CYP11B2/HSF1/SLC34A1	5	BP
GO:0070571	negative regulation of neuron projection	5/2734	14/17381	0.05574	0.738	0.72581	INPP5F/LRIG2/RGMA/RTN4RL1/THY1	5	BP
GO:2001212	regulation of vasculogenesis	5/2734	14/17381	0.05574	0.738	0.72581	ADM/HEY1/KDR/TMEM100/XDH	5	BP

GO:0010043	response to zinc ion	13/2734	52/17381	0.05576	0.738	0.72581	ALAD/ATP13A2/BGLAP/CRIP1/GGH/GLRA1/HAAO/KRT14/MT2A/OTC/SLC30A3/SLC39A13/TH	13	BP
GO:0038083	peptidyl-tyrosine autophosphoryl	13/2734	52/17381	0.05576	0.738	0.72581	ABL1/BLK/CSK/EGFR/FES/GRAP/KDR/LCK/MAPK3/PTK2B/TEC/TNK1/TNK2	13	BP
GO:0060113	inner ear receptor cell	13/2734	52/17381	0.05576	0.738	0.72581	BMP4/CDH23/FZD2/IFT20/JAG2/LHFPL5/LRTOMT/MYCN/MYO7A/NAGLU/NOTCH1/SCRIB/WDPCP	13	BP
GO:0035088	establishment or maintenance of apical/basal cell polarity	11/2734	42/17381	0.0559	0.738	0.72581	CDX2/CRB2/DLG4/EYA1/FSCN1/ILK/LRCH4/MARK2/SCRIB/WDR1/WNT11	11	BP
GO:0061245	establishment or maintenance of bipolar cell polarity	11/2734	42/17381	0.0559	0.738	0.72581	CDX2/CRB2/DLG4/EYA1/FSCN1/ILK/LRCH4/MARK2/SCRIB/WDR1/WNT11	11	BP
GO:0070316	regulation of G0 to G1	11/2734	42/17381	0.0559	0.738	0.72581	BRCA1/DAB2IP/DUX4/EHMT1/EHMT2/EPC1/FOXO4/MAX/PCGF2/PPP2R5B/UXT	11	BP
GO:1903170	negative regulation of calcium ion transmembrane	11/2734	42/17381	0.0559	0.738	0.72581	ADRA2A/CRHR1/DRD2/GNB5/GPR35/GSTM2/MIR328/RRAD/THADA/TLR9/TRDN	11	BP
GO:0002067	glandular epithelial cell	12/2734	47/17381	0.05606	0.738	0.72581	BAD/BMP4/FGFR2/IL13/IL31RA/MEN1/NOTCH1/RARA/RFX6/SIDT2/SMO/SPDEF	12	BP
GO:0006220	pyrimidine nucleotide metabolic	12/2734	47/17381	0.05606	0.738	0.72581	AK5/DHODH/ERH/MBD4/NEIL2/NME1/NME4/NT5M/OGG1/SMUG1/TBPL1/UCK1	12	BP
GO:0031529	ruffle organization	12/2734	47/17381	0.05606	0.738	0.72581	CCL21/CORO1B/CSF1R/DEF8/EPS8L2/EVL/HRAS/INPPL1/MTOR/PFN1/PLEKHM1/WDPCP	12	BP
GO:0032964	collagen biosynthetic process	12/2734	47/17381	0.05606	0.738	0.72581	BMP4/CIITA/COL1A1/ENG/MIR29A/MIR92A2/P3H3/PDGFRB/PPARD/SCX/SERPINF2/SERPINH1	12	BP

GO:0007631	feeding behavior	24/2734	110/17381	0.05618	0.738	0.72581	AGT/CNTFR/DMBX1/DRD2/EIF2AK4/EN1/GCG/GPR88/GRIN1/INS/LEPR/MMP17/MTOR/NPW/NPY4R/OPRD1/OPRM1/PYY/REN/STRA6/TACR1/TBR1/	24	BP
GO:0031333	negative regulation of protein complex	26/2734	121/17381	0.0568	0.738	0.72581	CAPZA3/CLU/CRYAB/EIF4EBP1/GBA/HIP1R/INS/LCMT1/LMOD1/OPRD1/PEX14/PEX5/PFN1/PRKCD/RAF1/SCIN/SMAD6/SOST/SPTAN1/SPTB/SPTBN2/STXBP1/THRA/TRAF3IP1/ULK1/VDAC2	26	BP
GO:0098656	anion transmembrane transport	53/2734	273/17381	0.05754	0.738	0.72581	ABCC3/ABCC4/ABCC5/ABCC6/ACACB/AGT/ANO1/ANO2/ANO4/ANO7/BEST3/BEST4/BSND/CLCN2/CLCNKA/CLCNKB/CLDN4/CLIC1/CPT2/GLRA1/GPR89A/MID1IP1/MTOR/OSR1/PQLC2/SLC12A4/SLC12A7/SLC12A9/SLC13A2/SLC1A7/SLC20A1/SLC20A2/SLC22A9/SLC25A20/SLC25A22/SLC26A1/SLC26A10/SLC26A6/SLC34A1/SLC35A2/SLC36A1/SLC36A3/SLC38A1/SLC38A10/SLC38A8/SLC4A9/SLC6A18/SLC6A6/SLC6A7/SLC6A8/SLC6A9/SLC7A	53	BP
GO:0050730	regulation of peptidyl-tyrosine phosphorylation	46/2734	233/17381	0.05757	0.738	0.72581	ABL1/ADRA2A/ADRA2B/ADRA2C/AGT/ARL2BP/ARRB2/CCL5/CD74/CD81/CLCF1/CSF1R/CSF3/DGKQ/DOK7/DVL2/EFNA1/EGFR/HSF1/IL13/IL18/IL31RA/INPP5F/INS/MIR221/MLST8/MTOR/NCF1/NRG1/PARP9/PDGFA/PPP2R5B/PRKCD/PTK2B/SH3BP5/SH3BP5L/SHC1/TDGF1/TEC/THY1/TNFRSF18/TNK2/TREM2/UNC119/VPS25/WNT3A	46	BP
GO:0008045	motor neuron axon guidance	7/2734	23/17381	0.05761	0.738	0.72581	ALCAM/CDK5/ERBB2/LHX1/LHX3/LHX4/SEMA3F	7	BP
GO:0044788	modulation by host of viral	7/2734	23/17381	0.05761	0.738	0.72581	CFL1/EIF2AK4/MIR221/MIR222/PC/PPIB/ZNF502	7	BP
GO:0051953	negative regulation of	7/2734	23/17381	0.05761	0.738	0.72581	ADRA2A/ADRA2B/ADRA2C/CHGA/DRD2/OSR1/TRH	7	BP
GO:0071157	negative regulation of	7/2734	23/17381	0.05761	0.738	0.72581	CDK4/CDK9/FOXE3/FZD9/GPER1/MLXIPL/PHOX2B	7	BP

GO:0001933	negative regulation of protein phosphorylation	79/2734	424/17381	0.05777	0.738	0.72581	ABL1/ARRB2/ATF3/BGN/BMP4/C1QL4/CAMK2N2/CASP3/CAV3/CDK5RAP3/CDKN2A/CHAD/CIB1/CISH/CRIPAK/CSK/CTDSP1/DAB2IP/DGKQ/DTNBP1/DUSP2/DUSP21/DUSP26/DUSP5/EFNA1/EIF4G1/ENG/F2RL1/FKTN/FOXM1/GBA/GPER1/HSPB1/IGFBP3/ILK/INCA1/INPP5F/LEMD2/LRRTM1/MAPK8IP1/MARVELD3/MEN1/MIR221/MIR92A2/MLXIPL/MTOR/NCOR1/NDRG2/PARD3/PARD6A/PBLD/PDCD4/PER1/PID1/PPP1R1B/PREX2/PRKAR1A/PRKAR1B/PRKCD/PRKRIP1/PSCA/PYDC1/RGS14/RTN4RL1/RTN4RL2/SFN/SH3BP5/SH3BP5L/SMAD6/SMYD3/TAF7/TARBP2/THY1/TRAF3IP1/UBE2B[illegible]	79	BP
GO:0006285	base-excision repair, AP site formation	4/2734	10/17381	0.05809	0.738	0.72581	MBD4/NEIL2/OGG1/SMUG1	4	BP
GO:0007168	receptor guanylyl cyclase	4/2734	10/17381	0.05809	0.738	0.72581	GUCA1B/GUCY2D/NPPA/PDZD3	4	BP
GO:0009629	response to gravity	4/2734	10/17381	0.05809	0.738	0.72581	BGLAP/FOSL1/PKM/STX1A	4	BP
GO:0021859	pyramidal neuron	4/2734	10/17381	0.05809	0.738	0.72581	DCLK2/DISC1/FGFR2/OGDH	4	BP
GO:0021932	hindbrain radial glia guided cell migration	4/2734	10/17381	0.05809	0.738	0.72581	CEND1/CTNNA2/DAB1/FLNA	4	BP
GO:0030259	lipid glycosylation	4/2734	10/17381	0.05809	0.738	0.72581	B4GALNT1/GBGT1/SLC35C1/ST3GAL4	4	BP
GO:0035878	nail development	4/2734	10/17381	0.05809	0.738	0.72581	FOXN1/HOXC13/KRT84/PRKAB1	4	BP
GO:0038003	opioid receptor signaling	4/2734	10/17381	0.05809	0.738	0.72581	OPRD1/OPRM1/PPP1R9B/SIGMAR1	4	BP
GO:0045217	cell-cell junction	4/2734	10/17381	0.05809	0.738	0.72581	CSF1R/F2RL1/KIFC3/PARD6A	4	BP

GO:0046501	protoporphyrinogen IX metabolic	4/2734	10/17381	0.05809	0.738	0.72581	ALAD/ALAS2/HMBS/UROS	4	BP
GO:0051006	positive regulation of lipoprotein	4/2734	10/17381	0.05809	0.738	0.72581	APOA5/GPIHBP1/LMF1/NR1H3	4	BP
GO:0060346	bone trabecula formation	4/2734	10/17381	0.05809	0.738	0.72581	CHAD/COL1A1/MMP2/THBS3	4	BP
GO:0060453	regulation of gastric acid secretion	4/2734	10/17381	0.05809	0.738	0.72581	HIP1R/KCNQ1/PTGER3/SGK1	4	BP
GO:0061469	regulation of type B pancreatic cell	4/2734	10/17381	0.05809	0.738	0.72581	MEN1/NR4A1/WDR13/WNT3A	4	BP
GO:0072584	caveolin-mediated	4/2734	10/17381	0.05809	0.738	0.72581	CAV3/MAPK3/PROM2/UNC119	4	BP
GO:0098792	xenophagy	4/2734	10/17381	0.05809	0.738	0.72581	LRSAM1/MAPK3/NOD1/TBK1	4	BP
GO:1990403	embryonic brain development	4/2734	10/17381	0.05809	0.738	0.72581	CC2D2A/EN1/IFT140/WNT1	4	BP
GO:2000586	regulation of platelet-derived growth factor receptor-beta signaling	4/2734	10/17381	0.05809	0.738	0.72581	HIP1R/LRP1/PDGFA/PDGFRB	4	BP
GO:0009636	response to toxic substance	44/2734	222/17381	0.05889	0.7465	0.73421	ADAMTS13/ALAD/AQP10/ASNS/BLMH/BPHL/CCL5/CDK4/CYP1A1/DDC/DNMT3A/DRD2/EHMT1/EPHX2/EPX/FAM213A/GCH1/GPX1/GPX2/GPX3/GSR/GSTM1/GSTM2/GSTZ1/HTR1D/KDM6B/LPO/MAPK3/MGST1/MGST3/MPO/NOS3/NUPR1/OPRD1/PDGFRB/PDZD3/PRDX1/PRDX6/SDC1/SESN1/SO	44	BP

GO:0043405	regulation of MAP kinase activity	64/2734	337/17381	0.05908	0.7473	0.73501	ADAM8/ADRA2A/ADRA2B/ARAF/AXIN1/BMP4/CAV3/CCL19/CD74/CD81/CDK5RAP3/CSK/DAB2IP/DTNBP1/DUSP2/DUSP21/DUSP5/DVL2/EGFR/ERBB2/ERN2/FZD8/GADD45G/GBA/GNG3/HACD3/HRAS/ILK/IRAK1/KARS/MADD/MAP2K3/MAP3K14/MAP3K15/MAP3K6/MAP4K2/MAPK3/MAPK8IP1/MAPK8IP3/MAPKAPK3/MIR92A2/NEK10/NOD1/NOX4/NRG1/PDCD4/PDGFA/PDGFRB/PEA15/PIK3R5/PIK3R6/PLA2G1B/PRKCD/PTK2B/PTPN11/RAF1/RGS14/SHC1/TAOK2/TDGF1/TLR9/TNIK/TRAF2/TRAF7	64	BP
GO:0072511	divalent inorganic cation transport	85/2734	460/17381	0.05972	0.7484	0.73612	ABL1/ADRA1A/ADRA2A/AGT/AHNAK/ARRB2/ATP13A2/ATP2A3/ATP2C2/BSPRY/CACNA1F/CACNA1G/CACNA1H/CACNA1S/CACNB1/CACNB3/CACNG1/CAMK2D/CATSPER1/CAV3/CCL19/CCL21/CCL5/CDH23/CDK5/CNNM2/CORO1A/CRACR2A/CRHR1/DRD2/FASLG/GCG/GCK/GNAO1/GNB5/GPER1/GPR35/GRIN1/GRIN2C/GSTM2/HSPA2/IL13/ITPR3/JPH3/LCK/LGALS3/MIR328/NIPA2/NIPAL1/NIPAL2/NOS3/NPPA/NPSR1/OPRD1/OPRM1/ORAI1/ORAI3/P2RX2/PACSIN3/PDE2A/PDGFRB/PKD1/PLA2G1B/PMPCA/PTK2B/RASA3/RRAD/SLC11A1/SLC25A25/SLC30A3/SLC39A13/SLC39A5/TFR2/THADA/THY1/TLR9/TMEM37/TRDN/TRPM5/TRPV2/TRPV4/TRPV6/WFS1[illegible]	85	BP
GO:0071887	leukocyte apoptotic process	22/2734	100/17381	0.06051	0.7484	0.73612	ADAM8/BCL6/BMP4/CCL19/CCL21/CCL5/CD27/CD74/CDKN2A/CTSL/DNAJA3/EFNA1/FASLG/HCAR2/IDO1/LGALS3/LGALS9/NOC2L/PDCD1/PRELID1/TSC22D3/TSC22D4	22	BP
GO:1990778	protein localization to cell periphery	49/2734	251/17381	0.06061	0.7484	0.73612	AP2M1/ARHGEF16/BLZF1/CACNB3/CAV3/CIB1/CNPY4/CSK/DPP6/EGFR/EPB41L2/FLNA/GAK/GPER1/GRIPAP1/IFT20/IKBKB/INS/KIF13A/LRP1/LYPD1/NKD2/NUBP1/NUMA1/PACS1/PID1/PKDCC/PKP3/RAB26/RAB34/RAB40C/RILPL1/RILPL2/SCRIB/STX4/STX8/STXBP1/TMBIM1/TMEM150A/TNIK/TREM2/TSPAN15/TSPAN33/TULP1/TULP3/VAMP5/VTI1B/WNT3A/ZFYV	49	BP
GO:0050891	multicellular organismal water	15/2734	63/17381	0.06126	0.7484	0.73612	AQP1/AVPR2/CLDN4/CYP11B2/FLG2/GBA/GRHL3/KRT16/PRKAR1A/PRKAR1B/SCNN1A/SFN/TMEM79/TRPV4/WFS1	15	BP
GO:0042303	molting cycle	24/2734	111/17381	0.06139	0.7484	0.73612	DNASE1L2/EGFR/FARP2/FGFR2/FOXN1/FOXQ1/HOXC13/KRT14/KRT16/KRT71/KRT84/KRTAP4-3/LDB1/NOTCH1/NSDHL/NUMA1/PDGFA/PER1/PTCH2/SHH/SMO/SPINK5/TMEM79/WNT10A	24	BP

GO:0042633	hair cycle	24/2734	111/17381	0.06139	0.7484	0.73612	DNASE1L2/EGFR/FARP2/FGFR2/FOXN1/FOXQ1/HOXC13/KRT14/KRT16/KRT71/KRT84/KRTAP4-3/LDB1/NOTCH1/NSDHL/NUMA1/PDGFA/PER1/PTCH2/SHH/SMO/SPINK5/TMEM79/WNT10A	24	BP
GO:0002026	regulation of the force of heart	8/2734	28/17381	0.06148	0.7484	0.73612	ADM/ADRA1A/ATP1A1/CAMK2D/CHGA/EDN2/MYL4/NOS3	8	BP
GO:0014912	negative regulation of smooth muscle	8/2734	28/17381	0.06148	0.7484	0.73612	APEX1/CORO1B/IGFBP3/ILK/LRP1/MIR15A/MIR503/PPARD	8	BP
GO:0032607	interferon-alpha production	8/2734	28/17381	0.06148	0.7484	0.73612	HAVCR2/IRF5/NMI/SETD2/STAT1/TBK1/TLR8/TLR9	8	BP
GO:0070207	protein homotrimerizati	8/2734	28/17381	0.06148	0.7484	0.73612	BRK1/CD247/EMILIN1/HSF1/MGST1/OTC/SIGMAR1/TRAF2	8	BP
GO:0006816	calcium ion transport	76/2734	408/17381	0.06194	0.7484	0.73612	ABL1/ADRA1A/ADRA2A/AGT/AHNAK/ARRB2/ATP13A2/ATP2A3/ATP2C2/BSPRY/CACNA1F/CACNA1G/CACNA1H/CACNA1S/CACNB1/CACNB3/CACNG1/CAMK2D/CATSPER1/CAV3/CCL19/CCL21/CCL5/CDH23/CDK5/CORO1A/CRACR2A/CRHR1/DRD2/FASLG/GCG/GCK/GNAO1/GNB5/GPER1/GPR35/GRIN1/GRIN2C/GSTM2/HSPA2/IL13/ITPR3/JPH3/LCK/LGALS3/MIR328/NOS3/NPPA/NPSR1/OPRD1/OPRM1/ORAI1/ORAI3/P2RX2/PACSIN3/PDE2A/PDGFRB/PKD1/PLA2G1B/PMPCA/PTK2B/RASA3/RRAD/SLC25A25/THADA/THY1/TLR9/TMEM37/TRDN/TRPM5/TRPV2/TRPV4/TRPV6/WFS1/WNT[illegible]	76	BP
GO:0032970	regulation of actin filament-based process	64/2734	338/17381	0.06205	0.7484	0.73612	ABL1/ARAP1/ARFIP1/ARHGEF10/ARPC1A/ARPC1B/ARPC2/ARPC4/ATP1A1/BAIAP2L1/BCAS3/BRK1/CAMK2D/CAPZA3/CAV3/CCL21/CDC42EP2/CDK5/CFL1/CORO1A/CORO1B/CSF1R/CSF3/CTTN/EVL/F2RL1/FES/FHOD1/FLNA/FSCN1/GATA4/GRHL3/HIP1R/HRAS/ILK/LIMK1/LMOD1/LRP1/MIR328/MLST8/MTOR/MYBPC3/MYO1C/MYOC/NOX4/PDGFA/PDGFRB/PFN1/PRKCD/PTK2B/RHOD/SCIN/SERPINF2/SH3PXD2B/SORBS3/SPTAN1/SPTB/SPTBN2/TACR1/TNNC1/WAS/WDR1/WHAMM/WNT11	64	BP
GO:0001947	heart looping	14/2734	58/17381	0.06257	0.7484	0.73612	C2CD3/CCDC103/CCDC40/ENG/GATA4/MICAL2/NKX2-5/NOTCH1/NOTO/SHH/SMO/SUFU/TBX2/WNT3A	14	BP

GO:0035082	axoneme assembly	14/2734	58/17381	0.06257	0.7484	0.73612	CC2D2A/CCDC103/CCDC40/CFAP157/CFAP74/DNAJB13/DNHD1/GAS8/HYDIN/LRRC6/SPAG16/TMEM141/TTLL5/UBE2B	14	BP
GO:0035278	miRNA mediated inhibition of	14/2734	58/17381	0.06257	0.7484	0.73612	EIF4G1/MIR106B/MIR15A/MIR16-1/MIR181B1/MIR212/MIR221/MIR222/MIR29A/MIR503/MIR92A2/MIR96/MIRLET7A2/RBM4	14	BP
GO:0040033	negative regulation of translation,	14/2734	58/17381	0.06257	0.7484	0.73612	EIF4G1/MIR106B/MIR15A/MIR16-1/MIR181B1/MIR212/MIR221/MIR222/MIR29A/MIR503/MIR92A2/MIR96/MIRLET7A2/RBM4	14	BP
GO:0045974	regulation of translation, ncRNA-	14/2734	58/17381	0.06257	0.7484	0.73612	EIF4G1/MIR106B/MIR15A/MIR16-1/MIR181B1/MIR212/MIR221/MIR222/MIR29A/MIR503/MIR92A2/MIR96/MIRLET7A2/RBM4	14	BP
GO:0043583	ear development	41/2734	206/17381	0.06276	0.7484	0.73612	ANP32B/ATP6V1B1/BMP4/C1QB/CDH23/DVL2/EYA1/FGFR2/FZD2/GBX2/GRHL3/HMX2/IFT20/JAG2/KCNQ1/KCNQ4/LHFPL5/LHX3/LRP10/LRTOMT/MAPK3/MCM2/MYCN/MYO3A/MYO7A/NAGLU/NKX3-2/NOTCH1/OSR1/PDGFRB/PHOX2B/PTPN11/SCRIB/SHH/SLC44A4/STRA6/TCAP/TMIE/WDPCP/WNT1/WNT3A	41	BP
GO:0051052	regulation of DNA metabolic process	73/2734	391/17381	0.06354	0.7484	0.73612	ACD/APBB1/ARRB2/BCL6/BMP4/BRCA1/BRCC3/CACYBP/CCT3/CDAN1/CDK9/CLCF1/E2F8/EGFR/EHMT2/ENDOG/EXOSC10/EYA1/FGFR4/FOXM1/GDF2/GPER1/HDAC8/HRAS/HSF1/HSP90AA1/INO80/INS/LIG3/MAPK15/MAPK3/MEN1/MIR221/MIR29A/MIR29C/MPHOSPH8/NOX4/NPAS2/NUDT16L1/NVL/OGG1/OTUB1/PARP3/PARP9/PAXIP1/PDGFA/PDGFRB/PDS5A/PFN1/PID1/PIF1/PLA2G1B/PRDM14/PRDM9/PRKCD/PTK2B/RECQL5/RFC2/RLF/SETD2/SH2B1/SHC1/SLF2/SMG5/SPI1/SUPT6H/TICRR/TIMELESS/TINF2[illegible]	73	BP
GO:0032456	endocytic recycling	9/2734	33/17381	0.06366	0.7484	0.73612	EHD1/INPP5F/PTPN23/RAB11FIP3/RAB17/SNF8/VPS51/VPS52/VPS53	9	BP
GO:0032633	interleukin-4 production	9/2734	33/17381	0.06366	0.7484	0.73612	CLECL1/EPX/HAVCR2/HLA-E/IL20RB/LGALS9/NOTCH1/RARA/SCGB1A1	9	BP
GO:0045746	negative regulation of Notch signaling	9/2734	33/17381	0.06366	0.7484	0.73612	ARRDC1/BCL6/CHAC1/DLK1/DLK2/EGFL7/HEY1/LFNG/SLC35C1	9	BP

GO:0045907	positive regulation of	9/2734	33/17381	0.06366	0.7484	0.73612	ADRA1A/ADRA1B/ADRA2C/AVPR2/EGFR/FGA/HRH2/SMTNL1/TACR1	9	BP
GO:0035065	regulation of histone	13/2734	53/17381	0.06367	0.7484	0.73612	BRCA1/CTBP1/HDAC8/KAT2A/MAPK3/MUC1/NOC2L/PAXIP1/PIWIL2/PYGO2/RPS6KA4/SPI1/TAF7	13	BP
GO:0046068	cGMP metabolic	13/2734	53/17381	0.06367	0.7484	0.73612	AIPL1/AQP1/FZD2/GUCA1B/GUCA2A/GUCA2B/GUCY2D/NOS3/NPPA/PDE2A/PDZD3/PTK2B/RUNDC3A	13	BP
GO:0042391	regulation of membrane potential	75/2734	403/17381	0.06435	0.7484	0.73612	ABCB5/ABL1/ADRA1A/ASIC2/ATP1A1/ATP1A4/ATPIF1/BAD/BOK/CACNA1F/CACNA1G/CACNA1H/CACNA1S/CACNB3/CAMK2D/CASP1/CATSPER1/CAV3/CDK5/CHRNA6/CHRNB1/CHRNB3/CLIC1/CNGA2/CNGB1/CNIH2/DGKI/DLG4/FLNA/FZD9/GBA/GLRA1/GPER1/GPR35/GPR88/GRIA3/GRIN1/GRIN2C/HCN3/KCND3/KCNH4/KCNH6/KCNJ5/KCNK12/KCNK13/KCNK17/KCNK4/KCNK7/KCNQ1/KDR/MIR328/MYOC/NLGN3/NPPA/OPRD1/OPRM1/P2RX2/PID1/PPA2/PPP2R3C/PRELID1/PTK2B/SCN11A/SLC26A1/SLC26A10/SLC26A6/SLC29A1/SLC34A1/STX1A/TACR1/TRDN/TUSC2/WDR1/WN[illegible]	75	BP
GO:0003044	regulation of systemic arterial blood pressure mediated by a chemical signal	12/2734	48/17381	0.06447	0.7484	0.73612	ADRA1A/ADRA1B/AGT/AVPR2/CYP11B2/EDN2/F2RL1/HSD11B2/NOS3/REN/SERPINF2/TACR1	12	BP
GO:0008542	visual learning	12/2734	48/17381	0.06447	0.7484	0.73612	B4GALT2/CDK5/DEAF1/DRD2/GRIN1/HRH2/IFT20/MEIS2/MTOR/NLGN3/PPP1R1B/RGS14	12	BP
GO:0043113	receptor clustering	12/2734	48/17381	0.06447	0.7484	0.73612	AGRN/CDK5/DLG4/DNAJA3/FLNA/FNTA/FZD9/ITGAL/NRXN2/SCRIB/SSNA1/THY1	12	BP
GO:1903307	positive regulation of regulated	12/2734	48/17381	0.06447	0.7484	0.73612	CACNA1G/CACNA1H/CDK5/CDK5R2/DOC2B/F2RL1/IL13/IL4R/ITGAM/STX1A/STX4/STXBP1	12	BP
GO:0001990	regulation of systemic arterial blood pressure by hormone	10/2734	38/17381	0.06467	0.7484	0.73612	AGT/AVPR2/CYP11B2/EDN2/F2RL1/HSD11B2/NOS3/REN/SERPINF2/TACR1	10	BP

GO:0035315	hair cell differentiation	10/2734	38/17381	0.06467	0.7484	0.73612	JAG2/LHFPL5/LRTOMT/MYCN/MYO7A/NOTCH1/SCRIB/SLC44A4/SPINK5/WDPCP	10	BP
GO:0061512	protein localization to	10/2734	38/17381	0.06467	0.7484	0.73612	BBS9/CC2D2A/CROCC/EHD1/IFT140/IFT20/RAB11FIP3/TBC1D32/TULP1/TULP3	10	BP
GO:0072350	tricarboxylic acid metabolic	10/2734	38/17381	0.06467	0.7484	0.73612	ACO1/CS/DLST/GLUD1/IDH1/IDH2/OGDH/OGDHL/PDHB/SLC34A1	10	BP
GO:1901020	negative regulation of calcium ion transmembrane	10/2734	38/17381	0.06467	0.7484	0.73612	ADRA2A/CRHR1/DRD2/GNB5/GPR35/GSTM2/RRAD/THADA/TLR9/TRDN	10	BP
GO:2000648	positive regulation of stem cell	10/2734	38/17381	0.06467	0.7484	0.73612	DISC1/DRD2/LTBP3/NOTCH1/OTP/PDCD2/SETD1A/SHH/SMO/THPO	10	BP
GO:0061097	regulation of protein tyrosine kinase activity	17/2734	74/17381	0.06499	0.7484	0.73612	ADRA2A/ADRA2B/ADRA2C/AGT/CCL5/CSF1R/DOK7/DVL2/EFNA1/NCF1/SH3BP5/SH3BP5L/SHC1/THY1/UNC119/VPS25/WNT3A	17	BP
GO:0061387	regulation of extent of cell	20/2734	90/17381	0.06507	0.7484	0.73612	ABL1/ANAPC2/ARHGAP4/BARHL2/CDK5/CTTN/DISC1/DRAXIN/FN1/ILK/ISLR2/L1CAM/LIMK1/NRG1/SEMA3F/SEMA7A/TNFRSF12A/TRPV2/WNT3	20	BP
GO:0006833	water transport	6/2734	19/17381	0.06522	0.7484	0.73612	AQP1/AQP10/AQP5/AQP8/MIP/PDZD3	6	BP
GO:0009219	pyrimidine deoxyribonucleotide metabolic process	6/2734	19/17381	0.06522	0.7484	0.73612	MBD4/NEIL2/NT5M/OGG1/SMUG1/TBPL1	6	BP
GO:0010002	cardioblast differentiation	6/2734	19/17381	0.06522	0.7484	0.73612	GATA4/NKX2-5/NOTCH1/NRG1/TBX2/WNT3A	6	BP
GO:0010592	positive regulation of lamellipodium	6/2734	19/17381	0.06522	0.7484	0.73612	AQP1/ARPC2/BRK1/FSCN1/MTOR/WNT1	6	BP
GO:0030220	platelet formation	6/2734	19/17381	0.06522	0.7484	0.73612	CASP3/CIB1/NBEAL2/PTPN11/WDR1/ZNF385A	6	BP

GO:0031114	regulation of microtubule depolymerizatio	6/2734	19/17381	0.06522	0.7484	0.73612	CIB1/KATNB1/MAP6D1/MID1IP1/TRIM54/TRPV4	6	BP
GO:0046475	glycerophospholipid catabolic process	6/2734	19/17381	0.06522	0.7484	0.73612	INPP5F/PLA2G15/PLA2G4B/PRDX6/SCARB1/SMPD4	6	BP
GO:0048026	positive regulation of mRNA splicing,	6/2734	19/17381	0.06522	0.7484	0.73612	HMX2/LMNTD2/NUP98/PRDX6/SF3B4/SLC39A5	6	BP
GO:0070230	positive regulation of lymphocyte	6/2734	19/17381	0.06522	0.7484	0.73612	ADAM8/CCL5/IDO1/LGALS9/PDCD1/PRELID1	6	BP
GO:0060562	epithelial tube morphogenesis	59/2734	310/17381	0.06528	0.7484	0.73612	ABL1/ADM/AGT/BMP4/C2CD3/CAV3/CC2D2A/CCDC103/CCDC40/CFL1/CSF1/CSF1R/CTSH/DEAF1/DVL2/ENG/ESRP2/EYA1/FGFR2/FZD2/GATA4/GBX2/GDF2/GRHL3/HHIP/HOXB7/ILK/IRX3/KAT2A/LHX1/LIAS/MICAL2/MTHFD1/MYCN/NKX2-5/NOTCH1/NOTO/OSR1/PFN1/PKD1/PLXND1/RARA/SCRIB/SETD2/SHH/SMO/SOX8/SPINT1/ST14/SUFU/TBX2/TCAP/TIMELESS/TNC/TULP3/WNT1/	59	BP
GO:0043542	endothelial cell migration	37/2734	184/17381	0.06532	0.7484	0.73612	ABL1/ADGRB1/AGT/BCAS3/BMP4/CDH13/CIB1/CORO1B/DAB2IP/EFNA1/EPHB4/FLT4/GDF2/GPX1/HSPB1/KDR/LOXL2/MIR10A/MIR16-1/MIR212/MIR221/MIR29C/MIR503/MIR92A2/NOS3/NOTCH1/NR4A1/PAXIP1/PLEKHG5/PLXND1/PRCP/PTK2B/PTP4A3/SCARB1/SEMA4A/TDGF1/W	37	BP
GO:0006475	internal protein amino acid acetylation	32/2734	156/17381	0.06565	0.7489	0.73657	APBB1/ATF2/BRCA1/BRPF1/CRTC2/CTBP1/EPC1/HCFC1/HDAC8/ING4/KAT2A/LDB1/MAPK3/MSL3/MUC1/MYOD1/NAA60/NAT8B/NOC2L/PAXIP1/PCGF2/PER1/PIWIL2/POLE4/POR/PYGO2/RPS6KA4/SPI1/TADA3/TAF1/TAF1	32	BP
GO:0048017	inositol lipid-mediated signaling	32/2734	156/17381	0.06565	0.7489	0.73657	AGT/CCL5/CSF1R/CSF3/DAB2IP/EGFR/ERBB2/F2RL1/GPER1/IL18/INPP5F/INS/KDR/LTK/MAPK3/MUC5AC/MYOC/NCF1/NRG1/NYAP1/PDGFA/PDGFRB/PI4KB/PIK3R5/PIK3R6/PLD2/PLXNB1/PPARD/PPP2R5B/PREX2/SELP/T	32	BP

GO:0070997	neuron death	60/2734	316/17381	0.06593	0.7489	0.73657	AARS/ABL1/ADAM8/AIMP2/AMBRA1/ATF2/ATN1/ATP13A2/BAD/BARHL1/BOK/CASP3/CCL5/CDK5/CHGA/CLCF1/CLU/CNTFR/CORO1A/CSF3/DPYSL4/DRAXIN/EGR1/EIF4G1/EN1/ENDOG/FASLG/FZD9/GAPDH/GBA/GPX1/GRIN1/HRAS/HSF1/HSPA5/IL13/ILK/ITGAM/LRP1/MAX/MTOR/NAIP/NES/NLRP1/NONO/NQO2/NSMF/PM20D1/PPP1R13B/PTK2B/RILPL1/SIGMAR1/SNCB/STXBP1/TBK1/TRAF2/WFS1/WNT1/WNT3A/ZPR1	60	BP
GO:0032944	regulation of mononuclear cell proliferation	41/2734	207/17381	0.06679	0.7489	0.73657	BCL6/BMP4/CARD11/CASP3/CCDC88B/CCL19/CCL5/CCR2/CD6/CD74/CD81/CLCF1/CLECL1/CORO1A/CSF1/DNAJA3/EFNB1/ERBB2/HAVCR2/HLA-E/IDO1/IL13/IL18/IL20RB/INPP5D/LGALS3/LGALS9/LMO1/MAD1L1/MZB1/PLA2G2F/PRKAR1A/SCGB1A1/SHH/TACR1/TIRAP/TLR9/TNFRSF13B/TNFRSF4/VSIG4/WNT3A	41	BP
GO:0006664	glycolipid metabolic process	26/2734	123/17381	0.06709	0.7489	0.73657	ARSE/ARSI/B4GALNT1/CLN6/CPTP/CREM/CWH43/DPM2/GAL3ST4/GBA/GBGT1/GLTP/GPAA1/HEXA/NEU3/PIGC/PIGG/PIGO/PIGQ/PIGY/PIGZ/PRKCD/PSAP/PYURF/SMPD4/ST3GAL4	26	BP
GO:1903509	liposaccharide metabolic process	26/2734	123/17381	0.06709	0.7489	0.73657	ARSE/ARSI/B4GALNT1/CLN6/CPTP/CREM/CWH43/DPM2/GAL3ST4/GBA/GBGT1/GLTP/GPAA1/HEXA/NEU3/PIGC/PIGG/PIGO/PIGQ/PIGY/PIGZ/PRKCD/PSAP/PYURF/SMPD4/ST3GAL4	26	BP
GO:0070588	calcium ion transmembrane transport	58/2734	305/17381	0.06795	0.7489	0.73657	ABL1/ADRA2A/AHNAK/ATP13A2/ATP2A3/ATP2C2/CACNA1F/CACNA1G/CACNA1H/CACNA1S/CACNB1/CACNB3/CACNG1/CAMK2D/CATSPER1/CAV3/CCL19/CCL21/CORO1A/CRACR2A/CRHR1/DRD2/FASLG/GNB5/GPER1/GPR35/GRIN1/GRIN2C/GSTM2/HSPA2/IL13/ITPR3/JPH3/LCK/MIR328/NPPA/NPSR1/OPRM1/ORAI1/ORAI3/PDE2A/PKD1/PMPCA/PTK2B/RASA3/RRAD/SLC25A25/THADA/THY1/TLR9/TMEM37/TRDN/TRPM5/TRPV2/TRPV4	58	BP
GO:0002064	epithelial cell development	39/2734	196/17381	0.06829	0.7489	0.73657	BAD/BMP4/CDH5/DACT2/DNASE1L2/E2F4/ENG/F2RL1/GPX1/HRH2/HYDIN/IKBKB/LHFPL5/LRTOMT/MARVELD2/MYO1E/MYO7A/NKX3-2/NOTCH1/NUP210L/PDE2A/PLOD3/RAB25/RARA/RILPL1/RILPL2/SCRIB/SDC1/SFN/SIDT2/SMO/SOX8/SPDEF/ST14/TMEM79/TNMD/VSIG1/WDPCP/	39	BP
GO:0030641	regulation of cellular pH	21/2734	96/17381	0.0689	0.7489	0.73657	ATP1A4/ATP6V0B/ATP6V0E2/ATP6V1B1/ATP6V1F/ATP6V1G1/ATP6V1G2/CA7/CLN6/FASLG/GPR89A/MAPK3/RAB20/RAB7A/SLC11A1/SLC26A1/SLC26A10/SLC26A6/SLC4A9/SLC9A3/SLC9A5	21	BP

GO:0043903	regulation of symbiosis, encompassing mutualism through	43/2734	219/17381	0.0693	0.7489	0.73657	BAD/CCL5/CD74/CDK9/CFL1/CHMP4C/DDB1/EIF2AK4/F2RL1/FKBP6/GAPDH/HACD3/IFITM2/ISG15/LAMP3/LGALS9/LTA/MIR221/MIR222/MPO/NELFB/NOTCH1/PARP10/PC/PFN1/POLR2G/POLR2L/PPIB/PPIE/PSMC3/RAB7A/RSF1/SNF8/STAT1/TARBP2/TIRAP/TRIM11/TRIM14/TRIM26/TRIM31/TRIM62/VPS37B/ZNF502	43	BP
GO:0031396	regulation of protein ubiquitination	51/2734	265/17381	0.06967	0.7489	0.73657	ABL1/ADGRB1/AIMP2/ANAPC15/ANAPC2/ARRB2/AVPR2/AXIN1/BRCA1/BUB1B/CAV3/CDK5/CDK5RAP3/CDK9/CHFR/CLU/COMMD1/DISC1/FBXO2/GOLGA2/GORASP1/HDAC8/HERPUD1/HSP90AA1/HSPA5/ISG15/KLHL40/LIMK1/MTA1/MTOR/NMI/OTUB1/PARP10/PAXIP1/PSMB11/PSMB6/PSMB7/PSMB8/PSMC3/PSMD13/PSMD3/PSMD5/PSMD7/PTK2B/SEPT4/SUFU/TA	51	BP
GO:0034330	cell junction organization	51/2734	265/17381	0.06967	0.7489	0.73657	ABL1/AGT/ANG/BCAS3/CDH12/CDH13/CDH15/CDH24/CDH5/CLDN6/COL16A1/COL17A1/CSF1R/CSK/CTTN/F2RL1/FBLIM1/FERMT2/FLNA/FLNC/FN1/FSCN1/GNPAT/IKBKB/ILK/KDR/KIFC3/KRT14/LAMC2/LDB1/LIMS2/MARVELD2/MARVELD3/MYO1C/MYOC/PARD3/PARD6A/PKP3/PTK2B/PTPN23/RASSF8/RHOD/TAOK2/THY1/TLN1/TRPV4/WDPCP/WDR1/WHAMM/	51	BP
GO:0098661	inorganic anion transmembrane transport	27/2734	129/17381	0.06972	0.7489	0.73657	ABCC3/ABCC4/ABCC5/ABCC6/ANO1/ANO2/ANO4/ANO7/BEST3/BEST4/BSND/CLCN2/CLCNKA/CLCNKB/CLDN4/CLIC1/GLRA1/MTOR/SLC12A4/SLC12A7/SLC12A9/SLC20A1/SLC20A2/SLC26A1/SLC26A10/SLC26A6/SLC34A	27	BP
GO:0072659	protein localization to plasma membrane	44/2734	225/17381	0.07048	0.7489	0.73657	AP2M1/ARHGEF16/BLZF1/CACNB3/CAV3/CIB1/CNPY4/CSK/DPP6/EGFR/FLNA/GAK/GPER1/GRIPAP1/IFT20/IKBKB/INS/KIF13A/LRP1/LYPD1/NKD2/PACS1/PID1/PKDCC/PKP3/RAB26/RAB34/RAB40C/RILPL1/RILPL2/SCRIB/STX4/STX8/STXBP1/TMBIM1/TMEM150A/TNIK/TREM2/TSPAN15/TSPAN33/VAMP5/VTI1B/WNT3A/ZFYVE27	44	BP
GO:0018394	peptidyl-lysine acetylation	32/2734	157/17381	0.07049	0.7489	0.73657	APBB1/ATF2/BRCA1/BRPF1/CRTC2/CTBP1/EPC1/HCFC1/HDAC8/ING4/KAT2A/LDB1/MAPK3/MSL3/MUC1/MYOD1/NAA60/NAT8B/NOC2L/PAXIP1/PCGF2/PER1/PIWIL2/POLE4/POR/PYGO2/RPS6KA4/SPI1/TADA3/TAF1/TAF1	32	BP

GO:0042493	response to drug	76/2734	411/17381	0.0706	0.7489	0.73657	ABCA2/ABCB5/ABCC3/ABCC4/ABCC6/ABCG5/ACACB/ADIRF/ADRA1A/ALAD/APEX1/AQP1/ATP1A1/BAD/BGLAP/BLMH/CARD9/CASP3/CD69/CDK4/CDK9/COL1A1/CSAG3/CYP1A1/CYP1A2/DAB1/DAD1/DDC/DHODH/DNMT3A/DPEP1/DRD2/EFTUD2/EGFR/EIF2B5/EMX1/ENG/FOSL1/GAD1/GATA4/GGH/GNAO1/GNPAT/HADHA/HSD11B2/HSF1/HSPA5/KCNQ1/LCK/LDHA/LTA/MGST1/MT2A/NME1/NPC1L1/OGG1/OTC/PDE2A/PEMT/POR/PTGR1/PTK2B/RECQL5/REN/RPP21/SCGB1A1/SCN11A/SLC22A1/SLC22A12/SL[illegible]	76	BP
GO:0006779	porphyrin-containing compound biosynthetic process	7/2734	24/17381	0.0707	0.7489	0.73657	ALAD/ALAS2/ATPIF1/COX10/HMBS/NFE2L1/UROS	7	BP
GO:0044030	regulation of DNA	7/2734	24/17381	0.0707	0.7489	0.73657	BRCA1/MIR29A/MIR29C/MPHOSPH8/PRDM14/RLF/SPI1	7	BP
GO:0045662	negative regulation of myoblast	7/2734	24/17381	0.0707	0.7489	0.73657	ANKRD2/BMP4/CMTM5/IL18/NOTCH1/PPARD/SOX8	7	BP
GO:0048103	somatic stem cell division	7/2734	24/17381	0.0707	0.7489	0.73657	DOCK7/FGFR2/HOXB4/NOTCH1/SOX5/WNT3A/WNT7A	7	BP
GO:0030198	extracellular matrix organization	63/2734	335/17381	0.07108	0.7489	0.73657	ABL1/ACAN/ADAM12/ADAM8/ADAMTS14/ADAMTS2/AGRN/AGT/BCAN/BGN/C6orf15/COL16A1/COL1A1/COL4A2/COL7A1/COL9A2/CTSL/DDR2/EFEMP2/ELN/ENG/FBLN2/FGA/FGFR4/FN1/FSCN1/FURIN/GFOD2/HSPG2/ITGA7/ITGAL/ITGAM/ITGAX/ITGB5/KDR/KIF9/KLKB1/LAMA2/LAMA4/LAMC2/LAMC3/LOXL1/LOXL2/LRP1/MMP15/MMP2/MMP3/MYO1E/NOTCH1/NOXO1/PDGFA/PLOD3/SCX/SERPINF2/SERPINH1/SH3PXD2B/SPINK5/SPI[illegible]	63	BP
GO:0016573	histone acetylation	30/2734	146/17381	0.07145	0.7489	0.73657	APBB1/ATF2/BRCA1/BRPF1/CRTC2/CTBP1/EPC1/HCFC1/HDAC8/ING4/KAT2A/LDB1/MAPK3/MSL3/MUC1/MYOD1/NAA60/NOC2L/PAXIP1/PCGF2/PER1/PIWIL2/POLE4/PYGO2/RPS6KA4/SPI1/TADA3/TAF1/TAF1L/TAF7	30	BP
GO:0001523	retinoid metabolic	20/2734	91/17381	0.07157	0.7489	0.73657	AGRN/ALDH8A1/APOB/APOC3/CLPS/CYP1A1/DGAT1/GPC1/GPC2/GPIHBP1/HSPG2/LRP1/OPN1MW/PLB1/PPARD/RARRES2/RBP1/RBP2/SDC1/STR	20	BP

GO:0006383	transcription from RNA polymerase III	13/2734	54/17381	0.07228	0.7489	0.73657	BRCA1/BRF1/CHD8/ERBB2/GTF3C1/MAF1/MTOR/POLR1D/POLR2L/POLR3D/SNAPC1/SNAPC5/ZNF76	13	BP
GO:0009066	aspartate family amino acid metabolic	13/2734	54/17381	0.07228	0.7489	0.73657	ASNS/BHMT2/CRYM/DLST/GNMT/GOT2/HYKK/MTHFD1/NMNAT3/OGDH/PHYKPL/PIPOX/PLOD3	13	BP
GO:0014015	positive regulation of	13/2734	54/17381	0.07228	0.7489	0.73657	BIN1/CCR2/CLCF1/CSF1/LTA/MIR221/MIR222/MTOR/NOTCH1/RNF112/SHH/SOX8/SPINT1	13	BP
GO:0097345	mitochondrial outer membrane permeabilizatio	13/2734	54/17381	0.07228	0.7489	0.73657	ATPIF1/BAD/BOK/DYNLL2/FZD9/HIP1R/MOAP1/PPP1R13B/RHOT2/SFN/SLC25A5/YWHAQ/ZNF205	13	BP
GO:0050808	synapse organization	50/2734	260/17381	0.07261	0.7489	0.73657	ABL1/ACHE/ADGRB1/AGRN/AMIGO1/ANAPC2/ASIC2/C1QL1/CACNB1/CACNB3/CDK5/CHRNB1/CLSTN1/CLSTN3/CTNNA2/DAB2IP/DISC1/DLG4/DNAJA3/DRD2/EIF4G1/EPHB3/ERBB2/FNTA/FZD9/GNPAT/GRIN1/GRIPAP1/KIRREL3/L1CAM/LINGO2/LRRTM1/NEUROD2/NLGN3/NRG1/NRXN2/P2RX2/PCDHB1/PCDHGC4/PCDHGC5/PDLIM5/PLXND1/RAB17/SLITRK3/SNC	50	BP
GO:0045995	regulation of embryonic development	26/2734	124/17381	0.07269	0.7489	0.73657	BMP4/CDX2/CRB2/DVL2/E4F1/FZD2/GRHL3/IL1RN/LAMA2/LAMA4/LFNG/LHX1/MIR221/NOTCH1/OSR1/PHLDA2/RBM19/SCX/SHH/SUFU/TBX2/TRAF3IP1/TULP3/WDPCP/WNT1/WNT3A	26	BP
GO:0060359	response to ammonium ion	26/2734	124/17381	0.07269	0.7489	0.73657	ABL1/AGRN/CASP3/CASP6/CDK5/CHRM1/DNMT3A/DRD2/EFTUD2/GNAO1/GRIN1/HCN3/HNMT/HOMER2/HSPA5/LYPD1/MAPK3/MTOR/OPRM1/PEA15/PPP1R1B/PPP1R9B/PTK2B/RGS10/SLC34A1/TACR1	26	BP
GO:0002031	G-protein coupled receptor	5/2734	15/17381	0.07314	0.7489	0.73657	ADM/ARRB2/DNM1/DRD2/NECAB2	5	BP
GO:0002830	positive regulation of type 2 immune	5/2734	15/17381	0.07314	0.7489	0.73657	CD74/IDO1/IL18/IL4R/RARA	5	BP
GO:0022038	corpus callosum development	5/2734	15/17381	0.07314	0.7489	0.73657	CDK5/EPHB3/RTN4RL1/RTN4RL2/TSKU	5	BP

GO:0048308	organelle inheritance	5/2734	15/17381	0.07314	0.7489	0.73657	GBF1/GOLGA2/MAPK3/RBSN/STX5	5	BP
GO:0050651	dermatan sulfate proteoglycan biosynthetic	5/2734	15/17381	0.07314	0.7489	0.73657	B3GAT3/BCAN/BGN/CHST12/DSE	5	BP
GO:0061577	calcium ion transmembrane transport via high voltage-gated calcium	5/2734	15/17381	0.07314	0.7489	0.73657	CACNB1/CACNB3/CAMK2D/MIR328/NPPA	5	BP
GO:0072176	nephric duct development	5/2734	15/17381	0.07314	0.7489	0.73657	BMP4/LHX1/OSR1/PKD1/WNT11	5	BP
GO:0072677	eosinophil migration	5/2734	15/17381	0.07314	0.7489	0.73657	ADAM8/CCL5/DAPK2/EPX/LGALS3	5	BP
GO:1902188	positive regulation of viral release	5/2734	15/17381	0.07314	0.7489	0.73657	CHMP4C/DDB1/PC/VPS37B/ZNF502	5	BP
GO:2000353	positive regulation of endothelial cell	5/2734	15/17381	0.07314	0.7489	0.73657	ECSCR/FASLG/GPER1/MIR15A/PDCD4	5	BP
GO:2000846	regulation of corticosteroid hormone	5/2734	15/17381	0.07314	0.7489	0.73657	AGT/C1QTNF1/CRHR1/PTPN11/REN	5	BP
GO:0007205	protein kinase C-activating G-protein coupled receptor signaling	8/2734	29/17381	0.07367	0.7489	0.73657	CHRM1/CISH/DGKI/DGKQ/DGKZ/LRP1/PARD3/PLPP1	8	BP

GO:0016056	rhodopsin mediated signaling	8/2734	29/17381	0.07367	0.7489	0.73657	CNGB1/FNTA/FNTB/GNAT1/GUCA1B/GUCY2D/OPN4/PPEF1	8	BP
GO:0042168	heme metabolic process	8/2734	29/17381	0.07367	0.7489	0.73657	ALAD/ALAS2/ATPIF1/COX10/HMBS/HMOX2/NFE2L1/UROS	8	BP
GO:0061900	glial cell activation	8/2734	29/17381	0.07367	0.7489	0.73657	CLU/EGFR/FAM19A3/IL13/ITGAM/LRP1/SMO/TLR8	8	BP
GO:0090023	positive regulation of neutrophil	8/2734	29/17381	0.07367	0.7489	0.73657	C3AR1/CAMK1D/CCL19/CCL21/CD74/DAPK2/PF4V1/TIRAP	8	BP
GO:2000108	positive regulation of leukocyte	8/2734	29/17381	0.07367	0.7489	0.73657	ADAM8/CCL5/CDKN2A/HCAR2/IDO1/LGALS9/PDCD1/PRELID1	8	BP
GO:0045806	negative regulation of	12/2734	49/17381	0.07367	0.7489	0.73657	APOC3/CD300LF/CSK/DLG4/LGALS3/LRRTM1/LRSAM1/NECAB2/NR1H3/PACSIN3/PROM2/UNC119	12	BP
GO:0045933	positive regulation of muscle	12/2734	49/17381	0.07367	0.7489	0.73657	ADRA1A/ADRA1B/ADRA2B/ATP1A1/CHGA/CTTN/EDN2/GPER1/KCNQ1/NPPA/TACR1/TACR2	12	BP
GO:0046579	positive regulation of Ras protein	12/2734	49/17381	0.07367	0.7489	0.73657	CSF1/DGKI/F2RL1/GPR17/GPR20/GPR35/HRAS/MMD2/NOTCH1/NRG1/PDGFRB/RASGEF1A	12	BP
GO:0030317	flagellated sperm motility	19/2734	86/17381	0.07431	0.7489	0.73657	APOB/ATP1A4/CATSPER1/CCDC40/CFAP157/DDX4/DNAH11/GAS8/LRRC6/PGAM4/PRDM14/RNASE9/SPAG16/TACR1/TACR2/TEKT4/TEKT5/TTLL5	19	BP
GO:0043062	extracellular structure organization	63/2734	336/17381	0.07451	0.7489	0.73657	ABL1/ACAN/ADAM12/ADAM8/ADAMTS14/ADAMTS2/AGRN/AGT/BCAN/BGN/C6orf15/COL16A1/COL1A1/COL4A2/COL7A1/COL9A2/CTSL/DDR2/EFEMP2/ELN/ENG/FBLN2/FGA/FGFR4/FN1/FSCN1/FURIN/GFOD2/HSPG2/ITGA7/ITGAL/ITGAM/ITGAX/ITGB5/KDR/KIF9/KLKB1/LAMA2/LAMA4/LAMC2/LAMC3/LOXL1/LOXL2/LRP1/MMP15/MMP2/MMP3/MYO1E/NOTCH1/NOXO1/PDGFA/PLOD3/SCX/SERPINF2/SERPINH1/SH3PXD2B/SPINK5/SPI[illegible]	63	BP
GO:0009583	detection of light stimulus	16/2734	70/17381	0.07465	0.7489	0.73657	AIPL1/ASIC2/CACNA1F/CNGB1/FNTA/FNTB/GNAT1/GUCA1B/GUCY2D/OPN1MW/OPN4/PITPNM1/PPEF1/SEMA5B/TULP1/UNC119	16	BP

GO:0010660	regulation of muscle cell apoptotic	16/2734	70/17381	0.07465	0.7489	0.73657	AGT/AMBRA1/APOPT1/ARRB2/CAMK2D/CDKN2A/EIF5A/HSF1/ILK/LTK/MIR16-1/MIR195/MIR92A2/NKX2-5/PDCD4/PTK2B	16	BP
GO:0051924	regulation of calcium ion transport	44/2734	226/17381	0.07468	0.7489	0.73657	ABL1/ADRA2A/AGT/AHNAK/ARRB2/CACNB1/CACNB3/CAMK2D/CATSPER1/CAV3/CCL5/CORO1A/CRACR2A/CRHR1/DRD2/GCG/GNAO1/GNB5/GPER1/GPR35/GRIN1/GSTM2/HSPA2/IL13/JPH3/LGALS3/MIR328/NOS3/NPPA/NPSR1/OPRD1/ORAI1/P2RX2/PACSIN3/PDGFRB/PLA2G1B/PTK2B/RRAD/THADA/THY1/TLR9/TRDN/TRPV2/WFS1	44	BP
GO:0032873	negative regulation of stress-activated	11/2734	44/17381	0.0747	0.7489	0.73657	F2RL1/FKTN/FOXM1/MAPK8IP1/MARVELD3/MEN1/MIR92A2/NCOR1/PDCD4/PER1/ZMYND11	11	BP
GO:0045023	G0 to G1 transition	11/2734	44/17381	0.0747	0.7489	0.73657	BRCA1/DAB2IP/DUX4/EHMT1/EHMT2/EPC1/FOXO4/MAX/PCGF2/PPP2R5B/UXT	11	BP
GO:0050999	regulation of nitric-oxide synthase	11/2734	44/17381	0.0747	0.7489	0.73657	CAV3/EGFR/GCH1/GCHFR/HSP90AA1/INS/NOD1/NOS3/NOSTRIN/PTK2B/SCARB1	11	BP
GO:0070303	negative regulation of stress-activated protein kinase	11/2734	44/17381	0.0747	0.7489	0.73657	F2RL1/FKTN/FOXM1/MAPK8IP1/MARVELD3/MEN1/MIR92A2/NCOR1/PDCD4/PER1/ZMYND11	11	BP
GO:0051258	protein polymerization	48/2734	249/17381	0.07472	0.7489	0.73657	ABL1/ANG/ANKRD53/ARFIP1/ARPC1A/ARPC1B/ARPC2/ARPC4/BAIAP2L1/BRK1/CAPZA3/CATIP/CAV3/CCL21/CDC42EP2/CKAP5/CORO1A/CORO1B/CSF3/CTTN/DNM1/EVL/FES/FGA/GOLGA2/HIP1R/LMOD1/MID1IP1/MLST8/MTOR/MYO1C/NUMA1/PFN1/PRKCD/PTK2B/SCIN/SPTAN1/SPTB/SPTBN2/TRPV4/TUBG1/TUBG2/TUBGCP2/TUBGCP3/TUBGCP5/VDAC2/WAS/W	48	BP
GO:0006361	transcription initiation from RNA polymerase I	9/2734	34/17381	0.07499	0.7489	0.73657	GTF2H4/MAPK3/POLR1A/POLR1D/POLR1E/POLR2L/TAF1A/TTF1/ZNRD1	9	BP

GO:0034383	low-density lipoprotein particle	9/2734	34/17381	0.07499	0.7489	0.73657	AP2A2/AP2M1/APOB/APOC3/CSK/EHD1/NPC2/SCARB1/SOAT2	9	BP
GO:0038202	TORC1 signaling	9/2734	34/17381	0.07499	0.7489	0.73657	CARD11/DGKQ/LARS/MLST8/MTOR/NPRL3/RRAGC/SESN1/TELO2	9	BP
GO:1905332	positive regulation of morphogenesis	9/2734	34/17381	0.07499	0.7489	0.73657	ABL1/AGT/BMP4/HOXB7/LHX1/MIR221/MTOR/SMO/SOX8	9	BP
GO:0002639	positive regulation of immunoglobuli	10/2734	39/17381	0.07522	0.7489	0.73657	CLCF1/HLA-E/IL13/IL4R/MZB1/PAXIP1/STX4/TLR9/TNFRSF4/TNFSF13	10	BP
GO:1901186	positive regulation of ERBB signaling	10/2734	39/17381	0.07522	0.7489	0.73657	ADRA2A/ADRA2B/ADRA2C/AGT/ARAP1/DOK1/FASLG/GPER1/HIP1R/NCF1	10	BP
GO:0044259	multicellular organismal macromolecule metabolic	27/2734	130/17381	0.07533	0.7489	0.73657	ADAMTS14/ADAMTS2/BMP4/CIITA/COL1A1/COL4A2/COL7A1/CTSD/CTSL/ENG/FURIN/MIR29A/MIR92A2/MMP15/MMP2/MMP3/MRC2/P3H1/P3H3/PDGFRB/PHYKPL/PLOD3/PPARD/SCX/SERPINF2/SERPINH1/TNS2	27	BP
GO:0030323	respiratory tube development	35/2734	175/17381	0.07597	0.7489	0.73657	ADAMTS2/AIMP2/BMP4/CCDC40/CTSH/CYP1A2/EDN2/EGFR/EIF4EBP1/ESRP2/EYA1/FGFR2/FLT4/HHIP/IL13/LHX3/MAPK3/MYCN/NKX2-8/NOS3/NOTCH1/NUMA1/PDGFA/PDGFRB/PKD1/PKDCC/PLOD3/SHH/SPDEF/STRA6/THRA/TIMELESS/TNC/TULP3/WNT11	35	BP
GO:0006801	superoxide metabolic	15/2734	65/17381	0.07705	0.7489	0.73657	AGT/CCS/EGFR/F2RL1/GCH1/ITGAM/MPO/NCF1/NOS3/NOX4/NOXO1/PRDX1/PRKCD/SH3PXD2B/SOD3	15	BP
GO:0030239	myofibril assembly	15/2734	65/17381	0.07705	0.7489	0.73657	ACTA1/CAV3/IGSF22/LMOD1/MYBPC1/MYBPC3/MYBPH/MYBPHL/NKX2-5/OBSCN/OBSL1/PDGFRB/PRKAR1A/TCAP/WDR1	15	BP
GO:0051881	regulation of mitochondrial membrane potential	15/2734	65/17381	0.07705	0.7489	0.73657	ABL1/ATPIF1/BAD/BOK/CASP1/CLIC1/FZD9/KDR/MYOC/OPRD1/PID1/PPA2/PPP2R3C/PRELID1/TUSC2	15	BP

GO:0051926	negative regulation of calcium ion	15/2734	65/17381	0.07705	0.7489	0.73657	ADRA2A/CAV3/CRHR1/DRD2/GNAO1/GNB5/GPR35/GSTM2/MIR328/NOS3/PACSIN3/RRAD/THADA/TLR9/TRDN	15	BP
GO:0010975	regulation of neuron projection development	78/2734	425/17381	0.07748	0.7489	0.73657	ABL1/AGT/AMIGO1/ANAPC2/APBB1/ARC/ARHGAP4/BARHL2/CAMK1D/CDK5/CFL1/CIB1/CPNE5/CPNE9/CTTN/DAB1/DAB2IP/DBN1/DGUOK/DISC1/DRAXIN/EFNA1/EPHB3/FES/FN1/FOXO6/GAK/GORASP1/GRIN1/HSPA5/ILK/INPP5F/ISLR2/KATNB1/KNDC1/L1CAM/LIMK1/LINGO1/LLPH/LRIG2/LRP1/LTK/MARK2/METRN/MIR221/MIR222/MTOR/NLGN3/NME1/NR2F1/NRG1/NSMF/OBSL1/PDLIM5/PLXNB1/PLXNB3/PLXND1/PPP2R5B/PTK2B/PTPRF/RAB17/RGMA/RTN4RL1/RTN4RL2/SCARF1/SEMA3F/SEMA7A/SERPINI1/TBR1/THY1/TLX2/TNFRSF12A/TNIK/TRPV2/TRPV4/WNT3A/WNT7[illegible]	78	BP
GO:0019884	antigen processing and presentation of exogenous	36/2734	181/17381	0.07762	0.7489	0.73657	ACTR1A/AP1S1/AP2A2/AP2M1/CAPZA3/CD74/CTSD/CTSE/CTSF/CTSL/DCTN2/DYNC1H1/DYNC1I1/DYNLL2/HLA-E/IKBKB/ITGB5/KIF23/KIF26A/KIF2B/KIF4A/KLC1/KLC2/NCF1/PSMB11/PSMB6/PSMB7/PSMB8/PSMC3/PSMD13/PSMD3/PSMD5/PSMD7/RAB7A/SPTB	36	BP
GO:0007612	learning	28/2734	136/17381	0.07782	0.7489	0.73657	ARC/B4GALT2/C1QL1/CDK5/DEAF1/DGKI/DLG4/DRD2/EIF2AK4/EN1/FOSL1/GPR88/GRIN1/HRH2/IFT20/JPH3/MEIS2/MTOR/NEUROD2/NLGN3/NRXN2/PPP1R1B/RGS14/STRA6/TACR1/TACR2/TBR1/TH	28	BP
GO:0043241	protein complex disassembly	52/2734	273/17381	0.0783	0.7489	0.73657	AXIN1/C12orf65/CAPZA3/CFL1/CHMP1A/CHMP7/CIB1/DVL2/EIF5A/ERAL1/F2RL1/FRAT1/FZD2/GAK/GBA/KATNB1/KIF2B/LMOD1/MAP6D1/MICAL2/MID1IP1/MRPL10/MRPL14/MRPL28/MRPL36/MRPL37/MRPL43/MRPL52/MRPL57/MRPL9/MRPS10/MRPS11/MRPS18A/MRPS18B/MRPS21/MRPS33/MRPS5/NCKAP5L/NES/PEX14/SCIN/SPTAN1/SPTB/SPTBN2/STMN4/TBC1D25/TECPR1/TRIM54/TRPV4/WDR1/WNT1/WNT3A	52	BP
GO:0045765	regulation of angiogenesis	52/2734	273/17381	0.0783	0.7489	0.73657	ABL1/ADGRB1/ADM/AGT/AQP1/BRCA1/C3AR1/CCR2/CIB1/COL4A2/CTSH/DAB2IP/ECM1/ECSCR/EFNA1/EFNA3/ENG/ERBB2/FASLG/FOXO4/GATA4/GDF2/HSPB1/HSPG2/HTATIP2/KDR/MIR106B/MIR10A/MIR15A/MIR16-1/MIR210/MIR212/MIR221/MIR222/MIR29C/MIR361/MIR451A/MIR503/MIR92A2/NOS3/NOTCH1/PIK3R6/PLXND1/PTK2B/RNH1/SARS/SEMA4A/SPINK5/STAT1/TIE1/TNFRSF12A/TNMD	52	BP

GO:0022617	extracellular matrix	20/2734	92/17381	0.07849	0.7489	0.73657	ACAN/ADAM8/BCAN/CTSL/DDR2/ELN/FGFR4/FN1/FSCN1/FURIN/HSPG2/KIF9/KLKB1/LAMC2/LRP1/MMP15/MMP2/MMP3/NOXO1/SH3PXD2B	20	BP
GO:2000379	positive regulation of reactive oxygen species	20/2734	92/17381	0.07849	0.7489	0.73657	ADGRB1/AGT/CLU/DUOXA1/EGFR/EIF5A/F2RL1/GRIN1/HSP90AA1/INS/ITGAM/MTOR/NOX4/NQO2/PDGFRB/PID1/PRKCD/PTK2B/XDH/ZNF205	20	BP
GO:0008637	apoptotic mitochondrial changes	26/2734	125/17381	0.0786	0.7489	0.73657	APOPT1/ARRB2/ATF2/ATPIF1/BAD/BOK/CCAR2/CLU/DYNLL2/FZD9/GPER1/GPX1/HIP1R/HRK/IFIT2/LMNA/MIR29A/MIR29C/MOAP1/PPP1R13B/PRELID1/RHOT2/SFN/SLC25A5/YWHAQ/ZNF205	26	BP
GO:0009799	specification of symmetry	26/2734	125/17381	0.0786	0.7489	0.73657	AXIN1/BMP4/C2CD3/CC2D2A/CCDC103/CCDC40/CFC1/DNAH11/ENG/GAS8/GATA4/IFT140/LRRC6/MICAL2/NBL1/NEK8/NKX2-5/NKX3-2/NOTCH1/NOTO/SHH/SMO/SUFU/TBC1D32/TBX2/WNT3A	26	BP
GO:0001952	regulation of cell-matrix	22/2734	103/17381	0.07907	0.7489	0.73657	ABL1/AJAP1/BCAS3/BCL6/CCL21/CDH13/CDKN2A/CIB1/COL16A1/CSF1/DISC1/ILK/KDR/LDB1/MIR29C/MIR92A2/MYOC/PLAU/PTK2B/RHOD/THY1/	22	BP
GO:0006885	regulation of pH	22/2734	103/17381	0.07907	0.7489	0.73657	ATP1A4/ATP6V0B/ATP6V0E2/ATP6V1B1/ATP6V1F/ATP6V1G1/ATP6V1G2/CA7/CLN6/FASLG/GPR89A/MAPK3/RAB20/RAB7A/RHCG/SLC11A1/SLC26A1/SLC26A10/SLC26A6/SLC4A9/SLC9A3/SLC9A5	22	BP
GO:0030032	lamellipodium assembly	14/2734	60/17381	0.07939	0.7489	0.73657	AQP1/ARHGEF4/ARPC2/BIN3/BRK1/CDH13/FSCN1/MTOR/PLXNB3/RHOD/SH2B1/WAS/WHAMM/WNT1	14	BP
GO:0030888	regulation of B cell	14/2734	60/17381	0.07939	0.7489	0.73657	BCL6/CARD11/CASP3/CD74/CD81/CLCF1/IL13/INPP5D/MZB1/TIRAP/TLR9/TNFRSF13B/TNFRSF4/WNT3A	14	BP
GO:0048857	neural nucleus development	14/2734	60/17381	0.07939	0.7489	0.73657	CASP5/CDK5R2/GLUD1/HOXB1/HOXB2/HSPA5/KIRREL3/MAPKAP1/NDRG2/PHOX2A/PHOX2B/SCRIB/SEC16A/YWHAQ	14	BP
GO:0097755	positive regulation of blood vessel	14/2734	60/17381	0.07939	0.7489	0.73657	ADM/AGT/EGFR/EPHX2/F2RL1/GCH1/GPER1/GPX1/INS/KNG1/MIR153-1/NOS3/PLOD3/PPARD	14	BP
GO:0042310	vasoconstriction	17/2734	76/17381	0.07999	0.7489	0.73657	ADM/ADRA1A/ADRA1B/ADRA2A/ADRA2B/ADRA2C/AGT/ASIC2/AVPR2/CHRM1/EDN2/EGFR/FGA/HRH2/HTR1D/SMTNL1/TACR1	17	BP

GO:0048872	homeostasis of number of cells	45/2734	233/17381	0.08016	0.7489	0.73657	ABL1/AHSP/ALAS2/ATPIF1/BCL6/BMP4/CACNA1F/CARD11/CASP3/CCNB2/CD74/CORO1A/CSF1/DNAJA3/EMX1/FOXN1/HBZ/HCAR2/IL20RB/INPP5D/ISG15/L3MBTL3/LDB1/LGALS9/LMO1/MAEA/MIR221/MIR222/MTHFD1/NFE2L1/NKX2-3/NLE1/PPP2R3C/PRDM14/PRDX1/PTPN11/RPS24/SETD1A/SMO/SPI1/STA	45	BP
GO:0030833	regulation of actin filament polymerization	29/2734	142/17381	0.08018	0.7489	0.73657	ARFIP1/ARPC1A/ARPC1B/ARPC2/ARPC4/BAIAP2L1/BRK1/CAPZA3/CCL21/CDC42EP2/CORO1A/CORO1B/CSF3/CTTN/EVL/HIP1R/LMOD1/MLST8/MTOR/MYO1C/PFN1/PRKCD/PTK2B/SCIN/SPTAN1/SPTB/SPTBN2/WAS/WH	29	BP
GO:0002093	auditory receptor cell	4/2734	11/17381	0.08025	0.7489	0.73657	LHFPL5/MYO7A/SCRIB/WDPCP	4	BP
GO:0006853	carnitine shuttle	4/2734	11/17381	0.08025	0.7489	0.73657	ACACB/CPT2/MID1IP1/SLC25A20	4	BP
GO:0007288	sperm axoneme assembly	4/2734	11/17381	0.08025	0.7489	0.73657	CFAP157/SPAG16/TTLL5/UBE2B	4	BP
GO:0007494	midgut development	4/2734	11/17381	0.08025	0.7489	0.73657	DAB1/EGFR/OTC/SMO	4	BP
GO:0009151	purine deoxyribonucleotide metabolic process	4/2734	11/17381	0.08025	0.7489	0.73657	AK5/DGUOK/NUDT1/NUDT18	4	BP
GO:0034244	negative regulation of transcription elongation from RNA	4/2734	11/17381	0.08025	0.7489	0.73657	AXIN1/NELFB/RECQL5/SHH	4	BP
GO:0034379	very-low-density lipoprotein	4/2734	11/17381	0.08025	0.7489	0.73657	APOB/APOC3/DGAT1/SOAT2	4	BP
GO:0042135	neurotransmitter catabolic	4/2734	11/17381	0.08025	0.7489	0.73657	ACHE/HNMT/LRTOMT/NAALAD2	4	BP

GO:0045945	positive regulation of transcription from RNA polymerase III	4/2734	11/17381	0.08025	0.7489	0.73657	BRF1/CHD8/ERBB2/MTOR	4	BP
GO:0045953	negative regulation of natural killer cell mediated	4/2734	11/17381	0.08025	0.7489	0.73657	ARRB2/HAVCR2/HLA-E/LGALS9	4	BP
GO:0060433	bronchus development	4/2734	11/17381	0.08025	0.7489	0.73657	BMP4/IL13/SPDEF/TULP3	4	BP
GO:0060856	establishment of blood-brain barrier	4/2734	11/17381	0.08025	0.7489	0.73657	ENG/MFSD2A/MXRA8/WNT7A	4	BP
GO:0060965	negative regulation of gene silencing	4/2734	11/17381	0.08025	0.7489	0.73657	DND1/NCOR1/NCOR2/ZC3H10	4	BP
GO:0061365	positive regulation of triglyceride	4/2734	11/17381	0.08025	0.7489	0.73657	APOA5/GPIHBP1/LMF1/NR1H3	4	BP
GO:0070914	UV-damage excision repair	4/2734	11/17381	0.08025	0.7489	0.73657	DDB1/DDB2/INO80/XPC	4	BP
GO:2000774	positive regulation of cellular	4/2734	11/17381	0.08025	0.7489	0.73657	CDKN2A/HMGA1/MIR10A/YPEL3	4	BP
GO:0036344	platelet morphogenesis	6/2734	20/17381	0.08125	0.7489	0.73657	CASP3/CIB1/NBEAL2/PTPN11/WDR1/ZNF385A	6	BP
GO:0043046	DNA methylation involved in	6/2734	20/17381	0.08125	0.7489	0.73657	DDX4/DNMT3A/FKBP6/PIWIL2/TDRD1/TDRD9	6	BP

GO:0060046	regulation of acrosome	6/2734	20/17381	0.08125	0.7489	0.73657	CACNA1H/GLRA1/PLB1/POMZP3/PRSS37/SPINK2	6	BP
GO:0061436	establishment of skin barrier	6/2734	20/17381	0.08125	0.7489	0.73657	CLDN4/FLG2/GRHL3/KRT16/SFN/TMEM79	6	BP
GO:0072087	renal vesicle development	6/2734	20/17381	0.08125	0.7489	0.73657	BMP4/LHX1/OSR1/SMO/SOX8/STAT1	6	BP
GO:0072111	cell proliferation involved in	6/2734	20/17381	0.08125	0.7489	0.73657	BMP4/EGR1/OSR1/PDGFRB/SHH/STAT1	6	BP
GO:0072234	metanephric nephron tubule development	6/2734	20/17381	0.08125	0.7489	0.73657	AQP1/OSR1/PKD1/POU3F3/SOX8/STAT1	6	BP
GO:1903077	negative regulation of protein localization to	6/2734	20/17381	0.08125	0.7489	0.73657	AP2M1/CSK/LYPD1/PID1/PKDCC/TMBIM1	6	BP
GO:2000757	negative regulation of peptidyl-lysine	6/2734	20/17381	0.08125	0.7489	0.73657	BRCA1/CTBP1/HDAC8/NOC2L/SPI1/TAF7	6	BP
GO:2000780	negative regulation of double-strand	6/2734	20/17381	0.08125	0.7489	0.73657	HSF1/MIR221/NUDT16L1/OGG1/OTUB1/RECQL5	6	BP
GO:0034394	protein localization to	13/2734	55/17381	0.08162	0.7489	0.73657	CAV3/COMMD1/FLNA/GBF1/GPIHBP1/LRIG2/NRG1/PTPRU/STX4/TAX1BP3/TOR1A/USP4/WNT11	13	BP
GO:0042130	negative regulation of T cell	13/2734	55/17381	0.08162	0.7489	0.73657	BMP4/CASP3/ERBB2/HAVCR2/IDO1/IL20RB/LGALS9/MAD1L1/PLA2G2F/PRKAR1A/SCGB1A1/SHH/VSIG4	13	BP
GO:2000243	positive regulation of reproductive	13/2734	55/17381	0.08162	0.7489	0.73657	CACNA1H/CIB1/GLRA1/INHBB/LFNG/NR5A1/PIWIL2/PLB1/POMZP3/PRDM9/PRSS37/SHH/UBE2B	13	BP

GO:0001843	neural tube closure	19/2734	87/17381	0.08165	0.7489	0.73657	ABL1/ADM/BMP4/CC2D2A/DEAF1/DVL2/FZD2/GRHL3/KAT2A/LIAS/MTHFD1/PFN1/RARA/SCRIB/SETD2/SPINT1/ST14/SUFU/TULP3	19	BP
GO:1901019	regulation of calcium ion transmembrane transporter	19/2734	87/17381	0.08165	0.7489	0.73657	ADRA2A/AHNAK/CACNB1/CACNB3/CAMK2D/CAV3/CRACR2A/CRHR1/DRD2/GNB5/GPR35/GSTM2/HSPA2/JPH3/NPPA/RRAD/THADA/TLR9/TRDN	19	BP
GO:0043280	positive regulation of cysteine-type endopeptidase activity	25/2734	120/17381	0.08204	0.7489	0.73657	ANP32B/APOPT1/BAD/BCAP31/BOK/CASP1/CASP3/CTSH/DAP/DAPK1/FASLG/GPER1/HIP1R/HSF1/LCK/LGALS9/MIR15A/MTCH1/NLRP1/NOD1/PDCD2/SOX7/TRAF2/WNT3A/XDH	25	BP
GO:0043491	protein kinase B signaling	47/2734	245/17381	0.08223	0.7489	0.73657	ADAM8/ARRB2/C1QTNF1/CAV3/CCL19/CCL21/CCL5/CIB1/CSF3/DRD2/EGFR/ERBB2/FGF17/FGF3/FGFR2/FGFR4/FOXO4/GPER1/GPX1/IL18/ILK/INPP5F/INS/KDR/LCK/LEMD2/MIR221/MIR222/MIR29A/MIR29C/MTOR/MYOC/NOX4/NR4A1/NRG1/PDGFA/PDGFRB/PHLDA3/PIK3R5/PPP2R5B/PTPN11/RPS6KB2/SFRP5/THPO/TMEM100/TNFAIP8L3/XDH	47	BP
GO:0016101	diterpenoid metabolic	21/2734	98/17381	0.08239	0.7489	0.73657	AGRN/ALDH8A1/APOB/APOC3/CLPS/CYP1A1/DGAT1/EGFR/GPC1/GPC2/GPIHBP1/HSPG2/LRP1/OPN1MW/PLB1/PPARD/RARRES2/RBP1/RBP2/SDC	21	BP
GO:0021510	spinal cord development	21/2734	98/17381	0.08239	0.7489	0.73657	DAB1/DRAXIN/EVX1/HOXB8/HOXC10/LHX1/LHX3/LHX4/LHX5/MTOR/NOTCH1/OLIG3/PHOX2A/PKD1/SHH/SMO/SUFU/TULP3/WNT1/WNT3A/ZPR	21	BP
GO:0071902	positive regulation of protein serine/threonine kinase activity	65/2734	350/17381	0.08273	0.7489	0.73657	ADAM8/ADRA2A/ADRA2B/ARAF/AXIN1/BMP4/CCL19/CCND3/CD74/CD81/CDK5R2/CIB1/CSF1R/CSK/DAB2IP/DUSP5/DVL2/EGFR/ERBB2/ERN2/FZD8/GADD45G/GNG3/HACD3/HRAS/HSPA2/ILK/IRAK1/KARS/MADD/MAP2K3/MAP3K14/MAP3K15/MAP3K6/MAP4K2/MAPK3/MAPK8IP3/MAPKAPK3/MAPRE3/MIR92A2/MLST8/NEK10/NOD1/NOX4/NRG1/PDGFA/PDGFRB/PEA15/PIK3R5/PIK3R6/PKD1/PLA2G1B/PTK2B/PTPN11/RAF1/RALB/SHC1/TAOK2/TDGF1/TELO2/TIRAP/TLR9/TNIK/TRAF2/TRAF7	65	BP
GO:0043409	negative regulation of MAPK cascade	33/2734	165/17381	0.08283	0.7489	0.73657	ABL1/ATF3/BMP4/C1QL4/CAV3/CDK5RAP3/CSK/DAB2IP/DUSP2/DUSP21/DUSP26/DUSP5/EFNA1/F2RL1/FKTN/FOXM1/GBA/GPER1/LEMD2/MAPK8IP1/MARVELD3/MEN1/MIR221/MIR92A2/NCOR1/NDRG2/PDCD4/PER1/PRKCD/PSCA/RGS14/WNK2/ZMYND11	33	BP

GO:0009064	glutamine family amino acid metabolic	16/2734	71/17381	0.08291	0.7489	0.73657	AGMAT/ALDH4A1/ASL/ASNS/FAH/GAD1/GLUD1/GLYATL1/GOT2/NAGS/NOS3/OAT/OTC/PADI4/SLC7A7/UROC1	16	BP
GO:1901879	regulation of protein depolymerizatio	16/2734	71/17381	0.08291	0.7489	0.73657	CAPZA3/CFL1/CIB1/F2RL1/KATNB1/LMOD1/MAP6D1/MID1IP1/NES/SCIN/SPTAN1/SPTB/SPTBN2/TRIM54/TRPV4/WDR1	16	BP
GO:1901214	regulation of neuron death	53/2734	280/17381	0.08319	0.7489	0.73657	AARS/ABL1/ADAM8/AIMP2/AMBRA1/ATF2/ATP13A2/BAD/BARHL1/BOK/CASP3/CCL5/CDK5/CHGA/CLCF1/CLU/CNTFR/CORO1A/CSF3/DRAXIN/EGR1/EIF4G1/EN1/FASLG/FZD9/GBA/GPX1/GRIN1/HRAS/HSF1/IL13/ILK/ITGAM/LRP1/MTOR/NAIP/NES/NONO/NQO2/NSMF/PM20D1/PPP1R13B/PTK2B/RILPL1/SIGMAR1/SNCB/STXBP1/TBK1/TRAF2/WFS1/WNT1/WNT3A/Z	53	BP
GO:0050708	regulation of protein secretion	77/2734	421/17381	0.08376	0.7489	0.73657	ABL1/ACHE/ADAM8/ADRA2A/ADRA2C/ANG/ANO1/APBB1/ARFIP1/ARL2BP/ATP13A2/BAD/BLK/BRSK2/CAPN10/CASP1/CASP5/CCL19/CCL5/CD58/CDK16/CSF1R/DOC2B/DRD2/EGFR/F2RL1/FGA/FN1/GAPDH/GCG/GCK/GLUD1/GPER1/GSDMD/HAVCR2/HCAR2/HLA-E/IDH2/IL13/IL4R/INHBB/INS/ITPR3/KARS/LGALS9/LLGL1/MAPK3/MTNR1B/MYO18A/NLRP1/NR1H3/OPRM1/ORM1/ORM2/P3H1/PFKM/PLA2G1B/PPARD/PTPN11/PTPN23/PYDC1/RAB11FIP3/RFX6/RHBDF1/SERGEF/SIDT2/SLC25A5/SSTR5/STX1A/STX4/TLR8/TLR9/TNFRSF4/TRAF2/TRH/TRPV4/[illegible]	77	BP
GO:0016053	organic acid biosynthetic process	60/2734	321/17381	0.08387	0.7489	0.73657	ABCC5/ACACB/ACADVL/ACOT7/ACSF3/ACSM6/ALDH8A1/APOA5/APOC3/ASL/ASNS/BCAN/BGN/BHMT2/BRCA1/CARNS1/CD74/CHST12/CYP1A1/DSE/EDN2/ELOVL1/ELOVL5/FADS2/FADS3/FGFR4/GAD1/GCH1/GGT6/GGTA1P/GLUD1/GOT2/GPT/HAAO/HACD1/HACD3/IDO1/LIAS/MGST3/MID1IP1/MLXIPL/MTHFD1/NAALAD2/NAGS/NR1H3/OAT/OLAH/OSBPL7/OTC/PHGDH/PKM/PLA2G1B/PLA2G5/PLOD3/PRKAB1/RBP1/SCAP/SHMT2/THNS[illegible]	60	BP
GO:0046394	carboxylic acid biosynthetic process	60/2734	321/17381	0.08387	0.7489	0.73657	ABCC5/ACACB/ACADVL/ACOT7/ACSF3/ACSM6/ALDH8A1/APOA5/APOC3/ASL/ASNS/BCAN/BGN/BHMT2/BRCA1/CARNS1/CD74/CHST12/CYP1A1/DSE/EDN2/ELOVL1/ELOVL5/FADS2/FADS3/FGFR4/GAD1/GCH1/GGT6/GGTA1P/GLUD1/GOT2/GPT/HAAO/HACD1/HACD3/IDO1/LIAS/MGST3/MID1IP1/MLXIPL/MTHFD1/NAALAD2/NAGS/NR1H3/OAT/OLAH/OSBPL7/OTC/PHGDH/PKM/PLA2G1B/PLA2G5/PLOD3/PRKAB1/RBP1/SCAP/SHMT2/THNS[illegible]	60	BP

GO:0050821	protein stabilization	31/2734	154/17381	0.08452	0.7489	0.73657	AHSP/CCT3/CLU/CPN2/CRYAB/CSN3/DNLZ/EFNA1/FLNA/GAPDH/GPIHBP1/HCFC1/HIP1R/HSP90AA1/NAA16/P3H1/PEX6/PFN1/PPIB/PRKCD/SMO/STXBP1/SYVN1/TAF1/TELO2/TNIP2/UBE2B/USP19/USP2/USP27X/WFS1	31	BP
GO:1905475	regulation of protein localization to	31/2734	154/17381	0.08452	0.7489	0.73657	AP2M1/ARHGEF16/BAD/CDK5/CIB1/CNPY4/CSK/DYNLL2/EGFR/ERBB2/FZD9/GPER1/INS/ITGAM/LRP1/LYPD1/MIEF2/MYO1C/NECAB2/NKD2/PID1/PKDCC/PPP1R13B/SFN/STX4/STX8/TMBIM1/TREM2/VTI1B/WNT3A/YWH	31	BP
GO:0021953	central nervous system neuron differentiation	34/2734	171/17381	0.08463	0.7489	0.73657	AXIN1/CDK5/CEND1/CSF1R/DCLK2/DISC1/DRAXIN/DRD2/EMX1/EPHB3/EVX1/FGFR2/GBX2/HOXC10/KNDC1/LDB1/LHX1/LHX3/LHX4/LHX5/OGDH/OLIG3/OTP/PHOX2A/PHOX2B/SHH/SMO/SUFU/TBR1/TSKU/TULP3/WN	34	BP
GO:0030324	lung development	34/2734	171/17381	0.08463	0.7489	0.73657	ADAMTS2/AIMP2/BMP4/CCDC40/CTSH/CYP1A2/EDN2/EGFR/EIF4EBP1/ESRP2/EYA1/FGFR2/FLT4/HHIP/IL13/LHX3/MAPK3/MYCN/NKX2-8/NOS3/NOTCH1/NUMA1/PDGFA/PDGFRB/PKD1/PKDCC/PLOD3/SHH/SPDEF/STRA6/THRA/TIMELESS/TNC/WNT11	34	BP
GO:0051896	regulation of protein kinase B signaling	41/2734	211/17381	0.08479	0.7489	0.73657	ADAM8/ARRB2/C1QTNF1/CAV3/CCL19/CCL21/CIB1/CSF3/DRD2/EGFR/ERBB2/FGF17/FGF3/FGFR2/FGFR4/GPER1/GPX1/IL18/ILK/INPP5F/INS/LCK/LEMD2/MIR221/MIR222/MIR29A/MIR29C/MTOR/MYOC/NOX4/NRG1/PDGFA/PDGFRB/PHLDA3/PIK3R5/PPP2R5B/PTPN11/SFRP5/THPO/TNFAIP8L3/	41	BP
GO:0001704	formation of primary germ layer	26/2734	126/17381	0.08484	0.7489	0.73657	AXIN1/BMP4/COL4A2/COL7A1/CRB2/DUSP2/DUSP5/EYA1/FGFR2/FN1/ITGA7/ITGB5/KDM6B/LHX1/MESP2/MMP15/MMP2/PRKAR1A/SCX/SETD2/SOX7/TLX2/TXNRD1/WLS/WNT11/WNT3A	26	BP
GO:0014065	phosphatidylinositol 3-kinase signaling	26/2734	126/17381	0.08484	0.7489	0.73657	AGT/CCL5/CSF3/DAB2IP/EGFR/ERBB2/F2RL1/GPER1/IL18/INS/KDR/LTK/MAPK3/MYOC/NCF1/NRG1/NYAP1/PDGFA/PDGFRB/PIK3R5/PIK3R6/PLXNB1/PPARD/PPP2R5B/PREX2/SELP	26	BP
GO:0090263	positive regulation of canonical Wnt	26/2734	126/17381	0.08484	0.7489	0.73657	AXIN1/CCAR2/COL1A1/DVL2/FGFR2/FZD9/ILK/LGR6/MIR222/NAIP/NLE1/PSMB11/PSMB6/PSMB7/PSMB8/PSMC3/PSMD13/PSMD3/PSMD5/PSMD7/RNF220/RSPO1/WLS/WNT1/WNT3A/WNT7A	26	BP

GO:0002237	response to molecule of bacterial origin	62/2734	333/17381	0.08499	0.7489	0.73657	ABL1/ADAMTS13/ADM/ALAD/APOB/CARD9/CASP1/CASP3/CCL5/CD180/CD27/CD6/CDK4/CSF3/CYP1A1/CYP1A2/DAB2IP/FASLG/FGFR2/GCH1/HAVCR2/HSF1/IDO1/IL13/IL18/IL18BP/IRAK1/IRF5/LGALS9/LIAS/LOXL1/LTA/LTBR/MAPK3/MAPKAPK3/MGST1/MPO/NFKBIL1/NOS3/NOTCH1/NR1H3/OPRM1/OTUD5/PDCD4/PF4V1/PPARD/PRPF8/RARA/RELT/REN/SCARB1/SCGB1A1/SELP/SLC11A1/SPON2/TH/TIRAP/TLR9/TNFRSF18/TNFRSF4/TN[illegible]	62	BP
GO:0051047	positive regulation of secretion	71/2734	386/17381	0.08528	0.7489	0.73657	ABL1/ACHE/ADAM8/AGT/ANG/ANO1/APBB1/AQP1/ATP13A2/BAD/BLK/C1QTNF1/CACNA1G/CACNA1H/CAPN10/CASP1/CASP5/CCL19/CD58/CDK5/CDK5R2/CSF1R/DOC2B/DRD2/DTNBP1/EGFR/F2RL1/FGA/GAPDH/GCG/GCK/GLUD1/GPER1/GSDMD/HAVCR2/HCAR2/HLA-E/IL13/IL4R/INHBB/INS/ITGAM/KARS/LGALS9/MAPK3/MYO18A/NLRP1/ORM1/ORM2/PFKM/PLA2G1B/PPARD/PTPN11/PTPN23/PYDC1/RAB7A/RFX6/SCIN/SDC1/SNF8/STX1A/STX4/STXBP1/TACR1/TACR2/TFR2/TLR8/TNF[illegible]	71	BP
GO:0002407	dendritic cell chemotaxis	7/2734	25/17381	0.08542	0.7489	0.73657	CCL19/CCL21/CCL5/CCR2/CXCR1/CXCR2/LGALS9	7	BP
GO:0003148	outflow tract septum morphogenesis	7/2734	25/17381	0.08542	0.7489	0.73657	BMP4/ENG/FGFR2/NKX2-5/RARA/SMAD6/TBX2	7	BP
GO:0006700	C21-steroid hormone biosynthetic process	7/2734	25/17381	0.08542	0.7489	0.73657	ADM/CACNA1H/CYP11A1/CYP11B2/DGKQ/EGR1/STARD3	7	BP
GO:0035902	response to immobilization stress	7/2734	25/17381	0.08542	0.7489	0.73657	CYP1A1/CYP1A2/FOXO4/HNMT/PTK2B/REN/TH	7	BP
GO:0071526	semaphorin-plexin signaling	7/2734	25/17381	0.08542	0.7489	0.73657	FARP2/FLNA/PLXNB1/PLXNB3/PLXND1/SEMA3F/SEMA4A	7	BP

GO:0044743	protein transmembrane import into intracellular organelle	11/2734	45/17381	0.08544	0.7489	0.73657	DNLZ/HSP90AA1/PEX14/PEX16/PEX3/PEX5/PEX6/TIMM17B/TIMM22/TIMM23B/TIMM9	11	BP
GO:0045824	negative regulation of innate immune	11/2734	45/17381	0.08544	0.7489	0.73657	ARRB2/DHX58/DRD2/HAVCR2/HLA-E/INS/LGALS9/NLRX1/NMI/NR1H3/SCRIB	11	BP
GO:0007368	determination of left/right symmetry	24/2734	115/17381	0.08564	0.7489	0.73657	AXIN1/C2CD3/CC2D2A/CCDC103/CCDC40/CFC1/DNAH11/ENG/GAS8/GATA4/IFT140/LRRC6/MICAL2/NEK8/NKX2-5/NKX3-2/NOTCH1/NOTO/SHH/SMO/SUFU/TBC1D32/TBX2/WNT3A	24	BP
GO:0048708	astrocyte differentiation	15/2734	66/17381	0.08585	0.7489	0.73657	ABL1/BIN1/CLCF1/DAB1/EGFR/EIF2B5/LAMC3/LRP1/MAPK3/MYCN/NOTCH1/PTPN11/SHH/SMO/SOX8	15	BP
GO:1903725	regulation of phospholipid metabolic	15/2734	66/17381	0.08585	0.7489	0.73657	AMBRA1/CCL19/CCL21/CD81/DAB2IP/EPHA8/IDH1/PDGFA/PDGFRB/PIK3R5/PIK3R6/PRKCD/PTK2B/SCARB1/TNFAIP8L3	15	BP
GO:0043543	protein acylation	42/2734	217/17381	0.08603	0.7489	0.73657	APBB1/ATF2/BRCA1/BRPF1/CRTC2/CTBP1/EPC1/HCFC1/HDAC8/ING4/KAT2A/LDB1/MAP6D1/MAPK3/MSL3/MUC1/MYOD1/NAA16/NAA60/NAT16/NAT8B/NOC2L/NUPR1/PAXIP1/PCGF2/PER1/PIWIL2/POLE4/POR/PORCN/PYGO2/RPS6KA4/SPI1/TADA3/TAF1/TAF1L/TAF7/ZDHHC12/ZDHHC14/ZD	42	BP
GO:0070663	regulation of leukocyte proliferation	42/2734	217/17381	0.08603	0.7489	0.73657	BCL6/BMP4/CARD11/CASP3/CCDC88B/CCL19/CCL5/CCR2/CD6/CD74/CD81/CLCF1/CLECL1/CORO1A/CSF1/DNAJA3/EFNB1/ERBB2/HAVCR2/HLA-E/IDO1/IL13/IL18/IL20RB/INPP5D/LGALS3/LGALS9/LMO1/MAD1L1/MIR181B1/MZB1/PLA2G2F/PRKAR1A/SCGB1A1/SHH/TACR1/TIRAP/TLR9/TNFRSF13B/TNFRSF4/VSIG4/WNT3A	42	BP
GO:0060485	mesenchyme development	47/2734	246/17381	0.08673	0.7489	0.73657	ACTA1/BMP4/CFL1/COL1A1/CRB2/CRELD1/DAB2IP/EFNA1/EFNB1/ENG/FGFR2/GATA4/GBX2/GLIPR2/HEY1/LOXL2/MAPK3/MIR221/MIR222/MTOR/NKX2-5/NOTCH1/NRG1/OSR1/PBLD/PDCD4/PDGFRB/PHOX2B/SCX/SEMA3F/SHH/SMO/SOX8/STAT1/TBX2/TGFB1I1/TMEM100/TRIM62/TSPY1/TSPY2/TSP	47	BP

GO:0001937	negative regulation of endothelial cell	10/2734	40/17381	0.08678	0.7489	0.73657	AIMP1/ATPIF1/GDF2/MIR16-1/MIR222/MIR29C/MIR503/STAT1/TNMD/XDH	10	BP
GO:0002833	positive regulation of response to	10/2734	40/17381	0.08678	0.7489	0.73657	CD180/DHX58/F2RL1/HAVCR2/LRSAM1/MAPK3/NCR3/NOD1/PGC/TBK1	10	BP
GO:0042088	T-helper 1 type immune	10/2734	40/17381	0.08678	0.7489	0.73657	CCL19/CCR2/HAVCR2/HLX/HRAS/IL18/IL18BP/IL4R/SEMA4A/SLC11A1	10	BP
GO:0042181	ketone biosynthetic process	10/2734	40/17381	0.08678	0.7489	0.73657	ADM/CACNA1H/COQ2/COQ4/CYP11B2/DGKQ/EGR1/HSD17B3/PDSS1/STARD3	10	BP
GO:0042461	photoreceptor cell	10/2734	40/17381	0.08678	0.7489	0.73657	CNGB1/GNAT1/IFT140/IFT20/MYO7A/NAGLU/NRL/TH/THY1/TULP1	10	BP
GO:0050706	regulation of interleukin-1 beta secretion	10/2734	40/17381	0.08678	0.7489	0.73657	CASP1/CASP5/CCL19/GSDMD/LGALS9/NLRP1/ORM1/ORM2/PYDC1/TLR8	10	BP
GO:0090184	positive regulation of kidney	10/2734	40/17381	0.08678	0.7489	0.73657	AGT/BMP4/EGR1/HOXB7/LHX1/PDGFA/PDGFRB/SHH/SMO/SOX8	10	BP
GO:0003016	respiratory system process	8/2734	30/17381	0.08721	0.7489	0.73657	ECEL1/FLT4/GLRA1/JAG2/NLGN3/PHOX2B/TLX3/TNNC1	8	BP
GO:0006099	tricarboxylic acid cycle	8/2734	30/17381	0.08721	0.7489	0.73657	ACO1/CS/DLST/IDH1/IDH2/OGDH/OGDHL/PDHB	8	BP
GO:0006658	phosphatidylserine metabolic	8/2734	30/17381	0.08721	0.7489	0.73657	OSBPL5/PLA2G16/PLA2G1B/PLA2G2F/PLA2G4B/PLA2G5/PTDSS1/PTDSS2	8	BP
GO:0036258	multivesicular body assembly	8/2734	30/17381	0.08721	0.7489	0.73657	CHMP1A/CHMP4C/CHMP7/SNF8/VPS25/VPS37B/VPS37C/VPS37D	8	BP
GO:0042491	auditory receptor cell	8/2734	30/17381	0.08721	0.7489	0.73657	JAG2/LHFPL5/LRTOMT/MYCN/MYO7A/NOTCH1/SCRIB/WDPCP	8	BP
GO:0043403	skeletal muscle tissue	8/2734	30/17381	0.08721	0.7489	0.73657	BCL9/BIN3/GJD4/GPX1/MYOD1/PKM/PPARD/WNT7A	8	BP

GO:0032496	response to lipopolysaccharide	59/2734	316/17381	0.08726	0.7489	0.73657	ABL1/ADAMTS13/ADM/ALAD/APOB/CASP1/CASP3/CCL5/CD180/CD27/CD6/CDK4/CSF3/CYP1A1/CYP1A2/DAB2IP/FASLG/FGFR2/GCH1/HAVCR2/HSF1/IDO1/IL13/IL18/IL18BP/IRAK1/LGALS9/LIAS/LOXL1/LTA/LTBR/MAPK3/MAPKAPK3/MGST1/MPO/NFKBIL1/NOS3/NOTCH1/NR1H3/OPRM1/OTUD5/PDCD4/PF4V1/PPARD/PRPF8/RARA/RELT/REN/SCARB1/SCGB1A1/SELP/SLC11A1/SPON2/TH/TIRAP/TNFRSF18/TNFRSF4/TNIP2/TREM2	59	BP
GO:0045471	response to ethanol	27/2734	132/17381	0.08747	0.7489	0.73657	ALAD/BAD/BGLAP/CD27/CSF3/DNMT3A/DRD2/EIF4EBP1/FGFR2/GGH/GLRA1/GOT2/GRIN1/IL13/OGG1/OPRM1/PEMT/PTK2B/RARA/SDF4/SETD7/SLC2A4/SPI1/TACR1/TH/TNC/TRH	27	BP
GO:0050921	positive regulation of chemotaxis	27/2734	132/17381	0.08747	0.7489	0.73657	ARTN/C3AR1/CAMK1D/CCL1/CCL19/CCL21/CCL5/CCR2/CD74/CDH13/CREB3/CSF1/DAPK2/EDN2/F2RL1/HSPB1/KARS/KDR/LGALS9/MAPK3/PDGFRB/PF4V1/PTK2B/RARRES2/STX4/TIRAP/TRPV4	27	BP
GO:0051017	actin filament bundle assembly	27/2734	132/17381	0.08747	0.7489	0.73657	ABL1/ARAP1/ARHGEF10/BAIAP2L1/CORO1B/ESPN/ESPNL/EVL/FHOD1/FSCN1/ITGB5/LIMK1/MTOR/MYOC/NOX4/PFN1/PHACTR1/PTK2B/RHOD/SERPINF2/SH3PXD2B/SHROOM1/SORBS3/TACR1/WAS/WNT11/ZYX	27	BP
GO:0007271	synaptic transmission, cholinergic	9/2734	35/17381	0.08748	0.7489	0.73657	ACHE/CHRM1/CHRNA6/CHRNB1/CHRNB3/LAMA2/LYPD1/TACR1/TACR2	9	BP
GO:0007603	phototransduction, visible light	9/2734	35/17381	0.08748	0.7489	0.73657	AIPL1/CNGB1/FNTA/FNTB/GNAT1/GUCA1B/GUCY2D/OPN4/PPEF1	9	BP
GO:0050892	intestinal absorption	9/2734	35/17381	0.08748	0.7489	0.73657	ABCG5/ACO1/ADRA2A/APOA5/KCNQ1/NPC1L1/SCARB1/SLC26A6/SOAT2	9	BP
GO:0072028	nephron morphogenesis	17/2734	77/17381	0.08828	0.7492	0.73686	AGT/BMP4/EYA1/HOXB7/ILK/IRX3/LHX1/OSR1/PDGFRB/PKD1/SHH/SMO/SOX8/STAT1/WNT1/WNT11/WNT6	17	BP
GO:0010948	negative regulation of cell cycle process	61/2734	328/17381	0.08839	0.7492	0.73686	ANAPC15/BCL6/BMP4/BRCA1/BUB1B/C10orf99/CCNF/CDK2AP2/CDK4/CDK5RAP3/CDK9/CHMP4C/CTDSP1/DAB2IP/DUX4/E2F4/E2F8/EHMT1/EHMT2/EPC1/FOXE3/FOXO4/FZD9/GPER1/GPR132/HORMAD1/LCMT1/MAD1L1/MAX/MEN1/MIIP/MIR10A/MIR15A/MIR16-1/MIR195/MIR29A/MIR29C/MIR503/MLXIPL/MUC1/NUBP1/PCBP4/PCGF2/PHOX2B/PPP2R5B/PRKAR1A/PSMB11/PSMB6/PSMB7/PSMB8/PSMC3/PSMD13/PSMD3/PSMD5/PSMD7/RINT1/SFN/TICRR/UXT/XRCC3/ZNF385A	61	BP

GO:0050670	regulation of lymphocyte proliferation	40/2734	206/17381	0.08849	0.7492	0.73686	BCL6/BMP4/CARD11/CASP3/CCDC88B/CCL19/CCL5/CCR2/CD6/CD74/CD81/CLCF1/CLECL1/CORO1A/DNAJA3/EFNB1/ERBB2/HAVCR2/HLA-E/IDO1/IL13/IL18/IL20RB/INPP5D/LGALS3/LGALS9/LMO1/MAD1L1/MZB1/PLA2G2F/PRKAR1A/SCGB1A1/SHH/TACR1/TIRAP/TLR9/TNFRSF13B/TNFRSF4/VSIG4/WNT3A	40	BP
GO:0002228	natural killer cell mediated	14/2734	61/17381	0.08879	0.7492	0.73686	ARRB2/CORO1A/HAVCR2/HLA-E/IL18/KLRC2/LGALS9/MICB/NCR3/PIK3R6/PRDX1/RAET1G/SLAMF7/UL	14	BP
GO:0032651	regulation of interleukin-1 beta production	14/2734	61/17381	0.08879	0.7492	0.73686	ARRB2/CASP1/CASP5/CCL19/EGR1/GSDMD/HSPB1/LGALS9/NLRP1/NOD1/ORM1/ORM2/PYDC1/TLR8	14	BP
GO:0060193	positive regulation of	14/2734	61/17381	0.08879	0.7492	0.73686	ADRA1A/AGT/ANG/APOA5/CCL5/EGFR/FGFR2/GPIHBP1/LMF1/NR1H3/PDGFRB/PLA2G1B/PLA2G5/PLCB2	14	BP
GO:0030004	cellular monovalent inorganic cation homeostasis	23/2734	110/17381	0.08941	0.7492	0.73686	AGT/ATP1A1/ATP1A4/ATP6V0B/ATP6V0E2/ATP6V1B1/ATP6V1F/ATP6V1G1/ATP6V1G2/CA7/CLN6/FASLG/GPR89A/MAPK3/RAB20/RAB7A/SLC11A1/SLC26A1/SLC26A10/SLC26A6/SLC4A9/SLC9A3/SLC9A5	23	BP
GO:0060606	tube closure	19/2734	88/17381	0.08946	0.7492	0.73686	ABL1/ADM/BMP4/CC2D2A/DEAF1/DVL2/FZD2/GRHL3/KAT2A/LIAS/MTHFD1/PFN1/RARA/SCRIB/SETD2/SPINT1/ST14/SUFU/TULP3	19	BP
GO:0097722	sperm motility	19/2734	88/17381	0.08946	0.7492	0.73686	APOB/ATP1A4/CATSPER1/CCDC40/CFAP157/DDX4/DNAH11/GAS8/LRRC6/PGAM4/PRDM14/RNASE9/SPAG16/TACR1/TACR2/TEKT4/TEKT5/TTLL5	19	BP
GO:1990748	cellular detoxification	21/2734	99/17381	0.08975	0.7492	0.73686	EPX/FAM213A/GCH1/GPX1/GPX2/GPX3/GSR/GSTM1/GSTM2/GSTZ1/LPO/MGST1/MGST3/MPO/NOS3/PRDX1/PRDX6/SESN1/SOD3/TXNRD1/TXNRD	21	BP
GO:1902903	regulation of supramolecular fiber organization	57/2734	305/17381	0.09018	0.7492	0.73686	ABL1/ANKRD53/ARAP1/ARFIP1/ARHGEF10/ARPC1A/ARPC1B/ARPC2/ARPC4/BAIAP2L1/BRK1/CAPZA3/CAV3/CCL21/CDC42EP2/CFL1/CIB1/CLU/CORO1A/CORO1B/CRYAB/CSF3/CTTN/EVL/F2RL1/FES/FHOD1/GPX1/HIP1R/KATNB1/LIMK1/LMOD1/MAP6D1/MID1IP1/MLST8/MTOR/MYO1C/MYOC/NOX4/NUMA1/PFN1/PRKCD/PTK2B/SCIN/SERPINF2/SH3PXD2B/SORBS3/SPTAN1/SPTB/SPTBN2/TACR1/TRIM54/TRPV4/WAS/WDR1/WHAMM/W	57	BP

GO:0033209	tumor necrosis factor-mediated signaling pathway	34/2734	172/17381	0.09019	0.7492	0.73686	CASP1/CCDC3/CD27/F2RL1/IKBKB/ILK/KARS/LTA/LTBR/MADD/MAP3K14/PELI3/PSMB11/PSMB6/PSMB7/PSMB8/PSMC3/PSMD13/PSMD3/PSMD5/PSMD7/PTK2B/PYDC1/RELT/RNF31/SHARPIN/STAT1/TNFRSF12A/TNFRSF13B/TNFRSF18/TNFRSF4/TNFSF13/TRAF2/TRAIP	34	BP
GO:0051702	interaction with symbiont	16/2734	72/17381	0.09173	0.7492	0.73686	AQP1/CCL5/CFL1/DDB1/EIF2AK4/F2RL1/FN1/GAPDH/GPX1/MIR221/MIR222/PC/PPIB/PSMC3/TUSC2/ZNF502	16	BP
GO:0008064	regulation of actin polymerization	32/2734	161/17381	0.09236	0.7492	0.73686	ARFIP1/ARPC1A/ARPC1B/ARPC2/ARPC4/BAIAP2L1/BRK1/CAPZA3/CCL21/CDC42EP2/CFL1/CORO1A/CORO1B/CSF3/CTTN/EVL/F2RL1/HIP1R/LMOD1/MLST8/MTOR/MYO1C/PFN1/PRKCD/PTK2B/SCIN/SPTAN1/SPTB/SPTB	32	BP
GO:0071496	cellular response to external stimulus	54/2734	288/17381	0.09252	0.7492	0.73686	AGT/AMBRA1/AQP1/ASGR1/ASNS/ATF3/ATP1A1/BAD/BGLAP/CASP1/CASP5/CHMP1A/COL1A1/DAP/DAPL1/EGFR/EHMT2/EIF2AK4/ENG/FOLR2/FOSL1/GBA/GSDMD/HSPA5/IL13/INHBB/KCNK4/KIF26A/LARS/LTBR/MAP3K14/MAPK3/MAX/MTOR/MYOD1/NPPA/NPRL3/NUDT1/PDE2A/PPP1R9B/PTPN11/RALB/RRAGC/SCX/SESN1/SIPA1/SLC39A5/TLR8/TMEM150C/TN	54	BP
GO:0072089	stem cell proliferation	24/2734	116/17381	0.09257	0.7492	0.73686	ARTN/DAGLB/DISC1/DOCK7/DRD2/EML1/FGFR2/FZD9/HHIP/LTBP3/MIR221/MIR222/NES/NOTCH1/OTP/PDCD2/SETD1A/SHH/SMO/SOX5/THPO/VAX1/WNT1/WNT3A	24	BP
GO:0030322	stabilization of membrane potential	5/2734	16/17381	0.09313	0.7492	0.73686	KCNK12/KCNK13/KCNK17/KCNK4/KCNK7	5	BP
GO:0032011	ARF protein signal	5/2734	16/17381	0.09313	0.7492	0.73686	GBF1/IQSEC2/PSD/PSD2/PSD4	5	BP
GO:0032012	regulation of ARF protein signal	5/2734	16/17381	0.09313	0.7492	0.73686	GBF1/IQSEC2/PSD/PSD2/PSD4	5	BP
GO:0032495	response to muramyl	5/2734	16/17381	0.09313	0.7492	0.73686	CARD9/IRF5/NLRP1/NOD1/NOTCH1	5	BP
GO:0034315	regulation of Arp2/3 complex-mediated actin	5/2734	16/17381	0.09313	0.7492	0.73686	ARFIP1/BRK1/CORO1B/HIP1R/WAS	5	BP

GO:0035930	corticosteroid hormone	5/2734	16/17381	0.09313	0.7492	0.73686	AGT/C1QTNF1/CRHR1/PTPN11/REN	5	BP
GO:0045064	T-helper 2 cell differentiation	5/2734	16/17381	0.09313	0.7492	0.73686	BCL6/HLX/IL18/IL4R/RARA	5	BP
GO:0045723	positive regulation of fatty acid biosynthetic	5/2734	16/17381	0.09313	0.7492	0.73686	APOA5/ELOVL5/MID1IP1/MLXIPL/NR1H3	5	BP
GO:0045989	positive regulation of striated muscle	5/2734	16/17381	0.09313	0.7492	0.73686	ADRA1A/ATP1A1/CHGA/KCNQ1/NPPA	5	BP
GO:0048532	anatomical structure arrangement	5/2734	16/17381	0.09313	0.7492	0.73686	DAB1/HOXB1/HOXB2/HSPA5/SEMA3F	5	BP
GO:0050655	dermatan sulfate proteoglycan	5/2734	16/17381	0.09313	0.7492	0.73686	B3GAT3/BCAN/BGN/CHST12/DSE	5	BP
GO:0050884	neuromuscular process controlling	5/2734	16/17381	0.09313	0.7492	0.73686	GCH1/GLRA1/PNKD/SLURP1/TMEM150C	5	BP
GO:0050908	detection of light stimulus involved in visual	5/2734	16/17381	0.09313	0.7492	0.73686	CACNA1F/CNGB1/GNAT1/SEMA5B/TULP1	5	BP
GO:0050962	detection of light stimulus involved in sensory	5/2734	16/17381	0.09313	0.7492	0.73686	CACNA1F/CNGB1/GNAT1/SEMA5B/TULP1	5	BP
GO:0051151	negative regulation of smooth muscle	5/2734	16/17381	0.09313	0.7492	0.73686	FOXO4/MIR221/PDCD4/RBPMS2/SHH	5	BP

GO:0060117	auditory receptor cell	5/2734	16/17381	0.09313	0.7492	0.73686	LHFPL5/LRTOMT/MYO7A/SCRIB/WDPCP	5	BP
GO:1901317	regulation of flagellated sperm motility	5/2734	16/17381	0.09313	0.7492	0.73686	PGAM4/PRDM14/RNASE9/TACR1/TACR2	5	BP
GO:0050886	endocrine process	18/2734	83/17381	0.0932	0.7492	0.73686	AGT/AQP1/AVPR2/C1QTNF1/CRHR1/CYP11B2/EDN2/F2RL1/HCAR2/HSD11B2/INHBB/NOS3/PTPN11/RAB11FIP3/REN/SERPINF2/TACR1/TACR2	18	BP
GO:0006575	cellular modified amino acid metabolic process	36/2734	184/17381	0.09348	0.7492	0.73686	ALDH1L1/ALDH4A1/BHMT2/CARNS1/CHAC1/CHDH/CRYM/DPEP1/DUOXA1/FOLR2/GCH1/GGH/GGT6/GLO1/GOT2/GPX1/GSR/GSTM1/GSTM2/GSTZ1/IDH1/MTHFD1/OSBPL5/PEMT/PIPOX/PLA2G16/PLA2G1B/PLA2G2F/PLA2G4B/PLA2G5/PLOD3/PTDSS1/PTDSS2/SHMT2/SLC6A8/VNN3	36	BP
GO:1901136	carbohydrate derivative catabolic process	36/2734	184/17381	0.09348	0.7492	0.73686	ACAN/AGRN/AMDHD2/BCAN/BGN/CDA/CHID1/CHIT1/FBXO2/FBXO6/GALT/GBA/GPC1/GPC2/HEXA/HINT1/HSPG2/IDS/MBD4/MGAT1/MIR181B1/NAGK/NAGLU/NEIL2/NEU3/NPL/NT5M/NUDT1/NUDT18/OGG1/PDE2A/PRKCD/SDC1/SGSH/SMUG1/SPACA3	36	BP
GO:0014020	primary neural tube formation	20/2734	94/17381	0.09365	0.7492	0.73686	ABL1/ADM/BMP4/CC2D2A/CFL1/DEAF1/DVL2/FZD2/GRHL3/KAT2A/LIAS/MTHFD1/PFN1/RARA/SCRIB/SETD2/SPINT1/ST14/SUFU/TULP3	20	BP
GO:0019233	sensory perception of	20/2734	94/17381	0.09365	0.7492	0.73686	ACPP/ADRA2C/ANO1/AQP1/ARRB2/ASIC3/CDK5/GRIN1/HOXB8/HOXD1/KCNK4/MAPK3/MTOR/OPRD1/OPRM1/PHF24/PRDM12/SCN11A/TACR1/T	20	BP
GO:0006626	protein targeting to mitochondrion	30/2734	150/17381	0.09446	0.7492	0.73686	ACSM6/AIP/ATPIF1/CYB5R1/DNAJC19/DNLZ/ELMOD1/GPHA2/HNMT/HSP90AA1/ITGAX/KAT2A/LRRC46/MFN2/MYBPC1/NBPF3/NRG1/OSCP1/PDE2A/PEMT/PMPCA/PSMB7/RNF31/TIMM17B/TIMM22/TIMM23B/TIMM9/TS	30	BP
GO:0019320	hexose catabolic	12/2734	51/17381	0.09448	0.7492	0.73686	ALDOA/BAD/FUT7/GALK1/GALM/GALT/GAPDH/GCK/GLYCTK/HK3/PFKM/PKM	12	BP
GO:0006026	aminoglycan catabolic	15/2734	67/17381	0.09527	0.7492	0.73686	ACAN/AGRN/BCAN/BGN/CHID1/CHIT1/GPC1/GPC2/HEXA/HSPG2/IDS/NAGLU/SDC1/SGSH/SPACA3	15	BP
GO:0006305	DNA alkylation	15/2734	67/17381	0.09527	0.7492	0.73686	BRCA1/DDX4/DNMT3A/EHMT1/EHMT2/FKBP6/MIR29A/MIR29C/MPHOSPH8/PIWIL2/PRDM14/RLF/SPI1/TDRD1/TDRD9	15	BP
GO:0006306	DNA methylation	15/2734	67/17381	0.09527	0.7492	0.73686	BRCA1/DDX4/DNMT3A/EHMT1/EHMT2/FKBP6/MIR29A/MIR29C/MPHOSPH8/PIWIL2/PRDM14/RLF/SPI1/TDRD1/TDRD9	15	BP

GO:0030104	water homeostasis	15/2734	67/17381	0.09527	0.7492	0.73686	AQP1/AVPR2/CLDN4/CYP11B2/FLG2/GBA/GRHL3/KRT16/PRKAR1A/PRKAR1B/SCNN1A/SFN/TMEM79/TRPV4/WFS1	15	BP
GO:1900076	regulation of cellular response to	15/2734	67/17381	0.09527	0.7492	0.73686	AGT/AHSG/BAIAP2L1/CCND3/CDK4/CISH/ESRRA/INS/MYO1C/MZB1/PID1/PRKCD/PTPN11/RARRES2/TRIM72	15	BP
GO:1903557	positive regulation of tumor necrosis factor superfamily	15/2734	67/17381	0.09527	0.7492	0.73686	ADAM8/CARD9/CCL19/CCR2/CLU/HAVCR2/HLA-E/HSPB1/LGALS9/NOD1/ORM1/ORM2/SPON2/TIRAP/TLR9	15	BP
GO:1904035	regulation of epithelial cell apoptotic	15/2734	67/17381	0.09527	0.7492	0.73686	ABL1/BOK/CAPN10/ECSCR/FASLG/FGA/GPER1/IL13/KDR/MIR15A/MTOR/PDCD4/SPOP/TNIP2/WFS1	15	BP
GO:0000302	response to reactive oxygen species	42/2734	219/17381	0.09613	0.7492	0.73686	ABL1/ADPRHL2/APEX1/AQP1/BAD/CASP3/CCL19/COL1A1/CRYAB/DPEP1/EGFR/ENDOG/FOSL1/GCH1/GNAO1/GPX1/HSF1/IL18BP/KDM6B/LDHA/MAPK3/MIR92A2/MMP3/MPO/NCF1/NOS3/PCGF2/PDGFRB/PRDX1/PRKCD/PSAP/PTK2B/SCGB1A1/SDC1/SESN1/SOD3/TACR1/TRAF2/TRAP1/TXN	42	BP
GO:0032868	response to insulin	47/2734	248/17381	0.09623	0.7492	0.73686	ADM/AGT/AHSG/ATP6V0B/ATP6V0E2/ATP6V1B1/ATP6V1F/ATP6V1G1/ATP6V1G2/BAIAP2L1/CAPN10/CCND3/CDK4/CISH/EGR1/EIF4EBP1/ESRRA/FOXO4/GCK/GGH/HADHA/HSD11B2/INHBB/INPPL1/INS/MAX/MTOR/MYO1C/MZB1/NPPA/OTC/PFKFB1/PID1/PKM/PLA2G1B/PRKCD/PTPN11/RARRES2/SCAP/SHC1/SLC2A4/SLC2A8/SRSF4/TRIM72/TRPV4/UCP3/VGF	47	BP
GO:0072330	monocarboxylic acid biosynthetic process	38/2734	196/17381	0.09644	0.7492	0.73686	ACACB/ACADVL/ACOT7/ACSF3/ACSM6/ALDH8A1/APOA5/APOC3/ASNS/BCAN/BGN/BRCA1/CD74/CHST12/CYP1A1/DSE/EDN2/ELOVL1/ELOVL5/FADS2/FADS3/FGFR4/GAD1/HACD1/HACD3/IDO1/LIAS/MID1IP1/MLXIPL/NR1H3/OLAH/OSBPL7/PKM/PLA2G1B/PRKAB1/RBP1/SCAP/THNSL2	38	BP

GO:1903522	regulation of blood circulation	54/2734	289/17381	0.09702	0.7492	0.73686	ADM/ADRA1A/ADRA1B/ADRA2A/ADRA2B/ADRA2C/AGT/ASIC2/ATP1A1/ATP1A4/ATP2A3/AVPR2/CACNA1F/CACNA1G/CACNA1H/CACNA1S/CACNB1/CACNB3/CACNG1/CAMK2D/CAV3/CHGA/CHRM1/DES/DRD2/EDN2/EGFR/F2RL1/FGA/FLNA/GATA4/GCH1/GNAO1/GSTM2/HRH2/HSPB7/ITPR3/KCND1/KCND3/KCNH6/KCNJ5/KCNQ1/MIR328/MIR92A2/MYL4/NKX2-5/NOS3/NPPA/SMTNL1/TACR1/TBX2/TH/THRA/TRDN	54	BP
GO:0006893	Golgi to plasma membrane transport	11/2734	46/17381	0.09708	0.7492	0.73686	BLZF1/COMMD1/CSK/KIF13A/LLGL1/OSBPL5/PKDCC/RAB26/RAB34/STEAP2/VAMP5	11	BP
GO:0050702	interleukin-1 beta secretion	11/2734	46/17381	0.09708	0.7492	0.73686	CASP1/CASP5/CCL19/F2RL1/GSDMD/LGALS9/NLRP1/ORM1/ORM2/PYDC1/TLR8	11	BP
GO:1901028	regulation of mitochondrial outer membrane permeabilization involved in apoptotic signaling	11/2734	46/17381	0.09708	0.7492	0.73686	ATPIF1/BAD/BOK/DYNLL2/FZD9/HIP1R/PPP1R13B/SFN/SLC25A5/YWHAQ/ZNF205	11	BP
GO:1900181	negative regulation of protein localization to	17/2734	78/17381	0.0971	0.7492	0.73686	CD27/DAB2IP/DCLK2/EMD/FAM89B/LZTS2/MTOR/NFKBIL1/PARP10/PBLD/PDE2A/PKD1/POLR1A/SFRP5/SUFU/THRA/TRIM40	17	BP
GO:0007044	cell-substrate junction	19/2734	89/17381	0.09773	0.7492	0.73686	ABL1/BCAS3/COL16A1/COL17A1/CTTN/FERMT2/FN1/KDR/KRT14/LAMC2/LDB1/MYOC/PTK2B/RHOD/TAOK2/THY1/TLN1/WDPCP/WHAMM	19	BP
GO:0022404	molting cycle process	19/2734	89/17381	0.09773	0.7492	0.73686	DNASE1L2/EGFR/FARP2/FGFR2/FOXN1/FOXQ1/HOXC13/KRT71/KRT84/LDB1/NOTCH1/NSDHL/NUMA1/PDGFA/SHH/SMO/SPINK5/TMEM79/WNT1	19	BP
GO:0022405	hair cycle process	19/2734	89/17381	0.09773	0.7492	0.73686	DNASE1L2/EGFR/FARP2/FGFR2/FOXN1/FOXQ1/HOXC13/KRT71/KRT84/LDB1/NOTCH1/NSDHL/NUMA1/PDGFA/SHH/SMO/SPINK5/TMEM79/WNT1	19	BP

GO:0043393	regulation of protein binding	39/2734	202/17381	0.0978	0.7492	0.73686	ABL1/AKTIP/ARRB2/BMP4/CDK5/CSF3/DAB2IP/DISC1/DTNBP1/GAS8/GCG/GOLGA2/GPSM1/HERPUD1/HIP1R/HSF1/HSPA5/IFIT2/LFNG/LRP1/MAPK3/MAPRE3/MEN1/NES/NRG1/PEX14/PHLDA2/PKD1/PLXND1/PRKCD/PTPRF/RALB/RFNG/TAF1/TIRAP/TRAF2/WFIKKN2/WNT3A/XIRP1	39	BP
GO:0048588	developmental cell growth	39/2734	202/17381	0.0978	0.7492	0.73686	ABL1/AGT/ALCAM/ANAPC2/ARHGAP4/BARHL2/CAV3/CDK5/CPNE5/CPNE9/CTTN/DISC1/DRAXIN/EIF2AK4/EMX1/FN1/GATA4/ILK/ISLR2/L1CAM/LIMK1/LLPH/MIR195/MTOR/NLGN3/NPPA/NRG1/NRN1L/PDLIM5/PLXNB1/PLXNB3/SEMA3F/SEMA5B/SEMA7A/TNFRSF12A/TRPV2/ULK1/WNT3A/	39	BP
GO:0009581	detection of external stimulus	26/2734	128/17381	0.09827	0.7492	0.73686	AIPL1/ANO1/ARRB2/ASIC2/ASIC3/CACNA1F/CAV3/CNGB1/FNTA/FNTB/GNAT1/GUCA1B/GUCY2D/KCNK4/LHFPL5/OPN1MW/OPN4/PHF24/PITPNM1/PKD1/PPEF1/PRDM12/SEMA5B/TCAP/TULP1/UNC119	26	BP
GO:0006457	protein folding	44/2734	231/17381	0.09833	0.7492	0.73686	AHSP/AIP/CCT3/CD74/CLU/CRYAB/DNAJA3/DNAJB13/DNAJB5/DNAJC19/DNLZ/FKBP10/FKBP6/FKBP9/GAK/GANAB/GNAO1/GNAT1/GNB3/GNB5/HSP90AA1/HSPA2/HSPA5/HSPB1/MPDU1/NPPA/NUDC/P3H1/PFDN1/PPIAL4A/PPIAL4C/PPIAL4D/PPIAL4E/PPIAL4F/PPIAL4G/PPIB/PPIE/SIL1/TOR1A/TOR1B/TOR2A/TRAP1/TTC1/WFS1	44	BP
GO:0097485	neuron projection guidance	44/2734	231/17381	0.09833	0.7492	0.73686	ALCAM/ARTN/BOC/CDK5/CELSR3/CSF1R/DAB1/DOK1/DOK2/DPYSL4/DRAXIN/EFNA1/EFNA3/EFNA4/EFNB1/EPHA8/EPHB3/ERBB2/EVL/GBX2/GPC1/HRAS/L1CAM/LAMA2/LHX1/LHX3/LHX4/LYPLA2/MAPK3/PDLIM7/PTPN11/RAP1GAP/SEMA3F/SEMA5B/SEMA6C/SHC1/SHH/SPON2/SPTAN1/	44	BP
GO:0030832	regulation of actin filament length	32/2734	162/17381	0.09847	0.7492	0.73686	ARFIP1/ARPC1A/ARPC1B/ARPC2/ARPC4/BAIAP2L1/BRK1/CAPZA3/CCL21/CDC42EP2/CFL1/CORO1A/CORO1B/CSF3/CTTN/EVL/F2RL1/HIP1R/LMOD1/MLST8/MTOR/MYO1C/PFN1/PRKCD/PTK2B/SCIN/SPTAN1/SPTB/SPTB	32	BP
GO:0072583	clathrin-dependent	14/2734	62/17381	0.09886	0.7492	0.73686	AP2A2/AP2M1/CDK5/CLTB/DNM1/GAK/HIP1R/INPP5F/NLGN3/SCRIB/STON2/TNK2/TOR1A/UNC119	14	BP
GO:0002052	positive regulation of neuroblast	6/2734	21/17381	0.09927	0.7492	0.73686	DISC1/DRD2/NOTCH1/OTP/SHH/SMO	6	BP
GO:0014821	phasic smooth muscle	6/2734	21/17381	0.09927	0.7492	0.73686	DRD2/EDN2/HTR1D/P2RX2/PTGER3/TACR2	6	BP

GO:0030825	positive regulation of cGMP	6/2734	21/17381	0.09927	0.7492	0.73686	FZD2/GUCA1B/GUCA2A/GUCA2B/NOS3/RUNDC3A	6	BP
GO:0032727	positive regulation of interferon-alpha	6/2734	21/17381	0.09927	0.7492	0.73686	IRF5/SETD2/STAT1/TBK1/TLR8/TLR9	6	BP
GO:0036150	phosphatidylserine acyl-chain remodeling	6/2734	21/17381	0.09927	0.7492	0.73686	OSBPL5/PLA2G16/PLA2G1B/PLA2G2F/PLA2G4B/PLA2G5	6	BP
GO:0002042	cell migration involved in sprouting angiogenesis	10/2734	41/17381	0.09936	0.7492	0.73686	ABL1/CIB1/EPHB4/KDR/MIR221/MIR29C/MIR503/NOTCH1/NR4A1/TDGF1	10	BP
GO:0006182	cGMP biosynthetic process	10/2734	41/17381	0.09936	0.7492	0.73686	AQP1/GUCA1B/GUCA2A/GUCA2B/GUCY2D/NOS3/NPPA/PDZD3/PTK2B/RUNDC3A	10	BP
GO:0030890	positive regulation of B cell	10/2734	41/17381	0.09936	0.7492	0.73686	BCL6/CARD11/CD74/CD81/CLCF1/IL13/TIRAP/TLR9/TNFRSF4/WNT3A	10	BP
GO:0032459	regulation of protein oligomerization	10/2734	41/17381	0.09936	0.7492	0.73686	CLU/CRYAB/GBA/HRK/INS/MIEF2/MMP3/OPRD1/PEX14/PEX5	10	BP
GO:0032147	activation of protein kinase activity	60/2734	325/17381	0.1005	0.7492	0.73686	ABL1/ADRA2A/ADRA2B/ADRA2C/AGT/ANG/ARAF/AXIN1/BMP4/CCL19/CCL5/CD74/CD81/DAB2IP/DGKQ/DRD2/DUSP5/EGFR/ERN2/GADD45G/GNG3/HACD3/IL18/INS/IRAK1/KARS/MADD/MAP2K3/MAP3K14/MAP3K15/MAP3K6/MAP4K2/MAPK3/MAPK8IP3/MAPKAP1/MAPKAPK3/MARK2/MLST8/MTOR/NOD1/NRG1/PEA15/PILRB/PLA2G1B/PPP2R3C/PRKAR1A/PRKAR1B/PRKCD/PTK2B/PTPN11/RAF1/SHC1/SLC11A1/TAOK2/TDGF1/TIRAP/[illegible]	60	BP

GO:0008277	regulation of G-protein coupled receptor protein signaling	27/2734	134/17381	0.10085	0.7492	0.73686	ACPP/ADM/ADRA2A/ARRB2/CCL5/CHGA/CNGB1/DGKQ/DNM1/DRD2/DTNBP1/FNTA/FNTB/GNAT1/GPER1/GRK6/GUCA1B/GUCY2D/HOMER2/NECAB2/PHF24/PPEF1/PPP1R9B/RGS14/RGS16/TULP3/USP20	27	BP
GO:0050806	positive regulation of synaptic	27/2734	134/17381	0.10085	0.7492	0.73686	ABL1/ADRA1A/ARC/ARRB2/CA7/CLSTN1/CLSTN3/DLG4/DRD2/DTNBP1/EGFR/EIF2AK4/GPER1/GRIN1/INS/ITPR3/LAMA2/LRRTM1/NLGN3/PTK2B/RGS14/STX1A/STX4/STXBP1/TACR1/TACR2/WNT7A	27	BP
GO:0003179	heart valve morphogenesis	9/2734	36/17381	0.10113	0.7492	0.73686	BMP4/EFNA1/FGFRL1/HEY1/MTOR/NOTCH1/SCX/SMAD6/STRA6	9	BP
GO:0014911	positive regulation of smooth muscle	9/2734	36/17381	0.10113	0.7492	0.73686	AGT/CCL5/DOCK7/FOXO4/MIR221/MIR451A/NOX4/P2RY6/PDGFRB	9	BP
GO:0045823	positive regulation of heart	9/2734	36/17381	0.10113	0.7492	0.73686	ADM/ADRA1A/ATP1A1/CHGA/EDN2/GCH1/KCNQ1/NKX2-5/NPPA	9	BP
GO:1900087	positive regulation of G1/S transition of mitotic cell	9/2734	36/17381	0.10113	0.7492	0.73686	APEX1/CDK10/CYP1A1/EIF4G1/MEPCE/MIR221/MIR222/MIR29A/UBE2E2	9	BP
GO:1901385	regulation of voltage-gated calcium channel activity	9/2734	36/17381	0.10113	0.7492	0.73686	AHNAK/CACNB1/CACNB3/CRHR1/DRD2/GNB5/GPR35/NPPA/RRAD	9	BP
GO:0051148	negative regulation of muscle cell	16/2734	73/17381	0.10113	0.7492	0.73686	ANKRD2/BMP4/CAV3/CMTM5/FOXO4/IL18/MIR221/MIR222/NKX2-5/NOTCH1/PDCD4/PPARD/RBPMS2/SHH/SOX8/TRIM72	16	BP

GO:0050769	positive regulation of neurogenesis	75/2734	414/17381	0.10154	0.7492	0.73686	ADRA2B/ADRA2C/AGT/AMIGO1/ANAPC2/APBB1/BCL6/BIN1/BMP4/CAMK1D/CCR2/CLCF1/CPNE5/CPNE9/CSF1/CYB5D2/DAB1/DAB2IP/DISC1/DRD2/DUOXA1/EIF4G1/FES/FLNA/FN1/FOXO6/GPER1/HOXD3/HSPA5/ILK/IRX3/ISLR2/KATNB1/L1CAM/LIMK1/LLPH/LTA/LTK/MARK2/METRN/MIR221/MIR222/MMD2/MTOR/NBL1/NEUROD2/NKX2-5/NME1/NOTCH1/NRG1/NSMF/OBSL1/OPRM1/OTP/PHOX2B/PLXNB1/PLXNB3/PLXND1/PPP2R5B/PTK2B/RARA/RGS14/RNF112/SCARF1/SEMA7A/SERPINI1/SHH/SMO/SOX8/SPEN/SPINT1/TNFRSF12A/TRPV2/WNT3A/ZFYV[illegible]	75	BP
GO:0022400	regulation of rhodopsin mediated signaling	7/2734	26/17381	0.10175	0.7492	0.73686	CNGB1/FNTA/FNTB/GNAT1/GUCA1B/GUCY2D/PPEF1	7	BP
GO:0042044	fluid transport	7/2734	26/17381	0.10175	0.7492	0.73686	AQP1/AQP10/AQP5/AQP8/MIP/PDZD3/SLC26A6	7	BP
GO:0060343	trabecula formation	7/2734	26/17381	0.10175	0.7492	0.73686	CAV3/CHAD/COL1A1/HEY1/MMP2/NKX2-5/THBS3	7	BP
GO:0061615	glycolytic process through fructose-6-	7/2734	26/17381	0.10175	0.7492	0.73686	ALDOA/GALK1/GAPDH/GCK/HK3/PFKM/PKM	7	BP
GO:0061620	glycolytic process through glucose-6-	7/2734	26/17381	0.10175	0.7492	0.73686	ALDOA/GALK1/GAPDH/GCK/HK3/PFKM/PKM	7	BP
GO:0072273	metanephric nephron morphogenesis	7/2734	26/17381	0.10175	0.7492	0.73686	BMP4/LHX1/PDGFRB/PKD1/SMO/SOX8/STAT1	7	BP
GO:1900027	regulation of ruffle assembly	7/2734	26/17381	0.10175	0.7492	0.73686	DEF8/EPS8L2/EVL/HRAS/PFN1/PLEKHM1/WDPCP	7	BP
GO:0009953	dorsal/ventral pattern	20/2734	95/17381	0.10189	0.7492	0.73686	BMP4/EN1/EVX1/HHIP/HOXB2/LHX1/LHX3/NBL1/NOTO/SHH/SMAD6/SMO/SOST/SUFU/TBC1D32/TRAF3IP1/TULP3/VAX2/WNT3A/WNT7A	20	BP
GO:0098869	cellular oxidant detoxification	20/2734	95/17381	0.10189	0.7492	0.73686	EPX/FAM213A/GCH1/GPX1/GPX2/GPX3/GSR/GSTM2/GSTZ1/LPO/MGST1/MGST3/MPO/NOS3/PRDX1/PRDX6/SESN1/SOD3/TXNRD1/TXNRD3	20	BP

GO:0052547	regulation of peptidase activity	77/2734	426/17381	0.10197	0.7492	0.73686	A2ML1/AGT/AHSG/ANP32B/APLP2/APOPT1/AQP1/ARRB2/ATP13A2/BAD/BCAP31/BOK/CASP1/CASP3/CD27/COL7A1/CRB2/CRYAB/CTSH/DAP/DAPK1/DPEP1/ECM1/EFNA1/EFNA3/FASLG/FN1/FURIN/GAPDH/GPER1/GPX1/HERPUD1/HIP1R/HSF1/ITIH4/ITIH6/KARS/KNG1/LAMP3/LCK/LGALS9/MAPK3/MIR15A/MIR195/MIR29C/MIR92A2/MTCH1/NAIP/NLE1/NLRP1/NOD1/PCOLCE/PDCD2/PEBP1/PI16/POR/PRELID1/PSMB8/RAF1/RPS6KA1/SERPINA2/SERPINA3/SERPINA4/SERPINA5/SERPINF2/SERPINH1/SERPINI1/SFN/SOX7/SPINK2/SPINK5/SPINT1/SPOCK2/TRAF2/WFIKKN2/WNT3A/XDH	77	BP
GO:0019319	hexose biosynthetic process	18/2734	84/17381	0.10199	0.7492	0.73686	ALDOA/ATF3/CHST15/CRTC2/DGKQ/G6PC3/GAPDH/GCG/GCK/GNMT/GOT2/INS/KAT2A/LEPR/MAEA/PC/PFKFB1/PGAM4	18	BP
GO:0060840	artery development	18/2734	84/17381	0.10199	0.7492	0.73686	APOB/BMP4/ENG/EYA1/HEY1/LOXL1/LRP1/MIR153-1/NOTCH1/NPRL3/PDGFRB/PLXND1/SHH/SMAD6/STRA6/SUFU/TBX2/WN	18	BP
GO:0015698	inorganic anion transport	34/2734	174/17381	0.10202	0.7492	0.73686	ABCC3/ABCC4/ABCC5/ABCC6/ANO1/ANO2/ANO4/ANO7/BEST3/BEST4/BSND/CA7/CLCN2/CLCNKA/CLCNKB/CLDN4/CLIC1/GLRA1/MTOR/P2RY6/SLC11A1/SLC12A4/SLC12A7/SLC12A9/SLC20A1/SLC20A2/SLC22A12/SLC22A13/SLC22A9/SLC26A1/SLC26A10/SLC26A6/SLC34A1/SLC4A9	34	BP
GO:0006363	termination of RNA polymerase I	8/2734	31/17381	0.10209	0.7492	0.73686	GTF2H4/POLR1A/POLR1D/POLR1E/POLR2L/TAF1A/TTF1/ZNRD1	8	BP
GO:0036257	multivesicular body	8/2734	31/17381	0.10209	0.7492	0.73686	CHMP1A/CHMP4C/CHMP7/SNF8/VPS25/VPS37B/VPS37C/VPS37D	8	BP
GO:0071353	cellular response to	8/2734	31/17381	0.10209	0.7492	0.73686	ADAMTS13/ALAD/CD300LF/CDK4/CORO1A/HSPA5/IL4R/MCM2	8	BP
GO:0051147	regulation of muscle cell differentiation	38/2734	197/17381	0.10211	0.7492	0.73686	ABL1/ADGRB1/ANKRD2/ARRB2/BMP4/BOC/CAV3/CDH15/CDK9/CMTM5/CTNNA2/EHD1/EIF5A/ENG/FGFR2/FOXO4/GPER1/IGFBP3/IL18/IL4R/ILK/MIR221/MIR222/MTOR/MYOD1/NKX2-5/NOTCH1/NRG1/PDCD4/PPARD/RBM4/RBPMS2/SHH/SOX8/SUPT6H/THRA/TRIM72/WNT3A	38	BP

GO:0001836	release of cytochrome c from	13/2734	57/17381	0.10249	0.7492	0.73686	APOPT1/ARRB2/BAD/BOK/CLU/FZD9/GPER1/GPX1/HRK/LMNA/MOAP1/PRELID1/SFN	13	BP
GO:0002637	regulation of immunoglobulin production	13/2734	57/17381	0.10249	0.7492	0.73686	BCL6/CLCF1/HLA-E/IL13/IL4R/MZB1/PAXIP1/STX4/SUPT6H/TLR9/TNFRSF4/TNFSF13/TRAF2	13	BP
GO:0007602	phototransduction	13/2734	57/17381	0.10249	0.7492	0.73686	AIPL1/ASIC2/CNGB1/FNTA/FNTB/GNAT1/GUCA1B/GUCY2D/OPN1MW/OPN4/PITPNM1/PPEF1/UNC119	13	BP
GO:0009584	detection of visible light	13/2734	57/17381	0.10249	0.7492	0.73686	AIPL1/CACNA1F/CNGB1/FNTA/FNTB/GNAT1/GUCA1B/GUCY2D/OPN1MW/OPN4/PPEF1/SEMA5B/TULP1	13	BP
GO:0042267	natural killer cell mediated cytotoxicity	13/2734	57/17381	0.10249	0.7492	0.73686	ARRB2/CORO1A/HAVCR2/HLA-E/IL18/LGALS9/MICB/NCR3/PIK3R6/PRDX1/RAET1G/SLAMF7/ULBP2	13	BP
GO:1903051	negative regulation of proteolysis involved in cellular protein	13/2734	57/17381	0.10249	0.7492	0.73686	ALAD/CCAR2/EFNA1/FHIT/KLHL40/LAMP3/OS9/PANO1/SHH/SUFU/TAF1/USP19/WNT1	13	BP
GO:2000756	regulation of peptidyl-lysine acetylation	13/2734	57/17381	0.10249	0.7492	0.73686	BRCA1/CTBP1/HDAC8/KAT2A/MAPK3/MUC1/NOC2L/PAXIP1/PIWIL2/PYGO2/RPS6KA4/SPI1/TAF7	13	BP
GO:0007224	smoothened signaling pathway	25/2734	123/17381	0.10277	0.7492	0.73686	ANKMY2/B9D1/BMP4/BOC/C2CD3/CC2D2A/EVC2/FGFR2/GPC2/HHIP/IFT140/IFT20/NSDHL/POR/PTCH2/SHH/SMO/SSNA1/SUFU/TBC1D32/TMEM17/TMEM231/TRAF3IP1/TULP3/WDPCP	25	BP

GO:0042326	negative regulation of phosphorylation	83/2734	462/17381	0.10292	0.7492	0.73686	ABL1/AHSG/ARRB2/ATF3/BGN/BMP4/C1QL4/CAMK2N2/CASP3/CAV3/CDK5RAP3/CDKN2A/CHAD/CIB1/CISH/COX11/CRIPAK/CSK/CTDSP1/DAB2IP/DGKQ/DTNBP1/DUSP2/DUSP21/DUSP26/DUSP5/EFNA1/EIF4G1/ENG/F2RL1/FKTN/FOXM1/GBA/GPER1/HSPB1/IGFBP3/ILK/INCA1/INPP5F/LEMD2/LRRTM1/MAPK8IP1/MARVELD3/MEN1/MIR221/MIR92A2/MLXIPL/MTOR/MYCNOS/NCOR1/NDRG2/PARD3/PARD6A/PBLD/PDCD4/PER1/PFKFB1/PID1/PPP1R1B/PREX2/PRKAR1A/PRKAR1B/PRKCD/PRKRIP1/PSCA/PYDC1/RGS14/RTN4RL1/RTN4RL2/SFN/SH3BP5/SH3BP5L/SMAD6/SMYD3/TAF7/[illegible]	83	BP
GO:0030041	actin filament polymerization	31/2734	157/17381	0.10293	0.7492	0.73686	ANG/ARFIP1/ARPC1A/ARPC1B/ARPC2/ARPC4/BAIAP2L1/BRK1/CAPZA3/CATIP/CCL21/CDC42EP2/CORO1A/CORO1B/CSF3/CTTN/EVL/HIP1R/LMOD1/MLST8/MTOR/MYO1C/PFN1/PRKCD/PTK2B/SCIN/SPTAN1/SPTB/SPTB	31	BP
GO:0010632	regulation of epithelial cell migration	39/2734	203/17381	0.10344	0.7492	0.73686	ABL1/ADGRB1/AGT/AQP1/BCAS3/BMP4/CIB1/CTSH/DAB2IP/DOCK1/EFNA1/EVL/FLT4/GDF2/GLIPR2/HSPB1/KDR/MARVELD3/MIR10A/MIR16-1/MIR212/MIR221/MIR222/MIR29C/MIR503/MIR92A2/MTOR/NOTCH1/PBLD/PFN1/PRCP/PTK2B/PTPN23/RAB25/SCARB1/SEMA4A/TACR1/TDGF1/W	39	BP
GO:0031334	positive regulation of protein complex assembly	44/2734	232/17381	0.1036	0.7492	0.73686	ANKRD53/ARFIP1/ARPC1A/ARPC1B/ARPC2/ARPC4/BAIAP2L1/BRK1/CAV3/CCL21/CDC42EP2/CLU/CORO1A/CORO1B/CSF3/CTTN/DDB1/DDB2/EIF4G1/EVL/FES/FOSL1/FSCN1/GTF2H4/HIP1R/HRK/LMOD1/MIEF2/MLST8/MMP3/MTOR/MYO1C/NKX2-5/NUMA1/PAXIP1/PFN1/PPP2R5B/PSMC3/PTK2B/SCIN/SLF2/TAF1/WAS/W	44	BP
GO:0007266	Rho protein signal transduction	35/2734	180/17381	0.10365	0.7492	0.73686	ABL1/ADRA1A/ADRA2A/APOC3/ARHGAP4/ARHGEF10/ARHGEF16/ARHGEF17/ARHGEF25/ARHGEF4/BCL6/CDC42EP2/CDH13/CFL1/EPS8L2/F2RL1/FARP2/GPR17/GPR20/GPR35/HACD3/LIMK1/MYOC/OBSCN/PDGFRB/PLEKHG4/PLEKHG5/PLEKHG6/PREX2/RAF1/RHOD/RTKN/TAX1BP3/TRIO/W	35	BP
GO:0051188	cofactor biosynthetic process	35/2734	180/17381	0.10365	0.7492	0.73686	ACACB/ACOT7/ACSF3/ALAD/ALAS2/ATPIF1/CIAPIN1/COQ2/COQ4/COX10/ELOVL1/ELOVL5/FAM96B/GCH1/HAAO/HACD1/HMBS/IDH2/ISCA2/LIAS/MPC1/MTHFD1/NARFL/NFE2L1/NMNAT3/NUBP1/NUBP2/PARP10/PARP9/PDHB/PDSS1/PPCDC/QPRT/SLC22A13/UROS	35	BP
GO:0036498	IRE1-mediated unfolded protein response	15/2734	68/17381	0.10531	0.7492	0.73686	ACADVL/DAB2IP/EXTL1/GOSR2/HSPA5/LMNA/PLA2G4B/PPP2R5B/SEC61A2/SHC1/SYVN1/TLN1/TSPYL2/WFS1/ZBTB17	15	BP

GO:0048663	neuron fate commitment	15/2734	68/17381	0.10531	0.7492	0.73686	AXIN1/BMP4/EVX1/EYA1/HOXC10/JAG2/LHX3/NOTCH1/NRG1/OLIG3/SHH/SUFU/TBR1/TLX3/WNT1	15	BP
GO:0001819	positive regulation of cytokine production	74/2734	409/17381	0.10532	0.7492	0.73686	ABL1/ADAM8/ADRA2A/AGT/ATF2/BRCA1/C3AR1/CARD11/CARD9/CASP1/CASP5/CCDC88B/CCL19/CCR2/CD58/CD6/CD74/CLU/CSF1R/DHX58/DRD2/EGR1/EPX/F2RL1/FLT4/GAPDH/GATA4/GDF2/GSDMD/HAVCR2/HILPDA/HLA-E/HRAS/HSPB1/IDO1/IL13/IL18/IL20RB/IL4R/INS/IRAK1/IRF5/KARS/LGALS9/LRRFIP1/LTA/LY9/MAPK3/MIR92A2/NLRP1/NLRX1/NOD1/ORM1/ORM2/POLR1D/POLR2L/POLR3D/PYDC1/RARA/SEMA7A/SERPINF2/SETD2/SL[illegible]	74	BP
GO:0032355	response to estradiol	26/2734	129/17381	0.10548	0.7492	0.73686	AGT/APOB/ARPC1B/BAD/CASP3/COL1A1/CRYAB/CYP1A2/DNMT3A/EGFR/ENDOG/ESRRA/GPER1/HSF1/MBD4/MMP15/MYOD1/OGG1/PDGFRB/RARA/SLC34A1/STXBP1/TACR1/TH/WNT7A/ZNF703	26	BP
GO:0035051	cardiocyte differentiation	29/2734	146/17381	0.10552	0.7492	0.73686	AGT/ARRB2/BMP4/CACYBP/CAV3/EGFR/GATA4/GPER1/IFT20/KAT2A/KDM6B/LMNA/MAPK3/MIR195/MIR222/MTOR/NKX2-5/NOTCH1/NOX4/NPPA/NRG1/OBSL1/PDCD4/PDGFRB/PDLIM5/RARA/TBX2/TCAP/WNT3A	29	BP
GO:1903362	regulation of cellular protein catabolic process	53/2734	285/17381	0.10583	0.7492	0.73686	ALAD/ANAPC15/ANAPC2/ARAF/ATP13A2/ATPIF1/AXIN1/BCAP31/BRSK2/BUB1B/CAV3/CCAR2/CD81/CDK5RAP3/CHFR/CLU/COMMD1/DAB2IP/DISC1/ECSCR/EFNA1/FHIT/FURIN/GBA/GPX1/HERPUD1/KLHL40/LAMP3/LRP1/NKD2/OS9/OSBPL7/PANO1/PKD1/PSMB11/PSMB6/PSMB7/PSMB8/PSMC3/PSMD13/PSMD3/PSMD5/PSMD7/PTK2B/RHBDF1/RNF166/RNF180/SH	53	BP
GO:0000022	mitotic spindle elongation	4/2734	12/17381	0.10593	0.7492	0.73686	CDC14A/KIF23/KIF4A/NUMA1	4	BP
GO:0002716	negative regulation of natural killer	4/2734	12/17381	0.10593	0.7492	0.73686	ARRB2/HAVCR2/HLA-E/LGALS9	4	BP
GO:0003084	positive regulation of systemic arterial	4/2734	12/17381	0.10593	0.7492	0.73686	ADRA1A/AVPR2/CYP11B2/HSD11B2	4	BP
GO:0008298	intracellular mRNA	4/2734	12/17381	0.10593	0.7492	0.73686	CASC3/EXOSC10/EXOSC2/ZNF385A	4	BP

GO:0010935	regulation of macrophage cytokine production	4/2734	12/17381	0.10593	0.7492	0.73686	CD74/CUEDC2/SEMA7A/SPON2	4	BP
GO:0014745	negative regulation of muscle	4/2734	12/17381	0.10593	0.7492	0.73686	LMNA/MIR25/MTOR/NOS3	4	BP
GO:0016558	protein import into peroxisome	4/2734	12/17381	0.10593	0.7492	0.73686	PEX14/PEX16/PEX5/PEX6	4	BP
GO:0018065	protein-cofactor linkage	4/2734	12/17381	0.10593	0.7492	0.73686	GAD1/GLRX5/HMBS/LIAS	4	BP
GO:0030208	dermatan sulfate biosynthetic	4/2734	12/17381	0.10593	0.7492	0.73686	BCAN/BGN/CHST12/DSE	4	BP
GO:0030497	fatty acid elongation	4/2734	12/17381	0.10593	0.7492	0.73686	ELOVL1/ELOVL5/HACD1/HACD3	4	BP
GO:0035090	maintenance of apical/basal cell polarity	4/2734	12/17381	0.10593	0.7492	0.73686	CRB2/LRCH4/WDR1/WNT11	4	BP
GO:0045199	maintenance of epithelial cell apical/basal polarity	4/2734	12/17381	0.10593	0.7492	0.73686	CRB2/LRCH4/WDR1/WNT11	4	BP
GO:0045351	type I interferon biosynthetic process	4/2734	12/17381	0.10593	0.7492	0.73686	NMI/TBK1/TLR8/TLR9	4	BP
GO:0045475	locomotor rhythm	4/2734	12/17381	0.10593	0.7492	0.73686	CIART/MTA1/NAGLU/USP2	4	BP

GO:0045741	positive regulation of epidermal growth factor-activated	4/2734	12/17381	0.10593	0.7492	0.73686	ADRA2A/ADRA2B/ADRA2C/NCF1	4	BP
GO:0048681	negative regulation of axon	4/2734	12/17381	0.10593	0.7492	0.73686	INPP5F/LRIG2/RGMA/RTN4RL1	4	BP
GO:0051127	positive regulation of	4/2734	12/17381	0.10593	0.7492	0.73686	BRK1/SCIN/WAS/WHAMM	4	BP
GO:0060272	embryonic skeletal joint morphogenesis	4/2734	12/17381	0.10593	0.7492	0.73686	AXIN1/BMP4/HOXC11/OSR1	4	BP
GO:0060670	branching involved in labyrinthine layer	4/2734	12/17381	0.10593	0.7492	0.73686	ADM/FGFR2/SPINT1/ST14	4	BP
GO:0072070	loop of Henle development	4/2734	12/17381	0.10593	0.7492	0.73686	AQP1/IRX3/PKD1/POU3F3	4	BP
GO:0090128	regulation of synapse maturation	4/2734	12/17381	0.10593	0.7492	0.73686	ANAPC2/DAB2IP/DISC1/NEUROD2	4	BP
GO:0090239	regulation of histone H4 acetylation	4/2734	12/17381	0.10593	0.7492	0.73686	BRCA1/CTBP1/MUC1/SPI1	4	BP
GO:2000543	positive regulation of	4/2734	12/17381	0.10593	0.7492	0.73686	LHX1/OSR1/SCX/WNT3A	4	BP
GO:0035904	aorta development	12/2734	52/17381	0.10609	0.7492	0.73686	ENG/EYA1/HEY1/LOXL1/LRP1/NOTCH1/NPRL3/PDGFRB/PLXND1/SMAD6/SUFU/TBX2	12	BP
GO:0048747	muscle fiber development	12/2734	52/17381	0.10609	0.7492	0.73686	ACTA1/BIN3/BMP4/CHRNB1/FLNC/GPX1/KLHL40/MYOD1/P2RX2/SMO/TCAP/WFIKKN2	12	BP

GO:2000027	regulation of organ morphogenesis	47/2734	250/17381	0.10641	0.7492	0.73686	ABL1/AGT/AJAP1/AMTN/AP2A2/AP2M1/ARRB2/BMP4/CAV3/CELSR2/CELSR3/CHAD/CSF1/DVL2/ENG/EYA1/FGFR2/FZD2/GRHL3/HOXB7/HOXC11/LHX1/LIMS2/NOTCH1/PARD6A/PDGFA/PFN1/POR/PSMB11/PSMB6/PSMB7/PSMB8/PSMC3/PSMD13/PSMD3/PSMD5/PSMD7/SHH/SMO/SOX8/STAT1/TBX2/WNT1/WNT10A/WNT11/WNT3A/WNT6	47	BP
GO:0007492	endoderm development	17/2734	79/17381	0.10645	0.7492	0.73686	ARC/BMP4/COL4A2/COL7A1/DUSP2/DUSP5/FN1/GATA4/HOXC11/ITGA7/ITGB5/LHX1/MMP15/MMP2/NOTCH1/SETD2/SOX7	17	BP
GO:0090277	positive regulation of peptide	19/2734	90/17381	0.10647	0.7492	0.73686	ANO1/BAD/BLK/CAPN10/DOC2B/DRD2/EGFR/FGA/GCG/GCK/GLUD1/GPER1/INS/PFKM/PPARD/RFX6/STX4/TFR2/TRH	19	BP
GO:0006006	glucose metabolic process	37/2734	192/17381	0.10663	0.7492	0.73686	ACACB/AIMP1/ALDOA/ATF3/BAD/BRAT1/C1QTNF1/COX11/CREM/CRTC2/DGKQ/G6PC3/GALM/GAPDH/GCG/GCK/GNMT/GOT2/HK3/IGFBP3/INPPL1/INS/KAT2A/LCMT1/LEPR/MAEA/MTOR/NISCH/PC/PDHB/PFKFB1/PFKM/PGAM4/PHLDA2/PKM/PPARD/RORC	37	BP
GO:0007626	locomotory behavior	37/2734	192/17381	0.10663	0.7492	0.73686	ARRB2/ASL/B4GALT2/C1QL1/CDH23/CEND1/CIART/CLN6/DAB1/DLG4/DMBX1/DNM1/DRD2/EN1/ESPN/GAD1/GLRA1/GNAO1/GPR88/GRIN1/HOXB8/IDO1/INPP5F/LRRTM1/MTA1/NAGLU/NRG1/OPRD1/OPRM1/PPP1R1B/PREX2/SLURP1/TH/TRH/USP2/VPS13A/ZNF385A	37	BP
GO:0050792	regulation of viral process	37/2734	192/17381	0.10663	0.7492	0.73686	CCL5/CD74/CDK9/CFL1/CHMP4C/DDB1/EIF2AK4/FKBP6/HACD3/IFITM2/ISG15/LAMP3/LGALS9/MIR221/MIR222/NELFB/NOTCH1/PARP10/PC/PFN1/POLR2G/POLR2L/PPIB/PPIE/PSMC3/RAB7A/RSF1/SNF8/STAT1/TARBP2/TRIM11/TRIM14/TRIM26/TRIM31/TRIM62/VPS37B/ZNF502	37	BP
GO:0051897	positive regulation of protein kinase B	30/2734	152/17381	0.10763	0.7546	0.74219	ADAM8/ARRB2/C1QTNF1/CCL19/CCL21/CSF3/EGFR/ERBB2/FGF17/FGF3/FGFR2/FGFR4/GPX1/IL18/ILK/INS/LCK/MIR221/MIR222/MIR29A/MTOR/MYOC/NOX4/NRG1/PDGFA/PDGFRB/PIK3R5/PTPN11/THPO/TNFAIP8L3	30	BP
GO:0051056	regulation of small GTPase mediated signal transduction	57/2734	309/17381	0.10817	0.7546	0.74219	ABL1/ADRA1A/APOC3/ARAP1/ARHGAP10/ARHGAP22/ARHGAP30/ARHGAP4/ARHGEF10/ARHGEF16/ARHGEF17/ARHGEF25/ARHGEF4/BCL6/CSF1/DAB2IP/DGKI/EPS8L2/F2RL1/FARP2/FOXM1/GBF1/GPR17/GPR20/GPR35/HRAS/IQSEC2/MADD/MAPKAP1/MFN2/MMD2/MYOC/NOTCH1/NRG1/OBSCN/PDGFRB/PLEKHG4/PLEKHG5/PLEKHG6/PREX2/PSD/PSD2/PSD4/RAF1/RALGPS1/RAP1GAP/RASA3/RASA4B/RASGEF1A/RHOD/RHOF/RHOT2/[illegible]	57	BP

GO:0010876	lipid localization	69/2734	380/17381	0.10828	0.7546	0.74219	ABCA2/ABCC3/ABCC4/ABCG5/ACACB/AGT/ANO4/ANO7/APOA5/APOB/APOC3/B4GALNT1/C1QTNF1/CLU/CPT2/CPTP/CRHR1/CROT/DGAT1/DRD2/EHD1/FITM1/GLTP/GLTPD2/GOT2/GPIHBP1/HEATR4/HILPDA/LCN12/LRP1/LRP10/MFSD2A/MID1IP1/MROH6/NCOR1/NME4/NPC1L1/NPC2/NR1H3/OSBPL5/OSBPL7/PITPNM1/PLA2G1B/PLA2G2F/PLA2G5/PNPLA2/PPARD/PRELID1/PRKCD/PSAP/PTPN11/REN/SCARB1/SERPINA5/SFTPA1/SHH/SIGMAR1/SLC22A9/SLC25A20/SLCO2B1/SOAT2/SPNS1/SPNS3/STARD3/STAR[illegible]	69	BP
GO:0019318	hexose metabolic process	44/2734	233/17381	0.10906	0.7546	0.74219	ACACB/AIMP1/ALDOA/ATF3/BAD/BRAT1/C1QTNF1/CHST15/COX11/CREM/CRTC2/DGKQ/FUOM/FUT7/G6PC3/GALK1/GALM/GALT/GAPDH/GCG/GCK/GLYCTK/GNMT/GOT2/HK3/IGFBP3/INPPL1/INS/KAT2A/LCMT1/LEPR/MAEA/MTOR/NISCH/PC/PDHB/PFKFB1/PFKM/PGAM4/PHLDA2/PKM/P	44	BP
GO:0034329	cell junction assembly	39/2734	204/17381	0.10928	0.7546	0.74219	ABL1/AGT/BCAS3/CDH5/COL16A1/COL17A1/CTTN/FBLIM1/FERMT2/FLNA/FLNC/FN1/FSCN1/GNPAT/IKBKB/ILK/KDR/KRT14/LAMC2/LDB1/LIMS2/MARVELD2/MARVELD3/MYO1C/MYOC/PARD3/PARD6A/PKP3/PTK2B/RHOD/TAOK2/THY1/TLN1/TRPV4/WDPCP/WDR1/WHAMM/WNT11/ZNF703	39	BP
GO:0002260	lymphocyte homeostasis	14/2734	63/17381	0.10961	0.7546	0.74219	ABL1/CACNA1F/CASP3/CCNB2/CD74/CORO1A/DNAJA3/FOXN1/LGALS9/LMO1/PPP2R3C/TNFRSF13B/TSC22D3/TSC22D4	14	BP
GO:0003143	embryonic heart tube morphogenesis	14/2734	63/17381	0.10961	0.7546	0.74219	C2CD3/CCDC103/CCDC40/ENG/GATA4/MICAL2/NKX2-5/NOTCH1/NOTO/SHH/SMO/SUFU/TBX2/WNT3A	14	BP
GO:0006360	transcription from RNA polymerase I	14/2734	63/17381	0.10961	0.7546	0.74219	ERBB2/FLNA/GTF2H4/MAPK3/MTOR/NOL11/POLR1A/POLR1D/POLR1E/POLR2L/TAF1/TAF1A/TTF1/ZNRD1	14	BP
GO:0043967	histone H4 acetylation	14/2734	63/17381	0.10961	0.7546	0.74219	APBB1/BRCA1/CTBP1/EPC1/HCFC1/ING4/KAT2A/MSL3/MUC1/MYOD1/NAA60/PER1/SPI1/TADA3	14	BP
GO:0061371	determination of heart left/right	14/2734	63/17381	0.10961	0.7546	0.74219	C2CD3/CCDC103/CCDC40/ENG/GATA4/MICAL2/NKX2-5/NOTCH1/NOTO/SHH/SMO/SUFU/TBX2/WNT3A	14	BP
GO:0071806	protein transmembrane transport	14/2734	63/17381	0.10961	0.7546	0.74219	AZGP1/CLU/DNLZ/GPIHBP1/HSP90AA1/PEX14/PEX16/PEX3/PEX5/PEX6/TIMM17B/TIMM22/TIMM23B/TIMM9	14	BP

GO:0008347	glial cell migration	11/2734	47/17381	0.10961	0.7546	0.74219	CCR2/CDK5/CDK5R2/CSF1/DAB1/DAB2IP/DISC1/IDH2/MIR221/MIR222/SCRIB	11	BP
GO:0010718	positive regulation of epithelial to mesenchymal	11/2734	47/17381	0.10961	0.7546	0.74219	BMP4/COL1A1/CRB2/ENG/GLIPR2/MIR221/MIR222/MTOR/NOTCH1/TGFB1I1/ZNF703	11	BP
GO:0034260	negative regulation of	11/2734	47/17381	0.10961	0.7546	0.74219	ARRB2/BCAS3/DAB2IP/DGKI/GPSM1/HRAS/PDE6D/PLXNB3/PTPRN2/RTKN/TNK2	11	BP
GO:1905268	negative regulation of chromatin	11/2734	47/17381	0.10961	0.7546	0.74219	BRCA1/CTBP1/HDAC8/HMGA1/NOC2L/OTUB1/PHF2/SPI1/SUPT6H/TAF7/UBE2B	11	BP
GO:0006473	protein acetylation	35/2734	181/17381	0.10989	0.7546	0.74219	APBB1/ATF2/BRCA1/BRPF1/CRTC2/CTBP1/EPC1/HCFC1/HDAC8/ING4/KAT2A/LDB1/MAPK3/MSL3/MUC1/MYOD1/NAA16/NAA60/NAT16/NAT8B/NOC2L/NUPR1/PAXIP1/PCGF2/PER1/PIWIL2/POLE4/POR/PYGO2/RPS6KA4/SPI1/TADA3/TAF1/TAF1L/TAF7	35	BP
GO:0009855	determination of bilateral symmetry	25/2734	124/17381	0.11037	0.7546	0.74219	AXIN1/C2CD3/CC2D2A/CCDC103/CCDC40/CFC1/DNAH11/ENG/GAS8/GATA4/IFT140/LRRC6/MICAL2/NBL1/NEK8/NKX2-5/NKX3-2/NOTCH1/NOTO/SHH/SMO/SUFU/TBC1D32/TBX2/WNT3A	25	BP
GO:0051591	response to cAMP	20/2734	96/17381	0.11057	0.7546	0.74219	APEX1/AQP1/AQP8/COL1A1/DGKQ/FOSL1/HSPA5/KCNQ1/LDHA/NME1/NOX4/PER1/PFKFB1/PLA2G5/PTK2B/REN/SDC1/SLC26A6/STAT1/VGF	20	BP
GO:0051051	negative regulation of transport	89/2734	500/17381	0.11091	0.7546	0.74219	ABCG5/ADRA2A/ADRA2B/ADRA2C/APOC3/AVPR2/BEST3/BOK/CAMK2D/CAV3/CCR2/CD27/CD300LF/CD74/CDK5/CHGA/CNIH2/COMMD1/CORO1A/CRHR1/CRYAB/CSK/DAB2IP/DLG4/DRD2/EMD/F2RL1/FAM89B/FN1/FZD9/GNAO1/GNB5/GPR35/GSTM2/IDH2/INHBB/INS/LGALS3/LGALS9/LRRTM1/LRSAM1/MIR153-1/MIR212/MIR328/MTOR/MZB1/NECAB2/NFKBIL1/NOS3/NOTCH1/NR1H3/NRG1/OAZ2/OPRM1/OS9/OSR1/PACSIN3/PARP10/PBLD/PDE2A/PEA15/PID1/PKD1/PKDCC/PNKD/PROM2/PTGER3/PTK2B/PTPN11/RAB11FIP3/RAB7A/RHBDF1/RRAD/SERGEF/SFRP5/SHH/SLC25A5/SNX12/SUFU/TACR2/T[illegible]	89	BP

GO:0003333	amino acid transmembrane transport	16/2734	74/17381	0.1111	0.7546	0.74219	AGT/OSR1/PQLC2/SLC1A7/SLC25A22/SLC36A1/SLC36A3/SLC38A1/SLC38A10/SLC38A8/SLC6A18/SLC6A6/SLC6A7/SLC6A8/SLC6A9/SLC7A7	16	BP
GO:0008593	regulation of Notch signaling pathway	16/2734	74/17381	0.1111	0.7546	0.74219	ARRDC1/BCL6/CHAC1/DLK1/DLK2/EGFL7/ERH/EYA1/HEY1/JAG2/LFNG/MIR212/NOTCH1/RFNG/SLC35C1/WNT1	16	BP
GO:0010657	muscle cell apoptotic	16/2734	74/17381	0.1111	0.7546	0.74219	AGT/AMBRA1/APOPT1/ARRB2/CAMK2D/CDKN2A/EIF5A/HSF1/ILK/LTK/MIR16-1/MIR195/MIR92A2/NKX2-5/PDCD4/PTK2B	16	BP
GO:0043627	response to estrogen	16/2734	74/17381	0.1111	0.7546	0.74219	AQP1/ARID5A/ARPC1B/ASH2L/BCAS3/BGLAP/BRCA1/CITED4/CRIPAK/GBA/IL4R/LDHA/PDGFRB/RARA/SMAD6/WNT7A	16	BP
GO:0051289	protein homotetramerization	16/2734	74/17381	0.1111	0.7546	0.74219	ACACB/ACPP/ALDOA/ATPIF1/CD247/CDA/CRTC2/EVL/GNMT/GOLGA2/GPX3/MIP/PEX14/PEX5/PKM/SHMT2	16	BP
GO:0006903	vesicle targeting	18/2734	85/17381	0.11128	0.7546	0.74219	CEP19/CNIH2/COL7A1/GBF1/GOLGA2/GORASP1/GOSR2/MAP4K2/SCRIB/SEC16A/SEC16B/STARD3/STX5/TRAPPC1/TRAPPC2L/TRAPPC3/TRAPPC4	18	BP
GO:0015837	amine transport	18/2734	85/17381	0.11128	0.7546	0.74219	ADRA2A/ADRA2B/ADRA2C/AGT/CHGA/CHRNA6/DDC/DRD2/DTNBP1/OSR1/RHCG/STX1A/STXBP1/SV2A/TACR2/TH/TOR1A/TRH	18	BP
GO:0043506	regulation of JUN kinase	18/2734	85/17381	0.11128	0.7546	0.74219	AXIN1/CCL19/DAB2IP/DTNBP1/DVL2/ERN2/FZD8/HACD3/MAP3K6/MAP4K2/MAPK8IP1/MAPK8IP3/MIR92A2/PDCD4/PTK2B/TLR9/TNIK/TRAF2	18	BP
GO:0051054	positive regulation of DNA metabolic process	41/2734	216/17381	0.11164	0.7546	0.74219	ACD/APBB1/ARRB2/BMP4/BRCA1/BRCC3/CACYBP/CCT3/CLCF1/E2F8/EGFR/ENDOG/EYA1/FGFR4/FOXM1/HRAS/HSF1/HSP90AA1/INO80/INS/MAPK15/MAPK3/NOX4/NPAS2/NVL/PARP3/PARP9/PAXIP1/PDGFA/PDGFRB/PFN1/PLA2G1B/PRDM9/PRKCD/PTK2B/RFC2/SHC1/SLF2/TIMELESS/TNFSF	41	BP
GO:0070268	cornification	23/2734	113/17381	0.11251	0.7546	0.74219	FURIN/KRT12/KRT14/KRT16/KRT23/KRT3/KRT32/KRT6A/KRT6C/KRT71/KRT75/KRT76/KRT79/KRT81/KRT84/KRT85/PKP3/SPINK5/SPRR1A/ST14/TGM1/TGM5/TMEM79	23	BP
GO:1903050	regulation of proteolysis involved in cellular protein catabolic	48/2734	257/17381	0.11256	0.7546	0.74219	ALAD/ANAPC15/ANAPC2/ARAF/ATPIF1/AXIN1/BCAP31/BRSK2/BUB1B/CAV3/CCAR2/CHFR/CLU/COMMD1/DAB2IP/DISC1/ECSCR/EFNA1/FHIT/GBA/GPX1/HERPUD1/KLHL40/LAMP3/NKD2/OS9/OSBPL7/PANO1/PKD1/PSMB11/PSMB6/PSMB7/PSMB8/PSMC3/PSMD13/PSMD3/PSMD5/PSMD7/PTK2B/RHBDF1/RNF166/RNF180/SHH/SUFU/TAF1/USP19/USP5/WNT1	48	BP

GO:0055067	monovalent inorganic cation homeostasis	29/2734	147/17381	0.11257	0.7546	0.74219	AGT/ATP1A1/ATP1A4/ATP6V0B/ATP6V0E2/ATP6V1B1/ATP6V1F/ATP6V1G1/ATP6V1G2/AVPR2/CA7/CLN6/CYP11B2/DRD2/FASLG/GPR89A/MAPK3/RAB20/RAB7A/RHCG/SCNN1A/SLC11A1/SLC26A1/SLC26A10/SLC26A6/SLC4A9/SLC9A3/SLC9A5/TACR1	29	BP
GO:0006284	base-excision repair	10/2734	42/17381	0.11294	0.7546	0.74219	APEX1/HMGA1/LIG3/MBD4/NEIL2/OGG1/PARP2/POLE/RECQL4/SMUG1	10	BP
GO:0043551	regulation of phosphatidylinositol 3-kinase	10/2734	42/17381	0.11294	0.7546	0.74219	AMBRA1/CCL19/CCL21/DAB2IP/EPHA8/PDGFRB/PIK3R5/PIK3R6/PTK2B/TNFAIP8L3	10	BP
GO:0072210	metanephric nephron development	10/2734	42/17381	0.11294	0.7546	0.74219	AQP1/BMP4/EGR1/LHX1/OSR1/PDGFRB/PKD1/SMO/SOX8/STAT1	10	BP
GO:2000351	regulation of endothelial cell apoptotic	10/2734	42/17381	0.11294	0.7546	0.74219	ABL1/ECSCR/FASLG/FGA/GPER1/IL13/KDR/MIR15A/PDCD4/TNIP2	10	BP
GO:0072175	epithelial tube formation	26/2734	130/17381	0.11302	0.7546	0.74219	ABL1/ADM/BMP4/CC2D2A/CFL1/DEAF1/DVL2/FGFR2/FZD2/GRHL3/IRX3/KAT2A/LIAS/MTHFD1/OSR1/PFN1/RARA/SCRIB/SETD2/SOX8/SPINT1/ST14/SUFU/TCAP/TULP3/WNT6	26	BP
GO:0051099	positive regulation of binding	33/2734	170/17381	0.1132	0.7546	0.74219	ABL1/ACD/AKTIP/BMP4/CDK5/CDK9/CSF3/EDF1/EIF4G1/GCG/GPSM1/HDAC8/HERPUD1/HIP1R/HSF1/KAT2A/LARP6/LFNG/LRP1/MAPRE3/MEN1/NME1/PARP9/PKD1/PLXND1/PYGO2/RALB/RARA/RFNG/TAF1/TIRAP/TRA	33	BP
GO:0043687	post-translational protein modification	79/2734	441/17381	0.11432	0.7546	0.74219	AHSG/ALPPL2/AMTN/ANKRD9/APLP2/APOA5/APOB/ARSE/ARSI/ASB11/ASB14/BMP4/CCNF/CISH/COMMD1/COPS6/CSF1/DCAF11/DCAF8/DDB1/DDB2/FBXL13/FBXL16/FBXL19/FBXL22/FBXL8/FBXO10/FBXO2/FBXO6/FBXW4/FGA/FN1/FOLR2/GP2/GPIHBP1/IGFBP3/KNG1/LCMT1/LY6D/LY6E/LY6H/LYPD1/LYPD2/LYPD6B/LYPD8/MEN1/MSLN/MXRA8/NEDD8/NRN1/NRN1L/NTM/NTNG2/OBSL1/P3H1/PNPLA2/PSCA/PSMB11/PSMB6/PSMB7/PSMB8/PSMC3/PSMD13/PSMD3/PSMD5/PSMD7/QSOX1/RAET1G/RTN4RL1/RTN4RL2/SPSB1/SPSB2/SPSB3/THY1/TNC/ULBP2/VGF/VNN3/WFS1	79	BP

GO:0048015	phosphatidylinositol-mediated signaling	30/2734	153/17381	0.11463	0.7546	0.74219	AGT/CCL5/CSF1R/CSF3/DAB2IP/EGFR/ERBB2/F2RL1/GPER1/IL18/INPP5F/INS/KDR/LTK/MAPK3/MUC5AC/MYOC/NCF1/NRG1/NYAP1/PDGFA/PDGFRB/PI4KB/PIK3R5/PIK3R6/PLXNB1/PPARD/PPP2R5B/PREX2/SELP	30	BP
GO:0110053	regulation of actin filament organization	44/2734	234/17381	0.11471	0.7546	0.74219	ABL1/ARAP1/ARFIP1/ARHGEF10/ARPC1A/ARPC1B/ARPC2/ARPC4/BAIAP2L1/BRK1/CAPZA3/CCL21/CDC42EP2/CFL1/CORO1A/CORO1B/CSF3/CTTN/EVL/F2RL1/FHOD1/HIP1R/LIMK1/LMOD1/MLST8/MTOR/MYO1C/MYOC/NOX4/PFN1/PRKCD/PTK2B/SCIN/SERPINF2/SH3PXD2B/SORBS3/SPTAN1/SPTB/SPTBN2/TACR1/WAS/WDR1/WHAMM/WNT11	44	BP
GO:0032103	positive regulation of response to external stimulus	52/2734	281/17381	0.11534	0.7546	0.74219	ADAM8/AGT/ARTN/C3AR1/CAMK1D/CCL1/CCL19/CCL21/CCL5/CCR2/CD180/CD6/CD74/CDH13/CREB3/CSF1/DAPK2/DHX58/EDN2/EGFR/F2RL1/HAVCR2/HSPB1/IDO1/IL18/KARS/KDR/KLKB1/LGALS9/LRSAM1/LTA/MAPK3/MIR221/MIR222/MIR92A2/NOD1/OPRM1/PDCD4/PDE2A/PDGFRB/PF4V1/PGC/PTGER3/PTK2B/RARRES2/SCARF1/SERPINF2/STX4/TBK1/TIRAP/TL	52	BP
GO:0061572	actin filament bundle organization	27/2734	136/17381	0.11549	0.7546	0.74219	ABL1/ARAP1/ARHGEF10/BAIAP2L1/CORO1B/ESPN/ESPNL/EVL/FHOD1/FSCN1/ITGB5/LIMK1/MTOR/MYOC/NOX4/PFN1/PHACTR1/PTK2B/RHOD/SERPINF2/SH3PXD2B/SHROOM1/SORBS3/TACR1/WAS/WNT11/ZYX	27	BP
GO:0046887	positive regulation of hormone	24/2734	119/17381	0.11552	0.7546	0.74219	ANO1/BAD/BLK/C1QTNF1/CAPN10/DOC2B/DRD2/EGFR/FGA/GCG/GCK/GLUD1/GPER1/HCAR2/INHBB/INS/PFKM/PPARD/PTPN11/RFX6/STX4/TACR1/TFR2/TRH	24	BP
GO:0001696	gastric acid secretion	5/2734	17/17381	0.11561	0.7546	0.74219	HIP1R/HRH2/KCNQ1/PTGER3/SGK1	5	BP
GO:0002076	osteoblast development	5/2734	17/17381	0.11561	0.7546	0.74219	ACHE/BGLAP/MEN1/SHH/TNN	5	BP
GO:0002183	cytoplasmic translational initiation	5/2734	17/17381	0.11561	0.7546	0.74219	EIF4B/EIF4EBP1/EIF4G1/RBM4/TICRR	5	BP
GO:0006625	protein targeting to	5/2734	17/17381	0.11561	0.7546	0.74219	PEX14/PEX16/PEX3/PEX5/PEX6	5	BP
GO:0014061	regulation of norepinephrine secretion	5/2734	17/17381	0.11561	0.7546	0.74219	ADRA2A/ADRA2B/ADRA2C/AGT/STX1A	5	BP

GO:0045056	transcytosis	5/2734	17/17381	0.11561	0.7546	0.74219	CD300LG/GPIHBP1/LRP1/MFSD2A/RAB17	5	BP
GO:0070293	renal absorption	5/2734	17/17381	0.11561	0.7546	0.74219	AQP1/CLDN4/KCNQ1/MAGED2/SGK1	5	BP
GO:0072224	metanephric glomerulus development	5/2734	17/17381	0.11561	0.7546	0.74219	AQP1/EGR1/LHX1/OSR1/PDGFRB	5	BP
GO:0072662	protein localization to	5/2734	17/17381	0.11561	0.7546	0.74219	PEX14/PEX16/PEX3/PEX5/PEX6	5	BP
GO:0072663	establishment of protein localization to	5/2734	17/17381	0.11561	0.7546	0.74219	PEX14/PEX16/PEX3/PEX5/PEX6	5	BP
GO:0090201	negative regulation of release of cytochrome c	5/2734	17/17381	0.11561	0.7546	0.74219	ARRB2/CLU/GPX1/LMNA/PRELID1	5	BP
GO:1901386	negative regulation of voltage-gated calcium channel	5/2734	17/17381	0.11561	0.7546	0.74219	CRHR1/DRD2/GNB5/GPR35/RRAD	5	BP
GO:0002534	cytokine production involved in inflammatory response	9/2734	37/17381	0.11592	0.7546	0.74219	CD6/CHID1/CUEDC2/IDO1/KARS/MIR221/MIR222/PDCD4/PER1	9	BP
GO:0043954	cellular component	9/2734	37/17381	0.11592	0.7546	0.74219	C1QL1/CSF1R/CTTN/F2RL1/INS/KIFC3/PARD6A/RASSF8/SUPT6H	9	BP

GO:0045197	establishment or maintenance of epithelial cell apical/basal polarity	9/2734	37/17381	0.11592	0.7546	0.74219	CDX2/CRB2/DLG4/ILK/LRCH4/MARK2/SCRIB/WDR1/WNT11	9	BP
GO:0060119	inner ear receptor cell	9/2734	37/17381	0.11592	0.7546	0.74219	CDH23/FZD2/IFT20/LHFPL5/LRTOMT/MYO7A/NAGLU/SCRIB/WDPCP	9	BP
GO:1901030	positive regulation of mitochondrial outer membrane permeabilization involved in apoptotic	9/2734	37/17381	0.11592	0.7546	0.74219	ATPIF1/BAD/BOK/DYNLL2/HIP1R/PPP1R13B/SFN/YWHAQ/ZNF205	9	BP
GO:1902622	regulation of neutrophil migration	9/2734	37/17381	0.11592	0.7546	0.74219	ADAM8/C3AR1/CAMK1D/CCL19/CCL21/CD74/DAPK2/PF4V1/TIRAP	9	BP
GO:0032945	negative regulation of mononuclear	15/2734	69/17381	0.11596	0.7546	0.74219	BMP4/CASP3/ERBB2/HAVCR2/IDO1/IL20RB/INPP5D/LGALS9/MAD1L1/PLA2G2F/PRKAR1A/SCGB1A1/SHH/TNFRSF13B/VSIG4	15	BP
GO:0035690	cellular response to	15/2734	69/17381	0.11596	0.7546	0.74219	CD69/DDC/DPEP1/EFTUD2/EGFR/EIF2B5/HSF1/HSPA5/KCNQ1/MT2A/NME1/PDE2A/RECQL5/REN/TH	15	BP
GO:0050672	negative regulation of lymphocyte	15/2734	69/17381	0.11596	0.7546	0.74219	BMP4/CASP3/ERBB2/HAVCR2/IDO1/IL20RB/INPP5D/LGALS9/MAD1L1/PLA2G2F/PRKAR1A/SCGB1A1/SHH/TNFRSF13B/VSIG4	15	BP
GO:0006721	terpenoid metabolic	22/2734	108/17381	0.11778	0.7546	0.74219	AGRN/ALDH8A1/APOB/APOC3/CLPS/CYP1A1/CYP1A2/DGAT1/EGFR/GPC1/GPC2/GPIHBP1/HSPG2/LRP1/OPN1MW/PLB1/PPARD/RARRES2/RBP1/RB	22	BP
GO:0097237	cellular response to	22/2734	108/17381	0.11778	0.7546	0.74219	EPX/FAM213A/GCH1/GPX1/GPX2/GPX3/GSR/GSTM1/GSTM2/GSTZ1/LPO/MGST1/MGST3/MPO/NOS3/OPRD1/PRDX1/PRDX6/SESN1/SOD3/TXNRD1/	22	BP
GO:0007031	peroxisome organization	8/2734	32/17381	0.11829	0.7546	0.74219	PEX11A/PEX14/PEX16/PEX3/PEX5/PEX6/PLA2G16/SEC16B	8	BP

GO:0071624	positive regulation of granulocyte	8/2734	32/17381	0.11829	0.7546	0.74219	C3AR1/CAMK1D/CCL19/CCL21/CD74/DAPK2/PF4V1/TIRAP	8	BP
GO:0090218	positive regulation of lipid kinase	8/2734	32/17381	0.11829	0.7546	0.74219	AMBRA1/CCL19/CCL21/CD81/EPHA8/PDGFRB/PTK2B/TNFAIP8L3	8	BP
GO:0007632	visual behavior	12/2734	53/17381	0.11851	0.7546	0.74219	B4GALT2/CDK5/DEAF1/DRD2/GRIN1/HRH2/IFT20/MEIS2/MTOR/NLGN3/PPP1R1B/RGS14	12	BP
GO:0002825	regulation of T-helper 1 type immune	6/2734	22/17381	0.1192	0.7546	0.74219	CCL19/CCR2/HAVCR2/HLX/IL4R/SLC11A1	6	BP
GO:0010884	positive regulation of	6/2734	22/17381	0.1192	0.7546	0.74219	ACACB/APOB/EHD1/FITM1/HILPDA/SCARB1	6	BP
GO:0021513	spinal cord dorsal/ventral patterning	6/2734	22/17381	0.1192	0.7546	0.74219	EVX1/LHX3/SHH/SMO/SUFU/TULP3	6	BP
GO:0032753	positive regulation of interleukin-4	6/2734	22/17381	0.1192	0.7546	0.74219	EPX/HAVCR2/HLA-E/IL20RB/LGALS9/RARA	6	BP
GO:0043302	positive regulation of leukocyte	6/2734	22/17381	0.1192	0.7546	0.74219	F2RL1/IL13/IL4R/ITGAM/STX4/STXBP1	6	BP
GO:0044381	glucose import in response to insulin stimulus	6/2734	22/17381	0.1192	0.7546	0.74219	AGT/MZB1/PID1/PTPN11/RARRES2/SLC2A4	6	BP
GO:0045738	negative regulation of	6/2734	22/17381	0.1192	0.7546	0.74219	HSF1/MIR221/NUDT16L1/OGG1/OTUB1/RECQL5	6	BP
GO:0051004	regulation of lipoprotein lipase activity	6/2734	22/17381	0.1192	0.7546	0.74219	APOA5/APOC3/FURIN/GPIHBP1/LMF1/NR1H3	6	BP

GO:0060969	negative regulation of	6/2734	22/17381	0.1192	0.7546	0.74219	DND1/HMGA1/NCOR1/NCOR2/PHF2/ZC3H10	6	BP
GO:0061082	myeloid leukocyte cytokine	6/2734	22/17381	0.1192	0.7546	0.74219	BCL6/CD74/CHGA/CUEDC2/SEMA7A/SPON2	6	BP
GO:0071800	podosome assembly	6/2734	22/17381	0.1192	0.7546	0.74219	BIN2/FARP2/FSCN1/KIF9/NCF1/SH3PXD2B	6	BP
GO:0072170	metanephric tubule	6/2734	22/17381	0.1192	0.7546	0.74219	AQP1/OSR1/PKD1/POU3F3/SOX8/STAT1	6	BP
GO:1901071	glucosamine-containing compound metabolic	6/2734	22/17381	0.1192	0.7546	0.74219	AMDHD2/CHID1/CHIT1/CHST5/CHST7/NAGK	6	BP
GO:1904376	negative regulation of protein localization to	6/2734	22/17381	0.1192	0.7546	0.74219	AP2M1/CSK/LYPD1/PID1/PKDCC/TMBIM1	6	BP
GO:1904886	beta-catenin destruction complex disassembly	6/2734	22/17381	0.1192	0.7546	0.74219	AXIN1/DVL2/FRAT1/FZD2/WNT1/WNT3A	6	BP
GO:0009065	glutamine family amino acid catabolic	7/2734	27/17381	0.11963	0.7546	0.74219	ALDH4A1/FAH/GAD1/GLUD1/GOT2/NOS3/PADI4	7	BP
GO:0021846	cell proliferation in	7/2734	27/17381	0.11963	0.7546	0.74219	DISC1/DOCK7/FGFR2/LHX5/POU3F3/WNT3A/WNT7A	7	BP
GO:0032673	regulation of interleukin-4 production	7/2734	27/17381	0.11963	0.7546	0.74219	EPX/HAVCR2/HLA-E/IL20RB/LGALS9/RARA/SCGB1A1	7	BP

GO:0033014	tetrapyrrole biosynthetic process	7/2734	27/17381	0.11963	0.7546	0.74219	ALAD/ALAS2/ATPIF1/COX10/HMBS/NFE2L1/UROS	7	BP
GO:0050685	positive regulation of mRNA	7/2734	27/17381	0.11963	0.7546	0.74219	HMX2/HSF1/LMNTD2/NUP98/PRDX6/SF3B4/SLC39A5	7	BP
GO:0051450	myoblast proliferation	7/2734	27/17381	0.11963	0.7546	0.74219	ANKRD2/ATF2/GPX1/MEIS2/MIR10A/MYOD1/PPARD	7	BP
GO:0071480	cellular response to gamma	7/2734	27/17381	0.11963	0.7546	0.74219	CRYAB/EGR1/HRAS/HSF1/HSPA5/NOX4/TMEM109	7	BP
GO:1902745	positive regulation of lamellipodium	7/2734	27/17381	0.11963	0.7546	0.74219	AQP1/ARPC2/BRK1/CORO1B/FSCN1/MTOR/WNT1	7	BP
GO:1904037	positive regulation of epithelial cell	7/2734	27/17381	0.11963	0.7546	0.74219	CAPN10/ECSCR/FASLG/GPER1/MIR15A/PDCD4/SPOP	7	BP
GO:0014066	regulation of phosphatidylinositol 3-kinase signaling	20/2734	97/17381	0.11969	0.7546	0.74219	AGT/CCL5/CSF3/DAB2IP/EGFR/F2RL1/GPER1/IL18/INS/KDR/MAPK3/MYOC/NCF1/NRG1/PDGFA/PDGFRB/PLXNB1/PPARD/PPP2R5B/SELP	20	BP
GO:0043534	blood vessel endothelial cell migration	20/2734	97/17381	0.11969	0.7546	0.74219	ABL1/CIB1/EFNA1/EPHB4/GDF2/GPX1/HSPB1/KDR/MIR10A/MIR212/MIR221/MIR29C/MIR503/MIR92A2/NOTCH1/NR4A1/PRCP/PTK2B/SCARB1/TDGF1	20	BP
GO:0050905	neuromuscular process	20/2734	97/17381	0.11969	0.7546	0.74219	AARS/ABL1/CDH23/CTNNA2/DLG4/DRD2/GCH1/GLRA1/GPR88/GRIN1/GRIN2C/HOXC10/IGDCC3/JPH3/MYO7A/NRG1/PNKD/SLURP1/STRA6/TME	20	BP

GO:0006869	lipid transport	63/2734	347/17381	0.12025	0.7574	0.74492	ABCA2/ABCC3/ABCC4/ABCG5/ACACB/AGT/ANO4/ANO7/APOA5/APOB/APOC3/C1QTNF1/CLU/CPT2/CPTP/CRHR1/CROT/DRD2/GLTP/GLTPD2/GOT2/GPIHBP1/HEATR4/LCN12/LRP1/LRP10/MFSD2A/MID1IP1/MROH6/NCOR1/NME4/NPC1L1/NPC2/NR1H3/OSBPL5/OSBPL7/PITPNM1/PLA2G1B/PLA2G2F/PLA2G5/PPARD/PRELID1/PRKCD/PSAP/PTPN11/REN/SCARB1/SERPINA5/SFTPA1/SHH/SIGMAR1/SLC22A9/SLC25A20/SLCO2B1/SOAT2/SPNS1/SPNS3/STARD3/STARD5/STOML1/STRA6/TNFAIP8L3/VPS51	63	BP
GO:0003014	renal system process	23/2734	114/17381	0.12096	0.7601	0.74756	ADM/ADRA1A/AGT/AQP1/AVPR2/BMP4/CLDN4/CYP11B2/DRD2/F2RL1/HSD11B2/KCNQ1/MAGED2/MCAM/MYO1E/PRKAR1A/PRKAR1B/PRKRIP1/REN/SERPINF2/SGK1/TACR1/WFS1	23	BP
GO:0042471	ear morphogenesis	23/2734	114/17381	0.12096	0.7601	0.74756	ATP6V1B1/DVL2/EYA1/FGFR2/FZD2/GBX2/GRHL3/HMX2/KCNQ4/LHFPL5/MAPK3/MYO3A/MYO7A/NAGLU/NKX3-2/OSR1/SCRIB/SLC44A4/TCAP/TMIE/WDPCP/WNT1/WNT3A	23	BP
GO:1903955	positive regulation of protein	18/2734	86/17381	0.12106	0.7601	0.74756	ACSM6/CYB5R1/ELMOD1/GPHA2/HNMT/ITGAX/KAT2A/LRRC46/MYBPC1/NBPF3/NRG1/OSCP1/PEMT/PSMB7/RNF31/TSGA13/UBL4B/ZBTB17	18	BP
GO:2001235	positive regulation of apoptotic signaling	34/2734	177/17381	0.12161	0.7609	0.74839	AGT/APOPT1/ATF3/ATPIF1/BAD/BCAP31/BOK/CTSH/DAB2IP/DYNLL2/FASLG/G0S2/GPER1/HIP1R/HRK/INCA1/INHBB/LCK/LGALS9/LTBR/MIR15A/MIR16-1/MOAP1/PEA15/PPP1R13B/PRKCD/SEPT4/SFN/SPOP/TNFRSF12A/TRAF2/	34	BP
GO:0051492	regulation of stress fiber	16/2734	75/17381	0.12164	0.7609	0.74839	ABL1/ARAP1/ARHGEF10/EVL/FHOD1/LIMK1/MTOR/MYOC/NOX4/PFN1/SERPINF2/SH3PXD2B/SORBS3/TACR1/WAS/WNT11	16	BP
GO:0072088	nephron epithelium	16/2734	75/17381	0.12164	0.7609	0.74839	AGT/BMP4/EYA1/HOXB7/ILK/IRX3/LHX1/OSR1/PKD1/SHH/SMO/SOX8/STAT1/WNT1/WNT11/WNT6	16	BP
GO:0033157	regulation of intracellular protein transport	69/2734	383/17381	0.12171	0.7609	0.74839	ACSM6/ANP32B/ATP13A2/ATPIF1/B3GAT3/BCAP31/BCAS3/BMP4/BRSK2/CCL19/CD27/CDK5/CHRM1/CIB1/CSF3/CYB5R1/DAB2IP/EGFR/ELMOD1/EMD/ERBB2/FAM89B/FLNA/GPHA2/HNMT/IL18/ITGAM/ITGAX/KAT2A/LGALS9/LRRC46/MIEF2/MTOR/MYBPC1/MYO1C/NBPF3/NFKBIL1/NRG1/OAZ2/OGG1/OS9/OSCP1/PARP10/PBLD/PDE2A/PEMT/PKD1/PRDX1/PRKCD/PSMB7/PTPN11/RBPMS/RNF31/SEC16B/SETD2/SFN/SFRP5/SHH/SMO/SUFU/SUPT6H/THRA/TLR9/TSGA13/UBL4B/WNT3A/ZBTB17/ZC3H3/ZPR1	69	BP

GO:0010506	regulation of autophagy	55/2734	300/17381	0.12223	0.7624	0.74986	ABL1/AMBRA1/ATG101/ATP13A2/ATP6V0B/ATP6V0E2/ATP6V1B1/ATP6V1G1/ATP6V1G2/ATPIF1/BAD/BOK/CASP1/CASP3/CDK5/CTTN/DAP/DAPK1/DAPK2/DAPL1/EIF2AK4/EIF4G1/GAPDH/GATA4/GBA/GOLGA2/GPSM1/HSPB1/IFT20/KAT2A/KDR/LAMP3/LARS/LEPR/LRSAM1/MAPK3/MFN2/MLST8/MTOR/NOD1/NPRL3/OSBPL7/POLDIP2/PRKAB1/PSAP/QSOX1/RALB/RRAGC/SPTLC1/TBC1D25/TBK1/TFEB/TRIM65/ULK1/ZBTB17	55	BP
GO:0009314	response to radiation	78/2734	437/17381	0.12277	0.7624	0.74986	ABCG5/ADIRF/AIPL1/ALAD/AQP1/ASIC2/ASNS/B4GALT2/BRAT1/BRCA1/BRCC3/CACNA1F/CASP3/CCAR2/CDK5/CNGB1/CRIP1/CRYAB/DDB1/DDB2/DEAF1/DNMT3A/DRD2/EGFR/EGR1/EIF2AK4/EYA1/FNTA/FNTB/GNAT1/GPX1/GRIN1/GUCA1B/GUCY2D/HRAS/HRH2/HSF1/HSPA5/IFT20/INIP/INO80/INTS3/KARS/KRT14/MBD4/MEIS2/MEN1/MTA1/MTOR/NLGN3/NOC2L/NOX4/OGG1/OPN1MW/OPN4/OPRM1/PAXIP1/PER1/PITPNM1/PPEF1/PPP1R1B/RBM4/RGS14/SCARA3/SDF4/SEMA5B/TAF1/TH/TICRR/TMEM109/[illegible]	78	BP
GO:0006644	phospholipid metabolic process	82/2734	461/17381	0.1229	0.7624	0.74986	ACHE/AMBRA1/APOA5/CCL19/CCL21/CD81/CDIPT/CPNE7/CSF1R/CWH43/DAB2IP/DGKQ/DOLPP1/DPM2/DRD2/EGFR/EPHA8/ERBB2/ETNK2/FGF17/FGF3/FGFR2/FGFR4/FITM1/GDPD1/GNPAT/GPAA1/HADHA/IDH1/INPP5D/INPP5E/INPP5F/INPPL1/LCK/MFSD2A/MTMR1/MTMR14/NRG1/OSBPL5/PDGFA/PDGFRB/PEMT/PHOSPHO1/PI4KB/PIGC/PIGG/PIGO/PIGQ/PIGY/PIGZ/PIK3R5/PIK3R6/PITPNM1/PLA2G15/PLA2G16/PLA2G1B/PLA2G2F/PLA2G4B/PLA2G5/PLB1/PLCB2/PLCD1/PLCH2/PLD2/PLPP1/PMVK/PPARD/PRDX6/PRKCD/PTDSS1/PTDSS2/PTK2B/PTPN11/PYURF/SCARB1/SGMS1/SLC4[illegible]	82	BP
GO:0006953	acute-phase response	11/2734	48/17381	0.12303	0.7624	0.74986	AHSG/FN1/IL1RN/INS/ITIH4/ORM1/ORM2/PTGER3/SERPINA3/SERPINF2/TFR2	11	BP
GO:0010463	mesenchymal cell	11/2734	48/17381	0.12303	0.7624	0.74986	BMP4/FBXW4/FGFR2/LMNA/MYCN/OSR1/PDGFA/SHH/SMO/STAT1/WNT11	11	BP
GO:0048662	negative regulation of smooth muscle	11/2734	48/17381	0.12303	0.7624	0.74986	ANG/GPER1/IGFBP3/ILK/MFN2/MIR15A/MIR503/MIR96/NDRG2/PPARD/SF1	11	BP
GO:0071825	protein-lipid complex subunit	11/2734	48/17381	0.12303	0.7624	0.74986	AGT/APOA5/APOB/APOC3/BIN1/DGAT1/GPIHBP1/MPO/PCDHGA3/SCARB1/SOAT2	11	BP

GO:0032271	regulation of protein polymerization	35/2734	183/17381	0.12308	0.7624	0.74986	ABL1/ANKRD53/ARFIP1/ARPC1A/ARPC1B/ARPC2/ARPC4/BAIAP2L1/BRK1/CAPZA3/CAV3/CCL21/CDC42EP2/CORO1A/CORO1B/CSF3/CTTN/EVL/FES/HIP1R/LMOD1/MLST8/MTOR/MYO1C/NUMA1/PFN1/PRKCD/PTK2B/SCIN/SPTAN1/SPTB/SPTBN2/VDAC2/WAS/WHAMM	35	BP
GO:0010770	positive regulation of cell morphogenesis involved in	27/2734	137/17381	0.12328	0.7624	0.74986	ABL1/AMIGO1/ANAPC2/ARPC2/CIB1/DISC1/DOCK1/FGA/FLNA/FN1/ILK/ISLR2/L1CAM/LIMK1/METRN/MYOC/NRG1/OBSL1/OLFM4/PLXNB1/PLXNB3/PLXND1/SEMA7A/TNFRSF12A/TRPV2/WNT3A/ZFYVE27	27	BP
GO:0090501	RNA phosphodiester bond hydrolysis	27/2734	137/17381	0.12328	0.7624	0.74986	ANG/APEX1/AZGP1/CPSF1/CPSF4/CSTF2/DIS3L/DIS3L2/ERN2/EXO1/EXOSC10/EXOSC2/FCF1/ISG20L2/NHP2/NOB1/PAN2/PIWIL2/PNLDC1/RNASE1/RNASE8/RNASEK/RPP21/SMG5/TSR1/ZC3H3/ZNRD1	27	BP
GO:0098754	detoxification	21/2734	103/17381	0.12336	0.7624	0.74986	EPX/FAM213A/GCH1/GPX1/GPX2/GPX3/GSR/GSTM1/GSTM2/GSTZ1/LPO/MGST1/MGST3/MPO/NOS3/PRDX1/PRDX6/SESN1/SOD3/TXNRD1/TXNRD	21	BP
GO:0046488	phosphatidylinositol metabolic process	41/2734	218/17381	0.12378	0.7627	0.75013	CDIPT/CSF1R/CWH43/DPM2/DRD2/EGFR/ERBB2/FGF17/FGF3/FGFR2/FGFR4/GPAA1/INPP5D/INPP5E/INPP5F/INPPL1/LCK/MTMR1/MTMR14/NRG1/PDGFA/PDGFRB/PI4KB/PIGC/PIGG/PIGO/PIGQ/PIGY/PIGZ/PIK3R5/PIK3R6/PITPNM1/PLA2G16/PLA2G1B/PLA2G2F/PLA2G5/PLCH2/PTPN11/PYURF/T	41	BP
GO:0030010	establishment of cell polarity	24/2734	120/17381	0.12389	0.7627	0.75013	BCAS3/BRSK2/CCL19/CCL21/CDH5/CFL1/DOCK7/EYA1/FAM89B/FSCN1/GBF1/MARK2/MARK3/MCPH1/MYO18A/NUMA1/PARD3/PKD1/PTK2B/SCRIB/SDCCAG8/SHH/WDPCP/WNT7A	24	BP
GO:0010810	regulation of cell-substrate adhesion	36/2734	189/17381	0.12446	0.7627	0.75013	ABL1/AJAP1/ARPC2/BCAS3/BCL6/CCL21/CDH13/CDKN2A/CIB1/COL16A1/COL1A1/CSF1/DISC1/DOCK1/FBLN2/FGA/FLNA/FN1/ILK/KDR/LDB1/MEN1/MIR29C/MIR503/MIR92A2/MYOC/NOTCH1/OLFM4/PLAU/PTK2B/RELL2/RHOD/SPOCK2/THY1/WDPCP/WNT1	36	BP
GO:0072655	establishment of protein localization to mitochondrion	36/2734	189/17381	0.12446	0.7627	0.75013	ACSM6/AIP/ATPIF1/BAD/CYB5R1/DNAJC19/DNLZ/DYNLL2/ELMOD1/GPHA2/HNMT/HSP90AA1/ITGAX/KAT2A/LRRC46/MFN2/MOAP1/MYBPC1/NBPF3/NRG1/OSCP1/PDE2A/PEMT/PMPCA/PPP1R13B/PSMB7/RNF31/SFN/TIMM17B/TIMM22/TIMM23B/TIMM9/TSGA13/UBL4B/YWHAQ/ZBTB17	36	BP

GO:0051453	regulation of intracellular pH	19/2734	92/17381	0.12535	0.7627	0.75013	ATP6V0B/ATP6V0E2/ATP6V1B1/ATP6V1F/ATP6V1G1/ATP6V1G2/CA7/CLN6/FASLG/GPR89A/RAB20/RAB7A/SLC11A1/SLC26A1/SLC26A10/SLC26A6/SLC4A9/SLC9A3/SLC9A5	19	BP
GO:0060021	palate development	19/2734	92/17381	0.12535	0.7627	0.75013	ANP32B/CSRNP1/EPHB3/FZD2/JAG2/MMP25/MSC/NPRL3/OSR1/PKDCC/PYGO2/SHH/TBX2/VAX1/WDPCP/WFIKKN2/WNT11/WNT3A/WNT7A	19	BP
GO:0072080	nephron tubule development	19/2734	92/17381	0.12535	0.7627	0.75013	AGT/AQP1/BMP4/EYA1/HOXB7/ILK/IRX3/LHX1/NOTCH1/OSR1/PKD1/POU3F3/SHH/SMO/SOX8/STAT1/WNT1/WNT11/WNT6	19	BP
GO:0008654	phospholipid biosynthetic process	52/2734	283/17381	0.12613	0.7627	0.75013	ACHE/CDIPT/CPNE7/CWH43/DGKQ/DOLPP1/DPM2/ETNK2/FGF17/FGF3/FGFR2/FGFR4/FITM1/GNPAT/GPAA1/IDH1/INPP5D/INPP5E/INPP5F/INPPL1/MFSD2A/MTMR1/MTMR14/PDGFA/PEMT/PHOSPHO1/PI4KB/PIGC/PIGG/PIGO/PIGQ/PIGY/PIGZ/PIK3R5/PIK3R6/PITPNM1/PLA2G16/PLA2G1B/PLA2G2F/PLA2G4B/PLA2G5/PLD2/PMVK/PPARD/PTDSS1/PTDSS2/PTPN11/PYU	52	BP
GO:0006633	fatty acid biosynthetic process	25/2734	126/17381	0.12659	0.7627	0.75013	ACACB/ACADVL/ACOT7/ACSF3/ACSM6/APOA5/APOC3/BRCA1/CD74/EDN2/ELOVL1/ELOVL5/FADS2/FADS3/HACD1/HACD3/LIAS/MID1IP1/MLXIPL/NR1H3/OLAH/PLA2G1B/PRKAB1/SCAP/THNSL2	25	BP
GO:0002688	regulation of leukocyte chemotaxis	22/2734	109/17381	0.12671	0.7627	0.75013	C3AR1/CAMK1D/CCL1/CCL19/CCL21/CCL5/CCR2/CD74/CREB3/CSF1/DAPK2/EDN2/F2RL1/KARS/LGALS9/MAPK3/NBL1/PF4V1/PTK2B/RARRES2/TIRAP/TRPV4	22	BP
GO:0006094	gluconeogenesis	17/2734	81/17381	0.12675	0.7627	0.75013	ALDOA/ATF3/CRTC2/DGKQ/G6PC3/GAPDH/GCG/GCK/GNMT/GOT2/INS/KAT2A/LEPR/MAEA/PC/PFKFB1/PGAM4	17	BP
GO:0045921	positive regulation of	17/2734	81/17381	0.12675	0.7627	0.75013	ATP13A2/CACNA1G/CACNA1H/CDK5/CDK5R2/DOC2B/F2RL1/FGA/IL13/IL4R/ITGAM/RAB7A/SDC1/SNF8/STX1A/STX4/STXBP1	17	BP
GO:0070098	chemokine-mediated signaling	17/2734	81/17381	0.12675	0.7627	0.75013	CCL1/CCL19/CCL21/CCL5/CCR2/CIB1/CXCR1/CXCR2/CXCR3/CXCR5/GPR17/GPR35/MPL/PF4V1/PTK2B/THPO/TREM2	17	BP
GO:0032611	interleukin-1 beta production	15/2734	70/17381	0.12723	0.7627	0.75013	ARRB2/CASP1/CASP5/CCL19/EGR1/F2RL1/GSDMD/HSPB1/LGALS9/NLRP1/NOD1/ORM1/ORM2/PYDC1/TLR8	15	BP
GO:0043507	positive regulation of JUN kinase	15/2734	70/17381	0.12723	0.7627	0.75013	AXIN1/CCL19/DAB2IP/DVL2/ERN2/FZD8/HACD3/MAP3K6/MAP4K2/MAPK8IP3/MIR92A2/PTK2B/TLR9/TNIK/TRAF2	15	BP

GO:0048199	vesicle targeting, to, from or within	15/2734	70/17381	0.12723	0.7627	0.75013	CEP19/CNIH2/COL7A1/GBF1/GOLGA2/GORASP1/GOSR2/SEC16A/SEC16B/STX5/TRAPPC1/TRAPPC2L/TRAPPC3/TRAPPC4/TRAPPC9	15	BP
GO:0002437	inflammatory response to antigenic	10/2734	43/17381	0.12751	0.7627	0.75013	CD6/GPR17/GPX1/IL20RB/IL31RA/IL5RA/KDM6B/LTA/NOTCH1/OPRM1	10	BP
GO:0031103	axon regeneration	10/2734	43/17381	0.12751	0.7627	0.75013	INPP5F/LRIG2/MIR221/MIR222/PTPRF/RGMA/RTN4RL1/RTN4RL2/SCARF1/TNC	10	BP
GO:0034198	cellular response to amino acid	10/2734	43/17381	0.12751	0.7627	0.75013	ATF3/DAP/DAPL1/EIF2AK4/LARS/MAPK3/MTOR/NPRL3/RRAGC/SESN1	10	BP
GO:0036465	synaptic vesicle recycling	10/2734	43/17381	0.12751	0.7627	0.75013	CDK5/DNM1/GAK/NLGN3/PLD2/SCRIB/STON2/TOR1A/WNT3A/WNT7A	10	BP
GO:0048483	autonomic nervous system development	10/2734	43/17381	0.12751	0.7627	0.75013	GBX2/HLX/HOXB1/HOXB2/KIF26A/PHOX2A/PHOX2B/SEMA3F/SOX8/TLX2	10	BP
GO:0072528	pyrimidine-containing compound biosynthetic process	10/2734	43/17381	0.12751	0.7627	0.75013	AK5/CDA/DHODH/ERH/MTOR/NME1/NME4/PUDP/TBPL1/UCK1	10	BP
GO:0008361	regulation of cell size	29/2734	149/17381	0.12757	0.7627	0.75013	ABL1/ANAPC2/AQP1/ARHGAP4/BARHL2/CAV3/CDK4/CDK5/CTTN/DISC1/DRAXIN/E2F4/FN1/ILK/ISLR2/L1CAM/LARS/LIMK1/MTOR/NRG1/SEMA3F/SEMA7A/SLC12A4/SLC12A7/TNFRSF12A/TRPV2/TRPV4/WNT3A/ZFYVE	29	BP
GO:0050678	regulation of epithelial cell proliferation	56/2734	307/17381	0.12801	0.7627	0.75013	A4GNT/AIMP1/ANG/ATF2/ATPIF1/BAD/BMP4/CCL5/CDH13/DAB2IP/DEAF1/ECM1/EGFL7/EGFR/ERBB2/ESRP2/EYA1/FGFR2/FLT4/FOXE3/GDF2/GPX1/HRAS/KDR/LIMS2/MARVELD3/MEN1/MIR16-1/MIR222/MIR29A/MIR29C/MIR503/MTOR/NKX2-8/NME1/NOTCH1/NR4A1/OSR1/PBLD/PLXNB3/PPARD/PYGO2/SFN/SHH/S	56	BP

GO:0043281	regulation of cysteine-type endopeptidase activity involved in	39/2734	207/17381	0.12809	0.7627	0.75013	ANP32B/APOPT1/AQP1/ARRB2/BAD/BCAP31/BOK/CASP1/CASP3/CD27/CRYAB/CTSH/DAP/DAPK1/DPEP1/FASLG/GPER1/GPX1/HERPUD1/HIP1R/HSF1/LAMP3/LCK/LGALS9/MIR15A/MTCH1/NAIP/NLE1/NLRP1/NOD1/PDCD2/POR/RAF1/RPS6KA1/SFN/SOX7/TRAF2/WNT3A/XDH	39	BP
GO:0060828	regulation of canonical Wnt signaling pathway	46/2734	248/17381	0.12813	0.7627	0.75013	ANKRD10/AXIN1/CCAR2/CHD8/COL1A1/DAB2IP/DRAXIN/DVL2/EGR1/EMD/FGFR2/FZD9/IFT20/IGFBP6/ILK/KREMEN2/LGR6/LZTS2/MIR222/NAIP/NKD2/NKX2-5/NLE1/NOTCH1/PSMB11/PSMB6/PSMB7/PSMB8/PSMC3/PSMD13/PSMD3/PSMD5/PSMD7/RNF220/RSPO1/SFRP5/SHH/SOST/SOX7/TNN/WLS/WNT1/	46	BP
GO:1903749	positive regulation of establishment of protein	23/2734	115/17381	0.12979	0.7627	0.75013	ACSM6/BAD/CYB5R1/DYNLL2/ELMOD1/GPHA2/HNMT/ITGAX/KAT2A/LRRC46/MYBPC1/NBPF3/NRG1/OSCP1/PEMT/PPP1R13B/PSMB7/RNF31/SFN/TSGA13/UBL4B/YWHAQ/ZBTB17	23	BP
GO:0050920	regulation of chemotaxis	35/2734	184/17381	0.13003	0.7627	0.75013	ARTN/C3AR1/CAMK1D/CCL1/CCL19/CCL21/CCL5/CCR2/CD74/CDH13/CORO1B/CREB3/CSF1/DAPK2/EDN2/F2RL1/HSPB1/KARS/KDR/LGALS9/MAPK3/MIR15A/MIR16-1/NBL1/NOTCH1/PDGFRB/PF4V1/PTK2B/RARRES2/SEMA3F/STX4/TBR1/TIRAP/TRPV4/WNT3A	35	BP
GO:0002446	neutrophil mediated immunity	88/2734	499/17381	0.13119	0.7627	0.75013	ACPP/ADAM8/ADGRG3/AHSG/ALAD/ALDOA/ANPEP/AP2A2/ARL8A/BIN2/C1orf35/C3AR1/CALML5/CD58/CD63/CD68/CDA/CHIT1/COTL1/CRACR2A/CTSD/CTSH/CXCR1/CXCR2/DDOST/DGAT1/DOK3/DPP7/DYNC1H1/EPX/F2RL1/FLG2/FRMPD3/GGH/GHDC/GSDMD/HK3/HMOX2/HSP90AA1/IDH1/IMPDH1/ITGAL/ITGAM/ITGAX/LGALS3/MGAM/MGST1/MMP25/MPO/NBEAL2/NPC2/OLFM4/OLR1/ORM1/ORM2/ORMDL3/PKM/PLA2G1B/PLAU/PPIE/PRCP/PRDX6/PRKCD/PSAP/PSMB7/PSMC3/PSMD13/PSMD3/PSMD7/PTPRN2/QSOX1/RAB24/RAB44/RAB7A/RHOF/SERPINA3/SLC11A1/SLC15A4/SPTAN1/SRP14/STK11IP/TMBIM1/TMEM63A/TRAPPC1/TUSC2/UBR4/VA[illegible]	88	BP

GO:0016050	vesicle organization	69/2734	385/17381	0.13124	0.7627	0.75013	ADPRHL1/AKTIP/ALS2CL/AP2M1/AQP1/CCDC136/CHMP1A/CHMP4C/CHMP7/CNIH2/COL7A1/CORO1A/DLG4/DNM1/DOC2A/DOC2B/DTNBP1/FAM160A2/FASLG/GBF1/GOLGA2/GORASP1/GOSR2/GRTP1/HPS1/KIF13A/MYO18A/MYO7A/NKD2/RAB20/RAB34/RAB7A/RABGAP1/RABGAP1L/RBSN/SAMD9L/SDC1/SEC16A/SEC16B/SGSM2/SNF8/SNX11/SNX12/STX1A/STX4/STX5/STX8/STXBP1/SYTL3/TAP1/TBC1D25/TBC1D28/TBC1D9/TBPL1/TRAPPC1/TRAPPC2L/TRAPPC3/TRAPPC4/TRAPPC9/USP6NL/VAMP1/VAMP5[illegible]	69	BP
GO:0001656	metanephros development	18/2734	87/17381	0.13135	0.7627	0.75013	APH1A/AQP1/BMP4/CTSH/EGR1/EYA1/HOXC11/IRX3/LHX1/OSR1/PDGFA/PDGFRB/PKD1/POU3F3/SHH/SMO/SOX8/STAT1	18	BP
GO:0001942	hair follicle development	18/2734	87/17381	0.13135	0.7627	0.75013	DNASE1L2/EGFR/FGFR2/FOXN1/FOXQ1/HOXC13/KRT71/KRT84/LDB1/NOTCH1/NSDHL/NUMA1/PDGFA/SHH/SMO/SPINK5/TMEM79/WNT10A	18	BP
GO:0002066	columnar/cuboidal epithelial cell	12/2734	54/17381	0.13172	0.7627	0.75013	BAD/BMP4/LHFPL5/LRTOMT/MYO7A/NKX3-2/RARA/SCRIB/SIDT2/SMO/SPDEF/WDPCP	12	BP
GO:0090183	regulation of kidney development	12/2734	54/17381	0.13172	0.7627	0.75013	AGT/BMP4/EGR1/HOXB7/LHX1/OSR1/PDGFA/PDGFRB/SHH/SMO/SOX8/STAT1	12	BP
GO:1904377	positive regulation of protein localization to	12/2734	54/17381	0.13172	0.7627	0.75013	ARHGEF16/CIB1/CNPY4/EGFR/EPB41L2/GPER1/LRP1/NKD2/NUMA1/STX4/TREM2/WNT3A	12	BP
GO:0001755	neural crest cell migration	9/2734	38/17381	0.13183	0.7627	0.75013	BMP4/CFL1/EFNB1/GBX2/PHOX2B/SEMA3F/SHH/SMO/SOX8	9	BP
GO:0014009	glial cell proliferation	9/2734	38/17381	0.13183	0.7627	0.75013	ASCL2/CLU/IDH2/LTA/MIR221/MIR222/MTOR/NOTCH1/PTK2B	9	BP
GO:0030225	macrophage differentiation	9/2734	38/17381	0.13183	0.7627	0.75013	BMP4/C1QC/CSF1/CSF1R/CSF3/IL31RA/L3MBTL3/NKX2-3/SPI1	9	BP
GO:0031057	negative regulation of histone	9/2734	38/17381	0.13183	0.7627	0.75013	BRCA1/CTBP1/HDAC8/NOC2L/OTUB1/SPI1/SUPT6H/TAF7/UBE2B	9	BP

GO:0050850	positive regulation of calcium-	9/2734	38/17381	0.13183	0.7627	0.75013	CDH13/CIB1/GSTM2/HINT1/NEUROD2/NRG1/P2RX2/TRDN/TREM2	9	BP
GO:0051932	synaptic transmission, GABAergic	9/2734	38/17381	0.13183	0.7627	0.75013	ADRA1A/CA7/CLSTN3/DNM1/DRD2/NISCH/PHF24/STXBP1/TACR1	9	BP
GO:0099622	cardiac muscle cell membrane repolarization	9/2734	38/17381	0.13183	0.7627	0.75013	ATP1A1/CAV3/FLNA/KCND3/KCNJ5/KCNQ1/MIR328/NPPA/WDR1	9	BP
GO:0006304	DNA modification	21/2734	104/17381	0.1328	0.7627	0.75013	ALKBH5/APEX1/BRCA1/DDX4/DNMT3A/EHMT1/EHMT2/FKBP6/MBD4/MIR29A/MIR29C/MPHOSPH8/NEIL2/OGG1/PIWIL2/PRDM14/RLF/SMUG1/SPI	21	BP
GO:0009247	glycolipid biosynthetic process	14/2734	65/17381	0.13309	0.7627	0.75013	B4GALNT1/CWH43/DPM2/GAL3ST4/GBGT1/GPAA1/PIGC/PIGG/PIGO/PIGQ/PIGY/PIGZ/PYURF/ST3GAL4	14	BP
GO:0032760	positive regulation of tumor necrosis	14/2734	65/17381	0.13309	0.7627	0.75013	CARD9/CCL19/CCR2/CLU/HAVCR2/HLA-E/HSPB1/LGALS9/NOD1/ORM1/ORM2/SPON2/TIRAP/TLR9	14	BP
GO:0048678	response to axon injury	14/2734	65/17381	0.13309	0.7627	0.75013	DRD2/INPP5F/LRIG2/MAX/MIR221/MIR222/MORN4/NAIP/PTPRF/RGMA/RTN4RL1/RTN4RL2/SCARF1/TNC	14	BP
GO:0061077	chaperone-mediated protein folding	14/2734	65/17381	0.13309	0.7627	0.75013	CCT3/CD74/CLU/FKBP10/FKBP6/FKBP9/GAK/HSPB1/P3H1/PPIB/TOR1A/TOR1B/TOR2A/TRAP1	14	BP
GO:0006352	DNA-templated transcription, initiation	45/2734	243/17381	0.13351	0.7627	0.75013	BRF1/CDK4/CDK9/ESR2/ESRRA/FOSL1/GTF2H4/GTF3C1/HIST1H4F/HIST2H4A/HIST2H4B/MAPK3/MED24/MED6/NKX2-5/NOTCH1/NPPA/NR1H3/NR2F1/NR4A1/NR5A1/NRBP1/PAXIP1/POLR1A/POLR1D/POLR1E/POLR2G/POLR2L/PPARD/PSMC3/RARA/RORC/RSF1/SNAPC5/TAF1/TAF1A/TAF1L/TAF3/TAF6/TAF7/TBPL1/TEAD4/THRA/TTF1/ZN	45	BP
GO:0006661	phosphatidylinositol biosynthetic	28/2734	144/17381	0.13353	0.7627	0.75013	CDIPT/CWH43/DPM2/FGF17/FGF3/FGFR2/FGFR4/GPAA1/INPP5D/INPP5E/INPP5F/INPPL1/MTMR1/MTMR14/PDGFA/PI4KB/PIGC/PIGG/PIGO/PIGQ/PIGY/PIGZ/PIK3R5/PIK3R6/PITPNM1/PTPN11/PYURF/TLR9	28	BP

GO:0010952	positive regulation of peptidase	33/2734	173/17381	0.13446	0.7627	0.75013	ANP32B/APOPT1/BAD/BCAP31/BOK/CASP1/CASP3/CTSH/DAP/DAPK1/EFNA1/EFNA3/FASLG/FN1/GPER1/HIP1R/HSF1/KARS/LCK/LGALS9/MAPK3/MIR15A/MIR92A2/MTCH1/NLRP1/NOD1/PCOLCE/PDCD2/PRELID1/SOX7/	33	BP
GO:0001894	tissue homeostasis	39/2734	208/17381	0.13479	0.7627	0.75013	ADAM8/AIPL1/AZGP1/BGLAP/CDH23/CDHR1/CIB2/CNGB1/CORO1A/CROCC/CSF1/CSF1R/CSK/CTSH/CUBN/DEF8/EGFR/HOMER2/HSPB1/IL20RB/INPP5D/LDB1/MUC2/MUC6/NCDN/NOX4/PBLD/PLEKHM1/PRDM14/PRDX1/PTK2B/PTPN11/RAB7A/SCX/SERPINA3/SMO/TLR9/TULP1/VSIG1	39	BP
GO:0002087	regulation of respiratory gaseous exchange by neurological	4/2734	13/17381	0.13479	0.7627	0.75013	GLRA1/NLGN3/PHOX2B/TLX3	4	BP
GO:0006012	galactose metabolic	4/2734	13/17381	0.13479	0.7627	0.75013	GALK1/GALM/GALT/SLC35A2	4	BP
GO:0007039	protein catabolic process in the	4/2734	13/17381	0.13479	0.7627	0.75013	ATP13A2/CD81/GBA/LRP1	4	BP
GO:0010885	regulation of cholesterol	4/2734	13/17381	0.13479	0.7627	0.75013	APOB/EHD1/NR1H3/SCARB1	4	BP
GO:0021520	spinal cord motor neuron cell fate	4/2734	13/17381	0.13479	0.7627	0.75013	HOXC10/LHX3/OLIG3/SUFU	4	BP
GO:0030205	dermatan sulfate	4/2734	13/17381	0.13479	0.7627	0.75013	BCAN/BGN/CHST12/DSE	4	BP
GO:0032688	negative regulation of interferon-beta	4/2734	13/17381	0.13479	0.7627	0.75013	NLRX1/NMI/TRAF3IP1/TRAIP	4	BP
GO:0032780	negative regulation of	4/2734	13/17381	0.13479	0.7627	0.75013	ATPIF1/THADA/TLR9/VCPKMT	4	BP

GO:0032785	negative regulation of DNA-templated transcription, elongation	4/2734	13/17381	0.13479	0.7627	0.75013	AXIN1/NELFB/RECQL5/SHH	4	BP
GO:0033127	regulation of histone phosphorylation	4/2734	13/17381	0.13479	0.7627	0.75013	CDK9/MAPK3/RPS6KA4/UBE2B	4	BP
GO:0034370	triglyceride-rich lipoprotein particle	4/2734	13/17381	0.13479	0.7627	0.75013	APOA5/APOB/APOC3/GPIHBP1	4	BP
GO:0042761	very long-chain fatty acid biosynthetic process	4/2734	13/17381	0.13479	0.7627	0.75013	ELOVL1/ELOVL5/HACD1/HACD3	4	BP
GO:0043970	histone H3-K9 acetylation	4/2734	13/17381	0.13479	0.7627	0.75013	BRCA1/CRTC2/HDAC8/PIWIL2	4	BP
GO:0044794	positive regulation by host of viral	4/2734	13/17381	0.13479	0.7627	0.75013	CFL1/PC/PPIB/ZNF502	4	BP
GO:0045342	MHC class II biosynthetic process	4/2734	13/17381	0.13479	0.7627	0.75013	CIITA/NFX1/SLC11A1/SPI1	4	BP
GO:0045579	positive regulation of B cell	4/2734	13/17381	0.13479	0.7627	0.75013	BAD/CD27/INPP5D/PPP2R3C	4	BP
GO:0051231	spindle elongation	4/2734	13/17381	0.13479	0.7627	0.75013	CDC14A/KIF23/KIF4A/NUMA1	4	BP

GO:0060149	negative regulation of posttranscriptional gene	4/2734	13/17381	0.13479	0.7627	0.75013	DND1/NCOR1/NCOR2/ZC3H10	4	BP
GO:0060252	positive regulation of glial cell	4/2734	13/17381	0.13479	0.7627	0.75013	LTA/MIR221/MIR222/MTOR	4	BP
GO:0060452	positive regulation of cardiac muscle	4/2734	13/17381	0.13479	0.7627	0.75013	ADRA1A/CHGA/KCNQ1/NPPA	4	BP
GO:0060967	negative regulation of gene silencing	4/2734	13/17381	0.13479	0.7627	0.75013	DND1/NCOR1/NCOR2/ZC3H10	4	BP
GO:0071280	cellular response to	4/2734	13/17381	0.13479	0.7627	0.75013	AQP1/CYP1A1/CYP1A2/HSF1	4	BP
GO:0072044	collecting duct development	4/2734	13/17381	0.13479	0.7627	0.75013	BMP4/DACT2/NOTCH1/PKD1	4	BP
GO:0072189	ureter development	4/2734	13/17381	0.13479	0.7627	0.75013	BMP4/OSR1/SHH/SOX8	4	BP
GO:0090335	regulation of brown fat cell differentiation	4/2734	13/17381	0.13479	0.7627	0.75013	FNDC5/INS/MTOR/TRPV4	4	BP
GO:0099133	ATP hydrolysis coupled anion transmembrane transport	4/2734	13/17381	0.13479	0.7627	0.75013	ABCC3/ABCC4/ABCC5/ABCC6	4	BP

GO:1902514	regulation of calcium ion transmembrane transport via high voltage-gated calcium	4/2734	13/17381	0.13479	0.7627	0.75013	CACNB1/CAMK2D/MIR328/NPPA	4	BP
GO:0050680	negative regulation of epithelial cell	25/2734	127/17381	0.13522	0.7627	0.75013	A4GNT/AIMP1/ATF2/ATPIF1/BMP4/DAB2IP/FGFR2/GDF2/LIMS2/MARVELD3/MEN1/MIR16-1/MIR222/MIR29C/MIR503/NKX2-8/PBLD/PPARD/SFN/SLURP1/STAT1/TINF2/TNMD/WDR13/XDH	25	BP
GO:0034766	negative regulation of ion transmembrane	19/2734	93/17381	0.1355	0.7627	0.75013	ADRA2A/CAMK2D/CAV3/COMMD1/CRHR1/DRD2/GNB5/GPR35/GSTM2/MIR153-1/MIR212/MIR328/MTOR/OSR1/RRAD/THADA/TLR9/TRDN/YWHAQ	19	BP
GO:1903828	negative regulation of cellular protein	29/2734	150/17381	0.1355	0.7627	0.75013	AP2M1/CAV3/CD27/CDK5/COMMD1/CSK/DAB2IP/DCLK2/EMD/FAM89B/FZD9/LYPD1/LZTS2/MTOR/NFKBIL1/OS9/PARP10/PBLD/PDE2A/PID1/PKD1/PKDCC/POLR1A/SFRP5/SUFU/TAX1BP3/THRA/TMBIM1/TRIM40	29	BP
GO:0007616	long-term memory	8/2734	33/17381	0.13577	0.7627	0.75013	ARC/DRD2/EIF2AK4/GRIN1/MTOR/RGS14/SGK1/TACR1	8	BP
GO:0030513	positive regulation of BMP signaling	8/2734	33/17381	0.13577	0.7627	0.75013	BMP4/CRB2/ENG/GATA4/GDF2/ILK/NOTCH1/NUMA1	8	BP
GO:0045987	positive regulation of smooth muscle	8/2734	33/17381	0.13577	0.7627	0.75013	ADRA1A/ADRA1B/ADRA2B/CTTN/EDN2/GPER1/TACR1/TACR2	8	BP
GO:0097009	energy homeostasis	8/2734	33/17381	0.13577	0.7627	0.75013	ACACB/EDN2/EIF4G1/LEPR/MLXIPL/PM20D1/PRCP/TRPV4	8	BP
GO:2000008	regulation of protein localization to	8/2734	33/17381	0.13577	0.7627	0.75013	CAV3/COMMD1/GBF1/LRIG2/NRG1/STX4/TAX1BP3/TOR1A	8	BP

GO:0060071	Wnt signaling pathway, planar cell polarity pathway	22/2734	110/17381	0.13604	0.7627	0.75013	ABL1/AP2A2/AP2M1/ARRB2/CELSR2/CELSR3/DVL2/FZD2/GRHL3/PARD6A/PFN1/PSMB11/PSMB6/PSMB7/PSMB8/PSMC3/PSMD13/PSMD3/PSMD5/PSMD7/WNT1/WNT11	22	BP
GO:0008154	actin polymerization or depolymerizatio	35/2734	185/17381	0.13723	0.7627	0.75013	ANG/ARFIP1/ARPC1A/ARPC1B/ARPC2/ARPC4/BAIAP2L1/BRK1/CAPZA3/CATIP/CCL21/CDC42EP2/CFL1/CORO1A/CORO1B/CSF3/CTTN/EVL/F2RL1/HIP1R/LMOD1/MICAL2/MLST8/MTOR/MYO1C/PFN1/PRKCD/PTK2B/SCIN/SPTAN1/SPTB/SPTBN2/WAS/WDR1/WHAMM	35	BP
GO:0061383	trabecula morphogenesis	11/2734	49/17381	0.13732	0.7627	0.75013	CAV3/CHAD/COL1A1/ENG/HEY1/MMP2/NKX2-5/NOTCH1/NRG1/PLXNB1/THBS3	11	BP
GO:0072577	endothelial cell apoptotic	11/2734	49/17381	0.13732	0.7627	0.75013	ABL1/DAB2IP/ECSCR/FASLG/FGA/GPER1/IL13/KDR/MIR15A/PDCD4/TNIP2	11	BP
GO:1903900	regulation of viral life cycle	26/2734	133/17381	0.13761	0.7627	0.75013	CCL5/CD74/CHMP4C/DDB1/EIF2AK4/FKBP6/HACD3/IFITM2/ISG15/LAMP3/LGALS9/MIR221/MIR222/NOTCH1/PARP10/PC/PPIB/PPIE/SNF8/TARBP2/TRIM11/TRIM26/TRIM31/TRIM62/VPS37B/ZNF502	26	BP
GO:0006919	activation of cysteine-type endopeptidase activity involved in	17/2734	82/17381	0.13769	0.7627	0.75013	ANP32B/BAD/BOK/CASP1/CASP3/CTSH/DAP/FASLG/HIP1R/LCK/MIR15A/MTCH1/NLRP1/NOD1/PDCD2/TRAF2/XDH	17	BP
GO:1904063	negative regulation of cation transmembrane	17/2734	82/17381	0.13769	0.7627	0.75013	ADRA2A/CAMK2D/CAV3/COMMD1/CRHR1/DRD2/GNB5/GPR35/GSTM2/MIR153-1/MIR212/MIR328/OSR1/RRAD/THADA/TLR9/TRDN	17	BP
GO:0006260	DNA replication	53/2734	291/17381	0.13798	0.7627	0.75013	ACHE/ATRIP/BCL6/BMP4/BRCA1/CACYBP/CDAN1/CDK2AP1/CDK9/CINP/DNAJA3/E2F8/E4F1/EGFR/EHMT2/EXO1/GDF2/HMGA1/HRAS/IGHMBP2/ING4/INO80/INS/LIG3/MCM2/MCM4/MCM9/MCMDC2/NT5M/NUP98/ORC6/PARP2/PARP3/PDGFA/PDS5A/PID1/PIF1/PLA2G1B/POLA2/POLD4/POLE/POLE4/RECQL4/RECQL5/RFC1/RFC2/RPAIN/SHC1/TICRR/TIMELESS/TSPY	53	BP

GO:1903320	regulation of protein modification by small protein conjugation or removal	53/2734	291/17381	0.13798	0.7627	0.75013	ABL1/ADGRB1/AIMP2/ANAPC15/ANAPC2/ARRB2/AVPR2/AXIN1/BRCA1/BUB1B/CAV3/CDK5/CDK5RAP3/CDK9/CHFR/CLU/COMMD1/DISC1/EGR1/FBXO2/GOLGA2/GORASP1/HDAC8/HERPUD1/HSP90AA1/HSPA5/ISG15/KLHL40/LIMK1/MTA1/MTOR/NMI/OTUB1/PARP10/PAXIP1/PSMB11/PSMB6/PSMB7/PSMB8/PSMC3/PSMD13/PSMD3/PSMD5/PSMD7/PTK2B/RWDD3/SEPT4/SUFU/TAF1/USP4/WFS1/WNT1/ZYG11A	53	BP
GO:0055007	cardiac muscle cell differentiation	23/2734	116/17381	0.139	0.7627	0.75013	AGT/ARRB2/BMP4/CACYBP/CAV3/GATA4/IFT20/KAT2A/KDM6B/LMNA/MIR195/MIR222/MTOR/NKX2-5/NOX4/NPPA/NRG1/OBSL1/PDGFRB/PDLIM5/RARA/TCAP/WNT3A	23	BP
GO:0002053	positive regulation of mesenchymal	7/2734	28/17381	0.13901	0.7627	0.75013	FBXW4/FGFR2/MYCN/PDGFA/SHH/SMO/STAT1	7	BP
GO:0006760	folic acid-containing compound metabolic	7/2734	28/17381	0.13901	0.7627	0.75013	ALDH1L1/FOLR2/GCH1/GGH/MTHFD1/PIPOX/SHMT2	7	BP
GO:0021602	cranial nerve morphogenesis	7/2734	28/17381	0.13901	0.7627	0.75013	HOXA3/HOXB1/HOXB2/HOXB3/HOXD3/PHOX2A/SEMA3F	7	BP
GO:0034367	macromolecular complex remodeling	7/2734	28/17381	0.13901	0.7627	0.75013	AGT/APOA5/APOB/APOC3/GPIHBP1/MPO/SCARB1	7	BP
GO:0034368	protein-lipid complex remodeling	7/2734	28/17381	0.13901	0.7627	0.75013	AGT/APOA5/APOB/APOC3/GPIHBP1/MPO/SCARB1	7	BP
GO:0034369	plasma lipoprotein	7/2734	28/17381	0.13901	0.7627	0.75013	AGT/APOA5/APOB/APOC3/GPIHBP1/MPO/SCARB1	7	BP
GO:0060441	epithelial tube branching involved in lung morphogenesis	7/2734	28/17381	0.13901	0.7627	0.75013	BMP4/CTSH/ESRP2/FGFR2/HHIP/SHH/TNC	7	BP

GO:0071108	protein K48-linked deubiquitination	7/2734	28/17381	0.13901	0.7627	0.75013	OTUB1/OTUD5/OTUD7A/USP19/USP20/USP27X/USP5	7	BP
GO:1902692	regulation of neuroblast proliferation	7/2734	28/17381	0.13901	0.7627	0.75013	DISC1/DRD2/NOTCH1/OTP/SHH/SMO/VAX1	7	BP
GO:1905476	negative regulation of protein localization to	7/2734	28/17381	0.13901	0.7627	0.75013	AP2M1/CSK/FZD9/LYPD1/PID1/PKDCC/TMBIM1	7	BP
GO:0007422	peripheral nervous system development	15/2734	71/17381	0.1391	0.7627	0.75013	ADGRB1/ARHGEF10/ARTN/ASIC2/CDK5/ERBB2/GPC1/ILK/LAMA2/MYOC/NRG1/PARD3/POU3F1/SERPINI1/SOX8	15	BP
GO:0043966	histone H3 acetylation	13/2734	60/17381	0.13924	0.7627	0.75013	BRCA1/BRPF1/CRTC2/HDAC8/ING4/KAT2A/LDB1/MYOD1/PER1/PIWIL2/POLE4/SPI1/TADA3	13	BP
GO:0034763	negative regulation of transmembrane	20/2734	99/17381	0.13925	0.7627	0.75013	ADRA2A/CAMK2D/CAV3/COMMD1/CRHR1/DRD2/GNB5/GPR35/GSTM2/MIR153-1/MIR212/MIR328/MTOR/OAZ2/OSR1/RRAD/THADA/TLR9/TRDN/YWHAQ	20	BP
GO:0071900	regulation of protein serine/threonine kinase activity	87/2734	495/17381	0.14009	0.7627	0.75013	ABL1/ADAM8/ADRA2A/ADRA2B/ARAF/AXIN1/BMP4/CASP3/CAV3/CCL19/CCND3/CD74/CD81/CDK5R2/CDK5RAP3/CDKN2A/CIB1/CSF1R/CSK/DAB2IP/DTNBP1/DUSP2/DUSP21/DUSP5/DVL2/EGFR/ERBB2/ERN2/FZD8/GADD45G/GBA/GNG3/HACD3/HRAS/HSPA2/HSPB1/ILK/INCA1/IRAK1/KARS/MADD/MAP2K3/MAP3K14/MAP3K15/MAP3K6/MAP4K2/MAPK3/MAPK8IP1/MAPK8IP3/MAPKAPK3/MAPRE3/MEN1/MIR92A2/MLST8/NEK10/NOD1/NOX4/NRG1/PDCD4/PDGFA/PDGFRB/PEA15/PIK3R5/PIK3R6/PKD1/PKMYT1/PLA2G1B/PPP1R1B/PRKAR1A/PRKAR1B/PRKCD/PTK2B/PTPN11/RAF1/RALB/RGS14/SFN/SHC1/TAOK2/TDGF1/TELO2/THY1/TIRAP/TLR9/TNIK/TRAF2/TRAF7	87	BP
GO:0000185	activation of MAPKKK activity	5/2734	18/17381	0.14037	0.7627	0.75013	DAB2IP/GADD45G/MAP4K2/TNIK/TRAF7	5	BP

GO:0006825	copper ion transport	5/2734	18/17381	0.14037	0.7627	0.75013	ATOX1/CCS/CUTC/HEPH/STEAP2	5	BP
GO:0006884	cell volume homeostasis	5/2734	18/17381	0.14037	0.7627	0.75013	AQP1/E2F4/SLC12A4/SLC12A7/TRPV4	5	BP
GO:0007026	negative regulation of microtubule	5/2734	18/17381	0.14037	0.7627	0.75013	CIB1/KATNB1/MAP6D1/MID1IP1/TRIM54	5	BP
GO:0009084	glutamine family amino acid biosynthetic	5/2734	18/17381	0.14037	0.7627	0.75013	ASL/GLUD1/NAGS/OAT/OTC	5	BP
GO:0031342	negative regulation of	5/2734	18/17381	0.14037	0.7627	0.75013	ARRB2/HAVCR2/HLA-E/IL13/LGALS9	5	BP
GO:0032928	regulation of superoxide anion	5/2734	18/17381	0.14037	0.7627	0.75013	AGT/EGFR/F2RL1/ITGAM/PRKCD	5	BP
GO:0034587	piRNA metabolic	5/2734	18/17381	0.14037	0.7627	0.75013	DDX4/FKBP6/PIWIL2/TDRD1/TDRD9	5	BP
GO:0042136	neurotransmitter biosynthetic process	5/2734	18/17381	0.14037	0.7627	0.75013	ACHE/DAGLB/GAD1/SLC44A4/TH	5	BP
GO:0043574	peroxisomal transport	5/2734	18/17381	0.14037	0.7627	0.75013	PEX14/PEX16/PEX3/PEX5/PEX6	5	BP
GO:0048261	negative regulation of receptor-mediated	5/2734	18/17381	0.14037	0.7627	0.75013	APOC3/DLG4/LRRTM1/NECAB2/UNC119	5	BP
GO:0048486	parasympathetic nervous system development	5/2734	18/17381	0.14037	0.7627	0.75013	HOXB1/HOXB2/PHOX2A/PHOX2B/SEMA3F	5	BP

GO:0051000	positive regulation of nitric-oxide	5/2734	18/17381	0.14037	0.7627	0.75013	GCH1/INS/NOD1/PTK2B/SCARB1	5	BP
GO:0051084	'de novo' posttranslational protein folding	5/2734	18/17381	0.14037	0.7627	0.75013	CD74/GAK/TOR1A/TOR1B/TOR2A	5	BP
GO:2000831	regulation of steroid hormone secretion	5/2734	18/17381	0.14037	0.7627	0.75013	AGT/C1QTNF1/CRHR1/PTPN11/REN	5	BP
GO:0002888	positive regulation of myeloid leukocyte	6/2734	23/17381	0.14094	0.7627	0.75013	F2RL1/IL13/IL4R/ITGAM/STX4/STXBP1	6	BP
GO:0033688	regulation of osteoblast proliferation	6/2734	23/17381	0.14094	0.7627	0.75013	ABL1/FGFR2/PLXNB1/SOX8/TMEM119/TNN	6	BP
GO:0042832	defense response to	6/2734	23/17381	0.14094	0.7627	0.75013	BATF2/CCDC88B/CLEC7A/HRAS/IL4R/SLC11A1	6	BP
GO:0043576	regulation of respiratory gaseous	6/2734	23/17381	0.14094	0.7627	0.75013	GLRA1/GRIN1/NLGN3/PHOX2A/PHOX2B/TLX3	6	BP
GO:0072243	metanephric nephron epithelium	6/2734	23/17381	0.14094	0.7627	0.75013	AQP1/OSR1/PKD1/POU3F3/SOX8/STAT1	6	BP
GO:0090189	regulation of branching involved in ureteric bud	6/2734	23/17381	0.14094	0.7627	0.75013	AGT/BMP4/HOXB7/LHX1/SMO/SOX8	6	BP
GO:1901984	negative regulation of protein	6/2734	23/17381	0.14094	0.7627	0.75013	BRCA1/CTBP1/HDAC8/NOC2L/SPI1/TAF7	6	BP

GO:2000291	regulation of myoblast proliferation	6/2734	23/17381	0.14094	0.7627	0.75013	ANKRD2/ATF2/MEIS2/MIR10A/MYOD1/PPARD	6	BP
GO:0014706	striated muscle tissue development	66/2734	369/17381	0.14103	0.7627	0.75013	ACTA1/AGT/ANKRD2/ARRB2/ATF3/BCL9/BIN3/BMP4/CACYBP/CAV3/CDK5/EGR1/EMD/ENG/EYA1/FGF3/FGFR2/GATA4/GPC1/GPX1/HIVEP3/HLX/IFT20/KAT2A/KDM6B/KLHL40/LEMD2/LMNA/LUC7L/MIR195/MIR222/MIR25/MSC/MTOR/MYBPC3/MYL6B/MYOD1/NKX2-5/NOTCH1/NOX4/NPPA/NPRL3/NRG1/NUPR1/OBSL1/P2RX2/PDGFRB/PDLIM5/PHOX2B/PITX1/PRKAR1A/RARA/SCX/SHH/SMO/SOX8/SRPK3/TBX2/TCAP/TNNC1/TSC22D3/USP19/VAMP5/VAX1/VGLL2/WNT3A	66	BP
GO:0045862	positive regulation of proteolysis	70/2734	393/17381	0.14127	0.7627	0.75013	ADAM8/ADRA2A/ANAPC15/ANAPC2/ANP32B/APOPT1/ATPIF1/AXIN1/BAD/BCAP31/BOK/BUB1B/CASP1/CASP3/CAV3/CHFR/CLN6/CLU/CTSH/DAB2IP/DAP/DAPK1/DISC1/ECSCR/EFNA1/EFNA3/FASLG/FGFR4/FN1/FURIN/GBA/GPER1/HERPUD1/HIP1R/HSF1/KARS/KLHL40/KLKB1/LCK/LGALS9/MAPK3/MIR15A/MIR92A2/MTCH1/NKD2/NLRP1/NOD1/OSBPL7/PACSIN3/PCOLCE/PDCD2/PRELID1/PSMB11/PSMB6/PSMB7/PSMB8/PSMC3/PSMD13/PSMD3/PSMD5/PSMD7/PTK2B/RNF166/RNF180/SOX7/TAF1/TRAF2/USP[illegible]	70	BP
GO:0042176	regulation of protein catabolic process	71/2734	399/17381	0.14129	0.7627	0.75013	ADAM8/ADGRB1/ADRA2A/ALAD/ANAPC15/ANAPC2/ARAF/ATP13A2/ATPIF1/AXIN1/BCAP31/BRSK2/BUB1B/CAV3/CCAR2/CD81/CDK5RAP3/CHFR/CLU/COMMD1/DAB2IP/DDB1/DISC1/ECSCR/EFNA1/EGFR/FHIT/FLNA/FURIN/GBA/GPX1/GRIN2C/HERPUD1/INS/KLHL40/LAMP3/LRIG2/LRP1/MIR181B1/NKD2/NRG1/OAZ2/OS9/OSBPL7/PACSIN3/PANO1/PKD1/PSMB11/PSMB6/PSMB7/PSMB8/PSMC3/PSMD13/PSMD3/PSMD5/PSMD7/PTK2B/RAB7A/RHBDF1/RNF166/RNF180/SHH/SNF8/SNX12/STX5/SUFU/TAF1/TRIM4[illegible]	71	BP
GO:0052548	regulation of endopeptidase activity	71/2734	399/17381	0.14129	0.7627	0.75013	A2ML1/AGT/AHSG/ANP32B/APLP2/APOPT1/AQP1/ARRB2/ATP13A2/BAD/BCAP31/BOK/CASP1/CASP3/CD27/COL7A1/CRB2/CRYAB/CTSH/DAP/DAPK1/DPEP1/EFNA1/EFNA3/FASLG/FURIN/GAPDH/GPER1/GPX1/HERPUD1/HIP1R/HSF1/ITIH4/ITIH6/KNG1/LAMP3/LCK/LGALS9/MIR15A/MIR195/MIR29C/MIR92A2/MTCH1/NAIP/NLE1/NLRP1/NOD1/PDCD2/PEBP1/POR/PRELID1/PSMB8/RAF1/RPS6KA1/SERPINA2/SERPINA3/SERPINA4/SERPINA5/SERPINF2/SERPINH1/SERPINI1/SFN/SOX7/SPINK2/SPINK5/SPINT1/SPOCK[illegible]	71	BP

GO:0090316	positive regulation of intracellular protein transport	48/2734	262/17381	0.14165	0.7627	0.75013	ACSM6/ANP32B/B3GAT3/BCAP31/BCAS3/BMP4/CCL19/CD27/CDK5/CHRM1/CIB1/CSF3/CYB5R1/EGFR/ELMOD1/EMD/ERBB2/FLNA/GPHA2/HNMT/IL18/ITGAM/ITGAX/KAT2A/LGALS9/LRRC46/MIEF2/MYBPC1/MYO1C/NBPF3/NRG1/OAZ2/OSCP1/PEMT/PRKCD/PSMB7/RBPMS/RNF31/SEC16B/SFN/SHH/SMO/TLR9/TSGA13/UBL4B/WNT3A/ZBTB17/ZPR1	48	BP
GO:0001736	establishment of planar polarity	24/2734	122/17381	0.14171	0.7627	0.75013	ABL1/AP2A2/AP2M1/ARRB2/CELSR2/CELSR3/DVL2/FZD2/GRHL3/IFT20/PARD6A/PFN1/PSMB11/PSMB6/PSMB7/PSMB8/PSMC3/PSMD13/PSMD3/PSMD5/PSMD7/WDR1/WNT1/WNT11	24	BP
GO:0001838	embryonic epithelial tube formation	24/2734	122/17381	0.14171	0.7627	0.75013	ABL1/ADM/BMP4/CC2D2A/CFL1/DEAF1/DVL2/FZD2/GRHL3/IRX3/KAT2A/LIAS/MTHFD1/OSR1/PFN1/RARA/SCRIB/SETD2/SOX8/SPINT1/ST14/SUFU/TULP3/WNT6	24	BP
GO:0007164	establishment of tissue polarity	24/2734	122/17381	0.14171	0.7627	0.75013	ABL1/AP2A2/AP2M1/ARRB2/CELSR2/CELSR3/DVL2/FZD2/GRHL3/IFT20/PARD6A/PFN1/PSMB11/PSMB6/PSMB7/PSMB8/PSMC3/PSMD13/PSMD3/PSMD5/PSMD7/WDR1/WNT1/WNT11	24	BP
GO:0032543	mitochondrial translation	24/2734	122/17381	0.14171	0.7627	0.75013	AARS2/C12orf65/COA3/ERAL1/GATB/MRPL10/MRPL14/MRPL28/MRPL36/MRPL37/MRPL43/MRPL52/MRPL57/MRPL9/MRPS10/MRPS11/MRPS18A/MRPS18B/MRPS21/MRPS33/MRPS5/RMND1/TARS2/YARS2	24	BP
GO:0051494	negative regulation of cytoskeleton	24/2734	122/17381	0.14171	0.7627	0.75013	ARAP1/BRCA1/CAPZA3/CAV3/CCNF/CIB1/CORO1A/CORO1B/ESPN/HIP1R/KATNB1/LMOD1/MAP6D1/MID1IP1/MYOC/NUBP1/PFN1/PRKCD/SCIN/SPTAN1/SPTB/SPTBN2/TRIM54/WAS	24	BP
GO:0014074	response to purine-containing	28/2734	145/17381	0.14185	0.7628	0.75021	APEX1/AQP1/AQP8/CACNA1S/CASP1/CIB2/COL1A1/DGKQ/DHODH/FOSL1/GSTM2/HSPA5/KCNQ1/LDHA/NME1/NOX4/P2RX2/PDE2A/PER1/PFKFB1/PLA2G5/PTK2B/REN/SDC1/SLC26A6/STAT1/TAF1/VGF	28	BP
GO:0006986	response to unfolded protein	33/2734	174/17381	0.14205	0.7628	0.75028	ACADVL/ASNS/ATF3/BOK/CDK5RAP3/CHAC1/CREB3/DAB2IP/DNAJB5/EXTL1/FBXO6/GOSR2/HERPUD1/HERPUD2/HSF1/HSP90AA1/HSPA2/HSPA5/HSPB1/HSPB7/LMNA/MFN2/PLA2G4B/PPP2R5B/SEC61A2/SERPINH1/SHC1/SYVN1/TLN1/TOR1B/TSPYL2/WFS1/ZBTB17	33	BP
GO:0070126	mitochondrial translational termination	18/2734	88/17381	0.14212	0.7628	0.75028	C12orf65/ERAL1/MRPL10/MRPL14/MRPL28/MRPL36/MRPL37/MRPL43/MRPL52/MRPL57/MRPL9/MRPS10/MRPS11/MRPS18A/MRPS18B/MRPS21/MRPS33/MRPS5	18	BP

GO:0015701	bicarbonate transport	10/2734	44/17381	0.14304	0.765	0.75246	AQP1/CA4/CA6/CA7/CYB5R1/CYB5R2/SLC26A1/SLC26A10/SLC26A6/SLC4A9	10	BP
GO:0021517	ventral spinal cord	10/2734	44/17381	0.14304	0.765	0.75246	DAB1/EVX1/HOXC10/LHX1/LHX3/LHX4/OLIG3/PHOX2A/SHH/SUFU	10	BP
GO:0044060	regulation of endocrine	10/2734	44/17381	0.14304	0.765	0.75246	AGT/C1QTNF1/CRHR1/F2RL1/HCAR2/INHBB/PTPN11/RAB11FIP3/REN/TACR2	10	BP
GO:1902743	regulation of lamellipodium organization	10/2734	44/17381	0.14304	0.765	0.75246	AQP1/ARPC2/BIN3/BRK1/CORO1B/FSCN1/MTOR/PLXNB3/WAS/WNT1	10	BP
GO:0070661	leukocyte proliferation	52/2734	286/17381	0.1435	0.7668	0.75417	ABL1/BCL6/BMP4/CARD11/CASP3/CCDC88B/CCL19/CCL5/CCND3/CCR2/CD180/CD6/CD74/CD81/CLCF1/CLECL1/CLU/CORO1A/CSF1/DNAJA3/EFNB1/ERBB2/F2RL1/HAVCR2/HLA-E/IDO1/IFNA5/IL13/IL18/IL20RB/IMPDH1/INPP5D/LGALS3/LGALS9/LMO1/MAD1L1/MIR181B1/MZB1/PLA2G2F/PRKAR1A/PRKCD/SCGB1A1/SHH/SLC11A1/TACR1/TBK1/TIRAP/TLR9/TNFRSF13B/TNFRSF4/VSIG4/WNT3A	52	BP
GO:0035567	non-canonical Wnt signaling pathway	29/2734	151/17381	0.14372	0.7673	0.75467	ABL1/AP2A2/AP2M1/ARRB2/CELSR2/CELSR3/DVL2/FZD2/FZD8/FZD9/GNAO1/GRHL3/MYOC/PARD6A/PFN1/PLCB2/PSMB11/PSMB6/PSMB7/PSMB8/PSMC3/PSMD13/PSMD3/PSMD5/PSMD7/SFRP5/WNT1/WNT11/WNT7A	29	BP
GO:0050807	regulation of synapse organization	25/2734	128/17381	0.14419	0.7691	0.75643	ABL1/ADGRB1/AGRN/AMIGO1/ANAPC2/ASIC2/CLSTN1/CLSTN3/CTNNA2/DAB2IP/DISC1/DRD2/EIF4G1/EPHB3/FZD9/GRIN1/GRIPAP1/LINGO2/LRRTM1/NEUROD2/NLGN3/PDLIM5/RAB17/SLITRK3/WNT7A	25	BP
GO:0032984	macromolecular complex disassembly	55/2734	304/17381	0.14452	0.7695	0.75684	AXIN1/C12orf65/CAPZA3/CFL1/CHMP1A/CHMP7/CIB1/DVL2/EIF5A/ERAL1/F2RL1/FRAT1/FZD2/GAK/GBA/HMGA1/KATNB1/KIF2B/KLC1/LMOD1/MAP6D1/MICAL2/MID1IP1/MRPL10/MRPL14/MRPL28/MRPL36/MRPL37/MRPL43/MRPL52/MRPL57/MRPL9/MRPS10/MRPS11/MRPS18A/MRPS18B/MRPS21/MRPS33/MRPS5/NCKAP5L/NES/PEX14/SCIN/SMARCD2/SPTAN1/SPTB/SPTBN2/STMN4/TBC1D25/TECPR1/TRIM54/TRPV4/WDR1/WNT1/WNT3	55	BP

GO:0042119	neutrophil activation	87/2734	496/17381	0.14463	0.7695	0.75684	ACPP/ADAM8/ADGRG3/AHSG/ALAD/ALDOA/ANPEP/AP2A2/ARL8A/BIN2/C1orf35/C3AR1/CALML5/CCL5/CD58/CD63/CD68/CDA/CHIT1/COTL1/CRACR2A/CTSD/CTSH/CXCR1/CXCR2/DDOST/DGAT1/DOK3/DPP7/DYNC1H1/EPX/F2RL1/FLG2/FRMPD3/GGH/GHDC/GSDMD/HK3/HMOX2/HSP90AA1/IDH1/IL18/IMPDH1/ITGAL/ITGAM/ITGAX/LGALS3/MGAM/MGST1/MMP25/MPO/NBEAL2/NPC2/OLFM4/OLR1/ORM1/ORM2/ORMDL3/PKM/PLAU/PPIE/PRCP/PRDX6/PRKCD/PSAP/PSMB7/PSMC3/PSMD13/PSMD3/PSMD7/PTPRN2/QSOX1/RAB24/RAB44/RAB7A/RHOF/SERPINA3/SLC11A1/SLC15A4/S[illegible]	87	BP
GO:1901215	negative regulation of neuron death	35/2734	186/17381	0.14465	0.7695	0.75684	AARS/ADAM8/AMBRA1/ATP13A2/BARHL1/BOK/CDK5/CHGA/CLCF1/CNTFR/CORO1A/CSF3/DRAXIN/EIF4G1/EN1/FZD9/GBA/GRIN1/HRAS/HSF1/IL13/ILK/LRP1/NAIP/NES/NONO/PM20D1/PTK2B/SNCB/STXBP1/TRAF2/W	35	BP
GO:0045216	cell-cell junction organization	43/2734	233/17381	0.14503	0.7695	0.75686	ABL1/AGT/ANG/BCAS3/CDH12/CDH13/CDH15/CDH24/CDH5/CLDN6/COL16A1/CSF1R/CSK/CTTN/F2RL1/FERMT2/FSCN1/GNPAT/IKBKB/KDR/KIFC3/LDB1/LIMS2/MARVELD2/MARVELD3/MYO1C/MYOC/PARD3/PARD6A/PKP3/PTK2B/PTPN23/RASSF8/RHOD/TAOK2/THY1/TLN1/TRPV4/WDPCP/	43	BP
GO:0001706	endoderm formation	12/2734	55/17381	0.1457	0.7695	0.75686	COL4A2/COL7A1/DUSP2/DUSP5/FN1/ITGA7/ITGB5/LHX1/MMP15/MMP2/SETD2/SOX7	12	BP
GO:0014888	striated muscle adaptation	12/2734	55/17381	0.1457	0.7695	0.75686	ACTA1/CAMTA2/INPP5F/LMNA/MIR25/MTOR/MYOC/MYOD1/NPPA/TCAP/TNNC1/TRIM63	12	BP
GO:0032768	regulation of monooxygenase activity	12/2734	55/17381	0.1457	0.7695	0.75686	CAV3/EGFR/GCH1/GCHFR/HSP90AA1/INS/NOD1/NOS3/NOSTRIN/POR/PTK2B/SCARB1	12	BP
GO:0051149	positive regulation of muscle cell	22/2734	111/17381	0.14576	0.7695	0.75686	ABL1/ADGRB1/ARRB2/BMP4/BOC/CAV3/CDH15/CTNNA2/EHD1/EIF5A/ENG/GPER1/IGFBP3/IL4R/ILK/MTOR/MYOD1/NRG1/RBM4/SHH/THRA/WNT3A	22	BP
GO:0006901	vesicle coating	14/2734	66/17381	0.14581	0.7695	0.75686	CNIH2/COL7A1/GBF1/GOLGA2/GORASP1/GOSR2/SEC16A/SEC16B/STX5/TRAPPC1/TRAPPC2L/TRAPPC3/TRAPPC4/TRAPPC9	14	BP
GO:0070585	protein localization to mitochondrion	36/2734	192/17381	0.14581	0.7695	0.75686	ACSM6/AIP/ATPIF1/BAD/CYB5R1/DNAJC19/DNLZ/DYNLL2/ELMOD1/GPHA2/HNMT/HSP90AA1/ITGAX/KAT2A/LRRC46/MFN2/MOAP1/MYBPC1/NBPF3/NRG1/OSCP1/PDE2A/PEMT/PMPCA/PPP1R13B/PSMB7/RNF31/SFN/TIMM17B/TIMM22/TIMM23B/TIMM9/TSGA13/UBL4B/YWHAQ/ZBTB17	36	BP

GO:0002791	regulation of peptide secretion	79/2734	448/17381	0.14582	0.7695	0.75686	ABL1/ACHE/ADAM8/ADRA2A/ADRA2C/ANG/ANO1/APBB1/ARFIP1/ARL2BP/ATP13A2/BAD/BLK/BRSK2/CAPN10/CASP1/CASP5/CCL19/CCL5/CD58/CD74/CDK16/CSF1R/DOC2B/DRD2/EGFR/F2RL1/FGA/FN1/GAPDH/GCG/GCK/GLUD1/GPER1/GSDMD/HAVCR2/HCAR2/HLA-E/IDH2/IL13/IL4R/INHBB/INS/ITPR3/KARS/LGALS9/LLGL1/MAPK3/MTNR1B/MYO18A/NLRP1/NR1H3/OPRM1/ORM1/ORM2/P3H1/PFKM/PLA2G1B/PPARD/PTPN11/PTPN23/PYDC1/RAB11FIP3/RFX6/RHBDF1/SERGEF/SIDT2/SLC25A5/SSTR5/STX1A/STX4/TFR2/TLR8/TLR9/TNFRSF4/TRAF2/TRH/TR[illegible]	79	BP
GO:0003012	muscle system process	79/2734	448/17381	0.14582	0.7695	0.75686	ACTA1/ADRA1A/ADRA1B/ADRA2A/ADRA2B/ADRA2C/AGT/ALDOA/ANKRD2/ATP1A1/CACNA1G/CACNA1H/CACNA1S/CACNG1/CAMK2D/CAMTA2/CAV3/CDK9/CHGA/CHRM1/CHRNB1/CRYAB/CTTN/DES/DRD2/EDN2/EMD/FLNA/GATA4/GLRA1/GNAO1/GPER1/GSTM2/HMCN2/HTR1D/IGSF22/INPP5F/ITGB5/KCND3/KCNJ5/KCNQ1/LMNA/LMOD1/LTB4R/MAP2K3/MIR153-1/MIR195/MIR25/MIR328/MTOR/MYBPC1/MYBPC3/MYBPH/MYBPHL/MYL4/MYL5/MYL6B/MYOC/MYOD1/NKX2-5/NOS3/NPPA/OBSL1/P2RX2/PDLIM5/PPP1R12B/PTGER3/SLC6A8/SORBS3[illegible]	79	BP
GO:0061326	renal tubule development	19/2734	94/17381	0.1461	0.7703	0.75764	AGT/AQP1/BMP4/EYA1/HOXB7/ILK/IRX3/LHX1/NOTCH1/OSR1/PKD1/POU3F3/SHH/SMO/SOX8/STAT1/WNT1/WNT11/WNT6	19	BP
GO:0009308	amine metabolic process	26/2734	134/17381	0.14646	0.7715	0.75886	AGMAT/AOC2/CHDH/CYP1A1/DDC/GDPD1/HAAO/HDC/HNMT/IDO1/INS/ITGAM/OAZ2/PAOX/PNKD/PSMB11/PSMB6/PSMB7/PSMB8/PSMC3/PSMD13/PSMD3/PSMD5/PSMD7/SLC7A7/TRH	26	BP
GO:0046467	membrane lipid biosynthetic process	27/2734	140/17381	0.14855	0.7777	0.76492	B4GALNT1/CWH43/DEGS2/DPM2/ELOVL1/ELOVL5/GAL3ST4/GBA/GBGT1/GPAA1/HACD1/HACD3/ORMDL3/PEMT/PIGC/PIGG/PIGO/PIGQ/PIGY/PIGZ/PLPP1/PRKCD/PYURF/SGMS1/SMPD4/SPTLC1/ST3GAL4	27	BP
GO:0018107	peptidyl-threonine phosphorylation	23/2734	117/17381	0.14858	0.7777	0.76492	AXIN1/CAMK1D/CAMK1G/CAMK2D/CAMKK1/CAMKV/CDK5/DAPK2/DCLK2/DGKQ/EIF4G1/GCG/MARK2/MTOR/OSR1/PARD3/PHKG1/PNCK/PRKCD/STK33/TAF1/TBK1/ULK1	23	BP
GO:0030049	muscle filament sliding	9/2734	39/17381	0.14881	0.7777	0.76492	ACTA1/DES/MYBPC1/MYBPC3/MYL4/MYL6B/TCAP/TNNC1/TPM2	9	BP

GO:0032965	regulation of collagen biosynthetic process	9/2734	39/17381	0.14881	0.7777	0.76492	BMP4/CIITA/ENG/MIR29A/MIR92A2/PDGFRB/PPARD/SCX/SERPINF2	9	BP
GO:0033275	actin-myosin filament sliding	9/2734	39/17381	0.14881	0.7777	0.76492	ACTA1/DES/MYBPC1/MYBPC3/MYL4/MYL6B/TCAP/TNNC1/TPM2	9	BP
GO:0033628	regulation of cell adhesion mediated by	9/2734	39/17381	0.14881	0.7777	0.76492	CCL21/CCL5/CIB1/EFNA1/EPHA8/FERMT3/MUC1/PLAU/PTPN11	9	BP
GO:0051150	regulation of smooth muscle cell	9/2734	39/17381	0.14881	0.7777	0.76492	BMP4/ENG/FGFR2/FOXO4/GPER1/MIR221/PDCD4/RBPMS2/SHH	9	BP
GO:1904705	regulation of vascular smooth muscle cell proliferation	9/2734	39/17381	0.14881	0.7777	0.76492	AGT/GPER1/MIR15A/MIR221/MIR222/MIR503/MIR96/MMP2/NQO2	9	BP
GO:1990874	vascular smooth muscle cell proliferation	9/2734	39/17381	0.14881	0.7777	0.76492	AGT/GPER1/MIR15A/MIR221/MIR222/MIR503/MIR96/MMP2/NQO2	9	BP
GO:0001776	leukocyte homeostasis	17/2734	83/17381	0.14915	0.7781	0.76534	ABL1/CACNA1F/CASP3/CCNB2/CD74/CORO1A/DNAJA3/FOXN1/HCAR2/LGALS9/LMO1/MTHFD1/NKX2-3/PPP2R3C/TNFRSF13B/TSC22D3/TSC22D4	17	BP
GO:0045445	myoblast differentiation	17/2734	83/17381	0.14915	0.7781	0.76534	ANKRD2/BMP4/BOC/CMTM5/HINFP/IGFBP3/IL18/ILK/MYOD1/NOTCH1/NRG1/PITX1/PPARD/SDC1/SHH/SOX8/TBX2	17	BP
GO:0007569	cell aging	20/2734	100/17381	0.14968	0.7802	0.76741	ABL1/BCL6/BGLAP/CDKN2A/DNAJA3/ENG/FOXM1/HMGA1/HRAS/ILK/LMNA/MIR10A/MTOR/NOX4/PDCD4/PRKCD/RWDD1/TBX2/WNT1/YPEL3	20	BP
GO:0009416	response to light stimulus	54/2734	299/17381	0.1502	0.7809	0.76804	AIPL1/AQP1/ASIC2/ASNS/B4GALT2/CACNA1F/CASP3/CCAR2/CDK5/CNGB1/CRIP1/DDB1/DDB2/DEAF1/DRD2/EGFR/EIF2AK4/FNTA/FNTB/GNAT1/GPX1/GRIN1/GUCA1B/GUCY2D/HRH2/IFT20/INO80/MEIS2/MEN1/MTA1/MTOR/NLGN3/NOC2L/OGG1/OPN1MW/OPN4/PER1/PITPNM1/PPEF1/PPP1R1B/RBM4/RGS14/SCARA3/SDF4/SEMA5B/TAF1/TH/TP53I13/TULP1/UBE2	54	BP

GO:0034332	adherens junction organization	24/2734	123/17381	0.15114	0.7809	0.76804	ABL1/ANG/BCAS3/CDH12/CDH13/CDH15/CDH24/CDH5/COL16A1/CSK/CTTN/FERMT2/KDR/LDB1/MYOC/PTK2B/PTPN23/RASSF8/RHOD/TAOK2/THY1/WDPCP/WHAMM/ZNF703	24	BP
GO:0001666	response to hypoxia	61/2734	341/17381	0.15147	0.7809	0.76804	ADAM8/ADM/ALAD/ALAS2/ALKBH5/ANG/AQP1/ASCL2/BAD/CASP1/CASP3/CRYAB/CYP1A1/DNMT3A/DRD2/EGR1/EIF4EBP1/ENDOG/HMOX2/HSD11B2/HSF1/IRAK1/LDHA/LMNA/LOXL2/LTA/MMP2/MTOR/NARFL/NOTCH1/NOX4/NPPA/OPRD1/P2RX2/PDLIM1/PKM/PLAU/PPARD/PSMB11/PSMB6/PSMB7/PSMB8/PSMC3/PSMD13/PSMD3/PSMD5/PSMD7/PTK2B/RAF1/RWDD3/SCAP/SLC29A1/SLC2A4/SLC2A8/SOD3/SUV39H1/TH/TRH/UCP3/[illegible]	61	BP
GO:0008306	associative learning	15/2734	72/17381	0.15156	0.7809	0.76804	B4GALT2/CDK5/DEAF1/DRD2/GRIN1/HRH2/IFT20/MEIS2/MTOR/NEUROD2/NLGN3/PPP1R1B/RGS14/TACR1/TBR1	15	BP
GO:0007517	muscle organ development	69/2734	389/17381	0.15169	0.7809	0.76804	ACHE/ACTA1/ADGRB1/AEBP1/ANKRD2/ARRB2/ATF3/BCL9/BIN3/BMP4/CACNA1H/CAV3/CDK5/CNTFR/COPRS/CRYAB/EGR1/EMD/ENG/FGF3/FGFR2/FGFRL1/FHL3/FKTN/FOXO4/FZD2/GPC1/GPX1/HIVEP3/HLX/ITGA7/KLHL40/LAMA2/LEMD2/LUC7L/MIR195/MIR222/MIR25/MSC/MTOR/MYBPC3/MYL6B/MYOD1/NKX2-5/NOTCH1/NRG1/NUPR1/P2RX2/PAX3/PHOX2B/PITX1/SCX/SHH/SMO/SOX8/SRPK3/STRA6/TBX2/TCAP/TEAD4/TNNC1/TRIM72/TSC22D3/USP19/U[illegible]	69	BP
GO:0070482	response to oxygen levels	66/2734	371/17381	0.15173	0.7809	0.76804	ADAM8/ADM/ALAD/ALAS2/ALKBH5/ANG/AQP1/ASCL2/ATP6V1G1/BAD/CASP1/CASP3/CDK4/COL1A1/CRYAB/CYP1A1/DNMT3A/DRD2/EGR1/EIF4EBP1/ENDOG/HMOX2/HSD11B2/HSF1/IRAK1/LDHA/LMNA/LOXL2/LTA/MMP2/MTOR/MYOD1/NARFL/NOTCH1/NOX4/NPPA/OPRD1/P2RX2/PDGFRB/PDLIM1/PKM/PLAU/PPARD/PSMB11/PSMB6/PSMB7/PSMB8/PSMC3/PSMD13/PSMD3/PSMD5/PSMD7/PTK2B/RAF1/RWDD3/SCAP/SLC29A1/SLC2A4/SLC2A8/SOD3/SUV39H1/TH/TRH/UCP3/USP19/VASN	66	BP
GO:0007369	gastrulation	35/2734	187/17381	0.15231	0.7809	0.76804	AXIN1/BMP4/CFC1/CFC1B/COL4A2/COL7A1/CRB2/DUSP2/DUSP5/DVL2/EYA1/FGFR2/FN1/IL1RN/ITGA7/ITGB5/KDM6B/LDB1/LHX1/MESP2/MIR221/MMP15/MMP2/NAT8B/OSR1/PRKAR1A/SCX/SETD2/SOX7/TLX2/TXNRD1/WLS/WNT11/WNT3A/ZBTB17	35	BP

GO:0010823	negative regulation of mitochondrion	11/2734	50/17381	0.15245	0.7809	0.76804	ARRB2/BOK/CLU/FZD9/GPX1/LIG3/LMNA/PID1/PRELID1/SLC25A5/VAT1	11	BP
GO:0045661	regulation of myoblast differentiation	11/2734	50/17381	0.15245	0.7809	0.76804	ANKRD2/BMP4/BOC/CMTM5/IGFBP3/IL18/ILK/MYOD1/NOTCH1/PPARD/SOX8	11	BP
GO:0048008	platelet-derived growth factor receptor signaling	11/2734	50/17381	0.15245	0.7809	0.76804	ABL1/CSRNP1/HIP1R/LRIG2/LRP1/MIR221/MYO1E/PDGFA/PDGFRB/PTPN11/RGS14	11	BP
GO:0070206	protein trimerization	11/2734	50/17381	0.15245	0.7809	0.76804	BRK1/C1QTNF1/CD247/CD74/COL1A1/EMILIN1/HSF1/MGST1/OTC/SIGMAR1/TRAF2	11	BP
GO:0003073	regulation of systemic arterial blood pressure	18/2734	89/17381	0.15337	0.7809	0.76804	ADM/ADRA1A/ADRA1B/AGT/ASIC2/AVPR2/CYP11B2/DRD2/EDN2/F2RL1/HSD11B2/NOS3/NPPA/P2RX2/PRCP/REN/SERPINF2/TACR1	18	BP
GO:0098773	skin epidermis development	18/2734	89/17381	0.15337	0.7809	0.76804	DNASE1L2/EGFR/FGFR2/FOXN1/FOXQ1/HOXC13/KRT71/KRT84/LDB1/NOTCH1/NSDHL/NUMA1/PDGFA/SHH/SMO/SPINK5/TMEM79/WNT10A	18	BP
GO:0006101	citrate metabolic	8/2734	34/17381	0.15448	0.7809	0.76804	ACO1/CS/DLST/IDH1/IDH2/OGDH/OGDHL/PDHB	8	BP
GO:0014037	Schwann cell differentiation	8/2734	34/17381	0.15448	0.7809	0.76804	ARHGEF10/CDK5/GPC1/ILK/LAMA2/MYOC/PARD3/POU3F1	8	BP
GO:0014904	myotube cell development	8/2734	34/17381	0.15448	0.7809	0.76804	ACTA1/BIN3/GPX1/KLHL40/MYOD1/P2RX2/SMO/SMYD3	8	BP
GO:0043171	peptide catabolic	8/2734	34/17381	0.15448	0.7809	0.76804	ADAMTS13/ANPEP/CHAC1/CTSH/GGT6/LOC440434/RNPEP/RNPEPL1	8	BP
GO:0043243	positive regulation of protein complex	8/2734	34/17381	0.15448	0.7809	0.76804	CFL1/EIF5A/F2RL1/GBA/KATNB1/NES/TRPV4/WDR1	8	BP
GO:0045070	positive regulation of viral genome	8/2734	34/17381	0.15448	0.7809	0.76804	CCL5/DDB1/FKBP6/HACD3/NOTCH1/PPIB/PPIE/TARBP2	8	BP

GO:0070670	response to interleukin-4	8/2734	34/17381	0.15448	0.7809	0.76804	ADAMTS13/ALAD/CD300LF/CDK4/CORO1A/HSPA5/IL4R/MCM2	8	BP
GO:0090022	regulation of neutrophil chemotaxis	8/2734	34/17381	0.15448	0.7809	0.76804	C3AR1/CAMK1D/CCL19/CCL21/CD74/DAPK2/PF4V1/TIRAP	8	BP
GO:0097178	ruffle assembly	8/2734	34/17381	0.15448	0.7809	0.76804	DEF8/EPS8L2/EVL/HRAS/INPPL1/PFN1/PLEKHM1/WDPCP	8	BP
GO:1900015	regulation of cytokine production involved in inflammatory response	8/2734	34/17381	0.15448	0.7809	0.76804	CD6/CHID1/CUEDC2/KARS/MIR221/MIR222/PDCD4/PER1	8	BP
GO:1900026	positive regulation of substrate adhesion-dependent cell	8/2734	34/17381	0.15448	0.7809	0.76804	ABL1/ARPC2/CIB1/DOCK1/FGA/FLNA/MYOC/OLFM4	8	BP
GO:0032943	mononuclear cell proliferation	49/2734	270/17381	0.15487	0.7809	0.76804	ABL1/BCL6/BMP4/CARD11/CASP3/CCDC88B/CCL19/CCL5/CCND3/CCR2/CD180/CD6/CD74/CD81/CLCF1/CLECL1/CORO1A/CSF1/DNAJA3/EFNB1/ERBB2/HAVCR2/HLA-E/IDO1/IFNA5/IL13/IL18/IL20RB/IMPDH1/INPP5D/LGALS3/LGALS9/LMO1/MAD1L1/MZB1/PLA2G2F/PRKAR1A/PRKCD/SCGB1A1/SHH/SLC11A1/TACR1/TBK1/TIRAP/TLR9/TNFRSF13B/TNFRSF4/VSIG4/WNT3A	49	BP
GO:0090175	regulation of establishment of planar polarity	22/2734	112/17381	0.15586	0.7809	0.76804	ABL1/AP2A2/AP2M1/ARRB2/CELSR2/CELSR3/DVL2/FZD2/GRHL3/PARD6A/PFN1/PSMB11/PSMB6/PSMB7/PSMB8/PSMC3/PSMD13/PSMD3/PSMD5/PSMD7/WNT1/WNT11	22	BP

GO:0016570	histone modification	78/2734	444/17381	0.15591	0.7809	0.76804	APBB1/ASH2L/ATF2/BAZ1B/BCL6/BRCA1/BRCC3/BRMS1/BRPF1/CAMK2D/CDK9/COPRS/CRTC2/CTBP1/DDB1/DDB2/EHMT1/EHMT2/EPC1/EYA1/FKBP10/GCG/HCFC1/HDAC11/HDAC8/HR/ING4/KAT2A/KDM4E/KDM6B/KDM8/LDB1/MAPK3/MEN1/MSL3/MTA1/MUC1/MYOD1/NAA60/NEK11/NOC2L/NTMT1/OTUB1/PADI3/PADI4/PADI6/PAXIP1/PCGF2/PER1/PHF19/PHF2/PIWIL2/POLE4/PPP1R1B/PRDM12/PRDM14/PRDM7/PRDM9/PRKCD/PYGO2/RLF/RPS6KA4/SETD1A/SETD2/SETD7/SMYD3/SPI1/SUPT6H/SUV39H1/TADA3/TAF1/TAF1L/TAF7/UBE2A/UBE2B/UBE2U/USP49/ZNHIT1	78	BP
GO:0006979	response to oxidative stress	75/2734	426/17381	0.1564	0.7809	0.76804	ABL1/ADPRHL2/ALAD/ANKRD2/APEX1/AQP1/ATOX1/ATP13A2/BAD/CASP3/CCL19/CCS/COL1A1/CRYAB/DAPK1/DPEP1/EGFR/ENDOG/EPX/FOSL1/GCH1/GNAO1/GPX1/GPX2/GPX3/GSR/HMOX2/HSF1/HSPB1/IDH1/IL18BP/INS/KDM6B/LDHA/LIAS/LPO/MAPK3/MGST1/MICB/MIR92A2/MMP3/MPO/NCF1/NFE2L1/NONO/NOS3/NOX4/NUDT1/NUDT2/OGG1/PCGF2/PDGFRB/PDLIM1/PRDX1/PRDX6/PRKCD/PSAP/PTK2B/RBPMS/RGS14/RWDD1/SCARA3/SCGB1A1/SDC1/SESN1/SOD3/STX4/TACR1/TOR1A/TRAF2/TRAP1[illegible]	75	BP
GO:0046902	regulation of mitochondrial membrane permeability	16/2734	78/17381	0.15663	0.7809	0.76804	ATF2/ATPIF1/BAD/BOK/DYNLL2/FZD9/HIP1R/MIR29A/MIR29C/MOAP1/PPP1R13B/RHOT2/SFN/SLC25A5/YWHAQ/ZNF205	16	BP
GO:0097581	lamellipodium organization	16/2734	78/17381	0.15663	0.7809	0.76804	AQP1/ARHGEF4/ARPC2/BIN3/BRK1/CDH13/CORO1B/CTTN/FSCN1/MTOR/PLXNB3/RHOD/SH2B1/WAS/WHAMM/WNT1	16	BP
GO:0051260	protein homooligomerization	56/2734	312/17381	0.15672	0.7809	0.76804	ACACB/ACPP/ALAD/ALDOA/ATPIF1/BRK1/C1QTNF1/CD247/CD79B/CDA/CLU/CRTC2/CRYAB/EHD1/EMILIN1/EVL/FGFRL1/GBA/GCH1/GLRA1/GNMT/GOLGA2/GPX3/GSDMD/HRK/HSF1/IGHMBP2/ITPR3/KCND1/KCND3/KCTD14/KCTD19/KCTD5/LY6G5B/MGST1/MIEF2/MIP/OLFM4/OTC/P2RX2/PEX14/PEX5/PKM/SEPT4/SHARPIN/SHMT2/SIGMAR1/SLC22A1/SLC34A1/TOR1A/TOR1B/TOR2A/TRAF2/TRIM72/TYSND1/VWF	56	BP
GO:0006643	membrane lipid metabolic process	40/2734	217/17381	0.15691	0.7809	0.76804	ARSE/ARSI/B4GALNT1/CLN6/CPTP/CREM/CWH43/DEGS2/DPM2/ELOVL1/ELOVL5/FUT7/GAL3ST4/GBA/GBGT1/GLTP/GPAA1/HACD1/HACD3/HEXA/NEU3/ORMDL3/PEMT/PIGC/PIGG/PIGO/PIGQ/PIGY/PIGZ/PLA2G15/PLPP1/PRKCD/PSAP/PYURF/SFTPB/SGMS1/SMPD4/SPTLC1/ST3GAL4/TH	40	BP

GO:0042472	inner ear morphogenesis	19/2734	95/17381	0.15715	0.7809	0.76804	ATP6V1B1/DVL2/EYA1/FGFR2/FZD2/GBX2/GRHL3/HMX2/KCNQ4/LHFPL5/MYO3A/MYO7A/SCRIB/SLC44A4/TCAP/TMIE/WDPCP/WNT1/WNT3A	19	BP
GO:1902476	chloride transmembrane transport	19/2734	95/17381	0.15715	0.7809	0.76804	ANO1/ANO2/ANO4/ANO7/BEST3/BEST4/BSND/CLCN2/CLCNKA/CLCNKB/CLDN4/CLIC1/GLRA1/SLC12A4/SLC12A7/SLC12A9/SLC26A1/SLC26A10/SLC26A6	19	BP
GO:0019216	regulation of lipid metabolic process	64/2734	360/17381	0.15731	0.7809	0.76804	ACACB/ACADVL/ADRA2A/AGT/AMBRA1/APOA5/APOB/APOC3/ATP1A1/BRCA1/CCDC3/CCL19/CCL21/CD81/CDK4/CPT2/CYP1A1/DAB2IP/DGKQ/EDF1/EGR1/ELOVL5/EPHA8/EPHX2/FGFR4/G0S2/GPER1/HCAR2/IDH1/INS/LMF1/MID1IP1/MLXIPL/MTOR/NCOR1/NCOR2/NPAS2/NPC2/NR1H3/NR5A1/ORMDL3/PDGFA/PDGFRB/PDHB/PEX11A/PIK3R5/PIK3R6/PLPP1/PMVK/PNPLA2/POR/PRKCD/PSAP/PTK2B/RARRES2/RORC/SCAP/SCARB1/SE [illegible]	64	BP
GO:0043433	negative regulation of DNA binding transcription	27/2734	141/17381	0.15759	0.7809	0.76804	ADGRG3/ARRB2/BRMS1/CDK5RAP3/CDKN2A/COMMD1/DAB2IP/DAP/EIF2AK4/FLNA/HAVCR2/IRAK1/MEN1/NFKBIL1/PARP10/PEX14/PYDC1/RWDD3/SFRP5/SPOP/SUFU/TAF3/TLR9/TNFRSF4/TRIM40/WFS1/WWP2	27	BP
GO:0007411	axon guidance	42/2734	229/17381	0.15827	0.7809	0.76804	ALCAM/ARTN/BOC/CDK5/CELSR3/CSF1R/DAB1/DOK1/DOK2/DRAXIN/EFNA1/EFNA3/EFNA4/EFNB1/EPHA8/EPHB3/ERBB2/EVL/GBX2/GPC1/HRAS/L1CAM/LAMA2/LHX1/LHX3/LHX4/LYPLA2/MAPK3/PDLIM7/PTPN11/RAP1GAP/SEMA3F/SEMA6C/SHC1/SHH/SPON2/SPTAN1/SPTB/SPTBN2/TB	42	BP
GO:0042446	hormone biosynthetic process	14/2734	67/17381	0.15915	0.7809	0.76804	ADM/ATP1A1/CACNA1H/CYP11A1/CYP11B2/DGKQ/EGR1/HSD17B1/HSD17B3/HSD17B7/HSD3B1/POR/SCARB1/STARD3	14	BP
GO:0060411	cardiac septum morphogenesis	14/2734	67/17381	0.15915	0.7809	0.76804	BMP4/ENG/FGFR2/FGFRL1/FZD2/GATA4/HEY1/NKX2-5/NOTCH1/RARA/SMAD6/SMO/TBX2/WNT11	14	BP
GO:0001890	placenta development	28/2734	147/17381	0.15939	0.7809	0.76804	ADM/ANG/ASCL2/CCNF/CDX2/DLX3/E2F8/EGFR/ETNK2/FGFR2/FOSL1/GCM2/GJB3/HEY1/HSF1/LHX3/LHX4/MEN1/NSDHL/PCDH12/PHLDA2/PKD1/PLCD1/PPARD/SETD2/SPINT1/ST14/TFEB	28	BP

GO:0046474	glycerophospholipid biosynthetic process	44/2734	241/17381	0.15941	0.7809	0.76804	ACHE/CDIPT/CPNE7/CWH43/DGKQ/DPM2/ETNK2/FGF17/FGF3/FGFR2/FGFR4/GNPAT/GPAA1/INPP5D/INPP5E/INPP5F/INPPL1/MFSD2A/MTMR1/MTMR14/PDGFA/PEMT/PHOSPHO1/PI4KB/PIGC/PIGG/PIGO/PIGQ/PIGY/PIGZ/PIK3R5/PIK3R6/PITPNM1/PLA2G1B/PLA2G2F/PLA2G4B/PLA2G5/PLD2/PTDSS1/PTDSS2/PTPN11/PYURF/SLC44A4/TLR9	44	BP
GO:0050704	regulation of interleukin-1 secretion	10/2734	45/17381	0.1595	0.7809	0.76804	CASP1/CASP5/CCL19/GSDMD/LGALS9/NLRP1/ORM1/ORM2/PYDC1/TLR8	10	BP
GO:0051353	positive regulation of oxidoreductase	10/2734	45/17381	0.1595	0.7809	0.76804	ABL1/AGT/CCS/EDN2/GCH1/INS/NOD1/POR/PTK2B/SCARB1	10	BP
GO:0090199	regulation of release of cytochrome c from	10/2734	45/17381	0.1595	0.7809	0.76804	APOPT1/ARRB2/BAD/CLU/GPER1/GPX1/HRK/LMNA/MOAP1/PRELID1	10	BP
GO:0000303	response to superoxide	7/2734	29/17381	0.15981	0.7809	0.76804	ADPRHL2/GCH1/MPO/NOS3/PRDX1/SOD3/UCP3	7	BP
GO:0030262	apoptotic nuclear changes	7/2734	29/17381	0.15981	0.7809	0.76804	CASP3/CDK5RAP3/ENDOG/ERN2/GPER1/HSF1/SHARPIN	7	BP
GO:0035066	positive regulation of histone	7/2734	29/17381	0.15981	0.7809	0.76804	BRCA1/KAT2A/MAPK3/MUC1/PAXIP1/PIWIL2/RPS6KA4	7	BP
GO:0035116	embryonic hindlimb morphogenesis	7/2734	29/17381	0.15981	0.7809	0.76804	ALX3/BMP4/NOTCH1/OSR1/PITX1/SHH/WNT7A	7	BP
GO:0035886	vascular smooth muscle cell differentiation	7/2734	29/17381	0.15981	0.7809	0.76804	ADM/ENG/EPC1/GPER1/MIR221/NOTCH1/PDCD4	7	BP
GO:0036336	dendritic cell migration	7/2734	29/17381	0.15981	0.7809	0.76804	CCL19/CCL21/CCL5/CCR2/CXCR1/CXCR2/LGALS9	7	BP

GO:0042554	superoxide anion	7/2734	29/17381	0.15981	0.7809	0.76804	AGT/EGFR/F2RL1/ITGAM/NCF1/NOX4/PRKCD	7	BP
GO:0043552	positive regulation of phosphatidylinositol 3-kinase	7/2734	29/17381	0.15981	0.7809	0.76804	AMBRA1/CCL19/CCL21/EPHA8/PDGFRB/PTK2B/TNFAIP8L3	7	BP
GO:0050690	regulation of defense response to	7/2734	29/17381	0.15981	0.7809	0.76804	AP1S1/AP2A2/AP2M1/CD247/LCK/PACS1/SCRIB	7	BP
GO:0060251	regulation of glial cell	7/2734	29/17381	0.15981	0.7809	0.76804	ASCL2/IDH2/LTA/MIR221/MIR222/MTOR/NOTCH1	7	BP
GO:0061036	positive regulation of cartilage	7/2734	29/17381	0.15981	0.7809	0.76804	BMP4/GDF2/LOXL2/PKDCC/POR/SCX/SOX5	7	BP
GO:0071604	transforming growth factor beta production	7/2734	29/17381	0.15981	0.7809	0.76804	ATF2/FURIN/IL13/LGALS9/LTBP3/SERPINF2/WNT11	7	BP
GO:0086011	membrane repolarization during action potential	7/2734	29/17381	0.15981	0.7809	0.76804	ATP1A1/CACNB3/FLNA/KCND3/KCNJ5/KCNQ1/MIR328	7	BP
GO:0007040	lysosome organization	12/2734	56/17381	0.16043	0.7809	0.76804	ACP2/AKTIP/CLN6/CORO1A/FAM160A2/HPS1/MYO7A/NAGLU/RAB20/RAB34/RAB7A/TFEB	12	BP
GO:0034381	plasma lipoprotein	12/2734	56/17381	0.16043	0.7809	0.76804	AP2A2/AP2M1/APOB/APOC3/CSK/CUBN/EHD1/GPIHBP1/LMF1/NPC2/SCARB1/SOAT2	12	BP
GO:0045453	bone resorption	12/2734	56/17381	0.16043	0.7809	0.76804	ADAM8/BGLAP/CSF1R/CSK/DEF8/EGFR/INPP5D/NCDN/NOX4/PLEKHM1/PTK2B/RAB7A	12	BP
GO:0046530	photoreceptor cell	12/2734	56/17381	0.16043	0.7809	0.76804	CNGB1/GNAT1/IFT140/IFT20/MYO7A/NAGLU/NOTCH1/NRL/SOX8/TH/THY1/TULP1	12	BP

GO:0051057	positive regulation of small GTPase mediated signal	12/2734	56/17381	0.16043	0.7809	0.76804	CSF1/DGKI/F2RL1/GPR17/GPR20/GPR35/HRAS/MMD2/NOTCH1/NRG1/PDGFRB/RASGEF1A	12	BP
GO:0070527	platelet aggregation	12/2734	56/17381	0.16043	0.7809	0.76804	C1QTNF1/CLIC1/CSRP1/FERMT3/FGA/FLNA/HSPB1/ILK/PRKCD/STXBP1/TLN1/WNT3A	12	BP
GO:0080171	lytic vacuole organization	12/2734	56/17381	0.16043	0.7809	0.76804	ACP2/AKTIP/CLN6/CORO1A/FAM160A2/HPS1/MYO7A/NAGLU/RAB20/RAB34/RAB7A/TFEB	12	BP
GO:0045446	endothelial cell differentiation	20/2734	101/17381	0.16054	0.7809	0.76804	BMP4/CDH5/EDF1/ENG/F2RL1/GDF2/GPX1/HEY1/IKBKB/KDM6B/MARVELD2/MIR10A/NOTCH1/NRG1/PDE2A/PLOD3/TMEM100/TNMD/WNT7A/XD	20	BP
GO:0018210	peptidyl-threonine modification	24/2734	124/17381	0.16093	0.7809	0.76804	AXIN1/CAMK1D/CAMK1G/CAMK2D/CAMKK1/CAMKV/CDK5/DAPK2/DCLK2/DGKQ/EIF4G1/GALNT16/GCG/MARK2/MTOR/OSR1/PARD3/PHKG1/PNCK/PRKCD/STK33/TAF1/TBK1/ULK1	24	BP
GO:0097530	granulocyte migration	24/2734	124/17381	0.16093	0.7809	0.76804	ADAM8/C3AR1/CAMK1D/CCL1/CCL19/CCL21/CCL5/CD74/CKLF/CSF1/CXCR2/DAPK2/EDN2/EPX/GBF1/KARS/LGALS3/MAPK3/PF4V1/PLA2G1B/RARRES2/TIRAP/TRPV4/WDR1	24	BP
GO:0035966	response to topologically incorrect protein	36/2734	194/17381	0.16119	0.7809	0.76804	ACADVL/ASNS/ATF3/BOK/CDK5RAP3/CHAC1/CLU/CREB3/DAB2IP/DNAJB5/EXTL1/FBXO6/GOSR2/HERPUD1/HERPUD2/HSF1/HSP90AA1/HSPA2/HSPA5/HSPB1/HSPB7/LMNA/MFN2/PLA2G4B/PPP2R5B/SDF2/SEC61A2/SERPINH1/SHC1/SYVN1/TLN1/TOR1A/TOR1B/TSPYL2/WFS1/ZBTB17	36	BP
GO:0051090	regulation of DNA binding transcription factor activity	69/2734	391/17381	0.16261	0.7809	0.76804	ADAM8/ADGRG3/AGT/ARRB2/ATF2/BRMS1/CAMK1D/CARD11/CDK5RAP3/CDKN2A/CIB1/CLU/COMMD1/CRTC2/DAB2IP/DAP/DDR2/DVL2/EIF2AK4/ESR2/FANCA/FLNA/FOSL1/FZD2/HAVCR2/IKBKB/INS/IRAK1/LGALS9/LRRFIP1/MAPK3/MEN1/NEUROD2/NFKBIL1/NOD1/OPRD1/PARP10/PEX14/PLA2G1B/PPP2R5B/PYDC1/RHEBL1/RNF31/RPS6KA4/RWDD1/RWDD3/SFRP5/SGK1/SHH/SMO/SPOP/SUFU/TAF1/TAF3/TAF6/TIRAP/TLR9/TNFRSF4/TRAF2/TRAPPC9/TRIM14/TRIM26/TRIM31/TRIM40/TRIM62/WFS1/WNT1/[illegible]	69	BP

GO:0060249	anatomical structure homeostasis	68/2734	385/17381	0.16273	0.7809	0.76804	ACD/ADAM8/AIPL1/ALDOA/APEX1/AZGP1/BGLAP/CAV3/CCT3/CDH23/CDHR1/CIB2/CNGB1/CORO1A/CROCC/CSF1/CSF1R/CSK/CTSH/CUBN/DEF8/EGFR/EXO1/EXOSC10/HAAO/HDAC8/HIST1H4F/HIST2H4A/HIST2H4B/HOMER2/HSP90AA1/HSPB1/IL20RB/INPP5D/LDB1/MAPK15/MAPK3/MUC2/MUC6/NCDN/NHP2/NOX4/PARP3/PBLD/PFKM/PIF1/PLEKHM1/POLA2/POLD4/POLE/POLE4/PRDM14/PRDX1/PTK2B/PTPN11/RAB7A/RECQL4/RFC1/RFC2/SCX/SERPINA3/SMG5/SMO/TINF2/TLR9/TULP1/VSIG1/XRCC3	68	BP
GO:2001056	positive regulation of cysteine-type endopeptidase	25/2734	130/17381	0.16313	0.7809	0.76804	ANP32B/APOPT1/BAD/BCAP31/BOK/CASP1/CASP3/CTSH/DAP/DAPK1/FASLG/GPER1/HIP1R/HSF1/LCK/LGALS9/MIR15A/MTCH1/NLRP1/NOD1/PDCD2/SOX7/TRAF2/WNT3A/XDH	25	BP
GO:0010811	positive regulation of cell-substrate	21/2734	107/17381	0.16359	0.7809	0.76804	ABL1/ARPC2/CCL21/CDH13/CIB1/COL16A1/CSF1/DISC1/DOCK1/FBLN2/FGA/FLNA/FN1/ILK/KDR/MYOC/OLFM4/PTK2B/RELL2/SPOCK2/THY1	21	BP
GO:0010720	positive regulation of cell development	85/2734	488/17381	0.16432	0.7809	0.76804	ABL1/ADRA2B/ADRA2C/AGT/AMIGO1/ANAPC2/APBB1/ARPC2/BAD/BCL6/BIN1/BMP4/CAMK1D/CCR2/CDH5/CIB1/CLCF1/CPNE5/CPNE9/CSF1/CYB5D2/DAB1/DAB2IP/DISC1/DOCK1/DRD2/DUOXA1/EIF4G1/FES/FGA/FLNA/FN1/FOXO6/GPER1/HOXD3/HSPA5/ILK/IRX3/ISLR2/KATNB1/L1CAM/LIMK1/LLPH/LTA/LTK/MARK2/METRN/MIR221/MIR222/MMD2/MTOR/MYOC/MYOD1/NBL1/NEUROD2/NKX2-5/NME1/NOTCH1/NRG1/NSMF/OBSL1/OLFM4/OPRM1/OTP/PHOX2B/PLXNB1/PLXNB3/PLXND1/PPP2R5B/PTK2B/RARA/RGS14/RNF112/SCARF1/SE[illegible]	85	BP
GO:0003071	renal system process involved in regulation of systemic arterial	6/2734	24/17381	0.16435	0.7809	0.76804	AGT/CYP11B2/F2RL1/HSD11B2/REN/SERPINF2	6	BP
GO:0007020	microtubule nucleation	6/2734	24/17381	0.16435	0.7809	0.76804	GOLGA2/TUBG1/TUBG2/TUBGCP2/TUBGCP3/TUBGCP5	6	BP
GO:0008211	glucocorticoid metabolic	6/2734	24/17381	0.16435	0.7809	0.76804	ATP1A1/CACNA1H/CYP11B2/DGKQ/HSD11B2/HSD3B1	6	BP

GO:0021511	spinal cord patterning	6/2734	24/17381	0.16435	0.7809	0.76804	EVX1/LHX3/SHH/SMO/SUFU/TULP3	6	BP
GO:0021871	forebrain regionalization	6/2734	24/17381	0.16435	0.7809	0.76804	AXIN1/BMP4/EMX1/LHX1/SHH/WNT1	6	BP
GO:0032196	transposition	6/2734	24/17381	0.16435	0.7809	0.76804	DDX4/L1TD1/PGBD5/PIWIL2/TDRD9/VRTN	6	BP
GO:0032528	microvillus organization	6/2734	24/17381	0.16435	0.7809	0.76804	CDHR5/FSCN1/MYO1A/PTPN11/RAP1GAP/TNIK	6	BP
GO:0035336	long-chain fatty-acyl-CoA metabolic	6/2734	24/17381	0.16435	0.7809	0.76804	ACOT7/ACSF3/DGAT1/ELOVL1/ELOVL5/HACD1	6	BP
GO:0046835	carbohydrate phosphorylation	6/2734	24/17381	0.16435	0.7809	0.76804	GALK1/GCK/HK3/NAGK/PFKFB1/PFKM	6	BP
GO:0060487	lung epithelial cell	6/2734	24/17381	0.16435	0.7809	0.76804	AIMP2/EYA1/IL13/NUMA1/SPDEF/THRA	6	BP
GO:0098868	bone growth	6/2734	24/17381	0.16435	0.7809	0.76804	DDR2/FGFR2/LEPR/POR/RARA/THBS3	6	BP
GO:2000463	positive regulation of excitatory postsynaptic	6/2734	24/17381	0.16435	0.7809	0.76804	DLG4/GRIN1/NLGN3/PTK2B/STX1A/WNT7A	6	BP
GO:0032652	regulation of interleukin-1 production	15/2734	73/17381	0.1646	0.7809	0.76804	ARRB2/CASP1/CASP5/CCL19/EGR1/GSDMD/HAVCR2/HSPB1/LGALS9/NLRP1/NOD1/ORM1/ORM2/PYDC1/TLR8	15	BP
GO:0035050	embryonic heart tube	15/2734	73/17381	0.1646	0.7809	0.76804	C2CD3/CCDC103/CCDC40/ENG/GATA4/MICAL2/NKX2-5/NOTCH1/NOTO/SHH/SMO/SUFU/TBX2/TRAF3IP1/WNT3A	15	BP
GO:0070664	negative regulation of leukocyte	15/2734	73/17381	0.1646	0.7809	0.76804	BMP4/CASP3/ERBB2/HAVCR2/IDO1/IL20RB/INPP5D/LGALS9/MAD1L1/PLA2G2F/PRKAR1A/SCGB1A1/SHH/TNFRSF13B/VSIG4	15	BP
GO:0072078	nephron tubule morphogenesis	15/2734	73/17381	0.1646	0.7809	0.76804	AGT/BMP4/EYA1/HOXB7/ILK/IRX3/LHX1/OSR1/PKD1/SHH/SMO/SOX8/WNT1/WNT11/WNT6	15	BP

GO:0046364	monosaccharide biosynthetic process	18/2734	90/17381	0.1651	0.7809	0.76804	ALDOA/ATF3/CHST15/CRTC2/DGKQ/G6PC3/GAPDH/GCG/GCK/GNMT/GOT2/INS/KAT2A/LEPR/MAEA/PC/PFKFB1/PGAM4	18	BP
GO:0090630	activation of GTPase activity	18/2734	90/17381	0.1651	0.7809	0.76804	ADPRHL1/ARHGEF10/ARHGEF16/BCAS3/CCL21/DOCK7/GRTP1/PLEKHG4/PTK2B/RABGAP1/RABGAP1L/SCRIB/SGSM2/TAX1BP3/TBC1D25/TBC1D28/TBC1D9/USP6NL	18	BP
GO:0050679	positive regulation of epithelial cell	32/2734	171/17381	0.16516	0.7809	0.76804	ANG/BAD/BMP4/CCL5/CDH13/ECM1/EGFL7/EGFR/ERBB2/ESRP2/EYA1/FGFR2/FLT4/FOXE3/GDF2/HRAS/KDR/MIR29A/MIR503/MTOR/NME1/NOTCH1/NR4A1/OSR1/PLXNB3/SHH/SMO/TACR1/TGM1/WNT3A/WNT7A/ZNF	32	BP
GO:0071156	regulation of cell cycle arrest	22/2734	113/17381	0.16634	0.7809	0.76804	BIN1/BRCA1/CDK4/CDK5/CDK9/DAB2IP/E2F4/E2F8/FOXE3/FOXM1/FOXO4/FZD9/GPER1/MLXIPL/MUC1/PCBP4/PHOX2B/PPP2R5B/RNF112/SFN/ZBT	22	BP
GO:0003188	heart valve formation	4/2734	14/17381	0.16642	0.7809	0.76804	EFNA1/HEY1/NOTCH1/SCX	4	BP
GO:0010934	macrophage cytokine production	4/2734	14/17381	0.16642	0.7809	0.76804	CD74/CUEDC2/SEMA7A/SPON2	4	BP
GO:0030207	chondroitin sulfate catabolic	4/2734	14/17381	0.16642	0.7809	0.76804	BCAN/BGN/HEXA/IDS	4	BP
GO:0030299	intestinal cholesterol absorption	4/2734	14/17381	0.16642	0.7809	0.76804	ABCG5/APOA5/NPC1L1/SOAT2	4	BP
GO:0032966	negative regulation of collagen biosynthetic	4/2734	14/17381	0.16642	0.7809	0.76804	CIITA/MIR29A/MIR92A2/PPARD	4	BP
GO:0034134	toll-like receptor 2	4/2734	14/17381	0.16642	0.7809	0.76804	F2RL1/IRAK1/TIRAP/TNIP2	4	BP
GO:0036445	neuronal stem cell division	4/2734	14/17381	0.16642	0.7809	0.76804	DOCK7/FGFR2/SOX5/WNT3A	4	BP

GO:0042487	regulation of odontogenesis of dentin-containing tooth	4/2734	14/17381	0.16642	0.7809	0.76804	AMTN/BMP4/CSF1/WNT10A	4	BP
GO:0043650	dicarboxylic acid biosynthetic	4/2734	14/17381	0.16642	0.7809	0.76804	GLUD1/GOT2/HAAO/MTHFD1	4	BP
GO:0044827	modulation by host of viral genome	4/2734	14/17381	0.16642	0.7809	0.76804	EIF2AK4/MIR221/MIR222/PPIB	4	BP
GO:0045721	negative regulation of	4/2734	14/17381	0.16642	0.7809	0.76804	GCK/INS/LEPR/MAEA	4	BP
GO:0046541	saliva secretion	4/2734	14/17381	0.16642	0.7809	0.76804	AQP1/AQP5/CHRM1/TACR1	4	BP
GO:0046710	GDP metabolic process	4/2734	14/17381	0.16642	0.7809	0.76804	CARD11/DLG4/NUDT18/SCRIB	4	BP
GO:0048313	Golgi inheritance	4/2734	14/17381	0.16642	0.7809	0.76804	GBF1/GOLGA2/MAPK3/STX5	4	BP
GO:0050667	homocysteine metabolic	4/2734	14/17381	0.16642	0.7809	0.76804	BLMH/DPEP1/MTHFD1/NOX4	4	BP
GO:0055057	neuroblast division	4/2734	14/17381	0.16642	0.7809	0.76804	DOCK7/FGFR2/SOX5/WNT3A	4	BP
GO:0060831	smoothened signaling pathway involved in dorsal/ventral neural tube	4/2734	14/17381	0.16642	0.7809	0.76804	SUFU/TBC1D32/TRAF3IP1/TULP3	4	BP
GO:0098856	intestinal lipid absorption	4/2734	14/17381	0.16642	0.7809	0.76804	ABCG5/APOA5/NPC1L1/SOAT2	4	BP

GO:1900424	regulation of defense response to	4/2734	14/17381	0.16642	0.7809	0.76804	F2RL1/HAVCR2/PGC/SPINK5	4	BP
GO:1990845	adaptive thermogenesis	4/2734	14/17381	0.16642	0.7809	0.76804	PM20D1/THADA/TRPV4/UCP3	4	BP
GO:2000781	positive regulation of double-strand	4/2734	14/17381	0.16642	0.7809	0.76804	FOXM1/PARP9/SLF2/TIMELESS	4	BP
GO:0003170	heart valve development	9/2734	40/17381	0.16681	0.7809	0.76804	BMP4/EFNA1/FGFRL1/HEY1/MTOR/NOTCH1/SCX/SMAD6/STRA6	9	BP
GO:0042398	cellular modified amino acid biosynthetic	9/2734	40/17381	0.16681	0.7809	0.76804	CARNS1/CHAC1/CHDH/GCH1/GGT6/MTHFD1/PLOD3/PTDSS1/PTDSS2	9	BP
GO:0061647	histone H3-K9 modification	9/2734	40/17381	0.16681	0.7809	0.76804	BRCA1/CRTC2/EHMT1/EHMT2/HDAC8/PIWIL2/PRDM12/SETD7/SUV39H1	9	BP
GO:1903169	regulation of calcium ion transmembrane transport	27/2734	142/17381	0.16694	0.7809	0.76804	ABL1/ADRA2A/AHNAK/CACNB1/CACNB3/CAMK2D/CAV3/CORO1A/CRACR2A/CRHR1/DRD2/GNB5/GPER1/GPR35/GSTM2/HSPA2/IL13/JPH3/MIR328/NPPA/NPSR1/PTK2B/RRAD/THADA/THY1/TLR9/TRDN	27	BP
GO:0031647	regulation of protein stability	45/2734	248/17381	0.16696	0.7809	0.76804	ACSM6/AHSP/CCAR2/CCT3/CD81/CHFR/CLU/CPN2/CRYAB/CSN3/CTSH/DNLZ/DPM2/EFNA1/ELMOD1/FLNA/GAPDH/GPIHBP1/HCFC1/HDAC8/HIP1R/HSP90AA1/KAT2A/LRRC46/MYCNOS/NAA16/P3H1/PANO1/PEX6/PFN1/PPIB/PRKCD/SMO/STXBP1/SYVN1/TADA3/TAF1/TELO2/TNIP2/UBE2B/U	45	BP
GO:0002029	desensitization of G-protein coupled receptor protein	5/2734	19/17381	0.16721	0.7809	0.76804	ADM/ARRB2/DNM1/DRD2/NECAB2	5	BP

GO:0006978	DNA damage response, signal transduction by p53 class mediator resulting in transcription of [illegible]	5/2734	19/17381	0.16721	0.7809	0.76804	BRCA1/FOXM1/ING4/MUC1/ZNF385A	5	BP
GO:0007597	blood coagulation,	5/2734	19/17381	0.16721	0.7809	0.76804	GP9/KLKB1/KNG1/PRCP/VWF	5	BP
GO:0008356	asymmetric cell division	5/2734	19/17381	0.16721	0.7809	0.76804	DOCK7/GOLGA2/PARD3/RGS14/SOX5	5	BP
GO:0022401	negative adaptation of signaling	5/2734	19/17381	0.16721	0.7809	0.76804	ADM/ARRB2/DNM1/DRD2/NECAB2	5	BP
GO:0030828	positive regulation of cGMP biosynthetic	5/2734	19/17381	0.16721	0.7809	0.76804	GUCA1B/GUCA2A/GUCA2B/NOS3/RUNDC3A	5	BP
GO:0046653	tetrahydrofolate metabolic	5/2734	19/17381	0.16721	0.7809	0.76804	ALDH1L1/GCH1/MTHFD1/PIPOX/SHMT2	5	BP
GO:0060294	cilium movement involved in cell	5/2734	19/17381	0.16721	0.7809	0.76804	CATSPER1/GAS8/SPAG16/TEKT4/TEKT5	5	BP
GO:0060438	trachea development	5/2734	19/17381	0.16721	0.7809	0.76804	BMP4/HYDIN/MAPK3/RARA/SHH	5	BP
GO:0071731	response to nitric oxide	5/2734	19/17381	0.16721	0.7809	0.76804	AQP1/CCL19/DPEP1/MMP3/TRAF2	5	BP
GO:0072074	kidney mesenchyme development	5/2734	19/17381	0.16721	0.7809	0.76804	BMP4/OSR1/PDGFRB/SHH/STAT1	5	BP

GO:0072077	renal vesicle morphogenesis	5/2734	19/17381	0.16721	0.7809	0.76804	BMP4/LHX1/SMO/SOX8/STAT1	5	BP
GO:0090190	positive regulation of branching involved in ureteric bud	5/2734	19/17381	0.16721	0.7809	0.76804	AGT/HOXB7/LHX1/SMO/SOX8	5	BP
GO:0006027	glycosaminoglycan catabolic	13/2734	62/17381	0.16725	0.7809	0.76804	ACAN/AGRN/BCAN/BGN/GPC1/GPC2/HEXA/HSPG2/IDS/NAGLU/SDC1/SGSH/SPACA3	13	BP
GO:0031398	positive regulation of protein ubiquitination	34/2734	183/17381	0.16737	0.7809	0.76804	AIMP2/ANAPC15/ANAPC2/ARRB2/AVPR2/AXIN1/BRCA1/BUB1B/CAV3/CDK5RAP3/CDK9/CHFR/CLU/COMMD1/DISC1/GOLGA2/GORASP1/HSPA5/KLHL40/MTA1/NMI/PAXIP1/PSMB11/PSMB6/PSMB7/PSMB8/PSMC3/PSMD13/PSMD3/PSMD5/PSMD7/PTK2B/SEPT4/WFS1	34	BP
GO:0043434	response to peptide hormone	72/2734	410/17381	0.16768	0.7817	0.76886	ADM/AGT/AHSG/APEX1/ATP6V0B/ATP6V0E2/ATP6V1B1/ATP6V1F/ATP6V1G1/ATP6V1G2/BAIAP2L1/CACYBP/CAPN10/CCND3/CDK4/CISH/COL1A1/CRHR1/CRHR2/CSK/CYC1/EGR1/EIF2B2/EIF2B5/EIF4EBP1/ESRRA/FOXO4/GCG/GCK/GGH/GNB3/GNG3/GPER1/HADHA/HSD11B2/HSF1/INHBB/INPPL1/INS/MAPK3/MAX/MEN1/MTOR/MYO1C/MZB1/NPPA/NR4A1/NR5A1/OTC/PFKFB1/PID1/PKM/PLA2G1B/POR/PRKAR1A/PRKAR1B/PRKCD/PTPN11/RARRES2/SCAP/SHC1/SLC2A4/SLC2A8/SLC34A1/SRSF4/STAT1/TH/[illegible]	72	BP
GO:0043550	regulation of lipid kinase	11/2734	51/17381	0.1684	0.7838	0.77087	AMBRA1/CCL19/CCL21/CD81/DAB2IP/EPHA8/PDGFRB/PIK3R5/PIK3R6/PTK2B/TNFAIP8L3	11	BP
GO:0016331	morphogenesis of embryonic epithelium	28/2734	148/17381	0.16859	0.7838	0.77087	ABL1/ADM/BMP4/CC2D2A/CFL1/DEAF1/DVL2/FGFR2/FZD2/GRHL3/IRX3/JAG2/KAT2A/LIAS/MTHFD1/OSR1/PFN1/RARA/SCRIB/SETD2/SHH/SOX8/SPINT1/ST14/SUFU/TGFB1I1/TULP3/WNT6	28	BP
GO:0051225	spindle assembly	19/2734	96/17381	0.16864	0.7838	0.77087	ARHGEF10/CDC14A/CHMP1A/CHMP4C/DYNC1H1/FLNA/GOLGA2/HAUS4/HAUS7/INO80/KIF23/KIF4A/NCOR1/NUMA1/SENP6/TUBGCP2/TUBGCP3/TUBGCP5/WRAP73	19	BP
GO:1903214	regulation of protein targeting to	19/2734	96/17381	0.16864	0.7838	0.77087	ACSM6/ATPIF1/CYB5R1/ELMOD1/GPHA2/HNMT/ITGAX/KAT2A/LRRC46/MYBPC1/NBPF3/NRG1/OSCP1/PEMT/PSMB7/RNF31/TSGA13/UBL4B/ZBTB17	19	BP

GO:0045598	regulation of fat cell differentiation	23/2734	119/17381	0.16882	0.784	0.77107	ADIRF/AXIN1/C1QL4/CCDC3/CCDC85B/DLK2/FNDC5/GPER1/INS/MEDAG/MTOR/PPARD/PTPRQ/RARRES2/RORC/SH3PXD2B/TGFB1I1/TRIO/TRPV4/WIF1/WNT1/WNT3A/ZNF385A	23	BP
GO:0097164	ammonium ion metabolic process	36/2734	195/17381	0.16921	0.7847	0.7718	ACHE/AGMAT/APOA5/CHDH/CROT/DDC/DEGS2/DRD2/GBA/GCH1/HDC/HNMT/ITGAM/MFSD2A/PAOX/PEMT/PHOSPHO1/PLA2G15/PLA2G16/PLA2G1B/PLA2G2F/PLA2G4B/PLA2G5/PLB1/PNKD/POR/PRKCD/RNF180/SCARB1/SGMS1/SLC44A4/SMPD4/SNCB/SPTLC1/TH/TRH	36	BP
GO:0048259	regulation of receptor-mediated	16/2734	79/17381	0.16937	0.7847	0.7718	APOA5/APOC3/ARRB2/CCL19/CCL21/CD63/DLG4/DRD2/HIP1R/LRRTM1/NECAB2/RSPO1/TNK2/TOR1A/UNC119/WNT3A	16	BP
GO:0072527	pyrimidine-containing compound metabolic	16/2734	79/17381	0.16937	0.7847	0.7718	ACPP/AK5/CDA/DHODH/ERH/MBD4/MTOR/NEIL2/NME1/NME4/NT5M/OGG1/PUDP/SMUG1/TBPL1/UCK1	16	BP
GO:1901652	response to peptide	83/2734	477/17381	0.17006	0.7868	0.77391	ADM/AGT/AHSG/APEX1/ATP6V0B/ATP6V0E2/ATP6V1B1/ATP6V1F/ATP6V1G1/ATP6V1G2/AVPR2/BAIAP2L1/CACNB1/CACYBP/CAPN10/CARD9/CCND3/CDK4/CDK5/CISH/COL1A1/CRHR1/CRHR2/CSK/CYC1/EGR1/EIF2B2/EIF2B5/EIF4EBP1/ESRRA/FOXO4/GCG/GCK/GGH/GNB3/GNG3/GPER1/HADHA/HSD11B2/HSF1/INHBB/INPPL1/INS/IRF5/LRP1/MAPK3/MAX/MEN1/MTOR/MYO1C/MZB1/NLRP1/NOD1/NOTCH1/NPPA/NR4A1/NR5A1/OTC/PFKFB1/PID1/PKM/PLA2G1B/POR/PRKAR1A/PRKAR1B/PRKCD/PTPN11/QRFPR/RARRES2/SCAP/SHC1/SLC2A4/SLC2A8/SLC34A1/SRSF4/STAT1/TH/[illegible]	83	BP
GO:0015992	proton transport	29/2734	154/17381	0.1701	0.7868	0.77391	ATP1A4/ATP5G1/ATP5I/ATP6V0B/ATP6V0E2/ATP6V1B1/ATP6V1F/ATP6V1G1/ATP6V1G2/COX10/COX11/COX8A/CYC1/IL13/NDUFA4L2/PM20D1/SLC15A3/SLC15A4/SLC25A14/SLC25A22/SLC2A6/SLC2A8/SLC35A2/SLC36A1/SLC36A3/SLC9A3/SLC9A5/UCP3/VPS9D1	29	BP
GO:0044106	cellular amine metabolic process	24/2734	125/17381	0.17106	0.7899	0.77695	AGMAT/CHDH/DDC/GDPD1/HAAO/HDC/HNMT/IDO1/INS/ITGAM/OAZ2/PAOX/PNKD/PSMB11/PSMB6/PSMB7/PSMB8/PSMC3/PSMD13/PSMD3/PSMD5/PSMD7/SLC7A7/TRH	24	BP
GO:0001823	mesonephros development	20/2734	102/17381	0.17181	0.7899	0.77695	AGT/BMP4/EYA1/FGFR2/HOXB7/ILK/LHX1/OSR1/PKD1/RARA/REN/SDC1/SHH/SIM1/SMAD6/SMO/SOX8/WNT1/WNT11/WNT6	20	BP

GO:0001841	neural tube formation	20/2734	102/17381	0.17181	0.7899	0.77695	ABL1/ADM/BMP4/CC2D2A/CFL1/DEAF1/DVL2/FZD2/GRHL3/KAT2A/LIAS/MTHFD1/PFN1/RARA/SCRIB/SETD2/SPINT1/ST14/SUFU/TULP3	20	BP
GO:0042180	cellular ketone metabolic process	42/2734	231/17381	0.17302	0.7899	0.77695	ACACB/ACADVL/ADM/APOA5/APOC3/BRCA1/CACNA1H/COQ2/COQ4/CYP11B2/CYP2W1/DGKQ/EGR1/ELOVL5/FGFR4/GLO1/GOT2/HSD17B3/IDO1/INS/MID1IP1/MLXIPL/MTOR/NCOR2/NR1H3/OAZ2/PDHB/PDSS1/PNKD/PSMB11/PSMB6/PSMB7/PSMB8/PSMC3/PSMD13/PSMD3/PSMD5/PSMD7/S	42	BP
GO:0031056	regulation of histone modification	25/2734	131/17381	0.17309	0.7899	0.77695	BCL6/BRCA1/CAMK2D/CDK9/CTBP1/GCG/HDAC8/KAT2A/MAPK3/MUC1/NOC2L/OTUB1/PAXIP1/PHF19/PIWIL2/PRDM12/PYGO2/RPS6KA4/SETD7/SPI1/SUPT6H/TADA3/TAF7/UBE2B/ZNHIT1	25	BP
GO:0046683	response to organophosphorus	25/2734	131/17381	0.17309	0.7899	0.77695	APEX1/AQP1/AQP8/CASP1/CIB2/COL1A1/DGKQ/FOSL1/HSPA5/KCNQ1/LDHA/NME1/NOX4/P2RX2/PDE2A/PER1/PFKFB1/PLA2G5/PTK2B/REN/SDC1/SLC26A6/STAT1/TAF1/VGF	25	BP
GO:0071901	negative regulation of protein serine/threonine	25/2734	131/17381	0.17309	0.7899	0.77695	ABL1/BMP4/CASP3/CAV3/CDK5RAP3/CDKN2A/CIB1/DAB2IP/DTNBP1/DUSP2/DUSP21/DUSP5/GBA/HSPB1/INCA1/MAPK8IP1/MEN1/MIR92A2/PDCD4/PPP1R1B/PRKAR1A/PRKAR1B/PRKCD/RGS14/SFN	25	BP
GO:1903533	regulation of protein targeting	25/2734	131/17381	0.17309	0.7899	0.77695	ACSM6/ATPIF1/CDK5/CIB1/CYB5R1/ELMOD1/ERBB2/GPHA2/HNMT/ITGAM/ITGAX/KAT2A/LRRC46/MIEF2/MYBPC1/MYO1C/NBPF3/NRG1/OSCP1/PEMT/PSMB7/RNF31/TSGA13/UBL4B/ZBTB17	25	BP
GO:0003341	cilium movement	14/2734	68/17381	0.17312	0.7899	0.77695	CATSPER1/CCDC103/CCDC40/DNAH11/DNAH17/DNHD1/GAS8/HYDIN/LRRC6/SPAG16/TEKT4/TEKT5/TMEM141/WDR66	14	BP
GO:0006970	response to osmotic stress	14/2734	68/17381	0.17312	0.7899	0.77695	AQP1/ATF2/BAD/CASP3/EGFR/HNMT/MARVELD3/OSR1/PTK2B/SLC2A4/TH/TRPV4/TSC22D2/TSC22D3	14	BP
GO:0032024	positive regulation of	14/2734	68/17381	0.17312	0.7899	0.77695	ANO1/BAD/BLK/CAPN10/DOC2B/GCG/GCK/GLUD1/GPER1/PFKM/PPARD/RFX6/STX4/TRH	14	BP
GO:0061053	somite development	17/2734	85/17381	0.17357	0.7899	0.77695	CDX2/CRB2/KAT2A/LFNG/LHX1/MESP2/MTHFD1/NLE1/NOTCH1/RIPPLY1/SCX/SHH/SMO/TCAP/WNT1/WNT11/WNT3A	17	BP
GO:0110020	regulation of actomyosin structure organization	17/2734	85/17381	0.17357	0.7899	0.77695	ABL1/ARAP1/ARHGEF10/CAV3/EVL/FHOD1/LIMK1/MTOR/MYOC/NOX4/PFN1/SERPINF2/SH3PXD2B/SORBS3/TACR1/WAS/WNT11	17	BP

GO:0048736	appendage development	32/2734	172/17381	0.17386	0.7899	0.77695	ACD/ALX3/B9D1/BMP4/C2CD3/EN1/FBXW4/FGFR2/FOXN1/HOXC10/HOXC11/HOXC13/IFT140/KREMEN2/KRT84/MYCN/NOTCH1/OSR1/PBX2/PITX1/PKDCC/PRKAB1/RARA/SCX/SHH/TBC1D32/TBX2/TMEM231/TRAF3IP1/	32	BP
GO:0060173	limb development	32/2734	172/17381	0.17386	0.7899	0.77695	ACD/ALX3/B9D1/BMP4/C2CD3/EN1/FBXW4/FGFR2/FOXN1/HOXC10/HOXC11/HOXC13/IFT140/KREMEN2/KRT84/MYCN/NOTCH1/OSR1/PBX2/PITX1/PKDCC/PRKAB1/RARA/SCX/SHH/TBC1D32/TBX2/TMEM231/TRAF3IP1/	32	BP
GO:0060537	muscle tissue development	68/2734	387/17381	0.1742	0.7899	0.77695	ACTA1/AGT/ANKRD2/ARRB2/ATF3/BCL9/BIN3/BMP4/CACYBP/CAV3/CDK5/EGR1/EMD/ENG/EYA1/FGF3/FGFR2/GATA4/GPC1/GPX1/HIVEP3/HLX/IFT20/KAT2A/KDM6B/KLHL40/LEMD2/LMNA/LUC7L/MIR195/MIR222/MIR25/MSC/MTOR/MYBPC3/MYL6B/MYOD1/NKX2-5/NOTCH1/NOX4/NPPA/NPRL3/NRG1/NUPR1/OBSL1/OSR1/P2RX2/PDGFRB/PDLIM5/PHOX2B/PITX1/PRKAR1A/RARA/SCX/SHH/SMO/SOX8/SRPK3/STRA6/TBX2/TCAP/TNNC1/TSC22D3/USP19/VAMP5/VAX1/VGLL2/WNT3[illegible]	68	BP
GO:0035909	aorta morphogenesis	8/2734	35/17381	0.17432	0.7899	0.77695	ENG/EYA1/HEY1/LRP1/NOTCH1/NPRL3/PDGFRB/TBX2	8	BP
GO:0048566	embryonic digestive tract development	8/2734	35/17381	0.17432	0.7899	0.77695	FGFR2/HLX/PKDCC/RARRES2/RBPMS2/SHH/STRA6/VPS52	8	BP
GO:0032946	positive regulation of mononuclear	26/2734	137/17381	0.17492	0.7899	0.77695	BCL6/CARD11/CCDC88B/CCL19/CCL5/CCR2/CD6/CD74/CD81/CLCF1/CLECL1/CORO1A/CSF1/DNAJA3/EFNB1/HAVCR2/HLA-E/IL13/IL18/LGALS9/SHH/TACR1/TIRAP/TLR9/TNFRSF4/WNT3A	26	BP
GO:0010038	response to metal ion	58/2734	327/17381	0.17558	0.7899	0.77695	ACO1/ACTA1/ADAMTS13/ALAD/APBB1/AQP1/ATP13A2/BAD/BGLAP/CACNA1G/CACNA1H/CACYBP/CAMK2D/CASP3/CDK4/CPNE7/CRIP1/CYP11B2/CYP1A1/CYP1A2/DNMT3A/DPEP1/DRD2/EGFR/EIF2B5/ENDOG/FGA/GGH/GLRA1/HAAO/HSF1/HSPA5/ITPR3/KRT14/LOXL2/MAPK3/MT2A/NCF1/NEUROD2/NUDT1/OGG1/OTC/PTK2B/RASGRP2/S100A16/SDC1/SHH/SLC30A3/SLC34A1/SLC39A13/SNCB/SOD3/SPAG16/TFR2/TH/TNNC1/TRPV6/U[illegible]	58	BP
GO:0006399	tRNA metabolic process	34/2734	184/17381	0.17583	0.7899	0.77695	AARS/AARS2/ADAT1/AIMP1/AIMP2/BRF1/C9orf64/CDKAL1/CPSF1/CPSF4/CSTF2/DALRD3/DARS/EXOSC2/GATB/GTF3C1/KARS/LARS/METTL2A/METTL2B/PARS2/PPA2/PUSL1/RPP21/SARS/TARS2/THUMPD2/TRMT10B/TRMT2B/TRMT44/TYW1/TYW1B/YARS/YARS2	34	BP

GO:0010517	regulation of phospholipase activity	12/2734	57/17381	0.17588	0.7899	0.77695	ABL1/ADRA1A/AGT/ANG/CCL5/EGFR/FGFR2/LRP1/PDGFRB/PLA2G1B/PLA2G5/PLCB2	12	BP
GO:0010812	negative regulation of cell-substrate	12/2734	57/17381	0.17588	0.7899	0.77695	AJAP1/BCAS3/BCL6/CDKN2A/COL1A1/MEN1/MIR29C/MIR503/MIR92A2/MYOC/NOTCH1/WNT1	12	BP
GO:0030166	proteoglycan biosynthetic process	12/2734	57/17381	0.17588	0.7899	0.77695	B3GAT3/BCAN/BGN/CHPF/CHPF2/CHST12/CHST13/CHST15/CHST7/DSE/GAL3ST4/XYLT2	12	BP
GO:0048762	mesenchymal cell differentiation	35/2734	190/17381	0.17668	0.7899	0.77695	BMP4/CFL1/COL1A1/CRB2/DAB2IP/EFNA1/EFNB1/ENG/FGFR2/GBX2/GLIPR2/HEY1/LOXL2/MAPK3/MIR221/MIR222/MTOR/NOTCH1/NRG1/OSR1/PBLD/PDCD4/PHOX2B/SEMA3F/SHH/SMO/SOX8/STAT1/TGFB1I1/TMEM100/TRIM62/VASN/WNT10A/WNT11/ZNF703	35	BP
GO:0097305	response to alcohol	35/2734	190/17381	0.17668	0.7899	0.77695	ALAD/BAD/BGLAP/BRCA1/CCL19/CCL21/CD27/CSF3/DNMT3A/DRD2/EIF4EBP1/FGFR2/GGH/GLRA1/GOT2/GRIN1/IL13/OGG1/OPRM1/OSBPL7/P2RY6/PEMT/PMVK/PTK2B/RARA/RECQL5/SDF4/SETD7/SLC2A4/SMO/SPI1/T	35	BP
GO:0010665	regulation of cardiac muscle cell apoptotic	10/2734	46/17381	0.17684	0.7899	0.77695	AGT/AMBRA1/CAMK2D/EIF5A/HSF1/ILK/LTK/MIR16-1/MIR195/NKX2-5	10	BP
GO:0050766	positive regulation of	10/2734	46/17381	0.17684	0.7899	0.77695	AHSG/C2/CAMK1D/CD300LF/F2RL1/LMAN2/MYO18A/SLC11A1/SPACA3/TULP1	10	BP
GO:0086009	membrane repolarization	10/2734	46/17381	0.17684	0.7899	0.77695	ATP1A1/CACNB3/CAV3/FLNA/KCND3/KCNJ5/KCNQ1/MIR328/NPPA/WDR1	10	BP
GO:1903510	mucopolysaccharide metabolic process	22/2734	114/17381	0.17719	0.7899	0.77695	ABCC5/ACAN/B3GAT3/B4GALT2/BCAN/BGN/CHPF/CHPF2/CHST12/CHST13/CHST15/CHST5/CHST7/DSE/HEXA/IDS/ITIH4/ITIH6/SLC35D2/SPOCK2/ST3GAL4/XYLT2	22	BP
GO:0001892	embryonic placenta development	18/2734	91/17381	0.17728	0.7899	0.77695	ADM/ASCL2/CDX2/E2F8/EGFR/FGFR2/GCM2/HEY1/HSF1/NSDHL/PCDH12/PHLDA2/PKD1/PLCD1/SETD2/SPINT1/ST14/TFEB	18	BP
GO:1904019	epithelial cell apoptotic	18/2734	91/17381	0.17728	0.7899	0.77695	ABL1/BOK/CAPN10/DAB2IP/DNMT3A/ECSCR/FASLG/FGA/GPER1/IL13/JAG2/KDR/MIR15A/MTOR/PDCD4/SPOP/TNIP2/WFS1	18	BP

GO:0010633	negative regulation of epithelial cell	15/2734	74/17381	0.1782	0.7899	0.77695	ADGRB1/DAB2IP/EVL/GDF2/MARVELD3/MIR10A/MIR16-1/MIR212/MIR221/MIR29C/MIR503/MIR92A2/NOTCH1/PBLD/PTPN23	15	BP
GO:0032088	negative regulation of NF-kappaB transcription	15/2734	74/17381	0.1782	0.7899	0.77695	ARRB2/BRMS1/CDK5RAP3/CDKN2A/COMMD1/DAB2IP/DAP/HAVCR2/IRAK1/NFKBIL1/PARP10/PYDC1/RWDD3/TLR9/TRIM40	15	BP
GO:0032413	negative regulation of ion transmembrane	15/2734	74/17381	0.1782	0.7899	0.77695	ADRA2A/CAMK2D/CAV3/CRHR1/DRD2/GNB5/GPR35/GSTM2/MIR153-1/MIR212/OSR1/RRAD/THADA/TLR9/TRDN	15	BP
GO:0043312	neutrophil degranulation	84/2734	485/17381	0.18022	0.7899	0.77695	ACPP/ADAM8/ADGRG3/AHSG/ALAD/ALDOA/ANPEP/AP2A2/ARL8A/BIN2/C1orf35/C3AR1/CALML5/CD58/CD63/CD68/CDA/CHIT1/COTL1/CRACR2A/CTSD/CTSH/CXCR1/CXCR2/DDOST/DGAT1/DOK3/DPP7/DYNC1H1/EPX/FLG2/FRMPD3/GGH/GHDC/GSDMD/HK3/HMOX2/HSP90AA1/IDH1/IMPDH1/ITGAL/ITGAM/ITGAX/LGALS3/MGAM/MGST1/MMP25/MPO/NBEAL2/NPC2/OLFM4/OLR1/ORM1/ORM2/ORMDL3/PKM/PLAU/PPIE/PRCP/PRDX6/PRKCD/PSAP/PSMB7/PSMC3/PSMD13/PSMD3/PSMD7/PTPRN2/QSOX1/RAB24/RAB44/RAB7A/RHOF/SERPINA3/SLC11A1/SLC15A4/SPTAN1/SRP14/STK11IP/TMBIM1/TMEM63A/TRAPPC1/UBR4/VAT1	84	BP
GO:0043244	regulation of protein complex disassembly	19/2734	97/17381	0.18057	0.7899	0.77695	CAPZA3/CFL1/CIB1/EIF5A/F2RL1/GBA/KATNB1/LMOD1/MAP6D1/MID1IP1/NES/SCIN/SPTAN1/SPTB/SPTBN2/TBC1D25/TRIM54/TRPV4/WDR1	19	BP
GO:0021537	telencephalon development	43/2734	238/17381	0.18101	0.7899	0.77695	AQP1/AVPR2/BAD/BCAN/BMP4/CASP3/CDK5/CDK5R2/CSF1R/DAB1/DAB2IP/DCLK2/DISC1/DRD2/EGFR/EIF2B5/EMX1/EPHB3/FLNA/GRIN1/KAT2A/KDM6B/KIRREL3/LHX1/LHX5/LRP1/MCPH1/MFSD2A/NME1/NRG1/OGDH/POU3F3/RARA/RTN4RL1/RTN4RL2/SEMA7A/SHH/SMO/TBR1/TH/TRAP	43	BP

GO:0044782	cilium organization	63/2734	358/17381	0.18125	0.7899	0.77695	ABCC4/ACTR1A/B9D1/BBS9/C2CD3/CATIP/CC2D2A/CCDC103/CCDC13/CCDC40/CDC14A/CELSR3/CEP164/CEP19/CEP70/CFAP126/CFAP157/CFAP74/CKAP5/CROCC/DCTN2/DISC1/DNAJB13/DNHD1/DYNC1H1/DYNLL2/E2F4/EHD1/FLNA/FOPNL/GAS8/HAUS4/HAUS7/HSP90AA1/HYDIN/IFT140/IFT20/IFT22/KIF17/LRRC6/NOTCH1/NOTO/PTPN23/RAB17/RILPL1/RILPL2/SDCCAG8/SEPT9/SPAG16/SSNA1/TBC1D32/TEKT4/TEKT5/TMEM138/TMEM141/TMEM17/TMEM231/TRAF3IP1/TTLL5/TUBG1/UBE2B/WDPCP/WRA [illegible]	63	BP
GO:0034219	carbohydrate transmembrane transport	7/2734	30/17381	0.1819	0.7899	0.77695	SLC2A4/SLC2A6/SLC2A8/SLC35A2/SLC45A1/SLC50A1/SLC5A2	7	BP
GO:0035767	endothelial cell chemotaxis	7/2734	30/17381	0.1819	0.7899	0.77695	CORO1B/HSPB1/KDR/MIR16-1/NOTCH1/NR4A1/PLEKHG5	7	BP
GO:0043114	regulation of vascular permeability	7/2734	30/17381	0.1819	0.7899	0.77695	ADM/FGFBP3/PDE2A/PTP4A3/TACR1/TACR2/TRPV4	7	BP
GO:0044068	modulation by symbiont of host cellular	7/2734	30/17381	0.1819	0.7899	0.77695	BAD/CPSF4/EIF2AK4/GAPDH/MIR221/MIR222/SCRIB	7	BP
GO:0046596	regulation of viral entry into	7/2734	30/17381	0.1819	0.7899	0.77695	CD74/IFITM2/LGALS9/TRIM11/TRIM26/TRIM31/TRIM62	7	BP
GO:2000758	positive regulation of peptidyl-lysine	7/2734	30/17381	0.1819	0.7899	0.77695	BRCA1/KAT2A/MAPK3/MUC1/PAXIP1/PIWIL2/RPS6KA4	7	BP
GO:0046651	lymphocyte proliferation	48/2734	268/17381	0.18211	0.7899	0.77695	ABL1/BCL6/BMP4/CARD11/CASP3/CCDC88B/CCL19/CCL5/CCND3/CCR2/CD180/CD6/CD74/CD81/CLCF1/CLECL1/CORO1A/DNAJA3/EFNB1/ERBB2/HAVCR2/HLA-E/IDO1/IFNA5/IL13/IL18/IL20RB/IMPDH1/INPP5D/LGALS3/LGALS9/LMO1/MAD1L1/MZB1/PLA2G2F/PRKAR1A/PRKCD/SCGB1A1/SHH/SLC11A1/TA	48	BP
GO:0050909	sensory perception of	13/2734	63/17381	0.18223	0.7899	0.77695	ASIC2/ASIC3/AZGP1/CA6/GNAT1/ITPR3/LPO/P2RX2/PLCB2/REEP2/SCNN1A/TAS2R3/TRPM5	13	BP

GO:0097193	intrinsic apoptotic signaling pathway	52/2734	292/17381	0.18238	0.7899	0.77695	ABL1/ANKRD2/APOPT1/BAD/BCAP31/BOK/BRCA1/BRSK2/CASP3/CCAR2/CD74/CHAC1/CLU/CREB3/CRIP1/DAB2IP/DAPK2/ERN2/FHIT/GPX1/HERPUD1/HINT1/HRAS/HSPB1/INS/LCK/MAPK8IP1/MIR15A/MIR16-1/MOAP1/MUC1/NOC2L/NONO/NUPR1/PHLDA3/PPP1R13B/PRKCD/SART1/SEPT4/SFN/SGMS1/SPOP/SYVN1/TMEM109/TRAF2/TRAP1/VDAC2/WFS1/ZNF346/ZNF385A/ZNF385C/ZNF385D	52	BP
GO:0010927	cellular component assembly	20/2734	103/17381	0.18349	0.7899	0.77695	ACTA1/CAV3/CCDC136/GNPAT/GPC1/IGSF22/ILK/LMOD1/MYBPC1/MYBPC3/MYBPH/MYBPHL/NKX2-5/OBSCN/OBSL1/PDGFRB/PRKAR1A/TBPL1/TCAP/WDR1	20	BP
GO:2001237	negative regulation of extrinsic apoptotic	20/2734	103/17381	0.18349	0.7899	0.77695	BMP4/BRCA1/CTTN/DAPK1/EYA1/FASLG/FGA/GPX1/LGALS3/LMNA/MIR221/MIR222/NOS3/PEA15/PELI3/RAF1/TMBIM1/TRAF2/ZC3HC1/ZMYND11	20	BP
GO:0043123	positive regulation of I-kappaB kinase/NF-	34/2734	185/17381	0.18452	0.7899	0.77695	ABL1/CARD11/CARD9/CASP1/CCL19/CCL21/CD27/CD74/ECM1/F2RL1/FASLG/FLNA/GPR89A/IKBKB/IL18/IRAK1/LGALS9/LTBR/MAP3K14/MIB2/NOD1/PLEKHG5/RNF31/SHARPIN/SLC20A1/TBK1/TIRAP/TLR9/TMEM101/TNIP2/TRAF2/TRIM62/UNC5CL/WLS	34	BP
GO:0001738	morphogenesis of a polarized epithelium	26/2734	138/17381	0.18503	0.7899	0.77695	ABL1/AJAP1/AP2A2/AP2M1/ARRB2/CELSR2/CELSR3/DVL2/FZD2/GRHL3/IFT20/PARD6A/PFN1/PSMB11/PSMB6/PSMB7/PSMB8/PSMC3/PSMD13/PSMD3/PSMD5/PSMD7/TRAF3IP1/WDR1/WNT1/WNT11	26	BP
GO:0042220	response to cocaine	11/2734	52/17381	0.18511	0.7899	0.77695	CDK5/DNMT3A/DRD2/EFTUD2/HNMT/HOMER2/HSPA5/MTOR/OPRM1/PPP1R1B/PTK2B	11	BP
GO:0050701	interleukin-1 secretion	11/2734	52/17381	0.18511	0.7899	0.77695	CASP1/CASP5/CCL19/F2RL1/GSDMD/LGALS9/NLRP1/ORM1/ORM2/PYDC1/TLR8	11	BP

GO:0002283	neutrophil activation involved in immune response	84/2734	486/17381	0.1856	0.7899	0.77695	ACPP/ADAM8/ADGRG3/AHSG/ALAD/ALDOA/ANPEP/AP2A2/ARL8A/BIN2/C1orf35/C3AR1/CALML5/CD58/CD63/CD68/CDA/CHIT1/COTL1/CRACR2A/CTSD/CTSH/CXCR1/CXCR2/DDOST/DGAT1/DOK3/DPP7/DYNC1H1/EPX/FLG2/FRMPD3/GGH/GHDC/GSDMD/HK3/HMOX2/HSP90AA1/IDH1/IMPDH1/ITGAL/ITGAM/ITGAX/LGALS3/MGAM/MGST1/MMP25/MPO/NBEAL2/NPC2/OLFM4/OLR1/ORM1/ORM2/ORMDL3/PKM/PLAU/PPIE/PRCP/PRDX6/PRKCD/PSAP/PSMB7/PSMC3/PSMD13/PSMD3/PSMD7/PTPRN2/QSOX1/RAB24/RAB44/RAB7A/RHOF/SERPINA3/SLC11A1/SLC15A4/SPTAN1/SRP14/S[illegible]	84	BP
GO:0038066	p38MAPK cascade	9/2734	41/17381	0.18579	0.7899	0.77695	CAV3/DAB2IP/GADD45G/KARS/LGALS9/MIR181B1/NCF1/PER1/XDH	9	BP
GO:0042304	regulation of fatty acid biosynthetic	9/2734	41/17381	0.18579	0.7899	0.77695	ACADVL/APOA5/APOC3/BRCA1/ELOVL5/MID1IP1/MLXIPL/NR1H3/SCAP	9	BP
GO:0042551	neuron maturation	9/2734	41/17381	0.18579	0.7899	0.77695	AGRN/C1QL1/EPHA8/FARP2/MTCH1/MTOR/MYOC/RND1/SCARF1	9	BP
GO:0044058	regulation of digestive system process	9/2734	41/17381	0.18579	0.7899	0.77695	ABCG5/APOA5/AQP1/HIP1R/KCNQ1/NR1H3/PTGER3/SGK1/TACR1	9	BP
GO:0055081	anion homeostasis	9/2734	41/17381	0.18579	0.7899	0.77695	DGAT1/FASLG/FGFR4/GCM2/INS/MLXIPL/OTC/PQLC2/SLC34A1	9	BP
GO:0098927	vesicle-mediated transport between	9/2734	41/17381	0.18579	0.7899	0.77695	AKTIP/CORO1A/FAM160A2/MAPK3/PTPN23/RAB7A/SNF8/SNX12/STX8	9	BP
GO:0006821	chloride transport	21/2734	109/17381	0.18609	0.7899	0.77695	ANO1/ANO2/ANO4/ANO7/BEST3/BEST4/BSND/CA7/CLCN2/CLCNKA/CLCNKB/CLDN4/CLIC1/GLRA1/P2RY6/SLC12A4/SLC12A7/SLC12A9/SLC26A1/SLC26A10/SLC26A6	21	BP
GO:0042058	regulation of epidermal growth factor receptor	17/2734	86/17381	0.18651	0.7899	0.77695	ADRA2A/ADRA2B/ADRA2C/AGT/ARAP1/CDH13/DAB2IP/DOK1/EGFR/FASLG/GPER1/HIP1R/NCF1/RAB7A/RHBDF1/SHC1/VPS25	17	BP

GO:0070125	mitochondrial translational elongation	17/2734	86/17381	0.18651	0.7899	0.77695	ERAL1/MRPL10/MRPL14/MRPL28/MRPL36/MRPL37/MRPL43/MRPL52/MRPL57/MRPL9/MRPS10/MRPS11/MRPS18A/MRPS18B/MRPS21/MRPS33/MRPS5	17	BP
GO:0090559	regulation of membrane permeability	17/2734	86/17381	0.18651	0.7899	0.77695	ATF2/ATPIF1/BAD/BOK/DYNLL2/FZD9/HIP1R/MIR29A/MIR29C/MOAP1/MTOR/PPP1R13B/RHOT2/SFN/SLC25A5/YWHAQ/ZNF205	17	BP
GO:0070665	positive regulation of leukocyte	27/2734	144/17381	0.18652	0.7899	0.77695	BCL6/CARD11/CCDC88B/CCL19/CCL5/CCR2/CD6/CD74/CD81/CLCF1/CLECL1/CORO1A/CSF1/DNAJA3/EFNB1/HAVCR2/HLA-E/IL13/IL18/LGALS9/MIR181B1/SHH/TACR1/TIRAP/TLR9/TNFRSF4/WNT3	27	BP
GO:0007416	synapse assembly	28/2734	150/17381	0.18786	0.7899	0.77695	ACHE/ADGRB1/AGRN/AMIGO1/ASIC2/CDK5/CLSTN1/CLSTN3/DRD2/EIF4G1/EPHB3/GNPAT/GRIN1/KIRREL3/LINGO2/LRRTM1/NLGN3/NRG1/NRXN2/PCDHB1/PDLIM5/PLXND1/RAB17/SLITRK3/SPOCK2/SPTBN2/WNT3A/	28	BP
GO:0060271	cilium assembly	61/2734	347/17381	0.18802	0.7899	0.77695	ABCC4/ACTR1A/B9D1/BBS9/C2CD3/CC2D2A/CCDC103/CCDC13/CCDC40/CDC14A/CELSR3/CEP164/CEP19/CEP70/CFAP157/CFAP74/CKAP5/CROCC/DCTN2/DISC1/DNAJB13/DNHD1/DYNC1H1/DYNLL2/E2F4/EHD1/FLNA/FOPNL/GAS8/HAUS4/HAUS7/HSP90AA1/HYDIN/IFT140/IFT20/IFT22/KIF17/LRRC6/NOTCH1/NOTO/PTPN23/RAB17/RILPL1/RILPL2/SDCCAG8/SEPT9/SPAG16/SSNA1/TBC1D32/TEKT4/TEKT5/TMEM138/TMEM141/TMEM17/TMEM231/TRAF3IP1/TTLL5/TUBG1/UBE2B/WDPCP/WRAP73	61	BP
GO:0050715	positive regulation of cytokine	22/2734	115/17381	0.1884	0.7899	0.77695	ABL1/CASP1/CASP5/CCL19/CD58/CSF1R/DRD2/F2RL1/GAPDH/GSDMD/HAVCR2/IL4R/INS/KARS/LGALS9/MAPK3/NLRP1/ORM1/ORM2/PYDC1/TLR8/TRPV4	22	BP
GO:0006818	hydrogen transport	29/2734	156/17381	0.18907	0.7899	0.77695	ATP1A4/ATP5G1/ATP5I/ATP6V0B/ATP6V0E2/ATP6V1B1/ATP6V1F/ATP6V1G1/ATP6V1G2/COX10/COX11/COX8A/CYC1/IL13/NDUFA4L2/PM20D1/SLC15A3/SLC15A4/SLC25A14/SLC25A22/SLC2A6/SLC2A8/SLC35A2/SLC36A1/SLC36A3/SLC9A3/SLC9A5/UCP3/VPS9D1	29	BP
GO:0010950	positive regulation of endopeptidase	29/2734	156/17381	0.18907	0.7899	0.77695	ANP32B/APOPT1/BAD/BCAP31/BOK/CASP1/CASP3/CTSH/DAP/DAPK1/EFNA1/EFNA3/FASLG/GPER1/HIP1R/HSF1/LCK/LGALS9/MIR15A/MIR92A2/MTCH1/NLRP1/NOD1/PDCD2/PRELID1/SOX7/TRAF2/WNT3A/XDH	29	BP

GO:0051650	establishment of vesicle localization	45/2734	251/17381	0.18914	0.7899	0.77695	CDK5/CEP19/CNIH2/COL7A1/CPLX1/DCTN2/DNM1/DOC2A/DOC2B/DTNBP1/DYNC1H1/DYNC1I1/GAK/GBF1/GOLGA2/GORASP1/GOSR2/KIF13A/KIF23/MAP4K2/MLPH/MOBP/MYO7A/NLGN3/PLD2/RAB17/SCRIB/SEC16A/SEC16B/STARD3/STON2/STX1A/STX4/STX5/STXBP1/SYTL3/TOR1A/TRAPPC1/TRAPPC2L/TRAPPC3/TRAPPC4/TRAPPC9/TRIM46/WNT3A/WNT7A	45	BP
GO:0045927	positive regulation of growth	46/2734	257/17381	0.18927	0.7899	0.77695	ACACB/AGRN/ANAPC2/BRAT1/CIB1/CPNE5/CPNE9/CSF1/DISC1/DNPH1/DRD2/EGFR/EIF4G1/ERBB2/EXOSC2/FGFR2/FN1/HLX/HSF1/ILK/INO80/INS/ISLR2/L1CAM/LIMK1/MIR222/MTOR/MYOD1/NOTCH1/NRG1/PPARD/PPIB/PTK2B/RPS6KA1/SEMA7A/SFN/SH3PXD2B/SLC44A4/SMO/TBX2/TNFRSF12A/TRPV2/WFS1/WNT3A/ZFYVE27/ZPR1	46	BP
GO:0001562	response to protozoan	6/2734	25/17381	0.18927	0.7899	0.77695	BATF2/CCDC88B/CLEC7A/HRAS/IL4R/SLC11A1	6	BP
GO:0003309	type B pancreatic cell	6/2734	25/17381	0.18927	0.7899	0.77695	BAD/BMP4/MEN1/RFX6/SIDT2/SMO	6	BP
GO:0006735	NADH regeneration	6/2734	25/17381	0.18927	0.7899	0.77695	ALDOA/GAPDH/GCK/HK3/PFKM/PKM	6	BP
GO:0010460	positive regulation of	6/2734	25/17381	0.18927	0.7899	0.77695	ADM/ADRA1A/EDN2/GCH1/KCNQ1/NPPA	6	BP
GO:0031112	positive regulation of microtubule polymerization	6/2734	25/17381	0.18927	0.7899	0.77695	ANKRD53/CAV3/FES/KATNB1/NUMA1/TRPV4	6	BP
GO:0032770	positive regulation of monooxygenase	6/2734	25/17381	0.18927	0.7899	0.77695	GCH1/INS/NOD1/POR/PTK2B/SCARB1	6	BP
GO:0033522	histone H2A ubiquitination	6/2734	25/17381	0.18927	0.7899	0.77695	DDB1/DDB2/OTUB1/PCGF2/UBE2A/UBE2B	6	BP
GO:0042219	cellular modified amino acid catabolic	6/2734	25/17381	0.18927	0.7899	0.77695	ALDH1L1/ALDH4A1/CHAC1/GGACT/GGT6/GOT2	6	BP

GO:0043501	skeletal muscle adaptation	6/2734	25/17381	0.18927	0.7899	0.77695	ACTA1/MTOR/MYOC/MYOD1/TNNC1/TRIM63	6	BP
GO:0060351	cartilage development involved in endochondral bone morphogenesis	6/2734	25/17381	0.18927	0.7899	0.77695	COL1A1/POR/RARA/SERPINH1/THBS3/TRPV4	6	BP
GO:0060479	lung cell differentiation	6/2734	25/17381	0.18927	0.7899	0.77695	AIMP2/EYA1/IL13/NUMA1/SPDEF/THRA	6	BP
GO:0060561	apoptotic process involved in	6/2734	25/17381	0.18927	0.7899	0.77695	CRYAB/JAG2/NKX2-5/NOTCH1/SCRIB/SPI1	6	BP
GO:0061621	canonical glycolysis	6/2734	25/17381	0.18927	0.7899	0.77695	ALDOA/GAPDH/GCK/HK3/PFKM/PKM	6	BP
GO:0061718	glucose catabolic process to	6/2734	25/17381	0.18927	0.7899	0.77695	ALDOA/GAPDH/GCK/HK3/PFKM/PKM	6	BP
GO:0072012	glomerulus vasculature development	6/2734	25/17381	0.18927	0.7899	0.77695	AQP1/BMP4/EGR1/NOTCH1/OSR1/PDGFRB	6	BP
GO:0097120	receptor localization to synapse	6/2734	25/17381	0.18927	0.7899	0.77695	CEP112/CNIH2/DLG4/GRIPAP1/KIF17/SCRIB	6	BP
GO:1904738	vascular associated smooth muscle cell migration	6/2734	25/17381	0.18927	0.7899	0.77695	AGT/DOCK7/MIR15A/MIR221/MIR451A/MIR503	6	BP

GO:1904752	regulation of vascular associated smooth muscle cell migration	6/2734	25/17381	0.18927	0.7899	0.77695	AGT/DOCK7/MIR15A/MIR221/MIR451A/MIR503	6	BP
GO:0070085	glycosylation	53/2734	299/17381	0.18932	0.7899	0.77695	A4GNT/ADAMTS13/ADAMTS7/ALG10/ALG3/ASGR1/B3GAT3/B3GNT6/B4GALNT1/B4GALT2/B4GALT7/DAD1/DDOST/DOLPP1/DPM2/EXTL1/FKTN/FUOM/FUT7/GALNT16/GALNT8/GALNT9/GBGT1/GGTA1P/GOLGA2/GORASP1/LFNG/LMF1/MGAT1/MGAT4B/MGAT5B/MUC1/MUC2/MUC3A/MUC5AC/MUC5B/MUC6/OST4/PARP10/PARP2/PARP3/PARP9/PIGZ/PLOD3/RFNG/RPN1/SDF2/SLC35C1/ST3GAL4/SYVN1/TINF2/TMEM115/XXYLT1	53	BP
GO:0045017	glycerolipid biosynthetic process	48/2734	269/17381	0.18943	0.7899	0.77695	ACHE/ANG/CDIPT/CPNE7/CWH43/DGAT1/DGKQ/DPM2/ETNK2/FGF17/FGF3/FGFR2/FGFR4/GNPAT/GPAA1/INPP5D/INPP5E/INPP5F/INPPL1/MFSD2A/MTMR1/MTMR14/NR1H3/PDGFA/PEMT/PHOSPHO1/PI4KB/PIGC/PIGG/PIGO/PIGQ/PIGY/PIGZ/PIK3R5/PIK3R6/PITPNM1/PLA2G1B/PLA2G2F/PLA2G4B/PLA2G5/PLD2/PTDSS1/PTDSS2/PTPN11/PYURF/SCARB1/SLC44A4/TLR	48	BP
GO:0010972	negative regulation of G2/M transition of mitotic cell	18/2734	92/17381	0.18991	0.7899	0.77695	BRCA1/CDK5RAP3/CHMP4C/FOXO4/GPR132/MIIP/MIR195/PSMB11/PSMB6/PSMB7/PSMB8/PSMC3/PSMD13/PSMD3/PSMD5/PSMD7/RINT1/TICRR	18	BP
GO:0016241	regulation of macroautophagy	30/2734	162/17381	0.19015	0.7899	0.77695	ATG101/ATP13A2/ATP6V0B/ATP6V0E2/ATP6V1B1/ATP6V1G1/ATP6V1G2/CASP3/CDK5/GAPDH/GBA/GPSM1/IFT20/KDR/LRSAM1/MAPK3/MFN2/MLST8/MTOR/NOD1/NPRL3/POLDIP2/PRKAB1/QSOX1/RALB/RRAGC/SPTL	30	BP
GO:0031960	response to corticosteroid	30/2734	162/17381	0.19015	0.7899	0.77695	ADM/ALAD/AQP1/BAD/BGLAP/CASP3/CCL1/COL1A1/EGFR/EIF4EBP1/ENG/FOSL1/GBA/GPER1/HEY1/HNMT/HSD11B2/IL1RN/MYOD1/NOTCH1/PFKFB1/PTPRU/SCGB1A1/SDC1/SMYD3/SSTR5/TH/TRH/TRIM63/UCP3	30	BP
GO:0009135	purine nucleoside diphosphate	23/2734	121/17381	0.19047	0.7899	0.77695	AK5/ALDOA/BAD/CARD11/DLG4/GALK1/GAPDH/GCK/HK3/INS/LDHA/MLXIPL/NCOR1/NUDT18/NUP210/NUP98/OGDH/OGDHL/PFKFB1/PFKM/PGAM4/PKM/SCRIB	23	BP

GO:0009179	purine ribonucleoside diphosphate metabolic	23/2734	121/17381	0.19047	0.7899	0.77695	AK5/ALDOA/BAD/CARD11/DLG4/GALK1/GAPDH/GCK/HK3/INS/LDHA/MLXIPL/NCOR1/NUDT18/NUP210/NUP98/OGDH/OGDHL/PFKFB1/PFKM/PGAM4/PKM/SCRIB	23	BP
GO:1903034	regulation of response to wounding	31/2734	168/17381	0.19112	0.7899	0.77695	ADRA2A/AJAP1/ASIC2/C1QTNF1/F2RL1/FGA/GJD4/HRAS/INPP5F/KLKB1/KNG1/LRIG2/MIR221/MIR222/MIR451A/MTOR/MYOD1/NOS3/PDGFA/PLAU/PPARD/PRKCD/PROZ/PTPRF/RGMA/RTN4RL1/SCARF1/SELP/SERPINF2	31	BP
GO:0021766	hippocampus development	15/2734	75/17381	0.19234	0.7899	0.77695	BCAN/CASP3/CDK5/CDK5R2/DCLK2/EIF2B5/KDM6B/KIRREL3/LHX5/MFSD2A/NME1/OGDH/RARA/SMO/WNT3A	15	BP
GO:0034109	homotypic cell-cell adhesion	15/2734	75/17381	0.19234	0.7899	0.77695	C1QTNF1/CCL5/CLIC1/CSRP1/FERMT3/FGA/FLNA/HSPB1/ILK/PRKCD/PTPRU/STXBP1/TLN1/WNT3A/ZNF703	15	BP
GO:0042632	cholesterol homeostasis	15/2734	75/17381	0.19234	0.7899	0.77695	ABCA2/ABCG5/APOA5/APOB/APOC3/CAV3/EHD1/EPHX2/FGFR4/GPIHBP1/IL18/NPC2/NR1H3/SCARB1/SOAT2	15	BP
GO:0055092	sterol homeostasis	15/2734	75/17381	0.19234	0.7899	0.77695	ABCA2/ABCG5/APOA5/APOB/APOC3/CAV3/EHD1/EPHX2/FGFR4/GPIHBP1/IL18/NPC2/NR1H3/SCARB1/SOAT2	15	BP
GO:0002040	sprouting angiogenesis	19/2734	98/17381	0.19291	0.7899	0.77695	ABL1/BMP4/CDH13/CIB1/E2F8/EPHB4/FLT4/KDR/LOXL2/MIR16-1/MIR221/MIR222/MIR29C/MIR503/MIR92A2/NOTCH1/NR4A1/PTK2B/TDG	19	BP
GO:0003279	cardiac septum development	19/2734	98/17381	0.19291	0.7899	0.77695	BMP4/CRELD1/ENG/FGFR2/FGFRL1/FZD2/GATA4/HEY1/NKX2-5/NOTCH1/NPRL3/PLXND1/RARA/SMAD6/SMO/STRA6/SUFU/TBX2/WNT	19	BP
GO:0072163	mesonephric epithelium development	19/2734	98/17381	0.19291	0.7899	0.77695	AGT/BMP4/EYA1/FGFR2/HOXB7/ILK/LHX1/OSR1/PKD1/RARA/SDC1/SHH/SIM1/SMAD6/SMO/SOX8/WNT1/WNT11/WNT6	19	BP
GO:0072164	mesonephric tubule development	19/2734	98/17381	0.19291	0.7899	0.77695	AGT/BMP4/EYA1/FGFR2/HOXB7/ILK/LHX1/OSR1/PKD1/RARA/SDC1/SHH/SIM1/SMAD6/SMO/SOX8/WNT1/WNT11/WNT6	19	BP
GO:0050803	regulation of synapse structure or	25/2734	133/17381	0.19396	0.7899	0.77695	ABL1/ADGRB1/AGRN/AMIGO1/ANAPC2/ASIC2/CLSTN1/CLSTN3/CTNNA2/DAB2IP/DISC1/DRD2/EIF4G1/EPHB3/FZD9/GRIN1/GRIPAP1/LINGO2/LRRTM1/NEUROD2/NLGN3/PDLIM5/RAB17/SLITRK3/WNT7A	25	BP

GO:0006650	glycerophospholipid metabolic process	62/2734	354/17381	0.19426	0.7899	0.77695	ACHE/APOA5/CDIPT/CPNE7/CSF1R/CWH43/DGKQ/DPM2/DRD2/EGFR/ERBB2/ETNK2/FGF17/FGF3/FGFR2/FGFR4/GNPAT/GPAA1/HADHA/INPP5D/INPP5E/INPP5F/INPPL1/LCK/MFSD2A/MTMR1/MTMR14/NRG1/OSBPL5/PDGFA/PDGFRB/PEMT/PHOSPHO1/PI4KB/PIGC/PIGG/PIGO/PIGQ/PIGY/PIGZ/PIK3R5/PIK3R6/PITPNM1/PLA2G15/PLA2G16/PLA2G1B/PLA2G2F/PLA2G4B/PLA2G5/PLB1/PLCH2/PLD2/PRDX6/PTDSS1/PTDSS2/PTPN11/PYURF/SC[illegible]	62	BP
GO:0032648	regulation of interferon-beta production	10/2734	47/17381	0.19501	0.7899	0.77695	IRF5/NLRX1/NMI/POLR3D/TBK1/TIRAP/TLR8/TLR9/TRAF3IP1/TRAIP	10	BP
GO:0033344	cholesterol efflux	10/2734	47/17381	0.19501	0.7899	0.77695	ABCG5/APOA5/APOB/APOC3/LRP1/NPC2/NR1H3/SCARB1/SHH/SOAT2	10	BP
GO:0035094	response to nicotine	10/2734	47/17381	0.19501	0.7899	0.77695	BAD/CASP3/CHRNA6/CHRNB1/CHRNB3/DRD2/IL13/LYPD1/TACR1/TH	10	BP
GO:0045739	positive regulation of	10/2734	47/17381	0.19501	0.7899	0.77695	APBB1/BRCA1/BRCC3/EGFR/EYA1/FOXM1/NPAS2/PARP9/SLF2/TIMELESS	10	BP
GO:0006040	amino sugar metabolic	8/2734	36/17381	0.19524	0.7899	0.77695	AMDHD2/CHID1/CHIT1/CHST5/CHST7/MGAT1/NAGK/NPL	8	BP
GO:0007257	activation of JUN kinase	8/2734	36/17381	0.19524	0.7899	0.77695	AXIN1/CCL19/DAB2IP/ERN2/HACD3/MAP3K6/MAP4K2/MAPK8IP3	8	BP
GO:0045124	regulation of bone resorption	8/2734	36/17381	0.19524	0.7899	0.77695	ADAM8/BGLAP/CSF1R/CSK/DEF8/EGFR/INPP5D/PLEKHM1	8	BP
GO:0045684	positive regulation of epidermis	8/2734	36/17381	0.19524	0.7899	0.77695	BMP4/FOXN1/NOTCH1/NUMA1/PPARD/PTCH2/SFN/TMEM79	8	BP
GO:0045747	positive regulation of Notch signaling	8/2734	36/17381	0.19524	0.7899	0.77695	ERH/EYA1/JAG2/LFNG/MIR212/NOTCH1/RFNG/WNT1	8	BP
GO:1900449	regulation of glutamate receptor	8/2734	36/17381	0.19524	0.7899	0.77695	ARC/CNIH2/DAPK1/DLG4/NECAB2/NLGN3/OPRM1/PTK2B	8	BP

GO:1900744	regulation of p38MAPK cascade	8/2734	36/17381	0.19524	0.7899	0.77695	CAV3/DAB2IP/GADD45G/KARS/MIR181B1/NCF1/PER1/XDH	8	BP
GO:0031397	negative regulation of protein	26/2734	139/17381	0.19543	0.7899	0.77695	ABL1/ADGRB1/ANAPC15/ANAPC2/ARRB2/BUB1B/CDK5/HDAC8/ISG15/KLHL40/LIMK1/MTOR/OTUB1/PARP10/PSMB6/PSMB7/PSMB8/PSMC3/PSMD13/PSMD3/PSMD5/PSMD7/SUFU/TAF1/USP4/WNT1	26	BP
GO:0001502	cartilage condensation	5/2734	20/17381	0.19586	0.7899	0.77695	BARX2/MYCN/PKD1/THRA/WNT7A	5	BP
GO:0006516	glycoprotein catabolic	5/2734	20/17381	0.19586	0.7899	0.77695	FBXO2/FBXO6/GPC1/MIR181B1/SGSH	5	BP
GO:0006925	inflammatory cell apoptotic	5/2734	20/17381	0.19586	0.7899	0.77695	CCL5/CDKN2A/CTSL/FASLG/HCAR2	5	BP
GO:0010804	negative regulation of tumor necrosis factor-mediated	5/2734	20/17381	0.19586	0.7899	0.77695	CCDC3/F2RL1/PELI3/PYDC1/TRAIP	5	BP
GO:0023058	adaptation of signaling	5/2734	20/17381	0.19586	0.7899	0.77695	ADM/ARRB2/DNM1/DRD2/NECAB2	5	BP
GO:0034123	positive regulation of toll-like receptor	5/2734	20/17381	0.19586	0.7899	0.77695	F2RL1/NR1H3/TIRAP/TLR9/TREML4	5	BP
GO:0034390	smooth muscle cell apoptotic	5/2734	20/17381	0.19586	0.7899	0.77695	APOPT1/ARRB2/CDKN2A/MIR92A2/PDCD4	5	BP
GO:0034391	regulation of smooth muscle cell apoptotic	5/2734	20/17381	0.19586	0.7899	0.77695	APOPT1/ARRB2/CDKN2A/MIR92A2/PDCD4	5	BP

GO:0042772	DNA damage response, signal transduction resulting in transcription	5/2734	20/17381	0.19586	0.7899	0.77695	BRCA1/FOXM1/ING4/MUC1/ZNF385A	5	BP
GO:0045603	positive regulation of endothelial cell	5/2734	20/17381	0.19586	0.7899	0.77695	BMP4/CDH5/GDF2/NOTCH1/TMEM100	5	BP
GO:0051900	regulation of mitochondrial depolarization	5/2734	20/17381	0.19586	0.7899	0.77695	BOK/FZD9/KDR/MYOC/PPP2R3C	5	BP
GO:0060008	Sertoli cell differentiation	5/2734	20/17381	0.19586	0.7899	0.77695	NUP210L/RARA/SCX/SDC1/SOX8	5	BP
GO:1902176	negative regulation of oxidative stress-induced intrinsic	5/2734	20/17381	0.19586	0.7899	0.77695	GPX1/HSPB1/INS/NONO/TRAP1	5	BP
GO:2000178	negative regulation of neural precursor	5/2734	20/17381	0.19586	0.7899	0.77695	CEND1/ILK/LIMS2/SPINT1/VAX1	5	BP
GO:2001273	regulation of glucose import in response to insulin stimulus	5/2734	20/17381	0.19586	0.7899	0.77695	AGT/MZB1/PID1/PTPN11/RARRES2	5	BP

GO:0051186	cofactor metabolic process	73/2734	421/17381	0.19631	0.7899	0.77695	ACACB/ACOT7/ACOT9/ACSF3/ACSM6/ALAD/ALAS2/ALDH1L1/ALDOA/ATPIF1/BHMT2/CIAPIN1/COQ2/COQ4/COX10/CROT/CYP1A1/CYP1A2/DGAT1/DLST/ELOVL1/ELOVL5/FAM96B/FOLR2/GALK1/GAPDH/GCH1/GCK/GGH/GNMT/HAAO/HACD1/HK3/HMBS/HMOX2/IDH1/IDH2/INS/ISCA2/LDHA/LIAS/LOC344967/MLXIPL/MPC1/MTHFD1/NARFL/NCOR1/NFE2L1/NMNAT3/NUBP1/NUBP2/NUP210/NUP98/OGDH/OGDHL/PARP10/PARP9/PC/PDHB/PDSS1/PEMT/PFKFB1/PFKM/PGAM4/PIPOX/PKM/PMVK/PPCDC/QPRT[illegible]	73	BP
GO:0007249	I-kappaB kinase/NF-kappaB signaling	48/2734	270/17381	0.1969	0.7899	0.77695	ABL1/AVPR2/CARD11/CARD9/CASP1/CCL19/CCL21/CD27/CD74/DAB2IP/ECM1/F2RL1/FASLG/FLNA/GPR89A/HACD3/HSPB1/IKBKB/IL18/IRAK1/LGALS9/LTBR/MAP3K14/MIB2/NFKBIL1/NKIRAS2/NLRX1/NOD1/OTUD7A/PER1/PLEKHG5/PRDX1/RNF31/SHARPIN/SLC20A1/STAT1/TBK1/TIAF1/TIRAP/TLR8/TLR9/TMEM101/TNIP2/TRAF2/TRIM62/UNC5CL/WLS/ZMYND1	48	BP
GO:0060326	cell chemotaxis	48/2734	270/17381	0.1969	0.7899	0.77695	ADAM8/ARHGEF16/ARRB2/BIN2/C3AR1/CAMK1D/CCL1/CCL19/CCL21/CCL5/CCR2/CD74/CHGA/CKLF/CORO1A/CORO1B/CREB3/CSF1/CXCR1/CXCR2/CXCR3/CXCR5/DAPK2/EDN2/F2RL1/GBF1/HOXB9/HSPB1/KARS/KDR/LGALS3/LGALS9/MAPK3/MIR15A/MIR16-1/NBL1/NOTCH1/NR4A1/PDGFRB/PF4V1/PLA2G1B/PLEKHG5/PLXNB3/PRKCD/PTK2B/RARRES2/TIRAP/TRPV4	48	BP
GO:0046785	microtubule polymerization	13/2734	64/17381	0.19783	0.7899	0.77695	ABL1/ANKRD53/CAV3/CKAP5/FES/GOLGA2/NUMA1/TRPV4/TUBG1/TUBG2/TUBGCP2/TUBGCP3/TUBGCP5	13	BP
GO:0048207	vesicle targeting, rough ER to cis-Golgi	13/2734	64/17381	0.19783	0.7899	0.77695	CNIH2/COL7A1/GOLGA2/GORASP1/GOSR2/SEC16A/SEC16B/STX5/TRAPPC1/TRAPPC2L/TRAPPC3/TRAPPC4/TRAPPC9	13	BP
GO:0048208	COPII vesicle coating	13/2734	64/17381	0.19783	0.7899	0.77695	CNIH2/COL7A1/GOLGA2/GORASP1/GOSR2/SEC16A/SEC16B/STX5/TRAPPC1/TRAPPC2L/TRAPPC3/TRAPPC4/TRAPPC9	13	BP
GO:0051145	smooth muscle cell	13/2734	64/17381	0.19783	0.7899	0.77695	ADM/BMP4/ENG/EPC1/FGFR2/FOXO4/GPER1/HEY1/MIR221/NOTCH1/PDCD4/RBPMS2/SHH	13	BP
GO:0051937	catecholamine transport	13/2734	64/17381	0.19783	0.7899	0.77695	ADRA2A/ADRA2B/ADRA2C/AGT/CHGA/CHRNA6/DRD2/DTNBP1/NISCH/SLC22A1/SLC6A2/STX1A/TOR1A	13	BP
GO:1901983	regulation of protein	13/2734	64/17381	0.19783	0.7899	0.77695	BRCA1/CTBP1/HDAC8/KAT2A/MAPK3/MUC1/NOC2L/PAXIP1/PIWIL2/PYGO2/RPS6KA4/SPI1/TAF7	13	BP

GO:0006493	protein O-linked glycosylation	21/2734	110/17381	0.1979	0.7899	0.77695	A4GNT/ADAMTS13/ADAMTS7/B3GNT6/DPM2/FKTN/GALNT16/GALNT8/GALNT9/LFNG/MUC1/MUC2/MUC3A/MUC5AC/MUC5B/MUC6/PLOD3/RFNG/SDF2/ST3GAL4/XXYLT1	21	BP
GO:0034101	erythrocyte homeostasis	21/2734	110/17381	0.1979	0.7899	0.77695	AHSP/ALAS2/ATPIF1/BCL6/BMP4/CASP3/HBZ/INPP5D/ISG15/L3MBTL3/LDB1/MAEA/MIR221/MIR222/NFE2L1/PRDX1/RPS24/SETD1A/SPI1/STAT1/T	21	BP
GO:0071621	granulocyte chemotaxis	21/2734	110/17381	0.1979	0.7899	0.77695	C3AR1/CAMK1D/CCL1/CCL19/CCL21/CCL5/CD74/CKLF/CSF1/CXCR2/DAPK2/EDN2/GBF1/KARS/LGALS3/MAPK3/PF4V1/PLA2G1B/RARRES2/TIRA	21	BP
GO:0001558	regulation of cell growth	68/2734	391/17381	0.19849	0.7899	0.77695	ABL1/AGT/ANAPC2/APBB1/ARHGAP4/BARHL2/BCL6/BLZF1/BRAT1/CAMK2D/CAV3/CCAR2/CCDC85B/CDA/CDK5/CDKN2A/CIB1/CISH/CLSTN1/CLSTN3/CPNE5/CPNE9/CRYAB/CTTN/DISC1/DNPH1/DRAXIN/EGFR/EIF4G1/ERBB2/ESR2/EXOSC2/FN1/GDF2/HTRA3/IGFBP3/IGFBP6/ILK/INO80/INS/ISLR2/KIF26A/L1CAM/LIMK1/MTOR/NPPA/NRG1/OSGIN1/PPARD/PPP1R9B/PTK2B/RASGRP2/RPS6KA1/SEMA3F/SEMA7A/SFN/SGK1/SIPA1/SLC44A4/TAOK2/TNFRSF12A/TNK1/TRIM40/TRPV2/TSPYL2/WNT11/WNT3A/Z[illegible]	68	BP
GO:0043900	regulation of multi-organism process	67/2734	385/17381	0.19891	0.7899	0.77695	BAD/CACNA1H/CCL5/CD180/CD74/CDK9/CFL1/CHMP4C/CIB1/DDB1/DHX58/EIF2AK4/F2RL1/FKBP6/GAPDH/GLRA1/HACD3/HAVCR2/IFITM2/INHBB/ISG15/LAMP3/LGALS9/LRSAM1/LTA/MAPK3/MICB/MIR221/MIR222/MPO/NELFB/NFKBIL1/NLRX1/NOD1/NOTCH1/PARP10/PARP9/PC/PFN1/PGC/PLB1/POLR2G/POLR2L/POMZP3/PPIB/PPIE/PPP2R3C/PRSS37/PSMC3/RAB7A/RSF1/SNF8/SPINK2/SPINK5/STAT1/TARBP2/TBK1/TCP11/TIRAP/TRAF3IP1/TRIM11/TRIM14/TRIM26/TRIM31/TRIM62/VPS37B/ZNF502	67	BP
GO:0003183	mitral valve morphogenesis	3/2734	10/17381	0.19905	0.7899	0.77695	EFNA1/NOTCH1/SMAD6	3	BP
GO:0006527	arginine catabolic	3/2734	10/17381	0.19905	0.7899	0.77695	FAH/NOS3/PADI4	3	BP
GO:0006924	activation-induced cell death of T cells	3/2734	10/17381	0.19905	0.7899	0.77695	DNAJA3/TSC22D3/TSC22D4	3	BP
GO:0007084	mitotic nuclear envelope reassembly	3/2734	10/17381	0.19905	0.7899	0.77695	EMD/LMNA/REEP4	3	BP

GO:0007440	foregut morphogenesis	3/2734	10/17381	0.19905	0.7899	0.77695	GATA4/NOTCH1/SHH	3	BP
GO:0010826	negative regulation of centrosome	3/2734	10/17381	0.19905	0.7899	0.77695	BRCA1/CCNF/NUBP1	3	BP
GO:0014010	Schwann cell proliferation	3/2734	10/17381	0.19905	0.7899	0.77695	ASCL2/MIR221/MIR222	3	BP
GO:0015816	glycine transport	3/2734	10/17381	0.19905	0.7899	0.77695	SLC36A1/SLC36A3/SLC6A9	3	BP
GO:0016102	diterpenoid biosynthetic process	3/2734	10/17381	0.19905	0.7899	0.77695	ALDH8A1/CYP1A1/RBP1	3	BP
GO:0018342	protein prenylation	3/2734	10/17381	0.19905	0.7899	0.77695	AIPL1/FNTA/FNTB	3	BP
GO:0019755	one-carbon compound transport	3/2734	10/17381	0.19905	0.7899	0.77695	AQP1/AQP10/AQP5	3	BP
GO:0021561	facial nerve development	3/2734	10/17381	0.19905	0.7899	0.77695	HOXB1/HOXB2/SEMA3F	3	BP
GO:0021604	cranial nerve structural organization	3/2734	10/17381	0.19905	0.7899	0.77695	HOXB1/HOXB2/SEMA3F	3	BP
GO:0021610	facial nerve morphogenesis	3/2734	10/17381	0.19905	0.7899	0.77695	HOXB1/HOXB2/SEMA3F	3	BP
GO:0021924	cell proliferation in external granule	3/2734	10/17381	0.19905	0.7899	0.77695	CEND1/GBX2/SHH	3	BP
GO:0021930	cerebellar granule cell precursor	3/2734	10/17381	0.19905	0.7899	0.77695	CEND1/GBX2/SHH	3	BP

GO:0032464	positive regulation of protein homooligomeri	3/2734	10/17381	0.19905	0.7899	0.77695	CLU/HRK/MIEF2	3	BP
GO:0032957	inositol trisphosphate metabolic	3/2734	10/17381	0.19905	0.7899	0.77695	GPER1/ITPK1/PTK2B	3	BP
GO:0033131	regulation of glucokinase activity	3/2734	10/17381	0.19905	0.7899	0.77695	BAD/COX11/PFKFB1	3	BP
GO:0033623	regulation of integrin	3/2734	10/17381	0.19905	0.7899	0.77695	FARP2/FBLIM1/SELP	3	BP
GO:0034145	positive regulation of toll-like receptor 4	3/2734	10/17381	0.19905	0.7899	0.77695	F2RL1/NR1H3/TIRAP	3	BP
GO:0034393	positive regulation of smooth muscle cell apoptotic	3/2734	10/17381	0.19905	0.7899	0.77695	APOPT1/CDKN2A/PDCD4	3	BP
GO:0035376	sterol import	3/2734	10/17381	0.19905	0.7899	0.77695	APOC3/SCARB1/STARD5	3	BP
GO:0035437	maintenance of protein localization in endoplasmic	3/2734	10/17381	0.19905	0.7899	0.77695	GPAA1/HSPA5/OS9	3	BP
GO:0036005	response to macrophage colony-stimulating	3/2734	10/17381	0.19905	0.7899	0.77695	CSF1/CSF1R/PDE2A	3	BP

GO:0036006	cellular response to macrophage colony-	3/2734	10/17381	0.19905	0.7899	0.77695	CSF1/CSF1R/PDE2A	3	BP
GO:0042178	xenobiotic catabolic	3/2734	10/17381	0.19905	0.7899	0.77695	CYP1A1/GSTM1/GSTM2	3	BP
GO:0043031	negative regulation of macrophage	3/2734	10/17381	0.19905	0.7899	0.77695	FAM19A3/IL31RA/NR1H3	3	BP
GO:0044793	negative regulation by host of viral	3/2734	10/17381	0.19905	0.7899	0.77695	EIF2AK4/MIR221/MIR222	3	BP
GO:0045059	positive thymic T cell selection	3/2734	10/17381	0.19905	0.7899	0.77695	CD74/FOXN1/SHH	3	BP
GO:0045324	late endosome to vacuole	3/2734	10/17381	0.19905	0.7899	0.77695	CHMP7/SNF8/VPS25	3	BP
GO:0045625	regulation of T-helper 1 cell differentiation	3/2734	10/17381	0.19905	0.7899	0.77695	CCL19/HLX/IL4R	3	BP
GO:0048143	astrocyte activation	3/2734	10/17381	0.19905	0.7899	0.77695	EGFR/LRP1/SMO	3	BP
GO:0048280	vesicle fusion with Golgi	3/2734	10/17381	0.19905	0.7899	0.77695	GOSR2/STX5/VTI1B	3	BP
GO:0048570	notochord morphogenesis	3/2734	10/17381	0.19905	0.7899	0.77695	CRB2/EFNA1/WNT11	3	BP
GO:0051256	mitotic spindle midzone	3/2734	10/17381	0.19905	0.7899	0.77695	CDC14A/KIF23/KIF4A	3	BP
GO:0060484	lung-associated mesenchyme development	3/2734	10/17381	0.19905	0.7899	0.77695	FGFR2/SHH/WNT11	3	BP

GO:0061140	lung secretory cell	3/2734	10/17381	0.19905	0.7899	0.77695	AIMP2/IL13/SPDEF	3	BP
GO:0070508	cholesterol import	3/2734	10/17381	0.19905	0.7899	0.77695	APOC3/SCARB1/STARD5	3	BP
GO:0071287	cellular response to	3/2734	10/17381	0.19905	0.7899	0.77695	ATP13A2/HSPA5/TH	3	BP
GO:0071600	otic vesicle morphogenesis	3/2734	10/17381	0.19905	0.7899	0.77695	EYA1/FGFR2/TCAP	3	BP
GO:0072110	glomerular mesangial cell proliferation	3/2734	10/17381	0.19905	0.7899	0.77695	BMP4/EGR1/PDGFRB	3	BP
GO:0072321	chaperone-mediated protein	3/2734	10/17381	0.19905	0.7899	0.77695	CLU/TIMM9/TOR1A	3	BP
GO:0097354	prenylation	3/2734	10/17381	0.19905	0.7899	0.77695	AIPL1/FNTA/FNTB	3	BP
GO:1904995	negative regulation of leukocyte adhesion to vascular	3/2734	10/17381	0.19905	0.7899	0.77695	CCL21/MIR221/MIR222	3	BP
GO:2000674	regulation of type B pancreatic cell	3/2734	10/17381	0.19905	0.7899	0.77695	CAPN10/SPOP/WFS1	3	BP
GO:2001214	positive regulation of	3/2734	10/17381	0.19905	0.7899	0.77695	ADM/KDR/TMEM100	3	BP
GO:0090174	organelle membrane fusion	30/2734	163/17381	0.19987	0.7899	0.77695	ADPRHL1/CORO1A/DOC2A/DOC2B/GOSR2/GRTP1/MFN1/NKD2/RAB20/RAB34/RAB7A/RABGAP1/RABGAP1L/RBSN/SAMD9L/SGSM2/STX1A/STX4/STX5/STX8/STXBP1/SYTL3/TAP1/TBC1D25/TBC1D28/TBC1D9/USP6NL/V	30	BP
GO:0021782	glial cell development	17/2734	87/17381	0.19991	0.7899	0.77695	ARHGEF10/CDK5/CLU/EGFR/EIF2B2/EIF2B5/HDAC11/ILK/LAMC3/LRP1/MXRA8/MYOC/PARD3/POU3F1/PRDM8/SHH/SMO	17	BP

GO:0030510	regulation of BMP signaling	17/2734	87/17381	0.19991	0.7899	0.77695	ABL1/BMP4/CRB2/ENG/GATA4/GDF2/HTRA3/ILK/LEMD2/NBL1/NOTCH1/NUMA1/RBPMS2/SFRP5/SMAD6/SOST/WNT1	17	BP
GO:0055088	lipid homeostasis	22/2734	116/17381	0.19996	0.7899	0.77695	ABCA2/ABCG5/APOA5/APOB/APOC3/CAV3/DGAT1/EHD1/EPHX2/FGFR4/GPIHBP1/IL18/INS/MLXIPL/NPC2/NR1H3/ORMDL3/PNPLA2/RBP1/SCARB	22	BP
GO:0001911	negative regulation of leukocyte mediated	4/2734	15/17381	0.20034	0.7899	0.77695	ARRB2/HAVCR2/HLA-E/LGALS9	4	BP
GO:0002827	positive regulation of T-helper 1 type	4/2734	15/17381	0.20034	0.7899	0.77695	CCL19/CCR2/HLX/SLC11A1	4	BP
GO:0007252	I-kappaB phosphorylation	4/2734	15/17381	0.20034	0.7899	0.77695	DAB2IP/IKBKB/TLR9/TRAF2	4	BP
GO:0010713	negative regulation of collagen	4/2734	15/17381	0.20034	0.7899	0.77695	CIITA/MIR29A/MIR92A2/PPARD	4	BP
GO:0010715	regulation of extracellular matrix	4/2734	15/17381	0.20034	0.7899	0.77695	DDR2/FGFR4/FSCN1/LRP1	4	BP
GO:0010878	cholesterol storage	4/2734	15/17381	0.20034	0.7899	0.77695	APOB/EHD1/NR1H3/SCARB1	4	BP
GO:0016973	poly(A)+ mRNA export from nucleus	4/2734	15/17381	0.20034	0.7899	0.77695	NXF2/NXF2B/NXF3/ZC3H3	4	BP
GO:0021794	thalamus development	4/2734	15/17381	0.20034	0.7899	0.77695	GBX2/OGDH/SHH/SMO	4	BP
GO:0036066	protein O-linked	4/2734	15/17381	0.20034	0.7899	0.77695	ADAMTS13/ADAMTS7/LFNG/RFNG	4	BP
GO:0036159	inner dynein arm assembly	4/2734	15/17381	0.20034	0.7899	0.77695	CCDC103/CCDC40/DNHD1/LRRC6	4	BP

GO:0045780	positive regulation of	4/2734	15/17381	0.20034	0.7899	0.77695	ADAM8/DEF8/EGFR/PLEKHM1	4	BP
GO:0046852	positive regulation of bone	4/2734	15/17381	0.20034	0.7899	0.77695	ADAM8/DEF8/EGFR/PLEKHM1	4	BP
GO:0050961	detection of temperature stimulus involved in sensory	4/2734	15/17381	0.20034	0.7899	0.77695	ANO1/ARRB2/ASIC3/PRDM12	4	BP
GO:0050965	detection of temperature stimulus involved in sensory	4/2734	15/17381	0.20034	0.7899	0.77695	ANO1/ARRB2/ASIC3/PRDM12	4	BP
GO:0051044	positive regulation of membrane protein ectodomain	4/2734	15/17381	0.20034	0.7899	0.77695	ADAM8/ADRA2A/FURIN/PACSIN3	4	BP
GO:0070828	heterochromatin organization	4/2734	15/17381	0.20034	0.7899	0.77695	CDKN2A/HMGA1/SETD7/TNRC18	4	BP
GO:0072075	metanephric mesenchyme development	4/2734	15/17381	0.20034	0.7899	0.77695	OSR1/PDGFRB/SHH/STAT1	4	BP
GO:0072109	glomerular mesangium development	4/2734	15/17381	0.20034	0.7899	0.77695	BMP4/EGR1/NOTCH1/PDGFRB	4	BP
GO:0072498	embryonic skeletal joint	4/2734	15/17381	0.20034	0.7899	0.77695	AXIN1/BMP4/HOXC11/OSR1	4	BP

GO:1903543	positive regulation of exosomal	4/2734	15/17381	0.20034	0.7899	0.77695	ATP13A2/RAB7A/SDC1/SNF8	4	BP
GO:1904467	regulation of tumor necrosis factor secretion	4/2734	15/17381	0.20034	0.7899	0.77695	HAVCR2/LGALS9/ORM1/ORM2	4	BP
GO:1904754	positive regulation of vascular associated smooth muscle	4/2734	15/17381	0.20034	0.7899	0.77695	AGT/DOCK7/MIR221/MIR451A	4	BP
GO:0036293	response to decreased oxygen levels	61/2734	349/17381	0.20121	0.7929	0.77984	ADAM8/ADM/ALAD/ALAS2/ALKBH5/ANG/AQP1/ASCL2/BAD/CASP1/CASP3/CRYAB/CYP1A1/DNMT3A/DRD2/EGR1/EIF4EBP1/ENDOG/HMOX2/HSD11B2/HSF1/IRAK1/LDHA/LMNA/LOXL2/LTA/MMP2/MTOR/NARFL/NOTCH1/NOX4/NPPA/OPRD1/P2RX2/PDLIM1/PKM/PLAU/PPARD/PSMB11/PSMB6/PSMB7/PSMB8/PSMC3/PSMD13/PSMD3/PSMD5/PSMD7/PTK2B/RAF1/RWDD3/SCAP/SLC29A1/SLC2A4/SLC2A8/SOD3/SUV39H1/TH/TRH/UCP3/[illegible]	61	BP
GO:0006898	receptor-mediated endocytosis	60/2734	343/17381	0.20156	0.7937	0.78067	ACHE/ADM/AP1S1/AP2A2/AP2M1/APOA5/APOB/APOC3/ARHGAP27/ARRB2/ASGR1/CAV3/CCL19/CCL21/CD5/CD5L/CD6/CD63/CD81/CDK5/CLTB/CTTN/CUBN/CXCR1/CXCR2/DLG4/DNM1/DRD2/FOLR2/GAK/HHIPL1/HIP1R/HSP90AA1/HSPG2/INPP5F/LMBR1L/LOXL2/LRP1/LRP10/LRRTM1/NECAB2/NLGN3/OLR1/PDLIM7/PI4KB/RSPO1/SCARA3/SCARB1/SCARF1/SCRIB/SSC4D/STON2/TFR2/TINAGL1/TMPRSS13/TMPRSS4/TNK2/TOR1A/UNC1[illegible]	60	BP
GO:0048732	gland development	73/2734	422/17381	0.20239	0.794	0.78091	ABL1/ASNS/BMP4/CAV3/CCDC40/CCNB2/CLCN2/CRIP1/CSF1/CSF1R/CSN3/CYP1A1/DEAF1/DHODH/DRD2/E2F8/EGFR/EPHB3/ESRP2/FGFR2/FOXN1/GCM2/GFER/GPX1/HLX/HOXA3/HOXB3/HOXB9/HOXD3/HRH2/KRT76/LHX3/LIMS2/MAD1L1/MAPK3/NKX2-3/NKX2-5/NKX2-8/NME1/NOTCH1/NR5A1/NRG1/OTC/OTP/PDGFA/PHF2/PITX1/PKD1/PKM/PLXND1/PSAP/PYGO2/RAF1/RARA/RPS6KA1/SCRIB/SERPINA5/SHH/SLC29A1/SMO/STRA6/TBX19/TBX2/TDGF1/THRA/TNC/WLS/WNT1/WNT10A/WNT11/WNT3A/XDH/ZNF703	73	BP

GO:0006576	cellular biogenic amine metabolic	11/2734	53/17381	0.20256	0.794	0.78091	AGMAT/CHDH/DDC/GDPD1/HAAO/HDC/HNMT/IDO1/OAZ2/PAOX/TRH	11	BP
GO:0006584	catecholamine metabolic	11/2734	53/17381	0.20256	0.794	0.78091	AOC2/DDC/DRD2/GCH1/HDC/ITGAM/LRTOMT/PNKD/RNF180/SNCB/TH	11	BP
GO:0009712	catechol-containing compound metabolic	11/2734	53/17381	0.20256	0.794	0.78091	AOC2/DDC/DRD2/GCH1/HDC/ITGAM/LRTOMT/PNKD/RNF180/SNCB/TH	11	BP
GO:0044273	sulfur compound	11/2734	53/17381	0.20256	0.794	0.78091	ACAN/ACOT7/BCAN/BGN/BLMH/CHAC1/GGT6/GPC1/HEXA/IDS/SGSH	11	BP
GO:0060688	regulation of morphogenesis of a branching structure	11/2734	53/17381	0.20256	0.794	0.78091	ABL1/AGT/BMP4/CAV3/FGFR2/HOXB7/LHX1/PDGFA/SHH/SMO/SOX8	11	BP
GO:2000779	regulation of double-strand break repair	11/2734	53/17381	0.20256	0.794	0.78091	FOXM1/HSF1/MIR221/NUDT16L1/OGG1/OTUB1/PARP9/RECQL5/SETD2/SLF2/TIMELESS	11	BP
GO:0019674	NAD metabolic process	14/2734	70/17381	0.20277	0.7943	0.78123	ALDOA/GAPDH/GCK/HAAO/HK3/LDHA/NMNAT3/OGDH/PARP10/PARP9/PFKM/PKM/QPRT/SLC22A13	14	BP
GO:0042100	B cell proliferation	18/2734	93/17381	0.20298	0.7946	0.7815	ABL1/BCL6/CARD11/CASP3/CD180/CD74/CD81/CLCF1/IFNA5/IL13/INPP5D/MZB1/PRKCD/TIRAP/TLR9/TNFRSF13B/TNFRSF4/WNT3A	18	BP
GO:0035023	regulation of Rho protein signal	24/2734	128/17381	0.20342	0.7949	0.78183	ABL1/ADRA1A/APOC3/ARHGEF10/ARHGEF16/ARHGEF17/ARHGEF25/ARHGEF4/BCL6/EPS8L2/F2RL1/FARP2/GPR17/GPR20/GPR35/MYOC/OBSCN/PDGFRB/PLEKHG4/PLEKHG5/PLEKHG6/PREX2/RAF1/TRIO	24	BP
GO:1903747	regulation of establishment of protein localization to	24/2734	128/17381	0.20342	0.7949	0.78183	ACSM6/ATPIF1/BAD/CYB5R1/DYNLL2/ELMOD1/GPHA2/HNMT/ITGAX/KAT2A/LRRC46/MYBPC1/NBPF3/NRG1/OSCP1/PEMT/PPP1R13B/PSMB7/RNF31/SFN/TSGA13/UBL4B/YWHAQ/ZBTB17	24	BP

GO:0033002	muscle cell proliferation	36/2734	199/17381	0.20346	0.7949	0.78183	ABCC4/AGT/ANG/BMP4/CCL5/CDH13/EGFR/FGFR2/GPER1/IGFBP3/IL13/IL18/ILK/IRAK1/MFN2/MIR15A/MIR221/MIR222/MIR503/MIR96/MMP2/MTOR/NDRG2/NKX2-5/NOTCH1/NQO2/PDGFRB/PPARD/PRKAR1A/RBPMS2/SERPINF2/SF1/SH	36	BP
GO:0006007	glucose catabolic	7/2734	31/17381	0.20518	0.7962	0.78307	ALDOA/BAD/GAPDH/GCK/HK3/PFKM/PKM	7	BP
GO:0006362	transcription elongation from RNA polymerase I	7/2734	31/17381	0.20518	0.7962	0.78307	GTF2H4/POLR1A/POLR1D/POLR1E/POLR2L/TAF1A/ZNRD1	7	BP
GO:0006536	glutamate metabolic	7/2734	31/17381	0.20518	0.7962	0.78307	ALDH4A1/ASL/GAD1/GLUD1/GOT2/NAGS/UROC1	7	BP
GO:0019359	nicotinamide nucleotide biosynthetic process	7/2734	31/17381	0.20518	0.7962	0.78307	HAAO/IDH2/NMNAT3/PARP10/PARP9/QPRT/SLC22A13	7	BP
GO:0019363	pyridine nucleotide biosynthetic	7/2734	31/17381	0.20518	0.7962	0.78307	HAAO/IDH2/NMNAT3/PARP10/PARP9/QPRT/SLC22A13	7	BP
GO:0042462	eye photoreceptor	7/2734	31/17381	0.20518	0.7962	0.78307	GNAT1/MYO7A/NAGLU/NRL/TH/THY1/TULP1	7	BP
GO:0043001	Golgi to plasma membrane protein	7/2734	31/17381	0.20518	0.7962	0.78307	BLZF1/CSK/KIF13A/PKDCC/RAB26/RAB34/VAMP5	7	BP
GO:0048741	skeletal muscle fiber	7/2734	31/17381	0.20518	0.7962	0.78307	ACTA1/BIN3/GPX1/KLHL40/MYOD1/P2RX2/SMO	7	BP
GO:0065005	protein-lipid complex	7/2734	31/17381	0.20518	0.7962	0.78307	APOA5/APOB/APOC3/BIN1/DGAT1/PCDHGA3/SOAT2	7	BP
GO:1903671	negative regulation of sprouting	7/2734	31/17381	0.20518	0.7962	0.78307	MIR16-1/MIR221/MIR222/MIR29C/MIR503/MIR92A2/NOTCH1	7	BP

GO:0006270	DNA replication	9/2734	42/17381	0.20565	0.7962	0.78307	MCM2/MCM4/MCM9/MCMDC2/ORC6/POLA2/POLE/POLE4/TICRR	9	BP
GO:0090342	regulation of cell aging	9/2734	42/17381	0.20565	0.7962	0.78307	ABL1/BCL6/CDKN2A/FOXM1/HMGA1/LMNA/MIR10A/WNT1/YPEL3	9	BP
GO:1903727	positive regulation of phospholipid	9/2734	42/17381	0.20565	0.7962	0.78307	AMBRA1/CCL19/CCL21/CD81/EPHA8/PDGFRB/PRKCD/PTK2B/TNFAIP8L3	9	BP
GO:1901184	regulation of ERBB signaling pathway	19/2734	99/17381	0.20566	0.7962	0.78307	ADRA2A/ADRA2B/ADRA2C/AGT/ARAP1/CDH13/DAB2IP/DOK1/EGFR/ERBB2/FASLG/GPER1/HIP1R/NCF1/NRG1/RAB7A/RHBDF1/SHC1/VPS25	19	BP
GO:0045787	positive regulation of cell cycle	65/2734	374/17381	0.20624	0.7979	0.78478	ABL1/APEX1/ASNS/BRCA1/CCND3/CDK10/CDK4/CDK9/CXCR5/CYP1A1/DAB2IP/DRD2/DYNC1H1/E2F4/E2F8/EGFR/EIF4EBP1/EIF4G1/FGFR2/FNTB/FOSL1/FOXE3/FOXO4/FZD9/GPER1/HCFC1/HSF1/HSPA2/INO80/INS/KIF23/LFNG/MAPRE3/MEIS2/MEPCE/MIR221/MIR222/MIR29A/MLXIPL/MUC1/NUMA1/OPN1MW/PCBP4/PDGFRB/PHOX2B/PIWIL2/PKD1/POLDIP2/PPP2R5B/PRDM9/PTPN11/RARA/RNF112/SFN/SH2B1/SLF2/SSTR5/TGM1/UBE2B/[illegible]	65	BP
GO:0048194	Golgi vesicle budding	15/2734	76/17381	0.207	0.7998	0.78664	CNIH2/COL7A1/GBF1/GOLGA2/GORASP1/GOSR2/MYO18A/SEC16A/SEC16B/STX5/TRAPPC1/TRAPPC2L/TRAPPC3/TRAPPC4/TRAPPC9	15	BP
GO:0070167	regulation of biomineral tissue	15/2734	76/17381	0.207	0.7998	0.78664	AHSG/AMTN/BGLAP/BMP4/DDR2/ECM1/FZD9/IFITM5/ISG15/OSR1/PHOSPHO1/PKDCC/PTK2B/TMEM119/WNT6	15	BP
GO:0051262	protein tetramerization	27/2734	146/17381	0.20724	0.8002	0.78706	ACACB/ACHE/ACPP/ALDOA/ATPIF1/CCL5/CD247/CD74/CDA/CNGB1/CRTC2/CUTC/DNM1/EVL/GNMT/GOLGA2/GPX3/GRIN1/HIST1H4F/HIST2H4A/HIST2H4B/MIP/PDSS1/PEX14/PEX5/PKM/SHMT2	27	BP
GO:0051668	localization within	20/2734	105/17381	0.208	0.8021	0.78895	AGRN/CDH13/CDK5/CEP112/CNIH2/DLG4/DNAJA3/FLNA/FNTA/FZD9/GRIPAP1/ITGAL/KIF17/NRXN2/REEP2/RILPL1/RILPL2/SCRIB/SSNA1/THY1	20	BP

GO:0048608	reproductive structure development	74/2734	429/17381	0.20801	0.8021	0.78895	ADM/ANG/ARRB2/ASCL2/BMP4/BOK/CCNF/CDX2/CRIP1/CSDE1/DLX3/DNAJC19/E2F8/EGFR/EIF2B2/EIF2B5/ETNK2/FANCA/FGFR2/FOSL1/GATA4/GCM2/GJB3/HEY1/HSD17B3/HSF1/IDH1/INHBB/LFNG/LHX1/LHX3/LHX4/LRRC6/MEN1/MGST1/NOS3/NOTCH1/NR5A1/NSDHL/NUDT1/NUP210L/NUPR1/OSR1/PCDH12/PDGFRB/PHLDA2/PKD1/PLCD1/PPARD/PSAP/PTPN11/RARA/REN/RXFP2/SCX/SDC1/SERPINA5/SETD2/SF1/SHH/SOX8/SPINT1/ST14/STRA6/TFEB/TNC/TSPY1/TSPY2/TSPY4/TSPY8/UTF1/VGF/WNT7A/Z[illegible]	74	BP
GO:0016266	O-glycan processing	12/2734	59/17381	0.2088	0.8042	0.79094	A4GNT/B3GNT6/GALNT8/GALNT9/MUC1/MUC2/MUC3A/MUC5AC/MUC5B/MUC6/ST3GAL4/XXYLT1	12	BP
GO:0090307	mitotic spindle assembly	12/2734	59/17381	0.2088	0.8042	0.79094	ARHGEF10/CDC14A/CHMP1A/CHMP4C/FLNA/GOLGA2/KIF23/KIF4A/TUBGCP2/TUBGCP3/TUBGCP5/WRAP73	12	BP
GO:0006906	vesicle fusion	29/2734	158/17381	0.20909	0.8042	0.79099	ADPRHL1/CORO1A/DOC2A/DOC2B/GOSR2/GRTP1/NKD2/RAB20/RAB34/RAB7A/RABGAP1/RABGAP1L/RBSN/SAMD9L/SGSM2/STX1A/STX4/STX5/STX8/STXBP1/SYTL3/TAP1/TBC1D25/TBC1D28/TBC1D9/USP6NL/VAMP1	29	BP
GO:1902275	regulation of chromatin organization	29/2734	158/17381	0.20909	0.8042	0.79099	BCL6/BRCA1/CAMK2D/CDK9/CTBP1/GCG/HDAC8/HMGA1/KAT2A/MAPK3/MUC1/NOC2L/OTUB1/PARP10/PAXIP1/PHF19/PHF2/PIWIL2/PRDM12/PYGO2/RPS6KA4/SETD1A/SETD7/SPI1/SUPT6H/TADA3/TAF7/UBE2B/ZNHIT	29	BP
GO:2000377	regulation of reactive oxygen species metabolic	31/2734	170/17381	0.21049	0.8046	0.79136	ADGRB1/AGT/BRCA1/CLU/CRYAB/DUOXA1/EGFR/EIF5A/F2RL1/FOXM1/GCH1/GRIN1/HSP90AA1/INS/ITGAM/MIR92A2/MMP3/MTOR/MYCN/NOX4/NOXO1/NQO2/PDGFRB/PID1/PRCP/PRKCD/PTK2B/TRAP1/TUSC2/XDH/ZNF205	31	BP
GO:0032410	negative regulation of transporter	16/2734	82/17381	0.21062	0.8046	0.79136	ADRA2A/CAMK2D/CAV3/CRHR1/DRD2/GNB5/GPR35/GSTM2/MIR153-1/MIR212/OSR1/RRAD/THADA/TLR9/TRDN/WWP2	16	BP
GO:0071214	cellular response to abiotic stimulus	52/2734	296/17381	0.21127	0.8046	0.79136	ADIRF/AGT/AQP1/ATP1A1/BAD/CASP1/CASP3/CASP5/CNGB1/COL1A1/CRIP1/CRYAB/DDB1/DDB2/EGFR/EGR1/EIF2AK4/ENG/FNTA/FNTB/GNAT1/GUCA1B/GUCY2D/HRAS/HSF1/HSPA5/IL13/INO80/KCNK4/LTBR/MAP3K14/MAPK3/NEUROD2/NOC2L/NOX4/NPPA/NSMF/OPN4/OSR1/PDE2A/PPEF1/PTPN11/SCX/SIPA1/SLC2A4/TAF1/TLR8/TMEM109/TMEM150C/TRPV4/	52	BP

GO:0104004	cellular response to environmental stimulus	52/2734	296/17381	0.21127	0.8046	0.79136	ADIRF/AGT/AQP1/ATP1A1/BAD/CASP1/CASP3/CASP5/CNGB1/COL1A1/CRIP1/CRYAB/DDB1/DDB2/EGFR/EGR1/EIF2AK4/ENG/FNTA/FNTB/GNAT1/GUCA1B/GUCY2D/HRAS/HSF1/HSPA5/IL13/INO80/KCNK4/LTBR/MAP3K14/MAPK3/NEUROD2/NOC2L/NOX4/NPPA/NSMF/OPN4/OSR1/PDE2A/PPEF1/PTPN11/SCX/SIPA1/SLC2A4/TAF1/TLR8/TMEM109/TMEM150C/TRPV4/	52	BP
GO:0032886	regulation of microtubule-based process	33/2734	182/17381	0.21153	0.8046	0.79136	ABL1/ANKRD53/BRCA1/CATSPER1/CAV3/CCDC40/CCNF/CDK2AP2/CHMP1A/CHMP4C/CIB1/CNIH2/DNAH11/DYNC1H1/ERBB2/FES/KATNB1/MAP6D1/MCPH1/MEMO1/MID1IP1/NUBP1/NUMA1/PARP3/PKD1/RASSF7/SENP6/STMN4/TRAF3IP1/TRIM54/TRPV4/WNT3A/XRCC3	33	BP
GO:0002700	regulation of production of molecular mediator of immune	22/2734	117/17381	0.21187	0.8046	0.79136	BCL6/CD74/CLCF1/CUEDC2/F2RL1/HLA-E/IL13/IL4R/KARS/MAPK3/MZB1/PAXIP1/PGC/SEMA7A/SPINK5/SPON2/STX4/SUPT6H/TLR9/TNFRSF4/TNFSF13/TRAF2	22	BP
GO:0009410	response to xenobiotic	22/2734	117/17381	0.21187	0.8046	0.79136	AIP/AKR7A3/AOC2/AS3MT/BPHL/CES2/CYP1A1/CYP1A2/CYP2W1/DPEP1/EPHX2/GPX1/GRIN1/GSTM1/GSTM2/LPO/MGST1/MGST3/NQO2/POR/RO	22	BP
GO:1902904	negative regulation of supramolecular	22/2734	117/17381	0.21187	0.8046	0.79136	ARAP1/CAPZA3/CAV3/CIB1/CLU/CORO1A/CORO1B/CRYAB/HIP1R/KATNB1/LMOD1/MAP6D1/MID1IP1/MYOC/PFN1/PRKCD/SCIN/SPTAN1/SPTB/SPTBN2/TRIM54/WAS	22	BP
GO:1903825	organic acid transmembrane transport	22/2734	117/17381	0.21187	0.8046	0.79136	ACACB/AGT/CPT2/MID1IP1/OSR1/PQLC2/SLC1A7/SLC22A12/SLC22A9/SLC25A20/SLC25A22/SLC36A1/SLC36A3/SLC38A1/SLC38A10/SLC38A8/SLC6A18/SLC6A6/SLC6A7/SLC6A8/SLC6A9/SLC7A7	22	BP
GO:0048284	organelle fusion	34/2734	188/17381	0.21194	0.8046	0.79136	ADPRHL1/CORO1A/DOC2A/DOC2B/GOSR2/GRTP1/MFN1/MFN2/NKD2/PCDHGA3/PID1/RAB20/RAB34/RAB7A/RABGAP1/RABGAP1L/RBSN/SAMD9L/SGSM2/STX1A/STX4/STX5/STX8/STXBP1/SYTL3/TAP1/TBC1D25/TBC1D28/TBC1D9/USP6NL/VAMP1/VAMP5/VAT1/VTI1B	34	BP
GO:0060047	heart contraction	48/2734	272/17381	0.2123	0.8046	0.79136	ADM/ADRA1A/ADRA1B/AGT/ATP1A1/ATP1A4/ATP2A3/CACNA1F/CACNA1G/CACNA1H/CACNA1S/CACNB1/CACNB3/CACNG1/CAMK2D/CAV3/CHGA/DES/DRD2/EDN2/FLNA/GATA4/GCH1/GNAO1/GPX1/GSTM2/HSPB7/ITPR3/KCND1/KCND3/KCNH6/KCNJ5/KCNQ1/MAP2K3/MIR328/MIR92A2/MTOR/MYBPC3/MYL4/NKX2-	48	BP

GO:0007601	visual perception	37/2734	206/17381	0.21276	0.8046	0.79136	ABCC6/AIPL1/AOC2/ARR3/BBS9/CACNA1F/CDH23/CLN6/CNGB1/COL1A1/DNAJC19/GLRA1/GNAT1/GUCA1B/GUCY2D/HPS1/KIFC3/KRT12/LAMC3/MIP/MYO3A/MYO7A/NOB1/NRL/OAT/OPN1MW/OPN4/PDE6D/RGS16/SEMA5B/SFRP5/TGFBI/TH/TULP1/UNC119/VAX2/WFS1	37	BP
GO:0044282	small molecule catabolic process	65/2734	375/17381	0.21288	0.8046	0.79136	ACACB/ACAD10/ACADVL/ACOXL/ACSF3/ALDH1L1/ALDH2/ALDH4A1/ALDOA/AMDHD2/BAD/BCKDK/BLMH/CARNS1/CPT2/CROT/CRYM/CYP1A1/DLST/ENTPD8/FAH/FUT7/GAD1/GALK1/GALM/GALT/GAPDH/GCK/GLUD1/GLYCTK/GOT2/GPT/GSTZ1/HAAO/HADHA/HAO2/HDC/HEXA/HK3/HNMT/HYKK/IDO1/INPP5E/MTOR/NAGK/NOS3/NPL/NUDT1/NUDT18/OGDH/OTC/PADI4/PFKM/PHYKPL/PIPOX/PKM/PLA2G15/PNKD/PPARD/QPR[illegible]	65	BP
GO:0015850	organic hydroxy compound transport	41/2734	230/17381	0.21311	0.8046	0.79136	ABCA2/ABCC3/ABCG5/ADRA2A/ADRA2B/ADRA2C/AGT/APOA5/APOB/APOC3/AQP1/AQP10/ASIC3/C1QTNF1/CHGA/CHRNA6/CLU/CRHR1/DRD2/DTNBP1/LRP1/NISCH/NPC1L1/NPC2/NR1H3/OSBPL5/OSBPL7/PTPN11/REN/SCARB1/SHH/SLC22A1/SLC26A6/SLC6A2/SLCO2B1/SOAT2/STARD3/ST[illegible]	41	BP
GO:0009185	ribonucleoside diphosphate metabolic	23/2734	123/17381	0.21345	0.8046	0.79136	AK5/ALDOA/BAD/CARD11/DLG4/GALK1/GAPDH/GCK/HK3/INS/LDHA/MLXIPL/NCOR1/NUDT18/NUP210/NUP98/OGDH/OGDHL/PFKFB1/PFKM/PGAM4/PKM/SCRIB	23	BP
GO:0031623	receptor internalization	17/2734	88/17381	0.21375	0.8046	0.79136	ACHE/ADM/AP2M1/ARRB2/CAV3/CD63/CD81/CXCR1/CXCR2/DLG4/DNM1/DRD2/LRP1/LRRTM1/NECAB2/RSPO1/WNT3A	17	BP
GO:0051341	regulation of oxidoreductase activity	17/2734	88/17381	0.21375	0.8046	0.79136	ABL1/AGT/CAV3/CCS/EDN2/EGFR/GCH1/GCHFR/HSP90AA1/IL13/INS/NOD1/NOS3/NOSTRIN/POR/PTK2B/SCARB1	17	BP
GO:0010518	positive regulation of phospholipase	10/2734	48/17381	0.21395	0.8046	0.79136	ADRA1A/AGT/ANG/CCL5/EGFR/FGFR2/PDGFRB/PLA2G1B/PLA2G5/PLCB2	10	BP
GO:0010662	regulation of striated muscle cell apoptotic	10/2734	48/17381	0.21395	0.8046	0.79136	AGT/AMBRA1/CAMK2D/EIF5A/HSF1/ILK/LTK/MIR16-1/MIR195/NKX2-5	10	BP
GO:0060986	endocrine hormone	10/2734	48/17381	0.21395	0.8046	0.79136	AGT/AQP1/C1QTNF1/CRHR1/HCAR2/INHBB/PTPN11/RAB11FIP3/REN/TACR2	10	BP

GO:1903078	positive regulation of protein localization to	10/2734	48/17381	0.21395	0.8046	0.79136	ARHGEF16/CIB1/CNPY4/EGFR/GPER1/LRP1/NKD2/STX4/TREM2/WNT3A	10	BP
GO:0003281	ventricular septum	13/2734	65/17381	0.21402	0.8046	0.79136	BMP4/FGFR2/FGFRL1/FZD2/GATA4/HEY1/NKX2-5/NOTCH1/NPRL3/SMAD6/STRA6/SUFU/WNT11	13	BP
GO:0009880	embryonic pattern	13/2734	65/17381	0.21402	0.8046	0.79136	C2CD3/EFNB1/FGFR2/LHX1/MESP2/NOTO/RIPPLY1/SEMA3F/SHH/SMAD6/TDGF1/WNT1/WNT7A	13	BP
GO:0042308	negative regulation of protein import	13/2734	65/17381	0.21402	0.8046	0.79136	CD27/DAB2IP/EMD/FAM89B/MTOR/NFKBIL1/PARP10/PBLD/PDE2A/PKD1/SFRP5/SUFU/THRA	13	BP
GO:0046782	regulation of viral	13/2734	65/17381	0.21402	0.8046	0.79136	CCL5/CDK9/NELFB/PFN1/POLR2G/POLR2L/PSMC3/RSF1/TARBP2/TRIM11/TRIM14/TRIM31/TRIM62	13	BP
GO:0048864	stem cell development	13/2734	65/17381	0.21402	0.8046	0.79136	BMP4/CFL1/EFNB1/GBX2/MAPK3/NRG1/PHOX2B/SEMA3F/SETD2/SHH/SMO/SOX8/WNT7A	13	BP
GO:0072091	regulation of stem cell	13/2734	65/17381	0.21402	0.8046	0.79136	DISC1/DRD2/LTBP3/MIR221/MIR222/NOTCH1/OTP/PDCD2/SETD1A/SHH/SMO/THPO/VAX1	13	BP
GO:0072171	mesonephric tubule morphogenesis	13/2734	65/17381	0.21402	0.8046	0.79136	AGT/BMP4/EYA1/HOXB7/ILK/LHX1/OSR1/SHH/SMO/SOX8/WNT1/WNT11/WNT6	13	BP
GO:1904590	negative regulation of	13/2734	65/17381	0.21402	0.8046	0.79136	CD27/DAB2IP/EMD/FAM89B/MTOR/NFKBIL1/PARP10/PBLD/PDE2A/PKD1/SFRP5/SUFU/THRA	13	BP
GO:0045580	regulation of T cell differentiation	24/2734	129/17381	0.21483	0.8046	0.79136	ABL1/ADAM8/BAD/BCL6/BMP4/CARD11/CCL19/CD27/CD74/ERBB2/FANCA/FOXN1/HLX/IL18/IL4R/LGALS9/PIK3R6/PRELID1/RARA/SART1/SHH/SOX13/SPINK5/TNFRSF18	24	BP
GO:0014044	Schwann cell development	6/2734	26/17381	0.21553	0.8046	0.79136	ARHGEF10/CDK5/ILK/MYOC/PARD3/POU3F1	6	BP
GO:0018195	peptidyl-arginine	6/2734	26/17381	0.21553	0.8046	0.79136	COPRS/NDUFAF7/PADI3/PADI4/PADI6/PRDM14	6	BP

GO:0019054	modulation by virus of host process	6/2734	26/17381	0.21553	0.8046	0.79136	BAD/CPSF4/EIF2AK4/MIR221/MIR222/SCRIB	6	BP
GO:0033687	osteoblast proliferation	6/2734	26/17381	0.21553	0.8046	0.79136	ABL1/FGFR2/PLXNB1/SOX8/TMEM119/TNN	6	BP
GO:0034243	regulation of transcription elongation from RNA polymerase II	6/2734	26/17381	0.21553	0.8046	0.79136	AXIN1/NELFB/RECQL5/SHH/SUPT6H/ZMYND11	6	BP
GO:0042481	regulation of odontogenesis	6/2734	26/17381	0.21553	0.8046	0.79136	AMTN/BMP4/CSF1/SHH/WNT10A/WNT6	6	BP
GO:0045577	regulation of B cell	6/2734	26/17381	0.21553	0.8046	0.79136	BAD/CARD11/CD27/INPP5D/PPP2R3C/TLR9	6	BP
GO:0045992	negative regulation of embryonic	6/2734	26/17381	0.21553	0.8046	0.79136	BMP4/LFNG/SUFU/TBX2/TRAF3IP1/TULP3	6	BP
GO:0046688	response to copper ion	6/2734	26/17381	0.21553	0.8046	0.79136	AQP1/CYP1A1/CYP1A2/HSF1/LOXL2/SOD3	6	BP
GO:0048011	neurotrophin TRK receptor signaling	6/2734	26/17381	0.21553	0.8046	0.79136	AGT/CASP3/PPP2R5B/PTPN11/RAF1/ZFYVE27	6	BP
GO:0061217	regulation of mesonephros development	6/2734	26/17381	0.21553	0.8046	0.79136	AGT/BMP4/HOXB7/LHX1/SMO/SOX8	6	BP
GO:0071450	cellular response to	6/2734	26/17381	0.21553	0.8046	0.79136	ADPRHL2/GCH1/MPO/NOS3/PRDX1/SOD3	6	BP
GO:0071451	cellular response to	6/2734	26/17381	0.21553	0.8046	0.79136	ADPRHL2/GCH1/MPO/NOS3/PRDX1/SOD3	6	BP

GO:0071875	adrenergic receptor signaling	6/2734	26/17381	0.21553	0.8046	0.79136	ADRA1A/ADRA1B/ADRA2A/ADRA2B/ADRA2C/CHGA	6	BP
GO:0072207	metanephric epithelium development	6/2734	26/17381	0.21553	0.8046	0.79136	AQP1/OSR1/PKD1/POU3F3/SOX8/STAT1	6	BP
GO:0086013	membrane repolarization during cardiac muscle cell action potential	6/2734	26/17381	0.21553	0.8046	0.79136	ATP1A1/FLNA/KCND3/KCNJ5/KCNQ1/MIR328	6	BP
GO:1903393	positive regulation of adherens junction	6/2734	26/17381	0.21553	0.8046	0.79136	ABL1/COL16A1/KDR/MYOC/PTPN23/THY1	6	BP
GO:0006282	regulation of DNA repair	18/2734	94/17381	0.21645	0.8065	0.79321	APBB1/BRCA1/BRCC3/CDK9/EGFR/EYA1/FOXM1/HSF1/MIR221/NPAS2/NUDT16L1/OGG1/OTUB1/PARP9/RECQL5/SETD2/SLF2/TIMELESS	18	BP
GO:1902750	negative regulation of cell cycle G2/M	18/2734	94/17381	0.21645	0.8065	0.79321	BRCA1/CDK5RAP3/CHMP4C/FOXO4/GPR132/MIIP/MIR195/PSMB11/PSMB6/PSMB7/PSMB8/PSMC3/PSMD13/PSMD3/PSMD5/PSMD7/RINT1/TICRR	18	BP
GO:2000736	regulation of stem cell differentiation	26/2734	141/17381	0.21709	0.8065	0.79321	ABL1/BMP4/GATA4/HIST1H4F/HIST2H4A/HIST2H4B/HOXB4/LDB1/LMO1/LTBP3/NKX2-5/NOTCH1/OCIAD1/PSMB11/PSMB6/PSMB7/PSMB8/PSMC3/PSMD13/PSMD3/PSMD5/PSMD7/SETD1A/SOX5/SPI1/WNT3A	26	BP
GO:0009395	phospholipid catabolic	8/2734	37/17381	0.21712	0.8065	0.79321	IDH1/INPP5F/PLA2G15/PLA2G4B/PRDX6/PRKCD/SCARB1/SMPD4	8	BP
GO:0014075	response to amine	8/2734	37/17381	0.21712	0.8065	0.79321	ADAMTS13/DRD2/GRIN1/HNMT/NME1/PPP1R1B/TH/UROS	8	BP
GO:0016572	histone phosphorylation	8/2734	37/17381	0.21712	0.8065	0.79321	BAZ1B/CDK9/MAPK3/NEK11/PPP1R1B/PRKCD/RPS6KA4/UBE2B	8	BP

GO:0031076	embryonic camera-type eye development	8/2734	37/17381	0.21712	0.8065	0.79321	IFT140/NES/RARA/STRA6/TBX2/TH/TRAF3IP1/TULP3	8	BP
GO:0070897	DNA-templated transcriptional preinitiation complex	8/2734	37/17381	0.21712	0.8065	0.79321	BRF1/MED6/POLR1E/PSMC3/TAF1/TAF1L/TAF7/THRA	8	BP
GO:0001701	in utero embryonic development	55/2734	315/17381	0.21763	0.8079	0.79459	ADM/APOB/ASCL2/B9D1/BRK1/C2CD3/CCNB2/CDX2/CHD8/DAD1/E2F8/EGFR/EMX1/ENDOG/ETNK2/FGFR2/FOSL1/GCM2/GJB3/HEY1/HINFP/HORMAD1/HSF1/IFITM5/JAG2/KAT2A/MFN2/MGAT1/MYO1E/NLE1/NOS3/NOTCH1/NSDHL/PCDH12/PCGF2/PHLDA2/PKD1/PLCD1/PLOD3/PRDM14/PSMC3/PYGO2/RARRES2/SETD2/SMO/SOX8/SPINT1/ST14/TFEB/TIE1/TMEM	55	BP
GO:0016125	sterol metabolic process	28/2734	153/17381	0.2188	0.809	0.7957	ACACB/ACADVL/APOA5/APOB/CLN6/CUBN/CYB5R1/CYB5R2/CYP11A1/CYP11B2/DGKQ/EBPL/EPHX2/HSD17B7/LEPR/LMF1/NPC1L1/NPC2/NSDHL/OSBPL5/PMVK/POR/PPARD/SCAP/SCARB1/SCARF1/SOAT2/STARD3	28	BP
GO:0031345	negative regulation of cell projection	28/2734	153/17381	0.2188	0.809	0.7957	ARHGAP4/CCL21/CDK5/CIB1/DAB1/DGUOK/DRAXIN/EFNA1/EVL/GAK/GORASP1/INPP5F/LINGO1/LRIG2/LRP1/NLGN3/NR2F1/PLXNB3/PRKCD/RAP1GAP/RGMA/RTN4RL1/RTN4RL2/SEMA3F/THY1/TLX2/TRPV4/WNT3A	28	BP
GO:0033673	negative regulation of kinase activity	48/2734	273/17381	0.22022	0.809	0.7957	ABL1/BGN/BMP4/CAMK2N2/CASP3/CAV3/CDK5RAP3/CDKN2A/CHAD/CIB1/CISH/COX11/CRIPAK/CSK/DAB2IP/DTNBP1/DUSP2/DUSP21/DUSP26/DUSP5/GBA/HSPB1/ILK/INCA1/LRRTM1/MAPK8IP1/MEN1/MIR92A2/MYCNOS/PDCD4/PPP1R1B/PREX2/PRKAR1A/PRKAR1B/PRKCD/PRKRIP1/PYDC1/RGS14/RTN4RL1/RTN4RL2/SFN/SH3BP5/SH3BP5L/SMYD3/TAF7/TARBP	48	BP
GO:0009100	glycoprotein metabolic process	74/2734	431/17381	0.22055	0.809	0.7957	A4GNT/ADAMTS13/ADAMTS7/ALG10/ALG3/ASGR1/B3GAT3/B3GNT6/B4GALT2/B4GALT7/BCAN/BGN/CCL19/CCL21/CHPF/CHPF2/CHST12/CHST13/CHST15/CHST7/DAD1/DDOST/DOLPP1/DPM2/DSE/EXTL1/FBXO2/FBXO6/FKTN/FUT7/GAL3ST4/GALNT16/GALNT8/GALNT9/GBGT1/GGTA1P/GOLGA2/GORASP1/GPC1/HEXA/IDS/LFNG/LMF1/MGAT1/MGAT4B/MGAT5B/MIR181B1/MUC1/MUC2/MUC3A/MUC5AC/MUC5B/MUC6/OS9/OST4/PARP10/PARP2/PARP3/PARP9/PLOD3/PORCN/PPARD/RFNG/RPN1/SDF2/SGSH/SLC34A1/SPOCK2/ST3GAL4/SYVN1/TINF2/TMEM115/XXYLT1/XYLT2	74	BP

GO:0021879	forebrain neuron	11/2734	54/17381	0.22068	0.809	0.7957	AXIN1/CSF1R/DCLK2/DISC1/DRD2/FGFR2/GBX2/LHX5/OGDH/OTP/TBR1	11	BP
GO:0006805	xenobiotic metabolic	20/2734	106/17381	0.22081	0.809	0.7957	AIP/AKR7A3/AOC2/AS3MT/BPHL/CES2/CYP1A1/CYP1A2/CYP2W1/DPEP1/EPHX2/GRIN1/GSTM1/GSTM2/LPO/MGST1/MGST3/NQO2/POR/RORC	20	BP
GO:2000058	regulation of protein ubiquitination involved in ubiquitin-dependent protein [illegible]	20/2734	106/17381	0.22081	0.809	0.7957	ANAPC15/ANAPC2/AXIN1/BUB1B/CAV3/CLU/DISC1/PSMB11/PSMB6/PSMB7/PSMB8/PSMC3/PSMD13/PSMD3/PSMD5/PSMD7/PTK2B/SUFU/TAF1/WNT1	20	BP
GO:0006469	negative regulation of protein kinase activity	45/2734	255/17381	0.22104	0.809	0.7957	ABL1/BGN/BMP4/CAMK2N2/CASP3/CAV3/CDK5RAP3/CDKN2A/CHAD/CIB1/CISH/CRIPAK/DAB2IP/DTNBP1/DUSP2/DUSP21/DUSP26/DUSP5/GBA/HSPB1/ILK/INCA1/LRRTM1/MAPK8IP1/MEN1/MIR92A2/PDCD4/PPP1R1B/PREX2/PRKAR1A/PRKAR1B/PRKCD/PRKRIP1/PYDC1/RGS14/RTN4RL1/RTN4RL2/SFN/SH3BP5/SH3BP5L/SMYD3/TAF7/TARBP2/THY1/VPS25	45	BP
GO:0030072	peptide hormone secretion	43/2734	243/17381	0.22146	0.809	0.7957	ADRA2A/ADRA2C/ANO1/AQP1/ARL2BP/BAD/BLK/BRSK2/CAPN10/CCL5/CDK16/CPLX1/CRHR1/DOC2B/DRD2/EGFR/FGA/GCG/GCK/GLUD1/GPER1/IL1RN/INHBB/INS/ITPR3/MAFA/MTNR1B/PFKM/PPARD/PTPN11/PTPRN2/RAF1/RFX6/SIDT2/SLC25A5/SSTR5/STX1A/STX4/TACR2/TFR2/TRH/TRPV	43	BP
GO:0033500	carbohydrate homeostasis	39/2734	219/17381	0.22192	0.809	0.7957	ADRA1B/ADRA2A/ANO1/BAD/BRSK2/CAV3/CDK16/CRTC2/ENDOG/FGFR4/GATA4/GCG/GCK/GPER1/HK3/INS/LEPR/MEN1/MIRLET7G/MLXIPL/MTNR1B/NME1/NOX4/PFKM/PRCP/PTPN11/PTPRN2/RAF1/RFX6/SIDT2/SLC29A1/SLC2A4/SPOP/SSTR5/STX4/TH/TRPV4/VGF/WFS1	39	BP
GO:0042593	glucose homeostasis	39/2734	219/17381	0.22192	0.809	0.7957	ADRA1B/ADRA2A/ANO1/BAD/BRSK2/CAV3/CDK16/CRTC2/ENDOG/FGFR4/GATA4/GCG/GCK/GPER1/HK3/INS/LEPR/MEN1/MIRLET7G/MLXIPL/MTNR1B/NME1/NOX4/PFKM/PRCP/PTPN11/PTPRN2/RAF1/RFX6/SIDT2/SLC29A1/SLC2A4/SPOP/SSTR5/STX4/TH/TRPV4/VGF/WFS1	39	BP
GO:0048332	mesoderm morphogenesis	15/2734	77/17381	0.22214	0.809	0.7957	AXIN1/BMP4/CRB2/EYA1/FGFR2/KDM6B/MESP2/PRKAR1A/SCX/SETD2/TLX2/TXNRD1/WLS/WNT11/WNT3A	15	BP

GO:0051952	regulation of amine transport	15/2734	77/17381	0.22214	0.809	0.7957	ADRA2A/ADRA2B/ADRA2C/AGT/CHGA/CHRNA6/DRD2/DTNBP1/OSR1/STX1A/STXBP1/SV2A/TACR2/TOR1A/TRH	15	BP
GO:0055006	cardiac cell development	15/2734	77/17381	0.22214	0.809	0.7957	AGT/BMP4/CAV3/GATA4/LMNA/MIR195/MTOR/NKX2-5/NOTCH1/NPPA/OBSL1/PDCD4/PDGFRB/PDLIM5/TCAP	15	BP
GO:0061333	renal tubule morphogenesis	15/2734	77/17381	0.22214	0.809	0.7957	AGT/BMP4/EYA1/HOXB7/ILK/IRX3/LHX1/OSR1/PKD1/SHH/SMO/SOX8/WNT1/WNT11/WNT6	15	BP
GO:1903038	negative regulation of leukocyte cell-	21/2734	112/17381	0.22257	0.809	0.7957	BCL6/BMP4/CASP3/CCL21/CD74/ERBB2/HAVCR2/HLX/IDO1/IL20RB/IL4R/LGALS3/LGALS9/MAD1L1/MIR221/MIR222/PLA2G2F/PRKAR1A/SCGB1A1/SHH/VSIG4	21	BP
GO:1905477	positive regulation of protein localization to	21/2734	112/17381	0.22257	0.809	0.7957	ARHGEF16/BAD/CDK5/CIB1/CNPY4/DYNLL2/EGFR/ERBB2/GPER1/ITGAM/LRP1/MIEF2/MYO1C/NECAB2/NKD2/PPP1R13B/SFN/STX4/TREM2/WNT3A/YWHAQ	21	BP
GO:0032612	interleukin-1 production	16/2734	83/17381	0.2253	0.809	0.7957	ARRB2/CASP1/CASP5/CCL19/EGR1/F2RL1/GSDMD/HAVCR2/HSPB1/LGALS9/NLRP1/NOD1/ORM1/ORM2/PYDC1/TLR8	16	BP
GO:0044728	DNA methylation or	16/2734	83/17381	0.2253	0.809	0.7957	APEX1/BRCA1/DDX4/DNMT3A/EHMT1/EHMT2/FKBP6/MIR29A/MIR29C/MPHOSPH8/PIWIL2/PRDM14/RLF/SPI1/TDRD1/TDRD9	16	BP
GO:0007617	mating behavior	5/2734	21/17381	0.22605	0.809	0.7957	FUOM/GRIN1/PPP1R1B/TH/THRA	5	BP
GO:0008535	respiratory chain complex IV assembly	5/2734	21/17381	0.22605	0.809	0.7957	COA3/COA4/COX10/COX11/SMIM20	5	BP
GO:0019370	leukotriene biosynthetic process	5/2734	21/17381	0.22605	0.809	0.7957	GGT6/GGTA1P/MGST3/PLA2G1B/PLA2G5	5	BP
GO:0034162	toll-like receptor 9	5/2734	21/17381	0.22605	0.809	0.7957	HAVCR2/IRAK1/TLR8/TLR9/TNIP2	5	BP
GO:0035929	steroid hormone secretion	5/2734	21/17381	0.22605	0.809	0.7957	AGT/C1QTNF1/CRHR1/PTPN11/REN	5	BP

GO:0036499	PERK-mediated unfolded protein response	5/2734	21/17381	0.22605	0.809	0.7957	ASNS/ATF3/BOK/HERPUD1/HSPA5	5	BP
GO:0045061	thymic T cell selection	5/2734	21/17381	0.22605	0.809	0.7957	CARD11/CD74/FOXN1/JAG2/SHH	5	BP
GO:0045624	positive regulation of T-helper cell	5/2734	21/17381	0.22605	0.809	0.7957	CCL19/HLX/IL18/IL4R/RARA	5	BP
GO:0050951	sensory perception of temperature	5/2734	21/17381	0.22605	0.809	0.7957	ANO1/ARRB2/ASIC3/KCNK4/PRDM12	5	BP
GO:0098743	cell aggregation	5/2734	21/17381	0.22605	0.809	0.7957	BARX2/MYCN/PKD1/THRA/WNT7A	5	BP
GO:0014823	response to activity	12/2734	60/17381	0.22619	0.809	0.7957	AGT/ALAD/BGLAP/FNDC5/HSF1/KDM6B/PERM1/PPARD/SLC25A25/TH/TNS2/UCP3	12	BP
GO:0031110	regulation of microtubule polymerization or	12/2734	60/17381	0.22619	0.809	0.7957	ABL1/ANKRD53/CAV3/CIB1/FES/KATNB1/MAP6D1/MID1IP1/NUMA1/STMN4/TRIM54/TRPV4	12	BP
GO:0071242	cellular response to	12/2734	60/17381	0.22619	0.809	0.7957	ABL1/AGRN/CASP3/CASP6/CHRM1/HCN3/LYPD1/MAPK3/OPRM1/PPP1R9B/RGS10/SLC34A1	12	BP
GO:0007528	neuromuscular junction development	9/2734	43/17381	0.22634	0.809	0.7957	AGRN/CACNB1/CACNB3/DNAJA3/ERBB2/FNTA/FZD9/P2RX2/TNC	9	BP
GO:1901224	positive regulation of NIK/NF-	9/2734	43/17381	0.22634	0.809	0.7957	EGFR/HAVCR2/ILK/IRAK1/NOD1/PDCD4/PTP4A3/TIRAP/TRAF2	9	BP
GO:0030048	actin filament-based movement	24/2734	130/17381	0.22654	0.809	0.7957	ACTA1/ATP1A1/CACNA1G/CAMK2D/CAV3/DES/FLNA/GATA4/KCND3/KCNJ5/KCNQ1/MIR328/MLPH/MOBP/MYBPC1/MYBPC3/MYL4/MYL6B/MYO1E/MYO7A/TCAP/TNNC1/TPM2/WAS	24	BP

GO:0061458	reproductive system development	74/2734	432/17381	0.22697	0.809	0.7957	ADM/ANG/ARRB2/ASCL2/BMP4/BOK/CCNF/CDX2/CRIP1/CSDE1/DLX3/DNAJC19/E2F8/EGFR/EIF2B2/EIF2B5/ETNK2/FANCA/FGFR2/FOSL1/GATA4/GCM2/GJB3/HEY1/HSD17B3/HSF1/IDH1/INHBB/LFNG/LHX1/LHX3/LHX4/LRRC6/MEN1/MGST1/NOS3/NOTCH1/NR5A1/NSDHL/NUDT1/NUP210L/NUPR1/OSR1/PCDH12/PDGFRB/PHLDA2/PKD1/PLCD1/PPARD/PSAP/PTPN11/RARA/REN/RXFP2/SCX/SDC1/SERPINA5/SETD2/SF1/SHH/SOX8/SPINT1/ST14/STRA6/TFEB/TNC/TSPY1/TSPY2/TSPY4/TSPY8/UTF1/VGF/WNT7A/Z[illegible]	74	BP
GO:0050671	positive regulation of lymphocyte	25/2734	136/17381	0.22751	0.809	0.7957	BCL6/CARD11/CCDC88B/CCL19/CCL5/CCR2/CD6/CD74/CD81/CLCF1/CLECL1/CORO1A/DNAJA3/EFNB1/HAVCR2/HLA-E/IL13/IL18/LGALS9/SHH/TACR1/TIRAP/TLR9/TNFRSF4/WNT3A	25	BP
GO:0009798	axis specification	17/2734	89/17381	0.228	0.809	0.7957	BMP4/C2CD3/CDX2/LDB1/LHX1/NOTCH1/RIPPLY1/SHH/SMAD6/SMO/SOST/TDGF1/VAX2/WLS/WNT1/WNT6/WNT7A	17	BP
GO:0043087	regulation of GTPase activity	81/2734	475/17381	0.22805	0.809	0.7957	ACAP1/ACAP3/ADAP1/ADPRHL1/AGAP3/AGRN/ALS2CL/ARAP1/ARFGAP2/ARHGAP10/ARHGAP22/ARHGAP27/ARHGAP30/ARHGAP4/ARHGEF10/ARHGEF16/ARRB2/AXIN1/BCAS3/BCL6/CCL1/CCL19/CCL21/CCL5/CDC42EP2/DAB2IP/DGKI/DOCK1/DOCK7/DVL2/ELMOD1/EPHB3/ERBB2/F2RL1/GNAO1/GNB5/GPSM1/GRHL3/GRTP1/HACD3/HRAS/LARS/LLGL1/MLST8/MTOR/NPRL3/PDE6D/PGAM5/PLEKHG4/PLEKHG6/PLXNB1/PLXNB3/PLXND1/PREX2/PROM2/PTK2B/PTPRN2/RABGAP1/RABGAP1L/RAP1GAP/RASA3/RASA4B/RASGRP2/RGS10/RGS14/RGS16/RIN3/RTKN/SCRIB/SGSM2/SIPA1/STARD8/TAX1BP3/TBC1D25/TBC1D28/TBC1D9/THY1/TNK2/USP6N[illegible]	81	BP
GO:0000187	activation of MAPK activity	26/2734	142/17381	0.22833	0.809	0.7957	ADRA2B/AXIN1/CCL19/CD74/CD81/DAB2IP/DUSP5/ERN2/GNG3/HACD3/IRAK1/KARS/MADD/MAP2K3/MAP3K6/MAP4K2/MAPK3/MAPK8IP3/MAPKAPK3/NOD1/NRG1/PEA15/PLA2G1B/PTPN11/SHC1/TDGF1	26	BP
GO:0048754	branching morphogenesis of an epithelial	27/2734	148/17381	0.22903	0.809	0.7957	ABL1/AGT/BMP4/CAV3/CSF1/CTSH/ENG/ESRP2/EYA1/FGFR2/GBX2/GDF2/HHIP/HOXB7/ILK/LHX1/MYCN/NOTCH1/PKD1/PLXND1/SHH/SMO/SOX8/TIMELESS/TNC/WNT1/WNT6	27	BP
GO:0003298	physiological muscle hypertrophy	7/2734	32/17381	0.22951	0.809	0.7957	AGT/CAV3/GATA4/MIR195/MTOR/NPPA/PDLIM5	7	BP

GO:0003301	physiological cardiac muscle hypertrophy	7/2734	32/17381	0.22951	0.809	0.7957	AGT/CAV3/GATA4/MIR195/MTOR/NPPA/PDLIM5	7	BP
GO:0006482	protein demethylation	7/2734	32/17381	0.22951	0.809	0.7957	ALKBH4/HR/KDM4E/KDM6B/KDM8/PHF2/UBE2B	7	BP
GO:0006691	leukotriene metabolic	7/2734	32/17381	0.22951	0.809	0.7957	DPEP1/GGT6/GGTA1P/MGST3/PLA2G1B/PLA2G5/PTGR1	7	BP
GO:0008214	protein dealkylation	7/2734	32/17381	0.22951	0.809	0.7957	ALKBH4/HR/KDM4E/KDM6B/KDM8/PHF2/UBE2B	7	BP
GO:0021522	spinal cord motor neuron differentiation	7/2734	32/17381	0.22951	0.809	0.7957	HOXC10/LHX3/LHX4/OLIG3/PHOX2A/SHH/SUFU	7	BP
GO:0032228	regulation of synaptic transmission, GABAergic	7/2734	32/17381	0.22951	0.809	0.7957	ADRA1A/CA7/DRD2/NISCH/PHF24/STXBP1/TACR1	7	BP
GO:0033120	positive regulation of	7/2734	32/17381	0.22951	0.809	0.7957	HMX2/LMNTD2/NUP98/PRDX6/SF3B4/SLC39A5/ZPR1	7	BP
GO:0048488	synaptic vesicle endocytosis	7/2734	32/17381	0.22951	0.809	0.7957	CDK5/DNM1/GAK/NLGN3/SCRIB/STON2/TOR1A	7	BP
GO:0051930	regulation of sensory perception of	7/2734	32/17381	0.22951	0.809	0.7957	ACPP/ADRA2C/MTOR/OPRD1/OPRM1/SCN11A/TMEM100	7	BP
GO:0051931	regulation of sensory	7/2734	32/17381	0.22951	0.809	0.7957	ACPP/ADRA2C/MTOR/OPRD1/OPRM1/SCN11A/TMEM100	7	BP
GO:0061049	cell growth involved in cardiac muscle cell	7/2734	32/17381	0.22951	0.809	0.7957	AGT/CAV3/GATA4/MIR195/MTOR/NPPA/PDLIM5	7	BP

GO:2001236	regulation of extrinsic apoptotic	28/2734	154/17381	0.22962	0.809	0.7957	AGT/ATF3/BMP4/BRCA1/CTTN/DAPK1/EYA1/FASLG/FGA/G0S2/GPER1/GPX1/LGALS3/LMNA/LTBR/MADD/MIR221/MIR222/NOS3/PEA15/PELI3/RAF1/STX4/TMBIM1/TNFRSF12A/TRAF2/ZC3HC1/ZMYND11	28	BP
GO:0030177	positive regulation of Wnt signaling	29/2734	160/17381	0.2301	0.809	0.7957	ABL1/AXIN1/CCAR2/COL1A1/DISC1/DVL2/FGFR2/FZD9/ILK/LGR6/MIR222/NAIP/NLE1/PSMB11/PSMB6/PSMB7/PSMB8/PSMC3/PSMD13/PSMD3/PSMD5/PSMD7/RNF220/RSPO1/SHH/WLS/WNT1/WNT3A/WNT7A	29	BP
GO:0035282	segmentation	18/2734	95/17381	0.23031	0.809	0.7957	ACD/BMP4/CDX2/CRB2/DVL2/IRX3/KAT2A/LFNG/LHX1/MESP2/NLE1/NOTCH1/OSR1/RIPPLY1/SEMA3F/TCAP/TDGF1/WNT3A	18	BP
GO:0048844	artery morphogenesis	13/2734	66/17381	0.23075	0.809	0.7957	APOB/BMP4/ENG/EYA1/HEY1/LRP1/MIR153-1/NOTCH1/NPRL3/PDGFRB/STRA6/TBX2/WNT11	13	BP
GO:0021761	limbic system development	19/2734	101/17381	0.23228	0.809	0.7957	BCAN/CASP3/CDK5/CDK5R2/DCLK2/DRD2/EIF2B5/FGFR2/KDM6B/KIRREL3/LHX5/MFSD2A/NME1/OGDH/OTP/RARA/SMO/TBR1/WNT3A	19	BP
GO:0043648	dicarboxylic acid metabolic	19/2734	101/17381	0.23228	0.809	0.7957	ACSF3/ALDH1L1/ALDH4A1/ASL/FOLR2/GAD1/GLUD1/GOT2/HAAO/IDH1/IDH2/MTHFD1/NAGS/NMNAT3/OGDH/QPRT/SHMT2/TH/UROC1	19	BP
GO:1903409	reactive oxygen species biosynthetic process	19/2734	101/17381	0.23228	0.809	0.7957	ADGRB1/AGT/CLU/CYP1A1/CYP1A2/EGFR/GBF1/GCH1/GCHFR/GRIN1/HSP90AA1/INS/MIR92A2/MPO/MTOR/NOS3/PTK2B/TRAP1/ZNF205	19	BP
GO:0010659	cardiac muscle cell apoptotic	10/2734	49/17381	0.2336	0.809	0.7957	AGT/AMBRA1/CAMK2D/EIF5A/HSF1/ILK/LTK/MIR16-1/MIR195/NKX2-5	10	BP
GO:0030195	negative regulation of blood	10/2734	49/17381	0.2336	0.809	0.7957	C1QTNF1/FGA/KLKB1/KNG1/NOS3/PDGFA/PLAU/PRKCD/PROZ/SERPINF2	10	BP
GO:0032608	interferon-beta production	10/2734	49/17381	0.2336	0.809	0.7957	IRF5/NLRX1/NMI/POLR3D/TBK1/TIRAP/TLR8/TLR9/TRAF3IP1/TRAIP	10	BP
GO:1900047	negative regulation of	10/2734	49/17381	0.2336	0.809	0.7957	C1QTNF1/FGA/KLKB1/KNG1/NOS3/PDGFA/PLAU/PRKCD/PROZ/SERPINF2	10	BP
GO:0051928	positive regulation of calcium ion	20/2734	107/17381	0.23397	0.809	0.7957	ABL1/ARRB2/CCL5/CRACR2A/GCG/GPER1/GRIN1/GSTM2/HSPA2/IL13/LGALS3/NPSR1/ORAI1/P2RX2/PDGFRB/PLA2G1B/THY1/TRDN/TRPV2/WFS1	20	BP

GO:1905039	carboxylic acid transmembrane transport	20/2734	107/17381	0.23397	0.809	0.7957	ACACB/AGT/CPT2/MID1IP1/OSR1/PQLC2/SLC1A7/SLC25A20/SLC25A22/SLC36A1/SLC36A3/SLC38A1/SLC38A10/SLC38A8/SLC6A18/SLC6A6/SLC6A7/SLC6A8/SLC6A9/SLC7A7	20	BP
GO:0001936	regulation of endothelial cell proliferation	21/2734	113/17381	0.2354	0.809	0.7957	AIMP1/ANG/ATPIF1/BMP4/CDH13/ECM1/EGFL7/FLT4/GDF2/KDR/MIR16-1/MIR222/MIR29A/MIR29C/MIR503/MTOR/NR4A1/PLXNB3/STAT1/TNMD/XDH	21	BP
GO:0045727	positive regulation of translation	21/2734	113/17381	0.2354	0.809	0.7957	BARHL2/CCL5/CDK4/COA3/DND1/EIF2AK4/EIF2B5/EIF5A/ERBB2/MAPK3/MIR16-1/MTOR/NCOR1/NCOR2/PIWIL2/POLR2G/PTK2B/RMND1/RPS6KB2/TARB	21	BP
GO:0003222	ventricular trabecula myocardium morphogenesis	4/2734	16/17381	0.23607	0.809	0.7957	ENG/NKX2-5/NOTCH1/NRG1	4	BP
GO:0006044	N-acetylglucosamine metabolic	4/2734	16/17381	0.23607	0.809	0.7957	AMDHD2/CHST5/CHST7/NAGK	4	BP
GO:0007379	segment specification	4/2734	16/17381	0.23607	0.809	0.7957	DVL2/IRX3/OSR1/RIPPLY1	4	BP
GO:0010744	positive regulation of macrophage derived foam	4/2734	16/17381	0.23607	0.809	0.7957	AGT/APOB/CSF1/IL18	4	BP
GO:0015074	DNA integration	4/2734	16/17381	0.23607	0.809	0.7957	GIN1/KRBA2/NYNRIN/RLF	4	BP
GO:0018904	ether metabolic process	4/2734	16/17381	0.23607	0.809	0.7957	CYP2W1/EPHX2/GNPAT/PLA2G16	4	BP
GO:0021783	preganglionic parasympathetic fiber	4/2734	16/17381	0.23607	0.809	0.7957	HOXB1/HOXB2/PHOX2A/SEMA3F	4	BP
GO:0030033	microvillus assembly	4/2734	16/17381	0.23607	0.809	0.7957	FSCN1/MYO1A/RAP1GAP/TNIK	4	BP

GO:0032793	positive regulation of CREB transcription	4/2734	16/17381	0.23607	0.809	0.7957	CAMK1D/CRTC2/OPRD1/RPS6KA4	4	BP
GO:0033599	regulation of mammary gland epithelial cell proliferation	4/2734	16/17381	0.23607	0.809	0.7957	DEAF1/GPX1/PYGO2/ZNF703	4	BP
GO:0035493	SNARE complex	4/2734	16/17381	0.23607	0.809	0.7957	STX1A/STX4/STXBP1/VAMP1	4	BP
GO:0036149	phosphatidylinositol acyl-chain remodeling	4/2734	16/17381	0.23607	0.809	0.7957	PLA2G16/PLA2G1B/PLA2G2F/PLA2G5	4	BP
GO:0042448	progesterone metabolic	4/2734	16/17381	0.23607	0.809	0.7957	ADM/DGKQ/EGR1/STARD3	4	BP
GO:0044065	regulation of respiratory system process	4/2734	16/17381	0.23607	0.809	0.7957	GLRA1/NLGN3/PHOX2B/TLX3	4	BP
GO:0044252	negative regulation of multicellular organismal	4/2734	16/17381	0.23607	0.809	0.7957	CIITA/MIR29A/MIR92A2/PPARD	4	BP
GO:0046339	diacylglycerol metabolic	4/2734	16/17381	0.23607	0.809	0.7957	ANG/DGAT1/DGKQ/DGKZ	4	BP
GO:0046885	regulation of hormone biosynthetic process	4/2734	16/17381	0.23607	0.809	0.7957	ATP1A1/DGKQ/EGR1/POR	4	BP
GO:0046931	pore complex assembly	4/2734	16/17381	0.23607	0.809	0.7957	BAD/CCT3/GSDMD/NUP98	4	BP

GO:0050774	negative regulation of dendrite	4/2734	16/17381	0.23607	0.809	0.7957	EFNA1/GORASP1/NLGN3/TLX2	4	BP
GO:0051883	killing of cells in other organism involved in symbiotic	4/2734	16/17381	0.23607	0.809	0.7957	BAD/F2RL1/GAPDH/TUSC2	4	BP
GO:0055003	cardiac myofibril	4/2734	16/17381	0.23607	0.809	0.7957	NKX2-5/OBSL1/PDGFRB/TCAP	4	BP
GO:0055070	copper ion homeostasis	4/2734	16/17381	0.23607	0.809	0.7957	ATOX1/COMMD1/CUTC/MT2A	4	BP
GO:0060390	regulation of SMAD protein import into	4/2734	16/17381	0.23607	0.809	0.7957	BMP4/FAM89B/PBLD/RBPMS	4	BP
GO:0071732	cellular response to	4/2734	16/17381	0.23607	0.809	0.7957	AQP1/DPEP1/MMP3/TRAF2	4	BP
GO:0072283	metanephric renal vesicle morphogenesis	4/2734	16/17381	0.23607	0.809	0.7957	BMP4/LHX1/SMO/STAT1	4	BP
GO:0090051	negative regulation of cell migration involved in sprouting	4/2734	16/17381	0.23607	0.809	0.7957	MIR221/MIR29C/MIR503/NOTCH1	4	BP
GO:0140115	export across plasma	4/2734	16/17381	0.23607	0.809	0.7957	ATP1A1/KCND3/KCNQ1/NPPA	4	BP
GO:1902001	fatty acid transmembrane transport	4/2734	16/17381	0.23607	0.809	0.7957	ACACB/CPT2/MID1IP1/SLC25A20	4	BP

GO:1903541	regulation of exosomal secretion	4/2734	16/17381	0.23607	0.809	0.7957	ATP13A2/RAB7A/SDC1/SNF8	4	BP
GO:1903830	magnesium ion transmembrane transport	4/2734	16/17381	0.23607	0.809	0.7957	CNNM2/NIPA2/NIPAL1/NIPAL2	4	BP
GO:1990774	tumor necrosis factor secretion	4/2734	16/17381	0.23607	0.809	0.7957	HAVCR2/LGALS9/ORM1/ORM2	4	BP
GO:0007034	vacuolar transport	22/2734	119/17381	0.23663	0.809	0.7957	AKTIP/CDX2/CHMP1A/CHMP4C/CHMP7/CLU/DENND3/FAM160A2/GAK/GOSR2/KIF13A/LARS/PCDHGA3/RAB7A/RBSN/SNF8/STX8/VPS25/VPS51/VPS52/VPS53/VTI1B	22	BP
GO:0045807	positive regulation of	22/2734	119/17381	0.23663	0.809	0.7957	AHSG/APOA5/ARRB2/C2/CALY/CAMK1D/CAV3/CCL19/CCL21/CD300LF/CD63/DRD2/F2RL1/LMAN2/LRP1/MYO18A/SLC11A1/SPACA3/TFR2/TOR1	22	BP
GO:0007050	cell cycle arrest	44/2734	251/17381	0.23838	0.809	0.7957	ABL1/APBB1/BIN1/BRCA1/CDC14A/CDK4/CDK5/CDK9/CDKN2A/DAB2IP/E2F4/E2F8/E4F1/EIF2AK4/ERN2/FOXE3/FOXM1/FOXO4/FZD9/GPER1/HRAS/ILK/ING4/MCPH1/MLST8/MLXIPL/MTOR/MUC1/NOTCH1/PCBP4/PHOX2B/PKD1/PPP1R9B/PPP2R3B/PPP2R5B/PRKAB1/RNF112/RRAGC/SART1/SFN/WHAMM/ZBTB17/ZBTB49/ZNF385A	44	BP
GO:0016054	organic acid catabolic process	44/2734	251/17381	0.23838	0.809	0.7957	ACACB/ACAD10/ACADVL/ACOXL/ACSF3/ALDH1L1/ALDH4A1/AMDHD2/BCKDK/BLMH/CARNS1/CPT2/CROT/CRYM/DLST/FAH/GAD1/GLUD1/GOT2/GPT/GSTZ1/HAAO/HADHA/HAO2/HDC/HEXA/HNMT/HYKK/IDO1/MTOR/NAGK/NOS3/NPL/OGDH/OTC/PADI4/PHYKPL/PIPOX/PLA2G15/PPAR	44	BP
GO:0046395	carboxylic acid catabolic process	44/2734	251/17381	0.23838	0.809	0.7957	ACACB/ACAD10/ACADVL/ACOXL/ACSF3/ALDH1L1/ALDH4A1/AMDHD2/BCKDK/BLMH/CARNS1/CPT2/CROT/CRYM/DLST/FAH/GAD1/GLUD1/GOT2/GPT/GSTZ1/HAAO/HADHA/HAO2/HDC/HEXA/HNMT/HYKK/IDO1/MTOR/NAGK/NOS3/NPL/OGDH/OTC/PADI4/PHYKPL/PIPOX/PLA2G15/PPAR	44	BP
GO:0002532	production of molecular mediator involved in inflammatory	11/2734	55/17381	0.23942	0.809	0.7957	CD6/CHID1/CUEDC2/IDO1/IL4R/INS/KARS/MIR221/MIR222/PDCD4/PER1	11	BP

GO:0051898	negative regulation of protein kinase B	11/2734	55/17381	0.23942	0.809	0.7957	ARRB2/CIB1/DRD2/GPER1/LEMD2/MIR29A/MIR29C/PHLDA3/PPP2R5B/SFRP5/XDH	11	BP
GO:0009132	nucleoside diphosphate metabolic	26/2734	143/17381	0.23983	0.809	0.7957	AK5/ALDOA/BAD/CARD11/DLG4/ENTPD8/GALK1/GAPDH/GCK/HK3/INS/LDHA/MLXIPL/NCOR1/NME1/NME4/NUDT18/NUP210/NUP98/OGDH/OGDHL/PFKFB1/PFKM/PGAM4/PKM/SCRIB	26	BP
GO:0010594	regulation of endothelial cell migration	26/2734	143/17381	0.23983	0.809	0.7957	ABL1/ADGRB1/AGT/BCAS3/BMP4/CIB1/DAB2IP/EFNA1/FLT4/GDF2/HSPB1/KDR/MIR10A/MIR16-1/MIR212/MIR221/MIR29C/MIR503/MIR92A2/NOTCH1/PRCP/PTK2B/SCARB1/SEMA4A/TDGF1/WNT7A	26	BP
GO:0002090	regulation of receptor internalization	8/2734	38/17381	0.23986	0.809	0.7957	ARRB2/CD63/DLG4/DRD2/LRRTM1/NECAB2/RSPO1/WNT3A	8	BP
GO:0006111	regulation of gluconeogenesi	8/2734	38/17381	0.23986	0.809	0.7957	DGKQ/GCG/GCK/GNMT/INS/KAT2A/LEPR/MAEA	8	BP
GO:0008156	negative regulation of DNA	8/2734	38/17381	0.23986	0.809	0.7957	BCL6/CDAN1/GDF2/LIG3/PDS5A/PID1/TSPYL2/TTF1	8	BP
GO:0032094	response to food	8/2734	38/17381	0.23986	0.809	0.7957	CLPS/CLPSL1/CYP1A1/HSD11B2/MPO/MTOR/OPRM1/SLC25A25	8	BP
GO:0032715	negative regulation of interleukin-6	8/2734	38/17381	0.23986	0.809	0.7957	ARRB2/CSK/GBA/HAVCR2/INPP5D/NLRX1/ORM1/TLR9	8	BP
GO:0043537	negative regulation of blood vessel endothelial cell	8/2734	38/17381	0.23986	0.809	0.7957	GDF2/MIR10A/MIR212/MIR221/MIR29C/MIR503/MIR92A2/NOTCH1	8	BP
GO:0045022	early endosome to late endosome	8/2734	38/17381	0.23986	0.809	0.7957	AKTIP/FAM160A2/MAPK3/PTPN23/RAB7A/SNF8/SNX12/STX8	8	BP

GO:0060412	ventricular septum	8/2734	38/17381	0.23986	0.809	0.7957	BMP4/FGFR2/FGFRL1/FZD2/HEY1/NKX2-5/NOTCH1/WNT11	8	BP
GO:0090224	regulation of spindle organization	8/2734	38/17381	0.23986	0.809	0.7957	ANKRD53/CHMP1A/CHMP4C/DYNC1H1/NUMA1/PARP3/PKD1/SENP6	8	BP
GO:0090311	regulation of protein deacetylation	8/2734	38/17381	0.23986	0.809	0.7957	BCL6/BRMS1/CAMK2D/CCAR2/CTBP1/FNTA/TADA3/ZNHIT1	8	BP
GO:1905209	positive regulation of cardiocyte	8/2734	38/17381	0.23986	0.809	0.7957	ARRB2/BMP4/GATA4/GPER1/MTOR/NKX2-5/NRG1/WNT3A	8	BP
GO:0030301	cholesterol transport	16/2734	84/17381	0.2404	0.809	0.7957	ABCA2/ABCG5/APOA5/APOB/APOC3/CLU/LRP1/NPC1L1/NPC2/NR1H3/OSBPL5/SCARB1/SHH/SOAT2/STARD3/STARD5	16	BP
GO:0048167	regulation of synaptic plasticity	28/2734	155/17381	0.24068	0.809	0.7957	ABL1/ADGRB1/AGT/ANAPC2/ARC/CDK5/DBN1/DGKI/DLG4/DRD2/EIF2AK4/GRIN1/HRAS/HRH2/INS/ITPR3/JPH3/LRRTM1/NCDN/NEUROD2/NLGN3/NSMF/PTK2B/RARA/RGS14/STX4/STXBP1/VGF	28	BP
GO:0030307	positive regulation of cell growth	29/2734	161/17381	0.24096	0.809	0.7957	ANAPC2/BRAT1/CIB1/CPNE5/CPNE9/DISC1/DNPH1/EGFR/EIF4G1/ERBB2/EXOSC2/FN1/ILK/INO80/INS/ISLR2/L1CAM/LIMK1/MTOR/NRG1/PTK2B/RPS6KA1/SEMA7A/SFN/SLC44A4/TNFRSF12A/TRPV2/WNT3A/ZFYVE27	29	BP
GO:0009914	hormone transport	54/2734	312/17381	0.24104	0.809	0.7957	ADM/ADRA2A/ADRA2B/ADRA2C/AGT/ANO1/AQP1/ARL2BP/BAD/BLK/BRSK2/C1QTNF1/CAPN10/CCL5/CDK16/CPLX1/CRHR1/CRYM/DOC2B/DRD2/EGFR/FGA/GCG/GCK/GLUD1/GPER1/HCAR2/IL1RN/INHBB/INS/ITPR3/MAFA/MTNR1B/PFKM/PPARD/PTPN11/PTPRN2/RAB11FIP3/RAF1/REN/RFX6/SIDT2/SLC22A1/SLC22A9/SLC25A5/SSTR5/STX1A/STX4/TACR1/TACR	54	BP
GO:0071772	response to BMP	30/2734	167/17381	0.24115	0.809	0.7957	ABL1/ADAMTS7/BMP4/CRB2/EGR1/ENG/GATA4/GDF2/HTRA3/ILK/LEFTY2/LEMD2/MAPK3/NBL1/NKX2-5/NOTCH1/NUMA1/PDCD4/PHOX2B/RBPMS2/RGMA/SCX/SFRP5/SLC39A5/SMAD6/SOST/SPINT1/TMEM100/TNMD/WNT1	30	BP

GO:0071773	cellular response to BMP stimulus	30/2734	167/17381	0.24115	0.809	0.7957	ABL1/ADAMTS7/BMP4/CRB2/EGR1/ENG/GATA4/GDF2/HTRA3/ILK/LEFTY2/LEMD2/MAPK3/NBL1/NKX2-5/NOTCH1/NUMA1/PDCD4/PHOX2B/RBPMS2/RGMA/SCX/SFRP5/SLC39A5/SMAD6/SOST/SPINT1/TMEM100/TNMD/WNT1	30	BP
GO:0030901	midbrain development	17/2734	90/17381	0.24265	0.809	0.7957	BARHL1/CASP5/EN1/FGFR2/GLUD1/HSPA5/KAT2A/MAPKAP1/NDRG2/PHOX2A/SEC16A/SHH/TAF1/WLS/WNT1/WNT3A/YWHAQ	17	BP
GO:0009101	glycoprotein biosynthetic process	62/2734	361/17381	0.24283	0.809	0.7957	A4GNT/ADAMTS13/ADAMTS7/ALG10/ALG3/ASGR1/B3GAT3/B3GNT6/B4GALT2/B4GALT7/BCAN/BGN/CCL19/CCL21/CHPF/CHPF2/CHST12/CHST13/CHST15/CHST7/DAD1/DDOST/DOLPP1/DPM2/DSE/EXTL1/FKTN/FUT7/GAL3ST4/GALNT16/GALNT8/GALNT9/GBGT1/GGTA1P/GOLGA2/GORASP1/LFNG/LMF1/MGAT1/MGAT4B/MGAT5B/MUC1/MUC2/MUC3A/MUC5AC/MUC5B/MUC6/OST4/PARP10/PARP2/PARP3/PARP9/PLOD3/RFNG/RPN1/SDF2/ST3GAL4/SYVN1/TINF2/TMEM115/XXYLT1/XYLT2	62	BP
GO:0050673	epithelial cell proliferation	62/2734	361/17381	0.24283	0.809	0.7957	A4GNT/AIMP1/ANG/ATF2/ATPIF1/BAD/BMP4/CCL5/CDH13/DAB2IP/DEAF1/ECM1/EGFL7/EGFR/ERBB2/ESRP2/EYA1/FGFR2/FLT4/FOXE3/FOXN1/GDF2/GPX1/HRAS/IGFBP3/KDR/LIMS2/LOXL2/MARVELD3/MEN1/MIR16-1/MIR222/MIR29A/MIR29C/MIR503/MTOR/NKX2-8/NME1/NOTCH1/NR4A1/OSR1/PBLD/PLXNB3/PPARD/PYGO2/RPS6KA1/SCARB1/SFN/SHH/SIDT2/SLURP1/SMO/STAT1/TACR1/TGM1/TINF2/TNMD/WDR13/WNT3A/WNT7A/XDH/ZNF703	62	BP
GO:0006929	substrate-dependent cell migration	6/2734	27/17381	0.24293	0.809	0.7957	ADAM8/CTTN/EPHA8/FMNL1/FN1/TNFRSF12A	6	BP
GO:0007628	adult walking behavior	6/2734	27/17381	0.24293	0.809	0.7957	ARRB2/CEND1/DAB1/DRD2/GLRA1/TRH	6	BP
GO:0008299	isoprenoid biosynthetic process	6/2734	27/17381	0.24293	0.809	0.7957	ALDH8A1/COQ2/CYP1A1/PDSS1/PMVK/RBP1	6	BP
GO:0009435	NAD biosynthetic	6/2734	27/17381	0.24293	0.809	0.7957	HAAO/NMNAT3/PARP10/PARP9/QPRT/SLC22A13	6	BP

GO:0015988	energy coupled proton transmembrane transport, against electrochemical	6/2734	27/17381	0.24293	0.809	0.7957	ATP1A4/ATP5G1/ATP6V0B/ATP6V0E2/ATP6V1B1/ATP6V1F	6	BP
GO:0015991	ATP hydrolysis coupled proton transport	6/2734	27/17381	0.24293	0.809	0.7957	ATP1A4/ATP5G1/ATP6V0B/ATP6V0E2/ATP6V1B1/ATP6V1F	6	BP
GO:0032201	telomere maintenance via semi-conservative	6/2734	27/17381	0.24293	0.809	0.7957	POLA2/POLD4/POLE/POLE4/RFC1/RFC2	6	BP
GO:0035411	catenin import into nucleus	6/2734	27/17381	0.24293	0.809	0.7957	AGT/DAB2IP/EGFR/EMD/SFRP5/WNT3A	6	BP
GO:0035883	enteroendocrine cell	6/2734	27/17381	0.24293	0.809	0.7957	BAD/BMP4/MEN1/RFX6/SIDT2/SMO	6	BP
GO:0036065	fucosylation	6/2734	27/17381	0.24293	0.809	0.7957	ADAMTS13/ADAMTS7/FUOM/FUT7/LFNG/RFNG	6	BP
GO:0036151	phosphatidylcholine acyl-chain remodeling	6/2734	27/17381	0.24293	0.809	0.7957	PLA2G16/PLA2G1B/PLA2G2F/PLA2G4B/PLA2G5/PLB1	6	BP
GO:0043372	positive regulation of CD4-positive, alpha-beta T	6/2734	27/17381	0.24293	0.809	0.7957	CCL19/HLX/IL18/IL4R/LGALS9/RARA	6	BP
GO:0060260	regulation of transcription initiation from RNA polymerase II	6/2734	27/17381	0.24293	0.809	0.7957	NKX2-5/PAXIP1/PSMC3/TAF1/TAF7/THRA	6	BP

GO:0061437	renal system vasculature development	6/2734	27/17381	0.24293	0.809	0.7957	AQP1/BMP4/EGR1/NOTCH1/OSR1/PDGFRB	6	BP
GO:0061440	kidney vasculature	6/2734	27/17381	0.24293	0.809	0.7957	AQP1/BMP4/EGR1/NOTCH1/OSR1/PDGFRB	6	BP
GO:0072378	blood coagulation, fibrin clot	6/2734	27/17381	0.24293	0.809	0.7957	FGA/GP9/KLKB1/KNG1/PRCP/VWF	6	BP
GO:1900078	positive regulation of cellular response to	6/2734	27/17381	0.24293	0.809	0.7957	AGT/ESRRA/INS/MYO1C/PTPN11/RARRES2	6	BP
GO:0002676	regulation of chronic inflammatory response	3/2734	11/17381	0.2436	0.809	0.7957	CCL5/IDO1/LTA	3	BP
GO:0003161	cardiac conduction system	3/2734	11/17381	0.2436	0.809	0.7957	NKX2-5/NOTCH1/NRG1	3	BP
GO:0003174	mitral valve development	3/2734	11/17381	0.2436	0.809	0.7957	EFNA1/NOTCH1/SMAD6	3	BP
GO:0003356	regulation of cilium beat	3/2734	11/17381	0.2436	0.809	0.7957	CATSPER1/CCDC40/DNAH11	3	BP
GO:0006702	androgen biosynthetic process	3/2734	11/17381	0.2436	0.809	0.7957	HSD17B3/HSD3B1/SCARB1	3	BP
GO:0006983	ER overload response	3/2734	11/17381	0.2436	0.809	0.7957	ATG10/HSPA5/WFS1	3	BP
GO:0007512	adult heart development	3/2734	11/17381	0.2436	0.809	0.7957	ADRA1A/NKX2-5/TCAP	3	BP

GO:0009048	dosage compensation by inactivation of X	3/2734	11/17381	0.2436	0.809	0.7957	BRCA1/EXOSC10/PRDM14	3	BP
GO:0014831	gastro-intestinal system smooth muscle	3/2734	11/17381	0.2436	0.809	0.7957	HTR1D/PTGER3/TACR2	3	BP
GO:0015747	urate transport	3/2734	11/17381	0.2436	0.809	0.7957	SLC22A12/SLC22A13/SLC22A9	3	BP
GO:0015780	nucleotide-sugar transport	3/2734	11/17381	0.2436	0.809	0.7957	SLC35A2/SLC35C1/SLC35D2	3	BP
GO:0015791	polyol transport	3/2734	11/17381	0.2436	0.809	0.7957	AQP1/AQP10/SLC26A6	3	BP
GO:0019883	antigen processing and presentation of endogenous	3/2734	11/17381	0.2436	0.809	0.7957	CD74/HLA-E/TAP1	3	BP
GO:0021521	ventral spinal cord interneuron	3/2734	11/17381	0.2436	0.809	0.7957	EVX1/LHX3/SUFU	3	BP
GO:0021534	cell proliferation in	3/2734	11/17381	0.2436	0.809	0.7957	CEND1/GBX2/SHH	3	BP
GO:0021681	cerebellar granular layer	3/2734	11/17381	0.2436	0.809	0.7957	CEND1/KNDC1/WNT7A	3	BP
GO:0030575	nuclear body organization	3/2734	11/17381	0.2436	0.809	0.7957	FAM118B/SF1/ZPR1	3	BP
GO:0031581	hemidesmosome assembly	3/2734	11/17381	0.2436	0.809	0.7957	COL17A1/KRT14/LAMC2	3	BP
GO:0032488	Cdc42 protein signal	3/2734	11/17381	0.2436	0.809	0.7957	ABL1/APOC3/WAS	3	BP

GO:0032494	response to peptidoglycan	3/2734	11/17381	0.2436	0.809	0.7957	CARD9/IRF5/TREM2	3	BP
GO:0033690	positive regulation of osteoblast	3/2734	11/17381	0.2436	0.809	0.7957	ABL1/SOX8/TMEM119	3	BP
GO:0035414	negative regulation of catenin import	3/2734	11/17381	0.2436	0.809	0.7957	DAB2IP/EMD/SFRP5	3	BP
GO:0038065	collagen-activated	3/2734	11/17381	0.2436	0.809	0.7957	COL1A1/COL4A2/DDR2	3	BP
GO:0042482	positive regulation of	3/2734	11/17381	0.2436	0.809	0.7957	AMTN/CSF1/WNT6	3	BP
GO:0046007	negative regulation of activated T cell	3/2734	11/17381	0.2436	0.809	0.7957	CASP3/LGALS9/PRKAR1A	3	BP
GO:0046689	response to mercury ion	3/2734	11/17381	0.2436	0.809	0.7957	ALAD/AQP1/SLC34A1	3	BP
GO:0048245	eosinophil chemotaxis	3/2734	11/17381	0.2436	0.809	0.7957	CCL5/DAPK2/LGALS3	3	BP
GO:0051024	positive regulation of immunoglobuli	3/2734	11/17381	0.2436	0.809	0.7957	HLA-E/STX4/TNFRSF4	3	BP
GO:0051103	DNA ligation involved in DNA repair	3/2734	11/17381	0.2436	0.809	0.7957	LIG3/PARP2/PARP3	3	BP
GO:0060379	cardiac muscle cell myoblast differentiation	3/2734	11/17381	0.2436	0.809	0.7957	NOTCH1/NRG1/TBX2	3	BP
GO:0060439	trachea morphogenesis	3/2734	11/17381	0.2436	0.809	0.7957	BMP4/MAPK3/SHH	3	BP

GO:0060573	cell fate specification involved in pattern	3/2734	11/17381	0.2436	0.809	0.7957	EVX1/LHX3/SUFU	3	BP
GO:0060736	prostate gland growth	3/2734	11/17381	0.2436	0.809	0.7957	FGFR2/PSAP/SHH	3	BP
GO:0061081	positive regulation of myeloid leukocyte cytokine production	3/2734	11/17381	0.2436	0.809	0.7957	CD74/SEMA7A/SPON2	3	BP
GO:0071372	cellular response to follicle-stimulating	3/2734	11/17381	0.2436	0.809	0.7957	EPHA8/NOTCH1/POR	3	BP
GO:0071639	positive regulation of monocyte chemotactic protein-1	3/2734	11/17381	0.2436	0.809	0.7957	LGALS9/MIR92A2/TRPV4	3	BP
GO:0071888	macrophage apoptotic	3/2734	11/17381	0.2436	0.809	0.7957	CCL5/CDKN2A/CTSL	3	BP
GO:0072567	chemokine (C-X-C motif) ligand 2	3/2734	11/17381	0.2436	0.809	0.7957	CD74/F2RL1/TIRAP	3	BP
GO:0097050	type B pancreatic cell apoptotic	3/2734	11/17381	0.2436	0.809	0.7957	CAPN10/SPOP/WFS1	3	BP

GO:1902306	negative regulation of sodium ion transmembrane	3/2734	11/17381	0.2436	0.809	0.7957	CAMK2D/COMMD1/OSR1	3	BP
GO:1903299	regulation of hexokinase	3/2734	11/17381	0.2436	0.809	0.7957	BAD/COX11/PFKFB1	3	BP
GO:1905214	regulation of RNA binding	3/2734	11/17381	0.2436	0.809	0.7957	CDK9/EIF4G1/LARP6	3	BP
GO:2000615	regulation of histone H3-K9 acetylation	3/2734	11/17381	0.2436	0.809	0.7957	BRCA1/HDAC8/PIWIL2	3	BP
GO:2000786	positive regulation of autophagosome	3/2734	11/17381	0.2436	0.809	0.7957	LRSAM1/RALB/ULK1	3	BP
GO:0006521	regulation of cellular amino acid metabolic	12/2734	61/17381	0.24414	0.8099	0.79658	INS/OAZ2/PSMB11/PSMB6/PSMB7/PSMB8/PSMC3/PSMD13/PSMD3/PSMD5/PSMD7/SLC7A7	12	BP
GO:0046365	monosaccharide catabolic	12/2734	61/17381	0.24414	0.8099	0.79658	ALDOA/BAD/FUT7/GALK1/GALM/GALT/GAPDH/GCK/GLYCTK/HK3/PFKM/PKM	12	BP
GO:1903829	positive regulation of cellular protein localization	69/2734	404/17381	0.24428	0.8099	0.79659	ACSM6/ANP32B/ARHGEF16/B3GAT3/BAD/BCAP31/BCAS3/BMP4/CCL19/CCT3/CD27/CDK5/CDK5RAP3/CHRM1/CIB1/CNPY4/CROCC/CSF3/CYB5R1/DYNLL2/EGFR/ELMOD1/EMD/EPB41L2/ERBB2/FLNA/GPER1/GPHA2/HNMT/IL18/INS/ITGAM/ITGAX/KAT2A/LGALS9/LRIG2/LRP1/LRRC46/MIEF2/MYBPC1/MYO1C/NBPF3/NECAB2/NKD2/NRG1/NUMA1/OAZ2/OSCP1/PARD6A/PARP9/PEMT/PPP1R13B/PRKCD/PSMB7/RBPMS/RNF31/SEC16B/SFN/SHH/SMO/STX4/TLR9/TREM2/TSGA13/UBL4B/WNT3A/YWHAQ/ZBTB[illegible]	69	BP
GO:0003015	heart process	48/2734	276/17381	0.24486	0.8114	0.79803	ADM/ADRA1A/ADRA1B/AGT/ATP1A1/ATP1A4/ATP2A3/CACNA1F/CACNA1G/CACNA1H/CACNA1S/CACNB1/CACNB3/CACNG1/CAMK2D/CAV3/CHGA/DES/DRD2/EDN2/FLNA/GATA4/GCH1/GNAO1/GPX1/GSTM2/HSPB7/ITPR3/KCND1/KCND3/KCNH6/KCNJ5/KCNQ1/MAP2K3/MIR328/MIR92A2/MTOR/MYBPC3/MYL4/NKX2-	48	BP

GO:0006415	translational termination	19/2734	102/17381	0.24612	0.8138	0.80038	C12orf65/EIF5A/ERAL1/MRPL10/MRPL14/MRPL28/MRPL36/MRPL37/MRPL43/MRPL52/MRPL57/MRPL9/MRPS10/MRPS11/MRPS18A/MRPS18B/MRPS21/MRPS33/MRPS5	19	BP
GO:0007052	mitotic spindle organization	19/2734	102/17381	0.24612	0.8138	0.80038	ANKRD53/ARHGEF10/CDC14A/CHMP1A/CHMP4C/DCTN2/DYNC1H1/EML1/FLNA/GOLGA2/KIF23/KIF4A/NUMA1/PARP3/PKD1/TUBGCP2/TUBGCP3/TUBGCP5/WRAP73	19	BP
GO:0030218	erythrocyte differentiation	19/2734	102/17381	0.24612	0.8138	0.80038	AHSP/ALAS2/ATPIF1/BCL6/BMP4/CASP3/HBZ/INPP5D/ISG15/L3MBTL3/LDB1/MAEA/MIR221/MIR222/NFE2L1/SETD1A/SPI1/STAT1/THRA	19	BP
GO:2000278	regulation of DNA biosynthetic	19/2734	102/17381	0.24612	0.8138	0.80038	ACD/ARRB2/CCT3/EXOSC10/FGFR4/GDF2/HSP90AA1/MAPK15/MAPK3/MEN1/NOX4/NVL/PDGFRB/PIF1/PTK2B/RFC2/SH2B1/SMG5/TINF2	19	BP
GO:0045666	positive regulation of neuron differentiation	57/2734	331/17381	0.24668	0.815	0.80155	ADRA2B/ADRA2C/AGT/AMIGO1/ANAPC2/APBB1/BCL6/BMP4/CAMK1D/CPNE5/CPNE9/CYB5D2/DAB1/DAB2IP/DISC1/DUOXA1/EIF4G1/FES/FN1/FOXO6/HOXD3/HSPA5/ILK/IRX3/ISLR2/KATNB1/L1CAM/LIMK1/LLPH/LTK/MARK2/METRN/MIR221/MIR222/MMD2/MTOR/NBL1/NEUROD2/NKX2-5/NME1/NRG1/OBSL1/PHOX2B/PLXNB1/PLXNB3/PLXND1/PPP2R5B/PTK2B/RARA/RNF112/SCARF1/SEMA7A/SERPINI1/TNFRSF12A/TRPV2/WNT3A [illegible]	57	BP
GO:0043200	response to amino acid	20/2734	108/17381	0.24745	0.815	0.80155	ALAD/ASNS/BAD/CASP3/CFL1/COL16A1/COL1A1/DHODH/DNMT3A/EGFR/GLRA1/GRIN1/HSF1/LARS/MMP2/MTOR/NAIP/NSMF/PEMT/RRAGC	20	BP
GO:0002886	regulation of myeloid leukocyte	9/2734	44/17381	0.24775	0.815	0.80155	CCR2/F2RL1/FES/IL13/IL4R/ITGAM/LGALS9/STX4/STXBP1	9	BP
GO:0006984	ER-nucleus signaling	9/2734	44/17381	0.24775	0.815	0.80155	ASNS/ATF3/ATG10/BOK/HERPUD1/HSPA5/LMNA/SCAP/WFS1	9	BP
GO:0007029	endoplasmic reticulum organization	9/2734	44/17381	0.24775	0.815	0.80155	GAK/REEP2/SEC16A/SEC16B/STX18/TOR1B/TRDN/UBL4A/ZFYVE27	9	BP
GO:0010712	regulation of collagen metabolic	9/2734	44/17381	0.24775	0.815	0.80155	BMP4/CIITA/ENG/MIR29A/MIR92A2/PDGFRB/PPARD/SCX/SERPINF2	9	BP

GO:0043300	regulation of leukocyte degranulation	9/2734	44/17381	0.24775	0.815	0.80155	CCR2/F2RL1/FES/IL13/IL4R/ITGAM/LGALS9/STX4/STXBP1	9	BP
GO:0046850	regulation of bone	9/2734	44/17381	0.24775	0.815	0.80155	ADAM8/BGLAP/CSF1R/CSK/DEF8/EGFR/INPP5D/LEPR/PLEKHM1	9	BP
GO:0099518	vesicle cytoskeletal trafficking	9/2734	44/17381	0.24775	0.815	0.80155	CNIH2/DTNBP1/DYNC1H1/DYNC1I1/KIF13A/KIF23/MLPH/MOBP/TRIM46	9	BP
GO:0007588	excretion	13/2734	67/17381	0.24799	0.815	0.80155	ABCG5/ADRA1A/AGT/AQP5/ATP6V1B1/AVPR2/CLCNKA/CLCNKB/DRD2/GUCA2B/POU3F3/TACR1/TACR2	13	BP
GO:0030500	regulation of bone	13/2734	67/17381	0.24799	0.815	0.80155	AHSG/BGLAP/BMP4/DDR2/ECM1/FZD9/IFITM5/ISG15/OSR1/PHOSPHO1/PKDCC/PTK2B/TMEM119	13	BP
GO:0042476	odontogenesis	22/2734	120/17381	0.24946	0.8193	0.80585	ADM/AMTN/AQP1/AQP5/ATF2/BGLAP/BMP4/COL1A1/CSF1/DLX3/FGFR2/JAG2/NKX2-	22	BP
GO:0048813	dendrite morphogenesis	23/2734	126/17381	0.25019	0.8213	0.80779	ANAPC2/ARC/CDK5/CELSR2/CFL1/CTNNA2/DLG4/DTNBP1/EFNA1/EPHB3/GORASP1/GRIN1/ILK/KNDC1/MAP6D1/NLGN3/NSMF/OBSL1/PDLIM5/PREX2/TLX2/TNIK/WNT7A	23	BP
GO:0050953	sensory perception of light stimulus	37/2734	210/17381	0.25044	0.8216	0.80813	ABCC6/AIPL1/AOC2/ARR3/BBS9/CACNA1F/CDH23/CLN6/CNGB1/COL1A1/DNAJC19/GLRA1/GNAT1/GUCA1B/GUCY2D/HPS1/KIFC3/KRT12/LAMC3/MIP/MYO3A/MYO7A/NOB1/NRL/OAT/OPN1MW/OPN4/PDE6D/RGS16/SEMA5B/SFRP5/TGFBI/TH/TULP1/UNC119/VAX2/WFS1	37	BP
GO:0007498	mesoderm development	24/2734	132/17381	0.25078	0.8218	0.80831	AXIN1/BMP4/CRB2/EYA1/FGFR2/IRX3/KDM6B/MESP2/OSR1/PRKAR1A/SCX/SETD2/SHH/SMO/TIE1/TLX2/TSPY1/TSPY2/TSPY4/TSPY8/TXNRD1/W	24	BP
GO:0044272	sulfur compound biosynthetic process	36/2734	204/17381	0.25079	0.8218	0.80831	ACACB/ACAN/ACSF3/B3GAT3/B4GALT2/BCAN/BGN/BHMT2/CHAC1/CHPF/CHPF2/CHST12/CHST13/CHST15/CHST5/CHST7/CSAD/DSE/ELOVL1/ELOVL5/GGT6/GGTA1P/GSTM1/GSTM2/GSTZ1/HACD1/LIAS/MGST1/MGST3/MPC1/MTHFD1/PDHB/SLC26A1/SLC35D2/ST3GAL4/XYLT2	36	BP
GO:0007045	cell-substrate adherens junction	14/2734	73/17381	0.25115	0.8218	0.80831	ABL1/BCAS3/COL16A1/CTTN/FERMT2/KDR/LDB1/MYOC/PTK2B/RHOD/TAOK2/THY1/WDPCP/WHAMM	14	BP

GO:0048041	focal adhesion assembly	14/2734	73/17381	0.25115	0.8218	0.80831	ABL1/BCAS3/COL16A1/CTTN/FERMT2/KDR/LDB1/MYOC/PTK2B/RHOD/TAOK2/THY1/WDPCP/WHAMM	14	BP
GO:0031099	regeneration	33/2734	186/17381	0.25163	0.8218	0.80831	ADM/APOA5/BCL9/BIN3/CDK4/EGFR/FZD9/GJD4/GPX1/INPP5F/LGR6/LRIG2/MIR221/MIR222/MUSTN1/MYOD1/NINJ2/NOTCH1/PFKFB1/PKM/PPARD/PTPRF/PTPRU/RGMA/RTN4RL1/RTN4RL2/SCARF1/TEC/THY1/TNC/UL	33	BP
GO:0002699	positive regulation of immune	31/2734	174/17381	0.25195	0.8218	0.80831	CCL19/CCR2/CD74/CLCF1/DHX58/F2RL1/FES/HLA-E/HLX/IL13/IL18/IL4R/ITGAM/KARS/LGALS9/LTA/MAPK3/MYO18A/MZB1/NCR3/PAXIP1/PGC/RARA/SEMA7A/SPON2/STX4/STXBP1/TLR9/TNFRSF	31	BP
GO:0048639	positive regulation of developmental	31/2734	174/17381	0.25195	0.8218	0.80831	ACACB/AGRN/ANAPC2/CPNE5/CPNE9/CSF1/DISC1/DRD2/FGFR2/FN1/HLX/HSF1/ILK/ISLR2/L1CAM/LIMK1/MIR222/MTOR/MYOD1/NOTCH1/NRG1/PPARD/PPIB/SEMA7A/SH3PXD2B/SMO/TBX2/TNFRSF12A/TRPV2/WNT3A	31	BP
GO:0033238	regulation of cellular amine metabolic	15/2734	79/17381	0.25375	0.8218	0.80831	INS/ITGAM/OAZ2/PAOX/PNKD/PSMB11/PSMB6/PSMB7/PSMB8/PSMC3/PSMD13/PSMD3/PSMD5/PSMD7/SLC7A7	15	BP
GO:0048814	regulation of dendrite morphogenesis	15/2734	79/17381	0.25375	0.8218	0.80831	ANAPC2/ARC/CDK5/CFL1/EFNA1/GORASP1/GRIN1/ILK/KNDC1/NLGN3/NSMF/OBSL1/PDLIM5/TLX2/TNIK	15	BP
GO:0097194	execution phase of apoptosis	15/2734	79/17381	0.25375	0.8218	0.80831	BCAP31/BOK/CAPN10/CASP3/CASP6/CDK5RAP3/ENDOG/ERN2/FNTA/GCG/GPER1/HSF1/MADD/PRKCD/SHARPIN	15	BP
GO:0060324	face development	10/2734	50/17381	0.25389	0.8218	0.80831	COL1A1/CSRNP1/HOXB3/MAPK3/MMP2/PTPN11/RAF1/RARA/SCX/STRA6	10	BP
GO:0032844	regulation of homeostatic process	80/2734	473/17381	0.25441	0.8218	0.80831	ABL1/ACD/ADAM8/ADRA2A/AGT/ANO1/AVPR2/BAD/BGLAP/BOK/BRSK2/CA7/CACNA1G/CAMK2D/CAV3/CCL19/CCL21/CCT3/CD74/CDK16/CORO1A/CSF1R/CSK/DEF8/DRD2/EGFR/EIF4G1/EXOSC10/FASLG/FGFR4/FZD9/GCG/GPER1/GRIN1/GSTM2/HCAR2/HDAC8/IL13/INPP5D/ISG15/ITPR3/JPH3/KDR/LCK/LDB1/LEPR/LGALS9/LMO1/MAPK15/MAPK3/MIR221/MIR222/MLXIPL/MYOC/NPSR1/NR1H3/P2RX2/PIF1/PLA2G1B/PLEKHM1/PPP2R3C/PTGER3/PTK2B/RASA3/RFX6/SETD1A/SIDT2/SLC30A3/SMG5/SPI1/STAT1/STX4/TACR1/THY1/TINF2/TRDN/TRPV4/TSC22D3/TSC22D4/WNK2	80	BP

GO:0051648	vesicle localization	46/2734	265/17381	0.25474	0.8218	0.80831	CDK5/CEP19/CNIH2/COL7A1/CPLX1/DCTN2/DNM1/DOC2A/DOC2B/DTNBP1/DYNC1H1/DYNC1I1/GAK/GBF1/GOLGA2/GORASP1/GOSR2/KIF13A/KIF23/MAP4K2/MLPH/MOBP/MYO1A/MYO7A/NLGN3/PLD2/RAB17/SCRIB/SEC16A/SEC16B/STARD3/STON2/STX1A/STX4/STX5/STXBP1/SYTL3/TOR1A/TRAPPC1/TRAPPC2L/TRAPPC3/TRAPPC4/TRAPPC9/TRIM46/WNT3A/W	46	BP
GO:0001941	postsynaptic membrane organization	7/2734	33/17381	0.25475	0.8218	0.80831	CHRNB1/DLG4/DNAJA3/FNTA/FZD9/NLGN3/NRXN2	7	BP
GO:0003338	metanephros morphogenesis	7/2734	33/17381	0.25475	0.8218	0.80831	BMP4/LHX1/PDGFRB/PKD1/SMO/SOX8/STAT1	7	BP
GO:0006221	pyrimidine nucleotide biosynthetic process	7/2734	33/17381	0.25475	0.8218	0.80831	AK5/DHODH/ERH/NME1/NME4/TBPL1/UCK1	7	BP
GO:0010574	regulation of vascular endothelial growth factor	7/2734	33/17381	0.25475	0.8218	0.80831	BRCA1/C3AR1/CCR2/FLT4/GATA4/NDRG2/SARS	7	BP
GO:0042311	vasodilation	7/2734	33/17381	0.25475	0.8218	0.80831	AGT/GCH1/GPX1/KNG1/MIR153-1/NOS3/PLOD3	7	BP
GO:0048147	negative regulation of fibroblast	7/2734	33/17381	0.25475	0.8218	0.80831	B4GALT7/C1QL4/DAB2IP/EMD/LTA/NUPR1/PARP10	7	BP
GO:0048665	neuron fate specification	7/2734	33/17381	0.25475	0.8218	0.80831	EVX1/EYA1/HOXC10/LHX3/OLIG3/SUFU/TLX3	7	BP
GO:0055090	acylglycerol homeostasis	7/2734	33/17381	0.25475	0.8218	0.80831	APOA5/APOC3/GPIHBP1/IL18/MLXIPL/NR1H3/SCARB1	7	BP
GO:0070328	triglyceride homeostasis	7/2734	33/17381	0.25475	0.8218	0.80831	APOA5/APOC3/GPIHBP1/IL18/MLXIPL/NR1H3/SCARB1	7	BP

GO:1902742	apoptotic process involved in	7/2734	33/17381	0.25475	0.8218	0.80831	CRYAB/JAG2/NKX2-5/NOTCH1/SCRIB/SPI1/ZPR1	7	BP
GO:1903053	regulation of extracellular matrix	7/2734	33/17381	0.25475	0.8218	0.80831	ABL1/AGT/DDR2/FGFR4/FSCN1/LRP1/NOTCH1	7	BP
GO:0010717	regulation of epithelial to mesenchymal transition	16/2734	85/17381	0.2559	0.8218	0.80831	BMP4/COL1A1/CRB2/DAB2IP/EFNA1/ENG/GLIPR2/MIR221/MIR222/MTOR/NOTCH1/PBLD/TGFB1I1/TRIM62/VASN/ZNF703	16	BP
GO:0045621	positive regulation of lymphocyte	16/2734	85/17381	0.2559	0.8218	0.80831	ADAM8/BAD/BCL6/CCL19/CD27/CD74/HLX/IL18/IL4R/INPP5D/LGALS9/PIK3R6/PPP2R3C/RARA/SART1/SHH	16	BP
GO:0048709	oligodendrocyte differentiation	16/2734	85/17381	0.2559	0.8218	0.80831	BOK/CDK5/CLU/CSK/EIF2B2/EIF2B5/ERBB2/HDAC11/MTOR/NLGN3/NOTCH1/NRG1/PRDM8/SHH/SOX8/WDR1	16	BP
GO:0015711	organic anion transport	77/2734	455/17381	0.25738	0.8218	0.80831	ABCC3/ABCC4/ABCC5/ACACB/AGT/APOA5/APOC3/AQP1/ASIC3/CA4/CA6/CA7/CPLX1/CPT2/CPTP/CROT/CYB5R1/CYB5R2/DRD2/FOLR2/GOT2/LCN12/MFSD2A/MID1IP1/NCOR1/NPC2/OSBPL5/OSR1/PITPNM1/PLA2G1B/PLA2G2F/PLA2G5/PPARD/PQLC2/PRELID1/PRKCD/PSAP/SCARB1/SLC11A1/SLC13A2/SLC16A13/SLC1A7/SLC22A1/SLC22A12/SLC22A13/SLC22A9/SLC25A20/SLC25A22/SLC25A25/SLC25A5/SLC26A1/SLC26A10/SLC26A6/SLC35A2/SLC36A1/SLC36A3/SLC38A1/SLC38A10/SLC38A8/SLC44A4/SLC4A9/SLC52A1/SLC52A2/SLC6A18/SLC6A6/SLC6A7/SLC6A8/SLC6A9/SLC7A7/SLCO2B1/SLCO5A1/STARD5/STX1A/STXBP1/SV2A/TNFAIP8L3/TRH	77	BP
GO:0003416	endochondral bone growth	5/2734	22/17381	0.25747	0.8218	0.80831	DDR2/FGFR2/POR/RARA/THBS3	5	BP
GO:0009067	aspartate family amino acid biosynthetic process	5/2734	22/17381	0.25747	0.8218	0.80831	ASNS/BHMT2/GOT2/MTHFD1/PLOD3	5	BP

GO:0010640	regulation of platelet-derived growth factor receptor signaling	5/2734	22/17381	0.25747	0.8218	0.80831	HIP1R/LRIG2/LRP1/PDGFA/PDGFRB	5	BP
GO:0032469	endoplasmic reticulum calcium ion	5/2734	22/17381	0.25747	0.8218	0.80831	BCAP31/CAMK2D/HERPUD1/THADA/WFS1	5	BP
GO:0032616	interleukin-13 production	5/2734	22/17381	0.25747	0.8218	0.80831	HLA-E/IL18/LGALS9/RARA/SCGB1A1	5	BP
GO:0034067	protein localization to	5/2734	22/17381	0.25747	0.8218	0.80831	GAK/GBF1/IFT20/OBSL1/PACS1	5	BP
GO:0035428	hexose transmembrane transport	5/2734	22/17381	0.25747	0.8218	0.80831	SLC2A4/SLC2A6/SLC2A8/SLC45A1/SLC5A2	5	BP
GO:0042537	benzene-containing compound metabolic	5/2734	22/17381	0.25747	0.8218	0.80831	GOT2/GSTM1/GSTM2/IDO1/TH	5	BP
GO:0042994	cytoplasmic sequestering of transcription	5/2734	22/17381	0.25747	0.8218	0.80831	CD27/NFKBIL1/PKD1/SUFU/THRA	5	BP
GO:0044458	motile cilium assembly	5/2734	22/17381	0.25747	0.8218	0.80831	CC2D2A/CCDC40/E2F4/LRRC6/NOTO	5	BP
GO:0045947	negative regulation of translational	5/2734	22/17381	0.25747	0.8218	0.80831	EIF2AK4/EIF2B5/EIF4EBP1/RARA/RBM4	5	BP
GO:0050687	negative regulation of defense	5/2734	22/17381	0.25747	0.8218	0.80831	DHX58/MICB/NLRX1/TARBP2/TRAF3IP1	5	BP

GO:0050995	negative regulation of lipid catabolic	5/2734	22/17381	0.25747	0.8218	0.80831	ACACB/ADRA2A/APOC3/HCAR2/INS	5	BP
GO:0051043	regulation of membrane protein ectodomain	5/2734	22/17381	0.25747	0.8218	0.80831	ADAM8/ADRA2A/FURIN/LRIG2/PACSIN3	5	BP
GO:0060445	branching involved in salivary gland	5/2734	22/17381	0.25747	0.8218	0.80831	ESRP2/FGFR2/PDGFA/PLXND1/SHH	5	BP
GO:0061213	positive regulation of mesonephros	5/2734	22/17381	0.25747	0.8218	0.80831	AGT/HOXB7/LHX1/SMO/SOX8	5	BP
GO:0072215	regulation of metanephros development	5/2734	22/17381	0.25747	0.8218	0.80831	BMP4/EGR1/PDGFA/PDGFRB/STAT1	5	BP
GO:1901685	glutathione derivative metabolic	5/2734	22/17381	0.25747	0.8218	0.80831	GSTM1/GSTM2/GSTZ1/MGST1/MGST3	5	BP
GO:1901687	glutathione derivative biosynthetic process	5/2734	22/17381	0.25747	0.8218	0.80831	GSTM1/GSTM2/GSTZ1/MGST1/MGST3	5	BP
GO:1904659	glucose transmembrane transport	5/2734	22/17381	0.25747	0.8218	0.80831	SLC2A4/SLC2A6/SLC2A8/SLC45A1/SLC5A2	5	BP
GO:1905950	monosaccharide transmembrane transport	5/2734	22/17381	0.25747	0.8218	0.80831	SLC2A4/SLC2A6/SLC2A8/SLC45A1/SLC5A2	5	BP
GO:0006900	vesicle budding from membrane	17/2734	91/17381	0.25767	0.8218	0.80831	CNIH2/COL7A1/DNM1/GBF1/GOLGA2/GORASP1/GOSR2/MYO18A/RAB7A/SEC16A/SEC16B/STX5/TRAPPC1/TRAPPC2L/TRAPPC3/TRAPPC4/TRAPP	17	BP

GO:0015918	sterol transport	17/2734	91/17381	0.25767	0.8218	0.80831	ABCA2/ABCG5/APOA5/APOB/APOC3/CLU/LRP1/NPC1L1/NPC2/NR1H3/OSBPL5/OSBPL7/SCARB1/SHH/SOAT2/STARD3/STARD5	17	BP
GO:2000060	positive regulation of protein ubiquitination involved in ubiquitin-dependent	17/2734	91/17381	0.25767	0.8218	0.80831	ANAPC15/ANAPC2/AXIN1/BUB1B/CAV3/CLU/DISC1/PSMB11/PSMB6/PSMB7/PSMB8/PSMC3/PSMD13/PSMD3/PSMD5/PSMD7/PTK2B	17	BP
GO:0003007	heart morphogenesis	41/2734	235/17381	0.258	0.8225	0.80894	ATF2/BMP4/C2CD3/CAV3/CCDC103/CCDC40/DVL2/EFNA1/ENG/EPHB4/EYA1/FGFR2/FGFRL1/FZD2/GATA4/HEY1/ILK/LEMD2/MESP2/MICAL2/MIR195/MTOR/MYBPC3/NKX2-5/NOTCH1/NOTO/NRG1/PDCD4/PLXND1/RARA/SHH/SMAD6/SMO/SUFU/TBX2/TCAP/TH/TMEM100/TNNC1/WNT11/WNT3A	41	BP
GO:0065002	intracellular protein transmembrane	11/2734	56/17381	0.25873	0.8244	0.81079	DNLZ/HSP90AA1/PEX14/PEX16/PEX3/PEX5/PEX6/TIMM17B/TIMM22/TIMM23B/TIMM9	11	BP
GO:0001657	ureteric bud development	18/2734	97/17381	0.25911	0.8251	0.81155	AGT/BMP4/EYA1/FGFR2/HOXB7/ILK/LHX1/OSR1/RARA/SDC1/SHH/SIM1/SMAD6/SMO/SOX8/WNT1/WNT11/WNT6	18	BP
GO:0014013	regulation of gliogenesis	19/2734	103/17381	0.26029	0.828	0.81437	ASCL2/BIN1/CCR2/CLCF1/CSF1/DAB1/EMX1/IDH2/LTA/MIR221/MIR222/MTOR/MYCN/NOTCH1/RNF112/SHH/SOX8/SPINT1/WDR1	19	BP
GO:0034968	histone lysine methylation	19/2734	103/17381	0.26029	0.828	0.81437	ASH2L/BRCA1/EHMT1/EHMT2/GCG/MEN1/PAXIP1/PHF19/PRDM12/PRDM7/PRDM9/PYGO2/RLF/SETD1A/SETD2/SETD7/SMYD3/SUPT6H/SUV39H	19	BP
GO:0072009	nephron epithelium	20/2734	109/17381	0.26124	0.8287	0.81503	AGT/AQP1/BMP4/EYA1/HOXB7/ILK/IRX3/LHX1/MYO1E/NOTCH1/OSR1/PKD1/POU3F3/SHH/SMO/SOX8/STAT1/WNT1/WNT11/WNT6	20	BP
GO:0001763	morphogenesis of a branching structure	34/2734	193/17381	0.26173	0.8287	0.81503	ABL1/ADM/AGT/BMP4/CAV3/CSF1/CTSH/DRD2/ENG/ESRP2/EYA1/FGFR2/GBX2/GDF2/HHIP/HOXB7/ILK/LHX1/MYCN/NOTCH1/PDGFA/PKD1/PLXND1/SETD2/SHH/SMO/SOX8/SPINT1/ST14/TDGF1/TIMELESS/TNC/WNT1/	34	BP

GO:0045665	negative regulation of neuron differentiation	34/2734	193/17381	0.26173	0.8287	0.81503	ARHGAP4/CDK5/CIB1/CTDSP1/DAB1/DGUOK/DRAXIN/EFNA1/EIF2AK4/FUOM/GAK/GORASP1/INPP5F/IRX3/LINGO1/LRIG2/LRP1/LSM1/NLGN3/NOTCH1/NR2F1/PHOX2B/RGMA/RTN4RL1/RTN4RL2/SEMA3F/SHH/SOX8/THY1/TLX2/TLX3/TRPV4/WNT3A/ZHX2	34	BP
GO:0001505	regulation of neurotransmitter levels	32/2734	181/17381	0.26251	0.8287	0.81503	ACHE/BAIAP3/CDK5/CPLX1/DAGLB/DGKI/DOC2A/DOC2B/DRD2/DTNBP1/GAD1/GCHFR/GPER1/HNMT/LRTOMT/NAALAD2/NRXN2/PNKD/PTPRN2/SCRIB/SLC38A1/SLC44A4/SLC6A9/STX1A/STX4/STXBP1/SYTL3/TACR2/TH/TOR1A/WNT7A/ZNF219	32	BP
GO:0010634	positive regulation of epithelial cell	22/2734	121/17381	0.26257	0.8287	0.81503	ABL1/AGT/AQP1/BCAS3/BMP4/CIB1/CTSH/DOCK1/FLT4/GLIPR2/HSPB1/KDR/MIR221/MIR222/MTOR/PFN1/PTK2B/RAB25/SCARB1/TACR1/TDGF1/WNT7A	22	BP
GO:0030858	positive regulation of epithelial cell	12/2734	62/17381	0.26259	0.8287	0.81503	BAD/BMP4/CDH5/FOXN1/GDF2/IL13/LHX1/NOTCH1/NUMA1/PTCH2/SFN/TMEM100	12	BP
GO:0030326	embryonic limb morphogenesis	23/2734	127/17381	0.263	0.8287	0.81503	ACD/ALX3/B9D1/BMP4/C2CD3/EN1/FBXW4/HOXC10/HOXC11/IFT140/MYCN/NOTCH1/OSR1/PBX2/PITX1/SHH/TBC1D32/TBX2/TMEM231/TRAF3IP1/TULP3/WDPCP/WNT7A	23	BP
GO:0031023	microtubule organizing center	23/2734	127/17381	0.263	0.8287	0.81503	ARHGEF10/BCAS3/BRCA1/C2CD3/CCNF/CHMP1A/CHMP4C/CKAP5/CROCC/E2F4/FES/GOLGA2/HAUS4/HAUS7/MCPH1/NUBP1/PARD6A/SDCCAG8/TUBGCP2/TUBGCP3/TUBGCP5/UXT/XRCC3	23	BP
GO:0035113	embryonic appendage morphogenesis	23/2734	127/17381	0.263	0.8287	0.81503	ACD/ALX3/B9D1/BMP4/C2CD3/EN1/FBXW4/HOXC10/HOXC11/IFT140/MYCN/NOTCH1/OSR1/PBX2/PITX1/SHH/TBC1D32/TBX2/TMEM231/TRAF3IP1/TULP3/WDPCP/WNT7A	23	BP
GO:0035270	endocrine system	23/2734	127/17381	0.263	0.8287	0.81503	BAD/BMP4/DRD2/GCM2/HOXA3/HOXB3/HOXD3/LHX3/MAPK3/MEN1/NKX2-	23	BP
GO:0034250	positive regulation of cellular amide metabolic	24/2734	133/17381	0.26329	0.8287	0.81503	BARHL2/CCL5/CDK4/CLU/COA3/DND1/EFNA1/EIF2AK4/EIF2B5/EIF5A/ERBB2/MAPK3/MIR16-1/MTOR/NCOR1/NCOR2/PIWIL2/POLR2G/PRKCD/PTK2B/RMND1/RPS6KB2/TARBP2/ZC3H10	24	BP

GO:0050773	regulation of dendrite development	24/2734	133/17381	0.26329	0.8287	0.81503	ANAPC2/ARC/CAMK1D/CDK5/CFL1/DAB2IP/DBN1/DISC1/EFNA1/FOXO6/GORASP1/GRIN1/ILK/KNDC1/LLPH/MTOR/NLGN3/NRG1/NSMF/OBSL1/PDLIM5/RAB17/TLX2/TNIK	24	BP
GO:0000266	mitochondrial fission	8/2734	39/17381	0.26336	0.8287	0.81503	COX10/DHODH/DNM1/KDR/MIEF2/MTFR1L/MYO19/STAT2	8	BP
GO:0003197	endocardial cushion development	8/2734	39/17381	0.26336	0.8287	0.81503	BMP4/CRELD1/ENG/GATA4/HEY1/NOTCH1/TBX2/TMEM100	8	BP
GO:0015682	ferric iron transport	8/2734	39/17381	0.26336	0.8287	0.81503	ATP6V0B/ATP6V0E2/ATP6V1B1/ATP6V1F/ATP6V1G1/ATP6V1G2/STEAP2/TFR2	8	BP
GO:0045026	plasma membrane	8/2734	39/17381	0.26336	0.8287	0.81503	CATSPER1/ERVFRD-1/SERPINA5/SPACA3/STX1A/STX4/STXBP1/TIE1	8	BP
GO:0045777	positive regulation of	8/2734	39/17381	0.26336	0.8287	0.81503	ADRA1A/ADRA1B/ADRA2B/AGT/AVPR2/CYP11B2/HSD11B2/TACR1	8	BP
GO:0072512	trivalent inorganic cation	8/2734	39/17381	0.26336	0.8287	0.81503	ATP6V0B/ATP6V0E2/ATP6V1B1/ATP6V1F/ATP6V1G1/ATP6V1G2/STEAP2/TFR2	8	BP
GO:0007051	spindle organization	28/2734	157/17381	0.26348	0.8287	0.81503	ANKRD53/ARHGEF10/CDC14A/CHMP1A/CHMP4C/CKAP5/DCTN2/DYNC1H1/EML1/FLNA/GOLGA2/HAUS4/HAUS7/INO80/KIF23/KIF4A/NCOR1/NTMT1/NUMA1/PARP3/PKD1/RGS14/SENP6/TUBG1/TUBGCP2/TUBGCP3/TU	28	BP
GO:0022408	negative regulation of cell-cell	28/2734	157/17381	0.26348	0.8287	0.81503	ABL1/BCL6/BMP4/C1QTNF1/CASP3/CCL21/CD74/ERBB2/HAVCR2/HLX/IDO1/IL1RN/IL20RB/IL4R/LGALS3/LGALS9/MAD1L1/MIR221/MIR222/PLA2G2F/PRKAR1A/PRKCD/SCGB1A1/SHH/TRPV4/VSIG4/WNT1/ZNF703	28	BP
GO:0072073	kidney epithelium development	25/2734	139/17381	0.26348	0.8287	0.81503	AGT/AQP1/BMP4/EYA1/FGFR2/HOXB7/ILK/IRX3/LHX1/MYO1E/NOTCH1/OSR1/PKD1/POU3F3/RARA/SDC1/SHH/SIM1/SMAD6/SMO/SOX8/STAT1/WNT1/WNT11/WNT6	25	BP
GO:0006261	DNA-dependent DNA replication	26/2734	145/17381	0.26356	0.8287	0.81503	BCL6/BMP4/CDK2AP1/CDK9/DNAJA3/E2F8/HMGA1/INO80/LIG3/MCM2/MCM4/MCM9/MCMDC2/ORC6/PARP2/PARP3/PID1/POLA2/POLD4/POLE/POLE4/RFC1/RFC2/RPAIN/TICRR/ZPR1	26	BP
GO:0030574	collagen catabolic	13/2734	68/17381	0.26569	0.8314	0.81776	ADAMTS14/ADAMTS2/COL1A1/COL4A2/COL7A1/CTSD/CTSL/FURIN/MMP15/MMP2/MMP3/MRC2/PHYKPL	13	BP

GO:0051851	modification by host of symbiont morphology or	13/2734	68/17381	0.26569	0.8314	0.81776	AQP1/CCL5/CFL1/EIF2AK4/F2RL1/GAPDH/MIR221/MIR222/PC/PPIB/PSMC3/TUSC2/ZNF502	13	BP
GO:0008016	regulation of heart contraction	42/2734	242/17381	0.26669	0.8314	0.81776	ADM/ADRA1A/ADRA1B/AGT/ATP1A1/ATP1A4/ATP2A3/CACNA1F/CACNA1G/CACNA1H/CACNA1S/CACNB1/CACNB3/CACNG1/CAMK2D/CAV3/CHGA/DES/DRD2/EDN2/FLNA/GATA4/GCH1/GNAO1/GSTM2/HSPB7/ITPR3/KCND1/KCND3/KCNH6/KCNJ5/KCNQ1/MIR328/MIR92A2/MYL4/NKX2-	42	BP
GO:0003151	outflow tract morphogenesis	14/2734	74/17381	0.26817	0.8314	0.81776	ATF2/BMP4/DVL2/ENG/EYA1/FGFR2/FZD2/ILK/NKX2-5/PLXND1/RARA/SMAD6/TBX2/WNT11	14	BP
GO:0045582	positive regulation of T cell	14/2734	74/17381	0.26817	0.8314	0.81776	ADAM8/BAD/BCL6/CCL19/CD27/CD74/HLX/IL18/IL4R/LGALS9/PIK3R6/RARA/SART1/SHH	14	BP
GO:0046823	negative regulation of nucleocytoplasmic transport	14/2734	74/17381	0.26817	0.8314	0.81776	CD27/CDK5/DAB2IP/EMD/FAM89B/MTOR/NFKBIL1/PARP10/PBLD/PDE2A/PKD1/SFRP5/SUFU/THRA	14	BP
GO:0022604	regulation of cell morphogenesis	73/2734	432/17381	0.26842	0.8314	0.81776	ABL1/ALDOA/AMIGO1/ANAPC2/ARAP1/ARC/ARHGAP4/ARPC2/BARHL2/CDC42EP2/CDK5/CFL1/CIB1/CORO1A/CORO1B/CPNE5/CPNE9/CSF1R/CTTN/DAB1/DISC1/DOCK1/DRAXIN/EFNA1/EPHB3/FBLIM1/FERMT2/FES/FGA/FLNA/FMNL1/FMNL2/FN1/GORASP1/GRIN1/ILK/ISLR2/ITGA7/KDR/KNDC1/L1CAM/LIMK1/LINGO1/MARK2/METRN/MYOC/NLGN3/NRG1/NSMF/OBSL1/OLFM4/PDLIM5/PLXNB1/PLXNB3/PLXND1/PTK2B/SEMA3F/SEMA4A/SEMA7A/STRIP1/TAOK2/TBR1/THY1/TLX2/TNFRSF12A/TNIK/TRPV2[illegible]	73	BP
GO:0010639	negative regulation of organelle organization	58/2734	340/17381	0.26929	0.8314	0.81776	ACD/ANAPC15/ARAP1/ARRB2/BMP4/BOK/BRCA1/BUB1B/CAPZA3/CAV3/CCNF/CIB1/CLU/CORO1A/CORO1B/CTBP1/ESPN/EXOSC10/FZD9/GPX1/HDAC8/HIP1R/HMGA1/HORMAD1/KATNB1/LCMT1/LIG3/LMNA/LMOD1/MAD1L1/MAP6D1/MID1IP1/MYOC/NOC2L/NUBP1/OTUB1/PFN1/PHF2/PID1/PIF1/PRELID1/PRKAR1A/PRKCD/RAB7A/SCIN/SLC25A5/SPI1/SPTAN1/SPTB/SPTBN2/SUPT6H/TAF7/TINF2/TRIM54/UBE2B/VAT1/WAS/XRCC3	58	BP

GO:0030199	collagen fibril organization	9/2734	45/17381	0.26982	0.8314	0.81776	ADAMTS14/ADAMTS2/COL1A1/DDR2/LOXL2/PLOD3/SCX/SERPINF2/SERPINH1	9	BP
GO:0044246	regulation of multicellular organismal metabolic	9/2734	45/17381	0.26982	0.8314	0.81776	BMP4/CIITA/ENG/MIR29A/MIR92A2/PDGFRB/PPARD/SCX/SERPINF2	9	BP
GO:0048538	thymus development	9/2734	45/17381	0.26982	0.8314	0.81776	ABL1/CCNB2/EPHB3/FOXN1/HOXA3/MAD1L1/MAPK3/RAF1/SHH	9	BP
GO:0048701	embryonic cranial skeleton morphogenesis	9/2734	45/17381	0.26982	0.8314	0.81776	ALX3/BMP4/FGFR2/IFT140/LHX1/MTHFD1/SETD2/TBX15/TULP3	9	BP
GO:0071827	plasma lipoprotein particle	9/2734	45/17381	0.26982	0.8314	0.81776	AGT/APOA5/APOB/APOC3/DGAT1/GPIHBP1/MPO/SCARB1/SOAT2	9	BP
GO:1901799	negative regulation of proteasomal protein	9/2734	45/17381	0.26982	0.8314	0.81776	ALAD/CCAR2/FHIT/KLHL40/LAMP3/OS9/PANO1/SHH/USP19	9	BP
GO:1902808	positive regulation of cell cycle G1/S	9/2734	45/17381	0.26982	0.8314	0.81776	APEX1/CDK10/CYP1A1/EIF4G1/MEPCE/MIR221/MIR222/MIR29A/UBE2E2	9	BP
GO:0045815	positive regulation of gene expression,	15/2734	80/17381	0.27017	0.8314	0.81776	BAZ1B/HIST1H4F/HIST2H4A/HIST2H4B/HMGA1/KAT2A/MYO1C/PHF2/POLR1A/POLR1D/POLR1E/POLR2L/SLC50A1/TAF1A/ZNRD1	15	BP

GO:0009991	response to extracellular stimulus	77/2734	457/17381	0.27099	0.8314	0.81776	ABCG5/ACACB/ACTA1/ADM/ALAD/AMBRA1/ASGR1/ASNS/ATF3/BCAS3/BGLAP/C2/CHMP1A/CLPS/CLPSL1/COL1A1/CYP1A1/DAD1/DAP/DAPL1/DHODH/DNMT3A/EGFR/EHMT2/EIF2AK4/EIF4EBP1/FOLR2/FOSL1/FOXO4/GATA4/GBA/GCG/GNPAT/GSDMD/HSD11B2/HSF1/HSPA5/INHBB/KAT2A/KIF26A/LARS/LDHA/LTA/MAPK3/MAX/MPO/MTOR/MYOD1/NPRL3/NUDT1/OGG1/OPRM1/OTC/PEMT/PFKFB1/PKM/POR/PPARD/PPP1R9B/PYY/RALB/RARA/RRAGC/SESN1/SIPA1/SLC25A25/SLC34A1/SLC39A5/TH/TNC/[illegible]	77	BP
GO:0000272	polysaccharide catabolic	6/2734	28/17381	0.27127	0.8314	0.81776	CHIT1/INS/MGAM/PFKM/PHKG1/PYGM	6	BP
GO:0007274	neuromuscular synaptic transmission	6/2734	28/17381	0.27127	0.8314	0.81776	CHRM1/CHRNA6/CHRNB1/CHRNB3/P2RX2/STXBP1	6	BP
GO:0016339	calcium-dependent cell-cell adhesion via plasma membrane cell adhesion	6/2734	28/17381	0.27127	0.8314	0.81776	CDH13/CDH16/CDH23/PCDH12/PCDHGB4/PCDHGC3	6	BP
GO:0018345	protein palmitoylation	6/2734	28/17381	0.27127	0.8314	0.81776	MAP6D1/ZDHHC12/ZDHHC14/ZDHHC15/ZDHHC18/ZDHHC7	6	BP
GO:0051220	cytoplasmic sequestering of protein	6/2734	28/17381	0.27127	0.8314	0.81776	CD27/FLNA/NFKBIL1/PKD1/SUFU/THRA	6	BP
GO:0060512	prostate gland morphogenesis	6/2734	28/17381	0.27127	0.8314	0.81776	BMP4/CRIP1/FGFR2/NOTCH1/SHH/TNC	6	BP
GO:0070076	histone lysine demethylation	6/2734	28/17381	0.27127	0.8314	0.81776	HR/KDM4E/KDM6B/KDM8/PHF2/UBE2B	6	BP
GO:0070536	protein K63-linked deubiquitination	6/2734	28/17381	0.27127	0.8314	0.81776	BRCC3/OTUD5/OTUD7A/SHMT2/USP20/USP27X	6	BP

GO:0071634	regulation of transforming growth factor beta production	6/2734	28/17381	0.27127	0.8314	0.81776	ATF2/FURIN/IL13/LGALS9/SERPINF2/WNT11	6	BP
GO:0090049	regulation of cell migration involved in sprouting	6/2734	28/17381	0.27127	0.8314	0.81776	ABL1/CIB1/MIR221/MIR29C/MIR503/NOTCH1	6	BP
GO:0090314	positive regulation of protein	6/2734	28/17381	0.27127	0.8314	0.81776	CDK5/CIB1/ERBB2/ITGAM/MIEF2/MYO1C	6	BP
GO:0090659	walking behavior	6/2734	28/17381	0.27127	0.8314	0.81776	ARRB2/CEND1/DAB1/DRD2/GLRA1/TRH	6	BP
GO:0099623	regulation of cardiac muscle cell membrane repolarization	6/2734	28/17381	0.27127	0.8314	0.81776	CAV3/FLNA/KCNQ1/MIR328/NPPA/WDR1	6	BP
GO:1905508	protein localization to microtubule	6/2734	28/17381	0.27127	0.8314	0.81776	C2CD3/DCTN2/DISC1/MCPH1/NUMA1/PARD6A	6	BP
GO:0017038	protein import	56/2734	328/17381	0.27133	0.8314	0.81776	AGT/BCL6/BMP4/CCL19/CD27/CLU/CSF3/DAB2IP/DNLZ/EGFR/EMD/FAM89B/FLNA/GPIHBP1/GPN2/HEATR3/HSP90AA1/IL18/IPO13/IPO4/LGALS9/LMNA/MTOR/NFKBIL1/NUP98/OGG1/OPRD1/PARP10/PBLD/PDE2A/PEX14/PEX16/PEX3/PEX5/PEX6/PKD1/POLA2/PPP1R10/PRDX1/PRKCD/RBPMS/RPAIN/SFRP5/SHH/SMO/SUFU/THRA/TIMM17B/TIMM22/TIMM23B/TIMM9/	56	BP
GO:1903322	positive regulation of protein modification by small protein conjugation or	35/2734	200/17381	0.27162	0.8314	0.81776	AIMP2/ANAPC15/ANAPC2/ARRB2/AVPR2/AXIN1/BRCA1/BUB1B/CAV3/CDK5RAP3/CDK9/CHFR/CLU/COMMD1/DISC1/GOLGA2/GORASP1/HSPA5/KLHL40/MTA1/NMI/PAXIP1/PSMB11/PSMB6/PSMB7/PSMB8/PSMC3/PSMD13/PSMD3/PSMD5/PSMD7/PTK2B/RWDD3/SEPT4/WFS1	35	BP

GO:0051961	negative regulation of nervous system development	46/2734	267/17381	0.27253	0.8314	0.81776	ARHGAP4/ASCL2/CDK5/CEND1/CIB1/CTDSP1/DAB1/DGUOK/DRAXIN/EFNA1/EIF2AK4/FUOM/GAK/GORASP1/IDH2/INPP5F/IRX3/LINGO1/LRIG2/LRP1/LSM1/MYCN/NEUROD2/NLGN3/NOTCH1/NR2F1/NRG1/PHOX2B/RGMA/RTN4RL1/RTN4RL2/SEMA3F/SHH/SOX8/SUFU/THY1/TLX2/TLX3/TRAF3IP1/TRIM11/TRPV4/TULP3/VAX1/WNT3A/WNT7A/ZHX2	46	BP
GO:0009791	post-embryonic development	17/2734	92/17381	0.27302	0.8314	0.81776	ABL1/ACO1/APOB/ASL/BMP4/CSRNP1/CYP1A2/EMX1/ETNK2/FGFR2/INPPL1/LHX1/MTOR/MYO1E/MYO7A/NKX2-3/PYGO2	17	BP
GO:0060993	kidney morphogenesis	17/2734	92/17381	0.27302	0.8314	0.81776	AGT/BMP4/EYA1/HOXB7/ILK/IRX3/LHX1/OSR1/PDGFRB/PKD1/SHH/SMO/SOX8/STAT1/WNT1/WNT11/WNT6	17	BP
GO:0003198	epithelial to mesenchymal transition involved in endocardial	4/2734	17/17381	0.27314	0.8314	0.81776	ENG/HEY1/NOTCH1/TMEM100	4	BP
GO:0006103	2-oxoglutarate metabolic	4/2734	17/17381	0.27314	0.8314	0.81776	GOT2/IDH1/IDH2/OGDH	4	BP
GO:0006206	pyrimidine nucleobase metabolic	4/2734	17/17381	0.27314	0.8314	0.81776	CDA/DHODH/MTOR/UCK1	4	BP
GO:0010832	negative regulation of myotube	4/2734	17/17381	0.27314	0.8314	0.81776	ANKRD2/NKX2-5/NOTCH1/TRIM72	4	BP
GO:0015693	magnesium ion transport	4/2734	17/17381	0.27314	0.8314	0.81776	CNNM2/NIPA2/NIPAL1/NIPAL2	4	BP
GO:0016075	rRNA catabolic process	4/2734	17/17381	0.27314	0.8314	0.81776	DIS3L/ERN2/EXOSC10/EXOSC2	4	BP
GO:0031268	pseudopodium organization	4/2734	17/17381	0.27314	0.8314	0.81776	CCL21/CDC42EP2/F2RL1/RAB25	4	BP

GO:0032225	regulation of synaptic transmission, dopaminergic	4/2734	17/17381	0.27314	0.8314	0.81776	ARRB2/DRD2/PNKD/TOR1A	4	BP
GO:0032354	response to follicle-stimulating	4/2734	17/17381	0.27314	0.8314	0.81776	ASNS/EPHA8/NOTCH1/POR	4	BP
GO:0036148	phosphatidylglycerol acyl-chain remodeling	4/2734	17/17381	0.27314	0.8314	0.81776	PLA2G1B/PLA2G2F/PLA2G4B/PLA2G5	4	BP
GO:0043651	linoleic acid metabolic	4/2734	17/17381	0.27314	0.8314	0.81776	ELOVL1/ELOVL5/FADS2/GSTM2	4	BP
GO:0044320	cellular response to	4/2734	17/17381	0.27314	0.8314	0.81776	GCK/INHBB/LEPR/PID1	4	BP
GO:0045063	T-helper 1 cell differentiation	4/2734	17/17381	0.27314	0.8314	0.81776	CCL19/HLX/IL4R/SEMA4A	4	BP
GO:0051023	regulation of immunoglobulin secretion	4/2734	17/17381	0.27314	0.8314	0.81776	HLA-E/STX4/TNFRSF4/TRAF2	4	BP
GO:0051446	positive regulation of meiotic cell	4/2734	17/17381	0.27314	0.8314	0.81776	LFNG/PIWIL2/PRDM9/UBE2B	4	BP
GO:0051782	negative regulation of	4/2734	17/17381	0.27314	0.8314	0.81776	C10orf99/CHMP4C/E2F8/ORC6	4	BP
GO:0055083	monovalent inorganic anion homeostasis	4/2734	17/17381	0.27314	0.8314	0.81776	FASLG/FGFR4/GCM2/SLC34A1	4	BP
GO:0055119	relaxation of cardiac muscle	4/2734	17/17381	0.27314	0.8314	0.81776	ATP1A1/CAMK2D/CHGA/GSTM2	4	BP

GO:0071636	positive regulation of transforming growth factor	4/2734	17/17381	0.27314	0.8314	0.81776	ATF2/LGALS9/SERPINF2/WNT11	4	BP
GO:0072488	ammonium transmembrane transport	4/2734	17/17381	0.27314	0.8314	0.81776	AQP1/RHCG/SLC22A1/SLC25A20	4	BP
GO:0090280	positive regulation of calcium ion	4/2734	17/17381	0.27314	0.8314	0.81776	GCG/LGALS3/PDGFRB/TRPV2	4	BP
GO:1900115	extracellular regulation of signal	4/2734	17/17381	0.27314	0.8314	0.81776	ESR2/IL18BP/NBL1/WFIKKN2	4	BP
GO:1900116	extracellular negative regulation of signal	4/2734	17/17381	0.27314	0.8314	0.81776	ESR2/IL18BP/NBL1/WFIKKN2	4	BP
GO:1904706	negative regulation of vascular smooth muscle cell	4/2734	17/17381	0.27314	0.8314	0.81776	GPER1/MIR15A/MIR503/MIR96	4	BP
GO:0051216	cartilage development	32/2734	182/17381	0.27338	0.8314	0.81776	ADAMTS7/BARX2/BMP4/COL1A1/ESRRA/FBXW4/GDF2/HOXA3/HOXB3/HOXC4/HOXD3/LOXL2/MAPK3/MUSTN1/MYCN/NKX3-2/OSR1/PITX1/PKD1/PKDCC/POR/RARA/SCIN/SCX/SERPINH1/SOX5/TGFBI/THBS3/THRA/TRPV4/WNT11/WNT7A	32	BP
GO:0010906	regulation of glucose metabolic	18/2734	98/17381	0.27401	0.8314	0.81776	ACACB/BAD/C1QTNF1/COX11/DGKQ/GCG/GCK/GNMT/IGFBP3/INS/KAT2A/LCMT1/LEPR/MAEA/MTOR/PFKFB1/PHLDA2/RORC	18	BP

GO:0001960	negative regulation of cytokine-mediated	10/2734	51/17381	0.27474	0.8314	0.81776	CCDC3/CCL5/ECM1/F2RL1/IL1RN/NR1H3/PELI3/PYDC1/SCRIB/TRAIP	10	BP
GO:0010658	striated muscle cell apoptotic	10/2734	51/17381	0.27474	0.8314	0.81776	AGT/AMBRA1/CAMK2D/EIF5A/HSF1/ILK/LTK/MIR16-1/MIR195/NKX2-5	10	BP
GO:0048168	regulation of neuronal synaptic	10/2734	51/17381	0.27474	0.8314	0.81776	AGT/ARC/DBN1/DLG4/DRD2/GRIN1/HRAS/JPH3/NCDN/NSMF	10	BP
GO:0050432	catecholamine secretion	10/2734	51/17381	0.27474	0.8314	0.81776	ADRA2A/ADRA2B/ADRA2C/AGT/CHGA/CHRNA6/DRD2/DTNBP1/NISCH/STX1A	10	BP
GO:0060350	endochondral bone morphogenesis	10/2734	51/17381	0.27474	0.8314	0.81776	BMP4/COL1A1/INPPL1/PHOSPHO1/POR/RARA/SCX/SERPINH1/THBS3/TRPV4	10	BP
GO:0021543	pallium development	29/2734	164/17381	0.27481	0.8314	0.81776	BAD/BCAN/CASP3/CDK5/CDK5R2/DAB1/DAB2IP/DCLK2/DISC1/EGFR/EIF2B5/EMX1/FLNA/GRIN1/KDM6B/KIRREL3/LHX5/LRP1/MCPH1/MFSD2A/NME1/OGDH/POU3F3/RARA/SMO/TBR1/TH/TRAPPC9/WNT3A	29	BP
GO:0007599	hemostasis	60/2734	353/17381	0.27511	0.8314	0.81776	ADAMTS13/ADRA2A/ADRA2B/ADRA2C/ARRB2/ASIC2/AVPR2/C1QTNF1/CLIC1/COL1A1/CSRP1/DGKI/DGKQ/DGKZ/DOCK1/DTNBP1/EHD1/F2RL1/FERMT3/FGA/FLNA/GATA4/GP9/HSPB1/IFNA5/ILK/ITPK1/ITPR3/KLKB1/KNG1/LCK/MAPK3/MFN2/NOS3/P2RX2/PDGFA/PF4V1/PIK3R5/PIK3R6/PLAU/PRCP/PRKAR1A/PRKAR1B/PRKCD/PROZ/PTPN11/RAF1/RBSN/SELP/SERPINA5/SERPINF2/SH2B1/SHH/STXBP1/TEC/TLN1/VWF/WAS/WNT3A/Z[illegible]	60	BP
GO:0071466	cellular response to xenobiotic	20/2734	110/17381	0.27531	0.8314	0.81776	AIP/AKR7A3/AOC2/AS3MT/BPHL/CES2/CYP1A1/CYP1A2/CYP2W1/DPEP1/EPHX2/GRIN1/GSTM1/GSTM2/LPO/MGST1/MGST3/NQO2/POR/RORC	20	BP
GO:0038127	ERBB signaling pathway	27/2734	152/17381	0.27551	0.8314	0.81776	ABL1/ADRA2A/ADRA2B/ADRA2C/AGT/ARAP1/CDH13/CSK/DAB2IP/DOK1/EGFR/ERBB2/FASLG/FES/GPER1/HIP1R/HRAS/HSP90AA1/MYOC/NCF1/NRG1/PTK2B/PTPN11/RAB7A/RHBDF1/SHC1/VPS25	27	BP

GO:0006914	autophagy	79/2734	470/17381	0.27559	0.8314	0.81776	ABL1/AMBRA1/ATG10/ATG101/ATG2A/ATP13A2/ATP6V0B/ATP6V0E2/ATP6V1B1/ATP6V1G1/ATP6V1G2/ATPIF1/BAD/BOK/CASP1/CASP3/CDK5/CHMP4C/CLU/CTSD/CTTN/DAP/DAPK1/DAPK2/DAPL1/DYNLL2/EIF2AK4/EIF4G1/GAPDH/GATA4/GBA/GOLGA2/GPSM1/HSP90AA1/HSPB1/IFT20/KAT2A/KDR/LAMP3/LARS/LEPR/LRSAM1/MAPK3/MARK2/MFN1/MFN2/MLST8/MTMR14/MTOR/NOD1/NPRL3/OSBPL7/PGAM5/PGC/PLEKHM1/POLDIP2/PRKAB1/PSAP/QSOX1/RAB24/RAB7A/RALB/RRAGC/SNF8/SPTLC1/TBC1D25/TBK1/TECPR1/TFEB/TRIM65/ULK1/VPS13A/VPS25/VPS37B/VPS[illegible]	79	BP
GO:0061919	process utilizing autophagic mechanism	79/2734	470/17381	0.27559	0.8314	0.81776	ABL1/AMBRA1/ATG10/ATG101/ATG2A/ATP13A2/ATP6V0B/ATP6V0E2/ATP6V1B1/ATP6V1G1/ATP6V1G2/ATPIF1/BAD/BOK/CASP1/CASP3/CDK5/CHMP4C/CLU/CTSD/CTTN/DAP/DAPK1/DAPK2/DAPL1/DYNLL2/EIF2AK4/EIF4G1/GAPDH/GATA4/GBA/GOLGA2/GPSM1/HSP90AA1/HSPB1/IFT20/KAT2A/KDR/LAMP3/LARS/LEPR/LRSAM1/MAPK3/MARK2/MFN1/MFN2/MLST8/MTMR14/MTOR/NOD1/NPRL3/OSBPL7/PGAM5/PGC/PLEKHM1/POLDIP2/PRKAB1/PSAP/QSOX1/RAB24/RAB7A/RALB/RRAGC/SNF8/SPTLC1/TBC1D25/TBK1/TECPR1/TFEB/TRIM65/ULK1/VPS13A/VPS25/VPS37B/VPS[illegible]	79	BP
GO:1902850	microtubule cytoskeleton organization involved in	22/2734	122/17381	0.27594	0.8314	0.81776	ANKRD53/ARHGEF10/CDC14A/CHMP1A/CHMP4C/DCTN2/DYNC1H1/EML1/EYA1/FLNA/GOLGA2/KIF23/KIF4A/MCPH1/NUMA1/PARP3/PKD1/SETD2/TUBGCP2/TUBGCP3/TUBGCP5/WRAP73	22	BP
GO:0010977	negative regulation of neuron projection	23/2734	128/17381	0.27605	0.8314	0.81776	ARHGAP4/CDK5/CIB1/DAB1/DGUOK/DRAXIN/EFNA1/GAK/GORASP1/INPP5F/LINGO1/LRIG2/LRP1/NLGN3/NR2F1/RGMA/RTN4RL1/RTN4RL2/SEMA3F/THY1/TLX2/TRPV4/WNT3A	23	BP
GO:0043122	regulation of I-kappaB kinase/NF-kappaB	42/2734	243/17381	0.27614	0.8314	0.81776	ABL1/CARD11/CARD9/CASP1/CCL19/CCL21/CD27/CD74/DAB2IP/ECM1/F2RL1/FASLG/FLNA/GPR89A/HSPB1/IKBKB/IL18/IRAK1/LGALS9/LTBR/MAP3K14/MIB2/NLRX1/NOD1/OTUD7A/PER1/PLEKHG5/PRDX1/RNF31/SHARPIN/SLC20A1/STAT1/TBK1/TIRAP/TLR9/TMEM101/TNIP2/TRAF2/TRIM6	42	BP
GO:0010596	negative regulation of endothelial cell	11/2734	57/17381	0.27855	0.8314	0.81776	ADGRB1/DAB2IP/GDF2/MIR10A/MIR16-1/MIR212/MIR221/MIR29C/MIR503/MIR92A2/NOTCH1	11	BP

GO:0033013	tetrapyrrole metabolic	11/2734	57/17381	0.27855	0.8314	0.81776	ALAD/ALAS2/ATPIF1/COX10/CUBN/CYP1A1/CYP1A2/HMBS/HMOX2/NFE2L1/UROS	11	BP
GO:1901880	negative regulation of protein	11/2734	57/17381	0.27855	0.8314	0.81776	CAPZA3/CIB1/KATNB1/LMOD1/MAP6D1/MID1IP1/SCIN/SPTAN1/SPTB/SPTBN2/TRIM54	11	BP
GO:1901987	regulation of cell cycle phase transition	75/2734	446/17381	0.28028	0.8314	0.81776	ACTR1A/ANAPC15/ANAPC2/APEX1/BRCA1/BUB1B/C10orf99/CDC14A/CDK10/CDK2AP2/CDK4/CDK5RAP3/CEP164/CEP70/CHMP4C/CKAP5/CTDSP1/CYP1A1/DCTN2/DDB1/DYNC1H1/E2F4/E2F8/EGFR/EIF4G1/FOXO4/GPR132/HAUS4/HAUS7/HSP90AA1/HSPA2/INO80/LCMT1/MAD1L1/MEN1/MEPCE/MIIP/MIR10A/MIR15A/MIR16-1/MIR195/MIR221/MIR222/MIR29A/MIR29C/MIR503/MUC1/NEK10/NEK11/PAXIP1/PCBP4/PHOX2B/PID1/PKD1/PPP1R9B/PSMB11/PSMB6/PSMB7/PSMB8/PSMC3/PSMD13/PSMD3/PSMD5/PSMD7/RINT1/SDCCAG8/SFN/SSNA[illegible]	75	BP
GO:0001953	negative regulation of cell-matrix	7/2734	34/17381	0.28074	0.8314	0.81776	AJAP1/BCAS3/BCL6/CDKN2A/MIR29C/MIR92A2/MYOC	7	BP
GO:0038179	neurotrophin signaling	7/2734	34/17381	0.28074	0.8314	0.81776	AGT/CASP3/CORO1A/PPP2R5B/PTPN11/RAF1/ZFYVE27	7	BP
GO:0042558	pteridine-containing compound metabolic	7/2734	34/17381	0.28074	0.8314	0.81776	ALDH1L1/FOLR2/GCH1/GGH/MTHFD1/PIPOX/SHMT2	7	BP
GO:0044091	membrane biogenesis	7/2734	34/17381	0.28074	0.8314	0.81776	CAV3/GAK/NLGN3/NRXN2/PEX11A/PEX16/PEX3	7	BP
GO:0072525	pyridine-containing compound biosynthetic	7/2734	34/17381	0.28074	0.8314	0.81776	HAAO/IDH2/NMNAT3/PARP10/PARP9/QPRT/SLC22A13	7	BP
GO:1901985	positive regulation of protein	7/2734	34/17381	0.28074	0.8314	0.81776	BRCA1/KAT2A/MAPK3/MUC1/PAXIP1/PIWIL2/RPS6KA4	7	BP

GO:0051298	centrosome duplication	12/2734	63/17381	0.2815	0.8314	0.81776	ARHGEF10/BRCA1/C2CD3/CCNF/CHMP1A/CHMP4C/CKAP5/NUBP1/TUBGCP2/TUBGCP3/TUBGCP5/XRCC3	12	BP
GO:0070988	demethylation	12/2734	63/17381	0.2815	0.8314	0.81776	ALKBH4/ALKBH5/APEX1/CYP1A1/CYP1A2/HR/KDM4E/KDM6B/KDM8/PHF2/POR/UBE2B	12	BP
GO:0021700	developmental maturation	44/2734	256/17381	0.28367	0.8314	0.81776	ACRBP/AGRN/ANAPC2/ANG/C1QL1/CCL19/CCL21/CD63/CDH5/CDK5R2/CEND1/DAB2IP/DISC1/DLG4/EPHA8/FARP2/GRIN1/HBZ/L3MBTL3/MAEA/MMP2/MTCH1/MTOR/MYOC/NEUROD2/PHOSPHO1/PLXNB1/PTK2B/REN/RNASE9/RND1/RXFP2/SCARF1/SEPT4/SLC26A6/SOX8/SPINK5/STXBP1/TCP11/THBS3/TMEM79/TUSC2/WNT1/ZDHHC15	44	BP
GO:0090114	COPII-coated vesicle budding	13/2734	69/17381	0.2838	0.8314	0.81776	CNIH2/COL7A1/GOLGA2/GORASP1/GOSR2/SEC16A/SEC16B/STX5/TRAPPC1/TRAPPC2L/TRAPPC3/TRAPPC4/TRAPPC9	13	BP
GO:0099131	ATP hydrolysis coupled ion transmembrane transport	13/2734	69/17381	0.2838	0.8314	0.81776	ABCC3/ABCC4/ABCC5/ABCC6/ATP13A2/ATP1A4/ATP2A3/ATP2C2/ATP5G1/ATP6V0B/ATP6V0E2/ATP6V1B1/ATP6V1F	13	BP
GO:0045861	negative regulation of proteolysis	59/2734	348/17381	0.28421	0.8314	0.81776	A2ML1/AGT/AHSG/ALAD/APLP2/AQP1/ARRB2/CCAR2/CD27/CDK5/CHAC1/COL7A1/CRB2/CRYAB/DPEP1/ECM1/EFNA1/FHIT/FURIN/GAPDH/GPX1/HERPUD1/INS/ITIH4/ITIH6/KLHL40/KNG1/LAMP3/LRIG2/MIR195/MIR29C/NAIP/NLE1/OS9/PANO1/PEBP1/PI16/POR/RAF1/RPS6KA1/SERPINA2/SERPINA3/SERPINA4/SERPINA5/SERPINF2/SERPINH1/SERPINI1/SFN/SHH/SNX12/SPINK2/SPINK5/SPINT1/SPOCK2/SUFU/TAF1/USP19/WFIKKN2/WN	59	BP
GO:0001707	mesoderm formation	14/2734	75/17381	0.28557	0.8314	0.81776	AXIN1/BMP4/CRB2/EYA1/FGFR2/KDM6B/MESP2/PRKAR1A/SCX/TLX2/TXNRD1/WLS/WNT11/WNT3A	14	BP
GO:0002479	antigen processing and presentation of exogenous peptide antigen via MHC class	14/2734	75/17381	0.28557	0.8314	0.81776	HLA-E/IKBKB/ITGB5/NCF1/PSMB11/PSMB6/PSMB7/PSMB8/PSMC3/PSMD13/PSMD3/PSMD5/PSMD7/TAP1	14	BP

GO:0090662	ATP hydrolysis coupled transmembrane transport	14/2734	75/17381	0.28557	0.8314	0.81776	ABCC3/ABCC4/ABCC5/ABCC6/ATP13A2/ATP1A1/ATP1A4/ATP2A3/ATP2C2/ATP5G1/ATP6V0B/ATP6V0E2/ATP6V1B1/ATP6V1F	14	BP
GO:0002685	regulation of leukocyte migration	30/2734	171/17381	0.28584	0.8314	0.81776	ADAM8/C3AR1/CAMK1D/CCL1/CCL19/CCL21/CCL5/CCR2/CD74/CREB3/CSF1/CXCR3/DAPK2/ECM1/EDN2/F2RL1/KARS/LGALS3/LGALS9/MAPK3/NBL1/PF4V1/PTK2B/RARRES2/SELP/TACR1/THY1/TIRAP/TNFRSF18/TRPV	30	BP
GO:0017157	regulation of exocytosis	30/2734	171/17381	0.28584	0.8314	0.81776	ADRA2A/ATP13A2/CACNA1G/CACNA1H/CCR2/CDK5/CDK5R2/CPLX1/DOC2A/DOC2B/F2RL1/FES/FGA/IL13/IL4R/ITGAM/LGALS9/LGI3/LLGL1/NOTCH1/RAB26/RAB7A/RALB/SDC1/SNF8/STX1A/STX4/STXBP1/SYTL3/TRP	30	BP
GO:1903035	negative regulation of response to	15/2734	81/17381	0.28693	0.8314	0.81776	AJAP1/C1QTNF1/FGA/INPP5F/KLKB1/KNG1/LRIG2/NOS3/PDGFA/PLAU/PRKCD/PROZ/RGMA/RTN4RL1/SERPINF2	15	BP
GO:2001242	regulation of intrinsic apoptotic signaling	28/2734	159/17381	0.2871	0.8314	0.81776	ANKRD2/BAD/BCAP31/BOK/CCAR2/CD74/CLU/CREB3/DAPK2/GPX1/HERPUD1/HSPB1/INS/LCK/MAPK8IP1/MIR15A/MIR16-1/MUC1/NOC2L/NONO/SEPT4/SGMS1/SPOP/SYVN1/TRAP1/VDAC2/WFS1/ZNF385A	28	BP
GO:0007520	myoblast fusion	8/2734	40/17381	0.2875	0.8314	0.81776	ADAM12/ADGRB1/CACNA1H/CAV3/EHD1/IL4R/MYOD1/WNT1	8	BP
GO:0046825	regulation of protein export from nucleus	8/2734	40/17381	0.2875	0.8314	0.81776	ANP32B/CDK5/EMD/PTPN11/SETD2/SFN/SUPT6H/ZC3H3	8	BP
GO:0050832	defense response to	8/2734	40/17381	0.2875	0.8314	0.81776	C10orf99/CHGA/COTL1/GAPDH/MPO/RARRES2/RNASE8/SPON2	8	BP
GO:0070169	positive regulation of biomineral	8/2734	40/17381	0.2875	0.8314	0.81776	AMTN/BMP4/FZD9/ISG15/OSR1/PKDCC/TMEM119/WNT6	8	BP
GO:0071248	cellular response to metal ion	27/2734	153/17381	0.28767	0.8314	0.81776	ALAD/AQP1/ATP13A2/CACNA1H/CACYBP/CAMK2D/CPNE7/CYP11B2/CYP1A1/CYP1A2/DPEP1/EGFR/ENDOG/GLRA1/HSF1/HSPA5/MAPK3/MT2A/NCF1/NEUROD2/OGG1/RASGRP2/SHH/SLC34A1/SPAG16/TFR2/TH	27	BP

GO:0032231	regulation of actin filament bundle	16/2734	87/17381	0.28795	0.8314	0.81776	ABL1/ARAP1/ARHGEF10/EVL/FHOD1/LIMK1/MTOR/MYOC/NOX4/PFN1/SERPINF2/SH3PXD2B/SORBS3/TACR1/WAS/WNT11	16	BP
GO:0051963	regulation of synapse	16/2734	87/17381	0.28795	0.8314	0.81776	ADGRB1/AGRN/AMIGO1/ASIC2/CLSTN1/CLSTN3/EIF4G1/EPHB3/GRIN1/LINGO2/LRRTM1/NLGN3/PDLIM5/RAB17/SLITRK3/WNT7A	16	BP
GO:0051384	response to glucocorticoid	26/2734	147/17381	0.28818	0.8314	0.81776	ADM/ALAD/AQP1/BAD/BGLAP/CASP3/CCL1/EGFR/EIF4EBP1/FOSL1/GBA/HEY1/HNMT/HSD11B2/IL1RN/MYOD1/PFKFB1/PTPRU/SCGB1A1/SDC1/SMYD3/SSTR5/TH/TRH/TRIM63/UCP3	26	BP
GO:0043271	negative regulation of ion transport	25/2734	141/17381	0.28863	0.8314	0.81776	ADRA2A/BEST3/CAMK2D/CAV3/COMMD1/CRHR1/DRD2/GNAO1/GNB5/GPR35/GSTM2/MIR153-1/MIR212/MIR328/MTOR/NOS3/OSR1/PACSIN3/PTK2B/RRAD/THADA/TLR9/TRDN/TRH/YWHAQ	25	BP
GO:0001101	response to acid chemical	55/2734	324/17381	0.28897	0.8314	0.81776	ALAD/APOB/AQP1/ASNS/ATF2/BAD/CASP3/CCL19/CCL21/CDK4/CFL1/COL16A1/COL1A1/CTSH/DHODH/DNMT3A/EGFR/EGR1/FGFR2/FOLR2/GJB3/GLRA1/GNPAT/GRIN1/HSF1/KCNK4/KDR/LARS/LTK/MICB/MIR92A2/MMP2/MTOR/NAIP/NME1/NSMF/OGG1/OSR1/OTC/P2RY6/PDGFRB/PEMT/PID1/PTK2B/RARA/RRAGC/SCGB1A1/SIPA1/TACR1/TH/TIE1/TNC/WNT11/	55	BP
GO:0008203	cholesterol metabolic process	24/2734	135/17381	0.28902	0.8314	0.81776	ACACB/ACADVL/APOA5/APOB/CLN6/CUBN/CYP11A1/DGKQ/EPHX2/HSD17B7/LEPR/LMF1/NPC1L1/NPC2/NSDHL/OSBPL5/PMVK/POR/PPARD/SCAP/SCARB1/SCARF1/SOAT2/STARD3	24	BP
GO:0007006	mitochondrial membrane organization	23/2734	129/17381	0.28933	0.8314	0.81776	ATF2/ATP5G1/ATP5I/ATPIF1/BAD/BOK/DYNLL2/FZD9/HIP1R/HSP90AA1/MFN1/MFN2/MIR29A/MIR29C/MOAP1/PPP1R13B/RHOT2/SFN/SLC25A5/TIMM22/TIMM9/YWHAQ/ZNF205	23	BP
GO:0000050	urea cycle	3/2734	12/17381	0.28949	0.8314	0.81776	ASL/NAGS/OTC	3	BP
GO:0002862	negative regulation of inflammatory response to	3/2734	12/17381	0.28949	0.8314	0.81776	GPR17/GPX1/IL20RB	3	BP

GO:0002903	negative regulation of B cell apoptotic	3/2734	12/17381	0.28949	0.8314	0.81776	BCL6/CD74/NOC2L	3	BP
GO:0003207	cardiac chamber formation	3/2734	12/17381	0.28949	0.8314	0.81776	NKX2-5/NOTCH1/TBX2	3	BP
GO:0006069	ethanol oxidation	3/2734	12/17381	0.28949	0.8314	0.81776	ADH1A/ADH1C/ALDH2	3	BP
GO:0006183	GTP biosynthetic	3/2734	12/17381	0.28949	0.8314	0.81776	IMPDH1/NME1/NME4	3	BP
GO:0006264	mitochondrial DNA	3/2734	12/17381	0.28949	0.8314	0.81776	DNAJA3/LIG3/PID1	3	BP
GO:0006705	mineralocorticoid biosynthetic process	3/2734	12/17381	0.28949	0.8314	0.81776	CACNA1H/CYP11B2/HSD3B1	3	BP
GO:0008212	mineralocorticoid metabolic	3/2734	12/17381	0.28949	0.8314	0.81776	CACNA1H/CYP11B2/HSD3B1	3	BP
GO:0008228	opsonization	3/2734	12/17381	0.28949	0.8314	0.81776	MYO18A/SFTPA1/SPON2	3	BP
GO:0008655	pyrimidine-containing compound	3/2734	12/17381	0.28949	0.8314	0.81776	CDA/PUDP/UCK1	3	BP
GO:0009162	deoxyribonucleoside monophosphate metabolic	3/2734	12/17381	0.28949	0.8314	0.81776	DGUOK/DNPH1/NT5M	3	BP
GO:0010896	regulation of triglyceride catabolic	3/2734	12/17381	0.28949	0.8314	0.81776	APOA5/APOC3/PNPLA2	3	BP
GO:0016114	terpenoid biosynthetic process	3/2734	12/17381	0.28949	0.8314	0.81776	ALDH8A1/CYP1A1/RBP1	3	BP

GO:0019532	oxalate transport	3/2734	12/17381	0.28949	0.8314	0.81776	SLC26A1/SLC26A10/SLC26A6	3	BP
GO:0019934	cGMP-mediated	3/2734	12/17381	0.28949	0.8314	0.81776	PDE2A/PDZD3/PTK2B	3	BP
GO:0021903	rostrocaudal neural tube patterning	3/2734	12/17381	0.28949	0.8314	0.81776	EN1/GBX2/WNT1	3	BP
GO:0031507	heterochromatin assembly	3/2734	12/17381	0.28949	0.8314	0.81776	CDKN2A/HMGA1/TNRC18	3	BP
GO:0034384	high-density lipoprotein particle	3/2734	12/17381	0.28949	0.8314	0.81776	APOC3/CUBN/SCARB1	3	BP
GO:0042416	dopamine biosynthetic process	3/2734	12/17381	0.28949	0.8314	0.81776	DDC/GCH1/TH	3	BP
GO:0043097	pyrimidine nucleoside	3/2734	12/17381	0.28949	0.8314	0.81776	CDA/PUDP/UCK1	3	BP
GO:0044090	positive regulation of vacuole	3/2734	12/17381	0.28949	0.8314	0.81776	LRSAM1/RALB/ULK1	3	BP
GO:0045161	neuronal ion channel	3/2734	12/17381	0.28949	0.8314	0.81776	AGRN/MTCH1/MYOC	3	BP
GO:0045346	regulation of MHC class II biosynthetic process	3/2734	12/17381	0.28949	0.8314	0.81776	CIITA/NFX1/SPI1	3	BP
GO:0045759	negative regulation of	3/2734	12/17381	0.28949	0.8314	0.81776	CAV3/GPR35/MIR328	3	BP
GO:0045836	positive regulation of meiotic nuclear	3/2734	12/17381	0.28949	0.8314	0.81776	PIWIL2/PRDM9/UBE2B	3	BP

GO:0046606	negative regulation of centrosome	3/2734	12/17381	0.28949	0.8314	0.81776	BRCA1/CCNF/NUBP1	3	BP
GO:0048711	positive regulation of astrocyte	3/2734	12/17381	0.28949	0.8314	0.81776	BIN1/CLCF1/NOTCH1	3	BP
GO:0050665	hydrogen peroxide biosynthetic	3/2734	12/17381	0.28949	0.8314	0.81776	CYP1A1/CYP1A2/ZNF205	3	BP
GO:0051255	spindle midzone	3/2734	12/17381	0.28949	0.8314	0.81776	CDC14A/KIF23/KIF4A	3	BP
GO:0051712	positive regulation of killing of cells	3/2734	12/17381	0.28949	0.8314	0.81776	BAD/F2RL1/GAPDH	3	BP
GO:0051873	killing by host of symbiont	3/2734	12/17381	0.28949	0.8314	0.81776	F2RL1/GAPDH/TUSC2	3	BP
GO:0055062	phosphate ion homeostasis	3/2734	12/17381	0.28949	0.8314	0.81776	FGFR4/GCM2/SLC34A1	3	BP
GO:0060442	branching involved in prostate gland	3/2734	12/17381	0.28949	0.8314	0.81776	BMP4/FGFR2/SHH	3	BP
GO:0060742	epithelial cell differentiation involved in prostate gland development	3/2734	12/17381	0.28949	0.8314	0.81776	FGFR2/NOTCH1/PSAP	3	BP
GO:0061684	chaperone-mediated autophagy	3/2734	12/17381	0.28949	0.8314	0.81776	ATP13A2/CLU/HSP90AA1	3	BP

GO:0070243	regulation of thymocyte apoptotic	3/2734	12/17381	0.28949	0.8314	0.81776	ADAM8/BMP4/EFNA1	3	BP
GO:0071257	cellular response to electrical	3/2734	12/17381	0.28949	0.8314	0.81776	GNAT1/NEUROD2/NSMF	3	BP
GO:0071340	skeletal muscle acetylcholine-gated channel clustering	3/2734	12/17381	0.28949	0.8314	0.81776	DNAJA3/FNTA/FZD9	3	BP
GO:0072017	distal tubule development	3/2734	12/17381	0.28949	0.8314	0.81776	NOTCH1/PKD1/POU3F3	3	BP
GO:0072178	nephric duct morphogenesis	3/2734	12/17381	0.28949	0.8314	0.81776	BMP4/LHX1/OSR1	3	BP
GO:0072182	regulation of nephron tubule epithelial cell differentiation	3/2734	12/17381	0.28949	0.8314	0.81776	LHX1/OSR1/STAT1	3	BP
GO:0072505	divalent inorganic anion	3/2734	12/17381	0.28949	0.8314	0.81776	FGFR4/GCM2/SLC34A1	3	BP
GO:0072506	trivalent inorganic anion	3/2734	12/17381	0.28949	0.8314	0.81776	FGFR4/GCM2/SLC34A1	3	BP
GO:0098787	mRNA cleavage involved in	3/2734	12/17381	0.28949	0.8314	0.81776	CPSF1/CPSF4/CSTF2	3	BP
GO:1900119	positive regulation of execution phase	3/2734	12/17381	0.28949	0.8314	0.81776	BOK/ENDOG/HSF1	3	BP

GO:1902993	positive regulation of amyloid precursor	3/2734	12/17381	0.28949	0.8314	0.81776	CLU/EFNA1/EFNA3	3	BP
GO:1903421	regulation of synaptic vesicle recycling	3/2734	12/17381	0.28949	0.8314	0.81776	CDK5/DNM1/TOR1A	3	BP
GO:1903587	regulation of blood vessel endothelial cell proliferation involved in sprouting	3/2734	12/17381	0.28949	0.8314	0.81776	MIR16-1/MIR222/MIR503	3	BP
GO:2000009	negative regulation of protein localization to	3/2734	12/17381	0.28949	0.8314	0.81776	CAV3/COMMD1/TAX1BP3	3	BP
GO:0046031	ADP metabolic process	19/2734	105/17381	0.28951	0.8314	0.81776	AK5/ALDOA/BAD/GALK1/GAPDH/GCK/HK3/INS/LDHA/MLXIPL/NCOR1/NUP210/NUP98/OGDH/OGDHL/PFKFB1/PFKM/PGAM4/PKM	19	BP
GO:0002223	stimulatory C-type lectin receptor	22/2734	123/17381	0.28955	0.8314	0.81776	CARD11/CARD9/CLEC7A/HRAS/IKBKB/MUC1/MUC2/MUC3A/MUC5AC/MUC5B/MUC6/PRKCD/PSMB11/PSMB6/PSMB7/PSMB8/PSMC3/PSMD13/PSMD3/PSMD5/PSMD7/RAF1	22	BP
GO:0071331	cellular response to	22/2734	123/17381	0.28955	0.8314	0.81776	ADRA2A/ANO1/BAD/BRSK2/CDK16/ENDOG/GATA4/GCG/GPER1/MEN1/MIRLET7G/MLXIPL/NME1/NOX4/PTPRN2/RAF1/RFX6/SIDT2/SLC26A6/SL	22	BP
GO:0001774	microglial cell activation	5/2734	23/17381	0.28985	0.8314	0.81776	CLU/FAM19A3/IL13/ITGAM/TLR8	5	BP
GO:0006297	nucleotide-excision repair, DNA gap filling	5/2734	23/17381	0.28985	0.8314	0.81776	LIG3/POLD4/POLE/RFC1/RFC2	5	BP

GO:0009220	pyrimidine ribonucleotide biosynthetic process	5/2734	23/17381	0.28985	0.8314	0.81776	AK5/DHODH/NME1/NME4/UCK1	5	BP
GO:0016338	calcium-independent cell-cell adhesion via plasma membrane cell-	5/2734	23/17381	0.28985	0.8314	0.81776	CLDN15/CLDN2/CLDN23/CLDN4/CLDN6	5	BP
GO:0021801	cerebral cortex radial glia guided	5/2734	23/17381	0.28985	0.8314	0.81776	CDK5/CDK5R2/DAB1/DAB2IP/DISC1	5	BP
GO:0022011	myelination in peripheral nervous system	5/2734	23/17381	0.28985	0.8314	0.81776	ARHGEF10/ILK/MYOC/PARD3/POU3F1	5	BP
GO:0022030	telencephalon glial cell	5/2734	23/17381	0.28985	0.8314	0.81776	CDK5/CDK5R2/DAB1/DAB2IP/DISC1	5	BP
GO:0032292	peripheral nervous system axon	5/2734	23/17381	0.28985	0.8314	0.81776	ARHGEF10/ILK/MYOC/PARD3/POU3F1	5	BP
GO:0032594	protein transport within	5/2734	23/17381	0.28985	0.8314	0.81776	GRIPAP1/REEP2/RILPL1/RILPL2/SCRIB	5	BP
GO:0033081	regulation of T cell differentiation	5/2734	23/17381	0.28985	0.8314	0.81776	ADAM8/BMP4/ERBB2/FOXN1/SHH	5	BP
GO:0042401	cellular biogenic amine biosynthetic	5/2734	23/17381	0.28985	0.8314	0.81776	AGMAT/DDC/HDC/OAZ2/PAOX	5	BP

GO:0042402	cellular biogenic amine catabolic	5/2734	23/17381	0.28985	0.8314	0.81776	CHDH/HAAO/HNMT/IDO1/PAOX	5	BP
GO:0045606	positive regulation of epidermal cell	5/2734	23/17381	0.28985	0.8314	0.81776	BMP4/NOTCH1/NUMA1/PTCH2/SFN	5	BP
GO:0046856	phosphatidylinositol dephosphorylati	5/2734	23/17381	0.28985	0.8314	0.81776	INPP5D/INPP5E/INPP5F/INPPL1/MTMR1	5	BP
GO:0048745	smooth muscle tissue	5/2734	23/17381	0.28985	0.8314	0.81776	BMP4/ENG/OSR1/SHH/STRA6	5	BP
GO:0090140	regulation of mitochondrial fission	5/2734	23/17381	0.28985	0.8314	0.81776	DHODH/KDR/MIEF2/MYO19/STAT2	5	BP
GO:0120033	negative regulation of plasma membrane bounded cell projection	5/2734	23/17381	0.28985	0.8314	0.81776	CCL21/EVL/PLXNB3/PRKCD/RAP1GAP	5	BP
GO:1904707	positive regulation of vascular smooth muscle cell	5/2734	23/17381	0.28985	0.8314	0.81776	AGT/MIR221/MIR222/MMP2/NQO2	5	BP
GO:0046580	negative regulation of Ras protein	9/2734	46/17381	0.29243	0.838	0.82426	ADRA1A/BCL6/DAB2IP/MAPKAP1/MFN2/MYOC/RASA3/RASA4B/TNK1	9	BP
GO:0072132	mesenchyme morphogenesis	9/2734	46/17381	0.29243	0.838	0.82426	ACTA1/ENG/HEY1/NOTCH1/OSR1/TBX2/TMEM100/WNT11/WNT3A	9	BP

GO:0046879	hormone secretion	51/2734	300/17381	0.29386	0.8417	0.82789	ADM/ADRA2A/ADRA2B/ADRA2C/AGT/ANO1/AQP1/ARL2BP/BAD/BLK/BRSK2/C1QTNF1/CAPN10/CCL5/CDK16/CPLX1/CRHR1/DOC2B/DRD2/EGFR/FGA/GCG/GCK/GLUD1/GPER1/HCAR2/IL1RN/INHBB/INS/ITPR3/MAFA/MTNR1B/PFKM/PPARD/PTPN11/PTPRN2/RAB11FIP3/RAF1/REN/RFX6/SIDT2/SLC25A5/SSTR5/STX1A/STX4/TACR1/TACR2/TFR2/TRH/TRPV4/VGF	51	BP
GO:0001818	negative regulation of cytokine production	42/2734	245/17381	0.29544	0.8459	0.83194	ARRB2/BCL6/CHID1/CSK/CUEDC2/DHX58/EPX/F2RL1/FN1/FURIN/GBA/HAVCR2/HSF1/IDO1/IL13/IL20RB/INHBB/INPP5D/ISG15/LGALS9/MIR221/MIR222/NDRG2/NFKBIL1/NLRX1/NMI/ORM1/OTUD5/PDCD4/RARA/RPS6KA4/SARS/SCGB1A1/SLC11A1/TBK1/TLR8/TLR9/TRAF3IP1/TRAIP/TUSC2/VS	42	BP
GO:0010524	positive regulation of calcium ion transport into	10/2734	52/17381	0.29608	0.8465	0.83256	ABL1/GPER1/GRIN1/GSTM2/IL13/NPSR1/P2RX2/PLA2G1B/THY1/TRDN	10	BP
GO:0030042	actin filament depolymerizatio	10/2734	52/17381	0.29608	0.8465	0.83256	CAPZA3/CFL1/F2RL1/LMOD1/MICAL2/SCIN/SPTAN1/SPTB/SPTBN2/WDR1	10	BP
GO:1903428	positive regulation of reactive oxygen species biosynthetic	10/2734	52/17381	0.29608	0.8465	0.83256	ADGRB1/AGT/CLU/EGFR/GRIN1/HSP90AA1/INS/MTOR/PTK2B/ZNF205	10	BP
GO:0071241	cellular response to inorganic	31/2734	178/17381	0.29656	0.8475	0.83351	ALAD/AQP1/ATP13A2/BAD/CACNA1H/CACYBP/CAMK2D/CPNE7/CYP11B2/CYP1A1/CYP1A2/DPEP1/EGFR/ENDOG/GLRA1/HSF1/HSPA5/MAPK3/MMP3/MT2A/NCF1/NEUROD2/OGG1/RASGRP2/SHH/SLC34A1/SPAG16/TF	31	BP
GO:0032102	negative regulation of response to external stimulus	48/2734	282/17381	0.2976	0.85	0.83601	AJAP1/C1QTNF1/CHID1/CORO1B/CTNNA2/CUEDC2/DHX58/DRD2/FGA/GBA/GPER1/GPR17/GPX1/GRIN1/HAVCR2/IL20RB/INPP5F/INS/KLKB1/KNG1/LRIG2/MICB/MIR15A/MIR16-1/MIR221/MIR222/MIR92A2/NBL1/NFKBIL1/NLRX1/NOS3/NOTCH1/NR1H3/PDCD4/PDGFA/PLAU/PPARD/PRKCD/PROZ/RGMA/RTN4RL1/SEMA3F/SERPINF2/SHARPIN/SPINK5/TARBP2/TRAF3IP1/WNT3A	48	BP

GO:0042110	T cell activation	78/2734	467/17381	0.29777	0.8501	0.8361	ABL1/ADAM8/BAD/BCL6/BMP4/CARD11/CASP3/CCDC88B/CCL19/CCL21/CCL5/CCND3/CCR2/CD247/CD27/CD5/CD6/CD74/CLEC7A/CLECL1/CORO1A/CSK/DDOST/DNAJA3/EFNB1/EGR1/EIF2AK4/ERBB2/F2RL1/FANCA/FOXN1/FUT7/FZD8/HAVCR2/HLA-E/HLX/IDO1/IFNA5/IL18/IL20RB/IL4R/INS/ITGAL/JAG2/LCK/LEPR/LFNG/LGALS3/LGALS9/LMO1/LY9/MAD1L1/MAP3K14/MICB/NKX2-3/PDCD1/PIK3R6/PLA2G2F/PRELID1/PRKAR1A/PSMB11/PTPN11/RARA/RORC/SART1/SCGB1A1/SEMA4A/SHH/SLC11A1/SOX13/SPINK5/THY1/TNFRSF18/TNFRSF4/TRAF2/VSIG4/WAS/WNT1	78	BP
GO:0010565	regulation of cellular ketone metabolic	29/2734	166/17381	0.29834	0.8513	0.8373	ACACB/ACADVL/APOA5/APOC3/BRCA1/DGKQ/EGR1/ELOVL5/FGFR4/INS/MID1IP1/MLXIPL/MTOR/NCOR2/NR1H3/OAZ2/PDHB/PSMB11/PSMB6/PSMB7/PSMB8/PSMC3/PSMD13/PSMD3/PSMD5/PSMD7/SCAP/SLC7A7/TYS	29	BP
GO:0051865	protein autoubiquitinati	11/2734	58/17381	0.2988	0.8514	0.83741	BRCA1/DDB2/LRSAM1/MTA1/RNF220/TAF1/TRAF2/UBE2A/UBE2B/UBE3D/WWP2	11	BP
GO:1905207	regulation of cardiocyte differentiation	11/2734	58/17381	0.2988	0.8514	0.83741	ARRB2/BMP4/CAV3/EGFR/GATA4/GPER1/MIR222/MTOR/NKX2-5/NRG1/WNT3A	11	BP
GO:0070507	regulation of microtubule cytoskeleton organization	27/2734	154/17381	0.30001	0.8514	0.83741	ABL1/ANKRD53/BRCA1/CAV3/CCNF/CDK2AP2/CHMP1A/CHMP4C/CIB1/DYNC1H1/FES/KATNB1/MAP6D1/MCPH1/MID1IP1/NUBP1/NUMA1/PARP3/PKD1/RASSF7/SENP6/STMN4/TRAF3IP1/TRIM54/TRPV4/WNT3A/XRCC3	27	BP
GO:0007176	regulation of epidermal growth factor-activated	6/2734	29/17381	0.30035	0.8514	0.83741	ADRA2A/ADRA2B/ADRA2C/NCF1/SHC1/VPS25	6	BP
GO:0021884	forebrain neuron	6/2734	29/17381	0.30035	0.8514	0.83741	DCLK2/DISC1/DRD2/FGFR2/GBX2/OGDH	6	BP
GO:0046134	pyrimidine nucleoside biosynthetic process	6/2734	29/17381	0.30035	0.8514	0.83741	CDA/DHODH/NME1/NME4/PUDP/UCK1	6	BP

GO:0046685	response to arsenic-containing	6/2734	29/17381	0.30035	0.8514	0.83741	ALAD/ATF3/CYP1A1/HSF1/RBM4/UROS	6	BP
GO:0051567	histone H3-K9 methylation	6/2734	29/17381	0.30035	0.8514	0.83741	BRCA1/EHMT1/EHMT2/PRDM12/SETD7/SUV39H1	6	BP
GO:0060285	cilium-dependent cell	6/2734	29/17381	0.30035	0.8514	0.83741	CATSPER1/DNAH17/GAS8/SPAG16/TEKT4/TEKT5	6	BP
GO:0080154	regulation of fertilization	6/2734	29/17381	0.30035	0.8514	0.83741	CACNA1H/GLRA1/PLB1/POMZP3/PRSS37/SPINK2	6	BP
GO:0006940	regulation of smooth muscle contraction	12/2734	64/17381	0.30081	0.8514	0.83741	ADRA1A/ADRA1B/ADRA2A/ADRA2B/ADRA2C/CHRM1/CTTN/EDN2/GPER1/MIR153-1/TACR1/TACR2	12	BP
GO:0009060	aerobic respiration	12/2734	64/17381	0.30081	0.8514	0.83741	ACO1/CHCHD5/COX10/CS/DLST/IDH1/IDH2/MTFR1L/OGDH/OGDHL/PDHB/SLC25A14	12	BP
GO:0021872	forebrain generation of neurons	12/2734	64/17381	0.30081	0.8514	0.83741	AXIN1/CSF1R/DCLK2/DISC1/DRD2/FGFR2/GBX2/LHX5/OGDH/OTP/TBR1/WNT3A	12	BP
GO:0060675	ureteric bud morphogenesis	12/2734	64/17381	0.30081	0.8514	0.83741	AGT/BMP4/EYA1/HOXB7/ILK/LHX1/SHH/SMO/SOX8/WNT1/WNT11/WNT6	12	BP
GO:0061035	regulation of cartilage development	12/2734	64/17381	0.30081	0.8514	0.83741	ADAMTS7/BMP4/GDF2/LOXL2/NKX3-2/PKDCC/POR/RARA/SCIN/SCX/SOX5/WNT11	12	BP
GO:0070373	negative regulation of ERK1 and	12/2734	64/17381	0.30081	0.8514	0.83741	ABL1/ATF3/C1QL4/CSK/DAB2IP/DUSP26/GPER1/MIR221/NDRG2/PSCA/RGS14/WNK2	12	BP
GO:2000573	positive regulation of DNA biosynthetic	12/2734	64/17381	0.30081	0.8514	0.83741	ACD/ARRB2/CCT3/FGFR4/HSP90AA1/MAPK15/MAPK3/NOX4/NVL/PDGFRB/PTK2B/RFC2	12	BP

GO:0001822	kidney development	45/2734	264/17381	0.3014	0.8527	0.83866	AGT/APH1A/AQP1/BMP4/CTSH/DACT2/EGR1/EYA1/FGFR2/HOXB7/HOXC11/IFT20/ILK/IRX3/KIRREL3/LHX1/LZTS2/MMP17/MYO1E/NLE1/NOTCH1/OSR1/PDGFA/PDGFRB/PKD1/POU3F3/PYGO2/RARA/REN/SDC1/SHH/SIM1/SLC34A1/SMAD6/SMO/SOX8/STAT1/STRA6/TNS2/TRAF3IP1/WDPCP/W	45	BP
GO:0001935	endothelial cell proliferation	23/2734	130/17381	0.30283	0.8554	0.84137	AIMP1/ANG/ATPIF1/BMP4/CDH13/ECM1/EGFL7/FLT4/GDF2/KDR/LOXL2/MIR16-1/MIR222/MIR29A/MIR29C/MIR503/MTOR/NR4A1/PLXNB3/SCARB1/STAT	23	BP
GO:0007173	epidermal growth factor receptor	23/2734	130/17381	0.30283	0.8554	0.84137	ABL1/ADRA2A/ADRA2B/ADRA2C/AGT/ARAP1/CDH13/CSK/DAB2IP/DOK1/EGFR/FASLG/FES/GPER1/HIP1R/HRAS/NCF1/PTK2B/PTPN11/RAB7A/RHBDF1/SHC1/VPS25	23	BP
GO:0007032	endosome organization	14/2734	76/17381	0.30332	0.8554	0.84137	AKTIP/ALS2CL/CHMP1A/CHMP4C/CHMP7/DNM1/FAM160A2/FASLG/RAB7A/SNF8/VPS25/VPS37B/VPS37C/VPS37D	14	BP
GO:0015844	monoamine transport	14/2734	76/17381	0.30332	0.8554	0.84137	ADRA2A/ADRA2B/ADRA2C/AGT/CHGA/CHRNA6/DRD2/DTNBP1/NISCH/SLC22A1/SLC29A4/SLC6A2/STX1A/TOR1A	14	BP
GO:0043535	regulation of blood vessel endothelial cell	14/2734	76/17381	0.30332	0.8554	0.84137	ABL1/CIB1/EFNA1/GDF2/HSPB1/KDR/MIR10A/MIR212/MIR221/MIR29C/MIR503/MIR92A2/NOTCH1/PRCP	14	BP
GO:0044243	multicellular organismal catabolic	14/2734	76/17381	0.30332	0.8554	0.84137	ADAMTS14/ADAMTS2/COL1A1/COL4A2/COL7A1/CTSD/CTSL/FURIN/MMP15/MMP2/MMP3/MRC2/PHYKPL/PLA2G1B	14	BP
GO:0071326	cellular response to monosaccharide	22/2734	124/17381	0.30338	0.8554	0.84137	ADRA2A/ANO1/BAD/BRSK2/CDK16/ENDOG/GATA4/GCG/GPER1/MEN1/MIRLET7G/MLXIPL/NME1/NOX4/PTPRN2/RAF1/RFX6/SIDT2/SLC26A6/SLC29A1/STX4/TH	22	BP
GO:0030073	insulin secretion	35/2734	203/17381	0.30357	0.8556	0.84149	ADRA2A/ADRA2C/ANO1/ARL2BP/BAD/BLK/BRSK2/CAPN10/CCL5/CDK16/CPLX1/DOC2B/DRD2/GCG/GCK/GLUD1/GPER1/IL1RN/INHBB/ITPR3/MAFA/MTNR1B/PFKM/PPARD/PTPN11/PTPRN2/RAF1/RFX6/SIDT2/SLC25A5/SSTR5/STX1A/STX4/TRH/VGF	35	BP
GO:0006275	regulation of DNA	21/2734	118/17381	0.30386	0.8556	0.8415	BCL6/BMP4/CACYBP/CDAN1/E2F8/EGFR/EHMT2/GDF2/HRAS/INO80/INS/LIG3/PDGFA/PDS5A/PID1/PLA2G1B/RFC2/SHC1/TICRR/TSPYL2/TTF1	21	BP

GO:0007098	centrosome cycle	21/2734	118/17381	0.30386	0.8556	0.8415	ARHGEF10/BRCA1/C2CD3/CCNF/CHMP1A/CHMP4C/CKAP5/CROCC/FES/GOLGA2/HAUS4/HAUS7/MCPH1/NUBP1/PARD6A/SDCCAG8/TUBGCP2/TUBGCP3/TUBGCP5/UXT/XRCC3	21	BP
GO:1903305	regulation of regulated secretory	20/2734	112/17381	0.30424	0.8562	0.84216	ADRA2A/CACNA1G/CACNA1H/CCR2/CDK5/CDK5R2/DOC2A/DOC2B/F2RL1/FES/IL13/IL4R/ITGAM/LGALS9/NOTCH1/STX1A/STX4/STXBP1/SYTL3/TRPV6	20	BP
GO:0001909	leukocyte mediated cytotoxicity	16/2734	88/17381	0.30444	0.8564	0.84232	ARRB2/CORO1A/CTSH/F2RL1/HAVCR2/HLA-E/IL18/LGALS9/MICB/NCR3/PIK3R6/PRDX1/RAET1G/SLAMF7/TUSC2/ULBP2	16	BP
GO:0006486	protein glycosylation	49/2734	289/17381	0.30542	0.8577	0.84358	A4GNT/ADAMTS13/ADAMTS7/ALG10/ALG3/ASGR1/B3GAT3/B3GNT6/B4GALT2/B4GALT7/DAD1/DDOST/DOLPP1/DPM2/EXTL1/FKTN/FUT7/GALNT16/GALNT8/GALNT9/GBGT1/GGTA1P/GOLGA2/GORASP1/LFNG/LMF1/MGAT1/MGAT4B/MGAT5B/MUC1/MUC2/MUC3A/MUC5AC/MUC5B/MUC6/OST4/PARP10/PARP2/PARP3/PARP9/PLOD3/RFNG/RPN1/SDF2/ST3GAL4	49	BP
GO:0043413	macromolecule glycosylation	49/2734	289/17381	0.30542	0.8577	0.84358	A4GNT/ADAMTS13/ADAMTS7/ALG10/ALG3/ASGR1/B3GAT3/B3GNT6/B4GALT2/B4GALT7/DAD1/DDOST/DOLPP1/DPM2/EXTL1/FKTN/FUT7/GALNT16/GALNT8/GALNT9/GBGT1/GGTA1P/GOLGA2/GORASP1/LFNG/LMF1/MGAT1/MGAT4B/MGAT5B/MUC1/MUC2/MUC3A/MUC5AC/MUC5B/MUC6/OST4/PARP10/PARP2/PARP3/PARP9/PLOD3/RFNG/RPN1/SDF2/ST3GAL4	49	BP
GO:0006730	one-carbon metabolic	7/2734	35/17381	0.30735	0.8577	0.84358	ALDH1L1/CA4/CA6/CA7/GNMT/MTHFD1/SHMT2	7	BP
GO:0006891	intra-Golgi vesicle-	7/2734	35/17381	0.30735	0.8577	0.84358	COG4/COG5/COPZ1/GOLGA3/GOSR2/PACS1/VTI1B	7	BP
GO:0010573	vascular endothelial growth factor	7/2734	35/17381	0.30735	0.8577	0.84358	BRCA1/C3AR1/CCR2/FLT4/GATA4/NDRG2/SARS	7	BP
GO:0010742	macrophage derived foam cell	7/2734	35/17381	0.30735	0.8577	0.84358	AGT/APOB/CSF1/IL18/NR1H3/SOAT2/STAT1	7	BP
GO:0019228	neuronal action potential	7/2734	35/17381	0.30735	0.8577	0.84358	CACNA1G/CACNA1H/GBA/GPER1/GPR35/GPR88/SCN11A	7	BP

GO:0043277	apoptotic cell clearance	7/2734	35/17381	0.30735	0.8577	0.84358	ADGRB1/C2/CD300LF/LRP1/NR1H3/RARA/SCARB1	7	BP
GO:0045687	positive regulation of glial cell	7/2734	35/17381	0.30735	0.8577	0.84358	BIN1/CLCF1/MTOR/NOTCH1/RNF112/SHH/SPINT1	7	BP
GO:0046839	phospholipid dephosphorylati	7/2734	35/17381	0.30735	0.8577	0.84358	EPHX2/INPP5D/INPP5E/INPP5F/INPPL1/MTMR1/PLPP1	7	BP
GO:0060236	regulation of mitotic spindle organization	7/2734	35/17381	0.30735	0.8577	0.84358	ANKRD53/CHMP1A/CHMP4C/DYNC1H1/NUMA1/PARP3/PKD1	7	BP
GO:0060306	regulation of membrane repolarization	7/2734	35/17381	0.30735	0.8577	0.84358	CACNB3/CAV3/FLNA/KCNQ1/MIR328/NPPA/WDR1	7	BP
GO:0072666	establishment of protein localization to	7/2734	35/17381	0.30735	0.8577	0.84358	CLU/GOSR2/LARS/RAB7A/SNF8/VPS25/VTI1B	7	BP
GO:0090077	foam cell differentiation	7/2734	35/17381	0.30735	0.8577	0.84358	AGT/APOB/CSF1/IL18/NR1H3/SOAT2/STAT1	7	BP
GO:0090503	RNA phosphodiester bond hydrolysis,	7/2734	35/17381	0.30735	0.8577	0.84358	DIS3L/DIS3L2/EXOSC10/EXOSC2/ISG20L2/PAN2/PNLDC1	7	BP
GO:1900274	regulation of phospholipase C activity	7/2734	35/17381	0.30735	0.8577	0.84358	ABL1/ADRA1A/AGT/ANG/EGFR/PDGFRB/PLCB2	7	BP
GO:2000826	regulation of heart	7/2734	35/17381	0.30735	0.8577	0.84358	BMP4/ENG/EYA1/NOTCH1/SMO/TBX2/WNT3A	7	BP
GO:0046890	regulation of lipid biosynthetic	29/2734	167/17381	0.31036	0.8596	0.84548	ACACB/ACADVL/APOA5/APOB/APOC3/ATP1A1/BRCA1/CCDC3/CDK4/DGKQ/EGR1/ELOVL5/FGFR4/GPER1/IDH1/INS/MID1IP1/MLXIPL/MTOR/NR1H3/NR5A1/ORMDL3/PDGFA/PMVK/POR/PRKCD/SCAP/SCARB1/SF1	29	BP

GO:0032846	positive regulation of homeostatic process	38/2734	222/17381	0.31051	0.8596	0.84548	ABL1/ACD/ADAM8/AGT/ANO1/BAD/CA7/CCT3/DEF8/DRD2/EGFR/EIF4G1/GCG/GPER1/GRIN1/GSTM2/HCAR2/IL13/INPP5D/ISG15/LGALS9/MAPK15/MAPK3/MIR221/MIR222/NPSR1/NR1H3/P2RX2/PLA2G1B/PLEKHM1/PTGER3/RFX6/STAT1/STX4/TACR1/THY1/TRDN/TRPV4	38	BP
GO:1900180	regulation of protein localization to nucleus	38/2734	222/17381	0.31051	0.8596	0.84548	BMP4/CCL19/CCT3/CD27/CDK5RAP3/CSF3/DAB2IP/DCLK2/EGFR/EMD/FAM89B/FLNA/IL18/INS/LGALS9/LMNA/LZTS2/MTOR/NFKBIL1/OGG1/PARP10/PARP9/PBLD/PDE2A/PKD1/POLR1A/PRDX1/PRKCD/RBPMS/SFRP5/SHH/SMO/SUFU/THRA/TLR9/TRIM40/WNT3A/ZPR1	38	BP
GO:0001522	pseudouridine synthesis	4/2734	18/17381	0.31107	0.8596	0.84548	NHP2/PUSL1/RPUSD2/TRUB2	4	BP
GO:0010544	negative regulation of platelet	4/2734	18/17381	0.31107	0.8596	0.84548	C1QTNF1/NOS3/PDGFA/PRKCD	4	BP
GO:0030011	maintenance of cell polarity	4/2734	18/17381	0.31107	0.8596	0.84548	CRB2/LRCH4/WDR1/WNT11	4	BP
GO:0030903	notochord development	4/2734	18/17381	0.31107	0.8596	0.84548	CRB2/EFNA1/NOTO/WNT11	4	BP
GO:0031468	nuclear envelope	4/2734	18/17381	0.31107	0.8596	0.84548	CHMP7/EMD/LMNA/REEP4	4	BP
GO:0032332	positive regulation of chondrocyte	4/2734	18/17381	0.31107	0.8596	0.84548	LOXL2/PKDCC/POR/SOX5	4	BP
GO:0034143	regulation of toll-like receptor 4	4/2734	18/17381	0.31107	0.8596	0.84548	DAB2IP/F2RL1/NR1H3/TIRAP	4	BP
GO:0035338	long-chain fatty-acyl-CoA biosynthetic process	4/2734	18/17381	0.31107	0.8596	0.84548	ACSF3/ELOVL1/ELOVL5/HACD1	4	BP

GO:0045653	negative regulation of megakaryocyte	4/2734	18/17381	0.31107	0.8596	0.84548	CIB1/HIST1H4F/HIST2H4A/HIST2H4B	4	BP
GO:0051818	disruption of cells of other organism involved in symbiotic	4/2734	18/17381	0.31107	0.8596	0.84548	BAD/F2RL1/GAPDH/TUSC2	4	BP
GO:0051969	regulation of transmission of nerve impulse	4/2734	18/17381	0.31107	0.8596	0.84548	AGT/GBA/GLRA1/GPR35	4	BP
GO:0060973	cell migration involved in heart	4/2734	18/17381	0.31107	0.8596	0.84548	BMP4/ENG/NOTCH1/PDGFRB	4	BP
GO:0070633	transepithelial transport	4/2734	18/17381	0.31107	0.8596	0.84548	AQP1/P2RY6/RHCG/SLC26A6	4	BP
GO:0071371	cellular response to gonadotropin	4/2734	18/17381	0.31107	0.8596	0.84548	EPHA8/NOTCH1/NSMF/POR	4	BP
GO:0090185	negative regulation of kidney	4/2734	18/17381	0.31107	0.8596	0.84548	BMP4/OSR1/SHH/STAT1	4	BP
GO:0097320	plasma membrane	4/2734	18/17381	0.31107	0.8596	0.84548	BIN2/PACSIN3/PLEKHM2/WHAMM	4	BP
GO:0099054	presynapse assembly	4/2734	18/17381	0.31107	0.8596	0.84548	EIF4G1/NLGN3/WNT3A/WNT7A	4	BP
GO:1905063	regulation of vascular smooth muscle cell differentiation	4/2734	18/17381	0.31107	0.8596	0.84548	ENG/GPER1/MIR221/PDCD4	4	BP

GO:0002285	lymphocyte activation involved in immune	28/2734	161/17381	0.31145	0.8602	0.84608	ABL1/BCL6/CCL19/CD180/CLCF1/CORO1A/EIF2AK4/EXO1/F2RL1/HAVCR2/HLX/IFNA5/IL18/IL4R/ITGAL/LFNG/LGALS3/LGALS9/LY9/NKX2-3/PAXIP1/PTK2B/RARA/RORC/SEMA4A/SLC11A1/SUPT6H/TNFSF13	28	BP
GO:0051098	regulation of binding	57/2734	339/17381	0.31168	0.8602	0.84608	ABL1/ACD/AKTIP/ARRB2/BMP4/CDK5/CDK9/CSF3/DAB2IP/DISC1/DTNBP1/EDF1/EIF4G1/GAS8/GCG/GOLGA2/GPSM1/HDAC8/HERPUD1/HEY1/HIP1R/HJURP/HSF1/HSPA5/IFIT2/KAT2A/LARP6/LFNG/LRP1/MAPK3/MAPRE3/MEN1/MEPCE/NES/NME1/NRG1/PARP9/PEX14/PHLDA2/PKD1/PLXND1/PRKCD/PTPRF/PYGO2/RALB/RARA/RFNG/RSF1/SMO/TAF1/TIRAP/TRAF2/	57	BP
GO:0046822	regulation of nucleocytoplasmic transport	37/2734	216/17381	0.31184	0.8602	0.84608	ANP32B/BMP4/CCL19/CD27/CDK5/CSF3/DAB2IP/EGFR/EMD/FAM89B/FLNA/IL18/LGALS9/MTOR/NFKBIL1/OGG1/PARP10/PBLD/PDE2A/PKD1/PRDX1/PRKCD/PTPN11/RBM4/RBPMS/SETD2/SFN/SFRP5/SHH/SMO/SUFU/SUPT6H/THRA/TLR9/WNT3A/ZC3H3/ZPR1	37	BP
GO:0017145	stem cell division	8/2734	41/17381	0.31215	0.8602	0.84608	CDK2AP2/DOCK7/FGFR2/HOXB4/NOTCH1/SOX5/WNT3A/WNT7A	8	BP
GO:0050913	sensory perception of	8/2734	41/17381	0.31215	0.8602	0.84608	AZGP1/CA6/GNAT1/ITPR3/LPO/PLCB2/REEP2/TAS2R3	8	BP
GO:0097028	dendritic cell differentiation	8/2734	41/17381	0.31215	0.8602	0.84608	BATF2/CCL19/F2RL1/LGALS9/LTBR/SPI1/TMEM176B/TREM2	8	BP
GO:0042129	regulation of T cell proliferation	27/2734	155/17381	0.31252	0.8608	0.84668	BMP4/CARD11/CASP3/CCDC88B/CCL19/CCL5/CCR2/CD6/CLECL1/CORO1A/DNAJA3/EFNB1/ERBB2/HAVCR2/HLA-E/IDO1/IL18/IL20RB/LGALS3/LGALS9/LMO1/MAD1L1/PLA2G2F/PRKAR1A/SCGB1A1/SHH/VSIG4	27	BP
GO:0016358	dendrite development	36/2734	210/17381	0.31316	0.8622	0.84803	ANAPC2/ARC/CAMK1D/CDK5/CELSR2/CFL1/CTNNA2/DAB1/DAB2IP/DBN1/DISC1/DLG4/DTNBP1/EFNA1/EPHB3/FOXO6/GORASP1/GRIN1/ILK/KNDC1/LLPH/MAP6D1/MTOR/NLGN3/NRG1/NSMF/OBSL1/PDLIM5/PREX2/RAB17/SCARF1/TLX2/TNIK/TRAPPC4/TULP1/WNT7A	36	BP
GO:0010212	response to ionizing radiation	26/2734	149/17381	0.31357	0.8629	0.84873	ABCG5/ALAD/BRAT1/BRCA1/BRCC3/CASP3/CRYAB/DNMT3A/EGR1/EYA1/GPX1/HRAS/HSF1/HSPA5/INIP/INO80/INTS3/KARS/KRT14/MEN1/MTA1/NOX4/PAXIP1/TICRR/TMEM109/XRCC3	26	BP

GO:1902749	regulation of cell cycle G2/M phase transition	35/2734	204/17381	0.3145	0.8651	0.85086	ACTR1A/BRCA1/CDK4/CDK5RAP3/CEP164/CEP70/CHMP4C/CKAP5/DCTN2/DYNC1H1/FOXO4/GPR132/HAUS4/HAUS7/HSP90AA1/HSPA2/MIIP/MIR195/NEK10/PAXIP1/PHOX2B/PSMB11/PSMB6/PSMB7/PSMB8/PSMC3/PSMD13/PSMD3/PSMD5/PSMD7/RINT1/SDCCAG8/SSNA1/TICRR/TUBG1	35	BP
GO:0045599	negative regulation of fat cell	9/2734	47/17381	0.3155	0.8672	0.85298	AXIN1/C1QL4/CCDC85B/GPER1/TGFB1I1/TRIO/TRPV4/WNT1/WNT3A	9	BP
GO:0140053	mitochondrial gene expression	24/2734	137/17381	0.31557	0.8672	0.85298	AARS2/C12orf65/COA3/ERAL1/GATB/MRPL10/MRPL14/MRPL28/MRPL36/MRPL37/MRPL43/MRPL52/MRPL57/MRPL9/MRPS10/MRPS11/MRPS18A/MRPS18B/MRPS21/MRPS33/MRPS5/RMND1/TARS2/YARS2	24	BP
GO:0071322	cellular response to carbohydrate	23/2734	131/17381	0.31652	0.8681	0.8538	ADRA2A/ANO1/BAD/BRSK2/CDK16/CLEC7A/ENDOG/GATA4/GCG/GPER1/MEN1/MIRLET7G/MLXIPL/NME1/NOX4/PTPRN2/RAF1/RFX6/SIDT2/SLC26A6/SLC29A1/STX4/TH	23	BP
GO:0000768	syncytium formation by plasma membrane	10/2734	53/17381	0.31783	0.8681	0.8538	ADAM12/ADGRB1/CACNA1H/CAV3/EHD1/ERVFRD-1/IL4R/MYOD1/TCTA/WNT1	10	BP
GO:0032722	positive regulation of chemokine	10/2734	53/17381	0.31783	0.8681	0.8538	CD74/CSF1R/EGR1/HAVCR2/IL4R/LGALS9/MIR92A2/TIRAP/TLR9/TRPV4	10	BP
GO:0003158	endothelium development	21/2734	119/17381	0.31826	0.8681	0.8538	BMP4/CDH5/EDF1/ENG/F2RL1/GDF2/GPX1/HEY1/IKBKB/KDM6B/KDR/MARVELD2/MIR10A/NOTCH1/NRG1/PDE2A/PLOD3/TMEM100/TNMD/WNT	21	BP
GO:0009746	response to hexose	32/2734	186/17381	0.31851	0.8681	0.8538	ADRA2A/ANO1/BAD/BRSK2/CASP3/CDK16/EGR1/EIF2B2/EIF2B5/ENDOG/GATA4/GCG/GCK/GPER1/LDHA/MAFA/MEN1/MIRLET7G/MLXIPL/NME1/NOX4/PPARD/PTK2B/PTPRN2/RAF1/RFX6/SIDT2/SLC26A6/SLC29A1/STX4	32	BP
GO:0007200	phospholipase C-activating G-protein coupled receptor signaling	20/2734	113/17381	0.31904	0.8681	0.8538	ADRA1A/ADRA1B/ADRA2A/AGT/ANO1/C3AR1/CHGA/CHRM1/CXCR2/DRD2/F2RL1/GPR17/GPR20/GPR35/LTB4R/OPRD1/OPRM1/P2RY6/PTGER3/TACR1	20	BP

GO:0001658	branching involved in ureteric bud	11/2734	59/17381	0.31942	0.8681	0.8538	AGT/BMP4/EYA1/HOXB7/ILK/LHX1/SHH/SMO/SOX8/WNT1/WNT6	11	BP
GO:0010803	regulation of tumor necrosis factor-mediated signaling	11/2734	59/17381	0.31942	0.8681	0.8538	CASP1/CCDC3/F2RL1/IKBKB/MADD/PELI3/PYDC1/RNF31/SHARPIN/TRAF2/TRAIP	11	BP
GO:0035773	insulin secretion involved in cellular response to	11/2734	59/17381	0.31942	0.8681	0.8538	ADRA2A/ANO1/BAD/BRSK2/CDK16/GCG/PTPRN2/RAF1/RFX6/SIDT2/STX4	11	BP
GO:0070059	intrinsic apoptotic signaling pathway in response to	11/2734	59/17381	0.31942	0.8681	0.8538	BOK/BRSK2/CHAC1/CREB3/DAB2IP/ERN2/HERPUD1/SPOP/SYVN1/TRAF2/WFS1	11	BP
GO:0061138	morphogenesis of a branching epithelium	31/2734	180/17381	0.31985	0.8681	0.8538	ABL1/ADM/AGT/BMP4/CAV3/CSF1/CTSH/ENG/ESRP2/EYA1/FGFR2/GBX2/GDF2/HHIP/HOXB7/ILK/LHX1/MYCN/NOTCH1/PDGFA/PKD1/PLXND1/SHH/SMO/SOX8/SPINT1/ST14/TIMELESS/TNC/WNT1/WNT6	31	BP
GO:0050868	negative regulation of T	18/2734	101/17381	0.32035	0.8681	0.8538	BCL6/BMP4/CASP3/CD74/ERBB2/HAVCR2/HLX/IDO1/IL20RB/IL4R/LGALS3/LGALS9/MAD1L1/PLA2G2F/PRKAR1A/SCGB1A1/SHH/VSIG4	18	BP
GO:0051899	membrane depolarization	18/2734	101/17381	0.32035	0.8681	0.8538	ABL1/ATPIF1/BOK/CACNA1F/CACNA1G/CACNA1H/CACNA1S/CACNB3/CAMK2D/CASP1/CATSPER1/CAV3/CHRNA6/FZD9/KDR/MYOC/PPP2R3C/	18	BP
GO:1990266	neutrophil migration	18/2734	101/17381	0.32035	0.8681	0.8538	ADAM8/C3AR1/CAMK1D/CCL1/CCL19/CCL21/CCL5/CD74/CKLF/CXCR2/DAPK2/EDN2/GBF1/LGALS3/PF4V1/PLA2G1B/TIRAP/WDR1	18	BP
GO:0014031	mesenchymal cell	12/2734	65/17381	0.32046	0.8681	0.8538	BMP4/CFL1/EFNB1/GBX2/MAPK3/NOTCH1/NRG1/PHOX2B/SEMA3F/SHH/SMO/SOX8	12	BP
GO:0017158	regulation of calcium ion-dependent exocytosis	12/2734	65/17381	0.32046	0.8681	0.8538	ADRA2A/CACNA1G/CACNA1H/CDK5/CDK5R2/DOC2A/DOC2B/NOTCH1/STX1A/STXBP1/SYTL3/TRPV6	12	BP

GO:1901607	alpha-amino acid biosynthetic	12/2734	65/17381	0.32046	0.8681	0.8538	ASL/ASNS/BHMT2/GLUD1/GOT2/MTHFD1/NAGS/OAT/OTC/PHGDH/PLOD3/SHMT2	12	BP
GO:2001243	negative regulation of intrinsic apoptotic	17/2734	95/17381	0.32084	0.8681	0.8538	CCAR2/CD74/CLU/CREB3/GPX1/HERPUD1/HSPB1/INS/MAPK8IP1/MUC1/NOC2L/NONO/SYVN1/TRAP1/VDAC2/WFS1/ZNF385A	17	BP
GO:0031644	regulation of neurological system process	13/2734	71/17381	0.32107	0.8681	0.8538	ACPP/ADRA2C/AGT/GBA/GLRA1/GPR35/MTOR/NRG1/OPRD1/OPRM1/PARD3/SCN11A/TMEM100	13	BP
GO:0055013	cardiac muscle cell	13/2734	71/17381	0.32107	0.8681	0.8538	AGT/BMP4/CAV3/GATA4/LMNA/MIR195/MTOR/NKX2-5/NPPA/OBSL1/PDGFRB/PDLIM5/TCAP	13	BP
GO:0098876	vesicle-mediated transport to the	13/2734	71/17381	0.32107	0.8681	0.8538	BLZF1/COMMD1/CSK/GRIPAP1/KIF13A/LLGL1/OSBPL5/PKDCC/RAB26/RAB34/SCRIB/STEAP2/VAMP5	13	BP
GO:0050866	negative regulation of cell activation	30/2734	174/17381	0.32119	0.8681	0.8538	BCL6/BMP4/C1QTNF1/CASP3/CCR2/CD300LF/CD74/ERBB2/FAM19A3/GPER1/HAVCR2/HLX/IDO1/IL20RB/IL31RA/IL4R/INPP5D/LGALS3/LGALS9/MAD1L1/NOS3/NR1H3/PDGFA/PLA2G2F/PRKAR1A/PRKCD/SCGB1A1/SHH/	30	BP
GO:0097006	regulation of plasma lipoprotein	16/2734	89/17381	0.3212	0.8681	0.8538	AGT/AP2A2/AP2M1/APOA5/APOB/APOC3/CSK/CUBN/DGAT1/EHD1/GPIHBP1/LMF1/MPO/NPC2/SCARB1/SOAT2	16	BP
GO:0008344	adult locomotory	14/2734	77/17381	0.32136	0.8681	0.8538	ARRB2/CEND1/DAB1/DMBX1/DNM1/DRD2/EN1/GLRA1/GRIN1/HOXB8/INPP5F/OPRD1/PREX2/TRH	14	BP
GO:2001022	positive regulation of response to DNA damage	14/2734	77/17381	0.32136	0.8681	0.8538	APBB1/BRCA1/BRCC3/CCAR2/EGFR/EYA1/FOXM1/NPAS2/PARP9/PAXIP1/PRKCD/SLF2/TIMELESS/ZNF385A	14	BP
GO:0006890	retrograde vesicle-mediated transport, Golgi	15/2734	83/17381	0.32138	0.8681	0.8538	ARFGAP2/COG4/COPZ1/GBF1/KIF23/KIF26A/KIF2B/KIF4A/KLC1/KLC2/LMAN2/RINT1/STX18/TMED3/TMEM115	15	BP

GO:0008652	cellular amino acid biosynthetic	15/2734	83/17381	0.32138	0.8681	0.8538	ASL/ASNS/BHMT2/GAD1/GLUD1/GOT2/GPT/MTHFD1/NAALAD2/NAGS/OAT/OTC/PHGDH/PLOD3/SHMT2	15	BP
GO:0034333	adherens junction	15/2734	83/17381	0.32138	0.8681	0.8538	ABL1/BCAS3/COL16A1/CTTN/FERMT2/KDR/LDB1/MYOC/PTK2B/RHOD/TAOK2/THY1/WDPCP/WHAMM/ZNF703	15	BP
GO:0009895	negative regulation of catabolic process	44/2734	260/17381	0.32213	0.8681	0.8538	ACACB/ADGRB1/ADRA2A/ALAD/APOC3/ATP13A2/CCAR2/CDK5RAP3/DAB2IP/DAP/DAPL1/EFNA1/EGFR/EIF4G1/FHIT/FLNA/FURIN/GATA4/GOLGA2/GRIN2C/HCAR2/INS/KLHL40/LAMP3/LARS/LEPR/LRIG2/MTOR/NRG1/OS9/PANO1/PFKFB1/PKP3/POLDIP2/QSOX1/SHH/SLC11A1/SNX12/SUFU	44	BP
GO:0000469	cleavage involved in rRNA	5/2734	24/17381	0.32287	0.8681	0.8538	EXOSC2/FCF1/NHP2/NOB1/TSR1	5	BP
GO:0002068	glandular epithelial cell	5/2734	24/17381	0.32287	0.8681	0.8538	BAD/BMP4/RARA/SIDT2/SMO	5	BP
GO:0007202	activation of phospholipase C activity	5/2734	24/17381	0.32287	0.8681	0.8538	ADRA1A/AGT/ANG/EGFR/PLCB2	5	BP
GO:0009309	amine biosynthetic	5/2734	24/17381	0.32287	0.8681	0.8538	AGMAT/DDC/HDC/OAZ2/PAOX	5	BP
GO:0019430	removal of superoxide radicals	5/2734	24/17381	0.32287	0.8681	0.8538	GCH1/MPO/NOS3/PRDX1/SOD3	5	BP
GO:0030517	negative regulation of	5/2734	24/17381	0.32287	0.8681	0.8538	ARHGAP4/CDK5/DRAXIN/SEMA3F/WNT3A	5	BP
GO:0032897	negative regulation of viral	5/2734	24/17381	0.32287	0.8681	0.8538	CCL5/TRIM11/TRIM14/TRIM31/TRIM62	5	BP
GO:0032967	positive regulation of collagen biosynthetic	5/2734	24/17381	0.32287	0.8681	0.8538	BMP4/ENG/PDGFRB/SCX/SERPINF2	5	BP

GO:0035412	regulation of catenin import into nucleus	5/2734	24/17381	0.32287	0.8681	0.8538	DAB2IP/EGFR/EMD/SFRP5/WNT3A	5	BP
GO:0036003	positive regulation of transcription from RNA polymerase II promoter in	5/2734	24/17381	0.32287	0.8681	0.8538	ATF3/HSF1/HSPA5/MUC1/NOTCH1	5	BP
GO:0042430	indole-containing compound	5/2734	24/17381	0.32287	0.8681	0.8538	DDC/HAAO/IDO1/RNF180/SLC34A1	5	BP
GO:0051894	positive regulation of focal adhesion	5/2734	24/17381	0.32287	0.8681	0.8538	ABL1/COL16A1/KDR/MYOC/THY1	5	BP
GO:0060122	inner ear receptor stereocilium	5/2734	24/17381	0.32287	0.8681	0.8538	CDH23/IFT20/LHFPL5/MYO7A/SCRIB	5	BP
GO:0060259	regulation of feeding	5/2734	24/17381	0.32287	0.8681	0.8538	EIF2AK4/INS/LEPR/MTOR/TRH	5	BP
GO:0071425	hematopoietic stem cell proliferation	5/2734	24/17381	0.32287	0.8681	0.8538	MIR221/MIR222/PDCD2/THPO/WNT1	5	BP
GO:0071677	positive regulation of mononuclear	5/2734	24/17381	0.32287	0.8681	0.8538	CCL1/CCL5/CCR2/CREB3/LGALS3	5	BP
GO:0090025	regulation of monocyte chemotaxis	5/2734	24/17381	0.32287	0.8681	0.8538	CCL1/CCL5/CCR2/CREB3/NBL1	5	BP
GO:0090103	cochlea morphogenesis	5/2734	24/17381	0.32287	0.8681	0.8538	DVL2/EYA1/FZD2/GRHL3/MYO3A	5	BP

GO:1905048	regulation of metallopeptidase activity	5/2734	24/17381	0.32287	0.8681	0.8538	KARS/MAPK3/MIR195/MIR29C/MIR92A2	5	BP
GO:0007178	transmembrane receptor protein serine/threonine kinase signaling pathway	54/2734	322/17381	0.32495	0.87	0.85573	ABL1/ARRB2/BMP4/CAV3/CDH5/CFC1/CRB2/DACT2/EGR1/ENG/FAM89B/FERMT2/FNTA/FURIN/GATA4/GDF2/HSPA5/HTRA3/ILK/INHBB/INHBE/IRAK1/ITGB5/LEFTY2/LEMD2/MAPK3/MEN1/MIR212/NBL1/NEDD8/NKX2-5/NOTCH1/NUMA1/PARD3/PARD6A/PBLD/PDCD4/RBPMS/RBPMS2/RGMA/SCX/SFRP5/SHH/SLC39A5/SMAD6/SOST/TGFB1I1/TMEM100/VASN/WFIKKN2/WNT1/ZC3H3/ZNF703/ZYX	54	BP
GO:0050863	regulation of T cell activation	54/2734	322/17381	0.32495	0.87	0.85573	ABL1/ADAM8/BAD/BCL6/BMP4/CARD11/CASP3/CCDC88B/CCL19/CCL21/CCL5/CCR2/CD247/CD27/CD5/CD6/CD74/CLECL1/CORO1A/CSK/DNAJA3/EFNB1/ERBB2/FANCA/FOXN1/HAVCR2/HLA-E/HLX/IDO1/IL18/IL20RB/IL4R/LCK/LGALS3/LGALS9/LMO1/MAD1L1/MAP3K14/PDCD1/PIK3R6/PLA2G2F/PRELID1/PRKAR1A/PTPN11/RARA/SART1/SCGB1A1/SHH/SOX13/SPINK5/THY1/TNFRSF18/TRAF2/VSIG4	54	BP
GO:0045619	regulation of lymphocyte differentiation	27/2734	156/17381	0.32519	0.87	0.85573	ABL1/ADAM8/BAD/BCL6/BMP4/CARD11/CCL19/CD27/CD74/ERBB2/FANCA/FOXN1/HLX/IL18/IL4R/INPP5D/LGALS9/PIK3R6/PPP2R3C/PRELID1/RARA/SART1/SHH/SOX13/SPINK5/TLR9/TNFRSF18	27	BP
GO:0007204	positive regulation of cytosolic calcium ion concentration	48/2734	285/17381	0.32544	0.87	0.85573	ABL1/ADM/ADRA1A/ADRA1B/AGT/BCAP31/C1QTNF1/C3AR1/CACNB3/CAMK2D/CAV3/CCL1/CCL19/CCL21/CCR2/CIB2/CORO1A/CXCR3/DLG4/DRD2/EDN2/EIF5A/F2RL1/FASLG/GNG3/GPER1/GPR17/GPR20/GPR35/GRIN1/GRIN2C/GSTM2/IL13/ITPR3/JPH3/KNG1/LCK/NPSR1/P2RX2/PKD1/PLA2G1B/PTK2B/RASA3/TACR1/THY1/TRDN/TRPV4/TRPV6	48	BP
GO:0071695	anatomical structure maturation	26/2734	150/17381	0.32651	0.87	0.85573	AGRN/ANG/C1QL1/CDH5/CDK5R2/CEND1/EPHA8/FARP2/GRIN1/HBZ/L3MBTL3/MAEA/MMP2/MTCH1/MTOR/MYOC/PHOSPHO1/PLXNB1/PTK2B/RND1/RXFP2/SCARF1/SPINK5/THBS3/TMEM79/WNT1	26	BP

GO:1903321	negative regulation of protein modification by small protein conjugation or	26/2734	150/17381	0.32651	0.87	0.85573	ABL1/ADGRB1/ANAPC15/ANAPC2/ARRB2/BUB1B/CDK5/HDAC8/ISG15/KLHL40/LIMK1/MTOR/OTUB1/PARP10/PSMB6/PSMB7/PSMB8/PSMC3/PSMD13/PSMD3/PSMD5/PSMD7/SUFU/TAF1/USP4/WNT1	26	BP
GO:0071346	cellular response to interferon-gamma	25/2734	144/17381	0.32782	0.87	0.85573	ADAMTS13/CAMK2D/CASP1/CCL1/CCL19/CCL21/CCL5/CD58/CIITA/DAPK1/GAPDH/HLA-E/IRF5/LGALS9/MT2A/NMI/NR1H3/PARP9/PRKCD/SLC26A6/STAT1/TDGF1/TRIM26/TRIM31/TRIM62	25	BP
GO:0001539	cilium or flagellum-dependent cell	6/2734	30/17381	0.32996	0.87	0.85573	CATSPER1/DNAH17/GAS8/SPAG16/TEKT4/TEKT5	6	BP
GO:0003401	axis elongation	6/2734	30/17381	0.32996	0.87	0.85573	BMP4/FGFR2/SHH/TNC/WNT11/WNT3A	6	BP
GO:0016577	histone demethylation	6/2734	30/17381	0.32996	0.87	0.85573	HR/KDM4E/KDM6B/KDM8/PHF2/UBE2B	6	BP
GO:0021799	cerebral cortex radially oriented cell migration	6/2734	30/17381	0.32996	0.87	0.85573	CDK5/CDK5R2/DAB1/DAB2IP/DISC1/POU3F3	6	BP
GO:0030851	granulocyte differentiation	6/2734	30/17381	0.32996	0.87	0.85573	C1QC/CSF3/INPP5D/L3MBTL3/RARA/SPI1	6	BP
GO:0032365	intracellular lipid transport	6/2734	30/17381	0.32996	0.87	0.85573	ABCA2/ACACB/CPT2/MID1IP1/NPC2/SLC25A20	6	BP
GO:0045730	respiratory burst	6/2734	30/17381	0.32996	0.87	0.85573	CAMK1D/INS/MPO/NCF1/NOXO1/SLC11A1	6	BP
GO:0051491	positive regulation of filopodium	6/2734	30/17381	0.32996	0.87	0.85573	AGRN/ARAP1/BCAS3/CCL21/ESPN/FSCN1	6	BP

GO:1900271	regulation of long-term synaptic	6/2734	30/17381	0.32996	0.87	0.85573	ABL1/ARC/DRD2/EIF2AK4/INS/NLGN3	6	BP
GO:0006354	DNA-templated transcription, elongation	23/2734	132/17381	0.33039	0.87	0.85573	AXIN1/CCAR2/CDK9/GTF2H4/LDB1/NELFB/POLR1A/POLR1D/POLR1E/POLR2G/POLR2L/RECQL5/SETD2/SHH/SUPT6H/TAF1/TAF1A/TAF1L/TAF3/TAF6/TAF7/ZMYND11/ZNRD1	23	BP
GO:0002220	innate immune response activating cell surface receptor signaling	22/2734	126/17381	0.33164	0.87	0.85573	CARD11/CARD9/CLEC7A/HRAS/IKBKB/MUC1/MUC2/MUC3A/MUC5AC/MUC5B/MUC6/PRKCD/PSMB11/PSMB6/PSMB7/PSMB8/PSMC3/PSMD13/PSMD3/PSMD5/PSMD7/RAF1	22	BP
GO:0009749	response to glucose	31/2734	181/17381	0.33171	0.87	0.85573	ADRA2A/ANO1/BAD/BRSK2/CASP3/CDK16/EGR1/EIF2B2/EIF2B5/ENDOG/GATA4/GCG/GCK/GPER1/LDHA/MAFA/MEN1/MIRLET7G/MLXIPL/NME1/NOX4/PPARD/PTK2B/PTPRN2/RAF1/RFX6/SIDT2/SLC29A1/STX4/TH/TRH	31	BP
GO:0042098	T cell proliferation	31/2734	181/17381	0.33171	0.87	0.85573	ABL1/BMP4/CARD11/CASP3/CCDC88B/CCL19/CCL5/CCND3/CCR2/CD6/CLECL1/CORO1A/DNAJA3/EFNB1/ERBB2/HAVCR2/HLA-E/IDO1/IL18/IL20RB/LGALS3/LGALS9/LMO1/MAD1L1/PLA2G2F/PRKAR1A/SCGB1A1/SHH/SLC11A1/TNFRSF4/VSIG4	31	BP
GO:0048863	stem cell differentiation	44/2734	261/17381	0.33201	0.87	0.85573	ABL1/BMP4/CFL1/EFNB1/FGFR2/FOXO4/GATA4/GBX2/HIST1H4F/HIST2H4A/HIST2H4B/HOXB4/HOXD4/LDB1/LMO1/LTBP3/MAPK3/NKX2-5/NOTCH1/NRG1/OCIAD1/OSR1/PHOX2B/PSMB11/PSMB6/PSMB7/PSMB8/PSMC3/PSMD13/PSMD3/PSMD5/PSMD7/SEMA3F/SETD1A/SETD2/SHH/SMO/SOX5/SOX8/SPI1/TBX2/WNT10A/WNT3A/WNT7A	44	BP
GO:0071333	cellular response to	21/2734	120/17381	0.33286	0.87	0.85573	ADRA2A/ANO1/BAD/BRSK2/CDK16/ENDOG/GATA4/GCG/GPER1/MEN1/MIRLET7G/MLXIPL/NME1/NOX4/PTPRN2/RAF1/RFX6/SIDT2/SLC29A1/ST	21	BP
GO:0000209	protein polyubiquitination	49/2734	292/17381	0.3332	0.87	0.85573	ANAPC2/BLMH/BRCA1/CCNF/CHFR/DDB2/FBXL13/FBXL16/FBXL19/FBXL22/FBXL8/FBXO10/FBXO2/FBXO6/FBXW4/HERC6/LRSAM1/MIB2/NMI/OTUB1/PARP10/PELI3/PSMB11/PSMB6/PSMB7/PSMB8/PSMC3/PSMD13/PSMD3/PSMD5/PSMD7/RNF166/RNF167/RNF180/RNF183/RNF31/SHARPIN/SPSB1/SYVN1/TAF1/TRAF2/UBE2A/UBE2B/UBE2E2/UBE2U/UBE3C/UBE3D	49	BP

GO:0000578	embryonic axis specification	7/2734	36/17381	0.33441	0.87	0.85573	C2CD3/LHX1/RIPPLY1/SMAD6/TDGF1/WNT1/WNT7A	7	BP
GO:0002720	positive regulation of cytokine production involved in	7/2734	36/17381	0.33441	0.87	0.85573	CD74/F2RL1/KARS/MAPK3/SEMA7A/SPON2/TRAF2	7	BP
GO:0008207	C21-steroid hormone metabolic	7/2734	36/17381	0.33441	0.87	0.85573	ADM/CACNA1H/CYP11A1/CYP11B2/DGKQ/EGR1/STARD3	7	BP
GO:0033572	transferrin transport	7/2734	36/17381	0.33441	0.87	0.85573	ATP6V0B/ATP6V0E2/ATP6V1B1/ATP6V1F/ATP6V1G1/ATP6V1G2/TFR2	7	BP
GO:2000142	regulation of DNA-templated transcription, initiation	7/2734	36/17381	0.33441	0.87	0.85573	FOSL1/NKX2-5/PAXIP1/PSMC3/TAF1/TAF7/THRA	7	BP
GO:0046486	glycerolipid metabolic process	73/2734	441/17381	0.33481	0.87	0.85573	ACHE/ANG/APOA5/APOB/APOC3/CAV3/CDIPT/CPNE7/CSF1R/CWH43/DGAT1/DGKQ/DGKZ/DPM2/DRD2/EGFR/ERBB2/ETNK2/FGF17/FGF3/FGFR2/FGFR4/GNPAT/GPAA1/GPX1/HADHA/INPP5D/INPP5E/INPP5F/INPPL1/LCK/LMF1/MFSD2A/MTMR1/MTMR14/NKX2-3/NR1H3/NRG1/OSBPL5/PDGFA/PDGFRB/PEMT/PHOSPHO1/PI4KB/PIGC/PIGG/PIGO/PIGQ/PIGY/PIGZ/PIK3R5/PIK3R6/PITPNM1/PLA2G15/PLA2G16/PLA2G1B/PLA2G2F/PLA2G4B/PLA2G5/PLB1/PLCH2/PLD2/PNPLA2/PRDX6/PTDSS1/PTDSS2/PTPN11/PYURF/SCARB1/SLC44A4/SMPD4/TLR9/TMEM1[illegible]	73	BP
GO:0061136	regulation of proteasomal protein catabolic	29/2734	169/17381	0.33484	0.87	0.85573	ALAD/ARAF/BCAP31/BRSK2/CCAR2/CHFR/CLU/COMMD1/DAB2IP/ECSCR/FHIT/GBA/GPX1/HERPUD1/KLHL40/LAMP3/NKD2/OS9/OSBPL7/PANO1/PKD1/PSMC3/RHBDF1/RNF166/RNF180/SHH/TAF1/USP19/USP5	29	BP
GO:0007030	Golgi organization	19/2734	108/17381	0.33518	0.87	0.85573	BCAS3/BLZF1/COG4/GAK/GBF1/GOLGA2/GORASP1/KIFC3/MAPK3/MYO18A/OBSL1/PDE4DIP/PLEKHM2/RBSN/STX18/STX5/TJAP1/USP6NL/VPS51	19	BP

GO:0007596	blood coagulation	58/2734	348/17381	0.33579	0.87	0.85573	ADAMTS13/ADRA2A/ADRA2B/ADRA2C/ARRB2/ASIC2/C1QTNF1/CLIC1/COL1A1/CSRP1/DGKI/DGKQ/DGKZ/DOCK1/DTNBP1/EHD1/F2RL1/FERMT3/FGA/FLNA/GATA4/GP9/HSPB1/IFNA5/ILK/ITPK1/ITPR3/KLKB1/KNG1/LCK/MAPK3/MFN2/NOS3/P2RX2/PDGFA/PF4V1/PIK3R5/PIK3R6/PLAU/PRCP/PRKAR1A/PRKAR1B/PRKCD/PROZ/PTPN11/RAF1/RBSN/SELP/SERPINA5/SERPINF2/SH2B1/SHH/STXBP1/TEC/TLN1/VWF/WAS/WNT3A	58	BP
GO:0003337	mesenchymal to epithelial transition involved in metanephros	3/2734	13/17381	0.3359	0.87	0.85573	BMP4/SMO/STAT1	3	BP
GO:0006596	polyamine biosynthetic process	3/2734	13/17381	0.3359	0.87	0.85573	AGMAT/OAZ2/PAOX	3	BP
GO:0006703	estrogen biosynthetic process	3/2734	13/17381	0.3359	0.87	0.85573	HSD17B1/HSD17B7/HSD3B1	3	BP
GO:0007171	activation of transmembrane receptor protein tyrosine kinase activity	3/2734	13/17381	0.3359	0.87	0.85573	DGKQ/NRG1/PILRB	3	BP
GO:0007549	dosage compensation	3/2734	13/17381	0.3359	0.87	0.85573	BRCA1/EXOSC10/PRDM14	3	BP
GO:0009650	UV protection	3/2734	13/17381	0.3359	0.87	0.85573	GPX1/SCARA3/SDF4	3	BP
GO:0010248	establishment or maintenance of transmembrane electrochemical gradient	3/2734	13/17381	0.3359	0.87	0.85573	ATP1A1/ATP1A4/SLC22A1	3	BP

GO:0010766	negative regulation of sodium ion	3/2734	13/17381	0.3359	0.87	0.85573	CAMK2D/COMMD1/OSR1	3	BP
GO:0021819	layer formation in cerebral	3/2734	13/17381	0.3359	0.87	0.85573	CDK5/CDK5R2/DAB2IP	3	BP
GO:0021978	telencephalon regionalization	3/2734	13/17381	0.3359	0.87	0.85573	BMP4/EMX1/SHH	3	BP
GO:0030002	cellular anion homeostasis	3/2734	13/17381	0.3359	0.87	0.85573	FASLG/GCM2/SLC34A1	3	BP
GO:0030157	pancreatic juice secretion	3/2734	13/17381	0.3359	0.87	0.85573	AQP1/AQP5/NR1H3	3	BP
GO:0030320	cellular monovalent inorganic anion homeostasis	3/2734	13/17381	0.3359	0.87	0.85573	FASLG/GCM2/SLC34A1	3	BP
GO:0030836	positive regulation of actin filament	3/2734	13/17381	0.3359	0.87	0.85573	CFL1/F2RL1/WDR1	3	BP
GO:0031223	auditory behavior	3/2734	13/17381	0.3359	0.87	0.85573	DRD2/NRXN2/STRA6	3	BP
GO:0031274	positive regulation of pseudopodium	3/2734	13/17381	0.3359	0.87	0.85573	CCL21/CDC42EP2/F2RL1	3	BP
GO:0032230	positive regulation of synaptic transmission,	3/2734	13/17381	0.3359	0.87	0.85573	ADRA1A/CA7/TACR1	3	BP
GO:0032530	regulation of microvillus organization	3/2734	13/17381	0.3359	0.87	0.85573	CDHR5/FSCN1/RAP1GAP	3	BP

GO:0032802	low-density lipoprotein particle receptor catabolic	3/2734	13/17381	0.3359	0.87	0.85573	AP2A2/AP2M1/FURIN	3	BP
GO:0034374	low-density lipoprotein particle	3/2734	13/17381	0.3359	0.87	0.85573	AGT/APOB/MPO	3	BP
GO:0035815	positive regulation of renal sodium	3/2734	13/17381	0.3359	0.87	0.85573	AGT/DRD2/TACR1	3	BP
GO:0036109	alpha-linolenic acid metabolic process	3/2734	13/17381	0.3359	0.87	0.85573	ELOVL1/ELOVL5/FADS2	3	BP
GO:0042976	activation of Janus kinase	3/2734	13/17381	0.3359	0.87	0.85573	AGT/CCL5/PTK2B	3	BP
GO:0043249	erythrocyte maturation	3/2734	13/17381	0.3359	0.87	0.85573	HBZ/L3MBTL3/MAEA	3	BP
GO:0045760	positive regulation of	3/2734	13/17381	0.3359	0.87	0.85573	ADRA1A/GBA/TACR1	3	BP
GO:0046479	glycosphingolipid catabolic	3/2734	13/17381	0.3359	0.87	0.85573	GBA/NEU3/PRKCD	3	BP
GO:0046500	S-adenosylmethionine metabolic process	3/2734	13/17381	0.3359	0.87	0.85573	BHMT2/GNMT/PEMT	3	BP
GO:0046519	sphingoid metabolic	3/2734	13/17381	0.3359	0.87	0.85573	DEGS2/GBA/SPTLC1	3	BP
GO:0046629	gamma-delta T cell activation	3/2734	13/17381	0.3359	0.87	0.85573	JAG2/MICB/SOX13	3	BP

GO:0046831	regulation of RNA export from nucleus	3/2734	13/17381	0.3359	0.87	0.85573	SETD2/SUPT6H/ZC3H3	3	BP
GO:0051709	regulation of killing of cells of other	3/2734	13/17381	0.3359	0.87	0.85573	BAD/F2RL1/GAPDH	3	BP
GO:0055089	fatty acid homeostasis	3/2734	13/17381	0.3359	0.87	0.85573	DGAT1/INS/MLXIPL	3	BP
GO:0060134	prepulse inhibition	3/2734	13/17381	0.3359	0.87	0.85573	CTNNA2/DRD2/GRIN1	3	BP
GO:0060601	lateral sprouting from an	3/2734	13/17381	0.3359	0.87	0.85573	BMP4/FGFR2/SHH	3	BP
GO:0061029	eyelid development in camera-type eye	3/2734	13/17381	0.3359	0.87	0.85573	EGFR/GRHL3/STRA6	3	BP
GO:0061548	ganglion development	3/2734	13/17381	0.3359	0.87	0.85573	PHOX2B/SEMA3F/TULP3	3	BP
GO:0070365	hepatocyte differentiation	3/2734	13/17381	0.3359	0.87	0.85573	CYP1A1/E2F8/WNT1	3	BP
GO:0071391	cellular response to estrogen	3/2734	13/17381	0.3359	0.87	0.85573	ARID5A/BCAS3/RARA	3	BP
GO:0090151	establishment of protein localization to mitochondrial	3/2734	13/17381	0.3359	0.87	0.85573	HSP90AA1/TIMM22/TIMM9	3	BP
GO:0090179	planar cell polarity pathway involved in	3/2734	13/17381	0.3359	0.87	0.85573	DVL2/FZD2/GRHL3	3	BP

GO:0090266	regulation of mitotic cell cycle spindle assembly	3/2734	13/17381	0.3359	0.87	0.85573	ANAPC15/LCMT1/XRCC3	3	BP
GO:0098903	regulation of membrane repolarization during action potential	3/2734	13/17381	0.3359	0.87	0.85573	CACNB3/FLNA/MIR328	3	BP
GO:1901160	primary amino compound metabolic	3/2734	13/17381	0.3359	0.87	0.85573	AGMAT/DDC/RNF180	3	BP
GO:1901213	regulation of transcription from RNA polymerase II promoter involved in [illegible]	3/2734	13/17381	0.3359	0.87	0.85573	BMP4/NOTCH1/TBX2	3	BP
GO:1902166	negative regulation of intrinsic apoptotic signaling pathway in response to [illegible]	3/2734	13/17381	0.3359	0.87	0.85573	CD74/MUC1/ZNF385A	3	BP
GO:1902358	sulfate transmembrane transport	3/2734	13/17381	0.3359	0.87	0.85573	SLC26A1/SLC26A10/SLC26A6	3	BP
GO:1903504	regulation of mitotic spindle checkpoint	3/2734	13/17381	0.3359	0.87	0.85573	ANAPC15/LCMT1/XRCC3	3	BP

GO:1904925	positive regulation of autophagy of mitochondrion in response to mitochondrial	3/2734	13/17381	0.3359	0.87	0.85573	ATPIF1/GBA/MFN2	3	BP
GO:2000010	positive regulation of protein localization to	3/2734	13/17381	0.3359	0.87	0.85573	LRIG2/NRG1/STX4	3	BP
GO:2000059	negative regulation of protein ubiquitination involved in ubiquitin-dependent	3/2734	13/17381	0.3359	0.87	0.85573	SUFU/TAF1/WNT1	3	BP
GO:2001032	regulation of double-strand break repair via nonhomologous end joining	3/2734	13/17381	0.3359	0.87	0.85573	HSF1/NUDT16L1/PARP9	3	BP
GO:2001044	regulation of integrin-mediated	3/2734	13/17381	0.3359	0.87	0.85573	CD63/FLNA/LIMS2	3	BP
GO:0006937	regulation of muscle contraction	28/2734	163/17381	0.33642	0.87	0.85573	ADRA1A/ADRA1B/ADRA2A/ADRA2B/ADRA2C/ATP1A1/CAMK2D/CAV3/CHGA/CHRM1/CTTN/EDN2/FLNA/GATA4/GPER1/GSTM2/KCNQ1/MIR153-1/MIR328/MYBPC3/MYBPH/MYL5/NKX2-5/NPPA/PPP1R12B/TACR1/TACR2/TNNC1	28	BP
GO:0001754	eye photoreceptor	8/2734	42/17381	0.33721	0.87	0.85573	GNAT1/MYO7A/NAGLU/NRL/SOX8/TH/THY1/TULP1	8	BP

GO:0002832	negative regulation of response to	8/2734	42/17381	0.33721	0.87	0.85573	DHX58/HAVCR2/MICB/NFKBIL1/NLRX1/SPINK5/TARBP2/TRAF3IP1	8	BP
GO:0010883	regulation of lipid storage	8/2734	42/17381	0.33721	0.87	0.85573	ACACB/APOB/EHD1/FITM1/HILPDA/NR1H3/PNPLA2/SCARB1	8	BP
GO:0034105	positive regulation of tissue	8/2734	42/17381	0.33721	0.87	0.85573	ADAM8/DEF8/EGFR/IL18/MIR16-1/MIR195/PLEKHM1/TMBIM1	8	BP
GO:0051489	regulation of filopodium assembly	8/2734	42/17381	0.33721	0.87	0.85573	AGRN/ARAP1/BCAS3/CCL21/ESPN/FSCN1/PRKCD/RAB17	8	BP
GO:0070849	response to epidermal growth factor	8/2734	42/17381	0.33721	0.87	0.85573	COL1A1/DAB2IP/EGFR/ERBB2/MAPK3/PTPN11/TDGF1/ZPR1	8	BP
GO:0002286	T cell activation involved in immune	17/2734	96/17381	0.33726	0.87	0.85573	BCL6/CCL19/EIF2AK4/F2RL1/HAVCR2/HLX/IFNA5/IL18/IL4R/ITGAL/LGALS3/LGALS9/LY9/RARA/RORC/SEMA4A/SLC11A1	17	BP
GO:0031279	regulation of cyclase activity	17/2734	96/17381	0.33726	0.87	0.85573	ADRA2A/AVPR2/CCR2/CRHR1/DRD2/GABBR1/GPER1/GUCA1B/GUCA2A/GUCA2B/MAPK3/NOS3/OPRM1/PDZD3/RAF1/RUNDC3A/WFS1	17	BP
GO:0030593	neutrophil chemotaxis	16/2734	90/17381	0.33818	0.87	0.85573	C3AR1/CAMK1D/CCL1/CCL19/CCL21/CCL5/CD74/CKLF/CXCR2/DAPK2/EDN2/GBF1/LGALS3/PF4V1/PLA2G1B/TIRAP	16	BP
GO:0009409	response to cold	9/2734	48/17381	0.33894	0.87	0.85573	ACADVL/ADM/EIF2AK4/HSP90AA1/HSPA2/THRA/TRH/UCP3/VGF	9	BP
GO:0046677	response to antibiotic	9/2734	48/17381	0.33894	0.87	0.85573	CASP3/CIITA/CRIP1/CYP1A1/ENDOG/HDAC8/HSP90AA1/HSPA5/UROS	9	BP
GO:0050433	regulation of catecholamine secretion	9/2734	48/17381	0.33894	0.87	0.85573	ADRA2A/ADRA2B/ADRA2C/AGT/CHGA/CHRNA6/DRD2/DTNBP1/STX1A	9	BP
GO:0060711	labyrinthine layer	9/2734	48/17381	0.33894	0.87	0.85573	ADM/CDX2/FGFR2/HEY1/NSDHL/PCDH12/PLCD1/SPINT1/ST14	9	BP

GO:1905515	non-motile cilium assembly	9/2734	48/17381	0.33894	0.87	0.85573	C2CD3/CC2D2A/CCDC13/DISC1/IFT140/SEPT9/TBC1D32/TMEM17/WRAP73	9	BP
GO:0034103	regulation of tissue	15/2734	84/17381	0.33899	0.87	0.85573	ADAM8/AGT/BGLAP/CSF1R/CSK/DEF8/EGFR/FLT4/IL18/INPP5D/LEPR/MIR16-1/MIR195/PLEKHM1/TMBIM1	15	BP
GO:0042475	odontogenesis of dentin-containing tooth	15/2734	84/17381	0.33899	0.87	0.85573	ADM/AMTN/ATF2/BMP4/CSF1/DLX3/JAG2/NKX2-3/ROGDI/SHH/SLC34A1/SMO/TNC/WNT10A/WNT6	15	BP
GO:0045930	negative regulation of mitotic cell cycle	51/2734	305/17381	0.33905	0.87	0.85573	ABL1/ANAPC15/ATF2/BCL6/BMP4/BRCA1/BUB1B/CDC14A/CDK2AP2/CDK5RAP3/CHFR/CHMP4C/CTDSP1/E2F4/E2F8/E4F1/EGFR/FOXO4/GPR132/HRAS/INTS3/LCMT1/MAD1L1/MCPH1/MIIP/MIR15A/MIR16-1/MIR195/MIR29A/MIR29C/MUC1/NEK11/NLE1/PCBP4/PRCC/PSMB11/PSMB6/PSMB7/PSMB8/PSMC3/PSMD13/PSMD3/PSMD5/PSMD7/RINT1/SCRIB/SFN/TICRR/XPC/XRCC3/ZNF385A	51	BP
GO:0042590	antigen processing and presentation of exogenous peptide antigen	14/2734	78/17381	0.33965	0.87	0.85573	HLA-E/IKBKB/ITGB5/NCF1/PSMB11/PSMB6/PSMB7/PSMB8/PSMC3/PSMD13/PSMD3/PSMD5/PSMD7/TAP1	14	BP
GO:0001885	endothelial cell development	10/2734	54/17381	0.33991	0.87	0.85573	CDH5/ENG/F2RL1/GPX1/IKBKB/MARVELD2/PDE2A/PLOD3/TNMD/WNT7A	10	BP
GO:0050819	negative regulation of	10/2734	54/17381	0.33991	0.87	0.85573	C1QTNF1/FGA/KLKB1/KNG1/NOS3/PDGFA/PLAU/PRKCD/PROZ/SERPINF2	10	BP
GO:0006487	protein N-linked	13/2734	72/17381	0.34014	0.87	0.85573	ASGR1/B4GALT7/DAD1/DDOST/DOLPP1/DPM2/GORASP1/MGAT4B/MGAT5B/OST4/RPN1/ST3GAL4/SYVN1	13	BP
GO:0031146	SCF-dependent proteasomal ubiquitin-dependent protein	13/2734	72/17381	0.34014	0.87	0.85573	CCNF/FBXO2/FBXO6/FBXW4/PSMB11/PSMB6/PSMB7/PSMB8/PSMC3/PSMD13/PSMD3/PSMD5/PSMD7	13	BP
GO:0045454	cell redox homeostasis	13/2734	72/17381	0.34014	0.87	0.85573	APEX1/GLRX5/GPX1/GSR/NCF1/NOS3/PRDX1/PRDX6/QSOX1/SLC11A1/TXNDC15/TXNRD1/TXNRD3	13	BP

GO:0010389	regulation of G2/M transition of mitotic cell cycle	33/2734	194/17381	0.34016	0.87	0.85573	ACTR1A/BRCA1/CDK4/CDK5RAP3/CEP164/CEP70/CHMP4C/CKAP5/DCTN2/DYNC1H1/FOXO4/GPR132/HAUS4/HAUS7/HSP90AA1/HSPA2/MIIP/MIR195/PHOX2B/PSMB11/PSMB6/PSMB7/PSMB8/PSMC3/PSMD13/PSMD3/PSMD5/PSMD7/RINT1/SDCCAG8/SSNA1/TICRR/TUBG1	33	BP
GO:0031281	positive regulation of	11/2734	60/17381	0.34035	0.87	0.85573	AVPR2/CRHR1/GPER1/GUCA1B/GUCA2A/GUCA2B/MAPK3/NOS3/RAF1/RUNDC3A/WFS1	11	BP
GO:0048645	animal organ formation	11/2734	60/17381	0.34035	0.87	0.85573	BMP4/EYA1/FGFR2/HOXA3/HOXC11/LEMD2/MAPK3/NKX3-2/SHH/TBR1/WNT3A	11	BP
GO:1901222	regulation of NIK/NF-kappaB	11/2734	60/17381	0.34035	0.87	0.85573	ADGRG3/EGFR/HAVCR2/ILK/IRAK1/NOD1/PDCD4/PTP4A3/TIRAP/TRAF2/TRIM40	11	BP
GO:0009166	nucleotide catabolic	12/2734	66/17381	0.34039	0.87	0.85573	DNPH1/GPX1/HINT1/MBD4/NEIL2/NT5M/NUDT1/NUDT18/OGG1/PDE2A/SMUG1/XDH	12	BP
GO:0043367	CD4-positive, alpha-beta T cell	12/2734	66/17381	0.34039	0.87	0.85573	BCL6/CCL19/FUT7/HLX/IL18/IL4R/LGALS9/LY9/NKX2-3/RARA/RORC/SEMA4A	12	BP
GO:0051965	positive regulation of synapse	12/2734	66/17381	0.34039	0.87	0.85573	ADGRB1/AGRN/AMIGO1/ASIC2/CLSTN1/CLSTN3/EPHB3/LINGO2/LRRTM1/NLGN3/SLITRK3/WNT7A	12	BP
GO:0045931	positive regulation of mitotic cell	25/2734	145/17381	0.34121	0.8714	0.85705	ABL1/APEX1/ASNS/CDK10/CDK4/CYP1A1/EIF4EBP1/EIF4G1/HSF1/INS/MEIS2/MEPCE/MIR221/MIR222/MIR29A/NUMA1/PDGFRB/PHOX2B/POLDIP2/PTPN11/SH2B1/SLF2/UBE2E2/USP2/XRCC3	25	BP
GO:0060402	calcium ion transport into cytosol	25/2734	145/17381	0.34121	0.8714	0.85705	ABL1/ADRA1A/CACNB3/CAMK2D/CCL19/CCL21/CORO1A/DRD2/FASLG/GPER1/GRIN1/GRIN2C/GSTM2/IL13/ITPR3/JPH3/LCK/NPSR1/P2RX2/PLA2G1B/PTK2B/RASA3/THY1/TRDN/TRPV6	25	BP
GO:0030217	T cell differentiation	38/2734	225/17381	0.34246	0.8742	0.85981	ABL1/ADAM8/BAD/BCL6/BMP4/CARD11/CCL19/CD27/CD74/DNAJA3/EGR1/ERBB2/FANCA/FOXN1/FUT7/FZD8/HLX/IL18/IL4R/JAG2/LCK/LEPR/LFNG/LGALS9/LY9/NKX2-3/PIK3R6/PRELID1/PSMB11/RARA/RORC/SART1/SEMA4A/SHH/SOX13/SPINK5/TNFRSF18/WNT1	38	BP

GO:0002262	myeloid cell homeostasis	23/2734	133/17381	0.34442	0.8759	0.86145	AHSP/ALAS2/ATPIF1/BCL6/BMP4/CASP3/HBZ/HCAR2/INPP5D/ISG15/L3MBTL3/LDB1/MAEA/MIR221/MIR222/MTHFD1/NFE2L1/PRDX1/RPS24/SETD1A/SPI1/STAT1/THRA	23	BP
GO:0090596	sensory organ morphogenesis	42/2734	250/17381	0.34564	0.8759	0.86145	AQP1/AQP5/ATP6V1B1/AXIN1/BMP4/DVL2/EYA1/FASLG/FGFR2/FOXE3/FZD2/GBX2/GNAT1/GRHL3/HMX2/HOXC13/KCNQ4/LHFPL5/LHX1/MAPK3/MFN2/MYO3A/MYO7A/NAGLU/NKX3-2/NRL/OSR1/SCRIB/SLC44A4/SOX8/STRA6/TBX2/TCAP/TH/THY1/TMIE/TSKU/TULP1/VAX2/WDPCP/WNT1/WNT3A	42	BP
GO:1903037	regulation of leukocyte cell-cell adhesion	57/2734	343/17381	0.34627	0.8759	0.86145	ABL1/ADAM8/BAD/BCL6/BMP4/CARD11/CASP3/CCDC88B/CCL19/CCL21/CCL5/CCR2/CD247/CD27/CD5/CD6/CD74/CLECL1/CORO1A/CSK/DNAJA3/EFNB1/ERBB2/FANCA/FOXN1/HAVCR2/HLA-E/HLX/IDO1/IL18/IL20RB/IL4R/LCK/LGALS3/LGALS9/LMO1/MAD1L1/MAP3K14/MIR221/MIR222/MIR92A2/PDCD1/PIK3R6/PLA2G2F/PRELID1/PRKAR1A/PTPN11/RARA/SART1/SCGB1A1/SHH/SOX13/SPINK5/THY1/TNFRSF1[illegible]	57	BP
GO:0006813	potassium ion transport	41/2734	244/17381	0.34752	0.8759	0.86145	ADRA2A/AMIGO1/AQP1/ATP1A1/ATP1A4/CAV3/CNGA2/CNGB1/DPP6/DRD2/FLNA/GCK/HCN3/KCNAB3/KCND1/KCND3/KCNH4/KCNH6/KCNJ18/KCNJ5/KCNJ9/KCNK12/KCNK13/KCNK17/KCNK4/KCNK7/KCNQ1/KCNQ4/KCNT1/MIR153-1/MIR212/NOS3/NPPA/PTK2B/SLC12A4/SLC12A7/SLC12A9/SLC9A3/SLC9	41	BP
GO:0071804	cellular potassium ion transport	35/2734	207/17381	0.348	0.8759	0.86145	AMIGO1/AQP1/ATP1A1/CAV3/CNGA2/CNGB1/DPP6/FLNA/HCN3/KCNAB3/KCND1/KCND3/KCNH4/KCNH6/KCNJ18/KCNJ5/KCNJ9/KCNK12/KCNK13/KCNK17/KCNK4/KCNK7/KCNQ1/KCNQ4/KCNT1/MIR153-1/MIR212/NPPA/SLC12A4/SLC12A7/SLC12A9/SLC9A3/SLC9A5/TRPM5/W	35	BP
GO:0071805	potassium ion transmembrane transport	35/2734	207/17381	0.348	0.8759	0.86145	AMIGO1/AQP1/ATP1A1/CAV3/CNGA2/CNGB1/DPP6/FLNA/HCN3/KCNAB3/KCND1/KCND3/KCNH4/KCNH6/KCNJ18/KCNJ5/KCNJ9/KCNK12/KCNK13/KCNK17/KCNK4/KCNK7/KCNQ1/KCNQ4/KCNT1/MIR153-1/MIR212/NPPA/SLC12A4/SLC12A7/SLC12A9/SLC9A3/SLC9A5/TRPM5/W	35	BP
GO:0018022	peptidyl-lysine methylation	20/2734	115/17381	0.34921	0.8759	0.86145	ASH2L/BRCA1/EHMT1/EHMT2/GCG/MEN1/PAXIP1/PHF19/PRDM12/PRDM7/PRDM9/PYGO2/RLF/SETD1A/SETD2/SETD7/SMYD3/SUPT6H/SUV39H	20	BP

GO:0002043	blood vessel endothelial cell proliferation involved in sprouting angiogenesis	4/2734	19/17381	0.34943	0.8759	0.86145	BMP4/MIR16-1/MIR222/MIR503	4	BP
GO:0002320	lymphoid progenitor cell differentiation	4/2734	19/17381	0.34943	0.8759	0.86145	BMP4/NOTCH1/SHH/SPI1	4	BP
GO:0003323	type B pancreatic cell	4/2734	19/17381	0.34943	0.8759	0.86145	BAD/BMP4/SIDT2/SMO	4	BP
GO:0006907	pinocytosis	4/2734	19/17381	0.34943	0.8759	0.86145	AHSG/MAPKAPK3/NR1H3/PROM2	4	BP
GO:0009148	pyrimidine nucleoside triphosphate biosynthetic process	4/2734	19/17381	0.34943	0.8759	0.86145	NME1/NME4/TBPL1/UCK1	4	BP
GO:0010288	response to lead ion	4/2734	19/17381	0.34943	0.8759	0.86145	ALAD/CDK4/DNMT3A/SLC34A1	4	BP
GO:0016048	detection of temperature stimulus	4/2734	19/17381	0.34943	0.8759	0.86145	ANO1/ARRB2/ASIC3/PRDM12	4	BP
GO:0032674	regulation of interleukin-5 production	4/2734	19/17381	0.34943	0.8759	0.86145	EPX/IL5RA/RARA/SCGB1A1	4	BP
GO:0035994	response to muscle stretch	4/2734	19/17381	0.34943	0.8759	0.86145	CAV3/NPPA/RAF1/TCAP	4	BP
GO:0042474	middle ear morphogenesis	4/2734	19/17381	0.34943	0.8759	0.86145	EYA1/NAGLU/NKX3-2/OSR1	4	BP

GO:0042693	muscle cell fate commitment	4/2734	19/17381	0.34943	0.8759	0.86145	MYOD1/NKX2-5/TBX2/WNT3A	4	BP
GO:0042953	lipoprotein transport	4/2734	19/17381	0.34943	0.8759	0.86145	APOB/CUBN/LRP1/UNC119	4	BP
GO:0043153	entrainment of circadian clock by photoperiod	4/2734	19/17381	0.34943	0.8759	0.86145	MTA1/PER1/RBM4/USP2	4	BP
GO:0043162	ubiquitin-dependent protein catabolic process via the multivesicular body sorting [illegible]	4/2734	19/17381	0.34943	0.8759	0.86145	PTPN23/SNF8/UBAP1L/VPS25	4	BP
GO:0043691	reverse cholesterol	4/2734	19/17381	0.34943	0.8759	0.86145	APOA5/APOC3/CLU/SCARB1	4	BP
GO:0044872	lipoprotein localization	4/2734	19/17381	0.34943	0.8759	0.86145	APOB/CUBN/LRP1/UNC119	4	BP
GO:0046597	negative regulation of viral entry into	4/2734	19/17381	0.34943	0.8759	0.86145	IFITM2/TRIM11/TRIM26/TRIM31	4	BP
GO:0046639	negative regulation of alpha-beta T	4/2734	19/17381	0.34943	0.8759	0.86145	BCL6/HLX/IL4R/SHH	4	BP
GO:0046641	positive regulation of alpha-beta T	4/2734	19/17381	0.34943	0.8759	0.86145	CCR2/HLA-E/IL18/LGALS9	4	BP
GO:0046655	folic acid metabolic	4/2734	19/17381	0.34943	0.8759	0.86145	ALDH1L1/FOLR2/MTHFD1/SHMT2	4	BP

GO:0048557	embryonic digestive tract morphogenesis	4/2734	19/17381	0.34943	0.8759	0.86145	FGFR2/HLX/RBPMS2/SHH	4	BP
GO:0055093	response to hyperoxia	4/2734	19/17381	0.34943	0.8759	0.86145	CDK4/COL1A1/CYP1A1/PDGFRB	4	BP
GO:0060074	synapse maturation	4/2734	19/17381	0.34943	0.8759	0.86145	ANAPC2/DAB2IP/DISC1/NEUROD2	4	BP
GO:0060602	branch elongation of an	4/2734	19/17381	0.34943	0.8759	0.86145	BMP4/FGFR2/SHH/TNC	4	BP
GO:0070734	histone H3-K27 methylation	4/2734	19/17381	0.34943	0.8759	0.86145	EHMT1/EHMT2/PHF19/SUPT6H	4	BP
GO:0072079	nephron tubule formation	4/2734	19/17381	0.34943	0.8759	0.86145	IRX3/OSR1/SOX8/WNT6	4	BP
GO:0090026	positive regulation of monocyte	4/2734	19/17381	0.34943	0.8759	0.86145	CCL1/CCL5/CCR2/CREB3	4	BP
GO:0090208	positive regulation of triglyceride	4/2734	19/17381	0.34943	0.8759	0.86145	APOA5/NR1H3/PNPLA2/SCARB1	4	BP
GO:0098780	response to mitochondrial depolarisation	4/2734	19/17381	0.34943	0.8759	0.86145	AMBRA1/ATPIF1/GBA/MFN2	4	BP
GO:1901741	positive regulation of	4/2734	19/17381	0.34943	0.8759	0.86145	ADGRB1/EHD1/IL4R/MYOD1	4	BP
GO:1902170	cellular response to reactive	4/2734	19/17381	0.34943	0.8759	0.86145	AQP1/DPEP1/MMP3/TRAF2	4	BP

GO:1902236	negative regulation of endoplasmic reticulum stress-induced intrinsic [illegible]	4/2734	19/17381	0.34943	0.8759	0.86145	CREB3/HERPUD1/SYVN1/WFS1	4	BP
GO:1904994	regulation of leukocyte adhesion to vascular endothelial cell	4/2734	19/17381	0.34943	0.8759	0.86145	CCL21/MIR221/MIR222/MIR92A2	4	BP
GO:1990182	exosomal secretion	4/2734	19/17381	0.34943	0.8759	0.86145	ATP13A2/RAB7A/SDC1/SNF8	4	BP
GO:2000369	regulation of clathrin-dependent	4/2734	19/17381	0.34943	0.8759	0.86145	HIP1R/TNK2/TOR1A/UNC119	4	BP
GO:2000505	regulation of energy homeostasis	4/2734	19/17381	0.34943	0.8759	0.86145	EIF4G1/LEPR/MLXIPL/TRPV4	4	BP
GO:0010959	regulation of metal ion transport	59/2734	356/17381	0.3513	0.8779	0.86349	ABL1/ADRA2A/AGT/AHNAK/AMIGO1/ARRB2/ATP1A1/CACNB1/CACNB3/CAMK2D/CATSPER1/CAV3/CCL5/CD63/COMMD1/CORO1A/CRACR2A/CRHR1/DPP6/DRD2/FLNA/GCG/GCK/GNAO1/GNB5/GPER1/GPR35/GRIN1/GSTM2/HSPA2/IL13/JPH3/KCNQ1/LGALS3/MIR153-1/MIR212/MIR328/NKX2-5/NOS3/NPPA/NPSR1/OPRD1/ORAI1/OSR1/P2RX2/PACSIN3/PDGFRB/PER1[illegible]	59	BP

GO:0002683	negative regulation of immune system process	68/2734	412/17381	0.35137	0.8779	0.86349	ARRB2/BCL6/BMP4/C1QC/CASP3/CCL21/CCR2/CD300LF/CD74/CIB1/CUEDC2/DAB2IP/DHX58/DRD2/ELF1/ERBB2/F2RL1/FAM19A3/GPER1/GPR17/GPX1/HAVCR2/HIST1H4F/HIST2H4A/HIST2H4B/HLA-E/HLX/HOXB8/IDO1/IL20RB/IL31RA/IL4R/INPP5D/INS/LDB1/LGALS3/LGALS9/MAD1L1/MEIS2/MICB/MIR221/MIR222/NBL1/NFKBIL1/NLRX1/NME1/NMI/NOTCH1/NR1H3/PDCD1/PLA2G2F/PRKAR1A/PTK2B/RARA/SCGB1A1/SCRIB/SHH/SPINK5/TARBP2/TCTA/THY1/TLR9/TMEM176B/TNFRSF13B/TRAF3IP1/TSC22D3/TSC22D4/VSIG4	68	BP
GO:0030111	regulation of Wnt signaling pathway	54/2734	325/17381	0.35186	0.8779	0.86349	ABL1/ANKRD10/AXIN1/CCAR2/CHD8/COL1A1/DAB2IP/DACT2/DISC1/DRAXIN/DVL2/EGR1/EMD/FGFR2/FZD9/IFT20/IGFBP6/ILK/KREMEN2/LGR6/LRP1/LZTS2/MIR222/NAIP/NKD2/NKX2-5/NLE1/NOTCH1/PSMB11/PSMB6/PSMB7/PSMB8/PSMC3/PSMD13/PSMD3/PSMD5/PSMD7/RNF220/RSPO1/SFRP5/SHH/SOST/SOX7/TAX1BP3/TCF7L1/TNN/TSKU/WIF1/WLS/WNT1/WNT11/WNT3A/WNT7A/ZNF703	54	BP
GO:0007568	aging	49/2734	294/17381	0.35214	0.8779	0.86349	ABL1/ADM/ADRA1A/AGT/APEX1/BCL6/BGLAP/CACYBP/CDKN2A/CRYAB/CYP1A1/DDC/DNAJA3/DNMT3A/EIF2B5/EIF5A/ENDOG/ENG/FOXM1/FOXO4/GNAO1/HMGA1/HRAS/ILK/INPP5D/IRAK1/KRT14/KRT16/KRTAP4-3/LMNA/LOXL2/LRP1/MIR10A/MPO/MTOR/NOX4/NR5A1/NUDT1/OGG1/PDCD4/PDGFRB/PRKCD/RWDD1/SCAP/TBX2/TH/UCP3/WNT1/YPEL3	49	BP
GO:0010508	positive regulation of	18/2734	103/17381	0.35234	0.8779	0.86349	AMBRA1/ATPIF1/BAD/DAPK1/EIF2AK4/GBA/GPSM1/KDR/LRSAM1/MAPK3/MFN2/NOD1/RALB/SPTLC1/TBK1/TFEB/TRIM65/ULK1	18	BP
GO:0018205	peptidyl-lysine modification	63/2734	381/17381	0.35236	0.8779	0.86349	APBB1/ASH2L/ATF2/BRCA1/BRPF1/CRTC2/CTBP1/EGR1/EHMT1/EHMT2/EIF5A/EPC1/EYA1/GCG/GLRX5/HCFC1/HDAC8/ING4/KAT2A/LDB1/LIAS/MAPK3/MEN1/MSL3/MTA1/MUC1/MYOD1/NAA60/NAT8B/NOC2L/NUP210/NUP98/P3H3/PAXIP1/PCGF2/PER1/PHF19/PIWIL2/PLOD3/POLE4/POR/PRDM12/PRDM7/PRDM9/PYGO2/RLF/RNF212/RPS6KA4/RWDD3/SENP3/SENP6/SETD1A/SETD2/SETD7/SMYD3/SPI1/SUPT6H/SUV39H1/TADA3/TAF1/T	63	BP

GO:0032259	methylation	58/2734	350/17381	0.35318	0.8779	0.86349	AS3MT/ASH2L/BHMT2/BRCA1/CIAPIN1/CMTR1/COPRS/CYP1A2/DDX4/DNMT3A/EHMT1/EHMT2/FKBP6/GCG/GNMT/HNMT/LCMT1/LRTOMT/MEN1/MEPCE/METTL17/METTL2A/METTL2B/METTL7B/MIR29A/MIR29C/MPHOSPH8/NDUFAF7/NSUN5/NSUN5P2/NTMT1/PAXIP1/PEMT/PHF19/PIWIL2/PRDM12/PRDM14/PRDM7/PRDM8/PRDM9/PYGO2/RLF/SETD1A/SETD2/SETD7/SETD9/SMYD3/SMYD5/SPI1/SUPT6H/SUV39H1/TDRD1/TDRD9/TH[illegible]	58	BP
GO:0044839	cell cycle G2/M phase transition	43/2734	257/17381	0.35397	0.8779	0.86349	ACTR1A/BRCA1/BRSK2/CCNB2/CDK4/CDK5RAP3/CEP164/CEP70/CHMP4C/CKAP5/DCTN2/DYNC1H1/FOXM1/FOXO4/GPR132/HAUS4/HAUS7/HSP90AA1/HSPA2/KDM8/LCMT1/MIIP/MIR195/NEK10/NES/PAXIP1/PHOX2B/PKMYT1/PPP1R12B/PSMB11/PSMB6/PSMB7/PSMB8/PSMC3/PSMD13/PSMD3/PSMD5/PSMD7/RINT1/SDCCAG8/SSNA1/TICRR/TUBG1	43	BP
GO:0015908	fatty acid transport	16/2734	91/17381	0.35536	0.8779	0.86349	ABCC4/ACACB/CPT2/CROT/DRD2/GOT2/LCN12/MFSD2A/MID1IP1/NCOR1/PLA2G1B/PLA2G2F/PLA2G5/PPARD/SLC22A9/SLC25A20	16	BP
GO:0022407	regulation of cell-cell adhesion	70/2734	425/17381	0.35572	0.8779	0.86349	ABL1/ADAM8/BAD/BCL6/BMP4/C1QTNF1/CARD11/CASP3/CCDC88B/CCL19/CCL21/CCL5/CCR2/CD247/CD27/CD5/CD6/CD74/CELSR2/CLECL1/CORO1A/CSK/DNAJA3/EFNB1/EPHB3/ERBB2/FANCA/FERMT3/FGA/FOXN1/HAVCR2/HLA-E/HLX/IDO1/IL18/IL1RN/IL20RB/IL4R/LCK/LGALS3/LGALS9/LMO1/MAD1L1/MAP3K14/MIR221/MIR222/MIR92A2/PDCD1/PIK3R6/PLA2G2F/PRELID1/PRKAR1A/PRKCD/PTPN11/PTPN23/RARA/SART1/SCGB1A1/SERPINF2/S[illegible]	70	BP
GO:0006836	neurotransmitter transport	31/2734	183/17381	0.35576	0.8779	0.86349	BAIAP3/CDK5/CPLX1/DDC/DGKI/DOC2A/DOC2B/DRD2/DTNBP1/GAD1/GPER1/NRXN2/PNKD/PTPRN2/SCRIB/SLC22A1/SLC38A1/SLC6A18/SLC6A6/SLC6A7/SLC6A8/SLC6A9/STX1A/STX4/STXBP1/SV2A/SYTL3/TACR2/TH/T[illegible]	31	BP
GO:0051348	negative regulation of transferase activity	61/2734	369/17381	0.35613	0.8779	0.86349	ABL1/ANAPC15/ANAPC2/BGN/BMP4/BUB1B/CAMK2N2/CASP3/CAV3/CDK5RAP3/CDKN2A/CHAD/CIB1/CISH/COX11/CRIPAK/CSK/DAB2IP/DTNBP1/DUSP2/DUSP21/DUSP26/DUSP5/GBA/HSPB1/ILK/INCA1/LIMK1/LRRTM1/MAPK8IP1/MEN1/MIR92A2/MYCNOS/PDCD4/PIF1/PPP1R1B/PREX2/PRKAR1A/PRKAR1B/PRKCD/PRKRIP1/PSMB6/PSMB7/PSMB8/PSMC3/PSMD13/PSMD3/PSMD5/PSMD7/PYDC1/RGS14/RTN4RL1/RTN4RL2/SFN/SH3BP5/S[illegible]	61	BP
GO:0001964	startle response	5/2734	25/17381	0.35627	0.8779	0.86349	CTNNA2/DRD2/GLRA1/GRIN1/NRG1	5	BP

GO:0003081	regulation of systemic arterial blood pressure by renin-	5/2734	25/17381	0.35627	0.8779	0.86349	AGT/F2RL1/REN/SERPINF2/TACR1	5	BP
GO:0009218	pyrimidine ribonucleotide metabolic	5/2734	25/17381	0.35627	0.8779	0.86349	AK5/DHODH/NME1/NME4/UCK1	5	BP
GO:0009299	mRNA transcription	5/2734	25/17381	0.35627	0.8779	0.86349	FLNA/FOXE3/HSF1/PPARD/SUPT6H	5	BP
GO:0009310	amine catabolic process	5/2734	25/17381	0.35627	0.8779	0.86349	CHDH/HAAO/HNMT/IDO1/PAOX	5	BP
GO:0010714	positive regulation of collagen	5/2734	25/17381	0.35627	0.8779	0.86349	BMP4/ENG/PDGFRB/SCX/SERPINF2	5	BP
GO:0010996	response to auditory	5/2734	25/17381	0.35627	0.8779	0.86349	DRD2/NRXN2/STRA6/TACR1/XPC	5	BP
GO:0032350	regulation of hormone metabolic	5/2734	25/17381	0.35627	0.8779	0.86349	ATP1A1/DGKQ/DUOXA1/EGR1/POR	5	BP
GO:0044253	positive regulation of multicellular organismal	5/2734	25/17381	0.35627	0.8779	0.86349	BMP4/ENG/PDGFRB/SCX/SERPINF2	5	BP
GO:0045922	negative regulation of fatty acid	5/2734	25/17381	0.35627	0.8779	0.86349	ACACB/ACADVL/APOC3/BRCA1/INS	5	BP
GO:0048169	regulation of long-term neuronal	5/2734	25/17381	0.35627	0.8779	0.86349	AGT/DLG4/DRD2/GRIN1/HRAS	5	BP

GO:0050974	detection of mechanical stimulus involved in sensory	5/2734	25/17381	0.35627	0.8779	0.86349	ASIC2/ASIC3/KCNK4/LHFPL5/PHF24	5	BP
GO:0061037	negative regulation of cartilage	5/2734	25/17381	0.35627	0.8779	0.86349	ADAMTS7/BMP4/NKX3-2/RARA/WNT11	5	BP
GO:0072202	cell differentiation involved in metanephros	5/2734	25/17381	0.35627	0.8779	0.86349	BMP4/OSR1/POU3F3/SMO/STAT1	5	BP
GO:0090169	regulation of spindle	5/2734	25/17381	0.35627	0.8779	0.86349	CHMP1A/CHMP4C/DYNC1H1/NUMA1/SENP6	5	BP
GO:1900745	positive regulation of p38MAPK	5/2734	25/17381	0.35627	0.8779	0.86349	GADD45G/KARS/MIR181B1/NCF1/XDH	5	BP
GO:0030534	adult behavior	24/2734	140/17381	0.35665	0.8779	0.86349	ARRB2/CDK5/CEND1/CHRNB1/DAB1/DMBX1/DNM1/DRD2/EN1/GLRA1/GRIN1/HOMER2/HOXB8/INPP5F/NLGN3/NRXN2/OPRD1/OPRM1/PPP1R1B/PREX2/RNF180/SPTBN2/TRH/ZFHX2	24	BP
GO:1902652	secondary alcohol metabolic	24/2734	140/17381	0.35665	0.8779	0.86349	ACACB/ACADVL/APOA5/APOB/CLN6/CUBN/CYP11A1/DGKQ/EPHX2/HSD17B7/LEPR/LMF1/NPC1L1/NPC2/NSDHL/OSBPL5/PMVK/POR/PPARD/SCAP/SCARB1/SCARF1/SOAT2/STARD3	24	BP
GO:0045785	positive regulation of cell adhesion	65/2734	394/17381	0.35695	0.8779	0.86349	ABL1/ADAM8/ARPC2/BAD/BCL6/CARD11/CCDC88B/CCL19/CCL21/CCL5/CCR2/CD247/CD27/CD5/CD6/CD74/CDH13/CIB1/CLECL1/COL16A1/CORO1A/CSF1/CSK/DISC1/DNAJA3/DOCK1/DUSP26/EFNB1/ERBB2/FBLN2/FGA/FLNA/FN1/HAVCR2/HLA-E/HLX/IL18/IL4R/ILK/KDR/LCK/LDB1/LGALS9/MAP3K14/MIP/MIR92A2/MYOC/OLFM4/PDCD1/PIK3R6/PTK2B/PTPN11/PTPN23/RARA/RELL2/RHOD/SART1/SERPINF2/SHH/SPOCK2/STX4/THY1/TNFRSF18/TRAF2/WNT3A	65	BP

GO:0048545	response to steroid hormone	65/2734	394/17381	0.35695	0.8779	0.86349	ABCA2/ACTA1/ADM/ALAD/AQP1/ARRB2/ATP1A1/AXIN1/BAD/BGLAP/BMP4/BRCA1/CALCOCO1/CASP3/CATSPER1/CCL1/CLDN4/COL1A1/CRIPAK/DDX54/EGFR/EIF4EBP1/ENG/ESR2/ESRRA/FOSL1/GBA/GPER1/HEY1/HNMT/HSD11B2/IDH1/IL1RN/MED24/MYOD1/NOTCH1/NR1H3/NR2F1/NR4A1/NR5A1/PAQR6/PAQR7/PAQR9/PER1/PFKFB1/PLPP1/PPARD/PTPRU/RARA/RORC/RWDD1/SCGB1A1/SDC1/SMYD3/SSTR5/TACR1/TADA3/TAF1/T [illegible]	65	BP
GO:0046427	positive regulation of JAK-STAT	14/2734	79/17381	0.35815	0.8779	0.86349	AGT/ARL2BP/CCL5/CLCF1/CSF1R/HSF1/IL13/IL18/IL31RA/MIR221/NOTCH1/PARP9/PTK2B/TNFRSF18	14	BP
GO:0046849	bone remodeling	14/2734	79/17381	0.35815	0.8779	0.86349	ADAM8/BGLAP/CSF1R/CSK/DEF8/EFNA4/EGFR/INPP5D/LEPR/NCDN/NOX4/PLEKHM1/PTK2B/RAB7A	14	BP
GO:1904894	positive regulation of	14/2734	79/17381	0.35815	0.8779	0.86349	AGT/ARL2BP/CCL5/CLCF1/CSF1R/HSF1/IL13/IL18/IL31RA/MIR221/NOTCH1/PARP9/PTK2B/TNFRSF18	14	BP
GO:0042542	response to hydrogen peroxide	23/2734	134/17381	0.35859	0.8779	0.86349	ABL1/APEX1/AQP1/BAD/CASP3/COL1A1/CRYAB/ENDOG/FOSL1/GNAO1/GPX1/HSF1/IL18BP/KDM6B/LDHA/MIR92A2/PCGF2/PDGFRB/PRKCD/PSAP/PTK2B/SDC1/TRAP1	23	BP
GO:0006687	glycosphingolipid metabolic	13/2734	73/17381	0.35942	0.8779	0.86349	ARSE/ARSI/B4GALNT1/CLN6/CPTP/CREM/GBA/GLTP/HEXA/NEU3/PRKCD/PSAP/SMPD4	13	BP
GO:0043462	regulation of ATPase activity	13/2734	73/17381	0.35942	0.8779	0.86349	ATPIF1/BRSK2/DYNC1I1/DYNLL2/HSPA2/MYBPC3/MYL4/PFN1/THADA/TLR9/TNNC1/TPM2/VCPKMT	13	BP
GO:0001569	branching involved in blood vessel	6/2734	31/17381	0.35991	0.8779	0.86349	ABL1/ENG/GBX2/GDF2/PLXND1/SHH	6	BP
GO:0001844	protein insertion into mitochondrial membrane involved in apoptotic [illegible]	6/2734	31/17381	0.35991	0.8779	0.86349	BAD/DYNLL2/MOAP1/PPP1R13B/SFN/YWHAQ	6	BP
GO:0009954	proximal/distal pattern	6/2734	31/17381	0.35991	0.8779	0.86349	EN1/HOXC10/HOXC11/IRX3/OSR1/PBX2	6	BP

GO:0015949	nucleobase-containing small molecule interconversion	6/2734	31/17381	0.35991	0.8779	0.86349	AK5/AK6/GSR/NME1/NME4/TXNRD1	6	BP
GO:0033028	myeloid cell apoptotic	6/2734	31/17381	0.35991	0.8779	0.86349	CCL5/CDKN2A/CTSL/HCAR2/MAEA/THRA	6	BP
GO:0040036	regulation of fibroblast growth factor receptor	6/2734	31/17381	0.35991	0.8779	0.86349	FGFBP3/FGFR2/GPC1/HHIP/MIR16-1/PRDM14	6	BP
GO:0048873	homeostasis of number of cells within a tissue	6/2734	31/17381	0.35991	0.8779	0.86349	CORO1A/CSF1/IL20RB/PRDM14/PTPN11/SMO	6	BP
GO:0051123	RNA polymerase II transcriptional preinitiation	6/2734	31/17381	0.35991	0.8779	0.86349	MED6/PSMC3/TAF1/TAF1L/TAF7/THRA	6	BP
GO:0060674	placenta blood vessel development	6/2734	31/17381	0.35991	0.8779	0.86349	FOSL1/HEY1/NSDHL/PKD1/PLCD1/SPINT1	6	BP
GO:0071276	cellular response to	6/2734	31/17381	0.35991	0.8779	0.86349	CYP1A2/EGFR/HSF1/MAPK3/NCF1/OGG1	6	BP
GO:0071470	cellular response to	6/2734	31/17381	0.35991	0.8779	0.86349	AQP1/BAD/CASP3/OSR1/SLC2A4/TRPV4	6	BP
GO:0072595	maintenance of protein localization in	6/2734	31/17381	0.35991	0.8779	0.86349	ARL2BP/GPAA1/HSPA5/NR5A1/OS9/TAF3	6	BP
GO:0090322	regulation of superoxide metabolic	6/2734	31/17381	0.35991	0.8779	0.86349	AGT/EGFR/F2RL1/GCH1/ITGAM/PRKCD	6	BP

GO:1901890	positive regulation of cell junction	6/2734	31/17381	0.35991	0.8779	0.86349	ABL1/AGT/COL16A1/KDR/MYOC/THY1	6	BP
GO:1902235	regulation of endoplasmic reticulum stress-induced intrinsic apoptotic [illegible]	6/2734	31/17381	0.35991	0.8779	0.86349	BOK/CREB3/HERPUD1/SPOP/SYVN1/WFS1	6	BP
GO:2000516	positive regulation of CD4-positive, alpha-beta T	6/2734	31/17381	0.35991	0.8779	0.86349	CCL19/HLX/IL18/IL4R/LGALS9/RARA	6	BP
GO:0006414	translational elongation	22/2734	128/17381	0.36055	0.8779	0.86349	ABTB1/C12orf65/DPH1/EIF5A/ERAL1/MRPL10/MRPL14/MRPL28/MRPL36/MRPL37/MRPL43/MRPL52/MRPL57/MRPL9/MRPS10/MRPS11/MRPS18A/MRPS18B/MRPS21/MRPS33/MRPS5/ZNF598	22	BP
GO:0032387	negative regulation of intracellular	22/2734	128/17381	0.36055	0.8779	0.86349	BOK/CD27/CDK5/CNIH2/CRYAB/DAB2IP/EMD/FAM89B/FZD9/LGALS9/MTOR/NFKBIL1/OS9/PARP10/PBLD/PDE2A/PKD1/SFRP5/SLC25A5/SNX12/SUFU/THRA	22	BP
GO:0051705	multi-organism behavior	12/2734	67/17381	0.36056	0.8779	0.86349	CNTFR/DLG4/EN1/GAD1/GRIN1/KIRREL3/MTOR/NLGN3/NRXN2/TACR1/TH/VPS13A	12	BP
GO:0043547	positive regulation of GTPase activity	67/2734	407/17381	0.36138	0.8779	0.86349	ACAP1/ACAP3/ADAP1/ADPRHL1/AGAP3/AGRN/ALS2CL/ARAP1/ARFGAP2/ARHGAP10/ARHGAP22/ARHGAP27/ARHGAP30/ARHGAP4/ARHGEF10/ARHGEF16/AXIN1/BCAS3/CCL1/CCL19/CCL21/CCL5/CDC42EP2/DAB2IP/DOCK1/DOCK7/DVL2/ELMOD1/ERBB2/F2RL1/GNAO1/GNB5/GRHL3/GRTP1/HACD3/HRAS/LARS/LLGL1/NPRL3/PGAM5/PLEKHG4/PLEKHG6/PLXNB1/PREX2/PTK2B/RABGAP1/RABGAP1L/RAP1GAP/RASA3/RASA4B/RASGRP2/RGS10/RGS14/RGS16/RIN3/SCRIB/SGSM2/SIPA1/STARD8/TAX1BP3/[illegible]	67	BP
GO:0014032	neural crest cell development	11/2734	61/17381	0.36152	0.8779	0.86349	BMP4/CFL1/EFNB1/GBX2/MAPK3/NRG1/PHOX2B/SEMA3F/SHH/SMO/SOX8	11	BP

GO:0006734	NADH metabolic	7/2734	37/17381	0.36177	0.8779	0.86349	ALDOA/GAPDH/GCK/HK3/OGDH/PFKM/PKM	7	BP
GO:0009112	nucleobase metabolic	7/2734	37/17381	0.36177	0.8779	0.86349	ACPP/CDA/DHODH/MTHFD1/MTOR/UCK1/XDH	7	BP
GO:0035137	hindlimb morphogenesis	7/2734	37/17381	0.36177	0.8779	0.86349	ALX3/BMP4/NOTCH1/OSR1/PITX1/SHH/WNT7A	7	BP
GO:0042417	dopamine metabolic	7/2734	37/17381	0.36177	0.8779	0.86349	DDC/DRD2/GCH1/ITGAM/PNKD/SNCB/TH	7	BP
GO:0045601	regulation of endothelial cell differentiation	7/2734	37/17381	0.36177	0.8779	0.86349	BMP4/CDH5/GDF2/IKBKB/NOTCH1/TMEM100/XDH	7	BP
GO:0047496	vesicle transport along	7/2734	37/17381	0.36177	0.8779	0.86349	CNIH2/DTNBP1/DYNC1H1/DYNC1I1/KIF13A/KIF23/TRIM46	7	BP
GO:0048286	lung alveolus development	7/2734	37/17381	0.36177	0.8779	0.86349	BMP4/EDN2/FGFR2/FLT4/PDGFA/PKDCC/STRA6	7	BP
GO:1901998	toxin transport	7/2734	37/17381	0.36177	0.8779	0.86349	CASP1/CCT3/COPZ1/CRTC2/DNM1/HRH2/HSPA5	7	BP
GO:1904036	negative regulation of epithelial cell	7/2734	37/17381	0.36177	0.8779	0.86349	ABL1/FGA/IL13/KDR/MTOR/TNIP2/WFS1	7	BP
GO:0034341	response to interferon-gamma	28/2734	165/17381	0.36191	0.8779	0.86349	ADAMTS13/CAMK2D/CASP1/CCL1/CCL19/CCL21/CCL5/CD58/CIITA/DAPK1/GAPDH/GCH1/HLA-E/IFITM2/IRF5/LGALS9/MT2A/NMI/NR1H3/PARP9/PRKCD/SLC11A1/SLC26A6/STAT1/TDGF1/TRIM26/TRIM31/TRIM62	28	BP
GO:0002294	CD4-positive, alpha-beta T cell differentiation involved in	10/2734	55/17381	0.36224	0.8779	0.86349	BCL6/CCL19/HLX/IL18/IL4R/LGALS9/LY9/RARA/RORC/SEMA4A	10	BP
GO:0006949	syncytium formation	10/2734	55/17381	0.36224	0.8779	0.86349	ADAM12/ADGRB1/CACNA1H/CAV3/EHD1/ERVFRD-1/IL4R/MYOD1/TCTA/WNT1	10	BP

GO:0022029	telencephalon cell migration	10/2734	55/17381	0.36224	0.8779	0.86349	CDK5/CDK5R2/DAB1/DAB2IP/DISC1/DRD2/EGFR/NRG1/OGDH/POU3F3	10	BP
GO:0033627	cell adhesion mediated by integrin	10/2734	55/17381	0.36224	0.8779	0.86349	CCL21/CCL5/CIB1/COL16A1/EFNA1/EPHA8/FERMT3/MUC1/PLAU/PTPN11	10	BP
GO:0060761	negative regulation of response to	10/2734	55/17381	0.36224	0.8779	0.86349	CCDC3/CCL5/ECM1/F2RL1/IL1RN/NR1H3/PELI3/PYDC1/SCRIB/TRAIP	10	BP
GO:0032784	regulation of DNA-templated transcription, elongation	8/2734	43/17381	0.36256	0.8779	0.86349	AXIN1/CCAR2/LDB1/NELFB/RECQL5/SHH/SUPT6H/ZMYND11	8	BP
GO:0045646	regulation of erythrocyte differentiation	8/2734	43/17381	0.36256	0.8779	0.86349	INPP5D/ISG15/LDB1/MIR221/MIR222/SETD1A/SPI1/STAT1	8	BP
GO:0050982	detection of mechanical stimulus	8/2734	43/17381	0.36256	0.8779	0.86349	ASIC2/ASIC3/CAV3/KCNK4/LHFPL5/PHF24/PKD1/TCAP	8	BP
GO:0051602	response to electrical	8/2734	43/17381	0.36256	0.8779	0.86349	DISC1/GNAT1/NEUROD2/NSMF/TACR1/TACR2/TH/TRIM63	8	BP
GO:0061912	selective autophagy	8/2734	43/17381	0.36256	0.8779	0.86349	AMBRA1/LRSAM1/MAPK3/MFN2/NOD1/RAB7A/SPTLC1/TBK1	8	BP
GO:0030514	negative regulation of BMP signaling	9/2734	49/17381	0.36264	0.8779	0.86349	ABL1/HTRA3/LEMD2/NBL1/NOTCH1/RBPMS2/SMAD6/SOST/WNT1	9	BP
GO:0030834	regulation of actin filament depolymerizatio	9/2734	49/17381	0.36264	0.8779	0.86349	CAPZA3/CFL1/F2RL1/LMOD1/SCIN/SPTAN1/SPTB/SPTBN2/WDR1	9	BP
GO:0032480	negative regulation of type I interferon	9/2734	49/17381	0.36264	0.8779	0.86349	DHX58/HAVCR2/ISG15/NLRX1/NMI/OTUD5/TBK1/TRAF3IP1/TRAIP	9	BP

GO:0046626	regulation of insulin receptor signaling	9/2734	49/17381	0.36264	0.8779	0.86349	AHSG/BAIAP2L1/CCND3/CDK4/CISH/INS/PID1/PRKCD/TRIM72	9	BP
GO:0050885	neuromuscular process controlling	9/2734	49/17381	0.36264	0.8779	0.86349	AARS/ABL1/CDH23/DLG4/GPR88/GRIN2C/IGDCC3/JPH3/MYO7A	9	BP
GO:1901293	nucleoside phosphate biosynthetic process	56/2734	339/17381	0.366	0.8857	0.87114	ACOT7/ADM/ADRA2A/AK5/AK6/ALDOA/AQP1/ATP5G1/ATP5I/AVPR2/CCR2/CRHR1/CYC1/DGUOK/DHODH/DRD2/ENTPD8/ERH/GABBR1/GCG/GPER1/GPHA2/GUCA1B/GUCA2A/GUCA2B/GUCY2D/HAAO/IDH2/IMPDH1/KARS/MTHFD1/NME1/NME4/NMNAT3/NOS3/NPPA/OPRM1/PARP10/PARP9/PDE2A/PDZD3/PID1/PKM/PPCDC/PTK2B/QPRT/RAF1/RUNDC3A/RXFP2/SLC22A13/SLC26A1/TBPL1/UCK1/UCN2/VPS9D1/WFS1	56	BP
GO:0070252	actin-mediated cell contraction	19/2734	110/17381	0.36655	0.8867	0.87209	ACTA1/ATP1A1/CACNA1G/CAMK2D/CAV3/DES/FLNA/GATA4/KCND3/KCNJ5/KCNQ1/MIR328/MYBPC1/MYBPC3/MYL4/MYL6B/TCAP/TNNC1/TP	19	BP
GO:0030278	regulation of ossification	31/2734	184/17381	0.36794	0.8896	0.87495	AHSG/BGLAP/BMP4/CLIC1/CSF1/DDR2/ECM1/ESRRA/FGFR2/FZD9/GDF2/HDAC8/IFITM5/ILK/ISG15/KREMEN2/MAPK3/MEN1/NOTCH1/OSR1/PDLIM7/PHOSPHO1/PKDCC/PTK2B/SMAD6/SOST/SUFU/TACR1/TMEM119/TN	31	BP
GO:0035107	appendage morphogenesis	25/2734	147/17381	0.36837	0.8896	0.87495	ACD/ALX3/B9D1/BMP4/C2CD3/EN1/FBXW4/FGFR2/HOXC10/HOXC11/IFT140/MYCN/NOTCH1/OSR1/PBX2/PITX1/PKDCC/SHH/TBC1D32/TBX2/TMEM231/TRAF3IP1/TULP3/WDPCP/WNT7A	25	BP
GO:0035108	limb morphogenesis	25/2734	147/17381	0.36837	0.8896	0.87495	ACD/ALX3/B9D1/BMP4/C2CD3/EN1/FBXW4/FGFR2/HOXC10/HOXC11/IFT140/MYCN/NOTCH1/OSR1/PBX2/PITX1/PKDCC/SHH/TBC1D32/TBX2/TMEM231/TRAF3IP1/TULP3/WDPCP/WNT7A	25	BP
GO:1903707	negative regulation of hemopoiesis	24/2734	141/17381	0.3706	0.8896	0.87495	BCL6/BMP4/C1QC/CD74/CIB1/ERBB2/HIST1H4F/HIST2H4A/HIST2H4B/HLX/HOXB8/IL4R/INPP5D/LDB1/MEIS2/MIR221/MIR222/NME1/NOTCH1/PTK2B/RARA/SHH/TCTA/TMEM176B	24	BP
GO:0006941	striated muscle contraction	29/2734	172/17381	0.37247	0.8896	0.87495	ADRA1A/ADRA1B/ALDOA/ATP1A1/CACNA1G/CAMK2D/CAV3/CHGA/CHRNB1/FLNA/GATA4/GSTM2/IGSF22/KCND3/KCNJ5/KCNQ1/MAP2K3/MIR328/MTOR/MYBPC1/MYBPC3/MYBPH/MYBPHL/MYL4/NKX2-	29	BP

GO:0042306	regulation of protein import into nucleus	29/2734	172/17381	0.37247	0.8896	0.87495	BMP4/CCL19/CD27/CSF3/DAB2IP/EGFR/EMD/FAM89B/FLNA/IL18/LGALS9/MTOR/NFKBIL1/OGG1/PARP10/PBLD/PDE2A/PKD1/PRDX1/PRKCD/RBPMS/SFRP5/SHH/SMO/SUFU/THRA/TLR9/WNT3A/ZPR1	29	BP
GO:0042594	response to starvation	29/2734	172/17381	0.37247	0.8896	0.87495	ADM/AMBRA1/ASNS/ATF3/BCAS3/DAP/DAPL1/DHODH/EHMT2/EIF2AK4/EIF4EBP1/GBA/GCG/GNPAT/HSPA5/INHBB/LARS/MAPK3/MAX/MTOR/MYOD1/NPRL3/PFKFB1/RALB/RRAGC/SESN1/SLC39A5/ULK1/ZFYVE1	29	BP
GO:0050796	regulation of insulin secretion	29/2734	172/17381	0.37247	0.8896	0.87495	ADRA2A/ADRA2C/ANO1/ARL2BP/BAD/BLK/BRSK2/CAPN10/CCL5/CDK16/DOC2B/DRD2/GCG/GCK/GLUD1/GPER1/INHBB/ITPR3/MTNR1B/PFKM/PPARD/PTPN11/RFX6/SIDT2/SLC25A5/SSTR5/STX1A/STX4/TRH	29	BP
GO:0002224	toll-like receptor signaling	23/2734	135/17381	0.37287	0.8896	0.87495	APOB/ARRB2/CD180/CD300LF/CNPY3/CTSL/DAB2IP/F2RL1/FGA/HAVCR2/IKBKB/IRAK1/ITGAM/MAPKAPK3/NFKBIL1/NR1H3/SFTPA1/TBK1/TIRAP/TLR8/TLR9/TNIP2/TREML4	23	BP
GO:0007611	learning or memory	39/2734	234/17381	0.37297	0.8896	0.87495	ARC/B4GALT2/C1QL1/CASP3/CDK5/DEAF1/DGKI/DLG4/DRD2/EGFR/EIF2AK4/EN1/FOSL1/FOXO6/FZD9/GPR88/GRIN1/HRH2/IFT20/ITPR3/JPH3/KCNK4/MEIS2/MTOR/NEUROD2/NLGN3/NQO2/NRXN2/PPP1R1B/PRKAR1B/RGS14/SGK1/STRA6/TACR1/TACR2/TBR1/TH/THRA/ZNF385A	39	BP
GO:0016571	histone methylation	22/2734	129/17381	0.37519	0.8896	0.87495	ASH2L/BRCA1/COPRS/EHMT1/EHMT2/GCG/MEN1/NTMT1/PAXIP1/PHF19/PRDM12/PRDM14/PRDM7/PRDM9/PYGO2/RLF/SETD1A/SETD2/SETD7/SMYD3/SUPT6H/SUV39H1	22	BP
GO:0055076	transition metal ion homeostasis	22/2734	129/17381	0.37519	0.8896	0.87495	ACO1/ALAS2/ATOX1/ATP13A2/ATP6V1G1/COMMD1/CUTC/GDF2/HEPH/HMOX2/LCK/MT2A/NEDD8/NUBP1/SFXN2/SFXN4/SLC11A1/SLC30A3/SLC39A13/SLC39A5/STEAP2/TFR2	22	BP
GO:0060048	cardiac muscle contraction	22/2734	129/17381	0.37519	0.8896	0.87495	ADRA1A/ADRA1B/ATP1A1/CACNA1G/CAMK2D/CAV3/CHGA/FLNA/GATA4/GSTM2/KCND3/KCNJ5/KCNQ1/MAP2K3/MIR328/MTOR/MYBPC3/MYL4/NKX2-5/NPPA/TCAP/TNNC1	22	BP
GO:0071229	cellular response to acid chemical	33/2734	197/17381	0.37543	0.8896	0.87495	APOB/AQP1/CDK4/COL16A1/COL1A1/DNMT3A/EGFR/EGR1/FGFR2/FOLR2/GJB3/GLRA1/HSF1/KCNK4/KDR/LARS/LTK/MIR92A2/MMP2/MTOR/NME1/NSMF/OSR1/P2RY6/PID1/PTK2B/RARA/RRAGC/SIPA1/TNC/WNT11/W	33	BP

GO:0002758	innate immune response-activating signal transduction	47/2734	284/17381	0.37574	0.8896	0.87495	APOB/ARRB2/CARD11/CARD9/CD180/CD300LF/CLEC7A/CNPY3/CTSL/DAB2IP/DHX58/F2RL1/FGA/HAVCR2/HRAS/IKBKB/IRAK1/ITGAM/MAPKAPK3/MUC1/MUC2/MUC3A/MUC5AC/MUC5B/MUC6/NFKBIL1/NLRX1/NOD1/NR1H3/PRKCD/PSMB11/PSMB6/PSMB7/PSMB8/PSMC3/PSMD13/PSMD3/PSMD5/PSMD7/RAF1/SFTPA1/TBK1/TIRAP/TLR8/TLR9/TNIP2/TREML4	47	BP
GO:0046470	phosphatidylcholine metabolic process	14/2734	80/17381	0.37682	0.8896	0.87495	ACHE/APOA5/MFSD2A/PEMT/PHOSPHO1/PLA2G15/PLA2G16/PLA2G1B/PLA2G2F/PLA2G4B/PLA2G5/PLB1/SCARB1/SLC44A4	14	BP
GO:0045165	cell fate commitment	42/2734	253/17381	0.37683	0.8896	0.87495	AXIN1/BARHL2/BMP4/CASP3/DOCK7/EVX1/EYA1/FGFR2/FOXN1/HOXC10/JAG2/KDM6B/LHX3/LY9/MYOD1/NKX2-5/NOTCH1/NRG1/OLIG3/PITX1/PRDM14/PTCH2/RARA/RORC/SH3PXD2B/SHH/SMO/SOX5/SOX8/SPDEF/SUFU/TBR1/TBX19/TBX2/TGFB1I1/TLX3/WNT1/WNT10A/WNT11/WNT3A/WNT6/WNT7A	42	BP
GO:0010675	regulation of cellular carbohydrate metabolic	21/2734	123/17381	0.37755	0.8896	0.87495	ACACB/BAD/C1QTNF1/COX11/DGKQ/GCG/GCK/GNMT/GPER1/IGFBP3/INS/KAT2A/LCMT1/LEPR/MAEA/MTOR/PFKFB1/PGAM4/PHLDA2/PTK2B/RORC	21	BP
GO:0045834	positive regulation of lipid metabolic	21/2734	123/17381	0.37755	0.8896	0.87495	AGT/AMBRA1/APOA5/CCDC3/CCL19/CCL21/CD81/ELOVL5/EPHA8/INS/MID1IP1/MLXIPL/MTOR/NR1H3/PDGFRB/PNPLA2/POR/PRKCD/PTK2B/SCARB1/TNFAIP8L3	21	BP
GO:0046939	nucleotide phosphorylation	21/2734	123/17381	0.37755	0.8896	0.87495	AK5/AK6/ALDOA/GALK1/GAPDH/GCK/HK3/INS/LDHA/MLXIPL/NCOR1/NME1/NME4/NUP210/NUP98/OGDH/OGDHL/PFKFB1/PFKM/PGAM4/PKM	21	BP
GO:0050867	positive regulation of cell activation	59/2734	359/17381	0.37756	0.8896	0.87495	ADAM8/BAD/BCL6/CARD11/CCDC88B/CCL19/CCL21/CCL5/CCR2/CD247/CD27/CD5/CD6/CD74/CD81/CLCF1/CLECL1/CORO1A/CSK/DNAJA3/EFNB1/F2RL1/FAM19A3/HAVCR2/HLA-E/HLX/IL13/IL18/IL4R/INPP5D/ITGAM/KARS/LCK/LGALS9/MAP3K14/MIR92A2/PAXIP1/PDCD1/PDGFRB/PIK3R6/PPP2R3C/PTPN11/RARA/RPS6KA1/SART1/SELP/SHH/SPACA3/STX4/STXBP1/TACR1/THY1/TIRAP/TLR9/TNFRSF4/TNFSF13/TNIP2/TRAF2/WNT3A	59	BP

GO:0034284	response to monosaccharide	32/2734	191/17381	0.37779	0.8896	0.87495	ADRA2A/ANO1/BAD/BRSK2/CASP3/CDK16/EGR1/EIF2B2/EIF2B5/ENDOG/GATA4/GCG/GCK/GPER1/LDHA/MAFA/MEN1/MIRLET7G/MLXIPL/NME1/NOX4/PPARD/PTK2B/PTPRN2/RAF1/RFX6/SIDT2/SLC26A6/SLC29A1/STX4	32	BP
GO:0032508	DNA duplex unwinding	13/2734	74/17381	0.37886	0.8896	0.87495	CHD8/DDB1/DDB2/GTF2H4/HMGA1/IGHMBP2/INO80/MCM2/MCM4/PIF1/RECQL4/RECQL5/XPC	13	BP
GO:1905897	regulation of response to endoplasmic reticulum stress	13/2734	74/17381	0.37886	0.8896	0.87495	BCAP31/BOK/BRSK2/CLU/CREB3/DAB2IP/HERPUD1/HSPA5/OS9/SPOP/SYVN1/USP19/WFS1	13	BP
GO:0050768	negative regulation of neurogenesis	41/2734	247/17381	0.37914	0.8896	0.87495	ARHGAP4/ASCL2/CDK5/CIB1/CTDSP1/DAB1/DGUOK/DRAXIN/EFNA1/EIF2AK4/FUOM/GAK/GORASP1/IDH2/INPP5F/IRX3/LINGO1/LRIG2/LRP1/LSM1/MYCN/NLGN3/NOTCH1/NR2F1/NRG1/PHOX2B/RGMA/RTN4RL1/RTN4RL2/SEMA3F/SHH/SOX8/THY1/TLX2/TLX3/TRIM11/TRPV4/VAX1/WNT3	41	BP
GO:0006165	nucleoside diphosphate phosphorylation	20/2734	117/17381	0.37996	0.8896	0.87495	AK5/ALDOA/GALK1/GAPDH/GCK/HK3/INS/LDHA/MLXIPL/NCOR1/NME1/NME4/NUP210/NUP98/OGDH/OGDHL/PFKFB1/PFKM/PGAM4/PKM	20	BP
GO:1903052	positive regulation of proteolysis involved in cellular protein	31/2734	185/17381	0.38021	0.8896	0.87495	ANAPC15/ANAPC2/ATPIF1/AXIN1/BCAP31/BUB1B/CAV3/CHFR/CLU/DAB2IP/DISC1/ECSCR/GBA/HERPUD1/KLHL40/NKD2/OSBPL7/PSMB11/PSMB6/PSMB7/PSMB8/PSMC3/PSMD13/PSMD3/PSMD5/PSMD7/PTK2B/RNF166/RNF180/TAF1/USP5	31	BP
GO:0032418	lysosome localization	12/2734	68/17381	0.38089	0.8896	0.87495	BORCS6/CHGA/DEF8/FES/IL13/IL4R/LGALS9/MAP6D1/PLEKHM1/PLEKHM2/RAB34/STXBP1	12	BP
GO:0050764	regulation of phagocytosis	12/2734	68/17381	0.38089	0.8896	0.87495	AHSG/C2/CAMK1D/CD300LF/CSK/F2RL1/LMAN2/MYO18A/SCARB1/SLC11A1/SPACA3/TULP1	12	BP
GO:0071300	cellular response to	12/2734	68/17381	0.38089	0.8896	0.87495	AQP1/COL1A1/FGFR2/GJB3/LTK/OSR1/PTK2B/RARA/TNC/WNT11/WNT3A/WNT6	12	BP
GO:0001921	positive regulation of receptor	3/2734	14/17381	0.38212	0.8896	0.87495	ARAP1/INPP5F/SCRIB	3	BP

GO:0002693	positive regulation of cellular	3/2734	14/17381	0.38212	0.8896	0.87495	ADAM8/CCR2/THY1	3	BP
GO:0002829	negative regulation of type 2 immune	3/2734	14/17381	0.38212	0.8896	0.87495	BCL6/CCR2/HLX	3	BP
GO:0003352	regulation of cilium	3/2734	14/17381	0.38212	0.8896	0.87495	CATSPER1/CCDC40/DNAH11	3	BP
GO:0006047	UDP-N-acetylglucosamine metabolic	3/2734	14/17381	0.38212	0.8896	0.87495	AMDHD2/MGAT1/NAGK	3	BP
GO:0006957	complement activation, alternative pathway	3/2734	14/17381	0.38212	0.8896	0.87495	C8A/CFH/VSIG4	3	BP
GO:0009415	response to water	3/2734	14/17381	0.38212	0.8896	0.87495	ATF2/SIPA1/TH	3	BP
GO:0010310	regulation of hydrogen peroxide	3/2734	14/17381	0.38212	0.8896	0.87495	MMP3/NOXO1/ZNF205	3	BP
GO:0019627	urea metabolic process	3/2734	14/17381	0.38212	0.8896	0.87495	ASL/NAGS/OTC	3	BP
GO:0020027	hemoglobin metabolic	3/2734	14/17381	0.38212	0.8896	0.87495	AHSP/ALAS2/LDB1	3	BP
GO:0031272	regulation of pseudopodium assembly	3/2734	14/17381	0.38212	0.8896	0.87495	CCL21/CDC42EP2/F2RL1	3	BP
GO:0032736	positive regulation of interleukin-13	3/2734	14/17381	0.38212	0.8896	0.87495	HLA-E/LGALS9/RARA	3	BP

GO:0033008	positive regulation of mast cell activation involved in	3/2734	14/17381	0.38212	0.8896	0.87495	IL13/IL4R/STXBP1	3	BP
GO:0033169	histone H3-K9 demethylation	3/2734	14/17381	0.38212	0.8896	0.87495	HR/KDM4E/PHF2	3	BP
GO:0034111	negative regulation of homotypic cell-	3/2734	14/17381	0.38212	0.8896	0.87495	C1QTNF1/PRKCD/ZNF703	3	BP
GO:0034433	steroid esterification	3/2734	14/17381	0.38212	0.8896	0.87495	AGT/APOA5/SOAT2	3	BP
GO:0034434	sterol esterification	3/2734	14/17381	0.38212	0.8896	0.87495	AGT/APOA5/SOAT2	3	BP
GO:0034435	cholesterol esterification	3/2734	14/17381	0.38212	0.8896	0.87495	AGT/APOA5/SOAT2	3	BP
GO:0038092	nodal signaling pathway	3/2734	14/17381	0.38212	0.8896	0.87495	CFC1/DACT2/SHH	3	BP
GO:0042754	negative regulation of circadian	3/2734	14/17381	0.38212	0.8896	0.87495	DRD2/PASD1/SUV39H1	3	BP
GO:0043306	positive regulation of mast cell	3/2734	14/17381	0.38212	0.8896	0.87495	IL13/IL4R/STXBP1	3	BP
GO:0044130	negative regulation of growth of	3/2734	14/17381	0.38212	0.8896	0.87495	LTA/MPO/TIRAP	3	BP
GO:0046325	negative regulation of	3/2734	14/17381	0.38212	0.8896	0.87495	MZB1/PEA15/PID1	3	BP
GO:0046514	ceramide catabolic	3/2734	14/17381	0.38212	0.8896	0.87495	GBA/NEU3/PRKCD	3	BP

GO:0051770	positive regulation of nitric-oxide synthase biosynthetic	3/2734	14/17381	0.38212	0.8896	0.87495	FNTB/KDR/TLR9	3	BP
GO:0051852	disruption by host of	3/2734	14/17381	0.38212	0.8896	0.87495	F2RL1/GAPDH/TUSC2	3	BP
GO:0060009	Sertoli cell development	3/2734	14/17381	0.38212	0.8896	0.87495	NUP210L/SDC1/SOX8	3	BP
GO:0060263	regulation of respiratory burst	3/2734	14/17381	0.38212	0.8896	0.87495	CAMK1D/INS/NOXO1	3	BP
GO:0060712	spongiotrophoblast layer development	3/2734	14/17381	0.38212	0.8896	0.87495	ADM/ASCL2/PHLDA2	3	BP
GO:0071361	cellular response to	3/2734	14/17381	0.38212	0.8896	0.87495	DNMT3A/GLRA1/SPI1	3	BP
GO:0071435	potassium ion export	3/2734	14/17381	0.38212	0.8896	0.87495	KCND3/KCNQ1/NPPA	3	BP
GO:0072160	nephron tubule epithelial cell differentiation	3/2734	14/17381	0.38212	0.8896	0.87495	LHX1/OSR1/STAT1	3	BP
GO:0072216	positive regulation of metanephros	3/2734	14/17381	0.38212	0.8896	0.87495	EGR1/PDGFA/PDGFRB	3	BP
GO:0090178	regulation of establishment of planar polarity involved in neural tube	3/2734	14/17381	0.38212	0.8896	0.87495	DVL2/FZD2/GRHL3	3	BP

GO:0090196	regulation of chemokine secretion	3/2734	14/17381	0.38212	0.8896	0.87495	CSF1R/F2RL1/IL4R	3	BP
GO:0099068	postsynapse assembly	3/2734	14/17381	0.38212	0.8896	0.87495	NLGN3/NRXN2/WNT7A	3	BP
GO:1901070	guanosine-containing compound biosynthetic process	3/2734	14/17381	0.38212	0.8896	0.87495	IMPDH1/NME1/NME4	3	BP
GO:1903350	response to dopamine	3/2734	14/17381	0.38212	0.8896	0.87495	ABL1/HCN3/MAPK3	3	BP
GO:1903351	cellular response to	3/2734	14/17381	0.38212	0.8896	0.87495	ABL1/HCN3/MAPK3	3	BP
GO:1904152	regulation of retrograde protein transport, ER to	3/2734	14/17381	0.38212	0.8896	0.87495	BCAP31/BRSK2/OS9	3	BP
GO:1904923	regulation of autophagy of mitochondrion in response to mitochondrial depolarization	3/2734	14/17381	0.38212	0.8896	0.87495	ATPIF1/GBA/MFN2	3	BP
GO:1905050	positive regulation of metallopeptidase activity	3/2734	14/17381	0.38212	0.8896	0.87495	KARS/MAPK3/MIR92A2	3	BP

GO:2000105	positive regulation of DNA-dependent DNA	3/2734	14/17381	0.38212	0.8896	0.87495	BMP4/E2F8/INO80	3	BP
GO:0045444	fat cell differentiation	35/2734	210/17381	0.38236	0.8896	0.87495	ADIRF/ATF2/AXIN1/BBS9/C1QL4/CCDC3/CCDC85B/CTBP1/DLK2/FNDC5/GPER1/GPX1/INHBB/INS/MEDAG/MTOR/NR4A1/PEX11A/PPARD/PSMB8/PTPRQ/RARRES2/RORC/SDF4/SH3PXD2B/SLC2A4/SMAD6/SOX8/TGFB1I1/TRIO/TRPV4/WIF1/WNT1/WNT3A/ZNF385A	35	BP
GO:0010830	regulation of myotube differentiation	11/2734	62/17381	0.38286	0.8896	0.87495	ADGRB1/ANKRD2/CAV3/EHD1/IL4R/MTOR/MYOD1/NKX2-5/NOTCH1/THRA/TRIM72	11	BP
GO:0071230	cellular response to amino acid	11/2734	62/17381	0.38286	0.8896	0.87495	COL16A1/COL1A1/DNMT3A/EGFR/GLRA1/HSF1/LARS/MMP2/MTOR/NSMF/RRAGC	11	BP
GO:1901988	negative regulation of cell cycle phase transition	39/2734	235/17381	0.38388	0.8896	0.87495	ANAPC15/BRCA1/BUB1B/C10orf99/CDK2AP2/CDK5RAP3/CHMP4C/CTDSP1/E2F4/E2F8/FOXO4/GPR132/LCMT1/MAD1L1/MEN1/MIIP/MIR10A/MIR15A/MIR16-1/MIR195/MIR29A/MIR29C/MIR503/MUC1/PCBP4/PSMB11/PSMB6/PSMB7/	39	BP
GO:0002287	alpha-beta T cell activation involved in immune	10/2734	56/17381	0.38475	0.8896	0.87495	BCL6/CCL19/HLX/IL18/IL4R/LGALS9/LY9/RARA/RORC/SEMA4A	10	BP
GO:0002293	alpha-beta T cell differentiation involved in	10/2734	56/17381	0.38475	0.8896	0.87495	BCL6/CCL19/HLX/IL18/IL4R/LGALS9/LY9/RARA/RORC/SEMA4A	10	BP
GO:0045740	positive regulation of DNA	10/2734	56/17381	0.38475	0.8896	0.87495	BMP4/CACYBP/E2F8/EGFR/HRAS/INO80/INS/PDGFA/PLA2G1B/SHC1	10	BP
GO:0046503	glycerolipid catabolic	10/2734	56/17381	0.38475	0.8896	0.87495	APOA5/APOB/APOC3/INPP5F/PLA2G15/PLA2G4B/PNPLA2/PRDX6/SCARB1/SMPD4	10	BP

GO:0035272	exocrine system development	9/2734	50/17381	0.38651	0.8896	0.87495	CLCN2/EGFR/ESRP2/FGFR2/PDGFA/PLXND1/RAB26/SHH/WLS	9	BP
GO:2001258	negative regulation of cation channel	9/2734	50/17381	0.38651	0.8896	0.87495	CRHR1/DRD2/GNB5/GPR35/GSTM2/MIR153-1/MIR212/RRAD/TRDN	9	BP
GO:0001678	cellular glucose homeostasis	23/2734	136/17381	0.38726	0.8896	0.87495	ADRA2A/ANO1/BAD/BRSK2/CDK16/ENDOG/GATA4/GCG/GCK/GPER1/HK3/MEN1/MIRLET7G/MLXIPL/NME1/NOX4/PTPRN2/RAF1/RFX6/SIDT2/SL	23	BP
GO:0046883	regulation of hormone secretion	42/2734	254/17381	0.38736	0.8896	0.87495	ADRA2A/ADRA2B/ADRA2C/AGT/ANO1/ARL2BP/BAD/BLK/BRSK2/C1QTNF1/CAPN10/CCL5/CDK16/CRHR1/DOC2B/DRD2/EGFR/FGA/GCG/GCK/GLUD1/GPER1/HCAR2/INHBB/INS/ITPR3/MTNR1B/PFKM/PPARD/PTPN11/RAB11FIP3/REN/RFX6/SIDT2/SLC25A5/SSTR5/STX1A/STX4/TACR1/TACR2/	42	BP
GO:0006096	glycolytic process	17/2734	99/17381	0.38751	0.8896	0.87495	ALDOA/GALK1/GAPDH/GCK/HK3/INS/LDHA/MLXIPL/NCOR1/NUP210/NUP98/OGDH/OGDHL/PFKFB1/PFKM/PGAM4/PKM	17	BP
GO:0003351	epithelial cilium movement	4/2734	20/17381	0.38782	0.8896	0.87495	CCDC103/CCDC40/GAS8/LRRC6	4	BP
GO:0006488	dolichol-linked oligosaccharide biosynthetic process	4/2734	20/17381	0.38782	0.8896	0.87495	ALG10/ALG3/DOLPP1/MPDU1	4	BP
GO:0009074	aromatic amino acid family catabolic	4/2734	20/17381	0.38782	0.8896	0.87495	FAH/GSTZ1/HAAO/IDO1	4	BP
GO:0014850	response to muscle activity	4/2734	20/17381	0.38782	0.8896	0.87495	AGT/FNDC5/PERM1/TNS2	4	BP
GO:0032211	negative regulation of telomere maintenance via	4/2734	20/17381	0.38782	0.8896	0.87495	ACD/EXOSC10/PIF1/TINF2	4	BP
GO:0032634	interleukin-5 production	4/2734	20/17381	0.38782	0.8896	0.87495	EPX/IL5RA/RARA/SCGB1A1	4	BP

GO:0032656	regulation of interleukin-13 production	4/2734	20/17381	0.38782	0.8896	0.87495	HLA-E/LGALS9/RARA/SCGB1A1	4	BP
GO:0034138	toll-like receptor 3	4/2734	20/17381	0.38782	0.8896	0.87495	F2RL1/HAVCR2/TIRAP/TNIP2	4	BP
GO:0034311	diol metabolic process	4/2734	20/17381	0.38782	0.8896	0.87495	DEGS2/GBA/GCH1/SPTLC1	4	BP
GO:0035455	response to interferon-alpha	4/2734	20/17381	0.38782	0.8896	0.87495	IFIT2/IFIT3/IFITM2/LAMP3	4	BP
GO:0035584	calcium-mediated signaling using intracellular	4/2734	20/17381	0.38782	0.8896	0.87495	BCAP31/HOMER2/KDR/SELP	4	BP
GO:0045663	positive regulation of myoblast	4/2734	20/17381	0.38782	0.8896	0.87495	BOC/IGFBP3/ILK/MYOD1	4	BP
GO:0046037	GMP metabolic process	4/2734	20/17381	0.38782	0.8896	0.87495	CARD11/DLG4/IMPDH1/SCRIB	4	BP
GO:0048148	behavioral response to cocaine	4/2734	20/17381	0.38782	0.8896	0.87495	CDK5/DRD2/HOMER2/PPP1R1B	4	BP
GO:0060261	positive regulation of transcription initiation from RNA	4/2734	20/17381	0.38782	0.8896	0.87495	NKX2-5/PAXIP1/PSMC3/TAF1	4	BP
GO:0060575	intestinal epithelial cell	4/2734	20/17381	0.38782	0.8896	0.87495	CDX2/GATA4/NKX3-2/SPDEF	4	BP
GO:0086014	atrial cardiac muscle cell action potential	4/2734	20/17381	0.38782	0.8896	0.87495	FLNA/KCNJ5/KCNQ1/MIR328	4	BP

GO:0086026	atrial cardiac muscle cell to AV node cell	4/2734	20/17381	0.38782	0.8896	0.87495	FLNA/KCNJ5/KCNQ1/MIR328	4	BP
GO:0086066	atrial cardiac muscle cell to AV node cell communication	4/2734	20/17381	0.38782	0.8896	0.87495	FLNA/KCNJ5/KCNQ1/MIR328	4	BP
GO:0097734	extracellular exosome biogenesis	4/2734	20/17381	0.38782	0.8896	0.87495	ATP13A2/RAB7A/SDC1/SNF8	4	BP
GO:0140112	extracellular vesicle	4/2734	20/17381	0.38782	0.8896	0.87495	ATP13A2/RAB7A/SDC1/SNF8	4	BP
GO:0016574	histone ubiquitination	8/2734	44/17381	0.38807	0.8896	0.87495	CDK9/DDB1/DDB2/OTUB1/PCGF2/UBE2A/UBE2B/UBE2U	8	BP
GO:0031050	dsRNA fragmentation	8/2734	44/17381	0.38807	0.8896	0.87495	BMP4/EGFR/MYCN/NCOR1/NCOR2/TARBP2/TSNAX/ZC3H10	8	BP
GO:0070918	production of small RNA involved in gene silencing	8/2734	44/17381	0.38807	0.8896	0.87495	BMP4/EGFR/MYCN/NCOR1/NCOR2/TARBP2/TSNAX/ZC3H10	8	BP
GO:0072665	protein localization to	8/2734	44/17381	0.38807	0.8896	0.87495	CD81/CLU/GOSR2/LARS/RAB7A/SNF8/VPS25/VTI1B	8	BP
GO:1904837	beta-catenin-TCF complex	8/2734	44/17381	0.38807	0.8896	0.87495	ASH2L/BCL9/HIST1H4F/HIST2H4A/HIST2H4B/MEN1/PYGO2/TCF7L1	8	BP
GO:0050817	coagulation	58/2734	354/17381	0.38865	0.8896	0.87495	ADAMTS13/ADRA2A/ADRA2B/ADRA2C/ARRB2/ASIC2/C1QTNF1/CLIC1/COL1A1/CSRP1/DGKI/DGKQ/DGKZ/DOCK1/DTNBP1/EHD1/F2RL1/FERMT3/FGA/FLNA/GATA4/GP9/HSPB1/IFNA5/ILK/ITPK1/ITPR3/KLKB1/KNG1/LCK/MAPK3/MFN2/NOS3/P2RX2/PDGFA/PF4V1/PIK3R5/PIK3R6/PLAU/PRCP/PRKAR1A/PRKAR1B/PRKCD/PROZ/PTPN11/RAF1/RBSN/SELP/SERPINA5/SERPINF2/SH2B1/SHH/STXBP1/TEC/TLN1/VWF/WAS/WNT3A	58	BP

GO:0007431	salivary gland development	7/2734	38/17381	0.3893	0.8896	0.87495	CLCN2/EGFR/ESRP2/FGFR2/PDGFA/PLXND1/SHH	7	BP
GO:0007618	mating	7/2734	38/17381	0.3893	0.8896	0.87495	EDDM3A/FUOM/GRIN1/PPP1R1B/TACR1/TH/THRA	7	BP
GO:0010831	positive regulation of myotube	7/2734	38/17381	0.3893	0.8896	0.87495	ADGRB1/CAV3/EHD1/IL4R/MTOR/MYOD1/THRA	7	BP
GO:0040019	positive regulation of embryonic	7/2734	38/17381	0.3893	0.8896	0.87495	LHX1/OSR1/RBM19/SCX/SHH/WNT1/WNT3A	7	BP
GO:0045581	negative regulation of T cell	7/2734	38/17381	0.3893	0.8896	0.87495	BCL6/BMP4/CD74/ERBB2/HLX/IL4R/SHH	7	BP
GO:0051281	positive regulation of release of sequestered calcium ion into	7/2734	38/17381	0.3893	0.8896	0.87495	ABL1/GPER1/GSTM2/IL13/NPSR1/THY1/TRDN	7	BP
GO:0071364	cellular response to epidermal growth factor	7/2734	38/17381	0.3893	0.8896	0.87495	COL1A1/DAB2IP/EGFR/ERBB2/PTPN11/TDGF1/ZPR1	7	BP
GO:0006882	cellular zinc ion homeostasis	5/2734	26/17381	0.38978	0.8896	0.87495	ATP13A2/LCK/SLC30A3/SLC39A13/SLC39A5	5	BP
GO:0009251	glucan catabolic process	5/2734	26/17381	0.38978	0.8896	0.87495	INS/MGAM/PFKM/PHKG1/PYGM	5	BP
GO:0031063	regulation of histone deacetylation	5/2734	26/17381	0.38978	0.8896	0.87495	BCL6/CAMK2D/CTBP1/TADA3/ZNHIT1	5	BP

GO:0033032	regulation of myeloid cell apoptotic	5/2734	26/17381	0.38978	0.8896	0.87495	CCL5/CDKN2A/HCAR2/MAEA/THRA	5	BP
GO:0034661	ncRNA catabolic	5/2734	26/17381	0.38978	0.8896	0.87495	DIS3L/DIS3L2/ERN2/EXOSC10/EXOSC2	5	BP
GO:0035025	positive regulation of Rho protein	5/2734	26/17381	0.38978	0.8896	0.87495	F2RL1/GPR17/GPR20/GPR35/PDGFRB	5	BP
GO:0036152	phosphatidylethanolamine acyl-chain	5/2734	26/17381	0.38978	0.8896	0.87495	PLA2G16/PLA2G1B/PLA2G2F/PLA2G4B/PLA2G5	5	BP
GO:0036296	response to increased oxygen levels	5/2734	26/17381	0.38978	0.8896	0.87495	ATP6V1G1/CDK4/COL1A1/CYP1A1/PDGFRB	5	BP
GO:0043567	regulation of insulin-like growth factor receptor	5/2734	26/17381	0.38978	0.8896	0.87495	IGFBP3/IGFBP6/MIR29C/TRIM72/WNT1	5	BP
GO:0044247	cellular polysaccharide catabolic	5/2734	26/17381	0.38978	0.8896	0.87495	INS/MGAM/PFKM/PHKG1/PYGM	5	BP
GO:0060571	morphogenesis of an epithelial	5/2734	26/17381	0.38978	0.8896	0.87495	BMP4/CFL1/EGFR/FGFR2/SHH	5	BP
GO:0060669	embryonic placenta morphogenesis	5/2734	26/17381	0.38978	0.8896	0.87495	ADM/FGFR2/SETD2/SPINT1/ST14	5	BP
GO:0060740	prostate gland epithelium morphogenesis	5/2734	26/17381	0.38978	0.8896	0.87495	BMP4/FGFR2/NOTCH1/SHH/TNC	5	BP
GO:0097066	response to thyroid	5/2734	26/17381	0.38978	0.8896	0.87495	CTSH/CTSL/EIF5A/GBA/SLC34A1	5	BP

GO:0000086	G2/M transition of mitotic cell cycle	41/2734	248/17381	0.38981	0.8896	0.87495	ACTR1A/BRCA1/BRSK2/CCNB2/CDK4/CDK5RAP3/CEP164/CEP70/CHMP4C/CKAP5/DCTN2/DYNC1H1/FOXM1/FOXO4/GPR132/HAUS4/HAUS7/HSP90AA1/HSPA2/KDM8/LCMT1/MIIP/MIR195/NES/PHOX2B/PKMYT1/PPP1R12B/PSMB11/PSMB6/PSMB7/PSMB8/PSMC3/PSMD13/PSMD3/PSMD5/PSMD7/RINT1/SDCCAG8/SSNA1/TICRR/TUBG1	41	BP
GO:0001963	synaptic transmission, dopaminergic	6/2734	32/17381	0.39	0.8896	0.87495	ARRB2/CDK5/DRD2/PNKD/TH/TOR1A	6	BP
GO:0003299	muscle hypertrophy in response to	6/2734	32/17381	0.39	0.8896	0.87495	CAMTA2/INPP5F/LMNA/MIR25/NPPA/TCAP	6	BP
GO:0009303	rRNA transcription	6/2734	32/17381	0.39	0.8896	0.87495	ANG/BRF1/GTF3C1/MTOR/NOL11/POLR1E	6	BP
GO:0010543	regulation of platelet	6/2734	32/17381	0.39	0.8896	0.87495	C1QTNF1/NOS3/PDGFA/PRKCD/SELP/TEC	6	BP
GO:0014887	cardiac muscle adaptation	6/2734	32/17381	0.39	0.8896	0.87495	CAMTA2/INPP5F/LMNA/MIR25/NPPA/TCAP	6	BP
GO:0014898	cardiac muscle hypertrophy in response to	6/2734	32/17381	0.39	0.8896	0.87495	CAMTA2/INPP5F/LMNA/MIR25/NPPA/TCAP	6	BP
GO:0032735	positive regulation of interleukin-12	6/2734	32/17381	0.39	0.8896	0.87495	CCL19/IDO1/IRF5/LGALS9/TIRAP/TLR9	6	BP
GO:0034142	toll-like receptor 4	6/2734	32/17381	0.39	0.8896	0.87495	DAB2IP/F2RL1/IRAK1/ITGAM/NR1H3/TIRAP	6	BP
GO:0040018	positive regulation of multicellular	6/2734	32/17381	0.39	0.8896	0.87495	CSF1/DRD2/HSF1/PPIB/SH3PXD2B/SMO	6	BP
GO:0042755	eating behavior	6/2734	32/17381	0.39	0.8896	0.87495	MTOR/OPRD1/OPRM1/TACR1/TH/TRH	6	BP

GO:0051954	positive regulation of	6/2734	32/17381	0.39	0.8896	0.87495	AGT/DRD2/STX1A/STXBP1/TACR2/TRH	6	BP
GO:0060317	cardiac epithelial to mesenchymal	6/2734	32/17381	0.39	0.8896	0.87495	EFNA1/ENG/HEY1/NOTCH1/PDCD4/TMEM100	6	BP
GO:0060325	face morphogenesis	6/2734	32/17381	0.39	0.8896	0.87495	COL1A1/CSRNP1/MMP2/PTPN11/SCX/STRA6	6	BP
GO:0061756	leukocyte adhesion to vascular endothelial cell	6/2734	32/17381	0.39	0.8896	0.87495	CCL21/MIR221/MIR222/MIR92A2/PODXL2/SELP	6	BP
GO:0034340	response to type I interferon	16/2734	93/17381	0.39014	0.8896	0.87495	EGR1/HLA-E/IFIT2/IFIT3/IFITM2/IFNA5/IRAK1/IRF5/ISG15/PSMB8/PTPN11/SCRIB/SETD2/SHMT2/STAT1/STAT2	16	BP
GO:1901990	regulation of mitotic cell cycle phase transition	68/2734	417/17381	0.3923	0.8942	0.87945	ACTR1A/ANAPC15/ANAPC2/APEX1/BRCA1/BUB1B/CDC14A/CDK10/CDK2AP2/CDK4/CDK5RAP3/CEP164/CEP70/CHMP4C/CKAP5/CTDSP1/CYP1A1/DCTN2/DDB1/DYNC1H1/E2F4/E2F8/EGFR/EIF4G1/FOXO4/GPR132/HAUS4/HAUS7/HSP90AA1/HSPA2/INO80/LCMT1/MAD1L1/MEPCE/MIIP/MIR15A/MIR16-1/MIR195/MIR221/MIR222/MIR29A/MIR29C/MUC1/NEK11/PCBP4/PHOX2B/PID1/PKD1/PPP1R9B/PSMB11/PSMB6/PSMB7/PSMB8/PSMC3/PSMD13/PS[illegible]	68	BP
GO:0046434	organophosphate catabolic	21/2734	124/17381	0.39267	0.8947	0.87995	DNPH1/GPX1/HINT1/IDH1/INPP5E/INPP5F/MBD4/NEIL2/NT5M/NUDT1/NUDT18/OGG1/PDE2A/PLA2G15/PLA2G4B/PRDX6/PRKCD/SCARB1/SMPD4/S	21	BP
GO:0002695	negative regulation of leukocyte	26/2734	155/17381	0.39314	0.895	0.88032	BCL6/BMP4/CASP3/CCR2/CD300LF/CD74/ERBB2/FAM19A3/GPER1/HAVCR2/HLX/IDO1/IL20RB/IL31RA/IL4R/INPP5D/LGALS3/LGALS9/MAD1L1/NR1H3/PLA2G2F/PRKAR1A/SCGB1A1/SHH/TNFRSF13B/VSIG4	26	BP
GO:0030509	BMP signaling pathway	26/2734	155/17381	0.39314	0.895	0.88032	ABL1/BMP4/CRB2/EGR1/ENG/GATA4/GDF2/HTRA3/ILK/LEFTY2/LEMD2/MAPK3/NBL1/NKX2-5/NOTCH1/NUMA1/PDCD4/RBPMS2/RGMA/SCX/SFRP5/SLC39A5/SMAD6/SOST/TMEM100/WNT1	26	BP

GO:2000116	regulation of cysteine-type endopeptidase activity	39/2734	236/17381	0.39486	0.8986	0.88383	ANP32B/APOPT1/AQP1/ARRB2/BAD/BCAP31/BOK/CASP1/CASP3/CD27/CRYAB/CTSH/DAP/DAPK1/DPEP1/FASLG/GPER1/GPX1/HERPUD1/HIP1R/HSF1/LAMP3/LCK/LGALS9/MIR15A/MTCH1/NAIP/NLE1/NLRP1/NOD1/PDCD2/POR/RAF1/RPS6KA1/SFN/SOX7/TRAF2/WNT3A/XDH	39	BP
GO:0009165	nucleotide biosynthetic process	55/2734	336/17381	0.39557	0.8999	0.88508	ACOT7/ADM/ADRA2A/AK5/AK6/ALDOA/AQP1/ATP5G1/ATP5I/AVPR2/CCR2/CRHR1/CYC1/DGUOK/DHODH/DRD2/ERH/GABBR1/GCG/GPER1/GPHA2/GUCA1B/GUCA2A/GUCA2B/GUCY2D/HAAO/IDH2/IMPDH1/KARS/MTHFD1/NME1/NME4/NMNAT3/NOS3/NPPA/OPRM1/PARP10/PARP9/PDE2A/PDZD3/PID1/PKM/PPCDC/PTK2B/QPRT/RAF1/RUNDC3A/RXFP2/SLC22A13/SLC26A1/TBPL1/UCK1/UCN2/VPS9D1/WFS1	55	BP
GO:0031348	negative regulation of defense	29/2734	174/17381	0.39801	0.905	0.89015	ARRB2/CHID1/CUEDC2/DHX58/DRD2/GBA/GPER1/GPR17/GPX1/HAVCR2/HLA-E/IL20RB/INS/LGALS9/MICB/MIR221/MIR222/MIR92A2/NLRX1/NMI/NR1H	29	BP
GO:0072001	renal system development	46/2734	280/17381	0.39814	0.905	0.89015	AGT/APH1A/AQP1/BMP4/CTSH/DACT2/EGR1/EYA1/FGFR2/HOXB7/HOXC11/IFT140/IFT20/ILK/IRX3/KIRREL3/LHX1/LZTS2/MMP17/MYO1E/NLE1/NOTCH1/OSR1/PDGFA/PDGFRB/PKD1/POU3F3/PYGO2/RARA/REN/SDC1/SHH/SIM1/SLC34A1/SMAD6/SMO/SOX8/STAT1/STRA6/TNS2/TRAF3IP1/WD	46	BP
GO:0051346	negative regulation of hydrolase activity	69/2734	424/17381	0.39837	0.9052	0.89033	A2ML1/ABL1/AGT/AHSG/AIP/APLP2/APOC3/AQP1/ARRB2/ATPIF1/BCAS3/CD27/COL7A1/CRB2/CRYAB/DAB2IP/DGKI/DPEP1/ECM1/FURIN/GAPDH/GCHFR/GNAT1/GPSM1/GPX1/HERPUD1/HRAS/IKBKB/ITIH4/ITIH6/KNG1/LAMP3/LEPR/MIR195/MIR29C/NAIP/NLE1/NOS3/PDE6D/PEBP1/PI16/PKMYT1/PLXNB3/POR/PPP1R1B/PTPRN2/RAF1/RPS6KA1/RTKN/SERPINA2/SERPINA3/SERPINA4/SERPINA5/SERPINF2/SERPINH1/SERPINI1/SFN/SPINK2/SPINK5/SPINT1/SPOCD1/SPOCK2/THADA/TIPRL/TLR9/TNK2/VCPKMT/[illegible]	69	BP
GO:0061041	regulation of wound healing	24/2734	143/17381	0.39878	0.9058	0.89091	ADRA2A/AJAP1/ASIC2/C1QTNF1/F2RL1/FGA/GJD4/HRAS/KLKB1/KNG1/MIR221/MIR451A/MTOR/MYOD1/NOS3/PDGFA/PLAU/PPARD/PRKCD/PROZ/SELP/SERPINF2/TEC/TNFRSF12A	24	BP

GO:0007159	leukocyte cell-cell adhesion	61/2734	374/17381	0.39953	0.9072	0.89224	ABL1/ADAM8/BAD/BCL6/BMP4/CARD11/CASP3/CCDC88B/CCL19/CCL21/CCL5/CCR2/CD247/CD27/CD5/CD6/CD74/CLECL1/CORO1A/CSK/DNAJA3/EFNB1/ERBB2/FANCA/FERMT3/FOXN1/HAVCR2/HLA-E/HLX/IDO1/IL18/IL20RB/IL4R/ITGAL/LCK/LGALS3/LGALS9/LMO1/MAD1L1/MAP3K14/MIR221/MIR222/MIR92A2/PDCD1/PIK3R6/PLA2G2F/PODXL2/PRELID1/PRKAR1A/PTPN11/RARA/SART1/SCGB1A1/SELP/SHH/SOX13/SPINK5/THY1/TNFRSF18/TRAF2/VSIG4	61	BP
GO:0008589	regulation of smoothened signaling	12/2734	69/17381	0.40132	0.91	0.89503	ANKMY2/C2CD3/FGFR2/HHIP/IFT140/POR/PTCH2/SHH/SMO/SUFU/TRAF3IP1/TULP3	12	BP
GO:0014033	neural crest cell differentiation	12/2734	69/17381	0.40132	0.91	0.89503	BMP4/CFL1/EFNB1/GBX2/MAPK3/NRG1/PHOX2B/SEMA3F/SHH/SMO/SOX8/WNT10A	12	BP
GO:0006939	smooth muscle contraction	18/2734	106/17381	0.40139	0.91	0.89503	ADRA1A/ADRA1B/ADRA2A/ADRA2B/ADRA2C/AGT/CHRM1/CTTN/DRD2/EDN2/GPER1/HMCN2/HTR1D/MIR153-1/P2RX2/PTGER3/TACR1/TACR2	18	BP
GO:1902600	hydrogen ion transmembrane transport	18/2734	106/17381	0.40139	0.91	0.89503	ATP1A4/ATP5G1/ATP5I/ATP6V0B/ATP6V0E2/ATP6V1B1/ATP6V1F/ATP6V1G1/COX10/COX11/COX8A/CYC1/NDUFA4L2/SLC36A1/SLC36A3/SLC9A3/SLC9A5/VPS9D1	18	BP
GO:0043524	negative regulation of neuron	23/2734	137/17381	0.40172	0.9104	0.89542	AARS/ADAM8/AMBRA1/BARHL1/BOK/CLCF1/CNTFR/CORO1A/DRAXIN/EN1/FZD9/GRIN1/HRAS/ILK/LRP1/NAIP/NES/NONO/PTK2B/SNCB/STXBP1/WFS1/ZPR1	23	BP
GO:0051480	regulation of cytosolic calcium ion concentration	52/2734	318/17381	0.40281	0.9122	0.89723	ABL1/ADM/ADRA1A/ADRA1B/AGT/BCAP31/BOK/C1QTNF1/C3AR1/CACNB3/CAMK2D/CAV3/CCL1/CCL19/CCL21/CCR2/CDH23/CIB2/CNGB1/CORO1A/CXCR3/DLG4/DRD2/EDN2/EIF5A/F2RL1/FASLG/FZD9/GNG3/GPER1/GPR17/GPR20/GPR35/GRIN1/GRIN2C/GSTM2/IL13/ITPR3/JPH3/KNG1/LCK/NPSR1/P2RX2/PKD1/PLA2G1B/PTK2B/RASA3/TACR1/THY1/TRDN/TRPV4	52	BP
GO:0097191	extrinsic apoptotic signaling pathway	36/2734	218/17381	0.40283	0.9122	0.89723	AGT/ATF3/BAD/BMP4/BOK/BRCA1/CASP3/CD27/CIB1/CTTN/DAB2IP/DAPK1/EYA1/FASLG/FGA/G0S2/GPER1/GPX1/LGALS3/LMNA/LTBR/MADD/MIR221/MIR222/MOAP1/NOS3/PARP2/PEA15/PELI3/RAF1/STX4/TMBIM1/TNFRSF12A/TRAF2/ZC3HC1/ZMYND11	36	BP

GO:0061025	membrane fusion	40/2734	243/17381	0.40318	0.9127	0.89766	ADPRHL1/CATSPER1/CORO1A/DNM1/DOC2A/DOC2B/ERVFRD-1/GOSR2/GRTP1/MFN1/MFN2/NKD2/PID1/RAB20/RAB34/RAB7A/RABGAP1/RABGAP1L/RBSN/SAMD9L/SERPINA5/SGSM2/SPACA3/STX18/STX1A/STX4/STX5/STX8/STXBP1/SYTL3/TAP1/TBC1D25/TBC1D28/TBC1D9/TIE1/USP6NL/VAMP1/VAMP5/VAT1/VTI1B	40	BP
GO:0002181	cytoplasmic translation	11/2734	63/17381	0.4043	0.9149	0.8998	DPH1/EIF4B/EIF4EBP1/EIF4G1/RBM4/RPL35A/RPL36A/RPL7/RWDD1/TICRR/ZNF385A	11	BP
GO:0006757	ATP generation from ADP	17/2734	100/17381	0.40448	0.9149	0.89987	ALDOA/GALK1/GAPDH/GCK/HK3/INS/LDHA/MLXIPL/NCOR1/NUP210/NUP98/OGDH/OGDHL/PFKFB1/PFKM/PGAM4/PKM	17	BP
GO:0009743	response to carbohydrate	35/2734	212/17381	0.40561	0.9171	0.90204	ADRA2A/ANO1/APOB/BAD/BRSK2/CASP3/CDK16/CLEC7A/EGR1/EIF2B2/EIF2B5/ENDOG/GATA4/GCG/GCK/GPER1/LDHA/MAFA/MEN1/MIRLET7G/MLXIPL/NME1/NOX4/P2RX2/PPARD/PTK2B/PTPRN2/RAF1/RFX6/SIDT2/SLC26A6/SLC29A1/STX4/TH/TRH	35	BP
GO:0006749	glutathione metabolic	10/2734	57/17381	0.40734	0.9196	0.90452	CHAC1/DPEP1/GGT6/GLO1/GPX1/GSR/GSTM1/GSTM2/GSTZ1/IDH1	10	BP
GO:0046686	response to cadmium ion	10/2734	57/17381	0.40734	0.9196	0.90452	ALAD/CYP1A2/EGFR/HAAO/HSF1/MAPK3/NCF1/NUDT1/OGG1/SLC34A1	10	BP
GO:0046847	filopodium assembly	10/2734	57/17381	0.40734	0.9196	0.90452	AGRN/ARAP1/ARHGEF4/BCAS3/CCL21/ESPN/FSCN1/PPP1R9B/PRKCD/RAB17	10	BP
GO:1903391	regulation of adherens junction	10/2734	57/17381	0.40734	0.9196	0.90452	ABL1/BCAS3/COL16A1/KDR/LDB1/MYOC/PTPN23/RHOD/THY1/WDPCP	10	BP
GO:0045185	maintenance of protein location	16/2734	94/17381	0.40768	0.9196	0.90452	ARL2BP/CD27/DBN1/FLNA/GPAA1/HSPA5/NBL1/NFKBIL1/NR5A1/OS9/PKD1/SCIN/SUFU/TAF3/THRA/TLN1	16	BP
GO:0030837	negative regulation of actin filament	9/2734	51/17381	0.41046	0.9196	0.90452	CAPZA3/HIP1R/LMOD1/PFN1/PRKCD/SCIN/SPTAN1/SPTB/SPTBN2	9	BP
GO:0060425	lung morphogenesis	9/2734	51/17381	0.41046	0.9196	0.90452	BMP4/CTSH/ESRP2/FGFR2/HHIP/MAPK3/PLOD3/SHH/TNC	9	BP
GO:0060997	dendritic spine morphogenesis	9/2734	51/17381	0.41046	0.9196	0.90452	ARC/CDK5/CFL1/DLG4/EFNA1/EPHB3/NLGN3/PDLIM5/WNT7A	9	BP

GO:1901068	guanosine-containing compound metabolic	9/2734	51/17381	0.41046	0.9196	0.90452	CARD11/DGUOK/DLG4/IMPDH1/MFN1/NME1/NME4/NUDT18/SCRIB	9	BP
GO:0008217	regulation of blood pressure	29/2734	175/17381	0.41086	0.9196	0.90452	ADM/ADRA1A/ADRA1B/ADRA2B/AGT/ANPEP/ASIC2/ATP1A1/AVPR2/CHGA/CYP11B2/DRD2/EDN2/EPHX2/F2RL1/GCH1/GNB3/GUCA2B/HSD11B2/NISCH/NOS3/NPPA/P2RX2/PRCP/REN/RNPEP/SERPINF2/SGK1/TACR1	29	BP
GO:1904589	regulation of protein import	29/2734	175/17381	0.41086	0.9196	0.90452	BMP4/CCL19/CD27/CSF3/DAB2IP/EGFR/EMD/FAM89B/FLNA/IL18/LGALS9/MTOR/NFKBIL1/OGG1/PARP10/PBLD/PDE2A/PKD1/PRDX1/PRKCD/RBPMS/SFRP5/SHH/SMO/SUFU/THRA/TLR9/WNT3A/ZPR1	29	BP
GO:0030258	lipid modification	45/2734	275/17381	0.41094	0.9196	0.90452	ACACB/ACAD10/ACADVL/ACOXL/AGT/APOA5/B4GALNT1/CPT2/CROT/CYP1A1/DGKI/DGKQ/DGKZ/EGFR/EPHX2/ERBB2/FGF17/FGF3/FGFR2/FGFR4/GBGT1/HADHA/HAO2/INPP5D/INPP5E/INPP5F/INPPL1/LCK/MTMR1/MTOR/NRG1/PDGFA/PDGFRB/PI4KB/PIK3R5/PIK3R6/PLPP1/POR/PPARD/PTPN11/SLC35C1/SOAT2/ST3GAL4/TMEM150A/TYSND1	45	BP
GO:0007212	dopamine receptor	8/2734	45/17381	0.41364	0.9196	0.90452	ARRB2/CALY/DRD2/DTNBP1/FLNA/GNAO1/GNB5/OPRM1	8	BP
GO:0018279	protein N-linked glycosylation	8/2734	45/17381	0.41364	0.9196	0.90452	ASGR1/DAD1/DDOST/DPM2/OST4/RPN1/ST3GAL4/SYVN1	8	BP
GO:0060976	coronary vasculature development	8/2734	45/17381	0.41364	0.9196	0.90452	BMP4/GPER1/NOTCH1/PDGFRB/PLXND1/SETD2/SMAD6/SUFU	8	BP
GO:1903115	regulation of actin filament-based	8/2734	45/17381	0.41364	0.9196	0.90452	ATP1A1/CAMK2D/CAV3/FLNA/GATA4/MIR328/MYBPC3/TNNC1	8	BP

GO:0031349	positive regulation of defense response	73/2734	451/17381	0.4138	0.9196	0.90452	ADAM8/AGT/APOB/ARRB2/CARD11/CARD9/CCL5/CCR2/CD180/CD300LF/CD6/CLEC7A/CNPY3/CTSL/DAB2IP/DHX58/EGFR/F2RL1/FGA/HAVCR2/HLA-E/HRAS/IDO1/IKBKB/IL18/IRAK1/ITGAM/KARS/KLKB1/LRSAM1/LTA/MAPK3/MAPKAPK3/MIR92A2/MMP2/MUC1/MUC2/MUC3A/MUC5AC/MUC5B/MUC6/NCR3/NFKBIL1/NLRX1/NOD1/NONO/NR1H3/PARP9/PDCD4/PDE2A/PGC/POLR3D/PRKCD/PSMB11/PSMB6/PSMB7/PSMB8/PSMC3/PSMD13/[illegible]	73	BP
GO:1901532	regulation of hematopoietic progenitor cell differentiation	19/2734	113/17381	0.41444	0.9196	0.90452	ABL1/HIST1H4F/HIST2H4A/HIST2H4B/LDB1/LMO1/NOTCH1/PDCD2/PSMB11/PSMB6/PSMB7/PSMB8/PSMC3/PSMD13/PSMD3/PSMD5/PSMD7/SETD1A/SPI1	19	BP
GO:0031667	response to nutrient levels	69/2734	426/17381	0.41493	0.9196	0.90452	ABCG5/ACACB/ADM/ALAD/AMBRA1/ASNS/ATF3/BCAS3/BGLAP/C2/CHMP1A/CLPS/CLPSL1/COL1A1/CYP1A1/DAD1/DAP/DAPL1/DHODH/DNMT3A/EGFR/EHMT2/EIF2AK4/EIF4EBP1/FOLR2/FOXO4/GATA4/GBA/GCG/GNPAT/HSD11B2/HSF1/HSPA5/INHBB/KAT2A/LARS/LDHA/LTA/MAPK3/MAX/MPO/MTOR/MYOD1/NPRL3/OGG1/OPRM1/OTC/PEMT/PFKFB1/PKM/POR/PPARD/PYY/RALB/RARA/RRAGC/SESN1/SLC25A25/SLC34A1/SLC39A5/TH/TNC/TRPV4/UCN2/UCP3/ULK1/VGF/WNT11/ZFYVE1	69	BP
GO:0072006	nephron development	23/2734	138/17381	0.41624	0.9196	0.90452	AGT/AQP1/BMP4/EGR1/EYA1/HOXB7/ILK/IRX3/KIRREL3/LHX1/MYO1E/NOTCH1/OSR1/PDGFRB/PKD1/POU3F3/SHH/SMO/SOX8/STAT1/WNT1/W[illegible]	23	BP
GO:0032924	activin receptor signaling	7/2734	39/17381	0.41685	0.9196	0.90452	CFC1/DACT2/GDF2/INHBB/MEN1/SHH/ZC3H3	7	BP
GO:0033683	nucleotide-excision repair, DNA incision	7/2734	39/17381	0.41685	0.9196	0.90452	DDB1/DDB2/GTF2H4/OGG1/POLD4/RFC1/RFC2	7	BP
GO:0038128	ERBB2 signaling	7/2734	39/17381	0.41685	0.9196	0.90452	EGFR/ERBB2/HRAS/HSP90AA1/MYOC/NRG1/SHC1	7	BP
GO:0051646	mitochondrion localization	7/2734	39/17381	0.41685	0.9196	0.90452	BRAT1/KAT2A/MARK2/MFN1/MFN2/RHOT2/UXT	7	BP
GO:0061028	establishment of endothelial	7/2734	39/17381	0.41685	0.9196	0.90452	CDH5/ENG/F2RL1/IKBKB/MARVELD2/PDE2A/WNT7A	7	BP

GO:0006665	sphingolipid metabolic process	27/2734	163/17381	0.41711	0.9196	0.90452	ARSE/ARSI/B4GALNT1/CLN6/CPTP/CREM/DEGS2/ELOVL1/ELOVL5/FUT7/GBA/GLTP/HACD1/HACD3/HEXA/NEU3/ORMDL3/PEMT/PLA2G15/PLPP1/PRKCD/PSAP/SFTPB/SGMS1/SMPD4/SPTLC1/TH	27	BP
GO:0002244	hematopoietic progenitor cell differentiation	31/2734	188/17381	0.4174	0.9196	0.90452	ABL1/BMP4/DACT2/EML1/HEATR9/HERC6/HIST1H4F/HIST2H4A/HIST2H4B/HOXB3/HOXB4/KRT75/LDB1/LMO1/NARFL/NOTCH1/PDCD2/PSMB11/PSMB6/PSMB7/PSMB8/PSMC3/PSMD13/PSMD3/PSMD5/PSMD7/SETD1A/SHH/SPI1/STON2/TNFRSF13B	31	BP
GO:0006446	regulation of translational initiation	13/2734	76/17381	0.41805	0.9196	0.90452	CCL5/EIF2AK4/EIF2B2/EIF2B5/EIF3B/EIF4B/EIF4EBP1/EIF4G1/HSPB1/POLR2G/RARA/RBM4/RPS6KB2	13	BP
GO:0009142	nucleoside triphosphate biosynthetic process	13/2734	76/17381	0.41805	0.9196	0.90452	AK5/ALDOA/ATP5G1/ATP5I/CYC1/IMPDH1/NME1/NME4/PID1/PKM/TBPL1/UCK1/VPS9D1	13	BP
GO:0043407	negative regulation of MAP kinase	13/2734	76/17381	0.41805	0.9196	0.90452	BMP4/CAV3/CDK5RAP3/DAB2IP/DUSP2/DUSP21/DUSP5/GBA/MAPK8IP1/MIR92A2/PDCD4/PRKCD/RGS14	13	BP
GO:0050777	negative regulation of immune	22/2734	132/17381	0.41962	0.9196	0.90452	ARRB2/BCL6/CCR2/CUEDC2/DHX58/DRD2/GPR17/GPX1/HAVCR2/HLA-E/HLX/IL20RB/IL4R/INPP5D/INS/LGALS3/LGALS9/NLRX1/NMI/NR1H3/SCRIB/SPINK5	22	BP
GO:0010863	positive regulation of phospholipase	6/2734	33/17381	0.42006	0.9196	0.90452	ADRA1A/AGT/ANG/EGFR/PDGFRB/PLCB2	6	BP
GO:0030224	monocyte differentiation	6/2734	33/17381	0.42006	0.9196	0.90452	BMP4/CD74/CSF1/CSF1R/IL31RA/INPP5D	6	BP
GO:0045622	regulation of T-helper cell differentiation	6/2734	33/17381	0.42006	0.9196	0.90452	BCL6/CCL19/HLX/IL18/IL4R/RARA	6	BP
GO:0051204	protein insertion into mitochondrial	6/2734	33/17381	0.42006	0.9196	0.90452	BAD/DYNLL2/MOAP1/PPP1R13B/SFN/YWHAQ	6	BP

GO:1900077	negative regulation of cellular response to	6/2734	33/17381	0.42006	0.9196	0.90452	AHSG/CISH/MZB1/PID1/PRKCD/TRIM72	6	BP
GO:1903131	mononuclear cell	6/2734	33/17381	0.42006	0.9196	0.90452	BMP4/CD74/CSF1/CSF1R/IL31RA/INPP5D	6	BP
GO:0002218	activation of innate immune response	49/2734	301/17381	0.4203	0.9196	0.90452	APOB/ARRB2/CARD11/CARD9/CD180/CD300LF/CLEC7A/CNPY3/CTSL/DAB2IP/DHX58/F2RL1/FGA/HAVCR2/HRAS/IKBKB/IRAK1/ITGAM/MAPKAPK3/MUC1/MUC2/MUC3A/MUC5AC/MUC5B/MUC6/NFKBIL1/NLRX1/NOD1/NONO/NR1H3/PRKCD/PSMB11/PSMB6/PSMB7/PSMB8/PSMC3/PSMD13/PSMD3/PSMD5/PSMD7/PSPC1/RAF1/SFTPA1/TBK1/TIRAP/TLR8/TLR9/TN	49	BP
GO:0007187	G-protein coupled receptor signaling pathway, coupled to	30/2734	182/17381	0.42054	0.9196	0.90452	ADRA1A/ADRA1B/ADRA2A/ADRA2B/ADRA2C/AGT/AVPR2/CHGA/CHRM1/CRHR1/CRHR2/DRD2/FLNA/GABBR1/GCG/GNAO1/GNAT1/GPER1/GPR1/HRH2/HTR1D/MTNR1B/OPRD1/OPRM1/PSAP/RXFP2/SSTR5/TSKU/UCN2/VIPR1	30	BP
GO:0060079	excitatory postsynaptic potential	17/2734	101/17381	0.42151	0.9196	0.90452	CDK5/CHRNA6/CHRNB1/CHRNB3/DGKI/DLG4/GLRA1/GRIA3/GRIN1/GRIN2C/NLGN3/OPRM1/P2RX2/PTK2B/SLC29A1/STX1A/WNT7A	17	BP
GO:0031338	regulation of vesicle fusion	12/2734	70/17381	0.42182	0.9196	0.90452	ADPRHL1/CORO1A/DOC2B/GRTP1/RABGAP1/RABGAP1L/SGSM2/STXBP1/TBC1D25/TBC1D28/TBC1D9/USP6NL	12	BP
GO:0051155	positive regulation of striated muscle	12/2734	70/17381	0.42182	0.9196	0.90452	ADGRB1/ARRB2/BMP4/CAV3/EHD1/IL4R/MTOR/MYOD1/NRG1/SHH/THRA/WNT3A	12	BP
GO:0038061	NIK/NF-kappaB signaling	21/2734	126/17381	0.42311	0.9196	0.90452	ADGRG3/EGFR/HAVCR2/ILK/IRAK1/MAP3K14/NOD1/PDCD4/PSMB11/PSMB6/PSMB7/PSMB8/PSMC3/PSMD13/PSMD3/PSMD5/PSMD7/PTP4A3/TIRAP/TRAF2/TRIM40	21	BP
GO:0001958	endochondral ossification	5/2734	27/17381	0.42316	0.9196	0.90452	BMP4/COL1A1/INPPL1/PHOSPHO1/SCX	5	BP

GO:0031111	negative regulation of microtubule polymerization	5/2734	27/17381	0.42316	0.9196	0.90452	CIB1/KATNB1/MAP6D1/MID1IP1/TRIM54	5	BP
GO:0036075	replacement ossification	5/2734	27/17381	0.42316	0.9196	0.90452	BMP4/COL1A1/INPPL1/PHOSPHO1/SCX	5	BP
GO:0042346	positive regulation of NF-kappaB	5/2734	27/17381	0.42316	0.9196	0.90452	CCL19/CD27/IL18/LGALS9/TLR9	5	BP
GO:0045648	positive regulation of erythrocyte	5/2734	27/17381	0.42316	0.9196	0.90452	INPP5D/ISG15/MIR221/MIR222/STAT1	5	BP
GO:0045745	positive regulation of G-protein coupled receptor protein signaling	5/2734	27/17381	0.42316	0.9196	0.90452	ACPP/CHGA/DRD2/GPER1/NECAB2	5	BP
GO:0045879	negative regulation of smoothened	5/2734	27/17381	0.42316	0.9196	0.90452	HHIP/PTCH2/SUFU/TRAF3IP1/TULP3	5	BP
GO:0045911	positive regulation of DNA	5/2734	27/17381	0.42316	0.9196	0.90452	CLCF1/PAXIP1/PRDM9/TNFSF13/UBE2B	5	BP
GO:0048710	regulation of astrocyte differentiation	5/2734	27/17381	0.42316	0.9196	0.90452	BIN1/CLCF1/DAB1/MYCN/NOTCH1	5	BP
GO:0071539	protein localization to	5/2734	27/17381	0.42316	0.9196	0.90452	C2CD3/DCTN2/DISC1/MCPH1/PARD6A	5	BP

GO:0099625	ventricular cardiac muscle cell membrane repolarization	5/2734	27/17381	0.42316	0.9196	0.90452	CAV3/KCND3/KCNJ5/KCNQ1/WDR1	5	BP
GO:1903432	regulation of TORC1	5/2734	27/17381	0.42316	0.9196	0.90452	DGKQ/LARS/RRAGC/SESN1/TELO2	5	BP
GO:2000144	positive regulation of DNA-templated transcription, initiation	5/2734	27/17381	0.42316	0.9196	0.90452	FOSL1/NKX2-5/PAXIP1/PSMC3/TAF1	5	BP
GO:2000352	negative regulation of endothelial cell	5/2734	27/17381	0.42316	0.9196	0.90452	ABL1/FGA/IL13/KDR/TNIP2	5	BP
GO:2000727	positive regulation of cardiac muscle	5/2734	27/17381	0.42316	0.9196	0.90452	ARRB2/BMP4/MTOR/NRG1/WNT3A	5	BP
GO:0090276	regulation of peptide hormone	33/2734	201/17381	0.42343	0.9196	0.90452	ADRA2A/ADRA2C/ANO1/ARL2BP/BAD/BLK/BRSK2/CAPN10/CCL5/CDK16/DOC2B/DRD2/EGFR/FGA/GCG/GCK/GLUD1/GPER1/INHBB/INS/ITPR3/MTNR1B/PFKM/PPARD/PTPN11/RFX6/SIDT2/SLC25A5/SSTR5/STX1A/STX4/	33	BP
GO:0010976	positive regulation of neuron projection	40/2734	245/17381	0.42505	0.9196	0.90452	AGT/AMIGO1/ANAPC2/APBB1/CAMK1D/CPNE5/CPNE9/DAB2IP/DISC1/FES/FN1/FOXO6/HSPA5/ILK/ISLR2/KATNB1/L1CAM/LIMK1/LLPH/LTK/MARK2/METRN/MIR221/MIR222/MTOR/NME1/NRG1/OBSL1/PLXNB1/PLXNB3/PLXND1/PPP2R5B/PTK2B/SCARF1/SEMA7A/SERPINI1/TNFRSF12A/TRPV	40	BP
GO:0015914	phospholipid transport	11/2734	64/17381	0.42578	0.9196	0.90452	APOA5/APOC3/CPTP/MFSD2A/NPC2/OSBPL5/PITPNM1/PRELID1/PRKCD/SCARB1/TNFAIP8L3	11	BP
GO:0042531	positive regulation of tyrosine phosphorylation	11/2734	64/17381	0.42578	0.9196	0.90452	ARL2BP/CCL5/CLCF1/CSF1R/HSF1/IL13/IL18/IL31RA/MIR221/PARP9/TNFRSF18	11	BP

GO:0043242	negative regulation of protein complex	11/2734	64/17381	0.42578	0.9196	0.90452	CAPZA3/CIB1/KATNB1/LMOD1/MAP6D1/MID1IP1/SCIN/SPTAN1/SPTB/SPTBN2/TRIM54	11	BP
GO:0046889	positive regulation of lipid biosynthetic	11/2734	64/17381	0.42578	0.9196	0.90452	APOA5/CCDC3/ELOVL5/INS/MID1IP1/MLXIPL/MTOR/NR1H3/POR/PRKCD/SCARB1	11	BP
GO:1903036	positive regulation of response to	11/2734	64/17381	0.42578	0.9196	0.90452	ADRA2A/HRAS/MIR221/MIR222/MIR451A/MTOR/MYOD1/PPARD/SCARF1/SELP/SERPINF2	11	BP
GO:2001021	negative regulation of response to DNA damage	11/2734	64/17381	0.42578	0.9196	0.90452	CCAR2/CD74/CLU/HSF1/MIR221/MUC1/NUDT16L1/OGG1/OTUB1/RECQL5/ZNF385A	11	BP
GO:0003181	atrioventricular valve morphogenesis	4/2734	21/17381	0.42589	0.9196	0.90452	EFNA1/HEY1/NOTCH1/SMAD6	4	BP
GO:0006490	oligosaccharide-lipid intermediate biosynthetic	4/2734	21/17381	0.42589	0.9196	0.90452	ALG10/ALG3/DOLPP1/MPDU1	4	BP
GO:0009595	detection of biotic stimulus	4/2734	21/17381	0.42589	0.9196	0.90452	NOD1/SCARB1/SMO/TREM2	4	BP
GO:0009713	catechol-containing compound biosynthetic process	4/2734	21/17381	0.42589	0.9196	0.90452	DDC/GCH1/HDC/TH	4	BP
GO:0014829	vascular smooth muscle	4/2734	21/17381	0.42589	0.9196	0.90452	ADRA2B/AGT/CHRM1/EDN2	4	BP

GO:0019373	epoxygenase P450 pathway	4/2734	21/17381	0.42589	0.9196	0.90452	CYP1A1/CYP1A2/CYP2W1/EPHX2	4	BP
GO:0034114	regulation of heterotypic cell-cell adhesion	4/2734	21/17381	0.42589	0.9196	0.90452	FGA/IL1RN/MIR221/THY1	4	BP
GO:0042423	catecholamine biosynthetic process	4/2734	21/17381	0.42589	0.9196	0.90452	DDC/GCH1/HDC/TH	4	BP
GO:0043496	regulation of protein homodimerization activity	4/2734	21/17381	0.42589	0.9196	0.90452	HSPA5/NRG1/TIRAP/TRAF2	4	BP
GO:0043555	regulation of translation in response to	4/2734	21/17381	0.42589	0.9196	0.90452	EIF2AK4/EIF2B5/RBM4/RPS6KA1	4	BP
GO:0044321	response to leptin	4/2734	21/17381	0.42589	0.9196	0.90452	GCK/INHBB/LEPR/PID1	4	BP
GO:0048305	immunoglobulin secretion	4/2734	21/17381	0.42589	0.9196	0.90452	HLA-E/STX4/TNFRSF4/TRAF2	4	BP
GO:0051497	negative regulation of stress fiber	4/2734	21/17381	0.42589	0.9196	0.90452	ARAP1/MYOC/PFN1/WAS	4	BP
GO:0051968	positive regulation of synaptic transmission,	4/2734	21/17381	0.42589	0.9196	0.90452	EGFR/NLGN3/PTK2B/STXBP1	4	BP
GO:0060713	labyrinthine layer	4/2734	21/17381	0.42589	0.9196	0.90452	ADM/FGFR2/SPINT1/ST14	4	BP
GO:0071168	protein localization to	4/2734	21/17381	0.42589	0.9196	0.90452	CHMP7/HDAC8/LEMD2/SETD2	4	BP

GO:0090344	negative regulation of	4/2734	21/17381	0.42589	0.9196	0.90452	ABL1/BCL6/FOXM1/WNT1	4	BP
GO:1901739	regulation of myoblast fusion	4/2734	21/17381	0.42589	0.9196	0.90452	ADGRB1/EHD1/IL4R/MYOD1	4	BP
GO:2000114	regulation of establishment of cell polarity	4/2734	21/17381	0.42589	0.9196	0.90452	BCAS3/CDH5/PTK2B/WDPCP	4	BP
GO:2000515	negative regulation of CD4-positive, alpha-beta T	4/2734	21/17381	0.42589	0.9196	0.90452	BCL6/HLX/IL4R/LGALS9	4	BP
GO:2000637	positive regulation of gene silencing	4/2734	21/17381	0.42589	0.9196	0.90452	BMP4/EGFR/EIF4G1/MYCN	4	BP
GO:0022900	electron transport chain	28/2734	170/17381	0.42709	0.9196	0.90452	AKR7A3/ALDH2/ALDH4A1/AOC2/COX10/COX11/COX8A/CYC1/CYP1A2/GLRX5/GPX2/GSR/HAAO/IDO1/LOXL2/NCF1/NDUFA2/NDUFA4L2/NDUFB5/NDUFC2-	28	BP
GO:0010256	endomembrane system organization	64/2734	396/17381	0.42713	0.9196	0.90452	AKTIP/ALS2CL/ANO4/ANO7/AQP1/BAIAP2L1/BCAS3/BIN2/BLZF1/CAV3/CCDC136/CCNB2/CHMP1A/CHMP4C/CHMP7/CLU/COG4/DNM1/DTNBP1/EMD/FAM160A2/FASLG/GAK/GBF1/GOLGA2/GORASP1/GPER1/KIFC3/LEMD2/LMNA/MAPK3/MYO18A/NUP210/NUP98/OBSL1/PACSIN3/PDE4DIP/PLEKHM2/PRKCD/RAB7A/RBSN/REEP2/REEP4/SEC16A/SEC16B/SNF8/STX18/STX5/TBPL1/TJAP1/TOR1A/TOR1B/TRDN/TRIM72/UBL4A/USP6NL/VPS25/VPS37B/VPS37C/VPS37D/VPS51/WHAMM/ZFYVE27/ZNF385A	64	BP
GO:0002115	store-operated calcium entry	3/2734	15/17381	0.42756	0.9196	0.90452	CRACR2A/ORAI1/ORAI3	3	BP
GO:0003417	growth plate cartilage development	3/2734	15/17381	0.42756	0.9196	0.90452	POR/RARA/THBS3	3	BP
GO:0006000	fructose metabolic	3/2734	15/17381	0.42756	0.9196	0.90452	ALDOA/GLYCTK/PFKFB1	3	BP

GO:0006241	CTP biosynthetic	3/2734	15/17381	0.42756	0.9196	0.90452	NME1/NME4/UCK1	3	BP
GO:0006388	tRNA splicing, via endonucleolytic cleavage and	3/2734	15/17381	0.42756	0.9196	0.90452	CPSF1/CPSF4/CSTF2	3	BP
GO:0007158	neuron cell-cell adhesion	3/2734	15/17381	0.42756	0.9196	0.90452	NINJ2/NLGN3/NRXN2	3	BP
GO:0007638	mechanosensory behavior	3/2734	15/17381	0.42756	0.9196	0.90452	DRD2/NRXN2/STRA6	3	BP
GO:0008272	sulfate transport	3/2734	15/17381	0.42756	0.9196	0.90452	SLC26A1/SLC26A10/SLC26A6	3	BP
GO:0009209	pyrimidine ribonucleoside triphosphate biosynthetic process	3/2734	15/17381	0.42756	0.9196	0.90452	NME1/NME4/UCK1	3	BP
GO:0010801	negative regulation of peptidyl-threonine	3/2734	15/17381	0.42756	0.9196	0.90452	DGKQ/EIF4G1/PARD3	3	BP
GO:0015936	coenzyme A metabolic	3/2734	15/17381	0.42756	0.9196	0.90452	ACOT7/CROT/PPCDC	3	BP
GO:0019377	glycolipid catabolic	3/2734	15/17381	0.42756	0.9196	0.90452	GBA/NEU3/PRKCD	3	BP
GO:0030852	regulation of granulocyte differentiation	3/2734	15/17381	0.42756	0.9196	0.90452	C1QC/INPP5D/RARA	3	BP

GO:0031629	synaptic vesicle fusion to presynaptic active zone	3/2734	15/17381	0.42756	0.9196	0.90452	STX1A/STX4/STXBP1	3	BP
GO:0032075	positive regulation of	3/2734	15/17381	0.42756	0.9196	0.90452	DAB2IP/HSPA5/PRKCD	3	BP
GO:0032239	regulation of nucleobase-containing compound transport	3/2734	15/17381	0.42756	0.9196	0.90452	SETD2/SUPT6H/ZC3H3	3	BP
GO:0033617	mitochondrial respiratory chain complex IV assembly	3/2734	15/17381	0.42756	0.9196	0.90452	COA3/COA4/SMIM20	3	BP
GO:0034349	glial cell apoptotic	3/2734	15/17381	0.42756	0.9196	0.90452	CASP3/PRKCD/TRAF2	3	BP
GO:0034356	NAD biosynthesis via nicotinamide riboside salvage	3/2734	15/17381	0.42756	0.9196	0.90452	PARP10/PARP9/SLC22A13	3	BP
GO:0034656	nucleobase-containing small molecule catabolic	3/2734	15/17381	0.42756	0.9196	0.90452	ENTPD8/NUDT1/NUDT18	3	BP
GO:0035269	protein O-linked	3/2734	15/17381	0.42756	0.9196	0.90452	DPM2/FKTN/SDF2	3	BP
GO:0044126	regulation of growth of symbiont in	3/2734	15/17381	0.42756	0.9196	0.90452	LTA/MPO/TIRAP	3	BP

GO:0044146	negative regulation of growth of symbiont involved in	3/2734	15/17381	0.42756	0.9196	0.90452	LTA/MPO/TIRAP	3	BP
GO:0045623	negative regulation of T-helper cell	3/2734	15/17381	0.42756	0.9196	0.90452	BCL6/HLX/IL4R	3	BP
GO:0046036	CTP metabolic process	3/2734	15/17381	0.42756	0.9196	0.90452	NME1/NME4/UCK1	3	BP
GO:0048521	negative regulation of	3/2734	15/17381	0.42756	0.9196	0.90452	DRD2/INS/TRH	3	BP
GO:0048569	post-embryonic animal organ development	3/2734	15/17381	0.42756	0.9196	0.90452	LDHA/MYO1E/MYO7A	3	BP
GO:0051546	keratinocyte migration	3/2734	15/17381	0.42756	0.9196	0.90452	KRT16/MTOR/PPARD	3	BP
GO:0051571	positive regulation of histone H3-K4	3/2734	15/17381	0.42756	0.9196	0.90452	BRCA1/GCG/PAXIP1	3	BP
GO:0060253	negative regulation of glial cell	3/2734	15/17381	0.42756	0.9196	0.90452	ASCL2/IDH2/NOTCH1	3	BP
GO:0060347	heart trabecula formation	3/2734	15/17381	0.42756	0.9196	0.90452	CAV3/HEY1/NKX2-5	3	BP
GO:0060579	ventral spinal cord interneuron fate	3/2734	15/17381	0.42756	0.9196	0.90452	EVX1/LHX3/SUFU	3	BP

GO:0060581	cell fate commitment involved in pattern	3/2734	15/17381	0.42756	0.9196	0.90452	EVX1/LHX3/SUFU	3	BP
GO:0071243	cellular response to arsenic-containing	3/2734	15/17381	0.42756	0.9196	0.90452	ATF3/HSF1/UROS	3	BP
GO:0071599	otic vesicle development	3/2734	15/17381	0.42756	0.9196	0.90452	EYA1/FGFR2/TCAP	3	BP
GO:0071850	mitotic cell cycle arrest	3/2734	15/17381	0.42756	0.9196	0.90452	CDC14A/E4F1/MCPH1	3	BP
GO:0071941	nitrogen cycle metabolic	3/2734	15/17381	0.42756	0.9196	0.90452	ASL/NAGS/OTC	3	BP
GO:0090036	regulation of protein kinase C signaling	3/2734	15/17381	0.42756	0.9196	0.90452	ADRA1A/FLT4/WNT11	3	BP
GO:0090177	establishment of planar polarity involved in neural tube	3/2734	15/17381	0.42756	0.9196	0.90452	DVL2/FZD2/GRHL3	3	BP
GO:0090231	regulation of spindle	3/2734	15/17381	0.42756	0.9196	0.90452	ANAPC15/LCMT1/XRCC3	3	BP
GO:0099500	vesicle fusion to plasma	3/2734	15/17381	0.42756	0.9196	0.90452	STX1A/STX4/STXBP1	3	BP
GO:1902165	regulation of intrinsic apoptotic signaling pathway in response to	3/2734	15/17381	0.42756	0.9196	0.90452	CD74/MUC1/ZNF385A	3	BP

GO:2000484	positive regulation of interleukin-8	3/2734	15/17381	0.42756	0.9196	0.90452	CD58/F2RL1/LGALS9	3	BP
GO:2000647	negative regulation of stem cell	3/2734	15/17381	0.42756	0.9196	0.90452	MIR221/MIR222/VAX1	3	BP
GO:2001224	positive regulation of neuron	3/2734	15/17381	0.42756	0.9196	0.90452	DAB2IP/FLNA/NSMF	3	BP
GO:2001275	positive regulation of glucose import in response to	3/2734	15/17381	0.42756	0.9196	0.90452	AGT/PTPN11/RARRES2	3	BP
GO:0021885	forebrain cell migration	10/2734	58/17381	0.42996	0.9238	0.9086	CDK5/CDK5R2/DAB1/DAB2IP/DISC1/DRD2/EGFR/NRG1/OGDH/POU3F3	10	BP
GO:0044786	cell cycle DNA replication	10/2734	58/17381	0.42996	0.9238	0.9086	BCL6/E2F8/INO80/POLA2/POLD4/POLE/POLE4/RFC1/RFC2/ZPR1	10	BP
GO:0045600	positive regulation of fat cell	10/2734	58/17381	0.42996	0.9238	0.9086	ADIRF/CCDC3/FNDC5/INS/MEDAG/PPARD/RARRES2/SH3PXD2B/WIF1/ZNF385A	10	BP
GO:0019935	cyclic-nucleotide-	19/2734	114/17381	0.43052	0.9243	0.90913	ADM/ADRA1A/ADRA1B/ADRA2A/ADRA2B/ADRA2C/CHGA/CRHR1/DGKQ/GPER1/KSR1/OPRM1/PDE2A/PDZD3/PTK2B/RXFP2/TCP11/UBE2B/UCN	19	BP
GO:0099565	chemical synaptic transmission,	19/2734	114/17381	0.43052	0.9243	0.90913	ARRB2/CDK5/CHRNA6/CHRNB1/CHRNB3/DGKI/DLG4/DRD2/GLRA1/GRIA3/GRIN1/GRIN2C/NLGN3/OPRM1/P2RX2/PTK2B/SLC29A1/STX1A/WNT7A	19	BP
GO:0071674	mononuclear cell migration	14/2734	83/17381	0.43334	0.9291	0.91381	C3AR1/CCL1/CCL19/CCL21/CCL5/CCR2/CREB3/CSF1/KARS/LGALS3/MAPK3/NBL1/RARRES2/TRPV4	14	BP
GO:0072332	intrinsic apoptotic signaling pathway by p53	14/2734	83/17381	0.43334	0.9291	0.91381	ANKRD2/BOK/CD74/FHIT/HINT1/MUC1/NUPR1/PHLDA3/PPP1R13B/TMEM109/ZNF346/ZNF385A/ZNF385C/ZNF385D	14	BP

GO:0030490	maturation of SSU-rRNA	9/2734	52/17381	0.4344	0.9291	0.91381	FCF1/MRPS11/NOB1/NOL11/PDCD11/RPS24/SRFBP1/TSR1/TSR2	9	BP
GO:0032615	interleukin-12 production	9/2734	52/17381	0.4344	0.9291	0.91381	ARRB2/CCL19/IDO1/IRF5/LGALS9/PRKCD/TIRAP/TLR8/TLR9	9	BP
GO:0035176	social behavior	9/2734	52/17381	0.4344	0.9291	0.91381	DLG4/EN1/GAD1/GRIN1/MTOR/NLGN3/NRXN2/TH/VPS13A	9	BP
GO:0046635	positive regulation of alpha-beta T	9/2734	52/17381	0.4344	0.9291	0.91381	CCL19/CCR2/HLA-E/HLX/IL18/IL4R/LGALS9/RARA/SHH	9	BP
GO:0051058	negative regulation of small GTPase mediated signal	9/2734	52/17381	0.4344	0.9291	0.91381	ADRA1A/BCL6/DAB2IP/MAPKAP1/MFN2/MYOC/RASA3/RASA4B/TNK1	9	BP
GO:0051703	intraspecies interaction between organisms	9/2734	52/17381	0.4344	0.9291	0.91381	DLG4/EN1/GAD1/GRIN1/MTOR/NLGN3/NRXN2/TH/VPS13A	9	BP
GO:0061178	regulation of insulin secretion involved in cellular	9/2734	52/17381	0.4344	0.9291	0.91381	ADRA2A/ANO1/BAD/BRSK2/CDK16/GCG/RFX6/SIDT2/STX4	9	BP
GO:0002698	negative regulation of immune	18/2734	108/17381	0.43445	0.9291	0.91381	ARRB2/BCL6/CCR2/CUEDC2/DHX58/HAVCR2/HLA-E/HLX/IL20RB/IL4R/INS/LGALS3/LGALS9/MICB/NLRX1/SPINK5/TARBP2/TRAF3IP1	18	BP
GO:0044070	regulation of anion transport	18/2734	108/17381	0.43445	0.9291	0.91381	AGT/ANO1/BSND/CA7/CLCN2/CLCNKA/CLCNKB/GPR89A/MTOR/NCOR1/OSR1/PRELID1/PRKCD/SLC34A1/STXBP1/SV2A/TRH/VDAC2	18	BP
GO:1903364	positive regulation of cellular protein catabolic	33/2734	202/17381	0.43553	0.93	0.91467	ANAPC15/ANAPC2/ATPIF1/AXIN1/BCAP31/BUB1B/CAV3/CD81/CHFR/CLU/DAB2IP/DISC1/ECSCR/GBA/HERPUD1/KLHL40/LRP1/NKD2/OSBPL7/PSMB11/PSMB6/PSMB7/PSMB8/PSMC3/PSMD13/PSMD3/PSMD5/PSMD7/PTK2B/RNF166/RNF180/TAF1/USP5	33	BP

GO:0051091	positive regulation of DNA binding transcription	40/2734	246/17381	0.43602	0.93	0.91467	ADAM8/AGT/ATF2/CAMK1D/CARD11/CIB1/CLU/CRTC2/DDR2/DVL2/ESR2/FOSL1/FZD2/IKBKB/INS/IRAK1/LGALS9/LRRFIP1/NEUROD2/NOD1/OPRD1/PLA2G1B/PPP2R5B/RHEBL1/RNF31/RPS6KA4/RWDD1/SHH/SMO/TAF1/TIRAP/TLR9/TRAF2/TRAPPC9/TRIM14/TRIM26/TRIM31/TRIM62/WNT1/W	40	BP
GO:0051402	neuron apoptotic process	36/2734	221/17381	0.43749	0.93	0.91467	AARS/ADAM8/AMBRA1/ATF2/ATN1/BARHL1/BOK/CASP3/CDK5/CLCF1/CNTFR/CORO1A/DRAXIN/EN1/FASLG/FZD9/GAPDH/GPX1/GRIN1/HRAS/HSPA5/ILK/LRP1/MAX/NAIP/NES/NLRP1/NONO/NQO2/NSMF/PTK2B/SIGMAR1/SNCB/STXBP1/WFS1/ZPR1	36	BP
GO:0031016	pancreas development	13/2734	77/17381	0.43769	0.93	0.91467	BAD/BMP4/CCDC40/MEIS2/MEN1/MSLN/NKX3-2/RFX6/SHH/SIDT2/SMO/WFS1/WLS	13	BP
GO:0055117	regulation of cardiac muscle contraction	13/2734	77/17381	0.43769	0.93	0.91467	ADRA1A/ADRA1B/ATP1A1/CAMK2D/CAV3/CHGA/FLNA/GATA4/GSTM2/KCNQ1/MIR328/NKX2-5/NPPA	13	BP
GO:0030098	lymphocyte differentiation	53/2734	328/17381	0.43832	0.93	0.91467	ABL1/ADAM8/ADGRG3/BAD/BCL6/BLNK/BMP4/CARD11/CCL19/CD27/CD74/CD79B/CLCF1/CMTM7/DNAJA3/EGR1/ERBB2/FANCA/FOXN1/FUT7/FZD8/FZD9/GON4L/HLX/IFNA5/IL18/IL4R/INPP5D/JAG2/LCK/LEPR/LFNG/LGALS9/LY6D/LY9/NKX2-3/PIK3R6/PPP2R3C/PRELID1/PSMB11/PTK2B/RARA/RORC/SART1/SEMA4	53	BP
GO:0046916	cellular transition metal ion homeostasis	17/2734	102/17381	0.43856	0.93	0.91467	ACO1/ALAS2/ATOX1/ATP13A2/ATP6V1G1/GDF2/HEPH/HMOX2/LCK/MT2A/NEDD8/NUBP1/SLC11A1/SLC30A3/SLC39A13/SLC39A5/TFR2	17	BP
GO:0016236	macroautophagy	46/2734	284/17381	0.4389	0.93	0.91467	AMBRA1/ATG10/ATG101/ATG2A/ATP13A2/ATP6V0B/ATP6V0E2/ATP6V1B1/ATP6V1G1/ATP6V1G2/CASP3/CDK5/CHMP4C/DYNLL2/GAPDH/GBA/GPSM1/IFT20/KDR/LRSAM1/MAPK3/MFN1/MFN2/MLST8/MTMR14/MTOR/NOD1/NPRL3/PGAM5/POLDIP2/PRKAB1/QSOX1/RAB7A/RALB/RRAGC/SNF8/SPTLC1/TBC1D25/TBK1/TECPR1/ULK1/VPS25/VPS37B/VPS37C/VPS3	46	BP
GO:0018196	peptidyl-asparagine modification	8/2734	46/17381	0.43915	0.93	0.91467	ASGR1/DAD1/DDOST/DPM2/OST4/RPN1/ST3GAL4/SYVN1	8	BP

GO:0032330	regulation of chondrocyte differentiation	8/2734	46/17381	0.43915	0.93	0.91467	ADAMTS7/BMP4/LOXL2/NKX3-2/PKDCC/POR/SCIN/SOX5	8	BP
GO:0032613	interleukin-10 production	8/2734	46/17381	0.43915	0.93	0.91467	EPX/F2RL1/IDO1/IL20RB/LGALS9/PRKCD/TLR9/TUSC2	8	BP
GO:0035987	endodermal cell differentiation	8/2734	46/17381	0.43915	0.93	0.91467	COL4A2/COL7A1/FN1/ITGA7/ITGB5/MMP15/MMP2/SETD2	8	BP
GO:0045776	negative regulation of	8/2734	46/17381	0.43915	0.93	0.91467	ADRA1A/DRD2/GCH1/GUCA2B/NOS3/NPPA/PRCP/RNPEP	8	BP
GO:0090329	regulation of DNA-dependent DNA	8/2734	46/17381	0.43915	0.93	0.91467	BCL6/BMP4/E2F8/INO80/LIG3/PID1/RFC2/TICRR	8	BP
GO:0030900	forebrain development	59/2734	366/17381	0.44025	0.93	0.91467	AQP1/AVPR2/AXIN1/BAD/BCAN/BMP4/CASP3/CDK5/CDK5R2/CSF1R/DAB1/DAB2IP/DCLK2/DISC1/DOCK7/DRAXIN/DRD2/EGFR/EIF2B5/EMX1/EPHB3/FGFR2/FLNA/GBX2/GNAO1/GRIN1/KAT2A/KDM6B/KIRREL3/LHX1/LHX3/LHX5/LRP1/MCPH1/MFSD2A/NME1/NOTCH1/NRG1/OGDH/OTP/PITX1/POU3F1/POU3F3/RARA/RTN4RL1/RTN4RL2/SEMA7A/SETD2/SHH/SMO/TBR1/TBX19/TH/TRAPPC9/TSKU/VAX2/WNT1/WNT3A/WNT7A	59	BP
GO:0031668	cellular response to extracellular stimulus	35/2734	215/17381	0.44079	0.93	0.91467	AMBRA1/ASGR1/ASNS/ATF3/BGLAP/CHMP1A/COL1A1/DAP/DAPL1/EHMT2/EIF2AK4/FOLR2/FOSL1/GBA/GSDMD/HSPA5/INHBB/KIF26A/LARS/MAPK3/MAX/MTOR/MYOD1/NPRL3/NUDT1/PPP1R9B/RALB/RRAGC/SESN1/SIPA1/SLC39A5/TNC/UCN2/ULK1/ZFYVE1	35	BP
GO:0050870	positive regulation of T cell activation	35/2734	215/17381	0.44079	0.93	0.91467	ADAM8/BAD/BCL6/CARD11/CCDC88B/CCL19/CCL21/CCL5/CCR2/CD247/CD27/CD5/CD6/CD74/CLECL1/CORO1A/CSK/DNAJA3/EFNB1/HAVCR2/HLA-E/HLX/IL18/IL4R/LCK/LGALS9/MAP3K14/PDCD1/PIK3R6/PTPN11/RARA/S	35	BP
GO:0009988	cell-cell recognition	12/2734	71/17381	0.44231	0.93	0.91467	ALDOA/CATSPER1/CCL19/CCL21/CCT3/CD6/HAVCR2/LGALS3/POMZP3/PRSS37/SPACA3/VDAC2	12	BP
GO:0030279	negative regulation of	12/2734	71/17381	0.44231	0.93	0.91467	AHSG/ECM1/HDAC8/KREMEN2/MEN1/NOTCH1/PTK2B/SMAD6/SOST/SUFU/TNN/TWIST2	12	BP

GO:0001959	regulation of cytokine-mediated	27/2734	165/17381	0.44393	0.93	0.91467	CASP1/CCDC3/CCL5/CD300LF/CD74/CSF1/ECM1/ELF1/F2RL1/IFNA5/IKBKB/IL1RN/IRAK1/MADD/NR1H3/PARP9/PELI3/PTPN11/PYDC1/RNF31/SCRIB/SHARPIN/STAT1/STAT2/TRAF2/TRAIP/TREM2	27	BP
GO:0001655	urogenital system development	51/2734	316/17381	0.44407	0.93	0.91467	ACD/AGT/APH1A/AQP1/BMP4/CRIP1/CTSH/DACT2/EGR1/EPHB3/EYA1/FGFR2/HOXB7/HOXC11/IFT140/IFT20/ILK/IRX3/KIRREL3/LHX1/LZTS2/MMP17/MYO1E/NLE1/NOTCH1/OSR1/PDGFA/PDGFRB/PKD1/POU3F3/PSAP/PYGO2/RARA/REN/SDC1/SHH/SIM1/SLC34A1/SMAD6/SMO/SOX8/STAT1/STRA6/TNC/TNS2/TRAF3IP1/WDPCP/WFS1/WNT1/WNT11/WNT6	51	BP
GO:0031330	negative regulation of cellular catabolic	34/2734	209/17381	0.44417	0.93	0.91467	ACACB/ALAD/APOC3/ATP13A2/CCAR2/CDK5RAP3/DAB2IP/DAP/DAPL1/EFNA1/EIF4G1/FHIT/FURIN/GATA4/GOLGA2/INS/KLHL40/LAMP3/LARS/LEPR/LRIG2/MTOR/OS9/PANO1/PKP3/POLDIP2/QSOX1/SHH/SLC11A1/SUFU/TAF1/TIRAP/USP19/WNT1	34	BP
GO:0021762	substantia nigra development	7/2734	40/17381	0.44429	0.93	0.91467	CASP5/GLUD1/HSPA5/MAPKAP1/NDRG2/SEC16A/YWHAQ	7	BP
GO:0033865	nucleoside bisphosphate metabolic	7/2734	40/17381	0.44429	0.93	0.91467	ABHD14B/ACOT7/CROT/PODXL2/PPCDC/SLC26A1/SULT6B1	7	BP
GO:0033875	ribonucleoside bisphosphate metabolic	7/2734	40/17381	0.44429	0.93	0.91467	ABHD14B/ACOT7/CROT/PODXL2/PPCDC/SLC26A1/SULT6B1	7	BP
GO:0034032	purine nucleoside bisphosphate	7/2734	40/17381	0.44429	0.93	0.91467	ABHD14B/ACOT7/CROT/PODXL2/PPCDC/SLC26A1/SULT6B1	7	BP
GO:0046638	positive regulation of alpha-beta T	7/2734	40/17381	0.44429	0.93	0.91467	CCL19/HLX/IL18/IL4R/LGALS9/RARA/SHH	7	BP
GO:1903555	regulation of tumor necrosis factor superfamily cytokine	19/2734	115/17381	0.44661	0.93	0.91467	ADAM8/ARRB2/CARD9/CCL19/CCR2/CLU/HAVCR2/HLA-E/HSF1/HSPB1/LGALS9/NFKBIL1/NOD1/ORM1/ORM2/RARA/SPON2/TIRAP/TLR9	19	BP

GO:0006879	cellular iron ion homeostasis	11/2734	65/17381	0.44723	0.93	0.91467	ACO1/ALAS2/ATP13A2/ATP6V1G1/GDF2/HEPH/HMOX2/NEDD8/NUBP1/SLC11A1/TFR2	11	BP
GO:0009201	ribonucleoside triphosphate biosynthetic process	11/2734	65/17381	0.44723	0.93	0.91467	ALDOA/ATP5G1/ATP5I/CYC1/IMPDH1/NME1/NME4/PID1/PKM/UCK1/VPS9D1	11	BP
GO:1902117	positive regulation of organelle	11/2734	65/17381	0.44723	0.93	0.91467	CROCC/DYNC1H1/FSCN1/LRSAM1/NUMA1/RALB/SDC1/SEPT9/STX18/ULK1/WRAP73	11	BP
GO:0015748	organophosphate ester transport	15/2734	90/17381	0.44741	0.93	0.91467	APOA5/APOC3/CPTP/G6PC3/MFSD2A/NPC2/OSBPL5/PITPNM1/PRELID1/PRKCD/SCARB1/SLC25A25/SLC25A5/SLC44A4/TNFAIP8L3	15	BP
GO:0030595	leukocyte chemotaxis	33/2734	203/17381	0.44765	0.93	0.91467	ADAM8/C3AR1/CAMK1D/CCL1/CCL19/CCL21/CCL5/CCR2/CD74/CHGA/CKLF/CORO1A/CREB3/CSF1/CXCR1/CXCR2/CXCR3/CXCR5/DAPK2/EDN2/F2RL1/GBF1/KARS/LGALS3/LGALS9/MAPK3/NBL1/PF4V1/PLA2G1B/PTK2B/RARRES2/TIRAP/TRPV4	33	BP
GO:0045089	positive regulation of innate immune response	56/2734	348/17381	0.44862	0.93	0.91467	ADAM8/APOB/ARRB2/CARD11/CARD9/CCL5/CD180/CD300LF/CLEC7A/CNPY3/CTSL/DAB2IP/DHX58/F2RL1/FGA/HAVCR2/HLA-E/HRAS/IKBKB/IRAK1/ITGAM/MAPKAPK3/MMP2/MUC1/MUC2/MUC3A/MUC5AC/MUC5B/MUC6/NCR3/NFKBIL1/NLRX1/NOD1/NONO/NR1H3/PARP9/POLR3D/PRKCD/PSMB11/PSMB6/PSMB7/PSMB8/PSMC3/PSMD13/PSMD3/PSMD5/PSMD7/PSPC1/RAF1/SFTPA1/TBK1/TIRAP/TLR8/TLR9/TNIP2	56	BP
GO:0001837	epithelial to mesenchymal transition	22/2734	134/17381	0.44944	0.93	0.91467	BMP4/COL1A1/CRB2/DAB2IP/EFNA1/ENG/FGFR2/GLIPR2/HEY1/LOXL2/MIR221/MIR222/MTOR/NOTCH1/PBLD/PDCD4/TGFB1I1/TMEM100/TRIM62/VASN/WNT11/ZNF703	22	BP
GO:0003156	regulation of animal organ formation	6/2734	34/17381	0.44991	0.93	0.91467	BMP4/EYA1/HOXC11/NOTCH1/SHH/WNT3A	6	BP
GO:0006084	acetyl-CoA metabolic	6/2734	34/17381	0.44991	0.93	0.91467	ACACB/DLST/MPC1/PDHB/PIPOX/PMVK	6	BP

GO:0006356	regulation of transcription from RNA polymerase I	6/2734	34/17381	0.44991	0.93	0.91467	ERBB2/FLNA/MTOR/NOL11/POLR2L/TAF1	6	BP
GO:0007435	salivary gland morphogenesis	6/2734	34/17381	0.44991	0.93	0.91467	EGFR/ESRP2/FGFR2/PDGFA/PLXND1/SHH	6	BP
GO:0010039	response to iron ion	6/2734	34/17381	0.44991	0.93	0.91467	ACO1/ALAD/APBB1/CYP1A1/DRD2/TFR2	6	BP
GO:0042269	regulation of natural killer cell mediated cytotoxicity	6/2734	34/17381	0.44991	0.93	0.91467	ARRB2/HAVCR2/HLA-E/LGALS9/NCR3/PIK3R6	6	BP
GO:0042339	keratan sulfate metabolic	6/2734	34/17381	0.44991	0.93	0.91467	ACAN/B4GALT2/CHST5/HEXA/SLC35D2/ST3GAL4	6	BP
GO:0061384	heart trabecula morphogenesis	6/2734	34/17381	0.44991	0.93	0.91467	CAV3/ENG/HEY1/NKX2-5/NOTCH1/NRG1	6	BP
GO:0072529	pyrimidine-containing compound catabolic	6/2734	34/17381	0.44991	0.93	0.91467	CDA/MBD4/NEIL2/NT5M/OGG1/SMUG1	6	BP
GO:0090313	regulation of protein targeting to	6/2734	34/17381	0.44991	0.93	0.91467	CDK5/CIB1/ERBB2/ITGAM/MIEF2/MYO1C	6	BP
GO:2000772	regulation of cellular	6/2734	34/17381	0.44991	0.93	0.91467	ABL1/BCL6/CDKN2A/HMGA1/MIR10A/YPEL3	6	BP
GO:0006690	icosanoid metabolic	18/2734	109/17381	0.451	0.93	0.91467	CD74/CES2/CYP1A1/CYP1A2/CYP2W1/DAGLB/DPEP1/EDN2/EPHX2/GGT6/GGTA1P/GPX1/MAPK3/MGST3/PLA2G1B/PLA2G4B/PLA2G5/PTGR1	18	BP
GO:0034446	substrate adhesion-dependent cell spreading	14/2734	84/17381	0.45221	0.93	0.91467	ABL1/ARPC2/CIB1/DOCK1/EFNA1/EPHB3/FERMT2/FERMT3/FGA/FLNA/FN1/ILK/MYOC/OLFM4	14	BP

GO:1904668	positive regulation of ubiquitin	14/2734	84/17381	0.45221	0.93	0.91467	ANAPC15/ANAPC2/BUB1B/GOLGA2/GORASP1/PSMB11/PSMB6/PSMB7/PSMB8/PSMC3/PSMD13/PSMD3/PSMD5/PSMD7	14	BP
GO:0032623	interleukin-2 production	10/2734	59/17381	0.45251	0.93	0.91467	ABL1/CARD11/CARD9/CCR2/HAVCR2/IL18/IL20RB/SLC11A1/TRAF2/VSIG4	10	BP
GO:0050918	positive chemotaxis	10/2734	59/17381	0.45251	0.93	0.91467	ARTN/BMP4/CCL5/CDH13/CORO1A/F2RL1/KDR/LGALS3/PLXNB3/SCRIB	10	BP
GO:0097553	calcium ion transmembrane import into	21/2734	128/17381	0.45366	0.93	0.91467	ABL1/CAMK2D/CCL19/CCL21/CORO1A/DRD2/FASLG/GPER1/GRIN1/GRIN2C/GSTM2/IL13/ITPR3/JPH3/LCK/NPSR1/PTK2B/RASA3/THY1/TRDN/TRPV6	21	BP
GO:0017156	calcium ion regulated exocytosis	17/2734	103/17381	0.4556	0.93	0.91467	ADRA2A/CACNA1G/CACNA1H/CDK5/CDK5R2/CPLX1/DOC2A/DOC2B/NOTCH1/SCIN/SCRIB/SDF4/STX1A/STX4/STXBP1/SYTL3/TRPV6	17	BP
GO:0019933	cAMP-mediated	17/2734	103/17381	0.4556	0.93	0.91467	ADM/ADRA1A/ADRA1B/ADRA2A/ADRA2B/ADRA2C/CHGA/CRHR1/DGKQ/GPER1/KSR1/OPRM1/PDE2A/RXFP2/TCP11/UBE2B/UCN2	17	BP
GO:1902036	regulation of hematopoietic stem cell differentiation	17/2734	103/17381	0.4556	0.93	0.91467	ABL1/HIST1H4F/HIST2H4A/HIST2H4B/LDB1/LMO1/PSMB11/PSMB6/PSMB7/PSMB8/PSMC3/PSMD13/PSMD3/PSMD5/PSMD7/SETD1A/SPI1	17	BP
GO:2000021	regulation of ion homeostasis	34/2734	210/17381	0.45612	0.93	0.91467	ABL1/AGT/AVPR2/BOK/CA7/CACNA1G/CAMK2D/CAV3/CCL19/CCL21/CORO1A/DRD2/FASLG/FZD9/GPER1/GRIN1/GSTM2/IL13/ITPR3/JPH3/KDR/LCK/MYOC/NPSR1/P2RX2/PLA2G1B/PPP2R3C/PTK2B/RASA3/SLC30A3/T	34	BP
GO:0001702	gastrulation with mouth forming second	5/2734	28/17381	0.45617	0.93	0.91467	CRB2/LDB1/LHX1/NAT8B/ZBTB17	5	BP
GO:0001782	B cell homeostasis	5/2734	28/17381	0.45617	0.93	0.91467	ABL1/CASP3/CD74/PPP2R3C/TNFRSF13B	5	BP
GO:0006623	protein targeting to	5/2734	28/17381	0.45617	0.93	0.91467	CLU/GOSR2/LARS/RAB7A/VTI1B	5	BP
GO:0008209	androgen metabolic	5/2734	28/17381	0.45617	0.93	0.91467	ADM/HSD17B3/HSD3B1/SCARB1/SHH	5	BP

GO:0009072	aromatic amino acid family metabolic	5/2734	28/17381	0.45617	0.93	0.91467	FAH/GSTZ1/HAAO/IDO1/TH	5	BP
GO:0014002	astrocyte development	5/2734	28/17381	0.45617	0.93	0.91467	EGFR/EIF2B5/LAMC3/LRP1/SMO	5	BP
GO:0032435	negative regulation of proteasomal ubiquitin-dependent	5/2734	28/17381	0.45617	0.93	0.91467	CCAR2/FHIT/KLHL40/PANO1/SHH	5	BP
GO:0034377	plasma lipoprotein	5/2734	28/17381	0.45617	0.93	0.91467	APOA5/APOB/APOC3/DGAT1/SOAT2	5	BP
GO:0035774	positive regulation of insulin secretion involved in cellular	5/2734	28/17381	0.45617	0.93	0.91467	ANO1/BAD/GCG/RFX6/STX4	5	BP
GO:0043252	sodium-independent organic anion transport	5/2734	28/17381	0.45617	0.93	0.91467	SLC22A12/SLC22A13/SLC22A9/SLCO2B1/SLCO5A1	5	BP
GO:0044818	mitotic G2/M transition checkpoint	5/2734	28/17381	0.45617	0.93	0.91467	BRCA1/CDK5RAP3/FOXO4/RINT1/TICRR	5	BP
GO:0045948	positive regulation of translational	5/2734	28/17381	0.45617	0.93	0.91467	CCL5/EIF2AK4/EIF2B5/POLR2G/RPS6KB2	5	BP
GO:0048240	sperm capacitation	5/2734	28/17381	0.45617	0.93	0.91467	ACRBP/RNASE9/SEPT4/SLC26A6/TCP11	5	BP
GO:0055069	zinc ion homeostasis	5/2734	28/17381	0.45617	0.93	0.91467	ATP13A2/LCK/SLC30A3/SLC39A13/SLC39A5	5	BP

GO:1904292	regulation of ERAD pathway	5/2734	28/17381	0.45617	0.93	0.91467	BCAP31/BRSK2/HERPUD1/OS9/USP19	5	BP
GO:0048024	regulation of mRNA splicing, via spliceosome	13/2734	78/17381	0.4573	0.93	0.91467	CELF6/HMX2/LMNTD2/MYOD1/NUP98/PRDX6/RBFOX1/RBFOX3/RBM4/RNPS1/SF3B4/SLC39A5/SRSF4	13	BP
GO:1901216	positive regulation of	13/2734	78/17381	0.4573	0.93	0.91467	ABL1/AIMP2/ATF2/BAD/CASP3/CDK5/CLU/EGR1/FASLG/ITGAM/MTOR/NQO2/PPP1R13B	13	BP
GO:0022412	cellular process involved in reproduction in multicellular organism	53/2734	330/17381	0.45742	0.93	0.91467	ACRBP/ANG/ARRB2/BMP4/CAPZA3/CATSPER1/CCDC136/CFAP157/CIB1/DDX4/DEAF1/DND1/DNMT3A/FANCA/FKBP6/FOSL1/HILS1/HORMAD1/HSF1/HSPA2/INHBB/M1AP/MCMDC2/MTOR/NUP210L/PAQR7/PIWIL2/PLPP1/POMZP3/PRDM14/PRKAR1A/PTK2B/PYGO2/RARA/RNASE9/RXFP2/SEPT4/SERPINA5/SLC26A6/SLC2A8/SOHLH2/SPACA3/SPAG16/TAF1L/TARBP2/TBPL1/TCP11/TDRD1/TDRD9/TMEM119/TSSK1B/TTLL5/UBE2B	53	BP
GO:0008033	tRNA processing	20/2734	122/17381	0.45807	0.93	0.91467	AARS/AARS2/ADAT1/C9orf64/CDKAL1/CPSF1/CPSF4/CSTF2/KARS/METTL2A/METTL2B/PUSL1/RPP21/SARS/THUMPD2/TRMT10B/TRMT2B/TRMT4	20	BP
GO:0002011	morphogenesis of an epithelial	9/2734	53/17381	0.45824	0.93	0.91467	DVL2/FLNA/HOXB2/HOXB4/MIR221/MTOR/NOTCH1/TOR1A/WNT7A	9	BP
GO:0031663	lipopolysaccharide-mediated signaling	9/2734	53/17381	0.45824	0.93	0.91467	CCL5/CD180/CD6/IL18/IRAK1/MAPK3/NFKBIL1/NOS3/SCARB1	9	BP
GO:0042093	T-helper cell differentiation	9/2734	53/17381	0.45824	0.93	0.91467	BCL6/CCL19/HLX/IL18/IL4R/LY9/RARA/RORC/SEMA4A	9	BP
GO:0090181	regulation of cholesterol metabolic	9/2734	53/17381	0.45824	0.93	0.91467	ACACB/ACADVL/APOB/DGKQ/EPHX2/LMF1/PMVK/POR/SCAP	9	BP
GO:0016042	lipid catabolic process	49/2734	305/17381	0.45997	0.93	0.91467	ACACB/ACAD10/ACADVL/ACOXL/ADRA2A/APOA5/APOB/APOC3/CDK4/CLPS/CLPSL1/CPT2/CROT/CYP1A2/DAGLB/GBA/HADHA/HAO2/HCAR2/IDH1/INPP5F/INS/MTOR/NEU3/PLA2G15/PLA2G16/PLA2G1B/PLA2G2F/PLA2G4B/PLA2G5/PLB1/PLBD2/PLCB2/PLCD1/PLCH2/PLD2/PLIN1/PNPLA2/PNPLA7/PPARD/PRDX6/PRKCD/RAB7A/RARRES2/SCARB1/SCARF1/SMPD4/	49	BP

GO:0014897	striated muscle hypertrophy	16/2734	97/17381	0.46045	0.93	0.91467	AGT/CAMK2D/CAMTA2/CAV3/CDK9/GATA4/INPP5F/LMNA/MIR195/MIR25/MTOR/MYOC/NPPA/PDLIM5/TCAP/TRIM63	16	BP
GO:0051339	regulation of lyase activity	16/2734	97/17381	0.46045	0.93	0.91467	ADRA2A/AVPR2/CCR2/CRHR1/DRD2/GABBR1/GPER1/GUCA1B/GUCA2A/GUCA2B/NOS3/OPRM1/PDZD3/RAF1/RUNDC3A/WFS1	16	BP
GO:1901568	fatty acid derivative metabolic	26/2734	160/17381	0.46141	0.93	0.91467	ACOT7/ACSF3/ACSS3/CD74/CES2/CYP1A1/CYP1A2/CYP2W1/DAGLB/DGAT1/DPEP1/EDN2/ELOVL1/ELOVL5/EPHX2/GGT6/GGTA1P/GPX1/HACD1/HMGCLL1/MAPK3/MGST3/PLA2G1B/PLA2G4B/PLA2G5/PTGR1	26	BP
GO:0097529	myeloid leukocyte migration	29/2734	179/17381	0.46259	0.93	0.91467	ADAM8/C3AR1/CAMK1D/CCL1/CCL19/CCL21/CCL5/CCR2/CD74/CHGA/CKLF/CREB3/CSF1/CXCR2/DAPK2/EDN2/EPX/GBF1/KARS/LGALS3/MAPK3/NBL1/PF4V1/PLA2G1B/PTK2B/RARRES2/TIRAP/TRPV4/WDR1	29	BP
GO:0046717	acid secretion	19/2734	116/17381	0.46269	0.93	0.91467	ABCC3/ABCC4/CPLX1/DRD2/HIP1R/HRH2/KCNQ1/PLA2G1B/PLA2G2F/PLA2G5/PTGER3/SGK1/SLC1A7/SLC26A6/SLC6A9/STX1A/STXBP1/SV2A/TR	19	BP
GO:0090502	RNA phosphodiester bond hydrolysis,	12/2734	72/17381	0.46274	0.93	0.91467	APEX1/CPSF4/EXO1/FCF1/NOB1/PIWIL2/RNASE1/RNASE8/RNASEK/RPP21/TSR1/ZC3H3	12	BP
GO:1901292	nucleoside phosphate catabolic	12/2734	72/17381	0.46274	0.93	0.91467	DNPH1/GPX1/HINT1/MBD4/NEIL2/NT5M/NUDT1/NUDT18/OGG1/PDE2A/SMUG1/XDH	12	BP
GO:0000717	nucleotide-excision repair, DNA duplex unwinding	4/2734	22/17381	0.46331	0.93	0.91467	DDB1/DDB2/GTF2H4/XPC	4	BP
GO:0002335	mature B cell differentiation	4/2734	22/17381	0.46331	0.93	0.91467	CMTM7/LFNG/NKX2-3/PTK2B	4	BP
GO:0002438	acute inflammatory response to	4/2734	22/17381	0.46331	0.93	0.91467	CD6/IL20RB/IL31RA/OPRM1	4	BP
GO:0003094	glomerular filtration	4/2734	22/17381	0.46331	0.93	0.91467	AQP1/F2RL1/MCAM/MYO1E	4	BP

GO:0003272	endocardial cushion	4/2734	22/17381	0.46331	0.93	0.91467	ENG/HEY1/NOTCH1/TMEM100	4	BP
GO:0006855	drug transmembrane transport	4/2734	22/17381	0.46331	0.93	0.91467	ABCB5/ABCC3/ABCG5/SLC22A1	4	BP
GO:0007035	vacuolar acidification	4/2734	22/17381	0.46331	0.93	0.91467	ATP6V0B/ATP6V0E2/CLN6/SLC11A1	4	BP
GO:0007141	male meiosis I	4/2734	22/17381	0.46331	0.93	0.91467	DDX4/HSPA2/SLC2A8/TDRD9	4	BP
GO:0030149	sphingolipid catabolic	4/2734	22/17381	0.46331	0.93	0.91467	GBA/NEU3/PRKCD/SMPD4	4	BP
GO:0031116	positive regulation of microtubule	4/2734	22/17381	0.46331	0.93	0.91467	ANKRD53/CAV3/FES/NUMA1	4	BP
GO:0032799	low-density lipoprotein receptor particle metabolic	4/2734	22/17381	0.46331	0.93	0.91467	AP2A2/AP2M1/FURIN/SCAP	4	BP
GO:0035812	renal sodium excretion	4/2734	22/17381	0.46331	0.93	0.91467	AGT/AVPR2/DRD2/TACR1	4	BP
GO:0035813	regulation of renal sodium	4/2734	22/17381	0.46331	0.93	0.91467	AGT/AVPR2/DRD2/TACR1	4	BP
GO:0045109	intermediate filament organization	4/2734	22/17381	0.46331	0.93	0.91467	DES/KRT14/KRT71/SHH	4	BP
GO:0045943	positive regulation of transcription from RNA polymerase I	4/2734	22/17381	0.46331	0.93	0.91467	ERBB2/MTOR/NOL11/TAF1	4	BP

GO:0046132	pyrimidine ribonucleoside biosynthetic process	4/2734	22/17381	0.46331	0.93	0.91467	DHODH/NME1/NME4/UCK1	4	BP
GO:0060055	angiogenesis involved in wound healing	4/2734	22/17381	0.46331	0.93	0.91467	GPX1/MCAM/MIR451A/PRCP	4	BP
GO:0060148	positive regulation of posttranscriptional gene	4/2734	22/17381	0.46331	0.93	0.91467	BMP4/EGFR/EIF4G1/MYCN	4	BP
GO:0090312	positive regulation of protein	4/2734	22/17381	0.46331	0.93	0.91467	BCL6/BRMS1/CTBP1/FNTA	4	BP
GO:1903306	negative regulation of regulated	4/2734	22/17381	0.46331	0.93	0.91467	ADRA2A/CCR2/LGALS9/NOTCH1	4	BP
GO:1905208	negative regulation of cardiocyte	4/2734	22/17381	0.46331	0.93	0.91467	CAV3/EGFR/MIR222/WNT3A	4	BP
GO:2001026	regulation of endothelial cell chemotaxis	4/2734	22/17381	0.46331	0.93	0.91467	HSPB1/KDR/MIR16-1/NOTCH1	4	BP
GO:0003254	regulation of membrane depolarization	8/2734	47/17381	0.46452	0.93	0.91467	BOK/CACNA1G/CAMK2D/CAV3/FZD9/KDR/MYOC/PPP2R3C	8	BP
GO:0051205	protein insertion into membrane	8/2734	47/17381	0.46452	0.93	0.91467	BAD/DYNLL2/EGFR/MOAP1/PPP1R13B/SFN/UBL4A/YWHAQ	8	BP
GO:0006942	regulation of striated muscle contraction	15/2734	91/17381	0.46558	0.93	0.91467	ADRA1A/ADRA1B/ATP1A1/CAMK2D/CAV3/CHGA/FLNA/GATA4/GSTM2/KCNQ1/MIR328/MYBPC3/MYBPH/NKX2-5/NPPA	15	BP

GO:0033273	response to vitamin	15/2734	91/17381	0.46558	0.93	0.91467	ALAD/BGLAP/COL1A1/CYP1A1/DNMT3A/EGFR/FOLR2/GATA4/OGG1/OTC/PEMT/PPARD/RARA/SLC34A1/TNC	15	BP
GO:0005996	monosaccharide metabolic process	44/2734	274/17381	0.46598	0.93	0.91467	ACACB/AIMP1/ALDOA/ATF3/BAD/BRAT1/C1QTNF1/CHST15/COX11/CREM/CRTC2/DGKQ/FUOM/FUT7/G6PC3/GALK1/GALM/GALT/GAPDH/GCG/GCK/GLYCTK/GNMT/GOT2/HK3/IGFBP3/INPPL1/INS/KAT2A/LCMT1/LEPR/MAEA/MTOR/NISCH/PC/PDHB/PFKFB1/PFKM/PGAM4/PHLDA2/PKM/P	44	BP
GO:0001756	somitogenesis	11/2734	66/17381	0.4686	0.93	0.91467	CDX2/CRB2/KAT2A/LFNG/LHX1/MESP2/NLE1/NOTCH1/RIPPLY1/TCAP/WNT3A	11	BP
GO:0007189	adenylate cyclase-activating G-protein coupled receptor	11/2734	66/17381	0.4686	0.93	0.91467	ADRA1A/ADRA1B/ADRA2A/ADRA2B/ADRA2C/CHGA/CRHR1/GPER1/OPRM1/RXFP2/UCN2	11	BP
GO:0060291	long-term synaptic	11/2734	66/17381	0.4686	0.93	0.91467	ABL1/ARC/DRD2/EIF2AK4/INS/ITPR3/LRRTM1/NLGN3/PTK2B/RGS14/STX4	11	BP
GO:0070509	calcium ion import	11/2734	66/17381	0.4686	0.93	0.91467	CACNA1G/CACNA1H/CAV3/CDK5/GCG/GCK/LGALS3/PDGFRB/TRPV2/TRPV4/TRPV6	11	BP
GO:0045732	positive regulation of protein catabolic process	43/2734	268/17381	0.46937	0.93	0.91467	ADAM8/ADRA2A/ANAPC15/ANAPC2/ATPIF1/AXIN1/BCAP31/BUB1B/CAV3/CD81/CHFR/CLU/DAB2IP/DDB1/DISC1/ECSCR/FURIN/GBA/HERPUD1/KLHL40/LRP1/MIR181B1/NKD2/OAZ2/OSBPL7/PACSIN3/PSMB11/PSMB6/PSMB7/PSMB8/PSMC3/PSMD13/PSMD3/PSMD5/PSMD7/PTK2B/RAB7A/RNF166/RNF180/SNF8/STX5/TAF1/USP5	43	BP
GO:0090305	nucleic acid phosphodiester bond hydrolysis	46/2734	287/17381	0.46966	0.93	0.91467	ANG/APEX1/AZGP1/C11orf80/CASP3/CPSF1/CPSF4/CSTF2/DDB1/DDB2/DIS3L/DIS3L2/DNASE1L2/ENDOG/ERN2/EXD3/EXO1/EXOSC10/EXOSC2/FCF1/GTF2H4/HORMAD1/HSF1/ISG20L2/NHP2/NOB1/OGG1/PAN2/PGBD5/PIWIL2/PNLDC1/POLD4/POLE/REXO4/RFC1/RFC2/RNASE1/RNASE8/RNASE9/RNASEK/RPP21/SMG5/SND1/TSR1/ZC3H3/ZNRD1	46	BP
GO:0071774	response to fibroblast growth factor	24/2734	148/17381	0.46997	0.93	0.91467	CCL5/COL1A1/ESRP2/FGF17/FGF3/FGFBP3/FGFR2/FGFR4/FGFRL1/GPC1/HHIP/LHX1/MAPK3/MIR15A/MIR16-1/NR4A1/POLR2G/POLR2L/PRDM14/PTPN11/SCGB1A1/SHCBP1/TDGF1/T	24	BP

GO:0043112	receptor metabolic process	27/2734	167/17381	0.47078	0.93	0.91467	ACHE/ADM/AP2A2/AP2M1/ARAP1/ARRB2/CAV3/CD63/CD81/CDK5/CXCR1/CXCR2/DLG4/DNM1/DRD2/FNTA/FURIN/INPP5F/LRP1/LRRTM1/NECAB2/NR1H3/NRG1/RSPO1/SCAP/SCRIB/WNT3A	27	BP
GO:0019217	regulation of fatty acid metabolic	14/2734	85/17381	0.47102	0.93	0.91467	ACACB/ACADVL/APOA5/APOC3/BRCA1/ELOVL5/INS/MID1IP1/MLXIPL/MTOR/NR1H3/PDHB/SCAP/TYSND1	14	BP
GO:0030193	regulation of blood	14/2734	85/17381	0.47102	0.93	0.91467	ASIC2/C1QTNF1/F2RL1/FGA/KLKB1/KNG1/NOS3/PDGFA/PLAU/PRKCD/PROZ/SELP/SERPINF2/TEC	14	BP
GO:0045069	regulation of viral genome	14/2734	85/17381	0.47102	0.93	0.91467	CCL5/DDB1/EIF2AK4/FKBP6/HACD3/IFITM2/ISG15/MIR221/MIR222/NOTCH1/PARP10/PPIB/PPIE/TARBP2	14	BP
GO:1900046	regulation of hemostasis	14/2734	85/17381	0.47102	0.93	0.91467	ASIC2/C1QTNF1/F2RL1/FGA/KLKB1/KNG1/NOS3/PDGFA/PLAU/PRKCD/PROZ/SELP/SERPINF2/TEC	14	BP
GO:0006733	oxidoreduction coenzyme metabolic	30/2734	186/17381	0.4714	0.93	0.91467	ALDOA/COQ2/COQ4/GALK1/GAPDH/GCK/HAAO/HK3/IDH1/IDH2/INS/LDHA/MLXIPL/MPC1/NCOR1/NMNAT3/NUP210/NUP98/OGDH/OGDHL/PARP10/PARP9/PDHB/PDSS1/PFKFB1/PFKM/PGAM4/PKM/QPRT/SLC22A13	30	BP
GO:0001961	positive regulation of cytokine-mediated	7/2734	41/17381	0.4715	0.93	0.91467	CASP1/CD300LF/CD74/CSF1/PARP9/TRAF2/TREM2	7	BP
GO:0006654	phosphatidic acid biosynthetic	7/2734	41/17381	0.4715	0.93	0.91467	DGKQ/GNPAT/PLA2G1B/PLA2G2F/PLA2G4B/PLA2G5/PLD2	7	BP
GO:0035196	production of miRNAs involved in gene silencing	7/2734	41/17381	0.4715	0.93	0.91467	BMP4/EGFR/MYCN/NCOR1/NCOR2/TARBP2/ZC3H10	7	BP
GO:0043370	regulation of CD4-positive, alpha-beta T cell	7/2734	41/17381	0.4715	0.93	0.91467	BCL6/CCL19/HLX/IL18/IL4R/LGALS9/RARA	7	BP

GO:0002739	regulation of cytokine secretion involved in	3/2734	16/17381	0.47175	0.93	0.91467	F2RL1/KARS/MAPK3	3	BP
GO:0003085	negative regulation of systemic arterial	3/2734	16/17381	0.47175	0.93	0.91467	ADRA1A/NPPA/PRCP	3	BP
GO:0006595	polyamine metabolic	3/2734	16/17381	0.47175	0.93	0.91467	AGMAT/OAZ2/PAOX	3	BP
GO:0006744	ubiquinone biosynthetic process	3/2734	16/17381	0.47175	0.93	0.91467	COQ2/COQ4/PDSS1	3	BP
GO:0007213	G-protein coupled acetylcholine receptor	3/2734	16/17381	0.47175	0.93	0.91467	AGRN/CHRM1/RGS10	3	BP
GO:0007342	fusion of sperm to egg plasma membrane involved in single	3/2734	16/17381	0.47175	0.93	0.91467	CATSPER1/SERPINA5/SPACA3	3	BP
GO:0009129	pyrimidine nucleoside monophosphate metabolic	3/2734	16/17381	0.47175	0.93	0.91467	DHODH/NT5M/UCK1	3	BP
GO:0009208	pyrimidine ribonucleoside triphosphate metabolic	3/2734	16/17381	0.47175	0.93	0.91467	NME1/NME4/UCK1	3	BP
GO:0009950	dorsal/ventral axis	3/2734	16/17381	0.47175	0.93	0.91467	SMAD6/SOST/VAX2	3	BP

GO:0010642	negative regulation of platelet-derived growth factor receptor	3/2734	16/17381	0.47175	0.93	0.91467	LRP1/PDGFA/PDGFRB	3	BP
GO:0018216	peptidyl-arginine	3/2734	16/17381	0.47175	0.93	0.91467	COPRS/NDUFAF7/PRDM14	3	BP
GO:0030252	growth hormone	3/2734	16/17381	0.47175	0.93	0.91467	CDK16/DRD2/PTPN11	3	BP
GO:0030889	negative regulation of B cell	3/2734	16/17381	0.47175	0.93	0.91467	CASP3/INPP5D/TNFRSF13B	3	BP
GO:0031000	response to caffeine	3/2734	16/17381	0.47175	0.93	0.91467	CACNA1S/DHODH/GSTM2	3	BP
GO:0031269	pseudopodium assembly	3/2734	16/17381	0.47175	0.93	0.91467	CCL21/CDC42EP2/F2RL1	3	BP
GO:0031573	intra-S DNA damage checkpoint	3/2734	16/17381	0.47175	0.93	0.91467	ATF2/NEK11/XPC	3	BP
GO:0031998	regulation of fatty acid beta-oxidation	3/2734	16/17381	0.47175	0.93	0.91467	ACACB/MTOR/TYSND1	3	BP
GO:0032515	negative regulation of phosphoprotein phosphatase	3/2734	16/17381	0.47175	0.93	0.91467	IKBKB/PPP1R1B/TIPRL	3	BP
GO:0033539	fatty acid beta-oxidation using acyl-CoA dehydrogenase	3/2734	16/17381	0.47175	0.93	0.91467	ACAD10/ACADVL/ACOXL	3	BP

GO:0033866	nucleoside bisphosphate biosynthetic process	3/2734	16/17381	0.47175	0.93	0.91467	ACOT7/PPCDC/SLC26A1	3	BP
GO:0034030	ribonucleoside bisphosphate biosynthetic process	3/2734	16/17381	0.47175	0.93	0.91467	ACOT7/PPCDC/SLC26A1	3	BP
GO:0034033	purine nucleoside bisphosphate biosynthetic	3/2734	16/17381	0.47175	0.93	0.91467	ACOT7/PPCDC/SLC26A1	3	BP
GO:0034397	telomere localization	3/2734	16/17381	0.47175	0.93	0.91467	LEMD2/NUP98/UBE2B	3	BP
GO:0036315	cellular response to	3/2734	16/17381	0.47175	0.93	0.91467	OSBPL7/RORC/SMO	3	BP
GO:0042790	transcription of nuclear large rRNA transcript from RNA polymerase I promoter	3/2734	16/17381	0.47175	0.93	0.91467	MTOR/NOL11/POLR1E	3	BP
GO:0043371	negative regulation of CD4-positive, alpha-beta T	3/2734	16/17381	0.47175	0.93	0.91467	BCL6/HLX/IL4R	3	BP
GO:0043508	negative regulation of JUN kinase	3/2734	16/17381	0.47175	0.93	0.91467	MAPK8IP1/MIR92A2/PDCD4	3	BP

GO:0043931	ossification involved in bone maturation	3/2734	16/17381	0.47175	0.93	0.91467	PHOSPHO1/PLXNB1/THBS3	3	BP
GO:0044144	modulation of growth of symbiont involved in interaction with	3/2734	16/17381	0.47175	0.93	0.91467	LTA/MPO/TIRAP	3	BP
GO:0045408	regulation of interleukin-6 biosynthetic process	3/2734	16/17381	0.47175	0.93	0.91467	CARD9/INPP5D/TIRAP	3	BP
GO:0045717	negative regulation of fatty acid biosynthetic	3/2734	16/17381	0.47175	0.93	0.91467	ACADVL/APOC3/BRCA1	3	BP
GO:0051457	maintenance of protein location in nucleus	3/2734	16/17381	0.47175	0.93	0.91467	ARL2BP/NR5A1/TAF3	3	BP
GO:0051957	positive regulation of amino acid	3/2734	16/17381	0.47175	0.93	0.91467	AGT/STXBP1/TRH	3	BP
GO:0060192	negative regulation of	3/2734	16/17381	0.47175	0.93	0.91467	ABL1/APOC3/POR	3	BP
GO:0060413	atrial septum morphogenesis	3/2734	16/17381	0.47175	0.93	0.91467	GATA4/NKX2-5/SMO	3	BP
GO:0060841	venous blood vessel development	3/2734	16/17381	0.47175	0.93	0.91467	ENG/NKX2-5/NOTCH1	3	BP
GO:0071236	cellular response to	3/2734	16/17381	0.47175	0.93	0.91467	CRIP1/HDAC8/HSPA5	3	BP

GO:0071404	cellular response to low-density lipoprotein	3/2734	16/17381	0.47175	0.93	0.91467	CD81/CDH13/MIR92A2	3	BP
GO:0072148	epithelial cell fate	3/2734	16/17381	0.47175	0.93	0.91467	JAG2/NOTCH1/RARA	3	BP
GO:0072531	pyrimidine-containing compound transmembrane transport	3/2734	16/17381	0.47175	0.93	0.91467	SLC28A1/SLC35A2/SLC35D2	3	BP
GO:0090083	regulation of inclusion body assembly	3/2734	16/17381	0.47175	0.93	0.91467	CLU/HSF1/HSPA2	3	BP
GO:0090195	chemokine secretion	3/2734	16/17381	0.47175	0.93	0.91467	CSF1R/F2RL1/IL4R	3	BP
GO:0097034	mitochondrial respiratory chain complex IV biogenesis	3/2734	16/17381	0.47175	0.93	0.91467	COA3/COA4/SMIM20	3	BP
GO:0098915	membrane repolarization during ventricular cardiac muscle	3/2734	16/17381	0.47175	0.93	0.91467	KCND3/KCNJ5/KCNQ1	3	BP
GO:1900273	positive regulation of long-term synaptic	3/2734	16/17381	0.47175	0.93	0.91467	DRD2/EIF2AK4/INS	3	BP

GO:1901663	quinone biosynthetic process	3/2734	16/17381	0.47175	0.93	0.91467	COQ2/COQ4/PDSS1	3	BP
GO:0006826	iron ion transport	10/2734	60/17381	0.47495	0.93	0.91467	ATP6V0B/ATP6V0E2/ATP6V1B1/ATP6V1F/ATP6V1G1/ATP6V1G2/HEPH/SLC11A1/STEAP2/TFR2	10	BP
GO:0009206	purine ribonucleoside triphosphate biosynthetic process	10/2734	60/17381	0.47495	0.93	0.91467	ALDOA/ATP5G1/ATP5I/CYC1/IMPDH1/NME1/NME4/PID1/PKM/VPS9D1	10	BP
GO:0016239	positive regulation of macroautophag	10/2734	60/17381	0.47495	0.93	0.91467	GPSM1/KDR/LRSAM1/MAPK3/MFN2/NOD1/RALB/SPTLC1/TBK1/ULK1	10	BP
GO:0051349	positive regulation of	10/2734	60/17381	0.47495	0.93	0.91467	AVPR2/CRHR1/GPER1/GUCA1B/GUCA2A/GUCA2B/NOS3/RAF1/RUNDC3A/WFS1	10	BP
GO:0001938	positive regulation of endothelial cell	13/2734	79/17381	0.47683	0.93	0.91467	ANG/BMP4/CDH13/ECM1/EGFL7/FLT4/GDF2/KDR/MIR29A/MIR503/MTOR/NR4A1/PLXNB3	13	BP
GO:0023061	signal release	67/2734	421/17381	0.47914	0.93	0.91467	ADM/ADRA2A/ADRA2B/ADRA2C/AGT/ANO1/AQP1/ARL2BP/BAD/BAIAP3/BLK/BRSK2/C1QTNF1/CAPN10/CCL5/CDK16/CDK5/CPLX1/CRHR1/DGKI/DOC2A/DOC2B/DRD2/DTNBP1/EGFR/FGA/GAD1/GCG/GCK/GLUD1/GPER1/HCAR2/IL1RN/INHBB/INS/ITPR3/MAFA/MTNR1B/NRXN2/OPRM1/PFKM/PNKD/PPARD/PTPN11/PTPN23/PTPRN2/RAB11FIP3/RAF1/REN/RFX6/SCRIB/SIDT2/SLC25A5/SLC6A9/SSTR5/STX1A/STX4/STXBP1/SYTL3/TACR [illegible]	67	BP
GO:0000291	nuclear-transcribed mRNA catabolic process,	6/2734	35/17381	0.47941	0.93	0.91467	DIS3L2/EXOSC2/LSM1/NT5C3B/PATL1/POLR2G	6	BP
GO:0003091	renal water homeostasis	6/2734	35/17381	0.47941	0.93	0.91467	AQP1/AVPR2/CYP11B2/PRKAR1A/PRKAR1B/WFS1	6	BP

GO:0030261	chromosome condensation	6/2734	35/17381	0.47941	0.93	0.91467	CHMP1A/ERN2/GPER1/HILS1/MCPH1/NCAPD3	6	BP
GO:0030501	positive regulation of bone	6/2734	35/17381	0.47941	0.93	0.91467	BMP4/FZD9/ISG15/OSR1/PKDCC/TMEM119	6	BP
GO:0034122	negative regulation of toll-like receptor	6/2734	35/17381	0.47941	0.93	0.91467	ARRB2/CD300LF/DAB2IP/F2RL1/NFKBIL1/TLR9	6	BP
GO:0045494	photoreceptor cell	6/2734	35/17381	0.47941	0.93	0.91467	CDH23/CDHR1/CIB2/CNGB1/CROCC/TULP1	6	BP
GO:0051180	vitamin transport	6/2734	35/17381	0.47941	0.93	0.91467	CUBN/FOLR2/SCARB1/SLC52A1/SLC52A2/STRA6	6	BP
GO:0090207	regulation of triglyceride metabolic	6/2734	35/17381	0.47941	0.93	0.91467	APOA5/APOC3/LMF1/NR1H3/PNPLA2/SCARB1	6	BP
GO:0000723	telomere maintenance	25/2734	155/17381	0.47955	0.93	0.91467	ACD/APEX1/CCT3/EXO1/EXOSC10/HDAC8/HIST1H4F/HIST2H4A/HIST2H4B/HSP90AA1/MAPK15/MAPK3/NHP2/PARP3/PIF1/POLA2/POLD4/POLE/POLE4/RECQL4/RFC1/RFC2/SMG5/TINF2/XRCC3	25	BP
GO:0046942	carboxylic acid transport	49/2734	307/17381	0.47983	0.93	0.91467	ABCC3/ABCC4/ACACB/AGT/CPLX1/CPT2/CROT/DRD2/FOLR2/GOT2/LCN12/MFSD2A/MID1IP1/NCOR1/OSR1/PLA2G1B/PLA2G2F/PLA2G5/PPARD/PQLC2/PSAP/SLC11A1/SLC13A2/SLC16A13/SLC1A7/SLC22A13/SLC22A9/SLC25A20/SLC25A22/SLC26A1/SLC26A10/SLC26A6/SLC36A1/SLC36A3/SLC38A1/SLC38A10/SLC38A8/SLC6A18/SLC6A6/SLC6A7/SLC6A8/SLC6A9/SLC7A7/SLCO2B1/STARD5/STX1A/STXBP1/SV2A/TRH	49	BP
GO:0010951	negative regulation of endopeptidase activity	40/2734	250/17381	0.48001	0.93	0.91467	A2ML1/AGT/AHSG/APLP2/AQP1/ARRB2/CD27/COL7A1/CRB2/CRYAB/DPEP1/FURIN/GAPDH/GPX1/HERPUD1/ITIH4/ITIH6/KNG1/LAMP3/MIR195/MIR29C/NAIP/NLE1/PEBP1/POR/RAF1/RPS6KA1/SERPINA2/SERPINA3/SERPINA4/SERPINA5/SERPINF2/SERPINH1/SERPINI1/SFN/SPINK2/SPINK5/SPI	40	BP
GO:0000186	activation of MAPKK	9/2734	54/17381	0.48191	0.93	0.91467	ARAF/BMP4/EGFR/MAP3K14/MAP3K15/MAP3K6/RAF1/TAOK2/TNIK	9	BP

GO:0009620	response to fungus	9/2734	54/17381	0.48191	0.93	0.91467	C10orf99/CARD9/CHGA/COTL1/GAPDH/MPO/RARRES2/RNASE8/SPON2	9	BP
GO:0051893	regulation of focal adhesion	9/2734	54/17381	0.48191	0.93	0.91467	ABL1/BCAS3/COL16A1/KDR/LDB1/MYOC/RHOD/THY1/WDPCP	9	BP
GO:0061005	cell differentiation involved in	9/2734	54/17381	0.48191	0.93	0.91467	BMP4/LHX1/MYO1E/NOTCH1/OSR1/POU3F3/SHH/SMO/STAT1	9	BP
GO:0090109	regulation of cell-substrate junction	9/2734	54/17381	0.48191	0.93	0.91467	ABL1/BCAS3/COL16A1/KDR/LDB1/MYOC/RHOD/THY1/WDPCP	9	BP
GO:0098900	regulation of action potential	9/2734	54/17381	0.48191	0.93	0.91467	ADRA1A/CACNB3/CAMK2D/CAV3/FLNA/GBA/GPR35/MIR328/TACR1	9	BP
GO:0031331	positive regulation of cellular catabolic process	63/2734	396/17381	0.48224	0.93	0.91467	ADAM8/ADRA2A/AMBRA1/ANAPC15/ANAPC2/APOA5/ATPIF1/AXIN1/BAD/BCAP31/BUB1B/CAV3/CD81/CHFR/CLU/DAB2IP/DAPK1/DISC1/ECSCR/EIF2AK4/ENDOG/FURIN/GBA/GPSM1/HERPUD1/HSF1/INS/KDR/KLHL40/LRP1/LRSAM1/MAPK3/MFN2/MIR181B1/MLXIPL/NKD2/NOD1/OSBPL7/PACSIN3/PFKFB1/PNPLA2/POLR2G/PRKCD/PSMB11/PSMB6/PSMB7/PSMB8/PSMC3/PSMD13/PSMD3/PSMD5/PSMD7/PTK2B/RALB/RNF166/RNF180/S[illegible]	63	BP
GO:0000290	deadenylation-dependent decapping of nuclear-transcribed mRNA	2/2734	10/17381	0.48231	0.93	0.91467	LSM1/PATL1	2	BP
GO:0000338	protein deneddylation	2/2734	10/17381	0.48231	0.93	0.91467	COPS6/TOR1A	2	BP
GO:0000730	DNA recombinase	2/2734	10/17381	0.48231	0.93	0.91467	MCMDC2/XRCC3	2	BP
GO:0001768	establishment of T cell polarity	2/2734	10/17381	0.48231	0.93	0.91467	CCL19/CCL21	2	BP

GO:0001840	neural plate development	2/2734	10/17381	0.48231	0.93	0.91467	C2CD3/DVL2	2	BP
GO:0001886	endothelial cell morphogenesis	2/2734	10/17381	0.48231	0.93	0.91467	PLOD3/TNMD	2	BP
GO:0002887	negative regulation of myeloid leukocyte	2/2734	10/17381	0.48231	0.93	0.91467	CCR2/LGALS9	2	BP
GO:0003211	cardiac ventricle	2/2734	10/17381	0.48231	0.93	0.91467	NKX2-5/NOTCH1	2	BP
GO:0006222	UMP biosynthetic	2/2734	10/17381	0.48231	0.93	0.91467	DHODH/UCK1	2	BP
GO:0006228	UTP biosynthetic	2/2734	10/17381	0.48231	0.93	0.91467	NME1/NME4	2	BP
GO:0006569	tryptophan catabolic	2/2734	10/17381	0.48231	0.93	0.91467	HAAO/IDO1	2	BP
GO:0006670	sphingosine metabolic	2/2734	10/17381	0.48231	0.93	0.91467	GBA/SPTLC1	2	BP
GO:0007028	cytoplasm organization	2/2734	10/17381	0.48231	0.93	0.91467	FOSL1/PADI6	2	BP
GO:0009173	pyrimidine ribonucleoside monophosphate metabolic	2/2734	10/17381	0.48231	0.93	0.91467	DHODH/UCK1	2	BP
GO:0009174	pyrimidine ribonucleoside monophosphate biosynthetic process	2/2734	10/17381	0.48231	0.93	0.91467	DHODH/UCK1	2	BP

GO:0009396	folic acid-containing compound biosynthetic process	2/2734	10/17381	0.48231	0.93	0.91467	GCH1/MTHFD1	2	BP
GO:0010452	histone H3-K36 methylation	2/2734	10/17381	0.48231	0.93	0.91467	PAXIP1/SETD2	2	BP
GO:0010820	positive regulation of T cell chemotaxis	2/2734	10/17381	0.48231	0.93	0.91467	CCL5/CCR2	2	BP
GO:0010988	regulation of low-density lipoprotein	2/2734	10/17381	0.48231	0.93	0.91467	APOC3/CSK	2	BP
GO:0015802	basic amino acid transport	2/2734	10/17381	0.48231	0.93	0.91467	PQLC2/SLC7A7	2	BP
GO:0015937	coenzyme A biosynthetic process	2/2734	10/17381	0.48231	0.93	0.91467	ACOT7/PPCDC	2	BP
GO:0016559	peroxisome fission	2/2734	10/17381	0.48231	0.93	0.91467	PEX11A/SEC16B	2	BP
GO:0018095	protein polyglutamylati	2/2734	10/17381	0.48231	0.93	0.91467	TTLL5/TTLL7	2	BP
GO:0021877	forebrain neuron fate	2/2734	10/17381	0.48231	0.93	0.91467	AXIN1/TBR1	2	BP
GO:0030432	peristalsis	2/2734	10/17381	0.48231	0.93	0.91467	DRD2/P2RX2	2	BP
GO:0032025	response to cobalt ion	2/2734	10/17381	0.48231	0.93	0.91467	ALAD/CASP3	2	BP
GO:0032328	alanine transport	2/2734	10/17381	0.48231	0.93	0.91467	SLC36A1/SLC36A3	2	BP

GO:0032352	positive regulation of hormone	2/2734	10/17381	0.48231	0.93	0.91467	EGR1/POR	2	BP
GO:0032525	somite rostral/caudal axis	2/2734	10/17381	0.48231	0.93	0.91467	LHX1/RIPPLY1	2	BP
GO:0033089	positive regulation of T cell differentiation	2/2734	10/17381	0.48231	0.93	0.91467	ADAM8/SHH	2	BP
GO:0034058	endosomal vesicle fusion	2/2734	10/17381	0.48231	0.93	0.91467	RBSN/SAMD9L	2	BP
GO:0034115	negative regulation of heterotypic cell-	2/2734	10/17381	0.48231	0.93	0.91467	IL1RN/MIR221	2	BP
GO:0034214	protein hexamerization	2/2734	10/17381	0.48231	0.93	0.91467	OAT/YME1L1	2	BP
GO:0034378	chylomicron assembly	2/2734	10/17381	0.48231	0.93	0.91467	APOB/APOC3	2	BP
GO:0034427	nuclear-transcribed mRNA catabolic process, exonucleolytic,	2/2734	10/17381	0.48231	0.93	0.91467	DIS3L2/EXOSC2	2	BP
GO:0035457	cellular response to	2/2734	10/17381	0.48231	0.93	0.91467	IFIT2/IFIT3	2	BP
GO:0035871	protein K11-linked deubiquitination	2/2734	10/17381	0.48231	0.93	0.91467	OTUD6A/OTUD7A	2	BP

GO:0035933	glucocorticoid secretion	2/2734	10/17381	0.48231	0.93	0.91467	CRHR1/PTPN11	2	BP
GO:0036500	ATF6-mediated unfolded protein response	2/2734	10/17381	0.48231	0.93	0.91467	HSPA5/WFS1	2	BP
GO:0042428	serotonin metabolic	2/2734	10/17381	0.48231	0.93	0.91467	DDC/RNF180	2	BP
GO:0042436	indole-containing compound	2/2734	10/17381	0.48231	0.93	0.91467	HAAO/IDO1	2	BP
GO:0043569	negative regulation of insulin-like growth factor receptor	2/2734	10/17381	0.48231	0.93	0.91467	MIR29C/TRIM72	2	BP
GO:0045003	double-strand break repair via synthesis-dependent strand annealing	2/2734	10/17381	0.48231	0.93	0.91467	MCMDC2/XRCC3	2	BP
GO:0045713	low-density lipoprotein particle receptor biosynthetic process	2/2734	10/17381	0.48231	0.93	0.91467	FURIN/SCAP	2	BP
GO:0045792	negative regulation of	2/2734	10/17381	0.48231	0.93	0.91467	CAV3/MTOR	2	BP
GO:0046049	UMP metabolic process	2/2734	10/17381	0.48231	0.93	0.91467	DHODH/UCK1	2	BP
GO:0046218	indolalkylamine catabolic	2/2734	10/17381	0.48231	0.93	0.91467	HAAO/IDO1	2	BP

GO:0048340	paraxial mesoderm	2/2734	10/17381	0.48231	0.93	0.91467	WNT11/WNT3A	2	BP
GO:0048703	embryonic viscerocranium morphogenesis	2/2734	10/17381	0.48231	0.93	0.91467	LHX1/MTHFD1	2	BP
GO:0048845	venous blood vessel morphogenesis	2/2734	10/17381	0.48231	0.93	0.91467	ENG/NOTCH1	2	BP
GO:0048875	chemical homeostasis within a tissue	2/2734	10/17381	0.48231	0.93	0.91467	CTSH/HOMER2	2	BP
GO:0050966	detection of mechanical stimulus involved in sensory	2/2734	10/17381	0.48231	0.93	0.91467	ASIC3/PHF24	2	BP
GO:0051901	positive regulation of mitochondrial	2/2734	10/17381	0.48231	0.93	0.91467	KDR/MYOC	2	BP
GO:0051956	negative regulation of amino acid	2/2734	10/17381	0.48231	0.93	0.91467	OSR1/TRH	2	BP
GO:0060159	regulation of dopamine receptor	2/2734	10/17381	0.48231	0.93	0.91467	DRD2/DTNBP1	2	BP
GO:0060174	limb bud formation	2/2734	10/17381	0.48231	0.93	0.91467	FGFR2/SHH	2	BP
GO:0060352	cell adhesion molecule production	2/2734	10/17381	0.48231	0.93	0.91467	MIR221/MIR222	2	BP

GO:0060394	negative regulation of pathway-restricted SMAD protein	2/2734	10/17381	0.48231	0.93	0.91467	PBLD/SMAD6	2	BP
GO:0060502	epithelial cell proliferation involved in lung morphogenesis	2/2734	10/17381	0.48231	0.93	0.91467	BMP4/FGFR2	2	BP
GO:0060513	prostatic bud formation	2/2734	10/17381	0.48231	0.93	0.91467	BMP4/SHH	2	BP
GO:0060546	negative regulation of necroptotic	2/2734	10/17381	0.48231	0.93	0.91467	BOK/FZD9	2	BP
GO:0060600	dichotomous subdivision of an epithelial terminal unit	2/2734	10/17381	0.48231	0.93	0.91467	CTSH/PLXND1	2	BP
GO:0060768	regulation of epithelial cell proliferation involved in prostate gland development	2/2734	10/17381	0.48231	0.93	0.91467	NOTCH1/SHH	2	BP
GO:0060923	cardiac muscle cell fate	2/2734	10/17381	0.48231	0.93	0.91467	NKX2-5/WNT3A	2	BP
GO:0070131	positive regulation of mitochondrial	2/2734	10/17381	0.48231	0.93	0.91467	COA3/RMND1	2	BP
GO:0070170	regulation of tooth	2/2734	10/17381	0.48231	0.93	0.91467	AMTN/WNT6	2	BP

GO:0070189	kynurenine metabolic	2/2734	10/17381	0.48231	0.93	0.91467	GOT2/IDO1	2	BP
GO:0070475	rRNA base methylation	2/2734	10/17381	0.48231	0.93	0.91467	NSUN5/NSUN5P2	2	BP
GO:0070587	regulation of cell-cell adhesion involved in	2/2734	10/17381	0.48231	0.93	0.91467	IL1RN/MIR221	2	BP
GO:0070970	interleukin-2 secretion	2/2734	10/17381	0.48231	0.93	0.91467	ABL1/CARD11	2	BP
GO:0072173	metanephric tubule	2/2734	10/17381	0.48231	0.93	0.91467	PKD1/SOX8	2	BP
GO:0072537	fibroblast activation	2/2734	10/17381	0.48231	0.93	0.91467	PDGFRB/RPS6KA1	2	BP
GO:0090735	DNA repair complex	2/2734	10/17381	0.48231	0.93	0.91467	MCMDC2/XRCC3	2	BP
GO:0097104	postsynaptic membrane assembly	2/2734	10/17381	0.48231	0.93	0.91467	NLGN3/NRXN2	2	BP
GO:0097499	protein localization to non-motile	2/2734	10/17381	0.48231	0.93	0.91467	TULP1/TULP3	2	BP
GO:0098598	learned vocalization behavior or vocal learning	2/2734	10/17381	0.48231	0.93	0.91467	NRXN2/STRA6	2	BP
GO:0098722	asymmetric stem cell	2/2734	10/17381	0.48231	0.93	0.91467	DOCK7/SOX5	2	BP

GO:0098969	neurotransmitter receptor transport to postsynaptic	2/2734	10/17381	0.48231	0.93	0.91467	GRIPAP1/SCRIB	2	BP
GO:0101023	vascular endothelial cell proliferation	2/2734	10/17381	0.48231	0.93	0.91467	MIR29A/MIR29C	2	BP
GO:1901748	leukotriene D4 metabolic	2/2734	10/17381	0.48231	0.93	0.91467	GGT6/GGTA1P	2	BP
GO:1901750	leukotriene D4 biosynthetic process	2/2734	10/17381	0.48231	0.93	0.91467	GGT6/GGTA1P	2	BP
GO:1901978	positive regulation of cell cycle	2/2734	10/17381	0.48231	0.93	0.91467	CCAR2/XRCC3	2	BP
GO:1902004	positive regulation of amyloid-beta	2/2734	10/17381	0.48231	0.93	0.91467	CLU/EFNA1	2	BP
GO:1902237	positive regulation of endoplasmic reticulum stress-induced intrinsic	2/2734	10/17381	0.48231	0.93	0.91467	BOK/SPOP	2	BP
GO:1902414	protein localization to	2/2734	10/17381	0.48231	0.93	0.91467	MARVELD3/SCRIB	2	BP
GO:1902668	negative regulation of	2/2734	10/17381	0.48231	0.93	0.91467	SEMA3F/WNT3A	2	BP

GO:1903025	regulation of RNA polymerase II regulatory region	2/2734	10/17381	0.48231	0.93	0.91467	TAF1/ZNF593	2	BP
GO:1903539	protein localization to postsynaptic	2/2734	10/17381	0.48231	0.93	0.91467	GRIPAP1/SCRIB	2	BP
GO:1903540	establishment of protein localization to postsynaptic	2/2734	10/17381	0.48231	0.93	0.91467	GRIPAP1/SCRIB	2	BP
GO:1903729	regulation of plasma membrane	2/2734	10/17381	0.48231	0.93	0.91467	PLEKHM2/PRKCD	2	BP
GO:1903897	regulation of PERK-mediated unfolded protein response	2/2734	10/17381	0.48231	0.93	0.91467	BOK/HSPA5	2	BP
GO:1903960	negative regulation of anion transmembrane	2/2734	10/17381	0.48231	0.93	0.91467	MTOR/OSR1	2	BP
GO:1904338	regulation of dopaminergic neuron differentiation	2/2734	10/17381	0.48231	0.93	0.91467	SHH/WNT3A	2	BP
GO:1904478	regulation of intestinal absorption	2/2734	10/17381	0.48231	0.93	0.91467	ABCG5/APOA5	2	BP

GO:1904861	excitatory synapse	2/2734	10/17381	0.48231	0.93	0.91467	NRXN2/WNT7A	2	BP
GO:1904896	ESCRT complex	2/2734	10/17381	0.48231	0.93	0.91467	CHMP1A/CHMP7	2	BP
GO:1904903	ESCRT III complex disassembly	2/2734	10/17381	0.48231	0.93	0.91467	CHMP1A/CHMP7	2	BP
GO:1905031	regulation of membrane repolarization during cardiac muscle cell action potential	2/2734	10/17381	0.48231	0.93	0.91467	FLNA/MIR328	2	BP
GO:1905562	regulation of vascular endothelial cell proliferation	2/2734	10/17381	0.48231	0.93	0.91467	MIR29A/MIR29C	2	BP
GO:2000480	negative regulation of cAMP-dependent	2/2734	10/17381	0.48231	0.93	0.91467	PRKAR1A/PRKAR1B	2	BP
GO:2000508	regulation of dendritic cell chemotaxis	2/2734	10/17381	0.48231	0.93	0.91467	CCL21/LGALS9	2	BP
GO:2000650	negative regulation of sodium ion transmembrane	2/2734	10/17381	0.48231	0.93	0.91467	CAMK2D/OSR1	2	BP
GO:2001198	regulation of dendritic cell differentiation	2/2734	10/17381	0.48231	0.93	0.91467	LGALS9/TMEM176B	2	BP

GO:0035019	somatic stem cell population maintenance	12/2734	73/17381	0.48307	0.9311	0.91577	ASCL2/BCL9/CDX2/LDB1/POLR2G/POLR2L/PRDM14/RAF1/SPI1/TDGF1/WNT7A/ZHX2	12	BP
GO:0010466	negative regulation of peptidase activity	42/2734	263/17381	0.48356	0.9311	0.91577	A2ML1/AGT/AHSG/APLP2/AQP1/ARRB2/CD27/COL7A1/CRB2/CRYAB/DPEP1/ECM1/FURIN/GAPDH/GPX1/HERPUD1/ITIH4/ITIH6/KNG1/LAMP3/MIR195/MIR29C/NAIP/NLE1/PEBP1/PI16/POR/RAF1/RPS6KA1/SERPINA2/SERPINA3/SERPINA4/SERPINA5/SERPINF2/SERPINH1/SERPINI1/SFN/SPINK2/SPINK5/SPINT1/SPOCK2/WFIKKN2	42	BP
GO:0007041	lysosomal transport	15/2734	92/17381	0.48367	0.9311	0.91577	AKTIP/CDX2/CLU/DENND3/FAM160A2/GAK/KIF13A/LARS/PCDHGA3/RAB7A/RBSN/STX8/VPS51/VPS52/VPS53	15	BP
GO:0046632	alpha-beta T cell	15/2734	92/17381	0.48367	0.9311	0.91577	ABL1/BCL6/CCL19/FUT7/HLX/IL18/IL4R/LGALS9/LY9/NKX2-3/PSMB11/RARA/RORC/SEMA4A/SHH	15	BP
GO:0090317	negative regulation of intracellular	15/2734	92/17381	0.48367	0.9311	0.91577	CD27/CDK5/DAB2IP/EMD/FAM89B/MTOR/NFKBIL1/OS9/PARP10/PBLD/PDE2A/PKD1/SFRP5/SUFU/THRA	15	BP
GO:0046834	lipid phosphorylation	18/2734	111/17381	0.48399	0.9311	0.91583	DGKI/DGKQ/DGKZ/EGFR/ERBB2/FGF17/FGF3/FGFR2/FGFR4/LCK/NRG1/PDGFA/PDGFRB/PI4KB/PIK3R5/PIK3R6/PTPN11/TMEM150A	18	BP
GO:0030968	endoplasmic reticulum unfolded	21/2734	130/17381	0.48414	0.9311	0.91583	ACADVL/ASNS/ATF3/BOK/CDK5RAP3/CREB3/DAB2IP/EXTL1/GOSR2/HERPUD1/HSPA5/LMNA/PLA2G4B/PPP2R5B/SEC61A2/SHC1/SYVN1/TLN1/TSPYL2/WFS1/ZBTB17	21	BP
GO:0035265	organ growth	27/2734	168/17381	0.48417	0.9311	0.91583	ACACB/ADRA1A/AGT/CAV3/DDR2/FGFR2/GATA4/HLX/LEPR/MIR195/MIR222/MIR25/MTOR/NKX2-5/NOTCH1/NPPA/PDGFRB/PDLIM5/POR/PRKAR1A/PSAP/PTPN11/RARA/SHH/SMO/TBX2/THBS3	27	BP
GO:0045088	regulation of innate immune response	67/2734	422/17381	0.48764	0.932	0.91666	ADAM8/APOB/ARRB2/CARD11/CARD9/CCL5/CD180/CD300LF/CLEC7A/CNPY3/CTSL/DAB2IP/DHX58/DRD2/F2RL1/FGA/HAVCR2/HLA-E/HRAS/IFNA5/IKBKB/INS/IRAK1/ITGAM/LGALS9/MAPKAPK3/MMP2/MUC1/MUC2/MUC3A/MUC5AC/MUC5B/MUC6/NCR3/NFKBIL1/NLRX1/NMI/NOD1/NONO/NR1H3/OTUD7A/PARP9/PIK3R6/POLR3D/PRKCD/PSMB11/PSMB6/PSMB7/PSMB8/PSMC3/PSMD13/PSMD3/PSMD5/PSMD7/PSPC1/PTPN11/RAF1/SCRIB/SFTPA1/STAT1/STAT2/TBK1/TIRAP/TLR8/TLR9/TNIP2/T[illegible]	67	BP

GO:1902806	regulation of cell cycle G1/S phase transition	29/2734	181/17381	0.48843	0.932	0.91666	APEX1/C10orf99/CDK10/CDK2AP2/CTDSP1/CYP1A1/E2F4/E2F8/EGFR/EIF4G1/INO80/MEN1/MEPCE/MIR10A/MIR15A/MIR16-1/MIR221/MIR222/MIR29A/MIR29C/MIR503/MUC1/PCBP4/PID1/PKD1/SFN/TAF1/UBE2E2/ZNF385A	29	BP
GO:0003203	endocardial cushion morphogenesis	5/2734	29/17381	0.48864	0.932	0.91666	ENG/HEY1/NOTCH1/TBX2/TMEM100	5	BP
GO:0006471	protein ADP-ribosylation	5/2734	29/17381	0.48864	0.932	0.91666	PARP10/PARP2/PARP3/PARP9/TINF2	5	BP
GO:0010719	negative regulation of epithelial to mesenchymal	5/2734	29/17381	0.48864	0.932	0.91666	DAB2IP/EFNA1/PBLD/TRIM62/VASN	5	BP
GO:0010743	regulation of macrophage derived foam cell	5/2734	29/17381	0.48864	0.932	0.91666	AGT/APOB/CSF1/IL18/NR1H3	5	BP
GO:0018146	keratan sulfate biosynthetic process	5/2734	29/17381	0.48864	0.932	0.91666	ACAN/B4GALT2/CHST5/SLC35D2/ST3GAL4	5	BP
GO:0030970	retrograde protein transport, ER to	5/2734	29/17381	0.48864	0.932	0.91666	BCAP31/BRSK2/HERPUD1/OS9/SYVN1	5	BP
GO:0034698	response to gonadotropin	5/2734	29/17381	0.48864	0.932	0.91666	ASNS/EPHA8/NOTCH1/NSMF/POR	5	BP
GO:0051955	regulation of amino acid	5/2734	29/17381	0.48864	0.932	0.91666	AGT/OSR1/STXBP1/SV2A/TRH	5	BP
GO:0090200	positive regulation of release of cytochrome c	5/2734	29/17381	0.48864	0.932	0.91666	APOPT1/BAD/GPER1/HRK/MOAP1	5	BP

GO:1900739	regulation of protein insertion into mitochondrial membrane involved in apoptotic	5/2734	29/17381	0.48864	0.932	0.91666	BAD/DYNLL2/PPP1R13B/SFN/YWHAQ	5	BP
GO:1900740	positive regulation of protein insertion into mitochondrial membrane involved in	5/2734	29/17381	0.48864	0.932	0.91666	BAD/DYNLL2/PPP1R13B/SFN/YWHAQ	5	BP
GO:1902230	negative regulation of intrinsic apoptotic signaling pathway in	5/2734	29/17381	0.48864	0.932	0.91666	CCAR2/CD74/CLU/MUC1/ZNF385A	5	BP
GO:1903513	endoplasmic reticulum to cytosol	5/2734	29/17381	0.48864	0.932	0.91666	BCAP31/BRSK2/HERPUD1/OS9/SYVN1	5	BP
GO:2000403	positive regulation of lymphocyte	5/2734	29/17381	0.48864	0.932	0.91666	ADAM8/CCL21/CCL5/CCR2/PTK2B	5	BP
GO:2001251	negative regulation of chromosome	20/2734	124/17381	0.48929	0.932	0.91666	ACD/ANAPC15/BRCA1/BUB1B/CTBP1/EXOSC10/HDAC8/HMGA1/LCMT1/MAD1L1/NOC2L/OTUB1/PHF2/PIF1/SPI1/SUPT6H/TAF7/TINF2/UBE2B/XRCC3	20	BP

GO:0006213	pyrimidine nucleoside metabolic	8/2734	48/17381	0.48963	0.932	0.91666	CDA/DHODH/ERH/NME1/NME4/NT5M/PUDP/UCK1	8	BP
GO:0030850	prostate gland development	8/2734	48/17381	0.48963	0.932	0.91666	BMP4/CRIP1/FGFR2/NOTCH1/PSAP/RARA/SHH/TNC	8	BP
GO:0048260	positive regulation of receptor-mediated	8/2734	48/17381	0.48963	0.932	0.91666	APOA5/ARRB2/CCL19/CCL21/CD63/DRD2/TOR1A/WNT3A	8	BP
GO:0018209	peptidyl-serine modification	49/2734	308/17381	0.48976	0.932	0.91666	ARAF/ARRB2/AXIN1/BGN/BRSK2/CAMK1D/CAMK1G/CAMK2D/CAMKK1/CAMKV/CDK5/CSF3/DAPK2/DCLK2/DGKQ/DOCK7/EGFR/EIF4G1/GALNT16/GCG/IFNA5/IKBKB/ILK/INPP5F/MAPK3/MAPKAPK3/MLXIPL/MTOR/NTMT1/OPRD1/PARP2/PHKG1/PKD1/PNCK/PPP1R1B/PRKCD/RAF1/RPS6KA4/SGK1/SMYD3/SPOCK2/STK32B/STK32C/STK33/TAF1/TBK1/TDGF1/U	49	BP
GO:0002312	B cell activation involved in immune	11/2734	67/17381	0.48982	0.932	0.91666	ABL1/BCL6/CD180/CLCF1/EXO1/LFNG/NKX2-3/PAXIP1/PTK2B/SUPT6H/TNFSF13	11	BP
GO:0007004	telomere maintenance via telomerase	11/2734	67/17381	0.48982	0.932	0.91666	ACD/CCT3/EXOSC10/HSP90AA1/MAPK15/MAPK3/NHP2/PIF1/RFC1/SMG5/TINF2	11	BP
GO:0060998	regulation of dendritic spine development	11/2734	67/17381	0.48982	0.932	0.91666	ARC/CDK5/CFL1/DISC1/EFNA1/FOXO6/LLPH/MTOR/NLGN3/NRG1/PDLIM5	11	BP
GO:0010817	regulation of hormone levels	77/2734	486/17381	0.49188	0.932	0.91666	ADM/ADRA2A/ADRA2B/ADRA2C/AGT/ALDH8A1/ANO1/AQP1/ARL2BP/ATP1A1/BAD/BLK/BRSK2/C1QTNF1/CACNA1H/CAPN10/CCL5/CDK16/CPLX1/CRHR1/CRYM/CYP11A1/CYP11B2/CYP1A1/DGAT1/DGKQ/DOC2B/DRD2/DUOXA1/EGFR/EGR1/FGA/FURIN/GCG/GCK/GLUD1/GPER1/HCAR2/HSD17B1/HSD17B3/HSD17B7/HSD3B1/IL1RN/INHBB/INS/ITPR3/MAFA/MTNR1B/NR5A1/PFKM/PLB1/POR/PPARD/PTPN11/PTPRN2/RAB11FIP3/RAF1/RBP1/REN/RFX6/SCARB1/SHH/SIDT2/SLC22A1/SLC22A9/SLC25A5/SOX8/SSTR5/STARD3/STX1A/STX4/TACR1/TACR2/TFR2/TRH/TRPV4/VGF	77	BP

GO:0007259	JAK-STAT cascade	28/2734	175/17381	0.4929	0.932	0.91666	AGT/ARL2BP/BGN/CCL5/CCR2/CHAD/CISH/CLCF1/CSF1R/DAB1/HSF1/IFNA5/IL13/IL18/IL31RA/INPP5F/LRRTM1/MAPK3/MIR221/NMI/NOTCH1/PARP9/PKD1/RTN4RL1/RTN4RL2/STAT1/STAT2/TNFRSF18	28	BP
GO:0097696	STAT cascade	28/2734	175/17381	0.4929	0.932	0.91666	AGT/ARL2BP/BGN/CCL5/CCR2/CHAD/CISH/CLCF1/CSF1R/DAB1/HSF1/IFNA5/IL13/IL18/IL31RA/INPP5F/LRRTM1/MAPK3/MIR221/NMI/NOTCH1/PARP9/PKD1/RTN4RL1/RTN4RL2/STAT1/STAT2/TNFRSF18	28	BP
GO:0006865	amino acid transport	22/2734	137/17381	0.49405	0.932	0.91666	AGT/CPLX1/OSR1/PQLC2/SLC11A1/SLC1A7/SLC25A22/SLC36A1/SLC36A3/SLC38A1/SLC38A10/SLC38A8/SLC6A18/SLC6A6/SLC6A7/SLC6A8/SLC6A9/SLC7A7/STX1A/STXBP1/SV2A/TRH	22	BP
GO:0034620	cellular response to unfolded	22/2734	137/17381	0.49405	0.932	0.91666	ACADVL/ASNS/ATF3/BOK/CDK5RAP3/CREB3/DAB2IP/EXTL1/GOSR2/HERPUD1/HSF1/HSPA5/LMNA/PLA2G4B/PPP2R5B/SEC61A2/SHC1/SYVN1/TLN1/TSPYL2/WFS1/ZBTB17	22	BP
GO:1905952	regulation of lipid localization	22/2734	137/17381	0.49405	0.932	0.91666	ABCA2/ABCG5/ACACB/AGT/APOA5/APOB/APOC3/C1QTNF1/CRHR1/EHD1/FITM1/HILPDA/LRP1/NCOR1/NR1H3/PNPLA2/PRELID1/PRKCD/PTPN11/REN/SCARB1/SHH	22	BP
GO:0030183	B cell differentiation	19/2734	118/17381	0.4947	0.932	0.91666	ABL1/ADGRG3/BAD/BCL6/BLNK/CARD11/CD27/CD79B/CLCF1/CMTM7/FZD9/GON4L/IFNA5/INPP5D/LFNG/NKX2-3/PPP2R3C/PTK2B/TLR9	19	BP
GO:0046718	viral entry into host cell	19/2734	118/17381	0.4947	0.932	0.91666	ANPEP/CD74/CD81/CLDN6/EGFR/IFITM2/ITGB5/LGALS9/MOG/SCARB1/SLC20A2/SLC52A1/SLC52A2/TNFRSF4/TRIM11/TRIM26/TRIM31/TRIM62/W	19	BP
GO:0099504	synaptic vesicle cycle	19/2734	118/17381	0.4947	0.932	0.91666	CDK5/CPLX1/DDC/DNM1/DOC2A/DOC2B/GAK/NLGN3/PLD2/SCRIB/STON2/STX1A/STX4/STXBP1/SYTL3/TH/TOR1A/WNT3A/WNT7A	19	BP
GO:0006637	acyl-CoA metabolic	16/2734	99/17381	0.49542	0.932	0.91666	ACACB/ACOT7/ACOT9/ACSF3/ACSM6/DGAT1/DLST/ELOVL1/ELOVL5/HACD1/LOC344967/MPC1/OGDH/PDHB/PIPOX/PMVK	16	BP
GO:0014896	muscle hypertrophy	16/2734	99/17381	0.49542	0.932	0.91666	AGT/CAMK2D/CAMTA2/CAV3/CDK9/GATA4/INPP5F/LMNA/MIR195/MIR25/MTOR/MYOC/NPPA/PDLIM5/TCAP/TRIM63	16	BP
GO:0035335	peptidyl-tyrosine	16/2734	99/17381	0.49542	0.932	0.91666	CDC14A/DUSP2/DUSP21/DUSP26/DUSP5/EYA1/MTMR1/MTMR14/PTP4A3/PTPN11/PTPN23/PTPN7/PTPRF/PTPRN2/PTPRQ/PTPRU	16	BP
GO:0035383	thioester metabolic	16/2734	99/17381	0.49542	0.932	0.91666	ACACB/ACOT7/ACOT9/ACSF3/ACSM6/DGAT1/DLST/ELOVL1/ELOVL5/HACD1/LOC344967/MPC1/OGDH/PDHB/PIPOX/PMVK	16	BP

GO:1903039	positive regulation of leukocyte cell-cell adhesion	36/2734	226/17381	0.49543	0.932	0.91666	ADAM8/BAD/BCL6/CARD11/CCDC88B/CCL19/CCL21/CCL5/CCR2/CD247/CD27/CD5/CD6/CD74/CLECL1/CORO1A/CSK/DNAJA3/EFNB1/HAVCR2/HLA-E/HLX/IL18/IL4R/LCK/LGALS9/MAP3K14/MIR92A2/PDCD1/PIK3R6/PTPN1	36	BP
GO:0015849	organic acid transport	50/2734	315/17381	0.49617	0.932	0.91666	ABCC3/ABCC4/ACACB/AGT/CPLX1/CPT2/CROT/DRD2/FOLR2/GOT2/LCN12/MFSD2A/MID1IP1/NCOR1/OSR1/PLA2G1B/PLA2G2F/PLA2G5/PPARD/PQLC2/PSAP/SLC11A1/SLC13A2/SLC16A13/SLC1A7/SLC22A12/SLC22A13/SLC22A9/SLC25A20/SLC25A22/SLC26A1/SLC26A10/SLC26A6/SLC36A1/SLC36A3/SLC38A1/SLC38A10/SLC38A8/SLC6A18/SLC6A6/SLC6A7/SLC6A8/SLC6A9/SLC7A7/SLCO2B1/STARD5/STX1A/STXBP1/SV2A/TRH	50	BP
GO:0010595	positive regulation of endothelial cell	13/2734	80/17381	0.49623	0.932	0.91666	ABL1/AGT/BCAS3/BMP4/CIB1/FLT4/HSPB1/KDR/MIR221/PTK2B/SCARB1/TDGF1/WNT7A	13	BP
GO:0032602	chemokine production	13/2734	80/17381	0.49623	0.932	0.91666	CD74/CSF1R/EGR1/F2RL1/HAVCR2/IL18/IL4R/LGALS9/MIR92A2/TIRAP/TLR9/TRPV4/TUSC2	13	BP
GO:0045833	negative regulation of lipid metabolic	13/2734	80/17381	0.49623	0.932	0.91666	ACACB/ACADVL/ADRA2A/APOC3/ATP1A1/BRCA1/DAB2IP/GPER1/HCAR2/INS/ORMDL3/PDGFA/SCAP	13	BP
GO:0050848	regulation of calcium-mediated	13/2734	80/17381	0.49623	0.932	0.91666	CAMK2D/CDH13/CIB1/GSTM2/HINT1/JPH3/NEUROD2/NRG1/P2RX2/PTK2B/TMEM100/TRDN/TREM2	13	BP
GO:0009145	purine nucleoside triphosphate biosynthetic	10/2734	61/17381	0.49719	0.932	0.91666	ALDOA/ATP5G1/ATP5I/CYC1/IMPDH1/NME1/NME4/PID1/PKM/VPS9D1	10	BP
GO:0045685	regulation of glial cell	10/2734	61/17381	0.49719	0.932	0.91666	BIN1/CLCF1/DAB1/MTOR/MYCN/NOTCH1/RNF112/SHH/SPINT1/WDR1	10	BP
GO:0007219	Notch signaling pathway	27/2734	169/17381	0.49752	0.932	0.91666	APH1A/ARRDC1/BCL6/CHAC1/DLK1/DLK2/DTX2/EGFL7/ERH/EYA1/HEY1/HOXD3/JAG2/LFNG/MESP2/MIB2/MIR212/NLE1/NOTCH1/PTP4A3/RFNG/RIPPLY1/SLC35C1/SPEN/TBX2/TMEM100/WNT1	27	BP

GO:0019362	pyridine nucleotide metabolic	27/2734	169/17381	0.49752	0.932	0.91666	ALDOA/GALK1/GAPDH/GCK/HAAO/HK3/IDH1/IDH2/INS/LDHA/MLXIPL/MPC1/NCOR1/NMNAT3/NUP210/NUP98/OGDH/OGDHL/PARP10/PARP9/PDHB/PFKFB1/PFKM/PGAM4/PKM/QPRT/SLC22A13	27	BP
GO:0046496	nicotinamide nucleotide metabolic	27/2734	169/17381	0.49752	0.932	0.91666	ALDOA/GALK1/GAPDH/GCK/HAAO/HK3/IDH1/IDH2/INS/LDHA/MLXIPL/MPC1/NCOR1/NMNAT3/NUP210/NUP98/OGDH/OGDHL/PARP10/PARP9/PDHB/PFKFB1/PFKM/PGAM4/PKM/QPRT/SLC22A13	27	BP
GO:0045429	positive regulation of nitric oxide biosynthetic	7/2734	42/17381	0.49835	0.932	0.91666	AGT/CLU/EGFR/HSP90AA1/INS/MTOR/PTK2B	7	BP
GO:0046473	phosphatidic acid metabolic	7/2734	42/17381	0.49835	0.932	0.91666	DGKQ/GNPAT/PLA2G1B/PLA2G2F/PLA2G4B/PLA2G5/PLD2	7	BP
GO:0050922	negative regulation of	7/2734	42/17381	0.49835	0.932	0.91666	CORO1B/MIR15A/MIR16-1/NBL1/NOTCH1/SEMA3F/WNT3A	7	BP
GO:1904407	positive regulation of nitric oxide	7/2734	42/17381	0.49835	0.932	0.91666	AGT/CLU/EGFR/HSP90AA1/INS/MTOR/PTK2B	7	BP
GO:0002696	positive regulation of leukocyte activation	55/2734	347/17381	0.49837	0.932	0.91666	ADAM8/BAD/BCL6/CARD11/CCDC88B/CCL19/CCL21/CCL5/CCR2/CD247/CD27/CD5/CD6/CD74/CD81/CLCF1/CLECL1/CORO1A/CSK/DNAJA3/EFNB1/F2RL1/FAM19A3/HAVCR2/HLA-E/HLX/IL13/IL18/IL4R/INPP5D/ITGAM/KARS/LCK/LGALS9/MAP3K14/PAXIP1/PDCD1/PIK3R6/PPP2R3C/PTPN11/RARA/SART1/SHH/SPACA3/STX4/STXBP1/TACR1/THY1/TIRAP/TLR9/TNFRSF4/TNFSF13/TNIP2/TRAF2/WNT	55	BP
GO:0030879	mammary gland development	21/2734	131/17381	0.4993	0.932	0.91666	BMP4/CAV3/CSF1/CSF1R/CSN3/DEAF1/DHODH/FGFR2/GPX1/HOXB9/NME1/NRG1/PYGO2/SCRIB/SLC29A1/SMO/TBX2/TDGF1/WNT3A/XDH/ZNF70	21	BP
GO:0000715	nucleotide-excision repair, DNA damage recognition	4/2734	23/17381	0.49983	0.932	0.91666	COPS6/DDB1/DDB2/XPC	4	BP
GO:0001919	regulation of receptor	4/2734	23/17381	0.49983	0.932	0.91666	ACHE/ARAP1/INPP5F/SCRIB	4	BP

GO:0002861	regulation of inflammatory response to antigenic	4/2734	23/17381	0.49983	0.932	0.91666	GPR17/GPX1/IL20RB/LTA	4	BP
GO:0007184	SMAD protein import into	4/2734	23/17381	0.49983	0.932	0.91666	BMP4/FAM89B/PBLD/RBPMS	4	BP
GO:0007263	nitric oxide mediated signal transduction	4/2734	23/17381	0.49983	0.932	0.91666	AGT/MAFA/NOS3/RASD1	4	BP
GO:0008053	mitochondrial fusion	4/2734	23/17381	0.49983	0.932	0.91666	MFN1/MFN2/PID1/VAT1	4	BP
GO:0009147	pyrimidine nucleoside triphosphate metabolic	4/2734	23/17381	0.49983	0.932	0.91666	NME1/NME4/TBPL1/UCK1	4	BP
GO:0009648	photoperiodism	4/2734	23/17381	0.49983	0.932	0.91666	MTA1/PER1/RBM4/USP2	4	BP
GO:0010226	response to lithium ion	4/2734	23/17381	0.49983	0.932	0.91666	ACTA1/EIF2B5/PTK2B/SHH	4	BP
GO:0016180	snRNA processing	4/2734	23/17381	0.49983	0.932	0.91666	EXOSC2/INTS10/INTS3/INTS5	4	BP
GO:0031639	plasminogen activation	4/2734	23/17381	0.49983	0.932	0.91666	FGA/KLKB1/PLAU/SERPINF2	4	BP
GO:0032878	regulation of establishment or maintenance of cell polarity	4/2734	23/17381	0.49983	0.932	0.91666	BCAS3/CDH5/PTK2B/WDPCP	4	BP
GO:0032925	regulation of activin receptor signaling	4/2734	23/17381	0.49983	0.932	0.91666	DACT2/MEN1/SHH/ZC3H3	4	BP

GO:0051647	nucleus localization	4/2734	23/17381	0.49983	0.932	0.91666	BIN1/CAV3/DOCK7/FHOD1	4	BP
GO:0070861	regulation of protein exit from endoplasmic	4/2734	23/17381	0.49983	0.932	0.91666	BCAP31/BRSK2/OS9/SEC16B	4	BP
GO:0095500	acetylcholine receptor signaling	4/2734	23/17381	0.49983	0.932	0.91666	AGRN/CHRM1/LYPD1/RGS10	4	BP
GO:0097205	renal filtration	4/2734	23/17381	0.49983	0.932	0.91666	AQP1/F2RL1/MCAM/MYO1E	4	BP
GO:1900101	regulation of endoplasmic reticulum unfolded	4/2734	23/17381	0.49983	0.932	0.91666	BOK/DAB2IP/HSPA5/WFS1	4	BP
GO:1903020	positive regulation of glycoprotein	4/2734	23/17381	0.49983	0.932	0.91666	CCL19/CCL21/GOLGA2/MIR181B1	4	BP
GO:1903831	signal transduction involved in cellular	4/2734	23/17381	0.49983	0.932	0.91666	AGRN/CHRM1/LYPD1/RGS10	4	BP
GO:1905144	response to acetylcholine	4/2734	23/17381	0.49983	0.932	0.91666	AGRN/CHRM1/LYPD1/RGS10	4	BP
GO:1905145	cellular response to	4/2734	23/17381	0.49983	0.932	0.91666	AGRN/CHRM1/LYPD1/RGS10	4	BP
GO:2000778	positive regulation of interleukin-6	4/2734	23/17381	0.49983	0.932	0.91666	F2RL1/LGALS9/TLR8/TRPV4	4	BP

GO:2001267	regulation of cysteine-type endopeptidase activity involved in apoptotic	4/2734	23/17381	0.49983	0.932	0.91666	FASLG/LGALS9/NLE1/TRAF2	4	BP
GO:0051271	negative regulation of cellular component movement	46/2734	290/17381	0.50037	0.932	0.91666	ADGRB1/APEX1/ARHGAP4/CCL21/CNIH2/CORO1B/DAB2IP/DPEP1/DRD2/ENG/EVL/GDF2/IDH2/IGFBP3/ILK/KRT16/LRP1/MARVELD3/MIIP/MIR10A/MIR15A/MIR16-1/MIR212/MIR221/MIR29A/MIR29C/MIR328/MIR503/MIR92A2/NBL1/NISCH/NOTCH1/NRG1/PBLD/PLXNB3/PPARD/PTPN23/PTPRU/SEMA3F/SHH/SL	46	BP
GO:0032680	regulation of tumor necrosis factor	18/2734	112/17381	0.50038	0.932	0.91666	ARRB2/CARD9/CCL19/CCR2/CLU/HAVCR2/HLA-E/HSF1/HSPB1/LGALS9/NFKBIL1/NOD1/ORM1/ORM2/RARA/SPON2/TIRAP/TLR9	18	BP
GO:0050684	regulation of mRNA	18/2734	112/17381	0.50038	0.932	0.91666	CDK9/CELF6/CPSF4/HMX2/HSF1/LMNTD2/MYOD1/NUP98/PRDX6/RBFOX1/RBFOX3/RBM4/RNPS1/SF3B4/SLC39A5/SRPK3/SRSF4/SUPT6H	18	BP
GO:0051209	release of sequestered calcium ion into cytosol	18/2734	112/17381	0.50038	0.932	0.91666	ABL1/CAMK2D/CCL19/CCL21/CORO1A/DRD2/FASLG/GPER1/GSTM2/IL13/ITPR3/JPH3/LCK/NPSR1/PTK2B/RASA3/THY1/TRDN	18	BP
GO:0051283	negative regulation of sequestering of	18/2734	112/17381	0.50038	0.932	0.91666	ABL1/CAMK2D/CCL19/CCL21/CORO1A/DRD2/FASLG/GPER1/GSTM2/IL13/ITPR3/JPH3/LCK/NPSR1/PTK2B/RASA3/THY1/TRDN	18	BP
GO:0007548	sex differentiation	43/2734	271/17381	0.50112	0.932	0.91666	ANG/ARRB2/BOK/CNTFR/CSDE1/DNAJC19/EIF2B2/EIF2B5/FANCA/GATA4/HSD17B3/IDH1/INHBB/LFNG/LHX1/LRRC6/MGST1/NOS3/NR5A1/NUDT1/NUP210L/NUPR1/OSR1/PDGFRB/PKD1/PTPN11/RARA/REN/RXFP2/SCX/SDC1/SF1/SHH/SOX8/STRA6/TSPY1/TSPY2/TSPY4/TSPY8/UTF1/VGF/WNT7	43	BP
GO:0052652	cyclic purine nucleotide metabolic	26/2734	163/17381	0.50232	0.932	0.91666	ADM/ADRA2A/AQP1/AVPR2/CCR2/CRHR1/DRD2/GABBR1/GCG/GPER1/GPHA2/GUCA1B/GUCA2A/GUCA2B/GUCY2D/NOS3/NPPA/OPRM1/PDE2A/PDZD3/PTK2B/RAF1/RUNDC3A/RXFP2/UCN2/WFS1	26	BP

GO:0071375	cellular response to peptide hormone stimulus	48/2734	303/17381	0.50322	0.932	0.91666	AGT/AHSG/APEX1/ATP6V0B/ATP6V0E2/ATP6V1B1/ATP6V1F/ATP6V1G1/ATP6V1G2/BAIAP2L1/CAPN10/CCND3/CDK4/CISH/CRHR1/CRHR2/CSK/EIF4EBP1/ESRRA/FOXO4/GCG/GCK/GNB3/GNG3/GPER1/HSF1/INHBB/INS/MAPK3/MAX/MEN1/MYO1C/MZB1/NR4A1/PID1/PKM/PLA2G1B/POR/PRKAR1A/PRKAR1B/PRKCD/PTPN11/RARRES2/SHC1/SLC2A4/SLC2A8/TRIM7	48	BP
GO:0042509	regulation of tyrosine phosphorylation of STAT	12/2734	74/17381	0.50324	0.932	0.91666	ARL2BP/CCL5/CLCF1/CSF1R/HSF1/IL13/IL18/IL31RA/INPP5F/MIR221/PARP9/TNFRSF18	12	BP
GO:0046209	nitric oxide metabolic	12/2734	74/17381	0.50324	0.932	0.91666	AGT/CLU/EGFR/GCH1/GCHFR/HSP90AA1/INS/MIR92A2/MTOR/NOS3/POR/PTK2B	12	BP
GO:0002831	regulation of response to biotic stimulus	20/2734	125/17381	0.5048	0.932	0.91666	CD180/DHX58/EIF2AK4/F2RL1/HAVCR2/LRSAM1/MAPK3/MICB/NCR3/NFKBIL1/NLRX1/NOD1/PARP9/PGC/PPP2R3C/SPINK5/STAT1/TARBP2/TBK1/TRAF3IP1	20	BP
GO:0032479	regulation of type I interferon production	20/2734	125/17381	0.5048	0.932	0.91666	DHX58/HAVCR2/IRAK1/IRF5/ISG15/LRRFIP1/NLRX1/NMI/OTUD5/POLR1D/POLR2L/POLR3D/SETD2/STAT1/TBK1/TIRAP/TLR8/TLR9/TRAF3IP1/TRAIP	20	BP
GO:0032869	cellular response to insulin stimulus	31/2734	195/17381	0.50481	0.932	0.91666	AGT/AHSG/ATP6V0B/ATP6V0E2/ATP6V1B1/ATP6V1F/ATP6V1G1/ATP6V1G2/BAIAP2L1/CAPN10/CCND3/CDK4/CISH/EIF4EBP1/ESRRA/FOXO4/GCK/INHBB/INS/MYO1C/MZB1/PID1/PKM/PLA2G1B/PRKCD/PTPN11/RARRES2/SHC1/SLC2A4/SLC2A8/TRIM72	31	BP
GO:0043523	regulation of neuron apoptotic	31/2734	195/17381	0.50481	0.932	0.91666	AARS/ADAM8/AMBRA1/ATF2/BARHL1/BOK/CASP3/CDK5/CLCF1/CNTFR/CORO1A/DRAXIN/EN1/FASLG/FZD9/GPX1/GRIN1/HRAS/ILK/LRP1/NAIP/NES/NONO/NQO2/NSMF/PTK2B/SIGMAR1/SNCB/STXBP1/WFS1/ZPR1	31	BP
GO:0007018	microtubule-based movement	42/2734	265/17381	0.50494	0.932	0.91666	AP2A2/AP2M1/CATSPER1/CCDC103/CCDC40/CNIH2/DNAH11/DNAH12/DNAH17/DNHD1/DTNBP1/DYNC1H1/DYNC1I1/DYNLL2/GAS8/HSPB1/HYDIN/IFT140/IFT20/IFT22/KIF13A/KIF17/KIF23/KIF26A/KIF2B/KIF4A/KIF9/KIFC3/KLC1/KLC2/LRRC6/PEX14/RHOT2/SPAG16/SSNA1/TEKT4/TEKT5/TMEM141/TRAF3IP1/TRIM46/UXT/WDR66	42	BP
GO:0010332	response to gamma	9/2734	55/17381	0.50532	0.932	0.91666	CRYAB/EGR1/GPX1/HRAS/HSF1/HSPA5/MEN1/NOX4/TMEM109	9	BP

GO:0090303	positive regulation of	9/2734	55/17381	0.50532	0.932	0.91666	ADRA2A/HRAS/MIR221/MIR451A/MTOR/MYOD1/PPARD/SELP/SERPINF2	9	BP
GO:1901862	negative regulation of muscle tissue	9/2734	55/17381	0.50532	0.932	0.91666	BMP4/CAV3/FGF3/LUC7L/MIR222/MIR25/SHH/TSC22D3/USP19	9	BP
GO:0043500	muscle adaptation	17/2734	106/17381	0.50638	0.932	0.91666	ACTA1/AGT/CAMK2D/CAMTA2/CAV3/CDK9/INPP5F/LMNA/MIR25/MTOR/MYOC/MYOD1/NOS3/NPPA/TCAP/TNNC1/TRIM63	17	BP
GO:0060401	cytosolic calcium ion transport	25/2734	157/17381	0.50729	0.932	0.91666	ABL1/ADRA1A/CACNB3/CAMK2D/CCL19/CCL21/CORO1A/DRD2/FASLG/GPER1/GRIN1/GRIN2C/GSTM2/IL13/ITPR3/JPH3/LCK/NPSR1/P2RX2/PLA2G1B/PTK2B/RASA3/THY1/TRDN/TRPV6	25	BP
GO:0055072	iron ion homeostasis	14/2734	87/17381	0.50832	0.932	0.91666	ACO1/ALAS2/ATP13A2/ATP6V1G1/GDF2/HEPH/HMOX2/NEDD8/NUBP1/SFXN2/SFXN4/SLC11A1/STEAP2/TFR2	14	BP
GO:0000462	maturation of SSU-rRNA from tricistronic rRNA transcript (SSU-rRNA, 5.8S rRNA, LSU-rRNA)	6/2734	36/17381	0.50841	0.932	0.91666	FCF1/MRPS11/PDCD11/RPS24/TSR1/TSR2	6	BP
GO:0002715	regulation of natural killer cell mediated	6/2734	36/17381	0.50841	0.932	0.91666	ARRB2/HAVCR2/HLA-E/LGALS9/NCR3/PIK3R6	6	BP
GO:0006298	mismatch repair	6/2734	36/17381	0.50841	0.932	0.91666	ABL1/EXO1/PMS2P3/POLD4/SETD2/XPC	6	BP
GO:0006458	'de novo' protein folding	6/2734	36/17381	0.50841	0.932	0.91666	CCT3/CD74/GAK/TOR1A/TOR1B/TOR2A	6	BP
GO:0031280	negative regulation of	6/2734	36/17381	0.50841	0.932	0.91666	ADRA2A/CCR2/DRD2/GABBR1/OPRM1/PDZD3	6	BP
GO:0031572	G2 DNA damage	6/2734	36/17381	0.50841	0.932	0.91666	BRCA1/BRCC3/CDK5RAP3/FOXO4/RINT1/TAOK2	6	BP

GO:0048536	spleen development	6/2734	36/17381	0.50841	0.932	0.91666	ABL1/HOXB4/NKX2-3/NKX2-5/NKX3-2/PPP2R3C	6	BP
GO:0050919	negative chemotaxis	6/2734	36/17381	0.50841	0.932	0.91666	NRG1/PDGFA/SEMA3F/SEMA4A/SEMA5B/SEMA7A	6	BP
GO:0051693	actin filament capping	6/2734	36/17381	0.50841	0.932	0.91666	CAPZA3/LMOD1/SCIN/SPTAN1/SPTB/SPTBN2	6	BP
GO:0086091	regulation of heart rate by cardiac	6/2734	36/17381	0.50841	0.932	0.91666	CACNA1G/CAMK2D/KCND3/KCNH6/KCNJ5/KCNQ1	6	BP
GO:0098815	modulation of excitatory postsynaptic potential	6/2734	36/17381	0.50841	0.932	0.91666	DLG4/GRIN1/NLGN3/PTK2B/STX1A/WNT7A	6	BP
GO:2000279	negative regulation of DNA biosynthetic	6/2734	36/17381	0.50841	0.932	0.91666	ACD/EXOSC10/GDF2/MEN1/PIF1/TINF2	6	BP
GO:2000241	regulation of reproductive process	22/2734	138/17381	0.5088	0.932	0.91666	BMP4/CACNA1H/CIB1/FGFR2/GLRA1/HORMAD1/INHBB/LFNG/NOTCH1/NR5A1/NUPR1/PHLDA2/PIWIL2/PLB1/POMZP3/PRDM9/PRKAR1A/PRSS37/SHH/SPINK2/TCP11/UBE2B	22	BP
GO:0030336	negative regulation of cell migration	41/2734	259/17381	0.50885	0.932	0.91666	ADGRB1/APEX1/ARHGAP4/CCL21/CORO1B/DAB2IP/DPEP1/DRD2/ENG/EVL/GDF2/IDH2/IGFBP3/ILK/KRT16/LRP1/MARVELD3/MIIP/MIR10A/MIR15A/MIR16-1/MIR212/MIR221/MIR29A/MIR29C/MIR503/MIR92A2/NBL1/NISCH/NOTC	41	BP
GO:0022612	gland morphogenesis	19/2734	119/17381	0.51058	0.932	0.91666	BMP4/CAV3/CRIP1/CSF1/CSF1R/EGFR/ESRP2/FGFR2/LIMS2/NKX2-3/NOTCH1/PDGFA/PLXND1/RPS6KA1/SCRIB/SHH/TBX2/TNC/WNT3A	19	BP
GO:0043620	regulation of DNA-templated transcription in response to	19/2734	119/17381	0.51058	0.932	0.91666	ANKRD2/ATF3/EGR1/HSF1/HSPA5/MUC1/NOTCH1/PSMB11/PSMB6/PSMB7/PSMB8/PSMC3/PSMD13/PSMD3/PSMD5/PSMD7/RGS14/RPS6KA1/TAF1	19	BP

GO:0071706	tumor necrosis factor superfamily cytokine	19/2734	119/17381	0.51058	0.932	0.91666	ADAM8/ARRB2/CARD9/CCL19/CCR2/CLU/HAVCR2/HLA-E/HSF1/HSPB1/LGALS9/NFKBIL1/NOD1/ORM1/ORM2/RARA/SPON2/TIRAP/TLR9	19	BP
GO:0033077	T cell differentiation in thymus	11/2734	68/17381	0.51084	0.932	0.91666	ADAM8/BMP4/CARD11/CD74/DNAJA3/ERBB2/FOXN1/FZD8/JAG2/SHH/WNT1	11	BP
GO:0090287	regulation of cellular response to growth factor	40/2734	253/17381	0.51285	0.932	0.91666	ABL1/AGT/BMP4/CAV3/CD63/CRB2/DAB2IP/ENG/FAM89B/FGFBP3/FGFR2/GATA4/GDF2/GPC1/HHIP/HSPA5/HTRA3/ILK/LEMD2/MEN1/MIR15A/MIR16-1/MYO1C/NBL1/NOTCH1/NUMA1/PBLD/PPP2R5B/PRDM14/PTP4A3/RBPM	40	BP
GO:0006732	coenzyme metabolic process	56/2734	355/17381	0.51358	0.932	0.91666	ACACB/ACOT7/ACOT9/ACSF3/ACSM6/ALDH1L1/ALDOA/BHMT2/COQ2/COQ4/CROT/DGAT1/DLST/ELOVL1/ELOVL5/FOLR2/GALK1/GAPDH/GCH1/GCK/GGH/GNMT/HAAO/HACD1/HK3/IDH1/IDH2/INS/LDHA/LIAS/LOC344967/MLXIPL/MPC1/MTHFD1/NCOR1/NMNAT3/NUP210/NUP98/OGDH/OGDHL/PARP10/PARP9/PDHB/PDSS1/PEMT/PFKFB1/PFKM/PGAM4/PIPOX/PKM/PMVK/PPCDC/QPRT/SHMT2/SLC22A13/VNN3	56	BP
GO:0000920	cell separation after cytokinesis	3/2734	17/17381	0.51431	0.932	0.91666	CHMP1A/CHMP4C/CHMP7	3	BP
GO:0001573	ganglioside metabolic	3/2734	17/17381	0.51431	0.932	0.91666	B4GALNT1/CLN6/NEU3	3	BP
GO:0001977	renal system process involved in regulation of	3/2734	17/17381	0.51431	0.932	0.91666	CYP11B2/F2RL1/HSD11B2	3	BP
GO:0002643	regulation of tolerance	3/2734	17/17381	0.51431	0.932	0.91666	HAVCR2/IDO1/PDCD1	3	BP
GO:0006450	regulation of translational	3/2734	17/17381	0.51431	0.932	0.91666	AARS/LARS/TARS2	3	BP
GO:0006577	amino-acid betaine	3/2734	17/17381	0.51431	0.932	0.91666	CHDH/CROT/POR	3	BP

GO:0006586	indolalkylamine metabolic	3/2734	17/17381	0.51431	0.932	0.91666	DDC/HAAO/IDO1	3	BP
GO:0006743	ubiquinone metabolic	3/2734	17/17381	0.51431	0.932	0.91666	COQ2/COQ4/PDSS1	3	BP
GO:0007567	parturition	3/2734	17/17381	0.51431	0.932	0.91666	CRHR1/CYP1A1/PLA2G4B	3	BP
GO:0007635	chemosensory behavior	3/2734	17/17381	0.51431	0.932	0.91666	GRIN1/OBP2B/WFS1	3	BP
GO:0009200	deoxyribonucleoside triphosphate	3/2734	17/17381	0.51431	0.932	0.91666	DGUOK/NUDT1/TBPL1	3	BP
GO:0010042	response to manganese ion	3/2734	17/17381	0.51431	0.932	0.91666	ATP13A2/HSPA5/TH	3	BP
GO:0010829	negative regulation of glucose	3/2734	17/17381	0.51431	0.932	0.91666	MZB1/PEA15/PID1	3	BP
GO:0021514	ventral spinal cord interneuron	3/2734	17/17381	0.51431	0.932	0.91666	EVX1/LHX3/SUFU	3	BP
GO:0030150	protein import into mitochondrial	3/2734	17/17381	0.51431	0.932	0.91666	DNLZ/TIMM17B/TIMM23B	3	BP
GO:0033005	positive regulation of mast cell	3/2734	17/17381	0.51431	0.932	0.91666	IL13/IL4R/STXBP1	3	BP
GO:0033630	positive regulation of cell adhesion mediated by	3/2734	17/17381	0.51431	0.932	0.91666	CCL21/CCL5/CIB1	3	BP
GO:0034312	diol biosynthetic	3/2734	17/17381	0.51431	0.932	0.91666	GBA/GCH1/SPTLC1	3	BP

GO:0035313	wound healing, spreading of epidermal cells	3/2734	17/17381	0.51431	0.932	0.91666	MIR221/MTOR/WNT7A	3	BP
GO:0035518	histone H2A monoubiquitina	3/2734	17/17381	0.51431	0.932	0.91666	DDB1/DDB2/PCGF2	3	BP
GO:0036158	outer dynein arm assembly	3/2734	17/17381	0.51431	0.932	0.91666	CCDC103/LRRC6/TMEM141	3	BP
GO:0040037	negative regulation of fibroblast growth factor receptor	3/2734	17/17381	0.51431	0.932	0.91666	GPC1/MIR16-1/PRDM14	3	BP
GO:0042095	interferon-gamma biosynthetic	3/2734	17/17381	0.51431	0.932	0.91666	IL18/TLR8/TLR9	3	BP
GO:0042249	establishment of planar polarity of embryonic epithelium	3/2734	17/17381	0.51431	0.932	0.91666	DVL2/FZD2/GRHL3	3	BP
GO:0043174	nucleoside salvage	3/2734	17/17381	0.51431	0.932	0.91666	CDA/PUDP/UCK1	3	BP
GO:0046337	phosphatidylethanolamine metabolic	3/2734	17/17381	0.51431	0.932	0.91666	ETNK2/PHOSPHO1/PLA2G15	3	BP
GO:0048490	anterograde synaptic vesicle transport	3/2734	17/17381	0.51431	0.932	0.91666	CNIH2/DTNBP1/TRIM46	3	BP
GO:0048535	lymph node development	3/2734	17/17381	0.51431	0.932	0.91666	CXCR5/LTA/NKX2-3	3	BP

GO:0055012	ventricular cardiac muscle cell	3/2734	17/17381	0.51431	0.932	0.91666	LMNA/NKX2-5/RARA	3	BP
GO:0060457	negative regulation of digestive	3/2734	17/17381	0.51431	0.932	0.91666	ABCG5/NR1H3/PTGER3	3	BP
GO:0060572	morphogenesis of an epithelial	3/2734	17/17381	0.51431	0.932	0.91666	BMP4/FGFR2/SHH	3	BP
GO:0060977	coronary vasculature morphogenesis	3/2734	17/17381	0.51431	0.932	0.91666	NOTCH1/PDGFRB/SETD2	3	BP
GO:0070242	thymocyte apoptotic	3/2734	17/17381	0.51431	0.932	0.91666	ADAM8/BMP4/EFNA1	3	BP
GO:0071605	monocyte chemotactic protein-1 production	3/2734	17/17381	0.51431	0.932	0.91666	LGALS9/MIR92A2/TRPV4	3	BP
GO:0071637	regulation of monocyte chemotactic protein-1 production	3/2734	17/17381	0.51431	0.932	0.91666	LGALS9/MIR92A2/TRPV4	3	BP
GO:0072574	hepatocyte proliferation	3/2734	17/17381	0.51431	0.932	0.91666	LIMS2/RPS6KA1/WNT3A	3	BP
GO:0072575	epithelial cell proliferation involved in liver	3/2734	17/17381	0.51431	0.932	0.91666	LIMS2/RPS6KA1/WNT3A	3	BP
GO:0097067	cellular response to thyroid	3/2734	17/17381	0.51431	0.932	0.91666	CTSH/CTSL/EIF5A	3	BP

GO:0099514	synaptic vesicle cytoskeletal transport	3/2734	17/17381	0.51431	0.932	0.91666	CNIH2/DTNBP1/TRIM46	3	BP
GO:0099517	synaptic vesicle transport along microtubule	3/2734	17/17381	0.51431	0.932	0.91666	CNIH2/DTNBP1/TRIM46	3	BP
GO:1902914	regulation of protein polyubiquitinati	3/2734	17/17381	0.51431	0.932	0.91666	NMI/OTUB1/PARP10	3	BP
GO:1904683	regulation of metalloendopeptidase activity	3/2734	17/17381	0.51431	0.932	0.91666	MIR195/MIR29C/MIR92A2	3	BP
GO:2000136	regulation of cell proliferation involved in	3/2734	17/17381	0.51431	0.932	0.91666	BMP4/EYA1/NOTCH1	3	BP
GO:2000641	regulation of early endosome to late endosome	3/2734	17/17381	0.51431	0.932	0.91666	MAPK3/PTPN23/SNX12	3	BP
GO:2000696	regulation of epithelial cell differentiation involved in kidney	3/2734	17/17381	0.51431	0.932	0.91666	LHX1/OSR1/STAT1	3	BP
GO:0008333	endosome to lysosome	8/2734	49/17381	0.51441	0.932	0.91666	AKTIP/CDX2/DENND3/FAM160A2/KIF13A/PCDHGA3/RAB7A/STX8	8	BP
GO:0021545	cranial nerve development	8/2734	49/17381	0.51441	0.932	0.91666	HOXA3/HOXB1/HOXB2/HOXB3/HOXD3/PHOX2A/PHOX2B/SEMA3F	8	BP

GO:0051055	negative regulation of lipid biosynthetic	8/2734	49/17381	0.51441	0.932	0.91666	ACADVL/APOC3/ATP1A1/BRCA1/GPER1/ORMDL3/PDGFA/SCAP	8	BP
GO:1900024	regulation of substrate adhesion-dependent cell spreading	8/2734	49/17381	0.51441	0.932	0.91666	ABL1/ARPC2/CIB1/DOCK1/FGA/FLNA/MYOC/OLFM4	8	BP
GO:0050829	defense response to Gram-negative	13/2734	81/17381	0.51547	0.932	0.91666	ADGRB1/CD160/CHGA/F2RL1/GSDMD/LYPD8/RARRES2/RNASE8/SELP/SLC11A1/SPACA3/TLR9/TUSC2	13	BP
GO:0009190	cyclic nucleotide biosynthetic	26/2734	164/17381	0.51584	0.932	0.91666	ADM/ADRA2A/AQP1/AVPR2/CCR2/CRHR1/DRD2/GABBR1/GCG/GPER1/GPHA2/GUCA1B/GUCA2A/GUCA2B/GUCY2D/NOS3/NPPA/OPRM1/PDE2A/PDZD3/PTK2B/RAF1/RUNDC3A/RXFP2/UCN2/WFS1	26	BP
GO:0051282	regulation of sequestering of calcium ion	18/2734	113/17381	0.51667	0.932	0.91666	ABL1/CAMK2D/CCL19/CCL21/CORO1A/DRD2/FASLG/GPER1/GSTM2/IL13/ITPR3/JPH3/LCK/NPSR1/PTK2B/RASA3/THY1/TRDN	18	BP
GO:0060218	hematopoietic stem cell differentiation	18/2734	113/17381	0.51667	0.932	0.91666	ABL1/HIST1H4F/HIST2H4A/HIST2H4B/HOXB4/LDB1/LMO1/PSMB11/PSMB6/PSMB7/PSMB8/PSMC3/PSMD13/PSMD3/PSMD5/PSMD7/SETD1A/SPI1	18	BP
GO:0002292	T cell differentiation involved in immune	10/2734	62/17381	0.51917	0.932	0.91666	BCL6/CCL19/HLX/IL18/IL4R/LGALS9/LY9/RARA/RORC/SEMA4A	10	BP
GO:0032272	negative regulation of protein	10/2734	62/17381	0.51917	0.932	0.91666	CAPZA3/HIP1R/LMOD1/PFN1/PRKCD/SCIN/SPTAN1/SPTB/SPTBN2/VDAC2	10	BP

GO:0002474	antigen processing and presentation of peptide antigen	15/2734	94/17381	0.5195	0.932	0.91666	BCAP31/HLA-E/IKBKB/ITGB5/NCF1/PSMB11/PSMB6/PSMB7/PSMB8/PSMC3/PSMD13/PSMD3/PSMD5/PSMD7/TAP1	15	BP
GO:0032091	negative regulation of	15/2734	94/17381	0.5195	0.932	0.91666	ARRB2/DISC1/DTNBP1/GOLGA2/GPSM1/HSPA5/IFIT2/MAPK3/NES/PEX14/PRKCD/PTPRF/RALB/WFIKKN2/XIRP1	15	BP
GO:0006164	purine nucleotide biosynthetic process	41/2734	260/17381	0.51961	0.932	0.91666	ACOT7/ADM/ADRA2A/AK5/ALDOA/AQP1/ATP5G1/ATP5I/AVPR2/CCR2/CRHR1/CYC1/DRD2/GABBR1/GCG/GPER1/GPHA2/GUCA1B/GUCA2A/GUCA2B/GUCY2D/IMPDH1/MTHFD1/NME1/NME4/NOS3/NPPA/OPRM1/PDE2A/PDZD3/PID1/PKM/PPCDC/PTK2B/RAF1/RUNDC3A/RXFP2/SLC26A1/UCN	41	BP
GO:0051701	interaction with host	33/2734	209/17381	0.52009	0.932	0.91666	ANPEP/BAD/CD74/CD81/CHMP4C/CLDN6/CPSF4/DDB1/EGFR/EIF2AK4/GAPDH/IFITM2/ITGB5/LGALS9/MIR221/MIR222/MOG/PC/RAB7A/SCARB1/SCRIB/SLC20A2/SLC52A1/SLC52A2/TNFRSF4/TRIM11/TRIM26/TRIM31/TRIM62/TUSC2/VPS37B/WWP2/ZNF502	33	BP
GO:0032606	type I interferon production	20/2734	126/17381	0.52022	0.932	0.91666	DHX58/HAVCR2/IRAK1/IRF5/ISG15/LRRFIP1/NLRX1/NMI/OTUD5/POLR1D/POLR2L/POLR3D/SETD2/STAT1/TBK1/TIRAP/TLR8/TLR9/TRAF3IP1/TR	20	BP
GO:0007094	mitotic spindle assembly checkpoint	5/2734	30/17381	0.52037	0.932	0.91666	ANAPC15/BUB1B/LCMT1/MAD1L1/XRCC3	5	BP
GO:0010092	specification of animal organ identity	5/2734	30/17381	0.52037	0.932	0.91666	BMP4/FGFR2/HOXC11/TBR1/WNT3A	5	BP
GO:0019098	reproductive behavior	5/2734	30/17381	0.52037	0.932	0.91666	FUOM/GRIN1/PPP1R1B/TH/THRA	5	BP
GO:0030201	heparan sulfate proteoglycan metabolic	5/2734	30/17381	0.52037	0.932	0.91666	B3GAT3/DSE/GPC1/SGSH/XYLT2	5	BP
GO:0031128	developmental induction	5/2734	30/17381	0.52037	0.932	0.91666	BMP4/HOXC11/SOX8/WNT1/WNT3A	5	BP

GO:0033006	regulation of mast cell activation involved in	5/2734	30/17381	0.52037	0.932	0.91666	FES/IL13/IL4R/LGALS9/STXBP1	5	BP
GO:0033198	response to ATP	5/2734	30/17381	0.52037	0.932	0.91666	CASP1/CIB2/DGKQ/P2RX2/TAF1	5	BP
GO:0040020	regulation of meiotic nuclear division	5/2734	30/17381	0.52037	0.932	0.91666	HORMAD1/PIWIL2/PRDM9/PRKAR1A/UBE2B	5	BP
GO:0043304	regulation of mast cell	5/2734	30/17381	0.52037	0.932	0.91666	FES/IL13/IL4R/LGALS9/STXBP1	5	BP
GO:0044319	wound healing, spreading of	5/2734	30/17381	0.52037	0.932	0.91666	FLNA/MIR221/MTOR/TOR1A/WNT7A	5	BP
GO:0045066	regulatory T cell	5/2734	30/17381	0.52037	0.932	0.91666	BCL6/FANCA/FUT7/LGALS9/TNFRSF18	5	BP
GO:0045920	negative regulation of	5/2734	30/17381	0.52037	0.932	0.91666	ADRA2A/CCR2/LGALS9/NOTCH1/RAB7A	5	BP
GO:0046627	negative regulation of insulin receptor	5/2734	30/17381	0.52037	0.932	0.91666	AHSG/CISH/PID1/PRKCD/TRIM72	5	BP
GO:0046636	negative regulation of alpha-beta T	5/2734	30/17381	0.52037	0.932	0.91666	BCL6/HLX/IL4R/LGALS9/SHH	5	BP
GO:0071173	spindle assembly	5/2734	30/17381	0.52037	0.932	0.91666	ANAPC15/BUB1B/LCMT1/MAD1L1/XRCC3	5	BP
GO:0071174	mitotic spindle checkpoint	5/2734	30/17381	0.52037	0.932	0.91666	ANAPC15/BUB1B/LCMT1/MAD1L1/XRCC3	5	BP
GO:0090505	epiboly involved in	5/2734	30/17381	0.52037	0.932	0.91666	FLNA/MIR221/MTOR/TOR1A/WNT7A	5	BP

GO:1902175	regulation of oxidative stress-induced intrinsic apoptotic	5/2734	30/17381	0.52037	0.932	0.91666	GPX1/HSPB1/INS/NONO/TRAP1	5	BP
GO:0043270	positive regulation of ion transport	38/2734	241/17381	0.52115	0.932	0.91666	ABL1/ADRA2A/AGT/AMIGO1/ARC/ARRB2/CCL5/CHRM1/CRACR2A/DRD2/FLNA/GCG/GPER1/GRIN1/GSTM2/HSPA2/IL13/KCNQ1/LGALS3/NKX2-5/NLGN3/NPPA/NPSR1/ORAI1/P2RX2/PDGFRB/PLA2G1B/PRELID1/PRKCD/SLC34A1/STXBP1/TACR2/THY1/TRDN/TRH/TRPV2/WFS1/WNK2	38	BP
GO:0006333	chromatin assembly or disassembly	30/2734	190/17381	0.52195	0.932	0.91666	ANP32B/BAZ1B/CDAN1/CDKN2A/CENPN/H1FX/HDAC8/HILS1/HIRIP3/HIST1H4F/HIST2H4A/HIST2H4B/HJURP/HMGA1/IPO4/M1AP/MCM2/NAA60/NOC2L/PADI4/PARP10/RSF1/SMARCD2/SMYD3/TNRC18/TSPY1/TSPY2/TSP	30	BP
GO:0032481	positive regulation of type I interferon	12/2734	75/17381	0.5232	0.932	0.91666	DHX58/IRAK1/IRF5/LRRFIP1/POLR1D/POLR2L/POLR3D/SETD2/STAT1/TBK1/TLR8/TLR9	12	BP
GO:0035710	CD4-positive, alpha-beta T cell activation	12/2734	75/17381	0.5232	0.932	0.91666	BCL6/CCL19/FUT7/HLX/IL18/IL4R/LGALS9/LY9/NKX2-3/RARA/RORC/SEMA4A	12	BP
GO:0009108	coenzyme biosynthetic process	22/2734	139/17381	0.52347	0.932	0.91666	ACACB/ACOT7/ACSF3/COQ2/COQ4/ELOVL1/ELOVL5/GCH1/HAAO/HACD1/IDH2/LIAS/MPC1/MTHFD1/NMNAT3/PARP10/PARP9/PDHB/PDSS1/PPCDC/QPRT/SLC22A13	22	BP
GO:0090288	negative regulation of cellular response to growth factor	22/2734	139/17381	0.52347	0.932	0.91666	ABL1/AGT/DAB2IP/FAM89B/GPC1/HSPA5/HTRA3/LEMD2/MIR15A/MIR16-1/NBL1/NOTCH1/PBLD/PRDM14/RBPMS2/SMAD6/SOST/TGFB1I1/VASN/WFIKKN2/WNT1/XDH	22	BP
GO:0010470	regulation of gastrulation	7/2734	43/17381	0.52476	0.932	0.91666	CRB2/IL1RN/LHX1/MIR221/OSR1/SCX/WNT3A	7	BP
GO:0021795	cerebral cortex cell migration	7/2734	43/17381	0.52476	0.932	0.91666	CDK5/CDK5R2/DAB1/DAB2IP/DISC1/EGFR/POU3F3	7	BP

GO:0030857	negative regulation of epithelial cell	7/2734	43/17381	0.52476	0.932	0.91666	FOXE3/IL13/NOTCH1/OSR1/SMO/STAT1/XDH	7	BP
GO:0031638	zymogen activation	7/2734	43/17381	0.52476	0.932	0.91666	BAD/CTSH/FGA/FURIN/KLKB1/PLAU/SERPINF2	7	BP
GO:0045744	negative regulation of G-protein coupled receptor protein signaling	7/2734	43/17381	0.52476	0.932	0.91666	ADM/ADRA2A/ARRB2/CCL5/DNM1/DRD2/NECAB2	7	BP
GO:0098659	inorganic cation import across plasma	7/2734	43/17381	0.52476	0.932	0.91666	ATP1A1/KCNJ5/SLC9A3/SLC9A5/STEAP2/TFR2/TRPV6	7	BP
GO:0099587	inorganic ion import across plasma	7/2734	43/17381	0.52476	0.932	0.91666	ATP1A1/KCNJ5/SLC9A3/SLC9A5/STEAP2/TFR2/TRPV6	7	BP
GO:2000725	regulation of cardiac muscle cell	7/2734	43/17381	0.52476	0.932	0.91666	ARRB2/BMP4/CAV3/MIR222/MTOR/NRG1/WNT3A	7	BP
GO:0007033	vacuole organization	24/2734	152/17381	0.52648	0.932	0.91666	ACP2/AKTIP/AMBRA1/ATG101/ATG2A/ATP13A2/CLN6/CORO1A/FAM160A2/GBA/HPS1/IFT20/LRSAM1/MFN2/MYO7A/NAGLU/NPRL3/RAB20/RAB34/RAB7A/RALB/RBSN/TFEB/ULK1	24	BP
GO:0060337	type I interferon signaling pathway	14/2734	88/17381	0.52673	0.932	0.91666	EGR1/HLA-E/IFIT2/IFIT3/IFITM2/IFNA5/IRAK1/IRF5/ISG15/PSMB8/PTPN11/SCRIB/STAT1/STAT2	14	BP
GO:0071357	cellular response to type I interferon	14/2734	88/17381	0.52673	0.932	0.91666	EGR1/HLA-E/IFIT2/IFIT3/IFITM2/IFNA5/IRAK1/IRF5/ISG15/PSMB8/PTPN11/SCRIB/STAT1/STAT2	14	BP

GO:1904029	regulation of cyclin-dependent protein kinase	14/2734	88/17381	0.52673	0.932	0.91666	CASP3/CCND3/CDK20/CDK5R2/CDK5RAP3/CDKN2A/EGFR/HSPA2/INCA1/MAPRE3/MEN1/PKD1/PKMYT1/SFN	14	BP
GO:0031497	chromatin assembly	26/2734	165/17381	0.5293	0.932	0.91666	ANP32B/CDAN1/CDKN2A/CENPN/H1FX/HILS1/HIST1H4F/HIST2H4A/HIST2H4B/HJURP/HMGA1/IPO4/M1AP/MCM2/NAA60/NOC2L/PADI4/PARP10/RSF1/SMYD3/TNRC18/TSPY1/TSPY2/TSPY4/TSPY8/TSPYL2	26	BP
GO:0033559	unsaturated fatty acid metabolic	16/2734	101/17381	0.52991	0.932	0.91666	CD74/CES2/CYP1A1/CYP1A2/CYP2W1/DAGLB/EDN2/ELOVL1/ELOVL5/EPHX2/FADS2/FADS3/GSTM2/MAPK3/PLA2G4B/PTGR1	16	BP
GO:0097061	dendritic spine organization	11/2734	69/17381	0.53161	0.932	0.91666	ARC/CDK5/CFL1/CTTN/DLG4/EFNA1/EPHB3/INS/NLGN3/PDLIM5/WNT7A	11	BP
GO:0032640	tumor necrosis factor	18/2734	114/17381	0.53282	0.932	0.91666	ARRB2/CARD9/CCL19/CCR2/CLU/HAVCR2/HLA-E/HSF1/HSPB1/LGALS9/NFKBIL1/NOD1/ORM1/ORM2/RARA/SPON2/TIRA	18	BP
GO:0031058	positive regulation of histone	13/2734	82/17381	0.53451	0.932	0.91666	BCL6/BRCA1/CDK9/CTBP1/GCG/KAT2A/MAPK3/MUC1/PAXIP1/PHF19/PIWIL2/PRDM12/RPS6KA4	13	BP
GO:0002092	positive regulation of receptor	4/2734	24/17381	0.53522	0.932	0.91666	ARRB2/CD63/DRD2/WNT3A	4	BP
GO:0002691	regulation of cellular extravasation	4/2734	24/17381	0.53522	0.932	0.91666	ADAM8/CCL21/CCR2/THY1	4	BP
GO:0003171	atrioventricular valve	4/2734	24/17381	0.53522	0.932	0.91666	EFNA1/HEY1/NOTCH1/SMAD6	4	BP
GO:0006266	DNA ligation	4/2734	24/17381	0.53522	0.932	0.91666	LIG3/PARP2/PARP3/POLD4	4	BP
GO:0006541	glutamine metabolic	4/2734	24/17381	0.53522	0.932	0.91666	ASL/ASNS/GLUD1/GLYATL1	4	BP

GO:0031646	positive regulation of neurological	4/2734	24/17381	0.53522	0.932	0.91666	GBA/MTOR/NRG1/PARD3	4	BP
GO:0032232	negative regulation of actin filament	4/2734	24/17381	0.53522	0.932	0.91666	ARAP1/MYOC/PFN1/WAS	4	BP
GO:0032461	positive regulation of protein	4/2734	24/17381	0.53522	0.932	0.91666	CLU/HRK/MIEF2/MMP3	4	BP
GO:0032800	receptor biosynthetic process	4/2734	24/17381	0.53522	0.932	0.91666	ACHE/FURIN/NR1H3/SCAP	4	BP
GO:0036314	response to sterol	4/2734	24/17381	0.53522	0.932	0.91666	OSBPL7/PMVK/RORC/SMO	4	BP
GO:0046640	regulation of alpha-beta T cell	4/2734	24/17381	0.53522	0.932	0.91666	CCR2/HLA-E/IL18/LGALS9	4	BP
GO:0051569	regulation of histone H3-K4 methylation	4/2734	24/17381	0.53522	0.932	0.91666	BRCA1/GCG/PAXIP1/PYGO2	4	BP
GO:0060632	regulation of microtubule-based	4/2734	24/17381	0.53522	0.932	0.91666	CATSPER1/CCDC40/CNIH2/DNAH11	4	BP
GO:0060914	heart formation	4/2734	24/17381	0.53522	0.932	0.91666	BMP4/EYA1/LEMD2/WNT3A	4	BP
GO:0070193	synaptonemal complex organization	4/2734	24/17381	0.53522	0.932	0.91666	HORMAD1/HSPA2/MCMDC2/UBE2B	4	BP
GO:1900117	regulation of execution phase of apoptosis	4/2734	24/17381	0.53522	0.932	0.91666	BOK/ENDOG/GCG/HSF1	4	BP

GO:1903319	positive regulation of protein	4/2734	24/17381	0.53522	0.932	0.91666	ADAM8/KLKB1/NKD2/TFR2	4	BP
GO:2000406	positive regulation of T	4/2734	24/17381	0.53522	0.932	0.91666	ADAM8/CCL21/CCL5/CCR2	4	BP
GO:0000301	retrograde transport, vesicle recycling within	2/2734	11/17381	0.53536	0.932	0.91666	COG4/PACS1	2	BP
GO:0001767	establishment of lymphocyte polarity	2/2734	11/17381	0.53536	0.932	0.91666	CCL19/CCL21	2	BP
GO:0003128	heart field specification	2/2734	11/17381	0.53536	0.932	0.91666	BMP4/WNT3A	2	BP
GO:0003157	endocardium development	2/2734	11/17381	0.53536	0.932	0.91666	NOTCH1/NRG1	2	BP
GO:0003264	regulation of cardioblast proliferation	2/2734	11/17381	0.53536	0.932	0.91666	EYA1/NOTCH1	2	BP
GO:0006531	aspartate metabolic	2/2734	11/17381	0.53536	0.932	0.91666	GOT2/NMNAT3	2	BP
GO:0006558	L-phenylalanine metabolic	2/2734	11/17381	0.53536	0.932	0.91666	FAH/GSTZ1	2	BP
GO:0006559	L-phenylalanine catabolic	2/2734	11/17381	0.53536	0.932	0.91666	FAH/GSTZ1	2	BP
GO:0006563	L-serine metabolic	2/2734	11/17381	0.53536	0.932	0.91666	PHGDH/SHMT2	2	BP
GO:0006662	glycerol ether metabolic	2/2734	11/17381	0.53536	0.932	0.91666	GNPAT/PLA2G16	2	BP
GO:0006707	cholesterol catabolic	2/2734	11/17381	0.53536	0.932	0.91666	SCARB1/SCARF1	2	BP

GO:0006751	glutathione catabolic	2/2734	11/17381	0.53536	0.932	0.91666	CHAC1/GGT6	2	BP
GO:0006768	biotin metabolic process	2/2734	11/17381	0.53536	0.932	0.91666	ACACB/PC	2	BP
GO:0007135	meiosis II	2/2734	11/17381	0.53536	0.932	0.91666	BUB1B/HORMAD1	2	BP
GO:0007195	adenylate cyclase-inhibiting dopamine	2/2734	11/17381	0.53536	0.932	0.91666	DRD2/FLNA	2	BP
GO:0009125	nucleoside monophosphate catabolic	2/2734	11/17381	0.53536	0.932	0.91666	DNPH1/NT5M	2	BP
GO:0009265	2'-deoxyribonucleotide biosynthetic	2/2734	11/17381	0.53536	0.932	0.91666	AK5/TBPL1	2	BP
GO:0010819	regulation of T cell chemotaxis	2/2734	11/17381	0.53536	0.932	0.91666	CCL5/CCR2	2	BP
GO:0010867	positive regulation of triglyceride biosynthetic	2/2734	11/17381	0.53536	0.932	0.91666	NR1H3/SCARB1	2	BP
GO:0010870	positive regulation of receptor biosynthetic	2/2734	11/17381	0.53536	0.932	0.91666	NR1H3/SCAP	2	BP
GO:0010872	regulation of cholesterol esterification	2/2734	11/17381	0.53536	0.932	0.91666	AGT/APOA5	2	BP

GO:0010960	magnesium ion homeostasis	2/2734	11/17381	0.53536	0.932	0.91666	CNNM2/EGFR	2	BP
GO:0010985	negative regulation of lipoprotein	2/2734	11/17381	0.53536	0.932	0.91666	APOC3/CSK	2	BP
GO:0014857	regulation of skeletal muscle cell	2/2734	11/17381	0.53536	0.932	0.91666	PPARD/SHH	2	BP
GO:0015867	ATP transport	2/2734	11/17381	0.53536	0.932	0.91666	SLC25A25/SLC25A5	2	BP
GO:0016127	sterol catabolic process	2/2734	11/17381	0.53536	0.932	0.91666	SCARB1/SCARF1	2	BP
GO:0017187	peptidyl-glutamic acid	2/2734	11/17381	0.53536	0.932	0.91666	BGLAP/PROZ	2	BP
GO:0018214	protein carboxylation	2/2734	11/17381	0.53536	0.932	0.91666	BGLAP/PROZ	2	BP
GO:0019042	viral latency	2/2734	11/17381	0.53536	0.932	0.91666	HCFC1/HMGA1	2	BP
GO:0021670	lateral ventricle development	2/2734	11/17381	0.53536	0.932	0.91666	AQP1/TSKU	2	BP
GO:0031987	locomotion involved in locomotory behavior	2/2734	11/17381	0.53536	0.932	0.91666	CLN6/DRD2	2	BP
GO:0032276	regulation of gonadotropin secretion	2/2734	11/17381	0.53536	0.932	0.91666	INHBB/TACR2	2	BP
GO:0032490	detection of molecule of bacterial origin	2/2734	11/17381	0.53536	0.932	0.91666	SCARB1/TREM2	2	BP

GO:0033004	negative regulation of mast cell	2/2734	11/17381	0.53536	0.932	0.91666	CD300LF/LGALS9	2	BP
GO:0033327	Leydig cell differentiation	2/2734	11/17381	0.53536	0.932	0.91666	MGST1/SF1	2	BP
GO:0034372	very-low-density lipoprotein	2/2734	11/17381	0.53536	0.932	0.91666	APOA5/APOC3	2	BP
GO:0034982	mitochondrial protein	2/2734	11/17381	0.53536	0.932	0.91666	PMPCA/YME1L1	2	BP
GO:0035330	regulation of hippo signaling	2/2734	11/17381	0.53536	0.932	0.91666	MARK3/NEK8	2	BP
GO:0035413	positive regulation of catenin import	2/2734	11/17381	0.53536	0.932	0.91666	EGFR/WNT3A	2	BP
GO:0035766	cell chemotaxis to fibroblast growth factor	2/2734	11/17381	0.53536	0.932	0.91666	MIR15A/MIR16-1	2	BP
GO:0039532	negative regulation of viral-induced cytoplasmic pattern recognition	2/2734	11/17381	0.53536	0.932	0.91666	DHX58/NLRX1	2	BP
GO:0042535	positive regulation of tumor necrosis factor	2/2734	11/17381	0.53536	0.932	0.91666	CCR2/HSPB1	2	BP
GO:0043116	negative regulation of vascular	2/2734	11/17381	0.53536	0.932	0.91666	ADM/PDE2A	2	BP

GO:0043201	response to leucine	2/2734	11/17381	0.53536	0.932	0.91666	LARS/MTOR	2	BP
GO:0043301	negative regulation of leukocyte	2/2734	11/17381	0.53536	0.932	0.91666	CCR2/LGALS9	2	BP
GO:0045472	response to ether	2/2734	11/17381	0.53536	0.932	0.91666	CDK4/TH	2	BP
GO:0045722	positive regulation of	2/2734	11/17381	0.53536	0.932	0.91666	GCG/KAT2A	2	BP
GO:0045955	negative regulation of calcium ion-dependent	2/2734	11/17381	0.53536	0.932	0.91666	ADRA2A/NOTCH1	2	BP
GO:0046051	UTP metabolic process	2/2734	11/17381	0.53536	0.932	0.91666	NME1/NME4	2	BP
GO:0046349	amino sugar biosynthetic process	2/2734	11/17381	0.53536	0.932	0.91666	AMDHD2/NAGK	2	BP
GO:0046385	deoxyribose phosphate biosynthetic process	2/2734	11/17381	0.53536	0.932	0.91666	AK5/TBPL1	2	BP
GO:0046950	cellular ketone body metabolic process	2/2734	11/17381	0.53536	0.932	0.91666	ACSS3/HMGCLL1	2	BP
GO:0051386	regulation of neurotrophin TRK receptor signaling	2/2734	11/17381	0.53536	0.932	0.91666	AGT/PPP2R5B	2	BP
GO:0051451	myoblast migration	2/2734	11/17381	0.53536	0.932	0.91666	BIN3/SMO	2	BP

GO:0051593	response to folic acid	2/2734	11/17381	0.53536	0.932	0.91666	FOLR2/OGG1	2	BP
GO:0060315	negative regulation of ryanodine-sensitive calcium-release	2/2734	11/17381	0.53536	0.932	0.91666	GSTM2/TRDN	2	BP
GO:0060525	prostate glandular acinus development	2/2734	11/17381	0.53536	0.932	0.91666	FGFR2/NOTCH1	2	BP
GO:0060767	epithelial cell proliferation involved in prostate gland development	2/2734	11/17381	0.53536	0.932	0.91666	NOTCH1/SHH	2	BP
GO:0060947	cardiac vascular smooth muscle cell	2/2734	11/17381	0.53536	0.932	0.91666	GPER1/NOTCH1	2	BP
GO:0061307	cardiac neural crest cell differentiation involved in heart	2/2734	11/17381	0.53536	0.932	0.91666	BMP4/MAPK3	2	BP
GO:0061308	cardiac neural crest cell development involved in heart	2/2734	11/17381	0.53536	0.932	0.91666	BMP4/MAPK3	2	BP
GO:0070391	response to lipoteichoic	2/2734	11/17381	0.53536	0.932	0.91666	TIRAP/TREM2	2	BP

GO:0070586	cell-cell adhesion involved in	2/2734	11/17381	0.53536	0.932	0.91666	IL1RN/MIR221	2	BP
GO:0071044	histone mRNA catabolic	2/2734	11/17381	0.53536	0.932	0.91666	EXOSC10/LSM1	2	BP
GO:0071223	cellular response to	2/2734	11/17381	0.53536	0.932	0.91666	TIRAP/TREM2	2	BP
GO:0072015	glomerular visceral epithelial cell	2/2734	11/17381	0.53536	0.932	0.91666	BMP4/MYO1E	2	BP
GO:0090050	positive regulation of cell migration involved in sprouting	2/2734	11/17381	0.53536	0.932	0.91666	ABL1/CIB1	2	BP
GO:0090084	negative regulation of inclusion body	2/2734	11/17381	0.53536	0.932	0.91666	HSF1/HSPA2	2	BP
GO:0090161	Golgi ribbon formation	2/2734	11/17381	0.53536	0.932	0.91666	GOLGA2/MYO18A	2	BP
GO:0090331	negative regulation of platelet	2/2734	11/17381	0.53536	0.932	0.91666	C1QTNF1/PRKCD	2	BP
GO:0090657	telomeric loop disassembly	2/2734	11/17381	0.53536	0.932	0.91666	RECQL4/XRCC3	2	BP
GO:0098877	neurotransmitter receptor transport to plasma	2/2734	11/17381	0.53536	0.932	0.91666	GRIPAP1/SCRIB	2	BP

GO:1900363	regulation of mRNA polyadenylation	2/2734	11/17381	0.53536	0.932	0.91666	CDK9/HSF1	2	BP
GO:1902221	erythrose 4-phosphate/phosphoenolpyruvate family amino acid metabolic	2/2734	11/17381	0.53536	0.932	0.91666	FAH/GSTZ1	2	BP
GO:1902222	erythrose 4-phosphate/phosphoenolpyruvate family amino acid catabolic	2/2734	11/17381	0.53536	0.932	0.91666	FAH/GSTZ1	2	BP
GO:1902713	regulation of interferon-gamma	2/2734	11/17381	0.53536	0.932	0.91666	ABL1/LGALS9	2	BP
GO:1902855	regulation of non-motile cilium assembly	2/2734	11/17381	0.53536	0.932	0.91666	SEPT9/WRAP73	2	BP
GO:1903726	negative regulation of phospholipid	2/2734	11/17381	0.53536	0.932	0.91666	DAB2IP/PDGFA	2	BP
GO:1904753	negative regulation of vascular associated smooth muscle	2/2734	11/17381	0.53536	0.932	0.91666	MIR15A/MIR503	2	BP
GO:1904847	regulation of cell chemotaxis to fibroblast growth factor	2/2734	11/17381	0.53536	0.932	0.91666	MIR15A/MIR16-1	2	BP

GO:1990440	positive regulation of transcription from RNA polymerase II promoter in response to endoplasmic [illegible]	2/2734	11/17381	0.53536	0.932	0.91666	ATF3/HSPA5	2	BP
GO:2000047	regulation of cell-cell adhesion mediated by	2/2734	11/17381	0.53536	0.932	0.91666	SERPINF2/WNT3A	2	BP
GO:2000425	regulation of apoptotic cell clearance	2/2734	11/17381	0.53536	0.932	0.91666	C2/CD300LF	2	BP
GO:2000574	regulation of microtubule motor activity	2/2734	11/17381	0.53536	0.932	0.91666	DYNC1I1/DYNLL2	2	BP
GO:2000818	negative regulation of myoblast	2/2734	11/17381	0.53536	0.932	0.91666	MIR10A/MYOD1	2	BP
GO:0002707	negative regulation of lymphocyte	6/2734	37/17381	0.53679	0.932	0.91666	ARRB2/BCL6/HAVCR2/HLA-E/IL20RB/LGALS9	6	BP
GO:0006296	nucleotide-excision repair, DNA incision, 5'-to lesion	6/2734	37/17381	0.53679	0.932	0.91666	DDB1/DDB2/GTF2H4/POLD4/RFC1/RFC2	6	BP
GO:0007340	acrosome reaction	6/2734	37/17381	0.53679	0.932	0.91666	CACNA1H/GLRA1/PLB1/POMZP3/PRSS37/SPINK2	6	BP

GO:0010824	regulation of centrosome duplication	6/2734	37/17381	0.53679	0.932	0.91666	BRCA1/CCNF/CHMP1A/CHMP4C/NUBP1/XRCC3	6	BP
GO:0015695	organic cation transport	6/2734	37/17381	0.53679	0.932	0.91666	RHCG/SLC22A1/SLC22A13/SLC25A20/SLC44A4/TACR2	6	BP
GO:0034405	response to fluid shear	6/2734	37/17381	0.53679	0.932	0.91666	MIR92A2/NOS3/PDGFRB/PKD1/PTK2B/SMAD6	6	BP
GO:0051154	negative regulation of striated muscle	6/2734	37/17381	0.53679	0.932	0.91666	ANKRD2/CAV3/MIR222/NKX2-5/NOTCH1/TRIM72	6	BP
GO:0071542	dopaminergic neuron differentiation	6/2734	37/17381	0.53679	0.932	0.91666	EN1/PHOX2A/PHOX2B/SHH/WNT1/WNT3A	6	BP
GO:1902803	regulation of synaptic vesicle transport	6/2734	37/17381	0.53679	0.932	0.91666	CDK5/CNIH2/DNM1/STX1A/STXBP1/TOR1A	6	BP
GO:1904031	positive regulation of cyclin-dependent	6/2734	37/17381	0.53679	0.932	0.91666	CCND3/CDK20/EGFR/HSPA2/MAPRE3/PKD1	6	BP
GO:0019932	second-messenger-mediated signaling	47/2734	300/17381	0.53695	0.932	0.91666	ADM/ADRA1A/ADRA1B/ADRA2A/ADRA2B/ADRA2C/AGT/BCAP31/CAMK2D/CDH13/CHGA/CIB1/CRHR1/CXCR3/DGKQ/EDN2/EGFR/GPER1/GRIN1/GRIN2C/GSTM2/HINT1/HOMER2/ITPR3/JPH3/KDR/KSR1/MAFA/NEUROD2/NOS3/NR5A1/NRG1/OPRM1/P2RX2/PDE2A/PDZD3/PLA2G4B/PTK2B/RASD1/RXFP2/SELP/TCP11/TMEM100/TRDN/TREM2/UBE2B/UCN2	47	BP
GO:0003300	cardiac muscle hypertrophy	15/2734	95/17381	0.53717	0.932	0.91666	AGT/CAMK2D/CAMTA2/CAV3/CDK9/GATA4/INPP5F/LMNA/MIR195/MIR25/MTOR/NPPA/PDLIM5/TCAP/TRIM63	15	BP
GO:0007043	cell-cell junction	15/2734	95/17381	0.53717	0.932	0.91666	AGT/CDH5/FSCN1/GNPAT/IKBKB/MARVELD2/MARVELD3/MYO1C/PARD3/PARD6A/PKP3/TLN1/TRPV4/WDR1/WNT11	15	BP

GO:0006605	protein targeting	62/2734	396/17381	0.53792	0.9329	0.9176	ACSM6/AIP/AP4S1/ATPIF1/CDK5/CIB1/CLU/CYB5R1/DNAJC19/DNLZ/ELMOD1/ERBB2/GOSR2/GPHA2/HNMT/HSP90AA1/ITGAM/ITGAX/KAT2A/KATNB1/LARS/LRRC46/MFN2/MIEF2/MYBPC1/MYO1C/NBPF3/NCF1/NRG1/OS9/OSCP1/PACS1/PARD3/PDE2A/PEMT/PEX14/PEX16/PEX3/PEX5/PEX6/PMPCA/PSMB7/RAB7A/RHOD/RNF31/RPL35A/RPL36A/RPL41/RPL7/RPS24/SRP14/SSR2/TAOK2/TIMM17B/TIMM22/TIMM23B/TIMM9/TSGA13/UBL4	62	BP
GO:0001508	action potential	22/2734	140/17381	0.53803	0.9329	0.9176	ADRA1A/ATP1A1/CACNA1F/CACNA1G/CACNA1H/CACNA1S/CACNB3/CAMK2D/CATSPER1/CAV3/FLNA/GBA/GLRA1/GPER1/GPR35/GPR88/KCND3/KCNJ5/KCNQ1/MIR328/SCN11A/TACR1	22	BP
GO:0032655	regulation of interleukin-12 production	8/2734	50/17381	0.53878	0.9334	0.91806	ARRB2/CCL19/IDO1/IRF5/LGALS9/TIRAP/TLR8/TLR9	8	BP
GO:0033260	nuclear DNA replication	8/2734	50/17381	0.53878	0.9334	0.91806	BCL6/INO80/POLA2/POLD4/POLE/POLE4/RFC1/RFC2	8	BP
GO:2001238	positive regulation of extrinsic apoptotic	8/2734	50/17381	0.53878	0.9334	0.91806	AGT/ATF3/G0S2/GPER1/LTBR/PEA15/TNFRSF12A/TRAF2	8	BP
GO:0019058	viral life cycle	49/2734	313/17381	0.539	0.9335	0.91818	ANPEP/CCL5/CD74/CD81/CHMP1A/CHMP4C/CHMP7/CLDN6/CTBP1/DDB1/EGFR/EIF2AK4/FKBP6/FURIN/HACD3/IFITM2/ISG15/ITGB5/LAMP3/LGALS9/LRSAM1/MIR221/MIR222/MOG/NOTCH1/NUP210/NUP98/PARP10/PC/PPIB/PPIE/RAB7A/SCARB1/SLC20A2/SLC52A1/SLC52A2/SNF8/TARBP2/TNFRSF4/TRIM11/TRIM26/TRIM31/TRIM62/USP6NL/VPS37B/VPS37C/VPS37	49	BP
GO:0006367	transcription initiation from RNA polymerase II	29/2734	185/17381	0.53953	0.9339	0.91849	CDK4/CDK9/ESR2/ESRRA/GTF2H4/MED24/MED6/NKX2-5/NOTCH1/NPPA/NR1H3/NR2F1/NR4A1/NR5A1/NRBP1/PAXIP1/POLR2G/POLR2L/PPARD/PSMC3/RARA/RORC/TAF1/TAF1L/TAF3/TAF6/TAF7/TEAD4/THRA	29	BP
GO:0060348	bone development	29/2734	185/17381	0.53953	0.9339	0.91849	BGLAP/BMP4/CHAD/COL1A1/ENG/FGFR2/HOXB4/IFITM5/INPPL1/MCPH1/MYOC/P3H1/PHOSPHO1/PLXNB1/POR/PPIB/PTPN11/RARA/RIPPLY1/SCX/SERPINH1/SH3PXD2B/SLC38A10/THBS3/TMEM119/TRPV4/TULP3/WNT1/	29	BP

GO:0019218	regulation of steroid metabolic	17/2734	108/17381	0.53965	0.9339	0.91849	ACACB/ACADVL/AGT/APOA5/APOB/ATP1A1/DGKQ/EGR1/EPHX2/FGFR4/LMF1/NR5A1/PMVK/POR/RORC/SCAP/SF1	17	BP
GO:0046425	regulation of JAK-STAT cascade	24/2734	153/17381	0.54041	0.9346	0.91919	AGT/ARL2BP/BGN/CCL5/CDK5/CHAD/CISH/CLCF1/CSF1R/DAB1/HSF1/IFNA5/IL13/IL18/IL31RA/INPP5F/LRRTM1/MIR221/NOTCH1/PARP9/PTK2B/RTN4RL1/RTN4RL2/TNFRSF18	24	BP
GO:1904892	regulation of STAT cascade	24/2734	153/17381	0.54041	0.9346	0.91919	AGT/ARL2BP/BGN/CCL5/CDK5/CHAD/CISH/CLCF1/CSF1R/DAB1/HSF1/IFNA5/IL13/IL18/IL31RA/INPP5F/LRRTM1/MIR221/NOTCH1/PARP9/PTK2B/RTN4RL1/RTN4RL2/TNFRSF18	24	BP
GO:0019915	lipid storage	10/2734	63/17381	0.54085	0.9346	0.91919	ACACB/APOB/B4GALNT1/DGAT1/EHD1/FITM1/HILPDA/NR1H3/PNPLA2/SCARB1	10	BP
GO:0032729	positive regulation of interferon-	10/2734	63/17381	0.54085	0.9346	0.91919	ABL1/CCR2/HAVCR2/HRAS/IL18/LGALS9/LTA/SLC11A1/TLR8/TLR9	10	BP
GO:0086003	cardiac muscle cell contraction	10/2734	63/17381	0.54085	0.9346	0.91919	ATP1A1/CACNA1G/CAMK2D/CAV3/FLNA/GATA4/KCND3/KCNJ5/KCNQ1/MIR328	10	BP
GO:0008286	insulin receptor signaling	19/2734	121/17381	0.54199	0.936	0.9206	AHSG/ATP6V0B/ATP6V0E2/ATP6V1B1/ATP6V1F/ATP6V1G1/ATP6V1G2/BAIAP2L1/CCND3/CDK4/CISH/EIF4EBP1/FOXO4/INS/PID1/PRKCD/SHC1/SL	19	BP
GO:0051291	protein heterooligomerization	19/2734	121/17381	0.54199	0.936	0.9206	C1QTNF1/CD74/CHRNB3/CNGB1/COL1A1/GCH1/GCHFR/GLRA1/GRIN1/HIST1H4F/HIST2H4A/HIST2H4B/HRAS/ILK/ITPR3/PDSS1/PRKAB1/SEPT9/TRAF2	19	BP
GO:0048469	cell maturation	26/2734	166/17381	0.54266	0.9368	0.92137	ACRBP/AGRN/ANG/C1QL1/CCL19/CCL21/EPHA8/FARP2/HBZ/L3MBTL3/MAEA/MTCH1/MTOR/MYOC/PTK2B/REN/RNASE9/RND1/RXFP2/SCARF1/SEPT4/SLC26A6/SOX8/TCP11/TMEM79/TUSC2	26	BP
GO:0006081	cellular aldehyde	12/2734	76/17381	0.54292	0.9368	0.92137	AKR7A3/ALDH4A1/ALDH8A1/CACNA1H/CYP11B2/GLO1/GLYCTK/GOT2/IDH1/IDH2/PGAM4/PNKD	12	BP

GO:0051437	positive regulation of ubiquitin-protein ligase activity involved in regulation of [illegible]	12/2734	76/17381	0.54292	0.9368	0.92137	ANAPC15/ANAPC2/BUB1B/PSMB11/PSMB6/PSMB7/PSMB8/PSMC3/PSMD13/PSMD3/PSMD5/PSMD7	12	BP
GO:0006611	protein export from nucleus	28/2734	179/17381	0.5448	0.9369	0.92153	ALKBH5/ANP32B/CASC3/CCHCR1/CDK5/CPSF1/CPSF4/DHX38/EIF5A/EMD/NOL6/NUP210/NUP98/NXF2/NXF2B/NXF3/PKD1/PTPN11/RNPS1/SETD2/SFN/SMG5/SRSF4/SUPT6H/XPO6/XPO7/ZC3H3/ZNF593	28	BP
GO:0060996	dendritic spine development	14/2734	89/17381	0.54494	0.9369	0.92153	ARC/CDK5/CFL1/DISC1/DLG4/EFNA1/EPHB3/FOXO6/LLPH/MTOR/NLGN3/NRG1/PDLIM5/WNT7A	14	BP
GO:0043414	macromolecule methylation	45/2734	288/17381	0.54499	0.9369	0.92153	ASH2L/BRCA1/CMTR1/COPRS/DDX4/DNMT3A/EHMT1/EHMT2/FKBP6/GCG/LCMT1/MEN1/MEPCE/METTL2A/METTL2B/MIR29A/MIR29C/MPHOSPH8/NDUFAF7/NSUN5/NSUN5P2/NTMT1/PAXIP1/PHF19/PIWIL2/PRDM12/PRDM14/PRDM7/PRDM9/PYGO2/RLF/SETD1A/SETD2/SETD7/SMYD3/SPI1/SUPT6H/SUV39H1/TDRD1/TDRD9/THUMPD2/TRMT10B/TRMT2B/TRMT4	45	BP
GO:1901991	negative regulation of mitotic cell cycle phase	35/2734	224/17381	0.54592	0.9369	0.92153	ANAPC15/BRCA1/BUB1B/CDK2AP2/CDK5RAP3/CHMP4C/CTDSP1/E2F4/E2F8/FOXO4/GPR132/LCMT1/MAD1L1/MIIP/MIR15A/MIR16-1/MIR195/MIR29A/MIR29C/MUC1/PCBP4/PSMB11/PSMB6/PSMB7/PSMB8/PSMC3/PSMD13/PSMD3/PSMD5/PSMD7/RINT1/SFN/TICRR/XRCC3/ZNF38	35	BP
GO:0032609	interferon-gamma	16/2734	102/17381	0.54691	0.9369	0.92153	ABL1/AVPR2/CCR2/F2RL1/HAVCR2/HRAS/IL18/IL20RB/ISG15/LGALS9/LTA/RARA/SCGB1A1/SLC11A1/TLR8/TLR9	16	BP
GO:0030308	negative regulation of cell growth	25/2734	160/17381	0.54832	0.9369	0.92153	AGT/APBB1/ARHGAP4/BCL6/CAV3/CCAR2/CCDC85B/CDA/CDK5/CDKN2A/CRYAB/DRAXIN/ESR2/GDF2/NPPA/OSGIN1/PPARD/PPP1R9B/SEMA3F/SIPA1/TNK1/TRIM40/TSPYL2/WNT11/WNT3A	25	BP

GO:0008544	epidermis development	71/2734	455/17381	0.55005	0.9369	0.92153	BMP4/C1orf68/CALML5/CASP3/COL17A1/COL7A1/CTSL/DNASE1L2/EGFR/EVPLL/FGFR2/FOXN1/FOXQ1/FURIN/GRHL3/HOXC13/JAG2/KRT12/KRT14/KRT16/KRT23/KRT3/KRT32/KRT6A/KRT6C/KRT71/KRT75/KRT76/KRT79/KRT81/KRT84/KRT85/KRTAP17-1/KRTAP4-3/KRTAP5-10/KRTAP5-3/KRTAP5-5/KRTAP5-7/KRTAP5-9/LAMC2/LCE3E/LDB1/LHFPL5/LRTOMT/MYCN/MYO7A/NOTCH1/NSDHL/NUMA1/PDGFA/PKP3/PLOD3/POU3F1/PPARD/PTCH2/RBP2/SCRIB/SFN/[illegible]	71	BP
GO:0007584	response to nutrient	27/2734	173/17381	0.55026	0.9369	0.92153	ABCG5/ALAD/BGLAP/C2/CHMP1A/COL1A1/CYP1A1/DAD1/DNMT3A/EGFR/FOLR2/GATA4/GNPAT/HSF1/LDHA/LTA/MTOR/OGG1/OTC/PEMT/PKM/POR/PPARD/RARA/SLC34A1/TNC/UCP3	27	BP
GO:0060759	regulation of response to cytokine	27/2734	173/17381	0.55026	0.9369	0.92153	CASP1/CCDC3/CCL5/CD300LF/CD74/CSF1/ECM1/ELF1/F2RL1/IFNA5/IKBKB/IL1RN/IRAK1/MADD/NR1H3/PARP9/PELI3/PTPN11/PYDC1/RNF31/SCRIB/SHARPIN/STAT1/STAT2/TRAF2/TRAIP/TREM2	27	BP
GO:0033574	response to testosterone	7/2734	44/17381	0.55062	0.9369	0.92153	BAD/BGLAP/CDK4/GBA/HSF1/NME1/RWDD1	7	BP
GO:0035850	epithelial cell differentiation involved in kidney	7/2734	44/17381	0.55062	0.9369	0.92153	BMP4/LHX1/MYO1E/NOTCH1/OSR1/SMO/STAT1	7	BP
GO:0051290	protein heterotetramerization	7/2734	44/17381	0.55062	0.9369	0.92153	CD74/CNGB1/GRIN1/HIST1H4F/HIST2H4A/HIST2H4B/PDSS1	7	BP
GO:0055078	sodium ion homeostasis	7/2734	44/17381	0.55062	0.9369	0.92153	AGT/ATP1A1/AVPR2/CYP11B2/DRD2/SCNN1A/TACR1	7	BP
GO:0070534	protein K63-linked ubiquitination	7/2734	44/17381	0.55062	0.9369	0.92153	OTUB1/PARP10/PELI3/TRAF2/UBE2B/UBE2E2/WWP2	7	BP
GO:0048489	synaptic vesicle transport	20/2734	128/17381	0.55067	0.9369	0.92153	CDK5/CNIH2/CPLX1/DNM1/DOC2A/DOC2B/DTNBP1/GAK/NLGN3/PLD2/SCRIB/STON2/STX1A/STX4/STXBP1/SYTL3/TOR1A/TRIM46/WNT3A/WNT7	20	BP

GO:0097480	establishment of synaptic vesicle localization	20/2734	128/17381	0.55067	0.9369	0.92153	CDK5/CNIH2/CPLX1/DNM1/DOC2A/DOC2B/DTNBP1/GAK/NLGN3/PLD2/SCRIB/STON2/STX1A/STX4/STXBP1/SYTL3/TOR1A/TRIM46/WNT3A/WNT7A	20	BP
GO:0099003	vesicle-mediated transport in	20/2734	128/17381	0.55067	0.9369	0.92153	CDK5/CNIH2/CPLX1/DNM1/DOC2A/DOC2B/DTNBP1/GAK/NLGN3/PLD2/SCRIB/STON2/STX1A/STX4/STXBP1/SYTL3/TOR1A/TRIM46/WNT3A/WNT7A	20	BP
GO:0008406	gonad development	34/2734	218/17381	0.55072	0.9369	0.92153	ANG/ARRB2/BOK/CSDE1/EIF2B2/EIF2B5/FANCA/GATA4/IDH1/INHBB/LFNG/LRRC6/MGST1/NOS3/NR5A1/NUDT1/NUP210L/NUPR1/OSR1/PDGFRB/RARA/REN/RXFP2/SCX/SDC1/SF1/SOX8/TSPY1/TSPY2/TSPY4/TSPY8/UTF	34	BP
GO:0055025	positive regulation of cardiac muscle	9/2734	57/17381	0.55111	0.9369	0.92153	ARRB2/BMP4/FGFR2/MIR222/MTOR/NOTCH1/NRG1/TBX2/WNT3A	9	BP
GO:0071277	cellular response to	9/2734	57/17381	0.55111	0.9369	0.92153	CACYBP/CAMK2D/CPNE7/DPEP1/ENDOG/HSPA5/NEUROD2/RASGRP2/SPAG16	9	BP
GO:0099132	ATP hydrolysis coupled cation transmembrane transport	9/2734	57/17381	0.55111	0.9369	0.92153	ATP13A2/ATP1A4/ATP2A3/ATP2C2/ATP5G1/ATP6V0B/ATP6V0E2/ATP6V1B1/ATP6V1F	9	BP
GO:0003209	cardiac atrium morphogenesis	5/2734	31/17381	0.55123	0.9369	0.92153	ENG/GATA4/NKX2-5/NOTCH1/SMO	5	BP
GO:0017004	cytochrome complex	5/2734	31/17381	0.55123	0.9369	0.92153	COA3/COA4/COX10/COX11/SMIM20	5	BP
GO:0030488	tRNA methylation	5/2734	31/17381	0.55123	0.9369	0.92153	METTL2A/METTL2B/THUMPD2/TRMT10B/TRMT44	5	BP
GO:0032689	negative regulation of interferon-	5/2734	31/17381	0.55123	0.9369	0.92153	HAVCR2/IL20RB/LGALS9/RARA/SCGB1A1	5	BP
GO:0032728	positive regulation of interferon-beta	5/2734	31/17381	0.55123	0.9369	0.92153	IRF5/POLR3D/TBK1/TLR8/TLR9	5	BP

GO:0034694	response to prostaglandin	5/2734	31/17381	0.55123	0.9369	0.92153	APOB/CCL19/CCL21/P2RY6/TNC	5	BP
GO:0045191	regulation of isotype	5/2734	31/17381	0.55123	0.9369	0.92153	BCL6/CLCF1/PAXIP1/SUPT6H/TNFSF13	5	BP
GO:0045841	negative regulation of mitotic metaphase/anap	5/2734	31/17381	0.55123	0.9369	0.92153	ANAPC15/BUB1B/LCMT1/MAD1L1/XRCC3	5	BP
GO:0046471	phosphatidylglycerol metabolic process	5/2734	31/17381	0.55123	0.9369	0.92153	HADHA/PLA2G1B/PLA2G2F/PLA2G4B/PLA2G5	5	BP
GO:0060603	mammary gland duct morphogenesis	5/2734	31/17381	0.55123	0.9369	0.92153	CAV3/CSF1/CSF1R/FGFR2/SCRIB	5	BP
GO:0070286	axonemal dynein complex	5/2734	31/17381	0.55123	0.9369	0.92153	CCDC103/CCDC40/DNHD1/LRRC6/TMEM141	5	BP
GO:0090504	epiboly	5/2734	31/17381	0.55123	0.9369	0.92153	FLNA/MIR221/MTOR/TOR1A/WNT7A	5	BP
GO:2000404	regulation of T cell migration	5/2734	31/17381	0.55123	0.9369	0.92153	ADAM8/CCL21/CCL5/CCR2/ECM1	5	BP
GO:0006809	nitric oxide biosynthetic process	11/2734	70/17381	0.55208	0.9369	0.92153	AGT/CLU/EGFR/GCH1/GCHFR/HSP90AA1/INS/MIR92A2/MTOR/NOS3/PTK2B	11	BP
GO:0061045	negative regulation of	11/2734	70/17381	0.55208	0.9369	0.92153	AJAP1/C1QTNF1/FGA/KLKB1/KNG1/NOS3/PDGFA/PLAU/PRKCD/PROZ/SERPINF2	11	BP
GO:1901992	positive regulation of mitotic cell cycle phase	11/2734	70/17381	0.55208	0.9369	0.92153	APEX1/CDK10/CDK4/CYP1A1/EIF4G1/MEPCE/MIR221/MIR222/MIR29A/PHOX2B/UBE2E2	11	BP

GO:0001764	neuron migration	22/2734	141/17381	0.55247	0.9369	0.92153	BARHL1/BARHL2/CDK5/CDK5R2/CELSR3/DAB1/DAB2IP/DISC1/DRD2/FLNA/KIRREL3/LHX1/LRIG2/MARK2/NRG1/NSMF/OLIG3/PHOX2B/SDCCAG8/TLX3/TRIM46/VAX1	22	BP
GO:0044344	cellular response to fibroblast growth factor	22/2734	141/17381	0.55247	0.9369	0.92153	CCL5/COL1A1/ESRP2/FGF17/FGF3/FGFBP3/FGFR2/FGFR4/FGFRL1/GPC1/HHIP/LHX1/MAPK3/MIR15A/MIR16-1/NR4A1/POLR2G/POLR2L/PRDM14/PTPN11/SHCBP1/TDGF1	22	BP
GO:0050804	modulation of chemical synaptic transmission	48/2734	308/17381	0.55272	0.9369	0.92153	ABL1/ACHE/ADGRB1/ADRA1A/AGT/ANAPC2/ARC/ARRB2/CA7/CDK5/CLSTN1/CLSTN3/DBN1/DGKI/DISC1/DLG4/DRD2/DTNBP1/EGFR/EIF2AK4/GPER1/GRIN1/HRAS/HRH2/INS/ITPR3/JPH3/LAMA2/LRRTM1/NCDN/NEUROD2/NISCH/NLGN3/NSMF/PHF24/PNKD/PPP1R9B/PTK2B/RARA/RGS14/STX1A/STX4/STXBP1/TACR1/TACR2/TOR1A/VGF/WNT7A	48	BP
GO:0001708	cell fate specification	13/2734	83/17381	0.5533	0.9369	0.92153	EVX1/EYA1/HOXC10/LHX3/NOTCH1/OLIG3/PRDM14/PTCH2/SHH/SMO/SUFU/TLX3/WNT3A	13	BP
GO:0070542	response to fatty acid	13/2734	83/17381	0.5533	0.9369	0.92153	ALAD/APOB/BAD/CCL19/CCL21/CDK4/GNPAT/KCNK4/MIR92A2/NME1/P2RY6/PID1/TNC	13	BP
GO:0006606	protein import into nucleus	43/2734	276/17381	0.55338	0.9369	0.92153	AGT/BCL6/BMP4/CCL19/CD27/CSF3/DAB2IP/EGFR/EMD/FAM89B/FLNA/GPN2/HEATR3/IL18/IPO13/IPO4/LGALS9/LMNA/MTOR/NFKBIL1/NUP98/OGG1/OPRD1/PARP10/PBLD/PDE2A/PKD1/POLA2/PPP1R10/PRDX1/PRKCD/RBPMS/RPAIN/SFRP5/SHH/SMO/SUFU/THRA/TLR9/TMCO6/TNPO3/WNT3A	43	BP
GO:0097711	ciliary basal body-plasma membrane	15/2734	96/17381	0.55463	0.9369	0.92153	ACTR1A/B9D1/C2CD3/CC2D2A/CEP164/CEP70/CKAP5/DCTN2/DYNC1H1/HAUS4/HAUS7/HSP90AA1/SDCCAG8/SSNA1/TUBG1	15	BP
GO:0000394	RNA splicing, via endonucleolytic cleavage and	3/2734	18/17381	0.55496	0.9369	0.92153	CPSF1/CPSF4/CSTF2	3	BP
GO:0002313	mature B cell differentiation involved in immune	3/2734	18/17381	0.55496	0.9369	0.92153	LFNG/NKX2-3/PTK2B	3	BP

GO:0002902	regulation of B cell apoptotic	3/2734	18/17381	0.55496	0.9369	0.92153	BCL6/CD74/NOC2L	3	BP
GO:0006271	DNA strand elongation involved in DNA	3/2734	18/17381	0.55496	0.9369	0.92153	PARP2/PARP3/POLE	3	BP
GO:0006349	regulation of gene expression by genetic	3/2734	18/17381	0.55496	0.9369	0.92153	BRCA1/DNMT3A/KCNQ1	3	BP
GO:0007250	activation of NF-kappaB-inducing kinase	3/2734	18/17381	0.55496	0.9369	0.92153	IRAK1/TIRAP/TRAF2	3	BP
GO:0007625	grooming behavior	3/2734	18/17381	0.55496	0.9369	0.92153	DLG4/DRD2/HOXB8	3	BP
GO:0016137	glycoside metabolic	3/2734	18/17381	0.55496	0.9369	0.92153	GBA/SLC34A1/TH	3	BP
GO:0030502	negative regulation of bone	3/2734	18/17381	0.55496	0.9369	0.92153	AHSG/ECM1/PTK2B	3	BP
GO:0032042	mitochondrial DNA metabolic process	3/2734	18/17381	0.55496	0.9369	0.92153	DNAJA3/LIG3/PID1	3	BP
GO:0032288	myelin assembly	3/2734	18/17381	0.55496	0.9369	0.92153	GNPAT/GPC1/ILK	3	BP
GO:0035435	phosphate ion transmembrane transport	3/2734	18/17381	0.55496	0.9369	0.92153	SLC20A1/SLC20A2/SLC34A1	3	BP
GO:0042226	interleukin-6 biosynthetic process	3/2734	18/17381	0.55496	0.9369	0.92153	CARD9/INPP5D/TIRAP	3	BP

GO:0043984	histone H4-K16 acetylation	3/2734	18/17381	0.55496	0.9369	0.92153	BRCA1/HCFC1/MSL3	3	BP
GO:0044117	growth of symbiont in	3/2734	18/17381	0.55496	0.9369	0.92153	LTA/MPO/TIRAP	3	BP
GO:0045655	regulation of monocyte differentiation	3/2734	18/17381	0.55496	0.9369	0.92153	CD74/CSF1/INPP5D	3	BP
GO:0045821	positive regulation of glycolytic	3/2734	18/17381	0.55496	0.9369	0.92153	INS/MLXIPL/PFKFB1	3	BP
GO:0046112	nucleobase biosynthetic process	3/2734	18/17381	0.55496	0.9369	0.92153	DHODH/MTHFD1/MTOR	3	BP
GO:0050860	negative regulation of T cell receptor	3/2734	18/17381	0.55496	0.9369	0.92153	ELF1/LGALS3/THY1	3	BP
GO:0051412	response to corticosterone	3/2734	18/17381	0.55496	0.9369	0.92153	FOSL1/TH/TRH	3	BP
GO:0051767	nitric-oxide synthase biosynthetic process	3/2734	18/17381	0.55496	0.9369	0.92153	FNTB/KDR/TLR9	3	BP
GO:0051769	regulation of nitric-oxide synthase biosynthetic	3/2734	18/17381	0.55496	0.9369	0.92153	FNTB/KDR/TLR9	3	BP
GO:0070977	bone maturation	3/2734	18/17381	0.55496	0.9369	0.92153	PHOSPHO1/PLXNB1/THBS3	3	BP
GO:0071294	cellular response to zinc	3/2734	18/17381	0.55496	0.9369	0.92153	ATP13A2/GLRA1/MT2A	3	BP

GO:0072576	liver morphogenesis	3/2734	18/17381	0.55496	0.9369	0.92153	LIMS2/RPS6KA1/WNT3A	3	BP
GO:0072643	interferon-gamma	3/2734	18/17381	0.55496	0.9369	0.92153	ABL1/F2RL1/LGALS9	3	BP
GO:0097242	amyloid-beta clearance	3/2734	18/17381	0.55496	0.9369	0.92153	CLU/ITGAM/LRP1	3	BP
GO:0098581	detection of external biotic stimulus	3/2734	18/17381	0.55496	0.9369	0.92153	NOD1/SCARB1/TREM2	3	BP
GO:1903055	positive regulation of extracellular	3/2734	18/17381	0.55496	0.9369	0.92153	AGT/DDR2/FSCN1	3	BP
GO:1904872	regulation of telomerase RNA localization to	3/2734	18/17381	0.55496	0.9369	0.92153	CCT3/EXOSC10/NHP2	3	BP
GO:1905276	regulation of epithelial tube formation	3/2734	18/17381	0.55496	0.9369	0.92153	DVL2/FZD2/GRHL3	3	BP
GO:2001269	positive regulation of cysteine-type endopeptidase activity involved in	3/2734	18/17381	0.55496	0.9369	0.92153	FASLG/LGALS9/TRAF2	3	BP
GO:0006353	DNA-templated transcription, termination	17/2734	109/17381	0.55604	0.938	0.92255	CASC3/CPSF1/CPSF4/CSTF2/DHX38/GTF2H4/MED18/POLR1A/POLR1D/POLR1E/POLR2L/RNPS1/SRSF4/TAF1A/TTF1/TTF2/ZNRD1	17	BP
GO:0071482	cellular response to	17/2734	109/17381	0.55604	0.938	0.92255	AQP1/CNGB1/CRIP1/DDB1/DDB2/EIF2AK4/FNTA/FNTB/GNAT1/GUCA1B/GUCY2D/INO80/NOC2L/OPN4/PPEF1/TAF1/XPC	17	BP

GO:0009152	purine ribonucleotide biosynthetic process	40/2734	257/17381	0.55605	0.938	0.92255	ACOT7/ADM/ADRA2A/AK5/ALDOA/AQP1/ATP5G1/ATP5I/AVPR2/CCR2/CRHR1/CYC1/DRD2/GABBR1/GCG/GPER1/GPHA2/GUCA1B/GUCA2A/GUCA2B/GUCY2D/IMPDH1/NME1/NME4/NOS3/NPPA/OPRM1/PDE2A/PDZD3/PID1/PKM/PPCDC/PTK2B/RAF1/RUNDC3A/RXFP2/SLC26A1/UCN2/VPS9D1/	40	BP
GO:0000075	cell cycle checkpoint	35/2734	225/17381	0.55735	0.9399	0.92445	ANAPC15/ATF2/ATRIP/BRCA1/BRCC3/BUB1B/CCAR2/CDK5RAP3/CHFR/CHMP4C/E2F4/E2F8/EIF2AK4/FBXO6/FOXO4/HINFP/HORMAD1/HRAS/INTS3/LCMT1/MAD1L1/MUC1/NEK11/PCBP4/PEA15/PRCC/PTPN11/RINT1/SFN/TAOK2/TICRR/TIPRL/XPC/XRCC3/ZNF385A	35	BP
GO:0009260	ribonucleotide biosynthetic process	42/2734	270/17381	0.55772	0.9403	0.9248	ACOT7/ADM/ADRA2A/AK5/ALDOA/AQP1/ATP5G1/ATP5I/AVPR2/CCR2/CRHR1/CYC1/DHODH/DRD2/GABBR1/GCG/GPER1/GPHA2/GUCA1B/GUCA2A/GUCA2B/GUCY2D/IMPDH1/NME1/NME4/NOS3/NPPA/OPRM1/PDE2A/PDZD3/PID1/PKM/PPCDC/PTK2B/RAF1/RUNDC3A/RXFP2/SLC26A1/UCK1	42	BP
GO:0006090	pyruvate metabolic	21/2734	135/17381	0.55891	0.942	0.92651	ALDOA/GALK1/GAPDH/GCK/GLO1/HK3/INS/LDHA/MLXIPL/MPC1/NCOR1/NUP210/NUP98/OGDH/OGDHL/PC/PDHB/PFKFB1/PFKM/PGAM4/PKM	21	BP
GO:0010498	proteasomal protein catabolic process	68/2734	437/17381	0.55993	0.9434	0.92793	ABTB1/ALAD/ANAPC15/ANAPC2/ARAF/ARRB2/AXIN1/BCAP31/BRSK2/BUB1B/CCAR2/CCNF/CHFR/CLU/COMMD1/DAB2IP/DDB1/ECSCR/FBXL19/FBXL22/FBXO2/FBXO6/FBXW4/FHIT/GBA/GPX1/HECTD3/HERPUD1/HSPA5/KCTD5/KLHL40/LAMP3/MAEA/MTA1/NKD2/OS9/OSBPL7/PANO1/PKD1/PSMB11/PSMB6/PSMB7/PSMB8/PSMC3/PSMD13/PSMD3/PSMD5/PSMD7/RHBDF1/RMND5B/RNF166/RNF180/SDF2/SHARPIN/SHH/SPOP/SYVN1/TAF1/TMUB1/TOR1A/TRIM72/UBE2A/UBE2B/UBE2U/USP19/USP5/WFS1/WW[illegible]	68	BP
GO:0007260	tyrosine phosphorylation of STAT	12/2734	77/17381	0.56234	0.9455	0.92995	ARL2BP/CCL5/CLCF1/CSF1R/HSF1/IL13/IL18/IL31RA/INPP5F/MIR221/PARP9/TNFRSF18	12	BP
GO:2001057	reactive nitrogen species metabolic	12/2734	77/17381	0.56234	0.9455	0.92995	AGT/CLU/EGFR/GCH1/GCHFR/HSP90AA1/INS/MIR92A2/MTOR/NOS3/POR/PTK2B	12	BP
GO:0009948	anterior/posterior axis	8/2734	51/17381	0.56265	0.9455	0.92995	BMP4/CDX2/LDB1/LHX1/RIPPLY1/SHH/TDGF1/WLS	8	BP

GO:0030809	negative regulation of nucleotide biosynthetic	8/2734	51/17381	0.56265	0.9455	0.92995	ADRA2A/CCR2/DRD2/GABBR1/OPRM1/PDE2A/PDZD3/PID1	8	BP
GO:0046148	pigment biosynthetic process	8/2734	51/17381	0.56265	0.9455	0.92995	ALAD/ALAS2/ATPIF1/COX10/HMBS/MTHFD1/NFE2L1/UROS	8	BP
GO:0046426	negative regulation of JAK-STAT	8/2734	51/17381	0.56265	0.9455	0.92995	BGN/CHAD/CISH/DAB1/INPP5F/LRRTM1/RTN4RL1/RTN4RL2	8	BP
GO:0050771	negative regulation of	8/2734	51/17381	0.56265	0.9455	0.92995	ARHGAP4/CDK5/DAB1/DRAXIN/LINGO1/SEMA3F/THY1/WNT3A	8	BP
GO:1900372	negative regulation of purine nucleotide biosynthetic	8/2734	51/17381	0.56265	0.9455	0.92995	ADRA2A/CCR2/DRD2/GABBR1/OPRM1/PDE2A/PDZD3/PID1	8	BP
GO:1904893	negative regulation of	8/2734	51/17381	0.56265	0.9455	0.92995	BGN/CHAD/CISH/DAB1/INPP5F/LRRTM1/RTN4RL1/RTN4RL2	8	BP
GO:0072524	pyridine-containing compound	27/2734	174/17381	0.5632	0.9455	0.92995	ALDOA/GALK1/GAPDH/GCK/HAAO/HK3/IDH1/IDH2/INS/LDHA/MLXIPL/MPC1/NCOR1/NMNAT3/NUP210/NUP98/OGDH/OGDHL/PARP10/PARP9/PDHB/PFKFB1/PFKM/PGAM4/PKM/QPRT/SLC22A13	27	BP
GO:0032526	response to retinoic acid	16/2734	103/17381	0.5637	0.9455	0.92995	AQP1/COL1A1/CTSH/FGFR2/GJB3/LTK/MICB/OSR1/PDGFRB/PTK2B/RARA/TIE1/TNC/WNT11/WNT3A/WNT6	16	BP
GO:0000096	sulfur amino acid metabolic	6/2734	38/17381	0.56445	0.9455	0.92995	BHMT2/BLMH/DPEP1/GNMT/MTHFD1/NOX4	6	BP
GO:0010677	negative regulation of cellular carbohydrate	6/2734	38/17381	0.56445	0.9455	0.92995	COX11/GCK/INS/LEPR/MAEA/PFKFB1	6	BP

GO:0014003	oligodendrocyte development	6/2734	38/17381	0.56445	0.9455	0.92995	CLU/EIF2B2/EIF2B5/HDAC11/PRDM8/SHH	6	BP
GO:0019048	modulation by virus of host morphology or physiology	6/2734	38/17381	0.56445	0.9455	0.92995	BAD/CPSF4/EIF2AK4/MIR221/MIR222/SCRIB	6	BP
GO:0033003	regulation of mast cell	6/2734	38/17381	0.56445	0.9455	0.92995	CD300LF/FES/IL13/IL4R/LGALS9/STXBP1	6	BP
GO:0042769	DNA damage response, detection of DNA damage	6/2734	38/17381	0.56445	0.9455	0.92995	DDB1/MRPS11/POLD4/RFC1/RFC2/UBE2B	6	BP
GO:0051350	negative regulation of	6/2734	38/17381	0.56445	0.9455	0.92995	ADRA2A/CCR2/DRD2/GABBR1/OPRM1/PDZD3	6	BP
GO:0060323	head morphogenesis	6/2734	38/17381	0.56445	0.9455	0.92995	COL1A1/CSRNP1/MMP2/PTPN11/SCX/STRA6	6	BP
GO:0061001	regulation of dendritic spine morphogenesis	6/2734	38/17381	0.56445	0.9455	0.92995	ARC/CDK5/CFL1/EFNA1/NLGN3/PDLIM5	6	BP
GO:0034754	cellular hormone	18/2734	116/17381	0.56464	0.9455	0.92995	ADM/ALDH8A1/CACNA1H/CYP11A1/CYP11B2/CYP1A1/DGAT1/DGKQ/EGR1/HSD17B1/HSD17B3/HSD17B7/HSD3B1/PLB1/RBP1/SCARB1/SHH/STA	18	BP
GO:0051208	sequestering of calcium ion	18/2734	116/17381	0.56464	0.9455	0.92995	ABL1/CAMK2D/CCL19/CCL21/CORO1A/DRD2/FASLG/GPER1/GSTM2/IL13/ITPR3/JPH3/LCK/NPSR1/PTK2B/RASA3/THY1/TRDN	18	BP
GO:0051053	negative regulation of DNA metabolic	20/2734	129/17381	0.56567	0.9459	0.9303	ACD/BCL6/CDAN1/EXOSC10/GDF2/GPER1/HSF1/LIG3/MEN1/MIR221/NUDT16L1/OGG1/OTUB1/PDS5A/PID1/PIF1/RECQL5/TINF2/TSPYL2/TTF1	20	BP
GO:0007188	adenylate cyclase-modulating G-protein coupled receptor	24/2734	155/17381	0.56789	0.9459	0.9303	ADRA1A/ADRA1B/ADRA2A/ADRA2B/ADRA2C/AVPR2/CHGA/CHRM1/CRHR1/CRHR2/DRD2/FLNA/GABBR1/GCG/GNAO1/GNAT1/GPER1/HTR1D/OPRD1/OPRM1/PSAP/RXFP2/TSKU/UCN2	24	BP

GO:0035967	cellular response to topologically	24/2734	155/17381	0.56789	0.9459	0.9303	ACADVL/ASNS/ATF3/BOK/CDK5RAP3/CREB3/DAB2IP/EXTL1/GOSR2/HERPUD1/HSF1/HSPA5/LMNA/PLA2G4B/PPP2R5B/SDF2/SEC61A2/SHC1/SYVN1/TLN1/TOR1A/TSPYL2/WFS1/ZBTB17	24	BP
GO:0050890	cognition	42/2734	271/17381	0.5681	0.9459	0.9303	ARC/B4GALT2/C1QL1/CASP3/CDK5/CHRM1/DEAF1/DGKI/DLG4/DRD2/EGFR/EIF2AK4/EN1/FOSL1/FOXO6/FZD9/GPR88/GRIN1/HRH2/IFT20/INS/ITPR3/JPH3/KCNK4/MEIS2/MTOR/NEUROD2/NLGN3/NQO2/NRXN2/PPP1R1B/PRKAR1B/RGS14/SGK1/ST3GAL4/STRA6/TACR1/TACR2/TBR1/TH/THR	42	BP
GO:0090090	negative regulation of canonical Wnt signaling	26/2734	168/17381	0.56905	0.9459	0.9303	AXIN1/CHD8/DAB2IP/DRAXIN/DVL2/EGR1/IGFBP6/KREMEN2/LZTS2/NKD2/NKX2-5/NOTCH1/PSMB11/PSMB6/PSMB7/PSMB8/PSMC3/PSMD13/PSMD3/PSMD5/PSMD7/SFRP5/SHH/SOST/TNN/WNT11	26	BP
GO:0001975	response to amphetamine	4/2734	25/17381	0.56931	0.9459	0.9303	DRD2/GRIN1/PPP1R1B/TH	4	BP
GO:0002507	tolerance induction	4/2734	25/17381	0.56931	0.9459	0.9303	HAVCR2/IDO1/LGALS9/PDCD1	4	BP
GO:0005980	glycogen catabolic	4/2734	25/17381	0.56931	0.9459	0.9303	INS/PFKM/PHKG1/PYGM	4	BP
GO:0006379	mRNA cleavage	4/2734	25/17381	0.56931	0.9459	0.9303	CPSF1/CPSF4/CSTF2/ZNRD1	4	BP
GO:0009649	entrainment of circadian clock	4/2734	25/17381	0.56931	0.9459	0.9303	MTA1/PER1/RBM4/USP2	4	BP
GO:0021591	ventricular system	4/2734	25/17381	0.56931	0.9459	0.9303	ANP32B/AQP1/HYDIN/TSKU	4	BP
GO:0034035	purine ribonucleoside bisphosphate metabolic	4/2734	25/17381	0.56931	0.9459	0.9303	ABHD14B/PODXL2/SLC26A1/SULT6B1	4	BP
GO:0034110	regulation of homotypic cell-cell adhesion	4/2734	25/17381	0.56931	0.9459	0.9303	C1QTNF1/CCL5/PRKCD/ZNF703	4	BP

GO:0044062	regulation of excretion	4/2734	25/17381	0.56931	0.9459	0.9303	AGT/AVPR2/DRD2/TACR1	4	BP
GO:0045736	negative regulation of cyclin-dependent protein	4/2734	25/17381	0.56931	0.9459	0.9303	CASP3/CDKN2A/INCA1/MEN1	4	BP
GO:0050427	3'-phosphoadenosine 5'-phosphosulfate	4/2734	25/17381	0.56931	0.9459	0.9303	ABHD14B/PODXL2/SLC26A1/SULT6B1	4	BP
GO:0050927	positive regulation of positive	4/2734	25/17381	0.56931	0.9459	0.9303	ARTN/CDH13/F2RL1/KDR	4	BP
GO:0060143	positive regulation of syncytium formation by plasma	4/2734	25/17381	0.56931	0.9459	0.9303	ADGRB1/EHD1/IL4R/MYOD1	4	BP
GO:0000079	regulation of cyclin-dependent protein serine/threonine	13/2734	84/17381	0.57181	0.9459	0.9303	CASP3/CCND3/CDK5R2/CDK5RAP3/CDKN2A/EGFR/HSPA2/INCA1/MAPRE3/MEN1/PKD1/PKMYT1/SFN	13	BP
GO:0032392	DNA geometric change	13/2734	84/17381	0.57181	0.9459	0.9303	CHD8/DDB1/DDB2/GTF2H4/HMGA1/IGHMBP2/INO80/MCM2/MCM4/PIF1/RECQL4/RECQL5/XPC	13	BP
GO:0051444	negative regulation of ubiquitin-	13/2734	84/17381	0.57181	0.9459	0.9303	ABL1/ANAPC15/ANAPC2/BUB1B/LIMK1/PSMB6/PSMB7/PSMB8/PSMC3/PSMD13/PSMD3/PSMD5/PSMD7	13	BP
GO:0002027	regulation of heart rate	15/2734	97/17381	0.57186	0.9459	0.9303	ADM/ADRA1A/AGT/CACNA1G/CAMK2D/CAV3/DRD2/EDN2/GCH1/KCND3/KCNH6/KCNJ5/KCNQ1/MIR328/NPPA	15	BP

GO:0048638	regulation of developmental growth	48/2734	310/17381	0.57217	0.9459	0.9303	ABL1/ACACB/AGRN/ANAPC2/ARHGAP4/BARHL2/BMP4/CAV3/CDK4/CDK5/CPNE5/CPNE9/CSF1/CTTN/DISC1/DRAXIN/DRD2/FGFR2/FN1/GJD4/HLX/HSF1/ILK/ISLR2/L1CAM/LIMK1/MIR222/MIR25/MTOR/MYOD1/NKX2-5/NOTCH1/NRG1/PHLDA2/POR/PPARD/PPIB/PTPN11/RAI1/SEMA3F/SEMA7A/SH3PXD2B/SMO/TBX2/TNFRSF12A/TRPV2/WNT3A/ZFYVE27	48	BP
GO:0042274	ribosomal small subunit	11/2734	71/17381	0.57221	0.9459	0.9303	ERAL1/FCF1/MRPS11/NOB1/NOL11/PDCD11/RPS24/SRFBP1/SURF6/TSR1/TSR2	11	BP
GO:0051436	negative regulation of ubiquitin-protein ligase activity	11/2734	71/17381	0.57221	0.9459	0.9303	ANAPC15/ANAPC2/BUB1B/PSMB6/PSMB7/PSMB8/PSMC3/PSMD13/PSMD3/PSMD5/PSMD7	11	BP
GO:0006406	mRNA export from nucleus	17/2734	110/17381	0.57222	0.9459	0.9303	ALKBH5/CASC3/CPSF1/CPSF4/DHX38/EIF5A/NUP210/NUP98/NXF2/NXF2B/NXF3/RNPS1/SETD2/SMG5/SRSF4/SUPT6H/ZC3H3	17	BP
GO:0071427	mRNA-containing ribonucleoprotein complex	17/2734	110/17381	0.57222	0.9459	0.9303	ALKBH5/CASC3/CPSF1/CPSF4/DHX38/EIF5A/NUP210/NUP98/NXF2/NXF2B/NXF3/RNPS1/SETD2/SMG5/SRSF4/SUPT6H/ZC3H3	17	BP
GO:1903670	regulation of sprouting angiogenesis	9/2734	58/17381	0.57336	0.9459	0.9303	ABL1/CIB1/MIR16-1/MIR221/MIR222/MIR29C/MIR503/MIR92A2/NOTCH1	9	BP
GO:0009267	cellular response to	21/2734	136/17381	0.57346	0.9459	0.9303	AMBRA1/ASNS/ATF3/DAP/DAPL1/EHMT2/EIF2AK4/GBA/HSPA5/INHBB/LARS/MAPK3/MAX/MTOR/MYOD1/NPRL3/RALB/RRAGC/SESN1/SLC39A5/	21	BP
GO:0060078	regulation of postsynaptic membrane potential	21/2734	136/17381	0.57346	0.9459	0.9303	CACNA1G/CACNA1H/CDK5/CHRNA6/CHRNB1/CHRNB3/DGKI/DLG4/GLRA1/GRIA3/GRIN1/GRIN2C/HCN3/NLGN3/OPRM1/P2RX2/PTK2B/SCN11A/SLC29A1/STX1A/WNT7A	21	BP
GO:0032757	positive regulation of interleukin-8	7/2734	45/17381	0.57585	0.9459	0.9303	CD58/F2RL1/GDF2/LGALS9/TIRAP/TLR8/TLR9	7	BP

GO:0044380	protein localization to	7/2734	45/17381	0.57585	0.9459	0.9303	C2CD3/DCTN2/DISC1/FAM83H/MCPH1/NUMA1/PARD6A	7	BP
GO:0060443	mammary gland morphogenesis	7/2734	45/17381	0.57585	0.9459	0.9303	BMP4/CAV3/CSF1/CSF1R/FGFR2/SCRIB/TBX2	7	BP
GO:0060760	positive regulation of response to	7/2734	45/17381	0.57585	0.9459	0.9303	CASP1/CD300LF/CD74/CSF1/PARP9/TRAF2/TREM2	7	BP
GO:0022409	positive regulation of cell-cell adhesion	40/2734	259/17381	0.57725	0.9459	0.9303	ADAM8/BAD/BCL6/CARD11/CCDC88B/CCL19/CCL21/CCL5/CCR2/CD247/CD27/CD5/CD6/CD74/CLECL1/CORO1A/CSK/DNAJA3/EFNB1/FGA/HAVCR2/HLA-E/HLX/IL18/IL4R/LCK/LGALS9/MAP3K14/MIR92A2/PDCD1/PIK3R6/PTPN1	40	BP
GO:0051224	negative regulation of protein	33/2734	214/17381	0.57895	0.9459	0.9303	ADRA2A/CD27/CDK5/CSK/DAB2IP/DRD2/EMD/F2RL1/FAM89B/FN1/IDH2/INHBB/INS/MTOR/NFKBIL1/NR1H3/OPRM1/OS9/PARP10/PBLD/PDE2A/PKD1/PKDCC/PTPN11/RAB11FIP3/RHBDF1/SERGEF/SFRP5/SNX12/SUFU/TH	33	BP
GO:0001654	eye development	53/2734	343/17381	0.58	0.9459	0.9303	ABCB5/ACHE/AQP1/AQP5/AXIN1/B9D1/BMP4/CC2D2A/CDK4/CLCN2/CRYAB/CYP1A1/EGFR/FASLG/FOXE3/GNAT1/GRHL3/IFT140/LAMC3/LHX1/MAX/MEIS2/MFN2/MIP/MYO7A/NAGLU/NES/NRL/PDGFRB/PRSS56/PYGO2/RARA/SH3PXD2B/SHH/SLC25A25/SLC39A5/SOX8/STRA6/TBC1D32/TBX2/TH/THY1/TMEM231/TRAF3IP1/TSKU/TTLL5/TULP1/TULP3/VAX1/VAX	53	BP
GO:0051817	modification of morphology or physiology of other organism involved in symbiotic interaction	16/2734	104/17381	0.58026	0.9459	0.9303	AQP1/BAD/CCL5/CFL1/CPSF4/EIF2AK4/F2RL1/GAPDH/MIR221/MIR222/PC/PPIB/PSMC3/SCRIB/TUSC2/ZNF502	16	BP
GO:0043154	negative regulation of cysteine-type endopeptidase activity	14/2734	91/17381	0.5806	0.9459	0.9303	AQP1/ARRB2/CD27/CRYAB/DPEP1/GPX1/HERPUD1/LAMP3/NAIP/NLE1/POR/RAF1/RPS6KA1/SFN	14	BP

GO:0050818	regulation of coagulation	14/2734	91/17381	0.5806	0.9459	0.9303	ASIC2/C1QTNF1/F2RL1/FGA/KLKB1/KNG1/NOS3/PDGFA/PLAU/PRKCD/PROZ/SELP/SERPINF2/TEC	14	BP
GO:0050830	defense response to Gram-positive	14/2734	91/17381	0.5806	0.9459	0.9303	C10orf99/CARD9/CHGA/GSDMD/HAVCR2/HLA-E/LTA/NOD1/PLA2G1B/RARRES2/RNASE8/SPACA3/TBK1/TIRAP	14	BP
GO:0016482	cytosolic transport	22/2734	143/17381	0.5809	0.9459	0.9303	AKTIP/AP1S1/CORO1A/DENND2A/FAM160A2/GAK/GBF1/GOSR2/MAPK3/PTPN23/RAB7A/RBSN/SGSM2/SNF8/SNX12/STX5/STX8/VPS13A/VPS51/VPS52/VPS53/VTI1B	22	BP
GO:0030856	regulation of epithelial cell differentiation	22/2734	143/17381	0.5809	0.9459	0.9303	AJAP1/BAD/BMP4/CDH5/CTSL/FOXE3/FOXN1/GDF2/IKBKB/IL13/KRT84/LHX1/MYCN/NOTCH1/NUMA1/OSR1/PTCH2/SFN/SMO/STAT1/TMEM100/XDH	22	BP
GO:0061337	cardiac conduction	22/2734	143/17381	0.5809	0.9459	0.9303	AGT/ATP1A1/ATP1A4/ATP2A3/CACNA1F/CACNA1G/CACNA1S/CACNB1/CACNB3/CACNG1/CAMK2D/FLNA/ITPR3/KCND1/KCND3/KCNH6/KCNJ5/KCNQ1/MIR328/NKX2-5/NPPA/TRDN	22	BP
GO:0008038	neuron recognition	5/2734	32/17381	0.58109	0.9459	0.9303	AMIGO1/CELSR3/EPHB3/NTM/PCDH12	5	BP
GO:0008089	anterograde axonal transport	5/2734	32/17381	0.58109	0.9459	0.9303	CNIH2/DTNBP1/HSPB1/KIF4A/TRIM46	5	BP
GO:0033363	secretory granule	5/2734	32/17381	0.58109	0.9459	0.9303	AQP1/CCDC136/DTNBP1/TBPL1/ZNF385A	5	BP
GO:0035115	embryonic forelimb morphogenesis	5/2734	32/17381	0.58109	0.9459	0.9303	ALX3/EN1/OSR1/SHH/WNT7A	5	BP
GO:0045923	positive regulation of fatty acid	5/2734	32/17381	0.58109	0.9459	0.9303	APOA5/ELOVL5/MID1IP1/MLXIPL/NR1H3	5	BP
GO:0050435	amyloid-beta metabolic	5/2734	32/17381	0.58109	0.9459	0.9303	APH1A/CLU/EFNA1/NAT8B/REN	5	BP
GO:0085029	extracellular matrix assembly	5/2734	32/17381	0.58109	0.9459	0.9303	AGT/EFEMP2/LAMC2/NOTCH1/PLOD3	5	BP

GO:0086019	cell-cell signaling involved in	5/2734	32/17381	0.58109	0.9459	0.9303	CACNA1G/FLNA/KCNJ5/KCNQ1/MIR328	5	BP
GO:0090075	relaxation of muscle	5/2734	32/17381	0.58109	0.9459	0.9303	ATP1A1/CAMK2D/CHGA/GSTM2/MIR153-1	5	BP
GO:1902100	negative regulation of metaphase/anaphase transition of cell cycle	5/2734	32/17381	0.58109	0.9459	0.9303	ANAPC15/BUB1B/LCMT1/MAD1L1/XRCC3	5	BP
GO:0015718	monocarboxylic acid transport	24/2734	156/17381	0.58143	0.9459	0.9303	ABCC3/ABCC4/ACACB/CPT2/CROT/DRD2/GOT2/LCN12/MFSD2A/MID1IP1/NCOR1/PLA2G1B/PLA2G2F/PLA2G5/PPARD/SLC16A13/SLC22A13/SLC22A9/SLC25A20/SLC26A6/SLCO2B1/STARD5/SV2A/TRH	24	BP
GO:0007215	glutamate receptor	12/2734	78/17381	0.58144	0.9459	0.9303	ARC/CNIH2/DAPK1/DLG4/GRIA3/GRIN1/GRIN2C/HOMER2/NECAB2/NLGN3/OPRM1/PTK2B	12	BP
GO:1903708	positive regulation of hemopoiesis	26/2734	169/17381	0.58205	0.9459	0.9303	ADAM8/BAD/BCL6/CCL19/CD27/CD74/CSF1/CSF3/FES/HLX/IL18/IL4R/INPP5D/ISG15/LGALS9/MIR221/MIR222/PDCD2/PIK3R6/PPP2R3C/RARA/SART1/SCIN/SHH/STAT1/THPO	26	BP
GO:0015807	L-amino acid transport	10/2734	65/17381	0.58306	0.9459	0.9303	AGT/PQLC2/SLC11A1/SLC1A7/SLC25A22/SLC36A1/SLC36A3/SLC38A1/SLC6A9/SLC7A7	10	BP
GO:1904888	cranial skeletal system development	10/2734	65/17381	0.58306	0.9459	0.9303	ALX3/BMP4/FGFR2/IFT140/LHX1/MTHFD1/SETD2/SH3PXD2B/TBX15/TULP3	10	BP
GO:0000244	spliceosomal tri-snRNP complex	2/2734	12/17381	0.58453	0.9459	0.9303	PRPF8/USP4	2	BP
GO:0001660	fever generation	2/2734	12/17381	0.58453	0.9459	0.9303	IL1RN/PTGER3	2	BP
GO:0002003	angiotensin maturation	2/2734	12/17381	0.58453	0.9459	0.9303	AGT/REN	2	BP

GO:0002834	regulation of response to tumor cell	2/2734	12/17381	0.58453	0.9459	0.9303	HAVCR2/NCR3	2	BP
GO:0002837	regulation of immune response to	2/2734	12/17381	0.58453	0.9459	0.9303	HAVCR2/NCR3	2	BP
GO:0003334	keratinocyte development	2/2734	12/17381	0.58453	0.9459	0.9303	DNASE1L2/SFN	2	BP
GO:0003376	sphingosine-1-phosphate signaling	2/2734	12/17381	0.58453	0.9459	0.9303	SPNS1/SPNS3	2	BP
GO:0006568	tryptophan metabolic	2/2734	12/17381	0.58453	0.9459	0.9303	HAAO/IDO1	2	BP
GO:0009086	methionine biosynthetic process	2/2734	12/17381	0.58453	0.9459	0.9303	BHMT2/MTHFD1	2	BP
GO:0009109	coenzyme catabolic	2/2734	12/17381	0.58453	0.9459	0.9303	ACOT7/ALDH1L1	2	BP
GO:0009886	post-embryonic animal morphogenesis	2/2734	12/17381	0.58453	0.9459	0.9303	MYO7A/NKX2-3	2	BP
GO:0010454	negative regulation of cell fate	2/2734	12/17381	0.58453	0.9459	0.9303	SPDEF/WNT3A	2	BP
GO:0010649	regulation of cell communication	2/2734	12/17381	0.58453	0.9459	0.9303	CAMK2D/TRDN	2	BP
GO:0010889	regulation of sequestering of triglyceride	2/2734	12/17381	0.58453	0.9459	0.9303	FITM1/PNPLA2	2	BP

GO:0014819	regulation of skeletal muscle contraction	2/2734	12/17381	0.58453	0.9459	0.9303	CAV3/GSTM2	2	BP
GO:0014856	skeletal muscle cell	2/2734	12/17381	0.58453	0.9459	0.9303	PPARD/SHH	2	BP
GO:0017121	phospholipid scrambling	2/2734	12/17381	0.58453	0.9459	0.9303	ANO4/ANO7	2	BP
GO:0018206	peptidyl-methionine modification	2/2734	12/17381	0.58453	0.9459	0.9303	NAA16/NAA60	2	BP
GO:0019471	4-hydroxyproline	2/2734	12/17381	0.58453	0.9459	0.9303	ALDH4A1/GOT2	2	BP
GO:0033197	response to vitamin E	2/2734	12/17381	0.58453	0.9459	0.9303	ALAD/COL1A1	2	BP
GO:0033631	cell-cell adhesion mediated by	2/2734	12/17381	0.58453	0.9459	0.9303	CCL5/FERMT3	2	BP
GO:0033700	phospholipid efflux	2/2734	12/17381	0.58453	0.9459	0.9303	APOA5/APOC3	2	BP
GO:0034063	stress granule assembly	2/2734	12/17381	0.58453	0.9459	0.9303	ATXN2L/DYNC1H1	2	BP
GO:0034116	positive regulation of heterotypic cell-	2/2734	12/17381	0.58453	0.9459	0.9303	FGA/THY1	2	BP
GO:0034969	histone arginine methylation	2/2734	12/17381	0.58453	0.9459	0.9303	COPRS/PRDM14	2	BP
GO:0035404	histone-serine phosphorylation	2/2734	12/17381	0.58453	0.9459	0.9303	PPP1R1B/RPS6KA4	2	BP
GO:0042340	keratan sulfate catabolic	2/2734	12/17381	0.58453	0.9459	0.9303	ACAN/HEXA	2	BP

GO:0043471	regulation of cellular carbohydrate catabolic	2/2734	12/17381	0.58453	0.9459	0.9303	INS/PGAM4	2	BP
GO:0045060	negative thymic T cell selection	2/2734	12/17381	0.58453	0.9459	0.9303	CD74/SHH	2	BP
GO:0045078	positive regulation of interferon-gamma biosynthetic	2/2734	12/17381	0.58453	0.9459	0.9303	TLR8/TLR9	2	BP
GO:0045591	positive regulation of regulatory T	2/2734	12/17381	0.58453	0.9459	0.9303	BCL6/LGALS9	2	BP
GO:0045779	negative regulation of	2/2734	12/17381	0.58453	0.9459	0.9303	CSK/INPP5D	2	BP
GO:0045835	negative regulation of meiotic nuclear	2/2734	12/17381	0.58453	0.9459	0.9303	HORMAD1/PRKAR1A	2	BP
GO:0051583	dopamine uptake involved in synaptic transmission	2/2734	12/17381	0.58453	0.9459	0.9303	DRD2/TOR1A	2	BP
GO:0051764	actin crosslink formation	2/2734	12/17381	0.58453	0.9459	0.9303	BAIAP2L1/FLNA	2	BP
GO:0051775	response to redox state	2/2734	12/17381	0.58453	0.9459	0.9303	NPAS2/VASN	2	BP
GO:0051798	positive regulation of hair follicle	2/2734	12/17381	0.58453	0.9459	0.9303	FOXN1/NUMA1	2	BP

GO:0051934	catecholamine uptake involved in synaptic transmission	2/2734	12/17381	0.58453	0.9459	0.9303	DRD2/TOR1A	2	BP
GO:0055015	ventricular cardiac muscle cell	2/2734	12/17381	0.58453	0.9459	0.9303	LMNA/NKX2-5	2	BP
GO:0060081	membrane hyperpolarizatio	2/2734	12/17381	0.58453	0.9459	0.9303	ATP1A1/CASP1	2	BP
GO:0060391	positive regulation of SMAD protein	2/2734	12/17381	0.58453	0.9459	0.9303	BMP4/RBPMS	2	BP
GO:0060628	regulation of ER to Golgi vesicle-	2/2734	12/17381	0.58453	0.9459	0.9303	RINT1/STX18	2	BP
GO:0061042	vascular wound healing	2/2734	12/17381	0.58453	0.9459	0.9303	MCAM/MIR451A	2	BP
GO:0070262	peptidyl-serine dephosphorylati	2/2734	12/17381	0.58453	0.9459	0.9303	DUSP26/PPP2R2B	2	BP
GO:0070493	thrombin-activated receptor	2/2734	12/17381	0.58453	0.9459	0.9303	DGKQ/F2RL1	2	BP
GO:0070863	positive regulation of protein exit from	2/2734	12/17381	0.58453	0.9459	0.9303	BCAP31/SEC16B	2	BP
GO:0070989	oxidative demethylation	2/2734	12/17381	0.58453	0.9459	0.9303	ALKBH5/CYP1A2	2	BP
GO:0071107	response to parathyroid hormone	2/2734	12/17381	0.58453	0.9459	0.9303	SLC34A1/SOST	2	BP

GO:0072310	glomerular epithelial cell development	2/2734	12/17381	0.58453	0.9459	0.9303	BMP4/MYO1E	2	BP
GO:0090197	positive regulation of chemokine	2/2734	12/17381	0.58453	0.9459	0.9303	CSF1R/IL4R	2	BP
GO:0090493	catecholamine uptake	2/2734	12/17381	0.58453	0.9459	0.9303	DRD2/TOR1A	2	BP
GO:0090494	dopamine uptake	2/2734	12/17381	0.58453	0.9459	0.9303	DRD2/TOR1A	2	BP
GO:0090520	sphingolipid mediated signaling	2/2734	12/17381	0.58453	0.9459	0.9303	SPNS1/SPNS3	2	BP
GO:0097286	iron ion import	2/2734	12/17381	0.58453	0.9459	0.9303	STEAP2/TFR2	2	BP
GO:0099072	regulation of postsynaptic membrane neurotransmitter receptor levels	2/2734	12/17381	0.58453	0.9459	0.9303	GRIPAP1/SCRIB	2	BP
GO:1901550	regulation of endothelial cell development	2/2734	12/17381	0.58453	0.9459	0.9303	CDH5/IKBKB	2	BP
GO:1901836	regulation of transcription of nuclear large rRNA transcript from RNA polymerase I promoter	2/2734	12/17381	0.58453	0.9459	0.9303	MTOR/NOL11	2	BP

GO:1901841	regulation of high voltage-gated calcium channel activity	2/2734	12/17381	0.58453	0.9459	0.9303	NPPA/RRAD	2	BP
GO:1902224	ketone body metabolic	2/2734	12/17381	0.58453	0.9459	0.9303	ACSS3/HMGCLL1	2	BP
GO:1903140	regulation of establishment of endothelial	2/2734	12/17381	0.58453	0.9459	0.9303	CDH5/IKBKB	2	BP
GO:1903894	regulation of IRE1-mediated unfolded protein response	2/2734	12/17381	0.58453	0.9459	0.9303	DAB2IP/HSPA5	2	BP
GO:1904181	positive regulation of membrane	2/2734	12/17381	0.58453	0.9459	0.9303	KDR/MYOC	2	BP
GO:1904953	Wnt signaling pathway involved in midbrain dopaminergic neuron [illegible]	2/2734	12/17381	0.58453	0.9459	0.9303	WNT1/WNT3A	2	BP
GO:1905049	negative regulation of metallopeptidase activity	2/2734	12/17381	0.58453	0.9459	0.9303	MIR195/MIR29C	2	BP
GO:1905065	positive regulation of vascular smooth muscle cell	2/2734	12/17381	0.58453	0.9459	0.9303	ENG/GPER1	2	BP

GO:0071560	cellular response to transforming growth factor	34/2734	221/17381	0.58512	0.9466	0.93098	ARRB2/CAV3/CDH5/COL1A1/COL4A2/ENG/FAM89B/FERMT2/FGFR2/FNTA/FURIN/HSPA5/HTRA3/ITGB5/LEFTY2/MEN1/MIR212/NEDD8/NOX4/PARD3/PARD6A/PBLD/PDE2A/SCX/SMAD6/SOX5/TGFB1I1/VASN/WFIKKN2/WNT1/WNT10A/WNT7A/ZNF703/ZYX	34	BP
GO:0032663	regulation of interleukin-2 production	8/2734	52/17381	0.58597	0.947	0.93142	ABL1/CARD11/CARD9/CCR2/HAVCR2/IL20RB/TRAF2/VSIG4	8	BP
GO:0021987	cerebral cortex development	17/2734	111/17381	0.58817	0.947	0.93142	BAD/CDK5/CDK5R2/DAB1/DAB2IP/DISC1/EGFR/EMX1/FLNA/GRIN1/LRP1/MCPH1/POU3F3/SMO/TBR1/TH/TRAPPC9	17	BP
GO:0032675	regulation of interleukin-6 production	17/2734	111/17381	0.58817	0.947	0.93142	ARRB2/CARD9/CSK/F2RL1/GBA/HAVCR2/INPP5D/LGALS9/MIR92A2/NLRX1/NOD1/ORM1/SPON2/TIRAP/TLR8/TLR9/TRPV4	17	BP
GO:0051153	regulation of striated muscle cell	17/2734	111/17381	0.58817	0.947	0.93142	ADGRB1/ANKRD2/ARRB2/BMP4/CAV3/EHD1/IL4R/MIR222/MTOR/MYOD1/NKX2-5/NOTCH1/NRG1/SHH/THRA/TRIM72/WNT3A	17	BP
GO:0046390	ribose phosphate biosynthetic process	42/2734	273/17381	0.58863	0.947	0.93142	ACOT7/ADM/ADRA2A/AK5/ALDOA/AQP1/ATP5G1/ATP5I/AVPR2/CCR2/CRHR1/CYC1/DHODH/DRD2/GABBR1/GCG/GPER1/GPHA2/GUCA1B/GUCA2A/GUCA2B/GUCY2D/IMPDH1/NME1/NME4/NOS3/NPPA/OPRM1/PDE2A/PDZD3/PID1/PKM/PPCDC/PTK2B/RAF1/RUNDC3A/RXFP2/SLC26A1/UCK1	42	BP
GO:0072522	purine-containing compound biosynthetic	42/2734	273/17381	0.58863	0.947	0.93142	ACOT7/ADM/ADRA2A/AK5/ALDOA/AQP1/ATP5G1/ATP5I/AVPR2/CCR2/CRHR1/CYC1/DGUOK/DRD2/GABBR1/GCG/GPER1/GPHA2/GUCA1B/GUCA2A/GUCA2B/GUCY2D/IMPDH1/MTHFD1/NME1/NME4/NOS3/NPPA/OPRM1/PDE2A/PDZD3/PID1/PKM/PPCDC/PTK2B/RAF1/RUNDC3A/RXFP2/SLC26	42	BP
GO:0042307	positive regulation of protein import	15/2734	98/17381	0.58883	0.947	0.93142	BMP4/CCL19/CD27/CSF3/EGFR/FLNA/IL18/LGALS9/PRKCD/RBPMS/SHH/SMO/TLR9/WNT3A/ZPR1	15	BP
GO:0048738	cardiac muscle tissue development	33/2734	215/17381	0.59043	0.947	0.93142	AGT/ARRB2/BMP4/CACYBP/CAV3/ENG/FGF3/FGFR2/GATA4/IFT20/KAT2A/KDM6B/LMNA/MIR195/MIR222/MIR25/MTOR/MYBPC3/NKX2-5/NOTCH1/NOX4/NPPA/NPRL3/NRG1/OBSL1/PDGFRB/PDLIM5/PRKAR1A/RARA/TBX2/TCAP/TNNC1/WNT3A	33	BP

GO:0001659	temperature homeostasis	6/2734	39/17381	0.59128	0.947	0.93142	ACADVL/DRD2/EDN2/GPX1/IL1RN/PTGER3	6	BP
GO:0030835	negative regulation of actin filament	6/2734	39/17381	0.59128	0.947	0.93142	CAPZA3/LMOD1/SCIN/SPTAN1/SPTB/SPTBN2	6	BP
GO:0035337	fatty-acyl-CoA metabolic	6/2734	39/17381	0.59128	0.947	0.93142	ACOT7/ACSF3/DGAT1/ELOVL1/ELOVL5/HACD1	6	BP
GO:0050873	brown fat cell differentiation	6/2734	39/17381	0.59128	0.947	0.93142	FNDC5/INS/MTOR/PEX11A/SLC2A4/TRPV4	6	BP
GO:1902475	L-alpha-amino acid transmembrane transport	6/2734	39/17381	0.59128	0.947	0.93142	AGT/PQLC2/SLC1A7/SLC25A22/SLC38A1/SLC7A7	6	BP
GO:0016126	sterol biosynthetic	11/2734	72/17381	0.59195	0.947	0.93142	ACACB/APOA5/APOB/CYB5R1/CYB5R2/HSD17B7/NPC1L1/NSDHL/PMVK/POR/SCAP	11	BP
GO:0051439	regulation of ubiquitin-protein ligase activity involved in	11/2734	72/17381	0.59195	0.947	0.93142	ANAPC15/ANAPC2/BUB1B/PSMB6/PSMB7/PSMB8/PSMC3/PSMD13/PSMD3/PSMD5/PSMD7	11	BP
GO:0106027	neuron projection	11/2734	72/17381	0.59195	0.947	0.93142	ARC/CDK5/CFL1/CTTN/DLG4/EFNA1/EPHB3/INS/NLGN3/PDLIM5/WNT7A	11	BP
GO:0016197	endosomal transport	41/2734	267/17381	0.59332	0.947	0.93142	AKTIP/AP1S1/CALY/CD63/CDX2/CHMP4C/CHMP7/CORO1A/DENND2A/DENND3/EHD1/FAM160A2/GBF1/GOSR2/GRIPAP1/INPP5F/KIF13A/MAPK3/PCDHGA3/PTPN23/RAB11FIP3/RAB17/RAB7A/RBSN/SCRIB/SGSM2/SNF8/SNX12/STX5/STX8/TINAGL1/VPS13A/VPS25/VPS37B/VPS37C/VPS37D/VP	41	BP
GO:0001710	mesodermal cell fate	3/2734	19/17381	0.5935	0.947	0.93142	BMP4/EYA1/WNT3A	3	BP
GO:0001759	organ induction	3/2734	19/17381	0.5935	0.947	0.93142	BMP4/HOXC11/WNT3A	3	BP

GO:0001991	regulation of systemic arterial blood pressure by circulatory renin-	3/2734	19/17381	0.5935	0.947	0.93142	AGT/F2RL1/REN	3	BP
GO:0003215	cardiac right ventricle morphogenesis	3/2734	19/17381	0.5935	0.947	0.93142	GATA4/NKX2-5/NOTCH1	3	BP
GO:0006895	Golgi to endosome	3/2734	19/17381	0.5935	0.947	0.93142	GBF1/RBSN/VPS13A	3	BP
GO:0007095	mitotic G2 DNA damage checkpoint	3/2734	19/17381	0.5935	0.947	0.93142	CDK5RAP3/FOXO4/RINT1	3	BP
GO:0010528	regulation of transposition	3/2734	19/17381	0.5935	0.947	0.93142	DDX4/PIWIL2/TDRD9	3	BP
GO:0010529	negative regulation of	3/2734	19/17381	0.5935	0.947	0.93142	DDX4/PIWIL2/TDRD9	3	BP
GO:0010560	positive regulation of glycoprotein biosynthetic	3/2734	19/17381	0.5935	0.947	0.93142	CCL19/CCL21/GOLGA2	3	BP
GO:0015669	gas transport	3/2734	19/17381	0.5935	0.947	0.93142	AQP1/AQP5/HBZ	3	BP
GO:0032331	negative regulation of chondrocyte	3/2734	19/17381	0.5935	0.947	0.93142	ADAMTS7/BMP4/NKX3-2	3	BP
GO:0032372	negative regulation of	3/2734	19/17381	0.5935	0.947	0.93142	ABCG5/APOC3/SHH	3	BP
GO:0032375	negative regulation of cholesterol	3/2734	19/17381	0.5935	0.947	0.93142	ABCG5/APOC3/SHH	3	BP

GO:0034104	negative regulation of tissue	3/2734	19/17381	0.5935	0.947	0.93142	AGT/CSK/INPP5D	3	BP
GO:0034389	lipid particle organization	3/2734	19/17381	0.5935	0.947	0.93142	FITM1/HILPDA/PNPLA2	3	BP
GO:0035024	negative regulation of Rho protein	3/2734	19/17381	0.5935	0.947	0.93142	ADRA1A/BCL6/MYOC	3	BP
GO:0042533	tumor necrosis factor biosynthetic	3/2734	19/17381	0.5935	0.947	0.93142	CARD9/CCR2/HSPB1	3	BP
GO:0042534	regulation of tumor necrosis factor biosynthetic	3/2734	19/17381	0.5935	0.947	0.93142	CARD9/CCR2/HSPB1	3	BP
GO:0042776	mitochondrial ATP synthesis coupled proton	3/2734	19/17381	0.5935	0.947	0.93142	ATP5G1/ATP5I/CYC1	3	BP
GO:0042789	mRNA transcription from RNA polymerase II	3/2734	19/17381	0.5935	0.947	0.93142	FLNA/FOXE3/SUPT6H	3	BP
GO:0043011	myeloid dendritic cell	3/2734	19/17381	0.5935	0.947	0.93142	BATF2/LTBR/SPI1	3	BP
GO:0044110	growth involved in symbiotic interaction	3/2734	19/17381	0.5935	0.947	0.93142	LTA/MPO/TIRAP	3	BP
GO:0044116	growth of symbiont involved in interaction with	3/2734	19/17381	0.5935	0.947	0.93142	LTA/MPO/TIRAP	3	BP

GO:0051194	positive regulation of cofactor	3/2734	19/17381	0.5935	0.947	0.93142	INS/MLXIPL/PFKFB1	3	BP
GO:0051197	positive regulation of coenzyme	3/2734	19/17381	0.5935	0.947	0.93142	INS/MLXIPL/PFKFB1	3	BP
GO:0051797	regulation of hair follicle development	3/2734	19/17381	0.5935	0.947	0.93142	FOXN1/NUMA1/SMO	3	BP
GO:0061162	establishment of monopolar cell polarity	3/2734	19/17381	0.5935	0.947	0.93142	FSCN1/GBF1/SCRIB	3	BP
GO:0071625	vocalization behavior	3/2734	19/17381	0.5935	0.947	0.93142	DLG4/NLGN3/NRXN2	3	BP
GO:0090670	RNA localization to	3/2734	19/17381	0.5935	0.947	0.93142	CCT3/EXOSC10/NHP2	3	BP
GO:0090671	telomerase RNA localization to	3/2734	19/17381	0.5935	0.947	0.93142	CCT3/EXOSC10/NHP2	3	BP
GO:0090672	telomerase RNA	3/2734	19/17381	0.5935	0.947	0.93142	CCT3/EXOSC10/NHP2	3	BP
GO:0090685	RNA localization to	3/2734	19/17381	0.5935	0.947	0.93142	CCT3/EXOSC10/NHP2	3	BP
GO:1903599	positive regulation of autophagy of	3/2734	19/17381	0.5935	0.947	0.93142	ATPIF1/GBA/MFN2	3	BP
GO:1905331	negative regulation of morphogenesis	3/2734	19/17381	0.5935	0.947	0.93142	BMP4/STAT1/TBX2	3	BP

GO:0034614	cellular response to reactive oxygen	24/2734	157/17381	0.5948	0.9483	0.9327	ABL1/ADPRHL2/APEX1/AQP1/DPEP1/EGFR/ENDOG/GCH1/HSF1/IL18BP/KDM6B/MAPK3/MIR92A2/MMP3/MPO/NCF1/NOS3/PCGF2/PRDX1/PRKCD/PSAP/SOD3/TRAF2/TRAP1	24	BP
GO:0018149	peptide cross-linking	9/2734	59/17381	0.59511	0.9483	0.9327	BGN/C1orf68/FN1/LCE3E/SPOCK2/SPRR1A/TGM1/TGM5/TGM7	9	BP
GO:0032507	maintenance of protein location in cell	9/2734	59/17381	0.59511	0.9483	0.9327	ARL2BP/DBN1/GPAA1/HSPA5/NR5A1/OS9/SCIN/TAF3/TLN1	9	BP
GO:0034121	regulation of toll-like receptor	9/2734	59/17381	0.59511	0.9483	0.9327	ARRB2/CD300LF/DAB2IP/F2RL1/NFKBIL1/NR1H3/TIRAP/TLR9/TREML4	9	BP
GO:0048247	lymphocyte chemotaxis	9/2734	59/17381	0.59511	0.9483	0.9327	ADAM8/CCL1/CCL19/CCL21/CCL5/CCR2/CKLF/CXCR3/PTK2B	9	BP
GO:0015931	nucleobase-containing compound transport	36/2734	235/17381	0.59692	0.9509	0.93528	ALKBH5/CASC3/CKAP5/CPSF1/CPSF4/DHX38/EIF5A/HSF1/MYO1C/NOL6/NUP210/NUP98/NXF2/NXF2B/NXF3/RBFOX1/RNPS1/SETD2/SIDT2/SLC25A25/SLC25A5/SLC28A1/SLC28A2/SLC29A1/SLC29A2/SLC29A3/SLC29A4/SLC35A2/SLC35C1/SLC35D2/SMG5/SRSF4/SUPT6H/XPO7/ZC3H3/ZNF593	36	BP
GO:0032649	regulation of interferon-gamma	14/2734	92/17381	0.598	0.9511	0.93541	ABL1/CCR2/HAVCR2/HRAS/IL18/IL20RB/ISG15/LGALS9/LTA/RARA/SCGB1A1/SLC11A1/TLR8/TLR9	14	BP
GO:0071347	cellular response to	14/2734	92/17381	0.598	0.9511	0.93541	ADAMTS7/CCL1/CCL19/CCL21/CCL5/DAB2IP/EGR1/IKBKB/IL1RN/IRAK1/MAPK3/OTUB1/RPS6KA4/TNIP2	14	BP
GO:1904666	regulation of ubiquitin protein ligase	14/2734	92/17381	0.598	0.9511	0.93541	ANAPC15/ANAPC2/BUB1B/GOLGA2/GORASP1/PSMB11/PSMB6/PSMB7/PSMB8/PSMC3/PSMD13/PSMD3/PSMD5/PSMD7	14	BP
GO:1901653	cellular response to peptide	53/2734	345/17381	0.59818	0.9511	0.93541	AGT/AHSG/APEX1/ATP6V0B/ATP6V0E2/ATP6V1B1/ATP6V1F/ATP6V1G1/ATP6V1G2/BAIAP2L1/CACNB1/CAPN10/CCND3/CDK4/CDK5/CISH/CRHR1/CRHR2/CSK/EIF4EBP1/ESRRA/FOXO4/GCG/GCK/GNB3/GNG3/GPER1/HSF1/INHBB/INS/LRP1/MAPK3/MAX/MEN1/MYO1C/MZB1/NOD1/NR4A1/PID1/PKM/PLA2G1B/POR/PRKAR1A/PRKAR1B/PRKCD/PTPN11/RARRES2/SHC1/SLC2A4/SLC2A8/TIMELESS/TRIM72/WNT1	53	BP

GO:2000146	negative regulation of cell motility	42/2734	274/17381	0.59877	0.9511	0.93541	ADGRB1/APEX1/ARHGAP4/CCL21/CORO1B/DAB2IP/DPEP1/DRD2/ENG/EVL/GDF2/IDH2/IGFBP3/ILK/KRT16/LRP1/MARVELD3/MIIP/MIR10A/MIR15A/MIR16-1/MIR212/MIR221/MIR29A/MIR29C/MIR503/MIR92A2/NBL1/NISCH/NOTC	42	BP
GO:0010771	negative regulation of cell morphogenesis involved in	12/2734	79/17381	0.60018	0.9511	0.93541	ARHGAP4/CDK5/DAB1/DRAXIN/EFNA1/GORASP1/LINGO1/NLGN3/SEMA3F/THY1/TLX2/WNT3A	12	BP
GO:0042147	retrograde transport, endosome to	12/2734	79/17381	0.60018	0.9511	0.93541	AP1S1/DENND2A/GBF1/GOSR2/RAB7A/RBSN/SGSM2/STX5/VPS51/VPS52/VPS53/VTI1B	12	BP
GO:0045682	regulation of epidermis development	12/2734	79/17381	0.60018	0.9511	0.93541	BMP4/CTSL/FOXN1/KRT84/MYCN/NOTCH1/NUMA1/PPARD/PTCH2/SFN/SMO/TMEM79	12	BP
GO:0001974	blood vessel remodeling	7/2734	46/17381	0.60039	0.9511	0.93541	AGT/BGN/CCR2/FLT4/HOXA3/NOS3/TMBIM1	7	BP
GO:0006195	purine nucleotide	7/2734	46/17381	0.60039	0.9511	0.93541	DNPH1/GPX1/HINT1/NUDT1/NUDT18/PDE2A/XDH	7	BP
GO:0007140	male meiotic nuclear division	7/2734	46/17381	0.60039	0.9511	0.93541	DDX4/FANCA/HSPA2/M1AP/SLC2A8/TAF1L/TDRD9	7	BP
GO:0042993	positive regulation of transcription factor import	7/2734	46/17381	0.60039	0.9511	0.93541	CCL19/CD27/CSF3/FLNA/IL18/LGALS9/TLR9	7	BP
GO:0044003	modification by symbiont of host morphology or	7/2734	46/17381	0.60039	0.9511	0.93541	BAD/CPSF4/EIF2AK4/GAPDH/MIR221/MIR222/SCRIB	7	BP

GO:0045620	negative regulation of lymphocyte	7/2734	46/17381	0.60039	0.9511	0.93541	BCL6/BMP4/CD74/ERBB2/HLX/IL4R/SHH	7	BP
GO:0051972	regulation of telomerase	7/2734	46/17381	0.60039	0.9511	0.93541	ACD/HSP90AA1/MAPK15/MAPK3/MEN1/NVL/PIF1	7	BP
GO:0090102	cochlea development	7/2734	46/17381	0.60039	0.9511	0.93541	DVL2/EYA1/FZD2/GRHL3/IFT20/MCM2/MYO3A	7	BP
GO:2001020	regulation of response to DNA damage	27/2734	177/17381	0.60127	0.9511	0.93541	ABL1/APBB1/BRCA1/BRCC3/CCAR2/CD74/CDK9/CLU/EGFR/EYA1/FOXM1/HSF1/MIR221/MUC1/NPAS2/NUDT16L1/OGG1/OTUB1/PARP9/PAXIP1/PRKCD/RECQL5/RINT1/SETD2/SLF2/TIMELESS/ZNF385A	27	BP
GO:0022406	membrane docking	25/2734	164/17381	0.60134	0.9511	0.93541	ACTR1A/B9D1/C2CD3/CC2D2A/CEP164/CEP70/CKAP5/DCTN2/DYNC1H1/HAUS4/HAUS7/HSP90AA1/PEX16/RAB26/RAB7A/RALB/SDCCAG8/SSNA1/STX1A/STX4/STX5/STX8/STXBP1/TUBG1/VTI1B	25	BP
GO:0006091	generation of precursor metabolites and energy	70/2734	455/17381	0.6014	0.9511	0.93541	ACADVL/ACO1/AKR7A3/ALDH2/ALDH4A1/ALDOA/AOC2/ATPIF1/CHCHD5/COX10/COX11/COX8A/CROT/CS/CYC1/CYP1A2/DLST/GALK1/GAPDH/GCK/GLRX5/GNMT/GPX2/GSR/HAAO/HK3/IDH1/IDH2/IDO1/INS/LDHA/LEPR/LOXL2/MLXIPL/MTFR1L/MTOR/NCF1/NCOR1/NDUFA2/NDUFA4L2/NDUFB5/NDUFC2-KCTD14/NOX4/NQO2/NUP210/NUP98/OGDH/OGDHL/PCDH12/PDHB/PFKFB1/PFKM/PGAM4/PHGDH/PHKG1/PHLDA2/PID1/PKM/POR/PPARD/PRELI[illegible]	70	BP
GO:0051249	regulation of lymphocyte activation	70/2734	455/17381	0.6014	0.9511	0.93541	ABL1/ADAM8/BAD/BCL6/BMP4/CARD11/CASP3/CCDC88B/CCL19/CCL21/CCL5/CCR2/CD247/CD27/CD5/CD6/CD74/CD81/CLCF1/CLECL1/CORO1A/CSK/DNAJA3/EFNB1/ERBB2/FANCA/FOXN1/HAVCR2/HLA-E/HLX/IDO1/IL13/IL18/IL20RB/IL4R/INPP5D/LCK/LGALS3/LGALS9/LMO1/MAD1L1/MAP3K14/MZB1/PAXIP1/PDCD1/PIK3R6/PLA2G2F/PPP2R3C/PRELID1/PRKAR1A/PTPN11/RARA/SART1/SCGB1A1/SHH/SOX13/SPINK5/SUPT6H/TACR1/THY1/TIRAP/TLR9/TNFRSF13B/TNFRSF18/TNFRSF4/TNFSF13[illegible]	70	BP
GO:2001257	regulation of cation channel activity	23/2734	151/17381	0.60157	0.9511	0.93541	AHNAK/ARC/CACNB1/CACNB3/CAMK2D/CNIH2/CRACR2A/CRHR1/DAPK1/DLG4/DRD2/GNB5/GPR35/GSTM2/JPH3/MIR153-1/MIR212/NLGN3/NPPA/OPRM1/PTK2B/RRAD/TRDN	23	BP

GO:0090092	regulation of transmembrane receptor protein serine/threonine kinase signaling pathway	33/2734	216/17381	0.60181	0.9511	0.93541	ABL1/BMP4/CAV3/CRB2/DACT2/ENG/FAM89B/GATA4/GDF2/HSPA5/HTRA3/ILK/INHBB/INHBE/LEFTY2/LEMD2/MEN1/NBL1/NOTCH1/NUMA1/PBLD/RBPMS/RBPMS2/SFRP5/SHH/SMAD6/SOST/TGFB1I1/VASN/WFIKKN2/WNT1/ZC3H3/ZNF703	33	BP
GO:0015985	energy coupled proton transport, down electrochemical gradient	4/2734	26/17381	0.60195	0.9511	0.93541	ATP5G1/ATP5I/CYC1/VPS9D1	4	BP
GO:0015986	ATP synthesis coupled proton transport	4/2734	26/17381	0.60195	0.9511	0.93541	ATP5G1/ATP5I/CYC1/VPS9D1	4	BP
GO:0033598	mammary gland epithelial cell proliferation	4/2734	26/17381	0.60195	0.9511	0.93541	DEAF1/GPX1/PYGO2/ZNF703	4	BP
GO:0035235	ionotropic glutamate receptor	4/2734	26/17381	0.60195	0.9511	0.93541	GRIA3/GRIN1/GRIN2C/PTK2B	4	BP
GO:0035640	exploration behavior	4/2734	26/17381	0.60195	0.9511	0.93541	DLG4/GAD1/JPH3/LRRTM1	4	BP
GO:0046466	membrane lipid catabolic	4/2734	26/17381	0.60195	0.9511	0.93541	GBA/NEU3/PRKCD/SMPD4	4	BP
GO:0048821	erythrocyte development	4/2734	26/17381	0.60195	0.9511	0.93541	BCL6/HBZ/L3MBTL3/MAEA	4	BP
GO:0050926	regulation of positive chemotaxis	4/2734	26/17381	0.60195	0.9511	0.93541	ARTN/CDH13/F2RL1/KDR	4	BP

GO:0060314	regulation of ryanodine-sensitive calcium-release channel activity	4/2734	26/17381	0.60195	0.9511	0.93541	CAMK2D/GSTM2/JPH3/TRDN	4	BP
GO:0032465	regulation of cytokinesis	10/2734	66/17381	0.60349	0.953	0.93731	ANKRD53/CHMP4C/CXCR5/DRD2/E2F8/KIF23/MYO19/OPN1MW/SETD2/SSTR5	10	BP
GO:0061180	mammary gland epithelium development	10/2734	66/17381	0.60349	0.953	0.93731	CAV3/CSF1/CSF1R/DEAF1/FGFR2/GPX1/PYGO2/SCRIB/SMO/ZNF703	10	BP
GO:0030282	bone mineralization	15/2734	99/17381	0.60551	0.9538	0.93814	AHSG/BGLAP/BMP4/DDR2/ECM1/FGFR2/FZD9/IFITM5/ISG15/OSR1/PHOSPHO1/PKDCC/PTK2B/TMEM119/WNT11	15	BP
GO:0046854	phosphatidylinositol	15/2734	99/17381	0.60551	0.9538	0.93814	EGFR/ERBB2/FGF17/FGF3/FGFR2/FGFR4/LCK/NRG1/PDGFA/PDGFRB/PI4KB/PIK3R5/PIK3R6/PTPN11/TMEM150A	15	BP
GO:0032845	negative regulation of homeostatic	30/2734	197/17381	0.60733	0.9538	0.93814	ABL1/ACD/ADRA2A/AVPR2/CAMK2D/CCL19/CCL21/CD74/CORO1A/CSK/DRD2/EXOSC10/FASLG/GPER1/GSTM2/IL13/INPP5D/ITPR3/JPH3/LCK/LDB1/NPSR1/PIF1/PTK2B/RASA3/THY1/TINF2/TRDN/TSC22D3/TSC22D4	30	BP
GO:0006694	steroid biosynthetic process	28/2734	184/17381	0.6074	0.9538	0.93814	ACACB/ADM/APOA5/APOB/ATP1A1/CACNA1H/CYB5R1/CYB5R2/CYP11A1/CYP11B2/DGKQ/EGR1/FGFR4/HSD11B2/HSD17B1/HSD17B3/HSD17B7/HSD3B1/NPC1L1/NR5A1/NSDHL/OSBPL7/PMVK/POR/SCAP/SCARB1/SF1/	28	BP
GO:0099531	presynaptic process involved in chemical synaptic	22/2734	145/17381	0.60863	0.9538	0.93814	BAIAP3/CDK5/CPLX1/DGKI/DOC2A/DOC2B/DRD2/DTNBP1/GAD1/GPER1/NRXN2/PNKD/PTPRN2/SCRIB/SLC6A9/STX1A/STX4/STXBP1/SYTL3/TACR2/TOR1A/WNT7A	22	BP
GO:0006301	postreplication repair	8/2734	53/17381	0.60867	0.9538	0.93814	BRCA1/FAAP20/ISG15/POLD4/RFC1/RFC2/UBE2A/UBE2B	8	BP
GO:0032210	regulation of telomere maintenance via telomerase	8/2734	53/17381	0.60867	0.9538	0.93814	ACD/CCT3/EXOSC10/MAPK15/MAPK3/PIF1/SMG5/TINF2	8	BP

GO:0045843	negative regulation of striated muscle	8/2734	53/17381	0.60867	0.9538	0.93814	BMP4/CAV3/FGF3/LUC7L/MIR222/MIR25/TSC22D3/USP19	8	BP
GO:0086002	cardiac muscle cell action potential involved in	8/2734	53/17381	0.60867	0.9538	0.93814	ATP1A1/CACNA1G/CAV3/FLNA/KCND3/KCNJ5/KCNQ1/MIR328	8	BP
GO:0098840	protein transport along	8/2734	53/17381	0.60867	0.9538	0.93814	DYNLL2/HSPB1/IFT140/IFT20/IFT22/KIF17/SSNA1/TRAF3IP1	8	BP
GO:0099118	microtubule-based protein	8/2734	53/17381	0.60867	0.9538	0.93814	DYNLL2/HSPB1/IFT140/IFT20/IFT22/KIF17/SSNA1/TRAF3IP1	8	BP
GO:1900408	negative regulation of cellular response to	8/2734	53/17381	0.60867	0.9538	0.93814	GPX1/HSPB1/INS/MIR92A2/NONO/PSAP/TRAP1/WNT1	8	BP
GO:1903202	negative regulation of oxidative stress-	8/2734	53/17381	0.60867	0.9538	0.93814	GPX1/HSPB1/INS/MIR92A2/NONO/PSAP/TRAP1/WNT1	8	BP
GO:0090150	establishment of protein localization to membrane	42/2734	275/17381	0.60882	0.9538	0.93814	BAD/BLZF1/CDK5/CIB1/CSK/DYNLL2/EGFR/ERBB2/GRIPAP1/HSP90AA1/ITGAM/KIF13A/MIEF2/MOAP1/MYO1C/NCF1/PARD3/PEX16/PEX3/PEX5/PKDCC/PPP1R13B/RAB26/RAB34/REEP2/RILPL1/RILPL2/RPL35A/RPL36A/RPL41/RPL7/RPS24/SCRIB/SFN/SRP14/SSR2/TAOK2/TIMM22/TIMM9/UBL4	42	BP
GO:1904062	regulation of cation transmembrane transport	44/2734	288/17381	0.60926	0.9538	0.93814	ABL1/ADRA2A/AGT/AHNAK/AMIGO1/ARC/CACNB1/CACNB3/CAMK2D/CAV3/CNIH2/COMMD1/CORO1A/CRACR2A/CRHR1/DAPK1/DLG4/DPP6/DRD2/FLNA/GNB5/GPER1/GPR35/GSTM2/HSPA2/IL13/JPH3/KCNQ1/MIR153-1/MIR212/MIR328/NLGN3/NPPA/NPSR1/OPRM1/OSR1/PTK2B/RRAD/THA	44	BP
GO:0016601	Rac protein signal	5/2734	33/17381	0.60984	0.9538	0.93814	BRK1/CDH13/FARP2/HACD3/NISCH	5	BP

GO:0031062	positive regulation of histone	5/2734	33/17381	0.60984	0.9538	0.93814	BRCA1/GCG/PAXIP1/PHF19/PRDM12	5	BP
GO:0046131	pyrimidine ribonucleoside metabolic	5/2734	33/17381	0.60984	0.9538	0.93814	CDA/DHODH/NME1/NME4/UCK1	5	BP
GO:1901976	regulation of cell cycle	5/2734	33/17381	0.60984	0.9538	0.93814	ANAPC15/CCAR2/LCMT1/RINT1/XRCC3	5	BP
GO:0046631	alpha-beta T cell activation	18/2734	119/17381	0.61082	0.9538	0.93814	ABL1/BCL6/CCL19/CCR2/FUT7/HLA-E/HLX/IL18/IL4R/INS/LGALS9/LY9/NKX2-	18	BP
GO:0046824	positive regulation of nucleocytoplasmic transport	18/2734	119/17381	0.61082	0.9538	0.93814	ANP32B/BMP4/CCL19/CD27/CSF3/EGFR/EMD/FLNA/IL18/LGALS9/PRKCD/RBPMS/SFN/SHH/SMO/TLR9/WNT3A/ZPR1	18	BP
GO:0032642	regulation of chemokine production	11/2734	73/17381	0.61126	0.9538	0.93814	CD74/CSF1R/EGR1/F2RL1/HAVCR2/IL4R/LGALS9/MIR92A2/TIRAP/TLR9/TRPV4	11	BP
GO:0046128	purine ribonucleoside metabolic	11/2734	73/17381	0.61126	0.9538	0.93814	ACPP/CARD11/DGUOK/DLG4/IMPDH1/MFN1/NME1/NME4/NUDT18/PEMT/SCRIB	11	BP
GO:0006368	transcription elongation from RNA polymerase II	16/2734	106/17381	0.6126	0.9538	0.93814	AXIN1/CDK9/GTF2H4/NELFB/POLR2G/POLR2L/RECQL5/SETD2/SHH/SUPT6H/TAF1/TAF1L/TAF3/TAF6/TAF7/ZMYND11	16	BP
GO:0007613	memory	16/2734	106/17381	0.6126	0.9538	0.93814	ARC/B4GALT2/DRD2/EIF2AK4/FOXO6/GRIN1/HRH2/ITPR3/JPH3/KCNK4/MTOR/NQO2/RGS14/SGK1/TACR1/TH	16	BP
GO:0016052	carbohydrate catabolic process	27/2734	178/17381	0.61367	0.9538	0.93814	ALDOA/BAD/CHIT1/FUT7/GALK1/GALM/GALT/GAPDH/GCK/GLYCTK/HK3/INS/LDHA/MGAM/MLXIPL/NCOR1/NEU3/NUP210/NUP98/OGDH/OGDHL/PFKFB1/PFKM/PGAM4/PHKG1/PKM/PYGM	27	BP
GO:0006968	cellular defense response	9/2734	60/17381	0.61632	0.9538	0.93814	CCR2/CD160/CD5L/CXCR2/FOSL1/KLRC2/KLRG1/NCF1/PTK2B	9	BP

GO:0060393	regulation of pathway-restricted SMAD protein	9/2734	60/17381	0.61632	0.9538	0.93814	BMP4/ENG/GDF2/INHBB/INHBE/LEFTY2/PBLD/RBPMS/SMAD6	9	BP
GO:0090398	cellular senescence	9/2734	60/17381	0.61632	0.9538	0.93814	ABL1/BCL6/CDKN2A/HMGA1/HRAS/MIR10A/PRKCD/TBX2/YPEL3	9	BP
GO:0000289	nuclear-transcribed mRNA poly(A) tail shortening	6/2734	40/17381	0.61722	0.9538	0.93814	EIF4B/EIF4G1/MIRLET7A2/PAN2/PNLDC1/POLR2G	6	BP
GO:0001709	cell fate determination	6/2734	40/17381	0.61722	0.9538	0.93814	BARHL2/BMP4/MYOD1/PTCH2/TBX2/WNT1	6	BP
GO:0035735	intraciliary transport involved in	6/2734	40/17381	0.61722	0.9538	0.93814	DYNLL2/IFT140/IFT20/IFT22/KIF17/TRAF3IP1	6	BP
GO:0086004	regulation of cardiac muscle cell contraction	6/2734	40/17381	0.61722	0.9538	0.93814	ATP1A1/CAMK2D/CAV3/FLNA/GATA4/MIR328	6	BP
GO:0098693	regulation of synaptic vesicle cycle	6/2734	40/17381	0.61722	0.9538	0.93814	CDK5/DNM1/NLGN3/STX1A/STXBP1/TOR1A	6	BP
GO:1903573	negative regulation of response to endoplasmic	6/2734	40/17381	0.61722	0.9538	0.93814	CLU/CREB3/HERPUD1/OS9/SYVN1/WFS1	6	BP
GO:0031145	anaphase-promoting complex-dependent catabolic	12/2734	80/17381	0.61852	0.9538	0.93814	ANAPC15/ANAPC2/BUB1B/PSMB11/PSMB6/PSMB7/PSMB8/PSMC3/PSMD13/PSMD3/PSMD5/PSMD7	12	BP

GO:0045137	development of primary sexual characteristics	34/2734	224/17381	0.61856	0.9538	0.93814	ANG/ARRB2/BOK/CSDE1/EIF2B2/EIF2B5/FANCA/GATA4/IDH1/INHBB/LFNG/LRRC6/MGST1/NOS3/NR5A1/NUDT1/NUP210L/NUPR1/OSR1/PDGFRB/RARA/REN/RXFP2/SCX/SDC1/SF1/SOX8/TSPY1/TSPY2/TSPY4/TSPY8/UTF	34	BP
GO:0018105	peptidyl-serine phosphorylation	44/2734	289/17381	0.61899	0.9538	0.93814	ARAF/ARRB2/AXIN1/BRSK2/CAMK1D/CAMK1G/CAMK2D/CAMKK1/CAMKV/CDK5/CSF3/DAPK2/DCLK2/DGKQ/DOCK7/EGFR/EIF4G1/GCG/IFNA5/IKBKB/ILK/INPP5F/MAPK3/MAPKAPK3/MLXIPL/MTOR/OPRD1/PHKG1/PKD1/PNCK/PPP1R1B/PRKCD/RAF1/RPS6KA4/SGK1/SMYD3/STK32B/STK32C/STK33/TAF1/TBK1/TDGF1/ULK1/WNT3A	44	BP
GO:0007272	ensheathment of neurons	17/2734	113/17381	0.61933	0.9538	0.93814	AMIGO1/ARHGEF10/CLU/EIF2B2/EIF2B5/ERBB2/GNPAT/GPC1/ILK/MTOR/MYOC/NRG1/PARD3/POU3F1/PPARD/RARA/ZPR1	17	BP
GO:0008366	axon ensheathment	17/2734	113/17381	0.61933	0.9538	0.93814	AMIGO1/ARHGEF10/CLU/EIF2B2/EIF2B5/ERBB2/GNPAT/GPC1/ILK/MTOR/MYOC/NRG1/PARD3/POU3F1/PPARD/RARA/ZPR1	17	BP
GO:0043618	regulation of transcription from RNA polymerase II promoter in	17/2734	113/17381	0.61933	0.9538	0.93814	ANKRD2/ATF3/EGR1/HSF1/HSPA5/MUC1/NOTCH1/PSMB11/PSMB6/PSMB7/PSMB8/PSMC3/PSMD13/PSMD3/PSMD5/PSMD7/TAF1	17	BP
GO:0051251	positive regulation of lymphocyte activation	48/2734	315/17381	0.61957	0.9538	0.93814	ADAM8/BAD/BCL6/CARD11/CCDC88B/CCL19/CCL21/CCL5/CCR2/CD247/CD27/CD5/CD6/CD74/CD81/CLCF1/CLECL1/CORO1A/CSK/DNAJA3/EFNB1/HAVCR2/HLA-E/HLX/IL13/IL18/IL4R/INPP5D/LCK/LGALS9/MAP3K14/PAXIP1/PDCD1/PIK3R6/PPP2R3C/PTPN11/RARA/SART1/SHH/TACR1/THY1/TIRAP/TLR9/TNF	48	BP
GO:0042752	regulation of circadian	15/2734	100/17381	0.62188	0.9538	0.93814	CCAR2/CREM/DRD2/MTA1/NONO/NR1H3/OPN4/PASD1/PER1/PSPC1/RBM4/RORC/SUV39H1/TIMELESS/USP2	15	BP
GO:0060419	heart growth	15/2734	100/17381	0.62188	0.9538	0.93814	ACACB/AGT/CAV3/FGFR2/GATA4/MIR195/MIR222/MIR25/MTOR/NKX2-5/NOTCH1/NPPA/PDLIM5/PRKAR1A/TBX2	15	BP

GO:0090100	positive regulation of transmembrane receptor protein serine/threonine kinase signaling	15/2734	100/17381	0.62188	0.9538	0.93814	BMP4/CRB2/ENG/GATA4/GDF2/ILK/INHBB/INHBE/LEFTY2/MEN1/NOTCH1/NUMA1/RBPMS/TGFB1I1/ZC3H3	15	BP
GO:1904591	positive regulation of	15/2734	100/17381	0.62188	0.9538	0.93814	BMP4/CCL19/CD27/CSF3/EGFR/FLNA/IL18/LGALS9/PRKCD/RBPMS/SHH/SMO/TLR9/WNT3A/ZPR1	15	BP
GO:0001510	RNA methylation	10/2734	67/17381	0.62344	0.9538	0.93814	CMTR1/MEPCE/METTL2A/METTL2B/NSUN5/NSUN5P2/THUMPD2/TRMT10B/TRMT2B/TRMT44	10	BP
GO:0031341	regulation of cell killing	10/2734	67/17381	0.62344	0.9538	0.93814	ARRB2/BAD/F2RL1/GAPDH/HAVCR2/HLA-E/IL13/LGALS9/NCR3/PIK3R6	10	BP
GO:0040014	regulation of multicellular organism	10/2734	67/17381	0.62344	0.9538	0.93814	CDK4/CSF1/DRD2/FGFR2/HSF1/PPIB/PTPN11/RAI1/SH3PXD2B/SMO	10	BP
GO:0051250	negative regulation of lymphocyte	20/2734	133/17381	0.62373	0.9538	0.93814	BCL6/BMP4/CASP3/CD74/ERBB2/HAVCR2/HLX/IDO1/IL20RB/IL4R/INPP5D/LGALS3/LGALS9/MAD1L1/PLA2G2F/PRKAR1A/SCGB1A1/SHH/TNFRSF13B/VSIG4	20	BP
GO:0097479	synaptic vesicle localization	20/2734	133/17381	0.62373	0.9538	0.93814	CDK5/CNIH2/CPLX1/DNM1/DOC2A/DOC2B/DTNBP1/GAK/NLGN3/PLD2/SCRIB/STON2/STX1A/STX4/STXBP1/SYTL3/TOR1A/TRIM46/WNT3A/WNT7	20	BP
GO:0034599	cellular response to oxidative stress	43/2734	283/17381	0.62375	0.9538	0.93814	ABL1/ADPRHL2/ANKRD2/APEX1/AQP1/ATP13A2/CCS/DAPK1/DPEP1/EGFR/ENDOG/GCH1/GPX1/GPX2/GPX3/GSR/HSF1/HSPB1/IL18BP/INS/KDM6B/MAPK3/MGST1/MIR92A2/MMP3/MPO/NCF1/NFE2L1/NONO/NOS3/NOX4/NUDT2/PCGF2/PRDX1/PRDX6/PRKCD/PSAP/RWDD1/SOD3/TRAF2/TRA	43	BP
GO:0000060	protein import into nucleus, translocation	7/2734	47/17381	0.62417	0.9538	0.93814	BCL6/IPO4/OGG1/OPRD1/PARP10/PDE2A/POLA2	7	BP
GO:0002704	negative regulation of leukocyte	7/2734	47/17381	0.62417	0.9538	0.93814	ARRB2/BCL6/CCR2/HAVCR2/HLA-E/IL20RB/LGALS9	7	BP

GO:0010656	negative regulation of muscle cell	7/2734	47/17381	0.62417	0.9538	0.93814	AMBRA1/ARRB2/HSF1/ILK/MIR92A2/NKX2-5/PTK2B	7	BP
GO:0032720	negative regulation of tumor necrosis	7/2734	47/17381	0.62417	0.9538	0.93814	ARRB2/HAVCR2/HSF1/LGALS9/NFKBIL1/ORM1/RARA	7	BP
GO:0048806	genitalia development	7/2734	47/17381	0.62417	0.9538	0.93814	DNAJC19/HSD17B3/LHX1/PKD1/PTPN11/SHH/STRA6	7	BP
GO:1904950	negative regulation of establishment of	33/2734	218/17381	0.62417	0.9538	0.93814	ADRA2A/CD27/CDK5/CSK/DAB2IP/DRD2/EMD/F2RL1/FAM89B/FN1/IDH2/INHBB/INS/MTOR/NFKBIL1/NR1H3/OPRM1/OS9/PARP10/PBLD/PDE2A/PKD1/PKDCC/PTPN11/RAB11FIP3/RHBDF1/SERGEF/SFRP5/SNX12/SUFU/TH	33	BP
GO:0043401	steroid hormone mediated signaling	29/2734	192/17381	0.62515	0.9538	0.93814	ARRB2/AXIN1/BMP4/BRCA1/CALCOCO1/CRIPAK/DDX54/ESR2/ESRRA/GPER1/MED24/NR1H3/NR2F1/NR4A1/NR5A1/PAQR6/PAQR7/PAQR9/PER1/PLPP1/PPARD/RARA/RORC/RWDD1/TADA3/TAF1/TAF7/TGFB1I1/THRA	29	BP
GO:0002367	cytokine production involved in immune	13/2734	87/17381	0.62534	0.9538	0.93814	BCL6/CD74/CHGA/CUEDC2/F2RL1/IL18/IL31RA/KARS/MAPK3/SEMA7A/SLC11A1/SPON2/TRAF2	13	BP
GO:0032635	interleukin-6 production	18/2734	120/17381	0.62573	0.9538	0.93814	ARRB2/CARD9/CSK/F2RL1/GBA/HAVCR2/IL18/INPP5D/LGALS9/MIR92A2/NLRX1/NOD1/ORM1/SPON2/TIRAP/TLR8/TLR9/TRPV4	18	BP
GO:0008643	carbohydrate transport	23/2734	153/17381	0.62815	0.9538	0.93814	AGT/AQP1/AQP10/CAPN10/INS/MZB1/PEA15/PID1/PLA2G1B/PPARD/PTPN11/RARRES2/SLC26A6/SLC2A4/SLC2A6/SLC2A8/SLC35A2/SLC35C1/SLC35D2/SLC45A1/SLC45A4/SLC50A1/SLC5A2	23	BP
GO:0040013	negative regulation of locomotion	46/2734	303/17381	0.62868	0.9538	0.93814	ADGRB1/APEX1/ARHGAP4/CCL21/CORO1B/DAB2IP/DPEP1/DRD2/ENG/EVL/GDF2/IDH2/IGFBP3/ILK/KRT16/LRP1/MARVELD3/MIIP/MIR10A/MIR15A/MIR16-1/MIR212/MIR221/MIR29A/MIR29C/MIR503/MIR92A2/NBL1/NISCH/NOTCH1/NRG1/PBLD/PLXNB3/PPARD/PTPN23/PTPRU/SEMA3F/SHH/SLURP1/T	46	BP

GO:0071559	response to transforming growth factor beta	34/2734	225/17381	0.62945	0.9538	0.93814	ARRB2/CAV3/CDH5/COL1A1/COL4A2/ENG/FAM89B/FERMT2/FGFR2/FNTA/FURIN/HSPA5/HTRA3/ITGB5/LEFTY2/MEN1/MIR212/NEDD8/NOX4/PARD3/PARD6A/PBLD/PDE2A/SCX/SMAD6/SOX5/TGFB1I1/VASN/WFIKKN2/WNT1/WNT10A/WNT7A/ZNF703/ZYX	34	BP
GO:0000212	meiotic spindle organization	2/2734	13/17381	0.62974	0.9538	0.93814	GOLGA2/TUBG1	2	BP
GO:0001780	neutrophil homeostasis	2/2734	13/17381	0.62974	0.9538	0.93814	HCAR2/MTHFD1	2	BP
GO:0001967	suckling behavior	2/2734	13/17381	0.62974	0.9538	0.93814	CNTFR/GRIN1	2	BP
GO:0001973	adenosine receptor signaling	2/2734	13/17381	0.62974	0.9538	0.93814	ACPP/NECAB2	2	BP
GO:0002002	regulation of angiotensin levels in blood	2/2734	13/17381	0.62974	0.9538	0.93814	AGT/REN	2	BP
GO:0006089	lactate metabolic	2/2734	13/17381	0.62974	0.9538	0.93814	LDHA/PNKD	2	BP
GO:0006878	cellular copper ion homeostasis	2/2734	13/17381	0.62974	0.9538	0.93814	ATOX1/MT2A	2	BP
GO:0006883	cellular sodium ion homeostasis	2/2734	13/17381	0.62974	0.9538	0.93814	AGT/ATP1A1	2	BP
GO:0007320	insemination	2/2734	13/17381	0.62974	0.9538	0.93814	EDDM3A/TACR1	2	BP
GO:0009437	carnitine metabolic	2/2734	13/17381	0.62974	0.9538	0.93814	CROT/POR	2	BP
GO:0010566	regulation of ketone biosynthetic process	2/2734	13/17381	0.62974	0.9538	0.93814	DGKQ/EGR1	2	BP

GO:0010623	programmed cell death involved in cell	2/2734	13/17381	0.62974	0.9538	0.93814	CASP5/FASLG	2	BP
GO:0010839	negative regulation of keratinocyte	2/2734	13/17381	0.62974	0.9538	0.93814	SFN/SLURP1	2	BP
GO:0014889	muscle atrophy	2/2734	13/17381	0.62974	0.9538	0.93814	MTOR/TRIM63	2	BP
GO:0015812	gamma-aminobutyric acid transport	2/2734	13/17381	0.62974	0.9538	0.93814	SV2A/TRH	2	BP
GO:0016082	synaptic vesicle priming	2/2734	13/17381	0.62974	0.9538	0.93814	STX1A/STXBP1	2	BP
GO:0016540	protein autoprocessing	2/2734	13/17381	0.62974	0.9538	0.93814	CASP1/CTSE	2	BP
GO:0021781	glial cell fate commitment	2/2734	13/17381	0.62974	0.9538	0.93814	NRG1/SOX8	2	BP
GO:0031643	positive regulation of	2/2734	13/17381	0.62974	0.9538	0.93814	NRG1/PARD3	2	BP
GO:0032536	regulation of cell projection	2/2734	13/17381	0.62974	0.9538	0.93814	CDHR5/WNT7A	2	BP
GO:0032740	positive regulation of interleukin-17	2/2734	13/17381	0.62974	0.9538	0.93814	IL18/LY9	2	BP
GO:0033262	regulation of nuclear cell cycle DNA	2/2734	13/17381	0.62974	0.9538	0.93814	BCL6/INO80	2	BP
GO:0035563	positive regulation of chromatin	2/2734	13/17381	0.62974	0.9538	0.93814	PARP9/PYGO2	2	BP

GO:0035641	locomotory exploration behavior	2/2734	13/17381	0.62974	0.9538	0.93814	DLG4/GAD1	2	BP
GO:0035723	interleukin-15-mediated signaling	2/2734	13/17381	0.62974	0.9538	0.93814	IL15RA/SHC1	2	BP
GO:0042228	interleukin-8 biosynthetic process	2/2734	13/17381	0.62974	0.9538	0.93814	NOD1/TLR8	2	BP
GO:0042415	norepinephrine metabolic	2/2734	13/17381	0.62974	0.9538	0.93814	RNF180/TH	2	BP
GO:0042635	positive regulation of	2/2734	13/17381	0.62974	0.9538	0.93814	FOXN1/NUMA1	2	BP
GO:0043247	telomere maintenance in response to DNA damage	2/2734	13/17381	0.62974	0.9538	0.93814	ACD/APEX1	2	BP
GO:0043248	proteasome assembly	2/2734	13/17381	0.62974	0.9538	0.93814	PSMD13/PSMD5	2	BP
GO:0043383	negative T cell selection	2/2734	13/17381	0.62974	0.9538	0.93814	CD74/SHH	2	BP
GO:0043568	positive regulation of insulin-like growth factor receptor	2/2734	13/17381	0.62974	0.9538	0.93814	IGFBP3/WNT1	2	BP
GO:0044406	adhesion of symbiont to	2/2734	13/17381	0.62974	0.9538	0.93814	CD81/SCARB1	2	BP
GO:0045838	positive regulation of membrane	2/2734	13/17381	0.62974	0.9538	0.93814	BAD/SLC34A1	2	BP

GO:0048266	behavioral response to pain	2/2734	13/17381	0.62974	0.9538	0.93814	P2RX2/TACR1	2	BP
GO:0048642	negative regulation of skeletal muscle	2/2734	13/17381	0.62974	0.9538	0.93814	TSC22D3/USP19	2	BP
GO:0048712	negative regulation of astrocyte	2/2734	13/17381	0.62974	0.9538	0.93814	DAB1/MYCN	2	BP
GO:0051503	adenine nucleotide	2/2734	13/17381	0.62974	0.9538	0.93814	SLC25A25/SLC25A5	2	BP
GO:0060123	regulation of growth hormone	2/2734	13/17381	0.62974	0.9538	0.93814	DRD2/PTPN11	2	BP
GO:0060177	regulation of angiotensin metabolic	2/2734	13/17381	0.62974	0.9538	0.93814	AGT/REN	2	BP
GO:0060456	positive regulation of digestive	2/2734	13/17381	0.62974	0.9538	0.93814	AQP1/TACR1	2	BP
GO:0060576	intestinal epithelial cell	2/2734	13/17381	0.62974	0.9538	0.93814	NKX3-2/SPDEF	2	BP
GO:0061000	negative regulation of dendritic spine	2/2734	13/17381	0.62974	0.9538	0.93814	EFNA1/NLGN3	2	BP
GO:0070208	protein heterotrimerizat	2/2734	13/17381	0.62974	0.9538	0.93814	C1QTNF1/COL1A1	2	BP
GO:0070986	left/right axis specification	2/2734	13/17381	0.62974	0.9538	0.93814	NOTCH1/SMO	2	BP
GO:0071027	nuclear RNA surveillance	2/2734	13/17381	0.62974	0.9538	0.93814	EXOSC10/EXOSC2	2	BP

GO:0071028	nuclear mRNA surveillance	2/2734	13/17381	0.62974	0.9538	0.93814	EXOSC10/EXOSC2	2	BP
GO:0071318	cellular response to	2/2734	13/17381	0.62974	0.9538	0.93814	CIB2/TAF1	2	BP
GO:0071350	cellular response to	2/2734	13/17381	0.62974	0.9538	0.93814	IL15RA/SHC1	2	BP
GO:0071397	cellular response to	2/2734	13/17381	0.62974	0.9538	0.93814	OSBPL7/SMO	2	BP
GO:0071712	ER-associated misfolded protein	2/2734	13/17381	0.62974	0.9538	0.93814	SDF2/TOR1A	2	BP
GO:0097296	activation of cysteine-type endopeptidase activity involved in apoptotic [illegible]	2/2734	13/17381	0.62974	0.9538	0.93814	FASLG/TRAF2	2	BP
GO:0098719	sodium ion import across plasma	2/2734	13/17381	0.62974	0.9538	0.93814	SLC9A3/SLC9A5	2	BP
GO:1900004	negative regulation of serine-type endopeptidase	2/2734	13/17381	0.62974	0.9538	0.93814	SPINK2/SPINK5	2	BP
GO:1900103	positive regulation of endoplasmic reticulum	2/2734	13/17381	0.62974	0.9538	0.93814	BOK/DAB2IP	2	BP
GO:1901077	regulation of relaxation of muscle	2/2734	13/17381	0.62974	0.9538	0.93814	CAMK2D/CHGA	2	BP

GO:1901298	regulation of hydrogen peroxide-mediated programmed	2/2734	13/17381	0.62974	0.9538	0.93814	ENDOG/TRAP1	2	BP
GO:1901722	regulation of cell proliferation involved in	2/2734	13/17381	0.62974	0.9538	0.93814	BMP4/EGR1	2	BP
GO:1902572	negative regulation of serine-type	2/2734	13/17381	0.62974	0.9538	0.93814	SPINK2/SPINK5	2	BP
GO:1905205	positive regulation of connective	2/2734	13/17381	0.62974	0.9538	0.93814	MIR16-1/MIR195	2	BP
GO:1905809	negative regulation of synapse	2/2734	13/17381	0.62974	0.9538	0.93814	FZD9/NEUROD2	2	BP
GO:2000194	regulation of female gonad development	2/2734	13/17381	0.62974	0.9538	0.93814	NR5A1/NUPR1	2	BP
GO:2001028	positive regulation of endothelial cell	2/2734	13/17381	0.62974	0.9538	0.93814	HSPB1/KDR	2	BP
GO:0051092	positive regulation of NF-kappaB transcription	21/2734	140/17381	0.62977	0.9538	0.93814	ADAM8/AGT/CARD11/CIB1/CLU/IKBKB/INS/IRAK1/LGALS9/LRRFIP1/NOD1/PLA2G1B/RHEBL1/RNF31/RPS6KA4/TIRAP/TLR9/TRAF2/TRAPPC9/TRIM14/TRIM62	21	BP

GO:0000466	maturation of 5.8S rRNA from tricistronic rRNA transcript (SSU-rRNA, 5.8S rRNA, LSU-rRNA)	3/2734	20/17381	0.6298	0.9538	0.93814	EXOSC2/FCF1/PDCD11	3	BP
GO:0002347	response to tumor cell	3/2734	20/17381	0.6298	0.9538	0.93814	HAVCR2/HNMT/NCR3	3	BP
GO:0002374	cytokine secretion involved in	3/2734	20/17381	0.6298	0.9538	0.93814	F2RL1/KARS/MAPK3	3	BP
GO:0003283	atrial septum development	3/2734	20/17381	0.6298	0.9538	0.93814	GATA4/NKX2-5/SMO	3	BP
GO:0006309	apoptotic DNA fragmentation	3/2734	20/17381	0.6298	0.9538	0.93814	CASP3/ENDOG/HSF1	3	BP
GO:0006465	signal peptide processing	3/2734	20/17381	0.6298	0.9538	0.93814	BGLAP/FURIN/PROZ	3	BP
GO:0006555	methionine metabolic	3/2734	20/17381	0.6298	0.9538	0.93814	BHMT2/GNMT/MTHFD1	3	BP
GO:0007413	axonal fasciculation	3/2734	20/17381	0.6298	0.9538	0.93814	AMIGO1/CELSR3/EPHB3	3	BP
GO:0009070	serine family amino acid biosynthetic	3/2734	20/17381	0.6298	0.9538	0.93814	MTHFD1/PHGDH/SHMT2	3	BP
GO:0009651	response to salt stress	3/2734	20/17381	0.6298	0.9538	0.93814	AQP1/TH/TRPV4	3	BP
GO:0010869	regulation of receptor biosynthetic process	3/2734	20/17381	0.6298	0.9538	0.93814	FURIN/NR1H3/SCAP	3	BP

GO:0010984	regulation of lipoprotein particle	3/2734	20/17381	0.6298	0.9538	0.93814	APOC3/CSK/GPIHBP1	3	BP
GO:0032506	cytokinetic process	3/2734	20/17381	0.6298	0.9538	0.93814	ALKBH4/BIN3/KIF23	3	BP
GO:0032703	negative regulation of interleukin-2	3/2734	20/17381	0.6298	0.9538	0.93814	HAVCR2/IL20RB/VSIG4	3	BP
GO:0033158	regulation of protein import into nucleus, translocation	3/2734	20/17381	0.6298	0.9538	0.93814	OGG1/PARP10/PDE2A	3	BP
GO:0033962	cytoplasmic mRNA processing body	3/2734	20/17381	0.6298	0.9538	0.93814	ATXN2L/DYNC1H1/PATL1	3	BP
GO:0034205	amyloid-beta formation	3/2734	20/17381	0.6298	0.9538	0.93814	APH1A/CLU/EFNA1	3	BP
GO:0042359	vitamin D metabolic	3/2734	20/17381	0.6298	0.9538	0.93814	CUBN/CYP11A1/CYP1A1	3	BP
GO:0045724	positive regulation of	3/2734	20/17381	0.6298	0.9538	0.93814	CROCC/SEPT9/WRAP73	3	BP
GO:0046716	muscle cell cellular	3/2734	20/17381	0.6298	0.9538	0.93814	ALDOA/CAV3/PFKM	3	BP
GO:0046827	positive regulation of protein export	3/2734	20/17381	0.6298	0.9538	0.93814	ANP32B/EMD/SFN	3	BP
GO:0051152	positive regulation of smooth muscle	3/2734	20/17381	0.6298	0.9538	0.93814	ENG/GPER1/SHH	3	BP
GO:0051187	cofactor catabolic	3/2734	20/17381	0.6298	0.9538	0.93814	ACOT7/ALDH1L1/HMOX2	3	BP

GO:0051570	regulation of histone H3-K9 methylation	3/2734	20/17381	0.6298	0.9538	0.93814	BRCA1/PRDM12/SETD7	3	BP
GO:0060065	uterus development	3/2734	20/17381	0.6298	0.9538	0.93814	LHX1/STRA6/WNT7A	3	BP
GO:0060231	mesenchymal to epithelial	3/2734	20/17381	0.6298	0.9538	0.93814	BMP4/SMO/STAT1	3	BP
GO:0060544	regulation of necroptotic process	3/2734	20/17381	0.6298	0.9538	0.93814	BOK/FZD9/TRAF2	3	BP
GO:0061339	establishment or maintenance of monopolar cell polarity	3/2734	20/17381	0.6298	0.9538	0.93814	FSCN1/GBF1/SCRIB	3	BP
GO:0070168	negative regulation of biomineral	3/2734	20/17381	0.6298	0.9538	0.93814	AHSG/ECM1/PTK2B	3	BP
GO:0106030	neuron projection	3/2734	20/17381	0.6298	0.9538	0.93814	AMIGO1/CELSR3/EPHB3	3	BP
GO:1902254	negative regulation of intrinsic apoptotic signaling	3/2734	20/17381	0.6298	0.9538	0.93814	CD74/MUC1/ZNF385A	3	BP
GO:2000311	regulation of AMPA receptor activity	3/2734	20/17381	0.6298	0.9538	0.93814	ARC/CNIH2/NLGN3	3	BP
GO:0006278	RNA-dependent DNA biosynthetic	11/2734	74/17381	0.63012	0.9538	0.93814	ACD/CCT3/EXOSC10/HSP90AA1/MAPK15/MAPK3/NHP2/PIF1/RFC1/SMG5/TINF2	11	BP

GO:0030433	ubiquitin-dependent ERAD pathway	11/2734	74/17381	0.63012	0.9538	0.93814	BCAP31/FBXO2/FBXO6/HERPUD1/HSPA5/OS9/PSMC3/SYVN1/TMUB1/USP19/WFS1	11	BP
GO:0045980	negative regulation of nucleotide	11/2734	74/17381	0.63012	0.9538	0.93814	ADRA2A/CCR2/CDA/DRD2/GABBR1/MLXIPL/OPRM1/PDE2A/PDZD3/PFKFB1/PID1	11	BP
GO:0046513	ceramide biosynthetic process	8/2734	54/17381	0.63072	0.9541	0.93844	B4GALNT1/DEGS2/GBA/ORMDL3/PRKCD/SGMS1/SMPD4/SPTLC1	8	BP
GO:0031669	cellular response to nutrient levels	28/2734	186/17381	0.63145	0.9541	0.93844	AMBRA1/ASNS/ATF3/BGLAP/CHMP1A/COL1A1/DAP/DAPL1/EHMT2/EIF2AK4/FOLR2/GBA/HSPA5/INHBB/LARS/MAPK3/MAX/MTOR/MYOD1/NPRL3/RALB/RRAGC/SESN1/SLC39A5/TNC/UCN2/ULK1/ZFYVE1	28	BP
GO:0036503	ERAD pathway	14/2734	94/17381	0.6318	0.9541	0.93844	BCAP31/BRSK2/FBXO2/FBXO6/HERPUD1/HSPA5/OS9/PSMC3/SDF2/SYVN1/TMUB1/TOR1A/USP19/WFS1	14	BP
GO:0055017	cardiac muscle tissue growth	14/2734	94/17381	0.6318	0.9541	0.93844	AGT/CAV3/FGFR2/GATA4/MIR195/MIR222/MIR25/MTOR/NKX2-5/NOTCH1/NPPA/PDLIM5/PRKAR1A/TBX2	14	BP
GO:0071219	cellular response to molecule of	26/2734	173/17381	0.63252	0.9541	0.93844	ABL1/ADAMTS13/CASP1/CCL5/CD180/CD6/CDK4/CSF3/DAB2IP/HAVCR2/HSF1/IL18/IRAK1/MAPK3/NFKBIL1/NOS3/NR1H3/PDCD4/PPARD/PRPF8/RARA/SCARB1/SPON2/TIRAP/TNIP2/TREM2	26	BP
GO:0010575	positive regulation of vascular endothelial	4/2734	27/17381	0.63304	0.9541	0.93844	BRCA1/C3AR1/FLT4/GATA4	4	BP
GO:0042634	regulation of hair cycle	4/2734	27/17381	0.63304	0.9541	0.93844	FOXN1/NUMA1/PER1/SMO	4	BP
GO:0042730	fibrinolysis	4/2734	27/17381	0.63304	0.9541	0.93844	FGA/KLKB1/PLAU/SERPINF2	4	BP
GO:0046633	alpha-beta T cell	4/2734	27/17381	0.63304	0.9541	0.93844	CCR2/HLA-E/IL18/LGALS9	4	BP

GO:0048643	positive regulation of skeletal muscle	4/2734	27/17381	0.63304	0.9541	0.93844	GPC1/MYOD1/SHH/WNT3A	4	BP
GO:0060142	regulation of syncytium formation by plasma membrane	4/2734	27/17381	0.63304	0.9541	0.93844	ADGRB1/EHD1/IL4R/MYOD1	4	BP
GO:0060706	cell differentiation involved in embryonic placenta	4/2734	27/17381	0.63304	0.9541	0.93844	ASCL2/E2F8/GCM2/ST14	4	BP
GO:0061099	negative regulation of protein tyrosine	4/2734	27/17381	0.63304	0.9541	0.93844	SH3BP5/SH3BP5L/THY1/VPS25	4	BP
GO:0070528	protein kinase C signaling	4/2734	27/17381	0.63304	0.9541	0.93844	ADRA1A/DGKQ/FLT4/WNT11	4	BP
GO:1902253	regulation of intrinsic apoptotic signaling pathway by p53	4/2734	27/17381	0.63304	0.9541	0.93844	ANKRD2/CD74/MUC1/ZNF385A	4	BP
GO:1904030	negative regulation of cyclin-dependent	4/2734	27/17381	0.63304	0.9541	0.93844	CASP3/CDKN2A/INCA1/MEN1	4	BP

GO:1904357	negative regulation of telomere maintenance via telomere	4/2734	27/17381	0.63304	0.9541	0.93844	ACD/EXOSC10/PIF1/TINF2	4	BP
GO:0045844	positive regulation of striated muscle	12/2734	81/17381	0.63644	0.9571	0.94134	ARRB2/BMP4/FGFR2/GPC1/MIR222/MTOR/MYOD1/NOTCH1/NRG1/SHH/TBX2/WNT3A	12	BP
GO:0048636	positive regulation of muscle organ	12/2734	81/17381	0.63644	0.9571	0.94134	ARRB2/BMP4/FGFR2/GPC1/MIR222/MTOR/MYOD1/NOTCH1/NRG1/SHH/TBX2/WNT3A	12	BP
GO:1903426	regulation of reactive oxygen species biosynthetic process	12/2734	81/17381	0.63644	0.9571	0.94134	ADGRB1/AGT/CLU/EGFR/GRIN1/HSP90AA1/INS/MIR92A2/MTOR/PTK2B/TRAP1/ZNF205	12	BP
GO:0002521	leukocyte differentiation	73/2734	479/17381	0.63687	0.9571	0.94134	ABL1/ADAM8/ADGRG3/BAD/BATF2/BCL6/BGLAP/BLNK/BMP4/C1QC/CARD11/CCL19/CD27/CD74/CD79B/CLCF1/CMTM7/CSF1/CSF1R/CSF3/DNAJA3/EFNA4/EGR1/ERBB2/ESRRA/F2RL1/FAM213A/FANCA/FARP2/FOXN1/FUT7/FZD8/FZD9/GLO1/GON4L/HLX/IFNA5/IL18/IL31RA/IL4R/INPP5D/JAG2/L3MBTL3/LCK/LEPR/LFNG/LGALS9/LTBR/LY6D/LY9/MTOR/NKX2-3/NME1/PIK3R6/PPP2R3C/PRELID1/PSMB11/PTK2B/RARA/RORC/SART1/SEMA4A/SHH/SOX13/SPI1/SPINK5/TCTA/TLR9/TMEM176B/TNFRSF18/TREM2/TUSC2/WNT1	73	BP
GO:0007194	negative regulation of adenylate	5/2734	34/17381	0.63741	0.9571	0.94134	ADRA2A/CCR2/DRD2/GABBR1/OPRM1	5	BP
GO:0009225	nucleotide-sugar metabolic	5/2734	34/17381	0.63741	0.9571	0.94134	AMDHD2/GALT/GMPPB/MGAT1/NAGK	5	BP
GO:0017144	drug metabolic process	5/2734	34/17381	0.63741	0.9571	0.94134	ADH1A/CYP1A1/CYP1A2/DPEP1/EPHX2	5	BP

GO:0018200	peptidyl-glutamic acid	5/2734	34/17381	0.63741	0.9571	0.94134	BGLAP/PROZ/TTLL10/TTLL5/TTLL7	5	BP
GO:0031577	spindle checkpoint	5/2734	34/17381	0.63741	0.9571	0.94134	ANAPC15/BUB1B/LCMT1/MAD1L1/XRCC3	5	BP
GO:0032369	negative regulation of	5/2734	34/17381	0.63741	0.9571	0.94134	ABCG5/APOC3/NR1H3/PTPN11/SHH	5	BP
GO:0045737	positive regulation of cyclin-dependent protein	5/2734	34/17381	0.63741	0.9571	0.94134	CCND3/EGFR/HSPA2/MAPRE3/PKD1	5	BP
GO:0048048	embryonic eye morphogenesis	5/2734	34/17381	0.63741	0.9571	0.94134	AXIN1/STRA6/TBX2/TH/VAX2	5	BP
GO:0097502	mannosylation	5/2734	34/17381	0.63741	0.9571	0.94134	ALG3/DPM2/FKTN/PIGZ/SDF2	5	BP
GO:2000816	negative regulation of mitotic sister chromatid	5/2734	34/17381	0.63741	0.9571	0.94134	ANAPC15/BUB1B/LCMT1/MAD1L1/XRCC3	5	BP
GO:2001222	regulation of neuron	5/2734	34/17381	0.63741	0.9571	0.94134	DAB2IP/FLNA/LRIG2/NRG1/NSMF	5	BP
GO:0042102	positive regulation of T cell	15/2734	101/17381	0.63792	0.9576	0.94184	CARD11/CCDC88B/CCL19/CCL5/CCR2/CD6/CLECL1/CORO1A/DNAJA3/EFNB1/HAVCR2/HLA-E/IL18/LGALS9/SHH	15	BP
GO:0006631	fatty acid metabolic process	54/2734	356/17381	0.63808	0.9576	0.94184	ACACB/ACAD10/ACADVL/ACOT7/ACOXL/ACSF3/ACSM6/APOA5/APOC3/BRCA1/CD74/CES2/CPT2/CREM/CROT/CYP1A1/CYP1A2/CYP2W1/DAGLB/EDN2/ELOVL1/ELOVL5/EPHX2/FADS2/FADS3/GPX1/GSTM2/HACD1/HACD3/HADHA/HAO2/INS/LIAS/LOC344967/LYPLA2/MAPK3/MID1IP1/MLXIPL/MTOR/NR1H3/OLAH/PDHB/PLA2G15/PLA2G1B/PLA2G4B/POR/PPARD/PRKAB1/PTGR1/SCAP/TH/THNSL2/TYSND1/UCP3	54	BP

GO:0071478	cellular response to radiation	25/2734	167/17381	0.63938	0.9593	0.94353	ADIRF/AQP1/CNGB1/CRIP1/CRYAB/DDB1/DDB2/EGR1/EIF2AK4/FNTA/FNTB/GNAT1/GUCA1B/GUCY2D/HRAS/HSF1/HSPA5/INO80/NOC2L/NOX4/OPN4/PPEF1/TAF1/TMEM109/XPC	25	BP
GO:0009896	positive regulation of catabolic process	68/2734	447/17381	0.63971	0.9596	0.94378	ADAM8/ADRA2A/AMBRA1/ANAPC15/ANAPC2/APOA5/ATPIF1/AXIN1/BAD/BCAP31/BUB1B/CAV3/CD81/CHFR/CLU/DAB2IP/DAPK1/DDB1/DISC1/ECSCR/EIF2AK4/ENDOG/FURIN/GBA/GPSM1/HERPUD1/HSF1/INS/KDR/KLHL40/LRP1/LRSAM1/MAPK3/MFN2/MIR181B1/MLXIPL/NKD2/NOD1/OAZ2/OSBPL7/PACSIN3/PFKFB1/PNPLA2/POLR2G/PRKCD/PSMB11/PSMB6/PSMB7/PSMB8/PSMC3/PSMD13/PSMD3/PSMD5/PSMD7/PTK2B/RAB7A/RALB/RNF166/RNF180/SNF8/SPTLC1/STX5/TAF1/TBK1/TFEB/TRIM65/ULK1/U[illegible]	68	BP
GO:0030178	negative regulation of Wnt signaling pathway	30/2734	200/17381	0.64201	0.9619	0.94604	AXIN1/CHD8/DAB2IP/DRAXIN/DVL2/EGR1/IGFBP6/KREMEN2/LRP1/LZTS2/NKD2/NKX2-5/NOTCH1/PSMB11/PSMB6/PSMB7/PSMB8/PSMC3/PSMD13/PSMD3/PSMD5/PSMD7/SFRP5/SHH/SOST/TAX1BP3/TNN/TSKU/WIF1/WNT11	30	BP
GO:0000381	regulation of alternative mRNA splicing, via spliceosome	6/2734	41/17381	0.64221	0.9619	0.94604	CELF6/MYOD1/RBFOX1/RBFOX3/RBM4/RNPS1	6	BP
GO:0006378	mRNA polyadenylation	6/2734	41/17381	0.64221	0.9619	0.94604	CDK9/CPSF1/CPSF4/CSTF2/HSF1/ZC3H3	6	BP
GO:0006509	membrane protein ectodomain	6/2734	41/17381	0.64221	0.9619	0.94604	ADAM8/ADRA2A/APH1A/FURIN/LRIG2/PACSIN3	6	BP
GO:0032527	protein exit from endoplasmic	6/2734	41/17381	0.64221	0.9619	0.94604	BCAP31/BRSK2/HERPUD1/OS9/SEC16B/SYVN1	6	BP
GO:0045912	negative regulation of carbohydrate	6/2734	41/17381	0.64221	0.9619	0.94604	COX11/GCK/INS/LEPR/MAEA/PFKFB1	6	BP

GO:0045638	negative regulation of myeloid cell	13/2734	88/17381	0.64244	0.962	0.94614	C1QC/CIB1/HIST1H4F/HIST2H4A/HIST2H4B/HOXB8/INPP5D/LDB1/MEIS2/NME1/PTK2B/RARA/TCTA	13	BP
GO:0010507	negative regulation of	10/2734	68/17381	0.64285	0.9623	0.94651	DAP/DAPL1/EIF4G1/GATA4/GOLGA2/LARS/LEPR/MTOR/POLDIP2/QSOX1	10	BP
GO:0000041	transition metal ion transport	16/2734	108/17381	0.64376	0.9635	0.94762	ATOX1/ATP6V0B/ATP6V0E2/ATP6V1B1/ATP6V1F/ATP6V1G1/ATP6V1G2/CCS/CUTC/HEPH/SLC11A1/SLC30A3/SLC39A13/SLC39A5/STEAP2/TFR2	16	BP
GO:0007093	mitotic cell cycle checkpoint	24/2734	161/17381	0.6465	0.9643	0.94841	ANAPC15/ATF2/BRCA1/BUB1B/CDK5RAP3/CHFR/CHMP4C/E2F4/E2F8/FOXO4/HRAS/INTS3/LCMT1/MAD1L1/MUC1/NEK11/PCBP4/PRCC/RINT1/SFN/TICRR/XPC/XRCC3/ZNF385A	24	BP
GO:0002448	mast cell mediated	7/2734	48/17381	0.64713	0.9643	0.94841	CHGA/FES/IL13/IL4R/LGALS9/SPON2/STXBP1	7	BP
GO:0006754	ATP biosynthetic	7/2734	48/17381	0.64713	0.9643	0.94841	ALDOA/ATP5G1/ATP5I/CYC1/PID1/PKM/VPS9D1	7	BP
GO:0009163	nucleoside biosynthetic process	7/2734	48/17381	0.64713	0.9643	0.94841	CDA/DHODH/IMPDH1/NME1/NME4/PUDP/UCK1	7	BP
GO:0010862	positive regulation of pathway-restricted SMAD protein	7/2734	48/17381	0.64713	0.9643	0.94841	BMP4/ENG/GDF2/INHBB/INHBE/LEFTY2/RBPMS	7	BP
GO:0030803	negative regulation of cyclic nucleotide biosynthetic	7/2734	48/17381	0.64713	0.9643	0.94841	ADRA2A/CCR2/DRD2/GABBR1/OPRM1/PDE2A/PDZD3	7	BP
GO:0033762	response to glucagon	7/2734	48/17381	0.64713	0.9643	0.94841	CYC1/GCG/GNB3/GNG3/PFKFB1/PRKAR1A/PRKAR1B	7	BP

GO:0046456	icosanoid biosynthetic process	7/2734	48/17381	0.64713	0.9643	0.94841	CD74/EDN2/GGT6/GGTA1P/MGST3/PLA2G1B/PLA2G5	7	BP
GO:0055010	ventricular cardiac muscle tissue	7/2734	48/17381	0.64713	0.9643	0.94841	ENG/FGFR2/MYBPC3/NKX2-5/NOTCH1/NRG1/TNNC1	7	BP
GO:1903706	regulation of hemopoiesis	68/2734	448/17381	0.64736	0.9643	0.94841	ABL1/ADAM8/ASH2L/BAD/BCL6/BGLAP/BMP4/C1QC/CARD11/CCL19/CD27/CD74/CIB1/CSF1/CSF3/ERBB2/ESRRA/FAM213A/FANCA/FES/FOXN1/HIST1H4F/HIST2H4A/HIST2H4B/HLX/HOXB8/IL18/IL4R/INPP5D/ISG15/LDB1/LGALS3/LGALS9/LMO1/MEIS2/MIR221/MIR222/MTOR/NME1/NOTCH1/PDCD2/PIK3R6/PPP2R3C/PRELID1/PSMB11/PSMB6/PSMB7/PSMB8/PSMC3/PSMD13/PSMD3/PSMD5/PSMD7/PTK2B/RARA/SART1/SCIN/SETD1A/SHH/SOX13/SPI1/SPINK5/STAT1/TCTA/THPO/TLR9/TMEM176B/TNFRSF18	68	BP
GO:0002062	chondrocyte differentiation	14/2734	95/17381	0.64815	0.9643	0.94841	ADAMTS7/BMP4/LOXL2/MUSTN1/NKX3-2/OSR1/PKDCC/POR/SCIN/SCX/SERPINH1/SOX5/TGFBI/WNT7A	14	BP
GO:0007009	plasma membrane	11/2734	75/17381	0.6485	0.9643	0.94841	ANO4/ANO7/BAIAP2L1/BIN2/CAV3/CLU/PACSIN3/PLEKHM2/PRKCD/TRIM72/WHAMM	11	BP
GO:1903779	regulation of cardiac	11/2734	75/17381	0.6485	0.9643	0.94841	AGT/ATP1A1/ATP1A4/ATP2A3/CAMK2D/FLNA/ITPR3/MIR328/NKX2-5/NPPA/TRDN	11	BP
GO:0032434	regulation of proteasomal ubiquitin-dependent protein	17/2734	115/17381	0.64935	0.9643	0.94841	ARAF/BCAP31/CCAR2/CHFR/CLU/COMMD1/FHIT/GBA/HERPUD1/KLHL40/NKD2/PANO1/RNF166/RNF180/SHH/TAF1/USP5	17	BP
GO:0007269	neurotransmitter secretion	20/2734	135/17381	0.65138	0.9643	0.94841	BAIAP3/CDK5/CPLX1/DGKI/DOC2A/DOC2B/DTNBP1/GAD1/GPER1/NRXN2/PNKD/PTPRN2/SCRIB/SLC6A9/STX1A/STX4/STXBP1/SYTL3/TACR2/WN	20	BP
GO:0099643	signal release from synapse	20/2734	135/17381	0.65138	0.9643	0.94841	BAIAP3/CDK5/CPLX1/DGKI/DOC2A/DOC2B/DTNBP1/GAD1/GPER1/NRXN2/PNKD/PTPRN2/SCRIB/SLC6A9/STX1A/STX4/STXBP1/SYTL3/TACR2/WN	20	BP
GO:0032200	telomere organization	25/2734	168/17381	0.65168	0.9643	0.94841	ACD/APEX1/CCT3/EXO1/EXOSC10/HDAC8/HIST1H4F/HIST2H4A/HIST2H4B/HSP90AA1/MAPK15/MAPK3/NHP2/PARP3/PIF1/POLA2/POLD4/POLE/POLE4/RECQL4/RFC1/RFC2/SMG5/TINF2/XRCC3	25	BP

GO:0032922	circadian regulation of gene expression	8/2734	55/17381	0.65206	0.9643	0.94841	CIART/DRD2/MTA1/NPAS2/PER1/RAI1/RORC/USP2	8	BP
GO:0048635	negative regulation of muscle organ	8/2734	55/17381	0.65206	0.9643	0.94841	BMP4/CAV3/FGF3/LUC7L/MIR222/MIR25/TSC22D3/USP19	8	BP
GO:0051310	metaphase plate congression	8/2734	55/17381	0.65206	0.9643	0.94841	ANKRD53/CHMP1A/CHMP4C/CHMP7/DYNC1H1/KIF2B/MAD1L1/NUMA1	8	BP
GO:0071398	cellular response to fatty	8/2734	55/17381	0.65206	0.9643	0.94841	APOB/CDK4/KCNK4/MIR92A2/NME1/P2RY6/PID1/TNC	8	BP
GO:0086065	cell communication involved in	8/2734	55/17381	0.65206	0.9643	0.94841	ATP1A1/CACNA1G/CAMK2D/FLNA/KCNJ5/KCNQ1/MIR328/TRDN	8	BP
GO:1902883	negative regulation of response to	8/2734	55/17381	0.65206	0.9643	0.94841	GPX1/HSPB1/INS/MIR92A2/NONO/PSAP/TRAP1/WNT1	8	BP
GO:0008630	intrinsic apoptotic signaling pathway in	15/2734	102/17381	0.65362	0.9643	0.94841	ABL1/BAD/BOK/BRCA1/CCAR2/CD74/CLU/CRIP1/MOAP1/MUC1/NUPR1/PHLDA3/SFN/TMEM109/ZNF385A	15	BP
GO:1901800	positive regulation of proteasomal protein	15/2734	102/17381	0.65362	0.9643	0.94841	BCAP31/CHFR/CLU/DAB2IP/ECSCR/GBA/HERPUD1/KLHL40/NKD2/OSBPL7/PSMC3/RNF166/RNF180/TAF1/USP5	15	BP
GO:0000077	DNA damage checkpoint	23/2734	155/17381	0.65389	0.9643	0.94841	ATF2/ATRIP/BRCA1/BRCC3/CCAR2/CDK5RAP3/E2F4/E2F8/EIF2AK4/FBXO6/FOXO4/HINFP/MUC1/NEK11/PCBP4/PEA15/PTPN11/RINT1/SFN/TAOK2/TIPRL/XPC/ZNF385A	23	BP
GO:0140056	organelle localization by membrane tethering	23/2734	155/17381	0.65389	0.9643	0.94841	ACTR1A/B9D1/C2CD3/CC2D2A/CEP164/CEP70/CKAP5/DCTN2/DYNC1H1/HAUS4/HAUS7/HSP90AA1/RAB26/RALB/SDCCAG8/SSNA1/STX1A/STX4/STX5/STX8/STXBP1/TUBG1/VTI1B	23	BP

GO:1901863	positive regulation of muscle tissue	12/2734	82/17381	0.65392	0.9643	0.94841	ARRB2/BMP4/FGFR2/GPC1/MIR222/MTOR/MYOD1/NOTCH1/NRG1/SHH/TBX2/WNT3A	12	BP
GO:1901888	regulation of cell junction	12/2734	82/17381	0.65392	0.9643	0.94841	ABL1/AGT/BCAS3/COL16A1/IKBKB/KDR/LDB1/MYO1C/MYOC/RHOD/THY1/WDPCP	12	BP
GO:0003206	cardiac chamber morphogenesis	18/2734	122/17381	0.65471	0.9643	0.94841	BMP4/CAV3/ENG/FGFR2/FGFRL1/FZD2/GATA4/HEY1/MYBPC3/NKX2-5/NOTCH1/NRG1/RARA/SMAD6/SMO/TBX2/TNNC1/WNT11	18	BP
GO:0006323	DNA packaging	31/2734	208/17381	0.65803	0.9643	0.94841	ANP32B/CDAN1/CDKN2A/CENPN/CHMP1A/ERN2/GPER1/H1FX/HILS1/HIST1H4F/HIST2H4A/HIST2H4B/HJURP/HMGA1/IPO4/M1AP/MCM2/MCPH1/NAA60/NCAPD3/NOC2L/PADI4/PARP10/RSF1/SMYD3/TNRC18/TSPY1/TSPY2/TSPY4/TSPY8/TSPYL2	31	BP
GO:0071897	DNA biosynthetic process	31/2734	208/17381	0.65803	0.9643	0.94841	ACD/ARRB2/BRCA1/CCT3/EXO1/EXOSC10/FAAP20/FGFR4/GDF2/HSP90AA1/ISG15/LIG3/MAPK15/MAPK3/MEN1/NHP2/NOX4/NVL/PDGFRB/PIF1/POLA2/POLD4/POLE/POLE4/PTK2B/RFC1/RFC2/SH2B1/SMG5/TINF2/XRCC	31	BP
GO:0072507	divalent inorganic cation homeostasis	69/2734	456/17381	0.65873	0.9643	0.94841	ABL1/ADM/ADRA1A/ADRA1B/AGT/ATP13A2/ATP6V1B1/BCAP31/BOK/C1QTNF1/C3AR1/CACNB3/CAMK2D/CAV3/CCL1/CCL19/CCL21/CCL5/CCR2/CDH23/CIB2/CNGB1/CNNM2/CORO1A/CXCR3/DISC1/DLG4/DRD2/EDN2/EGFR/EIF5A/EPHX2/F2RL1/FASLG/FATE1/FZD9/GCM2/GNG3/GPER1/GPR17/GPR20/GPR35/GRIN1/GRIN2C/GSTM2/HERPUD1/IL13/ITPR3/JPH3/KNG1/LCK/NPSR1/P2RX2/PKD1/PLA2G1B/PTK2B/RASA3/SLC11A1/SLC30A3/SLC39A13/SLC39A5/SV2A/TACR1/THADA/THY1/TRDN/TRPV4/TRPV6/WF[illegible]	69	BP
GO:2000045	regulation of G1/S transition of mitotic cell cycle	24/2734	162/17381	0.65891	0.9643	0.94841	APEX1/CDK10/CDK2AP2/CTDSP1/CYP1A1/E2F4/E2F8/EGFR/EIF4G1/INO80/MEPCE/MIR15A/MIR16-1/MIR221/MIR222/MIR29A/MIR29C/MUC1/PCBP4/PID1/PKD1/SFN/UBE2E2/ZNF385A	24	BP
GO:0042990	regulation of transcription factor import	13/2734	89/17381	0.65911	0.9643	0.94841	CCL19/CD27/CSF3/FLNA/IL18/LGALS9/MTOR/NFKBIL1/PKD1/PRDX1/SUFU/THRA/TLR9	13	BP
GO:0060333	interferon-gamma-mediated	13/2734	89/17381	0.65911	0.9643	0.94841	CAMK2D/CIITA/HLA-E/IRF5/MT2A/NMI/NR1H3/PARP9/PRKCD/STAT1/TRIM26/TRIM31/TRIM62	13	BP

GO:0060349	bone morphogenesis	13/2734	89/17381	0.65911	0.9643	0.94841	BMP4/COL1A1/FGFR2/IFITM5/INPPL1/PHOSPHO1/POR/RARA/RIPPLY1/SCX/SERPINH1/THBS3/TRPV4	13	BP
GO:1905269	positive regulation of chromatin	13/2734	89/17381	0.65911	0.9643	0.94841	BCL6/BRCA1/CDK9/CTBP1/GCG/KAT2A/MAPK3/MUC1/PAXIP1/PHF19/PIWIL2/PRDM12/RPS6KA4	13	BP
GO:0071216	cellular response to biotic stimulus	29/2734	195/17381	0.65964	0.9643	0.94841	ABL1/ADAMTS13/ATG10/CASP1/CCL5/CD180/CD6/CDK4/CSF3/DAB2IP/HAVCR2/HSF1/HSPA5/IL18/IRAK1/MAPK3/NFKBIL1/NOS3/NR1H3/PDCD4/PPARD/PRPF8/RARA/SCARB1/SPON2/TIRAP/TNIP2/TREM2/WFS1	29	BP
GO:0071426	ribonucleoprotein complex export from	19/2734	129/17381	0.65986	0.9643	0.94841	ALKBH5/CASC3/CPSF1/CPSF4/DHX38/EIF5A/NOL6/NUP210/NUP98/NXF2/NXF2B/NXF3/RNPS1/SETD2/SMG5/SRSF4/SUPT6H/ZC3H3/ZNF593	19	BP
GO:0007281	germ cell development	36/2734	241/17381	0.6601	0.9643	0.94841	ACRBP/ANG/BMP4/CAPZA3/CCDC136/CFAP157/CIB1/DEAF1/DND1/HILS1/HORMAD1/HSPA2/INHBB/MCMDC2/MTOR/NUP210L/PAQR7/PIWIL2/POMZP3/PRDM14/PTK2B/PYGO2/RARA/RNASE9/RXFP2/SEPT4/SLC26A6/SOHLH2/SPAG16/TARBP2/TBPL1/TCP11/TDRD1/TSSK1B/TTLL5/UBE2B	36	BP
GO:0002221	pattern recognition receptor	27/2734	182/17381	0.66153	0.9643	0.94841	APOB/ARRB2/CD180/CD300LF/CLEC7A/CNPY3/CTSL/DAB2IP/DHX58/F2RL1/FGA/HAVCR2/IKBKB/IRAK1/ITGAM/MAPKAPK3/NFKBIL1/NLRX1/NOD1/NR1H3/SFTPA1/TBK1/TIRAP/TLR8/TLR9/TNIP2/TREML4	27	BP
GO:0030802	regulation of cyclic nucleotide biosynthetic	22/2734	149/17381	0.66159	0.9643	0.94841	ADM/ADRA2A/AVPR2/CCR2/CRHR1/DRD2/GABBR1/GCG/GPER1/GPHA2/GUCA1B/GUCA2A/GUCA2B/NOS3/OPRM1/PDE2A/PDZD3/PTK2B/RAF1/RUNDC3A/RXFP2/WFS1	22	BP
GO:0021954	central nervous system neuron development	10/2734	69/17381	0.66171	0.9643	0.94841	CDK5/DCLK2/DISC1/DRD2/EPHB3/FGFR2/GBX2/OGDH/PHOX2B/TSKU	10	BP

GO:0051656	establishment of organelle localization	68/2734	450/17381	0.66244	0.9643	0.94841	ANKRD53/CDK5/CEP19/CHGA/CHMP1A/CHMP4C/CHMP7/CNIH2/COL7A1/CPLX1/CROCC/DCTN2/DNM1/DOC2A/DOC2B/DOCK7/DTNBP1/DYNC1H1/DYNC1I1/EYA1/FES/FHOD1/GAK/GBF1/GOLGA2/GORASP1/GOSR2/IL13/IL4R/KIF13A/KIF23/KIF2B/LEMD2/LGALS9/MAD1L1/MAP4K2/MCPH1/MLPH/MOBP/MYO7A/NLGN3/NUMA1/PEX14/PLD2/RAB17/RHOT2/SCRIB/SEC16A/SEC16B/STARD3/STON2/STX1A/STX4/STX5/STXBP1/SYTL3/TOR1A/TRAPPC1/TRAPPC2L/TRAPPC3/TRAPPC4/TRAPPC9/TRIM46/UBE2B/UXT[illegible]	68	BP
GO:0051169	nuclear transport	72/2734	476/17381	0.66251	0.9643	0.94841	AGT/ALKBH5/ANP32B/BCL6/BMP4/CASC3/CCHCR1/CCL19/CD27/CDK5/CPSF1/CPSF4/CSF3/DAB2IP/DHX38/EGFR/EIF5A/EMD/FAM89B/FLNA/GPN2/HEATR3/HMGA1/HTATIP2/IL18/IPO13/IPO4/LGALS9/LMNA/MTOR/NFKBIL1/NOL6/NUP210/NUP98/NXF2/NXF2B/NXF3/OGG1/OPRD1/PARP10/PBLD/PDE2A/PKD1/POLA2/PPP1R10/PRDX1/PRKCD/PTPN11/RBM4/RBPMS/RGS14/RNPS1/RPAIN/SETD2/SFN/SFRP5/SHH/SMG5/SMO/SRSF4/SUFU/SUPT6H/THRA/TLR9/TMCO6/TNPO3/WNT3A/XPO6/XPO7/ZC3H3/ZNF593/ZPR[illegible]	72	BP
GO:0000002	mitochondrial genome maintenance	4/2734	28/17381	0.66252	0.9643	0.94841	DNAJA3/LIG3/PID1/PIF1	4	BP
GO:0010107	potassium ion import	4/2734	28/17381	0.66252	0.9643	0.94841	ATP1A1/KCNJ18/KCNJ5/KCNJ9	4	BP
GO:0010259	multicellular organism aging	4/2734	28/17381	0.66252	0.9643	0.94841	DDC/INPP5D/NR5A1/TH	4	BP
GO:0010614	negative regulation of cardiac muscle	4/2734	28/17381	0.66252	0.9643	0.94841	CAV3/LMNA/MIR25/TRIM63	4	BP
GO:0010644	cell communication by electrical	4/2734	28/17381	0.66252	0.9643	0.94841	ATP1A1/CAMK2D/DBN1/TRDN	4	BP
GO:0018198	peptidyl-cysteine	4/2734	28/17381	0.66252	0.9643	0.94841	GAPDH/HMBS/MAP6D1/ZDHHC7	4	BP

GO:0031365	N-terminal protein amino acid	4/2734	28/17381	0.66252	0.9643	0.94841	MAP6D1/NAA16/NAA60/NTMT1	4	BP
GO:0045589	regulation of regulatory T cell	4/2734	28/17381	0.66252	0.9643	0.94841	BCL6/FANCA/LGALS9/TNFRSF18	4	BP
GO:0046320	regulation of fatty acid	4/2734	28/17381	0.66252	0.9643	0.94841	ACACB/ACADVL/MTOR/TYSND1	4	BP
GO:0051156	glucose 6-phosphate metabolic	4/2734	28/17381	0.66252	0.9643	0.94841	G6PC3/GCK/HK3/PGAM4	4	BP
GO:0051385	response to mineralocortico	4/2734	28/17381	0.66252	0.9643	0.94841	FOSL1/GPER1/TH/TRH	4	BP
GO:0051482	positive regulation of cytosolic calcium ion concentration involved in phospholipase C-activating G-protein coupled [illegible]	4/2734	28/17381	0.66252	0.9643	0.94841	F2RL1/GPR17/GPR20/GPR35	4	BP
GO:0061311	cell surface receptor signaling pathway involved in	4/2734	28/17381	0.66252	0.9643	0.94841	BMP4/HEY1/NOTCH1/WNT3A	4	BP
GO:0070979	protein K11-linked ubiquitination	4/2734	28/17381	0.66252	0.9643	0.94841	ANAPC2/UBE2A/UBE2B/UBE2E2	4	BP

GO:0043588	skin development	62/2734	411/17381	0.66259	0.9643	0.94841	ADAMTS2/C1orf68/CASP3/CLDN4/COL1A1/CTSL/DACT2/DNASE1L2/EGFR/FGFR2/FLG2/FOXN1/FOXQ1/FURIN/GBA/GJB3/GRHL3/HOXC13/KRT12/KRT14/KRT16/KRT23/KRT3/KRT32/KRT6A/KRT6C/KRT71/KRT75/KRT76/KRT79/KRT81/KRT84/KRT85/KRTAP17-1/KRTAP4-3/KRTAP5-10/KRTAP5-3/KRTAP5-5/KRTAP5-7/KRTAP5-9/LCE3E/LDB1/NOTCH1/NSDHL/NUMA1/PDGFA/PKD1/PKP3/POU3F1/PTC	62	BP
GO:0034504	protein localization to nucleus	56/2734	372/17381	0.66314	0.9643	0.94841	AGT/ARL2BP/BCL6/BMP4/CCL19/CCT3/CD27/CDK5RAP3/COL1A1/CSF3/DAB2IP/DCLK2/EGFR/EMD/FAM89B/FLNA/GPN2/HEATR3/IL18/INS/IPO13/IPO4/LGALS9/LMNA/LZTS2/MTOR/NFKBIL1/NR5A1/NUP98/OGG1/OPRD1/PARP10/PARP9/PBLD/PDE2A/PKD1/POLA2/POLR1A/PPP1R10/PRDX1/PRKCD/RBPMS/RPAIN/SFRP5/SHH/SMO/SUFU/TAF3/THRA/TLR9/TMCO6/TNP	56	BP
GO:0003230	cardiac atrium development	5/2734	35/17381	0.66375	0.9643	0.94841	ENG/GATA4/NKX2-5/NOTCH1/SMO	5	BP
GO:0031641	regulation of myelination	5/2734	35/17381	0.66375	0.9643	0.94841	MTOR/NRG1/PARD3/RARA/ZPR1	5	BP
GO:0048333	mesodermal cell differentiation	5/2734	35/17381	0.66375	0.9643	0.94841	BMP4/EYA1/FGFR2/KDM6B/WNT3A	5	BP
GO:0072348	sulfur compound	5/2734	35/17381	0.66375	0.9643	0.94841	SLC26A1/SLC26A10/SLC26A6/SLC44A4/SLC6A6	5	BP
GO:0090279	regulation of calcium ion	5/2734	35/17381	0.66375	0.9643	0.94841	CAV3/GCG/LGALS3/PDGFRB/TRPV2	5	BP
GO:0098801	regulation of renal system	5/2734	35/17381	0.66375	0.9643	0.94841	AGT/AVPR2/DRD2/F2RL1/TACR1	5	BP
GO:1905819	negative regulation of chromosome	5/2734	35/17381	0.66375	0.9643	0.94841	ANAPC15/BUB1B/LCMT1/MAD1L1/XRCC3	5	BP
GO:0000132	establishment of mitotic spindle orientation	3/2734	21/17381	0.66378	0.9643	0.94841	EYA1/MCPH1/NUMA1	3	BP
GO:0002021	response to dietary excess	3/2734	21/17381	0.66378	0.9643	0.94841	SLC25A25/TRPV4/VGF	3	BP

GO:0006067	ethanol metabolic	3/2734	21/17381	0.66378	0.9643	0.94841	ADH1A/ADH1C/ALDH2	3	BP
GO:0006144	purine nucleobase	3/2734	21/17381	0.66378	0.9643	0.94841	ACPP/MTHFD1/XDH	3	BP
GO:0006293	nucleotide-excision repair, preincision complex stabilization	3/2734	21/17381	0.66378	0.9643	0.94841	DDB1/DDB2/GTF2H4	3	BP
GO:0006295	nucleotide-excision repair, DNA incision, 3'-to lesion	3/2734	21/17381	0.66378	0.9643	0.94841	DDB1/DDB2/GTF2H4	3	BP
GO:0006622	protein targeting to	3/2734	21/17381	0.66378	0.9643	0.94841	CLU/LARS/RAB7A	3	BP
GO:0009226	nucleotide-sugar biosynthetic	3/2734	21/17381	0.66378	0.9643	0.94841	AMDHD2/GMPPB/NAGK	3	BP
GO:0035809	regulation of urine volume	3/2734	21/17381	0.66378	0.9643	0.94841	ADM/AVPR2/DRD2	3	BP
GO:0042094	interleukin-2 biosynthetic process	3/2734	21/17381	0.66378	0.9643	0.94841	CARD11/CARD9/IL18	3	BP
GO:0043586	tongue development	3/2734	21/17381	0.66378	0.9643	0.94841	EGFR/HOXC13/WNT10A	3	BP
GO:0048485	sympathetic nervous system development	3/2734	21/17381	0.66378	0.9643	0.94841	PHOX2A/PHOX2B/SEMA3F	3	BP
GO:0048846	axon extension involved in axon guidance	3/2734	21/17381	0.66378	0.9643	0.94841	ALCAM/SEMA3F/WNT3A	3	BP

GO:0050855	regulation of B cell receptor signaling	3/2734	21/17381	0.66378	0.9643	0.94841	BLK/CMTM3/ELF1	3	BP
GO:0050901	leukocyte tethering or	3/2734	21/17381	0.66378	0.9643	0.94841	CCL21/PODXL2/SELP	3	BP
GO:0060716	labyrinthine layer blood vessel	3/2734	21/17381	0.66378	0.9643	0.94841	HEY1/NSDHL/PLCD1	3	BP
GO:0060972	left/right pattern formation	3/2734	21/17381	0.66378	0.9643	0.94841	NKX2-5/NOTCH1/SMO	3	BP
GO:0070723	response to cholesterol	3/2734	21/17381	0.66378	0.9643	0.94841	OSBPL7/PMVK/SMO	3	BP
GO:0070932	histone H3 deacetylation	3/2734	21/17381	0.66378	0.9643	0.94841	HDAC11/HDAC8/PER1	3	BP
GO:1901623	regulation of lymphocyte chemotaxis	3/2734	21/17381	0.66378	0.9643	0.94841	CCL5/CCR2/PTK2B	3	BP
GO:1902284	neuron projection extension involved in neuron	3/2734	21/17381	0.66378	0.9643	0.94841	ALCAM/SEMA3F/WNT3A	3	BP
GO:1902667	regulation of axon guidance	3/2734	21/17381	0.66378	0.9643	0.94841	SEMA3F/TBR1/WNT3A	3	BP
GO:1902991	regulation of amyloid precursor protein	3/2734	21/17381	0.66378	0.9643	0.94841	CLU/EFNA1/EFNA3	3	BP
GO:1903579	negative regulation of ATP metabolic	3/2734	21/17381	0.66378	0.9643	0.94841	MLXIPL/PFKFB1/PID1	3	BP

GO:1903649	regulation of cytoplasmic transport	3/2734	21/17381	0.66378	0.9643	0.94841	MAPK3/PTPN23/SNX12	3	BP
GO:1904385	cellular response to	3/2734	21/17381	0.66378	0.9643	0.94841	AGT/HSF1/PRKCD	3	BP
GO:2000104	negative regulation of DNA-dependent DNA	3/2734	21/17381	0.66378	0.9643	0.94841	BCL6/LIG3/PID1	3	BP
GO:2000678	negative regulation of transcription regulatory	3/2734	21/17381	0.66378	0.9643	0.94841	HEY1/TAF1/ZNF593	3	BP
GO:0006913	nucleocytoplasmic transport	71/2734	470/17381	0.66613	0.9643	0.94841	AGT/ALKBH5/ANP32B/BCL6/BMP4/CASC3/CCHCR1/CCL19/CD27/CDK5/CPSF1/CPSF4/CSF3/DAB2IP/DHX38/EGFR/EIF5A/EMD/FAM89B/FLNA/GPN2/HEATR3/HTATIP2/IL18/IPO13/IPO4/LGALS9/LMNA/MTOR/NFKBIL1/NOL6/NUP210/NUP98/NXF2/NXF2B/NXF3/OGG1/OPRD1/PARP10/PBLD/PDE2A/PKD1/POLA2/PPP1R10/PRDX1/PRKCD/PTPN11/RBM4/RBPMS/RGS14/RNPS1/RPAIN/SETD2/SFN/SFRP5/SHH/SMG5/SMO/SRSF4/SUFU/SUPT6H/THRA/TLR9/TMCO6/TNPO3/WNT3A/XPO6/XPO7/ZC3H3/ZNF593/ZPR1	71	BP
GO:0006775	fat-soluble vitamin	6/2734	42/17381	0.66618	0.9643	0.94841	CUBN/CYP11A1/CYP1A1/PPARD/RBP1/RBP2	6	BP
GO:0034080	CENP-A containing nucleosome assembly	6/2734	42/17381	0.66618	0.9643	0.94841	CENPN/HIST1H4F/HIST2H4A/HIST2H4B/HJURP/RSF1	6	BP
GO:0042992	negative regulation of transcription factor import	6/2734	42/17381	0.66618	0.9643	0.94841	CD27/MTOR/NFKBIL1/PKD1/SUFU/THRA	6	BP

GO:0045668	negative regulation of osteoblast	6/2734	42/17381	0.66618	0.9643	0.94841	HDAC8/MEN1/NOTCH1/SUFU/TNN/TWIST2	6	BP
GO:0050434	positive regulation of viral	6/2734	42/17381	0.66618	0.9643	0.94841	CDK9/NELFB/PFN1/POLR2G/POLR2L/RSF1	6	BP
GO:0061641	CENP-A containing chromatin organization	6/2734	42/17381	0.66618	0.9643	0.94841	CENPN/HIST1H4F/HIST2H4A/HIST2H4B/HJURP/RSF1	6	BP
GO:0072698	protein localization to microtubule	6/2734	42/17381	0.66618	0.9643	0.94841	C2CD3/DCTN2/DISC1/MCPH1/NUMA1/PARD6A	6	BP
GO:0097300	programmed necrotic cell	6/2734	42/17381	0.66618	0.9643	0.94841	BOK/CASP1/FASLG/FZD9/PGAM5/TRAF2	6	BP
GO:1902017	regulation of cilium assembly	6/2734	42/17381	0.66618	0.9643	0.94841	CROCC/IFT140/IFT20/NOTO/SEPT9/WRAP73	6	BP
GO:0031295	T cell costimulation	11/2734	76/17381	0.66637	0.9643	0.94841	CARD11/CCL19/CCL21/CD247/CD5/CSK/EFNB1/LCK/MAP3K14/PDCD1/PTPN11	11	BP
GO:0042278	purine nucleoside	11/2734	76/17381	0.66637	0.9643	0.94841	ACPP/CARD11/DGUOK/DLG4/IMPDH1/MFN1/NME1/NME4/NUDT18/PEMT/SCRIB	11	BP
GO:0050000	chromosome localization	11/2734	76/17381	0.66637	0.9643	0.94841	ANKRD53/CHMP1A/CHMP4C/CHMP7/DYNC1H1/KIF2B/LEMD2/MAD1L1/NUMA1/NUP98/UBE2B	11	BP
GO:1905954	positive regulation of	11/2734	76/17381	0.66637	0.9643	0.94841	ACACB/APOB/C1QTNF1/EHD1/FITM1/HILPDA/LRP1/NR1H3/PRELID1/PRKCD/SCARB1	11	BP
GO:0003205	cardiac chamber development	23/2734	156/17381	0.66642	0.9643	0.94841	BMP4/CAV3/CRELD1/ENG/FGFR2/FGFRL1/FZD2/GATA4/HEY1/MYBPC3/NKX2-5/NOTCH1/NPRL3/NRG1/PLXND1/RARA/SMAD6/SMO/STRA6/SUFU/TBX2	23	BP
GO:0007218	neuropeptide signaling	15/2734	103/17381	0.66894	0.9643	0.94841	ECEL1/GLRA1/GPR1/GPR88/LTB4R/NPPA/NPSR1/NPW/NPY4R/NXPH4/OPRD1/OPRM1/PYY/QRFPR/TAC3	15	BP

GO:0051568	histone H3-K4 methylation	7/2734	49/17381	0.66925	0.9643	0.94841	ASH2L/BRCA1/GCG/PAXIP1/PYGO2/RLF/SETD1A	7	BP
GO:0071320	cellular response to	7/2734	49/17381	0.66925	0.9643	0.94841	APEX1/AQP1/AQP8/HSPA5/KCNQ1/NOX4/SLC26A6	7	BP
GO:0086010	membrane depolarization during action potential	7/2734	49/17381	0.66925	0.9643	0.94841	CACNA1F/CACNA1G/CACNA1H/CACNA1S/CATSPER1/CAV3/SCN11A	7	BP
GO:1903556	negative regulation of tumor necrosis factor superfamily	7/2734	49/17381	0.66925	0.9643	0.94841	ARRB2/HAVCR2/HSF1/LGALS9/NFKBIL1/ORM1/RARA	7	BP
GO:2000514	regulation of CD4-positive, alpha-beta T cell activation	7/2734	49/17381	0.66925	0.9643	0.94841	BCL6/CCL19/HLX/IL18/IL4R/LGALS9/RARA	7	BP
GO:0072503	cellular divalent inorganic cation homeostasis	66/2734	438/17381	0.66998	0.9643	0.94841	ABL1/ADM/ADRA1A/ADRA1B/AGT/ATP13A2/BCAP31/BOK/C1QTNF1/C3AR1/CACNB3/CAMK2D/CAV3/CCL1/CCL19/CCL21/CCL5/CCR2/CDH23/CIB2/CNGB1/CORO1A/CXCR3/DISC1/DLG4/DRD2/EDN2/EIF5A/EPHX2/F2RL1/FASLG/FATE1/FZD9/GCM2/GNG3/GPER1/GPR17/GPR20/GPR35/GRIN1/GRIN2C/GSTM2/HERPUD1/IL13/ITPR3/JPH3/KNG1/LCK/NPSR1/P2RX2/PKD1/PLA2G1B/PTK2B/RASA3/SLC11A1/SLC30A3/SLC39A13/SLC39A5/SV2A/[illegible]	66	BP
GO:0033044	regulation of chromosome organization	45/2734	301/17381	0.67019	0.9643	0.94841	ACD/ANAPC15/BCL6/BRCA1/BUB1B/CAMK2D/CCT3/CDK9/CTBP1/EXOSC10/GCG/HDAC8/HMGA1/KAT2A/LCMT1/MAD1L1/MAPK15/MAPK3/MCPH1/MUC1/NOC2L/NUMA1/OTUB1/PARP10/PAXIP1/PHF19/PHF2/PIF1/PIWIL2/PRDM12/PYGO2/RPS6KA4/SENP6/SETD1A/SETD7/SLF2/SMG5/SPI1/SUPT6H/TADA3/TAF7/TINF2/UBE2B/XRCC3/ZNHIT1	45	BP
GO:0002821	positive regulation of adaptive	12/2734	83/17381	0.67092	0.9643	0.94841	CCL19/CCR2/CLCF1/EIF2AK4/HLA-E/HLX/LTA/NCR3/PAXIP1/SLC11A1/TNFSF13/TRAF2	12	BP

GO:0000479	endonucleolytic cleavage of tricistronic rRNA transcript (SSU-rRNA, 5.8S rRNA, LSU-rRNA)	2/2734	14/17381	0.67101	0.9643	0.94841	FCF1/TSR1	2	BP
GO:0001675	acrosome assembly	2/2734	14/17381	0.67101	0.9643	0.94841	CCDC136/TBPL1	2	BP
GO:0002418	immune response to	2/2734	14/17381	0.67101	0.9643	0.94841	HAVCR2/NCR3	2	BP
GO:0002674	negative regulation of acute inflammatory	2/2734	14/17381	0.67101	0.9643	0.94841	IL20RB/INS	2	BP
GO:0006646	phosphatidylethanolamine biosynthetic process	2/2734	14/17381	0.67101	0.9643	0.94841	ETNK2/PHOSPHO1	2	BP
GO:0007501	mesodermal cell fate	2/2734	14/17381	0.67101	0.9643	0.94841	EYA1/WNT3A	2	BP
GO:0009263	deoxyribonucleotide biosynthetic	2/2734	14/17381	0.67101	0.9643	0.94841	AK5/TBPL1	2	BP
GO:0010875	positive regulation of cholesterol	2/2734	14/17381	0.67101	0.9643	0.94841	LRP1/NR1H3	2	BP
GO:0014854	response to inactivity	2/2734	14/17381	0.67101	0.9643	0.94841	DRD2/PKM	2	BP

GO:0015868	purine ribonucleotide transport	2/2734	14/17381	0.67101	0.9643	0.94841	SLC25A25/SLC25A5	2	BP
GO:0016246	RNA interference	2/2734	14/17381	0.67101	0.9643	0.94841	TARBP2/TSNAX	2	BP
GO:0019081	viral translation	2/2734	14/17381	0.67101	0.9643	0.94841	EIF2AK4/EIF3B	2	BP
GO:0019511	peptidyl-proline hydroxylation	2/2734	14/17381	0.67101	0.9643	0.94841	P3H1/P3H3	2	BP
GO:0032725	positive regulation of granulocyte macrophage colony-	2/2734	14/17381	0.67101	0.9643	0.94841	IL18/TLR9	2	BP
GO:0033033	negative regulation of myeloid cell	2/2734	14/17381	0.67101	0.9643	0.94841	CCL5/MAEA	2	BP
GO:0033147	negative regulation of intracellular estrogen	2/2734	14/17381	0.67101	0.9643	0.94841	BRCA1/CRIPAK	2	BP
GO:0042033	chemokine biosynthetic process	2/2734	14/17381	0.67101	0.9643	0.94841	EGR1/IL18	2	BP
GO:0042159	lipoprotein catabolic	2/2734	14/17381	0.67101	0.9643	0.94841	APOB/LYPLA2	2	BP
GO:0045141	meiotic telomere	2/2734	14/17381	0.67101	0.9643	0.94841	LEMD2/UBE2B	2	BP

GO:0045898	regulation of RNA polymerase II transcriptional preinitiation	2/2734	14/17381	0.67101	0.9643	0.94841	PSMC3/THRA	2	BP
GO:0046851	negative regulation of bone	2/2734	14/17381	0.67101	0.9643	0.94841	CSK/INPP5D	2	BP
GO:0048368	lateral mesoderm	2/2734	14/17381	0.67101	0.9643	0.94841	SHH/SMO	2	BP
GO:0051580	regulation of neurotransmitter uptake	2/2734	14/17381	0.67101	0.9643	0.94841	DRD2/TOR1A	2	BP
GO:0051917	regulation of fibrinolysis	2/2734	14/17381	0.67101	0.9643	0.94841	KLKB1/SERPINF2	2	BP
GO:0060312	regulation of blood vessel	2/2734	14/17381	0.67101	0.9643	0.94841	FLT4/TMBIM1	2	BP
GO:0060547	negative regulation of necrotic cell	2/2734	14/17381	0.67101	0.9643	0.94841	BOK/FZD9	2	BP
GO:0060911	cardiac cell fate commitment	2/2734	14/17381	0.67101	0.9643	0.94841	NKX2-5/WNT3A	2	BP
GO:0070672	response to interleukin-15	2/2734	14/17381	0.67101	0.9643	0.94841	IL15RA/SHC1	2	BP
GO:0071801	regulation of podosome assembly	2/2734	14/17381	0.67101	0.9643	0.94841	FSCN1/KIF9	2	BP
GO:0089711	L-glutamate transmembrane transport	2/2734	14/17381	0.67101	0.9643	0.94841	SLC1A7/SLC25A22	2	BP

GO:0090153	regulation of sphingolipid biosynthetic process	2/2734	14/17381	0.67101	0.9643	0.94841	ORMDL3/PRKCD	2	BP
GO:0090192	regulation of glomerulus development	2/2734	14/17381	0.67101	0.9643	0.94841	BMP4/EGR1	2	BP
GO:0097369	sodium ion import	2/2734	14/17381	0.67101	0.9643	0.94841	SLC9A3/SLC9A5	2	BP
GO:0099632	protein transport within plasma	2/2734	14/17381	0.67101	0.9643	0.94841	GRIPAP1/SCRIB	2	BP
GO:0099637	neurotransmitter receptor	2/2734	14/17381	0.67101	0.9643	0.94841	GRIPAP1/SCRIB	2	BP
GO:1900037	regulation of cellular response to	2/2734	14/17381	0.67101	0.9643	0.94841	RWDD3/USP19	2	BP
GO:1901223	negative regulation of NIK/NF-	2/2734	14/17381	0.67101	0.9643	0.94841	ADGRG3/TRIM40	2	BP
GO:1902187	negative regulation of viral release	2/2734	14/17381	0.67101	0.9643	0.94841	TRIM11/TRIM26	2	BP
GO:1904294	positive regulation of	2/2734	14/17381	0.67101	0.9643	0.94841	BCAP31/HERPUD1	2	BP
GO:1905038	regulation of membrane lipid metabolic	2/2734	14/17381	0.67101	0.9643	0.94841	ORMDL3/PRKCD	2	BP
GO:1905203	regulation of connective tissue	2/2734	14/17381	0.67101	0.9643	0.94841	MIR16-1/MIR195	2	BP

GO:2000188	regulation of cholesterol homeostasis	2/2734	14/17381	0.67101	0.9643	0.94841	FGFR4/NR1H3	2	BP
GO:2000303	regulation of ceramide biosynthetic process	2/2734	14/17381	0.67101	0.9643	0.94841	ORMDL3/PRKCD	2	BP
GO:2000345	regulation of hepatocyte proliferation	2/2734	14/17381	0.67101	0.9643	0.94841	LIMS2/WNT3A	2	BP
GO:0031060	regulation of histone	8/2734	56/17381	0.67267	0.9662	0.95031	BRCA1/GCG/PAXIP1/PHF19/PRDM12/PYGO2/SETD7/SUPT6H	8	BP
GO:0046637	regulation of alpha-beta T cell	8/2734	56/17381	0.67267	0.9662	0.95031	BCL6/CCL19/HLX/IL18/IL4R/LGALS9/RARA/SHH	8	BP
GO:0042552	myelination	16/2734	110/17381	0.6736	0.9671	0.95116	AMIGO1/ARHGEF10/CLU/EIF2B2/EIF2B5/ERBB2/GNPAT/GPC1/ILK/MTOR/MYOC/NRG1/PARD3/POU3F1/RARA/ZPR1	16	BP
GO:0090101	negative regulation of transmembrane receptor protein serine/threonine kinase signaling	16/2734	110/17381	0.6736	0.9671	0.95116	ABL1/DACT2/FAM89B/HSPA5/HTRA3/LEMD2/NBL1/NOTCH1/PBLD/RBPMS2/SMAD6/SOST/TGFB1I1/VASN/WFIKKN2/WNT1	16	BP
GO:0051100	negative regulation of	22/2734	150/17381	0.67423	0.9677	0.95182	ARRB2/DISC1/DTNBP1/GOLGA2/GPSM1/HEY1/HSPA5/IFIT2/MAPK3/MEPCE/NES/PEX14/PRKCD/PTPRF/RALB/RSF1/SMO/TAF1/WFIKKN2/XIRP1/Z	22	BP
GO:0055024	regulation of cardiac muscle tissue	13/2734	90/17381	0.67536	0.9691	0.95318	ARRB2/BMP4/CAV3/FGF3/FGFR2/MIR222/MIR25/MTOR/NKX2-5/NOTCH1/NRG1/TBX2/WNT3A	13	BP
GO:0015909	long-chain fatty acid transport	9/2734	63/17381	0.67628	0.9697	0.95379	ACACB/CPT2/DRD2/LCN12/MID1IP1/PLA2G1B/PLA2G2F/PLA2G5/SLC25A20	9	BP

GO:0032835	glomerulus development	9/2734	63/17381	0.67628	0.9697	0.95379	AQP1/BMP4/EGR1/KIRREL3/LHX1/MYO1E/NOTCH1/OSR1/PDGFRB	9	BP
GO:0060389	pathway-restricted SMAD protein	9/2734	63/17381	0.67628	0.9697	0.95379	BMP4/ENG/GDF2/INHBB/INHBE/LEFTY2/PBLD/RBPMS/SMAD6	9	BP
GO:0006874	cellular calcium ion homeostasis	62/2734	413/17381	0.67801	0.9716	0.95557	ABL1/ADM/ADRA1A/ADRA1B/AGT/ATP13A2/BCAP31/BOK/C1QTNF1/C3AR1/CACNB3/CAMK2D/CAV3/CCL1/CCL19/CCL21/CCL5/CCR2/CDH23/CIB2/CNGB1/CORO1A/CXCR3/DISC1/DLG4/DRD2/EDN2/EIF5A/EPHX2/F2RL1/FASLG/FATE1/FZD9/GCM2/GNG3/GPER1/GPR17/GPR20/GPR35/GRIN1/GRIN2C/GSTM2/HERPUD1/IL13/ITPR3/JPH3/KNG1/LCK/NPSR1/P2RX2/PKD1/PLA2G1B/PTK2B/RASA3/SV2A/TACR1/THADA/THY1/TRDN/TRPV4/TRP	62	BP
GO:0050871	positive regulation of B	17/2734	117/17381	0.67812	0.9716	0.95557	BAD/BCL6/CARD11/CD27/CD74/CD81/CLCF1/IL13/INPP5D/PAXIP1/PPP2R3C/TIRAP/TLR9/TNFRSF4/TNFSF13/TNIP2/WNT3A	17	BP
GO:0070555	response to interleukin-1	17/2734	117/17381	0.67812	0.9716	0.95557	ADAMTS7/CCL1/CCL19/CCL21/CCL5/DAB2IP/EGR1/HNMT/IKBKB/IL1RN/IRAK1/LGALS9/MAPK3/OTUB1/RPS6KA4/TNIP2/TRIM63	17	BP
GO:0006909	phagocytosis	47/2734	315/17381	0.6782	0.9716	0.95557	ABL1/ADGRB1/AHSG/ARPC1A/ARPC1B/ARPC2/ARPC4/BIN2/BRK1/C2/CAMK1D/CD247/CD300LF/CLEC7A/CORO1A/CSK/DOCK1/ELMO3/F2RL1/FCGR3A/HSP90AA1/ITGAL/ITGAM/LEPR/LIMK1/LMAN2/LRP1/MAPK3/MYO18A/MYO1C/MYO7A/NR1H3/PLD2/PRKCD/RAB20/RAB34/RAB7A/RARA/SCARB1/SFTPA1/SLC11A1/SPACA3/SPON2/TREM2/TULP1/TUSC2/WAS	47	BP
GO:0051235	maintenance of location	45/2734	302/17381	0.67911	0.9726	0.95662	ABL1/ACACB/APOB/ARL2BP/B4GALNT1/CAMK2D/CCL19/CCL21/CD27/CORO1A/DBN1/DGAT1/DRD2/EHD1/FASLG/FITM1/FLNA/GPAA1/GPER1/GSTM2/HILPDA/HSPA5/IL13/ITPR3/JPH3/LCK/NBL1/NFKBIL1/NPSR1/NR1H3/NR5A1/OS9/PKD1/PNPLA2/PTK2B/RASA3/SCARB1/SCIN/SLC30A3/SUF	45	BP
GO:0010522	regulation of calcium ion transport into cytosol	14/2734	97/17381	0.67968	0.9732	0.9572	ABL1/CAMK2D/CORO1A/GPER1/GRIN1/GSTM2/IL13/JPH3/NPSR1/P2RX2/PLA2G1B/PTK2B/THY1/TRDN	14	BP
GO:0051438	regulation of ubiquitin-protein	18/2734	124/17381	0.68249	0.977	0.96093	ABL1/ANAPC15/ANAPC2/AXIN1/BUB1B/GOLGA2/GORASP1/LIMK1/PSMB11/PSMB6/PSMB7/PSMB8/PSMC3/PSMD13/PSMD3/PSMD5/PSMD7/ZYG11A	18	BP

GO:0031570	DNA integrity checkpoint	24/2734	164/17381	0.68306	0.9772	0.96114	ATF2/ATRIP/BRCA1/BRCC3/CCAR2/CDK5RAP3/E2F4/E2F8/EIF2AK4/FBXO6/FOXO4/HINFP/MUC1/NEK11/PCBP4/PEA15/PTPN11/RINT1/SFN/TAOK2/TICRR/TIPRL/XPC/ZNF385A	24	BP
GO:0021536	diencephalon development	11/2734	77/17381	0.68371	0.9772	0.96114	BMP4/DRD2/GBX2/LHX3/OGDH/OTP/PITX1/SHH/SMO/TBX19/WNT1	11	BP
GO:0035249	synaptic transmission, glutamatergic	11/2734	77/17381	0.68371	0.9772	0.96114	CDK5/CLSTN3/CNIH2/DGKI/DISC1/DRD2/EGFR/GRIN1/NLGN3/PTK2B/STXBP1	11	BP
GO:0051279	regulation of release of sequestered calcium ion into cytosol	11/2734	77/17381	0.68371	0.9772	0.96114	ABL1/CAMK2D/CORO1A/GPER1/GSTM2/IL13/JPH3/NPSR1/PTK2B/THY1/TRDN	11	BP
GO:1904667	negative regulation of ubiquitin	11/2734	77/17381	0.68371	0.9772	0.96114	ANAPC15/ANAPC2/BUB1B/PSMB6/PSMB7/PSMB8/PSMC3/PSMD13/PSMD3/PSMD5/PSMD7	11	BP
GO:0051443	positive regulation of ubiquitin-	15/2734	104/17381	0.68389	0.9772	0.96114	ANAPC15/ANAPC2/AXIN1/BUB1B/GOLGA2/GORASP1/PSMB11/PSMB6/PSMB7/PSMB8/PSMC3/PSMD13/PSMD3/PSMD5/PSMD7	15	BP
GO:0098781	ncRNA transcription	15/2734	104/17381	0.68389	0.9772	0.96114	ANG/BRF1/CDK9/GTF3C1/INTS10/INTS3/INTS5/MTOR/NOL11/POLR1E/POLR2G/POLR2L/SNAPC1/SNAPC5/TAF6	15	BP
GO:0043010	camera-type eye development	44/2734	296/17381	0.68406	0.9772	0.96114	ACHE/AQP1/AQP5/B9D1/BMP4/CC2D2A/CDK4/CLCN2/CRYAB/CYP1A1/EGFR/FOXE3/GNAT1/GRHL3/IFT140/LAMC3/LHX1/MAX/MFN2/MIP/NES/PDGFRB/PRSS56/PYGO2/RARA/SHH/SLC25A25/SOX8/STRA6/TBC1D32/TBX2/TH/THY1/TMEM231/TRAF3IP1/TSKU/TTLL5/TULP1/TULP3/VAX1/VAX	44	BP
GO:0090257	regulation of muscle system process	35/2734	237/17381	0.68583	0.9772	0.96114	ADRA1A/ADRA1B/ADRA2A/ADRA2B/ADRA2C/AGT/ATP1A1/CAMK2D/CAV3/CDK9/CHGA/CHRM1/CTTN/EDN2/FLNA/GATA4/GPER1/GSTM2/KCNQ1/LMNA/MIR153-1/MIR25/MIR328/MTOR/MYBPC3/MYBPH/MYL5/NKX2-	35	BP
GO:0031214	biomineral tissue	19/2734	131/17381	0.68673	0.9772	0.96114	AHSG/AMTN/BGLAP/BMP4/COL1A1/DDR2/ECM1/FAM83H/FGFR2/FZD9/IFITM5/ISG15/OSR1/PHOSPHO1/PKDCC/PTK2B/TMEM119/WNT11/WNT6	19	BP

GO:0071166	ribonucleoprotein complex localization	19/2734	131/17381	0.68673	0.9772	0.96114	ALKBH5/CASC3/CPSF1/CPSF4/DHX38/EIF5A/NOL6/NUP210/NUP98/NXF2/NXF2B/NXF3/RNPS1/SETD2/SMG5/SRSF4/SUPT6H/ZC3H3/ZNF593	19	BP
GO:1900182	positive regulation of protein localization to	19/2734	131/17381	0.68673	0.9772	0.96114	BMP4/CCL19/CCT3/CD27/CDK5RAP3/CSF3/EGFR/FLNA/IL18/INS/LGALS9/PARP9/PRKCD/RBPMS/SHH/SMO/TLR9/WNT3A/ZPR1	19	BP
GO:0030799	regulation of cyclic nucleotide	25/2734	171/17381	0.68728	0.9772	0.96114	ADM/ADRA2A/AIPL1/AVPR2/CCR2/CHGA/CRHR1/DRD2/FZD2/GABBR1/GCG/GPER1/GPHA2/GUCA1B/GUCA2A/GUCA2B/NOS3/OPRM1/PDE2A/PDZD3/PTK2B/RAF1/RUNDC3A/RXFP2/WFS1	25	BP
GO:0009266	response to temperature stimulus	33/2734	224/17381	0.68776	0.9772	0.96114	ACADVL/ADM/ANO1/ARRB2/ASIC3/CAMK2D/CCAR2/CRYAB/DNAJA3/EIF2AK4/EIF2B2/EIF2B5/HSF1/HSP90AA1/HSPA2/IRAK1/KCNK4/MAPK3/MICB/MLST8/MTOR/NOS3/NUP210/NUP98/PRDM12/TACR1/TGFB1I1/THRA/TRH/TRPV2/TRPV4/UCP3/VGF	33	BP
GO:0001580	detection of chemical stimulus involved in sensory	5/2734	36/17381	0.6888	0.9772	0.96114	AZGP1/CA6/GNAT1/LPO/TAS2R3	5	BP
GO:0002755	MyD88-dependent toll-like receptor signaling	5/2734	36/17381	0.6888	0.9772	0.96114	CD300LF/IRAK1/TIRAP/TLR8/TLR9	5	BP
GO:0006656	phosphatidylcholine biosynthetic	5/2734	36/17381	0.6888	0.9772	0.96114	ACHE/MFSD2A/PEMT/PHOSPHO1/SLC44A4	5	BP
GO:0035307	positive regulation of protein	5/2734	36/17381	0.6888	0.9772	0.96114	DUSP26/GBA/NSMF/PDGFRB/PRKCD	5	BP
GO:0043094	cellular metabolic	5/2734	36/17381	0.6888	0.9772	0.96114	BHMT2/CDA/DGUOK/PUDP/UCK1	5	BP

GO:0043267	negative regulation of potassium ion	5/2734	36/17381	0.6888	0.9772	0.96114	CAV3/MIR153-1/MIR212/NOS3/PTK2B	5	BP
GO:0050856	regulation of T cell receptor signaling	5/2734	36/17381	0.6888	0.9772	0.96114	CACNA1F/ELF1/LCK/LGALS3/THY1	5	BP
GO:0051973	positive regulation of telomerase	5/2734	36/17381	0.6888	0.9772	0.96114	ACD/HSP90AA1/MAPK15/MAPK3/NVL	5	BP
GO:1905898	positive regulation of response to endoplasmic	5/2734	36/17381	0.6888	0.9772	0.96114	BCAP31/BOK/DAB2IP/HERPUD1/SPOP	5	BP
GO:0002204	somatic recombination of immunoglobulin genes	6/2734	43/17381	0.68912	0.9772	0.96114	BCL6/CLCF1/EXO1/PAXIP1/SUPT6H/TNFSF13	6	BP
GO:0002208	somatic diversification of immunoglobulins involved in	6/2734	43/17381	0.68912	0.9772	0.96114	BCL6/CLCF1/EXO1/PAXIP1/SUPT6H/TNFSF13	6	BP
GO:0021983	pituitary gland development	6/2734	43/17381	0.68912	0.9772	0.96114	BMP4/DRD2/LHX3/OTP/PITX1/TBX19	6	BP
GO:0042771	intrinsic apoptotic signaling pathway in response to	6/2734	43/17381	0.68912	0.9772	0.96114	CD74/MUC1/NUPR1/PHLDA3/TMEM109/ZNF385A	6	BP

GO:0043631	RNA polyadenylation	6/2734	43/17381	0.68912	0.9772	0.96114	CDK9/CPSF1/CPSF4/CSTF2/HSF1/ZC3H3	6	BP
GO:0045190	isotype switching	6/2734	43/17381	0.68912	0.9772	0.96114	BCL6/CLCF1/EXO1/PAXIP1/SUPT6H/TNFSF13	6	BP
GO:0001773	myeloid dendritic cell	4/2734	29/17381	0.69034	0.9772	0.96114	BATF2/HAVCR2/LTBR/SPI1	4	BP
GO:0001825	blastocyst formation	4/2734	29/17381	0.69034	0.9772	0.96114	CDX2/MFN2/NLE1/PRDM14	4	BP
GO:0002675	positive regulation of acute inflammatory	4/2734	29/17381	0.69034	0.9772	0.96114	ADAM8/KLKB1/MIR92A2/PTGER3	4	BP
GO:0006294	nucleotide-excision repair, preincision complex	4/2734	29/17381	0.69034	0.9772	0.96114	DDB1/DDB2/GTF2H4/XPC	4	BP
GO:0015893	drug transport	4/2734	29/17381	0.69034	0.9772	0.96114	ABCB5/ABCC3/ABCG5/SLC22A1	4	BP
GO:0042987	amyloid precursor protein	4/2734	29/17381	0.69034	0.9772	0.96114	APH1A/CLU/EFNA1/EFNA3	4	BP
GO:0048265	response to pain	4/2734	29/17381	0.69034	0.9772	0.96114	GCH1/P2RX2/SLC6A2/TACR1	4	BP
GO:0050482	arachidonic acid secretion	4/2734	29/17381	0.69034	0.9772	0.96114	DRD2/PLA2G1B/PLA2G2F/PLA2G5	4	BP
GO:0071295	cellular response to	4/2734	29/17381	0.69034	0.9772	0.96114	BGLAP/COL1A1/FOLR2/TNC	4	BP
GO:0071709	membrane assembly	4/2734	29/17381	0.69034	0.9772	0.96114	CAV3/GAK/NLGN3/NRXN2	4	BP
GO:1903963	arachidonate transport	4/2734	29/17381	0.69034	0.9772	0.96114	DRD2/PLA2G1B/PLA2G2F/PLA2G5	4	BP

GO:2000171	negative regulation of dendrite	4/2734	29/17381	0.69034	0.9772	0.96114	EFNA1/GORASP1/NLGN3/TLX2	4	BP
GO:0032371	regulation of sterol transport	7/2734	50/17381	0.6905	0.9772	0.96114	ABCA2/ABCG5/APOA5/APOC3/LRP1/NR1H3/SHH	7	BP
GO:0032374	regulation of cholesterol transport	7/2734	50/17381	0.6905	0.9772	0.96114	ABCA2/ABCG5/APOA5/APOC3/LRP1/NR1H3/SHH	7	BP
GO:0034113	heterotypic cell-cell adhesion	7/2734	50/17381	0.6905	0.9772	0.96114	CD58/FGA/IL1RN/ITGA7/ITGAX/MIR221/THY1	7	BP
GO:0042073	intraciliary transport	7/2734	50/17381	0.6905	0.9772	0.96114	DYNLL2/IFT140/IFT20/IFT22/KIF17/SSNA1/TRAF3IP1	7	BP
GO:0043392	negative regulation of	7/2734	50/17381	0.6905	0.9772	0.96114	HEY1/RSF1/SMO/TAF1/WFIKKN2/ZNF462/ZNF593	7	BP
GO:1901659	glycosyl compound biosynthetic process	7/2734	50/17381	0.6905	0.9772	0.96114	CDA/DHODH/IMPDH1/NME1/NME4/PUDP/UCK1	7	BP
GO:0042991	transcription factor import	13/2734	91/17381	0.69115	0.9772	0.96114	CCL19/CD27/CSF3/FLNA/IL18/LGALS9/MTOR/NFKBIL1/PKD1/PRDX1/SUFU/THRA/TLR9	13	BP
GO:0048525	negative regulation of	13/2734	91/17381	0.69115	0.9772	0.96114	CCL5/EIF2AK4/IFITM2/ISG15/MIR221/MIR222/PARP10/STAT1/TRIM11/TRIM14/TRIM26/TRIM31/TRIM62	13	BP
GO:1901570	fatty acid derivative biosynthetic process	13/2734	91/17381	0.69115	0.9772	0.96114	ACSF3/ACSS3/CD74/EDN2/ELOVL1/ELOVL5/GGT6/GGTA1P/HACD1/HMGCLL1/MGST3/PLA2G1B/PLA2G5	13	BP
GO:1902106	negative regulation of leukocyte	13/2734	91/17381	0.69115	0.9772	0.96114	BCL6/BMP4/C1QC/CD74/ERBB2/HLX/IL4R/INPP5D/NME1/RARA/SHH/TCTA/TMEM176B	13	BP

GO:0009755	hormone-mediated signaling pathway	34/2734	231/17381	0.69174	0.9772	0.96114	ARRB2/AXIN1/BMP4/BRCA1/CALCOCO1/CRIPAK/DDX54/ESR2/ESRRA/GPER1/MED24/NR1H3/NR2F1/NR4A1/NR5A1/PAQR6/PAQR7/PAQR9/PER1/PLPP1/PPARD/PRCP/PTPN11/RARA/REN/RORC/RWDD1/SSTR5/TADA3/TAF1/TAF7/TGFB1I1/THRA/TRH	34	BP
GO:0050728	negative regulation of inflammatory	17/2734	118/17381	0.69199	0.9772	0.96114	CHID1/CUEDC2/GBA/GPER1/GPR17/GPX1/IL20RB/INS/MIR221/MIR222/MIR92A2/NLRX1/NR1H3/PDCD4/PPARD/PRKCD/SHARPIN	17	BP
GO:0043486	histone exchange	8/2734	57/17381	0.69252	0.9772	0.96114	ANP32B/CENPN/HIST1H4F/HIST2H4A/HIST2H4B/HJURP/RSF1/ZNHIT1	8	BP
GO:0097306	cellular response to	8/2734	57/17381	0.69252	0.9772	0.96114	BRCA1/DNMT3A/GLRA1/OSBPL7/P2RY6/RECQL5/SMO/SPI1	8	BP
GO:0098739	import across plasma	8/2734	57/17381	0.69252	0.9772	0.96114	AGT/ATP1A1/KCNJ5/SLC9A3/SLC9A5/STEAP2/TFR2/TRPV6	8	BP
GO:2001244	positive regulation of intrinsic apoptotic	8/2734	57/17381	0.69252	0.9772	0.96114	BAD/BCAP31/BOK/LCK/MIR15A/MIR16-1/SEPT4/SPOP	8	BP
GO:0051170	nuclear import	44/2734	297/17381	0.69288	0.9772	0.96114	AGT/BCL6/BMP4/CCL19/CD27/CSF3/DAB2IP/EGFR/EMD/FAM89B/FLNA/GPN2/HEATR3/HTATIP2/IL18/IPO13/IPO4/LGALS9/LMNA/MTOR/NFKBIL1/NUP98/OGG1/OPRD1/PARP10/PBLD/PDE2A/PKD1/POLA2/PPP1R10/PRDX1/PRKCD/RBPMS/RPAIN/SFRP5/SHH/SMO/SUFU/THRA/TLR9/TMCO6/TNP	44	BP
GO:0044843	cell cycle G1/S phase transition	42/2734	284/17381	0.69424	0.9772	0.96114	APEX1/C10orf99/CDK10/CDK2AP2/CDK4/CDKN2A/CTDSP1/CYP1A1/E2F4/E2F8/EGFR/EIF4EBP1/EIF4G1/GPR132/HINFP/INO80/MAX/MCM2/MCM4/MEN1/MEPCE/MIR10A/MIR15A/MIR16-1/MIR221/MIR222/MIR29A/MIR29C/MIR503/MUC1/ORC6/PCBP4/PID1/PKD1/POLA2/POLE/POLE4/SFN/TAF1/UBE2E2/ZNF385A/ZPR1	42	BP
GO:0071222	cellular response to lipopolysacchari	24/2734	165/17381	0.69478	0.9772	0.96114	ABL1/ADAMTS13/CASP1/CCL5/CD180/CD6/CDK4/CSF3/DAB2IP/HAVCR2/HSF1/IL18/IRAK1/MAPK3/NFKBIL1/NOS3/NR1H3/PDCD4/PPARD/PRPF8/RARA/SCARB1/SPON2/TNIP2	24	BP

GO:0030148	sphingolipid biosynthetic process	14/2734	98/17381	0.69482	0.9772	0.96114	B4GALNT1/DEGS2/ELOVL1/ELOVL5/GBA/HACD1/HACD3/ORMDL3/PEMT/PLPP1/PRKCD/SGMS1/SMPD4/SPTLC1	14	BP
GO:0055008	cardiac muscle tissue morphogenesis	9/2734	64/17381	0.69495	0.9772	0.96114	ENG/FGFR2/MIR195/MYBPC3/NKX2-5/NOTCH1/NRG1/TCAP/TNNC1	9	BP
GO:1904427	positive regulation of calcium ion transmembrane	9/2734	64/17381	0.69495	0.9772	0.96114	ABL1/CRACR2A/GPER1/GSTM2/HSPA2/IL13/NPSR1/THY1/TRDN	9	BP
GO:0002544	chronic inflammatory response	3/2734	22/17381	0.69544	0.9772	0.96114	CCL5/IDO1/LTA	3	BP
GO:0006972	hyperosmotic response	3/2734	22/17381	0.69544	0.9772	0.96114	AQP1/HNMT/TRPV4	3	BP
GO:0010818	T cell chemotaxis	3/2734	22/17381	0.69544	0.9772	0.96114	CCL5/CCR2/CXCR3	3	BP
GO:0010954	positive regulation of protein	3/2734	22/17381	0.69544	0.9772	0.96114	ADAM8/KLKB1/NKD2	3	BP
GO:0016578	histone deubiquitination	3/2734	22/17381	0.69544	0.9772	0.96114	BRCC3/KAT2A/USP49	3	BP
GO:0030325	adrenal gland development	3/2734	22/17381	0.69544	0.9772	0.96114	NR5A1/STRA6/WNT11	3	BP
GO:0030728	ovulation	3/2734	22/17381	0.69544	0.9772	0.96114	IL4R/INHBB/NOS3	3	BP
GO:0035268	protein mannosylation	3/2734	22/17381	0.69544	0.9772	0.96114	DPM2/FKTN/SDF2	3	BP
GO:0035561	regulation of chromatin	3/2734	22/17381	0.69544	0.9772	0.96114	MEPCE/PARP9/PYGO2	3	BP

GO:0048799	animal organ maturation	3/2734	22/17381	0.69544	0.9772	0.96114	PHOSPHO1/PLXNB1/THBS3	3	BP
GO:0050858	negative regulation of antigen receptor-	3/2734	22/17381	0.69544	0.9772	0.96114	ELF1/LGALS3/THY1	3	BP
GO:0050996	positive regulation of lipid catabolic	3/2734	22/17381	0.69544	0.9772	0.96114	APOA5/PNPLA2/PRKCD	3	BP
GO:0070841	inclusion body assembly	3/2734	22/17381	0.69544	0.9772	0.96114	CLU/HSF1/HSPA2	3	BP
GO:0071379	cellular response to prostaglandin	3/2734	22/17381	0.69544	0.9772	0.96114	APOB/P2RY6/TNC	3	BP
GO:0071402	cellular response to lipoprotein	3/2734	22/17381	0.69544	0.9772	0.96114	CD81/CDH13/MIR92A2	3	BP
GO:0001895	retina homeostasis	10/2734	71/17381	0.69764	0.9778	0.96172	AIPL1/AZGP1/CDH23/CDHR1/CIB2/CNGB1/CROCC/HSPB1/PRDX1/TULP1	10	BP
GO:0006635	fatty acid beta-oxidation	10/2734	71/17381	0.69764	0.9778	0.96172	ACACB/ACAD10/ACADVL/ACOXL/CPT2/CROT/HADHA/MTOR/PPARD/TYSND1	10	BP
GO:0072384	organelle transport along	10/2734	71/17381	0.69764	0.9778	0.96172	CNIH2/DTNBP1/DYNC1H1/DYNC1I1/KIF13A/KIF23/PEX14/RHOT2/TRIM46/UXT	10	BP
GO:0002526	acute inflammatory response	31/2734	212/17381	0.70025	0.9778	0.96172	ADAM8/AHSG/C1QB/C1QC/C2/C3AR1/C8A/CD6/CD81/CFH/CLU/CPN2/FN1/IL1RN/IL20RB/IL31RA/INS/ITIH4/KLKB1/MIR92A2/NUPR1/OGG1/OPRM1/ORM1/ORM2/PRCP/PTGER3/SERPINA3/SERPINF2/TACR1/TFR2	31	BP
GO:0010833	telomere maintenance via telomere lengthening	11/2734	78/17381	0.7005	0.9778	0.96172	ACD/CCT3/EXOSC10/HSP90AA1/MAPK15/MAPK3/NHP2/PIF1/RFC1/SMG5/TINF2	11	BP

GO:0046323	glucose import	11/2734	78/17381	0.7005	0.9778	0.96172	AGT/CAPN10/INS/MZB1/PEA15/PID1/PTPN11/RARRES2/SLC2A4/SLC2A6/SLC2A8	11	BP
GO:0061418	regulation of transcription from RNA polymerase II promoter in response to	11/2734	78/17381	0.7005	0.9778	0.96172	EGR1/NOTCH1/PSMB11/PSMB6/PSMB7/PSMB8/PSMC3/PSMD13/PSMD3/PSMD5/PSMD7	11	BP
GO:0030099	myeloid cell differentiation	58/2734	390/17381	0.70196	0.9778	0.96172	AHSP/ALAS2/ASH2L/ATPIF1/BATF2/BCL6/BGLAP/BMP4/C1QC/CASP3/CCL19/CD74/CIB1/CSF1/CSF1R/CSF3/EFNA4/ESRRA/F2RL1/FAM213A/FARP2/FES/GLO1/HBZ/HIST1H4F/HIST2H4A/HIST2H4B/HOXB7/HOXB8/IL31RA/INPP5D/ISG15/L3MBTL3/LDB1/LGALS3/LTBR/MAEA/MEIS2/MIR221/MIR222/MTOR/NBEAL2/NFE2L1/NKX2-3/NME1/PTK2B/PTPN11/RARA/SCIN/SETD1A/SPI1/STAT1/TCTA/THPO/TH	58	BP
GO:0008543	fibroblast growth factor receptor	16/2734	112/17381	0.70201	0.9778	0.96172	ESRP2/FGF17/FGF3/FGFBP3/FGFR2/FGFR4/FGFRL1/GPC1/HHIP/MAPK3/MIR16-1/POLR2G/POLR2L/PRDM14/PTPN11/SHCBP1	16	BP
GO:0030801	positive regulation of cyclic nucleotide	16/2734	112/17381	0.70201	0.9778	0.96172	ADM/AVPR2/CHGA/CRHR1/FZD2/GCG/GPER1/GPHA2/GUCA1B/GUCA2A/GUCA2B/NOS3/RAF1/RUNDC3A/RXFP2/WFS1	16	BP
GO:1900371	regulation of purine nucleotide biosynthetic	23/2734	159/17381	0.70255	0.9778	0.96172	ADM/ADRA2A/AVPR2/CCR2/CRHR1/DRD2/GABBR1/GCG/GPER1/GPHA2/GUCA1B/GUCA2A/GUCA2B/NOS3/OPRM1/PDE2A/PDZD3/PID1/PTK2B/RAF1/RUNDC3A/RXFP2/WFS1	23	BP
GO:0001906	cell killing	20/2734	139/17381	0.70343	0.9778	0.96172	ARRB2/BAD/CHGA/CORO1A/CTSH/F2RL1/GAPDH/HAVCR2/HLA-E/IL13/IL18/LGALS9/MICB/NCR3/PIK3R6/PRDX1/RAET1G/SLAMF7/TUSC2	20	BP
GO:0008584	male gonad development	20/2734	139/17381	0.70343	0.9778	0.96172	BOK/CSDE1/FANCA/GATA4/LRRC6/MGST1/NR5A1/NUDT1/NUP210L/NUPR1/PDGFRB/RARA/REN/RXFP2/SCX/SDC1/SF1/SOX8/UTF1/ZFP42	20	BP
GO:0045778	positive regulation of	12/2734	85/17381	0.70346	0.9778	0.96172	BMP4/CLIC1/DDR2/FZD9/GDF2/ILK/ISG15/OSR1/PDLIM7/PKDCC/TACR1/TMEM119	12	BP

GO:0003231	cardiac ventricle	17/2734	119/17381	0.70552	0.9778	0.96172	BMP4/ENG/FGFR2/FGFRL1/FZD2/GATA4/HEY1/MYBPC3/NKX2-5/NOTCH1/NPRL3/NRG1/SMAD6/STRA6/SUFU/TNNC1/WNT11	17	BP
GO:0051592	response to calcium ion	17/2734	119/17381	0.70552	0.9778	0.96172	BAD/CACYBP/CAMK2D/CPNE7/DPEP1/EGFR/ENDOG/FGA/HSPA5/ITPR3/NEUROD2/PTK2B/RASGRP2/S100A16/SDC1/SPAG16/TRPV6	17	BP
GO:0048771	tissue remodeling	24/2734	166/17381	0.70626	0.9778	0.96172	ADAM8/AGT/BGLAP/BGN/CCR2/CSF1R/CSK/DEF8/EFNA4/EGFR/FLT4/HOXA3/IL18/INPP5D/LEPR/MIR16-1/MIR195/NCDN/NOS3/NOX4/PLEKHM1/PTK2B/RAB7A/TMBIM1	24	BP
GO:0035264	multicellular organism	21/2734	146/17381	0.70706	0.9778	0.96172	CDK4/CSF1/DRD2/EN1/ETNK2/FGFR2/HSF1/KAT2A/MTOR/PKDCC/PPIB/PTPN11/RAI1/RARA/SH3PXD2B/SLC25A25/SMO/SP2/SPTBN2/TARBP2/TNS	21	BP
GO:1901657	glycosyl compound metabolic	21/2734	146/17381	0.70706	0.9778	0.96172	ACPP/CARD11/CDA/DGUOK/DHODH/DLG4/ERH/GBA/IMPDH1/LCTL/MFN1/NME1/NME4/NT5M/NUDT18/PEMT/PUDP/SCRIB/SLC34A1/TH/UCK1	21	BP
GO:0006814	sodium ion transport	33/2734	226/17381	0.70763	0.9778	0.96172	ASIC2/ASIC3/ATP1A1/ATP1A4/CACNA1G/CACNA1H/CAMK2D/CAV3/COMMD1/DRD2/HCN3/MAGED2/NKX2-5/NOS3/OSR1/PER1/SCN11A/SCNN1A/SGK1/SLC13A2/SLC20A1/SLC20A2/SLC34A1/SLC38A1/SLC38A10/SLC38A8/SLC4A9/SLC5A2/SLC6A8/SLC9A3/SLC9A5/TRPM5/WNK2	33	BP
GO:0031424	keratinization	33/2734	226/17381	0.70763	0.9778	0.96172	FURIN/KRT12/KRT14/KRT16/KRT23/KRT3/KRT32/KRT6A/KRT6C/KRT71/KRT75/KRT76/KRT79/KRT81/KRT84/KRT85/KRTAP17-1/KRTAP4-3/KRTAP5-10/KRTAP5-3/KRTAP5-5/KRTAP5-7/KRTAP5-9/LCE3E/PKP3/SFN/SHARPIN/SPINK5/SPRR1A/ST14/TGM1/TGM5/TMEM7	33	BP
GO:0000478	endonucleolytic cleavage involved in rRNA	2/2734	15/17381	0.70846	0.9778	0.96172	FCF1/TSR1	2	BP
GO:0001325	formation of extrachromosomal circular	2/2734	15/17381	0.70846	0.9778	0.96172	EXO1/XRCC3	2	BP
GO:0001916	positive regulation of T cell mediated	2/2734	15/17381	0.70846	0.9778	0.96172	HLA-E/NCR3	2	BP

GO:0002922	positive regulation of humoral	2/2734	15/17381	0.70846	0.9778	0.96172	LTA/PGC	2	BP
GO:0006086	acetyl-CoA biosynthetic process from pyruvate	2/2734	15/17381	0.70846	0.9778	0.96172	MPC1/PDHB	2	BP
GO:0007076	mitotic chromosome condensation	2/2734	15/17381	0.70846	0.9778	0.96172	CHMP1A/NCAPD3	2	BP
GO:0009130	pyrimidine nucleoside monophosphate biosynthetic process	2/2734	15/17381	0.70846	0.9778	0.96172	DHODH/UCK1	2	BP
GO:0009812	flavonoid metabolic	2/2734	15/17381	0.70846	0.9778	0.96172	CYP1A1/POR	2	BP
GO:0010421	hydrogen peroxide-mediated programmed	2/2734	15/17381	0.70846	0.9778	0.96172	ENDOG/TRAP1	2	BP
GO:0010455	positive regulation of cell fate	2/2734	15/17381	0.70846	0.9778	0.96172	SPDEF/WNT3A	2	BP
GO:0010919	regulation of inositol phosphate biosynthetic	2/2734	15/17381	0.70846	0.9778	0.96172	GPER1/PTK2B	2	BP
GO:0014048	regulation of glutamate secretion	2/2734	15/17381	0.70846	0.9778	0.96172	STXBP1/TRH	2	BP

GO:0014733	regulation of skeletal muscle adaptation	2/2734	15/17381	0.70846	0.9778	0.96172	MTOR/TNNC1	2	BP
GO:0014874	response to stimulus involved in regulation of	2/2734	15/17381	0.70846	0.9778	0.96172	AGT/TRIM63	2	BP
GO:0015865	purine nucleotide	2/2734	15/17381	0.70846	0.9778	0.96172	SLC25A25/SLC25A5	2	BP
GO:0018298	protein-chromophore linkage	2/2734	15/17381	0.70846	0.9778	0.96172	OPN1MW/OPN4	2	BP
GO:0030730	sequestering of triglyceride	2/2734	15/17381	0.70846	0.9778	0.96172	FITM1/PNPLA2	2	BP
GO:0032274	gonadotropin secretion	2/2734	15/17381	0.70846	0.9778	0.96172	INHBB/TACR2	2	BP
GO:0032509	endosome transport via multivesicular body sorting pathway	2/2734	15/17381	0.70846	0.9778	0.96172	SNF8/VPS25	2	BP
GO:0033540	fatty acid beta-oxidation using acyl-CoA	2/2734	15/17381	0.70846	0.9778	0.96172	ACOXL/CROT	2	BP
GO:0042074	cell migration involved in gastrulation	2/2734	15/17381	0.70846	0.9778	0.96172	CRB2/WNT11	2	BP

GO:0042559	pteridine-containing compound biosynthetic process	2/2734	15/17381	0.70846	0.9778	0.96172	GCH1/MTHFD1	2	BP
GO:0043981	histone H4-K5 acetylation	2/2734	15/17381	0.70846	0.9778	0.96172	HCFC1/ING4	2	BP
GO:0043982	histone H4-K8 acetylation	2/2734	15/17381	0.70846	0.9778	0.96172	HCFC1/ING4	2	BP
GO:0044331	cell-cell adhesion mediated by	2/2734	15/17381	0.70846	0.9778	0.96172	SERPINF2/WNT3A	2	BP
GO:0044849	estrous cycle	2/2734	15/17381	0.70846	0.9778	0.96172	EGR1/OPRM1	2	BP
GO:0045116	protein neddylation	2/2734	15/17381	0.70846	0.9778	0.96172	NEDD8/TRIM40	2	BP
GO:0045618	positive regulation of keratinocyte	2/2734	15/17381	0.70846	0.9778	0.96172	NOTCH1/NUMA1	2	BP
GO:0045725	positive regulation of glycogen biosynthetic	2/2734	15/17381	0.70846	0.9778	0.96172	GCK/INS	2	BP
GO:0046184	aldehyde biosynthetic process	2/2734	15/17381	0.70846	0.9778	0.96172	CACNA1H/CYP11B2	2	BP
GO:0048149	behavioral response to ethanol	2/2734	15/17381	0.70846	0.9778	0.96172	DRD2/OPRM1	2	BP

GO:0050746	regulation of lipoprotein metabolic	2/2734	15/17381	0.70846	0.9778	0.96172	GBA/PEMT	2	BP
GO:0050755	chemokine metabolic	2/2734	15/17381	0.70846	0.9778	0.96172	EGR1/IL18	2	BP
GO:0050765	negative regulation of	2/2734	15/17381	0.70846	0.9778	0.96172	CD300LF/CSK	2	BP
GO:0051280	negative regulation of release of sequestered calcium ion into	2/2734	15/17381	0.70846	0.9778	0.96172	GSTM2/TRDN	2	BP
GO:0055075	potassium ion homeostasis	2/2734	15/17381	0.70846	0.9778	0.96172	ATP1A1/CYP11B2	2	BP
GO:0060026	convergent extension	2/2734	15/17381	0.70846	0.9778	0.96172	DVL2/WNT11	2	BP
GO:0060080	inhibitory postsynaptic potential	2/2734	15/17381	0.70846	0.9778	0.96172	GLRA1/NLGN3	2	BP
GO:0060788	ectodermal placode	2/2734	15/17381	0.70846	0.9778	0.96172	AXIN1/TBX2	2	BP
GO:0071025	RNA surveillance	2/2734	15/17381	0.70846	0.9778	0.96172	EXOSC10/EXOSC2	2	BP
GO:0071071	regulation of phospholipid biosynthetic process	2/2734	15/17381	0.70846	0.9778	0.96172	IDH1/PDGFA	2	BP
GO:0071697	ectodermal placode morphogenesis	2/2734	15/17381	0.70846	0.9778	0.96172	AXIN1/TBX2	2	BP

GO:0090141	positive regulation of mitochondrial	2/2734	15/17381	0.70846	0.9778	0.96172	KDR/MIEF2	2	BP
GO:0090220	chromosome localization to nuclear envelope involved in homologous chromosome	2/2734	15/17381	0.70846	0.9778	0.96172	LEMD2/UBE2B	2	BP
GO:0090656	t-circle formation	2/2734	15/17381	0.70846	0.9778	0.96172	EXO1/XRCC3	2	BP
GO:0090737	telomere maintenance via telomere	2/2734	15/17381	0.70846	0.9778	0.96172	EXO1/XRCC3	2	BP
GO:0097468	programmed cell death in response to reactive oxygen	2/2734	15/17381	0.70846	0.9778	0.96172	ENDOG/TRAP1	2	BP
GO:1900003	regulation of serine-type endopeptidase activity	2/2734	15/17381	0.70846	0.9778	0.96172	SPINK2/SPINK5	2	BP
GO:1901201	regulation of extracellular matrix assembly	2/2734	15/17381	0.70846	0.9778	0.96172	AGT/NOTCH1	2	BP
GO:1902571	regulation of serine-type peptidase	2/2734	15/17381	0.70846	0.9778	0.96172	SPINK2/SPINK5	2	BP

GO:1904874	positive regulation of telomerase RNA	2/2734	15/17381	0.70846	0.9778	0.96172	CCT3/NHP2	2	BP
GO:1990000	amyloid fibril formation	2/2734	15/17381	0.70846	0.9778	0.96172	CLU/CRYAB	2	BP
GO:2000773	negative regulation of cellular	2/2734	15/17381	0.70846	0.9778	0.96172	ABL1/BCL6	2	BP
GO:0043266	regulation of potassium ion transport	14/2734	99/17381	0.70953	0.9778	0.96172	ADRA2A/AMIGO1/CAV3/DPP6/DRD2/FLNA/GCK/KCNQ1/MIR153-1/MIR212/NOS3/NPPA/PTK2B/WWP2	14	BP
GO:0006302	double-strand break repair	31/2734	213/17381	0.71033	0.9778	0.96172	APBB1/BRCA1/BRCC3/C7orf49/CIB1/EYA1/FOXM1/HELQ/HIST1H4F/HIST2H4A/HIST2H4B/HSF1/INO80/LIG3/MCM9/MCMDC2/MIR221/MTA1/NUDT16L1/OGG1/OTUB1/PARP3/PARP9/PAXIP1/RECQL4/RECQL5/SETD2/SLF2/TIMELESS/XRCC3/ZFYVE26	31	BP
GO:0033059	cellular pigmentation	7/2734	51/17381	0.71083	0.9778	0.96172	CD63/DCTN2/DTNBP1/HPS1/KIF13A/MYO7A/RAB17	7	BP
GO:0035384	thioester biosynthetic process	7/2734	51/17381	0.71083	0.9778	0.96172	ACACB/ACSF3/ELOVL1/ELOVL5/HACD1/MPC1/PDHB	7	BP
GO:0071616	acyl-CoA biosynthetic process	7/2734	51/17381	0.71083	0.9778	0.96172	ACACB/ACSF3/ELOVL1/ELOVL5/HACD1/MPC1/PDHB	7	BP
GO:0019731	antibacterial humoral	6/2734	44/17381	0.71098	0.9778	0.96172	FGA/HLA-E/PGC/PLA2G1B/SPINK5/SPON2	6	BP
GO:0032653	regulation of interleukin-10 production	6/2734	44/17381	0.71098	0.9778	0.96172	EPX/IDO1/IL20RB/LGALS9/TLR9/TUSC2	6	BP
GO:0051445	regulation of meiotic cell	6/2734	44/17381	0.71098	0.9778	0.96172	HORMAD1/LFNG/PIWIL2/PRDM9/PRKAR1A/UBE2B	6	BP

GO:1901031	regulation of response to reactive oxygen species	6/2734	44/17381	0.71098	0.9778	0.96172	ENDOG/GCH1/MIR92A2/PSAP/SESN1/TRAP1	6	BP
GO:1903793	positive regulation of	6/2734	44/17381	0.71098	0.9778	0.96172	AGT/PRELID1/PRKCD/SLC34A1/STXBP1/TRH	6	BP
GO:0055074	calcium ion homeostasis	63/2734	424/17381	0.71101	0.9778	0.96172	ABL1/ADM/ADRA1A/ADRA1B/AGT/ATP13A2/ATP6V1B1/BCAP31/BOK/C1QTNF1/C3AR1/CACNB3/CAMK2D/CAV3/CCL1/CCL19/CCL21/CCL5/CCR2/CDH23/CIB2/CNGB1/CORO1A/CXCR3/DISC1/DLG4/DRD2/EDN2/EIF5A/EPHX2/F2RL1/FASLG/FATE1/FZD9/GCM2/GNG3/GPER1/GPR17/GPR20/GPR35/GRIN1/GRIN2C/GSTM2/HERPUD1/IL13/ITPR3/JPH3/KNG1/LCK/NPSR1/P2RX2/PKD1/PLA2G1B/PTK2B/RASA3/SV2A/TACR1/THADA/THY1/TRDN/[illegible]	63	BP
GO:0043124	negative regulation of I-kappaB kinase/NF-	8/2734	58/17381	0.71159	0.9778	0.96172	ABL1/CD27/DAB2IP/NLRX1/OTUD7A/PER1/STAT1/ZMYND11	8	BP
GO:0045604	regulation of epidermal cell differentiation	8/2734	58/17381	0.71159	0.9778	0.96172	BMP4/CTSL/KRT84/MYCN/NOTCH1/NUMA1/PTCH2/SFN	8	BP
GO:0048278	vesicle docking	8/2734	58/17381	0.71159	0.9778	0.96172	RAB26/RALB/STX1A/STX4/STX5/STX8/STXBP1/VTI1B	8	BP
GO:0051784	negative regulation of	8/2734	58/17381	0.71159	0.9778	0.96172	ANAPC15/BMP4/BUB1B/HORMAD1/LCMT1/MAD1L1/PRKAR1A/XRCC3	8	BP
GO:0009411	response to UV	19/2734	133/17381	0.71237	0.9778	0.96172	AQP1/CASP3/CCAR2/CRIP1/DDB1/DDB2/EGFR/EIF2AK4/GPX1/INO80/MEN1/NOC2L/SCARA3/SDF4/TAF1/TP53I13/UBE2A/UBE2B/XPC	19	BP
GO:0010970	transport along microtubule	19/2734	133/17381	0.71237	0.9778	0.96172	CNIH2/DTNBP1/DYNC1H1/DYNC1I1/DYNLL2/HSPB1/IFT140/IFT20/IFT22/KIF13A/KIF17/KIF23/KIF4A/PEX14/RHOT2/SSNA1/TRAF3IP1/TRIM46/UXT	19	BP
GO:0099111	microtubule-based transport	19/2734	133/17381	0.71237	0.9778	0.96172	CNIH2/DTNBP1/DYNC1H1/DYNC1I1/DYNLL2/HSPB1/IFT140/IFT20/IFT22/KIF13A/KIF17/KIF23/KIF4A/PEX14/RHOT2/SSNA1/TRAF3IP1/TRIM46/UXT	19	BP
GO:0015804	neutral amino acid transport	5/2734	37/17381	0.71255	0.9778	0.96172	SLC36A1/SLC36A3/SLC38A1/SLC6A7/SLC6A9	5	BP

GO:0032467	positive regulation of	5/2734	37/17381	0.71255	0.9778	0.96172	CXCR5/DRD2/KIF23/OPN1MW/SSTR5	5	BP
GO:0033048	negative regulation of mitotic sister chromatid	5/2734	37/17381	0.71255	0.9778	0.96172	ANAPC15/BUB1B/LCMT1/MAD1L1/XRCC3	5	BP
GO:0035329	hippo signaling	5/2734	37/17381	0.71255	0.9778	0.96172	CASP3/DVL2/MARK3/NEK8/TEAD4	5	BP
GO:0043536	positive regulation of blood vessel endothelial cell	5/2734	37/17381	0.71255	0.9778	0.96172	ABL1/CIB1/HSPB1/KDR/MIR221	5	BP
GO:0070266	necroptotic process	5/2734	37/17381	0.71255	0.9778	0.96172	BOK/FASLG/FZD9/PGAM5/TRAF2	5	BP
GO:0071392	cellular response to estradiol	5/2734	37/17381	0.71255	0.9778	0.96172	EGFR/GPER1/HSF1/MYOD1/ZNF703	5	BP
GO:1902229	regulation of intrinsic apoptotic signaling pathway in	5/2734	37/17381	0.71255	0.9778	0.96172	CCAR2/CD74/CLU/MUC1/ZNF385A	5	BP
GO:2000785	regulation of autophagosome assembly	5/2734	37/17381	0.71255	0.9778	0.96172	IFT20/LRSAM1/NPRL3/RALB/ULK1	5	BP
GO:0060964	regulation of gene silencing by miRNA	15/2734	106/17381	0.71259	0.9778	0.96172	BMP4/DND1/EGFR/EIF4G1/HIST1H4F/HIST2H4A/HIST2H4B/MYCN/NCOR1/NCOR2/NUP210/NUP98/POLR2G/POLR2L/ZC3H10	15	BP
GO:0071359	cellular response to	9/2734	65/17381	0.71294	0.9781	0.96196	BMP4/EGFR/MYCN/NCOR1/NCOR2/RALB/TARBP2/TSNAX/ZC3H10	9	BP

GO:0035637	multicellular organismal signaling	29/2734	200/17381	0.71339	0.9784	0.96235	AGT/ATP1A1/ATP1A4/ATP2A3/CACNA1F/CACNA1G/CACNA1H/CACNA1S/CACNB1/CACNB3/CACNG1/CAMK2D/FLNA/GBA/GLRA1/GPER1/GPR35/GPR88/ITPR3/KCND1/KCND3/KCNH6/KCNJ5/KCNQ1/MIR328/NKX2-	29	BP
GO:0030808	regulation of nucleotide biosynthetic process	23/2734	160/17381	0.71409	0.9792	0.96307	ADM/ADRA2A/AVPR2/CCR2/CRHR1/DRD2/GABBR1/GCG/GPER1/GPHA2/GUCA1B/GUCA2A/GUCA2B/NOS3/OPRM1/PDE2A/PDZD3/PID1/PTK2B/RAF1/RUNDC3A/RXFP2/WFS1	23	BP
GO:0046546	development of primary male sexual characteristics	20/2734	140/17381	0.7157	0.9807	0.96457	BOK/CSDE1/FANCA/GATA4/LRRC6/MGST1/NR5A1/NUDT1/NUP210L/NUPR1/PDGFRB/RARA/REN/RXFP2/SCX/SDC1/SF1/SOX8/UTF1/ZFP42	20	BP
GO:0000038	very long-chain fatty acid metabolic	4/2734	30/17381	0.71649	0.9807	0.96457	ELOVL1/ELOVL5/HACD1/HACD3	4	BP
GO:0001913	T cell mediated cytotoxicity	4/2734	30/17381	0.71649	0.9807	0.96457	CTSH/HLA-E/MICB/NCR3	4	BP
GO:0010939	regulation of necrotic cell	4/2734	30/17381	0.71649	0.9807	0.96457	BOK/FZD9/MIR92A2/TRAF2	4	BP
GO:0014741	negative regulation of muscle	4/2734	30/17381	0.71649	0.9807	0.96457	CAV3/LMNA/MIR25/TRIM63	4	BP
GO:0032733	positive regulation of interleukin-10	4/2734	30/17381	0.71649	0.9807	0.96457	IL20RB/LGALS9/TLR9/TUSC2	4	BP
GO:0032743	positive regulation of interleukin-2	4/2734	30/17381	0.71649	0.9807	0.96457	ABL1/CARD11/CCR2/TRAF2	4	BP

GO:0002824	positive regulation of adaptive immune response based on somatic recombination of immune receptors built from [illegible]	11/2734	79/17381	0.71673	0.9807	0.96457	CCL19/CCR2/CLCF1/HLA-E/HLX/LTA/NCR3/PAXIP1/SLC11A1/TNFSF13/TRAF2	11	BP
GO:0031294	lymphocyte costimulation	11/2734	79/17381	0.71673	0.9807	0.96457	CARD11/CCL19/CCL21/CD247/CD5/CSK/EFNB1/LCK/MAP3K14/PDCD1/PTPN11	11	BP
GO:0001649	osteoblast differentiation	30/2734	207/17381	0.71685	0.9807	0.96457	ACHE/BGLAP/BMP4/CLIC1/COL1A1/DDR2/ESRRA/FGFR2/GDF2/HDAC8/IGFBP3/ILK/MEN1/MRC2/MYOC/NOTCH1/PDLIM7/SEMA7A/SH3PXD2B/SHH/SMO/SND1/SOX8/SUFU/TMEM119/TNC/TNN/TWIST2/WNT11/WNT3A	30	BP
GO:0019722	calcium-mediated signaling	24/2734	167/17381	0.71748	0.9814	0.96521	BCAP31/CAMK2D/CDH13/CIB1/CXCR3/EDN2/EGFR/GRIN1/GRIN2C/GSTM2/HINT1/HOMER2/JPH3/KDR/NEUROD2/NR5A1/NRG1/P2RX2/PLA2G4B/PTK2B/SELP/TMEM100/TRDN/TREM2	24	BP
GO:0006672	ceramide metabolic	12/2734	86/17381	0.71896	0.9831	0.96698	B4GALNT1/CLN6/DEGS2/FUT7/GBA/NEU3/ORMDL3/PLA2G15/PRKCD/SGMS1/SMPD4/SPTLC1	12	BP
GO:0006066	alcohol metabolic process	47/2734	320/17381	0.72012	0.9839	0.96769	ACACB/ACADVL/ADH1A/ADH1C/ALDH2/APOA5/APOB/CLN6/COQ2/CUBN/CYP11A1/DEGS2/DGAT1/DGKQ/DPM2/EPHX2/GALK1/GBA/GCH1/GDPD1/GPER1/HSD17B7/INPP5D/INPP5E/INPPL1/ITPK1/LEPR/LMF1/NPC1L1/NPC2/NSDHL/OSBPL5/PLB1/PLCB2/PLCD1/PLCH2/PMVK/POR/PPARD/PTK2B/SCAP/SCARB1/SCARF1/SLC34A1/SOAT2/SPTLC1/STARD3	47	BP
GO:0042787	protein ubiquitination involved in ubiquitin-dependent protein [illegible]	31/2734	214/17381	0.72023	0.9839	0.96769	ABTB1/ANAPC15/ANAPC2/AXIN1/BUB1B/CAV3/CLU/DDB1/DISC1/FBXO10/KLHL29/MAEA/OS9/PSMB11/PSMB6/PSMB7/PSMB8/PSMC3/PSMD13/PSMD3/PSMD5/PSMD7/PTK2B/RMND5B/SPOP/SUFU/SYVN1/TAF1/UBR4/WNT1/WWP2	31	BP

GO:0071456	cellular response to hypoxia	28/2734	194/17381	0.72023	0.9839	0.96769	ADAM8/AQP1/BAD/DNMT3A/EGR1/EIF4EBP1/ENDOG/IRAK1/LMNA/MTOR/NOTCH1/OPRD1/PPARD/PSMB11/PSMB6/PSMB7/PSMB8/PSMC3/PSMD13/PSMD3/PSMD5/PSMD7/RWDD3/SLC29A1/SLC2A4/SUV39H1/USP19/V	28	BP
GO:0045926	negative regulation of growth	34/2734	234/17381	0.72066	0.9839	0.96769	AGT/APBB1/ARHGAP4/BCL6/BMP4/CAV3/CCAR2/CCDC85B/CDA/CDK5/CDKN2A/CRYAB/DRAXIN/ESR2/GDF2/ING4/LTA/MIR25/MPO/MT2A/NOTCH1/NPPA/OSGIN1/PPARD/PPP1R9B/RAI1/SEMA3F/SIPA1/TIRAP/TNK1/TRIM40/TSPYL2/WNT11/WNT3A	34	BP
GO:0007179	transforming growth factor beta receptor signaling	25/2734	174/17381	0.72081	0.9839	0.96769	ARRB2/CAV3/CDH5/ENG/FAM89B/FERMT2/FNTA/FURIN/HSPA5/HTRA3/ITGB5/LEFTY2/MEN1/MIR212/NEDD8/PARD3/PARD6A/PBLD/SMAD6/TGFB1I1/VASN/WFIKKN2/WNT1/ZNF703/ZYX	25	BP
GO:0009124	nucleoside monophosphate biosynthetic process	13/2734	93/17381	0.72133	0.9839	0.96769	AK5/ALDOA/ATP5G1/ATP5I/CYC1/DGUOK/DHODH/ENTPD8/IMPDH1/PID1/PKM/UCK1/VPS9D1	13	BP
GO:0030705	cytoskeleton-dependent intracellular transport	22/2734	154/17381	0.72219	0.9839	0.96769	CCDC88B/CNIH2/DTNBP1/DYNC1H1/DYNC1I1/DYNLL2/HSPB1/IFT140/IFT20/IFT22/KIF13A/KIF17/KIF23/KIF4A/MLPH/MOBP/PEX14/RHOT2/SSNA1/TRAF3IP1/TRIM46/UXT	22	BP
GO:0090068	positive regulation of cell cycle process	40/2734	274/17381	0.72243	0.9839	0.96769	APEX1/BRCA1/CDK10/CDK4/CXCR5/CYP1A1/DAB2IP/DRD2/DYNC1H1/E2F4/E2F8/EIF4G1/FOXO4/INO80/INS/KIF23/MEPCE/MIR221/MIR222/MIR29A/MUC1/NUMA1/OPN1MW/PCBP4/PDGFRB/PHOX2B/PIWIL2/PPP2R5B/PRDM9/RNF112/SFN/SH2B1/SLF2/SSTR5/UBE2B/UBE2E2/USP19/XRCC3/ZBT	40	BP
GO:0009119	ribonucleoside metabolic	14/2734	100/17381	0.7238	0.9839	0.96769	ACPP/CARD11/CDA/DGUOK/DHODH/DLG4/IMPDH1/MFN1/NME1/NME4/NUDT18/PEMT/SCRIB/UCK1	14	BP
GO:0030260	entry into host cell	19/2734	134/17381	0.72471	0.9839	0.96769	ANPEP/CD74/CD81/CLDN6/EGFR/IFITM2/ITGB5/LGALS9/MOG/SCARB1/SLC20A2/SLC52A1/SLC52A2/TNFRSF4/TRIM11/TRIM26/TRIM31/TRIM62/W	19	BP
GO:0044409	entry into host	19/2734	134/17381	0.72471	0.9839	0.96769	ANPEP/CD74/CD81/CLDN6/EGFR/IFITM2/ITGB5/LGALS9/MOG/SCARB1/SLC20A2/SLC52A1/SLC52A2/TNFRSF4/TRIM11/TRIM26/TRIM31/TRIM62/W	19	BP

GO:0051806	entry into cell of other organism involved in symbiotic	19/2734	134/17381	0.72471	0.9839	0.96769	ANPEP/CD74/CD81/CLDN6/EGFR/IFITM2/ITGB5/LGALS9/MOG/SCARB1/SLC20A2/SLC52A1/SLC52A2/TNFRSF4/TRIM11/TRIM26/TRIM31/TRIM62/WWP2	19	BP
GO:0051828	entry into other organism involved in symbiotic	19/2734	134/17381	0.72471	0.9839	0.96769	ANPEP/CD74/CD81/CLDN6/EGFR/IFITM2/ITGB5/LGALS9/MOG/SCARB1/SLC20A2/SLC52A1/SLC52A2/TNFRSF4/TRIM11/TRIM26/TRIM31/TRIM62/WWP2	19	BP
GO:0001504	neurotransmitter uptake	3/2734	23/17381	0.72478	0.9839	0.96769	DRD2/SLC38A1/TOR1A	3	BP
GO:0001783	B cell apoptotic process	3/2734	23/17381	0.72478	0.9839	0.96769	BCL6/CD74/NOC2L	3	BP
GO:0010586	miRNA metabolic	3/2734	23/17381	0.72478	0.9839	0.96769	DIS3L2/HRAS/TARBP2	3	BP
GO:0018126	protein hydroxylation	3/2734	23/17381	0.72478	0.9839	0.96769	P3H1/P3H3/PLOD3	3	BP
GO:0032660	regulation of interleukin-17 production	3/2734	23/17381	0.72478	0.9839	0.96769	IL18/LY9/TUSC2	3	BP
GO:0034453	microtubule anchoring	3/2734	23/17381	0.72478	0.9839	0.96769	CEP19/FOPNL/PEX14	3	BP
GO:0034505	tooth mineralization	3/2734	23/17381	0.72478	0.9839	0.96769	AMTN/COL1A1/WNT6	3	BP
GO:0035308	negative regulation of protein	3/2734	23/17381	0.72478	0.9839	0.96769	IKBKB/PPP1R1B/TIPRL	3	BP
GO:0036475	neuron death in response to oxidative stress	3/2734	23/17381	0.72478	0.9839	0.96769	ENDOG/NONO/WNT1	3	BP

GO:0042573	retinoic acid metabolic	3/2734	23/17381	0.72478	0.9839	0.96769	ALDH8A1/CYP1A1/RBP1	3	BP
GO:0045686	negative regulation of glial cell	3/2734	23/17381	0.72478	0.9839	0.96769	DAB1/MYCN/NOTCH1	3	BP
GO:0045830	positive regulation of isotype	3/2734	23/17381	0.72478	0.9839	0.96769	CLCF1/PAXIP1/TNFSF13	3	BP
GO:0045940	positive regulation of steroid	3/2734	23/17381	0.72478	0.9839	0.96769	AGT/APOA5/POR	3	BP
GO:0051560	mitochondrial calcium ion homeostasis	3/2734	23/17381	0.72478	0.9839	0.96769	BCAP31/DISC1/FATE1	3	BP
GO:0051904	pigment granule transport	3/2734	23/17381	0.72478	0.9839	0.96769	DCTN2/MYO7A/RAB17	3	BP
GO:0060049	regulation of protein glycosylation	3/2734	23/17381	0.72478	0.9839	0.96769	FKTN/GOLGA2/TINF2	3	BP
GO:0060307	regulation of ventricular cardiac muscle cell membrane repolarization	3/2734	23/17381	0.72478	0.9839	0.96769	CAV3/KCNQ1/WDR1	3	BP
GO:0071577	zinc II ion transmembrane transport	3/2734	23/17381	0.72478	0.9839	0.96769	SLC30A3/SLC39A13/SLC39A5	3	BP

GO:0086064	cell communication by electrical coupling involved in	3/2734	23/17381	0.72478	0.9839	0.96769	ATP1A1/CAMK2D/TRDN	3	BP
GO:0009408	response to heat	23/2734	161/17381	0.72535	0.9844	0.96824	ANO1/ASIC3/CAMK2D/CCAR2/CRYAB/DNAJA3/EIF2B2/EIF2B5/HSF1/HSP90AA1/HSPA2/IRAK1/MAPK3/MICB/MLST8/MTOR/NOS3/NUP210/NUP98/TACR1/TGFB1I1/TRPV2/TRPV4	23	BP
GO:0022898	regulation of transmembrane transporter activity	33/2734	228/17381	0.72677	0.9861	0.96991	ADRA2A/AHNAK/ARC/CACNB1/CACNB3/CAMK2D/CAV3/CNIH2/CRACR2A/CRHR1/DAPK1/DLG4/DRD2/GNB5/GPR35/GSTM2/HSPA2/INS/JPH3/MIR153-1/MIR212/NLGN3/NPPA/OPRM1/OSR1/PM20D1/PTK2B/RRAD/THADA/TLR	33	BP
GO:0043161	proteasome-mediated ubiquitin-dependent protein catabolic	57/2734	387/17381	0.72841	0.9879	0.97165	ABTB1/ANAPC15/ANAPC2/ARAF/ARRB2/AXIN1/BCAP31/BUB1B/CCAR2/CCNF/CHFR/CLU/COMMD1/DDB1/FBXL19/FBXL22/FBXO2/FBXO6/FBXW4/FHIT/GBA/HECTD3/HERPUD1/HSPA5/KCTD5/KLHL40/MAEA/MTA1/NKD2/OS9/PANO1/PSMB11/PSMB6/PSMB7/PSMB8/PSMC3/PSMD13/PSMD3/PSMD5/PSMD7/RMND5B/RNF166/RNF180/SHARPIN/SHH/SPOP/SYVN1/TAF1/TMUB1/TRIM72/UBE2A/UBE2B/UBE2U/USP19/USP5/WFS1/WWP2	57	BP
GO:0043901	negative regulation of multi-organism	24/2734	168/17381	0.72845	0.9879	0.97165	CCL5/DHX58/EIF2AK4/HAVCR2/IFITM2/ISG15/LTA/MICB/MIR221/MIR222/MPO/NFKBIL1/NLRX1/PARP10/SPINK5/STAT1/TARBP2/TIRAP/TRAF3IP1/TRIM11/TRIM14/TRIM26/TRIM31/TRIM62	24	BP
GO:0010721	negative regulation of cell development	42/2734	288/17381	0.72881	0.9879	0.97165	ARHGAP4/ASCL2/CAV3/CDK5/CIB1/CTDSP1/DAB1/DGUOK/DRAXIN/EFNA1/EIF2AK4/FUOM/GAK/GORASP1/IDH2/INPP5F/IRX3/LINGO1/LRIG2/LRP1/LSM1/MYCN/NLGN3/NOTCH1/NR2F1/NRG1/PHOX2B/RGMA/RTN4RL1/RTN4RL2/SEMA3F/SHH/SOX8/THY1/TLX2/TLX3/TRIM11/TRPV4/VAX1/	42	BP
GO:0019751	polyol metabolic	16/2734	114/17381	0.7289	0.9879	0.97165	COQ2/DEGS2/GALK1/GBA/GCH1/GPER1/INPP5D/INPP5E/INPPL1/ITPK1/PLCB2/PLCD1/PLCH2/PTK2B/SLC34A1/SPTLC1	16	BP
GO:0050729	positive regulation of inflammatory	16/2734	114/17381	0.7289	0.9879	0.97165	ADAM8/AGT/CCR2/CD6/EGFR/IDO1/IL18/KARS/KLKB1/LTA/MIR92A2/PDCD4/PDE2A/PTGER3/TLR9/TRPV4	16	BP

GO:0045428	regulation of nitric oxide biosynthetic	8/2734	59/17381	0.72986	0.9879	0.97169	AGT/CLU/EGFR/HSP90AA1/INS/MIR92A2/MTOR/PTK2B	8	BP
GO:0046622	positive regulation of	8/2734	59/17381	0.72986	0.9879	0.97169	ACACB/FGFR2/HLX/MIR222/MTOR/NOTCH1/SMO/TBX2	8	BP
GO:0050795	regulation of behavior	9/2734	66/17381	0.73022	0.9879	0.97169	DLG4/DRD2/EIF2AK4/HTR1D/INS/LEPR/MTOR/STRA6/TRH	9	BP
GO:0030800	negative regulation of cyclic nucleotide	7/2734	52/17381	0.73026	0.9879	0.97169	ADRA2A/CCR2/DRD2/GABBR1/OPRM1/PDE2A/PDZD3	7	BP
GO:0035036	sperm-egg recognition	7/2734	52/17381	0.73026	0.9879	0.97169	ALDOA/CATSPER1/CCT3/POMZP3/PRSS37/SPACA3/VDAC2	7	BP
GO:0070265	necrotic cell death	7/2734	52/17381	0.73026	0.9879	0.97169	BOK/CASP1/FASLG/FZD9/MIR92A2/PGAM5/TRAF2	7	BP
GO:0070830	bicellular tight junction	7/2734	52/17381	0.73026	0.9879	0.97169	IKBKB/MARVELD2/MARVELD3/MYO1C/PARD3/PARD6A/WNT11	7	BP
GO:1903146	regulation of autophagy of mitochondrion	7/2734	52/17381	0.73026	0.9879	0.97169	ATP13A2/ATPIF1/CTTN/GBA/KAT2A/MFN2/ZBTB17	7	BP
GO:0006283	transcription-coupled nucleotide-excision repair	10/2734	73/17381	0.73111	0.988	0.97172	COPS6/DDB1/GTF2H4/LIG3/POLD4/POLR2G/POLR2L/PPIE/RFC1/RFC2	10	BP
GO:0021675	nerve development	10/2734	73/17381	0.73111	0.988	0.97172	HOXA3/HOXB1/HOXB2/HOXB3/HOXD3/ILK/LRIG2/PHOX2A/PHOX2B/SEMA3F	10	BP
GO:0032755	positive regulation of interleukin-6	10/2734	73/17381	0.73111	0.988	0.97172	CARD9/F2RL1/LGALS9/MIR92A2/NOD1/SPON2/TIRAP/TLR8/TLR9/TRPV4	10	BP

GO:1900543	negative regulation of purine nucleotide	10/2734	73/17381	0.73111	0.988	0.97172	ADRA2A/CCR2/DRD2/GABBR1/MLXIPL/OPRM1/PDE2A/PDZD3/PFKFB1/PID1	10	BP
GO:1903201	regulation of oxidative stress-induced cell	10/2734	73/17381	0.73111	0.988	0.97172	ENDOG/GPX1/HSPB1/INS/MIR92A2/MMP3/NONO/PSAP/TRAP1/WNT1	10	BP
GO:0006479	protein methylation	25/2734	175/17381	0.7315	0.988	0.97172	ASH2L/BRCA1/COPRS/EHMT1/EHMT2/GCG/LCMT1/MEN1/NDUFAF7/NTMT1/PAXIP1/PHF19/PRDM12/PRDM14/PRDM7/PRDM9/PYGO2/RLF/SETD1A/SETD2/SETD7/SMYD3/SUPT6H/SUV39H1/VCPKMT	25	BP
GO:0008213	protein alkylation	25/2734	175/17381	0.7315	0.988	0.97172	ASH2L/BRCA1/COPRS/EHMT1/EHMT2/GCG/LCMT1/MEN1/NDUFAF7/NTMT1/PAXIP1/PHF19/PRDM12/PRDM14/PRDM7/PRDM9/PYGO2/RLF/SETD1A/SETD2/SETD7/SMYD3/SUPT6H/SUV39H1/VCPKMT	25	BP
GO:0031018	endocrine pancreas development	6/2734	45/17381	0.73177	0.988	0.97172	BAD/BMP4/MEN1/RFX6/SIDT2/SMO	6	BP
GO:0044275	cellular carbohydrate catabolic	6/2734	45/17381	0.73177	0.988	0.97172	INS/MGAM/PFKM/PGAM4/PHKG1/PYGM	6	BP
GO:0032436	positive regulation of proteasomal ubiquitin-dependent	11/2734	80/17381	0.73238	0.9886	0.97231	BCAP31/CHFR/CLU/GBA/HERPUD1/KLHL40/NKD2/RNF166/RNF180/TAF1/USP5	11	BP
GO:0016073	snRNA metabolic	12/2734	87/17381	0.73394	0.9891	0.97287	CDK9/EXOSC2/INTS10/INTS3/INTS5/MEPCE/NHP2/POLR2G/POLR2L/SNAPC1/SNAPC5/TAF6	12	BP
GO:0000183	chromatin silencing at	5/2734	38/17381	0.73499	0.9891	0.97287	HIST1H4F/HIST2H4A/HIST2H4B/PHF2/SUV39H1	5	BP
GO:0003009	skeletal muscle contraction	5/2734	38/17381	0.73499	0.9891	0.97287	CAV3/CHRNB1/GSTM2/TCAP/TNNC1	5	BP

GO:0031113	regulation of microtubule polymerization	5/2734	38/17381	0.73499	0.9891	0.97287	ABL1/ANKRD53/CAV3/FES/NUMA1	5	BP
GO:0050691	regulation of defense response to	5/2734	38/17381	0.73499	0.9891	0.97287	EIF2AK4/MICB/PARP9/STAT1/TARBP2	5	BP
GO:0002819	regulation of adaptive immune	19/2734	135/17381	0.73672	0.9891	0.97287	BCL6/CCL19/CCR2/CD48/CLCF1/EIF2AK4/HAVCR2/HLA-E/HLX/IL20RB/IL4R/LTA/NCR3/PAXIP1/SLC11A1/SUPT6H/TNFSF13/TRAF2/WAS	19	BP
GO:0030804	positive regulation of cyclic nucleotide biosynthetic	14/2734	101/17381	0.73762	0.9891	0.97287	ADM/AVPR2/CRHR1/GCG/GPER1/GPHA2/GUCA1B/GUCA2A/GUCA2B/NOS3/RAF1/RUNDC3A/RXFP2/WFS1	14	BP
GO:0008202	steroid metabolic process	45/2734	309/17381	0.73793	0.9891	0.97287	ACACB/ACADVL/ADM/AGT/APOA5/APOB/ATP1A1/CACNA1H/CLN6/CUBN/CYB5R1/CYB5R2/CYP11A1/CYP11B2/CYP1A1/CYP1A2/DGKQ/EBPL/EGR1/EPHX2/FGFR4/HSD11B2/HSD17B1/HSD17B3/HSD17B7/HSD3B1/LEPR/LMF1/NPC1L1/NPC2/NR5A1/NSDHL/OSBPL5/OSBPL7/PMVK/POR/PPARD/RORC/SCAP/SCARB1/SCARF1/SF1/SHH/SOAT2/STARD3	45	BP
GO:0008360	regulation of cell shape	20/2734	142/17381	0.73933	0.9891	0.97287	ALDOA/ARAP1/CDC42EP2/CORO1A/CSF1R/FBLIM1/FERMT2/FES/FMNL1/FN1/ITGA7/KDR/PLXNB1/PLXNB3/PLXND1/PTK2B/SEMA4A/TAOK2/WDP	20	BP
GO:1903311	regulation of mRNA metabolic process	40/2734	276/17381	0.73943	0.9891	0.97287	APEX1/CDK9/CELF6/CPSF4/EIF4G1/EXOSC2/HMX2/HSF1/HSPB1/LMNTD2/MTOR/MYOD1/NUP98/PCBP4/PKP3/POLR2G/PRDX6/PRKCD/PSMB11/PSMB6/PSMB7/PSMB8/PSMC3/PSMD13/PSMD3/PSMD5/PSMD7/RBFOX1/RBFOX3/RBM4/RNPS1/SCGB1A1/SF3B4/SLC11A1/SLC39A5/SRPK3/SRSF4/SU	40	BP
GO:0042089	cytokine biosynthetic process	15/2734	108/17381	0.73964	0.9891	0.97287	CARD11/CARD9/CCR2/EGR1/HSPB1/IL18/INHBB/INPP5D/MAP2K3/NMI/NOD1/TBK1/TIRAP/TLR8/TLR9	15	BP

GO:0009913	epidermal cell differentiation	51/2734	349/17381	0.74008	0.9891	0.97287	BMP4/C1orf68/CASP3/CTSL/DNASE1L2/FOXN1/FURIN/JAG2/KRT12/KRT14/KRT16/KRT23/KRT3/KRT32/KRT6A/KRT6C/KRT71/KRT75/KRT76/KRT79/KRT81/KRT84/KRT85/KRTAP17-1/KRTAP4-3/KRTAP5-10/KRTAP5-3/KRTAP5-5/KRTAP5-7/KRTAP5-9/LCE3E/LHFPL5/LRTOMT/MYCN/MYO7A/NOTCH1/NUMA1/PKP3/POU3F1/PTCH2/SCRIB/SFN/SHARPIN/SLC44A4/SPINK5/SPRR1A/ST14/TGM1/TGM5/TMEM79/WDPCP	51	BP
GO:0046039	GTP metabolic process	4/2734	31/17381	0.74098	0.9891	0.97287	IMPDH1/MFN1/NME1/NME4	4	BP
GO:0046949	fatty-acyl-CoA biosynthetic process	4/2734	31/17381	0.74098	0.9891	0.97287	ACSF3/ELOVL1/ELOVL5/HACD1	4	BP
GO:0048713	regulation of oligodendrocyte differentiation	4/2734	31/17381	0.74098	0.9891	0.97287	MTOR/NOTCH1/SHH/WDR1	4	BP
GO:0050869	negative regulation of B	4/2734	31/17381	0.74098	0.9891	0.97287	BCL6/CASP3/INPP5D/TNFRSF13B	4	BP
GO:0061462	protein localization to	4/2734	31/17381	0.74098	0.9891	0.97287	CD81/CLU/LARS/RAB7A	4	BP
GO:0070873	regulation of glycogen metabolic	4/2734	31/17381	0.74098	0.9891	0.97287	GCK/INS/MTOR/PHLDA2	4	BP
GO:0006639	acylglycerol metabolic	16/2734	115/17381	0.74176	0.9891	0.97287	ANG/APOA5/APOB/APOC3/CAV3/DGAT1/DGKQ/DGKZ/GPX1/LMF1/NKX2-3/NR1H3/PLA2G16/PNPLA2/PTPN11/SCARB1	16	BP
GO:0006109	regulation of carbohydrate metabolic	25/2734	176/17381	0.74194	0.9891	0.97287	ACACB/BAD/C1QTNF1/COX11/DGKQ/GCG/GCK/GNMT/GPER1/IGFBP3/INS/KAT2A/LCMT1/LEPR/MAEA/MLXIPL/MTOR/NCOR1/NUP210/NUP98/PFKFB1/PGAM4/PHLDA2/PTK2B/RORC	25	BP
GO:0032409	regulation of transporter activity	35/2734	243/17381	0.74196	0.9891	0.97287	ADRA2A/AHNAK/ARC/CACNB1/CACNB3/CAMK2D/CAV3/CNIH2/CRACR2A/CRHR1/DAPK1/DLG4/DRD2/GNB5/GPR35/GSTM2/HSPA2/INS/JPH3/MIR153-1/MIR212/NLGN3/NPPA/OPRM1/OSR1/PM20D1/PRKCD/PTK2B/RRAD/SGK	35	BP

GO:0006004	fucose metabolic	2/2734	16/17381	0.74228	0.9891	0.97287	FUOM/FUT7	2	BP
GO:0006474	N-terminal protein amino acid acetylation	2/2734	16/17381	0.74228	0.9891	0.97287	NAA16/NAA60	2	BP
GO:0006750	glutathione biosynthetic process	2/2734	16/17381	0.74228	0.9891	0.97287	CHAC1/GGT6	2	BP
GO:0007220	Notch receptor processing	2/2734	16/17381	0.74228	0.9891	0.97287	APH1A/JAG2	2	BP
GO:0016446	somatic hypermutation of immunoglobuli	2/2734	16/17381	0.74228	0.9891	0.97287	EXO1/PMS2P3	2	BP
GO:0021756	striatum development	2/2734	16/17381	0.74228	0.9891	0.97287	DRD2/OGDH	2	BP
GO:0031645	negative regulation of neurological	2/2734	16/17381	0.74228	0.9891	0.97287	GLRA1/GPR35	2	BP
GO:0032645	regulation of granulocyte macrophage colony-stimulating	2/2734	16/17381	0.74228	0.9891	0.97287	IL18/TLR9	2	BP
GO:0032695	negative regulation of interleukin-12	2/2734	16/17381	0.74228	0.9891	0.97287	ARRB2/TLR8	2	BP
GO:0035855	megakaryocyte development	2/2734	16/17381	0.74228	0.9891	0.97287	PTPN11/ZNF385A	2	BP
GO:0035988	chondrocyte proliferation	2/2734	16/17381	0.74228	0.9891	0.97287	DDR2/MUSTN1	2	BP

GO:0036037	CD8-positive, alpha-beta T cell activation	2/2734	16/17381	0.74228	0.9891	0.97287	HLA-E/PSMB11	2	BP
GO:0043584	nose development	2/2734	16/17381	0.74228	0.9891	0.97287	AXIN1/STRA6	2	BP
GO:0043968	histone H2A acetylation	2/2734	16/17381	0.74228	0.9891	0.97287	EPC1/MSL3	2	BP
GO:0045072	regulation of interferon-gamma biosynthetic	2/2734	16/17381	0.74228	0.9891	0.97287	TLR8/TLR9	2	BP
GO:0045986	negative regulation of smooth muscle	2/2734	16/17381	0.74228	0.9891	0.97287	ADRA2C/MIR153-1	2	BP
GO:0048841	regulation of axon extension involved in	2/2734	16/17381	0.74228	0.9891	0.97287	SEMA3F/WNT3A	2	BP
GO:0051284	positive regulation of sequestering of	2/2734	16/17381	0.74228	0.9891	0.97287	GSTM2/TRDN	2	BP
GO:0060850	regulation of transcription involved in cell fate	2/2734	16/17381	0.74228	0.9891	0.97287	EVX1/RORC	2	BP
GO:0071696	ectodermal placode development	2/2734	16/17381	0.74228	0.9891	0.97287	AXIN1/TBX2	2	BP
GO:0071786	endoplasmic reticulum tubular network organization	2/2734	16/17381	0.74228	0.9891	0.97287	REEP2/ZFYVE27	2	BP

GO:0090136	epithelial cell-cell adhesion	2/2734	16/17381	0.74228	0.9891	0.97287	ITGB5/KIFC3	2	BP
GO:1900017	positive regulation of cytokine production involved in inflammatory	2/2734	16/17381	0.74228	0.9891	0.97287	CD6/KARS	2	BP
GO:1902003	regulation of amyloid-beta formation	2/2734	16/17381	0.74228	0.9891	0.97287	CLU/EFNA1	2	BP
GO:1902074	response to salt	2/2734	16/17381	0.74228	0.9891	0.97287	HSF1/HSPA5	2	BP
GO:1903209	positive regulation of oxidative stress-	2/2734	16/17381	0.74228	0.9891	0.97287	ENDOG/MMP3	2	BP
GO:2000001	regulation of DNA damage checkpoint	2/2734	16/17381	0.74228	0.9891	0.97287	CCAR2/RINT1	2	BP
GO:0009187	cyclic nucleotide metabolic	29/2734	203/17381	0.74316	0.9891	0.97287	ADM/ADRA2A/AIPL1/AQP1/AVPR2/CCR2/CHGA/CRHR1/DRD2/FZD2/GABBR1/GCG/GPER1/GPHA2/GUCA1B/GUCA2A/GUCA2B/GUCY2D/NOS3/NPPA/OPRM1/PDE2A/PDZD3/PTK2B/RAF1/RUNDC3A/RXFP2/UCN2/WFS1	29	BP
GO:0009116	nucleoside metabolic	17/2734	122/17381	0.74393	0.9891	0.97287	ACPP/CARD11/CDA/DGUOK/DHODH/DLG4/ERH/IMPDH1/MFN1/NME1/NME4/NT5M/NUDT18/PEMT/PUDP/SCRIB/UCK1	17	BP
GO:0071103	DNA conformation change	42/2734	290/17381	0.74521	0.9891	0.97287	ANP32B/CDAN1/CDKN2A/CENPN/CHD8/CHMP1A/DDB1/DDB2/ERN2/GPER1/GTF2H4/H1FX/HILS1/HIST1H4F/HIST2H4A/HIST2H4B/HJURP/HMGA1/IGHMBP2/INO80/IPO4/M1AP/MCM2/MCM4/MCPH1/NAA60/NCAPD3/NOC2L/PADI4/PARP10/PIF1/RECQL4/RECQL5/RSF1/SMYD3/TNRC18/TSPY1/TS	42	BP

GO:0002718	regulation of cytokine production involved in immune	9/2734	67/17381	0.74679	0.9891	0.97287	BCL6/CD74/CUEDC2/F2RL1/KARS/MAPK3/SEMA7A/SPON2/TRAF2	9	BP
GO:0006695	cholesterol biosynthetic process	9/2734	67/17381	0.74679	0.9891	0.97287	ACACB/APOA5/APOB/HSD17B7/NPC1L1/NSDHL/PMVK/POR/SCAP	9	BP
GO:0035304	regulation of protein dephosphorylati	9/2734	67/17381	0.74679	0.9891	0.97287	DRD2/DUSP26/GBA/IKBKB/NSMF/PDGFRB/PPP1R1B/PRKCD/TIPRL	9	BP
GO:0042440	pigment metabolic	9/2734	67/17381	0.74679	0.9891	0.97287	ALAD/ALAS2/ATPIF1/COX10/HMBS/HMOX2/MTHFD1/NFE2L1/UROS	9	BP
GO:0043647	inositol phosphate	9/2734	67/17381	0.74679	0.9891	0.97287	GPER1/INPP5D/INPP5E/INPPL1/ITPK1/PLCB2/PLCD1/PLCH2/PTK2B	9	BP
GO:0006400	tRNA modification	10/2734	74/17381	0.74689	0.9891	0.97287	AARS/AARS2/C9orf64/CDKAL1/METTL2A/METTL2B/PUSL1/THUMPD2/TRMT10B/TRMT44	10	BP
GO:0042108	positive regulation of cytokine biosynthetic	8/2734	60/17381	0.74733	0.9891	0.97287	CARD11/CCR2/EGR1/HSPB1/TBK1/TIRAP/TLR8/TLR9	8	BP
GO:1904356	regulation of telomere maintenance via telomere lengthening	8/2734	60/17381	0.74733	0.9891	0.97287	ACD/CCT3/EXOSC10/MAPK15/MAPK3/PIF1/SMG5/TINF2	8	BP
GO:0071158	positive regulation of	12/2734	88/17381	0.74839	0.9891	0.97287	BRCA1/DAB2IP/E2F4/E2F8/FOXO4/MUC1/PCBP4/PPP2R5B/RNF112/SFN/ZBTB17/ZNF385A	12	BP
GO:0000380	alternative mRNA splicing, via spliceosome	7/2734	53/17381	0.74876	0.9891	0.97287	CELF6/ESRP2/MYOD1/RBFOX1/RBFOX3/RBM4/RNPS1	7	BP

GO:0003229	ventricular cardiac muscle tissue	7/2734	53/17381	0.74876	0.9891	0.97287	ENG/FGFR2/MYBPC3/NKX2-5/NOTCH1/NRG1/TNNC1	7	BP
GO:0006336	DNA replication-independent nucleosome	7/2734	53/17381	0.74876	0.9891	0.97287	CENPN/HIST1H4F/HIST2H4A/HIST2H4B/HJURP/IPO4/RSF1	7	BP
GO:0010676	positive regulation of cellular carbohydrate	7/2734	53/17381	0.74876	0.9891	0.97287	BAD/GCG/GCK/GPER1/INS/KAT2A/PFKFB1	7	BP
GO:0019369	arachidonic acid metabolic	7/2734	53/17381	0.74876	0.9891	0.97287	CYP1A1/CYP1A2/CYP2W1/DAGLB/EPHX2/MAPK3/PLA2G4B	7	BP
GO:0045071	negative regulation of viral genome	7/2734	53/17381	0.74876	0.9891	0.97287	CCL5/EIF2AK4/IFITM2/ISG15/MIR221/MIR222/PARP10	7	BP
GO:0046605	regulation of centrosome	7/2734	53/17381	0.74876	0.9891	0.97287	BRCA1/CCNF/CHMP1A/CHMP4C/MCPH1/NUBP1/XRCC3	7	BP
GO:0072523	purine-containing compound	7/2734	53/17381	0.74876	0.9891	0.97287	DNPH1/GPX1/HINT1/NUDT1/NUDT18/PDE2A/XDH	7	BP
GO:0050663	cytokine secretion	28/2734	197/17381	0.75003	0.9891	0.97287	ABL1/AGT/CARD11/CASP1/CASP5/CCL19/CD58/CLECL1/CSF1R/DRD2/F2RL1/FN1/GAPDH/GSDMD/HAVCR2/IL4R/INS/KARS/LGALS9/MAPK3/NLRP1/NOTCH1/ORM1/ORM2/PYDC1/TLR8/TLR9/TRPV4	28	BP
GO:0033138	positive regulation of peptidyl-serine	14/2734	102/17381	0.75098	0.9891	0.97287	ARAF/ARRB2/AXIN1/CSF3/DOCK7/EGFR/EIF4G1/GCG/IFNA5/OPRD1/RAF1/SMYD3/TBK1/WNT3A	14	BP
GO:0002279	mast cell activation involved in	6/2734	46/17381	0.75148	0.9891	0.97287	CHGA/FES/IL13/IL4R/LGALS9/STXBP1	6	BP

GO:0008631	intrinsic apoptotic signaling pathway in	6/2734	46/17381	0.75148	0.9891	0.97287	GPX1/HSPB1/INS/NONO/PRKCD/TRAP1	6	BP
GO:0030818	negative regulation of cAMP biosynthetic	6/2734	46/17381	0.75148	0.9891	0.97287	ADRA2A/CCR2/DRD2/GABBR1/OPRM1/PDE2A	6	BP
GO:0031055	chromatin remodeling at centromere	6/2734	46/17381	0.75148	0.9891	0.97287	CENPN/HIST1H4F/HIST2H4A/HIST2H4B/HJURP/RSF1	6	BP
GO:0032570	response to progesterone	6/2734	46/17381	0.75148	0.9891	0.97287	BAD/CATSPER1/CLDN4/FOSL1/NR1H3/TACR1	6	BP
GO:0043303	mast cell degranulation	6/2734	46/17381	0.75148	0.9891	0.97287	CHGA/FES/IL13/IL4R/LGALS9/STXBP1	6	BP
GO:0048512	circadian behavior	6/2734	46/17381	0.75148	0.9891	0.97287	CIART/DRD2/MTA1/NAGLU/TH/USP2	6	BP
GO:2000401	regulation of lymphocyte migration	6/2734	46/17381	0.75148	0.9891	0.97287	ADAM8/CCL21/CCL5/CCR2/ECM1/PTK2B	6	BP
GO:0000737	DNA catabolic process, endonucleolytic	3/2734	24/17381	0.75186	0.9891	0.97287	CASP3/ENDOG/HSF1	3	BP
GO:0006817	phosphate ion transport	3/2734	24/17381	0.75186	0.9891	0.97287	SLC20A1/SLC20A2/SLC34A1	3	BP
GO:0015012	heparan sulfate proteoglycan biosynthetic process	3/2734	24/17381	0.75186	0.9891	0.97287	B3GAT3/DSE/XYLT2	3	BP
GO:0034695	response to prostaglandin E	3/2734	24/17381	0.75186	0.9891	0.97287	CCL19/CCL21/P2RY6	3	BP

GO:0035588	G-protein coupled purinergic	3/2734	24/17381	0.75186	0.9891	0.97287	ACPP/NECAB2/P2RY6	3	BP
GO:0042451	purine nucleoside biosynthetic	3/2734	24/17381	0.75186	0.9891	0.97287	IMPDH1/NME1/NME4	3	BP
GO:0043457	regulation of cellular	3/2734	24/17381	0.75186	0.9891	0.97287	PRELID1/TRAP1/TRPV4	3	BP
GO:0046129	purine ribonucleoside biosynthetic process	3/2734	24/17381	0.75186	0.9891	0.97287	IMPDH1/NME1/NME4	3	BP
GO:0051642	centrosome localization	3/2734	24/17381	0.75186	0.9891	0.97287	FHOD1/IFT20/NUBP1	3	BP
GO:0051905	establishment of pigment granule localization	3/2734	24/17381	0.75186	0.9891	0.97287	DCTN2/MYO7A/RAB17	3	BP
GO:0060330	regulation of response to interferon-	3/2734	24/17381	0.75186	0.9891	0.97287	NR1H3/PARP9/STAT1	3	BP
GO:0060334	regulation of interferon-gamma-mediated	3/2734	24/17381	0.75186	0.9891	0.97287	NR1H3/PARP9/STAT1	3	BP
GO:1903792	negative regulation of	3/2734	24/17381	0.75186	0.9891	0.97287	MTOR/OSR1/TRH	3	BP
GO:1904646	cellular response to	3/2734	24/17381	0.75186	0.9891	0.97287	CACNB1/CDK5/LRP1	3	BP
GO:1990776	response to angiotensin	3/2734	24/17381	0.75186	0.9891	0.97287	AGT/HSF1/PRKCD	3	BP

GO:2000482	regulation of interleukin-8 secretion	3/2734	24/17381	0.75186	0.9891	0.97287	CD58/F2RL1/LGALS9	3	BP
GO:0030810	positive regulation of nucleotide biosynthetic	15/2734	109/17381	0.75254	0.9891	0.97287	ADM/AVPR2/CRHR1/GCG/GPER1/GPHA2/GUCA1B/GUCA2A/GUCA2B/NOS3/PID1/RAF1/RUNDC3A/RXFP2/WFS1	15	BP
GO:0031929	TOR signaling	15/2734	109/17381	0.75254	0.9891	0.97287	ARAF/CARD11/DGKQ/DISC1/EIF4EBP1/LARS/MAPKAP1/MLST8/MTOR/NPRL3/PREX2/RHEBL1/RRAGC/SESN1/TELO2	15	BP
GO:0032368	regulation of lipid transport	15/2734	109/17381	0.75254	0.9891	0.97287	ABCA2/ABCG5/AGT/APOA5/APOC3/C1QTNF1/CRHR1/LRP1/NCOR1/NR1H3/PRELID1/PRKCD/PTPN11/REN/SHH	15	BP
GO:0060147	regulation of posttranscriptional gene	15/2734	109/17381	0.75254	0.9891	0.97287	BMP4/DND1/EGFR/EIF4G1/HIST1H4F/HIST2H4A/HIST2H4B/MYCN/NCOR1/NCOR2/NUP210/NUP98/POLR2G/POLR2L/ZC3H10	15	BP
GO:0060966	regulation of gene silencing	15/2734	109/17381	0.75254	0.9891	0.97287	BMP4/DND1/EGFR/EIF4G1/HIST1H4F/HIST2H4A/HIST2H4B/MYCN/NCOR1/NCOR2/NUP210/NUP98/POLR2G/POLR2L/ZC3H10	15	BP
GO:1900373	positive regulation of purine nucleotide biosynthetic	15/2734	109/17381	0.75254	0.9891	0.97287	ADM/AVPR2/CRHR1/GCG/GPER1/GPHA2/GUCA1B/GUCA2A/GUCA2B/NOS3/PID1/RAF1/RUNDC3A/RXFP2/WFS1	15	BP
GO:0006289	nucleotide-excision repair	16/2734	116/17381	0.75421	0.9891	0.97287	COPS6/DDB1/DDB2/GTF2H2C/GTF2H4/LIG3/NEIL2/OGG1/POLD4/POLE/POLR2G/POLR2L/PPIE/RFC1/RFC2/XPC	16	BP
GO:0006638	neutral lipid metabolic	16/2734	116/17381	0.75421	0.9891	0.97287	ANG/APOA5/APOB/APOC3/CAV3/DGAT1/DGKQ/DGKZ/GPX1/LMF1/NKX2-3/NR1H3/PLA2G16/PNPLA2/PTPN11/SCARB1	16	BP
GO:1904064	positive regulation of cation transmembrane	17/2734	123/17381	0.75599	0.9891	0.97287	ABL1/AGT/AMIGO1/ARC/CRACR2A/FLNA/GPER1/GSTM2/HSPA2/IL13/KCNQ1/NLGN3/NPPA/NPSR1/THY1/TRDN/WNK2	17	BP

GO:0006636	unsaturated fatty acid biosynthetic	5/2734	39/17381	0.75613	0.9891	0.97287	CD74/EDN2/ELOVL5/FADS2/FADS3	5	BP
GO:0006904	vesicle docking involved in exocytosis	5/2734	39/17381	0.75613	0.9891	0.97287	RAB26/RALB/STX1A/STXBP1/VTI1B	5	BP
GO:0007339	binding of sperm to zona	5/2734	39/17381	0.75613	0.9891	0.97287	ALDOA/CCT3/POMZP3/PRSS37/VDAC2	5	BP
GO:0032941	secretion by tissue	5/2734	39/17381	0.75613	0.9891	0.97287	AQP1/AQP5/CHRM1/NR1H3/TACR1	5	BP
GO:0033046	negative regulation of sister chromatid	5/2734	39/17381	0.75613	0.9891	0.97287	ANAPC15/BUB1B/LCMT1/MAD1L1/XRCC3	5	BP
GO:0043616	keratinocyte proliferation	5/2734	39/17381	0.75613	0.9891	0.97287	CDH13/PPARD/SFN/SLURP1/TGM1	5	BP
GO:0048009	insulin-like growth factor receptor	5/2734	39/17381	0.75613	0.9891	0.97287	IGFBP3/IGFBP6/MIR29C/TRIM72/WNT1	5	BP
GO:0071377	cellular response to glucagon	5/2734	39/17381	0.75613	0.9891	0.97287	GCG/GNB3/GNG3/PRKAR1A/PRKAR1B	5	BP
GO:0051168	nuclear export	28/2734	198/17381	0.75951	0.9891	0.97287	ALKBH5/ANP32B/CASC3/CCHCR1/CDK5/CPSF1/CPSF4/DHX38/EIF5A/EMD/NOL6/NUP210/NUP98/NXF2/NXF2B/NXF3/PKD1/PTPN11/RNPS1/SETD2/SFN/SMG5/SRSF4/SUPT6H/XPO6/XPO7/ZC3H3/ZNF593	28	BP
GO:0000726	non-recombinational repair	11/2734	82/17381	0.76195	0.9891	0.97287	BRCA1/BRCC3/C7orf49/HIST1H4F/HIST2H4A/HIST2H4B/HSF1/NUDT16L1/OGG1/PARP9/PAXIP1	11	BP
GO:0043255	regulation of carbohydrate biosynthetic process	11/2734	82/17381	0.76195	0.9891	0.97287	DGKQ/GCG/GCK/GNMT/GPER1/INS/KAT2A/LEPR/MAEA/MTOR/PTK2B	11	BP

GO:0050810	regulation of steroid biosynthetic process	11/2734	82/17381	0.76195	0.9891	0.97287	ACACB/APOB/ATP1A1/DGKQ/EGR1/FGFR4/NR5A1/PMVK/POR/SCAP/SF1	11	BP
GO:0006303	double-strand break repair via nonhomologous end joining	10/2734	75/17381	0.76202	0.9891	0.97287	BRCA1/BRCC3/C7orf49/HIST1H4F/HIST2H4A/HIST2H4B/HSF1/NUDT16L1/PARP9/PAXIP1	10	BP
GO:0007193	adenylate cyclase-inhibiting G-protein coupled receptor	10/2734	75/17381	0.76202	0.9891	0.97287	ADRA2A/CHRM1/DRD2/FLNA/GABBR1/HTR1D/OPRD1/OPRM1/PSAP/RXFP2	10	BP
GO:0051303	establishment of chromosome localization	10/2734	75/17381	0.76202	0.9891	0.97287	ANKRD53/CHMP1A/CHMP4C/CHMP7/DYNC1H1/KIF2B/LEMD2/MAD1L1/NUMA1/UBE2B	10	BP
GO:0051304	chromosome separation	10/2734	75/17381	0.76202	0.9891	0.97287	ANAPC15/BUB1B/DIS3L2/LCMT1/M1AP/MAD1L1/NCAPD3/NUMA1/RECQL5/XRCC3	10	BP
GO:0042273	ribosomal large subunit	9/2734	68/17381	0.76264	0.9891	0.97287	HEATR3/MDN1/NHP2/NLE1/NOC2L/NOP16/RPL35A/RPL7/SURF6	9	BP
GO:1902653	secondary alcohol biosynthetic	9/2734	68/17381	0.76264	0.9891	0.97287	ACACB/APOA5/APOB/HSD17B7/NPC1L1/NSDHL/PMVK/POR/SCAP	9	BP
GO:0018958	phenol-containing compound	13/2734	96/17381	0.76297	0.9891	0.97287	AOC2/CRYM/DDC/DRD2/DUOXA1/GCH1/HDC/ITGAM/LRTOMT/PNKD/RNF180/SNCB/TH	13	BP
GO:0000460	maturation of 5.8S rRNA	4/2734	32/17381	0.76382	0.9891	0.97287	EXOSC10/EXOSC2/FCF1/PDCD11	4	BP

GO:0006335	DNA replication-dependent nucleosome	4/2734	32/17381	0.76382	0.9891	0.97287	HIST1H4F/HIST2H4A/HIST2H4B/IPO4	4	BP
GO:0010667	negative regulation of cardiac muscle cell apoptotic	4/2734	32/17381	0.76382	0.9891	0.97287	AMBRA1/HSF1/ILK/NKX2-5	4	BP
GO:0034723	DNA replication-dependent nucleosome	4/2734	32/17381	0.76382	0.9891	0.97287	HIST1H4F/HIST2H4A/HIST2H4B/IPO4	4	BP
GO:0035587	purinergic receptor	4/2734	32/17381	0.76382	0.9891	0.97287	ACPP/NECAB2/P2RX2/P2RY6	4	BP
GO:0043368	positive T cell selection	4/2734	32/17381	0.76382	0.9891	0.97287	CD74/FOXN1/LY9/SHH	4	BP
GO:0070911	global genome nucleotide-excision repair	4/2734	32/17381	0.76382	0.9891	0.97287	DDB1/DDB2/GTF2H4/XPC	4	BP
GO:1901021	positive regulation of calcium ion transmembrane	4/2734	32/17381	0.76382	0.9891	0.97287	CRACR2A/GSTM2/HSPA2/TRDN	4	BP
GO:1903205	regulation of hydrogen peroxide-induced cell	4/2734	32/17381	0.76382	0.9891	0.97287	ENDOG/MIR92A2/PSAP/TRAP1	4	BP
GO:0001824	blastocyst development	8/2734	61/17381	0.76399	0.9891	0.97287	CDX2/DAD1/HORMAD1/MFN2/NLE1/PRDM14/PSMC3/ZPR1	8	BP
GO:0043297	apical junction assembly	8/2734	61/17381	0.76399	0.9891	0.97287	IKBKB/MARVELD2/MARVELD3/MYO1C/PARD3/PARD6A/WDR1/WNT11	8	BP

GO:0051785	positive regulation of	8/2734	61/17381	0.76399	0.9891	0.97287	INS/NUMA1/PDGFRB/PIWIL2/PRDM9/SH2B1/SLF2/UBE2B	8	BP
GO:0071383	cellular response to steroid hormone stimulus	37/2734	259/17381	0.76427	0.9891	0.97287	AQP1/ARRB2/ATP1A1/AXIN1/BMP4/BRCA1/CALCOCO1/CRIPAK/DDX54/EGFR/EIF4EBP1/ESR2/ESRRA/GPER1/HEY1/MED24/MYOD1/NR1H3/NR2F1/NR4A1/NR5A1/PAQR6/PAQR7/PAQR9/PER1/PLPP1/PPARD/RARA/RORC/RWDD1/SMYD3/SSTR5/TADA3/TAF1/TAF7/TGFB1I1/THRA	37	BP
GO:0042107	cytokine metabolic	15/2734	110/17381	0.765	0.9891	0.97287	CARD11/CARD9/CCR2/EGR1/HSPB1/IL18/INHBB/INPP5D/MAP2K3/NMI/NOD1/TBK1/TIRAP/TLR8/TLR9	15	BP
GO:1903008	organelle disassembly	15/2734	110/17381	0.765	0.9891	0.97287	AMBRA1/ATG2A/ATP13A2/ATPIF1/CTTN/GBA/GBF1/GOLGA2/KAT2A/KIF9/KLC1/MARK2/MFN2/STX5/ZBTB17	15	BP
GO:1902115	regulation of organelle assembly	22/2734	158/17381	0.76565	0.9891	0.97287	ATXN2L/BRCA1/CAV3/CHMP1A/CHMP4C/CROCC/DYNC1H1/FSCN1/IFT140/IFT20/KIF9/LRSAM1/NOTO/NPRL3/NUMA1/RALB/SDC1/SENP6/SEPT9/STX18/ULK1/WRAP73	22	BP
GO:1902807	negative regulation of cell cycle G1/S	16/2734	117/17381	0.76627	0.9891	0.97287	C10orf99/CDK2AP2/CTDSP1/E2F4/E2F8/MEN1/MIR10A/MIR15A/MIR16-1/MIR29A/MIR29C/MIR503/MUC1/PCBP4/SFN/ZNF385A	16	BP
GO:0034508	centromere complex	7/2734	54/17381	0.76634	0.9891	0.97287	CENPN/HIST1H4F/HIST2H4A/HIST2H4B/HJURP/RSF1/SENP6	7	BP
GO:0034724	DNA replication-independent nucleosome	7/2734	54/17381	0.76634	0.9891	0.97287	CENPN/HIST1H4F/HIST2H4A/HIST2H4B/HJURP/IPO4/RSF1	7	BP
GO:0050854	regulation of antigen receptor-mediated	7/2734	54/17381	0.76634	0.9891	0.97287	BLK/CACNA1F/CMTM3/ELF1/LCK/LGALS3/THY1	7	BP
GO:0042445	hormone metabolic process	27/2734	192/17381	0.76653	0.9891	0.97287	ADM/AGT/ALDH8A1/ATP1A1/CACNA1H/CRYM/CYP11A1/CYP11B2/CYP1A1/DGAT1/DGKQ/DUOXA1/EGR1/FURIN/HSD17B1/HSD17B3/HSD17B7/HSD3B1/NR5A1/PLB1/POR/PTPN11/RBP1/REN/SCARB1/SHH/STARD3	27	BP

GO:0002703	regulation of leukocyte mediated	23/2734	165/17381	0.76766	0.9891	0.97287	ARRB2/BCL6/CCR2/CLCF1/F2RL1/FES/HAVCR2/HLA-E/IL13/IL20RB/IL4R/ITGAM/LGALS9/LTA/NCR3/PAXIP1/PIK3R6/STX4/STXBP1/SUPT6H/TNFSF13/TRAF2/WAS	23	BP
GO:0002822	regulation of adaptive immune response based on somatic recombination of immune receptors built from immunoglobuli[illegible]	17/2734	124/17381	0.76767	0.9891	0.97287	BCL6/CCL19/CCR2/CLCF1/HAVCR2/HLA-E/HLX/IL20RB/IL4R/LTA/NCR3/PAXIP1/SLC11A1/SUPT6H/TNFSF13/TRAF2/WAS	17	BP
GO:0071824	protein-DNA complex subunit organization	39/2734	273/17381	0.76914	0.9891	0.97287	ANP32B/BRF1/CENPN/DDB1/DDB2/GTF2H4/H1FX/HILS1/HIST1H4F/HIST2H4A/HIST2H4B/HJURP/HMGA1/IPO4/MCM2/MCMDC2/MED6/NAA60/PADI4/POLR1E/PSMC3/RSF1/SENP6/SETD2/SMARCD2/SMYD3/SUPT6H/TAF1/TAF1L/TAF7/THRA/TSPY1/TSPY2/TSPY4/TSPY8/TSPYL2/XPC/XRCC3/ZN	39	BP
GO:0001503	ossification	54/2734	373/17381	0.76929	0.9891	0.97287	ACHE/AHSG/ATP6V1B1/BGLAP/BMP4/CLIC1/COL1A1/CSF1/DDR2/ECM1/EGFR/ESRRA/FGFR2/FZD9/GDF2/HDAC8/IFITM5/IGFBP3/ILK/INPPL1/ISG15/KREMEN2/MAPK3/MEN1/MMP2/MRC2/MYOC/NOTCH1/OSR1/PDLIM7/PHOSPHO1/PKDCC/PLXNB1/PTK2B/SCX/SEMA7A/SH3PXD2B/SHH/SLC34A1/SMAD6/SMO/SND1/SOST/SOX8/SUFU/TACR1/THBS3/THRA/TMEM11	54	BP
GO:0050707	regulation of cytokine secretion	24/2734	172/17381	0.76968	0.9891	0.97287	ABL1/CASP1/CASP5/CCL19/CD58/CSF1R/DRD2/F2RL1/FN1/GAPDH/GSDMD/HAVCR2/IL4R/INS/KARS/LGALS9/MAPK3/NLRP1/ORM1/ORM2/PYDC1/TLR8/TLR9/TRPV4	24	BP
GO:0000281	mitotic cytokinesis	6/2734	47/17381	0.77011	0.9891	0.97287	ANKRD53/BIN3/CFL1/KIF23/KIF4A/UNC119	6	BP
GO:0007622	rhythmic behavior	6/2734	47/17381	0.77011	0.9891	0.97287	CIART/DRD2/MTA1/NAGLU/TH/USP2	6	BP
GO:0008088	axo-dendritic transport	6/2734	47/17381	0.77011	0.9891	0.97287	CNIH2/DTNBP1/HSPB1/KIF17/KIF4A/TRIM46	6	BP

GO:0016447	somatic recombination of immunoglobuli	6/2734	47/17381	0.77011	0.9891	0.97287	BCL6/CLCF1/EXO1/PAXIP1/SUPT6H/TNFSF13	6	BP
GO:0045058	T cell selection	6/2734	47/17381	0.77011	0.9891	0.97287	CARD11/CD74/FOXN1/JAG2/LY9/SHH	6	BP
GO:0045839	negative regulation of mitotic nuclear	6/2734	47/17381	0.77011	0.9891	0.97287	ANAPC15/BMP4/BUB1B/LCMT1/MAD1L1/XRCC3	6	BP
GO:0050732	negative regulation of peptidyl-tyrosine	6/2734	47/17381	0.77011	0.9891	0.97287	INPP5F/PRKCD/SH3BP5/SH3BP5L/THY1/VPS25	6	BP
GO:1905953	negative regulation of	6/2734	47/17381	0.77011	0.9891	0.97287	ABCG5/APOC3/NR1H3/PNPLA2/PTPN11/SHH	6	BP
GO:0006997	nucleus organization	20/2734	145/17381	0.77237	0.9891	0.97287	BIN1/CCNB2/CHMP1A/CHMP4C/CHMP7/EMD/FAM118B/GPER1/LEMD2/LMNA/NUMA1/NUP210/NUP98/PYGO2/REEP4/SF1/TBPL1/TOR1A/TOR1B/Z	20	BP
GO:0045981	positive regulation of nucleotide	20/2734	145/17381	0.77237	0.9891	0.97287	ADM/AVPR2/CHGA/CRHR1/FZD2/GCG/GPER1/GPHA2/GUCA1B/GUCA2A/GUCA2B/INS/MLXIPL/NOS3/PFKFB1/PID1/RAF1/RUNDC3A/RXFP2/WFS1	20	BP
GO:0048592	eye morphogenesis	20/2734	145/17381	0.77237	0.9891	0.97287	AQP1/AQP5/AXIN1/BMP4/FASLG/FOXE3/GNAT1/LHX1/MFN2/MYO7A/NAGLU/NRL/SOX8/STRA6/TBX2/TH/THY1/TSKU/TULP1/VAX2	20	BP
GO:1900544	positive regulation of purine nucleotide	20/2734	145/17381	0.77237	0.9891	0.97287	ADM/AVPR2/CHGA/CRHR1/FZD2/GCG/GPER1/GPHA2/GUCA1B/GUCA2A/GUCA2B/INS/MLXIPL/NOS3/PFKFB1/PID1/RAF1/RUNDC3A/RXFP2/WFS1	20	BP
GO:0000097	sulfur amino acid biosynthetic	2/2734	17/17381	0.77267	0.9891	0.97287	BHMT2/MTHFD1	2	BP

GO:0002566	somatic diversification of immune receptors via somatic	2/2734	17/17381	0.77267	0.9891	0.97287	EXO1/PMS2P3	2	BP
GO:0010155	regulation of proton transport	2/2734	17/17381	0.77267	0.9891	0.97287	IL13/PM20D1	2	BP
GO:0010224	response to UV-B	2/2734	17/17381	0.77267	0.9891	0.97287	CRIP1/XPC	2	BP
GO:0010888	negative regulation of	2/2734	17/17381	0.77267	0.9891	0.97287	NR1H3/PNPLA2	2	BP
GO:0031061	negative regulation of histone	2/2734	17/17381	0.77267	0.9891	0.97287	BRCA1/SUPT6H	2	BP
GO:0031065	positive regulation of histone	2/2734	17/17381	0.77267	0.9891	0.97287	BCL6/CTBP1	2	BP
GO:0031958	corticosteroid receptor signaling	2/2734	17/17381	0.77267	0.9891	0.97287	GPER1/PER1	2	BP
GO:0032366	intracellular sterol transport	2/2734	17/17381	0.77267	0.9891	0.97287	ABCA2/NPC2	2	BP
GO:0032367	intracellular cholesterol transport	2/2734	17/17381	0.77267	0.9891	0.97287	ABCA2/NPC2	2	BP
GO:0032604	granulocyte macrophage colony-stimulating	2/2734	17/17381	0.77267	0.9891	0.97287	IL18/TLR9	2	BP

GO:0032693	negative regulation of interleukin-10	2/2734	17/17381	0.77267	0.9891	0.97287	EPX/IDO1	2	BP
GO:0032891	negative regulation of organic acid	2/2734	17/17381	0.77267	0.9891	0.97287	OSR1/TRH	2	BP
GO:0033145	positive regulation of intracellular steroid hormone receptor	2/2734	17/17381	0.77267	0.9891	0.97287	RWDD1/TAF1	2	BP
GO:0034199	activation of protein kinase A activity	2/2734	17/17381	0.77267	0.9891	0.97287	PRKAR1A/PRKAR1B	2	BP
GO:0034375	high-density lipoprotein particle	2/2734	17/17381	0.77267	0.9891	0.97287	APOC3/SCARB1	2	BP
GO:0035089	establishment of apical/basal cell polarity	2/2734	17/17381	0.77267	0.9891	0.97287	FSCN1/SCRIB	2	BP
GO:0039535	regulation of RIG-I signaling	2/2734	17/17381	0.77267	0.9891	0.97287	DHX58/NLRX1	2	BP
GO:0043092	L-amino acid import	2/2734	17/17381	0.77267	0.9891	0.97287	AGT/SLC11A1	2	BP
GO:0043558	regulation of translational initiation in response to	2/2734	17/17381	0.77267	0.9891	0.97287	EIF2AK4/EIF2B5	2	BP
GO:0045683	negative regulation of epidermis	2/2734	17/17381	0.77267	0.9891	0.97287	NOTCH1/SMO	2	BP

GO:0048268	clathrin coat assembly	2/2734	17/17381	0.77267	0.9891	0.97287	CALY/HIP1R	2	BP
GO:0048339	paraxial mesoderm	2/2734	17/17381	0.77267	0.9891	0.97287	WNT11/WNT3A	2	BP
GO:0048714	positive regulation of oligodendrocyte	2/2734	17/17381	0.77267	0.9891	0.97287	MTOR/SHH	2	BP
GO:0051447	negative regulation of meiotic cell	2/2734	17/17381	0.77267	0.9891	0.97287	HORMAD1/PRKAR1A	2	BP
GO:0060219	camera-type eye photoreceptor cell	2/2734	17/17381	0.77267	0.9891	0.97287	SOX8/THY1	2	BP
GO:0060644	mammary gland epithelial cell differentiation	2/2734	17/17381	0.77267	0.9891	0.97287	SMO/ZNF703	2	BP
GO:0061323	cell proliferation involved in	2/2734	17/17381	0.77267	0.9891	0.97287	BMP4/EYA1	2	BP
GO:0070129	regulation of mitochondrial translation	2/2734	17/17381	0.77267	0.9891	0.97287	COA3/RMND1	2	BP
GO:0070816	phosphorylation of RNA polymerase II C-terminal	2/2734	17/17381	0.77267	0.9891	0.97287	CDK9/GTF2H4	2	BP
GO:0070875	positive regulation of glycogen	2/2734	17/17381	0.77267	0.9891	0.97287	GCK/INS	2	BP

GO:0070884	regulation of calcineurin-NFAT signaling	2/2734	17/17381	0.77267	0.9891	0.97287	CIB1/NRG1	2	BP
GO:0097062	dendritic spine maintenance	2/2734	17/17381	0.77267	0.9891	0.97287	CTTN/INS	2	BP
GO:0098810	neurotransmitter reuptake	2/2734	17/17381	0.77267	0.9891	0.97287	DRD2/TOR1A	2	BP
GO:0106056	regulation of calcineurin-mediated	2/2734	17/17381	0.77267	0.9891	0.97287	CIB1/NRG1	2	BP
GO:1902043	positive regulation of extrinsic apoptotic signaling	2/2734	17/17381	0.77267	0.9891	0.97287	ATF3/PEA15	2	BP
GO:1903204	negative regulation of oxidative stress-induced neuron	2/2734	17/17381	0.77267	0.9891	0.97287	NONO/WNT1	2	BP
GO:1903817	negative regulation of voltage-gated potassium	2/2734	17/17381	0.77267	0.9891	0.97287	MIR153-1/MIR212	2	BP
GO:1904355	positive regulation of telomere	2/2734	17/17381	0.77267	0.9891	0.97287	MAPK15/MAPK3	2	BP
GO:1904948	midbrain dopaminergic neuron differentiation	2/2734	17/17381	0.77267	0.9891	0.97287	WNT1/WNT3A	2	BP

GO:2000479	regulation of cAMP-dependent protein kinase	2/2734	17/17381	0.77267	0.9891	0.97287	PRKAR1A/PRKAR1B	2	BP
GO:0007589	body fluid secretion	12/2734	90/17381	0.77568	0.9891	0.97287	AQP1/AQP5/CHRM1/CSN3/DHODH/GUCA1B/GUCA2B/NME1/NR1H3/SLC29A1/TACR1/XDH	12	BP
GO:0002705	positive regulation of leukocyte	13/2734	97/17381	0.77586	0.9891	0.97287	CLCF1/F2RL1/HLA-E/IL13/IL4R/ITGAM/LTA/NCR3/PAXIP1/STX4/STXBP1/TNFSF13/TRAF2	13	BP
GO:0008585	female gonad development	13/2734	97/17381	0.77586	0.9891	0.97287	ANG/ARRB2/EIF2B2/EIF2B5/FANCA/IDH1/INHBB/LFNG/NOS3/NR5A1/NUPR1/VGF/ZFP42	13	BP
GO:0042035	regulation of cytokine biosynthetic process	13/2734	97/17381	0.77586	0.9891	0.97287	CARD11/CARD9/CCR2/EGR1/HSPB1/INHBB/INPP5D/MAP2K3/NMI/TBK1/TIRAP/TLR8/TLR9	13	BP
GO:0009156	ribonucleoside monophosphate biosynthetic process	11/2734	83/17381	0.77586	0.9891	0.97287	AK5/ALDOA/ATP5G1/ATP5I/CYC1/DHODH/IMPDH1/PID1/PKM/UCK1/VPS9D1	11	BP
GO:1900407	regulation of cellular response to	11/2734	83/17381	0.77586	0.9891	0.97287	ENDOG/GCH1/GPX1/HSPB1/INS/MIR92A2/MMP3/NONO/PSAP/TRAP1/WNT1	11	BP
GO:0014014	negative regulation of	5/2734	40/17381	0.77597	0.9891	0.97287	ASCL2/DAB1/IDH2/MYCN/NOTCH1	5	BP
GO:0015872	dopamine transport	5/2734	40/17381	0.77597	0.9891	0.97287	CHRNA6/DRD2/DTNBP1/SLC22A1/TOR1A	5	BP
GO:0031670	cellular response to	5/2734	40/17381	0.77597	0.9891	0.97287	BGLAP/CHMP1A/COL1A1/FOLR2/TNC	5	BP
GO:0035136	forelimb morphogenesis	5/2734	40/17381	0.77597	0.9891	0.97287	ALX3/EN1/OSR1/SHH/WNT7A	5	BP

GO:0051985	negative regulation of chromosome	5/2734	40/17381	0.77597	0.9891	0.97287	ANAPC15/BUB1B/LCMT1/MAD1L1/XRCC3	5	BP
GO:0098930	axonal transport	5/2734	40/17381	0.77597	0.9891	0.97287	CNIH2/DTNBP1/HSPB1/KIF4A/TRIM46	5	BP
GO:1903901	negative regulation of	10/2734	76/17381	0.77652	0.9891	0.97287	CCL5/EIF2AK4/IFITM2/ISG15/MIR221/MIR222/PARP10/TRIM11/TRIM26/TRIM31	10	BP
GO:0002230	positive regulation of defense response to	3/2734	25/17381	0.77675	0.9891	0.97287	EIF2AK4/PARP9/STAT1	3	BP
GO:0002360	T cell lineage commitment	3/2734	25/17381	0.77675	0.9891	0.97287	FOXN1/LY9/SHH	3	BP
GO:0008210	estrogen metabolic	3/2734	25/17381	0.77675	0.9891	0.97287	HSD17B1/HSD17B7/HSD3B1	3	BP
GO:0022616	DNA strand elongation	3/2734	25/17381	0.77675	0.9891	0.97287	PARP2/PARP3/POLE	3	BP
GO:0040001	establishment of mitotic spindle localization	3/2734	25/17381	0.77675	0.9891	0.97287	EYA1/MCPH1/NUMA1	3	BP
GO:0045932	negative regulation of muscle	3/2734	25/17381	0.77675	0.9891	0.97287	ADRA2C/MIR153-1/MIR328	3	BP
GO:0048596	embryonic camera-type eye morphogenesis	3/2734	25/17381	0.77675	0.9891	0.97287	STRA6/TBX2/TH	3	BP
GO:0051181	cofactor transport	3/2734	25/17381	0.77675	0.9891	0.97287	FOLR2/SLC25A20/SLC44A4	3	BP
GO:0051354	negative regulation of oxidoreductase	3/2734	25/17381	0.77675	0.9891	0.97287	CAV3/IL13/INS	3	BP

GO:0051788	response to misfolded	3/2734	25/17381	0.77675	0.9891	0.97287	CLU/SDF2/TOR1A	3	BP
GO:0060037	pharyngeal system	3/2734	25/17381	0.77675	0.9891	0.97287	BMP4/EYA1/NKX2-5	3	BP
GO:0061842	microtubule organizing center	3/2734	25/17381	0.77675	0.9891	0.97287	FHOD1/IFT20/NUBP1	3	BP
GO:1901017	negative regulation of potassium ion transmembrane	3/2734	25/17381	0.77675	0.9891	0.97287	CAV3/MIR153-1/MIR212	3	BP
GO:1901032	negative regulation of response to reactive oxygen	3/2734	25/17381	0.77675	0.9891	0.97287	MIR92A2/PSAP/TRAP1	3	BP
GO:1903206	negative regulation of hydrogen peroxide-	3/2734	25/17381	0.77675	0.9891	0.97287	MIR92A2/PSAP/TRAP1	3	BP
GO:1903672	positive regulation of sprouting	3/2734	25/17381	0.77675	0.9891	0.97287	ABL1/CIB1/MIR503	3	BP
GO:0034976	response to endoplasmic reticulum stress	39/2734	274/17381	0.77689	0.9891	0.97287	ACADVL/ASNS/ATF3/ATG10/BCAP31/BOK/BRSK2/CDK5RAP3/CHAC1/CLU/CREB3/DAB2IP/EIF2AK4/EIF2B5/ERN2/EXTL1/FBXO2/FBXO6/GOSR2/HERPUD1/HSPA5/LMNA/OS9/PLA2G4B/PPP2R5B/PSMC3/SDF2/SEC61A2/SHC1/SPOP/SYVN1/TLN1/TMUB1/TOR1A/TRAF2/TSPYL2/USP19/WFS1/ZB	39	BP
GO:0006369	termination of RNA polymerase II	9/2734	69/17381	0.77778	0.9891	0.97287	CASC3/CPSF1/CPSF4/CSTF2/DHX38/MED18/RNPS1/SRSF4/TTF2	9	BP

GO:0033555	multicellular organismal response to	9/2734	69/17381	0.77778	0.9891	0.97287	DEAF1/EIF4G1/GCH1/IDO1/LYPD1/NEUROD2/P2RX2/SLC6A2/TACR1	9	BP
GO:0043484	regulation of RNA splicing	16/2734	118/17381	0.77792	0.9891	0.97287	AHNAK/CELF6/ESRP2/HMX2/LMNTD2/MYOD1/NUP98/PRDX6/RBFOX1/RBFOX3/RBM4/RNPS1/SF3B4/SLC39A5/SRSF4/ZPR1	16	BP
GO:1902105	regulation of leukocyte differentiation	36/2734	254/17381	0.778	0.9891	0.97287	ABL1/ADAM8/BAD/BCL6/BGLAP/BMP4/C1QC/CARD11/CCL19/CD27/CD74/CSF1/ERBB2/ESRRA/FAM213A/FANCA/FOXN1/HLX/IL18/IL4R/INPP5D/LGALS9/MTOR/NME1/PIK3R6/PPP2R3C/PRELID1/RARA/SART1/SHH/SOX13/SPINK5/TCTA/TLR9/TMEM176B/TNFRSF18	36	BP
GO:0009451	RNA modification	19/2734	139/17381	0.78139	0.9891	0.97287	AARS/AARS2/ALKBH5/C9orf64/CDKAL1/CMTR1/MEPCE/METTL2A/METTL2B/NHP2/NSUN5/NSUN5P2/PUSL1/RPUSD2/THUMPD2/TRMT10B/TRM	19	BP
GO:0034764	positive regulation of transmembrane	19/2734	139/17381	0.78139	0.9891	0.97287	ABL1/AGT/AMIGO1/ARC/CRACR2A/FLNA/GPER1/GSTM2/HSPA2/IL13/KCNQ1/NLGN3/NPPA/NPSR1/SLC26A6/SLC34A1/THY1/TRDN/WNK2	19	BP
GO:0001910	regulation of leukocyte mediated cytotoxicity	7/2734	55/17381	0.78301	0.9891	0.97287	ARRB2/F2RL1/HAVCR2/HLA-E/LGALS9/NCR3/PIK3R6	7	BP
GO:0016233	telomere capping	7/2734	55/17381	0.78301	0.9891	0.97287	ACD/HIST1H4F/HIST2H4A/HIST2H4B/MAPK15/MAPK3/TINF2	7	BP
GO:2000378	negative regulation of reactive oxygen species	7/2734	55/17381	0.78301	0.9891	0.97287	BRCA1/CRYAB/INS/MIR92A2/MMP3/MYCN/TRAP1	7	BP
GO:0002697	regulation of immune effector process	58/2734	402/17381	0.785	0.9891	0.97287	ARRB2/BCL6/C1QB/C1QC/C2/C3AR1/C8A/CCL19/CCR2/CD74/CD81/CFH/CLCF1/CLU/CPN2/CUEDC2/DHX58/EIF2AK4/F2RL1/FES/HAVCR2/HLA-E/HLX/IL13/IL18/IL20RB/IL4R/INS/ITGAM/KARS/LGALS3/LGALS9/LTA/MAPK3/MICB/MYO18A/MZB1/NCR3/NLRX1/PARP9/PAXIP1/PGC/PIK3R6/RARA/SEMA7A/SPINK5/SPON2/STAT1/STX4/STXBP1/SUPT6H/TARBP2/TLR9/TNFRSF4/TNFSF13/TRAF2/TRAF3IP1/WAS	58	BP

GO:0001881	receptor recycling	4/2734	33/17381	0.78506	0.9891	0.97287	ACHE/ARAP1/INPP5F/SCRIB	4	BP
GO:0006308	DNA catabolic process	4/2734	33/17381	0.78506	0.9891	0.97287	CASP3/DNASE1L2/ENDOG/HSF1	4	BP
GO:0006370	7-methylguanosin	4/2734	33/17381	0.78506	0.9891	0.97287	CMTR1/GTF2H4/POLR2G/POLR2L	4	BP
GO:0019433	triglyceride catabolic	4/2734	33/17381	0.78506	0.9891	0.97287	APOA5/APOB/APOC3/PNPLA2	4	BP
GO:0032801	receptor catabolic	4/2734	33/17381	0.78506	0.9891	0.97287	AP2A2/AP2M1/CDK5/FURIN	4	BP
GO:0043276	anoikis	4/2734	33/17381	0.78506	0.9891	0.97287	BRMS1/DAPK2/MTOR/NOTCH1	4	BP
GO:0043928	exonucleolytic nuclear-transcribed mRNA catabolic process involved in [illegible]	4/2734	33/17381	0.78506	0.9891	0.97287	EXOSC2/LSM1/NT5C3B/PATL1	4	BP
GO:0061614	pri-miRNA transcription from RNA polymerase II	4/2734	33/17381	0.78506	0.9891	0.97287	FOSL1/PPARD/SMAD6/SPI1	4	BP
GO:0072604	interleukin-6 secretion	4/2734	33/17381	0.78506	0.9891	0.97287	F2RL1/LGALS9/TLR8/TRPV4	4	BP
GO:0098901	regulation of cardiac muscle cell action	4/2734	33/17381	0.78506	0.9891	0.97287	CAMK2D/CAV3/FLNA/MIR328	4	BP
GO:1903580	positive regulation of ATP metabolic	4/2734	33/17381	0.78506	0.9891	0.97287	INS/MLXIPL/PFKFB1/PID1	4	BP

GO:0036294	cellular response to decreased	28/2734	201/17381	0.78655	0.9891	0.97287	ADAM8/AQP1/BAD/DNMT3A/EGR1/EIF4EBP1/ENDOG/IRAK1/LMNA/MTOR/NOTCH1/OPRD1/PPARD/PSMB11/PSMB6/PSMB7/PSMB8/PSMC3/PSMD13/PSMD3/PSMD5/PSMD7/RWDD3/SLC29A1/SLC2A4/SUV39H1/USP19/V	28	BP
GO:0002712	regulation of B cell mediated	6/2734	48/17381	0.78768	0.9891	0.97287	BCL6/CLCF1/LTA/PAXIP1/SUPT6H/TNFSF13	6	BP
GO:0002889	regulation of immunoglobulin mediated immune	6/2734	48/17381	0.78768	0.9891	0.97287	BCL6/CLCF1/LTA/PAXIP1/SUPT6H/TNFSF13	6	BP
GO:0050879	multicellular organismal movement	6/2734	48/17381	0.78768	0.9891	0.97287	CAV3/CHRNB1/GSTM2/MTOR/TCAP/TNNC1	6	BP
GO:0050881	musculoskeletal movement	6/2734	48/17381	0.78768	0.9891	0.97287	CAV3/CHRNB1/GSTM2/MTOR/TCAP/TNNC1	6	BP
GO:0060421	positive regulation of	6/2734	48/17381	0.78768	0.9891	0.97287	ACACB/FGFR2/MIR222/MTOR/NOTCH1/TBX2	6	BP
GO:0098586	cellular response to	6/2734	48/17381	0.78768	0.9891	0.97287	BAD/CCL19/DHX58/GBF1/LGALS9/NLRX1	6	BP
GO:0006641	triglyceride metabolic	13/2734	98/17381	0.78825	0.9891	0.97287	APOA5/APOB/APOC3/CAV3/DGAT1/GPX1/LMF1/NKX2-3/NR1H3/PLA2G16/PNPLA2/PTPN11/SCARB1	13	BP
GO:0070301	cellular response to hydrogen	12/2734	91/17381	0.78852	0.9891	0.97287	ABL1/APEX1/AQP1/ENDOG/HSF1/IL18BP/KDM6B/MIR92A2/PCGF2/PRKCD/PSAP/TRAP1	12	BP
GO:1901989	positive regulation of cell cycle phase	11/2734	84/17381	0.78919	0.9891	0.97287	APEX1/CDK10/CDK4/CYP1A1/EIF4G1/MEPCE/MIR221/MIR222/MIR29A/PHOX2B/UBE2E2	11	BP
GO:0065004	protein-DNA complex assembly	34/2734	242/17381	0.78964	0.9891	0.97287	ANP32B/BRF1/CENPN/DDB1/DDB2/GTF2H4/H1FX/HILS1/HIST1H4F/HIST2H4A/HIST2H4B/HJURP/IPO4/MCM2/MCMDC2/MED6/NAA60/PADI4/POLR1E/PSMC3/RSF1/SENP6/SMYD3/TAF1/TAF1L/TAF7/THRA/TSPY1/TSPY2/TSPY4/TSPY8/TSPYL2/XPC/XRCC3	34	BP

GO:0006073	cellular glucan metabolic	10/2734	77/17381	0.79037	0.9891	0.97287	GCK/GNMT/INS/MGAM/MTOR/PCDH12/PFKM/PHKG1/PHLDA2/PYGM	10	BP
GO:0044042	glucan metabolic	10/2734	77/17381	0.79037	0.9891	0.97287	GCK/GNMT/INS/MGAM/MTOR/PCDH12/PFKM/PHKG1/PHLDA2/PYGM	10	BP
GO:0060415	muscle tissue morphogenesis	10/2734	77/17381	0.79037	0.9891	0.97287	ENG/FGFR2/FZD2/MIR195/MYBPC3/NKX2-5/NOTCH1/NRG1/TCAP/TNNC1	10	BP
GO:0001889	liver development	18/2734	133/17381	0.79073	0.9891	0.97287	ASNS/CCDC40/CYP1A1/E2F8/EGFR/GFER/HLX/LIMS2/NKX2-8/NOTCH1/OTC/PHF2/PKD1/PKM/RARA/RPS6KA1/WNT1/WNT3A	18	BP
GO:0034767	positive regulation of ion transmembrane	18/2734	133/17381	0.79073	0.9891	0.97287	ABL1/AGT/AMIGO1/ARC/CRACR2A/FLNA/GPER1/GSTM2/HSPA2/IL13/KCNQ1/NLGN3/NPPA/NPSR1/SLC34A1/THY1/TRDN/WNK2	18	BP
GO:0032412	regulation of ion transmembrane transporter	31/2734	222/17381	0.792	0.9891	0.97287	ADRA2A/AHNAK/ARC/CACNB1/CACNB3/CAMK2D/CAV3/CNIH2/CRACR2A/CRHR1/DAPK1/DLG4/DRD2/GNB5/GPR35/GSTM2/HSPA2/JPH3/MIR153-1/MIR212/NLGN3/NPPA/OPRM1/OSR1/PTK2B/RRAD/THADA/TLR9/TRDN/	31	BP
GO:0009301	snRNA transcription	9/2734	70/17381	0.79221	0.9891	0.97287	CDK9/INTS10/INTS3/INTS5/POLR2G/POLR2L/SNAPC1/SNAPC5/TAF6	9	BP
GO:0030512	negative regulation of transforming growth factor beta receptor	9/2734	70/17381	0.79221	0.9891	0.97287	FAM89B/HSPA5/HTRA3/PBLD/SMAD6/TGFB1I1/VASN/WFIKKN2/WNT1	9	BP
GO:0032637	interleukin-8 production	9/2734	70/17381	0.79221	0.9891	0.97287	CD58/F2RL1/GDF2/LGALS9/NOD1/PLA2G1B/TIRAP/TLR8/TLR9	9	BP
GO:0042795	snRNA transcription from RNA polymerase II	9/2734	70/17381	0.79221	0.9891	0.97287	CDK9/INTS10/INTS3/INTS5/POLR2G/POLR2L/SNAPC1/SNAPC5/TAF6	9	BP

GO:0016051	carbohydrate biosynthetic process	27/2734	195/17381	0.79347	0.9891	0.97287	ALDOA/ATF3/CHST10/CHST12/CHST13/CHST15/CRTC2/DGKQ/G6PC3/GAPDH/GCG/GCK/GNMT/GOT2/GPER1/INS/KAT2A/LEPR/MAEA/MPDU1/MTOR/PC/PFKFB1/PGAM4/PHKG1/PTK2B/ST3GAL4	27	BP
GO:0031952	regulation of protein autophosphorylation	5/2734	41/17381	0.79455	0.9891	0.97287	ENG/INS/NEK10/PDGFA/PPP2R5B	5	BP
GO:0042455	ribonucleoside biosynthetic process	5/2734	41/17381	0.79455	0.9891	0.97287	DHODH/IMPDH1/NME1/NME4/UCK1	5	BP
GO:0043666	regulation of phosphoprotein phosphatase activity	5/2734	41/17381	0.79455	0.9891	0.97287	DRD2/IKBKB/PDGFRB/PPP1R1B/TIPRL	5	BP
GO:0044088	regulation of vacuole organization	5/2734	41/17381	0.79455	0.9891	0.97287	IFT20/LRSAM1/NPRL3/RALB/ULK1	5	BP
GO:0002548	monocyte chemotaxis	8/2734	63/17381	0.79491	0.9891	0.97287	CCL1/CCL19/CCL21/CCL5/CCR2/CREB3/LGALS3/NBL1	8	BP
GO:0010611	regulation of cardiac muscle hypertrophy	8/2734	63/17381	0.79491	0.9891	0.97287	AGT/CAMK2D/CAV3/CDK9/LMNA/MIR25/MTOR/TRIM63	8	BP
GO:1904018	positive regulation of vasculature	23/2734	168/17381	0.79644	0.9891	0.97287	ABL1/ADM/AQP1/BRCA1/C3AR1/CIB1/CTSH/ECM1/EGR1/ENG/GATA4/GDF2/GPER1/HSPB1/KDR/MIR210/MIR451A/MIR503/NOS3/NOTCH1/PIK3R6/PTK2B/TMEM100	23	BP
GO:0072331	signal transduction by p53 class mediator	38/2734	270/17381	0.79727	0.9891	0.97287	ANKRD2/ATRIP/BOK/BRCA1/BRPF1/CD74/CDK5/CDK5RAP3/E2F4/E2F8/EHMT1/EHMT2/EXO1/FHIT/FOXM1/HINT1/ING4/MUC1/NOC2L/NUPR1/PAXIP1/PCBP4/PHLDA3/PPP1R13B/PRKAB1/RFC2/SETD9/SFN/TAF1/TAF1L/TAF3/TAF6/TAF7/TMEM109/ZNF346/ZNF385A/ZNF385C/ZNF385D	38	BP

GO:0051604	protein maturation	52/2734	364/17381	0.79754	0.9891	0.97287	ADAM8/ADAMTS13/ADAMTS2/AEBP1/AGT/AIP/APH1A/BAD/BGLAP/C1QB/C1QC/C2/C2CD3/C3AR1/C8A/CASP1/CASP3/CD81/CFH/CHAC1/CLU/CPN2/CTSE/CTSH/EIF5A/FGA/FURIN/GLRX5/ISCA2/KLKB1/LIAS/LMF1/NAA16/NAA60/NAIP/NKD2/PLAU/PMPCA/PROZ/PRSS37/REN/RHBDL2/SERPINF2/SERPINH1/SHH/SNX12/TFR2/TSPAN15/TSPAN33/TYSND1/WFS1/YM	52	BP
GO:0034728	nucleosome organization	24/2734	175/17381	0.79776	0.9891	0.97287	ANP32B/CENPN/H1FX/HILS1/HIST1H4F/HIST2H4A/HIST2H4B/HJURP/HMGA1/IPO4/MCM2/NAA60/PADI4/RSF1/SETD2/SMARCD2/SMYD3/SUPT6H/TSPY1/TSPY2/TSPY4/TSPY8/TSPYL2/ZNHIT1	24	BP
GO:0019748	secondary metabolic	7/2734	56/17381	0.79877	0.9891	0.97287	AS3MT/CYP1A1/CYP1A2/CYP2W1/DDC/PGAM4/TH	7	BP
GO:0045576	mast cell activation	7/2734	56/17381	0.79877	0.9891	0.97287	CD300LF/CHGA/FES/IL13/IL4R/LGALS9/STXBP1	7	BP
GO:0000188	inactivation of MAPK activity	3/2734	26/17381	0.79955	0.9891	0.97287	DUSP2/DUSP21/DUSP5	3	BP
GO:0000732	strand displacement	3/2734	26/17381	0.79955	0.9891	0.97287	BRCA1/EXO1/XRCC3	3	BP
GO:0001556	oocyte maturation	3/2734	26/17381	0.79955	0.9891	0.97287	ANG/PTK2B/RXFP2	3	BP
GO:0005979	regulation of glycogen biosynthetic process	3/2734	26/17381	0.79955	0.9891	0.97287	GCK/INS/MTOR	3	BP
GO:0006829	zinc II ion transport	3/2734	26/17381	0.79955	0.9891	0.97287	SLC30A3/SLC39A13/SLC39A5	3	BP
GO:0010962	regulation of glucan biosynthetic	3/2734	26/17381	0.79955	0.9891	0.97287	GCK/INS/MTOR	3	BP
GO:0031954	positive regulation of protein autophosphoryl	3/2734	26/17381	0.79955	0.9891	0.97287	INS/NEK10/PDGFA	3	BP

GO:0032069	regulation of nuclease	3/2734	26/17381	0.79955	0.9891	0.97287	DAB2IP/HSPA5/PRKCD	3	BP
GO:0032438	melanosome organization	3/2734	26/17381	0.79955	0.9891	0.97287	DTNBP1/HPS1/KIF13A	3	BP
GO:0032620	interleukin-17 production	3/2734	26/17381	0.79955	0.9891	0.97287	IL18/LY9/TUSC2	3	BP
GO:0051294	establishment of spindle	3/2734	26/17381	0.79955	0.9891	0.97287	EYA1/MCPH1/NUMA1	3	BP
GO:0051875	pigment granule localization	3/2734	26/17381	0.79955	0.9891	0.97287	DCTN2/MYO7A/RAB17	3	BP
GO:0060765	regulation of androgen receptor	3/2734	26/17381	0.79955	0.9891	0.97287	ARRB2/RWDD1/TAF1	3	BP
GO:0007127	meiosis I	15/2734	113/17381	0.7998	0.9891	0.97287	C11orf80/DDX4/HORMAD1/HSPA2/LEMD2/M1AP/MCMDC2/PIWIL2/PRDM9/PSMD13/RNF212/SLC2A8/TDRD9/UBE2B/XRCC3	15	BP
GO:0019079	viral genome replication	15/2734	113/17381	0.7998	0.9891	0.97287	CCL5/CTBP1/DDB1/EIF2AK4/FKBP6/HACD3/IFITM2/ISG15/MIR221/MIR222/NOTCH1/PARP10/PPIB/PPIE/TARBP2	15	BP
GO:0045667	regulation of osteoblast differentiation	15/2734	113/17381	0.7998	0.9891	0.97287	BMP4/CLIC1/DDR2/ESRRA/FGFR2/GDF2/HDAC8/ILK/MEN1/NOTCH1/PDLIM7/SUFU/TMEM119/TNN/TWIST2	15	BP
GO:0006123	mitochondrial electron transport, cytochrome c to	2/2734	18/17381	0.79989	0.9891	0.97287	COX10/COX8A	2	BP
GO:0006337	nucleosome disassembly	2/2734	18/17381	0.79989	0.9891	0.97287	HMGA1/SMARCD2	2	BP
GO:0006448	regulation of translational elongation	2/2734	18/17381	0.79989	0.9891	0.97287	DPH1/EIF5A	2	BP
GO:0006677	glycosylceramide metabolic	2/2734	18/17381	0.79989	0.9891	0.97287	GBA/PRKCD	2	BP

GO:0007350	blastoderm segmentation	2/2734	18/17381	0.79989	0.9891	0.97287	SEMA3F/TDGF1	2	BP
GO:0009312	oligosaccharide biosynthetic process	2/2734	18/17381	0.79989	0.9891	0.97287	MPDU1/ST3GAL4	2	BP
GO:0010866	regulation of triglyceride biosynthetic process	2/2734	18/17381	0.79989	0.9891	0.97287	NR1H3/SCARB1	2	BP
GO:0015813	L-glutamate transport	2/2734	18/17381	0.79989	0.9891	0.97287	SLC1A7/SLC25A22	2	BP
GO:0019184	nonribosomal peptide biosynthetic process	2/2734	18/17381	0.79989	0.9891	0.97287	CHAC1/GGT6	2	BP
GO:0030540	female genitalia development	2/2734	18/17381	0.79989	0.9891	0.97287	LHX1/STRA6	2	BP
GO:0032095	regulation of response to	2/2734	18/17381	0.79989	0.9891	0.97287	MTOR/OPRM1	2	BP
GO:0036342	post-anal tail morphogenesis	2/2734	18/17381	0.79989	0.9891	0.97287	TRAF3IP1/WNT3A	2	BP
GO:0045076	regulation of interleukin-2 biosynthetic process	2/2734	18/17381	0.79989	0.9891	0.97287	CARD11/CARD9	2	BP
GO:0051131	chaperone-mediated protein complex	2/2734	18/17381	0.79989	0.9891	0.97287	CLU/HSP90AA1	2	BP
GO:0060004	reflex	2/2734	18/17381	0.79989	0.9891	0.97287	ADRA1A/GLRA1	2	BP

GO:0061318	renal filtration cell	2/2734	18/17381	0.79989	0.9891	0.97287	BMP4/MYO1E	2	BP
GO:0072112	glomerular visceral epithelial cell	2/2734	18/17381	0.79989	0.9891	0.97287	BMP4/MYO1E	2	BP
GO:0072337	modified amino acid transport	2/2734	18/17381	0.79989	0.9891	0.97287	FOLR2/SLC6A8	2	BP
GO:0090330	regulation of platelet aggregation	2/2734	18/17381	0.79989	0.9891	0.97287	C1QTNF1/PRKCD	2	BP
GO:0097709	connective tissue	2/2734	18/17381	0.79989	0.9891	0.97287	MIR16-1/MIR195	2	BP
GO:1900409	positive regulation of cellular response to	2/2734	18/17381	0.79989	0.9891	0.97287	ENDOG/MMP3	2	BP
GO:2000726	negative regulation of cardiac muscle	2/2734	18/17381	0.79989	0.9891	0.97287	CAV3/MIR222	2	BP
GO:2000737	negative regulation of stem cell	2/2734	18/17381	0.79989	0.9891	0.97287	NOTCH1/WNT3A	2	BP
GO:0006476	protein deacetylation	13/2734	99/17381	0.80015	0.9891	0.97287	BCL6/BRMS1/CAMK2D/CCAR2/CTBP1/FNTA/HDAC11/HDAC8/MSL3/MTA1/PER1/TADA3/ZNHIT1	13	BP
GO:1902882	regulation of response to oxidative stress	12/2734	92/17381	0.80082	0.9891	0.97287	ENDOG/GCH1/GPX1/HSPB1/INS/MIR92A2/MMP3/NONO/PSAP/SESN1/TRAP1/WNT1	12	BP
GO:1905037	autophagosome organization	12/2734	92/17381	0.80082	0.9891	0.97287	AMBRA1/ATG101/ATG2A/ATP13A2/GBA/IFT20/LRSAM1/MFN2/NPRL3/RAB7A/RALB/ULK1	12	BP
GO:0051302	regulation of cell division	19/2734	141/17381	0.80168	0.9891	0.97287	ANKRD53/C10orf99/CDK2AP2/CHMP4C/CIB1/CXCR5/DRD2/E2F8/FGF3/FGFR2/KIF23/MYO19/OPN1MW/ORC6/PDGFA/SETD2/SFN/SHH/SSTR5	19	BP

GO:0050864	regulation of B cell activation	21/2734	155/17381	0.8034	0.9891	0.97287	BAD/BCL6/CARD11/CASP3/CD27/CD74/CD81/CLCF1/IL13/INPP5D/MZB1/PAXIP1/PPP2R3C/SUPT6H/TIRAP/TLR9/TNFRSF13B/TNFRSF4/TNFSF13/TNI	21	BP
GO:0043044	ATP-dependent chromatin remodeling	10/2734	78/17381	0.80358	0.9891	0.97287	ANP32B/CENPN/CHD8/HIST1H4F/HIST2H4A/HIST2H4B/HJURP/RSF1/SMARCD2/ZNHIT1	10	BP
GO:1900034	regulation of cellular response to heat	10/2734	78/17381	0.80358	0.9891	0.97287	CAMK2D/CCAR2/CRYAB/HSF1/HSP90AA1/MAPK3/MLST8/MTOR/NUP210/NUP98	10	BP
GO:0002381	immunoglobulin production involved in immunoglobulin mediated immune	6/2734	49/17381	0.8042	0.9891	0.97287	BCL6/CLCF1/EXO1/PAXIP1/SUPT6H/TNFSF13	6	BP
GO:0007131	reciprocal meiotic	6/2734	49/17381	0.8042	0.9891	0.97287	C11orf80/MCMDC2/PRDM9/RNF212/UBE2B/XRCC3	6	BP
GO:0007157	heterophilic cell-cell adhesion via plasma membrane cell	6/2734	49/17381	0.8042	0.9891	0.97287	ALCAM/AMIGO1/CD6/ITGAL/NCR3/SELP	6	BP
GO:0035825	homologous recombination	6/2734	49/17381	0.8042	0.9891	0.97287	C11orf80/MCMDC2/PRDM9/RNF212/UBE2B/XRCC3	6	BP
GO:0042345	regulation of NF-kappaB import into	6/2734	49/17381	0.8042	0.9891	0.97287	CCL19/CD27/IL18/LGALS9/PRDX1/TLR9	6	BP
GO:0042348	NF-kappaB import into	6/2734	49/17381	0.8042	0.9891	0.97287	CCL19/CD27/IL18/LGALS9/PRDX1/TLR9	6	BP
GO:0072678	T cell migration	6/2734	49/17381	0.8042	0.9891	0.97287	ADAM8/CCL21/CCL5/CCR2/CXCR3/ECM1	6	BP

GO:0046661	male sex differentiation	22/2734	162/17381	0.8044	0.9891	0.97287	BOK/CSDE1/FANCA/GATA4/HSD17B3/LRRC6/MGST1/NR5A1/NUDT1/NUP210L/NUPR1/PDGFRB/RARA/REN/RXFP2/SCX/SDC1/SF1/SHH/SOX8/UTF	22	BP
GO:0002714	positive regulation of B cell mediated	4/2734	34/17381	0.80474	0.9891	0.97287	CLCF1/LTA/PAXIP1/TNFSF13	4	BP
GO:0002891	positive regulation of immunoglobulin mediated	4/2734	34/17381	0.80474	0.9891	0.97287	CLCF1/LTA/PAXIP1/TNFSF13	4	BP
GO:0006692	prostanoid metabolic	4/2734	34/17381	0.80474	0.9891	0.97287	CD74/CES2/EDN2/PTGR1	4	BP
GO:0006693	prostaglandin metabolic	4/2734	34/17381	0.80474	0.9891	0.97287	CD74/CES2/EDN2/PTGR1	4	BP
GO:0009452	7-methylguanosin	4/2734	34/17381	0.80474	0.9891	0.97287	CMTR1/GTF2H4/POLR2G/POLR2L	4	BP
GO:0010907	positive regulation of glucose	4/2734	34/17381	0.80474	0.9891	0.97287	GCG/GCK/INS/KAT2A	4	BP
GO:0032212	positive regulation of telomere maintenance via	4/2734	34/17381	0.80474	0.9891	0.97287	ACD/CCT3/MAPK15/MAPK3	4	BP
GO:0036260	RNA capping	4/2734	34/17381	0.80474	0.9891	0.97287	CMTR1/GTF2H4/POLR2G/POLR2L	4	BP
GO:0036474	cell death in response to hydrogen	4/2734	34/17381	0.80474	0.9891	0.97287	ENDOG/MIR92A2/PSAP/TRAP1	4	BP
GO:0051293	establishment of spindle	4/2734	34/17381	0.80474	0.9891	0.97287	DYNC1H1/EYA1/MCPH1/NUMA1	4	BP

GO:0071868	cellular response to monoamine	4/2734	34/17381	0.80474	0.9891	0.97287	ABL1/HCN3/KCNQ1/MAPK3	4	BP
GO:0071870	cellular response to catecholamine	4/2734	34/17381	0.80474	0.9891	0.97287	ABL1/HCN3/KCNQ1/MAPK3	4	BP
GO:0009127	purine nucleoside monophosphate biosynthetic	9/2734	71/17381	0.80594	0.9891	0.97287	AK5/ALDOA/ATP5G1/ATP5I/CYC1/IMPDH1/PID1/PKM/VPS9D1	9	BP
GO:0009168	purine ribonucleoside monophosphate biosynthetic process	9/2734	71/17381	0.80594	0.9891	0.97287	AK5/ALDOA/ATP5G1/ATP5I/CYC1/IMPDH1/PID1/PKM/VPS9D1	9	BP
GO:0019226	transmission of nerve impulse	9/2734	71/17381	0.80594	0.9891	0.97287	AGT/CACNA1G/CACNA1H/GBA/GLRA1/GPER1/GPR35/GPR88/SCN11A	9	BP
GO:0086001	cardiac muscle cell action	9/2734	71/17381	0.80594	0.9891	0.97287	ATP1A1/CACNA1G/CAMK2D/CAV3/FLNA/KCND3/KCNJ5/KCNQ1/MIR328	9	BP
GO:0071453	cellular response to oxygen levels	30/2734	217/17381	0.80634	0.9891	0.97287	ADAM8/AQP1/ATP6V1G1/BAD/DNMT3A/EGR1/EIF4EBP1/ENDOG/IRAK1/LMNA/MTOR/MYOD1/NOTCH1/OPRD1/PPARD/PSMB11/PSMB6/PSMB7/PSMB8/PSMC3/PSMD13/PSMD3/PSMD5/PSMD7/RWDD3/SLC29A1/SLC2A4/	30	BP
GO:0071384	cellular response to corticosteroid	8/2734	64/17381	0.80919	0.9891	0.97287	AQP1/EGFR/EIF4EBP1/GPER1/HEY1/MYOD1/SMYD3/SSTR5	8	BP
GO:0000082	G1/S transition of mitotic cell cycle	37/2734	265/17381	0.80987	0.9891	0.97287	APEX1/CDK10/CDK2AP2/CDK4/CDKN2A/CTDSP1/CYP1A1/E2F4/E2F8/EGFR/EIF4EBP1/EIF4G1/GPR132/HINFP/INO80/MAX/MCM2/MCM4/MEPCE/MIR15A/MIR16-1/MIR221/MIR222/MIR29A/MIR29C/MUC1/ORC6/PCBP4/PID1/PKD1/POLA2	37	BP

GO:0016202	regulation of striated muscle tissue	19/2734	142/17381	0.81131	0.9891	0.97287	ARRB2/BMP4/CAV3/FGF3/FGFR2/GPC1/LUC7L/MIR222/MIR25/MTOR/MYOD1/NKX2-5/NOTCH1/NRG1/SHH/TBX2/TSC22D3/USP19/WNT3A	19	BP
GO:0007080	mitotic metaphase plate	5/2734	42/17381	0.81189	0.9891	0.97287	ANKRD53/CHMP1A/CHMP4C/CHMP7/MAD1L1	5	BP
GO:0007595	lactation	5/2734	42/17381	0.81189	0.9891	0.97287	CSN3/DHODH/NME1/SLC29A1/XDH	5	BP
GO:0009069	serine family amino acid metabolic	5/2734	42/17381	0.81189	0.9891	0.97287	MTHFD1/PHGDH/SARS/SHMT2/THNSL2	5	BP
GO:0010799	regulation of peptidyl-threonine	5/2734	42/17381	0.81189	0.9891	0.97287	AXIN1/DGKQ/EIF4G1/GCG/PARD3	5	BP
GO:0019985	translesion synthesis	5/2734	42/17381	0.81189	0.9891	0.97287	FAAP20/ISG15/POLD4/RFC1/RFC2	5	BP
GO:0045540	regulation of cholesterol biosynthetic process	5/2734	42/17381	0.81189	0.9891	0.97287	ACACB/APOB/PMVK/POR/SCAP	5	BP
GO:0050912	detection of chemical stimulus involved in	5/2734	42/17381	0.81189	0.9891	0.97287	AZGP1/CA6/GNAT1/LPO/TAS2R3	5	BP
GO:0001541	ovarian follicle development	7/2734	57/17381	0.81364	0.9891	0.97287	ANG/ARRB2/EIF2B2/EIF2B5/INHBB/LFNG/VGF	7	BP
GO:0016445	somatic diversification of	7/2734	57/17381	0.81364	0.9891	0.97287	BCL6/CLCF1/EXO1/PAXIP1/PMS2P3/SUPT6H/TNFSF13	7	BP
GO:0033619	membrane protein	7/2734	57/17381	0.81364	0.9891	0.97287	ADAM8/ADRA2A/APH1A/CTSH/FURIN/LRIG2/PACSIN3	7	BP

GO:0061640	cytoskeleton-dependent cytokinesis	7/2734	57/17381	0.81364	0.9891	0.97287	ALKBH4/ANKRD53/BIN3/CFL1/KIF23/KIF4A/UNC119	7	BP
GO:0006767	water-soluble vitamin metabolic	11/2734	86/17381	0.8141	0.9891	0.97287	ACACB/ACPP/ALDH1L1/CUBN/FOLR2/MTHFD1/PC/SHMT2/SLC52A1/SLC52A2/VNN3	11	BP
GO:0006998	nuclear envelope	11/2734	86/17381	0.8141	0.9891	0.97287	CCNB2/CHMP7/EMD/GPER1/LEMD2/LMNA/NUP210/NUP98/REEP4/TOR1A/TOR1B	11	BP
GO:0016575	histone deacetylation	11/2734	86/17381	0.8141	0.9891	0.97287	BCL6/BRMS1/CAMK2D/CTBP1/HDAC11/HDAC8/MSL3/MTA1/PER1/TADA3/ZNHIT1	11	BP
GO:0045639	positive regulation of myeloid cell	11/2734	86/17381	0.8141	0.9891	0.97287	CD74/CSF1/CSF3/FES/INPP5D/ISG15/MIR221/MIR222/SCIN/STAT1/THPO	11	BP
GO:0046634	regulation of alpha-beta T cell activation	10/2734	79/17381	0.81617	0.9891	0.97287	BCL6/CCL19/CCR2/HLA-E/HLX/IL18/IL4R/LGALS9/RARA/SHH	10	BP
GO:0003208	cardiac ventricle	9/2734	72/17381	0.81897	0.9891	0.97287	ENG/FGFR2/GATA4/HEY1/MYBPC3/NKX2-5/NOTCH1/NRG1/TNNC1	9	BP
GO:1903845	negative regulation of cellular response to transforming growth factor [illegible]	9/2734	72/17381	0.81897	0.9891	0.97287	FAM89B/HSPA5/HTRA3/PBLD/SMAD6/TGFB1I1/VASN/WFIKKN2/WNT1	9	BP
GO:0000098	sulfur amino acid catabolic	1/2734	10/17381	0.81948	0.9891	0.97287	BLMH	1	BP
GO:0001553	luteinization	1/2734	10/17381	0.81948	0.9891	0.97287	NR5A1	1	BP
GO:0001765	membrane raft assembly	1/2734	10/17381	0.81948	0.9891	0.97287	CAV3	1	BP

GO:0002182	cytoplasmic translational elongation	1/2734	10/17381	0.81948	0.9891	0.97287	DPH1	1	BP
GO:0002328	pro-B cell differentiation	1/2734	10/17381	0.81948	0.9891	0.97287	NOTCH1	1	BP
GO:0002475	antigen processing and presentation via	1/2734	10/17381	0.81948	0.9891	0.97287	HLA-E	1	BP
GO:0002524	hypersensitivity	1/2734	10/17381	0.81948	0.9891	0.97287	IL20RB	1	BP
GO:0002638	negative regulation of immunoglobuli	1/2734	10/17381	0.81948	0.9891	0.97287	BCL6	1	BP
GO:0002713	negative regulation of B cell mediated	1/2734	10/17381	0.81948	0.9891	0.97287	BCL6	1	BP
GO:0002890	negative regulation of immunoglobulin mediated	1/2734	10/17381	0.81948	0.9891	0.97287	BCL6	1	BP
GO:0002923	regulation of humoral immune response mediated by	1/2734	10/17381	0.81948	0.9891	0.97287	LTA	1	BP
GO:0002934	desmosome organization	1/2734	10/17381	0.81948	0.9891	0.97287	PKP3	1	BP
GO:0003263	cardioblast proliferation	1/2734	10/17381	0.81948	0.9891	0.97287	EYA1	1	BP
GO:0005984	disaccharide metabolic	1/2734	10/17381	0.81948	0.9891	0.97287	MGAM	1	BP

GO:0006307	DNA dealkylation involved in	1/2734	10/17381	0.81948	0.9891	0.97287	ALKBH5	1	BP
GO:0006828	manganese ion transport	1/2734	10/17381	0.81948	0.9891	0.97287	SLC11A1	1	BP
GO:0007016	cytoskeletal anchoring at plasma	1/2734	10/17381	0.81948	0.9891	0.97287	TLN1	1	BP
GO:0007191	adenylate cyclase-activating dopamine	1/2734	10/17381	0.81948	0.9891	0.97287	OPRM1	1	BP
GO:0008343	adult feeding behavior	1/2734	10/17381	0.81948	0.9891	0.97287	DMBX1	1	BP
GO:0009071	serine family amino acid catabolic	1/2734	10/17381	0.81948	0.9891	0.97287	THNSL2	1	BP
GO:0010838	positive regulation of keratinocyte	1/2734	10/17381	0.81948	0.9891	0.97287	TGM1	1	BP
GO:0014842	regulation of skeletal muscle satellite cell proliferation	1/2734	10/17381	0.81948	0.9891	0.97287	PPARD	1	BP
GO:0018202	peptidyl-histidine	1/2734	10/17381	0.81948	0.9891	0.97287	DPH1	1	BP
GO:0019336	phenol-containing compound	1/2734	10/17381	0.81948	0.9891	0.97287	LRTOMT	1	BP

GO:0021892	cerebral cortex GABAergic interneuron differentiation	1/2734	10/17381	0.81948	0.9891	0.97287	DRD2	1	BP
GO:0031652	positive regulation of	1/2734	10/17381	0.81948	0.9891	0.97287	PTGER3	1	BP
GO:0031848	protection from non-homologous end joining at	1/2734	10/17381	0.81948	0.9891	0.97287	ACD	1	BP
GO:0032070	regulation of deoxyribonuclease activity	1/2734	10/17381	0.81948	0.9891	0.97287	PRKCD	1	BP
GO:0032275	luteinizing hormone	1/2734	10/17381	0.81948	0.9891	0.97287	TACR2	1	BP
GO:0032836	glomerular basement membrane development	1/2734	10/17381	0.81948	0.9891	0.97287	MYO1E	1	BP
GO:0032905	transforming growth factor beta1	1/2734	10/17381	0.81948	0.9891	0.97287	FURIN	1	BP
GO:0032908	regulation of transforming growth factor beta1	1/2734	10/17381	0.81948	0.9891	0.97287	FURIN	1	BP
GO:0034770	histone H4-K20 methylation	1/2734	10/17381	0.81948	0.9891	0.97287	BRCA1	1	BP
GO:0035247	peptidyl-arginine omega-N-methylation	1/2734	10/17381	0.81948	0.9891	0.97287	NDUFAF7	1	BP

GO:0035646	endosome to melanosome transport	1/2734	10/17381	0.81948	0.9891	0.97287	CD63	1	BP
GO:0035768	endothelial cell chemotaxis to fibroblast growth factor	1/2734	10/17381	0.81948	0.9891	0.97287	MIR16-1	1	BP
GO:0039530	MDA-5 signaling	1/2734	10/17381	0.81948	0.9891	0.97287	DHX58	1	BP
GO:0042759	long-chain fatty acid biosynthetic	1/2734	10/17381	0.81948	0.9891	0.97287	ACOT7	1	BP
GO:0043144	snoRNA processing	1/2734	10/17381	0.81948	0.9891	0.97287	EXOSC2	1	BP
GO:0043485	endosome to pigment granule transport	1/2734	10/17381	0.81948	0.9891	0.97287	CD63	1	BP
GO:0044557	relaxation of smooth muscle	1/2734	10/17381	0.81948	0.9891	0.97287	MIR153-1	1	BP
GO:0044650	adhesion of symbiont to host cell	1/2734	10/17381	0.81948	0.9891	0.97287	CD81	1	BP
GO:0045080	positive regulation of chemokine biosynthetic	1/2734	10/17381	0.81948	0.9891	0.97287	EGR1	1	BP
GO:0045602	negative regulation of endothelial cell	1/2734	10/17381	0.81948	0.9891	0.97287	XDH	1	BP

GO:0045837	negative regulation of membrane	1/2734	10/17381	0.81948	0.9891	0.97287	PRELID1	1	BP
GO:0046085	adenosine metabolic	1/2734	10/17381	0.81948	0.9891	0.97287	ACPP	1	BP
GO:0046838	phosphorylated carbohydrate dephosphorylati	1/2734	10/17381	0.81948	0.9891	0.97287	INPP5E	1	BP
GO:0048262	determination of dorsal/ventral asymmetry	1/2734	10/17381	0.81948	0.9891	0.97287	NBL1	1	BP
GO:0048304	positive regulation of isotype switching to	1/2734	10/17381	0.81948	0.9891	0.97287	PAXIP1	1	BP
GO:0048715	negative regulation of oligodendrocyte	1/2734	10/17381	0.81948	0.9891	0.97287	NOTCH1	1	BP
GO:0048757	pigment granule maturation	1/2734	10/17381	0.81948	0.9891	0.97287	CD63	1	BP
GO:0051132	NK T cell activation	1/2734	10/17381	0.81948	0.9891	0.97287	IL18	1	BP
GO:0051292	nuclear pore complex	1/2734	10/17381	0.81948	0.9891	0.97287	NUP98	1	BP
GO:0051547	regulation of keratinocyte migration	1/2734	10/17381	0.81948	0.9891	0.97287	MTOR	1	BP
GO:0051561	positive regulation of mitochondrial calcium ion	1/2734	10/17381	0.81948	0.9891	0.97287	BCAP31	1	BP

GO:0051573	negative regulation of histone H3-K9	1/2734	10/17381	0.81948	0.9891	0.97287	BRCA1	1	BP
GO:0051918	negative regulation of	1/2734	10/17381	0.81948	0.9891	0.97287	SERPINF2	1	BP
GO:0060068	vagina development	1/2734	10/17381	0.81948	0.9891	0.97287	LHX1	1	BP
GO:0060339	negative regulation of type I interferon-mediated	1/2734	10/17381	0.81948	0.9891	0.97287	SCRIB	1	BP
GO:0061072	iris morphogenesis	1/2734	10/17381	0.81948	0.9891	0.97287	FOXE3	1	BP
GO:0061088	regulation of sequestering of zinc ion	1/2734	10/17381	0.81948	0.9891	0.97287	SLC30A3	1	BP
GO:0070544	histone H3-K36 demethylation	1/2734	10/17381	0.81948	0.9891	0.97287	KDM8	1	BP
GO:0070673	response to interleukin-18	1/2734	10/17381	0.81948	0.9891	0.97287	IL18	1	BP
GO:0070857	regulation of bile acid biosynthetic	1/2734	10/17381	0.81948	0.9891	0.97287	FGFR4	1	BP
GO:0071281	cellular response to iron	1/2734	10/17381	0.81948	0.9891	0.97287	TFR2	1	BP
GO:0071436	sodium ion export	1/2734	10/17381	0.81948	0.9891	0.97287	ATP1A1	1	BP
GO:0071474	cellular hyperosmotic response	1/2734	10/17381	0.81948	0.9891	0.97287	AQP1	1	BP

GO:0071803	positive regulation of podosome	1/2734	10/17381	0.81948	0.9891	0.97287	FSCN1	1	BP
GO:0075522	IRES-dependent viral translational	1/2734	10/17381	0.81948	0.9891	0.97287	EIF3B	1	BP
GO:0086070	SA node cell to atrial cardiac muscle cell communication	1/2734	10/17381	0.81948	0.9891	0.97287	CACNA1G	1	BP
GO:0090193	positive regulation of glomerulus	1/2734	10/17381	0.81948	0.9891	0.97287	EGR1	1	BP
GO:0090209	negative regulation of triglyceride	1/2734	10/17381	0.81948	0.9891	0.97287	APOC3	1	BP
GO:0090394	negative regulation of excitatory postsynaptic	1/2734	10/17381	0.81948	0.9891	0.97287	NLGN3	1	BP
GO:0098734	macromolecule depalmitoylatio	1/2734	10/17381	0.81948	0.9891	0.97287	LYPLA2	1	BP
GO:1900247	regulation of cytoplasmic translational elongation	1/2734	10/17381	0.81948	0.9891	0.97287	DPH1	1	BP
GO:1901033	positive regulation of response to reactive oxygen	1/2734	10/17381	0.81948	0.9891	0.97287	ENDOG	1	BP

GO:1901569	fatty acid derivative catabolic	1/2734	10/17381	0.81948	0.9891	0.97287	ACOT7	1	BP
GO:1901626	regulation of postsynaptic membrane organization	1/2734	10/17381	0.81948	0.9891	0.97287	FZD9	1	BP
GO:1902916	positive regulation of protein	1/2734	10/17381	0.81948	0.9891	0.97287	NMI	1	BP
GO:1902969	mitotic DNA replication	1/2734	10/17381	0.81948	0.9891	0.97287	BCL6	1	BP
GO:1903651	positive regulation of cytoplasmic	1/2734	10/17381	0.81948	0.9891	0.97287	PTPN23	1	BP
GO:1904321	response to forskolin	1/2734	10/17381	0.81948	0.9891	0.97287	HDAC8	1	BP
GO:1904322	cellular response to	1/2734	10/17381	0.81948	0.9891	0.97287	HDAC8	1	BP
GO:1904627	response to phorbol 13-acetate 12-myristate	1/2734	10/17381	0.81948	0.9891	0.97287	CDK4	1	BP
GO:1990001	inhibition of cysteine-type endopeptidase activity involved in	1/2734	10/17381	0.81948	0.9891	0.97287	NAIP	1	BP
GO:1990173	protein localization to	1/2734	10/17381	0.81948	0.9891	0.97287	CCT3	1	BP

GO:2000544	regulation of endothelial cell chemotaxis to fibroblast growth factor	1/2734	10/17381	0.81948	0.9891	0.97287	MIR16-1	1	BP
GO:2001054	negative regulation of mesenchymal cell apoptotic	1/2734	10/17381	0.81948	0.9891	0.97287	SHH	1	BP
GO:2001169	regulation of ATP biosynthetic	1/2734	10/17381	0.81948	0.9891	0.97287	PID1	1	BP
GO:2001225	regulation of chloride	1/2734	10/17381	0.81948	0.9891	0.97287	CA7	1	BP
GO:0030815	negative regulation of cAMP	6/2734	50/17381	0.81971	0.9891	0.97287	ADRA2A/CCR2/DRD2/GABBR1/OPRM1/PDE2A	6	BP
GO:0036092	phosphatidylinositol-3-phosphate biosynthetic	6/2734	50/17381	0.81971	0.9891	0.97287	FGF17/FGF3/FGFR2/FGFR4/PTPN11/TLR9	6	BP
GO:0045123	cellular extravasation	6/2734	50/17381	0.81971	0.9891	0.97287	ADAM8/CCL21/CCR2/PODXL2/SELP/THY1	6	BP
GO:0007292	female gamete generation	17/2734	129/17381	0.82034	0.9891	0.97287	ANG/FOSL1/HORMAD1/HSF1/IL4R/INHBB/M1AP/MCM9/MCMDC2/NOS3/PAQR7/PIWIL2/POMZP3/PRKAR1A/PTK2B/RXFP2/SOHLH2	17	BP
GO:0006515	protein quality control for misfolded or incompletely synthesized proteins	3/2734	27/17381	0.82036	0.9891	0.97287	SDF2/TOR1A/YME1L1	3	BP

GO:0006706	steroid catabolic process	3/2734	27/17381	0.82036	0.9891	0.97287	CYP1A2/SCARB1/SCARF1	3	BP
GO:0010874	regulation of cholesterol	3/2734	27/17381	0.82036	0.9891	0.97287	LRP1/NR1H3/SHH	3	BP
GO:0045822	negative regulation of heart	3/2734	27/17381	0.82036	0.9891	0.97287	ADRA1A/ATP1A1/MIR328	3	BP
GO:0048753	pigment granule organization	3/2734	27/17381	0.82036	0.9891	0.97287	DTNBP1/HPS1/KIF13A	3	BP
GO:0060292	long term synaptic	3/2734	27/17381	0.82036	0.9891	0.97287	DGKI/PTK2B/STXBP1	3	BP
GO:0071514	genetic imprinting	3/2734	27/17381	0.82036	0.9891	0.97287	BRCA1/DNMT3A/KCNQ1	3	BP
GO:1904645	response to amyloid-beta	3/2734	27/17381	0.82036	0.9891	0.97287	CACNB1/CDK5/LRP1	3	BP
GO:0061008	hepaticobiliary system development	18/2734	136/17381	0.82039	0.9891	0.97287	ASNS/CCDC40/CYP1A1/E2F8/EGFR/GFER/HLX/LIMS2/NKX2-8/NOTCH1/OTC/PHF2/PKD1/PKM/RARA/RPS6KA1/WNT1/WNT3A	18	BP
GO:0006405	RNA export from nucleus	19/2734	143/17381	0.8206	0.9891	0.97287	ALKBH5/CASC3/CPSF1/CPSF4/DHX38/EIF5A/NOL6/NUP210/NUP98/NXF2/NXF2B/NXF3/RNPS1/SETD2/SMG5/SRSF4/SUPT6H/ZC3H3/ZNF593	19	BP
GO:0048515	spermatid differentiation	19/2734	143/17381	0.8206	0.9891	0.97287	ACRBP/CAPZA3/CCDC136/CFAP157/CIB1/HSPA2/NUP210L/PYGO2/RNASE9/SEPT4/SLC26A6/SPAG16/TARBP2/TBPL1/TCP11/TMEM119/TSSK1B/TT	19	BP
GO:0051028	mRNA transport	20/2734	150/17381	0.82095	0.9891	0.97287	ALKBH5/CASC3/CPSF1/CPSF4/DHX38/EIF5A/HSF1/MYO1C/NUP210/NUP98/NXF2/NXF2B/NXF3/RNPS1/SETD2/SMG5/SRSF4/SUPT6H/XPO7/ZC3H3	20	BP
GO:0014743	regulation of muscle hypertrophy	8/2734	65/17381	0.8227	0.9891	0.97287	AGT/CAMK2D/CAV3/CDK9/LMNA/MIR25/MTOR/TRIM63	8	BP
GO:0046324	regulation of glucose import	8/2734	65/17381	0.8227	0.9891	0.97287	AGT/CAPN10/INS/MZB1/PEA15/PID1/PTPN11/RARRES2	8	BP

GO:0046888	negative regulation of hormone	8/2734	65/17381	0.8227	0.9891	0.97287	ADRA2A/ADRA2B/ADRA2C/DRD2/INHBB/PTPN11/RAB11FIP3/TACR2	8	BP
GO:0051588	regulation of neurotransmitter transport	8/2734	65/17381	0.8227	0.9891	0.97287	DRD2/DTNBP1/GPER1/PNKD/STX1A/STXBP1/TACR2/TOR1A	8	BP
GO:1903313	positive regulation of mRNA	8/2734	65/17381	0.8227	0.9891	0.97287	HMX2/HSF1/LMNTD2/NUP98/POLR2G/PRDX6/SF3B4/SLC39A5	8	BP
GO:0001662	behavioral fear response	4/2734	35/17381	0.82294	0.9891	0.97287	DEAF1/EIF4G1/LYPD1/NEUROD2	4	BP
GO:0002209	behavioral defense	4/2734	35/17381	0.82294	0.9891	0.97287	DEAF1/EIF4G1/LYPD1/NEUROD2	4	BP
GO:0002369	T cell cytokine production	4/2734	35/17381	0.82294	0.9891	0.97287	IL18/IL31RA/SLC11A1/TRAF2	4	BP
GO:0010664	negative regulation of striated muscle cell apoptotic	4/2734	35/17381	0.82294	0.9891	0.97287	AMBRA1/HSF1/ILK/NKX2-5	4	BP
GO:0060045	positive regulation of cardiac muscle	4/2734	35/17381	0.82294	0.9891	0.97287	FGFR2/MIR222/NOTCH1/TBX2	4	BP
GO:0071549	cellular response to dexamethasone	4/2734	35/17381	0.82294	0.9891	0.97287	AQP1/EGFR/EIF4EBP1/SMYD3	4	BP
GO:0086005	ventricular cardiac muscle cell action	4/2734	35/17381	0.82294	0.9891	0.97287	CAV3/KCND3/KCNJ5/KCNQ1	4	BP

GO:1903514	release of sequestered calcium ion into cytosol by endoplasmic reticulum	4/2734	35/17381	0.82294	0.9891	0.97287	CAMK2D/FASLG/GSTM2/TRDN	4	BP
GO:1903523	negative regulation of blood	4/2734	35/17381	0.82294	0.9891	0.97287	ADM/ADRA1A/ATP1A1/MIR328	4	BP
GO:0000028	ribosomal small subunit	2/2734	19/17381	0.82417	0.9891	0.97287	ERAL1/MRPS11	2	BP
GO:0007096	regulation of exit from	2/2734	19/17381	0.82417	0.9891	0.97287	CDC14A/PPP1R9B	2	BP
GO:0007097	nuclear migration	2/2734	19/17381	0.82417	0.9891	0.97287	DOCK7/FHOD1	2	BP
GO:0010523	negative regulation of calcium ion transport into	2/2734	19/17381	0.82417	0.9891	0.97287	GSTM2/TRDN	2	BP
GO:0010612	regulation of cardiac muscle adaptation	2/2734	19/17381	0.82417	0.9891	0.97287	LMNA/MIR25	2	BP
GO:0015800	acidic amino acid transport	2/2734	19/17381	0.82417	0.9891	0.97287	SLC1A7/SLC25A22	2	BP
GO:0031649	heat generation	2/2734	19/17381	0.82417	0.9891	0.97287	IL1RN/PTGER3	2	BP
GO:0032682	negative regulation of chemokine	2/2734	19/17381	0.82417	0.9891	0.97287	F2RL1/LGALS9	2	BP

GO:0034643	establishment of mitochondrion localization, microtubule-mediated	2/2734	19/17381	0.82417	0.9891	0.97287	RHOT2/UXT	2	BP
GO:0034755	iron ion transmembrane transport	2/2734	19/17381	0.82417	0.9891	0.97287	STEAP2/TFR2	2	BP
GO:0035743	CD4-positive, alpha-beta T cell cytokine production	2/2734	19/17381	0.82417	0.9891	0.97287	IL18/IL31RA	2	BP
GO:0036303	lymph vessel morphogenesis	2/2734	19/17381	0.82417	0.9891	0.97287	FLT4/PKD1	2	BP
GO:0042053	regulation of dopamine metabolic	2/2734	19/17381	0.82417	0.9891	0.97287	ITGAM/PNKD	2	BP
GO:0042069	regulation of catecholamine metabolic	2/2734	19/17381	0.82417	0.9891	0.97287	ITGAM/PNKD	2	BP
GO:0042454	ribonucleoside catabolic	2/2734	19/17381	0.82417	0.9891	0.97287	CDA/NUDT18	2	BP
GO:0043090	amino acid import	2/2734	19/17381	0.82417	0.9891	0.97287	AGT/SLC11A1	2	BP
GO:0045649	regulation of macrophage differentiation	2/2734	19/17381	0.82417	0.9891	0.97287	C1QC/CSF1	2	BP
GO:0047497	mitochondrion transport along microtubule	2/2734	19/17381	0.82417	0.9891	0.97287	RHOT2/UXT	2	BP

GO:0061050	regulation of cell growth involved in cardiac muscle	2/2734	19/17381	0.82417	0.9891	0.97287	CAV3/MTOR	2	BP
GO:0070987	error-free translesion synthesis	2/2734	19/17381	0.82417	0.9891	0.97287	RFC1/RFC2	2	BP
GO:0072311	glomerular epithelial cell differentiation	2/2734	19/17381	0.82417	0.9891	0.97287	BMP4/MYO1E	2	BP
GO:1901673	regulation of mitotic spindle assembly	2/2734	19/17381	0.82417	0.9891	0.97287	CHMP1A/CHMP4C	2	BP
GO:1903242	regulation of cardiac muscle hypertrophy in response to	2/2734	19/17381	0.82417	0.9891	0.97287	LMNA/MIR25	2	BP
GO:1903861	positive regulation of dendrite	2/2734	19/17381	0.82417	0.9891	0.97287	CPNE5/CPNE9	2	BP
GO:1905939	regulation of gonad development	2/2734	19/17381	0.82417	0.9891	0.97287	NR5A1/NUPR1	2	BP
GO:0006835	dicarboxylic acid transport	11/2734	87/17381	0.8257	0.9891	0.97287	CPLX1/FOLR2/SLC13A2/SLC1A7/SLC25A22/SLC26A1/SLC26A10/SLC26A6/STX1A/STXBP1/TRH	11	BP
GO:0030520	intracellular estrogen receptor	7/2734	58/17381	0.82765	0.9891	0.97287	AXIN1/BRCA1/CRIPAK/DDX54/ESR2/TADA3/TAF7	7	BP

GO:0006470	protein dephosphorylation	35/2734	254/17381	0.82773	0.9891	0.97287	CDC14A/CTDSP1/DRD2/DUSP2/DUSP21/DUSP26/DUSP5/EYA1/GBA/IKBKB/ILKAP/LCK/MTMR1/MTMR14/NSMF/PDGFRB/PGAM5/PLPP1/PPA2/PPEF1/PPP1R1B/PPP2R2B/PPP2R3B/PRKCD/PTP4A3/PTPN11/PTPN23/PTPN7/PTPRF/PTPRN2/PTPRQ/PTPRU/STYXL1/TIPRL/TNS2	35	BP
GO:0014047	glutamate secretion	5/2734	43/17381	0.82805	0.9891	0.97287	CPLX1/SLC1A7/STX1A/STXBP1/TRH	5	BP
GO:0042982	amyloid precursor protein	5/2734	43/17381	0.82805	0.9891	0.97287	ACHE/APH1A/CLU/EFNA1/EFNA3	5	BP
GO:0043268	positive regulation of potassium ion	5/2734	43/17381	0.82805	0.9891	0.97287	ADRA2A/AMIGO1/FLNA/KCNQ1/NPPA	5	BP
GO:0046326	positive regulation of	5/2734	43/17381	0.82805	0.9891	0.97287	AGT/CAPN10/INS/PTPN11/RARRES2	5	BP
GO:0000288	nuclear-transcribed mRNA catabolic process, deadenylation-	10/2734	80/17381	0.82814	0.9891	0.97287	EIF4B/EIF4G1/EXOSC2/LSM1/MIRLET7A2/NT5C3B/PAN2/PATL1/PNLDC1/POLR2G	10	BP
GO:1901861	regulation of muscle tissue development	19/2734	144/17381	0.82956	0.9891	0.97287	ARRB2/BMP4/CAV3/FGF3/FGFR2/GPC1/LUC7L/MIR222/MIR25/MTOR/MYOD1/NKX2-5/NOTCH1/NRG1/SHH/TBX2/TSC22D3/USP19/WNT3A	19	BP
GO:0000910	cytokinesis	18/2734	137/17381	0.82957	0.9891	0.97287	ALKBH4/ANKRD53/BIN3/CFL1/CHMP4C/CXCR5/DRD2/E2F8/KIF13A/KIF23/KIF4A/MYO19/OPN1MW/RAB11FIP3/SETD2/SSTR5/UNC119/ZFYVE26	18	BP
GO:0043488	regulation of mRNA stability	20/2734	151/17381	0.8297	0.9891	0.97287	APEX1/EIF4G1/EXOSC2/HSPB1/MTOR/PCBP4/PRKCD/PSMB11/PSMB6/PSMB7/PSMB8/PSMC3/PSMD13/PSMD3/PSMD5/PSMD7/SCGB1A1/SLC11A1/	20	BP
GO:0000018	regulation of DNA	9/2734	73/17381	0.83133	0.9891	0.97287	BCL6/CLCF1/PAXIP1/PRDM9/RECQL5/SETD2/SUPT6H/TNFSF13/UBE2B	9	BP
GO:0060420	regulation of heart growth	9/2734	73/17381	0.83133	0.9891	0.97287	ACACB/CAV3/FGFR2/MIR222/MIR25/MTOR/NKX2-5/NOTCH1/TBX2	9	BP

GO:0035601	protein deacylation	14/2734	109/17381	0.83168	0.9891	0.97287	BCL6/BRMS1/CAMK2D/CCAR2/CTBP1/FNTA/HDAC11/HDAC8/LYPLA2/MSL3/MTA1/PER1/TADA3/ZNHIT1	14	BP
GO:0046545	development of primary female sexual characteristics	13/2734	102/17381	0.83293	0.9891	0.97287	ANG/ARRB2/EIF2B2/EIF2B5/FANCA/IDH1/INHBB/LFNG/NOS3/NR5A1/NUPR1/VGF/ZFP42	13	BP
GO:0010171	body morphogenesis	6/2734	51/17381	0.83423	0.9891	0.97287	COL1A1/CSRNP1/MMP2/PTPN11/SCX/STRA6	6	BP
GO:0032890	regulation of organic acid transport	6/2734	51/17381	0.83423	0.9891	0.97287	AGT/NCOR1/OSR1/STXBP1/SV2A/TRH	6	BP
GO:0071479	cellular response to ionizing	8/2734	66/17381	0.83546	0.9891	0.97287	CRYAB/EGR1/HRAS/HSF1/HSPA5/INO80/NOX4/TMEM109	8	BP
GO:0030316	osteoclast differentiation	11/2734	88/17381	0.83674	0.9891	0.97287	BGLAP/CSF1/CSF1R/EFNA4/ESRRA/FAM213A/FARP2/GLO1/INPP5D/MTOR/TCTA	11	BP
GO:0009615	response to virus	43/2734	310/17381	0.83785	0.9891	0.97287	ABCF3/AIMP1/AP1S1/APOB/BAD/CARD9/CCL19/CCL5/CFL1/CLU/CYP1A1/DHX58/EIF2AK4/F2RL1/FOSL1/GBF1/HMGA1/HSPB1/IFIT2/IFIT3/IFITM2/IFNA5/IKBKB/IRF5/ISG15/LGALS9/MAP3K14/MICB/NLRX1/NPC2/PARP9/POLR3D/SETD2/SPACA3/SPON2/STAT1/STAT2/TARBP2/TBK1/TLR8/TRAF3	43	BP
GO:0035725	sodium ion transmembrane transport	20/2734	152/17381	0.83813	0.9891	0.97287	ASIC2/ASIC3/ATP1A1/CACNA1G/CACNA1H/CAMK2D/CAV3/COMMD1/HCN3/OSR1/SCN11A/SCNN1A/SLC20A1/SLC20A2/SLC34A1/SLC4A9/SLC9A3/SLC9A5/TRPM5/WNK2	20	BP
GO:0007286	spermatid development	18/2734	138/17381	0.83839	0.9891	0.97287	ACRBP/CAPZA3/CCDC136/CFAP157/CIB1/HSPA2/NUP210L/PYGO2/RNASE9/SEPT4/SLC26A6/SPAG16/TARBP2/TBPL1/TCP11/TSSK1B/TTLL5/UBE2	18	BP
GO:0006338	chromatin remodeling	22/2734	166/17381	0.83842	0.9891	0.97287	ANP32B/BAZ1B/CDKN2A/CENPN/CHD8/HILS1/HIST1H4F/HIST2H4A/HIST2H4B/HJURP/HMGA1/INO80/KAT2A/KDM6B/MSL3/PADI4/RSF1/SMARCD2/SUPT6H/TNRC18/TTF1/ZNHIT1	22	BP
GO:0060968	regulation of gene silencing	17/2734	131/17381	0.83878	0.9891	0.97287	BMP4/DND1/EGFR/EIF4G1/HIST1H4F/HIST2H4A/HIST2H4B/HMGA1/MYCN/NCOR1/NCOR2/NUP210/NUP98/PHF2/POLR2G/POLR2L/ZC3H10	17	BP

GO:0010390	histone monoubiquitina	3/2734	28/17381	0.8393	0.9891	0.97287	DDB1/DDB2/PCGF2	3	BP
GO:0014046	dopamine secretion	3/2734	28/17381	0.8393	0.9891	0.97287	CHRNA6/DRD2/DTNBP1	3	BP
GO:0014059	regulation of dopamine secretion	3/2734	28/17381	0.8393	0.9891	0.97287	CHRNA6/DRD2/DTNBP1	3	BP
GO:0042036	negative regulation of cytokine biosynthetic	3/2734	28/17381	0.8393	0.9891	0.97287	INHBB/INPP5D/NMI	3	BP
GO:0045880	positive regulation of smoothened	3/2734	28/17381	0.8393	0.9891	0.97287	POR/SHH/SMO	3	BP
GO:0055094	response to lipoprotein	3/2734	28/17381	0.8393	0.9891	0.97287	CD81/CDH13/MIR92A2	3	BP
GO:0072606	interleukin-8 secretion	3/2734	28/17381	0.8393	0.9891	0.97287	CD58/F2RL1/LGALS9	3	BP
GO:0097720	calcineurin-mediated	3/2734	28/17381	0.8393	0.9891	0.97287	CIB1/NR5A1/NRG1	3	BP
GO:1901380	negative regulation of potassium ion transmembrane	3/2734	28/17381	0.8393	0.9891	0.97287	CAV3/MIR153-1/MIR212	3	BP
GO:1903427	negative regulation of reactive oxygen species biosynthetic	3/2734	28/17381	0.8393	0.9891	0.97287	INS/MIR92A2/TRAP1	3	BP
GO:0008645	hexose transport	16/2734	124/17381	0.83939	0.9891	0.97287	AGT/CAPN10/INS/MZB1/PEA15/PID1/PLA2G1B/PPARD/PTPN11/RARRES2/SLC2A4/SLC2A6/SLC2A8/SLC45A1/SLC50A1/SLC5A2	16	BP

GO:0045761	regulation of adenylate cyclase activity	10/2734	81/17381	0.83951	0.9891	0.97287	ADRA2A/AVPR2/CCR2/CRHR1/DRD2/GABBR1/GPER1/OPRM1/RAF1/WFS1	10	BP
GO:0006739	NADP metabolic	4/2734	36/17381	0.8397	0.9891	0.97287	GCK/IDH1/IDH2/PGAM4	4	BP
GO:0032008	positive regulation of	4/2734	36/17381	0.8397	0.9891	0.97287	LARS/MLST8/RRAGC/TELO2	4	BP
GO:0032205	negative regulation of telomere	4/2734	36/17381	0.8397	0.9891	0.97287	ACD/EXOSC10/PIF1/TINF2	4	BP
GO:0046460	neutral lipid biosynthetic process	4/2734	36/17381	0.8397	0.9891	0.97287	ANG/DGAT1/NR1H3/SCARB1	4	BP
GO:0046463	acylglycerol biosynthetic process	4/2734	36/17381	0.8397	0.9891	0.97287	ANG/DGAT1/NR1H3/SCARB1	4	BP
GO:0048016	inositol phosphate-	4/2734	36/17381	0.8397	0.9891	0.97287	CIB1/EDN2/ITPR3/NRG1	4	BP
GO:0055026	negative regulation of cardiac muscle	4/2734	36/17381	0.8397	0.9891	0.97287	CAV3/FGF3/MIR222/MIR25	4	BP
GO:1901381	positive regulation of potassium ion transmembrane	4/2734	36/17381	0.8397	0.9891	0.97287	AMIGO1/FLNA/KCNQ1/NPPA	4	BP
GO:1904358	positive regulation of telomere maintenance via telomere	4/2734	36/17381	0.8397	0.9891	0.97287	ACD/CCT3/MAPK15/MAPK3	4	BP

GO:0007623	circadian rhythm	26/2734	194/17381	0.84016	0.9891	0.97287	CCAR2/CDK4/CIART/CLDN4/CREM/DDC/DRD2/EGFR/MTA1/MTNR1B/NAGLU/NCOR1/NONO/NPAS2/NR1H3/OPN4/PASD1/PER1/PSPC1/RAI1/RBM4/RORC/SUV39H1/TH/TIMELESS/USP2	26	BP
GO:0046660	female sex differentiation	15/2734	117/17381	0.84025	0.9891	0.97287	ANG/ARRB2/EIF2B2/EIF2B5/FANCA/IDH1/INHBB/LFNG/LHX1/NOS3/NR5A1/NUPR1/STRA6/VGF/ZFP42	15	BP
GO:0051966	regulation of synaptic transmission, glutamatergic	7/2734	59/17381	0.84081	0.9891	0.97287	DGKI/DISC1/DRD2/EGFR/NLGN3/PTK2B/STXBP1	7	BP
GO:0050727	regulation of inflammatory response	54/2734	385/17381	0.84127	0.9891	0.97287	ADAM8/AGT/AHSG/BCL6/C1QB/C1QC/C2/C3AR1/C8A/CASP1/CASP5/CCL5/CCR2/CD6/CD81/CFH/CHID1/CLU/CPN2/CUEDC2/DUOXA1/EGFR/FANCA/GBA/GPER1/GPR17/GPX1/IDO1/IL18/IL20RB/INS/KARS/KLKB1/LTA/MCPH1/MIR221/MIR222/MIR92A2/NLRP1/NLRX1/NR1H3/OTUD7A/PDCD4/PDE2A/PER1/PPARD/PRKCD/PTGER3/SCGB1A1/SEMA7A/SHARPIN/TLR9/T	54	BP
GO:0098732	macromolecule deacylation	14/2734	110/17381	0.8414	0.9891	0.97287	BCL6/BRMS1/CAMK2D/CCAR2/CTBP1/FNTA/HDAC11/HDAC8/LYPLA2/MSL3/MTA1/PER1/TADA3/ZNHIT1	14	BP
GO:0017015	regulation of transforming growth factor beta receptor signaling	13/2734	103/17381	0.8429	0.9891	0.97287	CAV3/ENG/FAM89B/HSPA5/HTRA3/MEN1/PBLD/SMAD6/TGFB1I1/VASN/WFIKKN2/WNT1/ZNF703	13	BP
GO:0000731	DNA synthesis involved in DNA repair	9/2734	74/17381	0.84302	0.9891	0.97287	BRCA1/EXO1/FAAP20/ISG15/POLD4/POLE/RFC1/RFC2/XRCC3	9	BP
GO:0034644	cellular response to UV	9/2734	74/17381	0.84302	0.9891	0.97287	AQP1/CRIP1/DDB1/DDB2/EIF2AK4/INO80/NOC2L/TAF1/XPC	9	BP
GO:0007077	mitotic nuclear envelope disassembly	5/2734	44/17381	0.84306	0.9891	0.97287	CCNB2/EMD/LMNA/NUP210/NUP98	5	BP
GO:0031343	positive regulation of	5/2734	44/17381	0.84306	0.9891	0.97287	BAD/F2RL1/GAPDH/HLA-E/NCR3	5	BP

GO:0032309	icosanoid secretion	5/2734	44/17381	0.84306	0.9891	0.97287	ABCC4/DRD2/PLA2G1B/PLA2G2F/PLA2G5	5	BP
GO:0055023	positive regulation of cardiac muscle	5/2734	44/17381	0.84306	0.9891	0.97287	FGFR2/MIR222/MTOR/NOTCH1/TBX2	5	BP
GO:0000423	mitophagy	2/2734	20/17381	0.84576	0.9891	0.97287	AMBRA1/MFN2	2	BP
GO:0006085	acetyl-CoA biosynthetic process	2/2734	20/17381	0.84576	0.9891	0.97287	MPC1/PDHB	2	BP
GO:0006862	nucleotide transport	2/2734	20/17381	0.84576	0.9891	0.97287	SLC25A25/SLC25A5	2	BP
GO:0007289	spermatid nucleus	2/2734	20/17381	0.84576	0.9891	0.97287	PYGO2/TBPL1	2	BP
GO:0007620	copulation	2/2734	20/17381	0.84576	0.9891	0.97287	EDDM3A/TACR1	2	BP
GO:0019400	alditol metabolic	2/2734	20/17381	0.84576	0.9891	0.97287	COQ2/GALK1	2	BP
GO:0031498	chromatin disassembly	2/2734	20/17381	0.84576	0.9891	0.97287	HMGA1/SMARCD2	2	BP
GO:0032373	positive regulation of	2/2734	20/17381	0.84576	0.9891	0.97287	LRP1/NR1H3	2	BP
GO:0032376	positive regulation of cholesterol	2/2734	20/17381	0.84576	0.9891	0.97287	LRP1/NR1H3	2	BP
GO:0032986	protein-DNA complex disassembly	2/2734	20/17381	0.84576	0.9891	0.97287	HMGA1/SMARCD2	2	BP

GO:0036120	cellular response to platelet-derived growth factor	2/2734	20/17381	0.84576	0.9891	0.97287	CORO1B/PDGFRB	2	BP
GO:0039529	RIG-I signaling pathway	2/2734	20/17381	0.84576	0.9891	0.97287	DHX58/NLRX1	2	BP
GO:0039531	regulation of viral-induced cytoplasmic pattern recognition receptor [illegible]	2/2734	20/17381	0.84576	0.9891	0.97287	DHX58/NLRX1	2	BP
GO:0042276	error-prone translesion synthesis	2/2734	20/17381	0.84576	0.9891	0.97287	RFC1/RFC2	2	BP
GO:0042403	thyroid hormone	2/2734	20/17381	0.84576	0.9891	0.97287	CRYM/DUOXA1	2	BP
GO:0048025	negative regulation of mRNA splicing,	2/2734	20/17381	0.84576	0.9891	0.97287	RNPS1/SRSF4	2	BP
GO:0050849	negative regulation of calcium-	2/2734	20/17381	0.84576	0.9891	0.97287	GSTM2/TRDN	2	BP
GO:0051654	establishment of mitochondrion localization	2/2734	20/17381	0.84576	0.9891	0.97287	RHOT2/UXT	2	BP
GO:0060039	pericardium development	2/2734	20/17381	0.84576	0.9891	0.97287	NOTCH1/SETD2	2	BP
GO:0060216	definitive hemopoiesis	2/2734	20/17381	0.84576	0.9891	0.97287	HOXB3/HOXB4	2	BP

GO:0071305	cellular response to	2/2734	20/17381	0.84576	0.9891	0.97287	BGLAP/TNC	2	BP
GO:0071711	basement membrane organization	2/2734	20/17381	0.84576	0.9891	0.97287	LAMC2/PLOD3	2	BP
GO:1901889	negative regulation of cell junction	2/2734	20/17381	0.84576	0.9891	0.97287	BCAS3/IKBKB	2	BP
GO:1902884	positive regulation of response to	2/2734	20/17381	0.84576	0.9891	0.97287	ENDOG/MMP3	2	BP
GO:1902895	positive regulation of pri-miRNA transcription from RNA	2/2734	20/17381	0.84576	0.9891	0.97287	SMAD6/SPI1	2	BP
GO:1990126	retrograde transport, endosome to plasma	2/2734	20/17381	0.84576	0.9891	0.97287	GRIPAP1/SCRIB	2	BP
GO:2000380	regulation of mesoderm development	2/2734	20/17381	0.84576	0.9891	0.97287	SHH/WNT3A	2	BP
GO:0043487	regulation of RNA stability	21/2734	160/17381	0.84616	0.9891	0.97287	APEX1/EIF4G1/EXOSC2/HSPB1/MTOR/PCBP4/PRKCD/PSMB11/PSMB6/PSMB7/PSMB8/PSMC3/PSMD13/PSMD3/PSMD5/PSMD7/SCGB1A1/SLC11A1/SMG5/TIRAP/TNFSF13	21	BP
GO:0050852	T cell receptor signaling pathway	23/2734	174/17381	0.84636	0.9891	0.97287	CACNA1F/CACNB3/CARD11/CD247/CSK/ELF1/HRAS/IKBKB/INPP5D/LCK/LGALS3/PSMB11/PSMB6/PSMB7/PSMB8/PSMC3/PSMD13/PSMD3/PSMD5/PSMD7/RNF31/THY1/WAS	23	BP
GO:0006334	nucleosome assembly	19/2734	146/17381	0.84648	0.9891	0.97287	ANP32B/CENPN/H1FX/HILS1/HIST1H4F/HIST2H4A/HIST2H4B/HJURP/IPO4/MCM2/NAA60/PADI4/RSF1/SMYD3/TSPY1/TSPY2/TSPY4/TSPY8/TSPYL	19	BP

GO:0036473	cell death in response to oxidative stress	11/2734	89/17381	0.84724	0.9891	0.97287	ENDOG/GPX1/HSPB1/INS/MIR92A2/MMP3/NONO/PRKCD/PSAP/TRAP1/WNT1	11	BP
GO:0048641	regulation of skeletal muscle tissue	6/2734	52/17381	0.84779	0.9891	0.97287	GPC1/MYOD1/SHH/TSC22D3/USP19/WNT3A	6	BP
GO:0051306	mitotic sister chromatid separation	6/2734	52/17381	0.84779	0.9891	0.97287	ANAPC15/BUB1B/DIS3L2/LCMT1/MAD1L1/XRCC3	6	BP
GO:1903018	regulation of glycoprotein metabolic	6/2734	52/17381	0.84779	0.9891	0.97287	CCL19/CCL21/FKTN/GOLGA2/MIR181B1/TINF2	6	BP
GO:0001833	inner cell mass cell	1/2734	11/17381	0.84789	0.9891	0.97287	ZPR1	1	BP
GO:0002664	regulation of T cell tolerance	1/2734	11/17381	0.84789	0.9891	0.97287	IDO1	1	BP
GO:0002863	positive regulation of inflammatory response to	1/2734	11/17381	0.84789	0.9891	0.97287	LTA	1	BP
GO:0003413	chondrocyte differentiation involved in endochondral bone morphogenesis	1/2734	11/17381	0.84789	0.9891	0.97287	SERPINH1	1	BP
GO:0006105	succinate metabolic	1/2734	11/17381	0.84789	0.9891	0.97287	GAD1	1	BP
GO:0006600	creatine metabolic	1/2734	11/17381	0.84789	0.9891	0.97287	SLC6A8	1	BP

GO:0007253	cytoplasmic sequestering of NF-kappaB	1/2734	11/17381	0.84789	0.9891	0.97287	CD27	1	BP
GO:0009642	response to light intensity	1/2734	11/17381	0.84789	0.9891	0.97287	GNAT1	1	BP
GO:0010764	negative regulation of fibroblast	1/2734	11/17381	0.84789	0.9891	0.97287	ARHGAP4	1	BP
GO:0010998	regulation of translational initiation by eIF2 alpha phosphorylation	1/2734	11/17381	0.84789	0.9891	0.97287	EIF2AK4	1	BP
GO:0014841	skeletal muscle satellite cell proliferation	1/2734	11/17381	0.84789	0.9891	0.97287	PPARD	1	BP
GO:0014870	response to muscle	1/2734	11/17381	0.84789	0.9891	0.97287	PKM	1	BP
GO:0031125	rRNA 3'-end processing	1/2734	11/17381	0.84789	0.9891	0.97287	EXOSC2	1	BP
GO:0031953	negative regulation of protein autophosphoryl	1/2734	11/17381	0.84789	0.9891	0.97287	ENG	1	BP
GO:0032754	positive regulation of interleukin-5	1/2734	11/17381	0.84789	0.9891	0.97287	RARA	1	BP
GO:0032769	negative regulation of monooxygenase	1/2734	11/17381	0.84789	0.9891	0.97287	CAV3	1	BP

GO:0033235	positive regulation of protein	1/2734	11/17381	0.84789	0.9891	0.97287	RWDD3	1	BP
GO:0033523	histone H2B ubiquitination	1/2734	11/17381	0.84789	0.9891	0.97287	CDK9	1	BP
GO:0034776	response to histamine	1/2734	11/17381	0.84789	0.9891	0.97287	DRD2	1	BP
GO:0035461	vitamin transmembrane transport	1/2734	11/17381	0.84789	0.9891	0.97287	SCARB1	1	BP
GO:0035747	natural killer cell chemotaxis	1/2734	11/17381	0.84789	0.9891	0.97287	CCL5	1	BP
GO:0040015	negative regulation of multicellular	1/2734	11/17381	0.84789	0.9891	0.97287	RAI1	1	BP
GO:0042532	negative regulation of tyrosine phosphorylation	1/2734	11/17381	0.84789	0.9891	0.97287	INPP5F	1	BP
GO:0043374	CD8-positive, alpha-beta T cell	1/2734	11/17381	0.84789	0.9891	0.97287	PSMB11	1	BP
GO:0043619	regulation of transcription from RNA polymerase II promoter in response to [illegible]	1/2734	11/17381	0.84789	0.9891	0.97287	ANKRD2	1	BP
GO:0044848	biological phase	1/2734	11/17381	0.84789	0.9891	0.97287	SPINK5	1	BP

GO:0045793	positive regulation of	1/2734	11/17381	0.84789	0.9891	0.97287	CDK4	1	BP
GO:0045899	positive regulation of RNA polymerase II transcriptional preinitiation	1/2734	11/17381	0.84789	0.9891	0.97287	PSMC3	1	BP
GO:0046133	pyrimidine ribonucleoside catabolic	1/2734	11/17381	0.84789	0.9891	0.97287	CDA	1	BP
GO:0046185	aldehyde catabolic	1/2734	11/17381	0.84789	0.9891	0.97287	PNKD	1	BP
GO:0048742	regulation of skeletal muscle fiber	1/2734	11/17381	0.84789	0.9891	0.97287	MYOD1	1	BP
GO:0050910	detection of mechanical stimulus involved in sensory perception of	1/2734	11/17381	0.84789	0.9891	0.97287	LHFPL5	1	BP
GO:0051315	attachment of mitotic spindle microtubules to kinetochore	1/2734	11/17381	0.84789	0.9891	0.97287	MAD1L1	1	BP
GO:0051532	regulation of NFAT protein import into	1/2734	11/17381	0.84789	0.9891	0.97287	MTOR	1	BP
GO:0060019	radial glial cell differentiation	1/2734	11/17381	0.84789	0.9891	0.97287	EMX1	1	BP

GO:0060033	anatomical structure regression	1/2734	11/17381	0.84789	0.9891	0.97287	SPI1	1	BP
GO:0060099	regulation of phagocytosis, engulfment	1/2734	11/17381	0.84789	0.9891	0.97287	F2RL1	1	BP
GO:0060211	regulation of nuclear-transcribed mRNA poly(A) tail shortening	1/2734	11/17381	0.84789	0.9891	0.97287	POLR2G	1	BP
GO:0060213	positive regulation of nuclear-transcribed mRNA poly(A)	1/2734	11/17381	0.84789	0.9891	0.97287	POLR2G	1	BP
GO:0070341	fat cell proliferation	1/2734	11/17381	0.84789	0.9891	0.97287	PID1	1	BP
GO:0070344	regulation of fat cell	1/2734	11/17381	0.84789	0.9891	0.97287	PID1	1	BP
GO:0070601	centromeric sister chromatid cohesion	1/2734	11/17381	0.84789	0.9891	0.97287	BUB1B	1	BP
GO:0071545	inositol phosphate	1/2734	11/17381	0.84789	0.9891	0.97287	INPP5E	1	BP
GO:0072393	microtubule anchoring at microtubule organizing	1/2734	11/17381	0.84789	0.9891	0.97287	CEP19	1	BP
GO:0072672	neutrophil extravasation	1/2734	11/17381	0.84789	0.9891	0.97287	ADAM8	1	BP

GO:0086016	AV node cell action potential	1/2734	11/17381	0.84789	0.9891	0.97287	CACNA1G	1	BP
GO:0086027	AV node cell to bundle of His cell signaling	1/2734	11/17381	0.84789	0.9891	0.97287	CACNA1G	1	BP
GO:0090009	primitive streak formation	1/2734	11/17381	0.84789	0.9891	0.97287	LHX1	1	BP
GO:0090370	negative regulation of cholesterol	1/2734	11/17381	0.84789	0.9891	0.97287	SHH	1	BP
GO:0097090	presynaptic membrane organization	1/2734	11/17381	0.84789	0.9891	0.97287	NLGN3	1	BP
GO:0097202	activation of cysteine-type endopeptidase activity	1/2734	11/17381	0.84789	0.9891	0.97287	BAD	1	BP
GO:1900452	regulation of long term synaptic	1/2734	11/17381	0.84789	0.9891	0.97287	DGKI	1	BP
GO:1901096	regulation of autophagosome maturation	1/2734	11/17381	0.84789	0.9891	0.97287	TBC1D25	1	BP
GO:1902563	regulation of neutrophil activation	1/2734	11/17381	0.84789	0.9891	0.97287	ITGAM	1	BP
GO:1902570	protein localization to	1/2734	11/17381	0.84789	0.9891	0.97287	POLR1A	1	BP

GO:1902894	negative regulation of pri-miRNA transcription from RNA	1/2734	11/17381	0.84789	0.9891	0.97287	PPARD	1	BP
GO:1904424	regulation of GTP binding	1/2734	11/17381	0.84789	0.9891	0.97287	GPSM1	1	BP
GO:1905902	regulation of mesoderm formation	1/2734	11/17381	0.84789	0.9891	0.97287	WNT3A	1	BP
GO:1905941	positive regulation of gonad	1/2734	11/17381	0.84789	0.9891	0.97287	NR5A1	1	BP
GO:2000392	regulation of lamellipodium morphogenesis	1/2734	11/17381	0.84789	0.9891	0.97287	CORO1B	1	BP
GO:0015758	glucose transport	15/2734	118/17381	0.84932	0.9891	0.97287	AGT/CAPN10/INS/MZB1/PEA15/PID1/PLA2G1B/PPARD/PTPN11/RARRES2/SLC2A4/SLC2A6/SLC2A8/SLC45A1/SLC5A2	15	BP
GO:2000117	negative regulation of cysteine-type endopeptidase	14/2734	111/17381	0.85069	0.9891	0.97287	AQP1/ARRB2/CD27/CRYAB/DPEP1/GPX1/HERPUD1/LAMP3/NAIP/NLE1/POR/RAF1/RPS6KA1/SFN	14	BP
GO:0033047	regulation of mitotic sister chromatid segregation	7/2734	60/17381	0.85316	0.9891	0.97287	ANAPC15/BUB1B/LCMT1/MAD1L1/NUMA1/SLF2/XRCC3	7	BP
GO:0045669	positive regulation of osteoblast	7/2734	60/17381	0.85316	0.9891	0.97287	BMP4/CLIC1/DDR2/GDF2/ILK/PDLIM7/TMEM119	7	BP

GO:0070646	protein modification by small protein removal	42/2734	306/17381	0.85388	0.9891	0.97287	ARRB2/AXIN1/BRCA1/BRCC3/COPS6/DDB2/FOXO4/HCFC1/INO80/KAT2A/NEDD8/NFRKB/NOD1/OTUB1/OTUD5/OTUD6A/OTUD7A/PSMB11/PSMB6/PSMB7/PSMB8/PSMC3/PSMD13/PSMD3/PSMD5/PSMD7/SENP3/SENP6/SHMT2/TADA3/TNIP2/TOR1A/TRAF2/USP19/USP2/USP20/USP27X/USP35/US	42	BP
GO:0002573	myeloid leukocyte differentiation	25/2734	189/17381	0.85401	0.9891	0.97287	BATF2/BGLAP/BMP4/C1QC/CCL19/CD74/CSF1/CSF1R/CSF3/EFNA4/ESRRA/F2RL1/FAM213A/FARP2/GLO1/IL31RA/INPP5D/L3MBTL3/LTBR/MTOR/NKX2-3/NME1/RARA/SPI1/TCTA	25	BP
GO:0034502	protein localization to	9/2734	75/17381	0.85407	0.9891	0.97287	ACD/BUB1B/CHMP7/HDAC8/LEMD2/PARP3/SETD2/SLF2/TINF2	9	BP
GO:0045766	positive regulation of	19/2734	147/17381	0.85445	0.9891	0.97287	ABL1/ADM/AQP1/BRCA1/C3AR1/CIB1/CTSH/ECM1/ENG/GATA4/GDF2/HSPB1/KDR/MIR210/MIR451A/MIR503/NOS3/PIK3R6/PTK2B	19	BP
GO:0048634	regulation of muscle organ development	19/2734	147/17381	0.85445	0.9891	0.97287	ARRB2/BMP4/CAV3/FGF3/FGFR2/GPC1/LUC7L/MIR222/MIR25/MTOR/MYOD1/NKX2-5/NOTCH1/NRG1/SHH/TBX2/TSC22D3/USP19/WNT3A	19	BP
GO:0001676	long-chain fatty acid metabolic process	12/2734	97/17381	0.85456	0.9891	0.97287	ACOT7/CYP1A1/CYP1A2/CYP2W1/DAGLB/ELOVL1/ELOVL5/EPHX2/FADS2/GSTM2/MAPK3/PLA2G4B	12	BP
GO:0043331	response to dsRNA	12/2734	97/17381	0.85456	0.9891	0.97287	BMP4/CARD9/EGFR/IFNA5/MAPK3/MYCN/NCOR1/NCOR2/RALB/TARBP2/TSNAX/ZC3H10	12	BP
GO:0051048	negative regulation of secretion	28/2734	210/17381	0.85505	0.9891	0.97287	ADRA2A/ADRA2B/ADRA2C/AVPR2/CCR2/CD74/CHGA/DRD2/F2RL1/FN1/IDH2/INHBB/INS/LGALS9/NOTCH1/NR1H3/NRG1/OPRM1/PNKD/PTGER3/PTPN11/RAB11FIP3/RAB7A/RHBDF1/SERGEF/TACR2/TLR8/TRH	28	BP
GO:0010453	regulation of cell fate	4/2734	37/17381	0.85511	0.9891	0.97287	BMP4/FGFR2/SPDEF/WNT3A	4	BP
GO:0010613	positive regulation of cardiac muscle	4/2734	37/17381	0.85511	0.9891	0.97287	AGT/CAMK2D/CDK9/MTOR	4	BP
GO:0014742	positive regulation of muscle	4/2734	37/17381	0.85511	0.9891	0.97287	AGT/CAMK2D/CDK9/MTOR	4	BP

GO:0030212	hyaluronan metabolic	4/2734	37/17381	0.85511	0.9891	0.97287	ABCC5/HEXA/ITIH4/ITIH6	4	BP
GO:0042596	fear response	4/2734	37/17381	0.85511	0.9891	0.97287	DEAF1/EIF4G1/LYPD1/NEUROD2	4	BP
GO:0071867	response to monoamine	4/2734	37/17381	0.85511	0.9891	0.97287	ABL1/HCN3/KCNQ1/MAPK3	4	BP
GO:0071869	response to catecholamine	4/2734	37/17381	0.85511	0.9891	0.97287	ABL1/HCN3/KCNQ1/MAPK3	4	BP
GO:1902107	positive regulation of leukocyte	17/2734	133/17381	0.85577	0.9891	0.97287	ADAM8/BAD/BCL6/CCL19/CD27/CD74/CSF1/HLX/IL18/IL4R/INPP5D/LGALS9/PIK3R6/PPP2R3C/RARA/SART1/SHH	17	BP
GO:0000083	regulation of transcription involved in G1/S transition of mitotic cell	3/2734	29/17381	0.85649	0.9891	0.97287	E2F4/HINFP/ZPR1	3	BP
GO:0010837	regulation of keratinocyte proliferation	3/2734	29/17381	0.85649	0.9891	0.97287	SFN/SLURP1/TGM1	3	BP
GO:0016242	negative regulation of macroautophag	3/2734	29/17381	0.85649	0.9891	0.97287	MTOR/POLDIP2/QSOX1	3	BP
GO:0021955	central nervous system neuron axonogenesis	3/2734	29/17381	0.85649	0.9891	0.97287	EPHB3/PHOX2B/TSKU	3	BP
GO:0035666	TRIF-dependent toll-like receptor	3/2734	29/17381	0.85649	0.9891	0.97287	CD300LF/IKBKB/TBK1	3	BP

GO:0048791	calcium ion-regulated exocytosis of neurotransmitte	3/2734	29/17381	0.85649	0.9891	0.97287	DOC2A/DOC2B/SYTL3	3	BP
GO:1901797	negative regulation of signal transduction by	3/2734	29/17381	0.85649	0.9891	0.97287	CD74/MUC1/ZNF385A	3	BP
GO:1902893	regulation of pri-miRNA transcription from RNA polymerase II	3/2734	29/17381	0.85649	0.9891	0.97287	PPARD/SMAD6/SPI1	3	BP
GO:0015749	monosaccharide transport	16/2734	126/17381	0.85675	0.9891	0.97287	AGT/CAPN10/INS/MZB1/PEA15/PID1/PLA2G1B/PPARD/PTPN11/RARRES2/SLC2A4/SLC2A6/SLC2A8/SLC45A1/SLC50A1/SLC5A2	16	BP
GO:0010559	regulation of glycoprotein biosynthetic process	5/2734	45/17381	0.85697	0.9891	0.97287	CCL19/CCL21/FKTN/GOLGA2/TINF2	5	BP
GO:0030071	regulation of mitotic metaphase/anaphase transition	5/2734	45/17381	0.85697	0.9891	0.97287	ANAPC15/BUB1B/LCMT1/MAD1L1/XRCC3	5	BP
GO:2000272	negative regulation of	5/2734	45/17381	0.85697	0.9891	0.97287	GSTM2/LYPD1/MIR212/TRDN/VPS25	5	BP
GO:0006112	energy reserve metabolic	11/2734	90/17381	0.85721	0.9891	0.97287	GCK/GNMT/INS/LEPR/MTOR/PCDH12/PFKM/PHKG1/PHLDA2/PID1/PYGM	11	BP

GO:0030216	keratinocyte differentiation	41/2734	300/17381	0.85843	0.9891	0.97287	C1orf68/CASP3/CTSL/DNASE1L2/FOXN1/FURIN/KRT12/KRT14/KRT16/KRT23/KRT3/KRT32/KRT6A/KRT6C/KRT71/KRT75/KRT76/KRT79/KRT81/KRT84/KRT85/KRTAP17-1/KRTAP4-3/KRTAP5-10/KRTAP5-3/KRTAP5-5/KRTAP5-7/KRTAP5-9/LCE3E/NOTCH1/NUMA1/PKP3/POU3F1/SFN/SHARPIN/SPINK5/SPRR1A/	41	BP
GO:0002088	lens development in camera-type eye	8/2734	68/17381	0.8588	0.9891	0.97287	BMP4/CDK4/CRYAB/FOXE3/MIP/PYGO2/TBC1D32/WNT7A	8	BP
GO:0055021	regulation of cardiac muscle tissue growth	8/2734	68/17381	0.8588	0.9891	0.97287	CAV3/FGFR2/MIR222/MIR25/MTOR/NKX2-5/NOTCH1/TBX2	8	BP
GO:1900006	positive regulation of dendrite	8/2734	68/17381	0.8588	0.9891	0.97287	ANAPC2/DAB2IP/FOXO6/ILK/LLPH/MTOR/NRG1/OBSL1	8	BP
GO:0051101	regulation of DNA binding	14/2734	112/17381	0.85956	0.9891	0.97287	ACD/EDF1/HDAC8/HEY1/HJURP/KAT2A/NME1/RSF1/SMO/TAF1/WFIKKN2/WNT3A/ZNF462/ZNF593	14	BP
GO:0032781	positive regulation of	6/2734	53/17381	0.86043	0.9891	0.97287	DYNC1I1/DYNLL2/HSPA2/MYBPC3/MYL4/PFN1	6	BP
GO:0046928	regulation of neurotransmitter secretion	6/2734	53/17381	0.86043	0.9891	0.97287	DTNBP1/GPER1/PNKD/STX1A/STXBP1/TACR2	6	BP
GO:0002028	regulation of sodium ion transport	10/2734	83/17381	0.86047	0.9891	0.97287	ATP1A1/CAMK2D/CAV3/COMMD1/DRD2/NKX2-5/NOS3/OSR1/PER1/WNK2	10	BP
GO:0002456	T cell mediated immunity	10/2734	83/17381	0.86047	0.9891	0.97287	CTSH/HLA-E/IL18/IL20RB/IL31RA/MICB/NCR3/SLC11A1/TRAF2/WAS	10	BP
GO:0048477	oogenesis	10/2734	83/17381	0.86047	0.9891	0.97287	ANG/HORMAD1/INHBB/MCMDC2/PAQR7/PIWIL2/POMZP3/PTK2B/RXFP2/SOHLH2	10	BP
GO:0048644	muscle organ morphogenesis	10/2734	83/17381	0.86047	0.9891	0.97287	ENG/FGFR2/FZD2/MIR195/MYBPC3/NKX2-5/NOTCH1/NRG1/TCAP/TNNC1	10	BP

GO:0019730	antimicrobial humoral	13/2734	105/17381	0.86147	0.9891	0.97287	ATOX1/CHGA/CLU/FGA/GAPDH/HLA-E/PGC/PLA2G1B/PPP2R3C/RARRES2/SLC11A1/SPINK5/SPON2	13	BP
GO:1903844	regulation of cellular response to transforming growth factor	13/2734	105/17381	0.86147	0.9891	0.97287	CAV3/ENG/FAM89B/HSPA5/HTRA3/MEN1/PBLD/SMAD6/TGFB1I1/VASN/WFIKKN2/WNT1/ZNF703	13	BP
GO:0019827	stem cell population maintenance	20/2734	155/17381	0.86156	0.9891	0.97287	ASCL2/BCL9/CDX2/DIS3L2/LDB1/LSM1/MCPH1/NOTCH1/PADI4/PIWIL2/POLR2G/POLR2L/PRDM14/RAF1/SETD1A/SMO/SPI1/TDGF1/WNT7A/ZHX2	20	BP
GO:0002440	production of molecular mediator of immune	29/2734	218/17381	0.86187	0.9891	0.97287	BCL6/CARD11/CD74/CHGA/CLCF1/CUEDC2/EXO1/F2RL1/HLA-E/IL13/IL18/IL31RA/IL4R/KARS/MAPK3/MZB1/PAXIP1/PGC/PMS2P3/SEMA7A/SLC11A1/SPINK5/SPON2/STX4/SUPT6H/TLR9/TNFRSF4/TNFSF13/TRAF2	29	BP
GO:0019395	fatty acid oxidation	12/2734	98/17381	0.86381	0.9891	0.97287	ACACB/ACAD10/ACADVL/ACOXL/CPT2/CROT/HADHA/HAO2/MTOR/POR/PPARD/TYSND1	12	BP
GO:0005977	glycogen metabolic	9/2734	76/17381	0.86448	0.9891	0.97287	GCK/GNMT/INS/MTOR/PCDH12/PFKM/PHKG1/PHLDA2/PYGM	9	BP
GO:0030101	natural killer cell activation	9/2734	76/17381	0.86448	0.9891	0.97287	CORO1A/HAVCR2/IFNA5/IL18/NCR3/PRDX1/SLAMF7/TUSC2/ULBP2	9	BP
GO:0061515	myeloid cell development	7/2734	61/17381	0.86472	0.9891	0.97287	BCL6/CSF3/HBZ/L3MBTL3/MAEA/PTPN11/ZNF385A	7	BP
GO:0071385	cellular response to glucocorticoid	7/2734	61/17381	0.86472	0.9891	0.97287	AQP1/EGFR/EIF4EBP1/HEY1/MYOD1/SMYD3/SSTR5	7	BP
GO:0007063	regulation of sister chromatid cohesion	2/2734	21/17381	0.86492	0.9891	0.97287	HDAC8/SLF2	2	BP
GO:0007130	synaptonemal complex	2/2734	21/17381	0.86492	0.9891	0.97287	HORMAD1/MCMDC2	2	BP

GO:0010971	positive regulation of G2/M transition of mitotic cell	2/2734	21/17381	0.86492	0.9891	0.97287	CDK4/PHOX2B	2	BP
GO:0021895	cerebral cortex neuron differentiation	2/2734	21/17381	0.86492	0.9891	0.97287	DRD2/EMX1	2	BP
GO:0031290	retinal ganglion cell axon	2/2734	21/17381	0.86492	0.9891	0.97287	ALCAM/EPHB3	2	BP
GO:0031440	regulation of mRNA 3'-end processing	2/2734	21/17381	0.86492	0.9891	0.97287	CDK9/HSF1	2	BP
GO:0031935	regulation of chromatin	2/2734	21/17381	0.86492	0.9891	0.97287	HMGA1/PHF2	2	BP
GO:0032958	inositol phosphate biosynthetic	2/2734	21/17381	0.86492	0.9891	0.97287	GPER1/PTK2B	2	BP
GO:0036119	response to platelet-derived growth factor	2/2734	21/17381	0.86492	0.9891	0.97287	CORO1B/PDGFRB	2	BP
GO:0043369	CD4-positive or CD8-positive, alpha-beta T cell lineage commitment	2/2734	21/17381	0.86492	0.9891	0.97287	LY9/SHH	2	BP
GO:0046135	pyrimidine nucleoside catabolic	2/2734	21/17381	0.86492	0.9891	0.97287	CDA/NT5M	2	BP
GO:0046697	decidualization	2/2734	21/17381	0.86492	0.9891	0.97287	MEN1/PPARD	2	BP

GO:0050857	positive regulation of antigen receptor-	2/2734	21/17381	0.86492	0.9891	0.97287	CMTM3/LCK	2	BP
GO:0070584	mitochondrion morphogenesis	2/2734	21/17381	0.86492	0.9891	0.97287	PID1/POLDIP2	2	BP
GO:0071498	cellular response to fluid shear	2/2734	21/17381	0.86492	0.9891	0.97287	MIR92A2/PTK2B	2	BP
GO:0072539	T-helper 17 cell differentiation	2/2734	21/17381	0.86492	0.9891	0.97287	LY9/RORC	2	BP
GO:1900120	regulation of receptor binding	2/2734	21/17381	0.86492	0.9891	0.97287	PHLDA2/PTPRF	2	BP
GO:1901798	positive regulation of signal transduction by	2/2734	21/17381	0.86492	0.9891	0.97287	CDK5RAP3/ZNF385A	2	BP
GO:1902932	positive regulation of alcohol biosynthetic	2/2734	21/17381	0.86492	0.9891	0.97287	GPER1/POR	2	BP
GO:1903010	regulation of bone	2/2734	21/17381	0.86492	0.9891	0.97287	POR/TMEM119	2	BP
GO:1903859	regulation of dendrite	2/2734	21/17381	0.86492	0.9891	0.97287	CPNE5/CPNE9	2	BP
GO:1904380	endoplasmic reticulum mannose	2/2734	21/17381	0.86492	0.9891	0.97287	OS9/SYVN1	2	BP
GO:2000810	regulation of bicellular tight junction	2/2734	21/17381	0.86492	0.9891	0.97287	IKBKB/MYO1C	2	BP

GO:0015696	ammonium transport	11/2734	91/17381	0.86665	0.9891	0.97287	AQP1/CHRNA6/DRD2/DTNBP1/RHCG/SLC22A1/SLC25A20/SLC44A4/TACR2/TAF7/TOR1A	11	BP
GO:1901615	organic hydroxy compound metabolic process	69/2734	492/17381	0.86879	0.9891	0.97287	ACACB/ACADVL/ADH1A/ADH1C/ALDH2/AOC2/APOA5/APOB/CACNA1H/CLN6/COQ2/CRYM/CUBN/CYB5R1/CYB5R2/CYP11A1/CYP11B2/CYP1A1/DDC/DEGS2/DGAT1/DGKQ/DPM2/DRD2/DUOXA1/EBPL/EPHX2/FGFR4/GALK1/GBA/GCH1/GDPD1/GPER1/HDC/HSD17B3/HSD17B7/INPP5D/INPP5E/INPPL1/ITGAM/ITPK1/LDHA/LEPR/LMF1/LRTOMT/NPC1L1/NPC2/NSDHL/OSBPL5/OSBPL7/PLB1/PLCB2/PLCD1/PLCH2/PMVK/PNKD/POR/PPARD/PTK2B/RNF180/SCAP/SCARB1/SCARF1/SLC34A1/SNCB/SOAT2/SPTLC1/ST[illegible]	69	BP
GO:0002823	negative regulation of adaptive immune response based on somatic recombination of immune receptors built from [illegible]	4/2734	38/17381	0.86925	0.9891	0.97287	BCL6/HAVCR2/IL20RB/IL4R	4	BP
GO:0007223	Wnt signaling pathway, calcium modulating	4/2734	38/17381	0.86925	0.9891	0.97287	FZD2/GNAO1/PLCB2/WNT11	4	BP
GO:0032881	regulation of polysaccharide metabolic	4/2734	38/17381	0.86925	0.9891	0.97287	GCK/INS/MTOR/PHLDA2	4	BP
GO:0046676	negative regulation of	4/2734	38/17381	0.86925	0.9891	0.97287	ADRA2A/DRD2/INHBB/PTPN11	4	BP
GO:0070296	sarcoplasmic reticulum calcium ion	4/2734	38/17381	0.86925	0.9891	0.97287	CACNG1/CAMK2D/GSTM2/TRDN	4	BP

GO:1901658	glycosyl compound catabolic	4/2734	38/17381	0.86925	0.9891	0.97287	CDA/GBA/NT5M/NUDT18	4	BP
GO:0045913	positive regulation of carbohydrate	8/2734	69/17381	0.86942	0.9891	0.97287	BAD/GCG/GCK/GPER1/INS/KAT2A/MLXIPL/PFKFB1	8	BP
GO:0050688	regulation of defense response to	8/2734	69/17381	0.86942	0.9891	0.97287	DHX58/EIF2AK4/MICB/NLRX1/PARP9/STAT1/TARBP2/TRAF3IP1	8	BP
GO:0002762	negative regulation of myeloid leukocyte	5/2734	46/17381	0.86983	0.9891	0.97287	C1QC/INPP5D/NME1/RARA/TCTA	5	BP
GO:0010501	RNA secondary structure unwinding	5/2734	46/17381	0.86983	0.9891	0.97287	DDX31/DDX4/DDX53/DDX54/HELQ	5	BP
GO:0060338	regulation of type I interferon-mediated	5/2734	46/17381	0.86983	0.9891	0.97287	IFNA5/PTPN11/SCRIB/STAT1/STAT2	5	BP
GO:0071548	response to dexamethasone	5/2734	46/17381	0.86983	0.9891	0.97287	AQP1/EGFR/EIF4EBP1/GBA/SMYD3	5	BP
GO:1902099	regulation of metaphase/anaphase transition of cell cycle	5/2734	46/17381	0.86983	0.9891	0.97287	ANAPC15/BUB1B/LCMT1/MAD1L1/XRCC3	5	BP
GO:1990090	cellular response to nerve growth	5/2734	46/17381	0.86983	0.9891	0.97287	CIB1/CORO1A/EHD1/HSPA5/KAT2A	5	BP

GO:0000070	mitotic sister chromatid segregation	18/2734	142/17381	0.87029	0.9891	0.97287	ANAPC15/ANKRD53/BUB1B/CDC14A/CHMP1A/CHMP4C/CHMP7/DIS3L2/INO80/KIF23/KIF4A/LCMT1/MAD1L1/NCAPD3/NUMA1/PDS5A/SLF2/XRCC3	18	BP
GO:0001682	tRNA 5'-leader removal	1/2734	12/17381	0.87183	0.9891	0.97287	RPP21	1	BP
GO:0001787	natural killer cell	1/2734	12/17381	0.87183	0.9891	0.97287	IL18	1	BP
GO:0001867	complement activation, lectin pathway	1/2734	12/17381	0.87183	0.9891	0.97287	MASP2	1	BP
GO:0002281	macrophage activation involved in immune	1/2734	12/17381	0.87183	0.9891	0.97287	HAVCR2	1	BP
GO:0002430	complement receptor mediated	1/2734	12/17381	0.87183	0.9891	0.97287	C3AR1	1	BP
GO:0002551	mast cell chemotaxis	1/2734	12/17381	0.87183	0.9891	0.97287	CHGA	1	BP
GO:0002921	negative regulation of humoral	1/2734	12/17381	0.87183	0.9891	0.97287	SPINK5	1	BP
GO:0003093	regulation of glomerular filtration	1/2734	12/17381	0.87183	0.9891	0.97287	F2RL1	1	BP
GO:0003306	Wnt signaling pathway involved in heart	1/2734	12/17381	0.87183	0.9891	0.97287	WNT3A	1	BP
GO:0006068	ethanol catabolic	1/2734	12/17381	0.87183	0.9891	0.97287	ALDH2	1	BP

GO:0006107	oxaloacetate metabolic	1/2734	12/17381	0.87183	0.9891	0.97287	GOT2	1	BP
GO:0008340	determination of adult lifespan	1/2734	12/17381	0.87183	0.9891	0.97287	INPP5D	1	BP
GO:0009113	purine nucleobase biosynthetic	1/2734	12/17381	0.87183	0.9891	0.97287	MTHFD1	1	BP
GO:0010457	centriole-centriole	1/2734	12/17381	0.87183	0.9891	0.97287	CROCC	1	BP
GO:0010755	regulation of plasminogen activation	1/2734	12/17381	0.87183	0.9891	0.97287	SERPINF2	1	BP
GO:0014067	negative regulation of phosphatidylinositol 3-kinase signaling	1/2734	12/17381	0.87183	0.9891	0.97287	DAB2IP	1	BP
GO:0016045	detection of bacterium	1/2734	12/17381	0.87183	0.9891	0.97287	NOD1	1	BP
GO:0016264	gap junction assembly	1/2734	12/17381	0.87183	0.9891	0.97287	AGT	1	BP
GO:0021559	trigeminal nerve development	1/2734	12/17381	0.87183	0.9891	0.97287	SEMA3F	1	BP
GO:0031650	regulation of heat generation	1/2734	12/17381	0.87183	0.9891	0.97287	PTGER3	1	BP
GO:0032119	sequestering of zinc ion	1/2734	12/17381	0.87183	0.9891	0.97287	SLC30A3	1	BP
GO:0032700	negative regulation of interleukin-17	1/2734	12/17381	0.87183	0.9891	0.97287	TUSC2	1	BP

GO:0033605	positive regulation of catecholamine	1/2734	12/17381	0.87183	0.9891	0.97287	STX1A	1	BP
GO:0034086	maintenance of sister chromatid cohesion	1/2734	12/17381	0.87183	0.9891	0.97287	SLF2	1	BP
GO:0034088	maintenance of mitotic sister chromatid cohesion	1/2734	12/17381	0.87183	0.9891	0.97287	SLF2	1	BP
GO:0034497	protein localization to pre-autophagosomal	1/2734	12/17381	0.87183	0.9891	0.97287	MFN2	1	BP
GO:0035246	peptidyl-arginine N-	1/2734	12/17381	0.87183	0.9891	0.97287	NDUFAF7	1	BP
GO:0035721	intraciliary retrograde transport	1/2734	12/17381	0.87183	0.9891	0.97287	IFT140	1	BP
GO:0035751	regulation of lysosomal lumen pH	1/2734	12/17381	0.87183	0.9891	0.97287	CLN6	1	BP
GO:0035970	peptidyl-threonine	1/2734	12/17381	0.87183	0.9891	0.97287	DUSP5	1	BP
GO:0036166	phenotypic switching	1/2734	12/17381	0.87183	0.9891	0.97287	MIR221	1	BP
GO:0042118	endothelial cell activation	1/2734	12/17381	0.87183	0.9891	0.97287	MIR92A2	1	BP
GO:0042738	exogenous drug catabolic	1/2734	12/17381	0.87183	0.9891	0.97287	CYP1A2	1	BP

GO:0043217	myelin maintenance	1/2734	12/17381	0.87183	0.9891	0.97287	CLU	1	BP
GO:0043455	regulation of secondary metabolic	1/2734	12/17381	0.87183	0.9891	0.97287	PGAM4	1	BP
GO:0043476	pigment accumulation	1/2734	12/17381	0.87183	0.9891	0.97287	CD63	1	BP
GO:0043482	cellular pigment accumulation	1/2734	12/17381	0.87183	0.9891	0.97287	CD63	1	BP
GO:0043922	negative regulation by host of viral	1/2734	12/17381	0.87183	0.9891	0.97287	CCL5	1	BP
GO:0044745	amino acid transmembrane import	1/2734	12/17381	0.87183	0.9891	0.97287	AGT	1	BP
GO:0045086	positive regulation of interleukin-2 biosynthetic	1/2734	12/17381	0.87183	0.9891	0.97287	CARD11	1	BP
GO:0045414	regulation of interleukin-8 biosynthetic process	1/2734	12/17381	0.87183	0.9891	0.97287	TLR8	1	BP
GO:0045651	positive regulation of macrophage	1/2734	12/17381	0.87183	0.9891	0.97287	CSF1	1	BP
GO:0045820	negative regulation of glycolytic	1/2734	12/17381	0.87183	0.9891	0.97287	PFKFB1	1	BP

GO:0046929	negative regulation of neurotransmitte	1/2734	12/17381	0.87183	0.9891	0.97287	PNKD	1	BP
GO:0048311	mitochondrion distribution	1/2734	12/17381	0.87183	0.9891	0.97287	KAT2A	1	BP
GO:0048505	regulation of timing of cell differentiation	1/2734	12/17381	0.87183	0.9891	0.97287	NOTCH1	1	BP
GO:0051195	negative regulation of cofactor	1/2734	12/17381	0.87183	0.9891	0.97287	PFKFB1	1	BP
GO:0051198	negative regulation of coenzyme	1/2734	12/17381	0.87183	0.9891	0.97287	PFKFB1	1	BP
GO:0051531	NFAT protein import into	1/2734	12/17381	0.87183	0.9891	0.97287	MTOR	1	BP
GO:0060707	trophoblast giant cell	1/2734	12/17381	0.87183	0.9891	0.97287	E2F8	1	BP
GO:0060900	embryonic camera-type eye formation	1/2734	12/17381	0.87183	0.9891	0.97287	STRA6	1	BP
GO:0086067	AV node cell to bundle of His cell	1/2734	12/17381	0.87183	0.9891	0.97287	CACNA1G	1	BP
GO:0086103	G-protein coupled receptor signaling	1/2734	12/17381	0.87183	0.9891	0.97287	CHGA	1	BP
GO:0089718	amino acid import across plasma	1/2734	12/17381	0.87183	0.9891	0.97287	AGT	1	BP

GO:1900121	negative regulation of	1/2734	12/17381	0.87183	0.9891	0.97287	PTPRF	1	BP
GO:1901678	iron coordination	1/2734	12/17381	0.87183	0.9891	0.97287	ASIC3	1	BP
GO:1904251	regulation of bile acid metabolic	1/2734	12/17381	0.87183	0.9891	0.97287	FGFR4	1	BP
GO:1905153	regulation of membrane invagination	1/2734	12/17381	0.87183	0.9891	0.97287	F2RL1	1	BP
GO:2000052	positive regulation of non-canonical Wnt signaling	1/2734	12/17381	0.87183	0.9891	0.97287	ABL1	1	BP
GO:2000121	regulation of removal of superoxide radicals	1/2734	12/17381	0.87183	0.9891	0.97287	GCH1	1	BP
GO:2001053	regulation of mesenchymal cell apoptotic	1/2734	12/17381	0.87183	0.9891	0.97287	SHH	1	BP
GO:0009164	nucleoside catabolic	3/2734	30/17381	0.87205	0.9891	0.97287	CDA/NT5M/NUDT18	3	BP
GO:0010880	regulation of release of sequestered calcium ion into cytosol by sarcoplasmic reticulum	3/2734	30/17381	0.87205	0.9891	0.97287	CAMK2D/GSTM2/TRDN	3	BP

GO:0050686	negative regulation of mRNA	3/2734	30/17381	0.87205	0.9891	0.97287	CDK9/RNPS1/SRSF4	3	BP
GO:1901661	quinone metabolic	3/2734	30/17381	0.87205	0.9891	0.97287	COQ2/COQ4/PDSS1	3	BP
GO:0043388	positive regulation of	6/2734	54/17381	0.87219	0.9891	0.97287	ACD/EDF1/HDAC8/KAT2A/NME1/WNT3A	6	BP
GO:1905818	regulation of chromosome separation	6/2734	54/17381	0.87219	0.9891	0.97287	ANAPC15/BUB1B/LCMT1/MAD1L1/NUMA1/XRCC3	6	BP
GO:0044706	multi-multicellular organism	29/2734	220/17381	0.87395	0.9891	0.97287	ADM/ADRA2B/ADRA2C/AGT/CLDN4/COL16A1/CRHR1/CYP1A1/DHODH/EDDM3A/FOSL1/HAVCR2/HSD11B2/HSF1/IDO1/LGALS9/MAGED2/MEN1/MMP2/MTOR/NPPA/PLA2G4B/PPARD/PRDM14/RARA/SCGB1A1/SH3PXD	29	BP
GO:0016079	synaptic vesicle exocytosis	9/2734	77/17381	0.87429	0.9891	0.97287	CDK5/CPLX1/DOC2A/DOC2B/SCRIB/STX1A/STX4/STXBP1/SYTL3	9	BP
GO:0051321	meiotic cell cycle	32/2734	241/17381	0.87473	0.9891	0.97287	BUB1B/C11orf80/DDX4/EXO1/FANCA/FKBP6/GOLGA2/HORMAD1/HSF1/HSPA2/LEMD2/LFNG/M1AP/MCMDC2/NCAPD3/NUMA1/PIWIL2/PRDM9/PRKAR1A/PSMD13/RNF212/SLC2A8/TAF1L/TDRD1/TDRD9/TUBG1/TUBGCP2/TUBGCP3/TUBGCP5/UBE2B/XRCC3/ZFP42	32	BP
GO:0048010	vascular endothelial growth factor receptor	11/2734	92/17381	0.87559	0.9891	0.97287	BRK1/DAB2IP/DOCK1/FLT4/HSP90AA1/HSPB1/KDR/MAPKAPK3/NCF1/PTK2B/SH2D2A	11	BP
GO:0072676	lymphocyte migration	11/2734	92/17381	0.87559	0.9891	0.97287	ADAM8/ARTN/CCL1/CCL19/CCL21/CCL5/CCR2/CKLF/CXCR3/ECM1/PTK2B	11	BP
GO:0098727	maintenance of cell number	20/2734	157/17381	0.87568	0.9891	0.97287	ASCL2/BCL9/CDX2/DIS3L2/LDB1/LSM1/MCPH1/NOTCH1/PADI4/PIWIL2/POLR2G/POLR2L/PRDM14/RAF1/SETD1A/SMO/SPI1/TDGF1/WNT7A/ZHX2	20	BP
GO:0006140	regulation of nucleotide metabolic process	36/2734	269/17381	0.87637	0.9891	0.97287	ADM/ADRA2A/AIPL1/ATPIF1/AVPR2/CCR2/CDA/CHGA/CRHR1/DRD2/FZD2/GABBR1/GCG/GCK/GPER1/GPHA2/GUCA1B/GUCA2A/GUCA2B/INS/MLXIPL/NCOR1/NOS3/NUP210/NUP98/OPRM1/PDE2A/PDZD3/PFKFB1/PGAM4/PID1/PTK2B/RAF1/RUNDC3A/RXFP2/WFS1	36	BP

GO:0006766	vitamin metabolic	16/2734	129/17381	0.88004	0.9891	0.97287	ACACB/ACPP/ALDH1L1/CUBN/CYP11A1/CYP1A1/FOLR2/MTHFD1/PC/PPARD/RBP1/RBP2/SHMT2/SLC52A1/SLC52A2/VNN3	16	BP
GO:1903531	negative regulation of secretion by cell	24/2734	186/17381	0.88013	0.9891	0.97287	ADRA2A/ADRA2B/ADRA2C/CCR2/CHGA/DRD2/F2RL1/FN1/IDH2/INHBB/INS/LGALS9/NOTCH1/NR1H3/OPRM1/PNKD/PTPN11/RAB11FIP3/RAB7A/RHBDF1/SERGEF/TACR2/TLR8/TRH	24	BP
GO:0009062	fatty acid catabolic	12/2734	100/17381	0.88091	0.9891	0.97287	ACACB/ACAD10/ACADVL/ACOXL/CPT2/CROT/HADHA/HAO2/MTOR/PLA2G15/PPARD/TYSND1	12	BP
GO:0034440	lipid oxidation	12/2734	100/17381	0.88091	0.9891	0.97287	ACACB/ACAD10/ACADVL/ACOXL/CPT2/CROT/HADHA/HAO2/MTOR/POR/PPARD/TYSND1	12	BP
GO:0061013	regulation of mRNA catabolic	22/2734	172/17381	0.88096	0.9891	0.97287	APEX1/EIF4G1/EXOSC2/HSPB1/MTOR/PCBP4/PKP3/POLR2G/PRKCD/PSMB11/PSMB6/PSMB7/PSMB8/PSMC3/PSMD13/PSMD3/PSMD5/PSMD7/SCGB1A1/SLC11A1/TIRAP/TNFSF13	22	BP
GO:0007091	metaphase/anaphase transition of mitotic cell	5/2734	47/17381	0.8817	0.9891	0.97287	ANAPC15/BUB1B/LCMT1/MAD1L1/XRCC3	5	BP
GO:0010828	positive regulation of glucose	5/2734	47/17381	0.8817	0.9891	0.97287	AGT/CAPN10/INS/PTPN11/RARRES2	5	BP
GO:0045762	positive regulation of adenylate	5/2734	47/17381	0.8817	0.9891	0.97287	AVPR2/CRHR1/GPER1/RAF1/WFS1	5	BP
GO:0050798	activated T cell proliferation	5/2734	47/17381	0.8817	0.9891	0.97287	ABL1/CASP3/IL18/LGALS9/PRKAR1A	5	BP
GO:0071715	icosanoid transport	5/2734	47/17381	0.8817	0.9891	0.97287	ABCC4/DRD2/PLA2G1B/PLA2G2F/PLA2G5	5	BP
GO:1901571	fatty acid derivative transport	5/2734	47/17381	0.8817	0.9891	0.97287	ABCC4/DRD2/PLA2G1B/PLA2G2F/PLA2G5	5	BP

GO:2000677	regulation of transcription regulatory region DNA	5/2734	47/17381	0.8817	0.9891	0.97287	HEY1/KAT2A/TAF1/WNT3A/ZNF593	5	BP
GO:0002717	positive regulation of natural killer	2/2734	22/17381	0.88187	0.9891	0.97287	HLA-E/NCR3	2	BP
GO:0010447	response to acidic pH	2/2734	22/17381	0.88187	0.9891	0.97287	ASIC2/ASIC3	2	BP
GO:0010881	regulation of cardiac muscle contraction by regulation of the release of sequestered calcium ion	2/2734	22/17381	0.88187	0.9891	0.97287	CAMK2D/GSTM2	2	BP
GO:0021544	subpallium development	2/2734	22/17381	0.88187	0.9891	0.97287	DRD2/OGDH	2	BP
GO:0032402	melanosome transport	2/2734	22/17381	0.88187	0.9891	0.97287	DCTN2/RAB17	2	BP
GO:0035590	purinergic nucleotide receptor signaling	2/2734	22/17381	0.88187	0.9891	0.97287	P2RX2/P2RY6	2	BP
GO:0043949	regulation of cAMP-mediated	2/2734	22/17381	0.88187	0.9891	0.97287	TCP11/UBE2B	2	BP
GO:0072010	glomerular epithelium development	2/2734	22/17381	0.88187	0.9891	0.97287	BMP4/MYO1E	2	BP

GO:0097150	neuronal stem cell population maintenance	2/2734	22/17381	0.88187	0.9891	0.97287	MCPH1/NOTCH1	2	BP
GO:1903203	regulation of oxidative stress-induced neuron death	2/2734	22/17381	0.88187	0.9891	0.97287	NONO/WNT1	2	BP
GO:1903429	regulation of cell maturation	2/2734	22/17381	0.88187	0.9891	0.97287	MTOR/TCP11	2	BP
GO:2000679	positive regulation of transcription regulatory	2/2734	22/17381	0.88187	0.9891	0.97287	KAT2A/WNT3A	2	BP
GO:2000765	regulation of cytoplasmic translation	2/2734	22/17381	0.88187	0.9891	0.97287	DPH1/ZNF385A	2	BP
GO:0000154	rRNA modification	4/2734	39/17381	0.88218	0.9891	0.97287	NHP2/NSUN5/NSUN5P2/RPUSD2	4	BP
GO:0007007	inner mitochondrial membrane	4/2734	39/17381	0.88218	0.9891	0.97287	ATP5G1/ATP5I/TIMM22/TIMM9	4	BP
GO:0045616	regulation of keratinocyte differentiation	4/2734	39/17381	0.88218	0.9891	0.97287	CTSL/KRT84/NOTCH1/NUMA1	4	BP
GO:0046461	neutral lipid catabolic	4/2734	39/17381	0.88218	0.9891	0.97287	APOA5/APOB/APOC3/PNPLA2	4	BP
GO:0046464	acylglycerol catabolic	4/2734	39/17381	0.88218	0.9891	0.97287	APOA5/APOB/APOC3/PNPLA2	4	BP
GO:0051653	spindle localization	4/2734	39/17381	0.88218	0.9891	0.97287	DYNC1H1/EYA1/MCPH1/NUMA1	4	BP

GO:0097035	regulation of membrane lipid distribution	4/2734	39/17381	0.88218	0.9891	0.97287	ANO4/ANO7/PRELID1/PRKCD	4	BP
GO:2001259	positive regulation of cation channel	6/2734	55/17381	0.88311	0.9891	0.97287	ARC/CRACR2A/GSTM2/NLGN3/NPPA/TRDN	6	BP
GO:0032204	regulation of telomere maintenance	9/2734	78/17381	0.88351	0.9891	0.97287	ACD/CCT3/EXOSC10/HDAC8/MAPK15/MAPK3/PIF1/SMG5/TINF2	9	BP
GO:0000375	RNA splicing, via transesterification reactions	44/2734	326/17381	0.8855	0.9891	0.97287	CASC3/CELF6/CPSF1/CPSF4/CSTF2/CWC15/CWC25/DHX38/EFTUD2/ESRP2/GPKOW/HMX2/HNRNPH3/LMNTD2/LSM1/LUC7L/MYOD1/NONO/NUP98/POLR2G/POLR2L/PPIE/PRCC/PRDX6/PRPF4/PRPF8/PSPC1/RBFOX1/RBFOX3/RBM4/RBPMS/RNPS1/SART1/SF1/SF3B4/SLC39A5/SNRNP25/SPEN/SRPK3/SRRM2/SRSF4/USP4/USP49/ZCCHC8	44	BP
GO:0002431	Fc receptor mediated stimulatory signaling	17/2734	137/17381	0.88557	0.9891	0.97287	ABL1/ARPC1A/ARPC1B/ARPC2/ARPC4/BRK1/CD247/CSK/DOCK1/FCGR3A/HSP90AA1/LIMK1/MAPK3/MYO1C/PLD2/PRKCD/WAS	17	BP
GO:0032677	regulation of interleukin-8 production	7/2734	63/17381	0.8856	0.9891	0.97287	CD58/F2RL1/GDF2/LGALS9/TIRAP/TLR8/TLR9	7	BP
GO:0045670	regulation of osteoclast differentiation	7/2734	63/17381	0.8856	0.9891	0.97287	BGLAP/CSF1/ESRRA/FAM213A/INPP5D/MTOR/TCTA	7	BP
GO:0044262	cellular carbohydrate metabolic	33/2734	250/17381	0.88567	0.9891	0.97287	ACACB/BAD/C1QTNF1/COQ2/COX11/DGKQ/GALK1/GCG/GCK/GNMT/GPER1/HK3/IDH1/IDH2/IGFBP3/INPP5E/INS/KAT2A/LCMT1/LEPR/MAEA/MGAM/MTOR/NAGK/PCDH12/PFKFB1/PFKM/PGAM4/PHKG1/PHLDA2/PTK2	33	BP
GO:0010458	exit from mitosis	3/2734	31/17381	0.88609	0.9891	0.97287	CDC14A/CHMP7/PPP1R9B	3	BP
GO:0021988	olfactory lobe development	3/2734	31/17381	0.88609	0.9891	0.97287	CSF1R/OGDH/SEMA7A	3	BP

GO:0032892	positive regulation of organic acid	3/2734	31/17381	0.88609	0.9891	0.97287	AGT/STXBP1/TRH	3	BP
GO:0097484	dendrite extension	3/2734	31/17381	0.88609	0.9891	0.97287	CPNE5/CPNE9/LLPH	3	BP
GO:0098534	centriole assembly	3/2734	31/17381	0.88609	0.9891	0.97287	BRCA1/C2CD3/E2F4	3	BP
GO:0042113	B cell activation	36/2734	271/17381	0.88649	0.9891	0.97287	ABL1/ADGRG3/BAD/BCL6/BLNK/CARD11/CASP3/CD180/CD27/CD74/CD79B/CD81/CLCF1/CMTM7/CXCR5/EXO1/FZD9/GON4L/IFNA5/IL13/INPP5D/LFNG/MZB1/NKX2-3/PAXIP1/PPP2R3C/PRKCD/PTK2B/SUPT6H/TIRAP/TLR9/TNFRSF13B/TNFRSF4/TNFSF13/TNIP2/WNT3A	36	BP
GO:0016311	dephosphorylation	58/2734	422/17381	0.88673	0.9891	0.97287	ACP2/ACPP/ALPPL2/APEX1/ATP1A1/CDC14A/CDK5RAP3/CTDSP1/DRD2/DUSP2/DUSP21/DUSP26/DUSP5/EPHX2/EYA1/G6PC3/GBA/IKBKB/ILKAP/INPP5D/INPP5E/INPP5F/INPPL1/ITPK1/LCK/MTMR1/MTMR14/NSMF/NT5C3B/NT5M/PDGFRB/PFKFB1/PGAM5/PHOSPHO1/PKMYT1/PLPP1/PPA2/PPEF1/PPP1R1B/PPP2R2B/PPP2R3B/PRKCD/PTP4A3/PTPN11/PTPN23/PTPN7/PTPRF/PTPRN2/PTPRQ/PTPRU/PUDP/SMG5/SPOCD1/STYXL1/THNSL2/TIP [illegible]	58	BP
GO:0033045	regulation of sister chromatid segregation	8/2734	71/17381	0.88871	0.9891	0.97287	ANAPC15/BUB1B/HDAC8/LCMT1/MAD1L1/NUMA1/SLF2/XRCC3	8	BP
GO:0040029	regulation of gene expression, epigenetic	67/2734	484/17381	0.89011	0.9891	0.97287	BAZ1B/BMP4/BRCA1/DND1/DNMT3A/EGFR/EIF4G1/EPC1/EXOSC10/GPX1/HILS1/HIST1H2AH/HIST1H4F/HIST2H4A/HIST2H4B/HMGA1/KAT2A/KCNQ1/MIR106B/MIR10A/MIR153-1/MIR15A/MIR16-1/MIR181B1/MIR210/MIR212/MIR221/MIR222/MIR25/MIR29A/MIR29C/MIR32/MIR328/MIR503/MIR582/MIR892B/MIR92A2/MIR96/MIRLET7A2/MSL3/MTA1/MYCN/MYO1C/NCOR1/NCOR2/NUP210/NUP98/PHF19/PHF2/POLR1A/POLR1D/POLR1E/POLR2G/POLR2L/PRDM14/RBM4/SLC50A1/SPI1/SUPT6H/SUV39H1/TAF1A/TARBP2/TNRC18/TSNAX/UBE2B/ZC3H10/ZNRD1	67	BP

GO:0031047	gene silencing by RNA	45/2734	334/17381	0.89047	0.9891	0.97287	BMP4/DDX4/DND1/EGFR/EIF4G1/FKBP6/HIST1H4F/HIST2H4A/HIST2H4B/MIR106B/MIR10A/MIR153-1/MIR15A/MIR16-1/MIR181B1/MIR210/MIR212/MIR221/MIR222/MIR25/MIR29A/MIR29C/MIR32/MIR328/MIR503/MIR582/MIR892B/MIR92A2/MIR96/MIRLET7A2/MYCN/NCOR1/NCOR2/NUP210/NUP98/PIWIL2/POLR2G/POLR2L/RBM4/SND1/TARBP2/TDRD1/TDRD9/TSNAX/ZC3H10	45	BP
GO:0044242	cellular lipid catabolic process	25/2734	195/17381	0.89139	0.9891	0.97287	ACACB/ACAD10/ACADVL/ACOXL/APOA5/APOB/APOC3/CPT2/CROT/GBA/HADHA/HAO2/IDH1/INPP5F/MTOR/NEU3/PLA2G15/PLA2G4B/PNPLA2/PPARD/PRDX6/PRKCD/SCARB1/SMPD4/TYSND1	25	BP
GO:0050657	nucleic acid transport	25/2734	195/17381	0.89139	0.9891	0.97287	ALKBH5/CASC3/CKAP5/CPSF1/CPSF4/DHX38/EIF5A/HSF1/MYO1C/NOL6/NUP210/NUP98/NXF2/NXF2B/NXF3/RBFOX1/RNPS1/SETD2/SIDT2/SMG5/SRSF4/SUPT6H/XPO7/ZC3H3/ZNF593	25	BP
GO:0050658	RNA transport	25/2734	195/17381	0.89139	0.9891	0.97287	ALKBH5/CASC3/CKAP5/CPSF1/CPSF4/DHX38/EIF5A/HSF1/MYO1C/NOL6/NUP210/NUP98/NXF2/NXF2B/NXF3/RBFOX1/RNPS1/SETD2/SIDT2/SMG5/SRSF4/SUPT6H/XPO7/ZC3H3/ZNF593	25	BP
GO:0000722	telomere maintenance via recombination	1/2734	13/17381	0.89201	0.9891	0.97287	XRCC3	1	BP
GO:0002517	T cell tolerance induction	1/2734	13/17381	0.89201	0.9891	0.97287	IDO1	1	BP
GO:0009143	nucleoside triphosphate catabolic	1/2734	13/17381	0.89201	0.9891	0.97287	NUDT1	1	BP
GO:0009313	oligosaccharide catabolic	1/2734	13/17381	0.89201	0.9891	0.97287	NEU3	1	BP
GO:0010459	negative regulation of	1/2734	13/17381	0.89201	0.9891	0.97287	ADRA1A	1	BP

GO:0010510	regulation of acetyl-CoA biosynthetic process from pyruvate	1/2734	13/17381	0.89201	0.9891	0.97287	PDHB	1	BP
GO:0010745	negative regulation of macrophage derived foam	1/2734	13/17381	0.89201	0.9891	0.97287	NR1H3	1	BP
GO:0010763	positive regulation of fibroblast	1/2734	13/17381	0.89201	0.9891	0.97287	DDR2	1	BP
GO:0017014	protein nitrosylation	1/2734	13/17381	0.89201	0.9891	0.97287	GAPDH	1	BP
GO:0018119	peptidyl-cysteine S-	1/2734	13/17381	0.89201	0.9891	0.97287	GAPDH	1	BP
GO:0030213	hyaluronan biosynthetic process	1/2734	13/17381	0.89201	0.9891	0.97287	ABCC5	1	BP
GO:0031054	pre-miRNA processing	1/2734	13/17381	0.89201	0.9891	0.97287	TARBP2	1	BP
GO:0031665	negative regulation of lipopolysaccharide-mediated signaling	1/2734	13/17381	0.89201	0.9891	0.97287	NFKBIL1	1	BP
GO:0032988	ribonucleoprotein complex disassembly	1/2734	13/17381	0.89201	0.9891	0.97287	KLC1	1	BP
GO:0034472	snRNA 3'-end processing	1/2734	13/17381	0.89201	0.9891	0.97287	EXOSC2	1	BP

GO:0035745	T-helper 2 cell cytokine production	1/2734	13/17381	0.89201	0.9891	0.97287	IL31RA	1	BP
GO:0035810	positive regulation of	1/2734	13/17381	0.89201	0.9891	0.97287	DRD2	1	BP
GO:0040034	regulation of development, heterochronic	1/2734	13/17381	0.89201	0.9891	0.97287	NOTCH1	1	BP
GO:0042574	retinal metabolic	1/2734	13/17381	0.89201	0.9891	0.97287	ALDH8A1	1	BP
GO:0042659	regulation of cell fate	1/2734	13/17381	0.89201	0.9891	0.97287	WNT3A	1	BP
GO:0043518	negative regulation of DNA damage response, signal transduction by	1/2734	13/17381	0.89201	0.9891	0.97287	CD74	1	BP
GO:0045073	regulation of chemokine biosynthetic process	1/2734	13/17381	0.89201	0.9891	0.97287	EGR1	1	BP
GO:0045605	negative regulation of epidermal cell	1/2734	13/17381	0.89201	0.9891	0.97287	NOTCH1	1	BP
GO:0046415	urate metabolic process	1/2734	13/17381	0.89201	0.9891	0.97287	SLC22A12	1	BP
GO:0046628	positive regulation of insulin receptor	1/2734	13/17381	0.89201	0.9891	0.97287	INS	1	BP

GO:0048302	regulation of isotype switching to	1/2734	13/17381	0.89201	0.9891	0.97287	PAXIP1	1	BP
GO:0050862	positive regulation of T cell receptor	1/2734	13/17381	0.89201	0.9891	0.97287	LCK	1	BP
GO:0050872	white fat cell differentiation	1/2734	13/17381	0.89201	0.9891	0.97287	CTBP1	1	BP
GO:0051645	Golgi localization	1/2734	13/17381	0.89201	0.9891	0.97287	DAB1	1	BP
GO:0060158	phospholipase C-activating dopamine receptor	1/2734	13/17381	0.89201	0.9891	0.97287	DRD2	1	BP
GO:0060732	positive regulation of inositol phosphate biosynthetic	1/2734	13/17381	0.89201	0.9891	0.97287	GPER1	1	BP
GO:0061051	positive regulation of cell growth involved in cardiac muscle	1/2734	13/17381	0.89201	0.9891	0.97287	MTOR	1	BP
GO:0070307	lens fiber cell development	1/2734	13/17381	0.89201	0.9891	0.97287	WNT7A	1	BP
GO:0071285	cellular response to	1/2734	13/17381	0.89201	0.9891	0.97287	SHH	1	BP
GO:0071872	cellular response to epinephrine	1/2734	13/17381	0.89201	0.9891	0.97287	KCNQ1	1	BP

GO:0072540	T-helper 17 cell lineage commitment	1/2734	13/17381	0.89201	0.9891	0.97287	LY9	1	BP
GO:0090148	membrane fission	1/2734	13/17381	0.89201	0.9891	0.97287	DNM1	1	BP
GO:0090399	replicative senescence	1/2734	13/17381	0.89201	0.9891	0.97287	CDKN2A	1	BP
GO:0097106	postsynaptic density organization	1/2734	13/17381	0.89201	0.9891	0.97287	NRXN2	1	BP
GO:0097152	mesenchymal cell apoptotic	1/2734	13/17381	0.89201	0.9891	0.97287	SHH	1	BP
GO:0097154	GABAergic neuron differentiation	1/2734	13/17381	0.89201	0.9891	0.97287	DRD2	1	BP
GO:0097284	hepatocyte apoptotic	1/2734	13/17381	0.89201	0.9891	0.97287	DNMT3A	1	BP
GO:0097531	mast cell migration	1/2734	13/17381	0.89201	0.9891	0.97287	CHGA	1	BP
GO:0098543	detection of other organism	1/2734	13/17381	0.89201	0.9891	0.97287	NOD1	1	BP
GO:0098703	calcium ion import across plasma	1/2734	13/17381	0.89201	0.9891	0.97287	TRPV6	1	BP
GO:0099084	postsynaptic specialization organization	1/2734	13/17381	0.89201	0.9891	0.97287	NRXN2	1	BP
GO:1902656	calcium ion import into	1/2734	13/17381	0.89201	0.9891	0.97287	TRPV6	1	BP
GO:1904293	negative regulation of	1/2734	13/17381	0.89201	0.9891	0.97287	OS9	1	BP

GO:2000095	regulation of Wnt signaling pathway, planar cell polarity	1/2734	13/17381	0.89201	0.9891	0.97287	ABL1	1	BP
GO:0030397	membrane disassembly	5/2734	48/17381	0.89263	0.9891	0.97287	CCNB2/EMD/LMNA/NUP210/NUP98	5	BP
GO:0035306	positive regulation of dephosphorylati	5/2734	48/17381	0.89263	0.9891	0.97287	DUSP26/GBA/NSMF/PDGFRB/PRKCD	5	BP
GO:0044784	metaphase/anaphase transition of cell cycle	5/2734	48/17381	0.89263	0.9891	0.97287	ANAPC15/BUB1B/LCMT1/MAD1L1/XRCC3	5	BP
GO:0051081	nuclear envelope	5/2734	48/17381	0.89263	0.9891	0.97287	CCNB2/EMD/LMNA/NUP210/NUP98	5	BP
GO:1901185	negative regulation of ERBB signaling	6/2734	56/17381	0.89323	0.9891	0.97287	DAB2IP/EGFR/ERBB2/NRG1/RAB7A/VPS25	6	BP
GO:0044774	mitotic DNA integrity checkpoint	13/2734	109/17381	0.8934	0.9891	0.97287	ATF2/CDK5RAP3/E2F4/E2F8/FOXO4/MUC1/NEK11/PCBP4/RINT1/SFN/TICRR/XPC/ZNF385A	13	BP
GO:0009268	response to pH	4/2734	40/17381	0.89398	0.9891	0.97287	ASIC2/ASIC3/GBA/KCNK4	4	BP
GO:0046189	phenol-containing compound biosynthetic	4/2734	40/17381	0.89398	0.9891	0.97287	DDC/GCH1/HDC/TH	4	BP
GO:0060999	positive regulation of dendritic spine	4/2734	40/17381	0.89398	0.9891	0.97287	FOXO6/LLPH/MTOR/NRG1	4	BP

GO:1902930	regulation of alcohol biosynthetic process	7/2734	64/17381	0.89498	0.9891	0.97287	ACACB/APOB/GPER1/PMVK/POR/PTK2B/SCAP	7	BP
GO:0006403	RNA localization	30/2734	231/17381	0.89554	0.9891	0.97287	ALKBH5/CASC3/CCT3/CKAP5/CPSF1/CPSF4/DHX38/EIF5A/EXOSC10/EXOSC2/HSF1/MYO1C/NHP2/NOL6/NUP210/NUP98/NXF2/NXF2B/NXF3/RBFOX1/RNPS1/SETD2/SIDT2/SMG5/SRSF4/SUPT6H/XPO7/ZC3H3/ZNF385A/ZN	30	BP
GO:0002706	regulation of lymphocyte mediated	15/2734	124/17381	0.89569	0.9891	0.97287	ARRB2/BCL6/CLCF1/HAVCR2/HLA-E/IL20RB/LGALS9/LTA/NCR3/PAXIP1/PIK3R6/SUPT6H/TNFSF13/TRAF2/WAS	15	BP
GO:0044773	mitotic DNA damage checkpoint	12/2734	102/17381	0.89624	0.9891	0.97287	ATF2/CDK5RAP3/E2F4/E2F8/FOXO4/MUC1/NEK11/PCBP4/RINT1/SFN/XPC/ZNF385A	12	BP
GO:0046620	regulation of organ growth	12/2734	102/17381	0.89624	0.9891	0.97287	ACACB/CAV3/FGFR2/HLX/MIR222/MIR25/MTOR/NKX2-5/NOTCH1/POR/SMO/TBX2	12	BP
GO:0000470	maturation of LSU-rRNA	2/2734	23/17381	0.89683	0.9891	0.97287	NHP2/RPL7	2	BP
GO:0001516	prostaglandin biosynthetic process	2/2734	23/17381	0.89683	0.9891	0.97287	CD74/EDN2	2	BP
GO:0001914	regulation of T cell mediated cytotoxicity	2/2734	23/17381	0.89683	0.9891	0.97287	HLA-E/NCR3	2	BP
GO:0007064	mitotic sister chromatid cohesion	2/2734	23/17381	0.89683	0.9891	0.97287	PDS5A/SLF2	2	BP
GO:0007530	sex determination	2/2734	23/17381	0.89683	0.9891	0.97287	NR5A1/SF1	2	BP

GO:0010894	negative regulation of steroid biosynthetic	2/2734	23/17381	0.89683	0.9891	0.97287	ATP1A1/SCAP	2	BP
GO:0021952	central nervous system projection neuron	2/2734	23/17381	0.89683	0.9891	0.97287	EPHB3/TSKU	2	BP
GO:0030539	male genitalia development	2/2734	23/17381	0.89683	0.9891	0.97287	HSD17B3/SHH	2	BP
GO:0031664	regulation of lipopolysaccharide-mediated signaling	2/2734	23/17381	0.89683	0.9891	0.97287	CD180/NFKBIL1	2	BP
GO:0032401	establishment of melanosome localization	2/2734	23/17381	0.89683	0.9891	0.97287	DCTN2/RAB17	2	BP
GO:0033233	regulation of protein sumoylation	2/2734	23/17381	0.89683	0.9891	0.97287	EGR1/RWDD3	2	BP
GO:0035456	response to interferon-beta	2/2734	23/17381	0.89683	0.9891	0.97287	IFITM2/STAT1	2	BP
GO:0046457	prostanoid biosynthetic process	2/2734	23/17381	0.89683	0.9891	0.97287	CD74/EDN2	2	BP
GO:0060444	branching involved in mammary gland duct	2/2734	23/17381	0.89683	0.9891	0.97287	CAV3/CSF1	2	BP

GO:0071218	cellular response to misfolded	2/2734	23/17381	0.89683	0.9891	0.97287	SDF2/TOR1A	2	BP
GO:0071985	multivesicular body sorting pathway	2/2734	23/17381	0.89683	0.9891	0.97287	SNF8/VPS25	2	BP
GO:0072538	T-helper 17 type immune	2/2734	23/17381	0.89683	0.9891	0.97287	LY9/RORC	2	BP
GO:0086012	membrane depolarization during cardiac muscle cell action potential	2/2734	23/17381	0.89683	0.9891	0.97287	CACNA1G/CAV3	2	BP
GO:0097186	amelogenesis	2/2734	23/17381	0.89683	0.9891	0.97287	AMTN/ATF2	2	BP
GO:2000209	regulation of anoikis	2/2734	23/17381	0.89683	0.9891	0.97287	BRMS1/NOTCH1	2	BP
GO:0002711	positive regulation of T cell mediated	3/2734	32/17381	0.89874	0.9891	0.97287	HLA-E/NCR3/TRAF2	3	BP
GO:0033144	negative regulation of intracellular steroid hormone receptor	3/2734	32/17381	0.89874	0.9891	0.97287	BRCA1/CRIPAK/PER1	3	BP

GO:0048285	organelle fission	60/2734	439/17381	0.89943	0.9891	0.97287	ANAPC15/ANKRD53/ARHGEF10/BMP4/BUB1B/C11orf80/CDC14A/CHMP1A/CHMP4C/CHMP7/COX10/DDX4/DHODH/DIS3L2/DNM1/FANCA/FLNA/GOLGA2/HORMAD1/HSF1/HSPA2/INO80/INS/KDR/KIF23/KIF4A/LCMT1/LEMD2/M1AP/MAD1L1/MCMDC2/MIEF2/MTFR1L/MYO19/NCAPD3/NUMA1/OBSL1/PDGFRB/PDS5A/PEX11A/PIWIL2/PKMYT1/PPP1R9B/PRDM9/PRKAR1A/PSMD13/RNF212/SEC16B/SH2B1/SLC2A8/SLF2/STAT2/TAF1L/TDRD9/TUBGCP2/TUBGCP3/TUBGCP5/UBE2B/WRAP73/XRCC3	60	BP
GO:0051983	regulation of chromosome segregation	11/2734	95/17381	0.89954	0.9891	0.97287	ANAPC15/BUB1B/DYNC1H1/HDAC8/HORMAD1/KIF2B/LCMT1/MAD1L1/NUMA1/SLF2/XRCC3	11	BP
GO:0002433	immune response-regulating cell surface receptor signaling pathway [illegible]	16/2734	132/17381	0.90021	0.9891	0.97287	ABL1/ARPC1A/ARPC1B/ARPC2/ARPC4/BRK1/CD247/DOCK1/FCGR3A/HSP90AA1/LIMK1/MAPK3/MYO1C/PLD2/PRKCD/WAS	16	BP
GO:0006171	cAMP biosynthetic process	16/2734	132/17381	0.90021	0.9891	0.97287	ADM/ADRA2A/AVPR2/CCR2/CRHR1/DRD2/GABBR1/GCG/GPER1/GPHA2/OPRM1/PDE2A/RAF1/RXFP2/UCN2/WFS1	16	BP
GO:0038096	Fc-gamma receptor signaling pathway	16/2734	132/17381	0.90021	0.9891	0.97287	ABL1/ARPC1A/ARPC1B/ARPC2/ARPC4/BRK1/CD247/DOCK1/FCGR3A/HSP90AA1/LIMK1/MAPK3/MYO1C/PLD2/PRKCD/WAS	16	BP
GO:0051651	maintenance of location in cell	9/2734	80/17381	0.90029	0.9891	0.97287	ARL2BP/DBN1/GPAA1/HSPA5/NR5A1/OS9/SCIN/TAF3/TLN1	9	BP
GO:1901379	regulation of potassium ion transmembrane transport	9/2734	80/17381	0.90029	0.9891	0.97287	AMIGO1/CAV3/DPP6/FLNA/KCNQ1/MIR153-1/MIR212/NPPA/WWP2	9	BP

GO:0000377	RNA splicing, via transesterification reactions with bulged	43/2734	323/17381	0.90215	0.9891	0.97287	CASC3/CELF6/CPSF1/CPSF4/CSTF2/CWC15/CWC25/DHX38/EFTUD2/ESRP2/GPKOW/HMX2/HNRNPH3/LMNTD2/LUC7L/MYOD1/NONO/NUP98/POLR2G/POLR2L/PPIE/PRCC/PRDX6/PRPF4/PRPF8/PSPC1/RBFOX1/RBFOX3/RBM4/RBPMS/RNPS1/SART1/SF1/SF3B4/SLC39A5/SNRNP25/SPEN/SRPK3/SRRM2/SRSF4/USP4/USP49/ZCCHC8	43	BP
GO:0000398	mRNA splicing, via spliceosome	43/2734	323/17381	0.90215	0.9891	0.97287	CASC3/CELF6/CPSF1/CPSF4/CSTF2/CWC15/CWC25/DHX38/EFTUD2/ESRP2/GPKOW/HMX2/HNRNPH3/LMNTD2/LUC7L/MYOD1/NONO/NUP98/POLR2G/POLR2L/PPIE/PRCC/PRDX6/PRPF4/PRPF8/PSPC1/RBFOX1/RBFOX3/RBM4/RBPMS/RNPS1/SART1/SF1/SF3B4/SLC39A5/SNRNP25/SPEN/SRPK3/SRRM2/SRSF4/USP4/USP49/ZCCHC8	43	BP
GO:0007565	female pregnancy	24/2734	190/17381	0.90259	0.9891	0.97287	ADM/ADRA2B/ADRA2C/AGT/CLDN4/COL16A1/CRHR1/DHODH/FOSL1/HAVCR2/HSD11B2/HSF1/IDO1/LGALS9/MAGED2/MEN1/MMP2/MTOR/NPPA/PPARD/PRDM14/RARA/SCGB1A1/TAC3	24	BP
GO:0002562	somatic diversification of immune receptors via germline recombination within a single	6/2734	57/17381	0.9026	0.9891	0.97287	BCL6/CLCF1/EXO1/PAXIP1/SUPT6H/TNFSF13	6	BP
GO:0016444	somatic cell DNA	6/2734	57/17381	0.9026	0.9891	0.97287	BCL6/CLCF1/EXO1/PAXIP1/SUPT6H/TNFSF13	6	BP
GO:0043525	positive regulation of neuron	5/2734	49/17381	0.90268	0.9891	0.97287	ATF2/CASP3/CDK5/FASLG/NQO2	5	BP
GO:1990089	response to nerve growth	5/2734	49/17381	0.90268	0.9891	0.97287	CIB1/CORO1A/EHD1/HSPA5/KAT2A	5	BP
GO:0035821	modification of morphology or physiology of other organism	18/2734	147/17381	0.903	0.9891	0.97287	AQP1/BAD/CCL5/CFL1/CHGA/CPSF4/EIF2AK4/F2RL1/GAPDH/GSDMD/MIR221/MIR222/PC/PPIB/PSMC3/SCRIB/TUSC2/ZNF502	18	BP

GO:0005976	polysaccharide metabolic	12/2734	103/17381	0.90327	0.9891	0.97287	CHIT1/CHST7/GCK/GNMT/INS/MGAM/MTOR/PCDH12/PFKM/PHKG1/PHLDA2/PYGM	12	BP
GO:0008037	cell recognition	23/2734	183/17381	0.90327	0.9891	0.97287	ADGRB1/ALDOA/AMIGO1/CATSPER1/CCL19/CCL21/CCT3/CD5/CD6/CELSR3/CLEC7A/EPHB3/HAVCR2/LGALS3/NCR3/NTM/PCDH12/POMZP3/PRSS37/SCARB1/SPACA3/TULP1/VDAC2	23	BP
GO:1903046	meiotic cell cycle process	23/2734	183/17381	0.90327	0.9891	0.97287	BUB1B/C11orf80/DDX4/FANCA/GOLGA2/HORMAD1/HSF1/HSPA2/LEMD2/M1AP/MCMDC2/NCAPD3/PIWIL2/PRDM9/PRKAR1A/PSMD13/RNF212/SLC2A8/TAF1L/TDRD9/TUBG1/UBE2B/XRCC3	23	BP
GO:0072413	signal transduction involved in mitotic cell	7/2734	65/17381	0.90371	0.9891	0.97287	E2F4/E2F8/MUC1/PCBP4/RINT1/SFN/ZNF385A	7	BP
GO:1902402	signal transduction involved in mitotic DNA	7/2734	65/17381	0.90371	0.9891	0.97287	E2F4/E2F8/MUC1/PCBP4/RINT1/SFN/ZNF385A	7	BP
GO:1902403	signal transduction involved in mitotic DNA	7/2734	65/17381	0.90371	0.9891	0.97287	E2F4/E2F8/MUC1/PCBP4/RINT1/SFN/ZNF385A	7	BP
GO:1901796	regulation of signal transduction by	22/2734	176/17381	0.90407	0.9891	0.97287	ANKRD2/ATRIP/BRCA1/BRPF1/CD74/CDK5/CDK5RAP3/EHMT1/EHMT2/EXO1/MUC1/NOC2L/PPP1R13B/PRKAB1/RFC2/SETD9/TAF1/TAF1L/TAF3/TAF6/TAF7/ZNF385A	22	BP
GO:0002820	negative regulation of adaptive	4/2734	41/17381	0.90472	0.9891	0.97287	BCL6/HAVCR2/IL20RB/IL4R	4	BP
GO:0010955	negative regulation of protein	4/2734	41/17381	0.90472	0.9891	0.97287	CHAC1/NAIP/SERPINF2/SNX12	4	BP

GO:1903318	negative regulation of protein	4/2734	41/17381	0.90472	0.9891	0.97287	CHAC1/NAIP/SERPINF2/SNX12	4	BP
GO:0051236	establishment of RNA localization	25/2734	198/17381	0.90701	0.9891	0.97287	ALKBH5/CASC3/CKAP5/CPSF1/CPSF4/DHX38/EIF5A/HSF1/MYO1C/NOL6/NUP210/NUP98/NXF2/NXF2B/NXF3/RBFOX1/RNPS1/SETD2/SIDT2/SMG5/SRSF4/SUPT6H/XPO7/ZC3H3/ZNF593	25	BP
GO:0000054	ribosomal subunit export from nucleus	1/2734	14/17381	0.90901	0.9891	0.97287	ZNF593	1	BP
GO:0002523	leukocyte migration involved in inflammatory	1/2734	14/17381	0.90901	0.9891	0.97287	ADAM8	1	BP
GO:0002726	positive regulation of T cell cytokine	1/2734	14/17381	0.90901	0.9891	0.97287	TRAF2	1	BP
GO:0002864	regulation of acute inflammatory response to	1/2734	14/17381	0.90901	0.9891	0.97287	IL20RB	1	BP
GO:0003214	cardiac left ventricle morphogenesis	1/2734	14/17381	0.90901	0.9891	0.97287	NOTCH1	1	BP
GO:0006122	mitochondrial electron transport, ubiquinol to	1/2734	14/17381	0.90901	0.9891	0.97287	CYC1	1	BP
GO:0006534	cysteine metabolic	1/2734	14/17381	0.90901	0.9891	0.97287	MTHFD1	1	BP

GO:0007175	negative regulation of epidermal growth factor-activated	1/2734	14/17381	0.90901	0.9891	0.97287	VPS25	1	BP
GO:0007183	SMAD protein complex	1/2734	14/17381	0.90901	0.9891	0.97287	SMAD6	1	BP
GO:0007216	G-protein coupled glutamate	1/2734	14/17381	0.90901	0.9891	0.97287	HOMER2	1	BP
GO:0010893	positive regulation of steroid biosynthetic	1/2734	14/17381	0.90901	0.9891	0.97287	POR	1	BP
GO:0014744	positive regulation of muscle	1/2734	14/17381	0.90901	0.9891	0.97287	MTOR	1	BP
GO:0016074	snoRNA metabolic	1/2734	14/17381	0.90901	0.9891	0.97287	EXOSC2	1	BP
GO:0016998	cell wall macromolecule catabolic	1/2734	14/17381	0.90901	0.9891	0.97287	SPACA3	1	BP
GO:0021984	adenohypophysis development	1/2734	14/17381	0.90901	0.9891	0.97287	DRD2	1	BP
GO:0030238	male sex determination	1/2734	14/17381	0.90901	0.9891	0.97287	SF1	1	BP
GO:0032048	cardiolipin metabolic	1/2734	14/17381	0.90901	0.9891	0.97287	HADHA	1	BP
GO:0032310	prostaglandin secretion	1/2734	14/17381	0.90901	0.9891	0.97287	ABCC4	1	BP

GO:0033750	ribosome localization	1/2734	14/17381	0.90901	0.9891	0.97287	ZNF593	1	BP
GO:0034975	protein folding in endoplasmic reticulum	1/2734	14/17381	0.90901	0.9891	0.97287	HSPA5	1	BP
GO:0035589	G-protein coupled purinergic nucleotide receptor	1/2734	14/17381	0.90901	0.9891	0.97287	P2RY6	1	BP
GO:0036124	histone H3-K9 trimethylation	1/2734	14/17381	0.90901	0.9891	0.97287	SUV39H1	1	BP
GO:0036295	cellular response to increased	1/2734	14/17381	0.90901	0.9891	0.97287	ATP6V1G1	1	BP
GO:0043173	nucleotide salvage	1/2734	14/17381	0.90901	0.9891	0.97287	UCK1	1	BP
GO:0043517	positive regulation of DNA damage response, signal transduction by	1/2734	14/17381	0.90901	0.9891	0.97287	ZNF385A	1	BP
GO:0046341	CDP-diacylglycerol metabolic	1/2734	14/17381	0.90901	0.9891	0.97287	CDIPT	1	BP
GO:0046459	short-chain fatty acid metabolic process	1/2734	14/17381	0.90901	0.9891	0.97287	THNSL2	1	BP
GO:0048291	isotype switching to	1/2734	14/17381	0.90901	0.9891	0.97287	PAXIP1	1	BP

GO:0048853	forebrain morphogenesis	1/2734	14/17381	0.90901	0.9891	0.97287	SMO	1	BP
GO:0050812	regulation of acyl-CoA biosynthetic	1/2734	14/17381	0.90901	0.9891	0.97287	PDHB	1	BP
GO:0050930	induction of positive chemotaxis	1/2734	14/17381	0.90901	0.9891	0.97287	ARTN	1	BP
GO:0051382	kinetochore assembly	1/2734	14/17381	0.90901	0.9891	0.97287	SENP6	1	BP
GO:0051481	negative regulation of cytosolic calcium ion	1/2734	14/17381	0.90901	0.9891	0.97287	DRD2	1	BP
GO:0051967	negative regulation of synaptic transmission,	1/2734	14/17381	0.90901	0.9891	0.97287	DRD2	1	BP
GO:0097340	inhibition of cysteine-type endopeptidase activity	1/2734	14/17381	0.90901	0.9891	0.97287	NAIP	1	BP
GO:0097341	zymogen inhibition	1/2734	14/17381	0.90901	0.9891	0.97287	NAIP	1	BP
GO:1904262	negative regulation of TORC1	1/2734	14/17381	0.90901	0.9891	0.97287	SESN1	1	BP
GO:2000651	positive regulation of sodium ion transmembrane	1/2734	14/17381	0.90901	0.9891	0.97287	WNK2	1	BP

GO:1900542	regulation of purine nucleotide metabolic	34/2734	262/17381	0.90901	0.9891	0.97287	ADM/ADRA2A/AIPL1/ATPIF1/AVPR2/CCR2/CHGA/CRHR1/DRD2/FZD2/GABBR1/GCG/GCK/GPER1/GPHA2/GUCA1B/GUCA2A/GUCA2B/INS/MLXIPL/NCOR1/NOS3/NUP210/NUP98/OPRM1/PDE2A/PDZD3/PFKFB1/PID1/PTK2B/RAF1/RUNDC3A/RXFP2/WFS1	34	BP
GO:0006851	mitochondrial calcium ion transmembrane transport	2/2734	24/17381	0.91001	0.9891	0.97287	PMPCA/YME1L1	2	BP
GO:0007214	gamma-aminobutyric acid signaling	2/2734	24/17381	0.91001	0.9891	0.97287	GABBR1/PHF24	2	BP
GO:0008334	histone mRNA metabolic	2/2734	24/17381	0.91001	0.9891	0.97287	EXOSC10/LSM1	2	BP
GO:0019682	glyceraldehyde-3-phosphate metabolic	2/2734	24/17381	0.91001	0.9891	0.97287	GLYCTK/PGAM4	2	BP
GO:0032098	regulation of appetite	2/2734	24/17381	0.91001	0.9891	0.97287	OPRM1/PYY	2	BP
GO:0032104	regulation of response to extracellular stimulus	2/2734	24/17381	0.91001	0.9891	0.97287	MTOR/OPRM1	2	BP
GO:0032107	regulation of response to nutrient levels	2/2734	24/17381	0.91001	0.9891	0.97287	MTOR/OPRM1	2	BP
GO:0042026	protein refolding	2/2734	24/17381	0.91001	0.9891	0.97287	HSP90AA1/HSPA2	2	BP
GO:0045910	negative regulation of DNA	2/2734	24/17381	0.91001	0.9891	0.97287	BCL6/RECQL5	2	BP

GO:0048520	positive regulation of	2/2734	24/17381	0.91001	0.9891	0.97287	MTOR/STRA6	2	BP
GO:0048668	collateral sprouting	2/2734	24/17381	0.91001	0.9891	0.97287	ABL1/WNT3A	2	BP
GO:0051984	positive regulation of chromosome	2/2734	24/17381	0.91001	0.9891	0.97287	NUMA1/SLF2	2	BP
GO:2000036	regulation of stem cell population	2/2734	24/17381	0.91001	0.9891	0.97287	PRDM14/SMO	2	BP
GO:0006409	tRNA export from nucleus	3/2734	33/17381	0.91011	0.9891	0.97287	NOL6/NUP210/NUP98	3	BP
GO:0009154	purine ribonucleotide catabolic	3/2734	33/17381	0.91011	0.9891	0.97287	HINT1/NUDT18/PDE2A	3	BP
GO:0010762	regulation of fibroblast migration	3/2734	33/17381	0.91011	0.9891	0.97287	ARHGAP4/DDR2/WDPCP	3	BP
GO:0015721	bile acid and bile salt	3/2734	33/17381	0.91011	0.9891	0.97287	ABCC3/SLCO2B1/STARD5	3	BP
GO:0032885	regulation of polysaccharide biosynthetic process	3/2734	33/17381	0.91011	0.9891	0.97287	GCK/INS/MTOR	3	BP
GO:0071431	tRNA-containing ribonucleoprotein complex	3/2734	33/17381	0.91011	0.9891	0.97287	NOL6/NUP210/NUP98	3	BP
GO:0000045	autophagosome assembly	10/2734	89/17381	0.9105	0.9891	0.97287	AMBRA1/ATG101/ATG2A/IFT20/LRSAM1/MFN2/NPRL3/RAB7A/RALB/ULK1	10	BP

GO:0000422	autophagy of mitochondrion	10/2734	89/17381	0.9105	0.9891	0.97287	AMBRA1/ATG2A/ATP13A2/ATPIF1/CTTN/GBA/KAT2A/MARK2/MFN2/ZBTB17	10	BP
GO:0061726	mitochondrion disassembly	10/2734	89/17381	0.9105	0.9891	0.97287	AMBRA1/ATG2A/ATP13A2/ATPIF1/CTTN/GBA/KAT2A/MARK2/MFN2/ZBTB17	10	BP
GO:0034308	primary alcohol metabolic	6/2734	58/17381	0.91124	0.9891	0.97287	ADH1A/ADH1C/ALDH2/DGAT1/GDPD1/PLB1	6	BP
GO:0140029	exocytic process	7/2734	66/17381	0.9118	0.9891	0.97287	RAB26/RALB/SCRIB/STX1A/STX4/STXBP1/VTI1B	7	BP
GO:0010965	regulation of mitotic sister chromatid separation	5/2734	50/17381	0.9119	0.9891	0.97287	ANAPC15/BUB1B/LCMT1/MAD1L1/XRCC3	5	BP
GO:0045840	positive regulation of mitotic nuclear	5/2734	50/17381	0.9119	0.9891	0.97287	INS/NUMA1/PDGFRB/SH2B1/SLF2	5	BP
GO:0046173	polyol biosynthetic	5/2734	50/17381	0.9119	0.9891	0.97287	GBA/GCH1/GPER1/PTK2B/SPTLC1	5	BP
GO:0070936	protein K48-linked ubiquitination	5/2734	50/17381	0.9119	0.9891	0.97287	NMI/SYVN1/UBE2A/UBE2B/UBE2E2	5	BP
GO:0033135	regulation of peptidyl-serine phosphorylation	16/2734	134/17381	0.91206	0.9891	0.97287	ARAF/ARRB2/AXIN1/CSF3/DOCK7/EGFR/EIF4G1/GCG/IFNA5/INPP5F/MLXIPL/OPRD1/RAF1/SMYD3/TBK1/WNT3A	16	BP
GO:0045814	negative regulation of gene expression,	16/2734	134/17381	0.91206	0.9891	0.97287	DNMT3A/EPC1/HILS1/HIST1H2AH/HIST1H4F/HIST2H4A/HIST2H4B/HMGA1/MSL3/PHF19/PHF2/SPI1/SUPT6H/SUV39H1/TNRC18/UBE2B	16	BP
GO:0016579	protein deubiquitination	38/2734	291/17381	0.91307	0.9891	0.97287	ARRB2/AXIN1/BRCA1/BRCC3/DDB2/FOXO4/HCFC1/INO80/KAT2A/NEDD8/NFRKB/NOD1/OTUB1/OTUD5/OTUD6A/OTUD7A/PSMB11/PSMB6/PSMB7/PSMB8/PSMC3/PSMD13/PSMD3/PSMD5/PSMD7/SHMT2/TADA3/TNIP2/TRAF2/USP19/USP2/USP20/USP27X/USP35/USP4/USP49/USP5/USP54	38	BP

GO:0014855	striated muscle cell	8/2734	74/17381	0.91314	0.9891	0.97287	FGFR2/MIR222/NKX2-5/NOTCH1/PPARD/PRKAR1A/SHH/TBX2	8	BP
GO:0045637	regulation of myeloid cell differentiation	31/2734	242/17381	0.91367	0.9891	0.97287	ASH2L/BGLAP/C1QC/CD74/CIB1/CSF1/CSF3/ESRRA/FAM213A/FES/HIST1H4F/HIST2H4A/HIST2H4B/HOXB8/INPP5D/ISG15/LDB1/LGALS3/MEIS2/MIR221/MIR222/MTOR/NME1/PTK2B/RARA/SCIN/SETD1A/SPI1/STAT1/TCT	31	BP
GO:0002792	negative regulation of peptide	15/2734	127/17381	0.91411	0.9891	0.97287	ADRA2A/CD74/DRD2/F2RL1/FN1/IDH2/INHBB/INS/NR1H3/OPRM1/PTPN11/RAB11FIP3/RHBDF1/SERGEF/TLR8	15	BP
GO:0050900	leukocyte migration	64/2734	471/17381	0.91488	0.9891	0.97287	ADAM8/AIMP1/APOB/ARTN/C3AR1/CAMK1D/CCL1/CCL19/CCL21/CCL5/CCR2/CD48/CD58/CD74/CHGA/CKLF/COL1A1/CORO1A/CREB3/CSF1/CXCR1/CXCR2/CXCR3/CXCR5/DAPK2/DOK2/ECM1/EDN2/EPX/F2RL1/FN1/FUT7/GBF1/GPC1/HRAS/INPP5D/ITGAL/ITGAM/ITGAX/KARS/L1CAM/LCK/LGALS3/LGALS9/MAPK3/NBL1/NKX2-3/OLR1/PF4V1/PLA2G1B/PODXL2/PTK2B/PTPN11/RARRES2/SDC1/SELP/SHC1/SLC7A7/TACR1/THY1/TIRAP/TNFRSF18/TRPV4/WDR1	64	BP
GO:0140013	meiotic nuclear division	21/2734	171/17381	0.91522	0.9891	0.97287	BUB1B/C11orf80/DDX4/FANCA/GOLGA2/HORMAD1/HSF1/HSPA2/LEMD2/M1AP/MCMDC2/PIWIL2/PRDM9/PRKAR1A/PSMD13/RNF212/SLC2A8/TAF1L/TDRD9/UBE2B/XRCC3	21	BP
GO:0031124	mRNA 3'-end processing	10/2734	90/17381	0.91716	0.9891	0.97287	CASC3/CDK9/CPSF1/CPSF4/CSTF2/DHX38/HSF1/RNPS1/SRSF4/ZC3H3	10	BP
GO:1901654	response to ketone	23/2734	186/17381	0.91796	0.9891	0.97287	AQP1/BAD/BGLAP/CATSPER1/CCL19/CCL21/CDK4/CLDN4/EGFR/EIF4EBP1/FOSL1/GBA/HDAC8/HSF1/NME1/NR1H3/P2RY6/RWDD1/SMYD3/TACR1/TH/TRH/UGT3A2	23	BP
GO:0006110	regulation of glycolytic	7/2734	67/17381	0.91931	0.9891	0.97287	GCK/INS/MLXIPL/NCOR1/NUP210/NUP98/PFKFB1	7	BP
GO:0030521	androgen receptor	7/2734	67/17381	0.91931	0.9891	0.97287	ARRB2/BRCA1/MED24/PLPP1/RWDD1/TAF1/TGFB1I1	7	BP
GO:0072401	signal transduction involved in DNA integrity	7/2734	67/17381	0.91931	0.9891	0.97287	E2F4/E2F8/MUC1/PCBP4/RINT1/SFN/ZNF385A	7	BP

GO:0072422	signal transduction involved in DNA damage	7/2734	67/17381	0.91931	0.9891	0.97287	E2F4/E2F8/MUC1/PCBP4/RINT1/SFN/ZNF385A	7	BP
GO:0031503	protein complex localization	15/2734	128/17381	0.91961	0.9891	0.97287	CCL19/CD27/DYNLL2/GPN2/IFT140/IFT20/IFT22/IL18/KIF17/LGALS9/PRDX1/RALB/SSNA1/TLR9/TRAF3IP1	15	BP
GO:0045333	cellular respiration	21/2734	172/17381	0.91998	0.9891	0.97287	ACO1/CHCHD5/COX10/COX8A/CS/CYC1/CYP1A2/DLST/IDH1/IDH2/MTFR1L/NDUFA2/NDUFB5/NDUFC2-KCTD14/OGDH/OGDHL/PDHB/PRELID1/SLC25A14/SLC25A25/TRAP1	21	BP
GO:0010827	regulation of glucose	8/2734	75/17381	0.9202	0.9891	0.97287	AGT/CAPN10/INS/MZB1/PEA15/PID1/PTPN11/RARRES2	8	BP
GO:0002701	negative regulation of production of molecular mediator of	3/2734	34/17381	0.9203	0.9891	0.97287	BCL6/CUEDC2/SPINK5	3	BP
GO:0009261	ribonucleotide catabolic	3/2734	34/17381	0.9203	0.9891	0.97287	HINT1/NUDT18/PDE2A	3	BP
GO:0010737	protein kinase A signaling	3/2734	34/17381	0.9203	0.9891	0.97287	AIP/GCG/TCP11	3	BP
GO:0014808	release of sequestered calcium ion into cytosol by sarcoplasmic reticulum	3/2734	34/17381	0.9203	0.9891	0.97287	CAMK2D/GSTM2/TRDN	3	BP
GO:0016486	peptide hormone	3/2734	34/17381	0.9203	0.9891	0.97287	AGT/FURIN/REN	3	BP
GO:0019432	triglyceride biosynthetic process	3/2734	34/17381	0.9203	0.9891	0.97287	DGAT1/NR1H3/SCARB1	3	BP

GO:0048854	brain morphogenesis	3/2734	34/17381	0.9203	0.9891	0.97287	CTNNA2/EMX1/SMO	3	BP
GO:0050775	positive regulation of dendrite	3/2734	34/17381	0.9203	0.9891	0.97287	ANAPC2/ILK/OBSL1	3	BP
GO:0001945	lymph vessel development	2/2734	25/17381	0.92159	0.9891	0.97287	FLT4/PKD1	2	BP
GO:0002719	negative regulation of cytokine production involved in	2/2734	25/17381	0.92159	0.9891	0.97287	BCL6/CUEDC2	2	BP
GO:0030194	positive regulation of blood	2/2734	25/17381	0.92159	0.9891	0.97287	SELP/SERPINF2	2	BP
GO:0030511	positive regulation of transforming growth factor beta receptor	2/2734	25/17381	0.92159	0.9891	0.97287	MEN1/TGFB1I1	2	BP
GO:0032400	melanosome localization	2/2734	25/17381	0.92159	0.9891	0.97287	DCTN2/RAB17	2	BP
GO:0033119	negative regulation of	2/2734	25/17381	0.92159	0.9891	0.97287	RNPS1/SRSF4	2	BP
GO:0033173	calcineurin-NFAT signaling	2/2734	25/17381	0.92159	0.9891	0.97287	CIB1/NRG1	2	BP
GO:0036507	protein demannosylatio	2/2734	25/17381	0.92159	0.9891	0.97287	OS9/SYVN1	2	BP
GO:0036508	protein alpha-1,2-	2/2734	25/17381	0.92159	0.9891	0.97287	OS9/SYVN1	2	BP

GO:0045939	negative regulation of steroid	2/2734	25/17381	0.92159	0.9891	0.97287	ATP1A1/SCAP	2	BP
GO:0055022	negative regulation of cardiac muscle	2/2734	25/17381	0.92159	0.9891	0.97287	CAV3/MIR25	2	BP
GO:0061117	negative regulation of	2/2734	25/17381	0.92159	0.9891	0.97287	CAV3/MIR25	2	BP
GO:1900048	positive regulation of	2/2734	25/17381	0.92159	0.9891	0.97287	SELP/SERPINF2	2	BP
GO:1902751	positive regulation of cell cycle G2/M	2/2734	25/17381	0.92159	0.9891	0.97287	CDK4/PHOX2B	2	BP
GO:1903846	positive regulation of cellular response to transforming growth factor	2/2734	25/17381	0.92159	0.9891	0.97287	MEN1/TGFB1I1	2	BP
GO:2000300	regulation of synaptic vesicle exocytosis	2/2734	25/17381	0.92159	0.9891	0.97287	STX1A/STXBP1	2	BP
GO:0030819	positive regulation of cAMP biosynthetic	9/2734	83/17381	0.92162	0.9891	0.97287	ADM/AVPR2/CRHR1/GCG/GPER1/GPHA2/RAF1/RXFP2/WFS1	9	BP
GO:0050709	negative regulation of protein	14/2734	121/17381	0.92195	0.9891	0.97287	ADRA2A/DRD2/F2RL1/FN1/IDH2/INHBB/INS/NR1H3/OPRM1/PTPN11/RAB11FIP3/RHBDF1/SERGEF/TLR8	14	BP
GO:0042698	ovulation cycle	12/2734	106/17381	0.92203	0.9891	0.97287	ANG/ARRB2/EGFR/EGR1/EIF2B2/EIF2B5/INHBB/LFNG/NOS3/NR5A1/OPRM1/VGF	12	BP

GO:0000819	sister chromatid segregation	29/2734	230/17381	0.92255	0.9891	0.97287	ANAPC15/ANKRD53/BUB1B/CDC14A/CENPN/CHMP1A/CHMP4C/CHMP7/CKAP5/DIS3L2/DYNC1H1/DYNC1I1/DYNLL2/HDAC8/HORMAD1/INO80/KIF23/KIF2B/KIF4A/LCMT1/MAD1L1/NCAPD3/NUDC/NUMA1/NUP98/PDS5	29	BP
GO:0038094	Fc-gamma receptor signaling	16/2734	136/17381	0.92272	0.9891	0.97287	ABL1/ARPC1A/ARPC1B/ARPC2/ARPC4/BRK1/CD247/DOCK1/FCGR3A/HSP90AA1/LIMK1/MAPK3/MYO1C/PLD2/PRKCD/WAS	16	BP
GO:0001829	trophectodermal cell	1/2734	15/17381	0.92333	0.9891	0.97287	CDX2	1	BP
GO:0002070	epithelial cell maturation	1/2734	15/17381	0.92333	0.9891	0.97287	TMEM79	1	BP
GO:0002467	germinal center formation	1/2734	15/17381	0.92333	0.9891	0.97287	BCL6	1	BP
GO:0006999	nuclear pore organization	1/2734	15/17381	0.92333	0.9891	0.97287	NUP98	1	BP
GO:0007351	tripartite regional	1/2734	15/17381	0.92333	0.9891	0.97287	TDGF1	1	BP
GO:0008595	anterior/posterior axis specification,	1/2734	15/17381	0.92333	0.9891	0.97287	TDGF1	1	BP
GO:0010561	negative regulation of glycoprotein biosynthetic	1/2734	15/17381	0.92333	0.9891	0.97287	TINF2	1	BP
GO:0015671	oxygen transport	1/2734	15/17381	0.92333	0.9891	0.97287	HBZ	1	BP
GO:0021542	dentate gyrus development	1/2734	15/17381	0.92333	0.9891	0.97287	SMO	1	BP

GO:0032968	positive regulation of transcription elongation from RNA	1/2734	15/17381	0.92333	0.9891	0.97287	SUPT6H	1	BP
GO:0034310	primary alcohol catabolic	1/2734	15/17381	0.92333	0.9891	0.97287	ALDH2	1	BP
GO:0034380	high-density lipoprotein particle	1/2734	15/17381	0.92333	0.9891	0.97287	APOA5	1	BP
GO:0034616	response to laminar fluid shear stress	1/2734	15/17381	0.92333	0.9891	0.97287	SMAD6	1	BP
GO:0035458	cellular response to	1/2734	15/17381	0.92333	0.9891	0.97287	STAT1	1	BP
GO:0035729	cellular response to hepatocyte growth factor	1/2734	15/17381	0.92333	0.9891	0.97287	TDGF1	1	BP
GO:0042737	drug catabolic process	1/2734	15/17381	0.92333	0.9891	0.97287	CYP1A2	1	BP
GO:0042921	glucocorticoid receptor signaling	1/2734	15/17381	0.92333	0.9891	0.97287	PER1	1	BP
GO:0046599	regulation of centriole replication	1/2734	15/17381	0.92333	0.9891	0.97287	BRCA1	1	BP
GO:0051589	negative regulation of neurotransmitte	1/2734	15/17381	0.92333	0.9891	0.97287	PNKD	1	BP

GO:0051895	negative regulation of focal adhesion	1/2734	15/17381	0.92333	0.9891	0.97287	BCAS3	1	BP
GO:0060397	JAK-STAT cascade involved in growth	1/2734	15/17381	0.92333	0.9891	0.97287	MAPK3	1	BP
GO:0070200	establishment of protein localization to	1/2734	15/17381	0.92333	0.9891	0.97287	ACD	1	BP
GO:0071360	cellular response to exogenous	1/2734	15/17381	0.92333	0.9891	0.97287	RALB	1	BP
GO:1901524	regulation of mitophagy	1/2734	15/17381	0.92333	0.9891	0.97287	MFN2	1	BP
GO:1903818	positive regulation of voltage-gated potassium	1/2734	15/17381	0.92333	0.9891	0.97287	NPPA	1	BP
GO:0010761	fibroblast migration	4/2734	43/17381	0.92336	0.9891	0.97287	ARHGAP4/DDR2/ILK/WDPCP	4	BP
GO:0019835	cytolysis	4/2734	43/17381	0.92336	0.9891	0.97287	BAD/C8A/GSDMD/MICB	4	BP
GO:0046006	regulation of activated T cell proliferation	4/2734	43/17381	0.92336	0.9891	0.97287	CASP3/IL18/LGALS9/PRKAR1A	4	BP
GO:0090278	negative regulation of peptide	4/2734	43/17381	0.92336	0.9891	0.97287	ADRA2A/DRD2/INHBB/PTPN11	4	BP
GO:0043502	regulation of muscle	10/2734	91/17381	0.9234	0.9891	0.97287	AGT/CAMK2D/CAV3/CDK9/LMNA/MIR25/MTOR/NOS3/TNNC1/TRIM63	10	BP

GO:0044264	cellular polysaccharide metabolic	10/2734	91/17381	0.9234	0.9891	0.97287	GCK/GNMT/INS/MGAM/MTOR/PCDH12/PFKM/PHKG1/PHLDA2/PYGM	10	BP
GO:0030817	regulation of cAMP biosynthetic process	15/2734	129/17381	0.92482	0.9905	0.97419	ADM/ADRA2A/AVPR2/CCR2/CRHR1/DRD2/GABBR1/GCG/GPER1/GPHA2/OPRM1/PDE2A/RAF1/RXFP2/WFS1	15	BP
GO:0043473	pigmentation	11/2734	99/17381	0.92541	0.9908	0.97448	CD63/DCTN2/DRD2/DTNBP1/EN1/HPS1/KIF13A/KRT76/MYO7A/RAB17/TH	11	BP
GO:0002200	somatic diversification of immune	7/2734	68/17381	0.92625	0.9908	0.97448	BCL6/CLCF1/EXO1/PAXIP1/PMS2P3/SUPT6H/TNFSF13	7	BP
GO:0070192	chromosome organization involved in meiotic cell	7/2734	68/17381	0.92625	0.9908	0.97448	BUB1B/HORMAD1/LEMD2/MCMDC2/NCAPD3/RNF212/UBE2B	7	BP
GO:0072395	signal transduction involved in cell	7/2734	68/17381	0.92625	0.9908	0.97448	E2F4/E2F8/MUC1/PCBP4/RINT1/SFN/ZNF385A	7	BP
GO:0043470	regulation of carbohydrate catabolic	8/2734	76/17381	0.92675	0.9908	0.97448	GCK/INS/MLXIPL/NCOR1/NUP210/NUP98/PFKFB1/PGAM4	8	BP
GO:0045652	regulation of megakaryocyte differentiation	8/2734	76/17381	0.92675	0.9908	0.97448	ASH2L/CIB1/HIST1H4F/HIST2H4A/HIST2H4B/SCIN/SETD1A/THPO	8	BP
GO:0009123	nucleoside monophosphate metabolic process	44/2734	337/17381	0.9274	0.9908	0.97448	AK5/AK6/ALDOA/ATP5G1/ATP5I/ATP6V1B1/ATPIF1/BAD/CARD11/COX10/COX8A/CYC1/DGUOK/DHODH/DLG4/DNPH1/ENTPD8/GALK1/GAPDH/GCK/HK3/IMPDH1/INS/LDHA/MLXIPL/NCOR1/NDUFA2/NDUFAF7/NDUFB5/NDUFC2-KCTD14/NT5M/NUP210/NUP98/OGDH/OGDHL/PFKFB1/PFKM/PGAM4/PID	44	BP

GO:0051783	regulation of nuclear division	22/2734	181/17381	0.92771	0.9908	0.97448	ANAPC15/BMP4/BUB1B/CDC14A/CHMP1A/CHMP4C/HORMAD1/INS/LCMT1/MAD1L1/NUMA1/OBSL1/PDGFRB/PIWIL2/PKMYT1/PPP1R9B/PRDM9/PRKAR1A/SH2B1/SLF2/UBE2B/XRCC3	22	BP
GO:0051781	positive regulation of	9/2734	84/17381	0.9278	0.9908	0.97448	CXCR5/DRD2/FGF3/FGFR2/KIF23/OPN1MW/PDGFA/SHH/SSTR5	9	BP
GO:0140014	mitotic nuclear division	32/2734	253/17381	0.92883	0.9908	0.97448	ANAPC15/ANKRD53/ARHGEF10/BMP4/BUB1B/CDC14A/CHMP1A/CHMP4C/CHMP7/DIS3L2/FLNA/GOLGA2/INO80/INS/KIF23/KIF4A/LCMT1/MAD1L1/NCAPD3/NUMA1/OBSL1/PDGFRB/PDS5A/PKMYT1/PPP1R9B/SH2B1/SLF2/TUBGCP2/TUBGCP3/TUBGCP5/WRAP73/XRCC3	32	BP
GO:0030816	positive regulation of cAMP	10/2734	92/17381	0.92923	0.9908	0.97448	ADM/AVPR2/CHGA/CRHR1/GCG/GPER1/GPHA2/RAF1/RXFP2/WFS1	10	BP
GO:0001912	positive regulation of leukocyte mediated	3/2734	35/17381	0.92943	0.9908	0.97448	F2RL1/HLA-E/NCR3	3	BP
GO:0010165	response to X-ray	3/2734	35/17381	0.92943	0.9908	0.97448	BRCC3/CASP3/KARS	3	BP
GO:0051031	tRNA transport	3/2734	35/17381	0.92943	0.9908	0.97448	NOL6/NUP210/NUP98	3	BP
GO:0060795	cell fate commitment involved in formation of primary germ	3/2734	35/17381	0.92943	0.9908	0.97448	BMP4/EYA1/WNT3A	3	BP
GO:0034605	cellular response to heat	13/2734	115/17381	0.9298	0.9908	0.97448	ANO1/CAMK2D/CCAR2/CRYAB/HSF1/HSP90AA1/IRAK1/MAPK3/MLST8/MTOR/NUP210/NUP98/TRPV4	13	BP

GO:0009144	purine nucleoside triphosphate metabolic process	39/2734	303/17381	0.93067	0.9908	0.97448	AK5/ALDOA/ATP5G1/ATP5I/ATP6V1B1/ATPIF1/BAD/COX10/COX8A/CYC1/DGUOK/GALK1/GAPDH/GCK/HK3/IMPDH1/INS/LDHA/MFN1/MLXIPL/NCOR1/NDUFA2/NDUFAF7/NDUFB5/NDUFC2-KCTD14/NME1/NME4/NUDT1/NUP210/NUP98/OGDH/OGDHL/PFKFB1/PFKM/PGAM4/PID1/PKM/SLC25A25/VPS9D1	39	BP
GO:0042059	negative regulation of epidermal growth factor receptor	4/2734	44/17381	0.93139	0.9908	0.97448	DAB2IP/EGFR/RAB7A/VPS25	4	BP
GO:0031167	rRNA methylation	2/2734	26/17381	0.93176	0.9908	0.97448	NSUN5/NSUN5P2	2	BP
GO:0033137	negative regulation of peptidyl-serine	2/2734	26/17381	0.93176	0.9908	0.97448	INPP5F/MLXIPL	2	BP
GO:0035510	DNA dealkylation	2/2734	26/17381	0.93176	0.9908	0.97448	ALKBH5/APEX1	2	BP
GO:0039528	cytoplasmic pattern recognition receptor signaling	2/2734	26/17381	0.93176	0.9908	0.97448	DHX58/NLRX1	2	BP
GO:0043921	modulation by host of viral transcription	2/2734	26/17381	0.93176	0.9908	0.97448	CCL5/PSMC3	2	BP
GO:0045671	negative regulation of osteoclast	2/2734	26/17381	0.93176	0.9908	0.97448	INPP5D/TCTA	2	BP
GO:0050820	positive regulation of	2/2734	26/17381	0.93176	0.9908	0.97448	SELP/SERPINF2	2	BP

GO:0052472	modulation by host of symbiont	2/2734	26/17381	0.93176	0.9908	0.97448	CCL5/PSMC3	2	BP
GO:0071354	cellular response to	2/2734	26/17381	0.93176	0.9908	0.97448	PID1/TDGF1	2	BP
GO:1904353	regulation of telomere	2/2734	26/17381	0.93176	0.9908	0.97448	MAPK15/MAPK3	2	BP
GO:0042770	signal transduction in response to	14/2734	123/17381	0.93209	0.9908	0.97448	ABL1/BRCA1/CD74/E2F4/E2F8/FOXM1/ING4/MUC1/PAXIP1/PCBP4/RINT1/SFN/WNT1/ZNF385A	14	BP
GO:0016458	gene silencing	57/2734	429/17381	0.93235	0.9908	0.97448	BMP4/CHMP1A/DDX4/DND1/DNMT3A/EGFR/EIF4G1/FKBP6/HILS1/HIST1H2AH/HIST1H4F/HIST2H4A/HIST2H4B/HMGA1/KCNQ1/MIR106B/MIR10A/MIR153-1/MIR15A/MIR16-1/MIR181B1/MIR210/MIR212/MIR221/MIR222/MIR25/MIR29A/MIR29C/MIR32/MIR328/MIR503/MIR582/MIR892B/MIR92A2/MIR96/MIRLET7A2/MSL3/MYCN/NCOR1/NCOR2/NUP210/NUP98/PHF2/PIWIL2/POLR2G/POLR2L/RBM4/SND1/SUPT6H/SUV39H1/TARBP2/TDRD1/TDRD9/TNRC18/TSNAX/UB[illegible]	57	BP
GO:0022602	ovulation cycle process	9/2734	85/17381	0.93356	0.9908	0.97448	ANG/ARRB2/EIF2B2/EIF2B5/INHBB/LFNG/NOS3/NR5A1/VGF	9	BP
GO:0048640	negative regulation of developmental	9/2734	85/17381	0.93356	0.9908	0.97448	ARHGAP4/BMP4/CAV3/CDK5/DRAXIN/MIR25/RAI1/SEMA3F/WNT3A	9	BP
GO:0030219	megakaryocyte differentiation	10/2734	93/17381	0.93467	0.9908	0.97448	ASH2L/CIB1/HIST1H4F/HIST2H4A/HIST2H4B/PTPN11/SCIN/SETD1A/THPO/ZNF385A	10	BP
GO:0032411	positive regulation of transporter	10/2734	93/17381	0.93467	0.9908	0.97448	ARC/CRACR2A/GSTM2/HSPA2/NLGN3/NPPA/PRKCD/SGK1/TRDN/WNK2	10	BP
GO:0060043	regulation of cardiac muscle cell	5/2734	53/17381	0.93511	0.9908	0.97448	FGFR2/MIR222/NKX2-5/NOTCH1/TBX2	5	BP

GO:0000076	DNA replication	1/2734	16/17381	0.9354	0.9908	0.97448	TICRR	1	BP
GO:0001946	lymphangiogenesis	1/2734	16/17381	0.9354	0.9908	0.97448	FLT4	1	BP
GO:0002295	T-helper cell lineage commitment	1/2734	16/17381	0.9354	0.9908	0.97448	LY9	1	BP
GO:0002689	negative regulation of leukocyte	1/2734	16/17381	0.9354	0.9908	0.97448	NBL1	1	BP
GO:0003159	morphogenesis of an	1/2734	16/17381	0.9354	0.9908	0.97448	BMP4	1	BP
GO:0006544	glycine metabolic	1/2734	16/17381	0.9354	0.9908	0.97448	SHMT2	1	BP
GO:0006590	thyroid hormone	1/2734	16/17381	0.9354	0.9908	0.97448	DUOXA1	1	BP
GO:0016556	mRNA modification	1/2734	16/17381	0.9354	0.9908	0.97448	CMTR1	1	BP
GO:0019372	lipoxygenase pathway	1/2734	16/17381	0.9354	0.9908	0.97448	GPX1	1	BP
GO:0030214	hyaluronan catabolic	1/2734	16/17381	0.9354	0.9908	0.97448	HEXA	1	BP
GO:0032516	positive regulation of phosphoprotein phosphatase	1/2734	16/17381	0.9354	0.9908	0.97448	PDGFRB	1	BP
GO:0032816	positive regulation of natural killer	1/2734	16/17381	0.9354	0.9908	0.97448	IL18	1	BP
GO:0042268	regulation of cytolysis	1/2734	16/17381	0.9354	0.9908	0.97448	BAD	1	BP

GO:0043101	purine-containing	1/2734	16/17381	0.9354	0.9908	0.97448	DGUOK	1	BP
GO:0044036	cell wall macromolecule metabolic	1/2734	16/17381	0.9354	0.9908	0.97448	SPACA3	1	BP
GO:0048820	hair follicle maturation	1/2734	16/17381	0.9354	0.9908	0.97448	SPINK5	1	BP
GO:0051923	sulfation	1/2734	16/17381	0.9354	0.9908	0.97448	CHST5	1	BP
GO:0061154	endothelial tube morphogenesis	1/2734	16/17381	0.9354	0.9908	0.97448	BMP4	1	BP
GO:0061298	retina vasculature development in	1/2734	16/17381	0.9354	0.9908	0.97448	PDGFRB	1	BP
GO:0070166	enamel mineralization	1/2734	16/17381	0.9354	0.9908	0.97448	AMTN	1	BP
GO:0071428	rRNA-containing ribonucleoprotein complex	1/2734	16/17381	0.9354	0.9908	0.97448	ZNF593	1	BP
GO:0071554	cell wall organization or biogenesis	1/2734	16/17381	0.9354	0.9908	0.97448	SPACA3	1	BP
GO:0071871	response to epinephrine	1/2734	16/17381	0.9354	0.9908	0.97448	KCNQ1	1	BP

GO:1900151	regulation of nuclear-transcribed mRNA catabolic process, deadenylation-	1/2734	16/17381	0.9354	0.9908	0.97448	POLR2G	1	BP
GO:1900153	positive regulation of nuclear-transcribed mRNA catabolic process,	1/2734	16/17381	0.9354	0.9908	0.97448	POLR2G	1	BP
GO:1903392	negative regulation of adherens junction	1/2734	16/17381	0.9354	0.9908	0.97448	BCAS3	1	BP
GO:2000811	negative regulation of	1/2734	16/17381	0.9354	0.9908	0.97448	NOTCH1	1	BP
GO:0002377	immunoglobulin production	16/2734	139/17381	0.93667	0.9917	0.9754	BCL6/CARD11/CLCF1/EXO1/HLA-E/IL13/IL4R/MZB1/PAXIP1/PMS2P3/STX4/SUPT6H/TLR9/TNFRSF4/TNFSF1	16	BP
GO:0030518	intracellular steroid hormone receptor	16/2734	139/17381	0.93667	0.9917	0.9754	ARRB2/AXIN1/BRCA1/CALCOCO1/CRIPAK/DDX54/ESR2/GPER1/MED24/PER1/PLPP1/RWDD1/TADA3/TAF1/TAF7/TGFB1I1	16	BP
GO:0000959	mitochondrial RNA metabolic process	3/2734	36/17381	0.93759	0.9917	0.9754	AARS2/TARS2/YARS2	3	BP
GO:0002931	response to ischemia	3/2734	36/17381	0.93759	0.9917	0.9754	CIB1/EGR1/EIF4EBP1	3	BP

GO:0007190	activation of adenylate cyclase activity	3/2734	36/17381	0.93759	0.9917	0.9754	AVPR2/CRHR1/RAF1	3	BP
GO:0030431	sleep	3/2734	36/17381	0.93759	0.9917	0.9754	DRD2/IL18/SLC29A1	3	BP
GO:0060416	response to growth	3/2734	36/17381	0.93759	0.9917	0.9754	CACYBP/MAPK3/SLC34A1	3	BP
GO:0048593	camera-type eye morphogenesis	12/2734	109/17381	0.93762	0.9917	0.9754	AQP1/AQP5/BMP4/FOXE3/LHX1/MFN2/SOX8/STRA6/TBX2/TH/THY1/TSKU	12	BP
GO:0019080	viral gene expression	23/2734	191/17381	0.93834	0.992	0.97571	CCL5/CDK9/EIF2AK4/EIF3B/FURIN/NELFB/NUP210/NUP98/PFN1/POLR2G/POLR2L/PSMC3/RPL35A/RPL36A/RPL41/RPL7/RPS24/RSF1/TARBP2/TRIM11/TRIM14/TRIM31/TRIM62	23	BP
GO:0048599	oocyte development	4/2734	45/17381	0.93866	0.992	0.97571	ANG/INHBB/PTK2B/RXFP2	4	BP
GO:1902373	negative regulation of mRNA	4/2734	45/17381	0.93866	0.992	0.97571	MTOR/PKP3/SLC11A1/TIRAP	4	BP
GO:0006977	DNA damage response, signal transduction by p53 class mediator resulting in cell	6/2734	62/17381	0.93949	0.992	0.97571	E2F4/E2F8/MUC1/PCBP4/SFN/ZNF385A	6	BP
GO:0016485	protein processing	39/2734	306/17381	0.93972	0.992	0.97571	ADAM8/ADAMTS13/ADAMTS2/AEBP1/AGT/APH1A/BAD/BGLAP/C1QB/C1QC/C2/C2CD3/C3AR1/C8A/CASP1/CASP3/CD81/CFH/CHAC1/CLU/CPN2/CTSE/CTSH/FGA/FURIN/KLKB1/NAIP/NKD2/PLAU/PMPCA/PROZ/PRSS37/REN/RHBDL2/SERPINF2/SHH/SNX12/TYSND1/YME1L1	39	BP

GO:0010882	regulation of cardiac muscle contraction by calcium ion signaling	2/2734	27/17381	0.94068	0.992	0.97571	CAMK2D/GSTM2	2	BP
GO:0032814	regulation of natural killer cell activation	2/2734	27/17381	0.94068	0.992	0.97571	HAVCR2/IL18	2	BP
GO:0034629	cellular protein complex localization	2/2734	27/17381	0.94068	0.992	0.97571	GPN2/RALB	2	BP
GO:0042501	serine phosphorylation of STAT	2/2734	27/17381	0.94068	0.992	0.97571	CDK5/IFNA5	2	BP
GO:0052312	modulation of transcription in other organism involved in symbiotic interaction	2/2734	27/17381	0.94068	0.992	0.97571	CCL5/PSMC3	2	BP
GO:0070198	protein localization to chromosome,	2/2734	27/17381	0.94068	0.992	0.97571	ACD/TINF2	2	BP
GO:2000134	negative regulation of G1/S transition of mitotic cell	12/2734	110/17381	0.94218	0.992	0.97571	CDK2AP2/CTDSP1/E2F4/E2F8/MIR15A/MIR16-1/MIR29A/MIR29C/MUC1/PCBP4/SFN/ZNF385A	12	BP

GO:0002449	lymphocyte mediated immunity	40/2734	314/17381	0.94231	0.992	0.97571	ARRB2/BCL6/C1QB/C1QC/C1RL/C2/C8A/CD27/CD74/CLCF1/CLU/CORO1A/CTSH/EXO1/HAVCR2/HLA-E/IL18/IL20RB/IL31RA/IL4R/INPP5D/KLRC2/LGALS9/LTA/MASP2/MICB/NCR3/PAXIP1/PIK3R6/PRDX1/PRKCD/RAET1G/SLAMF7/SLC11A1/SUPT6H/TLR8/TNFSF13/TRAF2/ULBP2/WAS	40	BP
GO:0007062	sister chromatid cohesion	15/2734	133/17381	0.94289	0.992	0.97571	BUB1B/CENPN/CKAP5/DYNC1H1/DYNC1I1/DYNLL2/HDAC8/HORMAD1/KIF2B/MAD1L1/NUDC/NUP98/PDS5A/PMF1/SLF2	15	BP
GO:0006612	protein targeting to	19/2734	163/17381	0.94331	0.992	0.97571	CDK5/CIB1/ERBB2/ITGAM/MIEF2/MYO1C/NCF1/PARD3/PEX16/PEX3/PEX5/RPL35A/RPL36A/RPL41/RPL7/RPS24/SRP14/SSR2/TAOK2	19	BP
GO:0006342	chromatin silencing	13/2734	118/17381	0.94363	0.992	0.97571	DNMT3A/HILS1/HIST1H2AH/HIST1H4F/HIST2H4A/HIST2H4B/HMGA1/MSL3/PHF2/SUPT6H/SUV39H1/TNRC18/UBE2B	13	BP
GO:0033108	mitochondrial respiratory chain complex	9/2734	87/17381	0.94388	0.992	0.97571	AARS2/COA3/COA4/FOXRED1/NDUFA2/NDUFAF6/NDUFAF7/NDUFB5/SMIM20	9	BP
GO:0009141	nucleoside triphosphate metabolic process	41/2734	322/17381	0.94479	0.992	0.97571	AK5/ALDOA/ATP5G1/ATP5I/ATP6V1B1/ATPIF1/BAD/COX10/COX8A/CYC1/DGUOK/GALK1/GAPDH/GCK/HK3/IMPDH1/INS/LDHA/MFN1/MLXIPL/NCOR1/NDUFA2/NDUFAF7/NDUFB5/NDUFC2-KCTD14/NME1/NME4/NUDT1/NUP210/NUP98/OGDH/OGDHL/PFKFB1/PFKM/PGAM4/PID1/PKM/SLC25A25/TBPL1/UCK1/VPS9D1	41	BP
GO:0018023	peptidyl-lysine trimethylation	3/2734	37/17381	0.94486	0.992	0.97571	SETD2/SUV39H1/VCPKMT	3	BP
GO:0042149	cellular response to glucose	3/2734	37/17381	0.94486	0.992	0.97571	ASNS/HSPA5/SESN1	3	BP
GO:0070423	nucleotide-binding oligomerization domain	3/2734	37/17381	0.94486	0.992	0.97571	IKBKB/IRAK1/NOD1	3	BP
GO:2001252	positive regulation of chromosome	18/2734	156/17381	0.94487	0.992	0.97571	ACD/BCL6/BRCA1/CCT3/CDK9/CTBP1/GCG/KAT2A/MAPK15/MAPK3/MUC1/NUMA1/PAXIP1/PHF19/PIWIL2/PRDM12/RPS6KA4/SLF2	18	BP

GO:0060038	cardiac muscle cell	6/2734	63/17381	0.94517	0.992	0.97571	FGFR2/MIR222/NKX2-5/NOTCH1/PRKAR1A/TBX2	6	BP
GO:0072431	signal transduction involved in mitotic G1	6/2734	63/17381	0.94517	0.992	0.97571	E2F4/E2F8/MUC1/PCBP4/SFN/ZNF385A	6	BP
GO:1902400	intracellular signal transduction involved in G1 DNA damage	6/2734	63/17381	0.94517	0.992	0.97571	E2F4/E2F8/MUC1/PCBP4/SFN/ZNF385A	6	BP
GO:0007129	synapsis	4/2734	46/17381	0.94521	0.992	0.97571	HORMAD1/MCMDC2/RNF212/UBE2B	4	BP
GO:0043330	response to exogenous dsRNA	4/2734	46/17381	0.94521	0.992	0.97571	CARD9/IFNA5/MAPK3/RALB	4	BP
GO:0046164	alcohol catabolic	4/2734	46/17381	0.94521	0.992	0.97571	ALDH2/INPP5E/SCARB1/SCARF1	4	BP
GO:0009199	ribonucleoside triphosphate metabolic process	38/2734	301/17381	0.94543	0.992	0.97571	AK5/ALDOA/ATP5G1/ATP5I/ATP6V1B1/ATPIF1/BAD/COX10/COX8A/CYC1/GALK1/GAPDH/GCK/HK3/IMPDH1/INS/LDHA/MFN1/MLXIPL/NCOR1/NDUFA2/NDUFAF7/NDUFB5/NDUFC2-KCTD14/NME1/NME4/NUP210/NUP98/OGDH/OGDHL/PFKFB1/PFKM/PGAM4/PID1/PKM/SLC25A25/UCK1/VPS9D1	38	BP
GO:0001832	blastocyst growth	1/2734	17/17381	0.94557	0.992	0.97571	ZPR1	1	BP
GO:0006071	glycerol metabolic	1/2734	17/17381	0.94557	0.992	0.97571	COQ2	1	BP
GO:0015732	prostaglandin transport	1/2734	17/17381	0.94557	0.992	0.97571	ABCC4	1	BP
GO:0022010	central nervous system	1/2734	17/17381	0.94557	0.992	0.97571	CLU	1	BP

GO:0031293	membrane protein intracellular	1/2734	17/17381	0.94557	0.992	0.97571	APH1A	1	BP
GO:0032291	axon ensheathment in central nervous	1/2734	17/17381	0.94557	0.992	0.97571	CLU	1	BP
GO:0032717	negative regulation of interleukin-8	1/2734	17/17381	0.94557	0.992	0.97571	TLR9	1	BP
GO:0035459	cargo loading into vesicle	1/2734	17/17381	0.94557	0.992	0.97571	KIF13A	1	BP
GO:0035728	response to hepatocyte growth factor	1/2734	17/17381	0.94557	0.992	0.97571	TDGF1	1	BP
GO:0036010	protein localization to	1/2734	17/17381	0.94557	0.992	0.97571	SCRIB	1	BP
GO:0043373	CD4-positive, alpha-beta T cell lineage commitment	1/2734	17/17381	0.94557	0.992	0.97571	LY9	1	BP
GO:0045019	negative regulation of nitric oxide biosynthetic	1/2734	17/17381	0.94557	0.992	0.97571	MIR92A2	1	BP
GO:0070935	3'-UTR-mediated mRNA	1/2734	17/17381	0.94557	0.992	0.97571	TIRAP	1	BP
GO:0071380	cellular response to prostaglandin E	1/2734	17/17381	0.94557	0.992	0.97571	P2RY6	1	BP

GO:0072673	lamellipodium morphogenesis	1/2734	17/17381	0.94557	0.992	0.97571	CORO1B	1	BP
GO:1904406	negative regulation of nitric oxide	1/2734	17/17381	0.94557	0.992	0.97571	MIR92A2	1	BP
GO:2000269	regulation of fibroblast apoptotic	1/2734	17/17381	0.94557	0.992	0.97571	CHD8	1	BP
GO:0034470	ncRNA processing	52/2734	400/17381	0.94655	0.9927	0.97633	AARS/AARS2/ADAT1/C9orf64/CDKAL1/CPSF1/CPSF4/CSTF2/DHX37/DIS3L/DIS3L2/EXOSC10/EXOSC2/FCF1/INTS10/INTS3/INTS5/ISG20L2/KARS/MDN1/METTL2A/METTL2B/MRPS11/NHP2/NOB1/NOL11/NOL6/NSUN5/NSUN5P2/PDCD11/PUSL1/RPL35A/RPL36A/RPL41/RPL7/RPP21/RPS24/RPUSD2/SARS/SART1/SENP3/SRFBP1/SUV39H1/TARBP2/THUMPD2/TRMT10B/TR	52	BP
GO:0015980	energy derivation by oxidation of organic	33/2734	266/17381	0.94717	0.9927	0.97633	ACADVL/ACO1/CHCHD5/COX10/COX8A/CS/CYC1/CYP1A2/DLST/GCK/GNMT/IDH1/IDH2/INS/LEPR/MTFR1L/MTOR/NDUFA2/NDUFB5/NDUFC2-KCTD14/OGDH/OGDHL/PCDH12/PDHB/PFKM/PHKG1/PHLDA2/PID1/PRELID1/PYGM/SLC25A14/SLC25A25/TRAP1	33	BP
GO:0046034	ATP metabolic process	33/2734	266/17381	0.94717	0.9927	0.97633	AK5/ALDOA/ATP5G1/ATP5I/ATP6V1B1/ATPIF1/BAD/COX10/COX8A/CYC1/GALK1/GAPDH/GCK/HK3/INS/LDHA/MLXIPL/NCOR1/NDUFA2/NDUFAF7/NDUFB5/NDUFC2-KCTD14/NUP210/NUP98/OGDH/OGDHL/PFKFB1/PFKM/PGAM4/PID1/PKM/SLC25A25/VPS9D1	33	BP
GO:0002709	regulation of T cell mediated	5/2734	55/17381	0.94737	0.9927	0.97633	HLA-E/IL20RB/NCR3/TRAF2/WAS	5	BP
GO:0009311	oligosaccharide metabolic	5/2734	55/17381	0.94737	0.9927	0.97633	GAL3ST4/MGAM/MPDU1/NEU3/ST3GAL4	5	BP
GO:0006376	mRNA splice site selection	2/2734	28/17381	0.94847	0.9927	0.97633	LUC7L/SF1	2	BP
GO:0007099	centriole replication	2/2734	28/17381	0.94847	0.9927	0.97633	BRCA1/C2CD3	2	BP

GO:0007143	female meiotic nuclear division	2/2734	28/17381	0.94847	0.9927	0.97633	HSF1/PRKAR1A	2	BP
GO:0010569	regulation of double-strand break repair via homologous recombination	2/2734	28/17381	0.94847	0.9927	0.97633	RECQL5/SETD2	2	BP
GO:0090162	establishment of epithelial cell polarity	2/2734	28/17381	0.94847	0.9927	0.97633	MYO18A/PARD3	2	BP
GO:1901522	positive regulation of transcription from RNA polymerase II promoter involved in cellular	2/2734	28/17381	0.94847	0.9927	0.97633	CREB3/NOTCH1	2	BP
GO:0051193	regulation of cofactor metabolic	9/2734	88/17381	0.9485	0.9927	0.97633	GCK/INS/MLXIPL/NCOR1/NUP210/NUP98/PDHB/PFKFB1/PGAM4	9	BP
GO:0051196	regulation of coenzyme metabolic	9/2734	88/17381	0.9485	0.9927	0.97633	GCK/INS/MLXIPL/NCOR1/NUP210/NUP98/PDHB/PFKFB1/PGAM4	9	BP
GO:0031100	animal organ regeneration	8/2734	80/17381	0.94851	0.9927	0.97633	ADM/CDK4/EGFR/NOTCH1/PFKFB1/PKM/PTPRU/WNT1	8	BP
GO:0050805	negative regulation of synaptic	7/2734	72/17381	0.94906	0.9931	0.97673	ACHE/DGKI/DRD2/NLGN3/PNKD/PTK2B/STXBP1	7	BP

GO:0035194	posttranscriptional gene silencing by RNA	39/2734	310/17381	0.95023	0.994	0.97767	BMP4/DND1/EGFR/EIF4G1/HIST1H4F/HIST2H4A/HIST2H4B/MIR106B/MIR10A/MIR153-1/MIR15A/MIR16-1/MIR181B1/MIR210/MIR212/MIR221/MIR222/MIR25/MIR29A/MIR29C/MIR32/MIR328/MIR503/MIR582/MIR892B/MIR92A2/MIR96/MIRLET7A2/MYCN/NCOR1/NCOR2/NUP210/NUP98/POLR2G/POLR2L/RBM4/TARBP2/TSNAX/	39	BP
GO:0009205	purine ribonucleoside triphosphate metabolic process	37/2734	296/17381	0.95082	0.994	0.97767	AK5/ALDOA/ATP5G1/ATP5I/ATP6V1B1/ATPIF1/BAD/COX10/COX8A/CYC1/GALK1/GAPDH/GCK/HK3/IMPDH1/INS/LDHA/MFN1/MLXIPL/NCOR1/NDUFA2/NDUFAF7/NDUFB5/NDUFC2-KCTD14/NME1/NME4/NUP210/NUP98/OGDH/OGDHL/PFKFB1/PFKM/PGAM4/PID1/PKM/SLC25A25/VPS9D1	37	BP
GO:0005978	glycogen biosynthetic process	4/2734	47/17381	0.95112	0.994	0.97767	GCK/INS/MTOR/PHKG1	4	BP
GO:0009250	glucan biosynthetic	4/2734	47/17381	0.95112	0.994	0.97767	GCK/INS/MTOR/PHKG1	4	BP
GO:0002756	MyD88-independent toll-like receptor	3/2734	38/17381	0.95135	0.994	0.97767	CD300LF/IKBKB/TBK1	3	BP
GO:0035872	nucleotide-binding domain, leucine rich repeat containing receptor [illegible]	3/2734	38/17381	0.95135	0.994	0.97767	IKBKB/IRAK1/NOD1	3	BP
GO:0048255	mRNA stabilization	3/2734	38/17381	0.95135	0.994	0.97767	MTOR/SLC11A1/TIRAP	3	BP
GO:0038093	Fc receptor signaling pathway	30/2734	246/17381	0.95141	0.994	0.97767	ABL1/ARPC1A/ARPC1B/ARPC2/ARPC4/BRK1/CARD11/CD247/DOCK1/FCGR3A/HRAS/HSP90AA1/IKBKB/LIMK1/MAPK3/MYO1C/PLD2/PRKCD/PSMB11/PSMB6/PSMB7/PSMB8/PSMC3/PSMD13/PSMD3/PSMD5/PSMD7/SHC	30	BP

GO:0016441	posttranscriptional gene silencing	39/2734	311/17381	0.95261	0.994	0.97767	BMP4/DND1/EGFR/EIF4G1/HIST1H4F/HIST2H4A/HIST2H4B/MIR106B/MIR10A/MIR153-1/MIR15A/MIR16-1/MIR181B1/MIR210/MIR212/MIR221/MIR222/MIR25/MIR29A/MIR29C/MIR32/MIR328/MIR503/MIR582/MIR892B/MIR92A2/MIR96/MIRLET7A2/MYCN/NCOR1/NCOR2/NUP210/NUP98/POLR2G/POLR2L/RBM4/TARBP2/TSNAX/	39	BP
GO:0032370	positive regulation of	5/2734	56/17381	0.95268	0.994	0.97767	C1QTNF1/LRP1/NR1H3/PRELID1/PRKCD	5	BP
GO:0035195	gene silencing by miRNA	38/2734	304/17381	0.9529	0.994	0.97767	BMP4/DND1/EGFR/EIF4G1/HIST1H4F/HIST2H4A/HIST2H4B/MIR106B/MIR10A/MIR153-1/MIR15A/MIR16-1/MIR181B1/MIR210/MIR212/MIR221/MIR222/MIR25/MIR29A/MIR29C/MIR32/MIR328/MIR503/MIR582/MIR892B/MIR92A2/MIR96/MIRLET7A2/MYCN/NCOR1/NCOR2/NUP210/NUP98/POLR2G/POLR2L/RBM4/TARBP2/ZC3H10	38	BP
GO:0002082	regulation of oxidative phosphorylation	1/2734	18/17381	0.95414	0.994	0.97767	MLXIPL	1	BP
GO:0002363	alpha-beta T cell lineage commitment	1/2734	18/17381	0.95414	0.994	0.97767	LY9	1	BP
GO:0006198	cAMP catabolic process	1/2734	18/17381	0.95414	0.994	0.97767	PDE2A	1	BP
GO:0032026	response to magnesium ion	1/2734	18/17381	0.95414	0.994	0.97767	SLC34A1	1	BP
GO:0034501	protein localization to	1/2734	18/17381	0.95414	0.994	0.97767	BUB1B	1	BP
GO:0042753	positive regulation of circadian	1/2734	18/17381	0.95414	0.994	0.97767	RORC	1	BP
GO:0045292	mRNA cis splicing, via spliceosome	1/2734	18/17381	0.95414	0.994	0.97767	SART1	1	BP

GO:0046174	polyol catabolic process	1/2734	18/17381	0.95414	0.994	0.97767	INPP5E	1	BP
GO:0048670	regulation of collateral	1/2734	18/17381	0.95414	0.994	0.97767	WNT3A	1	BP
GO:0051016	barbed-end actin filament	1/2734	18/17381	0.95414	0.994	0.97767	CAPZA3	1	BP
GO:0051307	meiotic chromosome separation	1/2734	18/17381	0.95414	0.994	0.97767	M1AP	1	BP
GO:0051383	kinetochore organization	1/2734	18/17381	0.95414	0.994	0.97767	SENP6	1	BP
GO:0097094	craniofacial suture	1/2734	18/17381	0.95414	0.994	0.97767	BMP4	1	BP
GO:1903019	negative regulation of glycoprotein	1/2734	18/17381	0.95414	0.994	0.97767	TINF2	1	BP
GO:0006513	protein monoubiquitina	6/2734	65/17381	0.9551	0.9942	0.97788	DDB1/DDB2/PCGF2/UBE2B/UBE3D/ZNF598	6	BP
GO:0000027	ribosomal large subunit	2/2734	29/17381	0.95529	0.9942	0.97788	MDN1/NLE1	2	BP
GO:0042745	circadian sleep/wake	2/2734	29/17381	0.95529	0.9942	0.97788	DRD2/TH	2	BP
GO:0009994	oocyte differentiation	4/2734	48/17381	0.95643	0.9942	0.97788	ANG/INHBB/PTK2B/RXFP2	4	BP
GO:2000649	regulation of sodium ion transmembrane transporter	4/2734	48/17381	0.95643	0.9942	0.97788	CAMK2D/CAV3/OSR1/WNK2	4	BP

GO:0000724	double-strand break repair via homologous recombination	11/2734	106/17381	0.9571	0.9942	0.97788	BRCA1/HELQ/INO80/LIG3/MCM9/MCMDC2/RECQL4/RECQL5/SETD2/XRCC3/ZFYVE26	11	BP
GO:0000387	spliceosomal snRNP	3/2734	39/17381	0.95711	0.9942	0.97788	PRPF8/SART1/USP4	3	BP
GO:0033146	regulation of intracellular estrogen receptor	3/2734	39/17381	0.95711	0.9942	0.97788	AXIN1/BRCA1/CRIPAK	3	BP
GO:0038111	interleukin-7-mediated signaling	3/2734	39/17381	0.95711	0.9942	0.97788	CISH/LCK/PTK2B	3	BP
GO:0035722	interleukin-12-mediated signaling	5/2734	57/17381	0.95749	0.9942	0.97788	AIP/CFL1/LMNB1/PDCD4/STAT1	5	BP
GO:0060395	SMAD protein signal	5/2734	57/17381	0.95749	0.9942	0.97788	BMP4/GDF2/INHBB/INHBE/LEFTY2	5	BP
GO:0071349	cellular response to	5/2734	57/17381	0.95749	0.9942	0.97788	AIP/CFL1/LMNB1/PDCD4/STAT1	5	BP
GO:0051607	defense response to virus	27/2734	227/17381	0.95851	0.9942	0.97788	ABCF3/AIMP1/CARD9/DHX58/EIF2AK4/F2RL1/IFIT2/IFIT3/IFITM2/IFNA5/IRF5/ISG15/MAP3K14/MICB/NLRX1/PARP9/POLR3D/SETD2/SPON2/STAT1/STAT2/TARBP2/TBK1/TLR8/TRAF3IP1/TRIM11/ZMYND11	27	BP

GO:0002460	adaptive immune response based on somatic recombination of immune receptors built from immunoglobuli [illegible]	39/2734	314/17381	0.95916	0.9942	0.97788	BCL6/C1QB/C1QC/C1RL/C2/C8A/CCL19/CCR2/CD27/CD74/CLCF1/CLU/CTSH/EXO1/HAVCR2/HLA-E/HLX/HRAS/IL18/IL18BP/IL20RB/IL31RA/IL4R/INPP5D/LTA/LY9/MASP2/MICB/NCR3/PAXIP1/PRKCD/RORC/SEMA4A/SLC11A1/SUPT6H/TLR8/TNFSF13/TRAF2/WAS	39	BP
GO:0046165	alcohol biosynthetic process	14/2734	130/17381	0.9592	0.9942	0.97788	ACACB/APOA5/APOB/GBA/GCH1/GPER1/HSD17B7/NPC1L1/NSDHL/PMVK/POR/PTK2B/SCAP/SPTLC1	14	BP
GO:1903578	regulation of ATP metabolic	9/2734	91/17381	0.96039	0.9942	0.97788	ATPIF1/GCK/INS/MLXIPL/NCOR1/NUP210/NUP98/PFKFB1/PID1	9	BP
GO:0000725	recombinational repair	11/2734	107/17381	0.96049	0.9942	0.97788	BRCA1/HELQ/INO80/LIG3/MCM9/MCMDC2/RECQL4/RECQL5/SETD2/XRCC3/ZFYVE26	11	BP
GO:0000280	nuclear division	50/2734	393/17381	0.96082	0.9942	0.97788	ANAPC15/ANKRD53/ARHGEF10/BMP4/BUB1B/C11orf80/CDC14A/CHMP1A/CHMP4C/CHMP7/DDX4/DIS3L2/FANCA/FLNA/GOLGA2/HORMAD1/HSF1/HSPA2/INO80/INS/KIF23/KIF4A/LCMT1/LEMD2/M1AP/MAD1L1/MCMDC2/NCAPD3/NUMA1/OBSL1/PDGFRB/PDS5A/PIWIL2/PKMYT1/PPP1R9B/PRDM9/PRKAR1A/PSMD13/RNF212/SH2B1/SLC2A8/SLF2/TAF1L/TDRD9/TUBGCP2/TUBGCP3/TUBGCP5/UBE2B/WRAP73/XRCC3	50	BP
GO:0032414	positive regulation of ion transmembrane	8/2734	83/17381	0.96085	0.9942	0.97788	ARC/CRACR2A/GSTM2/HSPA2/NLGN3/NPPA/TRDN/WNK2	8	BP
GO:0030814	regulation of cAMP metabolic	16/2734	146/17381	0.96112	0.9942	0.97788	ADM/ADRA2A/AVPR2/CCR2/CHGA/CRHR1/DRD2/GABBR1/GCG/GPER1/GPHA2/OPRM1/PDE2A/RAF1/RXFP2/WFS1	16	BP

GO:0002323	natural killer cell activation involved in immune	2/2734	30/17381	0.96123	0.9942	0.97788	CORO1A/IFNA5	2	BP
GO:0010800	positive regulation of peptidyl-threonine	2/2734	30/17381	0.96123	0.9942	0.97788	AXIN1/GCG	2	BP
GO:0021772	olfactory bulb development	2/2734	30/17381	0.96123	0.9942	0.97788	CSF1R/OGDH	2	BP
GO:0042407	cristae formation	2/2734	30/17381	0.96123	0.9942	0.97788	ATP5G1/ATP5I	2	BP
GO:0042572	retinol metabolic	2/2734	30/17381	0.96123	0.9942	0.97788	DGAT1/PLB1	2	BP
GO:0043516	regulation of DNA damage response, signal transduction by p53 class	2/2734	30/17381	0.96123	0.9942	0.97788	CD74/ZNF385A	2	BP
GO:0048384	retinoic acid receptor signaling	2/2734	30/17381	0.96123	0.9942	0.97788	CREM/RARA	2	BP
GO:0070306	lens fiber cell differentiation	2/2734	30/17381	0.96123	0.9942	0.97788	FOXE3/WNT7A	2	BP
GO:0070741	response to interleukin-6	2/2734	30/17381	0.96123	0.9942	0.97788	PID1/TDGF1	2	BP
GO:0002710	negative regulation of T cell mediated	1/2734	19/17381	0.96136	0.9942	0.97788	IL20RB	1	BP
GO:0006098	pentose-phosphate shunt	1/2734	19/17381	0.96136	0.9942	0.97788	PGAM4	1	BP

GO:0030859	polarized epithelial cell	1/2734	19/17381	0.96136	0.9942	0.97788	AJAP1	1	BP
GO:0044804	autophagy of nucleus	1/2734	19/17381	0.96136	0.9942	0.97788	ATG2A	1	BP
GO:0051238	sequestering of metal ion	1/2734	19/17381	0.96136	0.9942	0.97788	SLC30A3	1	BP
GO:1902259	regulation of delayed rectifier potassium channel activity	1/2734	19/17381	0.96136	0.9942	0.97788	NPPA	1	BP
GO:1902307	positive regulation of sodium ion transmembrane	1/2734	19/17381	0.96136	0.9942	0.97788	WNK2	1	BP
GO:0033143	regulation of intracellular steroid hormone receptor	7/2734	75/17381	0.9618	0.9942	0.97788	ARRB2/AXIN1/BRCA1/CRIPAK/PER1/RWDD1/TAF1	7	BP
GO:0010257	NADH dehydrogenase complex	5/2734	58/17381	0.96186	0.9942	0.97788	FOXRED1/NDUFA2/NDUFAF6/NDUFAF7/NDUFB5	5	BP
GO:0031640	killing of cells of other	5/2734	58/17381	0.96186	0.9942	0.97788	BAD/CHGA/F2RL1/GAPDH/TUSC2	5	BP
GO:0032981	mitochondrial respiratory chain complex I assembly	5/2734	58/17381	0.96186	0.9942	0.97788	FOXRED1/NDUFA2/NDUFAF6/NDUFAF7/NDUFB5	5	BP
GO:0044364	disruption of cells of other	5/2734	58/17381	0.96186	0.9942	0.97788	BAD/CHGA/F2RL1/GAPDH/TUSC2	5	BP
GO:0070671	response to interleukin-12	5/2734	58/17381	0.96186	0.9942	0.97788	AIP/CFL1/LMNB1/PDCD4/STAT1	5	BP

GO:0075733	intracellular transport of	5/2734	58/17381	0.96186	0.9942	0.97788	NUP210/NUP98/VPS37B/VPS37C/VPS37D	5	BP
GO:0097031	mitochondrial respiratory chain complex I biogenesis	5/2734	58/17381	0.96186	0.9942	0.97788	FOXRED1/NDUFA2/NDUFAF6/NDUFAF7/NDUFB5	5	BP
GO:1902305	regulation of sodium ion transmembrane transport	5/2734	58/17381	0.96186	0.9942	0.97788	CAMK2D/CAV3/COMMD1/OSR1/WNK2	5	BP
GO:0098760	response to interleukin-7	3/2734	40/17381	0.96223	0.9943	0.97792	CISH/LCK/PTK2B	3	BP
GO:0098761	cellular response to	3/2734	40/17381	0.96223	0.9943	0.97792	CISH/LCK/PTK2B	3	BP
GO:0019083	viral transcription	20/2734	177/17381	0.963	0.9949	0.97853	CCL5/CDK9/NELFB/NUP210/NUP98/PFN1/POLR2G/POLR2L/PSMC3/RPL35A/RPL36A/RPL41/RPL7/RPS24/RSF1/TARBP2/TRIM11/TRIM14/TRIM31/TRI	20	BP
GO:1903312	negative regulation of mRNA	7/2734	76/17381	0.96535	0.9953	0.97897	CDK9/MTOR/PKP3/RNPS1/SLC11A1/SRSF4/TIRAP	7	BP
GO:0032206	positive regulation of telomere	4/2734	50/17381	0.9655	0.9953	0.97897	ACD/CCT3/MAPK15/MAPK3	4	BP
GO:0036297	interstrand cross-link repair	4/2734	50/17381	0.9655	0.9953	0.97897	ATRIP/FAAP20/FANCA/XRCC3	4	BP
GO:0007059	chromosome segregation	43/2734	346/17381	0.96554	0.9953	0.97897	ANAPC15/ANKRD53/ARL8A/BRCA1/BUB1B/CDC14A/CENPN/CHMP1A/CHMP4C/CHMP7/CKAP5/DIS3L2/DYNC1H1/DYNC1I1/DYNLL2/FAM96B/HDAC8/HJURP/HORMAD1/INO80/KIF23/KIF2B/KIF4A/LCMT1/LEMD2/M1AP/MAD1L1/MCMDC2/NAA60/NCAPD3/NTMT1/NUDC/NUMA1/NUP98/PDS5A/PMF1/RECQL5/RGS14/RNF212/SLC25A5/SLF2/UBE2B/XRCC3	43	BP

GO:1901016	regulation of potassium ion transmembrane transporter	5/2734	59/17381	0.9658	0.9953	0.97897	CAV3/MIR153-1/MIR212/NPPA/WWP2	5	BP
GO:0010765	positive regulation of sodium ion	2/2734	31/17381	0.96642	0.9953	0.97897	NKX2-5/WNK2	2	BP
GO:1902116	negative regulation of organelle	2/2734	31/17381	0.96642	0.9953	0.97897	BRCA1/CAV3	2	BP
GO:0030330	DNA damage response, signal transduction by p53 class	11/2734	109/17381	0.96655	0.9953	0.97897	BRCA1/CD74/E2F4/E2F8/FOXM1/ING4/MUC1/PAXIP1/PCBP4/SFN/ZNF385A	11	BP
GO:0032006	regulation of TOR signaling	9/2734	93/17381	0.96688	0.9953	0.97897	ARAF/DGKQ/LARS/MLST8/NPRL3/PREX2/RRAGC/SESN1/TELO2	9	BP
GO:0002920	regulation of humoral immune	13/2734	125/17381	0.96707	0.9953	0.97897	C1QB/C1QC/C2/C3AR1/C8A/CD81/CFH/CLU/CPN2/LTA/PGC/PPP2R3C/SPINK5	13	BP
GO:0001779	natural killer cell	1/2734	20/17381	0.96745	0.9953	0.97897	TUSC2	1	BP
GO:0002089	lens morphogenesis in camera-type	1/2734	20/17381	0.96745	0.9953	0.97897	BMP4	1	BP
GO:0006346	methylation-dependent chromatin	1/2734	20/17381	0.96745	0.9953	0.97897	DNMT3A	1	BP
GO:0006491	N-glycan processing	1/2734	20/17381	0.96745	0.9953	0.97897	MGAT4B	1	BP

GO:0009083	branched-chain amino acid catabolic	1/2734	20/17381	0.96745	0.9953	0.97897	BCKDK	1	BP
GO:0032691	negative regulation of interleukin-1	1/2734	20/17381	0.96745	0.9953	0.97897	ARRB2	1	BP
GO:0044346	fibroblast apoptotic	1/2734	20/17381	0.96745	0.9953	0.97897	CHD8	1	BP
GO:0044550	secondary metabolite biosynthetic process	1/2734	20/17381	0.96745	0.9953	0.97897	CYP1A2	1	BP
GO:0045117	azole transport	1/2734	20/17381	0.96745	0.9953	0.97897	SLC44A4	1	BP
GO:0045954	positive regulation of natural killer cell mediated	1/2734	20/17381	0.96745	0.9953	0.97897	NCR3	1	BP
GO:0071467	cellular response to pH	1/2734	20/17381	0.96745	0.9953	0.97897	KCNK4	1	BP
GO:0080111	DNA demethylation	1/2734	20/17381	0.96745	0.9953	0.97897	APEX1	1	BP
GO:2000050	regulation of non-canonical Wnt signaling	1/2734	20/17381	0.96745	0.9953	0.97897	ABL1	1	BP
GO:2001014	regulation of skeletal muscle cell	1/2734	20/17381	0.96745	0.9953	0.97897	GPC1	1	BP

GO:0006401	RNA catabolic process	47/2734	376/17381	0.96792	0.9957	0.97928	APEX1/CASC3/CSDE1/DIS3L/DIS3L2/EIF4B/EIF4G1/ERN2/EXOSC10/EXOSC2/HSPB1/LSM1/MIRLET7A2/MTOR/NT5C3B/PAN2/PATL1/PCBP4/PKP3/PNLDC1/POLR2G/PRKCD/PSMB11/PSMB6/PSMB7/PSMB8/PSMC3/PSMD13/PSMD3/PSMD5/PSMD7/RNH1/RNPS1/RPL35A/RPL36A/RPL41/RPL7/RPS24/SCGB1A1/SIDT2/SLC11A1/SMG5/SND1/TIRAP/TNFSF13/ZHX2/ZPR1	47	BP
GO:1902369	negative regulation of RNA catabolic	4/2734	51/17381	0.96934	0.9962	0.97978	MTOR/PKP3/SLC11A1/TIRAP	4	BP
GO:0045143	homologous chromosome segregation	5/2734	60/17381	0.96937	0.9962	0.97978	HORMAD1/LEMD2/MCMDC2/RNF212/UBE2B	5	BP
GO:0060135	maternal process involved in	5/2734	60/17381	0.96937	0.9962	0.97978	HAVCR2/LGALS9/MEN1/MTOR/PPARD	5	BP
GO:0007088	regulation of mitotic nuclear division	17/2734	157/17381	0.96945	0.9962	0.97978	ANAPC15/BMP4/BUB1B/CDC14A/CHMP1A/CHMP4C/INS/LCMT1/MAD1L1/NUMA1/OBSL1/PDGFRB/PKMYT1/PPP1R9B/SH2B1/SLF2/XRCC3	17	BP
GO:1990823	response to leukemia inhibitory factor	9/2734	94/17381	0.96976	0.9962	0.97978	BSPRY/CACYBP/LAPTM5/MPC1/MSC/PCOLCE/PTP4A3/SLC25A5/SPOCK2	9	BP
GO:1990830	cellular response to leukemia	9/2734	94/17381	0.96976	0.9962	0.97978	BSPRY/CACYBP/LAPTM5/MPC1/MSC/PCOLCE/PTP4A3/SLC25A5/SPOCK2	9	BP
GO:1901655	cellular response to	8/2734	86/17381	0.97046	0.9962	0.97978	AQP1/EGFR/EIF4EBP1/HDAC8/P2RY6/RWDD1/SMYD3/UGT3A2	8	BP
GO:0006402	mRNA catabolic process	42/2734	342/17381	0.97059	0.9962	0.97978	APEX1/CASC3/CSDE1/DIS3L2/EIF4B/EIF4G1/EXOSC10/EXOSC2/HSPB1/LSM1/MIRLET7A2/MTOR/NT5C3B/PAN2/PATL1/PCBP4/PKP3/PNLDC1/POLR2G/PRKCD/PSMB11/PSMB6/PSMB7/PSMB8/PSMC3/PSMD13/PSMD3/PSMD5/PSMD7/RNH1/RNPS1/RPL35A/RPL36A/RPL41/RPL7/RPS24/SCGB1A1/SLC11A1/SMG5/TIRAP/TNFSF13/ZHX2	42	BP

GO:0043489	RNA stabilization	3/2734	42/17381	0.97079	0.9962	0.97978	MTOR/SLC11A1/TIRAP	3	BP
GO:0001893	maternal placenta	2/2734	32/17381	0.97093	0.9962	0.97978	MEN1/PPARD	2	BP
GO:0002385	mucosal immune	2/2734	32/17381	0.97093	0.9962	0.97978	PLA2G1B/RAB17	2	BP
GO:0016072	rRNA metabolic process	35/2734	292/17381	0.9715	0.9962	0.97978	ANG/BRF1/DHX37/DIS3L/DIS3L2/ERN2/EXOSC10/EXOSC2/FCF1/GTF3C1/ISG20L2/MDN1/MRPS11/MTOR/NHP2/NOB1/NOL11/NOL6/NSUN5/NSUN5P2/PDCD11/POLR1E/RPL35A/RPL36A/RPL41/RPL7/RPP21/RPS24/RPUSD2/SART1/SENP3/SRFBP1/SUV39H1/TSR1/TSR2	35	BP
GO:0002708	positive regulation of lymphocyte	7/2734	78/17381	0.97157	0.9962	0.97978	CLCF1/HLA-E/LTA/NCR3/PAXIP1/TNFSF13/TRAF2	7	BP
GO:0002761	regulation of myeloid leukocyte	11/2734	111/17381	0.97177	0.9962	0.97978	BGLAP/C1QC/CD74/CSF1/ESRRA/FAM213A/INPP5D/MTOR/NME1/RARA/TCTA	11	BP
GO:0009214	cyclic nucleotide	1/2734	21/17381	0.97257	0.9962	0.97978	PDE2A	1	BP
GO:0009235	cobalamin metabolic	1/2734	21/17381	0.97257	0.9962	0.97978	CUBN	1	BP
GO:0033141	positive regulation of peptidyl-serine phosphorylation	1/2734	21/17381	0.97257	0.9962	0.97978	IFNA5	1	BP
GO:0035162	embryonic hemopoiesis	1/2734	21/17381	0.97257	0.9962	0.97978	KDR	1	BP
GO:0045187	regulation of circadian sleep/wake cycle, sleep	1/2734	21/17381	0.97257	0.9962	0.97978	DRD2	1	BP

GO:0071459	protein localization to chromosome,	1/2734	21/17381	0.97257	0.9962	0.97978	BUB1B	1	BP
GO:0097503	sialylation	1/2734	21/17381	0.97257	0.9962	0.97978	ST3GAL4	1	BP
GO:0002753	cytoplasmic pattern recognition receptor	5/2734	61/17381	0.97259	0.9962	0.97978	DHX58/IKBKB/IRAK1/NLRX1/NOD1	5	BP
GO:0046794	transport of virus	5/2734	61/17381	0.97259	0.9962	0.97978	NUP210/NUP98/VPS37B/VPS37C/VPS37D	5	BP
GO:0007566	embryo implantation	4/2734	52/17381	0.97278	0.9962	0.97978	MMP2/PPARD/PRDM14/SCGB1A1	4	BP
GO:0009161	ribonucleoside monophosphate metabolic process	39/2734	322/17381	0.97297	0.9962	0.97978	AK5/ALDOA/ATP5G1/ATP5I/ATP6V1B1/ATPIF1/BAD/CARD11/COX10/COX8A/CYC1/DHODH/DLG4/GALK1/GAPDH/GCK/HK3/IMPDH1/INS/LDHA/MLXIPL/NCOR1/NDUFA2/NDUFAF7/NDUFB5/NDUFC2-KCTD14/NUP210/NUP98/OGDH/OGDHL/PFKFB1/PFKM/PGAM4/PID1/PKM/SCRIB/SLC25A25/UCK1/VPS9D1	39	BP
GO:0009566	fertilization	20/2734	182/17381	0.97363	0.9962	0.97978	ALDOA/APOB/ATP1A4/CACNA1H/CATSPER1/CCDC136/CCT3/GLRA1/PLB1/POMZP3/PRDM14/PRSS37/SERPINA5/SPACA3/SPINK2/TARBP2/TDRD9/TTLL5/TUBGCP3/VDAC2	20	BP
GO:0048511	rhythmic process	38/2734	316/17381	0.97472	0.9962	0.97978	ANG/ARRB2/CCAR2/CDK4/CDK5/CIART/CLDN4/CREM/DDC/DRD2/EGFR/EGR1/EIF2B2/EIF2B5/INHBB/LFNG/MTA1/MTNR1B/NAGLU/NCOR1/NONO/NOS3/NPAS2/NR1H3/NR5A1/OPN4/OPRM1/PASD1/PER1/PSPC1/RAI1/RBM4/RORC/SUV39H1/TH/TIMELESS/USP2/VGF	38	BP
GO:0046621	negative regulation of	2/2734	33/17381	0.97485	0.9962	0.97978	CAV3/MIR25	2	BP
GO:0098751	bone cell development	2/2734	33/17381	0.97485	0.9962	0.97978	PTPN11/ZNF385A	2	BP
GO:0042255	ribosome assembly	5/2734	62/17381	0.9755	0.9962	0.97978	ERAL1/MDN1/MRPS11/NLE1/TSR1	5	BP

GO:0010923	negative regulation of phosphatase	6/2734	71/17381	0.9759	0.9962	0.97978	IKBKB/PKMYT1/PPP1R1B/SPOCD1/TIPRL/ZFYVE1	6	BP
GO:0031571	mitotic G1 DNA damage checkpoint	6/2734	71/17381	0.9759	0.9962	0.97978	E2F4/E2F8/MUC1/PCBP4/SFN/ZNF385A	6	BP
GO:0038034	signal transduction in absence of	6/2734	71/17381	0.9759	0.9962	0.97978	BAD/BOK/CASP3/EYA1/MOAP1/ZC3HC1	6	BP
GO:0044819	mitotic G1/S transition checkpoint	6/2734	71/17381	0.9759	0.9962	0.97978	E2F4/E2F8/MUC1/PCBP4/SFN/ZNF385A	6	BP
GO:0097192	extrinsic apoptotic signaling pathway in	6/2734	71/17381	0.9759	0.9962	0.97978	BAD/BOK/CASP3/EYA1/MOAP1/ZC3HC1	6	BP
GO:1903317	regulation of protein	18/2734	168/17381	0.97597	0.9962	0.97978	ADAM8/C1QB/C1QC/C2/C3AR1/C8A/CD81/CFH/CHAC1/CLU/CPN2/KLKB1/NAIP/NKD2/PRSS37/SERPINF2/SNX12/TFR2	18	BP
GO:0009167	purine ribonucleoside monophosphate metabolic process	37/2734	310/17381	0.97642	0.9962	0.97978	AK5/ALDOA/ATP5G1/ATP5I/ATP6V1B1/ATPIF1/BAD/CARD11/COX10/COX8A/CYC1/DLG4/GALK1/GAPDH/GCK/HK3/IMPDH1/INS/LDHA/MLXIPL/NCOR1/NDUFA2/NDUFAF7/NDUFB5/NDUFC2-KCTD14/NUP210/NUP98/OGDH/OGDHL/PFKFB1/PFKM/PGAM4/PID1/PKM/SCRIB/SLC25A25/VPS9D1	37	BP
GO:0002227	innate immune response in mucosa	1/2734	22/17381	0.97689	0.9962	0.97978	PLA2G1B	1	BP
GO:0010842	retina layer formation	1/2734	22/17381	0.97689	0.9962	0.97978	LHX1	1	BP
GO:0021854	hypothalamus development	1/2734	22/17381	0.97689	0.9962	0.97978	OTP	1	BP

GO:0042762	regulation of sulfur metabolic	1/2734	22/17381	0.97689	0.9962	0.97978	PDHB	1	BP
GO:0060396	growth hormone receptor	1/2734	22/17381	0.97689	0.9962	0.97978	MAPK3	1	BP
GO:0002429	immune response-activating cell surface receptor signaling	53/2734	426/17381	0.97733	0.9962	0.97978	ABL1/ARPC1A/ARPC1B/ARPC2/ARPC4/BLK/BRK1/C3AR1/CACNA1F/CACNB3/CARD11/CARD9/CD247/CD79B/CLEC7A/CMTM3/CSK/DOCK1/ELF1/FCGR3A/HRAS/HSP90AA1/IKBKB/INPP5D/LCK/LGALS3/LIMK1/MAPK3/MICB/MUC1/MUC2/MUC3A/MUC5AC/MUC5B/MUC6/MYO1C/NCR3/PLD2/PRKCD/PSMB11/PSMB6/PSMB7/PSMB8/PSMC3/PSMD13/PSMD3/PSMD5/PS	53	BP
GO:0006413	translational initiation	21/2734	192/17381	0.97764	0.9962	0.97978	CCL5/EIF1AY/EIF2AK4/EIF2B2/EIF2B5/EIF3B/EIF3I/EIF4B/EIF4EBP1/EIF4G1/HSPB1/POLR2G/RARA/RBM4/RPL35A/RPL36A/RPL41/RPL7/RPS24/RPS6	21	BP
GO:0009126	purine nucleoside monophosphate metabolic process	37/2734	311/17381	0.97768	0.9962	0.97978	AK5/ALDOA/ATP5G1/ATP5I/ATP6V1B1/ATPIF1/BAD/CARD11/COX10/COX8A/CYC1/DLG4/GALK1/GAPDH/GCK/HK3/IMPDH1/INS/LDHA/MLXIPL/NCOR1/NDUFA2/NDUFAF7/NDUFB5/NDUFC2-KCTD14/NUP210/NUP98/OGDH/OGDHL/PFKFB1/PFKM/PGAM4/PID1/PKM/SCRIB/SLC25A25/VPS9D1	37	BP
GO:0072329	monocarboxylic acid catabolic process	12/2734	122/17381	0.97806	0.9962	0.97978	ACACB/ACAD10/ACADVL/ACOXL/CPT2/CROT/HADHA/HAO2/MTOR/PLA2G15/PPARD/TYSND1	12	BP
GO:1901616	organic hydroxy compound catabolic	5/2734	63/17381	0.97811	0.9962	0.97978	ALDH2/INPP5E/LRTOMT/SCARB1/SCARF1	5	BP
GO:0060041	retina development in camera-type eye	14/2734	138/17381	0.97811	0.9962	0.97978	ACHE/CLCN2/GNAT1/IFT140/LAMC3/LHX1/MAX/PDGFRB/SOX8/TBC1D32/THY1/TTLL5/TULP1/VAX2	14	BP
GO:0042254	ribosome biogenesis	39/2734	326/17381	0.9782	0.9962	0.97978	DDX31/DHX37/DIS3L/DIS3L2/ERAL1/EXOSC10/EXOSC2/FCF1/HEATR3/ISG20L2/MDN1/MRPL10/MRPS11/NHP2/NLE1/NOB1/NOC2L/NOL11/NOL6/NOP16/NSUN5/NSUN5P2/NVL/PDCD11/RPL35A/RPL36A/RPL41/RPL7/RPP21/RPS24/RPUSD2/SART1/SENP3/SRFBP1/SURF6/SUV39H1/TSR1/TSR2/ZNF5	39	BP

GO:0002251	organ or tissue specific immune	2/2734	34/17381	0.97826	0.9962	0.97978	PLA2G1B/RAB17	2	BP
GO:0045005	DNA-dependent DNA replication maintenance of	2/2734	34/17381	0.97826	0.9962	0.97978	CDK9/POLE	2	BP
GO:1901099	negative regulation of signal transduction in	2/2734	34/17381	0.97826	0.9962	0.97978	EYA1/ZC3HC1	2	BP
GO:2001240	negative regulation of extrinsic apoptotic signaling	2/2734	34/17381	0.97826	0.9962	0.97978	EYA1/ZC3HC1	2	BP
GO:0007338	single fertilization	15/2734	146/17381	0.9783	0.9962	0.97978	ALDOA/CACNA1H/CATSPER1/CCDC136/CCT3/GLRA1/PLB1/POMZP3/PRSS37/SERPINA5/SPACA3/SPINK2/TARBP2/TUBGCP3/VDAC2	15	BP
GO:0044783	G1 DNA damage	6/2734	72/17381	0.97834	0.9962	0.97978	E2F4/E2F8/MUC1/PCBP4/SFN/ZNF385A	6	BP
GO:0061844	antimicrobial humoral immune response mediated by	4/2734	54/17381	0.97861	0.9962	0.97978	GAPDH/PGC/PLA2G1B/SPINK5	4	BP
GO:2000242	negative regulation of reproductive	4/2734	54/17381	0.97861	0.9962	0.97978	BMP4/HORMAD1/NR5A1/PRKAR1A	4	BP
GO:0046058	cAMP metabolic	17/2734	162/17381	0.97887	0.9962	0.97978	ADM/ADRA2A/AVPR2/CCR2/CHGA/CRHR1/DRD2/GABBR1/GCG/GPER1/GPHA2/OPRM1/PDE2A/RAF1/RXFP2/UCN2/WFS1	17	BP

GO:0008380	RNA splicing	53/2734	428/17381	0.97941	0.9962	0.97978	AHNAK/ARL6IP4/CASC3/CCAR2/CELF6/CPSF1/CPSF4/CSTF2/CWC15/CWC25/DHX38/EFTUD2/ESRP2/GPKOW/HMX2/HNRNPH3/LGALS3/LMNTD2/LSM1/LUC7L/MYOD1/NONO/NUP98/POLR2G/POLR2L/PPIE/PPP1R9B/PRCC/PRDX6/PRPF4/PRPF8/PSPC1/RBFOX1/RBFOX3/RBM4/RBPMS/RNPS1/RRAGC/SART1/SF1/SF3B4/SLC39A5/SNRNP25/SPEN/SRPK3/SRRM2/SRSF4/	53	BP
GO:0032007	negative regulation of	3/2734	45/17381	0.98026	0.9962	0.97978	NPRL3/PREX2/SESN1	3	BP
GO:0006607	NLS-bearing protein import into nucleus	1/2734	23/17381	0.98053	0.9962	0.97978	IPO4	1	BP
GO:0006688	glycosphingolipid biosynthetic process	1/2734	23/17381	0.98053	0.9962	0.97978	B4GALNT1	1	BP
GO:0009081	branched-chain amino acid metabolic	1/2734	23/17381	0.98053	0.9962	0.97978	BCKDK	1	BP
GO:0031579	membrane raft organization	1/2734	23/17381	0.98053	0.9962	0.97978	CAV3	1	BP
GO:0033139	regulation of peptidyl-serine phosphorylation of STAT	1/2734	23/17381	0.98053	0.9962	0.97978	IFNA5	1	BP
GO:0042347	negative regulation of NF-kappaB	1/2734	23/17381	0.98053	0.9962	0.97978	CD27	1	BP
GO:0042749	regulation of circadian sleep/wake	1/2734	23/17381	0.98053	0.9962	0.97978	DRD2	1	BP
GO:0070199	establishment of protein localization to	1/2734	23/17381	0.98053	0.9962	0.97978	ACD	1	BP

GO:0071378	cellular response to growth	1/2734	23/17381	0.98053	0.9962	0.97978	MAPK3	1	BP
GO:0006517	protein deglycosylation	2/2734	35/17381	0.98122	0.9962	0.97978	OS9/SYVN1	2	BP
GO:0006699	bile acid biosynthetic process	2/2734	35/17381	0.98122	0.9962	0.97978	FGFR4/OSBPL7	2	BP
GO:0043467	regulation of generation of precursor metabolites and energy	12/2734	124/17381	0.98151	0.9962	0.97978	GCK/INS/MLXIPL/MTOR/NCOR1/NUP210/NUP98/PFKFB1/PHLDA2/PRELID1/TRAP1/TRPV4	12	BP
GO:0044766	multi-organism transport	6/2734	74/17381	0.98254	0.9962	0.97978	NUP210/NUP98/RAB7A/VPS37B/VPS37C/VPS37D	6	BP
GO:1902579	multi-organism localization	6/2734	74/17381	0.98254	0.9962	0.97978	NUP210/NUP98/RAB7A/VPS37B/VPS37C/VPS37D	6	BP
GO:0006120	mitochondrial electron transport, NADH to	3/2734	46/17381	0.98271	0.9962	0.97978	NDUFA2/NDUFB5/NDUFC2-KCTD14	3	BP
GO:0070972	protein localization to endoplasmic	12/2734	125/17381	0.98304	0.9962	0.97978	BCAP31/GBF1/GPAA1/HSPA5/OS9/PEX16/RPL35A/RPL36A/RPL41/RPL7/RPS24/SRP14	12	BP
GO:0006310	DNA recombination	29/2734	257/17381	0.98349	0.9962	0.97978	APEX1/BCL6/BRCA1/C11orf80/CLCF1/ENDOG/EXO1/HELQ/IGHMBP2/INO80/LIG3/MCM9/MCMDC2/NFRKB/NONO/PAXIP1/PIF1/PRDM9/RECQL4/RECQL5/RNF212/RPAIN/SETD2/SUPT6H/TNFSF13/UBE2B/VRTN/XRCC3/ZFY	29	BP
GO:0006364	rRNA processing	29/2734	257/17381	0.98349	0.9962	0.97978	DHX37/DIS3L/DIS3L2/EXOSC10/EXOSC2/FCF1/ISG20L2/MDN1/MRPS11/NHP2/NOB1/NOL11/NOL6/NSUN5/NSUN5P2/PDCD11/RPL35A/RPL36A/RPL41/RPL7/RPP21/RPS24/RPUSD2/SART1/SENP3/SRFBP1/SUV39H1/TSR1/TSR	29	BP

GO:0000729	DNA double-strand break processing	1/2734	24/17381	0.9836	0.9962	0.97978	BRCA1	1	BP
GO:0002724	regulation of T cell cytokine production	1/2734	24/17381	0.9836	0.9962	0.97978	TRAF2	1	BP
GO:0006312	mitotic recombination	1/2734	24/17381	0.9836	0.9962	0.97978	XRCC3	1	BP
GO:0007210	serotonin receptor	1/2734	24/17381	0.9836	0.9962	0.97978	HTR1D	1	BP
GO:0010738	regulation of protein kinase A signaling	1/2734	24/17381	0.9836	0.9962	0.97978	AIP	1	BP
GO:0032786	positive regulation of DNA-templated transcription, elongation	1/2734	24/17381	0.9836	0.9962	0.97978	SUPT6H	1	BP
GO:0043628	ncRNA 3'-end processing	1/2734	24/17381	0.9836	0.9962	0.97978	EXOSC2	1	BP
GO:0045672	positive regulation of osteoclast	1/2734	24/17381	0.9836	0.9962	0.97978	CSF1	1	BP
GO:0098664	G-protein coupled serotonin	1/2734	24/17381	0.9836	0.9962	0.97978	HTR1D	1	BP
GO:1901018	positive regulation of potassium ion transmembrane	1/2734	24/17381	0.9836	0.9962	0.97978	NPPA	1	BP

GO:2000191	regulation of fatty acid	1/2734	24/17381	0.9836	0.9962	0.97978	NCOR1	1	BP
GO:1901617	organic hydroxy compound biosynthetic process	25/2734	227/17381	0.98363	0.9962	0.97978	ACACB/APOA5/APOB/CACNA1H/CYB5R1/CYB5R2/CYP11B2/DDC/DGKQ/FGFR4/GBA/GCH1/GPER1/HDC/HSD17B3/HSD17B7/NPC1L1/NSDHL/OSBPL7/PMVK/POR/PTK2B/SCAP/SPTLC1/TH	25	BP
GO:0033280	response to vitamin D	2/2734	36/17381	0.98379	0.9962	0.97978	BGLAP/TNC	2	BP
GO:0097352	autophagosome maturation	2/2734	36/17381	0.98379	0.9962	0.97978	TBC1D25/TECPR1	2	BP
GO:0002673	regulation of acute inflammatory	15/2734	150/17381	0.98422	0.9963	0.97996	ADAM8/C1QB/C1QC/C2/C3AR1/C8A/CD81/CFH/CLU/CPN2/IL20RB/INS/KLKB1/MIR92A2/PTGER3	15	BP
GO:0070613	regulation of protein	17/2734	166/17381	0.98443	0.9963	0.97996	ADAM8/C1QB/C1QC/C2/C3AR1/C8A/CD81/CFH/CHAC1/CLU/CPN2/KLKB1/NAIP/NKD2/PRSS37/SERPINF2/SNX12	17	BP
GO:0006397	mRNA processing	61/2734	491/17381	0.98448	0.9963	0.97996	ALKBH5/ARL6IP4/CASC3/CCAR2/CDK9/CELF6/CMTR1/CPSF1/CPSF4/CSTF2/CWC15/CWC25/DHX38/EFTUD2/ERN2/ESRP2/GPKOW/GTF2H4/HMX2/HNRNPH3/HSF1/LGALS3/LMNTD2/LSM1/LUC7L/MYOD1/NONO/NUP98/PAN2/PDCD11/POLR2G/POLR2L/PPIE/PRCC/PRDX6/PRPF4/PRPF8/PSPC1/RBFOX1/RBFOX3/RBM23/RBM4/RBPMS/RNPS1/SART1/SF1/SF3B4/SLC39A5/SNRNP25/SPEN/SRPK3/SRRM2/SRSF4/SUPT6H/TRUB2/TTF2/USP4/USP4[illegible]	61	BP
GO:0044764	multi-organism cellular process	3/2734	47/17381	0.98486	0.9964	0.98001	DHX58/INHBB/NLRX1	3	BP
GO:0060042	retina morphogenesis in camera-type	3/2734	47/17381	0.98486	0.9964	0.98001	LHX1/SOX8/THY1	3	BP
GO:0035303	regulation of dephosphorylati	14/2734	143/17381	0.98547	0.9968	0.98045	CDK5RAP3/DRD2/DUSP26/GBA/IKBKB/NSMF/PDGFRB/PKMYT1/PPP1R1B/PRKCD/SMG5/SPOCD1/TIPRL/ZFYVE1	14	BP

GO:0098813	nuclear chromosome segregation	34/2734	297/17381	0.98602	0.9969	0.98049	ANAPC15/ANKRD53/BUB1B/CDC14A/CENPN/CHMP1A/CHMP4C/CHMP7/CKAP5/DIS3L2/DYNC1H1/DYNC1I1/DYNLL2/HDAC8/HORMAD1/INO80/KIF23/KIF2B/KIF4A/LCMT1/LEMD2/M1AP/MAD1L1/MCMDC2/NCAPD3/NUDC/NUMA1/NUP98/PDS5A/PMF1/RNF212/SLF2/UBE2B/XRCC3	34	BP
GO:0031123	RNA 3'-end processing	11/2734	119/17381	0.98607	0.9969	0.98049	CASC3/CDK9/CPSF1/CPSF4/CSTF2/DHX38/EXOSC2/HSF1/RNPS1/SRSF4/ZC3H3	11	BP
GO:0050802	circadian sleep/wake cycle, sleep	1/2734	25/17381	0.98618	0.9969	0.98049	DRD2	1	BP
GO:0060384	innervation	1/2734	25/17381	0.98618	0.9969	0.98049	LRIG2	1	BP
GO:0042742	defense response to bacterium	33/2734	290/17381	0.98639	0.9969	0.98051	ADGRB1/C10orf99/CARD9/CD160/CHGA/F2RL1/FGA/GSDMD/HAVCR2/HLA-E/ISG15/LPO/LTA/LYPD8/MPO/NLRP1/NOD1/PGC/PLA2G1B/PRKCD/RARRES2/RNASE8/SELP/SHC1/SLC11A1/SPACA3/SPINK5/SPON2/TBK1/TIRAP	33	BP
GO:0016925	protein sumoylation	8/2734	94/17381	0.98653	0.9969	0.98051	EGR1/EYA1/MTA1/NUP210/NUP98/RNF212/RWDD3/SENP6	8	BP
GO:0006910	phagocytosis, recognition	4/2734	58/17381	0.98691	0.9971	0.98072	ADGRB1/CLEC7A/SCARB1/TULP1	4	BP
GO:0000184	nuclear-transcribed mRNA catabolic process, nonsense-	11/2734	120/17381	0.98728	0.9973	0.98092	CASC3/EIF4G1/EXOSC10/PNLDC1/RNPS1/RPL35A/RPL36A/RPL41/RPL7/RPS24/SMG5	11	BP
GO:0002686	negative regulation of leukocyte	2/2734	38/17381	0.98794	0.9977	0.98133	CCL21/NBL1	2	BP
GO:0002063	chondrocyte development	1/2734	26/17381	0.98836	0.9977	0.98133	SERPINH1	1	BP

GO:0030947	regulation of vascular endothelial growth factor receptor	1/2734	26/17381	0.98836	0.9977	0.98133	DAB2IP	1	BP
GO:0032692	negative regulation of interleukin-1	1/2734	26/17381	0.98836	0.9977	0.98133	ARRB2	1	BP
GO:0072376	protein activation	18/2734	178/17381	0.98868	0.9979	0.98148	C1QB/C1QC/C1RL/C2/C3AR1/C8A/CD81/CFH/CLU/CPN2/FGA/GP9/KLKB1/KNG1/MASP2/PRCP/VSIG4/VWF	18	BP
GO:0030522	intracellular receptor signaling	32/2734	287/17381	0.98971	0.9983	0.98188	ARRB2/AXIN1/BRCA1/CALCOCO1/CREM/CRIPAK/DDX54/DHX58/ESR2/ESRRA/GPER1/IKBKB/IRAK1/MED24/NLRX1/NOD1/NR1H3/NR2F1/NR4A1/NR5A1/PER1/PLPP1/PPARD/PRCP/RARA/RORC/RWDD1/TADA3/TAF1/TA	32	BP
GO:0016064	immunoglobulin mediated immune	20/2734	195/17381	0.98972	0.9983	0.98188	BCL6/C1QB/C1QC/C1RL/C2/C8A/CD27/CD74/CLCF1/CLU/EXO1/IL4R/INPP5D/LTA/MASP2/PAXIP1/PRKCD/SUPT6H/TLR8/TNFSF13	20	BP
GO:0033692	cellular polysaccharide biosynthetic process	4/2734	60/17381	0.98981	0.9983	0.98188	GCK/INS/MTOR/PHKG1	4	BP
GO:0050851	antigen receptor-mediated	28/2734	257/17381	0.98989	0.9983	0.98188	ABL1/BLK/CACNA1F/CACNB3/CARD11/CD247/CD79B/CMTM3/CSK/ELF1/HRAS/IKBKB/INPP5D/LCK/LGALS3/PSMB11/PSMB6/PSMB7/PSMB8/PSMC3/PSMD13/PSMD3/PSMD5/PSMD7/RNF31/TEC/THY1/WAS	28	BP
GO:0045132	meiotic chromosome segregation	7/2734	88/17381	0.98992	0.9983	0.98188	BUB1B/HORMAD1/LEMD2/M1AP/MCMDC2/RNF212/UBE2B	7	BP
GO:0022410	circadian sleep/wake cycle process	1/2734	27/17381	0.99019	0.9984	0.98198	DRD2	1	BP

GO:0035305	negative regulation of dephosphorylati	6/2734	80/17381	0.99103	0.999	0.98258	IKBKB/PKMYT1/PPP1R1B/SPOCD1/TIPRL/ZFYVE1	6	BP
GO:0019724	B cell mediated immunity	20/2734	197/17381	0.99117	0.999	0.98258	BCL6/C1QB/C1QC/C1RL/C2/C8A/CD27/CD74/CLCF1/CLU/EXO1/IL4R/INPP5D/LTA/MASP2/PAXIP1/PRKCD/SUPT6H/TLR8/TNFSF13	20	BP
GO:0030449	regulation of complement activation	9/2734	107/17381	0.9913	0.999	0.98258	C1QB/C1QC/C2/C3AR1/C8A/CD81/CFH/CLU/CPN2	9	BP
GO:0010922	positive regulation of phosphatase	1/2734	28/17381	0.99174	0.9993	0.98285	PDGFRB	1	BP
GO:2000257	regulation of protein activation	9/2734	108/17381	0.99213	0.9995	0.98307	C1QB/C1QC/C2/C3AR1/C8A/CD81/CFH/CLU/CPN2	9	BP
GO:0099024	plasma membrane	7/2734	91/17381	0.99272	0.9996	0.98321	ADGRB1/ALKBH4/BIN2/DOCK1/F2RL1/ITGAM/TREM2	7	BP
GO:0006911	phagocytosis, engulfment	6/2734	82/17381	0.99285	0.9996	0.98321	ADGRB1/BIN2/DOCK1/F2RL1/ITGAM/TREM2	6	BP
GO:0031297	replication fork processing	1/2734	29/17381	0.99304	0.9996	0.98321	CDK9	1	BP
GO:0031648	protein destabilization	2/2734	42/17381	0.99337	0.9996	0.98321	CHFR/CTSH	2	BP
GO:0050853	B cell receptor signaling	7/2734	92/17381	0.99347	0.9996	0.98321	ABL1/BLK/CD79B/CMTM3/ELF1/LCK/TEC	7	BP
GO:0000956	nuclear-transcribed mRNA catabolic	21/2734	209/17381	0.99355	0.9996	0.98321	CASC3/CSDE1/DIS3L2/EIF4B/EIF4G1/EXOSC10/EXOSC2/LSM1/MIRLET7A2/NT5C3B/PAN2/PATL1/PNLDC1/POLR2G/RNPS1/RPL35A/RPL36A/RPL41/RPL7/RPS24/SMG5	21	BP
GO:0010921	regulation of phosphatase activity	9/2734	110/17381	0.99358	0.9996	0.98321	CDK5RAP3/DRD2/IKBKB/PDGFRB/PKMYT1/PPP1R1B/SPOCD1/TIPRL/ZFYVE1	9	BP

GO:0002768	immune response-regulating cell surface receptor signaling	54/2734	458/17381	0.99361	0.9996	0.98321	ABL1/ARPC1A/ARPC1B/ARPC2/ARPC4/BLK/BRK1/C3AR1/CACNA1F/CACNB3/CARD11/CARD9/CD247/CD79B/CLEC7A/CMTM3/CSK/DOCK1/ELF1/FCGR3A/HRAS/HSP90AA1/IKBKB/INPP5D/LCK/LGALS3/LIMK1/MAPK3/MICB/MUC1/MUC2/MUC3A/MUC5AC/MUC5B/MUC6/MYO1C/NCR3/PLD2/PRKCD/PSMB11/PSMB6/PSMB7/PSMB8/PSMC3/PSMD13/PSMD3/PSMD5/PS	54	BP
GO:0042104	positive regulation of activated T cell	1/2734	30/17381	0.99414	1	0.98355	IL18	1	BP
GO:0042775	mitochondrial ATP synthesis coupled	6/2734	84/17381	0.99432	1	0.98355	COX10/COX8A/CYC1/NDUFA2/NDUFB5/NDUFC2-KCTD14	6	BP
GO:0042773	ATP synthesis coupled electron	6/2734	85/17381	0.99494	1	0.98355	COX10/COX8A/CYC1/NDUFA2/NDUFB5/NDUFC2-KCTD14	6	BP
GO:0008608	attachment of spindle microtubules to kinetochore	1/2734	31/17381	0.99506	1	0.98355	MAD1L1	1	BP
GO:0034249	negative regulation of cellular amide metabolic process	47/2734	413/17381	0.99559	1	0.98355	ANG/BMP4/CLU/DAPK1/DND1/EGFR/EIF2AK4/EIF2B5/EIF4EBP1/EIF4G1/GAPDH/HIST1H4F/HIST2H4A/HIST2H4B/MIR106B/MIR10A/MIR153-1/MIR15A/MIR16-1/MIR181B1/MIR210/MIR212/MIR221/MIR222/MIR25/MIR29A/MIR29C/MIR32/MIR328/MIR503/MIR582/MIR892B/MIR92A2/MIR96/MIRLET7A2/MYCN/	47	BP
GO:0006613	cotranslational protein targeting to	7/2734	96/17381	0.99582	1	0.98355	RPL35A/RPL36A/RPL41/RPL7/RPS24/SRP14/SSR2	7	BP
GO:0017148	negative regulation of translation	45/2734	399/17381	0.99582	1	0.98355	ANG/BMP4/DAPK1/DND1/EGFR/EIF2AK4/EIF2B5/EIF4EBP1/EIF4G1/GAPDH/HIST1H4F/HIST2H4A/HIST2H4B/MIR106B/MIR10A/MIR153-1/MIR15A/MIR16-1/MIR181B1/MIR210/MIR212/MIR221/MIR222/MIR25/MIR29A/MIR29C/MIR32/MIR328/MIR503/MIR582/MIR892B/MIR92A2/MIR96/MIRLET7A2/MYCN/	45	BP

GO:0000245	spliceosomal complex	3/2734	57/17381	0.99614	1	0.98355	LUC7L/SF1/SRPK3	3	BP
GO:0000271	polysaccharide biosynthetic process	4/2734	68/17381	0.99635	1	0.98355	GCK/INS/MTOR/PHKG1	4	BP
GO:0008206	bile acid metabolic	2/2734	46/17381	0.99638	1	0.98355	FGFR4/OSBPL7	2	BP
GO:2001239	regulation of extrinsic apoptotic signaling pathway in	2/2734	46/17381	0.99638	1	0.98355	EYA1/ZC3HC1	2	BP
GO:0010324	membrane invagination	7/2734	99/17381	0.99702	1	0.98355	ADGRB1/ALKBH4/BIN2/DOCK1/F2RL1/ITGAM/TREM2	7	BP
GO:0050931	pigment cell differentiation	1/2734	34/17381	0.99705	1	0.98355	CD63	1	BP
GO:0003407	neural retina development	3/2734	59/17381	0.99708	1	0.98355	LHX1/SOX8/THY1	3	BP
GO:0050710	negative regulation of cytokine	3/2734	60/17381	0.99747	1	0.98355	F2RL1/FN1/TLR8	3	BP
GO:0006614	SRP-dependent cotranslational protein targeting to	6/2734	91/17381	0.99751	1	0.98355	RPL35A/RPL36A/RPL41/RPL7/RPS24/SRP14	6	BP
GO:0097421	liver regeneration	1/2734	35/17381	0.99751	1	0.98355	EGFR	1	BP
GO:0002763	positive regulation of myeloid leukocyte	2/2734	50/17381	0.99803	1	0.98355	CD74/CSF1	2	BP

GO:0034637	cellular carbohydrate biosynthetic process	4/2734	74/17381	0.99835	1	0.98355	GCK/INS/MTOR/PHKG1	4	BP
GO:0006119	oxidative phosphorylation	7/2734	105/17381	0.99851	1	0.98355	COX10/COX8A/CYC1/MLXIPL/NDUFA2/NDUFB5/NDUFC2-KCTD14	7	BP
GO:0061014	positive regulation of mRNA	1/2734	38/17381	0.99851	1	0.98355	POLR2G	1	BP
GO:0022613	ribonucleoprotein complex biogenesis	51/2734	464/17381	0.99877	1	0.98355	ATXN2L/DDX31/DHX37/DIS3L/DIS3L2/DYNC1H1/EIF4B/ERAL1/EXOSC10/EXOSC2/FCF1/HEATR3/HSP90AA1/ISG20L2/LUC7L/MDN1/MRPL10/MRPS11/NHP2/NLE1/NOB1/NOC2L/NOL11/NOL6/NOP16/NSUN5/NSUN5P2/NVL/PATL1/PDCD11/PRPF8/RPL35A/RPL36A/RPL41/RPL7/RPP21/RPS24/RPUSD2/SART1/SENP3/SF1/SRFBP1/SRPK3/SURF6/SUV39H1/TARBP2/TICRR/TSR	51	BP
GO:0038095	Fc-epsilon receptor signaling	15/2734	179/17381	0.99878	1	0.98355	CARD11/HRAS/IKBKB/MAPK3/PSMB11/PSMB6/PSMB7/PSMB8/PSMC3/PSMD13/PSMD3/PSMD5/PSMD7/SHC1/TEC	15	BP
GO:0006956	complement activation	12/2734	155/17381	0.99903	1	0.98355	C1QB/C1QC/C1RL/C2/C3AR1/C8A/CD81/CFH/CLU/CPN2/MASP2/VSIG4	12	BP
GO:0006959	humoral immune response	32/2734	321/17381	0.99903	1	0.98355	ATOX1/BLNK/C1QB/C1QC/C1RL/C2/C3AR1/C8A/CD81/CFH/CHGA/CLU/CPN2/EXO1/FGA/GAPDH/HLA-E/IFNA5/LTA/MASP2/NOTCH1/PDCD1/PGC/PLA2G1B/PPP2R3C/RARRES2/SLC11A1/SPINK5/SPON2/TFEB/TREM2/VSIG4	32	BP
GO:0045047	protein targeting to ER	6/2734	100/17381	0.99917	1	0.98355	RPL35A/RPL36A/RPL41/RPL7/RPS24/SRP14	6	BP
GO:0022618	ribonucleoprotein complex	18/2734	209/17381	0.99919	1	0.98355	ATXN2L/DYNC1H1/EIF4B/ERAL1/HSP90AA1/LUC7L/MDN1/MRPS11/NLE1/PATL1/PRPF8/SART1/SF1/SRPK3/TARBP2/TICRR/TSR1/USP4	18	BP
GO:0022904	respiratory electron transport chain	6/2734	102/17381	0.99935	1	0.98355	COX10/COX8A/CYC1/NDUFA2/NDUFB5/NDUFC2-KCTD14	6	BP

GO:0071826	ribonucleoprotein complex subunit	19/2734	221/17381	0.99941	1	0.98355	ATXN2L/DYNC1H1/EIF4B/ERAL1/HSP90AA1/KLC1/LUC7L/MDN1/MRPS11/NLE1/PATL1/PRPF8/SART1/SF1/SRPK3/TARBP2/TICRR/TSR1/USP4	19	BP
GO:0072599	establishment of protein localization to endoplasmic	6/2734	104/17381	0.9995	1	0.98355	RPL35A/RPL36A/RPL41/RPL7/RPS24/SRP14	6	BP
GO:0002455	humoral immune response mediated by	9/2734	135/17381	0.99958	1	0.98355	C1QB/C1QC/C1RL/C2/C8A/CLU/EXO1/LTA/MASP2	9	BP
GO:0048066	developmental pigmentation	1/2734	47/17381	0.99968	1	0.98355	CD63	1	BP
GO:0006958	complement activation, classical	7/2734	124/17381	0.99985	1	0.98355	C1QB/C1QC/C1RL/C2/C8A/CLU/MASP2	7	BP
GO:0007608	sensory perception of	11/2734	452/17381	1	1	0.98355	CNGA2/CNGB1/DRD2/FZD2/OBP2B/OR2B3/OR2C1/OR2F1/OR4C11/OR51B5/OR6A2	11	BP
GO:0050907	detection of chemical stimulus involved in	12/2734	471/17381	1	1	0.98355	ASIC3/AZGP1/CA6/GNAT1/LPO/OR2B3/OR2C1/OR2F1/OR4C11/OR51B5/OR6A2/TAS2R3	12	BP
GO:0050911	detection of chemical stimulus involved in	6/2734	426/17381	1	1	0.98355	OR2B3/OR2C1/OR2F1/OR4C11/OR51B5/OR6A2	6	BP
GO:0031225	anchored component of membrane	45/2879	156/18511	1.7E-05	0.012	0.01183	ACHE/ALPPL2/BCAN/CA4/CD160/CD48/CD58/CDH13/CFC1/CNTFR/DPEP1/EFNA1/EFNA3/EFNA4/FOLR2/GP2/GPC1/GPC2/GPIHBP1/LY6D/LY6E/LY6H/LYNX1/LYPD1/LYPD2/LYPD6B/LYPD8/MMP17/MMP25/MSLN/NRN1/NRN1L/NTM/NTNG2/PRSS22/PSCA/RAET1G/RGMA/RTN4RL1/RTN4RL2/SE	45	CC

GO:0016323	basolateral plasma membrane	54/2879	207/18511	6.1E-05	0.0208	0.02056	ABCC3/ABCC4/ABCC6/ADRA2A/AJAP1/AQP1/AQP5/ARRB2/ATP1A1/ATP6V1B1/BSND/CD300LG/CD81/CDH16/CLDN4/CNNM2/CTNNA2/DLG4/EGFR/ERBB2/GPIHBP1/HEPH/KCNQ1/KCNQ4/LEPR/LPO/LRCH4/LRP1/MAP4K2/MARVELD2/MYO1A/MYO1C/NAIP/NKD2/NOD1/OSCP1/P2RY6/PIANP/PKD1/PROM2/RAB17/RHCG/SCRIB/SLC22A1/SLC22A9/SLC26A1/SLC26A6/SLC29A1/SLC29A2/SLC39A5/SLC7A7/ST14/STX4/TLR9	54	CC
GO:0015629	actin cytoskeleton	100/2879	457/18511	0.00019	0.0424	0.04188	ABL1/ABLIM2/ACTA1/ACTR1A/ADAM8/AHNAK/ALDOA/ARC/ARPC1A/ARPC1B/ARPC2/ARPC4/BAIAP2L1/BIN1/BIN2/BIN3/CAPZA3/CATIP/CCDC102A/CFL1/CIB2/CORO1A/CORO1B/CORO6/CROCC/CRYAB/CTNNA2/CTTN/DAPK1/DBN1/DCTN2/DDR2/DENND2A/DNAJA3/DYNLL2/EPB41L2/ESPN/ESPNL/FBLIM1/FERMT2/FERMT3/FHL3/FHOD1/FILIP1/FLNA/FSCN1/HSPB7/IGSF22/ILK/IPP/KIF9/LLGL1/LMOD1/MAD1L1/MAEA/MARK2/MLPH/MOBP/MYBPC1/MYBPC3/MYBPH/MYBPHL/MYL4/MYL5/MYL6B/MYO18A/MYO19/MYO1A/MYO1C/MYO1E/MYO3A/MYO7A/MYO7B/MYZAP/NCF1/NOX4/OBSL1/PDLIM3/PDLIM5/PDLIM7/PKNOX2/RARA/RND1/SCIN/SEPT9/SH3PXD2B/SHROOM1/SPTAN1/SPTB/SPTBN2/STX1A/TAX1BP3/TMEM63B/TNNC1/TPM2/TRPV4/WAS/WDR1/XIRP1/ZYX	100	CC
GO:0043202	lysosomal lumen	28/2879	93/18511	0.00029	0.0504	0.04982	ACAN/ACP2/AGRN/APOB/ATP13A2/BCAN/BGN/CD74/CHID1/CTSD/CTSF/CTSL/CUBN/FASLG/GBA/GPC1/GPC2/HEXA/HSP90AA1/HSPG2/IDS/NAGLU/NPC2/PDGFRB/PLBD2/PSAP/SDC1/SGSH	28	CC
GO:0098862	cluster of actin-based cell projections	37/2879	142/18511	0.00083	0.1147	0.11331	AQP1/CA4/CDC14A/CDH23/CDHR5/CIB2/CLIC1/CUBN/DAB1/EPS8L2/ESPN/ESPNL/FLII/HOMER2/IDO1/IFT20/ITPR3/LCTL/LHFPL5/MORN4/MYO1A/MYO1C/MYO1E/MYO3A/MYO7A/MYO7B/NPC1L1/PDZD3/PEMT/RALGDS/SCIN/SLC22A12/SLC26A6/SLC34A1/SLC9A3/SOAT2/TPRN	37	CC
GO:0016324	apical plasma membrane	65/2879	292/18511	0.00146	0.1503	0.14849	ABCC6/ABCG5/AJAP1/ANO1/AQP1/AQP5/ATP1A1/ATP6V1B1/CA4/CACNB3/CD300LG/CDHR5/CFAP126/CIB1/CLDN4/CNTFR/CRB2/CUBN/DDR2/DPEP1/EGFR/ERBB2/FN1/GNAT1/GPIHBP1/HIP1R/ITPK1/LHFPL5/MARVELD2/MGAM/MIP/MUC1/MYO1A/MYO7A/NOD1/NOTCH1/NOX4/NPC1L1/P2RX2/P2RY6/PDGFRB/PFKM/PLB1/PROM2/RAB17/RHCG/SCNN1A/SLC22A12/SLC22A13/SLC26A6/SLC29A1/SLC29A4/SLC34A1/SLC44A4/SLC9A3/SLC9A3R2/SPTBN2/TDGF1/THY1/TLR9/TMEM114/TNIK/TRPV4/UPK1B/WDP[illegible]	65	CC

GO:0045177	apical part of cell	78/2879	363/18511	0.00153	0.1503	0.14849	ABCC6/ABCG5/AJAP1/ANO1/AQP1/AQP5/AQP8/ATP1A1/ATP6V1B1/CA4/CACNB3/CD300LG/CDHR5/CFAP126/CIB1/CLDN4/CNTFR/CRB2/CUBN/DAB1/DDR2/DPEP1/DVL2/EGFR/ERBB2/FN1/GNAT1/GPIHBP1/HIP1R/HOMER2/ITPK1/ITPR3/LHFPL5/MARVELD2/MGAM/MGST1/MIP/MUC1/MYO1A/MYO7A/MYO7B/NOD1/NOTCH1/NOX4/NPC1L1/NUMA1/P2RX2/P2RY6/PARD6A/PDGFRB/PDZD3/PFKM/PLB1/PROM2/RAB17/REN/RHCG/SCNN1A/SLC22A12/SLC22A13/SLC26A6/SLC29A1/SLC29A4/SLC34A1/SLC44A4/SLC4A9/SLC9A3/SLC9A3R2/SPTBN2/TDGF1/THY1/TLR9/TMEM114/TNIK/T[illegible]	78	CC
GO:0005775	vacuolar lumen	41/2879	170/18511	0.00228	0.1957	0.19336	ACAN/ACP2/AGRN/APOB/ATP13A2/BCAN/BGN/CD74/CHID1/CTSD/CTSF/CTSL/CUBN/DAPK2/DPP7/DYNC1H1/FASLG/GBA/GGH/GPC1/GPC2/HEXA/HSP90AA1/HSPG2/IDS/IMPDH1/MPO/NAGLU/NPC2/ORM2/PDGFRB/PLBD2/PRDX6/PRKCD/PSAP/SDC1/SERPINA3/SGSH/STK11IP/TRAPPC1/VAT	41	CC
GO:0005796	Golgi lumen	27/2879	103/18511	0.0036	0.2551	0.25208	ACAN/AGRN/BCAN/BGLAP/BGN/BLZF1/FURIN/GPC1/GPC2/HSPG2/INS/MUC1/MUC2/MUC3A/MUC5AC/MUC5B/MUC6/PDGFA/PODXL2/PROZ/SDC1/SDF4/SOD3/WNT1/WNT3A/WNT6/WNT7A	27	CC
GO:0032426	stereocilium tip	7/2879	15/18511	0.00443	0.2551	0.25208	EPS8L2/ESPN/ESPNL/HOMER2/LHFPL5/MORN4/MYO3A	7	CC
GO:0016459	myosin complex	20/2879	71/18511	0.00479	0.2551	0.25208	CCDC102A/DYNLL2/IGSF22/MYBPC1/MYBPC3/MYBPH/MYBPHL/MYL4/MYL5/MYL6B/MYO18A/MYO19/MYO1A/MYO1C/MYO1E/MYO3A/MYO7A/MYO7B/OBSL1/SHROOM1	20	CC
GO:0000930	gamma-tubulin complex	8/2879	19/18511	0.00511	0.2551	0.25208	BRCA1/CKAP5/TUBG1/TUBG2/TUBGCP2/TUBGCP3/TUBGCP5/UXT	8	CC
GO:0031430	M band	11/2879	31/18511	0.00521	0.2551	0.25208	ALDOA/CRYAB/IGSF22/MYBPC1/MYBPC3/MYBPH/MYBPHL/OBSCN/OBSL1/SMTNL1/TRIM63	11	CC
GO:0031672	A band	14/2879	44/18511	0.00534	0.2551	0.25208	ALDOA/CRYAB/IGSF22/KLHL40/MYBPC1/MYBPC3/MYBPH/MYBPHL/MYL4/OBSCN/OBSL1/PPP1R12B/SMTNL1/TRIM63	14	CC
GO:0017101	aminoacyl-tRNA synthetase multienzyme	6/2879	12/18511	0.00557	0.2551	0.25208	AIMP1/AIMP2/DARS/KARS/LARS/YARS	6	CC

GO:0044441	ciliary part	83/2879	414/18511	0.00785	0.3373	0.33332	AMBRA1/ARR3/B9D1/BBS9/C2CD3/CACNA1F/CC2D2A/CCDC103/CCDC40/CDHR1/CEP164/CEP19/CFAP126/CFAP157/CFAP74/CIB2/CLTB/CNGA2/CNGB1/CROCC/DISC1/DNAH11/DNAH17/DNAJB13/DNHD1/DRD2/DYNLL2/EHD1/EVC2/FOPNL/GAS8/GNAT1/GNB5/GUCA1B/GUCA2B/GUCY2D/HHIP/HYDIN/IFT140/IFT20/IFT22/INPP5E/KIF17/MOK/MYO7A/NEK8/ODF1/ODF4/OPN1MW/OPN4/PEX6/PFKM/PGAM4/PKD1/PRKAR1A/PRKAR1B/PROM2/PTPN23/RILPL1/RILPL2/SCNN1A/SEPT4/SEPT9/SLC26A6/SMO/SPAG16/SSNA1/SUFU/TACR1/TACR2/TCP11/TMEM141/TMEM17/TMEM231/TRAF3IP1/TUBA3C/TUBA3D/TUBG1/TULP1/TULP3/WDPCP/WDR66/WRAP73	83	CC
GO:0032421	stereocilium bundle	15/2879	51/18511	0.00894	0.3541	0.34994	CDC14A/CDH23/CIB2/EPS8L2/ESPN/ESPNL/HOMER2/IDO1/IFT20/LHFPL5/MORN4/MYO1C/MYO3A/MYO7A/TPRN	15	CC
GO:0030863	cortical cytoskeleton	22/2879	85/18511	0.00943	0.3541	0.34994	CAPZA3/CFL1/CIB2/CORO1A/CTTN/DBN1/DLG4/EPB41L2/FLNA/LLGL1/MAEA/MLPH/MOBP/MYO1A/MYZAP/NSMF/NUMA1/SPTAN1/SPTB/SPTBN2	22	CC
GO:0005605	basal lamina	8/2879	21/18511	0.01031	0.3541	0.34994	ACHE/AGRN/AMTN/ANG/FN1/LAMA2/LAMA4/LAMC2	8	CC
GO:0042827	platelet dense granule	8/2879	21/18511	0.01031	0.3541	0.34994	ABCC4/CD63/CTSW/ECM1/ITIH4/RARRES2/SELP/SERPINA4	8	CC
GO:0030425	dendrite	96/2879	495/18511	0.0115	0.3762	0.37181	ABL1/ADGRB1/ALCAM/AMIGO1/APBB1/ARC/ARRB2/ASIC2/BGLAP/CCR2/CDK5/CHRM1/CIB1/CLU/CNIH2/CPLX1/CRYAB/CTTN/DAB2IP/DBN1/DGKI/DLG4/DRD2/DTNBP1/EIF5A/ELOVL5/EPHB3/FBXO2/FLNA/GABBR1/GCHFR/GLRA1/GLRX5/GNAO1/GNB3/GNG3/GPER1/GRIN1/GRIPAP1/HCFC1/HCN3/HIP1R/HOMER2/IFT20/ILK/INPP5F/KCND1/KCND3/KIF17/KIRREL3/KNDC1/L1CAM/LAMA2/LRP1/LSM1/LYNX1/MAF1/MAPK8IP1/MAX/MLPH/MTOR/NCDN/NCF1/NECAB2/NRG1/NSMF/NUMA1/OPRD1/OPRM1/PPP1R9B/PRR7/PSD/PSD2/PTK2B/RAB17/RARA/RGS10/RGS14/RTN4RL2/SHARPIN/STAT1/STX4/SV2A/TACR1/TAOK2/TH/THY1/TMEM185A/TRAPPC[illegible]	96	CC
GO:0005903	brush border	24/2879	97/18511	0.01228	0.3834	0.3789	AQP1/CA4/CDHR5/CLIC1/CUBN/DAB1/ESPN/FLII/ITPR3/LCTL/MYO1A/MYO1C/MYO1E/MYO7B/NPC1L1/PDZD3/PEMT/RALGDS/SCIN/SLC22A12/SLC26A6/SLC34A1/SLC9A3/SOAT2	24	CC
GO:0032420	stereocilium	13/2879	44/18511	0.01386	0.4139	0.40906	CDH23/CIB2/EPS8L2/ESPN/ESPNL/HOMER2/IFT20/LHFPL5/MORN4/MYO1C/MYO3A/MYO7A/TPRN	13	CC

GO:0042641	actomyosin	17/2879	64/18511	0.01621	0.4639	0.45846	ACTA1/CORO1B/DBN1/FBLIM1/FERMT2/FHL3/FHOD1/FSCN1/ILK/MYO18A/NOX4/PDLIM7/SEPT9/STX1A/WDR1/XIRP1/ZYX	17	CC
GO:0031091	platelet alpha granule	22/2879	90/18511	0.01838	0.478	0.47244	AHSG/ALDOA/APLP2/CLU/CYB5R1/FERMT3/FGA/FN1/KNG1/LEFTY2/MAGED2/ORM1/ORM2/PDGFA/QSOX1/SELP/SERPINA3/SERPINA5/SERPINF2/STXBP1/VTI1B/VWF	22	CC
GO:0005938	cell cortex	51/2879	247/18511	0.01898	0.478	0.47244	ACTR1A/BIN2/CAPZA3/CFL1/CIB2/CORO1A/CTTN/DBN1/DLG4/EMD/EPB41L2/FBLIM1/FERMT2/FGA/FGFR2/FLNA/FMNL1/GAD1/GPSM1/HIP1R/HMCN2/LAMC2/LLGL1/MAEA/MLPH/MOBP/MYO1A/MYO7A/MYZAP/NRBP1/NSMF/NUMA1/OR2C1/PARD3/PARD6A/PDZD4/PFN1/PTK2B/RALB/SCIN/SEPT4/SEPT9/SPINK5/SPTAN1/SPTB/SPTBN2/TRAF2/TRPV4/USP2/WDP	51	CC
GO:0046658	anchored component of plasma	12/2879	41/18511	0.01908	0.478	0.47244	CA4/CD160/EFNA1/FOLR2/GPIHBP1/NRN1L/NTNG2/PRSS22/RTN4RL1/RTN4RL2/THY1/ULBP2	12	CC
GO:0002102	podosome	10/2879	32/18511	0.01975	0.478	0.47244	ADAM8/BIN2/CTTN/FERMT3/FSCN1/KIF9/NCF1/SCIN/SH3PXD2B/WDR1	10	CC
GO:0032580	Golgi cisterna membrane	19/2879	76/18511	0.02166	0.478	0.47244	APH1A/ARAP1/B4GALNT4/B4GALT2/B4GALT7/BCAP31/CHPF/CHPF2/FUT7/GAL3ST4/GGTA1P/GOLGA2/GOLGA3/GPR89A/INPP5E/PITPNM1/ST3GAL4/TMED3/TMEM115	19	CC
GO:0036452	ESCRT complex	9/2879	28/18511	0.022	0.478	0.47244	CHMP1A/CHMP4C/CHMP7/SNF8/UBAP1L/VPS25/VPS37B/VPS37C/VPS37D	9	CC
GO:0097546	ciliary base	9/2879	28/18511	0.022	0.478	0.47244	DISC1/IFT20/MOK/NEK8/PRKAR1A/PRKAR1B/SUFU/TRAF3IP1/TULP3	9	CC
GO:0031985	Golgi cisterna	23/2879	97/18511	0.0229	0.478	0.47244	APH1A/ARAP1/B4GALNT4/B4GALT2/B4GALT7/BCAP31/CHPF/CHPF2/FUT7/GAL3ST4/GGTA1P/GOLGA2/GOLGA3/GPR89A/INPP5E/LLGL1/PITPNM1/RAB34/SGMS1/ST3GAL4/TMED3/TMEM115/YIPF1	23	CC
GO:0031253	cell projection membrane	62/2879	312/18511	0.023	0.478	0.47244	AQP1/ARHGEF4/ARPC1A/ARPC2/BBS9/CA4/CDHR1/CDHR5/CFL1/CIB1/CLTB/CNGA2/CNGB1/CUBN/DPEP1/DRD2/EHD1/EPS8L2/EVC2/FERMT2/FSCN1/FZD9/GNAT1/GPER1/GUCA1B/GUCY2D/HHIP/HIP1R/HSP90AA1/KSR1/MYO1C/NME1/NPC1L1/NRG1/OPN1MW/OPN4/OPRD1/OPRM1/PEMT/PKD1/PLXND1/PPP1R9B/PROM2/PSD/PSD2/PSD4/RAB25/RASGRP2/SCARB1/SCNN1A/SLC22A12/SLC26A6/SLC34A1/SLC9A3/SMO/THY1/TIRAP/TLN1/	62	CC

GO:0005795	Golgi stack	29/2879	129/18511	0.02366	0.478	0.47244	APH1A/ARAP1/ARSE/B4GALNT4/B4GALT2/B4GALT7/BCAP31/CHPF/CHPF2/FUT7/GAL3ST4/GBF1/GGTA1P/GOLGA2/GOLGA3/GPR89A/INPP5E/LLGL1/LYPLA2/MARCH4/PITPNM1/RAB34/SGMS1/ST3GAL4/TMED3/TMEM115/TRAPPC4/YIPF1/ZFYVE1	29	CC
GO:0031093	platelet alpha granule lumen	17/2879	67/18511	0.02513	0.4932	0.48741	AHSG/ALDOA/CLU/FERMT3/FGA/FN1/KNG1/LEFTY2/MAGED2/ORM1/ORM2/PDGFA/QSOX1/SERPINA3/SERPINF2/VTI1B/VWF	17	CC
GO:0060170	ciliary membrane	20/2879	83/18511	0.02751	0.525	0.51886	BBS9/CDHR1/CLTB/CNGA2/CNGB1/DRD2/EHD1/EVC2/GNAT1/GUCA1B/GUCY2D/HHIP/OPN1MW/OPN4/PKD1/PROM2/SCNN1A/SMO/TMEM17/TM	20	CC
GO:0031674	I band	29/2879	132/18511	0.03144	0.5837	0.57683	ADRA1A/ALDOA/ANKRD2/BIN1/CACNA1S/CAV3/CRYAB/DES/FBXL22/FERMT2/FHL3/FLNA/FLNC/HSPB1/IGSF22/KLHL40/MYBPC1/MYBPC3/MYBPH/MYBPHL/MYZAP/OBSCN/OBSL1/PDLIM3/PPP1R12B/SMTNL1/TCAP/	29	CC
GO:0030864	cortical actin cytoskeleton	15/2879	59/18511	0.0335	0.6057	0.59856	CFL1/CIB2/CORO1A/EPB41L2/LLGL1/MAEA/MLPH/MOBP/MYO1A/MYZAP/SPTAN1/SPTB/SPTBN2/TRPV4/WDR1	15	CC
GO:0005788	endoplasmic reticulum lumen	58/2879	299/18511	0.04133	0.6841	0.67606	ADAMTS13/ADAMTS7/AHSG/AMTN/APLP2/APOA5/APOB/ARSE/ARSI/BGLAP/BMP4/CES2/CLN6/CNPY3/COL16A1/COL17A1/COL1A1/COL4A2/COL7A1/COL9A2/CSF1/CYP2W1/FGA/FKBP10/FN1/GANAB/GBF1/GCG/HSPA5/IGFBP3/INS/KNG1/MEN1/MSLN/MXRA8/MZB1/OS9/P3H1/PDGFA/PNPLA2/PPIB/PROZ/PTPRN2/QSOX1/SERPINH1/SHH/SIL1/TNC/TOR1A/TOR1B/TOR2A/TRDN/VGF/WFS1/WNT1/WNT3A/WNT6/WNT7A	58	CC
GO:0031941	filamentous actin	9/2879	31/18511	0.0417	0.6841	0.67606	ESPN/ESPNL/FERMT2/FLNA/FSCN1/MYO1A/MYO1C/MYO3A/XIRP1	9	CC
GO:0005859	muscle myosin complex	8/2879	27/18511	0.04781	0.6841	0.67606	IGSF22/MYBPC1/MYBPC3/MYBPH/MYBPHL/MYL5/MYL6B/OBSL1	8	CC
GO:0005884	actin filament	20/2879	88/18511	0.04829	0.6841	0.67606	ACTA1/BIN3/CORO1A/CORO1B/CTTN/DNAJA3/ESPN/ESPNL/FERMT2/FLNA/FSCN1/LMOD1/MARK2/MYO1A/MYO1C/MYO3A/TPM2/WAS/WDR1/X	20	CC
GO:0001725	stress fiber	13/2879	52/18511	0.05167	0.6841	0.67606	ACTA1/CORO1B/FBLIM1/FERMT2/FHL3/FHOD1/FSCN1/ILK/NOX4/PDLIM7/SEPT9/XIRP1/ZYX	13	CC
GO:0097517	contractile actin filament bundle	13/2879	52/18511	0.05167	0.6841	0.67606	ACTA1/CORO1B/FBLIM1/FERMT2/FHL3/FHOD1/FSCN1/ILK/NOX4/PDLIM7/SEPT9/XIRP1/ZYX	13	CC

GO:0034774	secretory granule lumen	61/2879	321/18511	0.05295	0.6841	0.67606	AHSG/ALAD/ALDOA/BIN2/C1orf35/CDA/CHIT1/CLU/COTL1/CRACR2A/CTSD/CTSH/CTSW/DPP7/DYNC1H1/ECM1/EPX/FERMT3/FGA/FN1/GCG/GGH/GHDC/GSDMD/HK3/HSP90AA1/IDH1/IMPDH1/INS/ITIH4/KNG1/LEFTY2/MAGED2/MPO/NPC2/OLFM4/ORM1/ORM2/PDGFA/PKM/PPIE/PRDX6/PRKCD/PSMB7/PSMC3/PSMD13/PSMD3/PSMD7/QSOX1/RARRES2/SERPINA3/SERPINA4/SERPINF2/SERPINI1/SPTAN1/SRP14/STK11IP/TRAPPC1/VAT1/[illegible]	61	CC
GO:0005736	DNA-directed RNA polymerase I	5/2879	14/18511	0.05345	0.6841	0.67606	POLR1A/POLR1D/POLR1E/POLR2L/ZNRD1	5	CC
GO:0030008	TRAPP complex	5/2879	14/18511	0.05345	0.6841	0.67606	TRAPPC1/TRAPPC2L/TRAPPC3/TRAPPC4/TRAPPC9	5	CC
GO:0031089	platelet dense granule lumen	5/2879	14/18511	0.05345	0.6841	0.67606	CTSW/ECM1/ITIH4/RARRES2/SERPINA4	5	CC
GO:0031983	vesicle lumen	64/2879	339/18511	0.05423	0.6841	0.67606	AHSG/ALAD/ALDOA/APOB/BIN2/C1orf35/CDA/CHIT1/CLU/COTL1/CRACR2A/CTSD/CTSH/CTSW/DPP7/DYNC1H1/ECM1/EGFR/EPX/FASLG/FERMT3/FGA/FN1/GCG/GGH/GHDC/GSDMD/HK3/HSP90AA1/IDH1/IMPDH1/INS/ITIH4/KNG1/LEFTY2/MAGED2/MPO/NPC2/OLFM4/ORM1/ORM2/PDGFA/PKM/PPIE/PRDX6/PRKCD/PSMB7/PSMC3/PSMD13/PSMD3/PSMD7/QSOX1/RARRES2/SERPINA3/SERPINA4/SERPINF2/SERPINI1/SPTAN1/SRP14/STK[illegible]	64	CC
GO:0055029	nuclear DNA-directed RNA polymerase complex	28/2879	133/18511	0.05501	0.6841	0.67606	EDF1/GTF2H2C/GTF2H4/INTS10/INTS3/INTS5/KAT2A/MED18/MED6/POLA2/POLR1A/POLR1D/POLR1E/POLR2G/POLR2J2/POLR2J3/POLR2L/POLR3D/RECQL5/TADA3/TAF1/TAF1L/TAF3/TAF6/TAF7/TBPL1/ZNF768/ZNRD1	28	CC
GO:0000813	ESCRT I complex	4/2879	10/18511	0.05604	0.6841	0.67606	UBAP1L/VPS37B/VPS37C/VPS37D	4	CC
GO:0016461	unconventional myosin	4/2879	10/18511	0.05604	0.6841	0.67606	DYNLL2/MYL6B/MYO1C/MYO7A	4	CC
GO:0045240	dihydrolipoyl dehydrogenase complex	4/2879	10/18511	0.05604	0.6841	0.67606	BCKDK/DLST/OGDH/OGDHL	4	CC

GO:0032432	actin filament bundle	14/2879	58/18511	0.05786	0.6841	0.67606	ACTA1/CORO1B/CRYAB/FBLIM1/FERMT2/FHL3/FHOD1/FSCN1/ILK/NOX4/PDLIM7/SEPT9/XIRP1/ZYX	14	CC
GO:0044665	MLL1/2 complex	8/2879	28/18511	0.05814	0.6841	0.67606	ASH2L/CHD8/HCFC1/MAX/SENP3/TAF1/TAF6/TAF7	8	CC
GO:0071339	MLL1 complex	8/2879	28/18511	0.05814	0.6841	0.67606	ASH2L/CHD8/HCFC1/MAX/SENP3/TAF1/TAF6/TAF7	8	CC
GO:0000428	DNA-directed RNA polymerase	28/2879	134/18511	0.0596	0.6841	0.67606	EDF1/GTF2H2C/GTF2H4/INTS10/INTS3/INTS5/KAT2A/MED18/MED6/POLA2/POLR1A/POLR1D/POLR1E/POLR2G/POLR2J2/POLR2J3/POLR2L/POLR3D/RECQL5/TADA3/TAF1/TAF1L/TAF3/TAF6/TAF7/TBPL1/ZNF768/ZNRD1	28	CC
GO:0001772	immunological synapse	9/2879	33/18511	0.05997	0.6841	0.67606	ALCAM/CARD11/CD6/CD81/CORO1A/HAVCR2/LCK/LGALS3/PRKAR1A	9	CC
GO:0016460	myosin II complex	9/2879	33/18511	0.05997	0.6841	0.67606	IGSF22/MYBPC1/MYBPC3/MYBPH/MYBPHL/MYL5/MYL6B/OBSL1/SHROOM1	9	CC
GO:0030426	growth cone	31/2879	151/18511	0.06066	0.6841	0.67606	ANG/APBB1/ARHGAP4/BOC/CDK5/CIB1/CTTN/DBN1/DCTN2/DISC1/DOCK7/DTNBP1/FSCN1/IGHMBP2/KATNB1/KLC1/L1CAM/LRIG2/LRRTM1/MAPK8IP1/NDRG2/PARD3/PTK2B/SIGMAR1/STMN4/THY1/TOR1A/TRPV2/TR	31	CC
GO:0042599	lamellar body	6/2879	19/18511	0.06228	0.6841	0.67606	CTSH/LAMP3/RAB7A/SFTPA1/SFTPB/SPINK5	6	CC
GO:0044449	contractile fiber part	41/2879	209/18511	0.06562	0.6841	0.67606	ACTA1/ADRA1A/AHNAK/ALDOA/ANKRD2/ARHGEF25/BIN1/CACNA1S/CAV3/CRYAB/DES/FBXL22/FERMT2/FHL3/FLNA/FLNC/HSPB1/IDO1/IGSF22/ILK/KLHL40/LMOD1/MMP2/MYBPC1/MYBPC3/MYBPH/MYBPHL/MYL4/MYL5/MYL6B/MYZAP/OBSCN/OBSL1/PDLIM3/PPP1R12B/SMTNL1/TCAP	41	CC
GO:0005640	nuclear outer membrane	7/2879	24/18511	0.06723	0.6841	0.67606	BOK/CPTP/EMD/GUCY2D/ITPR3/SIGMAR1/TMEM109	7	CC
GO:0034399	nuclear periphery	27/2879	130/18511	0.06747	0.6841	0.67606	ATF7/ATN1/CFL1/CHMP1A/DGKQ/DNMT3A/GFI1B/IPO4/KIF4A/LMNA/LMNB1/MAEA/MEN1/NCOR2/NONO/NSMF/NUMA1/NUP98/OGG1/PAXIP1/PRKCD/PSPC1/SUV39H1/TELO2/TGFB1I1/TINF2/ZNF703	27	CC

GO:0060205	cytoplasmic vesicle lumen	63/2879	338/18511	0.06872	0.6841	0.67606	AHSG/ALAD/ALDOA/APOB/BIN2/C1orf35/CDA/CHIT1/CLU/COTL1/CRACR2A/CTSD/CTSH/CTSW/DPP7/DYNC1H1/ECM1/EPX/FASLG/FERMT3/FGA/FN1/GCG/GGH/GHDC/GSDMD/HK3/HSP90AA1/IDH1/IMPDH1/INS/ITIH4/KNG1/LEFTY2/MAGED2/MPO/NPC2/OLFM4/ORM1/ORM2/PDGFA/PKM/PPIE/PRDX6/PRKCD/PSMB7/PSMC3/PSMD13/PSMD3/PSMD7/QSOX1/RARRES2/SERPINA3/SERPINA4/SERPINF2/SERPINI1/SPTAN1/SRP14/STK11IP/T[illegible]	63	CC
GO:0005905	clathrin-coated pit	16/2879	70/18511	0.06878	0.6841	0.67606	AP1S1/AP2A2/AP2M1/AP4S1/ARRB2/CDHR5/CLTB/CTTN/CUBN/EGFR/HIP1R/INPP5F/LRP1/LRP10/SLC2A4/TNK2	16	CC
GO:0030880	RNA polymerase complex	28/2879	136/18511	0.06959	0.6841	0.67606	EDF1/GTF2H2C/GTF2H4/INTS10/INTS3/INTS5/KAT2A/MED18/MED6/POLA2/POLR1A/POLR1D/POLR1E/POLR2G/POLR2J2/POLR2J3/POLR2L/POLR3D/RECQL5/TADA3/TAF1/TAF1L/TAF3/TAF6/TAF7/TBPL1/ZNF768/ZNRD1	28	CC
GO:0099568	cytoplasmic region	58/2879	309/18511	0.07007	0.6841	0.67606	ACTR1A/BIN2/CAPZA3/CFL1/CIB2/CORO1A/CTTN/DBN1/DLG4/DTNBP1/EMD/EPB41L2/FBLIM1/FERMT2/FGA/FGFR2/FLNA/FMNL1/GAD1/GPSM1/HIP1R/HMCN2/HSPB1/KIF17/KIF4A/LAMC2/LLGL1/MAEA/MLPH/MOBP/MYO1A/MYO7A/MYZAP/NRBP1/NSMF/NUMA1/OPRM1/OR2C1/PARD3/PARD6A/PDZD4/PFN1/PTK2B/RALB/SCIN/SEPT4/SEPT9/SPINK5/SPTAN1/SPTB/SPTBN2/TRAF2/TRIM46/TRPV4/USP2/WDPCP/WDR1/WLS	58	CC
GO:0060077	inhibitory synapse	5/2879	15/18511	0.07024	0.6841	0.67606	CEP112/GAD1/GLRA1/MAF1/NLGN3	5	CC
GO:0005667	transcription factor complex	59/2879	315/18511	0.0705	0.6841	0.67606	APEX1/ASCL2/BARX2/BCL9/BRF1/CDK4/CREM/CTBP1/DEAF1/DMBX1/E2F4/E2F8/EDF1/FOXE3/GATA4/GFI1B/GTF2H2C/GTF2H4/GTF3C1/HES6/HMGA1/HOXB9/KAT2A/LDB1/LHX3/MAX/MED18/MED6/MLXIPL/MYOD1/NAA16/NKX2-5/NONO/NPAS2/NR1H3/NR5A1/PBX2/PDLIM1/PITX1/PMF1/POU3F1/RCOR2/SCX/SMAD6/SNAI3/SNAPC1/SNF8/SOX8/TADA3/TAF1/TAF1A/TAF1L/T[illegible]	59	CC
GO:0001917	photoreceptor inner segment	10/2879	39/18511	0.0707	0.6841	0.67606	AIPL1/ARR3/CIB2/CROCC/DNM1/GNAT1/GNB5/GUCA1B/MYO7A/TULP1	10	CC

GO:0061695	transferase complex, transferring phosphorus-containing	50/2879	263/18511	0.07306	0.6971	0.68897	CCND3/CDK4/CDK9/EDF1/ENG/GTF2H2C/GTF2H4/HOXC10/IKBKB/INTS10/INTS3/INTS5/KAT2A/MED18/MED6/MTOR/NEK10/PFKFB1/PFKM/PHKG1/PIK3R5/PIK3R6/PKM/POLA2/POLD4/POLE/POLE4/POLR1A/POLR1D/POLR1E/POLR2G/POLR2J2/POLR2J3/POLR2L/POLR3D/PRKAB1/PRKAR1A/PYDC1/RECQL5/TADA3/TAF1/TAF1L/TAF3/TAF6/TAF7/TBPL1/TRIM40/ULK1	50	CC
GO:1904724	tertiary granule lumen	13/2879	55/18511	0.076	0.6996	0.69142	ALDOA/CDA/CHIT1/CTSD/CTSH/FLG2/GGH/GSDMD/IDH1/OLFM4/ORM1/QSOX1/SPTAN1	13	CC
GO:0005885	Arp2/3 protein complex	4/2879	11/18511	0.07754	0.6996	0.69142	ARPC1A/ARPC1B/ARPC2/ARPC4	4	CC
GO:0019774	proteasome core complex, beta-subunit	4/2879	11/18511	0.07754	0.6996	0.69142	PSMB11/PSMB6/PSMB7/PSMB8	4	CC
GO:0031094	platelet dense tubular network	4/2879	11/18511	0.07754	0.6996	0.69142	ATP2A3/EHD1/ITPR3/SERPINA5	4	CC
GO:0005604	basement membrane	20/2879	93/18511	0.07841	0.6996	0.69142	ACHE/AGRN/AMTN/ANG/COL17A1/COL4A2/COL7A1/EFEMP2/FN1/HMCN2/HSPG2/LAMA2/LAMA4/LAMC2/LAMC3/LOXL1/LOXL2/RELL2/TGFBI/	20	CC
GO:0043292	contractile fiber	43/2879	224/18511	0.08036	0.7078	0.69946	ACTA1/ADRA1A/AHNAK/ALDOA/ANKRD2/ARHGEF25/BIN1/CACNA1S/CAV3/CRYAB/DES/FBXL22/FERMT2/FHL3/FLNA/FLNC/HSPB1/IDO1/IGSF22/ILK/KLHL40/LMOD1/MMP2/MYBPC1/MYBPC3/MYBPH/MYBPHL/MYL4/MYL5/MYL6B/MYOD1/MYZAP/OBSCN/OBSL1/PDE4DIP/PDLIM3/PPP1R12B/SMTNL1/TCAP/TNNC1/TPM2/TRIM54/TRIM63	43	CC
GO:0030139	endocytic vesicle	52/2879	277/18511	0.08223	0.7151	0.70672	ADAM8/AMBRA1/AP1S1/AP2A2/AP2M1/APOB/ARRB2/ATP6V0B/ATP6V0E2/CAMK2D/CD74/CLTB/CORO1A/CUBN/DAB2IP/DLG4/DRD2/DVL2/EGFR/FMNL1/FZD2/GRIA3/HLA-E/HSP90AA1/INPP5F/ITGB5/LRP1/MYO1E/NCF1/NLGN3/NOS3/NOSTRIN/PLEKHG5/RAB17/RAB20/RAB34/RAB7A/SCARB1/SCARF1/SFTPA1/SFTPB/SLC11A1/SMO/STX4/TAP1/TIRAP/TLR9/WLS/WNT1/WNT3A/WNT6/WNT7	52	CC
GO:0005811	lipid droplet	16/2879	72/18511	0.08479	0.7282	0.71962	ATG2A/BCAP31/EHD1/G0S2/GAPDH/GBF1/HILPDA/IRAK1/NSDHL/PITPNM1/PLIN1/PNPLA2/RAB7A/RBP1/SIGMAR1/TRAP1	16	CC

GO:0030427	site of polarized growth	31/2879	156/18511	0.08621	0.7312	0.72263	ANG/APBB1/ARHGAP4/BOC/CDK5/CIB1/CTTN/DBN1/DCTN2/DISC1/DOCK7/DTNBP1/FSCN1/IGHMBP2/KATNB1/KLC1/L1CAM/LRIG2/LRRTM1/MAPK8IP1/NDRG2/PARD3/PTK2B/SIGMAR1/STMN4/THY1/TOR1A/TRPV2/TR	31	CC
GO:0044448	cell cortex part	27/2879	134/18511	0.09092	0.7575	0.74863	ACTR1A/CAPZA3/CFL1/CIB2/CORO1A/CTTN/DBN1/DLG4/EMD/EPB41L2/FLNA/LLGL1/MAEA/MLPH/MOBP/MYO1A/MYZAP/NSMF/NUMA1/RALB/SEPT4/SEPT9/SPTAN1/SPTB/SPTBN2/TRPV4/WDR1	27	CC
GO:0030017	sarcomere	37/2879	192/18511	0.0944	0.7575	0.74863	ACTA1/ADRA1A/ALDOA/ANKRD2/ARHGEF25/BIN1/CACNA1S/CAV3/CRYAB/DES/FBXL22/FERMT2/FHL3/FLNA/FLNC/HSPB1/IGSF22/ILK/KLHL40/LMOD1/MMP2/MYBPC1/MYBPC3/MYBPH/MYBPHL/MYL4/MYZAP/OBSCN/OBSL1/PDLIM3/PPP1R12B/SMTNL1/TCAP/TNNC1/TPM2/TRIM54/TRI	37	CC
GO:0016328	lateral plasma membrane	13/2879	57/18511	0.09573	0.7575	0.74863	ABCC6/ATP6V1B1/AXIN1/CLDN15/CLDN4/DRD2/DVL2/MARK2/MYO1A/MYO1C/NKD2/NUMA1/PKD1	13	CC
GO:0070820	tertiary granule	32/2879	164/18511	0.09973	0.7575	0.74863	ADAM8/ALDOA/AP2A2/ARL8A/CD58/CDA/CHIT1/CTSD/CTSH/DOK3/FLG2/FRMPD3/GGH/GSDMD/IDH1/ITGAM/ITGAX/LGALS3/MGAM/NBEAL2/OLFM4/OLR1/ORM1/PLAU/PRCP/PTPRN2/QSOX1/SLC11A1/SPTAN1/TMEM	32	CC
GO:0016591	DNA-directed RNA polymerase II,	22/2879	107/18511	0.09977	0.7575	0.74863	EDF1/GTF2H2C/GTF2H4/INTS10/INTS3/INTS5/KAT2A/MED18/MED6/POLR2G/POLR2J2/POLR2J3/POLR2L/RECQL5/TADA3/TAF1/TAF1L/TAF3/TAF6/TAF7/TBPL1/ZNF768	22	CC
GO:0008250	oligosaccharyltransferase	4/2879	12/18511	0.1025	0.7575	0.74863	DAD1/DDOST/OST4/RPN1	4	CC
GO:0031932	TORC2 complex	4/2879	12/18511	0.1025	0.7575	0.74863	MAPKAP1/MLST8/MTOR/TELO2	4	CC
GO:0045239	tricarboxylic acid cycle enzyme	4/2879	12/18511	0.1025	0.7575	0.74863	BCKDK/DLST/OGDH/OGDHL	4	CC
GO:0061702	inflammasome complex	4/2879	12/18511	0.1025	0.7575	0.74863	CASP1/CASP5/GSDMD/NLRP1	4	CC
GO:0005891	voltage-gated calcium channel complex	10/2879	42/18511	0.10665	0.7575	0.74863	CACNA1F/CACNA1G/CACNA1H/CACNA1S/CACNB1/CACNB3/CACNG1/CATSPER1/HSPA2/TRDN	10	CC

GO:0001750	photoreceptor outer segment	17/2879	80/18511	0.10768	0.7575	0.74863	ARR3/CACNA1F/CDHR1/CIB2/CNGB1/GNAT1/GNB5/GUCA1B/GUCA2B/GUCY2D/IFT140/IFT20/MYO7A/OPN1MW/OPN4/PEX6/TULP1	17	CC
GO:0005875	microtubule associated complex	29/2879	148/18511	0.10814	0.7575	0.74863	ACTR1A/DCTN2/DISC1/DNAH11/DNAH12/DNAH17/DNHD1/DYNC1H1/DYNC1I1/DYNLL2/EML1/FNTA/FNTB/HAUS4/HAUS7/KATNB1/KIF13A/KIF17/KIF23/KIF26A/KIF2B/KIF4A/KIF9/KIFC3/KLC1/KLC2/PEA15/RABGAP1/TR	29	CC
GO:0043204	perikaryon	23/2879	114/18511	0.11046	0.7575	0.74863	AMIGO1/BGLAP/CACNA1F/CCR2/CDK5/CPNE5/CRYAB/DRD2/ENDOG/GLRA1/GOT2/KNDC1/NAIP/NSMF/OPRM1/RBFOX3/RTN4RL1/RTN4RL2/SERPINI1/TH/TMEM100/TTLL7/ZPR1	23	CC
GO:0005765	lysosomal membrane	63/2879	349/18511	0.11152	0.7575	0.74863	ABCA2/ABCB9/ACP2/ACPP/AHNAK/ANPEP/AP1S1/AP2A2/AP2M1/ARL8A/ATP13A2/ATP6V1G1/BORCS6/C3AR1/CD63/CD68/CD74/CUBN/CYB561A3/DAGLB/DDOST/GBA/LAMP3/LAPTM5/LRP1/MGST1/MTOR/MYO7A/NPRL3/PLEKHM1/PNPLA7/PQLC2/PRCP/PSAP/RAB44/RAB7A/SCARB1/SIDT2/SLC12A4/SLC15A3/SLC15A4/SLC29A3/SLC2A8/SLC30A3/SLC36A1/SPNS1/SPNS3/SPPL2C/STARD3/STK11IP/STX8/TECPR1/TLR8/TLR9/TMBIM1/TMEM150C/TMEM63A/TMEM79/TMEM8A/TMEM9/VASN/VTI1B/ZFYVE26	63	CC
GO:0098852	lytic vacuole membrane	63/2879	349/18511	0.11152	0.7575	0.74863	ABCA2/ABCB9/ACP2/ACPP/AHNAK/ANPEP/AP1S1/AP2A2/AP2M1/ARL8A/ATP13A2/ATP6V1G1/BORCS6/C3AR1/CD63/CD68/CD74/CUBN/CYB561A3/DAGLB/DDOST/GBA/LAMP3/LAPTM5/LRP1/MGST1/MTOR/MYO7A/NPRL3/PLEKHM1/PNPLA7/PQLC2/PRCP/PSAP/RAB44/RAB7A/SCARB1/SIDT2/SLC12A4/SLC15A3/SLC15A4/SLC29A3/SLC2A8/SLC30A3/SLC36A1/SPNS1/SPNS3/SPPL2C/STARD3/STK11IP/STX8/TECPR1/TLR8/TLR9/TMBIM1/TMEM150C/TMEM63A/TMEM79/TMEM8A/TMEM9/VASN/VTI1B/ZFYVE26	63	CC
GO:0031312	extrinsic component of organelle membrane	8/2879	32/18511	0.11255	0.7575	0.74863	ATP6V1B1/DUSP21/GOLGA3/PSD/RAB7A/TOR1A/ULK1/ZFYVE1	8	CC

GO:0044297	cell body	88/2879	500/18511	0.11275	0.7575	0.74863	ABL1/ACTA1/ALCAM/AMIGO1/ANG/APBB1/APOB/ARC/ASIC2/ATP13A2/BGLAP/CACNA1F/CACYBP/CASP5/CCR2/CCT3/CDK5/CIB1/CPLX1/CPNE5/CRYAB/DAB1/DAB2IP/DDC/DGKI/DHODH/DISC1/DRD2/EIF5A/ELOVL5/ENDOG/FLNA/GLRA1/GLRX5/GNAO1/GNAT1/GNB3/GNG3/GOT2/HCFC1/HCN3/HIP1R/HOMER2/ILK/INPP5F/ITPR3/KATNB1/KCND1/KNDC1/L1CAM/LRIG2/LRP1/LSM1/MAPK8IP1/MTOR/NAIP/NCDN/NCF1/NLRP1/NRG1/NSMF/NUMA1/OPRM1/PARD3/PPP1R1B/PSD2/PTK2B/PTPRF/RAB17/RARA/RBFOX3/RGS10/RTN4RL1/RTN4RL2/S100A5/SERPINI1/SPTBN2/SV2A/T [illegible]	88	CC
GO:0044420	extracellular matrix component	24/2879	120/18511	0.11296	0.7575	0.74863	ACHE/AGRN/AMTN/ANG/COL17A1/COL1A1/COL4A2/COL7A1/EFEMP2/ELN/EMILIN1/FN1/HMCN2/HSPG2/LAMA2/LAMA4/LAMC2/LAMC3/LOXL1/LOXL2/MFAP1/RELL2/TGFBI/TNC	24	CC
GO:0000314	organellar small ribosomal	7/2879	27/18511	0.11428	0.7575	0.74863	MRPS10/MRPS11/MRPS18A/MRPS18B/MRPS21/MRPS33/MRPS5	7	CC
GO:0005763	mitochondrial small ribosomal subunit	7/2879	27/18511	0.11428	0.7575	0.74863	MRPS10/MRPS11/MRPS18A/MRPS18B/MRPS21/MRPS33/MRPS5	7	CC
GO:0030016	myofibril	40/2879	213/18511	0.11442	0.7575	0.74863	ACTA1/ADRA1A/AHNAK/ALDOA/ANKRD2/ARHGEF25/BIN1/CACNA1S/CAV3/CRYAB/DES/FBXL22/FERMT2/FHL3/FLNA/FLNC/HSPB1/IGSF22/ILK/KLHL40/LMOD1/MMP2/MYBPC1/MYBPC3/MYBPH/MYBPHL/MYL4/MYOD1/MYZAP/OBSCN/OBSL1/PDE4DIP/PDLIM3/PPP1R12B/SMTNL1/TCAP/T	40	CC
GO:0044438	microbody part	19/2879	92/18511	0.11562	0.7575	0.74863	ACOXL/CROT/CRYM/FNDC5/GNPAT/HAO2/IDH1/MGST1/PAOX/PEX11A/PEX14/PEX16/PEX3/PEX5/PEX6/PIPOX/PLA2G16/PXMP2/TTC1	19	CC
GO:0044439	peroxisomal part	19/2879	92/18511	0.11562	0.7575	0.74863	ACOXL/CROT/CRYM/FNDC5/GNPAT/HAO2/IDH1/MGST1/PAOX/PEX11A/PEX14/PEX16/PEX3/PEX5/PEX6/PIPOX/PLA2G16/PXMP2/TTC1	19	CC

GO:0005774	vacuolar membrane	71/2879	398/18511	0.11578	0.7575	0.74863	ABCA2/ABCB9/ACP2/ACPP/AHNAK/ANPEP/AP1S1/AP2A2/AP2M1/ARL8A/ATP13A2/ATP6V0B/ATP6V1B1/ATP6V1F/ATP6V1G1/ATP6V1G2/BORCS6/C3AR1/CD63/CD68/CD74/CUBN/CYB561A3/DAGLB/DDOST/GBA/LAMP3/LAPTM5/LRP1/MGST1/MTOR/MYO7A/NPRL3/PLEKHM1/PNPLA7/PQLC2/PRCP/PSAP/RAB44/RAB7A/RRAGC/SCARB1/SIDT2/SLC12A4/SLC15A3/SLC15A4/SLC29A3/SLC2A8/SLC30A3/SLC36A1/SLC36A3/SPNS1/SPNS3/SPPL2C/STARD3/STK11IP/STX8/TECPR1/TLR8/TLR9/TMBIM1/TMEM138/TMEM150C/TMEM63A/TMEM79/TMEM8A/TMEM9/ULK1/VASN/VTI1B/ZFYVE[illegible]	71	CC
GO:0070382	exocytic vesicle	32/2879	167/18511	0.11951	0.7745	0.76546	ATP6V1B1/CDK16/DBNDD1/DDC/DGKI/DISC1/DLG4/DNM1/DOC2A/DOC2B/DRD2/DTNBP1/GRIN1/LGI3/NKD2/PTPRN2/RAB26/RAB40C/SLC2A8/SLC30A3/STON2/STX1A/STX4/SV2A/SYTL3/TH/TMEM163/TOR1A/TRAPPC4/	32	CC
GO:0101002	ficolin-1-rich granule	35/2879	185/18511	0.12284	0.7778	0.76872	ADAM8/ALAD/ALDOA/AP2A2/ARL8A/BIN2/C1orf35/CALML5/CD58/CDA/COTL1/CTSD/CTSH/DOK3/GSDMD/HK3/HSP90AA1/IDH1/IMPDH1/ITGAX/LGALS3/MGAM/NBEAL2/PKM/PPIE/PRCP/PSMB7/PSMC3/PSMD13/PSMD3/PSMD7/PTPRN2/SLC11A1/SRP14/UBR4	35	CC
GO:0043025	neuronal cell body	77/2879	436/18511	0.12352	0.7778	0.76872	ABL1/ALCAM/AMIGO1/ANG/APBB1/APOB/ARC/ASIC2/ATP13A2/BGLAP/CACNA1F/CASP5/CCR2/CDK5/CIB1/CPLX1/CPNE5/CRYAB/DAB1/DAB2IP/DDC/DGKI/DHODH/DRD2/EIF5A/ELOVL5/ENDOG/FLNA/GLRA1/GLRX5/GNAT1/GOT2/HCFC1/HCN3/HIP1R/HOMER2/ILK/INPP5F/ITPR3/KATNB1/KCND1/KNDC1/L1CAM/LRIG2/LRP1/LSM1/MTOR/NAIP/NCDN/NCF1/NLRP1/NSMF/NUMA1/OPRM1/PARD3/PPP1R1B/PSD2/PTK2B/PTPRF/RAB17/RARA/RBFOX3/RGS10/RTN4RL1/RTN4RL2/S100A5/SERPINI1/SPTBN2/SV2[illegible]	77	CC
GO:0042383	sarcolemma	24/2879	122/18511	0.12961	0.7778	0.76872	ADRA1A/AHNAK/AQP1/ATP1A1/BGN/BIN1/CACNA1S/CACNB1/CACNB3/CAMK2D/CAV3/CIB1/CIB2/DES/DTNBP1/FLNC/KCND3/KCNJ5/LAMA2/OBSCN/OPRM1/PEMT/SLC2A4/TRIM72	24	CC
GO:0099023	tethering complex	15/2879	71/18511	0.12983	0.7778	0.76872	AKTIP/COG4/COG5/GOLGA3/RALB/RINT1/TMEM115/TRAPPC1/TRAPPC2L/TRAPPC3/TRAPPC4/TRAPPC9/VPS51/VPS52/VPS53	15	CC

GO:0005743	mitochondrial inner membrane	84/2879	480/18511	0.13018	0.7778	0.76872	ACADVL/ALAS2/ATAD3A/ATP5G1/ATP5I/ATPIF1/BOK/C14orf2/CHDH/COA3/COQ2/COQ4/COX10/COX11/COX8A/CPT2/CSDE1/CYC1/CYP11B2/DHODH/DNAJC19/DUSP21/ERAL1/FAHD1/FOXRED1/GOT2/HADHA/HSD3B1/IDH2/LETMD1/LGALS3/MPC1/MRPL10/MRPL14/MRPL28/MRPL36/MRPL37/MRPL43/MRPL52/MRPL57/MRPL9/MRPS10/MRPS11/MRPS18A/MRPS18B/MRPS21/MRPS33/MRPS5/MTCH1/MYOC/NDUFA2/NDUFA4L2/NDUFAF6/NDUFB5/NDUFC2-KCTD14/NME4/OCIAD2/OTC/PDE2A/PLA2G4B/PMPCA/SFXN2/SFXN4/SHMT2/SLC25A14/SLC25A20/SLC25A22/SLC25A25/SLC25A43/SLC25A45/SLC25A47/SLC25A48/SLC25A5/SMIM20/SPNS1/TIMM17B/TIMM22/TIMM23B/TIMM9/TMEM11/TRAP1/UCP3/VDAC2/YME1L1	84	CC
GO:0005578	proteinaceous extracellular matrix	65/2879	365/18511	0.13047	0.7778	0.76872	ACAN/ACHE/ADAMTS13/ADAMTS14/ADAMTS2/ADAMTS7/AGRN/AMTN/ANG/BCAN/BGN/BMP4/C6orf15/CHAD/COL16A1/COL17A1/COL1A1/COL4A2/COL7A1/COL9A2/ECM1/EFEMP2/ELN/EMILIN1/FBLN2/FN1/GFOD2/GPC1/GPC2/HAPLN3/HMCN2/HSPG2/LAMA2/LAMA4/LAMC2/LAMC3/LINGO1/LINGO2/LOXL1/LOXL2/MMP17/MMP2/MMP25/MMP3/MYOC/P3H1/PHOSPHO1/PRSS36/RELL2/SFTPA1/SHH/SOST/SPOCK2/SPON2/TGFBI/TLR9/TNC/TNN/VWF/WNT1/WNT10A/WNT11/WNT3A/WNT6/WNT7A	65	CC
GO:0017119	Golgi transport complex	4/2879	13/18511	0.13061	0.7778	0.76872	COG4/COG5/GOLGA3/TMEM115	4	CC
GO:0032045	guanyl-nucleotide exchange factor	4/2879	13/18511	0.13061	0.7778	0.76872	DGKI/DOCK1/KNDC1/ULK1	4	CC
GO:0036038	MKS complex	4/2879	13/18511	0.13061	0.7778	0.76872	B9D1/CC2D2A/TMEM17/TMEM231	4	CC
GO:0016363	nuclear matrix	21/2879	105/18511	0.13134	0.7778	0.76872	ATN1/CFL1/CHMP1A/DGKQ/DNMT3A/GFI1B/KIF4A/LMNB1/MAEA/MEN1/NCOR2/NONO/NSMF/NUMA1/OGG1/PAXIP1/PRKCD/PSPC1/TGFB1I1/TIN	21	CC
GO:0005665	DNA-directed RNA polymerase II,	5/2879	18/18511	0.13541	0.7951	0.78575	POLR2G/POLR2J2/POLR2J3/POLR2L/ZNF768	5	CC
GO:0000502	proteasome complex	14/2879	66/18511	0.13656	0.7951	0.78575	ALAD/HSPB1/PSMB11/PSMB6/PSMB7/PSMB8/PSMC3/PSMD13/PSMD3/PSMD5/PSMD7/PSMG3/UBE3C/WFS1	14	CC

GO:0008021	synaptic vesicle	29/2879	152/18511	0.13815	0.7971	0.78773	ATP6V1B1/CDK16/DBNDD1/DDC/DGKI/DISC1/DLG4/DNM1/DOC2A/DRD2/DTNBP1/GRIN1/LGI3/PTPRN2/RAB26/RAB40C/SLC2A8/SLC30A3/STON2/STX1A/STX4/SV2A/TH/TMEM163/TOR1A/TRAPPC4/VAMP1/VDAC2/VTI1	29	CC
GO:0005777	peroxisome	25/2879	129/18511	0.14039	0.7971	0.78773	ACOXL/CROT/CRYM/EPHX2/FNDC5/GBF1/GNPAT/HAO2/IDH1/IDH2/MGST1/PAOX/PEX11A/PEX14/PEX16/PEX3/PEX5/PEX6/PIPOX/PLA2G16/PMVK/PXMP2/TTC1/TYSND1/XDH	25	CC
GO:0042579	microbody	25/2879	129/18511	0.14039	0.7971	0.78773	ACOXL/CROT/CRYM/EPHX2/FNDC5/GBF1/GNPAT/HAO2/IDH1/IDH2/MGST1/PAOX/PEX11A/PEX14/PEX16/PEX3/PEX5/PEX6/PIPOX/PLA2G16/PMVK/PXMP2/TTC1/TYSND1/XDH	25	CC
GO:0101003	ficolin-1-rich granule	13/2879	61/18511	0.14371	0.8093	0.79979	ADAM8/AP2A2/ARL8A/CD58/DOK3/ITGAX/LGALS3/MGAM/NBEAL2/PRCP/PTPRN2/SLC11A1/UBR4	13	CC
GO:1905369	endopeptidase complex	14/2879	67/18511	0.14929	0.8339	0.82409	ALAD/HSPB1/PSMB11/PSMB6/PSMB7/PSMB8/PSMC3/PSMD13/PSMD3/PSMD5/PSMD7/PSMG3/UBE3C/WFS1	14	CC
GO:0030018	Z disc	23/2879	119/18511	0.15539	0.8358	0.82597	ADRA1A/BIN1/CAV3/CRYAB/DES/FBXL22/FHL3/FLNA/FLNC/HSPB1/IGSF22/MYBPC1/MYBPC3/MYBPH/MYBPHL/MYZAP/OBSCN/OBSL1/PDLIM3/PPP1R12B/TCAP/TRIM54/TRIM63	23	CC
GO:0031201	SNARE complex	11/2879	51/18511	0.1594	0.8358	0.82597	CPLX1/DOC2B/GOSR2/STX18/STX1A/STX4/STX5/STX8/VAMP1/VAMP5/VTI1B	11	CC
GO:0001673	male germ cell nucleus	4/2879	14/18511	0.16147	0.8358	0.82597	ACTL7A/ACTRT3/HILS1/HSPA2	4	CC
GO:0005847	mRNA cleavage and polyadenylation specificity factor complex	4/2879	14/18511	0.16147	0.8358	0.82597	CPSF1/CPSF4/CSTF2/ZC3H3	4	CC
GO:0033276	transcription factor TFTC	4/2879	14/18511	0.16147	0.8358	0.82597	KAT2A/TADA3/TAF6/TAF7	4	CC
GO:0036020	endolysosome membrane	4/2879	14/18511	0.16147	0.8358	0.82597	AP2A2/AP2M1/TLR8/TLR9	4	CC
GO:0097440	apical dendrite	4/2879	14/18511	0.16147	0.8358	0.82597	CLU/FLNA/NSMF/PTK2B	4	CC

GO:0016471	vacuolar proton-transporting V-type ATPase	5/2879	19/18511	0.16153	0.8358	0.82597	ATP6V0B/ATP6V1B1/ATP6V1F/ATP6V1G1/ATP6V1G2	5	CC
GO:0000313	organellar ribosome	17/2879	85/18511	0.16191	0.8358	0.82597	C12orf65/MRPL10/MRPL14/MRPL28/MRPL36/MRPL37/MRPL43/MRPL52/MRPL57/MRPL9/MRPS10/MRPS11/MRPS18A/MRPS18B/MRPS21/MRPS33/M	17	CC
GO:0005761	mitochondrial ribosome	17/2879	85/18511	0.16191	0.8358	0.82597	C12orf65/MRPL10/MRPL14/MRPL28/MRPL36/MRPL37/MRPL43/MRPL52/MRPL57/MRPL9/MRPS10/MRPS11/MRPS18A/MRPS18B/MRPS21/MRPS33/M	17	CC
GO:0030055	cell-substrate junction	70/2879	402/18511	0.16552	0.8358	0.82597	ADGRB1/AHNAK/ALCAM/ARHGAP22/ARPC1B/ARPC2/CCND3/CD81/CDH13/CFL1/CIB2/COL17A1/CORO1B/CSRP1/CTTN/DDR2/DOCK7/EGFR/ENG/EPB41L2/EVL/FAT1/FBLIM1/FERMT2/FES/FHL3/FLII/FLNA/FLNC/FZD2/GAK/HACD3/HMGA1/HSPA5/HSPB1/HSPG2/ILK/ITGB5/KIF23/L1CAM/LAYN/LIMK1/LIMS2/LRP1/MAPK3/MCAM/MRC2/NOX4/OPRM1/PDGFRB/PDLIM1/PDLIM7/PFN1/PLAU/PPIB/PTK2B/RPL7/SLC9A3R2/SORBS3/STARD8/TGFB1I1/THY1/TLN1/TNC/TNS2/TRPV4/TSPAN4/XIRP1/YWHAQ/ZYX	70	CC
GO:0098858	actin-based cell projection	35/2879	191/18511	0.16742	0.8358	0.82597	ACPP/ACTA1/APBB1/AQP5/ATP6V1B1/CDH23/CDHR5/CDK5/CIB1/CIB2/DPEP1/DYNC1H1/EPS8L2/ESPN/ESPNL/FAT1/FSCN1/FZD9/HOMER2/IFT20/INPPL1/LHFPL5/MORN4/MYO1A/MYO1C/MYO3A/MYO7A/MYO7B/PDGFA/PLEKHG6/PPP1R9B/PROM2/SCARB1/TPRN/TRPV4	35	CC
GO:0005802	trans-Golgi network	36/2879	197/18511	0.16777	0.8358	0.82597	AP1S1/AP4S1/ARAP1/ARFIP1/BOK/CA4/CD74/CHAC1/CHID1/CLTB/COG4/COG5/FURIN/GBF1/GPER1/KIF13A/LGR6/LLGL1/MARCH4/MYO18A/PLOD3/RBFOX1/SCAMP2/SMPD4/STX4/STX8/TGFBI/TJAP1/TMEM79/TRAPPC9/USP6NL/VPS51/VPS52/VPS53/WLS/YIPF1	36	CC
GO:0045095	keratin filament	19/2879	97/18511	0.16785	0.8358	0.82597	FAM83H/GPER1/KRT14/KRT3/KRT6A/KRT6C/KRT71/KRT75/KRT76/KRT79/KRT81/KRT84/KRT85/KRTAP4-3/KRTAP5-10/KRTAP5-3/KRTAP5-	19	CC

GO:0005759	mitochondrial matrix	78/2879	452/18511	0.17158	0.8358	0.82597	ACAD10/ACADVL/ACOT9/ACSF3/ACSM6/ACSS3/ADPRHL2/ALAS2/ALDH2/ALDH4A1/ARL2BP/ATAD3A/BCKDK/C12orf65/CCAR2/CHPF/CS/CYP11A1/DGUOK/DLST/DNAJA3/DUSP21/ERAL1/GLRX5/GLUD1/GOT2/GPX1/GSR/HADHA/HYKK/IDH2/ISCA2/KARS/LIAS/MRPL10/MRPL14/MRPL28/MRPL36/MRPL37/MRPL43/MRPL52/MRPL57/MRPL9/MRPS10/MRPS11/MRPS18A/MRPS18B/MRPS21/MRPS33/MRPS5/NAGS/NDUFAF7/NME4/NMNAT3/NT5M/NUDT1/NUDT2/OAT/OGDH/OGDHL/OTC/PARS2/PC/PDE2A/PDHB/PDSS1/PHYKPL/PMPCA/POLDIP2/PPA2/SHC1/SHMT2/SLC25A5/TARS2/TR[illegible]	78	CC
GO:0005924	cell-substrate adherens junction	69/2879	397/18511	0.17168	0.8358	0.82597	ADGRB1/AHNAK/ALCAM/ARHGAP22/ARPC1B/ARPC2/CCND3/CD81/CDH13/CFL1/CIB2/CORO1B/CSRP1/CTTN/DDR2/DOCK7/EGFR/ENG/EPB41L2/EVL/FAT1/FBLIM1/FERMT2/FES/FHL3/FLII/FLNA/FLNC/FZD2/GAK/HACD3/HMGA1/HSPA5/HSPB1/HSPG2/ILK/ITGB5/KIF23/L1CAM/LAYN/LIMK1/LIMS2/LRP1/MAPK3/MCAM/MRC2/NOX4/OPRM1/PDGFRB/PDLIM1/PDLIM7/PFN1/PLAU/PPIB/PTK2B/RPL7/SLC9A3R2/SORBS3/STARD8/TGFB1I1/THY1/TLN1/TNC/TNS2/TRPV4/TSPAN4/XIRP1/YWHAQ/ZYX	69	CC
GO:0005930	axoneme	21/2879	109/18511	0.17238	0.8358	0.82597	AMBRA1/CCDC103/CCDC40/CFAP74/DNAH11/DNAH17/DNAJB13/DNHD1/GAS8/HYDIN/IFT140/INPP5E/KIF17/PRKAR1A/SEPT9/SPAG16/TMEM141/TRAF3IP1/TULP3/WDPCP/WDR66	21	CC
GO:0043679	axon terminus	22/2879	115/18511	0.17424	0.8358	0.82597	ADRA2C/AP1S1/AQP1/CHRM1/CNGB1/CPLX1/DGKI/DRD2/GAD1/GPER1/GRIN1/HCN3/ILK/OPRD1/PTPRN2/RAB7A/RGS10/STXBP1/SV2A/TH/TULP	22	CC
GO:0009925	basal plasma membrane	7/2879	30/18511	0.17453	0.8358	0.82597	AQP1/AQP5/CLDN4/KCNQ4/MYO1A/MYO1C/OSCP1	7	CC
GO:0030667	secretory granule membrane	53/2879	301/18511	0.18009	0.8358	0.82597	ABCC4/ACPP/ACRBP/ADAM8/ADGRG3/ANPEP/AP2A2/APLP2/ARL8A/C3AR1/CA4/CCDC136/CD58/CD63/CD68/CXCR1/CXCR2/CYB5R1/DDOST/DGAT1/DOK3/FRMPD3/HMOX2/ITGAL/ITGAM/ITGAX/LAMP3/LGALS3/MGAM/MGST1/MMP25/NBEAL2/OLR1/ORMDL3/PLAU/PRCP/PSAP/PTPRN2/RAB24/RAB26/RAB40C/RAB44/RAB7A/RHOF/SELP/SERPINA5/SLC11A1/SLC15A4/SPACA3/TMBIM1/TMEM63A/UBR4/VAMP1	53	CC
GO:0005778	peroxisomal membrane	12/2879	58/18511	0.18165	0.8358	0.82597	FNDC5/GNPAT/MGST1/PEX11A/PEX14/PEX16/PEX3/PEX5/PEX6/PLA2G16/PXMP2/TTC1	12	CC

GO:0031903	microbody membrane	12/2879	58/18511	0.18165	0.8358	0.82597	FNDC5/GNPAT/MGST1/PEX11A/PEX14/PEX16/PEX3/PEX5/PEX6/PLA2G16/PXMP2/TTC1	12	CC
GO:0030125	clathrin vesicle coat	6/2879	25/18511	0.18233	0.8358	0.82597	AP1G2/AP1S1/AP2A2/AP2M1/CLTB/EGFR	6	CC
GO:0030666	endocytic vesicle membrane	31/2879	169/18511	0.183	0.8358	0.82597	AP1S1/AP2A2/AP2M1/APOB/ATP6V0B/ATP6V0E2/CAMK2D/CD74/CORO1A/DLG4/EGFR/FZD2/GRIA3/HLA-E/LRP1/NOS3/NOSTRIN/RAB20/RAB34/RAB7A/SCARB1/SCARF1/SLC11A1/SMO/STX4/TAP1/WLS/WNT1/WNT3A/WNT6/WNT7A	31	CC
GO:0097014	ciliary plasm	21/2879	110/18511	0.1836	0.8358	0.82597	AMBRA1/CCDC103/CCDC40/CFAP74/DNAH11/DNAH17/DNAJB13/DNHD1/GAS8/HYDIN/IFT140/INPP5E/KIF17/PRKAR1A/SEPT9/SPAG16/TMEM141/TRAF3IP1/TULP3/WDPCP/WDR66	21	CC
GO:0045178	basal part of cell	10/2879	47/18511	0.18554	0.8358	0.82597	AQP1/AQP5/CLDN4/DOCK7/KCNQ4/KRT14/MYO1A/MYO1C/OSCP1/PRCP	10	CC
GO:0036019	endolysosome	5/2879	20/18511	0.18947	0.8358	0.82597	AP2A2/AP2M1/CTSL/TLR8/TLR9	5	CC
GO:0005925	focal adhesion	68/2879	394/18511	0.18996	0.8358	0.82597	ADGRB1/AHNAK/ALCAM/ARHGAP22/ARPC1B/ARPC2/CCND3/CD81/CDH13/CFL1/CORO1B/CSRP1/CTTN/DDR2/DOCK7/EGFR/ENG/EPB41L2/EVL/FAT1/FBLIM1/FERMT2/FES/FHL3/FLII/FLNA/FLNC/FZD2/GAK/HACD3/HMGA1/HSPA5/HSPB1/HSPG2/ILK/ITGB5/KIF23/L1CAM/LAYN/LIMK1/LIMS2/LRP1/MAPK3/MCAM/MRC2/NOX4/OPRM1/PDGFRB/PDLIM1/PDLIM7/PFN1/PLAU/PPIB/PTK2B/RPL7/SLC9A3R2/SORBS3/STARD8/TGFB1I1/THY1 [illegible]	68	CC
GO:0031514	motile cilium	27/2879	146/18511	0.19042	0.8358	0.82597	ACTL7A/CATSPER1/CCDC103/DNAH11/DNAH17/DNAJB13/DRC7/DRD2/FOPNL/GAS8/IFT20/ODF1/ODF4/PFKM/PGAM4/PKD1/SCNN1A/SEPT4/SLC26A6/SPAG16/TACR1/TACR2/TCP11/TEKT4/TEKT5/TSSK1B/WDR66	27	CC
GO:0032585	multivesicular body membrane	3/2879	10/18511	0.19431	0.8358	0.82597	ATP13A2/CD300LG/CD63	3	CC
GO:0043020	NADPH oxidase	3/2879	10/18511	0.19431	0.8358	0.82597	NCF1/NOX4/NOXO1	3	CC
GO:0043083	synaptic cleft	3/2879	10/18511	0.19431	0.8358	0.82597	ACHE/C1QL1/GRIN1	3	CC

GO:0030424	axon	70/2879	407/18511	0.19446	0.8358	0.82597	ADRA2C/ALCAM/AMIGO1/AP1S1/APBB1/AQP1/BIN1/BOC/C1QL1/CDK5/CIB1/CNGB1/CORO1A/CPLX1/CRYAB/CTNNA2/DAB2IP/DDC/DISC1/DLG4/DNM1/DOCK7/DRD2/DTNBP1/GAD1/GPER1/GRIN1/GRIPAP1/HCFC1/HCN3/HSPB1/IGHMBP2/IL31RA/ILK/INPP5F/IRX3/KATNB1/KIF4A/KIRREL3/L1CAM/LLGL1/LRRTM1/LSM1/MAF1/MAPK8IP1/MYOC/NECAB2/NRG1/NRN1L/NTNG2/OPRM1/PARD3/PTK2B/PTPRN2/RAB7A/RTN4RL2/SCN11A/STAT1/STMN4/STX1A/STXBP1/SV2A/TH/THY1/TRIM46/TRPV2/ULK1/VAMP[illegible]	70	CC
GO:0005671	Ada2/Gcn5/Ada3 transcription activator	4/2879	15/18511	0.19465	0.8358	0.82597	HCFC1/KAT2A/POLE4/TADA3	4	CC
GO:0031235	intrinsic component of the cytoplasmic side of the plasma	4/2879	15/18511	0.19465	0.8358	0.82597	DAB2IP/RASA3/RASA4B/SPTB	4	CC
GO:0031362	anchored component of external side of plasma	4/2879	15/18511	0.19465	0.8358	0.82597	CA4/FOLR2/GPIHBP1/THY1	4	CC
GO:0038201	TOR complex	4/2879	15/18511	0.19465	0.8358	0.82597	MAPKAP1/MLST8/MTOR/TELO2	4	CC
GO:0032587	ruffle membrane	17/2879	88/18511	0.20029	0.8476	0.83762	ARHGEF4/CFL1/CIB1/EPS8L2/HIP1R/HSP90AA1/KSR1/MYO1C/NME1/PPP1R9B/PSD/PSD2/PSD4/RASGRP2/TIRAP/TLN1/TRPV4	17	CC
GO:0031965	nuclear membrane	52/2879	298/18511	0.20158	0.8476	0.83762	ABL1/ADRA1A/ADRA1B/AQP1/ATP2A3/BOK/CASC3/CDK4/CLIC1/CMTM3/CPTP/CUEDC2/DTX2/DUSP2/DUX4/EGFR/EMD/EPC1/GAPDH/GCH1/GCHFR/GUCY2D/HACD3/IL15RA/INTS5/IPO13/IPO4/ITPR3/LEMD2/LMNA/LMNB1/NR4A1/NRM/NSMF/NUDT1/NUP210/NUP98/P2RX2/PNPLA7/POMZP3/RRP12/SIGMAR1/SLC29A2/STX1A/TAF3/TMEM109/TMEM176B/TNPO3/TN	52	CC

GO:0098589	membrane region	65/2879	378/18511	0.20494	0.8476	0.83762	ADRA1A/ADRA1B/AHNAK/AP1S1/AP2A2/AP2M1/AP4S1/ARRB2/ATP1A1/CARD11/CASP3/CAV3/CD48/CDH13/CDH15/CDHR5/CLTB/CRB2/CSK/CTSD/CTTN/CUBN/EFNB1/EGFR/EIF5A/FASLG/FURIN/GPC1/HIP1R/IKBKB/INPP5D/INPP5F/ITGAM/KCNQ1/KDR/LCK/LRP1/LRP10/LTB4R/MAPK3/MYO1A/MYO1C/NOS3/OLR1/OPRD1/OPRM1/PRKAR1A/PROM2/PTK2B/RTN4RL1/RTN4RL2/SCARB1/SERPINH1/SHH/SLC25A5/SLC2A4/SLC34A1/SLC6A2/SMO/STOML1/STOML3/TDGF1/THY1/TNK2/TRAF2	65	CC
GO:0042470	melanosome	20/2879	106/18511	0.20583	0.8476	0.83762	ATP1A1/ATP6V1G2/CD63/CTSD/DTNBP1/GANAB/GCHFR/GGH/HSP90AA1/HSPA5/MYO7A/PPIB/PRDX1/RAB17/RAB7A/RPN1/SGSM2/SND1/TH/TRP	20	CC
GO:0048770	pigment granule	20/2879	106/18511	0.20583	0.8476	0.83762	ATP1A1/ATP6V1G2/CD63/CTSD/DTNBP1/GANAB/GCHFR/GGH/HSP90AA1/HSPA5/MYO7A/PPIB/PRDX1/RAB17/RAB7A/RPN1/SGSM2/SND1/TH/TRP	20	CC
GO:0033176	proton-transporting V-type ATPase complex	6/2879	26/18511	0.20792	0.8476	0.83762	ATP6V0B/ATP6V0E2/ATP6V1B1/ATP6V1F/ATP6V1G1/ATP6V1G2	6	CC
GO:0034451	centriolar satellite	6/2879	26/18511	0.20792	0.8476	0.83762	BBS9/C2CD3/CCDC13/FOPNL/PARD6A/SDCCAG8	6	CC
GO:0044306	neuron projection terminus	24/2879	130/18511	0.20978	0.8476	0.83762	ADRA2C/AP1S1/AQP1/CHRM1/CNGB1/CPLX1/DGKI/DLG4/DRD2/GAD1/GCH1/GPER1/GRIN1/HCN3/ILK/OPRD1/PTPRN2/RAB7A/RGS10/STXBP1/SV2A/TH/TULP1/VAMP1	24	CC
GO:0031968	organelle outer membrane	35/2879	196/18511	0.21066	0.8476	0.83762	ACACB/AMBRA1/ATF2/BAD/BOK/BPHL/CPTP/EMD/FATE1/GUCY2D/ITPR3/LETMD1/MFN1/MFN2/MGST1/MIEF2/MOAP1/MTOR/MYO19/MYOC/NLRX1/NME1/PDE2A/PGAM5/PI4KB/PPP2R2B/RAF1/RHOT2/SIGMAR1/SPATA19/TMEM109/ULK1/VAMP1/VAT1/VDAC2	35	CC
GO:0042581	specific granule	29/2879	160/18511	0.21142	0.8476	0.83762	ADAM8/ADGRG3/C3AR1/CHIT1/CRACR2A/CTSD/DGAT1/GGH/GHDC/GSDMD/HMOX2/ITGAL/ITGAM/MMP25/OLFM4/OLR1/ORM1/ORM2/ORMDL3/PLAU/QSOX1/RAB44/SLC15A4/SPTAN1/STX4/TMBIM1/TMEM63A/UBR4	29	CC
GO:0005801	cis-Golgi network	9/2879	43/18511	0.21644	0.8476	0.83762	ANGEL1/B3GAT3/BCL9/BOK/FKTN/GBF1/GOLGA2/IFT20/MAP6D1	9	CC
GO:0036064	ciliary basal body	20/2879	107/18511	0.21842	0.8476	0.83762	B9D1/C2CD3/CEP19/CFAP126/CFAP157/DISC1/FOPNL/GAS8/IFT140/IFT20/KIF17/PTPN23/RILPL1/RILPL2/SSNA1/TRAF3IP1/TUBA3C/TUBA3D/TUBG	20	CC

GO:0005838	proteasome regulatory	5/2879	21/18511	0.21897	0.8476	0.83762	PSMC3/PSMD13/PSMD3/PSMD5/PSMD7	5	CC
GO:0044295	axonal growth cone	5/2879	21/18511	0.21897	0.8476	0.83762	BOC/L1CAM/MAPK8IP1/PARD3/TRPV2	5	CC
GO:0005874	microtubule	67/2879	393/18511	0.22269	0.8476	0.83762	ARHGAP4/BCAS3/CCT3/CDK2AP2/CDK5RAP3/CEP170B/CKAP5/DCLK2/DCTN2/DISC1/DNAH11/DNAH12/DNAH17/DNM1/DYNC1H1/DYNC1I1/DYNLL2/EMD/EML1/EML3/EML5/GAS8/GOLGA2/HAUS4/HAUS7/INO80/KATNB1/KIF13A/KIF17/KIF23/KIF26A/KIF2B/KIF4A/KIF9/KIFC3/KLC1/KLC2/LZTS2/MAP6D1/MAPRE3/MID1IP1/MTA1/NCKAP5L/NCOR1/NEIL2/NUDC/NUMA1/REEP2/REEP4/RGS14/SEPT9/SHROOM1/TEKT4/TRIM54/TRIM63/TRPV4/TTLL5/TTLL7/TUBA3C/TUBA3D/TUBA3E/TUBG1/TUBG2/TUBGCP2/T[illegible]	67	CC
GO:0031228	intrinsic component of Golgi	10/2879	49/18511	0.22289	0.8476	0.83762	B4GALNT1/CHST12/CHST5/LFNG/QSOX1/RFNG/SGMS1/SLC39A13/STEAP2/TVP23B	10	CC
GO:0034707	chloride channel	10/2879	49/18511	0.22289	0.8476	0.83762	ANO1/ANO2/BEST3/BEST4/CLCN2/CLCNKA/CLCNKB/CLIC1/GLRA1/SLC26A6	10	CC
GO:0032588	trans-Golgi network membrane	16/2879	84/18511	0.22635	0.8476	0.83762	AP1S1/AP4S1/ARFIP1/BOK/CD74/COG4/COG5/KIF13A/LGR6/LLGL1/SCAMP2/TMEM79/USP6NL/VPS51/VPS52/VPS53	16	CC
GO:0005871	kinesin complex	11/2879	55/18511	0.22796	0.8476	0.83762	DISC1/KIF13A/KIF17/KIF23/KIF26A/KIF2B/KIF4A/KIF9/KIFC3/KLC1/KLC2	11	CC
GO:0035869	ciliary transition zone	11/2879	55/18511	0.22796	0.8476	0.83762	B9D1/BBS9/CC2D2A/GNAT1/IFT140/IFT20/KIF17/MYO7A/TMEM17/TMEM231/TRAF3IP1	11	CC
GO:0019867	outer membrane	35/2879	198/18511	0.22938	0.8476	0.83762	ACACB/AMBRA1/ATF2/BAD/BOK/BPHL/CPTP/EMD/FATE1/GUCY2D/ITPR3/LETMD1/MFN1/MFN2/MGST1/MIEF2/MOAP1/MTOR/MYO19/MYOC/NLRX1/NME1/PDE2A/PGAM5/PI4KB/PPP2R2B/RAF1/RHOT2/SIGMAR1/SPATA19/TMEM109/ULK1/VAMP1/VAT1/VDAC2	35	CC
GO:0005771	multivesicular body	8/2879	38/18511	0.23023	0.8476	0.83762	ATP13A2/CD300LG/CD63/CD74/CTSH/EGFR/SFTPB/SLC2A4	8	CC

GO:0043209	myelin sheath	29/2879	162/18511	0.23227	0.8476	0.83762	ACTR1A/ATP1A1/CCT3/CNRIP1/CRYAB/DLST/DNM1/ERBB2/FSCN1/GNAO1/GNB5/GOT2/HSP90AA1/HSPA2/HSPA5/ITPR3/MOBP/NME1/PKM/PRDX1/SCRIB/SEPT4/SLC25A5/STX4/STXBP1/TAGLN3/THY1/VDAC2/WDR1	29	CC
GO:0044798	nuclear transcription factor complex	28/2879	156/18511	0.23256	0.8476	0.83762	ASCL2/BCL9/BRF1/EDF1/GATA4/GTF2H2C/GTF2H4/GTF3C1/HMGA1/HOXB9/KAT2A/MAX/MED18/MED6/NKX2-5/NONO/NR1H3/NR5A1/SNAPC1/SOX8/TADA3/TAF1/TAF1A/TAF1L/TAF3/TAF6/TAF7/TBPL1	28	CC
GO:0060076	excitatory synapse	6/2879	27/18511	0.23467	0.8476	0.83762	DGKI/DLG4/FGFR2/GRIN1/LRRTM1/NLGN3	6	CC
GO:0030173	integral component of Golgi	9/2879	44/18511	0.23725	0.8476	0.83762	B4GALNT1/CHST12/LFNG/QSOX1/RFNG/SGMS1/SLC39A13/STEAP2/TVP23B	9	CC
GO:0030315	T-tubule	9/2879	44/18511	0.23725	0.8476	0.83762	ADRA1A/AHNAK/ATP1A1/BIN1/CACNA1S/CACNB3/CAV3/KCNJ5/SLC2A4	9	CC
GO:0045171	intercellular bridge	9/2879	44/18511	0.23725	0.8476	0.83762	ESRRA/KIF23/KIF4A/PKNOX2/QSOX1/RAB11FIP3/SNRNP25/SRA1/UNC119	9	CC
GO:0008385	IkappaB kinase complex	3/2879	11/18511	0.23811	0.8476	0.83762	IKBKB/PYDC1/TRIM40	3	CC
GO:0017146	NMDA selective glutamate	3/2879	11/18511	0.23811	0.8476	0.83762	GRIN1/GRIN2C/PTK2B	3	CC
GO:0034663	endoplasmic reticulum chaperone complex	3/2879	11/18511	0.23811	0.8476	0.83762	HSPA5/MZB1/PPIB	3	CC
GO:0035631	CD40 receptor complex	3/2879	11/18511	0.23811	0.8476	0.83762	IKBKB/RNF31/TRAF2	3	CC
GO:0042555	MCM complex	3/2879	11/18511	0.23811	0.8476	0.83762	MCM2/MCM4/MCM9	3	CC
GO:0035579	specific granule membrane	17/2879	91/18511	0.24252	0.8547	0.84466	ADAM8/ADGRG3/C3AR1/DGAT1/HMOX2/ITGAL/ITGAM/MMP25/OLR1/ORMDL3/PLAU/RAB44/SLC15A4/TMBIM1/TMEM63A/UBR4/VAMP1	17	CC

GO:0016469	proton-transporting two-sector ATPase	10/2879	50/18511	0.2426	0.8547	0.84466	ATP5G1/ATP5I/ATP6V0B/ATP6V0E2/ATP6V1B1/ATP6V1F/ATP6V1G1/ATP6V1G2/ATPIF1/C14orf2	10	CC
GO:0005839	proteasome core complex	5/2879	22/18511	0.24974	0.8561	0.84606	ALAD/PSMB11/PSMB6/PSMB7/PSMB8	5	CC
GO:0033116	endoplasmic reticulum-Golgi intermediate compartment membrane	13/2879	68/18511	0.2523	0.8561	0.84606	BCAP31/CNIH2/COL7A1/GOLGA2/GORASP1/GOSR2/INS/LMAN2/NAT8B/STX5/TAP1/TMED3/WHAMM	13	CC
GO:0098793	presynapse	60/2879	354/18511	0.25235	0.8561	0.84606	ADRA2C/AP1S1/APBB1/AQP1/ATP6V1B1/BAIAP3/C1QL1/CDK16/CDK5/CHRM1/CNGB1/CPLX1/DBNDD1/DDC/DGKI/DISC1/DLG4/DNM1/DOC2A/DOC2B/DRD2/DTNBP1/FOSL1/GABBR1/GAD1/GPER1/GRIN1/HCN3/IL31RA/ILK/LGI3/NRXN2/OPRD1/PDE2A/PLD2/PTPRN2/RAB26/RAB40C/RAB7A/RGS10/SCRIB/SLC2A8/SLC30A3/SLC6A9/STON2/STX1A/STX4/STXBP1/SV2A/SYTL3/TH/TMEM163/TOR1A/TRAPPC4/TULP1/VAMP1/VDAC2/VTI1B/[illegible]	60	CC
GO:0097730	non-motile cilium	23/2879	128/18511	0.25756	0.8561	0.84606	ANO2/ARR3/CACNA1F/CDC14A/CDHR1/CIB2/CNGB1/DRD2/GNAT1/GNB5/GUCA1B/GUCA2B/GUCY2D/IFT140/IFT20/KIF17/MYO7A/OPN1MW/OPN4/PEX6/SEPT9/TUBG1/TULP1	23	CC
GO:0030140	trans-Golgi network transport vesicle	6/2879	28/18511	0.2624	0.8561	0.84606	AP1G2/AP1S1/CLTB/FURIN/SLC2A4/STEAP2	6	CC
GO:0019005	SCF ubiquitin ligase complex	10/2879	51/18511	0.26289	0.8561	0.84606	ABTB1/CACYBP/CCNF/FBXL19/FBXL22/FBXO2/FBXO25/FBXO6/FBXW4/SPOP	10	CC
GO:0031526	brush border membrane	10/2879	51/18511	0.26289	0.8561	0.84606	AQP1/CA4/CDHR5/CUBN/NPC1L1/PEMT/SLC22A12/SLC26A6/SLC34A1/SLC9A3	10	CC
GO:0098802	plasma membrane receptor	31/2879	177/18511	0.26317	0.8561	0.84606	ALCAM/CARD11/CD247/CD6/CD79B/CHRNA6/CHRNB1/CHRNB3/CNIH2/CNTFR/DLG4/EMILIN1/ENG/GABBR1/GRIA3/GRIN1/GRIN2C/HTR3B/IKBKB/IRAK1/ITGA7/ITGAL/ITGAM/ITGAX/ITGB5/PORCN/PTK2B/RNF31/SC	31	CC

GO:0005779	integral component of peroxisomal	4/2879	17/18511	0.26608	0.8561	0.84606	PEX11A/PEX16/PEX3/PXMP2	4	CC
GO:0031143	pseudopodium	4/2879	17/18511	0.26608	0.8561	0.84606	F2RL1/MAPK3/RAB25/RAF1	4	CC
GO:0031231	intrinsic component of peroxisomal membrane	4/2879	17/18511	0.26608	0.8561	0.84606	PEX11A/PEX16/PEX3/PXMP2	4	CC
GO:0043186	P granule	4/2879	17/18511	0.26608	0.8561	0.84606	DDX4/PIWIL2/TDRD1/TDRD9	4	CC
GO:0045495	pole plasm	4/2879	17/18511	0.26608	0.8561	0.84606	DDX4/PIWIL2/TDRD1/TDRD9	4	CC
GO:0060293	germ plasm	4/2879	17/18511	0.26608	0.8561	0.84606	DDX4/PIWIL2/TDRD1/TDRD9	4	CC
GO:0090723	growth cone part	4/2879	17/18511	0.26608	0.8561	0.84606	APBB1/DISC1/TRPV2/ZFYVE27	4	CC
GO:0043235	receptor complex	55/2879	325/18511	0.26727	0.8561	0.84606	ABCG5/ADRA2A/AIP/ALCAM/CARD11/CD247/CD6/CD74/CD79B/CHRNA6/CHRNB1/CHRNB3/CNGB1/CNIH2/CNTFR/DLG4/EGFR/EMILIN1/ENG/ERBB2/FLT4/GABBR1/GPR20/GPR62/GRIA3/GRIN1/GRIN2C/HTR3B/IKBKB/IL4R/IRAK1/ITGA7/ITGAL/ITGAM/ITGAX/ITGB5/ITPR3/KLRC1/KLRC2/LEPR/LRP1/NOTCH1/NR1H3/OLR1/P2RX2/PLXNB1/PORCN/PTK2B/PTPRN2/RNF	55	CC
GO:0034702	ion channel complex	49/2879	288/18511	0.26796	0.8561	0.84606	AMIGO1/ANO1/ANO2/BEST3/BEST4/CACNA1F/CACNA1G/CACNA1H/CACNA1S/CACNB1/CACNB3/CACNG1/CAMK2D/CATSPER1/CHRNA6/CHRNB1/CHRNB3/CLCN2/CLCNKA/CLCNKB/CLIC1/CNGB1/CNIH2/CTTN/DLG4/DPP6/GLRA1/GRIA3/GRIN1/GRIN2C/HERPUD1/HSPA2/HTR3B/KCND1/KCND3/KCNH4/KCNJ5/KCNK4/KCNQ1/KCNQ4/KCNT1/PORCN/PTK2B/SCN11A/SCNN1A/SCRIB/SLC26A6/STX1A/TRDN	49	CC
GO:0034704	calcium channel complex	12/2879	63/18511	0.26823	0.8561	0.84606	CACNA1F/CACNA1G/CACNA1H/CACNA1S/CACNB1/CACNB3/CACNG1/CAMK2D/CATSPER1/HERPUD1/HSPA2/TRDN	12	CC

GO:0031904	endosome lumen	7/2879	34/18511	0.27086	0.8561	0.84606	AP4S1/APOB/CD63/CTSH/CTSL/INS/SFTPB	7	CC
GO:0005902	microvillus	15/2879	81/18511	0.27183	0.8561	0.84606	AQP5/ATP6V1B1/CDHR5/DPEP1/ESPN/FSCN1/IFT20/MYO1A/MYO1C/MYO7A/MYO7B/PDGFA/PLEKHG6/PROM2/SCARB1	15	CC
GO:1990351	transporter complex	56/2879	332/18511	0.27377	0.8561	0.84606	ABCA2/ABCG5/AMIGO1/ANO1/ANO2/ATP1A1/ATP1A4/BEST3/BEST4/CACNA1F/CACNA1G/CACNA1H/CACNA1S/CACNB1/CACNB3/CACNG1/CAMK2D/CATSPER1/CHRNA6/CHRNB1/CHRNB3/CLCN2/CLCNKA/CLCNKB/CLIC1/CNGB1/CNIH2/CTTN/CYC1/DLG4/DPP6/GLRA1/GRIA3/GRIN1/GRIN2C/HERPUD1/HSPA2/HTR3B/KCND1/KCND3/KCNH4/KCNJ5/KCNK4/KCNQ1/KCNQ4/KCNT1/PEX14/PORCN/PTK2B/SCN11A/SCNN1A/SCRIB/SLC2[illegible]	56	CC
GO:0044450	microtubule organizing center part	29/2879	166/18511	0.27657	0.8561	0.84606	ACTR1A/BBS9/BRCA1/C2CD3/CCDC13/CCDC146/CCHCR1/CCNF/CEP164/CEP19/CKAP5/CROCC/FOPNL/IFT20/KATNB1/LCK/NEURL4/NUBP1/NUBP2/PARD6A/PARP3/SDCCAG8/TUBG1/TUBG2/TUBGCP2/TUBGCP3/TUBGC	29	CC
GO:0032590	dendrite membrane	5/2879	23/18511	0.28151	0.8561	0.84606	GPER1/OPRD1/OPRM1/THY1/WLS	5	CC
GO:0097381	photoreceptor disc membrane	5/2879	23/18511	0.28151	0.8561	0.84606	GNAT1/GUCA1B/GUCY2D/OPN1MW/OPN4	5	CC
GO:0000815	ESCRT III complex	3/2879	12/18511	0.28331	0.8561	0.84606	CHMP1A/CHMP4C/CHMP7	3	CC
GO:0030130	clathrin coat of trans-Golgi network vesicle	3/2879	12/18511	0.28331	0.8561	0.84606	AP1G2/AP1S1/CLTB	3	CC
GO:0031616	spindle pole centrosome	3/2879	12/18511	0.28331	0.8561	0.84606	EMD/NUBP2/NUMA1	3	CC
GO:0033162	melanosome membrane	3/2879	12/18511	0.28331	0.8561	0.84606	DTNBP1/RAB7A/TH	3	CC
GO:0045009	chitosome	3/2879	12/18511	0.28331	0.8561	0.84606	DTNBP1/RAB7A/TH	3	CC
GO:0090741	pigment granule membrane	3/2879	12/18511	0.28331	0.8561	0.84606	DTNBP1/RAB7A/TH	3	CC

GO:1990023	mitotic spindle midzone	3/2879	12/18511	0.28331	0.8561	0.84606	CTTN/EML1/NUMA1	3	CC
GO:0031902	late endosome membrane	22/2879	124/18511	0.28434	0.8561	0.84606	ARL8A/ATP13A2/CD300LG/CD63/CHMP4C/CYB561A3/GOSR2/RAB7A/SLC11A1/SLC29A3/SLC30A3/SNF8/STARD3/STOML1/STX8/TMEM9/TSPAN15/VPS37B/VPS37C/VPS37D/VTI1B/YIPF1	22	CC
GO:1904813	ficolin-1-rich granule lumen	22/2879	124/18511	0.28434	0.8561	0.84606	ALAD/ALDOA/BIN2/C1orf35/CALML5/CDA/COTL1/CTSD/CTSH/GSDMD/HK3/HSP90AA1/IDH1/IMPDH1/PKM/PPIE/PSMB7/PSMC3/PSMD13/PSMD3/	22	CC
GO:0097731	9+0 non-motile cilium	18/2879	100/18511	0.28747	0.8561	0.84606	ARR3/CACNA1F/CDHR1/CIB2/CNGB1/GNAT1/GNB5/GUCA1B/GUCA2B/GUCY2D/IFT140/IFT20/KIF17/MYO7A/OPN1MW/OPN4/PEX6/TULP1	18	CC
GO:0097733	photoreceptor cell cilium	18/2879	100/18511	0.28747	0.8561	0.84606	ARR3/CACNA1F/CDHR1/CIB2/CNGB1/GNAT1/GNB5/GUCA1B/GUCA2B/GUCY2D/IFT140/IFT20/KIF17/MYO7A/OPN1MW/OPN4/PEX6/TULP1	18	CC
GO:0045334	clathrin-coated endocytic	13/2879	70/18511	0.28786	0.8561	0.84606	AP1S1/AP2A2/AP2M1/APOB/CD74/CLTB/DVL2/EGFR/FZD2/INPP5F/MYO1E/SFTPA1/SFTPB	13	CC
GO:0005635	nuclear envelope	73/2879	440/18511	0.29057	0.8605	0.85037	ABCF1/ABL1/ADRA1A/ADRA1B/AQP1/ATP2A3/BIN1/BOK/CACYBP/CASC3/CDK4/CHMP7/CLIC1/CMTM3/CPTP/CUEDC2/DTX2/DUSP2/DUX4/EGFR/EIF5A/EMD/EPC1/GAPDH/GCH1/GCHFR/GHDC/GPER1/GUCY2D/HACD3/HTATIP2/IL15RA/INTS5/IPO13/IPO4/ITPR3/LEMD2/LMNA/LMNB1/MAD1L1/MAPK3/MGST3/MTA1/MYO1C/NR4A1/NRM/NSMF/NUDT1/NUP210/NUP98/OIT3/P2RX2/PNPLA7/POMZP3/PTGER3/RNF180/ROGDI/RRP12/S100A6/SCGB1A1/SIGMAR1/SLC29A2/STX1A/TAF3/TMEM109/TMEM176B/TNMD/[illegible]	73	CC
GO:0044440	endosomal part	80/2879	484/18511	0.29232	0.8607	0.85062	ABCA2/ACAP1/AP1G2/AP2A2/AP2M1/AP4S1/APOB/ARL8A/ATP13A2/ATP6V0B/ATP6V0E2/BOK/CD300LG/CD63/CD68/CHMP1A/CHMP4C/CHMP7/COMMD1/CPTP/CTSH/CTSL/CUBN/CYB561A3/DTNBP1/EGFR/EHD1/EPHA8/ERBB2/FURIN/GOSR2/GRIPAP1/HLA-E/INPP5F/INS/IRAK1/KIF13A/KREMEN2/LLGL1/PLA2G4B/PLEKHM1/PLEKHM2/PSD/RAB11FIP3/RAB17/RAB7A/RBSN/RHOD/RHOV/RRAGC/SCAMP2/SFTPB/SLC11A1/SLC29A3/SLC30A3/SNF8/STARD3/STEAP2/STOML1/STX8/TBK1/TLR8/TLR9/TMBIM1/TMEM163/TMEM9/TSPAN15/UBAP1L/VPS25/VPS37B/VPS37C/VPS37D/VPS51/VPS52/VPS53/VTI1B/WLS/WNT3A/YI[illegible]	80	CC

GO:0030176	integral component of endoplasmic reticulum	25/2879	143/18511	0.29385	0.8607	0.85062	ABCB9/BCAP31/CD74/DOLPP1/DPM2/ELOVL1/ELOVL5/EMC8/FITM1/G6PC3/GPAA1/HACD1/HACD3/HLA-E/HSPA5/LRIT1/PIGG/PORCN/SGMS1/SPPL2C/SYVN1/TAP1/WFS1/XXYLT1/ZFYVE27	25	CC
GO:0090575	RNA polymerase II transcription	23/2879	131/18511	0.29659	0.8607	0.85062	ASCL2/BCL9/EDF1/GATA4/GTF2H2C/GTF2H4/HMGA1/HOXB9/KAT2A/MAX/MED18/MED6/NKX2-5/NONO/NR1H3/NR5A1/TADA3/TAF1/TAF1L/TAF3/TAF6/TAF7/TBPL1	23	CC
GO:0005669	transcription factor TFIID	7/2879	35/18511	0.29693	0.8607	0.85062	EDF1/GTF2H4/TAF1/TAF1L/TAF3/TAF6/TAF7	7	CC
GO:0016592	mediator complex	7/2879	35/18511	0.29693	0.8607	0.85062	CDK10/MED11/MED18/MED22/MED24/MED6/MED9	7	CC
GO:0030133	transport vesicle	59/2879	354/18511	0.30091	0.8685	0.85833	AIMP1/AP1G2/AP1S1/ATP13A2/ATP6V1B1/BGN/CA4/CD74/CDK16/CHGA/CLTB/CNIH2/COPZ1/DBNDD1/DDC/DGKI/DISC1/DLG4/DNM1/DOC2A/DOC2B/DRD2/DTNBP1/FGFR4/FGFRL1/FURIN/GOSR2/GRIN1/HLA-E/INS/LGI3/NKD2/PTPRN2/RAB26/RAB40C/SCAMP2/SCAP/SFTA2/SIPA1/SLC2A4/SLC2A8/SLC30A3/STEAP2/STON2/STX1A/STX4/STX5/SV2A/SYTL3/TH/TMED3/TMEM163/TOR1A/TRAPPC4/VAMP1/VDAC2/VGF/VTI1B/YIPF[illegible]	59	CC
GO:0030132	clathrin coat of coated pit	4/2879	18/18511	0.30341	0.8685	0.85833	AP2A2/AP2M1/CLTB/EGFR	4	CC
GO:0035371	microtubule plus-end	4/2879	18/18511	0.30341	0.8685	0.85833	CKAP5/MAPRE3/NCKAP5L/NUMA1	4	CC
GO:0010008	endosome membrane	73/2879	442/18511	0.3051	0.8697	0.85952	ABCA2/ACAP1/AP1G2/AP2A2/AP2M1/APOB/ARL8A/ATP13A2/ATP6V0B/ATP6V0E2/BOK/CD300LG/CD63/CD68/CHMP1A/CHMP4C/CHMP7/COMMD1/CPTP/CUBN/CYB561A3/DTNBP1/EGFR/EHD1/EPHA8/ERBB2/FURIN/GOSR2/GRIPAP1/HLA-E/INPP5F/IRAK1/KIF13A/KREMEN2/LLGL1/PLA2G4B/PLEKHM1/PLEKHM2/PSD/RAB11FIP3/RAB17/RAB7A/RBSN/RHOD/RHOV/SCAMP2/SLC11A1/SLC29A3/SLC30A3/SNF8/STARD3/STEAP2/STOML1/STX8/TBK1/TLR8/TLR9/TMBIM1/TMEM163/TMEM9/TSPAN15/UBAP1L/VPS25/VPS37B/VPS37C[illegible]	73	CC

GO:0022624	proteasome accessory complex	5/2879	24/18511	0.31398	0.8833	0.87293	PSMC3/PSMD13/PSMD3/PSMD5/PSMD7	5	CC
GO:0032839	dendrite cytoplasm	5/2879	24/18511	0.31398	0.8833	0.87293	DLG4/HIP1R/KIF17/OPRM1/WLS	5	CC
GO:0036379	myofilament	5/2879	24/18511	0.31398	0.8833	0.87293	ACTA1/LMOD1/MYBPC3/TNNC1/TPM2	5	CC
GO:0005793	endoplasmic reticulum-Golgi intermediate compartment	20/2879	114/18511	0.315	0.8833	0.87293	ANPEP/BCAP31/CA4/CNIH2/COL7A1/FN1/GBF1/GOLGA2/GORASP1/GOSR2/HSPA5/INS/LMAN2/MYO18A/NAT8B/SERPINH1/STX5/TAP1/TMED3/WHAMM	20	CC
GO:1902495	transmembrane transporter complex	54/2879	325/18511	0.31938	0.8919	0.88146	ABCA2/ABCG5/AMIGO1/ANO1/ANO2/ATP1A1/ATP1A4/BEST3/BEST4/CACNA1F/CACNA1G/CACNA1H/CACNA1S/CACNB1/CACNB3/CACNG1/CAMK2D/CATSPER1/CHRNA6/CHRNB1/CHRNB3/CLCN2/CLCNKA/CLCNKB/CLIC1/CNGB1/CNIH2/CTTN/CYC1/DLG4/DPP6/GLRA1/GRIA3/GRIN1/GRIN2C/HERPUD1/HSPA2/HTR3B/KCND1/KCND3/KCNH4/KCNJ5/KCNK4/KCNQ1/KCNQ4/KCNT1/PORCN/PTK2B/SCN11A/SCNN1A/SCRIB/SLC26A6/ST[illegible]	54	CC
GO:0031252	cell leading edge	61/2879	369/18511	0.32138	0.8929	0.88248	ABL1/ACTA1/APBB1/ARHGEF4/ARPC2/BCAS3/BRK1/BSPRY/CDC42BPG/CDK5/CFL1/CIB1/CORO1A/CORO1B/CTNNA2/CTTN/DGKZ/EPS8L2/EVL/FAM89B/FAT1/FERMT2/FSCN1/GBF1/GPER1/HIP1R/HSP90AA1/ILK/INPP5E/INPPL1/KSR1/LAYN/LDB1/LIMK1/MTMR14/MYO1C/NME1/NRBP1/NRG1/OPRD1/OPRM1/PARD6A/PDLIM7/PLEKHG5/PLXND1/PPP1R9B/PSD/PSD2/PSD4/PTK2B/RASGRP2/S100A6/SCRIB/STX4/THY1/TIRAP/TLN1/TNFRSF1[illegible]	61	CC
GO:0098878	neurotransmitter receptor	9/2879	48/18511	0.32635	0.8929	0.88248	CNIH2/DLG4/GRIA3/GRIN1/GRIN2C/HTR3B/PORCN/PTK2B/SCRIB	9	CC
GO:0045121	membrane raft	50/2879	301/18511	0.32814	0.8929	0.88248	ADRA1A/ADRA1B/AHNAK/ATP1A1/CARD11/CASP3/CAV3/CD48/CDH13/CDH15/CRB2/CSK/CTSD/EFNB1/EGFR/FASLG/FURIN/GPC1/IKBKB/INPP5D/ITGAM/KCNQ1/KDR/LCK/LTB4R/MAPK3/MYO1A/MYO1C/NOS3/OLR1/OPRD1/OPRM1/PRKAR1A/PROM2/PTK2B/RTN4RL1/RTN4RL2/SCARB1/SERPINH1/SHH/SLC25A5/SLC2A4/SLC34A1/SLC6A2/SMO/STOML1/STOML[illegible]	50	CC

GO:0030122	AP-2 adaptor complex	3/2879	13/18511	0.32912	0.8929	0.88248	AP2A2/AP2M1/EGFR	3	CC
GO:0042627	chylomicron	3/2879	13/18511	0.32912	0.8929	0.88248	APOA5/APOB/APOC3	3	CC
GO:0048188	Set1C/COMPASS complex	3/2879	13/18511	0.32912	0.8929	0.88248	ASH2L/HCFC1/SETD1A	3	CC
GO:0098857	membrane microdomain	50/2879	302/18511	0.33731	0.8929	0.88248	ADRA1A/ADRA1B/AHNAK/ATP1A1/CARD11/CASP3/CAV3/CD48/CDH13/CDH15/CRB2/CSK/CTSD/EFNB1/EGFR/FASLG/FURIN/GPC1/IKBKB/INPP5D/ITGAM/KCNQ1/KDR/LCK/LTB4R/MAPK3/MYO1A/MYO1C/NOS3/OLR1/OPRD1/OPRM1/PRKAR1A/PROM2/PTK2B/RTN4RL1/RTN4RL2/SCARB1/SERPINH1/SHH/SLC25A5/SLC2A4/SLC34A1/SLC6A2/SMO/STOML1/STOML	50	CC
GO:0043197	dendritic spine	23/2879	134/18511	0.33742	0.8929	0.88248	ADGRB1/APBB1/ARC/ARRB2/ASIC2/CNIH2/CRYAB/CTTN/DGKI/DLG4/DRD2/DTNBP1/FBXO2/GPER1/GRIN1/HIP1R/LAMA2/PPP1R9B/PSD/PTK2B/RGS10/RGS14/STX4	23	CC
GO:0005849	mRNA cleavage factor	4/2879	19/18511	0.34124	0.8929	0.88248	CPSF1/CPSF4/CSTF2/ZC3H3	4	CC
GO:0042101	T cell receptor complex	4/2879	19/18511	0.34124	0.8929	0.88248	ALCAM/CARD11/CD247/CD6	4	CC
GO:0043073	germ cell nucleus	4/2879	19/18511	0.34124	0.8929	0.88248	ACTL7A/ACTRT3/HILS1/HSPA2	4	CC
GO:0036126	sperm flagellum	14/2879	79/18511	0.34181	0.8929	0.88248	DNAJB13/DRD2/GAS8/ODF1/ODF4/PFKM/PGAM4/SEPT4/SLC26A6/TACR1/TACR2/TCP11/TEKT4/TEKT5	14	CC
GO:0097729	9+2 motile cilium	14/2879	79/18511	0.34181	0.8929	0.88248	DNAJB13/DRD2/GAS8/ODF1/ODF4/PFKM/PGAM4/SEPT4/SLC26A6/TACR1/TACR2/TCP11/TEKT4/TEKT5	14	CC
GO:0031227	intrinsic component of endoplasmic reticulum membrane	25/2879	147/18511	0.34602	0.8929	0.88248	ABCB9/BCAP31/CD74/DOLPP1/DPM2/ELOVL1/ELOVL5/EMC8/FITM1/G6PC3/GPAA1/HACD1/HACD3/HLA-E/HSPA5/LRIT1/PIGG/PORCN/SGMS1/SPPL2C/SYVN1/TAP1/WFS1/XXYLT1/ZFYVE27	25	CC

GO:0032806	carboxy-terminal domain protein kinase	5/2879	25/18511	0.3469	0.8929	0.88248	CDK9/GTF2H2C/GTF2H4/HOXC10/TAF7	5	CC
GO:0032153	cell division site	11/2879	61/18511	0.34706	0.8929	0.88248	HMCN2/MAEA/MEN1/PITPNM1/PLEKHG6/PSD/PSD2/RAB11FIP3/TUBGCP2/TUBGCP3/TUBGCP5	11	CC
GO:0032155	cell division site part	11/2879	61/18511	0.34706	0.8929	0.88248	HMCN2/MAEA/MEN1/PITPNM1/PLEKHG6/PSD/PSD2/RAB11FIP3/TUBGCP2/TUBGCP3/TUBGCP5	11	CC
GO:0015030	Cajal body	10/2879	55/18511	0.34849	0.8929	0.88248	AK6/EFTUD2/FAM118B/HINFP/HSPB7/NHP2/PRPF4/SART1/SRRM2/ZPR1	10	CC
GO:0031594	neuromuscular junction	10/2879	55/18511	0.34849	0.8929	0.88248	ACHE/CAV3/CDH15/CDK5/CIB2/DES/DNAJA3/NRG1/PRKAR1A/SV2A	10	CC
GO:0030990	intraciliary transport	6/2879	31/18511	0.34949	0.8929	0.88248	IFT140/IFT20/IFT22/KIF17/TRAF3IP1/TULP3	6	CC
GO:0051233	spindle midzone	6/2879	31/18511	0.34949	0.8929	0.88248	ARL8A/BUB1B/CTTN/EML1/NUMA1/UNC119	6	CC
GO:0005782	peroxisomal matrix	8/2879	43/18511	0.35034	0.8929	0.88248	ACOXL/CROT/CRYM/GNPAT/HAO2/IDH1/PAOX/PIPOX	8	CC
GO:0031907	microbody lumen	8/2879	43/18511	0.35034	0.8929	0.88248	ACOXL/CROT/CRYM/GNPAT/HAO2/IDH1/PAOX/PIPOX	8	CC
GO:0031301	integral component of organelle	28/2879	166/18511	0.35094	0.8929	0.88248	ATP13A2/B4GALNT1/CHST12/COA3/COQ2/LEMD2/LFNG/MFN1/MPC1/P2RX2/PEX11A/PEX16/PEX3/PQLC2/PXMP2/QSOX1/RFNG/RHOT2/SGMS1/SLC39A13/STEAP2/SV2A/TIMM17B/TIMM23B/TMEM11/TMEM163/TVP23B/	28	CC
GO:0035097	histone methyltransferase complex	12/2879	68/18511	0.3654	0.9045	0.89392	ASH2L/CHD8/HCFC1/MAX/MEN1/PAXIP1/PHF19/SENP3/SETD1A/TAF1/TAF6/TAF7	12	CC
GO:0044309	neuron spine	23/2879	136/18511	0.3654	0.9045	0.89392	ADGRB1/APBB1/ARC/ARRB2/ASIC2/CNIH2/CRYAB/CTTN/DGKI/DLG4/DRD2/DTNBP1/FBXO2/GPER1/GRIN1/HIP1R/LAMA2/PPP1R9B/PSD/PTK2B/RGS10/RGS14/STX4	23	CC
GO:0035580	specific granule lumen	11/2879	62/18511	0.36803	0.9045	0.89392	CHIT1/CRACR2A/CTSD/GGH/GHDC/GSDMD/OLFM4/ORM1/ORM2/QSOX1/SPTAN1	11	CC

GO:0043195	terminal bouton	11/2879	62/18511	0.36803	0.9045	0.89392	AP1S1/CNGB1/CPLX1/GRIN1/ILK/PTPRN2/RAB7A/STXBP1/SV2A/TH/VAMP1	11	CC
GO:0031300	intrinsic component of organelle membrane	30/2879	180/18511	0.37	0.9045	0.89392	ATP13A2/B4GALNT1/CHST12/CHST5/COA3/COQ2/LEMD2/LFNG/MFN1/MFN2/MPC1/P2RX2/PEX11A/PEX16/PEX3/PQLC2/PXMP2/QSOX1/RFNG/RHOT2/SGMS1/SLC39A13/STEAP2/SV2A/TIMM17B/TIMM23B/TMEM11/TMEM163/TVP23B/VAMP5	30	CC
GO:0031256	leading edge membrane	25/2879	149/18511	0.37291	0.9045	0.89392	ARHGEF4/CFL1/CIB1/EPS8L2/FERMT2/GPER1/HIP1R/HSP90AA1/KSR1/MYO1C/NME1/NRG1/OPRD1/OPRM1/PLXND1/PPP1R9B/PSD/PSD2/PSD4/RASGRP2/THY1/TIRAP/TLN1/TRPV4/WLS	25	CC
GO:0030286	dynein complex	9/2879	50/18511	0.37312	0.9045	0.89392	DCTN2/DISC1/DNAH11/DNAH12/DNAH17/DNHD1/DYNC1H1/DYNC1I1/DYNLL2	9	CC
GO:0005741	mitochondrial outer membrane	29/2879	174/18511	0.37313	0.9045	0.89392	ACACB/AMBRA1/ATF2/BAD/BOK/BPHL/FATE1/LETMD1/MFN1/MFN2/MGST1/MIEF2/MOAP1/MTOR/MYO19/MYOC/NLRX1/NME1/PDE2A/PGAM5/PI4KB/PPP2R2B/RAF1/RHOT2/SPATA19/ULK1/VAMP1/VAT1/VDAC2	29	CC
GO:0012510	trans-Golgi network transport vesicle	3/2879	14/18511	0.37484	0.9045	0.89392	AP1G2/AP1S1/CLTB	3	CC
GO:0030128	clathrin coat of endocytic	3/2879	14/18511	0.37484	0.9045	0.89392	AP2A2/AP2M1/EGFR	3	CC
GO:0034703	cation channel complex	35/2879	212/18511	0.37803	0.9045	0.89392	AMIGO1/CACNA1F/CACNA1G/CACNA1H/CACNA1S/CACNB1/CACNB3/CACNG1/CAMK2D/CATSPER1/CNGB1/CNIH2/CTTN/DLG4/DPP6/GRIA3/GRIN1/GRIN2C/HERPUD1/HSPA2/HTR3B/KCND1/KCND3/KCNH4/KCNJ5/KCNK4/KCNQ1/KCNQ4/KCNT1/PORCN/PTK2B/SCN11A/SCNN1A/STX1A/TR	35	CC
GO:0005790	smooth endoplasmic reticulum	6/2879	32/18511	0.37919	0.9045	0.89392	APOB/HSD3B1/HSPA5/PPIB/SYVN1/TH	6	CC
GO:0008305	integrin complex	6/2879	32/18511	0.37919	0.9045	0.89392	EMILIN1/ITGA7/ITGAL/ITGAM/ITGAX/ITGB5	6	CC
GO:0048786	presynaptic active zone	6/2879	32/18511	0.37919	0.9045	0.89392	DGKI/GAD1/GPER1/STX1A/STXBP1/SV2A	6	CC

GO:0000242	pericentriolar material	4/2879	20/18511	0.37919	0.9045	0.89392	BBS9/LCK/TUBG1/TUBG2	4	CC
GO:0030992	intraciliary transport particle B	4/2879	20/18511	0.37919	0.9045	0.89392	IFT20/IFT22/KIF17/TRAF3IP1	4	CC
GO:0031233	intrinsic component of external side of plasma	4/2879	20/18511	0.37919	0.9045	0.89392	CA4/FOLR2/GPIHBP1/THY1	4	CC
GO:0097225	sperm midpiece	4/2879	20/18511	0.37919	0.9045	0.89392	SLC26A6/TACR1/TACR2/TCP11	4	CC
GO:0005581	collagen trimer	15/2879	88/18511	0.39308	0.9344	0.92345	C1QB/C1QC/C1QL1/C1QL2/C1QL4/C1QTNF1/COL16A1/COL17A1/COL1A1/COL4A2/COL7A1/COL9A2/EMILIN1/SCARA3/SFTPA1	15	CC
GO:0019897	extrinsic component of plasma	25/2879	151/18511	0.40017	0.9408	0.92974	ABL1/BLK/CDK16/CSK/CUBN/DLG4/DNAJA3/FERMT2/FES/GNAO1/GNAT1/GNG3/GRAP/LCK/MYZAP/NUMA1/PRSS22/PTK2B/S100A6/ST14/SYTL3/TDGF1/TEC/TNK1/TNK2	25	CC
GO:0009898	cytoplasmic side of plasma membrane	28/2879	170/18511	0.40206	0.9408	0.92974	ABL1/AJAP1/BLK/CDK16/CSK/DAB2IP/DLG4/FERMT2/FES/GNAO1/GNAT1/GNG3/GRAP/IKBKB/LCK/MYZAP/PTK2B/PTPN7/RASA3/RASA4B/RNF31/S100A6/SPTB/TEC/TH/TNK1/TNK2/TRAF2	28	CC
GO:0098562	cytoplasmic side of membrane	31/2879	189/18511	0.40346	0.9408	0.92974	ABL1/AJAP1/BLK/CDK16/CSK/DAB2IP/DLG4/FERMT2/FES/GNAO1/GNAT1/GNG3/GRAP/IKBKB/LCK/MYZAP/PTK2B/PTPN7/RASA3/RASA4B/RBSN/RNF31/S100A6/SPPL2C/SPTB/TEC/TH/THADA/TNK1/TNK2/TRAF2	31	CC
GO:0000791	euchromatin	6/2879	33/18511	0.4089	0.9408	0.92974	ANKRD2/ASH2L/DNMT3A/HSF1/NSMF/SETD1A	6	CC
GO:0031304	intrinsic component of mitochondrial inner membrane	6/2879	33/18511	0.4089	0.9408	0.92974	COA3/COQ2/MPC1/TIMM17B/TIMM23B/TMEM11	6	CC
GO:0031305	integral component of mitochondrial	6/2879	33/18511	0.4089	0.9408	0.92974	COA3/COQ2/MPC1/TIMM17B/TIMM23B/TMEM11	6	CC

GO:0044447	axoneme part	6/2879	33/18511	0.4089	0.9408	0.92974	DNAH17/DNHD1/HYDIN/SPAG16/WDPCP/WDR66	6	CC
GO:1905368	peptidase complex	15/2879	89/18511	0.41101	0.9408	0.92974	ALAD/HSPB1/PSMB11/PSMB6/PSMB7/PSMB8/PSMC3/PSMD13/PSMD3/PSMD5/PSMD7/PSMG3/TADA3/UBE3C/WFS1	15	CC
GO:0031234	extrinsic component of cytoplasmic side of plasma membrane	18/2879	108/18511	0.41439	0.9408	0.92974	ABL1/BLK/CDK16/CSK/DLG4/FERMT2/FES/GNAO1/GNAT1/GNG3/GRAP/LCK/MYZAP/PTK2B/S100A6/TEC/TNK1/TNK2	18	CC
GO:0035577	azurophil granule	10/2879	58/18511	0.41521	0.9408	0.92974	ACPP/ARL8A/C3AR1/CD63/CD68/DDOST/MGST1/PRCP/PSAP/RAB44	10	CC
GO:0005753	mitochondrial proton-transporting ATP synthase	4/2879	21/18511	0.41689	0.9408	0.92974	ATP5G1/ATP5I/ATPIF1/C14orf2	4	CC
GO:0005865	striated muscle thin filament	4/2879	21/18511	0.41689	0.9408	0.92974	ACTA1/LMOD1/TNNC1/TPM2	4	CC
GO:0033177	proton-transporting two-sector ATPase complex, proton-[illegible]	4/2879	21/18511	0.41689	0.9408	0.92974	ATP5G1/ATP5I/ATP6V0B/ATP6V0E2	4	CC
GO:0000109	nucleotide-excision repair complex	3/2879	15/18511	0.41989	0.9408	0.92974	POLE/POLE4/XPC	3	CC
GO:0032039	integrator complex	3/2879	15/18511	0.41989	0.9408	0.92974	INTS10/INTS3/INTS5	3	CC
GO:0032433	filopodium tip	3/2879	15/18511	0.41989	0.9408	0.92974	CIB1/MORN4/MYO3A	3	CC

GO:0000315	organellar large ribosomal	9/2879	52/18511	0.4204	0.9408	0.92974	C12orf65/MRPL10/MRPL14/MRPL28/MRPL36/MRPL37/MRPL43/MRPL52/MRPL9	9	CC
GO:0005762	mitochondrial large ribosomal	9/2879	52/18511	0.4204	0.9408	0.92974	C12orf65/MRPL10/MRPL14/MRPL28/MRPL36/MRPL37/MRPL43/MRPL52/MRPL9	9	CC
GO:0008328	ionotropic glutamate receptor	8/2879	46/18511	0.42595	0.9501	0.93895	CNIH2/DLG4/GRIA3/GRIN1/GRIN2C/PORCN/PTK2B/SCRIB	8	CC
GO:0019898	extrinsic component of membrane	42/2879	261/18511	0.43066	0.9575	0.94627	ABL1/ATG2A/ATP6V1B1/BLK/CDK16/CNTFR/CSK/CUBN/DLG4/DNAJA3/DUSP21/FERMT2/FES/GNAO1/GNAT1/GNG3/GOLGA3/GRAP/LCK/MTOR/MYZAP/NCF1/NUMA1/OSR1/PIK3R5/PIK3R6/PRSS22/PSD/PTK2B/RAB7A/S100A6/SNX11/SNX12/ST14/SYTL3/TDGF1/TEC/TNK1/TNK2/TOR1A/ULK1	42	CC
GO:0070461	SAGA-type complex	5/2879	28/18511	0.44576	0.9819	0.97041	HCFC1/KAT2A/TADA3/TAF6/TAF7	5	CC
GO:0045259	proton-transporting ATP synthase	4/2879	22/18511	0.45403	0.9819	0.97041	ATP5G1/ATP5I/ATPIF1/C14orf2	4	CC
GO:0032589	neuron projection	7/2879	41/18511	0.45893	0.9819	0.97041	GPER1/MYO1C/NRG1/OPRD1/OPRM1/THY1/WLS	7	CC
GO:0032994	protein-lipid complex	7/2879	41/18511	0.45893	0.9819	0.97041	APOA5/APOB/APOC3/BIN1/CLU/GPIHBP1/PEX3	7	CC
GO:0019908	nuclear cyclin-dependent protein kinase holoenzyme	3/2879	16/18511	0.46378	0.9819	0.97041	CDK9/HOXC10/TAF7	3	CC
GO:0034045	pre-autophagosomal structure membrane	3/2879	16/18511	0.46378	0.9819	0.97041	ATG2A/RAB7A/ULK1	3	CC
GO:0044232	organelle membrane contact site	3/2879	16/18511	0.46378	0.9819	0.97041	FATE1/STARD3/ZFYVE1	3	CC

GO:0070822	Sin3-type complex	3/2879	16/18511	0.46378	0.9819	0.97041	BRMS1/NCOR1/PHF12	3	CC
GO:0001726	ruffle	26/2879	162/18511	0.46384	0.9819	0.97041	ARHGEF4/CFL1/CIB1/CTTN/EPS8L2/FSCN1/HIP1R/HSP90AA1/INPP5E/KSR1/LAYN/MTMR14/MYO1C/NME1/PARD6A/PDLIM7/PPP1R9B/PSD/PSD2/PSD4/RASGRP2/S100A6/TIRAP/TLN1/TNFRSF12A/TRPV4	26	CC
GO:0000792	heterochromatin	12/2879	73/18511	0.46635	0.9819	0.97041	BAZ1B/CDKN2A/DNMT3A/HMGA1/HSF1/MPHOSPH8/NCAPD3/PCGF2/SUV39H1/UBE2A/UBE2B/UBE2U	12	CC
GO:0070821	tertiary granule membrane	12/2879	73/18511	0.46635	0.9819	0.97041	ADAM8/FRMPD3/ITGAM/ITGAX/MGAM/NBEAL2/OLR1/PLAU/SLC11A1/TMEM63A/UBR4/VAMP1	12	CC
GO:0098636	protein complex involved in cell adhesion	6/2879	35/18511	0.46777	0.9819	0.97041	EMILIN1/ITGA7/ITGAL/ITGAM/ITGAX/ITGB5	6	CC
GO:0000151	ubiquitin ligase complex	47/2879	297/18511	0.47305	0.9819	0.97041	ABTB1/ANAPC15/ANAPC2/ASB14/BRCA1/BRCC3/BUB1B/CACYBP/CCNF/COMMD1/DCAF11/DCAF8/DDB1/DDB2/FBXL19/FBXL22/FBXO10/FBXO2/FBXO24/FBXO25/FBXO6/FBXW4/IPP/KBTBD2/KCTD5/KLHL29/KLHL35/KLHL40/MED11/MED18/MIB2/MPHOSPH8/OS9/PCGF2/RNF31/SHARPIN/SPOP/SYVN1/TRAF2/TRAF7/TSPAN17/UBE2A/UBE2B/UBE2U/UBE3D/WWP2	47	CC
GO:0097223	sperm part	29/2879	182/18511	0.47489	0.9819	0.97041	ACRBP/ALDOA/ARC/CAPN11/CAPZA3/CCDC136/DNAJB13/DRD2/GAS8/LOXL1/NOTCH1/NUDT1/ODF1/ODF4/PFKM/PGAM4/PRSS37/SEPT4/SERPINA5/SLC26A6/SPACA3/SPINK2/TACR1/TACR2/TCP11/TEKT4/TEKT5/TSSK1	29	CC
GO:0000276	mitochondrial proton-transporting ATP synthase complex, coupling factor [illegible]	2/2879	10/18511	0.47592	0.9819	0.97041	ATP5G1/ATP5I	2	CC
GO:0001741	XY body	2/2879	10/18511	0.47592	0.9819	0.97041	DNMT3A/UBE2B	2	CC
GO:0016442	RISC complex	2/2879	10/18511	0.47592	0.9819	0.97041	SND1/TARBP2	2	CC

GO:0031588	nucleotide-activated protein kinase	2/2879	10/18511	0.47592	0.9819	0.97041	PRKAB1/PRKAR1A	2	CC
GO:0033179	proton-transporting V-type ATPase, V0 domain	2/2879	10/18511	0.47592	0.9819	0.97041	ATP6V0B/ATP6V0E2	2	CC
GO:0036128	CatSper complex	2/2879	10/18511	0.47592	0.9819	0.97041	CATSPER1/HSPA2	2	CC
GO:0044233	ER-mitochondrion membrane	2/2879	10/18511	0.47592	0.9819	0.97041	FATE1/ZFYVE1	2	CC
GO:0070938	contractile ring	2/2879	10/18511	0.47592	0.9819	0.97041	ALKBH4/MAEA	2	CC
GO:0098554	cytoplasmic side of endoplasmic reticulum	2/2879	10/18511	0.47592	0.9819	0.97041	SPPL2C/THADA	2	CC
GO:1902911	protein kinase complex	16/2879	99/18511	0.47595	0.9819	0.97041	CCND3/CDK4/CDK9/ENG/GTF2H2C/GTF2H4/HOXC10/IKBKB/NEK10/PHKG1/PRKAB1/PRKAR1A/PYDC1/TAF7/TRIM40/ULK1	16	CC
GO:0090568	nuclear transcriptional repressor	5/2879	29/18511	0.47802	0.9832	0.9717	LIN52/PASD1/SUV39H1/TBX15/TNRC18	5	CC
GO:0009295	nucleoid	7/2879	42/18511	0.48563	0.9845	0.97291	ACADVL/ATAD3A/HADHA/POLDIP2/SHMT2/SLC25A5/VDAC2	7	CC
GO:0042645	mitochondrial nucleoid	7/2879	42/18511	0.48563	0.9845	0.97291	ACADVL/ATAD3A/HADHA/POLDIP2/SHMT2/SLC25A5/VDAC2	7	CC
GO:0000178	exosome (RNase	4/2879	23/18511	0.49035	0.9845	0.97291	DIS3L/DIS3L2/EXOSC10/EXOSC2	4	CC
GO:0030137	COPI-coated vesicle	4/2879	23/18511	0.49035	0.9845	0.97291	ACTR1A/COPZ1/PACS1/TMED3	4	CC

GO:0031528	microvillus membrane	4/2879	23/18511	0.49035	0.9845	0.97291	CDHR5/DPEP1/PROM2/SCARB1	4	CC
GO:0046540	U4/U6 x U5 tri-snRNP complex	4/2879	23/18511	0.49035	0.9845	0.97291	EFTUD2/PRPF4/PRPF8/SART1	4	CC
GO:0098533	ATPase dependent transmembrane transport	4/2879	23/18511	0.49035	0.9845	0.97291	ABCA2/ABCG5/ATP1A1/ATP1A4	4	CC
GO:1905354	exoribonuclease complex	4/2879	23/18511	0.49035	0.9845	0.97291	DIS3L/DIS3L2/EXOSC10/EXOSC2	4	CC
GO:0032279	asymmetric synapse	33/2879	209/18511	0.4918	0.9845	0.97291	ADGRB1/ARC/ARRB2/ATP1A1/CDK5/CHRM1/CLSTN1/CLSTN3/CNIH2/CRYAB/CTNNA2/DAB1/DGKI/DISC1/DLG4/DRD2/DTNBP1/GNG3/GPER1/GRIN1/GRIN2C/HIP1R/HOMER2/NLGN3/NSMF/PDLIM5/PSD/PTK2B/RGS14/SCRIB/SHARPIN/SIGMAR1/STX1A	33	CC
GO:0005657	replication fork	11/2879	68/18511	0.49471	0.9845	0.97291	BAZ1B/BCL6/PIF1/POLA2/POLD4/POLE/POLE4/RFC1/RFC2/UBE2B/XRCC3	11	CC
GO:0005798	Golgi-associated vesicle	25/2879	158/18511	0.49649	0.9845	0.97291	ACTR1A/AP1G2/AP1S1/CD74/CLTB/CNGA2/CNGB1/CNIH2/COL7A1/COPZ1/FURIN/GOLGA2/GOSR2/GPR89A/HLA-E/MAP6D1/PACS1/PKD1/SCAP/SLC2A4/SPPL2C/STEAP2/STX5/TMED3/VT	25	CC
GO:0030660	Golgi-associated	17/2879	107/18511	0.50289	0.9845	0.97291	AP1G2/AP1S1/CD74/CLTB/CNGA2/CNGB1/CNIH2/COPZ1/GOSR2/GPR89A/HLA-E/PKD1/SCAP/SPPL2C/STX5/TMED3/VTI1B	17	CC
GO:0005892	acetylcholine-gated channel complex	3/2879	17/18511	0.50615	0.9845	0.97291	CHRNA6/CHRNB1/CHRNB3	3	CC
GO:0031092	platelet alpha granule	3/2879	17/18511	0.50615	0.9845	0.97291	APLP2/CYB5R1/SELP	3	CC
GO:0042575	DNA polymerase	3/2879	17/18511	0.50615	0.9845	0.97291	POLD4/POLE/POLE4	3	CC
GO:0005913	cell-cell adherens	14/2879	88/18511	0.50843	0.9845	0.97291	AJAP1/BAIAP2L1/CDH5/CTNNA2/DES/FAT2/KIFC3/NCR3/PDLIM1/PDLIM5/PDLIM7/PKP3/SCRIB/ZYX	14	CC

GO:0014069	postsynaptic density	32/2879	204/18511	0.50893	0.9845	0.97291	ADGRB1/ARC/ARRB2/ATP1A1/CDK5/CHRM1/CLSTN1/CLSTN3/CNIH2/CRYAB/CTNNA2/DAB1/DGKI/DISC1/DLG4/DRD2/DTNBP1/GNG3/GPER1/GRIN1/GRIN2C/HIP1R/HOMER2/NSMF/PDLIM5/PSD/PTK2B/RGS14/SCRIB/SH	32	CC
GO:0030131	clathrin adaptor complex	5/2879	30/18511	0.50962	0.9845	0.97291	AP1G2/AP1S1/AP2A2/AP2M1/EGFR	5	CC
GO:0005813	centrosome	73/2879	469/18511	0.5172	0.9845	0.97291	ACTR1A/APEX1/ARHGEF10/ARL2BP/B9D1/BBS9/BRSK2/C2CD3/CAMK2N2/CCDC13/CCDC81/CCDC85B/CCDC88B/CCNB2/CCNF/CDC14A/CDK5RAP3/CEP112/CEP164/CEP19/CEP70/CIB1/CKAP5/CROCC/DCTN2/DIS3L/DISC1/DYNC1H1/DYNLL2/EMD/FOPNL/HAUS4/HAUS7/HOXB4/HSF1/IFT140/IFT20/IFT22/KATNB1/KIF13A/KIF23/KIFC3/LCK/MAD1L1/NCKAP5L/NME1/NUBP2/NUMA1/OBSL1/PARD6A/PDE4DIP/PPP2R3C/RAB11FIP3/RABGAP1/RGS14/RILPL1/RILPL2/SDCCAG8/SSNA1/TRAF3IP1/TTLL5/TUBG1/TUBG2/TUBGCP2/TUBGCP3/TUBGCP5/UBR4/UNC119/USP2/USP20/UXT/WRAP7[illegible]	73	CC
GO:0098552	side of membrane	75/2879	482/18511	0.51809	0.9845	0.97291	ABL1/AJAP1/ALCAM/ANPEP/BCAP31/BLK/CA4/CD27/CD5/CD69/CD74/CD79B/CDH13/CDH5/CDK16/CSK/CUBN/CXCR3/CXCR5/DAB2IP/DLG4/ENG/FASLG/FCGR3A/FCRL6/FERMT2/FES/FGA/FOLR2/GLRA1/GNAO1/GNAT1/GNG3/GPIHBP1/GRAP/GSR/HLA-E/IKBKB/IL13/IL31RA/ITGAM/KCNJ5/LCK/LY6G5B/MCAM/MYZAP/NRG1/PDCD1/PTK2B/PTPN7/RASA3/RASA4B/RBSN/RNF31/RTN4RL1/RTN4RL2/S100A6/SDC1/SELP/SEMA7A/SERPINA5/SLC2A4/SPPL2C/SPTB/TEC/TFR2/TH/THADA/THY1/TLR8/TNFRSF13B/TNFRSF18/TNK1/TNK2/TRAF2	75	CC
GO:0099572	postsynaptic specialization	32/2879	205/18511	0.52094	0.9845	0.97291	ADGRB1/ARC/ARRB2/ATP1A1/CDK5/CHRM1/CLSTN1/CLSTN3/CNIH2/CRYAB/CTNNA2/DAB1/DGKI/DISC1/DLG4/DRD2/DTNBP1/GNG3/GPER1/GRIN1/GRIN2C/HIP1R/HOMER2/NSMF/PDLIM5/PSD/PTK2B/RGS14/SCRIB/SH	32	CC
GO:1902562	H4 histone acetyltransferase complex	6/2879	37/18511	0.52493	0.9845	0.97291	EPC1/HCFC1/KAT2A/MSL3/POLE4/TADA3	6	CC
GO:0005844	polysome	8/2879	50/18511	0.52501	0.9845	0.97291	DIS3L2/EIF2AK4/EIF4B/EIF4G1/LARP6/PIWIL2/RPL7/RWDD1	8	CC

GO:0009897	external side of plasma membrane	42/2879	270/18511	0.52573	0.9845	0.97291	ALCAM/ANPEP/CA4/CD27/CD5/CD69/CD74/CD79B/CDH13/CDH5/CUBN/CXCR3/CXCR5/ENG/FASLG/FCGR3A/FCRL6/FGA/FOLR2/GLRA1/GPIHBP1/GSR/IL13/IL31RA/ITGAM/KCNJ5/LY6G5B/MCAM/NRG1/PDCD1/RTN4RL1/RTN4RL2/SDC1/SELP/SEMA7A/SERPINA5/SLC2A4/TFR2/THY1/TLR8/TNF	42	CC
GO:0098984	neuron to neuron synapse	33/2879	212/18511	0.52735	0.9845	0.97291	ADGRB1/ARC/ARRB2/ATP1A1/CDK5/CHRM1/CLSTN1/CLSTN3/CNIH2/CRYAB/CTNNA2/DAB1/DGKI/DISC1/DLG4/DRD2/DTNBP1/GNG3/GPER1/GRIN1/GRIN2C/HIP1R/HOMER2/NLGN3/NSMF/PDLIM5/PSD/PTK2B/RGS14/SCRIB/SHARPIN/SIGMAR1/STX1A	33	CC
GO:0002178	palmitoyltransferase complex	2/2879	11/18511	0.52877	0.9845	0.97291	ORMDL3/SPTLC1	2	CC
GO:0005744	mitochondrial inner membrane presequence translocase complex	2/2879	11/18511	0.52877	0.9845	0.97291	TIMM17B/TIMM23B	2	CC
GO:0005890	sodium:potassium-exchanging ATPase	2/2879	11/18511	0.52877	0.9845	0.97291	ATP1A1/ATP1A4	2	CC
GO:0019031	viral envelope	2/2879	11/18511	0.52877	0.9845	0.97291	ERV3-1/ERVFRD-1	2	CC
GO:0030688	preribosome, small subunit	2/2879	11/18511	0.52877	0.9845	0.97291	NOB1/TSR1	2	CC
GO:0030877	beta-catenin destruction complex	2/2879	11/18511	0.52877	0.9845	0.97291	AXIN1/CACYBP	2	CC
GO:0031332	RNAi effector complex	2/2879	11/18511	0.52877	0.9845	0.97291	SND1/TARBP2	2	CC
GO:0036338	viral membrane	2/2879	11/18511	0.52877	0.9845	0.97291	ERV3-1/ERVFRD-1	2	CC

GO:0045263	proton-transporting ATP synthase complex, coupling factor	2/2879	11/18511	0.52877	0.9845	0.97291	ATP5G1/ATP5I	2	CC
GO:0097038	perinuclear endoplasmic reticulum	2/2879	11/18511	0.52877	0.9845	0.97291	BCAP31/OSBPL7	2	CC
GO:0097542	ciliary tip	7/2879	44/18511	0.53775	0.9985	0.98677	DYNLL2/IFT140/IFT20/IFT22/SMO/SUFU/TRAF3IP1	7	CC
GO:0032391	photoreceptor connecting	5/2879	31/18511	0.54041	0.9998	0.98803	GNAT1/IFT140/IFT20/KIF17/MYO7A	5	CC
GO:0005766	primary lysosome	24/2879	155/18511	0.54385	0.9998	0.98803	ACPP/ARL8A/C3AR1/CD63/CD68/DDOST/DPP7/DYNC1H1/GGH/HEXA/IMPDH1/MGST1/MPO/NPC2/ORM2/PRCP/PRDX6/PRKCD/PSAP/RAB44/SERPINA3/STK11IP/TRAPPC1/VAT1	24	CC
GO:0042582	azurophil granule	24/2879	155/18511	0.54385	0.9998	0.98803	ACPP/ARL8A/C3AR1/CD63/CD68/DDOST/DPP7/DYNC1H1/GGH/HEXA/IMPDH1/MGST1/MPO/NPC2/ORM2/PRCP/PRDX6/PRKCD/PSAP/RAB44/SERPINA3/STK11IP/TRAPPC1/VAT1	24	CC
GO:0005666	DNA-directed RNA polymerase III	3/2879	18/18511	0.54669	0.9998	0.98803	POLR1D/POLR2L/POLR3D	3	CC
GO:0030120	vesicle coat	8/2879	51/18511	0.54887	0.9998	0.98803	AP1G2/AP1S1/AP2A2/AP2M1/CLTB/COPZ1/EGFR/TMED3	8	CC
GO:0002080	acrosomal membrane	4/2879	25/18511	0.55967	0.9998	0.98803	ACRBP/CCDC136/SERPINA5/SPACA3	4	CC
GO:0097431	mitotic spindle pole	4/2879	25/18511	0.55967	0.9998	0.98803	EML1/HSF1/MAD1L1/NUMA1	4	CC
GO:0005758	mitochondrial intermembrane space	12/2879	78/18511	0.56455	0.9998	0.98803	ARL2BP/CHCHD5/CIAPIN1/COA4/GFER/HSD3B1/MYOC/NME4/PRELID1/SHMT2/TIMM9/TRAP1	12	CC

GO:0000407	pre-autophagosomal structure	5/2879	32/18511	0.57027	0.9998	0.98803	ATG101/ATG2A/RAB7A/ULK1/ZFYVE1	5	CC
GO:1902554	serine/threonine protein kinase complex	13/2879	85/18511	0.57246	0.9998	0.98803	CCND3/CDK4/CDK9/ENG/GTF2H2C/GTF2H4/HOXC10/IKBKB/PHKG1/PYDC1/TAF7/TRIM40/ULK1	13	CC
GO:0045335	phagocytic vesicle	16/2879	105/18511	0.57712	0.9998	0.98803	ADAM8/AMBRA1/ATP6V0B/ATP6V0E2/CORO1A/FMNL1/HLA-E/ITGB5/NCF1/RAB20/RAB34/RAB7A/SLC11A1/STX4/TAP1/TLR9	16	CC
GO:0005833	hemoglobin complex	2/2879	12/18511	0.57786	0.9998	0.98803	AHSP/HBZ	2	CC
GO:0008540	proteasome regulatory particle, base	2/2879	12/18511	0.57786	0.9998	0.98803	PSMC3/PSMD5	2	CC
GO:0030914	STAGA complex	2/2879	12/18511	0.57786	0.9998	0.98803	KAT2A/TADA3	2	CC
GO:0031462	Cul2-RING ubiquitin ligase complex	2/2879	12/18511	0.57786	0.9998	0.98803	COMMD1/ZYG11A	2	CC
GO:0033391	chromatoid body	2/2879	12/18511	0.57786	0.9998	0.98803	PIWIL2/TDRD1	2	CC
GO:0042622	photoreceptor outer segment membrane	2/2879	12/18511	0.57786	0.9998	0.98803	CDHR1/GNAT1	2	CC
GO:0043194	axon initial segment	2/2879	12/18511	0.57786	0.9998	0.98803	BIN1/TRIM46	2	CC
GO:0016327	apicolateral plasma	3/2879	19/18511	0.58522	0.9998	0.98803	CLDN4/CLDN6/TMEM114	3	CC
GO:0043034	costamere	3/2879	19/18511	0.58522	0.9998	0.98803	AHNAK/FLNC/ILK	3	CC
GO:0031970	organelle envelope lumen	13/2879	86/18511	0.59042	0.9998	0.98803	ARL2BP/CACYBP/CHCHD5/CIAPIN1/COA4/GFER/HSD3B1/MYOC/NME4/PRELID1/SHMT2/TIMM9/TRAP1	13	CC

GO:1990752	microtubule end	4/2879	26/18511	0.59234	0.9998	0.98803	CKAP5/MAPRE3/NCKAP5L/NUMA1	4	CC
GO:0045211	postsynaptic membrane	34/2879	225/18511	0.60145	0.9998	0.98803	ADGRB1/APBB1/ARC/ARRB2/CDK5/CHRM1/CHRNA6/CHRNB1/CHRNB3/CLSTN1/CLSTN3/CNIH2/DISC1/DLG4/DNAJA3/DTNBP1/GABBR1/GLRA1/GRASP/GRIA3/GRIN1/GRIN2C/HOMER2/HTR3B/LRRTM1/NLGN3/NSMF/OPRD1/PDLIM5/PRR7/RGS14/SCRIB/SIGMAR1/TMUB1	34	CC
GO:0000152	nuclear ubiquitin ligase	6/2879	40/18511	0.60546	0.9998	0.98803	ANAPC15/ANAPC2/BRCA1/BRCC3/BUB1B/PCGF2	6	CC
GO:0055038	recycling endosome membrane	10/2879	67/18511	0.60826	0.9998	0.98803	ACAP1/BOK/EHD1/GRIPAP1/HLA-E/RAB11FIP3/RAB17/SCAMP2/VTI1B/ZFYVE27	10	CC
GO:0005942	phosphatidylinositol 3-kinase complex	3/2879	20/18511	0.62157	0.9998	0.98803	MTOR/PIK3R5/PIK3R6	3	CC
GO:0034361	very-low-density	3/2879	20/18511	0.62157	0.9998	0.98803	APOA5/APOB/APOC3	3	CC
GO:0034385	triglyceride-rich plasma lipoprotein	3/2879	20/18511	0.62157	0.9998	0.98803	APOA5/APOB/APOC3	3	CC
GO:0098644	complex of collagen trimers	3/2879	20/18511	0.62157	0.9998	0.98803	COL1A1/COL4A2/COL7A1	3	CC
GO:0000153	cytoplasmic ubiquitin ligase complex	2/2879	13/18511	0.62309	0.9998	0.98803	OS9/SYVN1	2	CC
GO:0000176	nuclear exosome	2/2879	13/18511	0.62309	0.9998	0.98803	EXOSC10/EXOSC2	2	CC
GO:0000177	cytoplasmic exosome (RNase	2/2879	13/18511	0.62309	0.9998	0.98803	DIS3L/EXOSC2	2	CC
GO:0000782	telomere cap complex	2/2879	13/18511	0.62309	0.9998	0.98803	ACD/TINF2	2	CC

GO:0000783	nuclear telomere cap	2/2879	13/18511	0.62309	0.9998	0.98803	ACD/TINF2	2	CC
GO:0005614	interstitial matrix	2/2879	13/18511	0.62309	0.9998	0.98803	C6orf15/TNC	2	CC
GO:0005675	holo TFIIH complex	2/2879	13/18511	0.62309	0.9998	0.98803	GTF2H2C/GTF2H4	2	CC
GO:0016580	Sin3 complex	2/2879	13/18511	0.62309	0.9998	0.98803	NCOR1/PHF12	2	CC
GO:0030126	COPI vesicle coat	2/2879	13/18511	0.62309	0.9998	0.98803	COPZ1/TMED3	2	CC
GO:0031045	dense core granule	2/2879	13/18511	0.62309	0.9998	0.98803	ADAM8/VPS13A	2	CC
GO:0043240	Fanconi anaemia nuclear	2/2879	13/18511	0.62309	0.9998	0.98803	FAAP20/FANCA	2	CC
GO:0055037	recycling endosome	24/2879	161/18511	0.62327	0.9998	0.98803	ACAP1/BOK/COMMD1/DYNC1I1/EHD1/GPER1/GRIPAP1/HLA-E/INPP5F/NISCH/RAB11FIP3/RAB17/SCAMP2/SNF8/STX8/TMUB1/TNIK/TUBG1/ULK1/VPS51/VPS52/VPS53/VTI1B/ZFYVE27	24	CC
GO:0032281	AMPA glutamate	4/2879	27/18511	0.62353	0.9998	0.98803	CNIH2/DLG4/GRIA3/PORCN	4	CC
GO:0034364	high-density lipoprotein	4/2879	27/18511	0.62353	0.9998	0.98803	APOA5/APOC3/CLU/GPIHBP1	4	CC
GO:0033267	axon part	27/2879	181/18511	0.62526	0.9998	0.98803	AP1S1/APBB1/BIN1/BOC/CNGB1/CPLX1/DLG4/DNM1/DTNBP1/GRIN1/HSPB1/ILK/KIF4A/L1CAM/MAPK8IP1/MYOC/NRG1/PARD3/PTPRN2/RAB7A/STXBP1/SV2A/TH/THY1/TRIM46/TRPV2/VAMP1	27	CC
GO:0072562	blood microparticle	27/2879	181/18511	0.62526	0.9998	0.98803	ACTA1/AGT/AHSG/APOA5/C1QB/C1QC/C8A/CD5L/CFH/CIB2/CLIC1/CLU/CPN2/ENG/FGA/FN1/GRIPAP1/HSPA2/ITIH4/KNG1/MIR503/ORM1/ORM2/PFN1/SERPINA3/SERPINF2/TMPRSS13	27	CC
GO:0044291	cell-cell contact zone	10/2879	68/18511	0.62784	0.9998	0.98803	AHNAK/AJAP1/ATP1A1/CAV3/DES/FGFRL1/FHOD1/OBSL1/SCRIB/VAMP5	10	CC

GO:0097060	synaptic membrane	44/2879	295/18511	0.64433	0.9998	0.98803	ADGRB1/APBB1/ARC/ARRB2/CDK5/CHRM1/CHRNA6/CHRNB1/CHRNB3/CLSTN1/CLSTN3/CNIH2/CRYAB/DGKI/DISC1/DLG4/DNAJA3/DTNBP1/FOSL1/GABBR1/GLRA1/GPER1/GRASP/GRIA3/GRIN1/GRIN2C/HIP1R/HOMER2/HTR3B/IL31RA/LRRTM1/NLGN3/NSMF/OPRD1/PDE2A/PDLIM5/PRR7/RGS14/SCRIB/SIGMAR1/STX1A/STXBP1/TMEM240/TMUB1	44	CC
GO:0030175	filopodium	14/2879	96/18511	0.64663	0.9998	0.98803	ACPP/ACTA1/APBB1/CDK5/CIB1/DYNC1H1/FAT1/FSCN1/FZD9/INPPL1/MORN4/MYO3A/PPP1R9B/TRPV4	14	CC
GO:0044853	plasma membrane raft	14/2879	96/18511	0.64663	0.9998	0.98803	ADRA1A/ADRA1B/ATP1A1/CAV3/CDH13/CDH15/FASLG/ITGAM/MAPK3/MYO1A/NOS3/PRKAR1A/SCARB1/SMO	14	CC
GO:0005901	caveola	11/2879	76/18511	0.6509	0.9998	0.98803	ADRA1A/ADRA1B/ATP1A1/CAV3/CDH13/CDH15/FASLG/MAPK3/NOS3/SCARB1/SMO	11	CC
GO:0000922	spindle pole	22/2879	150/18511	0.65252	0.9998	0.98803	ANKRD53/BRCC3/CDC14A/CEP19/CKAP5/DYNC1I1/EMD/EML1/GOLGA2/HSF1/KATNB1/MAD1L1/NUBP2/NUMA1/PLEKHG6/RGS14/TUBG1/TUBGCP2/TUBGCP3/TUBGCP5/UNC119/UXT	22	CC
GO:0017053	transcriptional repressor	12/2879	83/18511	0.65483	0.9998	0.98803	CDX2/CTBP1/LIN52/NCOR1/NCOR2/PASD1/PHF12/RCOR2/SPEN/SUV39H1/TBX15/TNRC18	12	CC
GO:0000159	protein phosphatase type 2A	3/2879	21/18511	0.65569	0.9998	0.98803	PPP2R2B/PPP2R3B/PPP2R5B	3	CC
GO:0005680	anaphase-promoting complex	3/2879	21/18511	0.65569	0.9998	0.98803	ANAPC15/ANAPC2/BUB1B	3	CC
GO:0005697	telomerase holoenzyme complex	3/2879	21/18511	0.65569	0.9998	0.98803	NHP2/NVL/SMG5	3	CC
GO:0032809	neuronal cell body membrane	3/2879	21/18511	0.65569	0.9998	0.98803	AMIGO1/DAB2IP/THY1	3	CC
GO:0035145	exon-exon junction	3/2879	21/18511	0.65569	0.9998	0.98803	CASC3/R3HCC1/RNPS1	3	CC
GO:0044298	cell body membrane	3/2879	21/18511	0.65569	0.9998	0.98803	AMIGO1/DAB2IP/THY1	3	CC

GO:0030136	clathrin-coated vesicle	26/2879	177/18511	0.65655	0.9998	0.98803	AP1G2/AP1S1/AP2A2/AP2M1/APOB/AVPR2/BCAP31/CD74/CLTB/DVL2/EGFR/FURIN/FZD2/GAD1/HIP1R/INPP5F/LRP1/MYO1E/SFTPA1/SFTPB/SLC2A4/SLC2A8/STEAP2/STON2/TNK2/VWF	26	CC
GO:0005882	intermediate filament	29/2879	197/18511	0.65697	0.9998	0.98803	DES/FAM83H/GPER1/KRT12/KRT14/KRT16/KRT23/KRT3/KRT32/KRT6A/KRT6C/KRT71/KRT75/KRT76/KRT79/KRT81/KRT84/KRT85/KRTAP17-1/KRTAP4-3/KRTAP5-10/KRTAP5-3/KRTAP5-5/KRTAP5-7/KRTAP5-	29	CC
GO:0034708	methyltransferase complex	13/2879	90/18511	0.65868	0.9998	0.98803	ASH2L/CHD8/ERH/HCFC1/MAX/MEN1/PAXIP1/PHF19/SENP3/SETD1A/TAF1/TAF6/TAF7	13	CC
GO:0044304	main axon	9/2879	63/18511	0.66239	0.9998	0.98803	APBB1/BIN1/DLG4/DNM1/MYOC/NRG1/PARD3/THY1/TRIM46	9	CC
GO:0098573	intrinsic component of mitochondrial membrane	9/2879	63/18511	0.66239	0.9998	0.98803	COA3/COQ2/MFN1/MFN2/MPC1/RHOT2/TIMM17B/TIMM23B/TMEM11	9	CC
GO:0005814	centriole	18/2879	124/18511	0.66301	0.9998	0.98803	ACTR1A/C2CD3/CCDC146/CCHCR1/CCNF/CEP164/CEP19/CROCC/FOPNL/IFT20/NEURL4/NUBP1/NUBP2/PARP3/SDCCAG8/TUBG1/TUBGCP3/WRAP	18	CC
GO:0005751	mitochondrial respiratory chain complex	2/2879	14/18511	0.66446	0.9998	0.98803	COX8A/NDUFA4L2	2	CC
GO:0005767	secondary lysosome	2/2879	14/18511	0.66446	0.9998	0.98803	ADAM8/NCF1	2	CC
GO:0010369	chromocenter	2/2879	14/18511	0.66446	0.9998	0.98803	PIWIL2/TINF2	2	CC
GO:0016528	sarcoplasm	10/2879	70/18511	0.6654	0.9998	0.98803	ATP2A3/CAMK2D/DTNBP1/FLNC/GSTM2/JPH3/RASD1/TMEM109/TRDN/XDH	10	CC
GO:0030027	lamellipodium	27/2879	185/18511	0.67184	0.9998	0.98803	ACTA1/APBB1/BRK1/CDK5/CFL1/CIB1/CORO1A/CORO1B/CTNNA2/CTTN/DGKZ/EVL/FAM89B/FAT1/FERMT2/FSCN1/ILK/INPPL1/LIMK1/NRBP1/PLEKHG5/PLXND1/PPP1R9B/PTK2B/SCRIB/STX4/TRPV4	27	CC
GO:0035578	azurophil granule lumen	13/2879	91/18511	0.67473	0.9998	0.98803	DPP7/DYNC1H1/GGH/IMPDH1/MPO/NPC2/ORM2/PRDX6/PRKCD/SERPINA3/STK11IP/TRAPPC1/VAT1	13	CC

GO:0005881	cytoplasmic microtubule	7/2879	50/18511	0.6784	0.9998	0.98803	BCAS3/NUMA1/REEP2/TRPV4/TUBG1/TUBG2/TUBGCP2	7	CC
GO:0070069	cytochrome complex	4/2879	29/18511	0.68117	0.9998	0.98803	COX10/COX8A/CYC1/NDUFA4L2	4	CC
GO:0071556	integral component of lumenal side of endoplasmic reticulum	4/2879	29/18511	0.68117	0.9998	0.98803	BCAP31/CD74/HLA-E/SPPL2C	4	CC
GO:0097526	spliceosomal tri-snRNP	4/2879	29/18511	0.68117	0.9998	0.98803	EFTUD2/PRPF4/PRPF8/SART1	4	CC
GO:0098553	lumenal side of endoplasmic reticulum membrane	4/2879	29/18511	0.68117	0.9998	0.98803	BCAP31/CD74/HLA-E/SPPL2C	4	CC
GO:0005791	rough endoplasmic	10/2879	71/18511	0.68333	0.9998	0.98803	BCAP31/BGLAP/CA4/CDKAL1/MYOC/NCF1/PI4KB/PLOD3/RPN1/SCGB1A1	10	CC
GO:0001669	acrosomal vesicle	16/2879	112/18511	0.68403	0.9998	0.98803	ACRBP/ARC/CAPN11/CAPZA3/CCDC136/DRD2/LOXL1/NOTCH1/NUDT1/PRSS37/SERPINA5/SPACA3/SPINK2/TCP11/TSSK1B/VDAC2	16	CC
GO:0005868	cytoplasmic dynein complex	3/2879	22/18511	0.68753	0.9998	0.98803	DYNC1H1/DYNC1I1/DYNLL2	3	CC
GO:0031258	lamellipodium membrane	3/2879	22/18511	0.68753	0.9998	0.98803	CFL1/FERMT2/PLXND1	3	CC
GO:0032982	myosin filament	3/2879	22/18511	0.68753	0.9998	0.98803	MYBPC1/MYBPC3/MYBPH	3	CC
GO:0042629	mast cell granule	3/2879	22/18511	0.68753	0.9998	0.98803	CHGA/CXCR2/NPPA	3	CC
GO:0097346	INO80-type complex	3/2879	22/18511	0.68753	0.9998	0.98803	INO80/NFRKB/ZNHIT1	3	CC

GO:0030669	clathrin-coated endocytic vesicle	7/2879	51/18511	0.69901	0.9998	0.98803	AP1S1/AP2A2/AP2M1/APOB/CD74/EGFR/FZD2	7	CC
GO:0032838	cell projection cytoplasm	9/2879	65/18511	0.69959	0.9998	0.98803	DLG4/DTNBP1/HIP1R/HSPB1/KIF17/KIF4A/OPRM1/TRIM46/WLS	9	CC
GO:0030670	phagocytic vesicle	10/2879	72/18511	0.70067	0.9998	0.98803	ATP6V0B/ATP6V0E2/CORO1A/HLA-E/RAB20/RAB34/RAB7A/SLC11A1/STX4/TAP1	10	CC
GO:0030285	integral component of synaptic vesicle	2/2879	15/18511	0.70209	0.9998	0.98803	SV2A/TMEM163	2	CC
GO:0031011	Ino80 complex	2/2879	15/18511	0.70209	0.9998	0.98803	INO80/NFRKB	2	CC
GO:0033202	DNA helicase complex	2/2879	15/18511	0.70209	0.9998	0.98803	INO80/NFRKB	2	CC
GO:0033268	node of Ranvier	2/2879	15/18511	0.70209	0.9998	0.98803	BIN1/MYOC	2	CC
GO:0035859	Seh1-associated complex	2/2879	15/18511	0.70209	0.9998	0.98803	NPRL3/SESN1	2	CC
GO:0090533	cation-transporting	2/2879	15/18511	0.70209	0.9998	0.98803	ATP1A1/ATP1A4	2	CC
GO:0043198	dendritic shaft	5/2879	37/18511	0.70255	0.9998	0.98803	CNIH2/FLNA/GPER1/ILK/KIRREL3	5	CC
GO:0030135	coated vesicle	38/2879	262/18511	0.70689	0.9998	0.98803	ACTR1A/AP1G2/AP1S1/AP2A2/AP2M1/APOB/AVPR2/BCAP31/CD74/CLTB/CNIH2/COL7A1/COPZ1/DVL2/EGFR/FURIN/FZD2/GAD1/GOLGA2/GOSR2/HIP1R/HLA-E/INPP5F/LRP1/MYO1E/PACS1/SCAP/SFTPA1/SFTPB/SLC2A4/SLC2A8/ST	38	CC
GO:0043601	nuclear replisome	4/2879	30/18511	0.70757	0.9998	0.98803	POLA2/POLD4/POLE/POLE4	4	CC
GO:0001891	phagocytic cup	3/2879	23/18511	0.7171	0.9998	0.98803	ADGRB1/BIN2/CORO1A	3	CC

GO:0030867	rough endoplasmic reticulum	3/2879	23/18511	0.7171	0.9998	0.98803	BCAP31/PI4KB/PLOD3	3	CC
GO:0031306	intrinsic component of mitochondrial outer membrane	3/2879	23/18511	0.7171	0.9998	0.98803	MFN1/MFN2/RHOT2	3	CC
GO:0035327	transcriptionally active	3/2879	23/18511	0.7171	0.9998	0.98803	BCAS3/EXOSC10/SUPT6H	3	CC
GO:0005876	spindle microtubule	7/2879	52/18511	0.71873	0.9998	0.98803	KIF4A/NCOR1/NEIL2/NUMA1/TUBG1/TUBG2/TUBGCP3	7	CC
GO:0032154	cleavage furrow	7/2879	52/18511	0.71873	0.9998	0.98803	HMCN2/MEN1/PITPNM1/PLEKHG6/PSD/PSD2/RAB11FIP3	7	CC
GO:0097610	cell surface furrow	7/2879	52/18511	0.71873	0.9998	0.98803	HMCN2/MEN1/PITPNM1/PLEKHG6/PSD/PSD2/RAB11FIP3	7	CC
GO:0044452	nucleolar part	25/2879	176/18511	0.72074	0.9998	0.98803	CDCA7L/DEAF1/ESRRA/FBLIM1/FHIT/MAP3K14/NHP2/NOL11/NOL6/ORC6/PEX14/POLR1A/POLR1D/POLR1E/POLR2L/PSPC1/RPAIN/RPP21/SELENBP1/SESN1/SURF6/TAF1A/TAX1BP3/TXNRD1/ZNRD1	25	CC
GO:0031461	cullin-RING ubiquitin ligase complex	25/2879	177/18511	0.7313	0.9998	0.98803	ABTB1/ANAPC15/ANAPC2/BUB1B/CACYBP/CCNF/COMMD1/DCAF11/DCAF8/DDB1/DDB2/FBXL19/FBXL22/FBXO2/FBXO25/FBXO6/FBXW4/IPP/KBTBD2/KCTD5/KLHL29/KLHL35/KLHL40/SPOP/ZYG11A	25	CC
GO:0005921	gap junction	4/2879	31/18511	0.73233	0.9998	0.98803	DBN1/GJB3/GJD4/MIP	4	CC
GO:0030672	synaptic vesicle membrane	11/2879	81/18511	0.73346	0.9998	0.98803	ATP6V1B1/DBNDD1/DOC2A/DRD2/DTNBP1/PTPRN2/SLC30A3/STX1A/SV2A/TMEM163/VAMP1	11	CC
GO:0099501	exocytic vesicle membrane	11/2879	81/18511	0.73346	0.9998	0.98803	ATP6V1B1/DBNDD1/DOC2A/DRD2/DTNBP1/PTPRN2/SLC30A3/STX1A/SV2A/TMEM163/VAMP1	11	CC
GO:0005639	integral component of nuclear inner	2/2879	16/18511	0.73613	0.9998	0.98803	LEMD2/P2RX2	2	CC

GO:0019012	virion	2/2879	16/18511	0.73613	0.9998	0.98803	ERV3-1/ERVFRD-1	2	CC
GO:0030663	COPI-coated vesicle	2/2879	16/18511	0.73613	0.9998	0.98803	COPZ1/TMED3	2	CC
GO:0030673	axolemma	2/2879	16/18511	0.73613	0.9998	0.98803	NRG1/THY1	2	CC
GO:0031229	intrinsic component of nuclear inner membrane	2/2879	16/18511	0.73613	0.9998	0.98803	LEMD2/P2RX2	2	CC
GO:0044423	virion part	2/2879	16/18511	0.73613	0.9998	0.98803	ERV3-1/ERVFRD-1	2	CC
GO:0045277	respiratory chain complex	2/2879	16/18511	0.73613	0.9998	0.98803	COX8A/NDUFA4L2	2	CC
GO:0005770	late endosome	33/2879	232/18511	0.73932	0.9998	0.98803	ANKRD13A/ARL8A/ATP13A2/CD300LG/CD63/CD74/CHID1/CHMP4C/CTSH/CYB561A3/EGFR/GOSR2/KCNQ1/MAPK3/RAB7A/SDF4/SFTPB/SLC11A1/SLC29A3/SLC2A4/SLC30A3/SNF8/STARD3/STOML1/STX8/TMEM9/TSPAN15/VAMP5/VPS37B/VPS37C/VPS37D/VTI1B/YIPF1	33	CC
GO:0030119	AP-type membrane coat adaptor	6/2879	46/18511	0.74113	0.9998	0.98803	AP1G2/AP1S1/AP2A2/AP2M1/AP4S1/EGFR	6	CC
GO:0030687	preribosome, large subunit	3/2879	24/18511	0.74446	0.9998	0.98803	MDN1/NOC2L/ZNF593	3	CC
GO:0033017	sarcoplasmic reticulum membrane	5/2879	39/18511	0.74672	0.9998	0.98803	ATP2A3/CAMK2D/JPH3/TMEM109/TRDN	5	CC
GO:0034358	plasma lipoprotein	5/2879	39/18511	0.74672	0.9998	0.98803	APOA5/APOB/APOC3/CLU/GPIHBP1	5	CC
GO:1990777	lipoprotein particle	5/2879	39/18511	0.74672	0.9998	0.98803	APOA5/APOB/APOC3/CLU/GPIHBP1	5	CC

GO:0045111	intermediate filament cytoskeleton	33/2879	233/18511	0.74823	0.9998	0.98803	BCAS3/DES/FAM83H/GPER1/INO80/KRT12/KRT14/KRT16/KRT23/KRT3/KRT32/KRT6A/KRT6C/KRT71/KRT75/KRT76/KRT79/KRT81/KRT84/KRT85/KRTAP17-1/KRTAP4-3/KRTAP5-10/KRTAP5-3/KRTAP5-5/KRTAP5-7/KRTAP5-9/LMNA/LMNB1/MDN1/NES/NME1/PADI6	33	CC
GO:0016529	sarcoplasmic reticulum	8/2879	61/18511	0.75235	0.9998	0.98803	ATP2A3/CAMK2D/GSTM2/JPH3/RASD1/TMEM109/TRDN/XDH	8	CC
GO:0030894	replisome	4/2879	32/18511	0.75548	0.9998	0.98803	POLA2/POLD4/POLE/POLE4	4	CC
GO:0080008	Cul4-RING E3 ubiquitin ligase complex	4/2879	32/18511	0.75548	0.9998	0.98803	DCAF11/DCAF8/DDB1/DDB2	4	CC
GO:0005852	eukaryotic translation initiation factor	2/2879	17/18511	0.76679	0.9998	0.98803	EIF3B/EIF3I	2	CC
GO:0005858	axonemal dynein complex	2/2879	17/18511	0.76679	0.9998	0.98803	DNAH17/DNHD1	2	CC
GO:0033178	proton-transporting two-sector ATPase complex,	2/2879	17/18511	0.76679	0.9998	0.98803	ATP6V1B1/ATP6V1F	2	CC
GO:0044453	nuclear membrane part	2/2879	17/18511	0.76679	0.9998	0.98803	LEMD2/P2RX2	2	CC
GO:0032592	integral component of mitochondrial	8/2879	62/18511	0.76858	0.9998	0.98803	COA3/COQ2/MFN1/MPC1/RHOT2/TIMM17B/TIMM23B/TMEM11	8	CC
GO:0005719	nuclear euchromatin	3/2879	25/18511	0.76966	0.9998	0.98803	ASH2L/NSMF/SETD1A	3	CC
GO:0034705	potassium channel	13/2879	98/18511	0.77426	0.9998	0.98803	AMIGO1/CTTN/DLG4/DPP6/KCND1/KCND3/KCNH4/KCNJ5/KCNK4/KCNQ1/KCNQ4/KCNT1/STX1A	13	CC

GO:0005819	spindle	45/2879	318/18511	0.77844	0.9998	0.98803	ANKRD53/ARL2BP/ARL8A/BRCC3/BUB1B/CDC14A/CEP19/CKAP5/CTTN/DYNC1I1/E4F1/EMD/EML1/FAM96B/GOLGA2/HAUS4/HAUS7/HSF1/HSPA2/HSPB1/KAT2A/KATNB1/KIF23/KIF2B/KIF4A/MAD1L1/MAEA/MAP7D1/NCOR1/NEIL2/NUBP2/NUMA1/PLEKHG6/PPP2R3C/RGS14/SHCBP1/SLC25A5/TADA3/TUBG1/TUBG2/TUBGCP2/TUBGCP3/TUBGCP5/UNC119/UXT	45	CC
GO:0000307	cyclin-dependent protein kinase holoenzyme	5/2879	41/18511	0.78583	0.9998	0.98803	CCND3/CDK4/CDK9/HOXC10/TAF7	5	CC
GO:0000803	sex chromosome	3/2879	26/18511	0.79279	0.9998	0.98803	DNMT3A/PCGF2/UBE2B	3	CC
GO:0044322	endoplasmic reticulum quality control compartment	3/2879	26/18511	0.79279	0.9998	0.98803	FBXO6/OS9/SYVN1	3	CC
GO:0099738	cell cortex region	3/2879	26/18511	0.79279	0.9998	0.98803	ACTR1A/EPB41L2/NUMA1	3	CC
GO:0046930	pore complex	2/2879	18/18511	0.79431	0.9998	0.98803	C8A/VDAC2	2	CC
GO:0042734	presynaptic membrane	9/2879	71/18511	0.79473	0.9998	0.98803	APBB1/FOSL1/GABBR1/GPER1/IL31RA/PDE2A/SCRIB/STX1A/STXBP1	9	CC
GO:0030118	clathrin coat	6/2879	49/18511	0.79492	0.9998	0.98803	AP1G2/AP1S1/AP2A2/AP2M1/CLTB/EGFR	6	CC
GO:0005911	cell-cell junction	60/2879	423/18511	0.80222	0.9998	0.98803	ADGRB1/AHNAK/AJAP1/AMTN/ATP1A1/BAIAP2L1/CAV3/CDH24/CDH5/CDK4/CFL1/CLDN15/CLDN2/CLDN23/CLDN4/CLDN6/COL17A1/CORO1A/CSK/CTNNA2/DBN1/DES/FAT1/FAT2/FGFR4/FGFRL1/FHOD1/FLNA/FSCN1/GJB3/GJD4/ILK/KIFC3/MARVELD2/MARVELD3/MIP/MYO1E/NCR3/OBSL1/PARD3/PARD6A/PCDH12/PCDHGA12/PDLIM1/PDLIM5/PDLIM7/PDZD3/PKP3/PLEKHG5/PRKCD/PTPRU/SCRIB/SDCCAG8/SV2A/TJAP1/TLN1/VAM[illegible]	60	CC
GO:0000795	synaptonemal complex	5/2879	42/18511	0.80355	0.9998	0.98803	BRCA1/FKBP6/HORMAD1/HSPA2/RNF212	5	CC

GO:0099086	synaptonemal structure	5/2879	42/18511	0.80355	0.9998	0.98803	BRCA1/FKBP6/HORMAD1/HSPA2/RNF212	5	CC
GO:0098794	postsynapse	60/2879	424/18511	0.80791	0.9998	0.98803	ABL1/ADGRB1/APBB1/ARC/ARRB2/ASIC2/ATP1A1/CDK5/CHRM1/CHRNA6/CHRNB1/CHRNB3/CLSTN1/CLSTN3/CNIH2/CRYAB/CTNNA2/CTTN/DAB1/DGKI/DISC1/DLG4/DNAJA3/DRD2/DTNBP1/FBXO2/GABBR1/GLRA1/GNG3/GPER1/GRASP/GRIA3/GRIN1/GRIN2C/GRIPAP1/HIP1R/HOMER2/HTR3B/LAMA2/LRRTM1/NLGN3/NSMF/OPRD1/OPRM1/P2RX2/PDLIM5/PPP1R9B/PRR7/PSD/PTK2B/RGS10/RGS14/SCRIB/SHARPIN/SIGMAR1/SLC29A1 [illegible]	60	CC
GO:0015935	small ribosomal subunit	9/2879	72/18511	0.80817	0.9998	0.98803	EIF4G1/MRPS10/MRPS11/MRPS18A/MRPS18B/MRPS21/MRPS33/MRPS5/RPS24	9	CC
GO:0014704	intercalated disc	6/2879	50/18511	0.81081	0.9998	0.98803	ATP1A1/CAV3/DES/FHOD1/OBSL1/VAMP5	6	CC
GO:0008076	voltage-gated potassium channel	12/2879	94/18511	0.81163	0.9998	0.98803	AMIGO1/CTTN/DLG4/DPP6/KCND1/KCND3/KCNH4/KCNJ5/KCNQ1/KCNQ4/KCNT1/STX1A	12	CC
GO:0001527	microfibril	1/2879	10/18511	0.81565	0.9998	0.98803	MFAP1	1	CC
GO:0005916	fascia adherens	1/2879	10/18511	0.81565	0.9998	0.98803	DES	1	CC
GO:0030681	multimeric ribonuclease P complex	1/2879	10/18511	0.81565	0.9998	0.98803	RPP21	1	CC
GO:0031080	nuclear pore outer ring	1/2879	10/18511	0.81565	0.9998	0.98803	NUP98	1	CC
GO:0031414	N-terminal protein acetyltransferas	1/2879	10/18511	0.81565	0.9998	0.98803	NAA16	1	CC
GO:0032591	dendritic spine membrane	1/2879	10/18511	0.81565	0.9998	0.98803	GPER1	1	CC
GO:0044224	juxtaparanode region of axon	1/2879	10/18511	0.81565	0.9998	0.98803	DLG4	1	CC

GO:0072546	ER membrane protein complex	1/2879	10/18511	0.81565	0.9998	0.98803	EMC8	1	CC
GO:0097539	ciliary transition fiber	1/2879	10/18511	0.81565	0.9998	0.98803	CEP164	1	CC
GO:0005720	nuclear heterochromatin	4/2879	35/18511	0.81565	0.9998	0.98803	DNMT3A/MPHOSPH8/NCAPD3/PCGF2	4	CC
GO:0008180	COP9 signalosome	4/2879	35/18511	0.81565	0.9998	0.98803	COPS6/DOCK7/EPB41L2/HSPA5	4	CC
GO:0005721	pericentric heterochromatin	2/2879	19/18511	0.81891	0.9998	0.98803	BAZ1B/NCAPD3	2	CC
GO:0035267	NuA4 histone acetyltransferase complex	2/2879	19/18511	0.81891	0.9998	0.98803	EPC1/MSL3	2	CC
GO:0043189	H4/H2A histone acetyltransferase complex	2/2879	19/18511	0.81891	0.9998	0.98803	EPC1/MSL3	2	CC
GO:0071682	endocytic vesicle lumen	2/2879	19/18511	0.81891	0.9998	0.98803	APOB/HSP90AA1	2	CC
GO:0000794	condensed nuclear	12/2879	95/18511	0.82279	0.9998	0.98803	BRCA1/BUB1B/CDX2/CHMP1A/FKBP6/HORMAD1/HSPA2/NCAPD3/NOL6/RNF212/SUV39H1/TUBG1	12	CC
GO:0005776	autophagosome	11/2879	88/18511	0.82551	0.9998	0.98803	AMBRA1/ATP13A2/DAPK2/OSBPL7/RAB24/RAB7A/RALB/TBC1D25/TECPR1/ULK1/ZFYVE1	11	CC
GO:0072686	mitotic spindle	10/2879	81/18511	0.82888	0.9998	0.98803	CDC14A/CTTN/EML1/GOLGA2/HSF1/KAT2A/KIF23/MAD1L1/NUMA1/TADA3	10	CC
GO:0012507	ER to Golgi transport vesicle membrane	7/2879	59/18511	0.83187	0.9998	0.98803	CD74/CNIH2/GOSR2/HLA-E/SCAP/STX5/VTI1B	7	CC
GO:0090734	site of DNA damage	4/2879	36/18511	0.83279	0.9998	0.98803	ATF2/PARP3/PARP9/SLF2	4	CC
GO:0030684	preribosome	9/2879	74/18511	0.83304	0.9998	0.98803	FCF1/MDN1/NOB1/NOC2L/NOL6/PDCD11/SRFBP1/TSR1/ZNF593	9	CC

GO:0043596	nuclear replication fork	5/2879	44/18511	0.83547	0.9998	0.98803	BAZ1B/POLA2/POLD4/POLE/POLE4	5	CC
GO:0005685	U1 snRNP	2/2879	20/18511	0.84083	0.9998	0.98803	LUC7L/RBPMS	2	CC
GO:0005922	connexin complex	2/2879	20/18511	0.84083	0.9998	0.98803	GJB3/GJD4	2	CC
GO:0071782	endoplasmic reticulum tubular network	2/2879	20/18511	0.84083	0.9998	0.98803	EMD/ZFYVE27	2	CC
GO:0016605	PML body	12/2879	97/18511	0.84361	0.9998	0.98803	ANKRD2/CDK9/CHFR/CIART/CIITA/HSF1/LRCH4/MAX/MTOR/PATL1/RGS14/RPAIN	12	CC
GO:0000778	condensed nuclear chromosome	1/2879	11/18511	0.84433	0.9998	0.98803	BUB1B	1	CC
GO:0031010	ISWI-type complex	1/2879	11/18511	0.84433	0.9998	0.98803	RSF1	1	CC
GO:0035253	ciliary rootlet	1/2879	11/18511	0.84433	0.9998	0.98803	CROCC	1	CC
GO:0061700	GATOR2 complex	1/2879	11/18511	0.84433	0.9998	0.98803	SESN1	1	CC
GO:0000118	histone deacetylase complex	7/2879	60/18511	0.84462	0.9998	0.98803	BRMS1/HDAC11/HDAC8/HINT1/NCOR1/NCOR2/PHF12	7	CC
GO:0030658	transport vesicle membrane	24/2879	183/18511	0.84585	0.9998	0.98803	AP1G2/AP1S1/ATP6V1B1/CA4/CD74/CHGA/CLTB/CNIH2/DBNDD1/DOC2A/DRD2/DTNBP1/GOSR2/HLA-E/PTPRN2/RAB26/SCAP/SLC30A3/STX1A/STX5/SV2A/TMEM163/VAMP1/	24	CC
GO:0000123	histone acetyltransferase complex	10/2879	83/18511	0.85068	0.9998	0.98803	BRPF1/EPC1/HCFC1/ING4/KAT2A/MSL3/POLE4/TADA3/TAF6/TAF7	10	CC
GO:1990204	oxidoreductase complex	12/2879	98/18511	0.85328	0.9998	0.98803	BCKDK/CYC1/DLST/FOXRED1/NCF1/NDUFA2/NDUFB5/NOX4/NOXO1/OGDH/OGDHL/PDHB	12	CC

GO:0098797	plasma membrane protein complex	32/2879	241/18511	0.85862	0.9998	0.98803	ABCA2/ABCG5/ADAM8/AP2A2/AP2M1/APH1A/C8A/CASP3/CAV3/CD74/CD79B/CLTB/EGFR/EMILIN1/EVC2/FZD8/GJB3/GJD4/GNAO1/GNAT1/GNG3/HLA-	32	CC
GO:0031082	BLOC complex	2/2879	21/18511	0.86032	0.9998	0.98803	DTNBP1/HPS1	2	CC
GO:0034706	sodium channel complex	2/2879	21/18511	0.86032	0.9998	0.98803	SCN11A/SCNN1A	2	CC
GO:0030665	clathrin-coated vesicle	13/2879	107/18511	0.86807	0.9998	0.98803	AP1G2/AP1S1/AP2A2/AP2M1/APOB/AVPR2/CD74/CLTB/EGFR/FZD2/GAD1/HIP1R/SLC2A8	13	CC
GO:0000124	SAGA complex	1/2879	12/18511	0.86856	0.9998	0.98803	TADA3	1	CC
GO:0000164	protein phosphatase type 1 complex	1/2879	12/18511	0.86856	0.9998	0.98803	PPP1R9B	1	CC
GO:0000940	condensed chromosome outer	1/2879	12/18511	0.86856	0.9998	0.98803	BUB1B	1	CC
GO:0005583	fibrillar collagen trimer	1/2879	12/18511	0.86856	0.9998	0.98803	COL1A1	1	CC
GO:0005750	mitochondrial respiratory chain complex	1/2879	12/18511	0.86856	0.9998	0.98803	CYC1	1	CC
GO:0005832	chaperonin-containing T-complex	1/2879	12/18511	0.86856	0.9998	0.98803	CCT3	1	CC
GO:0031464	Cul4A-RING E3 ubiquitin ligase complex	1/2879	12/18511	0.86856	0.9998	0.98803	DDB1	1	CC
GO:0031597	cytosolic proteasome complex	1/2879	12/18511	0.86856	0.9998	0.98803	PSMC3	1	CC

GO:0032797	SMN complex	1/2879	12/18511	0.86856	0.9998	0.98803	ZPR1	1	CC
GO:0033643	host cell part	1/2879	12/18511	0.86856	0.9998	0.98803	AQP1	1	CC
GO:0034709	methylosome	1/2879	12/18511	0.86856	0.9998	0.98803	ERH	1	CC
GO:0035102	PRC1 complex	1/2879	12/18511	0.86856	0.9998	0.98803	PCGF2	1	CC
GO:0036513	Derlin-1 retrotranslocation complex	1/2879	12/18511	0.86856	0.9998	0.98803	SYVN1	1	CC
GO:0045275	respiratory chain complex	1/2879	12/18511	0.86856	0.9998	0.98803	CYC1	1	CC
GO:0060198	clathrin-sculpted vesicle	1/2879	12/18511	0.86856	0.9998	0.98803	GAD1	1	CC
GO:0070971	endoplasmic reticulum exit	1/2879	12/18511	0.86856	0.9998	0.98803	APOB	1	CC
GO:0097504	Gemini of coiled bodies	1/2879	12/18511	0.86856	0.9998	0.98803	ZPR1	1	CC
GO:0098643	banded collagen fibril	1/2879	12/18511	0.86856	0.9998	0.98803	COL1A1	1	CC
GO:0015934	large ribosomal subunit	14/2879	115/18511	0.87344	0.9998	0.98803	C12orf65/MRPL10/MRPL14/MRPL28/MRPL36/MRPL37/MRPL43/MRPL52/MRPL9/RPL35A/RPL36A/RPL41/RPL7/SURF6	14	CC
GO:0031248	protein acetyltransferase complex	11/2879	93/18511	0.87491	0.9998	0.98803	BRPF1/EPC1/HCFC1/ING4/KAT2A/MSL3/NAA16/POLE4/TADA3/TAF6/TAF7	11	CC
GO:1902493	acetyltransferase complex	11/2879	93/18511	0.87491	0.9998	0.98803	BRPF1/EPC1/HCFC1/ING4/KAT2A/MSL3/NAA16/POLE4/TADA3/TAF6/TAF7	11	CC
GO:0016234	inclusion body	9/2879	78/18511	0.87517	0.9998	0.98803	CLU/DVL2/GPX1/HERPUD1/HOXD3/HSPB7/NUP98/SERGEF/SNCB	9	CC

GO:0030134	COPII-coated ER to Golgi transport vesicle	9/2879	78/18511	0.87517	0.9998	0.98803	CD74/CNIH2/COL7A1/GOLGA2/GOSR2/HLA-E/SCAP/STX5/VTI1B	9	CC
GO:0016235	aggresome	4/2879	39/18511	0.87638	0.9998	0.98803	DVL2/HOXD3/HSPB7/SERGEF	4	CC
GO:1904115	axon cytoplasm	4/2879	39/18511	0.87638	0.9998	0.98803	DTNBP1/HSPB1/KIF4A/TRIM46	4	CC
GO:0031307	integral component of mitochondrial	2/2879	22/18511	0.8776	0.9998	0.98803	MFN1/RHOT2	2	CC
GO:0097228	sperm principal piece	2/2879	22/18511	0.8776	0.9998	0.98803	PFKM/PGAM4	2	CC
GO:0098563	intrinsic component of synaptic vesicle membrane	2/2879	22/18511	0.8776	0.9998	0.98803	SV2A/TMEM163	2	CC
GO:0000421	autophagosome membrane	3/2879	31/18511	0.88109	0.9998	0.98803	RAB7A/TECPR1/ULK1	3	CC
GO:0030686	90S preribosome	3/2879	31/18511	0.88109	0.9998	0.98803	NOC2L/NOL6/SRFBP1	3	CC
GO:0000788	nuclear nucleosome	4/2879	40/18511	0.88854	0.9998	0.98803	HIST1H4F/HIST2H4A/HIST2H4B/MPHOSPH8	4	CC
GO:0030677	ribonuclease P complex	1/2879	13/18511	0.88902	0.9998	0.98803	RPP21	1	CC
GO:0032300	mismatch repair complex	1/2879	13/18511	0.88902	0.9998	0.98803	PMS2P3	1	CC
GO:0042405	nuclear inclusion body	1/2879	13/18511	0.88902	0.9998	0.98803	NUP98	1	CC
GO:0101031	chaperone complex	1/2879	13/18511	0.88902	0.9998	0.98803	CCT3	1	CC

GO:0090543	Flemming body	2/2879	23/18511	0.89289	0.9998	0.98803	CHMP4C/KIF23	2	CC
GO:0030662	coated vesicle membrane	21/2879	169/18511	0.89407	0.9998	0.98803	AP1G2/AP1S1/AP2A2/AP2M1/APOB/AVPR2/CD74/CLTB/CNIH2/COPZ1/EGFR/FZD2/GAD1/GOSR2/HIP1R/HLA-E/SCAP/SLC2A8/STX5/TMED3/VTI1B	21	CC
GO:0000790	nuclear chromatin	45/2879	340/18511	0.89921	0.9998	0.98803	ASH2L/BRMS1/CALCOCO1/CCAR2/DNMT3A/E2F4/EHMT2/HIST1H2AH/HIST1H4F/HIST2H4A/HIST2H4B/INO80/IPO4/KAT2A/LDB1/MAX/MEN1/MPHOSPH8/MUC1/MYOD1/NCAPD3/NCOR1/NCOR2/NFRKB/NR1H3/NSMF/PCGF2/PHF12/PHOX2A/PHOX2B/POLR3D/PPARD/RARA/RSF1/SETD1A/SMARCD2/SPI1/STAT1/TAF1/TIMELESS/UBE2A/UBE2B/UBE2U/ZNF385A/ZNHI	45	CC
GO:0005840	ribosome	30/2879	235/18511	0.9018	0.9998	0.98803	ABCF1/APEX1/C12orf65/EIF2AK4/EIF4G1/METTL17/MRPL10/MRPL14/MRPL28/MRPL36/MRPL37/MRPL43/MRPL52/MRPL57/MRPL9/MRPS10/MRPS11/MRPS18A/MRPS18B/MRPS21/MRPS33/MRPS5/NLE1/RPL35A/RPL36A/RPL41/RPL7/RPS24/SF1/SURF6	30	CC
GO:0044391	ribosomal subunit	23/2879	185/18511	0.90256	0.9998	0.98803	C12orf65/EIF4G1/MRPL10/MRPL14/MRPL28/MRPL36/MRPL37/MRPL43/MRPL52/MRPL9/MRPS10/MRPS11/MRPS18A/MRPS18B/MRPS21/MRPS33/MRPS5/RPL35A/RPL36A/RPL41/RPL7/RPS24/SURF6	23	CC
GO:0005637	nuclear inner membrane	6/2879	58/18511	0.90543	0.9998	0.98803	EMD/LEMD2/LMNB1/NRM/P2RX2/SIGMAR1	6	CC
GO:0001518	voltage-gated sodium channel complex	1/2879	14/18511	0.90629	0.9998	0.98803	SCN11A	1	CC
GO:0005845	mRNA cap binding	1/2879	14/18511	0.90629	0.9998	0.98803	LSM1	1	CC
GO:0030897	HOPS complex	1/2879	14/18511	0.90629	0.9998	0.98803	AKTIP	1	CC
GO:0034362	low-density lipoprotein	1/2879	14/18511	0.90629	0.9998	0.98803	APOB	1	CC
GO:0098799	outer mitochondrial membrane protein complex	1/2879	14/18511	0.90629	0.9998	0.98803	MFN1	1	CC

GO:0008287	protein serine/threonine phosphatase complex	5/2879	50/18511	0.90657	0.9998	0.98803	PPP1R10/PPP1R9B/PPP2R2B/PPP2R3B/PPP2R5B	5	CC
GO:1903293	phosphatase complex	5/2879	50/18511	0.90657	0.9998	0.98803	PPP1R10/PPP1R9B/PPP2R2B/PPP2R3B/PPP2R5B	5	CC
GO:0005769	early endosome	43/2879	329/18511	0.91077	0.9998	0.98803	APOB/APOC3/BOK/CHMP1A/COMMD1/CORO1A/EGFR/EHD1/EPHA8/F2RL1/GPER1/GRIPAP1/HAVCR2/HLA-E/INPP5F/KCNQ1/KDR/KREMEN2/LAMP3/LLGL1/LRP1/MAPK3/MIB2/NIPA2/NISCH/PLA2G4B/PTP4A3/PTPN23/RAB17/RAB20/RABGAP1L/RBSN/RHOD/RIN3/SAMD9L/SNX12/STEAP2/STX8/TMEM163/TRIM3/VTI1B/WLS/W	43	CC
GO:0001650	fibrillar center	15/2879	128/18511	0.91132	0.9998	0.98803	CDCA7L/DEAF1/ESRRA/FBLIM1/FHIT/MAP3K14/ORC6/PEX14/POLR1E/PSPC1/RPAIN/SELENBP1/SESN1/TAX1BP3/TXNRD1	15	CC
GO:0098800	inner mitochondrial membrane	14/2879	121/18511	0.9141	0.9998	0.98803	ATP5G1/ATP5I/ATPIF1/C14orf2/COX8A/CYC1/FOXRED1/NDUFA2/NDUFA4L2/NDUFB5/TIMM17B/TIMM22/TIMM23B/TIMM9	14	CC
GO:0032040	small-subunit processome	3/2879	34/18511	0.91631	0.9998	0.98803	FCF1/NOL6/PDCD11	3	CC
GO:0035861	site of double-strand break	3/2879	34/18511	0.91631	0.9998	0.98803	ATF2/PARP3/SLF2	3	CC
GO:0042611	MHC protein complex	2/2879	25/18511	0.91827	0.9998	0.98803	CD74/HLA-E	2	CC
GO:0016514	SWI/SNF complex	1/2879	15/18511	0.92087	0.9998	0.98803	SMARCD2	1	CC
GO:0045120	pronucleus	1/2879	15/18511	0.92087	0.9998	0.98803	HSF1	1	CC
GO:0030496	midbody	19/2879	160/18511	0.92322	0.9998	0.98803	ALKBH4/ARL2BP/ARL8A/CHMP4C/DTNBP1/ERH/HSPA5/KATNB1/KIF13A/KIF23/KIF4A/MAPRE3/PITPNM1/RAB11FIP3/RALB/SEPT1/SHCBP1/VPS37	19	CC
GO:1990391	DNA repair complex	4/2879	44/18511	0.92731	0.9998	0.98803	PMS2P3/POLE/POLE4/XPC	4	CC

GO:0031901	early endosome membrane	15/2879	132/18511	0.93193	0.9998	0.98803	BOK/EGFR/EHD1/EPHA8/GRIPAP1/HLA-E/INPP5F/KREMEN2/LLGL1/PLA2G4B/RBSN/TMEM163/VTI1B/WLS/WNT3	15	CC
GO:0030014	CCR4-NOT complex	1/2879	16/18511	0.93319	0.9998	0.98803	PATL1	1	CC
GO:0031083	BLOC-1 complex	1/2879	16/18511	0.93319	0.9998	0.98803	DTNBP1	1	CC
GO:0034518	RNA cap binding	1/2879	16/18511	0.93319	0.9998	0.98803	LSM1	1	CC
GO:0042613	MHC class II protein complex	1/2879	16/18511	0.93319	0.9998	0.98803	CD74	1	CC
GO:0071437	invadopodium	1/2879	16/18511	0.93319	0.9998	0.98803	FSCN1	1	CC
GO:0031463	Cul3-RING ubiquitin ligase complex	7/2879	70/18511	0.93376	0.9998	0.98803	IPP/KBTBD2/KCTD5/KLHL29/KLHL35/KLHL40/SPOP	7	CC
GO:0005834	heterotrimeric G-protein	3/2879	36/18511	0.9342	0.9998	0.98803	GNAO1/GNAT1/GNG3	3	CC
GO:1905360	GTPase complex	3/2879	36/18511	0.9342	0.9998	0.98803	GNAO1/GNAT1/GNG3	3	CC
GO:0097525	spliceosomal snRNP complex	6/2879	62/18511	0.93503	0.9998	0.98803	EFTUD2/LUC7L/PRPF4/PRPF8/RBPMS/SART1	6	CC
GO:0005689	U12-type spliceosomal complex	2/2879	27/18511	0.93791	0.9998	0.98803	SF3B4/SNRNP25	2	CC
GO:0030117	membrane coat	10/2879	95/18511	0.9392	0.9998	0.98803	AP1G2/AP1S1/AP2A2/AP2M1/AP4S1/CLTB/COPZ1/DNM1/EGFR/TMED3	10	CC
GO:0048475	coated membrane	10/2879	95/18511	0.9392	0.9998	0.98803	AP1G2/AP1S1/AP2A2/AP2M1/AP4S1/CLTB/COPZ1/DNM1/EGFR/TMED3	10	CC
GO:0000777	condensed chromosome kinetochore	11/2879	103/18511	0.93991	0.9998	0.98803	BOD1/BUB1B/CENPN/CKAP5/DYNC1I1/HJURP/HSF1/KIF2B/MAD1L1/PHF2/PMF1	11	CC

GO:0000800	lateral element	1/2879	17/18511	0.94359	0.9998	0.98803	BRCA1	1	CC
GO:0008074	guanylate cyclase	1/2879	17/18511	0.94359	0.9998	0.98803	GUCY2D	1	CC
GO:0031527	filopodium membrane	1/2879	17/18511	0.94359	0.9998	0.98803	FZD9	1	CC
GO:0071004	U2-type prespliceosome	1/2879	17/18511	0.94359	0.9998	0.98803	LUC7L	1	CC
GO:0001533	cornified envelope	6/2879	64/18511	0.94648	0.9998	0.98803	C1orf68/EVPLL/LCE3E/PKP3/SPRR1A/TGM1	6	CC
GO:0044445	cytosolic part	29/2879	240/18511	0.94711	0.9998	0.98803	AHSP/BORCS6/CASP1/CASP5/CCT3/DTNBP1/EIF2AK4/EIF4G1/FAM96B/GSDMD/GUCY2D/HBZ/HPS1/IKBKB/MRPS18A/NARFL/NLRP1/PFKFB1/PFKM/PSMC3/PYDC1/RPL35A/RPL36A/RPL41/RPL7/RPS24/SURF6/TRIM40/UB	29	CC
GO:0008023	transcription elongation factor complex	5/2879	56/18511	0.94922	0.9998	0.98803	CDK9/NELFB/SUPT6H/TAF7/TTF2	5	CC
GO:0098798	mitochondrial protein complex	16/2879	144/18511	0.94956	0.9998	0.98803	ATP5G1/ATP5I/ATPIF1/BCKDK/C14orf2/COX8A/CYC1/FOXRED1/MFN1/NDUFA2/NDUFA4L2/NDUFB5/TIMM17B/TIMM22/TIMM23B/TIMM9	16	CC
GO:0035098	ESC/E(Z) complex	1/2879	18/18511	0.95237	0.9998	0.98803	PHF19	1	CC
GO:1902555	endoribonuclease complex	2/2879	29/18511	0.95301	0.9998	0.98803	RPP21/TARBP2	2	CC
GO:1905348	endonuclease complex	2/2879	29/18511	0.95301	0.9998	0.98803	RPP21/TARBP2	2	CC
GO:0000932	P-body	8/2879	83/18511	0.95716	0.9998	0.98803	DIS3L2/LSM1/MEX3B/PAN2/PATL1/POLR2G/PSMC3/RBPMS	8	CC
GO:0005643	nuclear pore	7/2879	75/18511	0.95838	0.9998	0.98803	EIF5A/IPO4/MAD1L1/MYO1C/NUP210/NUP98/XPO7	7	CC
GO:0000779	condensed chromosome, centromeric	12/2879	116/18511	0.95976	0.9998	0.98803	BOD1/BUB1B/CENPN/CKAP5/DYNC1I1/HJURP/HSF1/KIF2B/MAD1L1/NCAPD3/PHF2/PMF1	12	CC

GO:0000784	nuclear chromosome, telomeric region	14/2879	132/18511	0.96058	0.9998	0.98803	ACD/APEX1/DDB1/HIST1H4F/HIST2H4A/HIST2H4B/MCM2/MCM4/MEN1/NHP2/PIF1/PPP1R10/TINF2/XRCC3	14	CC
GO:0018995	host	6/2879	68/18511	0.96411	0.9998	0.98803	AQP1/NUP210/NUP98/VPS37B/VPS37C/VPS37D	6	CC
GO:0043657	host cell	6/2879	68/18511	0.96411	0.9998	0.98803	AQP1/NUP210/NUP98/VPS37B/VPS37C/VPS37D	6	CC
GO:0000145	exocyst	1/2879	20/18511	0.96605	0.9998	0.98803	RALB	1	CC
GO:1904949	ATPase complex	9/2879	94/18511	0.96656	0.9998	0.98803	ABCA2/ABCG5/ATP1A1/ATP1A4/INO80/NFRKB/RSF1/SMARCD2/ZNHIT1	9	CC
GO:0044455	mitochondrial membrane part	22/2879	196/18511	0.96694	0.9998	0.98803	ATP5G1/ATP5I/ATPIF1/C14orf2/COA3/COQ2/COX8A/CYC1/DUSP21/FOXRED1/MFN1/MFN2/MPC1/NDUFA2/NDUFA4L2/NDUFB5/RHOT2/TIMM17B/TIMM22/TIMM23B/TIMM9/TMEM11	22	CC
GO:0030532	small nuclear ribonucleoprotein complex	6/2879	69/18511	0.96759	0.9998	0.98803	EFTUD2/LUC7L/PRPF4/PRPF8/RBPMS/SART1	6	CC
GO:0022625	cytosolic large ribosomal	5/2879	61/18511	0.97029	0.9998	0.98803	RPL35A/RPL36A/RPL41/RPL7/SURF6	5	CC
GO:0005923	bicellular tight junction	12/2879	120/18511	0.9708	0.9998	0.98803	CDH5/CDK4/CLDN15/CLDN2/CLDN23/CLDN4/CLDN6/MARVELD2/MARVELD3/PARD3/PARD6A/TJAP1	12	CC
GO:0016010	dystrophin-associated glycoprotein complex	1/2879	21/18511	0.97133	0.9998	0.98803	CAV3	1	CC
GO:0030904	retromer complex	1/2879	21/18511	0.97133	0.9998	0.98803	RAB7A	1	CC
GO:0090665	glycoprotein complex	1/2879	21/18511	0.97133	0.9998	0.98803	CAV3	1	CC
GO:0043296	apical junction complex	14/2879	137/18511	0.97307	0.9998	0.98803	CDH5/CDK4/CLDN15/CLDN2/CLDN23/CLDN4/CLDN6/KIFC3/MARVELD2/MARVELD3/PARD3/PARD6A/PDZD3/TJAP1	14	CC

GO:0036464	cytoplasmic ribonucleoprotein granule	20/2879	185/18511	0.97512	0.9998	0.98803	ATXN2L/CDK9/CTSH/DDX4/DIS3L2/DYNC1I1/LSM1/MEX3B/PAN2/PATL1/PITPNM1/PIWIL2/POLR2G/PSMC3/RBM4/RBPMS/SMG5/TDRD1/TDRD9/TSTD1	20	CC
GO:0000780	condensed nuclear chromosome,	1/2879	22/18511	0.9758	0.9998	0.98803	BUB1B	1	CC
GO:0005682	U5 snRNP	1/2879	22/18511	0.9758	0.9998	0.98803	PRPF8	1	CC
GO:0071010	prespliceosome	1/2879	22/18511	0.9758	0.9998	0.98803	LUC7L	1	CC
GO:0005747	mitochondrial respiratory chain complex I	3/2879	44/18511	0.97588	0.9998	0.98803	FOXRED1/NDUFA2/NDUFB5	3	CC
GO:0010494	cytoplasmic stress granule	3/2879	44/18511	0.97588	0.9998	0.98803	ATXN2L/RBM4/RBPMS	3	CC
GO:0030964	NADH dehydrogenase complex	3/2879	44/18511	0.97588	0.9998	0.98803	FOXRED1/NDUFA2/NDUFB5	3	CC
GO:0045271	respiratory chain complex I	3/2879	44/18511	0.97588	0.9998	0.98803	FOXRED1/NDUFA2/NDUFB5	3	CC
GO:0016607	nuclear speck	45/2879	372/18511	0.97614	0.9998	0.98803	ALKBH5/APBB1/APEX1/ARL6IP4/ATXN2L/CASC3/CEP164/CWC15/CWC25/DGKQ/DGKZ/EFTUD2/EHMT2/FNBP4/FNDC1/FOXO4/GTF2H2C/GTF2H4/HOXB2/LIMK1/LMNA/MBD4/NONO/OGG1/PASD1/PATL1/POLR3D/PPIE/PRCC/PRPF4/PRPF8/PSPC1/RAF1/RBM4/REXO4/RNPS1/SART1/SETD1A/SGK1/SNURF/SPOP/SRRM2/SRSF4/ZC3H13/ZC3H18	45	CC
GO:0070160	occluding junction	12/2879	123/18511	0.97721	0.9998	0.98803	CDH5/CDK4/CLDN15/CLDN2/CLDN23/CLDN4/CLDN6/MARVELD2/MARVELD3/PARD3/PARD6A/TJAP1	12	CC
GO:0022627	cytosolic small ribosomal	3/2879	45/18511	0.97881	0.9998	0.98803	EIF4G1/MRPS18A/RPS24	3	CC
GO:0035770	ribonucleoprotein granule	21/2879	196/18511	0.98014	0.9998	0.98803	ATXN2L/CDK9/CTSH/DDX4/DIS3L2/DYNC1I1/HSF1/LSM1/MEX3B/PAN2/PATL1/PITPNM1/PIWIL2/POLR2G/PSMC3/RBM4/RBPMS/SMG5/TDRD1/TD	21	CC

GO:0000776	kinetochore	13/2879	133/18511	0.9805	0.9998	0.98803	BOD1/BUB1B/CENPN/CKAP5/DCTN2/DYNC1I1/HJURP/HSF1/KIF2B/MAD1L1/NUP98/PHF2/PMF1	13	CC
GO:0044215	other organism	6/2879	74/18511	0.9808	0.9998	0.98803	AQP1/NUP210/NUP98/VPS37B/VPS37C/VPS37D	6	CC
GO:0044216	other organism cell	6/2879	74/18511	0.9808	0.9998	0.98803	AQP1/NUP210/NUP98/VPS37B/VPS37C/VPS37D	6	CC
GO:0044217	other organism part	6/2879	74/18511	0.9808	0.9998	0.98803	AQP1/NUP210/NUP98/VPS37B/VPS37C/VPS37D	6	CC
GO:0098803	respiratory chain complex	6/2879	74/18511	0.9808	0.9998	0.98803	COX8A/CYC1/FOXRED1/NDUFA2/NDUFA4L2/NDUFB5	6	CC
GO:0030057	desmosome	1/2879	25/18511	0.98543	0.9998	0.98803	PKP3	1	CC
GO:0000793	condensed chromosome	23/2879	216/18511	0.98544	0.9998	0.98803	BAZ1B/BOD1/BRCA1/BUB1B/CDX2/CENPN/CHMP1A/CKAP5/DYNC1I1/FKBP6/HJURP/HORMAD1/HSF1/HSPA2/KIF2B/MAD1L1/NCAPD3/NOL6/PHF2/PMF1/RNF212/SUV39H1/TUBG1	23	CC
GO:0005732	small nucleolar ribonucleoprotein complex	1/2879	26/18511	0.9877	0.9998	0.98803	NHP2	1	CC
GO:0070469	respiratory chain	7/2879	88/18511	0.98873	0.9998	0.98803	COX8A/CYC1/FOXRED1/NDUFA2/NDUFA4L2/NDUFB5/NDUFC2-KCTD14	7	CC
GO:0071013	catalytic step 2 spliceosome	8/2879	97/18511	0.98884	0.9998	0.98803	CWC15/DHX38/EFTUD2/PPIE/PRPF8/SART1/SRRM2/ZCCHC8	8	CC
GO:0070603	SWI/SNF superfamily-type complex	5/2879	70/18511	0.98927	0.9998	0.98803	INO80/NFRKB/RSF1/SMARCD2/ZNHIT1	5	CC
GO:0000781	chromosome, telomeric region	16/2879	167/18511	0.99111	0.9998	0.98803	ACD/APEX1/DDB1/HIST1H4F/HIST2H4A/HIST2H4B/MCM2/MCM4/MEN1/NHP2/PIF1/PPP1R10/RECQL4/TELO2/TINF2/XRCC3	16	CC
GO:0005746	mitochondrial respiratory	6/2879	82/18511	0.99201	0.9998	0.98803	COX8A/CYC1/FOXRED1/NDUFA2/NDUFA4L2/NDUFB5	6	CC
GO:0022626	cytosolic ribosome	9/2879	113/18511	0.99458	0.9998	0.98803	EIF2AK4/EIF4G1/MRPS18A/RPL35A/RPL36A/RPL41/RPL7/RPS24/SURF6	9	CC

GO:0000775	chromosome, centromeric	18/2879	191/18511	0.99524	0.9998	0.98803	BAZ1B/BOD1/BUB1B/CENPN/CKAP5/DCTN2/DNMT3A/DYNC1I1/HJURP/HSF1/KIF2B/MAD1L1/NCAPD3/NUP98/PDS5A/PHF2/PMF1/SUV39H1	18	CC
GO:0005681	spliceosomal complex	17/2879	184/18511	0.99571	0.9998	0.98803	CCAR2/CWC15/DHX38/EFTUD2/HNRNPH3/LGALS3/LUC7L/PPIE/PRPF4/PRPF8/SART1/SF1/SF3B4/SNRNP25/SRRM2/TTF2/ZCCHC8	17	CC
GO:0031519	PcG protein complex	2/2879	46/18511	0.99606	0.9998	0.98803	PCGF2/PHF19	2	CC
GO:0005684	U2-type spliceosomal complex	1/2879	35/18511	0.99732	0.9998	0.98803	LUC7L	1	CC
GO:0044815	DNA packaging complex	8/2879	112/18511	0.99771	0.9998	0.98803	H1FX/HILS1/HIST1H2AH/HIST1H4F/HIST2H4A/HIST2H4B/MPHOSPH8/NCAPD3	8	CC
GO:0032993	protein-DNA complex	18/2879	201/18511	0.99795	0.9998	0.98803	ACD/EYA1/H1FX/HILS1/HIST1H2AH/HIST1H4F/HIST2H4A/HIST2H4B/MAX/MPHOSPH8/NKX2-5/NUPR1/POLA2/POLD4/POLE/POLE4/TINF2/ZBTB17	18	CC
GO:0000786	nucleosome	7/2879	105/18511	0.99828	0.9998	0.98803	H1FX/HILS1/HIST1H2AH/HIST1H4F/HIST2H4A/HIST2H4B/MPHOSPH8	7	CC
GO:0098687	chromosomal region	35/2879	352/18511	0.99923	0.9998	0.98803	ACD/APEX1/BAZ1B/BOD1/BUB1B/CENPN/CKAP5/DCTN2/DDB1/DNMT3A/DYNC1I1/HIST1H4F/HIST2H4A/HIST2H4B/HJURP/HSF1/KIF2B/MAD1L1/MCM2/MCM4/MEN1/NCAPD3/NHP2/NUP98/PDS5A/PHF2/PIF1/PIWIL2/PMF1/PPP1R10/RECQL4/SUV39H1/TELO2/TINF2/XRCC3	35	CC
GO:0019814	immunoglobulin complex	1/2879	49/18511	0.99975	0.9998	0.98803	CD79B	1	CC
GO:0051015	actin filament binding	43/2726	156/17354	0.00011	0.0619	0.06088	ABL1/ARPC1A/ARPC1B/ARPC2/ARPC4/BIN1/CFL1/CORO1A/CORO1B/CORO6/CTNNA2/EGFR/ESPN/ESPNL/FLII/FLNA/FLNC/FMNL1/FSCN1/HIP1R/IGSF22/MYBPC1/MYBPC3/MYBPH/MYBPHL/MYL4/MYO18A/MYO1A/MYO1C/MYO1E/MYO7A/OBSL1/PKNOX2/SCIN/SHROOM1/SPTB/TAGLN3/TLN1/TNNC1/TPM2/TRPV4/TULP1/WDR1	43	MF
GO:0016597	amino acid binding	21/2726	58/17354	0.00011	0.0619	0.06088	AARS/AARS2/ALAS2/DDC/GAD1/GCHFR/GLRA1/GLUD1/GNMT/GOT2/GRIN1/KARS/NAGS/NOS3/OTC/PADI4/SESN1/SHMT2/TH/THNSL2/YARS2	21	MF

GO:0003779	actin binding	85/2726	403/17354	0.00228	0.4741	0.46665	ABL1/ABLIM2/ALDOA/ALKBH4/ANG/ARPC1A/ARPC1B/ARPC2/ARPC4/BAIAP2L1/BCL7B/BIN1/CAPZA3/CFL1/CORO1A/CORO1B/CORO6/COTL1/CROCC/CTNNA2/DBN1/EGFR/EMD/EPB41L2/EPS8L2/ESPN/ESPNL/EVL/FBXO25/FHL3/FHOD1/FLII/FLNA/FLNC/FMNL1/FMNL2/FSCN1/HIP1R/HOMER2/IGSF22/INO80/INPPL1/IPP/LMOD1/MAEA/MARCKSL1/MIB2/MICAL2/MLPH/MOBP/MYBPC1/MYBPC3/MYBPH/MYBPHL/MYL4/MYO18A/MYO19/MYO1A/MYO1C/MYO1E/MYO3A/MYO7A/MYO7B/NOS3/OBSL1/PDLIM5/PFN1/PHACTR1/PKNOX2/PPP1R9B/SCIN/SHROOM1/SPTAN1/SPTB/SPTBN2/TAGLN3/TLN1/TNNC1/TPM2/TRPV4/TULP1/WAS/WDR1/WHAMM/XIR[illegible]	85	MF
GO:0097493	structural molecule activity	8/2726	17/17354	0.00234	0.4741	0.46665	AHNAK/EMILIN1/IGSF22/MYBPC1/MYBPC3/MYBPH/MYBPHL/OBSL1	8	MF
GO:0031432	titin binding	7/2726	14/17354	0.00289	0.4741	0.46665	ANKRD2/CAMK2D/MYBPC1/MYBPC3/OBSCN/TCAP/TRIM63	7	MF
GO:1901505	carbohydrate derivative transmembrane transporter	12/2726	33/17354	0.00308	0.4741	0.46665	SLC25A25/SLC25A5/SLC28A1/SLC28A2/SLC29A1/SLC29A2/SLC29A3/SLC29A4/SLC35A2/SLC35C1/SLC35D2/SLC50A1	12	MF
GO:0030548	acetylcholine receptor regulator	6/2726	11/17354	0.00337	0.4741	0.46665	CDK5/FNTA/LYNX1/LYPD1/LYPD6B/SLURP1	6	MF
GO:0099602	neurotransmitter receptor regulator	6/2726	11/17354	0.00337	0.4741	0.46665	CDK5/FNTA/LYNX1/LYPD1/LYPD6B/SLURP1	6	MF
GO:0015294	solute:cation symporter activity	28/2726	108/17354	0.00419	0.5227	0.51447	SLC12A4/SLC12A7/SLC12A9/SLC13A2/SLC15A3/SLC15A4/SLC1A7/SLC20A1/SLC20A2/SLC25A22/SLC28A1/SLC28A2/SLC2A6/SLC2A8/SLC34A1/SLC35A2/SLC36A1/SLC36A3/SLC38A1/SLC45A4/SLC4A9/SLC5A2/SLC6A18/SLC6A2/SLC6A6/SLC6A7/SLC6A8/SLC6A9	28	MF

GO:0004714	transmembrane receptor protein tyrosine kinase activity	18/2726	62/17354	0.00571	0.5868	0.57757	CSF1R/DDR2/EFNA3/EFNA4/EGFR/EPHA8/EPHB3/EPHB4/EPHB6/ERBB2/FGFR2/FGFR4/FGFRL1/FLT4/KDR/LTK/PDGFRB/TIE1	18	MF
GO:0015932	nucleobase-containing compound transmembrane transporter	12/2726	36/17354	0.0069	0.5868	0.57757	SIDT2/SLC25A25/SLC25A5/SLC28A1/SLC28A2/SLC29A1/SLC29A2/SLC29A3/SLC29A4/SLC35A2/SLC35C1/SLC35D2	12	MF
GO:0031406	carboxylic acid binding	42/2726	184/17354	0.00694	0.5868	0.57757	AARS/AARS2/ACACB/ACAN/AGRN/ALAS2/BCAN/C6orf15/DDC/FOLR2/GAD1/GCHFR/GLRA1/GLUD1/GNMT/GOT2/GRIN1/HAPLN3/KARS/LAYN/LCN12/NAGS/NOS3/OGFOD2/OTC/P3H1/P3H3/PADI4/PC/PLA2G1B/PLOD3/PPARD/PSAP/RARA/SELP/SERPINA5/SESN1/SHMT2/STARD5/TH/THNSL2/	42	MF
GO:0043177	organic acid binding	42/2726	185/17354	0.00764	0.5868	0.57757	AARS/AARS2/ACACB/ACAN/AGRN/ALAS2/BCAN/C6orf15/DDC/FOLR2/GAD1/GCHFR/GLRA1/GLUD1/GNMT/GOT2/GRIN1/HAPLN3/KARS/LAYN/LCN12/NAGS/NOS3/OGFOD2/OTC/P3H1/P3H3/PADI4/PC/PLA2G1B/PLOD3/PPARD/PSAP/RARA/SELP/SERPINA5/SESN1/SHMT2/STARD5/TH/THNSL2/	42	MF
GO:0004683	calmodulin-dependent protein kinase	10/2726	28/17354	0.00775	0.5868	0.57757	CAMK1D/CAMK1G/CAMK2D/CAMKK1/CAMKV/DAPK1/MAPKAPK3/PHKG1/PNCK/PTK2B	10	MF
GO:0004602	glutathione peroxidase	8/2726	20/17354	0.00783	0.5868	0.57757	GPX1/GPX2/GPX3/GSTM2/GSTZ1/MGST1/MGST3/PRDX6	8	MF
GO:0005507	copper ion binding	16/2726	55/17354	0.00869	0.6105	0.60092	ANG/AOC2/ATOX1/ATP13A2/CCS/COMMD1/COX11/CUTC/GPC1/HEPH/LOXL1/LOXL2/S100A5/SNAI3/SNCB/SOD3	16	MF
GO:0102567	phospholipase A2 activity (consuming 1,2-dipalmitoylphosphatidylcholine)	8/2726	21/17354	0.01094	0.6787	0.66798	PLA2G16/PLA2G1B/PLA2G2F/PLA2G4B/PLA2G5/PLB1/PRDX6/TMEM8A	8	MF

GO:0102568	phospholipase A2 activity consuming 1,2-dioleoylphosphatidylethanolami	8/2726	21/17354	0.01094	0.6787	0.66798	PLA2G16/PLA2G1B/PLA2G2F/PLA2G4B/PLA2G5/PLB1/PRDX6/TMEM8A	8	MF
GO:0004935	adrenergic receptor activity	5/2726	10/17354	0.01201	0.6787	0.66798	ADRA1A/ADRA1B/ADRA2A/ADRA2B/ADRA2C	5	MF
GO:0005496	steroid binding	23/2726	92/17354	0.01386	0.6787	0.66798	APOA5/APOC3/ATP1A1/ESR2/ESRRA/GPER1/HSD11B2/HSD17B1/NPC2/NR1H3/OSBPL5/OSBPL7/PAQR6/PAQR7/PAQR9/PLA2G1B/PROM2/RORC/SCAP/SOAT2/STARD3/STARD5/TSPO2	23	MF
GO:0010181	FMN binding	6/2726	14/17354	0.01436	0.6787	0.66798	DHODH/HAO2/NOS3/POR/TYW1/TYW1B	6	MF
GO:0008308	voltage-gated anion channel activity	7/2726	18/17354	0.01508	0.6787	0.66798	ANO1/BSND/CLCN2/CLCNKA/CLCNKB/GPR89A/VDAC2	7	MF
GO:0005244	voltage-gated ion channel activity	43/2726	198/17354	0.01522	0.6787	0.66798	ANO1/BSND/CACNA1F/CACNA1G/CACNA1H/CACNA1S/CACNB1/CACNB3/CACNG1/CATSPER1/CLCN2/CLCNKA/CLCNKB/CLIC1/CNGA2/CNGB1/GPR89A/GRIN1/GRIN2C/HCN3/KCNAB3/KCND1/KCND3/KCNH4/KCNH6/KCNJ18/KCNJ5/KCNJ9/KCNK12/KCNK13/KCNK17/KCNK4/KCNK7/KCNQ1/KCNQ4/KCNT1/OPRM1/PTK2B/SCN11A/TMEM109/TMEM37/TRPM5/VD	43	MF
GO:0022832	voltage-gated channel activity	43/2726	198/17354	0.01522	0.6787	0.66798	ANO1/BSND/CACNA1F/CACNA1G/CACNA1H/CACNA1S/CACNB1/CACNB3/CACNG1/CATSPER1/CLCN2/CLCNKA/CLCNKB/CLIC1/CNGA2/CNGB1/GPR89A/GRIN1/GRIN2C/HCN3/KCNAB3/KCND1/KCND3/KCNH4/KCNH6/KCNJ18/KCNJ5/KCNJ9/KCNK12/KCNK13/KCNK17/KCNK4/KCNK7/KCNQ1/KCNQ4/KCNT1/OPRM1/PTK2B/SCN11A/TMEM109/TMEM37/TRPM5/VD	43	MF
GO:0038024	cargo receptor activity	18/2726	68/17354	0.01553	0.6787	0.66798	ASGR1/CD5/CD5L/CD6/FOLR2/HHIPL1/LOXL2/LRP1/LRP10/OLR1/SCARA3/SCARB1/SCARF1/SSC4D/TFR2/TINAGL1/TMPRSS13/TMPRSS4	18	MF
GO:0004177	aminopeptidase activity	13/2726	45/17354	0.01813	0.6787	0.66798	ANPEP/BLMH/CTSH/DPEP1/DPP7/LOC440434/MMP15/MMP17/NAALAD2/PRCP/PRSS16/RNPEP/RNPEPL1	13	MF

GO:0001161	intronic transcription regulatory region sequence-	5/2726	11/17354	0.0192	0.6787	0.66798	BCL6/HSF1/SMYD3/VAX1/VAX2	5	MF
GO:0008239	dipeptidyl-peptidase	5/2726	11/17354	0.0192	0.6787	0.66798	DPEP1/DPP7/NAALAD2/PRCP/PRSS16	5	MF
GO:0015250	water channel activity	5/2726	11/17354	0.0192	0.6787	0.66798	AQP1/AQP10/AQP5/AQP8/MIP	5	MF
GO:0032036	myosin heavy chain binding	5/2726	11/17354	0.0192	0.6787	0.66798	CORO1A/MYBPC3/MYL4/NKD2/STX1A	5	MF
GO:0044213	intronic transcription regulatory region DNA	5/2726	11/17354	0.0192	0.6787	0.66798	BCL6/HSF1/SMYD3/VAX1/VAX2	5	MF
GO:0005543	phospholipid binding	74/2726	375/17354	0.02034	0.6787	0.66798	ADAP1/ADGRB1/APOA5/APOB/APOC3/ARAP1/ARFIP1/ATP13A2/BAD/BIN2/CD300LF/COMMD1/CPTP/DAB2IP/DOC2A/DOC2B/DPEP1/FERMT2/FES/GBF1/GOT2/GPAA1/GSDMD/HIP1R/HS1BP3/ITPR3/KCNQ1/MAPKAP1/MYO1E/NCF1/NISCH/NME4/NOXO1/NR5A1/NUMA1/OSBPL5/OTC/PARD3/PEBP1/PEMT/PFN1/PHF12/PHLDA3/PITPNM1/PLA2G15/PLA2G4B/PLCD1/PLD2/PSAP/PSD/PSD2/PSD4/SCARB1/SCIN/SERPINA5/SH3PXD2B/SHC1/SNX11/SNX12/SNX15/SPTBN2/SYTL3/TEC/TECPR1/THY1/TIRAP/TLN1/TNFAI[illegible]	74	MF
GO:0016859	cis-trans isomerase	12/2726	41/17354	0.02051	0.6787	0.66798	FKBP10/FKBP6/FKBP9/GSTZ1/PPIAL4A/PPIAL4C/PPIAL4D/PPIAL4E/PPIAL4F/PPIAL4G/PPIB/PPIE	12	MF
GO:0051861	glycolipid binding	7/2726	19/17354	0.0207	0.6787	0.66798	DPEP1/GLTP/GPAA1/HSPA2/PSAP/SELP/THY1	7	MF
GO:0008307	structural constituent of muscle	13/2726	46/17354	0.02174	0.6787	0.66798	ANKRD2/IGSF22/MYBPC1/MYBPC3/MYBPH/MYBPHL/MYL5/MYL6B/OBSCN/OBSL1/PDLIM3/TCAP/TPM2	13	MF

GO:0016684	oxidoreductase activity, acting on peroxide as acceptor	13/2726	46/17354	0.02174	0.6787	0.66798	EPX/GPX1/GPX2/GPX3/GSTM2/GSTZ1/LPO/MGST1/MGST3/MPO/PRDX1/PRDX6/SESN1	13	MF
GO:0015295	solute:proton symporter	9/2726	28/17354	0.02335	0.6906	0.67973	SLC15A3/SLC15A4/SLC25A22/SLC2A6/SLC2A8/SLC35A2/SLC36A1/SLC36A3/SLC45A4	9	MF
GO:1904680	peptide transmembrane transporter	9/2726	28/17354	0.02335	0.6906	0.67973	ABCB9/AZGP1/GPIHBP1/SLC15A3/SLC15A4/TAP1/TIMM17B/TIMM22/TIMM23B	9	MF
GO:0048037	cofactor binding	54/2726	265/17354	0.02446	0.6993	0.68832	ACAD10/ACADVL/ACBD4/ACOT7/ACOXL/ALAS2/ALDH2/AOC2/ASNS/CHDH/CRYM/CSAD/CTBP1/DDC/DHODH/GAD1/GAPDH/GCH1/GFER/GLUD1/GOT2/GPT/GSR/HADHA/HAO2/HDC/HSD11B2/HSD17B1/IDH1/IDH2/LDHA/MICAL2/NOS3/NOX4/NQO2/OAT/OGDH/OGDHL/PHGDH/PHYKPL/POR/PYGM/SDSL/SHMT2/SOAT2/SPTLC1/TH/THNSL2/TXNRD1/TXNRD3/T	54	MF
GO:0001054	RNA polymerase I	5/2726	12/17354	0.02873	0.6993	0.68832	POLR1A/POLR1D/POLR1E/POLR2L/ZNRD1	5	MF
GO:0005247	voltage-gated chloride channel activity	5/2726	12/17354	0.02873	0.6993	0.68832	ANO1/BSND/CLCN2/CLCNKA/CLCNKB	5	MF
GO:0005372	water transmembrane transporter	5/2726	12/17354	0.02873	0.6993	0.68832	AQP1/AQP10/AQP5/AQP8/MIP	5	MF
GO:0008331	high voltage-gated calcium channel activity	5/2726	12/17354	0.02873	0.6993	0.68832	CACNA1F/CACNA1S/CACNB1/CACNB3/CATSPER1	5	MF
GO:0008656	cysteine-type endopeptidase activator activity involved in	6/2726	16/17354	0.02897	0.6993	0.68832	BAD/CASP1/CASP3/CTSH/NLRP1/NOD1	6	MF

GO:0051959	dynein light intermediate chain binding	9/2726	29/17354	0.02926	0.6993	0.68832	CCDC88B/DNAH11/DNAH17/DNHD1/DYNC1H1/DYNLL2/RAB11FIP3/RILPL1/RILPL2	9	MF
GO:0004601	peroxidase activity	12/2726	43/17354	0.02954	0.6993	0.68832	EPX/GPX1/GPX2/GPX3/GSTM2/GSTZ1/LPO/MGST1/MGST3/MPO/PRDX1/PRDX6	12	MF
GO:0015485	cholesterol binding	12/2726	43/17354	0.02954	0.6993	0.68832	APOA5/APOC3/NPC2/NR1H3/OSBPL5/OSBPL7/PROM2/SCAP/SOAT2/STARD3/STARD5/TSPO2	12	MF
GO:0032934	sterol binding	13/2726	48/17354	0.03051	0.6993	0.68832	APOA5/APOC3/NPC2/NR1H3/OSBPL5/OSBPL7/PROM2/RORC/SCAP/SOAT2/STARD3/STARD5/TSPO2	13	MF
GO:0030170	pyridoxal phosphate	14/2726	53/17354	0.03111	0.6993	0.68832	ALAS2/CSAD/DDC/GAD1/GOT2/GPT/HDC/OAT/PHYKPL/PYGM/SDSL/SHMT2/SPTLC1/THNSL2	14	MF
GO:0070279	vitamin B6 binding	14/2726	53/17354	0.03111	0.6993	0.68832	ALAS2/CSAD/DDC/GAD1/GOT2/GPT/HDC/OAT/PHYKPL/PYGM/SDSL/SHMT2/SPTLC1/THNSL2	14	MF
GO:0042887	amide transmembrane transporter	10/2726	34/17354	0.03191	0.7028	0.69175	ABCB9/AQP10/AZGP1/GPIHBP1/SLC15A3/SLC15A4/TAP1/TIMM17B/TIMM22/TIMM23B	10	MF
GO:0005326	neurotransmitter transporter	8/2726	25/17354	0.03262	0.7028	0.69175	CPLX1/SLC22A1/SLC6A18/SLC6A2/SLC6A6/SLC6A7/SLC6A8/SLC6A9	8	MF
GO:0022804	active transmembrane transporter activity	71/2726	367/17354	0.03375	0.7028	0.69175	ABCA2/ABCB5/ABCB9/ABCC12/ABCC3/ABCC4/ABCC5/ABCC6/ABCG5/ATP13A2/ATP1A1/ATP1A4/ATP2A3/ATP2C2/ATP5G1/ATP6V0B/ATP6V0E2/ATP6V1B1/ATP6V1F/ATP6V1G1/ATP6V1G2/MFSD2A/SLC11A1/SLC12A4/SLC12A7/SLC12A9/SLC13A2/SLC15A3/SLC15A4/SLC16A13/SLC1A7/SLC20A1/SLC20A2/SLC22A1/SLC22A12/SLC22A13/SLC22A9/SLC25A20/SLC25A22/SLC25A5/SLC26A1/SLC26A10/SLC26A6/SLC28A1/SLC28A2/SLC29A4/SLC2A6/SLC2A8/SLC34A1/SLC35A2/SLC36A1/SLC36A3/SLC38A1/SLC45A1/SLC45A4/SLC4A9/SLC5A2/SLC6A18/SLC6A2/SLC6A6/SLC6A7/SLC6A8/SLC6A9/SLC7A7/SLC9A3/SLC9A5/SLCO2B1/SLCO5A1/TAP1/TIMM17B/TIMM2[illegible]	71	MF
GO:0003755	peptidyl-prolyl cis-trans isomerase	11/2726	39/17354	0.03376	0.7028	0.69175	FKBP10/FKBP6/FKBP9/PPIAL4A/PPIAL4C/PPIAL4D/PPIAL4E/PPIAL4F/PPIAL4G/PPIB/PPIE	11	MF

GO:0015370	solute:sodium symporter	14/2726	54/17354	0.03612	0.7078	0.69664	SLC13A2/SLC1A7/SLC20A1/SLC25A22/SLC28A1/SLC28A2/SLC38A1/SLC5A2/SLC6A18/SLC6A2/SLC6A6/SLC6A7/SLC6A8/SLC6A9	14	MF
GO:0004693	cyclin-dependent protein serine/threonine	9/2726	30/17354	0.03615	0.7078	0.69664	CCNB2/CCND3/CDK10/CDK20/CDK4/CDK5/CDK9/CDKL4/MOK	9	MF
GO:0043014	alpha-tubulin binding	9/2726	30/17354	0.03615	0.7078	0.69664	ARL8A/C9orf24/CAV3/FNTA/INO80/SETD2/SLC6A2/TRPV4/TTLL7	9	MF
GO:0035091	phosphatidylinositol binding	45/2726	221/17354	0.03763	0.7078	0.69664	ADAP1/ARAP1/ARFIP1/ATP13A2/COMMD1/DAB2IP/DPEP1/FERMT2/FES/GBF1/GPAA1/GSDMD/HIP1R/HS1BP3/ITPR3/KCNQ1/MAPKAP1/MYO1E/NCF1/NISCH/NOXO1/NUMA1/OSBPL5/PARD3/PFN1/PHF12/PHLDA3/PITPNM1/PLCD1/PLD2/SCARB1/SCIN/SH3PXD2B/SNX11/SNX12/SNX15/TECPR1/THY1/TIRAP/TLN1/TNFAIP8L3/TULP1/TULP3/ZFYVE1/ZFYVE26	45	MF
GO:0005200	structural constituent of cytoskeleton	24/2726	106/17354	0.03791	0.7078	0.69664	ACTA1/ACTL7A/AGRN/ARPC1B/ARPC2/ARPC4/CTNNA2/DES/EPB41L2/KRT14/KRT16/KRT84/SORBS3/SPTAN1/SPTB/SPTBN2/TLN1/TUBA3C/TUBA3D/TUBA3E/TUBG1/TUBGCP2/TUBGCP3/TUBGCP5	24	MF
GO:0019842	vitamin binding	29/2726	133/17354	0.03853	0.7078	0.69664	ACACB/ALAS2/CSAD/CUBN/DDC/FOLR2/GAD1/GNMT/GOT2/GPT/HDC/OAT/OGDH/OGDHL/OGFOD2/OPN4/P3H1/P3H3/PC/PHYKPL/PLOD3/PYGM/RBP1/RBP2/RBP7/SDSL/SHMT2/SPTLC1/THNSL2	29	MF
GO:0140098	catalytic activity, acting on RNA	70/2726	364/17354	0.03902	0.7078	0.69664	AARS/AARS2/ALKBH5/ANG/APEX1/AZGP1/C12orf65/CDKAL1/CMTR1/CPSF4/DALRD3/DARS/DDX31/DDX4/DDX53/DDX54/DHX37/DHX38/DIS3L/DIS3L2/ERN2/EXO1/EXOSC10/EXOSC2/GATB/HELQ/IGHMBP2/ISG20L2/KARS/LARS/MEPCE/METTL2A/METTL2B/NOB1/NSUN5/NSUN5P2/PAN2/PARS2/PIF1/PIWIL2/PNLDC1/POLR1A/POLR1D/POLR1E/POLR2G/POLR2J2/POLR2J3/POLR2L/POLR3D/PRPF8/PTRH1/RNASE1/RNASE8/RNASEK/RPP21/RTCA/SARS/SMG5/TARS2/TDRD9/THUMPD2/TRMT10B/TRMT2B/TRMT[illegible]	70	MF
GO:0043178	alcohol binding	18/2726	75/17354	0.03981	0.7078	0.69664	ADAP1/APOA5/APOC3/CDIPT/ITPR3/NPC2/NR1H3/OSBPL5/OSBPL7/PROM2/RBP1/RBP2/RBP7/SCAP/SOAT2/STARD3/STARD5/TSPO2	18	MF
GO:0048019	receptor antagonist	5/2726	13/17354	0.04078	0.7078	0.69664	CCL5/ESR2/IL18BP/IL1RN/WFIKKN2	5	MF

GO:0005044	scavenger receptor activity	13/2726	50/17354	0.0416	0.7078	0.69664	CD5/CD5L/CD6/HHIPL1/LOXL2/LRP1/SCARA3/SCARB1/SCARF1/SSC4D/TINAGL1/TMPRSS13/TMPRSS4	13	MF
GO:0005085	guanyl-nucleotide exchange factor activity	62/2726	320/17354	0.04353	0.7078	0.69664	ALS2CL/ARHGEF10/ARHGEF16/ARHGEF17/ARHGEF25/ARHGEF4/ARTN/CAMK2D/DENND2A/DENND2C/DENND3/DLG4/DOCK1/DOCK7/EGFR/EIF2B2/EIF2B5/EPS8L2/ERBB2/FARP2/FGF17/FGF3/FGFR2/FGFR4/GBF1/GRIN1/GRIN2C/HPS1/IL5RA/IQSEC2/KNDC1/MADD/MON1A/NRG1/OBSCN/PDGFA/PDGFRB/PLEKHG4/PLEKHG5/PLEKHG6/PREX2/PSD/PSD2/PSD4/RALGDS/RALGPS1/RAPGEFL1/RASGEF1A/RASGEF1C/RASGRP2/RIN3/SERGEF/SHC1/SPTAN1/SPTB/SPTBN2/TRAPPC1/TRAPPC2L/TRAPPC3/TRAPPC[illegible]	62	MF
GO:0030332	cyclin binding	9/2726	31/17354	0.04408	0.7078	0.69664	CDK4/CDK5RAP3/CDK9/GAK/INCA1/KLHDC9/RBM4/UBE3D/USP2	9	MF
GO:0031369	translation initiation factor	9/2726	31/17354	0.04408	0.7078	0.69664	ANGEL1/EIF2B5/EIF3B/EIF4EBP1/EIF4G1/GCH1/POLR2G/RPS24/ZPR1	9	MF
GO:0097472	cyclin-dependent protein kinase	9/2726	31/17354	0.04408	0.7078	0.69664	CCNB2/CCND3/CDK10/CDK20/CDK4/CDK5/CDK9/CDKL4/MOK	9	MF
GO:0004713	protein tyrosine kinase activity	37/2726	179/17354	0.0454	0.7078	0.69664	ABL1/BAZ1B/BLK/CSF1R/CSK/DDR2/EFNA3/EFNA4/EGFR/EPHA8/EPHB3/EPHB4/EPHB6/ERBB2/FES/FGF17/FGF3/FGFR2/FGFR4/FGFRL1/FLT4/GRAP/HSP90AA1/IL5RA/KDR/LCK/LTK/MAP2K3/NRG1/PDGFRB/PKDCC/PRKCD/PTK2B/TEC/TIE1/TNK1/TNK2	37	MF
GO:0016799	hydrolase activity, hydrolyzing N-glycosyl	7/2726	22/17354	0.04583	0.7078	0.69664	ADPRHL1/APEX1/DNPH1/MBD4/NEIL2/OGG1/SMUG1	7	MF
GO:0015293	symporter activity	31/2726	146/17354	0.04599	0.7078	0.69664	MFSD2A/SLC12A4/SLC12A7/SLC12A9/SLC13A2/SLC15A3/SLC15A4/SLC16A13/SLC1A7/SLC20A1/SLC20A2/SLC25A22/SLC28A1/SLC28A2/SLC2A6/SLC2A8/SLC34A1/SLC35A2/SLC36A1/SLC36A3/SLC38A1/SLC45A1/SLC45A4/SLC4A9/SLC5A2/SLC6A18/SLC6A2/SLC6A6/SLC6A7/SLC6A8/SLC6A9	31	MF
GO:0009055	electron transfer activity	24/2726	108/17354	0.04607	0.7078	0.69664	AKR7A3/ALDH2/ALDH4A1/AOC2/COX10/COX11/COX8A/CYC1/CYP1A2/GLRX5/GPX2/GSR/HAAO/IDO1/LOXL2/NCF1/NDUFA4L2/NOX4/NQO2/PHGDH/POR/TXNRD1/TXNRD3/XDH	24	MF

GO:0004715	non-membrane spanning protein tyrosine kinase activity	13/2726	51/17354	0.04809	0.7078	0.69664	ABL1/BAZ1B/BLK/CSK/FES/GRAP/LCK/PKDCC/PRKCD/PTK2B/TEC/TNK1/TNK2	13	MF
GO:0005416	cation:amino acid symporter	6/2726	18/17354	0.05091	0.7078	0.69664	SLC25A22/SLC36A1/SLC36A3/SLC38A1/SLC6A7/SLC6A9	6	MF
GO:0030547	receptor inhibitor	6/2726	18/17354	0.05091	0.7078	0.69664	CCL5/ESR2/IL18BP/IL1RN/LYPD1/WFIKKN2	6	MF
GO:0051371	muscle alpha-actinin binding	6/2726	18/17354	0.05091	0.7078	0.69664	IGSF22/MYBPC1/MYBPC3/MYBPH/MYBPHL/OBSL1	6	MF
GO:0070300	phosphatidic acid binding	6/2726	18/17354	0.05091	0.7078	0.69664	ATP13A2/COMMD1/GSDMD/MAPKAP1/PITPNM1/PLCD1	6	MF
GO:1901681	sulfur compound binding	46/2726	232/17354	0.05315	0.7078	0.69664	ACACB/ACBD4/ACOT7/AGRN/ANG/APLP2/APOA5/APOB/BMP4/C6orf15/CFH/CHST12/CHST15/FGFBP3/FGFR2/FGFR4/FGFRL1/FN1/GAL3ST4/GLRA1/GPC1/GPC2/GSTM1/GSTM2/HADHA/KNG1/LAMC2/LRP1/MGST1/MPO/OGDH/OGDHL/PC/PCOLCE/PF4V1/PLA2G5/PTPRF/REG4/RSPO1/RTN4RL1/SELP/SERPINA5/SOAT2/SOD3/SOST/THBS3	46	MF
GO:0022838	substrate-specific channel activity	81/2726	435/17354	0.05452	0.7078	0.69664	ANO1/ANO2/ANO4/ANO7/AQP1/AQP10/AQP5/AQP8/ASIC2/ASIC3/BEST3/BEST4/BSND/CACNA1F/CACNA1G/CACNA1H/CACNA1S/CACNB1/CACNB3/CACNG1/CALHM2/CALHM3/CATSPER1/CHRNA6/CHRNB1/CHRNB3/CLCN2/CLCNKA/CLCNKB/CLDN4/CLIC1/CNGA2/CNGB1/DLG4/GLRA1/GPR89A/GRIA3/GRIN1/GRIN2C/HCN3/HTR3B/ITPR3/JPH3/KCNAB3/KCND1/KCND3/KCNH4/KCNH6/KCNJ18/KCNJ5/KCNJ9/KCNK12/KCNK13/KCNK17/KCNK4/KCNK7/KCNQ1/KCNQ4/KCNT1/MIP/OPRM1/ORAI1/ORAI3/P2RX2/PDE2A/PKD1/PTK2B/RASA3/SCN11A/SCNN1A/SLC26A1/SLC26A10/SLC26A6/TMEM109/TMEM150C/TMEM37/TRPM5/TRPV2/TRPV4/TRPV6/VDAC2	81	MF
GO:0005547	phosphatidylinositol-3,4,5-trisphosphate binding	10/2726	37/17354	0.05468	0.7078	0.69664	ADAP1/ARAP1/COMMD1/FERMT2/GBF1/HIP1R/MAPKAP1/PARD3/PHLDA3/ZFYVE1	10	MF

GO:0008375	acetylglucosaminyltransferase	13/2726	52/17354	0.05525	0.7078	0.69664	A4GNT/B3GNT6/EXTL1/HEXA/LFNG/MGAT1/MGAT4B/MGAT5B/PIGC/PIGQ/PIGY/RFNG/XYLT2	13	MF
GO:0019104	DNA N-glycosylase	5/2726	14/17354	0.05546	0.7078	0.69664	APEX1/MBD4/NEIL2/OGG1/SMUG1	5	MF
GO:0031434	mitogen-activated protein kinase	5/2726	14/17354	0.05546	0.7078	0.69664	ARAF/DAB2IP/MAPK8IP1/RAF1/TAOK2	5	MF
GO:0032794	GTPase activating	5/2726	14/17354	0.05546	0.7078	0.69664	FMNL1/GNAO1/PLCD1/PLXNB1/RGS14	5	MF
GO:0043274	phospholipase binding	5/2726	14/17354	0.05546	0.7078	0.69664	DGKQ/LMNB1/PTPN11/STXBP1/WAS	5	MF
GO:0050780	dopamine receptor binding	5/2726	14/17354	0.05546	0.7078	0.69664	ARRB2/DLG4/DNM1/PPP1R1B/PTPN11	5	MF
GO:0000182	rDNA binding	4/2726	10/17354	0.05784	0.7078	0.69664	GTF3C1/MAF1/MTOR/TSPYL2	4	MF
GO:0005522	profilin binding	4/2726	10/17354	0.05784	0.7078	0.69664	CTTN/DBN1/EVL/FMNL1	4	MF
GO:0031702	type 1 angiotensin	4/2726	10/17354	0.05784	0.7078	0.69664	AGT/ARAP1/ARRB2/GNG3	4	MF
GO:0051011	microtubule minus-end	4/2726	10/17354	0.05784	0.7078	0.69664	NUMA1/TUBGCP2/TUBGCP3/TUBGCP5	4	MF
GO:0046873	metal ion transmembrane transporter activity	83/2726	449/17354	0.06011	0.7078	0.69664	AQP1/ASIC2/ASIC3/ATOX1/ATP13A2/ATP1A1/ATP1A4/ATP2A3/ATP2C2/CACNA1F/CACNA1G/CACNA1H/CACNA1S/CACNB1/CACNB3/CACNG1/CATSPER1/CNGA2/CNGB1/CNNM2/GRIN1/GRIN2C/HCN3/ITPR3/JPH3/KCNAB3/KCND1/KCND3/KCNH4/KCNH6/KCNJ18/KCNJ5/KCNJ9/KCNK12/KCNK13/KCNK17/KCNK4/KCNK7/KCNQ1/KCNQ4/KCNT1/NIPA2/NIPAL1/NIPAL2/OPRM1/ORAI1/ORAI3/PDE2A/PKD1/RASA3/SCN11A/SCNN1A/SLC11A1/SLC12A4/SLC12A7/SLC12A9/SLC13A2/SLC1A7/SLC20A1/SLC20A2/SLC25A22/SLC28A1/SLC28A2/SLC30A3/SLC34A1/SLC38A1/SLC39A13/SLC39A5/SLC4A9/SLC5A2/SLC6A18/SLC6A2/SLC6A6/SLC6A7/SLC6A8/SLC6A9/SL[illegible]	83	MF

GO:0022836	gated channel activity	63/2726	332/17354	0.06012	0.7078	0.69664	ANO1/ANO2/ANO4/ANO7/AQP1/ASIC2/ASIC3/BSND/CACNA1F/CACNA1G/CACNA1H/CACNA1S/CACNB1/CACNB3/CACNG1/CATSPER1/CHRNA6/CHRNB1/CHRNB3/CLCN2/CLCNKA/CLCNKB/CLIC1/CNGA2/CNGB1/DLG4/GLRA1/GPR89A/GRIA3/GRIN1/GRIN2C/HCN3/HTR3B/ITPR3/JPH3/KCNAB3/KCND1/KCND3/KCNH4/KCNH6/KCNJ18/KCNJ5/KCNJ9/KCNK12/KCNK13/KCNK17/KCNK4/KCNK7/KCNQ1/KCNQ4/KCNT1/OPRM1/P2RX2/PTK2B/RASA3/SCN11A/SCNN1A/TMEM109/TMEM150C/TMEM37/TRPM5/TRPV4/[illegible]	63	MF
GO:0046875	ephrin receptor binding	8/2726	28/17354	0.06107	0.7078	0.69664	ABL1/AQP1/CDK5/EFNA1/EFNA3/EFNA4/EFNB1/SHC1	8	MF
GO:0019199	transmembrane receptor protein kinase activity	18/2726	79/17354	0.0623	0.7078	0.69664	CSF1R/DDR2/EFNA3/EFNA4/EGFR/EPHA8/EPHB3/EPHB4/EPHB6/ERBB2/FGFR2/FGFR4/FGFRL1/FLT4/KDR/LTK/PDGFRB/TIE1	18	MF
GO:0004623	phospholipase A2 activity	9/2726	33/17354	0.0632	0.7078	0.69664	PLA2G15/PLA2G16/PLA2G1B/PLA2G2F/PLA2G4B/PLA2G5/PLB1/PRDX6/TMEM8A	9	MF
GO:0030291	protein serine/threonine kinase inhibitor activity	9/2726	33/17354	0.0632	0.7078	0.69664	CASP3/CDKN2A/CIB1/HSPB1/INCA1/PPP1R1B/PRKAR1A/PRKAR1B/SFN	9	MF
GO:1990841	promoter-specific	9/2726	33/17354	0.0632	0.7078	0.69664	E2F4/EGR1/EHMT2/FOXO4/HSF1/NRL/NUP98/STAT1/WDR13	9	MF
GO:0045296	cadherin binding	58/2726	304/17354	0.06325	0.7078	0.69664	ABCF3/AHNAK/ALDOA/ARFIP1/ARHGEF16/ATXN2L/BAIAP2L1/CCNB2/CCS/CDH13/CKAP5/CLIC1/CORO1B/CTNNA2/CTTN/DAB2IP/DBN1/EGFR/EHD1/EIF4G1/EMD/EPS8L2/FLNA/FMNL2/FSCN1/GOLGA2/GOLGA3/H1FX/HCFC1/HSPA5/IDH1/KLC2/LDHA/LRRFIP1/LYPLA2/MARK2/NUDC/OLFM4/PDLIM1/PDLIM5/PFN1/PKM/PKP3/PRDX1/PRDX6/PSMB6/SCRIB/SEPT9/SFN/SH3GLB2/SLC9A3R2/SND1/SPTAN1/SPTBN2/STAT1/STX5/TLN1/VAS	58	MF
GO:0004812	aminoacyl-tRNA ligase	11/2726	43/17354	0.06433	0.7078	0.69664	AARS/AARS2/DALRD3/DARS/KARS/LARS/PARS2/SARS/TARS2/YARS/YARS2	11	MF
GO:0005003	ephrin receptor activity	6/2726	19/17354	0.06486	0.7078	0.69664	EFNA3/EFNA4/EPHA8/EPHB3/EPHB4/EPHB6	6	MF

GO:0005328	neurotransmitter:sodium symporter	6/2726	19/17354	0.06486	0.7078	0.69664	SLC6A18/SLC6A2/SLC6A6/SLC6A7/SLC6A8/SLC6A9	6	MF
GO:0016505	peptidase activator activity	6/2726	19/17354	0.06486	0.7078	0.69664	BAD/CASP1/CASP3/CTSH/NLRP1/NOD1	6	MF
GO:0017127	cholesterol transporter	6/2726	19/17354	0.06486	0.7078	0.69664	ABCG5/APOA5/APOB/NPC2/STARD3/STARD5	6	MF
GO:0005088	Ras guanyl-nucleotide exchange factor activity	48/2726	248/17354	0.06941	0.7267	0.71525	ALS2CL/ARHGEF10/ARHGEF16/ARHGEF17/ARHGEF25/ARHGEF4/ARTN/CAMK2D/DENND2A/DENND2C/DENND3/DLG4/DOCK1/EGFR/EPS8L2/ERBB2/FARP2/FGF17/FGF3/FGFR2/FGFR4/GRIN1/GRIN2C/IL5RA/KNDC1/MADD/NRG1/OBSCN/PDGFA/PDGFRB/PLEKHG4/PLEKHG5/PLEKHG6/PREX2/RALGPS1/RASGEF1A/RIN3/SERGEF/SHC1/SPTAN1/SPTB/SPTBN2/TRAPPC1/TRAPPC2L/TRAPPC3/TRAPPC4/TRAPPC9/TRIO	48	MF
GO:0003707	steroid hormone receptor activity	14/2726	59/17354	0.07	0.7267	0.71525	ESR2/ESRRA/GPER1/NR1H3/NR2F1/NR4A1/NR5A1/PAQR6/PAQR7/PAQR9/PPARD/RARA/RORC/THRA	14	MF
GO:0008022	protein C-terminus binding	36/2726	180/17354	0.07164	0.7267	0.71525	ABL1/BAIAP3/CALCOCO1/CAV3/CIB1/CIITA/CORO1A/CSK/CTBP1/DLG4/DNM1/ECM1/ERBB2/FN1/HRAS/HSPB7/HSPG2/KSR1/LCK/MID1IP1/MYO1C/NUMA1/OPRM1/PDZD3/PEX16/PEX6/PFKM/RBFOX1/SDC1/SIPA1/SLC9A3R2/SNF8/TAX1BP3/TRIM3/VGLL2/YWHAQ	36	MF
GO:0019215	intermediate filament	5/2726	15/17354	0.07278	0.7267	0.71525	EVPLL/FAM83H/KRT14/NES/NME1	5	MF
GO:0043236	laminin binding	8/2726	29/17354	0.07318	0.7267	0.71525	ACHE/AGRN/C6orf15/ECM1/GPC1/LGALS3/SHH/TINAGL1	8	MF
GO:0001227	transcriptional repressor activity, RNA polymerase II transcription regulatory region	44/2726	226/17354	0.07338	0.7267	0.71525	AEBP1/ARID5A/ASCL2/ATF3/BCL6/CDX2/CREM/DEAF1/DLX4/DMBX1/E2F8/E4F1/EN1/ESRRA/ETV7/FERD3L/FOXM1/FOXQ1/HES6/HINFP/HMX1/HSF1/KLF17/LRRFIP1/MAX/MLXIPL/MSC/NFX1/NKX3-2/NR2F1/PPARD/RORC/SNAI3/SP2/SPDEF/SPI1/TBX15/TBX2/VAX1/VAX2/ZNF205/ZNF219/ZNF668/ZNF692	44	MF

GO:0015267	channel activity	85/2726	466/17354	0.07438	0.7267	0.71525	ANO1/ANO2/ANO4/ANO7/AQP1/AQP10/AQP5/AQP8/ASIC2/ASIC3/BEST3/BEST4/BSND/CACNA1F/CACNA1G/CACNA1H/CACNA1S/CACNB1/CACNB3/CACNG1/CALHM2/CALHM3/CATSPER1/CHRNA6/CHRNB1/CHRNB3/CLCN2/CLCNKA/CLCNKB/CLDN4/CLIC1/CNGA2/CNGB1/DLG4/GJB3/GLRA1/GPR89A/GRIA3/GRIN1/GRIN2C/HCN3/HTR3B/ITPR3/JPH3/KCNAB3/KCND1/KCND3/KCNH4/KCNH6/KCNJ18/KCNJ5/KCNJ9/KCNK12/KCNK13/KCNK17/KCNK4/KCNK7/KCNQ1/KCNQ4/KCNT1/MIP/OPRM1/ORAI1/ORAI3/P2RX2/PDE2A/PKD1/PTK2B/RASA3/SCN11A/SCNN1A/SLC26A1/SLC26A10/SLC26A6/TIMM17B/TIMM22/TIMM23B/TMEM109/TMEM150C/TMEM37/TRPM5/TRPV2/TRPV4/TRPV6/VDAC2	85	MF
GO:0005516	calmodulin binding	38/2726	192/17354	0.07466	0.7267	0.71525	AEBP1/ATPIF1/CACNA1S/CAMK1D/CAMK1G/CAMK2D/CAMKK1/CAMKV/CNGA2/DAPK1/DAPK2/EDF1/EGFR/GRIN1/IQCF6/KCNQ1/MAP6D1/MAPKAPK3/MARCKSL1/MIP/MYO1A/MYO1C/MYO1E/MYO3A/MYO7A/NOS3/OBSCN/ORAI1/PHKG1/PNCK/RGS16/RRAD/SMTNL1/SPTAN1/TRPV4/TR	38	MF
GO:0016209	antioxidant activity	18/2726	81/17354	0.07631	0.7267	0.71525	EPX/FAM213A/GPX1/GPX2/GPX3/GSR/GSTM2/GSTZ1/LPO/MGST1/MGST3/MPO/PRDX1/PRDX6/SESN1/SOD3/TXNRD1/TXNRD3	18	MF
GO:0005518	collagen binding	15/2726	65/17354	0.07632	0.7267	0.71525	ACHE/C1QTNF1/C6orf15/CTSL/DDR2/FN1/MRC2/P3H1/PCOLCE/PDGFA/PPIB/RELL2/SERPINH1/TGFBI/VWF	15	MF
GO:0048029	monosaccharide binding	15/2726	65/17354	0.07632	0.7267	0.71525	ALDOA/ENG/FUOM/GALK1/GCK/HK3/LGALS9/LMAN2/OGFOD2/P3H1/P3H3/PFKM/PLOD3/SELP/SLC2A8	15	MF
GO:0015631	tubulin binding	57/2726	302/17354	0.07657	0.7267	0.71525	ALDOA/ARL8A/BCAS3/BRCA1/C9orf24/CAV3/CCDC88B/CCSER2/CFAP157/CKAP5/CRYAB/DNM1/DYNC1I1/EMD/EML1/EML3/EML5/FES/FNTA/GAPDH/GAS8/GOLGA2/GPAA1/INO80/KATNB1/KIF13A/KIF17/KIF23/KIF26A/KIF2B/KIF4A/KIF9/KIFC3/MAP6D1/MAPRE3/NEIL2/NME1/NUMA1/OGG1/PEX14/RABGAP1/REEP4/RGS14/SAXO2/SETD2/SLC6A2/STMN4/TPPP2/TRAF3IP1/TRIM54/TRPV4/TTLL7/TUBGCP2/TUBGCP3/TUBGCP5/UXT/WHA[illegible]	57	MF

GO:0022803	passive transmembrane transporter activity	85/2726	467/17354	0.07737	0.7267	0.71525	ANO1/ANO2/ANO4/ANO7/AQP1/AQP10/AQP5/AQP8/ASIC2/ASIC3/BEST3/BEST4/BSND/CACNA1F/CACNA1G/CACNA1H/CACNA1S/CACNB1/CACNB3/CACNG1/CALHM2/CALHM3/CATSPER1/CHRNA6/CHRNB1/CHRNB3/CLCN2/CLCNKA/CLCNKB/CLDN4/CLIC1/CNGA2/CNGB1/DLG4/GJB3/GLRA1/GPR89A/GRIA3/GRIN1/GRIN2C/HCN3/HTR3B/ITPR3/JPH3/KCNAB3/KCND1/KCND3/KCNH4/KCNH6/KCNJ18/KCNJ5/KCNJ9/KCNK12/KCNK13/KCNK17/KCNK4/KCNK7/KCNQ1/KCNQ4/KCNT1/MIP/OPRM1/ORAI1/ORAI3/P2RX2/PDE2A/PKD1/PTK2B/RASA3/SCN11A/SCNN1A/SLC26A1/SLC26A10/SLC26A6/TIMM17B/TIMM22/TIMM23B/TMEM109/TMEM150C/TMEM37/[illegible]	85	MF
GO:0015291	secondary active transmembrane transporter activity	46/2726	239/17354	0.07979	0.7267	0.71525	MFSD2A/SLC11A1/SLC12A4/SLC12A7/SLC12A9/SLC13A2/SLC15A3/SLC15A4/SLC16A13/SLC1A7/SLC20A1/SLC20A2/SLC22A1/SLC22A12/SLC22A13/SLC22A9/SLC25A20/SLC25A22/SLC25A5/SLC26A1/SLC26A10/SLC26A6/SLC28A1/SLC28A2/SLC2A6/SLC2A8/SLC34A1/SLC35A2/SLC36A1/SLC36A3/SLC38A1/SLC45A1/SLC45A4/SLC4A9/SLC5A2/SLC6A18/SLC6A2/SLC6A6/SLC6A7/SLC6A8/SLC6A9/SLC7A7/SLC9A3/SLC9A5/SLCO2B1/SLCO5A1	46	MF
GO:0016829	lyase activity	35/2726	176/17354	0.07982	0.7267	0.71525	ACO1/ALAD/ALDOA/APEX1/ASL/CA4/CA6/CA7/CHAC1/CSAD/DDC/FAHD1/GAD1/GGACT/GLO1/GUCY2D/HACD1/HACD3/HADHA/HDC/HMGA1/HMGCLL1/MGST3/NEIL2/NPL/OGG1/PHYKPL/PM20D1/PPCDC/SDSL/SHMT2/TYW1/TYW1B/UROC1/UROS	35	MF
GO:0004674	protein serine/threonine kinase activity	82/2726	450/17354	0.07982	0.7267	0.71525	AATK/ADCK5/ANKK1/ARAF/BCKDK/BRSK2/BUB1B/CAMK1D/CAMK1G/CAMK2D/CAMKK1/CAMKV/CCNB2/CCND3/CDC42BPG/CDK10/CDK16/CDK20/CDK4/CDK5/CDK9/CDKL4/DAPK1/DAPK2/DCLK2/EGFR/EIF2AK4/ERN2/GAK/GRK6/GTF2H4/IKBKB/ILK/IRAK1/LIMK1/MAP2K3/MAP3K14/MAP3K15/MAP3K6/MAP4K2/MAPK15/MAPK3/MAPKAPK3/MARK2/MARK3/MOK/MTOR/MYO3A/NEK10/NEK11/NEK8/NIM1K/NRBP1/OBSCN/OSR1/PELI3/PHKG1/PKMYT1/PNCK/PRKAB1/PRKCD/PTK2B/RAF1/RPS6KA1/RPS6KA4/RPS6KB2/SGK1/SRPK3/STK32B/STK32C/STK33/STKLD1/TAF1/TAF1[illegible]	82	MF

GO:0016813	hydrolase activity, acting on carbon-nitrogen (but not peptide)	4/2726	11/17354	0.07992	0.7267	0.71525	AGMAT/PADI3/PADI4/PADI6	4	MF
GO:0031701	angiotensin receptor binding	4/2726	11/17354	0.07992	0.7267	0.71525	AGT/ARAP1/ARRB2/GNG3	4	MF
GO:0033130	acetylcholine receptor binding	4/2726	11/17354	0.07992	0.7267	0.71525	DLG4/LYNX1/LYPD1/PSCA	4	MF
GO:0023023	MHC protein complex	6/2726	20/17354	0.08081	0.7267	0.71525	CD74/CD81/HSP90AA1/KLRC1/KLRC2/PKM	6	MF
GO:0051428	peptide hormone	6/2726	20/17354	0.08081	0.7267	0.71525	GNAO1/GPHA2/NPPA/PTPN11/RUNDC3A/UCN2	6	MF
GO:0070006	metalloaminopeptidase activity	6/2726	20/17354	0.08081	0.7267	0.71525	ANPEP/LOC440434/MMP15/MMP17/RNPEP/RNPEPL1	6	MF
GO:0072341	modified amino acid binding	16/2726	71/17354	0.0821	0.7313	0.71976	ADGRB1/CD300LF/DPEP1/FOLR2/GNMT/GSDMD/GSTM1/GSTM2/MGST1/NOX4/OSBPL5/PLCD1/SCARB1/SCIN/TLN1/TRIM72	16	MF
GO:0003774	motor activity	28/2726	137/17354	0.08263	0.7313	0.71976	CCDC102A/DCTN2/DNAH11/DNAH12/DNAH17/DNHD1/DYNC1H1/DYNC1I1/DYNLL2/GPR88/KIF13A/KIF17/KIF23/KIF26A/KIF2B/KIF4A/KIF9/KIFC3/KLC1/KLC2/MYL6B/MYO19/MYO1A/MYO1C/MYO1E/MYO3A/MYO7A/M	28	MF
GO:0080025	phosphatidylinositol-3,5-bisphosphate binding	7/2726	25/17354	0.08492	0.7449	0.73314	ATP13A2/COMMD1/GBF1/HIP1R/MAPKAP1/PHLDA3/SH3PXD2B	7	MF
GO:0016741	transferase activity, transferring one-carbon groups	43/2726	223/17354	0.08576	0.7449	0.73314	ALDH1L1/AS3MT/ASH2L/BHMT2/CIAPIN1/CMTR1/DNMT3A/EHMT1/EHMT2/GNMT/HNMT/LCMT1/LRTOMT/MEN1/MEPCE/METTL17/METTL2A/METTL2B/METTL7B/NDUFAF7/NSUN5/NSUN5P2/NTMT1/OTC/PEMT/PRDM12/PRDM14/PRDM7/PRDM8/PRDM9/SETD1A/SETD2/SETD7/SETD9/SHMT2/SMYD3/SMYD5/SUV39H1/THUMPD2/TRMT10B/TRMT2B/TRMT44/VC	43	MF
GO:0051539	4 iron, 4 sulfur cluster binding	10/2726	40/17354	0.08615	0.7449	0.73314	ACO1/CDKAL1/ISCA2/LIAS/NARFL/NUBP1/NUBP2/POLE/TYW1/TYW1B	10	MF

GO:0015197	peptide transporter activity	23/2726	110/17354	0.08836	0.7557	0.74383	ABCB9/AP1G2/AP1S1/AP2A2/AP4S1/AZGP1/CHMP7/GPIHBP1/IPO13/IPO4/LRP1/SLC15A3/SLC15A4/TAP1/TIMM17B/TIMM22/TIMM23B/TIMM9/TMCO6/TNPO3/TSNAX/XPO6/XPO7	23	MF
GO:0019210	kinase inhibitor activity	21/2726	99/17354	0.08875	0.7557	0.74383	AHSG/BGN/CAMK2N2/CASP3/CDKN2A/CHAD/CIB1/CISH/HSPB1/INCA1/LRRTM1/MAPK8IP1/PPP1R1B/PRKAR1A/PRKAR1B/PRKRIP1/RTN4RL1/RTN4RL2/SFN/SH3BP5/SH3BP5L	21	MF
GO:0004197	cysteine-type endopeptidase activity	18/2726	83/17354	0.09226	0.7701	0.75797	BLMH/CAPN10/CAPN11/CAPN15/CAPN8/CASP1/CASP3/CASP5/CASP6/CTSD/CTSF/CTSH/CTSL/CTSW/USP2/USP20/USP49/USP5	18	MF
GO:0022841	potassium ion leak channel	5/2726	16/17354	0.09269	0.7701	0.75797	KCNK12/KCNK13/KCNK17/KCNK4/KCNK7	5	MF
GO:0031435	mitogen-activated protein kinase kinase kinase	5/2726	16/17354	0.09269	0.7701	0.75797	DAB2IP/MAP4K2/MAPK8IP1/MARVELD3/TRAF2	5	MF
GO:0050840	extracellular matrix binding	12/2726	51/17354	0.09372	0.7701	0.75797	ACHE/AGRN/BGN/C6orf15/ECM1/FBLN2/GPC1/LGALS3/SHH/SPOCK2/TGFBI/TINAGL1	12	MF
GO:0005216	ion channel activity	77/2726	425/17354	0.09595	0.7701	0.75797	ANO1/ANO2/ANO4/ANO7/AQP1/ASIC2/ASIC3/BEST3/BEST4/BSND/CACNA1F/CACNA1G/CACNA1H/CACNA1S/CACNB1/CACNB3/CACNG1/CALHM2/CALHM3/CATSPER1/CHRNA6/CHRNB1/CHRNB3/CLCN2/CLCNKA/CLCNKB/CLDN4/CLIC1/CNGA2/CNGB1/DLG4/GLRA1/GPR89A/GRIA3/GRIN1/GRIN2C/HCN3/HTR3B/ITPR3/JPH3/KCNAB3/KCND1/KCND3/KCNH4/KCNH6/KCNJ18/KCNJ5/KCNJ9/KCNK12/KCNK13/KCNK17/KCNK4/KCNK7/KCNQ1/KCNQ4/KCNT1/OPRM1/ORAI1/ORAI3/P2RX2/PDE2A/PKD1/PTK2B/RASA3/SCN11A/SCNN1A/SLC26A1/SLC26A10/SLC26A6/TMEM109/TMEM15[illegible]	77	MF
GO:0016755	transferase activity, transferring amino-acyl	6/2726	21/17354	0.09875	0.7701	0.75797	CHAC1/GGACT/GGT6/TGM1/TGM5/TGM7	6	MF
GO:0019200	carbohydrate kinase activity	6/2726	21/17354	0.09875	0.7701	0.75797	GALK1/GCK/HK3/NAGK/PFKFB1/PFKM	6	MF

GO:0022884	macromolecule transmembrane transporter	6/2726	21/17354	0.09875	0.7701	0.75797	AZGP1/GPIHBP1/SIDT2/TIMM17B/TIMM22/TIMM23B	6	MF
GO:0070530	K63-linked polyubiquitin modification-dependent protein binding	6/2726	21/17354	0.09875	0.7701	0.75797	ATRIP/FAAP20/OTUD7A/PARP10/PRPF8/TNIP2	6	MF
GO:0004860	protein kinase inhibitor activity	20/2726	95/17354	0.10081	0.7701	0.75797	BGN/CAMK2N2/CASP3/CDKN2A/CHAD/CIB1/CISH/HSPB1/INCA1/LRRTM1/MAPK8IP1/PPP1R1B/PRKAR1A/PRKAR1B/PRKRIP1/RTN4RL1/RTN4RL2/SFN/SH3BP5/SH3BP5L	20	MF
GO:0001637	G-protein coupled chemoattractant	7/2726	26/17354	0.10116	0.7701	0.75797	CCR2/CXCR1/CXCR2/CXCR3/CXCR5/GPR17/GPR35	7	MF
GO:0004709	MAP kinase kinase kinase activity	7/2726	26/17354	0.10116	0.7701	0.75797	ARAF/EGFR/MAP3K14/MAP3K15/MAP3K6/RAF1/TAOK2	7	MF
GO:0004950	chemokine receptor activity	7/2726	26/17354	0.10116	0.7701	0.75797	CCR2/CXCR1/CXCR2/CXCR3/CXCR5/GPR17/GPR35	7	MF
GO:0070325	lipoprotein particle receptor	7/2726	26/17354	0.10116	0.7701	0.75797	AP2M1/APOA5/APOB/APOC3/CLU/HSPG2/LRP1	7	MF
GO:0017147	Wnt-protein binding	8/2726	31/17354	0.10145	0.7701	0.75797	FZD2/FZD8/FZD9/PORCN/SFRP5/SMO/WIF1/WLS	8	MF
GO:0004540	ribonuclease activity	21/2726	101/17354	0.10458	0.7701	0.75797	ANG/APEX1/AZGP1/CPSF4/DIS3L/DIS3L2/ERN2/EXO1/EXOSC10/EXOSC2/ISG20L2/NOB1/PAN2/PIWIL2/PNLDC1/RNASE1/RNASE8/RNASEK/RPP21/	21	MF
GO:0016830	carbon-carbon lyase activity	12/2726	52/17354	0.10526	0.7701	0.75797	ALDOA/CSAD/DDC/FAHD1/GAD1/HDC/HMGCLL1/NPL/PPCDC/SHMT2/TYW1/TYW1B	12	MF

GO:0016653	oxidoreductase activity, acting on NAD(P)H, heme protein as acceptor	4/2726	12/17354	0.10551	0.7701	0.75797	CYB5R1/CYB5R2/NOS3/POR	4	MF
GO:0038191	neuropilin binding	4/2726	12/17354	0.10551	0.7701	0.75797	ABL1/SEMA3F/SEMA5B/SEMA7A	4	MF
GO:0043422	protein kinase B binding	4/2726	12/17354	0.10551	0.7701	0.75797	ANKRD2/ARRB2/BAD/RARA	4	MF
GO:0050321	tau-protein kinase activity	4/2726	12/17354	0.10551	0.7701	0.75797	BRSK2/CDK5/MARK2/PHKG1	4	MF
GO:0070492	oligosaccharide binding	4/2726	12/17354	0.10551	0.7701	0.75797	CHID1/LGALS9/LOXL2/SELP	4	MF
GO:1901981	phosphatidylinositol phosphate binding	27/2726	135/17354	0.10668	0.7736	0.76147	ADAP1/ARAP1/ARFIP1/ATP13A2/COMMD1/DAB2IP/FERMT2/GBF1/GSDMD/HIP1R/KCNQ1/MAPKAP1/NCF1/OSBPL5/PARD3/PFN1/PHLDA3/PLCD1/SCIN/SH3PXD2B/SNX11/TECPR1/TIRAP/TULP1/TULP3/ZFYVE1/ZFYVE26	27	MF
GO:0018024	histone-lysine N-methyltransfera	11/2726	47/17354	0.1088	0.7839	0.77161	ASH2L/EHMT1/EHMT2/MEN1/PRDM7/PRDM9/SETD1A/SETD2/SETD7/SMYD3/SUV39H1	11	MF
GO:0051082	unfolded protein binding	23/2726	113/17354	0.11125	0.7965	0.78396	AHSP/AIP/AIPL1/CCT3/CRYAB/DNAJA3/DNAJB13/DNAJB5/HEATR3/HSP90AA1/HSPA2/HSPA5/MDN1/NUDC/PFDN1/PPIB/SCAP/SERPINH1/SIL1/SYVN1/TOR1A/TRAP1/TTC1	23	MF
GO:0043028	cysteine-type endopeptidase regulator activity involved in	10/2726	42/17354	0.11216	0.7979	0.78534	BAD/CASP1/CASP3/CD27/CTSH/DPEP1/NAIP/NLRP1/NOD1/RPS6KA1	10	MF
GO:0005123	death receptor binding	5/2726	17/17354	0.11508	0.8034	0.79078	CASP3/DAB2IP/FASLG/MADD/TMBIM1	5	MF
GO:0017081	chloride channel	5/2726	17/17354	0.11508	0.8034	0.79078	BSND/SGK1/STX1A/STX8/VTI1B	5	MF

GO:0046625	sphingolipid binding	5/2726	17/17354	0.11508	0.8034	0.79078	CD300LF/CPTP/PSAP/SELP/TRAF2	5	MF
GO:0051019	mitogen-activated protein kinase	7/2726	27/17354	0.11897	0.8254	0.81245	ABL1/ARRB2/ATF7/CDK5RAP3/DUSP2/GCH1/MAPKAPK3	7	MF
GO:0008757	S-adenosylmethionine-dependent methyltransferase activity	30/2726	154/17354	0.12038	0.8287	0.81571	AS3MT/ASH2L/CMTR1/DNMT3A/EHMT1/EHMT2/GNMT/HNMT/LCMT1/LRTOMT/MEN1/MEPCE/METTL2A/METTL2B/NDUFAF7/NSUN5/NSUN5P2/PEMT/PRDM7/PRDM9/SETD1A/SETD2/SETD7/SETD9/SMYD3/SUV39H1/THUMPD2/TRMT10B/TRMT2B/VCPKMT	30	MF
GO:0008168	methyltransferase activity	40/2726	212/17354	0.12092	0.8287	0.81571	AS3MT/ASH2L/BHMT2/CIAPIN1/CMTR1/DNMT3A/EHMT1/EHMT2/GNMT/HNMT/LCMT1/LRTOMT/MEN1/MEPCE/METTL17/METTL2A/METTL2B/METTL7B/NDUFAF7/NSUN5/NSUN5P2/NTMT1/PEMT/PRDM12/PRDM14/PRDM7/PRDM8/PRDM9/SETD1A/SETD2/SETD7/SETD9/SMYD3/SMYD5/SUV39H1/THUMPD2/TRMT10B/TRMT2B/TRMT44/VCPKMT	40	MF
GO:0008013	beta-catenin binding	17/2726	81/17354	0.12559	0.8427	0.82948	AJAP1/ASH2L/AXIN1/BCL9/CALCOCO1/CDH5/CDHR5/CHD8/DACT2/FOXO4/KDM6B/PTPRU/SETD1A/SLC9A3R2/SUFU/TAX1BP3/TCF7L1	17	MF
GO:0030695	GTPase regulator activity	57/2726	313/17354	0.12614	0.8427	0.82948	ACAP1/ACAP3/ADAP1/ADPRHL1/AGAP3/ALS2CL/ARAP1/ARFGAP2/ARHGAP10/ARHGAP22/ARHGAP27/ARHGAP30/ARHGAP4/ARL2BP/AXIN1/CDC42EP2/DAB2IP/DGKI/DNAJA3/DOCK1/ELMOD1/GNB5/GPSM1/GRTP1/HACD3/LARS/LLGL1/NPRL3/PDE6D/PGAM5/PLEKHG6/PLXNB1/PREX2/RABGAP1/RABGAP1L/RALGDS/RAP1GAP/RASA3/RASA4B/RGS10/RGS14/RGS16/RIN3/RTKN/RUNDC3A/SGSM2/SIPA1/STARD8/TBC1D25/TBC1D28/T	57	MF
GO:0017137	Rab GTPase binding	34/2726	178/17354	0.12691	0.8427	0.82948	ADPRHL1/ALS2CL/DENND2A/DENND2C/DENND3/EHD1/GAS8/GRTP1/IFT20/LLGL1/MADD/MLPH/MOBP/NPC1L1/PDE6D/RAB11FIP3/RABGAP1/RABGAP1L/RBSN/RIN3/SGSM2/SYTL3/TBC1D25/TBC1D28/TBC1D9/TRAPPC1/TRAPPC2L/TRAPPC3/TRAPPC4/TRAPPC9/ULK1/USP6NL/VPS52/YIPF1	34	MF
GO:0022853	active ion transmembrane transporter	26/2726	132/17354	0.1276	0.8427	0.82948	ABCC3/ABCC4/ABCC5/ABCC6/ATP13A2/ATP1A1/ATP1A4/ATP2A3/ATP2C2/ATP5G1/ATP6V0B/ATP6V0E2/ATP6V1F/ATP6V1G1/SLC12A4/SLC12A7/SLC12A9/SLC13A2/SLC20A1/SLC20A2/SLC34A1/SLC4A9/SLC6A6/SLC6A7/S	26	MF

GO:0050839	cell adhesion molecule binding	82/2726	463/17354	0.12883	0.8427	0.82948	ABCF3/ADAM8/ADAMTS13/AHNAK/ALDOA/ARFIP1/ARHGEF16/ATXN2L/BAIAP2L1/CCNB2/CCS/CDH13/CDHR5/CIB2/CKAP5/CLIC1/COL16A1/CORO1B/CTNNA2/CTTN/DAB2IP/DBN1/EGFR/EHD1/EIF4G1/EMD/EMILIN1/EPS8L2/FERMT3/FGA/FLNA/FMNL2/FN1/FSCN1/GOLGA2/GOLGA3/H1FX/HCFC1/HSPA5/IDH1/ILK/ITGAL/KDR/KLC2/LDHA/LRRFIP1/LYPLA2/MARK2/NCR3/NISCH/NLGN3/NRG1/NRXN2/NUDC/OLFM4/PDLIM1/PDLIM5/PFN1/PKM/PKP3/PRDX1/PRDX6/PSMB6/PTPN11/SCRIB/SEMA7A/SEPT9/SFN/SH3GLB2/SLC9A3R2/SND1/SPTAN1/SPTBN2/STAT1/STX5/TGFBI/THY1/TL[illegible]	82	MF
GO:0005484	SNAP receptor activity	9/2726	38/17354	0.131	0.8427	0.82948	GOSR2/STX18/STX1A/STX4/STX5/STX8/VAMP1/VAMP5/VTI1B	9	MF
GO:0032451	demethylase activity	9/2726	38/17354	0.131	0.8427	0.82948	ALKBH4/ALKBH5/CYP1A1/CYP1A2/HR/KDM4E/KDM6B/KDM8/PHF2	9	MF
GO:0140101	catalytic activity, acting on a tRNA	24/2726	121/17354	0.13117	0.8427	0.82948	AARS/AARS2/C12orf65/CDKAL1/DALRD3/DARS/GATB/KARS/LARS/METTL2A/METTL2B/PARS2/PTRH1/RPP21/SARS/TARS2/THUMPD2/TRMT10B/TRMT2B/TRMT44/TYW1/TYW1B/YARS/YARS2	24	MF
GO:0005261	cation channel activity	57/2726	314/17354	0.13148	0.8427	0.82948	ANO1/AQP1/ASIC2/ASIC3/CACNA1F/CACNA1G/CACNA1H/CACNA1S/CACNB1/CACNB3/CACNG1/CALHM2/CALHM3/CATSPER1/CHRNA6/CHRNB1/CHRNB3/CNGA2/CNGB1/GRIA3/GRIN1/GRIN2C/HCN3/HTR3B/ITPR3/JPH3/KCNAB3/KCND1/KCND3/KCNH4/KCNH6/KCNJ18/KCNJ5/KCNJ9/KCNK12/KCNK13/KCNK17/KCNK4/KCNK7/KCNQ1/KCNQ4/KCNT1/OPRM1/ORAI1/ORAI3/P2RX2/PDE2A/PKD1/PTK2B/RASA3/SCN11A/SCNN1A/TMEM[illegible]	57	MF
GO:0030246	carbohydrate binding	49/2726	267/17354	0.13389	0.8427	0.82948	ACAN/ALDOA/ASGR1/BCAN/CD69/CDIPT/CHID1/CLEC18A/CLEC2L/CLEC7A/CLECL1/ENG/FBXO2/FBXO6/FUOM/GALK1/GALM/GALNT16/GALNT8/GALNT9/GANAB/GCK/HK3/KLRC1/KLRC2/KLRG1/KLRG2/LAYN/LGALS3/LGALS9/LGALS9B/LGALS9C/LMAN2/LOXL2/MGAM/MRC2/OGFOD2/OLR1/OS9/P3H1/P3H3/PFKM/PKD1/PLOD3/REG4/SELP/SFTPA1/SLC2A8/TI[illegible]	49	MF
GO:0003906	DNA-(apurinic or apyrimidinic site) lyase	4/2726	13/17354	0.13428	0.8427	0.82948	APEX1/HMGA1/NEIL2/OGG1	4	MF

GO:0034595	phosphatidylinositol phosphate 5-phosphatase activity	4/2726	13/17354	0.13428	0.8427	0.82948	INPP5D/INPP5E/INPP5F/INPPL1	4	MF
GO:0043225	ATPase-coupled anion transmembrane transporter	4/2726	13/17354	0.13428	0.8427	0.82948	ABCC3/ABCC4/ABCC5/ABCC6	4	MF
GO:0060229	lipase activator activity	4/2726	13/17354	0.13428	0.8427	0.82948	APOA5/CASP3/CCL5/STX4	4	MF
GO:0004879	nuclear receptor activity	11/2726	49/17354	0.13636	0.8427	0.82948	ESR2/ESRRA/GPER1/NR1H3/NR2F1/NR4A1/NR5A1/PPARD/RARA/RORC/THRA	11	MF
GO:0098531	transcription factor activity, direct ligand regulated sequence-	11/2726	49/17354	0.13636	0.8427	0.82948	ESR2/ESRRA/GPER1/NR1H3/NR2F1/NR4A1/NR5A1/PPARD/RARA/RORC/THRA	11	MF
GO:0008320	protein transmembrane transporter	5/2726	18/17354	0.13976	0.8427	0.82948	AZGP1/GPIHBP1/TIMM17B/TIMM22/TIMM23B	5	MF
GO:0008569	ATP-dependent microtubule motor activity, minus-end-	5/2726	18/17354	0.13976	0.8427	0.82948	DNAH11/DNAH17/DNHD1/DYNC1H1/KIFC3	5	MF
GO:0070064	proline-rich region binding	5/2726	18/17354	0.13976	0.8427	0.82948	ABL1/APBB1/BAIAP2L1/CSK/PFN1	5	MF
GO:0071837	HMG box domain binding	5/2726	18/17354	0.13976	0.8427	0.82948	HOXA3/HOXC4/PAX3/POU3F3/UTF1	5	MF
GO:0030552	cAMP binding	6/2726	23/17354	0.14025	0.8427	0.82948	CNGA2/CNGB1/HCN3/PDE2A/PRKAR1A/PRKAR1B	6	MF

GO:0140103	catalytic activity, acting on a	6/2726	23/17354	0.14025	0.8427	0.82948	B3GNT6/B4GALNT4/B4GALT7/MGAT1/MGAT4B/MGAT5B	6	MF
GO:0015296	anion:cation symporter	12/2726	55/17354	0.14464	0.8427	0.82948	SLC12A4/SLC12A7/SLC12A9/SLC13A2/SLC20A1/SLC20A2/SLC34A1/SLC4A9/SLC6A6/SLC6A7/SLC6A8/SLC6A9	12	MF
GO:0097110	scaffold protein binding	12/2726	55/17354	0.14464	0.8427	0.82948	CACNA1G/CACNA1H/CASP5/DLG4/DYNLL2/IKBKB/KCNQ1/MAPK3/NLGN3/NLRP1/NOS3/TREM2	12	MF
GO:0015108	chloride transmembrane transporter	19/2726	94/17354	0.14473	0.8427	0.82948	ANO1/ANO2/ANO4/ANO7/BEST3/BEST4/BSND/CLCN2/CLCNKA/CLCNKB/CLDN4/CLIC1/GLRA1/SLC12A4/SLC12A7/SLC12A9/SLC26A1/SLC26A10/SLC26A6	19	MF
GO:0052689	carboxylic ester hydrolase activity	27/2726	140/17354	0.14687	0.8427	0.82948	AARS/ACHE/ACOT7/ACOT9/BPHL/C12orf65/CES2/CES4A/DAGLB/GDPD1/LARS/LOC344967/LYPLA2/NLGN3/PLA2G15/PLA2G16/PLA2G1B/PLA2G2F/PLA2G4B/PLA2G5/PLB1/PNPLA2/PNPLA7/PRDX6/PTRH1/TARS2/TMEM8	27	MF
GO:0001786	phosphatidylserine binding	9/2726	39/17354	0.1479	0.8427	0.82948	ADGRB1/CD300LF/GSDMD/OSBPL5/PLCD1/SCARB1/SCIN/TLN1/TRIM72	9	MF
GO:0008017	microtubule binding	41/2726	222/17354	0.14833	0.8427	0.82948	CCDC88B/CCSER2/CFAP157/CKAP5/CRYAB/DNM1/DYNC1I1/EML1/EML3/EML5/FES/FNTA/GAPDH/GAS8/GOLGA2/KATNB1/KIF13A/KIF17/KIF23/KIF26A/KIF2B/KIF4A/KIF9/KIFC3/MAP6D1/MAPRE3/NEIL2/NUMA1/OGG1/PEX14/REEP4/RGS14/SAXO2/TRAF3IP1/TRIM54/TRPV4/TUBGCP2/TUBGCP	41	MF
GO:0032947	protein complex scaffold activity	15/2726	72/17354	0.15033	0.8427	0.82948	AIMP2/ARRB2/AXIN1/CAV3/DISC1/EIF3B/EIF4G1/HOMER2/KSR1/MAPK8IP1/MAPK8IP3/PDE4DIP/RGS14/SLC9A3R2/TELO2	15	MF
GO:0017112	Rab guanyl-nucleotide exchange factor activity	11/2726	50/17354	0.15141	0.8427	0.82948	ALS2CL/DENND2A/DENND2C/DENND3/MADD/RIN3/TRAPPC1/TRAPPC2L/TRAPPC3/TRAPPC4/TRAPPC9	11	MF
GO:0005546	phosphatidylinositol-4,5-bisphosphate binding	13/2726	61/17354	0.15176	0.8427	0.82948	COMMD1/GSDMD/HIP1R/KCNQ1/MAPKAP1/PARD3/PFN1/PHLDA3/PLCD1/SCIN/TIRAP/TULP1/TULP3	13	MF

GO:0016278	lysine N-methyltransferase activity	13/2726	61/17354	0.15176	0.8427	0.82948	ASH2L/EHMT1/EHMT2/MEN1/PRDM7/PRDM9/SETD1A/SETD2/SETD7/SETD9/SMYD3/SUV39H1/VCPKMT	13	MF
GO:1902936	phosphatidylinositol bisphosphate	18/2726	89/17354	0.152	0.8427	0.82948	ATP13A2/COMMD1/GBF1/GSDMD/HIP1R/KCNQ1/MAPKAP1/NCF1/PARD3/PFN1/PHLDA3/PLCD1/SCIN/SH3PXD2B/TIRAP/TULP1/TULP3/ZFYVE1	18	MF
GO:0008376	acetylgalactosaminyltransferase activity	8/2726	34/17354	0.1536	0.8427	0.82948	B4GALNT1/B4GALNT4/CHPF/CHPF2/GALNT16/GALNT8/GALNT9/GBGT1	8	MF
GO:0019840	isoprenoid binding	8/2726	34/17354	0.1536	0.8427	0.82948	FNTB/LCN12/OPN4/RARA/RBP1/RBP2/RBP7/SERPINA5	8	MF
GO:0005201	extracellular matrix structural	16/2726	78/17354	0.15532	0.8427	0.82948	ACAN/BCAN/BGN/COL1A1/COL4A2/COL9A2/EFEMP2/ELN/EMILIN1/FBLN2/HAPLN3/LAMA4/MUC3A/MUC5AC/MUC6/TINAGL1	16	MF
GO:0004867	serine-type endopeptidase inhibitor	19/2726	95/17354	0.15571	0.8427	0.82948	A2ML1/AGT/APLP2/COL7A1/FURIN/ITIH4/ITIH6/PEBP1/SERPINA2/SERPINA3/SERPINA4/SERPINA5/SERPINF2/SERPINH1/SERPINI1/SPINK2/SPINK5/SPINT1/WFIKKN2	19	MF
GO:0008144	drug binding	20/2726	101/17354	0.15904	0.8427	0.82948	CHRM1/CHRNB3/CUBN/DHODH/DRD2/FKBP10/FKBP6/FKBP9/FNTB/FOLR2/FSCN1/MT2A/NPC1L1/PDE2A/PPARD/PPIE/RARA/SIGMAR1/SMO/TLR	20	MF
GO:0003777	microtubule motor activity	17/2726	84/17354	0.15973	0.8427	0.82948	DNAH11/DNAH12/DNAH17/DNHD1/DYNC1H1/DYNC1I1/DYNLL2/KIF13A/KIF17/KIF23/KIF26A/KIF2B/KIF4A/KIF9/KIFC3/KLC1/KLC2	17	MF
GO:0004190	aspartic-type endopeptidase activity	6/2726	24/17354	0.16358	0.8427	0.82948	CASP3/CTSD/CTSE/PGC/REN/SPPL2C	6	MF
GO:0043325	phosphatidylinositol-3,4-bisphosphate binding	6/2726	24/17354	0.16358	0.8427	0.82948	COMMD1/HIP1R/MAPKAP1/NCF1/PHLDA3/ZFYVE1	6	MF

GO:0016746	transferase activity, transferring acyl groups	46/2726	254/17354	0.1649	0.8427	0.82948	ACSM6/ALAS2/ATF2/BRPF1/CHAC1/CIITA/CPT2/CROT/CS/DGAT1/DLST/ELOVL1/ELOVL5/EPC1/GGACT/GGT6/GLYATL1/GNPAT/HADHA/HCFC1/KAT2A/NAA16/NAA60/NAGS/NAT16/NAT6/NAT8B/OLAH/PLA2G15/PLA2G16/PNPLA2/PORCN/SOAT2/SPTLC1/TADA3/TAF1/TAF1L/TGM1/TGM5/TGM7/ZDHHC11/ZDHHC12/ZDHHC14/ZDHHC15/ZDHHC18/ZDHHC7	46	MF
GO:0005095	GTPase inhibitor	4/2726	14/17354	0.16581	0.8427	0.82948	DGKI/PDE6D/RTKN/TNK2	4	MF
GO:0016918	retinal binding	4/2726	14/17354	0.16581	0.8427	0.82948	OPN4/RBP1/RBP2/RBP7	4	MF
GO:0031005	filamin binding	4/2726	14/17354	0.16581	0.8427	0.82948	DPYSL4/FBLIM1/HSPB7/OPRM1	4	MF
GO:0034236	protein kinase A catalytic subunit binding	4/2726	14/17354	0.16581	0.8427	0.82948	CSK/KCNQ1/PRKAR1A/PRKAR1B	4	MF
GO:0050700	CARD domain binding	4/2726	14/17354	0.16581	0.8427	0.82948	CARD11/CARD9/CASP1/NOD1	4	MF
GO:0005245	voltage-gated calcium channel activity	9/2726	40/17354	0.16582	0.8427	0.82948	CACNA1F/CACNA1G/CACNA1H/CACNA1S/CACNB1/CACNB3/CACNG1/CATSPER1/OPRM1	9	MF
GO:0019894	kinesin binding	9/2726	40/17354	0.16582	0.8427	0.82948	ARHGEF10/CLSTN1/CROCC/DISC1/KLC2/MAPK8IP1/MAPK8IP3/PLEKHM2/TOR1A	9	MF
GO:0015077	monovalent inorganic cation transmembrane transporter activity	65/2726	368/17354	0.16588	0.8427	0.82948	AQP1/ASIC2/ASIC3/ATP1A1/ATP1A4/ATP5G1/ATP5I/ATP6V0B/ATP6V0E2/ATP6V1B1/ATP6V1F/ATP6V1G1/CACNA1G/CACNA1H/CNGA2/CNGB1/COX10/COX11/COX8A/HCN3/KCNAB3/KCND1/KCND3/KCNH4/KCNH6/KCNJ18/KCNJ5/KCNJ9/KCNK12/KCNK13/KCNK17/KCNK4/KCNK7/KCNQ1/KCNQ4/KCNT1/NDUFA4L2/SCN11A/SCNN1A/SLC11A1/SLC12A4/SLC12A7/SLC12A9/SLC13A2/SLC1A7/SLC20A1/SLC20A2/SLC25A22/SLC28A1/SLC28A2/SLC34A1/SLC36A1/SLC36A3/SLC38A1/SLC4A9/SLC5A2/SLC6A18/SLC6A2/SLC6A6/SLC6A7/SLC6A8/SLC6A9/SLC9A3/SLC9A5/TRPM5	65	MF

GO:0016780	phosphotransferase activity, for other substituted	5/2726	19/17354	0.16651	0.8427	0.82948	CDIPT/PIGG/PIGO/PTDSS2/SGMS1	5	MF
GO:0022840	leak channel activity	5/2726	19/17354	0.16651	0.8427	0.82948	KCNK12/KCNK13/KCNK17/KCNK4/KCNK7	5	MF
GO:0022842	narrow pore channel activity	5/2726	19/17354	0.16651	0.8427	0.82948	KCNK12/KCNK13/KCNK17/KCNK4/KCNK7	5	MF
GO:0030506	ankyrin binding	5/2726	19/17354	0.16651	0.8427	0.82948	ATP1A1/FLNC/OBSCN/RHCG/SPTB	5	MF
GO:0052745	inositol phosphate phosphatase	5/2726	19/17354	0.16651	0.8427	0.82948	INPP5D/INPP5E/INPP5F/INPPL1/ITPK1	5	MF
GO:0005539	glycosaminoglycan binding	39/2726	213/17354	0.16913	0.8427	0.82948	ACAN/AGRN/ANG/APLP2/APOA5/APOB/BCAN/BGN/BMP4/C6orf15/CFH/ENG/FGFBP3/FGFR2/FGFR4/FGFRL1/FN1/HAPLN3/KNG1/LAMC2/LAYN/MPO/NOD1/PCOLCE/PF4V1/PLA2G5/PODXL2/PTPRF/REG4/RSPO1/RTN4RL1/SELP/SERPINA5/SHH/SOD3/SOST/SPOCK2/THBS3/TREM2	39	MF
GO:0015103	inorganic anion transmembrane transporter	26/2726	137/17354	0.17308	0.8427	0.82948	ANO1/ANO2/ANO4/ANO7/BEST3/BEST4/BSND/CLCN2/CLCNKA/CLCNKB/CLDN4/CLIC1/GLRA1/SLC12A4/SLC12A7/SLC12A9/SLC20A1/SLC20A2/SLC22A12/SLC22A13/SLC22A9/SLC26A1/SLC26A10/SLC26A6/SLC34A1/SLC4	26	MF
GO:0008200	ion channel inhibitor	8/2726	35/17354	0.17337	0.8427	0.82948	ANKRD36C/CAMK2D/CAV3/PACSIN3/PDZD3/STX1A/STX8/VTI1B	8	MF
GO:0048487	beta-tubulin binding	8/2726	35/17354	0.17337	0.8427	0.82948	ARL8A/BCAS3/EMD/PEX14/SLC6A2/TRPV4/TTLL7/UXT	8	MF
GO:0032813	tumor necrosis factor receptor superfamily binding	10/2726	46/17354	0.17575	0.8427	0.82948	CASP3/DAB2IP/FASLG/LTA/MADD/STAT1/TMBIM1/TNFSF13/TRAF2/TRAP1	10	MF

GO:0001076	transcription factor activity, RNA polymerase II transcription	29/2726	155/17354	0.17746	0.8427	0.82948	ATF2/BRCA1/CITED4/CTBP1/GATA4/GBX2/LHX3/MED11/MED18/MED22/MED24/MED6/MED9/NFE2L1/NKX2-5/NOTCH1/NUP98/OLIG3/PARP9/PHF12/PITX1/POU3F1/SPEN/TAF1/TAF1L/TBX15/TTF1/UXT/WWP2	29	MF
GO:0001664	G-protein coupled receptor binding	47/2726	262/17354	0.17937	0.8427	0.82948	ADM/ADRA2A/ADRA2C/AGT/ARAP1/ARRB2/CCL1/CCL19/CCL21/CCL5/CCR2/CKLF/DLG4/DNM1/DVL2/EDN2/FLNA/GCG/GNAO1/GNAT1/GNG3/GPHA2/HOMER2/MYOC/NECAB2/NPPA/NPW/PF4V1/PPP1R1B/PSAP/PTCH2/PTPN11/PYY/REEP2/RSPO1/SFTPB/UCN2/USP20/USP4/WLS/WNT1/WNT10A/WNT11/WNT3A/WNT6/WNT7A/YARS	47	MF
GO:0051536	iron-sulfur cluster binding	13/2726	63/17354	0.18093	0.8427	0.82948	ACO1/CDKAL1/CIAPIN1/GLRX5/ISCA2/LIAS/NARFL/NUBP1/NUBP2/POLE/TYW1/TYW1B/XDH	13	MF
GO:0051540	metal cluster binding	13/2726	63/17354	0.18093	0.8427	0.82948	ACO1/CDKAL1/CIAPIN1/GLRX5/ISCA2/LIAS/NARFL/NUBP1/NUBP2/POLE/TYW1/TYW1B/XDH	13	MF
GO:0000175	3'-5'-exoribonuclease activity	7/2726	30/17354	0.18099	0.8427	0.82948	DIS3L/DIS3L2/EXOSC10/EXOSC2/ISG20L2/PAN2/PNLDC1	7	MF
GO:0015248	sterol transporter	7/2726	30/17354	0.18099	0.8427	0.82948	ABCG5/APOA5/APOB/NPC2/OSBPL7/STARD3/STARD5	7	MF
GO:0099094	ligand-gated cation channel	16/2726	80/17354	0.18118	0.8427	0.82948	ASIC2/ASIC3/CHRNA6/CHRNB1/CHRNB3/GRIA3/GRIN1/GRIN2C/ITPR3/JPH3/KCNJ18/KCNJ5/KCNJ9/PTK2B/RASA3/SCNN1A	16	MF
GO:0050662	coenzyme binding	34/2726	185/17354	0.18232	0.8427	0.82948	ACAD10/ACADVL/ACBD4/ACOT7/ACOXL/ALAS2/ALDH2/CHDH/CRYM/CTBP1/GAPDH/GCH1/GFER/GLUD1/GSR/HADHA/HSD11B2/HSD17B1/IDH1/IDH2/LDHA/MICAL2/NOS3/NOX4/NQO2/OGDH/OGDHL/PHGDH/POR/SOAT2/TH/TXNRD1/TXNRD3/XDH	34	MF
GO:0003899	DNA-directed 5'-3' RNA polymerase activity	9/2726	41/17354	0.18472	0.8427	0.82948	POLR1A/POLR1D/POLR1E/POLR2G/POLR2J2/POLR2J3/POLR2L/POLR3D/ZNRD1	9	MF

GO:0005262	calcium channel activity	22/2726	115/17354	0.18663	0.8427	0.82948	CACNA1F/CACNA1G/CACNA1H/CACNA1S/CACNB1/CACNB3/CACNG1/CATSPER1/GRIN1/GRIN2C/ITPR3/JPH3/OPRM1/ORAI1/ORAI3/PDE2A/PKD1/RASA3/TMEM37/TRPV2/TRPV4/TRPV6	22	MF
GO:0070001	aspartic-type peptidase	6/2726	25/17354	0.18842	0.8427	0.82948	CASP3/CTSD/CTSE/PGC/REN/SPPL2C	6	MF
GO:0005109	frizzled binding	8/2726	36/17354	0.1942	0.8427	0.82948	DVL2/MYOC/WNT1/WNT10A/WNT11/WNT3A/WNT6/WNT7A	8	MF
GO:0016248	channel inhibitor	8/2726	36/17354	0.1942	0.8427	0.82948	ANKRD36C/CAMK2D/CAV3/PACSIN3/PDZD3/STX1A/STX8/VTI1B	8	MF
GO:0043531	ADP binding	8/2726	36/17354	0.1942	0.8427	0.82948	ACTA1/ATP1A1/GLUD1/MYO18A/MYO3A/MYO7A/PKM/TAP1	8	MF
GO:0008194	UDP-glycosyltransferase activity	27/2726	145/17354	0.19471	0.8427	0.82948	A4GNT/B3GAT3/B3GNT6/B4GALNT1/B4GALNT4/B4GALT2/B4GALT7/CHPF/CHPF2/EXTL1/GALNT16/GALNT8/GALNT9/GBGT1/HEXA/LFNG/MGAT1/MGAT4B/MGAT5B/PIGC/PIGQ/PIGY/PLOD3/RFNG/UGT3A2/XXYLT1/X	27	MF
GO:0005086	ARF guanyl-nucleotide exchange factor activity	5/2726	20/17354	0.19508	0.8427	0.82948	GBF1/IQSEC2/PSD/PSD2/PSD4	5	MF
GO:0043395	heparan sulfate proteoglycan binding	5/2726	20/17354	0.19508	0.8427	0.82948	AGRN/CFH/GPC1/GPC2/LRP1	5	MF
GO:1901338	catecholamine binding	5/2726	20/17354	0.19508	0.8427	0.82948	ADRA2A/ADRA2B/ADRA2C/DRD2/TH	5	MF
GO:0015081	sodium ion transmembrane transporter activity	29/2726	157/17354	0.19682	0.8427	0.82948	ASIC2/ASIC3/ATP1A1/ATP1A4/CACNA1G/CACNA1H/HCN3/SCN11A/SCNN1A/SLC13A2/SLC1A7/SLC20A1/SLC20A2/SLC25A22/SLC28A1/SLC28A2/SLC34A1/SLC38A1/SLC4A9/SLC5A2/SLC6A18/SLC6A2/SLC6A6/SLC6A7/SLC6A8/SLC6A9/SLC9A3/SLC9A5/TRPM5	29	MF

GO:0016820	hydrolase activity, acting on acid anhydrides, catalyzing transmembrane movement of	22/2726	116/17354	0.19812	0.8427	0.82948	ABCA2/ABCB5/ABCB9/ABCC12/ABCC3/ABCC4/ABCC5/ABCC6/ABCG5/ATP13A2/ATP1A1/ATP1A4/ATP2A3/ATP2C2/ATP5G1/ATP6V0B/ATP6V0E2/ATP6V1B1/ATP6V1F/ATP6V1G1/ATP6V1G2/TAP1	22	MF
GO:0004576	oligosaccharyl transferase	3/2726	10/17354	0.19847	0.8427	0.82948	DAD1/DDOST/RPN1	3	MF
GO:0005338	nucleotide-sugar transmembrane	3/2726	10/17354	0.19847	0.8427	0.82948	SLC35A2/SLC35C1/SLC35D2	3	MF
GO:0005436	sodium:phosphate symporter	3/2726	10/17354	0.19847	0.8427	0.82948	SLC20A1/SLC20A2/SLC34A1	3	MF
GO:0008190	eukaryotic initiation factor 4E binding	3/2726	10/17354	0.19847	0.8427	0.82948	ANGEL1/EIF4EBP1/EIF4G1	3	MF
GO:0016176	superoxide-generating NADPH oxidase	3/2726	10/17354	0.19847	0.8427	0.82948	NCF1/NOXO1/SH3PXD2B	3	MF
GO:0016857	racemase and epimerase activity, acting on carbohydrates	3/2726	10/17354	0.19847	0.8427	0.82948	DSE/FUOM/GALM	3	MF
GO:0036122	BMP binding	3/2726	10/17354	0.19847	0.8427	0.82948	ENG/NBL1/TCAP	3	MF
GO:0039706	co-receptor binding	3/2726	10/17354	0.19847	0.8427	0.82948	BMP4/TFR2/WNT3A	3	MF

GO:0070016	armadillo repeat domain binding	3/2726	10/17354	0.19847	0.8427	0.82948	AXIN1/CALCOCO1/CHD8	3	MF
GO:0097001	ceramide binding	3/2726	10/17354	0.19847	0.8427	0.82948	CD300LF/CPTP/PSAP	3	MF
GO:0140078	class I DNA-(apurinic or apyrimidinic site) lyase	3/2726	10/17354	0.19847	0.8427	0.82948	APEX1/NEIL2/OGG1	3	MF
GO:0140080	class III/IV DNA-(apurinic or apyrimidinic site) lyase	3/2726	10/17354	0.19847	0.8427	0.82948	APEX1/NEIL2/OGG1	3	MF
GO:1990380	Lys48-specific deubiquitinase activity	3/2726	10/17354	0.19847	0.8427	0.82948	OTUD5/USP19/USP27X	3	MF
GO:0004659	prenyltransferase activity	4/2726	15/17354	0.19964	0.8427	0.82948	COQ2/COX10/FNTA/FNTB	4	MF
GO:0005351	sugar:proton symporter	4/2726	15/17354	0.19964	0.8427	0.82948	SLC2A6/SLC2A8/SLC35A2/SLC45A4	4	MF
GO:0005402	cation:sugar symporter	4/2726	15/17354	0.19964	0.8427	0.82948	SLC2A6/SLC2A8/SLC35A2/SLC45A4	4	MF
GO:0016594	glycine binding	4/2726	15/17354	0.19964	0.8427	0.82948	ALAS2/GLRA1/GNMT/GRIN1	4	MF
GO:0030546	receptor activator	4/2726	15/17354	0.19964	0.8427	0.82948	CDK5/DGKQ/NRG1/SLURP1	4	MF
GO:0030676	Rac guanyl-nucleotide exchange factor activity	4/2726	15/17354	0.19964	0.8427	0.82948	ARHGEF4/EPS8L2/FARP2/PREX2	4	MF
GO:0070742	C2H2 zinc finger domain	4/2726	15/17354	0.19964	0.8427	0.82948	EHMT1/EHMT2/SRRM2/ZXDC	4	MF

GO:0097153	cysteine-type endopeptidase activity involved in	4/2726	15/17354	0.19964	0.8427	0.82948	CASP1/CASP3/CASP5/CASP6	4	MF
GO:0008565	protein transporter	19/2726	99/17354	0.20392	0.8427	0.82948	AP1G2/AP1S1/AP2A2/AP4S1/AZGP1/CHMP7/GPIHBP1/IPO13/IPO4/LRP1/TIMM17B/TIMM22/TIMM23B/TIMM9/TMCO6/TNPO3/TSNAX/XPO6/XPO7	19	MF
GO:0005158	insulin receptor binding	7/2726	31/17354	0.20419	0.8427	0.82948	DOK1/DOK2/DOK3/DOK7/INS/PTPN11/SHC1	7	MF
GO:0016417	S-acyltransferase	7/2726	31/17354	0.20419	0.8427	0.82948	DLST/ZDHHC11/ZDHHC12/ZDHHC14/ZDHHC15/ZDHHC18/ZDHHC7	7	MF
GO:0060589	nucleoside-triphosphatase regulator activity	61/2726	350/17354	0.20474	0.8427	0.82948	ACAP1/ACAP3/ADAP1/ADPRHL1/AGAP3/ALS2CL/ARAP1/ARFGAP2/ARHGAP10/ARHGAP22/ARHGAP27/ARHGAP30/ARHGAP4/ARL2BP/ATPIF1/AXIN1/BRSK2/CDC42EP2/DAB2IP/DGKI/DNAJA3/DOCK1/ELMOD1/GNB5/GPSM1/GRTP1/HACD3/LARS/LLGL1/MYBPC3/NPRL3/PDE6D/PFN1/PGAM5/PLEKHG6/PLXNB1/PREX2/RABGAP1/RABGAP1L/RALGDS/RAP1GAP/RASA3/RASA4B/RGS10/RGS14/RGS16/RIN3/RTKN/RUNDC3A/SGSM2/SIPA1/STARD8/TBC1D25/TBC1D28/TBC1D9/THY1/TNK2/USP6NL/VPS9D1/WAS/[illegible]	61	MF
GO:0017022	myosin binding	12/2726	59/17354	0.20744	0.8427	0.82948	ACTA1/CORO1A/MLPH/MOBP/MYBPC3/MYL4/MYO19/MYOC/NKD2/NPC1L1/RAB25/STX1A	12	MF
GO:0042054	histone methyltransferase activity	12/2726	59/17354	0.20744	0.8427	0.82948	ASH2L/EHMT1/EHMT2/MEN1/NTMT1/PRDM7/PRDM9/SETD1A/SETD2/SETD7/SMYD3/SUV39H1	12	MF
GO:0005254	chloride channel activity	16/2726	82/17354	0.20902	0.8427	0.82948	ANO1/ANO2/ANO4/ANO7/BEST3/BEST4/BSND/CLCN2/CLCNKA/CLCNKB/CLDN4/CLIC1/GLRA1/SLC26A1/SLC26A10/SLC26A6	16	MF
GO:0005343	organic acid:sodium symporter	6/2726	26/17354	0.21459	0.8427	0.82948	SLC13A2/SLC38A1/SLC6A6/SLC6A7/SLC6A8/SLC6A9	6	MF
GO:0005319	lipid transporter activity	26/2726	141/17354	0.21497	0.8427	0.82948	ABCC3/ABCG5/ANO4/ANO7/APOA5/APOB/CPTP/GLTP/GLTPD2/HEATR4/MFSD2A/MROH6/NPC2/OSBPL5/OSBPL7/PITPNM1/PRELID1/SFTPA1/SLC22A9/SLCO2B1/SPNS1/SPNS3/STARD3/STARD5/STRA6/TNFAIP8L3	26	MF

GO:0016504	peptidase activator	8/2726	37/17354	0.21601	0.8427	0.82948	BAD/CASP1/CASP3/CTSH/FN1/NLRP1/NOD1/PCOLCE	8	MF
GO:0072509	divalent inorganic cation transmembrane transporter	32/2726	177/17354	0.21853	0.8427	0.82948	ATP13A2/ATP2A3/ATP2C2/CACNA1F/CACNA1G/CACNA1H/CACNA1S/CACNB1/CACNB3/CACNG1/CATSPER1/CNNM2/GRIN1/GRIN2C/ITPR3/JPH3/NIPA2/NIPAL1/NIPAL2/OPRM1/ORAI1/ORAI3/PDE2A/PKD1/RASA3/SLC30A3/SLC39A13/SLC39A5/TMEM37/TRPV2/TRPV4/TRPV6	32	MF
GO:0004857	enzyme inhibitor activity	68/2726	395/17354	0.22091	0.8427	0.82948	A2ML1/AGT/AHSG/APLP2/APOC3/ATPIF1/BGN/CAMK2N2/CASP3/CCAR2/CD27/CDKN2A/CHAD/CIB1/CISH/COL7A1/CRB2/DGKI/DPEP1/FURIN/GAPDH/GCHFR/HSPB1/INCA1/ITIH4/ITIH6/KNG1/LRRTM1/MAPK8IP1/NAIP/NOTCH1/OAZ2/PARP9/PDE6D/PDZD3/PEBP1/PHACTR1/PI16/PIF1/PPP1R10/PPP1R1B/PPP1R9B/PRKAR1A/PRKAR1B/PRKRIP1/RNH1/RPS6KA1/RTKN/RTN4RL1/RTN4RL2/SCGB1A1/SERPINA2/SERPINA3/SERPINA4/SERPINA5/SERPINF2/SERPINH1/SERPINI1/SFN/SH3BP5/SH3BP5L/SNCB/SPINK2/S[illegible]	68	MF
GO:0008509	anion transmembrane transporter activity	55/2726	316/17354	0.22185	0.8427	0.82948	ABCC3/ABCC4/ABCC5/ABCC6/ANO1/ANO2/ANO4/ANO7/ASIC3/BEST3/BEST4/BSND/CLCN2/CLCNKA/CLCNKB/CLDN4/CLIC1/GLRA1/GPR89A/MPC1/PQLC2/SLC12A4/SLC12A7/SLC12A9/SLC13A2/SLC16A13/SLC1A7/SLC20A1/SLC20A2/SLC22A1/SLC22A12/SLC22A13/SLC22A9/SLC25A22/SLC25A25/SLC25A5/SLC26A1/SLC26A10/SLC26A6/SLC34A1/SLC36A1/SLC36A3/SLC38A1/SLC38A10/SLC38A8/SLC4A9/SLC6A18/SLC6A6/SLC6A7/SLC6A8/S[illegible]	55	MF
GO:0019838	growth factor binding	24/2726	130/17354	0.22445	0.8427	0.82948	COL1A1/EGFR/ENG/ERBB2/FGFBP3/FGFR2/FGFR4/FGFRL1/FLT4/FURIN/GPC1/HTRA3/IGFBP3/IGFBP6/IL1RN/KDR/LTBP3/NKD2/PDGFA/PDGFRB/RHBDF1/SHC1/VASN/WFIKKN2	24	MF
GO:0016279	protein-lysine N-methyltransfera	12/2726	60/17354	0.22476	0.8427	0.82948	ASH2L/EHMT1/EHMT2/MEN1/PRDM7/PRDM9/SETD1A/SETD2/SETD7/SMYD3/SUV39H1/VCPKMT	12	MF
GO:0015144	carbohydrate transmembrane transporter	9/2726	43/17354	0.22511	0.8427	0.82948	AQP1/AQP10/SLC2A4/SLC2A6/SLC2A8/SLC35A2/SLC45A4/SLC50A1/SLC5A2	9	MF
GO:0034062	5'-3' RNA polymerase activity	9/2726	43/17354	0.22511	0.8427	0.82948	POLR1A/POLR1D/POLR1E/POLR2G/POLR2J2/POLR2J3/POLR2L/POLR3D/ZNRD1	9	MF

GO:0097747	RNA polymerase	9/2726	43/17354	0.22511	0.8427	0.82948	POLR1A/POLR1D/POLR1E/POLR2G/POLR2J2/POLR2J3/POLR2L/POLR3D/ZNRD1	9	MF
GO:0004622	lysophospholipase activity	5/2726	21/17354	0.22518	0.8427	0.82948	GDPD1/PLA2G15/PLA2G4B/PLB1/PNPLA7	5	MF
GO:0016638	oxidoreductase activity, acting on the CH-NH2 group of donors	5/2726	21/17354	0.22518	0.8427	0.82948	AOC2/CRYM/GLUD1/LOXL1/LOXL2	5	MF
GO:0050750	low-density lipoprotein particle receptor	5/2726	21/17354	0.22518	0.8427	0.82948	AP2M1/APOA5/APOB/CLU/HSPG2	5	MF
GO:0070273	phosphatidylinositol-4-phosphate	5/2726	21/17354	0.22518	0.8427	0.82948	ARFIP1/DAB2IP/GSDMD/OSBPL5/SH3PXD2B	5	MF
GO:0015085	calcium ion transmembrane transporter	25/2726	136/17354	0.22537	0.8427	0.82948	ATP13A2/ATP2A3/ATP2C2/CACNA1F/CACNA1G/CACNA1H/CACNA1S/CACNB1/CACNB3/CACNG1/CATSPER1/GRIN1/GRIN2C/ITPR3/JPH3/OPRM1/ORAI1/ORAI3/PDE2A/PKD1/RASA3/TMEM37/TRPV2/TRPV4/TRPV6	25	MF
GO:0005501	retinoid binding	7/2726	32/17354	0.22844	0.8427	0.82948	LCN12/OPN4/RARA/RBP1/RBP2/RBP7/SERPINA5	7	MF
GO:0016896	exoribonuclease activity, producing 5'-phosphomonoesters	7/2726	32/17354	0.22844	0.8427	0.82948	DIS3L/DIS3L2/EXOSC10/EXOSC2/ISG20L2/PAN2/PNLDC1	7	MF
GO:0042287	MHC protein binding	7/2726	32/17354	0.22844	0.8427	0.82948	BCAP31/CD74/CLEC7A/FCRL6/HLA-E/PILRB/TAP1	7	MF
GO:0051879	Hsp90 protein binding	7/2726	32/17354	0.22844	0.8427	0.82948	FKBP6/HDAC8/HSF1/KDR/NPAS2/TELO2/USP19	7	MF

GO:0046934	phosphatidylinositol-4,5-bisphosphate 3-kinase activity	13/2726	66/17354	0.22923	0.8427	0.82948	EGFR/ERBB2/FGF17/FGF3/FGFR2/FGFR4/LCK/NRG1/PDGFA/PDGFRB/PIK3R5/PIK3R6/PTPN11	13	MF
GO:0005229	intracellular calcium activated chloride	4/2726	16/17354	0.23528	0.8427	0.82948	ANO1/ANO2/ANO4/ANO7	4	MF
GO:0017160	Ral GTPase binding	4/2726	16/17354	0.23528	0.8427	0.82948	FLNA/MYO1C/RAB34/RALGPS1	4	MF
GO:0023026	MHC class II protein complex binding	4/2726	16/17354	0.23528	0.8427	0.82948	CD74/CD81/HSP90AA1/PKM	4	MF
GO:0061778	intracellular chloride channel activity	4/2726	16/17354	0.23528	0.8427	0.82948	ANO1/ANO2/ANO4/ANO7	4	MF
GO:0070182	DNA polymerase	4/2726	16/17354	0.23528	0.8427	0.82948	ACD/CDK2AP1/HSP90AA1/SMG5	4	MF
GO:0016758	transferase activity, transferring hexosyl groups	37/2726	209/17354	0.23802	0.8427	0.82948	A4GNT/ALG10/ALG3/B3GAT3/B3GNT6/B4GALNT1/B4GALNT4/B4GALT2/B4GALT7/CHPF/CHPF2/DAD1/DDOST/DPM2/EXTL1/FUT7/GALNT16/GALNT8/GALNT9/GBGT1/GGTA1P/HEXA/LFNG/MGAT1/MGAT4B/MGAT5B/PIGC/PIGQ/PIGY/PIGZ/PLOD3/PYGM/RFNG/RPN1/SDF2/UGT3A2/XYLT2	37	MF
GO:0016409	palmitoyltransferase activity	8/2726	38/17354	0.23867	0.8427	0.82948	CPT2/SPTLC1/ZDHHC11/ZDHHC12/ZDHHC14/ZDHHC15/ZDHHC18/ZDHHC7	8	MF
GO:0043394	proteoglycan binding	8/2726	38/17354	0.23867	0.8427	0.82948	AGRN/CFH/CTSL/GPC1/GPC2/LRP1/PTPRF/TNC	8	MF
GO:0003785	actin monomer binding	6/2726	27/17354	0.24191	0.8427	0.82948	ABL1/CORO1A/MYL4/NOS3/PFN1/PKNOX2	6	MF

GO:0016645	oxidoreductase activity, acting on the CH-NH group of donors	6/2726	27/17354	0.24191	0.8427	0.82948	ALDH1L1/ALDH4A1/CRYM/MTHFD1/PAOX/PIPOX	6	MF
GO:0019706	protein-cysteine S-palmitoyltransferase activity	6/2726	27/17354	0.24191	0.8427	0.82948	ZDHHC11/ZDHHC12/ZDHHC14/ZDHHC15/ZDHHC18/ZDHHC7	6	MF
GO:0019707	protein-cysteine S-acyltransferase	6/2726	27/17354	0.24191	0.8427	0.82948	ZDHHC11/ZDHHC12/ZDHHC14/ZDHHC15/ZDHHC18/ZDHHC7	6	MF
GO:0033764	steroid dehydrogenase activity, acting on the CH-OH group of donors, NAD or NADP as	6/2726	27/17354	0.24191	0.8427	0.82948	HSD11B2/HSD17B1/HSD17B3/HSD17B7/HSD3B1/NSDHL	6	MF
GO:0005253	anion channel activity	18/2726	96/17354	0.24266	0.8427	0.82948	ANO1/ANO2/ANO4/ANO7/BEST3/BEST4/BSND/CLCN2/CLCNKA/CLCNKB/CLDN4/CLIC1/GLRA1/GPR89A/SLC26A1/SLC26A10/SLC26A6/VDAC2	18	MF
GO:0001055	RNA polymerase II	3/2726	11/17354	0.24293	0.8427	0.82948	POLR2J2/POLR2J3/POLR2L	3	MF
GO:0004143	diacylglycerol kinase activity	3/2726	11/17354	0.24293	0.8427	0.82948	DGKI/DGKQ/DGKZ	3	MF
GO:0004312	fatty acid synthase	3/2726	11/17354	0.24293	0.8427	0.82948	ELOVL1/ELOVL5/OLAH	3	MF
GO:0005068	transmembrane receptor protein tyrosine kinase adaptor activity	3/2726	11/17354	0.24293	0.8427	0.82948	BLNK/DOK2/SHC1	3	MF

GO:0005536	glucose binding	3/2726	11/17354	0.24293	0.8427	0.82948	GCK/HK3/SLC2A8	3	MF
GO:0016004	phospholipase activator	3/2726	11/17354	0.24293	0.8427	0.82948	CASP3/CCL5/STX4	3	MF
GO:0016290	palmitoyl-CoA hydrolase	3/2726	11/17354	0.24293	0.8427	0.82948	ACOT7/GNPAT/LOC344967	3	MF
GO:0017070	U6 snRNA binding	3/2726	11/17354	0.24293	0.8427	0.82948	EIF5A/PRPF4/PRPF8	3	MF
GO:0017154	semaphorin receptor activity	3/2726	11/17354	0.24293	0.8427	0.82948	PLXNB1/PLXNB3/PLXND1	3	MF
GO:0019531	oxalate transmembrane transporter	3/2726	11/17354	0.24293	0.8427	0.82948	SLC26A1/SLC26A10/SLC26A6	3	MF
GO:0031996	thioesterase binding	3/2726	11/17354	0.24293	0.8427	0.82948	ADRA2A/HAUS7/TRAF2	3	MF
GO:0035615	clathrin adaptor activity	3/2726	11/17354	0.24293	0.8427	0.82948	AP2A2/AP2M1/HIP1R	3	MF
GO:0043295	glutathione binding	3/2726	11/17354	0.24293	0.8427	0.82948	GSTM1/GSTM2/MGST1	3	MF
GO:0048407	platelet-derived growth factor binding	3/2726	11/17354	0.24293	0.8427	0.82948	COL1A1/PDGFA/PDGFRB	3	MF
GO:0052743	inositol tetrakisphosphate phosphatase activity	3/2726	11/17354	0.24293	0.8427	0.82948	INPP5D/INPPL1/ITPK1	3	MF
GO:0070411	I-SMAD binding	3/2726	11/17354	0.24293	0.8427	0.82948	AXIN1/SMAD6/TGFB1I1	3	MF
GO:0098748	endocytic adaptor activity	3/2726	11/17354	0.24293	0.8427	0.82948	AP2A2/AP2M1/HIP1R	3	MF

GO:0016903	oxidoreductase activity, acting on the aldehyde or oxo group of donors	9/2726	44/17354	0.24646	0.8524	0.83895	ALDH1L1/ALDH2/ALDH4A1/ALDH8A1/GAPDH/OGDH/OGDHL/PDHB/XDH	9	MF
GO:0016798	hydrolase activity, acting on glycosyl	23/2726	126/17354	0.24803	0.8552	0.8417	ADPRHL1/ADPRHL2/APEX1/CHID1/CHIT1/DNPH1/GANAB/GBA/GLB1L/GLB1L2/GUSBP3/HEXA/LCTL/MANEAL/MBD4/MGAM/NAGLU/NEIL2/NEU3/OGG1/PSAP/SMUG1/SPACA3	23	MF
GO:0016835	carbon-oxygen lyase activity	14/2726	73/17354	0.24948	0.8576	0.84406	ACO1/ALAD/APEX1/CA4/CA6/CA7/HACD1/HACD3/HADHA/HMGA1/NEIL2/OGG1/UROC1/UROS	14	MF
GO:0008408	3'-5' exonuclease	10/2726	50/17354	0.2525	0.8626	0.84906	APEX1/DIS3L/DIS3L2/EXD3/EXOSC10/EXOSC2/ISG20L2/PAN2/PNLDC1/POLE	10	MF
GO:0016796	exonuclease activity, active with either ribo- or deoxyribonucleic acids and producing 5'-phosphomonoes	10/2726	50/17354	0.2525	0.8626	0.84906	APEX1/DIS3L/DIS3L2/EXO1/EXOSC10/EXOSC2/ISG20L2/PAN2/PNLDC1/POLE	10	MF
GO:0051393	alpha-actinin binding	7/2726	33/17354	0.2536	0.8633	0.84969	IGSF22/MYBPC1/MYBPC3/MYBPH/MYBPHL/OBSL1/RARA	7	MF
GO:0004896	cytokine receptor activity	17/2726	91/17354	0.25579	0.8633	0.84969	CCR2/CD74/CNTFR/CXCR1/CXCR2/CXCR3/CXCR5/GPR17/GPR35/IL15RA/IL17RE/IL20RB/IL31RA/IL4R/IL5RA/LEPR/MPL	17	MF
GO:0000146	microfilament motor activity	5/2726	22/17354	0.25652	0.8633	0.84969	MYO19/MYO1C/MYO1E/MYO3A/MYO7A	5	MF
GO:0005540	hyaluronic acid binding	5/2726	22/17354	0.25652	0.8633	0.84969	ACAN/BCAN/C6orf15/HAPLN3/LAYN	5	MF
GO:0042165	neurotransmitter binding	5/2726	22/17354	0.25652	0.8633	0.84969	ACHE/CHRNA6/CHRNB1/CHRNB3/GRIN1	5	MF

GO:0001078	transcriptional repressor activity, RNA polymerase II core promoter proximal region sequence-[illegible]	24/2726	133/17354	0.26101	0.8757	0.86197	ASCL2/ATF3/DLX4/E2F8/EN1/ESRRA/FOXQ1/HINFP/HSF1/KLF17/LRRFIP1/MAX/MLXIPL/NFX1/NKX3-2/NR2F1/RORC/SP2/SPI1/TBX15/TBX2/ZNF219/ZNF668/ZNF692	24	MF
GO:0035004	phosphatidylinositol 3-kinase	14/2726	74/17354	0.26644	0.8822	0.86828	EGFR/ERBB2/FGF17/FGF3/FGFR2/FGFR4/LCK/NRG1/PDGFA/PDGFRB/PIK3R5/PIK3R6/PTPN11/TLR9	14	MF
GO:0050660	flavin adenine dinucleotide binding	14/2726	74/17354	0.26644	0.8822	0.86828	ACAD10/ACADVL/ACOXL/CHDH/GFER/GSR/MICAL2/NOS3/NOX4/NQO2/POR/TXNRD1/TXNRD3/XDH	14	MF
GO:0001530	lipopolysaccharide binding	6/2726	28/17354	0.27018	0.8822	0.86828	ADGRB1/CD6/SCARB1/SELP/SPON2/TREM2	6	MF
GO:0008175	tRNA methyltransferase activity	6/2726	28/17354	0.27018	0.8822	0.86828	METTL2A/METTL2B/THUMPD2/TRMT10B/TRMT2B/TRMT44	6	MF
GO:0004620	phospholipase activity	18/2726	98/17354	0.272	0.8822	0.86828	CCL5/CHRM1/GDPD1/PLA2G15/PLA2G16/PLA2G1B/PLA2G2F/PLA2G4B/PLA2G5/PLB1/PLCB2/PLCD1/PLCH2/PLD2/PNPLA7/PRDX6/SMPD4/TMEM8	18	MF
GO:0000062	fatty-acyl-CoA binding	4/2726	17/17354	0.27227	0.8822	0.86828	ACBD4/ACOT7/HADHA/SOAT2	4	MF
GO:0008484	sulfuric ester hydrolase	4/2726	17/17354	0.27227	0.8822	0.86828	ARSE/ARSI/IDS/SGSH	4	MF
GO:0015095	magnesium ion transmembrane transporter	4/2726	17/17354	0.27227	0.8822	0.86828	CNNM2/NIPA2/NIPAL1/NIPAL2	4	MF
GO:0030228	lipoprotein particle receptor	4/2726	17/17354	0.27227	0.8822	0.86828	LRP1/LRP10/OLR1/SCARB1	4	MF
GO:0042166	acetylcholine binding	4/2726	17/17354	0.27227	0.8822	0.86828	ACHE/CHRNA6/CHRNB1/CHRNB3	4	MF

GO:0005057	signal transducer activity, downstream of	22/2726	122/17354	0.27369	0.8822	0.86828	ARAF/DOK1/DOK2/EGFR/ENG/ERBB2/IL4R/IRAK1/KDR/MAP3K14/MAP3K15/MAP3K6/MAP4K2/MAPK15/MAPK3/PGAM5/RAF1/RGS14/SMAD6/TAOK2/TNIK/TRAIP	22	MF
GO:0016765	transferase activity, transferring alkyl or aryl (other than	11/2726	57/17354	0.27699	0.8822	0.86828	COQ2/COX10/FNTA/FNTB/GSTM1/GSTM2/GSTZ1/HMBS/MGST1/MGST3/PDSS1	11	MF
GO:0004532	exoribonuclease activity	7/2726	34/17354	0.27953	0.8822	0.86828	DIS3L/DIS3L2/EXOSC10/EXOSC2/ISG20L2/PAN2/PNLDC1	7	MF
GO:0016620	oxidoreductase activity, acting on the aldehyde or oxo group of donors, NAD or NADP as acceptor	7/2726	34/17354	0.27953	0.8822	0.86828	ALDH1L1/ALDH2/ALDH4A1/ALDH8A1/GAPDH/OGDH/PDHB	7	MF
GO:0052813	phosphatidylinositol bisphosphate	13/2726	69/17354	0.28208	0.8822	0.86828	EGFR/ERBB2/FGF17/FGF3/FGFR2/FGFR4/LCK/NRG1/PDGFA/PDGFRB/PIK3R5/PIK3R6/PTPN11	13	MF
GO:0016874	ligase activity	30/2726	171/17354	0.28315	0.8822	0.86828	AARS/AARS2/ACACB/ACSF3/ACSM6/ACSS3/ASNS/ATG10/CARNS1/DARS/GATB/KARS/LARS/LIAS/LIG3/MTHFD1/NFX1/PARP2/PARP3/PARS2/PC/RNF212/RTCA/SARS/TARS2/TTLL10/TTLL5/TTLL7/YARS/YARS2	30	MF
GO:0015399	primary active transmembrane transporter	22/2726	123/17354	0.28725	0.8822	0.86828	ABCA2/ABCB5/ABCB9/ABCC12/ABCC3/ABCC4/ABCC5/ABCC6/ABCG5/ATP13A2/ATP1A1/ATP1A4/ATP2A3/ATP2C2/ATP5G1/ATP6V0B/ATP6V0E2/ATP6V1F/ATP6V1G1/TAP1/TIMM17B/TIMM23B	22	MF
GO:0015405	P-P-bond-hydrolysis-driven transmembrane	22/2726	123/17354	0.28725	0.8822	0.86828	ABCA2/ABCB5/ABCB9/ABCC12/ABCC3/ABCC4/ABCC5/ABCC6/ABCG5/ATP13A2/ATP1A1/ATP1A4/ATP2A3/ATP2C2/ATP5G1/ATP6V0B/ATP6V0E2/ATP6V1F/ATP6V1G1/TAP1/TIMM17B/TIMM23B	22	MF

GO:0002161	aminoacyl-tRNA editing	3/2726	12/17354	0.28873	0.8822	0.86828	AARS/LARS/TARS2	3	MF
GO:0004861	cyclin-dependent protein serine/threonine kinase inhibitor	3/2726	12/17354	0.28873	0.8822	0.86828	CASP3/CDKN2A/INCA1	3	MF
GO:0005283	sodium:amino acid symporter	3/2726	12/17354	0.28873	0.8822	0.86828	SLC38A1/SLC6A7/SLC6A9	3	MF
GO:0015266	protein channel activity	3/2726	12/17354	0.28873	0.8822	0.86828	TIMM17B/TIMM22/TIMM23B	3	MF
GO:0015271	outward rectifier potassium	3/2726	12/17354	0.28873	0.8822	0.86828	KCND1/KCND3/KCNQ1	3	MF
GO:0030898	actin-dependent ATPase activity	3/2726	12/17354	0.28873	0.8822	0.86828	MYO1C/MYO3A/MYO7A	3	MF
GO:0051787	misfolded protein binding	3/2726	12/17354	0.28873	0.8822	0.86828	CLU/HSPA5/TOR1A	3	MF
GO:0086008	voltage-gated potassium channel activity involved in cardiac muscle cell action [illegible]	3/2726	12/17354	0.28873	0.8822	0.86828	KCND3/KCNJ5/KCNQ1	3	MF
GO:1900750	oligopeptide binding	3/2726	12/17354	0.28873	0.8822	0.86828	GSTM1/GSTM2/MGST1	3	MF
GO:0000983	transcription factor activity, RNA polymerase II core promoter	5/2726	23/17354	0.28882	0.8822	0.86828	HEY1/MAX/STAT1/TAF1/TAF1L	5	MF

GO:0015035	protein disulfide oxidoreductase activity	5/2726	23/17354	0.28882	0.8822	0.86828	CCS/GFER/GLRX5/TXNRD1/TXNRD3	5	MF
GO:0016769	transferase activity, transferring nitrogenous	5/2726	23/17354	0.28882	0.8822	0.86828	GAPDH/GOT2/GPT/OAT/PHYKPL	5	MF
GO:0017025	TBP-class protein binding	5/2726	23/17354	0.28882	0.8822	0.86828	BRF1/PSMC3/TAF1/TAF1L/THRA	5	MF
GO:0017134	fibroblast growth factor	5/2726	23/17354	0.28882	0.8822	0.86828	FGFBP3/FGFR2/FGFR4/FGFRL1/GPC1	5	MF
GO:0099604	ligand-gated calcium channel activity	5/2726	23/17354	0.28882	0.8822	0.86828	GRIN1/GRIN2C/ITPR3/JPH3/RASA3	5	MF
GO:1990939	ATP-dependent microtubule motor activity	9/2726	46/17354	0.291	0.8864	0.87246	DNAH11/DNAH17/DNHD1/DYNC1H1/DYNC1I1/DYNLL2/KIF17/KIF4A/KIFC3	9	MF
GO:0016887	ATPase activity	73/2726	436/17354	0.29285	0.8896	0.87563	ABCA2/ABCB5/ABCB9/ABCC12/ABCC3/ABCC4/ABCC5/ABCC6/ABCF1/ABCF3/ABCG5/ATP13A2/ATP1A1/ATP1A4/ATP2A3/ATP2C2/ATP5G1/ATP5I/ATP6V0B/ATP6V0E2/ATP6V1F/ATP6V1G1/CARNS1/CHD8/DDX31/DDX4/DDX53/DDX54/DHX37/DHX38/DNAH11/DNAH12/DNAH17/DNHD1/DYNC1H1/DYNC1I1/DYNLL2/GTF2H4/HELQ/HSP90AA1/HSPA5/IGHMBP2/INO80/KATNB1/KIF13A/KIF17/KIF23/KIF2B/KIF4A/KIF9/KIFC3/MCM4/MDN1/MYO19/MYO1C/MYO1E/MYO3A/MYO7A/PEX6/PIF1/PMS2P3/PSMC3/RECQL4/RECQL5/RFC1/RFC2/TAP1/TDRD9/TOR1A/TOR1B/TTF2/XRCC3/YME1[illegible]	73	MF
GO:0048365	Rac GTPase binding	12/2726	64/17354	0.29911	0.9016	0.8874	ARHGAP4/ARHGEF4/BRK1/DOCK7/DVL2/EPS8L2/FARP2/FLNA/FMNL1/PREX2/RAB7A/WAS	12	MF
GO:0016229	steroid dehydrogenase activity	6/2726	29/17354	0.29919	0.9016	0.8874	HSD11B2/HSD17B1/HSD17B3/HSD17B7/HSD3B1/NSDHL	6	MF

GO:0043548	phosphatidylinositol 3-kinase	6/2726	29/17354	0.29919	0.9016	0.8874	ATP1A1/CORO1A/DAB1/DAB2IP/LCK/PDGFRB	6	MF
GO:0002039	p53 binding	13/2726	70/17354	0.3005	0.9031	0.88889	CDK5/CHD8/DUSP26/EHMT1/EHMT2/MUC1/SETD7/TAF1/TAF3/ZNF346/ZNF385A/ZNF385C/ZNF385D	13	MF
GO:0008170	N-methyltransferase activity	17/2726	94/17354	0.30258	0.9069	0.89267	ASH2L/EHMT1/EHMT2/GNMT/HNMT/MEN1/NDUFAF7/PEMT/PRDM7/PRDM9/SETD1A/SETD2/SETD7/SETD9/SMYD3/SUV39H1/VCPKMT	17	MF
GO:0016790	thiolester hydrolase	7/2726	35/17354	0.30607	0.9144	0.90001	ACOT7/ACOT9/GNPAT/LOC344967/LYPLA2/OLAH/PNKD	7	MF
GO:0017048	Rho GTPase binding	28/2726	161/17354	0.30874	0.9144	0.90001	ARHGAP4/ARHGEF10/ARHGEF16/ARHGEF17/ARHGEF25/ARHGEF4/BRK1/CDC42EP2/DOCK1/DOCK7/DVL2/EPS8L2/FARP2/FLNA/FMNL1/FMNL2/OBSCN/PARD6A/PFN1/PLEKHG4/PLEKHG5/PLEKHG6/PREX2/RAB7A/RT	28	MF
GO:0016646	oxidoreductase activity, acting on the CH-NH group of donors, NAD or NADP as	4/2726	18/17354	0.31013	0.9144	0.90001	ALDH1L1/ALDH4A1/CRYM/MTHFD1	4	MF
GO:0031690	adrenergic receptor binding	4/2726	18/17354	0.31013	0.9144	0.90001	ADRA2A/ADRA2C/ARRB2/DLG4	4	MF
GO:0047617	acyl-CoA hydrolase	4/2726	18/17354	0.31013	0.9144	0.90001	ACOT7/ACOT9/GNPAT/LOC344967	4	MF
GO:0098641	cadherin binding involved in cell-	4/2726	18/17354	0.31013	0.9144	0.90001	BAIAP2L1/PDLIM1/PDLIM5/PKP3	4	MF
GO:0042805	actinin binding	8/2726	41/17354	0.31076	0.9144	0.90001	IGSF22/MYBPC1/MYBPC3/MYBPH/MYBPHL/OBSL1/PDLIM5/RARA	8	MF
GO:0016879	ligase activity, forming carbon-nitrogen bonds	10/2726	53/17354	0.31625	0.9205	0.90601	ACACB/ASNS/CARNS1/GATB/LIAS/MTHFD1/PC/TTLL10/TTLL5/TTLL7	10	MF

GO:0005089	Rho guanyl-nucleotide exchange factor activity	14/2726	77/17354	0.31945	0.9205	0.90601	ARHGEF10/ARHGEF16/ARHGEF17/ARHGEF25/ARHGEF4/DOCK1/EPS8L2/FARP2/OBSCN/PLEKHG4/PLEKHG5/PLEKHG6/PREX2/TRIO	14	MF
GO:0042625	ATPase coupled ion transmembrane	14/2726	77/17354	0.31945	0.9205	0.90601	ABCC3/ABCC4/ABCC5/ABCC6/ATP13A2/ATP1A1/ATP1A4/ATP2A3/ATP2C2/ATP5G1/ATP6V0B/ATP6V0E2/ATP6V1F/ATP6V1G1	14	MF
GO:0015079	potassium ion transmembrane transporter	28/2726	162/17354	0.3211	0.9205	0.90601	AQP1/ATP1A1/ATP1A4/CNGA2/CNGB1/HCN3/KCNAB3/KCND1/KCND3/KCNH4/KCNH6/KCNJ18/KCNJ5/KCNJ9/KCNK12/KCNK13/KCNK17/KCNK4/KCNK7/KCNQ1/KCNQ4/KCNT1/SLC12A4/SLC12A7/SLC12A9/SLC9A3/SLC	28	MF
GO:0005031	tumor necrosis factor-activated receptor activity	5/2726	24/17354	0.32178	0.9205	0.90601	CD27/LTBR/RELT/TNFRSF18/TNFRSF4	5	MF
GO:0005035	death receptor activity	5/2726	24/17354	0.32178	0.9205	0.90601	CD27/LTBR/RELT/TNFRSF18/TNFRSF4	5	MF
GO:0005452	inorganic anion exchanger	5/2726	24/17354	0.32178	0.9205	0.90601	SLC22A12/SLC22A13/SLC22A9/SLC26A6/SLC4A9	5	MF
GO:0015347	sodium-independent organic anion transmembrane transporter	5/2726	24/17354	0.32178	0.9205	0.90601	SLC22A12/SLC22A13/SLC22A9/SLCO2B1/SLCO5A1	5	MF
GO:0045503	dynein light chain binding	5/2726	24/17354	0.32178	0.9205	0.90601	DNAH11/DNAH17/DNHD1/DYNC1H1/DYNC1I1	5	MF
GO:0051183	vitamin transporter	5/2726	24/17354	0.32178	0.9205	0.90601	FOLR2/SLC44A4/SLC52A1/SLC52A2/STRA6	5	MF

GO:0001205	transcriptional activator activity, RNA polymerase II distal enhancer sequence-	6/2726	30/17354	0.32874	0.9205	0.90601	HCFC1/HMGA1/HOXC4/NFE2L1/SCX/SPI1	6	MF
GO:0052866	phosphatidylinositol phosphate phosphatase activity	6/2726	30/17354	0.32874	0.9205	0.90601	INPP5D/INPP5E/INPP5F/INPPL1/MTMR1/MTMR14	6	MF
GO:0097718	disordered domain specific	6/2726	30/17354	0.32874	0.9205	0.90601	AP2A2/FN1/GAPDH/HSP90AA1/HSPA2/NUMA1	6	MF
GO:0016747	transferase activity, transferring acyl groups other than amino-acyl	37/2726	218/17354	0.33012	0.9205	0.90601	ACSM6/ALAS2/ATF2/BRPF1/CPT2/CROT/DGAT1/DLST/ELOVL1/ELOVL5/EPC1/GLYATL1/GNPAT/HADHA/HCFC1/KAT2A/NAA16/NAA60/NAGS/NAT16/NAT6/NAT8B/OLAH/PLA2G15/PNPLA2/PORCN/SOAT2/SPTLC1/TADA3/TAF1/TAF1L/ZDHHC11/ZDHHC12/ZDHHC14/ZDHHC15/ZDHHC18/ZDHHC7	37	MF
GO:0061134	peptidase regulator activity	37/2726	218/17354	0.33012	0.9205	0.90601	A2ML1/AGT/AHSG/APLP2/BAD/CASP1/CASP3/CD27/COL7A1/CRB2/CTSH/DPEP1/FN1/FURIN/GAPDH/ITIH4/ITIH6/KNG1/NAIP/NLRP1/NOD1/PCOLCE/PEBP1/PI16/RPS6KA1/SERPINA2/SERPINA3/SERPINA4/SERPINA5/SERPINF2/SERPINH1/SERPINI1/SPINK2/SPINK5/SPINT1/SPOCK2/WFIKKN2	37	MF
GO:0042626	ATPase activity, coupled to transmembrane movement of	20/2726	114/17354	0.33169	0.9205	0.90601	ABCA2/ABCB5/ABCB9/ABCC12/ABCC3/ABCC4/ABCC5/ABCC6/ABCG5/ATP13A2/ATP1A1/ATP1A4/ATP2A3/ATP2C2/ATP5G1/ATP6V0B/ATP6V0E2/ATP6V1F/ATP6V1G1/TAP1	20	MF
GO:0008238	exopeptidase activity	19/2726	108/17354	0.33289	0.9205	0.90601	AEBP1/ANPEP/BLMH/CPA1/CPN2/CTSH/DPEP1/DPP7/GGH/GGT6/LOC440434/MMP15/MMP17/NAALAD2/PRCP/PRSS16/RNPEP/RNPEPL1/SCRN2	19	MF
GO:0051119	sugar transmembrane transporter	7/2726	36/17354	0.33307	0.9205	0.90601	SLC2A4/SLC2A6/SLC2A8/SLC35A2/SLC45A4/SLC50A1/SLC5A2	7	MF

GO:0004385	guanylate kinase activity	3/2726	13/17354	0.33507	0.9205	0.90601	CARD11/DLG4/SCRIB	3	MF
GO:0008253	5'-nucleotidase activity	3/2726	13/17354	0.33507	0.9205	0.90601	ACPP/NT5C3B/NT5M	3	MF
GO:0008271	secondary active sulfate transmembrane transporter	3/2726	13/17354	0.33507	0.9205	0.90601	SLC26A1/SLC26A10/SLC26A6	3	MF
GO:0009982	pseudouridine synthase	3/2726	13/17354	0.33507	0.9205	0.90601	PUSL1/RPUSD2/TRUB2	3	MF
GO:0015926	glucosidase activity	3/2726	13/17354	0.33507	0.9205	0.90601	GANAB/LCTL/MGAM	3	MF
GO:0017017	MAP kinase tyrosine/serine/threonine phosphatase	3/2726	13/17354	0.33507	0.9205	0.90601	DUSP2/DUSP21/DUSP5	3	MF
GO:0032454	histone demethylase activity (H3-K9 specific)	3/2726	13/17354	0.33507	0.9205	0.90601	HR/KDM4E/PHF2	3	MF
GO:0035497	cAMP response element binding	3/2726	13/17354	0.33507	0.9205	0.90601	ATF2/CREB3/E4F1	3	MF
GO:0050998	nitric-oxide synthase	3/2726	13/17354	0.33507	0.9205	0.90601	CAV3/CD74/DNM1	3	MF
GO:0016776	phosphotransferase activity, phosphate group as	8/2726	42/17354	0.33577	0.9205	0.90601	AK5/AK6/CARD11/DLG4/NME1/NME4/PMVK/SCRIB	8	MF
GO:0008234	cysteine-type peptidase activity	33/2726	194/17354	0.33708	0.9216	0.90709	BLMH/BRCC3/CAPN10/CAPN11/CAPN15/CAPN8/CASP1/CASP3/CASP5/CASP6/CTSD/CTSF/CTSH/CTSL/CTSW/KAT2A/OTUB1/OTUD5/OTUD6A/OTUD7A/SENP3/SENP6/TADA3/UFSP1/USP19/USP2/USP20/USP27X/USP35/USP	33	MF

GO:0016836	hydro-lyase activity	10/2726	54/17354	0.33827	0.9216	0.90709	ACO1/ALAD/CA4/CA6/CA7/HACD1/HACD3/HADHA/UROC1/UROS	10	MF
GO:0001190	transcriptional activator activity, RNA polymerase II transcription	11/2726	60/17354	0.33863	0.9216	0.90709	BRCA1/CITED4/GBX2/MED6/NFE2L1/NKX2-5/NOTCH1/NUP98/PITX1/POU3F1/WWP2	11	MF
GO:0001965	G-protein alpha-subunit	4/2726	19/17354	0.34843	0.9347	0.91998	F2RL1/OPRM1/RGS10/RGS14	4	MF
GO:0004970	ionotropic glutamate receptor activity	4/2726	19/17354	0.34843	0.9347	0.91998	GRIA3/GRIN1/GRIN2C/PTK2B	4	MF
GO:0042288	MHC class I protein binding	4/2726	19/17354	0.34843	0.9347	0.91998	BCAP31/HLA-E/PILRB/TAP1	4	MF
GO:0050431	transforming growth factor beta binding	4/2726	19/17354	0.34843	0.9347	0.91998	ENG/LTBP3/VASN/WFIKKN2	4	MF
GO:0099529	neurotransmitter receptor activity involved in regulation of postsynaptic membrane [illegible]	4/2726	19/17354	0.34843	0.9347	0.91998	CHRNA6/CHRNB1/CHRNB3/GRIN1	4	MF
GO:1904315	transmitter-gated ion channel activity involved in regulation of postsynaptic membrane [illegible]	4/2726	19/17354	0.34843	0.9347	0.91998	CHRNA6/CHRNB1/CHRNB3/GRIN1	4	MF

GO:0044389	ubiquitin-like protein ligase binding	52/2726	313/17354	0.35174	0.9398	0.92504	ABTB1/AKTIP/AMBRA1/ANAPC2/ARRB2/ARRDC1/ASB14/AXIN1/BLZF1/BOK/BRCA1/CACYBP/CASC3/CDK5RAP3/CLU/EGFR/FATE1/FHIT/FZD8/HSPA5/LTBR/MFN2/MOAP1/MPHOSPH8/MYOD1/NEDD8/NKD2/OTUB1/PARP9/PER1/PRDX6/PRKAR1A/PTK2B/RALB/RNF31/SCAMP3/SLC25A5/SLF2/SMAD6/SMG5/SPOP/STAT1/STAT2/STX8/TNK2/TRAF2/UBE2A/UBE2B/U	52	MF
GO:0016853	isomerase activity	25/2726	146/17354	0.35202	0.9398	0.92504	DSE/EBPL/FKBP10/FKBP6/FKBP9/FUOM/GALM/GSTZ1/HSD3B1/HYI/ITPK1/PBLD/PGAM4/PPIAL4A/PPIAL4C/PPIAL4D/PPIAL4E/PPIAL4F/PPIAL4G/PPIB/PPIE/PUSL1/QSOX1/RPUSD2/TRUB2	25	MF
GO:0019201	nucleotide kinase activity	5/2726	25/17354	0.35512	0.9436	0.92879	AK5/AK6/CARD11/DLG4/SCRIB	5	MF
GO:0071889	14-3-3 protein binding	5/2726	25/17354	0.35512	0.9436	0.92879	ARRB2/BAD/DAB2IP/PI4KB/YWHAQ	5	MF
GO:0015171	amino acid transmembrane transporter	14/2726	79/17354	0.35614	0.9441	0.92926	PQLC2/SLC1A7/SLC25A22/SLC36A1/SLC36A3/SLC38A1/SLC38A10/SLC38A8/SLC6A18/SLC6A6/SLC6A7/SLC6A8/SLC6A9/SLC7A7	14	MF
GO:0005164	tumor necrosis factor receptor binding	6/2726	31/17354	0.35863	0.9463	0.93137	FASLG/LTA/STAT1/TNFSF13/TRAF2/TRAP1	6	MF
GO:0015301	anion:anion antiporter	6/2726	31/17354	0.35863	0.9463	0.93137	SLC22A9/SLC25A5/SLC26A1/SLC26A10/SLC26A6/SLC4A9	6	MF
GO:0070888	E-box binding	7/2726	37/17354	0.36038	0.9482	0.93324	ASCL2/CIART/MAX/MYOD1/NEUROD2/PER1/SCX	7	MF
GO:0003678	DNA helicase activity	9/2726	49/17354	0.36104	0.9482	0.93324	CHD8/GTF2H4/IGHMBP2/INO80/MCM2/MCM4/PIF1/RECQL4/RECQL5	9	MF
GO:0022843	voltage-gated cation channel activity	23/2726	135/17354	0.37022	0.9491	0.93417	CACNA1F/CACNA1G/CACNA1H/CACNA1S/CACNB1/CACNB3/CACNG1/CATSPER1/CNGA2/CNGB1/HCN3/KCNAB3/KCND1/KCND3/KCNH4/KCNH6/KCNJ18/KCNJ5/KCNJ9/KCNQ1/KCNQ4/KCNT1/OPRM1	23	MF

GO:0003924	GTPase activity	47/2726	284/17354	0.37188	0.9491	0.93417	AGAP3/ARL8A/DNM1/EFTUD2/GCH1/GNAO1/GNAT1/GNB3/GNB5/GNG3/GPN2/HRAS/MFN1/MFN2/NKIRAS2/NUDT1/RAB17/RAB20/RAB24/RAB25/RAB26/RAB34/RAB40C/RAB44/RAB7A/RABL6/RALB/RAP1GAP/RASD1/RASL12/RHEBL1/RHOD/RHOF/RHOT2/RHOV/RND1/RNF112/RRAD/RRAGC/SEPT4/SEPT9/TSR1/TUBA3C/TUBA3D/TUBA3E/TUBG1/TUBG2	47	MF
GO:0005096	GTPase activator activity	47/2726	284/17354	0.37188	0.9491	0.93417	ACAP1/ACAP3/ADAP1/ADPRHL1/AGAP3/ALS2CL/ARAP1/ARFGAP2/ARHGAP10/ARHGAP22/ARHGAP27/ARHGAP30/ARHGAP4/AXIN1/CDC42EP2/DAB2IP/DOCK1/ELMOD1/GNB5/GRTP1/HACD3/LARS/LLGL1/NPRL3/PGAM5/PLEKHG6/PLXNB1/PREX2/RABGAP1/RABGAP1L/RAP1GAP/RASA3/RASA4B/RGS10/RGS14/RGS16/RIN3/SGSM2/SIPA1/STARD8/TBC1D25/TBC1	47	MF
GO:0008201	heparin binding	27/2726	160/17354	0.37429	0.9491	0.93417	ANG/APLP2/APOA5/APOB/BMP4/C6orf15/CFH/FGFBP3/FGFR2/FGFR4/FGFRL1/FN1/KNG1/LAMC2/MPO/PCOLCE/PF4V1/PLA2G5/PTPRF/REG4/RSPO1/RTN4RL1/SELP/SERPINA5/SOD3/SOST/THBS3	27	MF
GO:0004089	carbonate dehydratase activity	3/2726	14/17354	0.38123	0.9491	0.93417	CA4/CA6/CA7	3	MF
GO:0005041	low-density lipoprotein receptor activity	3/2726	14/17354	0.38123	0.9491	0.93417	LRP1/LRP10/OLR1	3	MF
GO:0005388	calcium-transporting ATPase activity	3/2726	14/17354	0.38123	0.9491	0.93417	ATP13A2/ATP2A3/ATP2C2	3	MF
GO:0005527	macrolide binding	3/2726	14/17354	0.38123	0.9491	0.93417	FKBP10/FKBP6/FKBP9	3	MF
GO:0005528	FK506 binding	3/2726	14/17354	0.38123	0.9491	0.93417	FKBP10/FKBP6/FKBP9	3	MF
GO:0015116	sulfate transmembrane transporter	3/2726	14/17354	0.38123	0.9491	0.93417	SLC26A1/SLC26A10/SLC26A6	3	MF
GO:0019206	nucleoside kinase activity	3/2726	14/17354	0.38123	0.9491	0.93417	AK5/DGUOK/UCK1	3	MF

GO:0019841	retinol binding	3/2726	14/17354	0.38123	0.9491	0.93417	RBP1/RBP2/RBP7	3	MF
GO:0030169	low-density lipoprotein particle binding	3/2726	14/17354	0.38123	0.9491	0.93417	CDH13/SCARB1/SCARF1	3	MF
GO:0033549	MAP kinase phosphatase activity	3/2726	14/17354	0.38123	0.9491	0.93417	DUSP2/DUSP21/DUSP5	3	MF
GO:0043024	ribosomal small subunit binding	3/2726	14/17354	0.38123	0.9491	0.93417	EIF4B/ERAL1/NME1	3	MF
GO:0047485	protein N-terminus	18/2726	105/17354	0.38258	0.9491	0.93417	EIF5A/GAD1/MEN1/MYO7A/NCOR2/PDLIM5/PEX14/PEX5/SNF8/SRRM2/STX1A/STX5/STXBP1/SUV39H1/TARBP2/VPS25/VWF/YWHAQ	18	MF
GO:0035064	methylated histone binding	10/2726	56/17354	0.38302	0.9491	0.93417	CHD8/ING4/MPHOSPH8/MSL3/NCAPD3/PHF19/PHF2/TAF1/TAF7/ZMYND11	10	MF
GO:0051287	NAD binding	10/2726	56/17354	0.38302	0.9491	0.93417	ALDH2/CTBP1/GAPDH/GLUD1/HADHA/HSD11B2/IDH1/IDH2/LDHA/PHGDH	10	MF
GO:0140034	methylation-dependent protein binding	10/2726	56/17354	0.38302	0.9491	0.93417	CHD8/ING4/MPHOSPH8/MSL3/NCAPD3/PHF19/PHF2/TAF1/TAF7/ZMYND11	10	MF
GO:0019205	nucleobase-containing compound kinase activity	9/2726	50/17354	0.38487	0.9491	0.93417	AK5/AK6/CARD11/DGUOK/DLG4/NME1/NME4/SCRIB/UCK1	9	MF
GO:0022824	transmitter-gated ion	9/2726	50/17354	0.38487	0.9491	0.93417	CHRNA6/CHRNB1/CHRNB3/GLRA1/GRIA3/GRIN1/GRIN2C/HTR3B/PTK2B	9	MF
GO:0022835	transmitter-gated channel	9/2726	50/17354	0.38487	0.9491	0.93417	CHRNA6/CHRNB1/CHRNB3/GLRA1/GRIA3/GRIN1/GRIN2C/HTR3B/PTK2B	9	MF
GO:0001223	transcription coactivator	4/2726	20/17354	0.38677	0.9491	0.93417	CDK9/PASD1/ZBTB17/ZBTB49	4	MF

GO:0004198	calcium-dependent cysteine-type endopeptidase	4/2726	20/17354	0.38677	0.9491	0.93417	CAPN10/CAPN11/CAPN15/CAPN8	4	MF
GO:0016289	CoA hydrolase activity	4/2726	20/17354	0.38677	0.9491	0.93417	ACOT7/ACOT9/GNPAT/LOC344967	4	MF
GO:0031418	L-ascorbic acid binding	4/2726	20/17354	0.38677	0.9491	0.93417	OGFOD2/P3H1/P3H3/PLOD3	4	MF
GO:0015662	ATPase activity, coupled to transmembrane movement of ions,	7/2726	38/17354	0.38786	0.9491	0.93417	ABCC4/ATP13A2/ATP1A1/ATP1A4/ATP2A3/ATP2C2/ATP6V1G1	7	MF
GO:0016675	oxidoreductase activity, acting on a heme group of donors	5/2726	26/17354	0.38858	0.9491	0.93417	COX10/COX11/COX8A/NDUFA4L2/POR	5	MF
GO:0030507	spectrin binding	5/2726	26/17354	0.38858	0.9491	0.93417	DCTN2/DYNC1I1/EPB41L2/GNB3/MYO7A	5	MF
GO:0005310	dicarboxylic acid transmembrane	6/2726	32/17354	0.38868	0.9491	0.93417	SLC13A2/SLC1A7/SLC25A22/SLC26A1/SLC26A10/SLC26A6	6	MF
GO:0042169	SH2 domain binding	6/2726	32/17354	0.38868	0.9491	0.93417	ABL1/INPPL1/LCK/SH3PXD2B/SHCBP1/TRPV4	6	MF

GO:0001228	transcriptional activator activity, RNA polymerase II transcription regulatory region	67/2726	411/17354	0.38979	0.9491	0.93417	ATF2/ATF3/BARHL1/BARHL2/BARX2/CAMTA2/CREB3/CSRNP1/DLX3/DUX4/E2F4/EGR1/EGR4/ELF1/ESRRA/ETV4/FOSL1/FOXN1/FOXO4/GATA4/GBX2/GCM2/GMEB1/GRHL3/HCFC1/HMGA1/HOXB1/HOXB2/HOXB3/HOXB7/HOXC10/HOXC11/HOXC13/HOXC4/HOXD3/HOXD4/IRF5/KLF14/LHX3/LHX4/MAFA/MEIS2/MESP2/MYCN/MYOD1/NEUROD2/NFE2L1/NKRF/NKX2-5/NKX2-8/NKX6-3/NR4A1/NRL/PBX2/PHOX2B/PITX1/RFX5/RFX6/SCX/SOHLH2/SPI1/TBPL [illegible]	67	MF
GO:0016757	transferase activity, transferring glycosyl groups	46/2726	280/17354	0.39425	0.9491	0.93417	A4GNT/ALG10/ALG3/B3GAT3/B3GNT6/B3GNTL1/B4GALNT1/B4GALNT4/B4GALT2/B4GALT7/CHPF/CHPF2/DAD1/DDOST/DNPH1/DPM2/EXTL1/FUT7/GALNT16/GALNT8/GALNT9/GBGT1/GGTA1P/HEXA/LFNG/MGAT1/MGAT4B/MGAT5B/PARP10/PARP2/PARP3/PARP9/PIGC/PIGQ/PIGY/PIGZ/PLOD3/PYGM/QPRT/RFNG/RPN1/SDF2/ST3GAL4/UGT3A2/XXYLT1/XYLT2	46	MF
GO:0008227	G-protein coupled amine receptor activity	9/2726	51/17354	0.40878	0.9491	0.93417	ADRA1A/ADRA1B/ADRA2A/ADRA2B/ADRA2C/CHRM1/HRH2/HTR1D/ZNF219	9	MF
GO:0008276	protein methyltransferase activity	15/2726	88/17354	0.4088	0.9491	0.93417	ASH2L/EHMT1/EHMT2/LCMT1/MEN1/NDUFAF7/NTMT1/PRDM7/PRDM9/SETD1A/SETD2/SETD7/SMYD3/SUV39H1/VCPKMT	15	MF
GO:0019903	protein phosphatase binding	19/2726	113/17354	0.41195	0.9491	0.93417	BAD/CDH5/CSF1R/CSK/DAB2IP/DLG4/EGFR/EIF4EBP1/ERBB2/FCRL6/FLT4/KAT2A/KCNQ1/LCK/PARD3/PHACTR1/PPP1R9B/SMG5/TRAF2	19	MF
GO:0005272	sodium channel activity	8/2726	45/17354	0.41205	0.9491	0.93417	ASIC2/ASIC3/CACNA1G/CACNA1H/HCN3/SCN11A/SCNN1A/TRPM5	8	MF
GO:0022839	ion gated channel activity	8/2726	45/17354	0.41205	0.9491	0.93417	ANO1/ANO2/ANO4/ANO7/ASIC2/CATSPER1/KCNT1/TRPM5	8	MF
GO:0031593	polyubiquitin modification-dependent protein binding	8/2726	45/17354	0.41205	0.9491	0.93417	ATRIP/BRCC3/FAAP20/OTUD7A/PARP10/PRPF8/SHARPIN/TNIP2	8	MF

GO:0005525	GTP binding	60/2726	370/17354	0.41513	0.9491	0.93417	ACSM6/AGAP3/ARL10/ARL8A/CIITA/DAPK1/DNM1/EFTUD2/EHD1/EIF2B2/ERAL1/GCH1/GLUD1/GMPPB/GNAO1/GNAT1/GPN2/GTPBP6/GUCY2D/HRAS/IFT22/MFN1/MFN2/NKIRAS2/NME1/NUDT2/RAB17/RAB20/RAB24/RAB25/RAB26/RAB34/RAB40C/RAB44/RAB7A/RABL6/RALB/RASD1/RASL12/RHEBL1/RHOD/RHOF/RHOT2/RHOV/RND1/RNF112/RRAD/RRAGC/RTKN/SEPT1/SEPT4/SEPT8/SEPT9/TSR1/TUBA3C/TUBA3D/TUBA3E/TUBG1[illegible]	60	MF
GO:0032266	phosphatidylinositol-3-phosphate	6/2726	33/17354	0.4187	0.9491	0.93417	DAB2IP/PARD3/PHLDA3/SH3PXD2B/TECPR1/ZFYVE26	6	MF
GO:0004702	signal transducer, downstream of receptor, with serine/threonine	12/2726	70/17354	0.41985	0.9491	0.93417	ARAF/EGFR/IRAK1/MAP3K14/MAP3K15/MAP3K6/MAP4K2/MAPK15/MAPK3/RAF1/TAOK2/TNIK	12	MF
GO:0001106	RNA polymerase II transcription corepressor	5/2726	27/17354	0.42192	0.9491	0.93417	CTBP1/OLIG3/PHF12/TBX15/UXT	5	MF
GO:0004364	glutathione transferase	5/2726	27/17354	0.42192	0.9491	0.93417	GSTM1/GSTM2/GSTZ1/MGST1/MGST3	5	MF
GO:0004435	phosphatidylinositol phospholipase	5/2726	27/17354	0.42192	0.9491	0.93417	CCL5/CHRM1/PLCB2/PLCD1/PLCH2	5	MF
GO:0045505	dynein intermediate chain binding	5/2726	27/17354	0.42192	0.9491	0.93417	DNAH11/DNAH17/DNHD1/DYNC1H1/DYNLL2	5	MF
GO:0003950	NAD+ ADP-ribosyltransferase activity	4/2726	21/17354	0.42479	0.9491	0.93417	PARP10/PARP2/PARP3/PARP9	4	MF

GO:0004298	threonine-type endopeptidase activity	4/2726	21/17354	0.42479	0.9491	0.93417	PSMB11/PSMB6/PSMB7/PSMB8	4	MF
GO:0005112	Notch binding	4/2726	21/17354	0.42479	0.9491	0.93417	CHAC1/JAG2/NCOR2/NOTCH1	4	MF
GO:0005355	glucose transmembrane transporter	4/2726	21/17354	0.42479	0.9491	0.93417	SLC2A4/SLC2A6/SLC2A8/SLC5A2	4	MF
GO:0008483	transaminase activity	4/2726	21/17354	0.42479	0.9491	0.93417	GOT2/GPT/OAT/PHYKPL	4	MF
GO:0042813	Wnt-activated receptor activity	4/2726	21/17354	0.42479	0.9491	0.93417	FZD2/FZD8/FZD9/SFRP5	4	MF
GO:0042923	neuropeptide binding	4/2726	21/17354	0.42479	0.9491	0.93417	GPR1/OPRD1/OPRM1/SSTR5	4	MF
GO:0070003	threonine-type peptidase	4/2726	21/17354	0.42479	0.9491	0.93417	PSMB11/PSMB6/PSMB7/PSMB8	4	MF
GO:0098960	postsynaptic neurotransmitter receptor	4/2726	21/17354	0.42479	0.9491	0.93417	CHRNA6/CHRNB1/CHRNB3/GRIN1	4	MF
GO:0004029	aldehyde dehydrogenase (NAD) activity	3/2726	15/17354	0.42663	0.9491	0.93417	ALDH1L1/ALDH2/ALDH4A1	3	MF
GO:0004708	MAP kinase kinase activity	3/2726	15/17354	0.42663	0.9491	0.93417	MAP2K3/MAPK3/MAPKAPK3	3	MF
GO:0005545	1-phosphatidylinositol binding	3/2726	15/17354	0.42663	0.9491	0.93417	SCARB1/SCIN/ZFYVE1	3	MF
GO:0008252	nucleotidase activity	3/2726	15/17354	0.42663	0.9491	0.93417	ACPP/NT5C3B/NT5M	3	MF
GO:0016854	racemase and epimerase	3/2726	15/17354	0.42663	0.9491	0.93417	DSE/FUOM/GALM	3	MF

GO:0000979	RNA polymerase II core promoter sequence-	10/2726	58/17354	0.42815	0.9491	0.93417	CDK9/GBX2/HOXB3/HSF1/SMYD3/SOX8/SPI1/STAT1/TAF1/TBR1	10	MF
GO:0004521	endoribonuclease activity	10/2726	58/17354	0.42815	0.9491	0.93417	APEX1/CPSF4/EXO1/NOB1/PIWIL2/RNASE1/RNASE8/RNASEK/RPP21/ZC3H3	10	MF
GO:0008235	metalloexopeptidase activity	10/2726	58/17354	0.42815	0.9491	0.93417	AEBP1/ANPEP/CPA1/CPN2/DPEP1/LOC440434/MMP15/MMP17/RNPEP/RNPEPL1	10	MF
GO:0000049	tRNA binding	9/2726	52/17354	0.43269	0.9491	0.93417	AARS/AARS2/AIMP1/EIF2AK4/IGHMBP2/KARS/RPL35A/YARS/YARS2	9	MF
GO:0008146	sulfotransferase activity	9/2726	52/17354	0.43269	0.9491	0.93417	CHST10/CHST12/CHST13/CHST15/CHST5/CHST7/GAL3ST4/HS3ST6/SULT6B1	9	MF
GO:0005048	signal sequence binding	8/2726	46/17354	0.43754	0.9491	0.93417	AP2M1/IPO13/IPO4/NUP98/PEX5/SRP14/TIMM22/TNPO3	8	MF
GO:0016782	transferase activity, transferring sulfur-	12/2726	71/17354	0.44031	0.9491	0.93417	CDKAL1/CHST10/CHST12/CHST13/CHST15/CHST5/CHST7/GAL3ST4/HS3ST6/LIAS/SULT6B1/TSTD1	12	MF
GO:0042562	hormone binding	11/2726	65/17354	0.44531	0.9491	0.93417	ATP1A1/CDH13/CRYM/CTSH/EGFR/GPER1/PKM/RXFP2/SHC1/THRA/UCN2	11	MF
GO:0000978	RNA polymerase II core promoter proximal region sequence-specific DNA binding	58/2726	361/17354	0.44759	0.9491	0.93417	ASCL2/ATF3/CREB3/DLX4/DUX4/E2F4/E2F8/ELF1/EN1/ESRRA/ETV4/FOSL1/FOXQ1/GMEB1/HINFP/HOXB1/HOXB3/HOXB7/HOXC11/HSF1/KLF17/LHX3/LRRFIP1/MAFA/MAX/MEIS2/MESP2/MLXIPL/MUC1/MYCN/MYOD1/NKRF/NKX2-5/NKX2-8/NKX3-2/NR2F1/NRL/PER1/PHOX2B/PITX1/RFX5/RFX6/RORC/SMAD6/SMARCD2/SNAI3/SP2/SPI1/STAT1/TBPL1/TBX15/TBX19/TBX2/ZBTB17/ZNF219/ZNF668/ZNF692/ZSCAN21	58	MF
GO:0008080	N-acetyltransferase activity	14/2726	84/17354	0.45003	0.9491	0.93417	ATF2/BRPF1/EPC1/HCFC1/KAT2A/NAA16/NAA60/NAGS/NAT16/NAT6/NAT8B/TADA3/TAF1/TAF1L	14	MF

GO:0032550	purine ribonucleoside binding	60/2726	374/17354	0.45087	0.9491	0.93417	ACSM6/AGAP3/ARL10/ARL8A/CIITA/DAPK1/DNM1/EFTUD2/EHD1/EIF2B2/ERAL1/GCH1/GLUD1/GMPPB/GNAO1/GNAT1/GPN2/GTPBP6/GUCY2D/HRAS/IFT22/MFN1/MFN2/NKIRAS2/NME1/NUDT2/RAB17/RAB20/RAB24/RAB25/RAB26/RAB34/RAB40C/RAB44/RAB7A/RABL6/RALB/RASD1/RASL12/RHEBL1/RHOD/RHOF/RHOT2/RHOV/RND1/RNF112/RRAD/RRAGC/RTKN/SEPT1/SEPT4/SEPT8/SEPT9/TSR1/TUBA3C/TUBA3D/TUBA3E/TUBG1[illegible]	60	MF
GO:0016791	phosphatase activity	43/2726	267/17354	0.45487	0.9491	0.93417	ACP2/ACPP/ALPPL2/ATP1A1/CDC14A/CTDSP1/DUSP2/DUSP21/DUSP26/DUSP5/EPHX2/EYA1/G6PC3/ILKAP/INPP5D/INPP5E/INPP5F/INPPL1/ITPK1/LCK/MTMR1/MTMR14/NT5C3B/NT5M/PFKFB1/PGAM5/PHOSPHO1/PLPP1/PPA2/PPEF1/PPP2R2B/PPP2R3B/PTP4A3/PTPN11/PTPN23/PTPN7/PTPRF/PTPRN2/PTPRQ/PTPRU/PUDP/STYXL1/TNS2	43	MF
GO:0005217	intracellular ligand-gated ion channel activity	5/2726	28/17354	0.4549	0.9491	0.93417	AQP1/CNGB1/ITPR3/JPH3/RASA3	5	MF
GO:0008519	ammonium transmembrane transporter	5/2726	28/17354	0.4549	0.9491	0.93417	AQP1/RHCG/SLC22A1/SLC25A20/SLC44A4	5	MF
GO:0032452	histone demethylase activity	5/2726	28/17354	0.4549	0.9491	0.93417	HR/KDM4E/KDM6B/KDM8/PHF2	5	MF
GO:0046966	thyroid hormone	5/2726	28/17354	0.4549	0.9491	0.93417	ARID5A/MED24/NCOR1/TAF7/ZNHIT3	5	MF
GO:0051059	NF-kappaB binding	5/2726	28/17354	0.4549	0.9491	0.93417	APEX1/BRMS1/CDK5RAP3/CDKN2A/PPARD	5	MF
GO:0060090	molecular adaptor activity	30/2726	185/17354	0.45545	0.9491	0.93417	AP2A2/AP2M1/ARHGAP4/ARPC4/ARRDC1/BIN3/BLNK/DAB2IP/DDB1/DOK2/DVL2/FGA/FSCN1/GRAP/HIP1R/KSR1/MAPK8IP1/MAPK8IP3/MEN1/OBSL1/PTPN11/SH2B1/SH2D2A/SHC1/SHF/SPRR1A/STX1A/TCAP/TIRAP/TRD	30	MF
GO:0043022	ribosome binding	9/2726	53/17354	0.4565	0.9491	0.93417	EIF5A/GTPBP6/HSPA5/IGHMBP2/LETMD1/MTOR/NAA16/SEC61A2/ZNF598	9	MF

GO:0031625	ubiquitin protein ligase binding	48/2726	299/17354	0.45901	0.9491	0.93417	ABTB1/AKTIP/AMBRA1/ANAPC2/ARRB2/ARRDC1/ASB14/AXIN1/BLZF1/BOK/BRCA1/CACYBP/CASC3/CLU/EGFR/FATE1/FHIT/FZD8/HSPA5/LTBR/MFN2/MOAP1/MPHOSPH8/MYOD1/NEDD8/NKD2/OTUB1/PER1/PRDX6/PRKAR1A/PTK2B/RALB/RNF31/SCAMP3/SLC25A5/SLF2/SMAD6/SMG5/SPOP/STX8/TNK2/TRAF2/UBE2A/UBE2B/UBE2U/USP19/USP2/WFS1	48	MF
GO:0015101	organic cation transmembrane transporter	4/2726	22/17354	0.46218	0.9491	0.93417	SLC22A1/SLC22A13/SLC25A20/SLC44A4	4	MF
GO:0015149	hexose transmembrane transporter	4/2726	22/17354	0.46218	0.9491	0.93417	SLC2A4/SLC2A6/SLC2A8/SLC5A2	4	MF
GO:0015464	acetylcholine receptor activity	4/2726	22/17354	0.46218	0.9491	0.93417	CHRM1/CHRNA6/CHRNB1/CHRNB3	4	MF
GO:0016881	acid-amino acid ligase activity	4/2726	22/17354	0.46218	0.9491	0.93417	CARNS1/TTLL10/TTLL5/TTLL7	4	MF
GO:0048027	mRNA 5'-UTR binding	4/2726	22/17354	0.46218	0.9491	0.93417	LARP6/MRPS11/RARA/RPL41	4	MF
GO:0051537	2 iron, 2 sulfur cluster binding	4/2726	22/17354	0.46218	0.9491	0.93417	CIAPIN1/GLRX5/ISCA2/XDH	4	MF
GO:0005080	protein kinase C binding	8/2726	47/17354	0.46288	0.9491	0.93417	ABL1/DACT2/HINT1/HSPB1/PARD6A/PDLIM5/TIRAP/TRPV4	8	MF
GO:0004866	endopeptidase inhibitor activity	28/2726	173/17354	0.46348	0.9491	0.93417	A2ML1/AGT/AHSG/APLP2/CD27/COL7A1/CRB2/DPEP1/FURIN/GAPDH/ITIH4/ITIH6/KNG1/NAIP/PEBP1/RPS6KA1/SERPINA2/SERPINA3/SERPINA4/SERPINA5/SERPINF2/SERPINH1/SERPINI1/SPINK2/SPINK5/SPINT1/SPOCK2	28	MF
GO:0005267	potassium channel activity	21/2726	129/17354	0.46622	0.9491	0.93417	AQP1/CNGA2/CNGB1/HCN3/KCNAB3/KCND1/KCND3/KCNH4/KCNH6/KCNJ18/KCNJ5/KCNJ9/KCNK12/KCNK13/KCNK17/KCNK4/KCNK7/KCNQ1/KCNQ4/KCNT1/TRPM5	21	MF
GO:0070063	RNA polymerase	11/2726	66/17354	0.46666	0.9491	0.93417	ANP32B/BIN1/BRCA1/CCAR2/ERBB2/MAF1/MIR16-1/MIR584/PPIB/RECQL5/SMYD3	11	MF

GO:0001191	transcriptional repressor activity, RNA polymerase II transcription	7/2726	41/17354	0.46996	0.9491	0.93417	CTBP1/OLIG3/PARP9/PHF12/SPEN/TBX15/UXT	7	MF
GO:1901618	organic hydroxy compound transmembrane transporter	7/2726	41/17354	0.46996	0.9491	0.93417	ABCC3/AQP1/AQP10/ASIC3/SLC22A1/SLC6A2/SLCO2B1	7	MF
GO:0001056	RNA polymerase III	3/2726	16/17354	0.47078	0.9491	0.93417	POLR1D/POLR2L/POLR3D	3	MF
GO:0003995	acyl-CoA dehydrogenase activity	3/2726	16/17354	0.47078	0.9491	0.93417	ACAD10/ACADVL/ACOXL	3	MF
GO:0005001	transmembrane receptor protein tyrosine phosphatase activity	3/2726	16/17354	0.47078	0.9491	0.93417	PTPRF/PTPRN2/PTPRU	3	MF
GO:0005159	insulin-like growth factor receptor binding	3/2726	16/17354	0.47078	0.9491	0.93417	INS/REN/SHC1	3	MF
GO:0015114	phosphate ion transmembrane transporter	3/2726	16/17354	0.47078	0.9491	0.93417	SLC20A1/SLC20A2/SLC34A1	3	MF
GO:0016641	oxidoreductase activity, acting on the CH-NH2 group of donors, oxygen as acceptor	3/2726	16/17354	0.47078	0.9491	0.93417	AOC2/LOXL1/LOXL2	3	MF

GO:0016668	oxidoreductase activity, acting on a sulfur group of donors,	3/2726	16/17354	0.47078	0.9491	0.93417	GSR/TXNRD1/TXNRD3	3	MF
GO:0016805	dipeptidase activity	3/2726	16/17354	0.47078	0.9491	0.93417	DPEP1/NAALAD2/SCRN2	3	MF
GO:0019198	transmembrane receptor protein phosphatase activity	3/2726	16/17354	0.47078	0.9491	0.93417	PTPRF/PTPRN2/PTPRU	3	MF
GO:0022848	acetylcholine-gated cation-selective channel activity	3/2726	16/17354	0.47078	0.9491	0.93417	CHRNA6/CHRNB1/CHRNB3	3	MF
GO:0016298	lipase activity	20/2726	123/17354	0.47107	0.9491	0.93417	CCL5/CHRM1/DAGLB/GDPD1/PLA2G15/PLA2G16/PLA2G1B/PLA2G2F/PLA2G4B/PLA2G5/PLB1/PLCB2/PLCD1/PLCH2/PLD2/PNPLA2/PNPLA7/PRDX	20	MF
GO:0032561	guanyl ribonucleotide binding	62/2726	389/17354	0.47169	0.9491	0.93417	ACSM6/AGAP3/ARL10/ARL8A/CIITA/CNGB1/DAPK1/DNM1/EFTUD2/EHD1/EIF2B2/ERAL1/GCH1/GLUD1/GMPPB/GNAO1/GNAT1/GPN2/GTPBP6/GUCY2D/HRAS/IFT22/MFN1/MFN2/NKIRAS2/NME1/NUDT2/PDE2A/RAB17/RAB20/RAB24/RAB25/RAB26/RAB34/RAB40C/RAB44/RAB7A/RABL6/RALB/RASD1/RASL12/RHEBL1/RHOD/RHOF/RHOT2/RHOV/RND1/RNF112/RRAD/RRAGC/RTKN/SEPT1/SEPT4/SEPT8/SEPT9/TSR1/TUBA3C/TUBA3D/TU[illegible]	62	MF
GO:0033218	amide binding	47/2726	294/17354	0.47241	0.9491	0.93417	ACACB/ACHE/ANG/ANPEP/AP2M1/APBB1/AVPR2/CD300LF/CD74/CLSTN1/CLTB/CLU/CPTP/CRIP1/FBXO2/FNTB/FOLR2/FURIN/GNMT/GPR1/GRIA3/GSTM1/GSTM2/HLA-E/IPO13/IPO4/LOC440434/MGST1/NQO2/NUP98/OPRD1/OPRM1/PC/PEX5/PPIE/PSAP/QRFPR/RNPEP/RNPEPL1/RPS6KB2/RXFP2/SCARB1/SRP14/SSTR5/TAP1/TIMM22/TNPO3	47	MF

GO:0042393	histone binding	32/2726	199/17354	0.47248	0.9491	0.93417	ANP32B/APBB1/BAZ1B/BCAS3/CHD8/COPRS/CTSL/HILS1/HINFP/HIST1H4F/HIST2H4A/HIST2H4B/HJURP/ING4/MCM2/MPHOSPH8/MSL3/NCAPD3/NOC2L/PARP9/PHF12/PHF19/PHF2/PYGO2/RSF1/STAT1/SUPT6H/TAF1/TAF1L/TAF7/USP49/ZMYND11	32	MF
GO:0004843	thiol-dependent ubiquitin-specific	13/2726	79/17354	0.47471	0.9491	0.93417	BRCC3/OTUB1/OTUD5/OTUD6A/OTUD7A/USP19/USP2/USP20/USP27X/USP35/USP4/USP49/USP5	13	MF
GO:0035591	signaling adaptor activity	13/2726	79/17354	0.47471	0.9491	0.93417	ARHGAP4/BLNK/DAB2IP/DOK2/GRAP/KSR1/MAPK8IP1/MAPK8IP3/PTPN11/SH2B1/SH2D2A/SHC1/SHF	13	MF
GO:0001104	RNA polymerase II transcription	16/2726	98/17354	0.47562	0.9491	0.93417	BRCA1/CITED4/CTBP1/MED11/MED18/MED22/MED24/MED6/MED9/NKX2-5/NUP98/OLIG3/PHF12/POU3F1/TBX15/UXT	16	MF
GO:0001883	purine nucleoside binding	60/2726	377/17354	0.47778	0.9491	0.93417	ACSM6/AGAP3/ARL10/ARL8A/CIITA/DAPK1/DNM1/EFTUD2/EHD1/EIF2B2/ERAL1/GCH1/GLUD1/GMPPB/GNAO1/GNAT1/GPN2/GTPBP6/GUCY2D/HRAS/IFT22/MFN1/MFN2/NKIRAS2/NME1/NUDT2/RAB17/RAB20/RAB24/RAB25/RAB26/RAB34/RAB40C/RAB44/RAB7A/RABL6/RALB/RASD1/RASL12/RHEBL1/RHOD/RHOF/RHOT2/RHOV/RND1/RNF112/RRAD/RRAGC/RTKN/SEPT1/SEPT4/SEPT8/SEPT9/TSR1/TUBA3C/TUBA3D/TUBA3E/TUBG1 [illegible]	60	MF
GO:0004003	ATP-dependent DNA helicase activity	6/2726	35/17354	0.47799	0.9491	0.93417	GTF2H4/IGHMBP2/MCM4/PIF1/RECQL4/RECQL5	6	MF
GO:0003713	transcription coactivator activity	48/2726	301/17354	0.47905	0.9491	0.93417	AIP/APEX1/ARL2BP/ATF2/BCL9/BRCA1/CALCOCO1/CIITA/CITED4/E4F1/EDF1/ESR2/GATA4/GMEB1/HCFC1/HINFP/HMGA1/HTATIP2/IL31RA/ING4/KAT2A/MAX/MED24/MED6/MTA1/MYOD1/NR1H3/NR2F1/NR5A1/NRL/NUP98/PDLIM1/PHF2/PMF1/POU3F1/PPARD/RARA/RBPMS/SMARCD2/SRA1/TADA3/TAF1/TAF7/TBPL1/TGFB1I1/UTF1/VGLL2/WNT3A	48	MF
GO:0015179	L-amino acid transmembrane transporter	9/2726	54/17354	0.48015	0.9491	0.93417	PQLC2/SLC1A7/SLC25A22/SLC36A1/SLC36A3/SLC38A1/SLC6A7/SLC6A9/SLC7A7	9	MF

GO:0019001	guanyl nucleotide binding	62/2726	390/17354	0.48051	0.9491	0.93417	ACSM6/AGAP3/ARL10/ARL8A/CIITA/CNGB1/DAPK1/DNM1/EFTUD2/EHD1/EIF2B2/ERAL1/GCH1/GLUD1/GMPPB/GNAO1/GNAT1/GPN2/GTPBP6/GUCY2D/HRAS/IFT22/MFN1/MFN2/NKIRAS2/NME1/NUDT2/PDE2A/RAB17/RAB20/RAB24/RAB25/RAB26/RAB34/RAB40C/RAB44/RAB7A/RABL6/RALB/RASD1/RASL12/RHEBL1/RHOD/RHOF/RHOT2/RHOV/RND1/RNF112/RRAD/RRAGC/RTKN/SEPT1/SEPT4/SEPT8/SEPT9/TSR1/TUBA3C/TUBA3D/TU[illegible]	62	MF
GO:0004518	nuclease activity	33/2726	206/17354	0.48057	0.9491	0.93417	ANG/APEX1/AZGP1/CPSF4/DIS3L/DIS3L2/DNASE1L2/ENDOG/ERN2/EXD3/EXO1/EXOSC10/EXOSC2/ISG20L2/MBD4/NME1/NOB1/OGG1/PAN2/PGBD5/PIWIL2/PNLDC1/POLE/REXO4/RNASE1/RNASE8/RNASE9/RNASEK/RPP21/SMG5/SND1/XRCC3/ZC3H3	33	MF
GO:0005178	integrin binding	18/2726	111/17354	0.48148	0.9491	0.93417	ADAMTS13/CIB2/COL16A1/EGFR/EMILIN1/FERMT3/FN1/ILK/KDR/NISCH/NRG1/SEMA7A/TGFBI/THY1/TLN1/TNN/TSPAN4/VWF	18	MF
GO:0004185	serine-type carboxypeptidase activity	2/2726	10/17354	0.48153	0.9491	0.93417	AEBP1/PRCP	2	MF
GO:0005072	transforming growth factor beta receptor, cytoplasmic mediator	2/2726	10/17354	0.48153	0.9491	0.93417	ENG/SMAD6	2	MF
GO:0005391	sodium:potassium-exchanging ATPase activity	2/2726	10/17354	0.48153	0.9491	0.93417	ATP1A1/ATP1A4	2	MF
GO:0008296	3'-5'-exodeoxyribonuclease activity	2/2726	10/17354	0.48153	0.9491	0.93417	APEX1/POLE	2	MF
GO:0008970	phosphatidylcholine 1-acylhydrolase	2/2726	10/17354	0.48153	0.9491	0.93417	PLA2G16/PLA2G4B	2	MF

GO:0015143	urate transmembrane transporter	2/2726	10/17354	0.48153	0.9491	0.93417	SLC22A12/SLC22A9	2	MF
GO:0015174	basic amino acid transmembrane	2/2726	10/17354	0.48153	0.9491	0.93417	PQLC2/SLC7A7	2	MF
GO:0015279	store-operated calcium channel activity	2/2726	10/17354	0.48153	0.9491	0.93417	ORAI1/ORAI3	2	MF
GO:0015467	G-protein activated inward rectifier potassium	2/2726	10/17354	0.48153	0.9491	0.93417	KCNJ5/KCNJ9	2	MF
GO:0016595	glutamate binding	2/2726	10/17354	0.48153	0.9491	0.93417	GAD1/GRIN1	2	MF
GO:0016801	hydrolase activity, acting on ether bonds	2/2726	10/17354	0.48153	0.9491	0.93417	EPHX2/RNPEP	2	MF
GO:0016846	carbon-sulfur lyase activity	2/2726	10/17354	0.48153	0.9491	0.93417	GLO1/MGST3	2	MF
GO:0016884	carbon-nitrogen ligase activity, with glutamine as amido-N-	2/2726	10/17354	0.48153	0.9491	0.93417	ASNS/GATB	2	MF
GO:0016892	endoribonuclease activity, producing 3'-phosphomonoesters	2/2726	10/17354	0.48153	0.9491	0.93417	RNASE1/RNASE8	2	MF
GO:0017128	phospholipid scramblase	2/2726	10/17354	0.48153	0.9491	0.93417	ANO4/ANO7	2	MF

GO:0017166	vinculin binding	2/2726	10/17354	0.48153	0.9491	0.93417	SORBS3/TLN1	2	MF
GO:0019203	carbohydrate phosphatase activity	2/2726	10/17354	0.48153	0.9491	0.93417	G6PC3/PFKFB1	2	MF
GO:0048156	tau protein binding	2/2726	10/17354	0.48153	0.9491	0.93417	APBB1/BIN1	2	MF
GO:0050308	sugar-phosphatase	2/2726	10/17354	0.48153	0.9491	0.93417	G6PC3/PFKFB1	2	MF
GO:0070513	death domain binding	2/2726	10/17354	0.48153	0.9491	0.93417	DAP/DAPL1	2	MF
GO:0097200	cysteine-type endopeptidase activity involved in execution phase	2/2726	10/17354	0.48153	0.9491	0.93417	CASP3/CASP6	2	MF
GO:1901702	salt transmembrane transporter	2/2726	10/17354	0.48153	0.9491	0.93417	SLC22A12/SLC22A9	2	MF
GO:0140030	modification-dependent protein binding	20/2726	124/17354	0.48664	0.9491	0.93417	ATRIP/BAZ1B/BRCC3/CHD8/FAAP20/ING4/MPHOSPH8/MSL3/NCAPD3/OTUD7A/PARP10/PHF19/PHF2/PRPF8/SHARPIN/TAF1/TAF1L/TAF7/TNIP2/ZMYND11	20	MF
GO:0032549	ribonucleoside binding	60/2726	378/17354	0.48675	0.9491	0.93417	ACSM6/AGAP3/ARL10/ARL8A/CIITA/DAPK1/DNM1/EFTUD2/EHD1/EIF2B2/ERAL1/GCH1/GLUD1/GMPPB/GNAO1/GNAT1/GPN2/GTPBP6/GUCY2D/HRAS/IFT22/MFN1/MFN2/NKIRAS2/NME1/NUDT2/RAB17/RAB20/RAB24/RAB25/RAB26/RAB34/RAB40C/RAB44/RAB7A/RABL6/RALB/RASD1/RASL12/RHEBL1/RHOD/RHOF/RHOT2/RHOV/RND1/RNF112/RRAD/RRAGC/RTKN/SEPT1/SEPT4/SEPT8/SEPT9/TSR1/TUBA3C/TUBA3D/TUBA3E/TUBG1 [illegible]	60	MF
GO:1990782	protein tyrosine kinase binding	11/2726	67/17354	0.48786	0.9491	0.93417	ARHGEF16/BLNK/CD247/DOK2/FNTA/HSP90AA1/MYOC/NRG1/PTPN11/SHC1/ZPR1	11	MF

GO:0005231	excitatory extracellular ligand-gated ion channel activity	8/2726	48/17354	0.48798	0.9491	0.93417	CHRNA6/CHRNB1/CHRNB3/GLRA1/GRIA3/GRIN1/GRIN2C/P2RX2	8	MF
GO:0001882	nucleoside binding	61/2726	385/17354	0.49249	0.9491	0.93417	ACSM6/AGAP3/ARL10/ARL8A/CDA/CIITA/DAPK1/DNM1/EFTUD2/EHD1/EIF2B2/ERAL1/GCH1/GLUD1/GMPPB/GNAO1/GNAT1/GPN2/GTPBP6/GUCY2D/HRAS/IFT22/MFN1/MFN2/NKIRAS2/NME1/NUDT2/RAB17/RAB20/RAB24/RAB25/RAB26/RAB34/RAB40C/RAB44/RAB7A/RABL6/RALB/RASD1/RASL12/RHEBL1/RHOD/RHOF/RHOT2/RHOV/RND1/RNF112/RRAD/RRAGC/RTKN/SEPT1/SEPT4/SEPT8/SEPT9/TSR1/TUBA3C/TUBA3D/TUBA3E/TU[illegible]	61	MF
GO:0008173	RNA methyltransferase activity	10/2726	61/17354	0.49532	0.9491	0.93417	CMTR1/MEPCE/METTL2A/METTL2B/NSUN5/NSUN5P2/THUMPD2/TRMT10B/TRMT2B/TRMT44	10	MF
GO:0005342	organic acid transmembrane transporter	24/2726	150/17354	0.49544	0.9491	0.93417	ABCC3/MPC1/PQLC2/SLC13A2/SLC16A13/SLC1A7/SLC22A12/SLC22A9/SLC25A22/SLC26A1/SLC26A10/SLC26A6/SLC36A1/SLC36A3/SLC38A1/SLC38A10/SLC38A8/SLC6A18/SLC6A6/SLC6A7/SLC6A8/SLC6A9/SLC7A7/SLCO2	24	MF
GO:0008514	organic anion transmembrane transporter activity	32/2726	201/17354	0.49699	0.9491	0.93417	ABCC3/ABCC5/ASIC3/MPC1/PQLC2/SLC13A2/SLC16A13/SLC1A7/SLC22A1/SLC22A12/SLC22A13/SLC22A9/SLC25A22/SLC25A25/SLC25A5/SLC26A1/SLC26A10/SLC26A6/SLC36A1/SLC36A3/SLC38A1/SLC38A10/SLC38A8/SLC4A9/SLC6A18/SLC6A6/SLC6A7/SLC6A8/SLC6A9/SLC7A7/SLCO2B1/SLCO5	32	MF
GO:0030414	peptidase inhibitor activity	29/2726	182/17354	0.49807	0.9491	0.93417	A2ML1/AGT/AHSG/APLP2/CD27/COL7A1/CRB2/DPEP1/FURIN/GAPDH/ITIH4/ITIH6/KNG1/NAIP/PEBP1/PI16/RPS6KA1/SERPINA2/SERPINA3/SERPINA4/SERPINA5/SERPINF2/SERPINH1/SERPINI1/SPINK2/SPINK5/SPINT1/SP	29	MF
GO:0005248	voltage-gated sodium channel activity	4/2726	23/17354	0.49868	0.9491	0.93417	CACNA1G/CACNA1H/HCN3/SCN11A	4	MF
GO:0015106	bicarbonate transmembrane transporter	4/2726	23/17354	0.49868	0.9491	0.93417	SLC26A1/SLC26A10/SLC26A6/SLC4A9	4	MF
GO:0019865	immunoglobulin binding	4/2726	23/17354	0.49868	0.9491	0.93417	CD300LG/FCGR3A/LGALS3/VWF	4	MF

GO:0030742	GTP-dependent protein binding	4/2726	23/17354	0.49868	0.9491	0.93417	GCH1/GCHFR/PARD6A/RAB34	4	MF
GO:0036442	hydrogen-exporting ATPase activity	4/2726	23/17354	0.49868	0.9491	0.93417	ATP6V0B/ATP6V0E2/ATP6V1F/ATP6V1G1	4	MF
GO:1901567	fatty acid derivative	4/2726	23/17354	0.49868	0.9491	0.93417	ACBD4/ACOT7/HADHA/SOAT2	4	MF
GO:1905030	voltage-gated ion channel activity involved in regulation of postsynaptic membrane [illegible]	4/2726	23/17354	0.49868	0.9491	0.93417	CACNA1G/CACNA1H/HCN3/SCN11A	4	MF
GO:0017124	SH3 domain binding	20/2726	125/17354	0.50214	0.9491	0.93417	ABL1/ADAM12/ARHGAP27/DAB2IP/DOCK1/EFS/ELMO3/ESPN/EVL/GPX1/HIP1R/ILK/INPP5D/INPPL1/MAPK15/NCF1/SH2D2A/SH3BP5/SH3BP5L/WA	20	MF
GO:0004402	histone acetyltransferase activity	9/2726	55/17354	0.50355	0.9491	0.93417	ATF2/BRPF1/EPC1/HCFC1/KAT2A/NAA60/TADA3/TAF1/TAF1L	9	MF
GO:0043621	protein self-association	9/2726	55/17354	0.50355	0.9491	0.93417	ACHE/ADAM8/CCL5/DVL2/FHOD1/HSF1/LDB1/ZFYVE27/ZNF496	9	MF
GO:0001221	transcription cofactor binding	6/2726	36/17354	0.50698	0.9491	0.93417	CDK9/FAM89B/PASD1/PHF12/ZBTB17/ZBTB49	6	MF
GO:0016831	carboxy-lyase activity	6/2726	36/17354	0.50698	0.9491	0.93417	CSAD/DDC/FAHD1/GAD1/HDC/PPCDC	6	MF
GO:0000149	SNARE binding	19/2726	119/17354	0.50798	0.9491	0.93417	ABL1/CAPN10/CPLX1/DAPK1/DOC2A/DOC2B/GOLGA2/GOSR2/HECTD3/STX1A/STX4/STX5/STX8/STXBP1/SYTL3/VAMP1/VAMP5/VPS52/VTI1B	19	MF
GO:0004725	protein tyrosine phosphatase activity	16/2726	100/17354	0.51036	0.9491	0.93417	CDC14A/DUSP2/DUSP21/DUSP26/DUSP5/EYA1/MTMR1/MTMR14/PTP4A3/PTPN11/PTPN23/PTPN7/PTPRF/PTPRN2/PTPRQ/PTPRU	16	MF

GO:0003951	NAD+ kinase activity	3/2726	17/17354	0.51332	0.9491	0.93417	DGKI/DGKQ/DGKZ	3	MF
GO:0010485	H4 histone acetyltransferase activity	3/2726	17/17354	0.51332	0.9491	0.93417	HCFC1/KAT2A/NAA60	3	MF
GO:0017049	GTP-Rho binding	3/2726	17/17354	0.51332	0.9491	0.93417	CDC42EP2/RTKN/WHAMM	3	MF
GO:0030275	LRR domain binding	3/2726	17/17354	0.51332	0.9491	0.93417	NRL/PMF1/ZXDC	3	MF
GO:0055102	lipase inhibitor activity	3/2726	17/17354	0.51332	0.9491	0.93417	APOC3/SCGB1A1/SNCB	3	MF
GO:1901677	phosphate transmembrane transporter	3/2726	17/17354	0.51332	0.9491	0.93417	SLC20A1/SLC20A2/SLC34A1	3	MF
GO:0019887	protein kinase regulator activity	30/2726	190/17354	0.51866	0.9491	0.93417	BGN/CAMK2N2/CASP3/CCL5/CDK20/CDK4/CDK5R2/CDKN2A/CHAD/CIB1/CISH/DGKQ/HSPB1/INCA1/LRRTM1/MADD/MAPK8IP1/MARK2/MLST8/NRG1/PPP1R1B/PRKAR1A/PRKAR1B/PRKRIP1/RTN4RL1/RTN4RL2/SFN/S	30	MF
GO:0004629	phospholipase C activity	5/2726	30/17354	0.51906	0.9491	0.93417	CCL5/CHRM1/PLCB2/PLCD1/PLCH2	5	MF
GO:0015276	ligand-gated ion channel activity	22/2726	139/17354	0.52067	0.9491	0.93417	AQP1/ASIC2/ASIC3/CHRNA6/CHRNB1/CHRNB3/CNGB1/DLG4/GLRA1/GRIA3/GRIN1/GRIN2C/HTR3B/ITPR3/JPH3/KCNJ18/KCNJ5/KCNJ9/P2RX2/PTK2B/RASA3/SCNN1A	22	MF
GO:0022834	ligand-gated channel activity	22/2726	139/17354	0.52067	0.9491	0.93417	AQP1/ASIC2/ASIC3/CHRNA6/CHRNB1/CHRNB3/CNGB1/DLG4/GLRA1/GRIA3/GRIN1/GRIN2C/HTR3B/ITPR3/JPH3/KCNJ18/KCNJ5/KCNJ9/P2RX2/PTK2B/RASA3/SCNN1A	22	MF
GO:0030971	receptor tyrosine kinase	9/2726	56/17354	0.52663	0.9491	0.93417	ARHGEF16/BLNK/DOK2/FNTA/MYOC/NRG1/PTPN11/SHC1/ZPR1	9	MF

GO:0042623	ATPase activity, coupled	56/2726	357/17354	0.52747	0.9491	0.93417	ABCA2/ABCB5/ABCB9/ABCC12/ABCC3/ABCC4/ABCC5/ABCC6/ABCG5/ATP13A2/ATP1A1/ATP1A4/ATP2A3/ATP2C2/ATP5G1/ATP6V0B/ATP6V0E2/ATP6V1F/ATP6V1G1/CHD8/DDX31/DDX4/DDX53/DDX54/DHX37/DHX38/DNAH11/DNAH17/DNHD1/DYNC1H1/DYNC1I1/DYNLL2/GTF2H4/HELQ/IGHMBP2/KATNB1/KIF17/KIF4A/KIFC3/MCM4/MYO1C/MYO1E/MYO3A/MYO7A/PEX6/PIF1/PSMC3/RECQL4/RECQL5/RFC1/RFC2/TAP1/TDRD9/TTF2 [illegible]	56	MF
GO:0016407	acetyltransferase activity	16/2726	101/17354	0.52753	0.9491	0.93417	ATF2/BRPF1/EPC1/GNPAT/HADHA/HCFC1/KAT2A/NAA16/NAA60/NAGS/NAT16/NAT6/NAT8B/TADA3/TAF1/TAF1L	16	MF
GO:0016410	N-acyltransferase	16/2726	101/17354	0.52753	0.9491	0.93417	ALAS2/ATF2/BRPF1/EPC1/GLYATL1/HCFC1/KAT2A/NAA16/NAA60/NAGS/NAT16/NAT6/NAT8B/TADA3/TAF1/TAF1L	16	MF
GO:0019955	cytokine binding	16/2726	101/17354	0.52753	0.9491	0.93417	CD74/CNTFR/CSF1R/CXCR1/CXCR2/CXCR3/ENG/IL18BP/IL1RN/IL20RB/IL31RA/LTBP3/NBL1/TCAP/VASN/WFIKKN2	16	MF
GO:0003714	transcription corepressor activity	35/2726	223/17354	0.53086	0.9491	0.93417	AEBP1/APEX1/ARID5A/ATF3/ATN1/CDX2/CRYM/CTBP1/DDX54/DRAP1/E2F8/E4F1/GON4L/HOXC6/HR/LDB1/LHX1/MEIS2/MSC/MTA1/NCOR1/NCOR2/NEUROD2/NOC2L/OLIG3/PEX14/PHF12/RARA/RCOR2/SF1/SUFU/TBX1	35	MF
GO:0000217	DNA secondary structure	4/2726	24/17354	0.53405	0.9491	0.93417	MEN1/RECQL4/XPC/XRCC3	4	MF
GO:0015145	monosaccharide transmembrane transporter	4/2726	24/17354	0.53405	0.9491	0.93417	SLC2A4/SLC2A6/SLC2A8/SLC5A2	4	MF
GO:0030159	receptor signaling complex	4/2726	24/17354	0.53405	0.9491	0.93417	AXIN1/HOMER2/MAPK8IP3/RGS14	4	MF
GO:0071813	lipoprotein particle binding	4/2726	24/17354	0.53405	0.9491	0.93417	CDH13/GPIHBP1/SCARB1/SCARF1	4	MF
GO:0071814	protein-lipid complex	4/2726	24/17354	0.53405	0.9491	0.93417	CDH13/GPIHBP1/SCARB1/SCARF1	4	MF
GO:0000295	adenine nucleotide transmembrane	2/2726	11/17354	0.53456	0.9491	0.93417	SLC25A25/SLC25A5	2	MF

GO:0001222	transcription corepressor binding	2/2726	11/17354	0.53456	0.9491	0.93417	FAM89B/PHF12	2	MF
GO:0004303	estradiol 17-beta-dehydrogenase	2/2726	11/17354	0.53456	0.9491	0.93417	HSD17B1/HSD17B7	2	MF
GO:0004523	RNA-DNA hybrid ribonuclease activity	2/2726	11/17354	0.53456	0.9491	0.93417	APEX1/EXO1	2	MF
GO:0004596	peptide alpha-N-acetyltransferas	2/2726	11/17354	0.53456	0.9491	0.93417	NAA16/NAA60	2	MF
GO:0004791	thioredoxin-disulfide reductase	2/2726	11/17354	0.53456	0.9491	0.93417	TXNRD1/TXNRD3	2	MF
GO:0005346	purine ribonucleotide transmembrane transporter	2/2726	11/17354	0.53456	0.9491	0.93417	SLC25A25/SLC25A5	2	MF
GO:0008093	cytoskeletal adaptor activity	2/2726	11/17354	0.53456	0.9491	0.93417	BIN3/OBSL1	2	MF
GO:0008349	MAP kinase kinase kinase kinase activity	2/2726	11/17354	0.53456	0.9491	0.93417	MAP4K2/TNIK	2	MF
GO:0008556	potassium-transporting ATPase activity	2/2726	11/17354	0.53456	0.9491	0.93417	ATP1A1/ATP1A4	2	MF
GO:0015386	potassium:proton antiporter	2/2726	11/17354	0.53456	0.9491	0.93417	SLC9A3/SLC9A5	2	MF

GO:0016175	superoxide-generating NADPH oxidase activity	2/2726	11/17354	0.53456	0.9491	0.93417	NCF1/NOX4	2	MF
GO:0019211	phosphatase activator	2/2726	11/17354	0.53456	0.9491	0.93417	B3GAT3/IGFBP3	2	MF
GO:0019855	calcium channel inhibitor	2/2726	11/17354	0.53456	0.9491	0.93417	PACSIN3/STX1A	2	MF
GO:0031078	histone deacetylase activity (H3-K14 specific)	2/2726	11/17354	0.53456	0.9491	0.93417	HDAC11/HDAC8	2	MF
GO:0032041	NAD-dependent histone deacetylase activity (H3-	2/2726	11/17354	0.53456	0.9491	0.93417	HDAC11/HDAC8	2	MF
GO:0042577	lipid phosphatase	2/2726	11/17354	0.53456	0.9491	0.93417	EPHX2/PLPP1	2	MF
GO:0043522	leucine zipper domain binding	2/2726	11/17354	0.53456	0.9491	0.93417	NRL/PMF1	2	MF
GO:0043560	insulin receptor substrate	2/2726	11/17354	0.53456	0.9491	0.93417	PRKCD/PTPN11	2	MF
GO:0046030	inositol trisphosphate phosphatase activity	2/2726	11/17354	0.53456	0.9491	0.93417	INPP5D/INPP5E	2	MF
GO:1901611	phosphatidylglycerol binding	2/2726	11/17354	0.53456	0.9491	0.93417	GSDMD/NME4	2	MF
GO:1990825	sequence-specific mRNA	2/2726	11/17354	0.53456	0.9491	0.93417	LARP6/SRSF4	2	MF

GO:0046943	carboxylic acid transmembrane transporter	22/2726	140/17354	0.53523	0.9491	0.93417	ABCC3/MPC1/PQLC2/SLC13A2/SLC16A13/SLC1A7/SLC25A22/SLC26A1/SLC26A10/SLC26A6/SLC36A1/SLC36A3/SLC38A1/SLC38A10/SLC38A8/SLC6A18/SLC6A6/SLC6A7/SLC6A8/SLC6A9/SLC7A7/SLCO2B1	22	MF
GO:0030551	cyclic nucleotide	6/2726	37/17354	0.53535	0.9491	0.93417	CNGA2/CNGB1/HCN3/PDE2A/PRKAR1A/PRKAR1B	6	MF
GO:0061135	endopeptidase regulator activity	28/2726	179/17354	0.54163	0.9587	0.94365	A2ML1/AGT/AHSG/APLP2/CD27/COL7A1/CRB2/DPEP1/FURIN/GAPDH/ITIH4/ITIH6/KNG1/NAIP/PEBP1/RPS6KA1/SERPINA2/SERPINA3/SERPINA4/SERPINA5/SERPINF2/SERPINH1/SERPINI1/SPINK2/SPINK5/SPINT1/SPOCK2	28	MF
GO:0061733	peptide-lysine-N-acetyltransferas	9/2726	57/17354	0.54933	0.9699	0.9546	ATF2/BRPF1/EPC1/HCFC1/KAT2A/NAA60/TADA3/TAF1/TAF1L	9	MF
GO:0098632	cell-cell adhesion	5/2726	31/17354	0.54991	0.9699	0.9546	BAIAP2L1/PDLIM1/PDLIM5/PKP3/PLXNB3	5	MF
GO:0019207	kinase regulator activity	33/2726	212/17354	0.55223	0.9699	0.9546	AHSG/BGN/CAMK2N2/CASP3/CCL5/CDK20/CDK4/CDK5R2/CDKN2A/CHAD/CIB1/CISH/DGKQ/HSPB1/INCA1/LRRTM1/MADD/MAPK8IP1/MARK2/MLST8/NRG1/PIK3R5/PIK3R6/PPP1R1B/PRKAR1A/PRKAR1B/PRKRIP1/RTN4RL1/RTN4RL2/SFN/SH3BP5/SH3BP5L/WNT11	33	MF
GO:0001972	retinoic acid binding	3/2726	18/17354	0.55396	0.9699	0.9546	LCN12/RARA/SERPINA5	3	MF
GO:0004550	nucleoside diphosphate kinase activity	3/2726	18/17354	0.55396	0.9699	0.9546	AK5/NME1/NME4	3	MF
GO:0008157	protein phosphatase 1 binding	3/2726	18/17354	0.55396	0.9699	0.9546	KCNQ1/PHACTR1/PPP1R9B	3	MF
GO:0015278	calcium-release channel activity	3/2726	18/17354	0.55396	0.9699	0.9546	ITPR3/JPH3/RASA3	3	MF

GO:0004842	ubiquitin-protein transferase activity	68/2726	437/17354	0.55497	0.9701	0.95485	AKTIP/ANAPC2/ASB14/BRCA1/CCNF/CHFR/DDB2/FBXL13/FBXL16/FBXL19/FBXL22/FBXL8/FBXO10/FBXO2/FBXO24/FBXO25/FBXO6/FBXW4/HECTD3/HERC6/IPP/KBTBD2/KLHL29/KLHL35/KLHL40/LRSAM1/MAEA/MARCH4/MED11/MED18/MIB2/MPHOSPH8/NAIP/NEURL3/PELI3/RMND5B/RNF166/RNF167/RNF180/RNF183/RNF220/RNF31/SHARPIN/SPSB1/SYVN1/TAF1/TRAF2/TRAF7/TRAIP/TRIM11/TRIM3/TRIM62/TRIM63/TRIM72/TSPAN17/UBE2A/UBE2B/UBE2E2/UBE2U/UBE3B/UBE3C/UBE3D/UBR4/UNKL/W[illegible]	68	MF
GO:0004527	exonuclease activity	12/2726	77/17354	0.56028	0.9706	0.95529	APEX1/DIS3L/DIS3L2/EXD3/EXO1/EXOSC10/EXOSC2/ISG20L2/PAN2/PNLDC1/POLE/REXO4	12	MF
GO:0016763	transferase activity, transferring pentosyl groups	8/2726	51/17354	0.56097	0.9706	0.95529	DNPH1/PARP10/PARP2/PARP3/PARP9/QPRT/XXYLT1/XYLT2	8	MF
GO:0046915	transition metal ion transmembrane	6/2726	38/17354	0.563	0.9706	0.95529	ASIC3/ATOX1/SLC11A1/SLC30A3/SLC39A13/SLC39A5	6	MF
GO:0098631	cell adhesion mediator	6/2726	38/17354	0.563	0.9706	0.95529	BAIAP2L1/EMILIN1/PDLIM1/PDLIM5/PKP3/PLXNB3	6	MF
GO:0043492	ATPase activity, coupled to movement of	20/2726	129/17354	0.56301	0.9706	0.95529	ABCA2/ABCB5/ABCB9/ABCC12/ABCC3/ABCC4/ABCC5/ABCC6/ABCG5/ATP13A2/ATP1A1/ATP1A4/ATP2A3/ATP2C2/ATP5G1/ATP6V0B/ATP6V0E2/ATP6V1F/ATP6V1G1/TAP1	20	MF
GO:0000030	mannosyltransferase activity	4/2726	25/17354	0.56814	0.9706	0.95529	ALG3/DPM2/PIGZ/SDF2	4	MF
GO:0004129	cytochrome-c oxidase activity	4/2726	25/17354	0.56814	0.9706	0.95529	COX10/COX11/COX8A/NDUFA4L2	4	MF
GO:0015002	heme-copper terminal oxidase activity	4/2726	25/17354	0.56814	0.9706	0.95529	COX10/COX11/COX8A/NDUFA4L2	4	MF

GO:0016676	oxidoreductase activity, acting on a heme group of donors, oxygen	4/2726	25/17354	0.56814	0.9706	0.95529	COX10/COX11/COX8A/NDUFA4L2	4	MF
GO:0043027	cysteine-type endopeptidase inhibitor activity involved in	4/2726	25/17354	0.56814	0.9706	0.95529	CD27/DPEP1/NAIP/RPS6KA1	4	MF
GO:0005125	cytokine activity	34/2726	220/17354	0.57028	0.9706	0.95529	AIMP1/BMP4/C10orf99/CCL1/CCL19/CCL21/CCL5/CKLF/CLCF1/CMTM1/CMTM3/CMTM5/CMTM7/CSF1/CSF3/FASLG/GDF2/IFNA5/IL13/IL18/IL1RN/IL32/INHBB/INHBE/LEFTY2/LTA/NRG1/PF4V1/SLURP1/THNSL2/THPO/TN	34	MF
GO:0016614	oxidoreductase activity, acting on CH-OH group of donors	21/2726	136/17354	0.57074	0.9706	0.95529	ABCC4/ADH1A/ADH1C/AKR7A3/CHDH/CTBP1/HADHA/HAO2/HSD11B2/HSD17B1/HSD17B3/HSD17B7/HSD3B1/IDH1/IDH2/IMPDH1/LDHA/NSDHL/PHGDH/PTGR1/XDH	21	MF
GO:0033293	monocarboxylic acid binding	9/2726	58/17354	0.57158	0.9706	0.95529	ACACB/LCN12/PC/PLA2G1B/PPARD/PSAP/RARA/SERPINA5/STARD5	9	MF
GO:0008138	protein tyrosine/serine/threonine phosphatase	7/2726	45/17354	0.57429	0.9706	0.95529	CDC14A/DUSP2/DUSP21/DUSP26/DUSP5/PTP4A3/STYXL1	7	MF
GO:0015078	hydrogen ion transmembrane transporter	16/2726	104/17354	0.57789	0.9706	0.95529	ATP5G1/ATP5I/ATP6V0B/ATP6V0E2/ATP6V1B1/ATP6V1F/ATP6V1G1/COX10/COX11/COX8A/NDUFA4L2/SLC11A1/SLC36A1/SLC36A3/SLC9A3/SLC9A5	16	MF
GO:0019829	cation-transporting	10/2726	65/17354	0.58119	0.9706	0.95529	ATP13A2/ATP1A1/ATP1A4/ATP2A3/ATP2C2/ATP5G1/ATP6V0B/ATP6V0E2/ATP6V1F/ATP6V1G1	10	MF
GO:0034212	peptide N-acetyltransferase activity	10/2726	65/17354	0.58119	0.9706	0.95529	ATF2/BRPF1/EPC1/HCFC1/KAT2A/NAA16/NAA60/TADA3/TAF1/TAF1L	10	MF

GO:0000987	core promoter proximal region sequence-specific DNA binding	58/2726	376/17354	0.58274	0.9706	0.95529	ASCL2/ATF3/CREB3/DLX4/DUX4/E2F4/E2F8/ELF1/EN1/ESRRA/ETV4/FOSL1/FOXQ1/GMEB1/HINFP/HOXB1/HOXB3/HOXB7/HOXC11/HSF1/KLF17/LHX3/LRRFIP1/MAFA/MAX/MEIS2/MESP2/MLXIPL/MUC1/MYCN/MYOD1/NKRF/NKX2-5/NKX2-8/NKX3-2/NR2F1/NRL/PER1/PHOX2B/PITX1/RFX5/RFX6/RORC/SMAD6/SMARCD2/SNAI3/SP2/SPI1/STAT1/TBPL1/TBX15/TBX19/TBX2/ZBTB17/ZNF219/ZNF668/ZNF692/ZSCAN21	58	MF
GO:0004065	arylsulfatase activity	2/2726	12/17354	0.58372	0.9706	0.95529	ARSE/ARSI	2	MF
GO:0004859	phospholipase inhibitor	2/2726	12/17354	0.58372	0.9706	0.95529	SCGB1A1/SNCB	2	MF
GO:0005542	folic acid binding	2/2726	12/17354	0.58372	0.9706	0.95529	FOLR2/GNMT	2	MF
GO:0008020	G-protein coupled photoreceptor	2/2726	12/17354	0.58372	0.9706	0.95529	OPN1MW/OPN4	2	MF
GO:0010314	phosphatidylinositol-5-phosphate	2/2726	12/17354	0.58372	0.9706	0.95529	PHLDA3/SH3PXD2B	2	MF
GO:0015216	purine nucleotide transmembrane	2/2726	12/17354	0.58372	0.9706	0.95529	SLC25A25/SLC25A5	2	MF
GO:0016783	sulfurtransferase activity	2/2726	12/17354	0.58372	0.9706	0.95529	LIAS/TSTD1	2	MF
GO:0016863	intramolecular oxidoreductase activity, transposing C=C bonds	2/2726	12/17354	0.58372	0.9706	0.95529	EBPL/HSD3B1	2	MF
GO:0019992	diacylglycerol binding	2/2726	12/17354	0.58372	0.9706	0.95529	CDIPT/RASGRP2	2	MF

GO:0031994	insulin-like growth factor I	2/2726	12/17354	0.58372	0.9706	0.95529	IGFBP3/IGFBP6	2	MF
GO:0042043	neurexin family protein binding	2/2726	12/17354	0.58372	0.9706	0.95529	NLGN3/SYTL3	2	MF
GO:0051010	microtubule plus-end	2/2726	12/17354	0.58372	0.9706	0.95529	CKAP5/NUMA1	2	MF
GO:0052629	phosphatidylinositol-3,5-bisphosphate 3-phosphatase activity	2/2726	12/17354	0.58372	0.9706	0.95529	MTMR1/MTMR14	2	MF
GO:0070410	co-SMAD binding	2/2726	12/17354	0.58372	0.9706	0.95529	GATA4/SMAD6	2	MF
GO:0072349	modified amino acid transmembrane transporter	2/2726	12/17354	0.58372	0.9706	0.95529	SLC25A20/SLC6A8	2	MF
GO:0019787	ubiquitin-like protein transferase activity	71/2726	460/17354	0.58505	0.9708	0.9555	AKTIP/ANAPC2/ASB14/ATG10/BRCA1/CCNF/CHFR/DDB2/FBXL13/FBXL16/FBXL19/FBXL22/FBXL8/FBXO10/FBXO2/FBXO24/FBXO25/FBXO6/FBXW4/HECTD3/HERC6/IPP/KBTBD2/KLHL29/KLHL35/KLHL40/LRSAM1/MAEA/MARCH4/MED11/MED18/MIB2/MPHOSPH8/NAIP/NEURL3/PELI3/RMND5B/RNF166/RNF167/RNF180/RNF183/RNF212/RNF220/RNF31/SHARPIN/SPSB1/SYVN1/TAF1/TRAF2/TRAF7/TRAIP/TRIM11/TRIM3/TRIM62/TRIM63/TRIM72/TSPAN17/UBE2A/UBE2B/UBE2E2/UBE2U/UBE3B/UBE3C/UBE3D/[illegible]	71	MF
GO:0140097	catalytic activity, acting on DNA	29/2726	189/17354	0.58604	0.9708	0.9555	APEX1/CHD8/DNASE1L2/DNMT3A/ENDOG/EXO1/GTF2H4/HMGA1/IGHMBP2/INO80/LIG3/MBD4/MCM2/MCM4/NEIL2/NME1/OGG1/PARP2/PARP3/PIF1/POLA2/POLD4/POLE/POLE4/RECQL4/RECQL5/SMUG1/VRTN/XRCC3	29	MF
GO:0004529	exodeoxyribonuclease activity	3/2726	19/17354	0.59249	0.9708	0.9555	APEX1/EXO1/POLE	3	MF

GO:0004653	polypeptide N-acetylgalactosaminyltransferase activity	3/2726	19/17354	0.59249	0.9708	0.9555	GALNT16/GALNT8/GALNT9	3	MF
GO:0005234	extracellularly glutamate-gated ion channel	3/2726	19/17354	0.59249	0.9708	0.9555	GRIA3/GRIN1/GRIN2C	3	MF
GO:0016895	exodeoxyribonuclease activity, producing 5'-phosphomonoesters	3/2726	19/17354	0.59249	0.9708	0.9555	APEX1/EXO1/POLE	3	MF
GO:0017075	syntaxin-1 binding	3/2726	19/17354	0.59249	0.9708	0.9555	CPLX1/DAPK1/STXBP1	3	MF
GO:0030296	protein tyrosine kinase activator activity	3/2726	19/17354	0.59249	0.9708	0.9555	CCL5/DGKQ/NRG1	3	MF
GO:0070577	lysine-acetylated	3/2726	19/17354	0.59249	0.9708	0.9555	BAZ1B/TAF1/TAF1L	3	MF
GO:0140033	acetylation-dependent protein binding	3/2726	19/17354	0.59249	0.9708	0.9555	BAZ1B/TAF1/TAF1L	3	MF
GO:0048306	calcium-dependent	9/2726	59/17354	0.59335	0.9708	0.9555	MASP2/NSMF/S100A6/SELP/STX1A/TNNC1/VPS37B/VPS37C/WFS1	9	MF
GO:0001158	enhancer sequence-specific DNA	14/2726	92/17354	0.5958	0.9734	0.95805	ATF2/CDX2/GATA4/HMGA1/HOXC4/LDB1/MSC/NFE2L1/NONO/NR5A1/SCX/SMARCD2/SPI1/TBX19	14	MF
GO:0008188	neuropeptide receptor activity	7/2726	46/17354	0.59884	0.9769	0.96154	NPSR1/NPY4R/OPRM1/QRFPR/SSTR5/TACR1/TACR2	7	MF
GO:0016866	intramolecular transferase	4/2726	26/17354	0.60078	0.9772	0.96185	PGAM4/PUSL1/RPUSD2/TRUB2	4	MF

GO:0043015	gamma-tubulin binding	4/2726	26/17354	0.60078	0.9772	0.96185	NME1/TUBGCP2/TUBGCP3/TUBGCP5	4	MF
GO:0043130	ubiquitin binding	10/2726	66/17354	0.60164	0.9772	0.96185	DDI2/FAAP20/HSPB1/OTUB1/RNF31/UBAP1L/UBE2A/UBTD1/USP5/WDR92	10	MF
GO:0016616	oxidoreductase activity, acting on the CH-OH group of donors, NAD or NADP as	18/2726	119/17354	0.60834	0.9826	0.96715	ABCC4/ADH1A/ADH1C/AKR7A3/CTBP1/HADHA/HSD11B2/HSD17B1/HSD17B3/HSD17B7/HSD3B1/IDH1/IDH2/IMPDH1/LDHA/NSDHL/PHGDH/PTGR1	18	MF
GO:0015175	neutral amino acid transmembrane	5/2726	33/17354	0.60853	0.9826	0.96715	SLC36A1/SLC36A3/SLC38A1/SLC6A7/SLC6A9	5	MF
GO:0001159	core promoter proximal region DNA binding	58/2726	379/17354	0.60868	0.9826	0.96715	ASCL2/ATF3/CREB3/DLX4/DUX4/E2F4/E2F8/ELF1/EN1/ESRRA/ETV4/FOSL1/FOXQ1/GMEB1/HINFP/HOXB1/HOXB3/HOXB7/HOXC11/HSF1/KLF17/LHX3/LRRFIP1/MAFA/MAX/MEIS2/MESP2/MLXIPL/MUC1/MYCN/MYOD1/NKRF/NKX2-5/NKX2-8/NKX3-2/NR2F1/NRL/PER1/PHOX2B/PITX1/RFX5/RFX6/RORC/SMAD6/SMARCD2/SNAI3/SP2/SPI1/STAT1/TBPL1/TBX15/TBX19/TBX2/ZBTB17/ZNF219/ZNF668/ZNF692/ZSCAN21	58	MF
GO:0001618	virus receptor activity	11/2726	73/17354	0.60932	0.9826	0.96715	ANPEP/CD81/CLDN6/EGFR/ITGB5/MOG/SCARB1/SLC20A2/SLC52A1/SLC52A2/TNFRSF4	11	MF
GO:0104005	hijacked molecular	11/2726	73/17354	0.60932	0.9826	0.96715	ANPEP/CD81/CLDN6/EGFR/ITGB5/MOG/SCARB1/SLC20A2/SLC52A1/SLC52A2/TNFRSF4	11	MF
GO:0042578	phosphoric ester hydrolase activity	55/2726	360/17354	0.61226	0.9829	0.96748	ACP2/ACPP/ALPPL2/APEX1/ATP1A1/CCL5/CDC14A/CHRM1/CTDSP1/DUSP2/DUSP21/DUSP26/DUSP5/EPHX2/EYA1/G6PC3/GDPD1/HDDC3/ILKAP/INPP5D/INPP5E/INPP5F/INPPL1/ITPK1/LCK/MTMR1/MTMR14/NT5C3B/NT5M/PDE2A/PDE6D/PFKFB1/PGAM5/PHOSPHO1/PLCB2/PLCD1/PLCH2/PLD2/PLPP1/PPA2/PPEF1/PPP2R2B/PPP2R3B/PTP4A3/PTPN11/PTPN23/PTPN7/PTPRF/PTPRN2/PTPRQ/PTPRU/PUDP/SMPD4/STYXL1/TNS2	55	MF

GO:0099106	ion channel regulator	14/2726	93/17354	0.61289	0.9829	0.96748	AMIGO1/ANKRD36C/CAMK2D/CAV3/DPP6/FLNA/KCNAB3/NRXN2/PACSIN3/PDZD3/SGK1/STX1A/STX8/VTI1B	14	MF
GO:0050661	NADP binding	7/2726	47/17354	0.62263	0.9829	0.96748	CRYM/GAPDH/GSR/HSD17B1/IDH1/NOS3/POR	7	MF
GO:0001871	pattern binding	3/2726	20/17354	0.6288	0.9829	0.96748	CLEC18A/REG4/TINAGL1	3	MF
GO:0016408	C-acyltransferase	3/2726	20/17354	0.6288	0.9829	0.96748	ACSM6/HADHA/SPTLC1	3	MF
GO:0030247	polysaccharide binding	3/2726	20/17354	0.6288	0.9829	0.96748	CLEC18A/REG4/TINAGL1	3	MF
GO:0031683	G-protein beta/gamma-subunit complex	3/2726	20/17354	0.6288	0.9829	0.96748	GNAO1/GNAT1/PIK3R5	3	MF
GO:0004535	poly(A)-specific ribonuclease activity	2/2726	13/17354	0.62893	0.9829	0.96748	PAN2/PNLDC1	2	MF
GO:0015245	fatty acid transporter	2/2726	13/17354	0.62893	0.9829	0.96748	MFSD2A/SLC22A9	2	MF
GO:0015385	sodium:proton antiporter	2/2726	13/17354	0.62893	0.9829	0.96748	SLC9A3/SLC9A5	2	MF
GO:0016423	tRNA (guanine) methyltransferase activity	2/2726	13/17354	0.62893	0.9829	0.96748	THUMPD2/TRMT10B	2	MF
GO:0031543	peptidyl-proline dioxygenase activity	2/2726	13/17354	0.62893	0.9829	0.96748	P3H1/P3H3	2	MF
GO:0035240	dopamine binding	2/2726	13/17354	0.62893	0.9829	0.96748	DRD2/TH	2	MF
GO:0043138	3'-5' DNA helicase activity	2/2726	13/17354	0.62893	0.9829	0.96748	RECQL4/RECQL5	2	MF

GO:0043142	single-stranded DNA-dependent	2/2726	13/17354	0.62893	0.9829	0.96748	PIF1/RFC2	2	MF
GO:0044548	S100 protein binding	2/2726	13/17354	0.62893	0.9829	0.96748	AHNAK/S100A6	2	MF
GO:0070696	transmembrane receptor protein serine/threonine kinase binding	2/2726	13/17354	0.62893	0.9829	0.96748	BMP4/SMAD6	2	MF
GO:0097617	annealing activity	2/2726	13/17354	0.62893	0.9829	0.96748	EIF4B/RECQL4	2	MF
GO:0005249	voltage-gated potassium channel activity	14/2726	94/17354	0.62963	0.9829	0.96748	CNGA2/CNGB1/HCN3/KCNAB3/KCND1/KCND3/KCNH4/KCNH6/KCNJ18/KCNJ5/KCNJ9/KCNQ1/KCNQ4/KCNT1	14	MF
GO:0001968	fibronectin binding	4/2726	27/17354	0.63189	0.9829	0.96748	C6orf15/CTSL/IGFBP3/MYOC	4	MF
GO:0008066	glutamate receptor activity	4/2726	27/17354	0.63189	0.9829	0.96748	GRIA3/GRIN1/GRIN2C/PTK2B	4	MF
GO:0008574	ATP-dependent microtubule motor activity, plus-end-	4/2726	27/17354	0.63189	0.9829	0.96748	DYNC1I1/DYNLL2/KIF17/KIF4A	4	MF
GO:0035035	histone acetyltransferase binding	4/2726	27/17354	0.63189	0.9829	0.96748	BCAS3/EGR1/STAT1/TAF7	4	MF
GO:0099516	ion antiporter activity	9/2726	61/17354	0.6352	0.9829	0.96748	SLC11A1/SLC22A9/SLC25A5/SLC26A1/SLC26A10/SLC26A6/SLC4A9/SLC9A3/SLC9A5	9	MF
GO:0004553	hydrolase activity, hydrolyzing O-glycosyl	15/2726	101/17354	0.63569	0.9829	0.96748	ADPRHL2/CHID1/CHIT1/GANAB/GBA/GLB1L/GLB1L2/GUSBP3/HEXA/LCTL/MGAM/NAGLU/NEU3/PSAP/SPACA3	15	MF

GO:0008536	Ran GTPase binding	5/2726	34/17354	0.63612	0.9829	0.96748	IPO13/IPO4/SERGEF/XPO6/XPO7	5	MF
GO:0030674	protein binding, bridging	25/2726	167/17354	0.63651	0.9829	0.96748	AP2A2/AP2M1/ARHGAP4/ARPC4/ARRDC1/BIN3/BLNK/DDB1/DOK2/DVL2/FGA/FSCN1/GRAP/HIP1R/MEN1/OBSL1/PTPN11/SH2D2A/SHC1/SHF/SPRR1A/STX1A/TCAP/TIRAP/TRDN	25	MF
GO:0004180	carboxypeptidase activity	6/2726	41/17354	0.6408	0.9829	0.96748	AEBP1/BLMH/CPA1/CPN2/NAALAD2/PRCP	6	MF
GO:0004722	protein serine/threonine phosphatase activity	10/2726	68/17354	0.64104	0.9829	0.96748	CDC14A/CTDSP1/ILKAP/LCK/MTMR14/PGAM5/PPA2/PPEF1/PPP2R2B/PPP2R3B	10	MF
GO:0004519	endonuclease activity	19/2726	128/17354	0.64349	0.9829	0.96748	ANG/APEX1/CPSF4/DNASE1L2/ENDOG/ERN2/EXO1/MBD4/NOB1/OGG1/PGBD5/PIWIL2/RNASE1/RNASE8/RNASE9/RNASEK/RPP21/XRCC3/ZC3H3	19	MF
GO:0042277	peptide binding	39/2726	260/17354	0.65019	0.9829	0.96748	ACHE/ANG/ANPEP/AP2M1/APBB1/AVPR2/CD74/CLSTN1/CLTB/CLU/CRIP1/FBXO2/FNTB/FURIN/GPR1/GRIA3/GSTM1/GSTM2/HLA-E/IPO13/IPO4/LOC440434/MGST1/NUP98/OPRD1/OPRM1/PEX5/PPIE/QRFPR/RNPEP/RNPEPL1/RPS6KB2/RXFP2/SCARB1/SRP14/SSTR5/TAP1/TIMM2	39	MF
GO:0030276	clathrin binding	9/2726	62/17354	0.65523	0.9829	0.96748	AP2A2/AP2M1/CALY/CLTB/DOC2A/DOC2B/HIP1R/LRP1/SYTL3	9	MF
GO:0051087	chaperone binding	13/2726	89/17354	0.65707	0.9829	0.96748	ATP1A1/CLU/DNAJB5/DNLZ/FN1/GAK/GNB5/HSPA5/OGDH/SYVN1/TIMM9/UBL4A/VWF	13	MF
GO:0051213	dioxygenase activity	13/2726	89/17354	0.65707	0.9829	0.96748	ALKBH4/ALKBH5/HAAO/IDO1/KDM4E/KDM6B/KDM8/OGFOD2/P3H1/P3H3/PHF2/PLOD3/POR	13	MF
GO:0003887	DNA-directed DNA polymerase	4/2726	28/17354	0.66139	0.9829	0.96748	POLA2/POLD4/POLE/POLE4	4	MF
GO:0008139	nuclear localization	4/2726	28/17354	0.66139	0.9829	0.96748	IPO13/IPO4/NUP98/TNPO3	4	MF
GO:0044183	protein binding involved in protein folding	4/2726	28/17354	0.66139	0.9829	0.96748	CCT3/CD74/HSPB1/PFDN1	4	MF

GO:0061631	ubiquitin conjugating enzyme activity	4/2726	28/17354	0.66139	0.9829	0.96748	TAF1/UBE2A/UBE2B/UBE2E2	4	MF
GO:0016779	nucleotidyltransferase activity	20/2726	136/17354	0.6623	0.9829	0.96748	ABL1/FAM46B/GALT/GMPPB/KARS/LIAS/NMNAT3/POLA2/POLD4/POLE/POLE4/POLR1A/POLR1D/POLR1E/POLR2G/POLR2J2/POLR2J3/POLR2L/PO	20	MF
GO:0001848	complement binding	3/2726	21/17354	0.6628	0.9829	0.96748	C8A/ITGAM/MASP2	3	MF
GO:0005242	inward rectifier potassium channel activity	3/2726	21/17354	0.6628	0.9829	0.96748	KCNJ18/KCNJ5/KCNJ9	3	MF
GO:0015299	solute:proton antiporter	3/2726	21/17354	0.6628	0.9829	0.96748	SLC11A1/SLC9A3/SLC9A5	3	MF
GO:0016922	ligand-dependent nuclear receptor	3/2726	21/17354	0.6628	0.9829	0.96748	NCOR1/NCOR2/TADA3	3	MF
GO:0046961	proton-transporting ATPase activity, rotational	3/2726	21/17354	0.6628	0.9829	0.96748	ATP6V0B/ATP6V0E2/ATP6V1F	3	MF
GO:0140104	molecular carrier activity	3/2726	21/17354	0.6628	0.9829	0.96748	ATOX1/CCS/HBZ	3	MF
GO:0004712	protein serine/threonine/tyrosine kinase activity	6/2726	42/17354	0.6648	0.9829	0.96748	MAP2K3/MAPK3/MAPKAPK3/RPS6KA1/SGK1/TNK2	6	MF
GO:0015036	disulfide oxidoreductase activity	6/2726	42/17354	0.6648	0.9829	0.96748	CCS/GFER/GLRX5/GSR/TXNRD1/TXNRD3	6	MF

GO:0004721	phosphoprotein phosphatase activity	26/2726	176/17354	0.66561	0.9829	0.96748	CDC14A/CTDSP1/DUSP2/DUSP21/DUSP26/DUSP5/EYA1/ILKAP/LCK/MTMR1/MTMR14/PGAM5/PPA2/PPEF1/PPP2R2B/PPP2R3B/PTP4A3/PTPN11/PTPN23/PTPN7/PTPRF/PTPRN2/PTPRQ/PTPRU/STYXL1/TNS2	26	MF
GO:0031490	chromatin DNA binding	15/2726	103/17354	0.66677	0.9829	0.96748	APEX1/BCL6/GRHL3/HR/HSF1/LEMD2/MYOD1/NOTCH1/PER1/PRDM14/RARA/SMARCD2/THRA/VAX1/VAX2	15	MF
GO:0005160	transforming growth factor beta receptor	7/2726	49/17354	0.66776	0.9829	0.96748	BMP4/ENG/GDF2/INHBB/INHBE/LEFTY2/SMAD6	7	MF
GO:0019905	syntaxin binding	12/2726	83/17354	0.66898	0.9829	0.96748	ABL1/CPLX1/DAPK1/DOC2A/DOC2B/GOLGA2/HECTD3/STX8/STXBP1/SYTL3/VAMP1/VPS52	12	MF
GO:0001846	opsonin binding	2/2726	14/17354	0.67021	0.9829	0.96748	ITGAM/MASP2	2	MF
GO:0003688	DNA replication	2/2726	14/17354	0.67021	0.9829	0.96748	MCM2/ORC6	2	MF
GO:0004551	nucleotide diphosphatase activity	2/2726	14/17354	0.67021	0.9829	0.96748	FHIT/NUDT2	2	MF
GO:0004707	MAP kinase activity	2/2726	14/17354	0.67021	0.9829	0.96748	MAPK15/MAPK3	2	MF
GO:0005092	GDP-dissociation	2/2726	14/17354	0.67021	0.9829	0.96748	GPSM1/RGS14	2	MF
GO:0005313	L-glutamate transmembrane transporter	2/2726	14/17354	0.67021	0.9829	0.96748	SLC1A7/SLC25A22	2	MF
GO:0005451	monovalent cation:proton antiporter	2/2726	14/17354	0.67021	0.9829	0.96748	SLC9A3/SLC9A5	2	MF
GO:0005523	tropomyosin binding	2/2726	14/17354	0.67021	0.9829	0.96748	LMOD1/S100A6	2	MF
GO:0008242	omega peptidase	2/2726	14/17354	0.67021	0.9829	0.96748	GGH/GGT6	2	MF

GO:0015172	acidic amino acid transmembrane	2/2726	14/17354	0.67021	0.9829	0.96748	SLC1A7/SLC25A22	2	MF
GO:0016405	CoA-ligase activity	2/2726	14/17354	0.67021	0.9829	0.96748	ACSM6/ACSS3	2	MF
GO:0017110	nucleoside-diphosphatase activity	2/2726	14/17354	0.67021	0.9829	0.96748	ENTPD8/NUDT18	2	MF
GO:0030280	structural constituent of epidermis	2/2726	14/17354	0.67021	0.9829	0.96748	KRT84/SPRR1A	2	MF
GO:0030553	cGMP binding	2/2726	14/17354	0.67021	0.9829	0.96748	CNGB1/PDE2A	2	MF
GO:0042834	peptidoglycan binding	2/2726	14/17354	0.67021	0.9829	0.96748	NOD1/TREM2	2	MF
GO:0070008	serine-type exopeptidase activity	2/2726	14/17354	0.67021	0.9829	0.96748	AEBP1/PRCP	2	MF
GO:0008528	G-protein coupled peptide receptor activity	19/2726	130/17354	0.67101	0.9829	0.96748	AVPR2/CCR2/CXCR1/CXCR2/CXCR3/CXCR5/F2RL1/GPR17/GPR35/LTB4R/NPSR1/NPY4R/OPRD1/OPRM1/QRFPR/SIGMAR1/SSTR5/TACR1/TACR2	19	MF
GO:0001540	amyloid-beta binding	8/2726	56/17354	0.67109	0.9829	0.96748	ACHE/APBB1/CD74/CLSTN1/CLU/FBXO2/GRIA3/SCARB1	8	MF
GO:0005548	phospholipid transporter	8/2726	56/17354	0.67109	0.9829	0.96748	ANO4/ANO7/CPTP/MFSD2A/OSBPL5/PITPNM1/PRELID1/TNFAIP8L3	8	MF
GO:0004386	helicase activity	22/2726	150/17354	0.67162	0.9829	0.96748	CHD8/DDX31/DDX4/DDX53/DDX54/DHX37/DHX38/DHX58/EIF4B/GTF2H4/HELQ/IGHMBP2/INO80/MCM2/MCM4/MCM9/PIF1/RAD54L2/RECQL4/RE	22	MF

GO:0000982	transcription factor activity, RNA polymerase II core promoter proximal region	60/2726	400/17354	0.6741	0.9853	0.96978	ASCL2/ATF2/ATF3/BARHL1/BARHL2/CAMTA2/CREB3/DLX3/DLX4/DUX4/E2F4/E2F8/EGR1/ELF1/EN1/ESRRA/ETV4/FOSL1/FOXQ1/GCM2/GMEB1/HINFP/HOXB1/HOXB3/HOXB7/HOXC11/HOXC13/HSF1/KLF17/LHX3/LRRFIP1/MAFA/MAX/MEIS2/MESP2/MLXIPL/MYCN/NFX1/NKRF/NKX2-5/NKX2-8/NKX3-2/NR2F1/NR4A1/NRL/PHOX2B/PITX1/RORC/SNAI3/SP2/SPI1/TBPL1/TBX1 [illegible]	60	MF
GO:0008026	ATP-dependent helicase activity	14/2726	97/17354	0.6776	0.9878	0.9723	DDX31/DDX4/DDX53/DDX54/DHX37/DHX38/GTF2H4/HELQ/IGHMBP2/MCM4/PIF1/RECQL4/RECQL5/TDRD9	14	MF
GO:0070035	purine NTP-dependent helicase activity	14/2726	97/17354	0.6776	0.9878	0.9723	DDX31/DDX4/DDX53/DDX54/DHX37/DHX38/GTF2H4/HELQ/IGHMBP2/MCM4/PIF1/RECQL4/RECQL5/TDRD9	14	MF
GO:0001653	peptide receptor activity	19/2726	131/17354	0.68433	0.9932	0.97758	AVPR2/CCR2/CXCR1/CXCR2/CXCR3/CXCR5/F2RL1/GPR17/GPR35/LTB4R/NPSR1/NPY4R/OPRD1/OPRM1/QRFPR/SIGMAR1/SSTR5/TACR1/TACR2	19	MF
GO:0005246	calcium channel regulator	5/2726	36/17354	0.68756	0.9932	0.97758	CAV3/NRXN2/PACSIN3/SGK1/STX1A	5	MF
GO:0036002	pre-mRNA binding	5/2726	36/17354	0.68756	0.9932	0.97758	LSM1/PRPF8/RBM4/SF1/TARBP2	5	MF
GO:0042162	telomeric DNA binding	5/2726	36/17354	0.68756	0.9932	0.97758	ACD/APEX1/PIF1/SMG5/TINF2	5	MF
GO:0016303	1-phosphatidylinositol-3-kinase activity	6/2726	43/17354	0.68776	0.9932	0.97758	FGF17/FGF3/FGFR2/FGFR4/PTPN11/TLR9	6	MF
GO:0008374	O-acyltransferase	7/2726	50/17354	0.68903	0.9932	0.97758	CPT2/CROT/DGAT1/GNPAT/PLA2G15/PNPLA2/SOAT2	7	MF
GO:0030515	snoRNA binding	4/2726	29/17354	0.68923	0.9932	0.97758	NHP2/NUDT1/NUDT16L1/TSR1	4	MF
GO:0061650	ubiquitin-like protein conjugating	4/2726	29/17354	0.68923	0.9932	0.97758	TAF1/UBE2A/UBE2B/UBE2E2	4	MF

GO:0071855	neuropeptide receptor binding	4/2726	29/17354	0.68923	0.9932	0.97758	EDN2/GNAO1/NPPA/UCN2	4	MF
GO:0005385	zinc ion transmembrane transporter	3/2726	22/17354	0.69448	0.9944	0.97874	SLC30A3/SLC39A13/SLC39A5	3	MF
GO:0008171	O-methyltransferase activity	3/2726	22/17354	0.69448	0.9944	0.97874	CMTR1/LCMT1/LRTOMT	3	MF
GO:0008198	ferrous iron binding	3/2726	22/17354	0.69448	0.9944	0.97874	HAAO/HEPH/TH	3	MF
GO:0016894	endonuclease activity, active with either ribo- or deoxyribonucleic acids and producing 3'-phosphomonoes	3/2726	22/17354	0.69448	0.9944	0.97874	RNASE1/RNASE8/XRCC3	3	MF
GO:0070034	telomerase RNA binding	3/2726	22/17354	0.69448	0.9944	0.97874	EXOSC10/NHP2/SMG5	3	MF
GO:0004438	phosphatidylinositol-3-phosphatase	2/2726	15/17354	0.70769	0.9952	0.97955	MTMR1/MTMR14	2	MF
GO:0004953	icosanoid receptor activity	2/2726	15/17354	0.70769	0.9952	0.97955	LTB4R/PTGER3	2	MF
GO:0005161	platelet-derived growth factor receptor binding	2/2726	15/17354	0.70769	0.9952	0.97955	PDGFA/PDGFRB	2	MF
GO:0008191	metalloendopeptidase inhibitor activity	2/2726	15/17354	0.70769	0.9952	0.97955	SPOCK2/WFIKKN2	2	MF

GO:0009881	photoreceptor activity	2/2726	15/17354	0.70769	0.9952	0.97955	OPN1MW/OPN4	2	MF
GO:0015929	hexosaminidase activity	2/2726	15/17354	0.70769	0.9952	0.97955	HEXA/NAGLU	2	MF
GO:0016722	oxidoreductase activity, oxidizing metal	2/2726	15/17354	0.70769	0.9952	0.97955	HEPH/STEAP2	2	MF
GO:0030955	potassium ion binding	2/2726	15/17354	0.70769	0.9952	0.97955	ATP1A1/PKM	2	MF
GO:0033612	receptor serine/threonine kinase binding	2/2726	15/17354	0.70769	0.9952	0.97955	BMP4/SMAD6	2	MF
GO:0042809	vitamin D receptor binding	2/2726	15/17354	0.70769	0.9952	0.97955	MED24/TAF7	2	MF
GO:0052744	phosphatidylinositol monophosphate phosphatase	2/2726	15/17354	0.70769	0.9952	0.97955	MTMR1/MTMR14	2	MF
GO:0106018	phosphatidylinositol-3,5-bisphosphate phosphatase activity	2/2726	15/17354	0.70769	0.9952	0.97955	MTMR1/MTMR14	2	MF
GO:0003743	translation initiation factor	7/2726	51/17354	0.70941	0.9952	0.97955	EIF1AY/EIF2B2/EIF2B5/EIF3B/EIF3I/EIF4B/EIF4G1	7	MF
GO:0043175	RNA polymerase core enzyme binding	7/2726	51/17354	0.70941	0.9952	0.97955	CCAR2/ERBB2/MAF1/MIR16-1/MIR584/RECQL5/SMYD3	7	MF
GO:0052742	phosphatidylinositol kinase	7/2726	51/17354	0.70941	0.9952	0.97955	FGF17/FGF3/FGFR2/FGFR4/PI4KB/PTPN11/TLR9	7	MF

GO:0035326	enhancer binding	15/2726	106/17354	0.71053	0.9952	0.97955	ATF2/CDX2/GATA4/HMGA1/HOXC4/LDB1/MSC/NFE2L1/NONO/NR5A1/RAI1/SCX/SMARCD2/SPI1/TBX19	15	MF
GO:0000980	RNA polymerase II distal enhancer sequence-	10/2726	72/17354	0.713	0.9952	0.97955	ATF2/CDX2/HOXC4/MSC/NFE2L1/NONO/NR5A1/SCX/SMARCD2/SPI1	10	MF
GO:0070405	ammonium ion binding	10/2726	72/17354	0.713	0.9952	0.97955	ACHE/APOA5/CHRNA6/CHRNB1/CHRNB3/DRD2/HTR1D/PITPNM1/SERPINA5/TH	10	MF
GO:0043021	ribonucleoprotein complex	16/2726	113/17354	0.71353	0.9952	0.97955	EIF1AY/EIF4B/EIF5A/ERAL1/GTPBP6/HSPA5/IGHMBP2/LETMD1/MTOR/NAA16/NME1/NVL/SEC61A2/SMG5/ZNF593/ZNF598	16	MF
GO:0005227	calcium activated cation channel activity	4/2726	30/17354	0.71541	0.9952	0.97955	ANO1/CATSPER1/KCNT1/TRPM5	4	MF
GO:0016538	cyclin-dependent protein serine/threonine kinase regulator	4/2726	30/17354	0.71541	0.9952	0.97955	CASP3/CDK4/CDKN2A/INCA1	4	MF
GO:0035250	UDP-galactosyltransferase activity	4/2726	30/17354	0.71541	0.9952	0.97955	B3GNT6/B4GALT2/B4GALT7/PLOD3	4	MF
GO:1901682	sulfur compound transmembrane	4/2726	30/17354	0.71541	0.9952	0.97955	SLC26A1/SLC26A10/SLC26A6/SLC6A6	4	MF
GO:0008135	translation factor activity, RNA binding	12/2726	86/17354	0.71713	0.9964	0.98068	ABCF1/ABTB1/C12orf65/EIF1AY/EIF2B2/EIF2B5/EIF3B/EIF3I/EIF4B/EIF4G1/EIF5A/GATB	12	MF

GO:0030545	receptor regulator activity	72/2726	485/17354	0.72038	0.9995	0.98379	ADM/AGT/AIMP1/ARTN/BMP4/C10orf99/CCL1/CCL19/CCL21/CCL5/CDC42EP2/CDK5/CKLF/CLCF1/CMTM1/CMTM3/CMTM5/CMTM7/CSF1/CSF3/DGKQ/EDN2/ESR2/FASLG/FGF17/FGF3/FNDC5/FNTA/GCG/GDF2/GFER/GPHA2/GUCA2A/IFNA5/IL13/IL18/IL18BP/IL1RN/IL32/INHBB/INHBE/INS/INS-IGF2/JAG2/LEFTY2/LGALS3/LTA/LYNX1/LYPD1/LYPD6B/NBL1/NPPA/NRG1/OSGIN1/PDGFA/PF4V1/PYY/SEMA3F/SHH/SLURP1/TDGF1/THNSL2/THPO/TNFSF13/TRH/UCN2/VGF/WFIKKN2/WNT1/WNT10A/WNT3A/WNT7[illegible]	72	MF
GO:0031210	phosphatidylcholine binding	3/2726	23/17354	0.72385	0.9995	0.98379	APOA5/PITPNM1/SERPINA5	3	MF
GO:0050997	quaternary ammonium group binding	3/2726	23/17354	0.72385	0.9995	0.98379	APOA5/PITPNM1/SERPINA5	3	MF
GO:0070412	R-SMAD binding	3/2726	23/17354	0.72385	0.9995	0.98379	MEN1/SMAD6/ZC3H3	3	MF
GO:0090484	drug transporter activity	3/2726	23/17354	0.72385	0.9995	0.98379	ABCB5/ABCC3/SLC44A4	3	MF
GO:0033613	activating transcription factor binding	9/2726	66/17354	0.72865	1	0.98427	ATF2/CIITA/DGKQ/DUSP26/GATA4/HCFC1/LDB1/NCOR1/WFS1	9	MF
GO:0005179	hormone activity	17/2726	121/17354	0.72937	1	0.98427	ADM/AGT/EDN2/FNDC5/GCG/GPHA2/GUCA2A/INHBB/INHBE/INS/INS-IGF2/NPPA/PYY/THPO/TRH/UCN2/VGF	17	MF

GO:0016706	oxidoreductase activity, acting on paired donors, with incorporation or reduction of molecular oxygen, 2-oxoglutarate as one donor, and incorporation of one atom each of oxygen into	6/2726	45/17354	0.73049	1	0.98427	ALKBH4/ALKBH5/KDM8/P3H1/P3H3/PLOD3	6	MF
GO:0017069	snRNA binding	5/2726	38/17354	0.73382	1	0.98427	CDK9/EIF5A/PRPF4/PRPF8/RBPMS	5	MF
GO:0035254	glutamate receptor binding	5/2726	38/17354	0.73382	1	0.98427	DLG4/DRD2/HOMER2/NECAB2/SCRIB	5	MF
GO:0000400	four-way junction DNA	2/2726	16/17354	0.74153	1	0.98427	MEN1/XRCC3	2	MF
GO:0008353	RNA polymerase II carboxy-terminal domain	2/2726	16/17354	0.74153	1	0.98427	CDK9/GTF2H4	2	MF
GO:0015125	bile acid transmembrane transporter	2/2726	16/17354	0.74153	1	0.98427	ABCC3/SLCO2B1	2	MF
GO:0015215	nucleotide transmembrane transporter	2/2726	16/17354	0.74153	1	0.98427	SLC25A25/SLC25A5	2	MF

GO:0017136	NAD-dependent histone deacetylase	2/2726	16/17354	0.74153	1	0.98427	HDAC11/HDAC8	2	MF
GO:0022821	potassium ion antiporter	2/2726	16/17354	0.74153	1	0.98427	SLC9A3/SLC9A5	2	MF
GO:0034185	apolipoprotein binding	2/2726	16/17354	0.74153	1	0.98427	LRP1/SCARB1	2	MF
GO:0045236	CXCR chemokine	2/2726	16/17354	0.74153	1	0.98427	PF4V1/YARS	2	MF
GO:0046527	glucosyltransferase activity	2/2726	16/17354	0.74153	1	0.98427	ALG10/PLOD3	2	MF
GO:0046965	retinoid X receptor binding	2/2726	16/17354	0.74153	1	0.98427	ARID5A/HMGA1	2	MF
GO:0048038	quinone binding	2/2726	16/17354	0.74153	1	0.98427	AOC2/DHODH	2	MF
GO:0050664	oxidoreductase activity, acting on NAD(P)H, oxygen as acceptor	2/2726	16/17354	0.74153	1	0.98427	NCF1/NOX4	2	MF
GO:1990381	ubiquitin-specific	2/2726	16/17354	0.74153	1	0.98427	PDZD3/SYVN1	2	MF
GO:0019783	ubiquitin-like protein-specific protease activity	18/2726	129/17354	0.74401	1	0.98427	BRCC3/KAT2A/OTUB1/OTUD5/OTUD6A/OTUD7A/SENP6/TADA3/UFSP1/USP19/USP2/USP20/USP27X/USP35/USP4/USP49/USP5/USP54	18	MF
GO:0004004	ATP-dependent RNA helicase activity	9/2726	67/17354	0.74526	1	0.98427	DDX31/DDX4/DDX53/DDX54/DHX37/DHX38/HELQ/IGHMBP2/TDRD9	9	MF
GO:0008094	DNA-dependent	11/2726	81/17354	0.74578	1	0.98427	CHD8/GTF2H4/IGHMBP2/MCM4/PIF1/RECQL4/RECQL5/RFC1/RFC2/TTF2/XRCC3	11	MF

GO:0042379	chemokine receptor binding	8/2726	60/17354	0.74588	1	0.98427	CCL1/CCL19/CCL21/CCL5/CCR2/CKLF/PF4V1/YARS	8	MF
GO:0019956	chemokine binding	3/2726	24/17354	0.75096	1	0.98427	CXCR1/CXCR2/CXCR3	3	MF
GO:0035198	miRNA binding	3/2726	24/17354	0.75096	1	0.98427	RBM4/ZC3H10/ZNF346	3	MF
GO:0035255	ionotropic glutamate receptor binding	3/2726	24/17354	0.75096	1	0.98427	DLG4/DRD2/SCRIB	3	MF
GO:0070566	adenylyltransferase activity	3/2726	24/17354	0.75096	1	0.98427	ABL1/KARS/NMNAT3	3	MF
GO:0048020	CCR chemokine	5/2726	39/17354	0.75499	1	0.98427	CCL1/CCL19/CCL21/CCL5/CCR2	5	MF
GO:0005230	extracellular ligand-gated ion channel activity	10/2726	75/17354	0.76046	1	0.98427	CHRNA6/CHRNB1/CHRNB3/GLRA1/GRIA3/GRIN1/GRIN2C/HTR3B/P2RX2/PTK2B	10	MF
GO:0003724	RNA helicase activity	9/2726	68/17354	0.76116	1	0.98427	DDX31/DDX4/DDX53/DDX54/DHX37/DHX38/HELQ/IGHMBP2/TDRD9	9	MF
GO:0008186	RNA-dependent ATPase activity	9/2726	68/17354	0.76116	1	0.98427	DDX31/DDX4/DDX53/DDX54/DHX37/DHX38/HELQ/IGHMBP2/TDRD9	9	MF
GO:0004536	deoxyribonuclease activity	8/2726	61/17354	0.76259	1	0.98427	APEX1/DNASE1L2/ENDOG/EXO1/MBD4/NME1/POLE/XRCC3	8	MF
GO:0016810	hydrolase activity, acting on carbon-nitrogen (but	20/2726	145/17354	0.77024	1	0.98427	ADAT1/AGMAT/AMDHD2/BRMS1/CDA/DARS/DPYSL4/GCH1/HDAC11/HDAC8/MTA1/MTHFD1/NAALAD2/NIT1/PADI3/PADI4/PADI6/PM20D1/VNN3/WDYHV1	20	MF
GO:0003746	translation elongation factor activity	2/2726	17/17354	0.77196	1	0.98427	ABTB1/EIF5A	2	MF
GO:0005149	interleukin-1 receptor binding	2/2726	17/17354	0.77196	1	0.98427	IL1RN/TLR9	2	MF

GO:0008329	signaling pattern recognition	2/2726	17/17354	0.77196	1	0.98427	CLEC7A/SCARB1	2	MF
GO:0031489	myosin V binding	2/2726	17/17354	0.77196	1	0.98427	NPC1L1/RAB25	2	MF
GO:0034979	NAD-dependent protein deacetylase	2/2726	17/17354	0.77196	1	0.98427	HDAC11/HDAC8	2	MF
GO:0038187	pattern recognition	2/2726	17/17354	0.77196	1	0.98427	CLEC7A/SCARB1	2	MF
GO:0042800	histone methyltransferase activity (H3-K4 specific)	2/2726	17/17354	0.77196	1	0.98427	ASH2L/SETD1A	2	MF
GO:0019902	phosphatase binding	22/2726	159/17354	0.77356	1	0.98427	BAD/CDH5/CSF1R/CSK/DAB2IP/DLG4/EGFR/EIF4EBP1/ERBB2/FCRL6/FLT4/KAT2A/KCNQ1/LCK/MAPK3/PARD3/PHACTR1/PPP1R9B/SLC9A3R2/SM	22	MF
GO:0016651	oxidoreductase activity, acting on NAD(P)H	14/2726	104/17354	0.77455	1	0.98427	CYB5R1/CYB5R2/GSR/NARFL/NCF1/NDUFA2/NDUFB5/NDUFC2-KCTD14/NOS3/NOX4/POR/TXNRD1/TXNRD3/WDR93	14	MF
GO:0008009	chemokine activity	6/2726	48/17354	0.78652	1	0.98427	CCL1/CCL19/CCL21/CCL5/CKLF/PF4V1	6	MF
GO:0015459	potassium channel	6/2726	48/17354	0.78652	1	0.98427	AMIGO1/CAV3/DPP6/FLNA/KCNAB3/SGK1	6	MF
GO:0051018	protein kinase A binding	6/2726	48/17354	0.78652	1	0.98427	CSK/DACT2/KCNQ1/PRKAR1A/PRKAR1B/RARA	6	MF
GO:0001046	core promoter sequence-specific DNA	15/2726	112/17354	0.78684	1	0.98427	CDK9/CIART/ESR2/GBX2/HOXB3/HSF1/MTA1/SARS/SMYD3/SOHLH2/SOX8/SPI1/STAT1/TAF1/TBR1	15	MF
GO:0015297	antiporter activity	11/2726	84/17354	0.78765	1	0.98427	SLC11A1/SLC22A9/SLC25A20/SLC25A5/SLC26A1/SLC26A10/SLC26A6/SLC4A9/SLC7A7/SLC9A3/SLC9A5	11	MF

GO:0003684	damaged DNA binding	8/2726	63/17354	0.79361	1	0.98427	APEX1/BRCA1/DDB1/DDB2/NEIL2/OGG1/RECQL4/XPC	8	MF
GO:0001047	core promoter binding	23/2726	168/17354	0.79428	1	0.98427	CALCOCO1/CDK9/CIART/E2F8/ESR2/GBX2/HOXB3/HSF1/MTA1/MYOD1/NONO/NOTCH1/NPAS2/PSPC1/SARS/SMYD3/SOHLH2/SOX8/SPI1/STAT1/TAF1/TBR1/ZBTB17	23	MF
GO:0036459	thiol-dependent ubiquitinyl hydrolase	16/2726	120/17354	0.79822	1	0.98427	BRCC3/KAT2A/OTUB1/OTUD5/OTUD6A/OTUD7A/TADA3/USP19/USP2/USP20/USP27X/USP35/USP4/USP49/USP5/USP54	16	MF
GO:0004181	metallocarboxypeptidase	3/2726	26/17354	0.79873	1	0.98427	AEBP1/CPA1/CPN2	3	MF
GO:0016878	acid-thiol ligase activity	3/2726	26/17354	0.79873	1	0.98427	ACSF3/ACSM6/ACSS3	3	MF
GO:0004745	retinol dehydrogenase activity	2/2726	18/17354	0.79921	1	0.98427	ADH1A/ADH1C	2	MF
GO:0097602	cullin family protein binding	2/2726	18/17354	0.79921	1	0.98427	DDB1/KCTD5	2	MF
GO:0008395	steroid hydroxylase activity	4/2726	34/17354	0.80382	1	0.98427	CYP11A1/CYP11B2/CYP1A1/CYP2W1	4	MF
GO:0015020	glucuronosyltransferase activity	4/2726	34/17354	0.80382	1	0.98427	B3GAT3/CHPF/CHPF2/UGT3A2	4	MF
GO:0016814	hydrolase activity, acting on carbon-nitrogen (but not peptide)	4/2726	34/17354	0.80382	1	0.98427	ADAT1/CDA/GCH1/MTHFD1	4	MF

GO:0044769	ATPase activity, coupled to transmembrane movement of ions, rotational	4/2726	34/17354	0.80382	1	0.98427	ATP5G1/ATP6V0B/ATP6V0E2/ATP6V1F	4	MF
GO:0101005	ubiquitinyl hydrolase	16/2726	121/17354	0.80871	1	0.98427	BRCC3/KAT2A/OTUB1/OTUD5/OTUD6A/OTUD7A/TADA3/USP19/USP2/USP20/USP27X/USP35/USP4/USP49/USP5/USP54	16	MF
GO:0001105	RNA polymerase II transcription	5/2726	42/17354	0.81089	1	0.98427	BRCA1/CITED4/MED6/NUP98/POU3F1	5	MF
GO:0030165	PDZ domain binding	11/2726	86/17354	0.81266	1	0.98427	ADGRB1/ARHGEF16/ASIC3/DLG4/FZD2/FZD8/GRASP/SLC22A12/SLC26A6/SLC34A1/SLC9A3	11	MF
GO:0051117	ATPase binding	10/2726	79/17354	0.8148	1	0.98427	ATOX1/ATP6V1G1/ATPIF1/BRSK2/LCK/NKD2/RALB/SYVN1/VCPKMT/WFS1	10	MF
GO:0004955	prostaglandin receptor activity	1/2726	10/17354	0.81901	1	0.98427	PTGER3	1	MF
GO:0005381	iron ion transmembrane transporter	1/2726	10/17354	0.81901	1	0.98427	ASIC3	1	MF
GO:0008199	ferric iron binding	1/2726	10/17354	0.81901	1	0.98427	TH	1	MF
GO:0008474	palmitoyl-(protein)	1/2726	10/17354	0.81901	1	0.98427	LYPLA2	1	MF
GO:0015643	toxic substance binding	1/2726	10/17354	0.81901	1	0.98427	EPHX2	1	MF
GO:0016018	cyclosporin A binding	1/2726	10/17354	0.81901	1	0.98427	PPIE	1	MF

GO:0016679	oxidoreductase activity, acting on diphenols and related substances as	1/2726	10/17354	0.81901	1	0.98427	CYP1A1	1	MF
GO:0016868	intramolecular transferase activity, phosphotransferases	1/2726	10/17354	0.81901	1	0.98427	PGAM4	1	MF
GO:0019911	structural constituent of myelin sheath	1/2726	10/17354	0.81901	1	0.98427	MOBP	1	MF
GO:0030215	semaphorin receptor binding	1/2726	10/17354	0.81901	1	0.98427	PLXNB1	1	MF
GO:0030881	beta-2-microglobulin binding	1/2726	10/17354	0.81901	1	0.98427	HLA-E	1	MF
GO:0031957	very long-chain fatty acid-CoA ligase activity	1/2726	10/17354	0.81901	1	0.98427	ACSF3	1	MF
GO:0034596	phosphatidylinositol phosphate 4-phosphatase activity	1/2726	10/17354	0.81901	1	0.98427	INPP5F	1	MF
GO:0035325	Toll-like receptor binding	1/2726	10/17354	0.81901	1	0.98427	TIRAP	1	MF
GO:0036312	phosphatidylinositol 3-kinase regulatory subunit binding	1/2726	10/17354	0.81901	1	0.98427	DAB2IP	1	MF

GO:0042975	peroxisome proliferator activated receptor binding	1/2726	10/17354	0.81901	1	0.98427	HMGA1	1	MF
GO:0043121	neurotrophin binding	1/2726	10/17354	0.81901	1	0.98427	FURIN	1	MF
GO:0045294	alpha-catenin binding	1/2726	10/17354	0.81901	1	0.98427	PKP3	1	MF
GO:0047372	acylglycerol lipase activity	1/2726	10/17354	0.81901	1	0.98427	DAGLB	1	MF
GO:0051864	histone demethylase activity (H3-K36 specific)	1/2726	10/17354	0.81901	1	0.98427	KDM8	1	MF
GO:0070679	inositol 1,4,5 trisphosphate binding	1/2726	10/17354	0.81901	1	0.98427	ITPR3	1	MF
GO:0098599	palmitoyl hydrolase	1/2726	10/17354	0.81901	1	0.98427	LYPLA2	1	MF
GO:0044325	ion channel binding	15/2726	115/17354	0.81923	1	0.98427	AP2M1/CAMK2D/CAV3/CDH5/CIB1/FLNA/HERPUD1/KCND3/KCNQ1/PACS1/PKD1/STX1A/TCAP/TRDN/YWHAQ	15	MF
GO:0034593	phosphatidylinositol bisphosphate phosphatase	3/2726	27/17354	0.81959	1	0.98427	INPP5E/MTMR1/MTMR14	3	MF
GO:0051721	protein phosphatase 2A binding	3/2726	27/17354	0.81959	1	0.98427	DAB2IP/EIF4EBP1/SMG5	3	MF
GO:0061630	ubiquitin protein ligase activity	31/2726	226/17354	0.82051	1	0.98427	AKTIP/ANAPC2/CHFR/FBXL22/FBXO6/HECTD3/HERC6/LRSAM1/MED11/MED18/MIB2/NEURL3/PELI3/RNF166/RNF180/RNF183/RNF220/SYVN1/TRAIP/TRIM11/TRIM3/TRIM72/UBE2A/UBE2B/UBE2U/UBE3B/UBE3C/UBE3D	31	MF

GO:0001784	phosphotyrosine residue binding	4/2726	35/17354	0.82206	1	0.98427	ABL1/LCK/MAPK3/SHC1	4	MF
GO:0008378	galactosyltransferase activity	4/2726	35/17354	0.82206	1	0.98427	B3GNT6/B4GALT2/B4GALT7/PLOD3	4	MF
GO:0015238	drug transmembrane transporter	2/2726	19/17354	0.82353	1	0.98427	ABCB5/ABCC3	2	MF
GO:0015605	organophosphate ester transmembrane transporter	2/2726	19/17354	0.82353	1	0.98427	SLC25A25/SLC25A5	2	MF
GO:0051184	cofactor transporter	2/2726	19/17354	0.82353	1	0.98427	FOLR2/SLC6A8	2	MF
GO:0016667	oxidoreductase activity, acting on a sulfur group of donors	7/2726	58/17354	0.82653	1	0.98427	CCS/GFER/GLRX5/GSR/QSOX1/TXNRD1/TXNRD3	7	MF
GO:0000993	RNA polymerase II	5/2726	43/17354	0.82709	1	0.98427	CCAR2/MIR16-1/MIR584/RECQL5/SMYD3	5	MF
GO:0001085	RNA polymerase II transcription	15/2726	116/17354	0.82918	1	0.98427	ANKRD2/ATF2/DUSP26/GATA4/GFI1B/LDB1/LHX3/MTA1/NCOR1/PITX1/SPEN/SPI1/SPOP/TEAD4/WWP2	15	MF
GO:0030145	manganese ion binding	6/2726	51/17354	0.83321	1	0.98427	ABL1/ATP13A2/B4GALT7/CDIPT/PPEF1/XXYLT1	6	MF
GO:0003725	double-stranded RNA binding	8/2726	66/17354	0.8343	1	0.98427	DHX58/EIF4B/LRRFIP1/PRKRIP1/SIDT2/TARBP2/TLR8/ZNF346	8	MF
GO:0008237	metallopeptidase activity	25/2726	187/17354	0.83766	1	0.98427	ADAM12/ADAM8/ADAMTS13/ADAMTS14/ADAMTS2/ADAMTS7/AEBP1/ANPEP/BRCC3/CHMP1A/CPA1/CPN2/DPEP1/ECEL1/LOC440434/MMP15/MMP17/MMP2/MMP25/MMP3/NAALAD2/PMPCA/RNPEP/RNPEPL1/YME1L1	25	MF
GO:0005520	insulin-like growth factor	3/2726	28/17354	0.83857	1	0.98427	HTRA3/IGFBP3/IGFBP6	3	MF

GO:0004864	protein phosphatase inhibitor	4/2726	36/17354	0.83887	1	0.98427	PHACTR1/PPP1R10/PPP1R1B/PPP1R9B	4	MF
GO:0019003	GDP binding	7/2726	59/17354	0.83975	1	0.98427	GNAT1/HRAS/RAB17/RAB40C/RAB7A/RALB/RRAGC	7	MF
GO:0030295	protein kinase activator	9/2726	74/17354	0.84183	1	0.98427	CCL5/CDK20/CDK5R2/DGKQ/MADD/MARK2/MLST8/NRG1/WNT11	9	MF
GO:0005212	structural constituent of eye lens	2/2726	20/17354	0.84517	1	0.98427	CRYAB/MIP	2	MF
GO:0008266	poly(U) RNA binding	2/2726	20/17354	0.84517	1	0.98427	DIS3L2/PATL1	2	MF
GO:0031420	alkali metal ion binding	2/2726	20/17354	0.84517	1	0.98427	ATP1A1/PKM	2	MF
GO:0071949	FAD binding	2/2726	20/17354	0.84517	1	0.98427	MICAL2/NQO2	2	MF
GO:0001206	transcriptional repressor activity, RNA polymerase II distal enhancer sequence- [illegible]	1/2726	11/17354	0.84746	1	0.98427	MSC	1	MF
GO:0004000	adenosine deaminase	1/2726	11/17354	0.84746	1	0.98427	ADAT1	1	MF
GO:0004526	ribonuclease P activity	1/2726	11/17354	0.84746	1	0.98427	RPP21	1	MF
GO:0004875	complement receptor activity	1/2726	11/17354	0.84746	1	0.98427	C3AR1	1	MF
GO:0004954	prostanoid receptor activity	1/2726	11/17354	0.84746	1	0.98427	PTGER3	1	MF

GO:0005172	vascular endothelial growth factor receptor binding	1/2726	11/17354	0.84746	1	0.98427	DAB2IP	1	MF
GO:0009931	calcium-dependent protein serine/threonine	1/2726	11/17354	0.84746	1	0.98427	MAPKAPK3	1	MF
GO:0019864	IgG binding	1/2726	11/17354	0.84746	1	0.98427	FCGR3A	1	MF
GO:0036041	long-chain fatty acid binding	1/2726	11/17354	0.84746	1	0.98427	PPARD	1	MF
GO:0042301	phosphate ion binding	1/2726	11/17354	0.84746	1	0.98427	OTC	1	MF
GO:0043176	amine binding	1/2726	11/17354	0.84746	1	0.98427	HTR1D	1	MF
GO:0047429	nucleoside-triphosphate diphosphatase activity	1/2726	11/17354	0.84746	1	0.98427	NUDT1	1	MF
GO:0051378	serotonin binding	1/2726	11/17354	0.84746	1	0.98427	HTR1D	1	MF
GO:0051400	BH domain binding	1/2726	11/17354	0.84746	1	0.98427	BOK	1	MF
GO:0060590	ATPase regulator	4/2726	37/17354	0.85433	1	0.98427	ATPIF1/BRSK2/MYBPC3/PFN1	4	MF
GO:0061659	ubiquitin-like protein ligase activity	31/2726	231/17354	0.85457	1	0.98427	AKTIP/ANAPC2/CHFR/FBXL22/FBXO6/HECTD3/HERC6/LRSAM1/MED11/MED18/MIB2/NEURL3/PELI3/RNF166/RNF180/RNF183/RNF220/SYVN1/TRAIP/TRIM11/TRIM3/TRIM72/UBE2A/UBE2B/UBE2U/UBE3B/UBE3C/UBE3D	31	MF
GO:0008081	phosphoric diester	11/2726	90/17354	0.85597	1	0.98427	APEX1/CCL5/CHRM1/GDPD1/PDE2A/PDE6D/PLCB2/PLCD1/PLCH2/PLD2/SMPD4	11	MF

GO:0019825	oxygen binding	5/2726	45/17354	0.8561	1	0.98427	CYP1A1/CYP1A2/HBZ/NOX4/TH	5	MF
GO:0031072	heat shock protein binding	13/2726	105/17354	0.86016	1	0.98427	DNAJA3/FKBP6/HDAC8/HSF1/IRAK1/ITGAM/KDR/LIMK1/LMAN2/NPAS2/OGDH/TELO2/USP19	13	MF
GO:0001077	transcriptional activator activity, RNA polymerase II core promoter proximal region sequence-[illegible]	37/2726	274/17354	0.86413	1	0.98427	ATF2/BARHL1/BARHL2/CAMTA2/CREB3/DLX3/DUX4/E2F4/EGR1/ELF1/ESRRA/ETV4/FOSL1/GCM2/GMEB1/HOXB1/HOXB3/HOXB7/HOXC11/HOXC13/LHX3/MAFA/MEIS2/MESP2/MYCN/NKRF/NKX2-5/NKX2-8/NR4A1/NRL/PHOX2B/PITX1/SPI1/TBPL1/TBX19/ZBTB17/ZSCAN21	37	MF
GO:0001614	purinergic nucleotide receptor activity	2/2726	21/17354	0.86436	1	0.98427	P2RX2/P2RY6	2	MF
GO:0015645	fatty acid ligase activity	2/2726	21/17354	0.86436	1	0.98427	ACSF3/ACSM6	2	MF
GO:0016502	nucleotide receptor activity	2/2726	21/17354	0.86436	1	0.98427	P2RX2/P2RY6	2	MF
GO:0000287	magnesium ion binding	26/2726	198/17354	0.8657	1	0.98427	ABL1/ADPRHL1/BRSK2/CDC42BPG/CIB2/DIS3L2/EPHX2/ERN2/IDH1/IDH2/ITPK1/MAP3K6/MARK2/NIM1K/NME1/NT5C3B/NUDT18/PIF1/PKM/PPA2/RPS6KA1/RPS6KA4/RRAGC/SEPT4/TSSK1B/XXYLT1	26	MF
GO:0019212	phosphatase inhibitor	4/2726	38/17354	0.86851	1	0.98427	PHACTR1/PPP1R10/PPP1R1B/PPP1R9B	4	MF
GO:0032182	ubiquitin-like protein binding	10/2726	84/17354	0.86899	1	0.98427	DDI2/FAAP20/HSPB1/OTUB1/RNF31/UBAP1L/UBE2A/UBTD1/USP5/WDR92	10	MF
GO:0020037	heme binding	16/2726	128/17354	0.87126	1	0.98427	CYB5D2/CYC1/CYP11A1/CYP11B2/CYP1A1/CYP1A2/CYP2W1/EPX/HBZ/HMOX2/IDO1/LPO/MPO/NOS3/NOX4/THAP4	16	MF

GO:0003705	transcription factor activity, RNA polymerase II distal enhancer sequence- [illegible]	12/2726	99/17354	0.87139	1	0.98427	ATF2/HCFC1/HMGA1/HOXC4/MSC/MYOD1/NFE2L1/NKX2-5/NR5A1/SCX/SPI1/VGLL2	12	MF
GO:0030594	neurotransmitter receptor	12/2726	99/17354	0.87139	1	0.98427	CHRM1/CHRNA6/CHRNB1/CHRNB3/DRD2/GLRA1/GRIA3/GRIN1/GRIN2C/HTR1D/HTR3B/PTK2B	12	MF
GO:0005184	neuropeptide hormone	3/2726	30/17354	0.8714	1	0.98427	NPPA/PYY/VGF	3	MF
GO:0015491	cation:cation antiporter	3/2726	30/17354	0.8714	1	0.98427	SLC11A1/SLC9A3/SLC9A5	3	MF
GO:0042056	chemoattractant activity	3/2726	30/17354	0.8714	1	0.98427	BMP4/CCL5/LGALS3	3	MF
GO:0004062	aryl sulfotransferase activity	1/2726	12/17354	0.87144	1	0.98427	SULT6B1	1	MF
GO:0004952	dopamine neurotransmitter receptor	1/2726	12/17354	0.87144	1	0.98427	DRD2	1	MF
GO:0005165	neurotrophin receptor binding	1/2726	12/17354	0.87144	1	0.98427	SHC1	1	MF
GO:0010857	calcium-dependent protein kinase	1/2726	12/17354	0.87144	1	0.98427	MAPKAPK3	1	MF
GO:0016273	arginine N-methyltransferase activity	1/2726	12/17354	0.87144	1	0.98427	NDUFAF7	1	MF

GO:0016274	protein-arginine N-methyltransferase activity	1/2726	12/17354	0.87144	1	0.98427	NDUFAF7	1	MF
GO:0016493	C-C chemokine receptor activity	1/2726	12/17354	0.87144	1	0.98427	CCR2	1	MF
GO:0016500	protein-hormone	1/2726	12/17354	0.87144	1	0.98427	RXFP2	1	MF
GO:0016889	endodeoxyribonuclease activity, producing 3'-phosphomonoesters	1/2726	12/17354	0.87144	1	0.98427	XRCC3	1	MF
GO:0030983	mismatched DNA binding	1/2726	12/17354	0.87144	1	0.98427	XPC	1	MF
GO:0035925	mRNA 3'-UTR AU-rich region binding	1/2726	12/17354	0.87144	1	0.98427	CPSF1	1	MF
GO:0043495	protein membrane	1/2726	12/17354	0.87144	1	0.98427	CIB1	1	MF
GO:0048185	activin binding	1/2726	12/17354	0.87144	1	0.98427	ENG	1	MF
GO:0099095	ligand-gated anion channel	1/2726	12/17354	0.87144	1	0.98427	GLRA1	1	MF
GO:0106019	phosphatidylinositol-4,5-bisphosphate phosphatase activity	1/2726	12/17354	0.87144	1	0.98427	INPP5E	1	MF

GO:0008028	monocarboxylic acid transmembrane transporter	5/2726	47/17354	0.88093	1	0.98427	ABCC3/MPC1/SLC16A13/SLC26A6/SLCO2B1	5	MF
GO:0009975	cyclase activity	2/2726	22/17354	0.88135	1	0.98427	GUCY2D/RTCA	2	MF
GO:0022829	wide pore channel activity	2/2726	22/17354	0.88135	1	0.98427	GJB3/VDAC2	2	MF
GO:0070717	poly-purine tract binding	2/2726	22/17354	0.88135	1	0.98427	PATL1/RBPMS	2	MF
GO:0001102	RNA polymerase II activating transcription	4/2726	39/17354	0.88148	1	0.98427	ATF2/DUSP26/LDB1/NCOR1	4	MF
GO:0034061	DNA polymerase	4/2726	39/17354	0.88148	1	0.98427	POLA2/POLD4/POLE/POLE4	4	MF
GO:0004222	metalloendopeptidase activity	14/2726	115/17354	0.88249	1	0.98427	ADAM12/ADAM8/ADAMTS13/ADAMTS14/ADAMTS2/ADAMTS7/ECEL1/MMP15/MMP17/MMP2/MMP25/MMP3/PMPCA/YME1L1	14	MF
GO:0019888	protein phosphatase regulator	9/2726	78/17354	0.88252	1	0.98427	B3GAT3/IGFBP3/PHACTR1/PPP1R10/PPP1R1B/PPP1R9B/PPP2R2B/PPP2R3B/PPP2R5B	9	MF
GO:0046906	tetrapyrrole binding	17/2726	137/17354	0.88426	1	0.98427	CUBN/CYB5D2/CYC1/CYP11A1/CYP11B2/CYP1A1/CYP1A2/CYP2W1/EPX/HBZ/HMOX2/IDO1/LPO/MPO/NOS3/NOX4/THAP4	17	MF
GO:0048018	receptor ligand activity	63/2726	456/17354	0.8844	1	0.98427	ADM/AGT/AIMP1/ARTN/BMP4/C10orf99/CCL1/CCL19/CCL21/CCL5/CDC42EP2/CKLF/CLCF1/CMTM1/CMTM3/CMTM5/CMTM7/CSF1/CSF3/EDN2/FASLG/FGF17/FGF3/FNDC5/GCG/GDF2/GFER/GPHA2/GUCA2A/IFNA5/IL13/IL18/IL1RN/IL32/INHBB/INHBE/INS/INS-IGF2/JAG2/LEFTY2/LGALS3/LTA/NBL1/NPPA/NRG1/OSGIN1/PDGFA/PF4V1/PYY/SEMA3F/SHH/SLURP1/TDGF1/THNSL2/THPO/TNFSF13/TRH/UCN2/VGF/WNT1/WNT10A/WNT3A/WNT7A	63	MF

GO:0003735	structural constituent of ribosome	20/2726	159/17354	0.88725	1	0.98427	MRPL10/MRPL14/MRPL28/MRPL36/MRPL37/MRPL43/MRPL52/MRPL57/MRPL9/MRPS11/MRPS18A/MRPS18B/MRPS21/MRPS33/MRPS5/RPL35A/RPL36A/RPL41/RPL7/RPS24	20	MF
GO:0008195	phosphatidate phosphatase activity	1/2726	13/17354	0.89165	1	0.98427	PLPP1	1	MF
GO:0008417	fucosyltransferase activity	1/2726	13/17354	0.89165	1	0.98427	FUT7	1	MF
GO:0015037	peptide disulfide oxidoreductase	1/2726	13/17354	0.89165	1	0.98427	GSR	1	MF
GO:0016775	phosphotransferase activity, nitrogenous group as	1/2726	13/17354	0.89165	1	0.98427	KCNH4	1	MF
GO:0031402	sodium ion binding	1/2726	13/17354	0.89165	1	0.98427	ATP1A1	1	MF
GO:0035613	RNA stem-loop binding	1/2726	13/17354	0.89165	1	0.98427	RBPMS	1	MF
GO:1904264	ubiquitin protein ligase activity involved in	1/2726	13/17354	0.89165	1	0.98427	SYVN1	1	MF
GO:0001098	basal transcription	6/2726	56/17354	0.89245	1	0.98427	CCAR2/LLPH/MIR16-1/MIR584/RECQL5/SMYD3	6	MF
GO:0001099	basal RNA polymerase II transcription machinery	6/2726	56/17354	0.89245	1	0.98427	CCAR2/LLPH/MIR16-1/MIR584/RECQL5/SMYD3	6	MF

GO:0003954	NADH dehydrogenase activity	4/2726	40/17354	0.89333	1	0.98427	NARFL/NDUFA2/NDUFB5/NDUFC2-KCTD14	4	MF
GO:0044390	ubiquitin-like protein conjugating	4/2726	40/17354	0.89333	1	0.98427	RNF166/RNF180/TRIM72/UBE3D	4	MF
GO:1903231	mRNA binding involved in posttranscriptional gene	19/2726	153/17354	0.8945	1	0.98427	MIR106B/MIR10A/MIR153-1/MIR15A/MIR16-1/MIR181B1/MIR210/MIR212/MIR221/MIR222/MIR25/MIR29A/MIR29C/MIR32/MIR328/MIR503/MIR582/MIR892B/MIR92A2	19	MF
GO:0070330	aromatase activity	2/2726	23/17354	0.89635	1	0.98427	CYP1A1/CYP1A2	2	MF
GO:0070840	dynein complex binding	2/2726	23/17354	0.89635	1	0.98427	KATNB1/NUMA1	2	MF
GO:0016891	endoribonuclease activity, producing 5'-phosphomonoesters	3/2726	32/17354	0.89818	1	0.98427	APEX1/EXO1/RPP21	3	MF
GO:0016247	channel regulator	16/2726	132/17354	0.89905	1	0.98427	AMIGO1/ANKRD36C/BSND/CAMK2D/CAV3/DPP6/FLNA/KCNAB3/NRXN2/PACSIN3/PDZD3/SGK1/SNF8/STX1A/STX8/VTI1B	16	MF
GO:0016705	oxidoreductase activity, acting on paired donors, with incorporation or reduction of [illegible]	19/2726	154/17354	0.90042	1	0.98427	ALKBH4/ALKBH5/CYP11A1/CYP11B2/CYP1A1/CYP1A2/CYP2W1/DEGS2/FADS2/HMOX2/KDM8/MICAL2/NOS3/OGFOD2/P3H1/P3H3/PLOD3/POR/TH	19	MF
GO:0005070	SH3/SH2 adaptor activity	6/2726	57/17354	0.90186	1	0.98427	ARHGAP4/BLNK/GRAP/PTPN11/SH2D2A/SHF	6	MF
GO:0019213	deacetylase activity	6/2726	57/17354	0.90186	1	0.98427	AMDHD2/BRMS1/CES2/HDAC11/HDAC8/MTA1	6	MF

GO:0016811	hydrolase activity, acting on carbon-nitrogen (but not peptide)	10/2726	88/17354	0.90247	1	0.98427	AMDHD2/BRMS1/DARS/HDAC11/HDAC8/MTA1/NAALAD2/PM20D1/VNN3/WDYHV1	10	MF
GO:0005126	cytokine receptor binding	35/2726	268/17354	0.90311	1	0.98427	BMP4/CASP3/CCL1/CCL19/CCL21/CCL5/CCR2/CD300LF/CKLF/CLCF1/CSF1/CSF3/DAB2IP/ECM1/ENG/FASLG/GDF2/IFNA5/IL13/IL1RN/INHBB/INHBE/LEFTY2/LTA/MADD/PF4V1/SHC1/SMAD6/STAT1/TLR9/TMBIM1/TNFSF	35	MF
GO:0004520	endodeoxyribonuclease activity	4/2726	41/17354	0.90412	1	0.98427	APEX1/EXO1/MBD4/XRCC3	4	MF
GO:0008083	growth factor activity	20/2726	162/17354	0.90482	1	0.98427	AGT/ARTN/BMP4/CLCF1/CSF1/CSF3/FGF17/FGF3/GDF2/GFER/INHBB/INHBE/JAG2/LEFTY2/NRG1/OSGIN1/PDGFA/TDGF1/THPO/VGF	20	MF
GO:0003727	single-stranded RNA binding	9/2726	81/17354	0.90704	1	0.98427	DHX58/DIS3L2/EIF4B/L1TD1/PATL1/POLR2G/RBPMS/THRA/TLR8	9	MF
GO:0019209	kinase activator activity	9/2726	81/17354	0.90704	1	0.98427	CCL5/CDK20/CDK5R2/DGKQ/MADD/MARK2/MLST8/NRG1/WNT11	9	MF
GO:0000900	translation repressor activity, nucleic acid binding	1/2726	14/17354	0.90868	1	0.98427	RARA	1	MF
GO:0001134	transcription factor activity, transcription factor recruiting	1/2726	14/17354	0.90868	1	0.98427	TTF1	1	MF
GO:0001608	G-protein coupled nucleotide	1/2726	14/17354	0.90868	1	0.98427	P2RY6	1	MF
GO:0005344	oxygen carrier activity	1/2726	14/17354	0.90868	1	0.98427	HBZ	1	MF
GO:0008409	5'-3' exonuclease	1/2726	14/17354	0.90868	1	0.98427	EXO1	1	MF

GO:0008499	UDP-galactose:beta-N-acetylglucosamine beta-1,3-galactosyltransf	1/2726	14/17354	0.90868	1	0.98427	B3GNT6	1	MF
GO:0045028	G-protein coupled purinergic nucleotide	1/2726	14/17354	0.90868	1	0.98427	P2RY6	1	MF
GO:0045504	dynein heavy chain binding	1/2726	14/17354	0.90868	1	0.98427	DYNC1I1	1	MF
GO:0047555	3',5'-cyclic-GMP phosphodiestera	1/2726	14/17354	0.90868	1	0.98427	PDE2A	1	MF
GO:0050811	GABA receptor binding	1/2726	14/17354	0.90868	1	0.98427	MAF1	1	MF
GO:0070403	NAD+ binding	1/2726	14/17354	0.90868	1	0.98427	GLUD1	1	MF
GO:0098847	sequence-specific single stranded DNA	1/2726	14/17354	0.90868	1	0.98427	HSF1	1	MF
GO:0004114	3',5'-cyclic-nucleotide phosphodiesterase activity	2/2726	24/17354	0.90957	1	0.98427	PDE2A/PDE6D	2	MF
GO:0008187	poly-pyrimidine tract binding	2/2726	24/17354	0.90957	1	0.98427	DIS3L2/PATL1	2	MF
GO:0005154	epidermal growth factor receptor binding	3/2726	33/17354	0.90959	1	0.98427	LINGO1/SHC1/TNK2	3	MF

GO:0019239	deaminase activity	3/2726	33/17354	0.90959	1	0.98427	ADAT1/CDA/RPUSD2	3	MF
GO:0004869	cysteine-type endopeptidase inhibitor	6/2726	58/17354	0.91055	1	0.98427	AHSG/CD27/DPEP1/KNG1/NAIP/RPS6KA1	6	MF
GO:0030374	ligand-dependent nuclear receptor transcription	5/2726	51/17354	0.91975	1	0.98427	CALCOCO1/HMGA1/MED24/SRA1/TADA3	5	MF
GO:0016709	oxidoreductase activity, acting on paired donors, with incorporation or reduction of molecular oxygen, NAD(P)H as one donor, and incorporation of [illegible]	3/2726	34/17354	0.91983	1	0.98427	CYP1A1/MICAL2/NOS3	3	MF
GO:0031624	ubiquitin conjugating enzyme binding	3/2726	34/17354	0.91983	1	0.98427	RNF166/RNF180/TRIM72	3	MF
GO:0004112	cyclic-nucleotide phosphodiestera	2/2726	25/17354	0.9212	1	0.98427	PDE2A/PDE6D	2	MF

GO:0016702	oxidoreductase activity, acting on single donors with incorporation of molecular oxygen, incorporation of [illegible]	2/2726	25/17354	0.9212	1	0.98427	HAAO/IDO1	2	MF
GO:0017091	AU-rich element binding	2/2726	25/17354	0.9212	1	0.98427	CPSF1/DND1	2	MF
GO:0030371	translation repressor	2/2726	25/17354	0.9212	1	0.98427	EIF4EBP1/RARA	2	MF
GO:0035586	purinergic receptor activity	2/2726	25/17354	0.9212	1	0.98427	P2RX2/P2RY6	2	MF
GO:0042974	retinoic acid receptor binding	2/2726	25/17354	0.9212	1	0.98427	ARID5A/HMGA1	2	MF
GO:0019208	phosphatase regulator	10/2726	91/17354	0.92262	1	0.98427	B3GAT3/IGFBP3/PHACTR1/PPP1R10/PPP1R12B/PPP1R1B/PPP1R9B/PPP2R2B/PPP2R3B/PPP2R5B	10	MF
GO:0003796	lysozyme activity	1/2726	15/17354	0.92304	1	0.98427	SPACA3	1	MF
GO:0004115	3',5'-cyclic-AMP phosphodiestera	1/2726	15/17354	0.92304	1	0.98427	PDE2A	1	MF
GO:0005243	gap junction channel activity	1/2726	15/17354	0.92304	1	0.98427	GJB3	1	MF
GO:0008143	poly(A) binding	1/2726	15/17354	0.92304	1	0.98427	RBPMS	1	MF
GO:0034483	heparan sulfate sulfotransferase activity	1/2726	15/17354	0.92304	1	0.98427	HS3ST6	1	MF

GO:0004175	endopeptidase activity	63/2726	469/17354	0.92697	1	0.98427	ADAM12/ADAM8/ADAMTS13/ADAMTS14/ADAMTS2/ADAMTS7/APH1A/BLMH/C1QB/C1QC/C1RL/C2/CAPN10/CAPN11/CAPN15/CAPN8/CASP1/CASP3/CASP5/CASP6/CTSD/CTSE/CTSF/CTSH/CTSL/CTSW/ECEL1/FURIN/HTRA3/KLKB1/MASP2/MMP15/MMP17/MMP2/MMP25/MMP3/PGC/PLAU/PMPCA/PROZ/PRSS22/PRSS36/PRSS37/PRSS56/PSMB11/PSMB6/PSMB7/PSMB8/REN/RHBDL2/SENP3/SPPL2C/ST14/TMPRSS13/TMPRSS4/TMPRSS7/TPS [illegible]	63	MF
GO:0015298	solute:cation antiporter	3/2726	35/17354	0.92899	1	0.98427	SLC11A1/SLC9A3/SLC9A5	3	MF
GO:0004407	histone deacetylase activity	4/2726	44/17354	0.93091	1	0.98427	BRMS1/HDAC11/HDAC8/MTA1	4	MF
GO:0045309	protein phosphorylated amino acid	4/2726	44/17354	0.93091	1	0.98427	ABL1/LCK/MAPK3/SHC1	4	MF
GO:0005104	fibroblast growth factor receptor binding	2/2726	26/17354	0.9314	1	0.98427	FGF17/FGF3	2	MF
GO:0016701	oxidoreductase activity, acting on single donors with incorporation of	2/2726	26/17354	0.9314	1	0.98427	HAAO/IDO1	2	MF

GO:0016712	oxidoreductase activity, acting on paired donors, with incorporation or reduction of molecular oxygen, reduced flavin or flavoprotein as one donor, and incorporation of [illegible]	2/2726	26/17354	0.9314	1	0.98427	CYP1A1/CYP1A2	2	MF
GO:0042605	peptide antigen binding	2/2726	26/17354	0.9314	1	0.98427	HLA-E/TAP1	2	MF
GO:0051219	phosphoprotein binding	8/2726	77/17354	0.93219	1	0.98427	ABL1/ARR3/LCK/MAPK3/MTOR/SFN/SHC1/TBK1	8	MF
GO:0004549	tRNA-specific ribonuclease activity	1/2726	16/17354	0.93514	1	0.98427	RPP21	1	MF
GO:0004697	protein kinase C activity	1/2726	16/17354	0.93514	1	0.98427	PRKCD	1	MF
GO:0048531	beta-1,3-galactosyltransferase activity	1/2726	16/17354	0.93514	1	0.98427	B3GNT6	1	MF
GO:0017171	serine hydrolase activity	35/2726	277/17354	0.93654	1	0.98427	ACHE/ADAM8/AEBP1/C1QB/C1QC/C1RL/C2/CTSD/CTSH/CTSL/DPP6/DPP7/FURIN/HTRA3/KLKB1/MASP2/MMP2/MMP3/NAALAD2/NUP98/PLAU/PRCP/PROZ/PRSS16/PRSS22/PRSS36/PRSS37/PRSS56/RHBDL2/ST14/TMPRSS13/TMPRSS4/TMPRSS7/TPSB2/TYSND1	35	MF
GO:0017080	sodium channel regulator	3/2726	36/17354	0.93718	1	0.98427	CAMK2D/CAV3/SGK1	3	MF

GO:0002020	protease binding	15/2726	132/17354	0.93797	1	0.98427	CASP3/COL1A1/ECM1/FN1/FURIN/INS/LRP1/NFRKB/OS9/PDZD3/SERPINA5/SERPINF2/SYVN1/TYSND1/VWF	15	MF
GO:0033558	protein deacetylase activity	4/2726	45/17354	0.93821	1	0.98427	BRMS1/HDAC11/HDAC8/MTA1	4	MF
GO:0008236	serine-type peptidase activity	34/2726	272/17354	0.94288	1	0.98427	ADAM8/AEBP1/C1QB/C1QC/C1RL/C2/CTSD/CTSH/CTSL/DPP6/DPP7/FURIN/HTRA3/KLKB1/MASP2/MMP2/MMP3/NAALAD2/NUP98/PLAU/PRCP/PROZ/PRSS16/PRSS22/PRSS36/PRSS37/PRSS56/RHBDL2/ST14/TMPRSS13/TMPRSS4/TMPRSS7/TPSB2/TYSND1	34	MF
GO:0000339	RNA cap binding	1/2726	17/17354	0.94533	1	0.98427	LSM1	1	MF
GO:0004383	guanylate cyclase activity	1/2726	17/17354	0.94533	1	0.98427	GUCY2D	1	MF
GO:0005132	type I interferon receptor binding	1/2726	17/17354	0.94533	1	0.98427	IFNA5	1	MF
GO:0005537	mannose binding	1/2726	17/17354	0.94533	1	0.98427	LMAN2	1	MF
GO:0008391	arachidonic acid monooxygenase activity	1/2726	17/17354	0.94533	1	0.98427	CYP2W1	1	MF
GO:0008392	arachidonic acid epoxygenase activity	1/2726	17/17354	0.94533	1	0.98427	CYP2W1	1	MF
GO:0035173	histone kinase activity	1/2726	17/17354	0.94533	1	0.98427	BAZ1B	1	MF
GO:0046933	proton-transporting ATP synthase activity, rotational	1/2726	17/17354	0.94533	1	0.98427	ATP5G1	1	MF

GO:0043425	bHLH transcription factor binding	2/2726	28/17354	0.94817	1	0.98427	SCX/TSC22D3	2	MF
GO:0005544	calcium-dependent phospholipid	4/2726	47/17354	0.95074	1	0.98427	DOC2A/DOC2B/PLA2G4B/SYTL3	4	MF
GO:0016860	intramolecular oxidoreductase activity	4/2726	47/17354	0.95074	1	0.98427	EBPL/HSD3B1/HYI/QSOX1	4	MF
GO:0008137	NADH dehydrogenase (ubiquinone) activity	3/2726	38/17354	0.95101	1	0.98427	NDUFA2/NDUFB5/NDUFC2-KCTD14	3	MF
GO:0050136	NADH dehydrogenase (quinone)	3/2726	38/17354	0.95101	1	0.98427	NDUFA2/NDUFB5/NDUFC2-KCTD14	3	MF
GO:0003730	mRNA 3'-UTR binding	6/2726	65/17354	0.95468	1	0.98427	CPSF1/PCBP4/RBM4/RNPS1/RPL41/ZNF385A	6	MF
GO:0016411	acylglycerol O-acyltransferase activity	2/2726	29/17354	0.95502	1	0.98427	DGAT1/PNPLA2	2	MF
GO:0005506	iron ion binding	18/2726	160/17354	0.95715	1	0.98427	CYP11A1/CYP11B2/CYP1A1/CYP1A2/CYP2W1/HAAO/HBZ/HEPH/ISCA2/NOS3/OGFOD2/P3H1/P3H3/PHF2/PLOD3/PPEF1/TH/XDH	18	MF
GO:0046332	SMAD binding	7/2726	74/17354	0.95748	1	0.98427	AXIN1/FLNA/GATA4/MEN1/SMAD6/TGFB1I1/ZC3H3	7	MF
GO:0051427	hormone receptor binding	18/2726	161/17354	0.95997	1	0.98427	ARID5A/BCAS3/BRCA1/DDX54/GNAO1/GPHA2/HMGA1/MED24/MYOD1/NCOR1/NPPA/PTPN11/RUNDC3A/STAT1/TAF7/TGFB1I1/UCN2/ZNHIT3	18	MF
GO:0043539	protein serine/threonine kinase activator activity	2/2726	30/17354	0.96099	1	0.98427	CDK5R2/MLST8	2	MF

GO:0008301	DNA binding, bending	1/2726	19/17354	0.96117	1	0.98427	CRIP1	1	MF
GO:0015269	calcium-activated potassium	1/2726	19/17354	0.96117	1	0.98427	KCNT1	1	MF
GO:0019789	SUMO transferase	1/2726	19/17354	0.96117	1	0.98427	RNF212	1	MF
GO:0034237	protein kinase A regulatory subunit binding	1/2726	19/17354	0.96117	1	0.98427	KCNQ1	1	MF
GO:0061783	peptidoglycan muralytic	1/2726	19/17354	0.96117	1	0.98427	SPACA3	1	MF
GO:0016627	oxidoreductase activity, acting on the CH-CH group of donors	5/2726	58/17354	0.96151	1	0.98427	ACAD10/ACADVL/ACOXL/DHODH/PTGR1	5	MF
GO:0016877	ligase activity, forming carbon-sulfur bonds	3/2726	40/17354	0.96195	1	0.98427	ACSF3/ACSM6/ACSS3	3	MF
GO:0016893	endonuclease activity, active with either ribo- or deoxyribonucleic acids and producing 5'-phosphomonoes	3/2726	40/17354	0.96195	1	0.98427	APEX1/EXO1/RPP21	3	MF

GO:0046982	protein heterodimerization activity	64/2726	495/17354	0.96558	1	0.98427	ABCG5/ADRA1A/ADRA1B/ADRA2A/ADRA2C/ANO2/ATF2/ATF3/BAD/BOK/CLCF1/DMBX1/DRAP1/DRD2/DYNLL2/EGFR/ERBB2/FZD9/GAD1/GPHA2/HEXA/HIP1R/HIST1H2AH/HIST1H4F/HIST2H4A/HIST2H4B/HOMER2/HSF1/IKBKB/IRAK1/ITGAL/ITGAM/KATNB1/LIMK1/MAFA/MAX/MLXIPL/MYOD1/NEUROD2/NKX2-5/NOTCH1/NR4A1/NRN1L/PDGFA/PDSS1/POLA2/POLE4/PPARD/RAF1/RARA/RRAGC/SCGB1D1/SCX/SIM1/SOX8/STX1A/TAF1/TAF6/TAF7/TBX15/TI[illegible]	64	MF
GO:0005487	nucleocytoplasmic transporter	2/2726	31/17354	0.9662	1	0.98427	NUP98/XPO7	2	MF
GO:0050699	WW domain binding	2/2726	31/17354	0.9662	1	0.98427	SCNN1A/TNK2	2	MF
GO:0099589	serotonin receptor activity	2/2726	31/17354	0.9662	1	0.98427	HTR1D/HTR3B	2	MF
GO:0003756	protein disulfide isomerase	1/2726	20/17354	0.96728	1	0.98427	QSOX1	1	MF
GO:0005251	delayed rectifier potassium channel activity	1/2726	20/17354	0.96728	1	0.98427	KCNQ1	1	MF
GO:0016864	intramolecular oxidoreductase activity, transposing S-S bonds	1/2726	20/17354	0.96728	1	0.98427	QSOX1	1	MF
GO:0033038	bitter taste receptor activity	1/2726	20/17354	0.96728	1	0.98427	TAS2R3	1	MF
GO:0070491	repressing transcription factor binding	5/2726	60/17354	0.96908	1	0.98427	CTBP1/MTA1/NOC2L/STAT1/ZNF703	5	MF
GO:0004806	triglyceride lipase activity	1/2726	21/17354	0.97243	1	0.98427	PNPLA2	1	MF

GO:0008327	methyl-CpG binding	1/2726	21/17354	0.97243	1	0.98427	ERH	1	MF
GO:0008373	sialyltransferase activity	1/2726	21/17354	0.97243	1	0.98427	ST3GAL4	1	MF
GO:0050681	androgen receptor binding	3/2726	43/17354	0.97414	1	0.98427	ARID5A/BRCA1/TGFB1I1	3	MF
GO:0016849	phosphorus-oxygen lyase activity	1/2726	22/17354	0.97676	1	0.98427	GUCY2D	1	MF
GO:0016917	GABA receptor activity	1/2726	22/17354	0.97676	1	0.98427	GABBR1	1	MF
GO:0017056	structural constituent of nuclear pore	1/2726	22/17354	0.97676	1	0.98427	NUP98	1	MF
GO:0003729	mRNA binding	42/2726	348/17354	0.9779	1	0.98427	ACO1/CPSF1/CSTF2/EIF4G1/ESRP2/LARP6/LSM1/LUC7L/MIR106B/MIR10A/MIR153-1/MIR15A/MIR16-1/MIR181B1/MIR210/MIR212/MIR221/MIR222/MIR25/MIR29A/MIR29C/MIR32/MIR328/MIR503/MIR582/MIR892B/MIR92A2/MRPS11/NUP98/NXF3/PCBP4/PIWIL2/RARA/RBFOX1/RBFOX3/RBM4/RBPMS2/RNPS1/RPL41/RPL7/S	42	MF
GO:0001671	ATPase activator	1/2726	23/17354	0.98042	1	0.98427	MYBPC3	1	MF
GO:0070851	growth factor receptor binding	13/2726	132/17354	0.9811	1	0.98427	CD300LF/CSF3/DAB2IP/ECM1/FGF17/FGF3/IL1RN/LINGO1/PDGFA/PDGFRB/SHC1/TLR9/TNK2	13	MF
GO:0042826	histone deacetylase binding	10/2726	108/17354	0.98189	1	0.98427	BRMS1/CAMTA2/HSP90AA1/KAT2A/NCOR1/NCOR2/RARA/SMG5/SP2/ZNHIT1	10	MF
GO:0004993	G-protein coupled serotonin	1/2726	24/17354	0.9835	1	0.98427	HTR1D	1	MF

GO:0090079	translation regulator activity, nucleic acid binding	1/2726	24/17354	0.9835	1	0.98427	RARA	1	MF
GO:0004252	serine-type endopeptidase activity	27/2726	244/17354	0.9852	1	0.98427	ADAM8/C1QB/C1QC/C1RL/C2/CTSD/CTSH/CTSL/FURIN/HTRA3/KLKB1/MASP2/MMP2/MMP3/PLAU/PROZ/PRSS22/PRSS36/PRSS37/PRSS56/RHBDL2/ST14/TMPRSS13/TMPRSS4/TMPRSS7/TPSB2/TYSND1	27	MF
GO:0030544	Hsp70 protein binding	2/2726	37/17354	0.9859	1	0.98427	DNAJA3/HDAC8	2	MF
GO:0016628	oxidoreductase activity, acting on the CH-CH group of donors, NAD or NADP as	1/2726	25/17354	0.98609	1	0.98427	PTGR1	1	MF
GO:0004497	monooxygenase activity	8/2726	94/17354	0.98635	1	0.98427	CYP11A1/CYP11B2/CYP1A1/CYP1A2/CYP2W1/MICAL2/NOS3/TH	8	MF
GO:0045182	translation regulator	3/2726	48/17354	0.98663	1	0.98427	EIF2AK4/EIF4EBP1/RARA	3	MF
GO:0004033	aldo-keto reductase (NADP)	1/2726	26/17354	0.98828	1	0.98427	AKR7A3	1	MF
GO:0008527	taste receptor activity	1/2726	26/17354	0.98828	1	0.98427	TAS2R3	1	MF
GO:0005504	fatty acid binding	1/2726	27/17354	0.99012	1	0.98427	PPARD	1	MF
GO:0030331	estrogen receptor binding	2/2726	40/17354	0.99097	1	0.98427	ARID5A/DDX54	2	MF

GO:0016655	oxidoreductase activity, acting on NAD(P)H, quinone or similar compound as	3/2726	51/17354	0.99108	1	0.98427	NDUFA2/NDUFB5/NDUFC2-KCTD14	3	MF
GO:0001103	RNA polymerase II repressing transcription	1/2726	29/17354	0.99299	1	0.98427	MTA1	1	MF
GO:0035257	nuclear hormone	12/2726	137/17354	0.99417	1	0.98427	ARID5A/BCAS3/BRCA1/DDX54/HMGA1/MED24/MYOD1/NCOR1/STAT1/TAF7/TGFB1I1/ZNHIT3	12	MF
GO:0031491	nucleosome binding	4/2726	65/17354	0.99454	1	0.98427	NOC2L/SMARCD2/SUPT6H/ZNHIT1	4	MF
GO:0003697	single-stranded DNA binding	8/2726	107/17354	0.99652	1	0.98427	HSF1/IGHMBP2/MCM4/NME1/PMS2P3/POLR2G/XPC/XRCC3	8	MF
GO:0017046	peptide hormone	1/2726	34/17354	0.99702	1	0.98427	RXFP2	1	MF
GO:0019843	rRNA binding	3/2726	59/17354	0.99705	1	0.98427	ANG/ERAL1/MRPS11	3	MF
GO:0034987	immunoglobulin receptor	2/2726	50/17354	0.99801	1	0.98427	FES/FLNA	2	MF
GO:0015026	coreceptor activity	1/2726	40/17354	0.99893	1	0.98427	RGMA	1	MF
GO:0031492	nucleosomal DNA binding	1/2726	45/17354	0.99955	1	0.98427	SMARCD2	1	MF
GO:0035258	steroid hormone receptor binding	4/2726	88/17354	0.99975	1	0.98427	ARID5A/BRCA1/DDX54/TGFB1I1	4	MF
GO:0005549	odorant binding	1/2726	94/17354	1	1	0.98427	OBP2B	1	MF

GO:0003823	antigen binding	5/2726	193/17354	1	1	0.98427	HLA-E/KLRC2/SPON2/TAP1/TSPAN4	5	MF
GO:0004984	olfactory receptor activity	6/2726	426/17354	1	1	0.98427	OR2B3/OR2C1/OR2F1/OR4C11/OR51B5/OR6A2	6	MF

标题	说明
ID	GO号
Description	GO对应的描述信息
GeneRatio	富集基因在该GO上的比例
BgRatio	背景基因在该GO上的比例
pvalue	P值
p.adjust	校正P值（BH方法校正后的P值）
qvalue	Q值（Q方法校正后的P值）
geneID	该GO上所有富集基因的基因名，用“/”分割
Count	该GO上所有富集基因的数量
