# Supplementary material for: Whole-Blood MicroRNA Sequence Profiling and Identification of Specific miR-21 for Adolescents With Postural Tachycardia Syndrome
Source: Front Neurosci. 2022 Jun 30;16:920477. doi: 10.3389/fnins.2022.920477 (PMC9281551; doi:10.3389/fnins.2022.920477)
Supplement: Table S1 — Known_MicrorRNA_Target_Gene_Prediction_Summary.pdf. [file Data_Sheet_1.ZIP › supp table 3 KEGG_Enrichment_Summary.pdf]

ID	Description	GeneRat	BgRati	pvalue	p.adjust	qvalu	geneID	Coun
hsa00532	Glycosaminoglycan biosynthesis - chondroitin sulfate /	10/1172	20/7493	0.00035	0.0543	0.05	B3GAT3/B4GALT7/CHPF/CHPF2/CHST12/CHST13/CHST15/CHST7/DSE/XYLT2	10
hsa05165	Human papillomavirus infection	75/1172	330/7493	0.00035	0.0543	0.05	ATP6V0B/ATP6V0E2/ATP6V1B1/ATP6V1F/ATP6V1G1/ATP6V1G2/AXIN1/BAD/BCAP31/CASP3/CCND3/CDK4/CHAD/COL1A1/COL4A2/COL9A2/CREB3/DVL2/EGFR/EIF4EBP1/FASLG/FN1/FZD2/FZD8/FZD9/HES6/HEY1/HLA-E/HRAS/IFNA5/IKBKB/ISG15/ITGA7/ITGB5/LAMA2/LAMA4/LAMC2/LAMC3/LFNG/LLGL1/MAPK3/MTOR/NFX1/NOTCH1/PARD3/PARD6A/PDGFRB/PKM/PPP2R2B/PPP2R3B/PPP2R3C/PPP2R5B/RAF1/RFNG/RPS6KB2/SCRIB/STAT1/STAT2/TADA3/TBK1/TBPL1/TCF7L1/THBS3/TNC/TNN/TUBG1/TUBG2/UBR4/VWF/WNT1/WNT10 [illegible]	75
hsa05217	Basal cell carcinoma	20/1172	63/7493	0.00103	0.1076	0.1	AXIN1/BMP4/DDB2/DVL2/FZD2/FZD8/FZD9/GADD45G/HHIP/PTCH2/SHH/SMO/SUFU/TCF7L1/WNT1/WNT10A/WNT11/WNT3A/WNT6/WNT7A	20
hsa04934	Cushing syndrome	38/1172	154/7493	0.00219	0.1709	0.16	AIP/AIPL1/ASH2L/ATF2/AXIN1/CACNA1F/CACNA1G/CACNA1H/CACNA1S/CAMK2D/CDK4/CDKN2A/CREB3/CRHR1/CRHR2/CYP11A1/DVL2/EGFR/FZD2/FZD8/FZD9/HSD3B1/ITPR3/MAPK3/MEN1/NR4A1/NR5A1/ORAI1/PLCB2/RASD1/SCARB1/TCF7L1/WNT1/WNT10A/WNT11/WNT3A/WNT6/WNT7A	38
hsa00310	Lysine degradation	17/1172	59/7493	0.00727	0.4272	0.4	ALDH2/DLST/EHMT1/EHMT2/HADHA/HYKK/OGDH/OGDHL/PHYKPL/PIPOX/PLOD3/PRDM7/PRDM9/SETD1A/SETD2/SETD7/SUV39H1	17
hsa03410	Base excision repair	11/1172	33/7493	0.00914	0.4272	0.4	APEX1/LIG3/MBD4/NEIL2/OGG1/PARP2/PARP3/POLD4/POLE/POLE4/SMUG1	11
hsa05205	Proteoglycans in cancer	44/1172	201/7493	0.01099	0.4272	0.4	ARAF/CAMK2D/CASP3/CAV3/CD63/CTSL/CTTN/EGFR/EIF4B/ERBB2/FASLG/FLNA/FLNC/FN1/FZD2/FZD8/FZD9/GPC1/HRAS/HSPG2/ITGB5/ITPR3/KDR/MAPK3/MIR10A/MMP2/MTOR/NUDT16L1/PDCD4/PLAU/PPP1R12B/PTPN11/RAF1/RPS6KB2/SDC1/SHH/SMO/TWIST2/WNT1/WNT10A/WNT11/WNT3A/WNT6/WNT7A	44
hsa04925	Aldosterone synthesis and secretion	24/1172	96/7493	0.01126	0.4272	0.4	ATF2/ATP1A1/ATP1A4/CACNA1F/CACNA1G/CACNA1H/CACNA1S/CALML3/CALML5/CAMK1D/CAMK1G/CAMK2D/CREB3/CYP11A1/CYP11B2/DAGLB/HSD3B1/ITPR3/KCNJ5/NR4A1/ORAI1/PDE2A/PLCB2/SCARB1	24
hsa01210	2-Oxocarboxylic acid metabolism	7/1172	18/7493	0.01467	0.4272	0.4	ACO1/CS/GOT2/GPT/IDH1/IDH2/NAGS	7

hsa04144	Endocytosis	51/1172	244/7493	0.01593	0.4272	0.4	ACAP1/ACAP3/AGAP3/AP2A2/AP2M1/ARAP1/ARFGAP2/ARPC1A/ARPC1B/ARPC2/ARPC4/ARRB2/BIN1/CAPZA3/CAV3/CHMP1A/CHMP4C/CHMP7/CLTB/CXCR1/CXCR2/DNM1/EGFR/EHD1/FGFR2/FGFR4/FOLR2/GBF1/GRK6/HLA-E/HRAS/HSPA2/IQSEC2/PARD3/PARD6A/PLD2/PSD/PSD2/PSD4/RAB11FIP3/RAB7A/RBSN/SH3GLB2/SNF8/SNX12/VPS25/VPS37B/VPS37C/VPS37D/WAS/ZFYVE27	51
hsa05100	Bacterial invasion of	19/1172	74/7493	0.01713	0.4272	0.4	ARHGAP10/ARPC1A/ARPC1B/ARPC2/ARPC4/CAV3/CLTB/CTNNA2/CTTN/DNM1/DOCK1/ELMO3/FN1/ILK/SEPT1/SEPT8/SEPT9/SHC1/WAS	19
hsa05224	Breast cancer	33/1172	147/7493	0.01779	0.4272	0.4	ARAF/AXIN1/BRCA1/CDK4/DDB2/DVL2/EGFR/ERBB2/ESR2/FGF17/FGF3/FLT4/FRAT1/FZD2/FZD8/FZD9/GADD45G/HEY1/HRAS/JAG2/MAPK3/MTOR/NOTCH1/RAF1/RPS6KB2/SHC1/TCF7L1/WNT1/WNT10A/WNT11/WNT3A/WNT6/WNT7A	33
hsa01230	Biosynthesis of amino acids	19/1172	75/7493	0.01968	0.4272	0.4	ACO1/ALDOA/ASL/ASNS/CS/GAPDH/GOT2/GPT/IDH1/IDH2/NAGS/OTC/PC/PFKM/PGAM4/PHGDH/PKM/SDSL/SHMT2	19
hsa05226	Gastric cancer	33/1172	149/7493	0.02149	0.4272	0.4	ARAF/AXIN1/CDX2/CTNNA2/DDB2/DVL2/EGFR/ERBB2/FGF17/FGF3/FGFR2/FRAT1/FZD2/FZD8/FZD9/GADD45G/HRAS/MAPK3/MTOR/MUC2/RAF1/REG4/RPS6KB2/SHC1/SHH/TCF7L1/TERC/WNT1/WNT10A/WNT11/WNT3A/WNT6/WNT7A	33
hsa00564	Glycerophospholipid metabolism	23/1172	97/7493	0.02386	0.4272	0.4	ACHE/CDIPT/DGKI/DGKQ/DGKZ/ETNK2/GNPAT/JMJD7-PLA2G4B/LYPLA2/PEMT/PHOSPHO1/PLA2G15/PLA2G16/PLA2G1B/PLA2G2F/PLA2G4B/PLA2G5/PLB1/PLD2/PLPP1/PNPLA7/PTDSS1/PTDSS2	23
hsa04512	ECM-receptor interaction	20/1172	82/7493	0.02531	0.4272	0.4	AGRN/CHAD/COL1A1/COL4A2/COL9A2/FN1/GP9/HSPG2/ITGA7/ITGB5/LAMA2/LAMA4/LAMC2/LAMC3/SDC1/SV2A/THBS3/TNC/TNN/VWF	20
hsa04915	Estrogen signaling pathway	30/1172	137/7493	0.03161	0.4272	0.4	ATF2/CALML3/CALML5/CREB3/CTSD/EGFR/ESR2/GABBR1/GNAO1/GPER1/HRAS/HSP90AA1/HSPA2/ITPR3/KCNJ5/KCNJ9/KRT12/KRT14/KRT16/KRT23/KRT32/MAPK3/MMP2/NOS3/OPRM1/PLCB2/PRKCD/RAF1/RARA/SHC1	30
hsa00563	Glycosylphosphatidylinositol (GPI)-anchor	8/1172	25/7493	0.03174	0.4272	0.4	DPM2/GPAA1/PIGC/PIGG/PIGO/PIGQ/PIGY/PIGZ	8
hsa00592	alpha-Linolenic acid metabolism	8/1172	25/7493	0.03174	0.4272	0.4	FADS2/JMJD7-PLA2G4B/PLA2G16/PLA2G1B/PLA2G2F/PLA2G4B/PLA2G5/PLB1	8

hsa04360	Axon guidance	38/1172	181/7493	0.03194	0.4272	0.4	ABL1/ABLIM2/BOC/CAMK2D/CDK5/CFL1/EFNA1/EFNA3/EFNA4/EFNB1/EPHA8/EPHB3/EPHB4/EPHB6/FES/HRAS/ILK/L1CAM/LIMK1/MAPK3/MYL5/NTNG2/PARD3/PARD6A/PLXNB1/PLXNB3/PTPN11/RAF1/RGMA/RHOD/RND1/SEMA3F/SEMA4A/SEMA5B/SEMA6C/SEMA7A/SHH/SMO	38
hsa00020	Citrate cycle (TCA cycle)	9/1172	30/7493	0.03511	0.4272	0.4	ACO1/CS/DLST/IDH1/IDH2/OGDH/OGDHL/PC/PDHB	9
hsa00220	Arginine biosynthesis	7/1172	21/7493	0.03511	0.4272	0.4	ASL/GLUD1/GOT2/GPT/NAGS/NOS3/OTC	7
hsa05214	Glioma	18/1172	75/7493	0.03793	0.4272	0.4	ARAF/CALML3/CALML5/CAMK1D/CAMK1G/CAMK2D/CDK4/CDKN2A/DDB2/EGFR/GADD45G/HRAS/MAPK3/MTOR/PDGFA/PDGFRB/RAF1/SHC1	18
hsa04142	Lysosome	27/1172	123/7493	0.03883	0.4272	0.4	ABCA2/ABCB9/ACP2/AP1G2/AP1S1/AP4S1/ATP6V0B/CD63/CD68/CLTB/CTSD/CTSE/CTSF/CTSH/CTSL/CTSW/GBA/HEXA/IDS/LAMP3/LAPTM5/NAGLU/NPC2/PLA2G15/PSAP/SGSH/SLC11A1	27
hsa04666	Fc gamma R-mediated phagocytosis	21/1172	91/7493	0.03929	0.4272	0.4	ARPC1A/ARPC1B/ARPC2/ARPC4/BIN1/CFL1/FCGR3A/INPP5D/INPPL1/LIMK1/MAPK3/MARCKSL1/NCF1/PLA2G4B/PLD2/PLPP1/PRKCD/RAF1/RPS6KB2/SCIN/WAS	21
hsa05230	Central carbon metabolism in cancer	16/1172	65/7493	0.03933	0.4272	0.4	EGFR/ERBB2/FGFR2/GCK/HK3/HRAS/IDH1/LDHA/MAPK3/MTOR/PDGFRB/PDHB/PFKM/PGAM4/PKM/RAF1	16
hsa00052	Galactose metabolism	9/1172	31/7493	0.04284	0.4272	0.4	B4GALT2/G6PC3/GALK1/GALM/GALT/GCK/HK3/MGAM/PFKM	9
hsa03020	RNA	9/1172	31/749	0.04284	0.4272	0.4	POLR1A/POLR1D/POLR1E/POLR2G/POLR2J2/POLR2J3/POLR2L/POLR3D/ZNRD1	9
hsa05221	Acute myeloid leukemia	16/1172	66/7493	0.04466	0.4272	0.4	ARAF/BAD/CSF1R/EIF4EBP1/HRAS/IKBKB/ITGAM/MAPK3/MPO/MTOR/PPARD/RAF1/RARA/RPS6KB2/SPI1/TCF7L1	16
hsa00350	Tyrosine metabolism	10/1172	36/7493	0.04478	0.4272	0.4	ADH1A/ADH1C/AOC2/DDC/FAH/FAHD1/GOT2/GSTZ1/LRTOMT/TH	10
hsa04975	Fat digestion and absorption	11/1172	41/7493	0.04588	0.4272	0.4	ABCG5/APOB/CLPS/DGAT1/GOT2/NPC1L1/PLA2G1B/PLA2G2F/PLA2G5/PLPP1/SCARB1	11
hsa05219	Bladder cancer	11/1172	41/749	0.04588	0.4272	0.4	ARAF/CDK4/CDKN2A/DAPK1/DAPK2/EGFR/ERBB2/HRAS/MAPK3/MMP2/RAF1	11
hsa00480	Glutathione metabolism	14/1172	56/7493	0.04603	0.4272	0.4	ANPEP/CHAC1/GGT6/GPX1/GPX2/GPX3/GSR/GSTM1/GSTM2/IDH1/IDH2/MGST1/MGST3/NAT8B	14

hsa04210	Apoptosis	29/1172	136/7493	0.04662	0.4272	0.4	BAD/CASP3/CASP6/CTSD/CTSF/CTSH/CTSL/CTSW/DAB2IP/ENDOG/FASLG/GADD45G/HRAS/HRK/IKBKB/ITPR3/LMNA/LMNB1/MAP3K14/MAPK3/PARP2/PARP3/RAF1/SEPT4/SPTAN1/TRAF2/TUBA3C/TUBA3D/TUBA3E	29
hsa04010	MAPK signaling pathway	57/1172	295/7493	0.04792	0.4272	0.4	ARAF/ARRB2/ATF2/CACNA1F/CACNA1G/CACNA1H/CACNA1S/CACNB1/CACNB3/CACNG1/CASP3/CSF1/CSF1R/DUSP2/DUSP5/EFNA1/EFNA3/EFNA4/EGFR/ERBB2/FASLG/FGF17/FGF3/FGFR2/FGFR4/FLNA/FLNC/FLT4/GADD45G/HRAS/HSPA2/HSPB1/IKBKB/INS/IRAK1/JMJD7-PLA2G4B/KDR/MAP2K3/MAP3K14/MAP3K6/MAP4K2/MAPK3/MAPK8IP1/MAPK8IP3/MAPKAPK3/MAX/NR4A1/PDGFA/PDGFRB/PLA2G4B/PTPN7/RAF1/RASGRP2/RPS6KA1/RPS6KA4/TAOK2/TRAF2	57
hsa04151	PI3K-Akt signaling pathway	67/1172	354/7493	0.05014	0.4345	0.41	ATF2/BAD/BRCA1/CCND3/CDK4/CHAD/CHRM1/COL1A1/COL4A2/COL9A2/CREB3/CRTC2/CSF1/CSF1R/CSF3/EFNA1/EFNA3/EFNA4/EGFR/EIF4B/EIF4EBP1/ERBB2/FASLG/FGF17/FGF3/FGFR2/FGFR4/FLT4/FN1/G6PC3/GNB3/GNB5/GNG3/HRAS/HSP90AA1/IFNA5/IKBKB/IL4R/INS/ITGA7/ITGB5/KDR/LAMA2/LAMA4/LAMC2/LAMC3/MAPK3/MLST8/MTOR/NOS3/NR4A1/PDGFA/PDGFRB/PIK3R5/PIK3R6/PPP2R2B/PPP2R3B/PPP2R3C/PPP2R5B/RAF1/RPS6KB2/SGK1/THBS3/TNC/TNN/VWF/YWH	67
hsa04146	Peroxisome	19/1172	83/7493	0.05186	0.4373	0.41	CROT/EPHX2/GNPAT/HAO2/HMGCLL1/IDH1/IDH2/PAOX/PEX11A/PEX14/PEX16/PEX3/PEX5/PEX6/PIPOX/PMVK/PRDX1/PXMP2/XDH	19
hsa01200	Carbon metabolism	25/1172	116/7493	0.05508	0.4522	0.42	ACO1/ALDOA/CS/DLST/GAPDH/GCK/GLUD1/GLYCTK/GOT2/GPT/HADHA/HAO2/HK3/IDH1/IDH2/OGDH/OGDHL/PC/PDHB/PFKM/PGAM4/PHGDH/PKM/SDSL/SH	25
hsa05120	Epithelial cell signaling in Helicobacter pylori infection	16/1172	68/7493	0.05684	0.4547	0.42	ATP6V0B/ATP6V0E2/ATP6V1B1/ATP6V1F/ATP6V1G1/ATP6V1G2/CASP3/CCL5/CSK/CXCR1/CXCR2/EGFR/IKBKB/MAP3K14/NOD1/PTPN11	16
hsa04610	Complement and coagulation cascades	18/1172	79/7493	0.05964	0.4652	0.43	C1QB/C1QC/C2/C3AR1/C8A/CFH/CLU/FGA/ITGAM/ITGAX/KLKB1/KNG1/MASP2/PLAU/SERPINA5/SERPINF2/VSIG4/VWF	18
hsa00520	Amino sugar and nucleotide sugar	12/1172	48/7493	0.06188	0.4709	0.44	AMDHD2/CHIT1/CYB5R1/CYB5R2/GALK1/GALT/GCK/GMPPB/HEXA/HK3/NAGK/NPL	12

hsa05225	Hepatocellular carcinoma	34/1172	168/7493	0.06385	0.4743	0.44	ARAF/AXIN1/BAD/CDK4/CDKN2A/DDB2/DVL2/EGFR/FRAT1/FZD2/FZD8/FZD9/GADD45G/GSTM1/GSTM2/HRAS/MAPK3/MGST1/MGST3/MTOR/RAF1/RPS6KB2/SHC1/SMARCD2/TCF7L1/TERC/TXNRD1/TXNRD3/WNT1/WNT10A/WNT11/WNT3A	34
hsa04014	Ras signaling pathway	45/1172	232/7493	0.06866	0.4859	0.45	ABL1/BAD/CALML3/CALML5/CSF1/CSF1R/EFNA1/EFNA3/EFNA4/EGFR/FASLG/FGF17/FGF3/FGFR2/FGFR4/FLT4/FOXO4/GNB3/GNB5/GNG3/GRIN1/HRAS/IKBKB/INS/JMJD7-PLA2G4B/KDR/KSR1/MAPK3/PDGFA/PDGFRB/PLA2G16/PLA2G1B/PLA2G2F/PLA	45
hsa00510	N-Glycan biosynthesis	12/1172	49/7493	0.07079	0.4859	0.45	ALG10/ALG3/B4GALT2/DAD1/DDOST/DOLPP1/DPM2/GANAB/MGAT1/MGAT4B/MGAT5B/RPN1	12
hsa00591	Linoleic acid metabolism	8/1172	29/7493	0.07147	0.4859	0.45	CYP1A2/JMJD7-PLA2G4B/PLA2G16/PLA2G1B/PLA2G2F/PLA2G4B/PLA2G5/PLB1	8
hsa04130	SNARE interactions in vesicular	9/1172	34/7493	0.07258	0.4859	0.45	GOSR2/STX18/STX1A/STX4/STX5/STX8/VAMP1/VAMP5/VTI1B	9
hsa04150	mTOR signaling pathway	31/1172	153/7493	0.0732	0.4859	0.45	ATP6V1B1/ATP6V1F/ATP6V1G1/ATP6V1G2/DVL2/EIF4B/EIF4EBP1/FZD2/FZD8/FZD9/HRAS/IKBKB/INS/MAPK3/MAPKAP1/MLST8/MTOR/NPRL3/RAF1/RPS6KA1/RPS6KB2/RRAGC/SGK1/TELO2/ULK1/WNT1/WNT10A/WNT11/WNT3A/WNT6/WNT	31
hsa00260	Glycine, serine and threonine metabolism	10/1172	40/7493	0.08389	0.5453	0.51	ALAS2/AOC2/CHDH/GLYCTK/GNMT/PGAM4/PHGDH/PIPOX/SDSL/SHMT2	10
hsa04721	Synaptic vesicle cycle	17/1172	78/7493	0.09263	0.5848	0.55	AP2A2/AP2M1/ATP6V0B/ATP6V0E2/ATP6V1B1/ATP6V1F/ATP6V1G1/ATP6V1G2/CLTB/CPLX1/DNM1/SLC1A7/SLC6A2/SLC6A7/SLC6A9/STX1A/STXBP1	17
hsa04930	Type II diabetes mellitus	11/1172	46/7493	0.09372	0.5848	0.55	CACNA1G/GCK/HK3/IKBKB/INS/MAFA/MAPK3/MTOR/PKM/PRKCD/SLC2A4	11
hsa04713	Circadian entrainment	20/1172	96/7493	0.10512	0.6431	0.6	CACNA1G/CACNA1H/CALML3/CALML5/CAMK2D/GNAO1/GNB3/GNB5/GNG3/GRIA3/GRIN1/GRIN2C/ITPR3/KCNJ5/KCNJ9/MAPK3/MTNR1B/PER1/PLCB2/RASD1	20
hsa05213	Endometrial cancer	13/1172	58/7493	0.1098	0.6502	0.61	ARAF/AXIN1/BAD/CTNNA2/DDB2/EGFR/ERBB2/GADD45G/HRAS/ILK/MAPK3/RAF1/TCF7L1	13
hsa04960	Aldosterone-regulated sodium	9/1172	37/7493	0.11255	0.6502	0.61	ATP1A1/ATP1A4/HSD11B2/INS/MAPK3/SCNN1A/SFN/SGK1/SLC9A3R2	9

hsa04927	Cortisol synthesis and	14/1172	64/7493	0.1164	0.6502	0.61	ATF2/CACNA1F/CACNA1G/CACNA1H/CACNA1S/CREB3/CYP11A1/HSD3B1/ITPR3/NR4A1/NR5A1/ORAI1/PLCB2/SCARB1	14
hsa05212	Pancreatic cancer	16/1172	75/7493	0.1166	0.6502	0.61	ARAF/BAD/CDK4/CDKN2A/DDB2/EGFR/ERBB2/GADD45G/IKBKB/MAPK3/MTOR/RAF1/RALB/RALGDS/RPS6KB2/STAT1	16
hsa04966	Collecting duct acid secretion	7/1172	27/7493	0.11671	0.6502	0.61	ATP6V0E2/ATP6V1B1/ATP6V1F/ATP6V1G1/ATP6V1G2/CLCNKB/SLC12A7	7
hsa04912	GnRH signaling pathway	19/1172	93/7493	0.12943	0.7033	0.66	CACNA1F/CACNA1S/CALML3/CALML5/CAMK2D/EGFR/EGR1/HRAS/ITPR3/JMJD7-PLA2G4B/MAP2K3/MAPK3/MMP2/PLA2G4B/PLCB2/PLD2/PRKCD/PTK2B/RAF1	19
hsa05231	Choline metabolism in cancer	20/1172	99/7493	0.13288	0.7033	0.66	DGKI/DGKQ/DGKZ/EGFR/EIF4EBP1/HRAS/JMJD7-PLA2G4B/MAPK3/MTOR/PDGFA/PDGFRB/PLA2G4B/PLD2/PLPP1/RAF1/RALGDS/RPS6KB2/SLC22A1/SLC44A4/WAS	20
hsa05030	Cocaine	11/1172	49/749	0.13299	0.7033	0.66	ATF2/CDK5/CREB3/DDC/DLG4/DRD2/GPSM1/GRIN1/GRIN2C/PPP1R1B/TH	11
hsa04964	Proximal tubule bicarbonate reclamation	6/1172	23/7493	0.13795	0.7173	0.67	AQP1/ATP1A1/ATP1A4/CA4/GLUD1/SLC9A3	6
hsa04145	Phagosome	29/1172	152/7493	0.1437	0.735	0.69	ATP6V0B/ATP6V0E2/ATP6V1B1/ATP6V1F/ATP6V1G1/ATP6V1G2/CLEC7A/CORO1A/CTSL/DYNC1H1/DYNC1I1/FCGR3A/HLA-E/ITGAM/ITGB5/MPO/MRC2/NCF1/OLR1/RAB7A/SCARB1/SEC61A2/SFTPA1/STX18/TAP1/THBS3/TUBA3C/TUBA3D/TUBA3E	29
hsa04015	Rap1 signaling pathway	38/1172	206/7493	0.15236	0.7556	0.71	CALML3/CALML5/CSF1/CSF1R/DRD2/EFNA1/EFNA3/EFNA4/EGFR/FARP2/FGF17/FGF3/FGFR2/FGFR4/FLT4/GNAO1/GRIN1/HRAS/INS/ITGAL/ITGAM/KDR/MAP2K3/MAPK3/PARD3/PARD6A/PDGFA/PDGFRB/PFN1/PLCB2/RAF1/RALB/RALGDS/RAP1GAP/RASGRP2/RGS14/SIPA1/TLN1	38
hsa04916	Melanogenesis	20/1172	101/7493	0.15355	0.7556	0.71	CALML3/CALML5/CAMK2D/CREB3/DVL2/FZD2/FZD8/FZD9/GNAO1/HRAS/MAPK3/PLCB2/RAF1/TCF7L1/WNT1/WNT10A/WNT11/WNT3A/WNT6/WNT7A	20
hsa02010	ABC	10/1172	45/749	0.15498	0.7556	0.71	ABCA2/ABCB5/ABCB9/ABCC12/ABCC3/ABCC4/ABCC5/ABCC6/ABCG5/TAP1	10
hsa00380	Tryptophan metabolism	9/1172	40/7493	0.16246	0.7556	0.71	ALDH2/CYP1A1/CYP1A2/DDC/HAAO/HADHA/IDO1/OGDH/OGDHL	9
hsa01521	EGFR tyrosine kinase inhibitor resistance	16/1172	79/7493	0.16308	0.7556	0.71	ARAF/BAD/EGFR/EIF4EBP1/ERBB2/FGFR2/HRAS/KDR/MAPK3/MTOR/NRG1/PDGFA/PDGFRB/RAF1/RPS6KB2/SHC1	16

hsa04978	Mineral	11/1172	51/749	0.16343	0.7556	0.71	ATOX1/ATP1A1/ATP1A4/CLCN2/HEPH/HMOX2/MT2A/SLC26A6/SLC9A3/STEAP2/	11
hsa04728	Dopaminergic synapse	25/1172	131/7493	0.16468	0.7556	0.71	ARRB2/ATF2/CALML3/CALML5/CALY/CAMK2D/CREB3/DDC/DRD2/GNAO1/GNB3/GNB5/GNG3/GRIA3/ITPR3/KCNJ5/KCNJ9/LRTOMT/PLCB2/PPP1R1B/PPP2R2B/PPP2R3B/PPP2R3C/PPP2R5B/TH	25
hsa00010	Glycolysis / Gluconeogenesis	14/1172	68/7493	0.16724	0.7562	0.71	ADH1A/ADH1C/ALDH2/ALDOA/G6PC3/GALM/GAPDH/GCK/HK3/LDHA/PDHB/PFKM/PGAM4/PKM	14
hsa05020	Prion diseases	8/1172	35/749	0.17015	0.7571	0.71	C1QB/C1QC/C8A/CCL5/EGR1/HSPA5/MAPK3/NOTCH1	8
hsa05215	Prostate cancer	19/1172	97/7493	0.17329	0.7571	0.71	ARAF/BAD/CREB3/EGFR/ERBB2/FGFR2/HRAS/HSP90AA1/IKBKB/INS/MAPK3/MMP3/MTOR/PDGFA/PDGFRB/PLAU/RAF1/SPINT1/TCF7L1	19
hsa04922	Glucagon signaling	20/1172	103/7493	0.1759	0.7571	0.71	ACACB/ATF2/CALML3/CALML5/CAMK2D/CREB3/CRTC2/G6PC3/GCG/GCK/ITPR3/LDHA/PDHB/PFKFB1/PGAM4/PHKG1/PKM/PLCB2/PRKAB1/PYGM	20
hsa04261	Adrenergic signaling in cardiomyocytes	27/1172	144/7493	0.17731	0.7571	0.71	ADRA1A/ADRA1B/ATF2/ATP1A1/ATP1A4/CACNA1F/CACNA1S/CACNB1/CACNB3/CACNG1/CALML3/CALML5/CAMK2D/CREB3/CREM/KCNQ1/MAPK3/MYL4/PIK3R5/PIK3R6/PLCB2/PPP2R2B/PPP2R3B/PPP2R3C/PPP2R5B/TNNC1/TPM2	27
hsa05132	Salmonella infection	17/1172	86/7493	0.17958	0.7571	0.71	ARPC1A/ARPC1B/ARPC2/ARPC4/CASP1/DYNC1H1/DYNC1I1/FLNA/FLNC/IL18/KLC1/KLC2/MAPK3/PFN1/PLEKHM2/RAB7A/WAS	17
hsa00565	Ether lipid metabolism	10/1172	47/7493	0.18986	0.7644	0.71	GDPD1/JMJD7-PLA2G4B/PLA2G16/PLA2G1B/PLA2G2F/PLA2G4B/PLA2G5/PLB1/PLD2/PLPP1	10
hsa03420	Nucleotide excision repair	10/1172	47/7493	0.18986	0.7644	0.71	DDB1/DDB2/GTF2H2C/GTF2H4/POLD4/POLE/POLE4/RFC1/RFC2/XPC	10
hsa03030	DNA replication	8/1172	36/749	0.19074	0.7644	0.71	MCM2/MCM4/POLA2/POLD4/POLE/POLE4/RFC1/RFC2	8
hsa04510	Focal adhesion	36/1172	199/7493	0.19192	0.7644	0.71	BAD/CAV3/CCND3/CHAD/COL1A1/COL4A2/COL9A2/DOCK1/EGFR/ERBB2/FLNA/FLNC/FLT4/FN1/HRAS/ILK/ITGA7/ITGB5/KDR/LAMA2/LAMA4/LAMC2/LAMC3/MAPK3/MYL5/PDGFA/PDGFRB/PPP1R12B/RAF1/SHC1/THBS3/TLN1/TNC/TNN/VWF	36
hsa04140	Autophagy - animal	24/1172	128/7493	0.19442	0.7644	0.71	AMBRA1/ATG10/ATG101/ATG2A/BAD/CTSD/CTSL/DAPK1/DAPK2/EIF2AK4/HRAS/INS/MAPK3/MLST8/MTMR14/MTOR/PRAP1/PRKCD/RAB7A/RAF1/RPS6KB2/RR	24
hsa04072	Phospholipase D signaling pathway	27/1172	146/7493	0.19748	0.7644	0.71	AVPR2/CXCR1/CXCR2/DGKI/DGKQ/DGKZ/DNM1/EGFR/HRAS/INS/JMJD7-PLA2G4B/MAPK3/MTOR/PDGFA/PDGFRB/PIK3R5/PIK3R6/PLA2G4B/PLCB2/PLD2/PLPP1/PTK2B/PTPN11/RAF1/RALB/RALGDS/SHC1	27

hsa04020	Calcium signaling pathway	34/1172	188/7493	0.20056	0.7644	0.71	ADRA1A/ADRA1B/ATP2A3/CACNA1F/CACNA1G/CACNA1H/CACNA1S/CALML3/CALML5/CAMK1D/CAMK1G/CAMK2D/CHRM1/EGFR/ERBB2/GRIN1/GRIN2C/HRH2/ITPR3/NOS3/ORAI1/ORAI3/P2RX2/PDGFRB/PHKG1/PLCB2/PLCD1/PTGER3/PTK2B/SLC25A5/TACR1/TACR2/TNNC1/VDAC2	34
hsa01523	Antifolate resistance	7/1172	31/7493	0.20091	0.7644	0.71	ABCC3/ABCC4/ABCC5/FOLR2/GGH/IKBKB/SHMT2	7
hsa05131	Shigellosis	13/1172	65/7493	0.20763	0.7744	0.72	ABL1/ARPC1A/ARPC1B/ARPC2/ARPC4/CTTN/DOCK1/ELMO3/IKBKB/MAPK3/NOD1/PFN1/WAS	13
hsa04330	Notch signaling pathway	10/1172	48/7493	0.2085	0.7744	0.72	APH1A/CTBP1/DTX2/DVL2/JAG2/KAT2A/LFNG/NCOR2/NOTCH1/RFNG	10
hsa05218	Melanoma	14/1172	72/7493	0.22752	0.8316	0.78	ARAF/BAD/CDK4/CDKN2A/DDB2/EGFR/FGF17/FGF3/GADD45G/HRAS/MAPK3/PDGFA/PDGFRB/RAF1	14
hsa04910	Insulin signaling pathway	25/1172	137/7493	0.22921	0.8316	0.78	ACACB/ARAF/BAD/CALML3/CALML5/EIF4EBP1/G6PC3/GCK/HK3/HRAS/IKBKB/INPPL1/INS/MAPK3/MTOR/PHKG1/PRKAB1/PRKAR1A/PRKAR1B/PTPRF/PYGM/RAF1/RPS6KB2/SHC1/SLC2A4	25
hsa05130	Pathogenic Escherichia coli infection	11/1172	55/7493	0.23326	0.8365	0.78	ABL1/ARPC1A/ARPC1B/ARPC2/ARPC4/CTTN/TUBA3C/TUBA3D/TUBA3E/WAS/YWHAQ	11
hsa01040	Biosynthesis of unsaturated fatty acids	6/1172	27/7493	0.23861	0.8404	0.79	ACOT7/ELOVL1/ELOVL5/FADS2/HACD1/HACD3	6
hsa00430	Taurine and hypotaurine metabolism	3/1172	11/7493	0.24079	0.8404	0.79	CSAD/GAD1/GGT6	3
hsa04979	Cholesterol metabolism	10/1172	50/7493	0.24788	0.8404	0.79	ABCG5/APOB/APOC3/LRP1/NPC2/OSBPL5/SCARB1/SOAT2/STARD3/VDAC2	10
hsa04022	cGMP-PKG signaling pathway	29/1172	163/7493	0.25186	0.8404	0.79	ADRA1A/ADRA1B/ADRA2A/ADRA2B/ADRA2C/ATF2/ATP1A1/ATP1A4/ATP2A3/BAD/CACNA1F/CACNA1S/CALML3/CALML5/CNGB1/CREB3/GATA4/INS/ITPR3/MAPK3/NOS3/OPRD1/PDE2A/PIK3R5/PIK3R6/PLCB2/RAF1/SLC25A5/VDAC2	29
hsa00620	Pyruvate metabolism	8/1172	39/7493	0.25798	0.8404	0.79	ACACB/ACYP1/ALDH2/GLO1/LDHA/PC/PDHB/PKM	8

hsa05031	Amphetamine addiction	13/1172	68/7493	0.2585	0.8404	0.79	ARC/ATF2/CALML3/CALML5/CAMK2D/CREB3/DDC/GRIA3/GRIN1/GRIN2C/PPP1R1B/STX1A/TH	13
hsa04918	Thyroid hormone	14/1172	74/7493	0.26063	0.8404	0.79	ASGR1/ATF2/ATP1A1/ATP1A4/CREB3/GPX1/GPX2/GPX3/GSR/HSPA5/ITPR3/PLCB2/TTF1/TTF2	14
hsa04310	Wnt signaling pathway	28/1172	158/7493	0.26375	0.8404	0.79	AXIN1/CACYBP/CAMK2D/CCND3/CHD8/CTBP1/DVL2/FOSL1/FRAT1/FZD2/FZD8/FZD9/LGR6/NKD2/PLCB2/PORCN/PPARD/RSPO1/SFRP5/SOST/TCF7L1/WIF1/WNT1/WNT10A/WNT11/WNT3A/WNT6/WNT7A	28
hsa03022	Basal transcription	9/1172	45/7493	0.26398	0.8404	0.79	GTF2H2C/GTF2H4/TAF1/TAF15/TAF1L/TAF3/TAF6/TAF7/TBPL1	9
hsa04921	Oxytocin signaling pathway	27/1172	152/7493	0.26431	0.8404	0.79	CACNA1F/CACNA1S/CACNB1/CACNB3/CACNG1/CALML3/CALML5/CAMK1D/CAMK1G/CAMK2D/EGFR/GNAO1/HRAS/ITPR3/JMJD7-PLA2G4B/KCNJ5/KCNJ9/MAPK3/MYL6B/NOS3/PIK3R5/PIK3R6/PLA2G4B/PLCB2/PPP1R12B/PRKAB1/RAF1	27
hsa01522	Endocrine resistance	18/1172	98/7493	0.26519	0.8404	0.79	ARAF/BAD/CDK4/CDKN2A/EGFR/ERBB2/ESR2/GPER1/HRAS/JAG2/MAPK3/MMP2/MTOR/NCOR1/NOTCH1/RAF1/RPS6KB2/SHC1	18
hsa04744	Phototransductio	6/1172	28/749	0.26667	0.8404	0.79	CALML3/CALML5/CNGB1/GNAT1/GUCA1B/GUCY2D	6
hsa00910	Nitrogen metabolism	4/1172	17/7493	0.26952	0.8409	0.79	CA4/CA6/CA7/GLUD1	4
hsa00340	Histidine metabolism	5/1172	23/7493	0.28556	0.8821	0.82	ALDH2/CARNS1/HDC/HNMT/UROC1	5
hsa04928	Parathyroid hormone synthesis, secretion and	19/1172	106/7493	0.29499	0.886	0.83	ARAF/ARRB2/ATF2/BGLAP/CREB3/EGFR/EGR1/GCM2/ITPR3/MAPK3/MMP15/MMP17/MMP25/NACA/PLCB2/PLD2/RAF1/SLC34A1/SOST	19
hsa05133	Pertussis	14/1172	76/7493	0.29534	0.886	0.83	C1QB/C1QC/C2/CALML3/CALML5/CASP1/CASP3/CFL1/IRAK1/ITGAM/MAPK3/NOD1/SFTPA1/TIRAP	14
hsa05220	Chronic myeloid leukemia	14/1172	76/7493	0.29534	0.886	0.83	ABL1/ARAF/BAD/CDK4/CDKN2A/CTBP1/DDB2/GADD45G/HRAS/IKBKB/MAPK3/PTPN11/RAF1/SHC1	14
hsa00250	Alanine, aspartate and glutamate	7/1172	35/7493	0.30196	0.8882	0.83	ALDH4A1/ASL/ASNS/GAD1/GLUD1/GOT2/GPT	7

hsa05143	African trypanosomiasis	7/1172	35/7493	0.30196	0.8882	0.83	F2RL1/FASLG/IDO1/IL18/LAMA4/PLCB2/TLR9	7
hsa04340	Hedgehog signaling	9/1172	47/7493	0.3092	0.8882	0.83	ARRB2/BOC/EVC2/HHIP/PTCH2/SHH/SMO/SPOP/SUFU	9
hsa04370	VEGF signaling pathway	11/1172	59/7493	0.31232	0.8882	0.83	BAD/HRAS/HSPB1/JMJD7-PLA2G4B/KDR/MAPK3/MAPKAPK3/NOS3/PLA2G4B/RAF1/SH2D2A	11
hsa05410	Hypertrophic cardiomyopathy (HCM)	15/1172	83/7493	0.31289	0.8882	0.83	CACNA1F/CACNA1S/CACNB1/CACNB3/CACNG1/DES/EMD/ITGA7/ITGB5/LAMA2/LMNA/MYBPC3/PRKAB1/TNNC1/TPM2	15
hsa04977	Vitamin digestion and	5/1172	24/7493	0.31834	0.8882	0.83	APOB/CUBN/PLB1/RBP2/SCARB1	5
hsa05206	MicroRNAs in cancer	50/1172	299/7493	0.32361	0.8882	0.83	ABL1/BRCA1/CASP3/CDKN2A/DNMT3A/EFNA3/EGFR/ERBB2/FSCN1/HRAS/IKBKB/KIF23/MIR106B/MIR10A/MIR135B/MIR15A/MIR16-1/MIR181B1/MIR183/MIR192/MIR194-1/MIR194-2/MIR195/MIR210/MIR215/MIR221/MIR222/MIR25/MIR29A/MIR29C/MIR30D/MIR32/MIR326/MIR451A/MIR92A2/MIR96/MIRLET7A2/MIRLET7G/MTOR/NOTCH1/PDC	50
hsa00062	Fatty acid elongation	6/1172	30/7493	0.32487	0.8882	0.83	ACOT7/ELOVL1/ELOVL5/HACD1/HACD3/HADHA	6
hsa00630	Glyoxylate and dicarboxylate metabolism	6/1172	30/7493	0.32487	0.8882	0.83	ACO1/CS/GLYCTK/HAO2/HYI/SHMT2	6
hsa04550	Signaling pathways regulating pluripotency of	24/1172	139/7493	0.33148	0.8882	0.83	AXIN1/BMP4/DVL2/FGFR2/FGFR4/FZD2/FZD8/FZD9/HOXB1/HRAS/INHBB/INHBE/LEFTY2/LHX5/MAPK3/PCGF2/POU5F1B/RAF1/WNT1/WNT10A/WNT11/WNT3A/WNT6/WNT7A	24
hsa05412	Arrhythmogenic right ventricular cardiomyopathy (ARVC)	13/1172	72/7493	0.33213	0.8882	0.83	CACNA1F/CACNA1S/CACNB1/CACNB3/CACNG1/CTNNA2/DES/EMD/ITGA7/ITGB5/LAMA2/LMNA/TCF7L1	13
hsa05223	Non-small cell lung cancer	12/1172	66/7493	0.33274	0.8882	0.83	ARAF/BAD/CDK4/CDKN2A/DDB2/EGFR/ERBB2/FHIT/GADD45G/HRAS/MAPK3/RAF1	12

hsa04730	Long-term depression	11/1172	60/7493	0.33306	0.8882	0.83	ARAF/CRHR1/GNAO1/GRIA3/HRAS/ITPR3/JMJD7-PLA2G4B/MAPK3/PLA2G4B/PLCB2/RAF1	11
hsa04726	Serotonergic synapse	20/1172	115/7493	0.33893	0.8957	0.84	ARAF/CACNA1F/CACNA1S/CASP3/DDC/GNAO1/GNB3/GNB5/GNG3/HRAS/HTR1D/HTR3B/ITPR3/JMJD7-PLA2G4B/KCNJ5/KCNJ9/MAPK3/PLA2G4B/PLCB2/RAF1	20
hsa00531	Glycosaminoglycan degradation	4/1172	19/7493	0.3453	0.8957	0.84	HEXA/IDS/NAGLU/SGSH	4
hsa04062	Chemokine signaling pathway	32/1172	190/7493	0.3522	0.8957	0.84	ARRB2/BAD/CCL1/CCL19/CCL21/CCL5/CCR2/CXCR1/CXCR2/CXCR3/CXCR5/GNB3/GNB5/GNG3/GRK6/HRAS/IKBKB/MAPK3/NCF1/PARD3/PF4V1/PIK3R5/PIK3R6/PLCB2/PRKCD/PTK2B/RAF1/RASGRP2/SHC1/STAT1/STAT2/WAS	32
hsa04720	Long-term potentiation	12/1172	67/7493	0.35274	0.8957	0.84	ARAF/CALML3/CALML5/CAMK2D/GRIN1/GRIN2C/HRAS/ITPR3/MAPK3/PLCB2/RAF1/RPS6KA1	12
hsa04913	Ovarian steroidogenesis	9/1172	49/7493	0.35597	0.8957	0.84	CYP11A1/CYP1A1/HSD17B1/HSD17B7/HSD3B1/INS/JMJD7-PLA2G4B/PLA2G4B/SCARB1	9
hsa05144	Malaria	9/1172	49/749	0.35597	0.8957	0.84	CD81/CSF3/IL18/ITGAL/LRP1/SDC1/SELP/THBS3/TLR9	9
hsa05340	Primary immunodeficien	7/1172	37/7493	0.35599	0.8957	0.84	BLNK/CIITA/LCK/ORAI1/RFX5/TAP1/TNFRSF13B	7
hsa05210	Colorectal cancer	15/1172	86/7493	0.36581	0.9131	0.85	ARAF/AXIN1/BAD/CASP3/DDB2/EGFR/GADD45G/HRAS/MAPK3/MTOR/RAF1/RALB/RALGDS/RPS6KB2/TCF7L1	15
hsa04070	Phosphatidylinositol signaling	17/1172	99/7493	0.37785	0.9284	0.87	CALML3/CALML5/CDIPT/DGKI/DGKQ/DGKZ/INPP5D/INPP5E/INPP5F/INPPL1/ITPK1/ITPR3/MTMR1/MTMR14/PI4KB/PLCB2/PLCD1	17
hsa05202	Transcriptional misregulation in cancer	31/1172	186/7493	0.37907	0.9284	0.87	BAIAP3/BCL6/CDK9/CSF1R/DDB2/ETV4/ETV7/EYA1/GADD45G/GRIA3/IGFBP3/ITGAM/LDB1/MAX/MEN1/MMP3/MPO/MYCN/NCOR1/NUPR1/PAX3/PDGFA/PLAU/PRCC/RARA/SPI1/SPINT1/TAF15/TLX3/TSPAN7/ZBTB17	31
hsa05160	Hepatitis C	26/1172	155/7493	0.3809	0.9284	0.87	ARAF/BAD/CASP3/CD81/CDK4/CLDN15/CLDN2/CLDN23/CLDN4/CLDN6/EGFR/EIF2AK4/FASLG/HRAS/IFNA5/IKBKB/MAPK3/NR1H3/PPP2R2B/RAF1/SCARB1/STAT1/STAT2/TBK1/TRAF2/YWHAQ	26
hsa04725	Cholinergic synapse	19/1172	112/7493	0.38806	0.9386	0.88	ACHE/CACNA1F/CACNA1S/CAMK2D/CHRM1/CHRNA6/CREB3/GNAO1/GNB3/GNB5/GNG3/HRAS/ITPR3/KCNQ1/KCNQ4/MAPK3/PIK3R5/PIK3R6/PLCB2	19
hsa04650	Natural killer cell mediated cytotoxicity	22/1172	131/7493	0.39352	0.9445	0.88	ARAF/CASP3/CD247/CD48/FASLG/FCGR3A/HLA-E/HRAS/IFNA5/ITGAL/KLRC1/KLRC2/LCK/MAPK3/MICB/NCR3/PTK2B/PTPN11/RAET1G/RAF1/SHC1/ULBP2	22

hsa00590	Arachidonic acid metabolism	11/1172	63/7493	0.3966	0.9446	0.88	EPHX2/GPX1/GPX2/GPX3/JMJD7-PLA2G4B/PLA2G16/PLA2G1B/PLA2G2F/PLA2G4B/PLA2G5/PLB1	11
hsa03050	Proteasome	8/1172	45/749	0.40713	0.9566	0.89	PSMB11/PSMB6/PSMB7/PSMB8/PSMC3/PSMD13/PSMD3/PSMD7	8
hsa04931	Insulin resistance	18/1172	107/7493	0.40779	0.9566	0.89	ACACB/CREB3/CRTC2/G6PC3/IKBKB/INS/MLXIPL/MTOR/NOS3/NR1H3/PRKAB1/PRKCD/PTPN11/PTPRF/PYGM/RPS6KA1/RPS6KB2/SLC2A4	18
hsa04621	NOD-like receptor signaling	29/1172	178/7493	0.4365	1	0.93	BRCC3/CARD9/CASP1/CASP5/CCL5/GSDMD/HSP90AA1/IFNA5/IKBKB/IL18/ITPR3/MAPK3/MFN1/MFN2/NAIP/NLRP1/NLRX1/NOD1/PLCB2/PRKCD/PYDC1/RNF31/SHARPIN/STAT1/STAT2/TBK1/TRAF2/TRPV2/VDAC2	29
hsa04115	p53 signaling pathway	12/1172	72/7493	0.45449	1	0.93	ADGRB1/CASP3/CCNB2/CCND3/CD82/CDK4/CDKN2A/DDB2/GADD45G/IGFBP3/SESN1/SFN	12
hsa00514	Other types of O-glycan	4/1172	22/7493	0.45873	1	0.93	B4GALT2/LFNG/PLOD3/RFNG	4
hsa00900	Terpenoid backbone biosynthesis	4/1172	22/7493	0.45873	1	0.93	FNTA/FNTB/PDSS1/PMVK	4
hsa00970	Aminoacyl-tRNA	11/1172	66/7493	0.46071	1	0.93	AARS/AARS2/DARS/GATB/KARS/LARS/PARS2/SARS/TARS2/YARS/YARS2	11
hsa04012	ErbB signaling pathway	14/1172	85/7493	0.46206	1	0.93	ABL1/ARAF/BAD/CAMK2D/EGFR/EIF4EBP1/ERBB2/HRAS/MAPK3/MTOR/NRG1/RAF1/RPS6KB2/SHC1	14
hsa00140	Steroid hormone biosynthesis	10/1172	60/7493	0.46744	1	0.93	CYP11A1/CYP11B2/CYP1A1/CYP1A2/HSD11B2/HSD17B1/HSD17B3/HSD17B7/HSD3B1/LRTOMT	10
hsa00860	Porphyrin and chlorophyll metabolism	7/1172	42/7493	0.49211	1	0.93	ALAD/ALAS2/COX10/HEPH/HMBS/HMOX2/UROS	7
hsa05222	Small cell lung cancer	15/1172	93/7493	0.49239	1	0.93	CASP3/CDK4/COL4A2/DDB2/FHIT/FN1/GADD45G/IKBKB/LAMA2/LAMA4/LAMC2/LAMC3/MAX/TRAF2/ZBTB17	15
hsa00562	Inositol phosphate	12/1172	74/7493	0.49497	1	0.93	CDIPT/INPP5D/INPP5E/INPP5F/INPPL1/ITPK1/MTMR1/MTMR14/PI4KB/PLCB2/PLCD1/PLCH2	12
hsa00515	Mannose type O-glycan	4/1172	23/7493	0.49518	1	0.93	B4GALT2/CHST10/FKTN/MGAT5B	4
hsa03430	Mismatch repair	4/1172	23/749	0.49518	1	0.93	EXO1/POLD4/RFC1/RFC2	4

hsa04614	Renin-angiotensin	4/1172	23/7493	0.49518	1	0.93	AGT/ANPEP/PRCP/REN	4
hsa04066	HIF-1 signaling pathway	16/1172	100/7493	0.50318	1	0.93	ALDOA/CAMK2D/EGFR/EIF4EBP1/ERBB2/GAPDH/HK3/INS/LDHA/LTBR/MAPK3/MTOR/NOS3/NPPA/PDHB/RPS6KB2	16
hsa00360	Phenylalanine metabolism	3/1172	17/7493	0.51032	1	0.93	AOC2/DDC/GOT2	3
hsa04152	AMPK signaling pathway	19/1172	120/7493	0.51596	1	0.93	ACACB/ADRA1A/CREB3/CRTC2/EIF4EBP1/G6PC3/INS/LEPR/MTOR/PFKFB1/PFKM/PPP2R2B/PPP2R3B/PPP2R3C/PPP2R5B/PRKAB1/RPS6KB2/SLC2A4/ULK1	19
hsa00534	Glycosaminoglycan biosynthesis - heparan sulfate / heparin	4/1172	24/7493	0.53055	1	0.93	B3GAT3/B4GALT7/EXTL1/XYLT2	4
hsa04270	Vascular smooth muscle contraction	19/1172	121/7493	0.53167	1	0.93	ADRA1A/ADRA1B/ARAF/CACNA1F/CACNA1S/CALML3/CALML5/ITPR3/JMJD7-PLA2G4B/MAPK3/MYL6B/PLA2G1B/PLA2G2F/PLA2G4B/PLA2G5/PLCB2/PPP1R12B/PRKCD/RAF1	19
hsa05110	Vibrio cholerae infection	8/1172	50/7493	0.5321	1	0.93	ATP6V0B/ATP6V0E2/ATP6V1B1/ATP6V1F/ATP6V1G1/ATP6V1G2/KCNQ1/SEC61A2	8
hsa04390	Hippo signaling pathway	24/1172	154/7493	0.54269	1	0.93	AXIN1/BMP4/CCND3/CRB2/CTNNA2/DLG4/DVL2/FZD2/FZD8/FZD9/LLGL1/PARD3/PARD6A/PPP2R2B/SCRIB/TCF7L1/TEAD4/WNT1/WNT10A/WNT11/WNT3A/WNT	24
hsa00240	Pyrimidine metabolism	9/1172	57/7493	0.54404	1	0.93	CDA/DHODH/ENTPD8/NME1/NME4/NT5C3B/NT5M/NUDT2/UCK1	9
hsa04917	Prolactin signaling	11/1172	70/7493	0.54427	1	0.93	CISH/ESR2/GALT/GCK/HRAS/INS/MAPK3/RAF1/SHC1/STAT1/TH	11
hsa04962	Vasopressin-regulated water reabsorption	7/1172	44/7493	0.5444	1	0.93	AVPR2/CREB3/DCTN2/DYNC1H1/DYNC1I1/DYNLL2/STX4	7
hsa00410	beta-Alanine metabolism	5/1172	31/7493	0.54599	1	0.93	ALDH2/AOC2/CARNS1/GAD1/HADHA	5
hsa00511	Other glycan degradation	3/1172	18/7493	0.55095	1	0.93	GBA/HEXA/NEU3	3

hsa04810	Regulation of actin cytoskeleton	33/1172	213/7493	0.55399	1	0.93	ARAF/ARHGEF4/ARPC1A/ARPC1B/ARPC2/ARPC4/BRK1/CFL1/CHRM1/DOCK1/EGFR/FGF17/FGF3/FGFR2/FGFR4/FN1/HRAS/INS/ITGA7/ITGAL/ITGAM/ITGAX/ITGB5/LIMK1/MAPK3/MYL5/PDGFA/PDGFRB/PFN1/PPP1R12B/RAF1/SCIN/WAS	33
hsa04974	Protein digestion and absorption	14/1172	90/7493	0.55414	1	0.93	ATP1A1/ATP1A4/COL17A1/COL1A1/COL4A2/COL7A1/COL9A2/CPA1/ELN/KCNQ1/PRCP/SLC36A1/SLC7A7/SLC9A3	14
hsa05414	Dilated cardiomyopathy (DCM)	14/1172	90/7493	0.55414	1	0.93	CACNA1F/CACNA1S/CACNB1/CACNB3/CACNG1/DES/EMD/ITGA7/ITGB5/LAMA2/LMNA/MYBPC3/TNNC1/TPM2	14
hsa05014	Amyotrophic lateral sclerosis	8/1172	51/7493	0.55602	1	0.93	BAD/CASP1/CASP3/CCS/GPX1/GRIN1/GRIN2C/MAP2K3	8
hsa05203	Viral carcinogenesis	31/1172	201/7493	0.56512	1	0.93	ATF2/BAD/CASP3/CCND3/CDK4/CDKN2A/CREB3/DDB1/DNAJA3/GTF2H2C/GTF2H4/HDAC11/HDAC8/HIST1H4F/HIST2H4A/HIST2H4B/HLA-E/HRAS/KAT2A/LTBR/MAD1L1/MAPK3/MRPS18B/PKM/SCIN/SCRIB/SND1/TBPL1/TRAF2/UBR4/YWHAQ	31
hsa04625	C-type lectin receptor signaling	16/1172	104/7493	0.571	1	0.93	CALML3/CALML5/CARD9/CASP1/CLEC7A/HRAS/IKBKB/ITPR3/KSR1/MAP3K14/MAPK3/PRKCD/PTPN11/RAF1/STAT1/STAT2	16
hsa03015	mRNA surveillance	14/1172	91/7493	0.57193	1	0.93	CASC3/CPSF1/CPSF4/CSTF2/NXF2/NXF2B/NXF3/PABPN1L/PPP2R2B/PPP2R3B/PPP2R3C/PPP2R5B/RNPS1/SMG5	14
hsa04260	Cardiac muscle contraction	12/1172	78/7493	0.57341	1	0.93	ATP1A1/ATP1A4/CACNA1F/CACNA1S/CACNB1/CACNB3/CACNG1/COX8A/CYC1/MYL4/TNNC1/TPM2	12
hsa04137	Mitophagy -	10/1172	65/749	0.57572	1	0.93	AMBRA1/HRAS/MFN1/MFN2/PGAM5/RAB7A/RHOT2/TBK1/TFEB/ULK1	10
hsa04924	Renin secretion	10/1172	65/749	0.57572	1	0.93	AGT/AQP1/CACNA1F/CACNA1S/CALML3/CALML5/ITPR3/ORAI1/PLCB2/REN	10
hsa04136	Autophagy -	5/1172	32/749	0.57589	1	0.93	ATG10/ATG101/ATG2A/MLST8/MTOR	5
hsa04911	Insulin secretion	13/1172	85/7493	0.58169	1	0.93	ATF2/ATP1A1/ATP1A4/CACNA1F/CACNA1S/CAMK2D/CREB3/GCG/GCK/INS/ITPR3/PLCB2/STX1A	13
hsa00100	Steroid biosynthesis	3/1172	19/7493	0.58951	1	0.93	HSD17B7/NSDHL/SOAT2	3
hsa00983	Drug metabolism -	12/1172	79/7493	0.59225	1	0.93	CDA/CES2/GSTM1/GSTM2/IMPDH1/MGST1/MGST3/MPO/NME1/NME4/UCK1/XDH	12

hsa05164	Influenza A	26/1172	171/7493	0.59618	1	0.93	ATF2/CASP1/CCL5/CIITA/CPSF4/EIF2AK4/FASLG/FURIN/HSPA2/IFNA5/IKBKB/IL18/MAP2K3/MAPK3/NLRX1/NUP98/NXF2/NXF2B/NXF3/PABPN1L/RAF1/STAT1/STAT2/TBK1/TMPRSS13/TMPRSS4	26
hsa04933	AGE-RAGE signaling pathway in diabetic	15/1172	99/7493	0.59671	1	0.93	CASP3/CDK4/COL1A1/COL4A2/EGR1/FN1/HRAS/MAPK3/MMP2/NOS3/NOX4/PLCB2/PLCD1/PRKCD/STAT1	15
hsa00790	Folate	4/1172	26/749	0.59735	1	0.93	ALPPL2/GCH1/GGH/TH	4
hsa04950	Maturity onset diabetes of the young	4/1172	26/7493	0.59735	1	0.93	GCK/INS/MAFA/RFX6	4
hsa04068	FoxO signaling pathway	20/1172	132/7493	0.59947	1	0.93	ARAF/BCL6/CCNB2/EGFR/FASLG/FBXO25/FOXO4/FOXO6/G6PC3/GADD45G/HOMER2/HRAS/IKBKB/INS/MAPK3/PRKAB1/RAF1/SETD7/SGK1/SLC2A4	20
hsa01212	Fatty acid metabolism	8/1172	53/7493	0.60221	1	0.93	ACADVL/CPT2/ELOVL1/ELOVL5/FADS2/HACD1/HACD3/HADHA	8
hsa01524	Platinum drug resistance	11/1172	73/7493	0.60373	1	0.93	BAD/BRCA1/CASP3/CDKN2A/ERBB2/FASLG/GSTM1/GSTM2/MAPK3/MGST1/MGST3	11
hsa00051	Fructose and mannose metabolism	5/1172	33/7493	0.60471	1	0.93	ALDOA/GMPPB/HK3/PFKFB1/PFKM	5
hsa05418	Fluid shear stress and	21/1172	139/7493	0.60577	1	0.93	BMP4/CALML3/CALML5/CAV3/CDH5/CTSL/GPC1/GSTM1/GSTM2/HSP90AA1/IKBKB/KDR/MGST1/MGST3/MIR10A/MMP2/NCF1/NOS3/PDGFA/SDC1/TRPV4	21
hsa04657	IL-17 signaling pathway	14/1172	93/7493	0.60663	1	0.93	CASP3/CSF3/FOSL1/HSP90AA1/IKBKB/IL13/IL17RE/MAPK15/MAPK3/MMP3/MUC5AC/MUC5B/TBK1/TRAF2	14
hsa05152	Tuberculosis	27/1172	179/7493	0.6145	1	0.93	ATP6V0B/BAD/CALML3/CALML5/CAMK2D/CARD9/CASP3/CD74/CIITA/CLEC7A/CORO1A/CTSD/FCGR3A/IFNA5/IL18/IRAK1/ITGAM/ITGAX/KSR1/MAPK3/MRC2/RAB7A/RAF1/RFX5/STAT1/TIRAP/TLR9	27
hsa00600	Sphingolipid metabolism	7/1172	47/7493	0.61818	1	0.93	DEGS2/GBA/NEU3/PLPP1/SGMS1/SMPD4/SPTLC1	7

hsa04961	Endocrine and other factor-regulated calcium	7/1172	47/7493	0.61818	1	0.93	AP2A2/AP2M1/ATP1A1/ATP1A4/CLTB/DNM1/PLCB2	7
hsa04724	Glutamatergic synapse	17/1172	114/7493	0.62542	1	0.93	DLG4/GNAO1/GNB3/GNB5/GNG3/GRIA3/GRIN1/GRIN2C/HOMER2/ITPR3/JMJD7-PLA2G4B/MAPK3/PLA2G4B/PLCB2/PLD2/SLC1A7/SLC38A1	17
hsa00670	One carbon pool by folate	3/1172	20/7493	0.62587	1	0.93	ALDH1L1/MTHFD1/SHMT2	3
hsa00061	Fatty acid biosynthesis	2/1172	13/7493	0.62655	1	0.93	ACACB/OLAH	2
hsa05034	Alcoholism	27/1172	180/7493	0.62674	1	0.93	ARAF/ATF2/CALML3/CALML5/CAMKK1/CREB3/DDC/DRD2/GNAO1/GNB3/GNB5/GNG3/GRIN1/GRIN2C/HDAC11/HDAC8/HIST1H2AH/HIST1H4F/HIST2H4A/HIST2H4B/HRAS/MAPK3/PPP1R1B/RAF1/SHC1/SLC29A1/TH	27
hsa05150	Staphylococcus aureus infection	10/1172	68/7493	0.63586	1	0.93	C1QB/C1QC/C2/C3AR1/CFH/FCGR3A/ITGAL/ITGAM/MASP2/SELP	10
hsa04217	Necroptosis	24/1172	162/7493	0.6486	1	0.93	CAMK2D/CASP1/CHMP1A/CHMP4C/CHMP7/FASLG/GLUD1/HIST1H2AH/HSP90AA1/IFNA5/JMJD7-PLA2G4B/PARP2/PARP3/PGAM5/PLA2G4B/PYGM/RNF31/SHARPIN/SLC25A5/SPATA2L/STAT1/STAT2/TRAF2/VDAC2	24
hsa04211	Longevity regulating	13/1172	89/7493	0.65136	1	0.93	ATF2/ATG101/CREB3/EHMT1/EHMT2/EIF4EBP1/HRAS/INS/MTOR/PRKAB1/RPS6KB2/SESN1/ULK1	13
hsa04919	Thyroid hormone	17/1172	116/7493	0.65516	1	0.93	ATP1A1/ATP1A4/BAD/BMP4/GATA4/HRAS/KAT2A/MAPK3/MED24/MTOR/NCOR1/NOTCH1/PLCB2/PLCD1/RAF1/STAT1/THRA	17
hsa05146	Amoebiasis	14/1172	96/7493	0.65614	1	0.93	C8A/CASP3/COL1A1/COL4A2/FN1/HSPB1/ITGAM/LAMA2/LAMA4/LAMC2/LAMC3/MUC2/PLCB2/RAB7A	14
hsa00650	Butanoate metabolism	4/1172	28/7493	0.6581	1	0.93	ACSM6/GAD1/HADHA/HMGCLL1	4
hsa05323	Rheumatoid arthritis	13/1172	90/7493	0.66777	1	0.93	ATP6V0B/ATP6V0E2/ATP6V1B1/ATP6V1F/ATP6V1G1/ATP6V1G2/CCL5/CSF1/CTSL/IL18/ITGAL/MMP3/TNFSF13	13
hsa04623	Cytosolic DNA-sensing pathway	9/1172	63/7493	0.66989	1	0.93	CASP1/CCL5/IFNA5/IKBKB/IL18/POLR1D/POLR2L/POLR3D/TBK1	9

hsa04514	Cell adhesion molecules	21/1172	144/7493	0.67302	1	0.93	ALCAM/CD58/CD6/CDH15/CDH5/CLDN15/CLDN2/CLDN23/CLDN4/CLDN6/HLA-E/ITGAL/ITGAM/L1CAM/NLGN3/NRXN2/NTNG2/PDCD1/PTPRF/SDC1/SELP	21
hsa04110	Cell cycle	18/1172	124/7493	0.67377	1	0.93	ABL1/ANAPC2/BUB1B/CCNB2/CCND3/CDC14A/CDK4/CDKN2A/E2F4/GADD45G/MAD1L1/MCM2/MCM4/ORC6/PKMYT1/SFN/YWHAQ/ZBTB17	18
hsa04612	Antigen processing and	11/1172	77/7493	0.67677	1	0.93	CD74/CIITA/CTSL/HLA-E/HSP90AA1/HSPA2/HSPA5/KLRC1/KLRC2/RFX5/TAP1	11
hsa04940	Type I diabetes mellitus	6/1172	43/7493	0.68391	1	0.93	FASLG/GAD1/HLA-E/INS/LTA/PTPRN2	6
hsa00500	Starch and sucrose	5/1172	36/7493	0.68402	1	0.93	G6PC3/GCK/HK3/MGAM/PYGM	5
hsa00330	Arginine and proline	7/1172	50/7493	0.68492	1	0.93	AGMAT/ALDH2/ALDH4A1/CARNS1/GOT2/NOS3/OAT	7
hsa05170	Human immunodeficiency virus 1 infection	31/1172	212/7493	0.6895	1	0.93	AP1G2/AP1S1/BAD/CALML3/CALML5/CASP3/CCNB2/CD247/CFL1/DDB1/FASLG/GNAO1/GNB3/GNB5/GNG3/HLA-E/HRAS/IFNA5/IKBKB/IRAK1/ITPR3/LIMK1/MAP2K3/MAPK3/MTOR/PTK2B/RAF1/RPS6KB2/TAP1/TBK1/TRAF2	31
hsa04662	B cell receptor signaling	10/1172	71/7493	0.69114	1	0.93	BLNK/CARD11/CD79B/CD81/HRAS/IKBKB/INPP5D/INPPL1/MAPK3/RAF1	10
hsa04976	Bile secretion	10/1172	71/749	0.69114	1	0.93	ABCC3/ABCC4/ABCG5/AQP1/AQP8/ATP1A1/ATP1A4/SCARB1/SLC22A1/SLC9A3	10
hsa04024	cAMP signaling pathway	29/1172	199/7493	0.69266	1	0.93	ABCC4/ATP1A1/ATP1A4/BAD/CACNA1F/CACNA1S/CALML3/CALML5/CAMK2D/CHRM1/CNGA2/CNGB1/CREB3/DRD2/GABBR1/GRIA3/GRIN1/GRIN2C/HCAR2/HCAR3/HHIP/HTR1D/MAPK3/ORAI1/PLD2/PPP1R1B/PTGER3/RAF1/SSTR5	29
hsa04670	Leukocyte transendothelial migration	16/1172	112/7493	0.69402	1	0.93	CDH5/CLDN15/CLDN2/CLDN23/CLDN4/CLDN6/CTNNA2/ITGAL/ITGAM/MMP2/MYL5/NCF1/PTK2B/PTPN11/SIPA1/THY1	16
hsa04750	Inflammatory mediator regulation of	14/1172	99/7493	0.70211	1	0.93	ASIC2/ASIC3/CALML3/CALML5/CAMK2D/F2RL1/ITPR3/JMJD7-PLA2G4B/MAP2K3/PLA2G4B/PLCB2/PRKCD/TRPV2/TRPV4	14
hsa04218	Cellular senescence	23/1172	160/7493	0.70492	1	0.93	CALML3/CALML5/CCNB2/CCND3/CDK4/CDKN2A/E2F4/EIF4EBP1/FOXM1/GADD45G/GATA4/HLA-E/HRAS/IGFBP3/ITPR3/LIN52/MAP2K3/MAPK3/MTOR/RAF1/SLC25A5/TRPV4/VD	23

hsa00603	Glycosphingolipid biosynthesis - globo and isoglobo series	2/1172	15/7493	0.70545	1	0.93	GBGT1/HEXA	2
hsa00604	Glycosphingolipid biosynthesis - ganglio series	2/1172	15/7493	0.70545	1	0.93	B4GALNT1/HEXA	2
hsa00071	Fatty acid degradation	6/1172	44/7493	0.70594	1	0.93	ACADVL/ADH1A/ADH1C/ALDH2/CPT2/HADHA	6
hsa04973	Carbohydrate digestion and absorption	6/1172	44/7493	0.70594	1	0.93	ATP1A1/ATP1A4/G6PC3/HK3/MGAM/PLCB2	6
hsa05145	Toxoplasmosis	16/1172	113/7493	0.70787	1	0.93	BAD/CASP3/CIITA/GNAO1/HSPA2/IKBKB/IRAK1/LAMA2/LAMA4/LAMC2/LAMC3/MAP2K3/MAPK3/PIK3R5/PIK3R6/STAT1	16
hsa05216	Thyroid cancer	5/1172	37/749	0.70793	1	0.93	DDB2/GADD45G/HRAS/MAPK3/TCF7L1	5
hsa04060	Cytokine-cytokine receptor interaction	43/1172	294/7493	0.71193	1	0.93	BMP4/CCL1/CCL19/CCL21/CCL5/CCR2/CD27/CLCF1/CNTFR/CSF1/CSF1R/CSF3/CXCR1/CXCR2/CXCR3/CXCR5/FASLG/GDF2/IFNA5/IL13/IL15RA/IL17RE/IL18/IL1RN/IL20RB/IL31RA/IL32/IL4R/IL5RA/INHBB/INHBE/LEPR/LTA/LTBR/MPL/PF4V1/RELT/THPO/TNFRSF12A/TNFRSF13B/TNFRSF18/TNFRSF4/TNFSF13	43
hsa00760	Nicotinate and nicotinamide metabolism	4/1172	30/7493	0.71235	1	0.93	NMNAT3/NT5C3B/NT5M/QPRT	4
hsa03060	Protein export	3/1172	23/749	0.7212	1	0.93	HSPA5/SEC61A2/SRP14	3
hsa00512	Mucin type O-glycan	4/1172	31/7493	0.737	1	0.93	B3GNT6/GALNT16/GALNT8/GALNT9	4
hsa04710	Circadian	4/1172	31/749	0.737	1	0.93	NPAS2/PER1/PRKAB1/RORC	4
hsa00730	Thiamine metabolism	2/1172	16/7493	0.7394	1	0.93	AK5/ALPPL2	2
hsa04530	Tight junction	24/1172	170/7493	0.74103	1	0.93	CDK4/CLDN15/CLDN2/CLDN23/CLDN4/CLDN6/CTTN/ERBB2/GATA4/LLGL1/MARVELD2/MARVELD3/MYL6B/PARD3/PARD6A/PPP2R2B/PRKAB1/SCRIB/TJAP1/TUBA3C/TUBA3D/TUBA3E/WAS/WHAMM	24

hsa04540	Gap junction	12/1172	88/749	0.74199	1	0.93	DRD2/EGFR/HRAS/ITPR3/MAPK3/PDGFA/PDGFRB/PLCB2/RAF1/TUBA3C/TUBA3	12
hsa04064	NF-kappa B signaling	13/1172	95/7493	0.74298	1	0.93	BLNK/CARD11/CCL19/CCL21/IKBKB/IRAK1/LCK/LTA/LTBR/MAP3K14/PLAU/TIRAP/TRAF2	13
hsa04926	Relaxin signaling	18/1172	130/7493	0.7504	1	0.93	ARRB2/ATF2/COL1A1/COL4A2/CREB3/EGFR/GNAO1/GNB3/GNB5/GNG3/HRAS/MAPK3/MMP2/NOS3/PLCB2/RAF1/RXFP2/SHC1	18
hsa05010	Alzheimer disease	24/1172	171/7493	0.75144	1	0.93	APBB1/APH1A/ATP2A3/ATP5G1/BAD/CACNA1F/CACNA1S/CALML3/CALML5/CASP3/CDK5/COX8A/CYC1/GAPDH/GRIN1/GRIN2C/ITPR3/LRP1/MAPK3/NDUFA2/NDUFA4L2/NDUFB5/NDUFC2-KCTD14/PLCB2	24
hsa04371	Apelin signaling pathway	19/1172	137/7493	0.75217	1	0.93	CALML3/CALML5/EGR1/GNB3/GNB5/GNG3/HRAS/ITPR3/MAPK3/MTOR/MYL4/NOS3/PIK3R5/PIK3R6/PLCB2/PLIN1/PRKAB1/RAF1/RPS6KB2	19
hsa04668	TNF signaling pathway	15/1172	110/7493	0.75825	1	0.93	ATF2/CASP3/CCL5/CREB3/CSF1/DAB2IP/IKBKB/LTA/MAP2K3/MAP3K14/MAPK3/MMP3/PGAM5/RPS6KA4/TRAF2	15
hsa00561	Glycerolipid metabolism	8/1172	61/7493	0.75882	1	0.93	ALDH2/DGAT1/DGKI/DGKQ/DGKZ/GLYCTK/PLPP1/PNPLA2	8
hsa00640	Propanoate metabolism	4/1172	32/7493	0.76001	1	0.93	ACACB/ACSS3/HADHA/LDHA	4
hsa05016	Huntington disease	27/1172	193/7493	0.76742	1	0.93	AP2A2/AP2M1/ATP5G1/CASP3/CLTB/COX8A/CREB3/CYC1/DCTN2/DLG4/DNAH11/DNAH12/DNAH17/GPX1/GRIN1/NDUFA2/NDUFA4L2/NDUFB5/NDUFC2-KCTD14/PLCB2/POLR2G/POLR2J2/POLR2J3/POLR2L/SLC25A5/TBPL1/VDAC2	27
hsa04970	Salivary secretion	12/1172	90/7493	0.76971	1	0.93	ADRA1A/ADRA1B/AQP5/ATP1A1/ATP1A4/CALML3/CALML5/ITPR3/LPO/MUC5B/PLCB2/TRPV6	12
hsa00450	Selenocompound metabolism	2/1172	17/7493	0.76994	1	0.93	TXNRD1/TXNRD3	2
hsa04742	Taste	11/1172	83/749	0.77011	1	0.93	ASIC2/GABBR1/GNB3/HTR1D/HTR3B/ITPR3/P2RX2/PLCB2/SCNN1A/TAS2R3/TRP	11
hsa04071	Sphingolipid signaling	16/1172	118/7493	0.77123	1	0.93	CTSD/DEGS2/HRAS/MAPK3/NOS3/OPRD1/PLCB2/PLD2/PPP2R2B/PPP2R3B/PPP2R3C/PPP2R5B/RAF1/SGMS1/SPTLC1/TRAF2	16
hsa04920	Adipocytokine signaling	9/1172	69/7493	0.77253	1	0.93	ACACB/G6PC3/IKBKB/LEPR/MTOR/PRKAB1/PTPN11/SLC2A4/TRAF2	9
hsa05134	Legionellosis	7/1172	55/749	0.77836	1	0.93	CASP1/CASP3/HSF1/HSPA2/IL18/ITGAM/NAIP	7
hsa04722	Neurotrophin signaling	16/1172	119/7493	0.7827	1	0.93	ABL1/BAD/CALML3/CALML5/CAMK2D/FASLG/HRAS/IKBKB/IRAK1/MAPK3/PRKCD/PTPN11/RAF1/RPS6KA1/SH2B1/SHC1	16

hsa04080	Neuroactive ligand-receptor interaction	39/1172	277/7493	0.7903	1	0.93	ADRA1A/ADRA1B/ADRA2A/ADRA2B/ADRA2C/AVPR2/C3AR1/CHRM1/CHRNA6/CHRNB1/CHRNB3/CRHR1/CRHR2/DRD2/F2RL1/GABBR1/GLRA1/GPR35/GRIA3/GRIN1/GRIN2C/HRH2/HTR1D/LEPR/LTB4R/MTNR1B/NPY4R/OPRD1/OPRM1/P2RX2/P2RY6/PARD3/PTGER3/RXFP2/SSTR5/TACR1/TACR2/THRA/VIPR1	39
hsa05161	Hepatitis B	21/1172	155/7493	0.79654	1	0.93	ATF2/BAD/CASP3/CREB3/DDB1/DDB2/FASLG/HRAS/HSPG2/IFNA5/IKBKB/IRAK1/MAP2K3/MAPK3/PTK2B/RAF1/STAT1/STAT2/TBK1/TIRAP/YWHAQ	21
hsa04630	JAK-STAT signaling	22/1172	162/7493	0.79743	1	0.93	CCND3/CISH/CNTFR/CSF3/EGFR/HRAS/IFNA5/IL13/IL15RA/IL20RB/IL4R/IL5RA/LEPR/MPL/MTOR/PDGFA/PDGFRB/PTPN11/RAF1/STAT1/STAT2/THPO	22
hsa05163	Human cytomegalovirus infection	31/1172	225/7493	0.80784	1	0.93	ATF2/CALML3/CALML5/CASP3/CCL5/CDK4/CDKN2A/CREB3/CXCR2/EGFR/EIF4EBP1/FASLG/GNAO1/GNB3/GNB5/GNG3/HLA-E/HRAS/IFNA5/IKBKB/ITPR3/MAPK3/MTOR/PLCB2/PTGER3/PTK2B/RAF1/RPS6KB2/TAP1/TBK1/TRAF2	31
hsa04520	Adherens	9/1172	72/749	0.81436	1	0.93	CTNNA2/EGFR/ERBB2/FARP2/MAPK3/PARD3/PTPRF/TCF7L1/WAS	9
hsa05168	Herpes simplex infection	25/1172	185/7493	0.81745	1	0.93	CASP3/CCL5/CD74/EIF2AK4/FASLG/HCFC1/HLA-E/IFNA5/IKBKB/LTA/NXF2/NXF2B/NXF3/PER1/PTPN11/SRSF4/STAT1/STAT2/TAF3/TAF6/TAP1/TBK1/TBPL1/TLR9/TRAF2	25
hsa00601	Glycosphingolipid biosynthesis - lacto and	3/1172	27/7493	0.81746	1	0.93	B4GALT2/FUT7/ST3GAL4	3
hsa00072	Synthesis and degradation of ketone bodies	1/1172	10/7493	0.81769	1	0.93	HMGCLL1	1
hsa00770	Pantothenate and CoA	2/1172	19/7493	0.82176	1	0.93	PPCDC/VNN3	2
hsa00230	Purine metabolism	17/1172	130/7493	0.82405	1	0.93	AK5/AK6/DGUOK/ENTPD8/FHIT/GUCY2D/HDDC3/IMPDH1/NME1/NME4/NT5C3B/NT5M/NUDT2/PDE2A/PDE6D/PKM/XDH	17
hsa04611	Platelet activation	16/1172	123/7493	0.82458	1	0.93	COL1A1/FERMT3/FGA/GP9/ITPR3/JMJD7-PLA2G4B/MAPK3/NOS3/ORAI1/PIK3R5/PIK3R6/PLA2G4B/PLCB2/RASGRP2/TLN1/	16
hsa05169	Epstein-Barr virus infection	27/1172	201/7493	0.83436	1	0.93	BLNK/CASP3/CCND3/CD247/CD58/CDK4/DDB2/ENTPD8/GADD45G/HLA-E/IFNA5/IKBKB/IRAK1/ISG15/ITGAL/MAP2K3/MAP3K14/NCOR2/PSMC3/PSMD13/PSMD3/PSMD7/STAT1/STAT2/TAP1/TBK1/TRAF2	27

hsa03320	PPAR signaling pathway	9/1172	74/7493	0.83883	1	0.93	APOA5/APOC3/CPT2/FADS2/ILK/NR1H3/OLR1/PLIN1/PPARD	9
hsa00130	Ubiquinone and other terpenoid-quinone biosynthesis	1/1172	11/7493	0.84624	1	0.93	COQ2	1
hsa04971	Gastric acid secretion	9/1172	75/7493	0.85008	1	0.93	ATP1A1/ATP1A4/CALML3/CALML5/CAMK2D/HRH2/ITPR3/KCNQ1/PLCB2	9
hsa00190	Oxidative phosphorylation	17/1172	133/7493	0.85074	1	0.93	ATP5G1/ATP5I/ATP6V0B/ATP6V0E2/ATP6V1B1/ATP6V1F/ATP6V1G1/ATP6V1G2/COX10/COX11/COX8A/CYC1/NDUFA2/NDUFA4L2/NDUFB5/NDUFC2-	17
hsa04350	TGF-beta signaling	11/1172	90/7493	0.85297	1	0.93	BMP4/E2F4/INHBB/INHBE/LEFTY2/MAPK3/MINOS1-NBL1/NBL1/RGMA/RPS6KB2/SMAD6	11
hsa04664	Fc epsilon RI signaling	8/1172	68/7493	0.85508	1	0.93	HRAS/IL13/INPP5D/JMJD7-PLA2G4B/MAP2K3/MAPK3/PLA2G4B/RAF1	8
hsa00980	Metabolism of xenobiotics by cytochrome	9/1172	76/7493	0.86071	1	0.93	ADH1A/ADH1C/AKR7A3/CYP1A1/CYP1A2/GSTM1/GSTM2/MGST1/MGST3	9
hsa05032	Morphine	11/1172	91/749	0.86263	1	0.93	ARRB2/GABBR1/GNAO1/GNB3/GNB5/GNG3/GRK6/KCNJ5/KCNJ9/OPRM1/PDE2A	11
hsa04380	Osteoclast differentiation	16/1172	128/7493	0.86817	1	0.93	BLNK/CSF1/CSF1R/FCGR3A/FOSL1/IKBKB/LCK/MAP3K14/MAPK3/NCF1/SPI1/STAT1/STAT2/TEC/TRAF2/TREM2	16
hsa00030	Pentose phosphate	3/1172	30/7493	0.86969	1	0.93	ALDOA/GLYCTK/PFKM	3
hsa04213	Longevity regulating pathway -	7/1172	62/7493	0.8723	1	0.93	CRYAB/HRAS/HSPA2/INS/MTOR/PRKAB1/RPS6KB2	7
hsa04622	RIG-I-like receptor	8/1172	70/7493	0.87607	1	0.93	DHX58/IFNA5/IKBKB/ISG15/NLRX1/OTUD5/TBK1/TRAF2	8
hsa04141	Protein processing in endoplasmic	21/1172	165/7493	0.87694	1	0.93	BCAP31/CRYAB/DAD1/DDOST/EIF2AK4/FBXO2/FBXO6/GANAB/HERPUD1/HSP90AA1/HSPA2/HSPA5/LMAN2/OS9/RPN1/SEC61A2/SIL1/SSR2/SYVN1/TRAF2/WFS1	21

hsa00270	Cysteine and methionine metabolism	5/1172	47/7493	0.87897	1	0.93	BHMT2/DNMT3A/GOT2/LDHA/SDSL	5
hsa04714	Thermogenesis	30/1172	231/7493	0.89091	1	0.93	ATF2/ATP5G1/ATP5I/COA3/COA4/COX10/COX11/COX8A/CPT2/CREB3/CYC1/GCG/HRAS/MAP2K3/MLST8/MTOR/NDUFA2/NDUFA4L2/NDUFAF6/NDUFAF7/NDUFB5/NDUFC2-	30
hsa05033	Nicotine	4/1172	40/749	0.89165	1	0.93	CHRNA6/GRIA3/GRIN1/GRIN2C	4
hsa05142	Chagas disease (American trypanosomiasis)	12/1172	102/7493	0.89281	1	0.93	C1QB/C1QC/CCL5/CD247/FASLG/GNAO1/IKBKB/IRAK1/MAPK3/PLCB2/PPP2R2B/TLR9	12
hsa04672	Intestinal immune network for IgA	5/1172	49/7493	0.90031	1	0.93	IL15RA/LTBR/MAP3K14/TNFRSF13B/TNFSF13	5
hsa05166	Human T-cell leukemia virus 1 infection	28/1172	219/7493	0.9015	1	0.93	ANAPC2/ATF2/BUB1B/CCNB2/CCND3/CDK4/CDKN2A/CREB3/CRTC2/EGR1/FOSL1/HLA-E/HRAS/IKBKB/IL15RA/ITGAL/KAT2A/LCK/LTA/LTBR/MAD1L1/MAP3K14/MAPK	28
hsa03440	Homologous recombination	4/1172	41/7493	0.90257	1	0.93	BRCA1/BRCC3/POLD4/XRCC3	4
hsa04620	Toll-like receptor	12/1172	104/7493	0.90684	1	0.93	CCL5/IFNA5/IKBKB/IRAK1/IRF5/MAP2K3/MAPK3/STAT1/TBK1/TIRAP/TLR8/TLR9	12
hsa00533	Glycosaminoglycan biosynthesis - keratan sulfate	1/1172	14/7493	0.90778	1	0.93	B4GALT2	1
hsa04215	Apoptosis - multiple species	3/1172	33/7493	0.90825	1	0.93	BOK/CASP3/SEPT4	3
hsa05416	Viral	6/1172	59/749	0.91701	1	0.93	ABL1/CASP3/EIF4G1/HLA-E/ITGAL/LAMA2	6
hsa05211	Renal cell carcinoma	7/1172	69/7493	0.93065	1	0.93	ARAF/BAD/HRAS/MAPK3/PRCC/PTPN11/RAF1	7
hsa04660	T cell receptor signaling	11/1172	101/7493	0.93381	1	0.93	CARD11/CD247/CDK4/HRAS/IKBKB/LCK/MAP3K14/MAPK3/PDCD1/RAF1/TEC	11

hsa04923	Regulation of lipolysis in adipocytes	5/1172	54/7493	0.93992	1	0.93	INS/PLA2G16/PLIN1/PNPLA2/PTGER3	5
hsa04723	Retrograde endocannabinoid signaling	17/1172	148/7493	0.94054	1	0.93	CACNA1F/CACNA1S/DAGLB/GNAO1/GNB3/GNB5/GNG3/GRIA3/ITPR3/KCNJ5/KCNJ9/MAPK3/NDUFA2/NDUFA4L2/NDUFB5/NDUFC2-KCTD14/PLCB2	17
hsa03018	RNA	8/1172	79/749	0.94187	1	0.93	DIS3L/EXOSC10/EXOSC2/LSM1/PAN2/PATL1/PFKM/PNLDC1	8
hsa05167	Kaposi sarcoma-associated herpesvirus infection	22/1172	186/7493	0.94397	1	0.93	CALML3/CALML5/CASP3/CDK4/GNB3/GNB5/GNG3/HLA-E/HRAS/IFNA5/IKBKB/ITPR3/MAPK3/MICB/MTOR/PIK3R5/PIK3R6/RAF1/STAT1/STAT2/TBK1/TRAF2	22
hsa04972	Pancreatic secretion	10/1172	96/7493	0.94708	1	0.93	ATP1A1/ATP1A4/ATP2A3/CPA1/ITPR3/KCNQ1/PLA2G1B/PLA2G2F/PLA2G5/PLCB2	10
hsa00982	Drug metabolism -	7/1172	72/7493	0.94746	1	0.93	ADH1A/ADH1C/CYP1A2/GSTM1/GSTM2/MGST1/MGST3	7
hsa04727	GABAergic synapse	9/1172	89/7493	0.95114	1	0.93	CACNA1F/CACNA1S/GABBR1/GAD1/GNAO1/GNB3/GNB5/GNG3/SLC38A1	9
hsa04640	Hematopoietic cell lineage	10/1172	97/7493	0.9513	1	0.93	ANPEP/CD5/CSF1/CSF1R/CSF3/GP9/IL4R/IL5RA/ITGAM/THPO	10
hsa03008	Ribosome biogenesis in eukaryotes	11/1172	105/7493	0.95176	1	0.93	AK6/FCF1/MDN1/NHP2/NOB1/NOL6/NVL/NXF2/NXF2B/NXF3/RMRP	11
hsa00280	Valine, leucine and isoleucine degradation	4/1172	48/7493	0.95525	1	0.93	ACSF3/ALDH2/HADHA/HMGCLL1	4
hsa05204	Chemical carcinogenesis	8/1172	82/7493	0.95562	1	0.93	ADH1A/ADH1C/CYP1A1/CYP1A2/GSTM1/GSTM2/MGST1/MGST3	8
hsa05140	Leishmaniasis	7/1172	74/749	0.95654	1	0.93	FCGR3A/IRAK1/ITGAM/MAPK3/MARCKSL1/NCF1/STAT1	7
hsa04914	Progesterone-mediated oocyte maturation	10/1172	99/7493	0.95885	1	0.93	ANAPC2/ARAF/CCNB2/HSP90AA1/INS/MAD1L1/MAPK3/PKMYT1/RAF1/RPS6KA1	10

hsa04658	Th1 and Th2 cell differentiation	9/1172	92/7493	0.96247	1	0.93	CD247/IKBKB/IL13/IL4R/JAG2/LCK/MAPK3/NOTCH1/STAT1	9
hsa05162	Measles	14/1172	132/7493	0.96353	1	0.93	CCND3/CDK4/DOK1/EIF2AK4/FASLG/HSPA2/IFNA5/IL13/IRAK1/STAT1/STAT2/TACR1/TBK1/TLR9	14
hsa05310	Asthma	2/1172	31/749	0.96565	1	0.93	EPX/IL13	2
hsa04114	Oocyte meiosis	13/1172	125/7493	0.96576	1	0.93	ANAPC2/CALML3/CALML5/CAMK2D/CCNB2/INS/ITPR3/MAD1L1/MAPK3/PKMYT1/PPP2R5B/RPS6KA1/YWHAQ	13
hsa05332	Graft-versus-host disease	3/1172	41/7493	0.9659	1	0.93	FASLG/HLA-E/KLRC1	3
hsa03460	Fanconi anemia pathway	4/1172	54/7493	0.97795	1	0.93	ATRIP/BRCA1/FANCA/TELO2	4
hsa04659	Th17 cell differentiation	10/1172	107/7493	0.97967	1	0.93	CD247/HSP90AA1/IKBKB/IL4R/LCK/MAPK3/MTOR/RARA/RORC/STAT1	10
hsa05321	Inflammatory bowel disease (IBD)	5/1172	65/7493	0.982	1	0.93	IL13/IL18/IL4R/RORC/STAT1	5
hsa04932	Non-alcoholic fatty liver disease	15/1172	149/7493	0.98216	1	0.93	CASP3/COX8A/CYC1/FASLG/IKBKB/INS/LEPR/MLXIPL/NDUFA2/NDUFA4L2/NDUFB5/NDUFC2-KCTD14/NR1H3/PRKAB1/TRAF2	15
hsa03013	RNA transport	17/1172	165/7493	0.98245	1	0.93	CASC3/EIF1AY/EIF2B2/EIF2B5/EIF3B/EIF3I/EIF4B/EIF4EBP1/EIF4G1/NUP210/NUP210L/NUP98/NXF2/NXF2B/NXF3/RNPS1/RPP21	17
hsa00830	Retinol	5/1172	67/749	0.9857	1	0.93	ADH1A/ADH1C/CYP1A1/CYP1A2/DGAT1	5
hsa03010	Ribosome	15/1172	153/7493	0.98712	1	0.93	MRPL10/MRPL14/MRPL28/MRPL36/MRPL9/MRPS10/MRPS11/MRPS18A/MRPS21/MRPS5/RPL35A/RPL36A/RPL41/RPL7/RPS24	15
hsa04120	Ubiquitin mediated	13/1172	137/7493	0.98734	1	0.93	ANAPC2/BRCA1/DDB1/DDB2/FBXO2/SYVN1/UBE2A/UBE2B/UBE2E2/UBE2U/UBE3B/UBE3C/WWP2	13
hsa05330	Allograft	2/1172	38/749	0.98761	1	0.93	FASLG/HLA-E	2
hsa00053	Ascorbate and aldarate metabolism	1/1172	27/7493	0.98996	1	0.93	ALDH2	1

hsa04392	Hippo signaling pathway - multiple species	1/1172	29/7493	0.99286	1	0.93	TEAD4	1
hsa05320	Autoimmune thyroid disease	3/1172	53/7493	0.99307	1	0.93	FASLG/HLA-E/IFNA5	3
hsa05012	Parkinson disease	12/1172	142/7493	0.99629	1	0.93	ATP5G1/CASP3/COX8A/CYC1/DRD2/NDUFA2/NDUFA4L2/NDUFB5/NDUFC2-KCTD14/SLC25A5/TH/VDAC2	12
hsa03040	Spliceosome	10/1172	134/7493	0.99861	1	0.93	CWC15/DHX38/EFTUD2/HSPA2/PPIE/PRPF4/PRPF8/SART1/SF3B4/SRSF4	10
hsa04216	Ferroptosis	1/1172	40/749	0.99891	1	0.93	VDAC2	1
hsa05322	Systemic lupus erythematosus	9/1172	133/7493	0.99944	1	0.93	C1QB/C1QC/C2/C8A/FCGR3A/HIST1H2AH/HIST1H4F/HIST2H4A/HIST2H4B	9
hsa04740	Olfactory transduction	15/1172	448/7493	1	1	0.93	ANO2/ARRB2/CALML3/CALML5/CAMK2D/CNGA2/CNGB1/GUCY2D/OR2B3/OR2C1/OR2F1/OR4C11/OR51B5/OR6A2/PDE2A	15

标题
ID
Description
GeneRatio
BgRatio
pvalue
p.adjust
qvalue
geneID
Count

说明
KO号
KO对应的描述信息
富集基因在该KO上的比例
背景基因在该KO上的比例
P值
校正P值（BH方法校正后的P值）
Q值（Q方法校正后的P值）
该KO上所有富集基因的基因名，用“/”分割
该KO上所有富集基因的数量
