# Supplementary material for: Whole-Blood MicroRNA Sequence Profiling and Identification of Specific miR-21 for Adolescents With Postural Tachycardia Syndrome
Source: Front Neurosci. 2022 Jun 30;16:920477. doi: 10.3389/fnins.2022.920477 (PMC9281551; doi:10.3389/fnins.2022.920477)
Supplement: Table S1 — Known_MicrorRNA_Target_Gene_Prediction_Summary.pdf. [file Data_Sheet_1.ZIP › supl Table 1 Known_MicrorRNA_Target_Gene_Prediction_Summary.pdf]

miRNA	NM_number	symbol	Miranda_Tot_score	Miranda_Tot_energy	rnahybrid_energy	rnahybrid_Pvalue
hsa-let-7f-5p	NM_001099274	TINF2	151	-22.03	-25.1	0.027774
hsa-let-7f-5p	NM_001251877	USP4	150	-25	-30.3	0.016054
hsa-let-7f-5p	NM_001285485	NEURL3	150	-21.84	-28.5	0.043184
hsa-let-7f-5p	NM_001285486	NEURL3	150	-21.84	-28.5	0.044635
hsa-let-7f-5p	NM_001300973	ANKRD42	152	-21.6	-24.4	0.049172
hsa-let-7f-5p	NM_002188	IL13	166	-26.83	-30.4	0.02694
hsa-let-7f-5p	NR_029513	MIR98	156	-23.7	-26.6	0.006684
hsa-let-7f-5p	NR_029628	MIR215	151	-19.12	-23.7	0.035755
hsa-let-7f-5p	NR_049873	MIR5690	162	-20.01	-23.1	0.023205
hsa-let-7f-5p	NR_073450	PFKFB1	176	-28.24	-31.4	0.046373
hsa-miR-15a-5p	NM_001031741	NEK10	164	-20.17	-29	0.016484
hsa-miR-15a-5p	NM_001130089	KARS	171	-21.4	-26.9	0.013154
hsa-miR-15a-5p	NM_001145026	PTPRQ	179	-27.54	-31	0.037455
hsa-miR-15a-5p	NM_001286407	RPUSD2	167	-21.39	-27.8	0.041152
hsa-miR-15a-5p	NM_001300930	NAALAD2	151	-23.56	-29.5	0.049994
hsa-miR-15a-5p	NM_001301680	DNASE1L2	167	-24.54	-29	0.010866
hsa-miR-15a-5p	NM_001304384	NEK10	164	-20.17	-29	0.016484
hsa-miR-15a-5p	NM_001374	DNASE1L2	167	-24.54	-29	0.010866
hsa-miR-15a-5p	NM_002811	PSMD7	166	-25.94	-29.2	0.029452
hsa-miR-15a-5p	NM_004305	BIN1	155	-25.81	-29.7	0.019289
hsa-miR-15a-5p	NM_005467	NAALAD2	151	-23.56	-29.5	0.049994
hsa-miR-15a-5p	NM_005548	KARS	171	-21.4	-26.9	0.013154
hsa-miR-15a-5p	NM_012337	CFAP45	160	-18.82	-24	0.03945
hsa-miR-15a-5p	NM_022117	TSPYL2	168	-30.06	-34	0.002823
hsa-miR-15a-5p	NM_024686	TTLL7	163	-24.85	-28.5	0.046255
hsa-miR-15a-5p	NM_138331	RNASE8	165	-21.27	-27.4	0.00296
hsa-miR-15a-5p	NM_139343	BIN1	155	-25.81	-29.7	0.019289
hsa-miR-15a-5p	NM_139344	BIN1	155	-25.81	-29.7	0.019289

hsa-miR-15a-5p	NM_139345	BIN1	155	-25.81	-29.7	0.019289
hsa-miR-15a-5p	NM_139346	BIN1	155	-25.81	-29.7	0.019289
hsa-miR-15a-5p	NM_139347	BIN1	155	-25.81	-29.7	0.019289
hsa-miR-15a-5p	NM_139348	BIN1	155	-25.81	-29.7	0.019289
hsa-miR-15a-5p	NM_139349	BIN1	155	-25.81	-29.7	0.019289
hsa-miR-15a-5p	NM_139350	BIN1	155	-25.81	-29.7	0.019289
hsa-miR-15a-5p	NM_139351	BIN1	155	-25.81	-29.7	0.019289
hsa-miR-15a-5p	NM_144715	EFHB	152	-23.64	-28.3	0.004759
hsa-miR-15a-5p	NM_145047	OSCP1	155	-21.15	-25.7	0.046609
hsa-miR-15a-5p	NM_152260	RPUSD2	167	-21.39	-27.8	0.041152
hsa-miR-15a-5p	NM_152284	CHMP4C	167	-28.62	-31.5	0.02226
hsa-miR-15a-5p	NM_152534	NEK10	164	-20.17	-29	0.016484
hsa-miR-15a-5p	NM_153361	NIM1K	161	-16.88	-23.8	0.044611
hsa-miR-15a-5p	NR_029486	MIR16-1	160	-19.13	-23.8	0.024593
hsa-miR-15a-5p	NR_029712	MIR195	157	-17.65	-23.9	0.022009
hsa-miR-15a-5p	NR_031711	MIR1322	162	-19.33	-24.3	0.010963
hsa-miR-16-5p	NM_000552	VWF	153	-17.82	-25.3	0.027458
hsa-miR-16-5p	NM_001031741	NEK10	168	-24.1	-27.7	0.03839
hsa-miR-16-5p	NM_001122752	SERPINI1	153	-22.43	-28.3	0.013594
hsa-miR-16-5p	NM_001301107	CCDC53	150	-23.65	-27.7	0.02224
hsa-miR-16-5p	NM_001304384	NEK10	168	-24.1	-27.7	0.03839
hsa-miR-16-5p	NM_003487	TAF15	163	-23.68	-28	0.022583
hsa-miR-16-5p	NM_005025	SERPINI1	153	-22.43	-28.3	0.013594
hsa-miR-16-5p	NM_016053	CCDC53	150	-23.65	-27.7	0.02224
hsa-miR-16-5p	NM_139215	TAF15	163	-23.68	-28	0.022583
hsa-miR-16-5p	NM_152534	NEK10	168	-24.1	-27.7	0.03839
hsa-miR-16-5p	NR_002786	CIDECP	156	-24.31	-30.6	0.029919
hsa-miR-16-5p	NR_029486	MIR16-1	179	-28.3	-29.5	0.000728
hsa-miR-16-5p	NR_029712	MIR195	179	-28.97	-32.9	0.000076
hsa-miR-16-5p	NR_029831	MIR106B	155	-15.93	-24.9	0.011813

hsa-miR-17-3p	NM_000196	HSD11B2	165	-27.35	-30.9	0.018285
hsa-miR-17-3p	NM_000513	OPN1MW	176	-30.35	-32.4	0.016839
hsa-miR-17-3p	NM_000747	CHRNB1	171	-26.24	-30.6	0.045021
hsa-miR-17-3p	NM_001085451	LNP1	151	-24.38	-29.4	0.023283
hsa-miR-17-3p	NM_001110556	FLNA	161	-22.27	-27.3	0.047542
hsa-miR-17-3p	NM_001145446	ZNF200	155	-30.91	-34.1	0.020498
hsa-miR-17-3p	NM_001145447	ZNF200	155	-30.91	-34.1	0.020498
hsa-miR-17-3p	NM_001145448	ZNF200	155	-30.91	-34.1	0.020498
hsa-miR-17-3p	NM_001146698	RBM19	160	-26.61	-29.4	0.045468
hsa-miR-17-3p	NM_001159484	NUP210L	167	-22.05	-25.5	0.032491
hsa-miR-17-3p	NM_001160372	TRAPPC9	182	-32.82	-37.2	0.001276
hsa-miR-17-3p	NM_001199957	NDUFB5	164	-25.44	-29.6	0.026488
hsa-miR-17-3p	NM_001199958	NDUFB5	164	-25.44	-29.6	0.031794
hsa-miR-17-3p	NM_001215	CA6	158	-28.01	-32.2	0.005025
hsa-miR-17-3p	NM_001270501	CA6	158	-28.01	-32.2	0.005025
hsa-miR-17-3p	NM_001270502	CA6	158	-28.01	-32.2	0.005025
hsa-miR-17-3p	NM_001278590	LIAS	162	-23.76	-29.6	0.033876
hsa-miR-17-3p	NM_001281447	GTF2IRD2	157	-20.33	-24.6	0.019397
hsa-miR-17-3p	NM_001456	FLNA	161	-22.27	-27.3	0.047542
hsa-miR-17-3p	NM_002083	GPX2	171	-25.45	-29.5	0.016369
hsa-miR-17-3p	NM_002492	NDUFB5	164	-25.44	-29.6	0.026488
hsa-miR-17-3p	NM_003454	ZNF200	155	-30.91	-34.1	0.020498
hsa-miR-17-3p	NM_006348	COG5	170	-28.82	-33.7	0.029302
hsa-miR-17-3p	NM_006551	SCGB1D2	162	-23.11	-27.3	0.006446
hsa-miR-17-3p	NM_006552	SCGB1D1	162	-22.73	-27.2	0.006852
hsa-miR-17-3p	NM_006617	NES	154	-23.34	-30.8	0.019867
hsa-miR-17-3p	NM_006859	LIAS	162	-23.76	-29.6	0.033876
hsa-miR-17-3p	NM_007058	CAPN11	170	-27.2	-31.6	0.009859
hsa-miR-17-3p	NM_014786	ARHGEF17	152	-26.96	-32.2	0.049524
hsa-miR-17-3p	NM_015722	CALY	159	-26.43	-29.9	0.003574

hsa-miR-17-3p	NM_016204	GDF2	150	-21.49	-29.7	0.027536
hsa-miR-17-3p	NM_016243	CYB5R1	154	-25.71	-30.3	0.032705
hsa-miR-17-3p	NM_020216	RNPEP	163	-28.54	-32.6	0.00503
hsa-miR-17-3p	NM_022917	NOL6	166	-25.76	-32.2	0.037478
hsa-miR-17-3p	NM_031466	TRAPPC9	182	-32.82	-37.2	0.001276
hsa-miR-17-3p	NM_032272	MAF1	150	-21.4	-28.7	0.048121
hsa-miR-17-3p	NM_130899	FAM71B	167	-21.67	-24.4	0.040844
hsa-miR-17-3p	NM_139235	NOL6	166	-25.76	-32.2	0.037478
hsa-miR-17-3p	NM_145648	SLC15A4	178	-28.18	-31.3	0.038481
hsa-miR-17-3p	NM_177435	PPARD	162	-22.33	-31.7	0.014592
hsa-miR-17-3p	NM_181733	COG5	170	-28.82	-33.7	0.029302
hsa-miR-17-3p	NM_194451	LIAS	162	-23.76	-29.6	0.037712
hsa-miR-17-3p	NM_198087	ZNF200	155	-30.91	-34.1	0.020498
hsa-miR-17-3p	NM_198088	ZNF200	155	-30.91	-34.1	0.020498
hsa-miR-17-3p	NM_207308	NUP210L	167	-22.05	-25.5	0.032491
hsa-miR-17-3p	NR_001553	FAM197Y2	157	-29.05	-31	0.013267
hsa-miR-17-3p	NR_039668	MIR3135B	154	-20.7	-27.9	0.001483
hsa-miR-17-3p	NR_039750	MIR4524A	157	-20.11	-25.8	0.006025
hsa-miR-17-3p	NR_039865	MIR4715	157	-19.91	-25.3	0.011224
hsa-miR-17-3p	NR_046300	FAM197Y5	157	-29.05	-31	0.013267
hsa-miR-17-3p	NR_046764	AOAH-IT1	156	-24.84	-29.7	0.035791
hsa-miR-18a-5p	NM_001043	SLC6A2	174	-29.48	-33	0.030774
hsa-miR-18a-5p	NM_001080527	MYO7B	157	-20.02	-27.8	0.036828
hsa-miR-18a-5p	NM_001172501	SLC6A2	174	-29.48	-33	0.030774
hsa-miR-18a-5p	NM_001172502	SLC6A2	174	-29.48	-33	0.030774
hsa-miR-18a-5p	NM_001198974	TMEM110-MUSTN1	160	-21.73	-26.5	0.037968
hsa-miR-18a-5p	NM_198456	FAM120C	158	-24.19	-28.1	0.03643
hsa-miR-18a-5p	NM_205853	MUSTN1	160	-21.73	-26.5	0.037968
hsa-miR-21-5p	NM_002981	CCL1	163	-20.41	-25.7	0.046319

hsa-miR-21-5p	NR_030329	SP1	166	-22	-26.6	0.004671
hsa-miR-22-3p	NM_000174	GP9	162	-22.26	-25.1	0.03386
hsa-miR-22-3p	NM_001085401	C6orf201	169	-27.74	-30.1	0.017915
hsa-miR-22-3p	NM_001113205	TSTD1	156	-21.07	-27.6	0.023268
hsa-miR-22-3p	NM_001144951	GLYCTK	163	-24.39	-30.1	0.025531
hsa-miR-22-3p	NM_001160408	TULP3	165	-23.01	-28.8	0.029553
hsa-miR-22-3p	NM_001166418	HDAC8	164	-21.48	-28.9	0.042696
hsa-miR-22-3p	NM_001256094	ATF2	161	-27.93	-31.8	0.006908
hsa-miR-22-3p	NM_001272095	STX4	171	-30.69	-33.8	0.000886
hsa-miR-22-3p	NM_001272096	STX4	171	-30.69	-33.8	0.000886
hsa-miR-22-3p	NM_001278612	EIF1AY	151	-22.18	-30.6	0.034394
hsa-miR-22-3p	NM_001300980	TMCO6	154	-24.15	-28.4	0.022392
hsa-miR-22-3p	NM_001300982	TMCO6	154	-24.15	-28.4	0.022392
hsa-miR-22-3p	NM_001301168	SHF	168	-23.57	-29.7	0.046002
hsa-miR-22-3p	NM_001301171	SHF	168	-23.57	-29.7	0.046002
hsa-miR-22-3p	NM_001344	DAD1	172	-25.17	-28.9	0.013763
hsa-miR-22-3p	NM_003593	FOXN1	155	-23.4	-30.4	0.031854
hsa-miR-22-3p	NM_004305	BIN1	165	-21.42	-29.7	0.025546
hsa-miR-22-3p	NM_004604	STX4	171	-30.69	-33.8	0.000886
hsa-miR-22-3p	NM_004681	EIF1AY	151	-22.18	-30.6	0.034394
hsa-miR-22-3p	NM_014317	PDSS1	168	-24.47	-29.8	0.011354
hsa-miR-22-3p	NM_015308	FNBP4	168	-26.71	-32.3	0.01799
hsa-miR-22-3p	NM_016209	TRAPPC2L	151	-18.03	-25.8	0.027153
hsa-miR-22-3p	NM_018486	HDAC8	164	-21.48	-28.9	0.042696
hsa-miR-22-3p	NM_018502	TMCO6	154	-24.15	-28.4	0.022392
hsa-miR-22-3p	NM_022363	LHX5	170	-24.5	-30.1	0.00842
hsa-miR-22-3p	NM_024410	ODF1	162	-24.09	-27.4	0.007955
hsa-miR-22-3p	NM_052905	FMNL2	174	-28.68	-33.9	0.026544
hsa-miR-22-3p	NM_138356	SHF	168	-23.57	-29.7	0.046002
hsa-miR-22-3p	NM_139343	BIN1	165	-21.42	-29.7	0.025546

hsa-miR-22-3p	NM_139344	BIN1	165	-21.42	-29.7	0.025546
hsa-miR-22-3p	NM_139345	BIN1	165	-21.42	-29.7	0.025546
hsa-miR-22-3p	NM_139346	BIN1	165	-21.42	-29.7	0.025546
hsa-miR-22-3p	NM_139347	BIN1	165	-21.42	-29.7	0.025546
hsa-miR-22-3p	NM_139348	BIN1	165	-21.42	-29.7	0.025546
hsa-miR-22-3p	NM_139349	BIN1	165	-21.42	-29.7	0.025546
hsa-miR-22-3p	NM_139350	BIN1	165	-21.42	-29.7	0.025546
hsa-miR-22-3p	NM_139351	BIN1	165	-21.42	-29.7	0.025546
hsa-miR-22-3p	NM_178190	ATPIF1	165	-24.95	-28.4	0.022141
hsa-miR-22-3p	NR_036218	MIR4252	152	-18.58	-25.1	0.006668
hsa-miR-22-3p	NR_039807	MIR4659B	169	-21.75	-28.4	0.001078
hsa-miR-23a-3p	NM_001127487	FLNC	172	-24.57	-29.8	0.038297
hsa-miR-23a-3p	NM_001458	FLNC	172	-24.57	-29.8	0.038297
hsa-miR-23a-3p	NM_005415	SLC20A1	151	-27.94	-30.7	0.026945
hsa-miR-23a-3p	NM_018462	BRK1	160	-28.73	-31.6	0.020705
hsa-miR-23a-3p	NM_152610	CCDC185	160	-25.82	-28.3	0.003102
hsa-miR-23a-3p	NR_030623	MIR216B	165	-21.79	-27.3	0.002296
hsa-miR-23a-3p	NR_104631	LINC01265	157	-27.09	-30.2	0.03462
hsa-miR-23a-3p	NR_120458	LOC100507195	152	-26.53	-30.2	0.020428
hsa-miR-26a-5p	NM_001195627	MLLT10	174	-27.7	-31.4	0.010037
hsa-miR-26a-5p	NM_001195628	MLLT10	174	-27.7	-31.4	0.002468
hsa-miR-26a-5p	NM_001195630	MLLT10	174	-27.7	-31.4	0.002468
hsa-miR-26a-5p	NM_001256129	ARL8A	159	-30.27	-34.8	0.006961
hsa-miR-26a-5p	NM_003904	ZPR1	165	-20.56	-27.3	0.049441
hsa-miR-26a-5p	NM_138795	ARL8A	159	-30.27	-34.8	0.006459
hsa-miR-26a-5p	NR_130722	TLX1NB	169	-30.63	-33.9	0.0244
hsa-miR-26a-5p	NR_130723	TLX1NB	169	-30.63	-33.9	0.023869
hsa-miR-26b-5p	NM_001195627	MLLT10	173	-24.7	-27.4	0.047903
hsa-miR-26b-5p	NM_001195628	MLLT10	173	-24.7	-27.4	0.013365
hsa-miR-26b-5p	NM_001195630	MLLT10	173	-24.7	-27.4	0.013365

hsa-miR-26b-5p	NM_138795	ARL8A	150	-26.07	-29.5	0.049796
hsa-miR-27a-3p	NM_000107	DDB2	151	-24.89	-28	0.034168
hsa-miR-27a-3p	NM_000759	CSF3	153	-28.52	-31	0.02846
hsa-miR-27a-3p	NM_001012959	DISC1	156	-18.47	-24.6	0.036059
hsa-miR-27a-3p	NM_001026	RPS24	152	-24.89	-28.6	0.001363
hsa-miR-27a-3p	NM_001040097	MOSPD3	160	-21.32	-26	0.033968
hsa-miR-27a-3p	NM_001040098	MOSPD3	160	-21.32	-26	0.033968
hsa-miR-27a-3p	NM_001040099	MOSPD3	160	-21.32	-26	0.033968
hsa-miR-27a-3p	NM_001135919	SLC46A3	171	-26.64	-30.9	0.030523
hsa-miR-27a-3p	NM_001136493	MFSD2A	164	-29.03	-32.5	0.003577
hsa-miR-27a-3p	NM_001142282	RPS24	152	-24.89	-28.6	0.002744
hsa-miR-27a-3p	NM_001142283	RPS24	152	-24.89	-28.6	0.003563
hsa-miR-27a-3p	NM_001142284	RPS24	152	-24.89	-28.6	0.003563
hsa-miR-27a-3p	NM_001178147	CSF3	153	-28.52	-31	0.02846
hsa-miR-27a-3p	NM_001220494	GLYATL1	174	-29.17	-31.7	0.027682
hsa-miR-27a-3p	NM_001220496	GLYATL1	174	-29.17	-31.7	0.027682
hsa-miR-27a-3p	NM_001282629	GBGT1	160	-28.87	-33.6	0.006666
hsa-miR-27a-3p	NM_001282632	GBGT1	160	-28.87	-33.6	0.004679
hsa-miR-27a-3p	NM_001287808	MFSD2A	164	-29.03	-32.5	0.003577
hsa-miR-27a-3p	NM_001287809	MFSD2A	164	-29.03	-32.5	0.003577
hsa-miR-27a-3p	NM_001288572	GBGT1	160	-28.87	-33.6	0.004679
hsa-miR-27a-3p	NM_001288573	GBGT1	160	-28.87	-33.6	0.004679
hsa-miR-27a-3p	NM_001300734	DDB2	151	-24.89	-28	0.034168
hsa-miR-27a-3p	NM_004422	DVL2	169	-26.69	-29.5	0.027712
hsa-miR-27a-3p	NM_004913	VPS9D1	159	-28.67	-30.8	0.024469
hsa-miR-27a-3p	NM_007364	TMED3	161	-29.1	-31.9	0.011208
hsa-miR-27a-3p	NM_013254	TBK1	165	-25.63	-29.6	0.04514
hsa-miR-27a-3p	NM_015104	ATG2A	164	-27.96	-32.1	0.005318
hsa-miR-27a-3p	NM_017818	WRAP73	151	-23.5	-26.7	0.025832
hsa-miR-27a-3p	NM_021221	LY6G5B	163	-20.59	-25.3	0.039275

hsa-miR-27a-3p	NM_021996	GBGT1	160	-28.87	-33.6	0.004679
hsa-miR-27a-3p	NM_023945	MS4A5	151	-15.58	-20.5	0.032983
hsa-miR-27a-3p	NM_023948	MOSPD3	160	-21.32	-26	0.033968
hsa-miR-27a-3p	NM_030652	EGFL8	164	-26.62	-28.6	0.020372
hsa-miR-27a-3p	NM_032793	MFSD2A	164	-29.03	-32.5	0.003577
hsa-miR-27a-3p	NM_033022	RPS24	152	-24.89	-28.6	0.002744
hsa-miR-27a-3p	NM_080661	GLYATL1	174	-29.17	-31.7	0.027682
hsa-miR-27a-3p	NM_138349	TP53I13	153	-26.89	-30.6	0.003489
hsa-miR-27a-3p	NM_138387	G6PC3	152	-24.75	-27.7	0.028451
hsa-miR-27a-3p	NM_172219	CSF3	153	-28.52	-31	0.02846
hsa-miR-27a-3p	NM_172220	CSF3	153	-28.52	-31	0.02846
hsa-miR-27a-3p	NR_029615	MIR183	153	-22.34	-24.2	0.0345
hsa-miR-27a-3p	NR_033866	LOC390705	170	-26.37	-31.4	0.007505
hsa-miR-27a-3p	NR_039642	MIR4440	150	-18.76	-31.1	0.000353
hsa-miR-27a-3p	NR_104664	LOC102467147	161	-26.99	-30.4	0.025305
hsa-miR-27a-3p	NR_110050	LOC101927418	156	-25.89	-28.2	0.037147
hsa-miR-27a-3p	NR_110147	LOC101927123	168	-27.47	-32.7	0.014166
hsa-miR-27a-3p	NR_125397	LINC01471	152	-26.77	-31.2	0.044404
hsa-miR-27a-3p	NR_125868	LOC102723831	165	-26.94	-32	0.018492
hsa-miR-28-5p	NM_000502	EPX	152	-23.48	-28.8	0.035334
hsa-miR-28-5p	NM_000537	REN	165	-22.1	-27.4	0.018553
hsa-miR-28-5p	NM_001012503	KRTAP5-7	156	-23.98	-27.6	0.047209
hsa-miR-28-5p	NM_001077663	URGCP	180	-31.32	-35.6	0.002886
hsa-miR-28-5p	NM_001077664	URGCP	180	-31.32	-35.6	0.002886
hsa-miR-28-5p	NM_001093728	TCP11	164	-25.39	-28.9	0.007738
hsa-miR-28-5p	NM_001098673	ATG101	159	-24.66	-28.6	0.02043
hsa-miR-28-5p	NM_001136571	ZAR1L	151	-22.14	-26.5	0.009364
hsa-miR-28-5p	NM_001145795	SH2B1	156	-22.15	-27.8	0.047661
hsa-miR-28-5p	NM_001161707	KIRREL3	169	-26.03	-32.2	0.004208
hsa-miR-28-5p	NM_001193343	MRPS18A	170	-25.87	-31.3	0.020488

hsa-miR-28-5p	NM_001197234	BTN2A1	161	-24.91	-28.8	0.040329
hsa-miR-28-5p	NM_001199933	SESN1	169	-28.32	-31.5	0.037285
hsa-miR-28-5p	NM_001199934	SESN1	169	-28.32	-31.5	0.037285
hsa-miR-28-5p	NM_001242946	EPDR1	173	-29.32	-33.1	0.034185
hsa-miR-28-5p	NM_001242948	EPDR1	173	-29.32	-33.1	0.028905
hsa-miR-28-5p	NM_001261817	TCP11	164	-25.39	-28.9	0.007738
hsa-miR-28-5p	NM_001261818	TCP11	164	-25.39	-28.9	0.007738
hsa-miR-28-5p	NM_001261819	TCP11	164	-25.39	-28.9	0.007738
hsa-miR-28-5p	NM_001261820	TCP11	164	-25.39	-28.9	0.007738
hsa-miR-28-5p	NM_001261821	TCP11	164	-25.39	-28.9	0.007738
hsa-miR-28-5p	NM_001272002	DOCK7	188	-31.83	-35.6	0.002253
hsa-miR-28-5p	NM_001290075	URGCP	180	-31.32	-35.6	0.002886
hsa-miR-28-5p	NM_001290076	URGCP	180	-31.32	-35.6	0.002886
hsa-miR-28-5p	NM_001304359	MUC5AC	151	-22.93	-28.4	0.041705
hsa-miR-28-5p	NM_001308293	SH2B1	156	-22.15	-27.8	0.047661
hsa-miR-28-5p	NM_002888	RARRES1	163	-24.47	-28.1	0.004807
hsa-miR-28-5p	NM_005068	SIM1	150	-23.99	-33.5	0.022801
hsa-miR-28-5p	NM_005373	MPL	184	-32.14	-34.3	0.01917
hsa-miR-28-5p	NM_005850	SF3B4	157	-21.44	-26.4	0.040146
hsa-miR-28-5p	NM_007227	GPR45	178	-29.59	-33.2	0.004218
hsa-miR-28-5p	NM_013241	FHOD1	160	-22.85	-31.5	0.002776
hsa-miR-28-5p	NM_014454	SESN1	169	-28.32	-31.5	0.037285
hsa-miR-28-5p	NM_017549	EPDR1	173	-29.32	-33.1	0.028905
hsa-miR-28-5p	NM_017670	OTUB1	176	-30.57	-35	0.004808
hsa-miR-28-5p	NM_017920	URGCP	180	-31.32	-35.6	0.002886
hsa-miR-28-5p	NM_018135	MRPS18A	170	-25.87	-31.3	0.014346
hsa-miR-28-5p	NM_018679	TCP11	164	-25.39	-28.9	0.007738
hsa-miR-28-5p	NM_021871	FGA	161	-23.53	-28	0.015377
hsa-miR-28-5p	NM_021908	ST7	177	-26.67	-30.7	0.041973
hsa-miR-28-5p	NM_021934	ATG101	159	-24.66	-28.6	0.02043

hsa-miR-28-5p	NM_022052	NXF3	157	-22.14	-26.3	0.043726
hsa-miR-28-5p	NM_033540	MFN1	156	-25.6	-31.8	0.03475
hsa-miR-28-5p	NM_144772	APOA1BP	159	-21.54	-26.2	0.041485
hsa-miR-28-5p	NM_145170	CFAP70	156	-23.7	-27	0.026972
hsa-miR-28-5p	NM_172114	CAMK2D	154	-17.29	-25.3	0.033543
hsa-miR-28-5p	NM_172115	CAMK2D	154	-17.29	-25.3	0.033543
hsa-miR-28-5p	NM_172129	CAMK2D	154	-17.29	-25.3	0.033543
hsa-miR-28-5p	NR_003077	SNORD99	152	-18.34	-24.4	0.018641
hsa-miR-28-5p	NR_029892	MIR151A	166	-23.86	-26.9	0.004912
hsa-miR-28-5p	NR_030598	MIR708	175	-31.24	-34.3	0.000042
hsa-miR-28-5p	NR_034139	LOC338963	168	-35.08	-38.7	0.001783
hsa-miR-28-5p	NR_047684	TMEM249	180	-28.97	-33.7	0.0112
hsa-miR-28-5p	NR_109960	BEAN1-AS1	151	-23.26	-28.6	0.04378
hsa-miR-28-5p	NR_125725	LINC00456	176	-26.3	-31.6	0.02087
hsa-miR-28-5p	NR_125997	LOC102724450	151	-24.87	-28	0.040355
hsa-miR-28-5p	NR_126415	FOXP4-AS1	152	-22.55	-29.3	0.040381
hsa-miR-32-3p	NR_029506	MIR32	163	-22.45	-26.2	0.001592
hsa-miR-93-3p	NM_000094	COL7A1	155	-24.93	-28.9	0.031454
hsa-miR-93-3p	NM_000581	GPX1	157	-21.53	-26.9	0.041886
hsa-miR-93-3p	NM_001009943	ANKRD16	161	-31.35	-31.4	0.001384
hsa-miR-93-3p	NM_001030287	ATF3	151	-20.8	-32.3	0.045267
hsa-miR-93-3p	NM_001080511	CLEC2L	166	-28.39	-32.8	0.013536
hsa-miR-93-3p	NM_001105565	SMTNL1	165	-26.67	-36	0.000154
hsa-miR-93-3p	NM_001135004	DNAJB5	158	-25.2	-32.4	0.040918
hsa-miR-93-3p	NM_001135005	DNAJB5	158	-25.2	-32.4	0.040918
hsa-miR-93-3p	NM_001143996	OGDHL	166	-25.58	-29.8	0.049328
hsa-miR-93-3p	NM_001143997	OGDHL	166	-25.58	-29.8	0.049328
hsa-miR-93-3p	NM_001144961	NFKBIL1	172	-27.31	-32.5	0.002119
hsa-miR-93-3p	NM_001144962	NFKBIL1	172	-27.31	-32.5	0.002119
hsa-miR-93-3p	NM_001144963	NFKBIL1	172	-27.31	-32.5	0.002119

hsa-miR-93-3p	NM_001159651	BOD1	151	-27.54	-32.4	0.038255
hsa-miR-93-3p	NM_001190918	THRA	172	-29.73	-34.7	0.003261
hsa-miR-93-3p	NM_001190919	THRA	172	-29.73	-34.7	0.003261
hsa-miR-93-3p	NM_001206484	ATF3	151	-20.8	-32.3	0.045267
hsa-miR-93-3p	NM_001206488	ATF3	151	-20.8	-32.3	0.045267
hsa-miR-93-3p	NM_001278248	RWDD3	155	-25.67	-28.6	0.04067
hsa-miR-93-3p	NM_001282394	WNK2	164	-27.63	-33.8	0.026748
hsa-miR-93-3p	NM_001674	ATF3	151	-20.8	-32.3	0.045267
hsa-miR-93-3p	NM_002607	PDGFA	170	-28.87	-33.8	0.026229
hsa-miR-93-3p	NM_003250	THRA	172	-29.73	-34.7	0.003261
hsa-miR-93-3p	NM_003801	GPAA1	161	-21.35	-24.8	0.029399
hsa-miR-93-3p	NM_003828	MTMR1	160	-26.05	-30.5	0.03652
hsa-miR-93-3p	NM_004346	CASP3	152	-20.8	-35.3	0.017415
hsa-miR-93-3p	NM_004813	PEX16	158	-32.89	-35.7	0.002819
hsa-miR-93-3p	NM_005007	NFKBIL1	172	-27.31	-32.5	0.002119
hsa-miR-93-3p	NM_012266	DNAJB5	158	-25.2	-32.4	0.040918
hsa-miR-93-3p	NM_015319	TNS2	151	-23.77	-29.6	0.042021
hsa-miR-93-3p	NM_015485	RWDD3	155	-25.67	-28.6	0.04067
hsa-miR-93-3p	NM_015594	TBC1D29	153	-23.47	-27.6	0.002593
hsa-miR-93-3p	NM_016360	TACO1	156	-24.58	-29.4	0.028139
hsa-miR-93-3p	NM_018228	VRTN	172	-23.7	-35.5	0.009364
hsa-miR-93-3p	NM_018245	OGDHL	166	-25.58	-29.8	0.049328
hsa-miR-93-3p	NM_032536	NTNG2	172	-25.8	-30.7	0.043934
hsa-miR-93-3p	NM_032991	CASP3	152	-20.8	-35.3	0.017415
hsa-miR-93-3p	NM_033023	PDGFA	170	-28.87	-33.8	0.025539
hsa-miR-93-3p	NM_057174	PEX16	158	-32.89	-35.7	0.001499
hsa-miR-93-3p	NM_138369	BOD1	151	-27.54	-32.4	0.039682
hsa-miR-93-3p	NM_152577	ZNF645	160	-23.46	-29.6	0.007869
hsa-miR-93-3p	NM_170754	TNS2	151	-23.77	-29.6	0.042021
hsa-miR-93-3p	NM_173625	C17orf78	159	-30.3	-36.4	0.00505

hsa-miR-93-3p	NM_182553	CNIH2	171	-31.13	-36	0.002687
hsa-miR-93-3p	NM_183387	EML5	150	-29.34	-33.1	0.023187
hsa-miR-93-3p	NM_194284	CLDN23	166	-31.67	-35.6	0.005493
hsa-miR-93-3p	NM_198316	TNS2	151	-23.77	-29.6	0.042021
hsa-miR-93-3p	NR_003704	SNORA84	158	-26.25	-26.5	0.022579
hsa-miR-93-3p	NR_003940	SNORD80	156	-18.61	-23.4	0.042317
hsa-miR-93-3p	NR_003943	SNORD77	162	-24.07	-27.9	0.001767
hsa-miR-93-3p	NR_024192	HILS1	151	-22.7	-32.5	0.026649
hsa-miR-93-3p	NR_024193	HILS1	151	-22.7	-32.5	0.033257
hsa-miR-93-3p	NR_027120	HSD52	174	-27.86	-31.9	0.043235
hsa-miR-93-3p	NR_028373	RNU6-42P	163	-20.75	-26.7	0.013106
hsa-miR-93-3p	NR_030527	MIR765	150	-22.01	-25.1	0.037843
hsa-miR-93-3p	NR_033742	FRMD8P1	176	-33.11	-36.2	0.014326
hsa-miR-93-3p	NR_036049	MIR1184-1	167	-31.33	-35.1	0.000072
hsa-miR-93-3p	NR_036186	MIR4300	156	-21.94	-25	0.028956
hsa-miR-93-3p	NR_036250	MIR4288	154	-24.23	-28.7	0.00134
hsa-miR-93-3p	NR_036259	MIR1184-2	167	-31.33	-35.1	0.000072
hsa-miR-93-3p	NR_036260	MIR1184-3	167	-31.33	-35.1	0.000072
hsa-miR-93-3p	NR_037424	MIR3651	158	-26.25	-26.5	0.01032
hsa-miR-93-3p	NR_038310	MIR497HG	166	-26.11	-32.3	0.02402
hsa-miR-93-3p	NR_039634	MIR4435-1	161	-20.36	-25.7	0.013105
hsa-miR-93-3p	NR_039636	MIR4435-2	161	-20.36	-25.7	0.011095
hsa-miR-93-3p	NR_046491	RNU6-33P	163	-20.75	-26.7	0.013106
hsa-miR-93-3p	NR_046492	RNU6-34P	163	-20.75	-26.7	0.012611
hsa-miR-93-3p	NR_046928	RNU6-53P	163	-20.75	-26.7	0.012858
hsa-miR-93-3p	NR_046936	RNU6-66P	163	-20.75	-26.7	0.012367
hsa-miR-93-3p	NR_046939	RNU6-69P	163	-20.75	-26.7	0.012367
hsa-miR-93-3p	NR_126335	LOC101927708	158	-26.14	-30.1	0.041762
hsa-miR-93-3p	NR_132997	LINC00680-GUSBP4	156	-21.2	-31.8	0.04436

hsa-miR-96-5p	NM_001010903	C6orf222	153	-17.38	-33.6	0.020076
hsa-miR-96-5p	NM_001039112	FER1L6	152	-22.54	-26.5	0.044368
hsa-miR-96-5p	NM_001252195	C9orf24	151	-20.93	-24.8	0.005442
hsa-miR-96-5p	NM_001278352	ACO1	157	-21.28	-29.3	0.048405
hsa-miR-96-5p	NM_002197	ACO1	157	-21.28	-29.3	0.048405
hsa-miR-96-5p	NM_005040	PRCP	169	-23.4	-29.7	0.02504
hsa-miR-96-5p	NM_138345	VWA5B2	159	-25.67	-31.8	0.002647
hsa-miR-96-5p	NM_147169	C9orf24	151	-20.93	-24.8	0.005442
hsa-miR-96-5p	NM_170726	ALDH4A1	150	-24.45	-29.6	0.016133
hsa-miR-96-5p	NM_183040	DTNBP1	156	-25.18	-31.6	0.020067
hsa-miR-96-5p	NM_199418	PRCP	169	-23.4	-29.7	0.02504
hsa-miR-96-5p	NM_201566	SLC16A13	158	-16.64	-25.4	0.032731
hsa-miR-96-5p	NR_003660	LINC00680-GUSBP4	165	-22.76	-29.6	0.045857
hsa-miR-96-5p	NR_027503	LOC100133050	165	-22.76	-29.6	0.039819
hsa-miR-96-5p	NR_029512	MIR96	175	-27.84	-33.6	0.000034
hsa-miR-96-5p	NR_030315	MIR548B	150	-15.06	-25	0.016048
hsa-miR-96-5p	NR_033831	LINC01121	162	-27.73	-31.3	0.033997
hsa-miR-96-5p	NR_033968	GUSBP9	165	-22.76	-29.6	0.032116
hsa-miR-96-5p	NR_034021	SMA5	174	-26.47	-33.4	0.025332
hsa-miR-96-5p	NR_036113	MIR3157	156	-17.17	-23.5	0.031929
hsa-miR-96-5p	NR_039977	LINC01093	165	-24.33	-31.2	0.045286
hsa-miR-96-5p	NR_132998	LINC00680-GUSBP4	165	-22.76	-29.6	0.039474
hsa-miR-96-5p	NR_133000	GUSBP4	165	-22.76	-29.6	0.025654
hsa-miR-98-5p	NM_000018	ACADVL	160	-23.53	-25.8	0.020842
hsa-miR-98-5p	NM_000996	RPL35A	158	-20.76	-24.7	0.008213
hsa-miR-98-5p	NM_001033859	ACADVL	160	-23.53	-25.8	0.020842
hsa-miR-98-5p	NM_001100	ACTA1	157	-24.48	-26.8	0.024036
hsa-miR-98-5p	NM_001270447	ACADVL	160	-23.53	-25.8	0.020842

hsa-miR-98-5p	NM_001270448	ACADVL	160	-23.53	-25.8	0.020842
hsa-miR-98-5p	NM_001316311	RPL35A	158	-20.76	-24.7	0.008213
hsa-miR-98-5p	NM_005926	MFAP1	153	-23.61	-28.3	0.039917
hsa-miR-98-5p	NM_178829	C7orf34	159	-24.14	-26.9	0.027683
hsa-miR-98-5p	NR_029513	MIR98	160	-24.56	-28.4	0.001947
hsa-miR-98-5p	NR_029628	MIR215	151	-18	-23.9	0.029324
hsa-miR-98-5p	NR_049873	MIR5690	161	-20.02	-23.4	0.017589
hsa-miR-29b-3p	NM_000426	LAMA2	175	-26.05	-28.6	0.008727
hsa-miR-29b-3p	NM_001079823	LAMA2	175	-26.05	-28.6	0.008727
hsa-miR-29b-3p	NM_001142474	NREP	169	-27.78	-33	0.025798
hsa-miR-29b-3p	NM_001142475	NREP	169	-27.78	-33	0.025798
hsa-miR-29b-3p	NM_001142476	NREP	169	-27.78	-33	0.025798
hsa-miR-29b-3p	NM_001142477	NREP	169	-27.78	-33	0.025798
hsa-miR-29b-3p	NM_001142478	NREP	169	-27.78	-33	0.025798
hsa-miR-29b-3p	NM_001142479	NREP	169	-27.78	-33	0.025798
hsa-miR-29b-3p	NM_001142480	NREP	169	-27.78	-33	0.025798
hsa-miR-29b-3p	NM_001142481	NREP	169	-27.78	-33	0.025798
hsa-miR-29b-3p	NM_001142482	NREP	169	-27.78	-33	0.025798
hsa-miR-29b-3p	NM_001142483	NREP	169	-27.78	-33	0.025798
hsa-miR-29b-3p	NM_001166533	DDX4	163	-25.55	-28.4	0.011486
hsa-miR-29b-3p	NM_001293312	DARS	179	-27.48	-31.4	0.045658
hsa-miR-29b-3p	NM_001349	DARS	179	-27.48	-31.4	0.045658
hsa-miR-29b-3p	NM_004688	NMI	179	-25.13	-27.8	0.014689
hsa-miR-29b-3p	NM_004772	NREP	169	-27.78	-33	0.025798
hsa-miR-29b-3p	NM_006303	AIMP2	168	-18.53	-24.5	0.044106
hsa-miR-29b-3p	NM_017417	GALNT8	161	-20.28	-25.4	0.021104
hsa-miR-29b-3p	NM_018663	PXMP2	177	-30.55	-31.5	0.003233
hsa-miR-29b-3p	NM_022304	HRH2	167	-24.54	-28.7	0.010068
hsa-miR-29b-3p	NR_002983	SNORA55	158	-22.52	-25.1	0.028419
hsa-miR-29b-3p	NR_029503	MIR29A	160	-22.6	-28	0.00081

hsa-miR-29b-3p	NR_029891	MIR326	154	-19.44	-25.9	0.008248
hsa-miR-29b-3p	NR_033244	LOC729080	172	-26.66	-31.1	0.034475
hsa-miR-29b-3p	NR_037436	MIR3663	159	-22.15	-27.3	0.003526
hsa-miR-29b-3p	NR_039790	MIR4647	153	-19.95	-23.1	0.03504
hsa-miR-16-2-3p	NR_029486	MIR16-1	175	-25.86	-28.2	0.001095
hsa-miR-16-2-3p	NR_029712	MIR195	187	-29.91	-31.9	0.000088
hsa-miR-16-2-3p	NR_029842	MIR301A	163	-18.12	-24.7	0.010378
hsa-miR-199a-3p	NM_000029	AGT	158	-28.76	-31.7	0.011599
hsa-miR-199a-3p	NM_001005783	HAO2	156	-24.63	-29	0.012814
hsa-miR-199a-3p	NM_001080435	WHAMM	154	-18.01	-32.1	0.031658
hsa-miR-199a-3p	NM_001163812	C10orf2	182	-29.18	-32.6	0.022716
hsa-miR-199a-3p	NM_001163813	C10orf2	182	-29.18	-32.6	0.01356
hsa-miR-199a-3p	NM_001163814	C10orf2	182	-29.18	-32.6	0.022716
hsa-miR-199a-3p	NM_001202431	PRDX1	152	-22.74	-28.4	0.018292
hsa-miR-199a-3p	NM_001282957	CFAP77	156	-25.98	-30.1	0.039683
hsa-miR-199a-3p	NM_002574	PRDX1	152	-22.74	-28.4	0.018292
hsa-miR-199a-3p	NM_002840	PTPRF	182	-31.31	-34.3	0.016067
hsa-miR-199a-3p	NM_003686	EXO1	154	-26.84	-29.4	0.023639
hsa-miR-199a-3p	NM_004419	DUSP5	172	-25.26	-31.5	0.032713
hsa-miR-199a-3p	NM_006826	YWHAQ	158	-28.88	-32.1	0.031449
hsa-miR-199a-3p	NM_016527	HAO2	156	-24.63	-29	0.012814
hsa-miR-199a-3p	NM_021619	PRDM12	160	-26.73	-32	0.03328
hsa-miR-199a-3p	NM_021830	C10orf2	182	-29.18	-32.6	0.01356
hsa-miR-199a-3p	NM_130440	PTPRF	182	-31.31	-34.3	0.016067
hsa-miR-199a-3p	NM_152416	NDUFAF6	155	-25.3	-31.2	0.021795
hsa-miR-199a-3p	NM_181696	PRDX1	152	-22.74	-28.4	0.018292
hsa-miR-199a-3p	NM_181697	PRDX1	152	-22.74	-28.4	0.018292
hsa-miR-199a-3p	NM_207417	CFAP77	156	-25.98	-30.1	0.039683
hsa-miR-7-1-3p	NR_039867	MIR3529	183	-30.06	-32.4	0.000068
hsa-miR-181a-5p	NM_001856	COL16A1	160	-23.74	-31.5	0.010783

hsa-miR-181a-5p	NM_020247	ADCK3	165	-27.7	-30.8	0.046124
hsa-miR-181a-5p	NM_032790	ORAI1	150	-25.6	-28.6	0.045514
hsa-miR-181c-5p	NM_001856	COL16A1	167	-27.45	-35.3	0.001071
hsa-miR-183-5p	NM_000154	GALK1	151	-20.16	-24.5	0.034038
hsa-miR-183-5p	NM_000180	GUCY2D	158	-23.86	-28.9	0.009564
hsa-miR-183-5p	NM_001018016	MUC1	163	-25.4	-28.9	0.015805
hsa-miR-183-5p	NM_001018017	MUC1	163	-25.4	-28.9	0.015805
hsa-miR-183-5p	NM_001044390	MUC1	163	-25.4	-28.9	0.015805
hsa-miR-183-5p	NM_001044391	MUC1	163	-25.4	-28.9	0.015805
hsa-miR-183-5p	NM_001044392	MUC1	163	-25.4	-28.9	0.015805
hsa-miR-183-5p	NM_001044393	MUC1	163	-25.4	-28.9	0.025914
hsa-miR-183-5p	NM_001143835	NFRKB	166	-26.38	-31.3	0.028995
hsa-miR-183-5p	NM_001197225	BEAN1	155	-20.82	-24.4	0.019986
hsa-miR-183-5p	NM_001204285	MUC1	163	-25.4	-28.9	0.015805
hsa-miR-183-5p	NM_001204286	MUC1	163	-25.4	-28.9	0.015805
hsa-miR-183-5p	NM_001204287	MUC1	163	-25.4	-28.9	0.015805
hsa-miR-183-5p	NM_001204288	MUC1	163	-25.4	-28.9	0.025914
hsa-miR-183-5p	NM_001204289	MUC1	163	-25.4	-28.9	0.015805
hsa-miR-183-5p	NM_001204290	MUC1	163	-25.4	-28.9	0.015805
hsa-miR-183-5p	NM_001204291	MUC1	163	-25.4	-28.9	0.015805
hsa-miR-183-5p	NM_001204292	MUC1	163	-25.4	-28.9	0.015805
hsa-miR-183-5p	NM_001204293	MUC1	163	-25.4	-28.9	0.025914
hsa-miR-183-5p	NM_001204294	MUC1	163	-25.4	-28.9	0.015805
hsa-miR-183-5p	NM_001204295	MUC1	163	-25.4	-28.9	0.015805
hsa-miR-183-5p	NM_001204296	MUC1	163	-25.4	-28.9	0.015805
hsa-miR-183-5p	NM_001204297	MUC1	163	-25.4	-28.9	0.015805
hsa-miR-183-5p	NM_002456	MUC1	163	-25.4	-28.9	0.015805
hsa-miR-183-5p	NM_006165	NFRKB	166	-26.38	-31.3	0.028995
hsa-miR-183-5p	NM_020142	NDUFA4L2	166	-28.56	-31.3	0.01631
hsa-miR-183-5p	NM_032752	ZNF496	155	-16.65	-28	0.011437

hsa-miR-183-5p	NM_130901	OTUD7A	151	-24.51	-26.2	0.023613
hsa-miR-183-5p	NM_173589	DNHD1	166	-25.65	-31.1	0.022439
hsa-miR-183-5p	NR_029688	MIR153-1	150	-23.51	-26.2	0.00665
hsa-miR-183-5p	NR_106786	MIR6728	155	-22.16	-27.3	0.003207
hsa-miR-199b-3p	NM_000029	AGT	158	-28.76	-31.7	0.011599
hsa-miR-199b-3p	NM_001005783	HAO2	156	-24.63	-29	0.012814
hsa-miR-199b-3p	NM_001080435	WHAMM	154	-18.01	-32.1	0.031658
hsa-miR-199b-3p	NM_001163812	C10orf2	182	-29.18	-32.6	0.022716
hsa-miR-199b-3p	NM_001163813	C10orf2	182	-29.18	-32.6	0.01356
hsa-miR-199b-3p	NM_001163814	C10orf2	182	-29.18	-32.6	0.022716
hsa-miR-199b-3p	NM_001202431	PRDX1	152	-22.74	-28.4	0.018292
hsa-miR-199b-3p	NM_001282957	CFAP77	156	-25.98	-30.1	0.039683
hsa-miR-199b-3p	NM_002574	PRDX1	152	-22.74	-28.4	0.018292
hsa-miR-199b-3p	NM_002840	PTPRF	182	-31.31	-34.3	0.016067
hsa-miR-199b-3p	NM_003686	EXO1	154	-26.84	-29.4	0.023639
hsa-miR-199b-3p	NM_004419	DUSP5	172	-25.26	-31.5	0.032713
hsa-miR-199b-3p	NM_006826	YWHAQ	158	-28.88	-32.1	0.031449
hsa-miR-199b-3p	NM_016527	HAO2	156	-24.63	-29	0.012814
hsa-miR-199b-3p	NM_021619	PRDM12	160	-26.73	-32	0.03328
hsa-miR-199b-3p	NM_021830	C10orf2	182	-29.18	-32.6	0.01356
hsa-miR-199b-3p	NM_130440	PTPRF	182	-31.31	-34.3	0.016067
hsa-miR-199b-3p	NM_152416	NDUFAF6	155	-25.3	-31.2	0.021795
hsa-miR-199b-3p	NM_181696	PRDX1	152	-22.74	-28.4	0.018292
hsa-miR-199b-3p	NM_181697	PRDX1	152	-22.74	-28.4	0.018292
hsa-miR-199b-3p	NM_207417	CFAP77	156	-25.98	-30.1	0.039683
hsa-miR-221-5p	NM_001029991	METTL17	159	-18	-26.8	0.010958
hsa-miR-221-5p	NM_001304813	TMEM101	165	-24.26	-29.2	0.045685
hsa-miR-221-5p	NM_001304814	TMEM101	165	-24.26	-29.2	0.045685
hsa-miR-221-5p	NM_005642	TAF7	159	-25.05	-29.3	0.023748
hsa-miR-221-5p	NM_006642	SDCCAG8	158	-26.05	-29.5	0.008813

hsa-miR-221-5p	NM_022734	METTL17	159	-18	-26.8	0.009963
hsa-miR-221-5p	NM_032376	TMEM101	165	-24.26	-29.2	0.045685
hsa-miR-221-5p	NR_029635	MIR221	152	-26.22	-28.6	0.001775
hsa-miR-221-5p	NR_037423	MIR3650	152	-18.6	-24	0.009105
hsa-miR-221-5p	NR_039687	MIR4476	152	-17.36	-22.4	0.037452
hsa-miR-221-5p	NR_120676	NEURL1-AS1	170	-25.48	-28.9	0.028359
hsa-let-7g-5p	NM_000375	UROS	150	-22.35	-26.8	0.037463
hsa-let-7g-5p	NM_001035235	SRA1	160	-26.99	-31	0.014826
hsa-let-7g-5p	NM_001166269	HAUS4	151	-21.91	-27.2	0.031268
hsa-let-7g-5p	NM_001166270	HAUS4	151	-21.91	-27.2	0.031268
hsa-let-7g-5p	NM_001178135	PUDP	161	-19.48	-24.2	0.035799
hsa-let-7g-5p	NM_001195010	GRHL3	151	-25.67	-29.7	0.043798
hsa-let-7g-5p	NM_001202509	CKLF-CMTM1	150	-21.5	-25.7	0.017343
hsa-let-7g-5p	NM_001204099	CKLF-CMTM1	150	-21.5	-25.7	0.017343
hsa-let-7g-5p	NM_001211	BUB1B	152	-24.68	-29	0.017605
hsa-let-7g-5p	NM_001253764	SRA1	160	-26.99	-31	0.014826
hsa-let-7g-5p	NM_001289174	VPS52	168	-24.95	-28.3	0.049955
hsa-let-7g-5p	NM_001289175	VPS52	168	-24.95	-28.3	0.049955
hsa-let-7g-5p	NM_001289176	VPS52	168	-24.95	-28.3	0.049955
hsa-let-7g-5p	NM_002188	IL13	170	-27.78	-31.2	0.020702
hsa-let-7g-5p	NM_002696	POLR2G	155	-22.52	-26.2	0.029465
hsa-let-7g-5p	NM_005212	CSN3	159	-24.22	-25.8	0.034926
hsa-let-7g-5p	NM_017815	HAUS4	151	-21.91	-27.2	0.031268
hsa-let-7g-5p	NM_021180	GRHL3	151	-25.67	-29.7	0.043798
hsa-let-7g-5p	NM_022553	VPS52	168	-24.95	-28.3	0.049955
hsa-let-7g-5p	NM_052999	CMTM1	150	-21.5	-25.7	0.017343
hsa-let-7g-5p	NM_181269	CMTM1	150	-21.5	-25.7	0.017343
hsa-let-7g-5p	NM_181271	CMTM1	150	-21.5	-25.7	0.017343
hsa-let-7g-5p	NM_181272	CMTM1	150	-21.5	-25.7	0.017343
hsa-let-7g-5p	NM_198173	GRHL3	151	-25.67	-29.7	0.043798

hsa-let-7g-5p	NR_029628	MIR215	151	-19.63	-24.6	0.023224
hsa-let-7g-5p	NR_029660	MIRLET7G	152	-27.84	-33.2	0.000047
hsa-let-7g-5p	NR_049873	MIR5690	161	-19.36	-23.7	0.017881
hsa-let-7g-3p	NM_001001710	FAM166A	161	-24.47	-27.8	0.005816
hsa-let-7g-3p	NM_001003694	BRPF1	169	-28.5	-30.8	0.027844
hsa-let-7g-3p	NM_001031740	MANEAL	158	-27.25	-32.9	0.043449
hsa-let-7g-3p	NM_001077241	SLC25A45	154	-30.25	-32.1	0.037259
hsa-let-7g-3p	NM_001077663	URGCP	169	-24.91	-30.3	0.044479
hsa-let-7g-3p	NM_001077664	URGCP	169	-24.91	-30.3	0.044479
hsa-let-7g-3p	NM_001098510	PELI3	157	-27.5	-32.6	0.028041
hsa-let-7g-3p	NM_001113482	MANEAL	158	-27.25	-32.9	0.031906
hsa-let-7g-3p	NM_001128228	TPRN	153	-29.32	-33.6	0.00415
hsa-let-7g-3p	NM_001131015	CIZ1	150	-25.44	-29.3	0.003081
hsa-let-7g-3p	NM_001131016	CIZ1	150	-25.44	-29.3	0.003081
hsa-let-7g-3p	NM_001131017	CIZ1	150	-25.44	-29.3	0.003081
hsa-let-7g-3p	NM_001131018	CIZ1	150	-25.44	-29.3	0.003081
hsa-let-7g-3p	NM_001145411	CCDC110	167	-25.42	-29.8	0.013361
hsa-let-7g-3p	NM_001201484	PIGO	166	-31.93	-32.8	0.004286
hsa-let-7g-3p	NM_001243135	PELI3	157	-27.5	-32.6	0.028041
hsa-let-7g-3p	NM_001243136	PELI3	157	-27.5	-32.6	0.028041
hsa-let-7g-3p	NM_001257975	CIZ1	150	-25.44	-29.3	0.003081
hsa-let-7g-3p	NM_001257976	CIZ1	150	-25.44	-29.3	0.003081
hsa-let-7g-3p	NM_001258390	LAYN	151	-24.87	-33.6	0.020442
hsa-let-7g-3p	NM_001258391	LAYN	151	-24.87	-33.6	0.020442
hsa-let-7g-3p	NM_001261414	SOX5	170	-33.11	-33.7	0.034927
hsa-let-7g-3p	NM_001261415	SOX5	170	-33.11	-33.7	0.034927
hsa-let-7g-3p	NM_001270428	LMO1	168	-31.16	-34.8	0.000888
hsa-let-7g-3p	NM_001272051	C16orf91	150	-23.41	-29.2	0.039287
hsa-let-7g-3p	NM_001278250	SLC25A45	154	-30.25	-32.1	0.037259
hsa-let-7g-3p	NM_001278251	SLC25A45	154	-30.25	-32.1	0.037259

hsa-let-7g-3p	NM_001282500	TTC1	153	-27.87	-31.3	0.012656
hsa-let-7g-3p	NM_001286527	ATP2C2	174	-34.21	-34.7	0.002015
hsa-let-7g-3p	NM_001290075	URGCP	169	-24.91	-30.3	0.044479
hsa-let-7g-3p	NM_001290076	URGCP	169	-24.91	-30.3	0.044479
hsa-let-7g-3p	NM_001291454	ATP2C2	174	-34.21	-34.7	0.002015
hsa-let-7g-3p	NM_001292032	ZNF76	173	-31.94	-35.4	0.003509
hsa-let-7g-3p	NM_001300820	SLC25A45	154	-30.25	-32.1	0.037259
hsa-let-7g-3p	NM_001308069	XXYLT1	157	-27.93	-32.1	0.047211
hsa-let-7g-3p	NM_001314017	CPNE5	151	-23.97	-29.3	0.012365
hsa-let-7g-3p	NM_001407	CELSR3	154	-32.89	-37.6	0.005354
hsa-let-7g-3p	NM_002213	ITGB5	161	-26.11	-32.7	0.044737
hsa-let-7g-3p	NM_002315	LMO1	168	-31.16	-34.8	0.000888
hsa-let-7g-3p	NM_003314	TTC1	153	-27.87	-31.3	0.012656
hsa-let-7g-3p	NM_003427	ZNF76	173	-31.94	-35.4	0.003509
hsa-let-7g-3p	NM_004634	BRPF1	169	-28.5	-30.8	0.027844
hsa-let-7g-3p	NM_006940	SOX5	170	-33.11	-33.7	0.034927
hsa-let-7g-3p	NM_012127	CIZ1	150	-25.44	-29.3	0.003081
hsa-let-7g-3p	NM_014698	TMEM63A	155	-30.85	-33.3	0.026746
hsa-let-7g-3p	NM_014712	SETD1A	163	-27.41	-33.3	0.007617
hsa-let-7g-3p	NM_014861	ATP2C2	174	-34.21	-34.7	0.002015
hsa-let-7g-3p	NM_017920	URGCP	169	-24.91	-30.3	0.044479
hsa-let-7g-3p	NM_032634	PIGO	166	-31.93	-32.8	0.004286
hsa-let-7g-3p	NM_032928	TMEM141	164	-21.66	-31.8	0.009994
hsa-let-7g-3p	NM_145065	PELI3	157	-27.5	-32.6	0.028041
hsa-let-7g-3p	NM_152496	MANEAL	158	-27.25	-32.9	0.031906
hsa-let-7g-3p	NM_152531	XXYLT1	157	-27.93	-32.1	0.046983
hsa-let-7g-3p	NM_152775	CCDC110	167	-25.42	-29.8	0.013361
hsa-let-7g-3p	NM_152850	PIGO	166	-31.93	-32.8	0.004286
hsa-let-7g-3p	NM_152989	SOX5	170	-33.11	-33.7	0.034927
hsa-let-7g-3p	NM_178010	SOX5	170	-33.11	-33.7	0.034927

hsa-let-7g-3p	NM_178834	LAYN	151	-24.87	-33.6	0.020442
hsa-let-7g-3p	NM_182556	SLC25A45	154	-30.25	-32.1	0.037259
hsa-let-7g-3p	NR_029477	MIRLET7A2	155	-25.95	-23.2	0.036384
hsa-let-7g-3p	NR_029660	MIRLET7G	159	-27.09	-30.6	0.000474
hsa-let-7g-3p	NR_046730	AGBL5-AS1	154	-28.6	-29	0.031028
hsa-let-7g-3p	NR_073006	LMO1	168	-31.16	-34.8	0.013737
hsa-let-7g-3p	NR_110179	LOC101060385	158	-28.7	-32.5	0.012981
hsa-miR-1-3p	NM_001199119	TRIM39-	172	-19.27	-23.2	0.006401
hsa-miR-1-3p	NM_001199120	RPP21	172	-19.27	-23.2	0.006401
hsa-miR-1-3p	NM_001199121	RPP21	172	-19.27	-23.2	0.023153
hsa-miR-1-3p	NM_005562	LAMC2	168	-26.33	-30.9	0.049015
hsa-miR-1-3p	NM_024839	RPP21	172	-19.27	-23.2	0.006401
hsa-miR-1-3p	NM_178532	RNF180	151	-19.37	-22.8	0.035471
hsa-miR-23b-3p	NM_001127487	FLNC	172	-24.57	-29.8	0.038954
hsa-miR-23b-3p	NM_001289055	PIGG	159	-25.55	-31.1	0.035608
hsa-miR-23b-3p	NM_001289057	PIGG	159	-25.55	-31.1	0.038216
hsa-miR-23b-3p	NM_001458	FLNC	172	-24.57	-29.8	0.038954
hsa-miR-23b-3p	NM_018462	BRK1	162	-28.73	-31.6	0.021085
hsa-miR-23b-3p	NM_152610	CCDC185	160	-21.54	-24.4	0.034961
hsa-miR-23b-3p	NR_030623	MIR216B	160	-21.06	-25.5	0.007588
hsa-miR-23b-3p	NR_120458	LOC100507195	154	-26.53	-30.2	0.020803
hsa-miR-27b-3p	NM_000531	OTC	152	-21.22	-27	0.048545
hsa-miR-27b-3p	NM_001012959	DISC1	161	-23.88	-28.4	0.00286
hsa-miR-27b-3p	NM_001026	RPS24	152	-24.89	-28.6	0.00107
hsa-miR-27b-3p	NM_001040097	MOSPD3	160	-21.23	-28	0.00875
hsa-miR-27b-3p	NM_001040098	MOSPD3	160	-21.23	-28	0.00875
hsa-miR-27b-3p	NM_001040099	MOSPD3	160	-21.23	-28	0.00875
hsa-miR-27b-3p	NM_001136493	MFSD2A	160	-24.87	-30.2	0.009951
hsa-miR-27b-3p	NM_001142282	RPS24	152	-24.89	-28.6	0.002181
hsa-miR-27b-3p	NM_001142283	RPS24	152	-24.89	-28.6	0.002845

hsa-miR-27b-3p	NM_001142284	RPS24	152	-24.89	-28.6	0.002845
hsa-miR-27b-3p	NM_001195626	MLLT10	151	-29.14	-32	0.038886
hsa-miR-27b-3p	NM_001282626	LMNA	153	-26.42	-30.3	0.041247
hsa-miR-27b-3p	NM_001282629	GBGT1	156	-24.63	-30.7	0.023239
hsa-miR-27b-3p	NM_001282632	GBGT1	156	-24.63	-30.7	0.016763
hsa-miR-27b-3p	NM_001287808	MFSD2A	160	-24.87	-30.2	0.009951
hsa-miR-27b-3p	NM_001287809	MFSD2A	160	-24.87	-30.2	0.009951
hsa-miR-27b-3p	NM_001288572	GBGT1	156	-24.63	-30.7	0.016763
hsa-miR-27b-3p	NM_001288573	GBGT1	156	-24.63	-30.7	0.016763
hsa-miR-27b-3p	NM_001288776	E4F1	164	-23.77	-29.7	0.019715
hsa-miR-27b-3p	NM_001300838	CIART	157	-19.54	-24.5	0.036819
hsa-miR-27b-3p	NM_001300839	CIART	157	-19.54	-24.5	0.036819
hsa-miR-27b-3p	NM_001300840	CIART	157	-19.54	-24.5	0.036819
hsa-miR-27b-3p	NM_001300841	CIART	157	-19.54	-24.5	0.036819
hsa-miR-27b-3p	NM_001306200	SETD7	159	-26.05	-30	0.019252
hsa-miR-27b-3p	NM_001308093	GATA4	153	-27.08	-31.5	0.04404
hsa-miR-27b-3p	NM_001308094	GATA4	153	-27.08	-31.5	0.04404
hsa-miR-27b-3p	NM_002052	GATA4	153	-27.08	-31.5	0.04404
hsa-miR-27b-3p	NM_004422	DVL2	171	-24.26	-29.8	0.019644
hsa-miR-27b-3p	NM_004641	MLLT10	151	-29.14	-32	0.040459
hsa-miR-27b-3p	NM_004913	VPS9D1	155	-25.81	-29	0.049954
hsa-miR-27b-3p	NM_007364	TMED3	163	-31.39	-31.4	0.01182
hsa-miR-27b-3p	NM_013254	TBK1	170	-29.85	-33.8	0.004522
hsa-miR-27b-3p	NM_015104	ATG2A	167	-30.11	-32.9	0.002782
hsa-miR-27b-3p	NM_016610	TLR8	178	-27.33	-32.2	0.016816
hsa-miR-27b-3p	NM_021221	LY6G5B	163	-22.19	-25.3	0.0328
hsa-miR-27b-3p	NM_021996	GBGT1	156	-24.63	-30.7	0.016763
hsa-miR-27b-3p	NM_023945	MS4A5	151	-15.58	-20.5	0.027447
hsa-miR-27b-3p	NM_023948	MOSPD3	160	-21.23	-28	0.00875
hsa-miR-27b-3p	NM_024789	TMEM180	151	-26.47	-31.3	0.028444

hsa-miR-27b-3p	NM_032793	MFSD2A	160	-24.87	-30.2	0.009951
hsa-miR-27b-3p	NM_033022	RPS24	152	-24.89	-28.6	0.002181
hsa-miR-27b-3p	NM_138636	TLR8	178	-27.33	-32.2	0.016816
hsa-miR-27b-3p	NM_144697	CIART	157	-19.54	-24.5	0.036819
hsa-miR-27b-3p	NM_145238	ZSCAN20	165	-27.18	-31.1	0.0252
hsa-miR-27b-3p	NM_170707	LMNA	153	-26.42	-30.3	0.041247
hsa-miR-27b-3p	NM_170708	LMNA	153	-26.42	-30.3	0.041247
hsa-miR-27b-3p	NR_033866	LOC390705	170	-25.46	-29.3	0.018722
hsa-miR-27b-3p	NR_039642	MIR4440	150	-18.81	-28.2	0.001753
hsa-miR-27b-3p	NR_104664	LOC102467147	162	-29.04	-33.4	0.004493
hsa-miR-27b-3p	NR_110050	LOC101927418	156	-22.1	-29.2	0.018138
hsa-miR-27b-3p	NR_110147	LOC101927123	164	-23.31	-30.4	0.035997
hsa-miR-27b-3p	NR_125868	LOC102723831	163	-25.1	-30.5	0.031948
hsa-miR-27b-3p	NR_126167	TMEM5-AS1	163	-25.7	-28.8	0.037223
hsa-miR-122-5p	NM_001104577	SLC52A1	159	-26.04	-30.4	0.009797
hsa-miR-122-5p	NM_001143962	CAPN8	161	-24.35	-27.5	0.021331
hsa-miR-122-5p	NM_001174092	TMEM185A	168	-25.72	-31.9	0.044316
hsa-miR-122-5p	NM_001253815	SLC52A2	151	-24.99	-26.9	0.040488
hsa-miR-122-5p	NM_001253816	SLC52A2	151	-24.99	-26.9	0.040488
hsa-miR-122-5p	NM_001270940	XPO6	165	-23.47	-30.7	0.018578
hsa-miR-122-5p	NM_002169	IFNA5	165	-25.57	-28.4	0.001091
hsa-miR-122-5p	NM_004312	ARR3	150	-19.23	-24.5	0.014288
hsa-miR-122-5p	NM_015171	XPO6	165	-23.47	-30.7	0.018578
hsa-miR-122-5p	NM_016146	TRAPPC4	167	-25.22	-27.9	0.024056
hsa-miR-122-5p	NM_017986	SLC52A1	159	-26.04	-30.4	0.009797
hsa-miR-122-5p	NM_024531	SLC52A2	151	-24.99	-26.9	0.040488
hsa-miR-122-5p	NM_032508	TMEM185A	168	-25.72	-31.9	0.044316
hsa-miR-122-5p	NM_203373	FBXL22	157	-23.86	-29.7	0.046571
hsa-miR-122-5p	NR_037435	MIR3662	151	-20.52	-24.2	0.028899
hsa-miR-122-5p	NR_126363	LINC01526	154	-28.56	-32.2	0.008473

hsa-miR-122-5p	NR_131248	LINCMD1	154	-22.37	-28	0.037023
hsa-miR-130a-3p	NM_001142625	RAB34	153	-23.94	-26.8	0.035767
hsa-miR-130a-3p	NM_001199829	HORMAD1	152	-25.04	-29.1	0.033747
hsa-miR-130a-3p	NM_001277372	KIAA2012	153	-18.41	-23.9	0.035934
hsa-miR-130a-3p	NM_001282433	KRT23	168	-22.51	-27.5	0.024491
hsa-miR-130a-3p	NM_001282474	AP1G2	154	-19.82	-25.1	0.043421
hsa-miR-130a-3p	NM_001282475	AP1G2	154	-19.82	-25.1	0.043421
hsa-miR-130a-3p	NM_001289807	CRYAB	155	-21.32	-25.7	0.018878
hsa-miR-130a-3p	NM_001289808	CRYAB	155	-21.32	-25.7	0.018878
hsa-miR-130a-3p	NM_001885	CRYAB	155	-21.32	-25.7	0.018878
hsa-miR-130a-3p	NM_003917	AP1G2	154	-19.82	-25.1	0.043421
hsa-miR-130a-3p	NM_004997	MYBPH	154	-21.72	-27.4	0.029036
hsa-miR-130a-3p	NM_015515	KRT23	168	-22.51	-27.5	0.024491
hsa-miR-130a-3p	NM_016491	MRPL37	160	-28.16	-33	0.000177
hsa-miR-130a-3p	NM_021174	CCAR2	152	-24.98	-29.4	0.044675
hsa-miR-130a-3p	NM_032132	HORMAD1	152	-25.04	-29.1	0.033747
hsa-miR-130a-3p	NR_002967	SNORA31	166	-19.79	-24.4	0.034391
hsa-miR-130a-3p	NR_029498	MIR25	161	-26.15	-32.8	0.000056
hsa-miR-130a-3p	NR_029842	MIR301A	152	-20.88	-24.6	0.013239
hsa-miR-130a-3p	NR_039642	MIR4440	150	-20.14	-23.1	0.045276
hsa-miR-135a-5p	NM_001142501	MON1A	172	-28.16	-30.6	0.000908
hsa-miR-135a-5p	NM_001146108	PTGR1	150	-24.07	-26.6	0.012096
hsa-miR-135a-5p	NM_001163991	FAM170A	152	-21.42	-25.3	0.033751
hsa-miR-135a-5p	NM_001168468	PDZD3	155	-16.23	-29.9	0.029846
hsa-miR-135a-5p	NM_001242890	DDC	152	-25.73	-28.2	0.015302
hsa-miR-135a-5p	NM_001244249	APEX1	169	-26.25	-28.6	0.010509
hsa-miR-135a-5p	NM_001258459	PHKG1	165	-26.16	-29.7	0.035855
hsa-miR-135a-5p	NM_001258460	PHKG1	165	-26.16	-29.7	0.035855
hsa-miR-135a-5p	NM_001303264	TSC22D2	155	-26.87	-32	0.030848
hsa-miR-135a-5p	NM_001641	APEX1	169	-26.25	-28.6	0.010509

hsa-miR-135a-5p	NM_006213	PHKG1	165	-26.16	-29.7	0.035855
hsa-miR-135a-5p	NM_012212	PTGR1	150	-24.07	-26.6	0.012096
hsa-miR-135a-5p	NM_014779	TSC22D2	155	-26.87	-32	0.030848
hsa-miR-135a-5p	NM_024791	PDZD3	155	-16.23	-29.9	0.029846
hsa-miR-135a-5p	NM_032355	MON1A	172	-28.16	-30.6	0.000908
hsa-miR-135a-5p	NM_052943	FAM46B	153	-28.68	-32.5	0.011691
hsa-miR-135a-5p	NM_080648	APEX1	169	-26.25	-28.6	0.010509
hsa-miR-135a-5p	NM_080649	APEX1	169	-26.25	-28.6	0.010509
hsa-miR-135a-5p	NM_182761	FAM170A	152	-21.42	-25.3	0.033751
hsa-miR-135a-5p	NR_029893	MIR135B	171	-24.45	-29	0.000959
hsa-miR-140-5p	NM_001018038	VPS13A	158	-29.78	-29.4	0.049128
hsa-miR-140-5p	NM_001044264	MSMP	152	-19.19	-26.2	0.010635
hsa-miR-140-5p	NM_001605	AARS	167	-22.39	-27.2	0.045487
hsa-miR-140-5p	NM_002960	S100A3	152	-23.01	-28.3	0.028191
hsa-miR-140-5p	NM_007359	CASC3	159	-18.99	-33.2	0.031412
hsa-miR-140-5p	NM_175062	RASGEF1C	163	-32.37	-33.5	0.00642
hsa-miR-140-5p	NR_030313	MIR586	165	-22.82	-28.3	0.002213
hsa-miR-140-3p	NM_000360	TH	162	-25.9	-28.5	0.026842
hsa-miR-140-3p	NM_001142572	ZNF669	152	-29.08	-30	0.020684
hsa-miR-140-3p	NM_001145114	RRP12	164	-30.33	-32.7	0.004739
hsa-miR-140-3p	NM_001172557	GOLGA3	153	-24.57	-30.6	0.002368
hsa-miR-140-3p	NM_001173513	TXNRD3	154	-29.36	-33.8	0.010693
hsa-miR-140-3p	NM_001199787	SLC35E2	159	-23.43	-30.3	0.030775
hsa-miR-140-3p	NM_001254755	CYB5D2	151	-28.7	-31.5	0.019194
hsa-miR-140-3p	NM_001254756	CYB5D2	151	-28.7	-31.5	0.019194
hsa-miR-140-3p	NM_001278197	CDK5RAP3	166	-30.77	-33.7	0.00147
hsa-miR-140-3p	NM_001278216	CDK5RAP3	166	-30.77	-33.7	0.00147
hsa-miR-140-3p	NM_001278217	CDK5RAP3	166	-30.77	-33.7	0.00147
hsa-miR-140-3p	NM_001284337	RRP12	164	-30.33	-32.7	0.004739
hsa-miR-140-3p	NM_001285523	OPRM1	150	-18.79	-24	0.010269

hsa-miR-140-3p	NM_001285528	OPRM1	150	-18.79	-24	0.010269
hsa-miR-140-3p	NM_001781	CD69	170	-28.14	-32	0.03167
hsa-miR-140-3p	NM_002027	FNTA	150	-24.01	-30.1	0.027619
hsa-miR-140-3p	NM_003459	SLC30A3	165	-31.61	-33.2	0.011273
hsa-miR-140-3p	NM_007117	TRH	161	-27.81	-31.9	0.020255
hsa-miR-140-3p	NM_015179	RRP12	164	-30.33	-32.7	0.004739
hsa-miR-140-3p	NM_017818	WRAP73	171	-27.21	-30	0.005897
hsa-miR-140-3p	NM_017943	FBXO34	169	-30.66	-33.8	0.015685
hsa-miR-140-3p	NM_021224	ZNF462	163	-25.58	-29.7	0.031485
hsa-miR-140-3p	NM_024789	TMEM180	165	-31.1	-34.3	0.012009
hsa-miR-140-3p	NM_024804	ZNF669	152	-29.08	-30	0.020684
hsa-miR-140-3p	NM_031290	CCDC70	152	-23.5	-27.8	0.011566
hsa-miR-140-3p	NM_032118	WDR54	155	-20.94	-25	0.006616
hsa-miR-140-3p	NM_032222	FAM188B	171	-29.05	-30.6	0.013339
hsa-miR-140-3p	NM_032871	RELT	165	-30.89	-33.2	0.049086
hsa-miR-140-3p	NM_052883	TXNRD3	154	-29.36	-33.8	0.010693
hsa-miR-140-3p	NM_144611	CYB5D2	151	-28.7	-31.5	0.018993
hsa-miR-140-3p	NM_152222	RELT	165	-30.89	-33.2	0.049086
hsa-miR-140-3p	NM_152231	FBXO34	169	-30.66	-33.8	0.005144
hsa-miR-140-3p	NM_176096	CDK5RAP3	166	-30.77	-33.7	0.00147
hsa-miR-140-3p	NM_199292	TH	162	-25.9	-28.5	0.026842
hsa-miR-140-3p	NM_199293	TH	162	-25.9	-28.5	0.026842
hsa-miR-140-3p	NM_213636	PDLIM7	162	-25.33	-28.7	0.017211
hsa-miR-140-3p	NR_002950	SNORA2A	154	-21.98	-26.1	0.023341
hsa-miR-140-3p	NR_030278	MIR552	153	-24.05	-25	0.023069
hsa-miR-140-3p	NR_031672	MIR1268A	159	-25.19	-28.9	0.000447
hsa-miR-140-3p	NR_106907	MIR6848	153	-24.48	-25.8	0.007154
hsa-miR-140-3p	NR_120519	LOC102724094	166	-29.87	-32.8	0.004805
hsa-miR-140-3p	NR_120520	LOC102724094	166	-29.87	-32.8	0.004307
hsa-miR-143-3p	NM_001105533	CPED1	154	-26.5	-27.7	0.017386

hsa-miR-143-3p	NM_001135746	NEIL2	162	-22.54	-30.6	0.042966
hsa-miR-143-3p	NM_001135747	NEIL2	162	-22.54	-30.6	0.042966
hsa-miR-143-3p	NM_001135748	NEIL2	162	-22.54	-30.6	0.042966
hsa-miR-143-3p	NM_001144982	CCDC169	161	-19.54	-26.7	0.013676
hsa-miR-143-3p	NM_001144983	CCDC169	161	-19.54	-26.7	0.013676
hsa-miR-143-3p	NM_001144984	CCDC169	161	-19.54	-26.7	0.013676
hsa-miR-143-3p	NM_001166243	FHIT	164	-22.79	-26.1	0.048635
hsa-miR-143-3p	NM_001198908	CCDC169	161	-19.54	-26.7	0.013676
hsa-miR-143-3p	NM_001242890	DDC	158	-19.24	-26.5	0.044605
hsa-miR-143-3p	NM_006708	GLO1	160	-26.48	-31.4	0.041926
hsa-miR-143-3p	NM_012368	OR2C1	157	-18.27	-21	0.040659
hsa-miR-143-3p	NM_145043	NEIL2	162	-22.54	-30.6	0.042966
hsa-miR-143-3p	NM_173846	TPPP2	158	-19.55	-24.1	0.024871
hsa-miR-143-3p	NM_203423	LOC389199	152	-22.93	-26.8	0.044464
hsa-miR-143-3p	NM_207373	C10orf99	171	-24.99	-28.5	0.041669
hsa-miR-143-3p	NR_002584	SNORA69	154	-24.81	-29.5	0.001677
hsa-miR-143-3p	NR_029578	MIR192	150	-22.84	-27.1	0.004964
hsa-miR-143-3p	NR_132338	NAT8B	174	-26.44	-31.5	0.018848
hsa-miR-145-5p	NM_000263	NAGLU	155	-28.72	-34.3	0.000835
hsa-miR-145-5p	NM_000274	OAT	155	-28.37	-32.1	0.020657
hsa-miR-145-5p	NM_000460	THPO	163	-32.63	-35.8	0.002939
hsa-miR-145-5p	NM_001015055	RTKN	177	-26.04	-30.2	0.022598
hsa-miR-145-5p	NM_001015056	RTKN	177	-26.04	-30.2	0.022598
hsa-miR-145-5p	NM_001018016	MUC1	152	-27.66	-28	0.048991
hsa-miR-145-5p	NM_001018017	MUC1	152	-27.66	-28	0.048991
hsa-miR-145-5p	NM_001039457	ATP6V0B	166	-31.31	-35.8	0.000771
hsa-miR-145-5p	NM_001044390	MUC1	152	-27.66	-28	0.048991
hsa-miR-145-5p	NM_001044391	MUC1	152	-27.66	-28	0.048991
hsa-miR-145-5p	NM_001044392	MUC1	152	-27.66	-28	0.048991
hsa-miR-145-5p	NM_001085401	C6orf201	162	-26.82	-32	0.012616

hsa-miR-145-5p	NM_001098637	PWWP2B	160	-34.77	-37.1	0.002713
hsa-miR-145-5p	NM_001128827	DLG4	161	-31.45	-35.8	0.005408
hsa-miR-145-5p	NM_001129765	NSDHL	150	-16.7	-29.5	0.015206
hsa-miR-145-5p	NM_001134462	NOTO	177	-27.09	-30.5	0.0115
hsa-miR-145-5p	NM_001171814	OAT	155	-28.37	-32.1	0.020657
hsa-miR-145-5p	NM_001177597	THPO	163	-32.63	-35.8	0.002939
hsa-miR-145-5p	NM_001177598	THPO	163	-32.63	-35.8	0.003762
hsa-miR-145-5p	NM_001193434	PLEKHS1	156	-31.71	-33.9	0.037365
hsa-miR-145-5p	NM_001193435	PLEKHS1	156	-31.71	-33.9	0.037365
hsa-miR-145-5p	NM_001204285	MUC1	152	-27.66	-28	0.048991
hsa-miR-145-5p	NM_001204286	MUC1	152	-27.66	-28	0.048991
hsa-miR-145-5p	NM_001204287	MUC1	152	-27.66	-28	0.048991
hsa-miR-145-5p	NM_001204289	MUC1	152	-27.66	-28	0.048991
hsa-miR-145-5p	NM_001204290	MUC1	152	-27.66	-28	0.048991
hsa-miR-145-5p	NM_001204291	MUC1	152	-27.66	-28	0.048991
hsa-miR-145-5p	NM_001204292	MUC1	152	-27.66	-28	0.048991
hsa-miR-145-5p	NM_001204294	MUC1	152	-27.66	-28	0.048991
hsa-miR-145-5p	NM_001204295	MUC1	152	-27.66	-28	0.048991
hsa-miR-145-5p	NM_001204296	MUC1	152	-27.66	-28	0.048991
hsa-miR-145-5p	NM_001204297	MUC1	152	-27.66	-28	0.048991
hsa-miR-145-5p	NM_001254750	CD6	153	-23.73	-33.5	0.023478
hsa-miR-145-5p	NM_001254751	CD6	153	-23.73	-33.5	0.023478
hsa-miR-145-5p	NM_001282190	ZC3HC1	157	-24.79	-29.5	0.031538
hsa-miR-145-5p	NM_001282191	ZC3HC1	157	-24.79	-29.5	0.031538
hsa-miR-145-5p	NM_001286496	PIF1	168	-26.87	-30.8	0.040864
hsa-miR-145-5p	NM_001286499	PIF1	168	-26.87	-30.8	0.040864
hsa-miR-145-5p	NM_001289997	THPO	163	-32.63	-35.8	0.003762
hsa-miR-145-5p	NM_001289998	THPO	163	-32.63	-35.8	0.002939
hsa-miR-145-5p	NM_001290003	THPO	163	-32.63	-35.8	0.002939
hsa-miR-145-5p	NM_001290022	THPO	163	-32.63	-35.8	0.002939

hsa-miR-145-5p	NM_001290026	THPO	163	-32.63	-35.8	0.003762
hsa-miR-145-5p	NM_001290027	THPO	163	-32.63	-35.8	0.003762
hsa-miR-145-5p	NM_001290028	THPO	163	-32.63	-35.8	0.002939
hsa-miR-145-5p	NM_001294333	ATP6V0B	166	-31.31	-35.8	0.006234
hsa-miR-145-5p	NM_001297609	MAP3K6	154	-28.64	-32.3	0.002523
hsa-miR-145-5p	NM_001300750	LMBR1L	150	-22.88	-29.8	0.046564
hsa-miR-145-5p	NM_001300751	LMBR1L	150	-22.88	-29.8	0.046564
hsa-miR-145-5p	NM_001304353	CHCHD5	160	-23.01	-25.7	0.04955
hsa-miR-145-5p	NM_001304354	CHCHD5	160	-23.01	-25.7	0.04955
hsa-miR-145-5p	NM_001365	DLG4	161	-31.45	-35.8	0.005408
hsa-miR-145-5p	NM_002452	NUDT1	159	-23.15	-26.8	0.02466
hsa-miR-145-5p	NM_002456	MUC1	152	-27.66	-28	0.048991
hsa-miR-145-5p	NM_004047	ATP6V0B	166	-31.31	-35.8	0.000771
hsa-miR-145-5p	NM_004418	DUSP2	157	-32.3	-35.9	0.003501
hsa-miR-145-5p	NM_004625	WNT7A	172	-35.62	-37.1	0.000605
hsa-miR-145-5p	NM_004672	MAP3K6	154	-28.64	-32.3	0.002523
hsa-miR-145-5p	NM_004673	ANGPTL1	163	-30.61	-33.6	0.040459
hsa-miR-145-5p	NM_004933	CDH15	155	-27.32	-30.2	0.018274
hsa-miR-145-5p	NM_006725	CD6	153	-23.73	-33.5	0.023478
hsa-miR-145-5p	NM_007144	PCGF2	171	-33.53	-36.6	0.008285
hsa-miR-145-5p	NM_014587	SOX8	152	-35.1	-36.3	0.012546
hsa-miR-145-5p	NM_014943	ZHX2	160	-29.94	-32.7	0.044033
hsa-miR-145-5p	NM_015922	NSDHL	150	-16.7	-29.5	0.015206
hsa-miR-145-5p	NM_016170	TLX2	157	-34.34	-36.6	0.004894
hsa-miR-145-5p	NM_016478	ZC3HC1	157	-24.79	-29.5	0.031538
hsa-miR-145-5p	NM_017560	ZNF853	171	-33.06	-34.2	0.023813
hsa-miR-145-5p	NM_018113	LMBR1L	150	-22.88	-29.8	0.046564
hsa-miR-145-5p	NM_024889	PLEKHS1	156	-31.71	-33.9	0.042995
hsa-miR-145-5p	NM_025049	PIF1	168	-26.87	-30.8	0.040864
hsa-miR-145-5p	NM_025108	C16orf59	163	-31.51	-34.2	0.001803

hsa-miR-145-5p	NM_030645	SH3BP5L	167	-30.11	-33.6	0.035405
hsa-miR-145-5p	NM_032309	CHCHD5	160	-23.01	-25.7	0.04955
hsa-miR-145-5p	NM_033046	RTKN	177	-26.04	-30.2	0.022598
hsa-miR-145-5p	NM_080596	HIST1H2AH	163	-19.85	-22.6	0.034085
hsa-miR-145-5p	NM_133639	RHOV	155	-27.56	-32	0.03321
hsa-miR-145-5p	NM_138499	PWWP2B	160	-34.77	-37.1	0.002901
hsa-miR-145-5p	NM_145253	UBALD1	163	-32.95	-35.5	0.005379
hsa-miR-145-5p	NM_198948	NUDT1	159	-23.15	-26.8	0.02466
hsa-miR-145-5p	NM_198949	NUDT1	159	-23.15	-26.8	0.02466
hsa-miR-145-5p	NM_198950	NUDT1	159	-23.15	-26.8	0.02466
hsa-miR-145-5p	NM_198952	NUDT1	159	-23.15	-26.8	0.02466
hsa-miR-145-5p	NM_198953	NUDT1	159	-23.15	-26.8	0.02466
hsa-miR-145-5p	NM_198954	NUDT1	159	-23.15	-26.8	0.02466
hsa-miR-145-5p	NM_199247	CACNB1	152	-21.89	-26.8	0.014414
hsa-miR-145-5p	NM_199248	CACNB1	152	-21.89	-26.8	0.014414
hsa-miR-145-5p	NR_001527	TTTY6	152	-23.18	-30.3	0.032428
hsa-miR-145-5p	NR_002175	TTTY6B	152	-23.18	-30.3	0.030466
hsa-miR-145-5p	NR_040089	ALOX12-AS1	150	-26.93	-30.7	0.041204
hsa-miR-145-5p	NR_104589	OCIAD2	157	-27.12	-30.7	0.03031
hsa-miR-145-5p	NR_106744	MIR378J	160	-21.81	-25.9	0.024365
hsa-miR-145-5p	NR_110900	ZNF213-AS1	160	-30.81	-33.3	0.028132
hsa-miR-145-5p	NR_125994	LOC102724312	150	-30.92	-34.5	0.017974
hsa-miR-145-5p	NR_126423	SAPCD1-AS1	167	-25.61	-30.7	0.039691
hsa-miR-145-5p	NR_132126	TH2LCRR	153	-30.91	-34.9	0.00221
hsa-miR-191-3p	NM_000904	NQO2	150	-25	-27.1	0.041056
hsa-miR-191-3p	NM_001053	SSTR5	163	-37.89	-41.3	0.001148
hsa-miR-191-3p	NM_001099336	C12orf42	154	-22.42	-26.2	0.041183
hsa-miR-191-3p	NM_001127350	MED18	156	-26.03	-36.3	0.005512
hsa-miR-191-3p	NM_001135211	FKBP6	163	-26.78	-32.1	0.01098
hsa-miR-191-3p	NM_001135704	ACBD4	152	-29.14	-32.4	0.012309

hsa-miR-191-3p	NM_001135705	ACBD4	152	-29.14	-32.4	0.01511
hsa-miR-191-3p	NM_001135706	ACBD4	152	-29.14	-32.4	0.012309
hsa-miR-191-3p	NM_001144063	OSBPL5	158	-31.1	-33.9	0.018976
hsa-miR-191-3p	NM_001161415	GPR17	154	-22.72	-33.2	0.018993
hsa-miR-191-3p	NM_001161416	GPR17	154	-22.72	-33.2	0.018993
hsa-miR-191-3p	NM_001161417	GPR17	154	-22.72	-33.2	0.018993
hsa-miR-191-3p	NM_001166356	SHMT2	150	-25.85	-31.1	0.025975
hsa-miR-191-3p	NM_001166357	SHMT2	150	-25.85	-31.1	0.025975
hsa-miR-191-3p	NM_001166358	SHMT2	150	-25.85	-31.1	0.025975
hsa-miR-191-3p	NM_001166359	SHMT2	150	-25.85	-31.1	0.025975
hsa-miR-191-3p	NM_001172560	SSTR5	163	-37.89	-41.3	0.001148
hsa-miR-191-3p	NM_001203258	MTA1	150	-22.02	-33	0.037363
hsa-miR-191-3p	NM_001278419	C12orf42	154	-22.42	-26.2	0.041183
hsa-miR-191-3p	NM_001278420	C12orf42	154	-22.42	-26.2	0.041183
hsa-miR-191-3p	NM_001281304	FKBP6	163	-26.78	-32.1	0.01098
hsa-miR-191-3p	NM_001286526	BCL7C	160	-20.21	-31.7	0.039357
hsa-miR-191-3p	NM_001290221	NQO2	150	-25	-27.1	0.041056
hsa-miR-191-3p	NM_001290222	NQO2	150	-25	-27.1	0.041056
hsa-miR-191-3p	NM_001301168	SHF	158	-30.9	-31	0.039691
hsa-miR-191-3p	NM_001301171	SHF	158	-30.9	-31	0.039691
hsa-miR-191-3p	NM_003602	FKBP6	163	-26.78	-32.1	0.01098
hsa-miR-191-3p	NM_003936	CDK5R2	156	-33.54	-35.9	0.009305
hsa-miR-191-3p	NM_004526	MCM2	163	-29.73	-33.1	0.012524
hsa-miR-191-3p	NM_005291	GPR17	154	-22.72	-33.2	0.018993
hsa-miR-191-3p	NM_005412	SHMT2	150	-25.85	-31.1	0.025975
hsa-miR-191-3p	NM_006651	CPLX1	161	-32.98	-35	0.019607
hsa-miR-191-3p	NM_014207	CD5	150	-33.35	-36.6	0.009927
hsa-miR-191-3p	NM_015916	CALHM2	154	-26.79	-29.2	0.032087
hsa-miR-191-3p	NM_017638	MED18	156	-26.03	-36.3	0.005512
hsa-miR-191-3p	NM_020896	OSBPL5	158	-31.1	-33.9	0.018976

hsa-miR-191-3p	NM_024589	ROGDI	154	-28.57	-30.9	0.022783
hsa-miR-191-3p	NM_024722	ACBD4	152	-29.14	-32.4	0.01511
hsa-miR-191-3p	NM_031866	FZD8	159	-29.9	-41	0.000753
hsa-miR-191-3p	NM_138356	SHF	158	-30.9	-31	0.039691
hsa-miR-191-3p	NM_145638	OSBPL5	158	-31.1	-33.9	0.018976
hsa-miR-191-3p	NM_148910	TIRAP	155	-20.48	-25	0.010686
hsa-miR-191-3p	NM_198179	QRFPR	150	-25.88	-30.4	0.046175
hsa-miR-191-3p	NM_198521	C12orf42	154	-22.42	-26.2	0.041183
hsa-miR-191-3p	NR_002837	UBE2MP1	162	-30.35	-34.1	0.014291
hsa-miR-191-3p	NR_027285	FLVCR1-AS1	157	-27.59	-32.2	0.033119
hsa-miR-191-3p	NR_027286	FLVCR1-AS1	157	-27.59	-32.2	0.020465
hsa-miR-191-3p	NR_027337	HIST2H2BA	165	-29.16	-32.6	0.010458
hsa-miR-191-3p	NR_027761	LOC100132831	155	-32.65	-35.6	0.021677
hsa-miR-191-3p	NR_034139	LOC338963	168	-30.2	-36.7	0.007634
hsa-miR-191-3p	NR_045724	CLTB	166	-30.19	-34.5	0.028304
hsa-miR-191-3p	NR_073497	ESR2	158	-32.88	-35.7	0.016201
hsa-miR-191-3p	NR_104602	LOC101929034	151	-29.14	-33.5	0.006215
hsa-miR-191-3p	NR_108078	MIR202HG	154	-33.04	-39.1	0.001047
hsa-miR-191-3p	NR_108079	MIR202HG	154	-33.04	-39.1	0.000713
hsa-miR-126-5p	NR_031645	MIR548F4	156	-20.36	-22.7	0.044748
hsa-miR-134-5p	NM_000223	KRT12	150	-29.06	-31.3	0.020256
hsa-miR-134-5p	NM_000835	GRIN2C	152	-30.08	-32.2	0.009922
hsa-miR-134-5p	NM_001003676	C11orf49	160	-31.51	-35.4	0.010439
hsa-miR-134-5p	NM_001012502	CFAP157	155	-23.73	-27.4	0.012575
hsa-miR-134-5p	NM_001017963	HSP90AA1	162	-30.06	-32.8	0.029976
hsa-miR-134-5p	NM_001025598	ARHGAP30	161	-29.31	-31	0.04831
hsa-miR-134-5p	NM_001040455	SIDT2	154	-32.11	-35.3	0.016557
hsa-miR-134-5p	NM_001042610	DBNDD1	162	-31.18	-33.9	0.033418
hsa-miR-134-5p	NM_001128918	MARK3	153	-26.46	-35.3	0.004272
hsa-miR-134-5p	NM_001128919	MARK3	153	-26.46	-35.3	0.004272

hsa-miR-134-5p	NM_001128920	MARK3	153	-26.46	-35.3	0.004272
hsa-miR-134-5p	NM_001128921	MARK3	153	-26.46	-35.3	0.004272
hsa-miR-134-5p	NM_001129765	NSDHL	171	-25.49	-28.1	0.033054
hsa-miR-134-5p	NM_001130438	SPTAN1	156	-24.52	-29.5	0.026419
hsa-miR-134-5p	NM_001135954	UCK1	162	-31.28	-33.7	0.033222
hsa-miR-134-5p	NM_001142572	ZNF669	153	-26.88	-30.3	0.025473
hsa-miR-134-5p	NM_001195532	SPTAN1	156	-24.52	-29.5	0.026419
hsa-miR-134-5p	NM_001201372	CCDC136	159	-23.39	-28.7	0.041301
hsa-miR-134-5p	NM_001242521	C1orf234	159	-23.54	-26.3	0.007185
hsa-miR-134-5p	NM_001256416	NBPF3	152	-31.43	-33.3	0.045061
hsa-miR-134-5p	NM_001256417	NBPF3	152	-31.43	-33.3	0.045061
hsa-miR-134-5p	NM_001256896	DOK7	154	-32.49	-32.9	0.028962
hsa-miR-134-5p	NM_001287600	ARHGAP30	161	-29.31	-31	0.04831
hsa-miR-134-5p	NM_001287602	ARHGAP30	161	-29.31	-31	0.04831
hsa-miR-134-5p	NM_001288708	DBNDD1	162	-31.18	-33.9	0.033418
hsa-miR-134-5p	NM_001288709	DBNDD1	162	-31.18	-33.9	0.033418
hsa-miR-134-5p	NM_002376	MARK3	153	-26.46	-35.3	0.004272
hsa-miR-134-5p	NM_003127	SPTAN1	156	-24.52	-29.5	0.026419
hsa-miR-134-5p	NM_003658	BARX2	172	-34.83	-37.8	0.002571
hsa-miR-134-5p	NM_005348	HSP90AA1	162	-30.06	-32.8	0.029976
hsa-miR-134-5p	NM_006401	ANP32B	151	-28.09	-30.3	0.035957
hsa-miR-134-5p	NM_015922	NSDHL	171	-25.49	-28.1	0.033054
hsa-miR-134-5p	NM_015960	CUTC	151	-31.95	-33.3	0.005382
hsa-miR-134-5p	NM_022742	CCDC136	159	-23.39	-28.7	0.041301
hsa-miR-134-5p	NM_024043	DBNDD1	162	-31.18	-33.9	0.033418
hsa-miR-134-5p	NM_024804	ZNF669	153	-26.88	-30.3	0.025473
hsa-miR-134-5p	NM_032264	NBPF3	152	-31.43	-33.3	0.045061
hsa-miR-134-5p	NM_057088	KRT3	159	-28.03	-29.3	0.033111
hsa-miR-134-5p	NM_147156	SGMS1	166	-32.39	-34.5	0.027991
hsa-miR-134-5p	NM_152889	CHST13	154	-32.49	-33.3	0.014701

hsa-miR-134-5p	NM_153266	TMEM151A	167	-29.02	-32.7	0.033074
hsa-miR-134-5p	NM_153339	PUSL1	151	-25.8	-28.6	0.035019
hsa-miR-134-5p	NM_173660	DOK7	154	-32.49	-32.9	0.028962
hsa-miR-134-5p	NM_175575	WFIKKN2	167	-30.9	-32.9	0.044729
hsa-miR-134-5p	NM_175834	KRT79	158	-28.42	-30	0.031059
hsa-miR-134-5p	NM_181720	ARHGAP30	161	-29.31	-31	0.04831
hsa-miR-134-5p	NR_030308	MIR582	157	-24.66	-25.8	0.021672
hsa-miR-134-5p	NR_120444	LOC646522	156	-26.69	-33.1	0.026224
hsa-miR-185-3p	NM_000054	AVPR2	153	-29.29	-32.6	0.009835
hsa-miR-185-3p	NM_000088	COL1A1	163	-37.35	-39.7	0.002702
hsa-miR-185-3p	NM_000113	TOR1A	157	-30.49	-34.7	0.014967
hsa-miR-185-3p	NM_000157	GBA	152	-24.68	-30	0.049382
hsa-miR-185-3p	NM_000162	GCK	157	-26.22	-31.6	0.046446
hsa-miR-185-3p	NM_000195	HPS1	154	-31.46	-33.9	0.032391
hsa-miR-185-3p	NM_000199	SGSH	162	-33.85	-36.7	0.007518
hsa-miR-185-3p	NM_000207	INS	151	-21.14	-24.8	0.02352
hsa-miR-185-3p	NM_000246	CIITA	155	-35.68	-35.3	0.013185
hsa-miR-185-3p	NM_000256	MYBPC3	159	-28.54	-32.5	0.006456
hsa-miR-185-3p	NM_000265	NCF1	155	-25.42	-28.4	0.024083
hsa-miR-185-3p	NM_000347	SPTB	158	-33.54	-33.2	0.002691
hsa-miR-185-3p	NM_000561	GSTM1	154	-26.2	-32.4	0.011369
hsa-miR-185-3p	NM_000577	IL1RN	166	-28.14	-32.9	0.038934
hsa-miR-185-3p	NM_000598	IGFBP3	157	-31.85	-34.7	0.028879
hsa-miR-185-3p	NM_000603	NOS3	156	-25.87	-30.9	0.021942
hsa-miR-185-3p	NM_000683	ADRA2C	150	-29.17	-32.8	0.012414
hsa-miR-185-3p	NM_000803	FOLR2	153	-19	-30.4	0.006357
hsa-miR-185-3p	NM_000848	GSTM2	154	-26.2	-32.4	0.011369
hsa-miR-185-3p	NM_000883	IMPDH1	154	-32.89	-35.5	0.005934
hsa-miR-185-3p	NM_000941	POR	154	-26.84	-30.6	0.020154
hsa-miR-185-3p	NM_000964	RARA	163	-31.27	-33.8	0.035615

hsa-miR-185-3p	NM_001001712	LCN10	158	-30.18	-32.9	0.049406
hsa-miR-185-3p	NM_001004106	GRK6	160	-32.87	-37.2	0.005095
hsa-miR-185-3p	NM_001004432	LINGO4	152	-24.92	-32.2	0.02338
hsa-miR-185-3p	NM_001005336	DNM1	176	-34.73	-39.9	0.000534
hsa-miR-185-3p	NM_001005417	B4GALT2	168	-34.13	-31.2	0.042871
hsa-miR-185-3p	NM_001005741	GBA	152	-24.68	-30	0.049382
hsa-miR-185-3p	NM_001005742	GBA	152	-24.68	-30	0.049382
hsa-miR-185-3p	NM_001006616	TSPAN17	175	-36.05	-41.1	0.001687
hsa-miR-185-3p	NM_001006617	MAPKAP1	180	-41.59	-45.1	0.000285
hsa-miR-185-3p	NM_001006619	MAPKAP1	180	-41.59	-45.1	0.000285
hsa-miR-185-3p	NM_001006620	MAPKAP1	180	-41.59	-45.1	0.000285
hsa-miR-185-3p	NM_001006621	MAPKAP1	180	-41.59	-45.1	0.000285
hsa-miR-185-3p	NM_001007072	ZSCAN2	153	-26.53	-32.6	0.003939
hsa-miR-185-3p	NM_001008226	SAXO2	175	-31.99	-35.7	0.023361
hsa-miR-185-3p	NM_001008710	RBPMS	160	-26.53	-37.3	0.010676
hsa-miR-185-3p	NM_001008711	RBPMS	160	-26.53	-37.3	0.011255
hsa-miR-185-3p	NM_001008800	CCT3	155	-31.83	-34.8	0.000981
hsa-miR-185-3p	NM_001010863	C10orf128	150	-27.93	-32.4	0.049388
hsa-miR-185-3p	NM_001012301	ARSI	151	-26.44	-33.4	0.02069
hsa-miR-185-3p	NM_001012959	DISC1	150	-26.32	-28.9	0.006716
hsa-miR-185-3p	NM_001013398	IGFBP3	157	-31.85	-34.7	0.028879
hsa-miR-185-3p	NM_001014794	ILK	162	-27.65	-33	0.003104
hsa-miR-185-3p	NM_001014795	ILK	162	-27.65	-33	0.003104
hsa-miR-185-3p	NM_001014839	NCDN	150	-33.44	-33	0.035919
hsa-miR-185-3p	NM_001014841	NCDN	150	-33.44	-33	0.035919
hsa-miR-185-3p	NM_001015881	TSC22D3	158	-35.08	-40.8	0.001451
hsa-miR-185-3p	NM_001024809	RARA	163	-31.27	-33.8	0.035615
hsa-miR-185-3p	NM_001024845	SLC6A9	152	-17.5	-34	0.021444
hsa-miR-185-3p	NM_001025205	AP2M1	152	-31.84	-30.2	0.036163
hsa-miR-185-3p	NM_001025295	IFITM5	156	-27.73	-29	0.031074

hsa-miR-185-3p	NM_001030015	OPN4	183	-34.92	-40.8	0.00047
hsa-miR-185-3p	NM_001032367	SPINT1	154	-33.97	-32.7	0.0195
hsa-miR-185-3p	NM_001034833	NHP2	152	-30.42	-32.7	0.005642
hsa-miR-185-3p	NM_001038	SCNN1A	160	-27.85	-33.4	0.026761
hsa-miR-185-3p	NM_001039350	DPP6	154	-29.12	-34.3	0.040392
hsa-miR-185-3p	NM_001039476	NPRL3	151	-30.8	-32	0.041107
hsa-miR-185-3p	NM_001040160	C16orf13	152	-24.38	-29.6	0.002569
hsa-miR-185-3p	NM_001040161	C16orf13	152	-24.38	-29.6	0.008059
hsa-miR-185-3p	NM_001040162	C16orf13	152	-24.38	-29.6	0.008059
hsa-miR-185-3p	NM_001040165	C16orf13	152	-24.38	-29.6	0.002569
hsa-miR-185-3p	NM_001040260	DCLK2	150	-28.47	-33.9	0.027166
hsa-miR-185-3p	NM_001040261	DCLK2	150	-28.47	-33.9	0.027166
hsa-miR-185-3p	NM_001040439	MAPK8IP3	164	-28.69	-33.8	0.038312
hsa-miR-185-3p	NM_001040661	SLC29A4	165	-30.11	-35.5	0.012163
hsa-miR-185-3p	NM_001042376	INS-IGF2	158	-25.99	-31.4	0.002963
hsa-miR-185-3p	NM_001042428	ZNF205	152	-25.29	-30.6	0.005947
hsa-miR-185-3p	NM_001053	SSTR5	151	-32.58	-35.1	0.021891
hsa-miR-185-3p	NM_001077350	NPRL3	151	-30.8	-32	0.041107
hsa-miR-185-3p	NM_001077351	RBM23	151	-23.31	-32.3	0.046932
hsa-miR-185-3p	NM_001077352	RBM23	151	-23.31	-32.3	0.046932
hsa-miR-185-3p	NM_001077525	MTMR14	155	-31.54	-31.2	0.018285
hsa-miR-185-3p	NM_001077526	MTMR14	155	-31.54	-31.2	0.018285
hsa-miR-185-3p	NM_001077621	VPS37D	162	-28.91	-34.4	0.009996
hsa-miR-185-3p	NM_001078650	TMEM134	153	-27.26	-27.9	0.044551
hsa-miR-185-3p	NM_001078651	TMEM134	153	-27.26	-27.9	0.044551
hsa-miR-185-3p	NM_001079518	MED24	153	-29.77	-34.3	0.004154
hsa-miR-185-3p	NM_001080424	KDM6B	158	-29.64	-36.1	0.01125
hsa-miR-185-3p	NM_001080511	CLEC2L	158	-28.41	-34.5	0.007639
hsa-miR-185-3p	NM_001080514	SCX	151	-26.94	-31.1	0.013383
hsa-miR-185-3p	NM_001080779	MYO1C	150	-33.98	-38	0.006567

hsa-miR-185-3p	NM_001080950	MYO1C	150	-33.98	-38	0.006567
hsa-miR-185-3p	NM_001083947	TMPRSS4	159	-34.07	-35.3	0.028976
hsa-miR-185-3p	NM_001097615	POLR2J3	155	-32.8	-36	0.011092
hsa-miR-185-3p	NM_001098510	PELI3	178	-32.84	-39.4	0.002255
hsa-miR-185-3p	NM_001098536	USP5	154	-25.73	-31.2	0.028567
hsa-miR-185-3p	NM_001098673	ATG101	154	-27.59	-31	0.011547
hsa-miR-185-3p	NM_001098835	MS4A15	153	-29.07	-33.2	0.028668
hsa-miR-185-3p	NM_001099335	PHYHIP	162	-32.62	-36.5	0.013927
hsa-miR-185-3p	NM_001099780	PSMB11	158	-28.98	-32	0.043234
hsa-miR-185-3p	NM_001100592	ATP6V0E2	156	-25.15	-35.5	0.010533
hsa-miR-185-3p	NM_001100600	MMD2	156	-32.81	-35.5	0.016905
hsa-miR-185-3p	NM_001100915	KCTD19	155	-26.56	-28.8	0.002463
hsa-miR-185-3p	NM_001102605	IMPDH1	154	-32.89	-35.5	0.005934
hsa-miR-185-3p	NM_001111322	DDX54	159	-28.41	-35.6	0.02144
hsa-miR-185-3p	NM_001113534	FOLR2	153	-19	-30.4	0.006357
hsa-miR-185-3p	NM_001113535	FOLR2	153	-19	-30.4	0.006357
hsa-miR-185-3p	NM_001113536	FOLR2	153	-19	-30.4	0.006357
hsa-miR-185-3p	NM_001114632	JMJD7	171	-37.24	-42	0.000092
hsa-miR-185-3p	NM_001114726	PRRT4	157	-24.67	-36.2	0.006199
hsa-miR-185-3p	NM_001122636	GALNT9	156	-24.27	-32.9	0.020091
hsa-miR-185-3p	NM_001127213	LY6E	158	-31.8	-33.2	0.013315
hsa-miR-185-3p	NM_001127214	ACSF3	167	-34.81	-33.5	0.049425
hsa-miR-185-3p	NM_001127370	CDCA7L	150	-27.55	-36.4	0.011629
hsa-miR-185-3p	NM_001127371	CDCA7L	150	-27.55	-36.4	0.011629
hsa-miR-185-3p	NM_001127464	ZNF469	151	-27.3	-33.6	0.039115
hsa-miR-185-3p	NM_001128203	PLA2G16	150	-27.87	-33.5	0.007273
hsa-miR-185-3p	NM_001128225	SLC39A13	153	-27.79	-32.5	0.045922
hsa-miR-185-3p	NM_001129981	ANKRD2	151	-25.59	-26.6	0.037554
hsa-miR-185-3p	NM_001130012	SLC9A3R2	155	-24.95	-33.3	0.028463
hsa-miR-185-3p	NM_001130029	RELL2	159	-29.86	-31	0.018125

hsa-miR-185-3p	NM_001130099	KIFC3	161	-31.13	-35.4	0.006029
hsa-miR-185-3p	NM_001130100	KIFC3	161	-31.13	-35.4	0.006029
hsa-miR-185-3p	NM_001130442	HRAS	152	-26.89	-32.3	0.00947
hsa-miR-185-3p	NM_001134395	C7orf50	155	-26.75	-32	0.024459
hsa-miR-185-3p	NM_001134396	C7orf50	155	-26.75	-32	0.024459
hsa-miR-185-3p	NM_001134878	KIF9	151	-24.75	-31.1	0.036177
hsa-miR-185-3p	NM_001135639	CNGB1	152	-26.45	-31.8	0.028775
hsa-miR-185-3p	NM_001135917	DOLPP1	163	-32.6	-35.6	0.0166
hsa-miR-185-3p	NM_001135943	MADD	151	-29.1	-32.8	0.027782
hsa-miR-185-3p	NM_001135944	MADD	151	-29.1	-32.8	0.027782
hsa-miR-185-3p	NM_001136108	R3HCC1	156	-24.29	-27.5	0.028698
hsa-miR-185-3p	NM_001136109	CASP5	156	-26.66	-30.4	0.002128
hsa-miR-185-3p	NM_001136110	CASP5	156	-26.66	-30.4	0.002128
hsa-miR-185-3p	NM_001136112	CASP5	156	-26.66	-30.4	0.002128
hsa-miR-185-3p	NM_001136215	ARTN	153	-26.19	-30.3	0.034466
hsa-miR-185-3p	NM_001139490	TRAF3IP1	170	-33.38	-36.5	0.018198
hsa-miR-185-3p	NM_001141973	ATP13A2	159	-26.55	-30.5	0.01142
hsa-miR-185-3p	NM_001142448	SPNS1	152	-24.33	-28.1	0.03602
hsa-miR-185-3p	NM_001142449	SPNS1	152	-24.33	-28.1	0.03602
hsa-miR-185-3p	NM_001142450	SPNS1	152	-24.33	-28.1	0.03602
hsa-miR-185-3p	NM_001142451	SPNS1	152	-24.33	-28.1	0.03602
hsa-miR-185-3p	NM_001142573	IMPDH1	154	-32.89	-35.5	0.005934
hsa-miR-185-3p	NM_001142574	IMPDH1	154	-32.89	-35.5	0.005934
hsa-miR-185-3p	NM_001142575	IMPDH1	154	-32.89	-35.5	0.005934
hsa-miR-185-3p	NM_001142576	IMPDH1	154	-32.89	-35.5	0.005934
hsa-miR-185-3p	NM_001142605	EFTUD2	159	-28.23	-33.1	0.028761
hsa-miR-185-3p	NM_001142853	HES6	152	-26.49	-30.9	0.033487
hsa-miR-185-3p	NM_001142928	LRRC61	152	-23.93	-32.7	0.012371
hsa-miR-185-3p	NM_001143760	EIF5A	160	-29.7	-32.5	0.020076
hsa-miR-185-3p	NM_001143761	EIF5A	160	-29.7	-32.5	0.020076

hsa-miR-185-3p	NM_001143762	EIF5A	160	-29.7	-32.5	0.020076
hsa-miR-185-3p	NM_001143888	BSDC1	153	-30.63	-34.4	0.043987
hsa-miR-185-3p	NM_001143889	BSDC1	153	-30.63	-34.4	0.043987
hsa-miR-185-3p	NM_001143890	BSDC1	153	-30.63	-34.4	0.043987
hsa-miR-185-3p	NM_001143944	LEMD2	157	-28.88	-33.6	0.038733
hsa-miR-185-3p	NM_001143993	RASSF7	156	-24.08	-30.6	0.047727
hsa-miR-185-3p	NM_001143994	RASSF7	156	-24.08	-30.6	0.027943
hsa-miR-185-3p	NM_001144825	RUNDC3A	162	-26.22	-32.4	0.010593
hsa-miR-185-3p	NM_001144889	SLC23A3	152	-27.2	-31.1	0.02145
hsa-miR-185-3p	NM_001144890	SLC23A3	152	-27.2	-31.1	0.02145
hsa-miR-185-3p	NM_001144956	RIIAD1	164	-32.63	-36.8	0.000167
hsa-miR-185-3p	NM_001145031	PLAU	158	-31.18	-32.3	0.037988
hsa-miR-185-3p	NM_001145146	CRHR1	155	-26.63	-34.4	0.017993
hsa-miR-185-3p	NM_001145147	CRHR1	155	-26.63	-34.4	0.017993
hsa-miR-185-3p	NM_001145148	CRHR1	155	-26.63	-34.4	0.017993
hsa-miR-185-3p	NM_001145301	RARA	163	-31.27	-33.8	0.035615
hsa-miR-185-3p	NM_001145302	RARA	163	-31.27	-33.8	0.035615
hsa-miR-185-3p	NM_001145440	TYW1B	154	-29.94	-35.1	0.011788
hsa-miR-185-3p	NM_001145548	ZDHHC7	169	-39.19	-42.2	0.001502
hsa-miR-185-3p	NM_001145657	RAP1GAP	150	-31.48	-35	0.014168
hsa-miR-185-3p	NM_001145658	RAP1GAP	150	-31.48	-35	0.014168
hsa-miR-185-3p	NM_001145961	SLC12A4	152	-22.08	-35.6	0.015483
hsa-miR-185-3p	NM_001145962	SLC12A4	152	-22.08	-35.6	0.015483
hsa-miR-185-3p	NM_001145963	SLC12A4	152	-22.08	-35.6	0.015483
hsa-miR-185-3p	NM_001145964	SLC12A4	152	-22.08	-35.6	0.015483
hsa-miR-185-3p	NM_001146151	AVPR2	153	-29.29	-32.6	0.022836
hsa-miR-185-3p	NM_001146289	P3H1	154	-28.73	-33.4	0.008326
hsa-miR-185-3p	NM_001146314	ABHD14B	150	-27.72	-34.4	0.014287
hsa-miR-185-3p	NM_001150	ANPEP	154	-27.78	-34	0.005272
hsa-miR-185-3p	NM_001159575	SCNN1A	160	-27.85	-33.4	0.026761

hsa-miR-185-3p	NM_001159576	SCNN1A	160	-27.85	-33.4	0.026761
hsa-miR-185-3p	NM_001159727	PLBD2	163	-31.15	-33.1	0.030453
hsa-miR-185-3p	NM_001161527	TNIP2	155	-26.08	-32.6	0.01658
hsa-miR-185-3p	NM_001163499	FBXO24	154	-30.9	-37.2	0.000225
hsa-miR-185-3p	NM_001164673	DOK7	154	-32.84	-34.1	0.04089
hsa-miR-185-3p	NM_001164716	PYGM	152	-22.02	-29	0.025995
hsa-miR-185-3p	NM_001164	APBB1	154	-27.38	-33.4	0.005891
hsa-miR-185-3p	NM_001165036	OGDH	162	-32.72	-35.6	0.010633
hsa-miR-185-3p	NM_001165937	STARD3	150	-20.5	-34.1	0.027447
hsa-miR-185-3p	NM_001165938	STARD3	150	-20.5	-34.1	0.027447
hsa-miR-185-3p	NM_001166212	CLCF1	160	-29.42	-33.2	0.026719
hsa-miR-185-3p	NM_001166424	PGC	153	-23.55	-27.9	0.013546
hsa-miR-185-3p	NM_001166426	WDR13	150	-29.5	-29.6	0.007324
hsa-miR-185-3p	NM_001167827	TFEB	150	-30.29	-33.3	0.012701
hsa-miR-185-3p	NM_001167947	TIMM17B	158	-25.08	-29	0.027999
hsa-miR-185-3p	NM_001168347	NSUN5	155	-25.72	-34.4	0.013457
hsa-miR-185-3p	NM_001168348	NSUN5	155	-25.72	-34.4	0.018407
hsa-miR-185-3p	NM_001171087	CLCN2	151	-28.37	-29.3	0.046238
hsa-miR-185-3p	NM_001171088	CLCN2	151	-28.37	-29.3	0.046238
hsa-miR-185-3p	NM_001171089	CLCN2	151	-28.37	-29.3	0.046238
hsa-miR-185-3p	NM_001171092	CLDN2	166	-31.07	-35.2	0.030475
hsa-miR-185-3p	NM_001171095	CLDN2	166	-31.07	-35.2	0.030475
hsa-miR-185-3p	NM_001171811	GBA	152	-24.68	-30	0.049382
hsa-miR-185-3p	NM_001171812	GBA	152	-24.68	-30	0.049382
hsa-miR-185-3p	NM_001172425	LEFTY2	163	-29.79	-33.5	0.018894
hsa-miR-185-3p	NM_001172560	SSTR5	151	-32.58	-35.1	0.021891
hsa-miR-185-3p	NM_001172668	ZNF668	159	-26.62	-29.7	0.012508
hsa-miR-185-3p	NM_001172669	ZNF668	159	-26.62	-29.7	0.013039
hsa-miR-185-3p	NM_001172670	ZNF668	159	-26.62	-29.7	0.013039
hsa-miR-185-3p	NM_001173551	TMPRSS4	159	-34.07	-35.3	0.028976

hsa-miR-185-3p	NM_001173552	TMPRSS4	159	-34.07	-35.3	0.028976
hsa-miR-185-3p	NM_001174100	PCBP4	152	-22.64	-31.8	0.024843
hsa-miR-185-3p	NM_001185012	NDUFA2	150	-18.83	-30	0.023156
hsa-miR-185-3p	NM_001185060	AQP1	150	-22.29	-34.9	0.033257
hsa-miR-185-3p	NM_001185061	AQP1	150	-22.29	-34.9	0.033257
hsa-miR-185-3p	NM_001185062	AQP1	150	-22.29	-34.9	0.033257
hsa-miR-185-3p	NM_001185097	INS	151	-21.14	-24.8	0.02352
hsa-miR-185-3p	NM_001185098	INS	151	-21.14	-24.8	0.02352
hsa-miR-185-3p	NM_001190415	TAF6	150	-22.82	-26.4	0.048837
hsa-miR-185-3p	NM_001190728	RALGPS1	167	-33.85	-37.4	0.000949
hsa-miR-185-3p	NM_001190729	RALGPS1	167	-33.85	-37.4	0.000949
hsa-miR-185-3p	NM_001190996	ARPC1A	171	-31.73	-38.3	0.000331
hsa-miR-185-3p	NM_001194958	KCNJ18	152	-27.24	-32.3	0.015439
hsa-miR-185-3p	NM_001199161	USP19	151	-30.81	-29.8	0.028928
hsa-miR-185-3p	NM_001199162	USP19	151	-30.81	-29.8	0.028928
hsa-miR-185-3p	NM_001199173	MLST8	152	-25.15	-31.1	0.028773
hsa-miR-185-3p	NM_001199174	MLST8	152	-25.15	-31.1	0.028773
hsa-miR-185-3p	NM_001199175	MLST8	152	-25.15	-31.1	0.028773
hsa-miR-185-3p	NM_001199653	PMF1	168	-31.21	-36.7	0.001624
hsa-miR-185-3p	NM_001199654	PMF1	158	-24.09	-32.1	0.014482
hsa-miR-185-3p	NM_001199797	PTPN7	163	-35.65	-34.9	0.026236
hsa-miR-185-3p	NM_001202558	CHURC1-	161	-30.84	-33.1	0.044653
hsa-miR-185-3p	NM_001202559	CHURC1-	161	-30.84	-33.1	0.044653
hsa-miR-185-3p	NM_001204426	LIMK1	156	-23.42	-33.2	0.037549
hsa-miR-185-3p	NM_001206559	POLR1D	154	-32.19	-37.4	0.007909
hsa-miR-185-3p	NM_001206878	CTDSP1	174	-33.2	-38.3	0.005625
hsa-miR-185-3p	NM_001207014	SERPINH1	155	-33.96	-39.7	0.000815
hsa-miR-185-3p	NM_001207071	FAM181A	156	-27.82	-31	0.022029
hsa-miR-185-3p	NM_001207072	FAM181A	156	-27.82	-31	0.022029
hsa-miR-185-3p	NM_001207073	FAM181A	156	-27.82	-31	0.022029

hsa-miR-185-3p	NM_001207074	FAM181A	156	-27.82	-31	0.022029
hsa-miR-185-3p	NM_001235	SERPINH1	155	-33.96	-39.7	0.000815
hsa-miR-185-3p	NM_001243108	PLD2	153	-26.91	-30.1	0.049853
hsa-miR-185-3p	NM_001243135	PELI3	178	-32.84	-39.4	0.002255
hsa-miR-185-3p	NM_001243136	PELI3	178	-32.84	-39.4	0.002255
hsa-miR-185-3p	NM_001243246	P3H1	154	-28.73	-33.4	0.010714
hsa-miR-185-3p	NM_001243247	NPRL3	151	-30.8	-32	0.041107
hsa-miR-185-3p	NM_001243248	NPRL3	151	-30.8	-32	0.041107
hsa-miR-185-3p	NM_001243249	NPRL3	151	-30.8	-32	0.041107
hsa-miR-185-3p	NM_001243259	HINFP	155	-34.98	-32.6	0.032066
hsa-miR-185-3p	NM_001243279	ACSF3	167	-34.81	-33.5	0.049425
hsa-miR-185-3p	NM_001243535	ANKRD65	160	-32.02	-33.4	0.018728
hsa-miR-185-3p	NM_001243536	ANKRD65	160	-32.05	-33.4	0.030351
hsa-miR-185-3p	NM_001243538	LOC283710	161	-31.24	-33.7	0.007583
hsa-miR-185-3p	NM_001243925	MAPKAPK3	158	-29.76	-33.1	0.040445
hsa-miR-185-3p	NM_001243926	MAPKAPK3	158	-29.76	-33.1	0.040445
hsa-miR-185-3p	NM_001244014	PIANP	159	-30.25	-36.7	0.01261
hsa-miR-185-3p	NM_001244015	PIANP	159	-30.25	-36.7	0.008279
hsa-miR-185-3p	NM_001244666	STX5	163	-27.98	-32.1	0.029912
hsa-miR-185-3p	NM_001251882	VIPR1	164	-28.35	-35.4	0.015534
hsa-miR-185-3p	NM_001251883	VIPR1	164	-28.35	-35.4	0.015534
hsa-miR-185-3p	NM_001251884	VIPR1	164	-28.35	-35.4	0.015534
hsa-miR-185-3p	NM_001251885	VIPR1	164	-28.35	-35.4	0.015534
hsa-miR-185-3p	NM_001252073	SLC9A3R2	155	-24.95	-33.3	0.028463
hsa-miR-185-3p	NM_001252075	SLC9A3R2	155	-24.95	-33.3	0.028463
hsa-miR-185-3p	NM_001252076	SLC9A3R2	155	-24.95	-33.3	0.028463
hsa-miR-185-3p	NM_001252607	THBS3	158	-30.68	-35.9	0.000627
hsa-miR-185-3p	NM_001252608	THBS3	158	-30.68	-35.9	0.000627
hsa-miR-185-3p	NM_001254750	CD6	153	-27.66	-32.3	0.045293
hsa-miR-185-3p	NM_001254751	CD6	153	-27.66	-32.3	0.045293

hsa-miR-185-3p	NM_001254753	ABHD14B	150	-27.72	-34.4	0.014287
hsa-miR-185-3p	NM_001256134	PROZ	153	-23.94	-28.8	0.030457
hsa-miR-185-3p	NM_001256160	ATF7IP2	159	-29.06	-33.8	0.047184
hsa-miR-185-3p	NM_001256185	SNX12	165	-33.57	-37.7	0.009261
hsa-miR-185-3p	NM_001256186	SNX12	165	-33.57	-37.7	0.009847
hsa-miR-185-3p	NM_001256187	SNX12	165	-33.57	-37.7	0.009847
hsa-miR-185-3p	NM_001256188	SNX12	165	-33.57	-37.7	0.009261
hsa-miR-185-3p	NM_001256264	FLII	153	-23.49	-30.4	0.017152
hsa-miR-185-3p	NM_001256265	FLII	153	-23.49	-30.4	0.017152
hsa-miR-185-3p	NM_001256299	MGC57346-CRHR1	155	-26.63	-34.4	0.017993
hsa-miR-185-3p	NM_001256615	LOC149373	158	-31.89	-35.3	0.009476
hsa-miR-185-3p	NM_001256672	STOML1	152	-24.96	-31.1	0.044514
hsa-miR-185-3p	NM_001256673	STOML1	152	-24.96	-31.1	0.044514
hsa-miR-185-3p	NM_001256674	STOML1	152	-24.96	-31.1	0.044514
hsa-miR-185-3p	NM_001256675	STOML1	152	-24.96	-31.1	0.044514
hsa-miR-185-3p	NM_001256676	STOML1	152	-24.96	-31.1	0.044514
hsa-miR-185-3p	NM_001256677	STOML1	152	-24.96	-31.1	0.044514
hsa-miR-185-3p	NM_001256799	GAPDH	152	-27.08	-32.3	0.002715
hsa-miR-185-3p	NM_001256896	DOK7	154	-32.84	-34.1	0.018201
hsa-miR-185-3p	NM_001257319	APBB1	154	-27.38	-33.4	0.005891
hsa-miR-185-3p	NM_001257320	APBB1	154	-27.38	-33.4	0.005891
hsa-miR-185-3p	NM_001257321	APBB1	154	-27.38	-33.4	0.005891
hsa-miR-185-3p	NM_001257323	APBB1	154	-27.38	-33.4	0.005891
hsa-miR-185-3p	NM_001257325	APBB1	154	-27.38	-33.4	0.005891
hsa-miR-185-3p	NM_001257326	APBB1	154	-27.38	-33.4	0.005891
hsa-miR-185-3p	NM_001257328	ARRB2	154	-27.77	-32.5	0.011287
hsa-miR-185-3p	NM_001257329	ARRB2	154	-27.77	-32.5	0.015981
hsa-miR-185-3p	NM_001257330	ARRB2	154	-27.77	-32.5	0.011287
hsa-miR-185-3p	NM_001257331	ARRB2	154	-27.77	-32.5	0.011287

hsa-miR-185-3p	NM_001258353	EFTUD2	159	-28.23	-33.1	0.028761
hsa-miR-185-3p	NM_001258354	EFTUD2	159	-28.23	-33.1	0.028761
hsa-miR-185-3p	NM_001261380	SLC6A9	152	-17.5	-34	0.021444
hsa-miR-185-3p	NM_001261835	BZRAP1	154	-29.9	-34.5	0.022011
hsa-miR-185-3p	NM_001267774	IFT20	158	-21.42	-29.4	0.029318
hsa-miR-185-3p	NM_001267775	IFT20	158	-21.42	-29.4	0.029318
hsa-miR-185-3p	NM_001267776	IFT20	158	-21.42	-29.4	0.029318
hsa-miR-185-3p	NM_001267777	IFT20	158	-21.42	-29.4	0.026085
hsa-miR-185-3p	NM_001267778	IFT20	158	-21.42	-29.4	0.040168
hsa-miR-185-3p	NM_001267782	AMBRA1	160	-28.62	-32.1	0.048145
hsa-miR-185-3p	NM_001267783	AMBRA1	160	-28.62	-32.1	0.048145
hsa-miR-185-3p	NM_001267797	MED24	153	-29.77	-34.3	0.004154
hsa-miR-185-3p	NM_001270375	MMD2	156	-32.81	-35.5	0.020724
hsa-miR-185-3p	NM_001270768	STRIP1	167	-31.44	-34.8	0.008204
hsa-miR-185-3p	NM_001271082	NKD2	172	-31.68	-34.9	0.013849
hsa-miR-185-3p	NM_001271711	SLC2A8	163	-35.39	-37.4	0.002231
hsa-miR-185-3p	NM_001271712	SLC2A8	163	-35.39	-37.4	0.001933
hsa-miR-185-3p	NM_001271734	COPZ1	152	-30.53	-33.6	0.03543
hsa-miR-185-3p	NM_001271735	COPZ1	152	-30.53	-33.6	0.03543
hsa-miR-185-3p	NM_001271736	COPZ1	152	-30.53	-33.6	0.03543
hsa-miR-185-3p	NM_001271856	GRASP	161	-30.82	-35	0.006809
hsa-miR-185-3p	NM_001271943	TFEB	150	-30.29	-33.3	0.012701
hsa-miR-185-3p	NM_001271944	TFEB	150	-30.29	-33.3	0.012701
hsa-miR-185-3p	NM_001271945	TFEB	150	-30.29	-33.3	0.012701
hsa-miR-185-3p	NM_001272049	TRAP1	153	-20.27	-25.1	0.03112
hsa-miR-185-3p	NM_001272103	HERPUD1	159	-25.38	-32.6	0.025396
hsa-miR-185-3p	NM_001275	CHGA	150	-19.98	-32.1	0.011309
hsa-miR-185-3p	NM_001276326	SLC22A12	157	-20.55	-35.9	0.005785
hsa-miR-185-3p	NM_001276327	SLC22A12	157	-20.55	-35.9	0.005785
hsa-miR-185-3p	NM_001276418	SEC16A	160	-29.38	-34.3	0.035848

hsa-miR-185-3p	NM_001278158	ZNF205	152	-25.29	-30.6	0.005947
hsa-miR-185-3p	NM_001278242	MS4A15	153	-29.07	-33.2	0.028668
hsa-miR-185-3p	NM_001278441	ILK	162	-27.65	-33	0.003104
hsa-miR-185-3p	NM_001278442	ILK	162	-27.65	-33	0.003104
hsa-miR-185-3p	NM_001278694	GPR132	161	-31.07	-34.2	0.035725
hsa-miR-185-3p	NM_001278695	GPR132	161	-31.07	-34.2	0.035725
hsa-miR-185-3p	NM_001278696	GPR132	161	-31.07	-34.2	0.035725
hsa-miR-185-3p	NM_001278919	KCNH6	159	-26.75	-34.1	0.013905
hsa-miR-185-3p	NM_001278920	KCNH6	159	-26.75	-34.1	0.013905
hsa-miR-185-3p	NM_001281987	SPOCD1	158	-27.67	-35.1	0.000453
hsa-miR-185-3p	NM_001281988	SPOCD1	158	-27.67	-35.1	0.000453
hsa-miR-185-3p	NM_001282166	SUV39H1	152	-20.05	-37.3	0.00805
hsa-miR-185-3p	NM_001282211	NDRG2	155	-28.28	-32.3	0.032041
hsa-miR-185-3p	NM_001282212	NDRG2	155	-28.28	-32.3	0.032041
hsa-miR-185-3p	NM_001282213	NDRG2	155	-28.28	-32.3	0.032041
hsa-miR-185-3p	NM_001282214	NDRG2	155	-28.28	-32.3	0.032041
hsa-miR-185-3p	NM_001282215	NDRG2	155	-28.28	-32.3	0.032041
hsa-miR-185-3p	NM_001282216	NDRG2	155	-28.28	-32.3	0.032041
hsa-miR-185-3p	NM_001282434	HES6	152	-26.49	-32.4	0.031891
hsa-miR-185-3p	NM_001282444	EHD1	179	-41.09	-43.2	0.000743
hsa-miR-185-3p	NM_001282445	EHD1	179	-41.09	-43.2	0.000743
hsa-miR-185-3p	NM_001282450	ESRRA	163	-30.66	-34.4	0.010905
hsa-miR-185-3p	NM_001282451	ESRRA	163	-30.66	-34.4	0.010905
hsa-miR-185-3p	NM_001282474	AP1G2	153	-26.57	-30.3	0.006571
hsa-miR-185-3p	NM_001282475	AP1G2	153	-26.57	-30.3	0.006571
hsa-miR-185-3p	NM_001282862	RASGEF1A	151	-30.71	-36.1	0.017432
hsa-miR-185-3p	NM_001283024	WDYHV1	174	-35.25	-42.5	0.000289
hsa-miR-185-3p	NM_001283027	WDYHV1	174	-35.25	-42.5	0.000289
hsa-miR-185-3p	NM_001284297	PLCB2	161	-28.58	-33.2	0.020681
hsa-miR-185-3p	NM_001284298	PLCB2	161	-28.58	-33.2	0.020681

hsa-miR-185-3p	NM_001284299	PLCB2	158	-25.97	-32.8	0.029881
hsa-miR-185-3p	NM_001284308	ADAP1	151	-23.04	-32.8	0.0345
hsa-miR-185-3p	NM_001284309	ADAP1	151	-23.04	-32.8	0.0345
hsa-miR-185-3p	NM_001284310	ADAP1	151	-23.04	-32.8	0.0345
hsa-miR-185-3p	NM_001284311	ADAP1	151	-23.04	-32.8	0.0345
hsa-miR-185-3p	NM_001284316	ACSF3	167	-34.81	-33.5	0.049425
hsa-miR-185-3p	NM_001284417	LYSMD4	154	-29.45	-33.7	0.048559
hsa-miR-185-3p	NM_001284418	LYSMD4	154	-29.45	-33.7	0.048559
hsa-miR-185-3p	NM_001284419	LYSMD4	154	-29.45	-33.7	0.048559
hsa-miR-185-3p	NM_001284420	LYSMD4	154	-29.45	-33.7	0.048559
hsa-miR-185-3p	NM_001284421	LYSMD4	154	-29.45	-33.7	0.048559
hsa-miR-185-3p	NM_001284422	LYSMD4	154	-29.45	-33.7	0.048559
hsa-miR-185-3p	NM_001286191	C12orf43	151	-27.22	-33.8	0.046893
hsa-miR-185-3p	NM_001286192	C12orf43	151	-27.22	-33.8	0.046893
hsa-miR-185-3p	NM_001286195	C12orf43	151	-27.22	-33.8	0.046893
hsa-miR-185-3p	NM_001286196	C12orf43	151	-27.22	-33.8	0.046893
hsa-miR-185-3p	NM_001286197	C12orf43	151	-27.22	-33.8	0.046893
hsa-miR-185-3p	NM_001286198	C12orf43	151	-27.22	-33.8	0.046893
hsa-miR-185-3p	NM_001286375	ITGAX	152	-21.62	-30.5	0.034048
hsa-miR-185-3p	NM_001286402	CIITA	155	-35.68	-35.3	0.013185
hsa-miR-185-3p	NM_001286403	CIITA	155	-35.68	-35.3	0.013185
hsa-miR-185-3p	NM_001286451	HDDC3	151	-28.39	-32.5	0.007177
hsa-miR-185-3p	NM_001286633	TRIM40	156	-23.39	-32.4	0.035646
hsa-miR-185-3p	NM_001286655	DLK2	158	-28.65	-33.2	0.001374
hsa-miR-185-3p	NM_001286656	DLK2	158	-28.65	-33.2	0.001374
hsa-miR-185-3p	NM_001287010	GLIPR2	159	-33.55	-35.3	0.018128
hsa-miR-185-3p	NM_001287011	GLIPR2	159	-33.55	-35.3	0.023685
hsa-miR-185-3p	NM_001287012	GLIPR2	159	-33.55	-35.3	0.019965
hsa-miR-185-3p	NM_001287013	GLIPR2	159	-33.55	-35.3	0.018128
hsa-miR-185-3p	NM_001287014	GLIPR2	159	-33.55	-35.3	0.018128

hsa-miR-185-3p	NM_001287045	SH3GLB2	151	-26.35	-34.2	0.014978
hsa-miR-185-3p	NM_001287181	CCDC33	155	-27.19	-33.5	0.02339
hsa-miR-185-3p	NM_001287595	SLC1A7	151	-29.53	-33.5	0.018442
hsa-miR-185-3p	NM_001287597	SLC1A7	151	-29.53	-33.5	0.020797
hsa-miR-185-3p	NM_001288710	C16orf13	152	-24.38	-29.6	0.008059
hsa-miR-185-3p	NM_001288737	DNM1	176	-34.73	-39.9	0.000534
hsa-miR-185-3p	NM_001288738	DNM1	176	-34.73	-39.9	0.002039
hsa-miR-185-3p	NM_001288739	DNM1	176	-34.73	-39.9	0.000408
hsa-miR-185-3p	NM_001288740	C10orf128	150	-27.93	-32.4	0.049802
hsa-miR-185-3p	NM_001288987	OBP2B	150	-20.8	-31.9	0.011757
hsa-miR-185-3p	NM_001289413	EHMT2	160	-29.09	-33.8	0.003095
hsa-miR-185-3p	NM_001289745	GAPDH	152	-27.08	-32.3	0.002715
hsa-miR-185-3p	NM_001289746	GAPDH	152	-27.08	-32.3	0.002715
hsa-miR-185-3p	NM_001289910	IDH2	157	-33.12	-31.2	0.009271
hsa-miR-185-3p	NM_001289937	ERBB2	151	-26.26	-32.6	0.034787
hsa-miR-185-3p	NM_001289990	ATP6V0E2	156	-25.15	-35.5	0.010533
hsa-miR-185-3p	NM_001290094	TMPRSS4	159	-34.07	-35.3	0.028976
hsa-miR-185-3p	NM_001290096	TMPRSS4	159	-34.07	-35.3	0.028976
hsa-miR-185-3p	NM_001290114	IDH2	157	-33.12	-31.2	0.009271
hsa-miR-185-3p	NM_001290252	DPP6	154	-29.12	-34.3	0.040392
hsa-miR-185-3p	NM_001290787	ECEL1	150	-25.35	-32.1	0.00744
hsa-miR-185-3p	NM_001291218	ANKRD2	151	-25.59	-26.6	0.037554
hsa-miR-185-3p	NM_001291219	ANKRD2	151	-25.59	-26.6	0.037554
hsa-miR-185-3p	NM_001291897	INS	151	-21.14	-24.8	0.02352
hsa-miR-185-3p	NM_001292016	TNIP2	155	-26.08	-32.6	0.01658
hsa-miR-185-3p	NM_001294337	C5orf38	160	-33.03	-39.1	0.000256
hsa-miR-185-3p	NM_001294340	ZC3H18	150	-24.89	-30.6	0.049924
hsa-miR-185-3p	NM_001297758	HELQ	167	-31.73	-36	0.012269
hsa-miR-185-3p	NM_001297759	HELQ	167	-31.73	-36	0.012269
hsa-miR-185-3p	NM_001300731	AMBRA1	160	-28.62	-32.1	0.048145

hsa-miR-185-3p	NM_001300810	KRT85	151	-27.36	-31.4	0.049297
hsa-miR-185-3p	NM_001300821	EIF4B	168	-34.8	-37.2	0.013607
hsa-miR-185-3p	NM_001300847	SLC29A4	165	-30.11	-35.5	0.012163
hsa-miR-185-3p	NM_001301071	DOK7	164	-32.69	-36.6	0.002871
hsa-miR-185-3p	NM_001301168	SHF	154	-29.77	-31.7	0.034791
hsa-miR-185-3p	NM_001301169	SHF	154	-29.77	-32.7	0.032661
hsa-miR-185-3p	NM_001301170	SHF	154	-29.77	-31.7	0.046566
hsa-miR-185-3p	NM_001301171	SHF	154	-29.77	-31.7	0.034791
hsa-miR-185-3p	NM_001301226	TPM2	152	-21.42	-28.8	0.003159
hsa-miR-185-3p	NM_001301650	R3HCC1	156	-24.29	-27.5	0.028698
hsa-miR-185-3p	NM_001301690	CHGA	150	-19.98	-32.1	0.011309
hsa-miR-185-3p	NM_001302862	SLC22A18AS	159	-32.1	-35.3	0.008159
hsa-miR-185-3p	NM_001302959	AIP	150	-23.45	-26.4	0.022558
hsa-miR-185-3p	NM_001302960	AIP	150	-23.45	-29.8	0.01478
hsa-miR-185-3p	NM_001303012	PLCH2	161	-31.4	-37.5	0.011255
hsa-miR-185-3p	NM_001303013	PLCH2	161	-31.4	-33.7	0.041915
hsa-miR-185-3p	NM_001303016	MGC57346-CRHR1	155	-26.63	-34.4	0.016737
hsa-miR-185-3p	NM_001303018	CRHR1	155	-26.63	-34.4	0.017993
hsa-miR-185-3p	NM_001303020	CRHR1	155	-26.63	-34.4	0.017993
hsa-miR-185-3p	NM_001303024	SSSCA1	159	-24.69	-29.4	0.007392
hsa-miR-185-3p	NM_001303039	C7orf26	158	-30.02	-32.7	0.014443
hsa-miR-185-3p	NM_001303481	ZNF282	150	-27.7	-35.6	0.026297
hsa-miR-185-3p	NM_001303	COX10	159	-28.25	-33.7	0.038983
hsa-miR-185-3p	NM_001304385	LSMEM2	151	-34.09	-32.1	0.040069
hsa-miR-185-3p	NM_001304478	FGF17	165	-31.98	-34.5	0.005996
hsa-miR-185-3p	NM_001304497	FOPNL	184	-37.57	-42.5	0.001134
hsa-miR-185-3p	NM_001304498	FOPNL	184	-37.57	-42.5	0.001218
hsa-miR-185-3p	NM_001304499	FOPNL	184	-37.57	-42.5	0.001134
hsa-miR-185-3p	NM_001304500	FOPNL	184	-37.57	-42.5	0.001134

hsa-miR-185-3p	NM_001304502	FOPNL	184	-37.57	-42.5	0.001218
hsa-miR-185-3p	NM_001304519	EVX1	176	-34.69	-35	0.020715
hsa-miR-185-3p	NM_001304520	EVX1	176	-34.69	-35	0.020715
hsa-miR-185-3p	NM_001304521	IMPDH1	154	-32.89	-35.5	0.005934
hsa-miR-185-3p	NM_001304763	ZNF687	153	-30.47	-34.8	0.007589
hsa-miR-185-3p	NM_001304764	ZNF687	153	-30.47	-34.8	0.007589
hsa-miR-185-3p	NM_001304833	OGFOD2	160	-28.97	-33.7	0.012663
hsa-miR-185-3p	NM_001304834	OGFOD2	160	-28.97	-33.7	0.012663
hsa-miR-185-3p	NM_001304835	OGFOD2	160	-28.97	-33.7	0.012663
hsa-miR-185-3p	NM_001304836	OGFOD2	160	-28.97	-33.7	0.012663
hsa-miR-185-3p	NM_001304837	OGFOD2	160	-28.97	-33.7	0.012663
hsa-miR-185-3p	NM_001304838	OGFOD2	160	-28.97	-33.7	0.012663
hsa-miR-185-3p	NM_001305275	AGRN	162	-27.76	-33.9	0.024853
hsa-miR-185-3p	NM_001306149	C5orf38	160	-33.03	-39.1	0.000295
hsa-miR-185-3p	NM_001307990	MAP4K2	156	-28.76	-31.1	0.020359
hsa-miR-185-3p	NM_001308044	RBM23	151	-23.31	-32.3	0.046932
hsa-miR-185-3p	NM_001308053	RAB26	150	-32.32	-34	0.012199
hsa-miR-185-3p	NM_001308152	ARHGEF10	166	-29.96	-35.1	0.0195
hsa-miR-185-3p	NM_001308153	ARHGEF10	166	-29.96	-35.1	0.0195
hsa-miR-185-3p	NM_001311198	AP2M1	152	-31.84	-30.2	0.036163
hsa-miR-185-3p	NM_001311345	HPS1	154	-31.46	-33.9	0.032391
hsa-miR-185-3p	NM_001314018	CPNE5	154	-26.4	-33.5	0.043421
hsa-miR-185-3p	NM_001314019	CPNE5	154	-26.4	-33.5	0.043421
hsa-miR-185-3p	NM_001314020	CPNE5	154	-26.4	-33.5	0.043421
hsa-miR-185-3p	NM_001335	CTSW	150	-22.04	-27.3	0.015392
hsa-miR-185-3p	NM_001408	CELSR2	159	-29.29	-34.3	0.037286
hsa-miR-185-3p	NM_001417	EIF4B	168	-34.8	-37.2	0.013607
hsa-miR-185-3p	NM_001619	ADRBK1	160	-29.17	-36.3	0.008209
hsa-miR-185-3p	NM_001651	AQP5	164	-33.01	-33	0.010352
hsa-miR-185-3p	NM_001702	ADGRB1	154	-32.51	-32.6	0.016264

hsa-miR-185-3p	NM_001711	BGN	152	-18.55	-32.3	0.047907
hsa-miR-185-3p	NM_001715	BLK	156	-27.69	-32.7	0.011408
hsa-miR-185-3p	NM_001852	COL9A2	160	-25.18	-31	0.043949
hsa-miR-185-3p	NM_001888	CRYM	153	-27.45	-30.4	0.012029
hsa-miR-185-3p	NM_001927	DES	153	-33.83	-38.6	0.001464
hsa-miR-185-3p	NM_001934	DLX4	156	-27.72	-34.2	0.018335
hsa-miR-185-3p	NM_001936	DPP6	154	-29.12	-34.3	0.040392
hsa-miR-185-3p	NM_001950	E2F4	161	-31.28	-34.9	0.009045
hsa-miR-185-3p	NM_001970	EIF5A	160	-29.7	-32.5	0.020076
hsa-miR-185-3p	NM_001989	EVX1	176	-34.69	-35	0.020715
hsa-miR-185-3p	NM_002018	FLII	153	-23.49	-30.4	0.017152
hsa-miR-185-3p	NM_002028	FNTB	161	-30.84	-33.1	0.044515
hsa-miR-185-3p	NM_002046	GAPDH	152	-27.08	-32.3	0.002715
hsa-miR-185-3p	NM_002082	GRK6	160	-32.87	-37.2	0.004801
hsa-miR-185-3p	NM_002131	HMGA1	152	-22.69	-35.9	0.013298
hsa-miR-185-3p	NM_002168	IDH2	157	-33.12	-31.2	0.009271
hsa-miR-185-3p	NM_002180	IGHMBP2	155	-33.41	-31.5	0.048979
hsa-miR-185-3p	NM_002213	ITGB5	152	-34.25	-38.3	0.006579
hsa-miR-185-3p	NM_002278	KRT32	174	-30.43	-39.4	0.00065
hsa-miR-185-3p	NM_002283	KRT85	151	-27.36	-31.4	0.049297
hsa-miR-185-3p	NM_002314	LIMK1	156	-23.42	-33.2	0.037549
hsa-miR-185-3p	NM_002332	LRP1	162	-31.35	-36.5	0.004244
hsa-miR-185-3p	NM_002346	LY6E	158	-31.8	-33.2	0.013315
hsa-miR-185-3p	NM_002452	NUDT1	151	-24.69	-30.1	0.004359
hsa-miR-185-3p	NM_002457	MUC2	156	-26.98	-31.4	0.002398
hsa-miR-185-3p	NM_002488	NDUFA2	150	-18.83	-30	0.011033
hsa-miR-185-3p	NM_002541	OGDH	162	-32.72	-35.6	0.010633
hsa-miR-185-3p	NM_002586	PBX2	151	-28.28	-34.2	0.037087
hsa-miR-185-3p	NM_002593	PCOLCE	165	-24.49	-30.2	0.000866
hsa-miR-185-3p	NM_002596	CDK18	164	-37.08	-41.9	0.001043

hsa-miR-185-3p	NM_002609	PDGFRB	160	-32.36	-35.3	0.028892
hsa-miR-185-3p	NM_002616	PER1	154	-34.4	-37.3	0.001776
hsa-miR-185-3p	NM_002658	PLAU	158	-31.18	-32.3	0.037988
hsa-miR-185-3p	NM_002663	PLD2	153	-26.91	-30.1	0.049853
hsa-miR-185-3p	NM_002714	PPP1R10	150	-27.95	-34.6	0.018502
hsa-miR-185-3p	NM_002804	PSMC3	156	-23.61	-29.2	0.001248
hsa-miR-185-3p	NM_002826	QSOX1	164	-28.74	-36	0.007932
hsa-miR-185-3p	NM_002832	PTPN7	163	-35.65	-34.9	0.026236
hsa-miR-185-3p	NM_002840	PTPRF	164	-36.16	-37.5	0.00923
hsa-miR-185-3p	NM_002885	RAP1GAP	150	-31.48	-35	0.014168
hsa-miR-185-3p	NM_002889	RARRES2	167	-33.88	-36.8	0.000081
hsa-miR-185-3p	NM_002939	RNH1	156	-29.58	-30.6	0.005169
hsa-miR-185-3p	NM_003145	SSR2	154	-27.42	-34.4	0.005014
hsa-miR-185-3p	NM_003164	STX5	163	-27.98	-32.1	0.021682
hsa-miR-185-3p	NM_003170	SUPT6H	154	-32.86	-32	0.02255
hsa-miR-185-3p	NM_003173	SUV39H1	152	-20.05	-37.3	0.00805
hsa-miR-185-3p	NM_003213	TEAD4	151	-27.94	-29.6	0.010826
hsa-miR-185-3p	NM_003240	LEFTY2	163	-29.79	-33.5	0.018894
hsa-miR-185-3p	NM_003456	ZNF205	152	-25.29	-30.6	0.005947
hsa-miR-185-3p	NM_003459	SLC30A3	163	-29.77	-35.9	0.005041
hsa-miR-185-3p	NM_003481	USP5	154	-25.73	-31.2	0.028567
hsa-miR-185-3p	NM_003575	ZNF282	150	-27.7	-35.6	0.019589
hsa-miR-185-3p	NM_003585	DOC2B	157	-28.9	-30.5	0.048958
hsa-miR-185-3p	NM_003673	TCAP	159	-25.58	-30.3	0.031076
hsa-miR-185-3p	NM_003680	YARS	154	-28.61	-33.5	0.015449
hsa-miR-185-3p	NM_003682	MADD	151	-29.1	-32.8	0.027782
hsa-miR-185-3p	NM_003695	LY6D	160	-28.58	-32.3	0.007223
hsa-miR-185-3p	NM_003710	SPINT1	154	-33.97	-32.7	0.0195
hsa-miR-185-3p	NM_003780	B4GALT2	168	-34.13	-31.2	0.042871
hsa-miR-185-3p	NM_003793	CTSF	151	-28.51	-32.1	0.014124

hsa-miR-185-3p	NM_003863	DPM2	159	-28.44	-31.4	0.031524
hsa-miR-185-3p	NM_003867	FGF17	165	-31.98	-34.5	0.005996
hsa-miR-185-3p	NM_003891	PROZ	153	-23.94	-28.8	0.030457
hsa-miR-185-3p	NM_003917	AP1G2	153	-26.57	-30.3	0.006571
hsa-miR-185-3p	NM_003954	MAP3K14	164	-33.3	-35.6	0.018101
hsa-miR-185-3p	NM_003977	AIP	150	-23.45	-26.4	0.022558
hsa-miR-185-3p	NM_004068	AP2M1	152	-31.84	-30.2	0.036163
hsa-miR-185-3p	NM_004089	TSC22D3	158	-35.08	-40.8	0.001451
hsa-miR-185-3p	NM_004140	LLGL1	164	-25.54	-31.8	0.049877
hsa-miR-185-3p	NM_004186	SEMA3F	166	-32.29	-36.5	0.005945
hsa-miR-185-3p	NM_004195	TNFRSF18	159	-29.59	-33.6	0.003676
hsa-miR-185-3p	NM_004247	EFTUD2	159	-28.23	-33.1	0.028761
hsa-miR-185-3p	NM_004313	ARRB2	154	-27.77	-32.5	0.011287
hsa-miR-185-3p	NM_004347	CASP5	156	-26.66	-30.4	0.002128
hsa-miR-185-3p	NM_004366	CLCN2	151	-28.37	-29.3	0.046238
hsa-miR-185-3p	NM_004382	CRHR1	155	-26.63	-34.4	0.017993
hsa-miR-185-3p	NM_004408	DNM1	176	-34.73	-39.9	0.000408
hsa-miR-185-3p	NM_004419	DUSP5	161	-28.26	-35.7	0.010882
hsa-miR-185-3p	NM_004429	EFNB1	165	-31.93	-34	0.035701
hsa-miR-185-3p	NM_004451	ESRRA	163	-30.66	-34.4	0.010905
hsa-miR-185-3p	NM_004517	ILK	162	-27.65	-33	0.003104
hsa-miR-185-3p	NM_004565	PEX14	150	-25.1	-32.6	0.024998
hsa-miR-185-3p	NM_004573	PLCB2	161	-28.58	-33.2	0.020681
hsa-miR-185-3p	NM_004579	MAP4K2	156	-28.76	-31.1	0.020359
hsa-miR-185-3p	NM_004615	TSPAN7	152	-28.79	-33	0.030076
hsa-miR-185-3p	NM_004624	VIPR1	164	-28.35	-35.4	0.015534
hsa-miR-185-3p	NM_004628	XPC	160	-26.93	-32.3	0.029758
hsa-miR-185-3p	NM_004635	MAPKAPK3	158	-29.76	-33.1	0.040445
hsa-miR-185-3p	NM_004693	KRT75	156	-31.33	-33.6	0.006239
hsa-miR-185-3p	NM_004732	KCNAB3	152	-29.67	-34.9	0.018249

hsa-miR-185-3p	NM_004740	TIAF1	153	-29.61	-29.6	0.027059
hsa-miR-185-3p	NM_004758	BZRAP1	154	-29.9	-34.5	0.022011
hsa-miR-185-3p	NM_004785	SLC9A3R2	155	-24.95	-33.3	0.028463
hsa-miR-185-3p	NM_004809	STOML1	152	-24.96	-31.1	0.044514
hsa-miR-185-3p	NM_004826	ECEL1	150	-25.35	-32.1	0.00744
hsa-miR-185-3p	NM_004884	IGDCC3	151	-26.96	-35.4	0.024877
hsa-miR-185-3p	NM_004959	NR5A1	167	-32.87	-36.2	0.014043
hsa-miR-185-3p	NM_005072	SLC12A4	152	-22.08	-35.6	0.015483
hsa-miR-185-3p	NM_005147	DNAJA3	162	-32.95	-35.9	0.011738
hsa-miR-185-3p	NM_005157	ABL1	155	-28.8	-34.4	0.044076
hsa-miR-185-3p	NM_005169	PHOX2A	156	-27.89	-34	0.010411
hsa-miR-185-3p	NM_005185	CALML3	150	-35.41	-35.2	0.006872
hsa-miR-185-3p	NM_005332	HBZ	160	-29.34	-25.8	0.026725
hsa-miR-185-3p	NM_005334	HCFC1	163	-31.92	-37.4	0.010714
hsa-miR-185-3p	NM_005343	HRAS	152	-26.89	-32.3	0.005256
hsa-miR-185-3p	NM_005439	MLF2	158	-28.15	-32.2	0.019177
hsa-miR-185-3p	NM_005456	MAPK8IP1	159	-27.42	-32.1	0.029609
hsa-miR-185-3p	NM_005526	HSF1	150	-24.38	-30.7	0.020757
hsa-miR-185-3p	NM_005550	KIFC3	161	-31.13	-35.4	0.005323
hsa-miR-185-3p	NM_005576	LOXL1	157	-31.5	-33.7	0.002803
hsa-miR-185-3p	NM_005582	CD180	157	-23.72	-37.7	0.001389
hsa-miR-185-3p	NM_005609	PYGM	152	-22.02	-29	0.025995
hsa-miR-185-3p	NM_005641	TAF6	150	-22.82	-26.4	0.048837
hsa-miR-185-3p	NM_005834	TIMM17B	158	-25.08	-29	0.027999
hsa-miR-185-3p	NM_005886	KATNB1	150	-25.28	-29.7	0.022274
hsa-miR-185-3p	NM_005956	MTHFD1	170	-31.21	-35.6	0.000734
hsa-miR-185-3p	NM_005960	MUC3A	178	-32.14	-35.3	0.01421
hsa-miR-185-3p	NM_005998	CCT3	155	-31.83	-34.8	0.000981
hsa-miR-185-3p	NM_006018	HCAR3	152	-34.67	-37	0.003602
hsa-miR-185-3p	NM_006092	NOD1	161	-33.3	-38.2	0.003526

hsa-miR-185-3p	NM_006349	ZNHIT1	155	-27.66	-34.3	0.00121
hsa-miR-185-3p	NM_006373	VAT1	155	-26.98	-35.3	0.019394
hsa-miR-185-3p	NM_006396	SSSCA1	159	-24.69	-29.4	0.007392
hsa-miR-185-3p	NM_006409	ARPC1A	171	-31.73	-38.3	0.000331
hsa-miR-185-3p	NM_006445	PRPF8	162	-29.8	-33.1	0.001224
hsa-miR-185-3p	NM_006480	RGS14	165	-30.62	-32.6	0.013323
hsa-miR-185-3p	NM_006492	ALX3	150	-26.15	-29.7	0.029316
hsa-miR-185-3p	NM_006634	VAMP5	154	-28.6	-29	0.019782
hsa-miR-185-3p	NM_006651	CPLX1	164	-27.85	-34.3	0.032432
hsa-miR-185-3p	NM_006671	SLC1A7	151	-29.53	-33.5	0.018442
hsa-miR-185-3p	NM_006709	EHMT2	160	-29.09	-33.8	0.003095
hsa-miR-185-3p	NM_006725	CD6	153	-27.66	-32.3	0.045293
hsa-miR-185-3p	NM_006779	CDC42EP2	155	-26.66	-32.5	0.03273
hsa-miR-185-3p	NM_006795	EHD1	179	-41.09	-43.2	0.000743
hsa-miR-185-3p	NM_006804	STARD3	150	-20.5	-34.1	0.027447
hsa-miR-185-3p	NM_006829	ADIRF	167	-34.4	-29.4	0.029318
hsa-miR-185-3p	NM_006833	COPS6	160	-27.15	-31.1	0.018372
hsa-miR-185-3p	NM_006867	RBPMS	160	-26.53	-37.3	0.012622
hsa-miR-185-3p	NM_006869	ADAP1	151	-23.04	-32.8	0.0345
hsa-miR-185-3p	NM_006934	SLC6A9	152	-17.5	-34	0.021444
hsa-miR-185-3p	NM_007021	C10orf10	158	-30.52	-33.1	0.03504
hsa-miR-185-3p	NM_007069	PLA2G16	150	-27.87	-33.5	0.007273
hsa-miR-185-3p	NM_007102	GUCA2B	156	-28.72	-33	0.002325
hsa-miR-185-3p	NM_007105	SLC22A18AS	159	-32.1	-35.3	0.008159
hsa-miR-185-3p	NM_007108	TCEB2	154	-28.89	-30.9	0.034977
hsa-miR-185-3p	NM_007112	THBS3	158	-30.68	-35.9	0.000627
hsa-miR-185-3p	NM_007162	TFEB	150	-30.29	-33.3	0.012701
hsa-miR-185-3p	NM_007221	PMF1	158	-24.09	-32.1	0.014482
hsa-miR-185-3p	NM_007313	ABL1	155	-28.8	-34.4	0.044076
hsa-miR-185-3p	NM_007368	RASA3	152	-22.73	-34.4	0.031645

hsa-miR-185-3p	NM_012064	MIP	162	-33.51	-34.8	0.031947
hsa-miR-185-3p	NM_012134	LMOD1	152	-23.7	-35.1	0.031235
hsa-miR-185-3p	NM_012166	FBXO10	170	-35.42	-39.2	0.004388
hsa-miR-185-3p	NM_012171	TSPAN17	175	-36.05	-41.1	0.001382
hsa-miR-185-3p	NM_012172	FBXO24	154	-30.9	-37.2	0.000225
hsa-miR-185-3p	NM_012387	PADI4	156	-31.63	-36.8	0.000364
hsa-miR-185-3p	NM_012396	PHLDA3	154	-30.87	-36	0.022063
hsa-miR-185-3p	NM_012455	PSD4	168	-33.61	-38.1	0.007242
hsa-miR-185-3p	NM_013246	CLCF1	160	-29.42	-33.2	0.026719
hsa-miR-185-3p	NM_013324	CISH	163	-29.85	-33.5	0.03022
hsa-miR-185-3p	NM_013337	TIMM22	159	-27.91	-32.8	0.036526
hsa-miR-185-3p	NM_013345	GPR132	161	-31.07	-34.2	0.035725
hsa-miR-185-3p	NM_013346	SNX12	165	-33.57	-37.7	0.009261
hsa-miR-185-3p	NM_013366	ANAPC2	150	-22.4	-26.4	0.040501
hsa-miR-185-3p	NM_014045	LRP10	156	-28.89	-33.3	0.010524
hsa-miR-185-3p	NM_014228	SLC6A7	164	-31.53	-34.8	0.024092
hsa-miR-185-3p	NM_014235	UBL4A	156	-29.28	-35.1	0.029129
hsa-miR-185-3p	NM_014275	MGAT4B	150	-31.74	-34.3	0.004423
hsa-miR-185-3p	NM_014284	NCDN	150	-33.44	-33	0.035919
hsa-miR-185-3p	NM_014353	RAB26	150	-32.32	-34	0.011639
hsa-miR-185-3p	NM_014555	TRPM5	170	-33.71	-29.5	0.041966
hsa-miR-185-3p	NM_014580	SLC2A8	163	-35.39	-37.4	0.001933
hsa-miR-185-3p	NM_014621	HOXD4	152	-25.28	-28.3	0.039305
hsa-miR-185-3p	NM_014629	ARHGEF10	166	-29.96	-35.1	0.0195
hsa-miR-185-3p	NM_014759	PHYHIP	162	-32.62	-36.5	0.013927
hsa-miR-185-3p	NM_014815	MED24	153	-29.77	-34.3	0.004154
hsa-miR-185-3p	NM_014866	SEC16A	160	-29.38	-34.3	0.035848
hsa-miR-185-3p	NM_015133	MAPK8IP3	164	-28.69	-33.8	0.038312
hsa-miR-185-3p	NM_015193	ARC	151	-28.33	-33.9	0.038877
hsa-miR-185-3p	NM_015319	TNS2	160	-33.22	-32.3	0.014086

hsa-miR-185-3p	NM_015425	POLR1A	154	-20.79	-32.2	0.049669
hsa-miR-185-3p	NM_015650	TRAF3IP1	170	-33.38	-36.5	0.018198
hsa-miR-185-3p	NM_015689	DENND2A	159	-28.5	-33.8	0.002438
hsa-miR-185-3p	NM_015720	PODXL2	151	-27.77	-30.1	0.020933
hsa-miR-185-3p	NM_016057	COPZ1	152	-30.53	-33.6	0.03543
hsa-miR-185-3p	NM_016240	SCARA3	178	-33.45	-37.5	0.00923
hsa-miR-185-3p	NM_016250	NDRG2	155	-28.28	-32.3	0.032041
hsa-miR-185-3p	NM_016292	TRAP1	153	-20.27	-25.1	0.03112
hsa-miR-185-3p	NM_016337	EVL	167	-30.95	-37.1	0.001379
hsa-miR-185-3p	NM_016464	TMEM138	154	-28.18	-32.8	0.02372
hsa-miR-185-3p	NM_016633	AHSP	154	-28.44	-31.6	0.001062
hsa-miR-185-3p	NM_017515	SLC35F2	152	-29.15	-34.3	0.034804
hsa-miR-185-3p	NM_017553	INO80	158	-24.61	-33.5	0.037545
hsa-miR-185-3p	NM_017622	BORCS6	174	-34.64	-36.2	0.004322
hsa-miR-185-3p	NM_017649	CNNM2	165	-32.87	-34.5	0.026074
hsa-miR-185-3p	NM_017740	ZDHHC7	169	-39.19	-42.2	0.001502
hsa-miR-185-3p	NM_017749	AMBRA1	160	-28.62	-32.1	0.048145
hsa-miR-185-3p	NM_017758	ALKBH5	153	-25.58	-33.4	0.048105
hsa-miR-185-3p	NM_017781	CYP2W1	157	-30.44	-36.2	0.005209
hsa-miR-185-3p	NM_017882	CLN6	164	-31.01	-34.7	0.017678
hsa-miR-185-3p	NM_017883	WDR13	150	-29.5	-29.6	0.007324
hsa-miR-185-3p	NM_017950	CCDC40	169	-28.23	-32.7	0.026958
hsa-miR-185-3p	NM_018024	WDYHV1	174	-35.25	-42.5	0.000289
hsa-miR-185-3p	NM_018044	NSUN5	155	-25.72	-34.4	0.018407
hsa-miR-185-3p	NM_018045	BSDC1	153	-30.63	-34.4	0.043987
hsa-miR-185-3p	NM_018107	RBM23	151	-23.31	-32.3	0.046932
hsa-miR-185-3p	NM_018264	TYW1	154	-29.94	-35.1	0.011788
hsa-miR-185-3p	NM_018426	TMEM63B	153	-28.66	-31.4	0.032222
hsa-miR-185-3p	NM_018645	HES6	152	-26.49	-30.9	0.033487
hsa-miR-185-3p	NM_018719	CDCA7L	150	-27.55	-36.4	0.011629

hsa-miR-185-3p	NM_018960	GNMT	152	-23.17	-29.6	0.008436
hsa-miR-185-3p	NM_019065	NECAB2	154	-30.87	-35.4	0.002371
hsa-miR-185-3p	NM_019894	TMPRSS4	159	-34.07	-35.3	0.028976
hsa-miR-185-3p	NM_020063	BARHL2	160	-28.35	-34.3	0.001923
hsa-miR-185-3p	NM_020064	BARHL1	164	-31.5	-32.9	0.019919
hsa-miR-185-3p	NM_020142	NDUFA4L2	159	-29.49	-32.8	0.017744
hsa-miR-185-3p	NM_020145	SH3GLB2	151	-26.35	-34.2	0.014978
hsa-miR-185-3p	NM_020349	ANKRD2	151	-25.59	-26.6	0.037554
hsa-miR-185-3p	NM_020384	CLDN2	166	-31.07	-35.2	0.030475
hsa-miR-185-3p	NM_020418	PCBP4	152	-22.64	-31.8	0.024843
hsa-miR-185-3p	NM_020438	DOLPP1	163	-32.6	-35.6	0.0166
hsa-miR-185-3p	NM_020439	CAMK1G	152	-28.77	-31.8	0.047626
hsa-miR-185-3p	NM_020655	JPH3	167	-30.25	-35.2	0.02105
hsa-miR-185-3p	NM_020807	ZNF319	154	-30.87	-35.6	0.024089
hsa-miR-185-3p	NM_020832	ZNF687	153	-30.47	-34.8	0.007589
hsa-miR-185-3p	NM_020939	CPNE5	154	-26.4	-33.5	0.043421
hsa-miR-185-3p	NM_021078	KAT2A	155	-28.62	-30.6	0.036274
hsa-miR-185-3p	NM_021198	CTDSP1	174	-33.2	-38.3	0.005625
hsa-miR-185-3p	NM_021727	FADS3	167	-30.85	-33.5	0.002926
hsa-miR-185-3p	NM_021808	GALNT9	156	-24.27	-32.9	0.020091
hsa-miR-185-3p	NM_021934	ATG101	154	-27.59	-31	0.011547
hsa-miR-185-3p	NM_022054	KCNK13	158	-33.14	-38.1	0.002341
hsa-miR-185-3p	NM_022076	DUSP21	151	-26.22	-29.5	0.009827
hsa-miR-185-3p	NM_022089	ATP13A2	159	-26.55	-30.5	0.01142
hsa-miR-185-3p	NM_022342	KIF9	151	-24.75	-31.1	0.036177
hsa-miR-185-3p	NM_022343	GLIPR2	159	-33.55	-35.3	0.018128
hsa-miR-185-3p	NM_022356	P3H1	154	-28.73	-33.4	0.004682
hsa-miR-185-3p	NM_022372	MLST8	152	-25.15	-31.1	0.028773
hsa-miR-185-3p	NM_022468	MMP25	176	-33.74	-38.3	0.006279
hsa-miR-185-3p	NM_022485	MTMR14	155	-31.54	-31.2	0.018285

hsa-miR-185-3p	NM_022772	EPS8L2	152	-26.94	-32.5	0.024731
hsa-miR-185-3p	NM_022773	LMF1	161	-25.83	-33	0.026034
hsa-miR-185-3p	NM_022779	DDX31	151	-27.24	-30.9	0.03507
hsa-miR-185-3p	NM_022895	C12orf43	151	-27.22	-33.8	0.046893
hsa-miR-185-3p	NM_023015	INTS3	158	-30.14	-33.7	0.016164
hsa-miR-185-3p	NM_023932	DLK2	158	-28.65	-33.2	0.001374
hsa-miR-185-3p	NM_023942	LRRC61	152	-23.93	-32.7	0.012371
hsa-miR-185-3p	NM_023946	LYNX1	152	-24.52	-29.6	0.019206
hsa-miR-185-3p	NM_024046	CAMKV	159	-37.27	-42.9	0.000601
hsa-miR-185-3p	NM_024067	C7orf26	158	-30.02	-32.7	0.014443
hsa-miR-185-3p	NM_024072	DDX54	159	-28.41	-35.6	0.02144
hsa-miR-185-3p	NM_024092	TMEM109	150	-24.42	-33.9	0.029444
hsa-miR-185-3p	NM_024112	C9orf16	152	-27.96	-30.2	0.018169
hsa-miR-185-3p	NM_024117	MAPKAP1	180	-41.59	-45.1	0.000285
hsa-miR-185-3p	NM_024164	TPSB2	176	-38.22	-38.9	0.000214
hsa-miR-185-3p	NM_024309	TNIP2	155	-26.08	-32.6	0.01658
hsa-miR-185-3p	NM_024418	BZRAP1	154	-29.9	-34.5	0.022011
hsa-miR-185-3p	NM_024623	OGFOD2	160	-28.97	-33.7	0.012663
hsa-miR-185-3p	NM_024630	ZDHHC14	162	-26.24	-31.7	0.041307
hsa-miR-185-3p	NM_024653	PRKRIP1	151	-28.75	-34.1	0.036184
hsa-miR-185-3p	NM_024658	IPO4	158	-26.84	-33.2	0.001997
hsa-miR-185-3p	NM_024706	ZNF668	159	-26.62	-29.7	0.013039
hsa-miR-185-3p	NM_024757	EHMT1	162	-35.03	-36.2	0.009399
hsa-miR-185-3p	NM_024759	NIPAL2	162	-30.48	-33.5	0.020619
hsa-miR-185-3p	NM_024832	RIN3	163	-27.37	-32.9	0.020177
hsa-miR-185-3p	NM_024871	MAP6D1	163	-32.88	-37	0.009519
hsa-miR-185-3p	NM_024874	KIAA0319L	157	-31.34	-36	0.013559
hsa-miR-185-3p	NM_024939	ESRP2	177	-36.73	-39.8	0.00347
hsa-miR-185-3p	NM_024997	ATF7IP2	159	-29.06	-33.8	0.034628
hsa-miR-185-3p	NM_025069	ZNF703	156	-30.6	-35.3	0.017279

hsa-miR-185-3p	NM_025124	TMEM134	153	-27.26	-27.9	0.044551
hsa-miR-185-3p	NM_025256	EHMT2	160	-29.09	-33.8	0.003095
hsa-miR-185-3p	NM_025268	TMEM121	159	-33.72	-29.3	0.044031
hsa-miR-185-3p	NM_030587	B4GALT2	168	-34.13	-31.2	0.042871
hsa-miR-185-3p	NM_030779	KCNH6	159	-26.75	-34.1	0.013905
hsa-miR-185-3p	NM_030798	WBSCR16	168	-31.89	-36.6	0.004823
hsa-miR-185-3p	NM_031283	TCF7L1	150	-26.51	-32	0.042834
hsa-miR-185-3p	NM_031902	MRPS5	168	-30.84	-27.5	0.020143
hsa-miR-185-3p	NM_031964	KRTAP17-1	160	-32.61	-34.4	0.003686
hsa-miR-185-3p	NM_032028	TSSK1B	159	-31.38	-36.3	0.009101
hsa-miR-185-3p	NM_032038	SPNS1	152	-24.33	-28.1	0.03602
hsa-miR-185-3p	NM_032125	TMEM222	151	-18.83	-34	0.017652
hsa-miR-185-3p	NM_032223	PCNXL3	154	-31.15	-34.2	0.004915
hsa-miR-185-3p	NM_032323	TMEM79	158	-38.88	-41.2	0.000551
hsa-miR-185-3p	NM_032350	C7orf50	155	-26.75	-32	0.024459
hsa-miR-185-3p	NM_032366	C16orf13	152	-24.38	-29.6	0.008059
hsa-miR-185-3p	NM_032515	BOK	188	-38.64	-36.9	0.012445
hsa-miR-185-3p	NM_032548	ABTB1	167	-26.9	-29.7	0.042419
hsa-miR-185-3p	NM_032552	DAB2IP	163	-28.04	-37.2	0.013597
hsa-miR-185-3p	NM_032630	CINP	153	-30.59	-28.8	0.029722
hsa-miR-185-3p	NM_032648	FAM167B	153	-31.11	-32.9	0.003172
hsa-miR-185-3p	NM_032750	ABHD14B	150	-27.72	-34.4	0.014287
hsa-miR-185-3p	NM_032822	FAM136A	175	-30.41	-34.4	0.02463
hsa-miR-185-3p	NM_032829	FAM222A	163	-28.12	-32.6	0.048451
hsa-miR-185-3p	NM_032840	SPRYD3	156	-25.19	-36.8	0.010771
hsa-miR-185-3p	NM_032882	PNMA6A	150	-31.66	-32.8	0.022033
hsa-miR-185-3p	NM_032959	POLR2J2	155	-32.8	-36	0.015143
hsa-miR-185-3p	NM_033008	PCBP4	152	-22.64	-31.8	0.024843
hsa-miR-185-3p	NM_033010	PCBP4	152	-22.64	-31.8	0.024843
hsa-miR-185-3p	NM_033045	KRT84	164	-27.92	-32.3	0.003195

hsa-miR-185-3p	NM_033088	STRIP1	167	-31.44	-34.8	0.008204
hsa-miR-185-3p	NM_033120	NKD2	172	-31.68	-34.9	0.003184
hsa-miR-185-3p	NM_033131	WNT3A	161	-28.93	-34.1	0.043787
hsa-miR-185-3p	NM_033282	OPN4	183	-34.92	-40.8	0.00047
hsa-miR-185-3p	NM_033375	MYO1C	150	-33.98	-38	0.006567
hsa-miR-185-3p	NM_033506	FBXO24	154	-30.9	-37.2	0.000225
hsa-miR-185-3p	NM_033507	GCK	157	-26.22	-31.6	0.046446
hsa-miR-185-3p	NM_033508	GCK	157	-26.22	-31.6	0.046446
hsa-miR-185-3p	NM_052924	RHPN1	159	-31.36	-34.4	0.031787
hsa-miR-185-3p	NM_053054	CATSPER1	151	-26.39	-29.3	0.006255
hsa-miR-185-3p	NM_054013	MGAT4B	150	-31.74	-34.3	0.004423
hsa-miR-185-3p	NM_057090	ARTN	153	-26.19	-30.3	0.034466
hsa-miR-185-3p	NM_057091	ARTN	153	-26.19	-30.3	0.034466
hsa-miR-185-3p	NM_078471	MYO18A	153	-29.61	-33.1	0.040854
hsa-miR-185-3p	NM_080588	PTPN7	163	-35.65	-34.9	0.026236
hsa-miR-185-3p	NM_080722	ADAMTS14	167	-34.59	-37.1	0.010139
hsa-miR-185-3p	NM_130440	PTPRF	164	-36.16	-37.5	0.00923
hsa-miR-185-3p	NM_130465	TSPAN17	175	-36.05	-41.1	0.001382
hsa-miR-185-3p	NM_130466	UBE3B	151	-30.29	-34.7	0.037103
hsa-miR-185-3p	NM_130470	MADD	151	-29.1	-32.8	0.027782
hsa-miR-185-3p	NM_130471	MADD	151	-29.1	-32.8	0.027782
hsa-miR-185-3p	NM_130472	MADD	151	-29.1	-32.8	0.027782
hsa-miR-185-3p	NM_130473	MADD	151	-29.1	-32.8	0.027782
hsa-miR-185-3p	NM_130474	MADD	151	-29.1	-32.8	0.033911
hsa-miR-185-3p	NM_130475	MADD	151	-29.1	-32.8	0.033911
hsa-miR-185-3p	NM_130476	MADD	151	-29.1	-32.8	0.027782
hsa-miR-185-3p	NM_130797	DPP6	154	-29.12	-34.3	0.040392
hsa-miR-185-3p	NM_134425	SLC26A1	163	-32.51	-36.7	0.000738
hsa-miR-185-3p	NM_138281	DLX4	156	-27.72	-34.2	0.018335
hsa-miR-185-3p	NM_138300	PYGO2	152	-29.53	-35.4	0.024305

hsa-miR-185-3p	NM_138344	FAM181A	156	-27.82	-31	0.022029
hsa-miR-185-3p	NM_138356	SHF	154	-29.77	-31.7	0.034791
hsa-miR-185-3p	NM_138410	CMTM7	152	-23.28	-31.9	0.022472
hsa-miR-185-3p	NM_138418	FAM195A	155	-30.44	-33	0.003611
hsa-miR-185-3p	NM_138440	VASN	171	-32.46	-38.6	0.001053
hsa-miR-185-3p	NM_138693	KLF14	152	-18.26	-31.4	0.022612
hsa-miR-185-3p	NM_138700	TRIM40	156	-23.39	-32.4	0.035646
hsa-miR-185-3p	NM_138706	B3GNT6	154	-30.48	-35.6	0.013563
hsa-miR-185-3p	NM_139025	ADAMTS13	159	-26.07	-27.4	0.04302
hsa-miR-185-3p	NM_139026	ADAMTS13	159	-26.07	-27.4	0.04302
hsa-miR-185-3p	NM_139027	ADAMTS13	159	-26.07	-27.4	0.04302
hsa-miR-185-3p	NM_139155	ADAMTS14	167	-34.59	-37.1	0.010139
hsa-miR-185-3p	NM_139315	TAF6	150	-22.82	-26.4	0.048837
hsa-miR-185-3p	NM_144569	SPOCD1	158	-27.67	-35.1	0.000453
hsa-miR-185-3p	NM_144585	SLC22A12	157	-20.55	-35.9	0.005785
hsa-miR-185-3p	NM_144600	FOPNL	184	-37.57	-42.5	0.001134
hsa-miR-185-3p	NM_144604	ZC3H18	150	-24.89	-30.6	0.049924
hsa-miR-185-3p	NM_144712	SLC23A3	152	-27.2	-31.1	0.02145
hsa-miR-185-3p	NM_145065	PELI3	178	-32.84	-39.4	0.002255
hsa-miR-185-3p	NM_145071	CISH	163	-29.85	-33.5	0.03022
hsa-miR-185-3p	NM_145214	TRIM11	170	-31.45	-37	0.005314
hsa-miR-185-3p	NM_145230	ATP6V0E2	156	-25.15	-35.5	0.016726
hsa-miR-185-3p	NM_145313	RASGEF1A	151	-30.71	-36.1	0.017432
hsa-miR-185-3p	NM_145689	APBB1	154	-27.38	-33.4	0.005891
hsa-miR-185-3p	NM_145899	HMGA1	152	-22.69	-35.9	0.013298
hsa-miR-185-3p	NM_145901	HMGA1	152	-22.69	-35.9	0.013298
hsa-miR-185-3p	NM_145902	HMGA1	152	-22.69	-35.9	0.013298
hsa-miR-185-3p	NM_145903	HMGA1	152	-22.69	-35.9	0.013298
hsa-miR-185-3p	NM_145905	HMGA1	152	-22.69	-35.9	0.013298
hsa-miR-185-3p	NM_146421	GSTM1	154	-26.2	-32.4	0.011369

hsa-miR-185-3p	NM_148902	TNFRSF18	159	-29.59	-33.6	0.003676
hsa-miR-185-3p	NM_148956	NSUN5	155	-25.72	-34.4	0.01387
hsa-miR-185-3p	NM_152264	SLC39A13	153	-27.79	-32.5	0.045922
hsa-miR-185-3p	NM_152268	PARS2	158	-33.35	-35.1	0.008961
hsa-miR-185-3p	NM_152335	TMEM266	163	-30.58	-33.7	0.011285
hsa-miR-185-3p	NM_152449	LYSMD4	154	-29.45	-33.7	0.048559
hsa-miR-185-3p	NM_152705	POLR1D	154	-32.19	-37.4	0.007909
hsa-miR-185-3p	NM_152717	MS4A15	153	-29.07	-33.2	0.028668
hsa-miR-185-3p	NM_152743	BRAT1	159	-26.07	-30.2	0.01298
hsa-miR-185-3p	NM_152901	PYDC1	163	-27.2	-34.6	0.000849
hsa-miR-185-3p	NM_153006	NAGS	170	-30.37	-35.1	0.003239
hsa-miR-185-3p	NM_153215	LSMEM2	151	-34.09	-32.1	0.040069
hsa-miR-185-3p	NM_153247	SLC29A4	165	-30.11	-35.5	0.012163
hsa-miR-185-3p	NM_153254	TTLL10	151	-22.27	-32	0.029556
hsa-miR-185-3p	NM_153350	FBXL16	163	-29.03	-34.6	0.034674
hsa-miR-185-3p	NM_153378	SLC22A12	157	-20.55	-35.9	0.005785
hsa-miR-185-3p	NM_153685	PIANP	159	-30.25	-36.7	0.008279
hsa-miR-185-3p	NM_153746	ZDHHC14	162	-26.24	-31.7	0.041307
hsa-miR-185-3p	NM_170754	TNS2	160	-33.22	-32.3	0.014086
hsa-miR-185-3p	NM_172027	ABTB1	167	-26.9	-29.7	0.042419
hsa-miR-185-3p	NM_173520	C9orf62	159	-32.39	-38.2	0.004646
hsa-miR-185-3p	NM_173542	PLBD2	163	-31.15	-33.1	0.030453
hsa-miR-185-3p	NM_173660	DOK7	154	-32.84	-34.1	0.018201
hsa-miR-185-3p	NM_173828	RELL2	159	-29.86	-31	0.018125
hsa-miR-185-3p	NM_173841	IL1RN	166	-28.14	-32.9	0.038934
hsa-miR-185-3p	NM_173842	IL1RN	166	-28.14	-32.9	0.038934
hsa-miR-185-3p	NM_173843	IL1RN	166	-28.14	-32.9	0.038934
hsa-miR-185-3p	NM_174887	IFT20	158	-21.42	-29.4	0.040168
hsa-miR-185-3p	NM_174917	ACSF3	167	-34.81	-33.5	0.049425
hsa-miR-185-3p	NM_175062	RASGEF1C	150	-21.17	-31.7	0.032875

hsa-miR-185-3p	NM_176795	HRAS	152	-26.89	-32.3	0.015881
hsa-miR-185-3p	NM_177458	LYNX1	152	-24.52	-29.6	0.01871
hsa-miR-185-3p	NM_177551	HCAR2	152	-34.67	-37	0.004239
hsa-miR-185-3p	NM_177964	LYPD6B	152	-29.72	-33.8	0.008103
hsa-miR-185-3p	NM_178435	LCE3E	159	-26.95	-30.1	0.011083
hsa-miR-185-3p	NM_178537	B4GALNT4	155	-22.64	-28.7	0.04122
hsa-miR-185-3p	NM_178569	C5orf38	160	-33.03	-39.1	0.000256
hsa-miR-185-3p	NM_181336	LEMD2	157	-28.88	-33.6	0.038733
hsa-miR-185-3p	NM_181472	CMTM7	152	-23.28	-31.9	0.022472
hsa-miR-185-3p	NM_181642	SPINT1	154	-33.97	-32.7	0.0195
hsa-miR-185-3p	NM_181711	GRASP	161	-30.82	-35	0.006809
hsa-miR-185-3p	NM_181715	CRTC2	161	-27.98	-33.2	0.007293
hsa-miR-185-3p	NM_182553	CNIH2	161	-29.82	-36.1	0.00333
hsa-miR-185-3p	NM_182642	CTDSP1	174	-33.2	-38.3	0.005625
hsa-miR-185-3p	NM_182791	CCDC33	155	-27.19	-33.5	0.02339
hsa-miR-185-3p	NM_182902	KIF9	151	-24.75	-31.1	0.036177
hsa-miR-185-3p	NM_183243	IMPDH1	154	-32.89	-35.5	0.005934
hsa-miR-185-3p	NM_183415	UBE3B	151	-30.29	-34.7	0.037103
hsa-miR-185-3p	NM_194293	XIRP1	153	-33.03	-35.5	0.005494
hsa-miR-185-3p	NM_198057	TSC22D3	158	-35.08	-40.8	0.001451
hsa-miR-185-3p	NM_198098	AQP1	150	-22.29	-34.9	0.033257
hsa-miR-185-3p	NM_198154	TMEM95	169	-32.09	-36.4	0.002636
hsa-miR-185-3p	NM_198316	TNS2	160	-33.22	-32.3	0.014086
hsa-miR-185-3p	NM_198403	MMD2	156	-32.81	-35.5	0.016905
hsa-miR-185-3p	NM_198474	OLFML1	163	-30.42	-34.5	0.020308
hsa-miR-185-3p	NM_198527	HDDC3	151	-28.39	-32.5	0.018718
hsa-miR-185-3p	NM_198576	AGRN	162	-27.76	-33.9	0.024853
hsa-miR-185-3p	NM_198948	NUDT1	151	-24.69	-30.1	0.004359
hsa-miR-185-3p	NM_198949	NUDT1	151	-24.69	-30.1	0.004359
hsa-miR-185-3p	NM_198950	NUDT1	151	-24.69	-30.1	0.004359

hsa-miR-185-3p	NM_198952	NUDT1	151	-24.69	-30.1	0.004359
hsa-miR-185-3p	NM_198953	NUDT1	151	-24.69	-30.1	0.004359
hsa-miR-185-3p	NM_198954	NUDT1	151	-24.69	-30.1	0.004359
hsa-miR-185-3p	NM_199004	ARRB2	154	-27.77	-32.5	0.011287
hsa-miR-185-3p	NM_199076	CNNM2	165	-32.87	-34.5	0.026074
hsa-miR-185-3p	NM_201441	TEAD4	151	-27.94	-29.6	0.010826
hsa-miR-185-3p	NM_201443	TEAD4	151	-27.94	-29.6	0.010826
hsa-miR-185-3p	NM_201535	NDRG2	155	-28.28	-32.3	0.032041
hsa-miR-185-3p	NM_201536	NDRG2	155	-28.28	-32.3	0.032041
hsa-miR-185-3p	NM_201537	NDRG2	155	-28.28	-32.3	0.032041
hsa-miR-185-3p	NM_201538	NDRG2	155	-28.28	-32.3	0.032041
hsa-miR-185-3p	NM_201539	NDRG2	155	-28.28	-32.3	0.032041
hsa-miR-185-3p	NM_201540	NDRG2	155	-28.28	-32.3	0.032041
hsa-miR-185-3p	NM_201541	NDRG2	155	-28.28	-32.3	0.032041
hsa-miR-185-3p	NM_201589	MAFA	152	-34.02	-36.6	0.009095
hsa-miR-185-3p	NM_201649	SLC6A9	152	-17.5	-34	0.021444
hsa-miR-185-3p	NM_203318	MYO18A	153	-29.61	-33.1	0.040854
hsa-miR-185-3p	NM_203383	RNH1	156	-29.58	-30.6	0.005169
hsa-miR-185-3p	NM_203384	RNH1	156	-29.58	-30.6	0.005169
hsa-miR-185-3p	NM_203385	RNH1	156	-29.58	-30.6	0.005169
hsa-miR-185-3p	NM_203386	RNH1	156	-29.58	-30.6	0.005169
hsa-miR-185-3p	NM_203387	RNH1	156	-29.58	-30.6	0.005169
hsa-miR-185-3p	NM_203388	RNH1	156	-29.58	-30.6	0.005169
hsa-miR-185-3p	NM_203389	RNH1	156	-29.58	-30.6	0.005169
hsa-miR-185-3p	NM_203412	UBL4B	158	-30	-33.7	0.0187
hsa-miR-185-3p	NM_203422	LRRN4CL	168	-33.06	-38.7	0.004106
hsa-miR-185-3p	NM_205545	LYPD2	152	-22.47	-26.5	0.008352
hsa-miR-185-3p	NM_206539	DLK2	158	-28.65	-33.2	0.001374
hsa-miR-185-3p	NM_207401	C1orf229	167	-29.91	-35.2	0.018129
hsa-miR-185-3p	NM_207510	LCNL1	158	-29.89	-33.4	0.016355

hsa-miR-185-3p	NM_207511	C9orf139	161	-31.21	-36.8	0.013124
hsa-miR-185-3p	NM_212481	ARID5A	154	-25.77	-31.7	0.008864
hsa-miR-185-3p	NM_212502	CDK18	164	-37.08	-41.9	0.001043
hsa-miR-185-3p	NM_212503	CDK18	164	-37.08	-41.9	0.001043
hsa-miR-185-3p	NM_213674	TPM2	152	-21.42	-28.8	0.003159
hsa-miR-185-3p	NR_001555	GOLGA2P2Y	162	-32.3	-36.4	0.00733
hsa-miR-185-3p	NR_002195	GOLGA2P3Y	162	-32.3	-36.4	0.00733
hsa-miR-185-3p	NR_002974	SNORA80E	151	-20.22	-25.7	0.044421
hsa-miR-185-3p	NR_021493	LINC00930	174	-31.97	-37.3	0.010918
hsa-miR-185-3p	NR_024166	ZNF205-AS1	161	-23.2	-34.3	0.023071
hsa-miR-185-3p	NR_024167	ZNF205-AS1	161	-23.2	-34.3	0.022931
hsa-miR-185-3p	NR_024420	LINC00937	161	-29.74	-32.3	0.022473
hsa-miR-185-3p	NR_026642	FAM99B	156	-28.51	-32.2	0.047834
hsa-miR-185-3p	NR_026740	LOC389033	155	-30.29	-30.8	0.020522
hsa-miR-185-3p	NR_026874	LOC100130417	163	-31.95	-34.7	0.026378
hsa-miR-185-3p	NR_026925	LOC151174	162	-29.32	-36.2	0.020315
hsa-miR-185-3p	NR_026926	LOC151174	162	-29.32	-36.2	0.005515
hsa-miR-185-3p	NR_027336	URAHP	151	-28.23	-33	0.048916
hsa-miR-185-3p	NR_027645	ATP1A1-AS1	150	-27.83	-31.8	0.028842
hsa-miR-185-3p	NR_028293	PTGER3	162	-28.23	-37.1	0.014171
hsa-miR-185-3p	NR_028503	MIR22HG	158	-31.6	-35.2	0.028465
hsa-miR-185-3p	NR_029623	MIR210	168	-29.99	-32.2	0.000718
hsa-miR-185-3p	NR_029887	MIR328	154	-30.31	-32.7	0.000201
hsa-miR-185-3p	NR_030714	MIR629	164	-25.1	-28.1	0.005938
hsa-miR-185-3p	NR_033946	LOC646268	159	-35.01	-37.6	0.002832
hsa-miR-185-3p	NR_034002	MCF2L-AS1	162	-35.72	-39	0.002636
hsa-miR-185-3p	NR_036198	MIR4313	153	-25.14	-25.1	0.037173
hsa-miR-185-3p	NR_036253	MIR4290	150	-28.37	-32.2	0.000502
hsa-miR-185-3p	NR_036691	LOC494127	152	-27.84	-34.4	0.025371
hsa-miR-185-3p	NR_038174	TRIM51HP	161	-30.22	-36	0.008048

hsa-miR-185-3p	NR_038260	HIVEP3	155	-31.5	-36.9	0.014349
hsa-miR-185-3p	NR_038261	HIVEP3	155	-31.5	-36.9	0.012348
hsa-miR-185-3p	NR_038357	ZSWIM8-AS1	158	-27.49	-33.3	0.045983
hsa-miR-185-3p	NR_038919	LOC100128993	158	-27.69	-33.8	0.021768
hsa-miR-185-3p	NR_038935	LOC100288846	151	-25.97	-34.7	0.022733
hsa-miR-185-3p	NR_046289	ATP2A1-AS1	155	-23.21	-29.9	0.049503
hsa-miR-185-3p	NR_046290	ATP2A1-AS1	155	-23.21	-29.9	0.049083
hsa-miR-185-3p	NR_046411	POLD4	166	-30.05	-34.9	0.030197
hsa-miR-185-3p	NR_046412	POLD4	166	-30.14	-36.4	0.015062
hsa-miR-185-3p	NR_046413	POLD4	166	-29.37	-34.9	0.024415
hsa-miR-185-3p	NR_046672	NAV2-AS2	172	-28.24	-32.9	0.025286
hsa-miR-185-3p	NR_049879	MIR5694	151	-25.31	-32	0.000319
hsa-miR-185-3p	NR_073080	PHLDA3	154	-30.87	-36	0.016868
hsa-miR-185-3p	NR_073388	ALG1L9P	152	-29.98	-33	0.030932
hsa-miR-185-3p	NR_073410	TMEM134	156	-31.77	-32.1	0.03791
hsa-miR-185-3p	NR_073411	TMEM134	153	-27.26	-32.1	0.042502
hsa-miR-185-3p	NR_073413	TMEM134	156	-31.77	-32.1	0.040771
hsa-miR-185-3p	NR_103485	PLPP1	151	-27.19	-34.3	0.040786
hsa-miR-185-3p	NR_103784	ITPKB-IT1	166	-32.97	-34.1	0.029116
hsa-miR-185-3p	NR_103840	LINC00539	151	-29.59	-35.7	0.007842
hsa-miR-185-3p	NR_103841	LINC00539	151	-29.59	-35.7	0.003695
hsa-miR-185-3p	NR_104027	SMARCA5-	155	-33.82	-35.8	0.010836
hsa-miR-185-3p	NR_104028	SORCS3-AS1	163	-29.99	-34.7	0.021151
hsa-miR-185-3p	NR_104182	LOC100506142	161	-26.42	-33.6	0.042689
hsa-miR-185-3p	NR_104197	SEPT4	154	-31.8	-36.3	0.01724
hsa-miR-185-3p	NR_104425	DACT2	158	-31.73	-36.4	0.019006
hsa-miR-185-3p	NR_104447	HDDC3	151	-28.39	-32.5	0.036595
hsa-miR-185-3p	NR_104602	LOC101929034	155	-30.2	-34.8	0.004143
hsa-miR-185-3p	NR_104636	LOC101926960	158	-29.76	-34.4	0.041632
hsa-miR-185-3p	NR_106841	MIR6783	151	-23.96	-28.4	0.001904

hsa-miR-185-3p	NR_108070	TNKS2-AS1	153	-28.3	-33.8	0.0124
hsa-miR-185-3p	NR_108071	TNKS2-AS1	153	-28.3	-33.8	0.004133
hsa-miR-185-3p	NR_109910	LOC101928651	152	-28.99	-38.3	0.006542
hsa-miR-185-3p	NR_109945	CTD-2350J17.1	167	-33.27	-34.6	0.002882
hsa-miR-185-3p	NR_109980	ST7-AS2	155	-28.49	-33.6	0.036337
hsa-miR-185-3p	NR_110013	LOC101927630	163	-32.41	-38	0.0007
hsa-miR-185-3p	NR_110553	LOC102724190	164	-31.02	-33.9	0.047991
hsa-miR-185-3p	NR_110842	LOC101927950	151	-25.64	-31.7	0.039989
hsa-miR-185-3p	NR_120528	LOC100507144	155	-26.09	-35	0.014636
hsa-miR-185-3p	NR_120651	ZNF503	162	-30.99	-35	0.015519
hsa-miR-185-3p	NR_120669	LOC101929646	152	-29.56	-30.9	0.04299
hsa-miR-185-3p	NR_121626	LOC100996263	158	-27.54	-33.7	0.005679
hsa-miR-185-3p	NR_122035	ARRDC1-AS1	166	-28.57	-33.2	0.049375
hsa-miR-185-3p	NR_122036	ARRDC1-AS1	166	-28.57	-33.2	0.03564
hsa-miR-185-3p	NR_122045	LOC100130417	167	-29.68	-34.7	0.02488
hsa-miR-185-3p	NR_125409	LOC102724699	162	-28.52	-33.9	0.04852
hsa-miR-185-3p	NR_125771	LINC01058	162	-27.17	-33.3	0.009887
hsa-miR-185-3p	NR_125823	LOC101929897	161	-29.87	-33.7	0.011314
hsa-miR-185-3p	NR_126006	LINC00524	156	-20.92	-33.1	0.007077
hsa-miR-185-3p	NR_126363	LINC01526	151	-29.03	-33.2	0.01104
hsa-miR-185-3p	NR_130110	RAET1G	157	-27.9	-32.6	0.040922
hsa-miR-185-3p	NR_131339	SETD7	161	-27.25	-34.5	0.033823
hsa-miR-185-3p	NR_131938	LRP1-AS	156	-23.5	-33.7	0.011198
hsa-miR-185-3p	NR_131939	LRP1-AS	156	-23.5	-33.7	0.005191
hsa-miR-185-3p	NR_132125	TH2LCRR	160	-27.32	-31.6	0.014177
hsa-miR-185-3p	NR_132318	SHH	159	-30.14	-34.4	0.014953
hsa-miR-185-3p	NR_132319	SHH	159	-30.14	-34.4	0.01324
hsa-miR-185-3p	NR_133006	OLFM5P	154	-28.24	-33.5	0.030563
hsa-miR-193a-5p	NM_001034077	HIST2H4B	156	-30.77	-31.9	0.000124
hsa-miR-193a-5p	NM_001039920	ZNF384	174	-28.23	-32.4	0.04841

hsa-miR-193a-5p	NM_001083612	HYKK	154	-22.97	-28.8	0.001708
hsa-miR-193a-5p	NM_001100431	VSIG4	171	-32.77	-33.6	0.007272
hsa-miR-193a-5p	NM_001128141	DPEP1	154	-26.6	-28.9	0.015415
hsa-miR-193a-5p	NM_001135734	ZNF384	174	-28.23	-32.4	0.04841
hsa-miR-193a-5p	NM_001135914	KCP	170	-29.27	-32.3	0.001745
hsa-miR-193a-5p	NM_001143833	IQCF6	162	-23.95	-25.8	0.007879
hsa-miR-193a-5p	NM_001145033	C11orf96	154	-31.15	-32.8	0.013814
hsa-miR-193a-5p	NM_001145099	SLC2A6	158	-29.39	-32.3	0.03353
hsa-miR-193a-5p	NM_001145467	NCR3	152	-17.5	-21.4	0.046859
hsa-miR-193a-5p	NM_001161415	GPR17	152	-20.43	-34.1	0.013689
hsa-miR-193a-5p	NM_001161416	GPR17	152	-20.43	-34.1	0.013689
hsa-miR-193a-5p	NM_001161417	GPR17	152	-20.43	-34.1	0.013689
hsa-miR-193a-5p	NM_001166222	CARNS1	150	-29.17	-32.7	0.031525
hsa-miR-193a-5p	NM_001184830	VSIG4	171	-32.77	-33.6	0.02399
hsa-miR-193a-5p	NM_001184831	VSIG4	171	-32.77	-33.6	0.02399
hsa-miR-193a-5p	NM_001193452	NUDT16L1	151	-29.64	-35.2	0.007086
hsa-miR-193a-5p	NM_001200050	NPL	158	-30.73	-34.6	0.02381
hsa-miR-193a-5p	NM_001200051	NPL	158	-30.73	-34.6	0.007478
hsa-miR-193a-5p	NM_001200052	NPL	158	-30.73	-34.6	0.028201
hsa-miR-193a-5p	NM_001200056	NPL	158	-30.73	-34.6	0.007478
hsa-miR-193a-5p	NM_001257359	SAMD14	150	-23.42	-33.8	0.037204
hsa-miR-193a-5p	NM_001257403	VSIG4	171	-32.77	-33.6	0.003403
hsa-miR-193a-5p	NM_001258311	PGBD5	155	-32.21	-34.2	0.036818
hsa-miR-193a-5p	NM_001261835	BZRAP1	180	-30.52	-34.9	0.016365
hsa-miR-193a-5p	NM_001267782	AMBRA1	151	-29.5	-33.4	0.024095
hsa-miR-193a-5p	NM_001267783	AMBRA1	151	-29.5	-33.4	0.024095
hsa-miR-193a-5p	NM_001287593	CLIC1	156	-25.63	-28.3	0.03141
hsa-miR-193a-5p	NM_001287594	CLIC1	156	-25.63	-28.3	0.03141
hsa-miR-193a-5p	NM_001288	CLIC1	156	-25.63	-28.3	0.03141
hsa-miR-193a-5p	NM_001292032	ZNF76	151	-33.41	-36.8	0.002873

hsa-miR-193a-5p	NM_001300731	AMBRA1	151	-29.5	-33.4	0.024095
hsa-miR-193a-5p	NM_001304799	NARFL	150	-21.95	-30.8	0.040519
hsa-miR-193a-5p	NM_001304993	SDSL	172	-30.05	-33.8	0.000955
hsa-miR-193a-5p	NM_001308027	MSANTD2	160	-26.97	-33.4	0.012358
hsa-miR-193a-5p	NM_001308347	CIAPIN1	151	-29.73	-32.9	0.028596
hsa-miR-193a-5p	NM_001308358	CIAPIN1	151	-29.73	-32.9	0.029532
hsa-miR-193a-5p	NM_001312919	MSANTD2	160	-26.97	-33.4	0.043325
hsa-miR-193a-5p	NM_001312920	MSANTD2	160	-26.97	-33.4	0.012358
hsa-miR-193a-5p	NM_001312921	MSANTD2	160	-26.97	-33.4	0.012358
hsa-miR-193a-5p	NM_001383	DPH1	156	-26.45	-31.3	0.046118
hsa-miR-193a-5p	NM_003427	ZNF76	151	-33.41	-36.8	0.002873
hsa-miR-193a-5p	NM_003548	HIST2H4A	156	-30.77	-31.9	0.000124
hsa-miR-193a-5p	NM_003585	DOC2B	162	-26.59	-30.9	0.036388
hsa-miR-193a-5p	NM_004413	DPEP1	154	-26.6	-28.9	0.015415
hsa-miR-193a-5p	NM_004455	EXTL1	151	-31.09	-33	0.032085
hsa-miR-193a-5p	NM_004758	BZRAP1	180	-30.52	-34.9	0.016365
hsa-miR-193a-5p	NM_005291	GPR17	152	-20.43	-34.1	0.013689
hsa-miR-193a-5p	NM_005430	WNT1	161	-27.96	-32.2	0.03766
hsa-miR-193a-5p	NM_005973	PRCC	155	-29.44	-32.7	0.005682
hsa-miR-193a-5p	NM_007268	VSIG4	171	-32.77	-33.6	0.007272
hsa-miR-193a-5p	NM_012396	PHLDA3	174	-31.03	-33.9	0.047537
hsa-miR-193a-5p	NM_014922	NLRP1	155	-24.46	-30.4	0.046016
hsa-miR-193a-5p	NM_017585	SLC2A6	158	-29.39	-32.3	0.03353
hsa-miR-193a-5p	NM_017749	AMBRA1	151	-29.5	-33.4	0.024095
hsa-miR-193a-5p	NM_020313	CIAPIN1	151	-29.73	-32.9	0.028596
hsa-miR-193a-5p	NM_020643	C11orf16	151	-27.44	-28.9	0.046
hsa-miR-193a-5p	NM_020811	CARNS1	150	-29.17	-32.7	0.031525
hsa-miR-193a-5p	NM_022493	NARFL	150	-21.95	-30.8	0.040519
hsa-miR-193a-5p	NM_022773	LMF1	151	-20.3	-32.3	0.031882
hsa-miR-193a-5p	NM_024418	BZRAP1	180	-30.52	-34.9	0.016365

hsa-miR-193a-5p	NM_024631	MSANTD2	160	-26.97	-33.4	0.012358
hsa-miR-193a-5p	NM_024653	PRKRIP1	171	-35.42	-38.5	0.004771
hsa-miR-193a-5p	NM_030769	NPL	158	-30.73	-34.6	0.02381
hsa-miR-193a-5p	NM_032349	NUDT16L1	151	-29.64	-35.2	0.005289
hsa-miR-193a-5p	NM_032428	FRMPD3	150	-34.15	-36.4	0.015477
hsa-miR-193a-5p	NM_032680	CRACR2A	174	-27.08	-32	0.016043
hsa-miR-193a-5p	NM_033004	NLRP1	155	-24.46	-30.4	0.046016
hsa-miR-193a-5p	NM_033006	NLRP1	155	-24.46	-30.4	0.046016
hsa-miR-193a-5p	NM_033007	NLRP1	155	-24.46	-30.4	0.046016
hsa-miR-193a-5p	NM_052813	CARD9	151	-27.1	-30.6	0.015632
hsa-miR-193a-5p	NM_133476	ZNF384	174	-28.23	-32.4	0.04841
hsa-miR-193a-5p	NM_138432	SDSL	172	-30.05	-33.8	0.000955
hsa-miR-193a-5p	NM_152643	KNDC1	155	-29.46	-33.6	0.038358
hsa-miR-193a-5p	NM_174920	SAMD14	150	-23.42	-33.8	0.037204
hsa-miR-193a-5p	NM_175834	KRT79	165	-22.52	-31	0.018179
hsa-miR-193a-5p	NM_175922	PRR18	152	-30.49	-33.6	0.04022
hsa-miR-193a-5p	NM_207373	C10orf99	163	-35.25	-38	0.000857
hsa-miR-193a-5p	NR_038435	HOXD-AS2	163	-34.78	-36.2	0.003393
hsa-miR-193a-5p	NR_046287	ATP2A1-AS1	152	-26.35	-30	0.048892
hsa-miR-193a-5p	NR_046288	ATP2A1-AS1	152	-26.35	-30	0.048516
hsa-miR-193a-5p	NR_046289	ATP2A1-AS1	152	-26.35	-30	0.042333
hsa-miR-193a-5p	NR_046290	ATP2A1-AS1	152	-26.35	-30	0.041967
hsa-miR-193a-5p	NR_047508	LINC00417	151	-30.69	-33.5	0.008071
hsa-miR-193a-5p	NR_049793	LOC100130476	152	-28.22	-33.9	0.047764
hsa-miR-193a-5p	NR_110259	LOC101929596	159	-29.49	-33.1	0.03133
hsa-miR-194-5p	NM_000862	HSD3B1	170	-25.78	-30.4	0.013708
hsa-miR-194-5p	NM_001038	SCNN1A	151	-27.79	-33.5	0.013353
hsa-miR-194-5p	NM_001142627	SEC61A2	152	-22.52	-30.1	0.032159
hsa-miR-194-5p	NM_001159575	SCNN1A	151	-27.79	-33.5	0.013353
hsa-miR-194-5p	NM_001159576	SCNN1A	151	-27.79	-33.5	0.013353

hsa-miR-194-5p	NM_001276290	PRKAR1A	151	-22.86	-25.8	0.043735
hsa-miR-194-5p	NM_001277335	RASA4B	152	-26.3	-28.8	0.036629
hsa-miR-194-5p	NR_003365	SUGT1P3	157	-25.01	-31	0.044659
hsa-miR-194-5p	NR_003667	SUGT1P1	157	-25.01	-31	0.031044
hsa-miR-194-5p	NR_029711	MIR194-1	174	-28.63	-32.5	0.000126
hsa-miR-194-5p	NR_029829	MIR194-2	154	-25.44	-29.9	0.00066
hsa-miR-194-5p	NR_037459	MIR3688-1	153	-15.77	-23.9	0.035457
hsa-miR-194-5p	NR_104048	TMPRSS11BN	161	-25.74	-33.9	0.017492
hsa-miR-194-5p	NR_106881	MIR6823	155	-24.28	-30.7	0.000161
hsa-miR-195-5p	NM_001122752	SERPINI1	153	-20.59	-28	0.010728
hsa-miR-195-5p	NM_001146257	ZDHHC15	161	-23.27	-28.4	0.020891
hsa-miR-195-5p	NM_001161630	KDM4E	162	-25.09	-28.9	0.024477
hsa-miR-195-5p	NM_001165994	GDPD1	151	-23.19	-25.4	0.015746
hsa-miR-195-5p	NM_001301107	CCDC53	153	-23.9	-28	0.012618
hsa-miR-195-5p	NM_003836	DLK1	167	-20.02	-25.2	0.049961
hsa-miR-195-5p	NM_005025	SERPINI1	153	-20.59	-28	0.010728
hsa-miR-195-5p	NM_006435	IFITM2	172	-23.89	-27.6	0.013448
hsa-miR-195-5p	NM_016053	CCDC53	153	-23.9	-28	0.012618
hsa-miR-195-5p	NM_138346	KIAA2013	163	-27.42	-29.7	0.027895
hsa-miR-195-5p	NM_173649	C2orf61	159	-27.75	-30.7	0.04908
hsa-miR-195-5p	NR_029486	MIR16-1	174	-29.97	-31.4	0.000121
hsa-miR-195-5p	NR_029712	MIR195	178	-30.51	-34.8	0.000012
hsa-miR-320a	NM_000964	RARA	158	-35.7	-37.6	0.004749
hsa-miR-320a	NM_001008563	USP20	172	-30.97	-34.5	0.018486
hsa-miR-320a	NM_001024809	RARA	158	-35.7	-37.6	0.004749
hsa-miR-320a	NM_001034077	HIST2H4B	152	-19	-24.1	0.014982
hsa-miR-320a	NM_001110303	USP20	172	-30.97	-34.5	0.018486
hsa-miR-320a	NM_001127699	SPINK5	158	-20.96	-25.2	0.020858
hsa-miR-320a	NM_001136036	ZNF692	159	-21.33	-27.2	0.030725
hsa-miR-320a	NM_001144825	RUNDC3A	156	-25.36	-28.8	0.045645

hsa-miR-320a	NM_001145301	RARA	158	-35.7	-37.6	0.004749
hsa-miR-320a	NM_001145302	RARA	158	-35.7	-37.6	0.004749
hsa-miR-320a	NM_001145961	SLC12A4	171	-29.63	-36	0.009199
hsa-miR-320a	NM_001145962	SLC12A4	171	-29.63	-36	0.009199
hsa-miR-320a	NM_001145963	SLC12A4	171	-29.63	-36	0.009199
hsa-miR-320a	NM_001145964	SLC12A4	171	-29.63	-36	0.009199
hsa-miR-320a	NM_001164741	ARHGAP4	162	-23.19	-29.6	0.02189
hsa-miR-320a	NM_001166621	TRAPPC1	157	-27.87	-32.8	0.002303
hsa-miR-320a	NM_001193328	ZNF692	159	-21.33	-27.2	0.030725
hsa-miR-320a	NM_001205271	PRSS46	159	-28.07	-32	0.008527
hsa-miR-320a	NM_001256125	CHIT1	168	-28.86	-32.6	0.019407
hsa-miR-320a	NM_001270509	CHIT1	168	-28.86	-32.6	0.019407
hsa-miR-320a	NM_001271856	GRASP	173	-28.49	-34.2	0.006947
hsa-miR-320a	NM_001288812	ARL17A	156	-25.57	-28.8	0.015808
hsa-miR-320a	NM_001301101	PPCDC	178	-31.51	-33.5	0.032008
hsa-miR-320a	NM_001301102	PPCDC	178	-31.51	-33.5	0.032008
hsa-miR-320a	NM_001301103	PPCDC	178	-31.51	-33.5	0.032008
hsa-miR-320a	NM_001301104	PPCDC	178	-31.51	-33.5	0.032008
hsa-miR-320a	NM_001301105	PPCDC	178	-31.51	-33.5	0.032008
hsa-miR-320a	NM_001302654	APOPT1	165	-24.48	-27.8	0.048412
hsa-miR-320a	NM_001303024	SSSCA1	155	-20.98	-25.5	0.047119
hsa-miR-320a	NM_001606	ABCA2	162	-25.83	-31.1	0.031149
hsa-miR-320a	NM_001666	ARHGAP4	162	-23.19	-29.6	0.02189
hsa-miR-320a	NM_003465	CHIT1	168	-28.86	-32.6	0.019407
hsa-miR-320a	NM_003540	HIST1H4F	151	-22.28	-27	0.002254
hsa-miR-320a	NM_003548	HIST2H4A	152	-19	-24.1	0.014982
hsa-miR-320a	NM_004467	FGL1	165	-23.28	-26.8	0.036604
hsa-miR-320a	NM_004701	CCNB2	159	-24.61	-26.1	0.039295
hsa-miR-320a	NM_005072	SLC12A4	171	-29.63	-36	0.009199
hsa-miR-320a	NM_006244	PPP2R5B	174	-29.16	-31.8	0.019257

hsa-miR-320a	NM_006396	SSSCA1	155	-20.98	-25.5	0.047119
hsa-miR-320a	NM_006676	USP20	172	-30.97	-34.5	0.022073
hsa-miR-320a	NM_014611	MDN1	151	-28.47	-32.4	0.039692
hsa-miR-320a	NM_017865	ZNF692	159	-21.33	-27.2	0.030725
hsa-miR-320a	NM_019896	POLE4	157	-24.65	-29.6	0.017488
hsa-miR-320a	NM_021128	POLR2L	164	-23.61	-31	0.029532
hsa-miR-320a	NM_021210	TRAPPC1	157	-27.87	-32.8	0.002303
hsa-miR-320a	NM_021823	PPCDC	178	-31.51	-33.5	0.032008
hsa-miR-320a	NM_052960	RBP7	154	-19.14	-26.7	0.03439
hsa-miR-320a	NM_054034	FN1	163	-23.42	-27.5	0.013163
hsa-miR-320a	NM_080431	ACTRT2	157	-23.03	-26.1	0.009924
hsa-miR-320a	NM_130899	FAM71B	159	-21.54	-24.8	0.040887
hsa-miR-320a	NM_147203	FGL1	165	-23.28	-26.8	0.036604
hsa-miR-320a	NM_152544	TRMT44	167	-28.01	-32.3	0.012072
hsa-miR-320a	NM_173493	PASD1	164	-23.76	-30.2	0.037865
hsa-miR-320a	NM_174958	ATP2A3	179	-27.96	-31.4	0.044477
hsa-miR-320a	NM_181711	GRASP	173	-28.49	-34.2	0.006947
hsa-miR-320a	NM_201552	FGL1	165	-23.28	-26.8	0.036604
hsa-miR-320a	NM_201553	FGL1	165	-23.28	-26.8	0.036604
hsa-miR-320a	NM_212533	ABCA2	162	-25.83	-31.1	0.031149
hsa-miR-320a	NM_212559	XKRX	177	-31.14	-33.8	0.012955
hsa-miR-320a	NR_030684	TRAPPC1	157	-27.87	-32.8	0.015551
hsa-miR-320a	NR_030697	TRAPPC1	157	-27.87	-32.8	0.013575
hsa-miR-320a	NR_036190	MIR4305	153	-24.08	-26.9	0.009438
hsa-miR-320a	NR_045039	SMUG1	176	-26.48	-31.1	0.034491
hsa-miR-320a	NR_125851	LOC101928191	155	-27.28	-31.6	0.044257
hsa-miR-106b-5p	NM_000296	PKD1	164	-21.12	-29.9	0.049525
hsa-miR-106b-5p	NM_000413	HSD17B1	159	-22.31	-27.1	0.026609
hsa-miR-106b-5p	NM_001009944	PKD1	164	-21.12	-29.9	0.049525
hsa-miR-106b-5p	NM_001039724	NOSTRIN	168	-23.61	-27.5	0.035078

hsa-miR-106b-5p	NM_001111101	CNRIP1	158	-22.99	-26.1	0.041299
hsa-miR-106b-5p	NM_001171631	NOSTRIN	168	-23.61	-27.5	0.035078
hsa-miR-106b-5p	NM_001171632	NOSTRIN	168	-23.61	-27.5	0.035078
hsa-miR-106b-5p	NM_001289058	STK33	174	-27.17	-29.5	0.030246
hsa-miR-106b-5p	NM_001289059	STK33	174	-27.17	-29.5	0.030246
hsa-miR-106b-5p	NM_001289061	STK33	174	-27.17	-29.5	0.030246
hsa-miR-106b-5p	NM_001294345	NINJ2	153	-20.37	-27.8	0.034671
hsa-miR-106b-5p	NM_001294346	NINJ2	153	-20.37	-27.8	0.034671
hsa-miR-106b-5p	NM_001310156	SPATA1	167	-20.77	-28.3	0.048256
hsa-miR-106b-5p	NM_014798	PLEKHM1	187	-31.88	-36	0.007386
hsa-miR-106b-5p	NM_015697	COQ2	158	-20.9	-27.6	0.034133
hsa-miR-106b-5p	NM_016533	NINJ2	153	-20.37	-27.8	0.034671
hsa-miR-106b-5p	NM_018646	TRPV6	168	-24.84	-28.3	0.038094
hsa-miR-106b-5p	NM_030906	STK33	174	-27.17	-29.5	0.030246
hsa-miR-106b-5p	NM_052946	NOSTRIN	168	-23.61	-27.5	0.035078
hsa-miR-106b-5p	NM_139266	STAT1	157	-23.88	-26.8	0.030513
hsa-miR-106b-5p	NM_147133	NFX1	175	-22.78	-27.5	0.03366
hsa-miR-106b-5p	NM_152326	ANKRD9	160	-22.71	-24.5	0.009962
hsa-miR-106b-5p	NM_172207	CAMKK1	174	-22.79	-31.7	0.012524
hsa-miR-106b-5p	NM_178510	ANKK1	167	-22.58	-25.9	0.017546
hsa-miR-106b-5p	NR_026740	LOC389033	151	-20.77	-29.4	0.014414
hsa-miR-106b-5p	NR_104662	LOC102467081	175	-22.59	-29.8	0.02003
hsa-miR-29c-5p	NM_001031715	IQCH	152	-28.59	-33	0.020562
hsa-miR-29c-5p	NM_001100400	PDS5A	154	-26.65	-32.5	0.00475
hsa-miR-29c-5p	NM_001284347	IQCH	152	-28.59	-33	0.020562
hsa-miR-29c-5p	NM_001284348	IQCH	152	-28.59	-33	0.024777
hsa-miR-29c-5p	NR_029503	MIR29A	156	-26.83	-29.6	0.000469
hsa-miR-29c-5p	NR_029832	MIR29C	156	-26.83	-29.6	0.001058
hsa-miR-29c-3p	NM_000426	LAMA2	171	-25.4	-27.9	0.010784
hsa-miR-29c-3p	NM_001079823	LAMA2	171	-25.4	-27.9	0.010784

hsa-miR-29c-3p	NM_001166533	DDX4	158	-23.85	-26.2	0.033692
hsa-miR-29c-3p	NM_001293312	DARS	175	-27.57	-31.6	0.035498
hsa-miR-29c-3p	NM_001349	DARS	175	-27.57	-31.6	0.035498
hsa-miR-29c-3p	NM_004688	NMI	176	-24.24	-27.6	0.013647
hsa-miR-29c-3p	NM_006303	AIMP2	168	-18.16	-24.5	0.036989
hsa-miR-29c-3p	NM_017417	GALNT8	161	-21.27	-25.4	0.017452
hsa-miR-29c-3p	NM_018663	PXMP2	168	-26.2	-29.1	0.010163
hsa-miR-29c-3p	NM_022304	HRH2	163	-25.32	-28.7	0.008283
hsa-miR-29c-3p	NM_175918	CRIPAK	158	-20.4	-24.4	0.0388
hsa-miR-29c-3p	NR_002983	SNORA55	156	-22.87	-27	0.007302
hsa-miR-29c-3p	NR_029503	MIR29A	156	-23.06	-27.8	0.000724
hsa-miR-29c-3p	NR_029832	MIR29C	160	-25.19	-29.1	0.000687
hsa-miR-29c-3p	NR_039790	MIR4647	153	-19.58	-23.1	0.029074
hsa-miR-29c-3p	NR_132762	SCARNA26A	152	-23.83	-26.4	0.01238
hsa-miR-29c-3p	NR_132767	SCARNA26B	152	-23.83	-26.4	0.01238
hsa-miR-130b-3p	NM_001005354	PRR13	168	-20.08	-28.8	0.048063
hsa-miR-130b-3p	NM_001166058	RXFP2	158	-24.32	-28.3	0.043807
hsa-miR-130b-3p	NM_001199829	HORMAD1	156	-26.64	-33.1	0.005585
hsa-miR-130b-3p	NM_001242821	DEF8	154	-24.93	-27.4	0.007191
hsa-miR-130b-3p	NM_001242822	DEF8	154	-24.93	-27.4	0.007191
hsa-miR-130b-3p	NM_001271828	PRELID1	160	-23.29	-28.7	0.026412
hsa-miR-130b-3p	NM_001289807	CRYAB	159	-25.14	-28.3	0.005092
hsa-miR-130b-3p	NM_001289808	CRYAB	159	-25.14	-28.3	0.005092
hsa-miR-130b-3p	NM_001885	CRYAB	159	-25.14	-28.3	0.005092
hsa-miR-130b-3p	NM_004997	MYBPH	154	-24.2	-27.3	0.038793
hsa-miR-130b-3p	NM_013237	PRELID1	160	-23.29	-28.7	0.026412
hsa-miR-130b-3p	NM_016491	MRPL37	156	-29.23	-29.6	0.001984
hsa-miR-130b-3p	NM_017702	DEF8	154	-24.93	-27.4	0.007191
hsa-miR-130b-3p	NM_018457	PRR13	168	-20.08	-28.8	0.048063
hsa-miR-130b-3p	NM_032132	HORMAD1	156	-26.64	-33.1	0.005585

hsa-miR-130b-3p	NM_054031	MRGPRX3	169	-26.17	-27.6	0.032285
hsa-miR-130b-3p	NM_130806	RXFP2	158	-24.32	-28.3	0.043807
hsa-miR-130b-3p	NR_002326	SNORA64	162	-19.64	-24.4	0.045805
hsa-miR-130b-3p	NR_002967	SNORA31	158	-16.76	-24.4	0.043344
hsa-miR-130b-3p	NR_003266	LOC220729	168	-27.93	-33	0.026808
hsa-miR-130b-3p	NR_029498	MIR25	157	-24.11	-27.6	0.002361
hsa-miR-130b-3p	NR_029842	MIR301A	168	-21.89	-27.7	0.00234
hsa-miR-130b-3p	NR_039649	MIR4447	167	-20.73	-25.1	0.013989
hsa-miR-130b-3p	NR_125395	LOC101927056	171	-27.01	-32.9	0.0102
hsa-miR-130b-3p	NR_125396	LOC101927056	171	-27.01	-32.9	0.004889
hsa-miR-361-5p	NM_001098831	MORN4	150	-31.98	-33.5	0.029173
hsa-miR-361-5p	NM_022839	MRPS11	157	-27.13	-31.4	0.01806
hsa-miR-361-5p	NM_176805	MRPS11	157	-27.13	-31.4	0.01806
hsa-miR-361-5p	NM_178832	MORN4	150	-31.98	-33.5	0.029173
hsa-miR-361-5p	NM_201651	SLC28A1	163	-27.7	-32.4	0.009618
hsa-miR-361-5p	NR_106796	MIR6738	158	-18.35	-22.4	0.044602
hsa-miR-361-5p	NR_106943	MIR6883	159	-29.4	-32	0.000141
hsa-miR-361-5p	NR_120612	NUTM2B-AS1	170	-26.68	-31.1	0.034414
hsa-miR-361-5p	NR_125397	LINC01471	154	-29.53	-32.1	0.034316
hsa-miR-361-3p	NM_000135	FANCA	178	-37.51	-39.1	0.001652
hsa-miR-361-3p	NM_000261	MYOC	152	-26.69	-30.7	0.026048
hsa-miR-361-3p	NM_000263	NAGLU	163	-29.37	-37.2	0.000154
hsa-miR-361-3p	NM_000359	TGM1	150	-31.23	-35.4	0.000365
hsa-miR-361-3p	NM_000494	COL17A1	159	-30.66	-36.1	0.005527
hsa-miR-361-3p	NM_000607	ORM1	152	-27.52	-30.1	0.002433
hsa-miR-361-3p	NM_000608	ORM2	152	-27.52	-30.1	0.002433
hsa-miR-361-3p	NM_000667	ADH1A	160	-27.3	-29.1	0.018765
hsa-miR-361-3p	NM_000892	KLKB1	160	-28	-31.7	0.004748
hsa-miR-361-3p	NM_000920	PC	151	-26.85	-30	0.022236
hsa-miR-361-3p	NM_000964	RARA	168	-28.67	-33.4	0.034824

hsa-miR-361-3p	NM_001001712	LCN10	156	-30.48	-32.9	0.040858
hsa-miR-361-3p	NM_001006944	RPS6KA4	150	-25.66	-31.4	0.033111
hsa-miR-361-3p	NM_001024024	GCH1	150	-30.56	-33.1	0.026325
hsa-miR-361-3p	NM_001024070	GCH1	150	-30.56	-33.1	0.026178
hsa-miR-361-3p	NM_001024071	GCH1	150	-30.56	-33.1	0.025154
hsa-miR-361-3p	NM_001024809	RARA	168	-28.67	-33.4	0.034824
hsa-miR-361-3p	NM_001024957	BRMS1	163	-29.64	-31.8	0.007324
hsa-miR-361-3p	NM_001029891	PGAM4	162	-32.58	-32.8	0.023994
hsa-miR-361-3p	NM_001031677	RAB24	163	-30.24	-35.2	0.003314
hsa-miR-361-3p	NM_001033113	ENTPD8	162	-28.22	-32.2	0.01425
hsa-miR-361-3p	NM_001039397	TBC1D28	158	-27.75	-31.7	0.041026
hsa-miR-361-3p	NM_001039517	RUSC1-AS1	167	-27.98	-31.1	0.00957
hsa-miR-361-3p	NM_001040716	PC	151	-26.85	-30	0.022236
hsa-miR-361-3p	NM_001079675	ETV4	176	-27.59	-30.6	0.042518
hsa-miR-361-3p	NM_001080397	SLC45A1	157	-25.88	-29.1	0.007074
hsa-miR-361-3p	NM_001080514	SCX	152	-26.13	-34.3	0.00208
hsa-miR-361-3p	NM_001093730	DYTN	152	-25.06	-27.5	0.027429
hsa-miR-361-3p	NM_001098670	RASGRP2	152	-27.02	-28	0.026892
hsa-miR-361-3p	NM_001098671	RASGRP2	152	-27.02	-28	0.026892
hsa-miR-361-3p	NM_001099784	FBXL19	165	-31.37	-34	0.03049
hsa-miR-361-3p	NM_001100118	XRCC3	155	-31.17	-33.4	0.026789
hsa-miR-361-3p	NM_001100119	XRCC3	155	-31.17	-33.4	0.026789
hsa-miR-361-3p	NM_001100878	MROH6	181	-36.28	-37.7	0.003122
hsa-miR-361-3p	NM_001105563	CCHCR1	158	-27.61	-27.8	0.014561
hsa-miR-361-3p	NM_001105564	CCHCR1	158	-27.61	-27.8	0.014561
hsa-miR-361-3p	NM_001114748	TMEM240	151	-27.8	-30.5	0.035441
hsa-miR-361-3p	NM_001128141	DPEP1	153	-25.76	-27.3	0.033904
hsa-miR-361-3p	NM_001130101	NR1H3	151	-25.33	-28.6	0.038277
hsa-miR-361-3p	NM_001130102	NR1H3	151	-25.33	-28.6	0.038277
hsa-miR-361-3p	NM_001130845	BCL6	169	-27.6	-34.7	0.013012

hsa-miR-361-3p	NM_001134738	BCL6	169	-27.6	-34.7	0.013012
hsa-miR-361-3p	NM_001136106	BEAN1	169	-31.18	-33.8	0.044418
hsa-miR-361-3p	NM_001142805	SLC6A8	162	-27.46	-34.2	0.023539
hsa-miR-361-3p	NM_001142806	SLC6A8	162	-27.46	-34.2	0.023539
hsa-miR-361-3p	NM_001143839	PDE2A	161	-27.87	-32.3	0.047091
hsa-miR-361-3p	NM_001145301	RARA	168	-28.67	-33.4	0.034824
hsa-miR-361-3p	NM_001145302	RARA	168	-28.67	-33.4	0.034824
hsa-miR-361-3p	NM_001145527	EHMT1	165	-28.91	-28.6	0.022008
hsa-miR-361-3p	NM_001145795	SH2B1	163	-30.23	-31.8	0.009767
hsa-miR-361-3p	NM_001145796	SH2B1	163	-30.23	-31.8	0.028189
hsa-miR-361-3p	NM_001145797	SH2B1	163	-30.23	-31.8	0.029172
hsa-miR-361-3p	NM_001145812	SH2B1	163	-30.23	-31.8	0.028189
hsa-miR-361-3p	NM_001146209	PDE2A	161	-27.87	-32.3	0.047091
hsa-miR-361-3p	NM_001160042	IQCC	158	-28.48	-32.1	0.017494
hsa-miR-361-3p	NM_001165977	PROM2	156	-26.04	-28.7	0.027519
hsa-miR-361-3p	NM_001166660	NLGN3	169	-30.81	-32	0.045516
hsa-miR-361-3p	NM_001178020	BEAN1	169	-31.18	-33.8	0.044418
hsa-miR-361-3p	NM_001191054	ANHX	165	-28.37	-30.9	0.028614
hsa-miR-361-3p	NM_001193452	NUDT16L1	152	-28.14	-31.1	0.043745
hsa-miR-361-3p	NM_001194946	EIF4G1	150	-20.8	-29.1	0.047129
hsa-miR-361-3p	NM_001194947	EIF4G1	150	-20.8	-29.1	0.047129
hsa-miR-361-3p	NM_001195001	PTPRU	150	-25.61	-33.6	0.023603
hsa-miR-361-3p	NM_001195139	COG4	152	-22.44	-32.5	0.008414
hsa-miR-361-3p	NM_001197216	ASGR1	159	-26.57	-28.2	0.02435
hsa-miR-361-3p	NM_001200016	NAT6	151	-25	-27.3	0.031676
hsa-miR-361-3p	NM_001200018	NAT6	151	-25	-27.3	0.031676
hsa-miR-361-3p	NM_001203261	NDUFC2-KCTD14	169	-31.09	-33.3	0.040165
hsa-miR-361-3p	NM_001205252	RNF223	151	-25.04	-24.1	0.02858
hsa-miR-361-3p	NM_001220482	MEIS2	159	-29	-33.3	0.027014

hsa-miR-361-3p	NM_001242487	ZSWIM8	150	-27.64	-27.7	0.044644
hsa-miR-361-3p	NM_001242780	LOC100506388	167	-30.22	-30.9	0.008412
hsa-miR-361-3p	NM_001242929	ANKS3	150	-26.28	-28.8	0.045145
hsa-miR-361-3p	NM_001243784	PDE2A	161	-27.87	-32.3	0.047091
hsa-miR-361-3p	NM_001251934	NR1H3	151	-25.33	-28.6	0.038277
hsa-miR-361-3p	NM_001251935	NR1H3	151	-25.33	-28.6	0.038277
hsa-miR-361-3p	NM_001254726	AP4S1	161	-32.09	-32.6	0.026613
hsa-miR-361-3p	NM_001254727	AP4S1	161	-32.09	-32.6	0.033325
hsa-miR-361-3p	NM_001256197	ARAF	153	-23.18	-30.1	0.047325
hsa-miR-361-3p	NM_001256526	C9orf69	164	-29.77	-37.5	0.008955
hsa-miR-361-3p	NM_001257357	RAB44	158	-31.85	-33.9	0.018963
hsa-miR-361-3p	NM_001257389	CD63	163	-28.76	-31.7	0.000884
hsa-miR-361-3p	NM_001257390	CD63	163	-28.76	-31.7	0.000884
hsa-miR-361-3p	NM_001257391	CD63	163	-28.76	-31.7	0.000884
hsa-miR-361-3p	NM_001257392	CD63	163	-28.76	-31.7	0.000884
hsa-miR-361-3p	NM_001257400	CD63	163	-28.76	-31.7	0.000884
hsa-miR-361-3p	NM_001257401	CD63	163	-28.76	-31.7	0.000884
hsa-miR-361-3p	NM_001261412	DCTN2	158	-27.91	-31.8	0.022057
hsa-miR-361-3p	NM_001261413	DCTN2	158	-27.91	-31.8	0.022057
hsa-miR-361-3p	NM_001261437	ETV4	176	-27.59	-30.6	0.042518
hsa-miR-361-3p	NM_001261438	ETV4	176	-27.59	-30.6	0.042518
hsa-miR-361-3p	NM_001261439	ETV4	176	-27.59	-30.6	0.042518
hsa-miR-361-3p	NM_001267698	CD63	163	-28.76	-31.7	0.000884
hsa-miR-361-3p	NM_001270364	ALDH1L1	166	-29.88	-30.9	0.005788
hsa-miR-361-3p	NM_001270365	ALDH1L1	166	-29.88	-30.9	0.005788
hsa-miR-361-3p	NM_001272095	STX4	162	-29.06	-33.4	0.002104
hsa-miR-361-3p	NM_001272096	STX4	162	-29.06	-33.4	0.002104
hsa-miR-361-3p	NM_001278674	LINC00452	164	-28.74	-32.5	0.002592
hsa-miR-361-3p	NM_001282351	FBXL19	165	-31.37	-34	0.03049
hsa-miR-361-3p	NM_001282406	KCTD14	169	-31.09	-33.3	0.01793

hsa-miR-361-3p	NM_001282434	HES6	155	-27.42	-31.2	0.045516
hsa-miR-361-3p	NM_001282624	LMNA	156	-25.95	-29.5	0.002321
hsa-miR-361-3p	NM_001282625	LMNA	156	-25.95	-29.5	0.002321
hsa-miR-361-3p	NM_001282983	FARP2	156	-28.19	-29.8	0.042612
hsa-miR-361-3p	NM_001284295	CHPF2	153	-23.02	-26	0.036777
hsa-miR-361-3p	NM_001286167	FANCA	178	-37.51	-39.1	0.001931
hsa-miR-361-3p	NM_001286679	LARP6	153	-29.64	-30.6	0.029085
hsa-miR-361-3p	NM_001289413	EHMT2	159	-27.73	-30.5	0.013505
hsa-miR-361-3p	NM_001290225	PTDSS1	168	-34.25	-37.3	0.003391
hsa-miR-361-3p	NM_001291157	EIF4G1	150	-20.8	-29.1	0.047129
hsa-miR-361-3p	NM_001293167	PANO1	157	-29.08	-32.2	0.031567
hsa-miR-361-3p	NM_001300802	RPS6KA4	150	-25.66	-31.4	0.033111
hsa-miR-361-3p	NM_001300913	C11orf24	156	-25.63	-29.3	0.023262
hsa-miR-361-3p	NM_001301186	LINGO1	157	-30.09	-31.6	0.048697
hsa-miR-361-3p	NM_001301187	LINGO1	157	-30.09	-31.6	0.048697
hsa-miR-361-3p	NM_001301189	LINGO1	157	-30.09	-31.6	0.048697
hsa-miR-361-3p	NM_001301191	LINGO1	157	-30.09	-31.6	0.048697
hsa-miR-361-3p	NM_001301192	LINGO1	157	-30.09	-31.6	0.048697
hsa-miR-361-3p	NM_001301194	LINGO1	157	-30.09	-31.6	0.048697
hsa-miR-361-3p	NM_001301195	LINGO1	157	-30.09	-31.6	0.048697
hsa-miR-361-3p	NM_001301197	LINGO1	157	-30.09	-31.6	0.048697
hsa-miR-361-3p	NM_001301198	LINGO1	157	-30.09	-31.6	0.048697
hsa-miR-361-3p	NM_001301199	LINGO1	157	-30.09	-31.6	0.048697
hsa-miR-361-3p	NM_001301200	LINGO1	157	-30.09	-31.6	0.048697
hsa-miR-361-3p	NM_001304385	LSMEM2	160	-25.29	-32.2	0.031567
hsa-miR-361-3p	NM_001308089	ANKS3	150	-26.28	-28.8	0.044967
hsa-miR-361-3p	NM_001308293	SH2B1	163	-30.23	-31.8	0.009767
hsa-miR-361-3p	NM_001308294	SH2B1	163	-30.23	-31.8	0.028189
hsa-miR-361-3p	NM_001308360	ADGRG3	168	-31.78	-34.1	0.013249
hsa-miR-361-3p	NM_001671	ASGR1	159	-26.57	-28.2	0.02435

hsa-miR-361-3p	NM_001706	BCL6	169	-27.6	-34.7	0.013012
hsa-miR-361-3p	NM_001780	CD63	163	-28.76	-31.7	0.000884
hsa-miR-361-3p	NM_001950	E2F4	162	-27.79	-35.7	0.004954
hsa-miR-361-3p	NM_001964	EGR1	153	-31.61	-33.4	0.028564
hsa-miR-361-3p	NM_001986	ETV4	176	-27.59	-30.6	0.042518
hsa-miR-361-3p	NM_002180	IGHMBP2	166	-31.14	-34.9	0.00839
hsa-miR-361-3p	NM_002399	MEIS2	159	-29	-33.3	0.038524
hsa-miR-361-3p	NM_002452	NUDT1	158	-25.81	-28.2	0.010311
hsa-miR-361-3p	NM_002518	NPAS2	160	-30.01	-33.8	0.024147
hsa-miR-361-3p	NM_002599	PDE2A	161	-27.87	-32.3	0.047091
hsa-miR-361-3p	NM_003102	SOD3	161	-29.78	-32.2	0.016491
hsa-miR-361-3p	NM_003280	TNNC1	157	-28.16	-30.8	0.003917
hsa-miR-361-3p	NM_003672	CDC14A	164	-29.9	-34.3	0.037966
hsa-miR-361-3p	NM_003942	RPS6KA4	150	-25.66	-31.4	0.033111
hsa-miR-361-3p	NM_004054	C3AR1	165	-30.44	-32.6	0.00715
hsa-miR-361-3p	NM_004095	EIF4EBP1	150	-22.87	-29.1	0.043919
hsa-miR-361-3p	NM_004413	DPEP1	153	-25.76	-27.3	0.033904
hsa-miR-361-3p	NM_004604	STX4	162	-29.06	-33.4	0.002104
hsa-miR-361-3p	NM_004905	PRDX6	177	-32.27	-35.7	0.006618
hsa-miR-361-3p	NM_004952	EFNA3	165	-31.72	-33.6	0.018945
hsa-miR-361-3p	NM_004953	EIF4G1	150	-20.8	-29.1	0.047129
hsa-miR-361-3p	NM_005432	XRCC3	155	-31.17	-33.4	0.026789
hsa-miR-361-3p	NM_005572	LMNA	156	-25.95	-29.5	0.002321
hsa-miR-361-3p	NM_005629	SLC6A8	162	-27.46	-34.2	0.023539
hsa-miR-361-3p	NM_005693	NR1H3	151	-25.33	-28.6	0.038277
hsa-miR-361-3p	NM_005704	PTPRU	150	-25.61	-33.6	0.023603
hsa-miR-361-3p	NM_005995	TBX10	155	-27.1	-31.1	0.008709
hsa-miR-361-3p	NM_006368	CREB3	158	-26.73	-28.8	0.025654
hsa-miR-361-3p	NM_006400	DCTN2	158	-27.91	-31.8	0.022057
hsa-miR-361-3p	NM_006442	DRAP1	151	-21.96	-26.8	0.02225

hsa-miR-361-3p	NM_006709	EHMT2	159	-27.73	-30.5	0.013505
hsa-miR-361-3p	NM_007077	AP4S1	161	-32.09	-32.6	0.026613
hsa-miR-361-3p	NM_012106	ARL2BP	168	-33.66	-35	0.016276
hsa-miR-361-3p	NM_012190	ALDH1L1	166	-29.88	-30.9	0.005788
hsa-miR-361-3p	NM_012191	NAT6	151	-25	-27.3	0.031676
hsa-miR-361-3p	NM_013392	NRBP1	152	-29	-30.2	0.026915
hsa-miR-361-3p	NM_014241	HACD1	160	-28.8	-29.3	0.036181
hsa-miR-361-3p	NM_014360	NKX2-8	172	-30.75	-36	0.005226
hsa-miR-361-3p	NM_014624	S100A6	158	-25.57	-26.4	0.013021
hsa-miR-361-3p	NM_014652	IPO13	151	-27.82	-31.6	0.008343
hsa-miR-361-3p	NM_014712	SETD1A	163	-29.33	-31.2	0.029375
hsa-miR-361-3p	NM_014754	PTDSS1	168	-34.25	-37.3	0.003391
hsa-miR-361-3p	NM_015037	ZSWIM8	150	-27.64	-27.7	0.044644
hsa-miR-361-3p	NM_015386	COG4	152	-22.44	-32.5	0.008414
hsa-miR-361-3p	NM_015399	BRMS1	163	-29.64	-31.8	0.017267
hsa-miR-361-3p	NM_015503	SH2B1	163	-30.23	-31.8	0.028189
hsa-miR-361-3p	NM_016086	STYXL1	170	-29.41	-30.1	0.002844
hsa-miR-361-3p	NM_016155	MMP17	155	-29.61	-32.4	0.010887
hsa-miR-361-3p	NM_016639	TNFRSF12A	164	-33.44	-29.7	0.048638
hsa-miR-361-3p	NM_016938	EFEMP2	159	-34.64	-36.5	0.001317
hsa-miR-361-3p	NM_018134	IQCC	158	-28.48	-32.1	0.017494
hsa-miR-361-3p	NM_018357	LARP6	153	-29.64	-30.6	0.029085
hsa-miR-361-3p	NM_018977	NLGN3	169	-30.81	-32	0.045516
hsa-miR-361-3p	NM_018982	YIPF1	155	-28.84	-30.6	0.031902
hsa-miR-361-3p	NM_019015	CHPF2	153	-23.02	-26	0.036777
hsa-miR-361-3p	NM_019052	CCHCR1	158	-27.61	-27.8	0.014561
hsa-miR-361-3p	NM_020526	EPHA8	174	-34.38	-35.7	0.018778
hsa-miR-361-3p	NM_021933	MIIP	162	-25.13	-29.6	0.012717
hsa-miR-361-3p	NM_022172	PC	151	-26.85	-30	0.022236
hsa-miR-361-3p	NM_022338	C11orf24	156	-25.63	-29.3	0.023262

hsa-miR-361-3p	NM_023083	CAPN10	161	-28.8	-30	0.028011
hsa-miR-361-3p	NM_023085	CAPN10	161	-28.8	-30	0.028011
hsa-miR-361-3p	NM_023930	KCTD14	169	-31.09	-33.3	0.01793
hsa-miR-361-3p	NM_024082	PRRG3	150	-24.9	-30.8	0.028802
hsa-miR-361-3p	NM_024571	SNRNP25	171	-28.03	-30.4	0.031863
hsa-miR-361-3p	NM_025256	EHMT2	159	-27.73	-30.5	0.013505
hsa-miR-361-3p	NM_031923	TAF3	164	-29.14	-28.9	0.027366
hsa-miR-361-3p	NM_032349	NUDT16L1	152	-28.14	-31.1	0.033888
hsa-miR-361-3p	NM_032536	NTNG2	165	-32.21	-30.9	0.040742
hsa-miR-361-3p	NM_032548	ABTB1	153	-24.58	-29.4	0.040816
hsa-miR-361-3p	NM_032808	LINGO1	157	-30.09	-31.6	0.048697
hsa-miR-361-3p	NM_033067	DMRTB1	160	-30.3	-32.1	0.028445
hsa-miR-361-3p	NM_130781	RAB24	163	-30.24	-35.2	0.003314
hsa-miR-361-3p	NM_133177	PTPRU	150	-25.61	-33.6	0.023603
hsa-miR-361-3p	NM_133178	PTPRU	150	-25.61	-33.6	0.023603
hsa-miR-361-3p	NM_133450	ANKS3	150	-26.28	-28.8	0.045145
hsa-miR-361-3p	NM_144597	C15orf40	169	-32.88	-32.2	0.04703
hsa-miR-361-3p	NM_144702	LRRC71	150	-26.54	-30.7	0.00162
hsa-miR-361-3p	NM_152568	NKX6-3	153	-24.49	-26.3	0.028392
hsa-miR-361-3p	NM_152742	GPC2	157	-28.46	-31.3	0.026989
hsa-miR-361-3p	NM_152833	C9orf69	164	-29.77	-37.5	0.008955
hsa-miR-361-3p	NM_153200	EDF1	152	-24.76	-27.6	0.027161
hsa-miR-361-3p	NM_153215	LSMEM2	160	-25.29	-32.2	0.031567
hsa-miR-361-3p	NM_153819	RASGRP2	152	-27.02	-28	0.026892
hsa-miR-361-3p	NM_170674	MEIS2	159	-29	-33.3	0.038524
hsa-miR-361-3p	NM_170675	MEIS2	159	-29	-33.3	0.027014
hsa-miR-361-3p	NM_170676	MEIS2	159	-29	-33.3	0.027014
hsa-miR-361-3p	NM_170677	MEIS2	159	-29	-33.3	0.038524
hsa-miR-361-3p	NM_170776	ADGRG3	168	-31.78	-34.1	0.013249
hsa-miR-361-3p	NM_172027	ABTB1	153	-24.58	-29.4	0.040816

hsa-miR-361-3p	NM_172315	MEIS2	159	-29	-33.3	0.038524
hsa-miR-361-3p	NM_172316	MEIS2	159	-29	-33.3	0.038524
hsa-miR-361-3p	NM_173541	C10orf91	154	-27.8	-27.6	0.041871
hsa-miR-361-3p	NM_173615	VWA3A	163	-31.03	-34.1	0.013987
hsa-miR-361-3p	NM_174922	ADCK5	154	-24.28	-27.7	0.017573
hsa-miR-361-3p	NM_178537	B4GALNT4	150	-20.58	-30.2	0.015854
hsa-miR-361-3p	NM_178828	SPATA31E1	153	-24.35	-27.4	0.004016
hsa-miR-361-3p	NM_181303	NLGN3	169	-30.81	-32	0.045516
hsa-miR-361-3p	NM_181715	CRTC2	154	-27.37	-31	0.017542
hsa-miR-361-3p	NM_182917	EIF4G1	150	-20.8	-29.1	0.047129
hsa-miR-361-3p	NM_194249	DND1	163	-26.3	-31.9	0.013935
hsa-miR-361-3p	NM_198241	EIF4G1	150	-20.8	-29.1	0.047129
hsa-miR-361-3p	NM_198242	EIF4G1	150	-20.8	-29.1	0.047129
hsa-miR-361-3p	NM_198244	EIF4G1	150	-20.8	-29.1	0.047129
hsa-miR-361-3p	NM_198585	ENTPD8	162	-28.22	-32.2	0.01425
hsa-miR-361-3p	NM_198948	NUDT1	158	-25.81	-28.2	0.010311
hsa-miR-361-3p	NM_198949	NUDT1	158	-25.81	-28.2	0.010311
hsa-miR-361-3p	NM_198950	NUDT1	158	-25.81	-28.2	0.010311
hsa-miR-361-3p	NM_198952	NUDT1	158	-25.81	-28.2	0.010311
hsa-miR-361-3p	NM_198953	NUDT1	158	-25.81	-28.2	0.010311
hsa-miR-361-3p	NM_198954	NUDT1	158	-25.81	-28.2	0.010311
hsa-miR-361-3p	NM_207344	SPRYD4	154	-29.99	-33.3	0.035989
hsa-miR-361-3p	NR_027715	BIN3-IT1	153	-31.61	-33.8	0.041999
hsa-miR-361-3p	NR_030598	MIR708	162	-20.49	-24.1	0.04344
hsa-miR-361-3p	NR_031695	MIR1282	156	-24.32	-28.5	0.004127
hsa-miR-361-3p	NR_036440	POU5F1P3	163	-27.12	-32.4	0.03706
hsa-miR-361-3p	NR_037652	ST20	157	-28.64	-31.9	0.021342
hsa-miR-361-3p	NR_037653	ST20	157	-28.64	-31.9	0.022504
hsa-miR-361-3p	NR_046287	ATP2A1-AS1	167	-32.66	-34.3	0.005672
hsa-miR-361-3p	NR_046288	ATP2A1-AS1	167	-32.66	-34.3	0.005621

hsa-miR-361-3p	NR_049883	MIR5698	157	-26.55	-31.6	0.000272
hsa-miR-361-3p	NR_103750	KIF25-AS1	165	-33.31	-34.6	0.020269
hsa-miR-361-3p	NR_106758	MIR6503	153	-26.8	-27.8	0.004411
hsa-miR-361-3p	NR_109789	RAB24	163	-32.72	-37.2	0.009022
hsa-miR-361-3p	NR_109790	RAB24	163	-32.72	-37.2	0.009705
hsa-miR-361-3p	NR_120650	LOC101929073	165	-33.12	-33.4	0.007952
hsa-miR-361-3p	NR_121606	LOC102723709	150	-27.52	-30.4	0.023925
hsa-miR-361-3p	NR_125749	TBX2-AS1	153	-28.79	-32	0.031824
hsa-miR-361-3p	NR_125750	TBX2-AS1	153	-28.79	-32	0.027205
hsa-miR-361-3p	NR_125751	TBX2-AS1	153	-28.79	-32	0.021102
hsa-miR-363-3p	NM_001173487	NKRF	162	-27.23	-31	0.036596
hsa-miR-363-3p	NM_001173488	NKRF	162	-27.23	-31	0.036596
hsa-miR-363-3p	NM_001301168	SHF	158	-25.96	-29.8	0.040293
hsa-miR-363-3p	NM_001301171	SHF	158	-25.96	-29.8	0.040293
hsa-miR-363-3p	NM_004958	MTOR	154	-27.04	-31.4	0.027563
hsa-miR-363-3p	NM_017544	NKRF	162	-27.23	-31	0.036596
hsa-miR-363-3p	NM_138356	SHF	158	-25.96	-29.8	0.040293
hsa-miR-370-3p	NM_000063	C2	160	-30.07	-32.4	0.007304
hsa-miR-370-3p	NM_000375	UROS	161	-26.38	-29.1	0.032778
hsa-miR-370-3p	NM_000425	L1CAM	160	-28.57	-34.7	0.02096
hsa-miR-370-3p	NM_000499	CYP1A1	154	-28.71	-34	0.021346
hsa-miR-370-3p	NM_000503	EYA1	160	-35.16	-37.4	0.01404
hsa-miR-370-3p	NM_000725	CACNB3	158	-35.23	-33.2	0.03502
hsa-miR-370-3p	NM_000781	CYP11A1	150	-25.36	-34	0.001417
hsa-miR-370-3p	NM_000795	DRD2	155	-34.2	-36.9	0.007824
hsa-miR-370-3p	NM_001001683	MED11	158	-25.63	-29.7	0.044823
hsa-miR-370-3p	NM_001001712	LCN10	159	-33.7	-38.7	0.004792
hsa-miR-370-3p	NM_001001891	ANO7	150	-29.76	-35.4	0.016979
hsa-miR-370-3p	NM_001005752	GJB3	157	-26.37	-31.6	0.04759
hsa-miR-370-3p	NM_001006641	SLC25A25	169	-32.22	-34.2	0.049267

hsa-miR-370-3p	NM_001006642	SLC25A25	169	-32.22	-34.2	0.049267
hsa-miR-370-3p	NM_001008938	CKAP5	152	-24.43	-30.2	0.044221
hsa-miR-370-3p	NM_001009565	CDKL4	167	-32.39	-36.9	0.002801
hsa-miR-370-3p	NM_001009905	B3GNTL1	176	-39.05	-28.6	0.040434
hsa-miR-370-3p	NM_001009991	SYTL3	166	-30.77	-38.2	0.000281
hsa-miR-370-3p	NM_001010938	TNK2	165	-33.59	-39.8	0.001219
hsa-miR-370-3p	NM_001015891	AK6	159	-25.38	-30.3	0.022959
hsa-miR-370-3p	NM_001032377	SULT6B1	150	-27.29	-28.5	0.008124
hsa-miR-370-3p	NM_001039659	IL18BP	166	-32.65	-35.3	0.00673
hsa-miR-370-3p	NM_001039660	IL18BP	166	-32.65	-35.3	0.00673
hsa-miR-370-3p	NM_001040059	CD68	162	-28.31	-30.6	0.049951
hsa-miR-370-3p	NM_001083314	CHMP1A	152	-26.72	-35.6	0.020731
hsa-miR-370-3p	NM_001093770	SFTPA1	168	-34.59	-36.4	0.012514
hsa-miR-370-3p	NM_001098616	C1orf43	159	-26.54	-32.2	0.033754
hsa-miR-370-3p	NM_001099773	CYP11A1	150	-25.36	-34	0.001417
hsa-miR-370-3p	NM_001100118	XRCC3	161	-28.83	-34.1	0.028317
hsa-miR-370-3p	NM_001100119	XRCC3	161	-28.83	-34.1	0.028317
hsa-miR-370-3p	NM_001103150	PNMA5	155	-36.88	-39.8	0.003239
hsa-miR-370-3p	NM_001103151	PNMA5	155	-36.88	-39.8	0.003239
hsa-miR-370-3p	NM_001105579	SYNDIG1L	173	-39.26	-42	0.001599
hsa-miR-370-3p	NM_001122646	FAM178B	158	-31.26	-35.2	0.002731
hsa-miR-370-3p	NM_001122819	KIF17	158	-28.63	-32.4	0.020033
hsa-miR-370-3p	NM_001127258	HHIPL1	161	-27.77	-33.8	0.02709
hsa-miR-370-3p	NM_001130083	ABLIM2	160	-31.34	-36.5	0.016999
hsa-miR-370-3p	NM_001130084	ABLIM2	160	-31.34	-36.5	0.016999
hsa-miR-370-3p	NM_001130085	ABLIM2	160	-31.34	-36.5	0.016999
hsa-miR-370-3p	NM_001130086	ABLIM2	160	-31.34	-36.5	0.016999
hsa-miR-370-3p	NM_001130087	ABLIM2	160	-31.34	-36.5	0.016999
hsa-miR-370-3p	NM_001130860	HEPH	158	-28.62	-32.5	0.027354
hsa-miR-370-3p	NM_001130969	NSMF	158	-26.26	-34.5	0.043839

hsa-miR-370-3p	NM_001130970	NSMF	158	-26.26	-34.5	0.043839
hsa-miR-370-3p	NM_001130971	NSMF	158	-26.26	-34.5	0.043839
hsa-miR-370-3p	NM_001134395	C7orf50	159	-33.37	-34.5	0.009049
hsa-miR-370-3p	NM_001134396	C7orf50	159	-33.37	-34.5	0.009049
hsa-miR-370-3p	NM_001134774	KLC2	162	-30.04	-32.8	0.033963
hsa-miR-370-3p	NM_001134775	KLC2	162	-30.04	-32.8	0.033908
hsa-miR-370-3p	NM_001134776	KLC2	162	-30.04	-32.8	0.033908
hsa-miR-370-3p	NM_001135196	C10orf71	154	-28.34	-31.8	0.031655
hsa-miR-370-3p	NM_001135	ACAN	156	-28.95	-34.9	0.012584
hsa-miR-370-3p	NM_001136041	HDAC11	160	-30.01	-35.4	0.028352
hsa-miR-370-3p	NM_001142272	RAB11FIP3	155	-21.72	-33.9	0.048824
hsa-miR-370-3p	NM_001142593	ITPK1	158	-30.61	-40.2	0.004116
hsa-miR-370-3p	NM_001142776	CHAC1	156	-31.14	-31.3	0.049909
hsa-miR-370-3p	NM_001143758	LRRC27	157	-31.74	-33.8	0.00655
hsa-miR-370-3p	NM_001143759	LRRC27	157	-31.74	-33.8	0.008533
hsa-miR-370-3p	NM_001143804	PHOSPHO1	155	-33.11	-38.4	0.002613
hsa-miR-370-3p	NM_001143963	L1CAM	160	-28.57	-34.7	0.02096
hsa-miR-370-3p	NM_001144063	OSBPL5	150	-29.6	-37.5	0.005642
hsa-miR-370-3p	NM_001145055	IL18BP	166	-32.65	-35.3	0.006136
hsa-miR-370-3p	NM_001145057	IL18BP	166	-32.65	-35.3	0.00673
hsa-miR-370-3p	NM_001145201	PRAP1	160	-28.58	-32.4	0.004411
hsa-miR-370-3p	NM_001145853	WFS1	155	-25.36	-33.2	0.023472
hsa-miR-370-3p	NM_001145903	C2	160	-30.07	-32.4	0.007304
hsa-miR-370-3p	NM_001159996	NRG1	166	-32.25	-34.4	0.029033
hsa-miR-370-3p	NM_001160004	NRG1	166	-32.25	-34.4	0.029033
hsa-miR-370-3p	NM_001161465	JMJD4	166	-30.19	-33.2	0.042838
hsa-miR-370-3p	NM_001161	NUDT2	158	-25.13	-29.1	0.029622
hsa-miR-370-3p	NM_001164644	SFTPA1	168	-34.59	-36.4	0.012514
hsa-miR-370-3p	NM_001164645	SFTPA1	168	-34.59	-36.4	0.012514
hsa-miR-370-3p	NM_001164646	SFTPA1	168	-34.59	-36.4	0.012514

hsa-miR-370-3p	NM_001164647	SFTPA1	168	-34.59	-36.4	0.012514
hsa-miR-370-3p	NM_001164758	PRKAR1B	156	-30.06	-34.4	0.026484
hsa-miR-370-3p	NM_001164759	PRKAR1B	156	-30.06	-34.4	0.026484
hsa-miR-370-3p	NM_001164760	PRKAR1B	156	-30.06	-34.4	0.026484
hsa-miR-370-3p	NM_001164761	PRKAR1B	156	-30.06	-34.4	0.026484
hsa-miR-370-3p	NM_001164762	PRKAR1B	156	-30.06	-34.4	0.026484
hsa-miR-370-3p	NM_001164	APBB1	151	-25.02	-31.5	0.017907
hsa-miR-370-3p	NM_001166237	GSDMD	156	-28.24	-32.1	0.00211
hsa-miR-370-3p	NM_001168214	C3orf80	164	-30.59	-36.2	0.021867
hsa-miR-370-3p	NM_001170460	CDK16	156	-32.34	-36.4	0.011053
hsa-miR-370-3p	NM_001172667	FAM178B	158	-31.26	-35.2	0.002731
hsa-miR-370-3p	NM_001178063	C2	160	-30.07	-32.4	0.007304
hsa-miR-370-3p	NM_001178064	NSMF	158	-26.26	-34.5	0.043839
hsa-miR-370-3p	NM_001184924	PNMA5	155	-36.88	-39.8	0.003239
hsa-miR-370-3p	NM_001190728	RALGPS1	158	-30.52	-34.3	0.00529
hsa-miR-370-3p	NM_001190729	RALGPS1	158	-30.52	-34.3	0.00529
hsa-miR-370-3p	NM_001190918	THRA	159	-32.42	-36.7	0.001958
hsa-miR-370-3p	NM_001190919	THRA	159	-32.42	-36.7	0.001958
hsa-miR-370-3p	NM_001193333	CORO1A	151	-28.34	-30.8	0.002073
hsa-miR-370-3p	NM_001197224	BEAN1	151	-30.78	-33.1	0.010203
hsa-miR-370-3p	NM_001198843	RBM4	159	-30.31	-34	0.005702
hsa-miR-370-3p	NM_001198845	RBM14-RBM4	159	-30.31	-34	0.00639
hsa-miR-370-3p	NM_001198846	RBM14-RBM4	159	-30.31	-34	0.005702
hsa-miR-370-3p	NM_001204299	ZNF664-FAM101A	157	-33.75	-37.7	0.008611
hsa-miR-370-3p	NM_001204744	CDH16	151	-29.54	-31.7	0.007736
hsa-miR-370-3p	NM_001204745	CDH16	151	-29.54	-31.7	0.007736
hsa-miR-370-3p	NM_001204746	CDH16	151	-29.54	-31.7	0.007736
hsa-miR-370-3p	NM_001206915	CACNB3	158	-35.23	-33.2	0.03502
hsa-miR-370-3p	NM_001206916	CACNB3	158	-35.23	-33.2	0.03502

hsa-miR-370-3p	NM_001206917	CACNB3	158	-35.23	-33.2	0.03502
hsa-miR-370-3p	NM_001242384	SYTL3	166	-30.77	-38.2	0.000281
hsa-miR-370-3p	NM_001242394	SYTL3	166	-30.77	-38.2	0.000281
hsa-miR-370-3p	NM_001242395	SYTL3	166	-30.77	-38.2	0.000281
hsa-miR-370-3p	NM_001242648	PHACTR1	153	-31.76	-34.2	0.013991
hsa-miR-370-3p	NM_001242704	ZNF385C	157	-26.97	-33.6	0.041149
hsa-miR-370-3p	NM_001242783	TRIM26	164	-35.18	-39.1	0.004341
hsa-miR-370-3p	NM_001242786	BRF1	162	-33.67	-37.5	0.007189
hsa-miR-370-3p	NM_001242787	BRF1	162	-33.67	-37.5	0.007189
hsa-miR-370-3p	NM_001242788	BRF1	162	-33.67	-37.5	0.007189
hsa-miR-370-3p	NM_001242789	BRF1	162	-33.67	-37.5	0.007189
hsa-miR-370-3p	NM_001243539	IL15RA	152	-25.68	-33.8	0.015376
hsa-miR-370-3p	NM_001243878	FHL3	168	-29.53	-37.7	0.002019
hsa-miR-370-3p	NM_001244390	NUDT2	158	-25.13	-29.1	0.029622
hsa-miR-370-3p	NM_001251	CD68	162	-28.31	-30.6	0.049951
hsa-miR-370-3p	NM_001254757	ST3GAL4	158	-27.37	-34.8	0.006966
hsa-miR-370-3p	NM_001254758	ST3GAL4	158	-27.37	-34.8	0.006966
hsa-miR-370-3p	NM_001254759	ST3GAL4	158	-27.37	-34.8	0.006966
hsa-miR-370-3p	NM_001256482	EPHX2	158	-28.51	-32.3	0.011629
hsa-miR-370-3p	NM_001256483	EPHX2	158	-28.51	-32.3	0.011629
hsa-miR-370-3p	NM_001256484	EPHX2	158	-28.51	-32.3	0.011629
hsa-miR-370-3p	NM_001256627	BRSK2	158	-36.28	-35.2	0.036402
hsa-miR-370-3p	NM_001256629	BRSK2	158	-36.28	-35.2	0.03052
hsa-miR-370-3p	NM_001256630	BRSK2	158	-36.28	-35.2	0.036402
hsa-miR-370-3p	NM_001256765	IL15RA	152	-25.68	-33.8	0.015376
hsa-miR-370-3p	NM_001256789	CACNA1F	162	-29.39	-31.9	0.000399
hsa-miR-370-3p	NM_001256790	CACNA1F	162	-29.39	-31.9	0.000399
hsa-miR-370-3p	NM_001256964	CCDC51	153	-23.94	-28	0.041123
hsa-miR-370-3p	NM_001256965	CCDC51	153	-23.94	-28	0.041123
hsa-miR-370-3p	NM_001256966	CCDC51	153	-23.94	-28	0.041123

hsa-miR-370-3p	NM_001256967	CCDC51	153	-23.94	-28	0.041123
hsa-miR-370-3p	NM_001256968	CCDC51	153	-23.94	-28	0.041123
hsa-miR-370-3p	NM_001256969	CCDC51	153	-23.94	-28	0.041123
hsa-miR-370-3p	NM_001257118	CASP1	167	-27.65	-31.5	0.045894
hsa-miR-370-3p	NM_001257119	CASP1	167	-27.65	-31.5	0.045894
hsa-miR-370-3p	NM_001257319	APBB1	151	-25.02	-31.5	0.017907
hsa-miR-370-3p	NM_001257320	APBB1	151	-25.02	-31.5	0.017907
hsa-miR-370-3p	NM_001257321	APBB1	151	-25.02	-31.5	0.017907
hsa-miR-370-3p	NM_001257323	APBB1	151	-25.02	-31.5	0.017907
hsa-miR-370-3p	NM_001257325	APBB1	151	-25.02	-31.5	0.017907
hsa-miR-370-3p	NM_001257326	APBB1	151	-25.02	-31.5	0.017907
hsa-miR-370-3p	NM_001265614	SLC25A25	169	-32.22	-34.2	0.049267
hsa-miR-370-3p	NM_001267721	NOXO1	153	-22.83	-27.1	0.024361
hsa-miR-370-3p	NM_001271849	CDK2AP2	168	-29.81	-34.9	0.004137
hsa-miR-370-3p	NM_001271856	GRASP	159	-35.02	-34.8	0.008979
hsa-miR-370-3p	NM_001276294	NISCH	164	-30.61	-32.4	0.04747
hsa-miR-370-3p	NM_001278116	L1CAM	160	-28.57	-34.7	0.02096
hsa-miR-370-3p	NM_001282062	DOC2A	164	-29.47	-36.3	0.004288
hsa-miR-370-3p	NM_001282063	DOC2A	164	-29.47	-36.3	0.004288
hsa-miR-370-3p	NM_001282068	DOC2A	164	-29.47	-36.3	0.004288
hsa-miR-370-3p	NM_001282141	HEPH	158	-28.62	-32.5	0.027354
hsa-miR-370-3p	NM_001282218	BRSK2	158	-36.28	-35.2	0.03052
hsa-miR-370-3p	NM_001282457	C2	160	-30.07	-32.4	0.007304
hsa-miR-370-3p	NM_001282458	C2	160	-30.07	-32.4	0.007304
hsa-miR-370-3p	NM_001282582	MXRA8	152	-27.07	-32.1	0.046994
hsa-miR-370-3p	NM_001282583	MXRA8	152	-27.07	-32.1	0.046994
hsa-miR-370-3p	NM_001282584	MXRA8	152	-27.07	-32.1	0.046994
hsa-miR-370-3p	NM_001282585	MXRA8	152	-27.07	-32.1	0.025783
hsa-miR-370-3p	NM_001283053	STMN4	169	-27.98	-34	0.045843
hsa-miR-370-3p	NM_001283054	STMN4	169	-27.98	-34	0.042295

hsa-miR-370-3p	NM_001283055	STMN4	169	-27.98	-34	0.045843
hsa-miR-370-3p	NM_001283060	C14orf105	158	-24.98	-28	0.038266
hsa-miR-370-3p	NM_001284497	FAM234A	175	-31.74	-35.7	0.012719
hsa-miR-370-3p	NM_001286205	GAS8	150	-20.6	-36	0.020007
hsa-miR-370-3p	NM_001286208	GAS8	150	-20.6	-36	0.020007
hsa-miR-370-3p	NM_001286209	GAS8	150	-20.6	-36	0.020007
hsa-miR-370-3p	NM_001286351	DACT2	160	-31.78	-36.3	0.009228
hsa-miR-370-3p	NM_001286526	BCL7C	155	-36.14	-33.3	0.027295
hsa-miR-370-3p	NM_001286561	NUMA1	151	-28.73	-31.8	0.033484
hsa-miR-370-3p	NM_001286688	ABLIM2	160	-31.34	-36.5	0.016999
hsa-miR-370-3p	NM_001287212	KIF17	158	-28.63	-32.4	0.020033
hsa-miR-370-3p	NM_001288574	EYA1	160	-35.16	-37.4	0.01404
hsa-miR-370-3p	NM_001288575	EYA1	160	-35.16	-37.4	0.01404
hsa-miR-370-3p	NM_001288974	ADAM12	155	-38.68	-40.9	0.000694
hsa-miR-370-3p	NM_001288975	ADAM12	155	-38.68	-40.9	0.000694
hsa-miR-370-3p	NM_001289032	SERPINA4	159	-31.34	-34.2	0.002972
hsa-miR-370-3p	NM_001289033	SERPINA4	159	-31.34	-34.2	0.002972
hsa-miR-370-3p	NM_001289082	CD300LF	152	-26.39	-33.5	0.027651
hsa-miR-370-3p	NM_001289083	CD300LF	152	-26.39	-33.5	0.027651
hsa-miR-370-3p	NM_001289086	CD300LF	152	-26.39	-33.5	0.027651
hsa-miR-370-3p	NM_001289087	CD300LF	152	-26.39	-33.5	0.027651
hsa-miR-370-3p	NM_001291904	LOC79999	163	-32.75	-35.5	0.019179
hsa-miR-370-3p	NM_001291905	LOC388436	163	-32.75	-35.5	0.019179
hsa-miR-370-3p	NM_001291992	SPATA19	156	-25.82	-29	0.017578
hsa-miR-370-3p	NM_001297717	C1orf43	159	-26.54	-32.2	0.046874
hsa-miR-370-3p	NM_001297718	C1orf43	159	-26.54	-32.2	0.033754
hsa-miR-370-3p	NM_001297720	C1orf43	159	-26.54	-32.2	0.033754
hsa-miR-370-3p	NM_001301687	GBX2	163	-30.71	-32.7	0.046445
hsa-miR-370-3p	NM_001308046	TNK2	165	-33.59	-39.8	0.001222
hsa-miR-370-3p	NM_001308230	ATXN2L	153	-29.13	-30.4	0.036606

hsa-miR-370-3p	NM_001308370	ESPNL	150	-21.78	-34.7	0.032449
hsa-miR-370-3p	NM_001309434	SRP14	160	-32.49	-36.6	0.00345
hsa-miR-370-3p	NM_001470	GABBR1	165	-28.59	-37.4	0.007983
hsa-miR-370-3p	NM_001481	GAS8	150	-20.6	-36	0.020007
hsa-miR-370-3p	NM_001519	BRF1	162	-33.67	-37.5	0.007189
hsa-miR-370-3p	NM_001715	BLK	155	-30.89	-34.3	0.006332
hsa-miR-370-3p	NM_001722	POLR3D	150	-31.28	-31.7	0.033936
hsa-miR-370-3p	NM_001979	EPHX2	158	-28.51	-32.3	0.011629
hsa-miR-370-3p	NM_002189	IL15RA	152	-25.68	-33.8	0.015376
hsa-miR-370-3p	NM_002689	POLA2	159	-29.14	-34.6	0.029663
hsa-miR-370-3p	NM_002735	PRKAR1B	156	-30.06	-34.4	0.026484
hsa-miR-370-3p	NM_002768	CHMP1A	152	-26.72	-35.6	0.023862
hsa-miR-370-3p	NM_002840	PTPRF	151	-25.98	-34.2	0.044068
hsa-miR-370-3p	NM_002896	RBM4	159	-30.31	-34	0.00639
hsa-miR-370-3p	NM_002917	RFNG	151	-34.73	-34.2	0.015741
hsa-miR-370-3p	NM_002989	CCL21	153	-26.55	-32.5	0.010043
hsa-miR-370-3p	NM_003134	SRP14	160	-32.49	-36.6	0.00345
hsa-miR-370-3p	NM_003204	NFE2L1	161	-31.52	-35.7	0.028357
hsa-miR-370-3p	NM_003250	THRA	159	-32.42	-36.7	0.001958
hsa-miR-370-3p	NM_003449	TRIM26	164	-35.18	-39.1	0.004341
hsa-miR-370-3p	NM_003502	AXIN1	157	-25.63	-32.2	0.029114
hsa-miR-370-3p	NM_003565	ULK1	159	-31.24	-36.6	0.018033
hsa-miR-370-3p	NM_003586	DOC2A	164	-29.47	-36.3	0.004288
hsa-miR-370-3p	NM_003957	BRSK2	158	-36.28	-35.2	0.029809
hsa-miR-370-3p	NM_004062	CDH16	151	-29.54	-31.7	0.007736
hsa-miR-370-3p	NM_004204	PIGQ	150	-28.25	-33.3	0.036194
hsa-miR-370-3p	NM_004346	CASP3	175	-34.19	-38.5	0.00662
hsa-miR-370-3p	NM_004443	EPHB3	155	-26.23	-32.9	0.026309
hsa-miR-370-3p	NM_004468	FHL3	168	-29.53	-37.7	0.002019
hsa-miR-370-3p	NM_004679	VCY	157	-23.43	-27.5	0.010568

hsa-miR-370-3p	NM_005018	PDCD1	154	-30.18	-35.3	0.016736
hsa-miR-370-3p	NM_005183	CACNA1F	162	-29.39	-31.9	0.000399
hsa-miR-370-3p	NM_005228	EGFR	159	-31.35	-36.7	0.016189
hsa-miR-370-3p	NM_005411	SFTPA1	168	-34.59	-36.4	0.012514
hsa-miR-370-3p	NM_005432	XRCC3	161	-28.83	-34.1	0.028317
hsa-miR-370-3p	NM_005507	CFL1	156	-30.41	-30.5	0.042451
hsa-miR-370-3p	NM_005632	CAPN15	152	-30.1	-32.9	0.044089
hsa-miR-370-3p	NM_005672	PSCA	157	-28.92	-34	0.010163
hsa-miR-370-3p	NM_005678	SNURF	171	-34.65	-40.1	0.001675
hsa-miR-370-3p	NM_005699	IL18BP	166	-32.65	-35.3	0.018848
hsa-miR-370-3p	NM_005736	ACTR1A	158	-35.88	-35.8	0.021918
hsa-miR-370-3p	NM_005781	TNK2	165	-33.59	-39.8	0.001219
hsa-miR-370-3p	NM_005851	CDK2AP2	168	-29.81	-34.9	0.004137
hsa-miR-370-3p	NM_005892	FMNL1	156	-26.25	-30.2	0.039977
hsa-miR-370-3p	NM_006005	WFS1	155	-25.36	-33.2	0.023472
hsa-miR-370-3p	NM_006185	NUMA1	151	-28.73	-31.8	0.033484
hsa-miR-370-3p	NM_006201	CDK16	156	-32.34	-36.4	0.011053
hsa-miR-370-3p	NM_006215	SERPINA4	159	-31.34	-34.2	0.002972
hsa-miR-370-3p	NM_006278	ST3GAL4	158	-27.37	-34.8	0.006966
hsa-miR-370-3p	NM_006492	ALX3	155	-26.32	-29.3	0.041828
hsa-miR-370-3p	NM_006513	SARS	155	-26.26	-29.6	0.026105
hsa-miR-370-3p	NM_006764	IFRD2	168	-26.46	-30.1	0.042059
hsa-miR-370-3p	NM_006816	LMAN2	162	-22.14	-32.7	0.015164
hsa-miR-370-3p	NM_007074	CORO1A	151	-28.34	-30.8	0.002073
hsa-miR-370-3p	NM_007079	PTP4A3	162	-26.79	-33.5	0.046962
hsa-miR-370-3p	NM_007148	RNF112	163	-28.45	-32.8	0.04494
hsa-miR-370-3p	NM_007241	SNF8	155	-27.77	-31.1	0.019065
hsa-miR-370-3p	NM_012106	ARL2BP	160	-31.56	-33.3	0.048929
hsa-miR-370-3p	NM_012173	FBXO25	157	-28.12	-33.4	0.033303
hsa-miR-370-3p	NM_012202	GNG3	168	-30.82	-34.4	0.004161

hsa-miR-370-3p	NM_012285	KCNH4	152	-25.63	-31.9	0.02295
hsa-miR-370-3p	NM_013227	ACAN	156	-28.95	-34.9	0.012584
hsa-miR-370-3p	NM_013960	NRG1	166	-32.25	-34.4	0.029033
hsa-miR-370-3p	NM_014216	ITPK1	158	-30.61	-40.2	0.004116
hsa-miR-370-3p	NM_014272	ADAMTS7	153	-27.25	-30.4	0.010157
hsa-miR-370-3p	NM_014506	TOR1B	164	-27.99	-34.7	0.036652
hsa-miR-370-3p	NM_014698	TMEM63A	161	-31.74	-34.2	0.034835
hsa-miR-370-3p	NM_014700	RAB11FIP3	155	-21.72	-33.9	0.048824
hsa-miR-370-3p	NM_014756	CKAP5	152	-24.43	-30.2	0.044221
hsa-miR-370-3p	NM_014798	PLEKHM1	153	-28.83	-35.3	0.034745
hsa-miR-370-3p	NM_014799	HEPH	158	-28.62	-32.5	0.027354
hsa-miR-370-3p	NM_015106	RAD54L2	155	-28.54	-33.2	0.001486
hsa-miR-370-3p	NM_015175	NBEAL2	151	-37.24	-39.4	0.000328
hsa-miR-370-3p	NM_015193	ARC	164	-29.86	-34.5	0.035323
hsa-miR-370-3p	NM_015319	TNS2	160	-29.21	-32	0.019309
hsa-miR-370-3p	NM_015449	C1orf43	159	-26.54	-32.2	0.033754
hsa-miR-370-3p	NM_015537	NSMF	158	-26.26	-34.5	0.043839
hsa-miR-370-3p	NM_015694	ZNF777	152	-37.89	-40.5	0.000223
hsa-miR-370-3p	NM_015944	AMDHD2	153	-22.21	-28.5	0.017097
hsa-miR-370-3p	NM_016283	AK6	159	-25.38	-30.3	0.022959
hsa-miR-370-3p	NM_016490	FAM178B	158	-31.26	-35.2	0.002731
hsa-miR-370-3p	NM_016574	DRD2	155	-34.2	-36.9	0.007824
hsa-miR-370-3p	NM_016578	RSF1	152	-26.53	-34.4	0.010746
hsa-miR-370-3p	NM_017433	MYO3A	152	-28.79	-33.8	0.010478
hsa-miR-370-3p	NM_017492	ATXN2L	153	-29.13	-30.4	0.036606
hsa-miR-370-3p	NM_017553	INO80	159	-36.35	-37	0.009796
hsa-miR-370-3p	NM_017622	BORCS6	156	-32.71	-34.6	0.010901
hsa-miR-370-3p	NM_017825	ADPRHL2	159	-28.58	-31.8	0.024773
hsa-miR-370-3p	NM_017894	ZSCAN2	166	-29.61	-32.3	0.007371
hsa-miR-370-3p	NM_018026	PACS1	184	-41.66	-45.7	0.000269

hsa-miR-370-3p	NM_018043	ANO1	155	-30.1	-35.4	0.023873
hsa-miR-370-3p	NM_018688	BIN3	158	-28.96	-33.5	0.028497
hsa-miR-370-3p	NM_019060	CRCT1	157	-27.41	-31.2	0.013705
hsa-miR-370-3p	NM_019886	CHST7	168	-28.72	-34.2	0.014078
hsa-miR-370-3p	NM_020655	JPH3	150	-33.52	-41.9	0.001421
hsa-miR-370-3p	NM_020764	CASKIN1	168	-29.67	-35.1	0.022785
hsa-miR-370-3p	NM_020798	USP35	153	-22.43	-32.7	0.035099
hsa-miR-370-3p	NM_020816	KIF17	158	-28.63	-32.4	0.020033
hsa-miR-370-3p	NM_020896	OSBPL5	150	-29.6	-37.5	0.005642
hsa-miR-370-3p	NM_020992	PDLIM1	151	-28.46	-32.3	0.00952
hsa-miR-370-3p	NM_021641	ADAM12	155	-38.68	-40.9	0.000694
hsa-miR-370-3p	NM_021903	GABBR1	165	-28.59	-37.4	0.007983
hsa-miR-370-3p	NM_021904	GABBR1	165	-28.59	-37.4	0.007983
hsa-miR-370-3p	NM_022049	GPR88	156	-33.46	-40.1	0.004546
hsa-miR-370-3p	NM_022450	RHBDF1	155	-29.26	-34.2	0.002329
hsa-miR-370-3p	NM_022460	HS1BP3	155	-30.02	-33.3	0.040083
hsa-miR-370-3p	NM_022749	FAM160B2	158	-25.86	-33.7	0.044811
hsa-miR-370-3p	NM_022822	KLC2	162	-30.04	-32.8	0.033908
hsa-miR-370-3p	NM_023007	JMJD4	166	-30.19	-33.2	0.042838
hsa-miR-370-3p	NM_024003	L1CAM	160	-28.57	-34.7	0.02096
hsa-miR-370-3p	NM_024009	GJB3	157	-26.37	-31.6	0.047417
hsa-miR-370-3p	NM_024111	CHAC1	156	-31.14	-31.3	0.049909
hsa-miR-370-3p	NM_024532	SPAG16	151	-24.38	-28.1	0.025498
hsa-miR-370-3p	NM_024562	TANGO6	155	-29.85	-34.9	0.028914
hsa-miR-370-3p	NM_024661	CCDC51	153	-23.94	-28	0.041123
hsa-miR-370-3p	NM_024705	DHRS12	170	-36.09	-39.8	0.002182
hsa-miR-370-3p	NM_024736	GSDMD	156	-28.24	-32.1	0.00211
hsa-miR-370-3p	NM_024827	HDAC11	160	-30.01	-35.4	0.028352
hsa-miR-370-3p	NM_024923	NUP210	156	-30.79	-33.7	0.044896
hsa-miR-370-3p	NM_025112	ZXDC	171	-28.08	-33	0.024564

hsa-miR-370-3p	NM_030645	SH3BP5L	162	-31.05	-34.6	0.030618
hsa-miR-370-3p	NM_030795	STMN4	169	-27.98	-34	0.042295
hsa-miR-370-3p	NM_030948	PHACTR1	153	-31.76	-34.2	0.013991
hsa-miR-370-3p	NM_032039	FAM234A	175	-31.74	-35.7	0.012719
hsa-miR-370-3p	NM_032348	MXRA8	152	-27.07	-32.1	0.046994
hsa-miR-370-3p	NM_032350	C7orf50	159	-33.37	-34.5	0.009049
hsa-miR-370-3p	NM_032432	ABLIM2	160	-31.34	-36.5	0.016999
hsa-miR-370-3p	NM_032536	NTNG2	170	-31.55	-34.5	0.011109
hsa-miR-370-3p	NM_032611	PTP4A3	162	-26.79	-33.5	0.046962
hsa-miR-370-3p	NM_032840	SPRYD3	158	-30.47	-33.6	0.049686
hsa-miR-370-3p	NM_032951	MLXIPL	154	-26.93	-31.2	0.044814
hsa-miR-370-3p	NM_032952	MLXIPL	154	-26.93	-31.2	0.044814
hsa-miR-370-3p	NM_032953	MLXIPL	154	-26.93	-31.2	0.044814
hsa-miR-370-3p	NM_032954	MLXIPL	154	-26.93	-31.2	0.044814
hsa-miR-370-3p	NM_032991	CASP3	175	-34.19	-38.5	0.00662
hsa-miR-370-3p	NM_033018	CDK16	156	-32.34	-36.4	0.011053
hsa-miR-370-3p	NM_033067	DMRTB1	156	-29.36	-32.7	0.030963
hsa-miR-370-3p	NM_033553	GUCA2A	154	-23.74	-29.8	0.013552
hsa-miR-370-3p	NM_052901	SLC25A25	169	-32.22	-34.2	0.049267
hsa-miR-370-3p	NM_052926	PNMA5	155	-36.88	-39.8	0.003239
hsa-miR-370-3p	NM_080865	GPR62	167	-30.75	-32.2	0.033285
hsa-miR-370-3p	NM_130440	PTPRF	151	-25.98	-34.2	0.044068
hsa-miR-370-3p	NM_138430	ADPRHL1	156	-28.2	-32.1	0.04459
hsa-miR-370-3p	NM_138737	HEPH	158	-28.62	-32.5	0.027354
hsa-miR-370-3p	NM_138740	C1orf43	159	-26.54	-32.2	0.033754
hsa-miR-370-3p	NM_139208	MASP2	154	-28.25	-36.1	0.000185
hsa-miR-370-3p	NM_144603	NOXO1	153	-22.83	-27.1	0.024361
hsa-miR-370-3p	NM_145202	PRAP1	160	-28.58	-32.4	0.004411
hsa-miR-370-3p	NM_145273	CD300LG	162	-33.47	-36.4	0.018314
hsa-miR-370-3p	NM_145638	OSBPL5	150	-29.6	-37.5	0.005642

hsa-miR-370-3p	NM_145685	BRF1	162	-33.67	-37.5	0.007189
hsa-miR-370-3p	NM_145689	APBB1	151	-25.02	-31.5	0.017907
hsa-miR-370-3p	NM_145714	ATXN2L	153	-29.13	-30.4	0.033765
hsa-miR-370-3p	NM_147172	NUDT2	158	-25.13	-29.1	0.029622
hsa-miR-370-3p	NM_147173	NUDT2	158	-25.13	-29.1	0.029622
hsa-miR-370-3p	NM_148414	ATXN2L	153	-29.13	-30.4	0.044221
hsa-miR-370-3p	NM_148415	ATXN2L	153	-29.13	-30.4	0.036606
hsa-miR-370-3p	NM_148416	ATXN2L	153	-29.13	-30.4	0.033765
hsa-miR-370-3p	NM_152335	TMEM266	154	-23.38	-31.6	0.035444
hsa-miR-370-3p	NM_170754	TNS2	160	-29.21	-32	0.019309
hsa-miR-370-3p	NM_172058	EYA1	160	-35.16	-37.4	0.01404
hsa-miR-370-3p	NM_172059	EYA1	160	-35.16	-37.4	0.01404
hsa-miR-370-3p	NM_172060	EYA1	160	-35.16	-37.4	0.01404
hsa-miR-370-3p	NM_172167	NOXO1	153	-22.83	-27.1	0.024361
hsa-miR-370-3p	NM_172168	NOXO1	153	-22.83	-27.1	0.024361
hsa-miR-370-3p	NM_172200	IL15RA	152	-25.68	-33.8	0.015376
hsa-miR-370-3p	NM_173042	IL18BP	166	-32.65	-35.3	0.00673
hsa-miR-370-3p	NM_173044	IL18BP	166	-32.65	-35.3	0.011196
hsa-miR-370-3p	NM_173561	UNC5CL	154	-30.15	-34	0.040362
hsa-miR-370-3p	NM_173689	CRB2	151	-36.53	-41.6	0.001995
hsa-miR-370-3p	NM_173847	SPACA3	153	-26.91	-31.3	0.001088
hsa-miR-370-3p	NM_174927	SPATA19	156	-25.82	-29	0.038651
hsa-miR-370-3p	NM_175630	DNMT3A	154	-30.01	-32.4	0.042547
hsa-miR-370-3p	NM_178500	PHOSPHO1	155	-33.11	-38.4	0.002613
hsa-miR-370-3p	NM_178545	TMEM52	164	-36.19	-38.5	0.000282
hsa-miR-370-3p	NM_181050	AXIN1	157	-25.63	-32.2	0.029114
hsa-miR-370-3p	NM_181457	PAX3	159	-29.88	-30.1	0.011182
hsa-miR-370-3p	NM_181709	FAM101A	157	-33.75	-37.7	0.008611
hsa-miR-370-3p	NM_181711	GRASP	159	-35.02	-34.8	0.008979
hsa-miR-370-3p	NM_181880	VCY1B	157	-23.43	-27.5	0.010568

hsa-miR-370-3p	NM_182925	FLT4	155	-29.3	-34.5	0.039445
hsa-miR-370-3p	NM_183420	FBXO25	157	-28.12	-33.4	0.033303
hsa-miR-370-3p	NM_183421	FBXO25	157	-28.12	-33.4	0.033303
hsa-miR-370-3p	NM_194249	DND1	180	-41.06	-42.5	0.000127
hsa-miR-370-3p	NM_194312	ESPNL	150	-21.78	-34.7	0.032449
hsa-miR-370-3p	NM_198153	TREML4	166	-30.71	-33.6	0.042973
hsa-miR-370-3p	NM_198316	TNS2	160	-29.21	-32	0.019309
hsa-miR-370-3p	NM_198443	NRN1L	155	-27.46	-28.1	0.009133
hsa-miR-370-3p	NM_199162	ADPRHL1	156	-28.2	-32.1	0.04459
hsa-miR-370-3p	NM_203347	LCN15	151	-32.93	-30.4	0.007181
hsa-miR-370-3p	NM_207117	SLC25A47	163	-29.84	-34.7	0.01039
hsa-miR-370-3p	NR_002836	PGM5P2	151	-29.23	-34.9	0.041331
hsa-miR-370-3p	NR_003062	SPRR2C	159	-27.85	-32.7	0.022946
hsa-miR-370-3p	NR_024525	FAM96B	161	-28.27	-32.3	0.039014
hsa-miR-370-3p	NR_026752	CROCCP2	160	-30.99	-34.2	0.047504
hsa-miR-370-3p	NR_026882	DKFZP434K02	151	-24.71	-32.7	0.037285
hsa-miR-370-3p	NR_026901	LOC644172	171	-31.74	-35.7	0.025404
hsa-miR-370-3p	NR_027045	PIK3CD-AS1	156	-24.82	-35.2	0.032838
hsa-miR-370-3p	NR_027075	LINC00928	157	-36.02	-40.1	0.002987
hsa-miR-370-3p	NR_027076	LINC00928	157	-36.02	-40.1	0.002068
hsa-miR-370-3p	NR_027077	LINC00928	157	-36.02	-40.1	0.002604
hsa-miR-370-3p	NR_027256	TREML3P	166	-33.09	-36.5	0.00939
hsa-miR-370-3p	NR_027269	SEPT7P9	152	-31.39	-37	0.003091
hsa-miR-370-3p	NR_027335	URAHP	151	-32.41	-34.1	0.034257
hsa-miR-370-3p	NR_027503	LOC100133050	150	-29.58	-31.6	0.039877
hsa-miR-370-3p	NR_029831	MIR106B	160	-24.06	-31.3	0.000741
hsa-miR-370-3p	NR_030732	WFDC21P	152	-26.45	-33.9	0.011437
hsa-miR-370-3p	NR_031607	MIR1203	156	-22.33	-28.6	0.004002
hsa-miR-370-3p	NR_036691	LOC494127	160	-31.47	-34.4	0.029859
hsa-miR-370-3p	NR_037468	MIR3907	155	-30.98	-34.6	0.00049

hsa-miR-370-3p	NR_037509	MIR3944	161	-28.02	-37.8	0.000034
hsa-miR-370-3p	NR_037918	PRH1-PRR4	154	-31.11	-35	0.030953
hsa-miR-370-3p	NR_038321	OR51B5	164	-23.82	-31.9	0.043707
hsa-miR-370-3p	NR_038389	C9orf173-AS1	164	-36.06	-39.1	0.005787
hsa-miR-370-3p	NR_038857	CPEB2-AS1	170	-29.32	-35.7	0.02278
hsa-miR-370-3p	NR_040054	IQCH-AS1	158	-32.36	-37	0.004155
hsa-miR-370-3p	NR_046109	FAM96B	161	-28.27	-32.3	0.033104
hsa-miR-370-3p	NR_046362	IL15RA	152	-25.68	-33.8	0.047384
hsa-miR-370-3p	NR_046552	RNASEH2B-AS1	168	-36.39	-38.6	0.001283
hsa-miR-370-3p	NR_046647	SLC6A1-AS1	160	-25.92	-31	0.039907
hsa-miR-370-3p	NR_073484	CDK2AP2	168	-29.81	-34.9	0.021829
hsa-miR-370-3p	NR_103995	REXO4	163	-30.09	-34.6	0.043689
hsa-miR-370-3p	NR_104089	DOC2A	164	-29.47	-36.3	0.02122
hsa-miR-370-3p	NR_104090	DOC2A	164	-29.47	-36.3	0.020195
hsa-miR-370-3p	NR_104616	LOC100506858	155	-27.96	-29.7	0.048298
hsa-miR-370-3p	NR_105049	C5orf66-AS1	151	-20.23	-33.5	0.022348
hsa-miR-370-3p	NR_105050	C5orf66-AS1	151	-20.23	-33.5	0.022146
hsa-miR-370-3p	NR_106808	MIR6750	150	-26.49	-27.4	0.006185
hsa-miR-370-3p	NR_106902	MIR6843	152	-27.75	-34	0.000682
hsa-miR-370-3p	NR_106951	MIR6891	162	-30.39	-32.5	0.0005
hsa-miR-370-3p	NR_106985	MIR7162	155	-28.83	-31.7	0.000375
hsa-miR-370-3p	NR_109767	LOC100131315	157	-31.85	-33.6	0.011915
hsa-miR-370-3p	NR_109944	LOC101929454	168	-34.54	-37.5	0.005481
hsa-miR-370-3p	NR_110214	LOC101927196	157	-30.21	-33.7	0.035215
hsa-miR-370-3p	NR_110218	LINC01473	163	-30.39	-35.2	0.00591
hsa-miR-370-3p	NR_110271	LOC101927701	167	-30.64	-33.3	0.019366
hsa-miR-370-3p	NR_110563	SERPINA2	158	-28.81	-35.2	0.012163
hsa-miR-370-3p	NR_110573	CATIP-AS1	152	-25.73	-33.1	0.01348
hsa-miR-370-3p	NR_111905	ADGRA1-AS1	154	-26.29	-33.6	0.008204

hsa-miR-370-3p	NR_120571	MIR4300HG	155	-26.7	-33.4	0.048508
hsa-miR-370-3p	NR_120586	LOC101928069	170	-42.4	-45.3	0.000107
hsa-miR-370-3p	NR_120615	PLCE1-AS2	158	-29.65	-35	0.030297
hsa-miR-370-3p	NR_120641	LINC01468	151	-29.94	-32.6	0.043206
hsa-miR-370-3p	NR_120642	LINC01468	151	-29.94	-32.6	0.041138
hsa-miR-370-3p	NR_120665	LOC102724009	157	-31.56	-37.3	0.002073
hsa-miR-370-3p	NR_120672	LOC102723377	154	-28.87	-31.1	0.023066
hsa-miR-370-3p	NR_121606	LOC102723709	167	-29.7	-32.6	0.01185
hsa-miR-370-3p	NR_121651	MIR3663HG	150	-27.33	-31.4	0.031598
hsa-miR-370-3p	NR_125868	LOC102723831	150	-30.55	-33.9	0.020127
hsa-miR-370-3p	NR_125911	LOC101929095	153	-30.09	-33.7	0.012854
hsa-miR-370-3p	NR_126027	EXTL3-AS1	157	-29.23	-33.5	0.019824
hsa-miR-370-3p	NR_126522	EXOC3-AS1	159	-32.31	-33.3	0.046931
hsa-miR-370-3p	NR_130745	LOC100288778	165	-30.14	-34.5	0.045518
hsa-miR-370-3p	NR_132966	SNORA110	152	-23.81	-27.6	0.042918
hsa-miR-370-3p	NR_133005	ADH1C	152	-28.68	-37.3	0.012506
hsa-miR-374a-5p	NR_030785	MIR374A	176	-26.86	-29.1	0.000238
hsa-miR-330-3p	NM_001024679	C1orf68	157	-25.1	-28.7	0.011201
hsa-miR-330-3p	NM_001098811	SEPT8	175	-27.45	-32.9	0.041544
hsa-miR-330-3p	NM_001145311	PLIN1	178	-30.91	-35.9	0.011284
hsa-miR-330-3p	NM_001145353	ELF1	176	-34.16	-37.5	0.007836
hsa-miR-330-3p	NM_001190438	NCOR1	156	-22.7	-29	0.028779
hsa-miR-330-3p	NM_001254718	MYBPC1	163	-24.85	-28.2	0.034346
hsa-miR-330-3p	NM_001254723	MYBPC1	163	-24.85	-28.2	0.023222
hsa-miR-330-3p	NM_001267	CHAD	167	-31.67	-33.8	0.008484
hsa-miR-330-3p	NM_001271899	PPP2R2B	152	-25.14	-32	0.013478
hsa-miR-330-3p	NM_001271900	PPP2R2B	152	-25.14	-32	0.013478
hsa-miR-330-3p	NM_001271948	PPP2R2B	152	-25.14	-32	0.013478
hsa-miR-330-3p	NM_001303050	MAPRE3	172	-25.76	-32	0.039255
hsa-miR-330-3p	NM_001308151	SLC36A1	166	-24.86	-30	0.004816

hsa-miR-330-3p	NM_002054	GCG	158	-27.52	-29.7	0.049221
hsa-miR-330-3p	NM_002666	PLIN1	178	-30.91	-35.9	0.011284
hsa-miR-330-3p	NM_003005	SELP	171	-27.81	-31.6	0.027813
hsa-miR-330-3p	NM_003357	SCGB1A1	154	-24.49	-28.5	0.006877
hsa-miR-330-3p	NM_003673	TCAP	171	-26.31	-32.1	0.012982
hsa-miR-330-3p	NM_004668	MGAM	175	-28.15	-32.1	0.03658
hsa-miR-330-3p	NM_012326	MAPRE3	172	-25.76	-32	0.039255
hsa-miR-330-3p	NM_014427	CPNE7	157	-24.05	-30.6	0.045728
hsa-miR-330-3p	NM_024042	METRN	158	-19.74	-26.3	0.016031
hsa-miR-330-3p	NM_030768	ILKAP	157	-22.55	-27.7	0.014756
hsa-miR-330-3p	NM_052955	TGM7	165	-23.84	-28.6	0.014762
hsa-miR-330-3p	NM_145910	NEK11	175	-32.2	-36.8	0.000824
hsa-miR-330-3p	NM_153636	CPNE7	157	-24.05	-30.6	0.045728
hsa-miR-330-3p	NM_172373	ELF1	176	-34.16	-37.5	0.007836
hsa-miR-330-3p	NM_181674	PPP2R2B	152	-25.14	-32	0.013478
hsa-miR-330-3p	NM_181675	PPP2R2B	152	-25.14	-32	0.013478
hsa-miR-330-3p	NM_181676	PPP2R2B	152	-25.14	-32	0.013478
hsa-miR-330-3p	NM_181677	PPP2R2B	152	-25.14	-32	0.013478
hsa-miR-330-3p	NM_181678	PPP2R2B	152	-25.14	-32	0.013478
hsa-miR-330-3p	NM_181726	ANKRD37	168	-24.54	-30.3	0.009117
hsa-miR-330-3p	NR_026740	LOC389033	152	-23.9	-29	0.049953
hsa-miR-330-3p	NR_026861	LINC00473	168	-26.7	-33.9	0.024348
hsa-miR-330-3p	NR_029973	MIR452	159	-22.76	-28.5	0.00357
hsa-miR-330-3p	NR_036113	MIR3157	156	-21.43	-24.6	0.036623
hsa-miR-330-3p	NR_036461	HIST2H2BC	160	-29.16	-33.5	0.010391
hsa-miR-330-3p	NR_038252	LINC00466	163	-33.98	-36.4	0.016715
hsa-miR-330-3p	NR_047683	BLNK	171	-27.11	-33.3	0.046263
hsa-miR-330-3p	NR_104057	LINC00380	167	-24.18	-29	0.045186
hsa-miR-342-5p	NM_001042478	AJAP1	158	-23.08	-30.1	0.016725
hsa-miR-342-5p	NM_001085474	LYPD8	150	-22.23	-27.2	0.018024

hsa-miR-342-5p	NM_001097612	GPR89A	154	-22.62	-28.2	0.040268
hsa-miR-342-5p	NM_001097613	GPR89A	154	-22.62	-28.2	0.040268
hsa-miR-342-5p	NM_001099685	RHOXF2B	162	-18.22	-26.5	0.042232
hsa-miR-342-5p	NM_001109	ADAM8	158	-25.66	-31	0.023778
hsa-miR-342-5p	NM_001123068	PPIAL4G	159	-25.16	-26.7	0.027461
hsa-miR-342-5p	NM_001127190	CSK	154	-23.09	-31.2	0.019028
hsa-miR-342-5p	NM_001135789	PPIAL4C	159	-25.16	-26.7	0.028645
hsa-miR-342-5p	NM_001143883	PPIAL4A	159	-25.16	-26.7	0.028645
hsa-miR-342-5p	NM_001144032	PPIAL4E	159	-25.16	-26.7	0.028645
hsa-miR-342-5p	NM_001164261	PPIAL4D	159	-25.16	-26.7	0.028645
hsa-miR-342-5p	NM_001164262	PPIAL4F	159	-25.16	-26.7	0.028645
hsa-miR-342-5p	NM_001164489	ADAM8	158	-25.66	-31	0.027869
hsa-miR-342-5p	NM_001164490	ADAM8	158	-25.66	-31	0.023778
hsa-miR-342-5p	NM_001167827	TFEB	163	-26.98	-30.7	0.020834
hsa-miR-342-5p	NM_001167985	INCA1	167	-28.09	-32.2	0.002108
hsa-miR-342-5p	NM_001167986	INCA1	167	-28.09	-32.2	0.002108
hsa-miR-342-5p	NM_001167987	INCA1	167	-28.09	-32.2	0.002108
hsa-miR-342-5p	NM_001171932	CDH23	150	-19.21	-24.2	0.045727
hsa-miR-342-5p	NM_001171990	SETD9	161	-22.64	-28.7	0.007683
hsa-miR-342-5p	NM_001178044	SLC44A4	155	-22.66	-28.3	0.039695
hsa-miR-342-5p	NM_001178045	SLC44A4	155	-22.66	-28.3	0.039695
hsa-miR-342-5p	NM_001191054	ANHX	162	-24.27	-29.3	0.037043
hsa-miR-342-5p	NM_001206855	TFR2	162	-28.46	-31.8	0.006551
hsa-miR-342-5p	NM_001242884	ZBTB17	151	-21.64	-29.8	0.000896
hsa-miR-342-5p	NM_001244678	THNSL2	163	-24.49	-29.2	0.048607
hsa-miR-342-5p	NM_001271804	PFKFB1	159	-21.9	-28.5	0.015717
hsa-miR-342-5p	NM_001271805	PFKFB1	159	-21.9	-28.5	0.015717
hsa-miR-342-5p	NM_001271943	TFEB	163	-26.98	-30.7	0.020834
hsa-miR-342-5p	NM_001271944	TFEB	163	-26.98	-30.7	0.020834
hsa-miR-342-5p	NM_001271945	TFEB	163	-26.98	-30.7	0.020834

hsa-miR-342-5p	NM_001276468	B4GALNT1	154	-27.58	-30.3	0.041309
hsa-miR-342-5p	NM_001278794	NPY4R	159	-24.43	-33.9	0.001871
hsa-miR-342-5p	NM_001278795	CH17-360D5.1	159	-24.43	-33.9	0.000737
hsa-miR-342-5p	NM_001286625	RNPS1	162	-30.2	-31.1	0.028012
hsa-miR-342-5p	NM_001286626	RNPS1	162	-30.2	-31.1	0.028012
hsa-miR-342-5p	NM_001286627	RNPS1	162	-30.2	-31.1	0.024647
hsa-miR-342-5p	NM_001287603	ZBTB17	151	-21.64	-29.8	0.000896
hsa-miR-342-5p	NM_001287604	ZBTB17	151	-21.64	-29.8	0.000896
hsa-miR-342-5p	NM_001291283	LYPD8	150	-22.23	-27.2	0.018024
hsa-miR-342-5p	NM_001293798	DUX4	152	-23.27	-28.1	0.023639
hsa-miR-342-5p	NM_001304360	CFAP74	154	-22.01	-27.1	0.049235
hsa-miR-342-5p	NM_001306068	DUX4	152	-23.27	-28.1	0.044179
hsa-miR-342-5p	NM_001308220	ERN2	158	-25.94	-30.9	0.014273
hsa-miR-342-5p	NM_001308267	PTPRN2	173	-27.95	-33	0.031179
hsa-miR-342-5p	NM_001308268	PTPRN2	173	-27.95	-33	0.031179
hsa-miR-342-5p	NM_001478	B4GALNT1	154	-27.58	-30.3	0.041309
hsa-miR-342-5p	NM_001692	ATP6V1B1	150	-22.37	-28.9	0.016309
hsa-miR-342-5p	NM_001785	CDA	151	-23.12	-28.1	0.02934
hsa-miR-342-5p	NM_002625	PFKFB1	159	-21.9	-28.5	0.015717
hsa-miR-342-5p	NM_002847	PTPRN2	173	-27.95	-33	0.031179
hsa-miR-342-5p	NM_003227	TFR2	162	-28.46	-31.8	0.006551
hsa-miR-342-5p	NM_003443	ZBTB17	151	-21.64	-29.8	0.000896
hsa-miR-342-5p	NM_004178	TARBP2	150	-22.19	-28.3	0.01923
hsa-miR-342-5p	NM_004383	CSK	154	-23.09	-31.2	0.019028
hsa-miR-342-5p	NM_005022	PFN1	155	-24.17	-26.6	0.039432
hsa-miR-342-5p	NM_005258	GCHFR	154	-23.13	-30.8	0.007827
hsa-miR-342-5p	NM_005972	NPY4R	159	-24.43	-33.9	0.001871
hsa-miR-342-5p	NM_006711	RNPS1	162	-30.2	-31.1	0.028012
hsa-miR-342-5p	NM_007162	TFEB	163	-26.98	-30.7	0.020834
hsa-miR-342-5p	NM_007275	TUSC2	170	-26.04	-33.5	0.015447

hsa-miR-342-5p	NM_012339	TSPAN15	150	-24.58	-33.2	0.007074
hsa-miR-342-5p	NM_013310	C2orf27A	156	-19.5	-26.2	0.034
hsa-miR-342-5p	NM_014555	TRPM5	153	-25.87	-28.2	0.03981
hsa-miR-342-5p	NM_016403	CWC15	163	-23.65	-29.6	0.049429
hsa-miR-342-5p	NM_018029	EBLN2	153	-25.76	-28.5	0.033929
hsa-miR-342-5p	NM_018271	THNSL2	163	-24.49	-29.2	0.0328
hsa-miR-342-5p	NM_025041	C3orf36	160	-26.24	-29.8	0.00265
hsa-miR-342-5p	NM_025257	SLC44A4	155	-22.66	-28.3	0.039695
hsa-miR-342-5p	NM_032498	RHOXF2	162	-18.22	-26.5	0.038852
hsa-miR-342-5p	NM_033266	ERN2	158	-25.94	-30.9	0.014273
hsa-miR-342-5p	NM_080594	RNPS1	162	-30.2	-31.1	0.028012
hsa-miR-342-5p	NM_130842	PTPRN2	173	-27.95	-33	0.031179
hsa-miR-342-5p	NM_130843	PTPRN2	173	-27.95	-33	0.031179
hsa-miR-342-5p	NM_134323	TARBP2	150	-22.19	-28.3	0.01923
hsa-miR-342-5p	NM_134324	TARBP2	150	-22.19	-28.3	0.01923
hsa-miR-342-5p	NM_145285	NKX2-3	152	-24.26	-31.4	0.022188
hsa-miR-342-5p	NM_198508	KLRG2	158	-24.1	-29.4	0.007988
hsa-miR-342-5p	NM_213726	INCA1	167	-28.09	-32.2	0.002108
hsa-miR-342-5p	NM_214461	C2orf27B	156	-19.5	-26.2	0.034
hsa-miR-342-5p	NR_103835	ANO1-AS2	159	-27.78	-30.8	0.029855
hsa-miR-342-5p	NR_125791	RORB-AS1	167	-28.86	-30.7	0.032546
hsa-miR-342-5p	NR_125931	LOC101929762	169	-27.09	-32.9	0.039742
hsa-miR-323a-3p	NM_005334	HCFC1	163	-30.12	-33.2	0.035957
hsa-miR-323a-3p	NM_016071	MRPS33	172	-29.22	-31.4	0.003677
hsa-miR-323a-3p	NM_053035	MRPS33	172	-29.22	-31.4	0.003677
hsa-miR-323a-3p	NR_036680	DPY19L1P1	163	-35.12	-36.2	0.010316
hsa-miR-151a-5p	NM_000040	APOC3	151	-25.23	-27.5	0.017445
hsa-miR-151a-5p	NM_000418	IL4R	170	-28.72	-32.9	0.017396
hsa-miR-151a-5p	NM_001008272	TAGLN3	160	-23.76	-27.6	0.04623
hsa-miR-151a-5p	NM_001008273	TAGLN3	160	-23.76	-27.6	0.04623

hsa-miR-151a-5p	NM_001018070	CORO1B	157	-25.46	-29.4	0.017237
hsa-miR-151a-5p	NM_001077663	URGCP	174	-36.47	-39.9	0.000417
hsa-miR-151a-5p	NM_001077664	URGCP	174	-36.47	-39.9	0.000417
hsa-miR-151a-5p	NM_001098673	ATG101	151	-25.26	-34.5	0.000972
hsa-miR-151a-5p	NM_001130848	PITPNM1	159	-22.57	-27.2	0.038358
hsa-miR-151a-5p	NM_001136044	TMUB1	161	-34.2	-35.7	0.001226
hsa-miR-151a-5p	NM_001145795	SH2B1	150	-28.91	-32.7	0.0042
hsa-miR-151a-5p	NM_001145796	SH2B1	150	-28.91	-32.7	0.013082
hsa-miR-151a-5p	NM_001145797	SH2B1	150	-28.91	-32.7	0.013573
hsa-miR-151a-5p	NM_001145812	SH2B1	150	-28.91	-32.7	0.013082
hsa-miR-151a-5p	NM_001242946	EPDR1	167	-35.45	-37	0.006836
hsa-miR-151a-5p	NM_001242948	EPDR1	167	-35.45	-37	0.005664
hsa-miR-151a-5p	NM_001257406	IL4R	170	-28.72	-32.9	0.017396
hsa-miR-151a-5p	NM_001257407	IL4R	170	-28.72	-32.9	0.017396
hsa-miR-151a-5p	NM_001257997	IL4R	170	-28.72	-32.9	0.017396
hsa-miR-151a-5p	NM_001272002	DOCK7	175	-37.41	-40.2	0.000273
hsa-miR-151a-5p	NM_001290075	URGCP	174	-36.47	-39.9	0.000417
hsa-miR-151a-5p	NM_001290076	URGCP	174	-36.47	-39.9	0.000417
hsa-miR-151a-5p	NM_001306082	STK32B	179	-34.94	-36.2	0.01138
hsa-miR-151a-5p	NM_001308293	SH2B1	150	-28.91	-32.7	0.0042
hsa-miR-151a-5p	NM_001308294	SH2B1	150	-28.91	-32.7	0.013082
hsa-miR-151a-5p	NM_004910	PITPNM1	159	-22.57	-27.2	0.038358
hsa-miR-151a-5p	NM_005373	MPL	175	-39.71	-42.4	0.000577
hsa-miR-151a-5p	NM_013241	FHOD1	154	-27.05	-35.2	0.000418
hsa-miR-151a-5p	NM_013259	TAGLN3	160	-23.76	-27.6	0.04623
hsa-miR-151a-5p	NM_015503	SH2B1	150	-28.91	-32.7	0.013082
hsa-miR-151a-5p	NM_017549	EPDR1	167	-35.45	-37	0.005664
hsa-miR-151a-5p	NM_017670	OTUB1	170	-35.38	-38.4	0.001093
hsa-miR-151a-5p	NM_017920	URGCP	174	-36.47	-39.9	0.000417
hsa-miR-151a-5p	NM_018401	STK32B	179	-34.94	-36.2	0.01138

hsa-miR-151a-5p	NM_020441	CORO1B	157	-25.46	-29.4	0.017237
hsa-miR-151a-5p	NM_021871	FGA	155	-33.08	-35.5	0.000262
hsa-miR-151a-5p	NM_021908	ST7	171	-32.26	-35.6	0.004646
hsa-miR-151a-5p	NM_021934	ATG101	151	-25.26	-34.5	0.000972
hsa-miR-151a-5p	NM_031434	TMUB1	161	-34.2	-35.7	0.001226
hsa-miR-151a-5p	NM_032428	FRMPD3	178	-30.43	-32.9	0.046948
hsa-miR-151a-5p	NM_144683	DHRS13	155	-27	-30.7	0.028335
hsa-miR-151a-5p	NM_152544	TRMT44	160	-27.11	-31	0.018422
hsa-miR-151a-5p	NR_029892	MIR151A	157	-32.41	-35	0.000035
hsa-miR-151a-5p	NR_125732	USP6NL	151	-29.54	-33.6	0.036585
hsa-miR-324-5p	NM_000162	GCK	159	-37.33	-40.9	0.000831
hsa-miR-324-5p	NM_000883	IMPDH1	156	-25.41	-32	0.035739
hsa-miR-324-5p	NM_001009566	CLSTN1	151	-28.75	-34.7	0.030672
hsa-miR-324-5p	NM_001009606	HS3ST6	157	-26.25	-31.2	0.002209
hsa-miR-324-5p	NM_001010972	ZYX	161	-25.34	-29.7	0.046505
hsa-miR-324-5p	NM_001014435	CA7	150	-28.82	-33.1	0.017977
hsa-miR-324-5p	NM_001014445	NLE1	169	-28.54	-33.9	0.028637
hsa-miR-324-5p	NM_001018009	SH3BP5	166	-30.88	-34.4	0.042831
hsa-miR-324-5p	NM_001030287	ATF3	151	-31.82	-35.1	0.019455
hsa-miR-324-5p	NM_001035267	RPL41	153	-23.54	-30.7	0.016574
hsa-miR-324-5p	NM_001039582	PNCK	153	-29.8	-34.9	0.003383
hsa-miR-324-5p	NM_001040619	ATF3	151	-31.82	-35.1	0.030544
hsa-miR-324-5p	NM_001077239	RNF214	170	-26.21	-33.4	0.01149
hsa-miR-324-5p	NM_001101311	TMEM176B	152	-21.91	-28.9	0.027418
hsa-miR-324-5p	NM_001101312	TMEM176B	152	-21.91	-28.9	0.027418
hsa-miR-324-5p	NM_001101314	TMEM176B	152	-21.91	-28.9	0.027418
hsa-miR-324-5p	NM_001102605	IMPDH1	156	-25.41	-32	0.035739
hsa-miR-324-5p	NM_001135740	PNCK	153	-29.8	-34.9	0.003383
hsa-miR-324-5p	NM_001142276	APLP2	150	-27.37	-33.8	0.039768
hsa-miR-324-5p	NM_001142277	APLP2	150	-27.37	-33.8	0.039768

hsa-miR-324-5p	NM_001142278	APLP2	150	-27.37	-33.8	0.039768
hsa-miR-324-5p	NM_001142573	IMPDH1	156	-25.41	-32	0.035739
hsa-miR-324-5p	NM_001142574	IMPDH1	156	-25.41	-32	0.035739
hsa-miR-324-5p	NM_001142575	IMPDH1	156	-25.41	-32	0.035739
hsa-miR-324-5p	NM_001142576	IMPDH1	156	-25.41	-32	0.035739
hsa-miR-324-5p	NM_001143919	LTB4R	178	-29.95	-35.5	0.018477
hsa-miR-324-5p	NM_001171706	RIPPLY1	162	-27.26	-32.5	0.025906
hsa-miR-324-5p	NM_001171740	C3orf18	158	-26.5	-34.2	0.043019
hsa-miR-324-5p	NM_001171741	C3orf18	158	-26.5	-34.2	0.043019
hsa-miR-324-5p	NM_001171743	C3orf18	158	-26.5	-34.2	0.043019
hsa-miR-324-5p	NM_001195007	PPIE	157	-25.27	-31.3	0.008577
hsa-miR-324-5p	NM_001195132	CDKN2A	159	-29.09	-36.6	0.003469
hsa-miR-324-5p	NM_001204084	NBL1	153	-27.16	-33.6	0.041718
hsa-miR-324-5p	NM_001204085	NBL1	153	-27.16	-33.6	0.041718
hsa-miR-324-5p	NM_001204086	NBL1	153	-27.16	-33.6	0.041718
hsa-miR-324-5p	NM_001204088	MINOS1-NBL1	153	-27.16	-33.6	0.041718
hsa-miR-324-5p	NM_001204089	MINOS1-NBL1	153	-27.16	-33.6	0.041718
hsa-miR-324-5p	NM_001206484	ATF3	151	-31.82	-35.1	0.019455
hsa-miR-324-5p	NM_001206486	ATF3	151	-31.82	-35.1	0.030544
hsa-miR-324-5p	NM_001206488	ATF3	151	-31.82	-35.1	0.019455
hsa-miR-324-5p	NM_001206878	CTDSP1	165	-35.86	-40.8	0.002339
hsa-miR-324-5p	NM_001243299	APLP2	150	-27.37	-33.8	0.039768
hsa-miR-324-5p	NM_001243794	CHST12	152	-25.84	-31.3	0.045821
hsa-miR-324-5p	NM_001243795	CHST12	152	-25.84	-31.3	0.045821
hsa-miR-324-5p	NM_001254750	CD6	160	-29.49	-32.8	0.042861
hsa-miR-324-5p	NM_001254751	CD6	160	-29.49	-32.8	0.042861
hsa-miR-324-5p	NM_001254755	CYB5D2	158	-23.93	-36.2	0.00393
hsa-miR-324-5p	NM_001254756	CYB5D2	158	-23.93	-36.2	0.00393
hsa-miR-324-5p	NM_001270420	CFC1	150	-30.61	-33.9	0.013841
hsa-miR-324-5p	NM_001270421	CFC1	150	-30.61	-33.9	0.014456

hsa-miR-324-5p	NM_001272011	MOK	150	-25.96	-31.5	0.022604
hsa-miR-324-5p	NM_001278164	NBL1	153	-27.16	-33.6	0.041718
hsa-miR-324-5p	NM_001278165	NBL1	153	-27.16	-33.6	0.041718
hsa-miR-324-5p	NM_001278166	NBL1	153	-27.16	-33.6	0.041718
hsa-miR-324-5p	NM_001278192	GANAB	167	-34.81	-32.3	0.04852
hsa-miR-324-5p	NM_001278193	GANAB	167	-34.81	-32.3	0.04852
hsa-miR-324-5p	NM_001278194	GANAB	167	-34.81	-32.3	0.04852
hsa-miR-324-5p	NM_001278249	RNF214	170	-26.21	-33.4	0.01149
hsa-miR-324-5p	NM_001282167	PORCN	152	-22.92	-33.3	0.005329
hsa-miR-324-5p	NM_001286086	C11orf98	151	-26.74	-29.8	0.016014
hsa-miR-324-5p	NM_001288729	DAPK1	161	-27.65	-33.6	0.039246
hsa-miR-324-5p	NM_001288730	DAPK1	161	-27.65	-33.6	0.039246
hsa-miR-324-5p	NM_001288731	DAPK1	161	-27.65	-33.6	0.039246
hsa-miR-324-5p	NM_001289413	EHMT2	150	-26.19	-34.7	0.002438
hsa-miR-324-5p	NM_001301302	OAZ2	164	-31.2	-37.1	0.007196
hsa-miR-324-5p	NM_001302883	CLSTN1	151	-28.75	-34.7	0.030672
hsa-miR-324-5p	NM_001303012	PLCH2	157	-29.17	-35	0.038014
hsa-miR-324-5p	NM_001304521	IMPDH1	156	-25.41	-32	0.035739
hsa-miR-324-5p	NM_001567	INPPL1	162	-27.6	-35.2	0.009462
hsa-miR-324-5p	NM_001642	APLP2	150	-27.37	-33.8	0.039768
hsa-miR-324-5p	NM_001674	ATF3	151	-31.82	-35.1	0.019455
hsa-miR-324-5p	NM_002224	ITPR3	163	-33.14	-35.5	0.008255
hsa-miR-324-5p	NM_002478	MYOD1	160	-26.46	-31.2	0.040621
hsa-miR-324-5p	NM_002537	OAZ2	164	-31.2	-37.1	0.007196
hsa-miR-324-5p	NM_002960	S100A3	156	-32.06	-33.9	0.004195
hsa-miR-324-5p	NM_003204	NFE2L1	156	-30.08	-36.4	0.021323
hsa-miR-324-5p	NM_003280	TNNC1	172	-33.15	-39.2	0.000067
hsa-miR-324-5p	NM_003356	UCP3	155	-25.94	-34.1	0.026801
hsa-miR-324-5p	NM_003461	ZYX	161	-25.34	-29.7	0.046505
hsa-miR-324-5p	NM_004260	RECQL4	155	-24.69	-31.7	0.002339

hsa-miR-324-5p	NM_004844	SH3BP5	166	-30.88	-34.4	0.042831
hsa-miR-324-5p	NM_004938	DAPK1	161	-27.65	-33.6	0.039246
hsa-miR-324-5p	NM_005182	CA7	150	-28.82	-33.1	0.017977
hsa-miR-324-5p	NM_005380	NBL1	153	-27.16	-33.6	0.041718
hsa-miR-324-5p	NM_006059	LAMC3	153	-25.68	-33.9	0.034594
hsa-miR-324-5p	NM_006709	EHMT2	150	-26.19	-34.7	0.002438
hsa-miR-324-5p	NM_006725	CD6	160	-29.49	-32.8	0.042861
hsa-miR-324-5p	NM_012171	TSPAN17	151	-18.33	-33.5	0.045024
hsa-miR-324-5p	NM_014020	TMEM176B	152	-21.91	-28.9	0.027418
hsa-miR-324-5p	NM_014226	MOK	150	-25.96	-31.5	0.022604
hsa-miR-324-5p	NM_014238	KSR1	164	-29.65	-35.4	0.028874
hsa-miR-324-5p	NM_014944	CLSTN1	151	-28.75	-34.7	0.030672
hsa-miR-324-5p	NM_015944	AMDHD2	154	-27.38	-32.9	0.001618
hsa-miR-324-5p	NM_016210	C3orf18	158	-26.5	-34.2	0.043019
hsa-miR-324-5p	NM_016321	RHCG	155	-23	-32.7	0.012862
hsa-miR-324-5p	NM_018096	NLE1	169	-28.54	-33.9	0.028637
hsa-miR-324-5p	NM_018426	TMEM63B	174	-33.96	-37.1	0.002708
hsa-miR-324-5p	NM_018641	CHST12	152	-25.84	-31.3	0.045821
hsa-miR-324-5p	NM_020765	UBR4	155	-24.04	-29.2	0.038451
hsa-miR-324-5p	NM_021078	KAT2A	158	-27.83	-32.3	0.019378
hsa-miR-324-5p	NM_021104	RPL41	153	-23.54	-30.7	0.016574
hsa-miR-324-5p	NM_021198	CTDSP1	165	-35.86	-40.8	0.002339
hsa-miR-324-5p	NM_022062	PKNOX2	163	-27.29	-35.8	0.029204
hsa-miR-324-5p	NM_022479	WBSCR17	153	-29.56	-34	0.039994
hsa-miR-324-5p	NM_022825	PORCN	152	-22.92	-33.3	0.005329
hsa-miR-324-5p	NM_024040	CUEDC2	156	-22.68	-28	0.026187
hsa-miR-324-5p	NM_024099	LBHD1	151	-26.74	-29.8	0.026573
hsa-miR-324-5p	NM_024667	VPS37B	160	-29.18	-35	0.034929
hsa-miR-324-5p	NM_024705	DHRS12	155	-28.09	-34.2	0.025659
hsa-miR-324-5p	NM_025232	REEP4	155	-22.69	-30.3	0.039866

hsa-miR-324-5p	NM_025256	EHMT2	150	-26.19	-34.7	0.002438
hsa-miR-324-5p	NM_032545	CFC1	150	-30.61	-33.9	0.014456
hsa-miR-324-5p	NM_033400	ZFHX2	169	-28.63	-35.3	0.015362
hsa-miR-324-5p	NM_033507	GCK	159	-37.33	-40.9	0.000831
hsa-miR-324-5p	NM_033508	GCK	159	-37.33	-40.9	0.000831
hsa-miR-324-5p	NM_053044	HTRA3	151	-21.3	-32.4	0.045523
hsa-miR-324-5p	NM_130465	TSPAN17	151	-18.33	-33.5	0.045024
hsa-miR-324-5p	NM_138382	RIPPLY1	162	-27.26	-32.5	0.025906
hsa-miR-324-5p	NM_144611	CYB5D2	158	-23.93	-36.2	0.003885
hsa-miR-324-5p	NM_144717	IL20RB	155	-29.52	-34.7	0.013596
hsa-miR-324-5p	NM_152718	VWCE	152	-24.05	-29.7	0.039932
hsa-miR-324-5p	NM_153350	FBXL16	170	-28.52	-36.9	0.01565
hsa-miR-324-5p	NM_173560	RFX6	161	-31.75	-36.8	0.003302
hsa-miR-324-5p	NM_178562	TSPAN33	165	-28.64	-34.6	0.042215
hsa-miR-324-5p	NM_178570	RTN4RL2	163	-30.06	-35.2	0.006431
hsa-miR-324-5p	NM_181657	LTB4R	178	-29.95	-35.5	0.018477
hsa-miR-324-5p	NM_182642	CTDSP1	165	-35.86	-40.8	0.002339
hsa-miR-324-5p	NM_182744	NBL1	153	-27.16	-33.6	0.041718
hsa-miR-324-5p	NM_183240	TMEM37	161	-27.68	-35.4	0.014156
hsa-miR-324-5p	NM_183243	IMPDH1	156	-25.41	-32	0.035739
hsa-miR-324-5p	NM_198334	GANAB	167	-34.81	-32.3	0.04852
hsa-miR-324-5p	NM_198335	GANAB	167	-34.81	-32.3	0.04852
hsa-miR-324-5p	NM_201651	SLC28A1	157	-24.23	-30.8	0.046379
hsa-miR-324-5p	NM_203347	LCN15	153	-24	-27.1	0.042552
hsa-miR-324-5p	NM_203456	PPIE	157	-25.27	-31.3	0.004043
hsa-miR-324-5p	NM_203473	PORCN	152	-22.92	-33.3	0.005329
hsa-miR-324-5p	NM_203474	PORCN	152	-22.92	-33.3	0.005329
hsa-miR-324-5p	NM_203475	PORCN	152	-22.92	-33.3	0.005329
hsa-miR-324-5p	NM_207343	RNF214	170	-26.21	-33.4	0.01149
hsa-miR-324-5p	NR_004428	EGOT	167	-27.2	-34.4	0.034003

hsa-miR-324-5p	NR_024471	MRPL23-AS1	167	-31.57	-38.1	0.010505
hsa-miR-324-5p	NR_029429	PGCP1	161	-26.24	-32.7	0.019552
hsa-miR-324-5p	NR_033308	RAB17	162	-28.56	-36.8	0.017286
hsa-miR-324-5p	NR_039790	MIR4647	150	-19.66	-25.7	0.020166
hsa-miR-324-5p	NR_040096	LOC643339	155	-27.53	-34	0.009798
hsa-miR-324-5p	NR_046204	LINC01242	159	-28.35	-33.5	0.040286
hsa-miR-324-5p	NR_104108	SIGMAR1	152	-27.11	-34.2	0.041808
hsa-miR-324-5p	NR_104161	LOC338694	153	-28.56	-32.4	0.046383
hsa-miR-324-5p	NR_104197	SEPT4	161	-29.5	-34.4	0.04517
hsa-miR-324-5p	NR_104656	LOC100505942	150	-33.12	-34.1	0.026041
hsa-miR-324-5p	NR_105007	LOC102477328	165	-28.2	-34	0.011579
hsa-miR-324-5p	NR_109855	LINC-PINT	159	-28.51	-34	0.043073
hsa-miR-324-5p	NR_110790	LOC101929406	150	-30.65	-35.8	0.01984
hsa-miR-324-5p	NR_120421	LOC101927437	156	-27.57	-36.4	0.002825
hsa-miR-324-5p	NR_120483	LINC01498	168	-26.39	-33.8	0.009871
hsa-miR-324-5p	NR_125933	LOC102723766	155	-26.42	-31.7	0.048533
hsa-miR-324-5p	NR_125934	LOC102723766	155	-26.42	-31.7	0.043914
hsa-miR-324-5p	NR_132337	DANT2	159	-24.11	-33.3	0.00527
hsa-miR-338-3p	NM_001002027	ATP5G1	152	-21.23	-24.1	0.027359
hsa-miR-338-3p	NM_001126105	SLC7A7	153	-24.15	-30	0.010926
hsa-miR-338-3p	NM_001126106	SLC7A7	153	-24.15	-30	0.010926
hsa-miR-338-3p	NM_001135239	LDHA	166	-27.56	-31.1	0.026913
hsa-miR-338-3p	NM_001137608	ZNF732	172	-24.95	-28.8	0.024419
hsa-miR-338-3p	NM_001165414	LDHA	166	-27.56	-31.1	0.026913
hsa-miR-338-3p	NM_001165415	LDHA	166	-27.56	-31.1	0.022798
hsa-miR-338-3p	NM_001165416	LDHA	166	-27.56	-31.1	0.033579
hsa-miR-338-3p	NM_001178133	SUFU	154	-26.23	-28.8	0.021377
hsa-miR-338-3p	NM_001278201	SLC43A3	168	-30.42	-31	0.02378
hsa-miR-338-3p	NM_001278206	SLC43A3	168	-30.42	-31	0.02378
hsa-miR-338-3p	NM_001278506	NUBP1	163	-23.89	-26.9	0.027113

hsa-miR-338-3p	NM_001286423	GLB1L	155	-25.47	-28.5	0.034381
hsa-miR-338-3p	NM_001286427	GLB1L	155	-25.47	-28.5	0.034381
hsa-miR-338-3p	NM_002484	NUBP1	163	-23.89	-26.9	0.027113
hsa-miR-338-3p	NM_005175	ATP5G1	152	-21.23	-24.1	0.027359
hsa-miR-338-3p	NM_005566	LDHA	166	-27.56	-31.1	0.026913
hsa-miR-338-3p	NM_014096	SLC43A3	168	-30.42	-31	0.02378
hsa-miR-338-3p	NM_017611	SLC43A3	168	-30.42	-31	0.02378
hsa-miR-338-3p	NM_017758	ALKBH5	165	-27.52	-32.8	0.025542
hsa-miR-338-3p	NM_017825	ADPRHL2	150	-25.27	-29.2	0.029991
hsa-miR-338-3p	NM_024506	GLB1L	155	-25.47	-28.5	0.034381
hsa-miR-338-3p	NM_024870	PREX2	158	-22.8	-27.8	0.02109
hsa-miR-338-3p	NM_032341	DDI2	162	-28.88	-31.5	0.03481
hsa-miR-338-3p	NM_139249	MS4A6E	160	-22.96	-26.9	0.018953
hsa-miR-338-3p	NM_152489	UBE2U	150	-23.45	-25.4	0.048656
hsa-miR-338-3p	NM_152637	METTL7B	170	-24.27	-30.2	0.018673
hsa-miR-338-3p	NM_152911	PAOX	151	-22.53	-27.4	0.019672
hsa-miR-338-3p	NM_199329	SLC43A3	168	-30.42	-31	0.02378
hsa-miR-338-3p	NM_207127	PAOX	151	-22.53	-27.4	0.044509
hsa-miR-338-3p	NM_207128	PAOX	151	-22.53	-27.4	0.008745
hsa-miR-338-3p	NR_030328	MIR598	164	-20.12	-25.3	0.011744
hsa-miR-338-3p	NR_102754	SPATA8-AS1	159	-27.29	-30.3	0.009504
hsa-miR-335-3p	NM_001007253	ERV3-1	163	-25.56	-29.4	0.043772
hsa-miR-335-3p	NM_001297713	OLFML2B	167	-25.94	-28.8	0.024157
hsa-miR-335-3p	NM_004888	ATP6V1G1	163	-25.56	-30.4	0.040107
hsa-miR-335-3p	NM_015441	OLFML2B	167	-25.94	-28.8	0.024157
hsa-miR-335-3p	NR_003036	SNORD6	160	-17.12	-22.4	0.032601
hsa-miR-196b-5p	NM_000632	ITGAM	160	-33.16	-34.4	0.010399
hsa-miR-196b-5p	NM_001012398	AKTIP	156	-27.74	-32.1	0.037855
hsa-miR-196b-5p	NM_001105214	ASH2L	155	-26.7	-31.8	0.02773
hsa-miR-196b-5p	NM_001145808	ITGAM	160	-33.16	-34.4	0.010399

hsa-miR-196b-5p	NM_001164545	DISC1	163	-28.48	-32.1	0.018965
hsa-miR-196b-5p	NM_001164548	DISC1	163	-28.48	-32.1	0.019974
hsa-miR-196b-5p	NM_001164556	DISC1	163	-28.48	-32.1	0.019974
hsa-miR-196b-5p	NM_001177388	LGALS3	150	-17.17	-21.5	0.022565
hsa-miR-196b-5p	NM_001261832	ASH2L	155	-26.7	-31.8	0.02773
hsa-miR-196b-5p	NM_001282272	ASH2L	155	-26.7	-31.8	0.02773
hsa-miR-196b-5p	NM_001286433	NME4	161	-26.21	-29.6	0.019816
hsa-miR-196b-5p	NM_001286435	NME4	161	-26.21	-29.6	0.03315
hsa-miR-196b-5p	NM_001286436	NME4	161	-26.21	-29.6	0.019816
hsa-miR-196b-5p	NM_001286438	NME4	161	-26.21	-29.6	0.019816
hsa-miR-196b-5p	NM_001286439	NME4	161	-26.21	-29.6	0.019816
hsa-miR-196b-5p	NM_001286440	NME4	161	-26.21	-29.6	0.019816
hsa-miR-196b-5p	NM_001286791	CWH43	158	-23.62	-26.1	0.034109
hsa-miR-196b-5p	NM_001308325	AKTIP	156	-27.74	-32.1	0.037855
hsa-miR-196b-5p	NM_003806	HRK	152	-27.9	-30.9	0.048261
hsa-miR-196b-5p	NM_004256	SLC22A13	156	-36.68	-39.5	0.000496
hsa-miR-196b-5p	NM_004674	ASH2L	155	-26.7	-31.8	0.02773
hsa-miR-196b-5p	NM_005009	NME4	161	-26.21	-29.6	0.019816
hsa-miR-196b-5p	NM_013291	CPSF1	170	-25.93	-29	0.001171
hsa-miR-196b-5p	NM_015670	SENP3	154	-28.13	-31.1	0.012062
hsa-miR-196b-5p	NM_017422	CALML5	152	-23.02	-30.3	0.007872
hsa-miR-196b-5p	NM_018207	TRIM62	172	-32.38	-34.1	0.022106
hsa-miR-196b-5p	NM_018655	LENEP	160	-26.74	-31.2	0.009659
hsa-miR-196b-5p	NM_022476	AKTIP	156	-27.74	-32.1	0.037855
hsa-miR-196b-5p	NM_022658	HOXC8	158	-32.01	-35.4	0.008303
hsa-miR-196b-5p	NM_024016	HOXB8	176	-34.62	-37.4	0.001406
hsa-miR-196b-5p	NM_024671	ZNF768	151	-24.99	-29.6	0.028766
hsa-miR-196b-5p	NM_025087	CWH43	158	-23.62	-26.1	0.034109
hsa-miR-196b-5p	NM_030968	C1QTNF1	176	-33.24	-35.3	0.012038
hsa-miR-196b-5p	NM_153372	C1QTNF1	176	-33.24	-35.3	0.012038

hsa-miR-196b-5p	NM_198593	C1QTNF1	176	-33.24	-35.3	0.012038
hsa-miR-196b-5p	NM_198594	C1QTNF1	176	-33.24	-35.3	0.012038
hsa-miR-196b-5p	NR_002992	SNORA7B	150	-24.04	-28	0.006265
hsa-miR-196b-5p	NR_029911	MIR196B	160	-25.99	-29	0.001074
hsa-miR-196b-5p	NR_122035	ARRDC1-AS1	179	-33.98	-36	0.007116
hsa-miR-196b-5p	NR_122036	ARRDC1-AS1	179	-33.98	-36	0.004851
hsa-miR-424-5p	NM_000190	HMBS	167	-26.23	-28.2	0.010839
hsa-miR-424-5p	NM_000491	C1QB	152	-18.88	-25.4	0.019412
hsa-miR-424-5p	NM_000552	VWF	151	-24.54	-26.6	0.008102
hsa-miR-424-5p	NM_001024382	HMBS	167	-26.23	-28.2	0.010839
hsa-miR-424-5p	NM_001253676	TBPL1	187	-27.5	-31.1	0.005871
hsa-miR-424-5p	NM_001258208	HMBS	167	-26.23	-28.2	0.010839
hsa-miR-424-5p	NM_001258209	HMBS	167	-26.23	-28.2	0.010839
hsa-miR-424-5p	NM_001276290	PRKAR1A	163	-30.04	-32.4	0.000446
hsa-miR-424-5p	NM_001278422	DYNC1I1	157	-21.14	-25.5	0.046671
hsa-miR-424-5p	NM_001288721	B3GAT3	159	-23.14	-27.2	0.039971
hsa-miR-424-5p	NM_001300886	RHOD	159	-25.42	-26.9	0.048956
hsa-miR-424-5p	NM_001308175	SH3PXD2B	155	-22.02	-23.5	0.026386
hsa-miR-424-5p	NM_004865	TBPL1	187	-27.5	-31.1	0.005871
hsa-miR-424-5p	NM_012200	B3GAT3	159	-23.14	-27.2	0.039971
hsa-miR-424-5p	NM_014239	EIF2B2	162	-21.89	-27.8	0.028531
hsa-miR-424-5p	NM_014578	RHOD	159	-25.42	-26.9	0.048956
hsa-miR-424-5p	NM_016172	UBAC1	163	-19.17	-27.1	0.046672
hsa-miR-424-5p	NM_178238	PILRB	171	-24.88	-28.5	0.023774
hsa-miR-424-5p	NM_198546	SPATA21	164	-22.71	-25.2	0.014595
hsa-miR-424-5p	NR_029599	MIR30D	150	-16.02	-22.1	0.036111
hsa-miR-424-5p	NR_031711	MIR1322	162	-24.65	-29.2	0.000283
hsa-miR-424-5p	NR_107007	MIR7853	155	-21.93	-29.2	0.001354
hsa-miR-18b-5p	NM_001037283	EIF3B	165	-22.33	-29.5	0.037481
hsa-miR-18b-5p	NM_001043	SLC6A2	173	-29.05	-32.5	0.037303

hsa-miR-18b-5p	NM_001105565	SMTNL1	153	-21.63	-25.3	0.046247
hsa-miR-18b-5p	NM_001172501	SLC6A2	173	-29.05	-32.5	0.037303
hsa-miR-18b-5p	NM_001172502	SLC6A2	173	-29.05	-32.5	0.037303
hsa-miR-18b-5p	NM_001256399	ELOVL1	160	-24.74	-29.3	0.037517
hsa-miR-18b-5p	NM_001256401	ELOVL1	160	-24.74	-29.3	0.037517
hsa-miR-18b-5p	NM_001256402	ELOVL1	160	-24.74	-29.3	0.037517
hsa-miR-18b-5p	NM_001281301	KIF23	155	-24.56	-29.6	0.042531
hsa-miR-18b-5p	NM_002422	MMP3	161	-25.69	-28.7	0.024269
hsa-miR-18b-5p	NM_002620	PF4V1	152	-24.9	-28.9	0.025317
hsa-miR-18b-5p	NM_003751	EIF3B	165	-22.33	-29.5	0.029755
hsa-miR-18b-5p	NM_003786	ABCC3	164	-25.52	-30.9	0.015817
hsa-miR-18b-5p	NM_004856	KIF23	155	-24.56	-29.6	0.042531
hsa-miR-18b-5p	NM_022821	ELOVL1	160	-24.74	-29.3	0.037517
hsa-miR-18b-5p	NM_033187	KRTAP4-3	168	-26.16	-27.6	0.048846
hsa-miR-18b-5p	NM_138555	KIF23	155	-24.56	-29.6	0.042531
hsa-miR-18b-5p	NM_198456	FAM120C	163	-27.27	-32.1	0.004198
hsa-miR-18b-5p	NR_027112	LOC285692	168	-29.08	-33.5	0.033692
hsa-miR-18b-5p	NR_120481	LOC101928030	163	-23.22	-28.8	0.022178
hsa-miR-18b-5p	NR_120519	LOC102724094	151	-25.64	-30	0.017617
hsa-miR-450a-5p	NM_001307960	TM2D3	175	-27.89	-31.5	0.009997
hsa-miR-450a-5p	NM_001308026	TM2D3	175	-27.89	-31.5	0.009997
hsa-miR-433-3p	NM_001135721	PIWIL2	159	-23.3	-29.8	0.048565
hsa-miR-433-3p	NM_001144913	FGFR2	163	-24.67	-31	0.010353
hsa-miR-433-3p	NM_001144919	FGFR2	163	-24.67	-31	0.010353
hsa-miR-433-3p	NM_001198773	PI4KB	151	-29.65	-33.4	0.023273
hsa-miR-433-3p	NM_001198774	PI4KB	151	-29.65	-33.4	0.023273
hsa-miR-433-3p	NM_001198775	PI4KB	151	-29.65	-33.4	0.023273
hsa-miR-433-3p	NM_001201536	TAF1A	159	-23.3	-29.8	0.028614
hsa-miR-433-3p	NM_001297721	C1orf43	163	-25.71	-31.6	0.038703
hsa-miR-433-3p	NM_001297723	C1orf43	163	-25.71	-31.6	0.038703

hsa-miR-433-3p	NM_002651	PI4KB	151	-29.65	-33.4	0.023273
hsa-miR-433-3p	NM_018068	PIWIL2	159	-23.3	-29.8	0.048565
hsa-miR-433-3p	NM_080866	SLC22A9	159	-23.54	-29.6	0.028356
hsa-miR-433-3p	NM_139352	TAF1A	159	-23.3	-29.8	0.028614
hsa-miR-433-3p	NM_182506	MAGEB10	163	-24.67	-31	0.03503
hsa-miR-433-3p	NM_198276	TMEM17	163	-26.34	-31.8	0.043604
hsa-miR-433-3p	NR_045559	UXT	171	-33.16	-36.5	0.002863
hsa-miR-433-3p	NR_045560	UXT	175	-37.91	-41.4	0.000242
hsa-miR-433-3p	NR_103804	PDLIM7	151	-24.65	-33.8	0.045497
hsa-miR-433-3p	NR_105009	LINC01335	168	-26.46	-31	0.014702
hsa-miR-329-3p	NM_001080501	TMEM223	165	-27.4	-31.9	0.002925
hsa-miR-329-3p	NM_001105565	SMTNL1	162	-22.21	-26	0.024369
hsa-miR-329-3p	NM_001174101	PRR7	166	-20.61	-25.5	0.033798
hsa-miR-329-3p	NM_001174102	PRR7	166	-20.61	-25.5	0.032318
hsa-miR-329-3p	NM_001256743	GRIA3	166	-22.88	-27.4	0.026345
hsa-miR-329-3p	NM_001271862	PNLDC1	168	-28.47	-32.4	0.000759
hsa-miR-329-3p	NM_001271937	RMND1	171	-25.22	-29.3	0.025754
hsa-miR-329-3p	NM_001300866	FILIP1	152	-17.37	-21.5	0.029728
hsa-miR-329-3p	NM_017909	RMND1	171	-25.22	-29.3	0.025754
hsa-miR-329-3p	NM_030567	PRR7	166	-20.61	-25.5	0.033798
hsa-miR-329-3p	NM_145306	C10orf35	167	-26.67	-30.9	0.014034
hsa-miR-329-3p	NM_173516	PNLDC1	168	-28.47	-32.4	0.000759
hsa-miR-329-3p	NR_106823	MIR6765	151	-18.29	-23.8	0.028096
hsa-miR-451a	NM_002259	KLRC1	160	-30.17	-33.6	0.002527
hsa-miR-451a	NM_002260	KLRC2	160	-30.17	-33.6	0.00249
hsa-miR-451a	NM_007328	KLRC1	160	-30.17	-33.6	0.002527
hsa-miR-451a	NM_213657	KLRC1	160	-30.17	-33.6	0.002527
hsa-miR-451a	NM_213658	KLRC1	160	-30.17	-33.6	0.002527
hsa-miR-451a	NR_029970	MIR451A	176	-28.94	-30.7	0.000152
hsa-miR-409-5p	NM_000449	RFX5	152	-33.3	-39.4	0.002352

hsa-miR-409-5p	NM_001025603	RFX5	152	-33.3	-39.4	0.002352
hsa-miR-409-5p	NM_001144070	ABCC3	152	-19.21	-25.6	0.014451
hsa-miR-409-5p	NM_001173977	LRRC16A	155	-26.53	-33.7	0.014394
hsa-miR-409-5p	NM_006829	ADIRF	153	-27.97	-31	0.009041
hsa-miR-409-5p	NM_012168	FBXO2	156	-23.4	-29.2	0.023907
hsa-miR-409-5p	NM_014957	DENND3	177	-27.25	-32.6	0.04958
hsa-miR-409-5p	NM_015656	KIF26A	157	-27.63	-31.7	0.045709
hsa-miR-409-5p	NM_017640	LRRC16A	155	-26.53	-33.7	0.014394
hsa-miR-409-5p	NM_033328	CAPZA3	150	-15.49	-22.9	0.002648
hsa-miR-409-5p	NM_080429	AQP10	176	-30.31	-35.4	0.005357
hsa-miR-409-5p	NR_026684	HPYR1	175	-27.85	-32.9	0.022103
hsa-miR-409-5p	NR_104332	CISTR	153	-26.91	-33.6	0.012846
hsa-miR-409-5p	NR_104333	CISTR	153	-26.91	-33.6	0.006713
hsa-miR-409-3p	NM_004839	HOMER2	155	-24.73	-31.4	0.028641
hsa-miR-409-3p	NM_199330	HOMER2	155	-24.73	-31.4	0.028641
hsa-miR-409-3p	NR_132980	SNORD141A	153	-23.48	-25.9	0.017599
hsa-miR-409-3p	NR_132981	SNORD141B	153	-23.48	-25.9	0.017599
hsa-miR-146b-3p	NM_000155	GALT	153	-20.31	-26.1	0.03821
hsa-miR-146b-3p	NM_000256	MYBPC3	172	-35.4	-38.1	0.000315
hsa-miR-146b-3p	NM_000501	ELN	159	-27.71	-33.7	0.02611
hsa-miR-146b-3p	NM_000639	FASLG	155	-25.79	-32.3	0.029534
hsa-miR-146b-3p	NM_000832	GRIN1	150	-36.27	-36	0.006034
hsa-miR-146b-3p	NM_000911	OPRD1	171	-33.29	-32.1	0.009915
hsa-miR-146b-3p	NM_000942	PPIB	154	-28.13	-31.4	0.003914
hsa-miR-146b-3p	NM_001004440	FAM19A3	164	-27.45	-31.7	0.019835
hsa-miR-146b-3p	NM_001006665	RPS6KA1	164	-27.14	-35.2	0.007328
hsa-miR-146b-3p	NM_001037281	PARD6A	160	-24.13	-27.2	0.016169
hsa-miR-146b-3p	NM_001040118	ARAP1	165	-27.97	-32.6	0.014462
hsa-miR-146b-3p	NM_001040454	SLC26A6	152	-26.92	-29.8	0.014404
hsa-miR-146b-3p	NM_001053	SSTR5	151	-33.48	-34.7	0.022939

hsa-miR-146b-3p	NM_001081752	ELN	159	-27.71	-33.7	0.02611
hsa-miR-146b-3p	NM_001081753	ELN	159	-27.71	-33.7	0.02611
hsa-miR-146b-3p	NM_001081754	ELN	159	-27.71	-33.7	0.02611
hsa-miR-146b-3p	NM_001081755	ELN	159	-27.71	-33.7	0.02611
hsa-miR-146b-3p	NM_001098509	SGSM2	152	-28.27	-33.1	0.047693
hsa-miR-146b-3p	NM_001101672	ZNF219	154	-22.89	-29.8	0.040852
hsa-miR-146b-3p	NM_001102454	ZNF219	154	-22.89	-29.8	0.040852
hsa-miR-146b-3p	NM_001113205	TSTD1	160	-24.57	-28	0.033171
hsa-miR-146b-3p	NM_001130082	PLXNB1	171	-35.57	-37.2	0.001766
hsa-miR-146b-3p	NM_001135190	ARAP1	165	-27.97	-32.6	0.014462
hsa-miR-146b-3p	NM_001135195	SLC39A5	155	-25.06	-26.8	0.015484
hsa-miR-146b-3p	NM_001136037	LIMS2	161	-29.88	-33	0.021566
hsa-miR-146b-3p	NM_001142674	CHID1	172	-32.25	-35.3	0.02691
hsa-miR-146b-3p	NM_001142675	CHID1	172	-32.25	-35.3	0.02691
hsa-miR-146b-3p	NM_001142676	CHID1	172	-32.25	-35.3	0.02691
hsa-miR-146b-3p	NM_001142677	CHID1	172	-32.25	-35.3	0.02691
hsa-miR-146b-3p	NM_001145099	SLC2A6	172	-28.41	-31.8	0.041712
hsa-miR-146b-3p	NM_001145657	RAP1GAP	151	-28.74	-34.1	0.018612
hsa-miR-146b-3p	NM_001145658	RAP1GAP	151	-28.74	-34.1	0.018612
hsa-miR-146b-3p	NM_001160102	LPO	164	-28.14	-30.2	0.037385
hsa-miR-146b-3p	NM_001160184	PLEKHN1	150	-25.53	-31.7	0.018394
hsa-miR-146b-3p	NM_001161403	LIMS2	161	-29.88	-33	0.021566
hsa-miR-146b-3p	NM_001161404	LIMS2	161	-29.88	-33	0.021566
hsa-miR-146b-3p	NM_001161707	KIRREL3	151	-25.21	-30.1	0.02238
hsa-miR-146b-3p	NM_001163257	PLXNB3	151	-30.45	-33.7	0.002519
hsa-miR-146b-3p	NM_001165937	STARD3	156	-27.81	-32.7	0.044809
hsa-miR-146b-3p	NM_001165938	STARD3	156	-27.81	-32.7	0.044809
hsa-miR-146b-3p	NM_001170760	SRPK3	150	-22.56	-27.1	0.041095
hsa-miR-146b-3p	NM_001170761	SRPK3	150	-22.56	-27.1	0.041095
hsa-miR-146b-3p	NM_001172560	SSTR5	151	-33.48	-34.7	0.022939

hsa-miR-146b-3p	NM_001185090	GRIN1	150	-36.27	-36	0.006034
hsa-miR-146b-3p	NM_001185091	GRIN1	150	-36.27	-36	0.006034
hsa-miR-146b-3p	NM_001195087	GGACT	170	-29.98	-34.1	0.04364
hsa-miR-146b-3p	NM_001202467	ZMYND11	156	-27.59	-33.2	0.01353
hsa-miR-146b-3p	NM_001202468	ZMYND11	156	-27.59	-33.2	0.01353
hsa-miR-146b-3p	NM_001242464	PPP1R1B	153	-27.08	-32.2	0.025808
hsa-miR-146b-3p	NM_001243536	ANKRD65	155	-25.38	-34.2	0.018751
hsa-miR-146b-3p	NM_001256542	LIMS2	161	-29.88	-33	0.021566
hsa-miR-146b-3p	NM_001258332	GALT	153	-20.31	-26.1	0.03821
hsa-miR-146b-3p	NM_001277335	RASA4B	163	-28.68	-31.3	0.017293
hsa-miR-146b-3p	NM_001278794	NPY4R	153	-30.04	-32.9	0.006527
hsa-miR-146b-3p	NM_001278912	ELN	159	-27.71	-33.7	0.02611
hsa-miR-146b-3p	NM_001278913	ELN	159	-27.71	-33.7	0.02611
hsa-miR-146b-3p	NM_001278914	ELN	159	-27.71	-33.7	0.02611
hsa-miR-146b-3p	NM_001278915	ELN	159	-27.71	-33.7	0.02611
hsa-miR-146b-3p	NM_001278916	ELN	159	-27.71	-33.7	0.02611
hsa-miR-146b-3p	NM_001278917	ELN	159	-27.71	-33.7	0.02611
hsa-miR-146b-3p	NM_001278918	ELN	159	-27.71	-33.7	0.02611
hsa-miR-146b-3p	NM_001278939	ELN	159	-27.71	-33.7	0.02611
hsa-miR-146b-3p	NM_001281732	SLC26A6	152	-26.92	-29.8	0.014404
hsa-miR-146b-3p	NM_001281733	SLC26A6	152	-26.92	-29.8	0.014404
hsa-miR-146b-3p	NM_001282164	P2RX2	159	-28.62	-31.8	0.011568
hsa-miR-146b-3p	NM_001282165	P2RX2	159	-28.62	-31.8	0.038473
hsa-miR-146b-3p	NM_001282471	PRORY	159	-25.08	-28.6	0.018432
hsa-miR-146b-3p	NM_001288814	MROH1	154	-23.42	-30.6	0.005589
hsa-miR-146b-3p	NM_001290137	HAGHL	151	-26.19	-31.3	0.006354
hsa-miR-146b-3p	NM_001290139	HAGHL	151	-26.19	-31.3	0.006354
hsa-miR-146b-3p	NM_001290145	POLDIP2	156	-29.61	-35.2	0.018409
hsa-miR-146b-3p	NM_001291366	PERM1	164	-29.84	-33.3	0.022709
hsa-miR-146b-3p	NM_001291367	PERM1	164	-29.84	-33.3	0.022709

hsa-miR-146b-3p	NM_001291815	HMCN2	158	-30.51	-32.2	0.010125
hsa-miR-146b-3p	NM_001293634	DEAF1	153	-27.94	-28.8	0.035665
hsa-miR-146b-3p	NM_001303029	PKP3	151	-30.11	-34.6	0.001928
hsa-miR-146b-3p	NM_001303124	ZC3H10	150	-26.36	-30.6	0.04088
hsa-miR-146b-3p	NM_001303125	ZC3H10	150	-26.36	-30.6	0.04088
hsa-miR-146b-3p	NM_001304385	LSMEM2	162	-32.09	-33.8	0.016359
hsa-miR-146b-3p	NM_001304512	HSD17B7	157	-26.19	-29	0.014094
hsa-miR-146b-3p	NM_001304513	HSD17B7	157	-26.19	-29	0.029356
hsa-miR-146b-3p	NM_001305182	RSPRY1	160	-24.28	-27.6	0.044242
hsa-miR-146b-3p	NM_002081	GPC1	161	-26.64	-35.1	0.025058
hsa-miR-146b-3p	NM_002318	LOXL2	154	-25.08	-32.4	0.043154
hsa-miR-146b-3p	NM_002588	PCDHGC3	157	-31.83	-33.9	0.042279
hsa-miR-146b-3p	NM_002673	PLXNB1	171	-35.57	-37.2	0.001766
hsa-miR-146b-3p	NM_002880	RAF1	151	-26.49	-33	0.023634
hsa-miR-146b-3p	NM_002885	RAP1GAP	151	-28.74	-34.1	0.018612
hsa-miR-146b-3p	NM_002953	RPS6KA1	164	-27.14	-35.2	0.007328
hsa-miR-146b-3p	NM_003088	FSCN1	150	-29.6	-32.5	0.043134
hsa-miR-146b-3p	NM_003508	FZD9	153	-35.39	-28.3	0.047468
hsa-miR-146b-3p	NM_003735	PCDHGA12	157	-31.83	-33.9	0.042343
hsa-miR-146b-3p	NM_003736	PCDHGB4	157	-31.83	-33.9	0.042343
hsa-miR-146b-3p	NM_004178	TARBP2	157	-27.72	-28	0.041661
hsa-miR-146b-3p	NM_004245	TGM5	167	-26.79	-31.1	0.029703
hsa-miR-146b-3p	NM_004443	EPHB3	161	-28.75	-32	0.029809
hsa-miR-146b-3p	NM_004783	TAOK2	160	-30.31	-30.5	0.049482
hsa-miR-146b-3p	NM_005125	CCS	165	-25.11	-29.9	0.005515
hsa-miR-146b-3p	NM_005262	GFER	152	-17.05	-33.7	0.043172
hsa-miR-146b-3p	NM_005393	PLXNB3	151	-30.45	-33.7	0.002519
hsa-miR-146b-3p	NM_005886	KATNB1	157	-21.91	-29.5	0.021736
hsa-miR-146b-3p	NM_005972	NPY4R	153	-30.04	-32.9	0.006527
hsa-miR-146b-3p	NM_006151	LPO	164	-28.14	-30.2	0.037385

hsa-miR-146b-3p	NM_006613	GRAP	159	-26.42	-34.2	0.022894
hsa-miR-146b-3p	NM_006779	CDC42EP2	161	-28.02	-31.3	0.049944
hsa-miR-146b-3p	NM_006804	STARD3	156	-27.81	-32.7	0.044809
hsa-miR-146b-3p	NM_007183	PKP3	151	-30.11	-34.6	0.001928
hsa-miR-146b-3p	NM_007245	ATXN2L	162	-35.64	-36.7	0.004797
hsa-miR-146b-3p	NM_007327	GRIN1	150	-36.27	-36	0.009717
hsa-miR-146b-3p	NM_012079	DGAT1	167	-30.73	-36.3	0.017272
hsa-miR-146b-3p	NM_012184	FOXD4L1	150	-28.48	-31.4	0.030685
hsa-miR-146b-3p	NM_012226	P2RX2	159	-28.62	-31.8	0.011568
hsa-miR-146b-3p	NM_012320	PLA2G15	168	-33.64	-37.3	0.006663
hsa-miR-146b-3p	NM_013323	SNX11	165	-30.32	-34.4	0.019821
hsa-miR-146b-3p	NM_014370	SRPK3	150	-22.56	-27.1	0.041095
hsa-miR-146b-3p	NM_014671	UBE3C	151	-24.82	-34.6	0.026922
hsa-miR-146b-3p	NM_014853	SGSM2	152	-28.27	-33.1	0.047693
hsa-miR-146b-3p	NM_015242	ARAP1	165	-27.97	-32.6	0.014462
hsa-miR-146b-3p	NM_015456	NELFB	164	-31.51	-33.1	0.012185
hsa-miR-146b-3p	NM_015584	POLDIP2	156	-29.61	-35.2	0.018409
hsa-miR-146b-3p	NM_016318	P2RX2	159	-28.62	-31.8	0.011568
hsa-miR-146b-3p	NM_016423	ZNF219	154	-22.89	-29.8	0.040852
hsa-miR-146b-3p	NM_016948	PARD6A	160	-24.13	-27.2	0.016169
hsa-miR-146b-3p	NM_017585	SLC2A6	172	-28.41	-31.8	0.041712
hsa-miR-146b-3p	NM_017670	OTUB1	150	-29.63	-34	0.013953
hsa-miR-146b-3p	NM_017980	LIMS2	161	-29.88	-33	0.021566
hsa-miR-146b-3p	NM_018912	PCDHGA1	157	-31.83	-33.9	0.042343
hsa-miR-146b-3p	NM_018913	PCDHGA10	157	-31.83	-33.9	0.042343
hsa-miR-146b-3p	NM_018914	PCDHGA11	157	-31.83	-33.9	0.042343
hsa-miR-146b-3p	NM_018915	PCDHGA2	157	-31.83	-33.9	0.042343
hsa-miR-146b-3p	NM_018916	PCDHGA3	157	-31.83	-33.9	0.042343
hsa-miR-146b-3p	NM_018917	PCDHGA4	157	-31.83	-33.9	0.042343
hsa-miR-146b-3p	NM_018918	PCDHGA5	157	-31.83	-33.9	0.042343

hsa-miR-146b-3p	NM_018919	PCDHGA6	157	-31.83	-33.9	0.042343
hsa-miR-146b-3p	NM_018920	PCDHGA7	157	-31.83	-33.9	0.042343
hsa-miR-146b-3p	NM_018921	PCDHGA9	157	-31.83	-33.9	0.042343
hsa-miR-146b-3p	NM_018922	PCDHGB1	157	-31.83	-33.9	0.042343
hsa-miR-146b-3p	NM_018923	PCDHGB2	157	-31.83	-33.9	0.042343
hsa-miR-146b-3p	NM_018924	PCDHGB3	157	-31.83	-33.9	0.042343
hsa-miR-146b-3p	NM_018925	PCDHGB5	157	-31.83	-33.9	0.042343
hsa-miR-146b-3p	NM_018926	PCDHGB6	157	-31.83	-33.9	0.042343
hsa-miR-146b-3p	NM_018927	PCDHGB7	157	-31.83	-33.9	0.042407
hsa-miR-146b-3p	NM_018928	PCDHGC4	157	-31.83	-33.9	0.042343
hsa-miR-146b-3p	NM_018929	PCDHGC5	157	-31.83	-33.9	0.042343
hsa-miR-146b-3p	NM_020879	CCDC146	150	-24.59	-28.4	0.045358
hsa-miR-146b-3p	NM_021008	DEAF1	153	-27.94	-28.8	0.035665
hsa-miR-146b-3p	NM_021569	GRIN1	150	-36.27	-36	0.009717
hsa-miR-146b-3p	NM_022167	XYLT2	169	-32.31	-35.6	0.006917
hsa-miR-146b-3p	NM_022911	SLC26A6	152	-26.92	-29.8	0.014404
hsa-miR-146b-3p	NM_023947	CHID1	172	-32.25	-35.3	0.02691
hsa-miR-146b-3p	NM_024589	ROGDI	152	-19.53	-30.5	0.029858
hsa-miR-146b-3p	NM_030819	GFOD2	161	-26.12	-30.6	0.040194
hsa-miR-146b-3p	NM_031208	FAHD1	170	-28.79	-36.5	0.005532
hsa-miR-146b-3p	NM_032088	PCDHGA8	157	-31.83	-33.9	0.042343
hsa-miR-146b-3p	NM_032092	PCDHGA11	157	-31.83	-33.9	0.042343
hsa-miR-146b-3p	NM_032129	PLEKHN1	150	-25.53	-31.7	0.018394
hsa-miR-146b-3p	NM_032192	PPP1R1B	153	-27.08	-32.2	0.025808
hsa-miR-146b-3p	NM_032304	HAGHL	151	-26.19	-31.3	0.006354
hsa-miR-146b-3p	NM_032403	PCDHGC3	157	-31.83	-33.9	0.042279
hsa-miR-146b-3p	NM_032450	MROH1	154	-23.42	-30.6	0.005589
hsa-miR-146b-3p	NM_032786	ZC3H10	150	-26.36	-30.6	0.04088
hsa-miR-146b-3p	NM_033110	GGACT	170	-29.98	-34.1	0.04364
hsa-miR-146b-3p	NM_033413	LRRC46	156	-27.5	-30	0.049635

hsa-miR-146b-3p	NM_052943	FAM46B	153	-29.57	-32	0.037924
hsa-miR-146b-3p	NM_057176	BSND	155	-21.35	-28.2	0.014209
hsa-miR-146b-3p	NM_080861	SPSB3	157	-26.71	-29.5	0.032275
hsa-miR-146b-3p	NM_130769	GPHA2	162	-26.74	-29.2	0.025076
hsa-miR-146b-3p	NM_133467	CITED4	150	-27.61	-32.7	0.010883
hsa-miR-146b-3p	NM_134263	SLC26A6	152	-26.92	-29.8	0.014404
hsa-miR-146b-3p	NM_134323	TARBP2	157	-27.72	-28	0.041661
hsa-miR-146b-3p	NM_134324	TARBP2	157	-27.72	-28	0.041661
hsa-miR-146b-3p	NM_134426	SLC26A6	152	-26.92	-29.8	0.014404
hsa-miR-146b-3p	NM_144669	GLT1D1	159	-30.15	-36.8	0.012958
hsa-miR-146b-3p	NM_144683	DHRS13	155	-30.52	-35.8	0.003735
hsa-miR-146b-3p	NM_147192	DMBX1	173	-36.04	-39.5	0.003539
hsa-miR-146b-3p	NM_152244	SNX11	165	-30.32	-34.4	0.019821
hsa-miR-146b-3p	NM_152393	KLHL40	159	-29.21	-30.6	0.02441
hsa-miR-146b-3p	NM_153215	LSMEM2	162	-32.09	-33.8	0.016359
hsa-miR-146b-3p	NM_170682	P2RX2	159	-28.62	-31.8	0.011568
hsa-miR-146b-3p	NM_170683	P2RX2	159	-28.62	-31.8	0.011568
hsa-miR-146b-3p	NM_172225	DMBX1	173	-36.04	-39.5	0.003539
hsa-miR-146b-3p	NM_173596	SLC39A5	155	-25.06	-26.8	0.015484
hsa-miR-146b-3p	NM_174872	P2RX2	159	-28.62	-31.8	0.011568
hsa-miR-146b-3p	NM_174873	P2RX2	159	-28.62	-31.8	0.011568
hsa-miR-146b-3p	NM_181505	PPP1R1B	153	-27.08	-32.2	0.025808
hsa-miR-146b-3p	NM_182626	C2orf48	155	-23.92	-34.9	0.0139
hsa-miR-146b-3p	NM_182632	SLC6A18	156	-26.53	-30.5	0.001682
hsa-miR-146b-3p	NM_182759	FAM19A3	164	-27.45	-31.7	0.022193
hsa-miR-146b-3p	NM_198082	CCDC57	157	-25.05	-29.5	0.011355
hsa-miR-146b-3p	NM_201631	TGM5	167	-26.79	-31.1	0.029703
hsa-miR-146b-3p	NM_203286	PVRL1	151	-19.76	-28.1	0.0223
hsa-miR-146b-3p	NM_212479	ZMYND11	156	-27.59	-33.2	0.01353
hsa-miR-146b-3p	NR_003005	SCARNA4	159	-23.82	-28	0.009925

hsa-miR-146b-3p	NR_027254	LINC00671	158	-30.16	-33.6	0.049424
hsa-miR-146b-3p	NR_029578	MIR192	170	-30.33	-33.8	0.000237
hsa-miR-146b-3p	NR_031670	MIR1266	152	-28.04	-30.9	0.000679
hsa-miR-146b-3p	NR_033308	RAB17	150	-32.16	-34	0.041843
hsa-miR-146b-3p	NR_033346	BOK-AS1	152	-28.34	-34	0.012748
hsa-miR-146b-3p	NR_034002	MCF2L-AS1	163	-29.31	-35.5	0.010939
hsa-miR-146b-3p	NR_037416	MIR3621	152	-31.67	-34.5	0.000078
hsa-miR-146b-3p	NR_038923	SSSCA1-AS1	155	-27.78	-37.4	0.004915
hsa-miR-146b-3p	NR_039719	MIR4498	152	-28.88	-34	0.000052
hsa-miR-146b-3p	NR_039737	MIR4512	156	-19.54	-23.9	0.039897
hsa-miR-146b-3p	NR_039956	MIR4793	158	-26.95	-29.7	0.001534
hsa-miR-146b-3p	NR_040089	ALOX12-AS1	155	-28.18	-31.8	0.02447
hsa-miR-146b-3p	NR_046362	IL15RA	164	-30.43	-36.8	0.009792
hsa-miR-146b-3p	NR_106766	MIR6511A1	167	-30.59	-33.3	0.000084
hsa-miR-146b-3p	NR_106775	MIR6511B1	167	-30.59	-33.3	0.000163
hsa-miR-146b-3p	NR_106965	MIR6511B2	167	-30.59	-33.3	0.000099
hsa-miR-146b-3p	NR_106969	MIR6511A2	167	-30.59	-33.3	0.000084
hsa-miR-146b-3p	NR_106970	MIR6511A3	167	-30.59	-33.3	0.000084
hsa-miR-146b-3p	NR_106971	MIR6511A4	167	-30.59	-33.3	0.000084
hsa-miR-146b-3p	NR_111905	ADGRA1-AS1	159	-28.77	-31.3	0.01839
hsa-miR-146b-3p	NR_111955	LOC103091866	151	-29.07	-33.8	0.022225
hsa-miR-146b-3p	NR_120421	LOC101927437	157	-25.28	-30.6	0.032612
hsa-miR-146b-3p	NR_120511	SEPT7-AS1	159	-26.18	-32.9	0.020425
hsa-miR-146b-3p	NR_120512	SEPT7-AS1	159	-26.18	-32.9	0.01698
hsa-miR-146b-3p	NR_121579	LOC101928525	152	-27.91	-33.1	0.030476
hsa-miR-146b-3p	NR_121680	LINC01398	164	-31.78	-34.4	0.017305
hsa-miR-146b-3p	NR_125786	LINC01055	154	-24.97	-31.5	0.031943
hsa-miR-146b-3p	NR_125945	LOC101927851	165	-34.74	-35	0.009024
hsa-miR-146b-3p	NR_130724	TLX1NB	152	-22.6	-33.9	0.025453
hsa-miR-146b-3p	NR_132966	SNORA110	159	-26.8	-28.1	0.024917

hsa-miR-501-3p	NM_000832	GRIN1	152	-28.05	-32.4	0.026609
hsa-miR-501-3p	NM_000941	POR	157	-24.92	-29.8	0.022361
hsa-miR-501-3p	NM_001003941	OGDH	156	-24.68	-29.1	0.031236
hsa-miR-501-3p	NM_001112726	CEP170B	162	-28.78	-33.8	0.037811
hsa-miR-501-3p	NM_001184740	CTTN	157	-23.75	-26.7	0.031354
hsa-miR-501-3p	NM_001185090	GRIN1	152	-28.05	-32.4	0.026609
hsa-miR-501-3p	NM_001185091	GRIN1	152	-28.05	-32.4	0.026609
hsa-miR-501-3p	NM_001199828	SPRR1A	174	-28.16	-33.5	0.002377
hsa-miR-501-3p	NM_001243925	MAPKAPK3	169	-26.56	-33	0.03186
hsa-miR-501-3p	NM_001243926	MAPKAPK3	169	-26.56	-33	0.03186
hsa-miR-501-3p	NM_004394	DAP	159	-23.18	-35.6	0.017527
hsa-miR-501-3p	NM_004635	MAPKAPK3	169	-26.56	-33	0.03186
hsa-miR-501-3p	NM_005987	SPRR1A	174	-28.16	-33.5	0.002377
hsa-miR-501-3p	NM_007079	PTP4A3	162	-26.83	-32.5	0.047102
hsa-miR-501-3p	NM_007327	GRIN1	152	-28.05	-32.4	0.040902
hsa-miR-501-3p	NM_014599	MAGED2	162	-20.47	-27.2	0.016065
hsa-miR-501-3p	NM_015005	CEP170B	162	-28.78	-33.8	0.037811
hsa-miR-501-3p	NM_021569	GRIN1	152	-28.05	-32.4	0.040902
hsa-miR-501-3p	NM_032611	PTP4A3	162	-26.83	-32.5	0.047102
hsa-miR-501-3p	NM_177433	MAGED2	162	-20.47	-27.2	0.016065
hsa-miR-501-3p	NM_201222	MAGED2	162	-20.47	-27.2	0.016065
hsa-miR-501-3p	NR_030361	MIR33B	151	-22.04	-25.6	0.018724
hsa-miR-501-3p	NR_036182	MIR4294	152	-21.77	-27.4	0.003741
hsa-miR-501-3p	NR_109767	LOC100131315	157	-27.33	-31	0.025603
hsa-miR-501-3p	NR_120586	LOC101928069	158	-26.43	-31.4	0.037312
hsa-miR-501-3p	NR_125753	DLEU1-AS1	168	-27.73	-31.6	0.027229
hsa-miR-501-3p	NR_131938	LRP1-AS	153	-26.64	-32.4	0.015216
hsa-miR-532-3p	NM_000117	EMD	150	-26.56	-31.1	0.012035
hsa-miR-532-3p	NM_000287	PEX6	154	-29.69	-32.5	0.011028
hsa-miR-532-3p	NM_000289	PFKM	157	-28.94	-30.8	0.047577

hsa-miR-532-3p	NM_000358	TGFBI	155	-29.34	-34.6	0.006143
hsa-miR-532-3p	NM_000682	ADRA2B	172	-34.55	-34.8	0.034718
hsa-miR-532-3p	NM_000749	CHRNB3	156	-33	-36	0.001866
hsa-miR-532-3p	NM_000759	CSF3	162	-32.67	-32.6	0.028323
hsa-miR-532-3p	NM_000817	GAD1	164	-31.3	-34.3	0.023817
hsa-miR-532-3p	NM_000864	HTR1D	154	-32.21	-36	0.010239
hsa-miR-532-3p	NM_001001673	RNASE9	156	-26.73	-30.2	0.022389
hsa-miR-532-3p	NM_001004055	LRRC29	169	-32.3	-33.7	0.004116
hsa-miR-532-3p	NM_001007102	L3MBTL3	156	-31.22	-34.4	0.034587
hsa-miR-532-3p	NM_001013625	CFAP126	165	-27.78	-28.4	0.027038
hsa-miR-532-3p	NM_001017956	OS9	163	-34.42	-35.3	0.005156
hsa-miR-532-3p	NM_001017957	OS9	163	-34.42	-35.3	0.005156
hsa-miR-532-3p	NM_001017958	OS9	163	-34.42	-35.3	0.005156
hsa-miR-532-3p	NM_001018003	SORBS3	153	-31.85	-35.4	0.006573
hsa-miR-532-3p	NM_001025205	AP2M1	157	-27.65	-31.3	0.020739
hsa-miR-532-3p	NM_001025234	TSPAN4	152	-28.99	-31.9	0.016098
hsa-miR-532-3p	NM_001025235	TSPAN4	152	-28.99	-31.9	0.016098
hsa-miR-532-3p	NM_001025236	TSPAN4	152	-28.99	-31.9	0.016098
hsa-miR-532-3p	NM_001025237	TSPAN4	152	-28.99	-31.9	0.016098
hsa-miR-532-3p	NM_001025238	TSPAN4	152	-28.99	-31.9	0.016098
hsa-miR-532-3p	NM_001025239	TSPAN4	152	-28.99	-31.9	0.016098
hsa-miR-532-3p	NM_001025593	ARFIP1	150	-30.41	-34.1	0.039142
hsa-miR-532-3p	NM_001025595	ARFIP1	150	-30.41	-34.1	0.039142
hsa-miR-532-3p	NM_001031702	SEMA5B	153	-31.55	-34.9	0.011747
hsa-miR-532-3p	NM_001033053	NLRP1	167	-34.06	-35.4	0.002167
hsa-miR-532-3p	NM_001033088	NGRN	168	-30.37	-33.7	0.005223
hsa-miR-532-3p	NM_001039780	CCNI2	174	-35.89	-40.8	0.001672
hsa-miR-532-3p	NM_001042616	PIGY	167	-31.29	-31.8	0.023539
hsa-miR-532-3p	NM_001057	TACR2	150	-29.5	-32.6	0.017358
hsa-miR-532-3p	NM_001077628	APH1A	163	-29.82	-32.6	0.022316

hsa-miR-532-3p	NM_001080513	CPN2	161	-22.74	-33.5	0.035048
hsa-miR-532-3p	NM_001098173	PRDM7	154	-31.87	-36.7	0.013281
hsa-miR-532-3p	NM_001098537	PNPLA7	162	-29.47	-34.5	0.003797
hsa-miR-532-3p	NM_001099434	DCDC2B	158	-29.69	-32.9	0.00408
hsa-miR-532-3p	NM_001099640	TMEM100	157	-30.29	-32.8	0.034548
hsa-miR-532-3p	NM_001099780	PSMB11	158	-36.73	-38.1	0.00265
hsa-miR-532-3p	NM_001100118	XRCC3	153	-33.87	-37.1	0.006195
hsa-miR-532-3p	NM_001100119	XRCC3	153	-33.87	-37.1	0.006195
hsa-miR-532-3p	NM_001105568	KIF13A	151	-28.49	-34.5	0.032555
hsa-miR-532-3p	NM_001110356	RNASE9	156	-26.73	-30.2	0.022389
hsa-miR-532-3p	NM_001110357	RNASE9	156	-26.73	-30.2	0.022389
hsa-miR-532-3p	NM_001110358	RNASE9	156	-26.73	-30.2	0.022389
hsa-miR-532-3p	NM_001110359	RNASE9	156	-26.73	-30.2	0.022389
hsa-miR-532-3p	NM_001110360	RNASE9	156	-26.73	-30.2	0.022389
hsa-miR-532-3p	NM_001110361	RNASE9	156	-26.73	-30.2	0.022389
hsa-miR-532-3p	NM_001111270	ARHGEF25	152	-27.11	-31.2	0.013037
hsa-miR-532-3p	NM_001113491	SEPT9	161	-35.9	-38.8	0.006488
hsa-miR-532-3p	NM_001113492	SEPT9	161	-35.9	-38.8	0.006488
hsa-miR-532-3p	NM_001113493	SEPT9	161	-35.9	-38.8	0.006488
hsa-miR-532-3p	NM_001113494	SEPT9	161	-35.9	-38.8	0.006488
hsa-miR-532-3p	NM_001113496	SEPT9	161	-35.9	-38.8	0.006488
hsa-miR-532-3p	NM_001130848	PITPNM1	159	-26.88	-29.4	0.019961
hsa-miR-532-3p	NM_001134368	SLC6A6	156	-29.39	-31.9	0.009824
hsa-miR-532-3p	NM_001135004	DNAJB5	156	-34.27	-37.7	0.004696
hsa-miR-532-3p	NM_001135005	DNAJB5	156	-34.27	-37.7	0.004696
hsa-miR-532-3p	NM_001136037	LIMS2	163	-29.33	-32	0.037568
hsa-miR-532-3p	NM_001136158	OTUD5	159	-23.33	-31.7	0.047551
hsa-miR-532-3p	NM_001144037	TMEM25	155	-31.81	-34.7	0.003587
hsa-miR-532-3p	NM_001144038	TMEM25	155	-31.81	-34.7	0.003587
hsa-miR-532-3p	NM_001144063	OSBPL5	152	-22.38	-36	0.008883

hsa-miR-532-3p	NM_001160184	PLEKHN1	158	-27.46	-32.1	0.016822
hsa-miR-532-3p	NM_001161403	LIMS2	163	-29.33	-32	0.037568
hsa-miR-532-3p	NM_001161404	LIMS2	163	-29.33	-32	0.037568
hsa-miR-532-3p	NM_001164883	CTNNA2	159	-28.47	-34.2	0.017224
hsa-miR-532-3p	NM_001166400	MAGEA8	152	-26.79	-30.7	0.04054
hsa-miR-532-3p	NM_001166401	MAGEA8	152	-26.79	-30.7	0.04054
hsa-miR-532-3p	NM_001166686	PFKM	157	-28.94	-30.8	0.047577
hsa-miR-532-3p	NM_001166687	PFKM	157	-28.94	-30.8	0.047577
hsa-miR-532-3p	NM_001166688	PFKM	157	-28.94	-30.8	0.047577
hsa-miR-532-3p	NM_001168214	C3orf80	168	-31.31	-37	0.012823
hsa-miR-532-3p	NM_001170585	PLB1	160	-30.4	-34.4	0.009552
hsa-miR-532-3p	NM_001171093	FAM131A	151	-30.38	-34.4	0.023808
hsa-miR-532-3p	NM_001171940	FNDC5	170	-30.63	-33.4	0.043451
hsa-miR-532-3p	NM_001178147	CSF3	162	-32.67	-32.6	0.028323
hsa-miR-532-3p	NM_001185	AZGP1	154	-22.07	-31.6	0.006862
hsa-miR-532-3p	NM_001190720	IKBKB	155	-35.21	-39.3	0.004158
hsa-miR-532-3p	NM_001191028	TNPO3	152	-30.12	-32.6	0.048317
hsa-miR-532-3p	NM_001194990	MEPCE	159	-30.2	-32.7	0.014358
hsa-miR-532-3p	NM_001194991	MEPCE	159	-30.2	-32.7	0.014358
hsa-miR-532-3p	NM_001194992	MEPCE	159	-30.2	-32.7	0.014358
hsa-miR-532-3p	NM_001195007	PPIE	151	-25.67	-28.6	0.027885
hsa-miR-532-3p	NM_001197260	DOK1	157	-28.18	-34.2	0.004329
hsa-miR-532-3p	NM_001197323	HIRIP3	156	-32.68	-35.2	0.012746
hsa-miR-532-3p	NM_001199159	PI16	172	-31.53	-35.3	0.002884
hsa-miR-532-3p	NM_001204426	LIMK1	167	-30.36	-32.6	0.047614
hsa-miR-532-3p	NM_001214906	ZNF48	157	-31.32	-34.6	0.014586
hsa-miR-532-3p	NM_001214907	ZNF48	157	-31.32	-34.6	0.014586
hsa-miR-532-3p	NM_001214909	ZNF48	157	-31.32	-34.6	0.014586
hsa-miR-532-3p	NM_001242778	IKBKB	155	-35.21	-39.3	0.004158
hsa-miR-532-3p	NM_001242831	ELOVL5	168	-35.46	-36.8	0.000202

hsa-miR-532-3p	NM_001243771	APH1A	163	-29.82	-32.6	0.042195
hsa-miR-532-3p	NM_001243772	APH1A	163	-29.82	-32.6	0.022316
hsa-miR-532-3p	NM_001256041	MYO1A	156	-26.26	-29.9	0.012671
hsa-miR-532-3p	NM_001256346	SEMA5B	153	-31.55	-34.9	0.011747
hsa-miR-532-3p	NM_001256347	SEMA5B	153	-31.55	-34.9	0.011747
hsa-miR-532-3p	NM_001256348	SEMA5B	153	-31.55	-34.9	0.011747
hsa-miR-532-3p	NM_001256409	LRRC42	167	-29.83	-31.8	0.004307
hsa-miR-532-3p	NM_001256542	LIMS2	163	-29.33	-32	0.037568
hsa-miR-532-3p	NM_001257180	SLC20A2	167	-27.17	-32.9	0.04745
hsa-miR-532-3p	NM_001257181	SLC20A2	167	-27.17	-32.9	0.04745
hsa-miR-532-3p	NM_001258290	PRSS36	165	-34.81	-33.6	0.001405
hsa-miR-532-3p	NM_001258291	PRSS36	165	-34.81	-33.6	0.001405
hsa-miR-532-3p	NM_001261420	OS9	163	-34.42	-35.3	0.005156
hsa-miR-532-3p	NM_001261421	OS9	163	-34.42	-35.3	0.005156
hsa-miR-532-3p	NM_001261422	OS9	163	-34.42	-35.3	0.005156
hsa-miR-532-3p	NM_001261423	OS9	163	-34.42	-35.3	0.005156
hsa-miR-532-3p	NM_001270765	CHST15	162	-27.57	-33.9	0.033703
hsa-miR-532-3p	NM_001271933	CEP164	154	-34.86	-37.3	0.005026
hsa-miR-532-3p	NM_001272004	EPC1	156	-28.43	-37.7	0.005877
hsa-miR-532-3p	NM_001272019	EPC1	156	-28.43	-37.7	0.005877
hsa-miR-532-3p	NM_001278789	GORASP1	162	-34.59	-38.5	0.005664
hsa-miR-532-3p	NM_001278790	GORASP1	162	-34.59	-38.5	0.005664
hsa-miR-532-3p	NM_001278919	KCNH6	162	-30.86	-35.3	0.007794
hsa-miR-532-3p	NM_001278920	KCNH6	162	-30.86	-35.3	0.007794
hsa-miR-532-3p	NM_001281296	M1AP	159	-32.52	-35.3	0.007904
hsa-miR-532-3p	NM_001282391	EPC1	156	-28.43	-37.7	0.005877
hsa-miR-532-3p	NM_001282597	CTNNA2	159	-28.47	-34.2	0.017224
hsa-miR-532-3p	NM_001282598	CTNNA2	159	-28.47	-34.2	0.017224
hsa-miR-532-3p	NM_001282599	CTNNA2	159	-28.47	-34.2	0.017224
hsa-miR-532-3p	NM_001282600	CTNNA2	159	-28.47	-34.2	0.017224

hsa-miR-532-3p	NM_001286277	TUBGCP3	152	-25.57	-33.1	0.028106
hsa-miR-532-3p	NM_001287252	CCNI2	174	-35.89	-40.8	0.001672
hsa-miR-532-3p	NM_001287253	CCNI2	174	-35.89	-40.8	0.001672
hsa-miR-532-3p	NM_001287431	ARFIP1	150	-30.41	-34.1	0.039142
hsa-miR-532-3p	NM_001287432	ARFIP1	150	-30.41	-34.1	0.039142
hsa-miR-532-3p	NM_001287433	ARFIP1	150	-30.41	-34.1	0.039142
hsa-miR-532-3p	NM_001287754	TRAF3IP3	163	-26.92	-29.1	0.021653
hsa-miR-532-3p	NM_001288966	CEP70	152	-25.74	-28.5	0.021725
hsa-miR-532-3p	NM_001289110	RNASE9	156	-26.73	-30.2	0.022389
hsa-miR-532-3p	NM_001289823	FURIN	155	-29.13	-34.2	0.034123
hsa-miR-532-3p	NM_001289824	FURIN	155	-29.13	-34.2	0.034123
hsa-miR-532-3p	NM_001291988	CPN2	161	-22.74	-33.5	0.035048
hsa-miR-532-3p	NM_001293695	SEPT9	161	-35.9	-38.8	0.006488
hsa-miR-532-3p	NM_001293696	SEPT9	161	-35.9	-38.8	0.006488
hsa-miR-532-3p	NM_001293697	SEPT9	161	-35.9	-38.8	0.006488
hsa-miR-532-3p	NM_001293698	SEPT9	161	-35.9	-38.8	0.006488
hsa-miR-532-3p	NM_001297576	PEA15	166	-23.79	-34	0.047405
hsa-miR-532-3p	NM_001297577	PEA15	166	-23.79	-34	0.047405
hsa-miR-532-3p	NM_001297578	PEA15	166	-23.79	-34	0.047405
hsa-miR-532-3p	NM_001297640	C1RL	152	-32.84	-34.4	0.038711
hsa-miR-532-3p	NM_001301186	LINGO1	160	-27.25	-32.1	0.045892
hsa-miR-532-3p	NM_001301187	LINGO1	160	-27.25	-32.1	0.045892
hsa-miR-532-3p	NM_001301189	LINGO1	160	-27.25	-32.1	0.045892
hsa-miR-532-3p	NM_001301191	LINGO1	160	-27.25	-32.1	0.045892
hsa-miR-532-3p	NM_001301192	LINGO1	160	-27.25	-32.1	0.045892
hsa-miR-532-3p	NM_001301194	LINGO1	160	-27.25	-32.1	0.045892
hsa-miR-532-3p	NM_001301195	LINGO1	160	-27.25	-32.1	0.045892
hsa-miR-532-3p	NM_001301197	LINGO1	160	-27.25	-32.1	0.045892
hsa-miR-532-3p	NM_001301198	LINGO1	160	-27.25	-32.1	0.045892
hsa-miR-532-3p	NM_001301199	LINGO1	160	-27.25	-32.1	0.045892

hsa-miR-532-3p	NM_001301200	LINGO1	160	-27.25	-32.1	0.045892
hsa-miR-532-3p	NM_001304763	ZNF687	166	-37.31	-31.5	0.034447
hsa-miR-532-3p	NM_001304764	ZNF687	166	-37.31	-31.5	0.034447
hsa-miR-532-3p	NM_001308360	ADGRG3	160	-29.24	-33	0.026298
hsa-miR-532-3p	NM_001311198	AP2M1	157	-27.65	-31.3	0.020739
hsa-miR-532-3p	NM_001316313	PEX6	154	-29.69	-32.5	0.011028
hsa-miR-532-3p	NM_001316332	MAP2K3	157	-31.15	-34.1	0.018493
hsa-miR-532-3p	NM_001381	DOK1	157	-28.18	-34.2	0.004329
hsa-miR-532-3p	NM_001407	CELSR3	165	-32.23	-37.6	0.009186
hsa-miR-532-3p	NM_001556	IKBKB	155	-35.21	-39.3	0.004158
hsa-miR-532-3p	NM_001622	AHSG	151	-34.43	-32.1	0.009907
hsa-miR-532-3p	NM_001716	CXCR5	172	-36.49	-38.7	0.00505
hsa-miR-532-3p	NM_001933	DLST	153	-30.92	-34	0.031897
hsa-miR-532-3p	NM_002314	LIMK1	167	-30.36	-32.6	0.047614
hsa-miR-532-3p	NM_002569	FURIN	155	-29.13	-34.2	0.034123
hsa-miR-532-3p	NM_002756	MAP2K3	157	-31.15	-34.1	0.018493
hsa-miR-532-3p	NM_002809	PSMD3	156	-22.24	-30.1	0.025761
hsa-miR-532-3p	NM_002950	RPN1	154	-36.92	-36.5	0.001414
hsa-miR-532-3p	NM_003271	TSPAN4	152	-28.99	-31.9	0.016098
hsa-miR-532-3p	NM_003459	SLC30A3	154	-28.97	-32	0.030008
hsa-miR-532-3p	NM_003609	HIRIP3	156	-32.68	-35.2	0.009158
hsa-miR-532-3p	NM_003768	PEA15	166	-23.79	-34	0.047405
hsa-miR-532-3p	NM_004068	AP2M1	157	-27.65	-31.3	0.020739
hsa-miR-532-3p	NM_004312	ARR3	156	-24.33	-27.3	0.005131
hsa-miR-532-3p	NM_004389	CTNNA2	159	-28.47	-34.2	0.017224
hsa-miR-532-3p	NM_004479	FUT7	151	-21.47	-32.6	0.013519
hsa-miR-532-3p	NM_004700	KCNQ4	167	-30.46	-34.1	0.046726
hsa-miR-532-3p	NM_004783	TAOK2	154	-29.89	-31.3	0.037541
hsa-miR-532-3p	NM_004910	PITPNM1	159	-26.88	-29.4	0.019961
hsa-miR-532-3p	NM_005148	UNC119	169	-33.86	-35.5	0.003971

hsa-miR-532-3p	NM_005216	DDOST	161	-27.12	-33.1	0.013272
hsa-miR-532-3p	NM_005220	DLX3	159	-31.19	-33.5	0.043278
hsa-miR-532-3p	NM_005364	MAGEA8	152	-26.79	-30.7	0.04054
hsa-miR-532-3p	NM_005379	MYO1A	156	-26.26	-29.9	0.012671
hsa-miR-532-3p	NM_005432	XRCC3	153	-33.87	-37.1	0.006195
hsa-miR-532-3p	NM_005654	NR2F1	150	-30.22	-35	0.007916
hsa-miR-532-3p	NM_005775	SORBS3	153	-31.85	-35.4	0.006573
hsa-miR-532-3p	NM_005814	GPA33	159	-36.96	-40	0.002558
hsa-miR-532-3p	NM_005973	PRCC	162	-25.31	-31.1	0.014123
hsa-miR-532-3p	NM_005994	TBX2	151	-25.22	-33.7	0.020551
hsa-miR-532-3p	NM_006142	SFN	152	-28.4	-30.1	0.039755
hsa-miR-532-3p	NM_006322	TUBGCP3	152	-25.57	-33.1	0.028106
hsa-miR-532-3p	NM_006370	VTI1B	160	-28.52	-33.4	0.001941
hsa-miR-532-3p	NM_006640	SEPT9	161	-35.9	-38.8	0.006488
hsa-miR-532-3p	NM_006749	SLC20A2	167	-27.17	-32.9	0.04745
hsa-miR-532-3p	NM_006812	OS9	163	-34.42	-35.3	0.005156
hsa-miR-532-3p	NM_007144	PCGF2	156	-33.57	-33.7	0.03305
hsa-miR-532-3p	NM_007245	ATXN2L	160	-34.18	-36.3	0.006459
hsa-miR-532-3p	NM_012163	LRRC29	169	-32.3	-33.7	0.004116
hsa-miR-532-3p	NM_012227	GTPBP6	151	-29.07	-31.5	0.009711
hsa-miR-532-3p	NM_012266	DNAJB5	156	-34.27	-37.7	0.004696
hsa-miR-532-3p	NM_012470	TNPO3	152	-30.12	-32.6	0.048317
hsa-miR-532-3p	NM_014424	HSPB7	153	-29.96	-33.9	0.037829
hsa-miR-532-3p	NM_014447	ARFIP1	150	-30.41	-34.1	0.039142
hsa-miR-532-3p	NM_014620	HOXC4	160	-31.57	-32.4	0.02945
hsa-miR-532-3p	NM_014680	KIAA0100	162	-31.55	-32.2	0.020963
hsa-miR-532-3p	NM_014863	CHST15	162	-27.57	-33.9	0.033703
hsa-miR-532-3p	NM_014956	CEP164	154	-34.86	-37.3	0.005026
hsa-miR-532-3p	NM_015080	NRXN2	164	-34	-36.7	0.005846
hsa-miR-532-3p	NM_015130	TBC1D9	156	-29.6	-34	0.031769

hsa-miR-532-3p	NM_015327	SMG5	166	-33.17	-35.6	0.015034
hsa-miR-532-3p	NM_015356	SCRIB	154	-27.73	-29	0.021101
hsa-miR-532-3p	NM_015597	GPSM1	164	-27.34	-31.4	0.034523
hsa-miR-532-3p	NM_015656	KIF26A	151	-29.63	-33.8	0.024354
hsa-miR-532-3p	NM_015714	G0S2	151	-24.67	-30.1	0.027461
hsa-miR-532-3p	NM_016022	APH1A	163	-29.82	-32.6	0.042195
hsa-miR-532-3p	NM_016333	SRRM2	161	-34.26	-36.5	0.002123
hsa-miR-532-3p	NM_016459	MZB1	155	-27.01	-29.6	0.010436
hsa-miR-532-3p	NM_016546	C1RL	152	-32.84	-34.4	0.038711
hsa-miR-532-3p	NM_016565	COA4	157	-27.65	-30.4	0.029098
hsa-miR-532-3p	NM_016930	STX18	172	-38.69	-40.2	0.001293
hsa-miR-532-3p	NM_017622	BORCS6	160	-27.21	-30.9	0.049122
hsa-miR-532-3p	NM_017980	LIMS2	163	-29.33	-32	0.037568
hsa-miR-532-3p	NM_018226	RNPEPL1	159	-31.45	-34.7	0.010561
hsa-miR-532-3p	NM_018286	TMEM100	157	-30.29	-32.8	0.034548
hsa-miR-532-3p	NM_019606	MEPCE	159	-30.2	-32.7	0.014358
hsa-miR-532-3p	NM_020227	PRDM9	151	-25.26	-31.5	0.044249
hsa-miR-532-3p	NM_020832	ZNF687	166	-37.31	-31.5	0.034447
hsa-miR-532-3p	NM_020896	OSBPL5	152	-22.38	-36	0.008883
hsa-miR-532-3p	NM_020897	HCN3	167	-29.96	-32.9	0.049599
hsa-miR-532-3p	NM_021630	PDLIM2	164	-27.59	-29.6	0.034134
hsa-miR-532-3p	NM_021939	FKBP10	156	-29.7	-34.2	0.012509
hsa-miR-532-3p	NM_022093	TNN	166	-28.47	-34.1	0.018129
hsa-miR-532-3p	NM_023009	MARCKSL1	158	-35.98	-38.5	0.001556
hsa-miR-532-3p	NM_023083	CAPN10	151	-29.68	-32.4	0.010186
hsa-miR-532-3p	NM_023085	CAPN10	151	-29.68	-32.4	0.010186
hsa-miR-532-3p	NM_024015	HOXB4	150	-27.21	-34.9	0.017184
hsa-miR-532-3p	NM_024605	ARHGAP10	158	-33.53	-37.1	0.002497
hsa-miR-532-3p	NM_024667	VPS37B	162	-31.51	-34.8	0.031425
hsa-miR-532-3p	NM_024697	ZNF385D	171	-34.94	-36.9	0.000249

hsa-miR-532-3p	NM_024789	TMEM180	160	-30.11	-33.7	0.023869
hsa-miR-532-3p	NM_025081	NYNRIN	161	-34.14	-36.2	0.018125
hsa-miR-532-3p	NM_025209	EPC1	156	-28.43	-37.7	0.005877
hsa-miR-532-3p	NM_025237	SOST	156	-24.59	-33.8	0.041555
hsa-miR-532-3p	NM_030665	RAI1	156	-26.87	-33.5	0.041631
hsa-miR-532-3p	NM_030779	KCNH6	162	-30.86	-35.3	0.007794
hsa-miR-532-3p	NM_031899	GORASP1	162	-34.59	-38.5	0.005664
hsa-miR-532-3p	NM_032129	PLEKHN1	158	-27.46	-32.1	0.016822
hsa-miR-532-3p	NM_032408	BAZ1B	154	-29.22	-33.2	0.040396
hsa-miR-532-3p	NM_032438	L3MBTL3	156	-31.22	-34.4	0.034587
hsa-miR-532-3p	NM_032588	TRIM63	157	-31.46	-34.1	0.007261
hsa-miR-532-3p	NM_032808	LINGO1	160	-27.25	-32.1	0.045892
hsa-miR-532-3p	NM_032906	PYURF	167	-31.29	-31.8	0.044626
hsa-miR-532-3p	NM_032966	CXCR5	172	-36.49	-38.7	0.00505
hsa-miR-532-3p	NM_052940	LRRC42	167	-29.83	-31.8	0.004307
hsa-miR-532-3p	NM_054035	UNC119	169	-33.86	-35.5	0.008446
hsa-miR-532-3p	NM_058163	TSR2	158	-33.68	-36.6	0.003591
hsa-miR-532-3p	NM_080677	DYNLL2	163	-29.05	-33.2	0.026469
hsa-miR-532-3p	NM_080861	SPSB3	151	-27.21	-31.9	0.010798
hsa-miR-532-3p	NM_138732	NRXN2	164	-34	-36.7	0.005846
hsa-miR-532-3p	NM_138734	NRXN2	164	-34	-36.7	0.005846
hsa-miR-532-3p	NM_138769	RHOT2	155	-27.11	-30.4	0.04231
hsa-miR-532-3p	NM_138804	M1AP	159	-32.52	-35.3	0.007904
hsa-miR-532-3p	NM_144635	FAM131A	151	-30.38	-34.4	0.023808
hsa-miR-532-3p	NM_144696	AXDND1	153	-28.88	-32.1	0.003264
hsa-miR-532-3p	NM_145109	MAP2K3	157	-31.15	-34.1	0.018493
hsa-miR-532-3p	NM_145291	ZBTB49	150	-29.62	-31.9	0.01522
hsa-miR-532-3p	NM_145638	OSBPL5	152	-22.38	-36	0.008883
hsa-miR-532-3p	NM_152286	PNPLA7	162	-29.47	-34.5	0.003797
hsa-miR-532-3p	NM_152335	TMEM266	156	-27.33	-31	0.038998

hsa-miR-532-3p	NM_152643	KNDC1	154	-29.45	-33.5	0.043671
hsa-miR-532-3p	NM_152652	ZNF48	157	-31.32	-34.6	0.014586
hsa-miR-532-3p	NM_152716	PATL1	156	-31.32	-33.6	0.047641
hsa-miR-532-3p	NM_153021	PLB1	160	-30.4	-34.4	0.009552
hsa-miR-532-3p	NM_153237	TMEM252	151	-28.45	-31.6	0.031531
hsa-miR-532-3p	NM_153370	PI16	172	-31.53	-35.3	0.002884
hsa-miR-532-3p	NM_153633	HOXC4	160	-31.57	-32.4	0.02945
hsa-miR-532-3p	NM_153710	STKLD1	150	-33.25	-36.1	0.003796
hsa-miR-532-3p	NM_170776	ADGRG3	160	-29.24	-33	0.026298
hsa-miR-532-3p	NM_172163	KCNQ4	167	-30.46	-34.1	0.046726
hsa-miR-532-3p	NM_172219	CSF3	162	-32.67	-32.6	0.028323
hsa-miR-532-3p	NM_172220	CSF3	162	-32.67	-32.6	0.028323
hsa-miR-532-3p	NM_173502	PRSS36	165	-34.81	-33.6	0.001405
hsa-miR-532-3p	NM_178545	TMEM52	165	-25.78	-30	0.017371
hsa-miR-532-3p	NM_182706	SCRIB	154	-27.73	-29	0.021101
hsa-miR-532-3p	NM_182947	ARHGEF25	152	-27.11	-31.2	0.013037
hsa-miR-532-3p	NM_198042	PDLIM2	164	-27.59	-29.6	0.049541
hsa-miR-532-3p	NM_203400	RPRML	165	-27.28	-32.6	0.011578
hsa-miR-532-3p	NM_203456	PPIE	151	-25.67	-28.6	0.013876
hsa-miR-532-3p	NM_207321	ACSM6	175	-30.55	-35.4	0.000218
hsa-miR-532-3p	NM_207330	NIPAL1	155	-28.65	-32.8	0.02757
hsa-miR-532-3p	NR_024418	LOC389332	152	-28.84	-33.7	0.046487
hsa-miR-532-3p	NR_024462	RAMP2-AS1	154	-33.98	-36	0.017321
hsa-miR-532-3p	NR_033958	LOC283440	172	-32.21	-35.9	0.00309
hsa-miR-532-3p	NR_038368	LINC00273	150	-27.68	-34.5	0.026616
hsa-miR-532-3p	NR_039698	MIR3155B	154	-27.18	-28	0.001706
hsa-miR-532-3p	NR_046533	CLDN10-AS1	153	-29.32	-31.6	0.047658
hsa-miR-532-3p	NR_052852	MARCKSL1	158	-35.98	-38.5	0.004084
hsa-miR-532-3p	NR_108031	LOC102577426	150	-24.58	-29.5	0.048143
hsa-miR-532-3p	NR_110305	SFXN4	165	-33.12	-34.1	0.032555

hsa-miR-532-3p	NR_110374	LINC01159	150	-36.07	-38.2	0.003236
hsa-miR-532-3p	NR_110398	PABPC1L2B-AS1	155	-28.54	-33.3	0.032774
hsa-miR-532-3p	NR_110702	SEMA3B-AS1	157	-28.56	-29.6	0.027338
hsa-miR-532-3p	NR_121645	RNVU1-8	156	-22.09	-29.8	0.004796
hsa-miR-532-3p	NR_126027	EXTL3-AS1	166	-34.67	-37.5	0.002549
hsa-miR-574-5p	NM_000701	ATP1A1	161	-22.85	-28.4	0.040372
hsa-miR-574-5p	NM_001002292	WLS	169	-29.25	-31.1	0.002828
hsa-miR-574-5p	NM_001004310	FCRL6	156	-26.03	-30.4	0.03773
hsa-miR-574-5p	NM_001004356	FGFRL1	169	-32.6	-33.9	0.031008
hsa-miR-574-5p	NM_001004358	FGFRL1	169	-32.6	-33.9	0.031008
hsa-miR-574-5p	NM_001012428	ASB11	165	-33.98	-38.1	0.006148
hsa-miR-574-5p	NM_001014839	NCDN	197	-39.15	-37.9	0.003087
hsa-miR-574-5p	NM_001014841	NCDN	197	-39.15	-37.9	0.003087
hsa-miR-574-5p	NM_001030015	OPN4	165	-22.1	-32.9	0.014975
hsa-miR-574-5p	NM_001031702	SEMA5B	197	-37.66	-39.3	0.001219
hsa-miR-574-5p	NM_001038633	RSPO1	176	-29.83	-34.3	0.022555
hsa-miR-574-5p	NM_001039548	KLHL35	156	-20.38	-26.9	0.033524
hsa-miR-574-5p	NM_001040181	CLDND1	155	-30.26	-33.2	0.028751
hsa-miR-574-5p	NM_001040182	CLDND1	155	-30.26	-33.2	0.028751
hsa-miR-574-5p	NM_001040183	CLDND1	155	-30.26	-33.2	0.028751
hsa-miR-574-5p	NM_001040199	CLDND1	155	-30.26	-33.2	0.028751
hsa-miR-574-5p	NM_001040200	CLDND1	155	-30.26	-33.2	0.028751
hsa-miR-574-5p	NM_001079528	ABCC6	161	-31.18	-34.6	0.002148
hsa-miR-574-5p	NM_001080395	AATK	176	-30.6	-34.1	0.017245
hsa-miR-574-5p	NM_001080506	TMEM150C	181	-39.11	-40.7	0.000779
hsa-miR-574-5p	NM_001080515	FAM163B	184	-30.46	-32	0.017976
hsa-miR-574-5p	NM_001082486	ACD	150	-20.17	-24.5	0.037859
hsa-miR-574-5p	NM_001082487	ACD	150	-20.17	-24.5	0.037859
hsa-miR-574-5p	NM_001082533	CA10	185	-33.79	-38.4	0.003067

hsa-miR-574-5p	NM_001082534	CA10	185	-33.79	-38.4	0.003067
hsa-miR-574-5p	NM_001099403	PRDM8	189	-37.04	-39	0.00101
hsa-miR-574-5p	NM_001100396	C2orf73	165	-29.08	-35.5	0.007932
hsa-miR-574-5p	NM_001104577	SLC52A1	167	-26.31	-32.4	0.00581
hsa-miR-574-5p	NM_001130010	C15orf41	181	-37.07	-39.5	0.002185
hsa-miR-574-5p	NM_001135196	C10orf71	165	-34.46	-30.6	0.038132
hsa-miR-574-5p	NM_001142415	AIMP1	185	-32.99	-35.4	0.015764
hsa-miR-574-5p	NM_001142416	AIMP1	185	-32.99	-35.4	0.015764
hsa-miR-574-5p	NM_001143682	CALCOCO1	192	-41.11	-37.2	0.002527
hsa-miR-574-5p	NM_001145211	SLCO2B1	167	-24.35	-36.2	0.014346
hsa-miR-574-5p	NM_001145212	SLCO2B1	167	-24.35	-36.2	0.014346
hsa-miR-574-5p	NM_001160233	ATP1A1	161	-22.85	-28.4	0.040372
hsa-miR-574-5p	NM_001160234	ATP1A1	161	-22.85	-28.4	0.040372
hsa-miR-574-5p	NM_001161444	SH2D2A	152	-25.59	-29.9	0.002556
hsa-miR-574-5p	NM_001166283	RGMA	184	-33.33	-37	0.008253
hsa-miR-574-5p	NM_001166286	RGMA	184	-33.33	-37	0.008253
hsa-miR-574-5p	NM_001166287	RGMA	184	-33.33	-37	0.008253
hsa-miR-574-5p	NM_001166288	RGMA	184	-33.33	-37	0.008253
hsa-miR-574-5p	NM_001166289	RGMA	184	-33.33	-37	0.008253
hsa-miR-574-5p	NM_001167740	SMYD3	154	-18.27	-27.1	0.041152
hsa-miR-574-5p	NM_001168347	NSUN5	177	-31.41	-32.5	0.025493
hsa-miR-574-5p	NM_001168348	NSUN5	177	-31.41	-32.5	0.034383
hsa-miR-574-5p	NM_001172632	OLR1	165	-34.47	-37.2	0.008489
hsa-miR-574-5p	NM_001172633	OLR1	165	-34.47	-37.2	0.008489
hsa-miR-574-5p	NM_001173513	TXNRD3	163	-23.57	-31.4	0.040325
hsa-miR-574-5p	NM_001198910	CCDC169-SOHLH2	155	-26.31	-31.2	0.04219
hsa-miR-574-5p	NM_001201583	ASB11	165	-33.98	-38.1	0.006148
hsa-miR-574-5p	NM_001202233	NR4A1	157	-27.89	-32.8	0.010758
hsa-miR-574-5p	NM_001202234	NR4A1	157	-27.89	-32.8	0.010758

hsa-miR-574-5p	NM_001202	BMP4	159	-33.67	-34.1	0.001684
hsa-miR-574-5p	NM_001220488	CDH13	161	-34.76	-38.6	0.004023
hsa-miR-574-5p	NM_001220489	CDH13	161	-34.76	-38.6	0.004023
hsa-miR-574-5p	NM_001220490	CDH13	161	-34.76	-38.6	0.004023
hsa-miR-574-5p	NM_001242791	FLJ45513	181	-36.89	-39.5	0.002617
hsa-miR-574-5p	NM_001242908	RSPO1	176	-29.83	-34.3	0.022555
hsa-miR-574-5p	NM_001242909	RSPO1	176	-29.83	-34.3	0.022555
hsa-miR-574-5p	NM_001242910	RSPO1	176	-29.83	-34.3	0.022555
hsa-miR-574-5p	NM_001243794	CHST12	159	-29.7	-33	0.01392
hsa-miR-574-5p	NM_001243795	CHST12	159	-29.7	-33	0.01392
hsa-miR-574-5p	NM_001253815	SLC52A2	172	-29.32	-30.3	0.010573
hsa-miR-574-5p	NM_001253816	SLC52A2	172	-29.32	-30.3	0.010573
hsa-miR-574-5p	NM_001256020	TRDN	181	-32.02	-34.7	0.00521
hsa-miR-574-5p	NM_001256346	SEMA5B	197	-37.66	-39.3	0.001219
hsa-miR-574-5p	NM_001256347	SEMA5B	197	-37.66	-39.3	0.001219
hsa-miR-574-5p	NM_001256348	SEMA5B	197	-37.66	-39.3	0.001219
hsa-miR-574-5p	NM_001256371	E2F8	165	-34.2	-34	0.006587
hsa-miR-574-5p	NM_001256372	E2F8	165	-34.2	-34	0.006587
hsa-miR-574-5p	NM_001256429	PDLIM5	169	-29.84	-33.3	0.010226
hsa-miR-574-5p	NM_001257971	CTSL	154	-24.94	-30.7	0.014406
hsa-miR-574-5p	NM_001257972	CTSL	154	-24.94	-30.7	0.014406
hsa-miR-574-5p	NM_001257973	CTSL	154	-24.94	-30.7	0.014406
hsa-miR-574-5p	NM_001257	CDH13	161	-34.76	-38.6	0.004023
hsa-miR-574-5p	NM_001258288	SELENBP1	171	-25.41	-28.3	0.024802
hsa-miR-574-5p	NM_001258289	SELENBP1	171	-25.41	-28.3	0.024802
hsa-miR-574-5p	NM_001267568	CREM	189	-36.04	-40.5	0.001396
hsa-miR-574-5p	NM_001267569	CREM	189	-36.04	-40.5	0.001396
hsa-miR-574-5p	NM_001284217	FCRL6	156	-26.03	-30.4	0.04551
hsa-miR-574-5p	NM_001286365	MAP7D1	168	-29.85	-32.4	0.014178
hsa-miR-574-5p	NM_001286366	MAP7D1	168	-29.85	-32.4	0.014178

hsa-miR-574-5p	NM_001286648	SLC45A4	156	-28.57	-30.9	0.007271
hsa-miR-574-5p	NM_001287010	GLIPR2	154	-24.64	-35.2	0.014899
hsa-miR-574-5p	NM_001287011	GLIPR2	154	-24.64	-35.2	0.019559
hsa-miR-574-5p	NM_001287012	GLIPR2	154	-24.64	-35.2	0.016437
hsa-miR-574-5p	NM_001287013	GLIPR2	154	-24.64	-35.2	0.014899
hsa-miR-574-5p	NM_001287014	GLIPR2	154	-24.64	-35.2	0.014899
hsa-miR-574-5p	NM_001288705	CSF1R	175	-28.52	-33.7	0.011939
hsa-miR-574-5p	NM_001290007	BIN2	169	-27.04	-31.4	0.015311
hsa-miR-574-5p	NM_001290008	BIN2	169	-27.04	-31.4	0.015311
hsa-miR-574-5p	NM_001290009	BIN2	169	-27.04	-31.4	0.015311
hsa-miR-574-5p	NM_001290223	DOCK1	165	-34.46	-37.2	0.004086
hsa-miR-574-5p	NM_001291307	NOP16	185	-31.5	-32	0.005019
hsa-miR-574-5p	NM_001291308	NOP16	185	-31.5	-32	0.007698
hsa-miR-574-5p	NM_001300855	FOSL1	176	-28.13	-32.2	0.043211
hsa-miR-574-5p	NM_001300975	ANKRD42	197	-39.15	-41.5	0.000287
hsa-miR-574-5p	NM_001302831	C3orf35	165	-34.2	-38.4	0.001196
hsa-miR-574-5p	NM_001302832	C3orf35	165	-34.2	-38.4	0.001196
hsa-miR-574-5p	NM_001303496	SAMD9L	197	-38.65	-41.1	0.00077
hsa-miR-574-5p	NM_001303497	SAMD9L	197	-38.65	-41.1	0.00077
hsa-miR-574-5p	NM_001303498	SAMD9L	197	-38.65	-41.1	0.00077
hsa-miR-574-5p	NM_001303500	SAMD9L	197	-38.65	-41.1	0.00077
hsa-miR-574-5p	NM_001303	COX10	174	-29.86	-32.7	0.048395
hsa-miR-574-5p	NM_001380	DOCK1	165	-34.46	-37.2	0.004086
hsa-miR-574-5p	NM_001702	ADGRB1	153	-29.69	-33.1	0.010061
hsa-miR-574-5p	NM_001909	CTSD	184	-30.54	-32.1	0.024014
hsa-miR-574-5p	NM_001910	CTSE	173	-33.07	-36.8	0.003682
hsa-miR-574-5p	NM_001912	CTSL	154	-24.94	-30.7	0.014406
hsa-miR-574-5p	NM_002135	NR4A1	157	-27.89	-32.8	0.010758
hsa-miR-574-5p	NM_002303	LEPR	181	-36.93	-39.2	0.000332
hsa-miR-574-5p	NM_002543	OLR1	165	-34.47	-37.2	0.007468

hsa-miR-574-5p	NM_002586	PBX2	180	-37.5	-40	0.002327
hsa-miR-574-5p	NM_003324	TULP3	160	-31.65	-34.2	0.030864
hsa-miR-574-5p	NM_003944	SELENBP1	171	-25.41	-28.3	0.024802
hsa-miR-574-5p	NM_004061	CDH12	197	-39.15	-41.5	0.000371
hsa-miR-574-5p	NM_004160	PYY	150	-25.26	-30.8	0.005295
hsa-miR-574-5p	NM_004757	AIMP1	185	-32.99	-35.4	0.015764
hsa-miR-574-5p	NM_005211	CSF1R	175	-28.52	-33.7	0.011939
hsa-miR-574-5p	NM_005519	HMX2	157	-24.79	-31.8	0.016815
hsa-miR-574-5p	NM_005568	LHX1	181	-35.78	-36.7	0.008448
hsa-miR-574-5p	NM_006160	NEUROD2	172	-28.77	-34.1	0.031885
hsa-miR-574-5p	NM_006368	CREB3	158	-25.34	-29.7	0.015595
hsa-miR-574-5p	NM_007256	SLCO2B1	167	-24.35	-36.2	0.014346
hsa-miR-574-5p	NM_012093	AK5	172	-33.49	-37.5	0.004773
hsa-miR-574-5p	NM_012387	PADI4	156	-20.58	-29.7	0.01209
hsa-miR-574-5p	NM_014228	SLC6A7	169	-34.26	-37.6	0.0055
hsa-miR-574-5p	NM_014284	NCDN	197	-39.15	-37.9	0.003087
hsa-miR-574-5p	NM_014398	LAMP3	157	-18.39	-34.9	0.026091
hsa-miR-574-5p	NM_014926	SLITRK3	197	-38.65	-41.1	0.000787
hsa-miR-574-5p	NM_015726	DCAF8	189	-34.18	-36.1	0.015676
hsa-miR-574-5p	NM_016293	BIN2	169	-27.04	-31.4	0.015311
hsa-miR-574-5p	NM_016391	NOP16	185	-31.5	-32	0.005019
hsa-miR-574-5p	NM_016564	CEND1	161	-28.91	-32.1	0.037768
hsa-miR-574-5p	NM_017409	HOXC10	156	-31.21	-32.9	0.019347
hsa-miR-574-5p	NM_017442	TLR9	157	-23.4	-25.9	0.033835
hsa-miR-574-5p	NM_017826	SOHLH2	155	-26.31	-31.2	0.04219
hsa-miR-574-5p	NM_017950	CCDC40	181	-29.29	-33.4	0.015487
hsa-miR-574-5p	NM_017986	SLC52A1	167	-26.31	-32.4	0.00581
hsa-miR-574-5p	NM_018044	NSUN5	177	-31.41	-32.5	0.034383
hsa-miR-574-5p	NM_018067	MAP7D1	168	-29.85	-32.4	0.014178
hsa-miR-574-5p	NM_018150	RNF220	161	-26.43	-35.4	0.007455

hsa-miR-574-5p	NM_018641	CHST12	159	-29.7	-33	0.01392
hsa-miR-574-5p	NM_018836	AJAP1	173	-32.46	-35.5	0.011833
hsa-miR-574-5p	NM_019895	CLDND1	155	-30.26	-33.2	0.028751
hsa-miR-574-5p	NM_019896	POLE4	168	-23.81	-28.7	0.030923
hsa-miR-574-5p	NM_020178	CA10	185	-33.79	-38.4	0.003067
hsa-miR-574-5p	NM_020211	RGMA	184	-33.33	-37	0.008253
hsa-miR-574-5p	NM_020226	PRDM8	189	-37.04	-39	0.00101
hsa-miR-574-5p	NM_020439	CAMK1G	168	-26.31	-33.3	0.019316
hsa-miR-574-5p	NM_020898	CALCOCO1	192	-41.11	-37.2	0.002532
hsa-miR-574-5p	NM_020928	ZSWIM6	157	-31.1	-33.9	0.039747
hsa-miR-574-5p	NM_020988	GNAO1	158	-30.8	-33.7	0.028362
hsa-miR-574-5p	NM_021637	TMEM35	181	-39.92	-42.6	0.000557
hsa-miR-574-5p	NM_021923	FGFRL1	169	-32.6	-33.9	0.031008
hsa-miR-574-5p	NM_022343	GLIPR2	154	-24.64	-35.2	0.014899
hsa-miR-574-5p	NM_022475	HHIP	169	-33.53	-36.5	0.003127
hsa-miR-574-5p	NM_022743	SMYD3	154	-18.27	-27.1	0.041152
hsa-miR-574-5p	NM_022914	ACD	150	-20.17	-24.5	0.037859
hsa-miR-574-5p	NM_024015	HOXB4	185	-33.39	-34.5	0.016606
hsa-miR-574-5p	NM_024531	SLC52A2	172	-29.32	-30.3	0.010573
hsa-miR-574-5p	NM_024680	E2F8	165	-34.2	-34	0.006587
hsa-miR-574-5p	NM_031457	MS4A8	161	-28.55	-31.7	0.010173
hsa-miR-574-5p	NM_032109	OTP	167	-25.26	-37.1	0.00759
hsa-miR-574-5p	NM_032271	TRAF7	169	-33.49	-35.4	0.016118
hsa-miR-574-5p	NM_032479	MRPL36	150	-25	-27.7	0.033642
hsa-miR-574-5p	NM_032499	C15orf41	181	-37.07	-39.5	0.003369
hsa-miR-574-5p	NM_033085	FATE1	151	-29.57	-32.1	0.009683
hsa-miR-574-5p	NM_033282	OPN4	165	-22.1	-32.9	0.014975
hsa-miR-574-5p	NM_033303	ADRA1A	164	-28.19	-30.8	0.019099
hsa-miR-574-5p	NM_052883	TXNRD3	163	-23.57	-31.4	0.040325
hsa-miR-574-5p	NM_080873	ASB11	165	-33.98	-38.1	0.006148

hsa-miR-574-5p	NM_130850	BMP4	159	-33.67	-34.1	0.001684
hsa-miR-574-5p	NM_130851	BMP4	159	-33.67	-34.1	0.001684
hsa-miR-574-5p	NM_138331	RNASE8	161	-26.21	-25.5	0.022628
hsa-miR-574-5p	NM_138345	VWA5B2	160	-23.74	-27.6	0.049155
hsa-miR-574-5p	NM_139278	LGI3	167	-31.97	-34.9	0.015916
hsa-miR-574-5p	NM_145305	SLC25A43	168	-30.6	-33.2	0.036805
hsa-miR-574-5p	NM_145918	CTSL	154	-24.94	-30.7	0.014406
hsa-miR-574-5p	NM_148956	NSUN5	177	-31.41	-32.5	0.026241
hsa-miR-574-5p	NM_148964	CTSE	173	-33.07	-36.8	0.003389
hsa-miR-574-5p	NM_152380	TBX15	161	-23.91	-33.5	0.041529
hsa-miR-574-5p	NM_152703	SAMD9L	197	-38.65	-41.1	0.00077
hsa-miR-574-5p	NM_153350	FBXL16	184	-35.14	-36.5	0.012131
hsa-miR-574-5p	NM_173157	NR4A1	157	-27.89	-32.8	0.010758
hsa-miR-574-5p	NM_173628	DNAH17	164	-21.59	-26.6	0.048189
hsa-miR-574-5p	NM_174858	AK5	172	-33.49	-37.5	0.004773
hsa-miR-574-5p	NM_175747	OLIG3	156	-16.79	-42.3	0.000438
hsa-miR-574-5p	NM_178310	SNAI3	169	-28.08	-31.1	0.039547
hsa-miR-574-5p	NM_178342	C3orf35	165	-34.2	-38.4	0.001196
hsa-miR-574-5p	NM_178344	C3orf35	165	-34.2	-38.4	0.001196
hsa-miR-574-5p	NM_182548	LHFPL5	197	-39.15	-41.5	0.000588
hsa-miR-574-5p	NM_182603	ANKRD42	197	-39.15	-41.5	0.000456
hsa-miR-574-5p	NM_198510	ITIH6	169	-29.71	-31.8	0.042051
hsa-miR-574-5p	NR_003191	GGTA1P	173	-37.31	-38	0.006194
hsa-miR-574-5p	NR_023387	ABCC6P2	161	-31.18	-34.6	0.006621
hsa-miR-574-5p	NR_024610	HINT1	150	-28.13	-34.6	0.009507
hsa-miR-574-5p	NR_024611	HINT1	150	-28.13	-34.6	0.006894
hsa-miR-574-5p	NR_027070	PISRT1	175	-29.16	-31.3	0.020332
hsa-miR-574-5p	NR_038453	UNC5B-AS1	168	-25.32	-31.1	0.030631
hsa-miR-574-5p	NR_040584	STAG3L2	197	-38.64	-41.4	0.001562
hsa-miR-574-5p	NR_045211	GGTA1P	173	-37.31	-38	0.004752

hsa-miR-574-5p	NR_046748	ARHGAP31-	180	-29.22	-32.4	0.015501
hsa-miR-574-5p	NR_046873	TPRG1-AS1	161	-30.05	-37	0.002049
hsa-miR-574-5p	NR_073488	HINT1	150	-28.13	-34.6	0.013006
hsa-miR-574-5p	NR_104332	CISTR	189	-34.76	-36.8	0.003255
hsa-miR-574-5p	NR_104333	CISTR	189	-34.76	-36.8	0.001608
hsa-miR-574-5p	NR_120327	LOC101928134	159	-26.48	-30	0.030994
hsa-miR-574-5p	NR_120402	LOC101928273	165	-32.73	-35.2	0.013457
hsa-miR-574-5p	NR_120490	LOC102724421	164	-25.68	-30.7	0.025394
hsa-miR-574-5p	NR_125763	LINC01167	168	-30.66	-32.9	0.011534
hsa-miR-574-5p	NR_125930	LOC101929741	173	-28.88	-32.8	0.049901
hsa-miR-576-5p	NM_000634	CXCR1	156	-30.04	-32.9	0.014766
hsa-miR-576-5p	NM_001080976	DSE	158	-28.6	-31.5	0.017311
hsa-miR-576-5p	NM_001376	DYNC1H1	155	-20.25	-26.8	0.020833
hsa-miR-576-5p	NM_013352	DSE	158	-28.6	-31.5	0.017311
hsa-miR-576-5p	NR_002958	SNORA17	153	-20.05	-23.8	0.044369
hsa-miR-548b-5p	NM_001004700	OR4C11	154	-20.12	-24.5	0.011448
hsa-miR-548b-5p	NM_001012991	KNOP1	167	-22.53	-28.2	0.03945
hsa-miR-548b-5p	NM_032834	ALG10	195	-28.78	-30.9	0.039885
hsa-miR-548b-5p	NM_182601	PLEKHS1	170	-19.13	-22.5	0.046004
hsa-miR-548b-5p	NR_030305	MIR579	161	-22.57	-26.4	0.004229
hsa-miR-548b-5p	NR_030315	MIR548B	160	-23.81	-27.5	0.001999
hsa-miR-548b-5p	NR_030330	MIR548A3	178	-19.07	-24.4	0.015411
hsa-miR-548b-5p	NR_031643	MIR548F2	180	-21.81	-23.9	0.021835
hsa-miR-548b-5p	NR_031662	MIR548G	183	-22.23	-23.3	0.026621
hsa-miR-548b-5p	NR_031666	MIR548N	182	-23.5	-25.9	0.003174
hsa-miR-548b-5p	NR_031669	MIR548O	188	-25.91	-31.8	0.00018
hsa-miR-548b-5p	NR_031677	MIR548H1	174	-17.28	-23.1	0.039709
hsa-miR-548b-5p	NR_031678	MIR548H2	186	-25.36	-28.6	0.000758
hsa-miR-548b-5p	NR_031679	MIR548H3	184	-21.13	-26.5	0.005979
hsa-miR-548b-5p	NR_031680	MIR548H4	188	-23.92	-27.1	0.003546

hsa-miR-548b-5p	NR_031686	MIR548P	180	-21.34	-26.5	0.002768
hsa-miR-548b-5p	NR_031752	MIR548Q	176	-18.66	-24.2	0.018708
hsa-miR-548b-5p	NR_036103	MIR548V	183	-26.49	-30	0.00023
hsa-miR-548b-5p	NR_037503	MIR548Y	180	-24.5	-25.9	0.007567
hsa-miR-548b-5p	NR_037515	MIR548Z	188	-27.89	-31.7	0.000125
hsa-miR-548b-5p	NR_039621	MIR548AC	179	-24.18	-27.9	0.001211
hsa-miR-548b-5p	NR_039639	MIR548AE2	184	-21.95	-27.6	0.000742
hsa-miR-548b-5p	NR_039673	MIR548AJ1	182	-21.52	-27.3	0.0011
hsa-miR-548b-5p	NR_039674	MIR548AJ2	182	-21.52	-27.3	0.002012
hsa-miR-548b-5p	NR_039692	MIR548X2	182	-20.27	-26.1	0.00539
hsa-miR-548b-5p	NR_039699	MIR548AK	186	-23.46	-27.2	0.000638
hsa-miR-548b-5p	NR_039762	MIR548AM	180	-23.41	-28.8	0.000422
hsa-miR-548b-5p	NR_049838	MIR548AQ	184	-23.2	-28.8	0.000215
hsa-miR-548b-5p	NR_049853	MIR548AU	160	-19.38	-24.7	0.003143
hsa-miR-548b-5p	NR_049865	MIR548AX	184	-25.48	-26.8	0.001605
hsa-miR-548b-5p	NR_110050	LOC101927418	184	-25.56	-28.7	0.018374
hsa-miR-548b-5p	NR_110632	LINC01361	196	-30.39	-32	0.027027
hsa-miR-548b-5p	NR_110633	LINC01361	196	-30.39	-32	0.026949
hsa-miR-548b-5p	NR_128708	MIR548BB	184	-24.9	-29.3	0.000218
hsa-miR-589-5p	NM_000781	CYP11A1	154	-22.96	-27.7	0.024619
hsa-miR-589-5p	NM_001099773	CYP11A1	154	-22.96	-27.7	0.024619
hsa-miR-589-5p	NM_001178030	SF1	161	-27.54	-31.5	0.035577
hsa-miR-589-5p	NM_001271644	THADA	160	-24.54	-27.4	0.036802
hsa-miR-589-5p	NM_001276271	MBD4	152	-24.26	-29.2	0.017554
hsa-miR-589-5p	NM_001304799	NARFL	177	-35.44	-36.2	0.002501
hsa-miR-589-5p	NM_006001	TUBA3C	152	-19.6	-27.5	0.005487
hsa-miR-589-5p	NM_006432	NPC2	152	-25.63	-28.8	0.030626
hsa-miR-589-5p	NM_012207	HNRNPH3	159	-28.47	-31.7	0.04969
hsa-miR-589-5p	NM_021644	HNRNPH3	159	-28.47	-31.7	0.04969
hsa-miR-589-5p	NM_022493	NARFL	177	-35.44	-36.2	0.002501

hsa-miR-589-5p	NM_080386	TUBA3D	152	-19.6	-27.5	0.004612
hsa-miR-589-5p	NM_145058	RILPL2	175	-27.9	-31.5	0.035701
hsa-miR-589-5p	NM_153274	BEST4	157	-25.56	-30.8	0.031029
hsa-miR-589-5p	NM_201995	SF1	161	-27.54	-31.5	0.031569
hsa-miR-589-5p	NM_201997	SF1	161	-27.54	-31.5	0.031569
hsa-miR-589-5p	NM_201998	SF1	161	-27.54	-31.5	0.035577
hsa-miR-589-5p	NM_207312	TUBA3E	152	-19.6	-27.5	0.004612
hsa-miR-589-5p	NR_030322	MIR591	151	-23.64	-26	0.014271
hsa-miR-589-5p	NR_037480	MIR3916	153	-20.42	-24.6	0.032487
hsa-miR-589-5p	NR_039860	MIR4710	152	-24.3	-28.7	0.000758
hsa-miR-589-5p	NR_040056	IQCH-AS1	165	-27.33	-29.9	0.014782
hsa-miR-589-5p	NR_110271	LOC101927701	160	-26.54	-30.4	0.04699
hsa-miR-618	NM_001134440	ZNF502	184	-31.94	-34.9	0.011541
hsa-miR-618	NM_001134441	ZNF502	184	-31.94	-34.9	0.011541
hsa-miR-618	NM_001134442	ZNF502	184	-31.94	-34.9	0.011541
hsa-miR-618	NM_001282880	ZNF502	184	-31.94	-34.9	0.011541
hsa-miR-618	NM_003057	SLC22A1	155	-21.09	-26.6	0.015542
hsa-miR-618	NM_025112	ZXDC	171	-28.34	-34.6	0.005168
hsa-miR-618	NM_033210	ZNF502	184	-31.94	-34.9	0.011541
hsa-miR-618	NM_153187	SLC22A1	155	-21.09	-26.6	0.022989
hsa-miR-624-5p	NM_001001998	EXOSC10	152	-21.28	-24.8	0.022813
hsa-miR-624-5p	NM_001136534	TMEM233	160	-28.55	-31.9	0.018837
hsa-miR-624-5p	NM_001174098	SLC29A3	159	-30.18	-32.2	0.035859
hsa-miR-624-5p	NM_001256526	C9orf69	174	-32.77	-35.1	0.014325
hsa-miR-624-5p	NM_002685	EXOSC10	152	-21.28	-24.8	0.022813
hsa-miR-624-5p	NM_006067	EMC8	151	-26.94	-30.7	0.047945
hsa-miR-624-5p	NM_007028	TRIM31	151	-28.38	-32.6	0.008245
hsa-miR-624-5p	NM_018344	SLC29A3	159	-30.18	-32.2	0.014136
hsa-miR-624-5p	NM_152833	C9orf69	174	-32.77	-35.1	0.014325
hsa-miR-624-5p	NR_030354	MIR624	180	-32.75	-34.4	0.000045

hsa-miR-624-5p	NR_037486	MIR3921	171	-21.54	-23	0.04647
hsa-miR-624-5p	NR_106910	MIR6851	151	-17.86	-22.7	0.035197
hsa-miR-624-5p	NR_120481	LOC101928030	150	-25.6	-31.5	0.004192
hsa-miR-628-5p	NM_001198542	HYDIN	167	-19.93	-26.5	0.034882
hsa-miR-628-5p	NM_001198543	HYDIN	167	-19.93	-26.5	0.034882
hsa-miR-628-5p	NM_001306206	CSTF2	166	-23.78	-29.4	0.045378
hsa-miR-628-5p	NM_001306209	CSTF2	166	-23.78	-29.4	0.045378
hsa-miR-628-5p	NM_022475	HHIP	170	-28.75	-31.8	0.012569
hsa-miR-628-5p	NM_173855	MORN3	159	-22.09	-26.2	0.041306
hsa-miR-628-5p	NR_049887	MIR5702	157	-20.65	-25.1	0.008796
hsa-miR-548d-5p	NM_000269	NME1	151	-22.72	-25.8	0.030341
hsa-miR-548d-5p	NM_000971	RPL7	151	-15.42	-21.6	0.039019
hsa-miR-548d-5p	NM_001130820	IFT22	188	-28.19	-32.1	0.025576
hsa-miR-548d-5p	NM_001130821	IFT22	188	-28.19	-32.1	0.025576
hsa-miR-548d-5p	NM_001130822	IFT22	188	-28.19	-32.1	0.025576
hsa-miR-548d-5p	NM_001287525	IFT22	188	-28.19	-32.1	0.025576
hsa-miR-548d-5p	NM_001287526	IFT22	188	-28.19	-32.1	0.025576
hsa-miR-548d-5p	NM_022777	IFT22	188	-28.19	-32.1	0.025576
hsa-miR-548d-5p	NM_032834	ALG10	190	-28.78	-30.9	0.032128
hsa-miR-548d-5p	NM_198175	NME1	151	-22.72	-25.8	0.030341
hsa-miR-548d-5p	NR_030305	MIR579	164	-24.66	-28.2	0.000964
hsa-miR-548d-5p	NR_030315	MIR548B	168	-28.31	-31.6	0.000095
hsa-miR-548d-5p	NR_030330	MIR548A3	176	-21.57	-26.7	0.002583
hsa-miR-548d-5p	NR_031642	MIR548F1	188	-22.34	-22.2	0.039664
hsa-miR-548d-5p	NR_031643	MIR548F2	188	-26.31	-28	0.001103
hsa-miR-548d-5p	NR_031644	MIR548F3	188	-23.66	-25.5	0.004527
hsa-miR-548d-5p	NR_031646	MIR548F5	183	-22.3	-23.5	0.017257
hsa-miR-548d-5p	NR_031662	MIR548G	178	-22.23	-23.3	0.021234
hsa-miR-548d-5p	NR_031666	MIR548N	188	-25.94	-29.3	0.000225
hsa-miR-548d-5p	NR_031669	MIR548O	180	-18.92	-25.6	0.007731

hsa-miR-548d-5p	NR_031677	MIR548H1	180	-21.53	-26.7	0.002904
hsa-miR-548d-5p	NR_031678	MIR548H2	192	-29.14	-32.7	0.000034
hsa-miR-548d-5p	NR_031679	MIR548H3	192	-25.63	-30.6	0.000311
hsa-miR-548d-5p	NR_031680	MIR548H4	196	-28.42	-30	0.000396
hsa-miR-548d-5p	NR_031686	MIR548P	188	-25.84	-30.6	0.000125
hsa-miR-548d-5p	NR_031687	MIR548I1	168	-21.12	-26.3	0.008646
hsa-miR-548d-5p	NR_031688	MIR548I2	168	-21.12	-26.3	0.008646
hsa-miR-548d-5p	NR_031689	MIR548I3	168	-21.12	-26.3	0.008646
hsa-miR-548d-5p	NR_031752	MIR548Q	184	-23.16	-28.3	0.000947
hsa-miR-548d-5p	NR_036103	MIR548V	188	-25.13	-27.9	0.000708
hsa-miR-548d-5p	NR_037503	MIR548Y	188	-29	-30	0.000387
hsa-miR-548d-5p	NR_037515	MIR548Z	196	-32.39	-35.8	0.000006
hsa-miR-548d-5p	NR_039621	MIR548AC	187	-28.68	-32	0.000055
hsa-miR-548d-5p	NR_039639	MIR548AE2	192	-26.45	-31.7	0.00003
hsa-miR-548d-5p	NR_039673	MIR548AJ1	188	-25.77	-30.9	0.000065
hsa-miR-548d-5p	NR_039674	MIR548AJ2	188	-25.77	-30.9	0.000131
hsa-miR-548d-5p	NR_039692	MIR548X2	188	-24.52	-29.7	0.00037
hsa-miR-548d-5p	NR_039699	MIR548AK	188	-23.83	-28.3	0.000212
hsa-miR-548d-5p	NR_039762	MIR548AM	188	-27.91	-32.9	0.000017
hsa-miR-548d-5p	NR_049838	MIR548AQ	192	-27.7	-32.9	0.000008
hsa-miR-548d-5p	NR_049853	MIR548AU	160	-17.21	-23	0.008286
hsa-miR-548d-5p	NR_049865	MIR548AX	192	-29.98	-30.9	0.000068
hsa-miR-548d-5p	NR_110050	LOC101927418	182	-23.6	-26.8	0.042109
hsa-miR-548d-5p	NR_128708	MIR548BB	192	-26.12	-28.3	0.00032
hsa-miR-660-3p	NM_000047	ARSE	151	-21.66	-26	0.036786
hsa-miR-660-3p	NM_000196	HSD11B2	169	-28.75	-30.5	0.018428
hsa-miR-660-3p	NM_000679	ADRA1B	165	-25.66	-30	0.021678
hsa-miR-660-3p	NM_001004019	FBLN2	152	-23.36	-28.4	0.046838
hsa-miR-660-3p	NM_001005783	HAO2	162	-21.55	-27.1	0.037961
hsa-miR-660-3p	NM_001012710	KRTAP5-10	160	-20.97	-27.9	0.046266

hsa-miR-660-3p	NM_001033583	ACOT9	169	-27.67	-31.5	0.002182
hsa-miR-660-3p	NM_001037171	ACOT9	169	-27.67	-31.5	0.002182
hsa-miR-660-3p	NM_001037501	NBPF8	169	-28.63	-33.9	0.019642
hsa-miR-660-3p	NM_001037675	NBPF9	169	-28.63	-33.9	0.019642
hsa-miR-660-3p	NM_001040125	PQLC2	154	-24	-29.4	0.042823
hsa-miR-660-3p	NM_001040126	PQLC2	154	-24	-29.4	0.042823
hsa-miR-660-3p	NM_001076674	TMUB2	173	-28.38	-31.6	0.022347
hsa-miR-660-3p	NM_001077657	SMCO1	154	-26.95	-30.6	0.045264
hsa-miR-660-3p	NM_001081	CUBN	170	-28.93	-32.7	0.016644
hsa-miR-660-3p	NM_001083600	NAA60	172	-28.25	-32.1	0.047611
hsa-miR-660-3p	NM_001083601	NAA60	172	-28.25	-32.1	0.047611
hsa-miR-660-3p	NM_001099456	NPW	153	-20.82	-25.6	0.022791
hsa-miR-660-3p	NM_001101404	SH2D7	172	-27.94	-32.4	0.006688
hsa-miR-660-3p	NM_001101663	NBPF11	169	-28.63	-33.9	0.0238
hsa-miR-660-3p	NM_001134999	FERMT2	167	-24.72	-30.8	0.04711
hsa-miR-660-3p	NM_001135721	PIWIL2	158	-24.94	-29.7	0.029184
hsa-miR-660-3p	NM_001143944	LEMD2	162	-24.53	-33.9	0.015971
hsa-miR-660-3p	NM_001144063	OSBPL5	156	-27.11	-31.8	0.028753
hsa-miR-660-3p	NM_001165035	FBLN2	152	-23.36	-28.4	0.046838
hsa-miR-660-3p	NM_001170629	CHD8	155	-26.67	-29.2	0.023032
hsa-miR-660-3p	NM_001170755	NBPF15	169	-28.63	-33.9	0.019714
hsa-miR-660-3p	NM_001185092	NIT1	166	-26.82	-31.2	0.048699
hsa-miR-660-3p	NM_001198963	FKTN	166	-29.33	-31.7	0.026343
hsa-miR-660-3p	NM_001199279	CHRNA6	152	-25.84	-30.1	0.022263
hsa-miR-660-3p	NM_001199629	MYL6B	160	-23	-26.6	0.00885
hsa-miR-660-3p	NM_001199787	SLC35E2	150	-19.37	-28.7	0.049622
hsa-miR-660-3p	NM_001242464	PPP1R1B	152	-21.78	-29.6	0.048345
hsa-miR-660-3p	NM_001242790	BRF1	154	-21.95	-28.1	0.008611
hsa-miR-660-3p	NM_001242	CD27	155	-26.51	-30.2	0.008651
hsa-miR-660-3p	NM_001256416	NBPF3	169	-28.63	-33.9	0.0177

hsa-miR-660-3p	NM_001256417	NBPF3	169	-28.63	-33.9	0.0177
hsa-miR-660-3p	NM_001258008	ZCWPW1	153	-21.2	-28.1	0.023769
hsa-miR-660-3p	NM_001258282	LINGO2	167	-25.17	-30.4	0.033047
hsa-miR-660-3p	NM_001271755	ITGBL1	172	-29.26	-33	0.009717
hsa-miR-660-3p	NM_001271756	ITGBL1	172	-29.26	-33	0.009717
hsa-miR-660-3p	NM_001277115	DNAH11	158	-24.24	-26.9	0.048254
hsa-miR-660-3p	NM_001277444	NBPF9	169	-28.63	-33.9	0.019642
hsa-miR-660-3p	NM_001278141	NBPF12	169	-28.63	-33.9	0.01777
hsa-miR-660-3p	NM_001278267	NBPF20	169	-28.63	-33.9	0.019696
hsa-miR-660-3p	NM_001282628	ARSE	151	-21.66	-26	0.036786
hsa-miR-660-3p	NM_001282631	ARSE	151	-21.66	-26	0.036786
hsa-miR-660-3p	NM_001286577	C2CD3	161	-24.72	-29.9	0.03453
hsa-miR-660-3p	NM_001286615	ANO4	150	-26.45	-29.8	0.046035
hsa-miR-660-3p	NM_001286616	ANO4	150	-26.45	-29.8	0.046384
hsa-miR-660-3p	NM_001287531	PQLC2	154	-24	-29.4	0.042823
hsa-miR-660-3p	NM_001300732	TMC7	162	-24.41	-27.1	0.04727
hsa-miR-660-3p	NM_001301227	TPM2	153	-21.04	-27.2	0.024543
hsa-miR-660-3p	NM_001303029	PKP3	150	-23.92	-28.9	0.018763
hsa-miR-660-3p	NM_001303533	RAPGEFL1	162	-28.83	-33.2	0.033058
hsa-miR-660-3p	NM_001303534	RAPGEFL1	162	-28.83	-33.2	0.033058
hsa-miR-660-3p	NM_001305115	DUSP26	160	-23.81	-29.6	0.041161
hsa-miR-660-3p	NM_001305116	DUSP26	160	-23.81	-29.6	0.041161
hsa-miR-660-3p	NM_001305	CLDN4	164	-26.04	-31.2	0.026895
hsa-miR-660-3p	NM_001310154	ANKRD36C	161	-28.35	-32.3	0.020663
hsa-miR-660-3p	NM_001834	CLTB	160	-26.45	-27.7	0.032244
hsa-miR-660-3p	NM_001998	FBLN2	152	-23.36	-28.4	0.046838
hsa-miR-660-3p	NM_002475	MYL6B	160	-23	-26.6	0.00885
hsa-miR-660-3p	NM_002809	PSMD3	157	-22.44	-27.7	0.042895
hsa-miR-660-3p	NM_002817	PSMD13	155	-23.75	-27.5	0.048649
hsa-miR-660-3p	NM_003289	TPM2	153	-21.04	-27.2	0.024543

hsa-miR-660-3p	NM_003974	DOK2	153	-24.94	-29	0.027538
hsa-miR-660-3p	NM_004198	CHRNA6	152	-25.84	-30.1	0.022263
hsa-miR-660-3p	NM_004699	FAM50A	153	-17.97	-26.3	0.037309
hsa-miR-660-3p	NM_004752	GCM2	171	-27.89	-32.6	0.011346
hsa-miR-660-3p	NM_004791	ITGBL1	172	-29.26	-33	0.009717
hsa-miR-660-3p	NM_006556	PMVK	162	-26.26	-28.6	0.02884
hsa-miR-660-3p	NM_006687	ACTL7A	152	-26.58	-30.9	0.00076
hsa-miR-660-3p	NM_006832	FERMT2	167	-24.72	-30.8	0.04711
hsa-miR-660-3p	NM_006848	CCDC85B	158	-26.7	-30	0.0055
hsa-miR-660-3p	NM_007097	CLTB	160	-26.45	-27.7	0.032244
hsa-miR-660-3p	NM_007183	PKP3	150	-23.92	-28.9	0.018763
hsa-miR-660-3p	NM_012230	POMZP3	153	-23.3	-26.2	0.045427
hsa-miR-660-3p	NM_012235	SCAP	161	-20.8	-27.1	0.012822
hsa-miR-660-3p	NM_014448	ARHGEF16	163	-24.96	-28.5	0.047723
hsa-miR-660-3p	NM_014638	PLCH2	157	-25.64	-29.3	0.013407
hsa-miR-660-3p	NM_015327	SMG5	179	-28.19	-32.6	0.0276
hsa-miR-660-3p	NM_015383	NBPF14	169	-28.63	-33.9	0.019588
hsa-miR-660-3p	NM_015488	PNKD	157	-23.4	-33	0.034646
hsa-miR-660-3p	NM_016078	TVP23B	162	-27.83	-31.6	0.034087
hsa-miR-660-3p	NM_016111	TELO2	159	-22.24	-32	0.007869
hsa-miR-660-3p	NM_016113	TRPV2	157	-22.52	-27.1	0.006381
hsa-miR-660-3p	NM_016339	RAPGEFL1	162	-28.83	-33.2	0.033058
hsa-miR-660-3p	NM_016527	HAO2	162	-21.55	-27.1	0.037961
hsa-miR-660-3p	NM_017765	PQLC2	154	-24	-29.4	0.042823
hsa-miR-660-3p	NM_017940	NBPF1	166	-28.36	-32	0.042375
hsa-miR-660-3p	NM_017984	ZCWPW1	153	-21.2	-28.1	0.00554
hsa-miR-660-3p	NM_018068	PIWIL2	158	-24.94	-29.7	0.029184
hsa-miR-660-3p	NM_020896	OSBPL5	156	-27.11	-31.8	0.028753
hsa-miR-660-3p	NM_020920	CHD8	155	-26.67	-29.2	0.023032
hsa-miR-660-3p	NM_022572	PNKD	157	-23.4	-33	0.034646

hsa-miR-660-3p	NM_024025	DUSP26	160	-23.81	-29.6	0.041161
hsa-miR-660-3p	NM_024107	TMUB2	173	-28.38	-31.6	0.022347
hsa-miR-660-3p	NM_024504	PRDM14	166	-24.92	-28.1	0.042026
hsa-miR-660-3p	NM_024845	NAA60	172	-28.25	-32.1	0.048457
hsa-miR-660-3p	NM_024848	MORN1	169	-28.36	-31.5	0.000579
hsa-miR-660-3p	NM_031965	GSG2	174	-24.81	-29.5	0.01556
hsa-miR-660-3p	NM_032192	PPP1R1B	152	-21.78	-29.6	0.048345
hsa-miR-660-3p	NM_032264	NBPF3	169	-28.63	-33.9	0.0177
hsa-miR-660-3p	NM_033199	UCN2	159	-27.88	-32	0.025392
hsa-miR-660-3p	NM_052929	FHAD1	166	-29.33	-31.6	0.016343
hsa-miR-660-3p	NM_053044	HTRA3	165	-25.2	-30.3	0.049784
hsa-miR-660-3p	NM_057179	TWIST2	150	-25.96	-28.9	0.036996
hsa-miR-660-3p	NM_145638	OSBPL5	156	-27.11	-31.8	0.028753
hsa-miR-660-3p	NM_145914	ZSCAN21	159	-25.18	-29.3	0.022297
hsa-miR-660-3p	NM_152570	LINGO2	167	-25.17	-30.4	0.033047
hsa-miR-660-3p	NM_152992	POMZP3	153	-23.3	-26.2	0.041007
hsa-miR-660-3p	NM_173092	KCNH6	162	-24.73	-26.9	0.025992
hsa-miR-660-3p	NM_173588	IGSF22	159	-23.7	-26.5	0.018921
hsa-miR-660-3p	NM_173638	NBPF15	169	-28.63	-33.9	0.019714
hsa-miR-660-3p	NM_175932	PSMD13	155	-23.75	-27.5	0.048649
hsa-miR-660-3p	NM_177441	TMUB2	173	-28.38	-31.6	0.022347
hsa-miR-660-3p	NM_178314	RILPL1	161	-24.16	-31.3	0.040737
hsa-miR-660-3p	NM_178826	ANO4	150	-26.45	-29.8	0.046035
hsa-miR-660-3p	NM_181336	LEMD2	162	-24.53	-33.9	0.015971
hsa-miR-660-3p	NM_181505	PPP1R1B	152	-21.78	-29.6	0.048345
hsa-miR-660-3p	NM_183372	NBPF11	169	-28.63	-33.9	0.0238
hsa-miR-660-3p	NM_205854	SFTA2	157	-22.76	-26.3	0.015411
hsa-miR-660-3p	NR_026818	FAM138A	154	-25.01	-30.9	0.046364
hsa-miR-660-3p	NR_026820	FAM138F	154	-25.01	-30.9	0.046364
hsa-miR-660-3p	NR_026822	FAM138C	154	-25.01	-30.9	0.046364

hsa-miR-660-3p	NR_026823	FAM138D	154	-25.01	-30.9	0.046129
hsa-miR-660-3p	NR_027761	LOC100132831	157	-29.27	-33.6	0.030006
hsa-miR-660-3p	NR_027941	COX11	165	-26.69	-31.8	0.030306
hsa-miR-660-3p	NR_027942	COX11	165	-26.69	-31.8	0.029747
hsa-miR-660-3p	NR_028335	LOC284009	159	-26.32	-31.7	0.010865
hsa-miR-660-3p	NR_030714	MIR629	151	-21.44	-24.3	0.026032
hsa-miR-660-3p	NR_039857	MIR4708	154	-20.72	-25.7	0.004565
hsa-miR-660-3p	NR_039866	MIR4716	179	-28.62	-32.1	0.000128
hsa-miR-660-3p	NR_046287	ATP2A1-AS1	167	-26.48	-29.2	0.039456
hsa-miR-660-3p	NR_046288	ATP2A1-AS1	167	-26.48	-29.2	0.039141
hsa-miR-660-3p	NR_046289	ATP2A1-AS1	167	-26.48	-29.2	0.033966
hsa-miR-660-3p	NR_046290	ATP2A1-AS1	167	-26.48	-29.2	0.03366
hsa-miR-660-3p	NR_102684	YTHDF3-AS1	175	-28.5	-32.6	0.010047
hsa-miR-660-3p	NR_103772	LOC100288162	159	-26.5	-30.7	0.047458
hsa-miR-660-3p	NR_110259	LOC101929596	174	-28.54	-34.4	0.008876
hsa-miR-660-3p	NR_120478	LOC100129940	165	-26.12	-31	0.035803
hsa-miR-660-3p	NR_125750	TBX2-AS1	158	-23.01	-29.9	0.043187
hsa-miR-660-3p	NR_126413	SCEL-AS1	154	-22.98	-28.3	0.041218
hsa-miR-660-3p	NR_132966	SNORA110	153	-24.62	-28.9	0.008136
hsa-miR-421	NM_001033083	PBLD	177	-29.71	-33.4	0.000479
hsa-miR-421	NM_001042771	LCK	189	-27.69	-31.1	0.011121
hsa-miR-421	NM_001083612	HYKK	172	-21.92	-24.7	0.0129
hsa-miR-421	NM_001199327	FPGT-TNNI3K	159	-22.34	-25.9	0.038426
hsa-miR-421	NM_001304437	UBE3D	173	-26.75	-31	0.020201
hsa-miR-421	NM_005356	LCK	189	-27.69	-31.1	0.011121
hsa-miR-421	NM_021930	RINT1	166	-22.61	-28	0.039472
hsa-miR-421	NM_145282	SLC25A48	180	-27.67	-32.1	0.009479
hsa-miR-421	NM_198920	UBE3D	173	-26.75	-31	0.020201
hsa-miR-421	NR_029511	MIR95	188	-26.91	-30.6	0.000379
hsa-miR-421	NR_030398	MIR421	168	-21.9	-27.3	0.003516

hsa-miR-758-3p	NM_001008739	C6orf226	152	-23.72	-27.9	0.019309
hsa-miR-758-3p	NM_001134734	C1orf94	154	-30.25	-31.1	0.008961
hsa-miR-758-3p	NM_001143766	ZNF438	158	-22.13	-28.4	0.029678
hsa-miR-758-3p	NM_001143767	ZNF438	158	-22.13	-28.4	0.029678
hsa-miR-758-3p	NM_001143768	ZNF438	158	-22.13	-28.4	0.029678
hsa-miR-758-3p	NM_001143769	ZNF438	158	-22.13	-28.4	0.029678
hsa-miR-758-3p	NM_001143770	ZNF438	158	-22.13	-28.4	0.029678
hsa-miR-758-3p	NM_001143771	ZNF438	158	-22.13	-28.4	0.029678
hsa-miR-758-3p	NM_001272007	ISCA2	158	-30.59	-32.3	0.015875
hsa-miR-758-3p	NM_032884	C1orf94	154	-30.25	-31.1	0.008961
hsa-miR-758-3p	NM_152635	OIT3	163	-30.41	-34	0.003657
hsa-miR-758-3p	NM_178829	C7orf34	163	-23.46	-28.1	0.027495
hsa-miR-758-3p	NM_182755	ZNF438	158	-22.13	-28.4	0.029678
hsa-miR-758-3p	NM_194279	ISCA2	158	-30.59	-32.3	0.01036
hsa-miR-758-3p	NR_046505	LINC00280	165	-24.13	-29.4	0.030089
hsa-miR-151b	NM_000040	APOC3	151	-25.23	-27.5	0.007557
hsa-miR-151b	NM_000418	IL4R	164	-28.63	-31.4	0.0169
hsa-miR-151b	NM_001008272	TAGLN3	160	-23.76	-27.6	0.021974
hsa-miR-151b	NM_001008273	TAGLN3	160	-23.76	-27.6	0.021974
hsa-miR-151b	NM_001018070	CORO1B	157	-25.16	-28.7	0.011315
hsa-miR-151b	NM_001077663	URGCP	164	-35.67	-39.2	0.000213
hsa-miR-151b	NM_001077664	URGCP	164	-35.67	-39.2	0.000213
hsa-miR-151b	NM_001098673	ATG101	151	-25.26	-28.8	0.009264
hsa-miR-151b	NM_001130848	PITPNM1	159	-22.57	-27.2	0.017832
hsa-miR-151b	NM_001136044	TMUB1	161	-27.87	-32.1	0.003291
hsa-miR-151b	NM_001242946	EPDR1	160	-33.24	-36.5	0.003779
hsa-miR-151b	NM_001242948	EPDR1	160	-33.24	-36.5	0.003093
hsa-miR-151b	NM_001257406	IL4R	164	-28.63	-31.4	0.0169
hsa-miR-151b	NM_001257407	IL4R	164	-28.63	-31.4	0.0169
hsa-miR-151b	NM_001257997	IL4R	164	-28.63	-31.4	0.0169

hsa-miR-151b	NM_001272002	DOCK7	160	-33.45	-36.8	0.000562
hsa-miR-151b	NM_001290075	URGCP	164	-35.67	-39.2	0.000213
hsa-miR-151b	NM_001290076	URGCP	164	-35.67	-39.2	0.000213
hsa-miR-151b	NM_001306082	STK32B	168	-29.3	-32.6	0.027863
hsa-miR-151b	NM_004910	PITPNM1	159	-22.57	-27.2	0.017832
hsa-miR-151b	NM_005373	MPL	160	-33.45	-36.8	0.003133
hsa-miR-151b	NM_006418	OLFM4	161	-28.02	-30.2	0.048794
hsa-miR-151b	NM_013241	FHOD1	154	-27.05	-35.2	0.000142
hsa-miR-151b	NM_013259	TAGLN3	160	-23.76	-27.6	0.021974
hsa-miR-151b	NM_017549	EPDR1	160	-33.24	-36.5	0.003093
hsa-miR-151b	NM_017670	OTUB1	160	-34.58	-37.7	0.000594
hsa-miR-151b	NM_017920	URGCP	164	-35.67	-39.2	0.000213
hsa-miR-151b	NM_018401	STK32B	168	-29.3	-32.6	0.027863
hsa-miR-151b	NM_020441	CORO1B	157	-25.16	-28.7	0.011315
hsa-miR-151b	NM_021908	ST7	156	-29.11	-33.1	0.0069
hsa-miR-151b	NM_021934	ATG101	151	-25.26	-28.8	0.009264
hsa-miR-151b	NM_024098	CCDC86	154	-25.13	-29	0.040338
hsa-miR-151b	NM_031434	TMUB1	161	-27.87	-32.1	0.003291
hsa-miR-151b	NM_032428	FRMPD3	168	-29.63	-32.2	0.032391
hsa-miR-151b	NM_152544	TRMT44	152	-25.91	-30.3	0.012074
hsa-miR-454-3p	NM_001042618	PARP2	166	-23.68	-27.4	0.004211
hsa-miR-454-3p	NM_005484	PARP2	166	-23.68	-27.4	0.004211
hsa-miR-454-3p	NM_019060	CRCT1	166	-23.14	-27.5	0.033384
hsa-miR-454-3p	NM_020945	WDFY4	172	-24.16	-27.9	0.035579
hsa-miR-454-3p	NR_029509	MIR92A2	173	-22.39	-24.5	0.012524
hsa-miR-450b-5p	NM_001307960	TM2D3	192	-28.46	-32.2	0.005361
hsa-miR-450b-5p	NM_001308026	TM2D3	192	-28.46	-32.2	0.005361
hsa-miR-744-5p	NM_000244	MEN1	162	-24.66	-32.3	0.030543
hsa-miR-744-5p	NM_000347	SPTB	156	-26.02	-31.4	0.006703
hsa-miR-744-5p	NM_000526	KRT14	156	-29.1	-32.2	0.001553

hsa-miR-744-5p	NM_000577	IL1RN	150	-27.92	-32.7	0.041508
hsa-miR-744-5p	NM_000934	SERPINF2	166	-33.27	-35.4	0.006058
hsa-miR-744-5p	NM_001001502	SNCB	157	-27.94	-30.8	0.038298
hsa-miR-744-5p	NM_001001734	ATP1A4	160	-29.16	-34.9	0.001252
hsa-miR-744-5p	NM_001005373	LRSAM1	156	-29.99	-35.7	0.003742
hsa-miR-744-5p	NM_001005374	LRSAM1	156	-29.99	-35.7	0.003742
hsa-miR-744-5p	NM_001006616	TSPAN17	158	-28.05	-34	0.035405
hsa-miR-744-5p	NM_001008404	C14orf180	157	-32.91	-38.3	0.00304
hsa-miR-744-5p	NM_001010938	TNK2	154	-35.01	-40.6	0.000658
hsa-miR-744-5p	NM_001012631	IL32	153	-27.54	-31.8	0.007778
hsa-miR-744-5p	NM_001012632	IL32	153	-27.54	-31.8	0.007778
hsa-miR-744-5p	NM_001012633	IL32	153	-27.54	-31.8	0.007778
hsa-miR-744-5p	NM_001012634	IL32	153	-27.54	-31.8	0.007778
hsa-miR-744-5p	NM_001012635	IL32	153	-27.54	-31.8	0.007778
hsa-miR-744-5p	NM_001012636	IL32	153	-27.54	-31.8	0.007778
hsa-miR-744-5p	NM_001012718	IL32	153	-27.54	-31.8	0.007778
hsa-miR-744-5p	NM_001013836	MAD1L1	155	-26.09	-30.9	0.009999
hsa-miR-744-5p	NM_001013837	MAD1L1	155	-26.09	-30.9	0.009999
hsa-miR-744-5p	NM_001017915	INPP5D	151	-31.18	-35.7	0.011758
hsa-miR-744-5p	NM_001030015	OPN4	153	-26.84	-32	0.028047
hsa-miR-744-5p	NM_001032221	STXBP1	151	-31.15	-34	0.04637
hsa-miR-744-5p	NM_001037501	NBPF8	151	-29.79	-35.9	0.017126
hsa-miR-744-5p	NM_001037675	NBPF9	151	-29.79	-35.9	0.017126
hsa-miR-744-5p	NM_001037806	NCKAP5L	158	-31.26	-34.4	0.008839
hsa-miR-744-5p	NM_001039803	CDK20	150	-33.91	-39.4	0.001712
hsa-miR-744-5p	NM_001039966	GPER1	159	-25.99	-33.8	0.019613
hsa-miR-744-5p	NM_001070	TUBG1	163	-32.54	-36.6	0.000236
hsa-miR-744-5p	NM_001077261	NCOR2	168	-28.9	-32.8	0.032288
hsa-miR-744-5p	NM_001077621	VPS37D	160	-28.41	-31.2	0.042822
hsa-miR-744-5p	NM_001080395	AATK	153	-28.03	-32.4	0.045086

hsa-miR-744-5p	NM_001080511	CLEC2L	163	-29.94	-33.2	0.013731
hsa-miR-744-5p	NM_001080527	MYO7B	153	-28.74	-31.9	0.007346
hsa-miR-744-5p	NM_001081559	CPSF4	150	-27.99	-35.5	0.007791
hsa-miR-744-5p	NM_001083600	NAA60	158	-31.81	-37.5	0.009263
hsa-miR-744-5p	NM_001083601	NAA60	158	-31.81	-37.5	0.009263
hsa-miR-744-5p	NM_001098201	GPER1	159	-25.99	-33.8	0.019613
hsa-miR-744-5p	NM_001099409	EHBP1L1	152	-25.13	-30.1	0.021796
hsa-miR-744-5p	NM_001101663	NBPF11	151	-29.79	-35.9	0.02071
hsa-miR-744-5p	NM_001112704	VAX1	157	-30.96	-38.8	0.001193
hsa-miR-744-5p	NM_001114108	TTC22	158	-25.53	-32.4	0.001551
hsa-miR-744-5p	NM_001114726	PRRT4	158	-29.41	-35.4	0.008684
hsa-miR-744-5p	NM_001114748	TMEM240	161	-30.99	-36.5	0.002392
hsa-miR-744-5p	NM_001130107	KLC1	158	-26.02	-30.6	0.017374
hsa-miR-744-5p	NM_001130967	ZNF385A	154	-29.98	-33.4	0.029812
hsa-miR-744-5p	NM_001130968	ZNF385A	154	-29.98	-33.4	0.029812
hsa-miR-744-5p	NM_001134434	SPOCK2	156	-34.56	-34.9	0.007493
hsa-miR-744-5p	NM_001134774	KLC2	157	-30.78	-36.6	0.005
hsa-miR-744-5p	NM_001134775	KLC2	157	-30.78	-36.6	0.004991
hsa-miR-744-5p	NM_001134776	KLC2	157	-30.78	-36.6	0.004991
hsa-miR-744-5p	NM_001139441	BCAP31	151	-29.7	-32.3	0.011402
hsa-miR-744-5p	NM_001139457	BCAP31	151	-29.7	-32.3	0.011402
hsa-miR-744-5p	NM_001142805	SLC6A8	165	-30.61	-35.2	0.018217
hsa-miR-744-5p	NM_001142806	SLC6A8	165	-30.61	-35.2	0.018217
hsa-miR-744-5p	NM_001142853	HES6	151	-26.7	-33.1	0.011446
hsa-miR-744-5p	NM_001142936	DAGLB	155	-28.34	-33.8	0.012495
hsa-miR-744-5p	NM_001143944	LEMD2	151	-28.58	-33.1	0.046881
hsa-miR-744-5p	NM_001144063	OSBPL5	158	-27.31	-35.7	0.010161
hsa-miR-744-5p	NM_001156474	CCDC81	150	-29.18	-33.8	0.004359
hsa-miR-744-5p	NM_001160102	LPO	171	-29.57	-31.8	0.019132
hsa-miR-744-5p	NM_001165036	OGDH	157	-28.52	-32.8	0.0361

hsa-miR-744-5p	NM_001165920	SERPINF2	166	-33.27	-35.4	0.006058
hsa-miR-744-5p	NM_001165921	SERPINF2	166	-33.27	-35.4	0.006058
hsa-miR-744-5p	NM_001167985	INCA1	159	-27.22	-30.9	0.009801
hsa-miR-744-5p	NM_001167986	INCA1	159	-27.22	-30.9	0.009801
hsa-miR-744-5p	NM_001167987	INCA1	159	-27.22	-30.9	0.009801
hsa-miR-744-5p	NM_001168241	GAREML	166	-33.47	-37.1	0.008185
hsa-miR-744-5p	NM_001170639	CDK20	150	-34.43	-39.6	0.001911
hsa-miR-744-5p	NM_001170640	CDK20	150	-34.43	-39.6	0.001911
hsa-miR-744-5p	NM_001170755	NBPF15	151	-29.79	-35.9	0.017188
hsa-miR-744-5p	NM_001177355	MSLN	160	-29.6	-35.8	0.000137
hsa-miR-744-5p	NM_001184957	C3orf20	150	-27.03	-31.8	0.002078
hsa-miR-744-5p	NM_001184958	C3orf20	150	-27.03	-31.8	0.002078
hsa-miR-744-5p	NM_001185080	CLDN15	157	-25.85	-28.7	0.03886
hsa-miR-744-5p	NM_001190723	LRSAM1	156	-29.99	-35.7	0.003742
hsa-miR-744-5p	NM_001191033	GAREML	166	-33.47	-37.1	0.008185
hsa-miR-744-5p	NM_001199096	BAIAP3	155	-25.85	-32.3	0.038487
hsa-miR-744-5p	NM_001199097	BAIAP3	155	-25.85	-32.3	0.038487
hsa-miR-744-5p	NM_001199098	BAIAP3	155	-25.85	-32.3	0.038487
hsa-miR-744-5p	NM_001199099	BAIAP3	155	-25.85	-32.3	0.038487
hsa-miR-744-5p	NM_001206654	NCOR2	168	-28.9	-32.8	0.032288
hsa-miR-744-5p	NM_001256447	BCAP31	151	-29.7	-32.3	0.011402
hsa-miR-744-5p	NM_001257359	SAMD14	150	-30.78	-35.7	0.018007
hsa-miR-744-5p	NM_001270453	WWP2	162	-33.86	-38.7	0.006041
hsa-miR-744-5p	NM_001270454	WWP2	162	-33.86	-38.7	0.006041
hsa-miR-744-5p	NM_001271803	REEP2	163	-28.65	-35.2	0.015108
hsa-miR-744-5p	NM_001271933	CEP164	153	-25.83	-36.4	0.007524
hsa-miR-744-5p	NM_001277444	NBPF9	151	-29.79	-35.9	0.017126
hsa-miR-744-5p	NM_001278141	NBPF12	151	-29.79	-35.9	0.015511
hsa-miR-744-5p	NM_001278267	NBPF20	151	-29.79	-35.9	0.017173
hsa-miR-744-5p	NM_001279349	REXO4	151	-26.09	-32.2	0.036396

hsa-miR-744-5p	NM_001279350	REXO4	151	-26.09	-32.2	0.036396
hsa-miR-744-5p	NM_001279351	REXO4	151	-26.09	-32.2	0.036396
hsa-miR-744-5p	NM_001282195	SLC25A14	157	-28.66	-33.9	0.004486
hsa-miR-744-5p	NM_001282196	SLC25A14	157	-28.66	-33.9	0.004486
hsa-miR-744-5p	NM_001282197	SLC25A14	157	-28.66	-33.9	0.004486
hsa-miR-744-5p	NM_001282198	SLC25A14	157	-28.66	-33.9	0.004486
hsa-miR-744-5p	NM_001282290	ARHGAP27	152	-33.97	-39.6	0.003235
hsa-miR-744-5p	NM_001282434	HES6	151	-26.7	-33.1	0.022637
hsa-miR-744-5p	NM_001282944	PMPCA	154	-31.93	-36.7	0.001617
hsa-miR-744-5p	NM_001282946	PMPCA	154	-31.93	-36.7	0.001617
hsa-miR-744-5p	NM_001282962	HJURP	150	-26.02	-34.2	0.014204
hsa-miR-744-5p	NM_001282963	HJURP	150	-26.02	-34.2	0.014204
hsa-miR-744-5p	NM_001284341	FKBP9	150	-30.28	-38.1	0.006366
hsa-miR-744-5p	NM_001284343	FKBP9	150	-30.28	-38.1	0.006366
hsa-miR-744-5p	NM_001284351	SLC9A3	159	-30.02	-34.1	0.000571
hsa-miR-744-5p	NM_001286400	C14orf180	157	-32.91	-38.3	0.00304
hsa-miR-744-5p	NM_001286451	HDDC3	154	-27.1	-30.8	0.016419
hsa-miR-744-5p	NM_001286464	BAIAP3	155	-25.85	-32.3	0.038487
hsa-miR-744-5p	NM_001287181	CCDC33	156	-28.52	-33.9	0.019048
hsa-miR-744-5p	NM_001288974	ADAM12	151	-32.03	-35.9	0.00548
hsa-miR-744-5p	NM_001288975	ADAM12	151	-32.03	-35.9	0.00548
hsa-miR-744-5p	NM_001290001	ZNF385A	154	-29.98	-33.4	0.029812
hsa-miR-744-5p	NM_001290002	ZNF385A	154	-29.98	-33.4	0.029812
hsa-miR-744-5p	NM_001290004	ZNF385A	154	-29.98	-33.4	0.029812
hsa-miR-744-5p	NM_001304482	PTPN23	152	-25.63	-29.7	0.016063
hsa-miR-744-5p	NM_001304523	MAD1L1	155	-26.09	-30.9	0.009999
hsa-miR-744-5p	NM_001304524	MAD1L1	155	-26.09	-30.9	0.009999
hsa-miR-744-5p	NM_001304525	MAD1L1	155	-26.09	-30.9	0.009999
hsa-miR-744-5p	NM_001305275	AGRN	154	-31.3	-33.6	0.027681
hsa-miR-744-5p	NM_001307990	MAP4K2	151	-30.35	-36.4	0.001453

hsa-miR-744-5p	NM_001308046	TNK2	154	-35.01	-40.6	0.000659
hsa-miR-744-5p	NM_001308078	IL32	153	-27.54	-31.8	0.015639
hsa-miR-744-5p	NM_001308152	ARHGEF10	155	-30.6	-34.6	0.023628
hsa-miR-744-5p	NM_001308153	ARHGEF10	155	-30.6	-34.6	0.023628
hsa-miR-744-5p	NM_001310155	FAM231B	153	-28.86	-32.7	0.009925
hsa-miR-744-5p	NM_001311160	THY1	151	-28.22	-33.7	0.038105
hsa-miR-744-5p	NM_001311162	THY1	151	-28.22	-33.7	0.038105
hsa-miR-744-5p	NM_001316327	PRKCD	156	-25.35	-29.5	0.04563
hsa-miR-744-5p	NM_001505	GPER1	159	-25.99	-33.8	0.019613
hsa-miR-744-5p	NM_001715	BLK	156	-26.21	-29.6	0.049541
hsa-miR-744-5p	NM_001795	CDH5	159	-34.09	-38.8	0.00488
hsa-miR-744-5p	NM_001846	COL4A2	150	-28.06	-34.7	0.010459
hsa-miR-744-5p	NM_002332	LRP1	161	-28.69	-31.7	0.03777
hsa-miR-744-5p	NM_002536	TBC1D25	161	-29.16	-34.2	0.035757
hsa-miR-744-5p	NM_002541	OGDH	157	-28.52	-32.8	0.0361
hsa-miR-744-5p	NM_002653	PITX1	158	-33.27	-32.1	0.047493
hsa-miR-744-5p	NM_002928	RGS16	157	-29.06	-34.5	0.032419
hsa-miR-744-5p	NM_003015	SFRP5	163	-28.6	-35	0.007847
hsa-miR-744-5p	NM_003085	SNCB	157	-27.94	-30.8	0.038298
hsa-miR-744-5p	NM_003110	SP2	158	-27.27	-32.9	0.037452
hsa-miR-744-5p	NM_003165	STXBP1	151	-31.15	-34	0.049682
hsa-miR-744-5p	NM_003327	TNFRSF4	152	-25.79	-27.5	0.041708
hsa-miR-744-5p	NM_003550	MAD1L1	155	-26.09	-30.9	0.009999
hsa-miR-744-5p	NM_003933	BAIAP3	155	-25.85	-32.3	0.038487
hsa-miR-744-5p	NM_004174	SLC9A3	159	-30.02	-34.1	0.000571
hsa-miR-744-5p	NM_004221	IL32	153	-27.54	-31.8	0.007778
hsa-miR-744-5p	NM_004305	BIN1	158	-29.31	-34.8	0.004173
hsa-miR-744-5p	NM_004322	BAD	155	-26.52	-33.5	0.004759
hsa-miR-744-5p	NM_004444	EPHB4	162	-30.93	-34.8	0.011196
hsa-miR-744-5p	NM_004455	EXTL1	155	-28.84	-32.8	0.0383

hsa-miR-744-5p	NM_004579	MAP4K2	151	-30.35	-36.4	0.001453
hsa-miR-744-5p	NM_004732	KCNAB3	162	-28.26	-33.3	0.035876
hsa-miR-744-5p	NM_004769	ASIC3	157	-31.8	-36.6	0.000046
hsa-miR-744-5p	NM_004839	HOMER2	159	-27.57	-35.7	0.005352
hsa-miR-744-5p	NM_004913	VPS9D1	152	-27.63	-32.2	0.026685
hsa-miR-744-5p	NM_004959	NR5A1	167	-29.92	-34.2	0.03238
hsa-miR-744-5p	NM_005098	MSC	157	-24.44	-33.5	0.028227
hsa-miR-744-5p	NM_005112	WDR1	162	-29.27	-34.5	0.016165
hsa-miR-744-5p	NM_005144	HR	167	-28.8	-34.2	0.025287
hsa-miR-744-5p	NM_005173	ATP2A3	158	-34.27	-35.9	0.016046
hsa-miR-744-5p	NM_005479	FRAT1	158	-30.13	-34	0.038532
hsa-miR-744-5p	NM_005541	INPP5D	151	-31.18	-35.7	0.011758
hsa-miR-744-5p	NM_005552	KLC1	155	-30.14	-34.5	0.006825
hsa-miR-744-5p	NM_005629	SLC6A8	165	-30.61	-35.2	0.018217
hsa-miR-744-5p	NM_005632	CAPN15	161	-30.56	-32.8	0.038496
hsa-miR-744-5p	NM_005698	SCAMP3	155	-33.99	-38.7	0.000197
hsa-miR-744-5p	NM_005745	BCAP31	151	-29.7	-32.3	0.011402
hsa-miR-744-5p	NM_005781	TNK2	154	-35.01	-40.6	0.000658
hsa-miR-744-5p	NM_005814	GPA33	157	-29.78	-36	0.014562
hsa-miR-744-5p	NM_005823	MSLN	160	-29.6	-35.8	0.000137
hsa-miR-744-5p	NM_005865	PRSS16	154	-32.46	-38.8	0.003437
hsa-miR-744-5p	NM_005894	CD5L	164	-32.4	-36	0.008377
hsa-miR-744-5p	NM_005953	MT2A	153	-24.41	-29.3	0.006541
hsa-miR-744-5p	NM_005961	MUC6	157	-27.99	-34.8	0.006257
hsa-miR-744-5p	NM_005995	TBX10	157	-24.35	-29.8	0.020406
hsa-miR-744-5p	NM_006142	SFN	153	-26.16	-31.2	0.023434
hsa-miR-744-5p	NM_006151	LPO	171	-29.57	-31.8	0.019132
hsa-miR-744-5p	NM_006231	POLE	157	-31.65	-37.8	0.003056
hsa-miR-744-5p	NM_006253	PRKAB1	174	-38.78	-40.2	0.001837
hsa-miR-744-5p	NM_006254	PRKCD	156	-25.35	-29.5	0.04563

hsa-miR-744-5p	NM_006288	THY1	151	-28.22	-33.7	0.038105
hsa-miR-744-5p	NM_006289	TLN1	159	-27.28	-33.2	0.00759
hsa-miR-744-5p	NM_006312	NCOR2	168	-28.9	-32.8	0.032288
hsa-miR-744-5p	NM_006586	CNPY3	151	-30.5	-30.3	0.034094
hsa-miR-744-5p	NM_006593	TBR1	158	-28.99	-32.5	0.028653
hsa-miR-744-5p	NM_006693	CPSF4	150	-27.99	-35.5	0.007791
hsa-miR-744-5p	NM_007014	WWP2	162	-33.86	-38.7	0.006041
hsa-miR-744-5p	NM_007224	NXPH4	154	-35.27	-41.1	0.000388
hsa-miR-744-5p	NM_007270	FKBP9	150	-30.28	-38.1	0.006366
hsa-miR-744-5p	NM_007368	RASA3	155	-26.37	-35.5	0.019263
hsa-miR-744-5p	NM_012119	CDK20	150	-33.91	-39.4	0.001712
hsa-miR-744-5p	NM_012171	TSPAN17	158	-28.05	-34	0.030084
hsa-miR-744-5p	NM_012205	HAAO	161	-25.89	-31.2	0.012842
hsa-miR-744-5p	NM_012320	PLA2G15	169	-38.33	-41.6	0.001132
hsa-miR-744-5p	NM_012476	VAX2	152	-30.88	-36	0.000517
hsa-miR-744-5p	NM_013404	MSLN	160	-29.6	-35.8	0.000137
hsa-miR-744-5p	NM_014062	NOB1	168	-30.49	-33.7	0.00651
hsa-miR-744-5p	NM_014343	CLDN15	157	-25.85	-28.7	0.03886
hsa-miR-744-5p	NM_014390	SND1	164	-29.53	-35.3	0.00384
hsa-miR-744-5p	NM_014629	ARHGEF10	155	-30.6	-34.6	0.023628
hsa-miR-744-5p	NM_014956	CEP164	153	-25.83	-36.4	0.007524
hsa-miR-744-5p	NM_014976	PDCD11	157	-28.02	-32.7	0.020488
hsa-miR-744-5p	NM_015160	PMPCA	154	-31.93	-36.7	0.001617
hsa-miR-744-5p	NM_015175	NBEAL2	154	-25.39	-29.7	0.032084
hsa-miR-744-5p	NM_015383	NBPF14	151	-29.79	-35.9	0.017079
hsa-miR-744-5p	NM_015466	PTPN23	152	-25.63	-29.7	0.016063
hsa-miR-744-5p	NM_015481	ZNF385A	154	-29.98	-33.4	0.029812
hsa-miR-744-5p	NM_015656	KIF26A	168	-31.57	-36.4	0.007635
hsa-miR-744-5p	NM_015720	PODXL2	162	-28.26	-29.9	0.022577
hsa-miR-744-5p	NM_015722	CALY	153	-24.02	-27	0.032835

hsa-miR-744-5p	NM_015831	ACHE	151	-28.87	-31.8	0.048456
hsa-miR-744-5p	NM_016176	SDF4	157	-33.05	-31.8	0.024042
hsa-miR-744-5p	NM_016547	SDF4	157	-33.05	-31.8	0.034387
hsa-miR-744-5p	NM_016564	CEND1	157	-30.9	-36.4	0.00664
hsa-miR-744-5p	NM_016606	REEP2	163	-28.65	-35.2	0.015108
hsa-miR-744-5p	NM_017422	CALML5	157	-30.34	-29.8	0.020523
hsa-miR-744-5p	NM_017491	WDR1	162	-29.27	-34.5	0.016165
hsa-miR-744-5p	NM_017817	RAB20	157	-27.68	-31.5	0.027339
hsa-miR-744-5p	NM_017940	NBPF1	151	-29.79	-35.9	0.01548
hsa-miR-744-5p	NM_018066	GPN2	151	-24.54	-31	0.010583
hsa-miR-744-5p	NM_018410	HJURP	150	-26.02	-34.2	0.014204
hsa-miR-744-5p	NM_018411	HR	167	-28.8	-34.2	0.025287
hsa-miR-744-5p	NM_018645	HES6	151	-26.7	-33.1	0.011446
hsa-miR-744-5p	NM_019034	RHOF	167	-31.53	-37.1	0.011397
hsa-miR-744-5p	NM_020201	NT5M	158	-28.67	-32.2	0.02601
hsa-miR-744-5p	NM_020246	SLC12A9	153	-32.08	-35.7	0.001797
hsa-miR-744-5p	NM_020385	REXO4	151	-26.09	-32.2	0.036396
hsa-miR-744-5p	NM_020655	JPH3	163	-31.46	-34.5	0.027752
hsa-miR-744-5p	NM_020896	OSBPL5	158	-27.31	-35.7	0.010161
hsa-miR-744-5p	NM_021025	TLX3	159	-29.08	-33.1	0.010595
hsa-miR-744-5p	NM_021641	ADAM12	151	-32.03	-35.9	0.00548
hsa-miR-744-5p	NM_021827	CCDC81	150	-29.18	-33.8	0.004359
hsa-miR-744-5p	NM_022119	PRSS22	151	-22.71	-30.9	0.0163
hsa-miR-744-5p	NM_022468	MMP25	159	-28.5	-33.5	0.047742
hsa-miR-744-5p	NM_022478	CDH24	156	-34.99	-40.2	0.000823
hsa-miR-744-5p	NM_022822	KLC2	157	-30.78	-36.6	0.004991
hsa-miR-744-5p	NM_024016	HOXB8	167	-31.02	-35.7	0.00688
hsa-miR-744-5p	NM_024501	HOXD1	154	-32.8	-37.6	0.00235
hsa-miR-744-5p	NM_024602	HECTD3	166	-34.46	-36.5	0.005618
hsa-miR-744-5p	NM_024832	RIN3	161	-28.65	-33.4	0.015596

hsa-miR-744-5p	NM_024845	NAA60	158	-31.81	-37.5	0.009446
hsa-miR-744-5p	NM_025092	ATHL1	151	-30.34	-36.6	0.005321
hsa-miR-744-5p	NM_025237	SOST	151	-27.17	-33.4	0.049224
hsa-miR-744-5p	NM_030649	ACAP3	165	-28.04	-33	0.037984
hsa-miR-744-5p	NM_031313	ALPPL2	158	-27.23	-31.8	0.039869
hsa-miR-744-5p	NM_031471	FERMT3	152	-27.38	-32.5	0.00853
hsa-miR-744-5p	NM_032137	C3orf20	150	-27.03	-31.8	0.002078
hsa-miR-744-5p	NM_032431	SYVN1	157	-29.76	-32.8	0.037662
hsa-miR-744-5p	NM_032532	FNDC1	152	-25.92	-31	0.040637
hsa-miR-744-5p	NM_032595	PPP1R9B	156	-34.27	-41.4	0.00155
hsa-miR-744-5p	NM_032799	ZDHHC12	157	-26.36	-29.6	0.02376
hsa-miR-744-5p	NM_032809	FAM73B	172	-31.17	-36.9	0.011099
hsa-miR-744-5p	NM_032989	BAD	155	-26.52	-33.5	0.004759
hsa-miR-744-5p	NM_032995	ARHGEF4	153	-32.33	-39.8	0.003487
hsa-miR-744-5p	NM_033131	WNT3A	162	-30.13	-36.5	0.015505
hsa-miR-744-5p	NM_033259	CAMK2N2	151	-25.31	-36.4	0.005859
hsa-miR-744-5p	NM_033282	OPN4	153	-26.84	-32	0.028047
hsa-miR-744-5p	NM_033400	ZFHX2	161	-28.22	-32.4	0.045144
hsa-miR-744-5p	NM_033448	KRT71	156	-26.69	-31.8	0.025244
hsa-miR-744-5p	NM_052837	SCAMP3	155	-33.99	-38.7	0.000197
hsa-miR-744-5p	NM_052838	SEPT1	164	-29.9	-28.4	0.041329
hsa-miR-744-5p	NM_053044	HTRA3	152	-29.66	-34.9	0.012242
hsa-miR-744-5p	NM_080388	S100A16	155	-26.1	-33.1	0.014394
hsa-miR-744-5p	NM_080861	SPSB3	161	-29.05	-34.1	0.003593
hsa-miR-744-5p	NM_130465	TSPAN17	158	-28.05	-34	0.030084
hsa-miR-744-5p	NM_130799	MEN1	162	-24.66	-32.3	0.030543
hsa-miR-744-5p	NM_130800	MEN1	162	-24.66	-32.3	0.030543
hsa-miR-744-5p	NM_130801	MEN1	162	-24.66	-32.3	0.030543
hsa-miR-744-5p	NM_130802	MEN1	162	-24.66	-32.3	0.030543
hsa-miR-744-5p	NM_130803	MEN1	162	-24.66	-32.3	0.030543

hsa-miR-744-5p	NM_130804	MEN1	162	-24.66	-32.3	0.030543
hsa-miR-744-5p	NM_138350	THAP3	151	-29.42	-38.2	0.005601
hsa-miR-744-5p	NM_138361	LRSAM1	156	-29.99	-35.7	0.003742
hsa-miR-744-5p	NM_139179	DAGLB	155	-28.34	-33.8	0.012495
hsa-miR-744-5p	NM_139343	BIN1	158	-29.31	-34.8	0.004173
hsa-miR-744-5p	NM_139344	BIN1	158	-29.31	-34.8	0.004173
hsa-miR-744-5p	NM_139345	BIN1	158	-29.31	-34.8	0.004173
hsa-miR-744-5p	NM_139346	BIN1	158	-29.31	-34.8	0.004173
hsa-miR-744-5p	NM_139347	BIN1	158	-29.31	-34.8	0.004173
hsa-miR-744-5p	NM_139348	BIN1	158	-29.31	-34.8	0.004173
hsa-miR-744-5p	NM_139349	BIN1	158	-29.31	-34.8	0.004173
hsa-miR-744-5p	NM_139350	BIN1	158	-29.31	-34.8	0.004173
hsa-miR-744-5p	NM_139351	BIN1	158	-29.31	-34.8	0.004173
hsa-miR-744-5p	NM_144699	ATP1A4	160	-29.16	-34.9	0.001252
hsa-miR-744-5p	NM_144985	CDH24	156	-34.99	-40.2	0.000823
hsa-miR-744-5p	NM_145255	MRPL10	152	-29.18	-33.2	0.024661
hsa-miR-744-5p	NM_145270	PRR35	153	-25.95	-29	0.017953
hsa-miR-744-5p	NM_145638	OSBPL5	158	-27.31	-35.7	0.010161
hsa-miR-744-5p	NM_145798	OSBPL7	150	-24.34	-32.1	0.039882
hsa-miR-744-5p	NM_148887	MRPL10	152	-29.18	-33.2	0.024661
hsa-miR-744-5p	NM_152421	FAM69B	159	-27.1	-36.2	0.00644
hsa-miR-744-5p	NM_152653	UBE2E2	152	-28.49	-33.3	0.025152
hsa-miR-744-5p	NM_153254	TTLL10	156	-29.47	-31.7	0.033128
hsa-miR-744-5p	NM_153265	EML3	154	-28.76	-35.1	0.000947
hsa-miR-744-5p	NM_153266	TMEM151A	152	-28.19	-34.8	0.013538
hsa-miR-744-5p	NM_172230	SYVN1	157	-29.76	-32.8	0.037662
hsa-miR-744-5p	NM_173086	KRT6C	164	-28.84	-31.8	0.020455
hsa-miR-744-5p	NM_173528	CFAP161	159	-32.3	-37.4	0.001874
hsa-miR-744-5p	NM_173587	RCOR2	162	-28.25	-30.8	0.042402
hsa-miR-744-5p	NM_173638	NBPF15	151	-29.79	-35.9	0.017188

hsa-miR-744-5p	NM_173841	IL1RN	150	-27.92	-32.7	0.041508
hsa-miR-744-5p	NM_173842	IL1RN	150	-27.92	-32.7	0.041508
hsa-miR-744-5p	NM_173843	IL1RN	150	-27.92	-32.7	0.041508
hsa-miR-744-5p	NM_174920	SAMD14	150	-30.78	-35.7	0.018007
hsa-miR-744-5p	NM_174953	ATP2A3	158	-34.27	-35.9	0.016046
hsa-miR-744-5p	NM_174954	ATP2A3	158	-34.27	-35.9	0.016215
hsa-miR-744-5p	NM_174955	ATP2A3	158	-34.27	-35.9	0.015374
hsa-miR-744-5p	NM_174956	ATP2A3	158	-34.27	-35.9	0.016215
hsa-miR-744-5p	NM_174957	ATP2A3	158	-34.27	-35.9	0.016046
hsa-miR-744-5p	NM_174958	ATP2A3	158	-34.27	-35.9	0.007718
hsa-miR-744-5p	NM_178432	CDK20	150	-33.91	-39.4	0.001712
hsa-miR-744-5p	NM_178443	FERMT3	152	-27.38	-32.5	0.00853
hsa-miR-744-5p	NM_178536	LCN12	152	-29.5	-27.2	0.010026
hsa-miR-744-5p	NM_178568	RTN4RL1	156	-33.05	-37.9	0.008633
hsa-miR-744-5p	NM_181336	LEMD2	151	-28.58	-33.1	0.046881
hsa-miR-744-5p	NM_181843	NUDT8	163	-24.41	-29.9	0.026978
hsa-miR-744-5p	NM_182528	C1QL2	152	-27.54	-31	0.031087
hsa-miR-744-5p	NM_182632	SLC6A18	155	-22.32	-28.6	0.005752
hsa-miR-744-5p	NM_182791	CCDC33	156	-28.52	-33.9	0.019048
hsa-miR-744-5p	NM_182923	KLC1	158	-27.29	-30.6	0.017719
hsa-miR-744-5p	NM_183372	NBPF11	151	-29.79	-35.9	0.02071
hsa-miR-744-5p	NM_198427	BCAN	170	-29.73	-32.4	0.013518
hsa-miR-744-5p	NM_198527	HDDC3	154	-27.1	-30.8	0.040536
hsa-miR-744-5p	NM_198576	AGRN	154	-31.3	-33.6	0.027681
hsa-miR-744-5p	NM_199282	ARHGAP27	152	-33.97	-39.6	0.003235
hsa-miR-744-5p	NM_199330	HOMER2	159	-27.57	-35.7	0.005352
hsa-miR-744-5p	NM_199424	WWP2	162	-33.86	-38.7	0.006041
hsa-miR-744-5p	NM_203400	RPRML	157	-25.43	-30.6	0.030488
hsa-miR-744-5p	NM_206918	DEGS2	154	-27.31	-28.9	0.040121
hsa-miR-744-5p	NM_206967	C16orf74	161	-26.52	-31.6	0.018873

hsa-miR-744-5p	NM_207334	FAM43B	163	-26.43	-32.4	0.042281
hsa-miR-744-5p	NM_207401	C1orf229	154	-27.79	-32.9	0.047937
hsa-miR-744-5p	NM_212539	PRKCD	156	-25.35	-29.5	0.04563
hsa-miR-744-5p	NM_213726	INCA1	159	-27.22	-30.9	0.009801
hsa-miR-744-5p	NR_002330	ST7-AS1	156	-28.85	-34.7	0.03533
hsa-miR-744-5p	NR_002556	LOC388242	171	-37.06	-41.8	0.001141
hsa-miR-744-5p	NR_002557	LOC613038	171	-37.06	-41.8	0.001141
hsa-miR-744-5p	NR_002837	UBE2MP1	153	-28.92	-33.7	0.020584
hsa-miR-744-5p	NR_026642	FAM99B	150	-27.91	-32.5	0.040946
hsa-miR-744-5p	NR_026901	LOC644172	154	-28.92	-34.6	0.033454
hsa-miR-744-5p	NR_027423	FAM66B	151	-31.71	-36.7	0.01003
hsa-miR-744-5p	NR_028349	LOC100287834	159	-30.07	-34.5	0.007607
hsa-miR-744-5p	NR_029612	MIR181B1	151	-22.09	-26.8	0.016017
hsa-miR-744-5p	NR_029625	MIR212	156	-25.51	-29.9	0.00265
hsa-miR-744-5p	NR_037694	LOC100286922	153	-30.72	-37.5	0.004126
hsa-miR-744-5p	NR_037695	LOC100286922	153	-30.72	-37.5	0.002757
hsa-miR-744-5p	NR_037696	LOC100286922	153	-30.72	-37.5	0.001977
hsa-miR-744-5p	NR_038853	STX17-AS1	154	-26.39	-32.4	0.015626
hsa-miR-744-5p	NR_039736	MIR4511	150	-25.95	-27.8	0.00544
hsa-miR-744-5p	NR_045039	SMUG1	151	-26.15	-31.9	0.031617
hsa-miR-744-5p	NR_046205	RBPMS-AS1	155	-26.8	-33.2	0.023708
hsa-miR-744-5p	NR_046289	ATP2A1-AS1	156	-22.54	-30.1	0.044006
hsa-miR-744-5p	NR_046290	ATP2A1-AS1	156	-22.54	-30.1	0.043627
hsa-miR-744-5p	NR_046411	POLD4	167	-31.42	-33.7	0.048858
hsa-miR-744-5p	NR_046412	POLD4	167	-31.42	-33.7	0.046265
hsa-miR-744-5p	NR_046413	POLD4	167	-31.42	-36.2	0.013582
hsa-miR-744-5p	NR_046696	IPO9-AS1	156	-29.95	-34.6	0.0111
hsa-miR-744-5p	NR_047040	LINC00424	150	-25.88	-29.7	0.04172
hsa-miR-744-5p	NR_047504	HOXC-AS1	152	-31.01	-36.9	0.001769
hsa-miR-744-5p	NR_073388	ALG1L9P	151	-31.71	-32.1	0.045063

hsa-miR-744-5p	NR_073484	CDK2AP2	151	-28.87	-32.9	0.043136
hsa-miR-744-5p	NR_073542	MOK	155	-24.72	-32.5	0.047987
hsa-miR-744-5p	NR_073543	MOK	155	-24.72	-32.5	0.045921
hsa-miR-744-5p	NR_074074	NOB1	168	-30.49	-33.7	0.044569
hsa-miR-744-5p	NR_102427	CIB1	153	-24.22	-32.3	0.040884
hsa-miR-744-5p	NR_103466	NBPF13P	151	-28.72	-34.8	0.016501
hsa-miR-744-5p	NR_104174	TUSC8	151	-27.34	-33.7	0.024408
hsa-miR-744-5p	NR_104586	ANKRD10	159	-26.69	-33.9	0.030496
hsa-miR-744-5p	NR_104598	EXD3	155	-29.61	-36	0.014344
hsa-miR-744-5p	NR_104599	EXD3	155	-29.61	-33.6	0.048063
hsa-miR-744-5p	NR_106895	MIR6836	153	-27.65	-30.3	0.000539
hsa-miR-744-5p	NR_108105	LOC100507468	155	-27.21	-32.3	0.016686
hsa-miR-744-5p	NR_109991	B3GAT3	151	-30.15	-34.8	0.027623
hsa-miR-744-5p	NR_110056	ERI3-IT1	152	-26.8	-33.4	0.008099
hsa-miR-744-5p	NR_110901	GLIS2-AS1	156	-30.35	-32.4	0.007415
hsa-miR-744-5p	NR_120508	ELFN1-AS1	150	-28.05	-33.7	0.022065
hsa-miR-744-5p	NR_120509	ELFN1-AS1	150	-28.05	-33.7	0.015779
hsa-miR-744-5p	NR_120510	ELFN1-AS1	150	-28.05	-33.7	0.011724
hsa-miR-744-5p	NR_122036	ARRDC1-AS1	155	-29.58	-32.6	0.045243
hsa-miR-744-5p	NR_125749	TBX2-AS1	160	-27.36	-34	0.015172
hsa-miR-744-5p	NR_125997	LOC102724450	154	-25.83	-29.1	0.042053
hsa-miR-744-5p	NR_126098	EDN2	154	-25.89	-34.8	0.016786
hsa-miR-744-5p	NR_126423	SAPCD1-AS1	155	-23.24	-30.5	0.047728
hsa-miR-744-5p	NR_130715	C3orf22	150	-32.49	-36.3	0.016541
hsa-miR-744-5p	NR_132318	SHH	161	-30.33	-34.1	0.016686
hsa-miR-744-5p	NR_132319	SHH	161	-30.33	-34.1	0.014788
hsa-miR-374b-5p	NR_030785	MIR374A	168	-20.24	-24.1	0.009497
hsa-miR-374b-3p	NM_001257329	ARRB2	157	-26.34	-28.5	0.032723
hsa-miR-374b-3p	NR_030620	MIR374B	190	-29.27	-33.1	0.000019
hsa-miR-374b-3p	NR_120422	LOC101928730	167	-27.55	-33.4	0.018671

hsa-miR-941	NM_000727	CACNG1	152	-28.77	-32.9	0.013736
hsa-miR-941	NM_001001349	NKIRAS2	153	-30.74	-34.7	0.037671
hsa-miR-941	NM_001010938	TNK2	165	-28.33	-31.8	0.045316
hsa-miR-941	NM_001105540	DGKZ	154	-31.77	-33.6	0.012597
hsa-miR-941	NM_001142807	ACOXL	162	-34.43	-34.6	0.003861
hsa-miR-941	NM_001144927	NKIRAS2	153	-30.74	-34.7	0.037671
hsa-miR-941	NM_001144928	NKIRAS2	153	-30.74	-34.7	0.037671
hsa-miR-941	NM_001144929	NKIRAS2	153	-30.74	-34.7	0.043146
hsa-miR-941	NM_001145348	KDM8	152	-35.66	-38.7	0.003022
hsa-miR-941	NM_001145657	RAP1GAP	152	-25.26	-33.7	0.02951
hsa-miR-941	NM_001145658	RAP1GAP	152	-25.26	-33.7	0.02951
hsa-miR-941	NM_001161465	JMJD4	161	-29.55	-35.5	0.015701
hsa-miR-941	NM_001170690	SCUBE2	165	-30.29	-36.1	0.017604
hsa-miR-941	NM_001199266	DGKZ	154	-31.77	-33.6	0.012597
hsa-miR-941	NM_001199267	DGKZ	154	-31.77	-33.6	0.012597
hsa-miR-941	NM_001199268	DGKZ	154	-31.77	-33.6	0.012597
hsa-miR-941	NM_001242786	BRF1	155	-29.2	-35.7	0.015529
hsa-miR-941	NM_001242787	BRF1	155	-29.2	-35.7	0.015529
hsa-miR-941	NM_001242788	BRF1	155	-29.2	-35.7	0.015529
hsa-miR-941	NM_001242789	BRF1	155	-29.2	-35.7	0.015529
hsa-miR-941	NM_001242837	AP2A2	155	-30.36	-36.9	0.013445
hsa-miR-941	NM_001270987	LTBR	152	-27	-33.7	0.012912
hsa-miR-941	NM_001282062	DOC2A	161	-26.44	-33.1	0.018725
hsa-miR-941	NM_001282063	DOC2A	161	-26.44	-33.1	0.018725
hsa-miR-941	NM_001282068	DOC2A	161	-26.44	-33.1	0.018725
hsa-miR-941	NM_001303473	GPR146	151	-30.99	-34.1	0.014148
hsa-miR-941	NM_001303474	GPR146	151	-30.99	-34.1	0.014148
hsa-miR-941	NM_001308046	TNK2	165	-28.33	-31.8	0.045396
hsa-miR-941	NM_001312660	GSTZ1	151	-27.06	-29.8	0.039452
hsa-miR-941	NM_001347	DGKQ	163	-31.61	-36.2	0.019983

hsa-miR-941	NM_001519	BRF1	155	-29.2	-35.7	0.015529
hsa-miR-941	NM_001606	ABCA2	150	-27.35	-32.9	0.021645
hsa-miR-941	NM_002115	HK3	150	-21.65	-28.3	0.027175
hsa-miR-941	NM_002342	LTBR	152	-27	-33.7	0.012912
hsa-miR-941	NM_002452	NUDT1	165	-29.18	-36.9	0.000123
hsa-miR-941	NM_002477	MYL5	157	-21.11	-25.4	0.003406
hsa-miR-941	NM_002885	RAP1GAP	152	-25.26	-33.7	0.02951
hsa-miR-941	NM_003170	SUPT6H	167	-31.17	-32.4	0.021993
hsa-miR-941	NM_003586	DOC2A	161	-26.44	-33.1	0.018725
hsa-miR-941	NM_003646	DGKZ	154	-31.77	-33.6	0.012597
hsa-miR-941	NM_004045	ATOX1	158	-27.09	-31.4	0.004309
hsa-miR-941	NM_005568	LHX1	173	-36.85	-38	0.007359
hsa-miR-941	NM_005781	TNK2	165	-28.33	-31.8	0.045316
hsa-miR-941	NM_012305	AP2A2	155	-30.36	-36.9	0.013445
hsa-miR-941	NM_015944	AMDHD2	152	-25.95	-33.3	0.001275
hsa-miR-941	NM_016321	RHCG	164	-24.01	-31.5	0.022463
hsa-miR-941	NM_016518	PIPOX	150	-23.3	-32.4	0.040815
hsa-miR-941	NM_017595	NKIRAS2	153	-30.74	-34.7	0.037671
hsa-miR-941	NM_018992	KCTD5	160	-31.39	-35.8	0.023016
hsa-miR-941	NM_020397	CAMK1D	152	-22.97	-35.4	0.007579
hsa-miR-941	NM_020974	SCUBE2	165	-30.29	-36.1	0.017604
hsa-miR-941	NM_023007	JMJD4	161	-29.55	-35.5	0.015701
hsa-miR-941	NM_024533	CHST5	169	-30.75	-34.2	0.00983
hsa-miR-941	NM_024773	KDM8	152	-35.66	-38.7	0.003022
hsa-miR-941	NM_025264	THUMPD2	161	-27.02	-31	0.049332
hsa-miR-941	NM_078471	MYO18A	157	-26.41	-33.5	0.039884
hsa-miR-941	NM_133640	MED22	153	-25.97	-31	0.041861
hsa-miR-941	NM_138445	GPR146	151	-30.99	-34.1	0.014148
hsa-miR-941	NM_144719	CCDC13	155	-26.35	-32.1	0.024765
hsa-miR-941	NM_145685	BRF1	155	-29.2	-35.7	0.015529

hsa-miR-941	NM_145870	GSTZ1	151	-27.06	-29.8	0.039452
hsa-miR-941	NM_145871	GSTZ1	151	-27.06	-29.8	0.039452
hsa-miR-941	NM_152635	OIT3	165	-27.16	-30.7	0.037453
hsa-miR-941	NM_173555	TYSND1	154	-32.89	-35	0.038486
hsa-miR-941	NM_198948	NUDT1	165	-29.18	-36.9	0.000123
hsa-miR-941	NM_198949	NUDT1	165	-29.18	-36.9	0.000123
hsa-miR-941	NM_198950	NUDT1	165	-29.18	-36.9	0.000123
hsa-miR-941	NM_198952	NUDT1	165	-29.18	-36.9	0.000123
hsa-miR-941	NM_198953	NUDT1	165	-29.18	-36.9	0.000123
hsa-miR-941	NM_198954	NUDT1	165	-29.18	-36.9	0.000123
hsa-miR-941	NM_201532	DGKZ	154	-31.77	-33.6	0.012597
hsa-miR-941	NM_201533	DGKZ	154	-31.77	-33.6	0.012597
hsa-miR-941	NM_203318	MYO18A	157	-26.41	-33.5	0.039884
hsa-miR-941	NM_212533	ABCA2	150	-27.35	-32.9	0.021645
hsa-miR-941	NR_024127	SNHG12	156	-26.24	-31.9	0.037484
hsa-miR-941	NR_030318	MIR589	153	-24.99	-33.1	0.000415
hsa-miR-941	NR_036239	MIR4281	155	-24.97	-29.7	0.000984
hsa-miR-941	NR_038923	SSSCA1-AS1	154	-24.24	-32.9	0.047863
hsa-miR-941	NR_039821	MIR4674	154	-26.04	-27.3	0.009177
hsa-miR-941	NR_046415	LOC649352	152	-29.61	-33.5	0.010296
hsa-miR-941	NR_108027	LINC01484	150	-28.4	-31.9	0.025214
hsa-miR-941	NR_110213	LOC101927142	161	-29.52	-34.9	0.01022
hsa-miR-941	NR_125435	CIB2	154	-31.13	-34	0.040197
hsa-miR-548k	NM_001270481	PSMB6	170	-23.45	-28.6	0.004312
hsa-miR-548k	NM_018164	ASUN	167	-24.34	-27	0.032587
hsa-miR-548k	NR_029848	MIR361	167	-16.92	-21.9	0.047709
hsa-miR-548l	NM_033219	TRIM14	155	-21.45	-26.9	0.006882
hsa-miR-548l	NM_138387	G6PC3	176	-20.43	-26	0.048081
hsa-miR-1304-3p	NM_000048	ASL	173	-32.33	-34.7	0.002908
hsa-miR-1304-3p	NM_000075	CDK4	151	-29.77	-33.8	0.013234

hsa-miR-1304-3p	NM_000085	CLCNKB	150	-27.48	-30	0.02789
hsa-miR-1304-3p	NM_000098	CPT2	171	-32.24	-34.8	0.005123
hsa-miR-1304-3p	NM_000260	MYO7A	157	-26.77	-31.1	0.025794
hsa-miR-1304-3p	NM_000379	XDH	182	-33.52	-41.8	0.001196
hsa-miR-1304-3p	NM_000569	FCGR3A	151	-17.1	-33.7	0.027548
hsa-miR-1304-3p	NM_000637	GSR	173	-31.62	-36.3	0.011005
hsa-miR-1304-3p	NM_000747	CHRNB1	167	-31.03	-34.7	0.010402
hsa-miR-1304-3p	NM_000890	KCNJ5	171	-32.24	-34.6	0.020149
hsa-miR-1304-3p	NM_001001413	GOLGA6L1	167	-30.42	-34.2	0.034973
hsa-miR-1304-3p	NM_001001661	ZNF425	171	-32.58	-35.6	0.006058
hsa-miR-1304-3p	NM_001001671	MAP3K15	179	-32.33	-37.1	0.002185
hsa-miR-1304-3p	NM_001002251	ARL6IP4	151	-24.79	-28.4	0.025022
hsa-miR-1304-3p	NM_001002252	ARL6IP4	151	-24.79	-28.4	0.04301
hsa-miR-1304-3p	NM_001003941	OGDH	171	-32.24	-34.7	0.002166
hsa-miR-1304-3p	NM_001004339	ZYG11A	167	-30.42	-33.2	0.049124
hsa-miR-1304-3p	NM_001007169	ZNF483	183	-37.9	-34.7	0.021581
hsa-miR-1304-3p	NM_001007214	CACYBP	179	-30.24	-34.4	0.037994
hsa-miR-1304-3p	NM_001007240	GP2	176	-37.97	-38.6	0.004889
hsa-miR-1304-3p	NM_001007241	GP2	176	-37.97	-38.6	0.004889
hsa-miR-1304-3p	NM_001007242	GP2	176	-37.97	-38.6	0.004889
hsa-miR-1304-3p	NM_001008228	MOG	167	-30.42	-33.2	0.031846
hsa-miR-1304-3p	NM_001008229	MOG	167	-30.42	-33.2	0.021896
hsa-miR-1304-3p	NM_001008274	TRIM72	171	-32.24	-34.8	0.002816
hsa-miR-1304-3p	NM_001010978	LDLRAD1	175	-30.9	-33.9	0.038484
hsa-miR-1304-3p	NM_001013703	EIF2AK4	182	-33.52	-38	0.000797
hsa-miR-1304-3p	NM_001014342	FLG2	167	-30.42	-34.5	0.034518
hsa-miR-1304-3p	NM_001017405	MAEA	150	-33.31	-34.8	0.011578
hsa-miR-1304-3p	NM_001017967	MARVELD3	167	-30.42	-34.2	0.014234
hsa-miR-1304-3p	NM_001018055	BRCC3	171	-36.12	-39.2	0.004608
hsa-miR-1304-3p	NM_001024074	HNMT	179	-31.04	-34.1	0.002349

hsa-miR-1304-3p	NM_001024215	FBLIM1	159	-24.34	-27.3	0.030235
hsa-miR-1304-3p	NM_001024216	FBLIM1	167	-30.66	-34.5	0.035916
hsa-miR-1304-3p	NM_001024674	LIN52	171	-32.24	-34.7	0.021736
hsa-miR-1304-3p	NM_001024844	CD82	167	-31.73	-34.2	0.007578
hsa-miR-1304-3p	NM_001024943	ASL	173	-32.33	-34.7	0.002908
hsa-miR-1304-3p	NM_001024944	ASL	173	-32.33	-34.7	0.002908
hsa-miR-1304-3p	NM_001024946	ASL	173	-32.33	-34.7	0.002908
hsa-miR-1304-3p	NM_001025242	IRAK1	183	-38.51	-40.8	0.001332
hsa-miR-1304-3p	NM_001025243	IRAK1	183	-38.51	-40.8	0.001332
hsa-miR-1304-3p	NM_001031683	IFIT3	167	-28.56	-31.2	0.047479
hsa-miR-1304-3p	NM_001031800	TIPRL	159	-24.13	-28.2	0.014709
hsa-miR-1304-3p	NM_001033113	ENTPD8	150	-28.89	-31.2	0.024943
hsa-miR-1304-3p	NM_001033553	SPECC1	191	-39.33	-42	0.000214
hsa-miR-1304-3p	NM_001033555	SPECC1	191	-39.33	-42	0.000214
hsa-miR-1304-3p	NM_001039211	ATAD3C	167	-30.42	-34.2	0.032289
hsa-miR-1304-3p	NM_001040059	CD68	171	-32.24	-34.8	0.005093
hsa-miR-1304-3p	NM_001040431	COA3	167	-29.7	-31.3	0.016656
hsa-miR-1304-3p	NM_001040436	YARS2	171	-27.83	-31.6	0.027476
hsa-miR-1304-3p	NM_001042467	MLPH	178	-32.59	-36.9	0.010973
hsa-miR-1304-3p	NM_001042692	WDPCP	187	-32.84	-36.1	0.002772
hsa-miR-1304-3p	NM_001042704	CLCNKA	150	-27.48	-30	0.025908
hsa-miR-1304-3p	NM_001079839	OCIAD1	180	-30.21	-34	0.01868
hsa-miR-1304-3p	NM_001079840	OCIAD1	180	-30.21	-34	0.01868
hsa-miR-1304-3p	NM_001079841	OCIAD1	180	-30.21	-34	0.020484
hsa-miR-1304-3p	NM_001079842	OCIAD1	180	-30.21	-34	0.01868
hsa-miR-1304-3p	NM_001080779	MYO1C	169	-27.51	-34.7	0.024039
hsa-miR-1304-3p	NM_001080950	MYO1C	169	-27.51	-34.7	0.024039
hsa-miR-1304-3p	NM_001081	CUBN	167	-30.42	-33.2	0.025062
hsa-miR-1304-3p	NM_001083946	NDUFAF7	155	-31.59	-33.9	0.012966
hsa-miR-1304-3p	NM_001098728	GTF2H2C	167	-29.35	-32.1	0.024016

hsa-miR-1304-3p	NM_001098786	HILPDA	168	-33.36	-36.1	0.006165
hsa-miR-1304-3p	NM_001099	ACPP	187	-38.41	-39.9	0.003702
hsa-miR-1304-3p	NM_001100592	ATP6V0E2	152	-29.4	-33.2	0.026035
hsa-miR-1304-3p	NM_001100598	ZNF707	162	-28.74	-32.3	0.029482
hsa-miR-1304-3p	NM_001100599	ZNF707	162	-28.74	-32.3	0.029482
hsa-miR-1304-3p	NM_001102416	KNG1	167	-29.35	-33.4	0.008826
hsa-miR-1304-3p	NM_001105206	LAMA4	183	-32.33	-36.1	0.012636
hsa-miR-1304-3p	NM_001105207	LAMA4	183	-32.33	-36.1	0.012636
hsa-miR-1304-3p	NM_001105248	TMC5	167	-32.2	-35.7	0.009876
hsa-miR-1304-3p	NM_001105249	TMC5	167	-32.2	-35.7	0.009876
hsa-miR-1304-3p	NM_001111038	FBXL13	167	-27.47	-30.1	0.034316
hsa-miR-1304-3p	NM_001112706	SCIN	170	-29.38	-33	0.022654
hsa-miR-1304-3p	NM_001114380	ITGAL	171	-32.24	-34.6	0.024636
hsa-miR-1304-3p	NM_001122837	SLC50A1	171	-32.24	-34.8	0.004752
hsa-miR-1304-3p	NM_001122839	SLC50A1	171	-32.24	-34.8	0.004752
hsa-miR-1304-3p	NM_001127180	MYO7A	157	-26.77	-31.1	0.025794
hsa-miR-1304-3p	NM_001127460	PAN2	167	-29.35	-33.4	0.036398
hsa-miR-1304-3p	NM_001127592	FCGR3A	151	-17.1	-33.7	0.027548
hsa-miR-1304-3p	NM_001127593	FCGR3A	151	-17.1	-33.7	0.027548
hsa-miR-1304-3p	NM_001127595	FCGR3A	151	-17.1	-33.7	0.027548
hsa-miR-1304-3p	NM_001127596	FCGR3A	151	-17.1	-33.7	0.027548
hsa-miR-1304-3p	NM_001130037	ELMOD1	175	-32.75	-34.7	0.024989
hsa-miR-1304-3p	NM_001131028	ATG10	167	-30.42	-33.2	0.037591
hsa-miR-1304-3p	NM_001135721	PIWIL2	171	-32.24	-34.7	0.00482
hsa-miR-1304-3p	NM_001137552	LRRFIP1	183	-32.33	-36.1	0.01615
hsa-miR-1304-3p	NM_001137553	LRRFIP1	183	-32.33	-36.1	0.01615
hsa-miR-1304-3p	NM_001142594	ITPK1	163	-30.86	-33.2	0.047118
hsa-miR-1304-3p	NM_001142674	CHID1	182	-33.52	-36	0.020007
hsa-miR-1304-3p	NM_001142675	CHID1	182	-33.52	-36	0.020007
hsa-miR-1304-3p	NM_001142676	CHID1	182	-33.52	-36	0.020007

hsa-miR-1304-3p	NM_001142677	CHID1	182	-33.52	-36	0.020007
hsa-miR-1304-3p	NM_001142684	ZMYM5	167	-30.42	-33.2	0.024504
hsa-miR-1304-3p	NM_001143905	C12orf65	167	-29.35	-33.1	0.023146
hsa-miR-1304-3p	NM_001143935	CROT	174	-28.91	-34.2	0.019516
hsa-miR-1304-3p	NM_001144037	TMEM25	182	-33.52	-36	0.001667
hsa-miR-1304-3p	NM_001144038	TMEM25	182	-33.52	-36	0.001667
hsa-miR-1304-3p	NM_001144913	FGFR2	167	-29.35	-35.7	0.000967
hsa-miR-1304-3p	NM_001144919	FGFR2	167	-29.35	-35.7	0.000967
hsa-miR-1304-3p	NM_001144929	NKIRAS2	171	-29.63	-34.2	0.040816
hsa-miR-1304-3p	NM_001161009	ARMC10	167	-30.42	-34.5	0.018384
hsa-miR-1304-3p	NM_001161010	ARMC10	167	-30.42	-34.5	0.018384
hsa-miR-1304-3p	NM_001161011	ARMC10	167	-30.42	-34.5	0.018384
hsa-miR-1304-3p	NM_001161012	ARMC10	167	-30.42	-34.5	0.018384
hsa-miR-1304-3p	NM_001161013	ARMC10	167	-30.42	-34.5	0.018384
hsa-miR-1304-3p	NM_001162900	TMEM217	171	-32.24	-34.6	0.008777
hsa-miR-1304-3p	NM_001163941	ABCB5	167	-30.42	-33.2	0.039868
hsa-miR-1304-3p	NM_001164720	CC2D2A	171	-32.24	-34.6	0.012664
hsa-miR-1304-3p	NM_001165945	CLCNKB	150	-27.48	-30	0.02789
hsa-miR-1304-3p	NM_001166279	PAN2	167	-29.35	-33.4	0.036398
hsa-miR-1304-3p	NM_001167970	TRMT2B	171	-32.24	-34.6	0.018157
hsa-miR-1304-3p	NM_001167971	TRMT2B	171	-32.24	-34.6	0.018157
hsa-miR-1304-3p	NM_001167972	TRMT2B	171	-32.24	-34.6	0.018157
hsa-miR-1304-3p	NM_001168254	OCIAD1	180	-30.21	-34	0.01868
hsa-miR-1304-3p	NM_001168298	CXCR2	167	-30.42	-34.5	0.022097
hsa-miR-1304-3p	NM_001170418	MOG	167	-30.42	-33.2	0.031846
hsa-miR-1304-3p	NM_001170543	PGAM5	175	-29.99	-36.6	0.014506
hsa-miR-1304-3p	NM_001170544	PGAM5	175	-29.99	-36.6	0.014506
hsa-miR-1304-3p	NM_001170553	VSIG1	171	-32.24	-34.8	0.02882
hsa-miR-1304-3p	NM_001170569	CXorf56	171	-32.24	-34.7	0.024898
hsa-miR-1304-3p	NM_001170570	CXorf56	171	-32.24	-34.7	0.024898

hsa-miR-1304-3p	NM_001171930	CDH23	180	-30.97	-36.1	0.000725
hsa-miR-1304-3p	NM_001173431	OBSL1	170	-32.04	-36.1	0.00501
hsa-miR-1304-3p	NM_001178005	BHMT2	167	-30.42	-34.2	0.028756
hsa-miR-1304-3p	NM_001178044	SLC44A4	167	-30.42	-34.5	0.003271
hsa-miR-1304-3p	NM_001178045	SLC44A4	167	-30.42	-34.5	0.003271
hsa-miR-1304-3p	NM_001185092	NIT1	183	-32.33	-36.1	0.009952
hsa-miR-1304-3p	NM_001190707	ALS2CL	184	-37.38	-38.7	0.00629
hsa-miR-1304-3p	NM_001193480	ALDH8A1	182	-32.73	-38	0.002894
hsa-miR-1304-3p	NM_001194995	C12orf65	167	-29.35	-33.1	0.023146
hsa-miR-1304-3p	NM_001195102	GSR	173	-31.62	-36.3	0.011005
hsa-miR-1304-3p	NM_001195103	GSR	173	-31.62	-36.3	0.011005
hsa-miR-1304-3p	NM_001195104	GSR	173	-31.62	-36.3	0.011005
hsa-miR-1304-3p	NM_001195131	TSTD3	167	-27.5	-32.9	0.048719
hsa-miR-1304-3p	NM_001199492	PDCD4	167	-31.03	-34.4	0.037199
hsa-miR-1304-3p	NM_001199787	SLC35E2	172	-32.38	-35.9	0.002782
hsa-miR-1304-3p	NM_001199972	RPL36A	171	-32.58	-36.6	0.015461
hsa-miR-1304-3p	NM_001201536	TAF1A	171	-32.24	-34.7	0.002582
hsa-miR-1304-3p	NM_001205296	TTF1	171	-32.24	-34.8	0.002026
hsa-miR-1304-3p	NM_001206673	ABHD12B	178	-27.86	-30.8	0.034967
hsa-miR-1304-3p	NM_001214906	ZNF48	187	-34.68	-37.1	0.004191
hsa-miR-1304-3p	NM_001214907	ZNF48	187	-34.68	-37.1	0.004191
hsa-miR-1304-3p	NM_001214909	ZNF48	187	-34.68	-37.1	0.004191
hsa-miR-1304-3p	NM_001226	CASP6	159	-33.75	-36.5	0.002982
hsa-miR-1304-3p	NM_001242640	BRCC3	171	-36.12	-39.2	0.004608
hsa-miR-1304-3p	NM_001242929	ANKS3	155	-30.71	-30.8	0.01782
hsa-miR-1304-3p	NM_001243194	INPP5F	179	-30.48	-32.8	0.046484
hsa-miR-1304-3p	NM_001243439	SPECC1	191	-39.33	-42	0.000214
hsa-miR-1304-3p	NM_001243526	HYI	158	-25.02	-31.7	0.007103
hsa-miR-1304-3p	NM_001243778	FAM213A	155	-31.59	-33.9	0.01525
hsa-miR-1304-3p	NM_001243779	FAM213A	155	-31.59	-33.9	0.01525

hsa-miR-1304-3p	NM_001243780	FAM213A	155	-31.59	-33.9	0.01525
hsa-miR-1304-3p	NM_001243781	FAM213A	155	-31.59	-33.9	0.01525
hsa-miR-1304-3p	NM_001243782	FAM213A	155	-31.59	-33.9	0.01525
hsa-miR-1304-3p	NM_001244705	CSAD	168	-35.54	-38.2	0.00189
hsa-miR-1304-3p	NM_001244706	CSAD	168	-35.54	-38.2	0.00189
hsa-miR-1304-3p	NM_001244926	PRPF4	167	-30.42	-34.2	0.021922
hsa-miR-1304-3p	NM_001251	CD68	171	-32.24	-34.8	0.005093
hsa-miR-1304-3p	NM_001253866	YME1L1	171	-27.83	-33.9	0.044255
hsa-miR-1304-3p	NM_001254726	AP4S1	176	-38.61	-39.9	0.000998
hsa-miR-1304-3p	NM_001254727	AP4S1	176	-38.61	-39.9	0.001318
hsa-miR-1304-3p	NM_001256124	TRIM65	169	-28.85	-33.6	0.048531
hsa-miR-1304-3p	NM_001256526	C9orf69	179	-30.83	-35.5	0.023031
hsa-miR-1304-3p	NM_001257139	CLCNKA	150	-27.48	-30	0.025908
hsa-miR-1304-3p	NM_001258390	LAYN	182	-33.52	-38	0.004254
hsa-miR-1304-3p	NM_001258391	LAYN	182	-33.52	-38	0.004254
hsa-miR-1304-3p	NM_001261841	TMC5	167	-32.2	-35.7	0.009876
hsa-miR-1304-3p	NM_001270427	PSMD5	167	-30.42	-34.2	0.038592
hsa-miR-1304-3p	NM_001271329	MARVELD3	167	-30.42	-34.2	0.030433
hsa-miR-1304-3p	NM_001271572	PEX11A	183	-32.33	-36.1	0.017104
hsa-miR-1304-3p	NM_001271573	PEX11A	183	-32.33	-36.1	0.017104
hsa-miR-1304-3p	NM_001271664	GOLGA6L22	167	-30.42	-34.2	0.034973
hsa-miR-1304-3p	NM_001271895	TARS2	171	-31.4	-34.5	0.004964
hsa-miR-1304-3p	NM_001271896	TARS2	171	-31.4	-34.5	0.004964
hsa-miR-1304-3p	NM_001271937	RMND1	167	-29.35	-33.1	0.007664
hsa-miR-1304-3p	NM_001276392	LDLRAD1	175	-30.9	-33.9	0.038484
hsa-miR-1304-3p	NM_001276393	LDLRAD1	175	-30.9	-33.9	0.038484
hsa-miR-1304-3p	NM_001276394	LDLRAD1	175	-30.9	-33.9	0.038484
hsa-miR-1304-3p	NM_001276395	LDLRAD1	175	-30.9	-33.9	0.043393
hsa-miR-1304-3p	NM_001278378	ARL6IP4	151	-24.79	-28.4	0.025022
hsa-miR-1304-3p	NM_001278379	ARL6IP4	151	-24.79	-28.4	0.025022

hsa-miR-1304-3p	NM_001278380	ARL6IP4	151	-24.79	-28.4	0.025022
hsa-miR-1304-3p	NM_001278590	LIAS	167	-33.07	-34.8	0.004163
hsa-miR-1304-3p	NM_001278736	CCL5	175	-34.4	-37.2	0.002589
hsa-miR-1304-3p	NM_001278791	RFC2	171	-32.24	-34.8	0.005153
hsa-miR-1304-3p	NM_001278792	RFC2	171	-32.24	-34.8	0.005153
hsa-miR-1304-3p	NM_001278793	RFC2	171	-32.24	-34.8	0.005153
hsa-miR-1304-3p	NM_001281473	MLPH	178	-32.59	-36.9	0.010973
hsa-miR-1304-3p	NM_001281474	MLPH	178	-32.59	-36.9	0.010973
hsa-miR-1304-3p	NM_001282379	TRIM46	167	-29.35	-33.1	0.000382
hsa-miR-1304-3p	NM_001282424	A2ML1	171	-32.24	-34.6	0.007767
hsa-miR-1304-3p	NM_001282459	C2	155	-31.31	-33.8	0.00874
hsa-miR-1304-3p	NM_001282588	SLAMF7	171	-32.24	-34.6	0.027802
hsa-miR-1304-3p	NM_001282589	SLAMF7	171	-32.24	-34.6	0.027492
hsa-miR-1304-3p	NM_001282590	SLAMF7	171	-32.24	-34.6	0.027492
hsa-miR-1304-3p	NM_001282591	SLAMF7	171	-32.24	-34.6	0.027492
hsa-miR-1304-3p	NM_001282592	SLAMF7	171	-32.24	-34.6	0.027802
hsa-miR-1304-3p	NM_001282593	SLAMF7	171	-32.24	-34.6	0.027802
hsa-miR-1304-3p	NM_001282594	SLAMF7	171	-32.24	-34.6	0.027492
hsa-miR-1304-3p	NM_001282595	SLAMF7	171	-32.24	-34.6	0.027492
hsa-miR-1304-3p	NM_001282596	SLAMF7	171	-32.24	-34.6	0.027802
hsa-miR-1304-3p	NM_001282708	EXOSC2	167	-29.35	-33.1	0.030595
hsa-miR-1304-3p	NM_001282709	EXOSC2	167	-29.35	-33.1	0.030595
hsa-miR-1304-3p	NM_001282766	POLR1E	169	-24.41	-31.2	0.021036
hsa-miR-1304-3p	NM_001282861	GON4L	167	-26.42	-29.8	0.032141
hsa-miR-1304-3p	NM_001282862	RASGEF1A	162	-32.49	-34.7	0.027941
hsa-miR-1304-3p	NM_001284406	TMEM40	179	-34.35	-37.7	0.004598
hsa-miR-1304-3p	NM_001284407	TMEM40	179	-34.35	-37.7	0.004598
hsa-miR-1304-3p	NM_001284408	TMEM40	179	-34.35	-37.7	0.004598
hsa-miR-1304-3p	NM_001286074	TAF1	167	-31.03	-34.7	0.033738
hsa-miR-1304-3p	NM_001286191	C12orf43	183	-32.33	-36.1	0.015564

hsa-miR-1304-3p	NM_001286192	C12orf43	183	-32.33	-36.1	0.015564
hsa-miR-1304-3p	NM_001286195	C12orf43	183	-32.33	-36.1	0.015564
hsa-miR-1304-3p	NM_001286196	C12orf43	183	-32.33	-36.1	0.015564
hsa-miR-1304-3p	NM_001286197	C12orf43	183	-32.33	-36.1	0.015564
hsa-miR-1304-3p	NM_001286198	C12orf43	183	-32.33	-36.1	0.015564
hsa-miR-1304-3p	NM_001286423	GLB1L	167	-31.03	-33.4	0.006634
hsa-miR-1304-3p	NM_001286427	GLB1L	167	-31.03	-33.4	0.006634
hsa-miR-1304-3p	NM_001286446	FAM65B	179	-32.39	-38.3	0.00397
hsa-miR-1304-3p	NM_001286447	FAM65B	179	-32.39	-38.3	0.00397
hsa-miR-1304-3p	NM_001286732	GRTP1	167	-29.35	-33.1	0.015007
hsa-miR-1304-3p	NM_001286769	ZNF34	167	-29.35	-33.1	0.027508
hsa-miR-1304-3p	NM_001286770	ZNF34	167	-29.35	-33.1	0.027508
hsa-miR-1304-3p	NM_001286950	TRMT10B	163	-30.86	-33.2	0.034898
hsa-miR-1304-3p	NM_001286951	TRMT10B	163	-30.86	-33.2	0.034898
hsa-miR-1304-3p	NM_001286952	TRMT10B	163	-30.86	-33.2	0.034898
hsa-miR-1304-3p	NM_001286953	TRMT10B	163	-30.86	-33.2	0.034898
hsa-miR-1304-3p	NM_001286954	TRMT10B	163	-30.86	-33.2	0.034898
hsa-miR-1304-3p	NM_001287150	FBXL13	167	-27.47	-30.1	0.034316
hsa-miR-1304-3p	NM_001287223	SCN11A	167	-26.45	-32	0.037984
hsa-miR-1304-3p	NM_001287586	SLC50A1	171	-32.24	-34.8	0.004752
hsa-miR-1304-3p	NM_001287587	SLC50A1	171	-32.24	-34.8	0.004752
hsa-miR-1304-3p	NM_001287588	SLC50A1	171	-32.24	-34.8	0.006415
hsa-miR-1304-3p	NM_001287589	SLC50A1	171	-32.24	-34.8	0.004752
hsa-miR-1304-3p	NM_001287590	SLC50A1	171	-32.24	-34.8	0.004752
hsa-miR-1304-3p	NM_001287591	SLC50A1	171	-32.24	-34.8	0.004752
hsa-miR-1304-3p	NM_001287592	SLC50A1	171	-32.24	-34.8	0.004752
hsa-miR-1304-3p	NM_001288805	ZNF707	162	-28.74	-32.3	0.029482
hsa-miR-1304-3p	NM_001288806	ZNF707	162	-28.74	-32.3	0.029482
hsa-miR-1304-3p	NM_001288807	ZNF707	162	-28.74	-32.3	0.029482
hsa-miR-1304-3p	NM_001288808	ZNF707	162	-28.74	-32.3	0.029482

hsa-miR-1304-3p	NM_001288809	ZNF707	162	-28.74	-32.3	0.029482
hsa-miR-1304-3p	NM_001289132	C15orf57	167	-29.35	-33.1	0.024291
hsa-miR-1304-3p	NM_001289758	IFIT3	167	-28.56	-31.2	0.047479
hsa-miR-1304-3p	NM_001289759	IFIT3	167	-28.56	-31.2	0.047479
hsa-miR-1304-3p	NM_001289990	ATP6V0E2	152	-29.4	-33.2	0.026035
hsa-miR-1304-3p	NM_001290117	TIMM23B	187	-34.68	-37.1	0.0106
hsa-miR-1304-3p	NM_001290118	TIMM23B	187	-34.68	-37.1	0.0106
hsa-miR-1304-3p	NM_001290131	PHF12	175	-30.9	-34.7	0.009044
hsa-miR-1304-3p	NM_001292037	ACPP	187	-38.41	-39.9	0.003702
hsa-miR-1304-3p	NM_001293167	PANO1	167	-30.42	-34.2	0.013616
hsa-miR-1304-3p	NM_001293285	SPATS2	171	-31.51	-34.5	0.017494
hsa-miR-1304-3p	NM_001293286	SPATS2	171	-31.51	-34.5	0.017494
hsa-miR-1304-3p	NM_001297430	MAEA	150	-33.31	-34.8	0.011578
hsa-miR-1304-3p	NM_001297431	MAEA	150	-33.31	-34.8	0.011578
hsa-miR-1304-3p	NM_001297432	MAEA	150	-33.31	-34.8	0.011578
hsa-miR-1304-3p	NM_001297433	MAEA	150	-33.31	-34.8	0.011578
hsa-miR-1304-3p	NM_001297609	MAP3K6	153	-20.3	-26.8	0.048177
hsa-miR-1304-3p	NM_001297721	C1orf43	151	-28.7	-32.7	0.024536
hsa-miR-1304-3p	NM_001297723	C1orf43	151	-28.7	-32.7	0.024536
hsa-miR-1304-3p	NM_001300732	TMC7	163	-27.47	-31.5	0.009043
hsa-miR-1304-3p	NM_001303404	CWF19L1	171	-32.24	-34.6	0.01171
hsa-miR-1304-3p	NM_001303405	CWF19L1	171	-32.24	-34.6	0.01171
hsa-miR-1304-3p	NM_001303406	CWF19L1	171	-32.24	-34.6	0.01171
hsa-miR-1304-3p	NM_001303407	CWF19L1	171	-32.24	-34.6	0.01171
hsa-miR-1304-3p	NM_001304358	ZFP42	191	-37.28	-39.9	0.001877
hsa-miR-1304-3p	NM_001304746	RHBDL2	163	-30.86	-32.9	0.016264
hsa-miR-1304-3p	NM_001304947	KRBA2	167	-30.42	-33.2	0.007514
hsa-miR-1304-3p	NM_001306146	HDC	157	-27.19	-28.2	0.043883
hsa-miR-1304-3p	NM_001307931	ZYG11A	167	-30.42	-33.2	0.049124
hsa-miR-1304-3p	NM_001308018	ELMOD1	175	-32.75	-34.7	0.024989

hsa-miR-1304-3p	NM_001308089	ANKS3	155	-30.71	-30.8	0.017744
hsa-miR-1304-3p	NM_001308161	TMC5	167	-32.2	-35.7	0.009876
hsa-miR-1304-3p	NM_001308220	ERN2	191	-39.33	-42	0.000119
hsa-miR-1304-3p	NM_001308421	PIGBOS1	159	-26.26	-31.1	0.018457
hsa-miR-1304-3p	NM_001308422	PIGBOS1	159	-26.26	-31.1	0.018457
hsa-miR-1304-3p	NM_001308423	PIGBOS1	159	-26.26	-31.1	0.018457
hsa-miR-1304-3p	NM_001311175	TNFAIP8L3	175	-29.99	-36.3	0.008994
hsa-miR-1304-3p	NM_001312686	LRIG2	173	-26.57	-33.3	0.027285
hsa-miR-1304-3p	NM_001361	DHODH	167	-29.35	-33.1	0.034546
hsa-miR-1304-3p	NM_001502	GP2	176	-37.97	-38.6	0.004889
hsa-miR-1304-3p	NM_001549	IFIT3	167	-28.56	-31.2	0.047479
hsa-miR-1304-3p	NM_001557	CXCR2	167	-30.42	-34.5	0.022097
hsa-miR-1304-3p	NM_001569	IRAK1	183	-38.51	-40.8	0.001332
hsa-miR-1304-3p	NM_001761	CCNF	168	-33.42	-35.6	0.021049
hsa-miR-1304-3p	NM_002098	GUCA1B	176	-34.93	-35.5	0.016831
hsa-miR-1304-3p	NM_002112	HDC	157	-27.19	-28.2	0.043883
hsa-miR-1304-3p	NM_002160	TNC	183	-30.68	-33.7	0.038426
hsa-miR-1304-3p	NM_002209	ITGAL	171	-32.24	-34.6	0.024636
hsa-miR-1304-3p	NM_002231	CD82	167	-31.73	-34.2	0.007578
hsa-miR-1304-3p	NM_002290	LAMA4	183	-32.33	-36.1	0.012636
hsa-miR-1304-3p	NM_002433	MOG	167	-30.42	-33.2	0.021896
hsa-miR-1304-3p	NM_002914	RFC2	171	-32.24	-34.8	0.005153
hsa-miR-1304-3p	NM_002985	CCL5	175	-34.4	-37.2	0.003251
hsa-miR-1304-3p	NM_003337	UBE2B	182	-33.52	-38	0.006998
hsa-miR-1304-3p	NM_003594	TTF2	182	-33.52	-38	0.004574
hsa-miR-1304-3p	NM_003666	BLZF1	170	-32.37	-36.3	0.003072
hsa-miR-1304-3p	NM_003757	EIF3I	167	-30.42	-33.2	0.00559
hsa-miR-1304-3p	NM_003847	PEX11A	183	-32.33	-36.1	0.017104
hsa-miR-1304-3p	NM_003869	CES2	167	-30.42	-34.2	0.017341
hsa-miR-1304-3p	NM_003920	TIMELESS	167	-30.42	-33.2	0.037989

hsa-miR-1304-3p	NM_004070	CLCNKA	150	-27.48	-30	0.025908
hsa-miR-1304-3p	NM_004103	PTK2B	157	-30.26	-32.2	0.032332
hsa-miR-1304-3p	NM_004435	ENDOG	153	-22.49	-25.8	0.008979
hsa-miR-1304-3p	NM_004486	GOLGA2	155	-31.31	-33.7	0.027131
hsa-miR-1304-3p	NM_004528	MGST3	179	-34.18	-36.3	0.002711
hsa-miR-1304-3p	NM_004536	NAIP	167	-30.42	-34.5	0.028206
hsa-miR-1304-3p	NM_004606	TAF1	167	-31.03	-34.7	0.033738
hsa-miR-1304-3p	NM_004672	MAP3K6	153	-20.3	-26.8	0.048177
hsa-miR-1304-3p	NM_004697	PRPF4	167	-30.42	-34.2	0.021922
hsa-miR-1304-3p	NM_004735	LRRFIP1	183	-32.33	-36.1	0.01615
hsa-miR-1304-3p	NM_004870	MPDU1	166	-27.83	-31.8	0.024884
hsa-miR-1304-3p	NM_005047	PSMD5	167	-30.42	-34.2	0.038592
hsa-miR-1304-3p	NM_005242	F2RL1	167	-30.42	-33.2	0.044876
hsa-miR-1304-3p	NM_005347	HSPA5	167	-30.42	-34.5	0.031266
hsa-miR-1304-3p	NM_005516	HLA-E	167	-29.35	-33.4	0.039114
hsa-miR-1304-3p	NM_005810	KLRG1	187	-36.77	-39.9	0.001628
hsa-miR-1304-3p	NM_005882	MAEA	150	-33.31	-34.8	0.011578
hsa-miR-1304-3p	NM_005897	IPP	175	-30.9	-33.9	0.024626
hsa-miR-1304-3p	NM_005914	MCM4	167	-30.42	-33.2	0.039507
hsa-miR-1304-3p	NM_005999	TSNAX	167	-30.42	-34.2	0.032011
hsa-miR-1304-3p	NM_006028	HTR3B	178	-28.33	-32.1	0.010383
hsa-miR-1304-3p	NM_006582	GMEB1	167	-28.54	-31.9	0.033879
hsa-miR-1304-3p	NM_006600	NUDC	167	-29.52	-35.2	0.005373
hsa-miR-1304-3p	NM_006859	LIAS	167	-33.07	-34.8	0.004163
hsa-miR-1304-3p	NM_006903	PPA2	167	-30.42	-33.2	0.012499
hsa-miR-1304-3p	NM_006952	UPK1B	167	-27.41	-33.4	0.029107
hsa-miR-1304-3p	NM_006991	ZNF197	168	-30.99	-34.2	0.016694
hsa-miR-1304-3p	NM_007077	AP4S1	176	-38.61	-39.9	0.000998
hsa-miR-1304-3p	NM_007198	PROSC	187	-36.77	-39.9	0.002817
hsa-miR-1304-3p	NM_007211	RASSF8	171	-32.24	-34.7	0.009081

hsa-miR-1304-3p	NM_007294	BRCA1	171	-32.24	-34.7	0.020545
hsa-miR-1304-3p	NM_007297	BRCA1	171	-32.24	-34.7	0.020545
hsa-miR-1304-3p	NM_007298	BRCA1	171	-32.24	-34.7	0.020545
hsa-miR-1304-3p	NM_007299	BRCA1	171	-32.24	-34.7	0.022894
hsa-miR-1304-3p	NM_007300	BRCA1	171	-32.24	-34.7	0.020545
hsa-miR-1304-3p	NM_007344	TTF1	171	-32.24	-34.8	0.002026
hsa-miR-1304-3p	NM_012396	PHLDA3	171	-30.93	-34.2	0.041588
hsa-miR-1304-3p	NM_013332	HILPDA	168	-33.36	-36.1	0.006165
hsa-miR-1304-3p	NM_013340	PCDHB1	160	-22.69	-26.1	0.001557
hsa-miR-1304-3p	NM_014046	MRPS18B	167	-29.96	-32.3	0.016928
hsa-miR-1304-3p	NM_014139	SCN11A	167	-26.45	-32	0.037984
hsa-miR-1304-3p	NM_014263	YME1L1	171	-27.83	-33.9	0.044255
hsa-miR-1304-3p	NM_014285	EXOSC2	167	-29.35	-33.1	0.030595
hsa-miR-1304-3p	NM_014298	QPRT	169	-27.51	-31.5	0.023638
hsa-miR-1304-3p	NM_014321	ORC6	167	-30.42	-34.2	0.011232
hsa-miR-1304-3p	NM_014326	DAPK2	183	-36.02	-37.8	0.00586
hsa-miR-1304-3p	NM_014412	CACYBP	179	-30.24	-34.4	0.037994
hsa-miR-1304-3p	NM_014456	PDCD4	167	-31.03	-34.4	0.037199
hsa-miR-1304-3p	NM_014813	LRIG2	173	-26.57	-33.3	0.027285
hsa-miR-1304-3p	NM_014871	PAN2	167	-29.35	-33.4	0.036398
hsa-miR-1304-3p	NM_014937	INPP5F	179	-30.48	-32.8	0.046484
hsa-miR-1304-3p	NM_015070	ZC3H13	177	-31.01	-34.5	0.02875
hsa-miR-1304-3p	NM_015395	TECPR1	171	-31.51	-35.8	0.02099
hsa-miR-1304-3p	NM_015688	FAM184B	167	-30.42	-33.2	0.006569
hsa-miR-1304-3p	NM_015910	WDPCP	187	-32.84	-36.1	0.002772
hsa-miR-1304-3p	NM_015962	FCF1	171	-32.24	-34.8	0.027792
hsa-miR-1304-3p	NM_015989	CSAD	168	-35.54	-38.2	0.00189
hsa-miR-1304-3p	NM_016371	HSD17B7	167	-30.42	-34.2	0.003228
hsa-miR-1304-3p	NM_016395	HACD3	175	-30.9	-33.9	0.047779
hsa-miR-1304-3p	NM_016433	GLTP	171	-32.24	-34.7	0.025671

hsa-miR-1304-3p	NM_016518	PIPOX	163	-27.47	-31.4	0.048884
hsa-miR-1304-3p	NM_016638	ARL6IP4	151	-24.79	-28.4	0.025022
hsa-miR-1304-3p	NM_017520	MPHOSPH8	175	-30.9	-34.9	0.022959
hsa-miR-1304-3p	NM_017556	FBLIM1	167	-30.66	-34.5	0.035916
hsa-miR-1304-3p	NM_017612	ZCCHC8	171	-30	-36.1	0.017808
hsa-miR-1304-3p	NM_017614	BHMT2	167	-30.42	-34.2	0.028756
hsa-miR-1304-3p	NM_017621	ALKBH4	173	-26.04	-33.1	0.032763
hsa-miR-1304-3p	NM_017676	GIN1	183	-37.9	-39.8	0.003646
hsa-miR-1304-3p	NM_017696	MCM9	187	-36.75	-38.7	0.003478
hsa-miR-1304-3p	NM_017821	RHBDL2	163	-30.86	-32.9	0.016264
hsa-miR-1304-3p	NM_017830	OCIAD1	180	-30.21	-34	0.01868
hsa-miR-1304-3p	NM_017909	RMND1	167	-29.35	-33.1	0.007664
hsa-miR-1304-3p	NM_018068	PIWIL2	171	-32.24	-34.7	0.00482
hsa-miR-1304-3p	NM_018128	TSR1	162	-32.07	-34.6	0.024356
hsa-miR-1304-3p	NM_018141	MRPS10	175	-33.85	-35.3	0.017605
hsa-miR-1304-3p	NM_018289	VPS53	171	-32.24	-34.7	0.004342
hsa-miR-1304-3p	NM_018294	CWF19L1	171	-32.24	-34.6	0.01171
hsa-miR-1304-3p	NM_018306	TMEM40	179	-34.35	-37.7	0.004598
hsa-miR-1304-3p	NM_018396	METTL2B	167	-30.42	-33.2	0.025025
hsa-miR-1304-3p	NM_018561	USP49	167	-30.42	-34.2	0.016496
hsa-miR-1304-3p	NM_018656	SLC35E3	159	-27.52	-32.3	0.047161
hsa-miR-1304-3p	NM_018694	ARL6IP4	151	-24.79	-28.4	0.025022
hsa-miR-1304-3p	NM_018712	ELMOD1	175	-32.75	-34.7	0.024989
hsa-miR-1304-3p	NM_018842	BAIAP2L1	167	-30.42	-34.5	0.034467
hsa-miR-1304-3p	NM_018845	SLC50A1	171	-32.24	-34.8	0.004752
hsa-miR-1304-3p	NM_018891	LAMC2	171	-29.86	-32.1	0.033273
hsa-miR-1304-3p	NM_018997	MRPS21	167	-30.44	-34.2	0.009942
hsa-miR-1304-3p	NM_020117	LARS	172	-30.76	-35.8	0.008586
hsa-miR-1304-3p	NM_020682	AS3MT	173	-29.55	-32.4	0.045935
hsa-miR-1304-3p	NM_020745	AARS2	170	-23.27	-33.7	0.046595

hsa-miR-1304-3p	NM_020785	CC2D2A	171	-32.24	-34.6	0.014159
hsa-miR-1304-3p	NM_020950	KIAA1614	171	-31.4	-34.5	0.004484
hsa-miR-1304-3p	NM_021151	CROT	174	-28.91	-34.2	0.019516
hsa-miR-1304-3p	NM_021181	SLAMF7	171	-32.24	-34.6	0.027492
hsa-miR-1304-3p	NM_021218	INIP	163	-29.49	-33.3	0.024599
hsa-miR-1304-3p	NM_021599	ADAMTS2	151	-28.7	-31.4	0.005371
hsa-miR-1304-3p	NM_022101	CXorf56	171	-32.24	-34.7	0.024898
hsa-miR-1304-3p	NM_022171	TCTA	167	-30.42	-34.2	0.0319
hsa-miR-1304-3p	NM_022373	HERPUD2	167	-29.35	-32.1	0.042361
hsa-miR-1304-3p	NM_022490	POLR1E	169	-24.41	-31.2	0.021036
hsa-miR-1304-3p	NM_022568	ALDH8A1	182	-32.73	-38	0.002894
hsa-miR-1304-3p	NM_022892	NAIP	167	-30.42	-34.5	0.028206
hsa-miR-1304-3p	NM_022895	C12orf43	183	-32.33	-36.1	0.015564
hsa-miR-1304-3p	NM_023071	SPATS2	171	-31.51	-34.5	0.017448
hsa-miR-1304-3p	NM_023947	CHID1	182	-33.52	-36	0.020007
hsa-miR-1304-3p	NM_024015	HOXB4	155	-31.33	-34.5	0.018499
hsa-miR-1304-3p	NM_024026	MRPL57	171	-32.24	-34.8	0.030011
hsa-miR-1304-3p	NM_024101	MLPH	178	-32.59	-36.9	0.010973
hsa-miR-1304-3p	NM_024332	BRCC3	171	-36.12	-39.2	0.004608
hsa-miR-1304-3p	NM_024336	IRX3	158	-28.92	-30.8	0.016915
hsa-miR-1304-3p	NM_024482	GMEB1	167	-28.54	-31.9	0.033879
hsa-miR-1304-3p	NM_024506	GLB1L	167	-31.03	-33.4	0.006634
hsa-miR-1304-3p	NM_024561	NAA16	171	-32.24	-34.6	0.0225
hsa-miR-1304-3p	NM_024715	TXNDC15	167	-30.42	-34.5	0.036426
hsa-miR-1304-3p	NM_024745	SHCBP1	182	-35.3	-39.6	0.001772
hsa-miR-1304-3p	NM_024758	AGMAT	171	-32.24	-34.7	0.033573
hsa-miR-1304-3p	NM_024780	TMC5	167	-32.2	-35.7	0.009876
hsa-miR-1304-3p	NM_024874	KIAA0319L	175	-34.4	-37	0.007632
hsa-miR-1304-3p	NM_024917	TRMT2B	171	-32.24	-34.6	0.018157
hsa-miR-1304-3p	NM_025150	TARS2	171	-31.4	-34.5	0.004964

hsa-miR-1304-3p	NM_025159	CXorf21	167	-28.54	-31.2	0.030575
hsa-miR-1304-3p	NM_025257	SLC44A4	167	-30.42	-34.5	0.003271
hsa-miR-1304-3p	NM_030580	ZNF34	167	-29.35	-33.1	0.027508
hsa-miR-1304-3p	NM_030628	INTS5	154	-23.99	-28.5	0.01318
hsa-miR-1304-3p	NM_031207	HYI	158	-25.02	-31.7	0.007103
hsa-miR-1304-3p	NM_031439	SOX7	171	-32.24	-34.8	0.032594
hsa-miR-1304-3p	NM_031482	ATG10	167	-30.42	-33.2	0.037591
hsa-miR-1304-3p	NM_031901	MRPS21	167	-30.44	-34.2	0.009942
hsa-miR-1304-3p	NM_031905	ARMC10	167	-30.42	-34.5	0.018384
hsa-miR-1304-3p	NM_032292	GON4L	167	-26.42	-29.8	0.032141
hsa-miR-1304-3p	NM_032307	C9orf64	192	-43.79	-34.3	0.019823
hsa-miR-1304-3p	NM_032333	FAM213A	155	-31.59	-33.9	0.01525
hsa-miR-1304-3p	NM_032338	LLPH	171	-27.83	-31.6	0.034836
hsa-miR-1304-3p	NM_032898	CEP19	170	-29.38	-35.3	0.013929
hsa-miR-1304-3p	NM_032930	C11orf70	168	-32.89	-35.3	0.015403
hsa-miR-1304-3p	NM_032992	CASP6	159	-33.75	-36.5	0.002982
hsa-miR-1304-3p	NM_033128	SCIN	170	-29.38	-33	0.022654
hsa-miR-1304-3p	NM_033266	ERN2	191	-39.33	-42	0.000119
hsa-miR-1304-3p	NM_033375	MYO1C	169	-27.51	-34.7	0.024039
hsa-miR-1304-3p	NM_052836	CDH23	167	-29.35	-32.1	0.023856
hsa-miR-1304-3p	NM_052903	TUBGCP5	179	-31.41	-33.9	0.008838
hsa-miR-1304-3p	NM_052929	FHAD1	167	-30.42	-33.2	0.014613
hsa-miR-1304-3p	NM_080656	CDKN2AIPNL	171	-32.24	-34.6	0.01011
hsa-miR-1304-3p	NM_133450	ANKS3	155	-30.71	-30.8	0.01782
hsa-miR-1304-3p	NM_133474	ZNF721	155	-31.59	-34.1	0.036118
hsa-miR-1304-3p	NM_133480	TADA3	167	-29.35	-33.1	0.021938
hsa-miR-1304-3p	NM_138458	WDR92	167	-29.35	-33.4	0.025669
hsa-miR-1304-3p	NM_138477	CDAN1	171	-32.24	-34.6	0.01167
hsa-miR-1304-3p	NM_138706	B3GNT6	167	-29.35	-33.1	0.035934
hsa-miR-1304-3p	NM_138801	GALM	171	-32.24	-34.6	0.017407

hsa-miR-1304-3p	NM_138923	TAF1	167	-31.03	-34.7	0.033738
hsa-miR-1304-3p	NM_139312	YME1L1	171	-27.83	-33.9	0.044255
hsa-miR-1304-3p	NM_139352	TAF1A	171	-32.24	-34.7	0.002582
hsa-miR-1304-3p	NM_144670	A2ML1	171	-32.24	-34.6	0.007767
hsa-miR-1304-3p	NM_144736	NDUFAF7	155	-31.59	-33.9	0.012966
hsa-miR-1304-3p	NM_144964	TRMT10B	163	-30.86	-33.2	0.034898
hsa-miR-1304-3p	NM_145032	FBXL13	167	-27.47	-30.1	0.034316
hsa-miR-1304-3p	NM_145230	ATP6V0E2	152	-29.4	-33.2	0.040149
hsa-miR-1304-3p	NM_145313	RASGEF1A	162	-32.49	-34.7	0.027941
hsa-miR-1304-3p	NM_145341	PDCD4	167	-31.03	-34.4	0.037199
hsa-miR-1304-3p	NM_145798	OSBPL7	169	-28.67	-31.9	0.03968
hsa-miR-1304-3p	NM_147129	ALS2CL	184	-37.38	-38.7	0.00629
hsa-miR-1304-3p	NM_152269	C12orf65	167	-29.35	-33.1	0.023146
hsa-miR-1304-3p	NM_152284	CHMP4C	171	-27.83	-31.6	0.048636
hsa-miR-1304-3p	NM_152411	ZNF786	175	-30.9	-34.9	0.00766
hsa-miR-1304-3p	NM_152546	SRFBP1	171	-32.24	-34.6	0.023564
hsa-miR-1304-3p	NM_152586	USP54	167	-30.42	-34.5	0.015697
hsa-miR-1304-3p	NM_152652	ZNF48	187	-34.68	-37.1	0.004191
hsa-miR-1304-3p	NM_152730	TBC1D32	167	-29.35	-33.4	0.032394
hsa-miR-1304-3p	NM_152833	C9orf69	179	-30.83	-35.5	0.023031
hsa-miR-1304-3p	NM_153260	LRRC57	171	-32.24	-33.2	0.046791
hsa-miR-1304-3p	NM_153368	GJD4	183	-34.95	-40.1	0.000092
hsa-miR-1304-3p	NM_153809	TAF1L	167	-31.03	-34.7	0.006064
hsa-miR-1304-3p	NM_170771	ALDH8A1	182	-32.73	-38	0.002894
hsa-miR-1304-3p	NM_173092	KCNH6	167	-28.54	-31.2	0.004835
hsa-miR-1304-3p	NM_173174	PTK2B	157	-30.26	-32.2	0.032332
hsa-miR-1304-3p	NM_173175	PTK2B	157	-30.26	-32.2	0.032332
hsa-miR-1304-3p	NM_173176	PTK2B	157	-30.26	-32.2	0.032332
hsa-miR-1304-3p	NM_173547	TRIM65	169	-28.85	-33.6	0.048531
hsa-miR-1304-3p	NM_173831	ZNF707	162	-28.74	-32.3	0.029482

hsa-miR-1304-3p	NM_174900	ZFP42	191	-37.28	-39.9	0.001877
hsa-miR-1304-3p	NM_174958	ATP2A3	167	-28.52	-32.7	0.029671
hsa-miR-1304-3p	NM_176866	PPA2	167	-30.42	-33.2	0.012499
hsa-miR-1304-3p	NM_176867	PPA2	167	-30.42	-33.2	0.012499
hsa-miR-1304-3p	NM_176869	PPA2	167	-30.42	-33.2	0.012499
hsa-miR-1304-3p	NM_177435	PPARD	171	-30.36	-32.8	0.013654
hsa-miR-1304-3p	NM_178324	SPTLC1	167	-30.42	-34.2	0.004942
hsa-miR-1304-3p	NM_178336	MRPL52	171	-31.4	-34.5	0.007972
hsa-miR-1304-3p	NM_178559	ABCB5	167	-30.42	-33.2	0.039868
hsa-miR-1304-3p	NM_178834	LAYN	182	-33.52	-38	0.004254
hsa-miR-1304-3p	NM_180982	MRPL52	171	-31.4	-34.5	0.007972
hsa-miR-1304-3p	NM_181304	MRPL52	171	-31.4	-34.5	0.007972
hsa-miR-1304-3p	NM_181305	MRPL52	171	-31.4	-34.5	0.007972
hsa-miR-1304-3p	NM_181306	MRPL52	171	-31.4	-34.5	0.007972
hsa-miR-1304-3p	NM_181307	MRPL52	171	-31.4	-34.5	0.00879
hsa-miR-1304-3p	NM_181471	RFC2	171	-32.24	-34.8	0.005153
hsa-miR-1304-3p	NM_181725	METTL2A	171	-32.24	-33.2	0.038827
hsa-miR-1304-3p	NM_181814	ABHD12B	178	-27.86	-30.8	0.034967
hsa-miR-1304-3p	NM_182607	VSIG1	171	-32.24	-34.8	0.02882
hsa-miR-1304-3p	NM_182746	MCM4	167	-30.42	-33.2	0.039507
hsa-miR-1304-3p	NM_182775	ALS2CL	184	-37.38	-38.7	0.00629
hsa-miR-1304-3p	NM_182922	HEATR3	167	-27.41	-33.1	0.005747
hsa-miR-1304-3p	NM_194451	LIAS	167	-33.07	-34.8	0.004708
hsa-miR-1304-3p	NM_198061	CES2	167	-30.42	-34.2	0.017341
hsa-miR-1304-3p	NM_198493	ANKRD45	171	-32.24	-34.7	0.030215
hsa-miR-1304-3p	NM_198564	DNAH12	183	-33.45	-35	0.003275
hsa-miR-1304-3p	NM_198585	ENTPD8	150	-28.89	-31.2	0.024943
hsa-miR-1304-3p	NM_198795	TDRD1	183	-30.68	-33.7	0.013559
hsa-miR-1304-3p	NM_198843	SFTPB	167	-30.42	-33.2	0.046669
hsa-miR-1304-3p	NM_199124	C11orf63	167	-30.42	-34.5	0.006869

hsa-miR-1304-3p	NM_206809	MOG	167	-30.42	-33.2	0.031846
hsa-miR-1304-3p	NM_206810	MOG	167	-30.42	-33.2	0.021896
hsa-miR-1304-3p	NM_206811	MOG	167	-30.42	-33.2	0.021896
hsa-miR-1304-3p	NM_206812	MOG	167	-30.42	-33.2	0.031846
hsa-miR-1304-3p	NM_206814	MOG	167	-30.42	-33.2	0.031846
hsa-miR-1304-3p	NM_207320	OTUD6A	167	-27.5	-33.2	0.01715
hsa-miR-1304-3p	NM_207351	PRRT3	167	-30.42	-34.4	0.007756
hsa-miR-1304-3p	NM_207381	TNFAIP8L3	175	-29.99	-36.3	0.008994
hsa-miR-1304-3p	NM_207412	C8orf86	167	-29.35	-33.1	0.048498
hsa-miR-1304-3p	NM_213597	KRBA2	167	-30.42	-33.2	0.007514
hsa-miR-1304-3p	NM_213605	ZNF517	173	-27.63	-34.7	0.007827
hsa-miR-1304-3p	NR_002916	SNORD8	162	-25.19	-28.1	0.006611
hsa-miR-1304-3p	NR_024440	LOC729609	183	-32.33	-36.1	0.002959
hsa-miR-1304-3p	NR_026858	ULK4P1	175	-33.13	-35.4	0.007335
hsa-miR-1304-3p	NR_027277	LOC344967	167	-30.42	-34.2	0.031872
hsa-miR-1304-3p	NR_027447	MAN1B1-AS1	183	-37.9	-34.8	0.030372
hsa-miR-1304-3p	NR_027470	ULK4P2	175	-33.13	-35.4	0.007335
hsa-miR-1304-3p	NR_029893	MIR135B	166	-27.07	-30	0.001655
hsa-miR-1304-3p	NR_030228	MIR503	152	-21.93	-24.5	0.023535
hsa-miR-1304-3p	NR_031639	MIR1304	196	-40.04	-43	0.000001
hsa-miR-1304-3p	NR_031675	MIR1273A	171	-32.24	-36	0.000054
hsa-miR-1304-3p	NR_033934	MRPL45P2	175	-30.9	-33.3	0.038403
hsa-miR-1304-3p	NR_033995	LOC389247	167	-29.35	-33.4	0.015404
hsa-miR-1304-3p	NR_034126	LINC01160	171	-30.93	-34.5	0.026266
hsa-miR-1304-3p	NR_034170	EIF3J-AS1	177	-32.06	-35.6	0.021263
hsa-miR-1304-3p	NR_034171	EIF3J-AS1	177	-32.06	-35.6	0.013872
hsa-miR-1304-3p	NR_036216	MIR4254	151	-23.18	-26.4	0.008443
hsa-miR-1304-3p	NR_038841	LOC100506746	167	-30.42	-34.2	0.041245
hsa-miR-1304-3p	NR_039614	MIR4419A	164	-27.29	-29	0.001757
hsa-miR-1304-3p	NR_039664	MIR4459	171	-32.24	-34.8	0.000031

hsa-miR-1304-3p	NR_073199	LINC00483	167	-30.42	-33.2	0.018435
hsa-miR-1304-3p	NR_104191	C2-AS1	171	-30.93	-33.2	0.016259
hsa-miR-1304-3p	NR_104636	LOC101926960	167	-32.2	-34.8	0.031209
hsa-miR-1304-3p	NR_106721	MIR6073	163	-23.93	-27.4	0.006485
hsa-miR-1304-3p	NR_107005	MIR7851	179	-33.44	-35.3	0.000254
hsa-miR-1304-3p	NR_110208	LOC101927043	155	-29.33	-31.6	0.04537
hsa-miR-1304-3p	NR_110272	LOC101927709	179	-28.46	-34.5	0.017403
hsa-miR-1304-3p	NR_110608	FILIP1	167	-33.07	-34.8	0.01098
hsa-miR-1304-3p	NR_110751	LOC101927274	175	-30.17	-35.3	0.021759
hsa-miR-1304-3p	NR_110889	LOC101928489	179	-32.21	-38.1	0.008029
hsa-miR-1304-3p	NR_120335	LOC101928414	171	-32.24	-34.6	0.006826
hsa-miR-1304-3p	NR_120571	MIR4300HG	167	-30.42	-34.5	0.022835
hsa-miR-1304-3p	NR_120678	LOC102724719	167	-26.32	-29.7	0.033638
hsa-miR-1304-3p	NR_126167	TMEM5-AS1	171	-31.4	-34	0.006703
hsa-miR-1304-3p	NR_126344	LOC100506302	171	-32.24	-34.8	0.019704
hsa-miR-1304-3p	NR_126410	LINC01386	156	-23.26	-30.6	0.029268
hsa-miR-1304-3p	NR_128706	MIR1273E	173	-29.67	-33.9	0.000184
hsa-miR-1304-3p	NR_133012	CASP6	159	-33.75	-36.5	0.011458
hsa-miR-1246	NM_001243266	MS4A4A	165	-23.28	-28.9	0.030977
hsa-miR-1246	NM_001271643	THADA	165	-24.48	-26.7	0.003829
hsa-miR-1246	NM_023070	ZFP69B	165	-16.83	-23.8	0.024515
hsa-miR-1246	NM_024021	MS4A4A	165	-23.28	-28.9	0.030977
hsa-miR-1246	NM_148975	MS4A4A	165	-23.28	-28.9	0.030977
hsa-miR-1246	NR_126391	LINC01054	153	-24.81	-28.9	0.006069
hsa-miR-1254	NM_000032	ALAS2	154	-25.32	-31	0.003607
hsa-miR-1254	NM_000034	ALDOA	156	-24.56	-29	0.027514
hsa-miR-1254	NM_000040	APOC3	154	-24.37	-29.9	0.014347
hsa-miR-1254	NM_000069	CACNA1S	155	-26.1	-29.8	0.037753
hsa-miR-1254	NM_000246	CIITA	168	-36.57	-38.6	0.005337
hsa-miR-1254	NM_000377	WAS	164	-26.09	-28.9	0.043085

hsa-miR-1254	NM_000562	C8A	171	-31.16	-35.9	0.004565
hsa-miR-1254	NM_000717	CA4	167	-30.12	-31.5	0.002559
hsa-miR-1254	NM_000725	CACNB3	154	-27.72	-33.2	0.0476
hsa-miR-1254	NM_000883	IMPDH1	164	-34.56	-35.7	0.009362
hsa-miR-1254	NM_000892	KLKB1	150	-26.41	-31.3	0.012718
hsa-miR-1254	NM_000929	PLA2G5	190	-37.72	-41.1	0.002059
hsa-miR-1254	NM_001001791	C10orf55	158	-34.6	-36.8	0.021411
hsa-miR-1254	NM_001002913	PTRH1	154	-24.64	-28.7	0.018338
hsa-miR-1254	NM_001005226	OR2B3	159	-26.02	-27.6	0.01466
hsa-miR-1254	NM_001005408	NEURL4	162	-29.07	-35.4	0.005482
hsa-miR-1254	NM_001005862	ERBB2	164	-28.18	-34.2	0.014028
hsa-miR-1254	NM_001006665	RPS6KA1	163	-35.84	-41	0.001103
hsa-miR-1254	NM_001006941	ALG3	152	-25.52	-32.1	0.004484
hsa-miR-1254	NM_001006946	SDC1	169	-34.48	-39.4	0.009076
hsa-miR-1254	NM_001007464	RWDD1	178	-28.69	-32.8	0.018911
hsa-miR-1254	NM_001009991	SYTL3	161	-27.95	-31.5	0.012564
hsa-miR-1254	NM_001013628	DCAF12L2	154	-25.39	-33.1	0.048996
hsa-miR-1254	NM_001014975	CFH	158	-25.75	-30.5	0.015845
hsa-miR-1254	NM_001017915	INPP5D	168	-29.19	-35.1	0.025843
hsa-miR-1254	NM_001024844	CD82	168	-27.68	-31.6	0.047472
hsa-miR-1254	NM_001033113	ENTPD8	152	-25.04	-33.4	0.016734
hsa-miR-1254	NM_001037325	CEP112	169	-26.45	-30.4	0.045511
hsa-miR-1254	NM_001037967	ALAS2	154	-25.32	-31	0.003607
hsa-miR-1254	NM_001037968	ALAS2	154	-25.32	-31	0.003607
hsa-miR-1254	NM_001039457	ATP6V0B	162	-27.09	-31.2	0.016596
hsa-miR-1254	NM_001039888	ANKRD34A	155	-31.34	-32.5	0.046448
hsa-miR-1254	NM_001042483	NUPR1	153	-25.54	-30.8	0.026144
hsa-miR-1254	NM_001042	SLC2A4	173	-36.61	-43.1	0.001164
hsa-miR-1254	NM_001053	SSTR5	177	-33.91	-38.4	0.009006
hsa-miR-1254	NM_001077527	JRK	163	-29.35	-34.8	0.036437

hsa-miR-1254	NM_001078175	SLC29A1	165	-30.92	-35.1	0.009451
hsa-miR-1254	NM_001078177	SLC29A1	165	-30.92	-35.1	0.009451
hsa-miR-1254	NM_001082959	SCARB1	158	-32.55	-38.4	0.003563
hsa-miR-1254	NM_001085474	LYPD8	157	-23.21	-30.9	0.009252
hsa-miR-1254	NM_001094	ASIC2	157	-27.4	-34.2	0.024156
hsa-miR-1254	NM_001098627	IRF5	157	-28.67	-34.4	0.035875
hsa-miR-1254	NM_001098629	IRF5	157	-28.67	-34.4	0.035875
hsa-miR-1254	NM_001098630	IRF5	157	-28.67	-34.4	0.035875
hsa-miR-1254	NM_001099681	MAGIX	156	-30.82	-35.8	0.033217
hsa-miR-1254	NM_001099682	MAGIX	156	-30.82	-35.8	0.033217
hsa-miR-1254	NM_001099780	PSMB11	176	-30.21	-33.8	0.031415
hsa-miR-1254	NM_001100818	PID1	168	-33.67	-40.7	0.004098
hsa-miR-1254	NM_001101648	NPC1L1	155	-24.08	-33.4	0.03611
hsa-miR-1254	NM_001102605	IMPDH1	164	-34.56	-35.7	0.009362
hsa-miR-1254	NM_001109891	MAPK3	160	-34.75	-39.8	0.001158
hsa-miR-1254	NM_001109	ADAM8	168	-26.8	-38.6	0.002621
hsa-miR-1254	NM_001112704	VAX1	156	-29.75	-33.9	0.020339
hsa-miR-1254	NM_001114633	PLA2G4B	168	-32.07	-37.6	0.00068
hsa-miR-1254	NM_001122671	CCBL1	160	-24.84	-31.7	0.028597
hsa-miR-1254	NM_001122672	CCBL1	160	-24.84	-31.7	0.028597
hsa-miR-1254	NM_001123041	CCR2	156	-29.19	-33.7	0.039461
hsa-miR-1254	NM_001127204	HMOX2	154	-25.3	-34.4	0.011912
hsa-miR-1254	NM_001127205	HMOX2	154	-25.3	-34.4	0.011912
hsa-miR-1254	NM_001127206	HMOX2	154	-25.3	-34.4	0.011912
hsa-miR-1254	NM_001127393	C14orf2	150	-22.89	-30.2	0.040815
hsa-miR-1254	NM_001127617	ALDOA	156	-24.56	-29	0.027514
hsa-miR-1254	NM_001129742	CALHM3	150	-22.72	-30.7	0.036505
hsa-miR-1254	NM_001130841	RTCA	166	-35.99	-38.2	0.007597
hsa-miR-1254	NM_001134434	SPOCK2	162	-32.72	-35.1	0.012021
hsa-miR-1254	NM_001135943	MADD	168	-32.33	-37.3	0.006274

hsa-miR-1254	NM_001135944	MADD	168	-32.33	-37.3	0.006274
hsa-miR-1254	NM_001142573	IMPDH1	164	-34.56	-35.7	0.009362
hsa-miR-1254	NM_001142574	IMPDH1	164	-34.56	-35.7	0.009362
hsa-miR-1254	NM_001142575	IMPDH1	164	-34.56	-35.7	0.009362
hsa-miR-1254	NM_001142576	IMPDH1	164	-34.56	-35.7	0.009362
hsa-miR-1254	NM_001142617	STRA6	152	-23.74	-31.5	0.046824
hsa-miR-1254	NM_001142618	STRA6	152	-23.74	-31.5	0.046824
hsa-miR-1254	NM_001142619	STRA6	152	-23.74	-31.5	0.046824
hsa-miR-1254	NM_001142853	HES6	172	-30.08	-35.7	0.006
hsa-miR-1254	NM_001143783	FES	157	-34.5	-41.4	0.000053
hsa-miR-1254	NM_001143784	FES	157	-34.5	-41.4	0.000053
hsa-miR-1254	NM_001143785	FES	157	-34.5	-41.4	0.000053
hsa-miR-1254	NM_001144033	STOML3	152	-22.75	-33.1	0.040377
hsa-miR-1254	NM_001145023	SCRN2	160	-28.42	-32.9	0.022316
hsa-miR-1254	NM_001145082	ZNF619	168	-31.37	-35.5	0.044422
hsa-miR-1254	NM_001145093	ZNF619	168	-31.37	-35.5	0.044422
hsa-miR-1254	NM_001145127	EVPLL	159	-24.35	-32.4	0.048664
hsa-miR-1254	NM_001145210	ANKRD65	160	-25.09	-32.2	0.039851
hsa-miR-1254	NM_001145408	NONO	155	-29.34	-33.7	0.041192
hsa-miR-1254	NM_001145409	NONO	155	-29.34	-33.7	0.041192
hsa-miR-1254	NM_001145410	NONO	155	-29.34	-33.7	0.041192
hsa-miR-1254	NM_001145975	SLC13A2	171	-30.86	-35	0.007078
hsa-miR-1254	NM_001146029	SEMA7A	161	-27.04	-34.8	0.034282
hsa-miR-1254	NM_001146030	SEMA7A	161	-27.04	-34.8	0.034282
hsa-miR-1254	NM_001160243	RPAIN	168	-31.73	-35.6	0.025977
hsa-miR-1254	NM_001161465	JMJD4	163	-25.2	-34.6	0.032197
hsa-miR-1254	NM_001161834	C7orf72	156	-25.19	-30.6	0.002031
hsa-miR-1254	NM_001163735	MYO19	165	-28.08	-35.6	0.005084
hsa-miR-1254	NM_001164410	CDK5	163	-31.04	-33.9	0.002002
hsa-miR-1254	NM_001164489	ADAM8	168	-26.8	-38.6	0.003129

hsa-miR-1254	NM_001164490	ADAM8	168	-26.8	-38.6	0.002621
hsa-miR-1254	NM_001165903	STX1A	166	-33.7	-38.3	0.0073
hsa-miR-1254	NM_001165937	STARD3	159	-24.41	-35.7	0.022507
hsa-miR-1254	NM_001165938	STARD3	159	-24.41	-35.7	0.022507
hsa-miR-1254	NM_001166283	RGMA	174	-35.61	-41.8	0.002432
hsa-miR-1254	NM_001166286	RGMA	174	-35.61	-41.8	0.002432
hsa-miR-1254	NM_001166287	RGMA	174	-35.61	-41.8	0.002432
hsa-miR-1254	NM_001166288	RGMA	174	-35.61	-41.8	0.002432
hsa-miR-1254	NM_001166289	RGMA	174	-35.61	-41.8	0.002432
hsa-miR-1254	NM_001170585	PLB1	156	-24.89	-31.9	0.049999
hsa-miR-1254	NM_001172560	SSTR5	177	-33.91	-38.4	0.009006
hsa-miR-1254	NM_001173988	RABL6	157	-29.43	-34.9	0.011513
hsa-miR-1254	NM_001185080	CLDN15	156	-22.89	-29.7	0.039413
hsa-miR-1254	NM_001185093	NIT1	154	-23.48	-31.5	0.027072
hsa-miR-1254	NM_001185094	NIT1	154	-23.48	-31.5	0.027072
hsa-miR-1254	NM_001190201	CES4A	163	-35.68	-40.9	0.000656
hsa-miR-1254	NM_001190202	CES4A	163	-35.68	-40.9	0.000823
hsa-miR-1254	NM_001193270	MSL3	153	-25.99	-33.6	0.021082
hsa-miR-1254	NM_001198588	JMJD7-PLA2G4B	168	-32.07	-37.6	0.001722
hsa-miR-1254	NM_001198622	TNFSF13	150	-33.69	-40.2	0.001394
hsa-miR-1254	NM_001198623	TNFSF13	150	-33.69	-40.2	0.001394
hsa-miR-1254	NM_001198624	TNFSF13	150	-33.69	-40.2	0.001394
hsa-miR-1254	NM_001199040	STRA6	152	-23.74	-31.5	0.046824
hsa-miR-1254	NM_001199041	STRA6	152	-23.74	-31.5	0.046824
hsa-miR-1254	NM_001199042	STRA6	152	-23.74	-31.5	0.046824
hsa-miR-1254	NM_001199165	CEP112	169	-26.45	-30.4	0.045511
hsa-miR-1254	NM_001199378	GBF1	156	-33.67	-36.8	0.003653
hsa-miR-1254	NM_001199379	GBF1	156	-33.67	-36.8	0.003653
hsa-miR-1254	NM_001199661	PMF1-BGLAP	150	-22.19	-28.6	0.045415

hsa-miR-1254	NM_001199663	PMF1-BGLAP	150	-22.19	-28.6	0.045415
hsa-miR-1254	NM_001200047	NMNAT3	154	-30.31	-36	0.010066
hsa-miR-1254	NM_001202475	CRHR2	177	-28.83	-35.3	0.033298
hsa-miR-1254	NM_001202481	CRHR2	177	-28.83	-35.3	0.033298
hsa-miR-1254	NM_001202482	CRHR2	177	-28.83	-35.3	0.033298
hsa-miR-1254	NM_001202483	CRHR2	177	-28.83	-35.3	0.038891
hsa-miR-1254	NM_001204414	TINAGL1	159	-35.59	-32.6	0.035362
hsa-miR-1254	NM_001204415	TINAGL1	159	-35.59	-32.6	0.035362
hsa-miR-1254	NM_001205138	LRTOMT	166	-36.01	-37.9	0.009774
hsa-miR-1254	NM_001206796	PKM	151	-27.91	-33	0.02431
hsa-miR-1254	NM_001206797	PKM	151	-27.91	-33	0.02431
hsa-miR-1254	NM_001206798	PKM	151	-27.91	-33	0.02431
hsa-miR-1254	NM_001206799	PKM	151	-27.91	-33	0.02431
hsa-miR-1254	NM_001206915	CACNB3	154	-27.72	-33.2	0.0476
hsa-miR-1254	NM_001206916	CACNB3	154	-27.72	-33.2	0.0476
hsa-miR-1254	NM_001206917	CACNB3	154	-27.72	-33.2	0.0476
hsa-miR-1254	NM_001207036	ETV7	158	-23.67	-30.5	0.046553
hsa-miR-1254	NM_001207037	ETV7	158	-23.67	-30.5	0.046553
hsa-miR-1254	NM_001207038	ETV7	158	-23.67	-30.5	0.046553
hsa-miR-1254	NM_001207040	ETV7	158	-23.67	-30.5	0.046553
hsa-miR-1254	NM_001207041	ETV7	158	-23.67	-30.5	0.046553
hsa-miR-1254	NM_001242384	SYTL3	161	-27.95	-31.5	0.012564
hsa-miR-1254	NM_001242394	SYTL3	161	-27.95	-31.5	0.012564
hsa-miR-1254	NM_001242395	SYTL3	161	-27.95	-31.5	0.012564
hsa-miR-1254	NM_001242452	IRF5	157	-28.67	-34.4	0.035875
hsa-miR-1254	NM_001242791	FLJ45513	182	-36.31	-38.9	0.007764
hsa-miR-1254	NM_001243088	FOXM1	163	-28.34	-37.1	0.007832
hsa-miR-1254	NM_001243089	FOXM1	163	-28.34	-37.1	0.007832
hsa-miR-1254	NM_001243177	ALDOA	156	-24.56	-29	0.027514
hsa-miR-1254	NM_001243749	C7orf49	170	-31.38	-33.9	0.039665

hsa-miR-1254	NM_001243751	C7orf49	170	-31.38	-33.9	0.039665
hsa-miR-1254	NM_001243752	C7orf49	170	-31.38	-33.9	0.039665
hsa-miR-1254	NM_001243753	C7orf49	170	-31.38	-33.9	0.039665
hsa-miR-1254	NM_001243925	MAPKAPK3	160	-30.68	-34	0.043688
hsa-miR-1254	NM_001243926	MAPKAPK3	160	-30.68	-34	0.043688
hsa-miR-1254	NM_001253725	KREMEN2	163	-29.82	-32.2	0.018098
hsa-miR-1254	NM_001253726	KREMEN2	163	-29.82	-32.2	0.014754
hsa-miR-1254	NM_001256024	ARHGAP22	160	-27.32	-35.7	0.002781
hsa-miR-1254	NM_001256025	ARHGAP22	160	-27.32	-35.7	0.002781
hsa-miR-1254	NM_001256026	ARHGAP22	160	-27.32	-35.7	0.002781
hsa-miR-1254	NM_001256324	CACNA1G	161	-27.75	-33.5	0.024397
hsa-miR-1254	NM_001256325	CACNA1G	161	-27.75	-33.5	0.024397
hsa-miR-1254	NM_001256326	CACNA1G	161	-27.75	-33.5	0.024397
hsa-miR-1254	NM_001256327	CACNA1G	161	-27.75	-33.5	0.024397
hsa-miR-1254	NM_001256328	CACNA1G	161	-27.75	-33.5	0.024397
hsa-miR-1254	NM_001256329	CACNA1G	161	-27.75	-33.5	0.024397
hsa-miR-1254	NM_001256330	CACNA1G	161	-27.75	-33.5	0.024397
hsa-miR-1254	NM_001256331	CACNA1G	161	-27.75	-33.5	0.024397
hsa-miR-1254	NM_001256332	CACNA1G	161	-27.75	-33.5	0.024397
hsa-miR-1254	NM_001256333	CACNA1G	161	-27.75	-33.5	0.024397
hsa-miR-1254	NM_001256334	CACNA1G	161	-27.75	-33.5	0.024397
hsa-miR-1254	NM_001256359	CACNA1G	161	-27.75	-33.5	0.024397
hsa-miR-1254	NM_001256360	CACNA1G	161	-27.75	-33.5	0.024397
hsa-miR-1254	NM_001256361	CACNA1G	161	-27.75	-33.5	0.024397
hsa-miR-1254	NM_001265	CDX2	156	-31.54	-34.5	0.027521
hsa-miR-1254	NM_001270428	LMO1	163	-26.25	-32.4	0.010294
hsa-miR-1254	NM_001270765	CHST15	158	-27.51	-34.2	0.048186
hsa-miR-1254	NM_001271471	LRTOMT	166	-36.01	-37.6	0.016437
hsa-miR-1254	NM_001272003	KCNT1	153	-27.82	-33.1	0.045927
hsa-miR-1254	NM_001272104	PAQR6	169	-30.65	-34.9	0.015692

hsa-miR-1254	NM_001272105	PAQR6	169	-30.65	-34.9	0.015692
hsa-miR-1254	NM_001272106	PAQR6	169	-30.65	-34.9	0.008148
hsa-miR-1254	NM_001272107	PAQR6	169	-30.65	-34.9	0.015692
hsa-miR-1254	NM_001272108	PAQR6	169	-30.65	-34.9	0.015692
hsa-miR-1254	NM_001272109	PAQR6	169	-30.65	-34.9	0.008148
hsa-miR-1254	NM_001272110	PAQR6	169	-30.65	-34.9	0.008148
hsa-miR-1254	NM_001272111	PAQR6	169	-30.65	-34.9	0.008148
hsa-miR-1254	NM_001272112	PAQR6	169	-30.65	-34.9	0.008148
hsa-miR-1254	NM_001272113	PAQR6	169	-30.65	-34.9	0.008148
hsa-miR-1254	NM_001277335	RASA4B	156	-22.78	-32.7	0.016867
hsa-miR-1254	NM_001278081	LOC388282	162	-23.76	-32.4	0.038386
hsa-miR-1254	NM_001278485	ANAPC15	156	-25.8	-31.8	0.012435
hsa-miR-1254	NM_001278486	ANAPC15	156	-25.8	-31.8	0.012435
hsa-miR-1254	NM_001278487	ANAPC15	156	-25.8	-31.8	0.012435
hsa-miR-1254	NM_001278488	ANAPC15	156	-25.8	-31.8	0.012435
hsa-miR-1254	NM_001278489	ANAPC15	156	-25.8	-31.8	0.012435
hsa-miR-1254	NM_001278490	ANAPC15	156	-25.8	-31.8	0.012435
hsa-miR-1254	NM_001278491	ANAPC15	156	-25.8	-31.8	0.012435
hsa-miR-1254	NM_001278492	ANAPC15	156	-25.8	-31.8	0.012435
hsa-miR-1254	NM_001278493	ANAPC15	156	-25.8	-31.8	0.012435
hsa-miR-1254	NM_001278494	ANAPC15	156	-25.8	-31.8	0.012435
hsa-miR-1254	NM_001282174	MSL3	153	-25.99	-33.6	0.021082
hsa-miR-1254	NM_001282434	HES6	172	-30.08	-35.7	0.012085
hsa-miR-1254	NM_001282614	BEST3	166	-23.31	-27.9	0.0387
hsa-miR-1254	NM_001283053	STMN4	175	-29.64	-36.4	0.023165
hsa-miR-1254	NM_001283054	STMN4	175	-29.64	-36.4	0.021293
hsa-miR-1254	NM_001283055	STMN4	175	-29.64	-36.4	0.023165
hsa-miR-1254	NM_001284297	PLCB2	168	-28.94	-32.5	0.045652
hsa-miR-1254	NM_001284298	PLCB2	168	-28.94	-32.5	0.045652
hsa-miR-1254	NM_001286220	GOT2	152	-30.65	-34.7	0.025022

hsa-miR-1254	NM_001286267	HMOX2	154	-25.3	-34.4	0.011912
hsa-miR-1254	NM_001286268	HMOX2	154	-25.3	-34.4	0.011912
hsa-miR-1254	NM_001286269	HMOX2	154	-25.3	-34.4	0.011912
hsa-miR-1254	NM_001286270	HMOX2	154	-25.3	-34.4	0.011912
hsa-miR-1254	NM_001286271	HMOX2	154	-25.3	-34.4	0.011912
hsa-miR-1254	NM_001286402	CIITA	168	-36.57	-38.6	0.005337
hsa-miR-1254	NM_001286403	CIITA	168	-36.57	-38.6	0.005337
hsa-miR-1254	NM_001286833	GAK	166	-29.54	-36.8	0.001897
hsa-miR-1254	NM_001287005	SUSD3	154	-29.43	-33.9	0.007375
hsa-miR-1254	NM_001287006	SUSD3	154	-29.43	-33.9	0.007375
hsa-miR-1254	NM_001287007	SUSD3	154	-29.43	-33.9	0.007375
hsa-miR-1254	NM_001287008	SUSD3	154	-29.43	-33.9	0.007375
hsa-miR-1254	NM_001287053	C5orf67	158	-26.44	-32.4	0.011244
hsa-miR-1254	NM_001287390	CCBL1	160	-24.84	-31.7	0.045722
hsa-miR-1254	NM_001287595	SLC1A7	172	-31.18	-36	0.010086
hsa-miR-1254	NM_001287597	SLC1A7	172	-31.18	-36	0.011409
hsa-miR-1254	NM_001288564	TMEM9	182	-38.64	-41.9	0.00085
hsa-miR-1254	NM_001288565	TMEM9	182	-38.64	-41.9	0.00085
hsa-miR-1254	NM_001288566	TMEM9	182	-38.64	-41.9	0.00085
hsa-miR-1254	NM_001288567	TMEM9	182	-38.64	-41.9	0.00085
hsa-miR-1254	NM_001288568	TMEM9	182	-38.64	-41.9	0.00085
hsa-miR-1254	NM_001288569	TMEM9	182	-38.64	-41.9	0.00085
hsa-miR-1254	NM_001288570	TMEM9	182	-38.64	-41.9	0.00085
hsa-miR-1254	NM_001288571	TMEM9	182	-38.64	-41.9	0.00085
hsa-miR-1254	NM_001289082	CD300LF	159	-32.72	-34.2	0.028078
hsa-miR-1254	NM_001289083	CD300LF	159	-32.72	-34.2	0.028078
hsa-miR-1254	NM_001289086	CD300LF	159	-32.72	-34.2	0.028078
hsa-miR-1254	NM_001289087	CD300LF	159	-32.72	-34.2	0.028078
hsa-miR-1254	NM_001289936	ERBB2	164	-28.18	-34.2	0.014028
hsa-miR-1254	NM_001289937	ERBB2	164	-28.18	-34.2	0.027825

hsa-miR-1254	NM_001291283	LYPD8	157	-23.21	-30.9	0.009252
hsa-miR-1254	NM_001291904	LOC79999	160	-30.38	-36	0.021496
hsa-miR-1254	NM_001291905	LOC388436	160	-30.38	-36	0.021496
hsa-miR-1254	NM_001294345	NINJ2	160	-28.51	-30.8	0.034168
hsa-miR-1254	NM_001294346	NINJ2	160	-28.51	-30.8	0.034168
hsa-miR-1254	NM_001300807	BATF2	179	-40.27	-41.9	0.001412
hsa-miR-1254	NM_001300808	BATF2	179	-40.27	-41.9	0.001412
hsa-miR-1254	NM_001301071	DOK7	174	-36.27	-36.2	0.006133
hsa-miR-1254	NM_001301101	PPCDC	172	-34.22	-38.6	0.008287
hsa-miR-1254	NM_001301102	PPCDC	172	-34.22	-38.6	0.008287
hsa-miR-1254	NM_001301103	PPCDC	172	-34.22	-38.6	0.008287
hsa-miR-1254	NM_001301104	PPCDC	172	-34.22	-38.6	0.008287
hsa-miR-1254	NM_001301105	PPCDC	172	-34.22	-38.6	0.008287
hsa-miR-1254	NM_001302777	BPHL	158	-30.62	-32.5	0.03059
hsa-miR-1254	NM_001302891	CEP112	169	-26.45	-30.4	0.045511
hsa-miR-1254	NM_001303029	PKP3	164	-28.59	-31.2	0.021259
hsa-miR-1254	NM_001303272	NOL11	157	-23.09	-29.9	0.041509
hsa-miR-1254	NM_001303450	ZNF500	150	-27.25	-33.2	0.03356
hsa-miR-1254	NM_001303512	PDZD4	152	-30.19	-36.5	0.014154
hsa-miR-1254	NM_001303513	PDZD4	152	-30.19	-36.5	0.014154
hsa-miR-1254	NM_001303514	PDZD4	152	-30.19	-36.5	0.014154
hsa-miR-1254	NM_001303515	PDZD4	152	-30.19	-36.5	0.014154
hsa-miR-1254	NM_001303516	PDZD4	152	-30.19	-36.5	0.014154
hsa-miR-1254	NM_001304462	SLC29A1	165	-30.92	-35.1	0.009451
hsa-miR-1254	NM_001304463	SLC29A1	165	-30.92	-35.1	0.009451
hsa-miR-1254	NM_001304465	SLC29A1	165	-30.92	-35.1	0.009451
hsa-miR-1254	NM_001304466	SLC29A1	165	-30.92	-35.1	0.009451
hsa-miR-1254	NM_001304521	IMPDH1	164	-34.56	-35.7	0.009362
hsa-miR-1254	NM_001307952	HAPLN3	172	-30.4	-35.1	0.011042
hsa-miR-1254	NM_001308267	PTPRN2	156	-31.51	-37.3	0.016671

hsa-miR-1254	NM_001308268	PTPRN2	156	-31.51	-37.3	0.016671
hsa-miR-1254	NM_001311	CRIP1	168	-30.78	-35.1	0.000421
hsa-miR-1254	NM_001316318	PKM	151	-27.91	-33	0.02431
hsa-miR-1254	NM_001361	DHODH	164	-32.98	-38.1	0.007468
hsa-miR-1254	NM_001831	CLU	184	-34.35	-38.6	0.006892
hsa-miR-1254	NM_001868	CPA1	152	-22.68	-28.7	0.007584
hsa-miR-1254	NM_001883	CRHR2	177	-28.83	-35.3	0.033298
hsa-miR-1254	NM_001916	CYC1	150	-32.97	-37	0.000375
hsa-miR-1254	NM_001965	EGR4	157	-25.57	-34.8	0.008213
hsa-miR-1254	NM_002005	FES	157	-34.5	-41.4	0.000053
hsa-miR-1254	NM_002080	GOT2	152	-30.65	-34.7	0.025022
hsa-miR-1254	NM_002098	GUCA1B	155	-26.52	-34.5	0.046757
hsa-miR-1254	NM_002134	HMOX2	154	-25.3	-34.4	0.011912
hsa-miR-1254	NM_002144	HOXB1	156	-22.35	-28.1	0.011768
hsa-miR-1254	NM_002231	CD82	168	-27.68	-31.6	0.047472
hsa-miR-1254	NM_002253	KDR	160	-37.47	-43	0.001621
hsa-miR-1254	NM_002281	KRT81	153	-27.52	-34.7	0.003969
hsa-miR-1254	NM_002315	LMO1	163	-26.25	-32.4	0.010294
hsa-miR-1254	NM_002458	MUC5B	150	-23.52	-34.1	0.012799
hsa-miR-1254	NM_002622	PFDN1	157	-28.81	-32.7	0.049738
hsa-miR-1254	NM_002654	PKM	151	-27.91	-33	0.02431
hsa-miR-1254	NM_002746	MAPK3	160	-34.75	-39.8	0.001158
hsa-miR-1254	NM_002847	PTPRN2	156	-31.51	-37.3	0.016671
hsa-miR-1254	NM_002953	RPS6KA1	163	-35.84	-41	0.001103
hsa-miR-1254	NM_002997	SDC1	169	-34.48	-39.4	0.009076
hsa-miR-1254	NM_003015	SFRP5	169	-27.39	-32.7	0.03785
hsa-miR-1254	NM_003577	UTF1	164	-24.82	-33.1	0.000965
hsa-miR-1254	NM_003612	SEMA7A	161	-27.04	-34.8	0.034282
hsa-miR-1254	NM_003682	MADD	168	-32.33	-37.3	0.006274
hsa-miR-1254	NM_003693	SCARF1	161	-28.46	-34.2	0.025106

hsa-miR-1254	NM_003729	RTCA	166	-35.99	-38.2	0.007597
hsa-miR-1254	NM_003808	TNFSF13	150	-33.69	-40.2	0.001394
hsa-miR-1254	NM_003954	MAP3K14	155	-22.39	-35.1	0.036143
hsa-miR-1254	NM_003984	SLC13A2	171	-30.86	-35	0.007078
hsa-miR-1254	NM_004047	ATP6V0B	162	-27.09	-31.2	0.016596
hsa-miR-1254	NM_004059	CCBL1	160	-24.84	-31.7	0.028597
hsa-miR-1254	NM_004193	GBF1	156	-33.67	-36.8	0.003653
hsa-miR-1254	NM_004332	BPHL	158	-30.62	-32.5	0.03059
hsa-miR-1254	NM_004448	ERBB2	164	-28.18	-34.2	0.014028
hsa-miR-1254	NM_004573	PLCB2	168	-28.94	-32.5	0.045652
hsa-miR-1254	NM_004594	SLC9A5	161	-29.39	-35.8	0.014306
hsa-miR-1254	NM_004603	STX1A	166	-33.7	-38.3	0.006717
hsa-miR-1254	NM_004626	WNT11	176	-33.33	-38.2	0.003127
hsa-miR-1254	NM_004635	MAPKAPK3	160	-30.68	-34	0.043688
hsa-miR-1254	NM_004894	C14orf2	150	-22.89	-30.2	0.040815
hsa-miR-1254	NM_004935	CDK5	163	-31.04	-33.9	0.002002
hsa-miR-1254	NM_005018	PDCD1	176	-32.71	-38.3	0.006496
hsa-miR-1254	NM_005090	JMJD7-PLA2G4B	168	-32.07	-37.6	0.00068
hsa-miR-1254	NM_005148	UNC119	151	-34.8	-38.8	0.001588
hsa-miR-1254	NM_005255	GAK	166	-29.54	-36.8	0.001906
hsa-miR-1254	NM_005419	STAT2	155	-21.78	-36.9	0.021835
hsa-miR-1254	NM_005541	INPP5D	168	-29.19	-35.1	0.025843
hsa-miR-1254	NM_005600	NIT1	154	-23.48	-31.5	0.027072
hsa-miR-1254	NM_005631	SMO	151	-26.95	-33.9	0.03776
hsa-miR-1254	NM_005702	ERAL1	151	-25.1	-32.6	0.020807
hsa-miR-1254	NM_005787	ALG3	152	-25.52	-32.1	0.004484
hsa-miR-1254	NM_005879	TRAIP	162	-24.78	-31.9	0.029353
hsa-miR-1254	NM_006177	NRL	154	-29.18	-34.8	0.027025
hsa-miR-1254	NM_006578	GNB5	182	-39.64	-44.1	0.00119

hsa-miR-1254	NM_006671	SLC1A7	172	-31.18	-36	0.010086
hsa-miR-1254	NM_006800	MSL3	153	-25.99	-33.6	0.021082
hsa-miR-1254	NM_006804	STARD3	159	-24.41	-35.7	0.022507
hsa-miR-1254	NM_006829	ADIRF	158	-24.15	-29.7	0.041202
hsa-miR-1254	NM_006898	HOXD3	164	-28.58	-32.4	0.048167
hsa-miR-1254	NM_007024	TMEM115	159	-32.22	-35.3	0.007969
hsa-miR-1254	NM_007118	TRIO	177	-34.82	-39.3	0.002846
hsa-miR-1254	NM_007183	PKP3	164	-28.59	-31.2	0.021259
hsa-miR-1254	NM_007363	NONO	155	-29.34	-33.7	0.041192
hsa-miR-1254	NM_007368	RASA3	189	-39.19	-47.3	0.00024
hsa-miR-1254	NM_012134	LMOD1	153	-31.4	-36.6	0.027075
hsa-miR-1254	NM_012197	RABGAP1	160	-33.51	-38.2	0.011447
hsa-miR-1254	NM_012310	KIF4A	162	-29.4	-35.1	0.010367
hsa-miR-1254	NM_012385	NUPR1	153	-25.54	-30.8	0.026144
hsa-miR-1254	NM_013389	NPC1L1	155	-24.08	-33.4	0.03611
hsa-miR-1254	NM_013975	LIG3	156	-30.98	-34.2	0.012051
hsa-miR-1254	NM_014042	ANAPC15	156	-25.8	-31.8	0.012435
hsa-miR-1254	NM_014070	C6orf15	162	-26.5	-33.2	0.001815
hsa-miR-1254	NM_014343	CLDN15	156	-22.89	-29.7	0.039413
hsa-miR-1254	NM_014564	LHX3	163	-27.97	-33.9	0.036885
hsa-miR-1254	NM_014675	CROCC	169	-34.55	-37	0.003019
hsa-miR-1254	NM_014863	CHST15	158	-27.51	-34.2	0.048186
hsa-miR-1254	NM_014922	NLRP1	153	-22.52	-32.9	0.02638
hsa-miR-1254	NM_015103	PLXND1	159	-28.69	-35.9	0.016267
hsa-miR-1254	NM_015117	ZC3H3	167	-31.26	-35.6	0.003461
hsa-miR-1254	NM_015193	ARC	158	-31.61	-37.1	0.016451
hsa-miR-1254	NM_015392	NPDC1	160	-28.97	-34.6	0.00357
hsa-miR-1254	NM_015462	NOL11	157	-23.09	-29.9	0.041509
hsa-miR-1254	NM_015952	RWDD1	178	-28.69	-32.8	0.018911
hsa-miR-1254	NM_016104	RWDD1	178	-28.69	-32.8	0.018911

hsa-miR-1254	NM_016135	ETV7	158	-23.67	-30.5	0.046553
hsa-miR-1254	NM_016194	GNB5	182	-39.64	-44.1	0.00119
hsa-miR-1254	NM_016456	TMEM9	182	-38.64	-41.9	0.00085
hsa-miR-1254	NM_016533	NINJ2	160	-28.51	-30.8	0.034168
hsa-miR-1254	NM_017766	CASZ1	160	-28.47	-33.6	0.017143
hsa-miR-1254	NM_017933	PID1	168	-33.67	-40.7	0.004098
hsa-miR-1254	NM_018019	MED9	151	-27.03	-34.9	0.046603
hsa-miR-1254	NM_018056	TMEM39B	162	-30.54	-33.4	0.002486
hsa-miR-1254	NM_018295	TMEM140	167	-36.18	-34.1	0.040297
hsa-miR-1254	NM_018358	ABCF3	161	-28.87	-33	0.007543
hsa-miR-1254	NM_018645	HES6	172	-30.08	-35.7	0.006
hsa-miR-1254	NM_018896	CACNA1G	161	-27.75	-33.5	0.024397
hsa-miR-1254	NM_020211	RGMA	174	-35.61	-41.8	0.002432
hsa-miR-1254	NM_020216	RNPEP	161	-30.06	-33.1	0.012868
hsa-miR-1254	NM_020439	CAMK1G	157	-32.65	-39.4	0.002775
hsa-miR-1254	NM_020769	RGAG1	172	-28.43	-33.1	0.047622
hsa-miR-1254	NM_020822	KCNT1	153	-27.82	-33.1	0.045927
hsa-miR-1254	NM_021226	ARHGAP22	160	-27.32	-35.7	0.002781
hsa-miR-1254	NM_021823	PPCDC	172	-34.22	-38.6	0.008287
hsa-miR-1254	NM_021953	FOXM1	163	-28.34	-37.1	0.007832
hsa-miR-1254	NM_022042	SLC26A1	158	-26.8	-39	0.004813
hsa-miR-1254	NM_022055	KCNK12	150	-23.6	-28.7	0.013848
hsa-miR-1254	NM_022151	MOAP1	166	-35.84	-37.8	0.006115
hsa-miR-1254	NM_022164	TINAGL1	159	-35.59	-32.6	0.035362
hsa-miR-1254	NM_022369	STRA6	152	-23.74	-31.5	0.046824
hsa-miR-1254	NM_022772	EPS8L2	159	-26.68	-32.6	0.038266
hsa-miR-1254	NM_022819	PLA2G2F	169	-28.36	-38.4	0.013765
hsa-miR-1254	NM_023007	JMJD4	163	-25.2	-34.6	0.032197
hsa-miR-1254	NM_023014	PRAMEF2	156	-20.7	-27.3	0.029023
hsa-miR-1254	NM_024098	CCDC86	159	-25.86	-33	0.033185

hsa-miR-1254	NM_024119	DHX58	154	-27.53	-34	0.005966
hsa-miR-1254	NM_024507	KREMEN2	163	-29.82	-32.2	0.018098
hsa-miR-1254	NM_024718	RABL6	157	-29.43	-34.9	0.011513
hsa-miR-1254	NM_024815	NUDT18	162	-25.26	-30.2	0.049398
hsa-miR-1254	NM_024859	MAGIX	156	-30.82	-35.8	0.033217
hsa-miR-1254	NM_024897	PAQR6	169	-30.65	-34.9	0.008148
hsa-miR-1254	NM_025109	MYO19	165	-28.08	-35.6	0.005084
hsa-miR-1254	NM_030783	PTDSS2	153	-26.59	-35.2	0.013669
hsa-miR-1254	NM_030795	STMN4	175	-29.64	-36.4	0.021293
hsa-miR-1254	NM_032251	CCDC88B	151	-22.59	-33.1	0.013323
hsa-miR-1254	NM_032266	C2orf16	160	-25.92	-28.5	0.031748
hsa-miR-1254	NM_032353	VPS25	171	-34.27	-41.4	0.00039
hsa-miR-1254	NM_032442	NEURL4	162	-29.07	-35.4	0.005482
hsa-miR-1254	NM_032489	ACRBP	151	-24.5	-30.3	0.013942
hsa-miR-1254	NM_032512	PDZD4	152	-30.19	-36.5	0.014154
hsa-miR-1254	NM_032643	IRF5	157	-28.67	-34.4	0.035875
hsa-miR-1254	NM_032829	FAM222A	157	-30.23	-34.9	0.028925
hsa-miR-1254	NM_032849	MEDAG	167	-29.55	-35.5	0.020291
hsa-miR-1254	NM_033004	NLRP1	153	-22.52	-32.9	0.02638
hsa-miR-1254	NM_033006	NLRP1	153	-22.52	-32.9	0.02638
hsa-miR-1254	NM_033007	NLRP1	153	-22.52	-32.9	0.02638
hsa-miR-1254	NM_033121	ANKRD13A	180	-35.88	-40.4	0.005617
hsa-miR-1254	NM_033212	CCDC102A	156	-28.01	-31.3	0.045075
hsa-miR-1254	NM_052902	STK11IP	176	-35.04	-32.7	0.006275
hsa-miR-1254	NM_054035	UNC119	151	-34.8	-38.8	0.003477
hsa-miR-1254	NM_078629	MSL3	153	-25.99	-33.6	0.021082
hsa-miR-1254	NM_130470	MADD	168	-32.33	-37.3	0.006274
hsa-miR-1254	NM_130471	MADD	168	-32.33	-37.3	0.006274
hsa-miR-1254	NM_130472	MADD	168	-32.33	-37.3	0.006274
hsa-miR-1254	NM_130473	MADD	168	-32.33	-37.3	0.006274

hsa-miR-1254	NM_130474	MADD	168	-32.33	-37.3	0.007804
hsa-miR-1254	NM_130475	MADD	168	-32.33	-37.3	0.007804
hsa-miR-1254	NM_130476	MADD	168	-32.33	-37.3	0.006274
hsa-miR-1254	NM_130842	PTPRN2	156	-31.51	-37.3	0.016671
hsa-miR-1254	NM_130843	PTPRN2	156	-31.51	-37.3	0.016671
hsa-miR-1254	NM_138350	THAP3	171	-34.11	-36.5	0.019951
hsa-miR-1254	NM_138440	VASN	160	-29.84	-35	0.010047
hsa-miR-1254	NM_138456	BATF2	179	-40.27	-41.9	0.001412
hsa-miR-1254	NM_139162	MIEF2	168	-26.41	-33.2	0.045898
hsa-miR-1254	NM_144962	PEBP4	153	-24.29	-31.2	0.002858
hsa-miR-1254	NM_145006	SUSD3	154	-29.43	-33.9	0.007375
hsa-miR-1254	NM_145285	NKX2-3	157	-27.26	-32.4	0.046352
hsa-miR-1254	NM_145286	STOML3	152	-22.75	-33.1	0.040377
hsa-miR-1254	NM_145309	LRTOMT	166	-36.01	-37.9	0.009774
hsa-miR-1254	NM_148886	MIEF2	168	-26.41	-33.2	0.045898
hsa-miR-1254	NM_152288	ORAI3	182	-34.74	-38	0.006571
hsa-miR-1254	NM_152491	PM20D1	173	-28.88	-31.9	0.03765
hsa-miR-1254	NM_152889	CHST13	158	-29.87	-33.9	0.02012
hsa-miR-1254	NM_153021	PLB1	156	-24.89	-31.9	0.049999
hsa-miR-1254	NM_153046	TDRD9	167	-27.42	-35.5	0.007087
hsa-miR-1254	NM_153480	IL17RE	162	-27.76	-31.7	0.040674
hsa-miR-1254	NM_153481	IL17RE	162	-27.76	-31.7	0.040674
hsa-miR-1254	NM_153483	IL17RE	162	-27.76	-31.7	0.040674
hsa-miR-1254	NM_172087	TNFSF13	150	-33.69	-40.2	0.001394
hsa-miR-1254	NM_172088	TNFSF13	150	-33.69	-40.2	0.000872
hsa-miR-1254	NM_172089	TNFSF12-TNFSF13	150	-33.69	-40.2	0.001394
hsa-miR-1254	NM_172229	KREMEN2	163	-29.82	-32.2	0.014754
hsa-miR-1254	NM_173484	KLF17	153	-22.15	-39.1	0.009723
hsa-miR-1254	NM_173649	C2orf61	164	-30.15	-35.9	0.02508

hsa-miR-1254	NM_173815	CES4A	163	-35.68	-40.9	0.000823
hsa-miR-1254	NM_173855	MORN3	156	-24.24	-34.6	0.002536
hsa-miR-1254	NM_174891	C14orf79	152	-29.12	-35.5	0.0109
hsa-miR-1254	NM_176782	FAM151A	157	-24.18	-32.5	0.000763
hsa-miR-1254	NM_176792	MRPL43	180	-35.1	-40.2	0.003983
hsa-miR-1254	NM_178138	LHX3	163	-27.97	-33.9	0.036885
hsa-miR-1254	NM_178177	NMNAT3	154	-30.31	-36	0.010066
hsa-miR-1254	NM_178232	HAPLN3	172	-30.4	-35.1	0.011042
hsa-miR-1254	NM_178553	PRR30	166	-24.86	-30.4	0.017939
hsa-miR-1254	NM_178831	GATS	150	-34.85	-40.5	0.005935
hsa-miR-1254	NM_182470	PKM	151	-27.91	-33	0.02431
hsa-miR-1254	NM_182471	PKM	151	-27.91	-33	0.02431
hsa-miR-1254	NM_182699	DDX53	163	-29.72	-31.6	0.003112
hsa-miR-1254	NM_183243	IMPDH1	164	-34.56	-35.7	0.009362
hsa-miR-1254	NM_183377	ASIC2	157	-27.4	-34.2	0.024156
hsa-miR-1254	NM_184041	ALDOA	156	-24.56	-29	0.027514
hsa-miR-1254	NM_184043	ALDOA	156	-24.56	-29	0.027514
hsa-miR-1254	NM_194272	RBPMS2	165	-30.27	-35.8	0.017278
hsa-miR-1254	NM_198332	STAT2	155	-21.78	-36.9	0.021835
hsa-miR-1254	NM_198376	CACNA1G	161	-27.75	-33.5	0.024397
hsa-miR-1254	NM_198377	CACNA1G	161	-27.75	-33.5	0.024397
hsa-miR-1254	NM_198378	CACNA1G	161	-27.75	-33.5	0.024397
hsa-miR-1254	NM_198379	CACNA1G	161	-27.75	-33.5	0.024397
hsa-miR-1254	NM_198380	CACNA1G	161	-27.75	-33.5	0.024397
hsa-miR-1254	NM_198382	CACNA1G	161	-27.75	-33.5	0.024397
hsa-miR-1254	NM_198383	CACNA1G	161	-27.75	-33.5	0.024397
hsa-miR-1254	NM_198384	CACNA1G	161	-27.75	-33.5	0.024397
hsa-miR-1254	NM_198385	CACNA1G	161	-27.75	-33.5	0.024397
hsa-miR-1254	NM_198386	CACNA1G	161	-27.75	-33.5	0.024397
hsa-miR-1254	NM_198387	CACNA1G	161	-27.75	-33.5	0.024397

hsa-miR-1254	NM_198388	CACNA1G	161	-27.75	-33.5	0.024397
hsa-miR-1254	NM_198396	CACNA1G	161	-27.75	-33.5	0.024397
hsa-miR-1254	NM_198406	PAQR6	169	-30.65	-34.9	0.015692
hsa-miR-1254	NM_198443	NRN1L	155	-26.09	-34.4	0.000405
hsa-miR-1254	NM_198585	ENTPD8	152	-25.04	-33.4	0.016734
hsa-miR-1254	NM_199173	BGLAP	150	-22.19	-28.6	0.023697
hsa-miR-1254	NM_199184	DNPH1	154	-26.68	-35.5	0.002558
hsa-miR-1254	NM_201546	CDCP2	154	-24.8	-35.7	0.005364
hsa-miR-1254	NM_201651	SLC28A1	152	-31.25	-38.2	0.002169
hsa-miR-1254	NM_202002	FOXM1	163	-28.34	-37.1	0.007832
hsa-miR-1254	NM_202003	FOXM1	163	-28.34	-37.1	0.007832
hsa-miR-1254	NM_203412	UBL4B	154	-27.83	-33.2	0.037939
hsa-miR-1254	NM_203425	C17orf82	159	-27.28	-31.1	0.04752
hsa-miR-1254	NM_207421	PADI6	165	-27.81	-31.5	0.011671
hsa-miR-1254	NM_213613	SLC26A1	158	-26.8	-39	0.004813
hsa-miR-1254	NR_001541	TTTY5	152	-26.2	-34.6	0.046112
hsa-miR-1254	NR_001566	TERC	155	-28.49	-32.6	0.017547
hsa-miR-1254	NR_003667	SUGT1P1	163	-32.23	-37.3	0.005572
hsa-miR-1254	NR_024158	PP7080	162	-26.48	-37.1	0.019369
hsa-miR-1254	NR_024471	MRPL23-AS1	167	-31.12	-35.9	0.035567
hsa-miR-1254	NR_026818	FAM138A	157	-23.94	-33.8	0.041266
hsa-miR-1254	NR_026820	FAM138F	157	-23.94	-33.8	0.041266
hsa-miR-1254	NR_026822	FAM138C	157	-23.94	-33.8	0.041266
hsa-miR-1254	NR_026823	FAM138D	157	-23.94	-33.8	0.041063
hsa-miR-1254	NR_027025	MTVR2	166	-31.18	-34.1	0.022475
hsa-miR-1254	NR_027113	LOC285740	159	-32.35	-35.6	0.03524
hsa-miR-1254	NR_027123	KTN1-AS1	161	-31.2	-38.4	0.0092
hsa-miR-1254	NR_027755	LINC00922	178	-29.79	-37.1	0.019638
hsa-miR-1254	NR_028386	LOC375196	164	-30.79	-35.6	0.017439
hsa-miR-1254	NR_029623	MIR210	150	-20.32	-26.6	0.031219

hsa-miR-1254	NR_029712	MIR195	151	-22.23	-29.6	0.003601
hsa-miR-1254	NR_030310	MIR584	154	-28.02	-29.2	0.005722
hsa-miR-1254	NR_033323	NSUN5P2	180	-36.38	-40.3	0.005335
hsa-miR-1254	NR_033969	PLCE1-AS1	167	-31.3	-34.5	0.04176
hsa-miR-1254	NR_036218	MIR4252	155	-24.91	-31.2	0.000655
hsa-miR-1254	NR_037718	EFEMP2	163	-32.29	-36.6	0.027464
hsa-miR-1254	NR_038435	HOXD-AS2	161	-27.19	-35.9	0.007839
hsa-miR-1254	NR_038908	SENCR	154	-27.92	-34.4	0.038927
hsa-miR-1254	NR_038914	LINC01349	167	-28.08	-37.6	0.00491
hsa-miR-1254	NR_040054	IQCH-AS1	158	-34.53	-35.1	0.01398
hsa-miR-1254	NR_040073	MIR181A1HG	181	-31.05	-34.8	0.01029
hsa-miR-1254	NR_040117	LOC392364	164	-29.16	-33	0.049749
hsa-miR-1254	NR_046211	ELK2AP	180	-35.27	-40.2	0.00145
hsa-miR-1254	NR_046836	HS1BP3-IT1	157	-27.12	-32.8	0.025227
hsa-miR-1254	NR_047700	MYO16-AS1	153	-24.68	-34.6	0.00956
hsa-miR-1254	NR_072989	MMD2	158	-35.17	-41.1	0.002498
hsa-miR-1254	NR_073097	TRAPPC3	151	-29.26	-34.8	0.035178
hsa-miR-1254	NR_073110	PSD	172	-32.94	-37.7	0.017896
hsa-miR-1254	NR_073407	DEFA8P	159	-36.83	-29.1	0.044201
hsa-miR-1254	NR_073408	DEFA9P	151	-20.63	-32	0.010663
hsa-miR-1254	NR_073421	DEFA11P	159	-26.64	-33.2	0.006519
hsa-miR-1254	NR_103794	LOC100996385	160	-25.11	-39.7	0.005571
hsa-miR-1254	NR_106723	MIR6075	151	-22.78	-32.2	0.001002
hsa-miR-1254	NR_107009	MIR7855	160	-25.92	-30	0.00124
hsa-miR-1254	NR_108078	MIR202HG	152	-28.83	-36.1	0.009372
hsa-miR-1254	NR_108079	MIR202HG	152	-28.83	-36.1	0.006737
hsa-miR-1254	NR_108092	LINC01099	174	-34.15	-41.6	0.001657
hsa-miR-1254	NR_109817	LINC00266-3	186	-36.15	-39.9	0.000543
hsa-miR-1254	NR_109920	TMEM9	182	-38.64	-41.9	0.002896
hsa-miR-1254	NR_110148	LOC285740	159	-32.35	-35.6	0.040809

hsa-miR-1254	NR_110149	LOC285740	159	-32.35	-35.6	0.028197
hsa-miR-1254	NR_110731	LINC01232	151	-29.8	-38.5	0.011621
hsa-miR-1254	NR_120310	LOC400553	165	-23.41	-30.4	0.042792
hsa-miR-1254	NR_120583	LINC01495	156	-27.89	-30.4	0.046477
hsa-miR-1254	NR_121579	LOC101928525	164	-28.3	-34	0.037324
hsa-miR-1254	NR_125727	LINC00680	171	-32.88	-36.7	0.027132
hsa-miR-1254	NR_125728	LINC00680	171	-32.88	-36.7	0.024275
hsa-miR-1254	NR_125771	LINC01058	170	-29.6	-38.1	0.001827
hsa-miR-1254	NR_125791	RORB-AS1	162	-28.26	-32.5	0.046129
hsa-miR-1254	NR_125808	LOC101929294	162	-36.64	-42.5	0.000647
hsa-miR-1254	NR_125854	LOC101928280	158	-29.67	-33	0.027686
hsa-miR-1254	NR_125952	LOC101928324	155	-23.96	-34.6	0.017058
hsa-miR-1254	NR_125953	LOC101928324	155	-23.96	-34.6	0.013082
hsa-miR-1254	NR_125995	LOC102724312	150	-25.06	-33.5	0.034094
hsa-miR-1254	NR_126167	TMEM5-AS1	153	-27.5	-31.4	0.043089
hsa-miR-1254	NR_126345	LINC01044	152	-27.89	-32.9	0.027722
hsa-miR-1254	NR_126424	F10-AS1	161	-24.27	-30.4	0.049398
hsa-miR-1254	NR_130112	SFT2D1	153	-27.26	-36.6	0.012365
hsa-miR-1254	NR_130113	SFT2D1	153	-27.26	-36.6	0.010775
hsa-miR-1254	NR_130114	SFT2D1	153	-27.26	-36.6	0.010626
hsa-miR-1254	NR_130721	KCNQ1-AS1	165	-27.26	-33.6	0.042419
hsa-miR-1254	NR_130894	RAI1-AS1	167	-26.82	-32.3	0.041288
hsa-miR-1254	NR_130917	CCDC26	169	-34.51	-38	0.01313
hsa-miR-1254	NR_130918	CCDC26	169	-34.51	-38	0.010679
hsa-miR-1254	NR_130919	CCDC26	169	-34.51	-38	0.012362
hsa-miR-1254	NR_130920	CCDC26	169	-34.51	-38	0.01255
hsa-miR-1254	NR_132114	SNHG19	163	-25.99	-33.3	0.008447
hsa-miR-1254	NR_132318	SHH	167	-34.28	-39.6	0.002571
hsa-miR-1254	NR_132989	MIR3945HG	170	-33.2	-35.9	0.035943
hsa-miR-1254	NR_132996	LINC00680	171	-32.88	-36.7	0.022927

hsa-miR-548i	NR_029885	MIR340	163	-20.57	-25.6	0.006343
hsa-miR-548i	NR_030305	MIR579	160	-22.3	-25.9	0.005573
hsa-miR-548i	NR_030315	MIR548B	160	-23.64	-27.4	0.002015
hsa-miR-548i	NR_030330	MIR548A3	180	-23.24	-28.2	0.001185
hsa-miR-548i	NR_031642	MIR548F1	184	-21.33	-23.5	0.019741
hsa-miR-548i	NR_031643	MIR548F2	184	-25.3	-29.3	0.000586
hsa-miR-548i	NR_031644	MIR548F3	184	-22.65	-26.8	0.002323
hsa-miR-548i	NR_031645	MIR548F4	184	-21.33	-23.5	0.031033
hsa-miR-548i	NR_031646	MIR548F5	179	-21.29	-24.8	0.00868
hsa-miR-548i	NR_031662	MIR548G	178	-24.94	-28.8	0.000641
hsa-miR-548i	NR_031666	MIR548N	192	-27.96	-31.2	0.000079
hsa-miR-548i	NR_031669	MIR548O	178	-22.12	-25.3	0.011437
hsa-miR-548i	NR_031677	MIR548H1	176	-21.29	-24.8	0.012541
hsa-miR-548i	NR_031678	MIR548H2	176	-21.96	-25.5	0.005712
hsa-miR-548i	NR_031679	MIR548H3	188	-25.16	-29	0.001126
hsa-miR-548i	NR_031680	MIR548H4	192	-26.06	-27.7	0.002269
hsa-miR-548i	NR_031686	MIR548P	184	-25.37	-29	0.000482
hsa-miR-548i	NR_031687	MIR548I1	164	-20.37	-25	0.023651
hsa-miR-548i	NR_031688	MIR548I2	164	-20.37	-25	0.023651
hsa-miR-548i	NR_031689	MIR548I3	164	-20.37	-25	0.023651
hsa-miR-548i	NR_031752	MIR548Q	168	-17.84	-22.7	0.047359
hsa-miR-548i	NR_037503	MIR548Y	184	-25.22	-30.6	0.000335
hsa-miR-548i	NR_037515	MIR548Z	192	-30.03	-33.5	0.000035
hsa-miR-548i	NR_039621	MIR548AC	183	-26.32	-29.7	0.00034
hsa-miR-548i	NR_039639	MIR548AE2	188	-25.98	-30.1	0.000122
hsa-miR-548i	NR_039673	MIR548AJ1	184	-25.3	-29.3	0.00026
hsa-miR-548i	NR_039674	MIR548AJ2	184	-25.3	-22.9	0.035369
hsa-miR-548i	NR_039692	MIR548X2	184	-24.05	-28.1	0.001364
hsa-miR-548i	NR_039699	MIR548AK	184	-21.77	-26	0.001411
hsa-miR-548i	NR_039762	MIR548AM	184	-27.44	-31.3	0.000071

hsa-miR-548i	NR_049838	MIR548AQ	188	-27.23	-31.3	0.000034
hsa-miR-548i	NR_049865	MIR548AX	188	-28.1	-31.5	0.000059
hsa-miR-1278	NM_001127582	ING4	150	-23.87	-28.8	0.041745
hsa-miR-1278	NM_001127583	ING4	150	-23.87	-28.8	0.041745
hsa-miR-1278	NM_001127584	ING4	150	-23.87	-28.8	0.041745
hsa-miR-1278	NM_001127585	ING4	150	-23.87	-28.8	0.041745
hsa-miR-1278	NM_001127586	ING4	150	-23.87	-28.8	0.047892
hsa-miR-1278	NM_001285403	AIPL1	157	-17.93	-29.4	0.021922
hsa-miR-1278	NM_016162	ING4	150	-23.87	-28.8	0.041745
hsa-miR-1278	NM_152755	CNPY4	170	-25.28	-28.9	0.03596
hsa-miR-1278	NR_030350	MIR619	163	-19.09	-24.6	0.016005
hsa-miR-1284	NM_001129	AEBP1	164	-30.59	-33.9	0.001072
hsa-miR-1284	NM_001131034	RNF212	156	-28.38	-32	0.039607
hsa-miR-1284	NM_001166136	EVC2	152	-27.74	-31.2	0.008931
hsa-miR-1284	NM_003052	SLC34A1	152	-27.13	-29.1	0.041316
hsa-miR-1284	NM_005585	SMAD6	155	-27.85	-31.6	0.008335
hsa-miR-1284	NM_013334	GMPPB	162	-31.85	-35.8	0.000304
hsa-miR-1284	NM_021971	GMPPB	162	-31.85	-35.8	0.000304
hsa-miR-1284	NM_033411	RWDD2A	160	-25.43	-28.6	0.015179
hsa-miR-1284	NM_147127	EVC2	152	-27.74	-31.2	0.008931
hsa-miR-1284	NM_194439	RNF212	156	-28.38	-32	0.045379
hsa-miR-1284	NM_198504	PAQR9	162	-28.1	-31.6	0.028124
hsa-miR-1284	NR_004388	SCARNA14	177	-28.39	-30.9	0.001029
hsa-miR-1284	NR_028335	LOC284009	152	-30.88	-30.3	0.023471
hsa-miR-1827	NM_000862	HSD3B1	154	-20.71	-25.9	0.04416
hsa-miR-1827	NM_000928	PLA2G1B	154	-19.99	-24.2	0.00524
hsa-miR-1827	NM_001005373	LRSAM1	162	-22	-27.7	0.029895
hsa-miR-1827	NM_001005374	LRSAM1	162	-22	-27.7	0.029895
hsa-miR-1827	NM_001010886	CLPSL1	151	-17.38	-22.5	0.006654
hsa-miR-1827	NM_001035235	SRA1	163	-23.66	-27.2	0.042748

hsa-miR-1827	NM_001098673	ATG101	153	-19.93	-25.4	0.035193
hsa-miR-1827	NM_001099274	TINF2	159	-18.83	-23.5	0.031831
hsa-miR-1827	NM_001129742	CALHM3	168	-26.18	-30.8	0.002485
hsa-miR-1827	NM_001130964	PLCD1	151	-19.23	-24.9	0.040091
hsa-miR-1827	NM_001145054	C2orf81	156	-18.17	-23.2	0.039169
hsa-miR-1827	NM_001190723	LRSAM1	162	-22	-27.7	0.029895
hsa-miR-1827	NM_001243689	LETMD1	167	-23.01	-28.5	0.042998
hsa-miR-1827	NM_001251877	USP4	162	-24.73	-27.9	0.025277
hsa-miR-1827	NM_001252402	TMEM249	155	-20.62	-26.6	0.002512
hsa-miR-1827	NM_001252404	TMEM249	155	-20.62	-26.6	0.000908
hsa-miR-1827	NM_001253764	SRA1	163	-23.66	-27.2	0.042748
hsa-miR-1827	NM_001280561	TMEM249	155	-20.62	-26.6	0.002512
hsa-miR-1827	NM_001286731	AMTN	153	-19.22	-24.9	0.040346
hsa-miR-1827	NM_001300765	LETMD1	167	-23.01	-28.5	0.042998
hsa-miR-1827	NM_001308117	RBFOX1	161	-19.18	-23.9	0.045126
hsa-miR-1827	NM_002696	POLR2G	162	-19.13	-24.3	0.034558
hsa-miR-1827	NM_006225	PLCD1	151	-19.23	-24.9	0.040091
hsa-miR-1827	NM_012067	AKR7A3	155	-19.34	-25.1	0.013713
hsa-miR-1827	NM_015416	LETMD1	167	-23.01	-28.5	0.042998
hsa-miR-1827	NM_018986	SH3TC1	154	-17.7	-23	0.041874
hsa-miR-1827	NM_021934	ATG101	153	-19.93	-25.4	0.035193
hsa-miR-1827	NM_024596	MCPH1	158	-24.67	-27.6	0.028863
hsa-miR-1827	NM_032559	KIF2B	156	-17.2	-23	0.046785
hsa-miR-1827	NM_033303	ADRA1A	154	-20.19	-26.2	0.041331
hsa-miR-1827	NM_138361	LRSAM1	162	-22	-27.7	0.029895
hsa-miR-1827	NM_152516	COMMD1	159	-23.77	-27.8	0.001273
hsa-miR-1827	NM_175724	IL5RA	156	-24.52	-29.2	0.006267
hsa-miR-1827	NM_175727	IL5RA	156	-24.52	-29.2	0.006267
hsa-miR-1827	NM_212557	AMTN	153	-19.22	-24.9	0.040346
hsa-miR-1827	NR_002983	SNORA55	150	-19.75	-23.2	0.031653

hsa-miR-1827	NR_029485	MIR15A	151	-17.92	-23.4	0.009547
hsa-miR-1827	NR_106942	MIR6882	155	-22.79	-28	0.000186
hsa-miR-2277-5p	NM_000034	ALDOA	162	-32.52	-37.6	0.000507
hsa-miR-2277-5p	NM_000118	ENG	152	-29.66	-35	0.024296
hsa-miR-2277-5p	NM_000578	SLC11A1	154	-36.62	-36.1	0.042391
hsa-miR-2277-5p	NM_001001795	C8orf82	162	-31.53	-35.2	0.03052
hsa-miR-2277-5p	NM_001014985	GLTPD2	171	-33.48	-37.7	0.001
hsa-miR-2277-5p	NM_001037283	EIF3B	153	-32.34	-37.4	0.003848
hsa-miR-2277-5p	NM_001039211	ATAD3C	157	-26.25	-36.1	0.035516
hsa-miR-2277-5p	NM_001042535	AGAP3	169	-37.99	-34.6	0.026272
hsa-miR-2277-5p	NM_001080441	TTC36	162	-28.1	-33.2	0.001172
hsa-miR-2277-5p	NM_001093	ACACB	150	-32.09	-35.8	0.047774
hsa-miR-2277-5p	NM_001098533	CDK10	158	-30.4	-37	0.006425
hsa-miR-2277-5p	NM_001098637	PWWP2B	151	-31.65	-35.8	0.01378
hsa-miR-2277-5p	NM_001099409	EHBP1L1	155	-27.4	-31.1	0.030669
hsa-miR-2277-5p	NM_001127617	ALDOA	162	-32.52	-37.6	0.000507
hsa-miR-2277-5p	NM_001130045	TTLL10	157	-26.43	-30.9	0.00242
hsa-miR-2277-5p	NM_001130136	ISLR2	151	-36.87	-38.1	0.018629
hsa-miR-2277-5p	NM_001130137	ISLR2	151	-36.87	-38.1	0.018629
hsa-miR-2277-5p	NM_001130138	ISLR2	151	-36.87	-38.1	0.018629
hsa-miR-2277-5p	NM_001134340	PSMG3	163	-34.32	-35.6	0.004134
hsa-miR-2277-5p	NM_001136044	TMUB1	151	-25.1	-32.3	0.028565
hsa-miR-2277-5p	NM_001145033	C11orf96	158	-32.09	-35.6	0.010138
hsa-miR-2277-5p	NM_001160367	CDK10	158	-30.4	-37	0.006425
hsa-miR-2277-5p	NM_001163424	MYEOV2	153	-28.85	-36.2	0.001217
hsa-miR-2277-5p	NM_001172663	RAB40C	151	-24.87	-35.9	0.039171
hsa-miR-2277-5p	NM_001172664	RAB40C	151	-24.87	-35.9	0.039171
hsa-miR-2277-5p	NM_001172665	RAB40C	151	-24.87	-35.9	0.039171
hsa-miR-2277-5p	NM_001172666	RAB40C	151	-24.87	-35.9	0.039171
hsa-miR-2277-5p	NM_001177428	TRPV4	160	-33.16	-37	0.00433

hsa-miR-2277-5p	NM_001177431	TRPV4	160	-33.16	-37	0.00433
hsa-miR-2277-5p	NM_001177433	TRPV4	160	-33.16	-37	0.00433
hsa-miR-2277-5p	NM_001189	NKX3-2	158	-33.69	-36.2	0.021263
hsa-miR-2277-5p	NM_001243177	ALDOA	162	-32.52	-37.6	0.000507
hsa-miR-2277-5p	NM_001256185	SNX12	157	-32.15	-36.8	0.030151
hsa-miR-2277-5p	NM_001256186	SNX12	157	-32.15	-36.8	0.03185
hsa-miR-2277-5p	NM_001256187	SNX12	157	-32.15	-36.8	0.03185
hsa-miR-2277-5p	NM_001256188	SNX12	157	-32.15	-36.8	0.030151
hsa-miR-2277-5p	NM_001256627	BRSK2	164	-33.23	-37.1	0.030923
hsa-miR-2277-5p	NM_001256630	BRSK2	164	-33.23	-37.1	0.030923
hsa-miR-2277-5p	NM_001256672	STOML1	156	-34.19	-38.8	0.003281
hsa-miR-2277-5p	NM_001256673	STOML1	156	-34.19	-38.8	0.003281
hsa-miR-2277-5p	NM_001256674	STOML1	156	-34.19	-38.8	0.003281
hsa-miR-2277-5p	NM_001256675	STOML1	156	-34.19	-38.8	0.003281
hsa-miR-2277-5p	NM_001256676	STOML1	156	-34.19	-38.8	0.003281
hsa-miR-2277-5p	NM_001256677	STOML1	156	-34.19	-38.8	0.003281
hsa-miR-2277-5p	NM_001272011	MOK	163	-28.65	-31.3	0.045341
hsa-miR-2277-5p	NM_001278710	NRN1	160	-29.18	-33.9	0.042904
hsa-miR-2277-5p	NM_001278711	NRN1	160	-29.18	-33.9	0.042904
hsa-miR-2277-5p	NM_001278942	SURF6	158	-29.03	-38.8	0.012374
hsa-miR-2277-5p	NM_001282658	CCDC3	156	-32.9	-35.7	0.047184
hsa-miR-2277-5p	NM_001284351	SLC9A3	158	-31.69	-29.1	0.02224
hsa-miR-2277-5p	NM_001289395	TULP1	152	-26.5	-36.1	0.00487
hsa-miR-2277-5p	NM_001301060	MORN1	163	-32.32	-37.6	0.000908
hsa-miR-2277-5p	NM_001301168	SHF	162	-29.57	-34.2	0.024945
hsa-miR-2277-5p	NM_001301169	SHF	162	-29.57	-34.2	0.036623
hsa-miR-2277-5p	NM_001301170	SHF	162	-29.57	-34.2	0.033424
hsa-miR-2277-5p	NM_001301171	SHF	162	-29.57	-34.2	0.024945
hsa-miR-2277-5p	NM_001302959	AIP	153	-30.24	-34.9	0.00053
hsa-miR-2277-5p	NM_001302960	AIP	153	-30.24	-34.9	0.002787

hsa-miR-2277-5p	NM_001304359	MUC5AC	166	-35.14	-38	0.001923
hsa-miR-2277-5p	NM_001305275	AGRN	157	-29.06	-34.6	0.039771
hsa-miR-2277-5p	NM_001305	CLDN4	151	-33.92	-34.9	0.02358
hsa-miR-2277-5p	NM_001308304	AGAP3	169	-37.99	-34.6	0.026272
hsa-miR-2277-5p	NM_001308305	AGAP3	169	-37.99	-34.6	0.026272
hsa-miR-2277-5p	NM_001426	EN1	157	-30.74	-35.9	0.012855
hsa-miR-2277-5p	NM_001715	BLK	156	-29.85	-33.6	0.017389
hsa-miR-2277-5p	NM_002226	JAG2	152	-34.94	-36.1	0.038063
hsa-miR-2277-5p	NM_002653	PITX1	161	-34.26	-35.4	0.025335
hsa-miR-2277-5p	NM_002699	POU3F1	159	-31.93	-39.1	0.009846
hsa-miR-2277-5p	NM_003322	TULP1	152	-26.5	-36.1	0.00487
hsa-miR-2277-5p	NM_003751	EIF3B	153	-32.34	-37.4	0.002963
hsa-miR-2277-5p	NM_003952	RPS6KB2	152	-26.87	-31.8	0.011872
hsa-miR-2277-5p	NM_003977	AIP	153	-30.24	-34.9	0.00053
hsa-miR-2277-5p	NM_004160	PYY	159	-30.77	-37.1	0.000714
hsa-miR-2277-5p	NM_004174	SLC9A3	158	-31.69	-29.1	0.02224
hsa-miR-2277-5p	NM_004259	RECQL5	158	-33.81	-42.9	0.000355
hsa-miR-2277-5p	NM_004809	STOML1	156	-34.19	-38.8	0.003281
hsa-miR-2277-5p	NM_005098	MSC	167	-35.7	-39.5	0.005096
hsa-miR-2277-5p	NM_005170	ASCL2	154	-31.65	-35.7	0.01068
hsa-miR-2277-5p	NM_005173	ATP2A3	170	-38.03	-42.7	0.002336
hsa-miR-2277-5p	NM_005245	FAT1	156	-30.92	-36.8	0.009497
hsa-miR-2277-5p	NM_005929	MFI2	159	-31.32	-36.7	0.028063
hsa-miR-2277-5p	NM_006688	C1QL1	170	-33.96	-33.5	0.01843
hsa-miR-2277-5p	NM_007046	EMILIN1	154	-30.08	-32.9	0.015852
hsa-miR-2277-5p	NM_012186	FOXE3	153	-26.43	-33.6	0.035162
hsa-miR-2277-5p	NM_013346	SNX12	157	-32.15	-36.8	0.030151
hsa-miR-2277-5p	NM_014226	MOK	163	-28.65	-31.3	0.045341
hsa-miR-2277-5p	NM_014716	ACAP1	150	-26.05	-30.6	0.002361
hsa-miR-2277-5p	NM_015103	PLXND1	156	-31.56	-35.6	0.025088

hsa-miR-2277-5p	NM_015720	PODXL2	155	-34.85	-31.5	0.02389
hsa-miR-2277-5p	NM_016209	TRAPPC2L	158	-25.46	-31.4	0.006256
hsa-miR-2277-5p	NM_016588	NRN1	160	-29.18	-33.9	0.042904
hsa-miR-2277-5p	NM_017688	BSPRY	153	-35.2	-40.4	0.003357
hsa-miR-2277-5p	NM_017766	CASZ1	151	-31.57	-37.3	0.004521
hsa-miR-2277-5p	NM_018953	HOXC5	166	-32.3	-38.1	0.006097
hsa-miR-2277-5p	NM_020376	PNPLA2	163	-31.06	-34	0.025862
hsa-miR-2277-5p	NM_020655	JPH3	154	-33.25	-37.5	0.018019
hsa-miR-2277-5p	NM_020764	CASKIN1	164	-30.64	-34.9	0.045248
hsa-miR-2277-5p	NM_020851	ISLR2	151	-36.87	-38.1	0.018629
hsa-miR-2277-5p	NM_021025	TLX3	157	-21.59	-33.1	0.024927
hsa-miR-2277-5p	NM_021168	RAB40C	151	-24.87	-35.9	0.039171
hsa-miR-2277-5p	NM_021619	PRDM12	155	-30.69	-35.2	0.038563
hsa-miR-2277-5p	NM_021625	TRPV4	160	-33.16	-37	0.00433
hsa-miR-2277-5p	NM_022055	KCNK12	157	-23.67	-27.9	0.028805
hsa-miR-2277-5p	NM_022363	LHX5	150	-26.55	-30	0.041645
hsa-miR-2277-5p	NM_030923	TMEM163	159	-35.41	-42.9	0.000934
hsa-miR-2277-5p	NM_031434	TMUB1	151	-25.1	-32.3	0.028565
hsa-miR-2277-5p	NM_031455	CCDC3	156	-32.9	-35.7	0.047184
hsa-miR-2277-5p	NM_032125	TMEM222	166	-34.81	-38.6	0.005556
hsa-miR-2277-5p	NM_032294	CAMKK1	173	-33.58	-38.7	0.015605
hsa-miR-2277-5p	NM_032302	PSMG3	163	-34.32	-35.6	0.004134
hsa-miR-2277-5p	NM_032536	NTNG2	163	-34.95	-35.6	0.013303
hsa-miR-2277-5p	NM_032546	TRIM54	157	-30.07	-33.3	0.02408
hsa-miR-2277-5p	NM_032595	PPP1R9B	150	-26.76	-36.6	0.02834
hsa-miR-2277-5p	NM_033260	FOXQ1	158	-32.95	-39.4	0.003321
hsa-miR-2277-5p	NM_052987	CDK10	158	-30.4	-37	0.005136
hsa-miR-2277-5p	NM_052988	CDK10	158	-30.4	-37	0.006425
hsa-miR-2277-5p	NM_130459	TOR2A	161	-31.32	-35.7	0.043786
hsa-miR-2277-5p	NM_133467	CITED4	160	-29.43	-34.7	0.01153

hsa-miR-2277-5p	NM_138349	TP53I13	158	-32.73	-36.5	0.001118
hsa-miR-2277-5p	NM_138356	SHF	162	-29.57	-34.2	0.024945
hsa-miR-2277-5p	NM_138499	PWWP2B	151	-31.65	-35.8	0.014615
hsa-miR-2277-5p	NM_144674	TEKT5	158	-35.47	-37.4	0.000051
hsa-miR-2277-5p	NM_145159	JAG2	152	-34.94	-36.1	0.038063
hsa-miR-2277-5p	NM_147204	TRPV4	160	-33.16	-37	0.00433
hsa-miR-2277-5p	NM_172206	CAMKK1	173	-33.58	-38.7	0.015605
hsa-miR-2277-5p	NM_174953	ATP2A3	170	-38.03	-42.7	0.002336
hsa-miR-2277-5p	NM_174954	ATP2A3	170	-38.03	-42.7	0.002363
hsa-miR-2277-5p	NM_174955	ATP2A3	170	-38.03	-42.7	0.002228
hsa-miR-2277-5p	NM_174956	ATP2A3	170	-38.03	-42.7	0.002363
hsa-miR-2277-5p	NM_174957	ATP2A3	170	-38.03	-42.7	0.002336
hsa-miR-2277-5p	NM_174958	ATP2A3	170	-38.03	-42.7	0.001041
hsa-miR-2277-5p	NM_175630	DNMT3A	158	-33.59	-38.8	0.005122
hsa-miR-2277-5p	NM_178562	TSPAN33	170	-35.1	-40.2	0.008166
hsa-miR-2277-5p	NM_182905	WASH1	152	-32.14	-32.3	0.013609
hsa-miR-2277-5p	NM_184041	ALDOA	162	-32.52	-37.6	0.000507
hsa-miR-2277-5p	NM_184043	ALDOA	162	-32.52	-37.6	0.000507
hsa-miR-2277-5p	NM_187841	TRIM54	157	-30.07	-33.3	0.02408
hsa-miR-2277-5p	NM_198545	DRAXIN	151	-26.17	-32.3	0.040426
hsa-miR-2277-5p	NM_198576	AGRN	157	-29.06	-34.6	0.039771
hsa-miR-2277-5p	NR_001566	TERC	150	-23.89	-33.7	0.014464
hsa-miR-2277-5p	NR_003051	RMRP	156	-34.2	-41.2	0.000152
hsa-miR-2277-5p	NR_003272	PSPC1	173	-33.01	-38	0.018524
hsa-miR-2277-5p	NR_015395	MIR4435-2HG	154	-34.84	-39.9	0.00243
hsa-miR-2277-5p	NR_024337	SERTAD4-AS1	156	-40.21	-36.4	0.011079
hsa-miR-2277-5p	NR_024373	MIR4435-2HG	154	-34.84	-39.9	0.001211
hsa-miR-2277-5p	NR_024418	LOC389332	170	-32.02	-39.4	0.010111
hsa-miR-2277-5p	NR_024454	NDUFB2-AS1	178	-36.32	-40	0.007616
hsa-miR-2277-5p	NR_024610	HINT1	152	-31.04	-35.9	0.015832

hsa-miR-2277-5p	NR_024611	HINT1	152	-31.04	-35.9	0.011705
hsa-miR-2277-5p	NR_027142	LOC440895	158	-28.69	-33.1	0.043246
hsa-miR-2277-5p	NR_027143	LOC440895	158	-28.69	-33.1	0.049851
hsa-miR-2277-5p	NR_027715	BIN3-IT1	153	-32.23	-35.9	0.045181
hsa-miR-2277-5p	NR_028308	BRE-AS1	159	-35.18	-38.3	0.015192
hsa-miR-2277-5p	NR_031764	UGT3A2	160	-33.76	-35.8	0.04744
hsa-miR-2277-5p	NR_033384	PDHB	158	-35.95	-40.5	0.005936
hsa-miR-2277-5p	NR_034105	CRNDE	167	-36.32	-35.1	0.02925
hsa-miR-2277-5p	NR_036750	LOC440434	164	-34.22	-35.8	0.04078
hsa-miR-2277-5p	NR_037627	LOC100288570	158	-28.69	-33.1	0.049851
hsa-miR-2277-5p	NR_037652	ST20	161	-36.61	-39.3	0.002175
hsa-miR-2277-5p	NR_037653	ST20	161	-36.61	-39.3	0.002309
hsa-miR-2277-5p	NR_037869	LOC653160	150	-31.53	-36.5	0.036993
hsa-miR-2277-5p	NR_038378	LOC441242	170	-31.98	-38.3	0.019283
hsa-miR-2277-5p	NR_045724	CLTB	160	-29.95	-35.9	0.041145
hsa-miR-2277-5p	NR_046090	LOC283214	152	-35.34	-37.5	0.018176
hsa-miR-2277-5p	NR_046205	RBPMS-AS1	162	-28.47	-34.7	0.027748
hsa-miR-2277-5p	NR_046706	MID1IP1-AS1	150	-29.61	-34.2	0.023062
hsa-miR-2277-5p	NR_047484	PDX1-AS1	151	-34.48	-37.5	0.027095
hsa-miR-2277-5p	NR_049718	COX10-AS1	153	-35.49	-36.2	0.043236
hsa-miR-2277-5p	NR_073110	PSD	156	-34.59	-38.3	0.019214
hsa-miR-2277-5p	NR_073189	HRK	151	-32.92	-37.8	0.021445
hsa-miR-2277-5p	NR_073484	CDK2AP2	162	-34.5	-39.5	0.006098
hsa-miR-2277-5p	NR_073488	HINT1	152	-31.04	-35.9	0.021248
hsa-miR-2277-5p	NR_103508	PHYKPL	172	-33.32	-38	0.017496
hsa-miR-2277-5p	NR_103746	LINC01356	157	-38.21	-39.4	0.011255
hsa-miR-2277-5p	NR_103772	LOC100288162	154	-29.28	-34.9	0.032501
hsa-miR-2277-5p	NR_103802	EBPL	153	-33.05	-40.7	0.002988
hsa-miR-2277-5p	NR_103803	EBPL	153	-33.05	-40.7	0.00216
hsa-miR-2277-5p	NR_105014	SCAMP1-AS1	153	-36.17	-38.9	0.010808

hsa-miR-2277-5p	NR_105049	C5orf66-AS1	152	-31.99	-34.8	0.023798
hsa-miR-2277-5p	NR_105050	C5orf66-AS1	152	-31.99	-34.8	0.023588
hsa-miR-2277-5p	NR_109789	RAB24	152	-25.07	-35.6	0.047707
hsa-miR-2277-5p	NR_109869	HMGCLL1	171	-36.65	-40.9	0.006984
hsa-miR-2277-5p	NR_109915	CTD-	163	-25.56	-38.8	0.011621
hsa-miR-2277-5p	NR_109951	OSMR-AS1	151	-32.81	-35.6	0.01144
hsa-miR-2277-5p	NR_110227	UTAT33	156	-30.05	-34.8	0.013051
hsa-miR-2277-5p	NR_110453	CRNDE	167	-36.32	-35.1	0.032725
hsa-miR-2277-5p	NR_110736	TMEM114	153	-29.48	-35.9	0.006874
hsa-miR-2277-5p	NR_119380	LOC102723385	162	-32.74	-38.4	0.002181
hsa-miR-2277-5p	NR_120508	ELFN1-AS1	162	-31.54	-37.4	0.010383
hsa-miR-2277-5p	NR_120509	ELFN1-AS1	162	-31.54	-37.4	0.007352
hsa-miR-2277-5p	NR_120510	ELFN1-AS1	162	-31.54	-37.4	0.005418
hsa-miR-2277-5p	NR_120576	LOC101929089	169	-33.96	-37.4	0.002806
hsa-miR-2277-5p	NR_121682	LINC01465	163	-38.45	-37.6	0.020516
hsa-miR-2277-5p	NR_125384	LOC100507437	154	-29.98	-35.6	0.041653
hsa-miR-2277-5p	NR_125385	LOC100507437	154	-29.98	-35.6	0.036926
hsa-miR-2277-5p	NR_125749	TBX2-AS1	152	-24.93	-34.7	0.025665
hsa-miR-2277-5p	NR_125791	RORB-AS1	167	-35.32	-41.1	0.001539
hsa-miR-2277-5p	NR_130110	RAET1G	150	-35.6	-39	0.005939
hsa-miR-2277-5p	NR_132969	LOC145694	155	-28.94	-36.2	0.041292
hsa-miR-2277-5p	NR_132970	LOC145694	155	-28.94	-36.2	0.038432
hsa-miR-2277-5p	NR_132971	LOC145694	155	-28.94	-36.2	0.036851
hsa-miR-3130-5p	NM_000386	BLMH	153	-29.91	-33.9	0.01274
hsa-miR-3130-5p	NM_000747	CHRNB1	151	-28.46	-33.2	0.019972
hsa-miR-3130-5p	NM_001004019	FBLN2	166	-29.3	-30.4	0.030559
hsa-miR-3130-5p	NM_001004333	RNASEK	155	-25.61	-31.9	0.004001
hsa-miR-3130-5p	NM_001005407	CACNA1H	163	-27.91	-34.3	0.00966
hsa-miR-3130-5p	NM_001012631	IL32	164	-26.23	-30.6	0.012313
hsa-miR-3130-5p	NM_001012632	IL32	164	-26.23	-30.6	0.012313

hsa-miR-3130-5p	NM_001012633	IL32	164	-26.23	-30.6	0.012313
hsa-miR-3130-5p	NM_001012634	IL32	164	-26.23	-30.6	0.012313
hsa-miR-3130-5p	NM_001012635	IL32	164	-26.23	-30.6	0.012313
hsa-miR-3130-5p	NM_001012636	IL32	164	-26.23	-30.6	0.012313
hsa-miR-3130-5p	NM_001012718	IL32	164	-26.23	-30.6	0.012313
hsa-miR-3130-5p	NM_001040167	LFNG	159	-29.47	-33	0.034843
hsa-miR-3130-5p	NM_001079530	CFC1B	150	-19.48	-24.3	0.046109
hsa-miR-3130-5p	NM_001080524	C16orf90	155	-22.15	-29.2	0.031444
hsa-miR-3130-5p	NM_001100598	ZNF707	162	-26.42	-31.2	0.046981
hsa-miR-3130-5p	NM_001100599	ZNF707	162	-26.42	-31.2	0.046981
hsa-miR-3130-5p	NM_001145265	SLC35C1	163	-28.09	-34.4	0.029999
hsa-miR-3130-5p	NM_001145266	SLC35C1	163	-28.09	-34.4	0.029999
hsa-miR-3130-5p	NM_001165035	FBLN2	166	-29.3	-30.4	0.030559
hsa-miR-3130-5p	NM_001166355	LFNG	159	-29.47	-33	0.034843
hsa-miR-3130-5p	NM_001171971	CDHR1	168	-27.76	-31	0.011941
hsa-miR-3130-5p	NM_001174136	TDGF1	168	-29.19	-34.5	0.017782
hsa-miR-3130-5p	NM_001178087	SERF1B	153	-25.34	-27.9	0.048393
hsa-miR-3130-5p	NM_001195129	PRSS56	155	-28.23	-34.7	0.000621
hsa-miR-3130-5p	NM_001197244	BCL7B	165	-36.15	-39.3	0.001326
hsa-miR-3130-5p	NM_001199161	USP19	158	-30.99	-33	0.004822
hsa-miR-3130-5p	NM_001199162	USP19	158	-30.99	-33	0.004822
hsa-miR-3130-5p	NM_001248006	TRIM3	167	-28.3	-32.9	0.006398
hsa-miR-3130-5p	NM_001248007	TRIM3	167	-28.3	-32.9	0.006398
hsa-miR-3130-5p	NM_001253357	TIE1	152	-24.68	-30.2	0.022054
hsa-miR-3130-5p	NM_001271641	MTCH1	154	-31.07	-35.7	0.004898
hsa-miR-3130-5p	NM_001286561	NUMA1	150	-19.12	-30.9	0.036995
hsa-miR-3130-5p	NM_001288721	B3GAT3	156	-23.57	-28.9	0.045166
hsa-miR-3130-5p	NM_001288805	ZNF707	162	-26.42	-31.2	0.046981
hsa-miR-3130-5p	NM_001288806	ZNF707	162	-26.42	-31.2	0.046981
hsa-miR-3130-5p	NM_001288807	ZNF707	162	-26.42	-31.2	0.046981

hsa-miR-3130-5p	NM_001288808	ZNF707	162	-26.42	-31.2	0.046981
hsa-miR-3130-5p	NM_001288809	ZNF707	162	-26.42	-31.2	0.046981
hsa-miR-3130-5p	NM_001293228	MYCN	150	-27.74	-31.4	0.046951
hsa-miR-3130-5p	NM_001293231	MYCN	150	-27.74	-31.4	0.046951
hsa-miR-3130-5p	NM_001300750	LMBR1L	156	-27.56	-32.7	0.010945
hsa-miR-3130-5p	NM_001300751	LMBR1L	156	-27.56	-32.7	0.010945
hsa-miR-3130-5p	NM_001301061	BCL7B	165	-36.15	-39.3	0.001326
hsa-miR-3130-5p	NM_001308078	IL32	164	-26.23	-30.6	0.024331
hsa-miR-3130-5p	NM_001707	BCL7B	165	-36.15	-39.3	0.001326
hsa-miR-3130-5p	NM_001998	FBLN2	166	-29.3	-30.4	0.030559
hsa-miR-3130-5p	NM_002304	LFNG	159	-29.47	-33	0.034843
hsa-miR-3130-5p	NM_002689	POLA2	162	-28.97	-34.1	0.02673
hsa-miR-3130-5p	NM_002917	RFNG	155	-28.05	-31.1	0.046604
hsa-miR-3130-5p	NM_003212	TDGF1	168	-29.19	-34.5	0.017782
hsa-miR-3130-5p	NM_003215	TEC	158	-31.56	-34.7	0.025298
hsa-miR-3130-5p	NM_003738	PTCH2	154	-25.73	-27.5	0.016564
hsa-miR-3130-5p	NM_004077	CS	171	-30.86	-33.9	0.028256
hsa-miR-3130-5p	NM_004221	IL32	164	-26.23	-30.6	0.012313
hsa-miR-3130-5p	NM_005378	MYCN	150	-27.74	-31.4	0.046951
hsa-miR-3130-5p	NM_005424	TIE1	152	-24.68	-30.2	0.022054
hsa-miR-3130-5p	NM_006156	NEDD8	153	-21.15	-28.9	0.023944
hsa-miR-3130-5p	NM_006177	NRL	154	-33.25	-34.9	0.013325
hsa-miR-3130-5p	NM_006185	NUMA1	150	-19.12	-30.9	0.036995
hsa-miR-3130-5p	NM_006458	TRIM3	167	-28.3	-32.9	0.006398
hsa-miR-3130-5p	NM_012200	B3GAT3	156	-23.57	-28.9	0.045166
hsa-miR-3130-5p	NM_013334	GMPPB	150	-25.19	-27.8	0.042352
hsa-miR-3130-5p	NM_014341	MTCH1	154	-31.07	-35.7	0.004898
hsa-miR-3130-5p	NM_014506	TOR1B	157	-31.42	-35.7	0.017384
hsa-miR-3130-5p	NM_014718	CLSTN3	150	-30.73	-31.3	0.044737
hsa-miR-3130-5p	NM_015001	SPEN	150	-30.35	-33.8	0.018943

hsa-miR-3130-5p	NM_015024	XPO7	158	-28.95	-35.2	0.017929
hsa-miR-3130-5p	NM_015421	TMEM186	156	-27.16	-36.6	0.003237
hsa-miR-3130-5p	NM_015528	RNF167	155	-23.32	-31.2	0.004051
hsa-miR-3130-5p	NM_016215	EGFL7	157	-37.75	-41.7	0.000067
hsa-miR-3130-5p	NM_016233	PADI3	164	-29.42	-33.9	0.021561
hsa-miR-3130-5p	NM_017567	NAGK	161	-25.6	-28.5	0.045706
hsa-miR-3130-5p	NM_018113	LMBR1L	156	-27.56	-32.7	0.010945
hsa-miR-3130-5p	NM_018389	SLC35C1	163	-28.09	-34.4	0.029999
hsa-miR-3130-5p	NM_018397	CHDH	152	-29.47	-36.3	0.010883
hsa-miR-3130-5p	NM_021098	CACNA1H	163	-27.91	-34.3	0.00966
hsa-miR-3130-5p	NM_021971	GMPPB	150	-25.19	-27.8	0.042352
hsa-miR-3130-5p	NM_022968	SERF1A	153	-25.34	-27.9	0.048899
hsa-miR-3130-5p	NM_024933	ANKRD53	152	-21.18	-33	0.021781
hsa-miR-3130-5p	NM_032111	MRPL14	150	-26.56	-29.1	0.012798
hsa-miR-3130-5p	NM_033278	TRIM3	167	-28.3	-32.9	0.006398
hsa-miR-3130-5p	NM_152898	FERD3L	167	-29.3	-30.9	0.000559
hsa-miR-3130-5p	NM_173831	ZNF707	162	-26.42	-31.2	0.046981
hsa-miR-3130-5p	NM_199349	KCP	159	-31.09	-33.3	0.002558
hsa-miR-3130-5p	NM_201446	EGFL7	157	-37.75	-41.7	0.000067
hsa-miR-3130-5p	NR_026776	LOC100270746	157	-27.53	-31.8	0.041129
hsa-miR-3130-5p	NR_027005	KHDC1	170	-32.25	-34.3	0.025274
hsa-miR-3130-5p	NR_027274	SRRM2-AS1	171	-29.63	-33.6	0.031754
hsa-miR-3130-5p	NR_027761	LOC100132831	162	-29.79	-35.2	0.026851
hsa-miR-3130-5p	NR_029636	MIR222	158	-24.11	-27.4	0.009567
hsa-miR-3130-5p	NR_036077	MIR3130-1	195	-49.27	-52	0
hsa-miR-3130-5p	NR_036682	BCL7B	165	-36.15	-39.3	0.003633
hsa-miR-3130-5p	NR_073410	TMEM134	151	-31.39	-32.7	0.02461
hsa-miR-3130-5p	NR_103547	FER1L6-AS2	151	-29.41	-34	0.037596
hsa-miR-3130-5p	NR_106933	MIR6873	153	-21.49	-25.5	0.009142
hsa-miR-3130-5p	NR_106964	MIR7114	154	-19.82	-23.1	0.038728

hsa-miR-3130-5p	NR_110868	LOC101929767	155	-27.17	-30.6	0.045788
hsa-miR-3074-5p	NM_000172	GNAT1	155	-28.99	-32.2	0.034476
hsa-miR-3074-5p	NM_001037633	SIL1	150	-24.81	-29.6	0.019055
hsa-miR-3074-5p	NM_001039966	GPER1	167	-25.69	-31.2	0.035347
hsa-miR-3074-5p	NM_001040118	ARAP1	158	-26.1	-29.8	0.033588
hsa-miR-3074-5p	NM_001080442	SLC38A8	151	-16.92	-24.1	0.047029
hsa-miR-3074-5p	NM_001083885	DFNB31	157	-24.16	-31.5	0.017641
hsa-miR-3074-5p	NM_001085347	TOR2A	156	-25.1	-29.1	0.037403
hsa-miR-3074-5p	NM_001098201	GPER1	167	-25.69	-31.2	0.035347
hsa-miR-3074-5p	NM_001099274	TINF2	151	-22.1	-27.2	0.012342
hsa-miR-3074-5p	NM_001102594	DTX2	153	-26.44	-31.6	0.006599
hsa-miR-3074-5p	NM_001102595	DTX2	153	-26.44	-31.6	0.006599
hsa-miR-3074-5p	NM_001102596	DTX2	153	-26.44	-31.6	0.006599
hsa-miR-3074-5p	NM_001105669	TTC24	150	-22.82	-27.5	0.020986
hsa-miR-3074-5p	NM_001122646	FAM178B	169	-27.33	-33.3	0.00269
hsa-miR-3074-5p	NM_001130992	RBP1	154	-26.17	-31.2	0.032245
hsa-miR-3074-5p	NM_001134450	TMEM130	153	-29.33	-32.9	0.032974
hsa-miR-3074-5p	NM_001134451	TMEM130	153	-29.33	-32.9	0.032974
hsa-miR-3074-5p	NM_001134774	KLC2	155	-29.36	-33.2	0.012428
hsa-miR-3074-5p	NM_001134775	KLC2	155	-29.36	-33.2	0.012405
hsa-miR-3074-5p	NM_001134776	KLC2	155	-29.36	-33.2	0.012405
hsa-miR-3074-5p	NM_001135190	ARAP1	158	-26.1	-29.8	0.033588
hsa-miR-3074-5p	NM_001142623	GHDC	154	-28.04	-31.9	0.021934
hsa-miR-3074-5p	NM_001172667	FAM178B	169	-27.33	-33.3	0.00269
hsa-miR-3074-5p	NM_001173425	DFNB31	157	-24.16	-31.5	0.017641
hsa-miR-3074-5p	NM_001204747	RFC1	159	-30.36	-33.7	0.017582
hsa-miR-3074-5p	NM_001252018	TOR2A	156	-25.1	-29.1	0.037403
hsa-miR-3074-5p	NM_001252021	TOR2A	156	-25.1	-29.1	0.037403
hsa-miR-3074-5p	NM_001257359	SAMD14	154	-31.29	-34.5	0.016212
hsa-miR-3074-5p	NM_001270517	APITD1-CORT	155	-26.23	-30.5	0.028158

hsa-miR-3074-5p	NM_001271022	ATRIP	154	-24.86	-27.6	0.004094
hsa-miR-3074-5p	NM_001271023	ATRIP	154	-24.86	-27.6	0.004094
hsa-miR-3074-5p	NM_001276293	NISCH	166	-28.26	-32.5	0.03329
hsa-miR-3074-5p	NM_001276294	NISCH	166	-28.26	-32.5	0.020692
hsa-miR-3074-5p	NM_001282582	MXRA8	150	-27.82	-30.7	0.041773
hsa-miR-3074-5p	NM_001282583	MXRA8	150	-27.82	-30.7	0.041773
hsa-miR-3074-5p	NM_001282584	MXRA8	150	-27.82	-30.7	0.041773
hsa-miR-3074-5p	NM_001282585	MXRA8	150	-27.82	-30.7	0.02244
hsa-miR-3074-5p	NM_001284417	LYSMD4	158	-26.77	-33.3	0.031483
hsa-miR-3074-5p	NM_001284418	LYSMD4	158	-26.77	-33.3	0.031483
hsa-miR-3074-5p	NM_001284419	LYSMD4	158	-26.77	-33.3	0.031483
hsa-miR-3074-5p	NM_001284420	LYSMD4	158	-26.77	-33.3	0.031483
hsa-miR-3074-5p	NM_001284421	LYSMD4	158	-26.77	-33.3	0.031483
hsa-miR-3074-5p	NM_001284422	LYSMD4	158	-26.77	-33.3	0.031483
hsa-miR-3074-5p	NM_001505	GPER1	167	-25.69	-31.2	0.035347
hsa-miR-3074-5p	NM_002027	FNTA	153	-28.71	-32	0.008425
hsa-miR-3074-5p	NM_002281	KRT81	156	-24.1	-28.7	0.023813
hsa-miR-3074-5p	NM_002913	RFC1	159	-30.36	-33.7	0.017582
hsa-miR-3074-5p	NM_005125	CCS	153	-27.19	-31.5	0.001161
hsa-miR-3074-5p	NM_005247	FGF3	163	-22.85	-27.6	0.041578
hsa-miR-3074-5p	NM_006705	GADD45G	159	-20.56	-30.7	0.014712
hsa-miR-3074-5p	NM_007364	TMED3	154	-25.77	-30.5	0.026166
hsa-miR-3074-5p	NM_014069	PSORS1C2	150	-27.45	-31.1	0.009514
hsa-miR-3074-5p	NM_015242	ARAP1	158	-26.1	-29.8	0.033588
hsa-miR-3074-5p	NM_015404	DFNB31	157	-24.16	-31.5	0.017641
hsa-miR-3074-5p	NM_015594	TBC1D29	163	-25.53	-29.1	0.000555
hsa-miR-3074-5p	NM_016113	TRPV2	154	-26.6	-31.5	0.00049
hsa-miR-3074-5p	NM_016490	FAM178B	169	-27.33	-33.3	0.00269
hsa-miR-3074-5p	NM_017715	ZNF3	155	-25.44	-30.7	0.043529
hsa-miR-3074-5p	NM_018228	VRTN	152	-27.58	-33.1	0.018137

hsa-miR-3074-5p	NM_020319	ANKMY2	155	-22.63	-31	0.04041
hsa-miR-3074-5p	NM_020892	DTX2	153	-26.44	-31.6	0.006599
hsa-miR-3074-5p	NM_022464	SIL1	150	-24.81	-29.6	0.019055
hsa-miR-3074-5p	NM_022822	KLC2	155	-29.36	-33.2	0.012405
hsa-miR-3074-5p	NM_024571	SNRNP25	153	-20.55	-29.4	0.033768
hsa-miR-3074-5p	NM_032166	ATRIP	154	-24.86	-27.6	0.004094
hsa-miR-3074-5p	NM_032348	MXRA8	150	-27.82	-30.7	0.041773
hsa-miR-3074-5p	NM_033413	LRRC46	162	-24.79	-29.8	0.033411
hsa-miR-3074-5p	NM_130384	ATRIP	154	-24.86	-27.6	0.004094
hsa-miR-3074-5p	NM_152449	LYSMD4	158	-26.77	-33.3	0.031483
hsa-miR-3074-5p	NM_152913	TMEM130	153	-29.33	-32.9	0.032974
hsa-miR-3074-5p	NM_153217	TMEM174	160	-26.66	-30.8	0.043933
hsa-miR-3074-5p	NM_174920	SAMD14	154	-31.29	-34.5	0.016212
hsa-miR-3074-5p	NM_175575	WFIKKN2	155	-27.35	-33	0.024762
hsa-miR-3074-5p	NM_199006	APITD1-CORT	155	-26.23	-30.5	0.024144
hsa-miR-3074-5p	NM_201412	LUC7L	154	-23.7	-29	0.00928
hsa-miR-3074-5p	NR_024192	HILS1	155	-31.38	-36.8	0.002176
hsa-miR-3074-5p	NR_024193	HILS1	155	-31.38	-36.8	0.002837
hsa-miR-3074-5p	NR_026752	CROCCP2	153	-27.84	-33.6	0.028514
hsa-miR-3074-5p	NR_036511	LOC100129917	158	-39.63	-43	0.000379
hsa-miR-3074-5p	NR_077236	LOC101241902	168	-33.38	-37.7	0.003541
hsa-miR-3074-5p	NR_102753	SPATA8-AS1	157	-21.6	-30.9	0.014298
hsa-miR-3074-5p	NR_103773	LOC100288866	154	-30.99	-33.1	0.020817
hsa-miR-3074-5p	NR_110573	CATIP-AS1	164	-27.58	-31.5	0.012449
hsa-miR-3074-5p	NR_120616	PLCE1-AS2	164	-31.42	-35.5	0.004529
hsa-miR-3074-5p	NR_126046	GLIDR	154	-28.24	-31.3	0.037855
hsa-miR-3074-5p	NR_126050	LOC103908605	154	-28.24	-31.3	0.012721
hsa-miR-323b-3p	NM_001466	FZD2	150	-27.96	-34.6	0.029062
hsa-miR-323b-3p	NM_014003	DHX38	155	-27.84	-29	0.041026
hsa-miR-323b-3p	NM_017622	BORCS6	152	-27.73	-31.9	0.024595

hsa-miR-323b-3p	NM_031479	INHBE	158	-32.39	-34	0.018784
hsa-miR-4326	NM_000171	GLRA1	151	-28.97	-30.6	0.001546
hsa-miR-4326	NM_000384	APOB	164	-28.16	-31.3	0.003934
hsa-miR-4326	NM_001007026	ATN1	153	-29.6	-33.9	0.003053
hsa-miR-4326	NM_001010873	TSPO2	151	-28.23	-30.3	0.002998
hsa-miR-4326	NM_001017915	INPP5D	163	-32.34	-32	0.029444
hsa-miR-4326	NM_001029885	CPTP	154	-29.33	-33.6	0.012735
hsa-miR-4326	NM_001039792	HRCT1	153	-18.92	-30.8	0.013424
hsa-miR-4326	NM_001083600	NAA60	168	-29.57	-34.1	0.018909
hsa-miR-4326	NM_001083601	NAA60	168	-29.57	-34.1	0.018909
hsa-miR-4326	NM_001098483	FUOM	161	-23.9	-27.6	0.016447
hsa-miR-4326	NM_001098514	C16orf89	150	-20.81	-26.6	0.031429
hsa-miR-4326	NM_001098831	MORN4	162	-30.53	-32.7	0.035897
hsa-miR-4326	NM_001135111	KCNK17	158	-26.88	-32.6	0.010787
hsa-miR-4326	NM_001136103	TMEM132C	169	-29.18	-33.1	0.027708
hsa-miR-4326	NM_001144059	NTM	151	-25.77	-29.2	0.016123
hsa-miR-4326	NM_001146040	GLRA1	151	-28.97	-30.6	0.001546
hsa-miR-4326	NM_001159726	TSPO2	151	-28.23	-30.3	0.002998
hsa-miR-4326	NM_001161476	WDR25	159	-25.36	-27.8	0.020957
hsa-miR-4326	NM_001164379	FAM180B	166	-28.64	-33	0.008846
hsa-miR-4326	NM_001170629	CHD8	153	-29.1	-32	0.005099
hsa-miR-4326	NM_001170689	MIB2	166	-22.99	-27.1	0.040913
hsa-miR-4326	NM_001171968	CDHR5	153	-27.42	-31.3	0.02165
hsa-miR-4326	NM_001174092	TMEM185A	158	-29.99	-32	0.03923
hsa-miR-4326	NM_001178075	ASNS	156	-29.5	-32.6	0.000352
hsa-miR-4326	NM_001178076	ASNS	156	-29.5	-32.6	0.000352
hsa-miR-4326	NM_001178077	ASNS	156	-29.5	-32.6	0.000352
hsa-miR-4326	NM_001195381	GPR35	153	-28.56	-31.5	0.005455
hsa-miR-4326	NM_001195382	GPR35	153	-28.56	-31.5	0.005455
hsa-miR-4326	NM_001199172	MGAT5B	158	-21.52	-32.2	0.039213

hsa-miR-4326	NM_001242628	GFOD1	154	-30.11	-31.6	0.044064
hsa-miR-4326	NM_001242630	GFOD1	154	-30.11	-31.6	0.044064
hsa-miR-4326	NM_001243013	ABCB9	155	-30.13	-32.6	0.014741
hsa-miR-4326	NM_001257967	CCDC129	158	-21.19	-33.1	0.010365
hsa-miR-4326	NM_001258426	SLC4A9	157	-28.97	-31	0.006405
hsa-miR-4326	NM_001258427	SLC4A9	157	-28.97	-31	0.008434
hsa-miR-4326	NM_001258428	SLC4A9	157	-28.97	-31	0.008434
hsa-miR-4326	NM_001260511	MGST1	150	-25.53	-29.4	0.016148
hsa-miR-4326	NM_001260512	MGST1	150	-25.53	-29.4	0.042064
hsa-miR-4326	NM_001267053	RIBC1	152	-20.11	-27.2	0.042389
hsa-miR-4326	NM_001270379	ANKRD46	158	-27.3	-30.6	0.032873
hsa-miR-4326	NM_001277174	EFS	162	-23.86	-30.2	0.039985
hsa-miR-4326	NM_001278201	SLC43A3	158	-28.34	-32.2	0.015433
hsa-miR-4326	NM_001278206	SLC43A3	158	-28.34	-32.2	0.015433
hsa-miR-4326	NM_001278942	SURF6	150	-24.07	-35.7	0.008689
hsa-miR-4326	NM_001282143	NLRX1	156	-30.19	-34.4	0.002666
hsa-miR-4326	NM_001282144	NLRX1	156	-30.19	-34.4	0.002666
hsa-miR-4326	NM_001282161	UBE2A	155	-27.97	-33	0.017898
hsa-miR-4326	NM_001282358	NLRX1	156	-30.19	-34.4	0.002666
hsa-miR-4326	NM_001282856	GON4L	162	-23.76	-30.3	0.045173
hsa-miR-4326	NM_001282858	GON4L	162	-23.76	-30.3	0.045173
hsa-miR-4326	NM_001282860	GON4L	162	-23.76	-30.3	0.045173
hsa-miR-4326	NM_001288721	B3GAT3	153	-31.67	-34.2	0.001482
hsa-miR-4326	NM_001288722	B3GAT3	153	-31.67	-34.2	0.006412
hsa-miR-4326	NM_001288723	B3GAT3	153	-31.67	-34.2	0.00661
hsa-miR-4326	NM_001290007	BIN2	151	-28.73	-32.3	0.005203
hsa-miR-4326	NM_001290008	BIN2	151	-28.73	-32.3	0.005203
hsa-miR-4326	NM_001290009	BIN2	151	-28.73	-32.3	0.005203
hsa-miR-4326	NM_001291281	FOXO6	153	-30.85	-33.2	0.010839
hsa-miR-4326	NM_001292000	GLRA1	151	-28.97	-30.6	0.001546

hsa-miR-4326	NM_001301827	FUOM	161	-23.9	-27.6	0.016447
hsa-miR-4326	NM_001301828	FUOM	161	-23.9	-27.6	0.016447
hsa-miR-4326	NM_001304792	SENP6	157	-28.87	-31.5	0.025823
hsa-miR-4326	NM_001408	CELSR2	156	-29.98	-33.4	0.026085
hsa-miR-4326	NM_001673	ASNS	156	-29.5	-32.6	0.000352
hsa-miR-4326	NM_001940	ATN1	153	-29.6	-33.9	0.003053
hsa-miR-4326	NM_002160	TNC	158	-29.27	-32.3	0.038502
hsa-miR-4326	NM_002178	IGFBP6	150	-34.06	-36.1	0.000077
hsa-miR-4326	NM_003336	UBE2A	155	-27.97	-33	0.017898
hsa-miR-4326	NM_003578	SOAT2	164	-27.38	-31.4	0.006914
hsa-miR-4326	NM_003696	OR6A2	151	-24.84	-29	0.00635
hsa-miR-4326	NM_003907	EIF2B5	161	-23.2	-29.1	0.018864
hsa-miR-4326	NM_004326	BCL9	154	-28.2	-33.3	0.017336
hsa-miR-4326	NM_004390	CTSH	156	-26.5	-28.2	0.03214
hsa-miR-4326	NM_004979	KCND1	168	-25.09	-34.3	0.010058
hsa-miR-4326	NM_005125	CCS	152	-26.43	-30.3	0.001952
hsa-miR-4326	NM_005247	FGF3	158	-25.01	-28.9	0.017994
hsa-miR-4326	NM_005301	GPR35	153	-28.56	-31.5	0.005455
hsa-miR-4326	NM_005541	INPP5D	163	-32.34	-32	0.029444
hsa-miR-4326	NM_005864	EFS	162	-23.86	-30.2	0.039985
hsa-miR-4326	NM_006240	PPEF1	155	-29.13	-32	0.005329
hsa-miR-4326	NM_006373	VAT1	157	-31.14	-32	0.039268
hsa-miR-4326	NM_006753	SURF6	150	-24.07	-35.7	0.004886
hsa-miR-4326	NM_007191	WIF1	156	-35.86	-38.2	0.000606
hsa-miR-4326	NM_012200	B3GAT3	153	-31.67	-34.2	0.001482
hsa-miR-4326	NM_014096	SLC43A3	158	-28.34	-32.2	0.015433
hsa-miR-4326	NM_015072	TTLL5	153	-29.65	-32.6	0.007953
hsa-miR-4326	NM_015727	TACR1	157	-28.58	-30.3	0.003354
hsa-miR-4326	NM_016293	BIN2	151	-28.73	-32.3	0.005203
hsa-miR-4326	NM_016321	RHCG	154	-27.79	-30.5	0.013854

hsa-miR-4326	NM_017611	SLC43A3	158	-28.34	-32.2	0.015433
hsa-miR-4326	NM_018088	FAM90A1	154	-27.1	-29.8	0.026711
hsa-miR-4326	NM_018988	GFOD1	154	-30.11	-31.6	0.044064
hsa-miR-4326	NM_019624	ABCB9	155	-30.13	-32.6	0.014741
hsa-miR-4326	NM_019625	ABCB9	155	-30.13	-32.6	0.014741
hsa-miR-4326	NM_020300	MGST1	150	-25.53	-29.4	0.016148
hsa-miR-4326	NM_020920	CHD8	153	-29.1	-32	0.005099
hsa-miR-4326	NM_021924	CDHR5	153	-27.42	-31.3	0.02165
hsa-miR-4326	NM_022152	TMBIM1	151	-27.94	-31.8	0.035807
hsa-miR-4326	NM_024017	HOXB9	154	-30.54	-34.8	0.014235
hsa-miR-4326	NM_024515	WDR25	159	-25.36	-27.8	0.020957
hsa-miR-4326	NM_024589	ROGDI	154	-22.9	-28.2	0.049691
hsa-miR-4326	NM_024618	NLRX1	156	-30.19	-34.4	0.002666
hsa-miR-4326	NM_024671	ZNF768	170	-25.68	-29.1	0.033232
hsa-miR-4326	NM_024845	NAA60	168	-29.57	-34.1	0.019274
hsa-miR-4326	NM_025163	PIGZ	164	-26.42	-29.9	0.045228
hsa-miR-4326	NM_031264	CDHR5	153	-27.42	-31.3	0.02165
hsa-miR-4326	NM_031457	MS4A8	162	-24.16	-27.5	0.049839
hsa-miR-4326	NM_031467	SLC4A9	157	-28.97	-31	0.006405
hsa-miR-4326	NM_032246	MEX3B	158	-28.46	-31.7	0.041829
hsa-miR-4326	NM_032415	CARD11	150	-33.63	-37.1	0.000633
hsa-miR-4326	NM_032459	EFS	162	-23.86	-30.2	0.039985
hsa-miR-4326	NM_032508	TMEM185A	158	-29.99	-32	0.03923
hsa-miR-4326	NM_032648	FAM167B	158	-26.33	-29.2	0.009411
hsa-miR-4326	NM_032854	CORO6	153	-27.07	-34.4	0.006803
hsa-miR-4326	NM_054035	UNC119	152	-28.3	-31.1	0.030338
hsa-miR-4326	NM_133436	ASNS	156	-29.5	-32.6	0.000352
hsa-miR-4326	NM_144677	MGAT5B	158	-21.52	-32.2	0.039213
hsa-miR-4326	NM_145764	MGST1	150	-25.53	-29.4	0.016148
hsa-miR-4326	NM_145791	MGST1	150	-25.53	-29.4	0.016148

hsa-miR-4326	NM_145792	MGST1	150	-25.53	-29.4	0.016148
hsa-miR-4326	NM_152224	PPEF1	155	-29.13	-32	0.005329
hsa-miR-4326	NM_152226	PPEF1	155	-29.13	-32	0.005329
hsa-miR-4326	NM_178832	MORN4	162	-30.53	-32.7	0.035897
hsa-miR-4326	NM_181762	UBE2A	155	-27.97	-33	0.017898
hsa-miR-4326	NM_183356	ASNS	156	-29.5	-32.6	0.000352
hsa-miR-4326	NM_194300	CCDC129	158	-21.19	-33.1	0.010365
hsa-miR-4326	NM_198472	FUOM	161	-23.9	-27.6	0.037617
hsa-miR-4326	NM_198955	MGAT5B	158	-21.52	-32.2	0.039213
hsa-miR-4326	NM_199329	SLC43A3	158	-28.34	-32.2	0.015433
hsa-miR-4326	NR_024530	RPL23AP7	161	-31.18	-30.6	0.029552
hsa-miR-4326	NR_026926	LOC151174	164	-23.44	-31	0.028134
hsa-miR-4326	NR_030340	MIR609	157	-20.07	-25.2	0.013541
hsa-miR-4326	NR_030646	MIR1225	153	-30.61	-32.7	0.0001
hsa-miR-4326	NR_033654	TAC3	160	-31.32	-34.3	0.006884
hsa-miR-4326	NR_033871	FLJ42351	165	-27.6	-28.6	0.042608
hsa-miR-4326	NR_034126	LINC01160	152	-33.97	-34.1	0.016285
hsa-miR-4326	NR_037694	LOC100286922	169	-29.96	-33.8	0.009921
hsa-miR-4326	NR_038310	MIR497HG	168	-26.7	-31.5	0.019944
hsa-miR-4326	NR_106918	MIR6859-1	150	-20.26	-22.1	0.049726
hsa-miR-4326	NR_107062	MIR6859-2	150	-20.26	-22.1	0.049726
hsa-miR-4326	NR_107063	MIR6859-3	150	-20.26	-22.1	0.049726
hsa-miR-4326	NR_109991	B3GAT3	153	-31.67	-34.2	0.016933
hsa-miR-4326	NR_110114	LOC101927668	157	-31.65	-35.2	0.003415
hsa-miR-4326	NR_110243	OBP2B	152	-25.11	-28.9	0.042339
hsa-miR-4326	NR_110702	SEMA3B-AS1	156	-22.28	-29.8	0.011124
hsa-miR-4326	NR_125409	LOC102724699	152	-33.97	-32.5	0.043242
hsa-miR-4326	NR_125846	LOC101927972	159	-24.92	-30.1	0.022002
hsa-miR-4326	NR_125847	LOC101927972	159	-24.92	-30.1	0.021804
hsa-miR-4326	NR_125848	LOC101927972	159	-24.92	-30.1	0.017122

hsa-miR-4326	NR_128720	MIR6859-4	150	-20.26	-22.1	0.049726
hsa-miR-4326	NR_131926	MEAT6	152	-29.23	-33.4	0.012803
hsa-miR-3613-5p	NM_021979	HSPA2	160	-21.27	-26.3	0.045939
hsa-miR-3613-5p	NM_197954	CLEC7A	163	-20.42	-25.6	0.002644
hsa-miR-3653-5p	NM_001031717	CRELD1	153	-26.83	-30	0.038547
hsa-miR-3653-5p	NM_001077415	CRELD1	153	-26.83	-30	0.028272
hsa-miR-3653-5p	NM_001145545	C16orf82	186	-30.57	-33.8	0.013254
hsa-miR-3653-5p	NM_015513	CRELD1	153	-26.83	-30	0.028272
hsa-miR-3653-5p	NM_033226	ABCC12	170	-30.23	-32.6	0.006099
hsa-miR-3653-5p	NR_132366	GRPEL2-AS1	158	-22.3	-29.3	0.011948
hsa-miR-3940-3p	NM_000180	GUCY2D	150	-26	-29	0.025265
hsa-miR-3940-3p	NM_000265	NCF1	151	-25.95	-31.4	0.00613
hsa-miR-3940-3p	NM_000738	CHRM1	165	-32.79	-34.4	0.021245
hsa-miR-3940-3p	NM_000904	NQO2	155	-38.71	-42.7	0.000017
hsa-miR-3940-3p	NM_000941	POR	150	-25.07	-30.3	0.028473
hsa-miR-3940-3p	NM_001006944	RPS6KA4	154	-39.01	-41.3	0.000526
hsa-miR-3940-3p	NM_001012708	KRTAP5-3	162	-31.3	-34.4	0.000231
hsa-miR-3940-3p	NM_001031738	TMEM150A	160	-29.95	-34.5	0.006809
hsa-miR-3940-3p	NM_001040078	LGALS9C	162	-31.67	-36	0.003925
hsa-miR-3940-3p	NM_001042685	LGALS9B	162	-31.67	-36	0.000107
hsa-miR-3940-3p	NM_001080524	C16orf90	158	-32.54	-33.7	0.004888
hsa-miR-3940-3p	NM_001098627	IRF5	151	-32.35	-35.3	0.018362
hsa-miR-3940-3p	NM_001098629	IRF5	151	-32.35	-35.3	0.018362
hsa-miR-3940-3p	NM_001098630	IRF5	151	-32.35	-35.3	0.018362
hsa-miR-3940-3p	NM_001098637	PWWP2B	158	-27.03	-32	0.040219
hsa-miR-3940-3p	NM_001098790	MID1IP1	153	-36.95	-39.1	0.002641
hsa-miR-3940-3p	NM_001098791	MID1IP1	153	-36.95	-39.1	0.002641
hsa-miR-3940-3p	NM_001098794	FAM160A2	152	-33.5	-35.1	0.000529
hsa-miR-3940-3p	NM_001124	ADM	164	-29.62	-33.1	0.023637
hsa-miR-3940-3p	NM_001129727	PLEKHG4	151	-26.32	-31.6	0.040349

hsa-miR-3940-3p	NM_001129728	PLEKHG4	151	-26.32	-31.6	0.040349
hsa-miR-3940-3p	NM_001129729	PLEKHG4	151	-26.32	-31.6	0.040349
hsa-miR-3940-3p	NM_001129731	PLEKHG4	151	-26.32	-31.6	0.040349
hsa-miR-3940-3p	NM_001130144	LTBP3	160	-30.96	-32.6	0.016295
hsa-miR-3940-3p	NM_001130969	NSMF	167	-33.2	-34.9	0.038352
hsa-miR-3940-3p	NM_001130970	NSMF	167	-33.2	-34.9	0.038352
hsa-miR-3940-3p	NM_001130971	NSMF	167	-33.2	-34.9	0.038352
hsa-miR-3940-3p	NM_001142593	ITPK1	162	-36.05	-40.3	0.004101
hsa-miR-3940-3p	NM_001142797	CXCR3	169	-25.49	-31.1	0.02606
hsa-miR-3940-3p	NM_001142946	C11orf21	151	-35.82	-36.6	0.016759
hsa-miR-3940-3p	NM_001161504	ALDH4A1	151	-18.24	-33.6	0.046642
hsa-miR-3940-3p	NM_001164266	LTBP3	160	-30.96	-32.6	0.016295
hsa-miR-3940-3p	NM_001165937	STARD3	152	-33.84	-37.9	0.006495
hsa-miR-3940-3p	NM_001165938	STARD3	152	-33.84	-37.9	0.006495
hsa-miR-3940-3p	NM_001170535	ATAD3A	153	-33.57	-36.4	0.003637
hsa-miR-3940-3p	NM_001170536	ATAD3A	153	-33.57	-36.4	0.003637
hsa-miR-3940-3p	NM_001171093	FAM131A	150	-31.27	-33.3	0.047377
hsa-miR-3940-3p	NM_001171166	CAMTA2	155	-28.43	-32.5	0.031167
hsa-miR-3940-3p	NM_001171167	CAMTA2	155	-28.43	-32.5	0.02813
hsa-miR-3940-3p	NM_001171168	CAMTA2	155	-28.43	-32.5	0.031167
hsa-miR-3940-3p	NM_001178064	NSMF	167	-33.2	-34.9	0.038352
hsa-miR-3940-3p	NM_001185080	CLDN15	151	-29.43	-31.1	0.014589
hsa-miR-3940-3p	NM_001195731	CHPF	154	-34.89	-30.3	0.036414
hsa-miR-3940-3p	NM_001242452	IRF5	151	-32.35	-35.3	0.018362
hsa-miR-3940-3p	NM_001256404	DENND2C	150	-26.95	-32.7	0.043741
hsa-miR-3940-3p	NM_001256617	TUBGCP2	174	-34.75	-39.4	0.002728
hsa-miR-3940-3p	NM_001256618	TUBGCP2	174	-34.75	-39.4	0.002728
hsa-miR-3940-3p	NM_001278789	GORASP1	167	-33.22	-35.2	0.029195
hsa-miR-3940-3p	NM_001278790	GORASP1	167	-33.22	-35.2	0.029195
hsa-miR-3940-3p	NM_001280794	EPHB6	168	-27.39	-30.2	0.008917

hsa-miR-3940-3p	NM_001280795	EPHB6	168	-27.39	-30.2	0.008917
hsa-miR-3940-3p	NM_001282394	WNK2	152	-38.22	-40.6	0.002162
hsa-miR-3940-3p	NM_001288721	B3GAT3	155	-36.31	-38	0.000762
hsa-miR-3940-3p	NM_001288722	B3GAT3	155	-36.31	-38	0.003346
hsa-miR-3940-3p	NM_001288723	B3GAT3	155	-36.31	-38	0.00345
hsa-miR-3940-3p	NM_001290221	NQO2	155	-38.71	-42.7	0.000017
hsa-miR-3940-3p	NM_001290222	NQO2	155	-38.71	-42.7	0.000017
hsa-miR-3940-3p	NM_001300802	RPS6KA4	154	-39.01	-41.3	0.000526
hsa-miR-3940-3p	NM_001301159	NHLRC4	163	-33.65	-38.3	0.004037
hsa-miR-3940-3p	NM_001301186	LINGO1	163	-29.33	-33.3	0.033453
hsa-miR-3940-3p	NM_001301187	LINGO1	163	-29.33	-33.3	0.033453
hsa-miR-3940-3p	NM_001301189	LINGO1	163	-29.33	-33.3	0.033453
hsa-miR-3940-3p	NM_001301191	LINGO1	163	-29.33	-33.3	0.033453
hsa-miR-3940-3p	NM_001301192	LINGO1	163	-29.33	-33.3	0.033453
hsa-miR-3940-3p	NM_001301194	LINGO1	163	-29.33	-33.3	0.033453
hsa-miR-3940-3p	NM_001301195	LINGO1	163	-29.33	-33.3	0.033453
hsa-miR-3940-3p	NM_001301197	LINGO1	163	-29.33	-33.3	0.033453
hsa-miR-3940-3p	NM_001301198	LINGO1	163	-29.33	-33.3	0.033453
hsa-miR-3940-3p	NM_001301199	LINGO1	163	-29.33	-33.3	0.033453
hsa-miR-3940-3p	NM_001301200	LINGO1	163	-29.33	-33.3	0.033453
hsa-miR-3940-3p	NM_001504	CXCR3	169	-25.49	-31.1	0.02606
hsa-miR-3940-3p	NM_002308	LGALS9	161	-29.8	-33.4	0.013281
hsa-miR-3940-3p	NM_002917	RFNG	168	-29.38	-33.8	0.019496
hsa-miR-3940-3p	NM_003748	ALDH4A1	151	-18.24	-33.6	0.046642
hsa-miR-3940-3p	NM_003942	RPS6KA4	154	-39.01	-41.3	0.000526
hsa-miR-3940-3p	NM_004445	EPHB6	168	-27.39	-30.2	0.008917
hsa-miR-3940-3p	NM_004998	MYO1E	164	-39.9	-42.4	0.001081
hsa-miR-3940-3p	NM_005018	PDCD1	154	-30.13	-32.9	0.048707
hsa-miR-3940-3p	NM_005146	SART1	156	-38.89	-41.7	0.000947
hsa-miR-3940-3p	NM_006039	MRC2	164	-38.36	-40.3	0.001878

hsa-miR-3940-3p	NM_006049	SNAPC5	154	-32.92	-35.9	0.002188
hsa-miR-3940-3p	NM_006659	TUBGCP2	174	-34.75	-39.4	0.002728
hsa-miR-3940-3p	NM_006804	STARD3	152	-33.84	-37.9	0.006495
hsa-miR-3940-3p	NM_007176	C14orf1	150	-32.59	-34.6	0.009598
hsa-miR-3940-3p	NM_009587	LGALS9	161	-29.8	-33.4	0.013281
hsa-miR-3940-3p	NM_012200	B3GAT3	155	-36.31	-38	0.000762
hsa-miR-3940-3p	NM_014216	ITPK1	162	-36.05	-40.3	0.004101
hsa-miR-3940-3p	NM_014343	CLDN15	151	-29.43	-31.1	0.014589
hsa-miR-3940-3p	NM_014448	ARHGEF16	165	-29.05	-32.7	0.015791
hsa-miR-3940-3p	NM_015099	CAMTA2	155	-28.43	-32.5	0.031167
hsa-miR-3940-3p	NM_015392	NPDC1	155	-29.95	-32.8	0.006128
hsa-miR-3940-3p	NM_015537	NSMF	167	-33.2	-34.9	0.038352
hsa-miR-3940-3p	NM_015597	GPSM1	156	-36.47	-40.4	0.000676
hsa-miR-3940-3p	NM_015656	KIF26A	172	-33.04	-36.2	0.010655
hsa-miR-3940-3p	NM_015658	NOC2L	156	-37.26	-34.8	0.005175
hsa-miR-3940-3p	NM_016170	TLX2	164	-35.98	-41.1	0.000953
hsa-miR-3940-3p	NM_016176	SDF4	154	-22.94	-35.4	0.005619
hsa-miR-3940-3p	NM_016417	GLRX5	159	-27.78	-32.5	0.021058
hsa-miR-3940-3p	NM_016547	SDF4	154	-22.94	-35.4	0.008339
hsa-miR-3940-3p	NM_017525	CDC42BPG	152	-30.22	-33.1	0.004301
hsa-miR-3940-3p	NM_018188	ATAD3A	153	-33.57	-36.4	0.003637
hsa-miR-3940-3p	NM_019892	INPP5E	155	-28.89	-34.5	0.020955
hsa-miR-3940-3p	NM_021070	LTBP3	160	-30.96	-32.6	0.016295
hsa-miR-3940-3p	NM_021195	CLDN6	152	-27.36	-32.6	0.023076
hsa-miR-3940-3p	NM_021242	MID1IP1	153	-36.95	-39.1	0.002641
hsa-miR-3940-3p	NM_021259	TMEM8A	151	-33.02	-35	0.020572
hsa-miR-3940-3p	NM_021978	ST14	162	-39.37	-41.8	0.000222
hsa-miR-3940-3p	NM_024536	CHPF	154	-34.89	-30.3	0.036414
hsa-miR-3940-3p	NM_031899	GORASP1	167	-33.22	-35.2	0.029195
hsa-miR-3940-3p	NM_032127	FAM160A2	152	-33.5	-35.1	0.000529

hsa-miR-3940-3p	NM_032364	DNAJC14	150	-31.46	-33.5	0.030522
hsa-miR-3940-3p	NM_032595	PPP1R9B	151	-38.58	-38.5	0.007005
hsa-miR-3940-3p	NM_032643	IRF5	151	-32.35	-35.3	0.018362
hsa-miR-3940-3p	NM_032789	PARP10	153	-26.59	-31.8	0.011109
hsa-miR-3940-3p	NM_032790	ORAI1	151	-22.46	-29.5	0.046075
hsa-miR-3940-3p	NM_032799	ZDHHC12	160	-25.67	-31.6	0.010923
hsa-miR-3940-3p	NM_032808	LINGO1	163	-29.33	-33.3	0.033453
hsa-miR-3940-3p	NM_032871	RELT	151	-31.46	-35.4	0.035014
hsa-miR-3940-3p	NM_080916	DGUOK	161	-28.69	-35	0.001013
hsa-miR-3940-3p	NM_080918	DGUOK	161	-28.69	-35	0.001013
hsa-miR-3940-3p	NM_133489	SLC26A10	150	-26.9	-29.5	0.047705
hsa-miR-3940-3p	NM_138440	VASN	162	-31.26	-35.8	0.005005
hsa-miR-3940-3p	NM_138499	PWWP2B	158	-27.03	-32	0.042498
hsa-miR-3940-3p	NM_138706	B3GNT6	153	-36.53	-39.4	0.003216
hsa-miR-3940-3p	NM_139280	ORMDL3	162	-39.95	-40.4	0.002848
hsa-miR-3940-3p	NM_144635	FAM131A	150	-31.27	-33.3	0.047377
hsa-miR-3940-3p	NM_152222	RELT	151	-31.46	-35.4	0.035014
hsa-miR-3940-3p	NM_174891	C14orf79	154	-32.77	-35.3	0.008671
hsa-miR-3940-3p	NM_176677	NHLRC4	163	-33.65	-38.3	0.004037
hsa-miR-3940-3p	NM_178537	B4GALNT4	152	-27.4	-32.6	0.007175
hsa-miR-3940-3p	NM_198459	DENND2C	150	-26.95	-32.7	0.043741
hsa-miR-3940-3p	NM_198559	CATIP	152	-27.93	-33.2	0.000785
hsa-miR-3940-3p	NM_203423	LOC389199	153	-32.03	-36.4	0.001005
hsa-miR-3940-3p	NR_002318	CATSPER2P1	158	-23.11	-34.2	0.044191
hsa-miR-3940-3p	NR_003594	REXO1L2P	150	-29.37	-33.8	0.02916
hsa-miR-3940-3p	NR_024121	LINC00235	164	-34.96	-38.4	0.0012
hsa-miR-3940-3p	NR_026740	LOC389033	152	-27.72	-30.7	0.026284
hsa-miR-3940-3p	NR_027123	KTN1-AS1	167	-30.91	-35.5	0.022991
hsa-miR-3940-3p	NR_027423	FAM66B	171	-32.83	-36.7	0.012748
hsa-miR-3940-3p	NR_031674	MIR1272	156	-29.56	-32.4	0.001205

hsa-miR-3940-3p	NR_031719	MIR1538	151	-25.8	-28.1	0.002604
hsa-miR-3940-3p	NR_033179	TMEM150A	160	-29.95	-34.5	0.03419
hsa-miR-3940-3p	NR_036223	MIR4265	160	-32.72	-36	0.00008
hsa-miR-3940-3p	NR_036440	POU5F1P3	162	-32.93	-36.2	0.010294
hsa-miR-3940-3p	NR_038997	ANKRD34C-AS1	175	-38.95	-33.6	0.016258
hsa-miR-3940-3p	NR_039730	MIR4507	152	-35.1	-29.1	0.000935
hsa-miR-3940-3p	NR_045492	LSM1	166	-27.33	-33.2	0.036187
hsa-miR-3940-3p	NR_046690	HDAC11-AS1	167	-33.38	-39	0.000746
hsa-miR-3940-3p	NR_073008	CDK2AP1	156	-38.75	-37.5	0.007551
hsa-miR-3940-3p	NR_073093	MPC1	151	-32.69	-34.9	0.021323
hsa-miR-3940-3p	NR_073386	ALG1L9P	171	-32.83	-36.7	0.014882
hsa-miR-3940-3p	NR_073388	ALG1L9P	171	-32.83	-36.7	0.007337
hsa-miR-3940-3p	NR_073409	TMEM134	160	-34.17	-35.1	0.01719
hsa-miR-3940-3p	NR_073412	TMEM134	160	-34.17	-35.1	0.016367
hsa-miR-3940-3p	NR_102746	ROPN1L-AS1	152	-31.99	-34.6	0.013055
hsa-miR-3940-3p	NR_104653	LOC100287592	152	-29.11	-33.7	0.01317
hsa-miR-3940-3p	NR_109991	B3GAT3	155	-36.31	-38	0.00895
hsa-miR-3940-3p	NR_110398	PABPC1L2B-AS1	156	-38.75	-37.5	0.006554
hsa-miR-3940-3p	NR_110752	POTEB3	153	-32.22	-36.6	0.021486
hsa-miR-3940-3p	NR_122076	LOC403323	156	-32.17	-34.5	0.032972
hsa-miR-3940-3p	NR_122077	LOC403323	156	-32.17	-34.5	0.020038
hsa-miR-3940-3p	NR_125761	LINC01461	151	-29.72	-32.6	0.018334
hsa-miR-3940-3p	NR_125994	LOC102724312	164	-31.72	-36.4	0.010975
hsa-miR-3940-3p	NR_126032	MEMO1	155	-30.03	-34.5	0.039246
hsa-miR-3940-3p	NR_130467	MIR4538	154	-37.51	-27.8	0.005514
hsa-miR-3940-3p	NR_133008	LOC105369532	150	-32.33	-35.3	0.003013
hsa-miR-548ab	NR_029885	MIR340	159	-19.05	-24.6	0.009634
hsa-miR-548ab	NR_030305	MIR579	156	-18.29	-23	0.030135

hsa-miR-548ab	NR_030315	MIR548B	160	-21.94	-26.4	0.002986
hsa-miR-548ab	NR_030330	MIR548A3	176	-19.58	-25.7	0.004795
hsa-miR-548ab	NR_031643	MIR548F2	180	-19.94	-22.8	0.034427
hsa-miR-548ab	NR_031662	MIR548G	174	-19.58	-22.3	0.039754
hsa-miR-548ab	NR_031666	MIR548N	188	-26.61	-30.6	0.000084
hsa-miR-548ab	NR_031678	MIR548H2	184	-24.26	-29.2	0.000347
hsa-miR-548ab	NR_031679	MIR548H3	184	-21.92	-27.7	0.001977
hsa-miR-548ab	NR_031680	MIR548H4	188	-24.04	-25.8	0.006058
hsa-miR-548ab	NR_031686	MIR548P	180	-22.13	-27.7	0.000865
hsa-miR-548ab	NR_031752	MIR548Q	176	-17.72	-25.6	0.005494
hsa-miR-548ab	NR_036103	MIR548V	184	-21.02	-23.8	0.011429
hsa-miR-548ab	NR_037503	MIR548Y	180	-22.63	-24.8	0.011537
hsa-miR-548ab	NR_037515	MIR548Z	188	-26.02	-30.6	0.000174
hsa-miR-548ab	NR_039621	MIR548AC	179	-22.31	-26.8	0.001804
hsa-miR-548ab	NR_039639	MIR548AE2	184	-20.08	-26.5	0.001123
hsa-miR-548ab	NR_039673	MIR548AJ1	180	-19.4	-25.7	0.002373
hsa-miR-548ab	NR_039674	MIR548AJ2	180	-19.4	-25.7	0.004249
hsa-miR-548ab	NR_039692	MIR548X2	180	-18.15	-24.5	0.011511
hsa-miR-548ab	NR_039699	MIR548AK	180	-19.75	-24.1	0.0042
hsa-miR-548ab	NR_039762	MIR548AM	180	-21.54	-27.7	0.000623
hsa-miR-548ab	NR_049838	MIR548AQ	184	-21.33	-27.7	0.000321
hsa-miR-548ab	NR_049865	MIR548AX	184	-23.61	-25.7	0.002455
hsa-miR-548ab	NR_128708	MIR548BB	184	-21.74	-24.1	0.005975
hsa-miR-4429	NM_001008563	USP20	171	-28.29	-35.9	0.005979
hsa-miR-4429	NM_001017403	LGR6	150	-30.64	-36.4	0.000881
hsa-miR-4429	NM_001017404	LGR6	150	-30.64	-36.4	0.000881
hsa-miR-4429	NM_001018070	CORO1B	155	-22.88	-28.1	0.026557
hsa-miR-4429	NM_001034077	HIST2H4B	152	-19.54	-26.5	0.001678
hsa-miR-4429	NM_001110303	USP20	171	-28.29	-35.9	0.005979
hsa-miR-4429	NM_001114107	PDLIM3	181	-29.98	-33.9	0.01946

hsa-miR-4429	NM_001127699	SPINK5	151	-16.97	-23.7	0.031925
hsa-miR-4429	NM_001145862	MTMR11	164	-22.34	-29.2	0.027877
hsa-miR-4429	NM_001166449	ITIH4	154	-22.7	-28.9	0.027448
hsa-miR-4429	NM_001204698	TAX1BP3	154	-26.37	-32.1	0.01674
hsa-miR-4429	NM_001205271	PRSS46	153	-25.4	-31.8	0.005594
hsa-miR-4429	NM_001243787	SMUG1	174	-26.66	-32.2	0.045983
hsa-miR-4429	NM_001243788	SMUG1	174	-26.66	-32.2	0.045983
hsa-miR-4429	NM_001256125	CHIT1	167	-26.75	-31.8	0.017773
hsa-miR-4429	NM_001257962	PDLIM3	181	-29.98	-33.9	0.01946
hsa-miR-4429	NM_001257963	PDLIM3	181	-29.98	-33.9	0.01946
hsa-miR-4429	NM_001270509	CHIT1	167	-26.75	-31.8	0.017773
hsa-miR-4429	NM_001271851	RRAGC	153	-27.85	-31.3	0.048924
hsa-miR-4429	NM_001288812	ARL17A	158	-27.21	-31	0.002717
hsa-miR-4429	NM_001303024	SSSCA1	155	-21.13	-24.6	0.049836
hsa-miR-4429	NM_002218	ITIH4	154	-22.7	-28.9	0.027448
hsa-miR-4429	NM_003465	CHIT1	167	-26.75	-31.8	0.017773
hsa-miR-4429	NM_003540	HIST1H4F	151	-22.76	-27.4	0.000907
hsa-miR-4429	NM_003548	HIST2H4A	152	-19.54	-26.5	0.001678
hsa-miR-4429	NM_004701	CCNB2	162	-24.63	-27.2	0.01279
hsa-miR-4429	NM_005632	CAPN15	174	-27.28	-31.2	0.038947
hsa-miR-4429	NM_006244	PPP2R5B	173	-26.86	-31.3	0.015251
hsa-miR-4429	NM_006396	SSSCA1	155	-21.13	-24.6	0.049836
hsa-miR-4429	NM_006426	DPYSL4	161	-23.76	-30.1	0.043494
hsa-miR-4429	NM_006428	MRPL28	156	-21.96	-27.7	0.02667
hsa-miR-4429	NM_006676	USP20	171	-28.29	-35.9	0.007264
hsa-miR-4429	NM_014311	SMUG1	174	-26.66	-32.2	0.045983
hsa-miR-4429	NM_014476	PDLIM3	181	-29.98	-33.9	0.01946
hsa-miR-4429	NM_014604	TAX1BP3	154	-26.37	-32.1	0.01674
hsa-miR-4429	NM_015613	LRIT1	154	-19.16	-27.8	0.044362
hsa-miR-4429	NM_019896	POLE4	156	-22.32	-26.8	0.048326

hsa-miR-4429	NM_020441	CORO1B	155	-22.88	-28.1	0.026557
hsa-miR-4429	NM_020992	PDLIM1	154	-22.71	-27.9	0.033948
hsa-miR-4429	NM_021128	POLR2L	168	-24.29	-32.2	0.010133
hsa-miR-4429	NM_021636	LGR6	150	-30.64	-36.4	0.000881
hsa-miR-4429	NM_022157	RRAGC	153	-27.85	-31.3	0.048924
hsa-miR-4429	NM_054034	FN1	162	-21.87	-25.1	0.032441
hsa-miR-4429	NM_080431	ACTRT2	157	-20.63	-24.1	0.020539
hsa-miR-4429	NM_130899	FAM71B	159	-19.41	-24.9	0.023675
hsa-miR-4429	NM_152544	TRMT44	159	-22.69	-28.7	0.045538
hsa-miR-4429	NM_198552	FAM89A	174	-27.72	-30.5	0.039679
hsa-miR-4429	NR_045039	SMUG1	174	-26.66	-32.2	0.012666
hsa-miR-548ad-	NR_030305	MIR579	161	-19.88	-22.9	0.015863
hsa-miR-548ad-	NR_030315	MIR548B	158	-22.21	-25.1	0.00321
hsa-miR-548ad-	NR_031642	MIR548F1	178	-20.21	-21.8	0.025133
hsa-miR-548ad-	NR_031643	MIR548F2	178	-20.21	-21.5	0.042751
hsa-miR-548ad-	NR_031644	MIR548F3	178	-20.21	-21.5	0.033577
hsa-miR-548ad-	NR_031645	MIR548F4	178	-20.21	-21.8	0.039754
hsa-miR-548ad-	NR_031666	MIR548N	178	-21.91	-25.1	0.001703
hsa-miR-548ad-	NR_031669	MIR548O	178	-18.58	-23.6	0.013385
hsa-miR-548ad-	NR_031678	MIR548H2	182	-23.04	-26.2	0.00114
hsa-miR-548ad-	NR_031679	MIR548H3	182	-21.6	-26.4	0.002037
hsa-miR-548ad-	NR_031680	MIR548H4	186	-26.29	-29.6	0.000184
hsa-miR-548ad-	NR_031686	MIR548P	178	-21.81	-26.4	0.000874
hsa-miR-548ad-	NR_031752	MIR548Q	174	-17.06	-21.8	0.036056
hsa-miR-548ad-	NR_036103	MIR548V	178	-23.99	-27.5	0.000342
hsa-miR-548ad-	NR_037503	MIR548Y	178	-22.9	-23.5	0.0133
hsa-miR-548ad-	NR_037515	MIR548Z	186	-26.29	-29.3	0.000157
hsa-miR-548ad-	NR_039621	MIR548AC	177	-22.58	-25.5	0.001898
hsa-miR-548ad-	NR_039639	MIR548AE2	182	-20.35	-25.2	0.001179
hsa-miR-548ad-	NR_039673	MIR548AJ1	182	-20.26	-25.3	0.001321

hsa-miR-548ad-	NR_039674	MIR548AJ2	182	-20.26	-25.3	0.002447
hsa-miR-548ad-	NR_039692	MIR548X2	182	-19.01	-24.1	0.007049
hsa-miR-548ad-	NR_039699	MIR548AK	186	-23.83	-28.3	0.000069
hsa-miR-548ad-	NR_039762	MIR548AM	178	-21.81	-26.4	0.000625
hsa-miR-548ad-	NR_049838	MIR548AQ	182	-21.6	-26.4	0.000317
hsa-miR-548ad-	NR_049865	MIR548AX	182	-23.88	-24.4	0.002679
hsa-miR-548ad-	NR_110050	LOC101927418	182	-23.6	-26.8	0.020793
hsa-miR-548ad-	NR_128708	MIR548BB	182	-24.98	-27.9	0.000147
hsa-miR-548ae-	NR_030305	MIR579	161	-19.88	-22.9	0.015863
hsa-miR-548ae-	NR_030315	MIR548B	158	-22.21	-25.1	0.00321
hsa-miR-548ae-	NR_031642	MIR548F1	178	-20.21	-21.8	0.025133
hsa-miR-548ae-	NR_031643	MIR548F2	178	-20.21	-21.5	0.042751
hsa-miR-548ae-	NR_031644	MIR548F3	178	-20.21	-21.5	0.033577
hsa-miR-548ae-	NR_031645	MIR548F4	178	-20.21	-21.8	0.039754
hsa-miR-548ae-	NR_031666	MIR548N	178	-21.91	-25.1	0.001703
hsa-miR-548ae-	NR_031669	MIR548O	178	-18.58	-23.6	0.013385
hsa-miR-548ae-	NR_031678	MIR548H2	182	-23.04	-26.2	0.00114
hsa-miR-548ae-	NR_031679	MIR548H3	182	-21.6	-26.4	0.002037
hsa-miR-548ae-	NR_031680	MIR548H4	186	-26.29	-29.6	0.000184
hsa-miR-548ae-	NR_031686	MIR548P	178	-21.81	-26.4	0.000874
hsa-miR-548ae-	NR_031752	MIR548Q	174	-17.06	-21.8	0.036056
hsa-miR-548ae-	NR_036103	MIR548V	178	-23.99	-27.5	0.000342
hsa-miR-548ae-	NR_037503	MIR548Y	178	-22.9	-23.5	0.0133
hsa-miR-548ae-	NR_037515	MIR548Z	186	-26.29	-29.3	0.000157
hsa-miR-548ae-	NR_039621	MIR548AC	177	-22.58	-25.5	0.001898
hsa-miR-548ae-	NR_039639	MIR548AE2	182	-20.35	-25.2	0.001179
hsa-miR-548ae-	NR_039673	MIR548AJ1	182	-20.26	-25.3	0.001321
hsa-miR-548ae-	NR_039674	MIR548AJ2	182	-20.26	-25.3	0.002447
hsa-miR-548ae-	NR_039692	MIR548X2	182	-19.01	-24.1	0.007049
hsa-miR-548ae-	NR_039699	MIR548AK	186	-23.83	-28.3	0.000069

hsa-miR-548ae-	NR_039762	MIR548AM	178	-21.81	-26.4	0.000625
hsa-miR-548ae-	NR_049838	MIR548AQ	182	-21.6	-26.4	0.000317
hsa-miR-548ae-	NR_049865	MIR548AX	182	-23.88	-24.4	0.002679
hsa-miR-548ae-	NR_110050	LOC101927418	182	-23.6	-26.8	0.020793
hsa-miR-548ae-	NR_128708	MIR548BB	182	-24.98	-27.9	0.000147
hsa-miR-4479	NM_000832	GRIN1	166	-31.08	-39.2	0.002837
hsa-miR-4479	NM_001004326	SLC22A20	159	-29.25	-34.4	0.004415
hsa-miR-4479	NM_001010911	CASC10	161	-33.95	-37.9	0.006784
hsa-miR-4479	NM_001013661	VSIG8	156	-31.73	-36.6	0.002412
hsa-miR-4479	NM_001014985	GLTPD2	164	-29.39	-37.2	0.000814
hsa-miR-4479	NM_001037984	SLC38A10	153	-35.61	-37	0.005223
hsa-miR-4479	NM_001042454	TGFB1I1	151	-26.48	-30.5	0.034706
hsa-miR-4479	NM_001042535	AGAP3	153	-26.48	-32.6	0.044691
hsa-miR-4479	NM_001053	SSTR5	166	-40.63	-43.5	0.001045
hsa-miR-4479	NM_001080524	C16orf90	150	-26.4	-31.8	0.016409
hsa-miR-4479	NM_001099409	EHBP1L1	150	-31.22	-33.3	0.007488
hsa-miR-4479	NM_001099456	NPW	154	-30.8	-33	0.001359
hsa-miR-4479	NM_001124	ADM	155	-28.49	-35.3	0.011535
hsa-miR-4479	NM_001128325	SPON2	156	-30.96	-35.6	0.005639
hsa-miR-4479	NM_001145815	AMDHD2	158	-32.32	-34	0.001748
hsa-miR-4479	NM_001164719	TGFB1I1	151	-26.48	-30.5	0.034706
hsa-miR-4479	NM_001170460	CDK16	150	-27.73	-34.3	0.036429
hsa-miR-4479	NM_001170535	ATAD3A	150	-34.77	-32.7	0.026366
hsa-miR-4479	NM_001170536	ATAD3A	150	-34.77	-32.7	0.026366
hsa-miR-4479	NM_001170689	MIB2	158	-24.31	-31.1	0.018159
hsa-miR-4479	NM_001172560	SSTR5	166	-40.63	-43.5	0.001045
hsa-miR-4479	NM_001178061	SEMA6C	170	-41.49	-42.9	0.000426
hsa-miR-4479	NM_001178062	SEMA6C	170	-41.49	-42.9	0.000426
hsa-miR-4479	NM_001185090	GRIN1	166	-31.08	-39.2	0.002837
hsa-miR-4479	NM_001185091	GRIN1	166	-31.08	-39.2	0.002837

hsa-miR-4479	NM_001190720	IKBKB	150	-36.58	-39.2	0.007518
hsa-miR-4479	NM_001193380	IL17RE	168	-25.85	-33.3	0.045635
hsa-miR-4479	NM_001197244	BCL7B	159	-27.32	-32.9	0.0456
hsa-miR-4479	NM_001199021	SPON2	156	-30.96	-35.6	0.005639
hsa-miR-4479	NM_001199461	PDCD2	150	-35.98	-36.9	0.002805
hsa-miR-4479	NM_001199463	PDCD2	150	-35.98	-36.9	0.013019
hsa-miR-4479	NM_001199464	PDCD2	150	-35.98	-36.9	0.002805
hsa-miR-4479	NM_001199989	RASD1	168	-26.2	-33.5	0.043104
hsa-miR-4479	NM_001242778	IKBKB	150	-36.58	-39.2	0.007518
hsa-miR-4479	NM_001256627	BRSK2	159	-39.52	-38.2	0.014367
hsa-miR-4479	NM_001256630	BRSK2	159	-39.52	-38.2	0.014367
hsa-miR-4479	NM_001278694	GPR132	168	-30.07	-34.8	0.042922
hsa-miR-4479	NM_001278695	GPR132	168	-30.07	-34.8	0.042922
hsa-miR-4479	NM_001278696	GPR132	168	-30.07	-34.8	0.042922
hsa-miR-4479	NM_001284308	ADAP1	160	-30.97	-37.2	0.008039
hsa-miR-4479	NM_001284309	ADAP1	160	-30.97	-37.2	0.008039
hsa-miR-4479	NM_001284310	ADAP1	160	-30.97	-37.2	0.008039
hsa-miR-4479	NM_001284311	ADAP1	160	-30.97	-37.2	0.008039
hsa-miR-4479	NM_001293167	PANO1	159	-32.12	-34.1	0.025851
hsa-miR-4479	NM_001293171	HOTS	160	-34.64	-40.1	0.000988
hsa-miR-4479	NM_001301061	BCL7B	159	-27.32	-32.9	0.0456
hsa-miR-4479	NM_001301168	SHF	174	-34.23	-38.6	0.002483
hsa-miR-4479	NM_001301169	SHF	174	-34.23	-38.6	0.003916
hsa-miR-4479	NM_001301170	SHF	174	-34.23	-38.6	0.003513
hsa-miR-4479	NM_001301171	SHF	174	-34.23	-38.6	0.002483
hsa-miR-4479	NM_001301867	BOC	159	-33.11	-38.4	0.002256
hsa-miR-4479	NM_001302084	C11orf80	152	-27.6	-29.4	0.035713
hsa-miR-4479	NM_001308093	GATA4	156	-34.25	-37.4	0.013757
hsa-miR-4479	NM_001308094	GATA4	156	-34.25	-37.4	0.013757
hsa-miR-4479	NM_001308304	AGAP3	153	-26.48	-32.6	0.044691

hsa-miR-4479	NM_001308305	AGAP3	153	-26.48	-32.6	0.044691
hsa-miR-4479	NM_001556	IKBKB	150	-36.58	-39.2	0.007518
hsa-miR-4479	NM_001707	BCL7B	159	-27.32	-32.9	0.0456
hsa-miR-4479	NM_001715	BLK	150	-22.73	-32	0.02548
hsa-miR-4479	NM_002052	GATA4	156	-34.25	-37.4	0.013757
hsa-miR-4479	NM_003378	VGF	150	-31.34	-31.3	0.034731
hsa-miR-4479	NM_004305	BIN1	156	-33.36	-37.5	0.00206
hsa-miR-4479	NM_005245	FAT1	152	-30.23	-35.8	0.010203
hsa-miR-4479	NM_005929	MFI2	159	-30.04	-34.6	0.047558
hsa-miR-4479	NM_006026	H1FX	159	-39.48	-34.2	0.009149
hsa-miR-4479	NM_006201	CDK16	150	-27.73	-34.3	0.036429
hsa-miR-4479	NM_006236	POU3F3	152	-24.63	-36.3	0.022347
hsa-miR-4479	NM_006869	ADAP1	160	-30.97	-37.2	0.008039
hsa-miR-4479	NM_007327	GRIN1	166	-31.08	-39.2	0.004646
hsa-miR-4479	NM_012445	SPON2	156	-30.96	-35.6	0.005639
hsa-miR-4479	NM_013239	PPP2R3B	153	-27.46	-31.5	0.026387
hsa-miR-4479	NM_013345	GPR132	168	-30.07	-34.8	0.042922
hsa-miR-4479	NM_015927	TGFB1I1	151	-26.48	-30.5	0.034706
hsa-miR-4479	NM_016547	SDF4	171	-36.18	-40.2	0.001303
hsa-miR-4479	NM_017410	HOXC13	150	-32.65	-37	0.012586
hsa-miR-4479	NM_017617	NOTCH1	158	-33.41	-37.3	0.015742
hsa-miR-4479	NM_018019	MED9	156	-27.72	-34.9	0.045408
hsa-miR-4479	NM_018188	ATAD3A	150	-34.77	-32.7	0.026366
hsa-miR-4479	NM_021138	TRAF2	155	-26.36	-33.7	0.021237
hsa-miR-4479	NM_021569	GRIN1	166	-31.08	-39.2	0.004646
hsa-miR-4479	NM_022117	TSPYL2	161	-26.91	-31.9	0.037033
hsa-miR-4479	NM_024016	HOXB8	159	-28.59	-34.2	0.022503
hsa-miR-4479	NM_024650	C11orf80	152	-27.6	-29.4	0.035713
hsa-miR-4479	NM_030661	HOXA3	150	-19.48	-35.6	0.034229
hsa-miR-4479	NM_030913	SEMA6C	170	-41.49	-42.9	0.000426

hsa-miR-4479	NM_033018	CDK16	150	-27.73	-34.3	0.036429
hsa-miR-4479	NM_138356	SHF	174	-34.23	-38.6	0.002483
hsa-miR-4479	NM_139343	BIN1	156	-33.36	-37.5	0.00206
hsa-miR-4479	NM_139344	BIN1	156	-33.36	-37.5	0.00206
hsa-miR-4479	NM_139345	BIN1	156	-33.36	-37.5	0.00206
hsa-miR-4479	NM_139346	BIN1	156	-33.36	-37.5	0.00206
hsa-miR-4479	NM_139347	BIN1	156	-33.36	-37.5	0.00206
hsa-miR-4479	NM_139348	BIN1	156	-33.36	-37.5	0.00206
hsa-miR-4479	NM_139349	BIN1	156	-33.36	-37.5	0.00206
hsa-miR-4479	NM_139350	BIN1	156	-33.36	-37.5	0.00206
hsa-miR-4479	NM_139351	BIN1	156	-33.36	-37.5	0.00206
hsa-miR-4479	NM_144781	PDCD2	150	-35.98	-36.9	0.013019
hsa-miR-4479	NM_153631	HOXA3	150	-19.48	-35.6	0.034229
hsa-miR-4479	NM_174937	TCERG1L	167	-34.19	-34.3	0.018303
hsa-miR-4479	NM_175885	FAM181B	153	-25.55	-30.5	0.0437
hsa-miR-4479	NM_178568	RTN4RL1	155	-35.24	-35.8	0.033944
hsa-miR-4479	NR_001543	TTTY14	161	-28.07	-31.7	0.044427
hsa-miR-4479	NR_002556	LOC388242	167	-26.54	-35.2	0.032584
hsa-miR-4479	NR_002557	LOC613038	167	-26.54	-35.2	0.032584
hsa-miR-4479	NR_003034	LOC441601	168	-33.05	-38.3	0.004949
hsa-miR-4479	NR_015381	TP53TG1	173	-35.66	-39.4	0.001633
hsa-miR-4479	NR_015450	LINC01096	159	-30.24	-35.2	0.040992
hsa-miR-4479	NR_023387	ABCC6P2	156	-29.25	-33.7	0.020869
hsa-miR-4479	NR_024127	SNHG12	152	-27.5	-32.1	0.045328
hsa-miR-4479	NR_024420	LINC00937	151	-33.67	-35.8	0.007309
hsa-miR-4479	NR_024533	ALG3	151	-29.97	-34.6	0.039914
hsa-miR-4479	NR_024534	ALG3	151	-29.97	-34.6	0.042332
hsa-miR-4479	NR_024610	HINT1	151	-32.52	-33.1	0.037608
hsa-miR-4479	NR_024611	HINT1	151	-32.52	-32.1	0.04391
hsa-miR-4479	NR_026740	LOC389033	159	-28.23	-33.2	0.010363

hsa-miR-4479	NR_026766	MYCNOS	150	-31.05	-33.7	0.023853
hsa-miR-4479	NR_026901	LOC644172	150	-21.85	-35.1	0.043036
hsa-miR-4479	NR_027391	SLC15A3	160	-37.5	-36.7	0.025942
hsa-miR-4479	NR_028386	LOC375196	160	-29.37	-34.2	0.030713
hsa-miR-4479	NR_033248	GCSHP3	163	-29.24	-36	0.013061
hsa-miR-4479	NR_033656	STX8	153	-32.8	-34.5	0.016482
hsa-miR-4479	NR_034003	LOC100130298	162	-26.99	-33	0.017234
hsa-miR-4479	NR_034018	TP53TG3HP	179	-36.87	-42.4	0.001307
hsa-miR-4479	NR_034019	TP53TG3HP	179	-36.87	-42.4	0.001087
hsa-miR-4479	NR_034031	LOC389906	169	-36.74	-39.2	0.008335
hsa-miR-4479	NR_036682	BCL7B	164	-35.56	-37.2	0.017393
hsa-miR-4479	NR_037438	MIR3665	163	-31.52	-37.9	0.000048
hsa-miR-4479	NR_037631	LOC100288911	162	-36.65	-40.6	0.00097
hsa-miR-4479	NR_038263	SOCS2-AS1	162	-33.59	-38.4	0.001739
hsa-miR-4479	NR_038368	LINC00273	156	-32.3	-34.7	0.039008
hsa-miR-4479	NR_038378	LOC441242	161	-28.91	-35.7	0.039129
hsa-miR-4479	NR_038997	ANKRD34C-AS1	166	-36.38	-38.8	0.002051
hsa-miR-4479	NR_039821	MIR4674	160	-28.89	-33.2	0.00042
hsa-miR-4479	NR_039955	MIR4792	160	-28.15	-34.3	0.000145
hsa-miR-4479	NR_045559	UXT	153	-28.22	-32.9	0.02997
hsa-miR-4479	NR_046320	GPX2	159	-34.88	-40.7	0.002658
hsa-miR-4479	NR_046321	GPX2	159	-34.88	-40.7	0.002655
hsa-miR-4479	NR_046625	MEIS1-AS2	169	-36.6	-37.2	0.004301
hsa-miR-4479	NR_073080	PHLDA3	152	-27.17	-36.6	0.020946
hsa-miR-4479	NR_073110	PSD	166	-32.27	-35.7	0.038996
hsa-miR-4479	NR_073189	HRK	169	-35.41	-39.8	0.006791
hsa-miR-4479	NR_073199	LINC00483	166	-31.8	-34.5	0.018652
hsa-miR-4479	NR_073488	HINT1	151	-32.52	-33.1	0.049646
hsa-miR-4479	NR_103803	EBPL	151	-27.64	-32.7	0.046799

hsa-miR-4479	NR_104040	SERGEF	161	-29.55	-36	0.027334
hsa-miR-4479	NR_104041	SERGEF	161	-29.55	-36	0.021581
hsa-miR-4479	NR_104128	IDS	154	-32.78	-36.8	0.017783
hsa-miR-4479	NR_104328	GATA3-AS1	160	-31.44	-38.3	0.002289
hsa-miR-4479	NR_104330	GATA3-AS1	160	-31.44	-38.3	0.002451
hsa-miR-4479	NR_104586	ANKRD10	165	-33.24	-39.8	0.004078
hsa-miR-4479	NR_105049	C5orf66-AS1	153	-33.31	-34.4	0.020145
hsa-miR-4479	NR_105050	C5orf66-AS1	153	-33.31	-34.4	0.019962
hsa-miR-4479	NR_107031	MIR8064	150	-26.07	-26.4	0.022174
hsa-miR-4479	NR_108031	LOC102577426	151	-36.84	-33.7	0.010642
hsa-miR-4479	NR_109767	LOC100131315	164	-31.22	-32.3	0.029233
hsa-miR-4479	NR_110736	TMEM114	153	-37.54	-40.2	0.000635
hsa-miR-4479	NR_110880	THCAT158	154	-25.23	-34.9	0.012018
hsa-miR-4479	NR_111908	LOC101928794	159	-43.96	-42.3	0.000486
hsa-miR-4479	NR_120572	LOC101929089	156	-31.89	-35.3	0.011001
hsa-miR-4479	NR_120573	LOC101929089	156	-31.89	-35.3	0.008499
hsa-miR-4479	NR_120574	LOC101929089	156	-31.89	-35.3	0.005759
hsa-miR-4479	NR_120575	LOC101929089	156	-31.89	-35.3	0.008953
hsa-miR-4479	NR_125425	LOC101927815	161	-33.4	-34.2	0.015806
hsa-miR-4479	NR_125426	LOC101927815	161	-33.4	-34.2	0.013273
hsa-miR-4479	NR_126167	TMEM5-AS1	168	-30.98	-35.5	0.00637
hsa-miR-4479	NR_126375	LINC01336	154	-24.59	-34.2	0.010588
hsa-miR-4479	NR_126394	ACYP1	154	-33	-35.7	0.008596
hsa-miR-4482-3p	NM_000137	FAH	176	-22.11	-28.6	0.027121
hsa-miR-4482-3p	NM_001001349	NKIRAS2	158	-29.38	-33.7	0.024712
hsa-miR-4482-3p	NM_001144927	NKIRAS2	158	-29.38	-33.7	0.024712
hsa-miR-4482-3p	NM_001144928	NKIRAS2	158	-29.38	-33.7	0.024712
hsa-miR-4482-3p	NM_001144929	NKIRAS2	158	-29.38	-33.7	0.028545
hsa-miR-4482-3p	NM_001204477	CDRT4	155	-31.18	-33.8	0.023874
hsa-miR-4482-3p	NM_001284241	NO	155	-20.19	-24.1	0.041776

hsa-miR-4482-3p	NM_001301107	CCDC53	151	-22.83	-27.4	0.029465
hsa-miR-4482-3p	NM_004159	PSMB8	152	-22.58	-28.1	0.016668
hsa-miR-4482-3p	NM_005553	KRTAP5-9	152	-22.98	-28.3	0.041567
hsa-miR-4482-3p	NM_016053	CCDC53	151	-22.83	-27.4	0.029465
hsa-miR-4482-3p	NM_017595	NKIRAS2	158	-29.38	-33.7	0.024712
hsa-miR-4482-3p	NM_024786	ZDHHC11	151	-25.62	-32.5	0.018265
hsa-miR-4482-3p	NM_148919	PSMB8	152	-22.58	-28.1	0.016668
hsa-miR-4482-3p	NR_036549	CCNT2-AS1	164	-30.73	-32.7	0.028483
hsa-miR-4482-3p	NR_103827	ANTXRLP1	155	-33.1	-37.1	0.002672
hsa-miR-4482-3p	NR_103828	ANTXRLP1	155	-33.1	-37.1	0.001989
hsa-miR-4482-3p	NR_125997	LOC102724450	158	-23.7	-29.2	0.020184
hsa-miR-4676-5p	NM_000196	HSD11B2	167	-26.2	-31.2	0.017274
hsa-miR-4676-5p	NM_001001480	KRTAP5-5	175	-31.35	-34	0.002068
hsa-miR-4676-5p	NM_001001891	ANO7	150	-25.67	-31.6	0.048745
hsa-miR-4676-5p	NM_001008270	PRSS37	156	-24.78	-27.7	0.006424
hsa-miR-4676-5p	NM_001008860	NIPA2	170	-28.55	-33.1	0.03437
hsa-miR-4676-5p	NM_001008892	NIPA2	170	-28.55	-33.1	0.03437
hsa-miR-4676-5p	NM_001008894	NIPA2	170	-28.55	-33.1	0.03437
hsa-miR-4676-5p	NM_001093771	TXNRD1	165	-28.39	-33	0.047681
hsa-miR-4676-5p	NM_001098520	HTATIP2	158	-27.08	-30.4	0.028007
hsa-miR-4676-5p	NM_001098521	HTATIP2	158	-27.08	-30.4	0.028007
hsa-miR-4676-5p	NM_001098522	HTATIP2	158	-27.08	-30.4	0.028007
hsa-miR-4676-5p	NM_001122890	GGT6	156	-28.31	-34.2	0.01239
hsa-miR-4676-5p	NM_001142614	EHBP1	156	-26.96	-30.9	0.049472
hsa-miR-4676-5p	NM_001142615	EHBP1	156	-26.96	-30.9	0.049472
hsa-miR-4676-5p	NM_001142616	EHBP1	156	-26.96	-30.9	0.049472
hsa-miR-4676-5p	NM_001142733	ASB14	170	-29.73	-34.2	0.016159
hsa-miR-4676-5p	NM_001145017	SLC36A3	170	-30.32	-32.1	0.03014
hsa-miR-4676-5p	NM_001145524	YPEL3	156	-25.1	-30.2	0.016397
hsa-miR-4676-5p	NM_001146102	PARP9	161	-26.04	-30.1	0.021874

hsa-miR-4676-5p	NM_001146103	PARP9	161	-26.04	-30.1	0.021874
hsa-miR-4676-5p	NM_001146104	PARP9	161	-26.04	-30.1	0.021874
hsa-miR-4676-5p	NM_001146105	PARP9	161	-26.04	-30.1	0.021874
hsa-miR-4676-5p	NM_001171740	C3orf18	157	-30.94	-37.5	0.005246
hsa-miR-4676-5p	NM_001171741	C3orf18	157	-30.94	-37.5	0.005246
hsa-miR-4676-5p	NM_001171743	C3orf18	157	-30.94	-37.5	0.005246
hsa-miR-4676-5p	NM_001171951	PRSS37	156	-24.78	-27.7	0.006424
hsa-miR-4676-5p	NM_001184888	NIPA2	170	-28.55	-33.1	0.03437
hsa-miR-4676-5p	NM_001184889	NIPA2	170	-28.55	-33.1	0.03437
hsa-miR-4676-5p	NM_001204078	ATP6V1G2	163	-33.06	-35.4	0.005727
hsa-miR-4676-5p	NM_001243925	MAPKAPK3	168	-25.59	-33.7	0.019381
hsa-miR-4676-5p	NM_001243926	MAPKAPK3	168	-25.59	-33.7	0.019381
hsa-miR-4676-5p	NM_001261445	TXNRD1	165	-28.39	-33	0.047681
hsa-miR-4676-5p	NM_001261446	TXNRD1	165	-28.39	-33	0.047681
hsa-miR-4676-5p	NM_001270707	NRM	151	-25.8	-29.7	0.043344
hsa-miR-4676-5p	NM_001270709	NRM	151	-25.8	-29.7	0.043344
hsa-miR-4676-5p	NM_001282766	POLR1E	161	-23.35	-29.9	0.027991
hsa-miR-4676-5p	NM_001286843	PHF19	158	-24.62	-29.1	0.021408
hsa-miR-4676-5p	NM_001288702	GGT6	156	-28.31	-34.2	0.01239
hsa-miR-4676-5p	NM_001288703	GGT6	156	-28.31	-34.2	0.017964
hsa-miR-4676-5p	NM_001288704	GGT6	156	-28.31	-34.2	0.017964
hsa-miR-4676-5p	NM_001291306	NOP16	151	-25.42	-31.4	0.021882
hsa-miR-4676-5p	NM_001304799	NARFL	162	-29.22	-30.7	0.029957
hsa-miR-4676-5p	NM_001306200	SETD7	162	-32.45	-37.2	0.00086
hsa-miR-4676-5p	NM_001447	FAT2	172	-27.93	-35.2	0.012578
hsa-miR-4676-5p	NM_001540	HSPB1	161	-25.8	-32.3	0.000466
hsa-miR-4676-5p	NM_002084	GPX3	153	-27.44	-30.5	0.049143
hsa-miR-4676-5p	NM_003330	TXNRD1	165	-28.39	-33	0.047681
hsa-miR-4676-5p	NM_004164	RBP2	154	-24.43	-29.2	0.01072
hsa-miR-4676-5p	NM_004635	MAPKAPK3	168	-25.59	-33.7	0.019381

hsa-miR-4676-5p	NM_005293	GPR20	169	-31.7	-34	0.001917
hsa-miR-4676-5p	NM_005554	KRT6A	167	-28.6	-32.3	0.009911
hsa-miR-4676-5p	NM_006410	HTATIP2	158	-27.08	-30.4	0.028007
hsa-miR-4676-5p	NM_006656	NEU3	169	-29.7	-33.1	0.024136
hsa-miR-4676-5p	NM_007243	NRM	151	-25.8	-29.7	0.043344
hsa-miR-4676-5p	NM_012472	LRRC6	151	-20.88	-28.7	0.029593
hsa-miR-4676-5p	NM_014360	NKX2-8	154	-29.77	-33.4	0.013132
hsa-miR-4676-5p	NM_015252	EHBP1	156	-26.96	-30.9	0.049472
hsa-miR-4676-5p	NM_015597	GPSM1	170	-24.6	-30.6	0.032483
hsa-miR-4676-5p	NM_016210	C3orf18	157	-30.94	-37.5	0.005246
hsa-miR-4676-5p	NM_017547	FOXRED1	164	-29.3	-33.7	0.003338
hsa-miR-4676-5p	NM_020387	RAB25	154	-23.42	-27.5	0.024179
hsa-miR-4676-5p	NM_020692	GALNT16	162	-27.11	-31.6	0.044343
hsa-miR-4676-5p	NM_021958	HLX	155	-25.99	-30.6	0.011022
hsa-miR-4676-5p	NM_022490	POLR1E	161	-23.35	-29.9	0.027991
hsa-miR-4676-5p	NM_022493	NARFL	162	-29.22	-30.7	0.029957
hsa-miR-4676-5p	NM_030922	NIPA2	170	-28.55	-33.1	0.03437
hsa-miR-4676-5p	NM_031458	PARP9	161	-26.04	-30.1	0.021874
hsa-miR-4676-5p	NM_031477	YPEL3	156	-25.1	-30.2	0.016541
hsa-miR-4676-5p	NM_032425	HHIPL1	151	-22.04	-27.9	0.043347
hsa-miR-4676-5p	NM_032782	HAVCR2	154	-26.2	-31.7	0.049129
hsa-miR-4676-5p	NM_032829	FAM222A	164	-27.75	-34.5	0.012901
hsa-miR-4676-5p	NM_032847	C8orf76	152	-20.61	-26.1	0.026044
hsa-miR-4676-5p	NM_033027	CSRNP1	173	-27.4	-33.1	0.024076
hsa-miR-4676-5p	NM_058200	SPAG11B	152	-24.22	-27.5	0.03344
hsa-miR-4676-5p	NM_058202	SPAG11B	152	-24.22	-27.5	0.03344
hsa-miR-4676-5p	NM_130387	ASB14	170	-29.73	-34.2	0.016159
hsa-miR-4676-5p	NM_130463	ATP6V1G2	163	-33.06	-35.4	0.005727
hsa-miR-4676-5p	NM_138282	ATP6V1G2	163	-33.06	-35.4	0.005727
hsa-miR-4676-5p	NM_145051	RNF183	163	-25.34	-32.7	0.007268

hsa-miR-4676-5p	NM_152383	DIS3L2	165	-27.15	-30.3	0.028616
hsa-miR-4676-5p	NM_153265	EML3	150	-29.54	-31.8	0.003122
hsa-miR-4676-5p	NM_153338	GGT6	156	-28.31	-34.2	0.01239
hsa-miR-4676-5p	NM_181774	SLC36A3	170	-30.32	-32.1	0.03014
hsa-miR-4676-5p	NM_182729	TXNRD1	165	-28.39	-33	0.047681
hsa-miR-4676-5p	NM_182742	TXNRD1	165	-28.39	-33	0.047681
hsa-miR-4676-5p	NM_182743	TXNRD1	165	-28.39	-33	0.047681
hsa-miR-4676-5p	NM_198571	NAT16	154	-32.69	-33.8	0.026326
hsa-miR-4676-5p	NR_030301	MIR575	158	-21	-26	0.011353
hsa-miR-4676-5p	NR_030593	MIR892B	153	-20.32	-24.2	0.022962
hsa-miR-4676-5p	NR_037504	MIR3939	157	-22.17	-26.5	0.010761
hsa-miR-4676-5p	NR_039747	MIR1269B	154	-21.18	-26	0.006943
hsa-miR-4676-5p	NR_039889	MIR4736	161	-24.43	-32.7	0.000023
hsa-miR-4676-5p	NR_073163	ATF7	166	-31.18	-34.1	0.030662
hsa-miR-4676-5p	NR_120310	LOC400553	158	-24.81	-30	0.019785
hsa-miR-4676-5p	NR_121579	LOC101928525	157	-24.9	-31.3	0.049137
hsa-miR-4676-5p	NR_122031	GAS8-AS1	166	-26.63	-31.3	0.04161
hsa-miR-4676-5p	NR_126542	CEP112	164	-26.86	-31.6	0.044177
hsa-miR-4676-5p	NR_130951	ZNF33B	168	-28.07	-32.7	0.023929
hsa-miR-4676-5p	NR_131339	SETD7	162	-32.45	-37.2	0.006352
hsa-miR-4707-3p	NM_000296	PKD1	152	-31.68	-36	0.012633
hsa-miR-4707-3p	NM_000319	PEX5	151	-33.81	-34.9	0.027857
hsa-miR-4707-3p	NM_000425	L1CAM	168	-34.28	-39.8	0.002985
hsa-miR-4707-3p	NM_000498	CYP11B2	150	-31.05	-35.1	0.030405
hsa-miR-4707-3p	NM_000624	SERPINA5	154	-35.46	-37.6	0.004982
hsa-miR-4707-3p	NM_000681	ADRA2A	164	-31.59	-36.5	0.018541
hsa-miR-4707-3p	NM_000682	ADRA2B	154	-32.49	-42.7	0.002083
hsa-miR-4707-3p	NM_000734	CD247	151	-25.09	-34	0.030981
hsa-miR-4707-3p	NM_001001795	C8orf82	157	-30.71	-35.7	0.016739
hsa-miR-4707-3p	NM_001004019	FBLN2	152	-29.84	-35.5	0.004902

hsa-miR-4707-3p	NM_001009944	PKD1	152	-31.68	-36	0.012633
hsa-miR-4707-3p	NM_001013661	VSIG8	160	-27.24	-33.4	0.010292
hsa-miR-4707-3p	NM_001014435	CA7	166	-31.49	-36.7	0.004406
hsa-miR-4707-3p	NM_001014794	ILK	152	-28.21	-30.8	0.01499
hsa-miR-4707-3p	NM_001014795	ILK	152	-28.21	-30.8	0.01499
hsa-miR-4707-3p	NM_001024215	FBLIM1	156	-30.62	-36.5	0.000412
hsa-miR-4707-3p	NM_001024844	CD82	155	-28.65	-34.8	0.010217
hsa-miR-4707-3p	NM_001024959	ARPC4	156	-35.7	-41.4	0.000947
hsa-miR-4707-3p	NM_001024960	ARPC4	156	-35.7	-41.4	0.000947
hsa-miR-4707-3p	NM_001029989	KIAA0101	155	-28.85	-34.8	0.020505
hsa-miR-4707-3p	NM_001032391	LCMT1	157	-27.23	-31.9	0.005478
hsa-miR-4707-3p	NM_001033054	AIPL1	161	-30.79	-35.5	0.03372
hsa-miR-4707-3p	NM_001033055	AIPL1	161	-30.79	-35.5	0.03372
hsa-miR-4707-3p	NM_001039469	MARK2	167	-34.13	-38.4	0.010807
hsa-miR-4707-3p	NM_001039582	PNCK	152	-26.14	-32	0.017581
hsa-miR-4707-3p	NM_001039702	OLAH	150	-27.2	-34.9	0.009358
hsa-miR-4707-3p	NM_001040167	LFNG	154	-28.4	-34.3	0.034126
hsa-miR-4707-3p	NM_001042535	AGAP3	154	-25.61	-32.9	0.03722
hsa-miR-4707-3p	NM_001042575	TMPRSS7	154	-22.16	-30.4	0.009614
hsa-miR-4707-3p	NM_001042663	PLEKHG5	158	-32.39	-35.8	0.023879
hsa-miR-4707-3p	NM_001042664	PLEKHG5	158	-32.39	-35.8	0.023879
hsa-miR-4707-3p	NM_001042665	PLEKHG5	158	-32.39	-35.8	0.023879
hsa-miR-4707-3p	NM_001077263	TMPRSS13	153	-30.93	-35.8	0.02686
hsa-miR-4707-3p	NM_001077416	TMEM231	157	-28.32	-36.9	0.021493
hsa-miR-4707-3p	NM_001077418	TMEM231	157	-28.32	-36.9	0.021493
hsa-miR-4707-3p	NM_001077663	URGCP	150	-22.9	-34.4	0.016626
hsa-miR-4707-3p	NM_001077664	URGCP	150	-22.9	-34.4	0.016626
hsa-miR-4707-3p	NM_001078171	FAM127A	154	-36.99	-41.5	0.000778
hsa-miR-4707-3p	NM_001078172	FAM127B	150	-34.43	-39.4	0.002042
hsa-miR-4707-3p	NM_001080547	SPI1	156	-27.31	-32.1	0.014312

hsa-miR-4707-3p	NM_001098475	TDRD10	150	-29.79	-35.2	0.007441
hsa-miR-4707-3p	NM_001098784	FAM89B	160	-30.42	-37.6	0.00312
hsa-miR-4707-3p	NM_001098785	FAM89B	160	-30.42	-37.6	0.00222
hsa-miR-4707-3p	NM_001099432	BCAS3	154	-32.05	-35.6	0.008532
hsa-miR-4707-3p	NM_001100818	PID1	152	-32.23	-37.6	0.013498
hsa-miR-4707-3p	NM_001110199	SRRM3	153	-31.02	-34.2	0.045321
hsa-miR-4707-3p	NM_001122890	GGT6	161	-30.59	-35.5	0.017384
hsa-miR-4707-3p	NM_001127190	CSK	159	-28.43	-34.1	0.015704
hsa-miR-4707-3p	NM_001127660	MFN2	172	-33.12	-38.1	0.013938
hsa-miR-4707-3p	NM_001130144	LTBP3	165	-28.1	-32.2	0.024144
hsa-miR-4707-3p	NM_001131023	PEX5	151	-33.81	-34.9	0.027857
hsa-miR-4707-3p	NM_001131024	PEX5	151	-33.81	-34.9	0.027857
hsa-miR-4707-3p	NM_001131025	PEX5	151	-33.81	-34.9	0.027857
hsa-miR-4707-3p	NM_001135740	PNCK	152	-26.14	-32	0.017581
hsa-miR-4707-3p	NM_001136158	OTUD5	154	-29.34	-34	0.025955
hsa-miR-4707-3p	NM_001136161	MCMDC2	163	-29.55	-34.8	0.005081
hsa-miR-4707-3p	NM_001136216	TMEM51	162	-33.2	-39.1	0.00186
hsa-miR-4707-3p	NM_001136217	TMEM51	162	-33.2	-39.1	0.00186
hsa-miR-4707-3p	NM_001136218	TMEM51	162	-33.2	-39.1	0.00186
hsa-miR-4707-3p	NM_001136538	ACAD10	151	-27.37	-31.9	0.037481
hsa-miR-4707-3p	NM_001143688	DIS3L	162	-27.77	-33.4	0.016794
hsa-miR-4707-3p	NM_001143905	C12orf65	156	-30.42	-36.5	0.008802
hsa-miR-4707-3p	NM_001143963	L1CAM	168	-34.28	-39.8	0.002985
hsa-miR-4707-3p	NM_001158	AOC2	157	-27.12	-31.5	0.01858
hsa-miR-4707-3p	NM_001161009	ARMC10	162	-27.57	-33.4	0.049379
hsa-miR-4707-3p	NM_001161010	ARMC10	162	-27.57	-33.4	0.049379
hsa-miR-4707-3p	NM_001161011	ARMC10	162	-27.57	-33.4	0.049379
hsa-miR-4707-3p	NM_001161012	ARMC10	162	-27.57	-33.4	0.049379
hsa-miR-4707-3p	NM_001161013	ARMC10	162	-27.57	-33.4	0.049379
hsa-miR-4707-3p	NM_001162371	LOC728392	150	-32.23	-37.2	0.008388

hsa-miR-4707-3p	NM_001163296	MARK2	167	-34.13	-38.4	0.010807
hsa-miR-4707-3p	NM_001163297	MARK2	167	-34.13	-38.4	0.010807
hsa-miR-4707-3p	NM_001164266	LTBP3	165	-28.1	-32.2	0.024144
hsa-miR-4707-3p	NM_001164835	L1TD1	156	-31.43	-36.8	0.008098
hsa-miR-4707-3p	NM_001165035	FBLN2	152	-29.84	-35.5	0.004902
hsa-miR-4707-3p	NM_001166355	LFNG	154	-28.4	-34.3	0.034126
hsa-miR-4707-3p	NM_001172700	SHROOM1	154	-23.87	-32.1	0.035984
hsa-miR-4707-3p	NM_001178044	SLC44A4	162	-28.49	-33.4	0.010171
hsa-miR-4707-3p	NM_001178045	SLC44A4	162	-28.49	-33.4	0.010171
hsa-miR-4707-3p	NM_001185060	AQP1	162	-32.41	-40	0.006112
hsa-miR-4707-3p	NM_001185061	AQP1	162	-32.41	-40	0.006112
hsa-miR-4707-3p	NM_001185062	AQP1	162	-32.41	-40	0.006112
hsa-miR-4707-3p	NM_001193380	IL17RE	152	-30.04	-34.5	0.025963
hsa-miR-4707-3p	NM_001194995	C12orf65	156	-30.42	-36.5	0.008802
hsa-miR-4707-3p	NM_001198780	ARPC4	156	-35.7	-41.4	0.000947
hsa-miR-4707-3p	NM_001199653	PMF1	161	-33.19	-36.4	0.003066
hsa-miR-4707-3p	NM_001199654	PMF1	161	-33.19	-36.4	0.002906
hsa-miR-4707-3p	NM_001201536	TAF1A	151	-24.92	-31.7	0.020194
hsa-miR-4707-3p	NM_001204414	TINAGL1	150	-27.73	-35.9	0.007349
hsa-miR-4707-3p	NM_001204415	TINAGL1	150	-27.73	-35.9	0.007349
hsa-miR-4707-3p	NM_001206789	TMPRSS13	153	-30.93	-35.8	0.02686
hsa-miR-4707-3p	NM_001242521	C1orf234	154	-23.41	-29.8	0.001496
hsa-miR-4707-3p	NM_001243211	IL18	162	-27.57	-33.4	0.00677
hsa-miR-4707-3p	NM_001253357	TIE1	151	-37.91	-41	0.000215
hsa-miR-4707-3p	NM_001256946	FAAP20	152	-30.31	-35.9	0.012133
hsa-miR-4707-3p	NM_001265592	PLEKHG5	158	-32.39	-35.8	0.023879
hsa-miR-4707-3p	NM_001265593	PLEKHG5	158	-32.39	-35.8	0.023879
hsa-miR-4707-3p	NM_001265594	PLEKHG5	158	-32.39	-35.8	0.024531
hsa-miR-4707-3p	NM_001271856	GRASP	162	-28.64	-36.1	0.006445
hsa-miR-4707-3p	NM_001278116	L1CAM	168	-34.28	-39.8	0.002985

hsa-miR-4707-3p	NM_001278441	ILK	152	-28.21	-30.8	0.01499
hsa-miR-4707-3p	NM_001278442	ILK	152	-28.21	-30.8	0.01499
hsa-miR-4707-3p	NM_001278562	LCTL	161	-30.59	-35.5	0.015804
hsa-miR-4707-3p	NM_001278619	PCDH11Y	161	-30.79	-35.5	0.021054
hsa-miR-4707-3p	NM_001278791	RFC2	157	-28.43	-33.1	0.020213
hsa-miR-4707-3p	NM_001278792	RFC2	157	-28.43	-33.1	0.020213
hsa-miR-4707-3p	NM_001278793	RFC2	157	-28.43	-33.1	0.020213
hsa-miR-4707-3p	NM_001282665	MICAL2	155	-31.69	-35	0.027015
hsa-miR-4707-3p	NM_001284209	MED6	156	-31.43	-36.8	0.017684
hsa-miR-4707-3p	NM_001284210	MED6	156	-31.43	-36.8	0.018969
hsa-miR-4707-3p	NM_001285399	AIPL1	161	-30.79	-35.5	0.03372
hsa-miR-4707-3p	NM_001285400	AIPL1	161	-30.79	-35.5	0.03372
hsa-miR-4707-3p	NM_001285401	AIPL1	161	-30.79	-35.5	0.03372
hsa-miR-4707-3p	NM_001285402	AIPL1	161	-30.79	-35.5	0.03372
hsa-miR-4707-3p	NM_001286648	SLC45A4	153	-26.94	-33.8	0.003324
hsa-miR-4707-3p	NM_001288702	GGT6	161	-30.59	-35.5	0.017384
hsa-miR-4707-3p	NM_001288703	GGT6	161	-30.59	-35.5	0.024764
hsa-miR-4707-3p	NM_001288704	GGT6	161	-30.59	-35.5	0.024764
hsa-miR-4707-3p	NM_001289032	SERPINA4	153	-35.15	-34.4	0.003563
hsa-miR-4707-3p	NM_001289033	SERPINA4	153	-35.15	-34.4	0.003563
hsa-miR-4707-3p	NM_001289395	TULP1	165	-35.81	-32	0.021156
hsa-miR-4707-3p	NM_001290075	URGCP	150	-22.9	-34.4	0.016626
hsa-miR-4707-3p	NM_001290076	URGCP	150	-22.9	-34.4	0.016626
hsa-miR-4707-3p	NM_001291831	SRRM3	153	-31.02	-34.2	0.046001
hsa-miR-4707-3p	NM_001291860	HSPG2	150	-34.71	-34	0.032847
hsa-miR-4707-3p	NM_001293228	MYCN	151	-30.58	-36	0.01062
hsa-miR-4707-3p	NM_001293231	MYCN	151	-30.58	-36	0.01062
hsa-miR-4707-3p	NM_001300789	PEX5	151	-33.81	-34.9	0.027857
hsa-miR-4707-3p	NM_001300913	C11orf24	178	-39.08	-43	0.000048
hsa-miR-4707-3p	NM_001303012	PLCH2	150	-30.93	-38.9	0.009706

hsa-miR-4707-3p	NM_001303013	PLCH2	150	-30.93	-38.9	0.007064
hsa-miR-4707-3p	NM_001303095	ISG20L2	156	-30.42	-36.5	0.017614
hsa-miR-4707-3p	NM_001303470	C7orf43	151	-26.67	-31.1	0.048362
hsa-miR-4707-3p	NM_001304376	ADGRG5	156	-33.07	-37.5	0.013954
hsa-miR-4707-3p	NM_001304968	TSGA13	151	-28.17	-30.2	0.035427
hsa-miR-4707-3p	NM_001308304	AGAP3	154	-25.61	-32.9	0.03722
hsa-miR-4707-3p	NM_001308305	AGAP3	154	-25.61	-32.9	0.03722
hsa-miR-4707-3p	NM_001308370	ESPNL	156	-33.96	-39.6	0.005329
hsa-miR-4707-3p	NM_001517	GTF2H4	153	-37.48	-29.5	0.006593
hsa-miR-4707-3p	NM_001562	IL18	162	-27.57	-33.4	0.00677
hsa-miR-4707-3p	NM_001923	DDB1	155	-31.03	-35.9	0.007037
hsa-miR-4707-3p	NM_001998	FBLN2	152	-29.84	-35.5	0.004902
hsa-miR-4707-3p	NM_002164	IDO1	155	-28.65	-32.1	0.031987
hsa-miR-4707-3p	NM_002231	CD82	155	-28.65	-34.8	0.010217
hsa-miR-4707-3p	NM_002304	LFNG	154	-28.4	-34.3	0.034126
hsa-miR-4707-3p	NM_002653	PITX1	165	-37.25	-41.4	0.001267
hsa-miR-4707-3p	NM_002914	RFC2	157	-28.43	-33.1	0.020213
hsa-miR-4707-3p	NM_003082	SNAPC1	156	-30.62	-36.5	0.017384
hsa-miR-4707-3p	NM_003088	FSCN1	151	-27.87	-35	0.024342
hsa-miR-4707-3p	NM_003120	SPI1	156	-27.31	-32.1	0.014312
hsa-miR-4707-3p	NM_003311	PHLDA2	152	-27.95	-32.1	0.016764
hsa-miR-4707-3p	NM_003322	TULP1	165	-35.81	-32	0.021156
hsa-miR-4707-3p	NM_003924	PHOX2B	164	-32.12	-40.1	0.005101
hsa-miR-4707-3p	NM_004097	EMX1	159	-32.08	-35.6	0.013249
hsa-miR-4707-3p	NM_004212	SLC28A2	154	-23.87	-32.1	0.017886
hsa-miR-4707-3p	NM_004383	CSK	159	-28.43	-34.1	0.015704
hsa-miR-4707-3p	NM_004450	ERH	150	-32.14	-36.2	0.002471
hsa-miR-4707-3p	NM_004455	EXTL1	150	-28.77	-36.6	0.01127
hsa-miR-4707-3p	NM_004517	ILK	152	-28.21	-30.8	0.01499
hsa-miR-4707-3p	NM_004783	TAOK2	156	-37.41	-35.4	0.008906

hsa-miR-4707-3p	NM_004952	EFNA3	163	-31.12	-33.5	0.036009
hsa-miR-4707-3p	NM_004954	MARK2	167	-34.13	-38.4	0.010807
hsa-miR-4707-3p	NM_005018	PDCD1	152	-29.76	-37.8	0.007326
hsa-miR-4707-3p	NM_005182	CA7	166	-31.49	-36.7	0.004406
hsa-miR-4707-3p	NM_005378	MYCN	151	-30.58	-36	0.01062
hsa-miR-4707-3p	NM_005424	TIE1	151	-37.91	-41	0.000215
hsa-miR-4707-3p	NM_005466	MED6	156	-31.43	-36.8	0.017684
hsa-miR-4707-3p	NM_005529	HSPG2	150	-34.71	-34	0.032847
hsa-miR-4707-3p	NM_005557	KRT16	157	-34.86	-39.1	0.000065
hsa-miR-4707-3p	NM_005632	CAPN15	155	-29.12	-34.3	0.030303
hsa-miR-4707-3p	NM_005688	ABCC5	170	-33.57	-35	0.029915
hsa-miR-4707-3p	NM_005714	KCNK7	152	-34.88	-30.9	0.049517
hsa-miR-4707-3p	NM_005718	ARPC4	156	-35.7	-41.4	0.000947
hsa-miR-4707-3p	NM_006215	SERPINA4	153	-35.15	-34.4	0.003563
hsa-miR-4707-3p	NM_006236	POU3F3	154	-28.7	-34.7	0.04081
hsa-miR-4707-3p	NM_006244	PPP2R5B	155	-28	-31.5	0.044837
hsa-miR-4707-3p	NM_006613	GRAP	150	-27.03	-35	0.027383
hsa-miR-4707-3p	NM_006816	LMAN2	150	-34.29	-37.2	0.002387
hsa-miR-4707-3p	NM_007046	EMILIN1	156	-32.58	-32.3	0.013796
hsa-miR-4707-3p	NM_007079	PTP4A3	165	-35.89	-39.3	0.005171
hsa-miR-4707-3p	NM_007221	PMF1	161	-33.19	-36.4	0.002906
hsa-miR-4707-3p	NM_009590	AOC2	157	-27.12	-31.5	0.01858
hsa-miR-4707-3p	NM_012079	DGAT1	153	-22.31	-38.2	0.013593
hsa-miR-4707-3p	NM_012285	KCNH4	154	-31.71	-36.5	0.003447
hsa-miR-4707-3p	NM_013265	VPS51	161	-29.71	-33.1	0.005077
hsa-miR-4707-3p	NM_014046	MRPS18B	154	-32.45	-38.1	0.002022
hsa-miR-4707-3p	NM_014238	KSR1	160	-30.8	-36.2	0.026047
hsa-miR-4707-3p	NM_014336	AIPL1	161	-30.79	-35.5	0.03372
hsa-miR-4707-3p	NM_014638	PLCH2	150	-30.93	-38.9	0.000385
hsa-miR-4707-3p	NM_014736	KIAA0101	155	-28.85	-34.8	0.021254

hsa-miR-4707-3p	NM_014874	MFN2	172	-33.12	-38.1	0.013938
hsa-miR-4707-3p	NM_015050	CMTR1	151	-29.85	-36.1	0.01886
hsa-miR-4707-3p	NM_015164	PLEKHM2	150	-26.18	-34.9	0.015038
hsa-miR-4707-3p	NM_015679	TRUB2	156	-30.72	-37	0.00183
hsa-miR-4707-3p	NM_015720	PODXL2	153	-26.1	-35.8	0.001924
hsa-miR-4707-3p	NM_016161	A4GNT	152	-33.89	-38	0.001776
hsa-miR-4707-3p	NM_016233	PADI3	160	-29.65	-33.3	0.048184
hsa-miR-4707-3p	NM_016309	LCMT1	157	-27.23	-31.9	0.005478
hsa-miR-4707-3p	NM_016458	HGH1	152	-29.3	-33.7	0.047618
hsa-miR-4707-3p	NM_017490	MARK2	167	-34.13	-38.4	0.010807
hsa-miR-4707-3p	NM_017518	HAUS7	167	-30.23	-35.6	0.000859
hsa-miR-4707-3p	NM_017560	ZNF853	159	-30.24	-37.1	0.011785
hsa-miR-4707-3p	NM_017679	BCAS3	154	-32.05	-35.6	0.008532
hsa-miR-4707-3p	NM_017748	CWC25	156	-31.43	-36.8	0.018756
hsa-miR-4707-3p	NM_017920	URGCP	150	-22.9	-34.4	0.016626
hsa-miR-4707-3p	NM_017933	PID1	152	-32.23	-37.6	0.013498
hsa-miR-4707-3p	NM_018022	TMEM51	162	-33.2	-39.1	0.00186
hsa-miR-4707-3p	NM_018275	C7orf43	151	-26.67	-31.1	0.048362
hsa-miR-4707-3p	NM_018324	OLAH	150	-27.2	-34.9	0.009358
hsa-miR-4707-3p	NM_018405	COPRS	154	-28.9	-32.9	0.005651
hsa-miR-4707-3p	NM_018438	FBXO6	156	-31.43	-36.8	0.002648
hsa-miR-4707-3p	NM_018942	HMX1	162	-32.5	-36.6	0.004586
hsa-miR-4707-3p	NM_018992	KCTD5	163	-37.53	-36.4	0.022988
hsa-miR-4707-3p	NM_018997	MRPS21	162	-27.77	-33.4	0.024792
hsa-miR-4707-3p	NM_019079	L1TD1	156	-31.43	-36.8	0.008098
hsa-miR-4707-3p	NM_020631	PLEKHG5	158	-32.39	-35.8	0.023879
hsa-miR-4707-3p	NM_020815	PCDH10	150	-30.54	-35.3	0.017797
hsa-miR-4707-3p	NM_021070	LTBP3	165	-28.1	-32.2	0.024144
hsa-miR-4707-3p	NM_022164	TINAGL1	150	-27.73	-35.9	0.007349
hsa-miR-4707-3p	NM_022338	C11orf24	178	-39.08	-43	0.000048

hsa-miR-4707-3p	NM_024003	L1CAM	168	-34.28	-39.8	0.002985
hsa-miR-4707-3p	NM_024015	HOXB4	159	-39.61	-42.4	0.001078
hsa-miR-4707-3p	NM_024715	TXNDC15	161	-30.59	-35.5	0.039815
hsa-miR-4707-3p	NM_024886	C10orf95	154	-22.36	-34.1	0.01337
hsa-miR-4707-3p	NM_024954	UBTD1	154	-25.22	-33.6	0.019754
hsa-miR-4707-3p	NM_025247	ACAD10	151	-27.37	-31.9	0.037481
hsa-miR-4707-3p	NM_025257	SLC44A4	162	-28.49	-33.4	0.010171
hsa-miR-4707-3p	NM_025268	TMEM121	158	-30.82	-36.3	0.002281
hsa-miR-4707-3p	NM_031208	FAHD1	156	-31.43	-36.8	0.008734
hsa-miR-4707-3p	NM_031901	MRPS21	162	-27.77	-33.4	0.024792
hsa-miR-4707-3p	NM_031905	ARMC10	162	-27.57	-33.4	0.049379
hsa-miR-4707-3p	NM_032515	BOK	163	-39.06	-36.7	0.020532
hsa-miR-4707-3p	NM_032548	ABTB1	159	-35.59	-39.2	0.000697
hsa-miR-4707-3p	NM_032611	PTP4A3	165	-35.89	-39.3	0.005171
hsa-miR-4707-3p	NM_032788	ZNF514	156	-30.62	-36.5	0.020654
hsa-miR-4707-3p	NM_032971	PCDH11Y	161	-30.79	-35.5	0.021054
hsa-miR-4707-3p	NM_032972	PCDH11Y	161	-30.79	-35.5	0.021054
hsa-miR-4707-3p	NM_033027	CSRNP1	151	-30.78	-38.9	0.004746
hsa-miR-4707-3p	NM_033131	WNT3A	162	-32.75	-35.2	0.040594
hsa-miR-4707-3p	NM_033259	CAMK2N2	154	-30.81	-34.4	0.022527
hsa-miR-4707-3p	NM_033347	KCNK7	152	-34.88	-39.3	0.000053
hsa-miR-4707-3p	NM_033348	KCNK7	152	-34.88	-39.3	0.00049
hsa-miR-4707-3p	NM_033455	KCNK7	152	-34.88	-30.9	0.039707
hsa-miR-4707-3p	NM_052858	MARVELD3	156	-30.62	-36.5	0.016626
hsa-miR-4707-3p	NM_052920	KLHL29	160	-36.82	-41.1	0.004099
hsa-miR-4707-3p	NM_052933	TSGA13	151	-28.17	-30.2	0.035427
hsa-miR-4707-3p	NM_080866	SLC22A9	162	-28.56	-34.8	0.004062
hsa-miR-4707-3p	NM_133375	DIS3L	162	-27.77	-33.4	0.016794
hsa-miR-4707-3p	NM_133456	SHROOM1	154	-23.87	-32.1	0.035984
hsa-miR-4707-3p	NM_138440	VASN	152	-27.91	-33.1	0.021551

hsa-miR-4707-3p	NM_139278	LGI3	163	-35.34	-37.6	0.009663
hsa-miR-4707-3p	NM_139352	TAF1A	151	-24.92	-31.7	0.020194
hsa-miR-4707-3p	NM_147133	NFX1	157	-28.43	-33.1	0.008456
hsa-miR-4707-3p	NM_147196	TMIE	161	-27.8	-34	0.039517
hsa-miR-4707-3p	NM_152269	C12orf65	156	-30.42	-36.5	0.008802
hsa-miR-4707-3p	NM_152643	KNDC1	157	-30.27	-36.2	0.021242
hsa-miR-4707-3p	NM_152832	FAM89B	160	-30.42	-37.6	0.00222
hsa-miR-4707-3p	NM_153338	GGT6	161	-30.59	-35.5	0.017384
hsa-miR-4707-3p	NM_153837	ADGRG5	156	-33.07	-37.5	0.013954
hsa-miR-4707-3p	NM_172027	ABTB1	159	-35.59	-39.2	0.000697
hsa-miR-4707-3p	NM_173092	KCNH6	161	-30.79	-35.5	0.00096
hsa-miR-4707-3p	NM_173520	C9orf62	169	-30.09	-36	0.018573
hsa-miR-4707-3p	NM_173564	NYAP1	160	-34.37	-38	0.004313
hsa-miR-4707-3p	NM_173573	LMNTD2	161	-33.44	-39.7	0.000029
hsa-miR-4707-3p	NM_175922	PRR18	166	-34.43	-38.3	0.009342
hsa-miR-4707-3p	NM_178170	NEK8	162	-27.77	-33.4	0.026211
hsa-miR-4707-3p	NM_178336	MRPL52	162	-27.57	-33.4	0.023041
hsa-miR-4707-3p	NM_178562	TSPAN33	156	-31.64	-37.3	0.017476
hsa-miR-4707-3p	NM_178839	LRRTM1	151	-25.04	-31.6	0.018538
hsa-miR-4707-3p	NM_180982	MRPL52	162	-27.57	-33.4	0.023041
hsa-miR-4707-3p	NM_181304	MRPL52	162	-27.57	-33.4	0.023041
hsa-miR-4707-3p	NM_181305	MRPL52	162	-27.57	-33.4	0.023041
hsa-miR-4707-3p	NM_181306	MRPL52	162	-27.57	-33.4	0.023041
hsa-miR-4707-3p	NM_181307	MRPL52	162	-27.57	-33.4	0.025196
hsa-miR-4707-3p	NM_181471	RFC2	157	-28.43	-33.1	0.020213
hsa-miR-4707-3p	NM_181711	GRASP	162	-28.64	-36.1	0.006445
hsa-miR-4707-3p	NM_182925	FLT4	158	-31.8	-37.4	0.015011
hsa-miR-4707-3p	NM_194312	ESPNL	156	-33.96	-39.6	0.005329
hsa-miR-4707-3p	NM_198053	CD247	151	-25.09	-34	0.030981
hsa-miR-4707-3p	NM_198098	AQP1	162	-32.41	-40	0.006112

hsa-miR-4707-3p	NM_198179	QRFPR	156	-31.23	-36.8	0.004486
hsa-miR-4707-3p	NM_198681	PLEKHG5	158	-32.39	-35.8	0.023879
hsa-miR-4707-3p	NM_198993	STAC2	158	-23.6	-37.8	0.013518
hsa-miR-4707-3p	NM_201589	MAFA	154	-39.47	-44.8	0.000428
hsa-miR-4707-3p	NM_203434	IER5L	150	-28.42	-33.9	0.043583
hsa-miR-4707-3p	NM_207320	OTUD6A	154	-23.87	-32.1	0.047204
hsa-miR-4707-3p	NM_207338	LCTL	161	-30.59	-35.5	0.015804
hsa-miR-4707-3p	NR_001543	TTTY14	160	-32.58	-31.4	0.048193
hsa-miR-4707-3p	NR_002226	INGX	161	-30.79	-35.5	0.010102
hsa-miR-4707-3p	NR_003034	LOC441601	154	-32.51	-35.9	0.013152
hsa-miR-4707-3p	NR_003129	RNF5P1	158	-31.51	-37.8	0.006408
hsa-miR-4707-3p	NR_026665	CD58	152	-30.53	-36	0.016033
hsa-miR-4707-3p	NR_026668	VPS13A-AS1	150	-23.58	-30.7	0.037379
hsa-miR-4707-3p	NR_027025	MTVR2	160	-30.42	-34.9	0.014392
hsa-miR-4707-3p	NR_027386	GUSBP3	156	-31.43	-36.8	0.017023
hsa-miR-4707-3p	NR_027391	SLC15A3	160	-33.33	-37.4	0.018479
hsa-miR-4707-3p	NR_027761	LOC100132831	159	-26.94	-36.2	0.030722
hsa-miR-4707-3p	NR_027767	TNIK	154	-32.23	-38.3	0.002783
hsa-miR-4707-3p	NR_033464	NT5C3B	162	-34.27	-38.4	0.008229
hsa-miR-4707-3p	NR_033465	NT5C3B	162	-34.27	-38.4	0.009583
hsa-miR-4707-3p	NR_033697	NDUFA2	154	-31.64	-35.4	0.012312
hsa-miR-4707-3p	NR_033742	FRMD8P1	154	-34.49	-37.1	0.018665
hsa-miR-4707-3p	NR_033827	KDM4A-AS1	156	-31.43	-36.8	0.017168
hsa-miR-4707-3p	NR_033995	LOC389247	151	-27.27	-32.7	0.035823
hsa-miR-4707-3p	NR_034106	CRNDE	158	-30.04	-37.3	0.006154
hsa-miR-4707-3p	NR_036512	LOC100129917	154	-23.87	-32.1	0.032876
hsa-miR-4707-3p	NR_036580	DPP10-AS1	156	-30.52	-36	0.007412
hsa-miR-4707-3p	NR_037593	CAHM	154	-26.57	-32.6	0.044927
hsa-miR-4707-3p	NR_037718	EFEMP2	162	-32.35	-37.9	0.014961
hsa-miR-4707-3p	NR_038361	APTR	164	-30.72	-35.7	0.037506

hsa-miR-4707-3p	NR_038368	LINC00273	161	-27.56	-34.5	0.040166
hsa-miR-4707-3p	NR_039856	MIR4707	156	-37.52	-40.2	0.000005
hsa-miR-4707-3p	NR_040054	IQCH-AS1	157	-32.64	-37.2	0.004991
hsa-miR-4707-3p	NR_045277	NACA	157	-29.98	-34.5	0.026464
hsa-miR-4707-3p	NR_045680	HEIH	160	-34.47	-37.5	0.014142
hsa-miR-4707-3p	NR_046593	EIF2B5-AS1	156	-30.62	-36.5	0.003862
hsa-miR-4707-3p	NR_049739	VCPKMT	152	-29.22	-35.4	0.039523
hsa-miR-4707-3p	NR_073008	CDK2AP1	152	-32.04	-37	0.011672
hsa-miR-4707-3p	NR_102427	CIB1	159	-31.67	-34.1	0.028028
hsa-miR-4707-3p	NR_103802	EBPL	161	-33.89	-38.4	0.005051
hsa-miR-4707-3p	NR_103803	EBPL	161	-33.89	-38.4	0.003672
hsa-miR-4707-3p	NR_103832	SDR16C6P	162	-27.57	-33.4	0.014032
hsa-miR-4707-3p	NR_103849	CASC9	164	-29.42	-34.8	0.034369
hsa-miR-4707-3p	NR_104108	SIGMAR1	151	-30.87	-36.6	0.019313
hsa-miR-4707-3p	NR_104425	DACT2	167	-34.45	-37.7	0.016796
hsa-miR-4707-3p	NR_105052	LOC101929696	165	-34.71	-37.9	0.002936
hsa-miR-4707-3p	NR_105053	LOC101929696	165	-34.71	-37.9	0.001091
hsa-miR-4707-3p	NR_105061	LOC102546298	151	-28.44	-34.4	0.005639
hsa-miR-4707-3p	NR_109934	KIAA0101	155	-28.85	-34.8	0.038794
hsa-miR-4707-3p	NR_109951	OSMR-AS1	155	-29.44	-35.5	0.007786
hsa-miR-4707-3p	NR_110454	CRNDE	158	-30.04	-37.3	0.005377
hsa-miR-4707-3p	NR_110752	POTEB3	156	-32.05	-37.7	0.016869
hsa-miR-4707-3p	NR_120372	CRTC3-AS1	162	-39.16	-43	0.000254
hsa-miR-4707-3p	NR_120406	NOX4	156	-27.3	-33.2	0.019773
hsa-miR-4707-3p	NR_121659	ECSCR	166	-33.64	-42.7	0.000673
hsa-miR-4707-3p	NR_123717	VAMP1	156	-31.48	-36.2	0.014686
hsa-miR-4707-3p	NR_123725	LOC729867	154	-27.63	-35.4	0.031109
hsa-miR-4707-3p	NR_125331	MRPL9	171	-34.18	-40	0.005138
hsa-miR-4707-3p	NR_125733	TTTY14	160	-32.58	-36.9	0.010103
hsa-miR-4707-3p	NR_125734	TTTY14	160	-32.58	-36.9	0.008997

hsa-miR-4707-3p	NR_125735	TTTY14	160	-32.58	-36.9	0.008003
hsa-miR-4707-3p	NR_125749	TBX2-AS1	155	-28.64	-34.7	0.017258
hsa-miR-4707-3p	NR_125750	TBX2-AS1	155	-28.64	-34.7	0.014669
hsa-miR-4707-3p	NR_125751	TBX2-AS1	155	-28.64	-34.7	0.011277
hsa-miR-4707-3p	NR_125759	PKNOX2-AS1	160	-32.94	-33	0.020163
hsa-miR-4707-3p	NR_126394	ACYP1	164	-36.41	-36.6	0.005386
hsa-miR-4707-3p	NR_126508	CYB5R2	155	-31.34	-35.3	0.029326
hsa-miR-4707-3p	NR_126522	EXOC3-AS1	154	-23.51	-35.9	0.019677
hsa-miR-4707-3p	NR_130123	RHEBL1	161	-32.69	-35.6	0.01813
hsa-miR-4707-3p	NR_132125	TH2LCRR	158	-30.51	-37.4	0.00134
hsa-miR-4707-3p	NR_132422	LOC105370333	150	-33.55	-34	0.02956
hsa-miR-4747-3p	NM_000218	KCNQ1	156	-30.97	-36.7	0.008317
hsa-miR-4747-3p	NM_000738	CHRM1	164	-38.51	-39.7	0.001987
hsa-miR-4747-3p	NM_001001523	RORC	168	-37.04	-41.7	0.001398
hsa-miR-4747-3p	NM_001012614	CTBP1	154	-36.14	-39.9	0.001134
hsa-miR-4747-3p	NM_001029885	CPTP	163	-32.67	-35.8	0.012802
hsa-miR-4747-3p	NM_001033667	LY9	166	-33.7	-36	0.007107
hsa-miR-4747-3p	NM_001038633	RSPO1	167	-33.86	-34.7	0.028342
hsa-miR-4747-3p	NM_001040138	CKLF	150	-23.11	-28.3	0.025397
hsa-miR-4747-3p	NM_001040661	SLC29A4	163	-32.78	-39.6	0.002418
hsa-miR-4747-3p	NM_001042465	PSAP	154	-24.15	-33.7	0.03237
hsa-miR-4747-3p	NM_001042466	PSAP	154	-24.15	-33.7	0.03237
hsa-miR-4747-3p	NM_001076680	LYRM9	154	-32.91	-34.3	0.02547
hsa-miR-4747-3p	NM_001077628	APH1A	155	-33.63	-38.2	0.002093
hsa-miR-4747-3p	NM_001080441	TTC36	157	-21.96	-26.3	0.025626
hsa-miR-4747-3p	NM_001080547	SPI1	152	-29.34	-29.6	0.038232
hsa-miR-4747-3p	NM_001082575	RBFOX3	170	-33.81	-39	0.005196
hsa-miR-4747-3p	NM_001098510	PELI3	150	-29.47	-35.8	0.013453
hsa-miR-4747-3p	NM_001100878	MROH6	154	-30.11	-35.2	0.014723
hsa-miR-4747-3p	NM_001109891	MAPK3	151	-26.86	-32	0.028896

hsa-miR-4747-3p	NM_001136041	HDAC11	159	-29.53	-34.1	0.048977
hsa-miR-4747-3p	NM_001142617	STRA6	171	-34	-37.1	0.002538
hsa-miR-4747-3p	NM_001142618	STRA6	171	-34	-37.1	0.002538
hsa-miR-4747-3p	NM_001142619	STRA6	171	-34	-37.1	0.002538
hsa-miR-4747-3p	NM_001142633	PIK3R5	159	-33.85	-37.9	0.009879
hsa-miR-4747-3p	NM_001142674	CHID1	156	-37.32	-41.4	0.002877
hsa-miR-4747-3p	NM_001142675	CHID1	156	-37.32	-41.4	0.002877
hsa-miR-4747-3p	NM_001142676	CHID1	156	-37.32	-41.4	0.002877
hsa-miR-4747-3p	NM_001142677	CHID1	156	-37.32	-41.4	0.002877
hsa-miR-4747-3p	NM_001142946	C11orf21	161	-28.39	-35.4	0.027027
hsa-miR-4747-3p	NM_001145432	SMIM20	157	-31.41	-40.7	0.000589
hsa-miR-4747-3p	NM_001162530	SH3D21	164	-36.32	-40.3	0.000058
hsa-miR-4747-3p	NM_001165974	UROC1	155	-34.28	-36.8	0.008812
hsa-miR-4747-3p	NM_001166175	NKX2-5	168	-34.97	-38.3	0.005988
hsa-miR-4747-3p	NM_001166176	NKX2-5	168	-34.97	-38.3	0.004955
hsa-miR-4747-3p	NM_001171815	RNF166	160	-28.79	-35.2	0.015313
hsa-miR-4747-3p	NM_001171816	RNF166	160	-28.79	-35.2	0.015313
hsa-miR-4747-3p	NM_001184785	PARD3	152	-27.94	-34.2	0.042257
hsa-miR-4747-3p	NM_001184786	PARD3	152	-27.94	-34.2	0.042257
hsa-miR-4747-3p	NM_001184787	PARD3	152	-27.94	-34.2	0.042257
hsa-miR-4747-3p	NM_001184788	PARD3	152	-27.94	-34.2	0.042257
hsa-miR-4747-3p	NM_001184789	PARD3	152	-27.94	-34.2	0.042257
hsa-miR-4747-3p	NM_001184790	PARD3	152	-27.94	-34.2	0.042257
hsa-miR-4747-3p	NM_001184791	PARD3	152	-27.94	-34.2	0.042257
hsa-miR-4747-3p	NM_001184974	PACSIN3	153	-29.21	-33	0.00867
hsa-miR-4747-3p	NM_001184975	PACSIN3	153	-29.21	-33	0.00867
hsa-miR-4747-3p	NM_001191060	SLC25A22	159	-36.06	-39.1	0.004479
hsa-miR-4747-3p	NM_001191061	SLC25A22	159	-36.06	-39.1	0.004479
hsa-miR-4747-3p	NM_001193570	CSRP1	155	-28.68	-35.7	0.014292
hsa-miR-4747-3p	NM_001193571	CSRP1	155	-28.68	-35.7	0.014292

hsa-miR-4747-3p	NM_001193572	CSRP1	155	-28.68	-35.7	0.014292
hsa-miR-4747-3p	NM_001198832	PDE4DIP	150	-27.98	-35.2	0.014462
hsa-miR-4747-3p	NM_001199040	STRA6	171	-34	-37.1	0.002538
hsa-miR-4747-3p	NM_001199041	STRA6	171	-34	-37.1	0.002538
hsa-miR-4747-3p	NM_001199042	STRA6	171	-34	-37.1	0.002538
hsa-miR-4747-3p	NM_001199787	SLC35E2	159	-27.97	-34	0.009709
hsa-miR-4747-3p	NM_001204084	NBL1	163	-36.47	-38.9	0.004273
hsa-miR-4747-3p	NM_001204085	NBL1	163	-36.47	-38.9	0.004273
hsa-miR-4747-3p	NM_001204086	NBL1	163	-36.47	-38.9	0.004273
hsa-miR-4747-3p	NM_001204088	MINOS1-NBL1	163	-36.47	-38.9	0.004273
hsa-miR-4747-3p	NM_001204089	MINOS1-NBL1	163	-36.47	-38.9	0.004273
hsa-miR-4747-3p	NM_001207011	CNTFR	164	-28.66	-35.2	0.006612
hsa-miR-4747-3p	NM_001242908	RSPO1	167	-33.86	-34.7	0.028342
hsa-miR-4747-3p	NM_001242909	RSPO1	167	-33.86	-34.7	0.028342
hsa-miR-4747-3p	NM_001242910	RSPO1	167	-33.86	-34.7	0.028342
hsa-miR-4747-3p	NM_001243135	PELI3	150	-29.47	-35.8	0.013453
hsa-miR-4747-3p	NM_001243136	PELI3	150	-29.47	-35.8	0.013453
hsa-miR-4747-3p	NM_001243771	APH1A	155	-33.63	-38.2	0.004403
hsa-miR-4747-3p	NM_001243772	APH1A	155	-33.63	-38.2	0.002093
hsa-miR-4747-3p	NM_001251851	PIK3R5	159	-33.85	-37.9	0.009879
hsa-miR-4747-3p	NM_001251852	PIK3R5	159	-33.85	-37.9	0.009879
hsa-miR-4747-3p	NM_001251853	PIK3R5	159	-33.85	-37.9	0.009879
hsa-miR-4747-3p	NM_001251855	PIK3R5	159	-33.85	-37.9	0.009879
hsa-miR-4747-3p	NM_001254757	ST3GAL4	164	-36.89	-39.2	0.00089
hsa-miR-4747-3p	NM_001254758	ST3GAL4	164	-36.89	-39.2	0.00089
hsa-miR-4747-3p	NM_001254759	ST3GAL4	164	-36.89	-39.2	0.00089
hsa-miR-4747-3p	NM_001256030	CD48	154	-24.98	-31.6	0.037818
hsa-miR-4747-3p	NM_001256526	C9orf69	154	-33.52	-37.8	0.011925
hsa-miR-4747-3p	NM_001270428	LMO1	162	-32.22	-33.7	0.003721
hsa-miR-4747-3p	NM_001276267	DUOXA1	167	-29.92	-34.4	0.043242

hsa-miR-4747-3p	NM_001276268	DUOXA1	167	-29.92	-34.4	0.043242
hsa-miR-4747-3p	NM_001277204	P2RY6	150	-30.24	-35.6	0.013726
hsa-miR-4747-3p	NM_001277205	P2RY6	150	-30.24	-35.6	0.013726
hsa-miR-4747-3p	NM_001277206	P2RY6	150	-30.24	-35.6	0.013726
hsa-miR-4747-3p	NM_001277207	P2RY6	150	-30.24	-35.6	0.013726
hsa-miR-4747-3p	NM_001277208	P2RY6	150	-30.24	-35.6	0.013726
hsa-miR-4747-3p	NM_001278164	NBL1	163	-36.47	-38.9	0.004273
hsa-miR-4747-3p	NM_001278165	NBL1	163	-36.47	-38.9	0.004273
hsa-miR-4747-3p	NM_001278166	NBL1	163	-36.47	-38.9	0.004273
hsa-miR-4747-3p	NM_001278295	CCDC108	150	-29.44	-33.7	0.023729
hsa-miR-4747-3p	NM_001278296	CCDC108	150	-29.44	-33.7	0.023729
hsa-miR-4747-3p	NM_001281501	FADS2	156	-32.01	-35.5	0.025745
hsa-miR-4747-3p	NM_001281502	FADS2	156	-32.01	-35.5	0.025745
hsa-miR-4747-3p	NM_001282424	A2ML1	156	-27.9	-33.2	0.02044
hsa-miR-4747-3p	NM_001284497	FAM234A	156	-28.08	-35.3	0.015289
hsa-miR-4747-3p	NM_001284502	NUBP2	165	-25.69	-29.7	0.0101
hsa-miR-4747-3p	NM_001285485	NEURL3	169	-32.03	-35.6	0.004932
hsa-miR-4747-3p	NM_001285486	NEURL3	169	-32.03	-35.6	0.00512
hsa-miR-4747-3p	NM_001286585	CDIPT	159	-28.77	-32.6	0.033109
hsa-miR-4747-3p	NM_001286586	CDIPT	159	-28.77	-32.6	0.033109
hsa-miR-4747-3p	NM_001289934	LRCH4	160	-36.73	-42.3	0.000026
hsa-miR-4747-3p	NM_001291980	FGFR4	151	-27.31	-30.9	0.029696
hsa-miR-4747-3p	NM_001297649	CD81	152	-22.39	-34.3	0.007864
hsa-miR-4747-3p	NM_001300847	SLC29A4	163	-32.78	-39.6	0.002418
hsa-miR-4747-3p	NM_001300868	SLC29A2	151	-33.38	-37.4	0.004462
hsa-miR-4747-3p	NM_001300869	SLC29A2	151	-33.38	-37.4	0.005747
hsa-miR-4747-3p	NM_001301302	OAZ2	158	-36.36	-39.5	0.002512
hsa-miR-4747-3p	NM_001304968	TSGA13	160	-30.36	-29.9	0.032898
hsa-miR-4747-3p	NM_001307930	RASL12	156	-32.74	-36.7	0.014152
hsa-miR-4747-3p	NM_001307990	MAP4K2	150	-28.66	-32.8	0.010684

hsa-miR-4747-3p	NM_001328	CTBP1	154	-36.14	-39.9	0.001134
hsa-miR-4747-3p	NM_001407	CELSR3	169	-36.83	-40.6	0.003222
hsa-miR-4747-3p	NM_001408	CELSR2	151	-30.53	-35.4	0.027803
hsa-miR-4747-3p	NM_001532	SLC29A2	151	-33.38	-37.4	0.004462
hsa-miR-4747-3p	NM_001547	IFIT2	151	-32.95	-36.8	0.018088
hsa-miR-4747-3p	NM_001842	CNTFR	164	-28.66	-35.2	0.006612
hsa-miR-4747-3p	NM_002011	FGFR4	151	-27.31	-30.9	0.029696
hsa-miR-4747-3p	NM_002315	LMO1	162	-32.22	-33.7	0.003721
hsa-miR-4747-3p	NM_002319	LRCH4	160	-36.73	-42.3	0.000692
hsa-miR-4747-3p	NM_002537	OAZ2	158	-36.36	-39.5	0.002512
hsa-miR-4747-3p	NM_002689	POLA2	158	-33.04	-37.1	0.010286
hsa-miR-4747-3p	NM_002746	MAPK3	151	-26.86	-32	0.028896
hsa-miR-4747-3p	NM_002778	PSAP	154	-24.15	-33.7	0.03237
hsa-miR-4747-3p	NM_003120	SPI1	152	-29.34	-29.6	0.038232
hsa-miR-4747-3p	NM_003959	HIP1R	151	-32.47	-34.6	0.02374
hsa-miR-4747-3p	NM_004078	CSRP1	155	-28.68	-35.7	0.014292
hsa-miR-4747-3p	NM_004265	FADS2	156	-32.01	-35.5	0.025745
hsa-miR-4747-3p	NM_004356	CD81	152	-22.39	-34.3	0.007864
hsa-miR-4747-3p	NM_004387	NKX2-5	168	-34.97	-38.3	0.000843
hsa-miR-4747-3p	NM_004390	CTSH	155	-31.22	-37.6	0.000788
hsa-miR-4747-3p	NM_004579	MAP4K2	150	-28.66	-32.8	0.010684
hsa-miR-4747-3p	NM_004959	NR5A1	170	-35.73	-39.6	0.003956
hsa-miR-4747-3p	NM_005060	RORC	168	-37.04	-41.7	0.001398
hsa-miR-4747-3p	NM_005231	CTTN	163	-38.48	-41.6	0.001497
hsa-miR-4747-3p	NM_005380	NBL1	163	-36.47	-38.9	0.004273
hsa-miR-4747-3p	NM_005516	HLA-E	163	-30.56	-33.5	0.049567
hsa-miR-4747-3p	NM_005814	GPA33	159	-33.97	-37.3	0.010295
hsa-miR-4747-3p	NM_006160	NEUROD2	167	-33.01	-37.5	0.011284
hsa-miR-4747-3p	NM_006172	NPPA	154	-33.83	-36.4	0.000873
hsa-miR-4747-3p	NM_006278	ST3GAL4	164	-36.89	-39.2	0.00089

hsa-miR-4747-3p	NM_006319	CDIPT	159	-28.77	-32.6	0.033109
hsa-miR-4747-3p	NM_006500	MCAM	160	-29.55	-33.6	0.04326
hsa-miR-4747-3p	NM_006598	SLC12A7	156	-31.81	-37.4	0.015023
hsa-miR-4747-3p	NM_006623	PHGDH	153	-26.09	-29.6	0.011588
hsa-miR-4747-3p	NM_006898	HOXD3	157	-30.96	-34.1	0.016609
hsa-miR-4747-3p	NM_007079	PTP4A3	157	-33.31	-37.1	0.010219
hsa-miR-4747-3p	NM_012339	TSPAN15	172	-38.07	-41.9	0.000339
hsa-miR-4747-3p	NM_013392	NRBP1	150	-25.88	-30.5	0.033392
hsa-miR-4747-3p	NM_014159	SETD2	155	-31.36	-33.7	0.014857
hsa-miR-4747-3p	NM_014212	HOXC11	162	-30	-34.3	0.020625
hsa-miR-4747-3p	NM_014308	PIK3R5	159	-33.85	-37.9	0.009879
hsa-miR-4747-3p	NM_014621	HOXD4	168	-34.21	-37.4	0.000452
hsa-miR-4747-3p	NM_014644	PDE4DIP	150	-27.98	-35.2	0.014462
hsa-miR-4747-3p	NM_014714	IFT140	160	-33.75	-32.9	0.013605
hsa-miR-4747-3p	NM_014798	PLEKHM1	158	-34.14	-40.9	0.00343
hsa-miR-4747-3p	NM_014926	SLITRK3	151	-34.51	-36.2	0.011404
hsa-miR-4747-3p	NM_016022	APH1A	155	-33.63	-38.2	0.004403
hsa-miR-4747-3p	NM_016223	PACSIN3	153	-29.21	-33	0.00867
hsa-miR-4747-3p	NM_016326	CKLF	150	-23.11	-28.3	0.045446
hsa-miR-4747-3p	NM_016563	RASL12	156	-32.74	-36.7	0.014152
hsa-miR-4747-3p	NM_016951	CKLF	150	-23.11	-28.3	0.045446
hsa-miR-4747-3p	NM_018142	INTS10	158	-22.95	-28.3	0.043848
hsa-miR-4747-3p	NM_018378	FBXL8	154	-26	-29.5	0.037558
hsa-miR-4747-3p	NM_019619	PARD3	152	-27.94	-34.2	0.042257
hsa-miR-4747-3p	NM_021025	TLX3	161	-26.6	-31.9	0.02307
hsa-miR-4747-3p	NM_021149	COTL1	166	-27.99	-33.2	0.04538
hsa-miR-4747-3p	NM_021958	HLX	169	-35.75	-38.4	0.000457
hsa-miR-4747-3p	NM_022039	FBXW4	156	-27.83	-32.2	0.025775
hsa-miR-4747-3p	NM_022369	STRA6	171	-34	-37.1	0.002538
hsa-miR-4747-3p	NM_022749	FAM160B2	169	-32.43	-35.6	0.020209

hsa-miR-4747-3p	NM_022963	FGFR4	151	-27.31	-30.9	0.029696
hsa-miR-4747-3p	NM_023947	CHID1	156	-37.32	-41.4	0.002877
hsa-miR-4747-3p	NM_024016	HOXB8	156	-29.41	-32.2	0.041172
hsa-miR-4747-3p	NM_024671	ZNF768	155	-32.49	-37.6	0.001447
hsa-miR-4747-3p	NM_024676	SH3D21	164	-36.32	-40.3	0.000058
hsa-miR-4747-3p	NM_024698	SLC25A22	159	-36.06	-39.1	0.004479
hsa-miR-4747-3p	NM_024827	HDAC11	159	-29.53	-34.1	0.048977
hsa-miR-4747-3p	NM_031475	ESPN	160	-28.2	-34.4	0.013786
hsa-miR-4747-3p	NM_031923	TAF3	156	-31.24	-35.2	0.001636
hsa-miR-4747-3p	NM_032039	FAM234A	156	-28.08	-35.3	0.015289
hsa-miR-4747-3p	NM_032283	ZDHHC18	154	-33.6	-37.7	0.01221
hsa-miR-4747-3p	NM_032429	LZTS2	170	-35.93	-38.5	0.001544
hsa-miR-4747-3p	NM_032611	PTP4A3	157	-33.31	-37.1	0.010219
hsa-miR-4747-3p	NM_033127	SEC16B	151	-31.53	-35.8	0.006598
hsa-miR-4747-3p	NM_033310	KCNK4	151	-30.48	-31	0.013888
hsa-miR-4747-3p	NM_052933	TSGA13	160	-30.36	-29.9	0.032898
hsa-miR-4747-3p	NM_133639	RHOV	156	-32.8	-31.8	0.048163
hsa-miR-4747-3p	NM_138342	GLB1L2	151	-28.39	-35.1	0.015613
hsa-miR-4747-3p	NM_138565	CTTN	163	-38.48	-41.6	0.001497
hsa-miR-4747-3p	NM_144639	UROC1	155	-34.28	-36.8	0.008812
hsa-miR-4747-3p	NM_144670	A2ML1	156	-27.9	-33.2	0.02044
hsa-miR-4747-3p	NM_145065	PELI3	150	-29.47	-35.8	0.013453
hsa-miR-4747-3p	NM_145260	OSR1	171	-33.78	-37.2	0.003797
hsa-miR-4747-3p	NM_147164	CNTFR	164	-28.66	-35.2	0.006612
hsa-miR-4747-3p	NM_152339	SPATA2L	153	-34.81	-38.7	0.00279
hsa-miR-4747-3p	NM_152493	ZNF362	175	-33.43	-34.1	0.046435
hsa-miR-4747-3p	NM_152833	C9orf69	154	-33.52	-37.8	0.011925
hsa-miR-4747-3p	NM_153247	SLC29A4	163	-32.78	-39.6	0.002418
hsa-miR-4747-3p	NM_153614	DNAJB13	165	-31.59	-35.7	0.000375
hsa-miR-4747-3p	NM_176796	P2RY6	150	-30.24	-35.6	0.013726

hsa-miR-4747-3p	NM_176797	P2RY6	150	-30.24	-35.6	0.013726
hsa-miR-4747-3p	NM_176798	P2RY6	150	-30.24	-35.6	0.013726
hsa-miR-4747-3p	NM_178172	GPIHBP1	163	-26.73	-35.7	0.023412
hsa-miR-4747-3p	NM_178568	RTN4RL1	153	-26.44	-34.3	0.047819
hsa-miR-4747-3p	NM_178841	RNF166	160	-28.79	-35.2	0.015313
hsa-miR-4747-3p	NM_181640	CKLF	150	-23.11	-28.3	0.045446
hsa-miR-4747-3p	NM_181641	CKLF	150	-23.11	-28.3	0.045446
hsa-miR-4747-3p	NM_181798	KCNQ1	156	-30.97	-36.7	0.008317
hsa-miR-4747-3p	NM_182538	SPNS3	160	-34.92	-40	0.000153
hsa-miR-4747-3p	NM_182744	NBL1	163	-36.47	-38.9	0.004273
hsa-miR-4747-3p	NM_203293	TRIM7	159	-32.7	-36.8	0.009552
hsa-miR-4747-3p	NM_203294	TRIM7	159	-32.7	-36.8	0.009552
hsa-miR-4747-3p	NM_203295	TRIM7	159	-32.7	-36.8	0.009552
hsa-miR-4747-3p	NM_203296	TRIM7	159	-32.7	-36.8	0.009552
hsa-miR-4747-3p	NM_203297	TRIM7	159	-32.7	-36.8	0.009552
hsa-miR-4747-3p	NM_206967	C16orf74	164	-26.98	-33.8	0.008089
hsa-miR-4747-3p	NM_207370	GPR153	156	-24.55	-35.1	0.038904
hsa-miR-4747-3p	NM_213647	FGFR4	151	-27.31	-30.9	0.029696
hsa-miR-4747-3p	NR_002314	SLC25A3P1	155	-36.94	-39.6	0.001694
hsa-miR-4747-3p	NR_002330	ST7-AS1	166	-35.12	-36.5	0.020286
hsa-miR-4747-3p	NR_015356	NPSR1-AS1	150	-28.87	-33.8	0.041661
hsa-miR-4747-3p	NR_015383	LOC340357	155	-27.24	-32.6	0.029549
hsa-miR-4747-3p	NR_023344	RNU6ATAC	162	-27.19	-33.6	0.000553
hsa-miR-4747-3p	NR_024127	SNHG12	159	-31.36	-34.1	0.013974
hsa-miR-4747-3p	NR_028059	PMS2P3	167	-33.46	-38.2	0.007034
hsa-miR-4747-3p	NR_029907	MIR346	154	-31.61	-36	0.000069
hsa-miR-4747-3p	NR_031600	MIR1234	154	-28.09	-36	0.000043
hsa-miR-4747-3p	NR_034106	CRNDE	151	-32.04	-36.1	0.008121
hsa-miR-4747-3p	NR_045215	LOC100652768	156	-30.06	-34.9	0.021803
hsa-miR-4747-3p	NR_046368	LINC01023	156	-29.48	-34.5	0.004709

hsa-miR-4747-3p	NR_046647	SLC6A1-AS1	158	-27.05	-31.3	0.035011
hsa-miR-4747-3p	NR_046683	ST3GAL6-AS1	150	-29.73	-32.9	0.025655
hsa-miR-4747-3p	NR_047493	LINC00563	150	-34.84	-40.2	0.002664
hsa-miR-4747-3p	NR_073006	LMO1	162	-32.22	-33.7	0.043922
hsa-miR-4747-3p	NR_073565	DNLZ	158	-27.58	-40.5	0.000763
hsa-miR-4747-3p	NR_103794	LOC100996385	165	-32.61	-34.3	0.038508
hsa-miR-4747-3p	NR_104346	NEURL3	169	-32.03	-35.6	0.024963
hsa-miR-4747-3p	NR_106988	MIR7641-2	153	-21.59	-24.4	0.013405
hsa-miR-4747-3p	NR_110454	CRNDE	151	-32.04	-36.1	0.007108
hsa-miR-4747-3p	NR_110901	GLIS2-AS1	159	-27.15	-31.6	0.013707
hsa-miR-4747-3p	NR_120618	LINC01475	163	-30.98	-32.4	0.031959
hsa-miR-4747-3p	NR_125759	PKNOX2-AS1	168	-31.03	-36.7	0.002812
hsa-miR-4747-3p	NR_126155	RBSN	174	-38.1	-41.9	0.000593
hsa-miR-4747-3p	NR_126355	LINC01389	164	-32.91	-36.5	0.006039
hsa-miR-4747-3p	NR_130975	C1orf35	167	-30.98	-35.5	0.024567
hsa-miR-4753-3p	NM_000595	LTA	155	-28.24	-32.4	0.006384
hsa-miR-4753-3p	NM_001006944	RPS6KA4	163	-25.57	-30.9	0.017984
hsa-miR-4753-3p	NM_001025232	CLLU1OS	171	-24.74	-29.1	0.015089
hsa-miR-4753-3p	NM_001035230	RABGAP1L	170	-25.37	-27	0.029521
hsa-miR-4753-3p	NM_001040138	CKLF	163	-27.38	-30.7	0.001658
hsa-miR-4753-3p	NM_001099625	MTFR1L	154	-17.18	-29.9	0.041833
hsa-miR-4753-3p	NM_001099626	MTFR1L	154	-17.18	-29.9	0.041833
hsa-miR-4753-3p	NM_001113205	TSTD1	171	-23.54	-26.9	0.024277
hsa-miR-4753-3p	NM_001135554	EPB41L2	168	-25.1	-31.7	0.027835
hsa-miR-4753-3p	NM_001135555	EPB41L2	168	-25.1	-31.7	0.027835
hsa-miR-4753-3p	NM_001142807	ACOXL	159	-21.41	-29.4	0.014528
hsa-miR-4753-3p	NM_001146316	SPSB2	150	-21.71	-25.9	0.045634
hsa-miR-4753-3p	NM_001159542	POU5F1B	169	-24.75	-28.3	0.011105
hsa-miR-4753-3p	NM_001159740	LTA	155	-28.24	-32.4	0.006384
hsa-miR-4753-3p	NM_001164471	TSPY4	162	-22.39	-25.2	0.039977

hsa-miR-4753-3p	NM_001167857	PPP1R12B	153	-20.25	-25	0.022036
hsa-miR-4753-3p	NM_001199388	EPB41L2	168	-25.1	-31.7	0.027835
hsa-miR-4753-3p	NM_001243721	TSPY8	162	-22.39	-25.2	0.039977
hsa-miR-4753-3p	NM_001243763	RABGAP1L	170	-25.37	-27	0.029521
hsa-miR-4753-3p	NM_001252660	EPB41L2	168	-25.1	-31.7	0.027835
hsa-miR-4753-3p	NM_001256024	ARHGAP22	151	-22.84	-29.1	0.014249
hsa-miR-4753-3p	NM_001256025	ARHGAP22	151	-22.84	-29.1	0.014249
hsa-miR-4753-3p	NM_001256026	ARHGAP22	151	-22.84	-29.1	0.014249
hsa-miR-4753-3p	NM_001256430	STON2	160	-27.76	-30.6	0.040562
hsa-miR-4753-3p	NM_001256799	GAPDH	150	-23.37	-28.8	0.005625
hsa-miR-4753-3p	NM_001267774	IFT20	156	-24.64	-28.3	0.01816
hsa-miR-4753-3p	NM_001267775	IFT20	156	-24.64	-28.3	0.01816
hsa-miR-4753-3p	NM_001267776	IFT20	156	-24.64	-28.3	0.01816
hsa-miR-4753-3p	NM_001267777	IFT20	156	-24.64	-28.3	0.016034
hsa-miR-4753-3p	NM_001267778	IFT20	156	-24.64	-28.3	0.025413
hsa-miR-4753-3p	NM_001267782	AMBRA1	151	-30.46	-33.8	0.007382
hsa-miR-4753-3p	NM_001267783	AMBRA1	151	-30.46	-33.8	0.007382
hsa-miR-4753-3p	NM_001277423	TCP11X2	159	-25.59	-28.9	0.004737
hsa-miR-4753-3p	NM_001282442	LOC100131303	172	-23.06	-28.9	0.043249
hsa-miR-4753-3p	NM_001284396	WDR93	162	-31.69	-34.1	0.00932
hsa-miR-4753-3p	NM_001288794	RMND5B	158	-22.5	-32.3	0.005066
hsa-miR-4753-3p	NM_001288795	RMND5B	158	-22.5	-32.3	0.005066
hsa-miR-4753-3p	NM_001289745	GAPDH	150	-23.37	-28.8	0.005625
hsa-miR-4753-3p	NM_001289746	GAPDH	150	-23.37	-28.8	0.005625
hsa-miR-4753-3p	NM_001297571	GNB3	162	-27.56	-32.2	0.004618
hsa-miR-4753-3p	NM_001300731	AMBRA1	151	-30.46	-33.8	0.007382
hsa-miR-4753-3p	NM_001300802	RPS6KA4	163	-25.57	-30.9	0.017984
hsa-miR-4753-3p	NM_001303531	C8orf86	162	-28.21	-33	0.022078
hsa-miR-4753-3p	NM_001310138	FAM231C	177	-22.92	-30.6	0.000891
hsa-miR-4753-3p	NM_001310155	FAM231B	177	-21.12	-27.5	0.04959

hsa-miR-4753-3p	NM_001316350	GNPAT	159	-23.8	-28	0.03425
hsa-miR-4753-3p	NM_001431	EPB41L2	168	-25.1	-31.7	0.027835
hsa-miR-4753-3p	NM_002046	GAPDH	150	-23.37	-28.8	0.005625
hsa-miR-4753-3p	NM_002075	GNB3	162	-27.56	-32.2	0.004618
hsa-miR-4753-3p	NM_002881	RALB	158	-27.32	-32	0.029823
hsa-miR-4753-3p	NM_003308	TSPY1	162	-22.39	-25.2	0.039977
hsa-miR-4753-3p	NM_003942	RPS6KA4	163	-25.57	-30.9	0.017984
hsa-miR-4753-3p	NM_004428	EFNA1	150	-25.99	-30.9	0.0224
hsa-miR-4753-3p	NM_004983	KCNJ9	152	-27.25	-31.5	0.043627
hsa-miR-4753-3p	NM_005101	ISG15	161	-21.02	-24.2	0.00768
hsa-miR-4753-3p	NM_005959	MTNR1B	156	-20.57	-28.4	0.03188
hsa-miR-4753-3p	NM_007053	CD160	172	-21.82	-29.8	0.03551
hsa-miR-4753-3p	NM_007264	GPR182	150	-22.36	-27.4	0.020144
hsa-miR-4753-3p	NM_012421	RLF	162	-25.94	-30.6	0.009987
hsa-miR-4753-3p	NM_012452	TNFRSF13B	162	-22.27	-27.8	0.042764
hsa-miR-4753-3p	NM_014236	GNPAT	159	-23.8	-28	0.03425
hsa-miR-4753-3p	NM_014857	RABGAP1L	170	-25.37	-27	0.029521
hsa-miR-4753-3p	NM_015421	TMEM186	160	-31.73	-36	0.001443
hsa-miR-4753-3p	NM_015698	GPKOW	159	-22.68	-26.5	0.028153
hsa-miR-4753-3p	NM_015871	ZNF593	161	-17	-28.7	0.003423
hsa-miR-4753-3p	NM_016326	CKLF	163	-27.38	-30.7	0.003399
hsa-miR-4753-3p	NM_016951	CKLF	163	-27.38	-30.7	0.003399
hsa-miR-4753-3p	NM_017749	AMBRA1	151	-30.46	-33.8	0.007382
hsa-miR-4753-3p	NM_019557	MTFR1L	154	-17.18	-29.9	0.041833
hsa-miR-4753-3p	NM_021226	ARHGAP22	151	-22.84	-29.1	0.014249
hsa-miR-4753-3p	NM_022573	TSPY2	162	-22.39	-25.2	0.0396
hsa-miR-4753-3p	NM_022762	RMND5B	158	-22.5	-32.3	0.005066
hsa-miR-4753-3p	NM_024026	MRPL57	166	-28.6	-33	0.026714
hsa-miR-4753-3p	NM_024560	ACSS3	153	-28.49	-32.5	0.010769
hsa-miR-4753-3p	NM_032641	SPSB2	150	-21.71	-25.9	0.045634

hsa-miR-4753-3p	NM_052858	MARVELD3	157	-27.61	-32	0.028385
hsa-miR-4753-3p	NM_144706	C2orf15	162	-27.95	-32.9	0.004933
hsa-miR-4753-3p	NM_153007	ODF4	152	-23.59	-28	0.008008
hsa-miR-4753-3p	NM_174887	IFT20	156	-24.64	-28.3	0.025413
hsa-miR-4753-3p	NM_181640	CKLF	163	-27.38	-30.7	0.003399
hsa-miR-4753-3p	NM_181641	CKLF	163	-27.38	-30.7	0.003399
hsa-miR-4753-3p	NM_182685	EFNA1	150	-25.99	-30.9	0.0224
hsa-miR-4753-3p	NM_207412	C8orf86	162	-28.21	-33	0.020563
hsa-miR-4753-3p	NR_003539	BRDTP1	160	-27.23	-29.4	0.045187
hsa-miR-4753-3p	NR_039908	MIR4753	176	-32.25	-35.4	0.000009
hsa-miR-4753-3p	NR_049810	MIR5087	166	-23.08	-25.2	0.006447
hsa-miR-4753-3p	NR_104027	SMARCA5-	168	-17.71	-31.4	0.028084
hsa-miR-4753-3p	NR_104136	MANEA-AS1	168	-21.12	-28	0.042794
hsa-miR-4753-3p	NR_106944	MIR6884	156	-20.99	-25	0.007814
hsa-miR-4753-3p	NR_109755	LINC01251	158	-23.01	-30.1	0.013636
hsa-miR-4753-3p	NR_109934	KIAA0101	157	-16.49	-32.8	0.022515
hsa-miR-4753-3p	NR_110034	LOC100996671	153	-25.58	-28.9	0.031229
hsa-miR-4753-3p	NR_110035	LOC100996671	153	-25.58	-28.9	0.020705
hsa-miR-4753-3p	NR_110036	LOC100996671	153	-25.58	-28.9	0.046419
hsa-miR-4753-3p	NR_110252	LOC101929452	176	-28.76	-33	0.003398
hsa-miR-4753-3p	NR_110994	PDE6D	155	-27.15	-31.2	0.026732
hsa-miR-4753-3p	NR_117101	CASC11	162	-19.89	-29.5	0.04737
hsa-miR-4753-3p	NR_117102	CASC11	155	-24.62	-29.5	0.04095
hsa-miR-4753-3p	NR_120582	LINC01395	150	-26.64	-29.9	0.046005
hsa-miR-4753-3p	NR_121620	LINC01605	152	-20.66	-32	0.035903
hsa-miR-4755-3p	NM_000031	ALAD	168	-29.89	-34.9	0.02725
hsa-miR-4755-3p	NM_000048	ASL	164	-26.4	-29.3	0.036783
hsa-miR-4755-3p	NM_000265	NCF1	167	-27.15	-31.5	0.00334
hsa-miR-4755-3p	NM_000626	CD79B	154	-26.77	-29.8	0.035105
hsa-miR-4755-3p	NM_000682	ADRA2B	166	-31.57	-35.3	0.021873

hsa-miR-4755-3p	NM_000795	DRD2	155	-25.93	-33.2	0.02557
hsa-miR-4755-3p	NM_001002261	ZFYVE27	165	-29.02	-34.4	0.025017
hsa-miR-4755-3p	NM_001002262	ZFYVE27	165	-29.02	-34.4	0.025017
hsa-miR-4755-3p	NM_001002841	MYL4	156	-23.16	-26.7	0.035163
hsa-miR-4755-3p	NM_001003931	PARP3	168	-25.61	-29.3	0.031029
hsa-miR-4755-3p	NM_001004128	QSOX1	152	-26.45	-32.5	0.016589
hsa-miR-4755-3p	NM_001007226	SPOP	164	-28.46	-35.3	0.015113
hsa-miR-4755-3p	NM_001007227	SPOP	164	-28.46	-35.3	0.015113
hsa-miR-4755-3p	NM_001007228	SPOP	164	-28.46	-35.3	0.015113
hsa-miR-4755-3p	NM_001007229	SPOP	164	-28.46	-35.3	0.015113
hsa-miR-4755-3p	NM_001007230	SPOP	164	-28.46	-35.3	0.015113
hsa-miR-4755-3p	NM_001008223	C1QL4	173	-31.16	-35.8	0.002921
hsa-miR-4755-3p	NM_001014796	DDR2	162	-22.32	-28.1	0.020491
hsa-miR-4755-3p	NM_001015072	UFSP1	176	-27.15	-33	0.00038
hsa-miR-4755-3p	NM_001024943	ASL	164	-26.4	-29.3	0.036783
hsa-miR-4755-3p	NM_001024944	ASL	164	-26.4	-29.3	0.036783
hsa-miR-4755-3p	NM_001024946	ASL	164	-26.4	-29.3	0.036783
hsa-miR-4755-3p	NM_001025158	CD74	150	-24.53	-30.1	0.045531
hsa-miR-4755-3p	NM_001025159	CD74	150	-24.53	-30.1	0.042114
hsa-miR-4755-3p	NM_001025295	IFITM5	163	-28.35	-34.7	0.001176
hsa-miR-4755-3p	NM_001031719	DHRS12	162	-26	-31.3	0.015253
hsa-miR-4755-3p	NM_001033667	LY9	155	-27.84	-32.5	0.022026
hsa-miR-4755-3p	NM_001037288	CMTM5	151	-24.74	-27.8	0.040476
hsa-miR-4755-3p	NM_001039888	ANKRD34A	162	-28.8	-31.3	0.038479
hsa-miR-4755-3p	NM_001039933	CD79B	154	-26.77	-29.8	0.035105
hsa-miR-4755-3p	NM_001039958	MESP2	151	-23.63	-29.2	0.036815
hsa-miR-4755-3p	NM_001040138	CKLF	166	-26.27	-31.2	0.003228
hsa-miR-4755-3p	NM_001042368	RALGDS	150	-28	-32.5	0.023604
hsa-miR-4755-3p	NM_001042498	SLC35A2	169	-33.45	-36.1	0.006655
hsa-miR-4755-3p	NM_001042771	LCK	159	-24.48	-28.9	0.048327

hsa-miR-4755-3p	NM_001077663	URGCP	165	-28.3	-34.8	0.006638
hsa-miR-4755-3p	NM_001077664	URGCP	165	-28.3	-34.8	0.006638
hsa-miR-4755-3p	NM_001080487	PABPN1L	150	-24.79	-30.9	0.015598
hsa-miR-4755-3p	NM_001080527	MYO7B	150	-22.66	-29.3	0.021487
hsa-miR-4755-3p	NM_001082959	SCARB1	156	-24.72	-31	0.045082
hsa-miR-4755-3p	NM_001084	PLOD3	156	-24.61	-31	0.008479
hsa-miR-4755-3p	NM_001085	SERPINA3	157	-24.6	-29.9	0.009722
hsa-miR-4755-3p	NM_001098426	SMARCD2	152	-27.4	-33.5	0.014528
hsa-miR-4755-3p	NM_001098520	HTATIP2	154	-26.18	-31	0.025547
hsa-miR-4755-3p	NM_001098521	HTATIP2	154	-26.18	-31	0.025547
hsa-miR-4755-3p	NM_001098522	HTATIP2	154	-26.18	-31	0.025547
hsa-miR-4755-3p	NM_001099686	NXF2B	150	-23.46	-26.8	0.039779
hsa-miR-4755-3p	NM_001099780	PSMB11	164	-25.47	-32.9	0.021904
hsa-miR-4755-3p	NM_001100875	MARCH10	151	-26.46	-29.7	0.025561
hsa-miR-4755-3p	NM_001111125	IQSEC2	164	-29.88	-32.6	0.0428
hsa-miR-4755-3p	NM_001113407	LDB1	152	-28.2	-31.7	0.015607
hsa-miR-4755-3p	NM_001114617	MGAT1	162	-29.72	-35.5	0.008481
hsa-miR-4755-3p	NM_001114618	MGAT1	162	-29.72	-35.5	0.008481
hsa-miR-4755-3p	NM_001114619	MGAT1	162	-29.72	-35.5	0.008481
hsa-miR-4755-3p	NM_001114620	MGAT1	162	-29.72	-35.5	0.008481
hsa-miR-4755-3p	NM_001122957	BCKDK	166	-29.47	-34	0.009436
hsa-miR-4755-3p	NM_001126105	SLC7A7	156	-24.95	-28.8	0.039924
hsa-miR-4755-3p	NM_001126106	SLC7A7	156	-24.95	-28.8	0.039924
hsa-miR-4755-3p	NM_001127891	MMP2	153	-26.68	-32.9	0.033795
hsa-miR-4755-3p	NM_001128325	SPON2	153	-27.23	-29.4	0.049743
hsa-miR-4755-3p	NM_001130478	LY6H	157	-23.57	-29.5	0.022122
hsa-miR-4755-3p	NM_001130993	RBP1	152	-22.92	-26.8	0.008842
hsa-miR-4755-3p	NM_001134395	C7orf50	153	-25.9	-31.3	0.025843
hsa-miR-4755-3p	NM_001134396	C7orf50	153	-25.9	-31.3	0.025843
hsa-miR-4755-3p	NM_001134875	C14orf80	154	-27.56	-30	0.014158

hsa-miR-4755-3p	NM_001134876	C14orf80	154	-27.56	-30	0.014158
hsa-miR-4755-3p	NM_001134877	C14orf80	154	-27.56	-30	0.014158
hsa-miR-4755-3p	NM_001135031	GFI1B	160	-27.82	-33.1	0.011106
hsa-miR-4755-3p	NM_001135655	LY6H	157	-23.57	-29.5	0.022122
hsa-miR-4755-3p	NM_001135943	MADD	150	-29.52	-32.6	0.023092
hsa-miR-4755-3p	NM_001135944	MADD	150	-29.52	-32.6	0.023092
hsa-miR-4755-3p	NM_001139441	BCAP31	153	-21.7	-32	0.010067
hsa-miR-4755-3p	NM_001139457	BCAP31	153	-21.7	-32	0.010067
hsa-miR-4755-3p	NM_001142807	ACOXL	159	-29.89	-31.6	0.010488
hsa-miR-4755-3p	NM_001143760	EIF5A	160	-25.71	-30.2	0.045276
hsa-miR-4755-3p	NM_001143761	EIF5A	160	-25.71	-30.2	0.045276
hsa-miR-4755-3p	NM_001143762	EIF5A	160	-25.71	-30.2	0.045276
hsa-miR-4755-3p	NM_001144856	PLEKHG6	163	-26.21	-31.7	0.008074
hsa-miR-4755-3p	NM_001144857	PLEKHG6	163	-26.21	-31.7	0.008074
hsa-miR-4755-3p	NM_001144996	ITGA7	161	-25.99	-30.2	0.028829
hsa-miR-4755-3p	NM_001144997	ITGA7	161	-25.99	-30.2	0.028829
hsa-miR-4755-3p	NM_001146016	TJAP1	171	-28.11	-33	0.013306
hsa-miR-4755-3p	NM_001146017	TJAP1	171	-28.11	-33	0.013306
hsa-miR-4755-3p	NM_001146018	TJAP1	171	-28.11	-33	0.013306
hsa-miR-4755-3p	NM_001146019	TJAP1	171	-28.11	-33	0.013306
hsa-miR-4755-3p	NM_001146020	TJAP1	171	-28.11	-33	0.013306
hsa-miR-4755-3p	NM_001146336	TMEM114	153	-23.85	-28.5	0.026085
hsa-miR-4755-3p	NM_001159352	REG4	170	-28.09	-33.5	0.008048
hsa-miR-4755-3p	NM_001160184	PLEKHN1	158	-21.62	-30.3	0.031211
hsa-miR-4755-3p	NM_001163484	DCAF11	163	-30.78	-36.9	0.011041
hsa-miR-4755-3p	NM_001164356	THAP4	153	-28.79	-31.5	0.002881
hsa-miR-4755-3p	NM_001164741	ARHGAP4	161	-28.28	-33.9	0.002483
hsa-miR-4755-3p	NM_001165036	OGDH	153	-26.49	-31.6	0.048615
hsa-miR-4755-3p	NM_001166237	GSDMD	150	-24.14	-31.1	0.002134
hsa-miR-4755-3p	NM_001166424	PGC	151	-21.01	-25.5	0.039814

hsa-miR-4755-3p	NM_001169	AQP8	158	-23.34	-29.2	0.040092
hsa-miR-4755-3p	NM_001170931	FOXO4	170	-28.49	-34	0.025714
hsa-miR-4755-3p	NM_001172668	ZNF668	150	-22.73	-28.2	0.020929
hsa-miR-4755-3p	NM_001172669	ZNF668	150	-22.73	-28.2	0.021791
hsa-miR-4755-3p	NM_001172670	ZNF668	150	-22.73	-28.2	0.021791
hsa-miR-4755-3p	NM_001174119	ZFYVE27	165	-29.02	-34.4	0.025017
hsa-miR-4755-3p	NM_001174120	ZFYVE27	165	-29.02	-34.4	0.025017
hsa-miR-4755-3p	NM_001174121	ZFYVE27	165	-29.02	-34.4	0.025017
hsa-miR-4755-3p	NM_001174122	ZFYVE27	165	-29.02	-34.4	0.025017
hsa-miR-4755-3p	NM_001190918	THRA	162	-29.84	-29.8	0.034765
hsa-miR-4755-3p	NM_001190919	THRA	162	-29.84	-29.8	0.034765
hsa-miR-4755-3p	NM_001198983	C14orf80	154	-27.56	-30	0.014158
hsa-miR-4755-3p	NM_001199021	SPON2	153	-27.23	-29.4	0.049743
hsa-miR-4755-3p	NM_001199173	MLST8	151	-27.15	-29.9	0.039161
hsa-miR-4755-3p	NM_001199174	MLST8	151	-27.15	-29.9	0.039161
hsa-miR-4755-3p	NM_001199175	MLST8	151	-27.15	-29.9	0.039161
hsa-miR-4755-3p	NM_001202858	ECM1	183	-31.95	-37	0.000408
hsa-miR-4755-3p	NM_001204698	TAX1BP3	156	-28.68	-32	0.029162
hsa-miR-4755-3p	NM_001204744	CDH16	150	-25.29	-27.3	0.048046
hsa-miR-4755-3p	NM_001204745	CDH16	150	-25.29	-27.3	0.048046
hsa-miR-4755-3p	NM_001204746	CDH16	150	-25.29	-27.3	0.048046
hsa-miR-4755-3p	NM_001204871	URGCP-MRPS24	152	-25.16	-30.1	0.026379
hsa-miR-4755-3p	NM_001206796	PKM	160	-27.45	-32	0.017664
hsa-miR-4755-3p	NM_001206797	PKM	160	-27.45	-32	0.017664
hsa-miR-4755-3p	NM_001206798	PKM	160	-27.45	-32	0.017664
hsa-miR-4755-3p	NM_001206799	PKM	160	-27.45	-32	0.017664
hsa-miR-4755-3p	NM_001242773	TMEM139	171	-28.41	-32	0.048815
hsa-miR-4755-3p	NM_001242774	TMEM139	171	-28.41	-32	0.048815
hsa-miR-4755-3p	NM_001242775	TMEM139	171	-28.41	-32	0.048815

hsa-miR-4755-3p	NM_001242832	ARFGAP2	173	-33.11	-36.6	0.005843
hsa-miR-4755-3p	NM_001243473	B9D1	153	-27.73	-28.6	0.042343
hsa-miR-4755-3p	NM_001243749	C7orf49	163	-26.62	-32.7	0.032058
hsa-miR-4755-3p	NM_001243751	C7orf49	163	-26.62	-32.7	0.032058
hsa-miR-4755-3p	NM_001243752	C7orf49	163	-26.62	-32.7	0.032058
hsa-miR-4755-3p	NM_001243753	C7orf49	163	-26.62	-32.7	0.032058
hsa-miR-4755-3p	NM_001243754	C7orf49	163	-26.62	-32.7	0.020132
hsa-miR-4755-3p	NM_001243755	C7orf49	163	-26.62	-32.7	0.020132
hsa-miR-4755-3p	NM_001243759	USP2	163	-26.46	-36.6	0.009622
hsa-miR-4755-3p	NM_001243770	SLF2	165	-27.46	-31.6	0.019408
hsa-miR-4755-3p	NM_001244678	THNSL2	156	-26.55	-30.4	0.042034
hsa-miR-4755-3p	NM_001251902	TNK1	165	-28.03	-32.1	0.019899
hsa-miR-4755-3p	NM_001254718	MYBPC1	151	-22.01	-27.2	0.043996
hsa-miR-4755-3p	NM_001254723	MYBPC1	151	-21.62	-27.2	0.029869
hsa-miR-4755-3p	NM_001256447	BCAP31	153	-21.7	-32	0.010067
hsa-miR-4755-3p	NM_001258304	CCDC74A	160	-25.87	-30.9	0.00706
hsa-miR-4755-3p	NM_001258305	CCDC74A	160	-25.87	-30.9	0.014142
hsa-miR-4755-3p	NM_001258306	CCDC74A	160	-25.87	-30.9	0.00706
hsa-miR-4755-3p	NM_001258307	CCDC74B	160	-25.87	-30.9	0.007261
hsa-miR-4755-3p	NM_001258357	HPCAL1	160	-22.7	-31.8	0.028722
hsa-miR-4755-3p	NM_001258358	HPCAL1	160	-22.7	-31.8	0.028722
hsa-miR-4755-3p	NM_001258359	HPCAL1	160	-22.7	-31.8	0.028722
hsa-miR-4755-3p	NM_001258450	PKMYT1	152	-21.26	-25.9	0.042883
hsa-miR-4755-3p	NM_001258451	PKMYT1	152	-21.26	-25.9	0.042883
hsa-miR-4755-3p	NM_001267551	PEMT	153	-23.05	-28.7	0.017035
hsa-miR-4755-3p	NM_001267552	PEMT	153	-23.05	-28.7	0.023176
hsa-miR-4755-3p	NM_001270965	PSD	153	-26.17	-32	0.01593
hsa-miR-4755-3p	NM_001270966	PSD	153	-26.17	-32	0.01593
hsa-miR-4755-3p	NM_001271774	RALGDS	150	-28	-32.5	0.023604
hsa-miR-4755-3p	NM_001271775	RALGDS	150	-28	-32.5	0.023604

hsa-miR-4755-3p	NM_001271776	RALGDS	150	-28	-32.5	0.023604
hsa-miR-4755-3p	NM_001271926	BCKDK	166	-29.47	-34	0.009436
hsa-miR-4755-3p	NM_001272095	STX4	156	-24.73	-29.3	0.016873
hsa-miR-4755-3p	NM_001272096	STX4	156	-24.73	-29.3	0.016873
hsa-miR-4755-3p	NM_001276405	DALRD3	152	-20.09	-25.9	0.039202
hsa-miR-4755-3p	NM_001278390	SLC38A1	163	-25.01	-29.2	0.034712
hsa-miR-4755-3p	NM_001278596	ANO2	159	-25.19	-31.1	0.029144
hsa-miR-4755-3p	NM_001278597	ANO2	159	-25.19	-31.1	0.029144
hsa-miR-4755-3p	NM_001281734	ZFYVE1	159	-27.95	-32.9	0.041223
hsa-miR-4755-3p	NM_001281735	ZFYVE1	159	-27.95	-32.9	0.041223
hsa-miR-4755-3p	NM_001282386	IDH1	168	-27.37	-31.5	0.036588
hsa-miR-4755-3p	NM_001282387	IDH1	168	-27.37	-31.5	0.036588
hsa-miR-4755-3p	NM_001282649	SLC35A2	169	-33.45	-36.1	0.006655
hsa-miR-4755-3p	NM_001282650	SLC35A2	169	-33.45	-36.1	0.006655
hsa-miR-4755-3p	NM_001282651	SLC35A2	169	-33.45	-36.1	0.006655
hsa-miR-4755-3p	NM_001282876	TMEM139	171	-28.41	-32	0.048815
hsa-miR-4755-3p	NM_001282877	TMEM139	171	-28.41	-32	0.048815
hsa-miR-4755-3p	NM_001286242	GTF3C1	155	-25.21	-30.4	0.049293
hsa-miR-4755-3p	NM_001286561	NUMA1	154	-24.3	-30.9	0.033218
hsa-miR-4755-3p	NM_001286796	NTMT1	158	-26.71	-31.9	0.020794
hsa-miR-4755-3p	NM_001286797	NTMT1	158	-26.71	-31.9	0.020794
hsa-miR-4755-3p	NM_001286798	NTMT1	158	-26.71	-31.9	0.020794
hsa-miR-4755-3p	NM_001286799	NTMT1	158	-26.71	-31.9	0.020794
hsa-miR-4755-3p	NM_001286800	NTMT1	158	-26.71	-31.9	0.028464
hsa-miR-4755-3p	NM_001286801	NTMT1	158	-26.71	-31.9	0.028464
hsa-miR-4755-3p	NM_001286802	NTMT1	158	-26.71	-31.9	0.020794
hsa-miR-4755-3p	NM_001286803	NTMT1	158	-26.71	-31.9	0.020794
hsa-miR-4755-3p	NM_001287045	SH3GLB2	174	-29.71	-35.8	0.00527
hsa-miR-4755-3p	NM_001288738	DNM1	157	-29.62	-33.6	0.024876
hsa-miR-4755-3p	NM_001288744	CMTM5	151	-24.74	-27.8	0.040476

hsa-miR-4755-3p	NM_001288745	CMTM5	151	-24.74	-27.8	0.040476
hsa-miR-4755-3p	NM_001288746	CMTM5	151	-24.74	-27.8	0.040476
hsa-miR-4755-3p	NM_001288779	MARCH10	151	-26.46	-29.7	0.025561
hsa-miR-4755-3p	NM_001289162	DRC7	156	-25.19	-28.7	0.012991
hsa-miR-4755-3p	NM_001289163	DRC7	156	-25.19	-28.7	0.012991
hsa-miR-4755-3p	NM_001289934	LRCH4	154	-23.86	-28.1	0.025968
hsa-miR-4755-3p	NM_001290075	URGCP	165	-28.3	-34.8	0.006638
hsa-miR-4755-3p	NM_001290076	URGCP	165	-28.3	-34.8	0.006638
hsa-miR-4755-3p	NM_001290095	TMEM114	153	-23.85	-28.5	0.026085
hsa-miR-4755-3p	NM_001290097	TMEM114	153	-23.85	-28.5	0.026085
hsa-miR-4755-3p	NM_001291702	VNN3	156	-26.93	-31.1	0.001631
hsa-miR-4755-3p	NM_001291703	VNN3	156	-26.93	-31.1	0.001631
hsa-miR-4755-3p	NM_001291980	FGFR4	152	-22.78	-30.5	0.023056
hsa-miR-4755-3p	NM_001294328	PABPN1L	150	-24.79	-30.9	0.014392
hsa-miR-4755-3p	NM_001297760	ETNK2	172	-31.69	-34.3	0.017075
hsa-miR-4755-3p	NM_001297761	ETNK2	172	-31.69	-34.3	0.015323
hsa-miR-4755-3p	NM_001297762	ETNK2	172	-31.69	-34.3	0.015323
hsa-miR-4755-3p	NM_001301097	KIRREL3	158	-29.22	-35.1	0.00998
hsa-miR-4755-3p	NM_001302508	MMP2	153	-26.68	-32.9	0.033795
hsa-miR-4755-3p	NM_001302509	MMP2	153	-26.68	-32.9	0.033795
hsa-miR-4755-3p	NM_001302510	MMP2	153	-26.68	-32.9	0.033795
hsa-miR-4755-3p	NM_001303050	MAPRE3	157	-29.21	-33.2	0.017151
hsa-miR-4755-3p	NM_001303496	SAMD9L	150	-26.95	-32	0.045646
hsa-miR-4755-3p	NM_001303497	SAMD9L	150	-26.95	-32	0.045646
hsa-miR-4755-3p	NM_001303498	SAMD9L	150	-26.95	-32	0.045646
hsa-miR-4755-3p	NM_001303500	SAMD9L	150	-26.95	-32	0.045646
hsa-miR-4755-3p	NM_001303512	PDZD4	168	-28.24	-34.1	0.018268
hsa-miR-4755-3p	NM_001303513	PDZD4	168	-28.24	-34.1	0.018268
hsa-miR-4755-3p	NM_001303514	PDZD4	168	-28.24	-34.1	0.018268
hsa-miR-4755-3p	NM_001303515	PDZD4	168	-28.24	-34.1	0.018268

hsa-miR-4755-3p	NM_001303516	PDZD4	168	-28.24	-34.1	0.018268
hsa-miR-4755-3p	NM_001304763	ZNF687	159	-27.74	-32.9	0.013796
hsa-miR-4755-3p	NM_001304764	ZNF687	159	-27.74	-32.9	0.013796
hsa-miR-4755-3p	NM_001305629	C7orf49	163	-26.62	-32.7	0.020132
hsa-miR-4755-3p	NM_001305942	COQ4	156	-27.83	-31.5	0.02134
hsa-miR-4755-3p	NM_001316318	PKM	160	-27.45	-32	0.017664
hsa-miR-4755-3p	NM_001520	GTF3C1	155	-25.21	-30.4	0.049293
hsa-miR-4755-3p	NM_001666	ARHGAP4	161	-28.28	-33.9	0.002483
hsa-miR-4755-3p	NM_001852	COL9A2	161	-29.31	-34	0.008043
hsa-miR-4755-3p	NM_001970	EIF5A	160	-25.71	-30.2	0.045276
hsa-miR-4755-3p	NM_002011	FGFR4	152	-22.78	-30.5	0.023056
hsa-miR-4755-3p	NM_002149	HPCAL1	160	-22.7	-31.8	0.028722
hsa-miR-4755-3p	NM_002206	ITGA7	161	-25.99	-30.2	0.028829
hsa-miR-4755-3p	NM_002347	LY6H	157	-23.57	-29.5	0.022122
hsa-miR-4755-3p	NM_002406	MGAT1	162	-29.72	-35.5	0.008481
hsa-miR-4755-3p	NM_002476	MYL4	156	-23.16	-26.7	0.035163
hsa-miR-4755-3p	NM_002541	OGDH	153	-26.49	-31.6	0.048615
hsa-miR-4755-3p	NM_002567	PEBP1	157	-26.41	-31.3	0.035539
hsa-miR-4755-3p	NM_002654	PKM	160	-27.45	-32	0.017664
hsa-miR-4755-3p	NM_002779	PSD	153	-26.17	-32	0.01593
hsa-miR-4755-3p	NM_002826	QSOX1	152	-26.45	-32.5	0.029156
hsa-miR-4755-3p	NM_002840	PTPRF	164	-32.56	-33	0.048483
hsa-miR-4755-3p	NM_003041	SLC5A2	175	-30.08	-34.4	0.000821
hsa-miR-4755-3p	NM_003250	THRA	162	-29.84	-29.8	0.034765
hsa-miR-4755-3p	NM_003280	TNNC1	152	-24.72	-27.2	0.026195
hsa-miR-4755-3p	NM_003563	SPOP	164	-28.46	-35.3	0.015113
hsa-miR-4755-3p	NM_003630	PEX3	165	-27.19	-32.9	0.039268
hsa-miR-4755-3p	NM_003682	MADD	150	-29.52	-32.6	0.023092
hsa-miR-4755-3p	NM_003878	GGH	159	-22.77	-27.3	0.042865
hsa-miR-4755-3p	NM_003893	LDB1	152	-28.2	-31.7	0.015607

hsa-miR-4755-3p	NM_003985	TNK1	165	-28.03	-32.1	0.019899
hsa-miR-4755-3p	NM_004062	CDH16	150	-25.29	-27.3	0.048046
hsa-miR-4755-3p	NM_004188	GFI1B	160	-27.82	-33.1	0.01082
hsa-miR-4755-3p	NM_004203	PKMYT1	152	-21.26	-25.9	0.042883
hsa-miR-4755-3p	NM_004205	USP2	163	-26.46	-36.6	0.009622
hsa-miR-4755-3p	NM_004355	CD74	150	-24.53	-30.1	0.042114
hsa-miR-4755-3p	NM_004395	DBN1	160	-26.74	-33.7	0.01232
hsa-miR-4755-3p	NM_004422	DVL2	151	-25.89	-30.2	0.032189
hsa-miR-4755-3p	NM_004425	ECM1	183	-31.95	-37	0.000408
hsa-miR-4755-3p	NM_004502	HOXB7	157	-26.44	-32.3	0.014795
hsa-miR-4755-3p	NM_004530	MMP2	153	-26.68	-32.9	0.033795
hsa-miR-4755-3p	NM_004604	STX4	156	-24.73	-29.3	0.016873
hsa-miR-4755-3p	NM_004813	PEX16	152	-29.58	-32.7	0.011388
hsa-miR-4755-3p	NM_004913	VPS9D1	178	-33.05	-37.4	0.001725
hsa-miR-4755-3p	NM_005149	TBX19	165	-30.26	-34.2	0.022968
hsa-miR-4755-3p	NM_005271	GLUD1	153	-27.5	-34.5	0.022702
hsa-miR-4755-3p	NM_005309	GPT	158	-22.93	-28.7	0.010009
hsa-miR-4755-3p	NM_005356	LCK	159	-24.48	-28.9	0.048327
hsa-miR-4755-3p	NM_005485	PARP3	168	-25.61	-29.3	0.031029
hsa-miR-4755-3p	NM_005626	SRSF4	166	-28.35	-31.8	0.022074
hsa-miR-4755-3p	NM_005745	BCAP31	153	-21.7	-32	0.010067
hsa-miR-4755-3p	NM_005881	BCKDK	166	-29.47	-34	0.004993
hsa-miR-4755-3p	NM_005886	KATNB1	160	-28.85	-32.5	0.003853
hsa-miR-4755-3p	NM_005892	FMNL1	156	-25.05	-32.4	0.008662
hsa-miR-4755-3p	NM_005896	IDH1	168	-27.37	-31.5	0.036588
hsa-miR-4755-3p	NM_005938	FOXO4	170	-28.49	-34	0.025714
hsa-miR-4755-3p	NM_005961	MUC6	154	-27.91	-34	0.006902
hsa-miR-4755-3p	NM_006182	DDR2	162	-22.32	-28.1	0.020491
hsa-miR-4755-3p	NM_006185	NUMA1	154	-24.3	-30.9	0.033218
hsa-miR-4755-3p	NM_006253	PRKAB1	163	-27.91	-33.9	0.022883

hsa-miR-4755-3p	NM_006266	RALGDS	150	-28	-32.5	0.023604
hsa-miR-4755-3p	NM_006289	TLN1	158	-28.02	-31.8	0.011526
hsa-miR-4755-3p	NM_006410	HTATIP2	154	-26.18	-31	0.025547
hsa-miR-4755-3p	NM_006923	SDF2	163	-27.42	-34.3	0.005984
hsa-miR-4755-3p	NM_007169	PEMT	153	-23.05	-28.7	0.017035
hsa-miR-4755-3p	NM_012326	MAPRE3	157	-29.21	-33.2	0.017151
hsa-miR-4755-3p	NM_012445	SPON2	153	-27.23	-29.4	0.049743
hsa-miR-4755-3p	NM_013306	SNX15	166	-30.04	-33.9	0.01033
hsa-miR-4755-3p	NM_013379	DPP7	150	-22.66	-26.2	0.016865
hsa-miR-4755-3p	NM_014045	LRP10	169	-32.42	-35.6	0.002498
hsa-miR-4755-3p	NM_014064	NTMT1	158	-26.71	-31.9	0.020794
hsa-miR-4755-3p	NM_014604	TAX1BP3	156	-28.68	-32	0.029162
hsa-miR-4755-3p	NM_015175	NBEAL2	155	-26.42	-31	0.012908
hsa-miR-4755-3p	NM_015261	NCAPD3	162	-27.75	-32.7	0.008759
hsa-miR-4755-3p	NM_015305	ANGEL1	160	-30.14	-33.8	0.043502
hsa-miR-4755-3p	NM_015407	ABHD14A	153	-22.74	-29	0.00945
hsa-miR-4755-3p	NM_015848	KRT76	176	-27.7	-31.7	0.017158
hsa-miR-4755-3p	NM_015963	THAP4	153	-28.79	-31.5	0.002881
hsa-miR-4755-3p	NM_016035	COQ4	156	-27.83	-31.5	0.012051
hsa-miR-4755-3p	NM_016176	SDF4	166	-26.75	-32.1	0.016083
hsa-miR-4755-3p	NM_016326	CKLF	166	-26.27	-31.2	0.006364
hsa-miR-4755-3p	NM_016547	SDF4	166	-26.75	-32.1	0.02332
hsa-miR-4755-3p	NM_016574	DRD2	155	-25.93	-33.2	0.02557
hsa-miR-4755-3p	NM_016943	TAS2R3	154	-24.48	-30.4	0.000845
hsa-miR-4755-3p	NM_016951	CKLF	166	-26.27	-31.2	0.006364
hsa-miR-4755-3p	NM_017547	FOXRED1	152	-25.46	-29	0.04474
hsa-miR-4755-3p	NM_017920	URGCP	165	-28.3	-34.8	0.006638
hsa-miR-4755-3p	NM_017966	VPS37C	178	-31.91	-37.9	0.005122
hsa-miR-4755-3p	NM_018173	PLEKHG6	163	-26.21	-31.7	0.008074
hsa-miR-4755-3p	NM_018208	ETNK2	172	-31.69	-34.3	0.015323

hsa-miR-4755-3p	NM_018688	BIN3	164	-27.63	-32.9	0.02405
hsa-miR-4755-3p	NM_020137	GRIPAP1	164	-24.14	-29.3	0.04274
hsa-miR-4755-3p	NM_020145	SH3GLB2	174	-29.71	-35.8	0.00527
hsa-miR-4755-3p	NM_020427	SLURP1	161	-29.53	-33.5	0.000933
hsa-miR-4755-3p	NM_020832	ZNF687	159	-27.74	-32.9	0.013796
hsa-miR-4755-3p	NM_021078	KAT2A	152	-23.71	-29.9	0.038937
hsa-miR-4755-3p	NM_021260	ZFYVE1	159	-27.95	-32.9	0.041223
hsa-miR-4755-3p	NM_021602	CD79B	154	-26.77	-29.8	0.035105
hsa-miR-4755-3p	NM_022053	NXF2	150	-23.46	-26.8	0.039779
hsa-miR-4755-3p	NM_022372	MLST8	151	-27.15	-29.9	0.039161
hsa-miR-4755-3p	NM_022664	ECM1	183	-31.95	-37	0.000408
hsa-miR-4755-3p	NM_022963	FGFR4	152	-22.78	-30.5	0.023056
hsa-miR-4755-3p	NM_023946	LYNX1	156	-22.91	-27.8	0.037219
hsa-miR-4755-3p	NM_024033	C7orf49	163	-26.62	-32.7	0.020132
hsa-miR-4755-3p	NM_024671	ZNF768	153	-22.89	-29.7	0.040228
hsa-miR-4755-3p	NM_024706	ZNF668	150	-22.73	-28.2	0.021791
hsa-miR-4755-3p	NM_024736	GSDMD	150	-24.14	-31.1	0.002134
hsa-miR-4755-3p	NM_025216	WNT10A	166	-27.88	-33.7	0.008552
hsa-miR-4755-3p	NM_025230	DCAF11	163	-30.78	-36.9	0.011041
hsa-miR-4755-3p	NM_025232	REEP4	160	-24.52	-30.6	0.022015
hsa-miR-4755-3p	NM_030649	ACAP3	151	-27.06	-32.1	0.044554
hsa-miR-4755-3p	NM_030916	PVRL4	159	-28.35	-33.5	0.039093
hsa-miR-4755-3p	NM_030968	C1QTNF1	174	-32.41	-36.8	0.009489
hsa-miR-4755-3p	NM_032044	REG4	170	-28.09	-33.5	0.008048
hsa-miR-4755-3p	NM_032129	PLEKHN1	158	-21.62	-30.3	0.031211
hsa-miR-4755-3p	NM_032269	DRC7	156	-25.19	-28.7	0.012991
hsa-miR-4755-3p	NM_032350	C7orf50	153	-25.9	-31.3	0.025843
hsa-miR-4755-3p	NM_032389	ARFGAP2	173	-33.11	-36.6	0.005843
hsa-miR-4755-3p	NM_032512	PDZD4	168	-28.24	-34.1	0.018268
hsa-miR-4755-3p	NM_032531	KIRREL3	158	-29.22	-35.1	0.00998

hsa-miR-4755-3p	NM_032656	DHX37	163	-28.84	-31.9	0.03729
hsa-miR-4755-3p	NM_033343	LHX4	150	-23.58	-29.7	0.035218
hsa-miR-4755-3p	NM_052843	OBSCN	165	-22.84	-29.6	0.0396
hsa-miR-4755-3p	NM_052903	TUBGCP5	154	-25.18	-30.1	0.046392
hsa-miR-4755-3p	NM_054022	GOSR2	156	-24.19	-30.5	0.048643
hsa-miR-4755-3p	NM_057174	PEX16	152	-29.58	-32.7	0.006376
hsa-miR-4755-3p	NM_080604	TJAP1	171	-28.11	-33	0.013306
hsa-miR-4755-3p	NM_080744	SSC4D	152	-25.29	-31.5	0.028971
hsa-miR-4755-3p	NM_080881	DBN1	160	-26.74	-33.7	0.01232
hsa-miR-4755-3p	NM_130440	PTPRF	164	-32.56	-33	0.048483
hsa-miR-4755-3p	NM_130470	MADD	150	-29.52	-32.6	0.023092
hsa-miR-4755-3p	NM_130471	MADD	150	-29.52	-32.6	0.023092
hsa-miR-4755-3p	NM_130472	MADD	150	-29.52	-32.6	0.023092
hsa-miR-4755-3p	NM_130473	MADD	150	-29.52	-32.6	0.023092
hsa-miR-4755-3p	NM_130474	MADD	150	-29.52	-32.6	0.028311
hsa-miR-4755-3p	NM_130475	MADD	150	-29.52	-32.6	0.028311
hsa-miR-4755-3p	NM_130476	MADD	150	-29.52	-32.6	0.023092
hsa-miR-4755-3p	NM_130769	GPHA2	154	-24.16	-30.9	0.008828
hsa-miR-4755-3p	NM_133639	RHOV	158	-26.41	-31.5	0.036079
hsa-miR-4755-3p	NM_134421	HPCAL1	160	-22.7	-31.8	0.028722
hsa-miR-4755-3p	NM_138460	CMTM5	151	-24.74	-27.8	0.040476
hsa-miR-4755-3p	NM_138574	HDGFL1	157	-22.17	-26.8	0.034559
hsa-miR-4755-3p	NM_138770	CCDC74A	160	-25.87	-30.9	0.00706
hsa-miR-4755-3p	NM_139021	MAPK15	158	-24.24	-28.9	0.013361
hsa-miR-4755-3p	NM_139170	C16orf71	162	-26.65	-31	0.033003
hsa-miR-4755-3p	NM_144588	ZFYVE27	165	-29.02	-34.4	0.025017
hsa-miR-4755-3p	NM_147777	SNX15	166	-30.04	-33.9	0.01033
hsa-miR-4755-3p	NM_148172	PEMT	153	-23.05	-28.7	0.017035
hsa-miR-4755-3p	NM_148173	PEMT	153	-23.05	-28.7	0.017035
hsa-miR-4755-3p	NM_152272	CHMP7	162	-25.84	-33.6	0.029175

hsa-miR-4755-3p	NM_152285	ARRDC1	159	-22.82	-28.3	0.020152
hsa-miR-4755-3p	NM_152429	FGFBP3	163	-34.53	-36.8	0.008683
hsa-miR-4755-3p	NM_152598	MARCH10	151	-26.46	-29.7	0.025561
hsa-miR-4755-3p	NM_152703	SAMD9L	150	-26.95	-32	0.045646
hsa-miR-4755-3p	NM_153345	TMEM139	171	-28.41	-32	0.048815
hsa-miR-4755-3p	NM_153372	C1QTNF1	174	-32.41	-36.8	0.009489
hsa-miR-4755-3p	NM_153635	CPNE9	166	-25.29	-30.4	0.006099
hsa-miR-4755-3p	NM_171997	USP2	163	-26.46	-36.6	0.009622
hsa-miR-4755-3p	NM_172207	CAMKK1	165	-26.28	-31.2	0.034564
hsa-miR-4755-3p	NM_173575	STK32C	157	-25.89	-29.5	0.044606
hsa-miR-4755-3p	NM_173664	ARL10	157	-31.71	-33	0.045971
hsa-miR-4755-3p	NM_177458	LYNX1	156	-22.91	-27.8	0.0363
hsa-miR-4755-3p	NM_178441	ZFYVE1	159	-27.95	-32.9	0.041223
hsa-miR-4755-3p	NM_178537	B4GALNT4	161	-25.08	-29.8	0.017886
hsa-miR-4755-3p	NM_178840	C1orf64	159	-26.51	-32.1	0.006171
hsa-miR-4755-3p	NM_178858	SFXN2	154	-28.83	-33.2	0.035775
hsa-miR-4755-3p	NM_181357	DCAF11	163	-30.78	-36.9	0.011041
hsa-miR-4755-3p	NM_181640	CKLF	166	-26.27	-31.2	0.006364
hsa-miR-4755-3p	NM_181641	CKLF	166	-26.27	-31.2	0.006364
hsa-miR-4755-3p	NM_182470	PKM	160	-27.45	-32	0.017664
hsa-miR-4755-3p	NM_182471	PKM	160	-27.45	-32	0.017664
hsa-miR-4755-3p	NM_198546	SPATA21	152	-19.72	-26.6	0.016972
hsa-miR-4755-3p	NM_198593	C1QTNF1	174	-32.41	-36.8	0.009489
hsa-miR-4755-3p	NM_198594	C1QTNF1	174	-32.41	-36.8	0.009489
hsa-miR-4755-3p	NM_203402	FITM1	154	-22.53	-27.2	0.000244
hsa-miR-4755-3p	NM_207310	CCDC74B	160	-25.87	-30.9	0.007261
hsa-miR-4755-3p	NM_207311	CCDC64	168	-32.93	-34.7	0.016643
hsa-miR-4755-3p	NM_213647	FGFR4	152	-22.78	-30.5	0.023056
hsa-miR-4755-3p	NR_002982	SNORA54	150	-23.58	-28.1	0.007178
hsa-miR-4755-3p	NR_026766	MYCNOS	163	-27.68	-32.6	0.018719

hsa-miR-4755-3p	NR_027992	NBEAP1	158	-26.96	-32.9	0.009237
hsa-miR-4755-3p	NR_028293	PTGER3	157	-30.04	-34.9	0.027173
hsa-miR-4755-3p	NR_028510	TYRO3P	169	-36.13	-39.8	0.00076
hsa-miR-4755-3p	NR_033347	SNORA70E	163	-31.45	-37.4	0.000039
hsa-miR-4755-3p	NR_033995	LOC389247	169	-25.76	-31.7	0.02924
hsa-miR-4755-3p	NR_037652	ST20	152	-29.6	-30.2	0.044447
hsa-miR-4755-3p	NR_037653	ST20	152	-29.6	-30.2	0.046732
hsa-miR-4755-3p	NR_039627	MIR4429	154	-25.5	-29.9	0.000722
hsa-miR-4755-3p	NR_045585	SDF2	163	-27.42	-34.3	0.018315
hsa-miR-4755-3p	NR_046368	LINC01023	155	-23.97	-29.3	0.03774
hsa-miR-4755-3p	NR_046836	HS1BP3-IT1	164	-27.64	-32.5	0.013098
hsa-miR-4755-3p	NR_047494	LINC00564	172	-27.51	-32.9	0.028756
hsa-miR-4755-3p	NR_047507	HOXC13-AS	168	-29.75	-34.1	0.023568
hsa-miR-4755-3p	NR_106807	MIR6749	151	-20.84	-25.5	0.010124
hsa-miR-4755-3p	NR_106828	MIR6770-1	160	-24.2	-29.5	0.000559
hsa-miR-4755-3p	NR_107060	MIR6770-2	160	-24.2	-29.5	0.000559
hsa-miR-4755-3p	NR_107061	MIR6770-3	160	-24.2	-29.5	0.000559
hsa-miR-4755-3p	NR_109774	LOC100506188	161	-26.74	-32.6	0.048054
hsa-miR-4755-3p	NR_109775	LOC100422556	157	-25.55	-30.2	0.02574
hsa-miR-4755-3p	NR_110092	LOC101928731	161	-26.04	-33	0.045267
hsa-miR-4755-3p	NR_110154	ID2-AS1	161	-25.88	-31.3	0.036151
hsa-miR-4755-3p	NR_110982	LOC103021295	154	-27.83	-34.5	0.003089
hsa-miR-4755-3p	NR_120676	NEURL1-AS1	172	-27.9	-29.7	0.037706
hsa-miR-4755-3p	NR_126159	NTM-AS1	163	-29.26	-32.4	0.016798
hsa-miR-4755-3p	NR_126349	LINC01195	156	-25.29	-29.1	0.043336
hsa-miR-4755-3p	NR_130118	TSC22D4	163	-27.12	-33.9	0.02925
hsa-miR-4755-3p	NR_130119	TSC22D4	163	-27.12	-33.9	0.029168
hsa-miR-4755-3p	NR_130972	PPP2R3C	164	-29.11	-33.7	0.036235
hsa-miR-5188	NM_001007553	CSDE1	157	-29.76	-32.2	0.044621
hsa-miR-5188	NM_001130523	CSDE1	157	-29.76	-32.2	0.044621

hsa-miR-5188	NM_001242891	CSDE1	157	-29.76	-32.2	0.044621
hsa-miR-5188	NM_001242892	CSDE1	157	-29.76	-32.2	0.044621
hsa-miR-5188	NM_001242893	CSDE1	157	-29.76	-32.2	0.044621
hsa-miR-5188	NM_007158	CSDE1	157	-29.76	-32.2	0.044621
hsa-miR-5582-3p	NM_001037281	PARD6A	155	-17.65	-23.8	0.0439
hsa-miR-5582-3p	NM_016948	PARD6A	155	-17.65	-23.8	0.0439
hsa-miR-6130	NM_000520	HEXA	159	-25.42	-28.5	0.03755
hsa-miR-6130	NM_000593	TAP1	159	-21.04	-26.5	0.045317
hsa-miR-6130	NM_000690	ALDH2	152	-24.22	-27.8	0.026273
hsa-miR-6130	NM_001001683	MED11	151	-22.96	-27.4	0.034504
hsa-miR-6130	NM_001010855	PIK3R6	150	-21.45	-30.4	0.010619
hsa-miR-6130	NM_001010985	MYBPHL	161	-18.73	-25.7	0.038327
hsa-miR-6130	NM_001029870	SOWAHB	156	-27.78	-29.2	0.01955
hsa-miR-6130	NM_001039548	KLHL35	164	-23	-29.1	0.002666
hsa-miR-6130	NM_001040661	SLC29A4	155	-26.57	-29.8	0.046397
hsa-miR-6130	NM_001101648	NPC1L1	158	-24.24	-29	0.049981
hsa-miR-6130	NM_001105515	ABCC4	159	-23.69	-28.4	0.015259
hsa-miR-6130	NM_001114101	C1QC	151	-23.82	-27.1	0.026247
hsa-miR-6130	NM_001134693	OST4	156	-20.87	-27	0.019844
hsa-miR-6130	NM_001136214	CLEC18A	164	-30.24	-33.9	0.002808
hsa-miR-6130	NM_001136509	ZNF843	169	-27.17	-31.5	0.001089
hsa-miR-6130	NM_001143676	SGK1	155	-29.31	-31.7	0.015358
hsa-miR-6130	NM_001143677	SGK1	155	-29.31	-31.7	0.015358
hsa-miR-6130	NM_001143678	SGK1	155	-29.31	-31.7	0.015358
hsa-miR-6130	NM_001144822	CD58	177	-24.49	-27.9	0.037778
hsa-miR-6130	NM_001161344	CHFR	156	-26.15	-31.1	0.026722
hsa-miR-6130	NM_001161345	CHFR	156	-26.15	-31.1	0.026722
hsa-miR-6130	NM_001161346	CHFR	156	-26.15	-31.1	0.026722
hsa-miR-6130	NM_001161347	CHFR	156	-26.15	-31.1	0.026722
hsa-miR-6130	NM_001165136	HERC6	155	-25	-28.5	0.037271

hsa-miR-6130	NM_001193300	SEMA4A	158	-21.57	-29.9	0.023573
hsa-miR-6130	NM_001193301	SEMA4A	158	-21.57	-29.9	0.023573
hsa-miR-6130	NM_001193302	SEMA4A	158	-21.57	-29.9	0.023573
hsa-miR-6130	NM_001204889	ALDH2	152	-24.22	-27.8	0.026273
hsa-miR-6130	NM_001258008	ZCWPW1	152	-19.31	-27.4	0.019312
hsa-miR-6130	NM_001265613	MYBPHL	161	-18.73	-25.7	0.038327
hsa-miR-6130	NM_001271197	CLEC18A	164	-30.24	-33.9	0.002808
hsa-miR-6130	NM_001278323	MOBP	165	-21.34	-25.2	0.018021
hsa-miR-6130	NM_001278785	ZNRD1	167	-26.18	-29.9	0.00218
hsa-miR-6130	NM_001278786	ZNRD1	167	-26.18	-29.9	0.00218
hsa-miR-6130	NM_001290211	PIK3R6	150	-21.45	-30.4	0.010619
hsa-miR-6130	NM_001291995	SGK1	155	-29.31	-31.7	0.015358
hsa-miR-6130	NM_001292022	TAP1	159	-21.04	-26.5	0.045317
hsa-miR-6130	NM_001300847	SLC29A4	155	-26.57	-29.8	0.046397
hsa-miR-6130	NM_001301830	ABCC4	159	-23.69	-28.4	0.015259
hsa-miR-6130	NM_001304333	PHF24	158	-20.88	-24.4	0.009958
hsa-miR-6130	NM_001304762	EVA1B	155	-21.48	-29.5	0.004416
hsa-miR-6130	NM_001310332	RNF31	152	-22.27	-24.3	0.011206
hsa-miR-6130	NM_002145	HOXB2	157	-26.8	-29.1	0.012905
hsa-miR-6130	NM_002933	RNASE1	151	-22.05	-25.7	0.03447
hsa-miR-6130	NM_002962	S100A5	157	-20.17	-23.8	0.044732
hsa-miR-6130	NM_004443	EPHB3	158	-27.25	-31.3	0.012232
hsa-miR-6130	NM_004980	KCND3	158	-21.97	-25.6	0.037344
hsa-miR-6130	NM_005451	PDLIM7	159	-21.45	-25.4	0.043447
hsa-miR-6130	NM_005627	SGK1	155	-29.31	-31.7	0.015358
hsa-miR-6130	NM_005720	ARPC1B	153	-23.24	-28.1	0.012059
hsa-miR-6130	NM_006049	SNAPC5	156	-26.04	-30.2	0.006969
hsa-miR-6130	NM_006522	WNT6	159	-22.9	-27.2	0.033174
hsa-miR-6130	NM_006688	C1QL1	161	-25.07	-28.1	0.030632
hsa-miR-6130	NM_007046	EMILIN1	160	-24.98	-27.9	0.021994

hsa-miR-6130	NM_013334	GMPPB	159	-25.47	-29.3	0.005387
hsa-miR-6130	NM_013389	NPC1L1	158	-24.24	-29	0.049981
hsa-miR-6130	NM_014596	ZNRD1	167	-26.18	-29.9	0.00218
hsa-miR-6130	NM_016437	TUBG2	151	-19.86	-24.9	0.041417
hsa-miR-6130	NM_016459	MZB1	152	-20	-24.7	0.043065
hsa-miR-6130	NM_017912	HERC6	155	-25	-28.5	0.037271
hsa-miR-6130	NM_017984	ZCWPW1	152	-19.31	-27.4	0.004254
hsa-miR-6130	NM_017999	RNF31	152	-22.27	-24.3	0.011206
hsa-miR-6130	NM_018166	EVA1B	155	-21.48	-29.5	0.004416
hsa-miR-6130	NM_018223	CHFR	156	-26.15	-31.1	0.026722
hsa-miR-6130	NM_021971	GMPPB	159	-25.47	-29.3	0.005387
hsa-miR-6130	NM_022367	SEMA4A	158	-21.57	-29.9	0.023573
hsa-miR-6130	NM_024954	UBTD1	151	-23.85	-28.9	0.034108
hsa-miR-6130	NM_152781	HEATR9	152	-18.48	-23.1	0.037078
hsa-miR-6130	NM_153247	SLC29A4	155	-26.57	-29.8	0.046397
hsa-miR-6130	NM_170783	ZNRD1	167	-26.18	-29.9	0.00218
hsa-miR-6130	NM_172198	KCND3	158	-21.97	-25.6	0.037344
hsa-miR-6130	NM_172369	C1QC	151	-23.82	-27.1	0.026247
hsa-miR-6130	NM_181900	STARD5	152	-27.59	-28.1	0.040711
hsa-miR-6130	NM_182619	CLEC18A	164	-30.24	-33.9	0.002808
hsa-miR-6130	NM_198232	RNASE1	151	-22.05	-25.7	0.03447
hsa-miR-6130	NM_198234	RNASE1	151	-22.05	-25.7	0.03447
hsa-miR-6130	NM_198235	RNASE1	151	-22.05	-25.7	0.03447
hsa-miR-6130	NM_203352	PDLIM7	159	-21.45	-25.4	0.043447
hsa-miR-6130	NR_031593	MIR1182	151	-16.73	-23.3	0.02676
hsa-miR-6130	NR_039600	MIR550A3	152	-21.2	-27.4	0.001679
hsa-miR-6130	NR_039679	MIR4469	151	-23.69	-27.7	0.000862
hsa-miR-6130	NR_052852	MARCKSL1	168	-27.35	-31.3	0.02847
hsa-miR-6130	NR_104646	LOC101927592	160	-28.71	-31	0.032904
hsa-miR-6130	NR_120509	ELFN1-AS1	167	-24.1	-28.7	0.048751

hsa-miR-6130	NR_126397	LINC01177	156	-22.98	-28.5	0.028187
hsa-miR-6130	NR_130899	EVA1B	155	-21.48	-29.5	0.037276
hsa-miR-6131	NM_000260	MYO7A	160	-26.14	-30.4	0.016542
hsa-miR-6131	NM_000934	SERPINF2	150	-24.94	-29.5	0.041863
hsa-miR-6131	NM_001007226	SPOP	152	-27.05	-32.5	0.029061
hsa-miR-6131	NM_001007227	SPOP	152	-27.05	-32.5	0.029061
hsa-miR-6131	NM_001007228	SPOP	152	-27.05	-32.5	0.029061
hsa-miR-6131	NM_001007229	SPOP	152	-27.05	-32.5	0.029061
hsa-miR-6131	NM_001007230	SPOP	152	-27.05	-32.5	0.029061
hsa-miR-6131	NM_001017920	DAPL1	150	-29.87	-35.9	0.000057
hsa-miR-6131	NM_001018100	MYZAP	165	-23.91	-30.5	0.033212
hsa-miR-6131	NM_001040708	HEY1	171	-29.59	-34	0.009932
hsa-miR-6131	NM_001102576	CSAG1	161	-27.84	-31.4	0.003418
hsa-miR-6131	NM_001105519	C2orf70	150	-24.06	-30.3	0.000375
hsa-miR-6131	NM_001122646	FAM178B	158	-27.78	-30.1	0.010967
hsa-miR-6131	NM_001127180	MYO7A	160	-26.14	-30.4	0.016542
hsa-miR-6131	NM_001129826	CSAG3	161	-27.84	-31.4	0.003418
hsa-miR-6131	NM_001129828	CSAG3	161	-27.84	-31.4	0.003418
hsa-miR-6131	NM_001135031	GFI1B	156	-22.37	-36.2	0.001147
hsa-miR-6131	NM_001135917	DOLPP1	162	-27.11	-32.4	0.02857
hsa-miR-6131	NM_001136017	CCND3	168	-30.14	-33.7	0.008869
hsa-miR-6131	NM_001136125	CCND3	168	-30.14	-33.7	0.008869
hsa-miR-6131	NM_001136126	CCND3	168	-30.14	-33.7	0.008869
hsa-miR-6131	NM_001142503	STARD8	150	-30.18	-37.2	0.002971
hsa-miR-6131	NM_001142504	STARD8	150	-30.18	-37.2	0.002971
hsa-miR-6131	NM_001142674	CHID1	153	-29.67	-33.3	0.030699
hsa-miR-6131	NM_001142675	CHID1	153	-29.67	-33.3	0.030699
hsa-miR-6131	NM_001142676	CHID1	153	-29.67	-33.3	0.030699
hsa-miR-6131	NM_001142677	CHID1	153	-29.67	-33.3	0.030699
hsa-miR-6131	NM_001145106	FIBCD1	154	-27.55	-33.2	0.023835

hsa-miR-6131	NM_001152	SLC25A5	151	-24.91	-28.1	0.024586
hsa-miR-6131	NM_001159651	BOD1	157	-31.59	-36.4	0.002878
hsa-miR-6131	NM_001160042	IQCC	156	-24.4	-28.6	0.04905
hsa-miR-6131	NM_001165920	SERPINF2	150	-24.94	-29.5	0.041863
hsa-miR-6131	NM_001165921	SERPINF2	150	-24.94	-29.5	0.041863
hsa-miR-6131	NM_001171	ABCC6	154	-27.56	-28.7	0.042285
hsa-miR-6131	NM_001172667	FAM178B	158	-27.78	-30.1	0.010967
hsa-miR-6131	NM_001172684	CELF6	163	-29.16	-34	0.016761
hsa-miR-6131	NM_001172685	CELF6	163	-29.16	-34	0.016761
hsa-miR-6131	NM_001184783	VDAC2	164	-26.89	-30	0.015169
hsa-miR-6131	NM_001184823	VDAC2	164	-26.89	-30	0.015169
hsa-miR-6131	NM_001198557	LMNB1	150	-25.35	-30.4	0.028251
hsa-miR-6131	NM_001204871	URGCP-MRPS24	160	-26.08	-32.2	0.004534
hsa-miR-6131	NM_001242659	C1orf233	152	-27.96	-31.7	0.035429
hsa-miR-6131	NM_001243146	NVL	165	-35.97	-39.5	0.000033
hsa-miR-6131	NM_001243147	NVL	165	-35.97	-39.5	0.000033
hsa-miR-6131	NM_001243281	ALCAM	151	-24.28	-28.5	0.048464
hsa-miR-6131	NM_001252597	CLPS	153	-23.66	-26.3	0.018094
hsa-miR-6131	NM_001252598	CLPS	153	-23.66	-26.3	0.018094
hsa-miR-6131	NM_001258210	TSKU	157	-23.69	-32	0.035974
hsa-miR-6131	NM_001281433	ZNHIT3	153	-24.07	-30.8	0.016205
hsa-miR-6131	NM_001281434	ZNHIT3	153	-24.07	-30.8	0.008361
hsa-miR-6131	NM_001282851	HEY1	171	-29.59	-34	0.009932
hsa-miR-6131	NM_001284501	NUBP2	153	-25.43	-28.2	0.041428
hsa-miR-6131	NM_001286990	SLC35D2	153	-25.44	-29.9	0.020822
hsa-miR-6131	NM_001287427	CCND3	168	-30.14	-33.7	0.008869
hsa-miR-6131	NM_001287434	CCND3	168	-30.14	-33.7	0.008869
hsa-miR-6131	NM_001289160	MICB	159	-29.88	-33.7	0.012047
hsa-miR-6131	NM_001289161	MICB	159	-29.88	-33.7	0.012047

hsa-miR-6131	NM_001300763	CYB561A3	157	-23.38	-29.6	0.046463
hsa-miR-6131	NM_001300934	NPSR1	151	-27.81	-31.6	0.003323
hsa-miR-6131	NM_001300935	NPSR1	151	-27.81	-31.6	0.004249
hsa-miR-6131	NM_001304359	MUC5AC	150	-27.08	-29.9	0.014794
hsa-miR-6131	NM_001308025	TICRR	151	-25.88	-29.9	0.049877
hsa-miR-6131	NM_001308213	ZNF346	165	-27.95	-33.9	0.020269
hsa-miR-6131	NM_001308219	ZNF346	165	-27.95	-33.9	0.018216
hsa-miR-6131	NM_001308223	ZNF346	165	-27.95	-33.9	0.020269
hsa-miR-6131	NM_001308370	ESPNL	165	-28.92	-31.6	0.046556
hsa-miR-6131	NM_001760	CCND3	168	-30.14	-33.7	0.008869
hsa-miR-6131	NM_001832	CLPS	153	-23.66	-26.3	0.018094
hsa-miR-6131	NM_002533	NVL	165	-35.97	-39.5	0.000033
hsa-miR-6131	NM_003375	VDAC2	164	-26.89	-30	0.015169
hsa-miR-6131	NM_003563	SPOP	152	-27.05	-32.5	0.029061
hsa-miR-6131	NM_004074	COX8A	157	-26.97	-26.7	0.025543
hsa-miR-6131	NM_004188	GFI1B	156	-22.37	-36.2	0.001112
hsa-miR-6131	NM_004418	DUSP2	152	-27.72	-33	0.005988
hsa-miR-6131	NM_004564	GATB	160	-23.02	-32.3	0.005636
hsa-miR-6131	NM_004637	RAB7A	153	-27.82	-31.9	0.034032
hsa-miR-6131	NM_004773	ZNHIT3	153	-24.07	-30.8	0.008361
hsa-miR-6131	NM_005573	LMNB1	150	-25.35	-30.4	0.028251
hsa-miR-6131	NM_005686	SOX13	155	-24.28	-31.6	0.048831
hsa-miR-6131	NM_005697	SCAMP2	151	-23.07	-28.1	0.011878
hsa-miR-6131	NM_005931	MICB	159	-29.88	-33.7	0.012047
hsa-miR-6131	NM_006274	CCL19	152	-26.13	-30	0.004826
hsa-miR-6131	NM_006946	SPTBN2	161	-28.22	-31.3	0.013049
hsa-miR-6131	NM_007001	SLC35D2	153	-25.44	-29.9	0.020822
hsa-miR-6131	NM_007100	ATP5I	153	-23.21	-26.3	0.002267
hsa-miR-6131	NM_012225	NUBP2	153	-25.43	-28.2	0.041428
hsa-miR-6131	NM_012258	HEY1	171	-29.59	-34	0.009932

hsa-miR-6131	NM_014228	SLC6A7	154	-29.03	-32.2	0.032187
hsa-miR-6131	NM_014725	STARD8	150	-30.18	-37.2	0.002971
hsa-miR-6131	NM_015117	ZC3H3	158	-22.78	-27.7	0.040625
hsa-miR-6131	NM_015516	TSKU	157	-23.69	-32	0.035974
hsa-miR-6131	NM_015722	CALY	155	-21.77	-29.6	0.002773
hsa-miR-6131	NM_016240	SCARA3	152	-27.74	-31.7	0.049099
hsa-miR-6131	NM_016490	FAM178B	158	-27.78	-30.1	0.010967
hsa-miR-6131	NM_016580	PCDH12	165	-29.11	-32.3	0.041454
hsa-miR-6131	NM_018134	IQCC	156	-24.4	-28.6	0.04905
hsa-miR-6131	NM_018965	TREM2	151	-25.29	-29.8	0.005802
hsa-miR-6131	NM_020376	PNPLA2	156	-26.34	-30.2	0.028196
hsa-miR-6131	NM_020438	DOLPP1	162	-27.11	-32.4	0.02857
hsa-miR-6131	NM_023947	CHID1	153	-29.67	-33.3	0.030699
hsa-miR-6131	NM_024508	ZBED2	163	-25.05	-29.3	0.038118
hsa-miR-6131	NM_024556	FAM118B	151	-29.8	-34	0.00576
hsa-miR-6131	NM_024712	ELMO3	150	-21.82	-25.2	0.028239
hsa-miR-6131	NM_024874	KIAA0319L	160	-31.8	-34.5	0.010392
hsa-miR-6131	NM_025092	ATHL1	157	-28.24	-32.5	0.014212
hsa-miR-6131	NM_025217	ULBP2	157	-27.49	-33.3	0.003515
hsa-miR-6131	NM_032843	FIBCD1	154	-27.55	-33.2	0.023835
hsa-miR-6131	NM_052840	CELF6	163	-29.16	-34	0.016761
hsa-miR-6131	NM_138369	BOD1	157	-31.59	-36.4	0.00301
hsa-miR-6131	NM_138370	PKDCC	159	-24.73	-33.8	0.006218
hsa-miR-6131	NM_145910	NEK11	160	-25.92	-30	0.009666
hsa-miR-6131	NM_152259	TICRR	151	-25.88	-29.9	0.049877
hsa-miR-6131	NM_152451	MYZAP	165	-23.91	-30.5	0.033212
hsa-miR-6131	NM_153453	VGLL2	173	-31.81	-35.8	0.003555
hsa-miR-6131	NM_153478	CSAG1	161	-27.84	-31.4	0.003418
hsa-miR-6131	NM_172212	CSF1	160	-21.36	-30.5	0.023451
hsa-miR-6131	NM_182645	VGLL2	173	-31.81	-35.8	0.003555

hsa-miR-6131	NM_182981	OSGIN1	152	-21.23	-28.9	0.014828
hsa-miR-6131	NM_194312	ESPNL	165	-28.92	-31.6	0.046556
hsa-miR-6131	NM_206840	NVL	165	-35.97	-39.5	0.000033
hsa-miR-6131	NM_207172	NPSR1	151	-27.81	-31.6	0.003323
hsa-miR-6131	NR_027071	BAALC-AS2	152	-26.41	-31.6	0.00751
hsa-miR-6131	NR_027398	GFOD2	157	-29.64	-34.3	0.015759
hsa-miR-6131	NR_027921	CCL15-CCL14	153	-25.82	-32.3	0.030946
hsa-miR-6131	NR_027922	CCL15-CCL14	153	-25.82	-32.3	0.029468
hsa-miR-6131	NR_036222	MIR4261	151	-20.4	-24.2	0.007454
hsa-miR-6131	NR_126421	C10orf25	168	-28.99	-34.8	0.00422
hsa-miR-6131	NR_130638	CSAG3	161	-27.84	-31.4	0.018419
hsa-miR-548ay-	NR_030305	MIR579	163	-21.14	-24.9	0.0056
hsa-miR-548ay-	NR_030315	MIR548B	163	-24.79	-28.3	0.000509
hsa-miR-548ay-	NR_030330	MIR548A3	173	-19.46	-23.4	0.015531
hsa-miR-548ay-	NR_031642	MIR548F1	183	-22.34	-22.2	0.026507
hsa-miR-548ay-	NR_031643	MIR548F2	183	-22.79	-24.7	0.006436
hsa-miR-548ay-	NR_031644	MIR548F3	183	-22.79	-24.7	0.004901
hsa-miR-548ay-	NR_031645	MIR548F4	183	-22.34	-22.2	0.041599
hsa-miR-548ay-	NR_031666	MIR548N	183	-24.49	-28.5	0.000221
hsa-miR-548ay-	NR_031669	MIR548O	178	-18.92	-23.6	0.018986
hsa-miR-548ay-	NR_031677	MIR548H1	179	-18.01	-23.4	0.017274
hsa-miR-548ay-	NR_031678	MIR548H2	187	-25.62	-29.4	0.000181
hsa-miR-548ay-	NR_031679	MIR548H3	187	-24.18	-29.8	0.000301
hsa-miR-548ay-	NR_031680	MIR548H4	191	-28.42	-30	0.000223
hsa-miR-548ay-	NR_031686	MIR548P	183	-24.39	-29.8	0.00012
hsa-miR-548ay-	NR_031687	MIR548I1	163	-17.6	-23	0.047628
hsa-miR-548ay-	NR_031688	MIR548I2	163	-17.6	-23	0.047628
hsa-miR-548ay-	NR_031689	MIR548I3	163	-17.6	-23	0.047628
hsa-miR-548ay-	NR_031752	MIR548Q	179	-19.64	-25	0.005469
hsa-miR-548ay-	NR_036103	MIR548V	183	-25.13	-27.9	0.000406

hsa-miR-548ay-	NR_037503	MIR548Y	183	-25.48	-26.7	0.002108
hsa-miR-548ay-	NR_037515	MIR548Z	191	-28.87	-32.5	0.000027
hsa-miR-548ay-	NR_039621	MIR548AC	182	-25.16	-28.7	0.000297
hsa-miR-548ay-	NR_039639	MIR548AE2	187	-22.93	-28.4	0.000172
hsa-miR-548ay-	NR_039673	MIR548AJ1	187	-22.25	-27.6	0.000379
hsa-miR-548ay-	NR_039674	MIR548AJ2	187	-22.25	-27.6	0.000726
hsa-miR-548ay-	NR_039692	MIR548X2	187	-21	-26.4	0.002067
hsa-miR-548ay-	NR_039699	MIR548AK	187	-23.83	-28.3	0.000115
hsa-miR-548ay-	NR_039762	MIR548AM	183	-24.39	-29.6	0.000096
hsa-miR-548ay-	NR_049838	MIR548AQ	187	-24.18	-29.6	0.000046
hsa-miR-548ay-	NR_049865	MIR548AX	187	-26.46	-27.6	0.000393
hsa-miR-548ay-	NR_110050	LOC101927418	182	-23.6	-26.8	0.028693
hsa-miR-548ay-	NR_128708	MIR548BB	187	-26.12	-28.3	0.000178
hsa-miR-6501-5p	NM_000761	CYP1A2	170	-32.14	-34.9	0.02021
hsa-miR-6501-5p	NM_001005339	RGS10	151	-23.46	-27.8	0.044446
hsa-miR-6501-5p	NM_001008223	C1QL4	150	-27.65	-33.4	0.010361
hsa-miR-6501-5p	NM_001010938	TNK2	152	-31.39	-35.1	0.007013
hsa-miR-6501-5p	NM_001012974	LRRC73	150	-29.02	-34.3	0.001346
hsa-miR-6501-5p	NM_001025091	ABCF1	159	-25.92	-31.7	0.035154
hsa-miR-6501-5p	NM_001040666	STEAP2	157	-31.35	-35.4	0.005348
hsa-miR-6501-5p	NM_001078172	FAM127B	153	-20.62	-32.3	0.026789
hsa-miR-6501-5p	NM_001080515	FAM163B	156	-27.29	-31.8	0.02063
hsa-miR-6501-5p	NM_001090	ABCF1	159	-25.92	-31.7	0.035154
hsa-miR-6501-5p	NM_001097577	ANG	154	-21.51	-26.8	0.0337
hsa-miR-6501-5p	NM_001102658	CT62	155	-26.97	-31.8	0.038211
hsa-miR-6501-5p	NM_001130040	SHC1	156	-29.23	-32.9	0.047948
hsa-miR-6501-5p	NM_001130041	SHC1	156	-29.23	-32.9	0.047948
hsa-miR-6501-5p	NM_001134321	FAM127B	153	-20.62	-32.3	0.007097
hsa-miR-6501-5p	NM_001135110	DNAJA3	165	-28.4	-37.2	0.005108
hsa-miR-6501-5p	NM_001135111	KCNK17	152	-26.58	-32.4	0.022158

hsa-miR-6501-5p	NM_001135211	FKBP6	156	-29.89	-34.5	0.003385
hsa-miR-6501-5p	NM_001135685	LTK	150	-27.26	-33.9	0.002797
hsa-miR-6501-5p	NM_001142805	SLC6A8	156	-30.09	-33.9	0.027411
hsa-miR-6501-5p	NM_001142806	SLC6A8	156	-30.09	-33.9	0.027411
hsa-miR-6501-5p	NM_001142853	HES6	161	-31.18	-36.9	0.001508
hsa-miR-6501-5p	NM_001144913	FGFR2	150	-25.26	-29.9	0.018105
hsa-miR-6501-5p	NM_001144919	FGFR2	150	-25.26	-29.9	0.018105
hsa-miR-6501-5p	NM_001145	ANG	154	-21.51	-26.8	0.0337
hsa-miR-6501-5p	NM_001146008	SLCO5A1	163	-27.5	-34	0.015058
hsa-miR-6501-5p	NM_001162900	TMEM217	152	-27.88	-31.9	0.029128
hsa-miR-6501-5p	NM_001163692	UBAP1L	176	-26.75	-30.4	0.001093
hsa-miR-6501-5p	NM_001166598	APOA5	156	-29.48	-35.2	0.005905
hsa-miR-6501-5p	NM_001190233	DNAJC19	163	-27.03	-31.4	0.047523
hsa-miR-6501-5p	NM_001190720	IKBKB	155	-30.52	-34.5	0.027856
hsa-miR-6501-5p	NM_001202859	SHC1	156	-29.23	-32.9	0.047948
hsa-miR-6501-5p	NM_001242778	IKBKB	155	-30.52	-34.5	0.027856
hsa-miR-6501-5p	NM_001242	CD27	151	-21.52	-30.8	0.011828
hsa-miR-6501-5p	NM_001244666	STX5	164	-30.52	-35.4	0.005263
hsa-miR-6501-5p	NM_001244945	STEAP2	157	-31.35	-35.4	0.005348
hsa-miR-6501-5p	NM_001244946	STEAP2	157	-31.35	-35.4	0.004832
hsa-miR-6501-5p	NM_001248006	TRIM3	150	-23.99	-30.9	0.017119
hsa-miR-6501-5p	NM_001248007	TRIM3	150	-23.99	-30.9	0.017119
hsa-miR-6501-5p	NM_001253750	CLECL1	160	-30.32	-34.3	0.013271
hsa-miR-6501-5p	NM_001267701	CLECL1	160	-30.32	-34.3	0.011933
hsa-miR-6501-5p	NM_001271068	MAX	156	-29.6	-36.1	0.001409
hsa-miR-6501-5p	NM_001271882	LRRC73	150	-29.02	-34.3	0.001346
hsa-miR-6501-5p	NM_001271933	CEP164	150	-30.07	-36.3	0.006581
hsa-miR-6501-5p	NM_001277423	TCP11X2	158	-19.55	-27.4	0.028312
hsa-miR-6501-5p	NM_001278719	SV2A	156	-33.4	-34.4	0.029577
hsa-miR-6501-5p	NM_001281304	FKBP6	156	-29.89	-34.5	0.003385

hsa-miR-6501-5p	NM_001282434	HES6	161	-31.18	-36.9	0.003279
hsa-miR-6501-5p	NM_001283	AP1S1	150	-27.06	-31.6	0.029024
hsa-miR-6501-5p	NM_001286516	DNAJA3	165	-28.4	-37.2	0.005108
hsa-miR-6501-5p	NM_001286823	EXD3	161	-19.52	-25.2	0.048169
hsa-miR-6501-5p	NM_001297570	IL31RA	161	-28.41	-30.2	0.030866
hsa-miR-6501-5p	NM_001297572	IL31RA	161	-28.41	-30.2	0.030866
hsa-miR-6501-5p	NM_001300844	FOSL1	154	-32.47	-35.6	0.004799
hsa-miR-6501-5p	NM_001300855	FOSL1	154	-32.47	-35.6	0.009781
hsa-miR-6501-5p	NM_001300856	FOSL1	154	-32.47	-35.6	0.004799
hsa-miR-6501-5p	NM_001300857	FOSL1	154	-32.47	-35.6	0.004799
hsa-miR-6501-5p	NM_001308046	TNK2	152	-31.39	-35.1	0.007027
hsa-miR-6501-5p	NM_001347	DGKQ	160	-31.53	-36	0.015344
hsa-miR-6501-5p	NM_001556	IKBKB	155	-30.52	-34.5	0.027856
hsa-miR-6501-5p	NM_002344	LTK	150	-27.26	-33.9	0.002797
hsa-miR-6501-5p	NM_002925	RGS10	151	-23.46	-27.8	0.044446
hsa-miR-6501-5p	NM_003029	SHC1	156	-29.23	-32.9	0.047948
hsa-miR-6501-5p	NM_003164	STX5	164	-30.52	-35.4	0.003654
hsa-miR-6501-5p	NM_003602	FKBP6	156	-29.89	-34.5	0.003385
hsa-miR-6501-5p	NM_004503	HOXC6	152	-24.99	-33.4	0.016906
hsa-miR-6501-5p	NM_004854	CHST10	161	-25.75	-34.6	0.020261
hsa-miR-6501-5p	NM_005140	CNGA2	153	-29.41	-33.6	0.021411
hsa-miR-6501-5p	NM_005147	DNAJA3	165	-28.4	-37.2	0.005361
hsa-miR-6501-5p	NM_005438	FOSL1	154	-32.47	-35.6	0.004799
hsa-miR-6501-5p	NM_005629	SLC6A8	156	-30.09	-33.9	0.027411
hsa-miR-6501-5p	NM_005686	SOX13	150	-28.76	-33.7	0.03722
hsa-miR-6501-5p	NM_005731	ARPC2	162	-24.54	-29.3	0.040643
hsa-miR-6501-5p	NM_005781	TNK2	152	-31.39	-35.1	0.007013
hsa-miR-6501-5p	NM_005929	MFI2	168	-35.65	-40.4	0.002037
hsa-miR-6501-5p	NM_006026	H1FX	162	-24.63	-30	0.035046
hsa-miR-6501-5p	NM_006244	PPP2R5B	176	-27.03	-33.7	0.0089

hsa-miR-6501-5p	NM_006382	CDRT1	158	-26.71	-29.2	0.024992
hsa-miR-6501-5p	NM_006458	TRIM3	150	-23.99	-30.9	0.017119
hsa-miR-6501-5p	NM_006762	LAPTM5	150	-24.21	-32.8	0.043548
hsa-miR-6501-5p	NM_007274	ACOT7	163	-24.18	-31.2	0.009569
hsa-miR-6501-5p	NM_012369	OR2F1	160	-23.53	-27.5	0.005993
hsa-miR-6501-5p	NM_013975	LIG3	151	-25.71	-31.9	0.017399
hsa-miR-6501-5p	NM_014798	PLEKHM1	164	-32.48	-34.6	0.033222
hsa-miR-6501-5p	NM_014849	SV2A	156	-33.4	-34.4	0.029577
hsa-miR-6501-5p	NM_014956	CEP164	150	-30.07	-36.3	0.006581
hsa-miR-6501-5p	NM_016240	SCARA3	170	-23.73	-35.8	0.015788
hsa-miR-6501-5p	NM_016829	OGG1	151	-24.57	-32.2	0.031098
hsa-miR-6501-5p	NM_016930	STX18	158	-30.23	-38.6	0.002189
hsa-miR-6501-5p	NM_017774	CDKAL1	170	-29.35	-34.2	0.023636
hsa-miR-6501-5p	NM_018645	HES6	161	-31.18	-36.9	0.001508
hsa-miR-6501-5p	NM_021080	DAB1	158	-20.11	-32.5	0.002648
hsa-miR-6501-5p	NM_022144	TNMD	154	-17.52	-32.3	0.001412
hsa-miR-6501-5p	NM_024060	AHNAK	172	-34.02	-39.5	0.000146
hsa-miR-6501-5p	NM_024768	EFCC1	151	-30.18	-35.1	0.008163
hsa-miR-6501-5p	NM_030661	HOXA3	160	-28.6	-36.1	0.014555
hsa-miR-6501-5p	NM_031460	KCNK17	152	-26.58	-32.4	0.008447
hsa-miR-6501-5p	NM_032289	PSD2	151	-29.96	-35.3	0.025219
hsa-miR-6501-5p	NM_033278	TRIM3	150	-23.99	-30.9	0.017119
hsa-miR-6501-5p	NM_052968	APOA5	156	-29.48	-35.2	0.005905
hsa-miR-6501-5p	NM_080601	PTPN11	155	-25.95	-35.2	0.000951
hsa-miR-6501-5p	NM_138458	WDR92	154	-27.52	-32.6	0.03469
hsa-miR-6501-5p	NM_144601	CMTM3	160	-26.64	-35.7	0.010702
hsa-miR-6501-5p	NM_145114	MAX	156	-29.6	-36.1	0.001409
hsa-miR-6501-5p	NM_145261	DNAJC19	163	-27.03	-31.4	0.047523
hsa-miR-6501-5p	NM_148920	PIGQ	157	-27.56	-32.2	0.01241
hsa-miR-6501-5p	NM_152347	EFCAB13	156	-26.17	-29.9	0.049776

hsa-miR-6501-5p	NM_152862	ARPC2	162	-24.54	-29.3	0.040643
hsa-miR-6501-5p	NM_153228	ANKFN1	159	-20.83	-24.7	0.037897
hsa-miR-6501-5p	NM_153631	HOXA3	160	-28.6	-36.1	0.014555
hsa-miR-6501-5p	NM_153693	HOXC6	152	-24.99	-33.4	0.016906
hsa-miR-6501-5p	NM_175882	SPPL2C	168	-20.87	-25.7	0.026246
hsa-miR-6501-5p	NM_177964	LYPD6B	164	-27.25	-30.2	0.037877
hsa-miR-6501-5p	NM_178422	PAQR7	176	-35.84	-40.1	0.001606
hsa-miR-6501-5p	NM_181553	CMTM3	160	-26.64	-35.7	0.010702
hsa-miR-6501-5p	NM_181724	TMEM119	178	-32.73	-36.8	0.010374
hsa-miR-6501-5p	NM_181864	ACOT7	163	-24.18	-31.2	0.009569
hsa-miR-6501-5p	NM_181865	ACOT7	163	-24.18	-31.2	0.009569
hsa-miR-6501-5p	NM_181866	ACOT7	163	-24.18	-31.2	0.009569
hsa-miR-6501-5p	NM_183001	SHC1	156	-29.23	-32.9	0.047948
hsa-miR-6501-5p	NM_198488	FAM83H	154	-19.41	-36.9	0.012725
hsa-miR-6501-5p	NM_206918	DEGS2	164	-22.62	-29.1	0.031133
hsa-miR-6501-5p	NM_206961	LTK	150	-27.26	-33.9	0.002797
hsa-miR-6501-5p	NR_002787	LOC154449	151	-23.95	-34	0.024883
hsa-miR-6501-5p	NR_024393	CASC8	167	-28.06	-34.1	0.025978
hsa-miR-6501-5p	NR_024440	LOC729609	169	-26.44	-31.8	0.021784
hsa-miR-6501-5p	NR_024547	TMEM11	161	-24.81	-34.3	0.030725
hsa-miR-6501-5p	NR_026737	C6orf52	159	-24.2	-30.3	0.035181
hsa-miR-6501-5p	NR_026860	LINC00473	159	-30.25	-33.6	0.046047
hsa-miR-6501-5p	NR_026861	LINC00473	159	-30.25	-33.6	0.023157
hsa-miR-6501-5p	NR_027320	CSNK1A1P1	152	-28.35	-35.5	0.018318
hsa-miR-6501-5p	NR_030330	MIR548A3	152	-23.59	-28.1	0.004804
hsa-miR-6501-5p	NR_033774	BASP1P1	156	-36.75	-40.2	0.001214
hsa-miR-6501-5p	NR_037652	ST20	152	-28.39	-34.8	0.005404
hsa-miR-6501-5p	NR_037653	ST20	152	-28.39	-34.8	0.005729
hsa-miR-6501-5p	NR_037869	LOC653160	160	-32.88	-43.3	0.00074
hsa-miR-6501-5p	NR_038270	SHARPIN	150	-30.76	-36.8	0.010365

hsa-miR-6501-5p	NR_047686	PCCA-AS1	167	-21.51	-30.8	0.021605
hsa-miR-6501-5p	NR_073138	MAX	156	-29.6	-36.1	0.004765
hsa-miR-6501-5p	NR_073450	PFKFB1	159	-36.37	-38.4	0.005046
hsa-miR-6501-5p	NR_103508	PHYKPL	162	-28.63	-35.8	0.015998
hsa-miR-6501-5p	NR_103772	LOC100288162	151	-26.56	-33	0.028473
hsa-miR-6501-5p	NR_104634	LOC100506159	151	-28.8	-35	0.009911
hsa-miR-6501-5p	NR_105052	LOC101929696	166	-28.68	-34.7	0.006547
hsa-miR-6501-5p	NR_109920	TMEM9	175	-30.15	-35.3	0.022197
hsa-miR-6501-5p	NR_110608	FILIP1	161	-24.51	-32.7	0.027011
hsa-miR-6501-5p	NR_110861	RARA-AS1	153	-34.13	-38.9	0.004569
hsa-miR-6501-5p	NR_120428	LINC00463	150	-16.73	-35.7	0.00833
hsa-miR-6501-5p	NR_120528	LOC100507144	154	-26.84	-32.6	0.035524
hsa-miR-6501-5p	NR_121682	LINC01465	155	-15.94	-33.9	0.036377
hsa-miR-6501-5p	NR_125339	FOSL1	154	-32.47	-35.6	0.013305
hsa-miR-6501-5p	NR_126003	LINC01032	150	-28.88	-31.7	0.012438
hsa-miR-6501-5p	NR_126359	NRIR	158	-25.88	-31.1	0.046399
hsa-miR-6501-5p	NR_130751	TIMM9	159	-25.77	-31.5	0.047603
hsa-miR-6501-5p	NR_131250	RHOXF1P1	155	-31.02	-38.7	0.00119
hsa-miR-6501-5p	NR_131967	LVCAT8	152	-27.1	-33.4	0.010553
hsa-miR-6501-5p	NR_132337	DANT2	150	-23.86	-28.4	0.042513
hsa-miR-6501-5p	NR_132980	SNORD141A	151	-18.2	-26.5	0.014713
hsa-miR-6501-5p	NR_132981	SNORD141B	151	-18.2	-26.5	0.014713
hsa-miR-6511a-	NM_000182	HADHA	167	-34.81	-41.4	0.000239
hsa-miR-6511a-	NM_000250	MPO	169	-34.22	-35.7	0.005801
hsa-miR-6511a-	NM_000387	SLC25A20	169	-29.86	-34.5	0.009862
hsa-miR-6511a-	NM_001007255	KLHDC9	150	-27.02	-30.3	0.01715
hsa-miR-6511a-	NM_001014445	NLE1	156	-30.48	-33.9	0.02236
hsa-miR-6511a-	NM_001014839	NCDN	155	-31.49	-35	0.013692
hsa-miR-6511a-	NM_001014841	NCDN	155	-31.49	-35	0.013692
hsa-miR-6511a-	NM_001080395	AATK	162	-40.31	-40	0.001421

hsa-miR-6511a-	NM_001098199	GPR1	176	-30.02	-34.9	0.009549
hsa-miR-6511a-	NM_001103150	PNMA5	154	-31.13	-34.5	0.02472
hsa-miR-6511a-	NM_001103151	PNMA5	154	-31.13	-34.5	0.02472
hsa-miR-6511a-	NM_001111125	IQSEC2	157	-30.54	-33.4	0.036533
hsa-miR-6511a-	NM_001127350	MED18	169	-30.37	-33.1	0.027113
hsa-miR-6511a-	NM_001128850	RRAD	157	-30.21	-29.3	0.039292
hsa-miR-6511a-	NM_001130997	FAM58A	151	-29.24	-31.7	0.014245
hsa-miR-6511a-	NM_001134233	C1orf204	170	-33.27	-35.9	0.017151
hsa-miR-6511a-	NM_001134878	KIF9	161	-27.33	-32.7	0.015797
hsa-miR-6511a-	NM_001139456	SVOPL	151	-31.39	-37.2	0.000496
hsa-miR-6511a-	NM_001145073	USP27X	151	-35.99	-38.9	0.001754
hsa-miR-6511a-	NM_001145638	GPSM1	163	-34.19	-36.6	0.00942
hsa-miR-6511a-	NM_001145639	GPSM1	163	-34.19	-36.6	0.00942
hsa-miR-6511a-	NM_001146029	SEMA7A	165	-29.86	-34	0.027885
hsa-miR-6511a-	NM_001146030	SEMA7A	165	-29.86	-34	0.027885
hsa-miR-6511a-	NM_001172684	CELF6	166	-33.27	-36	0.01587
hsa-miR-6511a-	NM_001172685	CELF6	166	-33.27	-36	0.01587
hsa-miR-6511a-	NM_001178003	WDR66	154	-29.34	-35.2	0.002694
hsa-miR-6511a-	NM_001184866	FCRLA	150	-28.68	-34	0.017373
hsa-miR-6511a-	NM_001184867	FCRLA	150	-28.68	-34	0.017373
hsa-miR-6511a-	NM_001184870	FCRLA	150	-28.68	-34	0.017373
hsa-miR-6511a-	NM_001184871	FCRLA	150	-28.68	-34	0.017373
hsa-miR-6511a-	NM_001184872	FCRLA	150	-28.68	-34	0.017373
hsa-miR-6511a-	NM_001184873	FCRLA	150	-28.68	-34	0.017373
hsa-miR-6511a-	NM_001184924	PNMA5	154	-31.13	-34.5	0.02472
hsa-miR-6511a-	NM_001200003	GPSM1	163	-34.19	-36.6	0.00942
hsa-miR-6511a-	NM_001242628	GFOD1	167	-27.37	-33	0.045489
hsa-miR-6511a-	NM_001242630	GFOD1	167	-27.37	-33	0.045489
hsa-miR-6511a-	NM_001243211	IL18	172	-28.09	-33.1	0.004563
hsa-miR-6511a-	NM_001261452	GPR1	176	-30.02	-34.9	0.009549

hsa-miR-6511a-	NM_001261453	GPR1	176	-30.02	-34.9	0.009549
hsa-miR-6511a-	NM_001261454	GPR1	176	-30.02	-34.9	0.009549
hsa-miR-6511a-	NM_001261455	GPR1	176	-30.02	-34.9	0.009549
hsa-miR-6511a-	NM_001261	CDK9	156	-27.78	-32.6	0.045758
hsa-miR-6511a-	NM_001267053	RIBC1	179	-39.38	-40.5	0.000092
hsa-miR-6511a-	NM_001282535	PNMA3	168	-33.05	-37.3	0.011479
hsa-miR-6511a-	NM_001282566	ZCCHC17	161	-32.61	-37.7	0.002084
hsa-miR-6511a-	NM_001282567	ZCCHC17	161	-32.61	-37.7	0.002084
hsa-miR-6511a-	NM_001282568	ZCCHC17	161	-32.61	-37.7	0.002084
hsa-miR-6511a-	NM_001282569	ZCCHC17	161	-32.61	-37.7	0.002084
hsa-miR-6511a-	NM_001282570	ZCCHC17	161	-32.61	-37.7	0.002084
hsa-miR-6511a-	NM_001282571	ZCCHC17	161	-32.61	-37.7	0.002084
hsa-miR-6511a-	NM_001282572	ZCCHC17	161	-32.61	-37.7	0.001946
hsa-miR-6511a-	NM_001282573	ZCCHC17	161	-32.61	-37.7	0.001946
hsa-miR-6511a-	NM_001282574	ZCCHC17	161	-32.61	-37.7	0.002812
hsa-miR-6511a-	NM_001297760	ETNK2	170	-35.95	-35.7	0.011287
hsa-miR-6511a-	NM_001297761	ETNK2	170	-35.95	-35.7	0.010102
hsa-miR-6511a-	NM_001297762	ETNK2	170	-35.95	-35.7	0.010102
hsa-miR-6511a-	NM_001301302	OAZ2	152	-27.64	-33.2	0.031137
hsa-miR-6511a-	NM_001301772	GPIHBP1	150	-21.08	-26.5	0.035298
hsa-miR-6511a-	NM_001302489	ACP2	159	-30.12	-31.7	0.037853
hsa-miR-6511a-	NM_001302490	ACP2	159	-30.12	-31.7	0.037853
hsa-miR-6511a-	NM_001302491	ACP2	159	-30.12	-31.7	0.037853
hsa-miR-6511a-	NM_001302492	ACP2	159	-30.12	-31.7	0.037853
hsa-miR-6511a-	NM_001308235	DOK3	152	-30.18	-30.1	0.010384
hsa-miR-6511a-	NM_001308236	DOK3	152	-30.18	-30.1	0.010384
hsa-miR-6511a-	NM_001311160	THY1	154	-35.44	-38.2	0.005113
hsa-miR-6511a-	NM_001311162	THY1	154	-35.44	-38.2	0.005113
hsa-miR-6511a-	NM_001314017	CPNE5	156	-24.74	-30.1	0.012982
hsa-miR-6511a-	NM_001562	IL18	172	-28.09	-33.1	0.004563

hsa-miR-6511a-	NM_001610	ACP2	159	-30.12	-31.7	0.037853
hsa-miR-6511a-	NM_002146	HOXB3	168	-30.65	-35.5	0.016592
hsa-miR-6511a-	NM_002193	INHBB	151	-34.18	-36.8	0.014307
hsa-miR-6511a-	NM_002411	SCGB2A2	160	-23	-26	0.049025
hsa-miR-6511a-	NM_002428	MMP15	159	-35.27	-36.3	0.011582
hsa-miR-6511a-	NM_002537	OAZ2	152	-27.64	-33.2	0.031137
hsa-miR-6511a-	NM_002642	PIGC	152	-28.66	-29.8	0.016129
hsa-miR-6511a-	NM_002799	PSMB7	154	-19.11	-27.8	0.012484
hsa-miR-6511a-	NM_003585	DOC2B	163	-29.2	-32	0.022572
hsa-miR-6511a-	NM_003612	SEMA7A	165	-29.86	-34	0.027885
hsa-miR-6511a-	NM_003673	TCAP	163	-24.84	-39.7	0.000276
hsa-miR-6511a-	NM_003731	SSNA1	156	-35.21	-36.7	0.001283
hsa-miR-6511a-	NM_004165	RRAD	157	-30.21	-29.3	0.039292
hsa-miR-6511a-	NM_004225	MFHAS1	155	-36.22	-40.8	0.001763
hsa-miR-6511a-	NM_005279	GPR1	176	-30.02	-34.9	0.009549
hsa-miR-6511a-	NM_005392	PHF2	157	-28.13	-34.4	0.038832
hsa-miR-6511a-	NM_006039	MRC2	151	-33.37	-36.7	0.006702
hsa-miR-6511a-	NM_006062	SMYD5	162	-31.81	-33.5	0.0327
hsa-miR-6511a-	NM_006288	THY1	154	-35.44	-38.2	0.005113
hsa-miR-6511a-	NM_007255	B4GALT7	162	-32.12	-38.1	0.001291
hsa-miR-6511a-	NM_007260	LYPLA2	176	-32.16	-35.9	0.005365
hsa-miR-6511a-	NM_012091	ADAT1	172	-29.33	-34.3	0.039238
hsa-miR-6511a-	NM_014262	P3H3	150	-27.86	-30.1	0.026023
hsa-miR-6511a-	NM_014284	NCDN	155	-31.49	-35	0.013692
hsa-miR-6511a-	NM_014652	IPO13	150	-33.75	-36.4	0.000823
hsa-miR-6511a-	NM_014718	CLSTN3	155	-34.64	-38.3	0.001942
hsa-miR-6511a-	NM_015024	XPO7	156	-28.22	-33.2	0.04631
hsa-miR-6511a-	NM_015346	ZFYVE26	159	-39.7	-41.1	0.002253
hsa-miR-6511a-	NM_016505	ZCCHC17	161	-32.61	-37.7	0.002084
hsa-miR-6511a-	NM_016520	C9orf78	172	-29	-34.2	0.013471

hsa-miR-6511a-	NM_017638	MED18	169	-30.37	-33.1	0.027113
hsa-miR-6511a-	NM_018056	TMEM39B	164	-34.16	-37.7	0.000115
hsa-miR-6511a-	NM_018096	NLE1	156	-30.48	-33.9	0.02236
hsa-miR-6511a-	NM_018208	ETNK2	170	-35.95	-35.7	0.010102
hsa-miR-6511a-	NM_018988	GFOD1	167	-27.37	-33	0.045489
hsa-miR-6511a-	NM_020703	AMIGO1	153	-29.76	-32.7	0.015021
hsa-miR-6511a-	NM_020814	MARCH4	159	-33	-36.5	0.010567
hsa-miR-6511a-	NM_022342	KIF9	161	-27.33	-32.7	0.015797
hsa-miR-6511a-	NM_022436	ABCG5	174	-30.55	-35.6	0.004173
hsa-miR-6511a-	NM_024335	IRX6	155	-33.22	-35.7	0.002095
hsa-miR-6511a-	NM_024589	ROGDI	161	-27.71	-32.1	0.014438
hsa-miR-6511a-	NM_024637	GAL3ST4	155	-29.15	-30.7	0.046556
hsa-miR-6511a-	NM_024872	DOK3	152	-30.18	-30.1	0.010384
hsa-miR-6511a-	NM_032487	ACTRT3	150	-27.86	-30.4	0.034061
hsa-miR-6511a-	NM_032738	FCRLA	150	-28.68	-34	0.017373
hsa-miR-6511a-	NM_033337	CAV3	154	-34.8	-39.2	0.001407
hsa-miR-6511a-	NM_052840	CELF6	166	-33.27	-36	0.01587
hsa-miR-6511a-	NM_052926	PNMA5	154	-31.13	-34.5	0.02472
hsa-miR-6511a-	NM_138693	KLF14	168	-29.67	-30.6	0.030841
hsa-miR-6511a-	NM_145039	CENPBD1	172	-29.37	-34.2	0.032625
hsa-miR-6511a-	NM_148903	GREB1	176	-30.02	-34.9	0.008015
hsa-miR-6511a-	NM_148920	PIGQ	157	-28.41	-31	0.024949
hsa-miR-6511a-	NM_152274	FAM58A	151	-29.24	-31.7	0.014245
hsa-miR-6511a-	NM_152718	VWCE	158	-27.71	-28.8	0.04866
hsa-miR-6511a-	NM_153200	EDF1	156	-23.59	-27.6	0.030393
hsa-miR-6511a-	NM_153747	PIGC	152	-28.66	-29.8	0.016129
hsa-miR-6511a-	NM_174959	SVOPL	151	-31.39	-37.2	0.000496
hsa-miR-6511a-	NM_178167	ZNF598	163	-30.28	-31.6	0.023573
hsa-miR-6511a-	NM_182902	KIF9	161	-27.33	-32.7	0.015797
hsa-miR-6511a-	NM_207582	ERVFRD-1	160	-28.47	-33.3	0.032698

hsa-miR-6511a-	NR_024464	LINC00426	158	-29.58	-33.3	0.048908
hsa-miR-6511a-	NR_027992	NBEAP1	163	-25.18	-32.6	0.013304
hsa-miR-6511a-	NR_029614	MIR182	150	-23.13	-26.4	0.019171
hsa-miR-6511a-	NR_038361	APTR	183	-45.74	-46.9	0.0002
hsa-miR-6511a-	NR_038368	LINC00273	150	-35.81	-37.3	0.007461
hsa-miR-6511a-	NR_039721	MIR4499	163	-25.89	-29.4	0.001118
hsa-miR-6511a-	NR_103772	LOC100288162	173	-34.48	-34.5	0.016194
hsa-miR-6511a-	NR_104139	LINC01081	171	-37.39	-38.5	0.001349
hsa-miR-6511a-	NR_106924	MIR6864	153	-19	-27.1	0.004827
hsa-miR-6511a-	NR_109951	OSMR-AS1	162	-32.95	-37.8	0.001519
hsa-miR-6511a-	NR_125749	TBX2-AS1	163	-27.2	-31.6	0.043083
hsa-miR-6511a-	NR_125774	LINC01170	151	-31.92	-34.2	0.010181
hsa-miR-6808-3p	NM_000197	HSD17B3	159	-24.77	-28.3	0.008422
hsa-miR-6808-3p	NM_001005920	JMJD8	167	-28.59	-31.9	0.040069
hsa-miR-6808-3p	NM_001079839	OCIAD1	174	-33.65	-37.3	0.002971
hsa-miR-6808-3p	NM_001079840	OCIAD1	174	-33.65	-37.3	0.002971
hsa-miR-6808-3p	NM_001079841	OCIAD1	174	-33.65	-37.3	0.003299
hsa-miR-6808-3p	NM_001079842	OCIAD1	174	-33.65	-37.3	0.002971
hsa-miR-6808-3p	NM_001080424	KDM6B	167	-27.86	-32.2	0.041929
hsa-miR-6808-3p	NM_001130969	NSMF	160	-30.33	-34.6	0.024001
hsa-miR-6808-3p	NM_001130970	NSMF	160	-30.33	-34.6	0.024001
hsa-miR-6808-3p	NM_001130971	NSMF	160	-30.33	-34.6	0.024001
hsa-miR-6808-3p	NM_001144951	GLYCTK	155	-28.92	-32.3	0.011389
hsa-miR-6808-3p	NM_001145348	KDM8	155	-27.63	-31.8	0.037219
hsa-miR-6808-3p	NM_001168254	OCIAD1	174	-33.65	-37.3	0.002971
hsa-miR-6808-3p	NM_001170931	FOXO4	152	-23.02	-34.4	0.018751
hsa-miR-6808-3p	NM_001178064	NSMF	160	-30.33	-34.6	0.024001
hsa-miR-6808-3p	NM_001220484	HEATR4	160	-24.77	-30.6	0.001339
hsa-miR-6808-3p	NM_001271719	SPINK2	162	-28.62	-30.7	0.009939
hsa-miR-6808-3p	NM_001271721	SPINK2	162	-28.62	-30.7	0.009939

hsa-miR-6808-3p	NM_001278506	NUBP1	156	-24.81	-32.4	0.002231
hsa-miR-6808-3p	NM_001286527	ATP2C2	163	-25.85	-31.4	0.012134
hsa-miR-6808-3p	NM_001291454	ATP2C2	163	-25.85	-31.4	0.012134
hsa-miR-6808-3p	NM_002484	NUBP1	156	-24.81	-32.4	0.002231
hsa-miR-6808-3p	NM_004717	DGKI	163	-26.84	-33.6	0.030388
hsa-miR-6808-3p	NM_005938	FOXO4	152	-23.02	-34.4	0.018751
hsa-miR-6808-3p	NM_006747	SIPA1	167	-28.86	-31.9	0.002005
hsa-miR-6808-3p	NM_013306	SNX15	157	-33.91	-36.1	0.003084
hsa-miR-6808-3p	NM_014861	ATP2C2	163	-25.85	-31.4	0.012134
hsa-miR-6808-3p	NM_015537	NSMF	160	-30.33	-34.6	0.024001
hsa-miR-6808-3p	NM_016320	NUP98	170	-30.34	-35.2	0.00908
hsa-miR-6808-3p	NM_017830	OCIAD1	174	-33.65	-37.3	0.002971
hsa-miR-6808-3p	NM_022492	TTC31	176	-33.03	-34.4	0.016761
hsa-miR-6808-3p	NM_024773	KDM8	155	-27.63	-31.8	0.037219
hsa-miR-6808-3p	NM_138477	CDAN1	150	-28.11	-31.3	0.039899
hsa-miR-6808-3p	NM_139132	NUP98	170	-30.34	-35.2	0.00908
hsa-miR-6808-3p	NM_145270	PRR35	161	-25.72	-28	0.020343
hsa-miR-6808-3p	NM_147777	SNX15	157	-33.91	-36.1	0.003084
hsa-miR-6808-3p	NM_153253	SIPA1	167	-28.86	-31.9	0.002005
hsa-miR-6808-3p	NM_178336	MRPL52	158	-27.58	-31	0.031053
hsa-miR-6808-3p	NM_180982	MRPL52	158	-27.58	-31	0.031053
hsa-miR-6808-3p	NM_181304	MRPL52	158	-27.58	-31	0.031053
hsa-miR-6808-3p	NM_181305	MRPL52	158	-27.58	-31	0.031053
hsa-miR-6808-3p	NM_181306	MRPL52	158	-27.58	-31	0.031053
hsa-miR-6808-3p	NM_181307	MRPL52	158	-27.58	-31	0.033954
hsa-miR-6808-3p	NM_198174	GRHL3	157	-24.36	-28	0.004932
hsa-miR-6808-3p	NM_203309	HEATR4	160	-24.77	-30.6	0.001339
hsa-miR-6808-3p	NR_029608	MIR10A	160	-24.09	-29.5	0.002038
hsa-miR-6808-3p	NR_039614	MIR4419A	154	-34.74	-34.3	0.000041
hsa-miR-6808-3p	NR_073417	SPINK2	162	-28.62	-30.7	0.026722

hsa-miR-6808-3p	NR_073418	SPINK2	156	-28.81	-30.3	0.03578
hsa-miR-6808-3p	NR_073419	SPINK2	162	-28.62	-30.7	0.029625
hsa-miR-6808-3p	NR_103488	TADA3	151	-31.03	-33.5	0.042551
hsa-miR-6808-3p	NR_106838	MIR6780A	158	-21.94	-25.2	0.009935
hsa-miR-6808-3p	NR_110121	LOC101928791	165	-27.09	-30.3	0.018949
hsa-miR-6808-3p	NR_130464	MIR4259	150	-17.94	-28.2	0.003691
hsa-miR-7706	NM_000199	SGSH	158	-29.7	-34.4	0.032715
hsa-miR-7706	NM_000934	SERPINF2	154	-31.42	-34.6	0.014811
hsa-miR-7706	NM_001008707	EML1	169	-33.07	-38.6	0.011918
hsa-miR-7706	NM_001015072	UFSP1	151	-21.63	-28.4	0.013292
hsa-miR-7706	NM_001033604	BBS9	158	-31.06	-36.5	0.007991
hsa-miR-7706	NM_001033605	BBS9	158	-31.06	-36.5	0.007991
hsa-miR-7706	NM_001037125	UNKL	159	-30.38	-33.6	0.014196
hsa-miR-7706	NM_001038603	MARVELD2	172	-27.71	-33.3	0.010341
hsa-miR-7706	NM_001077427	LYPD1	166	-29.65	-34.7	0.032699
hsa-miR-7706	NM_001079675	ETV4	164	-29.04	-34.5	0.013677
hsa-miR-7706	NM_001080495	TNRC18	172	-32.82	-37.9	0.008828
hsa-miR-7706	NM_001100625	CENPN	156	-30.07	-32.9	0.012559
hsa-miR-7706	NM_001113567	LRRC75A	170	-31.17	-35.9	0.026756
hsa-miR-7706	NM_001129742	CALHM3	152	-28.52	-33.6	0.008965
hsa-miR-7706	NM_001143888	BSDC1	156	-33.06	-36.6	0.027606
hsa-miR-7706	NM_001143889	BSDC1	156	-33.06	-36.6	0.027606
hsa-miR-7706	NM_001143890	BSDC1	156	-33.06	-36.6	0.027606
hsa-miR-7706	NM_001165920	SERPINF2	154	-31.42	-34.6	0.014811
hsa-miR-7706	NM_001165921	SERPINF2	154	-31.42	-34.6	0.014811
hsa-miR-7706	NM_001171083	SMPD4	165	-30.51	-35.6	0.019457
hsa-miR-7706	NM_001172663	RAB40C	156	-31.41	-34.9	0.042657
hsa-miR-7706	NM_001172664	RAB40C	156	-31.41	-34.9	0.042657
hsa-miR-7706	NM_001172665	RAB40C	156	-31.41	-34.9	0.042657
hsa-miR-7706	NM_001172666	RAB40C	156	-31.41	-34.9	0.042657

hsa-miR-7706	NM_001173988	RABL6	151	-21.9	-32.6	0.031283
hsa-miR-7706	NM_001198909	ATP6V1F	158	-23.36	-29.6	0.030918
hsa-miR-7706	NM_001244734	MARVELD2	172	-27.71	-33.3	0.010341
hsa-miR-7706	NM_001252294	SPDEF	150	-24.22	-34.1	0.009065
hsa-miR-7706	NM_001258397	CCDC103	174	-38.98	-40	0.003754
hsa-miR-7706	NM_001258398	CCDC103	174	-38.98	-40	0.003478
hsa-miR-7706	NM_001258399	CCDC103	174	-38.98	-40	0.003478
hsa-miR-7706	NM_001261437	ETV4	164	-29.04	-34.5	0.013677
hsa-miR-7706	NM_001261438	ETV4	164	-29.04	-34.5	0.013677
hsa-miR-7706	NM_001261439	ETV4	164	-29.04	-34.5	0.013677
hsa-miR-7706	NM_001286559	TEKT4	152	-26.35	-31.3	0.000521
hsa-miR-7706	NM_001303039	C7orf26	158	-27.92	-34.1	0.01226
hsa-miR-7706	NM_001986	ETV4	164	-29.04	-34.5	0.013677
hsa-miR-7706	NM_004231	ATP6V1F	158	-23.36	-29.6	0.030918
hsa-miR-7706	NM_004434	EML1	169	-33.07	-38.6	0.011918
hsa-miR-7706	NM_004610	TCP10	150	-25.99	-29.6	0.00287
hsa-miR-7706	NM_006683	EDDM3A	155	-27.91	-33.5	0.007363
hsa-miR-7706	NM_007349	PAXIP1	152	-25.12	-30.6	0.044304
hsa-miR-7706	NM_012391	SPDEF	150	-24.22	-34.1	0.009065
hsa-miR-7706	NM_014360	NKX2-8	153	-31.95	-33.5	0.032465
hsa-miR-7706	NM_014451	BBS9	158	-31.06	-36.5	0.007991
hsa-miR-7706	NM_014470	RND1	160	-30.55	-36.7	0.007616
hsa-miR-7706	NM_015316	PPP1R13B	160	-28.36	-34.1	0.04754
hsa-miR-7706	NM_015483	KBTBD2	165	-35.9	-38.6	0.005525
hsa-miR-7706	NM_015720	PODXL2	162	-28.57	-32.3	0.011524
hsa-miR-7706	NM_017751	SMPD4	165	-30.51	-35.6	0.019457
hsa-miR-7706	NM_017951	SMPD4	165	-30.51	-35.6	0.019457
hsa-miR-7706	NM_018045	BSDC1	156	-33.06	-36.6	0.027606
hsa-miR-7706	NM_021168	RAB40C	156	-31.41	-34.9	0.042657
hsa-miR-7706	NM_024067	C7orf26	158	-27.92	-34.1	0.01226

hsa-miR-7706	NM_024718	RABL6	151	-21.9	-32.6	0.031283
hsa-miR-7706	NM_025106	SPSB1	158	-33.99	-36.5	0.02809
hsa-miR-7706	NM_144586	LYPD1	166	-29.65	-34.7	0.032699
hsa-miR-7706	NM_144705	TEKT4	152	-26.35	-31.3	0.000521
hsa-miR-7706	NM_182689	EFNA4	154	-23.38	-30.9	0.028652
hsa-miR-7706	NM_182690	EFNA4	154	-23.38	-30.9	0.036383
hsa-miR-7706	NM_198428	BBS9	158	-31.06	-36.5	0.007991
hsa-miR-7706	NM_207387	LRRC75A	170	-31.17	-35.9	0.033483
hsa-miR-7706	NR_024116	RASA4CP	168	-31.06	-34.6	0.043405
hsa-miR-7706	NR_026910	ABHD11	168	-37.31	-37.2	0.013923
hsa-miR-7706	NR_026912	ABHD11	168	-37.31	-37.2	0.016448
hsa-miR-7706	NR_046320	GPX2	161	-31.1	-36.1	0.018433
hsa-miR-7706	NR_046321	GPX2	161	-31.1	-36.1	0.018412
hsa-miR-7706	NR_104108	SIGMAR1	159	-34.22	-35.1	0.037626
hsa-miR-7706	NR_120459	LINC01481	170	-31.05	-33.5	0.032041

标题
miRNA
NM_number
symbol
Miranda_Tot_score
Miranda_Tot_energy
rnahybrid_energy
rnahybrid_Pvalue

说明
已知的miRNA的名字
mRNA的转录本编号（来自Refseq数据库）
转录本对应的基因名称
miranda 软件预测的两者结合区域比对情况的打分值的总和
miranda软件预测的两者结合区域的自由能的总和
rnahybird 软件预测的二者结合区域的自由能
ahybird 软件为了控制结果的假阳性率，对每个预测的结合位点计算的p值
